
JOURNAL INFORMATION
==============================
NLM Title Abbreviation: Innov Aging
Journal ID: Innov Aging
NLM Journal ID: 101703706
Journal ID: innovateage

Innovation in Aging

EISSN: 2399-5300
Publisher: Oxford University Press

ARTICLE INFORMATION
==============================
PMCID: PMC6840740
DOI: 10.1093/geroni/igz038
Article ID: igz038
Article version: 1
Subjects: Full GSA 2019 Abstract Book Pdf

Program Abstracts from the GSA 2019 Annual Scientific Meeting “Strength in Age—Harnessing the Power of Networks”

Publication date: 2019 Nov
Electronic publication date: 2019 Nov 8
Volume: 3
Issue: Suppl 1
Issue title: Program Abstracts from the GSA 2019 Annual Scientific Meeting “Strength in Age—Harnessing the Power of Networks”
First page: NP
Last page: NP
Copyright: © The Author(s) 2019. Published by Oxford University Press on behalf of The Gerontological Society of America.
Copyright year: 2019
License: This is an Open Access article distributed under the terms of the Creative Commons Attribution License (http://creativecommons.org/licenses/by/4.0/), which permits unrestricted reuse, distribution, and reproduction in any medium, provided the original work is properly cited.
License URL: https://creativecommons.org/licenses/by/4.0/

Page count: 1

==============================
ISSN 2399-5300 | ONLINE
Vol. 3 | No. S1 • November 2019

### INNOVATION IN AGING®

An Open Access Journal of
The Gerontological Society of America

**Program Abstracts from the GSA 2019 Annual Scientific Meeting**
**"Strength in Age—Harnessing the Power of Networks"**

OXFORD UNIVERSITY PRESS

THE GERONTOLOGICAL SOCIETY OF AMERICA®

### INNOVATION IN AGING

### An Open Access Journal of The Gerontological Society of America

**Laura P. Sands, PhD, FGSA**
**Editor-in-Chief**
Virginia Tech
230 Grove Lane (0555)
Blacksburg, VA 24061
E-mail: lsands@vt.edu

**Steven M. Albert, PhD, FGSA**
**Deputy Editor-in-Chief**
University of Pittsburgh

**Christine E. Bishop, PhD, FGSA**
**Associate Editor, Economics of Aging**
Brandeis University

**Howard B. Degenholtz, PhD, FGSA**
**Associate Editor, Long-Term Services and Supports**
University of Pittsburgh

**Min-Ah Lee, PhD**
**Associate Editor, Relationships & Well-Being**
Chung-Ang University, South Korea

**Richard Pak, PhD**
**Associate Editor, Technology**
Clemson University

**Alison Phinney, PhD, RN**
**Associate Editor, Qualitative Research**
University of British Columbia, Canada

**J. Tina Savla, PhD, FGSA**
**Associate Editor, Methodology**
Virginia Tech

**Karen Jung, MSW**
**Managing Editor**
The Gerontological Society of America
1220 L Street, NW, Suite 901
Washington, DC 20005
E-mail: ia@geron.org

**J. Jill Suitor, PhD, FGSA**
**Deputy Editor-in-Chief**
Purdue University

**Jennifer Tehan Stanley, PhD**
**Associate Editor, Psychological Sciences**
University of Akron

**Roland J. Thorpe, Jr., PhD, FGSA**
**Associate Editor, Minority Health/Health Disparities**
Johns Hopkins Bloomberg School of Public Health

**Michael Weiner, MD, MPH**
**Associate Editor, Health Outcomes Research and Informatics**
Indiana University, Regenstrief Institute, Inc., and U.S. Department of Veterans Affairs

**Elaine C. Wiersma, PhD**
**Deputy Associate Editor, Qualitative Research**
Lakehead University, Canada

### ADVISORY BOARD

**Jacqueline L. Angel, PhD, FGSA**
The University of Texas at Austin

**Toni C. Antonucci, PhD, FGSA**
University of Michigan

**Kenneth Covinsky, MD**
University of California, San Francisco

**Kenneth F. Ferraro, PhD, FGSA**
Purdue University

**Karl Pillemer, PhD, FGSA**
Cornell University

**Wendy A. Rogers, PhD, FGSA**
University of Illinois Urbana-Champaign

**Richard Schulz, PhD, FGSA**
University of Pittsburgh

### PUBLICATIONS COMMITTEE

**Margie E. Lachman, PhD, FGSA, Chair**
Brandeis University

**Panayiotis D. Tsitouras, MD, FGSA, Past Chair**
University of Oklahoma

**Rozalyn M. Anderson, PhD, FGSA**
University of Wisconsin-Madison

**Debra Bakerjian, PhD, APRN, FAAN, FAANP, FGSA**
University of California, Davis

**Elizabeth J. Bergman, PhD**
Ithaca College

**Lisa K. Borrero, PhD**
University of Indianapolis

**J. Scott Brown, PhD, FGSA**
Miami University

**Deborah Carr, PhD, FGSA**
Boston University

**Tamas Fulop, MD, PhD, FGSA**
Université de Sherbrooke

**Judith L. Howe, PhD, DSW, FGSA**
Mount Sinai School of Medicine

**Derek M. Isaacowitz, PhD, FGSA**
Northeastern University

**Brian P. Kaskie, PhD**
University of Iowa

**Catheryn Koss, JD, PhD**
California State University, Sacramento

**David G. Le Couteur, FRACP, PhD**
The University of Sydney

**Skye N. Leedahl, PhD**
University of Rhode Island

**Robert T. Mankowski, PhD**
University of Florida

**Suzanne Meeks, PhD, FGSA**
University of Louisville

**Anne B. Newman, MD, MPH, FGSA**
University of Pittsburgh

**Kushang V. Patel, PhD, MPH, FGSA**
University of Washington

**Miriam S. Rose, M.Ed.**
Benjamin Rose Institute on Aging

**Laura P. Sands, PhD, FGSA**
Virginia Tech

**Amber Watts, PhD**
The University of Kansas

**Rong Yuan, MD, PhD**
SIU School of Medicine

**Fellow of The Gerontological Society of America (FGSA)**

### OUP PRODUCTION DEPARTMENT

**Katie Ostrowka**, *Journal Manager - Production*

### INNOVATION IN AGING

**An Open Access Journal of The Gerontological Society of America**

### EDITORIAL BOARD

**Kathleen Abrahamson, RN, PhD, FGSA**
Purdue University

**Tamara A. Baker, PhD, FGSA**
University of Kansas

**Scott R. Beach, PhD, FGSA**
University of Pittsburgh

**Sara J. Czaja, PhD, FGSA**
University of Miami

**Adam Davey, PhD, FGSA**
University of Delaware, Newark

**Nancy R. Gee, PhD**
Virginia Commonwealth University

**Megan Gilligan, PhD**
Iowa State University

**Mary Ann Johnson, PhD**
University of Nebraska Lincoln

**Yoshinori Kamo, PhD**
Louisiana State University

**Giyeon Kim, PhD, FGSA**
Chung-Ang University, South Korea

**Hui Liu, PhD, FGSA**
Michigan State University

**Mariana López-Ortega, PhD, MPP**
National Institute of Geriatrics, National Institutes of Health, Mexico

**Anne Collins McLaughlin, PhD**
North Carolina State University

**David A. Nace, MD, MPH**
University of Pittsburgh

**Marcia Ory, PhD, FGSA**
Texas A&M

**Karen A. Roberto, PhD, FGSA**
Virginia Tech

**Miles G. Taylor, PhD, FGSA**
Florida State University

**Robert Joseph Taylor, MSW, PhD**
University of Michigan

**Noah J. Webster, PhD**
University of Michigan

**Richard Winett, PhD**
Virginia Tech

**Jeremy B. Yorgason, PhD**
Brigham Young University

**Anna Zajacova, PhD, FGSA**
Western University, Canada

**Fellow of The Gerontological Society of America (FGSA)**

### Abstracts

### GSA Annual Scientific Meeting 2019

Abstracts are arranged numerically by session and in the order of presentation within each session.

Abstracts are published as received.

### SESSION 500 (PAPER)

### ADVANCEMENTS IN DEMENTIA AND ALZHEIMER'S DISEASE RESEARCH

### BRAIN FITNESS INTERVENTION ON KNOWLEDGE AND WORRIES ABOUT ALZHEIMER'S: A RANDOMIZED CONTROLLED TRIAL

Kaipeng Wang,[1] Fei Sun,[2] Qiuling An,[3] Yanfei Han,[3] and Yi Zhou[3], *1. Texas State University, San Marcos, Texas, United States, 2. Michigan State University, East Lansing, Michigan, United States, 3. East China Normal University, Putuo, Shanghai, China*

As one of the world's fastest aging countries, China's growing prevalence of Alzheimer's and related dementias (ADRD) poses concerns among older adults. Lack of knowledge about ADRD and excessive worries about ADRD can cause cumulative stress and threaten physical and psychological well-being of older adults. We conducted a randomized controlled trial to examine the effectiveness of a Brain Fitness Intervention (BFI) on the knowledge and worries about Alzheimer's at three senior residential care facilities in China. Ninety older adults aged 60 and above underwent randomization. Fifty participants in the intervention group received eight weekly BFI sessions, including Tai Chi exercise, experiential learning, and group discussions. The primary outcomes were the changes from baseline to Week 8 in the scores on the ADRD knowledge scale (ranging 5–50) and worry scale (ranging 5–45). Twenty-seven participants withdrew from the study. Intent-to-treat analysis showed that the estimated mean change in knowledge on ADRD was 4.26 in the treatment group and -1.52 in the control group ($p < 0.001$). The estimated mean change in worries about ADRD was -10.25 in the treatment group and -2.9 in the control group ($p < 0.001$). Results remained robust in sensitivity analysis adjusting for study sites and baseline characteristics. Heterogeneity analysis showed that the treatment effect became stronger with the increase of age. Findings support the effectiveness of BFI in increasing ADRD knowledge and reducing worries among Chinese older adults. Future trials with larger sample sizes will be needed for more conclusive results.

### MY MESSAGE: CUSTOMIZED VISUAL CUES FOR PERSONS WITH DEMENTIA USING ADAPTIVE DISPLAY TECHNOLOGY

Michael Skrajner,[1] John Zeisel,[2] gregg Gorzelle,[2] Tom Albright,[3] and Sergei Gepshtein[3], *1. Hearthstone Alzheimer Care, Woburn, Massachusetts, United States, 2. Hearthstone, Woburn, Massachusetts, United States, 3. Salk Institute, La Jolla, California, United States*

The My MESSAGE™ (MM) intervention visually conveys individualized messages to persons with dementia (PWD) on adaptive electronic displays in strategic locations activated by an approaching individual. MM aims to improve quality of life (QoL) and reduce challenging behaviors, especially repetitive questioning and wayfinding difficulties. For example, for a PWD who repeatedly asks when his/her daughter will visit, the monitors display a reassuring message, such as, "I love you and will visit soon." A PWD who has trouble finding his/her bedroom, receives directions on the monitor. MM was tested in a Phase 1 STTR. The sample consisted of 22 PWD: 10 in Experimental Group (EG) and 12 in Control Group (CG). MM had positive immediate impacts on EG participants, with 100% exhibiting positive affect, 80% reduced agitation, 60% reduced anxiety, and 50% improved mood. MM had positive longer-term/generalized impacts on PWD, as well. For the CG, QoL (based on the Dementia Quality of Life Scale) tended to decline from baseline to post-treatment for the CG ($p=0.053$); with no decline in the EG. Agitation (based on the Cohen-Mansfield Agitation Inventory) tended to increase in the CG ($p=0.057$); no change was found in the EG. There was a significant decrease in neuropsychiatric symptoms (based on the Neuropsychiatric Inventory-Nursing Home) for the EG ($p=.040$); no change was detected for the CG. The results suggest that MM is worthy of further development and evaluation in a planned Phase 2 study.

### OBJECTIVELY MEASURED SLEEP CHARACTERISTICS OF OLDER ADULTS WITH AND WITHOUT ALZHEIMER'S DISEASE

Alex Laffer,[1] Hilary J. Hicks,[1] Genna Losinski,[1] and Amber Watts[1], *1. University of Kansas, Lawrence, Kansas, United States*

Older adults commonly experience disturbed sleep such as difficulty initiating or maintaining sleep. Older adults who experience impaired sleep are at increased risk for cognitive decline or developing Alzheimer's disease (AD). Research has shown that people with AD experience changes in sleep patterns, however, these changes are not well characterized. To better understand sleep in an older adult population with and without AD, the present study aimed to describe and compare objective sleep characteristics in both. Participants were older adults (126 with and 41 without AD) who wore an ActiGraph GT9X monitor on their non-dominant wrist for 7 days in a free-living environment. Results suggest that, compared to those without AD, participants with AD spent significantly more time in bed, $t(165) = -4.37$, $p = .001$), slept for longer durations, $t(165) = -2.39$, $p = .044$), and had less efficient sleep, $t(165) = 2.71$, $p = .007$. Participants with AD also had significantly greater sleep onset latency, more time awake after sleep onset, longer awakening lengths, and tended to arise later in the morning (all $p \leq .016$). No differences were found between the groups in age, bedtime, or the number of awakenings during the night. These findings add to our understanding of the sleep disturbances experienced by older adults with and without AD. Significant group differences suggest that interventions may be necessary in treating sleep disturbances for older adults with and without AD. Future studies should examine sleep longitudinally to understand risk factors related to AD.

### TASK-SPECIFIC METACOGNITIVE ACCURACY DECLINES ACROSS THE DEMENTIA-ALZHEIMER'S TYPE SPECTRUM

Annalise M. Rahman-Filipiak,[1] Arijit Bhaumik,[2] Bruno Giordani,[3] Henry Paulson,[3] and Benjamin M. Hampstead[1], *1. University of Michigan, Ann Arbor, United States, 2. Michigan Alzheimer's Disease Center, Ann Arbor, Michigan, United States, 3. University of Michigan, Ann Arbor, Michigan, United States*

Subjective cognitive complaints (SCCs) remain part of the diagnostic criteria for amnestic mild cognitive impairment (aMCI), the prodromal stage of dementia - Alzheimer's type (DAT), despite weak relationships between self-reported and objectively-measured functioning. Most metacognitive measures focus on ratings of global retrospective memory rating only; greater subtlety in measurement of SCCs is required. Similarly, it is critical to identify the disease stage at which the clinical utility of SCCs is nullified by impaired insight. This study aims to evaluate group differences in (a) task-specific metacognitive ratings, and (b) the accuracy of these ratings in individuals diagnosed as cognitively intact (CI), with aMCI, or with DAT. 99 older adults (M-age = 69.43, SD-age = 6.98; M-edu = 15.54, SD-edu = 2.47; CI: n = 50, aMCI: n = 34, DAT: n = 15) enrolled in the University of Michigan Memory and Aging Project rated their performance on the Object Location Touchscreen Task (OLTT), an ecologically valid memory measure. One-way analysis of variance (ANOVA) revealed that individuals with aMCI-multiple domain or DAT rated their memory performance similarly to CI individuals, though the aMCI-single domain group rated themselves as more impaired. Bivariate Pearson's r correlations demonstrated a decline in the strength of the relationship between task-specific metacognitive ratings and actual OLTT memory performance with increasing diagnostic severity. These findings suggest a decline in insight on task-specific memory ratings across the DAT spectrum, and call into question the use of self-reported SCCs as a diagnostic tool in later stages of disease progression.

### THE LEAD GUIDE: LIFE PLANNING IN EARLY ALZHEIMER'S AND DEMENTIA

Kara B. Dassel,[1] Katherine Supiano,[1] Rebecca Utz,[1] Sara Bybee,[1] and Eli Iacob[1], *1. University of Utah, Salt Lake City, Utah, United States*

To address the characteristics of Alzheimer's disease and related dementias (ADRD) that complicate end-of-life (EOL), we created and validated a dementia-specific EOL planning instrument. This instrument can be used to facilitate discussions and provide documentation of EOL values and care preferences prior to loss of decisional capacity. Instrument development used a mixed-method design that included: a) conducting a series of focus groups (healthy adults, persons with early-stage ADRD, and dementia caregivers) to develop and confirm content, comprehensiveness, and usability, b) evaluation by content experts to verify instrument utility in clinical practice; and c) conducting a national survey of healthy older adults and adults with early-stage ADRD to evaluate instrument psychometric properties. We describe the expansion of the instrument from survey tool into a user-friendly guide: "The LEAD Guide" or Life-Planning in Early Alzheimer's and Dementias Guide. We describe the utility of the LEAD Guide for persons planning ahead for the possibility of ADRD, those with MCI and their families, for providers to use with patients and families, and for researchers studying the EOL preferences and values of persons in these populations. Instructions specific to each user group are health literacy appropriate, and include a glossary of terms. Age-friendly graphic design and availability in both print and e-versions enhance utility. The LEAD Guide can be utilized to help inform EOL care decisions and ensure that they align with the patient's values and may serve as the basis of an intervention to support choice in persons with dementia.

### SESSION 505 (PAPER)

### CURRENT WORKFORCE INITIATIVES IN GERIATRICS

### ARE YOU READY FOR CHANGE? FACTORS ASSOCIATED WITH COMMITMENT TO CHANGE IN CLINICAL PRACTICE

Jasleen K. Chahal,[1] Kimberly Northrip,[1] and Dena Silva[2], *1. UK HealthCare CECentral, Lexington, Kentucky, United States, 2. Incedo - University of North Texas Health Science Center, Fort Worth, Texas, United States*

Commitment to Change (CTC) has been shown to be an effective performance measure of continuing professional development (CPD) training, in that 50-67% of providers choosing CTC also made behavioral changes in clinical practice (Domino et al. 2011; Perkins et al., 2007). However, very little is known about the factors that are associated with a learner committing to practice change. This study presents findings from a retrospective observational study that compared trends across various health profession, demographics, and activity-type categories to identify learner characteristics that may lead to higher CTC responses. Learner data from 2014 -2017 was obtained from two different continuing education office databases. The combined dataset contained 68,365 evaluations from 26,508 learners, of which 22.8% wrote a CTC. At both sites, CTC was more likely if the activity was enduring or charged a fee and less likely for an RSS ($p<0.001$). Results indicate that CTC varies based on provider demographics, such as profession and specialty. Allied health providers (32.4-37.5%) and health educators (36%) were most likely to make a commitment, followed by nurses (28.6%), physicians (23.9%), pharmacists (14.9%), and dentists (9.1%). Physician specialties and provider region also varied with the highest CTC by geriatricians (50%) and occupational medicine providers (42.3%) and the lowest rates of CTC found in the southeast region. Results of the study will help inform the development and implementation of future CPD in order to successfully engage learners in committing to change clinical practices.

### ATRA COMPETENCY STUDY: PROMOTING DEVELOPMENT OF RECREATIONAL THERAPISTS SERVING OLDER ADULTS

Martha E. Kemeny,[1] Brent Hawkins,[2] and Heather Porter[3], *1. Slippery Rock University, Slippery Rock, Pennsylvania, United States, 2. Clemson University, Clemson, South*

*Carolina, United States, 3. Temple University, Philadelphia, Pennsylvania, United States*

Competency concerns the knowledge, skills, and abilities needed to perform specific job tasks. In the field of recreational therapy (RT), four documents from the certification (NCTRC), accreditation (CARTE), and professional association (ATRA) broadly identify areas of competency for RTs. While broad competencies are defined, no specific competencies (i.e., diagnoses, assessments, interventions, theories) are identified. Sixty-seven RT experts, including 10 verified older adult experts with greater than five years experience and peer-reviewed publications, participated in three rounds of a Delphi study to gain content consensus. After experts in each setting identified key terms in the first round, experts reviewed the consolidated list twice to generate the final competency list. Next, all certified recreational therapists were invited to rate the extent that each competency item is being used in RT practice; the extent of their knowledge/skill for each item; and the degree of interest in gaining more knowledge/skill. A total of 1377 recreational therapists participated in the study (88% females, 11% male, 1% non-disclosed) with participants ranging in age from 20-60+ years of age. The study's findings related to specific diagnostic populations served; interventions/modalities, techniques, standardized assessment tools, and theories utilized; education, training, and counseling topics in treatment; and on-the-horizon treatment and issues. This comprehensive two-part multi-year study is the first to explore current RT practice at a micro-competency level. In the effort to improve practice, these specific competencies necessary for RT practice with older adults are significant to both educators and practitioners in future curriculum and professional development efforts.

### BUILDING CAPACITY TO CARE FOR OLDER PEOPLE! HOW IS CARE OF THE OLDER PERSON TAUGHT IN AUSTRALIAN SCHOOLS OF NURSING?

Deirdre M. Fetherstonhaugh,[1] Jo-anne Rayner,[1] Elizabeth Beattie,[2] Ann Harrington,[3] Yun-Hee Jeon,[4] Wendy Moyle,[5] Deborah Parker,[6] and Chris Toye[7], *1. La Trobe University, Victoria, Australia, 2. Queensland University of Technology, Queensland, Australia, 3. Flinders University, South Australia, Australia, 4. Sydney University, New South Wales, Australia, 5. Griffith University, Queensland, Australia, 6. University of Technology, New South Wales, Australia, 7. Curtin University, Western Australia, Australia*

As the Australian population ages the demand for nursing care which focuses on responding to the needs of the older person will increase. Few newly graduated Registered Nurses (RNs) currently enter the aged care workforce and few select a career in caring for older people; yet older people are the largest patient group in most health care environments. This research, conducted by the Australian Hartford Consortium of Gerontological Nursing Excellence (AusHCGNE), explored how care of the older person is currently taught in Australian schools of nursing (SoN). The interview guide included questions about: whether care of the older person is taught in separate subjects or integrated across the curriculum; academics' qualifications; subject content; and aged care clinical placements. The head of each of the 33 Australian schools of nursing was contacted, invited to participate and asked to nominate the appropriate academics (undergraduate/curriculum co-ordinators) who would be the most appropriate person to participate in the interview. These academics were then contacted, written informed consent was obtained, interviews were scheduled and completed. This research is timely given the current Royal Commission into Aged Care Quality and Safety in Australia, one focus of which is nurses in residential aged care in respect to numbers, education and competence. This research will be completed by mid-2019. The results will be fed back to SoN to inform the development of their curricula and the preparation of future RNs who will undoubtably need to be expert in the care of older people across the health sector.

### ENHANCING PERSON-CENTERED DEMENTIA CARE BY NURSING HOME STAFF USING MICROLEARNING: LITTLE MESSAGE WITH A BIG IMPACT

Jenny Inker,[1] Christine J. Jensen,[2] and Sonya Barsness[3], *1. Virginia Commonwealth University, Richmond, Virginia, United States, 2. Riverside Center for Excellence in Aging and Lifelong Health, Williamsburg, Virginia, United States, 3. Sonya Barsness Consulting, LLC, Arlington, Virginia, United States*

Effective training is critical to providing quality care in long-term care environments, where many residents have dementia. Training has been linked to positive resident care outcomes and improved job satisfaction of staff. The aim of this study was to develop, pilot, and evaluate a Microlearning training curriculum, using short (5-10 minute) "bursts" of training available through an online platform on demand (i.e. 24/7). The expected outcomes were to improve staff knowledge, attitudes, and skills regarding person-centered dementia care and to increase job satisfaction. Researchers translated the Centers for Medicare and Medicaid Hand-in-Hand training curriculum into 52 weekly Microlearning lessons delivered via an online platform (accessible by computer, IPad or smart phone) followed by a short quiz. Using pre- and post-tests, nine focus groups, and fourteen telephone interviews, the researchers engaged with a convenience sample of staff (N = 244) working at all levels from direct care to leadership in nine nursing homes in Virginia. Pre- and post-tests comprised items from the Dementia Attitudes Scale and the Nursing Home Nurse Aide Job Satisfaction Scale. Results from a between subjects t-test demonstrated significant improvements in attitudes to people with dementia. Focus groups and interviews revealed high satisfaction with the training with a significant majority agreeing it was a helpful way to learn and that they were able to apply what they had learned to caring for residents. This pilot demonstrates a promising new practice for training long-term care staff. Further research using a control group receiving usual training is indicated.

### SESSION 510 (SYMPOSIUM)

### DISPARITIES IN HUMAN CAPITAL INVESTMENT OVER THE GENDERED LIFE COURSE: AN INTERNATIONAL COMPARISON

Chair: Phyllis Cummins, *Scripps Gerontology Center, Miami University, Oxford, Ohio, United States*

Co-Chair: Takashi Yamashita, *University of Maryland, Baltimore County, Baltimore, Maryland, United States*
Discussant: Christopher Phillipson, *The University of Manchester, Manchester, United Kingdom*

Income disparities by gender are a persistent problem throughout the world. These disparities place women at risk for economic insecurity both while working and in retirement. Education and continued skill upgrading are key to reducing income disparities, but it is well documented that both older men and women are less likely to participate in adult education and training (AET) than their younger counterparts. In this symposium we present gender and age-based differences in AET in Australia, Canada, England/Northern Ireland and the United States. Also, given the increasing use of technology, technology-related problem-solving skills are compared across these four nations. In addition, we discuss current, and potentially new, country level policies and practices that facilitate the provision of AET over the second half of the life course. Yamashita and colleagues use data from the Program for the International Assessment of Adult Competencies (PIAAC) to provide an overview of AET participation, income, and technology-related problem-solving skills by sex and age groups in the four countries. Vickerstaff and van der Horst use data from five different organizations in the United Kingdom to examine attitudes of older workers about participation in training and the extent to which these attitudes result from self-imposed ageism. Taylor presents survey data from Australia that analyzes types of training women are undertaking, factors associated with participation in training, and the importance of employer support. Finally, Harrington and Cummins use PIAAC data to analyze age variations in AET participation and gender differences in employer sponsored training in Canada.

### TRAINING AT WORK: THE IMPACT OF INTERNALIZED AGEISM

Sarah A. Vickerstaff,[1] and Mariska F. van der Horst[2], *1. University of Kent, Canterbury, Kent, United Kingdom, 2. Vrije Universiteit Amsterdam, Amsterdam, Noord-Holland, Netherlands*

It is well documented that seniors receive less work based training than younger counterparts. Studies have assessed the extent to which managers afford access to training because of stereotypes about older workers being less trainable or interested in training. Less is known about the degree to which individuals have internalised age stereotypes and self-stereotype which impacts their attitudes about training. In this work place based study, through analysis of interviews with older workers we examine the extent to which internalised ageism is an inhibitor for seeking further training. We examine the role of gender and work setting. The data includes 185 participants in five different UK organisations in blue collar, white collar, managerial, manufacturing and services sectors. There is some evidence that older workers may feel that they are less deserving and less capable of training and development with implications for policy in this field.

### OLDER AUSTRALIAN WOMEN'S HUMAN CAPITAL: PERCEPTIONS OF SKILL LEVELS AND PARTICIPATION IN LEARNING ACTIVITIES

Philip Taylor[1], *1. Federation University Australia, Melbourne, Victoria, Australia*

While business cases for older workers' employment stress their value it is well-known that they participate less in training activities than younger workers. Women are at particular risk of not accessing training opportunities. Drawing on a survey of 2500 women aged over 50 we report a fine-grained analysis of the types of training women were undertaking and the factors associated with participation in training. The analysis indicates that training is infrequently undertaken in preparation for a new job and large majorities of women see no need to and are not interested in retraining. This is observed across occupational groups, but more commonly among those with low educational levels. A lack of employer support is much less commonly reported as a barrier to older women's participating in training. The findings suggest that it is primarily at women themselves that efforts aimed at promoting human capital development need to be directed.

### ADULT EDUCATION AND TRAINING IN CANADA: OPPORTUNITIES, FUNDING, AND GENDER GAPS

A. Katherine Harrington,[1] and Phyllis Cummins[1], *1. Scripps Gerontology Center, Miami University, Oxford, Ohio, United States*

Labor force participation rates for middle-aged and older Canadians have increased substantially over the past two decades, with increases for women outpacing men. Given the importance of adult education and training (AET) to stay competitive in later career, we used a mixed methods approach to examine gender differences. Our analysis of the 2012 Program for the International Assessment of Adult Competencies (PIAAC) data indicated that, for ages 55-65, rates of AET participation are similar for both men and women. However, women are less likely than men to have AET funded by their employers. Findings suggest that women are more likely to need alternate funding sources for AET, such as other organizations or through self-funding. In addition, our review of literature, policy-related documents, and key informant interviews identified possible changes in policies and practices for the promotion of AET for middle-aged and older Canadians.

### AN INTERNATIONAL COMPARISON OF INCOME DISPARITIES BY PROBLEM-SOLVING SKILLS, GENDER, AND AGE

Nader Mehri,[1] Takashi Yamashita,[2] Roberto Millar,[2] and Phyllis Cummins[1], *1. Miami University, Oxford, Ohio, United States, 2. University of Maryland, Baltimore, Maryland, United States*

Income disparities by gender have been a persistent problem in economically-developed countries for decades, with income gaps often widening over the adult life course. We use data from the 2012 Program for the International Assessment of Adult Competencies (PIAAC) to examine relationships among problem solving skills in technology-rich environments (PSTRE), income, sex, and age in Australia, Canada, England/Northern Ireland and the United States. Women age 35 to 44 in the middle-to-high (i.e., 50th - 75th percentile) income group had significantly higher PSTRE scores than their male counterparts in Australia and Canada. For the same income group, women ages 55 to 65 had significantly higher. PSTRE scores than men in Canada and England/Northern Ireland. These results suggest that women with similar skills lagged their male counterparts in income in specific sub-populations in specific countries. We provide possible explanations for these differences and conclude with implications for policy and practice.

### SESSION 515 (PAPER)

### ECONOMIC OPPORTUNITIES AND CHALLENGES ACROSS THE LIFE COURSE

### INFLUENCE OF ECONOMIC WELL-BEING ON HEALTH, AND MODERATING EFFECT OF SOCIAL ENGAGEMENT AMONG OLDER CAMBODIANS

Kakada Kuy,[1] and Sojung Park[2], *1. WASHINGTON UNIVERSITY IN ST LOUIS, St. Louis, United States, 2. Brown School, Washington University in St. Louis, St. Louis, Missouri, United States*

Similar to other post-conflict nations in Southeast Asia, impacts of civil wars and violence, coupled with the present poverty, place older Cambodians in vulnerable health conditions. Older Cambodians have limited access to basic healthcare and decent living conditions. To date, little research has been conducted to understand their health and related determinants. This study aimed to examine the influence of economic wellbeing and the moderating effect of social engagement on the physical health of older Cambodians. Data came from Survey of Elderly Cambodia (2004), the only existing nationally representative dataset of older Cambodians age 60 and above (N=1,273). Economic wellbeing was measured in two dimensions: (1) subjective economic wellbeing with three indicators of self-rated economic satisfaction and (2) objective economic wellbeing with household item possession and current housing conditions. Social engagement was examined with four types of engagement in the community. For physical health, the number of health complaints was examined. Results of Poisson regression showed objective economic wellbeing, such as access to basic household items and decent housing conditions, was significantly related to physical health ($p < .001$). However, we found no role of social engagement in the association between the subjective economic wellbeing and health. Furthermore, social engagement has a significant moderating effect on the association between objective economic wellbeing and physical health ($p < .001$), controlling for all covariates. For anti-poverty and health policy development for older Cambodians, a promising intervention effort may focus on the ways to facilitate social engagement in later years.

### NATURALLY OCCURRING VARIABILITY IN FINANCIAL STRESS AND INFLAMMATORY BIOMARKERS AMONG OLDER ADULTS

Laura Samuel,[1] Sarah L. Szanton,[2] Neal Fedarko,[2] and Eleanor M. Simonsick[3], *1. Johns Hopkins University School of Nursing, Baltimore, Maryland, United States, 2. Johns Hopkins School of Medicine, Baltimore, Maryland, United States, 3. Longitudinal Studies Section, Intramural Research Program, National Institute on Aging, Baltimore, Maryland, United States*

Financial strain (i.e. difficulty making ends meet) is associated with earlier disability and mortality among older adults, but inflammatory response to financial stress is untested. This study leverages monthly increases in financial stress among older adults receiving Social Security payments monthly on the 3rd. The Health ABC Study randomly sampled black and white adults aged 70-79 free of disability from Memphis, TN and Pittsburgh, PA in 1997 and 1998. Baseline biomarkers included TNF α, IL-6, CRP, and soluble receptors were measured for about half the sample (IL-2, IL-6, and TNF α Type I and II). Days since Social Security payment was calculated using clinic visit date, which naturally varied in the sample. Restricted cubic spline models stratified by financial strain status tested hypothesized associations, adjusting for financial strain scores, food insecurity, employment status and monthly invariant potential confounders (age, gender, number of inflammatory conditions, waist circumference and anti-inflammatory medications). Among those with financial strain, each additional day since payment was associated with higher TNF-α levels during first week of the month (n=1,633, coefficient=0.0830, $p=0.030$) and higher IL-6 soluble receptor levels during the last week of the month (n=828, coefficient=12033.44, $p=0.037$), but not with other biomarkers nor during the middle of the month. Days since payment was not associated with biomarkers among those without financial strain. These results suggest upregulation of pro-inflammatory and not anti-inflammatory pathways during the beginning and end of the month among financially strained older adults. Financial strain is modifiable and these results call attention to addressing it.

### OLD-AGE DEPRESSIVE SYMPTOMS: THE ROLE OF EARLY-LIFE FINANCIAL STRAIN AND LATE-LIFE SOCIAL ENGAGEMENT

Federico Triolo,[1] Linnea Sjöberg,[1] Davide Vetrano,[1] Alexander Darin-Mattsson,[1] Marco Bertolotti,[2] Laura Fratiglioni,[1] and Serhiy Dekhtyar[1], *1. Aging Research Center, Department of Neurobiology, Care Sciences and Society, Karolinska Institutet and Stockholm University, Stockholm, Sweden, 2. Department of Biomedical, Metabolic and Neural Sciences, University of Modena and Reggio Emilia, Modena, Italy*

It remains unclear if childhood socioeconomic disadvantage is associated with depression in old age. This study aims to investigate the effect of childhood financial strain on depressive symptoms in old age, and to examine whether late-life social engagement modifies this association. Data from the Swedish National study of Aging and Care in Kungsholmen, a community-based longitudinal study of aging, spanning clinical assessments over 15 years of follow-up were used. Information on financial strain in childhood was collected at baseline. Repeated measures of the Montgomery–Åsberg Depression Rating Scale were used to define depressive trajectories. A social engagement index comprised information on baseline social network and leisure activities. Linear mixed models were used to estimate depressive trajectories. Childhood financial strain was associated with a higher level of depressive symptoms ($\beta = 0.36$; $p<0.05$), but not the rate of symptom accumulation over time. Relative to those with a combination of no financial strain and active social engagement, the level of depressive symptoms was progressively increased in those without financial strain but with inactive social engagement (β = 0.29; p<0.05), as well as in those with both financial strain and inactive engagement (β= 0.83; p<0.05). Individuals with financial strain who had active social engagement exhibited a similar burden of symptoms as those without financial strain and with rich social engagement. Early-life financial strain may have a lasting effect on old age depressive symptoms, although its detrimental consequences may be modified by active social engagement in late life.

### ROBBING PETER TO PAY PAUL: HANDLING FINANCIAL CHALLENGES AMONG LOW-INCOME OLDER ADULTS

Laura Samuel,[1] Rebecca Wright,[2] Melissa Spahr,[1] Laken C. Roberts,[2] Sarah L. Szanton[2], *1. Johns Hopkins University School of Nursing, Baltimore, Maryland, United States, 2. Johns Hopkins School of Nursing, Baltimore, Maryland, United States*

Low income older adults often face financial challenges which increase their risk for earlier disability and mortality. This study explored the social norms, beliefs and practices relevant to handling financial challenges among low-income community-dwelling older adults residing near Baltimore, MD whom we recruited using convenience and snowball sampling. Four vignette-based focus group sessions included 28 participants. Using hierarchical thematic analysis, three key themes emerged. First, the theme "Rob Peter to pay Paul" describes the consensus that individuals must prioritize financial needs, which required individuals to "work with a budget", apply for aid, "cry for [aid]" and, when needed, "work something out" with landlords and lenders. One participant described the amount of work by saying "We're retired but we're working for ourselves." Secondly, the theme "Your rent should be first" describes how low income older adults prioritize housing over food and other needs because "resources for housing is a problem" and because homelessness is both more permanent and socially stigmatizing than hunger - "Don't nobody know you're hungry unless you tell them, but everybody know when you outdoors." Finally, the theme "We need to put the word out" describes the consensus that public benefits and community resources should be made more visible and accessible. Many individuals only know about resources because they seek information ("you go and you find out"), but "it's hard to ask for help." These results can inform the development and improvement of financial and community programs and policies for low-income older adults addressing financial challenges.

### THE POWER OF LOOKING AHEAD? A FIXED-EFFECTS MODEL OF FUTURE ASPIRATIONS OVER THE LIFE COURSE AND INCOME

Ioana Sendroiu,[1] and Laura Upenieks[1], *1. University of Toronto, Toronto, Canada, Canada*

Perceived life trajectories are rooted in structural systems of advantage and disadvantage, but individuals also shape their futures through setting goals and expectations. "Future aspirations" have typically been used in life course research to refer to one's conception of their chances of success across life domains and can serve as a resource to help individuals persevere in the face of hardship. Taking a life course approach and using three waves of data from the MIDUS study, we utilize hybrid fixed effects models to assess the relationship between future aspirations and income. We find that, net of age, health, and a host of other time-varying factors, more positive future aspirations are indeed related to higher income over time, but that this relationship takes different shapes in different contexts. In particular, in lower quality neighborhoods, higher future aspirations lead to worse economic outcomes over the life course, while in higher quality neighborhoods, higher aspirations are indeed related to higher incomes. We thus argue that aspirations are only helpful in some contexts, and are inherently contextual not just in their sources but also in their effects.

### SESSION 520 (PAPER)

### END-OF-LIFE CARE

### CULTURAL COMPETENCE TRAINING FOR HOSPICE STAFF: FINDINGS FROM A NATIONAL HOSPICE SURVEY

Nathan Boucher,[1] Kimberly S. Johnson[2], *1. Durham VA Health System, Durham, North Carolina, United States, 2. Duke University, Durham, North Carolina, United States*

Compared to whites, racial/ethnic minorities are less likely to enroll in hospice and if they enroll, more likely to experience poor quality care. Building cultural competence (CC) among hospice staff is a strategy that may reduce these disparities. We conducted a national survey of hospices' practices to promote CC. A total of 197 hospices participated; most were not-for-profit (80%) with an average daily census over 100 (53%); 73% offered staff cultural competence training (CCT). There were no differences in characteristics of hospices who offered CCT and those that did not. Of hospices offering CCT, 54% held it annually. Most trainings were one hour (60%); content was delivered via web (58%) and/or lecture (57%). While over 90% of staff (i.e., nurses, social workers, and chaplains) completed CCT, a smaller proportion of medical directors (63%), senior leaders (70%) and board members (23%) did so. Most common (>70%) topics were: cross-cultural communication, death and illness beliefs, and spirituality's role, and healthcare disparities. The majority focused on African-Americans (83%), Hispanics (76%), and Asians (61%)—the most common U.S. minority groups. Almost 30% reported no assessment of effectiveness of CCT while 45% reported a quiz at the end. In this study, most hospices offered some CCT. CCT has been shown to improve healthcare providers' knowledge and skills in caring for diverse patients and is associated with increased patient satisfaction. Future research should evaluate effectiveness of CCT in improving the ability of hospices to deliver high quality end-of-life care to diverse groups of older adults.

### DEVELOPMENT AND PILOT TESTING OF THE END-OF-LIFE READINESS ASSESSMENT (ERA) SURVEY

Alexis M. Coulourides Kogan,[1] and Julie Taguchi[2], *1. University of Southern California, Keck School of Medicine, Los Angeles, California, United States, 2. Ridley-Tree Cancer Center, Santa Barbara, California, United States*

An emerging conceptual framework on the relationship between serious and sensitive end-of-life (EOL) discussions and patient hope points to the mediating role that patient readiness may play. Additional research has also found that among some patients, engaging in and EOL conversation before they are ready may actually cause harm. Presently, health practitioners do not have a way to measure patient readiness. Therefore, the purpose of this study was to develop a survey assessment tool, rooted in research, to measure patient readiness to engage in an EOL discussion. The 16-item survey was initially developed by a gerontological researcher and clinical oncologist, and tested for face reliability and appropriateness among a geriatrician and three patients with chronic illness. In May 2018, the final version of the survey was pilot tested among 168 patients attending their regularly scheduled oncology appointment. On average, most participants identified as Caucasian (74%) females (61%) in partnerships (58%) and having a cancer diagnosis (64%). Bivariate analyses revealed that older age (60+, p=0.008), Caucasian race (p=0.04), and reported greater knowledge of community and supportive services (p=0.049) was significantly associated with increased readiness to engage in an EOL discussion. Additionally, a higher score on the Advance Care Planning Engagement Survey was significantly associated with increased readiness (p<0.001). Findings reveal patient groups that might be more appropriate targets for interventions, education, and resources and highlight the role of interprofessionals in outpatient health settings to "prime the pump" for these types of conversations.

### HOSPICE VISIT INTENSITY BY PHYSICIANS AND NURSE PRACTITIONERS ON THE GENERAL INPATIENT LEVEL OF CARE

Thomas J. Christian,[1] Joan Teno,[2]
Pedro L. Gozalo,[3] and Michael Plotzke[1], *1. Abt Associates, Cambridge, Massachusetts, United States, 2. Oregon Health & Science University, Portland, Oregon, United States, 3. Brown University, Providence, Rhode Island, United States*

The Medicare Hospice Benefit's General Inpatient (GIP) level of care provides short-term services for pain and symptom management in an inpatient facility that cannot be managed in the patient's home. Relatively little is known about how beneficiaries utilize services during GIP care. Among a cohort of Medicare hospice beneficiaries utilizing GIP during Federal Fiscal Year 2014 (FY2014), we used 100% Medicare hospice and Part B claims to identify physician and nurse practitioner services concurrent with GIP dates. We estimated logistic regression models to determine the likelihood a beneficiary never receives physician or nurse practitioner services. We found that among the 1.5 million GIP days serviced in FY2014, more than half (52.4%) lacked any recorded physician or nurse practitioner services. Absence rates for these services were particularly high among hospice GIP days provided in inpatient facilities (69.1% missing services), long-term care hospitals (84.3% missing services), and skilled nursing facilities (85.3% missing services). Moreover, one in five hospice episodes having at least three sequential GIP days lacked any physician or nurse practitioner services. Relative to hospice inpatient units, rates of absence were higher among episodes beginning in long-term care hospitals [59.3% long-term care hospital vs. 11.5% hospice inpatient units; AOR 9.65 95% CI 7.47-12.46] and skilled nursing facilities [51.3% skilled nursing facility vs. 11.5% hospice inpatient units; AOR 5.98, 95% CI 5.63-6.36]. More in depth research and monitoring is needed to further understand dimensions of GIP care provision, to ensure that hospice beneficiaries are receiving adequate services regardless of their inpatient setting.

### IMPLEMENTATION OF THE GOALS OF CARE DECISION AID IN NURSING HOMES: AN EVIDENCE-BASED INTERVENTION

Latarsha Chisholm,[1] Laura Hanson,[2] Sheryl Zimmerman,[3]
Cherie Rosemond,[2] Bryan Weiner,[4] Eleanor S. McConnell[5], *1. University of Central Florida, Orlando, Florida, United States, 2. University of North Carolina, Chapel Hill, North Carolina, United States, 3. Cecil G. Sheps Center for Health Services Research, Chapel Hill, North Carolina, United States, 4. University of Washington, Seattle, Washington, United States, 5. Duke University, Durham, North Carolina, United States*

Nursing homes (NH) must implement best practices to improve care. The purpose of this study is to understand NH characteristics that help or hinder implementation of the Goals of Care (GOC) intervention, an evidence-based decision aid to guide decision-making in advanced dementia. Study design was a cross-sectional staff survey at 11 NHs in North Carolina that participated in the GOC trial. Questions measured the dependent variable of implementation effectiveness (IE) (the consistency and quality of use of the GOC intervention). NH organizational characteristics were measured using publicly available data and administrator surveys. Averages were obtained, IE and NH factors above the average were ranked as high and vice versa. Analysis consisted of pattern matching logic, predicted results are compared to actual results, using within-case and cross-case analyses. NHs with high IE were expected to have the majority (five or more) of NH characteristics to be high to confirm the within (within-case analysis) and between (cross-case analysis) relationships. Within-case analysis expected results were met in 5 NHs, with 3 highs (IE high and five or more characteristics were high) and 2 lows (IE low and five or more characteristics were low). Among three NHs with high IE, the following characteristics were high: Medicare and SNF/ICF beds. Across 6 of the 11 NHs, high total beds and percent of White residents were related to high IE. NHs with enhanced resources may have fewer challenges implementing innovations compared to resource constrained NHs. Implementation strategies should account for resource limitations to promote successful implementation.

### USING LONGITUDINAL HEALTH INFORMATION TO ENHANCE MORTALITY PREDICTION

Megan K. Beckett,[1] Marc N. Elliott,[1] Doug Ritenour,[2]
Laura A. Giordano,[2] and Debra Saliba[1], *1. RAND Corporation, Santa Monica, California, United States, 2. Health Services Advisory Group, Phoenix, Arizona, United States*

The Vulnerable Elders Survey-13-Health Outcomes Survey (VES-13-HOS) 2.5 is a simple, validated method to predict two-year mortality using older adults' responses to the Medicare HOS. We explore whether adding longitudinal function measures and expanded sociodemographic information significantly improves the performance of the VES-13-HOS 2.5. Using Medicare HOS data, we developed risk scores that used coefficients from three logistic regressions predicting two-year mortality (2013-2015): (1) standard 0-10 VES-HOS 2.5 inputs at 2013 (function and limited demographics), (2) expanded demographics at 2013 (adding gender, fine-grained age, race/ethnicity, marital status, missing income indicator, and Medicaid status), (3) expanded demographics at 2013 plus change in pre-baseline (2011) health score two years prior. Fine-grained age and sociodemographic information slightly improve VES mortality prediction. Holding the 2013 VES-HOS 2.5 0-18 enhanced-demographic risk score constant, seniors with worse 2011 than 2013 functioning had higher two-year mortality than seniors with better 2011 functioning. Adding this prior health status further improved model performance slightly- 80% of mortality prediction is explained by "current" health and function and 20% by status two years earlier; such weighted scoring could be employed. We find that prior health status measurements do not generally indicate a trajectory that is likely to continue. Rather, health and function information from two years prior reduces measurement error via a second assessment of health; those with much worse health and function two years earlier are at slightly higher mortality risk. The VES-HOS can be used to identify patients with high mortality risk and to guide their care.

### SESSION 525 (SYMPOSIUM)

### EXPANDING THE NETWORK: NONTRADITIONAL ANIMAL MODELS IN GEROSCIENCE

Chair: Alan A. Cohen, *University of Sherbrooke, Sherbrooke, Quebec, Canada*

Important insights into the biology of aging are coming from research on animals that have not been traditional staples of geroscience research. This symposium will highlight cutting-edge approaches to understand aging biology in the African killifish, exceptionally long-lived mammals such as the Naked Mole Rat, and across primate species, including the short-lived Marmoset.

### CONSERVATION OF INTEGRATIVE PHYSIOLOGICAL AGING MECHANISMS ACROSS PRIMATES

Alan A. Cohen,[1] Tina W. Wey,[1] Gabriel Dansereau,[1] Emy Roberge,[1] Véronique Legault,[1] Marie Brunet,[1] Joseph W. Kemnitz,[2] and Luigi Ferrucci[3], *1. Université de Sherbrooke, Sherbrooke, Quebec, Canada, 2. University of Wisconsin, Madison, Wisconsin, United States, 3. NIH, Baltimore, Maryland, United States*

Physiological dysregulation (PD) and integrated albunemia (IA) are organism-level aging mechanisms that can be measured using standard biomarkers, and in humans they have been shown to increase with age and predict health outcomes. Here, we use 10 species from the Internet Primate Aging Database (iPAD), a longitudinal database of biomarkers and mortality in captive primates, to analyze the generalizability of the role of PD and IA in aging, as well as the conservation of the underlying physiology. Human patterns are broadly but not universally replicated in primates. For example, PD increases with age in nine of eleven species, and predicts mortality in three of four. Both IA and PD can to some extent be cross-calibrated across species, indicating surprising conservation of underlying homeostatic norms; in the case of PD, the calibration weakens with phylogenetic distance.

### MECHANISMS OF LONGEVITY: LESSONS FROM LONG-LIVED MAMMALS

Vera Gorbunova[1], *1. University of Rochester, Rochester, New York, United States*

Animals have evolved a dramatic diversity of aging rates with lifespans ranging from 2 years to 200 years. This natural diversity of lifespan can be exploited to understand the mechanisms of longevity and develop anti-aging interventions. Our goal is to identify mechanisms that allow such exceptionally long-lived animals to live long and healthy lives and then use these mechanisms to benefit human health. Naked mole rat is the longest-lived rodent with the maximum lifespan of 32 years. We discovered that the mechanism of longevity and cancer resistance in the naked mole rat mediated by high molecular weight hyaluronan. I will discuss the mouse model we generated that mimics the naked mole rat and shows increased healthspan and lifespan. I will also the role of SIRT6 in mediating longevity across mammals and in human centenarians by improving DNA repair and silencing transposable elements.

### GENOME-WIDE RELAXATION OF SELECTIVE CONSTRAINTS UNDERLIES THE EVOLUTION OF SHORT LIFE SPAN IN AFRICAN KILLIFISHES

Dario Riccardo Valenzano,[1] and Rongfeng Cui[2], *1. Max Planck Institute for Biology of Ageing, Cologne, Germany, 2. Max Planck Institute for Biology of Ageing, Cologne, Nordrhein-Westfalen, Germany*

African killifishes independently evolved annual life cycles at least three times, offering a unique natural experiment of diversification of life history strategies. Using a comprehensive whole-genome sampling of 46 species of African killifishes, we found that genome size correlates with annual life style and climate. Annual species underwent genome-wide expansion of transposable elements, higher gene family turn-over rates and relaxed selection in genes in known aging pathways, such as mitochondrial replication and translation, mTOR pathway and DNA repair. Whole-genome resequencing in wild Nothobranchius populations showed bottle-necks and a genome-wide signature of relaxation of selection in populations evolved in dryer climates. In conclusion, evolution in ephemeral environments in African killifishes caused an extensive relaxation of selective constraints at genome-wide level. We discovered that, in African killifishes, ecology drove the evolution of short life span, associated to tens of thousands of slightly deleterious mutations driven to intermediate to high frequencies.

### MARMOSET MONKEYS AS A MODEL OF AGING

Suzette Tardif,[1] and Corinna Ross[1], *1. Southwest National Primate Research Center, San Antonio, Texas, United States*

Interest in the New World Monkey, the common marmoset, as a nonhuman primate aging model is growing. Because marmosets have a fast maturation and short life span compared with more commonly used Old World monkey models, the aging research community began to explore the potential of this model species. In addition, the relative ease with which marmosets can be bred in a barrier environment enhances their value as a life-span model. Since that time, efforts to better define what aging actually looks like in marmosets has intensified. Important findings of the past decade include: (1) a refined definition of lifespan in this species and what affects age-specific survival; (2) assessments of age-related pathological changes; (3) development of functional phenotyping relevant to aging, such as activiyy, strength, body composition, cytokine profiling; (4) support of studies using the marmoset as a preclinical model to test intervention that may modulate the aging process.

### SESSION 530 (SYMPOSIUM)

### HARNESSING THE POWER OF OLDER AMERICANS: INDEPENDENCE CENTER NETWORKS TO DEVELOP TECHNOLOGIES PROMOTING MOBILITY

Chair: Katherine S. Hall, *Durham Veterans Affairs Health Care System, Durham, North Carolina, United States*
Co-Chair: Kevin Caves, *Duke University, Durham, United States*
Discussant: Neil Alexander, *University of Michigan, Ann Arbor, Michigan, United States*

Information communication technology (ICT) refers to various technologies encompassing software, networking, the Internet, telecommunications, information systems, and more. As healthcare organizations adopt ICT devices and platforms, patients and providers will have more tools available to improve access to monitoring, telehealth, and timely interventions. The use of alternative methods of collecting, recording, and displaying data (e.g., smart speakers, chat bots, wearables) promise to improve health outcomes for the older adult population. As the U.S. population ages, opportunities for development designed specifically for older adults should be a focus for healthcare organizations. While there are challenges and barriers to enabling new technology within this population, research shows that older adults are adopting new technology. This symposium is focused on these emerging technologies and will showcase diverse examples of ICT implemented across various older adult populations and clinical application areas. The first paper describes the validation of Gaitbox, a walking speed measurement device. The second paper describes using multiple sensors to capture real-world loss of balance and recovery responses. The third paper reports the feasibility of using fitness gamification with a Virtual Reality Treadmill in older adults. The fourth paper describes a smartphone-based assessment of dual task standing and walking. The fifth paper describes wearable sensor-based assessment of falls risk of Timed Up-and-Go test. The Claude D. Pepper Centers maintain year-round coordination and collaboration through a national coordinating center. This powerful network, working towards the common goal of improving the lives of older Americans, has sparked technologic advances that will be highlighted here.

### CAPTURING REAL-WORLD LOSSES OF BALANCE AND RECOVERY RESPONSES IN OLDER ADULTS AT RISK FOR FALLS

Neil Alexander,[1] Shirley Handelzalts-Pereg,[2] Linda Nyquist,[1] Debbie Strasburg,[1] Nicholas Mastruserio,[1] and Lauro Ojeda[1], *1. University of Michigan, Ann Arbor, Michigan, United States, 2. Ben gurion University, Israel, Ann Arbor, Michigan, United States*

Losses of balance (LOBs) such as trips can lead to falls in older adults; what actually happens during real-world LOBs is unclear. With 4 wearable inertial measurement units (IMUs), we recorded feet, trunk and wrist movements over 2 weeks. Using a wrist voice recorder to report the LOBs, we applied our IMU processing algorithms and reconstructed the full body LOB and recovery motions. We recruited 7 at-risk older adults (M=76 yrs) who reported 114 LOBs of which we reconstructed over 90%. Using a rating system, 52% of the LOBs involved a significant trip, stumble, recovery step, and/or large trunk motion. 25% involved double or stutter steps and smaller trunk motions. The other 23% had less striking associated motions. These data suggest that most, but not all, self-reported real world LOBs involve substantial postural destabilization and near falls. Analyses of the voice-recorded context under which the LOBs occurred are ongoing.

### VALIDATION OF A WALKING SPEED MEASUREMENT DEVICE: GAITBOX

Leighanne Jarvis,[1] Matthew J. Peterson,[2] Miriam C. Morey,[3] and Kevin Caves[1], *1. Duke University, Durham, North Carolina, United States, 2. Campbell University, Buies Creek, North Carolina, United States, 3. Duke Center for Aging/OAIC & Durham VA GRECC, Durham, North Carolina, United States*

The NIH 4m Walk Test is a clinically validated tool to measure adult walking speed. Human reaction limitations can contribute to measurement error when manually timing gait speed. This is important considering a 0.10m/s decrease in walking speed is associated with a 12% decrease in life expectancy for older adults. The goal of this study was to validate a low cost, custom built device, Gait Box (GB), compared to human timer (HT) and a research grade Sprint Timing System (STS) with an older adult (mean 72.4 + 7.4 years of age) population (N = 35). Validity was assessed via accuracy (correlations), precision (mean differences), and bias (Bland-Altman plots). Results showed strong correlations between the GB and HT (0.99) and the GB and STS (0.98), with negligible mean differences. This demonstrates the GB can be used to accurately and precisely measure gait speed in clinical and research settings.

### FEASIBILITY OF VIRTUAL REALITY TREADMILL TRAINING IN OLDER ADULTS WITH MOBILITY AND FITNESS DEFICITS

Steven Castle,[1] Cathy C. Lee,[2] Erin H. Blanchard,[3] Christian K. Roberts,[3] Malcolm J. Jones,[4] E. T. Schroeder[5], *1. University of California, Los Angeles, Los Angeles, California, United States, 2. VA Greater Los Angeles/UCLA, Los Angeles, California, United States, 3. VA Greater Los Angeles Healthcare System, Los Angeles, California, United States, 4. Exercise Physiology, Division of Biokinesiology and Physical Therapy, University of Southern California, Los Angeles, California, United States, 5. Division of Biokinesiology and Physical Therapy, University of Southern California, Los Angeles, California, United States*

Only 23.6% of adults and <10% of adults age >75 years meet physical activity (PA) guidelines (aerobic & muscle-strengthening). Health benefit gym memberships has not improved participation. Therefore, Blue Goji developed a Virtual Reality (VR) treadmill to improve PA through fitness gamification with cognitive involvement in postural control to improve balance. Feasibility testing was done with seven older Veterans and one spouse, mean age of 81.3 years who participate in the VA Gerofit program & were near the 50th percentile (by age & gender) in strength, balance and endurance. Even those with lower levels of fitness, balance or chronic conditions (i.e., kyphosis or vision impairment) strongly supported this for enjoyment, benefit, comfort, safety and strongly recommended this (mean of 4.65 out of 5). The Gamification approach supports anti-ageism and intergenerational fitness activities. Further study on the additive effect on exercise intensity and improvement in fitness, balance and cognition is needed.

### SENSOR-BASED ASSESSMENT OF FALLS RISK OF THE TIMED UP AND GO IN REAL-WORLD SETTINGS

Charlene C. Quinn,[1] Barry R. Greene,[2] Killian McManus,[2] Stephen J. Redmond,[3] and Brian Caulfield[4], *1. University of Maryland School of Medicine, Baltimore, Maryland, United States, 2. Kinesis Health Technologies Ltd, Insight Centre for Data Analytics, Dublin, Dublin, Ireland, 3. School of Electrical and Electronic Engineering, Dublin, Dublin, Ireland, 4. Insight Centre for Data Analytics, Dublin, Dublin, Ireland*

Falls are the leading cause of older adult injury and cost $50bn annually. New digital technologies can quantitatively measure falls risk. Objective is to report on a validated wearable sensor-based Timed Up and Go (QTUG) assessment detailing 11 measures of falls risk, frailty and mobility impairment in older adults in six countries in 38 clinical and community settings. Second objective is to generate individual targeted falls prevention programs. 14,611 QTUG records from 8,521 participants (63% female) (72.7±10.7 years) available for analysis. QTUG time was 13.9±7.4 s; gait velocity was 101.9±32.5 cm/s. 25.8% of patients reported falling in previous 12 months; 26.2% of patients were at high fall risk. 21.5% not reporting a fall, were high fall risk. Participants had slow walking speed (29.8%); high gait variability (19.8%); problems with transfers (17.5%). Easily captured and interpreted sensor data is useful in a population-based approach to quantify falls risk stratification.

### VALIDITY AND RELIABILITY OF REMOTE, SMARTPHONE-BASED ASSESSMENT OF DUAL-TASK STANDING AND WALKING IN OLDER ADULTS

Brad Manor,[1] Wanting Yu,[2] On-Ye Lo,[2] Hao Zhu,[2] Thomas G. Travison,[2] Lewis Lipsitz,[2] and Junhong Zhou[2], *1. Hinda and Arthur Marcus Institute for Aging Research, Harvard Medical School, Boston, Massachusetts, United States, 2. Hinda and Arthur Marcus Institute for Aging Research, Roslindale, Massachusetts, United States*

Dual task walking assessments provide valuable insights into cognitive-motor function in aging. To date, such assessments have been limited primarily to laboratory-based settings. We thus created a smartphone App utilizing multi-media instructions and the phone's motion sensors to record movements during normal and dual task walking, with the phone placed in the user's pants pocket. Thirty younger and older adults completed two lab visits, during which walking data were simultaneously acquired by the App and the GAITRite mat. Participants also completed App-based assessments in their homes on three separate days. Across all detected strides in laboratory trials, gait metrics derived from the App correlated closely with those derived from the GAITRite mat ($r2>0.96$). Across trials, gait metrics demonstrated excellent test-retest reliability, both within and between laboratory visits and home-based assessments (ICC: 0.79–0.90). Remote, smartphone-based dual task walking assessments may therefore be feasible for relatively healthy younger and older adults.

### SESSION 535 (SYMPOSIUM)

### HARNESSING THE POWER OF PROFESSIONAL NETWORKS: IDENTIFYING FACILITATORS AND BARRIERS TO CAREERS IN GEROPSYCHOLOGY

Chair: Rebecca S. Allen, *The University of Alabama, Tuscaloosa, Alabama, United States*
Discussant: Brian Carpenter, *Washington University in St. Louis, St. Louis, Missouri, United States*

This symposium presents data from a mixed method study designed to explore how to harness the power of professional networks to increase the pipeline of trainees pursuing careers in academic and clinical geropsychology. Participants were recruited through professional websites, listserves, announcements at annual meetings, and emails from directors of clinical training at pre-and post-doctoral training sites. A total of 107 geropsychologists completed the survey, including 28 graduate students/interns and 76 post-doctoral psychologists ranging from early to late career. The mean age of respondents was 39.18 (SD = 12.05). The sample was largely female (71.7%) and Caucasian (88.7%), paralleling previous work. The first paper describes attractive and unattractive aspects of clinical and academic career options, including gender differences in perceptions of the feasibility of changing career foci. The second paper examines perceptions of clinically-focused and academic jobs, and discrepancies between professional psychologists' actual and ideal job activities. Examining content analysis of 28 qualitative transcripts, the third paper focuses on VA training and the convenience and comfort of transitioning into a VA job after training within this system, identifying benefits and challenges of work in the VA. The fourth explores these 28 transcripts to identify perceptions of mentorship and supervision during training, including time spent in the training sites (graduate school vs internship) and observations of how working environments impact future career choices. Discussion will address the critical shortage of geropsychologists in academic and clinical settings and strategies to improve the professional pipeline to increase the numbers of trainees pursuing these careers.

### THE PIPELINE OF GEROPSYCHOLOGY: INTEGRATION OF CLINICAL AND ACADEMIC CAREER TRAJECTORIES

Hillary Dorman,[1] Jessica V. Strong,[2] Caitlan A. Tighe,[3] Benjamin T. Mast,[4] Rebecca S. Allen[5], *1. University of Alabama, Tuscaloosa, Tuscaloosa, Alabama, United States, 2. VA Boston Healthcare System, Brockton, Massachusetts, United States, 3. VA Pittsburgh Healthcare System, Pittsburgh, Pennsylvania, United States, 4. University of Louisville, Louisville, Kentucky, United States, 5. The University of Alabama, Tuscaloosa, Alabama, United States*

The current exploratory study aimed to identify factors influencing gerospychologists' career trajectories, discerning what is "attractive" and "unattractive" across common career options. Participants rated career characteristics similarly (higher starting salary attractive, administrative tasks unattractive in a clinical VA; setting own hours attractive, few collaboration opportunities unattractive in a clinical non-VA; work variety attractive, publication expectations unattractive in academia). Interestingly, men found a clinical VA's set hours more unattractive ($t(50) = -2.59$, $p = .010$). Sex differences also were evident in career change feasibility. 50% of respondents would consider switching careers (61% clinical to academia, 79% academia to clinical). Men were significantly more likely to consider switching from clinical to academia ($\chi2(2) = 6.33$, $p = .042$) and find it feasible ($\chi2(2) = 8.92$, $p = .012$). Enhancing our understanding of what encourages/discourages careers in geropsychology is needed to address the workforce shortage; this study takes important steps in this direction.

### CAREER PERCEPTIONS AND IDEALS FOR CLINICALLY FOCUSED AND ACADEMIC PSYCHOLOGY CAREERS: A MIXED-METHODS STUDY

Jessica Strong,[1] Rebecca Allen,[2] Caitlan Tighe,[2] Mary Jacobs,[1] Hillary Dorman,[2] and Benjamin Mast[3], *1. VA Boston Healthcare System, Boston, Massachusetts, United States, 2. University of Alabama, Tuscaloosa, Alabama, United States, 3. University of Louisville, Louisville, Kentucky, United States*

More psychologists who have specialty training in geriatrics are needed to meet the growing demand. However, there is a shortage of individuals in academic geropsychology, which feeds the clinical geropsychology pipeline. Barriers to recruiting trainees into jobs in academia are not well understood. The current mixed-methods study examined trainees' perceptions of clinically-focused and academic jobs, and discrepancies between professional psychologists actual and ideal job activities. Results found that trainees have less accurate perceptions of the activities of academic compared to clinically-focused jobs. Interviews with trainees revealed negative perceptions of the university system, including bureaucracy, salary, and perceived workload. However, professional psychologists, both clinical and academic, reported high agreement between actual and ideal activities. Academic psychologists reported desiring more time in clinical work, without reducing research or teaching time. Clinically-focused psychologists desired and increase in research and teaching time without sacrificing clinical activities. Each group discussed struggles in obtaining work-life balance.

### REFLECTIONS ON VA TRAINING AND EMPLOYMENT FROM INTERVIEWS ON CAREER PATHS IN GEROPSYCHOLOGY

Elizabeth M. Shumaker,[1] M. Lindsey Jacobs,[2] Jessica V. Strong,[2] Rebecca S. Allen[3], *1. San Francisco VA Health Care System, San Francisco, California, United States, 2. VA Boston Healthcare System, Boston, Massachusetts, United States, 3. University of Alabama, Tuscaloosa, Alabama, United States*

The U.S. Department of Veterans Affairs (VA) is one of the largest trainers and employers of psychologists in the world and many clinical geropsychologists have worked for the VA for some portion if not all of their careers. The current qualitative analysis was conducted on phone interviews with 28 psychologists and psychology trainees regarding careers in geropsychology that focused on participants' reflections on factors that influenced their career paths. This presentation will summarize subthemes from the many participant responses that included statements about the VA. Examples include discussion of the convenience and comfort of transitioning to a VA job after training within this system as well as reflections about the timing of professional development conversations with supervisors and mentors on VA internships and fellowships given the proximity to entering the workforce. Identified benefits and challenges of work in the VA will also be discussed (e.g., varied roles and responsibilities).

### MENTORSHIP AND CAREER PATHS IN GEROPSYCHOLOGY

Lindsey Jacobs,[1] Rebecca S. Allen,[2] Jessica Strong,[1] and Elizabeth Shumaker[3], *1. VA Boston Healthcare System, Brockton, Massachusetts, United States, 2. University of Alabama, Tuscaloosa, Alabama, United States, 3. San Francisco VA Health Care System, San Francisco, California, United States*

Mentoring is a critical process for guiding the next generation of geropsychologists into career paths that fit their goals and needs. However, the context and content of mentorship in geropsychology is not well-documented. Understanding geropsychologists' experiences may provide some insight into how mentorship is executed in academia versus clinical settings and which factors are relevant to career decision-making. We conducted a qualitative analysis of 28 transcripts from individual telephone interviews with geropsychologists and geropsychology trainees, focusing on mentorship and how it influenced their career path. Themes were differences in the focus of mentorship (e.g., immediate projects versus career goals), frequency of career-focused mentorship, length of mentorship (years versus months), and mentors' openness to share opinions and experiences (e.g., academic mentors are more open about negative aspects of academia, while clinical mentors "guard" trainees from the negative aspects of clinical work). Subthemes and recommendations will be discussed.

### SESSION 540 (SYMPOSIUM)

### HEALTH INSURANCE AND HEALTH CARE DECISION-MAKING FOR ADULTS IN THE PRE-MEDICARE YEARS: FINDINGS FROM NATIONAL SURVEYS

Chair: Erica Solway, *Institute for Healthcare Policy and Innovation, University of Michigan, Ann Arbor, Michigan, United States*
Co-Chair: Renuka Tipirneni, *University of Michigan Medical School, Ann Arbor, Michigan, United States*
Discussant: Erica Solway, *Institute for Healthcare Policy and Innovation, University of Michigan, Ann Arbor, Michigan, United States*

As more Americans approach retirement age and eligibility for Medicare coverage, many face difficult decisions about their health insurance and health care. This session explores how adults age 50-64 are navigating these choices following implementation of the Affordable Care Act (ACA), presenting data from two nationally representative surveys: The University of Michigan's National Poll on Healthy Aging (NPHA) and the Health and Retirement Study (HRS). Erica Solway, Associate Director of the NPHA, will begin by presenting background information about the NPHA and an overview of critical health policy issues for adults age 50-64. Jamie Luster, Research Area Specialist at the University of Michigan, will then provide NPHA findings linking concerns about health insurance affordability with delayed/forgone health care. Next, Aaron Scherer, Associate of Internal Medicine at the University of Iowa Carver College of Medicine, will discuss NPHA findings on factors associated with adults' concern about affordability of health insurance in retirement but before Medicare eligibility begins at age 65. Finally, Renuka Tipirneni, Assistant Professor of Internal Medicine at the University of Michigan, will present findings based on the HRS on changes in health care utilization for adults age 55-64 since implementation of the ACA's Medicaid expansion. To conclude, Erica Solway will discuss current federal health care policy proposals for adults age 50-64, including the recent introduction of the Medicare at 50 bill, and how the perspectives and experiences of adults in this age group can help inform those policies.

### CONFIDENCE IN HEALTH INSURANCE AFFORDABILITY AND DELAYED OR FORGONE HEALTH CARE AMONG ADULTS APPROACHING RETIREMENT

Jamie Luster,[1] Renuka Tipirneni,[1] Erica Solway,[2] Preeti Malani,[1] Jeffrey Kullgren,[1] Matthias A. Kirch,[2] Dianne Singer,[3] Aaron M. Scherer[4], *1. University of Michigan Medical School, Ann Arbor, Michigan, United States, 2. Institute for Healthcare Policy and Innovation, University of Michigan, Ann Arbor, Michigan, United States, 3. Child Health Evaluation and Research Center, University of Michigan, Ann Arbor, Michigan, United States, 4. University of Iowa Carver College of Medicine, Iowa City, Iowa, United States*

Recent challenges to the ACA may add uncertainties to decision-making about health insurance. We sought to determine if health insurance affordability concerns were associated with delayed/forgone health care among adults approaching retirement age. In October 2018, the NPHA conducted an online survey of US adults age 50-64. 45% of respondents had little/no confidence in ability to afford health insurance when they retire, and 27% little/no confidence in this over the next year. In the past year, 13% had not gotten medical care and 12% had not filled a prescription because of cost concerns. Controlling for demographic and health characteristics, having little/no confidence in health insurance affordability in retirement/within the next year was associated with delaying/forgoing health care (aOR 2.80, p<0.001). Despite the ACA's coverage expansions and consumer protections, these findings suggest adults worry about the affordability of health insurance in retirement and may avoid needed health care for that reason.

### HEALTH INSURANCE LITERACY, HEALTH STATUS, AND CONCERNS ABOUT AFFORDABILITY OF HEALTH INSURANCE NEAR RETIREMENT

Aaron M. Scherer,[1] Erica Solway,[2] Preeti Malani,[3] Jamie Luster,[3] Jeffrey Kullgren,[3] Matthias A. Kirch,[2] Dianne Singer,[4] and Renuka Tipirneni[3], *1. University of Iowa Carver College of Medicine, Iowa City, Iowa, United States, 2. Institute for Healthcare Policy and Innovation, University of Michigan, Ann Arbor, Michigan, United States, 3. University of Michigan Medical School, Ann Arbor, Michigan, United States, 4. Child Health Evaluation and Research Center, University of MIchigan, Ann Arbor, Michigan, United States*

Results from the NPHA highlight the link between health insurance affordability concerns and delaying/forgoing health care near retirement. We sought to determine factors associated with health insurance affordability concerns for US adults age 50-64. We regressed little/no confidence in health insurance affordability in retirement/within the next year on health insurance literacy, age, gender, race/ethnicity, income, employment, education, and health status. Factors associated with greater health insurance affordability concerns included lower health insurance literacy—measured via confidence knowing health insurance terms (aOR=1.78, p=0.035), identifying covered services (aOR=1.81, p=0.038), and finding out service costs (aOR=2.69, p<0.001)—female gender (aOR=1.73, p=0.001), and fair/poor health (aOR=1.88, p=0.020). Factors associated with fewer health insurance affordability concerns included African-American race (aOR=0.55, p=0.038) and higher income (aOR=0.34, p<0.001). These results suggest that it may be possible to reduce health insurance affordability concerns and delayed/forgone care by improving adults' confidence in understanding and using health insurance.

### CHANGES IN HEALTH CARE UTILIZATION FOR LOW-SES ADULTS NEAR RETIREMENT AFTER THE ACA MEDICAID EXPANSION

Renuka Tipirneni,[1] Kenneth M. Langa,[1] Ryan J. McCammon,[1] Kara Zivin,[1] Jamie Luster,[1] John Z. Ayanian[1], *1. University of Michigan Medical School, Ann Arbor, Michigan, United States*

Low-SES Americans approaching retirement are experiencing rising morbidity and mortality. We examined longitudinal changes in health care access, utilization, and health for low-SES adults age 55-64 before (2010-2012) and after (2014-2016) ACA Medicaid expansion using the HRS. With a longitudinal difference-in-differences (DID) approach adjusting for demographics and the complex survey design, we found that low-SES adults age 55-64 had increased rates of Medicaid coverage (+10.7 percentage points [pp] in expansion states, +3.4 pp in non-expansion states, DID +7.3 pp) and increased likelihood of hospitalizations (+9.9 pp in expansion states, -1.2 pp in non-expansion states, DID +11.1 pp) in Medicaid expansion compared with non-expansion states. There were no other significant differences in access, utilization or health trends between expansion and non-expansion states. After Medicaid expansion, low-SES adults age 55-64 were more likely to be hospitalized, suggesting poorer baseline access to chronic disease management and associated pent-up demand for health care services.

### SESSION 545 (SYMPOSIUM)

### NIA HEALTH DISPARITIES RESEARCH NETWORK: APPROACHES AND FINDINGS FROM GERIATRICS AND CLINICAL GERONTOLOGY

Chair: Lyndon Joseph, *National Institute on Aging, Bethesda, Maryland, United States*
Co-Chair: CarlV Hill, *National Institute on Aging (NIA), Bethesda, Maryland, United States*

Health disparities are differences in the incidence, prevalence and burden of diseases, mortality rates and causes of death that exist among population groups. Health disparities are associated with a broad, complex, and interrelated array of factors that influence health, accelerate aging and reduce life expectancy. NIA's health disparities research goals are to understand environmental and sociocultural factors and related behavioral and biological mechanisms that diminish health and reduce life expectancy for vulnerable populations, explore the biological mechanisms through which disparities influence age-related change, and identify where disparities emerge in diagnosis, prognosis or treatment in geriatric conditions. Presentations will focus on whether structural-level discrimination may be a key factor in potentiating well known race-related health disparities especially those with an accelerated onset and may be associated with MRI-indicators of subclinical brain pathology; identifying biomarkers for early detection of cognitive and functional decline in high risk subpopulations and how ethnicity influences cerebral spinal fluid and imaging biomarkers link to early identification of cognitive and functional impairment ; effects of medication management and deprescribing among African American and Hispanic older adults with Alzheimer's disease and related dementias and multiple chronic conditions; examine the use of multi-level factors and technology to overcome the barriers to urban-rural health disparities in managing many chronic diseases such as hepatitis C virus infection and delivery of appropriate medical services; and understanding the racial and ethnic differences in the link between environmental exposures and auto-immune comorbid asthma.

### ASTHMA IN OLDER ADULTS: IDENTIFYING PHENOTYPES AND FACTORS IMPACTING OUTCOMES

Rodney Folz[1], *1. University Hospitals Cleveland Medical Center, Cleveland, Ohio, United States*

Asthma, of all chronic diseases, has the highest disease burden attributed to environmental exposures. Few studies have attempted to characterize the prevalence of co-existing auto-inflammatory disease and asthma, or to link environmental exposure as a factor that may increase asthmatic lung obstruction and racial disparity with auto-inflammatory comorbidity. While there is an increased risk for asthma development and severity linked to certain autoimmune diseases, there is a known racial disparity in the prevalence of these autoimmune diseases. Racial and ethnic differences in the link between environmental exposures and auto-immune comorbid asthma as a potential common trigger of inflammation is not well understood. This talk will focus on developing a model to longitudinally predict asthma control and quality of life associated with home environmental triggers and volatile organic chemical (VOC) exposure in older adults and investigate the direct and indirect effect of autoimmune disease in racial disparities of the longitudinal relationships of home environmental asthma triggers on airway obstruction and functional status in older adults with asthma.

### OPTIMAL MEDICATION MANAGEMENT IN ALZHEIMER'S DISEASE AND DEMENTIA

Ariel Green[1], *1. Johns Hopkins University, Baltimore, Maryland, United States*

For older individuals with Alzheimer's disease and related dementias (ADRD) and multiple chronic conditions (MCC), taking more medications is associated with greater risk of adverse drug events, drug interactions, treatment burden, and cognitive changes from medication side effects. Optimizing medication through deprescribing (the process of reducing or stopping the use of inappropriate medications or medications unlikely to be beneficial) can help avoid adverse drug effects and improve outcomes for MCC patients, particularly for those with ADRD. Findings to date are limited to primarily Caucasian patients. This talk will focus on work geared to elicit perspectives on medication use, communication about medication, and deprescribing among African American and Hispanic older adults with ADRD and MCC, their family members, and clinicians caring for these populations.

### USE OF NEW HEPATITIS C DRUGS IN MEDICARE: PERSISTENT URBAN-RURAL DISPARITIES AND POTENTIAL INTERVENTIONS

Ping Du[1], *1. Pennsylvania State University College of Medicine, Hershey, Pennsylvania, United States*

Chronic hepatitis C virus (HCV) infection, whose prevalence is concentrated among older adults, can bring serious health impacts and high financial burden. With the availability of highly effective and well tolerated direct-acting antiviral (DAA) therapy, treatment of chronic HCV infection has rapidly evolved, making HCV treatment less burdensome. However, the high cost of DAA and lack of clinical expertise are still important barriers for providing DAA therapy to rural patients, highlighting the urban-rural disparities in managing many chronic diseases for aging populations. Telehealth could serve as effective care-model to improve management of HCV infection for older, rural populations. This talk will present work that examines multi-level factors affecting HCV DAA treatment (focusing on urban-rural disparities), evaluates changes in urban-rural disparities in DAA utilization over time, and explores the role of a telehealth-based intervention in reducing urban-rural disparities in HCV DAA treatment in Medicare patients.

### UTILITY OF EXECUTIVE FUNCTION TO IDENTIFY EARLY COGNITIVE IMPAIRMENT IN AFRICAN AMERICANS

Stephanie Garrett, Felicia C. Goldstein, Yunyun Chen, Kirk Easley, Darius McDaniel, Janice Lea, Tiffany Thomas, Sabria Saleh, and Ihab Hajjar[1], *1. Emory University, Atlanta, Georgia, United States*

Within diverse cohorts, African Americans (AA) demonstrate higher rates of Alzheimer's dementia (AD) and Alzheimer's dementia combined with multiple comorbid conditions. AA are also two times more likely to develop late-onset AD than whites and less likely to be diagnosed. Yet, our understanding of this disparity in cognition remains limited. Cognitive impairment (CI) and dementia are both underdiagnosed and underreported in primary care patients. The lack of early detection of cognitive and functional decline in high risk populations results in failure to provide care and interventions to members of vulnerable groups. This talk will focus on research that seeks to identify a more sensitive cognitive marker for early identification of cognitive impairment for AA and advances this objective by linking the cognitive marker to AD cerebral spinal fluid biomarkers (Aβ1-42, p-tau) and testing whether this association differs between AA and whites.

### STRUCTURAL DISCRIMINATION AND MRI-ASSESSED BRAIN ENDPOINTS: HANDLS BRAINCHILD

Danielle L. Beatty Moody,[1] Rao P. Gullapalli,[2] Christos Davatzikos,[3] Shuyan Sun,[1] Lesile Katzel,[2] Alan Zonderman,[4] Michele Evans,[4] Shari R. Waldstein[1], *1. University of Maryland Baltimore County, Baltimore, Maryland, United States, 2. University of Maryland, School of Medicine, Baltimore, Maryland, United States, 3. University of Pennsylvania, Philadelphia, Pennsylvania, United States, 4. NIH/NIA, Intramural Research Program, Baltimore, Maryland, United States*

Emerging evidence demonstrates that exposure to race-related adversity, specifically, individual-level discrimination, in middle-age is adversely linked with white matter lesion volume, a prospective marker of future cerebrovascular disease as indicated on Magnetic Resonance Imaging (MRI). It remains unclear whether exposure to indices of neighborhood-level structural discrimination (e.g., residential segregation, % of population employed & with high school diploma/equivalency), are linked to MRI-assessed brain pathology and how these linkages may be patterned by key sociodemographic characteristics (e.g., race, age, sex, class). Knowledge of this linkage may help us further understand well-documented racial disparities in multiple clinical brain health endpoints including stroke, dementia, cognitive decline, functional disability, and subclinical brain pathology in adulthood. Thusly, this talk will focus on work that examines whether neighborhood-level structural discrimination is associated with MRI-brain assessed indicators of subclinical brain pathology and the role of key sociodemographic factors, with emphasis on the role of race.

### SESSION 550 (SYMPOSIUM)

### OLDER ADULTS' SOCIAL RELATIONSHIPS AND WELL-BEING IN A DIGITAL WORLD

Chair: Shannon T. Mejia, *University of Illinois, Urbana-Champaign, Champaign, Illinois, United States*
Discussant: Sara J. Czaja, *Weill Cornell Medicine, New York, New York, United States*

As adults age into a digitally connected world, communication technologies such as the internet, email, social media, and video chats offer new opportunities to connect with others. The implications of older adults' use of technology in the context of their social relationships—such as the implications for social integration, the relational circumstances of technology adoption, implications for daily experiences of well-being, and opportunities to form new relationships—are less understood. This symposium brings together diverse and complementary perspectives on the contribution of technology to older adults' social experiences. We begin with inquiry into implications of internet use for social integration. Hees and colleagues use data from the German Ageing Survey to examine how internet use is associated with change in loneliness over a three-year period in older adults who are either before or after retirement. Our symposium continues with papers on technology use within the context of older adults' existing close relationships. Chopik examines individual and dyadic predictors of technology adoption. Mejía and colleagues consider the implications for digital social interactions for older adult's well-being on that day. Our final paper discusses the potential for technology to aid in the development of new relationships. Rogers and colleagues describe findings from their OneClick.chat project, a web-based video chat application that connects older adults based on their shared interests. Our session concludes with a discussion led by Czaja, who will integrate the four papers and discuss the challenges and opportunities of using technology to support older adults' social relationships and well-being.

### INTERNET USE AND LONELINESS: CURE OR CAUSE? LONGITUDINAL ANALYSIS OF OLDER ADULTS' INTERNET USE

Elena Hees,[1] Clemens Tesch-Römer,[2] and Oliver Huxhold[2], *1. German Centre of Gerontology, Berlin, Germany, 2. German Centre of Gerontology, Berlin, Berlin, Germany*

The internet provides an indispensable platform for social interaction, entertainment and everyday tasks. Especially older adults might benefit from staying engaged online to counteract loneliness. Yet, current research on how internet use effects loneliness still paints a contradictory picture. The current study investigates the longitudinal influence of social internet use forms as opposed to general internet use on loneliness across three years (2014-2017) separately in two age groups (pre-retirement: 40-64 years and post-retirement: 65-85 years), using data from the German Ageing Survey (DEAS). Structural equation modelling shows, that general web use predicts an increase in loneliness in both age-groups. However, contacting friends and family online seems to protect against loneliness over and above the effect of overall internet use, at least for the younger age-group. Therefore, the current study underlines the importance of investigating what exactly people do online instead of seeing the internet as a homogenous tool.

### INDIVIDUAL AND DYADIC PREDICTORS OF TECHNOLOGY ADOPTION: IMPLICATIONS FOR HEALTH AND WELL-BEING

William J. Chopik,[1] Shelia R. Cotten[1], *1. Michigan State University, East Lansing, Michigan, United States*

Technology has the ability to enhance and enrich the lives of older adults by facilitating better relationships, health, and well-being. However, older adults vary in how often—and even whether—they use information and communication technologies (ICTs). Further, interactions and relationships with people in their immediate social networks might have implications for whether or not older adults adopt ICTs. In two studies of individuals (N=595 participants; Mage=67.09; 56% Female; 69.2% White) and couples (N=542 couples; Mage=63.65; 50% Female; 83.9% White), I examined individual and dyadic predictors of technology adoption among older adults. Among a wide array of individual difference constructs, the most reliable predictor of technology adoption in both individuals and their spouses was need for cognition ($.08 \leq r \leq .23$). The results will be discussed in the context of how individual differences modulate adoption and the benefits accrued from ICTs across the lifespan.

### THE IMPLICATIONS OF DIGITAL SOCIAL INTERACTIONS FOR OLDER ADULTS' EXPERIENCES OF WELL-BEING IN DAILY LIFE

Shannon T. Mejia,[1] Shelbie Turner,[2] and Karen Hooker[2], *1. University of Illinois, Urbana-Champaign, Champaign, Illinois, United States, 2. Oregon State University, Corvallis, Oregon, United States*

Digital communication technologies expand opportunities for social interactions and as a result have the potential to either amplify or dampen the coupling of social interactions with well-being in daily life. We use data from the 100-day Personal Understanding of Life and Social Experiences project (n = 99, age = 50 – 88) to examine variation in the sensitivity of older adults' daily reports of well-being to the quality of social interactions with their five closest social partners across digital (email/social media) and analogue (in person/by phone) interactions. Digital interactions were more common among less-close social partners. Multilevel random coefficient models showed days with more digital interactions than normal to be characterized by a) lower well-being and b) less sensitivity in well-being to the quality of social interactions with close social partners on that day. The implications of our findings are discussed within a lifespan perspective of social relationships and well-being.

### SOCIAL ENGAGEMENT THROUGH VIDEO CHAT FOR OLDER INDIVIDUALS WITH AND WITHOUT COGNITIVE IMPAIRMENT

Wendy Rogers,[1] Qiong Nie,[2] Lydia Nguyen,[2] Raksha Mudar,[2] Dillon Myers,[3] Alan Gibson,[3] and Chantal Kerssens[4], *1. University of Illinois, Urbana-Champaign, Champaign, Illinois, United States, 2. University of Illinois, Champaign, Illinois, United States, 3. OneClick.Chat, Philadelphia, Pennsylvania, United States, 4. Eyiapp, Atlanta, Georgia, United States*

Social engagement is a fundamental component of health and quality-of-life outcomes. However, there is a prevailing view that older adults primarily want to engage socially with current family and friends – that they are not interested in developing new relationships. That is an overgeneralization. We have found that older adults are interested in the opportunity to engage in social interactions with people who have shared interests. Technology can facilitate these interactions. We will describe our research with OneClick.chat, a web-based video chat system. We explored potential benefits of use by adults aged 70-85, including those with mild cognitive impairment (MCI), as well as barriers and facilitators to adoption. Participants saw value of this online social engagement platform and were able to use it with some initial training. They envisioned using OneClick not only for conversations but also for learning and doing activities with like-minded individuals.

### SESSION 555 (SYMPOSIUM)

### ONDRI: A PROVINCIAL INITIATIVE TO UNDERSTAND THE HEALTH SYSTEM IMPACT OF AGING AND NEURODEGENERATIVE DISEASES

Chair: Susan E. Bronskill, *Institute for Clinical Evaluative Sciences (ICES), Toronto, Ontario, Canada*
Co-Chair: Colleen J. Maxwell, *University of Waterloo, Waterloo, Ontario, Canada*
Discussant: Nathalie Jette, *Department of Population Health Science and Policy, Icahn School of Medicine at Mount Sinai, New York, New York, United States*

As populations worldwide are living longer, the impact of neurodegenerative diseases on health resource utilization is expected to increase. Providing care to older adults with neurodegenerative diseases is challenging, and requires adequate supports across multiple health sectors including community, acute care and nursing home settings to allow individuals to maximize their quality of life. The Ontario Neurodegenerative Disease Research Initiative (ONDRI) is a collaborative research program that aims to improve diagnosis, treatment and management of neurodegenerative diseases including Alzheimer's disease and related dementias, Parkinson's disease, Amyotrophic Lateral Sclerosis (ALS), and Vascular Cognitive Impairment. Using population-based linked health administrative and clinical databases--covering over 14 million individuals residing in the province of Ontario, Canada-- the ONDRI health services research platform will address knowledge gaps regarding the health service utilization and outcomes of older adults with neurodegenerative diseases and impacts on family and care partners. Access to over two decades of historical health administrative data on a large population of older individuals uniquely positions our collaborative to examine trajectories of health system use as well as rare neurodegenerative diseases which have been previously understudied. The health services research platform is embedded within a larger ONDRI network of biomedical researchers, provincial decision-makers and health system stakeholders. Our research findings will inform health system planning and interventions to support older adults to live independently in the community. This session will explore how health administrative databases may be used to address knowledge gaps regarding health service utilization and outcomes in older persons with neurodegenerative diseases.

### TRAJECTORIES OF HEALTH SYSTEM USE AND TRANSITIONS IN OLDER ADULTS WITH DEMENTIA

Susan E. Bronskill,[1] Laura C. Maclagan,[2] Jennifer Walker,[3] Jun Guan,[2] Xuesong Wang,[2] Ryan Ng,[2] Marian J. Vermeulen,[2] and Colleen J. Maxwell[4], *1. Institute for Clinical Evaluative Sciences (ICES), Toronto, Ontario, Canada, 2. ICES, Toronto, Ontario, Canada, 3. Laurentian University, Sudbury, Ontario, Canada, 4. University of Waterloo, Waterloo, Ontario, Canada*

Health systems strive to enable persons with Alzheimer's and related dementias to remain at home to maximize their quality of life. There is limited evidence describing long-term trajectories of health system use by persons with dementia as they remain in the community over time. A cohort of 62,622 community-dwelling older adults was followed for seven years and matched to persons without dementia (controls) based on age, sex and comorbidities. Overall, persons with dementia were more likely than controls to use health services, particularly home care and hospitalizations with discharge delay; and were more likely to be admitted to a nursing home. As they remained in the community over time, persons with dementia used home care at an increasingly intensive rate. Our approach to examine trajectories of health system use among persons with dementia is of particular value to capacity planning initiatives to anticipate future health service needs of this population.

### COMORBIDITY IN NEURODEGENERATIVE DISEASES AND MENTAL HEALTH CONDITIONS: IMPLICATIONS FOR HEALTH SYSTEM BURDEN

Colleen J. Maxwell,[1] Laura C. Maclagan,[2] Ruth Ann Marrie,[3] Luke Mondor,[2] Walter Wodchis,[4] David B. Hogan,[5] and Susan E. Bronskill[2], *1. University of Waterloo, Waterloo, Ontario, Canada, 2. ICES, Toronto, Ontario, Canada, 3. University of Manitoba, Winnipeg, Manitoba, Canada, 4. Institute of Health Policy, Management & Evaluation, University Of Toronto, Toronto, Ontario, Canada, 5. University of Calgary, Calgary, Alberta, Canada*

Research suggests that older adults with neurodegenerative diseases are at increased risk of developing a subsequent neurodegenerative or comorbid psychiatric disorder or both. Depression and other psychiatric conditions, though prevalent, are often under-diagnosed and under-treated among those with neurodegenerative conditions potentially leading to more rapid disease progression, poorer health outcomes and increased health care use. Few population-based studies have comprehensively examined the risk and temporal ordering of common neurodegenerative and psychiatric conditions, including whether these associations differ by age or sex. Initial findings regarding the incidence of ordered pairs of neurological conditions (including dementia, Parkinson's disease and stroke) and psychiatric disorders (including mood and anxiety disorders, and schizophrenia) will be summarized. This population-based retrospective cohort study will provide essential data to allow policymakers, planners and providers to better anticipate the prognosis and care needs of older adults with comorbid neurodegenerative and psychiatric conditions.

### CHALLENGES IN IDENTIFYING PERSONS WITH NEURODEGENERATIVE DISEASES USING HEALTH ADMINISTRATIVE DATABASES

Laura C. Maclagan,[1] Serena Soleimani,[2] Agessandro Abrahao,[3] Lorne Zinman,[3] Michael A. Campitelli,[4] Liisa Jaakkimainen,[4] Colleen J. Maxwell,[5] and Susan E. Bronskill[4], *1. Institute for Clinical Evaluative Sciences (ICES), Toronto, Ontario, Canada, 2. Institute of Medical Science, University of Toronto, Toronto, Ontario, Canada, 3. Sunnybrook Health Sciences Centre, Toronto, Ontario, Canada, 4. ICES, Toronto, Ontario, Canada, 5. University of Waterloo, Waterloo, Ontario, Canada*

Health administrative databases can be used to quantify prevalence and incidence of neurodegenerative diseases and their impact on health service utilization outcomes at the population level. Algorithms based on diagnosis codes and health service patterns can be used to identify persons suspected to have a neurodegenerative disease. Previous studies have developed and validated algorithms to identify persons with Alzheimer's and related dementias using primary care medical records as the reference standard, however, little previous work has focused on developing algorithms for rare neurodegenerative diseases including amyotrophic lateral sclerosis (ALS). This session will discuss challenges in developing algorithms to identify persons with neurodegenerative diseases accurately and opportunities to improve existing definitions using novel data sources including electronic medical record databases. Preliminary findings regarding the development of an ALS algorithm will be presented.

### UNDERSTANDING SEX DIFFERENCES IN OLDER PERSONS WITH NEURODEGENERATIVE DISEASES

Michael A. Campitelli,[1] Laura C. Maclagan,[2] Christina Diong,[2] Longdi Fu[2] Amy Y. Yu,[3] Lorne Zinman,[3] Rick Swartz[3] and Susan E. Bronskill[2], *1. Institute for Clinical Evaluative Sciences (ICES), Toronto, Ontario, Canada, 2. ICES, Toronto, Ontario, Canada, 3. Sunnybrook Health Sciences Centre, Toronto, Ontario, Canada*

Sex differences in the incidence, prevalence, and clinical presentation of neurodegenerative diseases have been previously documented, however, sex differences in how individuals with neurodegenerative diseases access the health system remain understudied. Clinical trials and observational studies often do not report data stratified by sex, which limits the understanding of sex-related differences in persons with neurodegenerative diseases. This session will highlight both opportunities and methodological challenges researchers face when undertaking sex and gender research in persons with neurodegenerative diseases using two case studies: 1) sex differences in health service utilization prior to a diagnosis of Amyotrophic Lateral Sclerosis (ALS); and 2) sex differences in care needs and survival among persons who are admitted to a nursing home after a stroke. The findings of these studies may support the development of guidelines and care plans to meet the needs of men and women with neurodegenerative disorders in varied care settings.

### WHAT'S OLD IS NEW: USING ARTIFICIAL INTELLIGENCE TO ACCELERATE DISCOVERY OF NEW TREATMENTS

Connie Marras[1] Laura C. Maclagan,[2] Yi Cheng[2] Naomi Visanji[3] Mina Tadrous[4] and Susan E. Bronskill[2], *1. Krembil Research Institute, University Health Network, Toronto, Ontario, Canada, 2. ICES, Toronto, Ontario, Canada, 3. Toronto Western Hospital, University Health Network, Toronto, Ontario, Canada, 4. St Michael's Hospital, Toronto, Ontario, Canada*

Given the high cost of drug development and low success rates, repurposing drugs already proven safe provides a promising avenue for identifying effective therapies with additional indications. The IBM Watson artificial intelligence system was used to search 1.3 million Medline abstracts to prioritize medications that may be potentially disease-modifying in Parkinson's disease. We assessed patterns of use of the top 50 Watson-ranked drugs among 14,866 adults with Parkinson's disease aged 70 and older who were matched to persons without Parkinson's disease on age, sex, and comorbidity. Sociodemographic characteristics, chronic conditions, and use of other medications were compared using standardized differences. Patterns of potentially disease-modifying drug use were examined prior to and following ascertainment of Parkinson's disease. Preliminary findings from multivariable conditional logistic regression models on the association between previous exposure to potentially disease-modifying drugs and Parkinson's disease diagnosis will be presented.

### SESSION 560 (SYMPOSIUM)

### PRODUCTIVE AND ACTIVE RURAL AGING: TOWARD CRITICAL PERSPECTIVES

Chair: Mark Skinner, *Trent University, Peterborough, Ontario, Canada*
Co-Chair: Rachel Winterton, *La Trobe University, Bendigo, Victoria, Australia*
Discussant: Kieran Walsh, *NUI Galway, Galway, Ireland, Ireland*

Despite global trends in rural population ageing, relatively little attention within research and policy has been directed to understanding what it means for rural people, communities and institutions to be at the forefront of twenty-first century demographic change. To build understanding of rural ageing, this symposium draws together papers from four countries to provide insights in the gaps in rural ageing research – specifically the in context of productive and active rural ageing by examining rural work, retirement and volunteering through the critical perspectives of citizenship, contestation and complexity. Winterton and Warburton will explore how active citizenship trends among rural older adults support or hinder the capacity of rural settings to support health ageing. Colibaba and Skinner will discuss the contestation of rural ageing by examining a volunteer-based rural library and the emergent 'contested spaces of older voluntarism' whereby older volunteer negotiate their rights and responsibilities associated with ageing and volunteering in rural communities. Duvvury and Ni Leime will examine the interactions between the twin phenomena of feminisation of agriculture and the feminisation of ageing in the consequent implications for rural women's work and retirement. Skinner and Joseph offer a critical perspective on voluntarism in ageing rural communities by examining volunteer leadership biographies as another means of understanding the contribution of older rural adults.

### CRITICAL PERSPECTIVES ON VOLUNTARISM IN AGING RURAL COMMUNITIES: VOLUNTEER LEADERSHIP BIOGRAPHIES

Mark Skinner[1] Alun Joseph[2], *1. Trent University, Peterborough, Ontario, Canada, 2. University Of Guelph, Guelph, Ontario, Canada*

Voluntarism has been portrayed as a productive and even transformative process whereby rural communities, households and older residents are able to meet the challenges of changing rural demographics. Yet, little attention has been paid to building a critical perspective on the complex and often-contested expectations placed on older rural volunteers. This paper focuses on the particular gap in understanding the contributions of older rural adults as a crucial resource in creating opportunities for aging in place and sustainable rural community development. Drawing on research into voluntarism in Canada's aging resource communities, this paper presents qualitative findings from innovative 'volunteer leadership biographies' with older residents who were involved in key voluntary sector initiatives to improve community development. The findings show how older volunteer leadership is embedded in both place (residency) and time (life course), revealing new dimensions to the problem of understanding volunteer leadership in an era of rural population change.

### DEFINING THE RELATIONSHIP BETWEEN ACTIVE CITIZENSHIP AND HEALTHY RURAL AGING: A CRITICAL APPROACH

Rachel Winterton[1], *1. La Trobe University, Bendigo, Victoria, Australia*

Active citizenship is romanticized in policy for its role in keeping older adults healthy, and rural communities sustainable. However, as proportions of older adults resident in rural communities continue to increase, the gerontological literature has begun to highlight challenges associated with both the capacity and desire of rural older adults to be active citizens. Through an integrative review of the international literature, this paper interrogates how active citizenship trends among rural older adults support or hinder healthy aging in rural settings. Findings indicate that active citizenship among older adults can increase rural age-friendliness and facilitate individual wellbeing. However, practices associated with active citizenship among this cohort can disenfranchise certain groups of older adults, through reshaping societal norms relating to citizenship, age-friendliness and rurality. These findings indicate that programs designed to promote active citizenship must both consider, and account for, diverse capacities and desires for active citizenship among rural older adults.

### RURAL LIBRARIES AS CONTESTED SPACES OF OLDER VOLUNTARISM IN AGING RURAL COMMUNITIES

Amber Colibaba,[1] and Mark Skinner[1], *1. Trent University, Peterborough, Ontario, Canada*

Recent efforts to better understand voluntarism as fundamental to how rural communities are meeting the challenges of population ageing have highlighted ageing rural volunteers, and the attendant burden of older voluntarism, as key issues for ageing in place of rural residents and ageing rural community sustainability. Drawing on a case study of a volunteer-based rural library in Ontario, Canada, this study examines the experiences of older volunteers, the challenges of sustaining volunteer programs, and the implications of older voluntarism for rural community development. Findings from interviews and focus groups with library volunteers, staff, board members and community stakeholders demonstrate how the experiences of older volunteers and challenges of older voluntarism affect rural community development. The results reveal how participation, well-being, conflict and territoriality associated with older voluntarism contributes to 'contested spaces of older voluntarism' whereby older volunteers negotiate their rights and responsibilities associated with ageing and volunteering in rural communities.

### RURAL WOMEN AND AGING: IMPLICATIONS FOR WORK AND RETIREMENT OF OLDER WOMEN

Aine Ni Leime,[1] Nata Duvvury,[1] and Tanya Watson[1],
*1. National University of Ireland, Galway, Galway, Galway, Ireland*

At least half of the world's female population live in rural areas, and many are ageing. For these rural women, agriculture and informal rural livelihoods are the primary sources of employment, posing critical challenges for them with regard to work and retirement. This paper focuses on the interaction between the twin phenomena of the feminisation of agriculture and the feminisation of ageing and the consequent implications for rural women's work and retirement. Drawing on qualitative interviews and focus groups with 48 older rural Irish women, the paper establishes the 'invisibility' of women's economic contribution in agriculture, limiting their pension accumulation and constraining their retirement planning. The study found that even women property owners, and designated 'farmers', had uncertainty about their pension or retirement income. A key conclusion is that rural women's pension rights are still not guaranteed posing increased risk of economic insecurity and wellbeing for older rural women.

### SESSION 565 (PAPER)

### RISKS OF FALLS AND INJURY

### CHRONIC PAIN, FEAR OF FALLING, AND RESTRICTED ACTIVITY DAYS IN AN OLDER POPULATION

Suzanne Leveille[1], *1. University of Massachusetts Boston, Boston, Massachusetts, United States*

Both chronic pain and fear of falling can lead to activity restriction and increased fall risk among vulnerable elders. Little is known about pain characteristics that may be associated with fear of falling, contributing to restricted activity. We studied 765 adults aged ≥65y (mean=78.9y) in the MOBILIZE Boston Study, to evaluate the cross-sectional relationship between pain characteristics and fear of falling measured using the Falls Efficacy Scale (FES). In addition, we examined the impact of pain and fear of falling on restricted activity. We measured 3 domains of global pain: pain distribution (none, single site or multisite pain), and Brief Pain Inventory subscales of pain severity and pain interference. Restricted activity days (RADs) refer to the count of self-reported days of reduced activity due to illness or injury in the previous 12 months. We performed multivariable logistic regressions predicting fear of falling (FES<90/100) adjusted for sociodemographics, fall history and fall risk factors. Participants with multisite pain or moderate-to-high pain interference ratings were more likely to have fear of falling (adj. OR 1.97, 95%CI 1.05-3.67; adj.OR 4.02, 95%CI 2.0-8.06, respectively). Pain severity was not associated with FES. Older adults with multisite pain and fear of falling reported significantly more RADs than those with multisite pain without fear of falling (79±135 and 26±74 RADs, respectively; test for pain x FES interaction, p=0.01). Older adults with chronic pain have greater fear of falling which may contribute to restricted activity. Efforts are needed to increase activity and falls efficacy among older adults with chronic pain.

### CROSS-SECTIONAL ASSOCIATION OF FALLS AND POST-TRAUMATIC STRESS IN CANADIANS ACROSS LEVELS OF FRAILTY

Olga Theou[1] Mario Ulises Pérez-Zepeda[2]
Joshua Armstrong[3] Judith Godin[4] Melissa Andrew,[1]
Susan Kirkland,[1] and Kenneth Rockwood[1], *1. Dalhousie University, Halifax, Nova Scotia, Canada, 2. Instituto Nacional de Geriatria, Ciudad de Mexico, Mexico, 3. Lakehead University, Thunder Bay, Ontario, Canada, 4. Division of Geriatric Medicine, Dalhousie University & Nova Scotia Health Authority, Halifax, Nova Scotia, Canada*

Frail older adults are vulnerable to stressors and are more likely to experience adverse outcomes. Post-traumatic stress is common in older adults, and can be related to common adverse outcomes, such as falls. We examined whether falls are associated with post-traumatic stress in middle-aged and older Canadians, by levels of frailty. We conducted cross-sectional analysis of the baseline assessment of the Canadian Longitudinal Study on Aging's tracking cohort, comprising 21,241 individuals, aged 45 to 85 years. We constructed a 60-item frailty index (FI) and defined post-traumatic stress using the primary care post-traumatic stress disorder four-item tool (score 3 as the cut-point). Logistic regressions with post-traumatic stress as the dependent variable and at least one fall in the past year as the independent variable, were adjusted for socio-demographic variables and stratified according to FI 0.1 groups. Prevalence of post-traumatic stress and falls was of 6.5% and 5.0%, respectively for the whole sample. Among those who did not fall prevalence of post-traumatic stress ranged across frailty levels from 3.2% (FI<0.1) to 24.5% (FI≥0.3). Among those who fell, post-traumatic stress ranged from 3.4% (FI<0.1) to 36.9% (FI≥0.3). Falls were not significantly associated with post-traumatic stress among people who had an FI<0.3, but among those with an FI≥0.3 the odds ratio for having post-traumatic stress for those who fell was 2.25 (95% CI 1.2-4.23, p=0.011) compared to non-fallers. In conclusion, high levels of frailty can impact how a stressor, such as a fall, can be associated with an adverse psychological outcome.

### EFFECT OF A HOME-BASED EXERCISE PROGRAM ON SUBSEQUENT FALLS IN SENIORS AFTER A FALL: A RANDOMIZED CLINICAL TRIAL

Teresa Liu-Ambrose[1] Jennifer C. Davis[2] John R. Best,[1]
Larry Dian[3] Wendy Cook[4] Kenneth Madden[5]
Chun Liang Hsu[6] and Karim M Khan[7], *1. University of British Columbia, Vancouver, British Columbia, Canada, 2. University of British Columbia - Okanagan Campus, Kelowna, British Columbia, Canada, 3. Department of Medicine, Division of Geriatric Medicine, Faculty of Medicine, University of British Columbia, British Columbia, Canada, 4. Division of Geriatric Medicine, University of British Columbia, Vancouver, Canada, 5. Gerontology*

*and Diabetes Laboratory, University of British Columbia, Vancouver, British Columbia, Canada, 6. University of British Columbia, Vancouver, British Columbia, Canada, 7. Center for Hip Health and Mobility, Vancouver Coastal Health Research Institute, Vancouver, Canada*

We assessed the efficacy of the home-based Otago Exercise Program (OEP) as a secondary falls prevention strategy in seniors referred to a falls prevention clinic after an index fall. We conducted a 12-month randomized controlled trial of 344 adults, aged 70 years and older, with = or > 1 fall resulting in medical attention in the prior 12 months. Participants were randomized to OEP or standard of care (CON). The OEP is a home-based strength and balance training program delivered by a physical therapist. All participants received AGS Guideline Care for falls prevention from a geriatrician. Differences in falls rate was tested with a negative binomial regression model. The rate of falls was lower in the OEP group vs the CON group (incident rate ratio [IRR] = 0.64, 95% CI 0.46 to 0.90). The estimated incidence rate of falls per person-year was 1.4 (95% CI 0.1 to 2.0) in the OEP group and 2.1 (95% CI 0.1 to 3.2) in the CON group, with an absolute incidence rate difference of 0.74 (95% CI 0.04 to 1.78) falls per person-year. DSST performance also increased in the OEP group by a mean change of 1.1 points (95% CI 0.02 to 2.1) vs the CON group. Improved DSST was associated with fewer falls (IRR = 0.80, 95% CI 0.68 to 0.95). These findings support the use of the OEP for secondary falls prevention.

### MULTIPLE MEDICATION USE AND RISK OF TREATED FALL INJURY: THE HEALTH ABC STUDY

Lingshu Xue[1] Robert M. Boudreau,[2] Julie M. Donohue[2] Janice C. Zgibor[3] Zachary A. Marcum[4] Tina Costacou,[2] Anne B. Newman,[2] and Elsa Strotmeyer[2], *1. University of Pittsburgh, Pittsburgh, United States, 2. University of Pittsburgh, Pittsburgh, Pennsylvania, United States, 3. University of South Florida, Tampa, Florida, United States, 4. University of Washington, Seattle, Washington, United States*

Multiple medication use within one year is associated with increased fall injury risk in older adults. However, chronically using multiple medications and treated fall injury have rarely been explored, particularly in cohort studies linked with claims data. We examined using >5 medications in 2 or more consecutive years (chronic medication use) as a risk factor for treated fall injury in 1,898 community-dwelling adults (age 73.6±2.9 years; 53% women; 37% black) with linked Medicare Fee-For-Service (FFS) claims from the Health, Aging and Body Composition Study since 1997/98 clinic visit. Incident fall injury (N=546) was the first claim from 1998/99 clinic visit to 12/31/08 with an ICD-9 fall code and non-fracture injury code, or fracture code with/without a fall code. Stepwise Cox models with a time-varying predictor of chronic medication use before fall injury or censoring (N=414) vs. not using >5 medications at the same time (N=1008) were adjusted for baseline demographics, lifestyle factors, fall history, quadriceps strength, cardiovascular disease (CVD), diabetes, sensory nerve impairment, and kidney function. Fall injury risk increased for chronic medication users (37%) vs. non-users (29%) (HR=1.25[1.00-1.57]), though was attenuated after adjustment for CVD and diabetes (HR=1.18[0.93-1.51]). Sensitivity analyses excluding fall-risk-increasing drugs (FRIDs) from medication counts (HR=1.32[0.54-3.20]), or including those using >5 medications non-chronically (N=365) in referent groups (HR=1.22[0.96-1.55]) had consistent findings. Unmeasured comorbidity differences may confound associations of chronic medication use and treated fall injury risk in older adults with Medicare FFS. Considering both chronic diseases and medication use in fall risk assessments is needed.

### PROGRAM CHARACTERISTICS ASSOCIATED WITH REDUCED FEAR OF FALLING: A SYSTEMATIC REVIEW AND META-ANALYSIS OF RCTS

Marlot Kruisbrink[1] Kim Delbaere[2] Gertrudis Kempen,[1] Rik Crutzen,[1] and G.A. Rixt Zijlstra[1], *1. Maastricht University, Care and Public Health Research Institute (CAPHRI), Department of Health Services Research, Maastricht, Netherlands, 2. Neuroscience Research Australia, School of Public Health and Community Medicine, UNSW, Randwick, New South Wales, Australia*

Fear of falling (FOF) is common among older people and can result in activity avoidance and decreased physical functioning. Different types of interventions have demonstrated significant small reductions in FOF. To optimize effect sizes, we sought to identify characteristics of interventions that were associated with a change in FOF. Five scientific databases were searched for articles using randomized controlled trial designs in community-dwelling older people without medical conditions. Data extraction included intervention type, setting, group format, type of supervision, provider, delivery format, duration, number of sessions, contact time, and risk of bias (assessed with the Cochrane Collaboration's Risk of Bias Tool). After screening of titles, abstracts, and full texts, 55 unique studies – reporting on 68 interventions – were systematically reviewed. The majority of interventions focused on exercise (n=50). Interventions were performed at home (n=21) or in a community setting (n=23), were delivered in a group (n=26) or individual (n=30) format, and were often supervised (n=60) and delivered face-to-face (n=56). Duration ranged from 1 to 52 weeks and total contact time with the provider from 2 to 56 hours. Results of 42 interventions were suitable for meta-analysis. Univariate meta-regressions to evaluate associations between intervention characteristics and intervention effects directly after the intervention yielded no significant results. Due to self-reported outcomes and difficulties with blinding, risk of bias was high in all studies. To conclude, intervention characteristics were not associated with changes in FOF in this study. Possible reasons for an absence of associations and future research directions will be discussed.

### SESSION 570 (SYMPOSIUM)

### SEDENTARY BEHAVIOR AND PHYSICAL ACTIVITY IN THE ADULT CHANGES IN THOUGHT (ACT) STUDY

Chair: Dori E. Rosenberg, *Kaiser Permanente Washington Health Research Institute, Seattle, Washington, United States*
Co-Chair: Andrea Z. LaCroix, *University of California San Diego, La Jolla, California, United States*
Discussant: Jack Guralnik, *University of Maryland School of Medicine, Baltimore, Maryland, United States*

Few epidemiologic studies have examined device-measured sitting patterns and health outcomes. Furthermore, there is a need to continue understanding the role of prospectively measured physical activity in relation to older adult health. The Adult Changes in Thought (ACT) study is an on-going epidemiologic study of adults age ≥65 years that began in 1994. Participants complete biennial assessments including a self-reported measure of physical activity. Starting in 2016, ACT participants could enroll in a physical activity sub-study that involved wearing a thigh-worn activPAL device and maintaining sleep logs for 7 days. Of those approached to participate in the sub-study, 64% agreed (N = 1139). A total of 961 had valid wear time (≥4 days with 10-20 hours of data per day) and completed survey collecting measures on pain and built environments (56% female, 57% > age 75, 89% non-Hispanic white). Participants who consented to the sub-study were generally younger and had fewer chronic conditions than those who did not consent. After removing sleep time, mean daily activPAL measures calculated included hours sitting and standing, number of sitting bouts lasting 30 minutes or more, number of breaks from sitting, and steps walked. The first session in this symposium will present historical self-reported physical activity trajectories in relation to cognitive function. The subsequent sessions will present novel cross-sectional data examining activPAL variables with measures of physical function, pain, and perceived built environments. This symposium will provide new insights on the roles of sedentary behavior and physical activity in aging and health.

### SITTING PATTERNS, PHYSICAL ACTIVITY, AND PHYSICAL FUNCTIONING IN OLDER ADULTS

Dori E. Rosenberg[1] Mikael Anne Greenwood-Hickman,[2] Rod walker,[2] KatieRose Richmire[2] Paul Crane[3] Eric Larson,[2] and Andrea LaCroix[4], *1. Kaiser Permanente Washington Health Research Institute, Seattle, Washington, United States, 2. KPWHRI, seattle, Washington, United States, 3. UW, Seattle, Washington, United States, 4. UC San Diego, La Jolla, Washington, United States*

We examined cross-sectional associations between physical function and device-based (activPAL) sedentary patterns and physical activity. Physical function tasks included time to complete 5 chair stands and walk a 10-foot gait speed course. We estimated associations using linear regression models adjusting for age and sex; coefficients represent estimated change in mean activPAL measures associated with each second increase in gait/chair stands time. Longer gait speed times were associated with more total sitting time (b=0.19, p < 0.01), fewer steps (b=-788.0, p<0.001), fewer sitting breaks (b=-1.7, p<0.01), and more prolonged sitting bouts (b=0.19, p<0.01). Longer chair stand times were associated with more total sitting time (b=0.06, p<0.001), less standing time (b=-0.04, p<0.01), fewer steps (b=-176.8, p<0.001), fewer sitting breaks (b=-0.45, p<0.01), and more prolonged sitting bouts (b=0.07, p<0.001). Prolonged patterns of sitting time and higher total sitting time, in addition to lower physical activity, were consistently associated with worse physical function.

### ASSOCIATION OF 10-YEAR WALKING TRAJECTORIES WITH COGNITIVE FUNCTION IN OLDER ADULTS: ADULT CHANGES IN THOUGHT STUDY

Paul Gardiner[1] Barbara J. Jefferis[2] KatieRose Richmere[3] Andrea Z. LaCroix[4] Paul K. Crane[5] Eric B. Larson,[3] and Dori E. Rosenberg[3], *1. The University of Queensland, Brisbane, Queensland, Australia, 2. University College London, London, England, United Kingdom, 3. Kaiser Permanente Washington Health Research Institute, Seattle, Washington, United States, 4. University of California San Diego, La Jolla, California, United States, 5. University of Washington, Seattle, Washington, United States*

We identified trajectories of older adults' walking and their associations with cognitive function. Data on walking (days/week) were collected at baseline of the Adult Changes in Thought study and every two years for 10 years. Cognitive function was assessed by the Cognitive Abilities Screening Instrument (CASI) at year 12. Group-based trajectory analyses identified trajectories among 763 participants (baseline age 70±5 years, 60% female). Regression models, adjusted for baseline sociodemographic and health factors, examined associations with cognitive function. Five walking trajectories were identified: consistently inactive (18.1%), medium active (21.9%), early decline (15.8%), late decline (18.4%), and consistently active (25.8%). Mean CASI score was 92.0 (SD 6.9). CASI scores were lower in early b = -1.66 (95%CI: -2.97, -0.35) and late decline b = -1.89 (-3.26, -0.51) groups, with no difference in consistently active and inactive groups, compared to the medium active trajectory group. Ten-year walking trajectories may determine late-life cognitive function.

### ASSOCIATIONS OF PAIN INTENSITY WITH SEDENTARY BEHAVIOR AND PHYSICAL ACTIVITY IN OLDER ADULTS

Kushang Patel[1] Andrea LaCroix[2] Paul Crane,[1] Rod L. Walker,[3] KatieRose Richmire,[3] Eric B. Larson,[3] Dori E. Rosenberg[3], *1. University of Washington, Seattle, Washington, United States, 2. University of California San Diego, La Jolla, California, United States, 3. Kaiser Permanente Washington Health Research Institute, Seattle, Washington, United States*

Exercise is recommended for several painful, age-associated conditions; however, relationships between pain intensity and objectively measured sedentary behavior and physical activity have not been investigated in older adults. Accordingly, we analyzed cross-sectional data on 936 older adults in the ACT Study who self-reported their pain intensity on a 0-10 rating scale (0=no pain; 1-3=mild pain; and 4-10=moderate/severe pain) and wore an activPAL accelerometer. A total of 181 (19.3%) reported no pain, while 564 (60.3%) and 191 (20.4%) reported mild and moderate/severe pain, respectively. Linear regression models adjusted for age and sex estimated that compared to those with no pain, participants with moderate/severe pain walked significantly fewer steps/day (b-coefficient=-778 [95%CI: -1377, -179]) and had fewer sit-to-stand transitions/day (b-coefficient=-2.9 [95%CI: -5.6, -0.1]). In contrast, there were no significant differences in these outcomes comparing no pain versus mild pain. Future research will examine effects of pain treatments (opioids) and diagnoses on accelerometer-measured outcomes.

### ASSOCIATIONS BETWEEN NEIGHBORHOOD CHARACTERISTICS AND SITTING PATTERNS IN OLDER ADULTS

MIkael Anne Greenwood-Hickman,[1]
Mikael Anne Greenwood-Hickman,[1] Rod L. Walker,[1] KatieRose Richmire[1] Andrea LaCroix[2] Eric B. Larson,[1] Paul K. Crane[3] and Dori E. Rosenberg[1], *1. Kaiser Permanente Washington Health Research Institute, Seattle, Washington, United States, 2. University of California San Diego, La Jolla, California, United States, 3. Department of Medicine, University of Washington, Seattle, Washington, United States*

Neighborhood characteristics are associated with self-reported sedentary behavior (SB) in older adults. However, self-report measures are not able to accurately assess total sitting time nor detailed patterns of SB. This analysis explores the relationship between device-based SB variables from activPAL and neighborhood characteristics (demographics) in the ACT cohort. Neighborhood characteristics were assessed with the modified Physical Activity Neighborhood Environment Scale (PANES; scored 1.0-4.0, higher score, higher walkability). Data were analyzed using linear regression models adjusted for demographic factors. Higher PANES score was associated with higher daily steps (+1180 daily steps/point on PANES, $p<0.001$) and sit-to-stand transitions (+2.7 daily transitions/point on PANES, $p=0.004$). Confirming other studies, neighborhood walkability promotes physical activity. A novel finding was that sitting interruptions, which can only be assessed with devices, were also associated with higher neighborhood walkability, while total sitting time was not.

### SESSION 575 (SYMPOSIUM)

### STUDIES ON ADJUSTMENT TO RETIREMENT: HOW OLDER EUROPEANS NAVIGATE THE RETIREMENT TRANSITION

Chair: van Solinge, *Netherlands Interdisciplinary Demographic Institute, The Hague, Netherlands*
Discussant: Olga Grunwald, *NIDI, Den Haag, South Holland, Netherlands*

Retirement is a significant life transition in late adult life that often brings about great changes in individuals' patterns of everyday activity, social networks as well as one's economic resources, requiring adjustment for both the retiree and other members of the household. Retirement is a process that starts with a preparatory stage, followed by the actual act of retirement and a post-retirement stage where retirees have to get used to the changing aspects of life that result from the work-retirement transition, and seek to achieve psychological comfort with their retirement life. This symposium brings together empirical research on the various stages of the retirement process, from different national backgrounds. The guiding question is how work and the loss of work affect well-being. Hence, the symposium will give insights into the circumstances under which retirement risks well-being and psychological comfort of older adults. Anna Wanka discusses under which conditions retirement feels right for German retirees, and how this feeling shifts and changes throughout the retirement process. Sarah Dury follows with the post-retirement stage by demonstrating a qualitative perspective of recently retired Belgians about their adjustment, role and activities they exert during post-retirement. Isabelle Hansson examines the role of personality for retirement adjustment in a Swedish sample of older adults. Hanna van Solinge explores the impact of agency in the work-retirement transition on adjustment to a longer working life /retirement and life satisfaction in a Dutch panel study.

### AGENCY IN LATE-CAREER TRANSITIONS: THE IMPACT ON ADJUSTMENT AND SATISFACTION

van Solinge[1], *1. Netherlands Interdisciplinary Demographic Institute, The Hague, Netherlands*

In research on late career transitions agency is implicitly assumed. The extent to which older adults are able to shape their late career in the face of external constraints, such as a rising state pension age, may however be limited. Constraint agency may have impact on well-being. Using data from a panel study among 5,300 older workers in the Netherlands, we examined the impact of agency in the work-retirement transition on adjustment and life satisfaction. Results show that adjustment to a the higher retirement age is more challenging than adjustment to retirement. Life satisfaction increased among those who retired, but not among those who remained working. One third experienced constrained agency (involuntary retirement or non-retirement). The negative association between constrained agency and life satisfaction was stronger for participants still in the labor force than for retirees. Our findings demonstrate that involuntary non-retirement has stronger implications for well-being than involuntary retirement.

### IS THERE A RIGHT TIME TO RETIRE? AFFECTIVE DIS/ENGAGEMENT FROM WORK IN A LONGITUDINAL QUALITATIVE STUDY FROM GERMANY

Anna Wanka[1], *1. Goethe University Frankfurt am Main, Frankfurt am Main, Germany*

Recent retirement research has argued that the once predictable pattern associated with retiring is becoming increasingly differentiated by the age at which it occurs, if it is gradual of abrupt, voluntary or involuntary, etc. (Moffatt & Heaven 2017). Even though research suggests that retirement legislation and statutory retirement ages influence the subjective perception of a 'right time to retire' (cf. Jansen, 2018), many people don't feel ready to retire when they should and hence retire involuntarily (Steiber & Kohli, 2017). This paper focuses on the dis/engagement processes that lead to feeling 'ready' to retire at a certain time, or not. Drawing on data from a qualitative longitudinal study in Germany, results suggest (1) that readiness to retire is a continuum, rather than a binary, which is influenced by a variety of actors, and (2) that it is and a process that changes multiple times in the retirement transition.

### A BELGIAN VIEW ON BEING RETIRED BUT NOT OUT OF WORK

Sarah Dury[1], *1. Vrije Universiteit Brussel, Brussels, Belgium*

Retirement is no longer merely the end of a productive life. This changing nature of retirement challenges the common definitions of retirement – that define retirement primarily by what it is not (i.e. no longer working). The aim of this paper is to gain insight into the activity patterns of individuals who recently retired from a full-time job in relation with their well-being. We use data from a qualitative study in which we conducted semi-structured interviews with 45 individuals who retired one to two years ago in Belgium. We used a hybrid approach of inductive and deductive thematic analysis. Our findings demonstrate that most of the people who are retired from their full-time job remain active within society. First, productive activities, including work and civic engagement. Second, consumer-oriented activities comprising leisure and social contacts. The results suggest that being active, regardless of the type of activity, contributes to well-being.

### THE ROLE OF PERSONALITY IN RETIREMENT ADJUSTMENT: LONGITUDINAL EFFECTS ON LIFE SATISFACTION

Isabelle Hansson[1] Georg Henning,[2] Sandra Buratti,[2] Magnus Lindwall,[2] Marie Kivi,[2] Boo Johansson,[2] and Anne Ingeborg Berg[2], *1. Department of Psychology and Centre for Ageing and Health (AgeCap), University of Gothenburg, Gothenburg, Sweden, 2. Department Of Psychology And Centre For Ageing And Health (AgeCap), University Of Gothenburg, Gothenburg, Vastra Gotaland, Sweden*

Research on the retirement transition suggests that personality can influence the adjustment process, but the mechanisms involved remain still largely unknown. In the present study we investigate direct and indirect associations between the Big Five personality traits and life satisfaction over the retirement transition. Indirect effects were evaluated through the role of personality for self-esteem, autonomy, social support, perceived physical and cognitive health, and financial satisfaction. The sample included 796 older adults and four annual measurement waves in the Swedish longitudinal HEARTS study. Results from multivariate latent growth curve models showed multiple indirect effects of personality. Extraversion was positively related to life satisfaction through increased self-esteem, autonomy, and social support. Neuroticism was negatively associated with life satisfaction through decreased self-esteem, autonomy, social support, and perceived cognitive ability. Our findings suggest that retirees with higher neuroticism are more likely to experience adjustment problems resulting from negative changes in key resources.

### SESSION 580 (SYMPOSIUM)

### TECHNOLOGY AND AGING FUTURES: DISCOVERING THE CONSUMER ELECTRONICS SHOW (CES) 2019

Chair: Stephen Katz, *Trent University, Toronto, Ontario, Canada*

The growing field of technology and aging or gerontechnology has largely been considered from a health perspective on technological intervention to ameliorate conditions of isolation, disconnection, inactivity, and loneliness, as well as provide efficient alert systems, transportation coordination, and emergency services. Contesting the image of a 'digital divide' separating younger from older generations, the recreational industry has also produced a seniors market of technological games, toys, apps, exercises, and social media. The four papers in this symposium, however, are individual critical reflections by a group of social scientists who visited the Consumer Electronics Show (CES) in January 2019 (Las Vegas) as part of an ethnographic project about the politics of the technical turn in gerontological studies. In particular, the authors gathered evidence from the CES to support their interests in four trends: a) The collecting, aggregating, and sharing of personal data by home surveillance, artificial intelligence monitoring, and self-tracking systems for commercial, insurance and work-place purposes, b) The popularization of healthy lifestyles based on technical and exclusionary models of 'smart', 'fit', and 'optimal' standards, c) The technical rhetoric that infuses designs for efficiency, speed, and convenience with anti-aging and ageist ideologies, d) The challenges to older people to manage their lives against the health risks, interventions, and expectations posed by technology-driven austerity programs. The papers have in common their creative interpretations of CES materials and shared concern about the many older groups whose insufficient access, skill, and resources will deny them participation in the technological imaginary of aging futures.

### PRIVATE PARTS: AGING, AI, AND THE ETHICS OF CONSENT IN SUBSCRIPTION-BASED ECONOMIES

Kim Sawchuk[1], *1. Concordia University, Montreal, Quebec, Canada*

This paper explores Artificial Intelligence (AI) as a technological design offered to assist elder-care based on tracking individual behavior amassed in data bases that are given predictive value through algorithm-identified normative patterns. Examples are drawn from ethnographic research conducted at the 2019 Consumer Electronics Show, including document analysis of product promotions and interviews conducted with company representatives, supplemented by interviews with older adults. The paper focuses on the ethical dilemmas of privacy, security, consent, and identity in home surveillance systems and financialization of personal data in AI subscription-based services. Theoretically the argument, informed by recent discussions on the datafication of self and quantified aging, emphasizes that such a subscription-based economy exploits older individuals by sharing their lifestyle profiles, health information, economic status, and consumer preferences within powerful corporate networks such as Google and Amazon. Conclusions question how the promise of AI is being tied to the future of population aging.

### THE RISE OF ROBOT PETS AND DISCOURSES OF TECHNO-COMPANIONSHIP IN LATER LIFE

Constance Lafontaine[1], *1. Concordia University, Montreal, Québec, Canada*

Robot pets of varying degrees of sophistication are advertised as ideal companions for older adults, with claims that they support their emotional and cognitive needs. A new generation of robot pets (e.g., Lovot) is emerging equipped with internet connections, recognition software, surveillance capacity, and AI platforms to mimic desired aspects of animal-human relationships. This paper draws upon promotional materials gathered at the Consumer Electronics Show 2019 and interviews with robot designers, to probe what the robotization of human-animal relationships tells us about shifting notions of companionship through the life course. The argument is that robot pets are inscribed within discourses that instrumentalize human-animal-technology entanglements and position digital technologies as idealized solutions to perceived age-related problems. Theoretical ideas from animals studies add a creative dimension to aging studies to explore what the societal enthusiasm for robot pets reveals about our understanding of the lived material and relational worlds of older adults.

### SMART LIFE, FITNESS, WELLNESS, AND THE PROMISE OF DIGITAL HEALTH TECHNOLOGIES FOR OLDER PEOPLE

Stephen Katz[1], *1. Trent University, Toronto, Ontario, Canada*

Based on the author's ethnographic observation and collection of product media kits, videos, and photographs from the Consumer Electronics Show 2019, this paper critiques the ways in which positive lifestyle concepts such as 'smart life', 'fitness' and 'wellness' are designed within products aimed at senior marketing to shape the older consumers as composites of health problems open to technological intervention. However helpful sensor clothing, home surveillance cameras, self-tracking appliances, robotic companions, or digital mobility devices may be, they are also opportunities to capitalize on shared personal data and subscription-based monitoring services. Discussion links these concepts to wider concerns about algorithimic standardization of health risks for older people, healthcare austerity programs, and social inequalities based on technical markers of successful aging and privileged life-course trajectories

### REPRESENTATIONS OF OLD AGE IN THE CONSUMER ELECTRONICS WORLD

Mireia Fernández Ardèvol[1], *1. Open University of Catalonia / Universitat Oberta de Catalunya, Barcelona, Catalonia, Spain*

This paper examines representations of old age at the Consumer Electronics Show 2019, identifying how explicit product discourses identify later life with Fourth Age dependency, fragility, decline, and care, as is the case with the home and companion-robot industry. While designers take a Fourth Age approach to the 'senior market', they liken their products with those for children, as both old and young are stereotyped as requiring surveillance based on their assumed weaknesses. Thus the technological depictions of old age neglect the diversities of older populations and reinforce dominant ageist and homogenizing narratives about older life as disempowering, passive, and digitally divided. Conclusions question why technological design aimed at helping older individuals are uninformed and misconceived about the realities of later life and what recommendations may be offered to resolve this resulting ageism.

### SESSION 585 (SYMPOSIUM)

### THE IMPORTANCE OF TRANSPARENCY AND OPEN SCIENCE IN GERONTOLOGY: PERSPECTIVES FROM EARLY-CAREER RESEARCHERS

Chair: Jennifer Lodi-Smith, *Canisius College, Buffalo, New York, United States*
Co-Chair: Eileen K. Graham, *Northwestern University, Chicago, Illinois, United States*

The past decade has seen rapid growth in conversations around and progress towards fostering a more transparent, open, and cumulative science. Best practices are being codified and established across fields relevant to gerontology from cancer science to psychological science. Many of the areas currently under development are of particular relevance to gerontologists such as best practices in balancing open science with participant confidentiality or best practices for preregistering archival, longitudinal data analysis. The present panel showcases one of the particular strengths of the open science movement - the contribution that early career researchers are making to these ongoing conversations on best practices. Early career researchers have the opportunity to blend their expertise with technology, their knowledge of their disciplines, and their vision for the future in shaping these conversations. In this panel, three early career researchers share their insights. Pfund presents an introduction to preregistration and the value of preregistration from the perspective of "growing up" within the open science movement. Seaman discusses efforts in and tool for transparency and reproducibility in neuroimaging of aging research. Ludwig introduces the idea of registered reports as a particularly useful form of publication for researchers who use longitudinal methods, and/or those who work with hard-to-access samples. The symposium will include time for the audience to engage the panel in questions and discussion about current efforts in and future directions for transparent, open, and cumulative science efforts in gerontology.

### GROWING UP WITH OPEN SCIENCE

Gabrielle N. Pfund[1], *1. Washington University in St. Louis, St. Louis, Missouri, United States*

While a novel concept for many, pre-registration is an outlet growing in prevalence that allows researchers to systematically work through future studies from start to finish by laying out research questions, relevant hypotheses, and study design. "Growing up" as a researcher with Open Science, I never felt that pre-registration limited me, my ability to think and ask questions, or any opportunities to explore new ideas with old data. Instead, it strengthened me as a researcher by giving me a methodical perspective to take when considering why I needed to ask a question and how I planned to answer it. In this talk, I will define what pre-registration is and discuss my experiences with it as an early career researcher. Furthermore, I will present different concerns and conceptions people have about it and expound upon the inherent benefits it provides to both critical thinking and study design.

### TRANSPARENCY AND REPRODUCIBILITY IN THE NEUROIMAGING OF AGING

Kendra L. Seaman[1], *1. Duke University, Durham, North Carolina, United States*

In concert with broader efforts to increase the reliability of social science research, there are several efforts to increase transparency and reproducibility in neuroimaging. The large-scale nature of neuroimaging data and constantly evolving analysis tools can make transparency challenging. I will describe emerging tools used to document, organize, and share behavioral and neuroimaging data. These tools include: (1) the preregistration of neuroimaging data sets which increases openness and protects researchers from suspicions of p-hacking, (2) the conversion of neuroimaging data into a standardized format (Brain Imaging Data Structure: BIDS) that enables standardized scripts to process and share neuroimaging data, and (3) the sharing of final neuroimaging results on Neurovault which allows the community to do rapid meta-analysis. Using these tools improves workflows within labs, improves the overall quality of our science and provides a potential model for other disciplines using large-scale data.

### REGISTERED REPORTS FOR ECRS: ENABLING SLOW SCIENCE ON TIGHT TIMELINES

Rita Ludwig[1], *1. University of Oregon, Eugene, Oregon, United States*

Early career researchers (ECRs) may experience tension between the ideal and actual amounts of time they have to complete a scientific project. Sometimes, a timeline is truncated because a citable product is required for applications or fellowship deadlines. This scenario is especially common for researchers who use longitudinal methods, and/or those who work with hard-to-access samples. Registered reports, an open science initiative, offer one resolution to this tension. In registered reports, the steps of analysis planning, manuscript writing, and peer review occur earlier than the traditional journal article publication process. If an in principle acceptance is earned, ECRs are afforded citable, peer-reviewed acknowledgement of their scientific thinking prior to the conclusion of a research project. This talk will serve as a primer on the registered report process. I will also discuss resources for writing registered reports, and provide a list of relevant participating journals in the field of gerontology.

### SESSION 590 (SYMPOSIUM)

### THE ROLE OF WORK AND RETIREMENT IN COGNITIVE AND BRAIN AGING

Chair: Gizem Hueluer, *University of Zurich, Zurich, Switzerland, Switzerland*
Discussant: George W. Rebok, *Johns Hopkins Bloomberg School of Public Health, Baltimore, Maryland, United States*

According to the "use it or lose it" hypothesis of cognitive aging, cognitive enrichment and cognitively engaging activities are associated with the maintenance of high levels of cognitive functioning in old age. Similar ideas have been brought forward with respect to characteristics of individuals' work environment, with more cognitively enriching work demands providing an optimal environment for cognitive development and maintenance. The goal of this research group is to showcase new developments in research on work, retirement and cognitive aging. Hülür et al. examine the role of perceived work environment for cohort differences in trajectories of cognitive change based on 56-year longitudinal data from the Seattle Longitudinal Study. Andel et al. use data from the Swedish Adoption/Twin Study of Aging to examine trajectories of cognitive aging before vs. after retirement with two-slope growth curve models. Zulka et al. conduct a systematic literature review on the association between retirement and cognition and examine the role of factors such as occupational experiences and the cognitive domain studied. Burzynska et al. investigate the relationship between stressful and stimulating occupational exposures and structural brain health and cognition in older age. The discussion by George Rebok will focus on how these findings contribute to our understanding of the role of occupational experiences for cognitive and brain aging and how they can be utilized to promote maintenance of cognitive functioning in old age.

### COHORT DIFFERENCES IN COGNITIVE AGING: THE ROLE OF PERCEIVED WORK ENVIRONMENT

Gizem Hueluer[1] Nilam Ram[2] Sherry L. Willis[3] K. Warner Schaie[4] and Denis Gerstorf[5], *1. University of Zurich, Zurich, Switzerland, Switzerland, 2. Pennsylvania State University, University Park, Pennsylvania, United States, 3. University of washington, Seattle, Washington, United States, 4. University of Washington, Seattle, Washington, United States, 5. Humboldt University, Berlin, Berlin, Germany*

Studies of historical change on cognitive aging generally document that later-born cohorts outperform earlier-born cohorts on tests of fluid cognitive performance. It is often noted how advances in educational attainment contribute to this finding. Over the last century, work demands and characteristics have changed profoundly, with shifts from a manufacturing to service and technical economy. We used data from the Seattle Longitudinal Study to compare trajectories of cognitive change between earlier-born (1901-1938) and later-born cohorts (1939-1966). Our findings show that (a) later-born cohorts had higher levels of performance on most cognitive tasks and exhibited less decline in word fluency, (b) had more enriched perceived work environment as indicated by higher levels of worker control and innovation, with no cohort differences in work autonomy (c) these experiences were associated with higher levels of cognitive performance independent of education and consistently across cohorts. We discuss potential mechanisms underlying these associations.

### THE ROLE OF RETIREMENT IN COGNITIVE AGING: RESULTS FROM THE SWEDISH ADOPTION/TWIN STUDY OF AGING

Ross Andel[1] Deborah Finkel[2] and Nancy L. Pedersen[3], *1. University of South Florida, Tampa, Florida, United States, 2. Indiana University Southeast, New Albany, Indiana, United States, 3. Karolinska Institutet, Stockholm, Stockholms Lan, Sweden*

Retirement is a major life transition that may influence the aging process. Using a two-slope growth curve model with retirement age as the pivot point, we studied change in major cognitive domains before and after retirement. Participants were 393 members of the Swedish Adoption/Twin Study of Aging who retired after the age of 50 (mean=63 years, range 51-75 years) and who were followed for over 20 years with seven testing occasions. After controlling for age, sex, and education, we observed no change in memory pre-retirement (p=.935) and significant memory decline post-retirement (Estimate=-0.17, p=.001); decline in speed, which more than doubled after retirement (Estimate=-0.20, p=.001

before, Estimate=0.54, p<.001 after); improvement in verbal abilities before (Estimate=0.15, p=.020) and decline in verbal abilities after retirement (Estimate=-0.11, p<.001); and some decline in spatial abilities before (Estimate=-0.18, p=.059) followed by accelerated decline after retirement (Estimate=-0.33, p<.001). Retirement may be a good target for intervention.

### REVIEW ON THE COMPLEX RELATION BETWEEN RETIREMENT AND COGNITION: UNRAVELING THE MIXED EVIDENCE

Linn Elena Zulka[1] Isabelle Hansson,[2] and
Linda B. Hassing[2], *1. Department of Psychology, University of Gothenburg, Gothenburg, Sweden, 2. Department of Psychology, University of Gothenburg, Gothenburg, Vastra Gotaland, Sweden*

A systematic review from 2017 revealed a great research gap concerning the question if retirement affects cognitive function. Since then, several longitudinal studies have been published, calling for an updated review. The aim of this review is to provide an update with a focus on different retirement operationalization, different cognitive outcomes, and potential mediators like occupational experiences. Twenty peer-reviewed studies with longitudinal designs were included. The results revealed no clear pattern regarding the association between retirement and the cognitive outcomes. Study results varied in relation to factors like occupational experiences, differences in study quality, and cognitive domains. To get an insight into mechanisms behind the relation between retirement and cognitive functioning, more complex study designs are needed that take into account the impact of pre-retirement factors, different retirement related aspects, and the varying effects depending on cognitive domain.

### ADULT-LIFE OCCUPATIONAL EXPOSURES: ENRICHED ENVIRONMENT OR A STRESSOR FOR THE AGING BRAIN?

Agnieszka Z. Burzynska[1], *1. Colorado State University, Fort Collins, Colorado, United States*

The positive association between midlife occupational complexity and cognitive functioning in older age is well documented. Much less is known about the underlying neural mechanisms and whether occupational stress may accelerate neurocognitive aging. According to the BOSS model (Brain aging: Occupational Stimulation and Stress), occupational exposures may serve as long-term protective and risk factors that influence the rate and extent of neurocognitive decline in aging (Burzynska, Jiao & Ganster 2018). We present findings from three independent samples linking different occupational exposures (e.g. work physical demands, innovation, autonomy, employer control) with brain volume in cognitively healthy older adults. We discuss the findings in the context of cognitive reserve and brain maintenance. Our findings suggest that occupational activities need to be acknowledged as an important factor in lifespan cognitive and brain development and warrant further research, with a possible outcome of workplace interventions aimed at optimizing neurocognitive aging.

### SESSION 595 (SYMPOSIUM)

### TOWARD A NETWORK OF CHANGE: PROMOTING AGE-FRIENDLY COMMUNITIES FROM THE INSIDE OUT

Chair: Shellae Versey, *Wesleyan University, Middletown, Connecticut, United States*
Co-Chair: Emily Greenfield, *Rutgers University, New Brunswick, New Jersey, United States*

The experience of aging is often framed by the communities in which we live, work and play. At the same time, these communities are also impacted by individuals as they age in place. This symposium presents research using community-partnered methods to highlight the agency of local actors—including older residents themselves—as they work to change their local communities. At the broadest level of geographic scale, Black illustrates how information from population surveys with older adults can be leveraged to mobilize public-private partnerships from the local to state level to advance policy and practice on housing and transportation to support aging in place. Focusing regionally in New Jersey, Greenfield and Reyes analyze longitudinal, qualitative interview data from leaders of age-friendly community initiatives to develop empirically-grounded theory on the range of roles of older adults in aging-friendly community change processes. The final two papers present depth in understanding how older adults actively construct their own sense of community vis-à-vis more micro-social processes. Yeh uses Photovoice methodology to understand how older adults aging in place manage the societal trends of urbanization and social inequalities as they manifest within their own city. Versey examines how older adults aging in place in the context of neighborhood gentrification mobilize networks to preserve a community "sense of place" when sociocultural resources are displaced. Consistent with a community gerontology framework, the presentations demonstrate how community-level dynamics around aging are shaped not only by macro-social influences, but also micro-social interactions including, and sometimes initiated by, older residents themselves.

### PLACEKEEPING AND SENSE OF PLACE: USING PHOTOVOICE TO HIGHLIGHT NEIGHBORHOOD DISPLACEMENT

Shellae Versey[1], *1. Wesleyan University, Middletown, Connecticut, United States*

There has been growing interest in the use of community-based participatory research (CBPR) in gerontology. Photovoice, one of several qualitative methods utilized in CBPR, pairs participants with photography to identify and represent issues of importance. This paper explores photovoice as a tool for meaning making and preserving a 'sense of place' in a gentrifying context in New York City. Older residents describe pending neighborhood displacement due to gentrification using photographs. Using these themes and a range of visual media, older adults mobilize preservation and resistance efforts to gentrification. The paper concludes with implications and directions for future research.

### FROM PROGRAM PARTICIPANT TO AGING ADVOCATE: THEORIZING OLDER ADULTS' ROLES IN AGE-FRIENDLY COMMUNITY INITIATIVES

Emily Greenfield[1] and Laurent Reyes[2], *1. Rutgers University, New Brunswick, New Jersey, United States, 2. Rutgers, The State University Of New Jersey, New Brunswick, New Jersey, United States*

Major professional organizations in health and aging have identified older adults' involvement as a defining feature of aging-friendly community initiatives (AFCIs), yet there is very little research on this aspect of the initiatives. Our study utilizes five waves of data from in-depth interviews with leaders of nine AFCIs across northern New Jersey. The study was conducted as part of a multi-year, community-partnered project on the development of these philanthropically supported initiatives. Multi-phase coding yielded four types of roles for older adults: (a) program participants, (b) informants and consultants, (c) volunteers assisting with programs, events, and administrative tasks, and (d) aging-friendly champions and advocates. Across role types, AFCI leaders reflected on the challenges of engaging particular subgroups of older adults, as well as how ambivalent age identities impeded involvement. We discuss implications for advancing research and evaluation on community-level interventions that seek to simultaneously serve and empower people as they age.

### PROMOTING AGING IN PLACE VIA RESEARCH, POLICY, AND PRACTICE IN AGE-FRIENDLY COMMUNITIES

Kathy Black[1], *1. University of South Florida, Sarasota -Manatee, Sarasota, Florida, United States*

The majority of Americans overwhelmingly prefer to age in place and in the communities in which they reside. Age-friendly communities support aging in place by focusing attention on features both inside and outside of the home. The global age-friendly community model provides a framework that requires assessing community-based older adults' needs and preferences about, and developing subsequent action towards, features of the social, service and built environment including housing and transportation which are considered essential to aging successfully at home. This presentation discusses the intersect between research, policy and practice in an age-friendly community which utilized micro-level findings from older adults (n = 1, 172) to enact macro-level collaborations across local and statewide government and professional groups to facilitate aging in place across the domains of housing and transportation.

### DOING AGING IN PLACE IN AN AGE-FRIENDLY CITY: AN APERTURE TO THE LIVED EXPERIENCES OF OLDER SAN FRANCISCANS

Jarmin C. Yeh[1], *1. University of California, San Francisco, California, United States*

Aging in place finds meaning through the quotidian. The mundanity of this work is the crux of its poignancy. This phenomenological study utilizes photovoice to explore how older adults manage to age in place in an age-friendly city. By interrogating micro- and macro-level realities, this study elicits the strategies seventeen informants use, including how their multiple identities and positionalities become implicated in the process of negotiating and navigating everyday environs, their acts of resistance and resilience, their articulations of hope or pressure to manage the future, as well as the risks and opportunities they encounter and the conditions shaping them, such as urbanization, discrimination, and distribution of resources between generations and groups. To "see" how informants do the "doing" of aging in place has implications for age-friendly community initiatives. It helps to capture the sociality of aging and demonstrates the way the materiality of inequality is sown through lived experience.

### SESSION 600 (SYMPOSIUM)

### CAN NEIGHBORHOOD AND LOCAL ENVIRONMENTS MODIFY COGNITIVE DECLINE? FINDINGS FROM THE REGARDS STUDY

Chair: Philippa J. Clarke, *University of Michigan, Ann Arbor, Michigan, United States*
Co-Chair: Jessica M. Finlay, *Social Environment and Health, Institute for Social Research, University of Michigan, Ann Arbor, Michigan, United States*

Environmental factors may significantly increase the risk of, or buffer against, age-related cognitive decline, yet policies and practices to improve cognitive health outcomes to date largely overlook the role of neighborhoods and socio-physical environmental contexts. Residence in socioeconomically advantaged neighborhoods may promote cognitive function through greater density of physical and social resources (e.g., libraries, parks, coffee shops, air conditioning, community centers) that promote physical activity, facilitate mental stimulation, and encourage social engagement. This symposium will identify natural, built, and social environmental factors linked to changes in cognitive function over time (assessed by animal naming and world list learning tests) based on secondary data analyses of a national, racially diverse (42% Black), population-based sample of over 30,000 Americans aged 45+ in the Reasons for Geographic and Racial Differences in Stroke (REGARDS) study followed annually since 2003. The first two papers investigate the roles of racial residential segregation and education on cognitive function disparities at the neighborhood and city scale. The third paper explores fast-food restaurants as socially interactive community spaces for older adults that may help buffer against cognitive decline. The fourth paper investigates effects of local air temperature on cognitive testing performance, and discusses how regional differences and seasonality may buffer or exacerbate temperature-cognition associations. Altogether, the symposium elucidates how cognitive health is impacted by a complex interplay of individual and geographic factors. The papers inform policy-making efforts to improve physical neighborhood environments and social community contexts, which are critical to the well-being of older adults aging in place.

### RACIAL RESIDENTIAL SEGREGATION AND COGNITIVE DECLINE AMONG OLDER ADULTS

Joy Bohyun Jang,[1] Margaret T. Hicken,[1] Philippa J. Clarke,[1] Ketlyne Sol,[1] Robert Melendez,[1] Min Hee Kim,[1] Megan Mullins[1] and Suzanne Judd[2], *1. University of Michigan, Ann Arbor, Michigan, United States, 2. University of Alabama at Birmingham, Birmingham, Alabama, United States*

Racial residential segregation may be a fundamental cause of health disparities in the U.S., and few studies employ objective measures of segregation to estimate its impacts on cognitive decline. Using data from 21,446 REGARDS participants in urban areas, we employed race-stratified growth curve models to examine how city racial segregation was associated with trajectories of cognitive decline over time. Controlling for demographics and health conditions/behaviors, higher segregation for blacks was marginally associated with lower cognitive function at baseline (b=-0.159, p<.10) while higher segregation for whites was associated with better cognitive function (b=0.158, p<.01). For both blacks and whites, there were no significant associations between segregation and rate of cognitive decline but neighborhood poverty was adversely related to cognitive function (b=-0.171, p<.01 for blacks, b=-0.289, p<.001 for whites). Further research into mechanisms that contributes to heterogeneity in associations between racial segregation and cognitive function is needed to develop effective prevention interventions.

### THE INTERACTIVE ASSOCIATION OF EDUCATION AND NEIGHBORHOOD ENVIRONMENTS ON COGNITIVE DECLINE

Michael H. Esposito,[1] Philippa Clarke,[1] Ketlyne Sol,[1] Jessica Finlay,[1] Margaret Hicken[1] Suzanne Judd,[2] and Virginia Wadley[2], *1. University of Michigan, Ann Arbor, Michigan, United States, 2. University of Alabama at Birmingham, Birmingham, Alabama, United States*

Education's ambiguous association with cognitive decline may be due to unmeasured effect heterogeneity, including variation across environmental contexts. In areas with more social and physical resources, education may play less of a role in shaping cognitive trajectories. In areas with fewer resources, educational capital may be more important for slowing cognitive decline. Using multilevel models, this paper examines whether education's impact on cognitive trajectories varies among neighborhoods defined by differential densities of social and physical resources. Findings suggest that education plays a consistent role in shaping cognition across contexts. Lower education is associated with lower cognitive function (High school vs College: b=-2.97; sd=0.16) and marginal differences in rates of decline (College vs High School: b=0.04; sd=0.03). However, these patterns are invariant across neighborhoods. Findings reiterate the importance of education for cognitive function in late life, and stimulate further research on other contextual factors that may affect rates of cognitive decline.

### FAST-FOOD RESTAURANTS: A NEIGHBORHOOD RESOURCE FOR COGNITIVE FUNCTION AMONG AGING AMERICANS?

Jessica M. Finlay[1] Philippa Clarke,[2] Mike Esposito,[2] Iris Gomez-Lopez[2] Suzanne Judd,[3] and Virginia Wadley[3], *1. Social Environment and Health, Institute for Social Research, University of Michigan, Ann Arbor, Michigan, United States, 2. Institute for Social Research, University of Michigan, Ann Arbor, Michigan, United States, 3. The University of Alabama at Birmingham, Birmingham, Alabama, United States*

In this exploratory mixed-methods sequential design study, interviews with 125 adults aged 55-92 (mean age 71) living in the Minneapolis (Minnesota) metropolitan area suggest that large-chain fast-food restaurants such as McDonald's may serve as reservoirs of cognitive function. Thematic analysis revealed perceived benefits of fast-food settings for older adults including familiarity and comfort; affordability; sociability with friends, family, staff, and customers; and entertainment (e.g., newspapers, crosswords). To further test these observations, we analyzed data from urban and suburban REGARDS participants. Preliminary multilevel regression models found that participants residing within 5 kilometers of a McDonald's restaurant exhibited higher cognitive function than similar individuals who live further from said organizations (b=0.31; se=0.12). The results complicate understanding of fast-food settings and prompt further research that tests whether restaurants can serve as community spaces for older adults to help buffer against cognitive decline by fostering social interaction and mental stimulation.

### TOO HOT OR TOO COLD? EXPOSURE TO EXTREME TEMPERATURES AND COGNITIVE FUNCTION IN OLDER ADULTS

Anam M. Khan[1] Philippa Clarke,[2] Jessica Finlay,[2] Carina Gronlund,[2] Robert Melendez,[2] Ketlyne Sol,[2] Suzanne Judd[2] and Virginia Wadley[3], *1. University of Michigan, Ann Arbor, Michigan, United States, 2. Department of Biostatistics, University of Alabama at Birmingham, Birmingham, Alabama, United States, 3. Department of Medicine, University of Alabama at Birmingham, Birmingham, Alabama, United States*

Research on temperature and cognition is sparse, including effects of outdoor air temperature on cognitive testing performance. Furthermore, little is known about the modifying role of region and seasonality in temperature-cognition associations. We linked daily temperature data from National Oceanic and Atmospheric Administration weather stations to REGARDS participants by cognitive assessment date. Controlling for season, generalized linear models including spline terms for temperature showed an adverse effect of hotter temperatures on cognition. At higher temperatures (30°C vs 0°C), there was a significant decrease in cognitive performance on the Word List Learning test (β=-0.68; 95% CI: -1.1, -0.25). Results also show regional differences in testing scores on hotter and colder days. The findings provide new understanding of cognitive susceptibility to extreme temperatures and factors that exacerbate or buffer this association. This can inform development of evidence-based public health guidelines and mitigation strategies aimed at reducing temperature-related morbidity in older adults.

### SESSION 605 (SYMPOSIUM)

### CONDUCTING CLINICAL TRIALS AMONG PERSONS WITH DEMENTIA: METHODS, CHALLENGES, AND OPPORTUNITIES FOR IMPROVEMENT

Chair: Justine S. Sefcik, *University of Pennsylvania School of Nursing, Philadelphia, Pennsylvania, United States*
Discussant: Nancy Hodgson, *University of Pennsylvania, Philadelphia, Pennsylvania, United States*

Evidence-based interventions for persons with dementia and their caregivers are important to improve both their quality of life and quality of care. Conducting rigorous clinical trials is essential to build evidence, guide research, and inform clinical practice. Clinical trials involve multifaceted phases which present opportunities and challenges to the research team. In this presentation, we discuss obtaining consent, gathering information to inform refinement of an intervention, adaptation of an intervention, and fidelity checks to ensure the rigor of intervention delivery. The first session will discuss initial challenges faced when consenting nursing home residents with dementia and tailored approaches to enhance clinical trial enrollment. The second session will share highlights of usability testing of a novel mobile app to refine and improve its ease in a future clinical trial of use for persons with dementia and their caregivers. The third session will describe findings from focus groups that generated recommendations for culturally adapting an intervention to promote quality of life among older Latinos with dementia. The final session will discuss the advantages, disadvantages and ethical considerations of in-person fidelity monitoring during a home-based intervention clinical trial involving persons with dementia and their caregivers. Together, these presentations describe the complex nature of implementing clinical trials and highlight the important lessons learned to strengthen study implementation.

### INFORMED CONSENT IN CLINICAL TRIALS FOR PERSONS WITH DEMENTIA

Joan G. Carpenter[1], *1. University of Pennsylvania School of Nursing, Philadelphia, Pennsylvania, United States*

Informed consent is one of the most important processes during the implementation of a clinical trial; special attention must be given to meeting the needs of persons with dementia in nursing homes who have impaired decision making capacity. We overcame several challenges during enrollment and consent of potential participants in a pilot clinical trial including: (1) the consent document was designed for legally authorized representatives however some potential participants were capable of making their own decisions; (2) the written document was lengthy yet all seven pages were required by the IRB; (3) the required legal wording was difficult to understand and deterred potential participants; and (4) the primary mode of communication was via phone. We tailored assent and informed consent procedures to persons with dementia and their legally authorized representative/surrogate decision maker to avoid risking an incomplete trial and to improve generalizability of trial results to all persons with dementia.

### USABILITY OF A MOBILE APP TO IMPROVE GOAL-DIRECTED BEHAVIOR: CHANGING APATHY INTO ACTION IN PERSONS WITH DEMENTIA

Lauren M. Massimo[1] Alexander Miller,[2] Daniel Schiffrin,[2] Sean Lydon,[2] and Dawn Mechanic-Hamilton[2], *1. University of Pennsylvania, Perelman School of Medicine, Philadelphia, Pennsylvania, United States, 2. University of Pennsylvania, Department of Neurology, Philadelphia, Pennsylvania, United States*

Impairment of goal-directed behavior, often labeled apathy, is the most common and disabling of the behavioral and psychological symptoms of dementia. Activi-daily is a mobile app that targets key and distinct components of goal-directed behavior (initiation, planning and motivation) found to contribute to apathy in persons with dementia. This presentation will focus on usability testing of Activi-daily in persons with dementia and their caregivers. After 1 week of at-home use, we gathered feedback via focus groups on features and usability of Activi-daily. Usability of Activi-daily and its' potential as an intervention for treating apathy were viewed favorably. Feedback regarding usability revealed that the app was easy for dyads to use and enhanced the patients' ability to complete their daily tasks with greater independence. Suggestions to improve app usability included adding a voice to text feature, a progress bar to visualize progression towards daily goals and gamification features to enhance motivation.

### CULTURALLY ADAPTING A TIMED ACTIVITY INTERVENTION FOR OLDER LATINOS WITH DEMENTIA AND THEIR CAREGIVERS

Adriana G. Perez[1], *1. University of Pennsylvania School of Nursing, Philadelphia, Pennsylvania, United States*

The number of Latinos living with Alzheimer's Disease (AD) is expected to grow from 379,000 in 2012 to 3.5 million in 2060. The purpose of this study was to generate recommendations for meaningful approaches to culturally adapt a timed-activity intervention, Healthy Patterns, to promote quality of life in this population. Consistent with a descriptive qualitative approach, community-based focus groups were conducted among a purposive sample of older Latinos with AD and their caregivers. Seventeen dyads participated. Surface structure issues included: need for linguistically appropriate intervention sessions; partnerships with local, trusted community organizations. Deep structure themes, included: 1) embedding social support through group sessions; 2) culturally relevant activities; and 3) building individual and caregiver motivation for participation. This study gives "voice" to older Latinos and caregivers in adapting a timed-activity intervention. Results may inform the design and implementation of AD interventions, including recruitment and retention of older Latinos in clinical trials.

### IN-PERSON FIDELITY MONITORING: HOME-BASED INTERVENTION DELIVERY FOR PERSONS WITH DEMENTIA AND THEIR CAREGIVERS

Justine S. Sefcik,[1] and Nancy A. Hodgson[1], *1. University of Pennsylvania, School of Nursing, Philadelphia, Pennsylvania, United States*

The gold standard for ensuring satisfactory delivery of an intervention remains fidelity monitoring. In-person observations for fidelity monitoring has received less attention in the literature compared to evaluating sessions via video and audio-recordings. This presentation focuses on a qualitative analysis of field notes from 15 scheduled fidelity visits of a home-based intervention involving persons with dementia and their caregivers (Healthy Patterns, NCT03682185). The findings of advantages (e.g., being able to complete observations in real time), disadvantages (e.g., additional time needed from staff to coordinate and attend visits), and ethical considerations (e.g., potential intrusiveness in the home when observing staff) with be discussed. Future research recommendation is to explore participant perspectives on having an in-person fidelity monitor in the home.

### SESSION 610 (SYMPOSIUM)

### CREATING STRENGTH IN AGE: HARNESSING THE POWER OF ARTS AND HUMANITIES NETWORKS

Chair: Gay P. Hanna, *Hanna Merrill Inc., Arlington, Viriginia, United States*
Co-Chair: Pamela Saunders, *Georgetown University, Washington, District of Columbia, United States*
Discussant: Niyati Dhokai, *George Mason University, Fairfax, Virginia, United States*

Arts and Humanities networks harness social capital in the service of older populations creating strength in age. This symposium will feature presentations in aging, arts, education, health and humanities exemplifying enormous and often underutilized resources readily available to engage older people across the spectrum of aging to combat decline and frailty at cognitive and physiological levels. Presenters will describe innovative partnership projects such as Sound Health, an initiative developed by the National Institutes of Health and the John F. Kennedy Center for the Performing Arts to expand knowledge and understanding of how listening, performing, or creating music could be harnessed for health and well-being; hybrid arts and humanities in health programs based within medical systems such as the Center for Performing Arts in Medicine at Texas Medical Center: Houston Methodist promoting research/evaluation of arts inventions to improve overall quality of patient care; and, MedStar Georgetown Lombardi Arts and Humanities Program providing a continuum of support for older patients and their caregivers from diagnoses through treatment processes. A Georgetown University case study will be presented on how arts, ethics and humanities are necessary and ideal components of an interdisciplinary master's degree program in aging studies to ensure understanding a diverse and intergenerational cohort and student's cultural value systems. The symposium will conclude with a presentation from the National Endowment of the Arts describing program service infrastructures across the country supporting arts engagement of older people, their families and caregivers focusing on lifelong learning; health and well-being; and age friendly design

### CREATING STRENGTH IN AGE: THE ROLE OF ARTS, ETHICS, AND HUMANITIES IN GERONTOLOGY EDUCATION

Pamela Saunders[1], *1. Georgetown University, Washington, District of Columbia, United States*

What are the necessary and ideal components of an interdisciplinary master's degree program in gerontology? What is the role of arts, humanities, and ethics to teach students and future leaders the power of social networks? This paper presents a case study of one academic program. Data are semi-structured interviews with students and faculty analyzed using content analysis to discover themes. In 2018, inspired by aging demographics, Georgetown University launched a one-year, interdisciplinary MS degree in Aging & Health. Informed by AGHE competencies including arts, ethics, and humanities, the program provides theoretically and scientifically grounded content, hands-on experience, and professional engagement. One core course specifically focuses on arts, ethics, and humanities, and provides a basis for students to see interdisciplinary links across the curriculum. Findings includes themes that illustrate the value of harnessing the power of arts, humanities, and ethics, including a diverse, and intergenerational cohort, and students' cultural value systems.

### SOUND HEALTH: HARNESSING MUSIC FOR HEALTH AND WELL-BEING IN SERVICE OF OLDER PEOPLE

Emmeline Edwards[1], *1. National Institutes of Health, National Center for Complementary and Integrative Health, Bethesda, Maryland, United States*

Music can get you moving, lift your mood, help you recall a memory, and can potentially improve your health. A partnership between the National Institutes of Health and the John F. Kennedy Center for the Performing Arts, called Sound Health is expanding current knowledge and understanding of how listening, performing, or creating music involves intricate circuitry in the brain that could be harnessed for health and wellness. The presentation will focus on research that shows that music provides cognitive, socio-emotional, motor benefits as well as evidence for neural plasticity. The potential for music as therapy for several neurological disorders associated with aging will be discussed. Dr. Edwards will also highlight research gaps and opportunities in basic/mechanistic and clinical research on music and the aging brain.

### HARNESSING ARTS IN MEDICINE FOR STRENGTH IN AGE: CASE STUDY, CENTER FOR PERFORMING ARTS MEDICINE, HOUSTON METHODIST

J. Todd Frazier[1], *1. Houston Methodist Hospital- Center for Performing Arts Medicine, Houston, Texas, United States*

In the 1970's hybrid arts and humanities programs began developing within healthcare systems across the country. Today, the National Organization for Arts in Health (NOAH) represents a network of hundreds of programs with a major focus of serving older people and their caregivers. This presentation will provide an overview of this rapidly developing network along with a multimedia overview of a comprehensive arts and medicine hospital based program in the Texas Medical Center: Houston Methodist Hospital's System Center for Performing Arts Medicine one of NOAH's founding members. The Center's expanding national, regional and community network of artist health, arts integration, creative arts therapy, research, and outreach programs serving older people will be described. Demonstrating how arts in health communicate and elevate value across disciplines through program evaluation (patient satisfaction and employee opinion), clinical research, and financial and outcome data will be explored as a case study.

### ARTS AND HUMANITIES BRIDGING ACROSS CONTINUUM OF CARE: CASE STUDY, GEORGETOWN LOMBARDI COMPREHENSIVE CENTER

Julia Langley[1], *1. Georgetown Lombardi Comprehensive Cancer Center, Washington, District of Columbia, United States*

How does a hospital-based arts and humanities program build towards a better healthcare system for older patients, caregivers and medical professionals? The Georgetown Lombardi Arts and Humanities Program (AHP) supports the continuum of care by encouraging a creative and constructive response to illness through the use of music, dance, expressive writing and visual arts. Artists-in-residence at MedStar Georgetown University Hospital work throughout the hospital, with in-patients and out-patients, in waiting rooms, intensive care units, and clinics. At the same time, the AHP recognizes the need to introduce the next generation of physicians, nurses, and medical professionals to the benefits of interacting with the arts to improve skills of observation, communication, empathy, and perspective-taking to serve all populations, especially older patients living with chronic illness. The AHP's educational programs at the National Gallery of Art, and the IONA Senior Day Center will be highlighted along with their supporting evidence base

### BUILDING RESOURCE NETWORKS IN SERVICE OF OLDER PEOPLE THROUGH STATE ARTS AGENCIES' COMMUNITIES OF PRACTICE

Beth Bienvenu[1], *1. National Endowment for the Arts, Washington, District of Columbia, United States*

National, state, and local arts networks help build the capacity of public and private sector organizations to serve older adults through quality arts engagement opportunities. The National Endowment for the Arts has worked closely with state arts agencies to build networks through a community of practice to facilitate partnerships with artists, arts organizations, aging services, and the healthcare system. With more than 40 states participating, the initiative has resulted in new state partnerships, new state grant initiatives, and new arts learning programs for older adults. Arts service organizations also have a role to play in this work. For example, the American Alliance of Museums is building a network of museums that will develop and implement high-quality, intensive arts learning opportunities for older adults across the United States. This presentation will address how these networks are helping build capacity across the country to improve the health and well-being of older adults

### SESSION 615 (SYMPOSIUM)

### CULTURAL AND SOCIAL RESILIENCE FACTORS ON HEALTH IN THE CONTEXT OF IMMIGRATION

Chair: XinQi Dong, *Rutgers Institute for Health, Health Care Policy and Aging Research, New Brunswick, United States*
Co-Chair: Mengting Li, *Rutgers, The State University of New Jersey, New Brunswick, New Jersey, United States*
Discussant: Man Guo, *University of Iowa, Iowa, United States*

Acculturation is a process whereby immigrants change their beliefs or behaviors in response to the prevailing norms and values in the host country. Acculturation may directly affect health outcomes, while it also operates through multi-level social factors, such as family relations, social network, and neighborhood cohesion, in shaping immigrants' health. Asian Americans are the fastest growing minority group in the United States. Chinese Americans constitute the largest segment of Asian Americans. The five studies aim to profile multi-level cultural and social resilience factors of older Asian Americans' health by analyzing the Asian American Quality of Life survey and the Population Study of Chinese Elderly in Chicago (PINE). Two studies, Acculturation and Cognitive Health and Factors Associated with Unmet Healthcare Needs demonstrated the direct effect of acculturation on health. Another two studies outlined a more complex mechanism between cultural and social determinants and health. Perceived Stress, Social Support, and Dry Mouth found the buffering effect of social support on the relationship between perceived stress and oral health. Neighborhood Social Integration, Social Network, and Cognitive Function identified micro- and macro-level resilience factors exert interaction effects on cognitive function. In addition, previous studies pay little attention to the dynamic nature of social relations. Transition in Family Relations in Immigrant Families took a typology approach to capture multifaceted family relations, with a longitudinal design to explore the transitions in family relations in the process of acculturation and its impact on mental health. This symposium will build an integrative resilience model for older Asian Americans.

### TRANSITION IN FAMILY RELATIONS IN IMMIGRANT FAMILIES: THE CASE OF CHINESE OLDER IMMIGRANTS IN THE UNITED STATES

Man Guo[1] Meredith stensland[2] Mengting Li,[3] and XinQi Dong[3], *1. University of Iowa, Iowa, United States, 2. university of Texas at Austin, Austin, Texas, United States, 3. Rutgers, The State University of New Jersey, New Brunswick, New Jersey, United States*

Using panel data of 2,604 Chinese older immigrants in Chicago over a two-year period, this study examined continuity and changes in intergenerational relationship patterns and their mental health implications. Latent transition analysis revealed five types of family relations: traditional, modified traditional, coresiding-unobligated, independent, and detached. Over the two years, about 43% of the respondents shifted to a different relationship type, with the most common changes being shifting into modified traditional or independent relations, or from detached relations. Controlling for baseline socio-demographic, acculturation, mental health variables, and variables representing life transitions over time, having detached relations was related to greater depressive symptoms in two years and having modified traditional relations was associated with better quality of life at the follow up. The findings revealed heterogeneity and fluidity of intergenerational relations among older immigrant populations and point to the important role of family relations in their well-being.

### NEIGHBORHOOD SOCIAL INTEGRATION, SOCIAL NETWORK, AND COGNITION: DOES THE INTERACTION EFFECT MATTER?

Jenhao Chen[1] Eileen Crimmins[2] Mengting Li,[3] and XinQi Dong[3], *1. Rutgers University, Camden NJ, United States, 2. University of Southern California, Los Angeles, California, United States, 3. Rutgers, The State University of New Jersey, New Brunswick, New Jersey, United States*

Social integration of neighborhoods and social network properties are associated with better cognitive function but the two factors are often investigated separately. This study examines the interaction between neighborhood social integration and quantity and composition of social network on cognitive domains by analyzing Population Study of Chinese Elderly, a population-based epidemiological study of over 3000 US Chinese older adults aged 60 and above in Chicago metropolitan. Regression results show that larger network size, volume of contact and smaller proportion kin and proportion co-resident were associated with higher level of global cognition. Higher sense of community was associated with higher level of global cognition. The interaction term of volume of contact and neighborhood cohesion was negative and statistically significant, suggesting the protective effect of volume of contact may decrease in high cohesion neighborhoods. Similar moderation effects were observed in specific cognitive domains, including episodic memory, working memory and executive function.

### PERCEIVED STRESS, SOCIAL SUPPORT, AND DRY MOUTH AMONG U.S. OLDER CHINESE ADULTS

Weiyu Mao,[1] Weiyu Mao[1] Yiwei Chen[2] Bei Wu[3] Shaoqing Ge[4] Wei Yang,[1] Iris Chi[5] and XinQi Dong[6], *1. University of Nevada, Reno, Reno, Nevada, United States, 2. Bowling Green State University, Bowling Green, Ohio, United States, 3. New York University, New York, New York, United States, 4. Duke University, Durham, North Carolina, United States, 5. University of Southern California, Los Angeles, California, United States, 6. Rutgers, The State University of New Jersey, New Brunswick, New Jersey, United States*

Dry mouth is a common condition among older adults that negatively influences oral health, general health, and quality of life. The role of psychosocial factors in oral health conditions and diseases remains largely unknown. We examined the relationship between perceived stress and dry mouth among US older Chinese adults and further investigated the moderating role of social support from different sources in the relationship. Data came from baseline of the Population Study of Chinese Elderly in Chicago between 2011 and 2013 (N = 3,157). Stepwise logistic regression models with interaction terms were used. More perceived stress was significantly associated with a higher likelihood of reporting dry mouth. Friend support was protective against dry mouth. The effect of perceived stress on dry mouth varied by levels of family and friend support. To prevent or reduce dry mouth, interventions need to consider perceived stress and social support in this growing population.

### FACTORS ASSOCIATED WITH UNMET HEALTHCARE NEEDS IN OLDER ASIAN AMERICANS

Hyunwoo Yoon[1] Hyunwoo Yoon[2] Yuri Jang[3] and Kwangyul Choi[4], *1. Texas State University, San Marcos, Texas, United States, 2. School of Social work, Texas state university, San Marcos, Texas, United States, 3. USC Suzanne Dworak-Peck School of Social Work University of Southern California, Los Angeles, California, United States, 4. aculty of Environmental Design, University of Calgary, Calgary, Alberta, Canada*

Older Asian Americans are the fast-growing but understudied population in health disparities research. Using a sample that reflects cultural/linguistic diversity, the present study explored general (health insurance, usual place for care, income) and immigrant-specific (nativity, length of stay in the US, English proficiency, acculturation) risk factors of unmet healthcare needs in older Asian Americans. Data were drawn from the Asian American Quality of Life survey (N = 533). With the inclusion of a considerable number of non-English-speaking individuals, the present sample presented a high rate of unmet healthcare needs. Those with a shorter stay in the US, limited English proficiency, and lower level of acculturation had increased odds of having unmet healthcare needs than their counterparts after controlling for background characteristics. Challenging the myth of model minority, findings highlight the importance of immigrant-specific factors in identifying risk groups of unmet healthcare needs and demonstrate vulnerability in access to healthcare.

### DIFFERENT ASPECTS OF ACCULTURATION AND COGNITIVE HEALTH AMONG MINORITY OLDER IMMIGRANTS

Bei Wang[1] XinQi Dong[2] Melissa A. Simon[3] and Mengting Li[2], *1. Institute for Health, Health Care Policy, & Aging Research, Rutgers University, New Brunswick, New Jersey, United States, 2. Rutgers, The State University of New Jersey, New Brunswick, New Jersey, United States, 3. Northwestern University, Chicago, Illinois, United States*

Immigration and acculturation process in host society have been closely linked to health consequences among the immigrant populations. However, it has been inadequately examined regarding its relationships with cognitive outcomes. Data were drawn from PINE Study. Linear regression analyses were used to examine associations between acculturation levels (media use, ethnic social relations, and language proficiency) and cognitive performance. After adjusting for potential confounding variables, higher levels of acculturation were associated with better cognitive performance. More specifically, higher levels of acculturation in aspects of media use and ethnic social relations were associated with better cognitive performance, whereas it has no significant association with the language preference aspect. Research and practice addressing health disparities and cognitive impairment should leverage efforts to provide culturally relevant resources to less acculturated populations in the US. More longitudinal studies are needed to clarify the influence of acculturation on cognitive performance and its mechanism.

### SESSION 620 (SYMPOSIUM)

### DESIGNING TECHNOLOGY TO SUPPORT HEALTHCARE FOR AGING ADULTS

Chair: Neil H. Charness, *Florida State University, Tallahassee, Florida, United States*
Discussant: Scott R. Beach, *University of Pittsburgh, Pittsburgh, Pennsylvania, United States*

Healthcare costs are rising in industrialized countries, partly as a function of managing costly chronic care conditions associated with aging populations. Of roughly 3 trillion USD expended in the U.S., almost 90% is spent on those with chronic conditions. Technology is touted as one tool to manage healthcare efficiently. However, human factors research has shown that technological systems that do not take human capabilities into account will fail to be adopted, or if adopted, will be abandoned by users. The Center for Research and Education on Aging and Technology Enhancement (CREATE) will describe research findings for four different facets of healthcare technology. Sara Czaja will provide an overview, describing technology for healthcare support. Caregiver needs are projected to rise rapidly, in part due to aging of the baby boom cohorts. We need new solutions for future generations of older adults as there will be insufficient numbers of caregivers to care for the increased number of older adults given changes in social structures. Wendy Rogers will discuss research on the design and use of televideo and robots to assist with healthcare. Neil Charness will discuss home monitoring technology, particularly practical issues around design, deployment, and maintenance, drawing on studies of heart failure patients and older adult controls. Walter Boot will discuss how gamification of healthcare interventions can help to address the adherence problem for behavior change. Scott Beach, Associate Director & Director of Survey Research Program, University Center for Social and Urban Research, University of Pittsburgh, will serve as discussant.

### TECHNOLOGY FOR HOME MONITORING

Neil H. Charness[1], *1. Florida State University, Tallahassee, Florida, United States*

Healthcare is increasingly moving away from expensive hospitals into the home. Although there are advantages to managing chronic health conditions in homes, there are also a number of disadvantages, beginning with aging adults' unfamiliarity with the technologies being deployed. Too often, designs and instructional materials are not developed with aging users' capabilities in mind. Health technology also confronts users with significant challenges including just-in-time learning when users are under significant stress. Technology support is often lacking. Also, care coordination for multiple chronic conditions is challenging. For instance, patients are often provided with multiple electronic health record portals. I discuss the reliability of and some of the practical challenges for a home health monitoring system used by older adults and those with heart failure over a 6-month interval. The system featured components such as a wrist-worn sensor package, daily tablet surveys, blood pressure cuff, weight scale, and bed sensors.

### THE HEALTHCARE CHALLENGE FOR AN AGING POPULATION: THE ROLE OF TECHNOLOGY

Sara J. Czaja[1], *1. Weill Cornell Medicine, New York, New York, United States*

Although many older adults enjoy relatively good health into their later years, many have one or more chronic conditions, diseases, or disabilities, and need help with disease management activities or activities important to independent living. With the increase in numbers of aging adults there is a concomitant strain on the healthcare system. Models of healthcare are also changing and moving towards a more partnership model where consumers are supposed to assume a more collaborative role in health decision making and a more active role in health management. This presentation will discuss the continuing and increasing role of technology in meeting the healthcare challenges for an aging population. The discussion will focus on technology applications to support the health and well-being of older adults as well as family caregivers. Challenges and barriers that currently limit the full potential of technology to be realized for these populations will also be discussed.

### THE POTENTIAL AND PITFALLS OF GAMIFICATION TO SUPPORT OLDER ADULTS' ADHERENCE TO HEALTHCARE INTERVENTIONS

Walter R. Boot,[1] and Neil Charness[1], *1. Florida State University, Tallahassee, Florida, United States*

The addition of video game-like elements to non-game activities, known as gamification, holds promise with respect to encouraging engagement with, and adherence to, health behaviors and healthcare interventions. Elements of gamification include the introduction of points systems, leaderboards, achievement badges, stories and themes, rewards, progress tracking, and challenges. However, a lack of enthusiasm for, and experience with, video game play by older adults has important implications for the effectiveness of these techniques across the lifespan. Specifically, the age-related "digital divide" must be considered before applying these approaches to improving the wellbeing, cognition, and health of older adults. This talk will build on the body of research conducted by the Center for Research and Education on Aging and Technology Enhancement (CREATE) focused on gaming and interventions to present best practice guidelines for implementing gamification with older adults.

### DESIGN AND USE OF ROBOTS TO ASSIST OLDER ADULTS WITH HEALTHCARE TASKS

Wendy Rogers,[1] and Megan Bayles[1], *1. University of Illinois Urbana-Champaign, Champaign, Illinois, United States*

There is much potential for robots to support the needs of older adults, in general, and particularly in healthcare. Older adults are quite open to the idea of interacting with robots, although they have preferences for the nature of the task they want the robot to do as well as what they want the robot to look like. These preferences should be considered in the process of design and deployment. Older adults should be involved throughout the design process from formative to summative evaluation and even beyond to the integration of the robot into their everyday activities. The extant research provides guidance regarding older adults' capabilities and limitations that might influence their ability to interact with a robot. Our goal in this presentation will be to focus on robots being designed to support older adults with healthcare tasks in the context of enhanced, instrumental, and basic activities of daily living.

### SESSION 625 (SYMPOSIUM)

### DNA METHYLATION: CAUSE OR CONSEQUENCE OF AGING?

Chair: Morgan E. Levine, *Yale University School of Medicine, New Haven, Connecticut, United States*
Co-Chair: Sara Hagg, *Karolinska Institutet, Stockholm, Sweden*

Epigenetic changes are one of the Hallmarks of Aging. DNA methylation is a key epigenetic mark that has been shown to change during aging. Several "clocks" have been developed whereby changes in DNA methylation can be used to predict chronological, and perhaps, biological age. This symposium will focus on recent advances in understanding how and why changes in DNA methylation occur during aging and whether these changes play a causal role in age-related functional declines and disease.

### A FUNCTIONAL EPIGENETIC CLOCK FOR RATS

Morgan E. Levine[1] Ross McDevitt,[2] Luigi Ferrucci,[2] and Rafael deCabo[2], *1. Yale University School of Medicine, New Haven, Connecticut, United States, 2. National Institute on Aging, Baltimore, Maryland, United States*

Evidence from humans suggests that incorporation of phenotypic aging measures in the development of epigenetic clocks leads to more functionally relevant biomarkers. As a result, the aim of this study was to utilize a deeply phenotyped rat cohort—that included data from rotarod, open field, frailty index, and FACS—to generate a novel epigenetic clock. DNA methylation was assessed via reduced representation bisulfite sequencing (RRBS) for n=142 male Fischer rats from NIA aging colony, ranging in age from 1 to 27 months. Phenotypic traits were combined to generate an multi-system aging measure that was then used to train the epigenetic clock. Using an independent validation sample, age-adjusted epigenetic clock measures were associated with numerous traits, including: open field time resting (p=0.005), open field time climbing (p=0.001), body weight (p=0.02), and rotarod max (p=0.04). In moving forward, it will be important to examine cross-species comparisons, longitudinal change, and functional enrichment.

### LONGITUDINAL TRAJECTORIES OF EPIGENETIC CLOCKS IN HUMANS AND EFFECTS OF MEDICATION USE

Sara Hagg[1], *1. Karolinska Institutet, Stockholm, Sweden*

We sought to investigate the longitudinal trajectories of the new generation epigenetic clock´s and how medication use is altering the DNA methylation age (DNAmAge). DNA methylation (Illumina 450k and EPIC) was assessed repeatedly up to six times (1992-2014) in whole blood (597 individuals, 1469 samples) from the Swedish Adoption/Twin Study of Aging (SATSA). DNAmAges were generated with the online calculator. Mean age at first measurement was 67 years (58% women). All clocks tested (Horvath, Hannum, Pheno, Grim, Skin&Blood) were correlated with chronological age ($\rho$=0.62-0.80). The steepest slope was found for Pheno while Horvath had the least steep slope. Correlations between the clocks ranged $\rho$=0.43-0.75. About 15% of the individuals started statin treatment during the follow-up, which changed the slopes to be less steep. Co-twin control analyses were confirmatory. Different DNAmAges are strongly correlated with each other in a longitudinal perspective. Treatment effects may alter the slopes of the DNAmAges.

### EPIGENETIC PREDICTORS OF LIFESPAN AND HEALTHSPAN

Ake T. Lu[1] and Steve Horvath[2], *1. Department of Human Genetics, University of California, Los Angeles, Los Angeles, California, United States, 2. Department of Human Genetics, UCLA, LOS ANGELES, California, United States*

Capturing aspects of biological age, DNA methylation based biomarkers collectively known as "epigenetic clock" can be used to measure the age of any human tissue, cell type, or fluid that contains DNA. Arguably the strongest predictor of lifespan, DNAmGrimAge, is a composite biomarker comprised of DNAm-based surrogates of plasma proteins and a DNAm-based estimate of smoking pack-years. Large-scale validation studies demonstrate that DNAmGrimAge stands out among existing epigenetic clocks when it comes to predicting time-to-death (P=2.0E-75), time-to-coronary heart disease (P=6.2E-24), and its strong relationship with computed tomography measures of fatty liver, and age-at-menopause (P=1.6E-12). DNAm-based estimates of plasma proteins and telomere length (TL) are attractive as well. For example, a DNAm based estimate of Plasminogen Activator Inhibitor 1 strongly relates to multi-morbidity and a DNAm based estimate of TL outperforms actual TL measurements in predicting lifespan. Overall, these epigenetic biomarkers are expected to find many applications including human anti-aging studies.

### ROBUST BIOMARKERS OF AGING AND THEIR BIOLOGICAL ORIGIN

Dmitriy Podolskiy,[1] Dmitriy Podolskiy,[1] and Vadim Gladyshev[1], *1. Brigham & Women's Hospital, Boston, Massachusetts, United States*

The biological origin of impressive accuracy of DNA methylation clocks, the most precise currently available biological markers of aging in humans and model animals, remains largely unclear. In addition, they sometimes suffer from uncontrollable precision loss out of sample. To address these two issues, we develop a novel method for constructing robust molecular markers of age based on network analysis of age-dependent omics data. The newly developed robust markers of aging in yeast, fruit fly, mouse and human are nearly free of batch effects and have a transparent biological nature related to tight control of translation-related processes and their disregulation associated with aging.

### SESSION 630 (SYMPOSIUM)

### HARNESSING THE POWER OF A GERIATRIC EXERCISE NETWORK TO IMPROVE HEALTH: THE VA GEROFIT WAY

Chair: Katherine S. Hall, *Durham Veterans Affairs Health Care System, Durham, North Carolina, United States*
Discussant: Janet Prvu-Bettger, *Duke University School of Medicine, Durham, North Carolina, United States*

The Gerofit Consortium is a clinical and research network that naturally evolved from the dissemination of a VA "Best Practice" exercise and health promotion program for older Veterans. Originally developed in the Durham VA, Gerofit has been disseminated to 15 VA Medical Centers using a successful "collaboratory" team network approach. This group has met regularly (biweekly) for five years; starting with four newly disseminated programs in 2014 and adding 2-3 new programs per year since, following a structured implementation process. We have learned that the sum is much greater than the parts. Each program shares important experiences and contributes to development of new program practices aimed at increasing access and adherence to physical activity for Veterans wherever they live, and improving their overall health and wellness. This consortium has served over 7000 Veterans. The first paper describes the impact of overweight/obesity on functional outcomes. The second abstract describes a newly developing use of live video connection from an exercise leader directly to the home for exercise. The third paper provides a case series on the impact of Gerofit on fall prevention. The fourth paper describes the impact of Gerofit on physical function among Veterans with arthritis. The fifth paper describes the impact of Gerofit on Post Traumatic Stress Disorder (PTSD) symptoms among participating Veterans with self-reported PSTD. This symposium highlights the impact of a highly synergistic network working together to improve the lives of older Veterans.

### GEROFIT: IMPACT ON PTSD SYMPTOMS AMONG PARTICIPATING VETERANS WITH SELF-REPORTED PTSD

Cathy Lee[1] Rebecca Melrose[2] Erin Blanchard[3] Stacy Wilkins,[2] Steven Castle,[2] Katherine Hall,[4] and Miriam Morey[4], *1. VA Greater Los Angeles Healthcare System GRECC, Los Angeles, California, United States, 2. VA Greater Los Angeles Healthcare System/UCLA, Los Angeles, California, United States, 3. VA Greater Los Angeles Healthcare System, Los Angeles, California, United States, 4. Durham VA Healthcare System/Duke, Durham, North Carolina, United States*

Post-traumatic stress disorder (PTSD) increases risk of medical comorbidities in aging. The Gerofit Program is an exercise program for older Veterans that shows efficacy for physical health. We sought to determine its impact on PTSD. Veterans in Gerofit completed a self-report questionnaire at 3 and 6 months assessing effect of Gerofit on: PTSD symptoms generally, disturbing dreams, avoidance, negative feelings, and irritability. Two hundred twenty-nine Veterans completed the questionnaire. Of these, 56 (24.5%) reported PTSD. None reported worsened PTSD following Gerofit participation. At 3 months, >50% of Veterans reported symptom improvement and this was maintained over 6 months for all items (p>0.05 paired t-test). There was an increase between 3 and 6 months in the percentage who reported "improved a lot" for overall symptoms (16.7% to 22.2%), negative feelings (5.6% to 11.1%) and irritability (0% to 11.1%). Gerofit may offer an effective intervention to improve PTSD symptoms in older Veterans.

### SIX MONTHS OF GEROFIT RESULTS IN MOBILITY IMPROVEMENTS IN OLDER VETERANS REGARDLESS OF BMI CLASSIFICATION

Odessa Addison[1] Jamie Giffuni[2] Monica Serra[3] and Leslie Katzel[2], *1. Baltimore GRECC VAMHCS, Baltimore, Maryland, United States, 2. Geriatric Research, Education, and clinical center, baltimore, Maryland, United States, 3. Atlanta VA, Atlanta, Georgia, United States*

Older Veterans represent a unique population at high risk for mobility limitations. They are also more likely to be overweight or obese when compared to the general population. We sought to compare changes in mobility function across the obesity spectrum in older Gerofit participants at six different sites. Two-hundred and seventy Veterans (mean age: 74 years) completed six-months of Gerofit participation and mobility assessments at baseline, three and six months. Our assessments included gait-speed, six-minute walk distance, 30-second chair stands, and the eight foot up and go. When comparing weight groups, we found no significant interaction of weight and time, however we found clinically significant (P<0.02) improvements of 7-20% across all mobility measures. Six-months of Gerofit participation appears to be one way to improve mobility function in older Veterans across the weight spectrum.

### MODERNIZING EXERCISE WITH TELE-VIDEO TO REACH A RURAL GERIATRIC POPULATION OF VETERANS

Stephen C. Jennings,[1] Kenneth Manning[1] Oliver Massey[2] Janet Prvu Bettger[3] Candace S. Brown[4] and Miriam C. Morey[5], *1. Durham VA Geriatric Research, Education, and Clinical Center (GRECC), Veterans Affairs Health Care System, Durham, North Carolina, United States, 2. Durham VA Veterans Affairs Health Care System, Durham, North Carolina, United States, 3. Duke Department of Orthopedics, Duke University Medical Center, Durham, North Carolina, United States, 4. Center for Cognitive Neuroscience, Motivated Cognition and Aging Brain Lab, Duke University, Durham, North Carolina Geriatric Research; Education, and Clinical Center (GRECC), Veterans Affairs Health Care System, Durham, North Carolina, United States, 5. Geriatric Research, Education, and Clinical Center (GRECC), Veterans Affairs Health Care System, Durham, North Carolina Center for the Study of Aging/OAIC, Duke University Medical Center, Durham, North Carolina, United States*

Rural Veterans often lack access to health care. Veterans Affairs (VA) supports telehealth technologies to provide services remotely that are comparable to onsite in-person care. We piloted VA Video Connect (VVC), to deliver an interactive exercise program for Veterans modeled on the VA Gerofit Program, a successful facility-based exercise program. VVC connects an exercise physiologist directly to the home with smart devices. Invitations to join Gerofit were mailed to 216 rural Veterans. Of 17 respondents, 7 (mean age 68) agreed to VVC tele-exercise 1x week for 12 weeks. Two Veterans were lost to follow-up prior to enrollment. Baseline VVC assessments (N=5) were indicative of high functional impairment in comparison to age-based norms: 2-minute step test (67.2 steps, 5th%tile), 30-second chair stands (12.4 stands, 26th%tile), and 30-second arm curls (15.3 curls, 25th%tile). Feasibility, barriers, and program impact will be discussed. Functional impairment indicates need for telehealth to reach Rural Veterans.

### THE IMPACT OF 3 MONTHS OF SUPERVISED EXERCISE ON CHANGE IN FUNCTION: COMPARISONS BY ARTHRITIS STATUS

Lauren M. Abbate[1] Katherine Hall,[2] Megan Pearson,[2] Richard Sloane,[2] Kelli D. Allen[2] Wendy M. Kohrt,[1] and Miriam C. Morey[2], *1. Rocky Mountain Regional VA Medical Center, Aurora, Colorado, United States, 2. Durham Veterans Affairs Health Care system, Durham, North Carolina, United States*

Physical activity is an established intervention for the management of arthritis. This study evaluated the effect of 3 months of participation in Gerofit on physical function by arthritis status. Participants, 519 Veterans aged ≥ 65 years self-reported either no arthritis (NA) (49%), upper body arthritis (UB) (8.2%), lower body arthritis (LB) (12.7%), or both upper and lower body arthritis (UB&LB) (30.4%) upon enrollment. Physical function measures [10-meter usual gait speed (m/s) (GS), arm curls (AC), chair stands (CS), and 6-minute walk (yards) (SMW)] were assessed at baseline and follow-up. Mean differences between time points were calculated. At baseline, compared to NA, LB and UB&LB had slower GS (1.10 and 1.06 vs 1.13) and shorter SMW distance (468.8 and 448.8 vs 490.7). All groups tended to increase physical function, with greatest improvement among LB (GS=0.27, AC=2.06, CS=2.52, SMW=42.53). Participation in Gerofit is associated with functional gains, regardless of burden of disease.

### SIX MONTHS OF FOCUSED EXERCISE IN GEROFIT LEADS TO REDUCED FALL RISK THROUGH IMPROVED BALANCE

Arti S. Tayade[1] Jody R. Colglazier[2] Alan D. Wesley[3] Paul J. Froehlich,[2] Elizabeth C. Hersch,[2] Anne P. Poppe,[3] David E. Hall,[3] and Suzanne M. Younker[4], *1. VA Puget Sound, Seattle, Washington, United States, 2. VA Puget Sound Health Care System, Lakewood, Washington, United States, 4. Greenriver Community College, Tacoma, Washington, United States, 3. VA Puget Sound Health Care System, Seattle, Washington, United States*

Many older Veterans are physically debilitated, with a reduced quality of life (QOL.) Use of assistive devices during ambulation is indicative of fall risk. We describe two case studies following six-months of participation in Gerofit, a structured exercise program. Ms. M., a 75 y/o Veteran with chronic PTSD and joint pain could walk 237 yards in six-minutes using a cane. Mr. D., an 86 y/o Veteran, came to Gerofit using a Front Wheeled Walker and could walk 307 yards in 6-minutes. After six-months of Gerofit, neither Veteran used assistive devices during testing, and both reported higher QOL. Ms. M. now walks 514 yards in six minutes, while Mr. D. can walk 375 yards in six minutes. Both Veterans exhibit improved balance and endurance. They tell us: "Gerofit gave me my life back!" and "Gerofit gets me out of the house and off the couch."

### SESSION 635 (PAPER)

### HEALTHCARE AND MANAGEMENT

### EDUCATIONAL INITIATIVE ABOUT MEDICATION MANAGEMENT FOR OLDER ADULTS

Manpreet Boparai,[1] Rosario Costas Muniz,[1] Natalie Gangai,[1] Ruth Manna,[1] Beatriz Korc-Grodzicki[1], *1. Memorial Sloan Kettering Cancer Center, New York, United States*

Older adult patients often have multiple comorbidities, see multiple providers, leading to poor medication management (MM). Educational seminars were conducted to health care providers (HCPs) and community-based organizations to deliver health programs to linguistically diverse communities by a pharmacist utilizing interpreters. Participant knowledge was assessed using matched pre-post surveys (translated to participants' respective languages). Behavioral intention was measured using a Likert scale. Results indicate participation from 136 community dwelling adults via 6 educational sessions; 44 caregivers via 5 sessions, and 22 HCPs (4 students and 22 residents) participated in one session. 91% of the community population were born outside of the US, 67% coming from South Asian countries, speaking over 7 different languages. Knowledge about medication management increased from an average of 44% correct responses pre-session to 48% correct post-session – a statistically significant change (t (135) =-2.26, p<.03). Most caregivers were females (82%) and born in a South Asian country (87%). Knowledge about medication management when caring for a loved one increased significantly from an average from pre (46%) to post (60%) (t (41) =-3.07, p< .01) Finally, most HCPs were females (53%), 63% Asian; 87% were born outside of the US, predominantly in East Asia. Providers had a significant increase on knowledge (t (21) =-3.03, p< .01) about prescribing for older adults. Detailed results of the programs will be presented. Despite challenges in diverse audience members, all three target populations indicated a statistically significant change in knowledge indicating the value in targeted and tailored health education topics.

### EFFECTS OF COGNITIVE DECLINE ON DIABETES SELF-MANAGEMENT EDUCATION AND CARE

Shinduk Lee[1] Matthew L. Smith[2] and Marcia G. Ory[3], *1. Center for Population Health and Aging, Texas A&M University, College Station, Texas, United States, 2. Texas A&M University, College Station, Texas, United States, 3. Texas A&M Center for Population Health and Aging, College Station, Texas, United States*

People with diabetes experience a faster cognitive decline and have a greater risk for future dementia diagnoses. Cognitive impairment can negatively influence diabetes management activities. Diabetes self-management education (DSME) can enhance diabetes control, but limited evidence exists about the differential effects of DSME based on cognitive status. This study examines the moderation effects of cognition on the relationship between participation in DSME and diabetes management among older adults using Georgia 2017 BRFSS data (N=496). Primary outcomes were diabetes self-management (e.g., self-blood glucose monitoring, self-feet check, and physical activity) and clinical care (e.g., seeing a health professional for diabetes and A1C, feet, and eye exams). Multiple logistic regression models examined the effects of DSME and self-reported cognitive decline on diabetes care. Based on the Anderson-and-Newman Framework, all regression models were adjusted for predisposing (age, sex, race, ethnicity, and education), enabling (income, marital status, and health plan), and need (insulin treatment) factors. About 48% of participants participated in a DSME, and about 16% reported experiencing cognitive decline. DSME participation was positively associated with self-blood glucose monitoring (p=0.014), physical activity (p=0.024), seeing a health professional for diabetes (p=0.002), and feet exam (p=0.043), but cognitive decline was not significantly associated with most diabetes care (p>0.05). Further, no significant difference in DSME impact on diabetes care based on reported cognitive decline was observed (p>0.05). Findings suggest that DSME can benefit diabetes care among people with and without cognitive decline. Future research can expand upon impacts of rates and degrees of cognitive decline on program benefits.

### IMPROVING PRIMARY CARE PROVIDER EDUCATION AND SATISFACTION VIA THE CATCH-ON LEARNING COMMUNITIES

Erin Emery-Tiburcio,[1] Magdalena Bednarczyk[1]
Febe Wallace[2] and Michelle Newman[1], *1. Rush University Medical Center, Chicago, Illinois, United States, 2. Cherokee Health Systems, Morristown, Tennessee, United States*

Nationally, there is a shortage of geriatric trained healthcare providers caring for older adults. As the population of older adults grows, health care systems and primary care providers struggle to provide high quality, cost effective care for older adults. Time for training is also limited in busy community health centers. The CATCH-ON Learning Communities (LCs) are telehealth educational interventions based on the ECHO model, modified to be less time intensive, thus decreasing cost to participating clinics. In the LC, geriatric specialists provide evidence-based, best practice training utilizing case discussions to illustrate pertinent learning points via monthly one hour video conferences. Practical, specific behavioral recommendations are offered for immediate implementation in each session. LCs are provided to interprofessional primary care teams. The first LC with a federally-qualified health center (FQHC) yielded consistently high satisfaction from participants, along with a 17% decrease in high risk medication prescriptions and 22% increase in falls screenings. Training the primary care workforce in evidence based geriatric interventions can improve the care of all older adults within each health system, improving healthcare access to help mitigate healthcare inequalities, slow adoption of best practices and rising costs of caring for complex older adults. The CATCH-ON Learning Community is an effective, low cost model of training the primary care work force without geographical or financial constraints that frequently limit access to specialized care.

### PERSON-CENTERED CARE RELATED TO RESOURCE USE, RESIDENT QUALITY OF LIFE, AND STAFF JOB STRAIN IN SWEDISH NURSING HOMES

Annica Backman,[1] Anders Sköldunger[1], *1. Department of Nursing Umea University, Umea, Sweden*

A critical challenge facing aged care systems throughout the world is to meet the complex care needs of a growing population of older persons. Although person-centred care has been advocated as the “gold standard” and a key component of high quality of care, the significance of care utilization in person-centred units as well as the impact of person-centred care on resident quality of life and staff job strain in nursing home care is yet to be explored. Thus, the aim was to explore person-centred care and its association to resource use, resident quality of life and staff job strain. The study is based on a cross-sectional national survey and data on 4831 residents and 3605 staff were collected by staff in 2014, deriving from nursing homes in 35 Swedish municipalities. In this study, descriptive statistics and regression modelling were used to explore this association. The preliminary results showed that person-centred care was positively associated to resource use (i.e care hours) and resident quality of life in Swedish nursing homes, when controlling for resident age, gender and cognitive status. Person-centred care was negatively associated to staff perception of job strain. This indicates that person-centred care provision seem to increase resource use (i.e. slightly more care hours utilized) but also beneficially impact resident quality of life as well as alleviate care burden in terms job strain among staff.

### WHOM DO WE SERVE? DIVERSITY OF OLDER COMMUNITY CARE RECIPIENTS’ FUNCTIONING ACROSS EUROPE

Annerieke Stoop[1] Manon Lette[2] Simone de Bruin,[1]
Giel Nijpels,[2] and Hein van Hout[2], *1. National Institute for Public Health and the Environment, Bilthoven, Netherlands, 2. Amsterdam UMC, Amsterdam, Netherlands*

Across Europe, an increasing number of older people with multiple health and social care needs stay in their own homes until old age. Community care aims to support them to live at home for as long as possible. Comparative studies showed that population characteristics of older community care recipients differ between European countries. This is due to differences in financing, delivery and governance of community care. However, little is known about differences in health, including physical, cognitive, mental and social functioning, of older community care recipients served across European countries. The aim of this study was to provide insight into these differences. We used data of the IBenC study, which was collected using the interRAI HC-Assessment among 2884 older community care recipients from six European countries: Belgium, Finland, Germany, Iceland, Italy and the Netherlands. We found that prevalences of impairments in different health domains were highest among Italian community care recipients followed by the Belgian population, and lowest among community care recipients from the Netherlands. Feelings of loneliness were lowest among the Italian and highest among the Dutch population. This variation between European countries may be explained by differences in eligibility for and access to formal community services and informal care provision as well as cultural diversity. Insight in these differences supports understanding of community care across Europe among European and national policy-makers and researchers.

### SESSION 640 (PAPER)

### INTERGENERATIONAL RELATIONS

### CUSTODIAL KNOWLEDGE AND LEGAL PERCEPTION AMONG GRANDPARENTS RAISING THEIR GRANDCHILDREN

Elizabeth Bownes,[1] Martha R. Crowther,[1] and
Jennifer Cox[1], *1. University of Alabama, Tuscaloosa, Alabama, United States*

The current study examines the ways in which grandparents raising their grandchildren (GRCs) understand custody, perceive the legal system, and access resources related to their grandchild(ren)'s welfare. Due to the detrimental impact of the opioid crisis over the last decade, the number of skipped generation households is growing significantly not only in Alabama, but across the U.S.. Many GRCs lack crucial information regarding custody arrangements in a general sense or as it applies to education, healthcare, mental health, and financial aid. Critical gaps remain present in the GRC literature necessary to aid in future intervention studies and promote more effective support, resources, and policy for this population. The present study sought to examine the unique needs and experiences of GRCs, and to specifically explore legal aspects associated to their grandchild(ren)'s welfare. Using a mixed methods approach, GRCs in Tuscaloosa, Alabama completed a quiz of custodial knowledge, a survey on legal perception, and a semi-structured interview. Quantitative data revealed the most and least commonly understood aspects of custody, as well as opinions on associated legal systems related to a child's "best interest." Qualitative data analysis revealed the common themes related to custody to be unexpected assistance, ineffective assistance, leniency for parental deviance, fear of losing custody, and time and cost demands.

### EFFECTS OF SOCIAL RELATIONS ON MORTALITY IN THE CONTEXT OF GRANDPARENTING

Heejung Jang,[1] and Fengyan Tang[1], *1. University of Pittsburgh, Pittsburgh, Pennsylvania, United States*

Issues of health and well-being have received considerable attention as a way to help grandparent caregivers. There is growing evidence that grandparenting is beneficial for grandparent caregivers' health, yet acting as grandparent caregiver also is detrimental to health and social relations when a grandparent provides an extensive level of care to grandchildren. The extent to which grandparent caregiving benefits or harms of the health of a grandparent is still unknown; mortality specifically has not been systematically studied. Moreover, although altruistic behaviors towards others have been shown to have beneficial effects on caregivers' health in general, there is little information regarding social relations of grandparent caregivers and their impact on mortality. This study aims to investigate the roles of different aspects of social relations among community-dwelling older adults, examining whether aspects of social relations, including social networks, received functional support aid, and perceived support quality, mediate the association between grandparent caregiving and mortality. The data were drawn from the 2008 and 2014 Health and Retirement Study (N=1,196). Results of survival analyses indicate that custodial and co-parenting grandparents were significantly associated with all-cause mortality over a 6-year period; however, the associations were marginally significant after health statuses were added into the model. Specifically, family-focused network groups were significantly associated with mortality. Received functional support and perceived positive support mediated the association between custodial grandparents and mortality. This study suggests that community-based support may be beneficial to older grandparents and perceived positive relationship quality could matter for older adults' well-being.

### OLDER ADULT WOMEN'S MULTIDIMENSIONAL RESPONSIBILITIES IN SKIPPED GENERATION HOUSEHOLDS IN NIGERIA

Mojirayo Afolabi[1], *1. Obafemi Awolowo University, Ile-Ife, Osun State, Nigeria*

This contribution studies the roles of grandparents in caring for grandchildren in skipped generation households, from gender perspective. Historical studies often focus on health and economic status of older adults generally, without distinguishing older adult women whose responsibilities are often undervalued. Such assessments assume that both grandparents engage in caring for grandchildren, being the joy of old age. Highlighting women's roles will ensure proper design and implementation of policies to enhance improvement in overall well-being of skipped generation households in Nigeria. The economic value of this is high. Using detailed data from three major states in Nigeria, - Imo, Lagos and Kano (representing each of the major ethnic groups) this study provides a detailed picture of the areas of women's responsibilities in skipped generation households, using both quantitative and qualitative methodologies. The quantitative study employed structured questionnaire to collect primary data while the qualitative technique employed the use of in-depth interviews and focus group discussions. The older adult participants are grouped into three; less than sixty five years, between sixty five and eighty and those above eighty years of age. The study reveals that social norms and expectations impact the lives of older adults, ensuring that responsibilities sharing in skipped generation households are strongly impacted by gender roles. The paper concludes that in order to ameliorate the difficulties and challenges faced in performing these roles, governments and other organizations need to put the realities in the skipped generation households into consideration during planning processes.

### THE ROLE OF AMBIVALENCE ON WELL-BEING OF AGING PARENTS WHO HAVE A DISABLED CHILD: MULTILEVEL MEDIATION APPROACH

Eun Ha Namkung[1], *1. Brandeis University, Waltham, Massachusetts, United States*

According to the family systems theory, strains from parenting an adult with disabilities may spillover to parents' relationships with their other children and disrupt family dynamics and their well-being in later life. This study examined whether parental ambivalence toward their non-disabled children is greater in families of adults with disabilities [developmental disabilities (DD) or serious mental illnesses (SMI)] than families without an adult child with disabilities. The study also investigated whether ambivalence mediates the associations of having an adult child with DD or SMI on parents' health. Data were from the 2011 Wisconsin Longitudinal Study in which aging parents (Mage = 71; n = 6,084) were asked about their relationship with each of their adult children. Multilevel regression models and multilevel structural equation models (MSEM) were estimated to analyze the data. Our findings showed that parents of an adult with SMI felt greater ambivalence toward their non-disabled adult children than comparison group parents of adults without disabilities, whereas no significant differences were found between parents of an adult with DD and comparison group parents. Parental ambivalence toward their non-disabled adult children played a significant indirect role in the negative association between having a child with SMI and parental physical and mental health, after adjusting for parent- and child-characteristics associated with parental health and/or ambivalence. The findings have implications for clinical practice with aging families of adults with disabilities and suggest the need for additional research to better understand intergenerational dynamics in these families.

### WHANAUNGATANGA AND FILIAL PIETY: INTERGENERATIONAL EXCHANGES IN CONTEMPORARY MAORI AND KOREAN CULTURES

Hong-Jae Park[1] and Jim Anglem[2], *1. Western Sydney University, Sydney, Australia, 2. University of Canterbury, Christchurch, New Zealand*

Every culture has its own tradition of intergenerational exchange based on accepted norms, while the meanings of traditional filial values have evolved over time. This paper aims to identify the various forms of filial care, support and respect for older people in Maori and Korean cultures, and reconceptualise current ways of intergenerational exchanges in both physical and virtual contexts. Data were collected through a qualitative inquiry framework consisting of 32 individual interviews and 5 ethnographic observations in New Zealand and South Korea. Thematic analysis of the data was used to identify themes and patterns from the participants' perspectives and experiences in the multilingual research context. In this cross-cultural study, for Māori participants, whanaungatanga (family relationships) was recognised as a core value that places whanau (family) at the centre of whakapapa (human and non-human relations). For Korean participants, their tradition of filial piety has continued to constitute a major component of familism mindsets and practices, while their ability to support their parents and maintain connections to their ancestors varied. Being knowledgeable about the traditional values of intergenerational solidarity helped generations feel connected and supported by each other, although both monetary and non-monetary support for one's elders has come under strain due to the impact of changes in family ties and social dynamics. Technological developments have reshaped traditional filial practices, offering new ways of intergenerational exchanges. Redefining whanaungatanga and filial piety can provide a theoretical basis for developing the concept of extended social work through avoiding excessive individualism and culture-blind approaches.

### SESSION 645 (SYMPOSIUM)

### IS IT LONELY AT THE TOP? BIOPSYCHOSOCIAL VARIANTS OF LONELINESS AMONG CENTENARIANS

Chair: Alex J. Bishop, *Oklahoma State University, Stillwater, Oklahoma, United States*
Discussant: Oscar Riberio, *University of Aveiro - Department of Education and Psychology | Center for Health Technology and Services Research (CINTESIS.UA), Aveiro, Portugal*

There is a growing body of evidence supporting the detrimental impact of loneliness on biological, psychological, and social functioning. Loneliness has been cited to contribute to social isolation, mental health disorders, and premature death in old age. In turn, the prevention of loneliness has emerged as a priority area in geriatric and gerontology research, practice, and policy. However, determination of whether persons living 100 or more years feel lonely or socially disconnected has remained limited within centenarian research. Such research has historically fostered translation of insights and secrets for living long and aging well. Centenarians represent persons who have managed to survive, delay, or escape varying biopsychosocial losses that might otherwise deteriorate emotional health, exacerbate feelings of isolation, and limit human longevity potentials. Guided by a biopsychosocial framework, this symposium will consider biological, psychological, and social variants that contribute to risk as well as resilience in loneliness in very old age. Of particular interest is the advancement of evidence-based research exposing the interplay between loneliness and nutritional health, impact of lifelong childlessness on feelings of solitude, role of personality traits and the expression of loneliness, and the intersection between active religious engagement and loneliness. Biopsychosocial attributes that reduce the threat of social isolation and loneliness, as well as improve emotional well-being in human longevity will be further discussed. Implications relevant for geriatric counseling and wellness programming for old-old adults will be highlighted.

### ALLEVIATING LONELINESS AT 100: IS RELIGION A SOLUTION?

Alex J. Bishop[1] and Kevin Randall[2], *1. Oklahoma State University, Stillwater, Oklahoma, United States, 2. Sam Houston State University, Huntsville, Texas, United States*

Data from N = 154 centenarians residing in Oklahoma were assessed using the Duke University Religious Index (DUREL). Items assessing religious salience ($\alpha$=.76) were employed to create a binary measure of high (N=56 or 36.4%; M= 29.77, SD=4.65) and low (N=49 or 31.8%; M=25.10, SD=6.58) religious salience (RS). A series of ANCOVA analyses were then conducted controlling for education, race, self-reported health, and self-care capacity relative to the binary outcome RS. Significant differences for both the corrected model and the pairwise comparisons using Bonferroni adjustment emerged in favor ($p \leq .001$) of the high RS group (M HI =29.60; M LO=25.29) for life satisfaction and social provisions (M HI =82.43; M LO=76.62). However, the RS group was also significantly higher (p =.004) in reported loneliness (M HI =34.56; M LO=31.63). Implications of the findings for reducing loneliness among centenarians reporting high religious engagement are further highlighted.

### THE UNIQUE CONNECTION BETWEEN PERSONALITY TRAITS AND LONELINESS AMONG CENTENARIANS

Jinmyoung Cho[1] and Alex J. Bishop[2], *1. Baylor Scott & White Health, Temple, Texas, United States, 2. Oklahoma State University, Stillwater, Oklahoma, United States*

Evidence suggests that personality traits contribute to risk and resilience in long-term survivorship. However, research examining the link between personality and loneliness among persons living 100 and more years has remained limited. The purpose of this study was to examine the association between Big 5 personality traits and loneliness among 154 centenarians residing in Oklahoma. Basic descriptive and multivariate regression analyses were conducted. Analyses of covariance (ANCOVA) indicated that mean level of loneliness was higher among centenarians possessing higher levels of extraversion, agreeableness, conscientiousness, and lower level of neuroticism compared to their counterparts. After controlling for demographic characteristics, physical health and cognitive functioning, neuroticism ($\beta=-.22$, $p<.05$) and agreeableness ($\beta=.40$, $p<.001$) were significantly associated with loneliness. It appears that experiencing emotional instability and being agreeable contributes to greater feelings of loneliness among centenarians. This has implications relative to further investigating how personality may uniquely contribute to loneliness after age 100.

### I MAY BE ALONE BUT I DON'T FEEL LONELY: INSIGHTS FROM THE ORAL NARRATIVES OF CHILDLESS CENTENARIANS

Melinda Heinz[1] Alex J. Bishop,[2] and Tanya Finchum[2], *1. Upper Iowa University, Fayette, Iowa, United States, 2. Oklahoma State University, Stillwater, Oklahoma, United States*

Oral history narratives of nine childless centenarians (7 women, 2 men) from the Oklahoma 100 Year Life Project were reviewed to investigate loneliness. Oral history narratives were assessed qualitatively, using content analysis to determine themes. We predicted that childless centenarians would feel lonely due to “elder orphanhood.” Findings revealed little indication of loneliness. Centenarians admitted they voluntarily chose to remain childless due to raising siblings earlier in life or delaying marriage. However, most remained socially well-adjusted and connected to extended family, particularly nieces. When confronted by social network losses due to death or relocation, most adapted by actively seeking and forming new relationships. In some cases, childless centenarians remained gainfully employed and working. Childlessness does not appear to make centenarians lonely. Rather, purposeful pursuit of intrapersonal and interpersonal sources of lifelong emotional contentment may render childless centenarians immune from conditions of loneliness.

### REMAINING SOCIALLY CONNECTED AT 100 AND BEYOND REDUCES IMPACT OF LONELINESS ON NUTRITIONAL STATUS

Seung Eun Jung[1] Alex bishop[2] Seoyoun Kim[3] and Janice Hermann[2], *1. University of Alabama, Tuscaloosa, Alabama, United States, 2. Oklahoma State University, Stillwater, Oklahoma, United States, 3. Texas State University, San Marcos, Texas, United States*

Understanding factors influencing centenarians' nutritional status can offer insight into effective nutrition interventions to improve quality of life among this population. This cross-sectional study was conducted to evaluate the moderating role of social support in the relationship between loneliness and nutritional status among Oklahoma centenarians ($n=140$). Nutritional status was assessed with the Mini Nutrition Assessment (MNA). Perceived social support was assessed with the 24-item Social Provisions Scale. Loneliness was examined with the 10-item UCLA loneliness scale. Ordinal logistic regression revealed that those who lacked social support were more likely to be at risk for malnutrition (OR=2.28, $p<.05$). Further, the interactive model revealed that centenarians who reported lack of support and loneliness were almost 2.8 times as likely to be at risk for malnutrition compared to their socially embedded counterparts ($p<.01$). Findings suggest that nutrition interventions offering centenarians opportunities to feel socially connected could improve their nutritional well-being.

### SESSION 650 (SYMPOSIUM)

### LONELINESS, ISOLATION, AND LIVING ALONE AMONG PEOPLE WITH DEMENTIA AND THEIR CARERS: INSIGHTS FROM THE IDEAL STUDY

Chair: Christina Victor, *Brunel University London, Uxbridge, Middlesex, United Kingdom*
Discussant: Elizabeth B. Fauth, *Utah State University, Logan, Utah, United States*

The IDEAL research programme is national nine-year (2014-2022) ESRC/NIHR/Alzheimer's Society UK funded longitudinal cohort study of 1547 people with mild to moderate dementia and 1283 family members or friends who provide support and aims to identify what promotes (or inhibits) people living well with dementia and their carers and how these change longitudinally. Loneliness and/or isolation are key indicators of quality of life and living well is posited as a factor which compromises wellbeing. Loneliness was measured using both the six-item de Jong Gierveld (DJG) scale (range 0-6) and isolation by the six-item Lubben social network scale (range 0 to 30). The three presentations in this symposium use data from the baseline assessment. Clare focuses upon the 18.5% of our participants who live alone and compares them with those living with others and suggests that there are few systematic differences in terms of cognition, psychological factors and well-being between these groups. Using a score of 5+ on the DJG scale, Victor reports that for people with dementia 5 % were severely lonely, which approximates to the national norm, compared with 17% for caregivers. For social isolation people with dementia had smaller social networks (mean =15.1) and higher levels of isolation as measured by a score of 12 or less on the Lubben scale (35%) compared with caregivers (mean network size=17.1 and 18% isolated). Victor and Clare use dyad data for 1089 pairs for loneliness and 1204 for isolation demonstrating congruence of 43% for loneliness and 68% for isolation

### HOW LONELY AND ISOLATED ARE OLDER PEOPLE WITH DEMENTIA AND THEIR CARERS: A DYADIC ANALYSIS

Christina Victor[1] Isla Rippon[2] Anthony Martyr[3] Fiona Mathews[4] and Linda Clare[3], *1. Brunel University London, Uxbridge, Middlesex, United Kingdom, 2. Brunel University London, Uxbridge, England, United Kingdom, 3. University of Exeter, Exeter, England, United Kingdom, 4. Newcastle University, Newcastle, England, United Kingdom*

Studies of loneliness and isolation have rarely explored is how these experiences are reported within couples or the wider households. The IDEAL study has collected details of loneliness, as measured by the de Jong Gierveld (DJG) scale (range 0-6) and a single-item self-report measure, and isolation, using the six-item Lubben social network scale (range 0-30) from both people with dementia and carers. Loneliness is classified into three groups: not lonely (score 0-2), moderately lonely (3-4) and severely lonely (5+) and isolation into two: not isolated (score of 13+) or isolated (12 or less). Of the 1547 people with dementia and 1283 carers interviewed at baseline we have 1089 dyads who provided complete data on loneliness and 1204 for social isolation. Loneliness ratings are congruent between 43.1% of dyads and for 67.8% for isolation highlighting the subjective evaluative nature of loneliness as compared with more objectively measured isolation.

### LIVING ALONE WITH DEMENTIA: FINDINGS FROM THE IDEAL COHORT

Linda Clare,[1] Linda Clare,[1] Anthony Martyr[1]
Catherine Quinn[2] Christina Victor[3] and Fiona Matthews[4], *1. University of Exeter, Exeter, England, United Kingdom, 2. Bradford University, Bradford, England, United Kingdom, 3. Brunel University London, Uxbridge, England, United Kingdom, 4. NewcCastle University, Newcastle, England, United Kingdom*

We aimed to better understand the profile of people living alone with mild-to-moderate dementia in the UK and to identify any systematic differences between those living alone and those living with others. We analysed cross-sectional data from 1541 people with mild-to-moderate dementia participating in the IDEAL cohort at the first wave of assessment. There were 285 participants (18.5%) living alone and 1256 (81.5%) living with others, usually a spouse/partner. Among those living alone, 145 (50.9%) had no care partner participating in the study, and 56 (19%) had received no help from a relative or friend in the past week. People living alone were older on average than those living with others, reported fewer functional difficulties, had slightly smaller social networks, engaged in fewer cultural activities, and experienced slightly more loneliness. People living alone had lower satisfaction with life scores, but quality of life scores did not differ between the groups.

### PREVALENCE OF LONELINESS AND ISOLATION AMONG PEOPLE WITH DEMENTIA AND THEIR CARERS

Isla Rippon[1] Catherine Quinn[2] Anthony Martyr[3]
Christina Victor,[1] Fiona Mathews[4] and Linda Clare[3], *1. Brunel University London, Uxbridge, Middlesex, United Kingdom, 2. University of Bradford, Bradford, England, United Kingdom, 3. University of Exeter, Exeter, England, United Kingdom, 4. Newcastle University, Newcastle, England, United Kingdom*

People with dementia and carers may be vulnerable to loneliness and isolation. The IDEAL study includes two loneliness measures: 6 item de Jong Gierveld (DJG) scale (range 0-6) and a single-item self-report measure and the six-item Lubben social network scale (range 0-30). Full data are available for 1533 people with dementia for self-rated loneliness and for 1455 for the DJG scale and 1232 and 1195 carers respectively. For isolation complete data are available for 1489 people with dementia and 1252 carers. The prevalence of severe loneliness for people with dementia were 10% (self-rated) and 5% (DJG score 5+), approximately the population norm, and 15% and 18% respectively for carers. Most people with dementia or carers did not rate themselves as lonely ((79% and 71%) compared with 65% and 39% using the DJG scale. One third, 35%, of people with dementia were at risk of isolation compared with 18% of carers.

### SESSION 655 (PAPER)

### MEMORY: BIOLOGICAL, PSYCHOSOCIAL, AND GENETIC FACTORS

### EXAMINING PSYCHOSOCIAL FACTORS, HEALTH BEHAVIORS, AND WHITE MATTER LESIONS IN OLDER ADULTS

Desiree Bygrave,[1] and Regina S. Wright[1], *1. University of Delaware, Newark, Delaware, United States*

Prior to the onset of dementia, subclinical indices of brain pathology may reliably predict cognitive decline, even among older adults with high cognitive reserve. Evidence suggests that positive psychosocial experiences and healthy behaviors buffer cognitive decline. However, their relationship with brain outcomes in cognitively intact older adults is not well understood. Therefore, the current study examined the cross-sectional association between perceived social support, generalized anxiety, psychosocial stress, physical activity, sleep quality, and magnetic resonance imaging (MRI)-assessed white matter lesions (WML), among a diverse sample of older adults. We also examined sex and race as effect modifiers. Data were analyzed from 129 participants (mean age=67.40y, 69% female, 43% African American) enrolled in the Healthy Heart & Mind Study. Participants completed psychosocial and health behavior measures and MRI-assessed periventricular and deep WML were ascertained. Multiple regression analyses assessed relations of psychosocial responses and physical activity to WML, adjusting for known covariates. Significant general anxiety x sex interactions on deep WML ($p<.05$), significant physical activity x race interactions on total WML, frontal lobe WML and deep WML, respectively, and total sleep quality x race interactions on deep WML, were observed ($p<.05$). Conditional effects showed greater physical activity and sleep quality were associated with lower WML in African-American women; greater social belonging was associated with lower WML in American-American men; and lower anxiety was associated with lower WML in African-American women and White men. Results suggest positive psychosocial factors and health behaviors may influence subclinical brain pathology via unique pathways.

### INVESTIGATING MODERATORS OF THE RELATIONSHIP BETWEEN SUBJECTIVE AND OBJECTIVE MEMORY

Francesca Falzarano,[1] Jillian Minahan,[1]
Karen L. Siedlecki,[1] and Timothy Salthouse[2], *1. Fordham University, Bronx, New York, United States, 2. University of Virginia, Charlottesville, Virginia, United States*

Subjective memory complaints (SMC) among older adults have been explored as an indicator of decline in objective memory functioning. While some research has found that SMC may be predictive of future cognitive impairment and dementia (Glodzik-Sobanska et al., 2007; Wang et al., 2004), others have suggested that SMC are common among healthy older adults (Cooper et al., 2011) and are not strongly related to objective memory performance. Researchers suggest that SMC may be more strongly related to affective factors (e.g., depression and anxiety; Rowell, Green, Teachman, & Salthouse, 2015). The current study examined the relationship between SMC, objective episodic memory performance (OEMP), along with depression and anxiety in a sample of 18-99 year olds (N = 5,430) from the Virginia Cognitive Aging Project (VCAP). Structural equation modeling with full information maximum likelihood estimation was used to investigate whether clinically-relevant depression and anxiety levels moderated the relationship between SMC and OEMP, controlling for age, education, gender, and health. OEMP was represented as a latent construct while the remaining variables were observed. Although depression and anxiety are significantly related to SMC (r's = .29, .17, respectively), they are not correlated with OEMP. Furthermore, depression, but not anxiety, moderated the relationship between SMC and OEMP, such that those at risk for depression had a stronger relationship between SMC and OEMP (-.07, p<.05) compared to those not at risk (-.02, p=.31). This suggests that SMC may not be a valid indicator of OEMP as it may reflect variance from other sources, such as depression.

### MEDIATORS OF GENETIC AND ENVIRONMENTAL MECHANISMS ON SELF-RATED HEALTH

Catalina M. Zavala,[1] Carol A. Prescott[1] and Susan Lapham[2], *1. University of Southern California, Los Angeles, California, United States, 2. American Institutes for Research, Washington, District of Columbia, United States*

Self-rated health (SRH), an individual's assessment of their own health status, is associated with older adults' chronic and acute health conditions, as well as mortality. Assessments of SRH indicate individual's global health is likely multifaceted. Level of education, particularly amount of post-secondary schooling, is associated with better SRH. Other indices of socioeconomic status (SES) such as income and wealth, have varying associations with SRH partly dependent on relative deprivation (e.g. Gini Index). The current study utilized data from 2,500 members of the Project Talent Twin and Sibling (PTTS) Study interviewed as adolescents in 1960 and followed up 54 years later. In 2014, participants were, on average, 70 years of age. Women comprised about 54% of the sample. We examined rearing family wealth, years of education, and functional independence as mediators of variance in SRH. Mean-level results indicated small positive associations between SES and SRH. Activities of Daily Living (ADL) accounted for about a quarter of variance in SRH, with higher functional independence predicting better SRH. Biometric analyses indicated that family wealth had small mediation effects on SRH via familial-environment (S) influences. Education mediated individual-specific (E) environmental influences. Functional independence (measured by ADL) mediated SRH via both additive genetic (A) and E influences. After adjusting for overall effects of sex, age, and specified mediators, a large portion of remaining variation in SRH was due to individual-specific (E) environmental influences. Current results suggest complex underlying genetic and environmental mechanisms contributing to an older adult's assessment of their own health.

### REDUCED ENDOTHELIAL FUNCTION IS ASSOCIATED WITH DEEP WHITE MATTER LESION VOLUMES AMONG HEALTHY OLDER ADULTS

Regina S. Wright,[1] and Desiree Bygrave[1], *1. University of Delaware, Newark, Delaware, United States*

Reduced endothelial function (EF) is a subclinical cardiovascular disease (CVD) risk factor and precursor to hypertension and atherosclerosis. Among older adults with CVD, reduced EF has been associated with poorer outcomes in a number of cognitive domains, partly explained by the presence of white matter lesion volumes (WMLV) detectable on brain magnetic resonance imaging (MRI). The role of EF as a key, early predictor of brain decrements among older adults without CVD, however, is not well understood. Therefore, the objective of the study was to examine associations between endothelial function and WMLV among cognitively intact older adults free of CVD. A diverse sample of 138 community-based older adults (30.4% male; mean age=68.54y) enrolled in the Healthy Heart & Mind Study underwent cognitive and psychosocial assessment, vascular testing, and brain MRI. Multiple regressions were run to examine associations between endothelial function, as measured by % change in brachial artery flow-mediated dilation (FMD), and MRI-assessed WMLV in brain regions of interest, after controlling for age, sex, race, education, depression, mean arterial pressure, total cholesterol, and hypertension medication use. Results showed a significant inverse association between % FMD change and deep WMLV (p<.05), but no other regions of interest. Results suggest that reduced EF is associated with greater deep WMLV, an outcome variable attributable to small vessel disease and linked to Alzheimer's disease in previous studies. The implications of this finding for predicting risk for cognitive impairment among healthy older adults will be discussed.

### SHRINKING AND GROWING SOCIAL NETWORKS IN THE SYDNEY MEMORY AND AGEING STUDY

Anne-Nicole S. Casey[1] Nicole A. Kochan,[2] Perminder Sachdev[2] and Henry Brodaty[3], *1. Centre for Healthy Brain Ageing, University of New South Wales (UNSW) Sydney, Sydney, New South Wales, Australia, 2. Centre for Healthy Brain Ageing, Sydney, New South Wales, Australia, 3. Centre for Healthy Brain Ageing (CHeBA), UNSW Sydney, Sydney, New South Wales, Australia*

Gerontological research suggests that shrinking social networks are characteristic of older age. Socioemotional selectivity theory suggests that older adults proactively reduce their networks over time. Core relationship networks consist of friends and family who are contacted at least monthly. Studies of perceived social support indicate that shrinking networks increase risk of isolation, loneliness, and associated ill health effects. Our research aimed to investigate change in size of older adults' core relationship network over time, and to explore associations between network size and participant characteristics, using data from the first four waves of the Sydney Memory and Ageing Study. Participants completed:

assessments of cognition, depression, personality; surveys of health-related quality of life, medical conditions, number of friends and relatives contacted at least monthly. Of 1037 participants aged 70 to 90 years surveyed at baseline 476 provided network data at all four time-points. Just over half of the participants (n=252, 52.9%) had smaller networks at wave 4 compared to baseline; nearly half of participants had stable (n=65, 13.7%) or larger networks (n=159, 33.4%), some doubling in size (n=65, 13.7%). Chi-square test indicated that more males (59.1%) had decreasing networks [X2(1, 476)=2.26, p=.02, phi=.11]. T-tests indicated: group differences in baseline neuroticism [t(276)=2.31, p=.02, Eta2=.02]; no significant difference in age, education, wave 4 global cognition, depression, quality of life, number of medical conditions. Results complement previous literature, yet challenge assumptions that shrinking social networks are a defining characteristic of older age. Future examination of our mental and physical engagement data may elucidate these differences.

### SESSION 660 (PAPER)

### RETIREMENT IN THE 21ST CENTURY

### MEASURING LOST-WORK OPPORTUNITY AT RETIREMENT AGE

Maren Wright Voss[1] Soham Al Snih[2] Wei Li[3] Man Hung[4] lorie Richards[3], *1. Utah State University, Logan, Utah, United States, 2. University of Texas Medical Branch at Galveston, Galveston, Texas, United States, 3. University of Utah, Salt Lake City, Utah, United States, 4. Roseman University of Health Sciences, South Jordan, Utah, United States*

There is uncertainty related to whether retirement negatively impacts health--possibly due to the complexity of retirement decisions. The role of lost work opportunity on retirement decisions may help clarify when retirement has a favorable or negative impact on health. Lost work opportunity can be defined as forced retirement or unemployment prompting an earlier than planned retirement. However, 17% of individuals retiring due to the loss of work opportunity (i.e., unemployment, temporary lay-offs, company buy-outs, forced relocations, etc.) do not report either unemployment or involuntary retirement in survey data. We propose a broader conceptualization of late-career unemployment. Using the Health and Retirement Study (HRS), a lost-work opportunity score (LOS) was computed from items indicating unemployment and forced or unplanned retirement. Correlations were computed between this LOS and all continuous variables in the RAND longitudinal compilation of the HRS to determine its convergent and discriminant validity. The LOS demonstrated a Chronbach's alpha of $\alpha$=0.82 and had convergent validity with constructs of employment (9 variables), finances (36 variables), and health (14 variables), as predicted by the literature on retirement timing. No other continuous variables in the HRS were identified with a moderate or strong correlation to LOS, demonstrating discriminant validity. Further research should explore whether a combination of variables in the HRS can improve the accuracy of measuring retirement voluntariness. Improved precision in measurement, through an expanded conceptualization of lost-work opportunity, may help explicate the retirement-related factors that impact health, to inform policy and support healthy aging decisions at a societal level.

### RETIRING AND CAREGIVING IN THE AGE OF STUDENT LOANS: THE IMPACT OF STUDENT DEBT ON RETIREMENT AND LONGEVITY PLANNING

Samantha Brady,[1] Julie Miller,[1] Alexa Balmuth,[1] Lisa D'Ambrosio,[1]and Joseph Coughlin[1], *1. MIT AgeLab, Cambridge, Massachusetts, United States*

Saving for retirement and the ability to provide care for a loved one can be dramatically affected by student loan debt. Currently, approximately 44 million people of all ages in the United States carry the weight of over 1.4 trillion dollars of student loan debt. Student loan borrowers of all ages may experience lower financial preparedness for retirement as well as decreased ability to provide care for family members, including aging parents. While older adults hold a relatively small proportion of student loans, they are the fastest growing subset of student loan borrowers and have disproportionately high rates of student loan defaults. As a result of their defaults, the Social Security retirement benefits of Americans ages 65 and older experienced a 500% increase in offsets over the last decade. This presentation will spotlight an MIT AgeLab mixed methods study about how student loan borrowers between the ages of 51 and 75 experience student loans within family systems and perceive and prioritize longevity planning in light of their student loans. Data collected for this study include focus groups and a large national survey. Preliminary findings suggest that for older borrowers, student loans are generally one of several financial constraints that can inform spending and saving decisions. For most, student loan payments are regarded as stunting overall retirement savings while the minority regard the two separately. Older borrowers also tend to have increased financial and familial responsibilities, including caring for aging parents, that compete for borrowers' limited financial and temporal resources.

### SPOUSAL INFLUENCE ON EARLY RETIREMENT DECISIONS: ORIGINS AND MECHANISMS

Maria Eismann[1] Kène Henkens[2] and Matthijs Kalmijn[3], *1. NIDI, The Hague, Netherlands, 2. Netherlands Interdisciplinary Demographic Institute, South-Holland, Netherlands, 3. University of Amsterdam, Amsterdam, Netherlands*

The interdependence between partners raises considerable interest in the sociology of life course, work and families. Partner influences play a particularly important role in the work domain, because each partner's work decisions have profound effects on the couple as a whole. In contrast to previous research, this study pays detailed attention to the role partners play in workers' labor market decisions by using the case of early retirement decisions. We hypothesize that partners' preferences for older workers' retirement originate from altruism and self-interest. For example, partners might prefer workers to retire early because the worker's job is highly stressful or partners might prefer workers to retire early to increase possibilities for joint leisure. Moreover, we expected that partners influence older workers' early retirement behavior via persuasion and pressure. So, partners might either convince workers to change their preferences, or they might pressure workers to act according to the partners' preferences, irrespective of workers' own preferences. To adequately estimate partners' and workers' preferences for workers' early retirement, we used an instrumental variable approach. This was possible due to the multi-actor longitudinal data available from a large representative sample of older workers and their partners in the Netherlands. The results support that partners' preferences originate in altruism and self-interest and that partners influence workers through persuasion and pressure. Gender differences were marginal, with stronger signs for altruistic origins among female than male partners.

### WHY DO OLDER WORKERS WITH CHRONIC HEALTH CONDITIONS PREFER TO RETIRE EARLY?

Anushiya Vanajan[1] Ute Bültmann[2] and Kène Henkens[1], *1. Netherlands Interdisciplinary Demographic Institute, South-Holland, Netherlands, 2. University Medical Center Groningen, Groningen, Netherlands*

Older workers experiencing chronic health conditions (CHCs) are more likely to retire early. Current literature, however, lacks knowledge on the different pathways through which CHCs stimulate retirement preference. Earlier research is highly fragmented. Some studies have found CHCs to impact vitality, work limitations, or subjective life expectancy. Others have found vitality, work limitations, or subjective life expectancy to predict retirement preferences. We present a comprehensive model in which we hypothesize that the effects of four CHCs - arthritis, cardiovascular disease, sleep disorders, and psychological disorders - on retirement preferences are differentially mediated by vitality, health-related work limitations, and subjective life expectancy. We analyzed data from 6,294 older workers (60 – 65 years) in the Netherlands. Effects of CHCs on older workers' retirement preferences were mediated by vitality, health-related work limitations, and subjective life expectancy. The main mediation pathway differed for each CHC. Severe health-related work limitations among older workers with arthritis (65.6% mediated) and cardiovascular disease (44.0%) predominantly guided their retirement preferences. Lower vitality levels mainly mediated retirement preferences of older workers with sleep (59.1%) and psychological disorders (52.9%). Lower subjective life expectancy was a significant mediation pathway (13.7%) for older workers with cardiovascular diseases. Extending working lives is a key public health and policy challenge. We show that health-related work limitations and vitality play a major role in determining retirement preferences of older workers experiencing CHCs. Since both mediators are modifiable, targeted interventions may not only extend the working lives of older workers, but also improve its quality.

### SESSION 665 (SYMPOSIUM)

### SENSORY LOSS AND THE HEALTH CARE SYSTEM

Chair: Nicholas Reed, *Johns Hopkins University, Baltimore, Maryland, United States*
Discussant: Charlotte Yeh, *AARP Services, Inc., Washington, District of Columbia, United States*

Communication is fundamental to patient-centered care. However, sensory impairment may limit communication among older adults. Specifically, hearing impairment strains communication via degraded auditory encoding while vision impairment distresses ability to read and interpret visual cues. The presence of dual sensory impairment, defined as concurrent hearing and vision impairment, may exacerbate these effects. The potential consequences of age-related sensory loss on health care interactions and outcomes are beginning to surface in epidemiologic studies demonstrating poorer patient-provider communication, higher incurred health care costs, increased risk of 30-day readmission, and longer length of stay when compared to individuals without sensory loss. Importantly, these associations may be amenable to intervention via sensory aids; however, uptake to sensory care is low. Notably, less than 20% of persons with hearing impairment have hearing aids and over 55% of Medicare Beneficiaries with reported vision problems have not had an eye examination in the prior year. Affordability and access may contribute to lack of sensory care uptake as Medicare explicitly excludes coverage of vision and hearing services. In this symposium, we will review current and new evidence for whether sensory loss affects health care outcomes, including satisfaction with care and medical costs, and present data on how persons with sensory loss interact with the health care system based on the need and reasons for accompaniment to care visits. Further, we will discuss and provide evidence for how sensory care may mitigate these associations. Lastly, we will place these results within the context of quality care and policy initiatives.

### HEARING LOSS AND HEALTH CARE SATISFACTION

Nicholas Reed,[1] Nicholas Reed,[1] Amber Willink,[1] Jennifer Deal,[1] and Frank R. lin[1], *1. Johns Hopkins University, Baltimore, Maryland, United States*

Hearing loss (HL) impacts two-thirds of adults over 70 years and affects patient-provider communication which could limit satisfaction. We used two cross-sectional cohorts, The Atherosclerosis Risk in Communities Study (ARIC, n=250) and the Medicare Current Beneficiaries Survey (MCBS, n=12,311) to examine the relationship between HL (subjective and objective measures) and self-report satisfaction with quality of health care using multivariable-adjusted logistic regression. In ARIC, there was an interaction between HL and age such that HL had a greater impact on odds of dissatisfaction as age increased. In an 85-year-old, for every 10 dB increase in HL, the odds of being dissatisfied increased 1.33 (95% Confidence Interval [CI]:0.96-1.83). In MCBS, compared to participants with no trouble hearing, those with a lot of trouble hearing had 1.7 times the odds ( 95% CI = 1.150-2.623) of being dissatisfied. This has implications for patient-centered care planning given that Medicare ties reimbursement to patient-reported satisfaction.

### COST-BENEFIT ANALYSIS OF HEARING CARE SERVICES AMONG MEDICARE BENEFICIARIES WITH HEARING AIDS

Amber Willink,[1] Amber Willink,[1] Nicholas S. Reed,[1] and Frank R. Lin[1], *1. Johns Hopkins University, Baltimore, Maryland, United States*

Hearing care services for older adults with hearing aids are underutilized and are not covered by the Medicare program. Little information exists to the value of hearing care services for older adults with hearing aids. Using the Medicare Current Beneficiary Survey 2013, we conducted a cross-sectional analysis of the impact of hearing care services use on Medicare spending among those with hearing aids. Older Medicare beneficiaries with hearing aids that received hearing care services in the previous 12 months were propensity score matched to those who did not receive services. Average annual Medicare spending was $8196 (CI:$6670-$9723) among Medicare beneficiaries who used hearing care services and $10,709 (CI:$8878-12541) among matched controls. Spending differences were driven by higher skilled nursing facility and home health spending among matched controls. Increasing access to hearing care services among Medicare beneficiaries with hearing aids may provide value to the health care system and Medicare program.

### IMPACT OF DUAL SENSORY IMPAIRMENT ON HEALTH CARE INTERACTION

Bonnielin Swenor[1] Bonnielin Swenor[2] Yasmeen Alshabasy[3] Emily Pedersen,[4] Jennifer Deal[4] Amber Willink[5] and Nicholas Reed[4], *1. The Wilmer Eye Institute, Johns Hopkins University School of Medicine, Baltimore, Maryland, United States, 2. The Wilmer Eye Institute, Johns Hopkins University School of Medicine, Department of Epidemiology; Johns Hopkins Bloomberg School of Public Health, Baltimore, Maryland, United States, 3. wesleyan University, Middletown, Connecticut, United States, 4. Cochlear Center for Hearing and Public Health, Johns Hopkins University Bloomberg School of Public Health, Baltimore, Maryland, United States, 5. Health Policy & Management and Cochlear Center for Hearing and Public Health, Johns Hopkins University Bloomberg School of Public Health, Baltimore, Maryland, United States*

Using data from 46,029,364 Medicare beneficiaries included in the 2015 Current Beneficiary Survey (MCBS), we examined the relationship between dual sensory impairment (DSI) – concurrent vision impairment (VI) and hearing impairment (HI) – and accompaniment to physician visits. Analyses examined reasons for accompaniment and self-reported sensory impairment was categorized as: no sensory impairment (89%), hearing impairment (HI) only (5%), vision impairment only (4%), and DSI (1%). There was no difference in odds of accompaniment among HI compared to those without sensory impairment (odds ratio [OR] = 1.04; 95% Confidence Interval [CI]: 0.84,1.29); however, VI and DSI were associated with accompaniment: (OR=2.14; [CI]:1.6,2.8 and [OR]= 2.70; [CI]:1.55,4.72, respectively). Our study further demonstrates that older adults with sensory impairment are accompanied to physician visits more often than those without sensory impairment, and transportation is the most frequently reported reason for accompaniment among adults with VI and communication for those with HI.

### ASSOCIATION OF SENSORY AND COGNITIVE IMPAIRMENT WITH HEALTHCARE UTILIZATION AND COST IN MEDICARE BENEFICIARIES

William Deardorff[1] Phillip liu[2] Richard Sloane[3] Courtney Van Houtven,[2] Susan N. Hastings,[2] Harvey J. Cohen,[3] and Heather E. Whitson[2], *1. Duke University School of Medicine, Durham, North Carolina, United States, 2. Duke University, Durham, North Carolina, United States, 3. Center for the Study of Aging, Duke University Medical Center, Durham, North Carolina, United States*

The combination of sensory and cognitive impairment is increasingly prevalent among older adults and may be an important driver of healthcare cost due to functional disability and reduced self-care. This presentation focuses on the relationship between hearing and/or vision impairment and cognitive impairment with hospital admissions and healthcare cost using data from the Medicare Current Beneficiary Survey, a nationally representative sample of community-dwelling adults. We show that the presence of sensory impairment is associated with increased risk of hospitalization regardless of dementia status. In adjusted models, annual total healthcare costs were generally higher among those with sensory impairments compared to those without sensory impairments. We will also discuss work related to the development of a prognostic model that provides estimates of hospitalization risk among older adults with self-reported hearing and/or vision impairment. This model may help inform allocation of health care resources to those at highest risk for adverse outcomes.

### SESSION 670 (SYMPOSIUM)

### SLEEP IN LATE LIFE: RECENT RESEARCH ON PSYCHIATRIC CORRELATES AND TREATMENT

Chair: Mary E. Dozier, *South Texas Veterans Health Care System, San Antonio, Texas, United States*

Sleep is an often overlooked health factor, particularly in older adults. Sleep disturbance is associated with increased functional impairment as well as poorer cognitive, mental, and physical health trajectories. Understanding the clinical impact of disturbed sleep, and the optimal targets for intervention, is critical for the promotion of health and well-being in older adults. This symposium will highlight recent findings that advance the extant knowledge on the interplay of sleep disturbance and physical and psychiatric co-morbidities in older adults across a variety of settings. Darina V. Petrovsky will discuss the impact of medical, demographic, and contextual factors on excessive daytime sleepiness in older adults receiving long-term services and supports. Kathi L. Heffner will present data on a recent study examining change in slow wave sleep, and subsequent change in osteoarthritis pain, following insomnia treatment. Courtney Bolstad will discuss the differential impact of onset, maintenance, and terminal insomnia on anxiety and depression symptoms in community-dwelling older adults. Eliza Davidson will present research on the association between sleep disturbance and hoarding symptoms in older adults engaged in behavioral interventions for hoarding disorder. Finally, Christina McCrae will discuss the relationship between sleep and cognition in older adults with insomnia.

### I CAN'T SLEEP IN MY BED: THE RELATIONSHIP BETWEEN SLEEP AND LATE-LIFE HOARDING

Eliza J. Davidson[1] Mary E. Dozier[2] Michael Nadorff[3] Catherine R. Ayers[1], *1. VA San Diego Healthcare System, San Diego, California, United States, 2. South Texas*

*Veterans Health Care System, San Antonio, Texas, United States, 3. Mississippi State University, Mississippi State, Mississippi, United States*

Hoarding disorder in late life has been associated with increased risk for medical conditions and decreased ability to perform activities of daily living in the home; however, no studies have yet examined the relationship between geriatric hoarding and sleep. This study represents a secondary data analysis of older adults who received 26 sessions of group behavioral treatment for hoarding disorder (n = 41; mean age 64, range 55-85). Baseline sleep disturbance was significantly associated with hoarding severity, even when controlling for inability to sleep in a bed due to household clutter level. However, no significant change in sleep disturbance was reported following completion of treatment and baseline sleep disturbance was not significantly predictive of change in hoarding symptom severity. Findings suggest that disturbed sleep quality is associated with greater hoarding symptom severity but does not preclude positive symptom change in treatment.

### DOES IMPROVING SLEEP IMPROVE COGNITION IN OLDER INDIVIDUALS WITH INSOMNIA?

Christina McCrae[1], *1. University of Missouri-Columbia, Columbia, Missouri, United States*

Late life insomnia is associated with worse cognitive performance. Behavioral/cognitive behavioral treatments for insomnia (BBT-I, CBT-I) improve sleep in older adults, but findings are mixed for cognition. This presentation examines the effects BBT-I and CBT-I on sleep and cognition across three RCTs involving older individuals (community-dwelling [N=62, Mage=69.45(SD=7.71)], chronic pain [N=64, Mage=53.2 (SD=13.7)], dementia caregiving [N=36, Mage=62.32 (SD=6.71]). Sleep was assessed using daily diaries and actigraphy for 1-2 weeks prior to randomization to treatment or control. Cognition was measured using standardized executive functioning, memory, and attention measures. Multiple regressions revealed improved executive functioning following treatment (caregivers), associations between improved executive performance and greater pain/sleep improvements (chronic pain), and associations between improved attention and processing speed and improved sleep 9-months following treatment (community-dwelling). BBT-I/CBT-I hold promise for improving cognition in older aged individuals with insomnia. Research is needed to determine what factors influence/which patients are most likely to experience cognitive benefits.

### THE EARLY BIRD GETS THE WORM, NOT ANXIETY OR DEPRESSION: HOW INSOMNIA SYMPTOMS PREDICT ANXIETY AND DEPRESSION

Courtney J. Bolstad,[1] Anisha L. Thomas,[1]
Michael R. Nadorff[1], *1. Mississippi State University, Mississippi State, Mississippi, United States*

Symptoms of insomnia are associated with symptoms of depression and anxiety in older adults, yet less is known about the impact of specific forms of insomnia (i.e. onset, maintenance, and terminal insomnia). We explored how insomnia type predicted symptoms of anxiety and depression in older adults (n = 133; mean age 69, range 65-89). We hypothesized that onset and maintenance insomnia would have stronger relations to depression and anxiety than terminal insomnia. Regression analyses indicated that onset insomnia was the only significant predictor of anxiety symptoms, and maintenance was the only significant predictor of depressive symptoms. Thus, our findings suggest that despite overlap between depression and anxiety, insomnia may have different mechanisms of affecting each disorder. Implications for the treatment of anxiety and depressive symptoms by addressing insomnia problems will be discussed.

### SLOW WAVE SLEEP AND PAIN AFTER BEHAVIORAL INSOMNIA TREATMENT IN ADULTS OVER AGE 50 WITH KNEE OSTEOARTHRITIS

Kathi L. Heffner,[1] Kathi L. Heffner[1]
Christopher R. France[2] and Wilfred R. Pigeon[1], *1. University of Rochester Medical Center, Rochester, New York, United States, 2. Ohio University, Athens, Ohio, United States*

Sleep disturbance can aggravate pain, and we recently found that insomnia treatment improved osteoarthritis (OA) pain, lowered inflammation, and improved quality of life in middle-to-older aged adults. Inadequate slow wave sleep (SWS), known as deep or restorative sleep, can decline with aging and is linked to pain and inflammation. We examined how insomnia treatment affects SWS, and the relationship between SWS and pain. In a pilot trial, 33 adults, ages 51 to 74 years with OA-related knee pain and insomnia, were randomized to 6-session CBTi (n=16) or a weekly phone contact control group (n=17). The CBT-I group showed significantly more laboratory-measured SWS across a study night than controls after controlling for baseline SWS. Greater SWS intensity was associated with lower OA-related pain among the CBT-I group, but not among controls. These preliminary data suggest that behavioral sleep treatment may strengthen the beneficial influence of restorative sleep on pain.

### CHANGES OVER TIME IN DAYTIME SLEEPINESS AMONG OLDER ADULTS RECEIVING LONG-TERM SERVICES AND SUPPORTS

Darina V. Petrovsky,[1] Karen B. Hirschman[1]
Glenna Brewster[2] Alexandra L. Hanlon[3] Liming Huang,[1]
Miranda Varrasse McPhillips,[1]
Nancy A. Hodgson,[1] and Mary D. Naylor[1], *1. University of Pennsylvania, Philadelphia, Pennsylvania, United States, 2. Nell Hodgson Woodruff School of Nursing Emory University, Atlanta, Georgia, United States, 3. Virginia Polytechnic Institute and State University, Roanoke, Virginia, United States*

The purpose of this study was to examine the predictors of excessive daytime sleepiness (EDS) over the first two years of long-term services and supports (LTSS) for 470 older adults in assisted living communities (ALCs), nursing homes (NHs), or their homes. Mixed effects linear regression modeling using a backward elimination process was used to build a final multivariable model. In the final model, being female ($p<0.001$) and fewer functional deficits ($p<0.001$) at baseline were associated with decreases in EDS, while higher baseline measures of BMI ($p=0.004$) and number of symptoms ($p<0.001$) were associated with higher EDS. Compared to older adults living in the community and receiving LTSS, those in NHs and ALCs had higher EDS ($p<0.001$). Greater feelings of belonging and depressive symptoms at baseline were associated with slower rates of increase in EDS over time (both p<0.001). Modifiable predictors of EDS and clinical implications will be discussed.

### SESSION 675 (SYMPOSIUM)

### STRATEGIES TO PREVENT FRAILTY: THE POWER OF EARLY DETECTION AND TRAINING OF PROFESSIONALS

Chair: Nienke Bleijenberg, *University of Applied Sciences Utrecht, Utrecht, Netherlands*
Discussant: Niek de Wit, *Julius Center for Health Sciences and Primary Care, University Medical Center Utrecht, Utrecht, Netherlands*

Aging in place is an important goal for both older adults as well as many health policies worldwide, as it is in the Netherlands. Within an aging society, the number of frail older people with complex care needs living at home is increasing. Despite the various definitions of frailty, it is important to early identify who is at risk in clinical practice in order to prevent functional decline, enhance quality of life, and reduce health care costs. Furthermore, an important requirement is effective collaboration between primary care professionals. Various factors are associated with frailty. However, early detection of frailty and its risk factors such as oral health, nutrition and medication related problems is not part of routine care of professionals. To recognize frailty and its risk factors we started a large proactive integrated primary care program that successfully identified frail older people living at home based on routine care data in the Netherlands. After two-year follow-up, a reduction in acute visits at the emergency department was observed. Next, we performed additional studies focusing on early detection and prevention of risk factors of frailty such as oral health, nutrition, and medication related problems among older people living at home. During this symposium we will present the results of the program, followed by our studies that investigated frailty or frailty related risk factors. Additionally, we will show how we enhanced and evaluated the knowledge and skills of professionals working with frail older people in primary care.

### A PROACTIVE CARE PROGRAM FOR FRAIL OLDER PEOPLE LIVING AT HOME: A 2-YEAR EVALUATION

Nienke Bleijenberg[1] Jonna Rijksen[2] Marieke J. Schuurmans[3] Niek J. de Wit[4], *1. University of Applied Sciences Utrecht, Utrecht, Netherlands, 2. Julius Center for Health Sciences and Primary Care, University Medical Center Utrecht, Utrecht, Utrecht, Netherlands, 3. Education Center, UMC Utrecht Academy, University Medical Center Utrecht, Utrecht, Utrecht, Netherlands, 4. Julius Center for Health Sciences and Primary Care, University Medical Center Utrecht, Utrecht, Overijssel, Netherlands*

This study presents the long-term evaluation and impact of a proactive primary care program that aims to reduce acute healthcare consumption and preservation of daily functioning of frail older people living at home. For nine years the program has been adapted and evaluated. We present the results of our implementation study with two-year follow-up. 53 general practices (GP) participated. They provided care to approximately 35000 people aged 65 years and older. Data was extracted from routine primary care data, hospital data and social care data from the municipality. Important outcomes were: number of GP visits, house visits by GP, out-of-hours primary care visits, emergency room (ER) visits, hospital admission, social support, self-sufficiency and wellbeing. After implementing the program, a significant reduction in acute visits (ER and out-of-hour visits) was observed. GP contacts and visits were also significantly increased. The program was well perceived by professionals.

### EXPLORING ASSOCIATIONS BETWEEN FRAILTY AND ORAL HEALTH IN COMMUNITY-DWELLING OLDER PEOPLE

Babette Everaars,[1] Katarina Jerković - Ćosić,[1] Nienke Bleijenberg[1] Niek J. de Wit[2] and Geert j. van der Heijden[3], *1. University of Applied Sciences Utrecht, Utrecht, Utrecht, Netherlands, 2. Julius Center for Health Sciences and Primary Care, University Medical Center Utrecht, Utrecht, Utrecht, Netherlands, 3. ACTA academisch Centrum Tandheelkunde Amsterdam, Amsterdam, Noord-Holland, Netherlands*

This study explores associations between frailty and oral health in cross-sectional data of 1,202 community-dwelling older people. Two dichotomous outcomes were used: 1. Potential frailty, using routine primary care data; 2. Self-reported frailty, using a questionnaire. Oral health concerned dental record data and self-reported oral problems. Following exploration of univariate associations, age and sex adjusted multivariate logistic regressions were performed. For potential frailty and self-reported frailty associations were found with dental emergency visit (odds ratio (OR)= 2.0, 95% confidence Interval (CI)=1.33;3.02 respectively OR=1.58, 95% CI=1.00;2.49), experiencing oral problems (resp. OR=2.07, 95% CI=1.52;2.81 and OR=2.87, 95% CI= 2.07), making dietary adjustments (resp. OR=2.66, 95% CI= 1.31;5.41 and OR=5.49, 95% CI= 3.01;10.01). Additional associations were found for self-reported frailty with wearing dental prosthesis (OR=3.33, 95% CI=1.49;7.44) and missing periodontal information (OR=1.56, 95% CI=1.05;2.32). The cross-sectional data of this study show that in community dwelling older people oral health is associated with frailty.

### POTENTIAL CLINICAL CONSEQUENCES OF ADMINISTRATIVE ISSUES REGARDING MEDICATION IN HOME CARE PATIENTS

Nienke E. Dijkstra[1] Carolien Sino[2] Marieke J. Schuurmans[3] Marcel L. Bouvy,[2] Aline Bouwes,[2] and Rob R. Heerdink[2], *1. University of Applied Sciences Utrecht, Utrecht, Netherlands, 2. University of Applied Sciences Utrecht, Utrecht, Utrecht, Netherlands, 3. Education Center, UMC Utrecht Academy, University Medical Center Utrecht, Utrecht, Utrecht, Netherlands*

Home care professionals observe drug-related problems (DRPs) as administrative problems (e.g. inconsistent registration of (changes in) drug prescription) and side effects which may have clinical consequences for older patients. This study aims to determine the potential clinical impact of administrative problems. A retrospective descriptive study was performed, using reports of home care professionals of the eHOME system (system that assist monitoring/reporting DRPs). Administrative problems of a one year period were assessed by three experts on potential discomfort/clinical deterioration using a 3-point scale. 309 DRPs of 120 out of 451 patients (age ≥65) were assessed. Problems involved undelivered medication administration record lists (n=103,33.3%), inconsistent registration of drug prescription (n=188,60.9%) and insufficient drug delivery (n=18,5.8%). 58.2% of the DRPs had the potential to cause moderate to severe discomfort or clinical deterioration. The results underlines the importance of the observation function of home care professionals and the need to improve pharmaceutical administration issues.

### THE EVALUATION OF INTERPROFESSIONAL EDUCATION IN PRIMARY CARE: A SOCIAL NETWORK ANALYSIS

Linda C. Smit[1] Jeroen Dikken[2] Nienke M. Moolenaar[3] Marieke J. Schuurmans[4] Niek J. De Wit[5] Nienke Bleijenberg[1], *1. University of Applied Sciences Utrecht, Utrech, Netherlands, 2. The Hague University of Applied Sciences, Den Haag, Zuid-Holland, Netherlands, 3. Social and Behavioural Sciences Utrecht University, Utrecht, Utrecht, Netherlands, 4. Education Center, UMC Utrecht Academy, University Medical Center Utrecht, Utrecht, Utrecht, Netherlands, 5. Julius Center for Health Sciences and Primary Care, University Medical Center Utrecht, Utrecht, Utrecht, Netherlands*

Effective, safe, person-centred care relies on skilled interprofessional collaboration (IPC) and practice. Little is known about interprofessional education (IPE) to increase IPC in the context of care for frail older people in the community. This study evaluates the effectiveness of IPE on IPC of primary health and social care providers providing care to frail older people in three districts in the Netherlands. A before-after study among 55 health care professionals using social network analysis was performed. The number of contacts increased on average with two contacts. The reciprocity in the districts increased with 15%, 2% and 13%. The diversity of contacts increased between 6% and 10% (p <.001; p .055; p .371). The IPE effectuated a larger, more collaborative, and diverse interprofessional network of health and social care professionals providing care to frail older people suggesting a ripple-effect of networked interventions.

### CHANGING NURSES' BEHAVIOR TOWARD NUTRITIONAL CARE WITH A SNACK-SIZED LEARNING INTERVENTION

Debbie Ten Cate[1] Jeroen Dikken[2] Roelof Ettema[3] Lisette Schoonhoven[4] and Marieke J. Schuurmans[5], *1. University of Applied Sciences Utrecht, Utrecht, Netherlands, 2. The Hague University of Applied Sciences, Den Haag, Zuid-Holland, Netherlands, 3. University of Applied Sciences Utrecht, Utrecht, Utrecht, Netherlands, 4. Julius Center for Health Sciences and Primary Care, University Medical Center Utrecht, Utrecht, Utrecht, Netherlands, 5. Education Center, UMC Utrecht Academy, University Medical Center Utrecht, Utrecht, Utrecht, Netherlands*

Nurses play an important role in the prevention and treatment of malnutrition in older adults. However, research shows that nurses lack the motivation to give adequate nutritional care. In order to change this motivation a learning intervention about nutrition in older adults targeted at nurses would be desirable. The aim of this study was to assess the development, validation, and reliability of a learning intervention about nutrition in older adults. The results show that this learning intervention has a good construct and content validity, and is psychometrically sound. Questions of the learning intervention can be presented at once or in a snack-sized way, where the questions are presented over a period of time. This learning intervention can be used for developers of similar interventions, and as part of educating programs for nursing professionals and nursing students about nutritional care for older adults.

### SESSION 680 (SYMPOSIUM)

### THE ROLE OF EDUCATORS AND EMPLOYERS IN REDUCING (OR PERPETUATING) AGEISM

Chair: Tina M. Kruger, *Indiana State University, Terre Haute, Indiana, United States*

As the population ages, increasing numbers of people are at risk of being harmed by ageism found in interpersonal interactions, medical settings, employment opportunities, and public policies. The way older people are talked to and about can facilitate the inclusion or exclusion/dismissal of the older population. Gerontology educators are well-positioned to combat ageism by discussing ageist beliefs with students and by teaching about stereotype development, aging stigma, and inclusive language and interactions. Aging services providers can address ageism by hiring the candidates most qualified to engage with older adults in a non-ageist manner. We explore these ideas in this symposium. First, information from the Gerontological Literacy Network's Sketches study regarding college students' (mis)perceptions of aging will be presented. Second, the Ageism First Aid online training modules, designed to reduce ageism, will be introduced. Third, the Disrupt Aging Classroom practice model, created by AARP CT, and Borrow My Glasses, used to transform attitudes about aging among college students, will be shared. Fourth, the disjoint between aging services job descriptions and how those who study aging/gerontology search for jobs will be reviewed. Finally, data from the GELS project, regarding where gerontology program graduates have gained employment, will be presented. Efforts to educate all college students and aging services providers about appropriate language and interaction styles, combined with modifying aging services employers' efforts to identify the most qualified candidates to hire, can ultimately reduce ageism and enhance quality of life for the fastest growing segment of the population.

### EXAMINING AGEISM IN UNDERGRADUATE STUDENTS THROUGH DRAWING

Katherina N. Terhune[1] Aaron Guest[2] Tina M. Kruger[3], *1. Northern Kentucky University, Highland Heights, Kentucky, United States, 2. University of Kentucky, Lexington, Kentucky, United States, 3. Indiana State University, Terre Haute, Indiana, United States*

Substantial demographic shifts in the U.S. will result in the growth of the aging population and the need for qualified professionals entering the field of aging. Yet, these emerging professionals have limited exposure to aging curricula. It is vital that gerontologists recognize the ageism present in how older adults and the aging process are viewed, as this ultimately impacts how they are treated. Data from 1,609 undergraduate surveys from the multi-institution, multi-year Gerontological Literacy Network can assist in our understanding emerging professionals' perspectives. Results indicate students incorrectly associate aging with loss of function and a reliance on assistive devices (canes 29.3% and glasses 19.6%), physical changes (balding 27.3% and wrinkles 30.3%), and greater likelihood of being male (32.5%). Aging is thought of as time passing (16.1%) and death (14.2%). Findings reveal a need to reframe students' understanding of aging from that of decline to recognizing the strengths associated with age.

### AGEISM FIRST AID: AN ONLINE COURSE DESIGNED TO ADVANCE GERONTOLOGY AND COMBAT AGEISM WITHIN THE HELPING PROFESSIONS

Laurinda Reynolds[1] Tina M. Kruger[2] Rebekah Knight[3], *1. American River College, Sacramento, California, United States, 2. Indiana State University, Terre Haute, Indiana, United States, 3. University of North Texas, Denton, Texas, United States*

Ageism First Aid is an online course designed to combat ageism, advance Gerontology, and generate revenue for Gerontology programs and GSA/AGHE activities. The project was funded by a grant from the Retirement Research Foundation and sponsored by the AGHE Academic Program Development Committee. The AFA course content is informed by developmental, social, and cognitive psychology, sociocultural and cognitive linguistics, speech-language science, and geriatrics. The content cultivates ageism awareness by replacing the common misconceptions about aging that underlie ageism with facts. The content also cultivates cultural consciousness and uses concise common language accessible to learners of diverse backgrounds. During this symposium, participants will be introduced to the course content and explore the benefits of this multidisciplinary approach to curriculum development. The results summary of the AFA course pilot will be presented as evidence of the course efficacy. Participants will leave able to utilize AFA within their institutions and local Aging Networks.

### FROM WRINKLES TO WISE: DISRUPTING AGING PERCEPTIONS AMONG COLLEGE STUDENTS

Katherine Bridges[1] Donna Fedus[2] Erica Michalowski[3], *1. AARP, Washington, District of Columbia, United States, 2. Borrow My Glasses, Inc., Madison, Connecticut, United States, 3. AARP-CT, Hartford, Connecticut, United States*

What words come to mind when students are asked about aging? Wrinkles, grumpy, gray. What words come to mind when students are asked about aging after Disrupt Aging Classroom? Wise, wisdom, opportunity. Disrupt Aging Classroom is a successful curriculum created by AARP Connecticut and Borrow My Glasses to help dispel the myths of aging among college students in multiple disciplines. In comparison to a control group, pre-and post-test results show college students' perceptions of aging change to a more positive frame after being exposed to this intervention administered in the classroom. There is also evidence to suggest that this program has a positive effect on behavior with students reporting they talked differently about aging and acted differently toward older adults since participating in this program. Presenters will describe the curriculum as well as the evaluation design and results collected in 2018-2019 from this successful intervention program.

### TERMINOLOGY USED BY EMPLOYERS AND JOB SEEKERS FOR AGING-RELATED POSITIONS

Leanne J. Clark-Shirley[1] Tina Kruger Newsham[2] and M. A. Guest[3], *1. AARP, Washington, District of Columbia, United States, 2. Indiana State University, Terre Haute, Indiana, United States, 3. University of Kentucky, Lexington, Kentucky, United States*

Our aging society calls for a workforce capable of meeting older adults' diverse needs. Yet the extent that employers seek out a workforce with aging-related training or education is unclear, as is how people with such backgrounds search for positions. We describe an exploratory content analysis of job postings to understand how employers are searching for applicants with aging-related backgrounds, and compare job posting keywords to terms used by a sample of aging-trained job seekers/employees. Results showed 35% of aging-related job postings used keywords expressing preference for applicants with aging-related backgrounds; the most commonly-occurring terms were "gerontology," "Assisted living" + "adult day" + "director" + "nursing home administrator," and "elderly." Job seekers also cited "gerontology" as a term used to search for positions, along with "aging," "older adults" and "seniors". Findings suggest that employers should use more positively-connoted terms to attract applicants with aging-related backgrounds, rather than terms like "elderly."

### WORKING WITH OR WITHOUT AGING SPECIALISTS: WHERE ARE THE GERONTOLOGISTS?

Heidi H. Ewen[1], *1. University of Indianapolis, University of Georgia, Indianapolis, Indiana, United States*

One question asked by generations of gerontology doctoral students is what types of employment can be secured after completing the PhD in Gerontology. The Gerontology Education Longitudinal Study (GELS) has surveyed graduate students and alumni of the various doctoral programs in order to understand the career trajectories of graduates. Of 102 alumni surveyed in 2014 (42% response rate), the majority (60%) were not working with other people who had degrees in gerontology, yet 51% report working with at least some people who have experience with aging and older adults. On the job, graduates say that their duties require knowledge and mastery of public policy issues, health and medical aspects related to aging, and psychological theories. As aging experts, it is inherent in their work to combat ageism and reduce age-related stereotypes. As such, gerontologists are using substantive content expertise within their careers and serving as experts in aging.

### SESSION 685 (SYMPOSIUM)

### UNDERSTANDING THE IMPACT OF OBESITY THROUGH THE LIFECOURSE ON LATER-LIFE FUNCTIONING AND DEPENDENCY

Chair: Carol Jagger, *Newcastle University, Newcastle upon Tyne, United Kingdom*

Populations worldwide are seeing rising levels of obesity and its health consequences, particularly diabetes. Levels of childhood obesity are particularly high with concerns of how this will affect individuals' health and functioning in mid and later life. Such research questions are difficult to answer as ideally they require longitudinal studies of cohorts from birth or childhood through to later life, with consistent measures of obesity and functioning throughout. The first two presentations in this session use the unique UK birth cohorts, the 1946 National Survey of Health and Development (1946-NSHD) and the 1958 National Child Development Study (1958-NCDS) with a focus on poor physical functioning (PF, i.e. the ability to perform physical tasks of daily living) in later life. Poor PF was defined as the lowest (gender and cohort-specific) 10% on the Short-form 36 subscale at 60-64y (1946-NSHD) and 50y (1958-NCDS). The presentations explore (i) how the timing of onset and duration of obesity, from childhood through to mid-life, affects later life PF, and (ii) whether the relationship between obesity and PF is mediated by physical inactivity. In the final presentation we utilise a new dynamic micro-simulation model, the Population Ageing and Care Simulation (PACSim) which simulates the ageing of a base population of individuals aged 35 years and over from three longitudinal studies (Understanding Society, the English Longitudinal Survey of Ageing, and the Cognitive Function and Ageing Study II) to examine the extent to which reducing obesity in mid life could potentially reduce later dependency and care needs.

### BIRTHWEIGHT, LIFETIME OBESITY, AND PHYSICAL FUNCTIONING IN MID-ADULTHOOD: A NATIONWIDE BIRTH COHORT STUDY

Nina Rogers[1], *1. University College London, London, United Kingdom*

Evidence is scant on long-term implications of body mass index (BMI) gains over the life-course for poor physical functioning (PF). Using the 1958 British birth cohort (N=8,674) we examine whether i) birthweight and BMI across the life-course; ii) BMI gains at specific life-stages; and iii) age of obesity onset, were associated with PF at 50y. At each adult age, obesity was associated with poor PF (e.g. for males at 23y adjusted-ORs for poor PF was 2.28(1.34,3.91)). BMI gains were associated with poor PF (e.g. for females, adjusted-OR per SD BMI gain 16-23y was 1.28(1.13,1.46)). Longer obesity duration was associated with poor PF (e.g. for males, adjusted-OR was 2.32(1.26,4.29) for childhood obesity onset, and 1.50(1.16,1.96) for mid-adulthood onset); associations were abolished with further adjustment for 50y BMI. Obesity, BMI gains, and earlier obesity onset were associated with poor PF in mid-adulthood reinforcing the importance of preventing obesity early in the lifecourse.

### OBESITY AND MIDLIFE PHYSICAL FUNCTIONING IN TWO BRITISH BIRTH COHORTS: THE MEDIATING ROLE OF PHYSICAL INACTIVITY

Snehal Pinto Pereira[1] Bianca L. De Stavola[2]
Nina T. Rogers[3] Rebecca Hardy[4] Rachel Cooper[5]
Chris Power[2], *1. University College London, London, England, United Kingdom, 2. UCL GOS Institute of Child Health, London, England, United Kingdom, 3. UCL Research Department of Epidemiology & Public Health, London, England, United Kingdom, 4. MRC Unit for Lifelong Health and Ageing, London, England, United Kingdom, 5. NA, London, England, United Kingdom*

Associations between obesity and physical inactivity are bi-directional. Both are associated with physical functioning (PF) but whether obesity influences PF via inactivity is unknown. We investigated whether mid-adult obesity trajectories were associated with subsequent PF and mediated by inactivity in the 1946 National Survey of Health and Development (1946-NSHD; N=2,427) and the 1958 National Child Development Study (1958-NCDS; N=8,674). Estimated randomised-interventional-analogue natural direct (rNDE), indirect (rNIE) and total (rTE=rNDExrNIE) effects of obesity trajectories on PF via inactivity are expressed as risk ratios. In 1946-NSHD, rTE of incident obesity at 43y (vs never) on poor PF=2.32(1.13,3.51); at 53y=1.53(0.91,2.15). rNIEs via inactivity were 1.02(0.97,1.07) and 1.02(0.99,1.04) respectively. Estimated rTE of persistent obesity from 36y=2.91(1.14,4.69), with rNIE of 1.03(0.96,1.10). Longer obesity duration was associated with increased risk of poor PF. Inactivity played a small mediating role. Findings reinforce the importance of preventing and delaying obesity onset to protect against poor PF.

### WHAT IS THE EFFECT OF REDUCING OBESITY ON LATER-LIFE DEPENDENCY? FINDINGS FROM THE PACSIM MODEL

Andrew Kingston[1] and Carol Jagger[2], *1. Newcastle University, Newcastle upon Tyne, United Kingdom, 2. Newcastle University, Newcastle upon Tyne, England, United Kingdom*

Understanding the extent to which reducing obesity will help maintain independence in later life is of key importance in the goal to achieve healthy ageing. To investigate this we use a unique dynamic microsimulation model, the Population Ageing and Care Simulation (PACSim) model, formed from three Longitudinal studies: Understanding Society, the English Longitudinal Study of Ageing, the Cognitive Function and Ageing Study II; with the base population of 303,589 individuals aged 35 years and over (a 1% random sample of the England population in 2014). PACSim simulates the characteristics (sociodemographic factors, health behaviours including overweight and obesity, chronic diseases and geriatric conditions) of individuals between 2014 and 2040, with transition probabilities for characteristics estimated by modelling state changes from baseline to two-year follow-up in the combined studies. We estimate the effect of different strategies for obesity reduction on years spent independent from age 55 between 2015 and 2030.

### SESSION 690 (SYMPOSIUM)

### WE READ HIS PAL CARD AT THE FUNERAL: EVALUATING THE IMPLEMENTATION OF A PERSON-CENTERED COMMUNICATION TOOL

Chair: Katherine M. Abbott, *Miami University, Oxford, Ohio, United States*
Discussant: Howard Degenholtz, *University of Pittsburgh, Pittsburgh, Pennsylvania, United States*

This symposium describes the development and implementation of an interdisciplinary and novel person-centered care (PCC) communication tool in nursing homes (NH). PCC is a philosophy that recognizes "knowing the person" and honoring individual preferences. The communication tool is based on an assessment of NH resident likes and dislikes via the Preferences for Everyday Living Inventory (PELI). The PELI is an evidenced-based, validated instrument that can be used to enhance the delivery of PCC. In 2016, the Ohio Department of Medicaid (ODM) mandated NHs use the PELI as one of the factors that determine the quality portion of their daily Medicaid reimbursement rate. The Preferences for Activity and Leisure (PAL) Card was developed to communicate important resident preferences across care team members. In 2018, the PAL Card Project was approved by the Ohio Department of Aging as a Quality Improvement Project. The first presentation will describe the implementation of PAL Cards with n=43 NH providers. The second presentation will present data regarding the acceptability, feasibility, and appropriateness of the communication tool as rated by providers. The final presentation explores provider qualitative responses regarding the characteristics of the PAL Card communication tool related to effective implementation. The Discussant, Dr. Howard Degenholtz will discuss the implications of initiatives to address the quality of resident care.

### PARTNERING WITH NURSING HOME PROVIDERS FOR PERSON-CENTERED QUALITY IMPROVEMENT

Abigail Hermesch[1] Da Jung Chang,[2] Chelsea Goldstein[2] Kimberly Van Haitsma[3] and Katherine Abbott[3], *1. Miami University, Oxford, Ohio, United States, 2. Scripps Gerontology Center at Miami University, Oxford, Ohio, United States, 3. The Pennsylvania State University, University Park, Pennsylvania, United States*

The purpose of this study was to assist Ohio NH providers in implementing PAL Cards. Providers were recruited to utilize the recreation and leisure items from the PELI to assess resident important preferences and create PAL cards for 15-20 residents. A total of n=43 providers registered and n=26 (60%) providers completed the project, which involved monthly coaching calls and an end of project telephone interview. Participating providers were not for profit 46% (n=12), for-profit 46% (n=12), and government-owned 8% (n=2). Their average bed size was 87 and 50% (n=13) of providers had five-star ratings with a range from 1-5. Participants attempted n=439 PAL card interviews with residents and completed n=414 (94.31%) PAL Cards. Providers received over 1,428 minutes of coaching support from the Project Manager over the phone. The majority of providers were successful in implementing PAL Cards for residents with substantial support from the project manager.

### THE FEASIBILITY, ACCEPTABILITY, AND APPROPRIATENESS OF A PERSON-CENTERED COMMUNICATION TOOL

Shamatree Shakya[1] Heshuo Yu,[2] Emily Mueller[2] Kimberly Van Haitsma[3] and Katherine Abbott[4], *1. Miami University, Oxford, Ohio, United States, 2. Scripps Gerontology Center at Miami University, Oxford, Ohio, United States, 3. The Pennsylvania State University, University Park, Pennsylvania, United States, 4. Miami University: Scripps Gerontology Center, Oxford, Ohio, United States*

The purpose of this study was to assess the acceptability, feasibility, and appropriateness of the PAL Card intervention. The data for this study came from monthly logs and final calls completed by n=26 NH providers. Staff from multiple departments contributed to the PAL card implementation. Common places for placing PAL cards were wheelchairs, walkers, doors, and in closets. Over 90 % of residents approved of the accuracy of information presented in PAL cards. From the providers' perspective, PAL cards' acceptability ranged from 96 to 100%, appropriateness ranged from 93.10 to 100 % and feasibility ranged from 90 to 100%. The total staff time estimated costs for PAL card implementation ranged from $180 to $3,236 depending on the individuals involved. The PAL card intervention was deemed acceptable, feasible, and appropriate by providers and accurate by residents. A discussion of the opportunity costs associated with implementing this intervention will be discussed.

### PROVIDER PERSPECTIVES ON IMPLEMENTING A PERSON-CENTERED COMMUNICATION TOOL

Alexandra Heppner,[1] Nytasia Hicks,[1] Sarina Poth[1] Kimberly Van Haitsma[2] and Katherine Abbott[3], *1. Miami University, Oxford, Ohio, United States, 2. The Pennsylvania State University, University Park, Pennsylvania, United States, 3. Scripps Gerontology Center at Miami University, Oxford, Ohio, United States*

The PAL Card QIP Project sought to understand the intervention characteristics associated with effective implementation. Telephone interviews were conducted with n=26 NH providers who completed the project. Calls were recorded, transcribed verbatim and checked for accuracy. The Consolidated Framework for Implementation Research was utilized as an a priori coding scheme to identify factors associated with effective implementation of the PAL Card project. Major themes emerging from the data related to the evidence strength and quality of the intervention as well as the relative advantage to not assessing preferences ("turns out she doesn't even like TV and we have just been having her watch TV"), adaptability (tailoring for their needs), trialability (expanding offering of intervention after initial success), and complexity of the intervention (sharing the work across departments, difficulty using unfamiliar technology). Providers reported positive intervention characteristics of the PAL cards, however, barriers remain that require additional strategies to successfully implement.

### SESSION 695 (SYMPOSIUM)

### POLICY SERIES: WOMEN'S PLANNING DECISIONS AND CHOICES AFFECT FINANCIAL WELLNESS IN LATER LIFE

### SESSION 700 (SYMPOSIUM)

### AN ECOLOGICAL MOMENTARY OUTLOOK ON SUBJECTIVE AGING

Chair: Dikla Segel-Karpas, *University of Haifa, Haifa, Israel, Israel*
Co-Chair: Amit Shrira, *Bar Ilan University, Ramat Gan, Israel, Israel*
Discussant: Margie E. Lachman, *Brandeis University, Waltham, Massachusetts, United States*

Cross-sectional and longitudinal studies showed that subjective age – individuals' perceptions of their own age as older or younger in relation to their actual age, is an important predictor of physical, cognitive, and mental health. Despite some initial findings suggesting that subjective aging responses to variations in the daily experiences, less is known about how daily and momentary experiences shape how old people feel, and how their perceived age affects their daily experiences. In this symposium, five studies using daily diaries and experience sampling methods will be presented and discussed to explore how subjective aging affects, and is affected by, daily changes. In the first presentation, Neupert will discuss her findings regarding the covariation of anticipatory next-day health-related stressors and coping with felt-age, suggesting that forecasting and coping with future stressors play a role in subjective aging. Presenting findings from his experience-moment-sampling study, Hughes will discuss the momentary association between subjective age and mind wandering. Zhang and Segel-Karpas will present findings from studies focusing on attitudes towards aging. Zhang will focus on state vs. trait subjective aging, exploring their association with daily variability in control and competence, while Segel-Karpas will focus on the moderating role of attitudes in the daily associations between subjective age and mental health. Finally, Shrira will present a study of rehabilitation patients, finding that subjective aging is related to physical and mental health, especially for patients with high age-awareness. Professor Lachman will lead a discussion.

### A MICROLONGITUDINAL ASSESSMENT OF SUBJECTIVE AGE AND MENTAL HEALTH DURING REHABILITATION

Amit Shrira[1] Yuval Palgi[2] Noemi Heyman[3]
Oleg Zaslavsky[4] and Ehud Bodner[5], *1. Bar Ilan University, Ramat Gan, Israel, Israel, 2. University of Haifa, Haifa, Hefa, Israel, 3. Shoham Geriatric Center, Pardes Hanna-Karkur, HaZafon, Israel, 4. University of Washington, Seattle, Washington, United States, 5. Bar-Ilan University, Ramat Gan, HaMerkaz, Israel*

Recent ecological momentary assessments focused on the concomitants of daily views on aging among community-dwelling participants, yet clinical samples are underexplored. Hence, this study examined the relationships between views on aging and daily mental health during rehabilitation following osteoporotic fractures and cerebrovascular events. Measures of daily subjective age, psychological distress, and mental health were assessed among 132 older adult patients (mean age=77.9, SD=7.5, 65.9% women). Multilevel models showed that on days patients felt younger, they reported lower psychological distress and higher mental health. Time lagged analyses further showed reciprocal effects between subjective age and mental health. Finally, the subjective age-mental health covariance was stronger among patients high on age awareness. The suddenness and brutality of acute medical events highlight subjective age as an important factor in patients' wellbeing, especially among those more attentive to their age. These findings suggest that practitioners should consider interventions focused on patients' age identity.

### DAILY FLUCTUATIONS IN SUBJECTIVE AGE AND MENTAL HEALTH: THE ROLES OF CHRONOLOGICAL AGE AND ATTITUDES TO AGING

Dikla Segel-Karpas[1] Amit Shrira,[2] and Ehud Bodner[2], *1. University of Haifa, Haifa, Israel, Israel, 2. Bar-Ilan University, Ramat Gan, HaMerkaz, Israel*

Studies indicate that subjective age – individuals' perception of their own age as older or younger than their chronological age, is related to their depressive symptoms. Less is known about the role that attitudes towards aging might play in this regard. 334 participants (age 30-90, M=58.15) reported their subjective age and depressive symptoms every day for a period of 14 days. Attitudes to aging were measured at baseline. Results indicated that daily subjective age was related to daily variation in depressive symptoms. Furthermore, we found that attitudes to aging (psychosocial losses, gains and physical changes) moderated the subjective age-depression relationship, such that it was stronger when psychosocial losses were high, and when physical changes and gains were low. The moderating effect of losses was especially prominent for older participants. This indicates that the general perception of aging moderates the toll that feeling old takes on mental health.

### FEEL YOUNGER, THINK YOUNGER? THE RELATIONSHIP BETWEEN MOMENTARY FLUCTUATIONS OF SUBJECTIVE AGE AND MIND-WANDERING

Matthew L. Hughes,[1] and Dayna Touron[1], *1. The University of North Carolina at Greensboro, Greensboro, North Carolina, United States*

Subjective age is susceptible to day-to-day influence from external factors (e.g., stress), but it is not known how it fluctuates throughout the day. Mind-wandering, when one's concentration is not on the task at hand, can be induced in a laboratory, but less is known about older adults' mind-wandering in everyday life. This pre-registered study used experience sampling to investigate the relationship between mind-wandering and subjective age. Participants ages 50 years and older carried iPods for 7 days. Participants received 8 daily probes asking questions about mind-wandering and subjective age. Subjective age was assessed using an unmarked sliding scale between 0 and 120 years. For the first time, we demonstrated subjective age fluctuation within each day, with an average coefficient of variation of 12.40 (range 9.73–47.56). Participants also reported mind wandering about 34% of the time. Multilevel modeling provides insight into the relationship between everyday fluctuations of subjective age and mind-wandering.

### FORECASTING AND COPING WITH FUTURE STRESSORS PREDICTS DAILY SUBJECTIVE AGE

Shevaun D. Neupert,[1] Shevaun D. Neupert,[1] and Xianghe Zhu[1], *1. North Carolina State University, Raleigh, North Carolina, United States*

Stressors are associated with higher subjective ages, but the role of forecasting and coping with future stressors is unknown. 223 adults (107 aged 18-36, 116 aged 60-90) reported their subjective age, forecasts of next-day health stressors, and anticipatory coping with next-day health stressors each day for eight consecutive days. There was no main effect of forecasting, but increases in plan rehearsal coping were associated with increases in felt age. In contrast, increases in problem analysis coping were associated with decreases in felt age. Daily forecasting and coping also interacted with each other. On days with low plan rehearsal or low problem analysis, there was no association between forecasting of health stressors and subjective age. However, on days with high plan rehearsal or high problem analysis, increases in forecasting ratings were associated with increases in subjective age. Forecasting and coping with future stressors may play a role in subjective aging.

### TRAIT AND STATE SUBJECTIVE AGING INTERACT TO PREDICT DAILY CONTROL BELIEFS

Shenghao Zhang,[1] Shevaun D. Neupert[1], *1. North Carolina State University, Raleigh, North Carolina, United States*

We examine trait and state subjective aging as antecedents of control beliefs in older adults with a daily diary design. Adults (n=116) ranging in age from 60 to 90 (M=64.71) completed a nine-day daily diary study online. Participants reported trait aging attitudes (Attitudes Towards Own Aging; ATOA) on Day 1 and daily Awareness of Age-Related Change (AARC) of loss and gain experiences and control beliefs (Locus of Control and Perceived Competence) on Days 2-9. Controlling for demographics and known antecedents of control beliefs (health, stressors, emotional well-being, and cognition), daily increases in AARC gain were associated with increases in both Locus of Control and Perceived Competence, and a cross-level interaction revealed that Locus of Control decreased for those with more positive ATOA on days when they reported more AARC losses. Discussion will focus on interpreting the interaction between trait and state subjective aging.

### SESSION 705 (PAPER)

### BENEFITS OF PETS AND THE ARTS

### EFFECTS OF AN ART GALLERY INTERVENTION IN PEOPLE LIVING WITH DEMENTIA: A PILOT STUDY

Nathan M. D'Cunha,[1] Andrew J. McKune,[1] Stephen Isbel[1] Ekavi N. Georgousopoulou[2] Jane Kellett,[1] and Nenad Naumovski[1], *1. University of Canberra, Bruce, ACT, Australia, 2. Australian National University, Garran, ACT, Australia*

Art gallery programs tailored to the needs of people living with dementia are becoming more popular worldwide. This study aimed to observe the effects of six consecutive weekly discussion-based small group visits to the National Gallery of Australia Art and Dementia program on the salivary cortisol (SC) diurnal rhythm, salivary interleukin-6, quality of life (QoL), depressive symptoms, and cognitive function. Twenty-five participants (17 female; mean age 84.6 ± 7.27 years) completed the study with data collection at baseline, post-intervention, and at a six-week follow-up. Statistical methods were selected based on data distribution. The waking to evening (WE) SC ratio was altered (p = 0.016) (Baseline: 1.35 (1.19, 1.64), Post-intervention: 1.72 (1.54, 1.96), Follow-up: 1.44 (1.22, 1.79)) in the 22 participants who provided viable saliva samples. The WE SC ratio was higher post-intervention compared with baseline (p = 0.011), indicating a more dynamic SC rhythm, but returned to baseline levels at follow-up (p = 0.020). Interleukin-6 levels were unchanged (p = 0.664). In the total sample, no improvements in QoL (Proxy) (p = 0.165) were observed. However, self-reported depressive symptoms differed (p = 0.006), decreasing post-intervention (2.00 (1.00, 2.00)) compared with baseline (3.00 (2.00, 4.50)) (p = 0.015), and verbal fluency was affected (p = 0.027), improving from baseline (2.00 (0.00, 3.00)) to post-intervention (2.00 (0.50, 4.00)) (p = 0.027). Art and Dementia programs appear to have quantifiable benefits, including improved hypothalamic-pituitary-adrenal axis function, justifying a longer controlled trial inclusive of physiological outcomes.

### MEASURING EFFECT OF PERSONALIZED MUSIC TO REDUCE BEHAVIORS IN NURSING HOME RESIDENTS WITH DEMENTIA

Ellen McCreedy[1] Xiaofei Yang[2] Rosa Baier,[1] Kali S. Thomas[3] James Rudolph[4] Vincent Mor[1], *1. Brown University, School of Public Health, Providence, Rhode Island, United States, 2. Brown University, Providence, Rhode Island, United States, 3. Providence VA Medical Center, Providence, Rhode Island, United States, 4. Providence VA Medical Center, Center of Innovation in Long Term Services and Supports, Providence, Rhode Island, United States*

The purpose of this six-month pilot study was to identify an optimal measurement strategy for assessing the effects of a personalized music program, MUSIC & MEMORY, on agitated and aggressive behaviors among 45 nursing home residents with moderate to severe dementia. Dementia-related behaviors were measured before and after the intervention with three methods 1) observationally using the Agitation Behavior Mapping Instrument (ABMI); 2) staff report using the Cohen-Mansfield Agitation Inventory (CMAI); and 3) administratively using Minimum Data Set - Aggressive Behavior Scale (MDS-ABS). ABMI score was 4.4 (standard deviation, SD: 2.3) while not listening to the music and 1.6 (SD: 1.5) while listening to music (p<.01). CMAI score was 61.24 (SD: 16.32) before the music and 51.24 (SD: 16.05) after the music (p<.01). MDS-ABS score was .8 (SD: 1.6) before music and .7 (SD: 1.4) after music (p=.59). Direct observations were most likely to capture behavioral responses, followed by staff interviews. No effect was found using exclusively available administrative data. There is growing interest in identifying and testing non-pharmaceutical alternatives to managing agitated and aggressive behaviors in nursing home residents with dementia. Measurement occurring closest in time to the intervention was most likely to capture responses, but was also most costly, least pragmatic, and most subject to confirmation bias. These findings will inform a large pragmatic trial, beginning Spring 2019.

### PET OWNERSHIP HISTORY AND SUCCESSFUL AGING OUTCOMES IN COMMUNITY-LIVING OLDER ADULTS

Erika Friedmann[1] Nancy R. Gee[2] Eleanor M. Simonsick[3] Stephanie Studenski[4] Erik Barr,[1] Barbara Resnick,[1] Melissa H. Kitner-Triolo[5] and Alisha Hackney[1], *1. University of Maryland School of Nursing, Baltimore, Maryland, United States, 2. SUNY Fredonia, Fredonia,*

*United States, 3. Longitudinal Studies Section, Intramural Research Program, National Institute on Aging, Baltimore, Maryland, United States, 4. NIA, Longitudinal Studies Section, Baltimore, Maryland, United States, 5. NIA, BRAIN AGING & BEHAVIOR SECTION, Baltimore, Maryland, United States*

Diminishing cognitive and physical function, worsening psychological symptoms and increased mortality risk and morbidity typically accompany aging. Health needs of the aging population will continue to increase as the proportion of the population aged 50 years and older increases. Pet ownership (PO) has been linked to better health outcomes in older adults, particularly those with chronic health conditions. However, much of the evidence is weak with little known about the contribution of PO to successful aging in community living older adults. This study examines associations between PO and cognitive performance, physical functioning, and psychological status in community living older adults. Participants in the Baltimore Longitudinal Study of Aging (>50 years old, N=378) completed physical functioning, cognitive and psychological testing, and a ten-year PO history. Most participants (89%) had owned pets at some point and 24% currently have pets. Fourteen percent have 1-4 dogs, 12% have 1-4 cats, and 3% have others. PO was lower with older age (p<.001). In regression analysis controlling for age decade, pet ownership within the past 10 years (PO10) independently predicted cognitive function [total verbal learning (p=.04), and short (p=.015) and long (p=.031) delay free recall, ] but not physical function or psychological status. PO was lower in older age groups as was cognitive, physical functional, and psychological status, while PO within the past 10 years was associated with better verbal learning and memory independent of age. Longitudinal analysis is required to disentangle the sequential associations between PO and change in health status over time..

### PETS PROVIDE SIGNIFICANT SUPPORT TO OLDER ADULTS LIVING ALONE: RESULTS FROM THE NATIONAL POLL ON HEALTHY AGING

Cathleen M. Connell[1] Erica Solway[2] Preeti Malani[3] Dianne Singer[4] Matthias Kirch,[3] Jeff Kullgren,[3] and Mary R. Janevic[1], *1. University of Michigan School of Public Health, Ann Arbor, Michigan, United States, 2. Institute for Healthcare Policy and Innovation, University of Michigan, Ann Arbor, Michigan, United States, 3. University of Michigan Medical School, Ann Arbor, Michigan, United States, 4. University of Michigan, Ann Arbor, Michigan, United States*

Human-animal interaction has been linked to health and social benefits for older adults. While pets can play a positive role in healthy aging, not all pet owners experience the same benefits. The present study uses data on how pets impact the well-being of older adults, including those living alone from the National Poll on Healthy Aging (NPHA), a nationally representative household survey conducted in October 2018. A randomly selected, stratified sample of adults age 50 to 80 (n=2,051) completed the survey online. Over half reported having a pet; the majority had dogs (70%) and cats (50%). Companionship was the main reason for getting a pet (52%); the majority believed their pets helped them enjoy life, reduce stress, and connect with others. Pet owners living alone were significantly more likely than pet owners living with others to report that their pet helps them cope with physical or emotional symptoms (72% vs. 58%), feel loved (94% vs. 85%), stick to a routine (76% vs. 60%), take their mind off pain (43% vs. 32%), and have a sense of purpose (82% vs. 72%; all p<.05). Respondents living alone were also more likely to report that pet care strains their budget (26% vs. 17%) and that their pet's needs take priority over their own (22% vs. 14%; p<.05). Given the important role that pets play in the lives of older adults, efforts to support this relationship (e.g., pet-friendly housing policies, low-cost and mobile veterinary clinics, pet walking and pet sitting services) are warranted.

### THE EFFECT OF AN ARTIST-IN-RESIDENCE PROGRAM ON SELF-REPORTED LONELINESS IN OLDER ADULTS

catherine Richmond-Cullen[1], *1. the university of Scranton, Scranton, Pennsylvania, United States*

The study, funded by the Pennsylvania Council on the Arts and the Pennsylvania Department of Aging, measured the effect that an artist in residence program (conducted by state-vetted professional teaching artists) had on self-reported loneliness in older adult. All participants were aged sixty years or older and participated in programming in state-funded adult community centers located in fourteen sites throughout the Commonwealth of Pennsylvania. Artists offered 10 sessions in creating and critiquing art to older citizens in the artists' respective art forms including performing arts, visual arts and multidisciplinary/interdisciplinary arts. Through pre and post-tests, changes in loneliness were measured using the Revised UCLA Loneliness Scale. The data revealed that there was a significant correlation between a self-reported decrease in feelings of loneliness and participation in a program conducted by professional artists. . It was proposed that findings from the study could influence the quality of programs provided by state-funded adult community centers in Pennsylvania and increase funding levels to adult community centers throughout the Commonwealth of Pennsylvania.

### SESSION 710 (PAPER)

### CANCER AND END-OF-LIFE CARE

### CAN PERSONS WITH DEMENTIA MEANINGFULLY PARTICIPATE IN ADVANCE CARE PLANNING CONVERSATIONS? A MIXED-METHODS STUDY

Mi-Kyung Song[1] Sandra Ward[2] Ken Hepburn[3] Sudeshna Paul,[1] Hyejin Kim[4] Darby Morhardt[5] and Raj C. Shah[6], *1. Nell Hodgson Woodruff School of Nursing, Emory University, Atlanta, Georgia, United States, 2. University of Wisconsin-Madison, Madison, Wisconsin, United States, 3. Emory University, Atlanta, Georgia, United States, 4. Emory School of Nursing, Atlanta, Georgia, United States, 5. Northwestern University, Chicago, Illinois, United States, 6. Rush University, Chicago, Illinois, United States*

Studies of advance care planning (ACP) in persons living with dementia (PLWDs) are rare. We conducted an intervention development study to adapt an efficacious ACP

intervention, SPIRIT (Sharing Patient's Illness Representations to Increase Trust), for PLWDs in early stages and their surrogates and assessed the feasibility/acceptability of the adapted SPIRIT. SPIRIT was adapted by the investigators and underwent expert panel review. The refined SPIRIT was then evaluated in a randomized trial with 23 dyads of PLWDs and their surrogates. Dyads were randomized to SPIRIT in-person (in a private room in a memory clinic) or SPIRIT remote (via videoconferencing from home). Participants completed preparedness outcome measures (dyad congruence on goals of care, patient decisional conflict, surrogate decision-making confidence) 2-3 days postintervention along with a semi-structured interview. PLWDs' levels of articulation of end-of-life wishes during SPIRIT sessions were rated (3 = expressed wishes very coherently, 2 = somewhat coherently, 1 = unable to express wishes coherently). Fourteen PLWDs had moderate dementia, but all 23 were able to articulate their end-of-life wishes very or somewhat coherently during the SPIRIT session. While decision-making capacity was higher in PLWDs who articulated their wishes very coherently, global cognitive function did not differ by articulation levels. PLWDs and surrogates perceived SPIRIT as beneficial, but the preparedness outcomes did not change from baseline to postintervention in either group. SPIRIT for PLWDs and surrogates engaged them in meaningful ACP discussions. Further research is warranted to test its efficacy and long-term outcomes with a larger and diverse sample.

### COMBINED EFFECT OF CMV SEROPOSITIVITY AND SYSTEMIC INFLAMMATION ON DEMENTIA PREVALENCE IN CANCER SURVIVORS

Sithara Vivek[1] Bharat Thyagarajan[2] Heather Nelson,[1] Anna Prizment,[1] Eileen Crimmins[3] and Jessica Faul[4], *1. University of Minnesota, Minneapolis, Minnesota, United States, 2. Department of Laboratory Medicine and Pathology University of Minnesota; Minneapolis, Minnesota, United States, 3. Davis School of Gerontology, University of Southern California, Los Angeles, California, United States, 4. University of Michigan, Ann Arbor, Michigan, United States*

Though cancer patients treated with multi-modal therapies demonstrate higher levels of systemic inflammation, which is associated with dementia, cancer survivors have not shown a consistent association with dementia. Since several studies reported an independent association between cytomegalovirus (CMV) infection, inflammation and dementia in non-cancer populations, we have evaluated whether CMV infection and systemic inflammation were associated with increased prevalence of dementia in cancer survivors in Health and Retirement Study (HRS). We evaluated prevalence of dementia (using score ≤7 on the 27-point scale) among 1607 cancer survivors, in whom we measured CMV seropositivity and two biomarkers of systemic inflammation: C-reactive protein (CRP) and neutrophil-lymphocyte ratio (NLR). The prevalence of CMV seropositivity was 68.26% (n=1097), while prevalence of increased systemic inflammation [CRP >5mg/L and NLR >4] was 4.23% (n=68). Using survey logistic regression, adjusted for age, race, gender, BMI (Body Mass Index) and sampling design, cancer survivors who were both CMV seropositive and had increased systemic inflammation had the highest odds of dementia compared to those who were CMV seronegative and had low levels of systemic inflammation (OR=6.59; 95% CI [2.81, 15.44]; p<.0001). Cancer survivors who were CMV seropositive without evidence of systemic inflammation had a lower but increased odds of dementia (OR=2.02; 95% CI [1.17, 3.47]; p=0.01). Odds of dementia among those who were CMV seronegative with elevated systemic inflammation was not significant (p=0.09). Our study demonstrates a possible role for ongoing CMV induced inflammation in determining dementia prevalence among cancer survivors that needs further confirmation.

### DISCORDANCE IN GOALS OF CARE BETWEEN PATIENTS WITH ADVANCED CANCER AND THEIR CAREGIVERS: DOES AGE PLAY A ROLE?

Amy R. Lipson,[1] and Sara Douglas[1], *1. Case Western Reserve University, Cleveland, Ohio, United States*

Cancer is considered a family disease as the caregivers (CG's) role extends beyond providing care as they can also help facilitate treatment decisions. While much has been reported in the literature about patient (PT) goals of care (GoC), little is known about discordance between PT and CG GoC and the impact of PT age. The variables of interest were PT and CG identified GoC using a 100-point visual analog scale (VAS) with anchors of quality of life (0) and survival (100). Discordance was defined as a > 40 point difference on the VAS. The GoC data reported here were those obtained at enrollment and prior to subject's death. A sample of 235 PTs and CGs of PTs diagnosed with advanced cancers were included in the study. Mean age for the PTs was 64.7 (SD=10.5, range =21-88) with 54% being > 65. At enrollment, 28.7% of the PT-CG pairs of those PTs 65 years (X2 (1)=1.06, p=.304). At death, 61.8% (X2 (1)=31.04 <.001, Φ=.49) with discord at enrollment had discord at death. For patients who were older, 66.7% who had discord at enrollment also had discord at death and for patients

### FAMILY CAREGIVERS' COMMUNICATION NEEDS AT THE END OF LIFE OF OLDER PATIENTS AT GERIATRIC FACILITIES

Rinat Cohen[1] Gal Maydan,[2] Shai Brill[2] and Jiska Cohen-Mansfield[1], *1. Tel Aviv University, Tel Aviv, Israel, Israel, 2. Beit Rivka Geriatric Medical Center, Petach Tikva, Israel, Israel*

Current literature on end-of-life communication (EOLC) between family caregivers (FCs) and health professionals (HPs) lacks reference to FCs' communication needs and primarily addresses its formal aspects of communication such as offering advance directives. We explored FC's communication needs and developed a questionnaire to evaluate the quality of EOLC. Interviews were conducted with 152 Israeli FCs of patients from nursing care, skilled nursing care, assisted ventilation, and dementia units within four facilities (nursing homes and geriatric medical centers). Most participants were women (61%), married (78%), and were children of the patients (77%), with a mean age of 57.5 (S.D.=12.01, range: 29-88). Qualitative analysis yielded several themes: FCs' concerns about the availability and accessibility of all types of HPs, information needs (e.g., the need for regular updates initiated by HPs), FCs' need for emotional support, and difficulties stemming from differences in language and culture.

The need for improved communication in these spheres extended to all stages of hospitalization. Based on these needs, we developed a questionnaire to evaluate the quality of EOLC. Reliability was measured in a different sample, and ranged from Cronbach alpha of .916 (41 items; 41 FCs) to .937 (41 items with discrimination index greater than .3; 78 FCs). Factor analysis yielded factors similar to the themes that emerged from the qualitative analysis. The findings highlight aspects of EOLC between FCs and HPs which should be addressed and improved. Thus, this study is a crucial first step toward improving the quality of care at the end-of-life.

### SESSION 715 (PAPER)

### DIET, EXERCISE, AND WELL-BEING

### AEROBIC TRAINING, THE DEFAULT MODE NETWORK, AND COGNITION IN OLDER ADULTS WITH MILD VASCULAR COGNITIVE IMPAIRMENT

Rachel A. Crockett,[1] Chun Liang Hsu,[1] Cindy Barha,[1] Ging-Yuek Robin Hsiung,[1] and Teresa Liu-Ambrose[1], *1. University of British Columbia, Vancouver, British Columbia, Canada*

Aerobic training has been shown to be effective at improving cognitive and brain outcomes in older adults with mild subcortical ischemic vascular cognitive impairment (SIVCI). However, uncertainty remains regarding the underlying neurobiological mechanisms by which exercise elicits these improvements in cognition. Increased aberrant functional connectivity of the default mode network has been highlighted as a factor contributing to cognitive decline in older adults with cognitive impairment. Greater connectivity of the DMN at rest is associated with poorer performance on attention-demanding tasks, indicative of a lack of ability to deactivate the network on task. Our previous work on a randomized controlled trial of participants with mild SIVCI, demonstrated that 6-months of thrice weekly aerobic training led to improved global cognitive function, as measured by Alzheimer's disease Assessment Scale-Cognitive subscale (ADAS-Cog), compared with a health education program. Thus, we conducted secondary analyses to investigate whether these changes in global cognitive function were associated with changes in resting state DMN connectivity. A subsample of 21 participants underwent a resting state functional magnetic resonance imaging (fMRI) scan before and after trial completion. Change in resting state DMN connectivity was found to significantly predict change in ADAS-Cog score ($\beta = -.442$, $p=.038$) after controlling for age, intervention group, and baseline functional capacity ($R2=.467$, $F(4,16)= 3.507$, $p=.031$). These findings suggest that functional connectivity of the DMN may underlie changes in global cognitive function. Furthermore, aerobic exercise is a promising intervention by which to elicit these changes in older adults with mild SIVCI.

### COMPONENTS OF A HEALTHFUL DIET ARE ASSOCIATED WITH WAIST CIRCUMFERENCE AMONG PRE- AND POSTMENOPAUSAL WOMEN

Lisa M. Troy[1] and Sarah Witkowski[2], *1. University of Massachusetts Amherst, Amherst, Massachusetts, United States, 2. Smith College, Northampton, Massachusetts, United States*

After menopause, women are at increased risk of diabetes and cardiovascular disease. A contributing factor to increased risk may be weight gain, especially visceral adiposity. Diet plays a role in maintaining weight at all ages but less is known about the specific contributions of a healthful dietary pattern after menopause. Therefore, we evaluated associations between diet and WC as a measure of visceral adiposity. We compared 869 pre- (aged 35-45 years) and 353 post-menopausal (aged 40-65 years) women from NHANES III (1988-94). Women were pre-menopausal if they self-reported menses in the past 2 months and postmenopausal if they reported no menses in past 12 months and were aged > 40 years. Compared to premenopausal women, postmenopausal women consumed fewer Calories (-200 kcal/d) and had a higher mean waist circumference (+4.43 cm, $p=0.007$), after adjusting for age, race-ethnicity, height, physical activity, and smoking. Higher intakes of dark green vegetables ($p=0.03$) and lower intakes of potatoes ($p=0.03$), refined grains ($p=0.001$), and meats ($p=0.04$) were associated with lower WC for all women. Higher intakes of nuts and seeds and fish high in Omega-3 fatty acids were associated with smaller WC while higher intakes of poultry and dairy products were associated with higher WC in post- but not pre-menopausal women. Our findings generally support a diet high in nuts and seeds, dark green vegetables, and fish, and low in potatoes, refined grains, and meats. After menopause it may be important to incorporate fatty fish, nuts and seeds into the diet for lower visceral adiposity.

### COMPUTERIZED COGNITIVE TRAINING, WITH OR WITHOUT EXERCISE, TO PROMOTE COGNITIVE FUNCTION: A RANDOMIZED TRIAL

Lisanne F. ten Brinke,[1] John R. Best,[1] Joey L. Chan,[1] and Cheyenne Ghag[1] Kirk I. Erickson[2] Todd C. Handy,[1] Teresa Liu-Ambrose[1], *1. University of British Columbia, Vancouver, British Columbia, Canada, 2. University of Pittsburgh, Pittsburgh, Pennsylvania, United States*

Given the world's aging population, it is important to identify strategies that promote healthy cognitive aging. Computerized cognitive training (CCT) may be a promising method to combat cognitive decline in older adults. Moreover, physical exercise immediately prior to CCT might provide additional cognitive benefits. We conducted a randomized controlled trial to examine the effect of a CCT intervention, alone or preceded by physical exercise, on memory and executive functions in older adults. 124 community-dwelling older adults aged 65-85 years were randomly assigned to either 8-weeks of: 1) 3x/week group-based CCT plus 3x/week CCT sessions at home; 2) 3x/week group-based CCT combined with a 15-minute brisk walk (Ex-CCT) plus 3x/week Ex-CCT sessions at home; or 3)3x/week group-based sham exercise and education sessions (CON). At baseline and 8-weeks standard neuropsychological tests of verbal memory and learning and executive functions were administered, including the Rey Auditory Verbal Learning Test (RAVLT), Stroop test, Flanker test, Trail Making Tests (TMT B-A), and Dimensional Change Card Sort (DCCS) Test. At trial completion, there were no differences in RAVLT performance. Compared with CON, FBT and Ex-FBT participants significantly improved performance on the Stroop test

(p = .001 and p = .023, respectively). Additionally, those randomized to Ex-CCT improved performance on the Flanker test (p = .002), TMT B-A (p = .047), and the DCCS Test (p = .023) compared with BAT. These findings suggest that an 8-week CCT program could benefit executive functions, and that implementing exercise immediately prior to CCT could provide broader benefits.

### NOVEL METABOLOMICS MARKERS PREDICT 18-MONTH DECLINE IN HAND GRIP STRENGTH IN COMMUNITY-DWELLING OLDER ADULTS

Ted Kheng Siang Ng[1] Jean-Paul Kovalik,[2]
Jianhong Ching[2] Angelique Chan[3] and David Matchar[4],
*1. DUKE-National University of Singapore, Singapore, Singapore, 2. Duke-NUS Medical School, Singapore, Singapore, 3. Centre for Ageing Research and Education, Duke-NUS Medical School, Singapore, Singapore, 4. Duke-NUS Medical School, Singapore, Singapore, Singapore*

Sarcopenia that accompanies aging necessitates early detection tools, ideally before the presentation of clinically evident symptoms. The acylcarnitines (ACs) are a class of metabolites generated by cellular fuel metabolism and their predictive utility in declining muscle strength in community-dwelling older adults is unknown. We aim to examine whether baseline acylcarnitines levels can predict changes in hand grip strength over 18 months in 121 community-dwelling older adults. We measured ACs by targeted plasma metabolomics profiling. We then performed a biologically-relevant classification of these markers. Hand grip strength was measured using a Smedley spring-type dynamometer. Multivariate linear regressions were performed to examine if: 1) there was an association between ACs and hand grip strength at baseline and 2) baseline ACs could significantly predict changes in hand grip strength over an 18-month period. At baseline, AC levels were not significantly associated with hand grip strength. We found an inverse association between baseline short-chain carboxyl and dihydroxl acylcarnitines (AC-DC/-OH) levels and 18-month changes in hand grip strength (p=0.047, $\beta$=-0.548, 95% CI=-1.088 to -0.008). Notably, a specific AC-DC/-OH species, C4-DC/C6-OH, accounts for the majority of the variance. The mean difference between Malay and Chinese ethnicity is 2.28kg (p=0.042, $\beta$=2.275, 95% CI=0.084 to 4.466). These findings suggest an association between metabolic markers and deterioration in hand grip strength. These results suggest that perturbations in fuel metabolism are detectable way before the emergence of clinically evident sarcopenia and frailty. The use of AC-DC/-OH panel as antecedent biomarkers may enable clinicians to risk stratify patients in the future.

### VITAMIN K ANTAGONISM AND CHONDROCALCINOSIS IN THE OSTEOARTHRITIS INITIATIVE

M. Kyla Shea[1] Richard F. Loeser[2]
Timothy E. McAlindon[3] Sarah L. Booth[1], *1. Tufts University USDA Human Nutrition Research Center on Aging, Boston, Massachusetts, United States, 2. University of North Carolina Chapel Hill, Chapel Hill, North Carolina, United States, 3. Tufts Medical Center, Boston, Massachusetts, United States*

Calcification of articular cartilage, known as chondrocalcinosis, becomes more prevalent with age. Although chondrocalcinosis can be characteristic of osteoarthritis, it has been associated with more joint pain and disability independent of osteoarthritis severity. Identifying novel modifiable risk factors for chondrocalcinosis may help reduce joint pain and disability. One potential risk factor involves vitamin K because vitamin K-dependent proteins that inhibit calcification are present in articular cartilage. To test the hypothesis that vitamin K antagonism is associated with more chondrocalcinosis, we evaluated the cross-sectional association between warfarin use and chondrocalcinosis prevalence in the Osteoarthritis Initiative (n=1472, 60% female, mean age 64 years). Warfarin is a vitamin K antagonist that interferes with vitamin K-dependent protein function. In a secondary analysis we evaluated whether the prevalence of chondrocalcinosis differed according to dietary vitamin K intake. Chondrocalcinosis of the knee, evaluated using plain x-rays, was detected in 8% of participants. It was three times more prevalent in warfarin users (n=20) compared to non-users [prevalence ratio (95% confidence interval) (PR(95%CI)): 3.02(1.42-6.46); p=0.004, adjusted for age, sex, race, BMI]. Chondrocalcinosis prevalence did not differ according to vitamin K intake [PR(95%CI), compared to tertile 3 (≥189 mcg/d): tertile 1 (<96 mcg/d) =1.30(0.82-2.07), tertile 2 (96-189 mcg/d) =1.17(0.76-1.81), p-trend=0.623, adjusted for age, sex, race, BMI, energy intake]. That chondrocalcinosis prevalence differed according to warfarin use, but not vitamin K intake, suggests vitamin K-dependent protein function may be involved in chondrocalcinosis development. Since warfarin was not commonly used in this cohort, additional studies are needed to substantiate our findings.

### SESSION 720 (SYMPOSIUM)

### EARLY-LIFE INFLUENCES ON LATER-LIFE HEALTH

Chair: Sara M. Moorman, *Boston College, Chestnut Hill, Massachusetts, United States*
Discussant: Jacqui Smith, *University of Michigan, Ann Arbor, Michigan, United States*

This symposium identifies risk and protective factors in childhood and adolescence that continue to influence the physical and cognitive health of older adults. Dimensions of early life inequality include geographic location, educational opportunities, and total amount of adversity faced. Papers in the symposium use data from three major U.S. longitudinal studies: the Health and Retirement Study (HRS), the MacArthur Study of Midlife in the U.S. (MIDUS), and the Wisconsin Longitudinal Study (WLS). Each paper focuses on the mechanisms (i.e., mediators and moderators) linking well-being in childhood and adolescence to health in older adulthood. Greenfield, Akincigil, and Moorman find that net of IQ, college education boosts later life cognition, especially for men who had a low probability of college attendance. Kemp and Montez further find that state economic policy and adult health behavior explain later life disparities in health by educational attainment. Herd, Sicinski, and Asthana continue the theme of geographic place, finding that rural children who were raised on farms are at a cognitive disadvantage in later life. Both Ferraro and Sauerteig and Homan and Kong generate indices of total childhood misfortune. Ferraro and Sauerteig conclude that childhood misfortune affects obesity by way of adult health behavior, specifically, physical activity. Homan and Kong find that childhood misfortune affects subjective and functional health by way of the psychological mechanism of purpose in life. Together, these five papers begin to identify the ways in which experiences in childhood and adolescence have long-lasting consequences for a variety of health outcomes in later life.

### COLLEGE COMPLETION AS A PROTECTIVE FACTOR FOR LATER-LIFE COGNITION: ISSUES OF SELECTION

Emily Greenfield[1] Ayse Akincigil[2] and Sara Moorman[3],
*1. Rutgers University, New Brunswick, New Jersey, United States, 2. Rutgers, The State University of New Jersey, New Brunswick, New Jersey, United States, 3. Boston College, Chestnut Hill, New Jersey, United States*

Additional years of education is considered a modifiable protective factor against Alzheimer's disease and related dementias. However, some empirical studies have suggested that linkages between educational attainment and later life cognition are largely a function of differential selection into higher education. Our study uses data from the Wisconsin Longitudinal Study, as one of the longest-running cohort studies in the U.S., to further probe how differential selection into higher education might influence associations between college completion and later life cognition. Using adjusted inverse probability weighting and with particular attention to adolescent IQ, we find evidence that college completion is associated with better language for both men and women at age 65, as well as with better memory for men. Examining heterogeneous treatment effects, we further find that associations between college completion and later life cognition are strongest for men who were least likely as adolescents to attend college.

### GEO-LIFE COURSE DETERMINANTS OF EDUCATIONAL DISPARITIES IN U.S. ADULT HEALTH

Blakelee Kemp,[1] Blakelee R. Kemp,[1] Jennifer K. Montez[1],
*1. Syracuse University, Syracuse, New York, United States*

Educational attainment is one of the strongest social determinants of adult health. However, recent studies show that it is a stronger determinant in some areas of the country than others. This study investigates geographic and life course contexts that may explain the pattern. We merge data on adults aged 50+ in the Health and Retirement Study (1998-2014) with contextual data on their state(s) of birth and residence. We examine: (1) how the education-health association varies across regions, and (2) how childhood (e.g., poverty, compulsory schooling) and adulthood experiences (e.g., smoking, minimum wage) explain the variation. Findings reveal that the education-health association varies across regions and is more pronounced for outcomes further along in the disablement process. Poor childhood health, adult behaviors, and states' economic policies partly explain why the association varies across regions. The findings underscore the importance of geographic and life course contexts for understanding educational disparities in health.

### GROWING UP ON A FARM AND COGNITIVE FUNCTIONING IN LATER LIFE

Pamela Herd[1] Sanjay Asthana,[2] and Kamil Sicinski[2],
*1. Georgetown University, Washington, District of Columbia, United States, 2. UW-Madison, Madison, Wisconsin, United States*

There is growing interest in rural disadvantage and the implications for health and well-being in later life. We examine the relationship between living in rural areas in childhood and cognitive outcomes later in life using the Wisconsin Longitudinal Study. The WLS has prospective childhood measures of geographic status, adolescent IQ, and detailed measures of socioeconomic status, combined with later life measures of health and cognitive functioning. We find a robust relationship between rurality and lower levels of cognitive functioning, but it is explained by growing up on a farm.

### REDUCING THE IMPACT OF CHILDHOOD MISFORTUNE: THE ROLE OF ADULT PHYSICAL ACTIVITY ON LATER OBESITY

Kenneth F. Ferraro,[1] Madison R. Sauerteig,[1] and Monica M. Williams-Farrelly[1], *1. Purdue University, West Lafayette, Indiana, United States*

This study investigates the effects of childhood misfortune and adult physical activity on later-life body mass index (BMI) and waist circumference. We use ordinary least squares regression to examine the impact of childhood misfortune (30 indicators), and adult physical activity (frequency and intensity) on waist circumference and BMI ($kg/m^2$) using data from the Health and Retirement Study (N=5,732). Results emphasize that experiencing childhood misfortune is associated with a larger waist circumference and BMI in later life, while adjusting for social status and lifestyle variables. Adjusting for adult physical activity decreases the effect of childhood misfortune on waist circumference, suggesting mediation. The analysis reveals that the effects of childhood misfortune on BMI and abdominal adiposity are remediable. Although childhood misfortune is associated with larger waist circumference and BMI in later life, regular physical activity reduces the risk on both indicators of obesity.

### LONGITUDINAL HEALTH CONSEQUENCES OF CHILDHOOD ADVERSITY: THE MEDIATING ROLE OF PURPOSE IN LIFE

Kristin J. Homan[1], *1. Grove City College, Grove City, Pennsylvania, United States*

Adverse childhood experiences have long-term detrimental effects on physical health. Although biological, behavioral, and social factors have been explored as intermediate mechanisms, little research has explored psychosocial factors as potential mediators. This study examined whether purpose in life longitudinally mediates the relationship between childhood adversity and two measures of adult health (self-rated health and functional limitations). Data were obtained from 3,871 participants in the Midlife in the United States (MIDUS) study. We tested a cross-lagged mediation model from childhood adversity to adult health via purpose in life, controlling for baseline measures of health and purpose in life. Good model fit was achieved indicating that childhood adversity is associated with poorer adult health through direct and mediated paths. Childhood adversity may restrict young people's search for purpose in life, and reduced purpose in life is ultimately associated with poorer subjective health and increased functional limitations.

### SESSION 725 (SYMPOSIUM)

### ESPO/BEHAVIORAL AND SOCIAL SCIENCES: SOCIAL RELATIONSHIPS, ISOLATION, AND WELL-BEING

Chair: Jeffrey E. Stokes, *University of Massachusetts Boston, Boston, Massachusetts, United States*
Co-Chair: Elliane Irani, *Case Western Reserve University, Frances Payne Bolton School of Nursing, Cleveland, Ohio, United States*
Discussant: Patricia A. Thomas, *Case Western Reserve University School of Medicine, Cleveland, Ohio, United States*

The purpose of this symposium is twofold: (1) To present innovative research linking social relationships, isolation, and well-being among older adults, and (2) To highlight new and emerging scholars in the Behavioral and Social Sciences section of GSA. The papers in this symposium examine the repercussions of numerous relationships for well-being in later life. Huo and colleagues examine the impacts of contact with close and not-close social partners on physical activity, highlighting differences by gender. Polenick and colleagues focus on perhaps the closest of relationships in later life: marriage, analyzing longitudinal associations between discordant chronic conditions and depressive symptoms among older couples. Upenieks takes an intergenerational perspective, examining the embeddedness of adult children in older adults' networks in the context of both depression onset and chronically high depressive symptomology. This paper also highlights the consequences of well-being for older adults' social isolation, and not merely the reverse. Hladek and colleagues explore the subjective side of isolation among older adults with chronic disease, noting links between loneliness and self-efficacy that may have clinical and interventional significance. Lastly, Meinertz and Gilligan explore potential gaps in service provision that may increase rural older adults' risk of isolation and abuse. Taken together, these five papers underscore the importance of various social relationships for older adults' well-being, and suggest implications for how best to promote healthy aging. As discussant, Thomas will assess the strengths and limitations of these papers, and consider the contributions these studies – and new scholars – can make to the field.

### HIS AND HER EVERYDAY LIFE: GENDER DIFFERENCES IN OLDER ADULTS' SOCIAL AND PHYSICAL ACTIVITY

Meng Huo,[1] Karen L. Fingerman,[1] Yee To Ng[1], *1. The University of Texas at Austin, Austin, Texas, United States*

The literature links social integration to better physical health, but little research asks how contact with diverse social partners influences older adults' physical activity in a daily context. We examined this link using the Daily Experiences and Well-being Study and explored whether this link varied by gender. The sample included 175 older women and 138 older men who reported their contact with close partners (e.g., family/friend) and not-close partners (e.g., acquaintances) throughout each day across 5 days. Participants also wore Actical accelerometers to track physical activity. Multilevel models revealed significant gender differences. Older men had reduced physical activity when having contact with close partners, whereas older women maintained physical activity during such contact. Both older men and women had increased physical activity when having contact with not-close partners, but this link was stronger for men. This study advances our understanding of gender differences in older adults' social experiences and well-being.

### DISCORDANT CHRONIC CONDITIONS AND DEPRESSIVE SYMPTOMS AMONG MIDDLE-AGED AND OLDER COUPLES

Courtney A. Polenick,[1] Kira S. Birditt,[1] Angela Turkelson,[1] Benjamin Bugajski,[1] and Helen C. Kales[1], *1. University of Michigan, Ann Arbor, Michigan, United States*

Discordant chronic conditions (i.e., those with competing management requirements) have adverse consequences for well-being, yet little is known about their implications among couples. We evaluated how depressive symptoms are linked to discordant conditions within individuals and between spouses across an 8-year period. The U.S. sample included 1,116 middle-aged and older couples from five waves (2006 – 2014) of the Health and Retirement Study. Longitudinal actor-partner interdependence models controlled for age, minority status, education, depressive symptoms in the previous wave, and each partner's report of baseline marital quality and number of chronic conditions in each wave. Wives and husbands with their own discordant conditions reported higher depressive symptoms, and this association intensified over time. Over and above this link, husbands had higher depressive symptoms when there were discordant conditions between spouses. Both individual-level and couple-level discordant chronic conditions appear to have enduring implications for depressive symptoms in middle and later life.

### HOW DO MENTAL HEALTH TRANSITIONS SHAPE THE ROLE OF ADULT CHILDREN IN OLDER ADULTS' SOCIAL NETWORKS?

Laura Upenieks[1], *1. University of Toronto, Toronto, Canada, Canada*

This study considers the role of adult children in the core networks of older adults undergoing mental health change. Taking a multidimensional perspective of the network system, I consider (a) presence of child(ren), (b) contact with children network members, and (c) embeddedness of children within the network using longitudinal data from the United States. Parameters were estimated with generalized estimating equations from the pooled panel data. There was no evidence that mental health transitions lead to systematic forms of child reshuffling or increased contact with child ties. Children that remained in networks, however, showed increased contact with other members of the network when the parent underwent depression onset, but became less embedded when their parents had chronically high levels of depression. These patterns may have far-reaching consequences for older people and their children, which could include increased feelings of loneliness and social isolation for parent and child alike.

### HIGHER COPING SELF-EFFICACY ASSOCIATED WITH LOW SELF-PERCEIVED LONELINESS IN OLDER ADULTS WITH CHRONIC DISEASE

Melissa D. Hladek,[1] Paula V. Nersesian[1] Thomas K. Cudjoe[2] Jessica M. Gill[3] and Sarah L. Szanton[1], *1. Johns Hopkins*

*University School of Nursing, Baltimore, Maryland, United States, 2. Johns Hopkins University School of Medicine, Baltimore, Maryland, United States, 3. NIH, NINR, Bethesda, Maryland, United States*

Loneliness is an emotional state involving social network perceptions and linked to worse health outcomes. Coping self-efficacy evaluates confidence in ability to manage problems effectively using problem-solving, emotional regulation and social coping. The purpose of this cross-sectional study (N=151 community dwelling adults ages ≥ 65) was to evaluate associations between loneliness and coping self-efficacy. All participants had at least one chronic condition and were cognitively intact. In this sample, 32.08% were lonely (score ≥ 5 on UCLA 3-item loneliness scale (range 3-9). Higher coping self-efficacy was significantly associated with low loneliness after adjustment for age, sex, race/ethnicity, social support, depressive symptoms, body mass index, and a chronic disease-function score (β= -0.03, p=0.014). Causality could not be assessed; higher loneliness may lead to lower self-efficacy or lower self-efficacy may lead to higher loneliness. Nonetheless, loneliness and self-efficacy are both modifiable with great potential for improvement, possibly bettering health outcomes.

### ADDRESSING OLDER ADULT SOCIAL ISOLATION AND ABUSE THROUGH RURAL OUTREACH AND INTER-ORGANIZATIONAL COLLABORATION

Naomi R. Meinertz,[1] Megan Gilligan,[1] Jeongeun Lee,[1] and Louise Peitz[1], *1. Iowa State University, Ames, Iowa, United States*

Elder abuse is commonly linked with social isolation and in a rural state, such as Iowa, older adults may be at increased risk of social isolation and elder abuse. A community-based needs assessment aimed to record the first-hand perspectives of service providers regarding the needs of older adults in rural areas across the state of Iowa, covering 54 of the 99 counties. Through a survey (N=202) and focus groups (N=24), service providers, including direct care, Area Agencies on Aging, law enforcement, and attorneys, offered ways in which to address the gaps in service provision and prevention of elder abuse. Based on survey and focus groups, suggestions included ways to decrease social isolation among older adults by improving service outreach, provider training, and inter-organization communication. Discussion will outline gaps in service outreach and address future inter-organizational collaboration and strategies to prevent social isolation and elder abuse in rural communities.

### SESSION 730 (PAPER)

### FALLS: PREDICTION, PREVENTION, AND FRAILTY

### LIFE COURSE ORIGINS OF FRAILTY IN LATER LIFE

Monica Williams-Farrelly,[1] Kenneth F. Ferraro[1], *1. Purdue University, West Lafayette, Indiana, United States*

Frailty, generally characterized as a clinical state of increased vulnerability resulting from age-related decline in reserve and function across multiple physiologic systems, has been gaining attention in recent years due to its high correlates with a number of poor health outcomes including falls, hospitalization, and mortality. Similar to other adult health outcomes, research on the etiology of frailty has begun to move from proximal risk factors only to those more distal in time. This research uses data from the Health and Retirement study (2004-2016) to examine whether childhood exposures predict developing frailty in later life. A series of ordinal logistic regression models were estimated to test whether six domains of childhood exposures (socioeconomic status, infectious disease, chronic disease, impairments, risky adolescent behavior, and risky parental behavior) were associated with frailty, composed of five components: unintentional weight loss, weakness, slowness, exhaustion, and low energy expenditure (Fried et al., 2001). After adjusting for demographic factors, experiencing multiple SES misfortunes or risky adolescent behaviors in childhood are associated with higher odds of frailty in later life (OR= 1.24 and 2.37, respectively), while experiencing any infectious diseases is associated with lower odds of frailty (OR= 0.67 and 0.72). After further adjusting for adult characteristics, experiencing 2 or more chronic diseases in childhood is associated with a 1.35 higher odds of incident frailty over an 8-year period. These results reveal some of the early exposures that may raise frailty risk in later life but also the mid-life factors that mediate those risks.

### PERCEPTIONS OF EXERGAMES FOR FALLS PREVENTION AMONG SENIORS AND THERAPISTS IN ASSISTED LIVING FACILITIES

Emma K. Stanmore[1] Lex D. de jong[2] Dawn A. Skelton[3] Wytske Meekes[4] Alexandra Mavroeidi[5] and Chris Todd[1], *1. University of Manchester, Manchester, United Kingdom, 2. Curtin University, Perth, Australia, 3. Glasgow Caledonian University, Glasgow, United Kingdom, 4. University of Tilburg, Tilburg, Netherlands, 5. University of Strathclyde, Glasgow, United Kingdom*

Strength and balance exercise programmes are effective in reducing the rate and risk of falls but uptake and adherence to community based programmes are low. Exergaming technology may be a promising tool to increase exercise uptake and adherence among seniors due to their motivational features. As yet, there is little published on the views of older people and therapists of their experiences of using Exergames to reduce falls in assisted living facilities that may inform implementation. This study is the qualitative component of a cluster randomized controlled trial that investigated the use of purpose-built strength and balance Exergames in community-dwelling older people in 18 assisted-living facilities in the UK. Data collection included 36 hours' observation, semi-structured interviews with 5 physiotherapists, assistants and researchers, and 13 focus groups with residents of assisted living facilities who had participated in a 12 week Exergame programme. Transcribed data were analysed using thematic content analysis to identify themes arising from older users' and therapists' experiences of using the Exergames. Findings suggest that the senior users enjoyed using the strength and balance Exergames and reported physical, psychological and social benefits. Although some games were generally favoured, there was no overall consensus on game preferences although social components, feedback, music and colourful animation appeared to increase the appeal of the Exergames. This is the first study exploring older users and therapists' perceptions of exergames in assisted living facilities and provides valuable insight into the barriers, facilitators, contextual factors and perceived benefits or drawbacks following 12 weeks use.

### PSYCHOLOGICAL PREDICTORS OF FALLS EFFICACY AMONG FILIPINO OLDER ADULTS IN AN ELDERLY DEVELOPMENT PROGRAM

Godfrey Josef R. Torres,[1] Katrina Marie Jolejole,[1] Nephtaly Joel Botor,[1] Teri-Marie Laude[1], *1. University of the Philippines - Los Banos, Los Banos, Laguna, Philippines*

Falls, common among aging persons, typically lead to catastrophic health consequences. Studies show several factors influencing an older person's risk to falling. Depression, a psychological condition, was identified as one of these factors. With the goal of determining potential psychosocial interventions for older persons, the present study explores what other psychological variables may explain falls efficacy, i.e., perceived concern about falling. 81 older adults who were participants in an elderly development program answered a socio-demographic survey and several scales (i.e., Satisfaction with Life Scale, Flourishing Scale, Geriatric Depression Scale – Short Form, Falls Efficacy Scale – International) to measure falls efficacy and other psychological variables. Bivariate correlation revealed that falls efficacy significantly increases as family problems (r=.228, p=.045), health concerns (r=.231, p=0.040), financial difficulties (r=.345, p=.002), and depression (r=.403, p<.001) increase. Conversely, it significantly decreases as psychological well-being (r=-.255, p=.022) and perceived resilience (r=-.459, p<.001) decrease. Multiple regression analysis confirmed that while depression is a significant positive predictor, F (1,79)=15.31, p<.001, R=.403, explaining 16.2% of falls efficacy variance, anxiety-provoking situations (i.e., family problems, health worries, financial worries) also explain additional falls efficacy (6.6% variance), F (4, 71)= 5.234, p<.001, R=.477, wherein financial worry is a significant positive predictor. Furthermore, entering psychological well-being and resilience in the model adds an additional variance of 6.6%, F (6, 69)=4.533, p<.001, R=.532, but only resilience is a significant negative predictor. This paper culminates with recommendations on potential research on the psychosocial dimension of falls and possible interventions to mitigate falls among older persons.

### SOCIAL AND BIOLOGICAL PREDICTORS OF HOSPITAL ADMISSIONS FOR A FALL IN THE ENGLISH LONGITUDINAL STUDY OF AGEING (ELSA)

Jessica G. Abell[1] Camille Lassale[2] Andrew Steptoe,[1] G. David Batty,[1] and Paola Zaninotto[1], *1. University College London, London, United Kingdom, 2. IMIM (Hospital del Mar Medical Research Institute), Barcelona, Spain*

Falls are the most frequent type of accidents among older people, with one in three people aged over 65 falling each year. Falls serious enough to result in hospital admission are especially problematic, since they can lead to an increased likelihood of future disability, loss of independence, and premature mortality. Understanding the factors that may determine the risk of experiencing a fall, which requires admission to hospital, is therefore an important priority. This paper seeks to examine this issue using Hospital Episode Statistics (HES) data – administrative data from English hospitals in the National Health Service (NHS). These data have recently been linked with the English Longitudinal Study of Ageing (ELSA). We examine the association between a range of predictors (demographic, social environment, physical and mental functioning) drawn from wave 4 of ELSA with the first occurrence of hospitalisation due to an accidental fall, identified using ICD-10 codes. Analysis using Cox regression suggest a range of factors are negatively associated with admission to hospital with diagnosis of a fall, such as living alone (HR=1.42; 95% CI: 1.19, 1.68), urinary incontinence (HR=1.33; 95% CI: 1.09, 1.61) and depressive symptoms (HR=1.50; 95% CI: 1.23, 1.82). High walking speed (HR=0.30; 95% CI: 0.23, 0.39) and good hand-grip strength (HR=0.97; 95% CI: 0.96, 0.98) were found to be protective. The prevention of serious falls amongst older people will require determinants to be identified and managed effectively by health and social care services.

### SESSION 735 (SYMPOSIUM)

### GERONTOLOGICAL PEDAGOGY: PASSION, PARADOX, AND PROMISE

Chair: Sarah J. Hahn, *Mercy College, Dobbs Ferry, New York, United States*
Discussant: Rona J. Karasik, *St. Cloud State University, Saint Cloud, Minnesota, United States*

Gerontology programs in the United States are on the decline despite the need for trained professionals who can serve a growing aging population (Pelham, Schafer, Abbott, & Estes, 2012). For many students, a gerontology course may be their only formal exposure to the concepts of aging. The development of gerontological pedagogy is important, but there is limited knowledge about what is being taught around the nation and to what extent course content reflects the current scope of the discipline. This symposium explores the role of individual instructors, the larger environment, and the efforts of organizational level criteria (i.e., AGHE standards to advance the field) to promote higher-quality gerontological education. The first presenter reports dissertation findings that examined college students' self-perceptions of aging and how stereotypes impact them even after taking an introduction to gerontology course. The second presenter discusses the need for service-learning and community engagement in gerontology, while reporting on theoretical and practical suggestions, as well as potential pitfalls to avoid. The third presenter reports on student evaluations in a Master of Science program before and after the implementation of the Association for Gerontology's proposed competencies. And our final presenter provides insights on applied perspectives and pedagogical approaches in and out of the classroom, including pitfalls and possibilities. Our discussant brings our ideas together to report on the discipline of gerontology and our potential to advance to the next level of pedagogical strategies.

### THE INFLUENCE OF REVISING AN ONLINE GERONTOLOGY PROGRAM ON THE STUDENT EXPERIENCE

Kara B. Dassel[1] Katarina Felsted,[2] and Jacqueline Eaton[2], *1. University of Utah, Salt Lake City, Utah, United States, 2.*

*University of Utah College of Nursing, Salt Lake City, Utah, United States*

The University of Utah's Gerontology Interdisciplinary Program (UUGIP), a fully online program recently aligned all courses to meet the 2014 Academy of Gerontology in Higher Education (AGHE) "Gerontology Competencies for Undergraduate and Graduate Education" and to meet best practices in online teaching. These efforts led to the GIP Masters of Science program being recognized in 2017 by AGHE as a Program of Merit as well as a publication in the AGHE Journal of Gerontology & Geriatrics Education (Dassel, Eaton, & Felsted, 2018). In an effort to further this work, we analyzed student evaluations in core Master of Science program courses prior to and following these program revisions, assessed by qualitative and quantitative evaluation data from two semesters immediately prior and two semesters immediately following revisions. This presentation will discuss results, implications, and future applications of this analysis.

### COMMUNITY ENGAGEMENT AND SERVICE LEARNING: PROMISING PEDAGOGICAL PRACTICES FOR TEACHING GERONTOLOGY

Tina M. Kruger[1], *1. Indiana State University, Terre Haute, Indiana, United States*

In terms of studying human development, gerontology is unique in that most college students have not experienced this aspect of the life course yet. While personal experience cannot be generalized, our students can at least relate to the idea of being a child, an adolescent, and a young adult. What they cannot do it relate to the experience of being old, and they may have limited contact with the older adult population, with the exception of grandparents, who tend to be viewed differently from older non-relatives. One way to facilitate students connecting with the older adult population is through community engagement or service-learning (CE/SL) projects. Such projects are ripe for facilitating learning, but there are also potential pitfalls to consider. Here we discuss the need for CE/SL in gerontology, theoretical and practical suggestions, and potential pitfalls to avoid.

### GERONTOLOGICAL EDUCATION: APPLIED PERSPECTIVES AND PEDAGOGICAL APPROACHES

Kelly Niles-Yokum[1], *1. University of La Verne, La Verne, California, United States*

The art and science of gerontological pedagogy is a balance of multidisciplinary paradigms that provide students with a pathway to the depth and breadth of the field of gerontology. This session will explore a variety of issues, including possibilities and challenges within the context of pedagogical approaches to gerontology education both in and out of the classroom. Topics include applied and theoretical perspectives and the what, why, and how gerontological educators do what they do and how it can impact the learning environment not only for students but for the community at large.

### WHEN I'M 75: COLLEGE STUDENTS' SELF-PERCEPTIONS OF AGING IN AN INTRODUCTORY GERONTOLOGY COURSE

Sarah J. Hahn[1] and Jennifer Kinney[2], *1. Mercy College, Dobbs Ferry, New York, United States, 2. Miami University, Oxford, Ohio, United States*

This presentation examines college students' self-perceptions of aging using written essays from the assignment "When I'm 75" that was assigned at the beginning and end of the semester in an introductory gerontology course. Despite robust literature on people's attitudes toward aging and older adults, far less is known about attitudes toward one's own aging, especially among college students. Interpretive Phenomenological Analysis was used to analyze the students' perception of their aging experience in their written assignment. Three overarching superordinate themes were identified: challenges of aging, proactive steps to avoid negative consequences of aging, and housing considerations. Findings suggest that after completing an introductory gerontology course, students demonstrated an understanding of some age-related changes yet still had a stereotypical understanding of what it is like to be age 75. This suggests the need to engage students in moving beyond stereotypes and to better link older age with their own future experience.

### SESSION 740 (PAPER)

### LONG-TERM CARE I

### A NATIONAL ASSESSMENT OF THE RELATIONSHIP BETWEEN NURSING HOME CULTURE CHANGE AND RESIDENT OUTCOMES

Margot L. Schwartz,[1] Julie C. Lima,[2] Pedro L. Gozalo,[2] Melissa A. Clark,[2] and Susan C. Miller[2], *1. Brown University School of Public Health, Providence, Rhode Island, United States, 2. Brown University, Providence, Rhode Island, United States*

Literature is mixed regarding the relationship between Nursing Home (NH) culture change and resident outcomes, and the majority of studies are limited to small samples. We evaluated this relationship separately for five unique domains of NH culture change (physical environment, resident care, staff empowerment, leadership, and family and community involvement practices) using a 2016/2017 survey administered to a stratified-random national sample of NHs; 74% of NH administrators responded (n=1,583). We assessed the relationship between each culture change domain and 8 outcomes (calculated with MDS 3.0 and Medicare claims data) using resident-level multivariable logistic regression models, that accounted for resident and NH characteristics, and were weighted by facility-level inverse probability weights (to address NH Selection). We found the relationship between NH culture change and resident outcomes varied by culture change domain. High scores on leadership practices (i.e., two-way communication, staff involvement, education/training, respect for workers, and coaching) were most strongly associated with outcomes. Compared to the lowest quartile, performance in the highest quartile (most implementation of practices) on the leadership domain was associated with 13% lower odds (OR: 0.87, 95%CI: 0.78, 0.96) of urinary tract infections, 15% lower odds (OR: 0.85, 95%CI: 0.80, 0.91) of worsened locomotion, and 41% lower odds (OR: 0.59, 95%CI: 0.42, 0.83) of physical restraint use. For the other domains the estimates (and statistical significance) of the relationship with outcomes varied more than observed for leadership. Our findings emphasize the importance of high-quality NH leadership. Investments in improved leadership practices may result in higher-quality resident outcomes.

### NURSING HOME CULTURE CHANGE AND QUALITY OF LIFE AND HEALTH DEFICIENCIES: NATIONWIDE PANEL STUDY

Michael J. Lepore,[1] Julie C. Lima,[2] Melissa A. Clark,[2] Pedro L. Gozalo,[2] and Susan C. Miller[2], *1. RTI International, Atlanta, Georgia, United States, 2. Brown University, Providence, Rhode Island, United States*

Transforming nursing home (NH) cultures—from impersonal institutions to enriching communities where residents and employees thrive—requires multifaceted change. NHs deploy an array of culture change (CC) practices, in three core domains: resident-centered care, staff empowerment, and physical environment. This study uses novel data on CC practice from a nationally representative panel of NHs (N=1,585) surveyed in 2009/2010 and 2016/2017. To understand how changes in practice adoption may relate to quality changes we linked longitudinal data on the CC practice domain scores for resident-centered care, staff empowerment, and physical environment and on NH deficiencies relating to health and quality of life (QoL) and combined these with Certification and Survey Provider Enhanced Reporting baseline data. Multinomial logistic regressions incorporating survey weights and inverse probability of weight (to address CC selection) estimated the relative risk ratios (RRR) of increased CC practice corresponding with NHs having fewer or no change in deficiencies, versus increased deficiencies. NHs with much increase in staff empowerment scores had a higher likelihood of no change (compared to an increase) in health deficiencies (RRR 2.0; 95% CI 1.15, 3.61) and severe health deficiencies (RRR 2.03; 95% CI 1.05, 3.93). With a RRR of 1.63, NHs with any improvement in resident-centered care scores appeared to have a higher likelihood of no change (compared to an increase) in QoL deficiencies but statistical significance was not reached ($p=0.11$). This study provides some support for the benefits of CC practices, and in particular, supports the importance of staff empowerment practices in NHs.

### PAYROLL-BASED STAFFING MEASURES FOR NURSING HOMES

Christianna Williams,[1] Qing Zheng,[1] and Alan White[1], *1. Abt Associates, Inc., Durham, North Carolina, United States*

The Centers for Medicare & Medicaid Services (CMS) developed the Payroll-Based Journal (PBJ) system for nursing homes to electronically submit direct care staffing information based on payroll and other auditable data. In spring 2018, CMS started reporting PBJ-based staffing measures on Nursing Home Compare. The objective of this research is to examine nursing home staffing patterns using PBJ data. We created measures of staffing hours per resident day, using PBJ staffing information and resident census calculated from MDS assessments. We examined how PBJ staffing levels varied for different types of nursing homes and the relationship between staffing and performance on other parts of CMS's Five-Star Quality Rating System. We also examined weekday/weekend variation in staffing levels. We tracked about 15,650 nursing homes from 2017 to 2018. The average staffing level was 3.85 hours per resident day, of which 0.66 hours were for RNs. Average staffing levels were higher for smaller, non-profit, and hospital-based facilities. They were also higher for facilities with higher health inspection and quality measure ratings. Staffing levels were about 17% lower on weekends than on weekdays, and RN staffing was 38% lower on weekends. About 20% of facilities had one or more weekend day without any RN staffing in the quarter, while only 8% of facilities had any weekday without RN staffing. The use of payroll-based staffing measures improves the accuracy of the staffing information reported on Nursing Home Compare, providing consumers with additional quality-related information that can help guide their nursing home placement decisions.

### SAFETY CLIMATE IN NURSING HOMES: ARE SENIOR MANAGERS IN AGREEMENT WITH DIRECT CARE STAFF?

Emma Quach,[1] Lewis Kazis,[2] Shibei Zhao,[3] Sarah McDannold,[3] and Christine W. Hartmann[3], *1. VA Center for Healthcare Organization and Implementation Research, Bedford, Massachusetts, United States, 2. Boston University School of Public Health, Boston, Massachusetts, United States, 3. Bedford VA Medical Center, Bedford, Massachusetts, United States*

Nursing home safety climate in nursing homes reflects norms and attitudes about the safety of residents and is a key driver of safety. We investigated views of direct care staff and senior managers in 56 Department of Veterans Affairs nursing homes, because prior hospital studies reveal incongruent views of these two groups, which compromises quality of care. Each domain of the previously validated CLC Employee Survey of Attitudes about Resident Safety (CESARS) served as a dependent variable, with occupation as the major independent variable distinguishing senior managers (the reference group) from licensed nurses, nursing assistants, and clinicians/specialists. Mixed random effect models controlled for job tenure, work shift, ≤ 40 weekly work hours or more, as well as clustering effects (by VA hospitals, VA service networks, and geographic regions). We analyzed responses of 1316 direct care staff and senior managers, a 26% response rate. Senior managers were more favorable about their co-worker interactions regarding safety and their nursing home globally than each direct care staff group (small to moderate effects or ⅓ to ½ of a standard deviation) ($p < 0.05$). Direct care staff had comparable ratings on these two safety climate domains. We found incongruence between senior managers and direct care staff in their perceptions of their facility's safety. Results imply that regular and open conversations between direct care staff and senior managers around safety may keep senior managers informed about frontline safety issues and direct care staff about high-level quality improvement initiatives, bringing them closer to a mutual understanding.

### SKILLED NURSING FACILITY VALUE-BASED PURCHASING PROGRAM: DISPARATE IMPACTS

Jennifer L. Gaudet Hefele,[1] Joyce Wang,[2] and Emily Lim[2], *1. University of Massachusetts Boston, Massachusetts, United States, 2. University of Massachusetts Boston, Boston, Massachusetts, United States*

In 2018, the federal government delivered the first round of incentive payments under the Skilled Nursing Facility (SNF) Value Based Purchasing (VBP) program. Incentive payments are based on post-acute readmissions performance. Facilities received payments that were either higher, on-par, or less-than typical. Our study sought to determine whether minority- and Medicaid-serving SNFs had the same likelihood of being among the top performers/incentive payment earners compared with SNFs serving more advantaged populations. We used publicly available data on SNF-VBP performance rankings available from CMS, linked to SNF characteristics publicly available from LTCFocus.org. Using logistic regression, our three analytic models examined the likelihood of being in the top 20% of SNF-VBP performance rankings among: SNFs in which the majority (>50%) of residents are African American(AA)/Black; SNFs in which the majority of residents are Hispanic/Latino; and SNFs in which 85% or more residents are Medicaid-pay. Results show that AA/Black-serving SNFs have two-thirds the odds of being in the top 20% of rankings compared to SNFs where the majority of residents are White (OR: 0.667 p<0.001). Hispanic/Latino-serving SNFs have less than half the odds (OR: 0.447 p<.01). High-Medicaid SNFs have less than one-third the odds of being in the top compared to SNFs with no Medicaid-pay residents (OR: 0.271 p<0.0001). Results are robust to alternate specifications of racial/ethnic and payer compositions. Lower payments to SNFs serving vulnerable populations is concerning, in that fewer resources may be available to these SNFs for patient care and quality improvement, which may result in creating or exacerbating disparities.

### SESSION 745 (SYMPOSIUM)

### NEIGHBORHOOD CONTEXT AND LATER-LIFE WELL-BEING: OBJECTIVE AND SUBJECTIVE INDICATORS

Chair: Noah J. Webster, *University of Michigan, Ann Arbor, Michigan, United States*
Co-Chair: Christine Mair, *University of Maryland, Baltimore County, Baltimore, Maryland, United States*
Discussant: Malcolm Cutchin, *Wayne State University, Detroit, Michigan, United States*

Neighborhoods are known to shape well-being across the life course, particularly in later life. Yet, neighborhoods remain an underutilized focus in public health interventions due to lack of measurement specificity and understanding of mechanisms across well-being outcomes. This symposium brings together four complementary papers from social work, sociology, and psychology that incorporate multiple (objective and/or subjective) indicators of neighborhood context from national to locally-based samples to predict diverse forms of well-being. Lehning and colleagues use objective (Census) indicators of neighborhoods from the National Neighborhood Change Database, combined with data from the National Health and Aging Trends Study (N=7,197), to explore links between changing neighborhood age composition and older adults' self-rated health.. Sharifian and colleagues examine the associations between perceived neighborhood characteristics (social cohesion, physical disorder) and cognition directly and indirectly through mental health pathways (depressive symptoms, anxiety) using the Health and Retirement Study. Webster and colleagues examine links between objective (Census) and subjective (perceived safety) neighborhood indicators and social well-being using data from the Detroit-based Social Relations Study (N=259). Mair et al. combine objective neighborhood-level data with individual-level data from the Healthy Aging in Neighborhoods of Diversity across the Life Span dataset to examine direct and indirect effects of neighborhood context on obesity risk among N=2,707 middle-age and older Baltimore City residents. These papers will be discussed by Malcolm Cutchin, a health geographer and social gerontologist, who will provide an interdisciplinary reflection of neighborhood measurement and use neighborhoods as a platform to promote diverse forms of well-being in later life.

### LINKS BETWEEN NEIGHBORHOOD CONTEXT AND ACCESS TO SOCIAL CAPITAL IN LATER LIFE

Noah J. Webster,[1] Kristine J. Ajrouch,[2] and Toni C. Antonucci[1], *1. University of Michigan, Ann Arbor, Michigan, United States, 2. Eastern Michigan University, Ypsilanti, Michigan, United States*

Social capital is essential for healthy living in later life, and evidence indicates the importance of neighborhood context in facilitating access to this resource. However, additional research is needed to compare how objective and subjective indicators of context shape this association. We examine links between objective (census tract-level population density and composition) and subjective (perceived safety and quiet) indicators of neighborhoods and social capital (contact frequency, network size, trust others in neighborhood). Data include participants age 65+ (N=259) from Wave 3 (2015) of the longitudinal Social Relations Study based in Detroit, Michigan. Multivariate analyses indicate respondents living in more densely populated neighborhoods reported more frequent contact with network members. Those perceiving their neighborhood as safe reported larger networks, more trust in and greater support exchanged among neighbors. Findings suggest both objective and subjective indicators of neighborhood context shape access to social capital, but perceptions play a larger role.

### NEIGHBORHOOD ENVIRONMENTS AND OBESITY: EXPLORING PATHWAYS TO RISK OF CARDIOVASCULAR DISEASE

Christine Mair,[1] Amanda Lehning,[2] Nicole Mattocks,[2] Kyeongmo Kim,[3] Roberto Millar,[1] Michele K. Evans,[4] Alan B. Zonderman,[4] and Shari Waldstein[1], *1. University of Maryland, Baltimore County, Baltimore, Maryland, United States, 2. University of Maryland, Baltimore, Baltimore, Maryland, United States, 3. Virginia Commonwealth University, Richmond, Virginia, United States, 4. National Institute on Aging, Baltimore, Maryland, United States*

Modifying neighborhood environments to target well-established risk factors for cardiovascular disease may reduce health disparities by complementing clinical services. Prior research, however, includes limited measures of neighborhoods and does not adequately account for individual-level processes known to mediate health outcomes. We combine baseline data from the Healthy Aging in Neighborhoods of Diversity Across the Life Span (HANDLS) dataset with neighborhood-level data to yield a diverse sample of Black and white middle-aged and older residents of Baltimore City (N=2707). We use structural equation modeling to examine associations between neighborhood environments and obesity (BMI>=30), focusing on individual-level mediators. Initial direct associations between neighborhoods and obesity (e.g., presence of businesses, $\beta$=-0.062) are mediated by healthcare access and health behaviors. Additional indirect pathways exist through health behaviors (e.g., neighborhood disorder, access to parks). These findings highlight the importance of considering indirect pathways to cardiovascular health promotion among aging adults in different neighborhood contexts.

### NEIGHBORHOOD AGE COMPOSITION AND SELF-RATED HEALTH: FINDINGS FROM A NATIONALLY REPRESENTATIVE STUDY

Amanda Lehning,[1] Amanda J. Lehning,[1] Nicole Mattocks,[1] Kyeongmo Kim,[2] and Richard J. Smith[3], *1. University of Maryland, Baltimore, Maryland, United States, 2. Virgina Commonwealth University, Richmond, Virginia, United States, 3. Wayne State University, Detroit, Michigan, United States*

Neighborhood age composition is an understudied area. Furthermore, existing empirical and conceptual work is conflicting, with some indicating neighborhoods with more older adults are beneficial and other scholarship suggesting it can be detrimental. Using data from 7,197 older adults from the first wave (2011) of the National Health & Aging Trends Study combined with census tract data from the National Neighborhood Change Database, we examined the association between neighborhood age composition and self-rated health. Findings from logistic regression models indicate those living in neighborhoods with a growing concentration of older residents are significantly more likely to report lower self-rated health compared to those living in a neighborhood in which older adults overall are declining ($\beta$=1.51, $p < .05$) or are becoming diluted by younger residents ($\beta$=.66, $p < .05$). Results have implications for interventions promoting aging in place, particularly for those who may be stuck in place in age-concentrated neighborhoods.

### ANXIETY AND DEPRESSIVE SYMPTOMS MEDIATE THE LINK BETWEEN PERCEIVED NEIGHBORHOOD CHARACTERISTICS AND COGNITION

Neika Sharifian,[1] Neika Sharifian,[1] Afsara B. Zaheed,[1] Briana N. Spivey,[2] and Laura B. Zahodne[1], *1. University of Michigan, Ann Arbor, Michigan, United States, 2. Spelman College, Lithonia, Georgia, United States*

Although prior research has linked perceived neighborhood characteristics to cognition, scant research has investigated underlying mechanisms regarding how neighborhood characteristics impact cognition. One pathway, in particular, may be through mental health outcomes. Poorer neighborhood characteristics have been independently linked to greater depressive and anxiety symptoms, which may, in turn, be risk factors for cognitive decline in later life. The current study examined direct and indirect effects of perceived neighborhood characteristics (social cohesion, physical disorder) on cognitive functioning (episodic memory, executive functioning) through anxiety and depressive symptoms using longitudinal data from the Health and Retirement Study (2010–2014). Results revealed that higher social cohesion was associated with better memory and executive functioning through lower anxiety and depressive symptoms. Physical disorder was associated with worse episodic memory and executive functioning through greater anxiety symptoms. These findings highlight the importance of neighborhood context for promoting both mental and cognitive health outcomes in older adulthood.

### SESSION 750 (SYMPOSIUM)

### OLD AND FORGOTTEN? CARE FOR ELDERS IN MEXICO AND THE U.S.

Chair: Emma Aguila, *University of Southern California, Los Angeles, California, United States*
Co-Chair: Jaqueline L. Angel, *The University of Texas at Austin, Austin, Texas, United States*
Discussant: Kyriakos Markides, *University of Texas Medical Branch, Galveston, Texas, United States*

The United States and Mexico differ greatly in the organization and financing of their old-age welfare states. They also differ politically and organizationally in government response at all levels to the needs of low-income and frail citizens. While both countries are aging rapidly, Mexico faces more serious challenges in old-age support that arise from a less developed old-age welfare state and economy. For Mexico, financial support and medical care for older low-income citizens are universal rights, however, limited fiscal resources for a large low-income population create inevitable competition among the old and the young alike. Although the United States has a more developed economy and well-developed Social Security and health care financing systems for the elderly, older Mexican-origin individuals in the U.S. do not necessarily benefit fully from these programs. These institutional and financial problems to aging are compounded in both countries by longer life spans, smaller families, as well as changing gender roles and cultural norms. In this interdisciplinary panel, the authors of five papers deal with the following topics: (1) an analysis of old age health and dependency conditions, the supply of aging and disability services, and related norms and policies, including the role of the government and the private sector; (2) a binational comparison of federal safety net programs for low-income elderly in U.S. and Mexico; (3) when strangers become family: the role of civil society in addressing the needs of aging populations; and (4) unmet needs for dementia care for Latinos in the Hispanic-EPESE.

### CARE DEPENDENCY IN OLD AGE IN MEXICO: SITUATIONAL ANALYSIS, IMPLICATIONS, AND RECOMMENDATIONS

Mariana López-Ortega[1] and
Luis Miguel F. Gutiérrez Robledo[2], *1. National Institute of Geriatrics, Mexico City, Mexico, 2. National Institute of Geriatrics, Mexico City, Distrito Federal, Mexico*

The demographic transition, population aging, and the epidemiological transition in Mexico have modified the burden of the disease profile, and impose important economic, social, and health care challenges, particularly old-age and disability-related care dependency. We describe old age health and dependency conditions, and analyze the supply of services to dependent population, including aging and disability norms and policies, the role of the government and the private sector (profit & non-profit) in the provision of related services. Based on the analysis, it is clear that public strategies for care dependency are practically non-existent and that policies aimed at these populations focus on guaranteeing access to basic health services, an economic income, and social integration. Also, there are no support strategies for family caregivers who currently carry most of the care work. Care dependency strategies to support those who need them as well as those who currently provide them are urgently needed.

### A BI-NATIONAL COMPARISON OF FEDERAL SAFETY NET PROGRAMS FOR LOW-INCOME ELDERLY IN THE U.S. AND MEXICO

Emma Aguila,[1] and Jaqueline L. Angel[2], *1. University of Southern California, Los Angeles, California, United States, 2. University of Texas at Austin, Austin, Texas, United States*

Population aging in Mexico as in the United States is expected to accelerate over the next thirty years, and the proportion of individuals 65 and older will triple to approximately 20 percent by 2050 in both nations. Older people of Mexican origin are at high risk of protracted periods of poor health, a reality exacerbated by poverty. We use the Health and Retirement Study (HRS 2012-2014, N= 2,575) and Mexican Health and Aging Study (MHAS 2012-2015; N=16,131) to compare profiles of older Mexican-origin recipients of income supplements. We find Mexican immigrants are lower-income, less healthy, and less likely to receive supplements than Mexican origin in U.S. In contrast, return migrants are more likely to receive supplements than non-migrants in Mexico. Income supplement recipients are more likely to receive Medicaid and Seguro Popular. We discuss implications of financing safety net programs and the potential dependency burden in two countries aging rapidly.

### WHEN STRANGERS BECOME FAMILY: THE ROLE OF CIVIL SOCIETY IN ADDRESSING THE NEEDS OF AGING POPULATION

Ronald J. Angel[1], *1. University of Texas, Austin, Austin, Texas, United States*

Mexico's rapidly aging population presents serious short and long-term challenges to the state and to families, since relatively few individuals have formal retirement plans. Although health care access is formally universal, and non-contributory retirement income is provided to all elderly in need, the Mexican old-age welfare remains limited. In this study we assess the potential role of civil society organizations (CSOs), a category that includes secular non-governmental organizations (NGOs), faith-based organizations (FBOs), in advocacy for and service provision to elders in need. Social and demographic changes, including the migration of children away from their parent's community, the need for women to work, smaller families, and more are undermining the capacity of the family to provide all of the care and support that frail aging parents need. Given the fact that the federal, state, and municipal governments are limited in what they can provide, the role of CSOs is potentially significant.

### MIDLIFE FACTORS, GENDER, AND MIGRATION ARE KEY PREDICTORS OF COGNITIVE HEALTH IN LATE LIFE

Sunshine Rote[1] and Jaqueline L. Angel[2], *1. UNIVERSITY OF LOUISVILLE, LOUISVILLE, Kentucky, United States, 2. University of Texas at Austin, Austin, Texas, United States*

Older Latinos are at 1.5 times greater risk for Alzheimer's disease and other dementias than non-Latino Whites (Wu et al., 2018) and there is evidence of high levels of cognitive impairment, dementia, and dementia-related neuropsychiatric symptoms among older Mexican Americans in particular (Rote et al., 2015). We use data from the Hispanic Established Population for the Epidemiologic Study of the Elderly (HEPESE, 1993/94- 2010/11, N=2,665), a national study of Mexican American 65 years and older residing in the southwestern U.S. Older adults who were in the paid labor force, except for those who worked in the agricultural sector, exhibit lower risk for cognitive impairment. The results hold for both women and men. For family size, number of children is associated with greater risk for cognitive impairment and this is especially evident among women and immigrant men. Midlife factors, gender, and migration are key predictors of cognitive health in late life.

### SESSION 755 (SYMPOSIUM)

### OPTIMAL HEALTH PROFESSIONAL STAFFING OF PRIMARY CARE AND GERIATRIC PRACTICES SERVING FRAIL OLDER ADULTS

Chair: Karen Donelan, *Massachusetts General Hospital, Boston, Massachusetts, United States*
Discussant: Joanne Spetz, *University of California, San Francisco, San Francisco, California, United States*

This symposium will include 3 papers that provide critical interprofessional and interdisciplinary perspectives on our work to understand and measure staffing in health care teams caring for older adults, and frail older adults. The Health Teams for Frail Elders project was funded by the Gordon and Betty Moore Foundation from August 2016 to October 2018. Dr. Karen Donelan, project Principal Investigator, will chair the session, providing a brief project overview of project aims and activities. A survey and health services researcher, Dr. Donelan will set the context for this large scale project. Dr. Barbara Roberge, a geriatric nurse practitioner who established one of the first senior health programs in the nation along with Dr. Kenneth Minaker at Mass General Hospital, was our primary care and nursing lead on our site visits. She will talk about the care settings we visited, her development of a site assessment tool that covered a range of frail elder needs, and will summarize professional roles and staffing observed within different site types. Dr. Julie Berrett-Abebe, a junior investigator on our team (PhD 2017), will present a paper on the competencies and roles of social workers and community health workers in primary and geriatric practices, as well as the roles of community health workers. Dr. David Auerbach, a national expert in health policy and workforce analysis, will present 4 models of staffing of practices, demonstrating efficiencies in optimizing services for frail elders while minimizing costs.Dr. Joanne Spetz will be the discussant of cross-cutting themes.

### OPTIMAL STAFFING MODELS TO CARE FOR FRAIL ELDERLY ADULTS IN PRIMARY CARE AND GERIATRIC PRACTICES

David I. Auerbach,[1] Carie Michael,[2] Douglas Levy,[2] Peter Maramaldi,[3] Robert Dittus,[4] Joann Spetz,[5] Peter Buerhaus,[6] and Karen Donelan[7], *1. Montana State University, Bozeman, Montana, United States, 2. Massachusetts General Hospital, Boston, Massachusetts, United States, 3. Simmons College School of Social Work, Boston, Massachusetts, United States, 4. Institute for Medicine and Public Health at Vanderbilt University, Nashville, Tennessee, United States, 5. Institute for Health Policy Studies at the University of California, San Francisco,, San Francisco, Massachusetts, United States, 6. Center for Interdisciplinary Health Workforce Studies at the College of Nursing, Montana State University, Bozeman, Montana, United States, 7. The Mongan Institute, Health Policy Research Center, Boston, Massachusetts, United States*

As the US population ages, primary care is expected to be the health care “home” for older adults, and several initiatives are aimed at helping to transform primary care practice to care for this population. Wide variation in staffing has been observed. Meyers et al proposed ideal models of primary care staffing for a general population and for a frail elderly population (2018). We developed the 2018 Survey of Primary Care and Geriatric Clinicians to measure optimal team configuration in clinical practices caring for older adults. A majority employed NPs, MDs and PAs, with [r = -.53] between % of clinician labor of NPs and physicians). High-NP practices are more likely located in states with full scope of practice, perform well for frail elders and are less expensive. Meyers' models, with fewer physicians, more SW and CHWs, more RNs, perform better for frail elders, and are less expensive.

### CLINICIAN PERCEPTIONS OF SOCIAL WORKERS AND COMMUNITY HEALTH WORKERS IN PRACTICES CARING FOR FRAIL ELDERS

Julie Berrett-Abebe,[1] Karen Donelan,[1] David I. Auerbach,[2] Peter Maramaldi,[3] and Barbara Berkman[4], *1. Massachusetts General Hospital, Boston, Massachusetts, United States, 2. Montana State University, Newton, Massachusetts, United States, 3. Simmons University, Boston, Massachusetts, United States, 4. Columbia University, Boston, Massachusetts, United States*

Social workers (SW) and community health workers (CHW) have emerged as key workforce personnel in efforts to care for elders in the U.S. However, little is known about the presence and roles of these professionals in outpatient practices. This paper presents findings from a nationally representative survey of geriatrics and primary care practices. Key findings include: reported challenges in meeting the social service needs of elders, underutilization of SW, and fuller utilization of social work competencies in practices in which both SW and CHW were present. These findings offer a unique perspective of SW on interprofessional teams and have implications for the future of the profession.

### PROFESSIONAL STAFFING AND ROLES IN CARE TEAMS SERVING FRAIL ELDERS LIVING IN THE COMMUNITY: 22 SITE VISITS

Barbara Roberge,[1] Carie Michael,[2] David I. Auerbach,[3] Peter Maramaldi,[4] and Karen Donelan[2], *1. Massachusetts General Hospital, Boston, Massachusetts, United States, 2. The Mongan Institute, Health Policy Research Center, Boston, Massachusetts, United States, 3. Massachusetts Health Policy Commission, Boston, Massachusetts, United States, 4. Simmons College School of Social Work, Boston, Massachusetts, United States*

In 2017, as part of a study to understand the evolving roles of nurses, physicians and social workers in leading and working in teams, our interprofessional team explored 22 sites of care for frail elderly adults in five US regions (Chicago IL, Denver CO, Tampa/Orlando FL, San Diego

CA, New England).. The purpose of these site visits was to understand the current range of models of care for frail elders living in community, the roles of health professionals within those care models, and to inform national measure development. We selected regions based on elder population density, scope of NP practice, and screened over 100 sites to identify physician, nurse and social work led teams. We included general primary care, PACE, academic geriatrics, home based primary care, assisted living, FQCHC, palliative care, mobile health. We interviewed 108 key informants. We found considerable variation in staffing/elders within each site type.

### SESSION 760 (SYMPOSIUM)

### POLICY SERIES: POLICY UPDATE: OLDER ADULT NUTRITION AND MALNUTRITION

Chair: Meredith Whitmire, *Matz, Blancato & Associates, Washington, District of Columbia, United States*
Co-Chair: Robert Blancato, *Matz, Blancato & Associates, Washington, District of Columbia, United States*

This symposium will provide an update on older adult nutrition policy, including background on the issues of older adult malnutrition and food insecurity. The federal policy update will include discussion of the Older Americans Act nutrition programs and their reauthorization progress, older adult programs under the US Department of Agriculture, and advances in nutrition services being made in healthcare programs such as Medicare Advantage and managed long-term care services and supports. It will also discuss funding for federal older adult nutrition programs and their sustainability moving forward.

### OLDER ADULT MALNUTRITION: A GROWING CRISIS

Meredith Whitmire[1], *1. Matz, Blancato and Associates, Washington, District of Columbia, United States*

Malnutrition, a caloric or nutrient imbalance, is particularly common in the older adult population, and food insecurity is a rising concern. This paper will discuss the incidence of malnutrition and food insecurity in the older adult population. It will also discuss federal and state programs in place that work to prevent and combat malnutrition. It will provide strategies for aging network members to engage in these policy discussions.

### FEDERAL NUTRITION POLICIES AND PROGRAMS

Robert Blancato[1], *1. Matz, Blancato & Associates, Washington, District of Columbia, United States*

This paper will discuss the Older Americans Act nutrition programs and their reauthorization progress, older adult programs under the US Department of Agriculture, and advances in nutrition services being made in healthcare programs such as Medicare Advantage and managed long-term care services and supports. It will also discuss funding for federal older adult nutrition programs and their sustainability moving forward.

### SESSION 765 (SYMPOSIUM)

### PROBING THE COMPLEX INTERACTIONS BETWEEN DIET, DISEASE, AND AGING

Chair: Rozalyn Anderson, *University of Wisconsin Madison, Madison, United States*

Nutrient response pathways are conserved modifiers of longevity, and dietary restriction is the most studied intervention for slowing aging in laboratory animals. For many years it was believed that lifespan extension from dietary restriction was tightly linked to total caloric intake. Recent evidence suggests that the interaction between diet and aging is more complex than this, however, with nutrient sensing, dietary composition, and circadian components all playing a role. This symposium will delve into some of the complex biological interactions linking food intake, lifespan, and diseases of aging.

### METABOLIC INTEGRITY – A FACTOR IN AGING AND A PLAYER IN THE MECHANISMS OF CR

Rozalyn Anderson[1], *1. University of Wisconsin Madison, Madison, United States*

An emerging paradigm in aging research identifies metabolic dysfunction as a root cause in age-related disease vulnerability. Several diseases of aging, including diabetes, cancer, and neurodegeneration, have an established metabolic component. Our studies have focused on links between metabolic status and disease vulnerability. Caloric restriction (CR) delays aging and the onset of age-related disease in diverse species, including nonhuman primates. Molecular profiling identifies CR responsive elements in the transcriptome, proteome, and metabolome that are highly enriched for metabolic pathways and in particular mitochondrial processes. These data show that improvements in health and survival are associated with maintenance of system wide metabolic homeostasis and preserved energy metabolism among tissues. Metabolic biomarkers identified in these studies may be clinically relevant for the early identification of elevated disease risk in humans and could even be potential targets for the development of novel strategies to lower disease vulnerability as a function of age.

### NOVEL METHIONINE-RELATED INTERVENTIONS THAT CONFER HEALTHSPAN BENEFITS TO YEAST AND RODENTS

Jay E. Johnson,[1] Jason D. Plummer,[1] Spike D. Postnikoff,[2] Jessica K. Tyler,[2] and Jay E. Johnson[1], *1. Orentreich Foundation for the Advancement of Science, Cold Spring, New York, United States, 2. Weill Cornell Medicine, New York, New York, United States*

Methionine restriction (MR) is one of only a few dietary manipulations known to robustly extend healthspan in mammals. Methionine-restricted rodents are up to 45% longer-lived than control-fed littermates and a number of studies suggest that humans may also benefit from MR. While a methionine-restricted human diet is technically feasible, compliance to such a regimen might not be practical or desirable. Therefore, an important goal is to identify and/or develop more facile dietary interventions, or preferably, pharmacological agents that mimic MR. Towards this end, we have made use of the yeast chronological lifespan and replicative lifespan assays, which serve as models of aging in quiescent and mitotic cells, respectively. Importantly, our lab and others have demonstrated that MR dramatically extends yeast lifespan. Here we show work aimed at developing novel MR-like interventions that extend yeast lifespan, as well as preliminary data demonstrating that such interventions significantly improve the healthspan of mice.

### A MEDITERRANEAN DIET INTERVENTION ALTERS AGE-ASSOCIATED PHYSIOLOGY IN A NOVEL NON-HUMAN PRIMATE MODEL

Noah Snyder-Mackler,[1] Carol Shively,[2] Corbin Johnson,[1] Kristopfer Michalson,[2] Susan Appt,[2] Daniel Belsky,[3] and Thomas Register[2], *1. University of Washington, Seattle, Washington, United States, 2. Wake Forest University, Winston-Salem, North Carolina, United States, 3. Columbia University Mailman School of Public Health, New York, New York, United States*

Diet modifications are some of the most well-established aging interventions. For decades we have known that caloric restriction can dramatically increase lifespan and healthspan in organisms ranging from yeast to primates. More recently, other dietary modifications, including varying nutrient composition, have been experimentally shown to alter healthspan and lifespan. However, limitations inherent in human trials, such as diet adherence and heterogeneity of other lifestyle factors, mitigate our ability to identify the mechanisms through which diet alters healthspan and lifespan. Here, we conducted a randomized, long-term, whole-diet manipulation in a nonhuman primate, where cynomolgus macaques consumed either a Mediterranean or Western diet for 15 months. We hypothesized that individuals fed a Western diet would exhibit accelerated rates of cellular and physiological aging relative to their Mediterranean-fed counterparts. Indeed, we found that Western diet-fed animals exhibited increases in physiological measures that also increase with age, including body weight, fasting insulin, and triglycerides. Animals eating a Mediterranean diet, on the other hand, had a more sensitive and tuned autonomic response, and reduced HPA responses to an acute stress challenge. Probing further, we found that diet strongly affected monocyte function, altering the expression of 40% of expressed genes, leading to a more proinflammatory monocyte phenotype in Western diet fed animals. Experiments are underway to explore effects of diet on other markers of biological aging. Together, these data provide the first controlled evidence that Western and Mediterranean diets can alter aging-associated function in a species with clear biological similarity and relevance to humans.

### A SCREEN OF WILD-DERIVED FLY STRAINS REVEALS CG34351 AS A REGULATOR OF TOXIC EFFECTS OF A RICH DIET

Rachel Brem[1], *1. Buck Institute for Research on Aging, Novato, California, United States*

Dietary restriction (DR) extends lifespan and healthspan, by mechanisms that remain incompletely understood. To identify new genes with a role in dietary response, we developed a screening strategy harnessing genetic variation among wild fruit flies. We quantified the lifespan of lines of the Drosophila Genetic Reference Panel on protein-rich and DR diets, and mapped polymorphisms associated with diet-dependent longevity. Among the hits was CG34351/decima, an ortholog of the mammalian GABA-receptor regulator RGS7BP. In validation experiments, pan-neuronal knockdown of decima extended lifespan and elicited repression of Drosophila insulin-like peptides, in flies on the rich diet but not on DR. Knockdown of decima in GABA neurons rescued fat storage and energy availability defects that derive from overnutrition. We propose that decima tunes signaling in these neurons upstream of the insulin pathway to rewire metabolism. These results establish decima as a determinant of the toxic effects of the rich diet in the fly.

### SESSION 770 (SYMPOSIUM)

### PROMOTING SUCCESSFUL CHRONIC DISEASE SELF-MANAGEMENT THROUGH COMMUNITY-BASED INTERVENTIONS

Chair: Tiffany R. Washington, *University of Georgia, Athens, Georgia, United States*
Co-Chair: Laura N. Gitlin, *Drexel University, Philadephia, Pennsylvania, United States*
Discussant: Matthew L. Smith, *Texas A&M University, College Station, Texas, United States*

Persistent chronic conditions are among the top leading causes of death in the U.S. The majority of older adults live with two or more chronic conditions. When poorly managed, chronic conditions can result in negative psychosocial and health outcomes such as low quality of life, diminished mood, workforce withdrawal, high healthcare utilization, and disability. Fortunately, chronic disease self-management interventions show promise in reducing the negative impact of chronic conditions on health status. This symposium will highlight formative work and recent findings of community-based interventions that promote successful self-management. To start, presenter one will describe findings from four studies that constitute the formative work necessary to inform implementation of self-management interventions in community and healthcare settings. Next, presenter two will report findings from an effectiveness trial of a technology-based intervention to support hypertension self-management. Then, presenter three will describe findings on the feasibility of a Health Passport tool to promote self-health management among individuals with physical limitations. Finally, presenter four will describe factors affecting attendance among African Americans with arthritis who participated in a national dissemination of Chronic Disease Self-Management Education programs. Collectively, these presentations will provide practical evidence and science-based recommendations for ways to increase successful chronic disease self-management and ultimately improve population health among older adults.

### SELF-MANAGEMENT INTERVENTIONS IN DIALYSIS FACILITIES

Tiffany R. Washington,[1] Chivon Mingo,[2] and Matthew L. Smith[3], *1. University of Georgia, Athens, Georgia, United States, 2. Georgia State University, Atlanta, Georgia, United States, 3. Texas A&M University, College Station, Texas, United States*

There have been few attempts to implement self-management interventions in dialysis facilities. This paper presentation describes four studies that constitute the formative work necessary to inform implementation. Studies one and two examined the relationship between psychosocial factors and kidney disease self-management, finding depression and cognitive decline to undermine self-management behaviors. Study three examined how dialysis patients define and think about self-management and their interest in participating in a self-management program. Among them, 78% affirmed they would participate in a self-management program if it were offered in their facility. Finally, when examining organizational readiness to implement a self-management intervention in dialysis facilities, staff perceived it to be consistent with facility values, and agreed they were well-positioned to implement the program. Taken together, these findings suggest a self-management intervention would be beneficial and supported, but would require thoughtful implementation considerations. These studies have implications for developmental self-management research with other chronic conditions.

### CHRONIC DISEASE SELF-MANAGEMENT PROGRAM ATTENDANCE AMONG AFRICAN AMERICANS WITH ARTHRITIS AND COMORBIDITIES

Chivon A. Mingo,[1] Tiffany R. Washington,[2] and Matthew L. Smith[3], *1. Georgia State University, Atlanta, Georgia, United States, 2. University Of Georgia, Athens, Georgia, United States, 3. Texas A&M University, College Station, Texas, United States*

African Americans (AA) are 17% less likely to be diagnosed with arthritis compared to Whites, yet disproportionately burdened by arthritis symptoms. AA are overrepresented in the diagnosis and burden of other chronic conditions (diabetes, heart disease), highlighting the need to engage them in evidence-based Chronic Disease Self-Management Education (CDSME) Programs. This study examines how disease profiles may influence program attendance. Using a multinomial logistic regression, data were analyzed from AA with arthritis (N=20,541) who attended CDSME programs in 48 states. Relative to those with only arthritis, participants with more complex disease profiles were less likely to attend an arthritis-specific program (P<0.001) and more likely to attend a diabetes-specific program (P<0.001). Those with more complex disease profiles were more likely to attend programs at healthcare organizations and residential facilities, and less likely to attend in faith-based organizations (P<0.001). Understanding barriers and facilitators to program attendance have policy and public health implications.

### A COMMUNITY AND TECH-BASED APPROACH FOR HYPERTENSION SELF-MANAGEMENT (COACHMAN)

Carolyn H. Still,[1] Shirley M. Moore,[2] Abdus Sattar,[3] and Jackson T. Wright Jr[3], *1. Case Western Reserve University, Cleveland, Ohio, United States, 2. Case Western Reserve University, Frances Payne Bolton School of Nursing, Cleveland, Ohio, United States, 3. Case Western Reserve University, School of Medicine, Cleveland, Ohio, United States*

African American (AAs) are disproportionately affected by hypertension. Developing effective outreach programs with community partners is a major public health priority and ideal to educate, empower, and offer support to self-manage hypertension in AAs. The purpose of this pilot is to investigate the effectiveness of a community outreach program using a technology-based intervention for hypertension self-management (COACHMAN) to improve blood pressure (BP) control. Forty AAs with hypertension will be randomly assigned to COACHMAN or enhanced usual care (EUC). COACHMAN is comprised of four components: self-monitoring of BP; web-based education; nurse counseling; and training on a medication management application. The primary outcome is change in BP from baseline to 3-months. We hypothesize that participants in COACHMAN (compared to EUC) will have better BP control. Findings from this study, if confirmed, will provide knowledge to the scarce literature available on technology-based interventions appropriate to help AAs self-manage hypertension and improve BP control.

### COMPREHENSIVE HEALTH ASSESSMENT AND MOTIVATION PROGRAM FOR PEOPLE AGING WITH DISABILITIES (CHAMP-D)

Patricia C. Heyn,[1] and James J. Carollo[2], *1. University of Colorado Denver Anschutz Medical Campus, Aurora, Colorado, United States, 2. Childrens Hospital Colorado, Aurora, Colorado, United States*

We developed a Comprehensive Health Assessment and Motivation program (CHAMP-D) focused on self-health promotion and management for people aging with disabilities. The goal of the CHAMP-D is to enhance healthy lifestyle, self-health management and communications with the care team by a person-centered Health Passport tool. All study participants underwent comprehensive health, physical, and blood laboratory evaluation. CHAMP-D results were formatted into an easy to follow health passport. A follow-up survey evaluated the CHAMP-D on 59 respondents and 77% found the recommendations to be achievable and reported improved quality of life (QoL). Other improvements were noted in in self-care (54%), physical activity (49%), and diet (24%). 51% of the participants shared the Health Passport with their PCP. Overall, the CHAMP-D had a positive impact on participants' self-reported health, self-care, and wellbeing. Empowering individuals with disabilities to take an active role in self-managing their health is feasible and can impact their QoL.

### SESSION 775 (SYMPOSIUM)

### PSYCHOSOCIAL AND CULTURAL PAIN EXPERIENCES AND SELF-MANAGEMENT ACROSS DIVERSE OLDER ADULT POPULATIONS

Chair: Staja Booker, *The University of Florida, Gainesville, Florida, United States*
Discussant: Keesha Roach, *The University of Florida, Gainesville, Florida, United States*

Racial/ethnic minority older adults experience a disproportionate burden of functionally-disabling chronic pain. However, minimal research in pain and aging has fully explicated the unique and endemic psychosocial and cultural factors that strongly influence appraisal, communication, and management/coping of pain. Yet, to fully engage with and care for diverse racial/ethnic older adults, intentional responsiveness to these factors is necessary. This symposium features under-represented racial/ethnic older adult populations and multiple methodologies, including advanced imaging techniques, to understand various psychosocial and cultural factors associated with chronic pain. Our first presenter, Dr. Lor, uses qualitative inquiry to examine pain-associated language and expression of pain in Hmong older adults, which is often laden with stress and misunderstanding. Following is Dr. Taylor who will discuss the mediating effect of stress and coping on bodily pain in inner-city Black older adults. Dr. Terry will present novel findings on the association between catastrophizing (i.e., negative cognitive and emotional response to actual or anticipated pain resulting in feelings of helplessness) and brain structure in non-Hispanic Black and White adults with or at high-risk for knee osteoarthritis pain. This presentation will segway into culturally-relevant pain self-management practices and the role of social support specifically for Blacks from urban Detroit, as presented by Dr. Janevic. We will conclude with Dr. Booker presenting mixed-level data on the lack of familial and social networks and provider support for osteoarthritis pain self-management in Southern-dwelling older Blacks. This symposium extends the knowledge on the nuanced complexity of biopsychosocial and cultural dynamics underlying the pain experience.

### OH, YOU JUST GOT ARTHRITIS: OLDER AFRICAN AMERICANS LACK FAMILIAL, SOCIAL, AND PROVIDER SUPPORT FOR PAIN MANAGEMENT

Staja Booker,[1] Keela Herr,[2] and Toni Tripp-Reimer[2], *1. The University of Florida, Gainesville, Florida, United States, 2. The University of Iowa College of Nursing, Iowa City, Iowa, United States*

Self-management support from family, friends, and providers is a crucial element in controlling osteoarthritis pain. 110 African-Americans (50-94 years) were surveyed regarding social and provider self-management support, and 18 of the African-American participants were also individually interviewed. This mixed-methods analysis unveiled that 77% were not receiving familial/social or provider support, and a conventional qualitative content analysis confirmed the lack of expected support for self-management with sentiments such as “I'm doin' this all on my own.” Nonetheless, older African-Americans respected providers' professional opinion, and 82% believed that treatment from a provider would be helpful. They desired more education and treatment options because they “need somebody to help with these joints and muscles”. However, participants were forced to learn how to care for osteoarthritis pain: “I was taking pain medication, but when I went to the doctor last time he told me to stop… Told me to deal with it [pain].”

### CHRONIC PAIN SELF-MANAGEMENT PRACTICES AND PREFERENCES AMONG URBAN AFRICAN AMERICAN OLDER ADULTS

Mary R. Janevic,[1] Mary Janevic,[1] Sheria Robinson-Lane,[2] Susan Murphy,[3] and John Piette[4], *1. University of Michigan School of Public Health, Ann Arbor, Michigan, United States, 2. University of Michigan School of Nursing, Ann Arbor, Michigan, United States, 3. University of Michigan Medical School, Ann Arbor, Michigan, United States, 4. University of Michigan Sch Public Health, ann Arbor, Michigan, United States*

African American older adults experience disproportionate burden from disabling chronic pain. Pain self-management interventions for this group are most effective when they integrate culturally-relevant preferences into intervention design. In the STEPS pilot trial, we collected focus group (n=23) and survey (n=57) data from African Americans age 60+ years about pain-management practices. Participants were recruited from the community and reported pain for 3+ months, with intensity >4 (0 to 10 scale). The most frequently-used pain-management strategies were exercise (75%) and prayer/Bible reading (74%). Also commonly used were healthy eating (61%), OTC medications (65%), and herbal supplements (51%). Focus group themes provided more nuanced information, including reasons for avoiding prescription pain medications, positive experiences with topical treatments, the value of movement, and the role of social support. Findings reveal strong engagement in pain self-care in this population. Interventions can build on existing practices by incorporating spirituality and appealing options for physical activity.

### THE RELATIONSHIP BETWEEN PAIN AND STRESS IN OLDER BLACKS

Janiece L. Taylor,[1] Janiece L. Taylor,[1] Lauren Parker,[2] Roland J. Thorpe Jr.,[2] and Keith E. Whitfield[3], *1. Johns Hopkins School of Nursing, Baltimore, Maryland, United States, 2. Johns Hopkins Bloomberg School of Public Health, Baltimore, Maryland, United States, 3. Wayne State University, Detroit, Michigan, United States*

Older Blacks have higher rates of undermanaged and undertreated pain than other racial/ethnic groups. Pain can lead to disability and poor quality of life. It is essential that we identify modifiable factors related to pain in this population. This study examined if stress was associated with pain among older Blacks. Data were taken from the Baltimore Study on Black Aging, (N=602, mean age 69 [SD= 9.76]). A total of 78% of participants reported bodily pain in the past month. Women had an increased odds of reporting bodily pain (OR 1.89, 95% CI 1.17, 3.07) compared to men. Using logistic regression controlling for age, self-rated health, sex, depressive symptoms, and chronic conditions, higher levels of stress were associated with increased odds of bodily pain (OR 1.04, 95% CI 1.00, 1.07). Identification of effective coping mechanisms to combat stress may lead to pain relief among older Blacks, particularly Black women.

### IT HURTS AS IF: PAIN LANGUAGE, VISUAL CHARACTERIZATION, AND STORY-TELLING IN HMONG OLDER ADULTS

Maichou Lor,[1] Xia Vang,[2] David Rabago,[2] Roger Brown,[3] and Miroslav Backonja[4], *1. Columbia University, New York, New York, United States, 2. University of Wisconsin-Madison, School of Medicine and Public Health, Department Family Medicine and Community Health, Madison, Wisconsin, United States, 3. University of Wisconsin-Madison, School of Nursing, Madison, Wisconsin, United States, 4. School of Medicine and Public Health, Department of Neurology, Madison, Wisconsin, United States*

Culture and language affect pain reporting, diagnosis, and treatment. Ethnic subgroup populations, such as the Hmong, are understudied in pain research. The study's purpose is to qualitatively understand older Hmong adults' pain expression and their pain communication with providers. Sixty-seven participants were recruited from one healthcare system and community. A directed content analysis revealed that all Hmong participants describe pain using stories with reference to the temporal context, causal attribution, co-occurring symptoms or related experiences, magnitude, and consequences of pain. Several participants also characterized their pain by associating it with visual metaphors as objects and animals. Some participants shared that their stories are often underappreciated by providers, and are therefore not understood by providers. This leads to subsequent feelings of stress, not receiving needed medication or other healthcare, and having less frequent contact with providers or switching providers. These findings have implications for more culturally attentive and appropriate pain care.

### PAIN-RELATED BRAIN STRUCTURE AND PAIN CATASTROPHIZING IN BLACKS AND WHITES WITH KNEE OSTEOARTHRITIS

Ellen Terry,[1] Ellen L. Terry,[1] Josue S. Cardoso,[1] Kimberly T. Sibille,[1] Jared J. Tanner,[1] Burel R. Goodin,[2] Catherine C. Price,[1] and Roger B. Fillingim[1], *1. University of Florida, Gainesville, Florida, United States, 2. University of Alabama at Birmingham (UAB), Birmingham, Alabama, United States*

Pain catastrophizing is a cognitive-affective response during painful experiences and is implicated in the facilitation of pain processing. Non-Hispanic blacks (NHB) more often engage in catastrophizing as a coping strategy compared to non-Hispanic whites (NHW). Hence, pain catastrophizing may contribute to poorer pain-related outcomes and greater disability in NHBs. Functional neuroimaging studies have linked high levels of catastrophizing with increased cerebral responses to pain in the insula and primary somatosensory cortex [S1], but associations between brain structure and catastrophizing remain largely unexplored. Moreover, no neuroimaging studies have investigated whether catastrophizing is differentially associated with pain-related brain structure across racial/ethnic groups. We examined the association between race/ethnicity, catastrophizing, and pain-related brain structure (insula, S1) among 176 participants with and without knee pain. Findings provide evidence for differing associations of catastrophizing with pain-related brain structures in NHBs and NHWs. It is therefore important to develop culturally-relevant, neural-mediated interventions targeting catastrophizing for NHBs.

### SESSION 780 (SYMPOSIUM)

### SOCIAL NETWORKS, HEALTH, AND WELL-BEING AMONG PEOPLE LIVING WITH DEMENTIA

Chair: Eleanor S. McConnell, *Duke University, Durham, North Carolina, United States*
Co-Chair: Kirsten Corazzini, *Duke University, Durham, North Carolina, United States*
Discussant: T. Robert Konrad, *University of North Carolina at Chapel Hill, Chapel Hill, North Carolina, United States*

Although the impact of dementia on the health and well-being of those living with Alzheimer's Disease and related Disorders (ADRD) and their care partners has been widely studied, less attention has been paid to how the disease impacts individuals within the context of their larger social networks. This symposium presents findings from a series of integrated studies aimed at strengthening measurement of health and well-being among older adults with living with dementia and well-being among members of their social networks. Findings will be presented from five studies: (1) a scoping review of social network measurement in older adults in chronic illness, including dementia, that emphasizes the use of technology in measuring older adults' social networks; (2) a simulation study to evaluate the feasibility and reliability of sensor technology to measure social interaction among a person living with dementia and others in their immediate surroundings; (3) development of a web-based application that allows older adults to map and activate their social networks; (4) a qualitative analysis of interviews from persons living with dementia, their unpaid caregivers, and paid caregivers from an adult day health program concerning well-being focused outcomes; and (5) a mixed methods analysis of the feasibility of using both traditional and novel measures of health and well-being deployed among networks of people living with dementia. Emerging technologies for measuring social networks health and well-being hold promise for advancing the study of the relationship-based nature of care for people living with dementia.

### MEASURING NETWORK WELL-BEING AND HEALTH AMONG PEOPLE LIVING WITH DEMENTIA: A MIXED-METHODS STUDY

Eleanor S. McConnell,[1] Sijia Wei,[1] Bada Kang,[1] Samantha Woog,[1] Kayla Wright-Freeman,[1] and Kirsten N. Corazzini[1], *1. Duke University, Durham, North Carolina, United States*

The feasibility and utility of measuring social networks of people living with mild to moderate stage dementia to improve care quality was examined by comparing information obtained using Antonucci's social network mapping approach and through information elicited through a series of open-ended questions regarding life story and well-being. Data were obtained from 24 interviews with 12 people belonging to one or more networks of people living with dementia receiving care in adult day programs. Concurrently we obtained measures of health and well-being using validated symptom checklists and the ICE-CAP suite of well-being measures. Parallel interviews were conducted with social network members who were in a care partner role, either paid or unpaid. Respondents were able to map social networks, and preferred open-ended questions to more standardized measures of quality of life and well-being. Findings from both sources were generally convergent, with open-ended questions providing richer information to guide care.

### MHEALTH PROTOTYPE AND PILOT PROTOCOL TO ENHANCE SOCIAL SUPPORT FOR PERSONS LIVING WITH DEMENTIA

Kirsten Corazzini,[1] Donald (chip) Bailey,[1]
Kayla Wright-Freeman,[1] and Eleanor McConnell[2], *1. Duke University, Durham, North Carolina, United States, 2. Duke, Hillsborough, North Carolina, United States*

An emerging component of mHealth is the use of tailored mobile applications (app) to facilitate self-management of chronic illnesses, including the mapping of social networks to assist adults living with chronic illnesses to help them be able to identify sources of social support. The purpose of this study is to describe a prototype app to support persons living with dementia (PLWD) in the community and their informal caregivers to map social networks and identify sources of emotional, instrumental, informational, and appraisal of social support. Adapting the Network Canvas open-source software and drawing upon a previously-developed mobile application for adults to self-manage chronic illnesses, we share the key specifications, including health care provider output, preliminary end user feedback, and the pilot protocol designed to test the feasibility. Findings illustrate the importance of leveraging social network data in novel ways to enhance self-management and well-being among PLWD and their caregivers

### MEASUREMENT OF SOCIAL NETWORKS OF ELDERS USING TECHNOLOGIES IN THE CONTEXT OF HEALTH AND SOCIAL CARE: A SCOPING REVIEW

Sijia Wei,[1] Eleanor S. McConnell,[2] Kayla Wright-Freeman,[3]
Amanda Woodward,[3] Bada Kang,[3] and
Kirsten N. Corazzini[3], *1. 1. Duke University 2. Duke University School of Nursing JBI-Affiliated Group, Durham, North Carolina, United States, 2. Duke University and Geriatric Research, Education, and Clinical Center, Durham Veterans Affairs Health Care System, Durham, North Carolina, United States, 3. Duke University, Durham, North Carolina, United States*

Social networks impact the health and wellbeing of older adults. The importance of social networks drives the need to reliably measure social networks. Advancements and innovations in the internet, electronic and digital devices, social media and health care technology enriches our ability to collect social network and health data to overcome limitations in social network measurement. This scoping review will review approaches utilizing technology to assist the measurement and analysis of social networks among older adults in the context of health and social care. Joanna Briggs Institute methodology was followed; PubMed (MEDLINE), Sociological Abstracts, SocINDEX, CINAHL, and Web of Science were searched for related articles. Conference abstracts and proceedings were included. We discuss the gaps and advances in measurement of social networks of older adults using technology and implications for future research in social networks of older adults as a lever for improving health and well-being.

### USE OF SENSOR TECHNOLOGY TO MAP THE SOCIAL NETWORKS OF PEOPLE LIVING WITH DEMENTIA: A FEASIBILITY STUDY

Kayla Wright-Freeman,[1] Sijia Wei,[1] Eleanor McConnell,[2]
Kevin Caves,[1] Leighanne Davis,[1] Adrienne Hawkes,[1]
Sarah Moninger,[1] and Kirsten N. Corazzini[1], *1. Duke University, Durham, North Carolina, United States, 2. Duke, Hillsborough, North Carolina, United States*

For older adults living with dementia, social network quality influences health outcomes. However, current social network measurement methods are time consuming and mentally draining for people living with dementia. This study aimed to accurately measure social networks using sensor technology. Bluetooth and radio-frequency identification (RFID) sensors were used to collect social network data in a simulation of a falling nursing home resident living with dementia. Participants wore sensors on their clothing, and video recordings were compared to sensor data. Bluetooth data reflected general direction of movement and instances of idling but were neither precise or accurate. RFID data was accurate after applying data filters. Both systems detected multiple sensors simultaneously. The Bluetooth system is not feasible for clinical use, but the RFID system shows potential for clinical application and accurate measurement of social network factors as interaction frequency and duration.

### RELATIONSHIP-CENTERED CARE: ADULT DAY CARE FOR PERSONS LIVING WITH DEMENTIA AND THE SENSES FRAMEWORK

Samantha Woog,[1] Eleanor McConnell,[2]
Deborah Gold,[1] and Kirsten Corazzini[1], *1. Duke University, Durham, North Carolina, United States, 2. Duke, Hillsborough, North Carolina, United States*

Relationship-centered dementia care (RCDC) has been related to improved quality of residential long-term care for persons living with dementia (PLWD). The senses framework supports accomplishing RCDC, whereby PLWD meet fundamental needs or senses through caregiving relationships. This study explored the application of the senses framework to a non-residential, long-term care setting, and included relationships across formal and informal caregivers. The study design is a qualitative, descriptive study of PLWD (N=3), with matched formal (N=3) and informal (N=3) caregivers in one adult day care setting in North Carolina. Semi-structured individual interviews explored each of the six senses of security, belonging, continuity, purpose, achievement, and significance. Interviews were analyzed using both inductive and deductive thematic analysis. Themes elucidate convergence and divergence of how senses are met or not met across triads of caregiving relationships. Findings inform our understanding of how to integrate the larger social network of PLWD for relationship-centered care.

### SESSION 785 (SYMPOSIUM)

### THE CARE RECIPIENT'S PERSPECTIVE ON QUALITY OF CARE: DIFFERENT APPROACHES IN NURSING HOMES

Chair: Hilde Verbeek, *Department of Health Services Research, Care and Public Health Research Institute (CAPHRI), Faculty of Health, Medicine and Life Sciences,, Maastricht, Netherlands*
Discussant: Kimberly Van Haitsma, *Penn State, University Park, Pennsylvania, United States*

In long-term care, there has been an ongoing shift focused on person-centered care, positioning the care recipient at the core of good quality of care. This has resulted in more emphasis on care recipients' preferences and experiences with the care they receive. In the Netherlands, Germany and the United States the need to focus on and assess quality from the care recipient's perspective has emerged. This symposium presents four different approaches to quality of care from the care recipient's perspective. The first speaker will focus on a narrative approach to assess experienced quality of care in nursing homes. The second speaker will present an observational method to assess the level of autonomy provided to people with dementia in nursing homes. The third speaker will present findings about the use of specialist health care in nursing homes as an indicator for quality of care. The last presentation will address the importance of preferences in quality of care. Defining, assessing and improving experienced quality of care from the care recipient's perspective is an ongoing challenge, as each care recipient's preferences and needs differ. It is important to assess in order to monitor that care is being tailored to the care recipient and to identify possible interventions that can enhance experienced quality of care.

### FACILITATING CARE: A NARRATIVE APPROACH TO ASSESS EXPERIENCED QUALITY OF CARE

Katya Sion,[1] Hilde Verbeek,[2] Gaby Odekerken-Schröder,[3] Sandra Zwakhalen,[2] Jos Schols,[2] and Jan Hamers[2], *1. Department of Health Services Research, Care and Public Health Research Institute (CAPHRI), Faculty of Health, Medicine and Life Sciences, Maastricht, Netherlands, 2. Department of Health Services Research, Care and Public Health Research Institute (CAPHRI), Faculty of Health, Medicine and Life Sciences, Maastricht, Limburg, Netherlands, 3. Department of Marketing and Supply Chain Management, Maastricht, Limburg, Netherlands*

This study aimed to develop a method to assess experienced quality of care (QoC) in nursing homes from the resident's perspective. A narrative approach "Facilitating Care" (FC) was developed based on the INDEXQUAL framework of experienced QoC and a needs assessment. FC assesses experienced QoC by training care professionals to perform individual conversations with residents, their family and their professional caregivers (triads) in another organization than where they are employed. FC consists of three phases: 1) training, 2) data collection and registration, and 3) analysis and reporting of the results. In 2018, 16 care professionals were trained and performed 148 conversations (47 residents, 44 family members, 57 professional caregivers) in 8 different nursing homes. Evaluation showed that FC teaches helpful conversation techniques and provides valuable insights into residents' experienced QoC. Whilst the process was considered time consuming, all participants emphasized the added value of taking time for FC conversations.

### THE SUPPORT OF AUTONOMY FOR NURSING HOME RESIDENTS WITH DEMENTIA: OBSERVATION OF MORNING CARE

Linda J. Hoek,[1] Hilde Verbeek,[2] Erica De Vries,[2] Jolanda C. Van Haastregt,[2] Ramona Backhaus,[2] and Jan P. Hamers[2], *1. Department of Health Services Research, Care and Public Health Research Institute (CAPHRI), Faculty of Health, Medicine and Life Sciences,, Maastricht, Netherlands, 2. Department of Health Service Research, Care and Public Health Research Institute (CAPHRI), Maastricht University, Netherlands, Maastricht, Limburg, Netherlands*

People with dementia in nursing homes need their social environment in supporting their autonomy. This study explored how this relational autonomy is supported by staff for residents with dementia during morning care in nursing homes. Structured observations (n=1815) were carried out to assess how resident choice is supported within staff-resident interaction. Observation of morning care consisted of four main categories: 'getting up', 'physical care', 'physical appearance' and 'breakfast'. In addition, qualitative field notes were taken to support observations. In total, 55 residents with dementia were included from eight nursing home wards in The Netherlands. Results indicated that resident autonomy during morning care was only limitedly supported. Individual staff members took over tasks, regardless of resident's individual capabilities to make a choice. Staff controlled resident's choice for almost all observed categories. The findings of this study implicate that person-centered care during morning routine can be improved by addressing individual needs

### THE USE OF SPECIALIST HEALTH CARE IN NURSING HOMES AS AN INDICATOR FOR QUALITY OF CARE

Karin Wolf-Ostermann,[1] Karin Wolf-Ostermann,[2] Annika Schmidt,[2] Daniel Gand,[2] Ansgar Gerhardus,[2] and Heinz Rothgang[2], *1. University of Bremen, Bremen, Germany, 2. University of Bremen, Bremen, Bremen, Germany*

Needs-based medical care is essential for maintaining and promoting health and quality of life in long-term care. But still knowledge of medical needs and of needs-based medical care of nursing home (NH) residents is limited. However, individual studies, primarily based on secondary data, show that NH residents have significantly less contact with specialists than community-dwelling elderly. The aim of the study is therefore to gain knowledge about medical needs of NH residents as well as about the use of (specialist) medical care especially with respect to vision, hearing and oral health. Besides sociodemographic and care-related data we evaluated health status and utilization of medical care by means of standardized assessments for n=500 NH-residents. Results show that there are inequalities in (specialist) medical care:

More experienced knowledge about unmet medical care needs will be used to generate ideas on how to improve the provision of medical care in nursing homes.

### CARE BASED ON PREFERENCES: EXPERIENCES OF RESIDENTS

Martina Roes,[1] Daniel Purwins,[2] Tobias Stacke,[2] Christina Manietta,[2] and Johannes Bergmann[2], *1. German Center for Neurodegenerative Diseases (DZNE), DZNE, Germany, 2. DZNE, Witten, Nordrhein-Westfalen, Germany*

To provide person-centered care, professional caregivers need to know about the individual preferences of the persons being cared for. Since there were no comparable instruments available, we translated the PELI (Preferences for Everyday Living Inventory) and tested the culturally translated version in German nursing homes. Besides testing for reliability and feasibility in German care settings, were are asking for satisfaction in fulfilling of the preferences and reasons for personal or institutional barriers that hinder adherence to the preferences. Furthermore, to determine the level of understanding and meaning of preferences, we interview a few residents and their close relatives in a cognitive interview. Preliminary results of the perspective of the care recipient will be presented.

### SESSION 795 (SYMPOSIUM)

### THE NATIONAL COLLABORATORY TO ADDRESS ELDER MISTREATMENT: COORDINATING NETWORKS OF CARE

Chair: Kristin Lees-Haggerty, *Education Development Center, Boston, Massachusetts, United States*
Co-Chair: Tony Rosen, *Weill Cornell Medical College / NewYork-Presbyterian Hospital, New York, New York, United States*
Discussant: Terry Fulmer, *The John A. Hartford Foundation, New York, New York, United States*

Elder mistreatment is a devastating and pervasive issue affecting an estimated one in ten older adults in the US. To effectively address elder mistreatment, it is essential for researchers, health care providers and communities to coordinate efforts and leverage each other's strengths. The National Collaboratory to Address Elder Mistreatment (NCAEM), funded by The John A. Hartford Foundation and Gordon and Betty Moore Foundation, is a group of elder mistreatment experts from across the country taking a collective impact approach to alleviating the burden of elder mistreatment on survivors, families, communities, and systems. NCAEM's goals are to provide hospital emergency departments with training and tools to address elder mistreatment and to develop networks of experts, health care, and community-based providers in a coordinated response to this complex problem. This symposium will include four presentations describing the NCAEM's collective impact approach, intersection with related work, and applicability to other initiatives. We will present our strategy for creating a team, moving beyond typical collaborative efforts through the development of a central infrastructure and streamlined processes to support experts from across the country as we work together toward common goals and shared measurement for addressing elder mistreatment. Following an overview of our approach, presentations will include specific examples of how the NCAEM worked with clinical partners to refine an elder mistreatment screening and response tool, case examples from NCAEM clinical test sites describing efforts to bridge hospital and community-based services, and learnings from NCAEM's strategic alignment with complimentary initiatives (the Geri-ED Accreditation and Collaborative).

### THE NATIONAL COLLABORATORY TO ADDRESS ELDER MISTREATMENT: A COLLECTIVE IMPACT APPROACH

Rebecca Jackson Stoeckle,[1] Kristin Lees-Haggerty,[2] Kim Dash,[2] Mark Lachs,[3] Laura Mosqueda,[4] Alice Bonner,[5] and Carmel Dyer[6], *1. Education Development Center, Boston, Massachusetts, United States, 2. Education Development Center, Waltham, Massachusetts, United States, 3. Weill Cornell, New York, New York, United States, 4. Keck School of Medicine University of Southern California, Los Angeles, California, United States, 5. IHI, Boston, Massachusetts, United States, 6. UT Health, Houston, Texas, United States*

The National Collaboratory to Address Elder Mistreatment is a team of experts who have come together to develop, test, and disseminate a care model for addressing elder mistreatment in emergency departments. In this presentation we will demonstrate how a collective impact approach, including a centralized infrastructure, dedicated staff, and structured process allowed the Collaboratory to develop and test the Elder Mistreatment Emergency Department Care Model. The model consists of four core elements: 1) The Elder Mistreatment Emergency Department Assessment Profile, 2) staff training modules, 3) brief screening and response tools adapted from a validated measure, and 4) a roadmap for leveraging community resources to support referral and follow up. We will explore the applicability of this approach for harnessing and integrating multidisciplinary expertise to develop, test, and disseminate innovative solutions to complex health care challenges.

### REFINING AN ELDER MISTREATMENT SCREENING AND RESPONSE TOOL TO IMPROVE ACCEPTABILITY IN AN EMERGENCY DEPARTMENT

Brian Tanksley,[1] Theresa Sivers-Teixeira,[2] Laura Mosqueda,[3] Bonnie Olsen,[2] Tim Platts-Mills,[4] and Kristin Lees-Haggerty[5], *1. USC Keck School of Medicine, Los Angeles, California, United States, 2. Keck School of Medicine USC, Alhambra, California, United States, 3. Keck School of Medicine USC, Los Angeles, California, United States, 4. University of North Carolina at Chapel Hill, Chapel Hill, North Carolina, United States, 5. Education Development Center, Waltham, Massachusetts, United States*

Elder mistreatment (EM) is a public health problem that is rarely recognized or addressed in emergency departments (ED) where a lack of evidence-based protocols leave clinicians to rely on intuition and inconsistent action plans. In this presentation we will share findings from focus groups and online surveys with ED clinicians and administrators to evaluate the perceived value and likelihood of adopting the National

Collaboratory's third core element: the EM Screening and Response Protocol (EM-SAR). Results indicated a strong support for the EM-SAR tool in general and highlighted specific considerations for refining the tool. Considerations include resistance to adding to the ED workload, need to clarify roles and responsibilities for administering the tool, hesitancy to rely on clinical judgement to assess EM, concerns over Adult Protective Services' ability to respond to increased reports, and a desire for cross-training and cooperation. These findings and implications for ongoing feasibility testing will be discussed.

### ELDER MISTREATMENT FOLLOW-UP: CONNECTING EMERGENCY DEPARTMENTS AND COMMUNITIES

Alice Bonner,[1] Kristin Lees-Haggerty,[2] Debi Lang,[3] Bree Cunningham,[4] Jason Burnett,[5] and Kathy Greenlee[6], *1. Institute for Health Care Improvement, Boston, Massachusetts, United States, 2. Education Development Center, Waltham, Massachusetts, United States, 3. UMASS Medical School, Shrewsbury, Massachusetts, United States, 4. MA Executive Office of Elder Affairs, Boston, Massachusetts, United States, 5. UT Health, Houston, Texas, United States, 6. Greenlee Global, Lenexa, Kansas, United States*

To effectively address elder mistreatment (EM) in the emergency department (ED) hospitals must have mechanisms that promote and, to the extent possible, ensure patient safety post-discharge. However, the realities of working within busy hospitals--limited staff time, financial resources, and EM-specific expertise--prevent many EDs from being able to dedicate staff for patient follow up or develop EM multi-disciplinary teams. The fourth core element of the NCAEM's ED Care Model aims to address this need with a roadmap for leveraging existing community resources. The roadmap provides streamlined tools to help hospitals assess their needs, identify existing teams and resources in their community, and connect with Adult Protective Services and other organizations. In this presentation we will present these tools and share case examples from beginning stages of feasibility testing in hospitals across the US. We will discuss specific strategies for implementing the model in hospitals of differing types, sizes, and resource levels.

### DISSEMINATION STRATEGIES: ALIGNMENT WITH GERI-ED

Tim Platts-Mills,[1] Tony Rosen,[2] Rebecca Jackson Stoeckle,[3] Kim Dash,[3] and Kristin Lees-Haggerty[3], *1. University of North Carolina, Chapel Hill, North Carolina, United States, 2. Weill Cornell Medical College, New York, New York, United States, 3. Education development Center, Waltham, Massachusetts, United States*

The National Collaboratory to Address Elder Mistreatment's model provides hospital emergency departments with training and tools to help recognize and respond to elder mistreatment. The Geriatric Emergency Department (GERI-ED) initiative, supported by The John A. Hartford Foundation and West Health, encourages hospitals to integrate best practices for older adults into their emergency departments by providing guidelines and options for accreditation (GED-A). Recognizing the importance of coordinating efforts to develop tools that can be shared and implemented widely, the Collaboratory has intentionally and strategically aligned itself with the GERI-ED by inviting members of the GED-A team to join the Collaboratory. In this presentation we will describe how aligning these two initiatives has the potential to achieve greater impact than either initiative alone. We will provide a case example of how the elder mistreatment care model is implemented in GERI-EDs, highlighting factors that facilitate and hinder model implementation in such settings.

### SESSION 800 (SYMPOSIUM)

### THE ROLE OF GERONTOLOGISTS IN ACHIEVING THE UN'S SUSTAINABLE DEVELOPMENT GOALS

Chair: Toni C. Antonucci, *University of Michigan, Ann Arbor, Michigan, United States*

The Sustainable Development Goals (SDG's) developed by the United Nations in 2015 have as their underlying theme, the pledge that no one will be left behind. The SDGs address global poverty, inequality, climate change, the environment, peace and justice. They are intended to be global benchmarks to be reached by 2030 to ensure well-being and prosperity while protecting and promoting human rights and freedoms (UN, 2015). They envision a world without poverty, where all persons can live with dignity and security in societies free of violence and discrimination based on the foundation of universal human rights. The 17 goals and their 169 targets cover a range of social and economic development issues from poverty and gender inequality to climate change and sustainable cities. These goals are both interrelated and indivisible with each important for individual and social well-being. For example, achieving gender equality can help eradicate poverty while improved health can contribute to increased individual productivity and economic growth. Unfortunately, the needs of older people are larger ignored. In this symposium we outline how gerontologists can and have contributed to the SDG goals. We provide input from four disciplines whose work directly addresses the needs of older people. The four disciplines are: health- how do we meet the health needs of older people, psychology – what are the mental health issues facing older people, public policy – how can\has governments assist through laws and policy, and social work – how can social work address the needs of the vulnerable old.

### THE ROLE OF GERONTOLOGICAL PSYCHOLOGY IN ACHIEVING THE UN'S SUSTAINABLE DEVELOPMENT GOALS

Toni C. Antonucci[1], *1. University of Michigan, Ann Arbor, Michigan, United States*

The United Nations has identified 17 Sustainable Development Goals (SDGs) designed to improve the health and well-being of the world's most vulnerable populations. This presentation will review the potential role psychology, in particular, illustrative theories and research, in achieving the SDGs of reducing poverty and achieving gender equality and empowerment. We consider life span (individual) developmental and life course (environmental structure) theories as useful for explaining how poverty and inequality influence the individual and community at one point in time and over time. Further, we use research evidence to illustrate how naturally occurring resources can be garnered to better explain, understand, identify and create successful intervention programs. We emphasize the importance psychology to achieving SDGs and emphasize that the application psychology to changing the behavior and expectations of individuals and societies to achieve sustainable development that contributes to a world that celebrates optimal and sustainable development for all.

### GOOD HEALTH AND WELL-BEING: ESSENTIAL TO ACHIEVING SDGS

Hans Stohrer[1], *1. Weill Cornell Medical University, New York, New York, United States*

The importance of good health for older persons is a human right, good public policy, and practical. We argue that it is fundamental to achieving the UNs SDGs. Older people can be important assets in the achievement of SDGs but need to be in good health so as to be valuable, productive and contributing members of society. This includes functional health, cognitive health and capacity, mental health, sustainable long term care for non-communicable diseases, and quality palliative care provided in the home at the end of life. This means not only access to health care services and access to safe and effective medicines and vaccines, but also access to rehabilitation and physical therapy, mental health care, long term care, and hospice and palliative care. In conclusion, to achieve the United Nations SDG on Health, it is important to consider explicitly the health of older persons, including the public policy needs.

### THE ROLE OF GERONTOLOGICAL PSYCHOLOGY IN ACHIEVING THE UN'S SUSTAINABLE DEVELOPMENT GOALS

Toni C. Antonucci[1], *1. University of Michigan, Ann Arbor, Michigan, United States*

The United Nations has identified 17 Sustainable Development Goals (SDGs) designed to improve the health and well-being of the world's most vulnerable populations. This presentation will review the potential role psychology, in particular, illustrative theories and research, in achieving the SDGs of reducing poverty and achieving gender equality and empowerment. We consider life span (individual) developmental and life course (environmental structure) theories as useful for explaining how poverty and inequality influence the individual and community at one point in time and over time. Further, we use research evidence to illustrate how naturally occurring resources can be garnered to better explain, understand, identify and create successful intervention programs. We emphasize the importance psychology to achieving SDGs and emphasize that the application psychology to changing the behavior and expectations of individuals and societies to achieve sustainable development that contributes to a world that celebrates optimal and sustainable development for all.

### THE ROLE OF GERONTOLOGISTS IN CONTRIBUTING TOWARD PUBLIC POLICY TO ACHIEVE THE UN'S SUSTAINABLE DEVELOPMENT GOALS

Ruth Finkelstein[1], *1. The Brookdale Center on Aging, Hunter College, New York City, United States*

The UN SDGs are aspirational goals whose achievement requires intentional policy-making at each level of government. Advocates for older people argued for goals specifically addressing older people, the fastest growing segment of the world's population. While unsuccessful, we did achieve a targets monitoring template that reports outcomes separately by age. This innovation will bring helpful attention to generational differences in needs and progress, but gerontologists must play a critical role for this innovation to be realized as a benefit. Gerontologists must actively prevent SDG implementation for older people from being siloed into aging services departments. Aging services agencies do not have the resources or skills to achieve the SDGs – they do not control policy levers to end poverty, ensure gender equity or sustainable urban development. Yet, too often, once a population is separately counted, responsibility for its wellbeing follows. Gerontologists must help governments adopt an "age in everything" lens.

### THE ROLE OF GERONTOLOGISTS IN ACHIEVING THE UN'S SUSTAINABLE DEVELOPMENT GOALS

Carole Cox,[1] and Carole B. Cox[1], *1. Fordham University, New York, New York, United States*

The Sustainable Development Goals (SDG's) developed by the United Nations in 2015 are global benchmarks for all countries to meet by 2030 to ensure well-being and prosperity while protecting and promoting human rights and freedoms. The underlying pledge is that no one will be left behind Globally, older adults are one of the most vulnerable populations, suffering from poverty and poor health and little social protection. Social workers can play key roles in assuring that the concerns and interests and rights of older adults are recognized in the SDGs and in the policies developed to meet them. This paper focuses on 6 of the SDG's that are most pertinent to the status and inclusion of older people and the implications they have for specific social work involvement.

### SESSION 805 (PAPER)

### WELL-BEING, PHYSICAL FUNCTIONING, COGNITIVE ABILITIES ACROSS DIVERSE POPULATIONS

### CAREGIVER TYPE AND ITS ASSOCIATION WITH MORTALITY AMONG OLDER ADULTS WITH DISABILITY IN CHINA

Chenkai Wu,[1] Haoxue Li,[1] Xiaoting liu,[2] and honglin chen[3], *1. Duke Kunshan Unviersity, Kunshan, Jiangsu, China, 2. Zhejiang University, Hangzhou, Zhejiang, China, 3. Fudan University, shanghai, China*

A large proportion of older adults in China needs assistance with activities of daily living (ADL). The quality of care for this population has gained growing attention. However, little is known about the patterns of their caregiver type or its association with mortality. We examined the patterns of primary caregiver type among community-dwelling older adults in China and to examine the association between caregiver type and mortality. Data were from the 2005, 2008, and 2011 waves of the China Longitudinal Health and Longevity Study. We included 437 married and 3,971 widowed participants. The average age was 96.7 years (SD=6.9) and 73.7% were female. For married persons, 55.1%, 25.2%, 12.1%,

2.3%, and 3.9% of the primary caregiver was spouse, son/daughter-in-law, daughter/son-in-law, grandchildren, and housekeeper. For widowed persons, 60.2%, 21.1%, 9.9%, and 5.9% of the primary caregiver was son/daughter-in-law, daughter/son-in-law, grandchildren, and housekeeper, respectively. Multivariable-adjusted model showed that, among married older adults, son/daughter-in-law and daughter/son-in-law as the primary caregiver was associated with 41% (95% confidence interval [CI]=6%-87%) and 67% (95%CI=10%-154%) higher mortality than spouse as the primary caregiver, respectively. For widowed persons, daughter/son-in-law and grandchildren as the primary caregiver was associated with 12% (95%CI=3%-20%) and 14% (95%CI=2%-24%) lower mortality than son/daughter-in-law as the primary caregiver, respectively. Majority of disabled older adults in China relied on their spouse and children to care for them. Type of primary caregiver was associated with death in both married and widowed persons. More resources need to be allocated to disabled Chinese older adults with poor survival outcomes.

### COGNITIVE AND HEARING IMPAIRMENTS IN OLDER ADULTS: EVIDENCE FROM THE HEALTH AND RETIREMENT STUDY

Jessica S. West,[1] and Scott Lynch[1], *1. Duke University, Durham, North Carolina, United States*

As the number of older adults increases, increased prevalence of cognitive and sensory impairments pose growing public health challenges. Research on the relationship between hearing impairment and cognition, however, is minimal and has yielded mixed results, with some studies finding that hearing impairment is associated with cognitive decline, and others reporting that the association is weak or non-existent. Most of this research has been conducted outside of the U.S., and the few U.S.-based longitudinal studies have relied mostly on small, non-representative samples involving short follow-up periods. Further, despite known gendered patterns in cognitive and hearing impairments, no studies to date have examined whether the relationship between the two varies by gender. Our study addresses these weaknesses in the literature by utilizing nine waves of the Health and Retirement Study (1998-2014; n=14,169), a large, nationally representative, longitudinal study that facilitates examination of long-term interrelationships between hearing and cognitive impairments. In this study, we use autoregressive latent trajectory (ALT) methods to model: 1) the relationship between hearing impairment and cognitive decline, and 2) sex differences in the relationship. ALT models enable us to determine whether hearing impairment and cognitive impairment are associated, net of their common tendency simply to co-trend with age. Results indicate that hearing and cognitive impairments are strongly interrelated processes that trend together over time. Moreover, hearing impairment has an increasing impact on cognitive impairment across age while the effect of cognitive impairment on hearing impairment levels out over time. Sex differences in these patterns are discussed.

### DEPRESSION, FUNCTIONAL DISABILITY, AND ACCESSING HEALTH CARE AMONG OLDER MEN AND WOMEN IN GHANA AND SOUTH AFRICA

Candidus C. Nwakasi,[1] and J. Scott Brown[1], *1. Miami University, Oxford, Ohio, United States*

Objectives. To inform a preventive approach to mild depression among older Ghanaians and South Africans, this study will investigate the association and possible variabilities between mild depression, functional disability, accessing health care, sociodemographic, and socioeconomic factors across genders in both countries. Methods. Cross-sectional wave 1 (2007-2010) data from WHO's Study on Global Ageing and Adult Health (SAGE) are used, and a sample of 3871 for Ghana and 3076 for South Africa are analyzed. Binary multiple logistic regression is used to identify the association between mild depression, functional disability status, socioeconomic and sociodemographic factors, and health status. Results. The proportion of mild depression (MD) is 3.78% and 8.15% for older Ghanaian men and women, and 2.29% and 11.91% for South African older men and women, respectively. At 95% CI, increased severity (mild and high levels) of functional disability are associated with increased odds of MD in Ghanaian and South African older men and women. Apart from South African older men, older people in the study who do not receive healthcare when needed have increased odds of MD. Sociodemographic and socioeconomic factors are also associated with MD. Discussion. An untreated, persistent MD may lead to worse conditions with fatal outcomes. Since, mental health care is lacking in both countries, this study recommends policies directed towards support for formal and informal long-term care, and healthcare access to reduce the risks of depression. Thus, this study's findings may provide relevant information for managing depression among older Ghanaians and South Africans.

### HEALTH BEHAVIORS AND PSYCHOLOGICAL WELL-BEING ALLEVIATE DIABETES-RELATED DISABILITY DETERIORATION

Yi-Hsuan Tsai,[1] and Ching-Ju Chiu[1], *1. Institute of Gerontology, National Cheng Kung University, Tainan, Taiwan*

Diabetes is known to increase the risk of disability, which may be buffered by health behaviors and psychological factors. However, few existing studies examine how these factors affect disability in diabetic patients over time. The present study assessed the extent to which diabetes affected disability with age and how that effects differed by health behaviors and psychological well-being in older Taiwanese. The data of 5131 adults aged 50 and older were drawn from the 1996-2007 Taiwan Longitudinal Study on Aging. A cohort sequential multilevel modeling was employed to explore the effects of sociodemographic, comorbidities, lifestyle, and psychosocial factors in mediating and moderating the link between diabetes and disability. Disability was measured by mobility limitation in 1999, 2003, and 2007, while health behaviors and psychological factors were measured in 1996, 1999, and 2003 to be lagged time-varying covariates in random effect model analyses. Our results showed that adults with diabetes had more mobility limitation ($\beta$(diabetes) =3.031, P<.001) and progressed each year with ageing ($\beta$(diabetes*age) =0.061, P<.005). Exercising more than four times a week reduced the risk of disability by 51 % in diabetic patients ($\beta$(diabetes*exercise≥4 times) =-1.220, P<0.05). Social participation ($\beta$(social participation)=-0.631, P<.005), stress ($\beta$(stress)=0.651, P<.001) and depression (β(depression)=0.144, P<.001) had independent effects on the risk of disability in adults, but the interaction with diabetes was not significant. To conclude, exercise is the most powerful factor to alleviate the risk of disability in diabetic patients.

### PREDICTORS OF CONCOMITANT COGNITIVE AND PHYSICAL FUNCTION DECLINE: RESULTS FROM THE HEALTH ABC STUDY

Elizabeth Handing,[1] Iris Leng,[1] Kathleen Hayden,[1] Caterina Rosano,[2] and Stephen Kritchevsky[1], *1. Wake Forest School of Medicine, Winston-Salem, North Carolina, United States, 2. University of Pittsburgh, PIttsburgh, Pennsylvania, United States*

Although many individual risk factors for cognitive and physical decline have been identified, less is known about factors that predict concomitant or “dual decline”. Utilizing data from the Health ABC study (n=2,553), we examined trajectories of decline based on repeated measures of the Modified Mini-Mental State Exam (3MS) and Short Physical Performance Battery (SPPB) across 5 years. Next, we calculated four mutually exclusive trajectories of decline derived from the slope: no decline (change in slope ≥ 0, n= 1,190), cognitive decline (lowest quartile of slope in 3MS; n=445), physical decline (lowest quartile of slope in SPPB; n= 407), and dual decline (lowest quartile for both cognitive and physical decline; n= 211). Logistic regression tested the association of 23 baseline risk factors with dual decline. Odds of dual decline where higher in relation with depression (CES-D >16) (Odds Ratio [OR]: OR:2.77, 95% Confidence Interval [95% CI] [1.25- 6.15]), and lower for individuals with a better score on the Digit Symbol Substitution Test (DSST) (OR: 0.97 [0.95-0.98]), grip strength (OR: 0.97 [0.94-1.00]), and 400m gait speed (OR: 0.11 [0.03-0.40]). Among the predictors identified, depressive mood at baseline nearly tripled the odds of developing dual decline, but was not associated with decline in either the cognitive or physical domains. Depressive mood is uniquely associated with dual decline; future studies should focus on the overlap between mood states and function, as mood may be modifiable.

### SESSION 810 (POSTER)

### AGE FRIENDLY COMMUNITIES

### NEIGHBORHOOD AND HEALTH AMONG CHINESE ADULTS: BEYOND THE URBAN AND RURAL DICHOTOMY

Yuekang Li,[1] and Yi Wang[2], *1. Washington University in St.Louis, St.Louis, Missouri, United States, 2. The University of Iowa School of Social Work, Iowa City, Iowa, United States*

The associations between physical frailty and depressive symptoms among older individuals were established in existing literature. Taking the person-environment perspective, we argue that neighborhood environment could either buffer the stress derived from being physically vulnerable or worsen it by adding another layer of stressors in the environmental context when physical health declined. The objectives of this study are to explore 1) to what extent the neighborhood-level characteristics moderate the relationship between physical frailty and depressive symptoms, 2) if there were rural-urban differences in the moderation effects, and 3) whether some of the environmental factors worked beyond the contextual influences of the rural-urban scope. Using the China Health and Retirement Longitudinal Study 2011 wave, 6,246 individuals ages 60 years and older were included for analyses. Multilevel mixed-effects models were fitted to examine the moderating effects of urbanicity and neighborhood-level socio-economic status (SES) on the relationship between frailty and depressive symptoms among older adults, controlling for individual-level characteristics. Results showed a stronger relationship between deterioration in physical health and depressive symptoms in rural neighborhoods and neighborhoods with lower SES, after controlling for individual-level SES. Also, the moderating effects of the neighborhood-level socioeconomic factors remained after controlling for urbanicity, indicating that neighborhood SES works beyond the rural-urban contexts. Findings from this study demonstrate the important roles of neighborhood socioeconomic characteristics in reshaping and the need to redefining China’s rural-urban dichotomy. The findings also identified neighborhoods with low SES as potential targets for policy and practice to reduce the stress associated with health decline.

### LAYING THE GROUNDWORK FOR BECOMING AN AGE-FRIENDLY UNIVERSITY: A MULTI-PHASE STUDY

Melissa L. Cannon,[1] Renata Kerwood,[1] Mandie Kondash,[1] Samuel Rowley,[1] and Hannah Wehr[1], *1. Western Oregon University, Monmouth, Oregon, United States*

As evidenced by the growing Age-Friendly University (AFU) global network, institutions of higher education are increasingly seeking ways to enhance the experiences of older adults who use them for reasons such as lifelong learning, career development, and intergenerational engagement. This multi-phase study explored the barriers and facilitators for older adults accessing a public university in a small Oregon town. The first phase of the study involved survey data collection from 46 members of the local senior center adjacent to the university campus. For the second phase, researchers recruited a sample from the survey respondents and used snowball sampling to conduct 12 interviews with senior center members, past and current senior center directors, and key contacts among university staff. The third phase of data collection paired student researchers with older adult research participants (N=14) in participatory action research to capture the unique perspectives of the research participants visiting the university campus using photovoice and a follow-up focus group. Quantitative data were analyzed using SPSS while qualitative data were analyzed using team coding and intensive group discussion to develop categories and themes. Findings revealed various opportunities to increase age-friendliness according to the principles outlined by the AFU initiative, such as developing a lifelong learning center on campus, strengthening the university-senior center partnership, and removing accessibility barriers in order to make the university campus friendlier for people of all ages and abilities. These findings are being used in a proposal to join the AFU network and to shape the university’s AFU vision.

### IS YOUR COMMUNITY-BASED HEALTH PROMOTION PROGRAM EVIDENCE BASED AND READY FOR DISSEMINATION? HERE'S HOW TO FIND OUT

Ellen C. Schneider,[1] Lesley Steinman,[2] and Casey Dicocco[3], *1. University of North Carolina at Chapel Hill, Chapel Hill, North Carolina, United States, 2. University of Washington Health Promotion Research Center, Seattle, Washington, United States, 3. U.S. Department of Health and Human Services, Administration on Aging/Administration for Community Living, Boston, Massachusetts, United States*

Evidence-based programs (EBPs) offer proven ways to promote health and prevent disease among older adults in their communities. EBPs are based on rigorous study of the effects of specific interventions or model programs, demonstrate consistently positive changes in important health-related and functional measures, and have tools in place to maintain program access, quality, and efficiency across diverse settings. The University of North Carolina at Chapel Hill's Center for Health Promotion and Disease Prevention (UNC HPDP), in partnership with the Evidence-Based Leadership Collaborative (EBLC), has established a review process and Review Council to identify new community programs that meet the evidence-based program criteria established by the Administration for Community Living (ACL), one of the chief U.S. federal agencies responsible for aging programs. Approved programs are then eligible for Older Americans Act Title III-D and other discretionary funding to support organizations that deliver EBPs to improve older adult health. The review process assesses the effectiveness, outcomes, and evaluation of the program, information about program implementation, training, and other key elements for successful program dissemination. The Review Council consists of national leaders with expertise in program research, evaluation, and implementation. The review process is supported by the ACL-funded National Chronic Disease Self-Management Education and Falls Prevention Resource Centers. This session will describe the ACL evidence-based health promotion program criteria that must be met for approval; an overview of the review process; and how researchers can submit their programs for review. Time will be allowed for questions, discussion, and research to practice implications.

### SESSION 815 (POSTER)

### AGEISM

### AGEISM IN HEALTH CARE: 72 IS NOT A DIAGNOSIS

Phyllis A. Greenberg,[1] and Tarynn Johnson[1], *1. St. Cloud State University, St. Cloud, Minnesota, United States*

This poster examines what value, if any, there is in using age as a predictor or impetus for testing, examining and diagnosing older adults. In a cross sectional survey (Davis et al. (2011) used the Expectations Regarding Aging Scale to assess primary care clinicians perceptions of aging in the domains of physical/mental health and cognitive functioning. Sixty-four percent of respondents agreed with the statement "Having more aches and pains is an accepted part of aging while 61% agreed that the "Human body is like a car when it gets old it gets worn out. And 51% agreed that one should expect to become more forgetful with age while 17% agreed that mental slowness is impossible to escape. How might these attitudes and biases effect how older adults are diagnosed, heard, spoken to, and treated (medical treatment as well as patient/professional interaction)? Are older patients/clients underserved or over served? Is forgetting where you put your keys always or even usually a sign of dementia? How helpful then is the use of age and are there other factors that should and can take precedence? What do we know and what don't we know if we know someone's age? Successful and innovative tools are explored that acknowledge age biases and strategies are presented to change age biases in education, training and practice.

### PERCEPTIONS OF PERPETRATORS OF AGEISM

Alison L. Chasteen,[1] Michelle Horhota,[2] and Jessica Crumley-Branyon[2], *1. University of Toronto, Toronto, Canada, 2. Furman University, Greenville, South Carolina, United States*

What are the consequences for perpetrators who engage in different types of ageism? We compared young (n=316), middle-aged (n=464), and older adults' (n=273) perceptions of a perpetrator who engaged in an ageist action. Participants read a vignette about a pedestrian (the perpetrator) offering unwanted help to an older woman crossing the street. We manipulated the ageism type (benevolent or hostile), the reaction of the older target (acceptance, moderate confrontation or strong confrontation) and assessed the overall impression of the perpetrator. Main effects emerged for Ageism Type and Age Group. Overall, participants rated the perpetrator more positively in the benevolent condition compared to the hostile condition. Middle-aged and older adults rated the perpetrator more positively than young adults did. A Time x Confront interaction suggested that the perpetrator's overall impression was not impacted when the target of the ageist act accepted the action or moderately confronted the perpetrator. In contrast, when the target confronted the perpetrator strongly, the overall impression of the perpetrator decreased. An Ageism Type x Age Group x Time interaction on overall impression also emerged. There were no age differences when the perpetrator committed a hostile act of ageism. In contrast, in the benevolent condition young and older adults perceived the perpetrator more negatively after the target's reaction, whereas middle-aged adults did not adjust their impression. Taken together, these results suggest that young and older adults may be less accepting of benevolent ageism compared to middle-aged adults.

### PERCEPTIONS OF INDIVIDUALS WHO CONFRONT AGEISM

Michelle Horhota,[1] Alison L. Chasteen,[2] and Jessica Crumley-Branyon[1], *1. Furman University, Greenville, South Carolina, United States, 2. University of Toronto, Toronto, Canada*

What are the consequences for older adults who confront ageism? We compared young (n=316), middle-aged (n=464), and older adults' (n=273) perceptions of an older target who confronts the perpetrator of an ageist action. Participants read a vignette about a pedestrian offering unwanted help to an older woman crossing the street. We manipulated the type of ageism (benevolent or hostile), the reaction of the older target (acceptance, moderate confrontation or strong confrontation)

and assessed how impressions of warmth, competence and overall impression of the target changed over time. Type of Ageism x Reaction x Time interactions emerged for all three variables. In the hostile condition, a strong confrontation resulted in the target being rated as less warm, more competent, and the overall impression decreased over time. In contrast, a moderate confrontation increased perceptions of warmth, competence and overall ratings of the target. In the benevolent condition, a strong confrontation decreased perceptions of the target's warmth, competence and overall impression. Moderate confrontation increased perceptions of target competence but did not change perceptions of warmth or overall impression. Targets that accepted the ageist act were rated lower on warmth for both hostile and benevolent conditions. Competence ratings were not affected. However, targets that accepted benevolent ageism experienced a cost to their overall impression. Taken together, these results suggest that when confronting ageism, older adults should take a moderate approach. When participants perceived the target's reaction to be incommensurate with the offer of help, the target was viewed more negatively overall.

### YOUNG ADULTS STEREOTYPE OLDER SPEAKERS WHO ADOPTED A POWER POSE AS LESS COMPETENT COMPARED TO SUBMISSIVE OR CONTROL

Jennifer R. Turner,[1] and Jennifer T. Stanley[1], *1. University of Akron, Akron, Ohio, United States*

Young adults (YA) frequently endorse age stereotypes (Levy, 2009). We examined whether older adult (OA) speakers influenced by embodied-cognition ("power posing"; Cuddy et al., 2015) would reduce YAs' stereotype-related judgments. Following the Stereotype Content Model (SCM; Fiske et al., 2002), we hypothesized that OA who held a power pose prior to giving their speech would be rated as higher in Competency, Performance, and Electability, but not Warmth. Sixty-three YA viewed and rated 9 videos of OA performing speeches after modeling a pose (power, submissive, control). Within-subjects ANOVAs revealed embodiment condition differences for Performance ($F2,124 = 207.76, \eta p2 = .77$). For ratings of Performance, speakers in the power condition were judged worse than either submissive or control ($ps < .001$). For Warmth ratings, power ($M = 4.81, SD = .62$) was worse than control ($M = 5.07, SD = .89, p = .003, d = .34$), but submissive ($M = 4.97, SD = .87$) was not significantly different from either group. These results suggest that YA may judge the Performance and Warmth of OA who adopted a power pose harsher because OA are not supposed to be powerful or adopt expansive postures (consistent with the SCM). In comparison, YA may be drawing upon the Representativeness Heuristic of OA in positions of power (e.g., Senators) when rating Electability and Competence.

### THE IMPACT OF MORTALITY SALIENCE ON COLLEGE STUDENTS' INTENT TO HELP OLDER ADULTS

Erika A. Fenstermacher,[1] Jessica Birg,[1]
Vincent Barbieri,[1] and Nathaniel Herr[1], *1. American University, Washington, District of Columbia, United States*

Terror Management Theory (TMT) states that the awareness of one's own death causes humans to experience intense anxiety, which must be continuously managed. Much of the research on TMT has focused on negative outcomes, rather than prosocial behavior, begging the question: "Can priming individuals with the thought of their own death trigger them to behave in ways that benefit others?". Jonas et al. (2002), found that when mortality salience was primed prosocial behavior increased. In line with TMT, they hypothesized that people may behave in a more prosocial manner as it fits in with their personal values. The present study recruited 108 students who were randomly assigned to a mortality salience (MS) or control condition. Participants also completed baseline self-reports, which included measures of ageism, social desirability, personality, and empathy. After the study seemed to end, participants were given a disguised measure of helping behavior, which they believed to be an interest survey for a student volunteer group. Preliminary analyses indicate that those in the MS condition were more willing to be contacted to volunteer with kids than being contacted to volunteer with older adults. We also found that those in the MS condition were more likely to be contacted to volunteer with kids than those in the control condition. Our findings are consistent with previous work showing that individuals favor their ingroup when primed with their death. This reflects the importance of focused efforts on encouraging young people to identify with older adults and on promoting prosocial behavior.

### KNOWLEDGE OF AGING, NEGATIVE AGE BIAS, AND POSITIVE AGE BIAS: AGE GROUP DIFFERENCES

Grace Caskie,[1] Anastasia E. Canell,[1] Hannah M. Bashian[1], *1. Lehigh University, Bethlehem, Pennsylvania, United States*

Attitudes towards aging include both positive and negative beliefs about older adults (Iverson et al., 2017; Palmore, 1999). Palmore's (1998) Facts on Aging Quiz, a widely used assessment of knowledge about aging, also identifies common societal misconceptions about aging. Findings regarding age group differences in attitudes toward aging are mixed (Bodner et al., 2012; Cherry & Palmore, 2008; Rupp et al., 2005). The current study compared knowledge of aging, negative age bias, and positive age bias between young adults (18-35 years, n=268) and middle-aged adults (40-55 years; n=277). Middle-aged adults reported significantly greater average knowledge of aging than young adults ($p=.019$), although both groups had relatively low knowledge (MA: $M=13.0$, YA: $M=12.2$). Middle-aged adults also showed significantly less negative age bias ($p<.001$) and significantly more positive age bias than young adults ($p=.026$). Although the total sample was significantly more likely to be incorrect than correct on 23 of the 25 facts ($p<.001$), young adults were significantly more likely than middle-aged adults ($p<.001$) to respond incorrectly for only 2 of 25 facts. Both facts reflected greater negative age bias among young adults than middle-aged adults. These facts concerned older adults' ability to work as effectively as young adults (fact 9) and frequency of depression in older adults (fact 13). Results demonstrate that age bias is not limited to young adults and may continue through midlife, though negative age bias in particular may be lower for individuals approaching older adulthood, which could have implications for their psychological and physical well-being.

### THE RELATIONSHIP BETWEEN PERSONAL AGING ANXIETY AND ATTITUDES TO OLDER PATIENTS AMONG HEALTHCARE PROFESSIONALS

Jenny Inker[1], *1. Virginia Commonwealth University, Richmond, Virginia, United States*

Nearly 20% of adults age 50 and older report experiencing age discrimination by a healthcare professional. Numerous harmful health consequences flow from ageism in healthcare encounters, including failure to accurately diagnose and respond to treatable conditions such as pain and depression. Despite this, little is known about ageism among healthcare professionals, including whether job role and work setting influences attitudes to older patients. This study aims to use relational ageism theory to explore the relationship between personal aging anxiety among healthcare professionals and their attitudes to older patients, including potential moderating factors of job role and work setting. A convenience sample of healthcare professionals working for a small regional health system in the US (N = 145) completed an online survey using the Aging Anxiety Scale to measure personal aging anxiety and the Geriatric Attitudes Scale to measure negative attitudes to older patients. Regression analyses demonstrated that personal aging anxiety significantly predicted attitudes to older patients, with greater anxiety associated with more negative attitudes. Job role significantly predicted attitudes to older patients with physicians' attitudes being more negative compared to other disciplines, while work setting was not predictive of attitudes toward older patients. This research confirms the need to provide ageism awareness training for healthcare professionals and to include in this training an exploration of internalized attitudes to aging. Further research with a larger, more representative sample is indicated to test replication and to better understand how to overcome job role risks of ageism among healthcare professionals.

### AGING KNOWLEDGE AND SELF-REPORTED AGEISM

Katie E. Cherry,[1] Marla J. Erwin,[1] Priscilla D. Allen[1], *1. Louisiana State University, Baton Rouge, Louisiana, United States*

The term, ageism, refers to any form of personal or institutional prejudice or discrimination based on chronological age. Ageism may encompass attitudes and prejudices, as well as behaviors, highlighting the complex nature of ageist behaviors observed among students and professionals alike (Allen, Cherry, & Palmore, 2009). We examined the prevalence of self-reported ageist behaviors in a sample of college students who ranged in age from 18 to 44 years to test the hypothesis that aging knowledge would be associated with self-reported ageist behaviors (positive and negative). The study sample was comprised of adults who were enrolled in classes at Louisiana State University (n = 110). Most of these students were traditional aged college students (18-25 years old). Participants completed the Relating to Older People Evaluation (ROPE; Cherry & Palmore, 2008), the Facts on Aging Quiz (FAQ; Palmore, 1998), and the Knowledge of Memory Aging Questionnaire (KMAQ: Cherry et al., 2003). Results indicated that positive ageist behaviors were more frequent than negative ageist behaviors. Men endorsed positive and negative ageism items more than women reported. Follow-up analyses on participants' responses to the two aging knowledge questionnaires showed that increased knowledge of aging was significantly correlated with diminished reports of negative ageist behaviors, after controlling for age and gender. These results imply that self-reported ageist behaviors are associated with aging knowledge. Strengthening college curricula by including course offerings in adult development and aging may improve self-reported ageist behaviors among college students.

### DEVELOPMENT OF A SCALE TO MEASURE CONTACT WITH OLDER ADULTS

Robert C. Intrieri,[1] and Maria Kurth[2], *1. Western Illinois University, Macomb, Illinois, United States, 2. OSU Oregon State University, Corvallis, Oregon, United States*

Allport (1954) hypothesized that intergroup contact would reduce prejudice that an in-group member would experience toward an out-group member. Allport held that positive effects of intergroup contact would occur when four conditions were met: (a) equal group status within the situation, (b) common goals, (c) intergroup cooperation, and (d) the support of authorities, law, or custom. Although contact with older adults is an important influence on attitudes toward older people, no psychometrically adequate measures of contact exist. Specifically, this study examined the factor structure of an instrument to measure contact with older adults. The convenience sample consisted of 188 women and 282 men (n = 470). Mean ages for men and women were 21.06 (SD = 2.28) and 20.88 (SD = 3.09), respectively (Mtotal = 20.99, SDtotal = 2.63). Participants were predominantly Caucasian (n=295, 62.6%), African American (n=67, 14.2%), Hispanic/Latino/a (n=63, 13.4%), and other minorities comprising the remaining 9.8%. Results of a confirmatory factor analysis showed the three factor model exhibited a reasonable fit to the data X2 (41, N = 471) = 281.81; p<.0001, CFI =.954; TLI =.938; RMSEA =. 000 (90% CI, 0.100-0.124) SRMS = .054. Results and further adjustments to the model will be discussed.

### GENDER DIFFERENCES ACROSS THE AGING SEMANTIC DIFFERENTIAL AND THE FRABONI SCALE OF AGEISM

Robert C. Intrieri,[1] and Rebecca A. Dunterman[1], *1. Western Illinois University, Macomb, Illinois, United States*

The purpose of this study was to compare attitudes about aging between groups of raters categorized by gender, using the Aging Semantic Differential (ASD; Rosencrantz & McNevin, 1969) and the Fraboni Scale of Ageism (FSA, Fraboni, Saltstone, & Hughes, 1990). The current study assesses the relationships between the four factors of Aging Semantic Differential (ASD; Instrumentality, Autonomy, Acceptability, and Integrity) as well as the three factors of the Fraboni Scale of Ageism (FSA; Antilocution, Discrimination, and Avoidance) across gender groups. The convenience sample consisted of 471 undergraduate students, with a mean age of 19.68 (SD = 2.28). The mean age for men was 19.88 (SD = 3.31) and for women was 19.56 (SD = 1.37). A series of four ANOVAs were conducted using the ASD factors. Results showed significance for the ASD-Instrumental (F(1, 470) = 4.922, p = .027); ASD-Acceptability F(1, 470) = 8.616, p = .003), and the ASD-Integrity factors F(1, 470) = 4.475, p = .035). Men endorsed more positive attitudes than women on both the Acceptability and Integrity factors. Women endorsed more positive attitudes on the Instrumental factor. Similar ANOVAs were conducted across the three Fraboni Scale of Ageism factors. Significant differences between men and women were only obtained across the Avoidance factor (F(1, 470) = 12.187, p = .001) with endorsing higher avoidance scores than women. Taken together these results show differential effects across three ASD factors between men and women while men demonstrated higher scores on the FSA Avoidance scale.

### QUALITY OF CONTACT WITH OLDER ADULTS AND KNOWLEDGE ABOUT AGING ARE ASSOCIATED WITH LOWER AGEISM AMONG YOUNG ADULTS

Jessica H. Helphrey,[1] Cassidy M. Adams,[2] Leah N. Smith,[1] Jennifer Sawyer,[1] Leigh A. Fierro,[3] Sarah Edzards,[1] Allyson M. Coldiron,[1] and Michael D. Barnett[1], *1. The University of Texas at Tyler, Tyler, Texas, United States, 2. University of North Texas, Denton, Texas, United States, 3. The University of Texas at Tyler, Texas, United States*

Ageism refers to stereotypes about and prejudice against individuals on the basis of age. Ageism among young adults may be different than other forms of intolerance simply because age changes; that is, young adults will grow older, and they will eventually become a member of what is presently an outgroup (i.e., older adults). The purpose of this study was to investigate whether ageism among young adults (N = 623) is more closely associated with future-oriented variables (i.e., optimism and fear of death) or whether ageism more closely resembles an outgroup attitude, which like other outgroup attitudes is mitigated by knowledge about and quality of contact with those outgroup members. Bivariate correlations found that knowledge of aging, quality of contact with older adults, and optimism were associated with lower ageism. In a multiple regression analysis, only knowledge about aging and quality of contact with older adults were associated with lower ageism. Overall, the results suggest that ageism represents more of an outgroup attitude rather than a future-oriented attitude. These results support the contact hypothesis in that knowledge of aging and quality of contact with older adults were associated with lower ageism among young adults. Education about aging and quality contact with older adults may be effective ways to reduce ageism among young adults.

### WHEN GRANDPA SAYS SOMETHING RACIST: THE ROLE OF AGEISM IN YOUNG ADULT RESPONSES

Jennifer Sawyer,[1] Jessica H. Helphrey,[1] Leah N. Smith,[1] Ben K. Mokhtari,[1] Allie M. Sandlin,[1] Christopher Reed,[1] Daniel Rodriguez,[1] and Michael D. Barnett[1], *1. The University of Texas at Tyler, Tyler, Texas, United States*

Previous research has found that older adults endorse higher levels of racist attitudes than younger adults. However, little extant research has explored how young adults may respond to an older adult expressing racist views. One factor that may drive young adults' responses is ageism, particularly stereotypes that older adults cannot handle disagreement or are incapable of changing their views. The purpose of this study was to investigate the relationships between ageism and young adults' likely responses to an older adult relative making a racist statement. College students (N = 110; 75.8% female) completed an online survey in which they were given a scenario in which an older adult relative makes a racist statement and rated how likely they would be to respond in different ways. Factor analysis of the likely response items found four facets: confront, agree, avoid, and leave. Bivariate correlations found that ageism was associated with higher likelihood of agreeing or avoiding, and lower likelihood of confronting the older adult relative. There was no association between ageism and likelihood of leaving the situation. Young adults higher in ageism may be more likely to agree or avoid because of ageist stereotypes that older adults cannot handle disagreement or are incapable of change, and they may be more likely to agree with the racist statement because they may have higher levels of intolerance toward both older adults and other ethnic groups. Ageism may play a role in how young adults respond to older adults expressing intolerant views.

### ATTACHMENT SECURITY AND VIEWS TOWARD AGING IN OLDER COUPLES: A DYADIC PERSPECTIVE

E-Shien Chang,[1] Seline O. Goktas,[2] and Joan K. Monin[1], *1. Yale School of Public Health, New Haven, Connecticut, United States, 2. Yale School of Medicine, New Haven, Connecticut, United States*

Research has shown that attachment security–feelings of emotional safety from interpersonal closeness and responsiveness– is associated with better appraisal of stressful situations. Individuals' views toward aging could be one avenue such appraisals are expressed that in turn contribute to better health in late life. However, no studies to our knowledge have examined the dyadic associations between attachment security and views towards aging in the context of close relationships. We hypothesized that attachment insecurity would be associated with individuals' own and partners' negative views toward aging in older married couples. The study sample was comprised of 77 older persons with a self-reported musculoskeletal condition and their caregiving spouses. The Experiences in Close Relationships Scale and the open-ended Image of Aging questions were used to measure attachment security and views toward aging. Data were analyzed with SPPS mixed models using the Actor Partner Interdependence Model. Mean age of care-recipients were 65.9 and 64.8 for their spouses. Contrary to our hypothesis, results showed no significant associations between each individual's attachment security and their own views toward aging. However, care-recipients reported particularly positive views toward aging when caregivers had low attachment anxiety (p=.03), and caregivers reported more negative views toward aging when care-recipients had low attachment avoidance (p=.02). Findings suggest that having a close partner who is securely attached may be protective of one's own views of aging, which may in turn have positive effects on health.

### SESSION 820 (POSTER)

### BIOLOGY OF AGING I

### DIFFERENTIAL EFFECTS OF VARYING DOSES OF DIETARY NITRATE ON MUSCLE FUNCTION AND BLOOD PRESSURE IN OLDER SUBJECTS

Andrew R. Coggan,[1] Edgar Gallardo,[1] Derrick A. Gray,[1] Richard Hoffman,[1] and Ranjani Moorthi[1], *1. Indiana University Purdue University Indianapolis, Indianapolis, Indiana, United States*

We have recently demonstrated that dietary nitrate, a source of nitric oxide via the enterosalivary pathway, can improve muscle contractile function in healthy older men and women. Nitrate ingestion has also been shown to reduce blood pressure in older individuals. However, the optimal dose for eliciting these beneficial effects is unknown. We therefore performed a randomized, double-blind, crossover study to determine the effects of ingesting 3.3 mL/kg of beetroot juice (BRJ) containing 0, 212, or 425 μmol/kg of nitrate in six healthy older (age 69±3 y) subjects. Maximal knee extensor speed (Vmax) and power (Pmax) were measured 2 h after BRJ ingestion using isokinetic dynamometry; blood pressure was monitored periodically throughout each study. Mean arterial pressure (in mmHg) was lower ($P<0.05$) after the high (80±4) vs. the low (84±3) or placebo (88±2) doses. Vmax (in rad/s), however, was higher ($P<0.05$) after the low dose (11.7±0.8), but not the high dose (10.8±1.0), compared to the placebo (10.5±1.0). Pmax (in W/kg) also tended to be higher ($P=0.11$) in the low (3.9±0.5) compared to the placebo (3.7±0.5) or high (3.7±0.5) trials. Five out of six subjects achieved a higher Vmax and Pmax after the low vs. the high dose. We conclude that dietary nitrate has differential effects on muscle function and blood pressure in older individuals. A high dose of nitrate intake further lowers blood pressure but does not enhance muscle contractility as much as a lower dose. Supported by Indiana University Purdue University Indianapolis and by the NIA (R21 AG053606)

### AGE-RELATED CHANGES TO MACROPHAGES AFFECT FRACTURE HEALING

Daniel Clark,[1] Theodore Miclau,[1] Mary Nakamura,[1] and Ralph Marcucio[1], *1. University of California San Francisco, San Francisco, California, United States*

Fracture healing follows a strict temporal sequence characterized by an initial inflammatory phase. Perturbation of the inflammatory phase may be responsible for the poorer fracture healing outcomes in older adults. Herein, we examine age-related changes to the macrophage during fracture healing. Macrophages regulate inflammation through pro-inflammatory (M1) and anti-inflammatory (M2) phenotypes. Anti-inflammatory activity is promoted via activation of triggering receptor expressed on myeloid cells 2 (TREM2). Tibia fractures were made in old (24 months) and young (3 months) mice. Immune cells from the fracture callus were analyzed via RNAseq and FACS, and fracture healing was evaluated histologically. Old mice demonstrated significantly delayed fracture healing compared to young ($p<0.05$). The quantity of infiltrating macrophages into the fracture callus was similar in young and old mice. However, 1222 genes were significantly differentially regulated (FDR<0.1) in callus macrophages from old mice compared to young, and old macrophages demonstrated a more pro-inflammatory phenotype. TREM2 expression was increased in macrophages after fracture in both groups but was significantly less in old mice compared to young via RNAseq and FACS (FDR<0.1, $p<0.05$). TREM2-/- mice demonstrated increased pro-inflammatory cytokine expression within the callus with resulting significant delays in fracture healing compared to age-matched controls ($p<0.05$). Inhibition of macrophage infiltration into the fracture callus significantly improved fracture healing in old mice compared to age-matched controls. Age-related changes to macrophages, including increased pro-inflammatory cytokine expression and dysregulated TREM2 expression, may explain fracture healing deficits observed in older adults. Therapeutically targeting macrophages may improve management of fractures in older adults.

### THE ROLE OF PERCEIVED SOCIAL SUPPORT AND PRUDENT DIET INTAKE ON ALLOSTATIC LOAD AMONG OLDER ADULTS

Danielle D'Amico,[1] Vivian Huang,[1] Geneva Millett,[1] Alexandra J. Fiocco[1], *1. Ryerson University, Toronto, Ontario, Canada*

Allostatic load (AL), an index of multisystem physiological dysregulation due to chronic stress, has been identified as a predictor of poor health outcomes in late life. Research suggests that perceived social support (PSS) improves health outcomes by buffering the negative effects of stress on wellbeing and increasing health promoting behaviours including consumption of a healthy prudent diet (i.e., fruits, vegetables, lean meats, nuts, and seeds). Research to date has independently demonstrated that higher PSS and prudent diet intake have an effect on AL. A paucity of research, however, has examined how dietary consumption and PSS interact to effect AL in older adults. The objective of this study was to examine the interaction between PSS and prudent diet pattern on AL in 164 non-demented, community-dwelling older adults (Mage= 68.5(.52), 64% female). PSS and diet intake were measured using the Perceived Social Support Scale and the EPIC-Norfolk Food Frequency Questionnaire, respectively. AL was composed of 16 biomarkers stemming from neuroendocrine, metabolic, inflammatory, and cardiovascular systems, stratified by sex. Controlling for age and usual daily energy intake, higher prudent diet consumption (B=-2.04, p=.001), but not PSS, was associated with lower AL. Moderation analysis revealed that higher prudent diet intake was associated with lower AL only for those with low PSS (B=-.83, p=.0006) and mean level of PSS (B=-.43, p=.02). These findings suggest that chronic biological stress may be mitigated by consuming a healthy diet specifically for older adults with lower social support and may further inform intervention strategies to promote healthy aging.

### METABOLOMICS IN MEN WITH DISTINCT INFLAMMATION TRAJECTORIES FOLLOWING HIP FRACTURE REVEAL RESPONSE MECHANISMS

Shabnam Salimi,[1] Alice Ryan,[1] Denise Orwig,[1] Ann Gruber-Baldini,[1] Jack Guralnik,[1] Jay S. Magaziner,[1] and March Hochberg[1], *1. University of Maryland Baltimore School of Medicine, Baltimore, Maryland, United States*

Introduction: Following hip fracture, men exhibit different trajectories of inflammation: high (HiInf) vs. low (LoInf). The purpose of this analyses was to compare metabolomics Between these groups. Methods: Seven men with HiInf and 7 with LoInf were randomly selected to quantify serum metabolites at baseline, 2, and 6 months following hip fracture in Baltimore Hip Study-7. We performed analysis of variance and mixed effects models. A $p <0.05$ was considered significant. Results: At baseline, men with HiInf had higher oxidative stress, lipid-related inflammatory markers, and antioxidant compounds compared to

LoInf group. These markers were reduced in both groups at follow-up, consistent with reduced stress. However, the attenuation level was significantly different between the two groups. Kynurenate, that indicates activation of the immune system, was higher in HiInf group and remained elevated over 6 months follow-up. Polyamines with anti-oxidant activity and boosting autophagy activities were declined more significantly in the LoInf group presumably indicating more response to bodily anti-oxidant activity compared to the HiInf group. Arachidonic acid-derived eicosanoids, mediators of the immune response, were significantly elevated at baseline in the LoInf while oxidative stress markers were increased more in HiInf group. Branched-chain amino acids (BCAAs), the essential amino acids abundant in muscle, were elevated at baseline in the LoInf compared to HiInf group. Conclusion: Both groups showed an increase in anti-oxidative stress while LoInf group showed more reduction in oxidative stress and immune cell activity. Reduced intermediate compounds of BCCA suggest that their catabolism was attenuated following hip fracture in LoInf.

### THE ASSOCIATION BETWEEN D3CR MUSCLE MASS AND MORTALITY IN COMMUNITY-DWELLING OLDER MEN

Peggy M. Cawthon,[1] Terri Blackwell,[1] Steven R. Cummings,[1] Eric S. Orwoll,[2] Kate A. Duchowny,[3] Kristine E. Ensrud,[4] Jane A. Cauley,[5] and William J. Evans[6], *1. California Pacific Medical Center Research Institute, San Francisco, California, United States, 2. Oregon Health & Science University, Portland, Oregon, United States, 3. University of California, San Francisco, San Francisco, California, United States, 4. University of Minnesota, Minneapolis, Minnesota, United States, 5. University of Pittsburgh, Pittsburgh, Pennsylvania, United States, 6. University of California, Berkeley, Berkeley, California, United States*

We have shown that men with low muscle mass assessed by D3Cr (deuterated creatine) dilution are more likely to have worse physical performance and incident fractures, injurious falls and disability. However, the relation between D3Cr muscle mass and mortality is unknown. With data from Year 14 Visit of the MrOS study (N=1400, mean age 84.2 yrs), proportional hazards models estimated the risk of mortality (hazard ratio and 95% CI) by quartiles of D3Cr muscle mass (standardized to body mass); we calculated p for trend across quartiles. Models were adjusted for age, race, clinical center, alcohol use, smoking status, comorbidities, activity, percent fat, exhaustion, and cognitive function. Cause of death was centrally adjudicated. Over 3.3±0.8 years of follow-up, 197 (14.1%) men died. Men in the lowest quartile of D3Cr muscle mass/wgt were 2.8-fold more likely to die than men in the highest quartile (HR: 2.8, 95% CI: 1.6, 4.9; p for trend<.001). The HRs for each cause-specific mortality outcome were of similar magnitude to the HR for overall mortality: cancer death (HR, Q1 vs Q4: 2.2, 95% CI: 0.7, 7.1; p trend =0.140); CVD death (HR, Q1 vs Q4: 3.7, 95% CI: 1.3, 10.5; p trend =0.008); or non-cancer non-CVD death (HR, Q1 vs Q4: 2.4, 95% CI: 1.0, 5.6; p trend=0.019). We conclude that low muscle mass assessed by D3Cr dilution is a strong risk factor for mortality in older men, providing additional evidence that low muscle mass is an important risk factor for adverse health outcomes.

### THE PROBLEM OF INTEGRATING OF BIOLOGICAL AND CLINICAL MARKERS OF AGEING

Arnold Mitnitski,[1] and Kenneth Rockwood[2], *1. Dalhousie University, Department of Medicine, Halifax, Nova Scotia, Canada, 2. Dalhousie University, Halifax, Nova Scotia, Canada*

The number of potential biological markers of ageing increases dramatically especially with the development omics technologies. These biomarkers are not generally independent from each other and also related to clinical markers of aging that also could be markers of some illnesses. We discuss three ways of integrating biological and clinical markers of ageing: a frailty index (FI), indices of biological age, and a statistical distance as a measure of physiological dysregulation. We shows that FI has a strong theoretical support in the complex dynamical network model of the ageing process. The theory allows to explain why the interdependence of variables (representing the attributes of health) is essential for understanding of the basic properties both of the FI and of ageing such as a Gompertz law of mortality. Further progress in the field will go hand-in-hand with the development of new technologies that allow more data to be collected and interpreted.

### THE ASSOCIATION BETWEEN MODERATE-TO-VIGOROUS PHYSICAL ACTIVITY AND MUSCLE OXIDATIVE CAPACITY IN OLDER ADULTS

Fatemeh Adelnia,[1] Jacek Urbanek,[2] Yusuke Osawa,[3] Michelle Shardell,[3] Eleanor M. Simonsick,[4] Jennifer A. Schrack,[5] and Luigi Ferrucci[3], *1. National Institute of Health, Baltimore, Maryland, United States, 2. John Hopkins University School of Medicine, Department of Medicine, Division of Geriatric Medicine and Gerontology, Center on Aging and Health, Baltimore, Maryland, United States, 3. Translational Gerontology Branch, Intramural Research Program, National Institute on Aging, National Institutes of Health, Baltimore, Maryland, United States, 4. Longitudinal Studies Section, Intramural Research Program, National Institute on Aging, Baltimore, Maryland, United States, 5. Johns Hopkins University, Baltimore, Maryland, United States*

Age-related decline in muscle oxidative capacity negatively affects muscle function and mobility, which may lead to disability and frailty. Whether exercise and other life-style practices reduce age-related decline in muscle oxidative capacity is unclear. We assessed whether, after accounting for age, higher daily physical activity levels are associated with greater muscle oxidative capacity. Participants included 384 adults (54.7% women) aged 22 to 92 years from the Baltimore Longitudinal Study of Aging. Muscle oxidative capacity was measured in vivo using phosphorous magnetic resonance spectroscopy. We determined the time constant for phosphocreatine recovery (τPCr, in seconds) after exercise, with lower values of τPCr reflecting greater oxidative capacity. Time spent in moderate-to-vigorous physical activity (MVPA) was assessed using accelerometers that participants wore for 5.9 ± 0.9 consecutive days in the free-living environment. In linear regression models, older age was associated with higher τPCr (β = 0.39, p-value <.001) after adjusting for sex, race, height and weight. After including MVPA as an independent variable, the standardized regression coefficient for age was attenuated by 40% to 0.22. p-value <.001). MVPA was strongly associated with lower τPCr (β = -0.33, p-value <.001) after adjusting for health status, education and smoking history and was only attenuated by 3% after additional adjustment for age. These results suggest that MVPA is strongly associated with muscle oxidative capacity independent of age, providing mechanistic insights into the health benefits of daily physical activity in older persons.

### HAND2 TRANSCRIPTION FACTOR ENHANCES NMJ ORGANIZATION AND FUNCTION IN OLD MICE

Anna Carolina Zaia Rodrigues,[1] Henry J. Bonilla,[1] Maria Laura Messi,[1] Zhong-Min Wang,[1] Willard M. Freeman,[2] and Osvaldo Delbono[1], *1. Wake Forest University, Winston-Salem, North Carolina, United States, 2. University of Oklahoma Health Science Center, Norman, Oklahoma, United States*

Over time, declining muscle force and power leads to mobility disability and impaired quality of life. In aging rodents and humans, a denervation and reinnervation process is strongly implicated in sarcopenia: the progressive decline of skeletal muscle mass, composition, and function. We propose that the concomitant decline in expression of Hand2, a key transcription factor (TF) for sympathetic neuron maintenance, induces motor pre- and postsynaptic neuromuscular junction (NMJ) instability and disorganization. To counter the deleterious effect of sympathetic denervation, we developed a novel viral vector (AAV9-Hand2-eGFP, Hand2) carrying Hand2 expression exclusively to sympathetic neurons. Male and female, 16-month-old mice, were examined for signs of muscle denervation and sarcopenia 6 months after IV injection with either Hand2 or control empty virus (AAV9-eGFP, EV). We found that Hand2 increased preterminal synaptic vesicle release, neurofilament phosphorylation (Neurite length: Hand2: 3732±496 µm, EV: 2674±165 µm; P <0.01), NMJ pre/postterminal co-localization, hindlimb muscle mass (EDL: 25%, soleus: 14%, tibialis anterior: 17% and gastrocnemius: 25%; n = 6-8 muscles per treatment group; P < 0.01), myofiber cross-sectional area, and protein kinase-A RIIα/RIα ratio (EV, RIIα:1.05±0.03, RIα:0.93±0.04, ratio: 1.13; Hand2, RIIα:1.81±0.03, RIα:0.94±0.03, ratio: 1.94; P<0.001) which contributes to stability of the NMJ. We also examined Hand2 gene methylation, and RNA-sequencing, muscle metabolomics, and whole body and muscle function with aging in EV and Hand2 injected mice. Our data indicate that expression of Hand2 significantly enhances skeletal muscle adrenergic receptor signaling through the canonical pathway, and prevents in NMJ transmission, and muscle mass and function decline with aging.

### FRAILTY ASSESSMENT BASED ON THE QUALITY OF DAILY WALKING

Danya Pradeep Kumar,[1] Nima Toosizadeh,[1] Jane Mohler,[1] and Kaveh Laksari[1], *1. Biomedical Engineering, University of Arizona, Tucson, Arizona, United States*

Frailty is an increasingly recognized geriatric syndrome resulting in age-related decline in reserve across multiple physiologic systems. An impaired physical function is a prime indicator of frailty. In this study, we aim to implement a body-worn sensor to characterize the quantity and quality of everyday walking, and establish associations between gait impairment and frailty. Daily physical activity was acquired for 48 hours from 125 older adults (≥65 years; 44 non-frail, 60 pre-frail, and 21 frail based on the Fried gold standard) using a tri-axial accelerometer motion-sensor. Continuous purposeful walks (≥60s) without pauses were identified from time-domain acceleration data. Power spectral density (PSD) analysis was performed to define higher gait variability, which was identified by a shorter and wider PSD peak. Association between frailty and gait parameters was assessed using multivariable nominal logistic models with frailty as the dependent variable, and demographic parameters along with the gait parameters as the independent variables. Stride times, PSD gait variability, and total and maximum continuous purposeful walking duration were significantly different between non-frail and pre-frail/frail groups (p<0.05). Using a step-wise model with the above qualitative and quantitative gait parameters as predictors, the pre-frail/frail group (vs. non-frail) was identified with 71.4% sensitivity and 75.4% specificity. Everyday walking characteristics were found to be accurate determinants of frailty. Along with quantitative measures of physical activity, qualitative measures are critical elements representing the stages of frailty. In-home gait analysis is advantageous over clinical gait analysis as it enables cost- and space-effective continuous monitoring.

### LOW-INTENSITY EXERCISE ENHANCES MUSCULAR ANDROGEN/ANDROGEN RECEPTOR TO INHIBIT MYOSTATIN PATHWAY

Bo-Kyung Son,[1] Masato Eto,[1] Miya Oura,[1] and Masahiro Akishita[1], *1. Department of Geriatric Medicine, Graduate School of Medicine, the University of Tokyo, Tokyo, Japan*

Background: Physical exercise is well documented to induce muscle size, strength, and energy metabolism. Although the contribution of systemic or local androgen in exercise-adapted muscle hypertrophy has been suggested, less is known about the molecular pathway of androgen in response to exercise. In the present study, we examined roles of androgen/androgen receptor (AR) after exercise, especially for the suppression of myostatin, a potent negative regulator of muscle mass. Methods and Results: To examine the effects of exercise, we employed low-intensity exercise in mice and electric pulse stimulation (EPS) in C2C12 myotubes. Both mRNA and protein levels of AR significantly increased in skeletal muscle of low-intensity exercised mice and C2C12 myotubes exposed to EPS. Production of testosterone and DHT from EPS-treated C2C12 myotubes was markedly increased. Of interest, we found that myostatin was clearly inhibited by EPS, and its inhibition was significantly abrogated by flutamide, a specific antagonist of AR. Furthermore, IL-6 and phospho-STAT3 (pSTAT3) expression, the downstream pathway of myostatin, were decreased by EPS and this was also reversed by flutamide. Similar downregulation of myostatin and IL-6 was seen in skeletal muscle of low-intensity exercised mice. Conclusion: Muscle AR expression and androgen production were increased by exercise and EPS treatment. As a mechanistical insight, it is suggested that AR inhibited myostatin expression transcriptionally, which downregulates IL-6/pSTAT3 pathway and thus contributes to the prevention of muscle degradation.

### THE EFFECTS OF FRAILTY, POLYPHARMACY, AND COGNITION ON HEALTH OUTCOMES: A STUDY ON INTERRAI RESIDENTIAL CARE DATA

Macy Zou,[1] Ronald Kelly,[2] Betty Chinda,[1] Mckenzie Braley,[1] Tony Zhang,[1] Tara Arvan,[1] Robert Mcdermid,[3] and Xiaowei Song[1], *1. Health Research and Innovation, Surrey Memorial Hospital, Fraser Health Authority, Surrey, British Columbia, Canada, 2. interRAI Evaluation & Research, Fraser Health Authority, Surrey, British Columbia, Canada, 3. Department of Emergency Medicine, Surrey Memorial Hospital, Fraser Health Authority, Surrey, British Columbia, Canada*

Frailty Index (FI), polypharmacy and cognition status are significant health concerns in older adults. We conducted this study to investigate the interplay of frailty, polypharmacy, and cognition, in determining health outcomes. InterRAI Residential Care (RAI-RC MDS2.0) data were retrieved from residential care homes in Surrey, BC, Canada. Older residents (65+ years) who had RAI-RC records between 2016 and 2018 were used in the analysis (n=976). A deficit accumulation-based FI was generated using 36 variables. Information on polypharmacy and cognition were obtained by accounting the total number of medications and the cognitive performance scale. Information on falls, emergency visits, and mortality were followed. Multivariate Cox proportional hazard models were used to examine the effects of these variables on different outcomes. The FI showed a near Gaussian distribution (median= 0.370 mean= 0.372 SD= 0.143), and increased linearly with age on a logarithm scale (R=0.75, p<0.001). Residents with cognitive impairment showed a higher level of the FI (KW= 863.3, p<0.001). A higher FI was associated with an increased risk of death (HR=15.2 p=0.006) and emergency visits (HR=2.72 p=0.048), adjusting for age, sex, medications, and education levels. Frailty, polypharmacy, and cognition levels are associated and have interactive effects on health outcomes. Ongoing research is to validate the findings with large samples in different health settings, and to understand the underlying processes of the effect. The close relationships between frailty, polypharmacy, and cognition with health outcomes call for effective integrated strategies for healthcare of older adults with multiple complex health problems.

### ANTIBIOTIC EXPOSURE IN LONG-TERM CARE FACILITIES IS ASSOCIATED WITH URINARY MICROBIOME PERTURBATIONS

Brent R. Schell,[1] Doyle V. Ward,[1] Evan S. Bradley,[1] Protiva Dutta,[1] and John P. Haran[1], *1. University of Massachusetts Medical School, Worcester, Massachusetts, United States*

The discovery of the human urinary microbiome, defined as the microbial communities which colonize the human urinary tract, has shed new light on the meaning and clinical significance of bacteriuria. The prevalence of asymptomatic bacteriuria has been reported to be as high as 50% in healthy older adults living in long term care facilities, yet the urinary microbiome of this population has not been reported. The aim of this pilot study was to describe the urinary microbiome of this population and explore the cross-sectional relationship with recent antibiotic exposure. Voided urine samples were obtained from healthy, institutionalized older adults (ages 79 to 95), including non-catheterized men and women without any urinary symptoms. The bacterial genomic content of each urine sample was assessed by 16S rRNA sequencing. Among the 77 genera found across 16 urine samples, there was no significant difference in the microbial diversity across age groups. When grouped by antibiotic exposure, those recently exposed had a significantly lower diversity (Shannon's index of 2.16 vs. 2.61, p = 0.029), and lower evenness (Pielou's evenness of 0.58 vs. 0.69, p= 0.017) relative to those who were not recently exposed. Enrichment analysis showed that recent antibiotic exposure was associated with an increased abundance of the genus Bacteroides and decreased abundance of the genus Streptococcus. To our knowledge, this is the first report describing the urinary microbiome of institutionalized older adults and suggests that recent antibiotic exposure should be accounted for in future studies of the urinary microbiome in this population.

### PROTEIN SUPPLEMENTATION DOES NOT RESCUE MUSCLE MASS AND FUNCTION–HEAVY RESISTANCE TRAINING IS REQUIRED

Lars Holm,[1] Rasmus Bechshoeft,[2] Soren Reitelseder,[2] Kenneth Mertz,[2] Jacob Bulow,[2] and Grith Hojfeldt[2], *1. University of Birmingham, Birmingham, UK, United Kingdom, 2. Institute of Sports Medicine, Bispebjerg Hospital, Copenhagen, Denmark, Denmark*

The requirement of an enhanced dietary protein intake to counteract the age-related loss of muscle mass is still debated. Further, the dinner meal generally contains the majority of protein and energy and since, the muscle of older adults responds less to protein intake than that of younger adults it is hypothesized that older adults would benefit from taking more protein in at other meals. The aim of this study was to investigate whether the provision of protein supplements for breakfast and lunch meals over the course of a year would make healthy, older, home-dwelling adults (N=136) take in more protein and whether that then would affect their muscle mass (primary outcome) and a number of metabolic health parameters, muscle strength parameters and functional capabilities. More than 77% ingested more than 75% of the provided supplements, irrespective of supplementation type (isocaloric carbohydrate; collagen hydrolysate low quality protein: whey hydrolysate high quality protein). Providing supplementation for a year among older adults makes them comply very well. However, provision of extra protein has no impact on the muscle mass or strength or on the functional parameters. Further, we studied the impact of adding resistance training on top of WHEY protein supplementation and found that heavy more than light-load resistance training affects fat-free mass and maximal-voluntary contraction. Daily protein intake can be enhanced by supplementation but do not impact muscle mass and function over the course of a year, where heavy resistance training on top benefits, but to a lesser than expected degree.

### CORRELATION BETWEEN FRAILTY AND DNA DAMAGE IN HEMATOPOIETIC STEM CELLS: A PILOT STUDY

Paolo Mazzola,[1] Silvia Bombelli,[2] Chiara Grasselli,[1] Maddalena Bolognesi,[1] Laura Antolini,[1] Giorgio Cattoretti,[1] Giorgio Annoni,[1] and Roberto Perego[1], *1. University of Milano-bicocca, School of medicine and surgery, Monza, Italy, 2. University of Milano-Bicocca, Department of Medicine and Surgery, Monza, Italy*

Frailty is an age-related syndrome characterized by a progressive impairment of multiple physiological systems and leading to poor clinical and functional outcomes. Our aim was to explore the DNA damage, as an effect of increased oxidative stress related to frailty, on peripheral blood mononuclear cells (PBMC) and circulating hematopoietic stem cells (cHSC). According to Fried's operating definition of frailty, we enrolled three groups of subjects: frail seniors (age >65 years, n=19), fit seniors (>65 years, n= 16) and young controls (age 25-35 years, n=19). We carried out a comprehensive assessment and obtained 3 vials of whole blood for cells and plasma separation. We separated PBMC by Ficoll-Paque and stained with specific conjugated antibodies leucocyte lineage and HSC. We evaluated DNA damage by FACS detection of γ-H2AX in the total PBMC and cHSC subpopulation. We observed an increased percentage of cells, although not significant, with DNA damage in PBMC from frail seniors (0.70%) compared to fit seniors (0.37%) and young controls (0.13%). The percentage of cells with DNA damage in cHSC of frail seniors (2.97%) was higher compared to fit seniors (1.66%, not significant) and young controls (0.46%, statistically significant). Moreover, cHSC present the statistically higher amount of DNA damage, measured as fluorescence intensity, compared to fit seniors and young controls. cHSC from frail seniors show the highest total DNA damage, compared to fit seniors and young controls. This is probably linked to an increase of oxidative stress related to frailty, which we are going to analyze in the near future.

### SUPPRESSION OF GHRELIN SIGNALING EXACERBATES ULCERATIVE COLITIS IN OLDER MICE

Chia-Shan Wu,[1] Jiyeon Noh,[1] Ellie Tuchaai,[1] Jennifer A. DeLuca,[1] Kimberly F. Allred,[1] Clinton D. Allred,[1] and Yuxiang Sun[1], *1. Texas A&M University, College Station, Texas, United States*

The aging process is characterized by increased chronic low-grade inflammation, aka inflamm-aging, which offend is accompanied by 'leaky gut' syndrome. Inflamm-aging is a highly significant risk factor for both morbidity and mortality in the older adult population (>65 years of age). In addition, there is a growing prevalence of inflammatory bowel disease (IBD), a chronic inflammatory condition of the gastrointestinal tract in the older adult population. The pathogenesis of late-onset IBD is suggested to be more complex compared with younger IBD patients; the causes determining the age of IBD onset remain unexplained. Ghrelin is a 28-amino-acid peptide hormone mainly produced by X/A-like cells of the stomach, with well-characterized functions in growth hormone secretion, food intake, adiposity and insulin resistance. Ghrelin's biological relevant receptor is Growth Hormone Secretagogue Receptor (GHS-R). Ghrelin and ghrelin mimetics have been considered viable candidates for treating cachexia, sarcopenia, and gastrointestinal disorders. As expected, we observed that the expression of tight junction proteins in colon mucosal layer decreases with age. When challenged with dextran sulfate sodium (DSS) to induce experimental ulcerative colitis, 18-months old male C57BL/6 mice exhibited exacerbated disease activity scores compared to young male mice (5-months), showing worsened pathology such as rectal bleeding and difficulty in defecation. DSS-induced colitis was exacerbated in both ghrelin-deficient (Ghrl-/-) and ghrelin receptor-deficient (Ghsr-/-) mice. Together, these data suggest endogenous ghrelin signaling contributes to susceptibility to colitis, and ghrelin signaling pathway may present a novel target for prevention and treatment of leaky gut syndrome in aging.

### GENOME-WIDE META-ANALYSIS SUPPORTS HETEROGENEITY IN POLYGENIC ARCHITECTURE OF DISABILITY

Alexander Kulminski,[1] Chansuk Kang,[1] Yury Loika,[1] Eric Stallard,[1] and Irina Culminskaya[1], *1. Duke University, Durham, North Carolina, United States*

Disability and frailty increase steeply with age after midlife. They comprise broad, non-specific aging-related health/well-being declines. Understanding the molecular mechanisms of these declines could help in combating processes related to intrinsic aging. We performed genome-wide meta-analysis of disability using data on N=12,550 disabled individuals of Caucasian ancestry from five independent studies with N=24,068 genotyped participants. Disability was measured using the Activities of Daily Living (ADL) scale. A subject was considered disabled if he/she had at least one ADL impairment. All subjects in our study were aged 50+ years. The analysis followed a discovery-replication strategy using two studies as discovery samples and three others as replication samples. At the discovery stage, we selected SNPs at nominal significance ($p<0.05$) and evaluated their associations with disability in the replication samples. Meta-analysis across all studies identified 30 SNPs (24 loci) at p<10e-4. SNPs from four loci (chromosomes 2, 8, and 12) attained suggestive significances at p<10-5. We constructed two polygenic risk scores (PGRS) using these 30 SNPs whose minor alleles were positively (19 SNPs) and negatively (11 SNPs) associated with disability, PGRS_p and PGRS_n, respectively. Meta-analysis across all studies identified strong effects for PGRS_p (beta=0.436, p=1.18×10e-32) and PGRS_n (beta=-0.426, p=8.74×10e-19). The associations for the PGRSs observed in two discovery studies were replicated in three independent studies. The lack of genome-wide significant effects of individual SNPs combined with the highly significant effects of the PGRSs shows that, in line with the heterogeneous origin of disability, its genetic architecture is of highly heterogeneous polygenic origin.

### WHOLE-BODY MRI TO ASSESS SUBCLINICAL CARDIOVASCULAR DISEASE AND FRAILTY DEVELOPMENT

Bharath Ambale-Venkatesh,[1] Jaclyn Sesso,[1] Jeremy Walston,[2] Karen Bandeen-Roche,[2] and Joao Lima[1], *1. Johns Hopkins University, Baltimore, Maryland, United States, 2. Johns Hopkins Bloomberg School of Public Health, Baltimore, Maryland, United States*

Cardiovascular disease (CVD) and frailty have been linked at the mechanistic and epidemiological levels. Our goal is to identify if subclinical markers such as atherosclerosis, body composition, and fibrofatty infiltration measured from non-contrast whole-body magnetic resonance imaging (MRI) are markers of physical frailty. Community dwelling older adults with frailty status ascertained by Fried measurement are being recruited from an aging studies registry. MRI is performed using a Canon Galan3T with dedicated coils. Preliminary analysis from 4 frail individuals (86±15 years, 3 female, BMI=22±3kg/m2) and 2 age-matched robust controls (86±1 years, 1 female, BMI=28±0.2kg/m2) is presented. Of 4 frail one had a prior heart attack; one was previously diagnosed with heart failure. Mean atheroma score from 28 vessel segments (0.42±0.26 vs 0.18±0.10) and aortic tortuosity (2.3±0.4 vs 2.1±0.1) were higher in frail compared to robust indicative of higher atherosclerotic burden and vascular stiffness. Mean subcutaneous and visceral adipose tissue volumes were lower in frail compared to robust. However, mean myocardial (1113±27 vs 1089±2), liver (729±92 vs 683±104) and skeletal muscle (1106±25 vs 1072±64) T1 times (milliseconds) were each higher indicative of greater diffuse interstitial fibrosis. Averaged intramuscular fat percent measured across the pelvis, forearm, pectus, thigh, and calf was higher in frail compared to robust (14.8±4.1% vs 8.5±2.3%) indicative of higher fatty infiltration. Although these early results do not reach statistical significance, they support further study to determine cardiovascular and tissue related differences between physically frail and robust older adults, which in turn may inform intervention developments for frailty and CVD.

### β-GPA: AN AMPK ACTIVATOR WITH POTENTIAL EFFECTS ON HEALTHSPAN AND FUNCTION

Jonathan Dorigatti,[1] Kevin Thyne,[1] Yuhong Liu,[2] and Adam B. Salmon[2], *1. University of Texas, Health Science Center at San Antonio, San Antonio, Texas, United States, 2. University of Texas Health at San Antonio, San Antonio, Texas, United States*

β-Guanidinopropionic acid (β-GPA) is a naturally occurring compound reported to activate AMP Activated Protein Kinase (AMPK) signaling in vivo. Acute administration of β-GPA in young animals has been reported to improve multiple functional measures including a switch to oxidative fatigue-resistant muscle fibers, improved glucose uptake, and increased mitochondrial biogenesis. However, it is unknown if β-GPA may promote healthy aging or prevent late-life functional decline. To address this knowledge gap, we tested the effects of β-GPA on mitochondrial energetics and cellular function in young and old genetically heterogenous mice (HET3). Both age groups were fed either 1% β-GPA or control chow for ~5 months and basic functional parameters including metabolism were assessed. β-GPA treatment decreased lean and fat mass in young males, but prevented late-life losses in these parameters in old animals. Notably, glycated hemoglobin (HbA1c) levels were lower in treated young and old males suggesting improved glucose homeostasis. Citrate synthase activity was also higher in old males fed β-GPA suggesting increased mitochondrial biogenesis. At the molecular level, mitochondrial Complex I expression decreased with β-GPA treatment in old males versus controls. High resolution respirometry revealed generally decreased respiration in old animals compared to young and decreased Complex 1 coupled respiration in soleus of β-GPA treated young but not old males. These findings indicate that the mitochondrial effects attributed to β-GPA may be mediated by its action on Complex I. While treatment outcomes varied in young and old males these results suggest β-GPA may prove beneficial in combating age-related declines in function.

### AGE, SEX, AND FRAILTY INFLUENCE AGE-DEPENDENT CHANGES IN VENTRICULAR STRUCTURE AND FUNCTION IN C57BL/6 MICE

Susan E. Howlett,[1] Alice Kane,[2] Elise Bisset,[1] and Kaitlyn Keller[1], *1. Dalhousie University, Halifax, Canada, 2. Paul F. Glenn Center for Biology of Aging Research at Harvard Medical School, Boston, Massachusetts, United States*

The heart undergoes maladaptive changes during aging that set the stage for cardiovascular diseases, but frail older individuals are most likely to develop such diseases. We investigated the impact of frailty on left ventricular (LV) remodeling in male and female mice (aged 9-23 mos). Ventricular function/structure and frailty were assessed with echocardiography (Vevo 2100) and a frailty index (FI) tool. Fractional shortening (systolic function) increased with age (9 vs 23 mos) in males (27.7±2.6 vs 38.4±1.6%; $p<0.05$) and females (26.9±1.4 vs 32.5±1.8%; $p<0.05$); similar results were seen with ejection fraction. Conversely, E/A ratios (diastolic function) declined with age in males (1.9±0.1 vs 1.3±0.1; $p<0.05$) and females (2.1±0.3 vs 1.6±0.1; $p<0.05$). LV mass and LV internal diameter (diastole) increased with age in females but not in males, while intraventricular septum (diastole) increased in males only. As age-dependent changes were heterogeneous, we stratified the data by FI scores. Interestingly, fractional shortening ($r=0.52$; $p=0.006$) and ejection fraction ($r=0.52$; $p<0.0001$) were graded by FI score, but only in males. By contrast, LV mass was graded by frailty, but only in females ($r=0.55$; $p<0.0001$). Thus, diastolic function declines with age in both sexes while systolic function actually increases. Aging females display increased LV mass and LV dilation whereas older males exhibit septal thickening. These maladaptive changes are graded by frailty, suggesting that cardiac aging is prominent in those with poor overall health. Age, sex and frailty influence cardiac aging, which may predispose frail older men and women to develop different cardiovascular diseases as they age.

### CASE STUDY EXEMPLAR OF DETECTING SEVERE DIASTOLIC DYSFUNCTION USING BALLISTOCARDIOGRAM

Laurel A. Despins,[1] Giovanna Guidoboni,[1] Marjorie Skubic,[1] Lorenzo Sala,[2] Moein Enayati,[1] James Keller,[1] and Mihail Popescu[1], *1. University of Missouri, Columbia, Missouri, United States, 2. Université de Strasbourg, Strasbourg Cedex, France*

The specific aim of this case study was to describe how monitoring ballistocardiogram (BCG) waveforms can detect early heart failure (HF) changes. HF significantly impairs quality of life and is the principal cause for hospital readmissions in older adults. HF prevalence in American adults aged

65 years and older is expected to increase over 70% by 2030. Detecting worsening HF is challenging. Invasive arterial waveforms display blood pressure changes with each heartbeat; BCG waveforms display repetitive body motions resulting from ejection of blood into the great vessels. BCG waveforms change as cardiac function changes. Currently, BCG signals can be captured non-invasively using sensors placed under a bed mattress and provide heart and respiratory rates. We have developed a new way to analyze the BCG waveform using an innovative closed-loop physiological model of the cardiovascular system. The subject, a 94-year old female with hypertension, presented to her physician with symptoms associated with a new diagnosis of acute mixed congestive HF. Mean heart and respiratory rate trends obtained from her bed sensor in the prior two months did not indicate HF. We simulated cardiac cycles using normal cardiac function data, mildly impaired diastolic function data, and the subject's echocardiography data. The results demonstrated BCG waveform changes that correlated with decreasing cardiac output related to worsening diastolic function. New methods for clinically interpreting BCG waveforms present a significant opportunity for improving early HF detection and improving outcomes. Working on a clinical problem from an engineering perspective merges two disciplines, creating a new methodology.

### GAIT SLOWING AMONG FRAIL OLDER ADULTS: IS HIGHER DOPAMINERGIC SIGNALING PROTECTIVE?

Caterina Rosano,[1] Stephanie Studenski,[2] Nicolaas Bohnen,[3] and Andrea Rosso[4], *1. University of Pittsburgh, PIttsburgh, Pennsylvania, United States, 2. NIA, Longitudinal Studies Section, Baltimore, Maryland, United States, 3. University of Michigan, Ann Arbor, Michigan, United States, 4. University of Pittsburgh, Department of Epidemiology, Pittsburgh, Pennsylvania, United States*

Strategies to reduce gait slowing in frail older adults are urgently needed. Higher dopaminergic (DA) signaling is emerging as a protecting factor against age-related gait slowing, in the absence of Parkinson's Disease (PD). DA signaling is potentially modifiable, thereby offering promising novel strategies to reduce gait slowing. In 3,752 PD-free participants of the Cardiovascular Health Study (72.3 years, 81% white, 39% male), we measured gait speed (usual pace, 15 feet), frailty (Fried definition), and genetic polymorphism of Catechol-O-methyltransferase (COMT, rs4680), an enzyme regulating tonic brain DA levels. Multivariable linear regression models of COMT predicting gait speed were adjusted for age, gender, BMI, ankle-arm index, vision, and arthritis. Strength, education, medications, pulmonary, cardio- and cerebro-vascular diseases, diabetes, mood, and cognition were considered as additional covariates. We examined the full cohort and the subgroup with frailty (n=222), without and with race-stratification to address racial differences in allele frequencies. Average (SE) gait speed was 0.88 (0.003) and 0.58 (0.01) m/sec in the full cohort and the frail subgroup, respectively. COMT was linearly associated with gait speed; gait was faster for met/met (higher DA signaling) and slower for val/val (lower DA signaling) participants. In adjusted models, differences between these two groups were: 0.02 (0.01) m/sec in the full cohort (p=0.4); 0.07(0.02) m/sec in the frail subgroup (p=0.02); 0.10 (0.02) m/sec in white with frailty (p=0.01). COMT genotyping may help identify frail adults who are less vulnerable to gait impairments. Studies of frailty should examine whether higher DA signaling offers resilience against age-related gait slowing.

### WHOLE-BODY TAU-KNOCKOUT MICE DEVELOP AGE-ASSOCIATED METABOLIC DYSFUNCTION AND OBESITY

Eric Baeuerle,[1] NING ZHANG,[2] Nicolas Musi,[2] and Miranda E. Orr[3], *1. Barshop Institute for Longevity and Aging Studies, UT Health San Antonio, San Antonio, Texas, United States, 2. Barshop Institute for Longevity and Aging Studies, University of Texas Health Science Center, San Antonio, Texas, United States, 3. GRECC, South Texas Veterans Health Care System, San Antonio, Texas, United States*

The microtubule associated tau protein (MAPT) is expressed in multiple tissues; however, the primary focus has been its role in neurodegenerative diseases such as Alzheimer's disease. Few efforts have been made to investigate tau protein function outside of the nervous system. We previously noted that aged (20 mo.) Mapt-KO mice had significantly greater body mass than either age-matched wild-type mice or a Mapt-KO/rTg4510 mouse model of late stage neurodegeneration. We hypothesize that endogenous tau contributes to normal metabolic function and sought to characterize potential metabolic alterations in whole body Mapt-KO mice. In 2-3 month-old wild type (WT) and Mapt-KO mice fed ad lib chow diet, we observed no significant difference in glucose or insulin tolerance. However, in 16 month-old Mapt-KO mice fed ad lib chow, we observed glucose intolerance ($p<0.05$) and increased body mass (1.45-fold, $p<0.001$) compared to WT. In this aged cohort, we evaluated body composition by quantitative magnetic resonance, spontaneous activity and running, grip strength and Rotarod performance, nest building, and performed high resolution respirometry in hippocampus and soleus. Mapt-KO shows significant increases in lean and fat mass (1.18-fold, 2.97-fold, $p<0.05$) and nest building (Deacon Score, $p<0.05$), and reductions in ambulatory activity ($p<0.01$) and rotarod balancing ($p<0.0001$) despite an ability to learn the task. No significant changes were seen in grip strength, spontaneous running, or mitochondrial respiration, although there was a trend of reduced maximum uncoupled respiration in Mapt-KO hippocampus ($p<0.1$). This study concludes that Mapt plays an important role in glucose homeostasis and body weight regulation.

### LOSS OF HYPOXIA SIGNALING LIMITS SKELETAL MUSCLE RESPONSE TO AEROBIC EXERCISE IN AGING

Yori Endo,[1] Bin Li,[2] Kodi Udeh,[3] Adriana C. Panayi,[3] Ron Neppl,[4] and Indranil Sinha[2], *1. Harvard Medical School, Boston, Massachusetts, United States, 2. Brigham and Women's Hospital, Division of Plastic Surgery, Boston, Massachusetts, United States, 3. Brigham and Women's Hospital, Harvard Medical School, Boston, Massachusetts, United States, 4. Brigham and Women's Hospital, Department of Orthopaedic Surgery, Boston, Massachusetts, United States*

Skeletal muscle function declines with aging. Physical activity improves muscle function, but may require upregulation of the hypoxia signalling pathway. In this study, we examine the effect of exercise on muscle function in young and old mice and explore the role of hypoxia pathway in mediating exercise-induced functional improvement. Young (3 months) and old (22-24 months) mice underwent either aerobic exercise training on a treadmill for 8 weeks (40 minutes per run, 3 times weekly, at 8 meters/min at a 15 degree inclination) or no exercise as a control. Maximum running speed and distance were measured pre- and post-exercise. Following training, there was a significant increase in both maximal speed (20%, $p<0.05$) and distance (2.5 fold) running in young mice which exercised versus young control ($p<0.01$). Old mice following exercise training did exhibit any improvement versus the old control group. Notably, protein levels of aryl hydrocarbon receptor nuclear translocator (ARNT), a critical regulator of hypoxia signalling, was 4.7-fold lower in the muscle of old mice after exercise as compared to young ($p<0.01$). To evaluate further whether loss of hypoxia signaling limits response to exercise, we developed a mouse with an inducible, skeletal muscle specific ARNT knockout (ARNT mKO). ARNT mKO mice exhibited no improvement in either maximal running speed or distance following exercise training. In contrast, littermate controls, with normal ARNT levels, exhibited improvement in both maximal speed and distance running following exercise. Our results suggest that an aging-associated decline ARNT and hypoxia signalling may limit aerobic exercise benefits.

### QUANTIFICATIONS OF BIOLOGICAL AGING PREDICT DISABILITY AND MORTALITY IN OLDER ADULTS IN THE DUKE EPESE

Daniel C. Parker,[1] Virginia B. Kraus,[2] and Daniel Belsky[3], *1. Duke University School of Medicine, Durham, North Carolina, United States, 2. Duke University, Durham, North Carolina, United States, 3. Columbia University Mailman School of Public Health, New York, New York, United States*

Methods to quantify biological aging have been proposed to measure age-related decline in system integrity for population surveillance and evaluation of geroprotective therapies. However, quantifications of biological aging have been little-studied in geriatric populations. We conducted analysis of three clinical-biomarker-algorithm methods to quantify biological aging, the Klemera-Doubal Method (KDM) Biological Age, homeostatic dysregulation (HD), and Levine Method (LM) Biological Age in a cohort of N=1,374 older adults aged 71-102 years (35% male, 52% African American), the Duke-EPESE. We parameterized algorithms from analysis of US NHANES data (N=36,207). We conducted criterion validity analyses using measures of disability and mortality as end-points. We analyzed counts of ADLs and iADLs using negative binomial regression. We analyzed time-to-death using Cox regression. Models were adjusted for age, sex, and race/ethnicity. We evaluated algorithms derived from analysis of different biomarker groupings. We also compared algorithms derived from analysis of mixed age and race/ethnicity samples to algorithms derived from older-age (65+) and individual race/ethnicity samples. Duke-EPESE participants with older KDM Biological Age reported dependence in more ADLs and iADLs, and were at increased risk of death (ADL IRR=1.19 [1.12, 1.27]; IADL IRR=1.18 [1.10, 1.26]; mortality HR=1.09 [1.06, 1.13]). Quantifications of biological aging derived from analysis of a mixed-age and race/ethnicity sample predicted disability and mortality in African-American and white older adults.

### SESSION 825 (POSTER)

### BIOLOGY OF AGING II

### UP-REGULATION OF LONG NON-CODING RNA MORBID ATTENUATES SENESCENCE

Zheng Kuai,[1] Meiting Chen,[2] yang yu,[2] Fan Yang,[2] and chunxiang Zhang[2], *1. Department of Geriatrics,Zhongshan Hospital, Fudan University, Shanghai, China, 2. the University of Alabama at Birmingham, Alabama, United States*

Aging is the inevitable, irreversible decline in function on the cellular and organ level leading to increased incidence of the most frequent diseases such as cancer and cardiovascular disease, that occurs over time. whereas the molecular mechanisms of senescence remain largely unknown. Here we identified that a novel long noncoding RNA, Morrbid was significantly decreased in different organs of aged mice, such as heart, liver, spleen, lung, kidney and brain. Interestingly, the telomeres length of Morrbid KO mice were significantly shorted than the WT mice at the same age. We also found that Morrbid was steeply decreased in a natural mouse cardiac myocyte senescence model. The senescence of mouse cardiac myocytes was effectively attenuated by Morrbid over-expression shown by the decreased β-galactosidase staining, increased telomere activity, decreased production of ROS and decreased cell apoptosis, but was enhanced by Morrbid knockdown. The results suggest that Morrbid is a critical regulator in senescence and could be used as a novel diagnostic biomarker for it, and a new therapeutic target for diverse diseases.

### BENEFICIAL EFFECTS OF A PLASMA FRACTION ON NEUROGENESIS FOR AGE-RELATED COGNITIVE DISORDERS

Ian Gallager,[1] Viktoria Kheifets,[1] S. Sakura Minami,[1] Eva Czirr,[1] scott lohr,[1] nina huber,[1] meghan Campbell,[1] and Steven P. Braithwaite[1], *1. Alkahest Inc., San Carlos, California, United States*

The process of aging is multifactorial and therefore single interventions may be unlikely to attenuate the myriad pathologies. Plasma contains many beneficial factors which have been shown in animal models to ameliorate multiple age-related deficits across varied organ systems, including the brain. We demonstrated that human plasma from young 18-22-year-old donors reverses age-related cognitive decline and enhances hippocampal neurogenesis and cell survival in aged immunocompromised mice, while plasma from aged individuals (62-68 years old) has detrimental effects in young mice. Utilizing cell-based assays we identified a human plasma fraction (PF) which enhanced neuronal outgrowth and synaptic connectivity. We demonstrate that PF administration provides benefits beyond those observed with whole plasma treatment, resulting in decreased brain inflammation, increased synaptic density and neuronal activation.

We evaluated longitudinal treatment of PF and observed no depletion in stem cell populations while maintaining an enhanced level of neurogenesis. We examined PF in an α-synuclein mouse model of Parkinson's disease, where treatment significantly reversed functional, inflammatory, and neuronal deficits. To further our understanding of interplay between multiple mechanisms inducing neurogenesis, we examined the effect of exercise on PF treated mice and observed a synergistic increase in neurogenesis. Proteomic analysis of mouse plasma following PF treatment demonstrates differential protein levels compared to running or the combination of running and PF, suggesting that PF is mechanistically different from the effect of running. In summary, we demonstrate that PF is a multifactorial and multimodal, clinically-relevant, intervention for the treatment of global changes induced by aging.

### NITRIC OXIDE SYNTHASE DYSFUNCTION UNDERLIES CEREBROVASCULAR DEFICITS IN A MOUSE MODEL OF TAUOPATHY

Candice E. Van Skike,[1] Stacy A. Hussong,[2] Andy Banh,[3] and Veronica Galvan[4], *1. Barshop Institute - UT Health San Antonio, San Antonio, Texas, United States, 2. University of Texas Health Science Center at San Antonio, San Antonio, Texas, United States, 3. Barshop Institute of Longevity and Aging Studies - UT Health San Antonio, San Antonio, Texas, United States, 4. Barshop Institute for Longevity and Aging Studies, San Antonio, Texas, United States*

We recently identified pathogenic soluble aggregated tau (tau oligomers) in the cerebral microvasculature of human patients with tauopathies, including Alzheimer's disease (AD). The functional consequences of cerebrovascular tau accumulation are not yet understood. The aim of the present study was to determine whether pathogenic tau accumulation leads to cerebrovascular dysfunction. To this end, we measured neurovascular coupling (NVC), a highly regulated process that synchronizes cerebral blood flow to neuronal activation, using the PS19(P301S) mouse model of tauopathy. The change in cerebral blood flow evoked by whisker stimulation was measured using Laser Doppler flowmetry in PS19 and wildtype control mice and the functional contribution of neuronal and endothelial nitric oxide synthase (nNOS and eNOS, respectively) was calculated. Vascular reactivity was assessed using topical acetylcholine to evoke endothelium-dependent vasodilation. To assess the direct impact of pathogenic tau on cell-specific NOS function, we treated N2a neuroblastoma cells or mouse brain vascular endothelial cells with soluble tau aggregates and measured activity of nNOS and eNOS. Our data indicate isolated overexpression of mutant tau impairs NVC responses, and this deficit is mediated by a reduction in nNOS activity in vivo. Further, our studies suggest tauopathy also impairs endothelium-dependent vasoreactivity in the cortex. Additionally, soluble tau aggregates inhibit the phosphorylation of NOS in primary cultured cells. Therefore, inhibition of NOS phosphorylation by pathogenic soluble tau aggregates may underlie cerebrovascular dysfunction in tauopathies. Thus, therapeutic modulation of pathogenic tau may mitigate brain microvascular deficits, which occur prior to clinical onset in Alzheimer's disease and potentially other tauopathies.

### IMPROVING BRAIN AGE PREDICTION MODELS: INCORPORATION OF AMYLOID STATUS IN ALZHEIMER'S DISEASE

Maria Ly,[1] Nishita Muppidi,[1] Helmet Karim,[1] Gary Yu,[1] Akiko Mizuno,[1] William Klunk,[1] and Howard Aizenstein[1], *1. University of Pittsburgh, Pittsburgh, Pennsylvania, United States*

Brain age prediction may serve as a promising, individualized biomarker of brain health and may help us understand the heterogeneous biological changes that occur in aging. Brain age prediction is a machine learning method that estimates an individual's chronological age from their neuroimaging scans. If predicted brain age is greater than chronological age, that individual may have an "older" brain than expected, suggesting that they may have experienced a higher cumulative exposure to brain insults or were more impacted by those pathological insults. However, contemporary brain age models include older participants with amyloid pathology in their training sets and thus may be confounded when studying Alzheimer's disease (AD). We showed that amyloid status is a critical feature for brain age prediction models by training a model on 808 individuals without significant amyloid pathology from the ADNI, OASIS-3, and IXI cohorts. Our model accurately predicted brain age in the training and independent test sets, comparable to previous published models: [$r(807) = 0.94$, $R2 = 0.88$, $p=0.001$, MAE = 4.9 years, $p=0.001$], [$r(39) = 0.67$, $R2 = 0.45$, and MAE = 4.6 years]. We demonstrated significant differences between AD diagnostic groups [$F(3,431)=30.7$, $p<0.001$], and our model was the first to delineate significant differences in brain age relative to chronological age between cognitively normal individuals with and without amyloid [mean difference, 95% CI; CN-Aβ(-) (-3.4, -4.9:-1.8), CN-Aβ(+) (-0.7, -1.9:0.5)]. Ultimately, incorporation of amyloid status in brain age prediction models improves the utility of brain age as a biomarker for aging and AD.

### TAU-INDUCED ASTROCYTE SENESCENCE: A NOVEL MECHANISM FOR NEURONAL DYSFUNCTION IN ALZHEIMER'S DISEASE

Angela Olson,[1] Stacy A. Hussong,[2] Rakez Kayed,[3] and Veronica Galvan[1], *1. Barshop Institute for Longevity and Aging Studies, San Antonio, Texas, United States, 2. University of Texas Health Science Center at San Antonio, San Antonio, Texas, United States, 3. Dept. of Neurology, University of Texas Medical Branch at Galveston, Galveston, Texas, United States*

Chronic sterile inflammation is a pathological feature of Alzheimer's disease (AD) and other neurodegenerative diseases. The mechanisms that drive neuroinflammation and its impact on AD progression are still incompletely understood. The accumulation of molecular damage in somatic cells can trigger cellular senescence, an irreversible state of cell cycle arrest accompanied by the expression of proinflammatory mediators known collectively as the "senescence-associated secretory phenotype" (SASP). Misfolded tau forms pathogenic soluble aggregates that are released extracellularly and are transmitted trans-neuronally, promoting native tau phosphorylation and aggregation in target cells. We recently showed that, in addition to neurons, pathogenic soluble extracellular tau aggregates propagate to brain microvascular endothelial cells, where microtubule destabilization triggers senescence/

SASP. Because astrocytes have a critical role in the regulation of both synaptic function and cerebral blood flow and are directly exposed to tau at its site of release at the tripartite synapse, we conducted studies to define whether soluble aggregated tau propagates to astrocytes, inducing astrocyte senescence/SASP and neuronal dysfunction/damage. Our studies indicate that tau can be propagated transcellularly to astrocytes, triggering cellular senescence/SASP. Our studies suggest that astrocyte senescence is detrimental to dendritic and synaptic structure and density, suggesting that pathogenic soluble tau-induced astrocyte senescence may contribute to synaptic dysfunction and loss in AD. Drugs that eliminate senescent cells are FDA-approved and antibody-based approaches to remove tau from brain are already in clinical trials. Our studies suggest that these interventions could be effective in the treatment of AD and other tauopathies.

### TYPE I INTERFERON-MEDIATED NEUROINFLAMMATORY PROGRAM AND SYNAPSE LOSS IN ALZHEIMER'S DISEASE

Ethan Roy,[1] Baiping Wang,[1] Hui Zheng,[1] and Wei Cao[1], *1. Baylor College of Medicine, Houston, Texas, United States*

The cytokine family type I interferon (IFN) is a major innate immune mediator extensively studied in the peripheral immune responses but largely under-investigated in AD. Previously, we established that innate immune cells readily produce IFN in response to amyloid fibrils containing nucleic acids as cofactor. Here, we investigated whether IFN pathway is associated with amyloidosis in AD brain and contributes to neuroinflammation. By systemically characterizing neuroinflammation in multiple murine AD models, we established a comprehensive core AD neuroinflammation profile that includes several key proinflammatory cytokine families, among which IFN pathway is consistently activated. When hippocampal slice culture was stimulated with different forms of amyloid fibrils, nucleic acid-containing amyloid fibrils, but not heparin-containing fibrils, potently activated IFN pathway and triggered comprehensive neuroinflammation. In addition, stereotaxic administration of IFNβ induced an immune response in the brain of wild type mice analogous to the core neuroinflammatory profile associated with Aβ pathology; whereas selective IFN receptor blockade significantly blunted the ongoing microgliosis in AD models in vivo. Furthermore, IFN promoted microglia-mediated synapse uptake from neurons, which depended on the induction of complement C3, and blockade of IFN signaling significantly abolished the pathogenic synapse loss in AD brain. Consistent with the findings in mice, we found that genes stimulated by IFN were grossly upregulated in human AD brains. Therefore, type I interferon constitutes a major pathway within the neuroinflammatory network of AD and may represent a molecular target to restrain the pathogenic inflammatory responses.

### METABOLOMICS OF DELIRIUM: A CASE-CONTROL STUDY

Bridget Tripp,[1] Simon Dillon,[2] Sarinnapha Vasunilashorn,[2] Long H. Ngo,[2] Tamara G. Fong,[2] Edward R. Marcantonio,[2] Towia A. Libermann,[2] and Hasan Otu[1], *1. University of Nebraska-Lincoln, Lincoln, Nebraska, United States, 2. Beth Israel Deaconess Medical Center, Boston, Massachusetts, United States*

Postoperative delirium complicates 15-50% of major surgery in older adults, resulting in poor patient outcomes and increased healthcare costs. Basic mechanisms of delirium are largely unknown. In this study we implemented a protocol that uses high-throughput metabolomics to explore potential biological mechanisms of delirium. We profiled the plasma metabolome of 10 delirium cases and 10 matched controls from the SAGES cohort of older adults without dementia undergoing major non-cardiac surgery. We used targeted mass spectrometry to measure 302 metabolites (features) preoperatively (PREOP) and on postoperative day 2 (POD2). Metabolomics studies are challenged by inherent technical variability and signal noise. With a small sample and a large feature count, signal noise diminishes statistical power and masks true biological signals. To address these challenges, we implemented quality control sample-based signal correction and normalization to internal standards (ISs). ISs were screened from a pool of 6 potential candidates, resulting in the removal of 3 that failed to perform well, while 3 were retained for our experiments. ISs also enabled successful concatenation of experiments run at different times. Prior to implementing quality control samples and customized ISs, no metabolites were identified as differentially expressed. After implementation, we identified one metabolite that was significantly differentially expressed at PREOP and 17 metabolites that were significantly differentially expressed at POD2 between delirium and controls (BH-corrected p-value < 0.05). In conclusion, integration of quality controls and normalization to internal standards enabled us to detect metabolites associated with postoperative delirium. Such methods should be considered for future metabolomics studies.

### ANTICHOLINERGIC DRUGS: CUT-OFF FOR IMPAIRED COGNITION AND MOBILITY IN SENIORS

Elpidio K. ATTOH-MENSAH,[1] Gilles Loggia,[2] Remy Morello,[3] Pascale Schumann-Bard,[1] Pablo Descatoire,[2] Christian Marcelli,[3] and Chantal Chavoix[4], *1. Normandie Univ, UNICAEN, INSERM, COMETE, 14000 Caen, France, Caen, France, Metropolitan, 2. CHU Caen, Department of Geriatrics, Caen, France, Caen, France, Metropolitan, 3. CHU de Caen, Department of Statistics and Clinical Research, 14000 Caen, France, Caen, France, 4. Normandie Univ, UNICAEN, INSERM, COMETE, 14000 Caen, France, Caen, France*

Background and Objectives: Anticholinergic drugs are commonly prescribed in older adults despite growing evidence of their adverse outcomes. We aimed to improve knowledge about deleterious effects of anticholinergic drugs on both cognition and mobility, in particular whether there is a threshold value for the number of anticholinergic drugs or for the anticholinergic burden leading to mobility or cognitive impairment. Methods: 177 community-dwelling individuals aged 55 years or over, with a fall history in the previous year, took part in the study. Anticholinergic drugs were identified using the Anticholinergic Drug Scale (ADS), and global cognition and mobility were assessed using the Mini Mental State Examination (MMSE) and the Time-Up-and-Go (TUG) test, respectively. Results: ROC (Receiver Operating Characteristics) curve analysis indicated that consumption of a single anticholinergic drug per day was a risk factor for impaired MMSE (p < .05) and TUG scores (p < .05). There was also a cut-off of anticholinergic burden of one for impaired MMSE scores (p < .05). Logistic regressions showed that impaired cognition induced by anticholinergic drugs were independent of confounding factors including comorbidities, while impaired mobility would be influenced by age and cardiac comorbidities. Conclusion: Daily consumption of a single anticholinergic drug, regardless of its anticholinergic burden, impairs both cognition and mobility community-dwelling seniors. Alternative solutions to anticholinergic drug prescription should thus be considered whenever possible.

### HYPERTENSION DOES NOT CONTRIBUTE TO MICROBLEEDS ONSET IN FEMALE EFAD MICE

Mafalda Cacciottolo,[1] Todd E. Morgan,[2] and Caleb E. Finch[2], *1. University of Southern California, Los Angeles, California, United States, 2. Leonard Davis School of Gerontology, University of Southern California, Los Angeles, California, United States*

Cerebral microbleeds (MBs) contribute to pre-clinical cognitive decline and are an additional clinical burden in Alzheimer Disease (AD). Hypertension is associated with MBs, with nearly 2-fold higher likelihood for MBs per SD increment in blood pressure (BP). We investigated the possible role of age-related hypertension in the EFAD mouse model (transgenic for carrying familial AD mutations and targeted replacement of human APO-E3 or -E4). MBs were detected by Prussian Blue histochemistry. We extended prior findings with observations that MBs arise early in life, by 2 months, and confirmed female excess for ApoE3 and-E4 carriers. Wildtype C57BL/6J mice also accumulated MBs, and a 10-fold lower level and more slowly up to 21mo of age. BP was measured by the tail-cuff method. All mice had BP in the normotensive range, <150 mm Hg, systolic. Longitudinal measurements of blood pressure at ages 2, 4, and 6 months showed few age changes, except for E3FAD females at 6 months (systolic, +20%, p<0.05; diastolic, +33%, p<0.05). A possible decrease in blood pressure was observed in EFAD mice (-33%, p<0.01) compared to C57BL/6J mice. A not statistical trend of increase was observed in older C57BL/6J mice up to 18 mo of age, consistent with previous reports. Older ages are required for complete negation of role of hypertension in the MB model. Ongoing studies will examine mice older than 6 months for potential relations of blood pressure and MB, and in relation to brain amyloid deposits which surrounded MBs in our prior study.

### NOVEL GAMMA-SECRETASE MODULATOR REGULATES APP PROCESSING AND INFLAMMATORY RESPONSES IN NPM-EXPOSED MICE

Carla D'Agostino,[1] Mafalda Cacciottolo,[1] Constantinos Sioutas,[2] Steven L Wagner,[3] Rudolph E Tanzi,[4] Todd E. Morgan,[5] and Caleb E. Finch[5], *1. University of Southern California, Los Angeles, California, United States, 2. Viterbi School of Engineering, University of Southern California, Los Angeles, California, United States, 3. Department of Neurosciences, University of California, San Diego, La Jolla, California, San Diego, United States, 4. Genetics and Aging Research Unit, Department of Neurology, Massachusetts General Hospital, Charlestown, Massachusetts, United States, 5. Leonard Davis School of Gerontology, University of Southern California, Los Angeles, California, United States*

Air pollution is associated with accelerated cognitive decline and increased risk of dementia in older populations (Cacciottolo et al 2017; Chen et al 2017). Rodent models of air pollution exposure also show Alzheimer-like changes including glial inflammatory responses and increased levels of endogenous amyloid beta (Aβ) peptides (Cacciottolo et al 2017 Levesque et al 2011). We hypothesized that pharmacological inhibition of Aβ production during nPM exposure would attenuate the amyloidogenic processing of APP and glial inflammatory responses. This hypothesis was tested using the γ-secretase modulator (GSM) (BPN-15606) which decreases Aβ42 levels in wild-type rodents (Wagner et al 2017; Kounnas et al. 2010). After nPM exposure of C57BL/6J male mice for 8 weeks, GSM-feeding attenuated pro-amyloidogenic and microglial inflammatory responses. Cerebral cortex levels of Aβ40 and 42 peptides were decreased by 35% and 45% respectively in mice exposed to filtered air and fed with GSM. Hippocampal levels of microglial Iba1 remained at control levels, a decrement of 50% below GSM treated mice. We suggest the Alzheimer candidate drug BPN-15606 has potential benefits to reducing the possible impact of urban air pollution in cognitive aging and Alzheimer risk. The attenuation of pro-amyloidogenic and microglial inflammatory responses is consistent with a role of endogenous Aβ levels in glial inflammatory responses and cognitive impairments from air pollution. Cacciottolo et al 2017, PMID 28140404; Chen H et al. 2017, PMID: 28917207; Levesque et al 2011, PMID 21864400; Wagner et al 2017, PMID 28416568; Kounnas 2010, PMID 20826309 Funding: P01 AG055367, R21AG050201, Cure Alzheimer's Fund

### BIOLOGICAL MARKERS OF AGING: CHRONIC STRESS AND COGNITIVE IMPAIRMENT IN RESPONDERS FROM THE WORLD TRADE CENTER

Erica D. Diminich,[1] Sean Clouston,[1] Stacey B. Scott,[2] Nikhil Palekar,[1] Erica D. santiago,[1] Evelyn Bromet,[1] and Benjamin Luft[1], *1. Stony Brook University, New York, United States, 2. Stony Brook University, Stony Brook, New York, United States*

Post-traumatic Stress Disorder (PTSD) is a stress related syndrome. Chronic PTSD has increasingly been associated with poor health outcomes, neurodegeneration and risk for cognitive impairment (CI). However, the biological mechanisms underlying the development and maintenance of symptoms and potential associations in accelerating aging are not well understood. The aim of this study was to evaluate whether specific biomarkers influence functional limitations and cognitive impairment in rescue and recovery workers (i.e. responders) from the attacks on the World Trade Center (WTC) in New York. Plasma biomarkers were collected during annual health and wellness visits at the WTC responder clinic between 2012 and 2014. Short Physical Performance Battery (SPPB) and clinical data were examined with prospective PTSD symptom scores collected during participant's initial enrollment into the parent study as early as 2002. We examined the relationship between cardiovascular (Diastolic Blood Pressure, Systolic Blood Pressure, pulse rate), metabolic (Total Cholesterol, HDL cholesterol, Triglycerides, Glucose, Body Mass Index) and inflammation markers (Albumin, White Blood Count) with Post-traumatic Stress Disorder (PTSD), cognitive functioning (Montreal Cognitive Assessment) and frailty (Short Physical Performance Battery) in responders from the World Trade Center (WTC). We first examined correlations between biomarkers, PTSD symptom severity, PTSD dimensions, cognitive functioning and frailty. We then conducted multivariate regression analyses. In models adjusted for potential confounders, among N=1,045 responders, elevated PTSD was strongly associated with increased frailty, cardiovascular dysregulation and mild cognitive impairment. Current work is ongoing to identify trajectories of change in cognition with frailty and biological factors.

### PARASYMPATHETIC INFLUENCE ON COGNITIVE AGING IS MODERATED BY SYMPATHETIC ACTIVITY, ESPECIALLY IN EARLY MIDLIFE

Erik L. Knight,[1] Ryan Giuliano,[2] Sean Shank,[1] Megan Clarke,[1] and David M. Almeida[1], *1. Pennsylvania State University, University Park, Pennsylvania, United States, 2. University of Manitoba, Manitoba, Winnipeg, Canada*

The two branches of the autonomic nervous system (ANS) have been individually linked to age-related changes in cognitive functioning: The parasympathetic nervous system (PNS) is thought to support healthy cognitive aging, whereas the sympathetic nervous system (SNS) has been linked to heightened cognitive decline. Despite these separate findings and despite the integrative nature of the ANS, little work has examined the two branches simultaneously to better understand their interactive effects on age-related cognitive changes. We examined cognitive change in two waves of the MIDUS cognitive project and indexed PNS and SNS activity from heart rate variability and epinephrine levels (respectively) from the MIDUS biomarker project (n = 764, 56% female, mean age = 54.1 years). Our findings indicate that higher PNS levels attenuate cognitive decline, but only among individuals with low SNS levels; at higher SNS levels, the beneficial effects of the PNS are blocked. Further, lower PNS levels can be somewhat compensated for by increased SNS levels. This pattern was most robust among individuals transitioning to mid-life (i.e., 35-40 years old at the initial cognitive test). These results suggest that interventions targeting the ANS as a modifiable factor in cognitive aging should consider both ANS branch's effects simultaneously, particularly in the early stages of midlife.

### MTOR-DRIVEN ALTERATIONS IN THE BRAIN MICROVASCULAR PROTEOME IN ALZHEIMER'S DISEASE

Andy Banh,[1] Carlos Pomilio,[2] Stacy A. Hussong,[3] and Veronica Galvan[4], *1. Barshop Institute of Longevity and Aging Studies - UT Health San Antonio, San Antonio, Texas, United States, 2. University of Buenos Aires, Buenos Aires, Argentina, Argentina, 3. University of Texas Health Science Center at San Antonio, San Antonio, Texas, United States, 4. Barshop Institute for Longevity and Aging Studies, San Antonio, Texas, United States*

Therapeutic interventions for Alzheimer's disease (AD) remain limited due to an incomplete understanding of the molecular mechanisms of its onset and progression. Cerebrovascular dysfunction occurs early during disease development. Attenuation of mTOR, a key regulator of aging, attenuates and reverses cerebrovascular deficits by restoring cerebral blood flow, brain vascular density, neurovascular coupling, and vascular amyloid-β clearance in hAPP(J20) mice expressing human amyloid precursor protein carrying two FAD-associated mutations. The mechanisms by which mTOR attenuation alleviates AD pathology are poorly understood. This study defined changes in the microvascular proteome of hAPP(J20) mice arising from mTOR attenuation. At 7 months of age, hAPP(J20) mice were fed vehicle- or rapamycin-supplemented diet (2.24 mg/kg/day) for 4 months. Mass spectrometry of collected brain microvasculature identified significant changes in 840 of 3361 proteins ($p<0.05$). mTOR attenuation led to significant changes in 26 of these proteins, some of which are involved in pathways including tight junction regulation, calcium signaling, and actin cytoskeleton regulation. Candidate mediators of mTOR-driven cerebrovascular dysfunction were identified by selecting proteins that were aberrantly altered in hAPP(J20) microvasculature and normalized by rapamycin. Examples include members of the heterogeneous nuclear ribonucleoprotein family (hnRNPA/B, hnRNPD) which regulate the mRNA stability of genes related to cellular cycle arrest and inflammatory cytokines as well as localization of crucial mRNA involved in nitric oxide signaling. Also included are nucleoporin 54 and vacuolar ATPase assembly factor, both of which are altered in aging and neurodegeneration. Subsequent studies will elucidate the role of these proteins in mTOR-driven cerebrovascular dysfunction in AD.

### A GENERIC METHODOLOGY FOR EFFECTIVE CREATION OF LABORATORY-TEST-BASED FRAILTY INDICES

Garrett M. Stubbings,[1] Arnold Mitnitski,[2] and Andrew Rutenberg[3], *1. Dalhousie University, Dartmouth, Nova Scotia, Canada, 2. Dalhousie University, Department of Medicine, Halifax, Nova Scotia, Canada, 3. Dalhousie University, Halifax, Nova Scotia, Canada*

Laboratory test-based frailty indices (FI) are known to be highly predictive of adverse health outcomes and mortality. However, these FI depend on the proper classification of continuous valued health measurements into binary deficits (healthy or unhealthy). This classification is not standardized and is often done by using thresholds for medical intervention or maximally predictive values from statistical tests. This work proposes a simple and generic methodology for the creation of FI from laboratory values and measures its performance against existing methods. The methodology is as follows: a direction of risk is determined for each measurement, individuals are then assigned a score based on their relative standing in the population, binarization is then done using a global cut-point which binarizes all measures based on a given quantile. This method is shown to outperform FI created with medical risk thresholds for a range of global cut-points in both the NHANES and CSHA studies. Furthermore, our method is shown to be more robust to cohort effects than FI created using cut-points determined by maximum information measures, such as maximal separation of survival curves.

### THE CEREBELLUM MAY MITIGATE OBESITY-DRIVEN COGNITIVE IMPAIRMENT IN LATE LIFE

Valentina R. Garbarino,[1] Eric Baeuerle,[2] and Miranda E. Orr[3], *1. UTHSA, San Antonio, Texas, United States, 2. Barshop Institute for Longevity and Aging Studies, UT Health San Antonio, San Antonio, Texas, United States, 3. GRECC, South Texas Veterans Health Care System, San Antonio, Texas, United States*

Mice that overexpress mutant human tau in forebrain neurons develop many features of Alzheimer's disease (AD), including behavioral impairments and neurodegeneration by 5 months of age. While an appropriate model to study AD-like pathology, the transgene's high neurotoxicity makes it difficult to investigate how aging impacts AD onset and progression. The removal of endogenous mouse tau decreases the transgene's neurotoxicity in young mice, which has allowed us to age mice to 20 months of age and investigate behavior at a more AD-relevant stage of life. Interestingly, the tau transgenic mice show increased discrimination between familiar and unfamiliar objects than non-transgenic littermates ($p = 0.02$) suggesting tau transgenic mice have better memory. The transgenic mice also displayed increased physical activity in the Open Field Test than non-transgenic littermates (distance traveled, $p = 0.0102$, and gait speed, $p = 0.0219$). Their improved behavioral performance occurred despite significant forebrain atrophy (20% smaller, $p=0.0003$). Interestingly, the non-transgenic control mice lacking endogenous mouse tau developed insulin resistance and obesity, and had significantly smaller cerebellum than transgenic mice (10% smaller, $p = 0.0007$). These data suggest that insulin resistance and obesity contribute more profoundly to poor behavioral performance than forebrain neurodegeneration. Moreover our study suggests that the cerebellum, recognized primarily for its role in coordination and motor function, may be an important mediator of late life cognitive function, especially in the presence of insulin resistance and obesity.

### PRIMING OF MICROGLIA ACTIVITY INCREASES SUSCEPTIBILITY TO DEPRESSION-LIKE BEHAVIORS

Tal Frolinger,[1] Umar Iqbal,[1] and Giulio M. Pasinetti[1], *1. Icahn School of Medicine at Mount Sinai, New York, New York, United States*

This study investigates the role of microglia activity in stress-induced depression and anxiety and the mechanisms associated with the role of certain microbiome derived anti-inflammatory polyphenols in attenuating stress-induced microglia immune priming and symptoms of depression. We implemented a chronic unpredictable stress (CUS) paradigm to exhibit priming of microglia innate immunity in the context of the onset of depression and anxiety phenotypes. Mechanistic studies related to prophylactic treatment using dietary microbiome derived polyphenols were also investigated in this model. Depression and anxiety phenotypes, gene expression in microglia and protein expression in the cortex of mice were measured following a primary exposure to short-term unpredictable stress (US) followed by CUS. We examined the long-term, persistent CUS induced changes at 4-weeks of post-stress rest following a secondary US exposure. We found depression phenotypes resulted from US only following exposure to CUS. This was accompanied by an increase and persistent upregulation of toll-like receptor 4 (TLR4), RAGE, and HMGB1 gene expression in isolated cortical microglia. Priming by CUS also amplified gene expression of IL-1β in microglia and protein IL-1β in the cerebral cortex following US re-exposure. Increased activity of NF-kB was also noted in the period following CUS. Furthermore, polyphenol treatment prevented stress-induced phenotypes, upregulation of HMGB1, IL-1B, and TLR4 gene expression, as well as upregulation of IL-1β and NF-kB. The study suggests that latent activity of the TLR4-NFkB-IL1β pathway contributes to immune priming and increases susceptibility to depression-like behaviors. Anti-depressant effects of polyphenols may result from their ability to attenuate microglia priming.

### TARGETING THE NLRP3 INFLAMMASOME IN MECHANISMS OF SLEEP DEPRIVATION-INDUCED NEUROINFLAMMATION

Chad Smith,[1] Kyle Trageser,[1] and Giulio M. Pasinetti[1], *1. Icahn School of Medicine at Mount Sinai, New York, New York, United States*

The purpose of this study was to determine if inhibitors of innate immunity in microglia could attenuate sleep deprivation (SD)-induced psychological impairment, which involve the assembly and activation of the NLRP3 inflammasome. In the study, CD-1 mice were immune-primed with chronic corticosterone treatment (20 mg/kg IP) for two weeks and were subsequently subjected to one bout of 6 hr SD. Mice were sacrificed immediately afterward to measure cytokine concentrations and caspase-1 activity. We found a significant upregulation of caspase-1 activity in the brain of both mice primed with corticosterone then subjected to sleep deprivation as well as mice only subjected to sleep deprivation ($p < 0.01$). Increased caspase-1 was NLRP3-dependent as treatment with MCC950 (20 mg/kg IP), an inhibitor of NLRP3 inflammasome assembly, completely attenuated SD-induced caspase-1 activity ($p < 0.01$). Additionally, in SD mice we observed increased microglia reactivity as quantified by IBA+ cells and an increased number of microglia that had reactive amoeboid morphologies, as measured through immunohistochemistry. The administration of the NLRP3 specific inhibitor MCC950 similarly prevented SD-induced changes in microglia morphology. The study established that consequential effects of SD- induced inflammasome activation and microglia activation could be prevented by a selective NLRP3 inhibitor. Given the preliminary beneficial effects of targeting NLRP3 in SD, future investigations will establish the clinical efficacy of microbiome-derived polyphenolic compounds, which we have shown provide protection against neuroinflammation in models of stress induced psychological impairment, to attenuate SD neuroinflammation by targeting the NLRP3 inflammasome.

### COMBINING MACHINE LEARNING WITH AUTOMATED NEMATODE LIFESPAN ANALYSIS TO IDENTIFY MODIFIERS OF ALZHEIMER'S DISEASE

Joshua Russell,[1] and Matt Kaeberlein[1], *1. University of Washington, Seattle, Washington, United States*

Here we present new computational and experimental methods to leverage the gene expression and neuropathology data collected from several large-scale studies of Alzheimer's disease . These data sets include diverse data types, including transcriptomics, neuropathology phenotypes such as quantification of amyloid beta plaques and tau tangles in different brain regions, as well as assessments of dementia prior to death. This meta-analysis is a complex undertaking because the available data are from different studies and/or brain regions involving study-specific confounders and/or region-specific biological processes. We have therefore taken neural network and probabilistic computational approaches that reduce the data dimensionality, allowing statistical comparison across all brain samples. These approaches identify gene expression changes that are significantly associated with clinical and neuropathological assessment of Alzheimer's disease. We then conduct in vivo validation of the genes through genetic screening of C. elegans models of Alzheimer's disease utilizing our automated robotic lifespan analysis platform. This approach allows for the greater leverage of existing Alzheimer's disease biobank data to identify deep genetic signatures that could help identify new clinical gene-expression markers and pharmacological targets for Alzheimer's disease.

### NEW APPROACH TO STUDYING COLLECTIVE EFFECTS OF GENETIC FACTORS ON ALZHEIMER'S DISEASE

Anatoliy Yashin,[1] Deqing Wu,[2] Konstantin Arbeev,[2] Igor Akushevich,[2] Arseniy Yashkin,[2] Matt Duan,[2] Eric Stallard,[1] and Svetlana Ukraintseva[1], *1. Duke University, Durham, North Carolina, United States, 2. Duke University, Durham, United States*

Despite the wide recognition of the multifactorial nature of Alzheimer's disease (AD), mechanisms of the collective influence of genetic and environmental factors on AD remain poorly understood. We used the Cardiovascular Health Study (CHS) and Framingham Heart Study (FHS) data to investigate the effects of genetic and gene-environment interactions on AD prevalence in the association analysis using logistic regression. Based on results of this analysis, we developed several new measures of integrated effects of SNPs, including for specific gene or group of genes, by constructing SNP-specific Interaction Polygenic Risk Scores (SIPRSs), Gene-specific Interaction Polygenic Risk Scores (GIPRS), and Trait-specific Interaction Polygenic Risk Scores (TIPRS), and tested them in the above data. We found strong interaction effects on AD among the SNPs in NRG3 gene and smoking, and among the SNPs in ATM and creatinine. In summary, we developed a new approach to measuring the collective impact of SNPs on complex traits, and discovered significant effects of the newly constructed SIPRS, GIPRS, and TIPRS on AD prevalence. The results of this study open a new opportunity of investigating the joint impacts of genetic and environmental factors on AD and other phenotypes of aging and health.

### GERIATRIC TRAUMATIC BRAIN INJURY AND ALZHEIMER'S DISEASE SHARE SIMILAR PATTERNS OF WHITE MATTER DEGRADATION

ANDREI IRIMIA,[1] Kenneth Rostowsky,[1] Nikhil Chaudhari,[1] Maria Calvillo,[1] and Sean Lee[1], *1. University of Southern California, Los Angeles, California, United States*

Although mild traumatic brain injury (mTBI) and Alzheimer's disease (AD) are associated with white matter (WM) degradation, the nature of these alterations and the outcomes of their comparison have not been elucidated. Diffusion tensor imaging (DTI) has been utilized in both conditions, and has uncovered decreases in the fractional anisotropy (FA) of the corpus callosum and cingulum bundle, compared to healthy control (HC) volunteers [1, 2]. Despite mTBI being a potential risk factor for AD, no systematic quantitative comparison has been drawn between their WM degradation patterns. Here we investigated WM FA differences using DTI and tract-based spatial statistics (TBSS) between age- and sex-matched adults: 33 chronic mTBI patients, 67 AD patients and 81 HC participants. T1-weighted magnetic resonance imaging (MRI) and DTI were acquired at 3T. mTBI patients were scanned acutely and ~6 months post-injury. FSL software was used for artefact correction, FA computation and TBSS implementation. Statistical comparison of WM FA patterns between mTBI and AD patients was achieved by two one-sided t tests (TOSTs) of statistical equivalence, with equivalence bounds defined where Cohen's $d < 0.3$. A significant difference was found between the FA means of mTBI vs. HC groups, and the AD vs. HC groups ($p < 0.01$, corrected). Mean FA differences between mTBI and AD were statistically equivalent in the corpus callosum and in the inferior longitudinal fasciculus ($p < 0.05$, corrected). Future research should focus on clarifying the similarities between mTBI and AD, potentially leading to novel hypotheses and improved AD diagnosis.

### NEURONAL-SPECIFIC PROTEASOME AUGMENTATION VIA EXTENDS LIFESPAN AND REDUCES AGE-RELATED NEURODEGENERATION

Andrew M. Pickering[1], *1. UT Health San Antonio, San Antonio, Texas, United States*

Cognitive function declines with age throughout the animal kingdom and increasing evidence shows that disruption of the proteasome system contributes to this decline. The proteasome has important roles in multiple aspects of the nervous system, including synapse function and plasticity, as well as preventing cell death and senescence. We report that augmentation of proteasome function, using overexpression of the proteasome $\beta5$ subunit, enhances proteasome assembly and function. Significantly, we go on to show neuronal-specific proteasome augmentation slows age-related declines in measures of learning, memory, and circadian rhythmicity. Surprisingly neuronal specific proteasome augmentation of proteasome function also produces a robust increase of lifespan in Drosophila melanogaster. Our findings appear specific to the nervous system; ubiquitous proteasome overexpression increases oxidative stress resistance but does not impact lifespan and is detrimental to some healthspan measures. These findings demonstrate a key role of the proteasome system in brain aging.

### SESSION 830 (POSTER)

### BIOLOGY OF AGING III

### PROLONGATION OF HUMAN LIFESPAN BY IMMATURE PEAR EXTRACT MEDIATED SIRTUIN-RELATED GENE EXPRESSION

Akira Ogita,[1] Wakae Murata,[1] Marina Hasegawa,[2] Ken Yamauchi,[3] Akiko Sakai,[4] Yoshihiro Yamaguchi,[1] Toshio Tanaka,[1] and Ken-ichi Fujita[1], *1. Graduate School of Sciences, Osaka City University., Osaka, Japan, 2. Department of Materials Science, National Institute of Technology, Yonago College., Yonago, Tottori, Japan, 3. Institute of Physical Education, Keio University, Kanagawa, Japan, 4. Research Center for Urban Health and Sports, Osaka City University., Osaka, Japan*

Demographics of the world are changing rapidly with older populations growing at an unprecedented rate. Cellular senescence, a decline of cellular function due to aging, causes gradual loss of physiological functions. Several cellular senescence-related chronic diseases, such as metabolic syndrome, cardiovascular disease, cancer, osteoporosis, diabetes, and hypertension, negatively affect the quality of human life. Intervention in the cellular senescence process may reduce these incidences and slow the progression of age-related diseases, while contributing to the longevity of healthy human lifespans. Saccharomyces cerevisiae, the budding yeast, is a simple model system that can provide significant insights into the human genetics and molecular biology of senescence and is considered suitable as a cellular model for research on mammalian cells. The aim of our study was to investigate the anti-aging effects of immature pear fruit extract (IPE) on yeast cells and its possible application to extend healthy lifespan in humans. Anti-aging effects of IPE were investigated using a chronological lifespan assay on S. cerevisiae cells. The chronological lifespan of the yeast treated with IPE at 1% (v/v) was significantly extended than that of untreated cells ($p < 0.05$). The expression of sirtuin-related genes, which regulate cellular senescence, was examined by reverse transcription-polymerase chain reaction and found to be significantly increased following IPE treatment. These results suggest that sirtuin-related genes have important roles in IPE-regulated lifespan extension, which provides a mechanism by which IPE could affect mammalian cells and potentially extend healthy human lifespans.

### CAENORHABDITIS ELEGANS AS A MODEL OF AIR POLLUTION TOXICITY DURING DEVELOPMENT AND LIFESPAN

Amin Haghani,[1] Hans M. Dalton,[1] Nikoo Safi,[2] Farimah Shirmohammadi,[3] Constantinos Sioutas,[3] Todd E. Morgan,[1] Caleb E. Finch,[1] and Sean P. Curran[1], *1. Leonard Davis School of Gerontology, University of Southern California, Los Angeles, California, United States, 2. Center for Cancer Prevention and Translational Genomics at the Samuel Oschin Comprehensive Cancer Institute, Cedars-Sinai Medical Center, Los Angeles, California, United States, 3. Viterbi School of Engineering, University of Southern California, Los Angeles, California, United States*

Air pollution (AirPoll) is among the leading human mortality risk factors and yet little is known about the molecular mechanisms of this global environmental toxin. Our recent studies using mouse models even showed genetic variation and sex can alter biological responses to air pollution. To expand genetic studies of AirPoll toxicity throughout the lifespan, we introduced Caenorhabditis elegans as a new AirPoll exposure model which has a short lifespan, high throughput capabilities and shared longevity pathways with mammals. Acute exposure of C. elegans to airborne nanosized AirPoll matter (nPM) caused similar gene expression changes to our prior findings in cell culture and mouse models. Initial C. elegans responses to nPM included antioxidant, inflammatory and Alzheimer homolog genes. The magnitude of changes was dependent on the developmental stage of the worms. Even short term exposure of C. elegans to nPM altered developmental and lifespan hormetic effects, with pathways that included skn-1/Nrf family antioxidant responses. We propose C. elegans as a new and complementary model for mouse and cultured cells to study AirPoll across the lifespan. Future chronic nPM exposure and high throughput genetic screening of C. elegans can identify other major regulators of the developmental and lifespan effects of air pollution. This work was supported by grants R01AG051521 (CEF); R21AG05020 (CEF); Cure Alzheimer's Fund (CEF); R01GM109028 (SPC), F31AG051382 (HMD) and T32AG000037 (HMD), T32AG052374 (AH).

### THE EFFECTS OF INDY ON FLY HEALTH

Blanka Rogina,[1] Pooja Patel,[1] Jacob Macro,[1] Michael Li,[1] and Ryan Rogers[2], *1. University of Connecticut Health Center, Farmington, Connecticut, United States, 2. Wentworth Institute of Technology, Boston, Massachusetts, United States*

Indy (I'm not dead yet) gene encodes a plasma membrane transporter of Krebs' cycle intermediates with highest affinity for citrate. Indy is the fly homolog of a mammalian mIndy (SLC13A5), which has the same physiological function. Reduced expression of the Indy gene extends longevity in fruit flies and worms. Genetic and pharmacological INDY reduction affects metabolism in flies, worms, mice, rats and non-human primates by affecting the levels of cytoplasmic citrate. In flies, INDY is predominantly expressed in the midgut, fat body and oenocytes, all tissues with a key role in metabolism. Our first goal was to examine our working hypothesis that INDY reduction in the midgut regulates citrate levels leading to metabolic changes that preserve intestinal stem cell (ISCs) homeostasis and slows aging by modifying Insulin/Insulin-like signaling (IIS). ISC homeostasis is vital for midgut homeostasis and contributes to health and longevity. We found that reduction of Indy preserves ISC homeostasis and intestinal integrity. The IIS is a key nutrient sensing pathway, which regulates growth, metabolism and longevity. Indy reduction is associated with decreased IIS activity. Our second goal was to examine the role of IIS in Indy mediated changes in ISC homeostasis and health. We found that at least some of INDY's beneficial effects on fly health are mediated by the IIS.

### THE ROLE OF RIBOSOMAL COMPOSITION IN SELECTIVE TRANSLATION OF LONGEVITY GENES UNDER DIETARY RESTRICTION

Jarod A. Rollins[1], *1. MDI Biological laboratory, Bar Harbor, Maine, United States*

Dietary restriction influences longevity through changes in gene expression on the level of transcription and translation. Our lab is investigating the changes occurring under dietary restriction in C. elegans on the level of individual ribosomal proteins (RPs) and changes in RP phosphorylation and how they shape gene expression and longevity. To look at the impact of changes in RP expression we used RNAi knockdowns starting at adulthood and assayed lifespan and health span. Two RPs knockdowns that increased longevity, rpl-7A and rpl-22, were then subjected to transcriptome and translatome analysis using polysome profiling and mRNA-seq. Both knockdowns resulted in similar transcriptomic changes with an up-regulation of genes related to translational fidelity and mitochondrion organization. In contrast, the translatome analysis showed minimal changes upon loss of rpl-7A and rpl-22 suggesting that they are not responsible for increase in selective translation previously observed under DR. To look at impact of changes in phosphorylation of rps-6 on DR, we generated mutant lines incapable of being phosphorylated by TOR signaling. These mutants were assayed for changes in lifespan, health span and selective translation. While phenotypically similar to controls under well fed conditions, the mutants were longer lived under DR. Furthermore, preliminary results indicated that the selective translation of the anti-longevity microchondrial electron transport genes cytochrome c oxidase and ubiquinol-cytochrome c reductase binding protein was repressed when rps-6 is not phosphorylated. In summary, our preliminary results that ribosomal protein phosphorylation but not protein composition is responsible for guiding selective translation pf longevity genes under DR.

### SENESCENCE-ASSOCIATED SECRETOME COMPONENTS AS BIOMARKERS FOR AGING

Xu Zhang,[1] Marissa Schafer,[1] Thomas White,[1] Sarah Jachim,[1] and Nathan LeBrasseur[2], *1. Mayo Clinic, Rochester, Minnesota, United States, 2. Robert and Arlene Kogod Center on Aging, Mayo Clinic, Rochester, Minnesota, United States*

Cellular senescence is a state of stable growth arrest in response to stress, which is a fundamental process of biological aging. They secrete products, the Senescence-Associated Secretory Phenotype (SASP), which consists of inflammatory cytokines, chemokines, growth factors and matrix remodeling proteins. Senescent cells accumulate with advancing age and partial elimination of senescent cells can reverse age-related dysfunction and increase mean lifespan in mice. However, it is not clear whether components of the SASP can be measured in human plasma and serve as aging biomarkers. Here we generated a candidate panel of senescence markers based on a multiplexed bead-based assay of proteins secreted by senescent preadipocytes, endothelial cells, fibroblasts, myoblasts, preadipocytes, and epithelial cells compared to non-senescent controls. The SASP undoubtedly varies by cell type; however, we observed that multiple components of the SASP are conserved. We then assessed circulating SASP components in human plasma samples from Mayo Clinic Biobank participants (n=280, 20 male and 20 female per decade, age 20-90) using the same method. We confirmed that components of the SASP can be quantified in human plasma with the multiplexed bead-based assay and observed several SASP components robustly increase with chronological age in humans. Our study illustrates that senescence-associated secretome components are detectable in human plasma and could potentially serve as biomarkers of systemic aging and senescent cell burden.

### SENESCENCE SECRETOME: BIOMARKERS OF BIOLOGICAL AGING AND POSTOPERATIVE OUTCOMES

Marissa Schafer,[1] Xu Zhang,[1] Amanika Kumar,[1] Thomas White,[1] Elizabeth Atkinson,[1] Michelle Burning,[1] and Nathan LeBrasseur[2], *1. Mayo Clinic, Rochester, Minnesota, United States, 2. Robert and Arlene Kogod Center on Aging, Mayo Clinic, Rochester, Minnesota, United States*

Senescent cells drive age-related tissue dysfunction through their potent secretome, termed the senescence associated secretory phenotype (SASP). Circulating concentrations of SASP factors may reflect biological age and serve as clinically useful biomarkers of surgical risk and ultimately, surrogate endpoints in clinical trials. However, they remain largely uncharacterized. We tested associations between circulating concentrations of SASP proteins and biological age, as determined by the accumulation of age-related health deficits, and/or postoperative outcomes in a sample of residents in Olmstead County, MN, age 60-90 years (n = 115) and cohorts of older adults undergoing surgery for severe aortic stenosis (prospective; n = 97) or ovarian cancer (case-control; n = 36). Circulating concentrations of SASP factors were associated with biological age and adverse postoperative outcomes, including risk of any adverse event or rehospitalization within the year following surgery (aortic stenosis group) or admission to an intensive care unit within 30 days of hospital discharge (ovarian cancer group). Gradient boosting machine modeling revealed a panel of SASP factors that predicted adverse outcomes across both surgical groups better than biological age or chronological age and sex. This suggests that the circulating SASP is a robust indicator of age-related health status and may help guide clinical decision making. Furthermore, circulating SASP factors may be harnessed as a readily quantifiable biomarkers in senescence-targeting interventional human studies.

### INCREASED HSP25 DRIVES THE TRANSITION FROM PROTEASOME TO AUTOPHAGY-MEDIATED DEGRADATION UNDER PROTEOTOXIC STRESS

Jessica L. Scheirer,[1] and Karl Rodriguez[2], *1. UT Health Science Center, San Antonio, San Antonio, Texas, United States, 2. UT Health San Antonio, San Antonio, Texas, United States*

Accumulation of protein aggregates are a common pathology in many neurodegenerative disorders. This accumulation may be due to a function decline in the protein homeostasis network known to occur during the aging process. Small heat shock proteins are a class of molecular chaperones that assist in protein folding and ameliorates the degradation activity of the proteasome and autolysosome thereby decreasing disease-associated aggregates. Prior work in rodents and C. elegans has shown expression levels of the small heat shock protein 25 (HSP25) correlates with maximum lifespan potential. Increased levels of HSP25 extends lifespan in a transgenic C. elegans model. This lifespan extension is dependent on skn-1 with evidence suggesting an enrichment in several skn-1-related pathways, such as lysosomal genes. Concomitantly, proteasome activity declines while autolysosome activity increases. This observation might suggest a switch from proteasome degradation to autophagy as the main driver of protein degradation in C. elegans in this transgenic model. To investigate if a reduction of proteasome function and elevated lysosomal gene activation during aging and under proteotoxic stress are modulated by HSP25 we have crossed our HSP25-transgenic worm with an aggregating and non-aggregating tau worm model. This work will elucidate a possible mechanism that explains the change in the protein degradation response pathways potentially modulated by HSP25 during increased protein misfolding.

### INDICES OF RESILIENCY IN CELLS FROM UM-HET3 MICE MAY CORRELATE WITH INDIVIDUAL FUTURE HEALTH OUTCOMES

Kennedy S. Mdaki,[1] Yuhong Liu,[1] and Adam B. Salmon[1], *1. University of Texas Health at San Antonio, San Antonio, Texas, United States*

The ability of an organism to respond to physical stresses and return to homeostasis (i.e. resilience) has been suggested to correlate with longevity. Here, we investigated whether this extends to resilience at a cellular level using primary fibroblasts isolated from tail skin of genetically heterogeneous young adult UM-HET3 mice. Cells isolated from each individual mouse (cell line) were tested in their response to concentrations of agents or conditions predicted to induce a cellular challenge, including paraquat, hydrogen peroxide, antimycin a, cadmium chloride, mdivi-1, thapsigargin, and nutrient starvation. Cell viability was monitored in real-time using an incucyte S3 live cell analysis system and we addressed the response following challenge as a marker of resilience. Cellular uptake of ethidium homodimer-1 was used to determine the loss of viability. Cellular bioenergetics were assessed using a seahorse XF24. We found that cell lines that were resistant to paraquat were also resistant to antimycin a, and hydrogen peroxide. Cell lines that were resistant to nutrient starvation were also resistant to mdivi-1. Indices of cellular bioenergetics status including ATP production rate and cell respiratory control ratio, revealed potential relationships with resiliency. Taken together, our data indicate that skin fibroblasts retain individual physiological programs that may in part explain the patterns of resiliency or sensitivity to a stressor at the organismal level. Since the cell lines tested in this study were obtained from living mice, future work will investigate whether these patterns of resiliency change with age and elucidate their utility in predicting future health outcome.

### THE ENDOPLASMIC RETICULUM PROTEIN QUALITY CONTROL ADAPTATION IN A LONG-LIVED C. ELEGANS PROTEASOMAL MUTANT

Meghna N. Chinchankar,[1] Karl Rodriguez,[1] and Alfred Fisher[2], *1. UT Health San Antonio, San Antonio, Texas, United States, 2. University of Nebraska Medical Center, Omaha, Nebraska, United States*

Protein degradation mechanisms are integral to protein homeostasis. Their reduced efficiency during aging leads to accumulation of misfolded and aggregated proteins which potentiate proteotoxic disorders. Paradoxically, our lab reported that the Caenorhabditis elegans rpn-10(ok1865) proteasome mutant possesses enhanced proteostasis and extended lifespan. RPN-10/PSMD4 is a ubiquitin receptor of the 26S proteasome that targets polyubiquitinated substrates to its catalytic core for degradation. Proteasome dysfunction of the rpn-10 mutant is characterized by reduced, not inhibited, ubiquitin fusion degradation. We ascertained that upregulated autophagy and SKN-1/Nrf-mediated responses partially contribute to the robust rpn-10 mutant phenotype. Further investigation of its underlying mechanism revealed that several ERQC genes are transcriptionally upregulated in the rpn-10 mutant. Thus, we hypothesized that the rpn-10 mutant exhibits improved ER proteostasis which mediates its elevated cellular stress resistance. Accordingly, the rpn-10 mutant shows increased ER stress resistance and altered ER homeostasis. Complementarily, attenuated expression of the aggregation-prone α-1 antitrypsin (ATZ) reporter proves that ER proteostasis is ameliorated in the rpn-10 mutant. Via a genetic screen for suppressors of decreased ATZ aggregation in the rpn-10 mutant, we identified novel player H04D03.3, which is a homolog of the proteasome adaptor ECM29. This suggests that assembly of the rpn-10 mutant proteasome itself critically regulates its ER proteostasis. Moreover, we observed that cytosolic proteostasis and longevity depend on ER master chaperone hsp-3/-4(BiP) and ER ATPase cdc-48.2(p97/VCP), further highlighting ERQC significance in the rpn-10 mutant. Altogether, it appears that mild proteasomal dysfunction induces ERQC adaptation that underlies proteostasis and longevity benefits of the rpn-10 mutant.

### THE LONGEVITY ASSOCIATED ALLELE OF FOXO3 PROTECTS AGAINST TELOMERE ATTRITION DURING AGING

Richard Allsopp,[1] Philip Davy,[1] Craig Willcox,[2] Randi Chen,[3] Michio Shimabukuro,[4] Immaculata de Vivo,[5] and Bradley J. Willcox[6], *1. University of Hawaii, Honolulu, Hawaii, United States, 2. Okinawa International University, Ginowan, Okinawa, Japan, 3. PHREI, Honolulu, Hawaii, United States, 4. Fukushima Medical University, Fukushima, Okinawa, Japan, 5. Harvard T. H. Chan School of Public Health, Boston, Massachusetts, United States, 6. Kuakini Medical Center, Honolulu, Hawaii, United States*

Telomere attrition in proliferative tissues is a hallmark feature of human aging. To date, the genetic influence on the rate of telomere attrition is poorly understood. Previously we discovered a variant of the FOXO3 gene that is strongly associated with human longevity, an observation that has been now reproduced in over a dozen independent studies. In the present study, we sought to assess the effect of the longevity associated variant of FOXO3 (rs2802292 - G allele) on the rate of telomere attrition during aging. The results from a cohort of Okinawan-Japanese (N=121), ranging in age from 25 – 94 years, demonstrates carriers of 1 or 2 copies of the longevity-associated G allele of FOXO3 showed markedly reduced rates of telomere loss in peripheral blood leucocytes as compared to carriers of the more common FOXO3 variant (TT – common genotype, m= -33bp/year, P=0.008). Interestingly, telomere shortening was not observed as a function of age for G allele carriers (m= -2bp/year, P>0.1). In an independent study of women (N=6,565) from the Nurses' Health Study cohort, ranging in age from 40 to 70 years, a similar observation was found. Notably, carriers of the TT or GT FOXO3 genotype showed a significant decline in telomere length with age (m= -15.5 bp/year, P0.1). These results mark the first validated longevity gene variant showing an association with negligible loss of telomere length with age in humans.

### PICOLINIC ACID, A TRYPTOPHAN METABOLITE, DOESN'T AFFECT BONE MINERAL DENSITY BUT UPREGULATES LIPID STORAGE GENES

Carlos Isales,[1] Meghan McGee-Lawrence,[1] Mark Hamrick,[1] William D. Hill,[2] Wendy Bollag,[1] Ke-Hong Ding,[1] Xing-ming Shi,[1] and Eileen Kennedy[3], *1. Augusta University, Augusta, Georgia, United States, 2. Medical University of South Carolina, Charleston, South Carolina, United States, 3. University of Georgia, Athens, Georgia, United States*

Tryptophan is an essential amino-acid broken down initially to kynurenine (kyn), an immunomodulatory metabolite that we have previously shown to promote bone loss. Kyn levels increase with aging and have also been associated with neurodegenerative disorders. Additional tryptophan metabolites include picolinic acid (PA); however, in contrast to kyn, PA is neuroprotective. Thus, we hypothesized that PA might be osteoprotective. In an IACUC-approved protocol, we fed PA to aged (23-month-old) C57BL/6 mice for eight weeks. In an effort to determine potential interactions of PA with dietary protein, we added PA to both a standard (18%) and a low protein diet (8%). The mice were divided into four groups: control (18% protein), +PA (700 ppm); low protein (8%), +PA (700 ppm). There was no difference in weight among the groups [36.1±4.1, 34.6±3.8, 32.8±3.2, 32.6±3.0 gm, (Means±SD, control vs +PA vs 8% vs +PA, p=ns; n=8-10/group). Mice were sacrificed and bones and stromal cells collected for analysis. We found that addition of PA to the diet had no impact on femoral BMD or BMC (BMD: 0.069±0.008 vs 0.075±0.007 vs 0.069±0.005 vs 0.070±007, p=ns). Addition of PA to the diet had no impact of % body fat as measured by DXA; however, stromal cells isolated from the PA-fed mice showed a significant increase in the expression of the lipid storage genes, Plin1 and Cidec. Thus, although PA is downstream of kyn, the kyn-induced detrimental effects on bone mass are no longer observed with PA but instead this kyn metabolite appears to impact energy balance.

### HETEROGENEITY OF SENESCENT RIBOSOME COMPLEX AFFECTS THE TRANSLATIONAL EFFICIENCY OF SENESCENCE RELATED MRNAS

Joon Kim,[1] Hag Dong Kim,[2] and Hee Woong Yang[1], *1. Korea University, Seoul, Korea, Republic of, 2. HAEL Lab, Seoul, Korea, Republic of*

The ribosome, a protein factory, has a lateral stalk known as the ribosomal P complex made up of rpLP0, rpLP1, and rpLP2. It plays an important role in translation by recruiting translational factors. One of these proteins, rpLP2, was decreased in translating ribosome when cellular senescence was induced. Additionally, Y-box binding protein-1 (YB-1), a multifunctional protein that regulates the transcription and translation, was also reduced in polysomal fraction of senescent cells. We have discovered that rpLP2 depletion in heterogeneous ribosome causes the detachment of YB-1 in polysomes and link to cellular senescence. Here, we also have found that a decrement of CK2α or GRK2 on senescent cells induced an increment of unphosphorylated rpLP2, resulting in the release of YB-1 from a ribosome complex. The heterogeneous senescent ribosome has different translational efficiency for some senescence related genes such as AHR, RAB27B, FEZ1, and DDIT4. Our results revealed that the decrease of rpLP1/rpLP2 and YB-1 in translating senescent ribosomes is not specific to cell type or stress type. Furthermore, the same phenomenon was observed in aged mouse liver. Taken together, our results suggest that the senescent ribosome complex appears to have low levels of rpLP1/ rpLP2 and YB-1, resulting in the alteration of translational efficiency for senescence related genes. (Journals of Gerontology: Biological Sciences, 2019 in press)

### PHYSICAL FRAILTY AND ITS ASSOCIATION WITH COGNITION: THE PREDICTIVE VALUE OF A SYNDROME BEYOND ITS COMPONENT PARTS

Nadia M Chu,[1] Karen Bandeen-Roche,[2] A. Richey Sharrett,[3] Michelle C. Carlson,[2] Qianli Xue,[4] and Alden Gross[5], *1. Department of Surgery, Johns Hopkins School of Medicine, Baltimore, Maryland, United States, 2. Johns Hopkins Bloomberg School of Public Health, Baltimore, Maryland, United States, 3. Johns Hopkins University, Baltimore, Maryland, United States, 4. Department of Medicine, School of Medicine, Johns Hopkins University, Baltimore, Maryland, United States, 5. Johns Hopkins University Bloomberg School of Public Health, Baltimore, Maryland, United States*

The extent to which frailty (PFP) affects cognitive performance and change beyond that expected from its component parts is uncertain. Leveraging NHATS, a nationally-representative cohort of U.S. Medicare beneficiaries, we quantified associations between each PFP criterion and global and domain-specific cognitive level and change (memory: immediate/ delayed word-list test, executive function: clock drawing test (CDT), orientation: date, time, president-vice-resident naming), using adjusted mixed effects models with random slopes (time) and intercepts (person). We tested whether presence of frailty was associated with excess cognitive vulnerability (synergistic/ excess effects, Cohen's d) above and beyond those found for its criteria by adding an interaction term between each PFP criterion and frailty. Among 7,439 community-dwelling older adults (mean age=75.2 years) followed for a weighted mean of 3.2 years (SE= 0.03), 14.1% were frail. The most prevalent PFP criteria were low activity (30.5%) and exhaustion (29.8%). Associations were strongest for executive function, where frailty added predictive value beyond its criteria (excess effects of cognitive vulnerability ranging from -0.38SD (SE-0.05) for slowness to -0.47SD (SE=0.06) for shrinking). Slowness was a strong predictor of cognitive change in both frail and non-frail participants, especially for executive function (frail: Cohen's d per year=-0.16, SE= 0.02; non-frail: Cohen's d per year=-0.15, SE= 0.02). PFP is an important measure of frailty that adds predictive value beyond its criteria, especially for cognitive levels. Additionally, gait speed remains an important predictor of change in executive function. These results suggest that frailty's contribution to cognitive performance amounts to more than the sum of its component parts.

### AGE-RELATED CHANGES IN THE MUSCLE SECRETOME

NING ZHANG,[1] Susan Weintraub,[2] and Nicolas Musi[1], *1. Barshop Institute for Longevity and Aging Studies, University of Texas Health Science Center, San Antonio, Texas, United States, 2. Graduate School of Biomedical Sciences, University of Texas Health Science Center, San Antonio, Texas, United States*

Skeletal muscle is one of the most abundant tissues in the body. In addition to its key roles in body support, movement and metabolic homeostasis, muscle also functions as an endocrine/secretory organ producing and releasing proteins into the circulation that modulate distant tissues (i.e. myokines). Considering that muscle mass and function changes with advancing age, here we tested the hypothesis that aging alters the muscle secretome profile. After euthanasia, soleus muscles from sedentary young and old mice were dissected, and incubated in oxygenated KRB buffer for 2 h. The buffer was subjected to in-gel trypsin-digestion and peptides analyzed by mass spectrometry. The concentration of 36 proteins were significantly ($P<0.05$) elevated in the young vs. the old group. In contrast, only 7 proteins were significantly elevated in the old group. Some notable differences include those in HSPA1B and HSPA5 that were detected only in the young group. HSPA8 also was significantly elevated by 1.8-fold ($P<0.05$) in the young versus the old group. Another prominent difference between groups involved translationally controlled tumor protein (TCTP), a critical regulator of apoptosis/carcinogenesis, that was elevated by 7-fold in the young vs. the old group ($P<0.05$). These results indicate that aging alters the muscle secretion profile. Identified differences in the muscle secretome could reflect intrinsic changes in muscle cells with age. Because these myokines are released into the circulation, it is also possible that myokine secretion is a regulated cellular process by which muscle communicates and modulates the aging process in distant tissues.

### 4-PHENYLBUTYRATE: MOLECULAR MECHANISMS AND AGING INTERVENTION POTENTIAL

Michael R. Bene,[1] Kevin Thyne,[1] Jonathan Dorigatti,[1] and Adam B. Salmon[2], *1. University of Texas Health Science Center at San Antonio, San Antonio, Texas, United States, 2. University of Texas Health at San Antonio, San Antonio, Texas, United States*

4-Phenylbutyrate (PBA) is a FDA approved drug for treating patients with urea cycle disorders. Additionally, PBA acts upon several pathways thought of as important modifiers of aging including: histone deacetylation, proteostasis as a chemical chaperone, and stress resistance by regulating expression of oxidative stress response proteins. PBA has also been shown to extend lifespan and improve markers of age-related health in Drosophila. Due to its wide range of effects PBA has been investigated for use in numerous age-related disorders including neurodegenerative and cardiovascular diseases. To better understand the effects of PBA on the molecular level, we used both in cellulo and in vivo studies. Treatment of primary mouse fibroblasts, C2C12 mouse muscle cells, and NCTC 1469 mouse liver cells with PBA demonstrated differential responses among cell lines to upregulation of oxidative stress response and histone acetylation. Specifically, upregulation of the oxidative stress response protein DJ-1 by PBA was found to have a corresponding dose response curve to histone H3 acetylation in primary fibroblasts. To study effects of PBA in vivo, four cohorts of HET3 mice were treated with PBA at different doses in drinking water for 4 weeks. PBA was well tolerated and led to different effects on body composition dependent on the sex of mice. We are currently investigating the molecular effects of PBA treatment in multiple tissues samples from these mice. The potential of PBA to alter many fundamental pathways, and specifically those related to stress responses, make it an attractive prospect for treatment of many age-related disorders.

### IN VIVO ANALYSIS OF REPORTER ALLELES REVEALS INCREASED VARIABILITY OF GENE EXPRESSION IN CELLS AND ANIMALS WITH AGE

Nikolay Burnaevskiy,[1] Bryan Sands,[1] Soo Yun,[1] and Alexander Mendenhall[1], *1. University of Washington, Seattle, Washington, United States*

As a major risk factor for a multitude of chronic diseases aging is being increasingly recognized as a necessary therapeutic target for preventive medicine. Yet, despite tremendous progress in our understanding of the genetic determinants of longevity, proximal causes of aging remain incompletely understood. In part, this may be due to a plethora of factors, such as various types of stochastic macromolecular damage that affect individual cells and individual animals. Indeed, recent studies point to an increase of cell-to-cell variability in gene expression within old tissues, supporting the idea that stochastic events contribute to the aging process. Therefore, more single-cell focused studies are needed for a complete understanding of biological aging. Here, we utilized quantitative microscopy for analysis of gene expression in individual aging cells, in vivo in C. elegans. Using transcriptional reporters, fluorescently tagged proteins and a quantitative analytical framework adapted from yeast, we have found that young C. elegans exhibit very little stochastic or signaling noise in gene expression. However, using quantitative microscopy, we directly observed dysregulation of gene expression with age in vivo. Specifically, the stoichiometric ratios of proteins that are tightly regulated among the youthful populace start deviating in a cell autonomous fashion. Importantly, we find that an increase of gene expression variation is a relatively early event in the aging of C. elegans, readily observed before median lifespan. Hence, we suggest that incoherent cell-to-cell variation in gene expression arising with age can be an immediate causal factor for age-related loss of robust tissue function.

### A SYSTEM TO IDENTIFY INHIBITORS OF MTOR SIGNALING USING HIGH-RESOLUTION GROWTH ANALYSIS IN S. CEREVISIAE

Michael Kiflezghi,[1] and Matt Kaeberlein[1], *1. University of Washington, Seattle, Washington, United States*

Age is the main risk factor for cancer, cardiovascular disease, neurodegeneration and other diseases prevalent in the world's aging population. These diseases increase the pain and suffering and result in billions of dollars in healthcare costs. Addressing the common risk factor may allow for simultaneous amelioration of these diseases providing personal and economic relief to people and societies around the globe. The mTOR signaling pathway has been shown to be a robust target in the aging process with its inhibition resulting in increased lifespan in model organisms from yeast to mice. Rapamycin is an FDA approved drug for use in transplant patients and is a potent and specific inhibitor of mTOR complex 1. Rapamycin's use as an anti-aging therapeutic in otherwise healthy individuals is complicated by the occurrence of side effects. As such, groups studying inhibition of the mTOR pathway are searching for alternative inhibitors that may be able decouple the deleterious effects of rapamycin administration from the lifespan extending effects. To address this issue, we have developed a high-throughput yeast-based assay for the identification of novel mTOR inhibitors. By utilizing mutant strains with differential sensitivity to mTOR inhibition, comparative growth kinetics of microcultures exposed to an inhibitor can be used to discern the mTOR inhibitory status of a compound. Furthermore, the assay can provide mechanistic insight on a compound's mode of inhibition providing a rich, fast readout of a compound's potential for inhibiting mTOR. This approach allows for the screening of large libraries of compounds speeding the discovery process.

### IDENTIFICATION OF ADAMTS7 AND CEACAM19 AS CANDIDATE HEALTHY AGING ASSOCIATED GENES

Daniel S. Evans,[1] and Steven R. Cummings[1], *1. California Pacific Medical Center Research Institute, San Francisco, California, United States*

The goals of the Longevity Genomics research group (www.longevitygenomics.org) are to develop translational strategies to promote healthy human aging based on findings from genomic studies of aging. Our investigation began with the previously reported and publicly available GWAS of the top 10% of parental lifespan in the UK Biobank, reported by Pilling et al. From five loci reported by Pilling et al., 61 candidate longevity-associated genes (LAGs) were identified using FUMA software. We then tested the 61 candidate genes for aging-related trait associations with tissue-specific predicted gene expression in cohort studies of elderly participants using PrediXcan. PrediXcan was applied to three longitudinal cohort studies of elderly individuals: Health ABC, MrOS, and SOF with a total N of 9893 participants. Aging outcomes tested for association were in the following categories: survival and lifespan, kidney function, cognitive function, physical performance tests, self-reported health and disability, diabetes-related traits, cardiovascular-related traits, lung function, and markers of inflammation. After correction for multiple testing, two genes were significantly associated with longevity-related outcomes. Predicted expression of CEACAM19 in the aorta was significantly associated with circulating C-reactive protein (CRP) levels. In addition, predicted expression of ADAMTS7 in the spleen was associated with a measure of lung function, the forced expiratory volume in the first second. Biological mechanisms for these associations are proposed, and our project provides funding opportunities for interested scientists to follow-up these results with laboratory studies (www.longevitygenomics.org/funding).

### A ROBOTIC SYSTEM FOR HIGH-THROUGHPUT AUTOMATED LIFESPAN ANALYSIS IN C. ELEGANS

Ben Blue,[1] Elena Vayndorf,[1] Matt Kaeberlein,[1] and Jason Pitt[1], *1. University of Washington, Seattle, Washington, United States*

Over the past decade, the identification of potential genetic and pharmacological modifiers of lifespan and age-related pathologies in C. elegans and other model organisms has yielded fruitful leads for follow-up investigation. A major limitation of such studies, however, is that they are often time-consuming and labor-intensive. The advent of affordable high-quality digital cameras, robotics systems, and 3D printers, as well as the decreasing costs of image storage and processing have allowed us to automate data capture and analysis at an unprecedented scale. To this end, our group developed a tool consisting of an unbiased, high-throughput, automated robotic system to perform genetic and pharmacological quantification of lifespan and health measures in C. elegans and related nematode species. The WormBot utilizes industry-standard, commercially available robotics components to position a digital camera over individual wells of standard 12-well culture plates, containing a small population of C. elegans per well. A high-resolution image is captured of each plate every 10 minutes throughout the course of the experiment. Our software processes the images for stabilization, compiles them into a time-lapse series for each well, and quantifies survival and mobility (paralysis) with minimal input. In addition, a short video is captured of each well once each day, to allow for quantitative analyses of activity and coordinated movement. We will describe this technology and present applications to screen genetic and pharmacological libraries in aging and age-related disease.

### LONGEVITY OVER THE COUNTER: INVESTIGATING THE EFFECTS OF COMMON DIETARY SUPPLEMENTS ON AGING

Ben Blue,[1] Elena Vayndorf,[1] and Matt Kaeberlein[1], *1. University of Washington, Seattle, Washington, United States*

C. elegans has been a workhorse within the field of aging biology for several decades due to its short lifespan, easy culturing, and robust genetic tools. However, the limiting factor in using C. elegans has been that throughput was constrained by the time and effort needed to manually check the worms for signs of life during longitudinal studies. By using the WormBot, a robotic image capture platform, we are able to successfully screen a wide array of compounds for their effects upon C. elegans lifespan. A single WormBot can monitor 144 individual experiments simultaneously and allows for accurate time of death calls. Here we present data generated with the WormBot that includes a screen of compounds from a wide array of natural and synthetic products that are often available as over-the-counter supplements. In order to better examine the effects of these widely-used compounds upon the aging process and an age-associated disease we examined longevity in a wildtype strain of C. elegans as well as an engineered strain that expresses human A$\beta$ protein in the body wall muscle. The age-related pathogenesis of the Aβ-expressing strain is a progressive paralysis that can be halted with treatment of known effectors of Alzheimer's disease. As such, we screened our battery of compounds with this strain to determine which compounds have a significant affect on delaying Aβ-associated paralysis. Lastly, using the WormBot's ability to capture video recording, we examine how each compound affects mobility as animals age.

### SESSION 835 (POSTER)

### BIOLOGY OF AGING IV

### EFFECT OF CALORIC RESTRICTION AND RAPAMYCIN ON OVARIAN AGING IN MICE

Driele Garcia,[1] Tatiana Saccon,[1] Joao Rincon,[1] Jorgea Pradiee,[1] Rafael Mondadori,[1] Michal Masternak,[2] Andrzej Bartke,[3] and Augusto Schneider[1], *1. Universidade Federal de Pelotas, Pelotas, RS, Brazil, 2. University of Central Florida, Orlando, Florida, United States, 3. Southern Illinois University School of Medicine, Springfield, Illinois, United States*

The ovarian follicular reserve of primordial follicle declines with aging in female mammals. Caloric restriction (CR) has been shown to increase the preservation of the ovarian follicular reserve. Likewise, rapamycin has similar effects to CR on the ovarian reserve. Therefore, the aim of our study was to evaluate the effects of rapamycin and CR on the metabolism and ovarian follicular reserve and gene expression in mice. Thirty-six female mice were used, and allocated into 3 groups: control, rapamycin (4mg/kg body weight every other day) and 30% CR. At 85 days of treatment, an insulin tolerance test (ITT) and glucose tolerance test (GTT) was performed. At 93 days ovaries were collected for analysis. CR females had lower body weight ($P<0.05$) and were more insulin sensitive ($P=0.003$), while rapamycin treated females did not change body weight ($P>0.05$) and were more resistant to insulin ($P<0.05$). Females from the CR and rapamycin groups had a twice higher number of primordial follicles ($P=0.02$ and 0.04) and half the number of primary, secondary and tertiary follicles ($P<0.05$). Both CR and rapamycin females had increased ovarian gene expression of Foxo3a mRNA ($P<0.05$). In conclusion, female mice from rapamycin and CR groups had an increased ovarian follicular reserve associated to higher expression of Foxo3a mRNA, despite divergent metabolic effects of the treatments.

### LATE-LIFE TIME-RESTRICTED FEEDING AND EXERCISE DIFFERENTIALLY ALTER HEALTHSPAN IN OBESITY

Marissa Schafer,[1] Daniel Mazula,[1] Thomas White,[1] Vesselina Pearsall,[1] Zaira Aversa,[1] Jordan Miller,[1] and Nathan LeBrasseur[2], *1. Mayo Clinic, Rochester, Minnesota, United States, 2. Robert and Arlene Kogod Center on Aging, Mayo Clinic, Rochester, Minnesota, United States*

Aging and obesity increase multimorbidity and disability risk, and determining interventions for reversing healthspan decline is a critical public health priority. Exercise and time restricted feeding (TRF) benefit multiple health parameters when initiated in early-life, but their efficacy and safety when initiated at older ages are uncertain. Here, we tested the effects of exercise versus TRF in diet-induced obese, aged mice from 20 to 24 months of age. We characterized healthspan across key domains: body composition, physical, metabolic, and cardiovascular function, activity of daily living (ADL) behavior, and pathology. We demonstrate that both exercise and TRF improved aspects of body composition. Exercise uniquely benefited physical function, and TRF uniquely benefited metabolism, ADL behavior, and circulating indicators of liver pathology. No adverse outcomes were observed in exercised mice, but in contrast, lean mass and cardiovascular maladaptations were observed following TRF. Through a composite index of benefits and risks, we conclude the net healthspan benefits afforded by exercise are more favorable than those of TRF. Extrapolating to obese older adults, exercise is a safe and effective option for healthspan improvement, but additional comprehensive studies are warranted before recommending TRF.

### GROWTH DIFFERENTIATION FACTOR 15 IS CORRELATED TO MARKERS OF IMMUNOSENESCENCE IN MONOCYTES

Brandt Pence,[1] and Johnathan Yarbro[1], *1. University of Memphis, Memphis, Tennessee, United States*

Immunosenescence is an age-associated decrease in function of immune cells precipitated by a variety of mechanisms and affecting nearly every immune cell subset. In myeloid cell subsets, aging reduces numbers of phagocytes and impairs their functional abilities, including antigen presentation, phagocytosis, and bacterial clearance. Recently, we have described an aging effect on several functions indicating immunosenescence in monocytes, including impaired mitochondrial function and reduced inflammatory cytokine gene expression during stimulation with lipopolysaccharide (LPS). We hypothesized that circulating factors altered by the aging process underly these changes. Growth/differentiation factor-15 (GDF-15) is a distant member of the transforming growth factor-beta superfamily that has known anti-inflammatory effects in macrophages and has recently been shown to be highly differentially expressed during aging. We used biobanked serum and plasma samples to assay circulating GDF-15 levels in subjects from our previous studies and examined correlations between GDF-15 levels and monocyte mitochondrial function and inflammatory responses. Monocyte interleukin-6 production due to LPS stimulation was negatively correlated to plasma GDF-15 levels ($p = 0.046$). Additionally, serum GDF-15 was positively correlated to circulating CD16+ monocyte proportions ($p = 0.021$) and negatively correlated to monocyte mitochondrial respiratory capacity ($p < 0.001$). Therefore, our data suggest that GDF-15 is a potential circulating factor affecting a variety of monocyte functions and promoting monocyte immunosenescence, and thus is an attractive candidate for therapeutic intervention to ameliorate this.

### AGE-RELATED DIFFERENCES IN IMMUNO-HEMATOLOGIC PROFILES AND THEIR ASSOCIATION WITH ALL-CAUSE MORTALITY

Jarrod E. Dalton,[1] David A. Zidar,[2] Nikolas I. Krieger,[3] Adam T. Perzynski,[4] and Douglas D. Gunzler[4], *1. Cleveland Clinic Lerner College of Medicine at Case Western Reserve University, Cleveland, Ohio, United States, 2. University*

*Hospitals Cleveland Medical Center, Cleveland, Ohio, United States, 3. Cleveland Clinic, Cleveland, Ohio, United States, 4. Case Western Reserve University at MetroHealth, Cleveland, Ohio, United States*

Immuno-hematologic function (IHF) is increasingly being recognized as a central component of health status in older age. In this study, we sought to identify homogeneous IHF profiles regarding their relationship to all-cause mortality. We then studied the distribution of these profiles among individuals over age 65. We used data on 30,828 NHANES participants, including 10 baseline complete blood count with differential components [e.g., lymphocytes, leukocytes, red cell distribution width (RDW)] and all-cause mortality. We used latent profile analysis (LPA) to simultaneously optimize intra-cluster homogeneity on CBC components and inter-cluster survival differences. LPA (using MPlus 8.2) allowed for the empirical comparison of different solutions based on goodness-of-fit criteria. After LPA model convergence, a 9-class solution balanced goodness-of-fit criteria and interpretability of the resulting classes. The largest 3 classes accounted for 83.7% of the sample, with classes 1, 2 and 3 comprising 32.1%, 28.6% and 23.6%. Class 2 had lower lymphocytes, monocytes, neutrophils and platelets relative to classes 1 and 3. Survival rates were different between classes 1 and 2 (Cox model hazard ratio, HR=0.85; P=0.012) and 2 vs 3 (HR=1.18; P=0.001). The remaining 6 classes, which generally shared in common characteristics of higher RDW and lower hemoglobin, also were involved with significant survival differences. Multinomial logistic regression revealed that, among the subset of 7,173 participants over 65, older age was significantly associated with membership in class 1 relative to classes 2 (P<0.001) and 3 (P<0.001). These results point toward the possibility of developing immune marker profile indicative of accelerated aging.

### EARLY TIME RESTRICTED FEEDING IMPROVES HIGH DENSITY LIPOPROTEIN FUNCTION IN GERIATRIC MONKEYS

Kylie Kavanagh,[1] Alexander Bashore,[1] Matthew Davis,[1] Chrissy Sherrill,[1] and John Parks[1], *1. Wake Forest School of Medicine, Winston Salem, North Carolina, United States*

Ageing conveys the greatest risk for cardiovascular disease (CVD) development, which is the dominant cause of mortality in developed nations. High density lipoprotein (HDL) particles mediate reverse cholesterol transport, are anti-inflammatory and their function predicts CVD. We observed lower plasma cholesterol efflux capacity in geriatric vervet monkeys (p=0.03) when consuming either healthy or Western diets. Adult (n=16) and geriatric (n=19) monkeys were stratified into groups fed Western diet on ad libitum (Ad Lib) or early time restricted feeding (eTFR) schedules. eTRF supplied excess food only between 6am to 2pm. Housing, seasonality and fasting conditions for data and sample collections were equivalent. After 6 months, cholesterol efflux to HDL was greater in eTRF monkeys (p=0.01), with no age by group interaction. Efflux media and plasma was chromatographically separated to confirm labelled cholesterol, and enzymatically measured cholesterol, respectively, was affiliated with HDL particles. eTRF monkeys had higher total plasma cholesterol levels (p=0.03) which was due to greater cholesterol amounts associated with only HDL, and resulted in HDL particles that were larger. eTRF resulted in robustly better HDL function such that measures from geriatric individuals were comparable to younger adults. Additionally, no differences in adiposity was observed in eTRF monkeys. Few interventions are known to raise HDL levels, and more importantly, are confirmed to improve HDL function. Our study is to date the largest, longest, controlled eTRF evaluation in primates and we show that positive biological effects are observable in HDL isolated from both adult and geriatric individuals independently of weight change.

### DEVELOPING THE COMMON MARMOSET AS A TRANSLATIONAL MODEL OF AGE-RELATED OSTEOARTHRITIS

Dennis M. Minton,[1] Angela J. Marolf,[2] Kelly S. Santangelo,[2] Adam B. Salmon,[3] and Adam R. Konopka[1], *1. University of Illinois at Urbana-Champaign, Urbana, Illinois, United States, 2. Colorado State University, Fort Collins, Colorado, United States, 3. University of Texas Health at San Antonio, San Antonio, Texas, United States*

Age is a primary risk factor for osteoarthritis (OA). The mechanisms that contribute to OA are poorly understood and disease modifying treatments have not been identified. A critical shortcoming in developing therapies is the limited number of translational models available to identify the causes of naturally occurring OA. Our goal is to use the common marmoset as a non-human primate (NHP) model of age-related OA. NHP are the closest evolutionary relative to humans and share many characteristics of human aging. The marmoset has advantages over other NHP for aging research because of their relatively short maximal lifespan and small size. Micro-computed tomography (uCT) was performed on whole-knee joints obtained from young (10 yrs, n=3) marmosets at necropsy. OA was evaluated using a clinical uCT scoring system and quantitative assessments of subchondral bone structure and ossified meniscal volume. Advancing age was positively correlated to increased uCT OA score (p<0.05, r=0.59 ), mainly through increased number and size of osteophytes and progressive subchondral bone sclerosis from the medial to both medial and lateral compartments. For marmosets displaying meniscal ossification, older marmosets had greater (p<0.05) ossified meniscal volume than middle-aged and younger marmosets, respectively. Trabecular (p=0.05) and cortical bone thickness (p<0.05) were also lower in older marmosets. These data are the first to indicate that the marmoset develops naturally occurring, age-related OA and support the pursuit of additional studies using the marmoset to identify OA mechanisms and test potential interventions.

### LIFETIME EXERCISE ATTENUATES AGE- AND WESTERN DIET-RELATED DECLINES IN PHYSICAL FUNCTION IN MICE

Zachary S. Clayton,[1] Rachel A. Gioscia-Ryan,[1] Jamie N. Justice,[2] Kara Lubieniecki,[1] Matthew Rossman,[1] Melanie Zigler,[1] and Douglas Seals[1], *1. University of Colorado Boulder, Boulder, Colorado, United States, 2. Wake Forest School of Medicine, Winston-Salem, North Carolina, United States*

Aging is associated with progressive declines in physical function. However, it is unknown if consumption of a western-style diet (WD; high-fat and sucrose, low fiber), compared with a non-WD (healthy diet), accelerates declines in physical function over the adult lifespan, and whether regular voluntary exercise attenuates age- and WD-associated declines in function. To determine this, we studied 4 cohorts of male C57BL/6 mice that consumed either normal chow [NC] or WD with or without access to voluntary running [VR] wheels beginning at 3 mo of age and assessed strength (grip strength normalized to body mass) and endurance (rota-rod distance) every 3 mo throughout life. WD decreased average lifespan by 30% (WD: 18.6±0.5 vs. NC: 26.7±0.8 mo); therefore, function was compared from 3-18 mo of age in all groups. Age-related declines (% change over 3-18 mo) in physical function were accelerated by WD (strength: WD -61.2±10.1%, NC -43.2±10.2%; endurance: WD -97.4±5.1%, NC -65.1±6.3%; all p<0.05 WD vs. NC). VR attenuated declines in physical function within the same diet group (strength: WDVR -34.7±5.1%, NCVR -18.6±5.2%; endurance: WDVR -48.5±5.2%, NCVR -41.4±4.7%; all p<0.05 versus same diet non-VR group). These unique data obtained from a lifelong study of aging in mice, indicate that: 1) consuming a WD reduces lifespan and accelerates age-related declines in physical function by 40-50% vs. a non-WD; regular voluntary exercise (wheel running) prevents this effect of WD on physical function; and 2) regular voluntary exercise also attenuates the age-associated decline in physical function by ~60-130% when consuming a healthy diet.

### METABOLIC FLEXIBILITY IN CLASSICAL MONOCYTES IS NOT AFFECTED BY AGE

Johnathan Yarbro,[1] and Brandt Pence[1], *1. University of Memphis, Memphis, Tennessee, United States*

Inflammaging is the chronic low-grade inflammation that occurs with age that contributes to the pathology of age-related diseases. Monocytes are innate immune cells that become dysregulated with age and which can contribute to inflammaging. Metabolism plays a key role in determining immune cell functions, with anti-inflammatory cells primarily relying on fatty acid oxidation and pro-inflammatory cells primarily relying on glycolysis. It was recently shown that lipopolysaccharide (LPS)-stimulated monocytes can compensate for a lack of glucose by utilizing fatty acid oxidation. Given that mitochondrial function decreases with age, we hypothesized that monocytes taken from aged individuals would have an impaired ability to upregulate oxidative metabolism and would have impaired effector functions. Aging did not impair LPS-induced oxygen consumption rate during glucose starvation as measured on a Seahorse XFp system. Additionally, aged monocytes maintained inflammatory gene expression responses and phagocytic capacity during LPS stimulation in the absence of glucose. In conclusion, aged monocytes maintain effector and metabolic functions during glucose starvation, at least in an ex vivo context.

### EFFECT OF TLR4 INHIBITION IN FAT-INDUCED INSULIN RESISTANCE IN HUMAN SUBJECTS

Hanyu Liang,[1] Nattapol Sathavarodom,[2]
Claudia Colmenares,[2] Vinutha Ganapathy,[2]
Beverly Orsak,[2] and Nicolas Musi[3], *1. Barshop Institute for Longevity and Aging Studies, UT Health San Antonio Geriatric Research Education Clinical Center, STVHCS, San Antonio, Texas, United States, 2. UT Health San Antonio, San Antonio, Texas, United States, 3. Barshop Institute for Longevity and Aging Studies, University of Texas Health Science Center, San Antonio, Texas, United States*

Older and obese subjects have increased levels of free fatty acids (FFA) in plasma that may mediate the inflammation and insulin resistance seen in these individuals. Data generated mostly in cells and rodents suggest that toll like receptor 4 (TLR4) mediates the inflammatory and insulin resistant states induced by FFA. In the present study, we tested the hypothesis that pharmacologic blockade of TLR4 would prevent lipid-induced insulin resistance. We recruited 10 lean, healthy subjects (Age: 51 ± 1 y, Sex: 6M/4F, BMI: 23.8 ± 0.7 kg/m2, Fasting plasma glucose (FPG): 5.5 ± 0.1 mmol/l). They were randomized to receive the following 72 h long i.v. treatments on separate occasions: saline (30 ml/h)+placebo (12 mg every 12 h); Intralipid (30 ml/h)+placebo; Intralipid (30 ml/h)+eritoran (12 mg every 12 h). After these infusions, insulin sensitivity was measured with an hyperinsulinemic clamp. Infusion of Intralipid significantly decreased insulin sensitivity (M value) by 14%. FPG and fasting plasma insulin concentrations increased with Intralipid infusion by 7% and 22%, respectively. Intralipid also caused a low-grade inflammatory state, evidenced by increases in plasma levels of TNFa (32%), lipopolysaccharide (14%), LPS binding protein (21%), and blood monocytes counts (15%). However, metabolic and inflammatory outcomes were not different between the Intralipid+placebo and the Intralipid+eritoran groups. We conclude that short-term TLR4 inhibition with eritoran fails to prevent lipid-induced inflammation and insulin resistance. Studies with longer acting TLR4 inhibitors may be needed to clarify the role of TLR4 on the pro-inflammatory and insulin resistant states seen with aging and obesity.

### CLONAL HEMATOPOIESIS IN A CENTENARIAN COHORT

Aparna Bhutkar,[1] Anastasia Gurinovich,[1] Thomas T. Perls,[2] Paola Sebastiani,[3] and Stefano Monti[1], *1. Boston University, Boston, Massachusetts, United States, 2. Boston University School of Medicine, Boston, Massachusetts, United States, 3. Boston University, Department of Biostatistics, Boston, Massachusetts, United States*

Mosaicism, the presence of two or more genotypically or karyotypically distinct populations of cells in a single individual, plays an important role in human disease. Mosaicism can result in mutations and/or chromosomal alterations such as loss, gain, or copy-number neutral loss of heterozygosity. Clonal mosaicism and its relationship to aging and cancer, has been previously studied, and earlier work suggests that clonal mosaicism tends to increase with age. The aim of our research is to use genotype data of centenarians to explore the relationship between extreme longevity and mosaic chromosomal alterations (mCAs). To this end, we analyzed genome-wide genotypes from blood-derived DNA of 338 individuals from the New England Centenarian Study. The participants in this dataset ranged from 45 to 112 years of age. For the detection of mCA events, we used MoChA (https://github.com/freeseek/mocha), a bcftools extension, that predicts mCAs based on B-allele frequency (BAF) and log2 intensity(R) ratio (LRR), and uses long-range phase information to increase sensitivity. Chromosomal alteration events, including whole chromosome events, were detected in 180 out of the 338 individuals. A total of 165 duplications, 97 deletions, and 9 copy-number neutral loss of heterozygosity were detected. Additionally, there were 42 events whose copy number state could not be determined with high confidence. 236 events out of the 313 were detected in individuals aged 100 and older. Our analysis of chromosomal alteration frequency by age indicates that, within centenarians, the proportion of individuals with mCAs significantly decreases with increased age ($p < 0.05$, correlation -0.73).

### INCREASED BETA2-ADRENERGIC RECEPTOR SIGNALING ENHANCES PROGRESSION OF HEPATOCELLULAR CARCINOMA

Jason Pizzini,[1] Hanzhou Wang,[2] Chih-Ko Yeh,[3] and Amrita Kamat[1], *1. University of Texas Health Science Center, San Antonio, Texas, United States, 2. University of Texas Health at San Antonio, Texas, United States, 3. Geriatric Research, Education and Clinical Center (GRECC) South Texas Veterans Health Care System (STVHCS), San Antonio, Texas, United States*

We investigated whether increased signaling by beta2-adrenergic receptors (β2-ARs), which mediate the action of catecholamines, enhances the progression of hepatocellular carcinoma (HCC). Mean age of patients with HCC, the most prolific form of liver cancer, has progressively increased over the last decade. Beta2-AR-mediated signaling in liver increases with age. We also observed increased β2-AR levels in liver tissues of patients with HCC compared to control subjects. We, therefore, hypothesized that increased β2-AR signaling enhances HCC progression while inhibition of β2-AR signaling by treatment with beta blockers suppresses its progression. To test this hypothesis, we used N-nitrosodiethylamine (DEN) to induce HCC in liver-specific β2-AR knockout (LKO) and control mice in the absence or presence of beta blocker propranolol. At the end of 25 weeks, we observed increased numbers of visible tumors, disarray of liver architecture, and mortality in DEN-induced control mice which was reduced by propranolol treatment. We also observed that DEN-treated LKO mice demonstrated reduced mortality, disarray of architecture, and phosphorylation of oncogene Src compared to DEN-treated control mice. Taken together, these results indicate that decreased β2-AR signaling because of a lack of receptors in the liver or inhibition of receptor action with propranolol reduces HCC progression. Studies are in progress to determine the β2-AR-mediated mechanisms involved in HCC progression. Our studies suggest that beta blocker propranolol, used to treat cardiovascular diseases, may be repurposed as a potential therapeutic option for treatment of HCC.

### LEFT VENTRICULAR REMODELING PROCEEDS FROM YOUNG ADULTHOOD INTO MIDLIFE IN INTRAUTERINE GROWTH RESTRICTION BABOONS

Geoffrey D. Clarke,[1] Hillary Huber,[2] Cun Li,[3] Anderson Kuo,[4] and Peter Nathanielsz[2], *1. UT Health San Antonio, San Antonio, Texas, United States, 2. University of Wyoming, Laramie, Wyoming, United States, 3. UNIVERSITY OF WYOMING, Laramie, Wyoming, United States, 4. Brigham and Women's Hospital, Boston, Massachusetts, United States*

Previous cross-sectional studies have shown young adult baboons (~5-6 y.o.), subjected to intrauterine growth restriction (IUGR) by maternal calorie restriction during pregnancy and lactation, exhibit ventricular remodeling with mildly impaired heart function relative to age/sex-matched controls (CTL). METHODS: In this longitudinal study cardiac MRI was performed on male IUGR baboons (n=7). A 3 Tesla, Siemens TIM Trio MRI system was used with phase-array coils with parallel imaging acquisition and breath-holding during the scan. Studies of IUGR animals occurred at 4.7 + 0.1 yr. intervals; the first scan (scan1) at 5.8 + 1.2 y (human equivalent - HE ~24 years) and the second (scan2) at 10.4 + 1.2 yr (HE~40 y). Scans on the CTL animals (N=4) occurred at 5.3 + 1.4 years and 10 + 1.4 years. RESULTS: Change in body weight over 4.7 years was less in the IUGR group (Δwt=6.3 + 6.1 kg) than in the control group (Δwt =11.5 + 8.2 kg). Left ventricular (LV) ejection fraction (EF) was significantly greater in IUGR animals for scan2 (+10.7%, p=0.03) but not in normal controls (+1.8%, p=0.75). Stroke volume and end-diastolic LV volume were normalized to body surface area (BSA). SV/BSA (17.6 + 4.9, 31.5 + 12.3 mL/sq.m; p=0.016) and EDV/BSA (47.3 + 13.6, 64.5 + 18.8 mL/sq.m; p=0.045) were also significantly increased in IUGR animals but not controls. In IUGR subjects, Δweight was significantly and positively correlated with ΔEF (r=0.86, p=0.01). CONCLUSIONS: In IUGR, but not in CTL baboons, cardiac function adaptations continue into midlife and are related to increases in body weight with aging. We conclude that IUGR programs cardiovascular function and that programmed changes continue into midlife.

### AGING INDUCES NLRP3 INFLAMMASOME DEPENDENT ADIPOSE B CELL EXPANSION TO IMPAIR METABOLIC HOMEOSTASIS

Christina Camell,[1] and Vishwa Deep Dixit[1], *1. Yale University, New Haven, Connecticut, United States*

Visceral adiposity in elderly is associated with alterations in adipose tissue immune cells leading to inflammation and metabolic dysfunction. The Nlrp3 inflammasome is a critical regulator of macrophage activation, inflammation, and immunometabolism in visceral adipose tissue during aging; however, the potential contribution of adipose tissue B cells is unexplored. Here, we show that aging expands adipose-resident B cells and fat-associated lymphoid clusters (FALCs) in visceral white adipose tissue of female mice. Adipose tissue B cells exhibit a memory-like B cell profile similar to the phenotype of aged B cells that are increased in spleen of old mice. Mechanistically, the age-induced FALC formation and adipose B cell expansion, but not B cell transcriptional program, is dependent on the Nlrp3 inflammasome. Furthermore, B cell depletion in aged mice improves insulin sensitivity and metabolic capacity of adipose tissue. These data reveal that inhibiting Nlrp3-dependent B cell accumulation can be targeted to reverse metabolic impairment in aging adipose tissue.

### METABOLIC CONSEQUENCES OF METHIONINE REDOX IN METHIONINE RESTRICTION

Kevin Thyne,[1] Yuhong Liu,[2] and Adam B. Salmon[2], *1. University of Texas, Health Science Center at San Antonio, San Antonio, Texas, United States, 2. University of Texas Health at San Antonio, San Antonio, Texas, United States*

While caloric restriction (CR) provides highly robust improvements to longevity and health, dietary restriction of the essential amino acid methionine can provide similar benefits including improved metabolic function and increased longevity. Despite these similarities between CR and methionine restriction (MR), there is growing evidence to suggest they may be mediated by different mechanisms that require further elucidation. The sulfur side-chain of methionine is highly prone to oxidation, even in vivo, with redox changes of these residues potentially altering protein function and interfering with its use as a substrate. An entire family of enzymes, methionine sulfoxide reductases, have evolved in aerobic organisms to regulate the redox status of methionine. We tested the role of methionine sulfoxide reductase A (MsrA) in the physiological and metabolic benefits of MR. After three months of MR, mice lacking MsrA (MsrA KO) showed significant loss of weight, including both fat and lean mass, in comparison to wild-type mice under MR. Both MsrA KO and wild-type mice responded to MR with improvements to both glucose and insulin tolerance. However, MR MsrA KO mice showed lower HbA1c and reduced leptin compared to MR wild-type mice. Overall, our results show mice lacking MsrA have a stronger response to MR suggesting that methionine redox may play an important role in some of the mechanisms responsible for these metabolic outcomes. Further studies clarify whether MsrA could also be a potential regulator of the longevity benefits of MR.

### AGE-RELATED PRESERVATION OF MOTOR NERVE CONDUCTION VELOCITY IN NEURONAL MTORC1 KNOCKDOWN MICE

Stacy A. Hussong,[1] and Veronica Galvan[2], *1. University of Texas Health Science Center at San Antonio, San Antonio, Texas, United States, 2. Barshop Institute for Longevity and Aging Studies, San Antonio, Texas, United States*

With age, peripheral nerves undergo demyelination along with overall decrease in peripheral nerve conduction velocity in both sensory and motor nerves. Loss of innervation in muscles is thought to be a major factor in causing age-related sarcopenia including a decrease in muscle function. Dietary restriction attenuates the detrimental effects of aging in mice. Reduction of mTOR signaling is hypothesized to have overlapping mechanisms with dietary restriction. Furthermore, inhibition of mTOR via rapamycin treatment is known to extend lifespan in mice as well as improve peripheral nerve myelination. Therefore, I hypothesized that reducing mTORC1 signaling in neurons would be able to ameliorate the deleterious effects of aging in peripheral nerves. An overall decrease in nerve conduction velocity was observed in both tail sensory and sural nerves with age (15 vs. 30 months). In neuronal mTORC1 KD animals, there was an age-related preservation of both sural and sciatic nerve conduction. Rapamycin treatment produced similar effects with a trend towards increased sciatic nerve conduction velocity in rapamycin-treated wild-type mice at 19 months. The preserve sciatic nerve conduction velocity could be partially explained by preserved myelination. Neuronal mTORC1 knockdown animals had more myelin in the sciatic nerve at 30 mo. as compared to age-matched controls. Overall, these data indicate that mTORC1 signaling plays a role in the age-related decline in peripheral nerve myelination as well as nerve conduction velocity. Future therapeutics could utilize rapamycin or other rapalogs to combat the decline in peripheral nerve function associated with age and other diseases as well.

### SKEWED MACROPHAGE POLARIZATION IN AGING SKELETAL MUSCLE

Chang-Yi Cui,[1] Riley Driscoll,[2] Yulan Piao,[2] Chee Chia,[2] Myriam Gorospe,[2] and Luigi Ferrucci[3], *1. Laboratory of Genetics & Genomics, National Institute on Aging, Baltimore, Maryland, United States, 2. National Institute on Aging, Baltimore, Maryland, United States, 3. Translational Gerontology Branch, Intramural Research Program, National Institute on Aging, National Institutes of Health, Baltimore, Maryland, United States*

Skeletal muscle aging is a major cause of disability and frailty in the elderly. The progressive impairment of skeletal muscle with aging was recently linked to a disequilibrium between damage and repair. Macrophages participate in muscle tissue repair first as pro-inflammatory M1 subtype and then as anti-inflammatory M2 subtype. However, information on the presence of macrophages in skeletal muscle is still sporadic and the effect of aging on macrophage phenotype remains unknown. In this study, we sought to characterize the polarization status of macrophages in human skeletal muscle at different ages. We found that most macrophages in human skeletal muscle are M2, and that this number increased with advancing age. On the contrary, M1 macrophages declined with aging, making the total number of macrophages invariant with older age. Notably, M2 macrophages co-localized with increasing intermuscular adipose tissue (IMAT) in aging skeletal muscle. Old BALB/c mice showed increased IMAT and regenerating myofibers in skeletal muscle, accompanied by elevated expression of adipocyte markers and M2 cytokines. Collectively, we report that polarization of macrophages to the major M2 subtype is associated with IMAT, and propose that increased M2 in aged skeletal muscle may reflect active repair of aging-associated muscle damage.

### AGE-ASSOCIATED INCREASE IN KYNURENINE SUPPRESSES AUTOPHAGY AND PROMOTES APOPTOSIS IN MESENCHYMAL STEM CELLS

Dmitry Kondrikov,[1] Ahmed Elmansi,[1] Robert T. Bragg,[2] Tanner Mobley,[2] Meghan mcGee-Lawrence,[2] Mark Hamrick,[2] Carlos Isales,[2] and William D. Hill[1], *1. Medical University of South Carolina, Charleston, South Carolina, United States, 2. Augusta University, Augusta, Georgia, United States*

The age-related increase of the tryptophan metabolite, kynurenine (KYN), has been associated with osteoporosis progression. Increased activity of by Indoleamine-(2,3)-dioxygenase(IDO), are responsible for the elevation of KYN levels in bone tissue. IDO activity is elevated with age and could be a promising therapeutic target forosteopenia and osteoporosis. Previously, our group has shown that the serum level of KYN is elevated with age and correlates with bone loss in vivo. Kynurenine suppress the expression and activity of chemokine CXCL12 essential for osteogenesis, bone marrow stem cells homing. Bone Marrow Stem Cells (BMSC) cultured in 1% FBS were treated with CXCL12

(100ng/ml) in the presence of saline control or the autophagic flux-inhibition agent chloroquine (CQ). CXCL12 treatment increased autophagy by upregulating the degree of LC3B-II by 20%. CXCL12 treatment also significantly increased co-localization of LC3B and LAMP-2 in serum starved cells. In the present study, we tested the theory that kynurenine plays an opposite role to CXCL12 by suppressing the autophagy cell survival pathway and by inducing apoptosis. Treatment of nutrient-deprived murine BMSCs with 10 or 100 µM of KYN suppresses autophagy in a dose dependent fashion while increasing cellular apoptosis. Treatment of BMSCs with KYN downregulated autophagic flux in BMSC preventing CQ-induced CL3B/LAMP-2 colocalization. KYN treatment prevented conversion of LC3B-I to LC3B-II in CQ-treated cells by 30 percent. At the same time, KYN treatment induces apoptosis, by increasing TUNEL-positive cells number by more than 50 percent. Additionally, KYN treatment significantly increased the levels of cleaved isoforms of PARP and caspase-3.

### AGED MICE ARE SUSCEPTIBLE TO CARDIAC HYPERTROPHY AFTER 1 WEEK OF CONSUMING A HIGH SUGAR DIET

Ana P. Valencia,[1] Jeremy Whitson,[1] Rudolph Stuppard,[1] Gustavo Valencia,[1] Peter Rabinovitch,[1] and David Marcinek[1], *1. University of Washington, Seattle, Washington, United States*

Over 80% of American adults exceed their daily recommended intake of sugar (<10% kcal). While habitual sugar consumption is associated with an increased risk for diabetes and cardiovascular disease, less is known about the effects of short-term sugar consumption on metabolic health, particularly in the elderly. The purpose of this study was to test whether aged hearts are more susceptible to pathology following a short-term high sucrose (HS) diet. Specific goals were to: A) determine the effects of a 1-week HS diet exposure on the hearts of 5 month-old and 24 month-old mice; and B) test if the mitochondrial targeted peptide SS-31 can protect against HS-diet induced effects. Male CB6F1 mice were placed either on standard chow or HS diet after 1 week of receiving saline (control) or SS-31 through osmotic pumps. Heart function was assessed in vivo through echocardiography before and after treatments. One week of HS induced significant cardiac hypertrophy in the old mice compared to age-matched chow controls. Treatment with SS-31 prevented this HS induced hypertrophy. Young hearts were smaller than in the old, but size was unaffected by diet or SS-31. We observed no effect of HS (with or without SS-31) on respiration or $H_2O_2$ production in isolated mitochondria from hearts using high-resolution respirometry. These data indicate that only 1-week exposure to HS diet is enough to exacerbate cardiac hypertrophy in aging mice, but factors other than heart mitochondrial ROS may mediate this effect.

### INTERVENTION WITH RAPAMYCIN TO IMPROVE HEALTHY AGING AND LONGEVITY IN A NON-HUMAN PRIMATE

Adam B. Salmon[1], *1. University of Texas Health at San Antonio, San Antonio, Texas, United States*

Interventions to extend lifespan and improve health with increasing age will have significant impact on a growing aged population. Several pharmaceutical interventions extend lifespan in laboratory rodent models with rapamycin, an inhibitor of mechanistic target of rapamycin (mTOR) being the most well studied. Bridging towards translation, we have an ongoing long-term study testing whether rapamycin treatment can extend lifespan and delay the progression of age-related disease in a short-lived non-human primate species, the common marmoset (Callithrix jacchus). We show that daily oral dosing of slow-releasing, encapsulated rapamycin will result in clinically effective concentrations of rapamycin in the blood and inhibit mTOR signaling. This treatment is well tolerated and does not dramatically promote known side effects of this drug, including altering clinical hematology, immune cell subsets, or promoting metabolic dysfunction including glucose intolerance in comparison to control aging marmosets. Unlike previous reports in rodents, rapamycin does not have clear effects on aging cardiovascular function in marmosets. However, in our oldest cohorts daily rapamycin treatment tends to prevent age-associated changes in body mass and composition and prevent decline in kidney function. Now more than three years after beginning treatment, we are now starting to assess the effects of rapamycin on marmoset longevity. When complete, this study will describe for the first time the potential for pharmaceutical intervention to extend longevity of a primate species with the ultimate goal of significant translational impact to human aging.

### SESSION 840 (POSTER)

### CAREGIVING

### A HEALTHY LIFE FOR AFRICAN AMERICAN WOMEN CARING FOR OLDER ADULTS: A CONCEPT MAPPING STUDY

Abiola O. Keller[1], *1. Marquette University, Milwaukee, Wisconsin, United States*

Achieving optimal health and well-being among African American older adults with chronic conditions requires addressing the needs of their caregivers. This study aimed to elucidate how African American females caring for older adults view health and the factors that influence health. We identified African American women ages 24 to 64 caring for an adult 60 years or older for group concept mapping (GCM), a mixed-methods approach. Participants (N=25) first completed idea generation by providing unlimited short, free-text responses to the focus prompt, "A healthy life for a caregiver includes: ___." The 512 identified factors were reduced to 99 unique ideas. Participants then sorted the 99 ideas into clusters based on conceptual similarity and rated each idea on desirability and familiarity. Ratings were recorded on a 5-point Likert scale, ranging from very undesirable to extremely desirable and not at all familiar to extremely familiar. Data were analyzed and mapped via CS Global Max software. A cluster map with 12 outcome domains best fits the data. Identified clusters included: (1) Spirituality, (2) Maintaining relationships, (3) Good character, (4) Action to cope, (5) Preserving self, (6) Support, (7) Personal empowerment, (8) Resources, (9) Release (10) Striving for peace, (11) Wellness, (12) Self-care. Seven of the 99 ideas (representing 5 of 12 domains)

were rated as desirable but unfamiliar ("go zone"). We identified elements necessary for health and wellbeing from the perspective of African American caregivers. Go-zone items represent opportunities to intervene to promote the health of African American women caring for older adults.

### CHOICE MODERATES RELATIONSHIPS BETWEEN LEVEL AND DURATION OF CARE AND THE HEALTH IMPACT OF CARING FOR AN OLDER PARENT

Robin Tarter,[1] Dena Hassouneh,[1] Allison Lindauer,[1] and Nathan Dieckmann[2], *1. Oregon Health and Science University, Portland, Oregon, United States, 2. Oregon Health & Science University School of Nursing, Portland, Oregon, United States*

The perception of choice in the caregiving role has emerged as a key theme in qualitative gerontological caregiving research but few studies have examined choice quantitatively. The aim of our study was to test whether perceived choice moderated the relation between level and duration of care and the health impact of caring for a parent over the age of 65. We tested these questions in a series of structural equation models using existing data from the National Alliance for Caregiving, Caregiving in the U.S. 2015 Survey. We found that for adult-child caregivers of parents who reported a lack of choice in taking on the caregiving role, greater responsibilities for assistance with activities of daily living (ADLs) ($p<0.01$) and instrumental ADLS ($p<0.01$), and greater time providing care ($p<0.05$) predicted the negative impact of caregiving on caregiver health. The number of ADLs performed also predicted the emotional stress of caregiving for parents ($p<0.01$). Conversely, for caregivers who reported that they did have a choice in taking on the caregiving role, level and duration of care were not significantly related to the impact of caregiving on caregiver health, or the emotional stress of caregiving. Women were significantly more likely to report a lack of choice than men ($p<0.05$). Additional research is needed to explore the meaning of choice, and the ways in which choice may be especially constrained for daughters who care for older adults, in order to develop interventions to ameliorate the potentially deleterious health effects of caregiving on adult-children.

### IMPACT OF END-OF-LIFE PLANNING ON SURROGATE DECISION-MAKER ANXIETY

April Aloiau,[1] Daniel L. Segal,[1] Alan Mouchawar,[2] and Melissa J. Benton[1], *1. University of Colorado at Colorado Springs, Colorado Springs, Colorado, United States, 2. Hoag Hospital, Newport Beach, California, United States*

At the end of life, adults with advanced illness frequently rely on surrogate decision makers to make health care decisions. Surrogate decision makers often have anxiety related to the difficulty and complexity of making end of life decisions. This project evaluated whether an educational intervention focused on creating a specific plan of care for hospice patients would reduce anxiety among their surrogate decision makers. The Geriatric Anxiety Scale (GAS), the State Trait Anxiety Inventory–State Anxiety Scale (STAI-S), and a single question about decision-making anxiety were used to measure surrogate decision maker anxiety before the intervention, immediately after the intervention, and 2 weeks following the intervention. After completing an informed consent, 12 patients (age 80 ± 14.7 years) and 18 surrogate decision makers (age 60 ± 12.9 years) from a Southern California hospice organization participated in the educational intervention. Immediately following the intervention surrogate decision maker anxiety decreased. Mean GAS anxiety scores decreased ($p = 0.003$) from 21.3 ± 9.8 to 16.6 ± 7.6 and STAI-S scores decreased ($p = 0.003$) from 43.3 ± 11.5 to 38.1 ± 9.9. However, when surrogate decision maker anxiety was measured 2 weeks post-intervention, anxiety had increased again, so that it was no longer significantly different from pre-intervention levels. Qualitative analyses showed high satisfaction, with 85% of decision makers reporting that the education was very or extremely helpful This project demonstrated that an educational intervention in the hospice setting can be effective in creating a short-term decrease in surrogate decision maker anxiety levels.

### BEHAVIORAL ACTIVATION FOR FAMILY CAREGIVERS OF PEOPLE WITH DEMENTIA: A SYSTEMATIC REVIEW AND META-ANALYSIS

XINYI XU,[1] Rick Kwan,[1] and Angela YM Leung[2], *1. The Hong Kong Polytechnic University, Hong Kong, Hong Kong, 2. Centre for Gerontological Nursing, School of Nursing, The Hong Kong Polytechnic University, Hong Kong, Hong Kong*

Behavioural activation (BA) aims to increase positive response-contingent environmental reinforcement and help caregivers to engage in pleasant and constructive activities, and therefore improve psychological and physical health among family caregivers of people with dementia (PWD). However, knowledge of the effectiveness of BA in this population remains limited. The current study applied a systematic review and meta-analysis in order to determine the effectiveness of BA among family caregivers of PWD. Literature was searched in PubMed, Medline, CINAHL, Cochrane, Embase and PsycINFO published from March 1988 to March 2018. Seven Randomized Control Trials (RCT)s evaluating the effects of BA in family caregivers of PWD were eligible to be included in this review. Cochrane's guideline was used in order to measure risk of bias and extract data. A random effects model was used to pool the effect size. Family caregivers of PWD receiving BA that only for caregivers demonstrated a statistically significant reduction in depression (n=3; 311 participants; Cohen'd=0.55; 95% CI: 0.30 to 0.81; $P<0.001$). BA also had a positive impact on interlukin-6, negative affect of caregiving, relationship satisfaction, dysfunctional thoughts and distress related to neuropsychiatric symptoms of PWD for family caregivers. The available evidence suggests that future studies are needed to focus on better ways of administering BA to family caregivers of PWD, to improve their physical and psychological health. Meanwhile, more RCTs to investigate the effects of BA on psychological and physical health for family caregivers of PWD is needed.

### DEMENTIA FAMILY CAREGIVERS' EXPERIENCES WITH A NURSE-LED MEMORY CARE CLINIC

Mariya A. Kovaleva,[1] Bonnie M. Jennings,[2] Carolyn Clevenger,[3] Mi-Kyung Song,[2] Patricia C. Griffiths,[4] and Ken Hepburn[3], *1. Vanderbilt University School of Nursing, Nashville, Tennessee, United States, 2. Nell Hodgson Woodruff School of Nursing,*

*Emory University, Atlanta, Georgia, United States, 3. Emory University, Atlanta, Georgia, United States, 4. Emory University School of Medicine, Atlanta, Georgia, United States*

The Integrated Memory Care Clinic (IMCC) at Emory Healthcare is a patient-centered medical home led by advanced practice registered nurses (APRNs) who provide both dementia care and primary care. We explored the experiences of informal caregivers of persons living with dementia at the IMCC during their first year post-enrollment. Twelve caregivers completed semi-structured telephone interviews that lasted 29 minutes on average. The data were analyzed via directed content analysis guided by attention only to caregivers' accounts of their experience at the IMCC. Caregivers' experiences clustered around two major considerations: the strengths of the IMCC, and ways to enhance the IMCC. Overall, caregivers' viewed the IMCC as their wished-for care model. Caregivers felt a sense of belonging to the IMCC team, as they understood that the IMCC personnel incorporate caregivers' input to deliver care. Participants valued APRNs' competence in dementia care and having direct telephone access to an on-duty APRN around the clock. Caregivers appreciated the care organization at the IMCC with adequate time dedicated for in-person visits. Areas for the IMCC improvement included clarifying the IMCC scope of practice, explaining dementia progression, involving physicians, and providing more medical and non-medical resources at the IMCC. Caregivers' willingness to have more resources provided by the IMCC emphasizes how many unmet needs caregivers and their persons have. Clarification of the clinics' scope of practice – what can be done to manage dementia, its symptoms, and comorbidities – highlighted the need to educate caregivers about ways in which dementia, albeit incurable, can be managed.

### EVALUATION OF THE RESCUE PROGRAM FOR REDUCING STROKE CAREGIVER STRESS AND IMPROVING PROBLEM-SOLVING ABILITIES

Meggan Jordan,[1] I. Magaly Freytes,[2] Tatiana Orozco,[2] Stuti Dang,[3] and Constance R. Uphold[4], *1. California State University Stanislaus, Turlock, California, United States, 2. North Florida/South Georgia Veterans Health System, Gainesville, Florida, United States, 3. Miami Veterans Affairs Healthcare System- GRECC, Miami, Florida, United States, 4. North Florida/South Georgia Veterans Health System, Gainesville, Florida, United States*

Research shows that caregiver interventions that combine problem-solving with psycho-education are the most effective for addressing stroke caregiver concerns. The Resources and Education for Stroke Caregivers' Understanding and Empowerment (RESCUE) program, designed to reduce caregiver stress, depression, and burden, developed as a result of this evidence. A 4-week telephone and web-based clinical demonstration project led by registered nurses was established as part of the VHA Office of Geriatrics and Extended Care's Non-Institutional Long Term Care Initiative. The goals of this clinical demonstration were to improve problem-solving skills and provide individualized support for stroke caregivers. A single-group pre and post-test design was used and 72 caregivers of veterans with stroke completed the intervention; qualitative and quantitative methods were used for evaluation. The outcome variables were caregiver depressive symptoms, problem-solving abilities, burden, health-related quality of life and care recipient functional abilities. Post-tests were conducted 2-6 weeks after the intervention. The evaluation found that there were statistically-significant decreases in caregiver depressive symptoms and burden from pre- to post-test assessments. Caregivers' negative problem orientation significantly decreased. The other components of problem-solving abilities did not change. Qualitative data revealed how the program increased caregivers' confidence in problem-solving which led to new strategies to relieve stress. Interviews also revealed how the intervention affected caregivers in unexpected ways, such as improved intimate relationships and new perspectives on caregiving. The preliminary effectiveness and barriers and facilitators of implementing a stroke caregiver program will be discussed.

### TRAJECTORIES AND CORRELATES OF POST-LOSS DEPRESSIVE SYMPTOMS IN FORMER DEMENTIA CAREGIVERS

Kristin L. Corey,[1] Karen Hirschman,[2] Lauren T. Starr,[1] and Salimah H. Meghani[1], *1. NewCourtland Center for Transitions and Health, University of Pennsylvania School of Nursing, Philadelphia, Pennsylvania, United States, 2. University of Pennsylvania, Philadelphia, Pennsylvania, United States*

Approximately 30-40% of family caregivers of relatives living with dementia report depression, compared to 9.5% of the general adult population. Studies suggest that depressive symptoms persist for many years following the care recipient's death, despite resolution of caregiving responsibilities. However, long-term patterns of post-loss depressive symptoms remain poorly understood. The aim of this integrative review was to examine longitudinal trajectories and correlates of depressive symptoms in dementia caregivers following the care recipient's death. A literature search was conducted using CINAHL, MEDLINE, and PsycINFO databases. Studies met the eligibility criteria if they were peer-reviewed, primary sources and reported research exploring correlates and/or longitudinal patterns of post-loss depressive symptoms in dementia caregivers. Data quality was evaluated using the widely-used quality appraisal tool developed by Hawker and colleagues (2002). Data were extracted, displayed in matrix format, and synthesized into themes. Fourteen studies met the eligibility criteria and were rated as high quality. Overall, depressive symptom severity trended down during the first year post-loss but did not reduce to levels reported by non-caregiving controls. Symptom trajectories varied among unique caregiver subgroups and included persistent-distressing symptoms, persistent-mild symptoms, and asymptomatic. Greater severity of post-loss depressive symptoms was associated with female gender, lower income, less social support, pre-loss depression, neurotic and optimistic personality traits, and avoidant coping style. The findings indicate that many caregivers could benefit from mental health screening and psychosocial support during the first year post-loss and underscore the need for longitudinal studies that explore depressive symptom trajectories beyond the first 1-2 years post-loss.

### DYADIC INTERVENTION FOR PEOPLE WITH DEMENTIA AND THEIR FAMILY CARERS AFTER THE DIAGNOSIS

Marleen Prins,[1] Marjolein Veerbeek,[1]
Claudia van der Velden,[1] and Bernadette Willemse[2], *1. Netherlands Institute of Mental Health Addiction (Trimbos-institute), Utrecht, Utrecht, Netherlands, 2. Trimbos-institute, Netherlands*

Adaptation to and coping with the diagnosis dementia is a complex process. The right support in this phase is important to find the right care and to (self-)manage future care wishes. However, people with dementia (PWD) and their family carers (FC) often experience support directly after the diagnosis as inadequate. Support is not provided until problems start to accumulate and available support is not focused on continuation of their life and the emotional impact of the diagnosis. Therefore, the intervention SHARE [Support, Health, Activities, Resources, Education] was developed and studied in the US and adjusted to and pilot-tested in the Netherlands with positive results. SHARE is innovative because it is designed for dyads dealing with early-stage dementia to enhance communication between PWD and their FC and to prepare them for the future. In this study, the Dutch SHARE intervention was adjusted because of new insights and an RCT is being conducted to evaluate the (cost)effectiveness of this version of the Dutch SHARE. The primary outcome measurement for the PWD is quality of life and self-efficacy for the FC. Secondary outcomes are stress, communication in the relationship and perspective taking (only FC). The design and procedures of the RCT will be presented in this poster session as well as the demographic characteristics of participating dyads. This study contributes to knowledge about psychosocial interventions for PWD and FC with special attention for preventive empowerment of the ability to cope with the disease and the capacity to deal with the situation.

### A STUDY ON THE COMPARISON OF STRESS INDEX OF DEMENTED OLDER ADULTS AND THE PAIN OF THE MAIN CAREGIVER

SUJIN KIM,[1] Eunhee Cho,[2] HWANG SINWOO,[1] and Eunji Kwon[1], *1. Yonsei University, Seoul, Korea, Republic of, 2. Professor, College of Nursing, Yonsei University, Seoul, Korea, Republic of*

The purpose of this study was to compare the stress and fatigue index between the demented older adults and the non-demented older adults and to identify the correlation between the behavioral psychological symptoms of the demented older adults and the pain of the main caregiver. A total of 100 participants(80 demented older adults and 20 non-demented older adults) were selected. The demented older adults, who visited hospital neurology as an outpatient, were paired up with the caregivers who provided the care for the demented older adults. The non-demented older adults who had normal cognitive function without limitation in a daily life and scored MMSE of 24 or higher were selected. The stress and fatigue index were measured by using an autonomic nervous system analyzer that measures heart rate variability(HRV), and structured questionnaires were used to examine behavioral psychological symptoms. The collected data were analyzed by using Independent t-test and Pearson's correlations analysis. The result of the analysis showed that average score of the demented older adults had higher stress index than the control group(non-demented older adults), which was statistically significant($t=3.350$, $p=.001$). In addition, the result indicated the statistically significant positive correlation between the behavioral psychological symptoms of demented older adults and the level of pain of main caregivers ($r=0.89$, $p=<.001$). The results of this study suggest that managing both the physical and psychological aspects of the stress in the main caregivers is necessary, as well as the development of stress management program for the demented older adults.

### STAYING ENGAGED: A TAILORED COMMUNITY PROGRAM FOR PEOPLE LIVING WITH DEMENTIA AND THEIR FAMILY CAREGIVERS

Gwen McGhan,[1] Kimberly Shapkin,[2]
Whitney Alpaugh,[2] and Jessica Power Cyr[3], *1. University of Calgary, Calgary, Alberta, Canada, 2. University of Calgary, Calgary, Canada, 3. The Glencoe Club, Calgary, Canada*

Tailored care strategies have potential to address declines in physical and cognitive functioning for people living with dementia (PLWD) while making a positive difference in their daily lives; however, these services are not commonplace. As dementia progresses, PLWD become more dependent upon caregivers, increasing caregiving strain and negatively affecting their ability to provide support. The purpose of this study is to examine the feasibility of a tailored community dementia program (TCDP) prescribed for individual abilities of PLWD and measure the impact on the caregiving dyad. A mixed method design was used for a 12-week TCDP with 8 dyads living in the community. Recreational therapy and exercise physiology specialists led the cognitive and physical components of the program. PLWD and caregivers completed assessments at baseline, 6, 12 and 18 weeks. Daily activity was measured in PLWD with no decline observed during the assessment period. Measurements for family caregivers included caregiver strain, satisfaction and assessment of the PLWD's abilities. Although quantitative findings were not significant, caregivers praised the program in the follow-up focus group with one commenting that "it is important for the mental health of the family and for the patient to keep them active…and (for) socialization". Another caregiver wrote in their journal "I feel he has really benefited from this program. It keeps him engaged with people and I do think he is more fit". Overall the TCPD shows promise as a meaningful intervention. Cohort 2 begins in summer 2019 and will implement changes suggested by the caregiving dyads.

### NEEDS AND EXPERIENCES OF CAREGIVERS TO PERSONS WITH DEMENTIA WHO HAVE LOST THEIR DRIVING PRIVILEGES

Stephanie Yamin,[1] and Cassandre Gratton[1], *1. Saint-Paul University, Ottawa, Ontario, Canada*

The ability to drive a motor vehicle for most older adults is associated with a sense of independence, well-being, quality of life and identity. For many older adults, driving cessation eventually becomes inevitable. This is especially the case for older adults with a diagnosis of dementia. Driving cessation has been shown to negatively impact individuals' mobility and, consequently, quality of life. Informal caregivers (i.e., family caregivers) can mitigate the negative consequences associated with driving cessation in persons with dementia (PWD) by meeting their mobility needs and by offering emotional support. The purpose of this study was to examine the experience and needs of informal caregivers of PWD who had recently lost their driving privileges. Ten informal caregivers of PWD were recruited from a tertiary memory disorders clinic. Semi-structured interviews were conducted and transcribed. Transcripts of interviews were thematically analyzed using a grounded theory approach. The major themes emerging from the experience of caregivers included being overwhelmed by responsibility, overwhelmed by the emotional response of their care recipient and feeling resentment towards their care recipient. Similarly, the major themes emerging from the needs of caregivers included having the need for mobility training, psychoeducation on how to best attend to the emotional needs of their care recipient and the need for coping strategies. These experiences and needs expressed by caregivers indicate that driving cessation of the care recipient is a difficult experience for caregivers and that a therapeutic intervention based on the reported needs may be beneficial.

### ADVERSE CONSEQUENCES OF UNMET NEEDS AMONG INDIVIDUALS WITH DEMENTIA: THE ROLE OF PAID CAREGIVING

Jennifer M. Reckrey,[1] Peiying Hua,[2] and Katherine Ornstein[1], *1. Icahn School of Medicine at Mount Sinai, New York, New York, United States, 2. Icahn School of Medicine at Mount Sinai, New York, United States*

Individuals with dementia may enlist help from paid caregivers (i.e. home health aides, personal care attendants) to address increasingly complex care needs. Yet it is unclear what specific tasks paid caregivers help with and how this is related to the individual's experience of unmet care needs. We used data from the 2015 National Health and Aging Trends Study (NHATS) to examine the association between type and intensity of individual paid caregiver tasks and adverse consequences of unmet needs among community-dwelling adults with dementia. Nearly one half (46%) of those with any functional impairment reported an adverse consequence of an unmet need (e.g., inability to go outside because of lack of help). Individuals who experienced adverse consequences were more likely to receive paid caregiving (37.9% vs. 21.4%, $p<0.01$). Those who received paid care with an individual task (e.g. toileting) were more likely to report an adverse consequence related to that task (e.g. remaining in wet or soiled clothes 50.2% vs. 41.0%, $p=0.21$). Paid caregivers provided a median of 15 hours of care per week. For those persons with dementia receiving less hours of care weekly, paid caregivers rarely helped with unscheduled or frequently recurring functional tasks like toileting or eating (<10% of the time). The help received by individuals with dementia was inadequate to meet their care needs. Paid caregiving will only be able to prevent adverse consequences of unmet care needs when the level of paid care provided is better matched to the care needs of the individual with dementia.

### SESSION 845 (POSTER)

### COGNITIVE IMPAIRMENT

### BRIDGING THE GAP BETWEEN COGNITIVE HEALTH AND FINANCIAL CAPACITY IN OLDER ADULTS: A SCOPING REVIEW

Jessica B. King McLaughlin,[1] Ashley Taeckens,[1] Jennifer C. Greenfield,[1] and Eric Chess[1], *1. University of Denver, Denver, Colorado, United States*

Significant declines in everyday functioning are often indicators of cognitive impairment. Although researchers agree that a diminished ability to carry out a variety of instrumental activities of daily living (IADL) tasks is a harbinger of cognitive impairment (CI), studies have repeatedly found that a compromised ability to successfully complete financial management tasks may be one of the initial IADLs affected by prodromal CI. Due to the combination of compromised financial functioning and the increased prevalence of CI, older adults as group are, therefore, put at a greater risk for financial abuse and exploitation. The aims of this scoping review were two-fold: 1) to synthesize current literature on CI as it relates to financial decision-making (FDM) and 2) to analyze the measures and instruments used to assess FDM in older adults. In a review of four databases, 39 studies were identified that met inclusion criteria. These studies used 22 different instruments to measure and assess financial functioning. Across the 39 studies, consistent themes emerged relevant to factors that impacted cognitive impairment and financial functioning. These themes included participants' education levels, premorbid financial literacy, and age. White participants were overrepresented in research samples from these studies. Future research should investigate how FDM and early signs of CI may interact in people of color. Additionally, research should be focused on how to make instrumentation more accessible and feasible for the general public to administer without extensive training. This would enable the lay population to more easily identify early signs of cognitive impairment.

### RESTING STATE NETWORK AND MINDFULNESS IN MOTION PLUS DASH DIET IN AFRICAN AMERICANS WITH MILD COGNITIVE IMPAIRMENT

Kathy D. Wright,[1] Klatt Maryanna,[2] Ingrid Adams,[3] Cady Block,[4] Todd Monroe,[1] Zhong-Lin Lu,[5] Doug Scharre,[6] and Lorraine Mion[1], *1. The Ohio State University College of Nursing, Columbus, Ohio, United States, 2. The Ohio State University College of Medicine, Columbus, Ohio, United States, 3. The Ohio State University College of Medicine, Ohio, United Arab Emirates, 4. The Ohio State Wexner Medical Center, Columbus, Ohio, United States, 5. The Ohio State University Department of Psychology, Columbus, Ohio, United States, 6. Center for Cognitive and Memory Disorders, Center for Neuromodulation, The Ohio State University Wexner Medical Center, Columbus, Ohio, United States*

The resting state network (RSN) is a target of interest in neurodegenerative research, with evidence linking functional connectivity of its constituent nodes with mild cognitive impairment and dementia. Given the emerging linkage between Alzheimer's disease and related dementia disorders (ADRD) and hypertension (HTN), non-pharmacological interventions that promote RSN connectivity and blood pressure are needed. The purpose of this pilot study protocol is to deliver a novel intervention, combining mindfulness and the Dietary Approaches to Stop Hypertension (DASH), to improve RSN connectivity and blood pressure in African American (AA) older adults with MCI and HTN. Thirty-six AAs aged 65 and older will be randomized to mindfulness plus DASH, attention control (non-health related education), or a control group. The Mindfulness in Motion (MIM) plus DASH intervention is delivered in 8-weekly group sessions of 6-10 participants. MIM includes mindful movements from chair/standing, breathing exercises and guided meditation. The DASH intervention uses a critical thinking approach that involves problem solving, goal setting, reflection, and developing self-efficacy. Both components are culturally tailored for older African Americans. Cognitive examination, diet and mindfulness practice surveys, blood pressure, and functional magnetic resonance imaging (RSN) data are collected at baseline and 3 months. Forty-eight AAs were screened and 17 were enrolled (women= 13; men= 4) to date. Of the 17 enrolled, 7 were eligible for neuroimaging. Findings from this pilot study may provide the preliminary evidence that MIM plus DASH may improve RSN connectivity and blood pressure in this population at risk for ADRD.

### PATTERNS OF SOCIAL ENGAGEMENT AMONG OLDER ADULTS WITH MILD COGNITIVE IMPAIRMENT

Takashi Amano,[1] Nancy Morrow-Howell,[2] and Sojung Park[1], *1. Brown School, Washington University in St. Louis, St. Louis, Missouri, United States, 2. Washington University, St. Louis, Missouri, United States*

Promoting engagement in social activities may be an intervention that prevents or delays cognitive impairment. Nevertheless, little is known about social engagement among people with mild cognitive impairment. We aim to examine patterns of social engagement among people with mild cognitive impairment and to assess whether factors under four domains of the WHO's ICF model (personal factors, environmental factors, body functions and structures, and health condition) are associated with patterns. Data were drawn from the 2010 Health and Retirement Study. The final sample comprised 1,227 people with Cognitive Impairment No Dementia (CIND). Latent class analysis and multinomial logistic regression were utilized. Three patterns of social engagement were identified: informal social engagement only, formal and informal social engagement, and low social engagement. At least one factor from each domain was associated with the probability of class membership. Our findings suggest that social engagement is heterogeneous among people with CIND and that some groups of people with CIND have possibilities of engaging in more social activities, especially in formal social activities. Results also indicate that providing informal social resources may be essential for social programs designed specifically for people with CIND to promote their formal social engagement. Future study is needed to examine possible differences in outcomes across groups with similar patterns of social engagement.

### OPTIMISM, QUALITY OF LIFE, AND COGNITION IN RECENT NURSING HOME RESIDENTS

Diana DiGasbarro,[1] Kimberly Van Haitsma,[2] Suzanne Meeks,[1] and Benjamin T. Mast[1], *1. University of Louisville, Louisville, Kentucky, United States, 2. Penn State, University Park, Pennsylvania, United States*

Dispositional optimism may be an important resource for well-being across the lifespan. However, the relationship between optimism and quality of life in recent nursing home residents with and without cognitive impairment has not been examined. The aim of this study is to fill this gap in a sample of 66 older adults with a mean age of 74.59 years old (SD=10.37) who were admitted to a nursing home within the previous 30 days. Sixty older adults completed measures of cognition, quality of life, and optimism, and thus were included in analysis for the current study. Participants were split into groups based on the presence or absence of cognitive impairment, and linear regressions were conducted to examine the relationship between optimism and quality of life. In recent nursing home residents without cognitive impairment (n=30), optimism did not predict quality of life and accounted for a very small amount of variance (R2=.042, p=.280). However, in recent nursing home residents with cognitive impairment (n=32), optimism accounted for 20.9% of the variance in quality of life (R2=.209, p=.009). Higher levels of optimism were associated with better quality of life. Future research should explore why a stable trait like dispositional optimism is a stronger predictor of quality of life in recent nursing home residents with cognitive impairment compared to those without cognitive impairment. This line of research would be synergistic with emerging research on the identification and encouragement of strengths in older adults with cognitive impairment.

### EFFECTS OF A STRENGTHS-BASED DYADIC PROGRAM ON THE HEALTH OF PERSONS WITH MILD COGNITIVE IMPAIRMENT AND THEIR FAMILY

Doris Yu,[1] Sheung-Tak Cheng,[2] Polly Li,[1] and Fan Zhang[2], *1. The Chinese University of Hong Kong, Hong Kong, Hong Kong, 2. The Education University of Hong Kong, Hong Kong, Hong Kong*

Mild cognitive impairment (MCI) is characterized by cognitive impairment cognition, neuro-psychiatric symptoms particularly depressed mood, and compromised self-image. This RCT evaluated the effects of a strength-based dyadic program, which supported MCI care dyads for effective symptom and disease management and active social engagement, on the dyadic health outcomes. From Dec 2017-Jan 2019, 103 care dyads were recruited in an elderly community center. By adopting the strength-based and person-centered approach, this 14-week program worked out a dyadic biographies for care dyads to increase identification and recognition of their own strengths. Then, six interactive educative-counseling sessions focusing on cognitive and behavioral symptom management, social interaction and gauge capability were delivered, with four sessions for the MCI clients in small groups and two sessions for the care dyads in their home environment. The control group received usual care. Dyadic health outcomes were measured at baseline, upon program completion and three months thereafter. The results of Generalized Estimating Equations showed that the strength-based dyadic program significantly improved the objective memory (post-test: β=-0.179, p=0.001), and subjective memory (post-test: β=7.542. p=0.007; 3-month: β=7.842. p=0.012), neuropsychiatric symptoms (post-test: β=2.822, p=0.001; 3-month: β=3.038. p=0.007), and depression (post-test: β=2.665, p=0.017; 3-month: β=3.556. p=0.007). It also improved the caregiver stress in symptom management at post-test (β=2.822. p=0.001) and depression at the 3-month endpoint (β=1.510. p=0.015). Identifying and mobilizing the strengths of the clients with MCI and their family caregivers not only promote a more assertive coping experience and positive self-image for the care dyads, but also improve their health outcomes.

### ENGAGEMENT MATTERS TO OPTIMIZE COGNITIVE BENEFITS OF COGNITIVELY IMPAIRED PEOPLE IN COGNITIVE STIMULATION THERAPY

Yan, Anna Zhang,[1] Gloria H.Y. Wong,[2] and Terry Lum[3], *1. The University of Hong Kong, Hong Kong, China, Hong Kong, 2. The University of Hong Kong, Hong Kong, Hong Kong, 3. Department of Social Work and Social Administration, PokFuLam, Hong Kong*

Background Cognitive stimulation therapy (CST), a 14-session themed groupwork for people with cognitive impairment, shows effectiveness in maintaining cognitive functioning, quality of life and communication. However, its mechanism of optimizing individual cognitive benefits is little known. Engagement, a state of being occupied by meaningful external stimuli, may be an overlooked link. Individual constructive engagement is defined as the verbal or motor individual behavior exhibited for the meaningful purposed activities. Objective To investigate the individual experience of engagement in CST and its effect on cognitive benefits. Methods A total of 108 participants were recruited from 8 community centers, 2 daycare centers and 2 residential care units in Hong Kong. Trained assessors not involved in CST delivery conducted the pre-and-post assessments using Alzheimer's Disease Assessment Scale-Cognitive Subscale (ADAS-Cog). Trained raters observed each participant's engagement under six 5-minute observation windows in total during CST sessions by time sampling using the adapted Myers Institute Engagement Scale. Results Paired t-test shows significant improvement in ADAS-Cog (Pretest: M=22.71, SD=12.63; Posttest: M=19.06, SD=10.93; MD=3.65, SD=7.38, t=5.138, p<.000). Individual constructive engagement was exhibited 23.3% of activity time averagely. It correlated with the change in ADAS-Cog positively(r=.292, p<.002). Greater individual constructive engagement predicted lower post-intervention ADAS-Cog score (B=-14.98, β=-.192, p<.001), indicating a better cognitive functioning, after controlling baseline ADAS-Cog score (B=.707, β=.816, p<.000) in the multiple linear regression analysis (R2 = .698, F (2,105) =121.38, p<.000). Conclusion Engagement is the potential mechanism to maximize individual cognitive benefits. Future studies can investigate contributing factors of engagement to improve intervention effectiveness

### EFFECTIVENESS OF DIFFERENT INTERGENERATIONAL LEARNING PROGRAMS FOR ELDERS WITH MILD COGNITIVE IMPAIRMENT

Li-Chan Lin[1], *1. Institute of Clinical Nursing, National Yang-Ming University, Taipei, Taiwan*

The purposes of this study was to investigate the efficacy of interpersonal relationships, cognition, instrumental activities of daily living, depression for elderly with cognitive impairment through intervention using the intergenerational somatosensory video game and company. The experimental design was used for this study. Eighty-nine elders with mild cognitive impairement and 180 adolescents were from nine junior and senior high school were recruited in this study. Eight day care centers were randomly assigned to the experimental group I (EGI) (5-week intergenerational somatosensory video game), the experimental group II (EGII) (8-week intergenerational somatosensory video game), the experimental group III (EGIII) (5-week intergenerational company), the experimental group IV (EGIV) (8-week intergenerational company) and control group for eight weeks of routine activities. Subjects were interviewed using structured instruments, including the Instrumental Activities of Daily Living (IADL), Token test, and Geriatric Depression Scale. Obtained data was analyzed using the Generalized Estimate Equation (GEE). The results revealed that there was no significant difference of eldsers' characteristics among five groups. After intervention, instrumental activities of daily living scores in 8-week intergenerational somatosensory video game (EGII) and 8-week intergenerational company groups (EGIV) were significantly better than the control group. Token test score in 8-week intergenerational company (EGIV) were significant higher than that in the control group; while depression in 5-week intergenerational company (EGIII) was significantly lower thatn that in the control group. Based on the research findings, to arrange intergenerational action video game and intergenerational company to enhance elders' attention, instrumental activities of daily living and decrease depression.

### PSYCHOLOGICAL WELL-BEING AND THE ONSET OF COGNITIVE IMPAIRMENT AMONG CHINESE OLDER ADULTS

Jiaan Zhang[1], *1. Fudan University, Shanghai, China*

Previous research has shown the beneficial effects of positive psychological assets on health, but more research is needed to confirm the prospective effects on cognitive function. The purpose of this study is to examine the relationship between psychological well-being and the earliest onset of cognitive impairment among Chinese older adults. Data came from 2000 to 2014 waves of the Chinese Longitudinal Healthy Longevity Survey. Study sample consisted of 6,225 older adults who were free from cognitive impairment in 2000. Psychological well-being was measured based on seven items that assessed optimism, conscientiousness, self-determination, happiness, self-esteem, pessimism, and loneliness, with responses ranging from "always (1)" to never (5)". Negative feelings items were reverse coded. Higher score indicated more positive psychological well-being. Cognitive impairment was measured by a Chinese version of the Mini-Mental State Examination. Respondents scored at or above 24 were regarded as having no cognitive impairment. A multi-category time-varying variable was used to capture four potential outcomes: (1) persistently free of cognitive impairment between waves, (2) onset of cognitive impairment, (3) death between waves, and (4) attrition. Socio-demographics, chronical diseases conditions, functional health status were served as controls. Multilevel multinomial logistic regression models that account for clustering of observations within a subject over time were employed for the study. Results show that more positive psychological well-being is significantly associated with reduced risk of cognitive impairment onset and death over time. Results suggest that developing more psychological resilience-based intervention programs among older adults may help them delay the onset of cognitive impairment.

### DEFINITIONS MATTER WHEN DIAGNOSING MILD COGNITIVE IMPAIRMENT

Carol Derby,[1] Charles B. Hall,[1] and Mindy Katz[1], *1. Albert Einstein College of Medicine, Bronx, New York, United States*

Prior studies have shown that when standard diagnostic criteria are applied, the majority of individuals diagnosed with aMCI do not progress to clinical dementia, with a much larger proportion reverting to normal cognition. This suggests that a prospective confirmation of aMCI diagnosis may improve the specificity of the classification. We examined the rates of aMCI reversion using two definitions: one based on a single annual assessment, and one requiring a diagnosis over two consecutive annual assessments within the population based Einstein Aging Study Cohort. Using the definition that used a single annual assessment resulted in 224 incident aMCI cases in 5,321 person years of follow-up, for an incidence rate of 4.21 cases per 100 person years. Requiring the confirmatory diagnosis resulted in only 94 incident aMCI cases in 5736 person years of follow-up, for an incidence rate of 1.64 cases per 100 person years. 41% of the persons diagnosed with aMCI using the single annual assessment were cognitively normal at the next follow-up. Only 14% of the persons diagnosed with incident aMCI using the definition requiring later confirmation ever returned to being cognitively normal. When the aMCI definition that required confirmation was used, a dramatic reduction in the incidence rate of aMCI was observed in persons born after 1930, similar to what has been reported in the same cohort for dementia, but there was no such difference for the definition based on a single annual assessment.

### SESSION 850 (POSTER)

### DEMENTIA

### PREDICTORS OF CHANGES IN WELL-BEING AMONG THE INTEGRATED MEMORY CARE CLINIC CLIENTS

Mariya A. Kovaleva,[1] Melinda Higgins,[2] Bonnie M. Jennings,[3] Mi-Kyung Song,[3] Carolyn Clevenger,[2] Patricia C. Griffiths,[4] and Ken Hepburn[2], *1. Vanderbilt University School of Nursing, Nashville, Tennessee, United States, 2. Emory university, Atlanta, Georgia, United States, 3. Nell Hodgson Woodruff School of Nursing, Emory University, Atlanta, Georgia, United States, 4. Emory University School of Medicine, Atlanta, Georgia, United States*

The Integrated Memory Care Clinic (IMCC) at Emory Healthcare is a patient-centered medical home led by advanced practice registered nurses who seamlessly provide dementia care and primary care. This analysis explored predictors of significant changes in clients' well-being and symptoms in clients' first-year experience at the IMCC (N=42 caregivers, three assessments over nine months). The significant changes were decreases in caregivers' distress regarding PLWDs' delusions (Delusions-Distress) and PLWDs' anxiety (Anxiety-Distress), and in PLWDs' severity of delusions, depression, and total symptom severity. Mixed linear models were used to determine significant predictors among baseline sociodemographic characteristics that correlated significantly with outcomes that changed significantly over time. Caregivers not employed outside home had lower baseline Delusions-Distress (p=0.006) and slower decline in Delusions-Distress (p=0.015). The longer PLWD needed care, the lower baseline Delusions-Distress caregivers reported (p=0.023). Caregivers not living with their PLWD reported higher baseline Anxiety-Distress (p=0.016). Caregivers not employed outside home reported lower baseline Delusions-Severity for their PLWD (p=0.006). Caregivers not employed outside home reported PLWDs' lower baseline depression severity (p=0.026). Older caregivers reported PLWDs' lower baseline total symptom severity (p=0.002). Increase in caregiver's age was associated with PLWDs' higher total symptom severity (p=0.049). For PLWD with male caregivers, total baseline symptom severity was lower compared to PLWD with female caregivers (p=0.01). These findings highlight that PLWDs' illness duration and caregivers' employment status, living arrangement, age, and gender may determine their perception of their PLWDs' symptoms. Clinicians may individualize caregiver education with the knowledge of such predictors.

### PREVENTABILITY OF DEMENTIA IN PRIMARY CARE SETTINGS

Anh Pham,[1] Neil Drummond,[2] Don Voaklander,[1] Adrian Wagg,[1] and Cliff Lindeman[1], *1. University of Alberta, Edmonton, Alberta, Canada, 2. University of Alberta, Edmonton. Alberta, Alberta, Canada*

Dementia is a long-term, chronic condition caused by a progressing physical damage in the brain. Evidence suggests that cardiovascular disease risk factors may contribute to the onset of dementia; however, the current literature on this association is inconsistent. To our knowledge, no study that has explored the occurrence of cardiovascular risk factors prior to a diagnosis of dementia using national primary care data in North America. We used electronic medical records from the Canadian Primary Care Sentinel Surveillance Network to create a Canadian cohort to conduct a retrospective analysis to (1) determine the number of incident diagnoses of dementia in primary care among community-dwelling seniors; (2) compare the risk of developing dementia in seniors (aged 65 and older) with and without modifiable cardiovascular risk factors. The cohort identified 21,628 patients who did not have a dementia diagnosis in 2008. During ten years of follow-up, 2,520 individuals developed dementia. The number of patients with dementia or cardiovascular risk factors increased slightly but steadily. Annually, the number of new cases of dementia increased from 0.5% in 2009 to 2.2% in 2017. Both Poisson regression and Cox's proportional hazard model showed statistically significant relationships between hypertension, diabetes, dyslipidemia and obesity and dementia onset ($p < 0.001$), hazard ratio equals to 0.82, 1.11, 0.68 and 0.58, respectively. In other words, people with hypertension, dyslipidemia and obesity being managed in primary care are less likely to develop dementia. These findings support the hypothesis that good control over chronic diseases may benefit cognitive health.

### ACUPUNCTURE AND ACUPRESSURE FOR DEMENTIA BEHAVIORAL AND PSYCHOLOGICAL SYMPTOMS: A SCOPING REVIEW

Melissa L. Harris,[1] Laura M. Struble,[1] and Marita Titler[1], *1. University of Michigan, Ann Arbor, Michigan, United States*

This scoping review synthesized the literature on acupuncture and acupressure therapy for treating behavioral and psychological symptoms of dementia (BPSD). BPSD affect most people with dementia and are overwhelming for caregivers and healthcare providers. Current evidence encourages use of nonpharmacologic interventions for BPSD due to the dangers of psychotropic medications. Acupuncture and acupressure present as alternative treatment options for BPSD. Systematic reviews examined acupuncture therapy for improving cognitive function, but not acupuncture and/or acupressure for BPSD. The methodology outlined by Joanna Briggs Institute Reviewers' Manual guided this scoping review. Databases searched were PubMed, CINAHL, Embase, PsychINFO and AgeLine. Study inclusion criteria were published in English, subjects with dementia, acupuncture or acupressure tested and outcome measures included at least one BPSD. Gray literature was excluded. 836 citations were screened by title and abstract. 57 full texts were reviewed to determine inclusion criteria and 15 studies were retained. Nine studies examined acupressure and 6 examined acupuncture. Eight studies were RCT, 11 were conducted in China and 10 were conducted in long-term care. The percent of studies with statistically significant improvements in BPSD outcomes measured were: ADL's (75%), agitation (100%), anxiety (67%), depression (100%), mood (100%), neuropsychological disturbances (67%) and sleep disturbances (100%). The therapies were safe and participant satisfaction was high across studies. Variations in research designs, outcome measures and incomplete descriptions of acupoints limit interpretations about effectiveness of these interventions for BPSD. Additional RCTs are needed to improve generalizability and to evaluate dosage/methods of acupressure and acupuncture for BPSD.

### NURSE-LED DEMENTIA MEDICAL HOME: WHAT IS IT LIKE FOR CLIENTS?

Mariya A. Kovaleva,[1] Melinda Higgins,[2] Bonnie M. Jennings,[2] Mi-Kyung Song,[2] Carolyn Clevenger,[3] Patricia C. Griffiths,[4] and Ken Hepburn[3], *1. Vanderbilt University School of Nursing, Nashville, Tennessee, United States, 2. Nell Hodgson Woodruff School of Nursing, Emory University, Atlanta, Georgia, United States, 3. Emory University, Atlanta, Georgia, United States, 4. Emory University School of Medicine, Atlanta, Georgia, United States*

The Integrated Memory Care Clinic (IMCC) at Emory Healthcare is a patient-centered medical home led by advanced practice registered nurses (APRNs) who provide both dementia care and primary care. In this prospective longitudinal cohort study we aimed to evaluate caregivers' and their persons' living with dementia (PLWD) (reported by caregivers) experiences at the IMCC. Changes in caregivers' psychological well-being and health status and in PLWDs' quality of life and neuropsychiatric symptoms were explored via three assessments during the clients' first year at the IMCC. Forty-nine caregivers completed baseline assessments, including a sociodemographic questionnaire and established instruments. Mixed linear models were used to examine changes in caregiver- and PLWD-centered variables. With time as the only predictor and with full baseline sample included, significant changes were observed in caregivers' distress regarding their PLWDs' delusions ($p=0.048$) and in caregivers' distress regarding their PLWDs' anxiety ($p=0.018$). Additionally, significant changes were observed in PLWDs' severity of delusions ($p=0.032$), depression ($p<0.001$), and total symptom severity ($p=0.005$). For outcomes that changed significantly over time, we explored whether time still significantly predicted changes when controlling for two variables deemed clinically important: the total number of PLWD's chronic comorbidities besides dementia at baseline and the total number of visits the dyads made to the clinic during the study. Controlling for the total number of visits to the IMCC cancelled significant improvement in caregivers' distress regarding PLWDs' delusions. While most variables remained unchanged, several important symptom-related outcomes improved rapidly upon clinic enrollment, indicating potentially efficacious symptom management at the IMCC.

### THE RELATIONSHIP BETWEEN IMMIGRANT STATUS AND UNDIAGNOSED DEMENTIA

Yujin Franco,[1] and Eun Young Choi[2], *1. University of Southern California, Los Angeles, California, United States, 2. Davis School of Gerontology, University Southern California, Los Angeles, California, United States*

In the U.S., the immigrant population is rising, and immigrants are more likely to develop dementia than the U.S.-born population. However, little is known about the rate of undiagnosed dementia among immigrants. This study investigates the relationship between immigrant status and undiagnosed dementia, using 2011 data from the National Health and Aging Trends Study. Data from 7,347 older adults aged 65 years and older (6,531 U.S.-born and 816 immigrants) were included in the analysis. Study participants were divided based on whether they had or had not been diagnosed with dementia, respectively. The results of binary logistic regression showed that being an immigrant was associated with two times higher odds (odds ratio [OR]: 2.00, 95% CI: 1.38-2.92) of undiagnosed dementia compared to US-born participants. Among immigrants, undiagnosed participants had significantly lower levels of depression ($t(166)=-2.60$, $p=.01$). Moreover, although marginally significant, the latter were younger ($t(166)=-1.90$, $p=.06$) and immigrated at an older age ($t(159)=1.87$, $p=.06$) than the diagnosed group. Thus, it is important to tailor dementia education and interventions to the immigrant population, as this may contribute to reducing health disparities in dementia outcomes within the older population.

### THE ROAD TO ACCEPTANCE OF DRIVER RETIREMENT FOR PATIENTS WITH DEMENTIA: PHYSICIANS' AND PATIENTS' PERSPECTIVES

Theresa L. Scott,[1] Jacki Liddle,[1] Nancy A. Pachana,[1] Elizabeth Beattie,[2] and Geoffrey Mitchell[1], *1. The University of Queensland, Queensland, Australia, 2. Queensland University of Technology, Queensland, Australia*

People living with Alzheimer's disease and related dementias (ADRD) must eventually stop driving. While some will voluntarily retire, many others will continue to drive until a crisis. In Australia, like many other countries, general physicians/practitioners ("GPs") play a key role in monitoring driving safety and driver retirement with their patients with ADRD. Advising patients about driving cessation is one of the most challenging aspects of clinical dementia care, complicated by limited time in consultations, lack of patient awareness and insight, and objective screening and assessment measures. We examined how to support best practice in relation to management of driving cessation with patients with ADRD through focus groups with 29 GPs and contrasted their perspectives with those of 11 retired drivers with ADRD. Focus groups and interviews were transcribed and thematically analysed. Themes discovered highlighted the importance of providing education about the effects of dementia on safe driving and incorporating regular assessment of driving safety into the care continuum. Key strategies that GPs successfully employed included acknowledging loss and encouraging continued community engagement, providing referral pathways, and deferring to other GPs within the practice in challenging circumstances. In conclusion, there is demand for an overhaul of the current system of management and a need to establish nationally aligned, standardized and evidence-based guidelines, in particular relating to assessment of safe driving. In the meantime, we can learn from these GPs who have implemented particular strategies that mitigate some of the challenges and complex driving related issues that present in primary care.

### SYSTEMATIC REVIEW AND GAP ANALYSIS ON ALZHEIMER'S DISEASE IN ASIAN AMERICANS, NATIVE HAWAIIANS, AND PACIFIC ISLANDERS

Sahnah Lim,[1] Timothy Roberts,[1] Jazmine Wong,[1] Sadia Mohaimin,[1] Young-Jin Sohn,[1] Ragavan Sivanesathurai,[1] Simona C. Kwon,[1] and Chau Trinh-Shevrin[1], *1. NYU School of Medicine, New York, New York, United States*

Background: The Asian American, Native Hawaiian, and Pacific Islander (AANHPI) aging population is rapidly growing and the burden of Alzheimer's disease and its related dementias (AD/ADRD) will likely mirror this demographic growth. AANHPIs face significant barriers in obtaining timely AD/ADRD diagnosis and services; yet little is known about AD/ADRD in this population. The study objective is to conduct a systematic review on the published literature on AD/ADRD among AANHPIs to identify gaps and priorities to inform future research and action plans. Methods: The systematic review was conducted following the PRISMA Protocol for Systematic Reviews. Co-author (TR), an experienced Medical Librarian, searched PubMed, EMBASE, PsycINFO, Cochrane Central of Clinical Trials, Ageline and Web of Science for peer-reviewed articles describing AD/ADRD among AANHPIs. The search was not limited by language or publication date. Each citation was reviewed by two trained independent reviewers. Conflicts were resolved through consensus. Results: The title/abstract and full texts of 1,447 unique articles were screened for inclusion, yielding 310 articles for analysis. Major research topics included prevalence, risk factors, comorbidities, interventions and outreach, knowledge/perceptions/attitudes, caregiving, and detection tools. A limited number of studies reported on national data, on NHPI communities generally, and on efficacy of interventions targeting AANHPI communities. Conclusion: To our knowledge, this is the first systematic review on AD/ADRD among AANHPI populations. Our review provides a first step in mapping the extant literature on AD/ADRD among this underserved and under-researched population and will serve as a guide for future research, policy and intervention.

### TELEPHONE-BASED COGNITIVE ASSESSMENTS IN A LARGE, MULTISITE RCT: THE COSMOS-MIND STUDY

Brad Caudle,[1] Mark M. Espeland,[1] Stephen R. Rapp,[1] Sally A. Shumaker,[1] Debbie Pleasants,[1] and Laura D. Baker[1], *1. Wake Forest School of Medicine, Winston-Salem, North Carolina, United States*

Identifying safe, affordable, and well-tolerated interventions that prevent or delay cognitive decline in older adults is of critical importance. There is growing evidence from basic science and small randomized trials that cocoa flavanols may provide protection against this decline. Funded by the NIA, COSMOS-Mind is an ancillary study of COSMOS and was designed to add cognitive outcomes to the parent study, a 2x2 factorial randomized controlled trial testing the effects of cocoa flavanols (600 mg/d) and a multivitamin with matching placebo on cardiovascular disease and cancer endpoints. A validated telephone-based protocol conducted at baseline and then annually for three years measures attention, memory, language, executive function, and global cognitive functioning in 2,262 women and men, ages 65 and older without insulin-dependent diabetes. Cases of mild cognitive impairment and Alzheimer's and related dementias will be centrally adjudicated. For participants who score below a pre-specified threshold on a test of global cognition, a study partner is interviewed to obtain additional information regarding cognitive and functional status. With >5,000 interviews completed, this presentation will describe the cognitive battery, operational procedures used to ensure high data fidelity, and strategies employed that have maintained retention at >90%. Our experiences in COSMOS-Mind can inform the design and implementation of other large, multisite RCTs and epidemiological studies. Telephone-based assessments of cognitive function are a cost-efficient method for assessing cognitive function.

### ASSESSING APATHY IN LONG-TERM CARE RESIDENTS WITH DEMENTIA: COMPARING SELF-EVALUATION WITH PROXY RATINGS

Ying-Ling Jao,[1] Kristine N. Williams,[2] and Diane Berish[1], *1. Pennsylvania State University, University Park, Pennsylvania, United States, 2. University of Kansas Medical Center, Kansas City, Kansas, United States*

Background: Apathy affects most individuals with dementia in long-term care. Apathy assessment is fundamental for appropriate treatment. Apathy involves subjective feelings thus individual's self-evaluation may offer important perspectives for assessment. However, it is unclear whether self-evaluation is a valid assessment approach for this population. This study compared apathy ratings from resident self-evaluation to assessments from family, clinicians, and research staff. Methods: This pilot study enrolled 8 residents from two long-term care facilities in Pennsylvania. One family member, one certified nursing assistant (CNA), and one nurse or activity staff were also enrolled for each resident. Researchers interviewed each resident using the Apathy Evaluation Scale (AES) and rated their apathy levels. Family, CNAs, and nurses/activity staff independently rated the resident's apathy level using the AES. Direct observations were conducted by researchers using the Person-Environment Apathy Rating (PEAR). Results: Correlation analysis revealed a discrepancy across raters in assessing apathy. While self-evaluation and family ratings where moderately positively correlated (r=0.48, p=.23), there was a moderate correlation in the opposite direction between self-evaluation and CNA ratings (r=-0.64, p=.09). Resident self-evaluation did not correlate with nurses/activity staff ratings (r=0.01, p=.99) or researcher observations (r=-0.08, p=.86). Discussion: These findings may reflect residents' cognitive impairment and lack of insights, family and clinicians' lack of understanding of apathy, or nurses' and researchers' lack of acquaintance with the resident. It remains undetermined whether self-evaluation provides valid information for apathy assessment for this population. Additional research is necessary to identify the most valid assessment approach for long-term care residents with dementia.

### ASKING THE PEOPLE WHO MATTER THE MOST: DESIGNING A VALUE-BASED DEMENTIA SPECIALTY CLINIC

Alyssa Aguirre,[1] Christopher Ulack,[1] Joel Suarez,[2] Kathleen Carberry,[2] Justin Rousseau,[1] Scott Wallace,[2] and Robin Hilsabeck[2], *1. University of Texas Dell Medical School, Austin, Texas, United States, 2. University of Texas, Austin, Texas, United States*

This presentation will highlight our research which uses a qualitative methodology to incorporate the voices and experiences of people impacted by dementia into the value-based health model. This model is characterized by a team-based approach as well as the measurement of outcomes. The aim of value-based care is to provide individuals meaningful and compassionate care that helps them achieve the health outcomes that matter most to them. Foundational to creating this person-centered model is the incorporation of the perspectives of individuals with dementia and their care partners. Experience Groups offer an opportunity for those affected by dementia to share their expertise and describe their daily challenges and successes so we are able to learn from their experiences and better understand unmet and unarticulated needs. The findings of this research—consisting of 41 patients and 11 care partners—enabled the development of outcome measurement tools implemented at the clinical level, and the design of a care delivery model that addresses unmet needs. Some of the key findings from the research that have been implemented at the Cognitive Disorders Clinic and that will be highlighted in this poster are: 1. Care partners would like more emotional support from their medical team; 2. Individuals want more information about the trajectory of the disease and an actionable "roadmap-of-care"; 3. Care partners and those with early stage memory loss desire counseling and team-based care versus strictly physician-provided care.

### ALZHEIMER'S DISEASE AND RELATED DEMENTIAS IN MEXICAN AMERICAN MEDICARE BENEFICIARIES

Soham Al Snih,[1] Lin-Na Chou,[2] Brian Downer,[3] Mukaila Raji,[2] Yong-fang Kuo,[2] Kyriakos Markides,[2] and Kenneth Ottenbacher[2], *1. University of Texas Medical Branch at Galveston, Galveston, Texas, United States, 2. The University of Texas Medical Branch, Galveston, Texas, United States, 3. University of Texas Medical Branch, Galveston, TX, Galveston, Texas, United States*

Objective: To determine the prevalence and incidence of Alzheimer's Disease and Related Dementias (ADRD), and to identify the socio-demographic and health characteristics of Mexican-American older adults with ADRD. Methods: Data are from wave 5 (2004/05) of the Hispanic Established Population for the Epidemiological Study of the Elderly linked with Centers for Medicare and Medicaid Services files. We studied 1166 participants of which 927 did not have an ADRD diagnosis before wave 5 interview and followed until 2016. Measures included socio-demographics, medical conditions, depression, physical function, Mini-Mental-State- Examination (MMSE), body mass index (BMI), disability, and ICD-9-CM codes for ADRD. Results: A total of 424 participants had an index diagnosis of ADRD during 11-years. The total prevalence rate ranged from 31.6% in 2006 to 72.8% in 2016, and the total incidence rate ranged from 9.3% in 2006 to 15.8% in 2016. The prevalence rate ranged from 30.3% to 69.7% in men and 32.5% to 74.1% in women. The incidence rate ranged from 8.5% to 12.9% in men and 9.8% to 12.9% in women. Those with ADRD were significantly more likely to be older (82.1 versus 81.6 years; p-value=0.024) and to have a lower score in the MMSE (21.1 versus 21.7; p-value=0.013) compared with whole sample (N=927). Non-significant differences were observed by sex, education, medical conditions, BMI, depression, physical function or disability compared with whole sample. Conclusions: The prevalence and incidence rates of ADRD in Mexican-American Beneficiaries is high. These findings underscore the need for clinical services and caregiving resources in this population.

### PROTECTIVE EFFECT OF LIFETIME EXPERIENCES ON FUNCTIONAL STATUS IN YOUNG-ONSET DEMENTIA

Tram N. Pham,[1] Lauren Massimo,[1] and Katheryn A. Cousins[1], *1. University of Pennsylvania, Philadelphia, Pennsylvania, United States*

Patients with Frontotemporal degeneration (FTD), a common form of young-onset dementia, experience decline in cognitive, social and daily functioning as the disease progresses. Research shows that lifestyle factors may be an important modifiable risk factor for dementia, but this has not been well studied in FTD. In this study, we test the hypothesis that lifetime experiences, including education, occupation, and leisure activities, are associated with better functional status in individuals with FTD. We also evaluated the relationship between timing of experiences (early, mid-life, and late-life) and functional status. Thirty-five patients (mean age 61.6 ± 8.7; 74% male; mean disease duration 3.4 ± 2.6; mean MMSE 24.0 ± 5.5) completed the Lifetime of Experiences Questionnaire (LEQ), a comprehensive assessment of lifelong cognitive lifestyle, and the Clinical Dementia Rating Scale (CDR), which was used to assess functional status. Linear regression tested the relationship between cognitive lifestyle and functional status, with age and disease duration included as covariates. Higher total LEQ score was associated with better functional status (lower score on CDR) ($\beta$ = -0.047, p = 0.009). While Young Adulthood LEQ score was not significantly associated with total CDR ($\beta$ = -0.047, p = 0.176), both Mid-life ($\beta$ = -0.117, p = 0.011) and Late-life ($\beta$ = -0.133, p = 0.013) LEQ score significantly contributed to functional status. Our results indicate that functional status is mediated in part by cognitive lifestyle and that experiences accumulated in mid-life and late-life have a greater effect on functional status at time of diagnosis.

### LONG-TERM EXPOSURE TO AIR POLLUTION AND THE RISK OF DEMENTIA: THE ROLE OF CARDIOVASCULAR DISEASES

Debora Rizzuto,[1] Giulia Grande,[1] Petter Ljungman,[2] and Tom Bellander[2], *1. Aging Research Center, Department of Neurobiology, Care Sciences and Society, Karolinska Institutet and Stockholm University, Stockholm, Sweden, 2. Institute of Environmental Medicine (IMM), Karolinska Institutet, Sweden, Sweden*

Aim: We aimed to investigate the association between long-term air pollution and cognitive decline and dementia, and to clarify the role of CVD on the studied association. Methods: We examined 3150 dementia-free 60+ year-olds in the Swedish National study on Aging and Care in Kungsholmen, Stockholm for up to 13 years, during which 363 persons developed dementia. Outdoor air pollution levels at the home address were assessed yearly for all participants, using a dispersion model for nitrogen oxides (NOX), mainly emitted from road traffic. Mixed-effect linear regression models were used to quantify the association between air pollution and cognitive decline (with the Mini Mental State Examination). The risk of dementia, in keeping with the Diagnostic and Statistical Manual of Mental Disorders IV edition, was estimated using competing-risks models, considering death as competing event, and considering an exposure window 0-5 years before a year at risk. Stratified analyses by CVD were also performed. Results: Higher levels of traffic-related residential air pollution were associated with steeper cognitive decline over the follow-up period. After controlling for potential confounders, higher levels of air pollution were associated with increased risk of dementia (HR: 1.13, 95%CI: 1.05-1.22, for an µg/m3 unit increase NOX). The stratified analyses showed that the presence of CVD enhanced the effect of air pollution on dementia risk. Conclusion: Long-term exposure to traffic-related air pollution was associated with a higher risk of dementia. Cardiovascular disease might have played a role in this association.

### MULTIPLE PERSPECTIVES ON WHAT (IF ANY) IS AN OPTIMAL TIME FOR PEOPLE WITH DEMENTIA TO MOVE TO A CARE HOME

Kritika Samsi,[1] Laura Cole,[1] and Jill Manthorpe[1], *1. King's College London, London, United Kingdom*

Deciding an 'optimal' time for a person with dementia to move to a care home may be difficult for people with dementia, family carers, and professionals who support them; but there is currently limited evidence to help make this decision. Using phenomenology, we carried out qualitative interviews with 20 family carers, 5 people with dementia, 20 care home managers and 20 social workers, about their experiences, views and attitudes regarding timing of a move to a care home. Social workers indicated that managing risks and safety of person with dementia living in their own home were paramount when considering where person with dementia should live. These concerns included mishandling gas and electrical equipment at home, wandering and getting lost outside, and breakdown of family care. They and care home managers valued wishes of the person with dementia, and minimising any emotional distress to them when a move did come about. Family carers reported feeling stressed, and guilty around decision-making and ultimate move of their relative to a care home. Many described weighing up various risks when reaching 'tipping point' and making trade-offs between available options or uncertain future choices. Participants with dementia recognised they had struggled to cope at home and needed more support; however, many found the move difficult as they relocated nearer to family, away from their home and friends, and resigned themselves to less independence. Most people with dementia reported that their carers initiated discussions about timing of move, and that family discussions about this were common.

### GREATER DEMENTIA SEVERITY IS ASSOCIATED WITH INCREASED RISK OF POTENTIALLY PREVENTABLE READMISSIONS DURING HOME HEALTH CARE

Sara Knox,[1] Brian Downer,[2] Allen Haas,[3] Addie Middleton,[4] and Kenneth Ottenbacher[3], *1. MGH Institute of Health Professions, Boston, Massachusetts, United States, 2. University of Texas Medical Branch, Galveston, TX, Galveston, Texas, United States, 3. University of Texas Medical Branch, Galveston, Texas, United States, 4. Medical College of South Carolina, Charleston, South Carolina, United States*

Approximately 14.0% of Medicare beneficiaries are readmitted to a hospital within 30-days of home health admission. Individuals with dementia account for 30% of all home health care admissions and are at high-risk for rehospitalizations. Our primary objective was to determine the association between dementia severity at admission to home health and 30-day potentially preventable readmissions (PPR) during home health care. A secondary objective was to develop a dementia severity category from OASIS items based on the Functional Assessment Staging Tool (FAST). Retrospective cohort study of 124,119 Medicare beneficiaries receiving home health (7/2013 – 6/2015) and diagnosed with dementia (ICD-9 codes). The primary outcome was 30-day PPR during home health. The predictor variable of dementia severity was categorized into six levels (non-affected to severe). The overall rate of 30-day PPR was 7.6% (95% CI 7.4, 7.7) but varied by patient and health care utilization characteristics. After adjusting for sociodemographic and clinical characteristics, patients classified as stage 6 and stage 7 had 1.36 (95% CI 1.28, 1.45) and 1.90 (95% CI 1.59, 2.26) times higher odds to experience a 30-day PPR compared to patients classified as stage 1-2. Dementia severity in the later stages is associated with increased risk for PPR. Development of a dementia severity category based on OASIS items and the FAST is feasible. Future research is needed to determine effective strategies for decreasing PPR during home health for individuals with severe dementia. Future research is needed to validate the proposed dementia severity categories used in this study.

### COMPARATIVE EFFECTIVENESS OF CANINE- OR EQUINE-ASSISTED THERAPY FOR RESIDENTS WITH DEMENTIA

Martha E. Kemeny,[1] Deborah Hutchins,[1] and Courtney Gramlich[2], *1. Slippery Rock University, Slippery Rock, Pennsylvania, United States, 2. Slippery Rock University Storm Harbor Equestrian Center, Slippery Rock, Pennsylvania, United States*

Older adults with dementia may manifest symptoms such as apathy, withdrawal, and aggressive actions (NIA, 2019). Even surrounded by people in LTC, residents with dementia may lack social engagement. Non-pharmacologic approaches are promising (Brodaty & Arasaratnam, 2012) and are reflected in the newer CMS F-tag guidelines (CMS, 2017), but there remains a gap in research about the most effective approaches for LTC residents. Limited research exists on animal-assisted interventions (AAI) as a non-pharmacological approach to increase engagement in people with dementia (Friedman, Thomas & Chung, 2015; Huff-Mercer, 2015) and no known research compares equine- and canine-assisted therapy protocols. This study employed a within-subject alternating-treatments design for three single subjects. Completing the similar tasks of grooming, walking, and interaction, the independent variables are two conditions: 1) Condition A: Equine-assisted activity 2) Condition B: Canine-assisted activity. Using the Dementia Interview Rating (Strauss & Sperry, 2002), apathy was measured before and after each phase. During each session, three outcomes were measured: 1) Engagement in Preferred Activities Scale (Nelson et al., 2014) was used to measure the level of engagement in the interaction; 2) Heart rate variability, a measure of coherence, was measured using the Emwave pro (Heart Math Institute, 2018), and 3) targeted observed social responsiveness (initiation, verbal response, non-verbal response, motor response to one-step instructions) using a smart tablet application. Preliminary results suggest past experience/preference impact the individual client's response to differing protocols. Effective non-pharmacological interventions for older adults with dementia are an essential alternative to current practice.

### SOCIAL COGNITION AND AWARENESS IN ALZHEIMER'S DISEASE

Marcia Dourado,[1] Tatiana Belfort,[1] and José Simões Neto[2], *1. Institute of Psychiatry - Universidade Federal do Rio de Janeiro, Rio de Janeiro, Brazil, 2. Sociology and Political Science Department, Federal University of Santa Catarina,, Florianópolis, Santa Catarina, Brazil*

Social cognition is the capacity to interpret and predict another's behavior according to beliefs, intentions, and emotions, and the ability to decode environmental stimuli in order to be better able to adapt to new situations. A key question is the relationship between social cognition and awareness in dementia. This study aimed to investigate the relation between social and emotional functioning (SEF) and awareness in Alzheimer's disease (AD). In a cross-sectional design, a consecutive series of 50 people with mild to moderate AD and their 50 family caregivers were assessed. The study variables were awareness, SEF, neuropsychiatric symptoms, cognition, working memory, quality of life, functional activities, presence of depressive symptoms, and caregivers' burden and cognition. We found a significant difference between self-rated SEF and informant-rated SEF. In 56% of the cases, self-rated SEF was lower than the informant-rated SEF. People with AD mostly (56%) had mildly impaired awareness of disease, 20% had moderate impaired awareness of disease, and 6% were unaware of the disease. A multivariate linear regression examined the association between informant-rated SEF score and the variables. The social functioning and relationship domain of awareness and informant-rated QoL of people with AD were significantly associated with informant-rated SEF. Conclusion: The relationship between informant-rated SEF and awareness of social functioning and relationship supports the multidimensional nature of awareness. SEF and awareness of social functioning shows that they are comprised of judgments related to perceptions about oneself and values qualitatively different from awareness of memory or functionality, which can be directly observed.

### SCREENING FOR VISION AND HEARING LOSS IN OLDER ADULTS WITH DEMENTIA: A FEASIBILITY STUDY

Katherine McGilton,[1] Fiona Höbler,[1] Marilyn Reed,[2] Tammy Labreche,[3] M. Kathleen Pichora-Fuller,[4] Valerie Shoemaker,[3] and Walter Wittich[5], *1. KITE, Toronto Rehab, University Health Network, Toronto, Ontario, Canada, 2. Baycrest Health Sciences, Toronto, Ontario, Canada, 3. School of Optometry and Vision Science, University of Waterloo, Waterloo, Ontario, Canada, 4. Department of Psychology, University of Toronto, Toronto, Ontario, Canada, 5. School of Optometry - Université de Montréal, Montréal, Québec, Canada*

Sensory loss accounts for one of the most common chronic conditions among older adults, with hearing loss affecting half of adults aged over 65 years and vision loss almost one fifth of those aged 70 years and over. Together, dual sensory loss is found to be most prevalent in older adults with dementia. The highest prevalence is found in long-term care (LTC) settings. For this reason, we conducted a multi-stage study to identify the most effective vision and hearing screening tools for use with older adults living with dementia and to evaluate their feasibility of use by nurses working in LTC. We first conducted a comprehensive review of the literature, and supplemented this with an environmental scan of healthcare professionals and sensory specialists working with older adults who have dementia. Following this extensive review and consultative decision-making process, a package of vision and hearing screening tools was selected for use by nurses working in LTC. On-site training was provided by two experienced audiologists and optometrists, after which the feasibility of sensory screening by three nurses of 17 residents under their care was evaluated. We report on the six measures of hearing and seven measures of vision that were piloted for screening of older adults with dementia living in LTC, and on the findings for their feasibility of use by nurses working in this setting. Recommendations regarding the feasibility and reliability of screening for vision and hearing loss in older adults with dementia are discussed.

### IDENTIFYING PATIENTS WITH PROBABLE DELIRIUM AND DEMENTIA FROM ROUTINE HOME HEALTH CARE ASSESSMENTS

Olga F. Jarrín,[1] Abner Nyandege,[2] Robert J. Rosati,[3] and Tami J. Videon[3], *1. Rutgers, The State University of New Jersey, New Brunswick, New Jersey, United States, 2. Rutgers University, New Brunswick, New Jersey, United States, 3. VNA Health Group, Holmdel, New Jersey, United States*

Timely education of patients and their family caregivers on dementia disease management and health behavior changes and referral to additional resources is an essential part of age-friendly and high quality care. Nearly one out-of-three home health care patients has been diagnosed with dementia, however, family members and front-line health care providers are frequently unaware that a diagnosis of dementia has made, and what to do. The aim of this study was to identify a small set of questions that are routinely collected during home health care assessment which can be used to rapidly identify patients with suspected dementia, who may benefit from additional screening and services. We developed the preliminary model from 100% national home health care assessment data from 2014 (4.1 million people). We validated the model with a sample of nearly 27,000 patients who received a new (start-of-care) home health assessment in 2016, from four home health agencies that share a common data warehouse. The final model consisting of five questions, performed well in national data (AUC 0.85) predicting any diagnosis of dementia contained in the Medicare Chronic Conditions warehouse or home health record. The final model performed similarly (AUC 0.87) in the validation sample, predicting a diagnosis of dementia or history of dementia medication during a 3-year window of time from clinical data warehouse. The potential applications of this model have the potential to accelerate timely identification of patients with probable dementia or delirium, patient and family caregiver education, and referral to rehabilitative and supportive services.

### TARGETED TOOL TO MEASURE DEMENTIA LITERACY IN PREVENTION AND TREATMENT AMONG SEXUAL AND GENDER MINORITY OLDER ADULTS

Maritza Dowling,[1] and Dana Hines[2], *1. George Washington University, Washington, District of Columbia, United States, 2. George Washington University, Washington, United States*

Dementia prevalence among sexual minority adults age 60+ in the US is approximately 7.4%. This represents over 200,000 sexual minority older adults living with Alzheimer's disease (AD). Compared to the general aging population, LGBTQ older adults face unique challenges throughout their life-course that may increase exposure to risk factors associated with neurocognitive dysfunction and progression to dementia. Culturally-competent instruments to measure dementia literacy are needed to inform the design of targeted educational interventions for early diagnosis and improved health outcomes. We sought to develop and test a measure of dementia literacy in a community sample of LBGTQ older adults. Following a comprehensive analysis of existing scales, items were adapted resulting in a 35-item survey containing subjective and objective literacy measures. Participants were recruited through community centers in Washington, DC and Maryland. Forty LGBTQ older adults (50-80 years) completed the questionnaire. The analysis of these data was used to modify the original survey. A final 25-item questionnaire was administered to another sample of 50 individuals. 60% correctly identified dementia symptoms from vignettes. Nearly-half believed that dementia symptoms could be reduced by lifestyle changes and drug therapies. Commonly identified risks for dementia included aging, family history, genetics, stroke, and brain injuries. Frequently-endorsed methods for risk reduction included physical activity, intellectual/brain training and social activities. Education level, age, and socioeconomic variables were strongly associated with literacy. Most respondents did not associate cardiovascular/metabolic risk factors with dementia risk. Knowledge of risk factors underlying AD may help underserved populations close current health disparities gaps.

### PATIENT PRIORITIES CARE: GOAL-ALIGNED CARE FOR VETERANS WITH DEMENTIA AND THEIR CAREGIVERS

Jennifer Freytag,[1] Lilian Dindo,[1] Angela Catic,[2] and Aanand Naik[1], *1. Center for Innovations in Quality, Effectiveness and Safety, Houston, Texas, United States, 2. Michael E DeBakey VA Medical Center, Houston, Texas, United States*

Patient Priorities Care (PPC) is an approach to clinical conversations that focuses on aligning care with patient priorities. This approach involves identifying and incorporating patient values, goals, and preferences into care for patients with multiple comorbid conditions. We have previously described how this clinically feasible and sustainable approach has been implemented in VA and private healthcare settings, and how this approach results in documented care aligned with patient goals. In this paper, we describe the way PPC has been adapted and implemented in a VA geriatrics clinic for Veterans with dementia and their caregivers. We discuss results from a funded pilot project in which we investigated the role of caregivers in patient goal setting; assessed patient functioning using self-reported and sensor measures; and examined clinical conversations and interviews. We use these results to discuss implementation strategies targeting: (1) caregiver activation and involvement in clinical conversation and decision-making, (2) patient and caregiver education about the disease trajectory of dementia, (3) life space function and using physical sensor data in dementia care, (4) measures of community integration for patients and caregivers, and (5) features of successful clinical conversations in this context.

### SESSION 855 (POSTER)

### DISEASE MANAGEMENT | FALLS | HEALTH PROMOTION

### GERIATRIC ASSESSMENT ADOPTION IN COMMUNITY CANCER CENTERS: TRENDS, BARRIERS, AND RECOMMENDATIONS

Elana Plotkin,[1] Lorna Lucas,[1] Peggy Sue Burhenn,[2] Ginah Nightingale,[3] Kah Poh Loh,[4] Priscilla D. Allen,[5] and Efrat Dotan[6], *1. Association of Community Cancer Centers, Rockville, Maryland, United States, 2. City of Hope, Duarte, California, United States, 3. Jefferson Health, Philadelphia, Pennsylvania, United States, 4. University of Rochester, Rochester, New York, United States, 5. Louisiana State University, Baton Rouge, Louisiana, United States, 6. Fox Chase Cancer Center, Philadelphia, Pennsylvania, United States*

Addressing the needs of older adults with cancer is critical for the delivery of high-quality, patient-centered care. The Association of Community Cancer Centers (ACCC) has identified barriers and best practices for serving this growing patient population in order to help support the multidisciplinary team in understanding and proactively preparing for this large subgroup of patients. A survey was administered to 332 cancer professionals. 95% agreed that their older adult patients would benefit from a comprehensive geriatric assessment, yet only 17% of respondents are performing CGAs. 74% of respondents are not using any screening tool to identify high risk patients. The top three barriers to this were time/personnel and limited familiarity with available, validated tools. 61% are not focused on increasing older adult participation in clinical trials which leads to a disparity in care. Techniques for evaluating fitness, cognitive status, psychological status, comorbidities, and toxicity risk were often informal and not recorded in an EMR. Three in-depth focus groups were completed at programs demonstrating effective, yet different models of care for an older population. City of Hope Cancer Center is running a Senior Adult program under a grant where patients receive care in concordance with a score (CARG toxicity calculator) and a team review with a geriatrician. Sidney Kimmel Cancer Center has a consultative clinic where patients attend a 2-hour appointment to complete a comprehensive geriatric assessment with oncology, geriatrics, and specialists including pharmacy and nutrition. ACCC has recommended resources to address deficits in care, particularly in the community setting.

### CHOOSE TO MOVE: IMPLEMENTATION OF A PHYSICAL ACTIVITY INTERVENTION AT SCALE ACROSS BRITISH COLUMBIA, CANADA

Joanie Sims Gould,[1] Heather McKay,[1] Samantha Gray,[1] Adrian Bauman,[2] Lindsay Nettlefold,[1] and Christa Hoy[1], *1. University of British Columbia, Vancouver, British Columbia, Canada, 2. University of Sydney, Sydney, NSW, Australia*

Despite the many benefits of physical activity (PA), older adults remain among the least active Canadians. Regular PA effectively enhances social connectedness which in turn, is linked to positive health benefits. PA also promotes older adult's physical mobility which is "the best guarantee of retaining independence and being able to cope" in later years. Although effective PA interventions exist, all but five were conducted at small scale. None were effectively scaled up and sustained over the longer term. To improve population health, effective interventions must be scaled-up. In 2015, BC Ministry of Health released a PA strategy and action plan--older adults were identified as one priority area. In partnership with government and community stakeholders we were entrusted to co-design, implement and evaluate a 6 month, evidence- and choice-based PA intervention (Choose to Move; CTM) across BC, Canada. Implementation and adaptation frameworks and processes we adopted were embedded within socioecological models. We evaluated CTM at scale-up in 26 communities with 458 low active older adults. Our implementation evaluation showed that relationships and infrastructure were key facilitators to delivering CTM at scale. Our impact evaluation showed that PA and social connectedness were enhanced; mental health (loneliness/happiness), grip strength and mobility all improved following participation in CTM. A flexible, adaptable PA model, designed with scalability in mind is key to enhance health indicators in low active older adults. Effectively engaging stakeholders at multiple levels in the implementation process is essential to success.

### INDOOR AND OUTDOOR FALLS AMONG ADULTS AGING WITH A PHYSICAL DISABILITY

Thomas J. Eagen,[1] Tracy Mroz,[1] Tracy Chippendale,[2] Carrie Karvonen-Gutierrez,[3] Sam Battalio,[1] Katie Singsank,[1] and Ivan Molton[1], *1. University of Washington, Seattle, Washington, United States, 2. New York University, New York, New York, United States, 3. University of Michigan, Ann Arbor, Michigan, United States*

Falls among community-dwelling adults are a significant public health concern. Adults aging with a physical disability report a high number of falls, recurrent falls, and injuries caused by falls. Prevention strategies are needed to reduce the incidence of falls among this population; however, the location of the fall may influence which strategies will be most effective. The purpose of this project was to examine falling indoors versus outdoors was associated with fall related psychological concerns (e.g., self-efficacy), self-reported physical activity levels, physical function and sociodemographic characteristics (e.g., sex, age, education, employment), using survey data of adults aging with four conditions: muscular dystrophy (MD), multiple sclerosis (MS), post-polio syndrome (PPS), and spinal cord injury (SCI). Of the 1381 participants who completed the survey in 2017, 52% (n=719) reported at least one fall in the past 6 months. When asked about their worst fall, 32% of falls (n=233) occurred outdoors and 68% (n=486) occurred indoors. Participants with MS were significantly more likely to report falling outdoors (MS=35%, MD = 21%, PPS = 21%, SCI = 24%). Factors significantly associated with outdoor falls included living in an urban environment (OR = 1.59; 95%CI:1.06, 2.39), being more physically active (OR = 1.01; 95%CI:1.001, 1.02) and having better physical function (OR = 1.05; 95%CI:1.03, 1.08). These results fill a critical gap in the falls literature on fall location and have important implications for tailoring fall prevention interventions for individuals aging with a physical disability.

### PERSONAL FACTORS ASSOCIATED WITH DROPOUT RATE IN SHORT-TERM FALL PREVENTION PROGRAM FOR OLDER ADULTS

Qiwei Li,[1] and Becky P. Knight[2], *1. University of North Texas, Denton, United States, 2. University of North Texas, Denton, Texas, United States*

Fall prevention programs towards older adults are beneficial, however, drop-out rate among older adults in short-term fall prevention programs are relatively high (one out of three). With older adults dropping out, the programs benefit less for their target populations and achieve a lower level of cost-effectiveness as reported to funders. Literature review has revealed that previous studies on attrition frequently focus on longitudinal research, leaving the factors causing older adults to quit short-term (e.g., 8 weeks) fall prevention programs understudied. This study aimed to explore the association between demographic factors, educational level, health status, fall history, and the completion of the short-term fall prevention program named A Matter of Balance. A total of 691 older adults (526 females, 76.1%) 65 and older participated in the program were included and a Descriptive Discrimination Analysis (DDA) was conducted to analyze the multivariate relationship stated above. The results have identified that fall frequency, educational level, fear of falls, health status, perceived limitations due to fall, injury cases resulted from falls, and having arthritis are the most statistically significant factors that are associated with the completions among older adults for the short-term fall prevention program. The implications of this study include suggestions to modify future recruitment eligibilities in order to include the most suitable participants in certain programs, as well as adaptive modifications for short-term fall prevention programs that aim to serve larger aging populations with individualized conditions. Networking of evidence-based research outcomes should be shared for increased success of future programs.

### GOALS OF CARE IN END-STAGE RENAL DISEASE: THE INFLUENCE OF AGE AND MULTIMORBIDITY

Deborah P. Waldrop,[1] Patricia Denny,[2] Sandra Lauer,[2] Kathleen Grimm,[2] Phyllis Murawski,[2] and Mandip Panesar[2], *1. University at Buffalo School of Social Work, Buffalo, New York, United States, 2. Erie County Medical Center, Buffalo, New York, United States*

The number of people with End Stage Renal Disease (ESRD) who need dialysis treatment has increased sharply among adults age 75+. Older adults on dialysis have lower rates of advance care planning and higher treatment intensity, hospitalization and intensive care than people other chronic illnesses. Comprehensive care of older adults with ESRD includes advance care planning that addresses goals of care and not just specific medical treatments. The purpose of this study was to explore the nature of symptom burden and advance care planning in dialysis patients. The study design was exploratory, descriptive and cross-sectional. Quantitative and qualitative data were collected during in-person chairside interviews with people having dialysis treatments. Categorical questions focused on demographics and advance directives. The Dialysis Symptom Inventory was used to measure symptom burden. Open-ended questions addressed the trajectory of illness and goals of care. Thirty-five interviews were conducted. Participants' Mage=55.8 years (range 27-84); 51 % were >60. A distinctive pattern of difference by age emerged. Participants >60 demonstrated greater multimorbidity and lower symptom burden (MDSI=30.13; Range 11-63) compared with those <60 (MDSI=36.31; Range 3-78). Goals of care also varied with age. Older adults' goals were: (1) Functional (e.g. to walk better, drive); and (2) Existential (e.g. maintaining, surviving, enjoying). Goals of participants <60 were: (1) Transplantation; and (2) Engagement (e.g. work, school, travel). The results suggest that the illness experience and goals are influenced by age and multimorbidity. Implications: ESRD-specific advance care planning conversations with a focus on goals of care are important.

### PREVALENCE AND MANAGEMENT OF HYPERTENSION AMONG MIDDLE-AGED AND OLDER ADULTS IN CHINA

Jianjia Cheng,[1] Jungtae Choi,[1] and Lydia Li[2], *1. School of Social Work, University of Michigan, Ann Arbor, Michigan, United States, 2. University of Michigan, Ann Arbor, Michigan, United States*

The study aimed to examine hypertension prevalence and management in China. Data were from a national survey of a probability sample of 45+ in China (N = 17,047). Self-reported hypertension diagnosis and readings from electronic blood pressure monitors were used to create four variables: diagnosed, measured (>=140/90 as high), undiagnosed, and overall (diagnosed + undiagnosed) hypertension. Respondents with diagnosed hypertension were asked about medication use, blood pressure monitoring, and lifestyle advice from doctors; and were considered inadequate blood pressure control if having measured hypertension. Weighted descriptive statistics and multivariate logistic regression were conducted. The prevalence of diagnosed, measured, undiagnosed and overall hypertension was 27%, 37%, 14%, and 51%, respectively. Across all four, older age adults, women, and urban residents had higher rates. Among hypertensive patients, 82% took anti-hypertensive medications, 91% monitored blood pressure, 60% received lifestyle advice, and 53% had inadequate blood pressure control. Compared to the 45-54 years old, the 75+ was less likely to receive lifestyle advice (OR=0.63, 95% CI=0.43-0.95) and the 65-74 was less likely to have adequate control (OR=0.75, 95% CI=0.73-0.98). Men were less likely to use medications (OR=0.77, 95% CI=0.63-0.95) but more likely to receive lifestyle advice (OR=1.48, 95% CI=1.23-1.78) than women. More education and urban (vs. rural) were associated with better hypertension management and control. In conclusion, hypertension affects half of the middle-aged and older population in China. More than half of hypertensive patients have inadequate blood pressure control. People who are older, women, low-educated, and rural residents are disadvantaged in hypertension management.

### PARTICIPATION IN TWO EVIDENCE-BASED FALLS PREVENTION PROGRAMS AMONG OLDER ADULTS WITH AND WITHOUT A DISABILITY

Thomas J. Eagen,[1] Ellen McGough,[1] Tracy Mroz,[1] Deborah Kartin,[1] Anjum Hajat,[1] and Ivan Molton[1], *1. University of Washington, Seattle, Washington, United States*

Older adults with a disability are at greater risk for falls and injury due to falling compared to those without a disability. Evidence-based falls prevention programs (EBFPPs) have been developed and disseminated broadly, however individuals with disabilities were excluded from original research on effectiveness. Using data from the National Falls Prevention Database from the National Council on Aging, we compared the reach and effectiveness of two EBFPPs, A Matter of Balance (MOB) or Stepping On, between those with and without a disability. Program reach was measured using attendance percentage. Program effectiveness was measured using change in fear of falling (FOF), fall-related activity restriction (FAR), and falls self-efficacy (FSE) post-program. A total of 12,667 participants were analyzed. Participants were, on average, 76 years old (M = 76.18, SD = 9.86), largely female (75%), well educated (80% some college or higher), and white (90%). Nearly half self-reported a disability (40%). Older adults with a disability were as likely to attend (M = 0.88, SD = 0.14) the program compared to those without a disability (M = 0.88, SD = 0.14, p =.30). Older adults with a disability reported greater FOF and FAR and lower FSE compared to participants without a disability at baseline. Significant improvements were made across effectiveness measures, irrespective of disability status. MOB and Stepping On are effective programs, well attended by older adults with and without disabilities, however older adults with a disability continued to report higher FOF and FAR, and lower FSE compared to those without a disability.

### EXERCISE EFFECTS ON WELL-BEING ON CANADIAN WOMEN OVER THE AGE OF 65

Holly A. Bardutz,[1] and Constance Corley[2], *1. University of Regina, Regina, Saskatchewan, Canada, 2. Fielding Graduate University, Santa Barbara, California, United States*

Two groups of Canadian women, over the age of 65, were interviewed (n = 20) in a study focused on brain health. The first group (n = 12) regularly attended exercise classes and met the exercise standards of the Canadian Society of Exercise Physiologists for the age group over 65 years for at least six months. Women in the comparison group (n = 8) had been taking adult education classes twice a week or more for at least six months. Thematic coding was used to analyze the results. Both groups reported benefits from their participation in their respective groups. However, the results show that the group who exercised regularly consistently reported improved mood, increased mental alertness, a better ability to handle stress, less pain, and improved sleep. These factors were not reported by the non-exercise group, which did benefit by gaining new knowledge, making new friends and feeling good because they were learning new things. This study suggests that Canadian women over the age of 65 who have been exercising regularly report many of the effects of exercise on the brain that are beneficial to their well-being. They did not specifically mention the new brain cells being made (neurogenesis) nor did they note neuronal rewiring (neuroplasticity), however they did self-report some psychological benefits that the Comparison Group did not report, as noted above. This research has implications for both practice and research.

### EFFECT OF FREQUENCY OF PARTICIPATING IN THE REGION ACTIVITY ON FUNCTIONAL DECLINE

Masashi Yasunaga,[1] Hisashi Kawai,[1] Hirohiko Hirano,[1] Hiroyuki Suzuki,[1] Yoshinori Fujiwara,[1] and shuichi Obuchi[1], *1. Tokyo Metropolitan Institute of Gerontology, Tokyo, Japan*

This 3-year prospective study was conducted to explore whether frequency of participating in the region activity exert independent effect on preventing functional decline among urban Japanese older adults after controlling for potential confounders. We examined a prospective cohort of 2,524 community-dwelling persons, aged 65 years or older, who responded to the baseline mail survey in Toshima ward, Tokyo, Japan in 2014. They were followed for the subsequent 3 years in terms of functional status. Multiple logistic regression models were used to analyze independent effects of frequency of participating in the region activity, such as 1) no participation, 2) no participation in the past year, 3) less than one day per month, 4) few days per month, 5) over one day per week, on functional status, controlling for potential confounders such as age, gender, self-rated health, chronic conditions and social capital at baseline. At baseline, the mean age of 1,261 participants who completely responded to follow-up survey in 2018 was72.1 years (SD=5.0), and 56.9% were women. As results of analyzing, only "over one day per week" was significant predictors of preventing subsequent functional decline even after adjustment for confounders (odds ratios .361; 95% CI .180–.725). Frequency of participating in the region activity over one day per week have effect on preventing functional decline among urban Japanese older adults after controlling for potential confounders.

### SESSION 860 (POSTER)

### ECONOMICS

### CONSEQUENCES OF ECONOMIC HARDSHIP AND SELF-RATED HEALTH AMONG OLDER ADULTS

Yang Li,[1] and Jan Mutchler[1], *1. University of Massachusetts Boston, Boston, Massachusetts, United States*

Adequate economic resources ensure that older adults' basic needs are met and facilitate a healthier lifestyle. Hardship signals unfulfilled needs experienced by individuals lacking adequate economic resources. Despite well-documented associations between indicators of hardship and self-rated health, little is known about whether hardship has the same impact on self-rated health across age groups. The purpose of this study was to investigate the association between hardship and self-rated health among older adults and determine whether this association differed by age. Employing data from the 2014 Survey of Income and Program Participation, we conducted logistic regression analysis to examine the association between hardship and self-rated health among adults age 55 and older in the United States, and the moderating effect of age on this relationship. Analyses were weighted using replicate weights provided by the survey. Indicators of hardship were dichotomized (1 = experienced hardship, 0 = no hardship). Analyses indicated that individuals who were unable to pay utility bills, unable to pay rent or mortgage, or who experienced food insecurity had lower odds of reporting good/very good/excellent health relative to those not experiencing these hardships. The association between hardship and self-rated health was shown to be less substantial among the oldest cohort (age 75 and older) relative to younger adults. Hardship is directly relevant to health outcomes as it represents the consequence of unfulfilled needs experienced by individuals lacking adequate economic resources. This study contributes to our understanding of the role of age in the association between hardship and self-rated health.

### ASSOCIATION OF ATTITUDES WITH ECONOMIC WELL-BEING AND HEALTH: A FOUNDATION FOR PUBLIC POLICY

Sarah B. Laditka,[1] James N. Laditka,[1] and Laura Gunn[1], *1. University of North Carolina at Charlotte, Charlotte, North Carolina, United States*

The 1960s War on Poverty was based on expectations that certain attitudes could improve health and economic well-being: aspiration and ambition, propensity to plan, personal efficacy, avoidance of unnecessary risk, connectedness to information and help, and trust. If true, promoting those attitudes might improve lives. The nationally representative Panel Study of Income Dynamics (PSID) developed scales to repeatedly measure associations of those attitudes with income and well-being. After five annual measurements, researchers found few associations. Acknowledging more data might be needed, researchers concluded that changing attitudes was unlikely to help. We studied those same associations using five decades of PSID measures on income and work disability, physical or "nervous" health problems limiting work (1968-present; n=5,027; 170,583 person-years; mean baseline age 34.2), with multinomial logistic Markov models and dynamic microsimulation, modelling three levels of each outcome plus death. We also examined persistence of the attitudes (measurement reliability). Results suggested the attitudes were persistent (intraclass correlations > 0.87). Controlling for age, sex, race, education, and baseline income, attitudes were strongly associated with the outcomes. For example, with above-median baseline income, 19.4% in the top baseline self-efficacy quintile had incomes below 150% of poverty at age 70, compared with 27.0% in quintile 1 (p<0.001). Similarly, 5.5% in the highest quintile reported severe work disability (could not work, or limited "a lot") at age 70 compared to 23.7% in the lowest (p<0.001). Other attitudes showed similar significant patterns. Attitudes in early- to mid-adulthood may contribute importantly to economic well-being and health throughout later life.

### THE LFDSS DETECTS HIGH-RISK FINANCIAL DECISION-MAKING IN COGNITIVELY VULNERABLE OLDER ADULTS

Rebecca J. Campbell,[1] and Peter Lichtenberg[1], *1. Wayne State University, Detroit, Michigan, United States*

The Lichtenberg Financial Decision-Making Screening Scale (LFDSS) is a brief screening tool that quantifies an individual's informed decision making and risk for exploitation in a real-world financial transaction. Previous literature found that the ideal cut-off score for those at higher risk of financial exploitation was five and above. The purpose of the present study was to examine the utility of the LFDSS as a screening tool for financial decision-making ability in a high-risk sample of people with cognitive impairment. The sample was obtained from the Michigan Alzheimer's Disease Center and was comprised of both cognitively healthy individuals (n=73) and those with cognitive impairment (n=45). All participants completed the LFDSS as part of a larger test battery. A Pearson chi-square analysis was used to examine group differences in financial decision-making risk between cognitively healthy individuals and those with cognitive impairment. A Pearson chi-square analysis found that those who had cognitive impairment were significantly more likely to score above the cut-off for high-risk financial decision-making compared to those who were cognitively healthy ($\chi 2(1)=4.61$, p=.032). The base rate of high-risk financial decision-making was 3.2x greater for those with cognitive impairment compared (17.7%) to those who were cognitively healthy (5.5%). These results demonstrate the utility of the LFDSS to detect high-risk decision-making in individuals with cognitive impairment. This tool serves a need in many professional settings (e.g. doctor's offices and Adult Protective Services) for a brief, standardized assessment measure of financial decision-making and exploitation risk for a real-world, significant financial transaction.

### DEBT AMONG OLDER ADULTS: DATA FROM AN EXPLORING STUDY IN SOUTH BRAZIL

Johannes Doll,[1] Francieli Conte,[1] and Karen Lima[1], *1. School of Education, Universidade Federal do Rio Grande do Sul, Porto Alegre, Brazil*

In recent years, Brazil has experienced significant increase in indebtedness rates amongst older adults. This is partly due to prominent forms of credit such as credit cards and payroll deductible credit, a type of credit that accounts for up to 35% of pensions. In addition, indebtedness is aggravated by high interest rates which are charged in Brazil. Exploratory study on the propensity for indebtedness amongst older people in Rio Grande do Sul, Southern Brazil (n = 406; age 46-97 years old; average 68,7 years old) aims to analyze reasons and factors which interfere in the financial-related issue. In general, indebtedness is a complex process that is often triggered by critical life events such as illness, death, separation, accident and unemployment. And, it is mediated by a number of behavioral and sociodemographic factors. The present work aims to analyze the relation between financial problems, income, schooling, age, materialism, consuming habits as well as altruism in two different social groups: a middle class (SESC) and a lower middle-class (FASC). Concerning theoretical reference, data discussion is based on Ronald Inglehart's theory on Materialism and Postmaterialism. Data analysis has shown differences in factors and relations among the two social groups. Consuming habits and altruism have a significant correlation with financial problems in both groups. However, income, education and materialism has shown a significant correlation only in the lower middle-class group. In the middle-class group, no relation between income, education, materialism and financial problems has been found.

### HETEROGENEITY IN PROGRESSION OF COGNITIVE IMPAIRMENT AND VARIATIONS IN HEALTH EXPENDITURES AND HEALTH SERVICES USE

Wassim Tarraf,[1] and Hector M. González[2], *1. Wayne State University, Detroit, Michigan, United States, 2. University of California, San Diego, San Diego, California, United States*

Cognitive aging and disease (e.g. dementia) are leading public health issues as longevity increases and the US population ages. We fit generalized linear models using data from the longitudinal Health and Retirement Study (2008-2014) on (Unweighted N=1,884) participants 70-years and older who met criteria for cognitive impairment not dementia (CIND), based on Aging, Demographics, and Memory Study specification, at baseline (2008) to test how impairment reversion, stability, and transition to dementia over 8-years affect change in biennial hospitalizations, nursing-home use, and out-of-pocket expenditures (OOP). Over 8-years, 13% reverted to normal cognition, 20% remained as CIND, 21% transitioned to dementia, and 46% died. In these groups, average OOP spending at baseline was $2311 (SE=$225), $2722 (SE=$278), $2180 (SE=$228), and $3653 (SE=$322), respectively. Average OOP spending increased to $3,095, $4,720, and $11,548 by the 8th year for those that reverted, stayed stable, and transitioned, respectively. Average OOP spending at the wave preceding death was $11,600. We observed substantial increases in nursing home use that were particularly pronounced among those that transitioned to dementia (Baseline Probability=0.04 increasing to 0.37 over 8-years) or died (0.09 increasing to 0.35 over 6-years), and similar but less pronounced differences in patterns of inpatient hospitalizations. Estimates were only slightly modified through adjustments to sociodemographic characteristics and comorbid conditions. We discuss how healthcare policy and clinical interventions focusing on early identification of impairment can potentially lead to improved and more efficient healthcare if better understanding of heterogeneities in impairment and cognitive disease progression is achieved.

### WHO ANSWERS THE HOUSEHOLD FINANCIAL QUESTIONS: ANALYSIS OF STABILITY AND CHANGE

Angela L. Curl,[1] and Chido Mudzonga[1], *1. Miami University, Oxford, Ohio, United States*

There is limited research on who acts as household financial respondents (FR) for couples who are responding to surveys. The purpose of this study was to determine the amount of stability and change in FR, as well as predictors of FR status and change of FR status. Methodology: This study analyzed data from Health Retirement Study from 2006 to 2014. The sample comprised of 6,755 households of couples over the age of 50. Data were analyzed using Hierarchical Linear Modeling (HLM). Results: The results indicated that 18.13% households experienced one or more transitions of the financial respondent. Black non-Hispanics, males, those with higher education, earning a greater percent of the household's income and those with higher cognitive ability were more likely to be the financial respondent than their counterparts. Higher depression (OR=1.22, p< .01) and lower cognitive ability (OR= 1.06, p<.01) were factors that predicted changes in the household's financial respondent. Health (number of chronic conditions, self-rated health) and percentage of the household earnings are not significant in the change of financial respondent. Discussion: Change of respondent may result in differences in reporting of household financial resources in longitudinal studies. Changes in respondent may also be indicative of a more Egalitarian household, or serve as an early sign of changes in family dynamics or roles. As changes in mental health and cognitive ability may prompt a change of financial respondent, this highlights the importance of both spouses being financially literate and aware of the family's economic resources, investments and obligations.

### ECONOMIC HARDSHIP INDICATORS ARE DIFFERENTIALLY ASSOCIATED WITH ADVERSE HEALTH OUTCOMES IN OLDER ADULTS

Loretta Anderson[1], *1. Johns Hopkins Bloomberg School of Public Health, Baltimore, Maryland, United States*

Previous studies have shown that higher levels of economic hardship in older adults is associated with increased odds of adverse health outcomes such as insomnia, anxiety, and depressive symptoms. The objective of this study was to determine if there was a differential association between individual measures of economic hardship and aforementioned adverse health outcomes. Cross-sectional analysis was conducted using data from the 2012 National Health and Aging Trends Study (NHATS). Logistic models were developed to assess the association between each of the four measures of economic hardship and three previously reported adverse health outcomes. Participants were asked if in the last month they did not have enough money for food, utility bills, mortgage/rent, or medical bills/prescription drugs. Measures of adverse health outcomes were symptoms of depression, anxiety, and insomnia. There were 7,075 community dwelling older adults aged 65 and older in the 2012 NHATS data. Results indicated that those who skipped meals were more likely to have depression, anxiety, and insomnia symptoms than those who did not skip meals. After adjusting for race, age, gender, education, and comorbid health conditions, skipping meals was associated with depression (OR=4.11, p<.000), anxiety (OR=2.81, p<.01), and insomnia (OR=2.16, p<.05). These results were higher and more statistically significant than the other measures of economic hardship. These findings are relevant to population-based efforts of nutrition interventions to improve quality of life in aging populations.

### SESSION 865 (POSTER)

### EMPLOYMENT AND RETIREMENT

### PHASED RETIREMENT IN OLDER AMERICAN WORKERS

Shinae Choi,[1] and Genevieve Smith[1], *1. The University of Alabama, Tuscaloosa, Alabama, United States*

Older workers are engaging in "phased retirement" in which they transition from full-time working status to part-time working and eventually retire at a later age. This study investigated whether phased retirement was financially and psychologically beneficial for middle-aged and older adults in the United States. The current study examined data on financial and psychological well-being and retirement transitions (i.e., immediate retirement and phased retirement) from the Health and Retirement Study (HRS) at two time points, four years apart. We analyzed 5,106 middle-aged and older adults from the 2010 and 2014 waves of the HRS data set using chi-square and one-way analysis of variance tests. Our results showed that 66.8% of respondents remained full-time working, while 12.7% of respondents chose phased retirement and 15.2% of respondents jumped straight into retirement. Our findings suggest that phased retirement is beneficial for older Americans financially and psychologically. Specifically, the level of total household financial wealth was significantly higher for those who chose phased retirement than immediate retirees from workforce. In terms of psychological perspectives, immediate retirees experienced more depressive symptoms than those who chose phased retirement. Our findings could help individuals and households to be better equipped when preparing for retirement. Our findings could also provide a basis for further research into phased retirement and its impact on well-being in middle-aged and older Americans. Furthermore, policymakers could be better informed about retirement trends and create policies based on our findings to better help older individuals and households be financially and psychologically prepared for retirement.

### A VIEW OF OLDER ADULTS' DEMOGRAPHIC, HEALTH, AND ECONOMIC PROFILES BY RETIREMENT CHOICE STATUS

Jane L. Tavares,[1] and Marc A. Cohen[2], *1. University of Massachusetts Boston Gerontology Institute, LeadingAge LTSS Center, Boston, Massachusetts, United States, 2. University of Massachusetts Boston Gerontology Institute, LeadingAge LTSS Center @UMass Boston, Boston, Massachusetts, United States*

Gerontologist, economists, and other scientists have documented that involuntary or forced retirement is a more common occurrence among middle-aged adults than previously thought. Prevailing research has further indicated that involuntary retirement and off-time transitions are associated with poorer physical and mental well-being in later life. However, few studies have sought to gain a comprehensive understanding of who compromises the population of involuntary retirees and how these individuals compare to voluntary retirees. This study used the 2014 wave of the Health and Retirement Study (N=9,409) to analyze a United States representative sample of fully retired individuals aged 55 and older in order to develop a demographic, health, and economic profile of voluntary versus involuntary retirees. Analyses showed that that male, younger age group, minority race, separated/divorced, less educated, and south and west region residing individuals have significantly greater representation in the involuntary retirement group compared to the voluntary retirement group. Further, involuntary retirees reported significantly poorer physical and mental health and fewer financial resources than voluntary retirees. A supplementary longitudinal analyses also revealed that while health steadily declines for both of the retirement choice status groups, involuntary retirees maintained significantly poorer health than voluntary retirees over time. Observations from this study highlight the importance of identifying predictors of involuntary retirement and utilizing longitudinal analyses to gain greater insight into how health and economic characteristics factor into retirement decisions as well as how retirement decisions impact future health and economic characteristics.

### UNFULFILLED PROMISES OF WORKING IN LATER LIFE OF NEW ZEALAND WOMEN

Christine G. Unson[1], *1. Southern Connecticut State University, New Haven, Connecticut, United States*

This critical study on women and work-life extension compared motivations, needs satisfaction, barriers and resources faced by ethnically diverse women in high and low status occupations. Twenty-seven women (mean age = 64; age range 55 to 79 years) who at 55 years or older, had changed careers or places of employment, or who had retired and currently volunteer were interviewed. Four were Asian/Pacific Islanders, seven Māori and 16 European New Zealanders. Themes were compared between women in low and high status occupations. Instrumental motivations were most common among women in lower-status occupations and entrepreneurs whereas idealistic motivations were more prevalent with higher- status occupations and volunteer posts. Themes related to needs satisfaction show that work met basic and security needs among lower-status occupations but self-actualization needs were expressed more frequently in higher-status occupation. Feelings of belonging and a sense of achievement were cited by most participants. Themes relating to barriers faced by participants in lower-status occupations and by minority women include inadequate skills and access to information, and racial, ageist, and sexist discrimination. Themes also revealed multiple sources of supports: employers provided training and managerial support, and for some non-profits, a collaborative working atmosphere and co-workers who met older workers' needs for belonging and sense of achievement, and who potentially, as a unit, could advocate for better working conditions for older workers. Work-life extension has the potential for creating classes of older female workers with differential access to work opportunities and level of aspirations achievable.

### AGE AND OCCUPATIONAL TIME PERSPECTIVE ARE ASSOCIATED WITH PREFERENCE FOR HELPING OTHERS AT WORK

Yochai Z. Shavit,[1] and Laura L. Carstensen[1], *1. Stanford University, Stanford, California, United States*

Workforces today are more age-diverse than ever before. Despite widespread beliefs that older workers are less productive than younger workers, when productivity is measured at the team level, the presence of older workers appears to positively contribute to productivity. Underlying reasons for this finding have been poorly understood. Socioemotional selectivity theory (SST) posits that goals change with age and that when time-horizons grow increasingly limited people prefer activities that hold meaning in their pursuit, such as helping others. Reasoning from SST, we hypothesized that age is associated with a preference for helping colleagues. We further hypothesized that expanding time-horizons would reduce age differences such that both younger and older people would prefer projects that help colleagues. 555 workers, aged 20-75, were assigned to one of three experimental conditions where time-horizons were expanded, constrained or not mentioned (control), and were asked to indicate their preferences for projects that benefitted themselves or others. Results from multinomial logistic regression show that collapsing across conditions, age was positively associated with a preference for projects helping friends or colleagues over ones that were career advancing, and participants were more likely to work on a helping project over one that would take the company in a new direction regardless of age. We further found effects of manipulating time horizons- in both the expanded and limited occupational time-horizons conditions workers were less likely to prefer helping compared to advancing their careers, and the positive association between age and preference for helping was attenuated.

### PSYCHOLOGICAL MECHANISMS THAT UNDERLIE GOAL EXPECTANCY AMONG RETIREES

Cindy Tsotsoros,[1] Anna Mooney,[2] Joanne Earl,[2] and Douglas Hershey[1], *1. Oklahoma State University, Stillwater, Oklahoma, United States, 2. Macquarie University, Sydney, Australia, Australia*

The way individuals envision, formulate, and strive to meet retirement goals is poorly understood. In particular, few studies have focused on the goal setting process of individuals who have already retired. In this investigation, the authors replicate and extend Hershey and Jacobs-Lawson's (2009) model of retirement goal expectancy across five retirement resource domains. Their (path) model posits individuals' perceptions of the consequence of failing to achieve a goal determines the perceived importance of the goal. Perceived goal importance, in turn, determines the effort individuals allocate toward achieving the goal (goal striving). And goal striving, in turn, predicts the perceived likelihood the goal will be achieved (goal expectancy). This basic model was empirically tested across five key retirement resource domains (health, physical, social, cognitive and emotional) identified by Wang and Shi (2013). The sample of 698 American retirees (Mage=77.14 years, SD=6.00) was divided into four subgroups: males and females, aged 66-77 and 78-94. Twenty theoretically-driven path analysis models were tested using AMOS (i.e., four subgroup models across five retirement domains). Differences were observed across subgroups and domains in terms of the magnitude of path coefficients and the amount of accounted variance in goal expectancy criterion measures (R2 values ranged from .26-.67). Overall, the path model was effective at capturing variability in retirement goal expectancy. Findings not only provide a synthesis of the 2009 goal model with Wang and Shi's Dynamic Resource Theory, but they also suggest areas in which retirement intervention specialists can intercede to increase the likelihood of goal attainment.

### ASSOCIATION OF AGE DISCRIMINATION, JOB STRESS, AND DEPRESSION AMONG OLDER WORKERS: ROLE OF SOCIAL NETWORKS

Abhijit Visaria,[1] Angelique Chan,[2] Grand Cheng,[1] Rahul Malhotra,[3] and Truls Ostbye[4], *1. Duke-NUS Medical School, Singapore, Singapore, 2. Centre for Ageing Research and Education, Duke-NUS Medical School, Singapore, Singapore, 3. Duke-NUS Medical School, Singapore, Singapore, Singapore, 4. Duke University Medical School, Durham, North Carolina, United States*

In this study, we examine the association between perceived age discrimination at the workplace and job stress, with depressive symptoms among currently working late-middle-aged adults. In particular, we investigate whether the association between these work-related factors and depression varies by the strength of their social networks. We use data from the Panel on Ageing and Transitions in Health Survey (PATHS) a national study of 1654 Singaporean citizens and permanent residents aged 50 to 59 years, conducted in 2016-2017. We account for age and other sociodemographic characteristics, measures of economic status, occupation, early retirement intentions, personality traits, as well as multiple physical health measures. Our findings indicate that age discrimination at work and job stress are both positively associated with higher depressive symptom scores. The relationship between age discrimination and depressive symptoms however varies by social networks, with age discrimination negatively associated with depressive symptom scores among those with larger friends-based social networks. Our findings indicate that while psychological wellbeing of late middle-aged workers is adversely affected by age discrimination and job stress, these workers benefit from wider and deeper social connections with friends. Our results provide empirical support to previous research that suggests that friends-based social networks yield distinct benefits in terms of subjective well-being and increased self-worth.

### CONTINUITY OF CAREER OVER THE LIFE COURSE ON LIFE SATISFACTION AND DEPRESSIVE SYMPTOMS IN LATER LIFE

Melanie S. Hill,[1] James E. Hill,[2] Stephanie Richardson,[1] Jessica Brown,[1] Jeremy B. Yorgason,[1] and Jeff Hill[1], *1. Brigham Young University, Provo, Utah, United States, 2. University of Utah, Salt Lake City, Utah, United States*

Identity scholars have suggested that having a unified sense of past, present, and future is related to positive well-being outcomes (Whitbourne, Sneed & Skultety, 2009). One's occupation can have a profound influence on an individual's identity throughout the life course (Nazar & van der Heijden, 2012). Research has looked at career mobility among younger age groups (Baiyun, Ramkissoon, Greenwood, & Hoyte, 2018); however, less is known about the impact of career stability later in life. Consistency in career choice over the life course may have positive outcomes down the line as career becomes part of an individual's identity. The current study uses the Life and Family Legacies dataset, a longitudinal state-representative sample of 3,348, to examine individual's careers at three points in the life course: high school (projected career choice), early adulthood, and later life. Results revealed that a match of desired career in high school and actual career in early adulthood was not predictive of life satisfaction or depressive symptoms in later life. However, a match of career in early adulthood and later life was significantly related to better life satisfaction and less depressive symptoms, which was explained through higher levels of job satisfaction. This study highlights the importance of acquiring and maintaining a career that is fulfilling to the individual over the course of early adulthood to later life.

### WORKING LONGER FOR ACADEMICS IN THE U.S. AND IRELAND: A GENDERED LIFECOURSE ANALYSIS

Aine Ni Leime[1], *1. National University of Ireland, Galway Ireland, Galway, Ireland, Ireland*

Many governments including the US and Ireland have been advocating longer working lives for all workers to ensure pension sustainability in the light of population ageing. Policy changes encouraging increased social security/state pensions age reflect this. However, there has been limited investigation of how the gender implications of these policy changes. While longer working lives may be attractive for some workers, there is evidence that women and men have profoundly different work-life trajectories and women may be more financially disadvantaged approaching retirement age. There is a need to explore how this affects their ability and/or desire to continue working past traditional retirement age and their financial security. This presentation is based on analysis of evidence from an EU-funded cross-national research project involving work-life history interviews conducted with forty older workers in academia in the US and Ireland in 2016 and 2017. A lifecourse approach is used to analyse interview data from ten male and ten female academics in Ireland and ten male and ten female academics in the US, aged 50 or over. Participants discussed early influences, work-life history and health concerns. The paper uses a cumulative disadvantage perspective to analyse how gender, family and health trajectories across the life course affect and can limit options around late work and retirement. It concludes that gender differences regarding norms of caregiving are important and that extending working life is more likely to be caused by financial necessity for women. The implications for future research and policy are discussed.

### THE IMPACT OF RETIREMENT ON VOLUNTEERING FREQUENCY: EVIDENCE FROM A NEW PANEL STUDY

Olga Grunwald,[1] Marleen Damman,[1] and Kène Henkens[2], *1. NIDI, Den Haag, South Holland, Netherlands, 2. Netherlands Interdisciplinary Demographic Institute, South-Holland, Netherlands*

OBJECTIVE: Retirement is a major life transition. People gain a considerable amount of free time, but also loose the benefits from work. Volunteering has been identified as a gratifying way to fill that time and to compensate some retirement-related losses, but is stratified by educational level. Research on how retirement changes volunteering behavior, particularly among different educational groups, is scarce. This study aims to fill this gap. Based on role theory, we hypothesize that the transition into retirement expands levels of volunteering, and that this effect will be relatively strong for the higher educated. METHODS: We use two-wave panel data that were collected in 2015 and 2018 among 5,312 Dutch individuals who were aged 60-65 and employed at baseline. Around half has retired at follow-up (N=2,618). RESULTS: Descriptive findings show that the share of frequent volunteers (i.e., at least once a week) was around 18% at baseline. At follow-up, the share of frequent volunteers rose to 36% among those who retired, but did not change among those who remained employed. Conditional change models show that transitioning into retirement significantly increases volunteering frequency, when controlling for demographic factors and individual resources. As hypothesized, the effect of retirement is relatively strong for the higher educated. DISCUSSION: To deal with the life changes upon retirement, volunteering appears to be an often-used strategy, in particular among the higher educated. Whether this is motivated by work role loss (compensation), or reflects having more time to 'do good' (opportunity) is an important question for future research.

### RETIREMENT ADJUSTMENT: COMPARISON AMONG CHINESE, JAPANESE, AND KOREAN RETIREES

Dannii Yeung,[1] Takeshi Nakagawa,[2] and Jinmyoung Cho[3], *1. City University of Hong Kong, Hong Kong, Hong Kong, 2. National Center for Geriatrics and Gerontology, Aichi, Japan, 3. Baylor Scott & White Health, Temple, Texas, United States*

Moving into retirement is a stressful life event. However, recent research findings suggest that not every retiree experiences maladjustment. This paper investigated longitudinal changes in depressive symptoms during the retirement transition utilizing harmonized data of CHARLS, JSTAR, and KLoSA. Participants were selected for examination if meeting the following criteria: They were working in Wave 1 and had retired in Wave 2 (China=1053; Japan=184; Korea=706). These participants were categorized into two subgroups based on their status in Wave 3, either remained retired or reemployed. The proportion of retirees who were reemployed in Wave 3 is significantly higher in Korea (49.3%) and China (41.1%) than in Japan (17.4%). In each wave, the level of depressive symptoms was measured by the 10-item CES-D scale. Results of the repeated measures analyses show that, even after controlling for gender, mean level of depression increased over time among Korean retirees [$F(2,702)=3.65, p=.026, \eta2=.010$], whereas Japanese retirees' depressive symptoms only increased in Wave 2 (Mean Difference =.83,SE=.39,p=.034) but not in Wave 3. Among Chinese, depressive symptoms did not significantly worsen after retirement, but the changes varied between retired persons and those who were reemployed in Wave 3 [$F(1,1030)=4.25$, $p=.040$, $\eta2=.004$]. Specifically, individuals who were reemployed after retirement experienced a reduction in depressive symptoms between Wave 1 and 3, whereas a reverse pattern is shown among retirees. This suggests that reemployment is beneficial to the well-being of Chinese retirees. The effects of socioeconomic factors (e.g., private and public health insurance and pension support) on depressive symptoms will be discussed.

### EMPLOYMENT AFTER NORMAL RETIREMENT AGE: EMPLOYERS' MOTIVES AND EXPERIENCES

Jaap Oude Mulders[1], *1. Netherlands Interdisciplinary Demographic Institute, The Hague, Netherlands*

Due to population aging, older workers in developed countries are working much longer than previous cohorts. Some older workers even extend their careers beyond normal retirement age – or the age that is traditionally associated with retirement. While earlier work has studied employees' motives and experiences while working after normal retirement age, motives and experiences of employers remain unexplored. Understanding employers' perspectives is imperative for a better grasp of employees' opportunity structures and labor market dynamics. This is especially relevant in countries with mandatory retirement systems, since here employer and employee need to negotiate a new contract after normal retirement age. I study employers' motives to and experiences with employing older workers after normal retirement age using data from a 2017 survey among 1,312 Dutch employers. The Netherlands has mandatory retirement regulations but is also seeing an increase in employment rates after normal retirement age. Results show that 54% of employers have, in recent years, employed one or more older workers beyond their normal retirement age. This is especially common in education. 70% of employers are very positive about their previous experiences with employing older workers after normal retirement age, mostly because they had rehired older workers with unique knowledge and experience. However, employers also hardly ever took the initiative for such employment arrangements, instead leaving it to the older workers to show the desire to continue working. Although employers are largely positive, they see it as a limited phenomenon, and do not consider it a solution to labor shortages.

### CONSEQUENCES OF AGE DISCRIMINATION IN THE WORKPLACE

Dannii Yeung[1], *1. City University of Hong Kong, Hong Kong, Hong Kong*

Despite a growing number of middle-aged employees in the workforce, Hong Kong still lags behind other developed countries in implementing age-friendly policies to protect older workers from being discriminated in recruitment, performance appraisal, and other personnel decisions. This paper therefore aims to investigate the effects of age discrimination on work-related outcomes and well-being. A total of 333 Hong Kong Chinese employees aged 40 years and above (Mage = 46.6 years, SD = 6.21, Range = 40-68; 60% female) completed an online survey on work experiences. Self-perceived age discrimination in the workplace (Furunes & Mykletun, 2010), perception of occupational future time (Ho & Yeung, 2017; Zacher & Frese, 2009), work engagement (Schaufeli et al., 2017), work stress (Cohen et al., 1983), and psychological distress (Shek, 1989) were measured. Almost 53% of the participants reported that older workers were less likely to get the same training opportunity for new technology as their younger peers, and 46.8% perceived that older workers did not get the same promotion opportunity as their younger peers. Regression analyses further revealed that employees' perceived age discrimination in the workplace indirectly influenced their levels of work engagement (B = -.038, SE = .017), work stress (B = .016, SE = .005), and psychological distress (B = .008, SE = .005) through perception of occupational future time. The findings of this study unveil the severity of workplace age discrimination in Hong Kong and the negative impacts of age discrimination on work-related outcomes. Recommendations for organizational practices will be discussed.

### THE EFFECT OF RETIREMENT TIMING ON HEALTH AND WELL-BEING OF HISPANICS COMPARED TO NON-HISPANIC WHITES LIVING IN THE U.S.

Antonia E. Diaz-Valdes Iriarte[1], *1. Boston College School of Social Work, MA, United States*

In the context of an aging society, where the proportion of older adults is rapidly increasing, ensuring healthier longer lives is key for individuals, families, policy makers and the population as a whole. In this context the productive aging framework has gained increased importance. There is evidence showing that engagement is related to late-life well-being and health (i.e., Hinterlong, 2006; Everard et al, 2000; Rozario et al, 2004; Matz-Costa et al, 2012). However, the productive aging framework lacks cultural sensitivity and evidence about the association between the effect of retirement on health and well-being in late-life is mixed. The current study seeks to contribute to this gap by exploring the consequences of the discrepancies between planned and actual retirement age on subjective health and well-being, comparing Hispanics and non-Hispanic Whites. A series of regression models were conducted to explore the effect of the discrepancy between planned and actual retirement age on retirement satisfaction, self-rated health and mental health (CESD). Results indicates that native born Hispanics presented more differences when compared to foreign born Hispanic than non-Hispanic Whites, which could indicate the effect of acculturation and its fading effect on cultural attitudes, such as familismo. Hispanic tend to have higher retirement satisfaction than non-Hispanics which is aligned with the happiness paradox found by Calvo and collagues (2017). Additionally, SES has a significant effect on health for non-Hispanic Whites but not among Hispanics. Finally, retirement timing predicted mental health among foreign born Hispanic but among native born Hispanics and non-Hispanic Whites.

### CULTURAL DIMENSION OF THE DISCREPANCY OF PLANNED AND ACTUAL RETIREMENT AGE IN THE U.S.: HISPANICS VERSUS NON-HISPANICS

Antonia E. Diaz-Valdes Iriarte[1], *1. Boston College School of Social Work, MA, United States*

The sustainability problems to the SS has led to a glowing debate about what the full retirement age should be and if working longer is a plausible option for everyone or just for those who have some control over their retirement decisions (e.g., Munnell & Sass, 2008; McNamara & Williamson, 2013; Munnell et al, 2016). All ethno-racial groups have increased their average retirement age over the last years. However, Hispanics' retirement age is still lower even if they stated they plan to continue to work at retirement (EBRI 2008; Diaz-Valdes, 2018). Most studies about retirement timing have focused on middle-class Whites, and the prediction of planned or actual retirement separately. One of the lesser studied complexities of the retirement conundrum concerns ethno-racial differences and cultural-related predictors of retirement timing (Lytle et al, 2015). This study seeks to extend the understanding of differences between Hispanics and non-Hispanics regarding the timing of retirement relative to when they thought they would retire by including a broad array of cultural and family related predictors. Multinomial regression models were used. The results indicate significant differences between Hispanics and non-Hispanic Whites. Taking care of grandchildren was a significant predictor among Hispanics but not among non-Hispanic Whites. For Hispanics taking care of grandchildren, for over 20 hrs., was associated with a decreased probability of stating they will never retire. The increase of one dependent was associated with an increased on the probability of retiring earlier than planned. The effect of one additional dependent was larger for non-Hispanics.

### EVALUATING THE FEASIBILITY AND EFFICACY OF A PILOT INTERVENTION TARGETING UNEMPLOYED OLDER WORKERS

Jie Yang[1], *1. East Carolina University School of Social Work, Greenville, North Carolina, United States*

The great recession of 2007–2009 has led to historically high unemployment rates, and almost half of the unemployed population was long-term unemployed in 2010. Both discriminations against the long-term unemployed and ageism hinders older unemployed workers reentering the workforce, and they risk severe mental health deterioration. However, traditional job training programs are shown to be ineffective in protecting their participants' mental health. Hence, adopting Jahoda's Latent Deprivation Theory, the author worked with a non-profit in the Greater Boston area on developing an innovative intervention targeting older unemployed older workers' mental health and income generation outcomes. The pilot intervention (lasting three months) created a quasi-office environment where participants can have a time structure, engage with peers, work on projects, and receive training. The current study evaluates the effect of this pilot program on multiple mental health outcomes. Pre-and-post comparisons were conducted using quantitative analysis (N=13). Participants' depressive symptoms dropped significantly, and they reported having a better time structure of the day as well as higher scores in extraversion in terms of personality change. Qualitative analyses were conducted to analyze the pre-and-post interviews. Participants reported overwhelming approval of the intervention in helping them better cope with unemployment and being productive in seeking jobs or generating incomes. One example of quotes is "…compared to three months ago, I'm in a very different place. Before the Collaboratory, I had become extremely depressed and withdrawn…The Collaboratory has helped make a massive difference in my sense of well being right now."

### AFFECT MODERATES THE ASSOCIATION BETWEEN SOCIAL SUPPORT AND RETIREMENT SATISFACTION OVER TIME

Kaleena Odd[1], *1. University of Nebraska Omaha, Omaha, Nebraska, United States*

Retirement is becoming more important for today's older adults because they are living longer than before. Recently, research has started to explore how different individual resources (e.g., health or finances) and social resources (e.g., social support or social network size) influence retirement outcomes such as retirement satisfaction. Moreover, the current study sought to examine the influence of time, satisfaction with social support, and affect (i.e., positive or negative) as predictors of retirement satisfaction. Data was obtained from a longitudinal study that explored how older adults in Montreal, Canada adjusted to life in retirement over the course of three years. Hypotheses were tested using a structural equation model that investigated retirement satisfaction as predicted by time, satisfaction with social support, positive affect, and negative affect. Gender differences were also explored. Overall, there was no change over time among the variables. Satisfaction with social support, positive affect, and negative affect were all associated with retirement satisfaction in the expected directions. Positive affect moderated the association between satisfaction with social support and retirement satisfaction, such that the association was stronger for those low in positive affect. Also, negative affect moderated the association between satisfaction with social support and retirement satisfaction as a function of gender. This study extended the literature by exploring how multiple predictors interacted to influence retirement satisfaction over time. Future research should examine how individual and social resources can interact with each other to better understand retirement satisfaction.

### PRECARIOUS PATHWAYS INTO RETIREMENT AND NEW RISKS FOR GENDERED ECONOMIC EXCLUSION IN SWEDEN, 1990-2015

Andreas Motel-Klingebiel,[1] and Susanne Kelfve[1], *1. Linkoping University, Linkoping University, Sweden, Sweden*

The ability and disposition of ageing people to maintain their labour market activity and/or to retire from work structurally depend on pension systems, activation policies, ageism, changing for labor demand and economic shifts. Structural conditions are changing, but social change does not mature homogeneously and neither do the institutional shifts induced by it. Gains in opportunities and resources do not benefit all people, groups and even societies in the same way. Changes increase insecurities and life course inhomogeneity, create unequally distributed challenges and show asynchrony in shifts and outcomes. They generate new precarity in ageing and socially structured risks for exclusion in work and retirement and refer to existing later life inequalities by cohort, gender, region, education, class and ethnicity. From this perspective of ageing and social change, the paper deals with shifts in late work and retirement patterns and later-life outcomes under changing institutional conditions, focusing on gendered risks for economic exclusion and later life precarity in Sweden. Swedish registry data comprising individual work and health histories as well as employer, regional and neighborhood information on the total population 50+ ever living in Sweden 1990-2015 is used in a cohort sequential perspective. Analyses focus on gender inequalities and concentrate on occupational activities, retirement transitions and pension revenues under changing social conditions. Models find increasingly heterogeneous preretirement and transition patterns, new gender gaps and increasing risks of economic exclusion in retirement with disadvantaged groups as forerunners in overall relative declines in later-life economic positions.

### SESSION 870 (POSTER)

### END OF LIFE ISSUES

### BEHAVIORAL DESIGN: A NOVEL APPROACH TO CREATING ADVANCE DIRECTIVE INTERVENTIONS

Elise Tarbi,[1] Brianna Morgan,[1] and Jason Sloan[2], *1. University of Pennsylvania School of Nursing, Philadelphia, Pennsylvania, United States, 2. University of Pennsylvania Law School, Philadelphia, Pennsylvania, United States*

The 1.7 million older adults receiving long-term services often do not receive end of life care consistent with their wishes. Advance directives (ADs) can help, yet only one-third of Americans have completed ADs. The limited effectiveness of traditional interventions to increase AD completion may be because they do not address the behavioral aspects of advance care planning. Behavioral Design is an innovative approach that combines design thinking and behavioral economics to identify predictable behavioral bottlenecks and create real-time solutions. This study used Behavioral Design to address low AD completion rates of long-term care residents. Consistent with the Behavioral Design process, an interdisciplinary team compiled evidence from 10 diverse data sources to identify behavioral bottlenecks to AD completion. These barriers were analyzed using the cognitive bias codex to determine behavioral levers for intervention. Informed by these findings, the study team designed multicomponent interventions to address behavioral aspects of AD completion. Four behavioral bottlenecks incorporating ten behaviorally mediated causes for lack of AD completion were identified. For example, AD completion is affected by complexity mediated by hassle factor, choice overload, and ambiguity effect. Three interventions were designed to address these behaviorally mediated causes. For example, the intervention HeAD Start would provide a simple, easy to read AD (addressing choice overload) to residents upon admission (addressing hassle factor) with scheduled follow-up by trained staff (addressing ambiguity effect). Behavioral Design incorporates design thinking and leverages behavioral economic principles to create behaviorally mediated AD interventions. Next steps include testing behaviorally informed designs in practice.

### ASPIRIN BENEFIT IS NOT AFFECTED BY BODY WEIGHT IN HEALTHY OLDER PERSONS

Robyn L.. Woods,[1] Galina Polekhina,[1] Mark Nelson,[2] Rory Wolfe,[3] Michael E. Ernst,[4] Anne M. Murray,[5] and John McNeil[6], *1. Monash University, Melbourne, Victoria, Australia, 2. University of Tasmania, Hobart, Tasmania, Australia, 3. Monash University, Melbourne, Australia, 4. University of Iowa College of Pharmacy, Iowa City, Iowa, United States, 5. Berman Center for Outcomes and Clinical Research, Minneapolis, Minnesota, United States, 6. Department of Epidemiology & Preventive Medicine, Monash University, Melbourne VIC, Australia, Melbourne VIC, Australia, Australia*

Recent meta-analysis (Rothwell et al, Lancet 2018;392:387-99) of aspirin trials in predominantly middle-aged people suggested that aspirin's effects on clinical outcomes vary according to dose and body weight, concluding that under dosing in heavier people may be responsible for reduced efficacy of aspirin. Data from ASPREE (ASPirin in Reducing Events in the Elderly; randomized primary prevention trial of low dose aspirin versus placebo in 19,114 healthy older participants, mainly aged 70+ years) were analyzed for interaction of body habitus on the main outcomes after 4.7 years of study treatment. Increases in body weight, BMI or waist circumference (WC) did not influence cardiovascular endpoints or incident cancers in the aspirin group compared with placebo. In ASPREE men, an increase of 10 kg body weight elevated the risk of major hemorrhage with aspirin (HR 1.20; 95% confidence interval 1.01-1.43; P=0.04) and 10 cm increase in WC elevated all-cause mortality by 23% with aspirin (HR 1.23; 95% confidence interval 105-1.44; P=0.01), driven by cancer-related deaths (HR 1.39; 95% confidence interval 1.11-1.73; P=0.004). These effects of increased abdominal girth were not seen in women. Evidence from ASPREE does not support increasing the dose of aspirin in larger older people to improve the drug's efficacy. Our results point to a link between central adiposity, hemorrhage risk and cancer-related death in older men taking aspirin.

### A HOSPICE COLLABORATIVE NETWORK TO IMPROVE SERIOUS ILLNESS CARE IN A LARGE HEALTHCARE SYSTEM

Richard E. Leiter,[1] Charles T. Pu,[2] Emanuele Mazzola,[1] and Rachelle E. Bernacki[1], *1. Dana-Farber Cancer Institute, Boston, Massachusetts, United States, 2. Massachusetts General Hospital, Boston, Massachusetts, United States*

The quality of hospice care in the United States varies significantly, yet healthcare systems lack methods to comprehensively evaluate and stimulate quality improvement in organizations that serve their patients. Partners HealthCare, an integrated healthcare system located in Eastern Massachusetts, sought to create a high-quality hospice collaborative network based on objective and quantitative criteria obtained from public reporting as well as the hospice itself. Through a modified Delphi procedure, clinicians, administrators, and data scientists developed a set of criteria and a scoring system focused on three areas: organizational information, clinical care quality indicators, and training and satisfaction. All Medicare-certified hospices in good-standing in Massachusetts were eligible to participate in a request for information (RFI) process. We blinded all hospice data prior to scoring and invited hospices scoring above the 15th percentile to join the collaborative for a 2-year initial term. Of 72 eligible hospices, the majority (53%) responded to the RFI, of which 60% submitted completed surveys. Hospices could receive up to 23.75 points with scores ranging from 2.25 to 19.5. The median score was 13.62 (IQR: 10.5-16.75). For the 19 hospices scoring above the 15th percentile, scores ranged from 10.0-19.5 (median: 14, IQR: 11.1-16.9). There was no association between quality score and continuous (Spearman's correlation 0.24, p=0.27) or dichotomous (Wilcoxon rank sum test p=0.13) measures of hospice size. The hospice collaborative network is one healthcare system's initial attempt to effectively leverage its influence and relationships to improve hospice quality for the benefit of its seriously ill patients and their families.

### TRENDS AND FACTORS ASSOCIATED WITH PLACE OF DEATH AMONG INDIVIDUALS WITH CARDIOVASCULAR DISEASE IN THE UNITED STATES

Sarah H. Cross,[1] Brystana G. Kaufman,[2] and Haider Warraich[3], *1. Duke University, Durham, North Carolina, United States, 2. Duke-Margolis Center for Health Policy, Durham, North Carolina, United States, 3. Duke University, North Carolina, United States*

While most patients prefer to die at home, trends and factors associated with place of death for patients dying of cardiovascular disease (CVD) remain unknown. Using data from the National Center for Health Statistics from 2003-2017, we described trends and conducted multivariable logistic regression to evaluate associations between demographic characteristics and place of death among CVD patients in the United States. From

2003-2017, the rate of CVD deaths occurring at home increased from 21.3% to 30.9%, and rate of hospice facility deaths increased from practically none to 6.0%. Over the same period, the rate of hospital deaths decreased from 36.5% to 27.3%, and nursing facility deaths decreased from 25.1% to 20.6%. With the exception of conduction disorders, temporal trends in place of death were consistent across CVD diagnosis subgroups: ischemic heart disease, hypertensive heart disease, heart failure/cardiomyopathy, cerebrovascular disease, aortic stenosis, and all other CVDs. Differences between demographic groups persisted over the study period, with reduced odds of home death among Hispanic versus non-Hispanic (OR=.942; 95% CI .929-.955) decedents, Black versus White (OR=.837; CI .809-.866) decedents and greater odds of home death among decedents with some college education or more (OR=1.08; CI 1.06-1.09) versus decedents with a high-school education or less. In 2014, home surpassed hospital as the most common place of death for CVD patients. CVD patients often have acute and intense needs at the end of life that are challenging to manage in the home and the quality of care these patients receive should be further investigated.

### ETHNIC AND DEMOGRAPHIC DIFFERENCES IN THE UTILIZATION OF ANCILLARY HOSPICE SERVICES

Anne D. Halli-Tierney,[1] Gregg Bell,[1] and Rebecca S. Allen[2], *1. The University of Alabama College of Community Health Sciences, Tuscaloosa, Alabama, United States, 2. The University of Alabama, Tuscaloosa, Alabama, United States*

Background: Research shows that ethnic differences exist in hospice service election, with fewer African American patients enrolling in hospice and having lower satisfaction with care compared to Non-Hispanic Whites. Hospice is interdisciplinary, with nursing care and “ancillary” services (social work, chaplain, nurses’ aide, volunteer). Little research exists about whether disparities exist in ancillary service election, and if patient demographics correlate with service utilization. We examined if ethnic or demographic differences exist among hospice beneficiaries in utilization of hospice services. Methods: Mixed-methods data collection took place from two community hospices. The quantitative arm involved retrospective chart review on new admissions from 2012 to 2016: acceptance of ancillary services and demographic data were collected as well as code status and outcome of hospice admission. The qualitative arm collected interview data from hospice personnel about thoughts on hospice care, which patients they think might decline ancillary services, and why. Results: Chart review was completed on 491 patient charts: interviews of hospice staff are ongoing. Sample was 55% female, 77% white, average age 77.8 years. Initial analysis on demographic data did not show statistical significance in utilization. Declination was lowest for social work (3.4% declined) and highest for hospice volunteer (88.9% declined). Initial interview themes involved need for patient education and role of health literacy. Discussion: Initial research does not show statistical ethnic or demographic differences in ancillary service utilization. However, broad utilization differences exist between services. Data can identify areas where hospices can improve care accessibility through patient education and personalization of services.

### ALIVIADO DEMENTIA CARE-HOSPICE EDITION: FEASIBILITY AND ACCEPTABILITY RESULTS OF A TWO-PHASED PILOT STUDY

Shih-Yin Lin,[1] Alycia A. Bristol,[1] Catherine E. Schneider,[2] Kimberly Convery,[1] Victor Sotelo,[1] and Abraham A. Brody[3], *1. NYU Rory Meyers College of Nursing, New York, New York, United States, 2. New York University, New York, New York, United States, 3. NYU Rory Meyers College of Nursing, New York, United States*

Limited work has been performed in helping hospice agencies to care for persons with dementia (PWD) and their caregivers in an evidence-based manner despite the increasing number of PWD cared for in this setting. To change the culture of care for PWD and their caregivers receiving hospice, we adapted Aliviado Dementia Care, an evidenced-based interdisciplinary quality improvement program, for use in hospice. The purpose of this pilot study is to examine feasibility and applicability of implementing the Aliviado Dementia Care-Hospice Edition sequentially in 2 hospice agencies in preparation for a nation-wide pragmatic trial. In the first pilot, concluded in March 2019, seven hospice interdisciplinary clinicians were trained as program champions and completed a two-day in-person intensive training on dementia symptom assessment and management, and quality improvement processes. Additionally, 47 interdisciplinary team (IDT) members were provided training via a 5-hour, online program covering dementia symptom assessment and management. All champions trained (100%) reported being satisfied/very satisfied with the program and agreed that the training is applicable to hospice practices. All IDT members who completed the online training (100%) reported being satisfied/very satisfied with the program quality, or agreed/strongly agreed that the content was relevant. The high rates of satisfaction and applicability, reported by the hospice champions and IDT members, provided preliminary evidence supporting the feasibility and applicability of the Aliviado Dementia Care-Hospice Edition.

### CHRONIC DISEASE AND TERMINAL DECLINE IN VERY OLD MEN: THE MANITOBA FOLLOW-UP STUDY

Robert B. Tate,[1] Philip St. John,[2] Audrey Swift,[1] and Edward H. Thompson[3], *1. University of Manitoba, Winnipeg, Manitoba, Canada, 2. University of Manitoba, Winnipeg, Canada, 3. College of the Holy Cross, Worcester, Massachusetts, United States*

The Manitoba Follow-up Study is in its 71st year of continuous operation. Since 1948, 3,983 aircrew recruits to the Royal Canadian Air Force during the Second World War have submitted routine medical examinations and completed questionnaires. On May 1, 2006, 1001 of these men (25%) were alive mean age of 86 years. The effects of 7 chronic diseases (CDs) diagnosed before 2006 were modeled with multinomial logistic regression to predict the pattern of decline of living and dying through an 11 year window to 2017. By 2017, 11% were still alive, 10% died very early in the window, 44% experienced a slow decline of a least three years to death, 17% experienced a step decline to death, and 18% experienced a terminal drop, death within six months of decline in functioning. Only 30% were free of CD in 2006; 36% had 1 CD, and 34% had more than 1 CD. As the number of CDs increased, the probability of remaining alive by 2017 decreased: 18% alive if no CD, 10% if 1 CD, 8% if 2 CDs, and 3% of >2 CDs. The chance of death with a terminal drop decreased: 22% if no CD, 20% if 1 CD, 14% if 2 CD, 11% if >2 CD. Conversely, the percent with a gradual decline to death increased with more CDs. Among old men are already in their 80s, a key determinant of the trajectory to end of life is the number of CDs.

### PALLIATIVE CARE NEEDS ASSESSMENT OF A RURAL AND FRONTIER STATE

Catherine Carrico,[1] Andrea Slosser,[1] Robin A. Barry,[1] and Christine McKibbin[1], *1. University of Wyoming, Laramie, Wyoming, United States*

Background: Palliative Care (PC) is a valuable tool for improving the lives of people living with chronic illness. However, access to PC is limited in rural areas. The purpose of this study is to describe the current PC needs and barriers to care in a rural state. Methods: An online survey was disseminated through professional organizations, licensing boards, and the University of Wyoming. Rurality of counties was classified using Rural Urban Continuum Codes (RUCC). Descriptive statistics were calculated using SPSS. Results: Responses were received from 336 individuals across 20 of 23 counties (i.e., RUCC range 3 - 9; 1=most metropolitan, 9=most rural). The majority worked in healthcare or social service sectors (n = 265, 78.8%). Approximately one-half (n = 119, 50.6%) of these individuals endorsed providing PC (i.e., typically symptom management, supportive resources, and family support). Over one-half of respondents (n = 173, 51.5%) rated availability of PC services in their communities as "poor" or "somewhat good." Key barriers to providing PC were lack of patient information and knowledge (n = 215), access to PC specialists (n = 183), and funding and reimbursement (n = 181). Approximately one third of healthcare professionals (n = 78, 32.8%) had received formal training in PC. A majority of healthcare and social service respondents (n = 139, 59.1%) endorsed interest in PC continuing education. Conclusions: This study provides insight into the state of PC across a rural state. Results highlight the need to design accessible education and implement system transformation to improve PC access.

### SESSION 875 (POSTER)

### ENGAGING STUDENTS IN AGING STUDIES: CASES FROM THE FIELD

### AGING IN PLACE: EFFECTIVE INTERPROFESSIONAL COLLABORATION AND ENGAGEMENT WITH OLDER ADULTS

Elizabeth Fine-Smilovich,[1] Diana L. Morris,[2] David M. Rosenberg,[3] Elizabeth O'Toole,[4] Cynthia Booth-Lord,[1] Klara K. Papp,[1] and Patricia A. Thomas[1], *1. Case Western Reserve University School of Medicine, Cleveland, Ohio, United States, 2. Case Western Reserve University, Cleveland, Ohio, United States, 3. Case Western University School of Medicine, Cleveland, Ohio, United States, 4. MetroHealth/Case Western Reserve University School of Medicine, Cleveland, United States*

Background: An innovative educational program addresses two gaps in health professions education: lack of an emerging workforce comfortable caring for older adults and proficiency in working in an interprofessional (IP) setting. We sought to explore whether AIP provides grounding in pillars of IPE and geriatric competencies through experiential learning in IP teams with older adults in a community setting. Methods: Early health profession students n=37 (MD, MSN, PA, SW), working in teams of 3, made monthly visits to older adults' residences over a one-year period. Workshops on core geriatric and IPE principles defined expected learning goals for client visits. Visits were followed by: 1) written field notes; 2) reflections based on pre-determined learning prompts; and 3) debriefing sessions with faculty members. Students completed pre and post program questionnaires including Attitudes Towards Social Issues in Medicine, Geriatrics Attitude Scale, ICCAS, and RIPLS. Pre-post results were analyzed using t-tests and qualitative analysis of comments. Results: 25 (68%) students completed pre-post questionnaires. Responses on the interprofessional collaboration scale significantly increased following the program (t=2.09; p = 0.047) and 94% responded that they could "well" or "very well" describe issues that impact older adults' health, quality of life, and convey appreciation toward older adults. Discussion: Students, engaging with older adults longitudinally in a community setting learned pillars of IPE, geriatric care competencies, and gained insights into this population. An interprofessional, experiential learning program is feasible and effective way to increase interest and self-efficacy in working with older adult populations.

### THE FALL OF THE BERLIN WALL AND STEREOTYPES OF AGING: AN INTERGENERATIONAL STUDY ABROAD EXPERIENCE

Suzie Macaluso[1], *1. Abilene Christian University, Abilene, Texas, United States*

One of the biggest barriers to encouraging a new generation of students to consider careers in aging are the ageist attitudes that they hold and the negative images of aging that they are bombarded with through the media. Sociologists have learned that an effective way to combat ageism is to bring together individuals from different cultural groups, including different age cohorts, to improve social attitudes. In a 2018 AGHE presentation, Jill J. Naar, explored the idea of promoting age-friendly universities by creating intergenerational education tourism programs. In this presentation I share my experience in leading an intergenerational study abroad in Germany that included five generations studying the creation of public memory on the eve of the 30th anniversary of the fall of the Berlin Wall. I will share some of the logistical considerations along with the way that the study abroad helped to meet some of the AGHE competencies for undergraduate programs in Gerontology.

### SESSION 880 (POSTER)

### FAMILY CAREGIVING ISSUES

### HEALTH INTERVIEWING IMPROVES THE MEDICAID HOME AND COMMUNITY-BASED SERVICES HOME CARE AIDE-CLIENT RELATIONSHIP

Margaret Danilovich,[1] Rebecca E. Johnson,[2] Laura Diaz,[2] and Lara Boyken[1], *1. Northwestern University, Chicago, Illinois, United States, 2. Northwestern, Chicago, Illinois, United States*

We investigated the feasibility of a Medicaid Home and Community-based Services home care aide (HCA) led health interview with clients (n=21) during usual care services provided by a single provider. We provided interview training in English and Spanish and HCAs (n=21) conducted five interviews using a card sort methodology to elicit client care preferences. The interviews consisted of five topics relative to care: 1) food and drink,

2) physical activity and mobility, 3) self-care, 4) home environment, and 5) how I spend my time. HCAs audio-recorded interviews and photographed card sorts for analysis. We conducted semi-structured interviews by telephone with clients and focus groups with HCAs, to evaluate the health interviewing experience. We transcribed interview recordings and evaluated fidelity to the health interview script. We administered the Your Health Orientation, Willingness to Communicate, and PROMIS-global health to clients and the Active Empathetic Listening Scale to HCAs. We used t-tests to investigate changes in survey outcomes pre and post interviews. Results show it is feasible to train English and Spanish speaking HCAs in a simple, health interviewing technique to elicit care preferences from clients. Doing so contributes new knowledge on client preferences. Clients desire HCAs who provide empathy, compassion, and motivation, and HCAs observed that interviewing clients helped them to better understand their care recipient's needs. Future work should determine how embedding health communications training as part of orientation to client care, would influence HCA retention rates, as well as modify client health outcomes.

### EXPLORING THE HEALTH BURDEN AND SOCIOECONOMIC COSTS OF DEPENDENT ELDERLY CARE IN NSUKKA, NIGERIA

Peter C. Ezeah[1], *1. NNAMDI AZIKIWE UNIVERSITY, AWKA, ANAMBRA, Nigeria*

It is projected that by 2030, 6 percent of Nigeria's present population of 180 million will be 60 years and above. However, the extent to which the traditional systems of family support and security can manage the care of the increasing number of older people in the country is not clear as limited studies are available in the country regarding the health burden and Socioeconomic costs of caring for dependent older people. This study is therefore aimed at assessing the health burden and costs of caring for dependent older people in Nsukka, Nigeria. This cross sectional survey involved 1030 randomly selected elderly persons in Nsukka, Nigeria (Mage=70.15, SD=12.23). Structured questionnaire and Focus Group Discussion Guide (FGD) provided the data for the study. The qualitative data were analyzed with descriptive statistics, while regression analysis formed the basis for predicting effects of the variables in the study. The qualitative data from the FGD were analyzed thematically. The findings show that the Nigeria government was largely uninvolved in the care and support for older dependent people; leaving families to negotiate a 'journey without maps'. Families carried the health burden of care for the elderly with attendant socioeconomic costs. The traditional role of female relatives as caregivers was beginning to give way to paid caregivers. An innovative policy frame work targeted at the needs of older persons in health care, social protection and other forms of intergenerational support is required to supplement inputs from families of the aged in Nigeria.

### WORK-RELATED OPPORTUNITY COSTS OF PROVIDING UNPAID FAMILY CARE

Stipica Mudrazija[1], *1. Urban Institute, Washington, District of Columbia, United States*

Older Americans living in the community who need help with basic activities of daily living overwhelmingly rely on unpaid care provided most commonly by working-age family members. Because unpaid family care limits the demand for nursing facilities and reduces expenses paid by Medicaid and other government programs, previous estimates of its economic value have mostly focused on estimating the benefits of unpaid family care. However, to assess accurately the overall economic value of unpaid family care and define better the scope for policy intervention, it is also important to account for the costs of such care, yet our knowledge of their magnitude remains limited. This study assesses the impact of unpaid family caregiving on the likelihood of working and hours worked for caregivers, and calculates the related cost of forgone earnings today and in 2050. To do so, it matches family caregivers from the National Study of Caregiving with non-caregivers from the Panel Study of Income Dynamics, and uses projections from the Urban Institute's DYNASIM microsimulation model to inform calculations of future costs of foregone earnings. Results suggest that the cost of foregone earnings attributable to caregiving is currently about $67 billion. By mid-century, it will likely more than double, outpacing the growth of disabled older population as the share of better-educated caregivers with higher earning capacity increases. Policymakers can use these results to inform their current and future policy efforts aimed at assisting family caregivers who are facing the challenge of balancing work and caregiving responsibilities.

### UNDERSTANDING THE ROLE OF SOCIAL SUPPORTS AND SOCIAL NETWORK FOR DEMENTIA CAREGIVERS' MENTAL HEALTH

Carmen Morano,[1] daejun park,[2] and Andrea Savage[3], *1. School of Social Welfare, University of Albany, State University of New York, Albany, New York, United States, 2. University at Albany, School of Social Welfare, Albany, New York, United States, 3. Silberman School of Social Work, New York, United States*

This paper explores the associations of depressive symptoms with social supports and social networks among dementia caregivers. It has been well documented that dementia caregivers are at greater risk of experiencing negative mental health and poorer physical health than non-caregivers. This paper describes a collaborative process between two universities and a community-based provider in designing a Social Network Analyses to examine the network structures used by dementia caregivers participating in a community-based support program. The relationship between the caregiver support networks and depressive symptoms, were analyzed using multivariate regression models. Given the small sample size and missing data multiple imputation was applied to the data. The findings suggest the effects of a variety of supports in the caregiver network on mental health and depressive symptoms. Among the findings it was found that the presence of financial support (B= - 0.58; p = .01) and frequency of contacts (B = -0.58; p = .01) support resulted in a decrease in depressive symptoms and better mental health than for caregivers without similar supports in their networks. This paper will conclude with a discussion of potential uses of social network analysis to better understand how the structure of caregivers' network can address the concrete physical, emotional and financial needs of dementia caregivers.

### FEDERAL POLICY EFFORTS TO SUPPORT FAMILY CAREGIVERS: PAST, PRESENT, AND FUTURE

Lauren R. Bangerter,[1] Nicole Ruggiano,[2] Joan M. Griffin,[1] and Kelly A. O Malley[3], *1. Mayo Clinic, Rochester, United States, 2. University of Alabama, Tuscaloosa, Alabama, United States, 3. New England GRECC, Boston VA Healthcare System, Boston, Massachusetts, United States*

Family caregivers are major providers of long-term care and support for family members with age-related decline and disability. Caregiving is a demanding and complex undertaking that has demonstrated extensive physical, emotional, relational, and financial burden on families. These factors underscore the urgency of addressing the needs of family caregivers at the policy level. This presentation focuses on the origins, goals, and mandates of federal policy efforts that support family caregivers. This includes an analysis on how federal support for caregivers has evolved over time, most notably through the National Family Caregiver Support Program, part of the Older Americans Act. It will also discuss the passage of more recent legislation (e.g., RAISE Family caregiver Act and the Grandparents Raising Grandchildren Act) that will contribute to a national strategy to support family caregivers. Moreover, through the discussion of these policies, we will articulate specific areas where gerontology research and public policy can and should intersect in order to optimize the effectiveness of policy efforts to support family caregivers.

### REACTIONS TO AMYLOID PET SCAN RESULTS AND LEVELS OF ANXIETY AND DEPRESSION AMONG CARE PARTNERS: CARE IDEAS STUDY

Emmanuelle Belanger,[1] Jessica D'Silva,[1] Courtney H. Van Houtven,[2] Megan Shepherd-Banigan,[3] Valerie Smith,[3] and Terrie Wetle[1], *1. Brown University, School of Public Health, Providence, Rhode Island, United States, 2. Durham VA HSR&D, Durham, North Carolina, United States, 3. Duke University, Durham, North Carolina, United States*

Few studies have examined caregiver reactions to their loved ones receiving the results of an Amyloid PET scan which can be indicative of Alzheimer's disease. Therefore, we examine: 1) What are care partner's reactions to their loved one receiving negative or positive amyloid PET scan results?, and 2) To what extent are scan results and diagnostic category (dementia vs. mild cognitive impairment) associated with care partner depressive symptoms (PHQ-2) and anxiety (STAI-6)? Using data from 1,799 care partners in the CARE IDEAS study, we applied a sequential mixed-methods design and explored the reactions of 192 care partners who answered open-ended interview questions after learning about the Amyloid PET scan results. We first conducted qualitative content analysis of transcripts from open-ended questions to explore caregivers' emotional responses after their loved one received an Amyloid PET scan result. The qualitative data suggest that when the scan results fit care partner's expectations, i.e. positive scan when the patient has dementia and negative scan when the patient has mild impairment, care partners report satisfaction with this information and relief, rather than shock and frustration. Adjusted logistic regression models of survey responses support this finding; having dementia and a positive scan both increased the likelihood of care partners having high levels of anxiety, and a significant interaction indicated that a positive scan was associated with high anxiety among care partners of patients with mild cognitive impairment but not dementia. Only lower education and higher impairment in everyday cognitive function were associated with high depressive symptoms.

### PLACE MATTERS: HOW LOCATION AT DEATH INFLUENCES CAREGIVER WELL-BEING IN BEREAVEMENT

Deborah P. Waldrop,[1] and Jacqueline M. McGinley[2], *1. University at Buffalo School of Social Work, Buffalo, New York, United States, 2. Buffalo State College, Buffalo, New York, United States*

Most older adults express the preference to die at home, but the desire for home death may go unfulfilled when the dying process become burdensome. Little is known about the congruence between older adults' and their caregivers' desired locations at death. The purpose of this study was to explore how the congruence between caregiver-care recipients desired and actual location at death influenced well-being in bereavement. This exploratory study utilized simultaneous qualitative and quantitative methods. Interviews were conducted with 108 bereaved caregivers about 4 months after the care recipient died while receiving hospice care. Care recipients' ages ranged from 43-101 (M=79.6); caregivers from 32-88 (M=61.5). Quantitative data included categorical variables about demographics, advance care planning and location at death. The Core Bereavement Items and CDC HRQOL–14 "Healthy Days Measure" scales were used. Qualitative data involved open-ended questions about the illness trajectory, desired location and perceptions of care at life's end. Quantitative analysis included comparison of group differences using both Independent Samples t-tests and One-way ANOVA. Of the 92 care recipients who had an advance directive, N=49 (45%) were in the location they desired and for N=49 (45%) there was caregiver/care recipient congruence about location. Caregivers who experienced incongruence reported poorer physical and emotional well-being and higher, more intense bereavement symptoms. Three overarching themes illuminated caregivers' experiences: (1) Caregiver-recipient congruence; (2) Caregiver-recipient incongruence; (3) Incongruence-influenced bereavement. Results suggest that incongruence between desired and actual location of death affects well-being in bereavement. Implications: Communication about location at death is an essential consideration.

### DOES CAREGIVING MATTER? THE RELATIONSHIPS BETWEEN CAREGIVING EXPERIENCE AND ADVANCE CARE PLANNING IN OLD AGE

Huei-wern Shen[1], *1. Graduate Institute of Social Work, National Taiwan Normal University, Taipei, Taiwan*

Advanced care planning (ACP) is encouraged as the completion of ACP increases the likelihood for patients to receive their preferable end-of-life care and for caregivers to be less stressed. Common approaches to increase the engagement of ACP target on intervention or information provision to patients in the very late stage of life. Arguing that caregiving experience may influence how people plan their own end-of-life care, the present study focuses on caregiving roles. Using seven waves of data (2002, 2004, 2006, 2008, 2010, 2012, and 2014) from the Health and Retirement Study (HRS), 863 older people who were 65+ and alive in 2012 but passed away prior to 2014 were included in this study to examine the relationships between an individual's caregiving experience (2002-2012) and his/her completion of ACP (2014). Findings from logistic regression showed that caregiving experience did not influence older adults' (65+) ACP completion in 2014. When considering different types of caregiving experience (none, spousal, parental) among older cohort (75+), the odds of completing ACP for those with spousal caregiving experience were 2.47 times more likely than those without any caregiving experience. However, no relationship was detected among those with parental caregiving experience. Other factors relating to ACP completion were poorer health, death being expected, death due to cancer, older age and being racial minorities. Practitioners and policymakers could consider encouraging older adults (65+) who provide spousal care to engage in ACP. Future research taking longitudinal approaches to identify reasons behind the existence of the relationship is urged.

### IDENTITY AND RESPONSIBILITY: WHAT IT MEANS TO BE A CAREGIVER AND ITS IMPLICATIONS FOR POLICY AND HEALTH CARE

Minakshi Raj,[1] Jodyn Platt,[2] Tom Fitzgerald,[2] Denise Anthony,[1] and Shoou-Yih Daniel Lee[1], *1. University of Michigan School of Public Health, Ann Arbor, Michigan, United States, 2. University of Michigan Medical School, Ann Arbor, Michigan, United States*

Improving health care and quality of life for older adults in the U.S. requires increased attention to the informal caregivers supporting aging relatives. Studies estimate that over 40 million unpaid caregivers provide more than $400 billion of unpaid care; however, there is little research examining the scope and variety of support caregivers provide. Less research has examined how caregivers conceptualize their experience beyond “caregiver burden”. A more comprehensive understanding of the types of support caregivers provide and caregiver perceptions of their role and purpose are critical to enhancing policies, e.g., the Family and Medical Leave Act, to be more responsive to caregiver needs. This study seeks to (a) identify the ways informal caregivers provide support and (b) describe what it means to be a caregiver from the perspective of informal caregivers. We conducted four focus groups with informal caregivers (relatives or partners) in Southeastern Michigan (n=18) and conducted qualitative thematic analyses. Support that caregivers provide ranges from financial planning to medical decision-making to social engagement. While caregivers face frustration and isolation consistent with “caregiver burden,” they also recognize positive attributes of being a caregiver such as overcoming adversity and helping elderly relatives maintain dignity. These attributes contribute to their identity of what it means to be a caregiver and perceived capacity to improve their relative’s health. Our findings suggest that we are underestimating the scope of work involved in care-giving and that a wider range of activities should be considered when developing workplace policies and resources to promote caregiver wellness.

### VALUE-BASED HEALTHCARE AS A FRAMEWORK OF CARE FOR OLDER ADULTS AND THEIR FAMILY CAREGIVERS

Gwen McGhan,[1] Fiona Clement,[1] Natalie Ludlow,[1] Jessica Lee,[1] Michelle Cheng,[1] and Deirdre McCaughey[1], *1. University of Calgary, Calgary, Alberta, Canada*

Value-based healthcare (VBHC) is a term synonymous with the pursuit of greater value in healthcare, however, the term “value” lacks conceptual clarity. Using Porter’s 2010 seminal VBHC shows promise for placing the older adult and their family caregiver at the center of care models, which is a central tenet in healthcare value. The purpose of our study is to conceptualize how Porter’s VBHC is defined, operationalized, and implemented to address the need for person-and-family centered care models that improve outcomes while being cost effective. A literature search in six academic databases was conducted to identify articles examining VBHC; specifically studies with a Porter-based patient-centric focus. 1,001 articles were retrieved for initial review. Using a consensus-based logic model for inclusion/exclusion, 802 met the inclusion criteria for full text review. Articles were examined using the following objectives: 1) conceptually map the VBHC literature, 2) identify application of Porter’s equation, and 3) identify the methodologies used to measure outcomes, costs, and value. Findings were cross-compared and emergent themes organized to Porter facets of value (outcomes, costs, and value). Our review found the three facets of Porter’s VBHC are established across the literature, however, most studies examine only one or two facets and fail to specify or define all three. Although applied in research, there is a lack of consistency in the actual use of Porter’s definition. Five recommendations for future research using Porter’s VBHC include: (1) pre-selection of outcomes/costs, (2) operationalizing outcomes/costs, (3) value framework creation, (4) data collection, and (5) value calculation.

### INTERNET USE, MENTAL HEALTH, SOCIAL SUPPORT, AND CARE BURDEN OF INFORMAL CAREGIVERS

Eun-Hye G. Yi,[1] and Margaret E. Adamek[1], *1. Indiana University School of Social Work, Indianapolis, Indiana, United States*

Given the psychological stress and health difficulties that stem from caregiver burden and lack of support, various technology-based supports have been introduced. This study aimed to understand the relationships of internet and social network use on informal caregivers’ health status, social support, and care burden. Outcomes were compared for three groups: Alzheimer’s caregivers (AC), other types of caregivers (OC), and non-caregivers (NC). A secondary data analysis of national data from the Health Information National Trends Survey 5-cycle for 2018 was conducted (N=3,297; NC= 2,918, OC=443; AC=113). Using Stata SE 15.1, various statistical analyses (Chi-square, ANOVA, logistic regression) with jackknife bias correction were used to compare the 3 groups. In general, OC experienced the most serious depression/anxiety. More OC (32%) were diagnosed with depression/anxiety by health professionals than NC (22.64%) and AC (19.82%) ($F(2,48)=5.58$, $p<.001$). The Other Caregiver group also had higher scores in PHQ-4 ($M=2.42$, $SD=3.07$) than NC ($M=1.75$, $SD=2.71$) and AC ($M=1.63$, $SD=2.42$) ($F(2,48)=3.97$, $p<.05$). Regarding care burden, AC provided more support for their care recipients for ADLs ($M=2.93$, $SD=1.65$) and IADLs ($M=3.75$, $SD=2.00$ for AC) than OC ($F(1,49)=4.39$, $p<.05$ for ADLs; $F(1,49)=3.17$, $p<.10$ for IADLs). OC ($M=2.57$, $SD=2.43$) and AC (2.96, $SD=2.39$) had fewer social supports than NC ($M=3.41$, $SD=2.38$) who can instantly assist them ($F=11.60$, $p<.001$). OC (21.68%) and AC (29.00%) more frequently than NC (12.01%) participated in social network ($F(2,48)=5.72$, $p<.01$) and online support groups (OC: 13.68%; AC=12.73%; NC=4.68%) ($F(2,48)=9.84$, $p<.001$). Developing practical online approaches for informal caregivers can supplement their limited in-person social support.

### CAREGIVER SUPPORT RATIO IN EUROPE

Oscar Riberio,[1] Laetitia Teixeira,[2] Lia Araujo,[3] and Constança Paúl[4], *1. University of Aveiro - Department of Education and Psychology | Center for Health Technology and Services Research (CINTESIS.UA), Aveiro, Portugal, 2. University of Porto - Institute of Biomedical Sciences Abel Salazar | Center for Health Technology and Services Research (CINTESIS), Porto, Portugal, 3. Escola Superior de Educação - Instituto Politécnico de Viseu | Center for Health Technology and Research (CINTESIS), Viseu, Portugal, 4. CINTESIS – Center for Health Technology and Services Research, Oporto, Portugal*

The caregiver support ratio (CSR) has been defined as the number of potential caregivers aged between 45 and 64 (the most common caregiving age range) for each person aged 80 and over (subgroup of older adults most at risk of needing long term services and support). In 2010, for the USA, this number was calculated to be 7 to 1, a ratio that was projected to shrink to 4 to 1 in 2030, and to 3 to 1 in 2015 according to the AARP Public Policy Institute. In this study we used data from CENSUS HUB to calculate the CSR in Europe considering a total of 27 countries. Main results revealed that a group of Mediterranean countries (Italy, Greece, Spain and Portugal), along with France, Belgium and Sweden have the lowest CSR (5 to 1); on the other hand, the countries with the highest CSR are Slovakia (9 to 1), and Ireland, Poland, Cyprus and Malta (8 to 1). In average, for the 27 countries, the estimated number of caregivers per frail older person today is 6 to 1. These findings reveal important differences between countries and may inform EU policy decisions regarding long-term care (LTC). Given that informal care forms a cornerstone of all LTC systems in Europe, and that this continent faces a rapidly increasing number of people in very advanced age with extended years of disability living at home, estimating the CSR for the next decades is of crucial importance.

### WHAT SHAPES ADULT SONS' INCENTIVE FOR PARENT CARE IN JAPAN? EFFECTS OF WORK AND FAMILY CIRCUMSTANCES

Tomoko Wakui,[1] Ryo Hirayama,[1] Emily M. Agree,[2] and Ichiro Kai[3], *1. Tokyo Metropolitan Institute of Gerontology, Tokyo, Japan, 2. Johns Hopkins University, Baltimore, Maryland, United States, 3. The University of Tokyo, Tokyo, Japan*

In Japan, because of marked sociodemographic shifts such as a growing number of older adults in need of care and fewer siblings in younger generations, adult sons are increasingly likely to be called upon to meet parental care needs while at working age. Using data from 1,183 employed men (M age = 44.6), our aim in this study was to explore work- and family-related factors that affect their incentives for parental caregiving, a potential component of their psychological preparedness for fulfilling care responsibility. Specifically, focusing on these sons' workplace environment, living arrangements, and the quality of relationship with parents, we sought to identify whether and how these factors influenced their desire to provide different types of care (e.g., domestic help, ADL assistance). Regression analysis revealed differential associations between work- and family-related factors and sons' desire for parental caregiving depending on type of care and parents' gender. For their desire to provide domestic help and attend parents when seeing a doctor, live-in sons were more likely to desire to provide care regardless of parents' gender. Whereas the quality of parent-child relationships was significantly associated with their desire to care for both parents, whether they had a supervisor to talk with about family issues had a significant effect only on that for their mother. On the basis of these findings, we discuss workplace needs for support to help sons continue work when they need to provide care.

### SESSION 885 (POSTER)

### HEALTH AND SOCIAL SERVICES INTERVENTIONS

### SURROGATES' DECISION-MAKING CONFIDENCE AND COLLABORATIVE WILLINGNESS ON THE POST: THE ROLE OF SOCIAL NORMS

Rachael Spalding,[1] Jenna Wilson,[1] and Barry Edelstein[1], *1. West Virginia University, Morgantown, West Virginia, United States*

When patients become incapacitated due to illness or frailty, "surrogates" work with patients' providers to make medical decisions on their behalf. In surrogate decision-making situations, surrogates' decision-making confidence predicts collaborative willingness, or the extent to which they are willing to work with the patient's providers when making decisions (Spalding & Edelstein, under review). In an attempt to explain this finding, the current study examined whether perceived social norms for patient-physician collaboration and another psychological variable, consideration of future consequences, mediated the relation between decision-making confidence and collaborative willingness. Participants (n= 172) from Amazon's Mechanical Turk completed self-report measures and a hypothetical surrogate decision-making task. A parallel multiple mediation analysis using 5000 bootstrapped samples with the PROCESS macro (Hayes, 2013) was conducted. The overall model explained 43.4% of the variance in collaborative willingness, $F(4, 166) = 9.59$, $p < .001$. There was a significant indirect effect of decision-making confidence on collaborative willingness through perceived social norms ($b = .068$, $SE = .034$, 95% CI [.014, .154]). There was not a significant indirect effect through consideration of future consequences. After including the significant indirect path through perceived social norms, the direct effect ($b = .348$, $p < .001$) of decision-making confidence on collaborative willingness was reduced ($b = .243$, $p = .003$). Thus, perceptions of social norms partially accounted for the relation between decision-making confidence and collaborative willingness. This finding illustrates how social perceptions of patient-provider collaboration can facilitate desirable medical decision-making behaviors, such as collaboration.

### HOUSE CALLS AND BEHAVIORAL HEALTH INTEGRATION: ADDRESSING MENTAL HEALTH LITERACY AND DEPRESSION CARE

Elyse Perweiler,[1] Lisa Bodenheimer,[2] Gail Belfer,[3] Jennifer DeGennaro,[1] Anita Chopra,[1] and Marla Meyers[3], *1. Rowan School of Osteopathic Medicine, Stratford, New Jersey, United States, 2. Rowan University School of Osteopathic Medicine, Stratford, New Jersey, United States, 3. Jewish Family & Children's Service of Southern New Jersey, Cherry Hill, New Jersey, United States*

The House Calls and Behavioral Health Integration project was an inter-agency collaboration to address depression in the older adult population by increasing knowledge and awareness of the condition and providing better access to care. Rowan Medicine, New Jersey Institute for Successful Aging (NJISA) and Jewish Family and Children Services (JFCS) partnered to deliver a comprehensive education, screening and referral program for residents of 3 senior housing facilities serving an estimated 300 elderly and disadvantaged residents. The goal of this year long project was to provide depression education for both housing residents and staff; offer on-going access to free depression screens (using PHQ-9) for residents; and refer residents to an appropriate resource (counseling/case management/house calls) depending on depression screen outcome. Depression education was provided to 15 housing staff and 78 older adult residents. A total of 34 depression screens were completed (82% female, average age=82 years); further evaluation of symptoms was indicated for 59% (n=20) of those screened. Seventeen of the 20 residents accepted a recommendation for a referral to supportive counseling; 3 declined. The average PHQ-9 score was 8.45 (SD=4.42; Range 1-24). Referrals to case management and house calls (n=4) were also made. Feedback from both housing residents and staff was positive and the project team learned valuable lessons about serving older adults in congregate living settings which has informed other programming. The project was successful in providing education, screening and referrals to residents who might not otherwise have access to specialized geriatric behavioral health interventions.

### EFFECTIVENESS OF A COMMUNITY HEALTH WORKER-BASED INTERVENTION TO IMPROVE DIETARY HABITS: A CROSSOVER TRIAL IN JAPAN

Hiroshi Murayama,[1] Atsuko Taguchi,[2] and Takuhiro Yamaguchi[3], *1. Institute of Gerontology, The University of Tokyo, Tokyo, Japan, 2. Department of Public Health Nursing, Tohoku University Graduate School of Medicine, Sendai, Miyagi, Japan, 3. Division of Biostatistics, Tohoku University Graduate School of Medicine, Sendai, Miyagi, Japan*

Background: Community health workers (CHWs), often called "health promotion volunteers" in Japan, are individuals who act as a natural helping resource in the community. This study tested the effectiveness of a CHW-based intervention in improving dietary habits among community-dwelling older people in Japan, using a controlled, cross-over design. Methods: Seventy-eight people aged 65–74 years with poor dietary variety living in four districts in Hikone City (Shiga Prefecture, Japan) were allocated to an immediate-intervened group (IIG; n = 41) or a delayed-intervened group (DIG; n = 37). Participants joined a bi-weekly, four-session program (120 minutes/session), comprising "CHWs' drama-style lectures," "group discussion among participants and CHWs," "tasting of dishes," and "take-home practical activities." For the initial 2-month period, the IIG received the intervention and the DIG did not. The groups were crossed over for the subsequent 2-month period. The primary outcome measure was the participants' dietary variety scale score. Results: The dietary variety scale score in the IIG significantly increased in the initial 2-month period compared with the DIG (effect size 1.60 points; 95% confidence interval 0.75, 2.45). The intervention had a similar effect in the DIG in the subsequent 2-month period. Moreover, an analysis within the IIG showed that the intervention effects persisted for at least 2 months after the intervention. Conclusions: The CHW-based intervention improved dietary habits among older people. Our findings provide evidence that a CHW-based natural helping approach is a possible solution to promote healthy aging in the community.

### TRANSLATIONAL ADVISORY BOARDS IN RESEARCH: EXPERIENCES FROM A PRAGMATIC RESEARCH TRIAL

Keith A. Anderson,[1] Holly Dabelko-Schoeny,[2] Laura N. Gitlin,[3] Joseph E. Gaugler,[4] Sokha Koeuth,[3] and Katherine A. Marx[5], *1. University of Montana, Missoula, Montana, United States, 2. The Ohio State University, Columbus, Ohio, United States, 3. Drexel University, Philadephia, Pennsylvania, United States, 4. University of Minnesota - School of Public Health, Division of Health Policy and Management, Minneapolis, Minnesota, United States, 5. Johns Hopkins University, Baltimore, Maryland, United States*

Translational Advisory Boards (TABs) are select groups of researchers, practitioners, and, in some cases, service recipients (e.g., patients, caregivers) that convene regularly to advise researchers on ongoing studies. TABs provide direction and support, knowledge and insight, alternative points of view, and suggestions for overcoming obstacles and improving research functions. TABs are especially valuable in applied research in which "real world" conditions create challenges ranging from the anticipated (e.g., participant drop out) to the unanticipated (e.g., government shutdown). In this presentation, the researchers evaluate one TAB involved with an ongoing pragmatic research trial of Adult Day Services Plus (ADS Plus), an intervention for family caregivers to persons with dementia. Including individuals with diverse points of view was critical in the composition of the TAB. The TAB consisted of four seasoned researchers, four ADS industry professionals, and six program directors from the ADS Plus treatment and control groups. Creating meetings that were productive was imperative. TAB sessions were held bi-annually and had highly structured agendas soliciting guidance on specific issues. Qualitative analysis of the TAB sessions revealed three themes: assistance with recruitment; fidelity of the intervention; and sustainability of the intervention. This guidance helped the researchers to shift directions in recruitment and to consider further refinement of the intervention to increase sustainability. In looking at costs, the annual research personnel, TAB personnel, and overhead expenses were estimated at $2,500-$3,000 and $4,000-$5,500, respectively. In conclusion, TABs can be an effective tool to support research and should be considered for use in large-scale projects.

### CONSIDERING AN INTERDISCIPLINARY DEMENTIA INTERVENTION, COMBINING BEHAVIORAL AND OCCUPATIONAL THERAPY

Lou Frankenstein[1], *1. Chemnitz University of Technology, Institut for Applied Gerontopsychology and Cognition, Germany, Chemnitz, Germany*

Behavioral therapy for people diagnosed with dementia and their informal caregivers can enhance the quality of life and the accomplishment of daily routines. The effectiveness of occupational therapy in dementia has also been proven several times. Still those therapies are often not part of the regular treatment. Through literature analyses and three focus group discussions with experts of both professions four occupational (ESP, WHEDA, Ergodem and HED-I) and two behavioral interventions (CBTAC, cordial-program) were compared to clarify how much and in what ways behavioral and occupational therapy in dementia overlap and differ. The interventions are similar with respect to intervention characteristics: They are non-pharmacological, client-centered, put a major emphasis on involving the caregivers and are similarly structured. Whilst occupational programs focus on practical issues, such as empowerment to perform daily activities or adjustment of the environment, cognitive-behavioral interventions specialize on planning activities, communication and reminiscence. These differences result from the theoretical basis and the primary goals of the professions. Behavioral therapy developed from learning theories and cognitive techniques. In contrast occupational therapy is based on environmental theories and the idea of empowerment. In addition two activating programs were included (CST, MAKS-active) to promote physical activity and social interactions since both positively influence the course of the disease. The focus group participants supported the idea of an interdisciplinary cooperation. The results suggest, that an intervention offered by both professions, ideally in cooperation, encompassing key elements identified in the studied programs would be an advance in dementia therapy.

### DOES THE ASSOCIATION BETWEEN AGE AND MAJOR ILLNESS VARY BY NATIONAL HEALTHCARE QUALITY?

Matthew A. Andersson,[1] Lindsay R. Wilkinson,[1] and Markus H. Schafer[2], *1. Baylor University, Waco, Texas, United States, 2. University of Toronto, Toronto, Ontario, Canada*

Though the risk of chronic disease and disability accelerates once adults are in their 60s, 70s, and 80s, researchers have long suspected that economic, social, and institutional variation — even among high-income Western nations — may powerfully influence the likelihood that people remain healthy at advanced ages. This study builds on comparative research into global aging, by offering a multiple-indicator test of whether national healthcare quality modifies the association between age and major illness. Recent individual-level data on morbidity among respondents aged 50 or older (16 countries; 2014 European Social Survey) are merged with nation-level healthcare indicators. Healthcare quality is assessed using a subjective, evaluation-based approach (based on the 2011 International Social Survey Programme) and an objective, attributable-mortality approach (2010 Healthcare Access and Quality, based on the Global Burden of Disease Study). Lagged nation-level economic and health indicators are controlled to help isolate healthcare effects. Multilevel logistic and linear regression models of any major health condition and morbidity reveal that while older individuals showed approximately a 10% reduction in probability of major illness when residing in countries with higher healthcare quality, associations between age and morbidity indices combining number and severity of illness showed greater modification by healthcare quality, with reductions around 18%. Results across subjective and objective approaches to healthcare quality are strikingly consistent. Taken together, results are suggestive of healthcare's protective role in reducing age-related illness and disability. Future research should illuminate pathways by which healthcare quality may lead to differences in healthy aging among advanced nations.

### PROMOTING OLDER ADULTS' INTERGENERATIONAL COMMUNICATION AND MOTIVATION TO REGULAR EXERCISE THROUGH EXERGAMING

Jinhui Li,[1] Chen Li,[1] Bing Xun Chia,[1] Xinran Chen,[1] Tan Phat Pham,[1] Charles T. Salmon,[1] and YIn Leng Theng[2], *1. Nanyang Technological University, Singapore, Singapore, 2. Ageing Research Institute for Society and Education, Singapore, Singapore*

Exergaming has become an important part of community programs to promote regular exercise and improve well-being for healthy aging. This study examines how different types of social playing and competitive information in exergaming affect older adults' inter-generational communication with youth, as well as their motivation to regular exercise through exergaming. A 2 (time: pre-test vs. post-test) * 3 (social play: play alone vs. play with elderly vs. play with youth) * 2 (competition: competition informed vs. non-competition informed) mixed experiment with 319 Singaporean older adults conducted over six weeks in 2018, analyzed through a series of three-way repeated ANOVAs. Results show significant three-way interaction effects among time, social type, and competitive information on older adults' inter-generation communication ($F (2, 300) = 3.206$, $p = .042$, $\eta 2 = .021$), extrinsic motivation ($F (2, 301) = 3.364$, $p = .036$, $\eta 2 = .022$) and intrinsic motivation ($F (2, 303) = 4.528$, $p = .012$, $\eta 2 = .029$). The inter-generational communication of participants in play with youth shows significant improvement over time in both competition conditions, while those in play with elderly was only significantly improved in competitive informed condition. Social play is found to significantly affect the changes of both intrinsic and extrinsic motivation over time, while competitive information only affects intrinsic motivation significantly. The findings make contributions to aging research and understanding of potential factors that promote inter-generational communication and adherence to regular exercise via exergaming.

### BARRIERS TO CARE FOR OLDER ADULTS IN MEDICATION-ASSISTED TREATMENT FOR OPIOID USE DISORDER

Alexis A. Bender,[1] Maggi N. Robert,[1] Nathan S. Quan,[1] Emma M. Klein,[1] and Molly M. Perkins[2], *1. Emory University, Atlanta, Georgia, United States, 2. Emory University School of Medicine, Atlanta, Georgia, United States*

Over the last decade, the number of older adults (people over the age of 50) who misuse opioids doubled and continues to increase. People over the age of 50 also represent one of the fastest growing groups entering into and sustaining medication assisted treatment (MAT) (i.e., methadone and buprenorphine) for opioid use disorder (OUD). Despite increasing awareness of this growing at-risk population, significant knowledge gaps regarding their support and care needs persist. To begin to address these gaps, we conducted interviews with 20 treatment staff, focus groups with 18 patients and surveys with 100 patients over the age of 50 at eight diverse Opioid Treatment Programs (OTPs) participating in a 1-year pilot study (Bender, PI) funded by the Georgia Clinical and Translation Science Alliance supported by the National Center Advancing Translational Sciences. Patients in this study do not always disclose their use of MAT to non-OTP providers. When they do, participants reported numerous negative experiences with non-OTP providers, including perceived discrimination, stigma, and misunderstanding by providers about MAT. These negative experiences potentially contribute to an over reliance on OTP providers to manage age-related health conditions (e.g., COPD, hypertension). Providers report minimal training about aging and varied levels of confidence to manage these conditions. We present the experiences of patients and providers with suggestions for improving care coordination. We conclude with recommendations to improve communication among providers working with older adults in recovery from OUD.

### OLDER ADULT KIDNEY TRANSPLANT RECIPIENTS: THE LIVED EXPERIENCE OF ADAPTATION AND INTEGRATION

Laura L. Kimberly,[1] Denise Burnette,[2] Ellen Lukens,[1] and Bruce Gelb[3], *1. Columbia University, New York, New York, United States, 2. Virginia Commonwealth University, Richmond, Virginia, United States, 3. NYU Langone Health, New York, New York, United States*

As the prevalence of end-stage renal disease increases in the United States, a growing number of adults aged 65 and over are receiving kidney transplants. While older adult recipients tend to fare well from a clinical standpoint, far less is known about their psychosocial wellbeing following transplantation. This study seeks to better understand the lived experience of older adult kidney transplant recipients, focusing on the 'liminal' period of adaptation following organ transplantation that has been reported elsewhere in the literature. Applying the hermeneutic phenomenology of philosopher Paul Ricoeur, the study explores the lived experience of 10 deceased donor kidney transplant recipients aged 65 and over. Guided by Ricoeur's conceptual approach to identity as constituted through two forms, idem and ipse, preliminary findings suggest that despite expressing some distress around the 'strangeness' of integrating part of another into oneself (disruption of the idem sense of self), participants also constructed powerful narratives of resilience and coping that were rooted in the continuity of a deeply held ipse sense of identity over the life course. In particular, participants emphasized their ability to overcome adversity as an anchor of their ipse sense of self that enabled them to navigate the idem corporeal changes of transplantation. Moreover, they described kidney transplantation as a form of liberation, ultimately restoring their idem sense of self that had been profoundly disrupted by 'machine life' (time spent on dialysis). These findings will have significant implications for ensuring the provision of optimal support to older adult kidney transplant recipients.

### SESSION 890 (POSTER)

### HEALTH PROMOTION

### FEASIBILITY OF A TELEHEALTH-DELIVERED LIFESTYLE INTERVENTION FOR LATE-MIDLIFE LATINOS LIVING IN RURAL CALIFORNIA

Stacey L. Schepens Niemiec,[1] Jeanine Blanchard,[1] Cheryl Vigen,[1] Jenny Martínez,[2] and Mike Carlson[1], *1. University of Southern California Chan Division of Occupational Science and Occupational Therapy, Los Angeles, California, United States*
*2. Jefferson College of Rehabilitation Sciences Department of Occupational Therapy, Philadelphia, Pennsylvania, United States*

Late-midlife Latinos (50–64 years old) living in rural regions experience significant health disparities, oftentimes exacerbated by limited access to healthcare services. In a previous pilot study, we observed psychosocial, behavioral, and cardiometabolic health improvements sustained over 12-months in late-midlife Latinos who participated in ¡Vivir Mi Vida! (¡VMV!), a culturally tailored lifestyle intervention led by a Latino community health worker (promotor) and occupational therapist team. For the present study, we assessed the feasibility of telehealth-delivered ¡VMV! modules. Participants (N=10) received an abbreviated three-week version of ¡VMV! consisting of an in-person promotor-led orientation and two one-hour telehealth sessions. Telehealth opinions/experiences were assessed at baseline and follow-up using study-specific questionnaires and by interview. Patient-identified health quality was measured pre-post intervention using the Measure Yourself Medical Outcome Profile (MYMOP2) and a single-item stress index. Participants generally agreed that telehealth session quality was equivalent to in-person sessions, and demonstrated confidence in their ability to communicate freely with the promotor and actively participate in telehealth sessions. We observed significant improvements in nearly all MYMOP2 components and a trend in stress reduction. The intervening promotor reflected that telehealth ¡VMV! extended healthcare to patients impacted by risk factors such as geographic isolation, lack of available services, and hesitancy to access in-person services due to fear of discrimination or deportation. Participants provided highly positive feedback, highlighting the practicality and convenience of the telehealth program. Feasibility of delivering ¡VMV! via telehealth to late-midlife rural-dwelling Latinos and its potential for positive effect was supported.

### WECHAT, WERUN: A GROUP-BASED WALKING STUDY TO ENHANCE PHYSICAL ACTIVITY AMONG COMMUNITY-DWELLING OLDER ADULTS

Yujun Liu,[1] and Margie E. Lachman[2], *1. Brandeis University, Waltham, United States, 2. Brandeis University, Waltham, Massachusetts, United States*

The benefits of physical activity (PA) and social engagement for older adults are well established, yet the majority of adults over age 50 in the U.S. do not engage in regular exercise. We conducted a 4-week pre-post study with a one-month follow-up using a smart phone application called WeChat WeRun to explore the mechanisms of social engagement and competition to increase PA among older adults. Participants (N=40, mean age=65.7) were randomly assigned to one of two conditions. Participants in the activity competition condition used the WeRun to track their daily walking steps and interact via text with their group members for 4 weeks. The daily walking steps were displayed to the group and the participant with the highest daily steps in the group was indicated as the champion each day. Participants in the control group only used WeRun to track their own walking steps. Outcome variables included average weekly steps, exercise self-efficacy, and social engagement. The two-way repeated measures ANOVA results revealed that participants in the competition condition had higher average weekly steps and level of social engagement at the posttest, compared to those in the control group. Post hoc tests using the Bonferroni corrections revealed that the intervention increased their average weekly steps and social engagement from pretest to post-test, while these outcomes did not change in the control group. The effects were maintained at the one-month follow up. Discussion will consider the motivational role of competition and social interactions in increasing PA among older adults.

### THE EFFECT OF SENSE OF COHERENCE ENHANCEMENT PROGRAM BASED ON SALUTOGENESIS FOR ELDERLY WITH METABOLIC SYNDROME

Jungmi Yun,[1] and Hyoung Sook Park[1], *1. College of Nursing, Pusan National University, Busan, Korea, Republic of*

This study aimed to develop and verify a sense of coherence (SOC) enhancement program based on salutogenesis for elderly with metabolic syndrome. This study was a quasi-experimental study. The inclusion criteria were patients with metabolic syndrome aged 60 or over who visited the outpatient department of a university hospital and a public health center located in an urban area. The intervention group (n=24) attended the SOC enhancement program. The health promotion theoretical framework, salutogenesis, provided the program structure and conceptual framework of this study. This program included comprehensibility, manageability, and meaningfulness which were core concepts of salutogenesis and consisted of multiple tailored behavior interventions composed of education, Tai-Chi exercises, individual activities, self-monitoring, self-help groups, and individual counseling. This program was provided once a week over 10 weeks, but Tai-Chi exercise was conducted twice a week. The control group (n=25) was provided with general health information once. The interactions between groups and measurement time showed significant differences in self-efficacy ($p=.001$), perceived social support ($p<.001$), SOC ($p<.001$), health behavior ($p=.010$), and the physical health-related quality of life ($p=.001$). The metabolic-related physiological indicators showed significant improvement in total cholesterol ($p=.004$) and LDL-cholesterol ($p=.009$). This study showed that the SOC enhancement program strengthened the self-efficacy, social support, and SOC in the elderly with metabolic syndrome, improved the health behavior, and was effective in improving some metabolic-related physiological indicators. Based on the results of this study, I suggest to expand the scope of the sense of coherence program and conduct the intervention longer.

### RESILIENCE, RESOURCEFULNESS, AND MINDFULNESS: DISTINCT BUT COMPLEMENTARY INTERVENTIONS

Carol M. Musil,[1] Alexandra Jeanblanc,[1] McKenzie Wallace,[1] Christopher J. Burant,[1] and Jaclene Zauszniewski[1], *1. Case Western Reserve University, Cleveland, Ohio, United States*

Stress and coping theory guides our interest in three related concepts: resilience (the capacity to bounce back and cope with stress), resourcefulness (cognitive and behavioral strategies to manage stress and adversity), and mindfulness (strategies of present moment awareness to reduce stress). These are theoretically distinct but related concepts relevant to improving health and social outcomes in older adults. Our aim was to evaluate the theoretical distinctions among these concepts in 236 grandmother caregivers. Measures of resilience (Connor-Davidson Scale), mindfulness (Decentering Scale) and resourcefulness (Resourcefulness Scale) were collected from a longitudinal, online study of 236 grandmother caregivers. To evaluate construct validity, we examined criterion validity and conducted exploratory factor analysis using Principal Axis Factoring with direct oblimin rotation in SPSS. Factor Analyses were conducted on each scale separately and with all items combined. Inter-correlations ranged from $r= .28$ (resourcefulness and resilience) to $r= .75$ (resilience and mindfulness). Factor analyses and scree plots indicated unidimensional factors for resilience and for mindfulness, and three factors for resourcefulness (personal resourcefulness aimed at either emotion regulation or planful problem solving, and social resourcefulness by external help seeking). When items from all measures were analyzed together, the five distinct factors remained. Additional construct validation with the CES-D and Duke Social Support scales supported convergent and discriminant validity of resilience, resourcefulness and mindfulness. Our results confirm the theoretical distinctions among resilience, resourcefulness, and mindfulness, providing support for the use of these concepts collectively or individually in interventions to improve health outcomes.

### PARTICIPANTS' PERCEPTIONS OF A COMMUNITY-BASED DANCE PROGRAM

Megan R. Bewernitz,[1] and Cari Freiberger[1], *1. Jacksonville University, Jacksonville, Florida, United States*

Parkinson's disease (PD) is a progressive disease that targets many aspects of the nervous system, both motor and non-motor functions. Dance as an intervention provides a creative outlet that can be more motivating than other interventions by allowing people an opportunity for not only increased movement and activity, but also socialization. Creative expression through dance also has the potential to improve not only motor capabilities like balance, but also non-motor aspects like self-efficacy, cognition, and quality of life. For the community program described in this presentation, dance faculty led the dance and choreography components of the program while an occupational therapist (OT) served to maximize options for adaptations, safety, and accessibility. OT faculty also led efforts in gathering and analyzing data regarding community participation and program design utilizing a qualitative approach by conducting semi-structured interviews with five participants (four with PD, one with hydrocephalus, all over age 65) in the program. Content analysis of the data suggests some emerging themes which include: 1) forming common bonds and making friendships within the class due to PD diagnosis and similar enjoyment of the class, 2) increased appreciation and enjoyment of music, 3) appreciation of dance instruction, and 4) how transportation options, or lack thereof, impact participation in the class. These themes will assist in informing future program delivery with the hopes of reaching a larger contingent of the community who may also be interested in dance.

### MAINTAINING HEALTHY LIFESTYLE: RACIAL AND GENDER DIFFERENCES IN PHYSICIANS' ADVICE TO OLDER ADULTS

Asif Iqbal,[1] Abolade Oladimeji,[1] Eva Kahana,[1] and Boaz Kahana[2], *1. Case Western Reserve University, Cleveland, Ohio, United States, 2. Cleveland State University, Cleveland, Ohio, United States*

Older adults are often excluded from physicians' preventive health promotion recommendations. The influence of patient race and gender on the physician-patient relationship in maintaining good health has been extensively researched, but little is known about the influence of patient race and gender on physicians' health promotion advice to the elderly. This study explored whether patient race and gender influence primary care physicians' health promotion advice to older adults. The sample of 536 respondents (µage = 75.51, 75.2% female) was obtained from a NCI funded study of "Health Care Partnerships in Cancer Prevention and Care of Aged. Respondents were randomly selected community dwelling older adults who attend Senior Center programs, sponsored by Area Agencies on Aging (AAA) (Kahana, Lee, Kahana, Langendoerfer, & Marshall, 2015). Multivariate logistic regression analyses were used to control for sociodemographic and health factors while accounting for a complex sampling design. African American patients had greater odds than whites for receiving recommendations to eat a healthy diet (OR = 1.62) and exercise more (OR = 1.83). Elderly women reported fewer recommendations to eat a healthy diet (OR = .81) & exercise more (OR = .45) than did men. Notably, respondents of both races and genders believed that maintaining a healthy lifestyle is very effective. These findings demonstrate that African American and female older adults receive differential health promotional advice from physician and suggest the need for raising physician awareness about the value of prevention advice for all patients.

### GENDER DIFFERENCES IN HEALTH-RELATED BEHAVIORS OF RETIRED PEOPLE IN TAIWAN

NUAN-CHING HUANG,[1] Susan C. Hu,[2] and Ying-Wei Wang[3], *1. Healthy Cities Research Center, National Cheng Kung University, Tainan City, Taiwan, 2. National Cheng Kung University, Tainan City, Taiwan, 3. Health Promotion Administration, Ministry of Health and Welfare, Taipei, Taiwan*

Many studies have indicated that lifestyles and health behaviors are important factors associated with elderly health. However, few studies have focused on such issues in Taiwan. The purposes of this study were to examine the gender differences in health-related behaviors of retired people in Taiwan. We used face-to-face interviews to collect 20 health-related behaviors among retired people aged 50-74 in Taiwan. A total of 3131 retired sample was collected including 1754 male (56.0%) and 1377 female (44.0%). The health behaviors were designed as binary variables. If the retirees executed exactly on the behavior within a month, they got 5 scores, otherwise, they got 0 scores. Then, we used factor analysis with Varimax Rotation to detect factors associated with the 20 health behaviors. Results showed that six factors were related to these 20 behaviors after conducting factor analysis. These six factors were named as 1) No tobacco, alcohol and betel nut, 2) Periodic health examination, 3) Correct medication, 4) Good habits, 5) Normal sleep and no pressure, (6) No high-fat and pickled foods. Gender differences were found in three factors: 1, 5 and 6. More female practiced every behavior in factor 1 and factor 6 than male (68.7%, 55.5% vs. 43.6%, 50.3%, p<.001, respectively). However, the male had better behaviors in factor 5 than female (54.0% vs. 47.3%, p<.001). We hope these findings could help design different health promotion programs for retirees of different genders.

### SOCIAL SUPPORT AND HEALTHCARE-SEEKING BEHAVIOR OF OLDER PERSONS IN NIGERIA

FUNMI TOGONU-BICKERSTETH,[1] Joshua O. Aransiola,[1] Catherine O. Oyetunji-Alemede,[2] OPEYEMI EKUNDAYO,[3] and OLUWASEGUN OLUWALEIMU[4], *1. Obafemi Awolowo University, Ile-Ife, Ile-Ife, Osun State, Nigeria, 2. Obafemi Awolowo University, Ile-Ife, Osun State, Nigeria, 3. OBAFEMI AWOLOWO UNIVERSITY, ILE IFE, OSUN, Nigeria, 4. Obafemi Awolowo University, Ile-Ife, Osun State, Nigeria*

Abstract The choice of healthcare facility by older persons is an important factor in their healthcare seeking behavior, and this can be associated with a number of factors. This study investigated the relationship between social support and healthcare facility choice of older persons in Nigeria. Other factors associated with the choice of healthcare facilities by older persons were also identified. Quantitative data were collected from a sample of 3,696 elderly aged 60 years above (55.6% male; 44.4% females; mean age = 69.2, SD = 8.60) who were selected through multi-stage systematic random sampling. Binary logistic regression analysis revealed that older persons who received social support were more likely to seek treatment in formal healthcare facilities, while older persons who did not receive any form of social support were more likely to seek treatment in informal healthcare facilities. Membership of social or religious groups was found to be a predictor of health seeking behavior among the older adults. Sex, age, level of education, and ability to handle activities of daily living (ADL), and ease of access to the nearest health facility, were found to be significantly associated with choice of healthcare facilities. The article concludes that there is need for conscious planning to provide formal supports to ease access of older persons to available health facilities. Such facilitation should include financial support and removal of existing physical and cultural barriers to health care utilization by older persons.

### THE IMPACT OF NEGATIVE SOCIAL EXCHANGES ON ADULT PHYSICAL AND MENTAL HEALTH

Abigail M. Nehrkorn-Bailey,[1] Julie Hicks Patrick,[1] and Madeline M. Marello[1], *1. West Virginia University, Morgantown, West Virginia, United States*

As some health components may change across adulthood (CDC, 2019), social support for aging adults may be one way to optimize physical and mental health (U.S. Department of Health and Human Services, 2018). When social encounters are negative, however, physical and mental health may be negatively affected (Chen & Feeley, 2013; Hawkley & Cacioppo, 2010). Negative social exchanges (NSE) have been linked to an increase in negative affect and a decrease in positive affect (Newsom et al., 2003), along with an increase in physical symptoms (Edwards et al., 2001). In order to examine the relations between age, NSE, and two components of health (chronic health conditions and mental health) two moderated regression analyses were conducted using data from 848 adults (Mage = 32.5 years). Studying chronic health conditions, the overall model was significant, [$F(3, 838) = 40.31$, $p < .001$; $R2 = .36$]. Significant main effects emerged for NSE and age, along with a significant interaction between age and NSE ($b = 0.010$, $p < 0.05$). As NSE increased, the number of chronic health conditions increased, especially for older adults. For mental health, the overall model was significant [$F(3, 845) = 52.96$, $p < .001$; $R2 = 0.16$]. A significant main effect emerged for NSE, but neither the main effect for age nor the interaction were significant. Thus, although NSE can have deleterious effects on both mental and physical health, special attention needs to focus on the physical health of older adults who experience a higher number of NSE.

### AN ACTING AND IMPROV CLASS: WELL-BEING AND COMMUNITY BELONGING FOR OLDER AFRICAN AMERICANS IN LOW-INCOME HOUSING

Laura Sutherland,[1] Ruth E. Dunkle,[2] Garrett T. Pace,[2] Ariel Kennedy,[2] and Pat Baldwin[3], *1. Wayne State University, Detroit, Michigan, United States, 2. University of Michigan, Ann Arbor, Michigan, United States, 3. Hannan Center, Detroit, Michigan, United States*

Arts-based interventions can enhance the quality of life of older adults, but community-dwelling older adults may have reduced access to such interventions. The purpose of this study was to examine whether a creative arts program can improve the overall health and well-being of older adults in low-income housing. A university social work department and community agency collaborated in establishing a professionally run theater group of older adults in two low-income housing buildings in an urban area. All residents were encouraged to participate. The study consisted of three twelve-week acting and improvisation courses, focusing on either staged reading of monologues and dialogues, co-writing a script, or staged reading of a complete play. Each class culminated in a public performance for the building. Fourteen participants enrolled, all African American (11 women, 3 men; mean age 66 years; mean ADL = 1.4 (range of 1-2.5)). Attendance on average was 8 classes. Data were collected through pre- and post-test questionnaires, participant observation of class sessions, semi-structured interviews with building managers, and post-session participant reflections. Thematic analysis was performed, and revealed key themes of increased community belonging, increased confidence, and increased daily coping abilities. Additional themes included the gaining of new artistic skills and interest in continuing classes. Barriers to participation included difficulty in recruitment and absence due to ongoing health conditions and caregiving responsibilities. This project has implications for the potential of arts-based programming to increase well-being for underrepresented community-dwelling older adults.

### ACCEPTABLE CHARACTERISTICS OF INTERVENTIONS FOR MIDDLE-AGED HISPANIC MEN: RESULTS FROM KEY INFORMANT INTERVIEWS

Cheryl DerAnanian,[1] Sonia Vega-Lopez,[2] Hector Valdez,[1] Shandel Vega-Soto,[1] Ferdinand Delgado,[1] and Steven Hooker[3], *1. Arizona State University, Phoenix, Arizona, United States, 2. Arizona State University, Phoenix, United States, 3. San Diego State University, San Diego, California, United States*

Hispanics are the fastest growing segment of the aging population. Little is known about acceptable intervention approaches for Hispanic men. Purpose: To explore perceptions of acceptable intervention characteristics for middle-aged Hispanic men. Methods: Eighteen key informants (KIs) with expertise in delivering programs to the Hispanic community participated in semi-structured interviews. All interviews were audio-recorded and transcribed verbatim. Themes were identified using a grounded theory approach. Results: “Cultural sensitivity/competency” or “biculturalism” were fundamental characteristics of an acceptable intervention (n=17 KIs). KIs indicated program facilitators need to understand and relate to the culture, and to establish the trust of the community by demonstrating mutual understanding, respect and dignity. Facilitators must be bilingual and use the same level of language as the participants. KIs highlighted the importance of the literacy level of the materials and indicated intervention content should tap into the participants’ unique cultural experiences. Including the family in the intervention, especially the spouse or partner, was considered essential for engaging the men (n=18 KIs). The KIs consistently indicated an intervention should be in-person, in either a group (n=7 KIs), one-on-one (n=5 KIs) or hybrid (group and individual; n=6) format. Only text messaging (n=10 KIs), phone calls (n=5 KIs), and Fitbits (n= 7KIs) were perceived as acceptable forms of technology for an intervention. Finally, the intervention needs to be appealing (N=5KIs), interactive (n=8KIs), and safe, fun and supportive. Conclusion: The KIs identified several important culturally relevant considerations when developing interventions for Hispanic men. Funded by the National Institute on Aging (R21 AG050084-01A1).

### A COMMUNITY HEALTH EDUCATION PROGRAM TO ENCOURAGE EXERCISE AND PROPER NUTRITION IN LATINOS

Karen Kopera-Frye,[1] Ashley Graboski-Bauer,[1] and Ana Luisa Gonzalez-Marquez[1], *1. New Mexico State University, Las Cruces, New Mexico, United States*

New Mexico now has the 32nd highest adult obesity rate (28.8% for 2015). Thirty one percent of this obesity rate encompasses Latinos; rates are the highest among the 26-44 years age group. Obesity rates in NM for children aged 2-11 range from 11 – 14%. Healthy Kids New Mexico adopted the 5-2-1-0 challenge. (American Academy of Pediatrics) which refers to eating 5+ vegetables, reducing screen time to 2 hours, 1 hour+ daily activity, and daily water intake (H20). Therefore, project I'M HIP2's main goal was to educate parents and children, within an intergenerational context, on improving diet and activity levels to reduce obesity. The Intergenerational Mentoring on Health Information Pathways 2 (I'M HIP2) program involved 74 Hispanic families including one target child, one parent, and one other relative, e.g., aunt or grandparent. Monthly educational sessions focused on physical activity and adapting meals to be healthy. Project outcomes included exercise frequency, Body Mass Index (BMI), and a knowledge quiz assessing healthy meal facts, exercise knowledge via a 10-item quiz; all assessments pre- and post-program. Paired t-test analyses revealed significant changes in knowledge quiz total scores (t 70 = 5.03, $p < .0001$), increased exercise frequency (t 72 = 2.106, $p < .05$); no significant change in BMI from pre- to post-assessments. The families reported overwhelmingly positive responses to how the program had changed their eating styles and activity levels. This study has implications for how we can harness this invaluable resource of the generations to affect health impacts on Hispanic families.

### HEALTH PROMOTION

### A NEW APPROACH TO DEVELOP GERONTOLOGICAL NURSING CARE QUALITY INDICATORS

Satomi Kitamura,[1] Ayumi Igarashi,[1] Asa Inagaki,[1] Manami Takaoka,[1] Maiko Noguchi-Watanabe,[1] Mariko Sakka,[1] Takashi Naruse,[2] and Noriko Yamamoto-Mitani[1], *1. Department of Gerontological Home Care and Long-term Care Nursing/ Palliative Care Nursing, Division of Health Sciences and Nursing, Graduate School of Medicine, The University of Tokyo, Tokyo, Japan, 2. Department of Community Health Nursing, Division of Health Sciences and Nursing, Graduate School of Medicine, The University of Tokyo, Tokyo, Japan*

While the inter-RAI, a comprehensive geriatric assessment tool, contains standardized system for assessing quality of care, there are limitations for its everyday use. Limitations include large number of items, lack of apparent process indicators, and limited symptoms and disease-related information. A new approach was introduced to develop gerontological nursing quality indicators that were targeted at long-term care. We plan to develop staff-friendly indicators, which can be extracted from regular routine client records. A group of nurse researchers discussed essential domains of elderly persons' life quality, based on nursing theory literature, that nurses strive to maintain. Several outcome indicators were derived out of the domains, and process quality indicators were developed based on literature review. We identified nine domains based on Gordon's functional health patterns: 1) minimizing symptoms and disease deterioration, 2) maintaining nutritional status, 3) controlling bowel movements, 4) encouraging physical activities, 5) promoting sound sleep, 6) minimizing dementia symptoms, 7) maintaining dignity, and 8) reducing family stress. We then developed 17 outcome indicators; each domain included one to four outcome indicators (e.g., maintaining nutritional status has three indicators: no aspiration, no weight loss by 3% or more, and no dehydration). Process indicators that covered regular assessment (e.g., swallowing evaluation), preventive interventions (e.g., adjusting body positions during a meal), and interventions for the problems (e.g., food texture modification) were determined. These indicators may be useful to assess gerontological nursing quality. We are planning to conduct expert panel and individual client surveys to assess its usability.

### THE BURDEN OF ENGAGING IN VOLUNTEER ATTENUATE THE RELATIONSHIP BETWEEN VOLUNTEER ENGAGEMENT AND HEALTH OUTCOMES

Yuta Nemoto,[1] Ryota Sakurai,[1] Masami Hasebe,[1] Kumiko Nonaka,[1] Hiroko Mtsunaga,[1] and Yoshinori Fujiwara[2], *1. Tokyo Metropolitan Institute of Gerontology, Itabashi, Tokyo, Japan, 2. Tokyo Metropolitan Institute of Gerontology, Tokyo, Japan*

This study examined the interaction of participating in volunteer activity and its burden on health outcomes. A community-based cross-sectional study was conducted in 2018. Of 8426 older adults aged 65 and over, 5232 individuals were included in the analyses (response rate: 62.1%). Health outcomes included self-rated health (SRH), mental health (The World Health Organization Five Well-Being Index: WHO-5), and Instrumental Activities of Daily Living (Tokyo Metropolitan Institute of Gerontology Index of Competence: TMIG-IC) as dependent variables. Independent variables included engagement in volunteer activity and its burden. These variables were combined and classified into three groups: non-participants; participants with burden; and participants without burden. Covariates included age, gender, educational attainments, economic status, and living arrangement. Logistic regression analysis and analysis of covariates were conducted to examine the interaction of volunteer activity and its burden on health outcomes. Of 5232 older adults, 76.3% of subjects were non-participants, 3.4% were participants with burden, and 20.3% were participants without burden. Multivariate analysis showed that non-participants were more likely to have poor health outcomes compared with participants with burden. Moreover, participants without burden were more likely to have better health outcomes (SRH: Odds Ratio [OR] = 1.92, 95% Confidence Interval [CI] = 1.70 to 2.17, WHO-5: OR = 1.69, 95% CI = 1.51 to 1.88, TMIG-IC: Coefficient = 0.36, 95% CI = 0.10 to 0.62). Our findings suggest that volunteer activity is related to better health regardless of their burden. However, burden of volunteer engagement might attenuate the relationships between volunteer activity and health outcomes.

### GERI-VET: A PROGRAM FOR OLDER VETERANS SEEN IN THE EMERGENCY ROOM

Denise M. Kresevic,[1] Albert Lee,[2] Mustafa Ascha,[2] and Todd Smith[2], *1. Northeast Ohio VAMC, Cleveland, Ohio, United States, 2. Northeast Ohio VA Health System, Cleveland, Ohio, United States*

Older adults made over 21 million Emergency Room (ER) visits accounting for nearly 46% of all ER hospital admissions in 2015. The ER setting provides not only a unique opportunity to assess patients' health, functional status and social issues, but also provide recommendations to help coordinate care. Geriatric ER assessments have been associated with reduced avoidable hospitalizations, functional decline, and institutionalization. However, few ER clinicians including physicians, nurses and technicians have received adequate training to perform geriatric screenings and implement timely referrals. In 2014 American College of Emergency physicians and American Geriatric Society published guidelines for care. Based on these guidelines A" Geri-Vet Bootcamp" Program was developed and piloted at the Northeast Ohio VAMC. This program included: simulation emphasizing standardized screenings, and the use of decision support aides for management and referrals for older adults seen in the ER. Following this multi-modal education program, 91% clinicians reported greater ability to apply knowledge learned, 82% clinicians were able to more accurately identify geriatric syndromes, and 86% were able to identify additional resources. Of the patients screened over one year, 73% of patients were identified as being at high risk for falls, 32% had high family caregiver burden, 15% had moderate to severe dementia, and 14% had positive delirium screens. Those veterans screened by Geri Vet trained Staff received significantly more referrals than usual care staff, home care 28.7% vs.15.6%, geriatric clinic 20.5% vs. 11.7% and caregiver support 5.0% vs. 1.3%. Data show hospital admissions have decreased 5-7%. Education and dissemination continues

### DOES PARTICIPATING IN SUPPLEMENTAL NUTRITION ASSISTANCE PROGRAM IMPROVE THE SLEEP OF OLDER ADULTS?

Queendaleen C. Chukwurah,[1] and M. Pia Chaparro[2], *1. Tulane Center for Aging, Tulane University, New Orleans, Louisiana, United States*

*2. Department of Global Community Health Behavioral Sciences, Tulane University, New Orleans, Louisiana, United States*

Food insecurity among lower income adults is associated with adverse health outcomes including sleep disorders. Using data from the National Health and Nutrition Examination Survey (NHANES), 2005-2008, we explored the association between participation in the Supplemental Nutrition Assistance Program (SNAP), a safety net program addressing food insecurity, and sleep outcomes among older adults. Total sleep duration (<7 hours inadequate sleep, ≥7 hours adequate sleep) and sleep latency (10-20 mins normal sleep latency, ≤ 9 mins and ≥ 21mins abnormal sleep latency) were available for 805 participants 50 years and older and eligible for SNAP participation (≤130% federal poverty level). SNAP participation (yes/no) was assessed for the previous year. Sleep inadequacy was higher among SNAP participants (46.5%) compared to SNAP-eligible non-participants (36.3%), whereas the corresponding numbers for abnormal sleep latency were 71.8% and 64.1%. Binary logistic regression analysis showed no significant association between SNAP participation and inadequate sleep duration (OR=1.19, 95% CI 0.84-1.70) after adjusting for age, gender, race/ethnicity, education, and food security status. SNAP participation was also not associated with abnormal sleep latency (adjusted OR=1.49, 95% CI 1.00-2.23). SNAP participation was not protective against poor sleep outcomes among a sample of low-income, at risk of food insecurity older adults.

### AN ACADEMIC-COMMUNITY PARTNERSHIP FOR IMPROVING ORAL HEALTH OF ADULTS 75-PLUS YEARS IN RURAL SOUTHEASTERN OREGON

tamara rose,[1] and darcy mize[1], *1. Oregon Health & Science University Regional Campus, Klamath Falls, Oregon, United States*

This presentation describes a series of research projects undertaken by a school of nursing to develop and test a model for interprofessional (IPE) practice and education. A pilot study and two funded projects have been completed and one funded project is currently underway. A goal has been to build an academic-community partnership to address health disparities for people 75 years and older living in rural areas of the state. This population is growing and more apt to be living in poverty. Low oral health literacy and limited dental health services in rural areas contribute to the likelihood of oral health problems impacting overall health. IPE supports the development of teamwork to improve health outcomes for patients by raising awareness about the relationship of oral health to overall health. This evolving IPE model has joined baccalaureate nursing and dental hygiene students in shared learning and practice. After classroom and simulation-based learning, student teams provided screening and education in primary care settings. The current study has expanded team membership by adding medical, dental and/or nutrition students. They are training and practicing together to be a "mobile oral health team" for older adults living in a rural community 100 miles from campus. Community stakeholders are helping to organize a day-long oral health screening clinic. Looking forward, this IPE model supported by an academic-community partnership will routinely visit chronically underserved areas with a mobile oral health team. Funding for a fully-equipped mobile unit are being sought to sustain this effort.

### STRATEGIES TO INCREASE MINORITY PARTICIPATION IN A HEALTHY AGING AND NEIGHBORHOOD STUDY

Linda C. Churchill,[1] Rachel Siden,[1] Annabella Aguirre,[1] Marline Ruiz,[1] Jie Cheng,[1] Anthony Clarke,[1] Kevin Kane,[1] and Wenjun Li[1], *1. University of Massachusetts Medical School, Worcester, Massachusetts, United States*

Adequate minority participation is critical to health disparity research. Conventional direct mails are less effective in minority recruitment. The Healthy Aging and Neighborhood Study developed a multifaceted, community-engaged, culturally and linguistically appropriate method to recruit community-living older adults (≥65y) in Worcester County, Massachusetts. The research team is bilingual, racially and culturally diverse. A direct mail campaign was conducted in a geographically diverse random sample of residents from neighborhoods with high concentrations of minorities, stratified by rurality. To increase minority participation, the mailings included an invitational letter or a flyer with a graphic that portrays diverse racial/ethnic background. We engaged communities by presenting the study at senior and community centers, and faith-based organizations that are frequented by minorities and by posting study information in minority social media groups (e.g., Chinese resident associations). Participants promoted the study to friends while staff promoted through professional or social networks. To recruit non-English speaking minorities, all materials were printed in age-friendly large fonts in Spanish or Chinese, and interviews were conducted using their preferred language. Within 9 months, we enrolled 326 participants, including 216 Whites, 57 Hispanics, 21 Blacks, 31 Asians, and 1 Native American. An additional 38 Asians are on the waiting list. Blacks were more likely to respond to community presentations. Hispanics were most likely to respond to the colorful flyer. Older minorities (>76y) were more likely to respond to presentations (57%) while the younger (<75y) to the mailings (60%). In summary, this multifaceted recruitment approach is effective in minority recruitment.

### GERIATRICS PREVENTABLE ADMISSIONS CARE TEAM (GERIPACT): A HIGH-RISK INTENSIVE AMBULATORY GERIATRICS PROGRAM

Stephanie W. Chow,[1] Lizette Munoz,[2] Susana Lavayen,[2] Shamsi Fani,[2] Blair MacKenzie,[2] and Audrey Chun[1], *1. Icahn School of Medicine at Mount Sinai, New york, New York, United States, 2. Mount Sinai Hospital, New York, New York, United States*

The Geriatrics Preventable Admissions Care Team (GERIPACT) is an inter-professional team of 2 clinicians, 1 social worker, and 1 care coordinator, dedicated to offering temporary intensive ambulatory care services to complex older patients at high-risk for incurring expensive health care (ie. frequent emergency room visits or hospitalizations). GERIPACT services include frequent office visits for medical and social work needs, frequent telephone contact, home visits, specialty visit accompaniment, and a 24/7 telephone hotline. Use of this innovative model aims to serve communities lacking in geriatrician and geriatric social work providers, with a main goal of serving the highest risk older population. We reviewed the healthcare utilization of GERIPACT enrollees 6 months prior-to-enrollment and compared with 6 months following graduation from GERIPACT from 2016 to 2018. 78 patients were evaluated, with 49 total ED visits prior to enrollment and 35 post-graduation, saving 14 ED visits for a ratio of 18 saved ED visits per 100 GERIPACT patients. There were 45 hospitalizations prior to enrollment with 29 hospitalizations post-graduation, saving 16 hospitalizations, or 20 hospitalizations per 100 GERIPACT patients. Hospital days were reduced by 237 days post-graduation. An intensive ambulatory program for high risk geriatrics patients may be shown to be an efficient model of care for targeting those older patients who potentially incur greater expenses to the health care system. This focused team may be deployed to primary care communities with complex elderly patients in need of geriatricians and geriatric social workers, and may reduce unnecessary emergency room visits and inpatient stays.

### ORAL FRAILTY IS ASSOCIATED WITH MULTIFACETED FRAILTY IN ELDERLY OUTPATIENTS AT COMMUNITY DENTAL CLINICS

Misa Nishimoto,[1] Tomoki Tanaka,[2] Yutaka Watanabe,[3] Hirohiko Hirano,[3] Takeshi Kikutani,[4] Tetsuro Sato,[5] Kazuko Nakajo,[6] and Katsuya Iijima[1], *1. Institute of Gerontology, The University of Tokyo, Tokyo, Japan, 2. Institute of Gerontology, The University of Tokyo, Japan, Tokyo, Japan, 3. Tokyo Metropolitan Institute of Gerontology, Tokyo, Japan, 4. Division of Clinical Oral Rehabilitation, Nippon Dental University Graduate School of Life Dentistry at Tokyo, Tokyo, Japan, 5. Kanagawa Dental Association, Kanagawa, Japan, 6. Kanagawa Prefectural Government, Japan, Kanagawa, Japan*

Aim For achieving healthy aging, frailty prevention is essential. Because it is reported that accumulated declines in multiple oral functions (i.e. oral frailty) could lead to frailty progression, detailed countermeasures for oral frailty are currently required. However, dentists of community dental clinics don't even know a prevalence of oral frailty among outpatients. Thus, we aimed to identify the prevalence of oral frailty and to examine the association with frailty in outpatients at community dental clinics. Methods The subjects were elderly outpatients at dental clinics in Kanagawa, Japan. Frailty was assessed using the Kihon checklists (KCL); those with ≥8 KCL score were classified as frailty. Furthermore, multiple functions (physical, nutrition, and oral) were assessed using subscale of the KCL. Oral frailty was defined as ≥3 deteriorations out of 5 oral status (remaining teeth, chewing ability, articulatory oral motor skill, subjective difficulties in eating and swallowing). Results Of 1,699 outpatients (mean age, 75 ± 6.3 years old; 40% men), 12% were frailty and 21% were oral frailty. When adjusted by confounding factors such as age and sex, those with oral frailty were associated with higher prevalence of frailty (OR, 3.25; 95%CI, 2.34-4.53), decreased physical and oral functions (OR, 1.53; 95%CI, 1.07-2.16: OR, 8.14; 95%CI, 6.05-10.95, respectively). Conclusions Oral frailty was associated with multi-faceted frailty in outpatients at community dental clinics. In addition to the importance of maintenance of whole oral functions including treating teeth, our findings suggest that it is also indispensable to consider the multi-faceted frailty for elderly patients.

### LIFE SATISFACTION AND SELF-PERCEPTION OF AGING IN CONTRIBUTING TO SUCCESSFUL AGING IN A CHINESE CONTEXT

Sunny H.W. Chan,[1] Yao Pan,[2] Yuebin Xu,[2] and Ka Ching Yeung[3], *1. The Hong Kong Polytechnic University, Hong Kong, Hong Kong, 2. Beijing Normal University, Beijing, China, 3. The University of Hong Kong, Hong Kong, Hong Kong*

Research indicated that life satisfaction and a positive self-perception of aging are vital during the aging process which contribute significantly to successful aging. Discovering the underlying determinants should provide a novel insight into the mechanism involved in achieving successful aging. A representative random sample of 2161 Chinese older people aged 60 years or above was surveyed by face-to-face interview. Sociodemographic factors were measured by age and educational level. Physical functioning was identified in terms of self-perceived health, basic and instrumental activities of daily living, and number of chronic illnesses. Social functioning was characterized in terms of number of people living together, social support network, and sense of loneliness. Hierarchical multiple linear regressions were performed to identify significant determinants of life satisfaction and self-perception of aging. Results showed that people in older age with lower educational level had a higher level of life satisfaction; whereas people in younger age with higher educational level had a more positive self-perception of aging. Moreover, social functioning took precedence over physical functioning in contributing to life satisfaction. In contrast, physical functioning outweighed social functioning in promoting a positive self-perception of aging. A sense of companionship and a supportive social network are imperative in enhancing life satisfaction, whereas perceived physical health and functional independence are essential in facilitating a positive self-perception of aging. This study provides empirical support to improve understanding of the primary mechanism of achieving successful aging. It also lays important groundwork for future tailored-made interventions for promoting successful aging in a Chinese context.

### THE IMPACT OF SOCIOECONOMIC STATUS ON AGE TRAJECTORY OF BODY MASS INDEX AMONG MIDDLE-AGED AND OLDER ADULTS

YU-CHUN LIN,[1] and YU-HUNG CHANG[2], *1. China Medical University Hospital, Taichung, Taiwan, 2. China Medical University, Taichung City, Taiwan*

Body weight tends to decrease along with age. Weight loss and low body mass index (BMI) in the elderly, associated with socioeconomic status, are both strong predictors of subsequent mortality.This study aims to investigate the relation between income and BMI changes in later life. We used data from the Taiwan Longitudinal Study in Aging (TLSA) from 1999 to 2007. There were 5,131 participants aged 50 and over, who were excluded for those without primary study variables. Income was evaluated by asking the amount of annual income, including salary, pension, rent, interest, welfare benefit, etc. Participants' BMI were assessed in each survey. General estimating equation models were performed to examine the association between age, annual income, and their interaction with BMI adjusting for covariates including sex, education, marital status, smoking, exercise frequency, appetite, and number of comorbidities. Totaling 11,350 person-times was in three follow-up surveys, which left 9,723 person-times of observations after exclusion. After adjusted for covariates, the low income group compared to the higher income, would have higher estimated BMI at age of 50 (BMI= 24.75 kg/m2 and 24.19 kg/m2 respectively), and more rapid reduction (-0.08 kg/m2 per year), while relatively stable BMI was found in higher income group (0.01 kg/m2 per year, slope difference= 0.10 kg/m2 per year, 95% confidence interval [CI] = 0.03-0.17). In conclusion, compared to invariable BMI observed among individuals with higher financial status, the economically disadvantaged experienced BMI decline with age among middle-aged and older adults.

### LIFE HABITS AND LIPID PEROXIDATION OF WOMEN OVER 40 YEARS OLD

Renata F. Oliveira,[1] Maria Mota,[2] Jorge Soares,[2] Zirlene Santos,[2] Bianca Rosa,[3] Caroline Santana,[3] Dalton Pessoa-Filho,[4] and Cassiano M. Neiva[4], *1. Universidade de Trás-os-Montes e Alto Douro, Vila Real, Portugal, Portugal, 2. UTAD, Vila Real, Portugal, Portugal, 3. UNIFENAS, Divinópolis, Minas Gerais, Brazil, 4. UNESP, Bauru, São Paulo, Brazil*

Oxidative stress is involved in degenerative processes, aging, and diseases. Lifestyle can be change oxidative stress. One of reactive oxygen species targets is polyunsaturated fatty acid, an important cellular membrane component. The aim of this study is to analyse the contribute of lifestyle in lipid peroxidation in over fourty years old women. Were included 60 women whith age ranged between 41 and 82 years old (53.3 ± 9.1 years). Lifestyle were explored doing a food frequency questionnaire, Perceptive Stress Scale (PSS) and International Physical Activity Questionnaire (IPAQ). The functional capacity was determinate by 6-min walking test (cardiorespiratory capacity) and Squat-jump (leg strength and power). Parameters of oxidative stress were determinate from plasma during fasting, lipid peroxidation was evaluated by TBARs, and antioxidant capacity was evaluated by catalase activity and ABTS. Spearman correlation and Multiple Linear Regression model, through Stepwise method, considering TBARs as dependent variable, and age, weight, body mass index, waist circumference, stress perception, physical activity level, total antioxidant capacity, catalase activity, cardiorespiratory capacity, leg strength and power, daily caloric intake, daily fruit, vegetables, coffee/tea, vitamin E and alcohol intake, as independent variable, was performed. Negative correlations were obtained between TBARs and cardiorespiratory capacity (r= -0.35; p=0.026) and between TBARS and ABTS (r= -0.33; p=0.038). Total antioxidant capacity was the model's first variable (F= 5.50; p = 0.013), explaining 15.3% of TBARS, then cardiorespiratory capacity (F= 5.50; p = 0.047), explaining 10.5% of TBARs The results revealed total antioxidant capacity and cardiorespiratory capacity as predictors to lipid peroxidation damage.

### INDIRECT EFFECT OF PARTICIPATION ON THE ASSOCIATION BETWEEN FUNCTIONAL VISION AND SELF-RATED HEALTH OF OLDER ADULTS

Bernard A. Steinman,[1] and Casandra Mittlieder[1], *1. University of Wyoming, Laramie, Wyoming, United States*

Older adults with visual impairments experience barriers to participation that could impact their own health and wellbeing. Participation limitations that are associated with poor vision and unsupportive environments have been associated with objective health outcomes, including chronic disease and secondary outcomes such as fall injuries. In this study, we assessed the association between functional vision impairment and self-rated health (SRH). We also tested the mediating role of participation in that relationship. We conducted analysis with covariates representing six domains included in the International Classification of Functioning, Disability, and Health (ICF). We computed ordinal logistic regression models using two waves (2011 and 2016) of the National Health and Aging Trends Study (NHATS). Vision status was assessed via self-reported measures of visual functioning (e.g., ability to recognize someone across the street). "Participation" was operationalized using 6 items that assessed participation in social and community-based activities (e.g., visiting friends, participating in classes). SRH (measured in 2016) was assessed using a single item asking participants to rate their health on a scale ranging from excellent to poor. We assessed key relationships while holding ICF's other health dimensions constant. Functional vision status was statistically associated with SRH in models containing all covariates. Participation variables reduced but did not eliminate the effects of vision, suggesting a partial mediating effect—that is, part of the association between vision and SRH was explained by participation factors. These results point to the importance of developing community support and reducing barriers to participation by older adults with functional vision impairments.

### FOOD ASSISTANCE PROGRAMS AND REDUCED HOSPITALIZATIONS FOR OLDER ADULTS WITH DIABETES

Monyca L. Johnson,[1] and Sarah E. Walsh[1], *1. Eastern Michigan University, Ypsilanti, Michigan, United States*

Diabetes is a an increasingly common and costly condition for older adults. Each year, as many as 1 in 3 Medicare dollars is spent to treat and manage diabetes and associated comorbidities for people with diabetes. To control health care spending in the US, it is imperative that we identify factors for reducing hospitalizations for these individuals. We used data from round five of the National Health and Aging Trends Study to identify predictors of hospitalization in the past 12 months for Medicare recipients ages 65 and older with diabetes. Previous research on the social determinants of health has demonstrated that social stressors like poverty and exposure to racism are associated with poorer health outcomes overall, but we did not find a statistically-significant association between race, gender or Medicaid dual-eligibility and hospitalization for our study population. Notably, receipt of SNAP benefits, Meals on Wheels services or other food assistance was associated with a 43% reduction in the risk of hospitalization in the past 12 months. As previous research has linked food insecurity with poorer medication adherence among individuals with Type II diabetes, food assistance programs appear to be an effective strategy for reducing hospitalizations associated with diabetes and its comorbidities.

### USE OF COMPLEMENTARY THERAPIES BY SEXUAL MINORITY OLDER ADULTS: FINDINGS FROM THE NATIONAL HEALTH INTERVIEW SURVEY

Joel G. Anderson,[1] Jason D. Flatt,[2] Jennifer Jabson,[1] and Whitney Wharton[3], *1. University of Tennessee, Knoxville, Knoxville, Tennessee, United States, 2. University of California, San Francisco, San Francisco, California, United States, 3. Emory University, Atlanta, Georgia, United States*

Sexual minority (lesbian, gay, bisexual; LGB) older adults age 50+ experience a higher prevalence of chronic disease and disability, as well as a poorer physical and mental health status. Many adults use complementary and integrative therapies, particularly mind-body therapies, as health-enhancing approaches and to support wellbeing. However, no study to date has examined the use of mind-body therapies among sexual minority older adults. We examined data from the 2017 National Health Interview Survey to determine the use of mind-body therapies by sexual minority older adults (aged 50+), as well as the influence of health and wellbeing characteristics on mind-body therapy use, compared with their non-LGB counterparts. Sexual minority older adults overall reported higher usage (36%) of mind-body therapies compared with their non-LGB counterparts (22%), with lesbians reporting the highest use (41%). Sexual minority identity was a significant predictor of mind-body therapy use, with LGB adults roughly two times more likely to use a mind-body therapy after controlling for chronic disease status and other wellbeing measures. Future research is needed to explore the reasons sexual minority older adults use complementary and integrative therapies, as well as potential development of mind-body interventions targeted toward this population to address stress and quality of life.

### RACIAL DIFFERENCES IN CONTEMPLATIVE PRACTICES IN LATER LIFE: EXAMINING THE ROLE OF SES AND HEALTH DISADVANTAGE

Nirmala Lekhak,[1] Tirth Bhatta,[2] and Timothy Goler[3], *1. University of Nevada Las Vegas, Las Vegas, Nevada, United States, 2. UNLV, Las Vegas, Nevada, United States, 3. Case Western Reserve University, Cleveland, Ohio, United States*

Substantial scholarly attention has been placed on prayer as a buffer of life events' adverse influences on well-being in later life. The disproportionate distribution of adverse life events among Black adults has also attracted scholarly interest in racial differences in contemplative practices. Black adults have been found to more likely engage in private prayer than White adults, whereas studies have observed an opposite pattern for meditation. The contribution of stratification in socioeconomic status and health to racial differences in contemplative practices, especially in meditation has received relatively less attention. Drawing from a subsample from Health and Retirement Survey (N = 1102), this study takes a next necessary step to assess the contribution of socioeconomic status, multimorbidity, and depressive symptoms to racial differences in both prayer and meditation use in later life. Consistent with prior studies, the odds of engaging in private prayer (OR=2.78, p<0.01) was higher among Blacks than White older adults. Our findings of higher odds (OR=2.92, p<0.001) of meditation among Black older adults than White older adults, however, do not align with previous studies. The disadvantage in socioeconomic status, health, and psychological well-being completely explain racial differences in prayer, but this difference in meditation persist even after adjusting for those factors. Our findings call for further research on contextually influenced underlying individual motivations that drive older adults of different racial and social economic groups to engage in various contemplative practices. Further research is also warranted on how older adults, particularly Blacks differentiate between private prayer and meditation.

### IMPLEMENTING THE AGE-FRIENDLY HEALTH SYSTEM INTO CVSMINUTE CLINICS

Mary A. Dolansky,[1] and Anne pohnert[2], *1. Case Western Reserve University, Cleveland, Ohio, United States, 2. CVSMinute Clinic, Rhode Island, United States*

Each day, 10,000 individuals in the United States turn 65 and nearly 30% of our nation's 884 million ambulatory care visits involve patients ages 65 and older. With this trend, the use of convenient care settings for treatment for chronic conditions associated with aging such as diabetes and hypertension is emerging. The need for the delivery of age-friendly care in convenient care settings is essential to ensure high quality and safe care. CVS MinuteClinic® and the Institute for Healthcare Improvement (IHI) will present the plan and preliminary findings for the extension of the IHI's Age-Friendly Health Systems initiative into the CVS MinuteClinic. The Age-Friendly Health System model includes 4 M's: What Matters, Medications, Mentation, and Mobility as essential components for care delivery. The presentation will include (1) the partnership with the IHI's Age-Friendly Health System Action Community, (2) a demonstration of the Age-friendly Virtual Clinic and learning platform that includes training modules and practice-based tools to facilitate assessment and action steps for the implementation of the 4Ms based on Age-Friendly Health System content, and (3) the evaluation logic model that will be implemented. The implementation plan will provide the framework to embed age-friendly process and outcome measures into the CVS MinuteClinic electronic health record platform, Epic. The project will impact all 1,100 CVS MinuteClinic locations across the nation.

### SESSION 900 (POSTER)

### INTERDISCIPLINARY EDUCATION IN GERONTOLOGY AND GERIATRICS

### SIGNIFICANT PREDICTORS OF INTEREST IN GERIATRICS AMONG LOW, MIDDLE, AND HIGH INCOME COUNTRIES

Grace Karikari,[1] David K. Lohrmann,[1] and Lesa L. Huber[1], *1. Indiana University School of Public Health, Bloomington, Indiana, United States*

Even though the demands for physicians with geriatric related specialties are higher than the available experts around the world, some countries and economies seem to be more impacted by the workforce shortage than others. This systematic review consolidated scientific evidence reporting the significant predictors of interest in pursuing a geriatric career among medical students' in different countries. A 20-year (1998 – 2018) systematic literature review of high-quality primary research articles was conducted using PubMed, ERIC (EBSCO), Embase and Cochrane Library. Eleven articles (n =11) met the eligibility and quality assessment criteria. For analysis, studies were categorized into (i) low- and middle-income countries and (ii) high income countries, based on the WHO and the World Bank's income grouping for the 2019 fiscal year. Medical students involved in this review were n = 1,683 representing students from different fields of medicine and year groups. The two most significant predictors of interest in geriatrics in the high-income countries were (1) positive attitudes towards the elderly and (ii) participation in a geriatric related intervention. Lack of published peer-reviewed articles from the low and middle-income countries limited the researchers' ability to evaluate the similarities and differences between the two income groups. There is an overall need to stimulate interest in geriatric specialization among medical students. Leaders of the LMICs should invest in geriatric education and research in order to promote interest in the field, increase the geriatric workforce, and ultimately, improve the quality of life of the elderly in their respective countries

### DEVELOPMENT AND IMPLEMENTATION OF AN INTERDISCIPLINARY HONORS COURSE ON AGING

Mary C. DiBartolo[1], *1. Salisbury University, Salisbury, Maryland, United States*

With the aging of Baby Boomers, 2030 will mark the first time in U.S. history that those aged 65 and older will outnumber children. This population shift is expected to place unprecedented demands on the healthcare system in terms of both volume and complexity of care. Given these population shifts and emphasis on an interdisciplinary approach to care, a 4-credit Honors aging course was developed for Honors students in nursing and other health-related majors. Aging Reexamined, Reimagined is offered in a discussion/seminar format with limited enrollment to allow for deep reflective discourse about pertinent issues affecting older adults. Topics include physical/cognitive changes, ageism, Alzheimer's disease, sexuality, aging in place, polypharmacy, addiction, depression, caregiving, elder justice, and end-of-life care. Guest speakers share their expertise on selected issues, otherwise students alternate leading discussions on remaining topics. There are three focused reflections on assigned experiences which include conducting a videotaped interview with a retired community-based older adult, attending a support group or senior center activity, and visiting a center to view various physical/technological adaptive aids that maintain mobility and independence in the home. There is also a culminating research paper on an issue of their choice. Student evaluations are overwhelmingly positive; comments include gaining in-depth knowledge about the unique needs of this population and the importance of healthy aging with an emphasis on a positive, inter-professional approach to care. It is incumbent upon educators to better prepare students to recognize ageist attitudes, as well as address the significant impact of this "longevity revolution".

### THE IMPACT OF AN INTERPROFESSIONAL SIMULATION ON TEAM COMMUNICATION AT THE END OF LIFE

Anne Halli-Tierney,[1] Megan E. Lippe,[1] Alexandra Stanley,[1] Allison Ricamato,[1] and Robert E. McKinney[1], *1. University of Alabama, Tuscaloosa, Alabama, United States*

It is increasingly important that healthcare professionals understand how to care for patients at the end of life, especially as 75% of hospital deaths occur in the geriatric population. Many new healthcare profession graduates feel under-prepared to provide end-of-life care. Healthcare education programs must provide education on interprofessional communication and end-of-life care. This study explored the effects of an interprofessional end-of-life simulation on medical, social work, and nursing students' communication skills. Across three simulation phases, teams were required to communicate with the patient (high-fidelity manikin), family members (scripted actors), and team members about treatment option, change in patient's condition, and withdrawal of life-sustaining measures. A total of 16 teams participated in the simulation, with each team comprised of nursing students (n=7-8), social work students (n=1), and medical students or residents (n=1). Team communication skills were assessed using the Gap Kalamazoo Communication Skills Assessment Form. The average communication score for teams reflected fair to good communication (M=28.81, SD=5.55). The best communication domains were "Shares information" (n=8), "Communicates accurate information" (n=6), and "Builds a relationship" (n=5). The worst domains were "Demonstrates empathy" (n=7), "Provides closure" (n=6), and "Builds a relationship" (n=5). This simulation provides an interactive educational mechanism by which to educate interprofessional healthcare students on communication and care of patients at the end of life.

### AGING IS VERY PERSONAL: INTERPROFESSIONAL GERONTOLOGY COURSE IMPROVING STUDENT ATTITUDES TOWARD OLDER ADULTS

Jason T. Garbarino[1], *1. University of Vermont, Burlington, Vermont, United States*

Educational programs that foster the development of a robust healthcare workforce committed to the provision of exemplary care of older adults is vital. The Aging is Very Personal (AIVP) service learning gerontology course has demonstrated the ability to foster future student interest and improved attitudes towards working with older adults. The AIVP program provides mutual benefit for undergraduate students from a variety of health science majors and older adult resident volunteers at local senior living facilities. For students, AIVP serves as direct insight into the lived experience of aging among community older adults. Students are provided the opportunity to practice communication skills, relationship-building skills, and gain an understanding of the multitude of diverse needs within this population. Older adults who volunteer to participate in the activity are provided with the opportunity to speak to and actively engage with students and feel empowered by the opportunity to provide valuable life guidance. This presentation will provide a curricular overview of the steps required to construct, implement, and evaluate an interprofessional gerontology course. A review of student learning objectives, service-learning program construction, selected course topics, and student assignments will be presented. Attitudes and future interest in working with older adults measured in the initial interprofessional student cohort (n=106) will be presented. A pre-established, validated tool utilized to effectively measure student attitudes and interest pre/post-course participation will be reviewed.

### GAINING INTERPROFESSIONAL COMPETENCE THROUGH A GERIATRICS CASE CONFERENCE

Amber S. McIlwain,[1] Danielle Backus,[1]
Kristine Marcus,[1] and Jeff Fortner[1], *1. Pacific University, Hillsboro, Oregon, United States*

There is increased demand to provide health professions students with interprofessional education and practice experience. Interprofessional Case Conferences (ICC) allow students to work in teams to learn about different professions while exploring a topic through the lens of an interprofessional core competency. The goal of this particular ICC was to provide students an experience to witness and discuss team-based, person-centered care for a common geriatric disorder. The case was designed to develop the interprofessional competency of teams and teamwork. Students were divided into teams and observed live vignettes of a care conference involving an 80-year old female admitted to a rehabilitation facility following a hip fracture and replacement. Students witnessed how patients, families, and healthcare providers work together during a stressful time. After each vignette, students discussed questions related to miscommunications, motivations of the different actors involved, and how the healthcare team should respond. In the first offering, 93 students participated, increasing to 150 in the next year. Students completed a post-survey to determine if the session delivered a positive interprofessional experience. The average positive response rate was 92.5% (92-94%, n = 53) in year-1 and 93.5% (90-97%, n = 71) in year-2. By allowing students to witness a simulated live care conference, they had a tangible event to dissect instead of discussing hypotheticals. By discussing a geriatrics case in a rehabilitation setting, students witnessed how numerous healthcare professions coordinate care for a patient and her family, thereby demonstrating competence in teamwork.

### IMPACT OF GERIATRIC INTERPROFESSIONAL TRAINING ON ADVANCE CARE PLANNING IN GERIATRIC PRIMARY CARE

Maureen E. Barrientos,[1] Anna Chodos,[1] Alicia Neumann,[1] Yvonne Troya,[2] and Pei Chen[1], *1. University of California San Francisco, San Francisco, California, United States, 2. University of California Hastings College of the Law, San Francisco, California, United States*

Currently, an important measure of Advance Care Planning (ACP), Advance Health Care Directives (AHCD) documentation rate, is at 33% for older adults in the United States. To address this disparity, geriatric faculty in an academic geriatric primary care practice aimed to train geriatrics fellows and other interprofessional (IP) learners to engage patients in ACP. As part of a Geriatric Workforce Enhancement Program funded by the Health Resources and Services Administration, geriatrics faculty and the Medical Legal Partnership for Seniors based at University of California Hastings College of Law provided ACP training to fellows and IP learners, including social work interns. In practice, the fellows and social work interns collaborated to incorporate ACP into patient visits and follow-up telephone calls. To monitor ACP progress, research staff reviewed patients' electronic health records and performed descriptive analysis of the data. In 21 months, 4 geriatrics fellows built a panel of 59 patients who on average had 3 office visits and 7 telephone calls per person. Prior to clinic enrollment, 12 (20.3%) patients had preexisting AHCD, and 47 lacked AHCD documentation. After ACP intervention, 42 of 47 patients without AHCD documentation engaged in ACP discussion. Of those who engaged in ACP discussion, 24 completed AHCD, raising AHCD completion rate to 61%, or 36 patients in the panel of 59. ACP is a complex process that benefits from skilled communication among interprofessional providers and patients. Findings underscore the potential advantages of IP training and engaging patients in ACP discussion in an academic primary care setting.

### PILOTING AN INTERPROFESSIONAL GERIATRICS COURSE FOCUSED ON SYSTEMS-BASED PRACTICE

Jennifer J. Severance,[1] and Sarah Ross[1], *1. University of North Texas Health Science Center, Fort Worth, Texas, United States*

Health professionals must be able to work together in the care of older adults to provide competent, value-based care within dynamic healthcare systems. Previous studies cite the need for health professions education addressing systems-based competencies and the interconnected components of health and aging services. With an aim to provide a foundation for systems-based interprofessional practice in the care of older adults, an academic medical center piloted a competency-based curriculum introducing different settings of care for older adults and exploring special topics in geriatric patient care and gerontology. Applying a framework of health and aging services, interprofessional faculty developed a curriculum delivered over six one-hour sessions during the Spring 2019 semester. Session topics included dementia care, end-of-life care, medication safety, activity, and financial and regulatory considerations across the continuum of care. Teaching strategies included didactics, case-based discussions, online reflection questions, shadowing of geriatric encounters in clinic and long-term care settings, and student presentations of a patient case. Post-session evaluation surveys were administered electronically to measure knowledge, skills and confidence using Likert-scale and open-ended questions. Eleven health professions students (medical [n=6], physician assistant [n=3], physical therapy [n=2]) participated. Preliminary findings show improved knowledge about healthcare and community-based options for older adults, value-based care, and the driving forces affecting health care delivery. Analysis of qualitative feedback identified key themes related to teaching methods, course materials, and course improvement opportunities. Interprofessional faculty collaboration can lead to quality health professions student training and increase competency in systems-based practice in the care of older adults.

### PREPARING FUTURE AGING PROFESSIONALS FOR A RACIALLY, INTERDISCIPLINARY, AND INTERGENERATIONALLY DIVERSE WORKFORCE

Laura K. Donorfio,[1] Kathryn Elliott,[1] Phyllis A. Greenberg,[2] Rona J. Karasik,[2] and Kyoko Kishimoto[2], *1. Minnesota State University, Mankato, Minnesota, United States, 2. St. Cloud State University, St. Cloud, Minnesota, United States*

Workforce development is a growing concern for educators and agencies serving older adults as both workers and aging clientele become more diverse (Obhi, et al., 2017; Spetz et al., 2015). Teaching courses on aging to traditionally-aged college students brings to light ageism, the continued need for cultural competence, and intergenerational experience. Typical pedagogical approaches of lecture and discussion only prepare future aging professionals for what Bloom (1956) calls the "cognitive domain". Absent is the "affective domain" - how students interpret aging, the lifespan, and diversity in all its forms. For example, issues of power in the aging experience need to be analyzed to understand root causes of racial disparities, as well as the impact of past, current, and future policies. Preparing students for an intergenerational workforce is also essential, as there can be upwards to five generations working in paid positions together. With each generation comes differences in workplace expectations, modes of interacting (e.g., communicating, pace), and the impact of societal policies (e.g., institutional racism, ageism). Drawing upon the multiple disciplinary perspectives of its authors, this poster outlines the most prevalent differences among and between workforce generations, and identifies methods of pedagogy, andragogy, and geragogy needed to prepare future generations of aging professionals. Successful strategies and tools are highlighted including various experiential learning techniques (e.g., service-learning, simulations, vignettes, and anti-racist pedagogy).

### AN INTERDISCIPLINARY PEDAGOGICAL APPROACH THAT HARNESSES COLLABORATIVE NETWORKS

Tobi A. Abramson,[1] and Martha Siegel[2], *1. New York City Department for the Aging, New York, New York, United States, 2. New York Institute of Technology, Old Westbury, New York, United States*

Opportunities abound in healthcare and non-healthcare for innovative pedagogy, products, and services to address the growth of the aging population and ageism. 'Aging-in-place' homes and healthcare settings, often ill-prepared to meet aging needs, require redesign to incorporate designs that are responsive to an older or disabled person's needs. This pedagogical approach used interprofessional/cross-disciplinary collaborative learning to incorporate a person-centered approach to developing evidence-based interior design solutions to transform homes, healthcare environments, and communities. This pedagogical collaboration built networks across disciplines, provided a unique learning environment by focusing on student learning through engagement of cross-discipline undergraduate and graduate students and external collaborators (older adults, healthcare systems). All students participated in the live project within and outside of the classroom utilizing print materials, didactic learning, experiential activities, research, technology, site visits, client interviewing, recording data, team meetings, and designing and presenting a solution to a panel of judges. Assessments indicated students' appreciation of the project for their professional development and an enhanced understanding of teamwork strategies and cross-disciplinary classrooms for interprofessional learning. This pedagogical approach broke down academic silos and deserves more attention in academia to prepare students with the skills to work with an aging population. As the population ages, it is essential that college courses include approaches to decrease ageism and lead to the building of the workforce. Collaborative learning builds the foundation and networks that will help emerging professionals meet the needs of an aging population.

### SESSION 905 (POSTER)

### INTERGENERATIONAL ARRANGEMENTS AND PROGRAMS

### ADULT CHILDREN AND THEIR BOOMER PARENTS: THE DYNAMICS OF INTERGENERATIONAL LIVING ARRANGEMENT AND LIFE EVENTS

Chiu-hua Huang,[1] and Ju-ping Lin[2], *1. Shih Chien University, Taipei, Taiwan, 2. National Taiwan Normal University, Taipei, Taiwan*

Taiwan became an aged society in 2018. As Baby Boomers enter late life, relationships with family members gain importance.This research aimed to examine the intergenerational living arrangement between adult children and their baby boomer parents. Taking the perspectives of adult children, five waves of data (2008, 2009, 2010, 2011, and 2012) from the Panel Study of Family Dynamics (PSFD) were analyzed. Latent class growth analysis (LCGA) was used to develop changes types of intergenerational living arrangements. Focus on the life events of the two generations, this research examined the effect on intergenerational living arrangements changes pattern. The main findings are as follow. First, The proportion of intergenerational co-residence is high, especially between adult sons and their parents. There are different types of changes of intergenerational living arrangements. The four types of changes of intergenerational living arrangements for adult sons and daughters are the same: "continuous co-residence," "continuous non-co-residence," "from co-residence to non-co-residence," and "from non-co-residence to co-residence." Second, Adult children's life events such as getting married and having children affect changes of intergenerational living arrangements. After marrying, the intergenerational living arrangement between adult children and their parents is inclined to be the "continuous non- co-residence" type. When adult sons have newborn babies, the living arrangement is inclined to be "from co-residence to non-co-residence."

### AGING IN RURAL ETHIOPIA: IMPACT ON FILIAL RESPONSIBILITY AND INTERGENERATIONAL SOLIDARITY

Margaret E. Adamek,[1] Messay Gebremariam,[2] and Abraham Zelalem[3], *1. Indiana University School of Social Work, Indianapolis, Indiana, United States, 2. Addis Ababa University, Addis Ababa, Ethiopia, Ethiopia, 3. Ambo University, Ambo, Ethiopia, Ethiopia*

As migration and urbanization continue to progress in developing nations, the filial support and traditional support mechanisms that serve as buffers against the plight of older people are diminishing. Agrarian families find themselves in a rapidly changing world that severely limits their ability to assume caregiving roles for elders. With these trends in mind, a phenomenological approach was used to explore the lived experiences of 10 rural elders in Ethiopia. Prominent themes in the elders' narratives was the nostalgia of filial responsibility and intergenerational solidarity in the "good old days" and a strong sense of devaluation. Elders expressed feeling devalued by their children, grandchildren, and youth in general. As one elder shared, "These days, there is no respect for an older person. We are treated like a broken utensil thrown away which is considered as useless anymore." Compared to how they treated their own parents, elders believed that their children's sense of moral obligation was weak and unreliable. Interactions with children and grandchildren were described as abusive, undermining, and embarrassing, triggering deep sorrow. Elders were pessimistic about the prospect of reliable caregivers, even expressing a wish to die before they become dependent on others for care. Despite the challenges they face in their daily lives, all participants viewed aging as a privilege that should be celebrated. As traditional family support structures in developing nations continue to deteriorate, new models of community-based care are needed to ensure that elders can expect adequate care throughout their lives.

### A PHENOMENOLOGICAL PERSPECTIVE OF INTERGENERATIONAL ENGAGEMENT, SOCIAL ISOLATION, AND AGE SEGREGATION

Andrew Steward,[1] and Kimberly McDevitt[1], *1. University of Denver, Denver, Colorado, United States*

Intergenerational engagement and its impact on social isolation is an area of critical importance within the broader topic of healthy aging. This phenomenological study involved individual qualitative interviews with older adults (60+ years of age without cognitive impairment). Participants (N=9) were recruited using fliers and referrals from assisted living staff. Purposive sampling was utilized, and researchers attempted to diversify the sample by selecting participants from multiple settings and cultural backgrounds. This study was guided by two overarching research aims: 1) To understand the nature of intergenerational engagement and its impact on reducing social isolation, and 2) To understand how intergenerational experiences have changed over time. Interviews were conducted by the study PI, lasted 60-minutes and took place in participants' homes or agreed-upon public settings. Two researchers (the PI and an MSW intern) participated in the data collection and analysis process, which helped strengthen inter-coder reliability. Two important themes emerged from the data. First, participants often described intergenerational engagement as stimulating and stated that they enjoy feeling "younger" or "childlike" when engaging with younger generations. Second, participants reflected that intergenerational connections were more naturally built into their communities as young people and that society today appears to be increasingly age-segregated. Further development of internalized ageism measures may lead to a better understanding of both the positive and negative aspects of internalized ageism. Future studies should also explore, from the perspective of older adults, recommendations for re-building intentionally intergenerational communities that facilitate authentic engagement and enhanced well-being for all generations.

### EXAMINING THE SOCIAL AND TECHNOLOGICAL BENEFITS OF AN INTERGENERATIONAL PROGRAM FOR OLDER ADULTS

Skye N. Leedahl,[1] and Itza Serrano[1], *1. University of Rhode Island, Kingston, Rhode Island, United States*

Intergenerational programs are increasingly being implemented across the country. Much of the research on these programs has shown beneficial outcomes for students related to reductions in aging stereotypes, but less in-depth research has been done examining older adult outcomes (see Andreoletti & Howard, 2016 for a review). Currently, faculty and students from the University of Rhode Island (URI) are implementing the Engaging Generations: Cyber-Seniors Program, which connects older adults with student mentors to provide technological support to older adults, help students build communication and teaching skills, and overall build positive intergenerational relations. The aim of this study was to evaluate the outcomes of intergenerational programming participation for older adults (N=41) related to social isolation, loneliness, social engagement, and digital competency, measured using pre and post surveys given to participants. SPSS software was used to conduct descriptive analysis, t Tests, and one-way ANOVAs. Thematic analysis was used to analyze open-ended participant responses. Results showed significant improvements on items of the digital competency scale, particularly in relation to social media and for those who started with lower levels of digital competence, but not for other measures. Qualitative analysis showed that the older adults valued the technological knowledge gained, and the pleasant interactions and pedagogy. This study advances the engagement theory principle of problem-based learning. Much can be learned from both the significant and non-significant results to help inform intergenerational programming and technology support programs for older adults.

### UNDERSTANDING INTERGENERATIONALITY: THEORIES, REFLECTIONS, AND EXPERIENCES

Stephanie Hatzifilalithis,[1] and Amanda M. Grenier[2], *1. McMaster University, Ontario, Canada, 2. McMaster University, Health Aging and Society, Ontario, Canada, Canada*

Research into different aspects of intergenerationalities continues to develop at a considerable pace for individuals, communities, and society more generally. A number of programs and practices for older people are organized around the presumed benefits of intergenerational interaction between younger and older people, with intergenerational programming operating as a taken-for-granted practice. However, the merits of this approach, the models that inform practice, and the learning that takes place between older and younger people, remain under-theorized. This poster reviews and discusses dominant theoretical frameworks including reflections and experiences from intergenerational learning programs in Canada (e.g. Co-Housing). It documents how the field of intergenerationality is conceptualized and executed in the realms of theory and practice; how models retain age and stage-based assumptions, including the polarizing discourses of 'decline' and 'activity'; and discusses the implications for methodology, application, and outcome measures. By understanding the underlying assumptions utilized in the field of intergenerational learning, this poster makes an important contribution to the theoretical foundations, methods, and approaches, that are required to build more appropriate intergenerational landscapes.

### MORE THAN JUST A ROOM: RESULTS FROM AN INTERGENERATIONAL HOME SHARING PROGRAM IN TORONTO

Raza M. Mirza,[1] Laura Martinez,[1] Andrea Austen,[1] Lynn McDonald,[1] Christopher Klinger,[1] Jessica Hsieh,[1] and Tonya Salomons[1], *1. National Initiative for the Care of the Elderly (NICE), Toronto, Ontario, Canada*

Older adults prefer to live in their own homes for as long as possible — to 'age in place' — but for myriad reasons may be unable to do so. To address this, a number of housing alternatives have been explored, including homesharing, or homeshare, an exchange-based shared housing approach with the potential to empower older adults to age in place by enabling them to obtain additional income, companionship, and assistance with completing household tasks in exchange for renting out a room in their home. An intergenerational homesharing pilot program was launched in Toronto, matching older adults (55+) with postsecondary students. With limited research in the area, a mixed methods research study was embedded within the pilot project with the goals of: 1) conducting a scoping review to map and synthesize the literature related to outcomes of homeshare participation for this population, 2) conducting in-depth interviews with homeshare participants (N=22) to learn about their experiences, and 3) conduct a full evaluation and exit survey to better understand the implications of the project. Results were organized around the following themes: (1) benefits and challenges of participating in homeshare for older adults; (2) intergenerational engagement as social exchange; and (3) the key role of agency facilitation as a determinant of the experience of homesharing for older adults. Results spoke to the unique benefits and challenges of participating in homeshare for this population. Findings were used to derive implications for policy and practice, as well as highlight areas for future research.

### SESSION 910 (POSTER)

### LONG-TERM CARE: PRACTICES AND POLICIES

### HOME CARE NURSES CLAIM MEDICARE IGNORES SOCIAL DETERMINANTS OF HEALTH

William D. Cabin[1], *1. Temple University, Philadelphia, Pennsylvania, United States*

There is significant literature on the importance of addressing social determinants of health (SDOH) in order to improve health care outcomes. In response, the Centers for Medicare and Medicaid Services (CMS) has expanded Medicare Advantage plans ability to cover SDOH-related services. Medicare home health does not cover SDOH-related services. A literature review indicates no studies on the nature, significance, or impacts of the lack of SDOH coverage in Medicare home health. The current study is an initial, exploratory study to address the literature gap, based on interviews of a convenience sample of 37 home care nurses between January 2013 and May 2014 in the New York City metropolitan area. Results indicate nurses believe the lack of SDOH coverage in Medicare home health results in exacerbation of existing patient conditions; creation of new, additional patient conditions; increased home care readmissions and re-hospitalizations; increased caregiver burden; and exacerbation of patients' mental health and substance abuse needs.

### ADULT DAY SERVICES IN A RURAL REGION: CHALLENGES AND OPPORTUNITIES

Sandra S. Butler,[1] Jennifer Crittenden,[2] Dyan Walsh,[3] and Lenard Kaye[1], *1. University of Maine, Orono, Maine, United States, 2. UMaine Center on Aging, University of Maine, Bangor, Maine, United States, 3. Eastern Area Agency on Aging, Bangor, Maine, United States*

Adult day services (ADS) programs provide stimulation and socialization for older adults with cognitive and physical disabilities, and much needed respite for family caregivers. Like many services for older adults, ADS programs are far less available in rural regions of the country than in more urban settings. This paper reports on a needs assessment for an ADS program in a small city, which serves as a health and human services hub for a large rural area; a particular focus of the study was to assess the feasibility and interest in intergenerational programming. Family caregivers were surveyed (n = 84) about their use and knowledge of and interest in ADS. Less than one in five respondents were using or had ever used ADS. Cost (20%) and ignorance of such programs (20%) were primary reasons for not using ADS; reduction of stress was the most frequently cited reason for using ADS (73%). Ten in-person interviews were conducted with ADS program directors and service providers who refer clients to ADS. Funding issues emerged as the key challenge given lack of private insurance coverage and poor reimbursement levels from public insurance programs. Challenges around transportation, stigma, and marketing of services also surfaced in the interviews. Nonetheless, all ten informants spoke of the positive impact of ADS for both consumers and their caregivers, and generally endorsed intergenerational activities, though with caveats. Implications will be discussed, including the need for greater financial support for this valuable aspect of our long-term supports and services system.

### INS, OUTS, AND UNINTENDED CONSEQUENCES OF THE CMS NURSING HOME QUALITY MEASURES

Heather Davila,[1] Tetyana P. Shippee,[2] Young Shin Park,[2] Daniel Brauner,[3] and R Tamara Konetzka[3], *1. VA Boston Healthcare System, Boston, Massachusetts, United States, 2. University of Minnesota, Minneapolis, Minnesota, United States, 3. University of Chicago, Chicago, Illinois, United States*

Ongoing concerns about the quality of care provided to nursing home (NH) residents have led the federal government to develop quality measures (QMs) for NHs. Many of these QMs are included in the NH 5-star ratings and reported online via Nursing Home Compare. However, we know little about how NH providers view the QMs, challenges they experience in addressing the measures, and strategies they use to achieve better scores. As part of a broader mixed-methods study to understand how NHs are responding to the 5-star ratings, we conducted interviews with NH personnel (n=110) and observed organizational processes in 12 NHs in three states. We also interviewed policy and industry leaders (n=34) to gain their perspectives. Interviews focused on perceptions of the 5-star ratings, organizational strategies to improve 5-star scores, experiences with the survey/regulatory process, and perceptions and responses to individual QMs. Key themes show that a) NH providers view the QMs as important indicators of quality, but there is variability across indicators; b) providers face challenges related to measurement and definitions for certain QMs (e.g., pain, restraints); and c) there are potentially conflicting goals, where some QMs aim to promote safety at the expense of resident autonomy and quality of life and vice versa. This work provides organizational context to the 5-star measures and the balancing act providers engage in to assess and improve their scores. The findings also identify several potentially unintended consequences related to the QMs, which can adversely affect residents, particularly those with more complex care needs.

### THE ROLE OF POLICY CONTEXT IN INDIVIDUAL LONG-TERM CARE DECISIONS IN OECD COUNTRIES

Selena Caldera[1], *1. LBJ School of Public Affairs - University of Texas at Austin, Austin, Texas, United States*

While family caregiving of the elderly has long been part of the cultural life of most OECD countries, longer life expectancy combined with low fertility rates has increased the share of the population dependent on current workers and minimized the available population of informal caregivers. The demand for expanded public provision of long-term care (LTC) resulting from this demographic shift prompted reforms in many OECD countries in the 1990s and 2000s. Differences in these reforms provide an opportunity to examine how individual choices between formal and informal care types are shaped by the policy context. I use longitudinal data on elders in three OECD countries, Sweden, Germany, and Japan, to examine LTC decisions under three varied approaches to population aging. The direction of LTC reforms in each country has been shaped by the existing model of care provision and financial constraints. In response to cost pressures, Sweden introduced need-based provision, financial devolution, and market-based approaches to its universal care model. Germany and Japan, in contrast, widely expanded restricted LTC coverage through public LTC insurance models. I use three multinomial logistic models of the LTC decision to test how differing policy schemes influence choices between formal and informal care. Using longitudinal Global Gateway to Aging data for each country, I model the LTC decision in each country as a factor of demographic and need characteristics of the elder experiencing limitations, characteristics of their family, and eligibility for publicly-provided LTC.

### LOOKING UPSTREAM: THE ASSOCIATION BETWEEN AFFORDABLE HOUSING, NURSING HOME USE, AND UNMET CARE NEEDS IN THE COMMUNITY

Meghan Jenkins Morales,[1] and Stephanie Robert[1], *1. University of Wisconsin-Madison, Madison, Wisconsin, United States*

In the U.S., population aging is coinciding with a growing affordable housing crisis. Evidence suggests that housing security contributes to health, but less is known about how affordable housing affects aging in place. We use a nationally representative sample (n=5,117) of older community-dwelling Medicare beneficiaries from the 2015 National Health and Aging Trends Study to test the association between housing cost burden (HCB) and moving to a nursing home, death, or remaining in the community by 2017. Among 2017 community-stayers (n=4,836), we also test the association between HCB and unmet care need, defined as experiencing a consequence related to 12 mobility (e.g., stayed in bed), self-care (e.g., skipped meals) and household (e.g., no clean laundry) activities. HCB is the proportion of income spent on rent or mortgage: low (<30%), moderate (30-50%), severe (≥50%), or home paid off (referent). Among nursing home movers, 26% had moderate or severe HCB in 2015 compared to 16% of community-stayers. Informed by the person-environment fit perspective, weighted stepwise regression models (multinomial and logistic) adjust for race, age, sex (Model 1), self-rated health, probable dementia (Model 2), living with others and high income (Model 3).

Severe HCB is significantly associated with nursing home entry (RRR=2.66, SE=0.89) and this association is only partially mediated by health factors (RRR=2.16, SE=0.72) and resources (RRR=1.95, SE=0.64). Among community-stayers, severe HCB is significantly associated with unmet care need across all models. This study suggests that affordable housing is an important protective factor for older adults to age well in the community.

### BARRIERS AND FACILITATORS TO IMPLEMENTING THE BALANCING INCENTIVE PROGRAM (BIP)

Lisa Beauregard,[1] and Edward A. Miller[1], *1. University of Massachusetts Boston, Boston, Massachusetts, United States*

The Balancing Incentive Program (BIP) was an optional Medicaid program within the Affordable Care Act. States spending less than 50% of Medicaid long-term services and supports on home and community-based services (HCBS) were eligible for the program and could participate from 2011 to 2015. Participating states received an enhanced federal match in exchange for rebalancing LTSS spending and adopting structural changes to their long-term services and supports system. The purpose of this study is to understand the barriers and facilitators to implementing the BIP in two states. Data was collected through semi-structured interviews with individuals involved in HCBS policy nationally and in Maryland and Texas, including government bureaucrats, consumer advocates, and provider representatives. Findings indicate that factors that facilitated Maryland and Texas' implementation of the BIP were regular communication with the Centers for Medicare and Medicaid Services and their consultants, Mission Analytics Group, merging the BIP with existing HCBS programs, and the substantial amount of funding associated with the program. On the other hand, the short duration of the BIP presented a challenge for states because they needed to enact multiple changes within a limited period of time. In addition, state procurement and contracting processes impeded the speed with which BIP requirements could be met. Key stakeholders, including consumer advocacy and provider organizations, often felt as though their state implemented the BIP with minimal input from interested groups. The findings indicate that the structure of the Balancing Incentive Program as well as internal state factors influenced the program's implementation.

### THE IMPACT OF CULTURE CHANGE ON FINANCIAL PERFORMANCE OF HIGH MEDICAID NURSING HOMES

Justin C. Lord,[1] Ganisher K. Davlyatov,[2] Akbar Ghiasi,[2] and Robert Weech-Maldonado[2], *1. Louisiana State University at Shreveport, Shreveport, United States, 2. University of Alabama at Birmingham, Birmingham, Alabama, United States*

This study examines the association between culture change artifacts and financial performance among under-resourced nursing homes (70% or higher Medicaid census). Culture change represents a transformational process to become person-centered, through staff and resident empowerment. Cultural artifacts represent the physical evidences that culture change is occurring. In this study, we focus on the workplace (nurse staffing consistent assignments) and leadership (residents engagement) artifacts to assess the relationship between culture change practices and performance. Survey data came from 387 nursing home directors from 2016- 2018, merged with secondary data from LTCFocus, Area Health Resource File, and Medicare Cost Reports. The dependent variable consisted of the total profit margin (%), while the independent variables comprised composite scores for leadership (0-25) and workplace artifacts (0-15). Control variables included organizational-level (ownership, chain affiliation, size, occupancy rate, and Medicare and Medicaid payer mix), and county-level factors (Medicare Advantage penetration, per capita income, educational level, unemployment rate, poverty level and competition). Multivariate regression was used to model the relationship between cultural change artifacts and financial performance. Workplace artifacts in nursing homes were found to be associated with significantly higher profit margin ($\beta = 0.30$, $p < 0.05$), while leadership artifacts were not. Culture change practices aimed at improving nursing staff consistent assignments are associated with better financial performance. Given increasing nursing home market competition and declining resources for high Medicaid nursing homes, facilities with a greater emphasis on workplace culture may be able to perform better financially among these under-resourced facilities.

### NAO TAKES A BOW: USING SOCIAL ROBOTS TO ENHANCE THE MOOD OF OLDER ADULTS LIVING IN RESIDENTIAL CARE SETTINGS

Noelle L. Fields,[1] Julienne Greer,[1] and Ling Xu[1], *1. University of Texas at Arlington, Arlington, Texas, United States*

Social robots have been utilized in research with older adults, however, few studies have integrated participatory arts (e.g. theatre) into social robotic platforms with this population. An interdisciplinary team designed an intervention integrating theater and social robotics with the aim of improving the mood of older adults. A purposive sample of persons age 65 and older (N = 15) participated in this 3-session pilot study that involved a Shakespeare activity using the robot, NAO. Mixed methods included interview questions as well as short survey measures of depression, loneliness, and a simplified face scale for mood pre and post each session. Results from Repeated Measurement Analysis of Variance (ANOVA) showed that face scale scores significantly decreased across six time periods ($F = 2.72$ (5, 50), $p <. 05$) and this decrease marginally differed between participants with dementia ($M = 2.50$, $SD = 1.73$) and those without dementia ($M = 1.62$, $SD = 0.52$). In addition, depression scores marginally significantly decreased after intervention ($F = 2.28$ (5, 40), $p <. 10$) and these declines were also marginally significantly different for participants with ($M = 0.67$, $SD = 0.58$) or without dementia ($M = 0.86$, $SD = 0.69$). Qualitative findings suggest that participants were highly engaged and responsive to the intervention. We discuss the promising aspects of using social robotics as a platform for participatory arts interventions with older adults and offer recommendations for future interdisciplinary studies involving the use of innovative technology in residential care settings.

### PERCEPTIONS OF LONG-TERM CARE RESIDENTS AND THEIR FAMILY MEMBERS ABOUT USING THE CONVERSATION STARTER KIT

Sharon Kaasalainen,[1] and Tamara Sussman[2], *1. McMaster University, Burlington, Ontario, Canada, 2. McGill University, Montreal, Quebec, Canada*

The need for a palliative approach in long term care (LTC) is widely recognized. However, advance care planning (ACP) is still rare. The purpose of this study was to explore the perceptions of LTC residents and their families about using an ACP tool called The Conversation Starter Kit (CSK). This study utilized a mixed methods approach. Data was collected in four LTC homes in Ontario, Canada from 31 residents and family members during an interview after they had completed the CSK. Data was analyzed using thematic analysis and descriptive statistics. All participants read all sections but only 73% completed all sections of the toolkit. Participants spent an average of 36 minutes discussing it with their family members and/or LTC staff. Participants reported: a better understanding of ACP after using the tool (80%), that the tool helped clarify the available resources and/or choices (53%), and that they felt less apprehensive about ACP after using the tool (60%). Qualitative findings revealed many strengths (e.g., usefulness, ability to start difficult conversations, content and clarification), and weaknesses of the tool (e.g., redundant information, difficulty understanding the content and lack of information regarding medically assisted dying). Family members noted that the toolkit would have been helpful to receive earlier on in their family members' disease trajectory, perhaps before being admitted into LTC. These study findings support the CSK for residents and family members to have ACP discussions in LTC. Future work is needed to evaluate the effectiveness of the tool with a larger sample.

### LGBTQ PROGRAMMING AT SENIOR CENTERS IN MASSACHUSETTS

Ceara Somerville,[1] Saralyn Collins,[1] and Caitlin E. Coyle[2], *1. University of Massachusetts Boston, Boston, Massachusetts, United States, 2. Gerontology Institute, University of Massachusetts Boston, Boston, Massachusetts, United States*

LGBTQ seniors have some different needs for programs and services, are at a higher risk of social isolation, and are often underserved in the community. Senior centers serve as a hub of resources in a community and are purposefully situated to address the needs and interests of all seniors in a community; they are a natural outlet for targeted programming for LGBTQ seniors. The purpose of this project is to demonstrate what municipal senior centers across Massachusetts are doing to meet the needs of their LGBTQ seniors. A total of 24 senior centers were identified by the Massachusetts Association of Councils on Aging (MCOA) as providing LGBTQ programming. Semi-structured interviews were conducted with 14 senior center directors or programming staff from different communities across Massachusetts to learn more about their specific programming. For almost all senior centers in this study, the main LGBTQ-specific programming was a congregate meal with an activity. Activities included both recreational activities like a film-screening and educational engagements such as guest speakers or specialists on housing, legal services, and health promotion. Distinguishing characteristics included whether or not programming had an intergenerational component, type of recruitment methods, and geographic clustering of programs. For example, two regions emerged as having shared activities for LGBTQ seniors. Results from this study will be used to illustrate models of best practice when it comes to LGBTQ programming for older people.

### LTSS IS LOCAL, EVEN IN COUNTRIES WITH NATIONAL LTSS PROGRAMS

Pamela Nadash[1], *1. University of Massachusetts Boston, Boston, Massachusetts, United States*

This study reports the results of a cross-national qualitative assessment of how different countries structure their provision of long term services and supports (LTSS). It emphasizes the universality of the local role, even in countries that offer some form of universal coverage for LTSS. At minimum, countries devolve the responsibility for administration and eligibility determination to sub-national units, variously called provinces, départements, Länder, or other terms. However, many countries do much more than that: subnational units can be responsible for the safety net welfare programs that pick up the costs that the universal programs do not cover. They may also run other programs that affect the ability of people with LTSS needs to live good lives, such as housing and health programs; again, the role of sub-national governments often focuses on those least able to pay. In addition, in some countries, local governments have a role in helping to finance the national program as well. Differing abilities to support these responsibilities across regions can result in geographic disparities in access to care – so, too, can differences in administration and eligibility determination, resulting in many of the same issues that we in the US confront regarding access to LTSS through the Medicaid program. Thus, even countries with strong national programs for LTSS experience many of the same tensions between national and sub-national units of government that we in the US do.

### DISPARITIES IN ACCESSING DIFFERENT TYPES OF LONG-TERM CARE SERVICES IN TAIWAN

Ya-Mei Chen,[1] Shih-Chi Lin,[2] Hsiao-Wei Yu,[3] Shwu-Chong Wu,[1] Shih-Cyuan Wu,[1] Ying-Chieh Wang,[1] and Tung-Liang Chiang[1], *1. National Taiwan University, Taipei, Taiwan, 2. National Yang-Ming University, Taipei, Taiwan, 3. Chang Gung University of Science and Technology, Taipei, Taiwan*

A growing body of evidence documents pervasive social and demographic factors relating to disparities in long-term care (LTC). In 2007, Taiwan implemented its Ten-year Long-Term Care Plan version 1.0 (TLTCP 1.0) that aimed to develop a home-and community-based (HCBS) LTC system. In 2016, Taiwan began to implement TLTCP 2.0. To continue providing effective LTC, this study aimed to assess the disparities in access to LTC services using Taiwan's LTC claim database from 2010 to 2013. A total of 87,438 older adults who had applied for LTC services from the TLTCP 1.0 were included. The study assessed LTC disparities related to five sociodemographic factors, including age, gender, living status, urbanization, and income status. Sixteen types of LTC services, including HCBS, home-based professional care, meal services, transportation, and institutional services were assessed. After controlling for the level of disability, we found that those who were older-old (age 80> over), male, and low-income were less likely to use HCBS, but more likely to use institutions services ($p < 0.001$). We also found that those who lived in the city were more likely to use HCBS and transportation services ($p < 0.001$). Yet, older adults living alone were more likely to use home care and meal services but not other types of LTC services ($p < 0.001$). In conclusion, the social disparities in access to LTC services in Taiwan remains, suggesting LTC 2.0 should continue monitoring and placing the LTC equity issue on the top priority.

### ADVANCE DIRECTIVES: STATE REQUIREMENTS, PRACTICES, AND PREVALENCE IN ADULT DAY SERVICES CENTERS

Jessica P. Lendon,[1] Christine Caffrey,[1] and Denys Lau[1], *1. National Center for Health Statistics, Hyattsville, Maryland, United States*

Advance directives (ADs) are documents that express a person's healthcare preferences if he/she is unable to make decisions. Adult day service centers (ADSC) may serve as an entrée to advance care planning for many people. This study examined the relationships among: 1-state requirements on ADSCs to provide information on ADs; 2-ADSC's awareness of their state requirement; 3-ADSC's practice in providing AD information; and 4-the percentage of ADSC participants with an AD. From the 2016 National Study of Long-Term Care Providers, 3,300 ADSCs reported that they maintained documentation of ADs in participants' files. Nine states required ADSCs to provide information on ADs; 22% of ADSCs were located in these states. About 24% of ADSCs did not know if their states had requirements; among the 76% of ADSCs that reported knowing, 62% were correct and 37% were incorrect. About 80% of ADSCs provided AD information to their participants, while 41% of ADSC participants had an AD on file. Regression models controlled for size, chain and profit statuses, Medicaid-licensing, medical or social care model, electronic health records use, and Census region. Having state requirements was not independently associated with ADSCs' practice of providing AD information or with the percentage of participants with an AD. Instead, ADSCs that thought their state had a requirement had greater odds of providing information on ADs, regardless of state requirements. Similarly, ADSCs that thought their state had a requirement and that provided AD information had a higher percentage of participants with an AD, independent of state requirements.

### STATE VARIATION IN NURSING HOME CIVIL MONEY PENALTY ENFORCEMENT ACTIONS FOR QUALITY DEFICIENCIES

Denise L. Gammonley,[1] Xiaochuan (Sharon) Wang,[1] and Felicia Bender[1], *1. University of Central Florida, Orlando, Florida, United States*

States vary in their overall rates of nursing home deficiency citations as well as deficiencies for actual harm or jeopardy (Harrington et al., 2018). Civil Money Penalty (CMP) fines collected by the Centers for Medicare and Medicaid Services (CMS) are one enforcement action imposed to promote nursing home compliance with regulations. Collected CMP funds are redistributed to states for the sole purpose of improving nursing home resident care and quality of life through reinvestment in quality improvement projects. Using CASPER data available for US skilled nursing homes in 2015 and 2016 through the CMS QCOR database we examined the distribution of quality of care (QOC) and quality of life (QOL) deficiencies and CMP enforcement action across states. Guided by the systems framework for evaluating nursing home quality (Unruh & Wan, 2004) we further explored how contextual factors such as state spending for nursing home care, structural characteristics of facilities in states, and inadequate care processes indicated by deficiencies contribute to CMP enforcement actions and fines. Findings indicate that 27% of enforcement actions resulting in a CMP between 2015 and 2016 were imposed for a QOL deficiency while 61.7% represented QOC deficiencies. QOL deficiencies represented only 8% of the highest severity deficiency category but 81.7% of enforcement actions for QOC were for those causing immediate harm or jeopardy. QOC deficiencies are a focus of enforcement actions as they represent critical care processes influencing resident basic needs for hydration, ambulation, skin integrity and care for other special physical and behavioral needs.

### ANTICIPATED NEED FOR NURSING HOME PLACEMENT AMONG LESBIAN, GAY, BISEXUAL ADULTS AGES 50-64: FINDINGS FROM THE HRS

Mekiayla Singleton,[1] Zach Gassoumis,[1] and Susan Enguidanos[2], *1. Leonard Davis School of Gerontology, University of Southern California, Los Angeles, California, United States, 2. University of Southern California, Los Angeles, California, United States*

By 2030, the population of LGBTQ older adults is expected to exceed 6 million. Yet little is known about the expected use of nursing homes (NH) among LGBTQ older adults. Prior research has found NHs lack cultural sensitivity, and that LGBTQ NH residents are going "back into the closet" and not disclosing their sexual orientation due to discrimination and quality of care concerns. Using data from 2016 HRS, we describe bivariate differences between the LGB and heterosexual population, ages 50 to 64, and conduct a linear regression to determine the impact of LGB status on self-reported chance of moving to a NH in the future. Compared with the heterosexual population (n=4,049), these LGB adults (n=158) had a higher mean self-reported chance of moving to a NH ($p<.01$), fewer children ($p<.01$) and reported a slightly higher health rating ($p<.05$). LGB adults ages 50-64 also were more likely to be unmarried (71%, $p< .001$), white (59%, $p< .001$) and have a college degree (51%, $p<.001$). After controlling for sociodemographic variables, there were no significant differences between LGB and heterosexual adults' self-reported chance of moving to a NH. Although anticipated chance of moving to a NH is no different for LGB adults ages 50-64 when controlling for their sociodemographic profiles, as a group they have a higher anticipated chance than heterosexual adults. These findings support the need for improved education, training, and structural changes within long-term care settings to better serve the growing older adult LGB population.

### RURAL-URBAN DISPARITIES IN UNMET LONG-TERM CARE NEED AND COMMUNITY CARE SERVICES EXPECTATION AMONG ELDERLY IN CHINA

Yanbing Zeng,[1] lixia wang,[1] and Ya Fang[2], *1. Xiamen University, Xiamen, China, 2. School of Public Health, Xiamen University, Xiamen, China*

Objective This study aimed to examine the urban-rural differences of unmet needs and their expected LTC services among community-dwelling old people. Methods The data comes from the Chinese Longitudinal Health Longevity Survey (CLHLS) in 2014. A total of 1587 community residents aged 65+ with disability of activities of daily life (ADL) were included in this study. Binary logistic regression was used to estimate correlates of unmet need in LTC. And chi-square test was used to examine the differences of expected community-based LTC services between urban and rural area. Results Over half (55.07%) of the participants reported their need were unmet. For both rural and urban residents, poorer economic status and reluctant caregivers (ORs>1, $P < 0.01$) seriously affected the unmet need. Besides, of urban older adults, people who were male and lonely(ORs>1, $P < 0.05$) reported more unmet need. While of rural old ones, people who were with severe ADL disability and poorer self-rated health(ORs>1, $P < 0.01$) reported more unmet need. And people with available medication and home visit services(ORs<1, $P < 0.01$) reported more met need. However, the supplies for community LTC care services were far below the demands. Conclusion The risk of having unmet need associated with ADL disabilities in LTC is largely determined by their economic status and caregivers' willingness to provide care for both rural and urban old people. There is a need for an overall improvement in the planning, provision and financing of long-term care services for elderly individuals in China.

### EFFECTIVENESS OF HOME- AND COMMUNITY-BASED SERVICES IN DECREASING HEALTH CARE EXPENDITURE IN TAIWAN

Ya-Mei Chen,[1] Hsiao-Wei Yu,[2] and Ying-Chieh Wang[3], *1. National Taiwan University, Taipei, Taiwan, 2. Chang Gung University of Science and Technology, Taipei, Taiwan, 3. Institute of Health Policy and Management, Taipei, Taiwan*

Ideally, continuum of care involves wide-ranging health and long-term care (LTC) services. Taiwan's National Health Insurance scheme and 10-Year Long-term Care Plan attempts to provide universal and fundamental services of continuum care. However, the accessibility of these services for care recipients remains unclear. This study aims to examine the effectiveness of continuum care in decreasing the healthcare expenditure of LTC recipients using home- and community-based services (HCBS). Data collated from the 2010–2013 Long-Term Care Service Management System (N = 77,251) were subjected to latent class analysis to identify subgroups of recipients using HCBS. Subsequently, the 1-year primary care expenditure after receiving HCBS was compared through generalized linear modeling. Three discrete HCBS subgroups were found: home-based personal care (HP), home-based health care (HH), and community-based care (CC). No difference in the number of visits to doctors and the average primary care expenses was observed between the HP and HH subgroups. However, considering physical and psychosocial confounders, care recipients in the CC subgroup recorded a higher number of visits to doctors ($\beta = 3.05$, SD = 0.25, $p < 0.05$) and lower primary care expenditure ($\beta = -98.15$, SD = 43.17, $p = 0.02$) than the other two subgroups. These findings suggest that LTC recipients in Taiwan may obtain better continuum care only for CC service recipients. Additionally, community-based LTC services may lower the cost of health expenditure after 1 year.

### AWRAMBA COMMUNITY-BASED CARE FOR ELDERS LACKING FAMILY SUPPORT IN ETHIOPIA

Margaret E. Adamek,[1] and Setegn Ali[2], *1. Indiana University School of Social Work, Indianapolis, Indiana, United States, 2. University of Gondar, Gondar, Ethiopia, Ethiopia*

In rural Ethiopia, land fragmentation, poverty, rural-urban migration, and the expansion of market forces are negatively impacting family support for older persons. This study explored an innovative community-based support system that uses its own wealth redistribution mechanism to support congregate residential care for older persons. The Awramba Community in northwest Ethiopia has different social norms and values that make it unique from surrounding communities. To explore this new approach to meeting the needs of rural elders, primary data were obtained from in-depth interviews with 8 elders who reside in the older adult center, 3 focus group discussions, and personal observations. In addition, key informant interviews were conducted with a full-time caregiver, two community leaders, and members of the Older Persons' Support Committee. Interviews explored the types of services provided to older persons, the interactions of older residents within the community, and the benefits of congregate living. Community members (N=403) must agree to 4 guiding principles relating to gender equality, respecting the rights of children, discouraging dishonesty, lying and stealing, and helping the less fortunate, ill and older persons. The Older Person's Support Committee is responsible for coordinating the provision of care for the older persons who live in the congregate center. Residing in a common residential home benefited the elders in multiple ways, including opportunities for socialization with peers and access to a full-time caregiver. As traditional family support wanes and the population of elders increases in developing nations, such alternative support strategies are critically needed.

### REDUCING ANTIPSYCHOTIC PRESCRIBING IN NURSING HOMES: FACILITATORS AND BARRIERS FOR HIGH-IMPACT STATE INITIATIVES

Stephen Crystal,[1] Richard Hermida,[2] Olga F. Jarrín,[1] Sheree Neese-Todd,[1] Beth Angell,[3] and Marsha Rosenthal[1], *1. Rutgers, The State University of New Jersey, New Brunswick, New Jersey, United States, 2. Rutgers University, New Brunswick, New Jersey, United States, 3. Virginia Commonwealth University, Richmond, Virginia, United States*

In conjunction with the National Partnership to Improve Dementia Care in Nursing Homes initiated in 2012, states implemented initiatives to reduce antipsychotic use. All achieved substantial reductions, but improvement varied across states. By 2018, several states had achieved reductions of more than 45%, including several of the largest states.

These reductions are noteworthy given the challenging nature of behavioral symptoms of dementia, and difficulties encountered historically and internationally in changing strongly-rooted clinical practices. How were these successful interventions achieved in high-performing state initiatives? What were the barriers encountered and facilitators that helped overcome these barriers? What does this experience suggest for sustainability of change? To address these questions, we draw on a mixed-methods study of antipsychotic prescribing in nursing homes incorporating analyses of prescribing data, state policy case studies, and facility case studies. Successful states integrated large-scale educational initiatives with strong regulatory action, often focusing especially on laggard facilities. Texas' initiative was particularly noteworthy, achieving a 56.5% reduction across its far-flung network of nearly 100,000 residents and 1,200 facilities. Texas used metrics to identify facilities that achieved notable reductions in antipsychotic prescribing, and encouraged them to share their strategies with "late adopters". The state deployed a designated Quality Monitoring Program (QMP), distinct from the survey process, to provide on-site technical assistance to laggard facilities, and provided education for all levels of staff and assistance in implementing data-driven improvement strategies. Successful state initiatives achieved considerable buy-in on the need to reduce antipsychotic use, a key factor in achieving successful system change.

### SOCIAL FUNCTIONING AS AN IMPORTANT CLINICAL TARGET DURING CARE TRANSITIONS FROM SKILLED NURSING FACILITIES

Kelsey Simons,[1] Whitney L. Mills,[2] Emily S. Bower,[1] Suzanne Gillespie,[3] and Kimberly Van Orden[4], *1. VISN 2 Center of Excellence for Suicide Prevention, Canandaigua, New York, United States, 2. Center for Innovation in Long-Term Services and Supports, Providence VA Medical Center, Providence, Rhode Island, United States, 3. Canandaigua VA Medical Center, Canandaigua, New York, United States, 4. University of Rochester Medical Center, Rochester, New York, United States*

Care transitions from skilled nursing facilities (SNF) to home signify a period of medical risk for older adults. They also present opportunities for clinical interventions to reduce these risks and to enhance or maintain patients' quality of life. A substantial body of research has been published on improving late life care transitions (CTs), including the development of standardized CT models for acute care. However, such models typically focus on improved coordination of medical services; overlook the need to address psychosocial well-being and social connectedness; and have rarely been implemented in SNFs. This poster will present a conceptual model of social functioning in older adults that draws on constructs from the World Health Organization's World Report on Ageing and Health (2015). We propose that social functioning is a key part of overall functioning among older adults who use SNFs and is simultaneously influenced by physical, psychological, and cognitive functioning. To illustrate our model, we will present results of a qualitative study (n= 21) that describes declines in social functioning following care transitions to the community from VA SNFs. Implications for clinical practice include the need to better integrate social functioning in clinical assessments, goal setting, discharge planning, and coordination of care activities. The need for additional research on this topic will also be addressed. Our project is highly relevant to the overall conference theme "Harnessing the Power of Networks" as it presents a conceptual model and study findings related to social connectedness and social functioning in older adults who use SNFs.

### DOES THE PATIENT HEALTH QUESTIONNAIRE MEASURE MOOD-RELATED QUALITY OF LIFE IN NURSING FACILITY RESIDENTS?

Weiwen Ng,[1] Yinfei Duan,[2] and Tetyana P. Shippee[1], *1. University of Minnesota, Minneapolis, Minnesota, United States, 2. School of Nursing, University of Minnesota, Minneapolis, Minnesota, United States*

The Patient Health Questionnaire-9 (PHQ-9) is a depressive symptom questionnaire administered to nursing facility (NF) residents in the Minimum Data Set (MDS). Does the PHQ-9 measure mood-related aspects of quality of life (QoL)? We assessed the PHQ-9's convergent validity with negative and positive mood items from Minnesota's QoL survey, which is administered annually to a random sample of residents. We also examined if scores on both instruments were associated with various psychiatric diagnoses on the MDS. Using item response theory (IRT) models, we estimated that depressive symptoms (PHQ-9) had a correlation of 0.546 with negative mood and -0.425 with positive mood. With explanatory IRT modeling, we estimated that diagnoses of anxiety, depression, and bipolar disorder were respectively associated with 0.261, 0.339, and 0.301 (all $p < 0.001$) standard deviation increases in (SD) depressive symptoms, and with 0.235, 0.261, and 0.306 SD increases in negative mood (all $p < 0.001$), thus indicating convergent validity. For positive mood, depression and bipolar disorder had associations of similar magnitude as the other two constructs. However, anxiety disorders were not associated with lower positive mood (-0.014 SD, $p = 0.636$). Thus, the PHQ-9 can measure mood-related aspects of QoL. However, the PHQ-9 appears to be sensitive to relatively serious depression, whereas the Minnesota items are more sensitive to lower levels of negative mood. Also, the PHQ-9 does not measure positive mood directly. Thus, the PHQ-9 is a more limited measure of mood-related QoL than the Minnesota items.

### WHEN A LITTLE GOES A LONG WAY: EXPANDING HOME CARE SERVICES TO ADULTS WITH DISABILITIES

Serena Hasworth,[1] Jaclyn Winfree,[2] Ozcan Tunalilar,[2] and Diana White[2], *1. Portland State University Institute on Aging, Portland, Oregon, United States, 2. Portland State University, Portland, Oregon, United States*

Policy makers are increasingly interested in reducing public spending while maintaining quality of life. Since 1975, Oregon Project Independence (OPI) has supported community-based adults aged 60 and older to avoid or delay the need for residential long-term care services by increasing access to personal and home care services. The program also aims to prevent the need for Medicaid by optimizing personal resources and natural supports. In 2014, the OPI Expansion (OPI-E) pilot project began to serve adults aged 18-59 with disabilities in seven of Oregon's seventeen Area Agencies on Aging (AAAs). This poster describes the evaluation of the expansion using three data sources: administrative data about consumer characteristics compiled by the AAAs and State of Oregon from 2015-2017 (N=3,824 traditional consumers, N= 581 OPI-E consumers), qualitative interviews conducted with AAA directors and OPI-E case managers (N=23), and a survey of current OPI-E consumers (N=126). Compared to traditional OPI consumers, OPI-E consumers were somewhat more likely to be men and people of color. Interviews with AAA staff highlighted the need for outreach, lack of service provider capacity, unique characteristics of younger consumers, and issues related to data management and rural access. Staff reported valuing the program, noting how "even low levels of service go a long way." Qualitative and quantitative consumer responses showed consumers found OPI-E services invaluable. The majority stressed their appreciation for the program, with several describing it as "lifesaving." These three sources informed recommendations for expanding the OPI-E program statewide.

### FEDERAL POLICY SUPPORTS AND GAPS IN ADDRESSING RACIAL-ETHNIC HEALTH DISPARITIES IN U.S. LONG-TERM CARE FACILITIES

Rebecca L. Mauldin,[1] Kathy Lee,[1] and Antwan Williams[1], *1. University of Texas at Arlinton, Arlington, Texas, United States*

Older adults from racial and ethnic minority groups face health inequities in long-term care facilities such as nursing homes and assisted living facilities just as they do in the United States as a whole. In spite of federal policy to support minority health and ensure the well-being of long-term care facility residents, disparities persist in residents' quality of care and quality of life. This poster presents current federal policy in the United States to reduce racial and ethnic health disparities and to support long-term care facility residents' health and well-being. It includes legislation enacted by the Patient Protection and Affordable Care Act of 2010 (ACA), regulations of the U.S. Department of Health and Human Services (DHHS) for health care facilities receiving Medicare or Medicare funds, and policies of the Long-term Care Ombudsman Program. Recommendations to address threats to or gaps in these policies include monitoring congressional efforts to revise portions of the ACA, revising DHHS requirements for long-term care facilities staff training and oversight, and amending requirements for the Long-term Care Ombudsman Program to mandate collection, analysis, and reporting of resident complaint data by race and ethnicity.

### SERVICE ENVIRONMENTS AND PSYCHOLOGICAL WELL-BEING AMONG RESIDENTIAL CARE FACILITY AND NURSING HOME RESIDENTS

Yeon Jin Choi[1], *1. University of Southern California, Los Angeles, California, United States*

One's competence, or ability to cope, becomes lower as people age due to multiple factors, such as decreased physical and cognitive functioning, social isolation, and reduced income. Considering this change, providing appropriate services may help older adults to maintain their independence and improve their psychological well-being and quality of life. Therefore, this study aimed to investigate services that are positively associated with psychological well-being (i.e., mood, psychological health, self-efficacy) of older adults. For the analysis, observations were derived from the National Health and Aging Trends Study (NHATS), which includes a nationally representative sample of Medicare beneficiaries ages 65 and older. Service environments were assessed by (1) reflect availability and usage of 13 services (i.e., service unavailable, service available/ not being used, service available/ being used by residents) and (2) number of services available within three categories (i.e., help with ADL/IADL, transportation services, social and health-related services) Both having social events and activities available and participating in it were positively associated with older residents' mood, while using housekeeping services and walking areas (e.g., outdoor walking path) were associated with better psychological health and self-efficacy. When models were estimated with service categories, having more social and/or health-related services available were associated with better psychological health and self-efficacy. Findings of this study suggests that social events and activities, housekeeping services, and areas to walk for pleasure or exercise would improve older residents' psychological well-being. For Not only providing those services, residential facilities should encourage older residents to participation in or use the services.

### SESSION 915 (POSTER)

### PERSONALITY, PSYCHOSOCIAL, AND EMOTIONAL ELEMENTS

### THE DOCTOR-PATIENT RELATIONSHIP, PERSONALITY, MOOD, AND FUNCTIONING IN OLDER ADULTS

Lauren S. Atlas,[1] and Richard Zweig[2], *1. Yeshiva University, Bronx, New York, United States, 2. Yeshiva University, New York, United States*

Personality pathology has been tied to mental and physical health in older adulthood. Less is known regarding the combined impact of personality and the doctor-patient relationship on mental health outcomes. This study examined relationships between personality, mood, and trust in physicians. Participants (N=170) were a sample of primary care older adults ages 60-99 (M = 70.73, SD = 7.054) who completed self-report measures of personality traits (NEO-FFI), processes (IIP-PD-25), depression (GDS-30; PHQ-9), social adjustment (SAS-SR) and trust in one's physician (GTIP). Medical burden data (CIRS) were retrieved from medical records. After adjusting for relevant covariates such as age, perceived health, cumulative illness burden, and income security there were several significant predictive relationships. In combined models more neuroticism (NEO-N, ß = .082, $p < .000$) and lower trust (GTIP, ß = -.025, $p = .014$) but not agreeableness (NEO-A, ß = -.006) or interpersonal problems (IIP-25, ß = .254) predicted depression. In combined models, higher neuroticism (NEO-N, ß = .018, $p < .000$) and interpersonal problems (IIP-25, ß = .186, $p = .002$) but not agreeableness (NEO-A, ß = -.003) or trust (GTIP, ß = -.002) predicted social adjustment. The results are consistent with previous findings that neuroticism predicts both depression and social adjustment in older adults. In addition, lower trust augmented neuroticism to predict depression. Results suggest that apart from general personality risk factors, situational personality processes such as trust in physicians may affect mood state, whereas personality processes such as interpersonal problems contribute to longer term functional impairment.

### THE MEDIATING ROLES OF HEALTH LOCUS OF CONTROL AND COPING ON THE PAIN-DEPRESSION PATHWAY

Kyrsten Costlow,[1] Patricia A. Parmelee,[2] and Tina Harralson[3], *1. University of Alabama, Tuscaloosa, Alabama, United States, 2. The University of Alabama, Tuscaloosa, Alabama, United States, 3. Tridiuum, Philadelphia, Pennsylvania, United States*

The literature on health locus of control (HLC) suggests that individuals who believe that their health is internally determined are more likely to use active coping strategies than those who believe their health is determined by chance or powerful others (Brosschot, Gebhardt, & Godaert, 1994; Gibson & Helme, 2000). Coping strategies (Klapow et al., 1995) and HLC (Campbell, Hope, & Dunn, 2017) have been found to influence the relation between chronic pain and depression. We hypothesized that the relation between osteoarthritis pain and depression would be serially mediated by HLC and coping. Self-report measures of osteoarthritis pain (Meenan, Mason, Anderson, Guccione, & Kazis, 1992; Parmelee, Katz, & Lawton), HLC (Wallston, Wallston, & DeVellis, 1978), coping strategies (Felton & Revenson, 1984; Rosenstiel & Keefe, 1983), and depression (Radloff, 1977) were examined in 367 older adults with osteoarthritis of the knee. Hayes' (2013) PROCESS macro was used to test the hypothesized serial multiple mediation for three subscales of HLC: internality (IHLC), chance (CHLC), and powerful others (PHLC). After controlling for age, the hypothesized serial mediation was statistically significant for IHLC and CHLC but not PHLC. More specifically, osteoarthritis pain significantly increased CHLC, which increased negative coping and depression in turn. Osteoarthritis pain significantly decreased IHLC, which was associated with both positive and negative coping strategies in a complex serial mediation. These findings suggest that interventions targeting HLC and/or coping strategies may be able to alter the pain-depression pathway for older adults with chronic osteoarthritis pain. (Supported by R01-MH51800, P. Parmelee, PI).

### POWER OF SOCIAL SUPPORT AGAINST DEPRESSIVE SYMPTOMS OF OLDER AFRICAN AMERICANS

Seungjong Cho[1], *1. Case Western Reserve University, Cleveland, Ohio, United States*

Depressive symptomatology is one of the most prevalent mental health problems. About 20% of Americans experience depressive symptoms in their lives (Gotlib & Hammen, 2014). Social support, on the other hand, was proved to be a powerful buffer against depressive symptoms of older adults (Kim & Ross, 2009; Sheiman & Meersman, 2004). Few studies have explored this association exclusively among older African Americans who had a culture of powerful social support. Therefore, the purpose of this study is to compare the effects of different sources of social support (from spouse/partner, children, relatives, and friends) against depressive symptoms among older African Americans. This study analyzed the 2014 Health and Retirement Study (HRS; N = 187). Depressive symptomatology was operationalized as a count outcome (number of having symptoms; CES-D8 scale). A negative binomial regression model of depressive symptoms showed that higher levels of spousal support were associated with lower levels of depressive symptoms (coefficient = –.179, $p < .001$). For each additional score in spousal support, the expected log count of the number of depressive symptoms was decreased by .179. Other sources of social support were not significant predictors of depressive symptoms among older African Americans in this study. Among the covariates, self-rated health (coefficient = .358, $p < .001$) and household income (logged; coefficient = –.275, $p = .014$) were significant. The current study supported the results of previous studies showing the power of positive spousal interactions against depressive symptomatology, especially among a nationally representative sample of older African Americans.

### ASSOCIATIONS BETWEEN DSM-5 PERSONALITY PATHOLOGY AND HORNEY'S INTERPERSONAL TRENDS AMONG OLDER ADULTS

Daniel L. Segal,[1] Lisa E. Stone,[1] Frederick L. Coolidge,[2] and Gabrielle Krus[2], *1. University of Colorado at Colorado Springs, Colorado Springs, Colorado, United States, 2. University of Colorado, Colorado Springs, Colorado, United States*

Introduction: The Personality Inventory for DSM-5 (PID-5) is a measure of the alternative model of personality disorders with limited evidence of validity among older adults. This study examined validity of the model through associations with the Horney-Coolidge Tridimensional Inventory (HCTI). Method: Older adults (N=125) completed the PID-5 and the HCTI. Results: Zero-order correlations were computed between the PID-5's five domains (Negative Affect, Detachment, Antagonism, Disinhibition, and Psychoticism) and the HCTI's three domains (Compliance, Aggression, and Detachment). Compliance was moderately negatively correlated with Detachment ($r = -.27$), as expected. Aggression was significantly positively related to all five PID-5 domains and was most strongly correlated with Antagonism ($r = .56$), Psychoticism ($r = .48$), and Disinhibition ($r = .32$). As predicted, Horney's Detachment was most strongly related to the PID-5's Detachment ($r = .48$). Regression analyses were also conducted with PID-5 domains predicting each HCTI type. The Compliance model was significant, with PID-5 domains predicting 13% of variability in Compliance. Negative Affect (positive) and Detachment (negative) were significant predictors. The Aggression model was also significant, with the PID-5 domains accounting for 40% of variability. Antagonism was the only significant positive predictor. Lastly, the Detachment model was significant, with the PID-5 domains predicting 29% of variance in Detachment scores. Negative Affect (negative) and Detachment (positive) were significant predictors. Discussion: Results indicate that the two measures of personality pathology generally converge regarding theoretically similar constructs and diverge around dissimilar domains, providing evidence of validity of the PID-5 for its ability to capture personality traits.

### INDIVIDUAL PREDICTORS OF VOLUNTEERING IN OLDER ADULTS USING STRUCTURAL EQUATION MODELING

Zolana J. Baumel,[1] and Christopher M. Kelly[1], *1. University of Nebraska - Omaha, Omaha, Nebraska, United States*

Why do some older Americans volunteer while others do not? Personality theory is used to explain volunteering behavior. This study used the Big 5 personality domains. Volunteering is also explained by resource theory, which includes personal, social, and cultural capital. Personal capital was measured by age, sex, race, marital status, health, and education (a proxy for socioeconomic status). Social capital was measured by frequency of email/write interactions and interactions with friends. Cultural capital was measured as church attendance. The purpose was to measure the extent to which social and cultural capital mediates between personality and personal capital, and volunteering outcomes. Participants were the 2010 Core Health and Retirement Study who also completed the leave-behind self-administered questionnaire (N=4,299). Structural equation modeling (SEM) tested personality and resource theory in one model. Exploratory factor analysis, followed by confirmatory factor analysis supported development of the final SEM. Extraversion directly predicted volunteering. Age was inversely correlated with volunteering. Health and education directly predicted volunteering. Church attendance and interactions with friends directly predicted volunteering. Conscientiousness predicted church attendance. Open to new experience was inversely related to church attendance and directly predicted frequency of email/write interactions. Extraversion predicted interactions with friends. Measuring direct, indirect, and total effects of predictors on volunteering showed that personality influences volunteering both directly and indirectly. Future applied research could test whether extraversion, which directly influences interactions with friends, would directly predict recruiting outcomes. It could also test whether open to new experience, which directly influences email/write interactions, would directly predict retention outcomes.

### CHANGES IN COGNITION ARE RELATED TO PERSONALITY CHANGES IN MIDDLE AND LATER ADULTHOOD

Yujun Liu,[1] and Margie E. Lachman[2], *1. Brandeis University, Waltham, United States, 2. Brandeis University, Waltham, Massachusetts, United States*

Previous studies have identified an association between personality and cognition in later life. Those with more stable personalities have better cognitive functioning. And those with more cognitive decline have higher neuroticism. We examined the association between changes in both personality and cognition in the same model, which has not been systematically investigated. Data were from the Midlife in the United States (MIDUS) study, a national survey that included 4268 participants ages 35 to 85 at the second wave. The Big Five Personality traits and cognitive function variables (episodic memory and executive functioning) were from waves two and three. The analysis included a latent change score model and a cross-lagged panel design using Mplus. The results show that personality changes and cognitive changes over 9 years are correlated. Cross-lagged findings indicate that cognitive functioning is positively related to changes in conscientiousness, agreeableness, openness, and extraversion and negatively related to changes in neuroticism. The findings advance our understanding of the association between changes in personality and cognition. The impact of cognitive change on personality stability and the role of personality traits for maintaining cognitive function in later life are discussed. The results have implications for developing interventions to maintain or enhance cognitive functioning and personality in middle and later adulthood.

### WHEN LIFE DOESN'T GO AS PLANNED: UNMET EXPECTATIONS ABOUT RETIREMENT TIMING AND SUBSEQUENT DEPRESSIVE SYMPTOMS

Leah R. Abrams,[1] and Neil Mehta[1], *1. University of Michigan School of Public Health, Ann Arbor, Michigan, United States*

The 2008 Great Recession affected American's retirement timing, but it remains unclear how unfulfilled expectations about retirement timing influence psychological well-being. This study examines how unmet expectations about working at age 62 relate to subsequent depressive symptoms, with special attention to sociodemographic differences in unmet expectations and their consequences. We use longitudinal data from 10,557 adults ages 51+ in the Health and Retirement Study (1994-2014). Mean expected probability of working full time at age 62 (ranging 0-100) was 40.5 (SD=54.65). We created quartiles: no chance (0 probability, 35% of sample), unlikely (1-30, 16%), unsure (33-80, 28%), and very likely (85-100, 21%). Expected probability and the association between expectations and reality were significantly lower for racial minorities compared to whites, low education compared to high, and pre-baby boomers compared to baby boomers. Those who were working at age 62 but expected to be retired did not experience elevated depressive symptoms compared to those who correctly expected to be working. In contrast, those who were unexpectedly not working experienced significantly higher depressive symptoms compared to those who correctly expected to be retired (Unsure: IRR=1.16 p=0.024, Very likely: IRR=1.19, p=0.010). This association was slightly attenuated after adjusting for declines in functioning, suggesting partial, but not complete, mediation by health status. The association was consistent by race, education, and birth cohort, but was larger in men than women. Taken together, our findings indicate that unexpected continued employment does not harm psychological well-being, but earlier than expected retirement may result in higher depressive symptoms.

### SOCIAL PARTICIPATION AND GENDER DISPARITY ON DEPRESSIVE SYMPTOMS AMONG INDONESIAN ELDERLY

Riska D. Astuti,[1] and Bondan Sikoki[2], *1. SurveyMETER institute, Yogyakarta, Indonesia, Indonesia, 2. SurveyMETER, Yogyakarta, Indonesia, Indonesia*

A voluminous literature empirically proves the significant relationship between social participation and elderly mental health. Further analysis discovers the different behavior between rural and urban elderly. Despite the importance of taking into account regional inequality, gender disparity which explains the inconsistent empirical results across countries, is sometimes negligible from the discussion. This study aims to investigate the link of social participation in voluntary activity and community regular meeting to depressive symptom among the elderly in Indonesia. Separated analysis based on gender is also conducted to examine the extent to which social activities could explain the depressive symptom disparity between male and female elderly. Data from Indonesia Family Life Survey 2007 and 2014 were analyzed using logistic regression. Sample of 2994 and 2917 respondents aged 60 and over in 2007 and 2014 respectively are combined as pooled cross-sectional data instead of panel data to deal with the large reduction of sample size due to mortality. To minimize the potential endogeneity, covariates are included in the model such as residence location, living arrangement, socio-economic status, and health condition. The results indicate that economic condition, chronic disease, and difficulty on instrumental activity daily living (IADL) play a significant role in depressive symptom among Indonesian elderly, regardless of the gender. Surprisingly, social participation that is widely believed in strengthening mental health is statistically significant for female sample only. Moreover, the contrast sign of voluntary participation and community regular meeting coefficients indicate a special behavior between these two activities.

### WHAT DOES MY PERSONALITY SAYS ABOUT MY FRIENDSHIPS IN LATER LIFE?

Emily Lim,[1] Elizabeth A. Gallagher,[1] Cindy N. Bui,[1] Celeste Beaulieu,[1] Elizabeth Simpson,[1] Laura Driscoll,[1] and Jeffrey Burr[1], *1. University of Massachusetts Boston, Boston, Massachusetts, United States*

Personality traits, such as those identified in the Big Five Personality Model (i.e., openness to experience, conscientiousness, extraversion, agreeableness, and neuroticism), may be associated with different aspects of friendship among older adults. Additionally, men and women form and maintain their friendships differently, which may result in gender differences in their friendships. This study examined the relationship between specific personality traits and friendship characteristics, including friendship quantity, frequency of social interactions with friends, positive and negative friendship quality. The study also explored whether gender moderates the relationship between personality traits and friendships in later life. This study used data from 7,250 community-dwelling older adults, aged 65 years and above (M=75.4 years old, SD=6.91), who participated in 2012 and 2014 Leave-Behind Questionnaire of the Health and Retirement Study. Results from the linear regression analysis indicated significant main effects for the different personality traits and friendship quantity, quality and social interaction frequency, but no main effect for gender was found. However, the moderating effect of gender was significant for the relationship between specific personality traits (i.e. openness to experience, agreeableness and extraversion) and social interaction with friends, as well as for positive and negative friendship quality. For example, older women who scored high on openness to experience reported significantly lower social contact frequency with friends (B=-.16, p<.05) and higher negative friendship quality (B=.08, p<.05) than men who scored high on openness to experience. Study results provide insights for understanding better how personality traits and gender play a role in friendships in later life.

### ASSOCIATION BETWEEN SOCIAL SUPPORT AND ANXIETY IN OLDER ADULTS NEW TO LONG-TERM SERVICES AND SUPPORTS

Yeji Hwang,[1] Nancy Hodgson,[1] Justine Sefcik,[1] Augustine Boateng,[1] Anjali Rajpara,[1] Kierra Foley,[1] and Karen Hirschman[1], *1. University of Pennsylvania, Philadelphia, Pennsylvania, United States*

Anxiety symptoms are common among older adults and are often associated with adverse outcomes. Thus, it is important to examine modifiable factors and manage anxiety symptoms in this population. While many biological and psychological factors related to anxiety symptoms in older adults have been found, little is known about social factors which are essential in one's mental health. The purpose of this study was to examine the prevalence of anxiety symptoms among older adults new to long-term services and supports (LTSS) and to investigate the relationship between social support and presence of anxiety. This was a secondary data analysis from a study funded to examine health related quality of life in older adults new to LTSS. Anxiety was assessed using a single item, "Recently, how often have you felt anxious?" and the answers were dichotomized into "anxiety" (ratings: 'very often', 'often', 'sometimes', and 'seldom') and "no anxiety" (rating: 'never'). Social support was measured by Medical Outcomes Study Social Support Scale. Prevalence of anxiety symptoms in this sample was 82.7% (n=225). In multivariate logistic regression, adjusting for age, gender, LTSS type, cognitive status, physical and emotional health, and depressive symptoms, older adults with more tangible social supports had lower odds of having anxiety symptoms (Odds ratio=0.515; 95% CI: 0.289-0.919, p=0.025). Improving access to tangible social supports for older adults at the start of LTSS may impact anxiety in older adults. Implications for future research and intervention development to provide tangible social support to older adults in LTSS will be discussed.

### IMPROVING MOOD THROUGH PHYSICAL ACTIVITY FOR CARERS AND CARE RECIPIENTS TRIAL (IMPACCT): RESULTS OF A RCT

Briony Dow,[1] Susan (Sue) Malta,[2] Ellen Gaffy,[1] Melissa Russell,[3] Sue Williams,[1] Kirsten J. Moore,[4] Nicola Lautenschlager,[3] and Samantha Loi[3], *1. National Ageing Research Institute, Parkville, Australia, 2. University of Melbourne, Victoria, Australia, 3. University of Melbourne, Parkville, Australia, 4. University College London, London, United Kingdom*

The aim of this Australian study was to investigate effects on depression of a 6-month individually tailored home-based exercise program for caregivers, designed to be done with the person they care for. Ninety-one caregiver-care recipient dyads and 30 caregiver-only participants (caregivers scoring ≥4 on the 15 item Geriatric Depression Scale (GDS-15)) were randomized into one of three groups: exercise intervention (n=50, 34 dyads and 16 caregiver only), social support control (n= 50, 42 dyads and 8 caregiver only) or usual care control (n= 21, 15 dyads and 6 caregiver only). The exercise group completed an individualised program based on the Otago-plus. The primary outcome was the proportion of participants with GDS-15 ≤4. Outcome assessors were blinded to group assignment. There were no significant difference in depression between the physical activity intervention group and the social control (OR 1.06, 95% Confidence Interval (CI) 0.44, 2.56) and the physical activity intervention group and the usual care control (OR 1.51 95% CI 0.46, 4.94) at six months or at 12-months. However, more than 50% of caregivers in all three groups no longer had a GDS-15 score >4 at 6 months. Sub-group analysis revealed that after 6 months caregivers in the exercise group caring for someone with an MMSE ≥24 were significantly less depressed than those caring for someone with an MMSE score of <24 compared with social (p value <0.02) and usual care groups (p value < 0.02). A dyad exercise intervention may be beneficial for those caring for someone without cognitive decline.

### DON'T WORRY, BE HAPPY NOW, PET OWNERS: THE RELATION BETWEEN PET OWNERSHIP AND ANXIETY AND DEPRESSION IN LATE LIFE

Courtney J. Bolstad,[1] Carolyn E. Adams-Price,[1] and Michael R. Nadorff[1], *1. Mississippi State University, Mississippi State, Mississippi, United States*

Pets can provide older adults a means of social support, which can combat problems faced in later life including loneliness, anxiety, and depression. However, current research findings in this area are mixed. The current study explored the differences in anxiety and depression between pet owners and non-pet owners and how pet ownership was associated with these symptoms after accounting for other established correlates. We hypothesized pet owners would endorse fewer symptoms of anxiety and depression than non-pet owners and owning a pet would be associated with these symptoms even after accounting for other common correlates. Participants included 608 older adults aged 70 to 95 that were included in the University of Alabama at Birmingham Study of Aging. As hypothesized, results indicated that pet owners endorsed significantly fewer symptoms of anxiety and depression than non-pet owners. Hierarchical regressions indicated that owning a pet explained a significant amount of variance in anxiety symptoms even after controlling for depression, self-reported health, and demographics. However, owning a pet did not have a significant association with depressive symptoms after accounting for anxiety, self-reported health, and demographics. These results suggest that lower rates of anxiety and depression are related to owning a pet and that pet ownership is associated with fewer anxiety symptoms, but not depressive symptoms, independent of several established correlates of anxiety. Future research is needed to determine the specific mechanisms of pet ownership that comprise this relationship as well as whether pet ownership may longitudinally reduce or buffer against anxiety in late life.

### LATENT PROFILE ANALYSIS OF ANXIETY, DEPRESSION, ANGER, AND ADHD IN OLDER ADULTS

Sarah M. Israel,[1] Erica Szkody,[1] Michael R. Nadorff,[1] and Daniel L. Segal[2], *1. Mississippi State University, Mississippi State, Mississippi, United States, 2. University of Colorado at Colorado Springs, Colorado Springs, Colorado, United States*

Older adults are generally happier, less likely to have depression or anxiety, and have better emotion regulation abilities than earlier in life. While older age predicts more hostile beliefs about others, older adults report less hostile behavior and no difference in covert hostility, compared to other age groups. However, brain regions associated with executive function and emotion regulation are impacted by even normal aging. Using latent profile analysis (LPA) we aimed to better understand what factors contribute to a dysregulated profile in older adults and how age altered the dysregulation profile. The current archival study includes data from 518 older adults between the ages of 60 and 95 years (M = 70.73, SD = 7.34). Participants completed the Coolidge Axis II Inventory (CATI) database. The CATI is a 250-item psychopathology and neuropsychological inventory that assesses over 40 clinical and neuropsychological disorders utilizing official DSM-5 criteria. A Dysregulated Profile was identified using an LPA of diagnosis subscales (i.e., Anxiety, Depression, Anger, and ADHD) that have been previously associated with dysregulation in children and young adults. Results demonstrated that female participants reported more ADHD symptoms (more impairment in executive function) than men. Furthermore, the dysregulated profile (high on all subscales) and age interacted such that, as age increased, scores on the Depression and Anger subscales decreased. No significant differences were found for any other interactions. Our findings are consistent with existing literature. Even in the dysregulated profile, participants reported less anger and depression with older age.

### OSTEOARTHRITIS AND DEPRESSION IN A MALE VA POPULATION

Christopher J. Burant,[1] Gregory Graham,[2] Denise M. Kresevic,[3] Gary Deimling,[1] Said Ibrahim,[4] and Kent Kwoh[5], *1. Case Western Reserve University, Cleveland, Ohio, United States, 2. Frances Payne Bolton School of Nursing , Case Western Reserve University, Cleveland, Ohio, United States, 3. Northeast Ohio VAMC, Cleveland, Ohio, United States, 4. Weill Cornell Medicine, New York, New York, United States, 5. University of Arizona Arthritis Center, Tucson, Arizona, United States*

Osteoarthritis (OA) is a leading cause of disability among older adults. By 2050, approximately 60 million will suffer from arthritis adding up to a total societal cost of $65 billion. Chronic illnesses resulting in pain, and functional decline have been associated with depression in previous studies. The primary goal of this study is to investigate whether OA severity, as measured by the Western Ontario McMasters Arthritis Composite (WOMAC), impacts reported levels of depression and to what degree clinical and sociodemographic variables play a part. A causal model was developed and tested examining the antecedents of OA disease severity and depression. Information on clinical, demographic, socioeconomic, and psychosocial variables was collected on 596 male Veterans with moderate to severe symptomatic OA of the knee\hip. A Confirmatory Factor Analysis was conducted to determine the factor structure of the WOMAC. A 2nd order three factor solution (pain, stiffness, and function) fit the data well (TLI of .94, a CFI of .94 and a RMSEA of .058). The results of the Structural Equation Model reveal a final model that fit the data well (TLI of .95, a CFI of .97 and a RMSEA of .047). Depression was predicted by higher WOMAC scores (beta=.37 , p<.01); higher levels of comorbidity (beta= .11, p<.05); younger age (beta= -.29, p<.01); being white (beta=-.11, p<.05); lower levels of income (beta= -.12, p<.05); lower levels of religiosity (beta= 11, p<.05). Clinicians should be aware of the impact of disease severity when treating OA patients with depression.

### DIRECTIONALITY BETWEEN COGNITION AND DEPRESSIVE SYMPTOMS: A LONGITUDINAL CROSS-LAGGED PANEL ANALYSIS

Randolph Chan,[1] Jennifer Y.M. Tang,[2] Tianyin Liu,[3] and Gloria H.Y. Wong[3], *1. The Education University of Hong Kong, Hong Kong, Hong Kong, 2. Sau Po Centre on Ageing, The University of Hong Kong, Pok Fu Lam, Hong Kong, 3. The University of Hong Kong, Hong Kong, Hong Kong*

Background and Objectives: The relationship between objective and subjective cognitive function and depressive symptoms is complex and potentially multidirectional. This longitudinal prospective study examined the directionality of their relationship among a community sample of older people with no known diagnosis or treatment for dementia or depression. Research Design and Methods: We examined the temporal relationship between objective cognitive functioning, subjective cognitive complaints, and depressive symptoms in 1,814 community-dwelling older people at baseline and one-year follow-up using regression and two-wave cross-lagged panel analyses, after controlling for demographic and health confounders. Results: Cross-lagged analysis showed that depressive symptoms at follow-up were directly predicted by baseline subjective cognitive complaints, but not baseline objective cognitive functioning. The effect differed across objective cognitive functioning levels. In people with clinically significant cognitive impairment at baseline, objective cognitive decline but not baseline subjective cognitive complaints predicted depressive symptoms. In people with mild objective cognitive impairment at baseline, baseline subjective complaints but not objective cognitive decline predicted depressive symptoms. Discussion and Implications: The effects of objective and subjective cognitive decline on depressive symptoms varied across older people with different levels of cognitive impairment. Awareness and insight of one's cognitive status may contribute to the development/progression in depressive symptom in people with mild cognitive impairment. Mechanisms unrelated to appraisal may be involved in increased depressive symptoms among older persons with significant objective cognitive impairment.

### SELF-RATED HEALTH AS A PREDICTOR OF DEPRESSION AND ANXIETY IN OLDER ADULT INMATES

Carlyn E. Vogel,[1] and Lisa C. Barry[2], *1. University of South Florida, Tampa, Florida, United States, 2. University of Connecticut School of Medicine, Farmington, Connecticut, United States*

Inmates age ≥50 years (older inmates) are a rapidly growing population within the U.S. correctional system with the highest suicide rate among adult prisoners. Although depression and anxiety are strong precursors of subsequent suicide, little is known regarding factors associated with these outcomes in older inmates. To inform suicide prevention efforts in this high-risk population, we evaluated the role of older inmates' self-rated health (SRH) in relation to depression and anxiety. We utilized data from the ongoing Aging Inmates Suicidal Ideation and Depression study (Aging INSIDE). Participants (N=175) included men age ≥50 (M=56.5, SD=6.3, range=50-79 years) from eight correctional facilities in Connecticut who completed face-to-face interviews. The outcomes, depression and anxiety, were assessed using the PHQ-9 (range 0-27) and GAD-7 (range 0-21); higher scores on each scale indicated worsening severity. SRH, operationalized as a pseudo-continuous variable (1=excellent; 5=Poor), was correlated with depression (r=0.379; p <.001) and anxiety (r=0.260; p =.001) in unadjusted analyses. Two linear regression models were conducted to determine if SRH was associated with depression and/or anxiety after controlling for age, race (white versus non-white), years of education, visitors (yes versus no), and number of chronic conditions. Increasingly worse SRH was significantly associated with more depressive symptoms ($\beta$=1.92, SE=.43, p <.001) and higher anxiety scores ($\beta$=1.41, SE=.41, p=.001). SRH explained 10.0% and 6.2% of the variance in depression and anxiety scores, respectively. SRH may be useful for identifying older inmates who are more likely to have depression or anxiety, and thus may be at higher risk for suicide.

### NEGATIVE SOCIAL SUPPORTS AND DEPRESSIVE SYMPTOMS AMONG OLDER ADULTS: A CROSS-LAGGED ANALYSIS

Nicholas Cone,[1] Gina Lee,[1] and Peter Martin[1], *1. Iowa State University, Ames, Iowa, United States*

The purpose of the study was to examine how negative social supports and depressive symptoms affect older adults over time. A subsample of participants (N = 3,084) from the Health and Retirement Study was used in this study. Summary scores for each negative social supports (spouse, children, family members, and friends) and the Center for Epidemiologic Studies Depression Scale (CES-D) were used to conduct two cross-lagged regression analyses for each negative social support type from waves 2010 and 2014. Covariate variables for this study included gender, years of education, self-report of health, and age. Results were computed for two age groups (i.e., 65 to 79, and 80+). Results from both age groups indicated high stability for negative social supports and depressive symptoms from waves 1 to 2. The younger age group showed no significant cross-lag or interaction effects when stabilities were included or excluded in the analyses. However, in the older group, wave 2 negative child and family member social support was predicted by wave 1 depression scores. Moreover, the older age group showed significant interaction effects of age by CESD scores on negative child and family member social supports. In conclusion, initial depressive symptoms predict higher negative social supports in children and family members at a second time point in the older age group. Future research could examine whether depressive symptoms continue to predict negative social supports in new waves. In addition, other factors, such as loneliness, or anxiety, may provide further understanding into older adults' negative social supports.

### HEARING IMPAIRMENT, DEPRESSION, AND THE ROLE OF SOCIAL ACTIVITY AMONG CHINESE OLDER ADULTS

Shu Xu,[1] Haowei Wang,[1] and Caitlin Connelly[1], *1. University of Massachusetts Boston, Boston, Massachusetts, United States*

Studies suggest that depression is closely linked to hearing impairment, which is highly prevalent among older adults in the United States. There is evidence that social engagement may be impacted by hearing impairment in older adults. However, there is relatively little research on these associations among Chinese older adults. This study examines the relationships between hearing impairment, social activities, and depressive symptoms among older adults in China. Using nationally representative data from the China Health and Retirement Longitudinal Study 2011, we conducted cross-sectional analysis on adults age 60 years and older (n=10,994). Depressive symptoms were assessed by the 10-item Center for Epidemiologic Studies Depression scale and we considered self-reported hearing status (if participants wear a hearing aid and how they would rate their hearing), and social activities (i.e., volunteering, dancing, attending courses, etc.). Models were controlled for age, gender, education, and other covariates. Descriptive analysis showed that 9% of older adults experienced hearing impairment. Multiple linear regression analyses revealed that hearing impairment was positively associated with depressive symptoms among older Chinese adults ($\beta$=1.32, p<.001). Social activities were found to partially mediate the relationship between hearing status and depressive symptoms. Respondents with hearing impairment were less likely to engage in social activities (OR=.78, p<.01) and those who did not participate in social activities reported more depressive symptoms ($\beta$=1.28, p<.001). These findings suggest that Chinese older adults experiencing hearing loss are at greater risk of depression and that social activities play an important role in the relationship between hearing status and depression.

### DEPRESSION SEVERITY AND OBSTRUCTIVE SLEEP APNEA IN OLDER ADULTS

Nicholas C. Boston,[1] Ryan Bennett,[1] Nikolas Cirillo,[1] Andrew Solow,[2] Nicole Fornalski,[1] and Ashely M. Stripling[3], *1. Nova Southeastern University, Davie, Florida, United States, 2. Nova Southeastern University, Fort Lauderdale, Florida, United States, 3. Nova Southeastern University, Fort Lauderdale, United States*

Objective: The connection between obstructive sleep apnea and depression in older adults is well documented; however, to date the relationship between severity of these depressive symptoms in this population remains under-explored. As such, the current analysis examined a potential relationship between varying levels of depression severity among older adults with sleep apnea. Participants and Methods: Data was derived from a de-identified database of older adults (age>=65) from the National Alzheimer's Coordinating Center (NACC). The sample (N=90; 50% female; 97.8% Caucasian; Mage=77 years; SDage=10.4 years) was sorted into three groups using the Neuropsychiatric Inventory Questionnaire (NPI-Q): 1) Mild Depression [n=56], 2) Moderate Depression [n=29], and 3) Severe Depression [n=5]. Results: A univariate analysis revealed an overall significant omnibus effect between sleep apnea and depression severity (F[2,4041])=16.231, p<.001), while controlling for age, race, and sex. Post-hoc comparison found that those with severe depression had significantly higher levels of sleep apnea compared to those with mild (Mdif =-.499, p = .029) and moderate (Mdif =-.597, p = .009). Conclusions: These data support the possible association between depression severity and obstructive sleep apnea. Results may be attributable to two different theories: that low serotonin levels may simultaneously influence depression, respiratory muscle-tone, and sleep disturbance, and that intermittent hypoxia may create a cascade effect of neurovascular pathology resulting in depressive symptoms. Implications of the current findings suggest it may prove beneficial to keep in mind the risks associated with sleep apnea, and more severe depression, should an individual present with either.

### SESSION 920 (POSTER)

### PHYSICAL ACTIVITY, EXERCISE, AND REHABILITATIVE CARE

### WHEN AND HOW I WANT: FEASIBILITY AND EFFECTS OF EMBEDDING PHYSICAL ACTIVITY INTO NURSING HOME CARE

Eva Barrett,[1] Paddy Gillespie,[1] John Newell,[1] and Dympna Casey[1], *1. National University of Ireland Galway, Galway, Ireland*

Physical activity (PA) is essential to maintaining health into older age. However, older adults living in nursing homes (NHs) remain highly inactive. This study tested the feasibility of a PA programme embedded into NH care and its potential effects on older adults' function and quality of life (QOL). A cluster-randomised controlled pilot feasibility study, including qualitative and economic components, was conducted. Intervention participants (n=18) performed Morning Movement (morning-time walking and sit-to-stand exercises) and Activity Bursts (bouts of activity throughout the day in standing), 3 times weekly for 12-weeks. Participants in the control NH (n=16) received usual care. At baseline and 12-weeks, feasibility and economic data were collected, function was measured using the Timed Up and Go (TUG) and 10-Metre Walk Test (10MWT) and QOL was measured with the Nottingham Health Profile (NHP) and Investigating Choice Experiments for the Preferences of Older People-CAPability (ICECAP-O). Semi-structured interviews were conducted with staff and participants at mid- and post-intervention and analysed thematically. The PA programme was acceptable to staff and participants and study procedures were feasible. Mean TUG improved by 10.2 (±21.6) seconds in the intervention group and was unchanged -0.2 (9.5) seconds in the control group (95% confidence interval of between-group difference in improvement -2.5 to 23.3 seconds). 10MWT scores stayed stable in the intervention group and disimproved in the control group. ICECAP-O and NHP scores were unchanged. While this study contained a small sample, it demonstrated a feasible, acceptable and potentially effective NH PA intervention and provides guidance for a definitive trial.

### CLINICAL FACTORS ASSOCIATED WITH INCREASED SEDENTARY TIME IN VERY ACTIVE OLDER ADULTS

Kenneth Madden,[1] and Jocelyn Chase[2], *1. Gerontology and Diabetes Laboratory, University of British Columbia, Vancouver, British Columbia, Canada, 2. Division of Geriatric Medicine, University of British Columbia, Vancouver, British Columbia, Canada*

Sedentary behavior (such as sitting) has been shown to be an independent risk factor for increased frailty and less successive aging, even in active individuals. Our study examined the clinical factors most associated with higher sedentary times (ST) in very active older adults. We recruited 54 adults from a Master's ski team (Whistler, British Columbia; mean age 71.5±0.6 years, 55% female). Activity levels were measured using an accelerometer (SenseWear) worn continuously for 7 days. ST was defined as a lack of activity when not in the supine position, in order to exclude time spent sleeping. Potential predictor variables consisted of metabolic syndrome criteria (blood pressure, high density lipoprotein, waist circumference, triglyceride levels, fasting blood glucose), age, biological sex and heart rate. Predictors associated with ST ($p<0.10$) were entered into a stepwise multivariate regression model. Our subjects were extremely active, engaging in moderate to vigorous physical activity for 2.6±0.2 hours per day, greatly exceeding current activity guidelines. Despite these high activity levels, they were also sedentary for an average of 9.4±0.2 hours per day. Our final minimum effective model showed that waist circumference had a significant association with ST (Standardized $\beta = 0.36\pm0.13$, $p=0.007$), explaining 18% of the variation in ST. People are often subjectively unaware of how long they spend sedentary. Our study suggests, that in addition to promoting leisure time physical activity, physicians should also objectively measure ST in highly active older patients with high waist circumferences.

### RACIAL DIFFERENCES IN PHYSICAL ACTIVITY IN NURSING HOME RESIDENTS WITH COGNITIVE IMPAIRMENT

Nicole A. Viviano,[1] Elizabeth Galik,[2] and Barbara Resnick[2], *1. University of Maryland, Baltimore, Baltimore, Maryland, United States, 2. University of Maryland School of Nursing, Baltimore, Maryland, United States*

INTRODUCTION: Nursing home residents with moderate to severe cognitive impairment are mostly sedentary. It is more likely that African-American (AA) older adults tend to be more sedentary than their white counterparts. The purpose of this study was to examine racial differences in overall time spent in physical activity (PA), time in sedentary, light intensity, and moderate levels of PA, and participation in activities of daily living (ADLs) among cognitively impaired nursing home residents. METHODS: This was a secondary data analysis from the Function and Behavior Focused Care Intervention study. The sample included 336 cognitively impaired residents from 12 nursing homes. RESULTS: The mean age of the residents was 86.2 (SD=10.1) with an average MMSE score of 7.8 (SD=5.0) where 41% were AA and 59% white. White and AA participants engaged in only 51.2 and 46.1 minutes of light and 1.5 and 1.1 minutes of moderate level PA, respectively. There was a significant difference in time spent in light-intensity PA with whites spending more time in this level of activity [$F(4, 242) = 3.360$, $p = .01$]. Conversely, AAs had better functional ability than white residents [$F(4, 242) = 4.754$, $p < .001$]. There were no significant racial differences in time in sedentary, or moderate level PA. DISCUSSION: These findings are consistent with prior research showing that AAs had lower PA levels compared to their white counterparts. Future research should focus on increasing PA among nursing home residents and consider specific interventions to increase activity among AA residents.

### INTERGENERATIONAL OCCUPATIONAL THERAPY PROGRAMMING FOR COMMUNITY-DWELLING OLDER ADULTS AND SCHOOL-AGE CHILDREN

Robin L. Chilton[1], *1. Cleveland State University, Cleveland, Ohio, United States*

Community Intergenerational Action (CIA) was a four-week pilot program designed to bring community dwelling older adults and fourth-grade students together to engage in meaningful activities within a supportive group context under the facilitation of Robin Chilton, MBA, OTR/L, and four Master of Occupational Therapy Students from Cleveland State University. A phenomenological, qualitative research design study was conducted to explore the impact and meaning of this intergenerational occupational therapy programming on the social and emotional well-being of older adults. Participant observation, journaling, and in-depth interviews were used to determine the meaning of the program to the participants. CIA was conducted using ten female elders, and fifteen children ages nine to ten years old. Each week a new theme was introduced to assist the participants in solving a mystery and included an occupation-based activity such as horticulture and crafts. The CIA program was developed in a way that would allow it to be replicated in other intergenerational settings. Students involved in this study identified helping and cooperating with the older adults throughout the program as very important to them. The students began to feel a sense of empathy and increased self-awareness after spending time with the older adults. The program allowed the older adults to reminisce about their past, and feel a sense of generativity, or contribution to the younger generation. Findings provide an opportunity for others to use similar programs to engage older adults and children in meaningful occupation that will contribute to their overall sense of social and emotional well-being.

### CAN GOLF INFLUENCE GAIT AND COGNITION IN OLDER ADULTS?

Nicole Marcione,[1] Andrea Du Bois,[1] Steven Castle,[2] and George Salem[1], *1. University of Southern California, Los Angeles, California, United States, 2. University of California, Los Angeles, Los Angeles, California, United States*

Gait speed and cognition are important predictors of successful aging. Both slow gait speeds and cognitive decline are associated with poor health outcomes, including hospitalization, falls, institutionalization and death. Exercise interventions can improve both gait and cognitive performance in older adults. Golf is a multimodal, cognitively-challenging physical activity. The purpose of the present study was to examine the influence of a 12-week golf intervention on walking performance and cognition in older adults. Gait speed and cognition were measured in intervention (INT) and control (CON) groups (n=20) before and after a 12-week period. The INT participated in a 12-week golf training program (2 x weekly; 90 min per session). All participants completed six-minute walk test (6MWT), fast single-task gait speed (STGS), fast dual-task gait speed (DTGS) with a subtraction by 3s task, California Verbal Learning Test

(CVLT) and National Institute Health Toolbox-Cognition (NIH-C). 2x2 (Time*Group) Mixed ANOVA revealed significant time*group interactions for 6MWT (p=0.05), STGS (p=0.02), DTGS (p=0.01), CVLT (p=0.01), and NIH-C Fluid Cognition (p=0.06) had a trend towards significance. Post-hoc t-tests demonstrated that INT significantly improved their pre-to-post 6MWT, STGS, DTGS, CVLT, and NIH-C Fluid. CON had no significant pre-to-post intervention changes. Participants in the 12-week golf training program improved gait and cognitive performance, compared to CON. These results provide evidence that golf, as a cognitively-challenging physical activity, may improve physical and cognitive function, leading to attenuated risk for poor health outcomes, maintaining independence and improved quality of life.

### TIME SPENT IN SEDENTARY BEHAVIOR DOMAINS AND PHYSICAL FUNCTION IN U.S. OLDER ADULTS

Nancy M. Gell,[1] Erin D. Bouldin,[2] and Dori Rosenberg[3], *1. University of Vermont, Burlington, Vermont, United States, 2. Appalachian State University, Boone, North Carolina, United States, 3. Kaiser Permanente Washington Health Research Institute, Seattle, Washington, United States*

Previous studies have reported associations of sedentary time with worse health outcomes in older adults. Yet, little is known about the relationships between the contexts of sedentary time and health outcomes. The purpose of this study was to examine associations of physical function with time spent in a variety of sedentary behavior domains. We analyzed data from the 2016 National Health and Aging Trends Study, a nationally representative sample of community-dwelling Medicare beneficiaries. Estimated time being sedentary by domains (e.g., TV watching, computer use, resting/napping, eating, transportation, socializing, sitting and doing hobbies) were collected from a subset of the sample population (N=2157). The Short Physical Performance Battery (SPPB) measured physical function. Linear regression models were conducted adjusting for sociodemographics, health conditions, pain, and dementia. More time watching TV and resting/napping was significantly associated total SPPB scores ($p < 0.01$). In adjusted models, lower SPPB scores were significantly associated with more time/day spent sitting and watching television or resting ($\beta$ =-0.16 hours; 95% Confidence Interval (CI): -.024, -0.08 for TV watching and $\beta$ =-0.63 hours; 95% CI: -0.80, -0.46 for resting). Average time in computer use, eating, transportation, hobbies, or social activities did not differ by physical function level. Associations between physical function and sedentary time vary by the context. Social or engaging sedentary activities do not appear to be associated with physical function limitations in the same way as passive sedentary domains like television viewing and resting. Context should be considered in evaluating relationships of sedentary time with health outcomes.

### AN MHEALTH BEHAVIORAL INTERVENTION FOR PROMOTING PHYSICAL ACTIVITY AND SLEEP IN COMMUNITY-DWELLING OLDER ADULTS

JUNXIN LI,[1] Sarah Szanton,[2] Minhui Liu,[2] Nada Lukkahatai,[3] Junxin Li,[3] and Nalaka Gooneratne[4], *1. Johns Hopkins School of Nursing, Baltimore, Maryland, United States, 2. Johns Hopkins University School of Nursing, Baltimore, Maryland, United States, 3. John Hopkins University, Baltimore, Maryland, United States, 4. University of Pennsylvania, Philadelphia, Pennsylvania, United States*

Evidence suggests physical activity (PA) improves sleep in older adults. This study examined the preliminary effect of a personalized mHealth behavioral intervention on PA and sleep in older adults. We conducted a randomized controlled pilot trial in 21 community-dwelling older adults with sleep complaints. The 24-week mHealth behavioral intervention included a 2-hour in person training session, personalized exercise prescription, real time PA self-monitoring, interactive prompts, phone consultation, and weekly financial incentives. PA and sleep were measured objectively using Actiwatch 2.0 and subjectively using questionnaires. Peripheral blood was drawn for measuring Plasma inflammatory biomarkers [interleukin 1$\beta$, 6, 8, Tumor Necrosis Factor- alpha (TNF-$\alpha$), and c-reactive protein (CRP)]. Data were collected at baseline, 8-week, 16-week, and post intervention. Repeated measures ANOVA (time*group) was used to examine differences of PA and sleep across times between the two groups. Majority of participants are women (71.4%) with mean age of 73.7 (SD = 6.9). Repeated measure ANOVA showed significant ($p <0.05$) improvement of objective and subjective PA, objective nocturnal sleep duration, self-report sleep quality (measured by Pittsburg Sleep Quality Index and Insomnia Severity Index) and decreasing of sedentary time over times in the intervention group (n=11), compared to the control group. The intervention group showed significant reduction of plasma TNF-$\alpha$ and CRP levels at 16-week and post intervention. Interventions combining personalized PA and mHealth strategies may positively affect physical activity and sleep in older adults. A larger study is needed to test the efficacy of this intervention and the mechanisms associated with it.

### EARLY INPATIENT REHABILITATION IN GERIATRIC PATIENTS: A SYSTEMATIC REVIEW OF OUTCOME MEASURES

Klaus Hauer,[1] Patrick Heldmann,[2] and Christian Werner[1], *1. Agaplesion Bethanien Hospital, Geriatric Center at the Heidelberg University, Heidelberg, Germany, 2. Network Aging Research (NAR), Heidelberg University, Heidelberg, Germany*

Selecting appropriate outcome measures for multimorbid, acutely hospitalized geriatric patients poses specific challenges, which may have caused inconsistent findings of previous intervention trials on early inpatient rehabilitation. The objective of this review was to describe primary outcome measures used in randomized controlled trials (RCTs) on early rehabilitation in older hospital patients, to analyze their matching to intervention programs, and to evaluate the effects of matching on the main findings of these RCTs. A systematic literature search was conducted in PubMed, Cochrane CENTRAL, CINAHL, and PEDro databases. Inclusion criteria were: RCT, patients aged ≥ 65 years, admission to hospital, physical exercise intervention, and primary outcome measure during hospitalization. Two independent reviewers extracted the data, assessed the methodological quality, and analyzed the matching of primary outcome measures to the intervention, study sample, and setting. Main study findings were related to the results of the matching procedure. In 28 included articles, 33 different primary outcome measures were identified, which we grouped into six categories: functional status, mobility status, hospital outcomes, adverse clinical events, psychological status, and cognitive functioning. Outcome measures differed considerably within each category showing a large heterogeneity in their matching to the intervention, study sample, and setting. Outcome measures that specifically matched the intervention contents were more likely to document intervention-induced benefits. Mobility instruments seemed to be the most sensitive outcome measures to reveal such benefits. High specificity (optimized match) of outcome measures and intervention contents is a key factor to reveal benefits of early rehabilitation in acutely hospitalized geriatric patients.

### MISMATCH BETWEEN PERCEIVED AND OBJECTIVE MOTOR IMPROVEMENTS WITH ADAPTED TANGO INTERVENTION IN OLDER ADULTS

Crystal Bennett,[1] Nathalie Angel,[2] and Madeleine Hackney[2], *1. University of West Florida, Pensacola, Florida, United States, 2. Emory University, Atlanta, Georgia, United States*

The purpose of this secondary analysis was to assess the relationship between objective and subjective perceptions of motor function measures in older adults following a 12 week adapted tango or health education intervention. A quasi-experimental, two-group, repeated-measures design was used. The study took place in diverse senior independent living communities in an urban metropolitan area. 74 older adults participated (Tango: n= 62, age: 82.3 (8.81) years; Education: n=12, age: 84.1 (7.86) years). Participants were assigned to 20 sessions of 90-minute tango (n = 62) or health education (n = 12) classes over 12 weeks. Motor function, depression, mental and physical quality of life were measured before and after intervention. At post-test, participants indicated their level of agreement with statements that they improved in the objectively measured domains of motor function. Correlations were performed between subjectively rated agreement, and changes in motor function and depression/quality of life. Tango subjective ratings were negatively correlated with empirically observed improvements in balance (r= -.423) and endurance (r= -.241); although their ratings moderately correlated positively with coordination (r=.319) and minimally correlated positively with lower body strength (r=.188). In Tango, decreased depression was positively correlated with self-perceived improved lower body strength (r=.271) and endurance (r= .254). Improved mental function was moderately (r=.423) positively correlated with self-perceived improved balance and coordination (r=.306). After rehabilitation, even in the presence of improved depression and quality of life, older adults may not perceive empirically observed motor function improvements, particularly in balance and lower body strength.

### THE INTERPLAY OF FUNCTIONAL PERFORMANCE AND COGNITION ON WALKING SPEED RESERVE

Nicole T. Dawson,[1] and Ashleigh Trapuzzano[1], *1. University of Central Florida, Orlando, Florida, United States*

Domains of functional performance (strength, balance) and cognition (executive function, processing speed) have been shown to independently predict both comfortable and gait speed. Walking speed reserve (WSR) reflects an individual's ability to increase their walking speed when needed to adjust to environmental demands. Increasing speed requires a higher demand of neuromuscular control and cognitive resources. Few studies have investigated WSR; however, poorer cognitive status has been shown to be associated with a smaller WSR. Understanding mechanisms in WSR could help with intervention development. The purpose of this study was to investigate the interplay of functional performance and cognition on WSR. Sixty-seven community-dwelling older adults (mean age 80.57; 71% female) completed assessments including: global cognition (Mini Mental State Examination), leg strength (30 Second Chair Stand), balance (Functional Reach), executive function (Trail Making Test Part-B, clock-drawing test, Flanker Task), simple reaction time, processing speed (Digit Symbol Substitution Test), and gait speed (comfortable and fast) using the GAITRite® system. WSR (fast-comfortable gait speed) was the dependent variable. Independent variables in the regression model were selected using Pearson correlation values <0.05 (strength (r=.332, p=.006) and processing speed (r=.361, p=.003)). Linear regression revealed that the strength and processing speed explained 16.3% of the variance (adjusted r2) of WSR and were both unique predictors of WSR indicating individuals with greater strength (B=.79, p=0.036) and faster processing speed (B=.44, p=0.015) have a greater capacity to alter their gait speed. Further, the interdependence between cognition and physical performance emphasizes the importance of interprofessional care for older adults.

### POOR PHYSICAL FUNCTION EXPLAINS INCREASED WRIST FRACTURE RISK IN COMMUNITY DWELLING OBESE ICELANDIC ADULTS

Alfons Ramel[1], *1. University of Iceland, Reykjavik, Capital area, Iceland*

Background: The associations between obesity, physical function, falls and fractures are unclear. Aim: The aim was to investigate whether obese adults have poorer sensory function, physical function as well as higher fracture risk than normal weight peers; and whether confounders explain potential differences between body mass index (BMI) categories. Methods: A case-control study was conducted using 98 wrist fracture cases (50-75 years) and 48 matched controls. Measurements included among others: anthropometrics, sensory function (Semmes-Weinstein-Monofilaments), physical function (Five-times-sit-to-stand test (FTSTS), questionnaires on previous fractures and fall history, the Activities-Specific-Balance-Confidence (ABC) and the Dizziness-Handicap-Inventory (DHI) scales. Results: Obese participants had lower physical function (FTSTS: +2.8sec,P<0.001), lower tactile sensitivity (monofilaments:+3.4 g,P=0.013), poorer ABC score (14.0,P<0.001), higher DHI score (11.6,P<0.001) and experienced more falls the previous 12 months (+0.8,P=0.006) compared to normal weight subjects. According to crude logistic regression analysis, the hazard ratios for wrist fracture in overweight and obese subjects were 2.7 (P=0.012) and 5.6 (P=0.002), respectively. When FTSTS was included in the statistical model (HR:1.7,P=0.004), it reduced the hazard ratios of overweight and obesity close to 1. Lifetime fractures (HR:5.5,P<0.001) and fall history (HR:4.3,P<0.001) were associated with an increased fracture risk independently from the BMI categories. Conclusion: Obese individuals have poorer physical function, higher risk of falls and increased wrist fracture risk compared to normal weight peers. Lower physical function is the main driver for the increased risk in obese subjects. Lifetime fractures and fall history are associated with an fracture independently from obesity.

### INERTIAL LOAD SPRINT TRAINING IMPROVES NEUROMUSCULAR POWER IN OLDER ADULTS

Jakob R. Allen,[1] Remzi Satiroglu,[1] Emre Vardarli,[2] Anthony Laico,[1] Anthony Wolfe,[1] and Edward Coyle[1], *1. University Of Texas at Austin, Austin, Texas, United States, 2. University of Texas Medical Branch at Galveston, Galveston, Texas, United States*

PURPOSE: Maximal strength and neuromuscular power decline after the fourth decade of life. The physiological cause of this progression is partly a selective atrophy of Type II or "Fast-twitch" muscle fibers. The aim of this study was to determine the efficacy of a novel, time-efficient form of exercise training involving repeated sprints lasting 4 s on an inertial load cycle ergometer, to promote increased neuromuscular power in males and females aged 50-70y. METHODS: Three days a week, forty older adults performed 15, 20, and 30 sprints per day over weeks 1, 2-4 and 5-8 of the study respectively. Rest intervals were progressively reduced from 56s, 41s, and 26s over the same time periods. Subjects began each sprint while stationary and then were instructed to pedal as hard and as fast as possible for 4 s. Maximal power was reached after 1-4 s of sprinting and measured after a familiarization day (PRE), and then post-training (POST). RESULTS: The average increase in maximal power was 10.5±1.4% from PRE to POST (616±41 to 684±48 watts) ($p<0.01$). CONCLUSION: Only ~2 min of cycle sprinting per training session was able to increase maximal neuromuscular power, an important physiological component of tasks of daily living throughout the lifespan.

### LIFESTYLE PHYSICAL ACTIVITY IN OLDER WOMEN: ASSOCIATIONS OF CHANGE, SELF-EFFICACY, AND WELL-BEING

Leanne L. Lefler,[1] Shelly Y. Lensing,[2] and Kimberly K. Garner[3], *1. University of Arkansas for Medical Sciences, Arkansas, United States, 2. University of Arkansas for Medical Sciences, Little Rock, Arkansas, United States, 3. Geriatric Research, Education and Clinical Center, Central Arkansas Veterans Healthcare, North Little Rock, Arkansas, United States*

Reduction of cardiovascular disease risk in undeserved populations, such as older women, is a top priority of the U.S. Our innovative trial tested a new approach to PA promotion for older women—motivational interviewing (MI), shifting the paradigm from structured exercise to self-selected activities. We present data comparing stage of change (SOC), self-efficacy for exercise (SEE), and well-being: 8 dimensions (physical, social, role limitations, emotional, general mental health, vitality, health perceptions and pain) and associations with physical activity outcomes in the Lifestyle Physical Activity for Women (LPAW) clinical trial. Methods: 106 women, > 60 years old, who did not engage in regular PA, and were not frail, participated in a clinical trial of a tailored MI intervention to increase PA. We report baseline, 3 and 6 month repeated measures and PA associations with SOC, SEE, and well-being (SF36). Results: Of 106 women, 36% were Black and 63% White, with a mean age of 69. Significant improvement in SOC in both arms noted but the proportion in action/maintenance was significantly higher in the PA arm at 3 mos (78% vs. 55%, $P=0.045$) and 6 mos (79% vs. 50%, $P= 0.019$). A decrease in SEE for control ($p=.001$), but not for PA arm ($p=.45$); at 6 months, The PA arm had greater SEE compared to control. There were significant arm difference for physical component scores of SF36 ($p=.02$), but not for mental scores. Associations with PA will be tabulated. Conclusions: Preliminary results support the PA intervention, more data to be presented.

### GENDER DIFFERENCES IN THE EFFECT OF DIFFERENT EXERCISE DOSES ON PREVENTING DEPRESSIVE SYMPTOMS IN OLDER ADULTS

Mei-Lan Chen,[1] Yu-Chen Chang,[2] Ying-Yu Chao,[3] Douglas S. Gardenhire,[1] and Elisabeth O. Burgess[4], *1. Byrdine F. Lewis College of Nursing and Health Professions, Georgia State University, Atlanta, Georgia, United States, 2. Department of Community Health, Chia-Yi Christian Hospital, Chiayi, Taiwan, 3. School of Nursing, Rutgers, the State University of New Jersey, Newark, New Jersey, United States, 4. Gerontology Institute, Georgia State University, Atlanta, Georgia, United States*

Regular exercise is potentially an effective way to prevent or reduce depressive symptoms for older adults. However, little research has focused on gender differences in exercise dose effects on depressive symptoms in older people. The purpose of this study was to test gender differences in the preventive effects of different exercise doses on depressive symptoms among community-dwelling older adults in Taiwan. This study was a secondary analysis of a longitudinal cohort study in a sample of older Taiwanese adults ($N = 2,673$; mean age 74.2 ± 5.7 years). Four different doses of moderate-intensity exercise were examined including three 15-min sessions/week, three 30-min sessions/week, six 15-min sessions/week, and six 30-min sessions/week. Descriptive statistics and generalized linear mixed models were used to analyze characteristics of the sample and hypotheses testing. All analysis models were adjusted according to age, gender, education, marital status, smoking, social participation, and chronic conditions. The results indicated that regular exercise with at least 15 min per session, 3 times a week of moderate intensity was significantly associated with lower levels of depressive symptoms for women, but there were no significant preventive effects on depressive symptoms for men. This study suggests that moderate-intensity exercise may play a protective role in depression prevention for older women, even with a very low dose (three 15-min sessions/week). Gender differences should be considered for future research and clinical practice when designing exercise interventions on preventing depression for older adults.

### THE ROLE OF S100B IN AEROBIC TRAINING EFFICACY IN OLDER ADULTS WITH MILD VASCULAR COGNITIVE IMPAIRMENT

Rachel A. Crockett,[1] Cindy Barha,[1] Ging-Yuek Robin Hsiung,[1] and Teresa Liu-Ambrose[1], *1. University of British Columbia, Vancouver, British Columbia, Canada*

Aerobic training improves cognitive and brain outcomes across different populations and neurocognitive disorders of aging, including mild subcortical ischemic vascular cognitive impairment (SIVCI). However, little is known of the underlying mechanisms through which aerobic training exerts its beneficial effects on the brain. Recently, S100 calcium-binding protein B (S100B) has been proposed as a possible mediator of aerobic training. At low levels, S100B is neurotrophic but at higher levels it is neurotoxic. Elevated levels of S100B have been associated with decreased performance on measures of global cognitive function. Thus, we conducted a secondary analysis of data collected from the proof-of-concept single-blind randomized controlled trial (NCT01027858) in older adults with mild SIVCI to determine whether the beneficial effects of 6-months, thrice weekly, moderate intensity aerobic training on cognitive performance is related to changes in S100B levels. In a subsample of 45 participants, blood samples were collected both before and after trial completion. Global cognitive function was assessed using Mini Mental State Examination (MMSE). At trial completion, aerobic training decreased circulating levels of S100B compared with usual care plus education ($F(1,41) = 6.673$, $p = 0.013$, $\eta p2 = 0.140$; Figure 1). Furthermore, reduced S100B levels were associated with improved global cognitive function in those who received the aerobic exercise intervention (partial $r = -0.519$, $p = 0.023$). Together these findings suggest that S100B is a promising target mediating the beneficial effects of moderate-intensity aerobic training on brain health in older adults with mild SIVCI.

### ACTIVITY EFFORT, SELF-MANAGEMENT AND INFLAMMATION IN OLDER MEXICAN AMERICANS WITH OSTEOARTHRITIS

Tracie C. Harrison,[1] Lauren Thill,[2] Bianca R. Schmidt,[3] and Shelley Blozis[4], *1. The University of Texas at Austin, Austin, Texas, United States, 2. The University of TX, Austin, Texas, United States, 3. The University of Texas, Austin, Texas, United States, 4. University of California, Davis, California, United States*

We extend our ongoing investigations of the health effects of activity effort among Mexican Americans (MA) with mobility limitations, specifically those with osteoarthritis (OA) (Harrison, 2009). Our previous research linked activity effort with co-morbidity and social participation in women with mobility limitations, finding significant variations between Non-Hispanic White and MA with physical disabilities. This bio-behavioral study takes the next step by examining the relationships between inflammatory measures (TNF-alpha & CRP), Mexican American-specific self-management behaviors (MA-SM), and activity effort (AE) in a sample of MA men and women. Over 5 months, 62 men and women, age 40 to 83, provided survey responses, blood, and saliva for analysis. After ensuring reliability of measures, we used Pearson correlations to provide initial associations. Findings indicated a significant negative correlation between AE and TNF-alpha (-.376, 0.005), which linked behaviors to inflammatory response; and between MA-SM and AE (-.254, 0.05), which linked the self-management to the behavior. These findings provide support for the biological impact of perceived activity effort on inflammation, as well as the positive effects that Mexican American specific self-management activities might have on health.

### AGE-RELATED FUNCTIONAL RESERVE DECLINE IS NOT SEEN IN PHARYNGEAL SWALLOWING PRESSURES

Corinne A. Jones,[1] Melanie Looper,[1] and Timothy McCulloch[2], *1. The University of Texas at Austin, Austin, Texas, United States, 2. University of Wisconsin - Madison, Madison, Wisconsin, United States*

Age-related decline in functional reserve has been described in tongue strength: tongue pressure during swallowing does not change with age, but maximal-effort isometric tongue pressure decreases with age. Healthy persons show a slight increase in pharyngeal swallowing pressure with age, but it is unknown if there is a similar decline in functional reserve. Fifty-six healthy adults (n=38 60 years) underwent pharyngeal high-resolution manometry during effortful and normal-effort thin liquid swallows. Repeated measures ANOVAs were performed on maximum pressures, pharyngeal contractile integral (PCI), pharyngeal pressure gradients, and upper esophageal sphincter minimum pressures. We hypothesized that older individuals would generate a less-robust pressure increase with effortful swallowing than younger individuals. Maximum pressures, PCI, and gradients increase during effortful swallowing ($p<0.001$), but there was no interaction effect with age, suggesting a lack of age-related functional reserve decline. Older individuals had greater UES minimum pressures than younger individuals in the effortful swallowing task ($p=0.03$), which may stem from reduced muscular compliance in this area. These findings do not align with those reported in tongue pressures, suggesting that muscle properties and pressure generation may be fundamentally different between the pharynx and the oral tongue. Alternatively, the effortful swallowing task may not elicit maximum contractility of the pharyngeal musculature. The preserved ability to increase pharyngeal pressure during effortful swallowing may support the use of the effortful swallow exercise in older adults with swallowing disorders.

### SESSION 925 (POSTER)

### SOCIAL NETWORKS AND SUPPORT

### CHATTIER WITH FRIENDS: OLDER ADULTS' DAILY SOCIAL CONTACT AND CONVERSATION

Yee To Ng,[1] Meng Huo,[2] and Karen Fingerman[1], *1. University of Texas, Austin, Austin, Texas, United States, 2. The University of Texas at Austin, Austin, Texas, United States*

Studies suggest conversation improves cognitive skills among older adults. While contact with family members is common in late life, contact with friends and acquaintances is relatively less frequent. Yet, we know little about how often older adults engage in conversation when they have contact with different social partners. This study used data from the Daily Experiences and Well-being Study to investigate how older adults talk with different social partners on a daily basis. Participants (N = 303) completed an initial interview about their social partners and reported on their contact with each social partner in ecological momentary assessments every 3 hours across 5 to 6 days. Participants also wore Electronically Activated Recorders (EAR), which captured snippets of their daily conversation. Findings revealed that contact with family members (e.g., spouse, children, siblings) occurred most often, with less frequent contact with other social partners (e.g., acquaintances, neighbors), and then friends. Multilevel models also revealed that participants talked more (i.e., saying more words in each 30-second snippet and had a greater proportion of snippets when they talked) when they had contact with their friends than when they had contact with family members or other social partners. Results from these multiple methods suggest that daily contact with friends could potentially encourage conversation that may facilitate cognitive functioning among older adults.

### PRESENCE AND PREDICTORS OF FAMILIAL AND NON-FAMILIAL AGE-INTEGRATED SOCIAL NETWORKS

Carly Roman,[1] Christopher Beam,[2] and Elizabeth M. Zelinski[3], *1. USC Leonard Davis School of Gerontology, Los Angeles, California, United States, 2. USC Dornsife College of Letters, Arts and Sciences, Los Angeles, California, United States, 3. Davis School of Gerontology, University of Southern California, Los Angeles, California, United States*

A growing older adult population living longer provides opportunities for greater age-integration, which includes reducing age-related structural barriers and increasing cross-age interactions (Riley & Riley, 2000). While research on the theoretical construct of age-integration is prevalent, empirical evidence of age-integration in older adults' social networks is lacking (Hagestad & Uhlenburg, 2005). This study uses the National Health and Aging Trends Study to quantify and characterize age-integrated social networks in the United States, and to understand the sociodemographic predictors of these age-integrated relationships. Participants' social networks, comprised of respondents' spouses, household members, children, helpers, care recipients, and up to five individuals they share important things with were considered age-integrated if individuals were at least 10 years younger than the respondent. About 96% of respondents reported at least one person 10+ years younger than them. Further, these relationships were coded as familial (i.e., spouse, children, grandchildren, parents, siblings, and other relatives) and non-familial relationships (i.e., other non-relatives) and analyses predicting age-integrated relationships as a function of sociodemographic characteristics were stratified by relationship type. Weighted multilevel logistic regression analyses suggest that females have lower odds of familial and non-familial age-integration than males; compared to white and married individuals, Black and Hispanic individuals have greater odds of familial and non-familial age-integration; compared to married individuals, separated, divorced, and widowed individuals have greater odds of familial age-integration, and those who were never married have greater odds of non-familial age-integration. This foundational study reveals that sociodemographic factors differentially predict familial and non-familial age-integrated social networks.

### SOCIAL NETWORKS AND NEIGHBORHOOD SATISFACTION OF AFRICAN AMERICAN OLDER ADULTS: AN ATLANTA STUDY OF RELOCATION

Nia Reed[1], *1. Georgia State University, Atlanta, Georgia, United States*

Atlanta was the first major city to offer federally-funded public housing and it is one of the first to demolish it. Unlike other cities undergoing public housing transformation through demolition under Housing for People Everywhere Program (HOPE VI), the Atlanta Housing Authority (AHA) targeted senior housing as part of the demolition process. Investigators conducting the Urban Health Initiative (UHI) study collected three waves of data (baseline, 6-month post-relocation, and 24-month post-relocation) from relocated seniors and a comparison group of seniors who aged-in-place. To understand the interactions between public housing residents and varied components of their environments, including social networks and neighborhood satisfaction, I will use place attachment theory to frame my research, as sense of place is rooted within the interplay of community cultural wealth components. I will also use aging-in-place theory, which refers to individuals' ability to grow old in their own homes and communities, while adjusting to needed modifications associated with aging and mobility. Analysis of Covariance will be applied to understand the relationship between social networks, relocation, and neighborhood satisfaction among older adults who age-in-place, compared to those who relocated.

### INITIATION OF INTERACTION AS THE BEGINNING OF SOCIAL PARTICIPATION: CONVERSATION ANALYSIS OF A JAPANESE SENIOR CLUB

Eri Sakai,[1] Akihiko Kamesawa,[2] Riko Nakayama,[3] Jihoon Kim,[4] Akizuki Yuri,[5] Yingxue Yang,[3] Ryogo Ogino,[6] and Jun Goto[7], *1. Department of Sociology, Graduate School of Humanities and Sociology, The University of Tokyo, Tokyo, Japan, 2. The University of Tokyo, Bunkyo-ku, Tokyo, Japan, 3. Department of Integrated Education and Science, Graduate School of Education, The University of Tokyo, Tokyo, Japan, 4. the University of Tokyo, Tokyo, Japan, 5. Department of Urban Engineering, Graduate School of Engineering, The University of Tokyo, Tokyo, Japan, 6. Institute of Gerontology, Tokyo, Japan, 7. Institute of Gerontology, The University of Tokyo, Tokyo, Japan*

The rate of social participation of senior citizens in a senior club's activities is not equal to the rate of desire for the said participation. Earlier studies mainly examined personal and social factors which influence the participation rate, overlooking the practical methods by which senior citizens can overcome barriers to participating in club activities. Our study aims to clarify the features of a club activity as a resource by analyzing the activity's interactions. Our study is based on data extracted from videotaped recordings of a senior calligraphy club in Kanto, Japan. In September 2018, one lecturer and 11 participants were videotaped for 3 hours, and the video underwent conversation analysis, which elucidates how people organize activities under specific circumstances. We analyzed how a female newcomer to the activity initiated face-to-face interaction, which is considered the first step of social participation. She talked to other participants who were familiar with the exercise several times by inquiring how to read kanjis on teaching materials. These findings suggest that visualization of skill relative to the other creates an environment for initiating face-to-face interaction. In this case, the newcomer utilized the difference in skill denoted by teaching materials and was given the rational reason to talk to the others already engaging in the activity. Therefore, designing teaching materials that assign the learning level of each participant may be effective in promoting social participation in senior study clubs.

### SOCIAL NETWORK SIZE AND FREQUENCY OF ELECTRONIC COMMUNICATION: THE RELATIONSHIP TO INTERPERSONAL STRESS

Xin Yao Lin,[1] and Margie E. Lachman[1], *1. Brandeis University, Waltham, Massachusetts, United States*

Social relationships are beneficial for psychological wellbeing, but they are also associated with interpersonal stress. With the growing usage of multiple forms of electronic communications (EC) including phone calls, text messages, video chat, and internet among adults of all ages, it was of interest to explore the relationship between social network size (SNS), in-person communication (PC), and EC, and whether the relationship between SNS and frequency of communication is associated with interpersonal stress. A daily diary study was conducted over seven days for 142 participants ages 22 to 94. SNS was assessed with the social convoy model. Frequency of PC and EC, along with interpersonal stress, were assessed daily. As expected, multiple regression analysis results showed that older adults had smaller SNS and less frequent technology communication (text messages, video chat, internet) compared to younger adults. With regard to effects on interpersonal stress, there were no main effects for frequency of PC, EC, or SNS. However, the frequency of EC moderated the relationship between SNS and interpersonal stress, controlling for amount of PC. Among those with a smaller SNS, having more frequent EC was associated with less interpersonal stress compared to those with less frequent EC. For those with a larger SNS, having more frequent EC was associated with more interpersonal stress compared to those with less EC, but PC was not related to interpersonal stress. The discussion will consider implications of the findings for developing interventions to minimize stress from interpersonal communications, especially those that involve EC.

### BUILDING SOCIAL CAPITAL: LESSONS FROM A SWISS ACTION-RESEARCH INTERVENTION

Annahita Ehsan[1], *1. University of Lausanne, Lausanne, Switzerland*

Social capital interventions to promote healthy aging seem promising, but recent evidence has questioned how social capital is constructed. In order to understand how social capital is built among older adults, this study draws from the diagnostic phase of one 'Neighbourhoods in Solidarity' (NS) intervention, which uses action research to promote wellbeing for older adults (55+) in Swiss communities. These findings arose from ethnographic fieldwork with 77 hours of observation during group gatherings and informal interviews with participants who identified and debated issues in their community. It became evident that the geographic space and the sense of identity that citizens attached to it (herein referred to as 'place') played a role in how the NS intervention developed. The community was divided into two groups with distinct identities: one in the north and one in the south. The sense of place for both groups was simultaneously disrupted when outsiders moved to both areas, exacerbating tensions. The NS brought the two groups together and helped develop social capital between them. This was highlighted by the changing willingness of citizens to navigate unfamiliar spaces, to create social ties, and to trust others. The NS helped create a new sense of place for citizens, which ultimately facilitated the creation of social capital in the community. The findings suggest that identities are dynamic and play a role in constructing social capital, as well as who benefits from social capital and who may be excluded. Lessons from this research may inform future social capital interventions.

### SOCIAL SUPPORT AND SOCIAL RESOURCES PREDICTING LIFE SATISFACTION AMONG CENTENARIANS AND OCTOGENARIANS

Rotem Arieli,[1] Peter Martin,[1] and Leonard Poon[2], *1. Iowa State University, Ames, Iowa, United States, 2. University of Georgia, Athens, Georgia, United States*

Although much research has assessed the relationship between social support and life satisfaction for older adults, there is little information on how social support predicts life satisfaction over and above social resources among very old people. The purpose of this research was to determine pathways from demographic variables, social resources, and social support to life satisfaction. Data from 208 cognitively-intact centenarians and octogenarians of the Georgia Centenarian Study (GCS) were analyzed using multiple regression analyses to evaluate pathways from social resources via social support to life satisfaction. Three different models were analyzed in the GCS sample: one with a combined group of octogenarians and centenarians, one with only octogenarians, and one with only centenarians. Path models included: demographic variables (gender, ethnicity, residential type, and age in years) to social resources to social provisions to life satisfaction. Results in the combined older adult group showed that residence type significantly predicted social resources, $\beta = -.26$, $p < .01$, social resources significantly predicted social provisions, $\beta = .15$, $p < .05$, and social provisions significantly predicted life satisfaction, $\beta = .15$, $p < .05$. Results in the centenarian sample showed that both residence type and age significantly predicted social resources, $\beta = -.19$, $p < .05$, and $\beta = -.17$, $p = .05$, respectively, and social resources significantly predicted social provisions, $\beta = .18$, $p = .05$. Overall, results indicate the uniqueness of the centenarian population and their paths to high life satisfaction through social resources and support.

### SELECTIVE NARROWING OF PERIPHERAL SOCIAL NETWORKS PREDICTS POOR LONG-TERM COGNITION IN OLD AGE

Hsiao-Wen Liao,[1] Yochai Z. Shavit,[1] Laura L. Carstensen[1], *1. Stanford University, Stanford, California, United States*

Socioemotional selectivity theory posits that emotionally meaningful goals such as spending precious time with close family and friends are prioritized in late life as a function of limited future time horizons. Research documents that older individuals include a smaller proportion of peripheral social partners than younger individuals in their social networks, and that this selectivity is associated with better daily emotional experience (English & Carstensen, 2014). Such limitation of social partners, however, might adversely affect cognitive function in the long run, since exposure to novel and cognitively stimulating environments has been tied to better cognitive functioning (Park et al., 2014). The current study examined the long-term association between proportions of peripheral social partners in older adults' social networks and cognitive performance. Sixty-one older participants (Mage = 71.53) reported the size of their inner, middle, and outer social circles using the Social Convoy Questionnaire (Kahn & Antonucci, 1980) and completed Digit Span Backward, Digit Span Forward, and Digit Symbol tasks at baseline and five years later. Results of multiple regression analysis show that participants who had a smaller proportion of social partners in their outer social circle at baseline performed poorer on the Backward Span task assessed five years later than those with a larger outer circle proportion. Results hold controlling baseline cognition, physical health, age, SES, education, and trait openness. We discuss the findings in terms of potential tradeoffs between the age-related social selection and working memory in the long run.

### KIN NETWORK DYNAMICS OVER THE LIFE COURSE AND HISTORICAL TIME IN 19TH-CENTURY ORKNEY, SCOTLAND

Julia Jennings[1], *1. University at Albany, Albany, New York, United States*

Kin are important sources of social, instrumental, and financial assistance for older adults. Support from kin is associated with improved wellbeing and longer lives among this age group, yet few longitudinal studies examine information on the composition and structure of kin networks beyond dyadic relationships, such as those between spouses or parents and their children. This study examines the dynamics of non-dyadic measures of kin networks among adults over age 60 using multiple longitudinal linked data sources from North Orkney, Scotland, 1851-1911. Reconstructed individual life courses (N=4,946) and genealogies, in combination in spatial information concerning the proximity non-coresident kin, are used to examine change in kin availability and propinquity over the life course and across historical time. Orkney provides an interesting case study; as information is available on individual-level change in kin availability with a long period of follow up during a time of population change. The study period covers the early stages of population aging and depopulation of the islands, which began in the 1870s in this community. A descriptive analysis of kin network change is presented. Kin availability is associated with longer lives in this sample. The presence of co-resident kin is associated with economic status, after controlling for other factors. Older adults who receive poor relief are significantly more likely to live alone and less likely to live with kin, and the association is stronger for men than for women.

### EXPANDING SOCIAL TIES AS A GOAL IN LATER LIFE: THE ROLE OF SOCIAL NETWORK CHARACTERISTICS

Danielle Oleskiewicz,[1] and Karen Rook[1], *1. University of California, Irvine, Irvine, California, United States*

Older adults often winnow their social ties to focus on emotionally rewarding ties (Charles & Carstensen, 2010). Some older adults, however, have small social networks that preclude much winnowing or aversive social ties from which disengagement is difficult. These individuals might be motivated to expand, rather than contract, their social ties. The current study sought to extend knowledge regarding potential links between social network characteristics and older adults' interest, effort, and success in creating new social ties. We expected that small social networks and negative social ties might motivate interest and effort directed toward forming new social ties but that positive social ties might foster success in efforts to form new ties. In-person interviews were conducted with participants (N = 351, Mean age = 74.16) in a larger study of older adults' social networks and well-being. The interviews assessed participants' social networks, as well as their interest, effort, and success in making new social ties. Participants' social network composition, rather than size, was associated with greater motivation to establish new social ties. Negative social ties were associated with greater interest and effort directed toward forming new social ties. Positive social ties were related to greater success (due, in part, to their support provision) and, unexpectedly, were also related to greater interest and effort directed toward forming new ties. Older adults sometimes seek to expand, rather than contract, their social ties, and characteristics of their social networks appear to play a role in fueling and influencing the success of such efforts.

### FREE TRANSPORTATION SERVICE RECIPIENTS MAY HAVE BETTER QUALITY OF LIFE BUT STILL LACK IN IMPROVING HEALTH

Machiko R. Tomita[1], *1. University at Buffalo, Buffalo, New York, United States*

This study aimed to determine if service recipients (SRs) of free transportation services experience better quality of life, health, and function compared to pre-service recipients (PSRs). We conducted a cross-sectional study using personal interviews with 43 PSRs and 30 SRs belonged to a volunteer organization. Outcome measures were Older People's Quality of Life (QoL), Center for Epidemiology Study-Depression, and Instrumental Activities of Daily Living (IADL). Total sample (N=73) had a mean age of 78.5 years and mostly female (86.3%). The majority of PSRs wanted to go to Drs' offices (74.4%) and Grocery stores (60.5%), followed by Drug stores (44.2%), when the service becomes available. The figures were substantially smaller among SR (40.0%, 30%, and 13.3%, respectively). In PSRs, 67.4% expected to improve health once they start receiving the service, and 70. 0% of SRs said it did with the service. Using independent t-tests, SRs were significantly better in depression (p<.001), IADL (p=0.29) and most QoL items (life overall, social relationship, home and neighborhood, psychological and emotional well-being and leisure and activities; p=.047-p=.001), except for perceived health and finance. SRs (100%) were very satisfied with the service and drivers, but 80% of SRs said they wished to use more driving services than the allowable four times per month maximum. This limitation was due to the insufficient number of volunteers compared to a large number of people in need. Availability of more volunteer drivers will likely improve SRs health. Effective approaches to increase the number of driving volunteers are necessary.

### THE EFFECT OF OLDER ADULTS' VOLUNTEERING ON THE SOCIAL SUPPORT

Meeryoung Kim[1], *1. Daegu University, Daegu, Korea, Republic of*

With increasing longevity, older adults need activities after retirement. Volunteering can be alternate for substituting role after retirement. Social capital is an important resource to start volunteering. Vice-versa, volunteering can increase their social network and social support. This study examined how the factors of volunteering affect emotional, instrumental and esteem support of older adults. This study used the 6th additional wave of the Korean Retirement and Income Study (2016). The sample size was 202 and target population were adults age 65+. Multiple regressions were used for data analysis.. Demographic variables (e.g. gender, age, etc) were controlled. Independent variables included volunteer time, how many places they volunteered at, whether volunteers were professionals providing pro-bono services or not, whether they were self-motivated or asked by others. For dependent variables, social support such as emotional, instrumental and esteem support were used. If volunteers were asked by others, emotional and esteem support were increased. If volunteers were self-motivated, it affected esteem support. If they were volunteering in multiple places, instrumental and esteem support were higher than volunteering in only one place. Length of volunteering time spent affected the instrumental support negatively. There were differences between those who were professionals versus nonprofessional volunteering affecting instrumental support. Nonprofessional volunteering affected instrumental support more than professional volunteering. These findings implied social support motivation, time, and whether the volunteer was a professional or not affected different kinds of social support differently. Findings show the importance of older adults doing volunteering to enhance social support.

### #GERIATRICS: A 7-YEAR TWITTER ANALYSIS

Brenna M. Parker,[1] Megan Walker,[2] and Jeanette Ross[3], *1. UTHSA Long School of Medicine, San Antonio, Texas, United States, 2. UT Health Science Center San Antonio, San Antonio, Texas, United States, 3. South Texas Veterans Health Care System, San Antonio, Texas, United States*

The use of social media platforms as an educational tool to promote awareness has become increasingly popular as technology advances. Twitter is a microblogging, social media platform in which users share short, text-based posts ("tweets") that can contain hyperlinked articles, web-pages, pictures, and more. 79% of the 336 million current monthly twitter users are international, suggesting Twitter serves as a tool allowing international connection via the rapid spread of information worldwide. Simplur Signals (Simplur LLC) was used to perform a retrospective analysis of the use of #Geriatrics on Twitter. Data was collected from Oct. 13th, 2010 through Jun. 5th, 2018. Spam and unknown accounts were excluded from the data set before analysis. Manual analysis was performed to qualitatively assess tweet content of the top 200 Retweets by Impressions. A total of 65,002 tweets were shared during the selected time frame. Tweet activity rose to a high in Year 5 (17,206) but has declined since. The majority of the top 100 influencers were doctors (57.4%). Regarding tweet content, most discussions focus on increasing awareness and promoting advocacy (30%) as well as sharing research related to the practice of geriatrics (23.5%). With its widespread use and lack of international boundaries, Twitter serves as an effective platform in informing and increasing awareness about geriatrics and other medical specialties.

### DO OLDER ADULTS WITH HIV HAVE A SOCIAL NETWORK DEFICIT? EVIDENCE FROM AGINCOURT, SOUTH AFRICA

Markus H. Schafer,[1] Laura Upenieks,[1] and Julia DeMaria[1], *1. University of Toronto, Toronto, Ontario, Canada*

HIV/AIDS has had a substantial social and economic impact on Sub-Saharan Africa, and research is only beginning to examine the prevalence and consequences of HIV infection among older adults in this region. Though informal social networks provide crucial resources for older people managing health problems, little is known about how the form and function of such networks differs by HIV status. Drawing from theories of health stigma and network mobilization, we use egocentric network data from HAALSI, the Health and Aging in Africa: A Longitudinal Study of an INDEPTH Community in South Africa (N=5,059). HAALSI is a community-based study centered in Agincourt, South Africa, and focuses on adults ≥40 years of age. Approximately 12% of this sample is HIV positive. Results of multivariable logistic and Poisson regression reveal three main findings. First, relative to those without HIV, infected older adults have larger personal networks—including more kin and more non-kin network members. Second, HIV status has no discernible impact on whether people receive regular emotional support from those in their networks. Third, older adults who have disclosed their HIV status have a relatively high proportion of kin members in the close networks relative to those not infected with HIV and to those with HIV who have not disclosed their disease. Our findings point to the need for further research on the implications of social networks for outcomes such as well-being and health care delivery among older AIDS patients in the Global South.

### A COMPARATIVE ANALYSIS OF THE SOCIAL SUPPORT IN SOLITARY SENIORS WHO ATTEND CLUB ACTIVITY OR GO OUT TO SEE FRIENDS

Mai Takase,[1] Ryogo Ogino,[2] Jun Goto,[3] and Junichiro Okata[4], *1. The University of Tokyo, Institute of Gerontology, Bunkyo-ku, Tokyo, Japan, 2. Institute of Gerontology, Tokyo, Japan, 3. Institute of Gerontology, The University of Tokyo, Tokyo, Japan, 4. The University of Tokyo, Institute of Gerontology, Tokyo, Japan, Japan*

The Toyoshiki-dai housing complex, constructed in 1960s, is located in one of the commuter towns in a metropolitan area of Japan. Those who moved to this area as youngsters are now aged over 75 and many live a solitary life; making social support vital to prevent social frailty. The municipalities and universities have been hosting interventions, e.g. club activities, to make social connection but the effects remain unclear. In this study, the relationship between activity participation and the size of social support was explored to develop an effective method to increase social connections. A cross-sectional study was conducted in October 2018 and 200 questionnaires were distributed at a lunch event at the housing complex. The participants were solitary and independent seniors over the age of 70 (M:F=14.4:67.5). The frequency of going out to participate in club activity, see friends, and the geriatric social support scale were used for analysis. As a result, compared to seniors who went out to see their friends, the size of social support was smaller in groups that remained isolated (N = 161, odds ratio = 0.26, 95% CI=0.11-0.60). On the contrary, seniors who joined club activities had similar size of social support despite the frequency of participation. Clubs in Japan are often closed groups with limited membership, which may have restricted the addition of new social connection. Future intervention researches should focus on modifying the membership system of clubs and opening of a public space where seniors can casually access and talk to acquaintances.

### AGE DIFFERENCES IN COGNITIVE PERSONAL NETWORKS

Christopher S. Marcum,[1] Jielu Lin,[2] and Laura M. Koehly[3], *1. National Institutes of Health, Bethesda, Maryland, United States, 2. Northern Arizona University, Flagstaff, Arizona, United States, 3. National Human Genome Research Institute, National Institutes of Health, Bethesda, Maryland, United States*

Previous research has found a negative association between network size and age, suggesting that people experience greater isolation with advancing age. In this paper, we evaluate age differences in how individuals perceive their social worlds to be structured, rather than focusing solely on network size. A nationally represented sample of respondents (n=1,824) reported on their own ties to their close personal network members (i.e., ego-alter ties) as well as their perceptions of acquaintanceship between those members (i.e., alter-alter ties). We used social network analysis to assess how the structure of these relationships vary by respondent age. We find a positive association between respondent age and personal network size and a negative association between network members' ages and the number of ties respondents' perceive their members to have to each other. This effect significantly weakens as respondent age increases. Moreover, we find evidence of age-homophily, intergenerational contact spanning three generations in both ego-alter and alter-alter ties, and age differences in ego network composition. Our results suggest that the evolution of our social worlds across the life course shifts in terms of size and structure. While contemporary close personal networks may grow slightly with age, perceived social ties among one's network members become less cohesive and less diverse with age. We discuss these results in the context of recent findings that suggest aging uniformly insulates individuals from social contact from both structural and symbolic perspectives.

### DOES MOVING LATER IN LIFE INFLUENCE IN-PERSON CONTACT WITH CHILDREN, FAMILY, AND FRIENDS?

Joonyoung Cho,[1] and Jacqui Smith[1], *1. University of Michigan, Ann Arbor, Michigan, United States*

Relocation is common in later life and older adults differ the distance they move. One possible consequence of relocation is that in-person contact frequency with social network members changes. To date, relatively little is known about how older adults' in-person contact frequency with their children, family members, and friends is influenced by the distance they move and if this differs by age group (50-64, 65-74, 75+). To examine this, we used information from the Health and Retirement Study about geographic mobility and social network contact frequency. The sample was restricted to respondents over age 50 in the 2006 wave with data on contact frequency with children, family members, and friends in 2006 and 2014 (N=5159). Distance moved from 2006 to 2014 was categorized as: stayer, <5 miles, 5–49.9 miles, and ≥50 miles. Linear regressions with covariates controlled revealed that moving ≥50 miles was significantly associated with less frequent in-person contact with family members (β=-0.31, 95%CI [- 0.46, -0.16], p≤0.001) and friends (β =-0.32, 95%CI [-0.46, -0.18], p≤0.001). Interestingly, the 50-64 group who moved ≥50 miles was less likely to meet up with their children (β=-.0.36, 95%CI [-0.56, -0.15], p≤0.001), family members (β=-0.31, 95%CI [-0.52, -0.11], p≤0.01) and friends (β=-0.21, 95%CI [-0.39, -0.03], p ≤ 0.05). In contrast, the 75+ group who moved ≥50 miles were significantly less likely to have in-person frequency only with their friends (β=-0.58, 95%CI [-1.04, -0.14], p≤0.05). These findings are discussed in relation to theories about social support and emotional well-being in old age.

### SOCIAL AND EMOTIONAL LONELINESS AS PREDICTORS OF COGNITION IN LATER LIFE

Bert Hayslip[1], *1. university of north texas, Denton, Texas, United States*

To explore the viability of a model illustrating the potential relationship between both social and emotional loneliness and both psychometric and everyday cognition in later life, 575 older adults (M = 73.49) completed measures of crystallized (Gc) and fluid (Gf) ability as well as indicators of self-rated participation in 84 everyday cognitive activities, self-rated stimulatory value of each activity, attitudinal predisposition toward an engaged lifestyle and everyday cognitive failures. Measures of social support, caregiving stress, needs for cognition and cognitive self-efficacy were treated as mediators of the loneliness-psychometric/everyday cognition relationship, controlling for age, gender, health, and education. Hierarchical regression analyses suggested that social loneliness predicted ($p < .04$) Gc as mediated by social support, cognitive self-efficacy and need for cognition, whereas emotional loneliness similarly predicted ($p < .04$) Gf. Parallel analyses indicated that social loneliness predicted ($p < .04$) everyday cognitive failures and that both social and emotional loneliness predicted ($p < .03$) engaged lifestyle attitudes. In each case, the overall model was statistically significant ($p < .01$). For everyday cognitive activities and the stimulation values of such, neither type of loneliness was predictive, though lifestyle attitudes and lifestyle activity were moderately interrelated. These findings support a model incorporating distinct dimensions of loneliness as a predictor of diverse aspects of both psychometric and everyday cognition. This suggests that not only are the antecedents of cognition in late adulthood social/emotional in nature, but also that interventions targeting the prevention of loneliness may enhance cognitive functioning in later life.

### (OLDER) PEOPLE REMEMBER HOW YOU MAKE THEM FEEL: AGE DIFFERENCES IN THE EFFECTS OF SOCIAL EXCHANGES

Madeline M. Marello,[1] Julie Hicks Patrick,[1] and Abigail M. Nehrkorn-Bailey[1], *1. West Virginia University, Morgantown, West Virginia, United States*

Socioemotional Selectivity Theory poses that as we age our motivations transition from knowledge focused to emotionally focused (Carstensen, 1995). This shift to emotional motivation increases the relevance of relationships and social interactions for older adults. We examined different aspects of social support: frequency of positive/negative social interactions, satisfaction with positive social interactions, and bothered by social interactions -- to investigate these effects on one's global well-being. Negative and positive social exchanges are linked to psychological health (Newsom et al., 2005), however one's perceptions of those social interactions are important to consider as well -- being satisfied or bothered by social interactions shows a better perspective of the individual's experience than simply recording frequency. The results of our multi-group path analysis show that there are different effects of social supports on global well-being contingent on age, consistent with socioemotional selectivity theory. For adults under 30 years old (Mage = 24.0, range 18 to 29) social support did not significantly relate to well-being. For adults over 30 and under 50 (Mage = 38.9) frequency of positive social interactions is significantly related to well-being (B = .201). For adults over 50 (Mage = 58.8, range 50 to 87) the perception of social exchanges, not their frequency, are what influence well-being: both satisfaction with positive social interactions (B = .402) and being bothered by negative social interactions predict well-being (B = -.193). It is important to know that older adult's perceptions of social exchanges effect their well-being, future directions are discussed.

### FEELING AND LIVING WELL: NETWORK COMPOSITION AND PREDICTING SELF-RATED HEALTH FOR RACIAL AND ETHNIC MINORITIES

Eli X. Ornelas[1], *1. University of Nebraska-Lincoln, Lincoln, Nebraska, United States*

Racial and ethnic inequities in health among older adults in the U.S. are well documented. A substantial amount of the health literature focuses on physical and mental disparities; however, less research has examined racial and ethnic differentials in subjective, self-rated health (SRH). Prior research has documented racial and ethnic inequities in SRH, though mechanisms by which these disparities occur are still largely unknown. One potential mechanism by which these disparities may arise is through unequal access to psychosocial resources through variability in social networks. Utilizing data from the 2006 Health and Retirement Study (HRS) along with the 2006 HRS Psychosocial and Lifestyle Questionnaire, the current study seeks to explore racial and ethnic differentials in SRH from a social network perspective. Ordinal logistic regression is used to predict SRH by race and Hispanic ethnicity potentially mediated by possession and number of friends, frequency of contact with friends, and psychosocial and subjective well-being. Results indicate that older black adults are less likely to rate their health in a higher category of SRH than older white adults, and this relationship is not significant for other racial groups and Hispanics. Additionally, psychosocial and subjective well-being and frequent written-communication with friends are found to significantly predict better SRH. The results suggest that feeling well mentally is crucial for SRH, but equally important is regular contact with friends. Further research should employ more robust measures of social networks to elucidate the role that network composition plays in predicting SRH for racial and ethnic minorities.

### VOLUNTEER ACTIVITY CHANGES AFTER MIDDLE YEARS OLD AND THE INFLUENCES OF VOLUNTEERING ON WELL-BEING

Hyunkee Lee[1], *1. Korean Gerontological Society, Suwon, South Korea, Korea, Republic of*

The objectives of this paper are to examine volunteer activity changes over the life courses and the long-term influences of volunteer activity on the well-being. The paper analyzes the KLoSA data from wave 1 to wave 5, and selects 10,254 persons over 45 years old as study samples in the base year. For the statistical analysis, the two-way connected line plot and transition probability analysis techniques are employed and the GEE method is used for the multivariate regression estimation of the coefficients. The results show that the volunteer activities increase highest in one's late 40s and decrease abruptly from the age, and slow down 50s to one's late 70s, showing a repeating rise and fall pattern. Entering one's 80s, the volunteer activities come to be stable relatively than the previous years. And, the probability of continuing the volunteer status after 2 years is about 31.2 percent and the probability of stopping volunteer activity comes to be around 68.9 percent. As a last, the regression analysis results show that there is a positive relationship between volunteerism and well-being variables. Expecially the interaction term is statistically significant, showing a negative sign of the coefficient. This implies that the volunteerism contributes to increasing the well-being but decreasing by engaging excessively. The results support the previous study outcomes that excessive volunteer activities are not good for the health.

### TYPES OF SOCIAL NETWORKS AND THEIR ASSOCIATION WITH MOBILITY AND DISABILITY IN LATE LIFE

Talha Ali,[1] Michael Elliott,[1] Toni C. Antonucci,[1] Belinda Needham,[1] Jonathan Zelner,[1] Carlos Mendes de Leon[1], *1. University of Michigan, Ann Arbor, Michigan, United States*

Social networks are critical in maintaining late-life functional health, but, previous studies have focused on isolated dimensions of social networks. We examined whether network types, representing multiple interrelated network characteristics, are associated with mobility and disability among older adults in America. Data are from the National Social Life, Health, and Aging Project, a nationally representative study of 3,005 adults aged 57-85 years at baseline (2005-2006). In a previous analysis, five social network types were derived at baseline, based on nine observed network characteristics. Functional outcomes were examined during two follow-up waves in 2010-2011 and 2015-2016. Mobility-related function was assessed as the time (in seconds) to complete a 6-meter walk. Disability was defined as experiencing any difficulty in performing one of six activities of daily living (ADLs). We estimated the effect of network types on risk of ADL disability onset using logistic regression, and on mobility using generalized linear mixed models. Social network type was associated with mobility over time, such that older adults in the "restricted" network had significantly slower walking times than those in the "diverse" network. There was no association between network types and risk of disability onset in the primary analysis. However, sensitivity analyses showed a protective effect of the "partner-centered" network on a 5-year, but not a 10-year, risk of disability onset. Network types can elucidate older adults' varied interpersonal and caregiving networks, and identify adults at risk of being socially isolated. However, the utility of network types in predicting late-life functional health may be limited.

### STRENGTHS OF GRANDPARENTING: ASSOCIATIONS WITH DAILY WELL-BEING

Tina Vo,[1] Helena Sidrak,[2] Elizabeth Munoz,[1] and Chandra A. Reynolds[1], *1. University of California Riverside, Riverside, California, United States, 2. Loma Linda University, Loma Linda, California, United States*

Potential long-term health benefits may be afforded to grandparents in close contact with their grandchildren, although whether such benefits are visible on a day-to-day basis and among others in similar caretaking roles is unclear. We investigated how the quality and quantity of social contacts, as well as caretaker or grandparenting roles, may mediate symptom perceptions in day-to-day context in a consecutive six-day period. Older adults were recruited using an online survey service aged 59-88 years (Mage= 64.8, 55.8% grandparents, 67.8% female). Participants completed a baseline survey (N=152) followed by up to six daily surveys (N=85 of 152). Measures included daily positive and negative affect, and overall frequency of physical health symptoms. Daily social contacts were rated by participants in terms of importance/closeness of the contact. Last, participants indicated the degree of regular contact and non-custodial caretaking roles of children and their grandparent status. Findings indicated that grandparents tended to report daily contacts with closer social convoy members (B=1.40 (.437); p = .002). Moreover, a trend of reduced symptom reporting across days for grandparents was observed (B=-0.145 (.073), p=.048) adjusting for sex and age. Last, grandparents who regularly took care of their grandchildren and reported increased daily positive affect, reported fewer symptoms throughout the week (B=-0.326 (.139), p=0.02). Although modest, results indicate potentially important health benefits of grandparenting in terms of daily physical functioning that may play out over the longer term to impact health and well-being.

### INTERGENERATIONAL RELATIONSHIPS AMONG OLDER ADULTS AND GRANDCHILDREN: SUPPORTIVE AND CONFLICTUAL RELATIONSHIPS

Sofia von Humboldt,[1] Ana Monteiro,[2] and Isabel Leal[1], *1. William James Center for Research, ISPA - Instituto Universitario, Lisbon, Portugal, 2. ISPA - Instituto Universitario, Lisbon, Portugal*

Objectives: To analyze how older adults conceptualize these intergenerational relationships. Methods: In this qualitative study, in-depth interviews were carried out with 316 older adults, aged 65-102, from three different nationalities who lived at home. Verbatim transcripts were examined. Results: Data analysis generated six themes representing intergenerational relationships: affection and reward; interest and integration; grandparent-grandchild interaction quality; privacy and boundaries definition; provision of support; and obligation of providing childcare, on two dimensions of ambivalence concerning their intergenerational relationships (supportive and conflictual). Conclusions: The empirical findings from this research indicate how ambivalence in intergenerational relationships is experienced by older adults and stress the contradictory expectations of older adults with grandchildren. Keywords: Ambivalence; conflict; intergenerational relationships; older adults; support.

### INTERGENERATIONAL RELATIONSHIPS AMONG OLDER ADULTS AND ADULT CHILDREN: AMBIVALENT FEELINGS

Sofia von Humboldt,[1] and Isabel Leal[1], *1. William James Center for Research, ISPA - Instituto Universitario, Lisbon, Portugal*

Objectives: The relationship of older adults with their adult children involves great emotional complexity and the quality of these relationships is associated with older adults' well-being. This qualitative study aims to examine how older adults conceptualize intergenerational relationships with adult children. Methods: The present study on qualitative data collected from in-depth interviews was conducted with English and Portuguese older adults living in the community, designed to address their perspectives on intergenerational relations with adult children. 316 older adults participated in our study. The mean age of this group was 71.2 years. 65.3% were women, and a majority (54.7%) had a partner. Results: Content analysis generated four themes: affection and integration; satisfaction in the relationship; privacy and boundaries; financial support. Conclusions: Intergenerational relationships are experienced by older adults with ambivalence and and stress the contradictory expectations of older adults with grandchildren.

### THREE-YEAR EVALUATION OF THE DETECTION OF ELDER ABUSE THROUGH EMERGENCY CARE TECHNICIANS PROJECT

Michael B. Cannell,[1] Doug Livingston,[2] and Megin Parayil[1], *1. University of Texas Health Science Center at Houston School of Public Health, Dallas, Texas, United States, 2. Emory University School of Public Health, Atlanta, Georgia, United States*

EA is difficult to detect and often goes unreported. To address this important public health issue we developed the DETECT screening tool, which assists paramedics and

EMT's (medics) with determining whether or not to report a case of potential EA to APS while in the field providing medical services. In the current study, we investigate the effects of the DETECT screening tool on changes in reports of EA to APS over a period of approximately 3 years (January 2015 to March 2018). We used a differences in differences in differences (DDD) design to estimate the effect of the DETECT tool on both the number of reports for EA reports made each week as well as the probability of a report being validated by APS. After adjusting for changes in the number of EA reports non-medics and medics outside the service area, there were on average three times as many reports among medics after the implementation of the DETECT screening tool (RR=3.03, 95% CI:[2.06, 4.46]). No differences were seen in the probability of a valid report attributable to DETECT.

### SOCIAL NETWORK STRENGTHS: AN EXPLORATORY ANALYSIS OF A MEASURE OF TIES AMONG OLDER PEOPLE

Raeann G. LeBlanc,[1] Lisa Chiodo,[1] and Cynthia Jacelon[1], *1. University of Massachusetts Amherst, Amherst, Massachusetts, United States*

Purpose: To explore a self-report measure of social network features among a sample of older people living with multiple chronic conditions based on the conceptual model of Social Network Influences on Health. Design: A cross-sectional descriptive study design using a telephone survey methodology was used. Methods: An exploratory principle component analysis with a varimax rotation was performed on items that measured the identified components (reciprocity, size, proximity, density, general activation, activation when sick, duration, closeness, involvement in health, interaction frequency) of social networks. All items were standardized prior to analyses. Results: Self-report social network data were collected from eighty-four older people living in the community and managing multiple chronic conditions. The principal components model, operationalized as tie strength, contained six items based on factorability: reciprocity, social network size, proximity, density and perception of the activation in general and when sick of close social network members. Results yielded acceptable factorability (KMO = 0.781, Bartlett p 0.70). Two components that had eigenvalues greater than 1.0, explained 61.7% of the total variance. The first factor was interpreted as total social network resources, while the second factor was identified as social network availability. Conclusion: Exploratory principal component analysis supports a measure of social network features, tie strength, that can be tested in future studies. Assessing these variables is useful in identifying specific relationship features critical to managing chronic conditions in older age and advances current measurement of social networks important to living well in older age.

### WHEN NETWORKS FAIL: ISOLATION AND INDEPENDENCE AMONG OLDER ADULTS IN PUBLIC HOUSING

Brooke Wagen,[1] Whitney Williams,[1] Jan Bennett,[2] and Elizabeth A. Jacobs[3], *1. Dell Medical School at the University of Texas at Austin, Austin, Texas, United States, 2. none, New Mexico, United States, 3. The University of Texas at Austin Dell Medical School, Austin, Texas, United States*

In the coming decades, the population of adults over 65 in the US will increase dramatically. Many older adults live at or below the poverty level, and the growing lack of affordable housing combined with fixed incomes promises to increase the number of older adults facing combined housing and health challenges. Despite their vulnerability, little is known about the lived experiences of older adults aging in place in public housing. We conducted semi-structured qualitative interviews with 27 older adults at two public housing sites in Austin, Texas to gain an understanding of their thoughts on health, aging, home, community, and problem solving. We conducted interviews in Spanish (n=10) and English (n=17) with 16 female and 11 male interviewees with a mean age of 71.7 years (range 65-85 years). We systematically coded transcribed interviews and used grounded theory to analyze the data. Participants described feeling isolated due to language barriers, cultural perceptions about neighbors, and previous problematic experiences with neighbors leading to intentional isolation for safety. Some, however, spoke of how they acted as community connectors or responded to connectors in the community in ways that reduced their isolation. Participants framed individual problem-solving and personal choices as central to health and wellness. Our findings suggest a way forward for housing authorities, communities, and health systems working together to provide services to these adults. Incorporating their points of view and even co-creating interventions to enhance their health and well-being will make these interventions more successful and welcome.

### AGING DISPLAYED IN SELF-PORTRAITS OF ARTISTS

Manfred Gogol[1], *1. Heidelberg University, Heidelberg, Germany*

A self-portrait is a common representation of an artist with different techniques by that artist and emerged since the 15th century. Albrecht Dürer was one of the first artists who performed various self-portraits during his life. The fascinating aspect from a gerontological point of view is that artists show themselves throughout the aging process as well as sometimes with manifest signs of diseases. The poster show self-portraits over the life course from Rembrandt van Rijn (1606-69), Vincent van Gogh (1853-90), Ferdinand Hodler (1853-1918), Lovis Corinth (1858-1925), Helene Schjerfbeck (1862-1946), Edvard Munch (1863-1944), Käthe Kollwitz (1867-1945), Pablo Picasso (1881-1973), and Max Beckmann (1884-1950).

### SESSION 1000 (SYMPOSIUM)

### INTEREST GROUP SESSION—HOSPICE, PALLIATIVE AND END-OF-LIFE CARE: ADDRESSING ADVANCED ILLNESS CHALLENGES THROUGH SYNERGY, PLANNING, COLLABORATION, AND ADVOCACY

Chair Discussant: Faith P. Hopp, *Wayne State University, Detroit, Michigan, United States*
Co-Chair: Frances R. Nedjat-Haiem, *San Diego State University, School of Social Work, San Diego, California, United States*

Advanced illness occurs when health conditions become serious, general health and functioning declines, and treatments begin to lose their impact. Advanced care planning (ACP) is critical, but challenges such as communication barriers, system fragmentation, and a lack of formal and informal supports can negatively influence ACP and quality of life. How can diverse groups of persons with advanced illness, their family members and friends have greater synergy when planning for care in the face of serious illness? What are the best practices for planning and provision of a spectrum of needed services and support during the dying process and afterwards? We extend a conversation important for formal and informal network members to transform the culture of ACP, death, dying, and bereavement for diverse vulnerable populations. For this symposium, we will present information on ACP and care for LGBT populations and older Latinos, those living in long-term care settings, and persons receiving Adult Protective Services (APS). We will discuss strategies for synergy, collaboration, and advocacy among persons with advanced illness and their formal and informal care networks as a means of reducing health disparities and promoting self-determination in the midst of advanced illness challenges.

### NAVIGATIONAL BARRIERS TO ADVANCE DIRECTIVE COMPLETION AMONG OLDER LATINOS WITH CHRONIC ILLNESS

Frances R. Nedjat-Haiem[1], *1. San Diego State University, School of Social Work, San Diego, California, United States*

This study examined navigational barriers to advance care planning (ACP) and advance directive (AD) completion among older Latinos with chronic illness. Older Latinos over the age of 50 were randomized to education only versus education plus counseling (N=61; education only n=31, education/counseling n=30). Exploratory factor analyses and logistic regression show relationships between navigational barriers, depression, anxiety and AD documentation. Barriers to ACP included difficulty understanding the doctor, fears about hearing bad news, worries about illness getting worse, and difficulties due to language, family conflict, and limited family discussions about dying. Treatment group was most significant contributing factor to AD completion (OR=4.213, CI=1.126 – 15.760, p=<.01). Findings indicate the importance of examining a variety of factors that could influence AD documentation. This study contributes to the literature by identifying the need for targeted and culturally relevant interventions that promote ACP education, counseling, communication, support, and AD documentation among older Latinos.

### CHANGING THE CULTURE OF ADVANCE CARE PLANNING, DEATH, DYING, AND BEREAVEMENT CARE IN NURSING HOMES

Toni Miles,[1] Amanda Brown,[1] and Pamela O'Rouke[2], *1. University of Georgia College of Public Health, Athens, Georgia, United States, 2. CHSGa, Gray, Georgia, United States*

Cultural change, using an Eden Alternative metaphor, means that palliative care becomes 'the servant of genuine human caring, not its master'. This project, funded through the Georgia Civil Monetary Penalty Fund Reinvestment Program (CMPRP), aimed to gather information from multiple stakeholders to promote changes in the culture of advance care planning, death, dying, and bereavement care in nursing homes. Staff, residents and families from nine nursing homes provided perspectives through qualitative interviews (n=70). Participants were drawn from both small facilities and corporate entities with multiple sites, and included residents in both short and long-stay beds. Qualitative analysis of interview data resulted in the publication and dissemination of Best Practice in Bereavement Care Guides, with separate versions for residents, families, and staff. This presentation will describe this project in terms of the process of consensuses building and obtaining funding, as well as development of the guides through qualitative interview data.

### RISKS AND VULNERABILITIES AFFECTING ADVANCED CARE PLANNING FOR OLDER ADULTS INVOLVED IN ADULT PROTECTIVE SERVICES

Joy S. Ernst[1], *1. Wayne State University School of Social Work, Detroit, Michigan, United States*

Adult protective services (APS) workers regularly engage in advanced care planning (ACP). This qualitative content analysis of 21 APS cases sought to extend knowledge and uncover characteristics that influenced the ACP process and increased vulnerability of older adults in cases of neglect. The case narratives were examined to identify themes of vulnerability and risk related to ACP. Vulnerabilities of the older adults included multimorbidities, geriatric syndromes, and functional limitations. Risks involving caregivers included health problems and cognitive impairment. Caregivers were overwhelmed or lacked insight into the older adults' needs. Involvement of other family members was detrimental or non-existent while some advocated for improvements where the caregiver refused assistance. Findings support the development of methods of ACP, including the facilitation of difficult conversations, that would respect the autonomy and dignity of the older adult while meeting the multiple needs of caregivers and family members.

### ADVANCE CARE PLANNING AMONG LGBT PEOPLE: AN INTEGRATIVE REVIEW AND ANALYSIS

Luisa Kcomt,[1] and Kevin M. Gorey[1], *1. University of Windsor, Windsor, Ontario, Canada*

Lesbian, gay, bisexual, and transgender people (LGBT) with advanced illness need culturally competent advanced care planning (ACP) services but often encounter structural and communication barriers. The aim of this study was to examine the ACP behaviors of LGBT people. An integrative rapid review method was used to search electronic databases for peer reviewed and non-peer reviewed publications between 2010 to 2017. Eight survey instruments comprising 30 prevalence estimates were analyzed. ACP discussions between LGBT people and their primary health care providers were rare, with an overall prevalence of 10%. Transgender people were 50–70% less likely than their LGB counterparts to have a living will or to have appointed a healthcare proxy. These results suggest there is a critical need for greater cultural competency among health care providers serving LGBT populations. Social workers can play a key role in advocacy and social justice for LGBT individuals with advanced illness.

### SESSION 1005 (SYMPOSIUM)

### INTEREST GROUP SESSION—BEHAVIORAL INTERVENTIONS FOR OLDER ADULTS: ADDRESSING CLINICAL SYMPTOMS OF DEMENTIA: PRELIMINARY RESULTS FROM DEMENTIA BEHAVIOR TRIAL

Chair: Katherine A. Marx, *Johns Hopkins University, Baltimore, Maryland, United States*
Co-Chair: Laura N. Gitlin, *Drexel University, Philadephia, Pennsylvania, United States*
Discussant: Joseph E. Gaugler, *University of Minnesota - School of Public Health, Division of Health Policy and Management, Minneapolis, Minnesota, United States*

Currently, just under six million people living in America are diagnosed with Alzheimer's disease or related dementia. Most people with dementia live in a community setting and are cared for by a family member. Persons living with dementia almost universally experience behavioral and psychological symptoms (BPSD), such as agitation, aggression, and rejection of care as well as functional dependence. These symptoms are related to negative outcomes for both the person living with dementia and the family caregiver. Prior research shows that nonpharmacologic interventions such as meaningful activities, education, and multicomponent interventions have promise in managing behaviors. This symposium focuses on preliminary outcomes from the Dementia Behavior Study (DBS), a Randomized Control study that examined the effect of the Tailored Activity Program (TAP) in a community setting on BPSD and functional dependence in persons living with dementia, and caregiver wellbeing (e.g. depression, burden, perceived change). Gitlin et al will present outcomes of the primary aim (BPSD) and secondary aims (functional dependence and caregiver wellbeing) of the DBS. Pizzi et al explore the cost analysis of the TAP intervention versus the active control group. Scerpella et al describe the alerts and adverse events that were associated with the DBS. Marx et al present the relationship between race and caregiver readiness to participate in TAP. Regier et al explore the BPSD Rejection of Care and the association to caregiver burden. Tailoring interventions, such as activities may improve quality of life for both the person with dementia and the family caregiver.

### FAMILY CAREGIVER READINESS: RACIAL DIFFERENCES AND RELIABILITY OF THE CAREGIVER READINESS SCALE

Katherine A. Marx,[1] Katherine A. Marx,[1] Lauren J. Parker,[1] Jin Huang,[1] and Daniel Scerpella,[1] Karen Rose,[2] Catherine V. Piersol,[3] and Laura N. Gitlin[4], *1. Johns Hopkins University, Baltimore, Maryland, United States, 2. Widener University, Chester, Pennsylvania, United States, 3. Thomas Jefferson University, Philadelphia, Pennsylvania, United States, 4. Drexel University, Philadelphia, Pennsylvania, United States*

The success of any intervention for caregivers of persons with dementia is dependent on the caregiver's readiness to enact the strategies. This presentation explores the reliability of the new 17-item Caregiver Readiness Scale (CRS) and also to examine the differences by race in readiness. Participants were caregivers in the Dementia Behavior Study who completed the CRS at baseline (n=129). Caregivers were on average 65.8 years old (sd = 12.2, range 28-88), the majority reported their race as non-Hispanic white (64.3%, n=83) and 33.4% (n=43) reported their race as African American or other. The average CRS score was 57.63 (sd=5.72, 40-68) ($\alpha$=0.73). The only significant interaction with race was negative communication (p=0.026) with negative communication scores having little effect on readiness in whites, but in non-white caregivers, there was an inverse relationship. Knowing the caregiver's level of readiness and communication style may help improve the acceptability and success of an intervention.

### ADDRESSING DEMENTIA CLINICAL SYMPTOMS USING NONPHARMACOLOGICAL STRATEGIES: WHAT WORKS AND FOR WHOM?

Laura N. Gitlin,[1] Laura N. Gitlin,[1] Katherine A. Marx,[2] Catherine Verrier Piersol,[3] Nancy A. Hodgson,[4] Jin Huang,[2] David Roth,[2] and Constantine G. Lyketsos[5], *1. Drexel University, Philadelphia, Pennsylvania, United States, 2. Johns Hopkins University, Baltimore, Maryland, United States, 3. Thomas Jefferson University, Philadelphia, Pennsylvania, United States, 4. University of Pennsylvania, Philadelphia, Pennsylvania, United States, 5. Johns Hopkins Bayview, Baltimore, Maryland, United States*

People living with dementia experience behavioral symptoms and functional decline and their caregivers (CG), reduced wellbeing. In an RCT (N=250 dyads), we tested whether tailoring activities to interests/abilities and providing CGs with instruction in their use (Tailored Activity Program, TAP) reduced clinically significant agitation/aggression (main outcome), functional decline and improved CG wellbeing (secondary outcomes) compared to CG education/support alone; with both groups receiving 8-sessions over 3-months. At 3-months,TAP had no effects on agitation/aggression compared to CG education/support but reduced functional decline (p=0.03), improved CG wellbeing (p=0.01) and confidence using activities (p=0.02). In secondary analyses, black vs. white CGs reported reduced agitation/aggression (p=0.01); female CGs reported reduced burden with TAP whereas male CGs reported reduced burden from education/support (p=0.04); spouses vs. non-spouses reported slower functional decline in participants (p=0.01). This trial suggests outcomes vary by subgroups. Different nonpharmacological approaches are needed for specific clinical characteristics: one size will not fit all.

### TAILORED ACTIVITY PROGRAM (TAP) COSTS: OPPORTUNITIES TO STREAMLINE DELIVERY AND PURSUE REIMBURSEMENT

Laura T. Pizzi,[1] Laura T. Pizzi,[1] Katherine M. Prioli,[1] Eric Jutkowitz,[2] Jing Yuan,[1] and Laura N. Gitlin[3], *1. Rutgers University, Piscataway, New Jersey, United States, 2. Brown University, Providence, Rhode Island, United States, 3. Drexel University, Philadelphia, Pennsylvania, United States*

TAP intervention costs captured alongside the randomized controlled study included labor of program staff, mileage, supplies and materials. Staff time costs were converted to $US 2017 by multiplying hours spent by the appropriate wage rate plus fringe benefits; mileage was costed using the federal reimbursement rate. Research costs were excluded to approximate real world implementation. Costs varied based on number of visits required but mean component costs per dyad were: occupational therapist training ($133.50), home visit time ($527.57), travel ($718.02), work outside of intervention delivery ($57.14), program screening for eligibility ($3.73), supervision for quality assurance ($250.23), activity supplies ($51.64), and program materials ($29.32). Findings will be compared to a 2008 TAP pilot study post hoc cost analysis to identify how broader scale implementation impacts intervention costs. Opportunities to streamline program delivery will be discussed as well as potential reimbursement mechanisms via Medicare part B, Medicare Advantage, and Medicaid waiver programs.

### REPORTING OF ADVERSE EVENTS IN BEHAVIORAL INTERVENTION TRIALS: THE TAILORED ACTIVITY PROGRAM

Daniel Scerpella,[1] Danny L. Scerpella,[2]
Katherine A. Marx,[2] and Laura N. Gitlin[3], *1. Johns Hopkins University Center for Innovative Care in Aging, Baltimore, Maryland, United States, 2. Johns Hopkins University, Baltimore, Maryland, United States, 3. Drexel University, Philadelphia, Pennsylvania, United States*

Adverse events (AE) are not regularly reported in behavioral research, especially when compared to similar reporting in pharmacological research. AE reporting is crucial for understanding the potential risks and outcomes of behavioral interventions as well as characterizing the study population. This presentation presents AE findings of a behavioral intervention to reduce neuropsychiatric behaviors in persons living with dementia, the Tailored Activity Program. A total of 250 dyads (persons living with dementia and caregivers) living in a community setting participated in this intervention, reporting 126 adverse events and 37 alerts. None of the events were attributable to the intervention, with the majority (46%) being related to the health of the person living with dementia or high depression scores in caregivers (19%). These results are analyzed in relation to outcomes and the potential frailty of this study population. Reporting AEs in behavioral trials is the best opportunity to inform treatment options.

### REJECTION OF CARE IN PERSONS WITH DEMENTIA

Natalie G. Regier,[1] Scott Choi,[2] and Laura N. Gitlin[3], *1. Johns Hopkins University, Baltimore, Maryland, United States, 2. Towson University Department of Nursing, Towson, Maryland, United States, 3. Drexel College of Nursing and Health Professions, Philadelphia, Pennsylvania, United States*

Most individuals with dementia develop significant behavioral problems, also known as neuropsychiatric symptoms (NPS). One problem that continues to plague measurement of NPS is inconsistency of terminology used to describe NPS. For example, in the Neuropsychiatric Inventory-Clinician Rating Scale (NPI-C), a gold standard for measuring NPS, rejection of care (rejection) is not differentiated from agitation or aggression. Rather, behaviors indicative of rejection are categorized as agitation. Using data from 250 persons with dementia who participated in the Dementia Behavior Study, principle components analysis of the NPI-C domain of Agitation identified four behavioral clusters: 1=rejection of care, 2=restlessness, 3=exiting behaviors, and 4=hiding/hoarding. Rejection was associated with a more distant relationship with the caregiver, lower cognitive status, and more negative caregiver communication style. Rejection was predictive of higher levels of caregiver burden. Findings support the argument that rejection is a clinically distinct NPS, and likely requires different nonpharmacological management than agitation.

### SESSION 1010 (PAPER)

### AGE-FRIENDLY COMMUNITIES

### DEVELOPING AGE-FRIENDLY COMMUNITIES: EVIDENCE FROM MULTIPLE CASE STUDIES

Patricia A. Oh[1], *1. University of Massachusetts, Boston, Boston, Massachusetts, United States*

The age-friendly community movement is gaining momentum in the United States. More than 325 communities have joined the AARP Network of Age-Friendly States and Communities or the WHO Global Network of Age-Friendly States and Communities. The purpose of this multiple case study was to explore what influences municipal decision-making about joining a network and how communities mobilize the resources at their disposal to make age-friendly changes after joining. The conceptual model that guided this exploratory study incorporated Kingdon's policy change model to explore municipal decision-making about joining a formal age-friendly network and resource mobilization theory to explore factors that influence implementation of age-friendly changes after a community joins an age-friendly network. Data was gathered in three in-depth case studies of age-friendly communities in New England--Brookline, Massachusetts; Newport, Vermont; and Ellsworth Maine. In these three cases, the policy entrepreneur was key to municipal decision-making. Kingdon posits that a single problem definition increases the likelihood that a policy is adopted. However, in these cases, the policy entrepreneur used selective framing to advocate with local organizations and municipal government, a departure from Kingdon's model. Implications for age-friendly policy adoption will be discussed. Resource mobilization theory posits that implementation of change is dependent on resources and collaborations. Each case had access to different resources, but partnerships were key to moving the work forward (with or without collaborations). The primary resources utilized were relational and ideological. Material resources were less likely to move the work forward. Implications will be discussed.

### NAVIGATING STREET AND SHELTER LIFE: THE CONSEQUENCES OF FORCED MOBILITY FOR OLDER HOMELESS WOMEN

Judith G. Gonyea[1], *1. Boston University, Boston, Massachusetts, United States*

Older homeless women have largely been an invisible population. Two co-occurring trends however are bringing them into the public spotlight—the aging and the feminization of the adult homeless population. Yet despite the steep increase in their numbers, relatively little research exists about how gender and age intersect to shape the homeless experience. Such information is critical if we are to transform our nation's homeless system, which is based largely on a male model of homelessness, to better support women at risk or experiencing homelessness. In this presentation, we therefore share findings from our qualitative study of homeless older urban women. Using a phenomenological approach, we conducted and recorded semi-structured, in depth interviews with fifteen chronically homeless women in their fifties. Our analysis process was inductive and iterative with the culminating phases being the generation and interpretation of themes. Our analysis revealed the links between place, social connection, sense of belonging, and identity. The women's narratives uncovered how the time-space discontinuity, created through homelessness, shaped the struggles they faced in trying to survive in degraded or threatening environments, altering their identities and impacting self-esteem. Also revealed was that mobility is a key factor to maintaining the place-identity connection. The women's narratives highlighted how forced mobility with constrained choice not only led to their pathways into homelessness but also dominated their daily navigation of street and shelter life. We conclude by exploring the question of how we might redesign policies and programs to disrupt homelessness for women in later life.

### USING PHOTOVOICE TO EXPLORE THE SALIENCY OF NEIGHBORHOOD LANDMARKS FOR PERSONS LIVING WITH DEMENTIA

Kishore Seetharaman,[1] and Mardelle Shepley[2], *1. Simon Fraser University, Vancouver, British Columbia, Canada, 2. Cornell University, Ithaca, New York, United States*

This study demonstrates the potential of Photovoice, a participatory action research method involving participant-generated photo-elicitation, to explore how persons living with dementia (PLWDs) perceive neighborhood landmarks. Previous research has highlighted the role of well-designed, stable geographical landmarks in improving the navigability of neighborhoods for PLWDs. However, the specific attributes that render landmarks salient have not yet been sufficiently explored, resulting in inadequate evidence-based environmental design guidelines for dementia-friendly communities (DFCs). To address this gap, a Photovoice study was conducted with five community-dwelling PLWDs and their care partners, as part of a dementia-friendly neighborhood walking program in the city of Seattle, USA. Photovoice facilitated the exploration of saliency of neighborhood landmarks from an emic perspective by empowering PLWDs to identify and take photos of salient landmarks during the group walk and interpret and reflect on attributes that contributed to saliency using the photos as visual aids in a focus group discussion and survey questionnaire. PLWDs associated the saliency of landmarks not only with objective physical attributes, e.g., size, shape, color, texture, but also with subjective factors linked to their past, passions, hobbies, and emotions related to having dementia. Findings suggest that the design of outdoor landmarks should satisfy universal design principles, as well as aspects of familiarity, recognizability, and memorability, to ensure that the neighborhood physical environment provides navigational support to PLWDs. The study proposes using Photovoice to facilitate community engagement in the planning and design of DFCs and mobilize people's lived experience to generate more robust dementia-friendly environmental design guidelines.

### SESSION 1015 (SYMPOSIUM)

### AGING STRONG 2020: INTERVENTIONS TO IMPROVE LONELINESS AMONG OLDER ADULTS

Chair: Charlotte Yeh, *AARP Services, Inc., Washington, District of Columbia, United States*
Co-Chair: Daniel Russell, *Iowa State University, Ames, Iowa, United States*
Discussant: James Schaeffer, *Optum, Ann Arbor, Michigan, United States*

Research confirms serious and concerning health implications for lonely and socially isolated older adults. Studies consistently demonstrate that older adults who are lonely or socially isolated have higher rates of depression, more health conditions, and greater mortality. AARP Services, Inc. (ASI) and UnitedHealthcare (UHC) are committed to the health and well-being of insureds in AARP® Medicare Supplement Plans insured by UnitedHealthcare Insurance Company (for New York certificate holders, UnitedHealthcare of New York), recognizing that health and wellness should be promoted on a holistic level to ensure successful aging. As part of this commitment, a research initiative entitled Aging Strong 2020 has been developed. Its purpose is to impact insureds' personal and social investments in their well-being Thus a related series of interventions are aiming to increase resilience by focusing on enhanced purpose in life, social connectedness, and optimism. This symposium will specifically discuss these efforts related to social connectedness and how they have improved well-being among lonely older adults. First discussed is the prevalence and outcomes of loneliness in a large national survey. Interventions include use of animatronic pets, a telephonic reminiscent memory program, and an online self-compassion mindfulness program. Findings from these initiatives demonstrate that interventions designed to improve loneliness and well-being among lonely older adults can contribute to the holistic model of health.

### LIFEBIO: PARTICIPANTS' EXPERIENCES IN A TELEPHONIC-BASED REMINISCENCE PROGRAM

Janella Hudson,[1] Rifky Tkatch,[1] Karen Keown,[2] Michael McGinn,[1] and Ellen Wicker[3], *1. Optum, Ann Arbor, Michigan, United States, 2. UnitedHealthcare, Minneapolis, Minnesota, United States, 3. AARP Services, Inc., Washington, District of Columbia, United States*

Older adults facing age-related transitions are at increased risk for depression and loneliness. Reminiscence therapy has demonstrated positive outcomes for older adults, including improved socialization and reduced depression. A program known as LifeBio was designed as a group intervention to engage participants by capturing their life stories to positively impact wellness through reminiscence. This program was adapted to a telephonic format for the Aging Strong 2020 initiative. Semi-structured interviews eliciting feedback about participants' experiences in the program were conducted with 24 participants. Respondent feedback indicated that those with limited mobility and fewer social connections reported the greatest benefit. Many identified the value of journaling as a form of recordkeeping for family members, but reported varying degrees of journal completion. Suggestions for improvement included future facilitation of two-way communication with other participants and opportunities for face-to-face interaction in group settings.

### LONELINESS AND ASSOCIATED OUTCOMES IN A LARGE SURVEY OF OLDER ADULTS

Timothy Barnes,[1] Gandhi Bhattarai,[1] Sandra Kraemer,[2] Shirley Musich,[1] James Schaeffer,[1] and Charlotte S. Yeh[3], *1. Optum, Ann Arbor, Michigan, United States, 2. UnitedHealthcare, Minneapolis, Minnesota, United States, 3. AARP Services, Inc., Washington, District of Columbia, United States*

Loneliness and social isolation are major risk factors for poor physical and mental health among older adults. Studies demonstrate that loneliness is associated with depression, impaired cognitive performance, increased chronic disease, and mortality. However, the impact on other psychosocial constructs and healthcare outcomes remain understudied. The purpose of this study was to estimate the prevalence of loneliness and examine associations with socio-demographic, medical, and psychosocial characteristics in a large national survey of older adults (N=4,525). Overall, 43% of participants reported either moderate or severe loneliness. Older age, female gender, income, depression, hearing difficulty, and poorer health were all associated with loneliness. Purpose of life, resilience, optimism, and a diverse social network were associated with low loneliness. On average, lonely participants had a higher rate of emergency department visits, inpatient admissions, and medical costs. Based on findings, interventions should aim to alleviate loneliness among older adults.

### ONLINE MINDFULNESS PROGRAM FOR LONELY OLDER ADULTS

Rachel Ungar,[1] Lizi Wu,[1] Karen Keown,[2] James Schaeffer,[1] and Ellen Wicker[3], *1. Optum, Ann Arbor, Michigan, United States, 2. UnitedHealthcare, Minneapolis, Minnesota, United States, 3. AARP Services, Inc., Washington, District of Columbia, United States*

Mindfulness meditation is a cognitive state of self-awareness that promotes emotional regulation and change in self-perspective. Mindfulness has been applied to address loneliness, stress, and anxiety, demonstrating consistent health benefits. The purpose of this study was to test the feasibility of an online mindfulness program and to measure its impact on well-being among lonely older adults. The intervention consisted of seven one-hour weekly online modules led by a trained facilitator via WebEx. Engagement was high with 63% of participants attending four or more sessions. Pre/post survey data (N=42) found decreased anxiety, stress, and improvement in mindfulness, purpose in life, and resilience. This program demonstrates that online mindfulness programs may be of great benefit for lonely older adults. Future research will include larger samples to investigate further impacts.

### REDUCING LONELINESS AMONG OLDER ADULTS WITH ANIMATRONIC PETS

Rifky Tkatch,[1] Lizi Wu,[1] Laurie Albright,[2] James Murphy,[2] James Schaeffer,[1] Ellen Wicker,[3] and Charlotte S. Yeh[3], *1. Optum, Ann Arbor, Michigan, United States, 2. UnitedHealthcare, Minneapolis, Minnesota, United States, 3. AARP Services, Inc., Washington, District of Columbia, United States*

Pet ownership has been examined as a solution for loneliness. However, multiple challenges of pet ownership exist for older adults. Therefore, research efforts are considering the use of animatronic pets to reduce loneliness. The purpose of this study was to determine if ownership of animatronic pets would decrease loneliness and improve well-being among lonely older adults. Individuals were identified as lonely through a prior survey. Participants were provided with their choice of either an animatronic cat or dog and completed T1, T2, and T3 surveys. Response rates were high; 167 (63%) completed T1 and T2, and 125 (48%) also completed T3. T2 data indicated that loneliness decreased, while mental well-being, resilience, purpose in life, and optimism improved. At T3 mental well-being and purpose and life continued to improve. Animatronic pets appear to provide significant benefits for the well-being of lonely older adults.

### SESSION 1020 (SYMPOSIUM)

### BUILDING NETWORKS TO ADDRESS AT-RISK OLDER ADULTS

Chair: Max Zubatsky, *Saint Louis University, Saint Louis, United States*
Discussant: Nina Tumosa, *Health Resources and Services Administration, Rockville, Maryland, United States*

With the rise of older adults and the number of chronic health issues in this population, comes the need for greater collaboration across organizations and health care settings. Age-friendly health systems offer the benefits of providing the best care possible to individuals and families, connect people to specific community resources, and optimize the best access to services and programs. The Gateway Geriatric Workforce Enhancement Program (GWEP) has combined the efforts of Saint Louis University and a rural, critical access hospital to establish a care network across Missouri. Together, this partnership has created a number of services, initiatives, and projects to help older adults maintain independence and offer families ways to take of their loved ones in more effective ways. In this symposium, presenters from Social Work, Marriage and Family Therapy, Geriatric Medicine, Psychology and Nursing disciplines will introduce several areas of this age-friendly network. The four abstracts for this symposium include: 1.) Assessing At-Risk Older Adults through the Rapid Geriatric Assessment, 2.) Cognitive Stimulation Therapy for Individuals with Memory Loss, 3.) Predictors of Falls in Older Adults Across Partner Settings, and 4.) Development of program initiatives such as the Rapid Geriatric Assessment screening, Cognitive Stimulation Therapy, Falls Assessment in Seniors, and Care for Persons with Dementia in their Environments will be covered in detail. At the end of the four presentations, the presenters will highlight the importance of this collaborative network and ways for audience members to consider building an age-friendly network in their community.

### COGNITIVE STIMULATION THERAPY FOR INDIVIDUALS WITH MEMORY LOSS

Max Zubatsky[1], *1. Saint Louis University, Saint Louis, United States*

Cognitive Stimulation Therapy (CST) is a non-pharmacological approach for individuals with mild to moderate dementia. It is one of the age friendly community activities of the GWEP to engage older adults with memory challenges. We explored the impact of caregiver-assisted CST (CA-CST) on participants' overall memory, mood, and retention in the groups. We also investigated the impact of this CST format on the overall well-being of dementia caregivers. Four CST groups (N=28) entered the CA-CST groups meeting once per week for 14 weeks in a university setting. 61 % of participants who completed the group (n=17) showed a .5 point improvement on the Saint Louis University Mental Status Exam (SLUMS), a 2-point decrease on the Cornell Scale for Depression in Dementia (p<.01), and 1.5 point increase in overall caregiver well-being. Continued psychosocial interventions for dementia are needed not just in university settings, but healthcare organizations and other age-friendly settings.

### ASSESSING AT-RISK OLDER ADULTS THROUGH THE RAPID GERIATRIC ASSESSMENT

Marla Berg-Weger,[1] and John Morley[1], *1. Saint Louis University, St. Louis, Missouri, United States*

With the need to increase gerontological competency among all health/social service professions, the Rapid Geriatric Assessment (RGA) (Morley, 2017) is a tool that can be used in most healthcare settings. Designed as a rapid screening, the validated RGA assesses frailty, sarcopenia, anorexia, cognitive function, and advance care planning. Developed in 2015 through the Geriatric Workforce Enhancement Program (GWEP), 3,489 students and 5,643 faculty and practitioners have been trained in its use and have completed 10,881 RGAs in case-finding, screening, nursing home, PACE, and hospital and residential settings. Non-population-based findings show higher-then-national prevalence in all four health condition areas across settings: frailty (n=3,140; 30%), sarcopenia (n=4,458; 42.1%), weight loss risk (n=3,012; 28.4%), and cognitive impairment (n=2,509; 23.7%). Only 34.8% of the total sample had completed advance care plans. Data comparing results by gender, age, race/ethnicity, and setting will be presented along with strategies for curricular and practice integration.

### PREDICTORS OF FALLS IN OLDER ADULTS ACROSS PARTNER SETTINGS

Helen W. Lach,[1] and Theodore Malmstrom[2], *1. Saint Louis University, Missouri, United States, 2. Saint Louis University, St. Louis, Missouri, United States*

Falls cause significant morbidity and mortality in older adults. We explored predictors of falls in older adults completing Rapid Geriatric Assessments in Missouri (N=7796). Assessments were completed by health professionals and students at community sites (n= 1817), physician practices/clinics (n= 4441), nursing homes (n=525), and hospitals (n=1013). Multinomial regression analyses were used to identify predictors of falls from: age, gender, frailty score, self-reported walking ability, and cognition. Participants had a mean age of 78.4±8.4, were 65.2% female, 41.7% fallers, 28.1% frail, and 40.5% pre-frail. All variables except gender were associated with falls. For the total sample, frailty (OR 2.11 for 1-3 falls, OR 3.74 for 4+ falls; p<.001) and self-reported walking difficulty (OR 1.85 for 1-3 falls, OR 2.36 for 4+ falls; p<.001) had the highest odds ratios and were consistent predictors of falls in site-specific analyses. Self-rated walking, age, and cognition were risk factors in some settings.

### BUILDING AN AGE-FRIENDLY HEALTH CARE SYSTEM

Janice Lundy[1], *1. Perry County Memorial Hospital, Perryville, Misouri, United States*

Gateway GWEP has partnered with a rural Missouri critical access hospital to establish an age-friendly health system in their community. Program innovations include development and training of: 1) electronic health records integration of the Rapid Geriatric Assessment (RGA) for all patients 65+ years old; 2) RGA-based protocol for Medicare Annual Wellness Visits (MAWV); 3) Multidisciplinary health care assessment team 4) Evidence-based or Evidence Informed treatment interventions, including Cognitive Stimulation Therapy (CST), exercise and strengthening program for persons participating in CST, Care of Persons with Dementia in their Environments (COPE) and caregiver support. Since 2015, 1,200 RGAs, 338 MAWVs have been completed; 165 persons with dementia participated in CST; and 74 have participated in exercise and strengthening. Data supports positive outcomes in functional independence, cognitive status, and care-giver dementia management skills and well-being. Successes and lessons learned regarding strategies to develop an age-friendly health systems will be discussed.

### SESSION 1025 (SYMPOSIUM)

### CAREGIVING CULTURES: RACE, REGION, AND RELATIONS

Chair: Jyoti Savla, Co-Chair: Karen A. Roberto, and Discussant: Rosemary Blieszner

Although families have always cared for their aging members, changes in contemporary society have added to the stress and challenges of providing daily care and support for relatives experiencing physical and/or cognitive decline. Personal characteristics, geographic location, and family structures and relationships influence beliefs about family care; thus, recognizing differences within and across families is crucial for developing culturally informed caregiving programs and practices. This symposium focuses on four diverse groups of caregivers. Recognition of caregiving cultures and the barriers caregivers encounter is the central theme of all four papers. Using daily diary interviews and GIS data, Savla and colleagues discuss the unique cultural and geographical challenges caregivers in rural Appalachian Virginia face when caring for a family member with dementia. J. Angel employs quantitative and qualitative data from two nationally representative datasets to discuss the effects of immigration on family structures and caregiving for Mexican-American older adults with dementia. Miyawaki and her colleagues provide a profile of caregivers of older Vietnamese refugees, the resources they use and support structures they rely on. Finally, Roberto and Savla expand the definition of family caregivers to include extended and fictive kin who are providing dementia care and provide an in-depth view of the circumstances that influence the responsibilities they assumed, the type of care they provide, and the coping strategies they use. Dr. Rosemary Blieszner will discuss the presenters' collective findings considering their unique caregiving practices and beliefs as well as the common grounds between the different races, regions and relationships.

### CAREGIVING AND SERVICE USE: CULTURAL INFLUENCES, REGIONAL BARRIERS, AND FAMILY RELATIONS

Jyoti Savla,[1] Karen A. Roberto,[1] and Rosemary Blieszner[1], *1. Virginia Tech, Blacksburg, Virginia, United States*

Elder care in rural Appalachia is challenging due to poor socioeconomic conditions, geographical isolation, and lack of services and transportation. Certain aspects of Appalachian culture, namely self-reliance, traditionalism, and strong family ties, also create unique barriers for using services to help care for persons with dementia (PwD). Quantitative and qualitative data from 85 caregivers of PWD with moderate to high care needs were explored to examine caregivers' use of personal care services, identification with their community, attitudes towards service use, and geographical distance from the nearest service location. Results suggest that although services such as adult day centers, food banks, meal delivery, and support groups are widely dispersed throughout rural Appalachian counties, use or nonuse of services was driven by lack of economic resources, care preferences of the PwD, and dissatisfaction with previous service use. Discussion focuses on suggestions for uptake of services by caregivers of PwD in rural Appalachia.

### CAREGIVERS OF OLDER VIETNAMESE REFUGEES: FINDINGS FROM THE VIETNAMESE AGING AND CARE SURVEY (VACS)

Christina E. Miyawaki,[1] Nai-Wei Chen,[2] Oanh L. Meyer,[3] Mindy T. Tran,[4] and Kyriakos S. Markides[5], *1. Graduate College of Social Work, University of Houston, Houston, Texas, United States, 2. Beaumont Research Institute, Beaumont Health, Royal Oak, Michigan, United States, 3. University of California, Davis School of Medicine, Sacrameto, California, United States, 4. University of Houston, College of Natural Sciences and Mathematics, Houston, Texas, United States, 5. University of Texas Medical Branch, Galveston, Texas, United States*

Since the fall of Saigon, over 1.3 million Vietnamese immigrated to the U.S. making Vietnamese the 4th largest Asian ethnic but most vulnerable group to disparities. There is a paucity of knowledge on the health of elders and their caregivers. The Vietnamese Aging and Care Survey (VACS) was developed, and health data on 67 caregivers were collected in Houston, Texas. Adult-child caregivers (n=44) were on average, 45.3 years old, married (64%), working (91%), female (61%) in good/excellent health (90%). Spousal caregivers (n=23) were 70.6 years-old, retired (57%), female (78%) in fair/good health (73%). Adult-child received more help (43%) than spousal caregivers (29%), however, felt more caregiver burden (p=0.01) and perceived stress (p=0.05). Living in a multi-generation household, sharing caregiving, and working may alleviate their financial burden and provide psychological support. Findings suggest healthcare professionals to encourage caregivers to utilize available culturally-relevant social services to further ease their caregiving experiences.

### LATE-LIFE CAREGIVING IN MEXICAN AMERICAN FAMILIES

Jaqueline L. Angel[1], *1. The University of Texas at Austin, Austin, Texas, United States*

This study explores how sociological triangulation can be used to examine Mexican-American families in late-life caregiving. We examine the importance of household structure in providing dementia care. The movement away from traditional residential arrangements that result from neoliberal market reforms and international migration means that intergenerational relationship norms and exchanges will inevitably change and affect Mexican-American families caring for their elders. For many aging Mexican-Americans, a severe lack of resources and health limitations introduce major uncertainties about their futures. We employ the H-EPESE and Sacramento Area Longitudinal Study of Aging to document the nature, extent, and quality of dementia caregiving in the Southwestern United States. Qualitative results uncover how the changing meaning of social relationships impacts family life for older parents with dementia. Multivariate analyses reveal that the late-life migration (after 50yrs) undermines resources and opportunities for community-based care. Implications of the findings for informal and formal support are discussed.

### EXPANDING RELATIONAL BOUNDARIES OF DEMENTIA CARE

Karen A. Roberto,[1] and J. Tina Savla[1], *1. Virginia Tech, Blacksburg, Virginia, United States*

Rapid changes in family structures have expanded caregiving boundaries beyond the level of lineal kin to include extended and fictive kin. Guided by stress process and health behavior models, we analyzed semi-structured interviews with 120 family caregivers of persons with dementia (PWD) in rural Appalachia to explore personal/family circumstances that influence the responsibilities nonlineal kin assumed to meet the needs of PwD. Compared to spouse and adult children caregivers, nonlineal caregivers reported that PWD had similar behavioral problems, but greater ADL limitations. They also expressed greater burden, overload and role captivity; yet, they reported higher personal mastery, and perseverance. Although sisters and nieces did not report using any paid services to care for PwD, grandchildren and fictive kin used paid services such as meal delivery, personal care, and respite services. Findings provide new insights into a more elaborated conception of caregiving that considers the transformations occurring in family life today.

### SESSION 1030 (SYMPOSIUM)

### CAREGIVING SURVEILLANCE AT THE CDC: PAST, PRESENT, AND FUTURE

Chair: Valerie J. Edwards, *Centers for Disease Control and Prevention, Atlanta, GA, Antigua and Barbuda*
Discussant: C. Grace Whiting, *National Alliance for Caregiving, Bethesda, Maryland, United States*

As the U.S. population ages, caregiving has emerged as an important public health issue affecting an increasing proportion of American families. In 2015, an estimated 17.7 million people provided assistance to family members and friends. Although caregiving can have positive aspects, many studies have found that caregivers report more health difficulties than non-caregivers. The importance of population-based information is central to public health's ability to respond effectively to this growing public health problem. The Alzheimer's Disease and Healthy Aging Program at the Centers for Disease Control and Prevention (CDC) has made surveillance of caregivers a priority area. To this end, the development and use of a caregiving module for the Behavioral Risk Factor Surveillance System (BRFSS) was undertaken. The BRFSS is one of the largest telephone-based health surveillance system in the world, and collects information from the public across a broad range of health topics. This platform therefore provides a unique opportunity to capture health status data from caregivers as well as the option of comparing caregivers to non-caregivers. The Caregiver Module consists of 9 questions that address the characteristics of care and the type of assistance provided. The objective of this symposium is to describe the development of the current caregiving module (Dr. Bouldin), present relevant findings from the previous three years of surveillance data (Drs. Edwards and Taylor), and to discuss future directions for caregiver surveillance and CDC-developed resources to facilitate date utilization (Dr. McGuire). The discussant will describe the impact and status of national-level surveillance data

### DEMOGRAPHIC CHARACTERISTICS OF CAREGIVERS, BRFSS, 2015-2017

Valerie J. Edwards,[1] Lisa C. McGuire,[2]
Erin D. Bouldin,[3] and Christopher A. Taylor[2], *1. Centers for Disease Control and Prevention, Atlanta, GA, Antigua and Barbuda, 2. Alzheimer's Disease and healthy aging program, centers for disease control & prevention, atlanta, Georgia, United States, 3. Appalachian State University, Boone, North Carolina, United States*

As caregiving is becoming a leading public health issue, we used the BRFSS to better understand who caregivers are as well as features of their caregiving experience. We combined BRFSS data from three years (2015-2017) to examine demographic characteristics including gender, race, age, education, employment, and marital status. Slightly more than one in five BRFSS respondents (20.7%) were caregivers. The majority were women, had at least some college, were employed, and married or living with a partner. Most caregivers were under 45 years old, but a substantial proportion (20.7%) were 65 years or older. There was significant state variation by every demographic characteristic. These data underscore that caregiving is a common feature of U.S. family life, and caregivers' concerns should be considered in healthcare, employment, and service provision. Important state-level variations uncovered by use of the BRFSS Caregiving Module could lead to better targeting of caregiver support programs.

### HISTORY OF CAREGIVING SURVEILLANCE AT THE CDC, 2005 TO PRESENT

Erin D. Bouldin[1], *1. Appalachian State University, Boone, North Carolina, United States*

Research on caregiving has been ongoing for decades, but little systematic data collection at the population level occurred until relatively recently. Surveillance data is critical because it provides an evidence base upon which to make informed decisions about allocating resources, targeting programs, and developing policy. In 2005, CDC and the Association for Prevention Teaching and Research funded Dr. Elena Andresen's proposal to develop a set of questions about caregiving to be used on the BRFSS. This Caregiver Module was piloted in North Carolina and has been used – with some modifications – ever since as an optional module. The Caregiver Module has provided state-level data on the prevalence and types of caregiving provided by community-dwelling adults age 18 and older to people with a variety of conditions and needs. In 2009, the caregiver screening question was included on the BRFSS core, yielding a national estimate of caregiving prevalence of 24.7%.

### SUBJECTIVE COGNITIVE DECLINE IN CAREGIVERS: 21 STATES, PUERTO RICO, AND DC, 2015-2017

Christopher A. Taylor,[1] Valerie J. Edwards,[2]
Kenneth A. Knapp,[3] Erin D. Bouldin,[4] and
Lisa C. McGuire[5], *1. Centers for Disease Control and Prevention, Atlanta, Georgia, United States, 2. Alzheimer's Disease and Healthy Aging Program, Centers for Disease Control and Prevention, Atlantta, Georgia, United States, 3. New York Medical College, Valhalla, New York, United States, 4. Appalachian State Unversity, Boone, North Carolina, United States, 5. Alzheimer's Disease and Healthy Aging Program, Centers for Disease Control & Prevention, Atlanta, Georgia, United States*

Informal caregivers can provide assistance that can help family members and friends live in the community longer but can place caregivers at increased risk for poorer health outcomes. Subjective cognitive decline (SCD) is the self-reported experience of worsening or more frequent confusion or memory loss. The objective of this study is to describe SCD in caregivers. Data were analyzed from 21 states, Puerto Rico, and District of Columbia who administered both the Caregiver and Cognitive Decline modules of the Behavioral Risk Factor Surveillance System in the same year for 2015–2017. A higher proportion of caregivers reported SCD (13.4%) compared to non-caregivers (10.2%). Of those who did need assistance with daily activities due to SCD, 1 in 8 non-caregivers were unable to the necessary assistance compared to 1 in 4 caregivers. SCD among caregivers is of particular concern because it affects both the caregiver and care recipient.

### LOOKING FORWARD: BEHAVIORAL RISK FACTOR SURVEILLANCE SYSTEMS (BRFSS) CAREGIVING DATA

Lisa C. McGuire[1], *1. CDC, Atlanta, Georgia, United States*

The Centers for Disease Control and Prevention (CDC), through its Behavioral Risk Factor Surveillance System (BRFSS), collects data on caregivers and the caregiving situation as well as many health behaviors, annually. The BRFSS is the world's largest ongoing health survey, administered in all 50 U.S. states, as well as the District of Columbia and the three U.S. territories, with data collected from more than 400,000 respondents. In 2015-2017, the 9-item caregiving module was administered in 44 states, DC, and Puerto Rico on the BRFSS. CDC's Alzheimer's Disease and Healthy Aging Program has developed many data for action resources for use by states and other partners to help identify populations and communities most at need. This presentation will describe future enhancements with the BRFSS caregiving module and the series CDC developed resources to facilitate date utilization, including state-specific infographics, data briefs, and the online data portal.

### SESSION 1035 (SYMPOSIUM)

### CLARK TIBBITTS AWARD AND HIRAM J. FRIEDSAM MENTORSHIP AWARD LECTURES

Chair: Cynthia Hancock, *University of North Carolina, Charlotte, Charlotte, North Carolina, United States*

AGHE's Clark Tibbits Award was established in 1980 and named for an architect of the field of gerontological education. The award is given each year to an individual or organization that has made an outstanding contribution to the advancement of gerontology and geriatrics education. The Clark Tibbits Award lecture will feature an address by the 2019 award recipient, David Burdick, PhD, of Stockton University. Hiram J. Friedsam was the professor, co-founder, and director of the Center for Studies in Aging and dean of the School of Community Service at the University of North Texas. Dr. Friedsam was an outstanding teacher, researcher, colleague, and mentor to students, faculty, and administrators, as well as a past president of AGHE. The purpose of this award is to recognize those who emulate Dr. Friedsam's excellence in mentorship. The Hiram J. Friedsam Award lecture will feature an address by the 2019 award recipient, Bradley Fisher, PhD, of Missouri State University.

### SESSION 1040 (SYMPOSIUM)

### INTEREST GROUP SESSION—CANCER AND AGING: CURRENT CHALLENGES IN CANCER SCREENING AMONG OLDER MINORITY POPULATIONS

Chair: Chien-Ching Li, *Rush University, Chicago, Illinois, United States*
Discussant: Darren Liu, *Des Moines University, Des Moines, Iowa, United States*

Cancer is an important public issue around the world. Among types of cancer, lung and colorectal cancer are the most common in men while breast and cervical cancer are the most common in women. Detection of early stage cancer via screening can significantly reduce the mortality and prolong life. Although cancer prevention and control has been served as the national priority, individual's utilization of cancer screening services is low due to limited knowledge of cancer screening and ineffective patient-provider commutation, especially in minority populations. In this symposium, we will examine three scenarios that highlight the challenges of cancer screenings in minority populations. First, we will share the results from a mixed method study that investigate the knowledge and attitudes towards Low Dose Computed Tomography lung cancer (LDCT) screening and assess the smoking cessation needs for African Americans who receive LDCT screening in an effort to reduce the health burden of lung cancer. The next study will discuss how the characteristics of older Chinese adults from the United States and Taiwan are associated cancer screening communication with physicians (i.e., whether doctor recommended screenings and whether communicated screenings with doctor). Lastly, we will share the results from a cross-sectional study that analyzed 10 years data of National Health Interview Survey to examine the difference in LDCT screening eligibility among Asian American (i.e., Chinese, Filipino, and other Asian) smokers. The discussant will summarize with an overview of the topic, and comment on the disparities of cancer screening in older minority populations.

### ELIGIBILITY FOR LOW-DOSE COMPUTED TOMOGRAPHY LUNG CANCER SCREENING IN OLDER ASIAN AMERICAN SMOKERS

Chien-Ching Li,[1] Kelsey Choi,[1] Alicia Matthews,[2] and Raj Shah[1], *1. Rush University, Chicago, Illinois, United States, 2. University of Illinois at chicago, Chicago, Illinois, United States*

Lung cancer is the leading cause of cancer-related deaths in Asian Americans. Low-dose computed tomography lung cancer (LDCT) screening is an effective way to decrease lung cancer mortality. This study aimed to examine the difference in LDCT screening eligibility among Asian American subgroups. The National Health Interview Survey data (2006-2016) was analyzed. The U.S. Preventive Services Task Force guideline was used to determine the LDCT eligibility. A higher and statistically significant proportion of current Filipino smokers (35.4%) met LDCT screening eligibility criteria compared to Chinese (26.5%) and other Asian smokers (22.7%) ($p=0.02$). Hierarchical logistic regression results further showed that Filipino were more likely to meet LDCT screening criteria than other Asian while adjusting demographics ($OR=1.87$; $p=0.01$). The differences in LDCT screening eligibility no longer existed after additionally adjusting socioeconomic factors as well as perceived health status. Future targeted outreach and intervention research is needed for Filipinos with lower socioeconomic status.

### CHARACTERISTICS OF CHINESE ADULTS ON CANCER SCREENING COMMUNICATIONS WITH PHYSICIANS

Su-I Hou[1], *1. The University of Central Florida, Orlando, Florida, United States*

This study examined characteristics of Chinese adults on cancer screening communication with physicians. Whether doctor recommended screenings and whether communicated screenings with doctor were used to assess cancer communication. Participants were recruited from 9 Chinese churches (5 in U.S. and 4 in Taiwan; N=372). Mean age was 44.31, 60% males, 72% married, 85% college education, and 54% had family history. Overall 35.2% reported doctor recommended screenings and 27.7% talked with doctors about screenings (27.7%). Regressions showed Chinese 40+ years (OR=2.66 & 2.49), had annual health exam (OR=3.43 & 4.41), and been a primary cancer caregiver (OR=2.12 & 2.29) were more likely to report doctor recommended screenings (p<.001; 69% correct classification) and communicated with doctors about screenings (p<.001; 76% correct classification). There were no significant relationships between family history, gender, perceived cancer risk or health, and screening communications. Findings have implication on designing effective doctor-patient cancer communication programs among Chinese adults.

### KNOWLEDGE AND ATTITUDES TOWARD LDCT LUNG CANCER SCREENING AND SMOKING AMONG AFRICAN AMERICANS: A MIXED METHODS STUDY

Tung-Sung Tseng[1], *1. Tulane University, New Orleans, Louisiana, United States*

The purpose of this study is to investigate knowledge, attitudes, and smoking cessation needs for African Americans who receive Low Dose Computed Tomography (LDCT) in an effort to reduce the health burden of lung cancer. A mixed method study was conducted among African Americans who received LDCT. The sample size for both the quantitative and qualitative approach was fifteen. The results showed that 73% of participants were male, the mean age was 61.8(SD=4.6) years old. Smoking history was long but 64% of the patients had a low nicotine dependence. Participants had a moderate/lower knowledge score (Mean=4.3 SD=2.6), and most had a positive attitude. Similar findings were also observed in the qualitative analysis. Understanding the factors associated with smoking cessation among at-risk African American smokers will help reduce disparities in lung cancer burden, and is important to improve health for medically underserved minority populations.

### SESSION 1045 (SYMPOSIUM)

### DETERMINANTS OF NEUROCOGNITIVE IMPAIRMENT AND DEMENTIA IN ENGLAND AND JAPAN

Chair: Dorina Cadar, *University College London, London, United Kingdom*
Co-Chair: Kokoro Shirai, *Graduate School of Medicine Osaka University, Suita, Osaka, Japan*

Dementia is one of the major contributors to disability and dependency amongst the elderly populations and a significant public health concern. Even though the prevalence of dementia in the UK is rising due to higher numbers of people surviving into older ages, recent evidence suggests that the UK is experiencing a decline in dementia incidence. By contrast, Japan has witnessed a different trend, with increases in both incidence and prevalence. This difference could be related to diagnostic practices within each country, or to the cultural variability in the risk and protective factors driving these emerging forecasts that remain fundamentally different between the UK and Japan. Research in this field has been dominated by clinical studies of dementia mostly conducted in the UK and US, and the current evidence lacks reliable national data on dementia incidence. Socioeconomic inequalities and social determinants of neurocognitive health and dementia risk in two longitudinal studies of ageing: the English Longitudinal Study of Ageing (ELSA) from the UK and Japan Gerontological Evaluation Study of Aging (JAGES). These studies are ideally placed for addressing pivotal research questions in gerontology: 1. What are the biopsychosocial determinants of cognitive impairment and dementia in England and Japan? 2. What are the potential exploratory mechanisms related to the divergent trends in dementia incidence observed in England and Japan? 3. What are the critical differences between the social determinants of dementia in England and Japan?

### BIOPSYCHOSOCIAL DETERMINANTS OF NEUROCOGNITIVE IMPAIRMENT IN THE ENGLISH LONGITUDINAL STUDY OF AGEING

Dorina Cadar,[1] Dorina Cadar,[2] Jessica Abell,[2] David J. Llewellyn,[3] and Andrew Steptoe[4], *1. University College London, London, United Kingdom, 2. University College London,, London, England, United Kingdom, 3. University of Exeter Medical School, Exeter, England, United Kingdom, 4. University College London, london, England, United Kingdom*

Biological and psychosocial risk factors, particularly those that are malleable across the life course, are important determinants of neurocognitive health in later life. We investigated several determinants of cognitive impairment using the Mini-Mental Status Examination (MMSE), as part of the Harmonised Cognitive Assessment Protocol in 1,200 individuals aged ≥65 years from the English Longitudinal Study of Ageing. More than half the participants (55%) were married, 15% had diabetes, 12% had CHD, and fewer than 10% had a stroke. A longitudinal investigation of various risk factors measured at wave 6 (2012-13) was conducted in relation to neurocognitive impairment ascertained with the MMSE ≤24 in 2018. Our results indicate that certain environmental compensatory factors such as education, a marker of cognitive reserve, wealth and psychological wellbeing are relevant determinants of subsequent neurocognitive impairment six years later. These findings are highly informative for the development of interventions aiming to maintain neurocognitive health.

### LONELINESS, SOCIAL ISOLATION, AND DOMAINS OF COGNITIVE IMPAIRMENT IN THE ENGLISH LONGITUDINAL STUDY OF AGEING

Jessica G. Abell,[1] Jessica Abell,[2] Dorina Cadar,[2] David J. Llewellyn,[3] and Andrew Steptoe[2], *1. University College London, London, United Kingdom, 2. University College London, London, England, United Kingdom, 3. University of Exeter Medical School, Exeter, England, United Kingdom*

Globally the numbers of older people who live alone and those who may experience certain risk factors have risen. In this study, we aim to examine associations between social isolation and loneliness with different domains of cognitive impairment. Data are from the English Longitudinal Study of Ageing (ELSA). Social isolation and loneliness were measured in 2012-2013 and cognition in 2017-2018, using the Harmonised Cognitive Assessment Protocol (HCAP) in 1,200 men and women aged ≥65 years. General cognitive impairment was measured using the Mini-Mental Status Examination (MMSE); memory was assessed using the CERAD word list, attention & speed using the Symbol-Digit Modalities Test and executive function by a number series test. Loneliness, measured using the UCLA scale, was associated with a higher risk of neurocognitive impairment (MMSE<24), lower memory scores, poorer attention and executive function. However, social isolation was only found only to be associated with lower levels of memory.

### POSITIVE PSYCHOLOGICAL DETERMINANTS OF DEMENTIA IN JAPAN

Kokoro Shirai,[1] Kokoro Shirai,[2] Hiroyasu Iso,[2] Ryoto Sakaniwa,[2] and Katsunori Kondo[3], *1. Graduate School of Medicine Osaka University, Suita, Osaka, Japan, 2. Graduate School of Medicine Osaka University, Osaka, Osaka, Japan, 3. Center for Preventive Medical Science, Chiba University, chiba, Chiba, Japan*

We examined the effects of sustained levels of happiness on dementia incidence among Japanese elderly from the Japan Gerontological Evaluation Study. Data were from 12,051 community-dwelling participants aged 65 and over, free from dementia or other chronic conditions at baseline (2010). A survey was conducted in 31 administrative districts from Japan. Participants were followed-up for six years, and the incidence of dementia or death was assessed through National Public Long-Term Care Insurance and Resident Registry, Japan. We conducted survival analysis using the Cox proportional hazard model with a competing risk analysis accounting for death. The adjusted differences in days for dementia onset were estimated using Laplace regression models. In this analytical sample, we observed 520 men and 693 women with dementia and found that higher levels of sustained happiness were associated with reduced risks of onset of dementia among Japanese elderly. These findings suggest a protective role of psychological mood.

### CHANGES IN SOCIOECONOMIC STATUS ACROSS THE LIFE COURSE AND DEMENTIA ONSET IN JAPAN GERONTOLOGICAL EVALUATION STUDY

Ryoto Sakaniwa,[1] Ryoto Sakaniwa,[2] Kokoro Shirai,[2] hiroyasu iso,[3] and Katsunori Kondo[4], *1. Osaka University, Osaka University, Japan, 2. Graduate School of Medicine Osaka University, osaka, Osaka, Japan, 3. osaka university graduate school of medicine, osaka, Osaka, Japan, 4. Center for Preventive Medical Sciences, Chiba University, Chiba, Chiba, Japan*

Socioeconomic status (SES) is associated with dementia onset, but its transition throughout the life-course is poorly understood. In a prospective cohort of 40,041 participants, aged over 65 years without dementia, we identified ten optimal classifications of life-course SES transitions and their associated impact on dementia. Our results showed a clear significant dose-response pattern with the highest risk of dementia from 1) impoverished SES throughout, 2) impoverished childhood SES with normal adult SES, 3) impoverished adult SES with self-employed in adulthood, 4) high educated with poor adult SES, 5) never having a job, 6) low educated but high SES (self- employed), 7) technician, 8) high educated with poor adult SES, 9) average SES throughout, and 10) high SES throughout. These results suggest that life-course SES history is strongly associated with dementia risk and did not interact with single scale measurement. Further causal pathways and theories are relevant for disease modification.

### SESSION 1050 (SYMPOSIUM)

### DISCRIMINATION, STRESS, AND HEALTH ACROSS THE LIFE COURSE

Chair: Roland J. Thorpe, *Johns Hopkins Bloomberg School of Public Health, Baltimore, Maryland, United States*
Discussant: Carl V Hill, *NIH/National Institute on Aging, Bethesda, Maryland, United States*

There is a paucity of research that seeks to understand why race disparities in health across the life course remain elusive. Two such explanations that have been garnering attention is stress and discrimination. This symposium contains papers seeking to address the impact of discrimination or stress on health or health disparities across the life course. First, Brown and colleagues examine black-white differences in the number of reported chronic stressors across five domains their appraised stressfulness, and their varying associations with anxiety and depression among a diverse sample of older adults using data from 6,019 adults ages 52+ from the 2006 HRS. Race and appraisal interactions show that blacks and whites report similar increases in anxiety and depressive symptoms with appraisal. Second, Tobin and colleagues investigate the impact of early life racial discrimination (ELRD) on mental health among Black adults. Using data from 618 participants in the Nashville Stress and Health Study, these investigators found that childhood and adolescent ELRD were positively associated with adult distress. Also, individuals who experienced childhood ERLD had 88% lower odds of adult MDD than individuals with no ELRD. Cobb and colleagues examine the cross-sectional association between everyday discrimination and kidney function among older adults in HRS. The authors report that after adjusting for demographic characteristics, everyday discrimination was associated with lower mean eGFR. The relationship between everyday discrimination and kidney function was not explained by biospsychosocial factors. This collection of papers provides insights into how discrimination or stress impacts health in middle to late life.

### BLACK-WHITE DIFFERENCES IN CHRONIC STRESS: DOES APPRAISAL MATTER FOR ANXIETY AND DEPRESSIVE SYMPTOMS?

Lauren Brown,[1] Leah Abrams,[2] Uchechi Mitchell,[3] and Jennifer Ailshire[4], *1. University of Michigan, Ann Arbor, Michigan, United States, 2. University of Michigan School of Public Health, Ann Arbor, Michigan, United States, 3. School of Public Health, University of Illinois at Chicago, Chicago, Illinois, United States, 4. University of Southern California, Los Angeles, California, United States*

Prior research has suggested that exposure to objectively stressful events contributes to mental health disparities in older adulthood. Yet, in order to understand the extent to which some groups bear a disproportionate stress and mental health burden, we consider black-white differences in not only stress exposure, but also stress appraisal—how upsetting the exposures are perceived to be across five domains (health, financial, residential, relationship and caregiving). Data come from 6,019 adults ages 52+ from the 2006 Health and Retirement Study. Fully adjusted models show stress exposure and appraisal significantly and independently predicted anxiety and depressive symptoms. Race and stress exposure interactions show that exposure differently predicts anxiety and depressive symptoms while race and appraisal interactions show blacks and whites report similar increases in anxiety and depressive symptoms. Findings suggest stress exposure has varying consequences for mental health of whites and blacks, while stress appraisals have similar consequences across groups.

### DOES EARLY-LIFE RACIAL DISCRIMINATION EXPLAIN A MENTAL HEALTH PARADOX AMONG BLACK ADULTS?

Courtney S. Thomas Tobin,[1] and Myles D. Moody[2], *1. UCLA Fielding School of Public Health, Los Angeles, California, United States, 2. Department of sociology, University of Kentucky, Lexington, Kentucky, United States*

To evaluate the impact of early life racial discrimination (ELRD) on mental health among Black adults. Data were from the Nashville Stress and Health Study (n=618). OLS regression models examined the relationship between ELRD and adult psychological distress; logistic regression estimated the probability of past-year major depressive disorder (MDD). We also assessed whether ELRD moderated the relationship between adult discrimination and mental health. Childhood (b=1.07, SE=0.51, p=0.04) and adolescent ELRD (b=1.32, SE=0.42, p=0.002) were associated with adult distress. Individuals who experienced childhood ERLD had 88% lower odds of adult MDD than individuals with no ELRD. Significant interaction analyses showed that ELRD was generally protective against adult discrimination. While ELRD importantly shapes distress and MDD among Black adults, patterns vary by outcome. Results indicate that adult distress and MDD develop through cumulative adversity processes that are further influenced by sensitive periods in the life course.

### EVERYDAY DISCRIMINATION AND KIDNEY FUNCTION AMONG OLDER ADULTS: EVIDENCE FROM THE HEALTH AND RETIREMENT STUDY

Ryon J. Cobb,[1] Roland J. Thorpe,[2] and Keith C. Norris[3], *1. University of Texas at Arlington, Arlington, Texas, United States, 2. The John Hopkins University, Baltimore, Maryland, United States, 3. UCLA School of Medicine, Los angeles, California, United States*

Background: The current study examines the cross-sectional association between everyday discrimination and kidney function among older adults. Methods: We use cross-sectional data from a nationally representative sample of older adults to examine this relationship. Our measure of kidney function derives from the estimated glomerular filtration rate (eGFR) obtained by the Chronic Kidney Disease Epidemiology Collaboration equation, while our indicator of everyday discrimination is drawn from self-reports. Results: Results from our ordinary least squared regression models reveals that, after adjusting for demographic characteristics, everyday discrimination was associated with lower mean eGFR (β=-.79; S.E.: .34). The relationship between everyday discrimination and kidney function was not explained by cardiovascular, metabolic, or economic factors. Conclusions: Findings suggest this study suggest that everyday discrimination may be a unique risk factor for poorer kidney function among older adults. Because these findings are cross-sectional, additional research is needed to determine whether the observed associations persist over time.

### SESSION 1055 (PAPER)

### ECONOMIC AND EMPLOYMENT INTERESTS OF OLDER ADULTS

### FINANCIAL EXPLOITATION OF OLDER ADULTS: PRELIMINARY RESULTS FROM A PROSPECTIVE LONGITUDINAL STUDY

Scott R. Beach,[1] Sara J. Czaja,[2] Richard Schulz,[1] David Loewenstein,[3] and Peter Lichtenberg[4], *1. University of Pittsburgh, Pittsburgh, Pennsylvania, United States, 2. Weill Cornell Medicine, New York, New York, United States, 3. University of Miami, Miller School of Medicine, Miami, Florida, United States, 4. Wayne State University, Detroit, Michigan, United States*

This paper presents study design and preliminary results from a new study funded by the National Institute on Aging that is examining financial exploitation (FE) among 720 White, African-American and Hispanic adults age 60+ (240 per group; 120 age 60-79; 120 age 80+). A conceptual model linking sociodemographics, physical health, social support / integration, cognitive function, financial skills / supports, and psychosocial factors to FE is being evaluated. Three assessments (baseline, 12; 24 mos.) include: a detailed cognitive battery, web-based banking simulation tasks, scam scenarios, and a standardized battery of self-report measures assessing socio-demographic and psychosocial variables. Preliminary baseline results from ~200 participants show support for the proposed model. Exposure to sales, remote purchasing behavior, and telemarketer receptivity (scam exposure); and scam vulnerability as measured by credibility ratings of "legitimate" and "fake" scam scenarios are positively associated with reports of both stranger-initiated and trusted other FE. Older adults with smaller social networks and less social support were more likely to report both exposure and vulnerability to scams. Higher general cognitive abilities, financial skills, and numeracy; and better performance on online banking tasks correlate with less scam exposure and vulnerability. Preliminary analyses of psychosocial factors also show that more depressed, impulsive, and trusting older adults report more exposure and scam vulnerability. The paper will present updated analyses of ~500 baseline participants. Understanding multiple pathways to FE is important to advance theory and for the development of interventions to minimize risk.

### NATIONAL ELDER MISTREATMENT STUDY WAVE II: MENTAL HEALTH CORRELATES OF FINANCIAL MISTREATMENT

Melba Hernandez-Tejada,[1] Ron Acierno,[1] Jordan Watkins,[1] Gabrielle Frook,[1] Wendy Muzzy,[1] Georgia Anetzberger,[2] and Mara Steedley[1], *1. Medical University of South Carolina, Charleston, South Carolina, United States, 2. Case Western Reserve University, Cleveland, Ohio, United States*

Objective: Whereas prevalence of elder financial mistreatment has received increased attention over the past decade, health and mental health correlates are rarely studied. Thus, the potential relevance of financial abuse to mental health and perceived health is relatively unknown, and the objective of this article is to illustrate this relationship. Method: The second wave of the National Elder Mistreatment Study used random digit dialing telephone survey methodology to assess both recent financial mistreatment and its potential mental health correlates (i.e., diagnoses of depression, post-traumatic stress disorder [PTSD], generalized anxiety disorder [GAD], and self-ratings of physical health) in 774 older adults. Results: The study indicated that past-year Wave II financial mistreatment was associated with significantly increased likelihood of depression, PTSD, GAD, and poor self-rated health; and financial mistreatment perpetrated by family members was associated with particularly increased risk of depression. Discussion: Assessment of mental health is relevant and important in cases of financial abuse.

### SUPPORTING AN OLDER WORKFORCE BY CREATING AGE-FRIENDLY WORKPLACES: INTRODUCING A NEW MULTIDIMENSIONAL MODEL

Raphael Eppler Hattab,[1] Ilan Meshoulam,[1] and Israel Doron[1], *1. University of Haifa, Israel, Israel*

Creating age-friendly workplace environments is considered a central organizational approach for addressing the challenges of supporting an aging and older workforce. However, there are no concrete definitions or theoretical frameworks that explain the full meaning, assumptions and basic processes of this concept. This paper critically reviews the conceptualizations of the age-friendly workplace in the fields of organizational psychology and gerontology, and proposes (a) a new working definition of the concept, and (b) a multidimensional model that consists of a typology of age-friendly dimensions, representing the implications of human resource policies and practices that demonstrate the ways in which organizational climate and organizational culture support aging workers. This framework enables a better understanding of the organizational-occupational realities within an aging and older labor market, and thus serves as an effective foundation upon which future organizational measurements can be constructed. This presentation is based on the article: Eppler-Hattab, R., Meshoulam, I., & Doron, I. (2019). Conceptualizing Age-Friendliness in Workplaces: Proposing a New Multidimensional Model. The Gerontologist. 10.1093/geront/gny184.

### UNDERSTANDING RISK AND PROTECTIVE FACTORS FOR ELDER FAMILY FINANCIAL EXPLOITATION: A PREVENTIVE APPROACH

Virginia B. Vincenti,[1] and Ashton Chapman[2], *1. University of Wyoming, Laramie, Wyoming, United States, 2. Social Grove, Inc., Joplin, Missouri, United States*

This paper presents qualitative data from a phenomenological study of 10 men and 38 women (N=48) within families with designated power of attorney (POA) agents. Participants were from 18 states nationwide, aged 20 to 73 (M = 46.6), and had varying educational and income levels. Eighteen individuals alleged that EFFE occurred in their families, 27 did not, and three didn't know. The study explored EFFE risk and protective factors within Bronfenbrenner's PPCT model (Tudge, 2018). Data were analyzed using a thematic, inductive approach. Specifically, person characteristics (e.g. perpetrators' personality and victims' cognitive functioning), proximal processes (e.g. family patterns of communication and resource sharing), context (e.g. geographic location), and time (e.g. prevailing legal, economic, and cohort factors) were associated with EFFE recognition and intervention. Implications and applications of the PPCT model for helping professionals (e.g., practitioners such as healthcare providers, attorneys, therapists) will be discussed to raise awareness of risk and protective factors within families that may increase or decrease the likelihood that elder financial exploitation by family members will occur. The goal is to improve prevention through helping families address risk factors before older relatives become dependent, encouraging more proactive planning in development of POA and other end-of-life documents, and detect exploitation early.

### WORKPLACE SUPPORTS FOR WORKING LONGER: A CASE FOR THE BLACK LOW-WAGE WORKER

Kendra Jason[1], *1. University of North Carolina at Charlotte, Charlotte, North Carolina, United States*

Government-sponsored retirement programs and employer benefits are directly tied to individual employment history and wages. Consequently, Black workers disproportionally face challenges in labor market compensation and retirement benefits. Due to a history of racial discrimination and economic oppression, Black workers earn less income over the life course and are less likely to have insurance support in comparison to their white counterparts in older age. This leads Black adults to remain in the workforce longer for financial support and presents unique physical and psychosocial challenges balancing work obligations and family responsibilities. Further, Black adults also suffer from more chronic illnesses, poor health outcomes, and death at higher rates compared to nearly all other racial groups. Drawing on data derived from a workplace case-study with interviews from 15 low-wage Black workers aged 50+ years, with multiple chronic conditions in the Southern United States. My aims are to (1) understand what workplace supports enable vulnerable workers to remain in the workforce, and (2) identity other buffers (i.e., resilience) to working with chronic conditions that enable prolonged work engagement. Findings suggest that workplaces can better support low-wage workers who cannot afford to retire by offering better pay and health benefits. Supervisor and coworker supports, flexible work arrangements and scheduling, and less stressful work environments also enable sustained work engagement. Research, policy and practice implications of this research include identifying workplace attributes and determining strategies to strengthen them, which is paramount to addressing disparities in work and health outcomes in the vulnerable communities.

### SESSION 1060 (SYMPOSIUM)

### EXPLORING THE IMPACT OF TECHNOLOGY ON OLDER ADULTS' WELL-BEING

Chair: Walter R. Boot, *Florida State University, Tallahassee, Florida, United States*

Recent research had indicated clear links between social isolation and loneliness, and a host of negative consequences, including poor mental and physical health, cognitive decline, and even increased mortality risk. These consequences make clear that social isolation is a significant and urgent public health concern that requires attention and action. Increasingly, researchers have begun to explore the role technology can play in reducing social isolation and increasing social support. This symposium contains five talks that address this question directly, presenting a nuanced picture of the potential effects of technology on well-being among older adults. The first presentation will highlight the potential positive side of internet use on wellbeing using longitudinal data from the Health and Retirement Study. The next talk explores how the internet can support older adults undergoing significant life transitions. Importantly, the link between internet use and well-being appears to be context-dependent, with internet use being associated with positive or negative effects depending on other contextual factors. This presentation is followed by another, finding that technology use can have varying effects depending on how and what the technology is used for. This is followed by a presentation that directly compares technology-based communication to in-person communication, and the different effects of each on well-being. The session concludes with a presentation on how novel robotic technology might provide emotional and social support. In answer to the question of whether technology can reduce social isolation and loneliness, and improve social support: It's complicated.

### INTERNET USE AND LONELINESS OF OLDER ADULTS OVER TIME: THE MEDIATION EFFECT OF SOCIAL CONTACT

Kexin Yu,[1] Kexin Yu,[1] Shinyi Wu,[1] and Iris Chi[1], *1. University of Southern California, Los Angeles, California, United States*

Internet is increasingly popular among older adults and have changed interpersonal interactions. However, it remains controversial whether older people are more or less lonely with internet use. This paper tests the longitudinal association of internet use and loneliness among older people. One pathway that explains the association, the mediation effect of social contact, was examined. Data from the 2006, 2010 and 2014 waves of Health and Retirement Study was used. Hierarchical liner modeling results showed internet use was related to decreased loneliness over 12-year period of time ($b=-0.044$, $p<.001$). Internet use was associated with more social contact with family and friends overtime ($b=0.261$, $p<.001$), social contact was related to less perceived loneliness longitudinally ($b=0.097$, $p<.001$). The total effect of internet use on loneliness is -0.054 and the mediated effect is -0.025. The findings imply that online activities can be effective for reducing loneliness for older people through increased social contact.

### INTERNET USE AND SOCIAL ISOLATION: THE SIGNIFICANCE OF LIFE TRANSITIONS

Hyunju Shim,[1] Jennifer A. Ailshire,[2] and Eileen M. Crimmins[2], *1. University of Southern California, Los Angeles, United States, 2. University of Southern California, Los Angeles, California, United States*

Social isolation has emerged as a significant public health risk for older people, and can be triggered by a number of life transitions in old age, such as retirement, bereavement, and divorce. Using the Health and Retirement Study (HRS), the current study aims to examine how internet use is associated with significant life transitions in marital and employment status in a longitudinal analysis. Results showed that using the internet was associated with lower social isolation and depression for people who became separated or divorced, but no significant interaction was observed for people who became widowed. On the other hand, becoming unemployed and using the internet was associated with more depressive symptoms. The results indicate that the link between psychological well-being and use of internet differs depending on the context of life transitions and may not have universal benefits for reducing social isolation.

### THE GOOD, THE BAD, AND THE UGLY: EXAMINING OUTCOMES OF TECHNOLOGY USE AMONG OLDER ADULTS

Shelia Cotten,[1] Shelia R. Cotten,[1] and Travis Kadylak[1], *1. Michigan State University, East Lansing, Michigan, United States*

Older adults are increasingly using information and communication technologies (ICTs) to communicate with social ties, gather information to make decisions, and for entertainment purposes. Research is increasingly showing that using ICTs has a range of potential benefits for older adults. However, less research examines the potential negative outcomes of ICT use for older adults. Data from a nationally representative sample of older adults in the United States is used to examine positive and negative outcomes of ICT use. Traditional well-being and social connection outcomes are examined as well as new stressors associated with mobile phone use. Our findings suggest that ICT use has varying effects on older adults, depending upon the type, level, and purposes of use. Implications are discussed for entities seeking to encourage ICT use to enhance health and quality of life among older adults.

### SOCIAL INTERACTIONS AND WELL-BEING: THE ROLE OF COMMUNICATION METHOD

Xin Yao Lin,[1] Xin Yao Lin,[1] and Margie E. Lachman[1], *1. Brandeis University, Waltham, Massachusetts, United States*

Given the increased usage of technology for social interactions, it is important to consider whether the method of communication makes a difference in one's daily well-being. We conducted a diary study, over seven days with 142 participants, to examine the role that communication method (in-person, phone, technology) plays in daily stress exposure, stress reactivity, and positive and negative affect among adults ages 22 to 94. Multilevel modeling results revealed that on days with higher use of technology (text, video, internet) communication than their weekly average, individuals had more negative outcomes (greater stress exposure and negative affect). On days with more in-person communication than their weekly average, individuals had more positive affect. On days with more phone communication than their weekly average, individuals had less negative affect. The discussion highlights the benefits of in-person and phone communications for well-being, while also considering the potential negative outcomes associated with frequent technology communication.

### EXPLORING THE PERCEPTION OF PATIENTS WITH DEMENTIA ABOUT A SOCIAL ROBOT PARO IN A HOSPITAL SETTING

Lillian Hung,[1] mario Gregorio,[2] Jim Mann,[2] Neil Horne,[2] Christine Wallsworth,[2] Annette Berndt,[2] and Habib Chaudhury[1], *1. Simon Fraser University, Vancouver, British Columbia, Canada, 2. CEAN, Vancouver, British Columbia, Canada*

PARO, a robotic pet, was designed to provide emotional and social support for older people with dementia. This project aims to explore the perception of persons with dementia about the role of PARO in a hospital setting. Video-ethnographic methods were applied. Patient and family partners were involved in the fieldwork of data collection and analysis. We conducted conversational interviews with ten patients with dementia staying in a geriatric unit and video observations. Thematic analysis yielded three substantive themes: (a) “it’s like a buddy”, the robot helps persons with dementia to uphold a sense of self in the world, (b) “it’s a conversation piece”, the baby seal facilitates social connection, and (c) “it’s all about love”, PARO transforms and humanizes the clinical setting. Our findings contribute to providing a better understanding of the direct perspectives of patients with dementia on the use of the social robot.

### SESSION 1065 (SYMPOSIUM)

### GLOBAL PUBLISHING OPPORTUNITIES IN GERONTOLOGY: A VIEW FROM THE EDITORS' DESKS

Chair: Edward A. Miller, *University of Massachusetts Boston, Boston, Massachusetts, United States*
Co-Chair: Elizabeth Simpson, *University of Massachusetts Boston, Boston, Massachusetts, United States*
Discussant: Michael Gusmano, *Rutgers: The State University of New Jersey, New Brunswick, New Jersey, United States*

Global aging has proceeded at an unprecedented and accelerating rate. The aging of the population creates both opportunities and challenges for elders, their families, and society in general. Importantly, there is substantial variation in the effects of and response to global aging both within and across nations depending, in part, on prevailing cultural expectations and values, political and economic imperatives, and social and demographic characteristics. Thus, while some regions and countries have responded with innovative policies and programs to better enable the growing cohort of older adults to remain active and engaged in the community, other regions and countries have struggled with their response or barely begun to plan for the rising population of elders. This symposium assembles editors at five leading gerontological journals to demonstrate the role that peer-reviewed scholarship can play in disseminating knowledge that informs gerontological research, policy, and practice internationally. Editors include: Jeffrey Burr, PhD, Research on Aging; Deborah Carr, PhD, Journal of Gerontology: Social Sciences; Edward Alan Miller, PhD, Journal of Aging & Social Policy; Julie Hicks Patrick, PhD, International Journal of Aging & Human Development; and Julie Robison, PhD, The Journal of Applied Gerontology. Each presenter will review the scope, content, and focus of their journals and the role and opportunities for international scholarship. Michael Gusmano, PhD, a leading expert on the economic, political, and social consequences of global aging and International Editor of the Journal of Aging & Social Policy, will serve as discussant.

### THE ROLE OF GLOBAL SCHOLARSHIP IN THE JOURNAL OF AGING & SOCIAL POLICY

Edward A. Miller,[1] Michael Gusmano,[2] Elizabeth Simpson,[1] and Pamela Nadash[1], *1. University of Massachusetts Boston, Boston, Massachusetts, United States, 2. Rutgers: The State University of New Jersey, New Brunswick, New Jersey, United States*

Policymakers, practitioners, and researchers need a balanced, thoughtful, and analytical resource to meet the challenge of global aging at a rate that’s historically unprecedented. The Journal of Aging & Social Policy (JASP), which was founded in 1989, serves this role by drawing contributions from an international panel of policy analysts and scholars who assume an interdisciplinary perspective in examining and analyzing critical phenomena that affect aging and the development and implementation of programs for elders from a global perspective. Study settings extend beyond the United States to include Europe, the Middle East, Australia, Latin America, Asia, and the Asia-Pacific rim. This presentation will document the scope, content, and focus of JASP, including the rise of international submissions, which now account for approximately half of articles published. Opportunities for publishing in JASP will be discussed; so too will strategies for navigating the peer-review process successfully.

### RESEARCH ON AGING: THE INTERNATIONAL VIEW FROM THE EDITORS' DESKS

Jeffrey Burr,[1] Kyungmin Kim,[1] and Sae Hwang Han[1], *1. University of Massachusetts Boston, Boston, Massachusetts, United States*

We review the scope, content, and focus of the peer-reviewed journal, Research on Aging (SAGE), publishing its 41st volume this year. We will discuss how scholarship produced from researchers around the globe has changed over the years. Data on submissions, acceptance rates, and the important role of an international editorial board will be presented. The review process will be described, along with suggestions on how to increase chances of success when submitting original research. Although Research on Aging is sometimes considered to focus primarily on social gerontology, the scope in recent years has widened considerably, with manuscripts in aging studies published from such fields as economics, psychology, demography, public health, and public policy, as well as from sociology, and social work, among others. One of several special issues forthcoming in the journal will be described to demonstrate the possibilities for international impact.

### THE JOURNAL OF GERONTOLOGY: SOCIAL SCIENCES: GLOBAL SCHOLARSHIP CHALLENGES AND OPPORTUNITIES

Deborah Carr[1], *1. Boston University, Boston, Massachusetts, United States*

Journal of Gerontology: Social Sciences aims to publish the highest quality social scientific research on aging and the life course in the U.S. and worldwide. The disciplinary scope is broad, encompassing scholarship from demography, economics, psychology, public health, and sociology. A key substantive focus is identifying the social, economic, and cultural contexts that shape aging experiences worldwide. In the coming decade, social gerontology research is poised to present many opportunities for cross-national and cross-cultural scholarship – driven in part by the proliferation of large parallel data sets from many nations in Europe, Latin America, and Asia. I will discuss the role that peer-reviewed cross-national scholarship can play in disseminating knowledge that informs gerontological research, policy, and practice internationally. I will also identify under-researched areas that will be of great interest to scholars in the coming decade, including LGBT older adults, aging in the Global South, reconfigured families, and centenarians.

### THE JOURNAL OF APPLIED GERONTOLOGY: AN INTERNATIONAL PERSPECTIVE

Julie Robison[1], *1. University of Connecticut, Farmington, Connecticut, United States*

The mission of applied gerontology is to bridge science and practice to benefit the health and well-being of older persons, their families, their communities, and other contexts. This presentation will provide insights from the Journal of Applied Gerontology and its attempts to publish and disseminate scholarship that has international application. Following an overview of the growing internationalization of peer-reviewed submissions to the Journal of Applied Gerontology on a variety of topics and from a range of perspectives, the presentation will highlight key achievements as well as ongoing concerns and opportunities to better achieve the goals of applying gerontological scholarship to aging contexts worldwide. Concluding comments will examine how outlets for dissemination and authors themselves can better position their work to enhance their influence on aging in an international context.

### TIPS FOR TIMELY (AND POSITIVE) REVIEWS: THE INTERNATIONAL JOURNAL OF AGING & HUMAN DEVELOPMENT

Julie Hicks Patrick[1], *1. West Virginia University, Morgantown, West Virginia, United States*

For more than 40 years, under the leadership of four editors and two publishers, The International Journal of Aging and Human Development (IJAHD) has featured multidisciplinary scholarship related to aging processes and older adults. With the publication of eight issues a year, with over 800 pages of scientific content, the IJAHD places emphasis upon psychological and social studies of aging and the aged. However, the Journal also publishes research that integrates observations from other disciplines that illuminate the "human" side of gerontology. A more recent focus includes midlife development, as well. About half (47%) of the publications in the IJAHD are from international colleagues. This presentation will discuss tips for both international and US-based scholars for ensuring timely reviews and positive decisions for manuscript submissions, including such areas as key words, suggesting unbiased reviewers, formatting, writing mechanics, clearly-articulated methods, and a sound theoretical basis.

### SESSION 1070 (SYMPOSIUM)

### HEALTH AND WELL-BEING AFTER DISPLACEMENT IN LATE LIFE AMONG ASIANS

Chair: Wenjun Li, *University of Massachusetts Medical School, Worcester, Massachusetts, United States*
Co-Chair: Allen Glicksman, *Philadelphia Corporation for Aging, Philadelphia, Pennsylvania, United States*
Discussant: Shantha Balaswamy, *The Ohio State University, Columbus, Ohio, United States*

Displacement in late life may cause social and health issues. Displaced older adults may experience language and cultural barriers, loss of social networks, difficulties in navigation in new physical and social environments, limited options in transportation and mobility, delayed access to health care, and thus sudden or gradual loss of their autonomy, and increasing dependence on their adult children. These issues are known but not well understood, and effective interventions are yet to do developed. This symposium brings together four studies that address several critical social and health issues among late-life Asian immigrants. Dr. Inoue discusses use of person-centered care to reduce social isolation and loneliness among old and ill Asian immigrants in long term care settings. Ms. Ring examines racial differences in navigation and access to long term aging services and social supports (LTSS), and evaluates the use of Social Interaction Modeling to help connect limited English-speaking minorities in need to the formal LTSS system. Dr. Torres from Sweden provides a systematic review of research into racial/ethnic differences in health and social care, and from an international perspective, advocates a research agenda that is both diversity-astute and injustice-aware. In settings where self-report data are likely unreliable, Dr. Li demonstrates an innovative method for objectively measuring spatiotemporal patterns of physical and social activities and use of neighborhood resources among non-English speaking late-life immigrants. Together these studies demonstrate that existing methods can be adapted and new methods can be created to answer important health and social issues among late-life Asian immigrants.

### MEASURING SPACE AND TIME USE, PHYSICAL AND SOCIAL ACTIVITIES AMONG NON-ENGLISH SPEAKING LATE-LIFE ASIAN IMMIGRANTS

Wenjun Li,[1] Linda Churchill,[1] Jie Cheng,[1] Rachel Siden,[1] Annabella Aguirre,[1] and Kevin Kane[1], *1. University of Massachusetts Medical School, Worcester, Massachusetts, United States*

Little is known about the health and health care needs of non-English Speaking late-life Asian immigrants. Due to language barriers and memory issues, self-report data are unreliable for investigating activity patterns in this population. In the ongoing NIA-funded Healthy Aging and Neighborhood Study, we developed a novel method to objectively measure space and time use, location- and time-specific physical and social activities using accelerometer (ACC) and Global Positioning System (GPS) devices. The study has recruited over 150 Caucasians and 150 minorities including 50 non-English speaking late-life Asian immigrants. The participants answered surveys in their preferred language (English, Spanish, traditional or simplified Chinese) and wore ACC/GPS devices for 7 to 10 full days. Activity levels and geographic locations are recorded every 30 seconds. Using the combined ACC/GPS data, time- and location-specific activity amounts, time use and mobility patterns are objectively measured. Baseline findings will be reported at the GSA conference.

### PERSON-CENTERED CARE TO DECREASE LONELINESS AND SOCIAL ISOLATION IN LONG-TERM CARE SETTINGS

Megumi Inoue,[1] Mai Hasegawa,[2]
Catherine Tompkins,[1] and Catherine Donnelly[1], *1. George Mason University, Fairfax, Virginia, United States, 2. JB Line, Inc., Arlington, Massachusetts, United States*

The older foreign/immigrant population is predicted to increase in the U.S. As a whole, this population faces greater challenges associated with loneliness and social isolation due to their smaller social networks, language and cultural barriers. Person-centered care should be considered as a potential solution to decrease loneliness and social isolation in long term care settings. In 2017, researchers conducted a thorough examination on the effects of a senior companion program on a Japanese woman with advanced dementia. Results suggest that the client benefitted from the program in regard to her physical wellbeing, emotional wellbeing, language communication and cultural support. Given the support of Japanese companions, the client was able to express her needs and health symptoms effectively and the staff were subsequently able to provide culturally-sensitive care. This study implies that the companion program may benefit other foreign/immigrant older adult populations in reducing social isolation and declining health.

### ACCESSING LONG-TERM SERVICES AND SUPPORTS: HOW DO ETHNIC MINORITIES NAVIGATE THE SYSTEM?

Lauren Ring,[1] and Allen Glicksman[1], *1. Philadelphia Corporation for Aging, Philadelphia, Pennsylvania, United States*

The decision to seek Long Term Services and Supports (LTSS) can be challenging for older adults and family members. These challenges can be greater for members of certain ethnic/cultural minority communities who are not fluent in English. Our study examines the ways in which older adults in limited English-speaking communities (Spanish / Mandarin Chinese) navigate the use of LTSS. The findings will be used to evaluate disparities in service perception and access experienced by these populations. Our research examines the ways in which information is shared among community members and how they identify trusted sources of information. Ultimately, we wish to examine how these social networks and trusted neighborhood institutions do, or do not, connect older adults in need to the formal LTSS system. We use a modeling technique called Social Interaction Modeling (SIM), which allows for the inclusion of both conceptual and data based elements, to frame this process.

### ETHNICITY, RACE, HEALTH, AND SOCIAL CARE: STUDYING INEQUALITIES IN AN INJUSTICE-OBLIVIOUS WAY

Sandra Torres[1], *1. Uppsala Universitet, UPPSALA, Sweden*

Scholarship on ethnicity and old age is at a crossroad now that increased diversity is a given in older populations. The same holds true for the study of inequalities in old age as it relates to ethnicity and race. This presentation relies on a scoping review of scholarship published between 1998 - 2017 (n=336) that brings attention to the ways in which ethnicity/ race – as grounds for stratification and disadvantage - are made sense of in this scholarship. The presentation will describe the topics that the review divulged when it comes to the study of health and social care (i.e. access and usage; attitudes, preferences and experiences; assessment of programs suitability and self-care practices). In doing so, this presentation will argue that if we are to address the inequalities that older ethnic minorities face we need not only a diversity-astute research agenda but also an injustice-aware one.

### SESSION 1075 (PAPER)

### HUMANITIES AND ARTS: INTERVENTIONS AND WELLNESS

### A NARRATIVE STUDY TO CONNECT THE GERONTOLOGY: THE ELDERLY CHARACTERS IN NURSING HOMES FIRE NEWS IN TAIWAN

Sheng-Chin HSU[1], *1. Ming Chuan University, Taipei, Taiwan*

There are more than 30 civilian deaths in 10 fire accidents in nursing homes since 2008 in Taiwan. Due to the collective space of nursing homes, the damages of fires in a facility are much worse than in a personal house. Since the type of family changed after The Chinese Civil War, the elderly members, retired from the labor market, like to choose to live in nursing homes, or hospitals. According to the Ministry of Interior, R.O.C., Taiwan turned into an Aged society in 2018. In those fire news, the duties of families are seldom discussed, but the duties of care center are emphasized. The narrative of a fire accident in nursing homes is composed of many elements. These elements have been connected with old people and various arrangements. It is important to understand how the duties shifting from families to nursing homes, and what characters and relationship disappear in a fire accident. This study adopts the narrative theory to analyze the characters in fire report about Nursing Homes. Three findings are discovered: first, the duties of taking care of elders in a nursing home fire are connecting to Care Facilities but not families nor government. Second, the role of taking care of older people is played by Care-Staff more than family members in a fire narrative. Finally, the duty of fire and the duty of taking care of elders are interwoven into a complicated narrative: the role of Long-Term Care Center emerges but family and government disappear.

### COMMUNITY BELONGING THROUGH THEATER: CREATIVE ARTS INTERVENTION FOR LOW-INCOME OLDER ADULTS

Ruth E. Dunkle,[1] Laura Sutherland,[2] Garrett T. Pace,[1] Ariel Kennedy,[1] and Patricia Baldwin[3], *1. University of Michigan, Ann Arbor, Michigan, United States, 2. Wayne State University, Detroit, Michigan, United States, 3. Hannan Foundation, Detroit, Michigan, United States*

Creative arts can promote social contact and possibly reduce isolation. A professionally run theater group comprised of low-income older adults met for 12 weeks to learn basic skills and perform a play. Using a pre-post questionnaire, data were gathered from the treatment group (n=14) who participated in the class and a non-participating comparison group (n=5) to identify potential program effects on measures of social isolation, community belonging, and social exclusion. Participants were African American living in low-income housing in an urban area. The average age of the sample was 65 years, 21% were men, 83% had at least high school degree, 71% reported good to excellent health, and 58% reported at least one ADL. Regression analyses showed that a sense of community belonging was significantly greater for the treatment group than the comparison group at time 2.This was not the case when considering social isolation or social exclusion. When controls were added (age, health, and previous theater experience), the significant difference remained with higher age predicting a sense of community belonging. The greater number of class sessions attended was also associated with a greater sense of community belonging for the treatment group. Through the shared experience of theater, participants can gain a sense of community, but this activity does not seem to be related to social isolation or social exclusion. It could be that theater participation fosters a sense of belonging due to group dynamics but is not a significant enough activity to reduce a sense of isolation or exclusion.

### FINDINGS FROM THE ALZHEIMER'S POETRY PROJECT LONG-TERM CARE IMPLEMENTATION STUDY

Daniel B. Kaplan,[1] and Gary Glazner[2], *1. Adelphi University, Garden City, New York, United States, 2. The Alzheimer's Poetry Project, New York, New York, United States*

Dementia interventions grounded in group participation in the cultural arts (e.g., poetry, storytelling, music, and dance) have been growing in reach in recent decades. Yet this growth has been stymied by a lack of empirical evidence to demonstrate measurable outcomes. In the current IRB-approved research study, the Alzheimer's Poetry Project (APP), in partnership with 15 Wisconsin nursing homes, provided staff training in an innovative non-pharmacological intervention for people living with dementia. The goal of APP is to facilitate creative self-expression, social and intellectual stimulation, respectful acceptance, validation of personhood, and valued inclusion of people living with Alzheimer's disease and other dementias through the performance and creation of poetry. APP's proven track record at over 500 facilities in 34 states and six countries internationally, serving over 40,000 people worldwide, demonstrates APP's ability to bring high-quality creative arts programming to people living with dementia. Through the implementation of APP in three groups of five nursing homes in Wisconsin, activities and care staff were trained to deliver the intervention over three-month periods. After training was completed, researchers evaluated and compared APP to non-arts programming in the same facilities. A novel dementia arts mapping technique was created to document observable impacts of interventions on social engagement, alertness, vocalization, vocabulary, affect, and self-expression. 35 interventions were documented, and variability in participant experiences reveal significantly greater impacts on personhood and creativity during the 11 APP interventions than during the 24 non-arts interventions. Implications for promoting dementia care programming grounded in group participatory arts will be discussed.

### GROUP-BASED PARTICIPATORY ARTS INTERVENTIONS: PRODUCING ART AND VALIDATION OF PERSONHOOD

Daniel B. Kaplan,[1] and Gary Glazner[2], *1. Adelphi University, Garden City, New York, United States, 2. The Alzheimer's Poetry Project, New York, New York, United States*

Participatory arts-based interventions for people living with dementia involve the collaborative creation and performance of poetry, story, song, dance, and visual arts. These programs are designed to support self-expression and productive collaboration among people living with dementia while stimulating positive social interactions and feelings of empowerment and validation. This paper explores the use and potential benefits of validation in the implementation of person-centered participatory arts interventions in the context of dementia care. The authors offer a theoretical framework for understanding validation as a common intervention method and participant experience during these interventions, organized into five themes: collaboration, connection, creation, communication, and confirmation. Basic validation techniques are easily learned and can be incorporated into a broad diversity of dementia care strategies and interventions including activity programs designed to stimulate cognitive, physical, and communicative abilities. Participatory arts programs offer tools for connecting to personhood in a population for whom such connections are under constant threat of erosion. As activity programs based in creative arts help to support self-expression among participants and serve as a vehicle for generating feelings of self-efficacy, such activities are well suited to fostering the person-centered goals of dementia care programming. Clinicians and other transdisciplinary care providers are encouraged to understand, use, and teach these and other validation-focused arts interventions with persons living with dementia. These validation experiences are suggested to offer direct clinical benefits for participants as well as indirect benefits when modeled in the presence of formal care providers and family members.

### POETRY INTERVENTION IMPACTS PERSPECTIVES ON DEMENTIA AND CAPABILITIES AMONG YOUNG VOLUNTEERS

Daniel B. Kaplan,[1] and Gary Glazner[2], *1. Adelphi University, Garden City, New York, United States, 2. The Alzheimer's Poetry Project, New York, New York, United States*

Poetry for Life (PFL), is a teaching and learning initiative that brings students together with older adults in meaningful community service workshops. PFL capitalizes on the skills and passions of young poets by offering opportunities to serve elders by leading poetry workshops at settings where older adults receive care. This study examines measurable impacts of training, exposure, and experience in poetry-based intergenerational workshops on students' knowledge, attitudes, and values. Participating groups of students receive instruction in performing and creating poetry in group settings. They visit local elder care settings to facilitate PFL workshops and then write reflections on their experiences. Students agree to complete pre- and post-program surveys to document the impacts of PFL experiences on students' social/emotional health and on their knowledge, attitudes, and values related to older adults, dementia and dementia care, poetry and arts-based interventions, and careers in healthcare, aging fields, and the arts. To date, 33 young people from one middle school, one high school, and one graduate college program have volunteered to participate in the program and completed the study. Findings reveal significant impacts on students' perceived capabilities working and communicating with people with dementia as well as leading poetry activities. Additionally, significant positive impacts were demonstrated on 12 of 20 items on the Dementia Attitudes Scale across participating students. The PFL experience did not, however, lead to significant impacts on student self-esteem or work interests. These findings suggest benefits and limitations of this service-learning experience. Implications for future programming will be discussed.

### SESSION 1080 (SYMPOSIUM)

### INNOVATIVE STRATEGIES TO SUPPORT OLDER ADULTS AND THEIR FAMILIES IN HOSPICE AND PALLIATIVE CARE

Chair: George Demiris, *University of Pennsylvania, Philadelphia, Pennsylvania, United States*
Discussant: Karen Hirschman

In order to better support older adults with life-limiting illness and their families, many initiatives utilize information technology and other innovative platforms to increase access to supportive services and bridge geographic distance. Such technologies cover a broad range of systems ranging from smart phone applications to wearables and traditional telehealth platforms. There is a growing evidence base for such interventions but technical, clinical and ethical challenges remain when utilizing technology in the context of hospice and palliative care especially for older adults, including the concerns for caregiver burden, privacy, security, confidentiality, obtrusiveness and accessibility. In this symposium we provide an overview of innovative tools available for interventions in palliative and hospice care designed for patients and/or family caregivers in urban and rural settings. We provide lessons learned from three NIH funded studies testing different technology-based interventions in various settings including home hospice and outpatient palliative care. Discussion will follow focused on the clinical, ethical and practical challenges of innovation and the unique considerations for technology-mediated intervention design in a variety of palliative and hospice care settings. This symposium aims to provide: 1. an overview of existing technology-based interventions for older adults and their families in palliative care and hospice 2. evidence-based recommendations resulting from clinical trials in urban and rural settings for the design and implementation of innovative tools in hospice and palliative care 3. a discussion of challenges and opportunities for the use of technology to support older adults and their families

### USING TECHNOLOGY TO FACILITATE SUPPORTIVE SERVICES FOR HOSPICE FAMILY CAREGIVERS: FINDINGS FROM THE PISCES TRIAL

George Demiris[1], *1. University of Pennsylvania, Philadelphia, Pennsylvania, United States*

Family caregivers of hospice patients play an essential role in the delivery of care but have multiple needs during this often stressful time. Cost-effective interventions supporting caregivers are greatly needed. We conducted a four-year randomized clinical trial of a problem solving therapy intervention called PISCES to support hospice caregivers. We recruited 514 caregivers (75% female, mean age 60.3 years) who were randomly assigned to either a control group (usual care) or a face to face group (where the intervention was delivered in three in-person sessions) or a telehealth group (where the intervention was delivered using technology). While the intervention was effective in reducing caregiver anxiety and improving overall quality of life, the intervention was found more effective in the face-to-face group than the telehealth group. We discuss challenges with the technology use and recommendations for the design of future telehealth systems targeting older adults in the hospice setting.

### TECHNOLOGICALLY MEDIATED INTERVENTIONS IN HOSPICE AND PALLIATIVE CARE: CONSIDERATIONS FOR RURAL ELDERS

Karla T. Washington[1], *1. University of Missouri, Columbia, Missouri, United States*

Rural communities are home to a disproportionate number of older adults, many of whom are living with a serious illness or providing care to a seriously ill family member or friend. For these individuals, hospice and palliative care services can provide much-needed biopsychosocial and spiritual support, leading to an enhanced quality of life. Technologically-mediated interventions hold promise as a strategy to bridge geographic distances between healthcare providers and rural elders; however, the effect of such interventions may be greater when they are designed in a manner congruent with rural culture and compatible with the technological infrastructure common in rural areas. Informed by lessons learned during a pilot study of a telehealth intervention in one Midwestern state (R21CA191165), this presentation includes a discussion of strategies to more effectively recruit, engage, and retain rural older adults into studies testing technologically-mediated interventions for hospice and palliative care patients and their families.

### DEVELOPING SOCIAL CONVOY PALLIATIVE CARE MOBILE HEALTH FOR OLDER ADULTS

Jennifer D. Portz[1], *1. University of Colorado, Aurora, Colorado, United States*

Social Convoy Theory is well established in the gerontology literature and provides a framework for understanding the relationships of individuals in a group of people with whom they give and receive social support over the life-cycle. Previous research substantiates a link between the importance of social convoys and health outcomes among older adults with advanced illness. However, the concept of social convoy has not been applied to mobile health (mHealth) for palliative care. Often mHealth systems are designed for individual users, lacking innovative solutions for multiple, age-diverse, simultaneous users to maximize benefit. Therefore, the Social Convoy Palliative Care (Convoy-Pal) mobile application (K76AG059934) was developed using a rigorous iterative process with older patients, their social convoy, and health providers, to foster effective integration of the social convoy in palliative care-specific mHealth. Based on these findings, this presentation discusses strategies to design mHealth for a convoy of users in geriatric palliative care.

### SESSION 1085 (SYMPOSIUM)

### MAXWELL A. POLLACK AWARD LECTURE

Chair: Robert Harootyan, *Senior Service America Inc., Silver Spring, Maryland, United States*

The Maxwell A. Pollack Award for Productive Aging recognizes instances of practice informed by research and analysis, research that directly improved policy or practice, and distinction in bridging the worlds of research and practice. The award lecture will be presented by the 2018 recipient, Karen Fredriksen-Goldsen, PhD, University of Washington. This award is generously funded by The New York Community Trust.

### LINKING LIVES: PROMOTING HEALTH EQUITY TO DISRUPT THE CYCLE OF SOCIAL ISOLATION

Karen Fredriksen Goldsen[1], *1. University of Washington, Seattle, Washington, United States*

Resulting from social, economic, and environmental disadvantage in vulnerable communities, health inequities have systematically created greater obstacles to aging. Despite the adversity of their lives, or perhaps because of it, older adults from vulnerable populations embody an Iridescent Life Course, displaying remarkable resilience and resistance, yet are at greater risk of health disparities and social isolation in later life. In the U.S. social isolation has reached epidemic proportions, creating a largely invisible public health crisis with few resources for an aging population. In her presentation, Professor Karen Fredriksen Goldsen will share her landmark longitudinal research on LGBT older adults, who have historically confronted social exclusion, with divergent pathways leading to social isolation. She will discuss the development and testing of community evidence-based interventions and how these findings can provide effective alternatives for anyone living in isolation as well as promote health equity, creating a future for full aging potential.

### SESSION 1090 (SYMPOSIUM)

### INTEREST GROUP SESSION—HUMAN-ANIMAL INTERACTION: PETS IN OLDER ADULTS' SOCIAL NETWORKS: HUMAN-ANIMAL INTERACTION, INTERPERSONAL CONNECTIONS, AND HEALTHY AGING

Chair: Jessica Bibbo, *Benjamin Rose Institute on Aging, Cleveland, Ohio, United States*
Co-Chair: Sandy Branson, *Cizik School of Nursing at UTHealth, Houston, Texas, United States*
Discussant: Jessica Bibbo, *Benjamin Rose Institute on Aging, Cleveland, Ohio, United States*

Empirical evidence supports positive associations between social support, interpersonal connections, and health as people age. This symposium addresses how human-animal interaction may facilitate connection throughout later life. Each talk presents unique ways pets: fit into social networks; expand interpersonal connections; and thereby, impact health and wellbeing. The first talk presents longitudinal associations of a history of pet ownership and marital status on health, in particular cognitive functioning, over time. The second talk presents qualitative evidence for how pets fit into older adults' social network and quantitative evidence for the impact of animal and interpersonal companionship on overall and functional health. The third talk builds upon the literature linking dog walking with older adults' physical health, by providing evidence for the positive impact of dog walking improving interpersonal connections with neighbors. The fourth talk discusses the influence of a unique intergenerational human-animal interaction service-learning course on university students' attitudes towards older adults and those with disabilities. Enrolled students provide pet care (e.g., brushing, dog walking, delivering pet food and supplies) to low-income pet owners ages 60 and older and disabled adults. Students reported decreased biases towards older adults and those with disabilities after completing the course. The final talk is the first study to focus on the influence of pets in LGBTQ older adults living in rural southern Appalachia. Identifying as a LGBTQ person and living in a rural environment can present unique challenges and these qualitative results provide insight into pets' influence on aging in this understudied population.

### OLDER ADULT PET OWNERSHIP HISTORY, MARITAL STATUS, AND HEALTH OUTCOMES: THE WISCONSIN LONGITUDINAL STUDY (WLS)

Alisha Hackney,[1] and Erika Friedmann[1], *1. University of Maryland School of Nursing, Baltimore, Maryland, United States*

We explore the association of PO and marital status to changes in health during aging. This secondary analysis uses 1992, 2004, and 2011 data from healthy participants (N=2,319) in the WLS. Health outcomes (symptoms, illnesses, days in bed due to illness, depression, and cognition) and demographic/ health variables (age, marital status, sex, smoking, BMI, engagement with friends/relatives). PO (ever owned a pet/dog/cat) was assessed in 2011. In linear mixed models, PO, dog ownership, and cat ownership (CO) independently predicted changes in health outcomes.

Marital status moderated the association of CO to changes in cognition[F(1,012.85)=4.09,p=0.043]. Both marriage and CO were associated with increased cognition from ages 53 to 64; being unmarried without CO was associated with reduced cognition during aging with the lowest cognition by age 70. Results provide insight into how PO and social support influence healthy aging. Longitudinal PO and health/social data are necessary to better understand these relationships.

### THE HUMAN-ANIMAL BOND: SOCIAL NETWORK COMPOSITIONS, ANIMAL COMPANIONS, AND HEALTH

Raeann G. LeBlanc[1], *1. University of Massachusetts Amherst, Amherst, Massachusetts, United States*

Animal companionship has been found to be positively related to health, though less is known about the features of social networks that include animal companions and how these relate to health outcomes. The purpose of this study was to explore the relationship between social network composition including animal companionship and health. A mixed methods cross-sectional descriptive, correlation study design was used. Eighty-nine people age sixty-five and older, living in the community, managing multiple chronic conditions, participated in telephone interviews. Animal companionship was common (42.7%) among the sample (66% lived alone) with at least one animal companion (M=1.57, SD=.903) and associated with improved health function (IADL scores) (r=.234, p=.028). Animal companionship correlated positively with health (SF12 General Health Scores) (r=.210, p=.048). Animal companionship is an important feature in social networks of older people that influences health. Social supports maintain these relationships and the animal human bond.

### DOG WALKING MEDIATES THE RELATIONSHIP BETWEEN DOG OWNERSHIP AND NEIGHBORHOOD SOCIAL INTERACTION

Katie Potter,[1] Hachem Saddiki,[2] and Laura B. Balzer[2], *1. University of Massachusetts Dept of Kinesiology, Amherst, Massachusetts, United States, 2. Department of Biostatistics and Epidemiology, University of Massachusetts Amherst, Amherst, Massachusetts, United States*

Social interaction may be facilitated by dog ownership. We surveyed 421 pet owners about neighborhood social interactions. Dog owners also completed a dog walking questionnaire. Among adults aged 55+ (n=99; 62.2±5.6 years; 90% female), we tested our hypotheses that (1) dog owners were more likely to meet neighbors than non-dog owners, and (2) increased dog walking frequency was associated with increased neighborhood social interaction. Inverse probability weighting was used to control for differences in age and neighborhood type (rural, suburban/urban) between groups. The probability of meeting neighbors was 2.4x higher (95%CI: 1.5-3.9) for dog than cat owners, after controlling for age and neighborhood type. Among dog owners, the odds of meeting a neighbor were 1.7x higher (95%CI: 0.9-3.1) with each unit increase in dog walking frequency (unit=5walks/week). Our findings suggest that programming to support dog ownership and dog walking among older adults may help reduce social isolation.

### PARTICIPATION IN AN INTERGENERATIONAL SERVICE-LEARNING COURSE AND IMPLICIT BIASES

Lori Kogan,[1] Lori Kogan,[1] Regina Schoenfeld-Tacher,[2] and James Oxley[3], *1. Colorado State University, Fort Collins, Colorado, United States, 2. North Carolina State University, Raleigh, North Carolina, United States, 3. NA, measham, England, United Kingdom*

Biases against older adults and people with disabilities can lead to discriminatory behaviors. One way to better understand attitudes towards these populations is through the examination of implicit (unconscious) factors. This paper utilizes The Implicit Association Test, a computer-based categorization task designed to assess implicit or unconscious attitudes, to assess the impact of an intergenerational service-learning course created to support the human animal bond between vulnerable pet owners and their companion animals. This study, using the Wilcoxon signed-rank test, assessed the impact of college students' interactions with older pet owners on these students' implicit attitudes. Pre- and post-assessment of participating students found statistically significant decreased biases towards older people and people with disabilities after completing the course (p=.032). Results from this study suggest that participating in an intergenerational service-learning course centered around the human animal bond can positively affect implicit attitudes towards older adults or people with disabilities.

### LGBT OLDER ADULTS AND THEIR PETS IN SOUTHERN APPALACHIA: RESULTS OF QUALITATIVE RESEARCH AND FUTURE RESEARCH DIRECTIONS

Maureen MacNamara[1], *1. Appalachian State University, Boone, North Carolina, United States*

This session reports on older LGBT adults' perspectives of roles that pets played in relation to older adults' aging-in-place experiences. The goal of this study is to advance understanding of the role of pets in the lives of older LGBT adults living in rural communities. The qualitative study used individual, semi-structured interviews with 11 older adults residing in rural Appalachia. Individuals were recruited using purposive sampling techniques, including recruitment through LGBT friendly service providers, a lesbian listserv, and snowball sampling. The majority of respondents reported that pets played a role in social support and some in social capital. One respondent described a utilitarian relationship with animals that is important to understanding the roles animals may play in rural communities in terms of "belongingness". After reviewing study findings, we will provide recommendations for including information about pets in formal social service delivery systems and conclude with implications for future research.

### SESSION 1095 (PAPER)

### PHYSICAL ACTIVITY AND HEALTH

### BARRIERS, FACILITATORS, BENEFITS, AND ENJOYMENT OF PHYSICAL ACTIVITY IN MIDDLE-AGED HISPANIC MEN

Ferdinand Delgado,[1] Cheryl DerAnanian,[1] Sonia Vega-Lopez,[2] Shandel Vega-Soto,[1] Hector Valdez,[1] and Steven Hooker[3], *1. Arizona State*

*University, Phoenix, Arizona, United States, 2. Arizona State University, Phoenix, United States, 3. San Diego State University, San Diego, California, United States*

Little is known about middle-aged Hispanic men's perceptions of physical activity (PA). Purpose: To examine perceived barriers, facilitators, and benefits of PA and what types of PA are enjoyable. Methods: Seven focus groups (FGs) were conducted with middle-aged Hispanic men (mean age 51.6±6.1 years; n=32) who primarily self-identified as Mexican (78.1%). All FGs were audio-recorded, transcribed, and translated into English. A grounded theory approach was used to identify themes. Results: Competing responsibilities (N=7 FGs), laziness/apathy (N=6 FGs), and a lack of habit/routine (N=5 FGs) were identified as the primary barriers to PA. Jobs were perceived as a PA barrier because of physical labor involved, long hours, having multiple jobs, and/or an inconsistent schedule. Laziness/apathy involved the notion that people have time to exercise, but choose not to. Not having a routine resulted in procrastination and not achieving exercise goals. Social support was the primary facilitator to PA, which included having a friend (N=5 FGs), family member (N=5 FGs), or spouse/partner (N=6 FGs) with whom they could participate in PA. Sports were mentioned in all FGs as enjoyable, including soccer (N=5 FGs), basketball (N=5 FGs), tennis/racquetball/handball (N=3 FGs), and baseball (N=2 FGs). Walking/running (N=6 FGs) and hiking (N=5 FGs) were also considered enjoyable. The primary perceived benefits of PA included increased energy after exercising (N=6 FGs) and improved overall health (N=6 FGs). Conclusion: Hispanic men realize the importance of PA and what may be hindrances or facilitators to increasing PA. Funded by the National Institute on Aging (R21 AG050084-01A1).

### CHANGE IN UNMET NEEDS FOR DAILY MOBILITY AND SELF-CARE ACTIVITIES AND FUTURE HEALTH OUTCOMES

Laura P. Sands,[1] Jyoti Savla,[1] Madhav Erraguntla,[2] and Mark Lawley[2], *1. Virginia Tech, Blacksburg, Virginia, United States, 2. Texas A and M, College Station, Texas, United States*

Among community-living older adults, unmet needs for help with daily self-care or mobility activities is associated with poor health outcomes. Few studies have examined whether change in unmet needs influences future health outcomes. Using 2011 – 2015 National Health and Aging Trends Study data, we studied 2,052 participants (3,654 pairs of observations) who had: (a) at least one limitation in mobility or self-care daily activities; (b) data from two or more adjacent years, and (c) retrospective data about falls and hospitalization ruing the year following the two adjacent years of data. We computed mixed effects models to assess health outcomes for four transitions (met-met, met-unmet, unmet-met, unmet-unmet). The models controlled for demographic characteristics, health conditions, change in daily activities limitations, and prior falls or hospitalization. We determined that those who changed from met to unmet needs for mobility limitations were significantly more likely to report hospitalization and falling in the following year than those with met needs in both years (OR=1.4; 95% CI=1.1 – 1.8; OR=1.4; 95% CI=1.1 – 1.0, respectively). Similar findings were found for those with self-care limitations. Respondents with unmet needs for mobility limitations in both years were significantly more likely to fall in the year after the interval (OR=1.4; 95% CI=1.0 – 1.9) than those who changed from unmet needs to met needs. The findings provide evidence that onset of unmet needs increases risk for future falls and hospitalization, and resolution of unmet needs decreases risk for falls in the subsequent year.

### LAGGED ASSOCIATIONS BETWEEN DAILY SYMPTOMS OF OSTEOARTHRITIS AND PHYSICAL ACTIVITY LEVEL

Dylan M. Smith,[1] and Patricia A. Parmelee[2], *1. Stony Brook University, Stony Brook, New York, United States, 2. The University of Alabama, Tuscaloosa, Alabama, United States*

Fatigue is commonly reported by persons with lower limb osteoarthritis (OA) and uniquely predicts worse functioning and decreased activity. The current research used a combination of wrist and waist accelerometry along with experience sampling methodology (ESM) in a diverse sample to examine the relationship between reports of fatigue and pain and subsequent physical activity among older adults with knee OA. Three hundred twenty-one participants with physician-diagnosed knee OA completed a baseline interview and mobility testing followed by a one-week observation period using accelerometry at both wrist and waist. During this week, participants also completed an ESM protocol assessing fatigue and pain 4 times daily. Multilevel models examined lagged within-subjects patterns of symptoms and their association with subsequent physical activity using three approaches: 1) wrist accelerometry alone, 2) waist accelerometry alone, and 3) a combined approach using both measures. These models also examined between-subjects predictors including measures of overall functioning, mobility testing, and demographics. Level of fatigue reported in the previous interval was the strongest and most consistent predictor of subsequent lowered physical activity. Other significant predictors included age and functional mobility. Waist and wrist actigraphy estimates were modestly correlated with each other, yet multi-level models showed consistent results regardless of placement at wrist versus waist versus a combined waist/wrist approach. In conclusion, fatigue symptoms are a significant factor in predicting subsequent decreased physical activity in OA. Waist and wrist accelerometry methods produced different estimates of activity level in this population, but similar associations with arthritis symptoms.

### PHYSICAL ACTIVITY INTERVENTION IN HOME CARE: COMPARING FAMILY AND NON-FAMILY CAREGIVING DYADS

Naoko Muramatsu,[1] and Lijuan Yin[1], *1. University of Illinois at Chicago School of Public Health, Chicago, Illinois, United States*

An increasing number of states pay family members who care for older adults in Medicaid-funded home care. Previous research documented pros and cons of hiring family members as home care providers. However, little is known about whether family and non-family caregiving dyads function differently when they participate in health promotion interventions in home care. Using data collected in a pilot study of a gentle physical activity program delivered by home care workers in a Medicare home care program, this study compared 18 family and 32 non-family caregiving dyads in client outcomes (self-reported and performance-based function) and process outcomes (exercise-related social support provided by home care aides) before and after the intervention. Linear mixed models indicated that client outcomes improved after the 4-month intervention ($p<0.05$), controlling for clients' age, gender, and number of chronic conditions. Compared to family caregiving dyads, greater improvement in exercise-related support was observed in non-family caregiving dyads ($p<0.05$), where care providers offered less exercise-related support at baseline. The intervention program was received well by both family and non-family dyads, as expressed by one of the caregivers: "It makes my client feel good about herself. I also feel good for my client." Results suggest that empowering caregivers with health promotion skills is a promising strategy, especially in non-family caregiving dyads. Further research is warranted to produce evidence-based health promotion programs for family and non-family caregiving dyads in home care.

### SOCIAL RELATIONSHIPS AND OBESITY IN LATER LIFE: A LONGITUDINAL ANALYSIS

Jane L. Tavares,[1] Jeffrey Burr,[2] and Edward A. Miller[2], *1. University of Massachusetts Boston Gerontology Institute, LeadingAge LTSS Center, Boston, Massachusetts, United States, 2. University of Massachusetts Boston, Boston, Massachusetts, United States*

The majority of U.S. older adults are overweight or obese. Social relationships are a key factor linked to obesity among younger age groups, but there are no known investigations of these modifiable risk factors older for adults. This study examined the association between quantitative and qualitative indicators of social relationships and waist circumference among middle-aged and older adults. We also examined whether psychosocial and health behavior characteristics mediate and/or moderate these relationships. Using the 2006 and 2010 waves of the Health and Retirement Study, a series of regression models were estimated to examine the longitudinal association between social relationships and waist circumference in age stratified samples (age 50 to 64; age 65 and older). Results for those age 50 to 64 indicated that higher positive social support and lower negative social support were associated with lower waist circumference over time. For those age 65 and older, lower negative social support and higher loneliness were associated with lower waist circumference over time. Further, daily exercise and anxiety were observed to be mediators of these associations; both variables also acted as moderators. Findings from this study highlight the need for healthcare providers to assess older adults' levels of social support and loneliness in order to achieve a more comprehensive understanding of wellness. The results also underscore the importance of developing intervention programs that provide widespread and ample opportunities for older adults to engage socially, particularly those that incorporate or encourage physical activity and healthy eating.

### SESSION 1100 (SYMPOSIUM)

### POLICY SERIES: REEFER MADNESS, SADNESS, OR GLADNESS? THE CANNABIS AND OLDER PERSONS STUDY

Chair: Brian P. Kaskie, *University of Iowa, College of Public Health, Iowa City, Iowa, United States*
Discussant: Julie Bobitt, *University of Illinois, Champaign-Urbana, Illinois, United States*

In 2016, we began our examination of the intersection between cannabis and older persons by convening focus groups with 163 older adults from senior centers and dispensaries in nine states with varying levels of cannabis legalization. Since then, we have secured competitive research grants and contracts to examine cannabis use among older persons in California, Colorado, Illinois and Iowa. Our work is guided by the primary hypothesis that cannabis use among older persons is shaped by an individual's calculations concerning risk (e.g., developing a cannabis use disorder, law-breaking) and reward (e.g., relaxation, symptom relief), and individuals living in a state with a legal cannabis program may perceive less risk and also may be receiving more information about the benefits of cannabis. We also hypothesize that older adults' access to and use of cannabis is shaped by where they live, particularly defined by local cannabis program implementation efforts and relevant contextual conditions. In this symposium, we will examine our latest work concerning (a) life-span attitudes toward cannabis, (b) clinical perspectives on counseling and certifying older persons for medical cannabis, (c) provider perspectives on state cannabis policy and program implementation, (d) cannabis use among a sample of dementia caregivers and (e) outcomes experienced by older persons who use cannabis for medical or recreational purposes. Our discussion focuses on implication for policy development and program implementation.

### EXPLORING STRUCTURAL BARRIERS TO MEDICAL CANNABIS ACCESS AMONG OLDER ADULTS

Kanika Arora,[1] Kanika Arora,[2] Julie Bobitt,[3] and Brian Kaskie[2], *1. University of Iowa, College of Public Health, Iowa City, Iowa, United States, 2. University of Iowa, Iowa City, Iowa, United States, 3. University of Illinois at Urbana Champaign, Champaign, Illinois, United States*

A growing number of older persons are using cannabis to treat medical conditions and symptoms. Preliminary work examining survey responses from persons over age 60 in Colorado and revealed the presence of structural barriers in safe and effective access to MC. In particular, older adults reported a gap in their expectations as a patient and current healthcare provider practice in counseling and educating them on MC use. To examine this from the provider perspective, we aim to survey Illinois state physicians and assess current knowledge, training needs, attitudes and practices associated with MC and older adults. Multivariate regression analysis will be conducted to predict current practice associated with MC (for example, whether physician ever certified a patient for MC) as a function of provider characteristics, as well as their knowledge and attitudes on the topic. We will also conduct sub-group analysis to understand practice patterns by specialty and experience.

### LIFESPAN CANNABIS USE PATTERNS IN OLDER USERS

Sara H. Qualls,[1] Kanika arora,[2] Julie Bobitt,[3] and Brian Kaskie[2], *1. University of Colorado Colorado Springs, Colorado Springs, Colorado, United States, 2. University of Iowa, Iowa City, Iowa, United States, 3. University of Illlinois at Urbana-Champaign, Champaign, Illinois, United States*

The rapidly rising rates of cannabis use among older adults may reflect a rise in late-onset users, re-engagement after a period without use, or a continuous use pattern since young adulthood that is more visible after legalization of cannabis. Older (age 60+) cannabis users (n=82) provided retrospective ratings on their frequency of use across adulthood. Approximately 28% were not using cannabis when young adults, with a larger percentage (40%) reporting non-use while ages 31-49 and 37% reported non-use when ages 50-64. Approximately 21% of older users were first time users, with 60% low frequency and 35% daily/weekly users. High frequency users generally were high frequency users throughout adulthood, but the pattern varied substantially by gender and mode of consumption. Women were more likely first-time users than men, and more likely non-smokers. Among non-smokers, about 40% were first-time users. Implications are explored for research, policy, and clinical practice.

### ASSESSING HEALTH-RELATED OUTCOMES OF MEDICAL CANNABIS USE AMONG OLDER PERSONS IN COLORADO AND ILLINOIS

J. Alton Croker,[1] Julie Bobitt,[2] Kanika Arora,[1] and Brian Kaskie[1], *1. University of Iowa, Iowa City, Iowa, United States, 2. UNIVERSITY OF ILLINOIS AT URBANA-CHAMPAIGN, Champaign, Illinois, United States*

The use of cannabis is increasing among older Americans. A growing body of evidence shows cannabis and cannabinoids effective for treating chronic pain, spasticity, nausea, and sleep disturbances. States seeking to respond to the treatment needs of specific patient populations have legalized the use of cannabis for medical purposes. Few instruments offer standard outcomes for understanding the use of medial cannabis from the patient perspective, particularly focusing on older persons. Using cross-sectional survey data from a sample of older persons in Colorado and Illinois, we validate two scales to consistently measure patient-reported health related outcomes of medical cannabis use. We confirmed the validity of two separate, reliable outcome scales: a three-factor scale for measuring global health outcomes and a single-factor scale for capturing adverse health events. The COPS questionnaire revealed strong construct validity and internal consistency, and a lack of meaningful factor variance.

### CANNABIS USE BY CAREGIVERS OF PERSONS WITH DEMENTIA

Freddi Segal-Gidan[1], *1. University of Southern California, Los Angeles, California, United States*

Thirty three states have legalized marijuana for medical or recreational use. This increased access to cannabis products has heightened interest in its use among family caregivers. Clinicians anecdotally report frequent questions from families about the use of these products, but lack sound information to provide an informed response. Some studies suggest cannabis could help to manage behavioral symptoms of dementia,. No studies to date have explored marijuana or cannabis product use by caregivers for themselves or for the person with dementia. Caregivers of persons with diagnosed dementia were invited to take part in a study that included completion of a survey and participation in a focus group that explored self care practices, including the use of marijuana and cannabidiol (CBD) products for themselves and the person with dementia. Results of the survey and trends from the focus groups will be presented, with a comparison between English and Spanish speaking individuals.

### MARIJUANA USE AMONG OLDER ADULTS: HARMS MAY OUTWEIGH BENEFITS

Namkee G. Choi,[1] Diana M. DiNitto,[1] and Stephan Arndt[2], *1. University of Texas at Austin, Austin, Texas, United States, 2. University of Iowa, Iowa City, Iowa, United States*

The US Epidemiologic data show that past-year marijuana use rates among the 50+ age group rose more than 300% between 2002 and 2017. In 2017, 10.2% of those aged 50-64 years and 3.7% of those aged 65+ years were past-year users, and 15% of these users reported all or part of their use to have been for medical purposes. In this symposium, using findings from previous research and recent epidemiologic data, we will present both positive and adverse effects of late-life marijuana use. Marijuana may be useful for the treatment of pain (particularly neuropathic pain) and chemotherapy-induced nausea and vomiting. However, older marijuana users are often long-term users and significant proportions suffer from marijuana use disorder along with other substance (nicotine, alcohol, prescription and illicit drugs) use and/or mental disorders. Adverse effects also include dizziness, confusion, impairments in memory and attention, somnolence, and hallucinations, which may outweigh purported positive effects.

### SESSION 1105 (SYMPOSIUM)

### SOCIAL AND PSYCHOLOGICAL FACTORS AND COGNITIVE FUNCTION: FINDINGS FROM INTERNATIONAL SURVEYS

Chair: HANZHANG XU, *Duke University, Durham, North Carolina, United States*
Discussant: Bei Wu, *New York University, New York, New York, United States*

This symposium examines how social and psychological factors including formal schooling, subjective memory, and neuropsychological symptoms impact cognitive function among older adults in China and the U.S. The first paper used the WHO's Study on global AGEing and adult health Wave-1 data to examine the relationship between subjective cognitive function, perceived memory decline, and objective cognitive function among older adults in China. The results showed worse subjective cognitive function was associated with poorer working memory and verbal fluency, whereas greater perceived memory decline was associated only with poorer working memory. Furthermore, using data from the Health and Retirement Study, the second paper applied group-based trajectory modeling to assess dual trajectories of subjective memory impairment and objective cognitive decline. Four distinct dual-trajectory typologies were identified, suggesting complex co-occurring changes in subjective memory and objective cognition in older adults. The third paper characterized the trajectories of three neuropsychological symptoms (pain, insomnia, and depression) prior to dementia onset. Using data from the National Health and Aging Trends Study, the study found older adults with dementia exhibit distinct trajectory of depression before dementia onset than those without dementia. Trajectories of pain and insomnia did not differ before dementia onset. The last paper examined the effect of education on cognitive decline among lower educated older adults using data from the Longitudinal Study of Older Adults in Anhui Province, China. Results suggest that older adults with some formal schooling had slower cognitive decline; the gap in cognition between the literate and illiterate widened with age.

### ASSOCIATION BETWEEN SUBJECTIVE AND OBJECTIVE COGNITIVE FUNCTION IN CHINA

Bada Kang,[1] Hanzhang Xu,[2] Eleanor S. McConnell,[3] and Bei Wu[4], *1. Duke University School of Nursing, Durham, North Carolina, United States, 2. Duke University Department of Community & Family Medicine, Durham, North Carolina, United States, 3. Duke University School of nursing Geriatric Research; Education and Clinical Center, Durham Department of Veterans Affairs Medical Center, Durham, North Carolina, United States, 4. New York University College of nursing, New York, New York, United States*

Although subjective cognitive decline is considered as a potential symptomatic indicator of cognitive decline, little is known regarding the relationships in older adults in China. Using the World Health Organization Study on global AGEing and adult health (SAGE) Wave 1 data, we examined the association between subjective cognitive function, perceived memory decline, and objective cognitive function among adults aged 50 or older (N=13,367) in China. Objective cognitive function was measured by immediate and delayed recall test, digit span test, and verbal fluency test. Multivariate linear regression models were used to account for sociodemographic, psychosocial, and health-related factors. We found worse subjective cognitive function was associated with poorer working memory and verbal fluency. Greater perceived memory decline was also associated with poorer working memory but not with verbal fluency. Psychosocial factors including social cohesion and social support attenuated the relationships between subjective cognitive function, perceived memory decline, and objective cognitive performance.

### TRAJECTORIES OF SUBJECTIVE MEMORY IMPAIRMENT AND OBJECTIVE COGNITIVE DECLINE IN U.S. OLDER ADULTS

HANZHANG XU,[1] Matthew E. Dupre,[2] and Bei Wu[3], *1. Duke University, Durham, North Carolina, United States, 2. Duke University School of Medicine, durham, North Carolina, United States, 3. new York University Rory Meyers College of Nursing, New York, New York, United States*

We examined the dual trajectories of subjective memory impairment (SMI) and objective cognitive decline and their associated factors in U.S. older adults. We used data from the Health and Retirement Study which includes a nationally representative sample of 19,408 Americans age 65 and older from 1998 to 2016. Trajectories of SMI and objective cognitive decline were simultaneously characterized using a group-based trajectory model and multinomial logistic regressions were used to assess factors associated with the dual-trajectory typologies. Four dual-trajectories were identified: "minimal SMI and stable-low cognitive decline" (33.1% of respondents); "minimal SMI with accelerated cognitive decline" (28.2%); "significant SMI with moderate cognitive decline" (21.0%); and "moderate SMI with steady cognitive decline" (17.6%). Being male, minority, low educated, living alone, and having comorbidities were associated with trajectories featuring greater SMI or more rapid deterioration in cognition. The results suggest complex co-occurring changes in subjective memory and objective cognition in older adults.

### TRAJECTORIES OF PAIN, INSOMNIA, AND DEPRESSIVE SYMPTOMS BEFORE ONSET OF DEMENTIA

Minhui Liu,[1] Hanzhang Xu,[2] Junxin Liu,[3] Nancy A. Perrin,[3] and Sarah L. Szanton[3], *1. Johns Hopkins University School of Nursing, Baltimore, Maryland, United States, 2. Duke University School of Medicine, Durham, North Carolina, United States, 3. Johns Hopkins School of Nursing, Baltimore, Maryland, United States*

This study examined the trajectories of pain, insomnia, and depression during a four-year period in community-dwelling participants aged 65 or older (N=2,486) from the National Health and Aging Trends Study. Self-reported pain, insomnia, and depressive symptoms were collected at each wave. Dementia status was determined using a modified previously validated algorithm. We analyzed retrospective trajectories of each symptom using mix-effect models and a backward timescale. We also tested for differences in each symptom trajectory between those with and without dementia. Results showed that the trajectory of depression significantly differed by dementia status (Wald $\chi2=388.34$; $P<.001$). Participants with dementia had persistently higher depression level than those without dementia, particularly during the year before dementia onset. Trajectories of pain and insomnia did not differ before dementia onset. Depression increases risks of dementia and studies with longer length of follow-up are needed to detect significant trajectories of pain and insomnia before dementia onset.

### COGNITIVE DECLINE AMONG OLDER ADULTS IN RURAL CHINA: THE PROTECTIVE ROLE OF FORMAL SCHOOLING

Yaolin Pei,[1] Bei Wu,[2] Zhen Cong,[3] and Mengyao Hu[2], *1. New York University, New York, New York, United States, 2. Rory Meyers College of Nursing, New York University, New York, New York, United States, 3. School of Social Work, University of Texas at arlington, Arlington, Texas, United States*

Evidence shows that education is strongly associated with cognitive functioning; however, few studies have examined the effect of education on cognitive decline among older adults with very limited education. Our study analyzed six waves of panel data (2001, 2003 2006, 2009, 2012 and 2015) from the

Longitudinal Study of Older Adults in Anhui Province, China. We estimated two-level multilevel models of cognitive functioning for older adults age 60+, sampled using probability sampling strategy. We found that having formal schooling was positively associated with better cognitive functioning. Older adults with formal schooling had slower decline in cognition and the gap in cognition between the literate and illiterate widened with age. These findings highlight the role of early life experience in affecting cognitive function in later life and suggest that disadvantages in cognitive functioning accumulate throughout the life course for persons with no formal education.

### SESSION 1110 (SYMPOSIUM)

### INTEREST GROUP SESSION—FAMILY CAREGIVING: SUPPORTING FAMILY CAREGIVERS WITHIN SYSTEMS OF CARE DELIVERY

Chair: Jennifer L. Wolff, *Johns Hopkins University, Maryland, United States*
Discussant: Lynn F. Feinberg, *AARP Public Policy Institute, Washington, District of Columbia, United States*

Family and other unpaid caregivers have a foundational role in supporting the health and well-being of older adults with complex health needs and disabilities and the demands imposed on them can be significant. The availability and adequacy of support provided by family and other unpaid caregivers has profound consequences for quality and outcomes of care delivery, but they are not well-supported in treatment decisions and care planning. Given population aging, the shift of long-term services and supports from nursing homes toward community settings, and technological advances that allow patients to be served in the community with higher acuity of care, there is a pressing need to develop systems-level processes to identify, engage, and support family caregivers in systems of care. This symposium will feature 5 presentations that provide novel insight regarding family caregivers' experience within systems of care. We focus on family caregivers to older adults living in the community and receiving home and community-based services, primary care, or Medicare skilled home health services. Individual presentations will describe 1) differences in access to services and experiences of family caregivers by under-represented minority status; 2) a framework for health systems to include family caregivers as part of health care teams; 3) family caregivers' capacity to help during the course of Medicare-funded skilled home health care; 4) perceived communication with health professionals, using a validated measure of family caregiver capacity; and 5) the feasibility of implementing a family caregiver screening instrument in primary care.

### RACIAL DIFFERENCES IN CHARACTERISTICS AND PERCEPTIONS OF CAREGIVERS TO BLACK AND WHITE OLDER ADULTS

Chanee D. Fabius,[1] Chanee Fabius,[1] Jennifer Wolff,[1] and Judith Kasper[1], *1. Johns Hopkins University, Baltimore, Maryland, United States*

Persistent racial differences in health, socioeconomic characteristics, and service utilization of older adults likely result in differential effects for the circumstances and experiences of family and unpaid caregivers. Utilization of community-based services has been found to alleviate caregiver burden, but the extent to which supports and informal help affect race differences in caregiver perceptions (e.g. positive/negative feelings associated with caregiving) and engagement in activities such as religious services, volunteering, or visiting family and friends, is less understood. Using data from the 2015 National Health and Aging Trends Study (NHATS; Round 5) and the National Study of Caregiving (NSOC; Round 2), nationally representative studies of Medicare beneficiaries aged 65 and older and their caregivers, this presentation will discuss the association between sociodemographic characteristics, use of assistance from others or supportive services, and perceived gains, difficulties, and social engagement among caregivers to older black and white adults.

### FAMILY CAREGIVER CONTRIBUTIONS TO HOME HEALTH FOR OLDER ADULTS: PREDICTORS OF ADEQUATE SUPPORT

Julia Burgdorf,[1] Julia Burgdorf,[1] Judith D. Kasper,[1] Jennifer L. Wolff[1], *1. Johns Hopkins Bloomberg School of Public Health, Baltimore, Maryland, United States*

Family caregivers play a crucial role in supporting older adults through home health (HH) episodes; yet no prior research examines factors affecting the adequacy of caregiver support during HH. We use a novel dataset linking nationally-representative survey data with HH assessment data for community-dwelling older adults (n=2,128) to identify older adult characteristics associated with adequate caregiver support in four task categories (Activities of Daily Living, medication management, medical tasks, and safety/oversight) during a subsequent HH episode. Weighted, multivariable logistic regression is used to model the likelihood of adequate caregiver support in each category. Multiple older adult characteristics prior to the HH episode were associated with greater likelihood of adequate caregiver support during HH, including: prior caregiver assistance with mobility, self-care, and health care tasks, living with others, and cognitive impairment. These findings reveal new risk factors for consideration in risk assessment and payment adjustments related to social determinants of health.

### QUALITY OF COMMUNICATION WITH THE HEALTH CARE TEAM FOR CARE PARTNERS OF PERSONS WITH COGNITIVE IMPAIRMENT

Courtney H. Van Houtven,[1] Steven Lippmann,[2] Emmanuelle Belanger,[3] Valerie A. Smith,[2] Hailey James,[4] Megan Shepherd-Banigan,[2] and Brenda Plassman[2], *1. Durham VA HSR&D, Durham, North Carolina, United States, 2. Duke University, Durham, North Carolina, United States, 3. Brown University, Providence, Rhode Island, United States, 4. Duke University/UNC, Durham, North Carolina, United States*

The CAregiver Perceptions About CommunIcaTion with Clinical Team members (CAPACITY) instrument measures perceived quality of communication with the health care team and the extent to which caregivers believe that the health care team considers their capacity and preferences in decision-making. A higher score reflects higher perceived quality. This presentation highlights features of CAPACITY scores in a national survey of care partners of persons with unexplained cognitive impairment who sought an Amyloid PET scan. A positive scan (presence of amyloid) indicates probably Alzheimer's (n=1746). In addition to presenting its psychometric properties in this new sample, discussion focuses on the relationship between CAPACITY scores and a process measure of care that matters – accurate reporting of a new diagnosis. The CAPACITY score reflects an important domain of care quality that could be used more broadly to reflect the caregiver-centeredness of health care experiences and to predict patient outcomes.

### FAMILY CAREGIVER SCREENING IN PRIMARY CARE: CLINICIAN AND CAREGIVER PERSPECTIVES

Catherine Riffin[1], *1. Weill Cornell Medicine, New York, New York, United States*

Despite broad appreciation of family caregivers' relevance to older adults' health care, few primary care-based interventions have incorporated mechanisms to facilitate systematic caregiver identification, screening, and support. Actionable knowledge regarding how such interventions may be incorporated into clinical practice is remarkably limited. This study used in-depth interviews to elucidate clinicians' (N=25) and caregivers' (N=20) perspectives on and suggestions for integrating caregiver screening into primary care practice. Transcripts were analyzed using qualitative content analysis. Participants emphasized the importance of tailoring the caregiver screening intervention to local circumstances and to patient and caregiver preferences. They advocated for an action-oriented approach that would link identified risks with a concrete plan for follow-up (e.g., referral to training) and outcomes relevant to the patient's care plan. Overall, participants advised that integrating the intervention into practice would require the support of multidisciplinary practice staff, stronger connections between medical and community-based services, and appropriate reimbursement for clinicians.

### INCLUDING FAMILY CAREGIVERS IN SERIOUSLY ILL VETERANS' CARE: A MIXED-METHODS STUDY

Nina R. Sperber,[1] Nathan A. Boucher,[1] Roxana Delgado,[2] Megan Shepherd-Banigan,[1] Kevin McKenna,[1] Madison Moore,[3] Margaret Kabat,[4] and Courtney H. Van Houtven[1], *1. Duke University, DURHAM, North Carolina, United States, 2. University of Texas Health San Antonio, San Antonio, Texas, United States, 3. The Elizabeth Dole Foundation, Washington, District of Columbia, United States, 4. Department of Veterans Affairs, Washington, District of Columbia, United States*

Family caregivers often serve as unpaid members of the home- and community-based care workforce for individuals with serious illness; as key partners in the home-clinic continuum, they should be included in healthcare teams. Without formal recognition, inconsistent identification of caregivers will remain, engendering communication gaps between caregivers and providers and difficulty connecting with supportive services. The Campaign for Inclusive Care is an initiative within the Veterans Administration Health Care System to improve practices for including caregivers in treatment planning and decisions. We define inclusive care using literature review, provider interviews, and a caregiver survey. We found that inclusive care involves clear definition of caregiver role, system policies for inclusion, assessment of caregiver capacity, explicit involvement of caregivers, and mutuality in caregiver/provider communication. We recommend solutions based on the definition, informative for development of a national caregiver strategy, required by the 2018 Recognize, Assist, Include, Support, and Engage Family Caregivers Act.

### SESSION 1115 (SYMPOSIUM)

### SYSTEMS BIOLOGY OF AGING

Chair: Vyacheslav Labunskyy, *Boston University School of Medicine, Boston, Massachusetts, United States*

The biology of aging is an immensely complex process that impacts function at the sub-cellular, cellular, tissue, organ, and whole organism levels. New technologies and systems-level approaches provide an opportunity to greatly enhance our understanding of this complexity.

### SYSTEMS LEVEL UNDERSTANDING OF AGING

Vyacheslav Labunskyy[1], *1. Boston University School of Medicine, Boston, Massachusetts, United States*

Aging is a complex trait is controlled by multiple genetic and environmental factors. Genome-wide screens in invertebrate organisms have led to the identification of hundreds of genes, whose deletion or knockdown can dramatically increase lifespan. Many of these genes represent conserved signaling pathways and biological processes suggesting that they might be involved in the regulation of mammalian aging. Nevertheless, the mechanistic basis by which the majority of these long-lived gene deletions extend lifespan remains poorly understood. Using yeast Saccharomyces cerevisiae as a model system, we employ systems biology network analysis of transcriptomic and protein expression data to build a detailed translational regulatory network of aging in yeast and identify genetic interactions among various aging pathways. Through these studies we expect to gain understanding how the regulatory networks are perturbed with aging, which could provide important insights into human disorders associated with the old age.

### HOW MANY MOLECULAR MECHANISMS MUST BE ALTERED SIMULTANEOUSLY TO SLOW AGING AND EXTEND LIFESPAN?

Nicholas Stroustrup[1], *1. Centre for Genomic Regulation, Barcelona, Spain*

Most anti-aging therapeutics are designed to target single molecules, single molecular mechanisms, or single cell types. Yet, to produce a substantial lifespan extension, these interventions would need to act promiscuously and delay the onset of many or all causes of death. In invertebrate models, several molecular-level interventions are known to act this way, yet their downstream action on multiple causes of death remains poorly understood. Recently, we identified a strong mathematical constraint in the way that many different interventions in aging alter all-cause mortality in the nematode Caenorhabditis elegans. Interventions including suppression of IGF/insulin signaling, disruption of the hsf-1 heat shock factor and the hif-1 hypoxia-inducible factor, as well as changes in diet and body temperature, all produce a temporal scaling of lifespan. This temporal scaling suggests a common physiologic path through which diverse, evolutionary-conserved, molecular mechanisms can simultaneously influence all causes of death.

### WADDINGTON'S LANDSCAPE OF CELL AGING

Nan Hao,[1] Yang Li,[1] Yanfei Jiang,[1] Julie Paxman,[1] Richard O'Laughlin,[1] Lorraine Pillus,[1] Lev Tsimring,[1] and Jeff Hasty[1], *1. UC San Diego, La Jolla, California, United States*

Cellular aging is a complex process that involves many interwoven molecular processes. Studies in model organisms have identified many individual genes and factors that have profound effects on lifespan. However, how these genes and factors interact and function collectively to drive the aging process remains unclear. We investigated single-cell aging dynamics throughout the replicative lifespans of S. cerevisiae, and found that isogenic cells diverge towards two aging paths, with distinct phenotypic changes and death forms. We further identified specific molecular pathways driving each aging fate and revealed that these pathways interact and operate dynamically to enable an early-life switch that governs the aging fate decision and the progression towards death. Our work uncovers the interconnected molecular pathways that drives the aging process and opens up the possibility of designing interventions that simultaneously target multiple network nodes, instead of single genes, to more effectively extend the healthspan.

### DISRUPTION OF CPG ISLAND-MEDIATED CHROMATIN ARCHITECTURE AND TRANSCRIPTIONAL HOMEOSTASIS DURING AGING

Samuel Beck,[1] and Junyeong Lee[1], *1. MDI Biological Laboratory, Bar Harbor, Maine, United States*

Aging causes the global disorganization of nuclear chromatin architecture. In a normal young nucleus, silent heterochromatin is associated with the nuclear lamina layer underlying nuclear envelope, thus spatially separated from euchromatin at the nuclear center. Notably, aging causes the disruption of nuclear lamina and the decondensation of associated heterochromatin. However, it is not clearly understood how these changes of chromatin architectures contribute to age-related diseases. Through large-scale computational analyses, we present that CpG islands (CGIs) give important clues to answering this question. CGIs are DNA elements with high Cytosine-phosphate-Guanine dinucleotide frequencies. In human, about 60% of total genes contain CGIs at their promoters (CGI+ genes) and are broadly expressed throughout the body. The other 40% of genes that do not have CGIs (CGI- genes) exhibit tissue-restricted expression patterns. Our results demonstrate that, in normal young nuclei, only CGI- genes can reside within lamina-associated heterochromatin when transcriptionally inactive, while CGI+ genes associate with nuclear central euchromatin even when they are repressed. In parallel, we show that age-associated heterochromatin decondensation can specifically de-repress tissue-specific CGI- genes leading to their uncontrolled expressions. Our results further demonstrate that global misregulation of CGI- genes increases the noise in gene transcription that, in turn, causes the loss of cellular identities during aging. Taken together, our study establishes critical implication of CGI-mediated chromatin architecture in age-associated degenerative changes and loss of tissue homeostasis.

### SESSION 1120 (SYMPOSIUM)

### THE LONGEVITY CONSORTIUM: MULTI-OMICS INTEGRATIVE APPROACH TO DISCOVERING HEALTHY AGING AND LONGEVITY DETERMINANTS

Chair: Thomas T. Perls, *Boston University School of Medicine, Boston, Massachusetts, United States*
Co-Chair: Daniel S. Evans, *California Pacific Medical Center Research Institute, San Francisco, California, United States*
Discussant: Evan Hadley, *National Institute on Aging, National Institutes of Health, Bethesda, Maryland, United States*

The Longevity Consortium (LC), a NIA-Cooperative project, is an integrated multi-disciplinary effort with cutting edge bioinformatic, systems biology and chemoinformatics approaches exploiting multiple omics data generated from multiple well-phenotyped aging cohorts, Study of Osteoporotic Fractures, MrOS (fractures in men), Cardiovascular Health Study, the Long Life Family Study and the Centenarian Project to discover pharmacologically targetable protective pathways that promote healthy aging. Omics studies of mice treated with candidate drugs and of multiple species with varying life spans further informs the LC efforts. We describe the integration of the above efforts and the LC goals as well as opportunities for interested investigators to access shared results as well as opportunity funds for pilot projects. Early successes are described in 4 presentations: (1) A genome-wide association study including 1317 centenarians discovered 8 new loci in chromosomes 3, 6, 7, 9, 10, 14 and 15. The list includes new serum pQTLs that suggest a new biological mechanism involved in extreme longevity. (2) Novel, high-throughput discovery-proteomics of serum from 2,473 MrOs participants identified 25 proteins, mostly found in inflammatory pathways, associated with living beyond the 90th percentile birth-cohort survival. (3) A biological age estimation algorithm utilizing multi-omics assays significantly differentiated between 3,558 wellness program participants and controls. (4) Mediation analyses were used to test causal relationships between many candidate aging-modulating drugs and compounds, expression levels of gene and protein variants (eQTLs and pQTLs) and aging and longevity phenotypes. This high throughput method shows promise as a means of discovering candidate drugs for healthy aging.

### THE LONGEVITY CONSORTIUM VISION

Daniel S. Evans,[1] Daniel S. Evans,[2] Steven R. Cummings,[3] and Nicholas Schork[4], *1. California Pacific Medical Center Research Institute, San Francisco, California, United States, 2. California Pacific Medical Center Research Institute, UCSF, San Francisco, California, United States, 3. San Francisco Coordinating Center, CPMCRI, UCSF, San Francisco, California, United States, 4. TGen, Phoenix, Arizona, United States*

Molecular factors and pathways promoting human longevity and healthy aging can potentially delay or prevent multiple chronic diseases and conditions, but identifying such factors that can be pharmacologically targeted requires an integrated multidisciplinary approach. We describe the design of the five research projects and three cores of the

Longevity Consortium (LC) and how their cooperative research is designed to discover molecular factors and pathways that can predict healthy human aging and longevity, associate with extreme human lifespan, show relevance to chronic age-related conditions, respond to interventions to slow aging in mice, and show evidence for association with lifespan across species. A systems biology approach is undertaken to identify common molecular features across human traits and organisms, and a chemoinformatics approach to link molecular targets to candidate healthy aging interventions. The LC results are made publicly available and we provide funding opportunities to the scientific community to support pilot projects.

### GENOME-WIDE ASSOCIATION STUDY OF EXTREME HUMAN LONGEVITY DISCOVERS UNCOMMON LONGEVITY VARIANTS

Anastasia Gurinovich,[1] Anastasia Gurinovich,[2] Zeyuan Song,[3] Stacy L. Andersen,[4] Thomas T. Perls,[4] and Paola Sebastiani[3], *1. Boston University, Boston, Massachusetts, United States, 2. Bioinformatics Program, Boston University, Boston, Massachusetts, United States, 3. Department of Biostatistics, Boston University School of Public Health, Boston, Massachusetts, United States, 4. Department of Medicine, Boston University School of Medicine, Boston, Massachusetts, United States*

The strong heritability of extreme human longevity supports the hypothesis that this is a genetically-regulated trait. However, association studies focused on common genetic variants have discovered a limited number of longevity-associated genes. We conducted a genome-wide association study of 4,216 individuals including 1317 centenarians from the New England Centenarian Study (median age = 104 years) using >9M genetic variants imputed to the HRC panel of ~65,000 haplotypes. The set included approximately 5M uncommon variants. The associations were tested using a mixed effect logistic regression model with genotype-based kinship covariance of the random effects to adjust for cryptic relations using the package GENESIS. The analysis discovered 45 genome-wide significant SNPs (p< 5E-08) including 8 new loci in chromosomes 3, 6, 7, 9, 10, 14 and 15 in addition to the APOE locus. The list includes new pQTLs in serum that suggest a new biological mechanism involved in extreme human longevity.

### THE PROTEOMICS OF LONGEVITY

Eric Orwoll,[1] Jack Wiedrick,[2] Steven R. Cummings,[3] Dan Evans,[3] Wanlin Zheng,[3] Cory Funk,[4] Nathan Price,[4] and Jodi Lapidus[2], *1. Oregon Health & Sciences University, Oregon, United States, 2. OHSU, Portland, Oregon, United States, 3. San Francisco Coordinating Center, San Francisco, California, United States, 4. Institute for Systems Biology, Seattle, Washington, United States*

The biological underpinnings of longevity are poorly elucidated in humans. We used a novel, high-throughput discovery-proteomics approach to identify serum proteins associated with longevity (living beyond 90th percentile of survival) in community-dwelling men age ≥65 years in the MrOs Study. Baseline serum from 2473 men was analyzed using liquid chromatography-ion mobility-mass spectrometry. >21,000 peptides and 2931 proteins were recognized. Twenty-five proteins significantly associated with attainment of longevity over 15 yrs of observation were identified using rigorous statistical methods. 25 proteins were significantly associated; all were lower in long lived men than in men dying earlier. Most longevity-associated proteins were from inflammatory pathways; some have multifunctional biological roles potentially reflecting other mechanisms. Pathway analyses suggest important upstream regulators may be causally responsible for the associations. These results provide the opportunity to evaluate these proteins as biomarkers, and highlight the potential importance of their biological pathways in the origins of long life.

### MULTI-OMIC BIOLOGICAL AGE ESTIMATION, CORRELATION WITH WELLNESS, DISEASE PHENOTYPES: LONGITUDINAL SAMPLE OF 3558

Nathan Price,[1] Nathan Price,[1] John Earls,[1] Noa Rappaport,[1] Laura Heath,[1] Tomasz Wilmanski,[1] Jennifer Lovejoy,[1] and Leroy Hood[1], *1. Institute for Systems Biology, Seattle, Washington, United States*

Biological age (BA) has been shown to be a better predictor of mortality and disease than chronological age (CA). Aging's diverse effects are visible across many data types. We investigated BA from deep phenotyping of 3558 wellness program participants and controls. The Klemera-Doubal BA estimation algorithm was applied to genetic and longitudinal clinical laboratory, metabolomic, and proteomic assay data. BA trajectories were calculated using Generalized Estimating Equations. BA of individuals with Type-2 Diabetes averaged 6+ years greater than CA. Wellness program participation decreased individuals' rate of aging (coefficient: -0.16, 95% CI: -0.45, 0.19), with effects dependent on sex, initial BA, and CA. Measures of metabolic health, inflammation, and toxin bioaccumulation were strong predictors across data types and sex. Deep phenotyping enabled calculation of a BA measure and a wellness program improved BA, either in absolute terms or relative to CA, showing it is modifiable.

### GENETIC SUPPORT FOR INTERVENTIONS THOUGHT TO PROMOTE HEALTHY AGING

Nicholas J. Schork,[1] Richard Miller,[2] and Thomas Girke[3], *1. The Translational Genomics Research Institute (TGen), Phoenix, Arizona, United States, 2. University of Michigan, Ann Arbor, Michigan, United States, 3. University of California Riverside, Riverside, California, United States*

We consider the human genetic targets of many proven and hypothesized pharmacologic interventions meant to promote healthy-aging and explored not only how polymorphic these targets are in the human population, but also whether these polymorphisms have been shown to impact longevity and age-related phenotypes in general. We also determined if polymorphism in the genes or proteins modulated by healthy-aging interventions have been shown to also modulate the expression levels of those genes and proteins (i.e., act as gene or protein expression quantitative trait loci – eQTLs or pQTLs, respectively) and whether they have been subjected to mediation analyses testing their potential causal relationships to aging and longevity phenotypes. We compare our results to studies of other classes of drugs used to treat specific conditions, such as hypertension.

### SESSION 1125 (SYMPOSIUM)

### THE NUTS AND BOLTS OF NIH PEER REVIEW: THE CENTER FOR SCIENTIFIC REVIEW

Chair: Elia Femia, *National Institutes of Health, Bethesda, Massachusetts, United States*
Co-Chair: Dana Plude, *National Institute on Aging/National Institutes of Health, Bethesda, Maryland, United States*
Discussant: George W. Rebok, *Johns Hopkins Bloomberg School of Public Health, Baltimore, Maryland, United States*

The National Institutes of Health is the largest public funder of biomedical and bio-behavioral research in the United States. The mission is to enhance health, lengthen life, and reduce illness and disability. To achieve this mission, the NIH provides support for cutting-edge research and technology development in a variety of fields, ranging from translation of innovative ideas in technology to basic science on major health challenges and disease. There are many types of research and training opportunities and technology development programs that are supported by the NIH across the 24 institutes and centers that provide funding. The majority of grant applications are reviewed by the NIH Center for Scientific Review (CSR). In this symposium, attendees will get 1) an overview of the types of applications submitted to the NIH for support; 2) the basics of the NIH peer review process and criteria and scoring system for evaluating applications, and 3) tips for writing a more successful grant application. Peer review is the cornerstone of the NIH grants process, and an insider's view can lead to a better understanding of how the most meritorious projects are identified that lead to innovative re-search in the biomedical and bio-behavioral sciences.

### THE REVIEW PROCESS AND TAKING STEPS TOWARD A SUCCESSFUL APPLICATION

Elia Femia,[1] and Dana Plude[2], *1. National Institutes of Health, Bethesda, Massachusetts, United States, 2. National Institute on Aging, Bethesda, Maryland, United States*

Applications reviewed by the Center for Scientific Review go through a process that starts with assignment to a review panel and ends with the assignment of a priority score and the production of a written evaluation (summary statement). This process is overseen by a Scientific Review Officer, who enlists expert reviewers to evaluate the scientific and technical merit, and the potential contribution and impact of the application to the research field. The goal is to ensure that applications are expertly and fairly evaluated in accordance to the NIH policies of rigor, reproducibility, transparency, and research integrity. In this presentation, learn about the criteria for review of an application, how reviewers shape their evaluation, and what applicants should be addressing in their grant applications. From the field, hear tips from successful applicants and experienced reviewers about what they do and what they look for in a well-written application.

### TYPES OF APPLICATIONS AND PUTTING YOUR BEST FOOT FORWARD

Dana Plude,[1] Dana Plude,[2] and Elia Femia[3], *1. National Institute on Aging/National Institutes of Health, Bethesda, Maryland, United States, 2. National Institute on Aging, Bethesda, Maryland, United States, 3. National Institutes of Health, Bethesda, Maryland, United States*

The majority of peer review is conducted through the NIH Center for Scientific Review (CSR), which works closely with the institutes and centers who ultimately fund projects of high scientific merit and high potential impact. CSR conducts the review of 90% of R01s, 85% of Fellowships, and 95% of SBIR applications as well as many other research and training opportunity activities. The playing field for successful funding from NIH is highly competitive. Understanding about different application types, who to talk to about your application, finding the right review panel, and learning about the policies pertaining to review are important steps in preparing an application. In this presentation, learn about the important aspects of the grant submission process and how CSR conducts the review of application in close coordination with NIH's 24 institutes and centers.

### SESSION 1130 (SYMPOSIUM)

### PRESIDENTIAL SYMPOSIUM: THE TIES THAT BIND: THE INFLUENCE OF SOCIAL MEDIA AND TECHNOLOGY IN THE LIVES OF OLDER ADULTS

Chair: Tamara A. Baker, *University of Kansas, Lawrence, Kansas, United States*
Discussant: Lewina O. Lee, *Boston University School of Medicine, Boston, Massachusetts, United States*

Data show that seven out of every ten adults, over the age of 50, own a smartphone, with one out of ten owning a tablet. While traditional activities dictate the use of technology among this cohort, there is growing evidence that adults similarly use devices to also manage their medical care and to learn online. This increase has guided scholars in recognizing the utility of technology from designing interventions to understanding how technology may serve as a barrier and/or facilitator to one's general well-being. This symposium features four presentations from nationally recognized scholars that will expand traditional perspectives on technology use, and how it influences social ties among older adults. Dr. Charness will examine the population-level trends in social network use by aging adults and discuss a recent CREATE intervention study (PRISM), that used a computer-based platform to reduce social isolation and loneliness among older adults. Dr. Czaja will similarly present findings from CREATE, and other trials, on the access to and use of email, social media sites, and online support groups among older adults, and the resultant impact on social connectivity, loneliness and social support. Dr. Rogers will discuss technologies that currently exist (e.g., apps, mobile devices, social networking) or are being developed (e.g., robotics, telepresence, virtual reality) to support social engagement. Dr. Antonucci will examine aspects of new technologies and their influence on health and well-being, while underscoring the perspective that new and emerging technologies hold great promise in overcoming traditional barriers to maintaining social contact and exchange.

### THE ROLE OF SOCIAL MEDIA NETWORKS FOR AGING ADULTS: PANACEA, PLAGUE, OR BEEN THERE, DONE THAT?

Neil H. Charness[1], *1. Florida State University, Tallahassee, Florida, United States*

Billions of people around the world rely on (or are addicted to) technology-based social networks such as Facebook. At first glance, particularly for aging adults with mobility challenges, internet-based networking seems like a panacea. At second glance, navigating through the thicket of 'bots', fraudsters and "fake friends" may turn out to be a plague. At third glance, technology-based interaction platforms are not that new (telegraph, telephone) and not that unusual. I examine the population-level trends in social network use by aging adults and discuss a recent CREATE intervention study, PRISM, that used a computer-based platform to try to reduce social isolation and loneliness in older adults at risk for social isolation.

### OLDER PEOPLE AND TECHNOLOGY: THE GOOD, THE BAD, AND THE UGLY

Toni C. Antonucci[1], *1. University of Michigan, Ann Arbor, Michigan, United States*

The use of multiple technologies in the service of social relations in widely evident. It is not at all clear, however, that we recognize the fundamental changes in social relations that are occurring as a result. Some changes are quite positive, e.g., low cost maintenance of geographically distant but emotionally close relationships. Others can be quite negative, e.g., the lost ability to gauge emotional reactions through face-to-face contact, often resulting in unnecessarily hurtful behaviors. Preliminary data indicate that people selectively use different forms of communication under positive circumstances, e.g. to transmit good news; or negative circumstances, e.g. resolve a dispute/express anger and dependent on the nature or closeness of the relationship e.g., parent, spouse, child. We need to be mindful of the good, the bad, and the ugly of technology; and, its specific effect on the relationships of and with older people.

### TECHNOLOGY DESIGN TO SUPPORT SOCIAL ENGAGEMENT FOR OLDER ADULTS

Wendy Rogers[1], *1. University of Illinois, Urbana-Champaign, Champaign, Illinois, United States*

Evidence that loneliness and isolation are precursors to myriad negative health issues is accumulating. For older adults, social engagement may be particularly important due to life-changing events that can accompany older adulthood, including retirement, disease, or mobility limitations. Individuals vary in their levels of social engagement, and these differences may have consequential effects on quality of life. Technology has the potential to create and enhance social engagement opportunities for older adults at risk for social isolation. For such technology innovations to be effective and widely adopted, designers must consider the unique needs, capabilities, limitations, and preferences of older adults. I will describe technologies that currently exist (e.g., apps, mobile devices, social networking) or are being developed (e.g., robotics, telepresence, virtual reality) to support social engagement, connectedness, and community participation. I will discuss the potential of these technologies as well as the design and training challenges unique to older adults.

### SOCIAL ISOLATION AND OLDER ADULTS: WHAT ROLE CAN TECHNOLOGY PLAY?

Sara J. Czaja[1], *1. Weill Cornell Medicine, New York, New York, United States*

Social isolation and loneliness are prevalent among older adults especially those who live in rural locations, have mobility restrictions, are in the older cohorts, live alone or live in residential institutions such as assisted living facilities or nursing homes. The detrimental consequences of isolation and loneliness on physical, cognitive, and emotional health are well documented. Technology applications such as the email, social media sites and online support groups hold promise in terms of enhancing engagement and providing support to older people and mitigating the negative impact of isolation and enhancing quality of life. Recent data indicate that use of these types of applications is increasing among older adults but there is still an age-related digital decline. This presentation will present findings from CREATE and other trials regarding the access to and use of these applications among older adults and the resultant impact on the social connectivity, loneliness and social support.

### SESSION 1135 (SYMPOSIUM)

### VACCINATION TO PROMOTE HEALTH THROUGHOUT LIFE AS A HEALTHY AGING STRATEGY

Chair: Leonard Friedland, *GSK Vaccines, Philadelphia, Pennsylvania, United States*

This symposium addresses the role of vaccination to promote healthy aging, the process of developing and maintaining the functional ability that enables wellbeing in older age. Life-span immunization of adults across all age categories can help to reduce morbidity and mortality. Healthy aging is critical for our global society to counter the surge in healthcare costs that is coming as a result of the demographic shift to older age. Immune system function and response to vaccination declines with advancing age. Generating effective immune responses against new infectious disease targets can be difficult in older individuals. Important progress has been made in understanding the mechanisms underlying immunosenescence, the age-related decline of the immune response to infections and vaccinations. Innovative research and the development of new technologies, such as adjuvants, substances that can enhance and shape the immune response to the target antigen(s), has facilitated the development of vaccines specially tailored for adults. This evidence-based approach to the development of innovative vaccines addressing immunosenescence is an important clinically relevant healthy aging strategy to promote health throughout life.

### ADJUVANTED VACCINES TO ADDRESS IMMUNOSENESCENCE AND PROMOTE HEALTH THROUGHOUT LIFE

Leonard Friedland[1], *1. GSK Vaccines, Philadelphia, Pennsylvania, United States*

Immunosenescence creates a challenge in developing vaccines tailored for older adults. Recent advances in immunology, molecular biology and systems vaccinology have enabled greater understanding of the innate and adaptive immune mechanisms behind vaccine responses in adults. Novel approaches to vaccine design for this population include adjuvant technology. New knowledge and accumulating experience enables evidence-based selection of the right antigen and adjuvant combination leading to the discovery and development of better and/or newer innovative vaccines specifically tailored to protect against pathogens responsible for infectious diseases resulting in significant morbidity and mortality in older adults. For example, the recent licensure of several vaccines formulated with a new generation of adjuvants to help protect older adults against influenza, hepatitis B and herpes zoster (shingles). This evidence-based approach to the development of adjuvanted vaccines addressing immunosenescence is a primary prevention strategy to develop and maintain the functional ability that enables wellbeing in older age.

### IMMUNIZATION AS A HEALTHY AGING STRATEGY

Eduardo De Gomensoro[1], *1. GSK Vaccines, Rockville, Maryland, United States*

The burden of infectious disease is heavier at the extremes of life: the youngest and the oldest typically have the highest incidence of infectious diseases along with morbidity and mortality. Demographic studies show by the next decade, adults over 65 will outnumber children under 5 years of age. Unfortunately, vaccination coverage in older adults even where permissive recommendations exist is universally lower than in infants or children. Key reasons are a lack of knowledge and understanding of the benefits of vaccination, and inconsistent recommendations by providers. Recently, the concept of 'healthy aging' – regular vaccination and lifestyle modification including exercise and diet – has been proposed to go beyond disease prevention and address quality of life issues such as the ability to remain in work, and to live independently. Public and healthcare provider education to ascertain the value of older adult's immunization are critical for the fulfilment of this agenda.

### UNDERSTANDING AND ADDRESSING IMMUNOSENESCENCE

Robbert Van Der Most[1], *1. GSK Vaccines, Rixensart, Belgium*

Aging comes with an increased impact of infectious disease in terms of hospitalization, morbidity and mortality. This increased susceptibility to infection appears to be linked to age-related changes in the immune system and its capacity to respond to infection and vaccination. Importantly, this phenomenon occurs despite existence of pre-existing immune memory. The age-related weakening of the immune response is referred to as "immunosenescence". Immunosenescence operates at several levels of the immune system and is multifactorial. Recent advances in systems immunology have shed new light on the immunological processes that may drive the age-related changes in immune response to infection and vaccination. However, gaps in our understanding still exist at basic and translational research levels. One approach to counteract this is the development and implementation of innovative vaccines against the pathogens with particular risks for older adults. The use of innovative immune adjuvants holds promise for the development of such vaccines.

### SESSION 1140 (SYMPOSIUM)

### WOMEN'S EXPERIENCES OF AGING AND SOCIAL CONNECTEDNESS

Chair: Nicky J. Newton, *Wilfrid Laurier University, Waterloo, Canada*
Discussant: Jamila Bookwala, *Lafayette College, Easton, Pennsylvania, United States*

Models of aging, such as the successful aging framework outlined by Rowe & Kahn (1987; 2015) should be holistic, necessitating the inclusion of health, psychosocial factors, and social connectedness. Even at the oldest ages, life expectancy and rates of survival are increasing, yet these longer lives are accompanied with disease and disability, especially among women (Crimmins & Beltrán-Sánchez, 2010); thus, maximizing health and well-being during these post-retirement years, which can often span decades, is a high priority. However, models of age-related change, such as those relating to age-related transitions, are predominantly based on men's experiences; less is known about how women navigate later life (Calasanti, 2010; Kim & Moen, 2002). The presentations in this symposium provide quantitative and qualitative data from women of a broad age range concerning their experiences of aging, with the shared theme of social relationships. Sherman examines the relationship between personality and social support for well-being outcomes in Native American, African American, and European American women (Mage = 57). Conceptualizing aging as the quintessential life transition, Newton outlines the diverse themes of physical, psychological, and social aging from interviews with older women (Mage = 72). Fuller and Toyama find that for older women (Mage = 80), friendships mattered more than family, and counting on neighbors could even be detrimental in terms of life satisfaction and stress. Taken together, these presentations provide a varied picture of what it means for women to 'age well', suggesting nuanced ways in which we might conceptualize theories of aging for women.

### INTERACTIONS OF OPTIMISM WITH SOCIAL RELATIONS FOR WOMEN'S WELL-BEING

Aurora M. Sherman[1], *1. Oregon State University, Corvallis, Oregon, United States*

The impact of personality on the relationship between social relations and well-being has been understudied. We assessed optimism, social support, and social strain in association with self-esteem, depressive symptoms and life satisfaction for a sample of 247 women (Mean age = 57.56, range 45-89 years) from three race groups (42% Native American, 34% African American, 24% European American). PROCESS models revealed significant interactions between optimism and support suggesting that high support buffers the risk of low optimism for all three dependent variables, and two interactions of optimism with social strain, showing that low optimism exacerbated the negative impact of high strain for CES-D and self-esteem scores. The full models accounted for 30-50% of the variance explained in each outcome. We discuss important resources for resilience shown by the women in the sample.

### OLDER WOMEN'S EXPERIENCES OF AGING: THE PHYSICAL, THE PSYCHOLOGICAL, AND THE SOCIAL

Nicky J. Newton[1], *1. Wilfrid Laurier University, Waterloo, Canada*

According to the life course perspective (Settersten, 2003), major life transitions are embedded in contexts shaped by personal history and social circumstances "as natural as the changing seasons" (Miller, 2010, p.663). Aging itself is perhaps the epitome of all transitions: a relatively measured movement through a series of situations, conditions, and social roles

(Hettich, 2010); a transition that particularly lends itself to a life course approach. In this qualitative interview study, 37 women (Mage = 72.27) responded to questions regarding their experiences of the physical, psychological and social aspects of aging. While themes of inevitability and physical health were evident, the highly-personalized nature of aging was also underscored through individual themes of invisibility, freedom from expectations, fear of cognitive decline, and the quality and maintenance of friendships. Similarities and differences in women's experience of aging are compared; the need to contextualize aging within the life course is discussed.

### IMPLICATIONS OF RELATIONSHIPS WITH FAMILY, FRIENDS, AND NEIGHBORS FOR WOMEN'S WELL-BEING IN LATE LIFE

Heather Fuller,[1] Heather Fuller,[1] and Masahiro Toyama[1], *1. North Dakota State University, Fargo, North Dakota, United States*

Social support is well documented as promoting women's well-being across the lifespan, yet implications vary depending on the source and type of support. The present study examined whether relationships with family, friends, and neighbors (both satisfaction with and number in social network) affected well-being over two years. Midwestern women (N=188, mean age = 80) were sampled from two waves of the Social Integration and Aging Study (2013, 2015). Hierarchical regression models indicated that satisfaction with friends predicted better life satisfaction, but satisfaction with family and neighbors did not predict well-being. In contrast, number of neighbors in social network predicted lower life satisfaction and greater stress, while number of family and friends were not associated with well-being. Moreover, differential effects emerged between older and younger women. Findings highlight unique facets of older women's social relationships and suggest that future research and interventions addressing age and the source of support are warranted.

### SESSION 1145 (SYMPOSIUM)

### ARCHIVES OF GERONTOLOGY AND GERIATRICS: MEN'S HEALTH AND AGING

Chair: Roland J. Thorpe, *Johns Hopkins Bloomberg School of Public Health, Baltimore, Maryland, United States*

Historically men typically have had more opportunities, privileges, and power; yet men die sooner and have earlier onset of health conditions compared to women. This is largely because there is a paucity of research focusing on the complex interaction that exists between social, behavioral, biological, and psychosocial factors among men. This symposium contains a collection of papers in the latest Volume of the Archives of Gerontology and Geriatrics that discuss some key factors that can provide insights to advance our understanding of men's health and aging. Kelley and colleagues bring together several important concepts from sociology and gerontology to provide an explanatory framework for older men's differential health profiles within and between cohorts, and over time. Wilmoth and colleagues discuss the complexities of understanding the health and well-being of male veterans in late life by providing critical insight on next steps that are needed on specific war-era cohorts to identify the mechanisms that shape veteran status differences in late-life health and mortality. Taylor and Taylor focus on social isolation and loneliness among a diverse sample of older men including understanding how social isolation and loneliness impact health outcomes. Bruce and Thorpe focus on how faith has implications for socio-biologic interactions associated with elevated risk for disease and premature death among this marginalized population. These presentations collectively will bolster our knowledge on men's health and aging.

### SOCIAL CHANGE, SOCIAL INSTITUTIONS, AND COHORTS: CONTEXTUALIZING MEN'S HEALTH IN LATER LIFE

Jessica Kelley[1], *1. Case Western Reserve University, Cleveland, Ohio, United States*

Research links men's health to their participation in, and access to, social institutions such as marriage, education, and work. However, these institutions have undergone significant social change in the past century, altering their scope and influence on men's health. We tie together several important concepts from sociology and gerontology to provide an explanatory framework for older men's differential health profiles within and between cohorts, and over time. First, we address the gendered life course which are the structural and social arrangements that create distinctive experiences over the life course for men and women. Second, we employ the concept of cohort analysis to capture social changes in the institutionalized life course. Finally, we utilize the cumulative dis/advantage framework to help understand within-cohort differentiation in health status as men age. Taken together, we can better understand health, longevity, and disability profiles for older men and how these have shifted over time.

### SOCIAL ISOLATION, LONELINESS, AND MEN'S HEALTH

Harry O. Taylor[1], *1. Washington University in St. Louis, St. Louis, Missouri, United States*

Strong and fulfilling relationships are important components of men's health and well-being across the life course; however, social isolation and loneliness are important but under-assessed conditions among older men. This is important to note because older men often subscribe to common masculinity themes regarding independence and self-sufficiency which places them at greater risk for social isolation and loneliness in comparison to older women. The purpose of this presentation is to review the social isolation and loneliness literature specifically among older men by 1) discussing gender differences, and the potential mechanisms behind these differences, in social isolation and loneliness, 2) examining health, behavioral and physiological effects of social isolation and loneliness specifically among older men, and 3) providing future research directions for understanding social isolation and loneliness among older men including understanding social isolation and loneliness and their associative outcomes among diverse samples of older men.

### STRESS, FAITH, AND HEALTH AMONG BLACK MEN IN MIDDLE AND LATE LIFE

Marino A. Bruce,[1] and Roland J. Thorpe[2], *1. Vanderbilt University, Nashville, Tennessee, United States, 2. Johns*

*Hopkins Bloomberg School of Public Health, Baltimore, Maryland, United States*

African American men experience extremely high levels of social and psychological stress from unfavorable social and economic circumstances emerging from institutional discrimination and unfair treatment. Stress has been linked to disproportionate risks for illness, disease and premature mortality among this population. But, few studies have examined how African American men manage or cope with stress, and even fewer have assessed how their coping responses have implications for their health. Faith has been considered a stress coping strategy and a growing number of studies explore how religiosity and spirituality have implications for health outcomes. No studies to our knowledge have examined how faith impacts stress and its influence on the health among African American men. The purpose of this chapter is to demonstrate how faith has implications for socio-biologic interactions associated with elevated risk for disease and premature death among this marginalized population.

### THE HEALTH OF MALE VETERANS IN LATER LIFE

Janet M. Wilmoth,[1] Scott D. Landes,[1] and Andrew S. London[1], *1. Syracuse University, Syracuse, New York, United States*

Veterans have the opportunity to accrue health-promoting "military capital," but they are also at risk of experiencing a "military hazard" effect that undermines later-life health and mortality outcomes. Given these possibly competing effects, there is substantial heterogeneity in physical and mental health among older male veterans. The health and mortality outcomes of older veterans who were not substantially harmed during military service appear to be just as good as, if not better than, those of nonveterans. However, older veterans who served in-theater, were exposed to combat or hazardous chemicals, and/or were physically or psychologically harmed during service tend to have worse health and higher mortality than non¬veterans. Some older veterans with these experiences struggle with life-long or late-onset PTSD, while others exhibit resilience and posttraumatic growth. Additional population-level, life-course research is needed on specific war-era cohorts to identify the mechanisms that shape veteran status differences in late-life health and mortality.

### SESSION 1150 (PAPER)

### BENEFITS OF SOCIAL AND PHYSICAL ACTIVITY IN LATER LIFE

### ACTIVITIES ACROSS AMERICA: EVALUATING GEOGRAPHIC DIFFERENCES IN ACTIVITY ENGAGEMENT

Brittany P. Trubenstein,[1] Robin Corley,[2] Kyle D. Gebelin,[1] Sergio Rey,[1] Sally Wadsworth,[2] and Chandra A. Reynolds[1], *1. University of California - Riverside, Riverside, California, United States, 2. University of Colorado – Boulder, Boulder, Colorado, United States*

Rurality is associated with cognitive health disparities. We investigated proximal and distal indices of rurality, activity engagement and cognitive performance in the ongoing Colorado Adoption/Twin Study of Lifespan behavioral development and cognitive aging (CATSLife; N = 979; 47% female). The Index of Relative Rurality (IRR) (0 = Urban to 1= Rural) was calculated using population density, population, percent urban, and remoteness at the census tract (IRRtract; M=0.40,SD =.05) and county levels (IRRcounty; M=0.53, SD=.09), which were moderately correlated (r = .21, p = .000). Individuals reported weekly-hours of engagement in 19 activities, classified into social (M=6.85, SD=4.03), physical (M=6.53, SD=4.76), family (M=10.76, SD=7.06), sedentary (M=11.84, SD=5.83), or cognitive (M=4.63, SD=3.74) domains. Social activities correlated with IRRcounty (r=0.091, p = .005) but not with IRRtract (r=-0.004). WAIS-III IQ scores were available. Social activities modestly correlated with IQ, particularly Verbal-IQ (r = .063, p = .049). Cognitive activities correlated with all IQ measures (r's = .17 to .25, p < .000). While IRRcounty correlated positively with IQ (r's=0.057 to 0.094, p's = .079 to .000), IRRtract correlated negatively but not significantly with IQ (r's=-0.053 to -0.062, p's = .104 - .054). Analyses accounting for family nesting, sex, and age suggested compensatory associations between IRRcounty versus IRRtract and Full-Scale-IQ (p < .019), with similar patterns for Verbal-IQ and Performance-IQ. Social activities did not uniquely contribute. Further investigation is warranted to better understand the complex relationships between proximal and distal rurality and the implications that these relationships have on activity engagement and cognitive performance.

### COGNITIVE TRAINING ATTENUATES AGE-RELATED DECLINE IN PHYSICAL FUNCTION ACROSS 10 YEARS

Briana N. Sprague,[1] Christine B. Phillips,[2] and Lesley A. Ross[1], *1. The Pennsylvania State University, University Park, Pennsylvania, United States, 2. Arizona State University, Arizona State University, United States*

Poor physical function is associated with negative health and cognitive outcomes. Although nine studies demonstrate that cognitive training reduces age-related declines in physical function, only one has examined effects beyond immediate posttest changes. The current study assessed the impact of three cognitive training programs on physical function measures across 10 years. Using data from the Advanced Cognitive Training for Independent and Vital Elderly (ACTIVE) trial, older adults randomized to a no-contact control condition (n = 698) were compared to those randomized to processing speed (n = 702), memory (n = 703), or reasoning (n = 694) training. Intention-to-treat and treatment-received analyses were conducted for grip strength, Digit Symbol Copy, and Turn 360. There were no significant effects of being assigned to processing speed, memory, or reasoning training to any physical function outcome (p > .05). Treatment-received models indicated that processing speed training attenuated age-related declines in Digit Symbol Copy (b = -.005, p < .01) and Turn 360 (b = -.011, p < .001), memory training attenuated age-related declines in Digit Symbol Copy (b = -.009, p < .001) and Turn 360 (b = -.011, p < .001), and reasoning training attenuated age-related declines in Digit Symbol Copy (b = -.012, p < .001) and Turn 360 (b = -.012, p < .001). There was no significant transfer to grip strength. This is the first study to demonstrate beneficial effects of cognitive training to some physical functions across 10 years. Future work should examine moderators and mediators of transfer effects.

### GOALS OF THE ENDGAME

Michelle Silver[1], *1. University of Toronto, Toronto, Ontario, Canada*

At all stages of life, the body can be considered an occupational resource that interacts with social structures in identity formation and complicates personal adaptation to life transitions. As the body declines, the economic and social standing it confers also tends to decline, leading to socially embedded fears about physical decline and marginalization. This paper applies theoretical work from embodiment theory and the life course perspective to examine how perceptions of aging and life experience with sport (or lack thereof) influence exercise participation and athletic identity. Using a narrative approach, I examine in-depth interviews I conducted with elite athletes, masters' athletes, coaches, athletic program directors, mature adults. Some participants struggled to exercise regularly, and others are exercising more in their later years than at any other point in their lives. Three key themes emerged: 1) bodily identity is tremendously important in relation to other forms of identity when it is affected by aging, ill-health, or other physical processes; 2) physical functional mobility becomes increasingly important with age; and 3) experiences with sports and athletic identity (or lack thereof) influence engagement in exercise in later life in surprising ways. The paper challenges society's focus on youth in sports and elite athletes, to discuss how our greater longevity means that we must place more emphasis on identifying ways to keep physically active and mobile throughout adulthood.

### SOCIAL ACTIVITIES AND DEPRESSIVE SYMPTOMS AMONG OLDER ADULTS IN MEXICO: IMPLICATIONS OF GENDER AND PHYSICAL HEALTH

Maria Monserud[1], *1. University of Houston, Houston, Texas, United States*

Studies in developed countries indicate that social activities can make a difference in mental health in later life. Yet, research on potential benefits of social activities for older adults in developing countries, including Mexico, has been scarce. This study uses the two most recent waves (2012, 2015) of the Mexican Health and Aging Study to investigate the impact of social activities on depressive symptoms among older men (n = 4, 749) and women (n = 6,527), aged 50+, in Mexico. The results of Ordinary Least Squares regressions indicate that it is important to differentiate among specific social activities in later life. Particularly, not only group-based but also solitary social activities were predictive of better mental health. Moreover, the findings demonstrate several gender differences and similarities. Participation in clubs, communication with relatives and friends, physical exercise, and watching television were beneficial for mental health among men, whereas volunteering, playing games, and making crafts were associated with fewer depressive symptoms among women. At the same time, reading as well as doing household chores were related to better mental health among older Mexicans, regardless of gender. Furthermore, this study shows that self-reported health, functional limitations, chronic conditions, and frequent pain might shape the implications of social activities for depressive symptoms among older adults in Mexico. The insights from this study can be helpful for intervention programs that are being developed to promote benefits of group-based and solitary social activities for mental health among older men and women with different levels of physical health.

### SOCIAL COHESION, TRANSPORTATION, AND PARTICIPATION IN SOCIAL ACTIVITIES AMONG OLDER ADULTS

Kenzie Latham-Mintus,[1] and Keith Miller[2], *1. IUPUI, Indianapolis, Indiana, United States, 2. Indiana University, Indianapolis, Indiana, United States*

Objectives: The purpose of this research is to examine the role that social cohesion and access (conceptualized as mobility and transportation) plays on participation in social activities (i.e., visiting friends/family, attending religious services, participating in organizations, and going out for enjoyment). Participation in valued, social activities promotes of well-being through social interactions and the maintenance of personally meaningful relationships and lifestyles. Methods: Data from the National Health and Aging Trends (NHATS) study were used. The NHATS is representative of U.S. Medicare recipients ages 65 and older. The NHATS collects information on health and participation as well as detailed environmental measures, which makes it well suited for this research. Results: Higher ratings of social cohesion were associated with higher cumulative odds of participating in social activities among older adults, net of sociodemographic characteristics, personal network size, neighborhood disorder, and health factors. Taking public transportation services and walking places were associated with higher cumulative odds of participating in social activities. An interaction between social cohesion and walking places was significant (p=0.002). Older adults who reported high levels of social cohesion and walked to get around their community were more likely to participate in social activities compared with those reporting low social cohesion and walking as a transportation. Discussion: This research provides evidence that socially cohesive neighborhoods enable greater access to social activities through transportation services. Offering a range of transportation services is only piece of creating an age-friendly community—older adults must also feel comfortable using these options.

### SESSION 1155 (SYMPOSIUM)

### BEYOND FUNCTIONAL SUPPORT: PAID CAREGIVERS AND THE HEALTH OF OLDER ADULTS

Chair: Jennifer M. Reckrey, *Icahn School of Medicine at Mount Sinai, New York, New York, United States*
Discussant: Barbara Bowers, *University of Wisconsin-Madison, School of Nursing, Madison, Wisconsin, United States*

This symposium will explore role that paid caregivers (e.g. home health aides, personal care attendants, and other direct care workers) play in the health and team-based healthcare of older adults living in the community. The large and growing workforce of paid caregivers witness the changes in health status, chronic health needs, and psychosocial stressors of the older adults they care for. Yet existing research on paid caregivers has largely been limited to workforce issues such as recruitment, retention, and job satisfaction. The unique potential of paid caregivers to impact the health of their clients remains largely unstudied. The first presenter will describe results from qualitative interviews with seriously ill older adults and their long-time paid caregivers that found that paid caregivers perform a wide variety of health-related tasks in the course of their routine care. The second presenter will describe results from focus groups with paid caregivers that found that though not a part of the official care plan, paid caregivers provided deliberate cognitive, emotional, and social care that sought to improve their clients' "total" health. The third presenter will outline the rationale for and development of an educational intervention aiming to improve paid caregiver's ability to provide care to patients with heart failure. Finally, the fourth presenter will discuss the limited role paid caregivers currently play in the healthcare team. She will then highlight key policy, educational, and clinical recommendations to promote further paid caregiver integration in the healthcare team.

### THE HEALTH-RELATED TASKS PAID CAREGIVERS IN NEW YORK STATE PERFORM IN THE HOME

Jennifer M. Reckrey,[1] Emma Tsui,[2] R. S. Morrison,[1] Emma Geduldig,[1] Robyn Stone,[3] Katherine Ornstein,[1] and Alex Federman[1], *1. Icahn School of Medicine at Mount Sinai, New York, New York, United States, 2. CUNY Graduate School of Public Health and Health Policy, New York, New York, United States, 3. Leading Age, Washington, District of Columbia, United States*

Paid caregivers (e.g. home health aides, personal care attendants) are formally tasked with helping older adults with functional impairment meet their basic needs at home. This study used semi-structured interviews (n=30) with dyads of patients or their proxies and their paid caregivers in New York City to 1) understand the range of health-related tasks paid caregivers perform in the home and 2) determine if these tasks are taught in the New York State government's Department of Health curricula. We found that patients, proxies, and paid caregivers all described that paid caregivers performed a wide range of health-related tasks that were often not a part of their formal training. Creating clear competencies for paid caregivers that reflect the full breadth of health-related tasks they may perform at home will help maximize the potentially positive impact of the paid caregiver workforce on the lives of patients living at home with functional impairment.

### YOU'RE THE ONLY PERSON THERE FOR THEM: LEVERAGING HOME HEALTH AIDES' EXPERTISE TO SUPPORT TOTAL CLIENT HEALTH

Emily C. Franzosa[1], *1. CUNY Graduate School of Public Health and Health Policy, New York, New York, United States*

Formally, home health aides provide physical, non-clinical care to support the health and safety of older and disabled individuals. But in practice, both workers and clients report that the often unrecognized relational care aides provide is also central to clients' well-being. In focus groups with New York City-based home health aides, aides described their conception and delivery of "total" care, which included specific and deliberate cognitive, emotional and social strategies to support clients' mental and physical well-being. However, since this work was not included in formal care plans and invisible to those outside the care team, aides felt unprepared and unsupported in performing it. Realigning the definition of home care to address "total" care and better integrating aides into the care team has the potential to both improve job satisfaction and patient care.

### HEART-FAILURE TRAINING FOR HOME CARE WORKERS IN NEW YORK CITY

Madeline Sterling,[1] and Peggy Leung[1], *1. Weill Cornell Medical College, New York, New York, United States*

Home care workers (HCWs), which include home health aides and personal care aides, are increasingly being used by community dwelling adults with heart failure (HF) for long-term assistance and post-acute care. Findings from our prior research suggest that HCWs are deeply involved in many aspects of HF patients' self-care, including HF maintenance and management, but the majority have not received any HF training or HF-specific resources. Due to this, many HCWs do not feel confident caring for their clients with HF. In this symposium, we will present the findings of a qualitative study that used a nominal group technique to elicit the educational needs of 40 English and Spanish speaking agency-employed HCWs caring for HF patients in New York City. We will also present an overview of the HF training course that was developed from this data and its effect on HCWs' HF knowledge and caregiving self-efficacy.

### CHALLENGES AND OPPORTUNITIES FOR TRAINING AND SUPPORTING AIDES AS MEMBERS OF HOME-BASED CARE TEAMS

Robyn Stone,[1] and Natasha Bryant[1], *1. LeadingAge, Washington, District of Columbia, United States*

Despite home heath/home care aides being the informal "eyes and ears" of the health system, team-based home care initiatives have not incorporated this workforce into their programs. This presentation summarizes barriers to their inclusion: a basic lack of understanding on the part of clinical team members of the complex tasks these caregivers perform, inadequate investments in competency-based aide training and education, and variation in state nurse delegation laws that limit aides' scope of practice and their ability to work effectively in teams. This is followed by a review of several programs that have successfully included aides as key members of home care teams. The presentation concludes with recommendations on how federal and state policymakers, educators and health systems and providers can support inclusion of aides in team-based care through standardization of competency-based training programs, expansion of nurse delegation nationwide, and support for piloting, evaluation, dissemination and replication of promising models.

### SESSION 1160 (PAPER)

### CAREGIVING, COGNITIVE FUNCTIONING, AND BEHAVIORAL AND SOCIAL DETERMINANTS

### AWARENESS OF HEALTH RISKS OF CAREGIVING AMONG PRIMARY PHYSICIANS AND THE CAREGIVERS THEMSELVES

Sara Carmel,[1] Yaacov Bachner,[2] and Aya Biderman[2], *1. Ben Gurion University of the Negev, Bee Sheva, Israel, 2. Faculty of Health Sicences, Ben Gurion University of the Negev, Beer-Sheva, Israel, Israel*

Literature indicates that family primary caregivers (FPC) to severely-ill patients are at high risk to incidence of physical and mental morbidity, especially if they are old themselves. The purpose of these studies was to examine the FPC's awareness to their own health risks, and primary physicians' awareness to the importance of locating, following and providing preventive treatment to FPC in their clinic. Participants included 202 FPC older spouses with average age 70.7 (SD=8.33), and 68% women, and primary physicians (N=201) with average age of 48.5 (SD=11.22) and 53.5% women. Among the FPC, awareness to the health risks of caregiving was higher the greater was the caregiving burden, the worse was their self-rated health, the severer was the patient's disease, as well as the lower was the number of children and among women and spouses. Awareness to importance of medical surveillance was low. Among physicians, awareness to the risks of caregiving was highest among physicians who received their diploma in Israeli universities, and to specialists in family medicine. Their awareness was higher to the FPC's susceptibility to mental risks rather than to physical risks. Physician's awareness to the efficiency of medical surveillance of the FPC was high but their actual performance of it was low. This finding is in accordance with the FPC's report about a low level of interest in their own health among their primary physicians. The importance of awareness to location, surveillance and preventive treatment of FPC should be promoted in medical education, and primary medical services.

### THE ALZHEIMER'S ASSOCIATION NATIONAL HELPLINE: EFFECTS ON FAMILY CAREGIVERS

Nancy Hodgson,[1] Darina V. Petrovsky,[1] Beth Kallmyer,[2] Joanne Pike,[2] and Sam Fazio[2], *1. University of Pennsylvania, Philadelphia, Pennsylvania, United States, 2. Alzheimer's Association Home Office, Chicago, Illinois, United States*

Family caregivers of persons with dementia experience high rates of social isolation and limited access to emotional support. The Alzheimer's Association National Helpline is an accessible and free resource available 24 hours/day, 365 days/year in which master's-level clinicians offer confidential emotional support and information on resources in the form of "action steps." We evaluated the preliminary effectiveness of the Helpline for family caregivers of persons with dementia. Between January and October 2018, 185 non-crisis, caregiver calls to the Helpline were assessed at the time of call, one week, and 1 month post-call for effects on caregivers' self-reported emotional distress, ability to manage anxiety, implement plan of action and access of services. The mean age of callers was 56 years; 22% were non white; 79% were women. Callers reported significantly ($p < .05$) improved caregiver emotional distress (27% net improvement) and ability to manage anxiety (29% net improvement). At one week, 70% of callers had put action steps in place, and by 1 month 80% of callers had put action steps into place. Over 80% of callers reported action steps were "helpful" and 65% reported that they had accessed additional dementia support services. This study suggests that support provided via the Alzheimer's Association National Helpline is effective at reducing caregiver emotional distress and improving the ability of callers to "take action". The results provide support for a larger study investigating caller characteristics and core content of the calls that provided sustained benefit to standardize the key elements of Helpline calls.

### THE EFFECT OF CAREGIVING EXPERIENCE ON CARE RECIPIENT OLDER ADULTS' MORTALITY: A SURVIVAL ANALYSIS

Teja Pristavec,[1] and Elizabeth A. Luth[2], *1. University of Virginia, Biocomplexity Institute and Initiative, Arlington, Virginia, United States, 2. Weill Cornell Medicine, New York, New York, United States*

Health and demographic mortality risk factors among older adults are well documented. However, less is known about the dyadic relationship between caregiver characteristics and care recipient mortality outcomes. In a nationally representative sample of older adults, we prospectively explore 1) whether and how having an informal caregiver is associated with care recipient mortality, and 2) among those with caregivers, how caregivers' experiences of burden and benefits relate to care recipient mortality. We match 6 waves of National Health and Aging Trends Study (2011-2016) with 2011 National Study of Caregivers data. We conduct survival analysis on 7,369 older adults and a subsample of 1,341 older adult-informal caregiver dyads to address our research questions. First, we find that simply having an informal caregiver increases mortality risk by 71% ($p<0.001$) over the 6-year time period, even when adjusting for key demographic, economic and health factors. Second, we find that older adults whose caregivers perceive burden have a significantly higher mortality risk. This risk is reduced if the caregiver also perceives caregiving benefits. The risk of death is 41% higher for older adults whose caregivers report burden but no benefit compared to those with caregivers who report neither burden nor benefit. Further research should investigate possible reasons why merely having a caregiver increases older adults' mortality risk. Interventions to increase caregivers' sense of benefit and reduce their burden may be an effective way of decreasing mortality risk for older adults with declining health and functional ability.

### THE IMPACT OF AN EARLY-STAGE DYADIC CARE PLANNING INTERVENTION ON CAREGIVER BURDEN

Silvia Orsulic-Jeras,[1] Carol Whitlatch,[1] and Justin Johnson[1], *1. Benjamin Rose Institute on Aging, Cleveland, Ohio, United States*

Evidence supports the development of proactive, dyadic interventions for used in early-stage dementia. This type of intervention leads to more effective decision making which can reduce subsequent caregiver stress and burden. SHARE (Support, Health, Activities, Resources, Education), a six-session counseling-based intervention, encourages and supports care dyads to have important discussions about health care preferences that are often delayed or avoided until later-stage dementia. Typically, both the PWD and caregiver assume that most of the help will be delivered by the caregiver. SHARE aims to expand the network of care by evenly distributing care task responsibilities from the caregiver alone to other potential sources of support: family/friends and paid service providers. Early-stage dyads (n=63) successfully created a balanced and manageable plan of care with the help of their SHARE Counselor over a 6-month period. Follow-up interviews assessed care dyads from 1.5 to 2 years after their participation in SHARE ended. Analyses determined change in PWD ADL needs from enrollment (T1) to two years later (T2) and impact on caregiver task burden. Paired samples t-tests indicated a significant increase in PWD need for assistance with 18 ADLs, from a T1 mean of 8.76 to a T2 mean of 11.34 (t=-6.72, p=.000). This paper examines: 1) the benefits and challenges of creating a Care Plan in the early stages of dementia, 2) how SHARE dyads utilized their Care Plans when PWDs needed assistance, and 3) if reliance on family/friend and paid service provider options were realistic over time.

### WORK ENVIRONMENTS AMONG OLDER WORKING CAREGIVERS

Dawn C. Carr,[1] Miles G. Taylor,[1] Kendra Jason,[2] Chivon A. Mingo,[3] and Tiffany R. Washington[4], *1. Florida State University, Tallahassee, Florida, United States, 2. University of North Carolina at Charlotte, Charlotte, North Carolina, United States, 3. Georgia State University, Atlanta, Georgia, United States, 4. University of Georgia, Athens, Georgia, United States*

Many older workers balance paid work with care work. Working caregivers face unique challenges that make them more likely to leave the work force. However work environments may be more or less accommodating to their needs, and in addition, they may need to work for financial reasons. Current research on working caregivers has not explored: a) the work environments of older working caregivers; b) whether particular work environments are likely to influence whether caregivers stop working; and c) whether these effects vary by type of care work (spousal versus parental). This study addresses these gaps. Using data drawn from the 2008-2014 waves of the Health and Retirement Study, we used latent class analysis to develop a typology of work environments of individuals 51-75 who are engaged in paid work. Four classes of work environments emerged: A) balanced, supportive work environments (34%); B) average environments with high job lock (30%); C) poor, unsupportive work environments (21%); and D) highly accommodating, stressful jobs (14%). Logistic regression results showed those in group D were less likely than all other groups to leave their jobs. In addition, relative to spousal caregivers, parental caregivers in class A were significantly more likely to leave the labor force. Results suggest that caregivers may be more likely to continue engaging in paid work in supportive work environments, and work environments may be more likely to retain older working caregivers by identifying ways to help them meet their work needs and maintain their caregiving roles.

### SESSION 1170 (PAPER)

### EMERGING TOPICS IN FAMILY CAREGIVING

### A PROFILE OF CAREGIVING AMONG SEPSIS SURVIVORS RECEIVING POST-ACUTE HOME HEALTH CARE

Jo-Ana D. Chase,[1] Christina R. Whitehouse,[2] Lizeyka Jordan,[3] and Kathryn H. Bowles[4], *1. University of Missouri, Columbia, Missouri, United States, 2. Villanova University M. Louise Fitzpatrick College of Nursing, Villanova, Pennsylvania, United States, 3. Visiting Nurse Service of New York, New York, New York, United States, 4. University of Pennsylvania School of Nursing, Philadelphia, Pennsylvania, United States*

Sepsis survivors transitioning from hospital-to-home are clinically complex. Family caregivers can face challenges managing patients' care needs; however, skilled home health care (HHC) can serve as an important resource during this care transition. This study's purpose was to describe caregiving needs among older sepsis survivors receiving post-acute HHC, and identify sources of unmet caregiving needs. We conducted a retrospective analysis of a national dataset of Medicare beneficiaries starting a new HHC episode who were after hospital discharge for sepsis between 2013 and 2014 (n=165,228). All patients received at least one HHC visit the first week after hospital discharge. Caregiving activities included seven items from the start of care Outcome and Assessment Information Set. Descriptive statistics were used to examine types of caregiving activities and needs, demographics, and clinical information. Proportions of patients with unmet caregiving needs ranged from 9%-29%, with the largest proportion of unmet needs in activities of daily living (ADL) assistance (29%), medication administration (28%), and medical procedures/treatments (25%). Unmet caregiving needs across activities were largely due to a caregiver needing training/supportive services (75%-88%), suggesting that many sepsis survivors receiving HHC have caregivers who are available to help, but who lack the knowledge and skills to manage patients' complex care needs. Thus, HHC providers should address caregiving training and support needs, especially related to assistance with ADLs, medication administration, and medical procedures/treatments. Future research is needed to determine specific educational strategies for caregiver training and support, especially related to skills and knowledge assessment, and training delivery and monitoring.

### ADMINISTRATIVE INDICATORS OF FAMILY SUPPORT AMONG COMPLEX PATIENTS

Elvira E. Jimenez,[1] Ranak Trivedi,[2] Alexis Huynh,[3] Taona Haderlein,[3] Marian Katz,[3] and Evelyn Chang[3], *1. Center for the Study of Healthcare Innovation, Implementation & Policy (CSHIIP), V.A. Greater Los Angeles Healthcare System, Los Angeles, California, United States, 2. Center for Innovation to Implementation, VA Palo Alto Health Care System, Menlo Park, California, United States, 3. Center for the Study of Healthcare Innovation, Implementation & Policy (CSHIIP), V.A. Greater Los Angeles Healthcare System, Los Angeles, CA, Los Angeles, California, United States*

Greater access to family support has been shown to positively affect the management of complex patients (i.e., multiple chronic conditions and psychosocial needs). However, patients' availability of family support is not easily obtainable from medical records. We aim to identify administrative variables that can be used as indicators of family support. We investigated secondary next-of-kin (i.e., patient identified two next-of-kin) and marital status as administratively defined family support availability in a Veteran sample (n=2210). A subsample (n=329) was further evaluated with documented responses to questions "Are there any friends/

family members you would like to involve in any aspect of your health care?" (i.e., "actual availability") and "Does anyone help you with your daily activities?" (i.e., "obtained" availability). We performed a logistic regression analysis to evaluate the association between administratively defined and "actual" or "obtained" availability of support controlling for age, race/ethnicity, and gender. The sample was 90% male, mean age 63 years, 50%White and 44% African American. We found that 32.9% had administratively defined availability by being married, and 32.5% by listing secondary next-of kin. Married Veterans were significantly more likely to report greater actual availability (p=0.01) and obtained (p=0.04) support. Veterans listing a secondary next-of-kin were significantly more likely to report "actual" availability (p= 0.04) but not on "obtained" (p=0.08) support. Marital status may be a useful proxy of actual family support and listing a secondary next-of kin may be an alternate indicator for complex patients. Our study provides guidance on the use of administrative data in understanding caregivers.

### DEMENTIA CAREGIVING NEGATIVELY AFFECTS THE HEALTH OF CAREGIVER AND CARE RECIPIENT. CAREGIVING TRANSITIONS STUDY

Orla C. Sheehan,[1] William E. Haley,[2] Virginia Howard,[3] Jin Huang,[4] J. David Rhodes,[5] and David L. Roth[4], *1. Center on Aging and Health, Johns Hopkins University School of Medicine, Baltimore, Maryland, United States, 2. University of South Florida, Tampa, Florida, United States, 3. University of Alabama at Birmingham, Birmingham, Alabama, United States, 4. Johns Hopkins University School of Medicine, Baltimore, Maryland, United States, 5. School of Public Health, Department of Biostatistics, Birmingham, Alabama, United States*

Dementia is one of the most common reasons for needing a caregiver (CG). Few studies have compared dementia and non-dementia caregivers who have transitioned into family caregiving roles. Participants in the REasons for Geographic and Racial Differences in Stroke (REGARDS) study who transitioned into a significant caregiving role were recruited to participate in the Caregiving Transitions Study (CTS). Of 11,483 REGARDS participants who were not caregivers at baseline, 1229 (11%) transitioned into a family caregiving role. Eligibility criteria were met by 251 and they were enrolled along with 251 demographically-matched noncaregiving controls. Enrolled caregivers are 65% female; 36% African American; 71.8 + 8.1 years of age; caring for a spouse/partner (51%), parent (25%), or another person (24%). 47% are caring for a person with dementia. Dementia CGs provide more hours of care per day (9.3 hours versus 6.7 hours), report being under more stress and twice as much strain as non-dementia CGs (p<0.03 for all). They feel more burdened by the care recipient's treatment (p=0.01) and report that the burden leads to delays in the care recipient receiving medical care (p<0.007). Dementia CGs are more than twice as likely as non-caregivers to report that their caregiving makes them worse at taking care of their own health (33.9% versus 15.4%, p=0.003). This prospective, population-based study confirms previous cross-sectional findings from convenience samples on the greater care burden experienced by dementia caregivers and extends this work to new measures of treatment burden and treatment delay.

### FAMILY CAREGIVER FACTORS AND SUBSEQUENT EMERGENCY DEPARTMENT UTILIZATION AMONG OLDER ADULTS WITH DISABILITY

Julia Burgdorf,[1] John Mulcahy,[2] Halima Amjad,[3] Judith D. Kasper,[1] Kenneth Covinsky,[4] and Jennifer L. Wolff[5], *1. Johns Hopkins Bloomberg School of Public Health, Baltimore, Maryland, United States, 2. Johns Hopkins University Bloomberg School of Public Health, Baltimore, Maryland, United States, 3. Johns Hopkins University School of Medicine, Baltimore, Maryland, United States, 4. University of California San Francisco, San Francisco, California, United States, 5. Johns Hopkins University, Maryland, United States*

Community-living older adults with disability are frequent Emergency Department (ED) users and most rely on family caregiver support. However, no prior research has examined associations between caregiver characteristics and subsequent ED utilization among older adults. We draw on a sample of 2,521 community-living older adults with mobility/self-care disability and their primary family caregivers to identify caregiver characteristics associated with all-cause or potentially preventable ED use. We use Cox proportional hazards regression to separately model the likelihood of all-cause and potentially preventable ED use as a function of caregiver characteristics. Models account for competing risk of mortality and adjust for measures of older adults' socio-demographic characteristics, health status, and survey wave. About half (52.5%) of older adults incurred 1+ ED visit and 26.8% incurred 1+ potentially preventable ED visit within 12 months of interview. Adjusting for survey wave and older adult sociodemographic characteristics and health status, older adults were at greater risk of all-cause ED use if their primary caregiver provided greater than 40 hours of care per week (HR: 1.22, 95% CI: 1.04-1.43; p=0.02), helped with health care tasks (HR: 1.26; 95% CI: 1.08-1.46; p<0.01), or experienced physical strain (HR: 1.18; 95% CI: 1.03-1.36; p=0.02). Older adults were at greater risk of potentially preventable ED use if their primary caregiver helped with health care tasks (HR: 1.25; 95% CI: 1.02-1.54; p=0.03). Findings highlight the relevance of caregiver factors to older adults' ED use and suggest the need for assessment and support of family caregivers in the care delivery setting.

### IDENTIFYING UNMET NEED IN INFORMAL CAREGIVING: DISPARITIES BY GENDER, EMPLOYMENT STATUS, AND RACE-ETHNICITY

Steven A. Cohen,[1] Furong Xu,[1] Marissa R. Meucci,[1] Symone Woodham,[1] Mary L. Greaney[1], *1. University of Rhode Island, Kingston, Rhode Island, United States*

Older adults, including those with dementia and other types of cognitive decline, often report a desire to remain in their homes. Over 50 million informal caregivers in the US provide needed in-home assistance to those in need, and there are well-documented disparities in informal caregiving responsibilities by sociodemographic factors , yet little is known about "unmet need" in informal caregiving. Therefore, the study's objective is to examine discrepancies in unmet caregiving-related need by race/ethnicity, gender, and employment status. We abstracted data about caregivers from the 2017 National Study of Caregiving and linked these data to participants in the National Health and Aging Trends Study on caregivers of older adults (n=993). Generalized linear models were used to model the discrepancies between the number of activities of daily living for which the care recipient required assistance and the number of tasks caregivers provide, by race/ethnicity, gender, and employment status, accounting for confounders and complex sampling. Care recipients whose primary informal caregivers were employed were 69% more likely than those whose informal caregivers were not employed to experience unmet caregiving need (OR 1.69, 95%CI 1.19-2.41). A similar association between employment and unmet caregiving was observed among White caregivers (OR=1.79, 95% CI 1.16-2.69), while the association was not significant among Black caregivers (p=0.228). These findings suggest potentially addressable disparities in informal caregiving duties between Black and White caregivers, and can be used to inform and develop of policies and programs designed to improve caregiver health and reduce undue strain on caregiver health and wellbeing.

### SESSION 1175 (SYMPOSIUM)

### FACTORS INFLUENCING SELF-REPORTED COGNITION OVER TIME

Chair: Nikki Hill, *Penn State University, University Park, Pennsylvania, United States*
Discussant: Mindy Katz, *Albert Einstein College of Medicine, Bronx, New York, United States*

Self-reported cognitive problems among cognitively intact older adults are often associated with an increased risk of future cognitive decline and Alzheimer's disease (AD). However, cross-sectional evidence suggests that self-reported cognition may be more influenced by factors such as personality or affective symptoms than concurrent objective cognitive performance. Furthermore, self-reported cognition is measured using a variety of items that assess different constructs (e.g., current memory performance, perceived decline over time), which may be differentially influenced by individual characteristics or item interpretation. The purpose of this symposium is to present findings from multiple analyses that examined the influence of individual characteristics (i.e., personality, perceived stress, and family history of dementia) on self-reported cognitive problems, and to further describe how item type influences older adults' responses to questions about their memory. First, we present the results of an investigation that examined the influence of personality on three types self-reported memory, with a specific focus on how these associations may differ in Black and White older adults. Second, we extend this discussion with results of an examination of associations among personality, family history of AD, and memory self-report. Our third presentation explores bidirectional associations between perceived stress and memory complaints over time. And finally, we present the results of a factor analysis of self-reported cognition items that distinguishes those that tend to travel together over time from those that are better at discriminating between individuals.

### THE INFLUENCE OF PERSONALITY ON MEMORY SELF-REPORT AMONG BLACK AND WHITE OLDER ADULTS

Nikki Hill,[1] Jacqueline Mogle,[2] Sakshi Bhargava,[2] Tyler Bell,[2] and Rachel Wion[2], *1. Penn State University, University Park, Pennsylvania, United States, 2. Penn State College of Nursing, University Park, Pennsylvania, United States*

Personality traits, particularly neuroticism, have been associated with self-reported memory problems, but little is known regarding differences across racial groups. Community-dwelling older adults (n=425; M(SD) = 76.7(4.7) years; 62.6% female; 72.0% White) without cognitive impairment completed up to 11 annual comprehensive medical and neuropsychological examinations as part of the Einstein Aging Study. Multilevel modeling tested: 1) the association of neuroticism, conscientiousness, extraversion, openness, and agreeableness with three types of self-reported memory problems (frequency, one-year decline, and ten-year decline), and 2) whether these associations differed by race, specifically Black and White. Neuroticism predicted self-reported frequency of memory problems and perceived one-year decline when considered alone; however, this did not remain significant after including all personality traits. Conscientiousness influenced perceived ten-year memory decline in Black older adults but not White. Our findings suggest that the influence of personality on self-reported memory problems may not be consistent across racial groups.

### PERSONALITY AND FAMILY HISTORY OF ALZHEIMER'S DISEASE AS PREDICTORS OF OLDER ADULTS' SELF-REPORTED MEMORY PROBLEMS

Sakshi Bhargava,[1] Nikki Hill,[2] Jacqueline Mogle,[2] Tyler R. Bell,[2] and Rachel Wion[2], *1. Penn State College of Nursing, University Park, Pennsylvania, United States, 2. The Pennsylvania State University, University Park, Pennsylvania, United States*

Understanding individual factors (e.g., personality) associated with self-reported memory problems is important to refine identification of individuals at a higher risk of developing Alzheimer's disease (AD). Using multilevel modeling, we examined the association of family history of AD and personality traits with self-reported memory problems in older adults (n = 421; 72.21% White; 62.95% female; Mage = 76.69). Results showed that individuals with a family history of AD reported more frequent memory problems and greater one-year memory decline. Similar findings were reported for individuals with higher extraversion scores. Further, older adults with higher neuroticism scores reported greater one- and ten-year memory decline. Neuroticism was positively related to frequency of memory problems, but only among participants with a family history of AD. Findings suggest that higher neuroticism and lower extraversion may increase older adults' reports of memory problems. Family history of AD may further exacerbate this tendency.

### LONGITUDINAL ASSOCIATIONS BETWEEN PERCEIVED STRESS AND MEMORY COMPLAINTS

Tyler Bell,[1] Tyler Bell,[2] Jacqueline Mogle,[2] and Nikki Hill[2], *1. Penn State College of Nursing, University Park, Pennsylvania, United States, 2. Pennsylvania State University, University Park, Pennsylvania, United States*

Memory complaints increase cognitive decline but show weak concurrent associations with objective memory. Instead, affect might underlie some memory complaints and their impact on future cognition. Perceived stress influences cognitive performance, but temporal associations with memory complaints is unknown. We therefore explored longitudinal relationships between perceived stress and memory complaints among cognitively normal older adults. From the Einstein Aging Study (n=507, Mage=77.88, 63.30% female; 73.21% White), multilevel models examined bi-directional concurrent and one-year-lagged associations between within-person changes in perceived stress and memory complaints (frequency of forgetting, perceived one-year memory decline, perceived ten-year memory decline). Perceived stress positively covaried with memory complaints. Looking at lagged effects, only frequency of forgetting predicted next-year perceived stress. Higher frequency of forgetting thus increases perceived stress while perceived decline associates with current perceived stress. Reframing perceptions of forgetfulness might reduce stress in cognitively intact older adults, which in turn may benefit cognition long term.

### THE FACTOR STRUCTURE OF MEMORY COMPLAINTS OVER TIME

Jacqueline Mogle,[1] Nikki Hill,[1] Sakshi Bhargava,[1] Tyler Bell,[1] and Emily Whitaker[1], *1. Penn State University, University Park, Pennsylvania, United States*

Although memory complaints are assessed with a variety of items to track change in individuals as they age, it remains unclear which items best capture change. Adults aged 70 to 104 (n=1,344, 38% Male) completed six memory complaints items annually for up to 11 years: frequency of problems, one year decline, ten year decline, seriousness of problems, forgetting important things, and current functioning compared to functioning at age 30. Using multilevel exploratory factor analysis, the best fitting model indicated one factor fit the between person structure with all items loading significantly. Across time, items required two factors. Items about decline loaded together while the item about functioning compared to functioning at age 30 dominated a second factor. Remaining items did not load on either factor across time. This suggests these items assessing memory complaints are better at discriminating across persons rather than tracking changes within a person across time.

### SESSION 1180 (SYMPOSIUM)

### GENETICS AND MECHANISMS OF HUMAN AGING AND FRAILTY

Chair: David Melzer, *University of Exeter, Exeter, Devon, United Kingdom*
Discussant: Luigi Ferrucci, *Translational Gerontology Branch, Intramural Research Program, National Institute on Aging, National Institutes of Health, Baltimore, Maryland, United States*

Great progress has been made recently in identifying the genetic and likely biological mechanisms of aging traits in humans, thanks to the very large UK Biobank cohort of 500,000 persons. This symposium will discuss new results for known genetic loci influencing aging, including in-depth analysis of the APOE e2 "protective" allele and aging outcomes, and will highlight new pathways for follow-up. Identifying genetic variants associated with frailty (accumulation of deficits) is implicating specific pathways with possible causal effects on premature frailty, with particular emphasis on neurological traits. Large-scale analysis of physical frailty – here parametrized as muscle weakness – is shedding light on specific mechanisms that are divergent from those associated with the Rockwood-like analysis, with evidence for sex-specific pathways. At the end of this symposium, the audience should be able to better understand the processes that potentially drive aging and frailty in older adults, and the possibilities for future diagnostic and treatment modalities for delaying or reversing aging and frailty in older adults.

### CONVERGENT AGING LOCI: PATHWAYS THAT TRANSCEND INDIVIDUAL DISEASES

Luke C. Pilling,[1] Luigi Ferrucci,[2] and David Melzer[3], *1. University of Exeter, Exeter, United Kingdom, 2. NIA, Baltimore, Maryland, United States, 3. University of Exeter, Exeter, England, United Kingdom*

Thousands of loci across the genome have been identified for specific diseases in genome-wide association studies (GWAS), yet very few are associated with lifespan itself. We hypothesized that specific biological pathways transcend individual diseases and affect health and lifespan more broadly. Using the published results for the most recent GWAS for 10 key age-related diseases (including coronary artery disease, type-2 diabetes, and several cancers) we identified 22 loci with a strong genetic association with at least three of the diseases. These multi-trait aging loci include known genes affecting multiple diverse health end points, such as CDKN2A/B (9p21.3) and APOE. There are also novel multi-trait genes including SH2B3 and CASC8, likely involved in hallmark pathways of aging biology, including telomere shortening and inflammation. Several of these loci involve trade-offs between chronic disease risk and cancer.

### APOE2 AND AGING-RELATED OUTCOMES IN 261,000 UK BIOBANK OLDER ADULTS

Chia-Ling Kuo,[1] Luke C. Pilling,[2] George A. Kuchel,[3] and David Melzer[2], *1. University of Connecticut, Farmington, Connecticut, United States, 2. University of Exeter, Exeter, England, United Kingdom, 3. UConn Health, Farmington, Connecticut, United States*

ApoeE2 (e2) has both protective and adverse effects on health outcomes, which needs to be considered when targeting e2. We aimed to understand the role of e2 in aging via associations between e2 and a selection of aging traits using UK older adults of European descent. e2e3 (n=32,262) and e2e2 (n=1,629) were compared to e3e3 (n=153,567), adjusted for sex, age, genotyping arrays, assessment centers, and the first five genetic principal components. We found that e2 was associated with both parents top 10% survival (OR=1.14, 95% CI: 1.06-1.24), reduced risks of hypertension (OR=0.93, 95% CI: 0.91-0.96) and coronary heart disease (OR=0.86, 95% CI: 0.82-0.89), but red cell width distribution above the reference range (OR=1.17, 95% CI: 1.09-1.26). Additionally, e2 was minimally associated with frailty, cognitive function, and physical measures, suggesting that its association with parental extreme longevity may be controlled by mechanisms underlying diseases.

### CAUSES OF FRAILTY: GENETIC ANALYSIS IN 164,000 UK BIOBANK PARTICIPANTS

Janice Atkins,[1] Luke C. Pilling,[2] Dylan Williams,[3] Juulia Jylhävä,[3] and David Melzer[2], *1. University of Exeter, Exeter, Devon, United Kingdom, 2. University of Exeter, Exeter, England, United Kingdom, 3. Karolinska Institutet, Stockholm, Stockholms Lan, Sweden*

Frailty is an important and common geriatric syndrome, yet the mechanisms are multifactorial and not well understood. The Frailty Index (FI) is based on the Rockwood Index for accumulation of deficits, and we aimed to use genetics to gain mechanistic insights for interventions to prevent and delay frailty in older people. We performed a genome-wide association study in 60 to 70 year old UK Biobank participants of European descent (n=164,610). We identified 26 independent genetic signals at 24 loci associated with the FI. Several of these signals have previously been associated with traits such as cardiovascular disease, intelligence, and educational attainment, but 6 of the signals appear to be novel. We will also present the results of ongoing work both to identify causal risk factors for FI using Mendelian randomization methods, and replication and functional follow-up in the TwinGene cohort, at the meeting.

### GENOME-WIDE ANALYSIS OF LOW STRENGTH IN OLDER PEOPLE: META-ANALYSIS OF >250,000 VOLUNTEERS IN 16 COHORTS

Garan Jones,[1] Luke C. Pilling,[2] and David Melzer[2], *1. University of Exeter, Exeter, Devon, United Kingdom, 2. University of Exeter, Exeter, England, United Kingdom*

Dynapenia or muscle weakness is a core feature of sarcopenia and frailty, and leads to significant functional impairment in older adults. We aimed to identify genetic variants associated with dynapenia in older adults, to shed light on the underlying mechanisms. A large-scale meta-analysis of >250,000 volunteers of European descent from 16 cohorts in the CHARGE and GEFOS consortia aged 60+ years at assessment is underway, using maximum hand grip strength to define dynapenia. Preliminary analysis in UK Biobank found that dynapenia is associated with nine genomic loci at genome wide significance (p<5x10-8), with differences between men and women, suggesting sex-specific routes to dynapenia. These results suggest that there are specific genetic determinants of low strength in older adults, and these differ from the determinants of normal-range strength differences. The full meta-analysis of all cohorts will extend these findings. Understanding the biological mechanisms may lead to geroscience interventions.

### SESSION 1185 (SYMPOSIUM)

### GERONTOLOGISTS' CONSIDERATIONS IN CHOOSING AND EXPERIENCING LATER-LIFE RELOCATION AND RESIDENCE

Chair: Helen Q. Kivnick, *University of Minnesota School of Social Work, Saint Paul, Minnesota, United States*
Co-Chair: Anne Wyatt-Brown, *University of Florida, Gainesville, Gainesville Florida, United States*
Discussant: Helen Q. Kivnick, *University of Minnesota School of Social Work, Saint Paul, Minnesota, United States*

Increasing numbers of Baby Boomers and their older cohorts are now confronting challenges of aging that have long been research topics and practice domains of gerontologists. Choosing later-life relocation and residence is one such challenge. However, large-scale, population-based research runs the risk of making unwarranted assumptions about individuals based on group membership, alone. As people continue to develop through older adulthood, residential preferences and priorities can change over time – based on financial pressures, health concerns, family circumstances, and more. No solution works for all people. And for many, a choice made early in older adulthood may prove unworkable in the longer term. As Stephen Golant put it in Aging in the Right Place (2015), it is important to try to match the place -- be it home, community, or institution -- with the temperament and the finances of the person who will be living in it. Each older adult needs information about different options and appropriate self-knowledge, in order make a decision that gives them pleasure for an extended amount of time. In this session, aging gerontologists (aged 69-90+) discuss their personal considerations in anticipating and making these major decisions and in experiencing their consequences – as influenced by the unique disciplinary and theoretical perspectives afforded by a lifetime of work in gerontology. Presenters will share their personal later-life housing stories, as they are influenced by theories, concepts, findings, personal circumstances, and styles of observing, thinking, and acting – all developed through their personal careers in gerontology.

### PERSISTENT THEMES IN WHERE WE LIVE AS WE ARE LIVING OUR VERY OLD LIVES

Miriam S. Moss,[1] and Sidney Z. Moss[2], *1. Arcadia University, Glenside, Pennsylvania, Glenside, Pennsylvania, United States, 2. Arcadia University, Glenside, PA, Northampton, Massachusetts, United States*

After living in our suburban house in Pennsylvania for over five decades, as long term gerontologists we moved to Massachusetts. First, we rented a house for three years; then the homeowner decided to sell and we declined to buy. Now in our 90's, we have been residents for four years of a town house in a retirement community in Massachusetts that emphasizes independence over dependency; it provides no meals or direct health care services. It is now our home and we have found that several themes persevere: (1) We adapt to the increasing presence of vulnerability in ourselves and in other older persons. (2) We try to maximize our sense of residential comfort and mastery (Golant, 2015) by asserting environmental proactivity ( Lawton , 1987). This reflects the continuity (Atchley, 1989) of our lives. (3) We recognize the ever-present complexities in our responses to personal and societal themes of ageism.

### POTENTIAL BENEFITS FROM LIVING IN A CCRC

Anne Wyatt-Brown[1], *1. University of Florida, Gainesville, Gainesville Florida, United States*

Unlike many people who hope to postpone moving until late in life, my husband and I had to move quickly because he could no longer manage the stairs in our condo. We chose a nearby CCRC where my parents had lived in the 1980s. My husband loved our choice, and gradually I adjusted to life in an institution. When he died in 2012, I had to start over again as a single woman. Fortunately, people were very kind to me, which made adjusting much easier. Other residents were equally supportive when I had breast cancer, which involved chemo and radiation. I realized recently that co-residents have taught me to make supportive comments when people tell me that they can't remember names. Acknowledging that we all have declines to deal with makes it easier for us all to accept aging's inevitable changes while using our housing community for support.

### THE FLUID COMPLEXITY OF RESIDENTIAL CHOICES IN LATER LIFE

Jaber F. Gubrium[1], *1. University of Missouri, Columbia, Missouri, United States*

Serious attention to the fluid complexity of lived experience throws intriguing light on residential choices in later life. Fluidity comes in the form of time, which shifts in both novel and patterned ways as one ages. Decisions in time not only follow well-documented patterns, but interpersonal and intersectional considerations, both retrospectively and prospectively, can alter the essential meaning and consequences of ostensible patterns in the matter. Complexity emerges when the interconnected meanings of individual variables are taken into account. Working together, fluidity and complexity turn what is otherwise well-documented into what might be called forms of "assemblage" of accountability and decision-making in residential choices.

### DO WHAT I SAY, NOT WHAT I DO? CHALLENGING CONTEMPORARY WISDOM ABOUT RESIDENCE IN OLD AGE

Graham D. Rowles[1], *1. University of Kentucky, Lexington, Kentucky, United States*

An environmental gerontologist teaching about "aging and environment" for 30 years, I had it all figured out. Downsize and divest, stair free environment, universal design, environmental centralization, location close to services. At 60, I was consulting with an architect to design an age friendly home and searching for a level plot. But then, serendipitously, we found our current home, a residence defying all of these principles. An irresponsible denial of aging and pending frailty? Thirteen years later, I'm not so sure. In this presentation, I explain why, focusing on processes of "making and remaking home" and "being in place" that have been central themes in my research career. I explore ways in which as my wife and I grow older, harnessing modern technologies, newly emergent living arrangements and contemporary communication, we anticipate being able to age in place far longer than has been possible for previous generations.

### SESSION 1190 (SYMPOSIUM)

### HOW TO PUBLISH: FROM START TO FINISH AND BEYOND

Chair: Laura P. Sands, *Virginia Tech, Blacksburg, Virginia, United States*

This symposium, organized by the Gerontological Society of America's (GSA) Publications Committee, will provide information on the publication process from the perspective of several editors of GSA's scientific journals that publish diverse types of gerontological research, basic to applied across multiple disciplines. This session is comprised of three parts including: 1) Podium presentations from editors-in-chief from GSA's The Gerontologist, Innovation in Aging (GSA's new open access journal), and Journal of Gerontology-Series B, Social Sciences. Editors will describe how to prepare your manuscript for submission, choose the right journal, and revise the manuscript for resubmission; 2) A presentation about how to assess and maximize the impact of published work given by a representative from Oxford University Press (OUP); and 3) Round table discussions with editors from the Journals of Gerontology-Series A (Medical Sciences) and B (Psychological and Social Sciences), The Gerontologist, and Innovation in Aging and OUP representatives. Editors will answer questions related to the podium presentations and other questions specific to each journal. Intended audiences include emerging and international scholars, and authors interested in learning more about best practices and tips for getting their scholarly work published. Current and future published authors will also gain information about how to leverage already published work.

### HOW TO CHOOSE THE RIGHT JOURNAL FOR YOUR MANUSCRIPT

Laura P. Sands[1], *1. Virginia Tech, Blacksburg, Virginia, United States*

Article submissions to The Gerontological Society of America's high impact scientific journals continue to increase each year which has led to editors becoming more selective about which articles are accepted for publication. The purpose of this session is to describe how to efficiently and successfully navigate the process of determining which journal is the best fit for your manuscript. The specific objectives are to: (1) increase understanding the differences in scope and features of each journal; (2) inform how to determine which journal is most appropriate for the topic and methods of your manuscript; and (3) describe how to convey the scientific contribution of your article to the journal.

### GETTING OVER THE FIRST HURDLE: PREPARING YOUR MANUSCRIPT FOR A POSITIVE REVIEW

Suzanne Meeks[1], *1. University of Louisville, Louisville, Kentucky, United States*

In this section of the symposium, I will talk about manuscript preparation for maximizing the likelihood that your work will be sent for review. I will talk about common author errors that usually guarantee an immediate reject decision such as not reading and following the Instructions to Authors. I will emphasize the importance of plain, good writing. Editors of high impact journals receive 10 or more new manuscripts per week, and due to limited page space, have to reject 80-90% of them. Regardless of scholarly quality, if the point and contribution are not clear in a quick scan of the paper, it likely will not be reviewed favorably. I will provide tips for strong scientific writing that are commonly violated in manuscript submissions, and provide references for additional writing support.

### HOW TO SUCCESSFULLY NAVIGATE A REVISE-AND-RESUBMIT DECISION AND HANDLE REJECTIONS

Deborah Carr[1], *1. Boston University, Boston, Massachusetts, United States*

Anyone who has ever submitted a manuscript to a peer-review journal has experienced rejection. Rejection can be demoralizing, for emerging and senior scholars alike, but is especially so for those on the job market and tenure-track. This presentation will explain the manuscript review process and suggest strategies for addressing decisions that include: (1) rejection without peer review (desk reject); (2) revise and resubmit invitations; and (3) rejection after peer review. Fixable problems (e.g., editorial, conceptual, minor methodological issues) will be distinguished from non-fixable problems (e.g., small sample size, flawed design). Advice will focus on how to successfully revise and resubmit a manuscript; how to write an effective revision memo; how to develop a "plan B" for a rejected article; and how to manage the emotional sting of rejection.

### HOW TO MAXIMIZE THE REACH OF YOUR PUBLISHED WORK

Sara McNamara[1], *1. Oxford University Press, New York, New York, United States*

Part 2 of this symposium will discuss how to maximize the reach and impact of your published work. Researchers are under increasing pressure to demonstrate the impact of their work. First, it is important to clearly articulate the translational impact of your work in the Discussion of your manuscript and briefly in the Abstract. In addition, it is important to know how to increase visibility of your manuscript because university administrators and funding agencies rely heavily on numerical metrics for decision-making. This presentation will look at the needs that metrics serve and provide an overview of how the dominant metric, the Impact Factor, is determined (along with a discussion of the metric's flaws). The value of newer methodologies and alternative metrics (Altmetrics) will also be discussed. The presentation will also cover practical steps for how researchers can increase the reach and potential impact of their work.

### SESSION 1195 (SYMPOSIUM)

### I-CONECT PROJECT: CAN SOCIAL INTERACTION IMPROVE COGNITIVE FUNCTIONS AMONG SOCIALLY ISOLATED OLDER ADULTS?

Chair: Hiroko H. Dodge, *University of Michigan, Ann Arbor, Michigan, United States*
Co-Chair: Karen Hooker, *Oregon State University, Corvallis, Oregon, United States*
Discussant: Toni C. Antonucci, *University of Michigan, Ann Arbor, Michigan, United States*

Epidemiological studies have demonstrated that larger social networks or more frequent social interactions may have protective effects against cognitive decline and the incidence of dementia. Therefore, increasing social interaction could be a promising intervention for improving cognitive well-being in socially isolated older adults. We have conducted multiple NIH-funded randomized controlled trials (RCT) over 10 years, examining whether conversational interactions through webcam and internet can improve cognitive functions and enhance cognitive reserve. In this symposium, we will introduce this series of behavioral intervention trials through 4 presentations. First, Dodge will provide background and results of their previous RCT where they showed efficacy of conversational intervention on domain-specific cognitive functions and introduce the ongoing larger project called I-CONECT (https://www.i-conect.org). Second, Lindsey will introduce technological innovations used in the I-CONECT project including development of user-friendly video-chat devices, recording of audio and video data and innovative recruitment efforts. Third, Asgari will share results on how speech utterance and characteristics collected through the project could distinguish those with mild cognitive impairment from those with normal cognition using machine learning modelling approaches. Finally, Cerino and his team will show results of the study which examined whether cognitive improvements through conversation-based intervention depend on an individual's personality, laying the groundwork for a personalized intervention trial in the future. The symposium is of interest for those who study social isolation and its prevention, the link among cognition, social isolation and personality, as well as those who focus on technology as a tool for improving well-being of older adults.

### I-CONECT: CHALLENGES AND OPPORTUNITIES

Hiroko H. Dodge,[1] Jeffrey Kaye,[1] Elena Goodrich,[1] Jacob Lindsley,[1] Mattie MacDonald,[1] Nora Mattek,[1] Meysam Asgari,[1] and Lisa Silbert[2], *1. University of Michigan, Ann Arbor, Michigan, United States, 2. Oregon Health & Science University, Portland, Oregon, United States*

In our previous NIH-funded randomized controlled behavioral clinical trial, we developed a conversation-based social interaction cognitive stimulation protocol delivered by trained interviewers through webcams and a user-friendly interactive Internet interface. Daily 30 minute face-to-face video-chats were conducted for 6 weeks. Despite a short duration, this proof of concept study demonstrated high adherence among older adults (mean age 80 years) and showed improvement in cognitive domains which tap language-based executive functions and semantic memory among the experimental group compared to the control group who did not engage in any video-chats. Building on these results, we are now conducting two NIH-funded projects (https://www.i-conect.org), targeting socially isolated older adults who are less likely to participate in clinical trials despite their high risk of cognitive decline. In this presentation, we introduce a series of projects outlined above and share the challenges and opportunities identified in our behavioral intervention trials focused on social interaction.

### LINGUISTIC MEASURES OF SPOKEN UTTERANCES FOR DETECTING MILD COGNITIVE IMPAIRMENT

Meysam Asgari,[1] Jeffrey Kaye,[1] and Hiroko Dodge[1], *1. Oregon Health & Science University, Portland, Oregon, United States*

Studies have shown that speech characteristics can aid in early-identification of those with mild cognitive impairment (MCI). We performed a linguistic analysis on spoken utterances of 41 participants (15 MCI, 26 healthy controls)

from conversations with a trained interviewer using the Term Frequency-Inverse Document Frequency (TF-IDF) method. Data came from a randomized controlled behavioral clinical trial (ClinicalTrials.gov: NCT01571427) to examine effects of conversation-based cognitive stimulation on cognitive functions among older adults with normal cognition or MCI, which served as a pilot study for I-CONECT. From the collected spoken utterances we first constructed a fixed-dimensional feature vector using TF-IDF. Next, to distinguish between MCI and healthy controls, we trained a support vector machine (SVM) classifier on per-subject feature vectors according to 5-fold cross-validation procedure. Our results verify the effectiveness of TF-IDF features in this classification task with Receiver Operating Characteristic Area Under Curve of 81%, well above chance at 65%.

### OVERCOMING THE CHALLENGES OF TECHNOLOGY IN GERONTOLOGICAL RESEARCH

Jacob Lindsey,[1] Elena Goodrich,[1] Khoa Nguyen,[1] Cierra Leon Guerrero,[1] Colton Scavone,[1] Supriya Pandya,[1] Tanner Dorsey,[1] and Hiroko Dodge[1], *1. Oregon Health & Science University, Portland, Oregon, United States*

Many older adults express a lack of confidence in using technology and this can become a barrier to participating in technology heavy research. This presentation will introduce the face-to-face digital communication, electronic medication adherence tracking, and online recruitment technology used in the I-CONECT study (ClinicalTrials.gov: NCT02871921). In particular, we will demonstrate how the project has simplified enterprise video conferencing for in-home use, removed obstacles related to remote hardware troubleshooting, and further discuss how it has been received by older adults who have participated thus far. Finally we will cover particular hurdles related to I-CONECT (e.g., targeting social isolated older adults, aiming to recruit 50% of participants being African American older subjects living in the Detroit Metropolitan area). Our experience indicates that such high tech gerontological research is feasible given a creative and solution-focused research approaches and multi-disciplinary team.

### PERSONALITY MODERATES INTERVENTION EFFECTS ON COGNITIVE FUNCTION: A 6-WEEK CONVERSATION-BASED INTERVENTION

Eric S. Cerino,[1] Karen Hooker,[2] Elena Goodrich,[3] and Hiroko H. Dodge[4], *1. Oregon State University, Corvallis, United States, 2. Oregon state University, Corvallis, Oregon, United States, 3. Layton Aging and Alzheimer's Disease Center, Oregon Health and Science University, Portland, Oregon, United States, 4. Department of Neurology, Layton Aging and Alzheimer's Disease Center, Oregon Health and Science University, Portland, Oregon, United States*

Conversation-based interventions have positive effects on cognitive health, though determining who benefits most is still unclear, and individuals' personality may play a role. We utilized data from a 6-week randomized controlled trial to determine if conversation-based intervention effects were moderated by personality traits in 83 older adults (Mean age = 80.51 years, 49 cognitively intact, 34 with mild cognitive impairment). The intervention group participated in daily 30-minute face-to-face semi-structured conversations with trained interviewers for six weeks. Baseline psychosocial questionnaires and a neuropsychological battery were completed. Intervention group participants with high agreeableness, conscientiousness, and extraversion exhibited significantly more improvement in language-based executive function tasks compared to a control group (ps<.05). An opposite pattern for delayed recall memory and working memory tasks emerged among highly extraverted participants (ps<.05). Findings suggest the adaptive role of personality traits in conversation-based cognitive interventions and offer evidence for personalized approaches to cognitive health in late life.

### SESSION 1200 (SYMPOSIUM)

### INEQUALITIES IN FRAILTY BY SOCIO-ECONOMIC POSITION: IT'S ALL IN THE TIMING

Chair: Carol Jagger, *Newcastle University, Newcastle upon Tyne, United Kingdom*
Discussant: Mark D. Hayward, *University of Texas at Austin, Austin, Texas, United States*

A number of studies have found that higher socio-economic position (SEP) appears protective of becoming frail. However, not only can SEP be defined in early, mid or late life, by education, occupational status or income/material disadvantage respectively, but frailty may occur in the young old as well as the very old. Do the same measures of SEP reflect inequalities in frailty in the young old as the very old? Does it matter when in the lifecourse SEP is measured? Have inequalities in frailty between SEP groups changed across the generations of older people? We seek to answer such questions from cohorts across the spectrum of later life. The first presentation, from the 1958 British Birth Cohort Study, examines the association between early-life SEP and frailty at age 50 and whether this association is due to continued disadvantage into mid-life. The second presentation moves to very old age, examining the role of early, mid and late life disadvantage on the progression of frailty between ages 85 and 90 in the Newcastle 85+ cohort. The third presentation, based on the electronic health records of adults aged 75 years and over in England, focuses on whether SEP modifies frailty trajectories in the last year of life. The final presentation examines whether SEP inequalities in frailty have changed over different generations of older people, and utilises data from the Cognitive Function and Ageing Studies. Together these presentations increase understanding of which SEP groups should be targeted for interventions to reduce frailty throughout later life.

### SOCIOECONOMIC INEQUALITIES IN TRANSITIONS BETWEEN FRAILTY STATES IN THE VERY OLD: THE NEWCASTLE 85+ STUDY

Carol Jagger,[1] Nuno Mendonca,[2] Andrew Kingston,[2] Louise Robinson,[2] Mohammad E. Yadegarfar,[3] Helen Hanson,[4] and Rachel Duncan[2], *1. Newcastle University, Newcastle upon Tyne, United Kingdom, 2. Newcastle University, Newcastle upon Tyne, England, United Kingdom, 3. University of Leeds, Leeds, England, United Kingdom, 4. Institute of Cellular Medicine, Newcastle upon Tyne, England, United Kingdom*

Early-life socio-economic position (SEP), defined by education, remains a significant factor in disability progression in very old age, but there is less evidence for its effect on frailty progression. We used the Newcastle 85+ Study, a longitudinal cohort of people born in 1921 and aged 85 at first assessment in 2006, and followed up at 18, 36, and 60 months. Of the 845 participants at baseline, the Fried frailty status (FFS) was available for 696 participants at baseline of whom 60% (n=414) were women. The effects of SEP in early, mid (occupation) and late-life (area deprivation) on frailty transitions between age 85 and 90 were investigated in multi-state models. We found no significant effect of education on any frailty transitions. However those living in less deprived areas were less likely to die when frail (HR: 0.60, 95%CI: 0.40-0.89), and this remained significant after further adjustment for education and morbidity count.

### EARLY-LIFE SOCIOECONOMIC POSITION AND ACCUMULATION OF HEALTH-RELATED DEFICITS IN MIDLIFE

Nina Rogers,[1] Chris Power,[2] and Snehal P. Pereira[2], *1. University College London, London, United Kingdom, 2. University College London, London, England, United Kingdom*

Improved understanding of predictors of frailty is key to delaying its onset. Yet, few studies have examined whether early-life socioeconomic position (SEP) predicts frailty in midlife. In the 1958 British Birth Cohort (n=7601), we examined (i) associations between early-life SEP and frailty at 50y and (ii) whether associations were due to continuities in disadvantage into mid-adulthood. Frailty was measured using an index composed of 37 health-deficits. Associations between early-life SEP and frailty were examined using linear regression. Lower early-life SEP was associated with higher frailty, e.g. compared to professional/managerial class, the frailty index was higher by 3.09% (95% CI:-0.65%, 6.84%) for skilled non-manual, 10.8% (8.20%, 13.4%) for skilled manual and 14.2% (11.1%, 17.2%) for partly skilled/unskilled. After adjustment for adult disadvantage, the trend remained, albeit weaker. Findings suggest that interventions in mid-adulthood targeted to those exposed to early-life disadvantage could reduce the risk of developing frailty when entering later life.

### HOW DOES SOCIAL DEPRIVATION INFLUENCE END-OF-LIFE FRAILTY TRAJECTORIES? EVIDENCE FROM ELECTRONIC HEALTH RECORDS

Daniel Stow,[1] Barbara Hanratty,[2] and Fiona E. Matthews[2], *1. Institute of Health and Society, Newcastle upon Tyne, United Kingdom, 2. Newcastle University, Newcastle Upon Tyne, England, United Kingdom*

Recent research has highlighted inequalities in frailty status, driven by social deprivation over the life course. However, little is known about the interaction between deprivation and frailty over clinically relevant timescales. We used an electronic frailty index, generated automatically in health records at monthly intervals for one year in 13,000 people age >75 who died (cases), matched to 13,000 people with no record of death (controls). We used Index of Multiple Deprivation deciles and latent growth curve models to investigate the impact of deprivation on frailty trajectories. Greater deprivation was associated with higher baseline frailty, but did not influence the rate of change in frailty. We observed greater deprivation driven differences in frailty at baseline in controls than in cases, suggesting that a degree of convergence occurs as people near end-of-life. We discuss these results in light of end-of-life care policy and planning.

### SOCIO-DETERMINANTS OF FRAILTY: HAVE THEY CHANGED OVER TIME?

Fiona Matthews,[1] andria Mousa,[2] and Carol Jagger[3], *1. Newcastle University, Newcastle, United Kingdom, 2. Department of Infectious Diseases, London, England, United Kingdom, 3. Newcastle University, Newcastle, England, United Kingdom*

Socioeconomic inequalities are important drivers of negative health outcomes. This study investigates the effect of social determinants on health using frailty from two studies 20-year apart and examines whether socio-economic differences are widening. A 30-item deficit accumulation score from the baseline data from the two Cognitive Function and Ageing Studies, 1991 (n=7,635) and 2011 (n=7,762) was calculated. For each of the two cohorts separately, binomial regression investigated the relationship between frailty and social determinants. Deprivation was most strongly associated with frailty among the other social determinants. The effect of high deprivation, when compared the lowest tertile, increased in CFAS II (CFAS I: RR=1.21, 95%CI:1.11-1.31; CFAS II: RR=1.47(95%CI: 1.36-1.59). Inequalities in frailty have increased over time, particularly in terms of deprivation. This has important implications for health policy as focusing policies including specific interventions and resources in more deprived areas could greatly improve health outcomes for those areas.

### SESSION 1205 (SYMPOSIUM)

### LEVERAGING COMMUNITIES AS NETWORKS TO REDUCE MALNUTRITION IN OLDER ADULTS

Chair: Tina Sadarangani, *New York University Rory Meyers College of Nursing, New York, New York, United States*
Co-Chair: Jeannette Beasley, *New York University Langone Health, New York, New York, United States*
Discussant: Shannon E. Jarrott, *The Ohio State University, Columbus, Ohio, United States*

Malnutrition in older adults, while ubiquitous, remains largely underrecognized and undertreated. In community-dwelling older adults, 25% of those at risk of over or under nutrition do not receive any dietary interventions; routine screenings for malnutrition are not typically required in community-based settings. In this interdisciplinary symposium, we explore issues focused on the delivery of evidence-based nutrition interventions to meet the needs of community-dwelling older adults. Using national survey data, we begin by underscoring the importance of treating the complex needs of adults at risk of malnutrition by examining health sequelae, specifically hospitalizations, in community-dwelling adults receiving home-delivered meals. We subsequently examine approaches to malnutrition screening in community-based settings, focusing on the utility of the DETERMINE checklist. We explore barriers and facilitators of providing person-centered nutrition to ethnically diverse

Asian American older adults in the adult day healthcare setting. Finally, we shift our focus to overnutrition, discussing the dissemination of a telehealth diabetes prevention program, BRInging the Diabetes prevention program to GEriatric populations (BRIDGE) among older adult meal program recipients. Older adults in community-based health settings are at risk of malnutrition, and among them, those who are prone to social isolation, are at highest risk for adverse outcomes. While congregate settings can facilitate social interaction, honoring food preferences and facilitating choice to address undernutrition, is challenging. Conversely, telehealth interventions may present a feasible approach for addressing overnutrition. We conclude by discussing how current and future research can inform innovative person-centered community-based approaches to identify and treat malnutrition.

### BARRIERS AND FACILITATORS TO DELIVERING PERSON-CENTERED NUTRITION FOR ASIAN AMERICANS IN ADULT DAY HEALTH SETTINGS

Tina Sadarangani,[1] Chau Trinh-Shevrin,[2] and Abraham Brody[1], *1. New York University Rory Meyers College of Nursing, New York, New York, United States, 2. New York university School of Medicine, New York, New York, United States*

Malnutrition is a growing problem in community-based long-term care settings. Delivering person-centered nutrition is particularly important in congregate settings serving ethnically diverse older adults who have strong culturally-derived preferences around food. We conducted in-depth semi-structured multi-stakeholder interviews (N = 13) in an adult day health center (ADHC) serving Asian immigrants to explore the ADHC's capacity to deliver person-centered nutrition interventions. Thematic analysis showed ADHCs successfully promoted social interaction at mealtime. However, participants had limited choice and restrictions on additives, like sodium, making it difficult to honor participants' cultural preferences. Lack of flavor, limited choice, and rushed mealtimes, driven by center policies and procedures, disproportionately affected persons with dementia. Among those with dementia, clinicians disagreed whether nutrition should be used to manage chronic illness or whether a more palliative approach was warranted. One potential way to address this challenge would be to enable greater choice within a supportive ADHC mealtime environment.

### PARTNERING WITH NUTRITION SERVICES PROGRAM PROVIDERS TO DISSEMINATE EVIDENCE-BASED PROGRAMS USING TELE-HEALTH

Jeannette Beasley,[1] Judith Wylie-Rosett,[2] Mary Ann Sevick,[1] Laura DeLuca,[3] and Joshua Chodosh[1], *1. New York University Langone Health, New York, New York, United States, 2. Albert Einstein College of Medicine, Bronx, New York, United States, 3. Yeshiva University, Bronx, New York, United States*

Among adults ≥ age 65, 48% have prediabetes and are eligible to participate in the Medicare-covered Diabetes Prevention Program (DPP). We conducted a six-week pilot study to evaluate the feasibility and acceptability of a telehealth-adapted DPP for Nutrition Services Program (NSP) older adult meal program recipients. We enrolled NSP recipients (n=16) from a New York City senior center. These DPP participants attended weekly interactive DPP webinars and completed questionnaires covering lifestyle, physical activity, quality of life, and food records, and wore physical activity trackers. Retention was 75%; attendance averaged 80%; and weight loss was 2.9% (p=0.001). Our six-week pilot data suggest that a tele-adapted DPP intervention can achieve the Medicare reimbursement goals for attendance and 5% weight loss. We are surveying NSP recipients, who receive home-delivered meals, to evaluate the acceptability and feasibility of conducting a larger scale tele-adapted DPP intervention trial among NSP participants.

### MENTAL HEALTH AND HUNGER: RISK FACTORS FOR HOSPITALIZATION AMONG HOME-DELIVERED MEAL PARTICIPANTS

Lisa Juckett[1], *1. The Ohio State University, Columbus, Ohio, United States*

Home-delivered meals (HDMs) provided through the Older Americans Act (OAA) are intended to reduce hunger, promote socialization, and maximize wellness. However, HDM recipients are at increased likelihood of being hospitalized due to their complex health needs and risk for social isolation and depression. Drawing data from the OAA-HDM National Survey, we evaluated the predictors of hospitalization among HDM recipients in 2017. From our sample (n = 578), we conducted random forest classification analyses to identify the most important risk factors related to HDM recipient hospitalization. Our random forest model yielded an accuracy rate of 66.3% with risk factors most indicative of hospitalization being attributed to number of co-morbidities, depressive symptoms, and feelings of social isolation. These findings indicate that although HDMs may help alleviate hunger among older adults, innovate strategies are warranted to address the unmet mental health needs of HDM recipients.

### ASSESSING NUTRITIONAL RISK IN ADULT DAY SERVICES: UTILITY OF THE DETERMINE CHECKLIST

Shannon E. Jarrott[1], *1. The Ohio State University, Columbus, Ohio, United States*

Older adults attending adult day services (ADS) often possess risk factors for malnutrition, such as chronic disease, physical disability, and cognitive impairment. We explored the utility of administering to ADS participants the DETERMINE Checklist - a measure of nutritional status. Among eleven participants (M age=77.3 years), 82% (n = 9) presented high nutritional risk. The three most common risk factors were: difficulty shopping, cooking, and/or feeding themselves (100%), making health-related dietary changes (63.7%), and taking three or more daily medications (63.7%). Our preliminary findings indicate that ADS participants may be at moderate-high risk of malnutrition; however, the DETERMINE Checklist may require modification for an ADS population. For example, the checklist may be more reliable if completed jointly by a participant and informal caregiver. We present recommendations for adaptions based on our pilot data as well as implications for ADS staff and clinicians.

### SESSION 1210 (SYMPOSIUM)

### LINKING ETHNIC MINORITY ELDERS TO PROGRAMS AND SERVICES: CHALLENGES TO BUILDING NETWORKS

Chair: Allen Glicksman, *Philadelphia Corporation for Aging, Philadelphia, Pennsylvania, United States*
Co-Chair: Lauren Ring, *Philadelphia Corporation for Aging, Philadelphia, Pennsylvania, United States*
Discussant: Sandra Torres, *Uppsala Universitet, UPPSALA, Sweden*

Building networks that are effective in linking older adults to supportive programs and services often involves challenges related to access, eligibility requirements, the elder's ability to understand enrollment processes, and lack of trust in service providers. For ethnic minority elders these challenges are often greater due to additional linguistic and cultural barriers. The four presentations on this panel address challenges to building effective service networks for ethnic minority elders using data derived from focus groups with members of these communities and those tasked with providing their care. The first presentation (Graham and Tseng) examines the Village model, a model designed to empower older adults, and asks why more Latino, African American and Asian elders do not participate. The second paper (Ågård) looks at communication difficulties as a source for understanding the nature of cross-cultural discussions around end of life issues with ethnic minority patients. The third paper (Ajrouch, Janevic, and Antonucci) explores how caregiving programs for Alzheimer's Disease patients can be modified to better serve Arabic speaking caregivers. The final paper (Ring, Liebman, Glicksman and Rodriguez) uses data collected among Spanish and Chinese (Mandarin) speaking elders to design a conceptual model which describes how ethnic minority and other elderly navigate the Long Term Care Services and Supports network. Our respondent will place these papers within the growing theoretical work on diversity and care support.

### BARRIERS TO VILLAGE MEMBERSHIP AMONG MINORITY SENIORS

Carrie Graham,[1] and Winston Tseng[2], *1. University of California, Berkeley, California, United States, 2. University of California, Berkeley, Berkeley, California, United States*

Villages are a relatively new consumer-driven model that promotes aging in place for community-dwelling seniors. Villages promote social engagement, civic engagement, member-to-member-support, and collectively bargain for services of their members. Members report improved social support and more confidence aging in their own homes. Currently, there are over 200 operational villages nationwide and the model is proliferating rapidly. Most Villages members are white, well-educated, and well resourced. Researchers at UC Berkeley conducted 6 focus groups with Latino, African American and Asian seniors (N=58) who have not joined Villages in their regions. Focus group findings describe a lack of awareness of the Village model among underrepresented groups; and barriers to membership including the cost of membership, lack of language inclusion, and lack of diversity. The national anti-immigrant discourse emerged as a barrier to membership for non-white seniors. Participants describe how Villages could make programmatic changes to attract a more diverse membership.

### EXPLORING HOW CARE PROVIDERS TALK ABOUT COMMUNICATION DIFFICULTIES WITH ETHNIC MINORITY PATIENTS

Pernilla Ågård[1], *1. Deparment of Sociology, Uppsala University, Uppsala, Sweden*

Previous research into cross-cultural interactions in health care settings shows that care providers experience communicating with elderly ethnic minority patients as problematic. According to the social constructionist framework upon which this presentation draws, people negotiate the characteristics they ascribe to the world around them through talk. It is against this backdrop that the presentation – which is based on a focus group study with end-of-life care providers (n=60) – sets out to explore how care providers talk about communication difficulties with elderly ethnic minority patients. The presentation demonstrates how the study of communication difficulties can illustrative the challenges of cross-cultural interaction in end-of-life care settings. Through the attention on how communication difficulties are discussed, this presentation shifts the focus from the elderly ethnic minority patients and the reasons for why they are experienced as problematic when it comes to communication, to the actual process where these problems are negotiated.

### A FAMILY-CENTERED APPROACH TO DEMENTIA CAREGIVING AMONG ARAB AMERICANS

Kristine Ajrouch,[1] Kristine Ajrouch,[1]
Mary Janevic,[2] and Toni C. Antonucci[2], *1. Eastern Michigan University, Ypsilanti, Michigan, United States, 2. University of Michigan, Ann Arbor, Michigan, United States*

This paper presents the process by which we adapted an existing Alzheimer's Disease and Related Dementia (ADRD) caregiver support intervention that is directed at multiple family caregivers and culturally-responsive to the needs of the Arab American community. Three focus group discussions with Arab American families caring for a family member with ADRD were organized in partnership with the Arab Community Center for Economic and Social Services (ACCESS). Focus group discussions included two members from each family to gather data on needs of Arab American ADRD caregivers, role of family in caregiving and use of technology for caregiving information. Results underscored the lack of and desire for knowledge around ADRD, and the perception by Arab Americans that they differ from non-Arab Americans in approaches to caregiving (e.g., person with ADRD often moves from one child's house to another). These data confirm the need for caregiving interventions responsive to Arab American needs/preferences.

### CREATING NEW MODELS FOR AGING IN PLACE: DIVERSE COMMUNITIES AND THE NAVIGATION OF LONG-TERM SERVICES AND SUPPORTS

Lauren Ring,[1] Michael Liebman,[2] Allen Glicksman,[1] and Misha Rodriguez[3], *1. Philadelphia Corporation for Aging, Philadelphia, Pennsylvania, United States, 2. IPQ Analytics, LLC, Kennett Square, Pennsylvania, United States, 3. Asociación Puertorriqueños en Marcha, Philadelphia, Pennsylvania, United States*

There is a growing interest among aging services providers to better understand the pathways through which older adults and their caregivers navigate LTSS. Although there have been attempts at modeling this process they are often dependent on the quality of existing data, which can result in models which are incomplete and study samples that homogenize diverse older adult populations. These models face two challenges – 1) the data may not include information about important elements of the LTSS navigation process, and 2) the actions of ethnic/cultural sub-groups may not be captured. This study uses a conceptual method called Social Interaction Modeling (SIM) to examine how older adults in two limited English-speaking communities (Spanish / Mandarin Chinese) navigate the use of LTSS and to evaluate disparities in service access. The findings will help to build a more comprehensive model which looks at service navigation among all older adults in Philadelphia.

### SESSION 1215 (SYMPOSIUM)

### MARGRET M. AND PAUL B. BALTES FOUNDATION IN BEHAVIORAL AND SOCIAL GERONTOLOGY AWARD LECTURE

Chair: Nancy Pedersen, *Karolinska Institutet, Stockholm, Sweden*

The Margret M. and Paul B. Baltes Foundation Award in Behavioral and Social Gerontology recognizes outstanding early-career contributions in behavioral and social gerontology. The lecture will be given by the 2018 Baltes Award recipient, Frank Infurna, PhD, of Arizona State University. This session will also include the presentation of the 2019 Baltes Award. The 2019 Baltes Award recipient is Allison Bielak, PhD, of Colorado State University. Supported by the Margret M. and Paul B. Baltes Foundation.

### UTILIZING PRINCIPLES OF LIFESPAN DEVELOPMENTAL PSYCHOLOGY TO EXAMINE RESILIENCE TO ADVERSITY

Frank J. Infurna[1], *1. Arizona State University, Tempe, Arizona, United States*

Lifespan developmental psychology has many guiding principles. My past, current, and future research has been influenced by the lifespan developmental principles of multidimensionality and multidirectionality and the premise that development is not entirely bound to chronological age, but to historical-, contextual-, non-normative event-, pathology- and mortality-related processes. The first part of my talk will discuss ways I have utilized these principles in my research examining individual's ability to be resilient to diverse types of adversities (cancer diagnosis, unemployment, and bereavement) and resources they are drawing on to promote more positive outcomes, such as social support. The second part of my talk will focus on future research directions that aim to advance the conceptual and methodological significance of this research. Future directions include explicitly studying individuals in midlife, inclusion of outcomes beyond that of mental health and well-being, such as character strengths, and longitudinal research designs that assess people more frequently.

### SESSION 1220 (SYMPOSIUM)

### MEDICAID MANAGED LONG-TERM CARE PROGRAMS VS. THE TRADITIONAL MODEL: COMPARATIVE COSTS AND OUTCOMES

Chair: Larry Polivka, *Florida State University, Tallahassee, Florida, United States*

Several states have adopted Medicaid Managed Long-Term Care (MLTC) programs over the last several years. At this point at least 30 states are either administering such models or have plans to in the near future. We do not, however, know much yet about the relative cost-effectiveness of the MLTC model when compared to the traditional non-profit model of Medicaid LTC. Is the for-profit MLTC model actually generating savings in the Medicaid program while improving the quality of care? This symposia is designed to address the question through three presentations on experiences with MLTC programs in the states of Ohio, Texas and Pennsylvania and a fourth presentation offering a national overview and critique of Medicaid MLTC in comparison to the traditional Medicaid LTC program still administered through non-profit Aging Network organizations. The state focused presentations describe the current status and results of MLTC in three states that vary in their specific features, extent of formal accountability for outcomes and the political contexts in which the programs currently function. The presentations also include discussions of the implications of each states experiences for the future of Medicaid LTC policy at the state and federal levels. The fourth presentation is a critical analysis of the main differences between the traditional non-profit model of Medicaid LTC services and the for-profit MLTC programs in terms of commonly accepted criteria of cost-effective LTC services, such as access, quality of care and per-person costs and differences in the roles of advocacy and accountability.

### MEDICAID-MANAGED LTC: A NATIONAL OVERVIEW

Larry Polivka[1], *1. Florida State University, Tallahassee, Florida, United States*

Most of the states now have Medicaid LTC programs administered by corporate HMOs. Several states, however, still have programs administered by non-profit community based organizations, most of which are members of long standing Aging Networks which grew out of the Older Americans Act in the 1970s. This paper will offer a comparative overview of these models of LTC administration including a typology designed to identify major cultural and political differences between the states with and without corporate managed LTC models and an analysis of the available information regarding their comparative costs and outcomes, mainly access to care and quality of care. The paper will conclude with an assessment of the implications of the information presented for the future of Medicaid LTC policy and politics at the state and federal levels and for the future of LTC advocacy and accountability across the states.

### INTEGRATING ACUTE AND LONG-TERM SERVICES: DOES IT WORK, CAN IT WORK, WILL IT WORK?

Robert A. Applebaum,[1] and Jennifer Heston[2], *1. Miami University, Scripps Gerontology Center, Oxford, Ohio, United States, 2. Miami University Scripps Geron, OXFORD, Ohio, United States*

The expansion of managed long-term services and supports has generated considerable interest over the last decade. However, studies on the impact of these efforts have produced mixed findings. Additionally, there is limited information about the care management models used in implementation. This lack of data makes it impossible to assess whether differences in managed care plan approaches have an impact on participants. Our study sought to gain better understanding of the integrated care management models being implemented in Ohio's MyCare Demonstration. Through qualitative interviews with 50 respondents, including area agency care managers, managed care staff, and service providers, we documented strengths and weaknesses of one integrated care management model used in Ohio's demonstration. Understanding what is inside the black box of managed care/care management model implementation is key to gaining insights into whether such an approach can ultimately improve the health and long-term service systems for older people with disability.

### MANDATORY MEDICAID MANAGED LONG-TERM SERVICES AND SUPPORTS IN PENNSYLVANIA: EVALUATING POLICY CHANGE

Howard Degenholtz[1], *1. University of Pittsburgh, Pittsburgh, Pennsylvania, United States*

The Commonwealth of Pennsylvania is implementing a mandatory Medicaid managed Long-Term Services and Supports (LTSS) program that covers people age 21 and older who are fully eligibly for both Medicare and Medicaid, living in a nursing facility paid for by Medicaid, or in an aged or physical disability home and community based services (HCBS) waiver. The overall program goals are to: Enhance Opportunities for Community Living; Improve Service Coordination; Enhance Quality and Accountability; Advance Program and Innovation; and Increase Efficiency. The program will be administered by 3 managed care organizations (MCOs) that are obligated to coordinate with Medicare Advantage and D-SNP plans. This major policy change affects the traditional roles and responsibilities of the aging network by shifting the locus of control to insurance companies. This presentation will describe the policy change, the implications for the aging network, and the multi-method evaluation designed to assess the implementation and outcomes.

### SESSION 1225 (SYMPOSIUM)

### NOTES FROM THE FIELD: TAPPING INTO RESILIENCE THROUGH REFLECTIVE WRITING

Chair: Holly M. Holmes, *UT Houston McGovern Medical School, Houston, Texas, United States*
Discussant: Thomas R. Cole, *UT Houston McGovern Medical School, Houston, Texas, United States*

Reflective writing is a powerful tool that can help healthcare providers address burnout and access inner strength. We describe the formation and functioning of a writing group for palliative care and geriatrics physicians at McGovern Medical School. The group has served several functions, including the promotion professional and personal growth. Using reflective writing and prompts, our group has explored issues of compassion, caregiving, grief, and loss. Group writing has provided a safe space for processing and letting go of professional and personal stressors related to caring for patients and to demands in our daily lives. The positive impact of the writing group has extended to caregiving and to other writing, including technical writing. This symposium is designed for healthcare professionals in all disciplines who are interested in exploring the use of reflective writing in regular practice.

### EXPERIENCES IN CREATING A WRITING GROUP

Renee J. Flores,[1] and Nahid J. Rianon[1], *1. UT Houston McGovern Medical School, Houston, Texas, United States*

Writing and publication in an academic setting is vital for advancing careers and knowledge. Attempting to increase scholarly productivity, our division created a physician-writing group, led by a prolific humanities expert to hone our writing skills. An unexpected outcome was realized. Using a mix of reflective, intent-driven, impromptu writing exercises and group sharing we discovered new opportunities for personal and professional growth through empathy. During these 1-hour sessions, several organic themes emerged. These included gaining greater inner-personal insight and recognizing inter-personal similarities in career paths and provider benevolence as motivation to continue when experiencing emotional fatigue and burnout. Ultimately, while honing our professional writing skills we also stimulated compassion to ourselves and our colleagues, opening new sources of resilience. We plan to continue these sessions exploring the potential multifaceted impacts on professional/academic growth these sorts of writing groups can have for geriatric and palliative medicine professionals and other healthcare providers.

### USING REFLECTIVE WRITING TO PROMOTE RESILIENCE

Ana L. Leech,[1] and Anson J. Koshy[1], *1. UT Houston McGovern Medical School, Houston, Texas, United States*

Patients invite us, rather reluctantly sometimes, into their lives during some of their most difficult moments; these encounters change us even if it is not readily apparent. Gathering for this writing group, we have found that creating a permanent record of our deepest thoughts and feelings enables further analysis and discussion in a collegial and supportive environment. In this paper, I relate one clinical story as an example. I will never forget caring for Mr. R. during what I can only call an ordeal, but writing it down, exploring my chosen words, and sharing them with the team brought validation and closure to what would have been a deep wound in my soul. The scar remains, being remodeled by time, experience, and other wounds, softened through writing and sharing, becoming part of me. We explore one way in which supportive writing groups can foster professional relationships and growth.

### SESSION 1230 (SYMPOSIUM)

### POLICY SERIES: OLDER AMERICANS ACT: REAUTHORIZATION PROCESS AND OUTCOMES

Chair: Brian W. Lindberg, *The Gerontological Society of America, Washington, District of Columbia, United States*

Discussant: Patricia M. D'Antonio, *The Gerontological Society of America, Washington, District of Columbia, United States*

The Older Americans Act (OAA) reauthorization looks like the perfect bipartisan bill supporting older adults to move forward in 2019. This session provides an DC-insiders look at the process and outcomes, including analysis of proposed revisions to the law, and the roles of the House, Senate, and the Administration. Key players in the process make up the panel and will share their insights and predications on the Act's future.

### OAA AND THE GOAL TO END MALNUTRITION

Robert Blancato[1], *1. Matz, Blancato & Associates, Washington, District of Columbia, United States*

Home delivered and congregate meals programs are a foundation part of the OAA and are a part of each reauthorization process.

### THE AGING NETWORK AND THE OAA REAUTHORIZATION

Amy Gotwals[1], *1. National Association of Area Agencies on Aging, Washington, District of Columbia, United States*

Area Agencies on Aging and the aging network are the key service organizers and providers across the nation. This session will address the goals of the aging network for the OAA reauthorization and the trends and innovations in community-based service delivery.

### FAMILY CAREGIVING AND THE OLDER AMERICANS ACT

C. Grace Whiting[1], *1. National Alliance for Caregiving, Bethesda, Maryland, United States*

This session will focus on the challenges faced by millions of family caregivers and the innovative approaches that the OAA and other programs are using to be supportive of caregivers.

### ADVANCED ILLNESS AND THE AGING NETWORK

Andrew MacPherson[1], *1. Coalition to End Social Isolation & Loneliness, Washington, District of Columbia, United States*

This session will provide insights on the many ways that the aging network and the Older Americans Act is able to assist individuals, and their families, who are dealing with a serious or advanced illness.

### SESSION 1235 (SYMPOSIUM)

### PARTICIPANT AND CAREGIVER VIEWS OF SELF-DIRECTION OF HOME AND COMMUNITY SERVICES 15 YEARS AFTER CASH & COUNSELING

Chair: Kevin J. Mahoney, *Boston College School of Social Work National Resource Center (NRCPDS), Chestnut Hill, Massachusetts, United States*

Discussant: Michelle Putnam, *Simmons College School of Social Work, Boston, Massachusetts, United States*

Results of the Cash and Counseling controlled experiment are now more than ten years old. This symposium, based on a recent Special Issue of the Journal of Gerontological Social Work on self direction of home and community-based services and supports for people with disabilities, begins with an overview of the status of self-direction in the United States where now over 1.1 million people are managing their own services and supports. After summarizing the six research studies in this Special Issue presenting feedback on the self-direction model from participants, their caregivers, and unpaid representatives followed by participant views on remaining unmet needs, the ideal and undesired characteristics of support brokers, and a research study to develop modules for training care managers and support brokers on person-centered planning and self-direction, papers will be presented focusing on two of these studies highlighting improvements needed in the self-direction approach if it is to become available to all persons with disabilities. The first paper is titled, "Unmet Needs Even When People Have Control of the HCBS Budget"; the second deals with the "Tasks and Characteristics of Supportive Support Brokers"; while the third paper looks at "Present Efforts and Recommendations for Training Support Brokers on the Principles, Values, and Skills to Assist People with Disabilities Who Wish to Direct Their Own Supports". The session ends with a presentation on the program and policy implications of this research for federal agencies.

### UNMET NEEDS IN SELF-DIRECTED HCBS PROGRAMS

Kevin J. Mahoney,[1] Ellen k. Mahoney,[2] Carmen Morano,[3] and Andrew DeVellis[4], *1. Boston College School of Social Work National Resource Center (NRCPDS), Chestnut Hill, Massachusetts, United States, 2. Connell School of Nursing, Boston College, Chestnut Hill, Massachusetts, United States, 3. School of Social Welfare, University at Albany, State University of New York, Albany, New York, United States, 4. Simmons School of Social Work, Boston, Massachusetts, United States*

Unmet Need for long-term services and supports has been linked to a variety of harmful health outcomes. One suggested strategy for ameliorating unmet need is to give participants control of the budget and let them construct individualized plans. The evaluation of the Cash and Counseling controlled experiment documented a marked reduction in unmet need when compared to traditional agency-based solutions, but it also showed significant unmet needs remained. This paper, drawing from 76 ethnographic case studies of Cash and Counseling participants, gives us an understanding of what those unmet needs are, who sees them, and what participants and their family caregivers see as possible remedies. Certain areas of unmet need identified in this study stand out. These included health-related problems, environmental issues, and the caregivers' need for relied. The paper concludes with implications for care integration and the training of support brokers as warnings about reducing budgets.

### PARTICIPANT VIEWS OF THE TASKS AND CHARACTERISTICS OF SUPPORTIVE SUPPORT BROKERS IN SELF-DIRECTION PROGRAMS

Ellen K. Mahoney,[1] Grace Oh,[1] Carmen Morano,[2] Kevin J. Mahoney,[3] and Andrew DeVellis[4], *1. Connell School of Nursing, Boston College, Chestnut Hill, Massachusetts, United States, 2. School of Social Welfare,*

*University at Albany, State University of New York, Albany, New York, United States, 3. Boston College School of Social Work National Resource Center (NRCPDS), Lauderdale-by-the-Sea, Florida, United States, 4. Simmons School of Social Work, Boston, Massachusetts, United States*

This qualitative study draws on 76 ethnographic case studies with Cash and Counseling participants, examines what participants and their caregivers saw the support broker doing, and looks at what the participants found helpful and less than helpful. Participants and their caregivers saw support broker duties as Coaching, Problem Solving, Advocacy and Monitoring. Equally important was how the support broker performed these roles. Six attributes that mattered to participants were: Familiarity, Supportive Relationship, Proactive Engagement, Responsiveness, Knowldge and Cultural Friendliness. These findings from the participant and caregiver perspective have great import for the training of present and future care managers and support broker, and have implications for regulatory and even licensure requirements. These results can be a first step in constructing a quality framework for self-directed supports and services.

### WORK FORCE COMPETENCIES AND TRAINING FOR SELF-DIRECTED SERVICE PROGRAMS

Mark Sciegaj,[1] and Nancy Hooyeman[2], *1. Penn State University, University Park, Pennsylvania, United States, 2. University of Washington, Seattle, Washington, United States*

In 2017 over one million individuals of all ages were enrolled in approximately 260 self-directed long-term services and support programs nationwide. Research conducted by the National Resource Center for Participant-Directed Care (NRCPDS) and the Council for Social Work Education identified training gaps among current aging and disability network professionals and within social work education. Believing that both self-directing individuals and their family caregivers would benefit from a workforce that has the knowledge and skills to implement the principles of self-directed care, NRCPDS and CSWE working with national professional organizations and government agencies have identified workforce competency domains and developed a number of training resources that can be used in both academic and professionals settings. This presentation will review the work of NRCPDS and CSWE in workforce competencies, training resources, and recommendations for self-directed services training.

### RESEARCH CAN CHANGE PROGRAMS AND POLICY

Lori Gerhard[1], *1. U.S. Administration for Community Living, Washington, District of Columbia, United States*

In addition to increasing knowledge, research is meant to improve practice and policy. The papers presented in this symposium draw from the experiences and insights of actual participants and their caregivers in major government-sponsored program options for people with disabilities wanting to remain in the community. Many of these programs are administered by our Administration for Community Living. For all, ACL serves as a main source of information for people in the community. These papers give us first-hand knowledge of what participants like and what they want improved. They give us guidance on how consumers define quality; the results can guide efforts to improve program design and the training of support brokers and representatives who assist people who want to manage their own supports and services. I will give a few examples starting with the paper on the Veterans-Directed Care Program and drawing ideas from the other papers.

### SESSION 1240 (SYMPOSIUM)

### PERCEIVED MENTAL FATIGABILITY: NOVEL INSIGHTS INTO SOCIOBEHAVIORAL CORRELATES AND HERITABILITY

Chair: Nancy W. Glynn, *University of Pittsburgh, Department of Epidemiology, Pittsburgh, Pennsylvania, United States*
Discussant: Eleanor M. Simonsick, *Longitudinal Studies Section, Intramural Research Program, National Institute on Aging, Baltimore, Maryland, United States*

Fatigue, a common patient-reported outcome, is a unique risk factor associated with both cognitive and physical function. Perceived mental fatigability, a self-report measure of cognitive fatigue anchored to activities of fixed intensity and duration, eliminates self-pacing bias, and therefore is a more sensitive measure of the degree to which cognitive tiredness limits activity. Higher perceived mental fatigability has been associated with functional decline and lower grey matter brain volumes in older adults. We developed the Pittsburgh Fatigability Scale (PFS), a self-administered, 10-item tool to assess perceived physical and mental fatigability across a range of activities, which is widely used internationally. We previously validated the PFS physical subscale. Using a large multicenter international cohort, the Long Life Family Study, we will present the validation of the PFS mental subscale, examine its epidemiology, and explore genetic and socio-behavioral factors associated with perceived mental fatigability in older adults. Specifically, Ms. Renner will share the results of the validation of the PFS mental subscale; Ms. Meinhardt will present heritability and prevalence of higher perceived mental fatigability across age strata and sex; and Ms. Gmelin will consider the link between stress and coping styles on perceived mental fatigability. Further, using a smaller methodological study, the Developmental Epidemiologic Cohort Study, Ms. Graves will explore whether diurnal patterns of physical activity using accelerometry differ in older adults with higher versus lower perceived mental fatigability. Dr. Simonisick, our Discussant, will critically review the presentations and share future directions to inform potential interventions aimed at lowering perceived mental fatigability.

### THE PITTSBURGH FATIGABILITY SCALE: VALIDATION OF THE MENTAL SUBSCALE IN THE LONG LIFE FAMILY STUDY

Sharon W. Renner,[1] Patrick J. Brown,[2] Todd M. Bear,[3] Stacy L. Andersen,[4] Stephanie Cosentino,[5] Robert M. Boudreau,[6] Adam J. Santanasto,[6] and Nancy W. Glynn[6], *1. University of Pittsburgh Graduate School of Public Health, Pittsburgh, Pennsylvania, United States, 2. Department of Clinical Psychology in Psychiatry, College of Physicians & Surgeons, Columbia University, New York, New York, United States, 3. Department of*

*Behavioral and Community Health Sciences, University of Pittsburgh Graduate School of Public Health, Pittsburgh, Pennsylvania, United States, 4. Department of Medicine, Boston Medical Center, University School of Medicine, Boston, Massachusetts, United States, 5. Department of Neurology, Columbia University Medical Center, New York, New York, United States, 6. Department of Epidemiology, University of Pittsburgh Graduate School of Public Health, Pittsburgh, Pennsylvania, United States*

We previously validated the physical, but not the mental subscale of the Pittsburgh Fatigability Scale (PFS). Thus, we aimed to validate the PFS mental subscale in 1,738 individuals aged ≥60 from the Long Life Family Study (55.5% female, age 74.8±11.1 years, PFS mental score 7.1±10.1, range 0-50). Confirmatory factor analysis with promax rotation showed all 10 items loaded on two factors: social and physical activities (SRMR=0.07, RMSEA=0.13, CFI=0.90). PFS mental score had strong internal consistency (Cronbach's α=0.90) and demonstrated moderate concurrent and construct validity using Pearson correlations against measures of cognition (Trail Making A (r=0.26) and B (r=0.29) time), gait speed (r=-0.30), and the Center for Epidemiologic Studies Depression Scale (r=0.35), p<0.0001 for all. In conclusion, by accounting for self-pacing inherent in common fatigue questionnaires, the validated PFS mental subscore may be a more sensitive tool to examine perceived mental fatigability as an important contributor to cognitive and physical function.

### PREVALENCE AND HERITABILITY OF PERCEIVED MENTAL FATIGABILITY IN THE LONG LIFE FAMILY STUDY

Alexa J. Meinhardt,[1] Theresa Gmelin,[2] Allison L. Kuipers,[2] Stacy L. Andersen,[3] Stephanie Cosentino,[4] Mary K. Wojczynski,[5] Kaare Christensen,[6] and Nancy W. Glynn[2], *1. University of Pittsburgh, Department of Epidemiology, Pittsburgh, Pennsylvania, United States, 2. University of Pittsburgh Graduate School of Public Health, Pittsburgh, Pennsylvania, United States, 3. Boston University School of Medicine, Boston, Massachusetts, United States, 4. Columbia University Department of Neurology, New York, New York, United States, 5. Washington University School of Medicine in St. Louis Department of Genetics, St. Louis, Missouri, United States, 6. University of Southern Denmark, Odense, Nordjylland, Denmark*

We examined the prevalence and heritability of perceived mental fatigability among older adults enrolled in the Long Life Family Study. Participants (N=2342; 55% female) self-administered the Pittsburgh Fatigability Scale (PFS; scores range 0-50; higher score=greater fatigability). Using the PFS mental subscale, we evaluated differences across age strata (adjusted for family structure and field center) and estimated genetic heritability using the variance covariance methods implemented in SOLAR to determine genetic heritability (adjusted for age, sex, and field center). PFS mental score (mean±SD) and prevalence of higher mental fatigability (PFS ≥13) was greater across age strata: 60-69 (N=996, 5.9± 6.5, 14.5%), 70-79 (N=830, 6.8 ±7.6, 18.7%), 80-89 (N=251, 11.7±10.8, 41.8%), and ≥90 (N=265, 20.2±13.6, 67.2%), p<0.0001. Only among those ≥90, females (21.7±13.5) had greater mental fatigability than males (18.0±13.5), p=0.03. Residual heritability of mental fatigability was 0.17, p<0.0001. Future analyses will evaluate correlates of mental fatigability to identify potential avenues for intervention.

### ROLE OF COPING STYLES AND NEGATIVE LIFE EVENTS ON HIGHER PERCEIVED MENTAL FATIGABILITY IN OLDER ADULTS

Theresa Gmelin,[1] Stacy L. Andersen,[2] Robert M. Boudreau,[3] Kaare Christensen,[4] Mary K. Wojczynski,[5] Stephanie Cosentino,[6] and Nancy W. Glynn[3], *1. University of Pittsburgh. Department of Epidemiology, Pittsburgh, Pennsylvania, United States, 2. Boston University School of Medicine, Boston, Massachusetts, United States, 3. University of Pittsburgh, Pittsburgh, Pennsylvania, United States, 4. University of Southern Denmark, Odense, Syddanmark, Denmark, 5. Washington University School of Medicine in St. Louis, St Louis, Missouri, United States, 6. Columbia University, New York, New York, United States*

Older adults are vulnerable to negative recent life events (RLE) which deplete attentional resources and leads to cognitive exhaustion. Adaptive coping styles reduce perceived stress severity but their role on cognitive tiredness is unknown. We examined RLE and coping styles on perceived mental fatigability (Pittsburgh Fatigability Scale (PFS), 0-50pts, higher=greater fatigability) in the Long Life Family Study (N=1464, age=74.7±12.6, female=57.7%, 43.9% ≥1 major RLE past 6 months, 27.8% higher mental fatigability≥13). All analyses adjusted for family structure, field center, age, and sex. PFS mental scores correlated with all NEO-FFI (60-item, 5-domain) personality traits representing maladaptive (neuroticism r=0.25 p<.0001) and adaptive (conscientiousness r=-0.18, extraversion r=-0.24, p<.00001) coping. Having ≥1RLE was associated with higher mental fatigability (OR=1.4, 95% CI:1.2,1.8, p=.0004); adjustment for neuroticism (OR=1.3, 95% CI:0.9,1.7, p=.06) attenuated the association. Education on adaptive coping may be a modifiable skill that allows older adults to maintain lower perceived mental fatigability despite stressful events.

### IS VARIABILITY OF FREE-LIVING ACTIVITY ASSOCIATED WITH PHYSICAL AND MENTAL FATIGABILITY IN OLDER ADULTS?

Jessica L. Graves,[1] Robert T. Krafty,[2] Jaroslaw Harezlak,[3] Eric J. Shiroma,[4] and Nancy W. Glynn[2], *1. University of Pittsburgh, Department of Epidemiology, Pittsburgh, Pennsylvania, United States, 2. Department of Biostatistics, Graduate School of Public Health, University of Pittsburgh, Pittsburgh, Pennsylvania, United States, 3. Department of Epidemiology and Biostatistics, School of Public Health, Indiana University, Bloomington, Indiana, United States, 4. Laboratory of Epidemiology and Population Science, National Institute on Aging, Bethesda, Maryland, United States*

Greater fatigability in older adults may be moderated by physical activity (PA). However, what features of PA timing are most strongly related to fatigability remains unknown. We examined the relationship between variability of free-living activity patterns and perceived physical and mental fatigability using the Pittsburgh Fatigability Scale (PFS, 0-50pts, higher=greater fatigability) in older adults from the Developmental Epidemiologic Cohort Study (DECOS, n=57, age=70-91yrs, 61% female). We assessed PA using ActiGraph GT3X+ over 7 days. Mean activity, standard deviation (SD) of mean activity across days, and relative activity [(mean at each bin)/(total mean)] were calculated across 24-hours in 4-hour bins , adjusting for estimated rise-time. Lower SD of PA from 0-4 hours after rising was associated with greater PFS physical scores (r=-0.27, p=0.05). No measures of PA correlated with PFS mental scores. In older adults with lower physical fatigability, associations with greater variability in activity may indicate larger energy reserves.

### SESSION 1245 (SYMPOSIUM)

### PRESIDENTIAL SYMPOSIUM: EXPANDING THE GEROSCIENCE NETWORK

Chair: Matt Kaeberlein, *University of Washington, Seattle, Washington, United States*

In keeping with the 2019 GSA Annual Meeting theme of "Strength in Age—Harnessing the Power of Networks", the Biological Sciences Presidential Symposium focuses on cutting edge approaches to understand the biology of aging using network and systems approaches.

### OMICS IN AGING RESEARCH: FROM BIOMARKERS TO SYSTEMS BIOLOGY

Daniel Promislow[1], *1. University of Washington, Seattle, Washington, United States*

Advances in whole genome sequencing have dramatically increased our potential to understand what shapes variation in rates of aging and age-related disease in natural populations, but we are still far from realizing this potential. Researchers have identified thousands of genetic markers associated with complex human traits. However, these markers typically explain a very small fraction of the observed variance, leaving an enormous explanatory gap between genotype and phenotype. I will present data from diverse species to illustrate the power of so-called endophenotypes—the epigenome, transcriptome, proteome, and metabolome—to bridge the genotype-phenotype gap. Using multivariate and network models that integrate genetic information with other endophenotype variation, we are closer than ever to understanding the mechanisms that account for natural variation in aging and age-related disease, and the evolutionary forces that have shaped that variation.

### HETEROGENEITY OF AGING IN HUMAN POPULATIONS

Jing-Dong Jackie Han[1], *1. CAS-MPG Partner Institute for Computational Biology, Shanghai, China, China*

Recently by analyzing the 3D facial images, we generated the first comprehensive mapping of the aging human facial phenome. We constructed a robust age predictor and found that on average people of the same chronological age differ by +/-6 years in facial age, with the deviations increasing after age 40. Using this predictor we identified slow- and fast-agers that are significantly supported by health indicators. We further profiled blood cell mRNA and lncRNA expression by RNA-seq of this cohort and computationally predict their regulatory networks and their contributions to the variation in aging rate among different individuals, and those that are modifiable by their lifestyles. By extending the study to a large Northern Chinese cohort of 10,000 people we can now use deep learning AI approaches to precisely estimate aging status based on 3D facial images and their associations with individuals' health and medical history.

### EPIGENETIC AND METABOLIC REGULATION OF AGING

Anne Brunet[1], *1. Stanford University, Stanford, California, United States*

Aging is accompanied by a decline in the regenerative potential of most tissues. The mammalian brain contains regenerative neurogenic niches composed of neural stem cells (NSCs), neural progenitors, and other cells, including microglia, and endothelial cells. Neurogenic niches become less functional with increasing age. This deterioration could underlie cognitive and sensory restriction with age, although the exact age at which it occurs is still debated in humans. How the neurogenic niche changes during aging, and whether new cell types arise in older individuals, is not known. Our lab has embarked on a global characterization of the neurogenic niche during aging. This work provides a global understanding of the old neurogenic niche and suggests possible cause for NSC decline during aging. Results from these studies could open new avenues to counter age-related decline in the neurogenic niche and brain aging.

### SESSION 1250 (SYMPOSIUM)

### PSYCHOLOGICAL AND HEALTH CONSEQUENCES OF HELPING OTHERS: INNOVATIVE METHODS TO UNDERSTAND STRAINS AND GAINS

Chair: William E. Haley, *University of South Florida, Tampa, Florida, United States*
Discussant: Karl Pillemer, *Cornell University, Ithaca, New York, United States*

Older adults are often involved in prosocial behaviors including volunteering, informal assistance to family members, or extensive caregiving for family with chronic disease or disability. Many studies find that volunteering and providing informal support can enhance health and well-being, but family caregiving has generally been characterized as being highly stressful and harmful to health and well-being. Recent research has suggested that involvement in prosocial activities, including caregiving, can actually build resilience and buffer the impacts of stress, and that the commonalities across different types of prosocial behaviors in older adults deserve greater attention. This symposium brings together researchers who are using innovative methods to study prosocial behaviors, including measuring daily experiences and their linkages with affect, epidemiological methods, and use of health outcomes including serum biomarkers of inflammation and immunity, activity tracking, and mortality. Results across the presentations show that the effects of helping others can be considered as mixed blessings, with potentially harmful and helpful effects depending on contextual factors. Factors including a history of adverse childhood experiences, and dementia caregiving, can create particular challenges. The Discussant, Dr. Karl Pillemer, will discuss implications for future research on volunteering, informal assistance to family, and family caregiving. He will also address ways that gerontological researchers can present a more balanced public narrative about how stressful experiences such as caregiving can produce not only negative affect, but also potentially positive health benefits, resilience to stress, and personal growth.

### DESIGNING FAMILY CAREGIVER STUDIES THAT BALANCE STRESS PROCESS AND HELPING RELATIONSHIP PERSPECTIVES

David L. Roth,[1] William Haley,[2] Orla Sheehan,[3] Jeremy Walston,[3] David Rhodes,[4] and Virginia Howard[4], *1. Johns Hopkins University School of Medicine, Baltimore, Maryland, United States, 2. University of South Florida, Tampa, Florida, United States, 3. Johns Hopkins University, Baltimore, Maryland, United States, 4. University of Alabama at Birmingham, Birmingham, Alabama, United States*

Family caregiving is often characterized as a chronically stressful situation, and stress process models have been the dominant conceptual foundation underlying caregiving studies for decades. Recently, this perspective has been augmented with more positive views that emphasize potentially healthy and prosocial aspects of caregiving. Replicated findings from population-based studies show that caregivers have lower mortality rates than noncaregivers, consistent with the more balanced conceptual approach. The Caregiving Transitions Study is investigating 251 participants who transitioned into a caregiving role at some point between two blood samples taken 10 years apart in a national epidemiological study and 251 matched controls. Preliminary analyses confirm that caregiving leads to increased psychological distress. Ongoing analyses are examining changes in inflammatory biomarkers, health status, and positive aspects of caregiving. Findings will be examined alongside our recent meta-analysis of convenience samples that found caregiving to have small and inconsistent relationships with biomarkers of inflammation and immunity.

### EFFECT OF ADVERSE CHILDHOOD EXPERIENCES ON DAILY SUPPORT TO FAMILY AND EMOTIONAL WELL-BEING IN ADULTHOOD

Jooyoung Kong,[1] Yin Liu,[2] and David Almeida[3], *1. University of Wisconsin-Madison, Madison, United States, 2. Utah State University, Logan, Utah, United States, 3. Penn State University, University Park, Pennsylvania, United States*

Extensive evidence suggests that adverse childhood experiences (ACEs) can lead to negative health effects across a lifetime. This study examines the impact of ACEs on the frequency of providing daily support (i.e., unpaid assistance, emotional support, and disability-related assistance) to family members and the moderating effects of ACEs in the association between providing daily support to family and daily negative affect. Using the National Study of Daily Experiences II, we analyzed a total of 14,912 daily interviews from 2,022 respondents aged 56 on average. Key results showed that a greater number of ACEs were associated with providing more frequent emotional support to family. We also found the significant interaction effect that adults with more ACEs showed greater negative affect on the days when they provided assistance to family members with disabilities. The findings underscore the long-term negative impact of ACEs on daily well-being in the context of family relationships.

### HELPING OTHERS IS A MIXED BLESSING: IMPLICATIONS FOR DAILY WELL-BEING

Meng Huo,[1] Yee Ng,[2] and Karen Fingerman[2], *1. The University of Texas at Austin, Austin, Texas, United States, 2. University of Texas at austin, Austin, Texas, United States*

The literature documents mixed findings regarding how helping others influences individuals' mental and physical health. We assessed various types of support that older adults offered (e.g., emotional, practical, advice) and examined how helping others was associated with older adults' daily mood and physical activity. This study utilized data from the Daily Experiences and Well-being Study, where 293 participants aged 65+ reported on their helping behaviors and mood at the end of each day across 5 days. Participants also wore Actical accelerometers to track physical activity. Multilevel models revealed that older adults reported greater negative mood and less physical activity on days when they provided emotional support. Yet, giving advice was associated with increased positive mood that day. Moreover, older adults spent less time being sedentary on days when they offered practical help. This study offers insights into psychological and health consequences of helping others by examining older adults' everyday lives.

### STRESS-BUFFERING EFFECTS OF VOLUNTEERING ON DAILY WELL-BEING: RESULTS FROM THE NATIONAL STUDY OF DAILY EXPERIENCES

Saehwang Han,[1] Kyungmin Kim,[2] and Jeffrey Burr[2], *1. University of Texas at Austin, Austin, Texas, United States, 2. University of Massachusetts Boston, Boston, Massachusetts, United States*

Based on theory and empirical evidence linking volunteering and health, we investigated the associations between daily engagements in formal volunteering, stressors, and negative affective well-being, focusing on the stress-buffering effect of volunteering. Using eight days of daily diary data from the second wave of the National Study of Daily Experiences (participants, N = 1,320; participant-day observations, N = 8,277), we estimated a series of multilevel models to assess the within-person associations between daily volunteering, stressors, and affect. Results indicated there were no direct associations between daily volunteering and negative affect. However, we found the association between daily stressors and negative affect (but not positive affect) was weaker on days when volunteering was performed compared to days volunteering was not performed. Taken together, our findings suggested that short-term health benefits associated with daily volunteering were largely based on the stress-buffering effects of helping others, rather than through a direct effect.

### SESSION 1255 (SYMPOSIUM)

### THE ASSISTED LIVING SETTING: CLINICAL CARE AND OUTCOMES

Chair: Barbara Resnick[1], *1. University of Maryland School of Nursing, Baltimore, Maryland, United States*

Although the description of assisted living (AL) varies by state this term generally refers to residences that provide housing and supportive services, 24-hour supervision, and at least two meals a day to meet the individual needs of residents. Approximately 37% of residents in AL need help with three or more activities of daily living (ADLs), 42% have some cognitive impairment and 39% need skilled nursing services. Approximately 15 to 50% of older adults living in AL communities experience a fall over a 6 to 24 month period. The cause of these falls involves multiple factors at the resident and facility level. In addition to a high rate of falls there is a high rate of polypharmacy, using the polypharmacy definition of taking five or more medications daily. Polypharmacy results in negative outcomes such as falls and hospitalizations among AL residents. Along with high rates of falls and polypharmacy there is also a high incidence of pain among AL residents and concerns that some pain goes unreported and untreated. Pain, polypharmacy and falls can all influence life satisfaction along with other factors such as the environment itself. The purpose of this symposium is to describe the incidence and factors that influence falls, polypharmacy, pain and pain management and the impact of these care concerns, among others, on life satisfaction drawn from 508 residents from 54 nursing homes participating in the first two cohorts of the study testing the implementation of Function Focused Care for Assisted Living (FFC-AL-EIT).

### THE INCIDENCE AND IMPACT OF POLYPHARMACY IN AL RESIDENTS

Elizabeth Galik[1], *1. University of Maryland School of Nursing, Baltimore, Maryland, United States*

The purpose of this study was to describe polypharmacy in AL settings. We hypothesized that: (1) age, gender, race, setting, multimorbidity and cognitive status would influence polypharmacy; and (2) polypharmacy would be associated with falls, emergency room visits and hospitalizations. This was a descriptive study using data from the first cohort of the FFC-AL-EIT Study. A total of 242 participants from 26 AL settings were included. Participants had a mean age of 86.86 (SD=7.0), the majority were women 179(74%) and white (N=232, 96%) with 5 (SD=2) comorbidities. The mean number of drugs was 7 (SD=3.56) and 51% were exposed to polypharmacy, 24% fell at least once, 9% were sent to the hospital and 13% to the emergency room. Neither hypothesis was supported. Continued research is needed to explore the factors that influence polypharmacy and to identify if there are negative outcomes associated with polypharmacy in this population.

### FACTORS THAT CONTRIBUTE TO FALLS AMONG AL RESIDENTS

Marie Boltz[1], *1. Pennsylvania State University, University Park, Pennsylvania, United States*

Many nurses, patients, and families continue to believe that physical activity increases the risk of falling. The purpose of this study was to test the hypothesis that residents who are exposed to Function Focused Care for Assisted Living (FFC-AL-EIT) and engage in moderate levels of physical activity would not be more likely to fall. This was a secondary data analysis using data from the first two cohorts of the FFC-AL-EIT study. The study included 508 residents the majority of whom were female (70%), white (97%), with a mean age of 87.72 (SD=7.47). Those who engaged in more moderate intensity physical activity were 1% less likely to fall (b = -.01, Wald =6.13, p =.01). There was no association between exposure to function focused care and falling (Beta =.41, Wald =2.35, p=.13). Further, engaging in moderate level physical activity was noted to be slightly protective of falling.

### PAIN, PAIN MANAGEMENT, AND CONSEQUENCES OF PAIN AMONG AL RESIDENTS

Barbara Resnick[1], *1. University of Maryland School of Nursing, Baltimore, Maryland, United States*

The purpose of this study was to describe the incidence, management and impact of pain on function, agitation, and resistance to care. This was a descriptive study using baseline data from 260 residents in the second cohort of the FFC-AL-EIT study. The majority of the sample was female (71%) and white (96%) with a mean age of 87 (SD=7). Fifty-two residents (20%) reported pain based on objective (PAINAD) or subjective (verbal descriptive scale, VDS) pain assessments. A total of 75 residents (29%) received pain medication and 22 (42%) individuals reporting pain were not getting pain medication. Controlling for age, gender and cognition, PAINAD was significantly associated with agitation, function, and resistance to care and the VDS was only associated with function. Pain assessments should include objective and subjective measures and management of pain should be considered as it may help to optimize function and decrease behavioral symptoms among AL residents.

### FACTORS THAT INFLUENCE LIFE SATISFACTION IN AL RESIDENTS

Sarah Holmes[1], *1. University of Maryland, Baltimore, Maryland, United States*

Life satisfaction is a multidimensional concept that addresses a personal judgment of quality from the resident's perspective. Components of life satisfaction include satisfaction related to health, the physical environment, relationships and activities. The purpose of this study was to test if there was a relationship between demographic factors, pain, falls, and use of psychotropics with life satisfaction. The sample included the first two cohorts from the FFC-AL-EIT study including 508 residents from 54 settings across Maryland, Pennsylvania, and Massachusetts. The majority of the participants were female (70%), white (97%) and the mean age was 87.72 (SD=7.47) . Based on a stepwise linear regression analysis there was a significant association between pain (r=-.20, p=.003) and psychotropic use (r=-.19, p=.003) and the model explained 11% of the variance in life satisfaction. Ongoing research is needed to consider the impact of the environment and staff-resident interactions on life satisfaction.

### SESSION 1260 (SYMPOSIUM)

### THE EXAMINATION OF DETERMINANTS AND BARRIERS TO END-OF-LIFE DECISION MAKING AND PLANNING

Chair: Brittany E. Gaines, *University of Massachusetts Boston, Boston, Massachusetts, United States*
Discussant: Debra J. Dobbs, *University of South Florida, Tampa, Florida, United States*

As individuals are living longer, in many cases with chronic diseases, there is an increased focus on end-of-life

(EOL) planning and decision making. This includes a broad spectrum of choices including advance care planning (ACP) and turning to palliative care or hospice care. Although there has been an increase in palliative and hospice care enrollment and ACP engagement over the past decade, participation remains low for certain subgroups of the population. The purpose of this symposium is to offer insight into reasons for these varying rates of engagement by exploring determinants and barriers to EOL decision making and planning and by examining caregiver knowledge of EOL decision making and planning from the service provider perspective. The first three studies examine various types of influences in EOL decision making and planning. Inoue and colleagues explore factors associated with the length of hospice stay, and Gaines and colleagues examine the impact of environmental characteristics in ACP. Ornstein and colleagues use Denmark registry data to assess the role of kinlessness at the time of death in EOL decision making and healthcare utilization. The final presentation by Noh and colleagues examines how service providers in rural areas perceive community residents' knowledge of ACP and palliative care. The discussion following these presentations will compare findings across different forms of EOL decision making and planning, consider the impact of the varying methodological approaches used, and highlight implications of these works for potential interventions and policies related to EOL decision making and planning.

### THE ROLE OF THE ENVIRONMENTAL CONTEXT IN ADVANCE CARE PLANNING AMONG OLDER ADULTS

Brittany E. Gaines,[1] Kathrin Boerner,[1] Kyungmin Kim,[1] and Sara Moorman[2], *1. University of Massachusetts Boston, boston, Massachusetts, United States, 2. Boston College, Chestnut Hill, Massachusetts, United States*

Little is known about how environmental context shapes individuals' advance care planning (ACP). We combined ACP information from the Wisconsin Longitudinal Study with county-level characteristics from the Area Health Resource File, Dartmouth Atlas, and US Census. Multilevel logistic regression models showed that local sociodemographic characteristics (e.g., rurality, racial/ethnic makeup, age composition, and prevalence of one-person households) and healthcare characteristics (e.g., number of hospice agencies, Medicare reimbursement rates) were related to rates of ACP. Additionally, the following environmental factors were moderated by both individual household income and educational attainment, Medicare physician reimbursement rate, racial/ethnic makeup, age composition, median household income, rurality, and the number of hospice agencies. These findings suggest that the environmental context of an individual's residence can impact their engagement in ACP. Evidence from this study may be used to target areas for, and guide the design of, effective intervention strategies to help increase ACP at an environmental level.

### FAMILY CAREGIVERS' ADVANCE CARE PLANNING FOR COGNITIVELY IMPAIRED OLDER ADULTS: SERVICE PROVIDERS' PERSPECTIVES

Hyunjin Noh,[1] and Temilade A. Aladeokin[1], *1. The University of Alabama School of Social Work, Tuscaloosa, Alabama, United States*

An increasing number of family caregivers face challenges of advance care planning (ACP) for their cognitively impaired older adults. The purpose of this study was to understand service providers' views of ACP knowledge and needs among such family caregivers. Purposive sampling was used to recruit 10 service providers who serve older adults and their family caregivers in community settings of West Alabama. Individual, face-to-face interviews were conducted guided by a semi-structured questionnaire, asking about their experiences with and views of family caregivers' ACP for their older adults. Thematic analysis of the qualitative data revealed several findings: family caregivers' lack of knowledge about ACP and end-of-life care, discomfort in end-of-life discussions, uncertainty about their older adult's end-of-life preferences, frustration with the surrogate decision-making role, family conflicts in ACP process, and logistical barriers to access ACP resources. Tailored services should be developed to address these barriers to promote ACP among this population.

### KINLESSNESS AT THE TIME OF DEATH: IMPLICATIONS FOR END-OF-LIFE DECISION-MAKING

Katherine Ornstein,[1] Christine Mair,[2] Marie Skov Kristensen,[3] Melissa Aldridge,[4] and Lau Caspar Thygesen[3], *1. Icahn School of Medicine at Mount Sinai, New York, New York, United States, 2. University of Maryland, Baltimore County (UMBC), Baltimore, Maryland, United States, 3. National Institute of Public Health, University of Southern Denmark, København, Hovedstaden, Denmark, 4. Icahn School of Medicine at Mount Sinai, New York, New York, United States*

As our society continues to age and family size decreases, there is increasing concern about lack of caregiver availability. This may be especially important in the context of end-of-life decision-making. The goal of this study was to characterize the size and composition of the family network of adults at the time of death using a population-based register study. All adults in Denmark who died of natural causes 2009-2016 (n= 401,000) were linked to living adult family members (parents, children, spouses, sibling, great/grandchildren). While the majority of decedents were linked to multiple family members (median =5), 21.6% had no identified living family at the time of their death. Kinlessness was especially common among older women and those with dementia. In addition to supporting caregiving families at the end-of-life, we must also recognize that there are many kinless individuals who may benefit from early formal care planning services to facilitate end-of-life decision-making.

### FACTORS ASSOCIATED WITH LENGTH OF HOSPICE STAY

Megumi Inoue,[1] Matthew G. Kestenbaum,[2] and Cameron Muir[3], *1. George Mason University, Fairfax, Virginia, United States, 2. Capital Caring, Falls Church, Virginia, United States, 3. Capital Caring, Washington DC, District of Columbia, United States*

The benefits of early referral to hospice services have been well documented. However, late admissions and short hospice stays are ongoing issues that are often barriers to improving terminally-ill persons' and the caregivers' quality of life and the quality of care. Using ordered logistic regression, this study analyzed the length of stay of 7,307 patients who died in 2015 while receiving care in the largest hospice agency in the DC metro region. Cancer diagnoses and residence in a higher median income neighborhood were associated with shorter lengths of stay. Female sex, older age, and residence in a lower median income neighborhood were associated with longer lengths of stay. The findings indicate that differences in demographic and diagnostic characteristics likely affect hospice length of stay.

### SESSION 1265 (SYMPOSIUM)

### THE INTERSECTION OF ETHICS AND VULNERABLE ELDERS

Chair: Pamela B. Teaster, *Virginia Tech, Blacksburg, Virginia, United States*
Discussant: Georgia Anetzberger, *Case Western Reserve University, Cleveland, Ohio, United States*

Researchers, practitioners and policymakers are daily confronted with multiple and competing situations regarding vulnerable older adults and the complex issues that they face in all aspects of their lives. Challenges can arise in the provision of social services, dispensing justice, conducting research, or addressing legal issues. The purpose of this symposium is to discuss dilemmas that vulnerable older adults and concerned others face by elucidating current and future challenges facing this population, particularly in the realms of compromised health (cognitive impairment); effective status (gender); care arrangements (home and community-based services); and abuse, neglect, and exploitation. Teaster and Anetzberger discuss relevant ethical theories and principles as well as a definition of vulnerability. Santos and Nichols-Hadeed report on ethical issues embedded in vulnerable elders' cognitive status. Bowland and Halaas highlight the intersection of ethics, gender and vulnerable elders. Niles-Yokum and Beaumaster discuss the nexus of ethics and the provision of home and community based services for vulnerable older adults. Heisler considers vulnerabilities of older adults and ethical challenges when addressing elder abuse. Throughout the papers, we weave the ethical principles of autonomy, beneficence, nonmaleficence, and justice.

### ETHICAL DILEMMAS PRESENTED BY COGNITIVE IMPAIRMENT AND VULNERABLE ELDERS

Elizabeth J. Santos,[1] and Corey A. Nichols-Hadeed[2], *1. University of Rochester School of Medicine & Dentistry, Rochester, New York, United States, 2. University of Rochester Medical Center, Rochester, New York, United States*

Identification and assessment for cognitive impairment is a difficult task further complicated by the need to determine capacity. Issues related to cognitive impairment and capacity create ethical dilemmas potentially spanning all four ethical principles: autonomy, beneficence, non-malfeasance, and justice. This paper uses a case scenario to describe different types of cognitive impairment and demonstrate ethical issues that commonly arise when treating patients with cognitive impairment in the clinical setting. The authors also recognize the complexity of capacity as an issue that spans both the medical and legal fields and provides explanations and distinctions. The overall goal of this paper is to raise awareness of the impact of cognitive impairment on the vulnerability of older adults, describe the complex ethical issues that cognitive impairment and capacity raise and the importance of defining capacity in the context of the legal and medical fields.

### A PRACTICE FRAMEWORK FOR CONSIDERATION OF GENDER IN ETHICAL DECISION-MAKING

Sharon Bowland,[1] and Beth Halaas[3], *1. University of North Texas, Denton, Texas, United States, 3. Eastern Washington University, Cheney, Washington, United States*

Gender role stereotypes, social norms and social policies negatively influence health and well-being for marginalized groups. These inequalities are embedded in the fabric of our society and are often unquestioned and hidden. Practitioners frequently use an ethical lens that does not consider the influence of gender on life course decision-making. We developed the Practice Framework for Older Persons (PFOP) to assess past and current realities that take gendered experiences into consideration. By contextualizing means and opportunities, a more complete picture can be drawn about a person's unique gender experiences. Subsequently, we can better understand their decision-making processes, wants, needs, and desires. This type of assessment may be particularly beneficial for women and transgender persons given ethical demands for practice paradigms which consider gender fluidity and development of a sense of personal agency.

### THE INTERSECTION OF VULNERABILITY AND OLD AGE: ETHICAL CONSIDERATIONS FOR LONG-TERM SERVICES AND SUPPORTS

Kelly Niles-Yokum[1], *1. University of La Verne, La Verne, California, United States*

This session focuses on ethical considerations in the context of long-term services and supports (LTSS) for vulnerable older adults. Long-term supports for vulnerable older adults can no longer adhere to a "one-sized fits all" solution. We will explore the intersection of vulnerability, old age, and community which present a myriad of ethical issues in both the planning and delivery of supports for older adults. The quest for a just society goes beyond understanding and considering the critical issues of the vulnerability of older adults in our society in that this pursuit provides a pathway to develop and implement programs and services that allow all of us the opportunity to live in a world that both protects and can provide the opportunity for self determination and dignity.

### ETHICAL DILEMMAS, VULNERABLE ELDERS, AND ELDER ABUSE

Candace J. Heisler[1], *1. Heisler and Associates, San Bruno, California, United States*

Elder abuse is a growing concern worldwide. It is described across multiple professional disciplines: as a social justice issue by social workers; as a medical syndrome and public health issue by health care providers; and as a violation of human rights and criminal laws by courts, legislators, and the justice system professionals. Elder abuse assumes different forms, including physical, emotional, and sexual abuse, financial exploitation, neglect, and abandonment. Forms often co-occur in a variety of settings. This presentation explores key ethical conundrums emerging when different professions address elder abuse. Specifically examined is how the ethical principles of autonomy and non-maleficence conflict with mandatory reporting laws, for example, if their purpose is to incarcerate older offenders who are ill and vulnerable serving lengthy mandated prison terms. The presentation also explores the rights of perpetrators, including how rights of crime victims are weighed against those of perpetrators in a just society.

### SESSION 1270 (SYMPOSIUM)

### INTEREST GROUP SESSION—TECHNOLOGY AND AGING: THE POTENTIAL AND PITFALLS OF EXTENDED REALITY SOLUTIONS FOR SUPPORTING AND ASSESSING OLDER ADULTS

Chair: Neil Charness, *Florida State University, Tallahassee, Florida, United States*
Discussant: Balaji Narasimhan, *Iowa State University, Ames, Iowa, United States*

Extended Reality (XR), which encompasses Virtual Reality (VR) and Augmented Reality (AR), holds a great deal of promise for improving the health and well-being of older adults. These opportunities include providing rehabilitation, physical exercise, skills training, leisure opportunities, and support for instrumental activities of daily living. Further, XR presents novel assessment opportunities. This session will explore the potential of XR solutions, and also crucial barriers to XR implementation, adoption, and engagement, particularly with respect to the "digital divide." Some older adults, for a number of reasons, experience greater challenges adopting and using newer technologies. This session will start with a broad overview of issues related to XR solutions and will identify critical research needs, with an emphasis on the needs of older adults. This will be followed by a presentation of older adults' perceptions of XR using data derived from a large, nationally representative sample. While some older adults reported not being ready for XR solutions, many older adults reported being willing to accept them to support optimal aging. Next, a study is presented that directly compares older adults' perceptions of presence and immersion in virtual spaces. Using VR to assess wayfinding and navigation abilities of older adults is discussed next. The final talk will present VR usability issues derived from interview and focus group data. The session discussant will bring an interdisciplinary perspective to these important issues.

### PERSONALIZED AND ADAPTIVE VR/AR FOR OLDER ADULTS: EIGHT CRITICAL NEEDS

Stephen B. Gilbert[1], *1. Iowa State University, Ames, Iowa, United States*

In the past decade there has been considerable progress in the technology associated with virtual reality and augmented reality, along with decreased costs. But with these advances come a new set of research questions. In July 2017, the National Science Foundation hosted a VR/AR visioning workshop to explore these challenges. The workshop focused on the research needed to achieve individual personalization and adaptation in the areas of visual, auditory and tactile perception, as well as the social, behavioral and cognitive patterns key to adaptation. These discussions yielded eight priorities for upcoming research for VR/AR. In this paper, we will describe these eight priorities from the perspective of the gerontology community, looking at the research gaps that must be addressed to apply VR/AR successfully with older adults.

### GERONTECHNOLOGY PERCEPTIONS AND POTENTIAL ROLE OF VR/AR IN OPTIMAL AGING

Jennifer Margrett,[1] Wally Boot,[2] Neil Charness,[2] Christopher Hertzog,[3] Mack Shelley,[1] and Balaji Narasimhan[1], *1. Iowa State University, Ames, Iowa, United States, 2. Florida State University, Tallahassee, Florida, United States, 3. Georgia Institute of Technology, Atlanta, Georgia, United States*

Technology presents opportunities to optimize whole person wellness and functioning. To understand tech readiness and the potential role of virtual (VR) and augmented reality (AR) to support optimal aging, we surveyed 604 participants from the nationally representative RAND American Life Panel. Participant age ranged from 50-90+, 51.5% were female, and 50% reported bachelor's education or higher. Overall, 8% of the sample identified as Hispanic, with 15% of individuals also identifying as Black, Asian, or Asian Indian or Alaskan Native. Males reported greater optimism and technology innovation and adults aged 50-64 were the most optimistic. Overall, 80% of the sample reported VR familiarity compared to 33% AR. Regarding future needs, 75% of the participants expressed specific concerns about future ADL ability. Almost half of the respondents indicated willingness to use VR and AR to maintain or improve functioning with age and increased personalization of optimal aging emerged as a significant predictor.

### EXPLORING OLDER ADULTS' PERCEPTIONS OF PRESENCE AND IMMERSION IN DIVERSE VIRTUAL ENVIRONMENTS

Walter R. Boot,[1] Andrew Dilanchian,[1] and Ronald Andringa[1], *1. Florida State University, Tallahassee, Florida, United States*

Virtual Reality (VR) holds great promise for enhancing the health, well-being, and skills of older adults. However, VR solutions must consider the age-related "digital divide;"many older adults have less experience and proficiency with a number of newer technologies, which may serve as a barrier. Older adults especially have less experience with virtual environments, an experience many younger adults have acquired through video gaming. This study compared younger and older adults' perceptions of immersion and presence in a series of diverse virtual environments using the HTC Vive. Participants experienced a VR meditation task, "indoor" and "outdoor" navigation tasks, and a fast-paced action game. Importantly, younger and older adults reported similarly high experiences of immersion and presence within virtual environments, and contrary to expectations, older adults reported fewer symptoms of cybersickness. Results suggest VR as a promising tool to promote the health and well-being of older adults.

### USING VR FOR NAVIGATION ASSESSMENT IN OLDER ADULTS

Scott Moffat[1], *1. Georgia Institute of Technology, Atlanta, Georgia, United States*

There has been a long tradition of wayfinding and orienteering studies in humans but these have mostly neglected possible age-related differences in navigation. This field of inquiry is experiencing something of a resurgence of interest due to the development of VR technology which has brought the systematic study of large scale navigation into the laboratory and into the MRI scanning environment. Empirical studies to date identify navigation as an aspect of cognition that is vulnerable to the aging process. Functional and structural neuroimaging studies in humans suggest that age-related changes in the brain's "navigation circuit" may underlie these behavioral age differences. Older adults also adopt unique spatial strategies and knowledge of these strategy preferences could enlighten both basic science research in spatial cognition and also inform the development of age-specific technological assistance that may extend functional independence of older adults into later life.

### VIRTUAL REALITY AND OLDER ADULTS: A USABILITY STUDY

Julie A. Brown[1], *1. Ohio University, Athens, Ohio, United States*

Within the past two years there has been a very small but growing number of scholarly articles that highlight the potential benefit of mobile Virtual Reality (VR) platforms among older adult populations. Yet, it is critical to assess older adult user needs and preferences, as well as ethical considerations, before utilizing VR in applied contexts. This pilot study investigated perceptions of VR use and its potential application by individually interviewing ten community-dwelling older adults (ages 63 to 89) both before and after trying the Samsung Gear VR and followed with two focus group discussions. Themes identified from the transcripts include 1) usability, 2) video subject matter preferences, and 3) implications with use. These themes highlighted both the challenges and opportunities of VR use among a wide range of older populations, and provided greater insight with its exploration and application in future studies, particularly with individuals with functional limitations.

### SESSION 1285 (SYMPOSIUM)

### INTEREST GROUP SESSION—COMMUNITY COLLEGE: THE ROLE OF COMMUNITY COLLEGES IN WORKFORCE DEVELOPMENT AND TRANSFER WITHIN GERONTOLOGY

Chair: Jan Abushakrah, *Portland Community College, Portland, Oregon, United States*
Discussant: Michael A. Faber, *Portland Community College, Portland, Oregon, United States*

This symposium will highlight how innovative and often non-traditional Community College Gerontology students are motivated to seek career shifts and intentional training and comprehensive education. Understanding and applying this approach allows students to build on their prior skills, especially caregiving experience, to advance to more professional roles in the field of Gerontology. During this symposium we will focus on the Applied Gerontology aspect of Community College programs, including short-term certificates and other approaches related to and informed by the rapidly evolving workforce development in the field of aging. Selected Community College and University representatives, who understand and are leaders in the field of Applied Gerontology, will highlight models of existing Community College and University partnerships/collaboration that work, as well as provide models of other applied approaches. In addition, there will be an opportunity for robust dialog between Community Colleges and four-year Colleges and Universities – both on developing other effective Applied Gerontology approaches, and on creating even better partnerships and collaboration. This symposium will appeal to professionals working in both two and four-year systems of higher education. Colleges and Universities desiring to develop or enhance relationships with area Community Colleges will find this session especially helpful.

### TOOLS FROM OUR APPLIED GERONTOLOGY TOOLBOX: FLIPPING MINDSETS AND PROPELLING INNOVATION

Roger A. Anunsen,[1] Roger Anunsen,[1] and Jan Abushakrah[1], *1. Portland Community College, Portland, Oregon, United States*

PCC's associate degree program has long embraced an applied approach to gerontology education through its career pathway model. This model incorporates a rock-solid theoretical base, experiential learning, and skill-development, all grounded in the foundational, interactional and contextual AGHE Gerontology Competencies. The Competencies infuse all our courses, our five certificates and our degree to effectively prepare graduates for their next step, whether a transfer a specialized career. Our toolbox includes robust, wrap-around supports that include empowering advising and career planning, intentional internships, mentoring, networking opportunities, and our newest tool, The Flipping Mindsets Project. We will demonstrate how these evidence-based approaches provide students the knowledge, skills and confidence propelled by a positive mindset, to go boldly into any field of aging as innovators within existing jobs and programs or as ground-breaking entrepreneurs.

### ADDRESSING THE SUCCESSFUL TRANSITION OF COMMUNITY COLLEGE GERONTOLOGY STUDENTS TO UNIVERSITY SETTINGS

Margaret M. Manoogian[1], *1. Western Oregon University, Monmouth, Oregon, United States*

Developed in 2012, our gerontology program has tracked enrollment, learning outcomes, student competency development, and career paths post-graduation. Enrolled students tend to be older, transferring from community colleges, reentering college due to work and family commitments, and retooling career paths. Our efforts have focused on career integration across all courses culminating in a two-term practicum program. Additionally, through discussions with community college faculty and students, alumni, and community partners, we have adapted our curriculum to address student needs for work, family care, and academic engagement through flexible course delivery, syllabus construction, applied project development, and direct contact with professionals in and outside the classroom. Developing strategies to ensure transfer student success is critical, as well as offering strong career preparation for older students entering the workforce. Comprehensive placement data and an overview of the needs and challenges for university programs to partner with community college programs will be highlighted.

### APPLIED GERONTOLOGY: HOW STUDENTS ADDRESS LONELINESS IN THE LOCAL COMMUNITY

Eric Schoenmakers[1], *1. Fontys University of Applied Sciences, Eindhoven, Netherlands*

Fontys University of Applied Sciences offers an Undergraduate program in Applied Gerontology. Full-time and part-time students are trained in gerontology and in applying this knowledge in developing and implementing products and services in order to improve quality of life of older adults. In our vision, students learn in practice. Therefore, the educational program largely consists of authentic projects for real organizations in the local community. In one of these ongoing projects, students research loneliness in the local community. A network of organizations, which communicates intensively with the University, is involved. For these organizations, students study how clients and employee's think about (coping with) loneliness, write testimonials about interventions, and advise in improving the services of organizations. For students, these kind of projects offer opportunities to learn in practice. For the community, students' involvement means extra manpower to further develop services and society as a whole.

### WORKFORCE TRAINING FOR MIDDLE-AGED AND OLDER ADULTS: THE ROLE OF COMMUNITY COLLEGES

Phyllis Cummins[1], *1. Scripps Gerontology Center, Miami University, Oxford, Ohio, United States*

In recent years, occupational changes have become increasingly common for mid-and later-life (MLL) workers. The aging population in the U.S. has resulted in increased opportunities in health-related related occupations and many MLLs seeking to re-career choose these professions. Because of their convenient locations and open access policies, community colleges are an ideal educational setting for MLLs to seek training. This presentation will discuss the results of a research project funded by the U.S. Department of Education that included focus groups and key informant interviews at community colleges in Ohio. More specifically, we will discuss reasons MLL students enroll at a community college, their process for choosing a program of study, services that are important to successful completion, and barriers they face. Finally, we will discuss policies and practices that can improve MLL student outcomes.

### THE ROLE OF COMMUNITY COLLEGES IN PARTNERSHIPS WITH UNIVERSITIES IN PRODUCING QUALIFIED GERONTOLOGY PROFESSIONALS

Karen Kopera-Frye[1], *1. New Mexico State University, Las Cruces, New Mexico, United States*

Today is especially critical for community colleges and universities to work together to allow for pathways in educating professionals in the field of gerontology. At New Mexico State University, Public Health Sciences Department, we have partnered with Dona Ana Community College and Central New Mexico College through articulation agreements to allow transfer of credits and an accelerated pathway to a Gerontology Minor for workforce development. This paper will discuss the steps we took in bridging these educational opportunities as an example for other institutions to consider. By incorporating joint Advisory Boards, faculty and administrators entering into articulation agreements, and coordinated course offerings, we were able to systematize the curriculum and allow for transfer of up to 12 credits from the community colleges into our Public Health Bachelors degree program, with attainment of the Gerontology minor along the way. Lessons learned during this process will be shared.

### SESSION 1290 (SYMPOSIUM)

### UNDERSTANDING AND MEETING THE NEEDS OF RESIDENTS IN LONG-TERM CARE FACILITIES: A MULTIDISCIPLINARY PERSPECTIVE

Chair: Barbara Hanratty, *Newcastle University, Newcastle upon Tyne, United Kingdom*
Co-Chair: Karen Spilsbury, *University of Leeds, Leeds, United Kingdom*

Long-term care facilities play a vital role in the care of older people. Across the world, service providers face common challenges to the delivery of high quality care to residents. Rising levels of morbidity and dependency, recruiting and retaining a skilled workforce, and separation from mainstream services are some of the issues that make this one of the most precarious care sectors. In this symposium, we will consider the evidence underlying some of these challenges, along with current and possible future service responses. The first presentation will look at factors that increase the risk of transition to dependency in the Newcastle 85+ cohort study. This is followed by an analysis of trends over time in health, morbidity and disability in the UK care home population, drawing on data from three later life cohorts. Having considered the characteristics and needs of residents, the next presentations move onto care services. Findings will be presented from a mixed methods study on the relationship between care home staffing and quality of care, followed by a study of the organisation of primary care for long term care facilities. This session will end by looking to the future, with findings from rapid syntheses of international evidence on technology, and evaluation methods, in animated format. Together, these presentations will enhance our understanding of the relationships between the needs of residents in long-term care facilities, demands on service providers and quality of care. We aim to stimulate debate and discussion on future directions for research and practice.

### ORGANIZING PRIMARY CARE FOR RESIDENTS IN LONG-TERM CARE FACILITIES: A MULTI-METHOD APPROACH

Barbara Hanratty,[1] Katie Brittain,[2] Rachel Stocker,[3] and Karen Spilsbury[4], *1. Newcastle University, Newcastle upon Tyne, United Kingdom, 2. Northumbria university, Newcastle upon Tyne, England, United Kingdom, 3. University of Newcastle, newcastle upon Tyne, England,*

*United Kingdom, 4. University of Leeds, Leeds, England, United Kingdom*

This multi-method research explores the challenges family practitioners and long-term care facilities face when they work together. It seeks to understand how different responses to these challenges may influence the delivery of care. Whilst different services have their own values, aims, structures and processes, all are contending with constrained resources and frequent organisational change. Our findings from a large qualitative study and analysis of routine health data are organised around the micro (individual), meso (organisational) and macro (system) factors that influence the organisation and delivery of resident care. In this presentation, we draw out the interplay between these levels, and how each shapes and is shaped by the changing demands and nature of care. This presentation will bring new insights into primary care for long-term care facilities, through the perspectives of those who experience and provide care in that setting.

### FACILITATORS OF DEPENDENCY IN THE VERY OLD: RESULTS FROM THE NEWCASTLE 85+ STUDY

A Kingston,[1] Louise A. Robinson,[2]
Rachel Duncan,[3] and Carol Jagger[3], *1. Newcastle University, Newcastle upon Tyne, United Kingdom, 2. University of Newcastle, Newcastle upon Tyne, England, United Kingdom, 3. Newcastle University, Newcastle upon tyne, England, United Kingdom*

In order for governments to plan health and social care strategies to help people maintain independence, evidence is required to show how risk factors are associated with progression in dependency. We use a transparent measure of dependency, based on help needed with activities of daily living, incontinence and cognitive impairment, categorised as: high (24-hour care); medium (daily care); low (less than daily) and independent, then characterise changes over ten years (age 85-95) using the Newcastle 85+ Study while exploring how eight disease groups, multimorbidity and impairments interact to increase care needs. Stroke and diabetes confer an increased risk of low-level dependency. Complex multimorbidity, or three or more falls engendered the greatest risk of transitions to substantial dependency. There should be a focus on prevention of, and appropriate and efficient service provision for those with complex multimorbidity with emphasis on stroke, diabetes and falls, to maintain the independence of older people

### HEALTH AND WELL-BEING OF OLDER ADULTS IN LONG-TERM CARE FACILITIES: LESSONS FROM LONGITUDINAL STUDIES.

Robert O. Barker,[1] Andrew Kingston,[2]
Fiona Matthews,[2] and Barbara Hanratty[2], *1. Newcastle University, Newcastle upon Tyne, United Kingdom, 2. Newcastle University, Newcastle upon Tyne, England, United Kingdom*

Older adults in long-term care facilities (LTCF) have complex needs for health care and support. There is a perception that residents' needs are increasing over time, but little research evidence to back this up. In this study we brought together data on 1640 residents in LTCFs from three longitudinal studies, and conducted repeated cross-sectional analyses across a 25 year period. We found that the prevalence of severe disability amongst residents has increased from 56% to 80% over a 20 year period, driven by increases in difficulties in bathing and dressing. The prevalence of multimorbidity also increased from 29% to 56% between 2006 and 2014. A growth in the number of people with dementia, cardiovascular and cerebrovascular diseases contributed to this. We conclude that residents in LTCFs have become a selected subset of the population, characterised by increasing needs for support. This poses an important challenge for future care provision.

### RELATIONSHIP BETWEEN CARE HOME STAFFING AND QUALITY OF CARE: A MIXED-METHODS APPROACH

Karen Spilsbury,[1] Andy Charlwood,[2]
Danat Valizade,[2] and Kirsty Haunch[2], *1. University of Leeds, Leeds, United Kingdom, 2. University of Leeds, Leeds, England, United Kingdom*

Beyond broad recognition that 'staff influence quality', little is known about the care home workforce and its relationship to quality. Our study examines this relationship for the first time in the UK. Quality is a complex, contested and dynamic concept: we have operationalised this concept using data collected at national and organisational levels to measure quality, as well as considering the views of quality of different stakeholders. We will present interim findings from this study's work packages (due to complete in July 2020), including: (1) our review which has developed theoretical explanations of how, why and in what circumstances staffing promotes quality for residents and relatives; and (2) our observational study using routine administrative data sets at national and provider level. Key findings include the importance of experience and stability of care hoe staff, as well as how care is delivered (individual commitment, team reciprocity and organisational mandate).

### NEW MODELS OF CARE FOR LONG-TERM CARE FACILITIES: WHAT DOES THE EVIDENCE TELL US?

Katie Brittain,[1] Dawn Craig,[2] Karen Spilsbury,[3]
Paul Wilson,[4] Katie Brittain,[5] and John Vines[5], *1. Northumbria University, Newcastle, United Kingdom, 2. University of Newcastle, Newcastle upon Tyne, England, United Kingdom, 3. University of Leeds, Leeds, England, United Kingdom, 4. University of Manchester, Manchester, England, United Kingdom, 5. Northumbria University, Newcastle upon Tyne, England, United Kingdom*

Models of care are evolving to meet the demands of an ageing population in long-term care facilities. Syntheses of current evidence are an essential to inform future change. In this project we conducted a rapid synthesis of evidence relating to enhancing health in long term care facilities across technology and evaluation. Mapping reviews were conducted on the uses, benefits and challenges of technology in care homes and approaches to evaluation of new models of care. Systematic evidence syntheses addressed the questions of which technologies have a positive impact on resident health and well-being and which measurement tools have been validated for use in UK care homes. Key findings will be presented in animated format, including that the most promising interventions appear to be games that promote physical activity and enhance mental health. This presentation will highlight the benefits and importance of evidence synthesis to the development of models of care.

### SESSION 1295 (SYMPOSIUM)

### USING ACCESSIBLE TECHNOLOGY TO SUPPORT CAREGIVERS OF PERSONS WITH DEMENTIA

Chair: Katie Maslow, *Gerontological Society of America, Washington, District of Columbia, United States*
Co-Chair: Scott A. Trudeau, *American Occupational Therapy Association, Bethesda, Maryland, United States*
Discussant: Sara J. Czaja, *Weill Cornell Medicine, New York, New York, United States*

There is widespread enthusiasm about the potential of technology in general to support persons living with dementia and their families and other caregivers. At the same time, recommendations from the 2017 National Research Summit on Care, Services, and Supports for Persons with Dementia and their Caregivers emphasize the need for research to develop, evaluate, and disseminate specific technologies that can achieve meaningful benefits for well-defined subgroups of persons living with dementia and their caregivers, including individuals from diverse populations and individuals who live and receive care in various settings. This symposium focuses on specific home-based technologies to help family caregivers of community-living persons with dementia. Our three speakers will talk about research results for three different technology-related interventions, including: use of home video telehealth visits to help family caregivers provide effective dementia care and provide medical management; use of home video assessments by occupational therapists to help family caregivers increase home safety for community-living persons with dementia; and approaches for making a self-paced Home Safety Toolkit available to family caregivers of community-living veterans with dementia. Each speaker will report both positive outcomes, including family caregiver satisfaction, and barriers encountered in delivering the interventions. Such barriers include difficulties with the technologies as well as caregiver reluctance to change and costs that were not covered by health care insurance or health systems. Our discussant will respond to the presentations and solicit audience questions and discussion.

### DEMENTIA CAREGIVER EMBRACE OF HOME VIDEO TELEHEALTH VISITS

Lauren Moo[1], *1. Geriatric Research Education & Clinical Center, Bedford, Massachusetts, United States*

Bringing people with dementia to in-person medical visits can be logistically challenging for family caregivers, especially when they themselves are older adults with their own health or mobility challenges, when they live far from the clinic, or when they have to combat inclement weather. Our dementia management clinic has successfully trialed video visits into the home. Video sessions have been welcomed by many dementia caregivers citing reduced travel and less disruption of daily routine as the primary benefits of participating. Caregivers report equivalent visit satisfaction compared to in-person visits. While technical issues have been common, most were just brief audio or video lags. Expansion of HIPAA compliant telemedicine software options across devices is increasing the population of caregivers who are able to participate in home video visits. (127 words)

### CAREGIVER EXPERIENCE OF TELEHEALTH-DELIVERED HOME SAFETY EVALUATIONS

Megan Gately[1], *1. Bedford VA Medical Center Geriatric Research, Education & Clinical Center, Bedford, Massachusetts, United States*

People with dementia are living in the community, necessitating in-home supports for their day-to-day needs. Given geriatrics work force shortages, innovative strategies that increase the reach of extant providers while maintaining quality are needed. Home-based video telehealth may increase access to specialty care such as a dementia-focused home safety evaluation by an occupational therapist; however, little is known about the technological demands and caregiver experience of a home safety evaluation delivered by telehealth. Our study employed video telehealth to deliver a dementia-focused home safety evaluation compared to in-person evaluation for caregivers (n=10) of veterans with dementia. Most video visits encountered technological problems. Caregiver experience between the video and in-person evaluations differed. Our findings reflect the highly dynamic, complex nature of in-home video telehealth which requires maximal collaboration with caregivers. By explicating the resource demands and potential burden of video telehealth for caregivers, development of effective in-home telehealth evaluation is enhanced

### IMPLEMENTING HOME SAFETY TOOLKIT TO CAREGIVERS OF VETERANS WITH DEMENTIA

Scott A. Trudeau[1], *1. American Occupational Therapy Association, Bethesda, Maryland, United States*

The purpose of this project was to study the processes necessary to make a Home Safety Toolkit (HST) for Veterans with dementia accessible to veterans and their caregivers. This Type 3 Implementation–Effectiveness Hybrid Research Design, included diagnostic analyses of the current processes by which Veterans receive home safety items, and identification of modifications necessary in order to provide the HST to Veterans with dementia. Two Veterans Health Administration Networks, one in the Northeast and one in the Mid-Atlantic region, participated. A formative evaluation used semi-structured interviews with key staff informants and caregivers identified facilitators and barriers to successful acquisition and use of home safety items. Qualitative data analysis reveals key barriers of time and cost, selection of best items, and caregiver reluctance to change. There was resounding support from caregivers regarding the potential benefits of self-paced toolkit including education and home safety items to implement for their veteran.

### SESSION 1297 (SYMPOSIUM)

### EAST MEETS WEST: IMPROVING HEALTH AND LONG-TERM CARE FOR OLDER ADULTS IN THE U.S. AND CHINA

Chair: Bei Wu, *New York University, New York, New York, United States*

Co-Chair: Jie Hua Lu, *Department of Sociology, Peking University, Beijing, Beijing, China*

As the number of older adults in the U.S. and China continues to increase, promoting healthy aging is essential for individuals, family, and society. Both countries face many similar issues due to their aging populations, including prolonging healthy life expectancy and providing quality of care. However, the change in demographics brings with it unique challenges for both the U.S and China. This forum invites scholars and researchers from these two countries to share their knowledge and insights on promoting healthy aging and improving care for older adults. This forum includes five presentations and one-panel discussion. Two presentations will focus on long-term care (LTC) in China, one is to forecast the needs of LTC in the next five decades, and the other is to evaluate the current LTC needs and discuss LTC policy. Using the data from the Health and Retirement Study, the third presentation aims to re-conceptualize spousal caregiving as a dyad-level phenomenon and provides a dynamic view of the spousal caregiving experiences. The last two presentations will focus on promoting healthy aging through clinical interventions. The fourth one is to evaluate the effectiveness of adaptive computer-based cognitive training among community-dwelling older adults in China. The last presentation provides some examples of using pragmatic clinical trials to improve the care of older adults in skilled nursing facilities in the U.S. After the five presentations, the three panelists will provide feedback to the presentations and share their views on healthy aging with the audience.

### THE FORECASTING OF POPULATION SUPER-AGING AND POLICY SIMULATION OF LONG-TERM CARE IN CHINA (2019-2070)

Chen Chen[1], *1. Institute for Population and Development Studies, Zhejiang University, Hangzhou, Zhejiang, China*

The purpose of this paper is to provide a policy simulation of Long-Term Care based on the aging population forecast for China, focusing on the super-aging and oldest-old segments of the population. As a developing country, it is a challenge for China to increase its wealth with the financial implications of an aging nation. The study identified two significant turning points for population development: 1) The multi-pilot program of long-term care policy reform must be executed between 2016 – 2020. This reform is time dependent because the multi-state longevity development period is from 2016 to 2030 and in the year 2030, the oldest-old population will be part of China's society. 2) The year 2060 will be representative of a stable period in China, as the age of China's population will no longer be accelerating.

### LONG-TERM CARE NEEDS BY CHINESE ELDERLY AND POLICY PRIORITIES IN CHINA

Juan Juan Sun,[1] and Haichao Wu[1], *1. Institute of Gerontology School of Sociology and Population Studies, Renmin University of China, Beijing, Beijing, China (People's Republic)*

With the life expectancy in China continuing to increase, age-dependent chronic diseases are also likely to increase, as is the number of people with long-term care needs. This study evaluated the Long Term Care (LTC) needs of the Chinese older population and introduced related policy priorities. Using the 2014 and 2016 "China Longitudinal Aging Social Survey", this study assessed the physical functions of older adults by measuring their ability to perform Activities of Daily Living independently, compared changes within the two years, and explored other related indicators including, Instrumental Activities of Daily Living, major chronic disease, and mental health conditions. The study also discussed the development of long-term care policies in China and highlighted the priorities of these policies.

### RE-CONCEPTUALIZING SPOUSAL CAREGIVING FROM A DYADIC PERSPECTIVE IN THE U.S.

Yuekang Li,[1] Huiying Liu,[2] Yi Wang,[1] and Nancy Morrow-Howell[1], *1. Washington University in St.Louis, St.Louis, Missouri, United States, 2. The university of Hong Kong, Hong Kong, Not Applicable, Hong Kong*

Existing literature on later-life spousal caregiving tends to focus on one member of the marital dyads, assuming a priori distinction between the caregiver and care-receiver. Theoretically and empirically, this individualistic role-related (caregiver-receiver) approach is inaccurate, as the concept of spousal care intrinsically involves two people within a marital dyad. Therefore, this paper used a social exchange perspective to re-conceptualize spousal caregiving as a dyad-level phenomenon. Using the 2014 wave of the Health and Retirement Study, 6,500 individuals (3,250 couples) aged 65 and above and their spouse were selected. Based on each partner's need for care, receipt of spousal care, and provision of spousal care, the study identified five distinctive caregiving typologies. Household-level factors such as the availability of other types of informal care were associated with these typologies. This paper offers a broader and more dynamic perspective of the spousal caregiving experiences.

### EFFECTS OF ADAPTIVE COMPUTER-BASED COGNITIVE TRAINING ON COMMUNITY-DWELLING ELDERS

Guifang Guo,[1] and Huijuan Gongzhi[2], *1. School of Nursing, Peking University, Beijing, Beijing, China, 2. Qingdao Municipal Hospital, Qingdao, Shandong, China (People's Republic)*

This quasi-experimental designed study analyzed the effects of adaptive computer-based cognitive training among community-dwelling older adults. A 6-week (5 times/week) program was implemented with an intervention group (Difficulty Adaptive Training) and control group (Difficulty Fixed Training). General cognitive, memory, executive and attention functions were evaluated before (T1), completion (T2), and one month after intervention (T3). Sixty-one participants completed data collection. (1) General cognitive function: improved in both groups at T2, and T3, intervention group had better effect; (2) Memory function: improved in both groups in immediate, short and long-delayed recalls at T2 and T3, and recognition at T2. (3) Executive function: improved in both groups. Time of simple information processing was shortened at T2 and T3 in intervention group, at T3 in control group; TMT response inhibition was shortened at T2 and T3 in both groups. (4) Attention function: digit span forward was improved at T2 in intervention group.

### PROMOTING HEALTHY AGING THROUGH PRAGMATIC CLINICAL TRIALS

Joshua Chodosh,[1] Mary P. Cadogan,[2] and Jennifer L. Martin[3], *1. New York University Langone*

*Health, New York, New York, United States, 2. UCLA School of Nursing, Los Angeles, California, United States, 3. VA Greater Los Angeles Healthcare System, UCLA, North Hills, California, United States*

Promoting healthy aging does not end when people enter skilled nursing facilities (SNF) where the demands for clinical and psychosocial care are likely to be greatest. Many chronic conditions present opportunities for better SNF care and thus, healthier aging. Such conditions cannot wait for the often-long path to discovery that is typical of most traditional randomized controlled clinical trials. Conversely, pragmatic clinical trials are real-world investigations that offer the possibility of immediate benefit while answering important research questions. Depression and disrupted sleep are two examples of treatable conditions with opportunities for immediate benefit through pragmatic trials and applied best practices. How best to support best-practice integration has received increasing attention but identifying the most effective strategies continues to evolve. We report two different SNF-mentorship models utilizing Minimum Data Set (MDS) data for depression and environmental (noise-level) data for disrupted sleep, which have supported better SNF practices and presumably, healthier aging.

### SESSION 1300 (POSTER)

### AGEISM | DISPARITIES | DIVERSITY

### RACIAL-ETHNIC DISPARITY IN DENTAL CARE IN NURSING HOMES

Zhiqiu Ye,[1] and Bei Wu[2], *1. University of Rochester Medical Center, Rochester, New York, United States, 2. New York University, New York, New York, United States*

Minority older adults are at higher risks of poor oral health. Little is known about the extent of and the contributing factors to racial/ethnic disparity in dental care quality in the long-term care settings. Previous studies suggest that organizational and system-level factors are key determinants of oral health among minority older adults. We examined the racial/ethnic disparity in dental care delivery in nursing homes (NHs) by facility and market characteristics. We analyzed the 2000-2016 national Inspection Survey data for all certified-NHs (n=248,975 facility-years). Two designated deficiency citations were used to measure dental care performance. Generalized estimating equations were used to compare the rates of deficiency citations among NHs in different quartiles of the share of minority residents, adjusting for facility characteristics, market characteristics, year and state fixed effects. Overall, compared to NHs in the lowest quartile of the share of minority residents (average % minority residents =0.24%), NHs in the highest quartile of the share of minority residents (average % minority residents = 46.5%) and those in the second highest share (average % minority residents=13.9%) had 46.8% and 31.2% higher odds of receiving dental care citations($p<0.001$ for both), respectively. The increased citation rates persisted over time ($p=0.40$) and were greater among for-profit NHs ($p=0.02$). Our study suggests that minority older adults in NHs are disproportionately affected by poorer dental care performance. There is a great need to improve quality of dental care in NHs, particularly for those that are for-profit and those that disproportionately serve minority residents.

### SCALES FOR MEASURING AGEISM AS EXPERIENCED BY OLDER ADULTS: LITERATURE REVIEW AND METHODOLOGICAL CRITIQUE

Hyun Kang[1], *1. University of Kansas, Lawrence, Kansas, United States*

A growing body of research shows that ageism negatively affects older adults' psychological well-being and even physical functioning. However, the tools to measure ageism as experienced by older adults are not well developed. This study reviewed the literature on ageism scale with an emphasis on the methodological issues. Most standardized ageism scales have focused on younger people's attitudes and beliefs toward older adults. We found only one standardized scale that examined how older adults felt and thought about their experiences being treated as a stereotype. However, the scale is incomplete because it does not fully measure ageism and it has received far less rigorous analysis. Many studies have adopted and revised ageism scales that were developed specifically to measure younger people's attitudes toward older adults, meaning that the scales' validity has been problematic when administered to older adults. Furthermore, many studies that discussed older adults' experience of ageism used uni-dimensional or simple measures. Although significant efforts have been made to outline ageism's various dimensions and constructs, these efforts have not led to a common consensus on ageism and its characteristics. Lack of consensus, in turn, makes it harder to develop a standardized scale. Finally, existing scales are more suitable for Western societies. Socio-cultural uniqueness has not been considered when developing scales, nor has the scales' cross-cultural reliability and validity been tested. Our findings suggest that a new scale that applies only to measuring ageism as perceived by older adults and corresponds to the significant dimensions of ageism must be developed.

### LONGITUDINAL MENTAL HEALTH CONSEQUENCES OF PHYSICAL DISABILITIES: THE MEDIATING ROLE OF PERCEIVED DISCRIMINATION

Eun Ha Namkung,[1] and Deborah Carr[2], *1. Brandeis University, Waltham, Massachusetts, United States, 2. Boston University, Boston, Massachusetts, United States*

Individuals with disabilities have been historically mistreated by discrimination. The detrimental mental health effects of self-reported interpersonal discrimination are well established. However, little empirical attention has been given to the role of perceived discrimination in the adverse mental health outcomes of adults with physical disabilities. This study aims to examine whether daily interpersonal discrimination (i.e., microaggression) mediates the prospective association between having a functional impairment and subsequent changes in the individuals' mental health outcomes over their midlife and old age. To address this question, this study used data from two waves of a population-based national study, the National Survey of Midlife Development in the United States, covering a 7- to 9-year period (n= 2,503; Mage at baseline = 57, SDage = 11). Physical disability or functional impairment was assessed with items adapted from the SF-36, capturing difficulty with nine activities of daily living. Having functional impairment at the baseline assessment was associated with increases in depressive symptoms and negative affect over the study period. Daily interpersonal discrimination partially mediated this longitudinal association, explaining 7.4% (for depressive symptoms) to 8.1% (for negative affect) of the total effects. Exposure to discrimination and its mental health consequences were also more pronounced at younger ages. Disability-related perceived discrimination is an under-recognized mechanism that is likely to contribute to mental health inequities in later life. Professionals in health and disability policy, research, and practice need to concentrate efforts on developing policy and programs that reduce discrimination experienced by US adults with disabilities.

### CORRELATES OF BREAST CANCER SCREENING BEHAVIORS AMONG INDIGENOUS WOMEN

Soonhee Roh,[1] Yeon-Shim Lee,[2] Kyoung Hag Lee,[3] and Jung Sim Jun[4], *1. University of South Dakota, Sioux Falls, South Dakota, United States, 2. San Francisco State University, San Francisco, California, United States, 3. Wichita State University, Wichita, Kansas, United States, 4. Kansas State University, MANHATTAN, Kansas, United States*

Cervical cancer remains a significant cause of morbidity and mortality among women globally; yet cancer burden is unevenly distributed among racial/ethnic groups. With 12,820 new cases in 2017 in the U.S., cervical cancer is the top cause of death among Indigenous women. Indeed, cervical cancer mortality rates among AI women in South Dakota are five times the national average and 79% higher compared to Whites in that region. This study examined predictive models of utilization of mammograms among Indigenous women adapting Andersen's behavioral model. Using a sample of 285 Indigenous women residing in South Dakota, nested logistic regression analyses were conducted to assess predisposing (age and marital status), need (personal and family cancer history), and enabling factors (education, monthly household income, mammogram screening awareness, breast cancer knowledge, self-rated health, and cultural practice to breast cancer screening). Results indicated that only 55.5% of participants reported having had a breast cancer screening within the past 2 years, whereas 21.0% never had a mammogram test. After controlling for predisposing and need factors, higher education, greater awareness of mammogram, and higher utilization of traditional Native American approaches were significant predictors of mammogram uptake. The results provide important implications for intervention strategies aimed at improving breast cancer screening and service use among Indigenous women. Educating health professionals and Indigenous community members about the importance of breast cancer screening is highly needed. It is critical to assess a woman's level of traditional beliefs and practices and its possible influence on screening participation and future screening intention.

### WE CAN'T AVOID IT. IT'S THERE! AGEISM EXPERIENCED BY DIVERSE CULTURAL GROUPS IN OTTAWA, CANADA

Caroline D. Bergeron,[1] and Martine Lagacé[2], *1. Bexar County Community Health Collaborative, San Antonio, Texas, United States, 2. University of Ottawa, Ottawa, Ontario, Canada*

Introduction: Discrimination based on age is pervasive across Canada. Little is known about the experiences of ageism among diverse cultural groups. The purpose of this pilot study was to explore the perceptions of ageism among culturally diverse older adults in Ottawa, Canada. Methods: Three focus groups were conducted with Chinese, Arab, and Indian older adults in Ottawa in June 2016. An 8-item protocol was developed to guide the discussions. Qualitative data were analyzed using open, axial, and selective coding. Results: Twenty-five culturally diverse older adults (9 Chinese, 6 Arab, and 10 Indian) participated in the focus groups. All described personal positive and negative examples of discrimination based on their age without being familiar with the term "ageism". Several described their experiences with the intersection of age, race, and gender, although these interpretations varied by cultural group. Ageism in the media was also easily recognized. Participants recommended using specific content, communication channels, and organizations to counteract ageism. Discussion: This pilot study helped to illustrate that ageism is a societal problem that requires a societal solution. As Canada's population becomes older and more diverse, important efforts are needed to raise awareness of ageism.

### OLDER ADULT HEALTH IN THE CITY OF CHICAGO

Brittney S. Lange Maia,[1] Emily Laflamme,[2] Fernando DeMaio,[3] and Raj C. Shah[4], *1. Rush University Medical Center, Chicago, Illinois, United States, 2. Chicago Department of Public Health, Chicago, Illinois, United States, 3. DePaul University, Chicago, Illinois, United States, 4. Rush University, Chicago, Illinois, United States*

In 2012, Chicago was designated as an Age Friendly City. However, city-wide data on the health and health disparities experienced by older adults have been scarce. In order to address this knowledge gap, the Chicago Department of Public Health (CDPH) partnered with the Center for Community Health Equity at Rush and DePaul Universities to create a report describing health status among adults age 65+. Data were from the Healthy Chicago Survey—a population-based health survey conducted by CDPH, the American Community Survey, Hospital Discharge Data, and State Vital Records. The report highlights considerable racial/ethnic diversity in Chicago, as 38% of older adults are white, 37% black, 18% Latinx, and 7% are Asian. Encouraging results exist regarding healthcare access; 96% have a personal health care provider and 89% report being able to get care needed through their health plan. Several areas of improvement are needed regarding root causes of health. More older adults live below the federal poverty level (15.9%) compared to the overall U.S (9.3%), and 45.8% would be unable to pay for an unexpected $400 expense. Disparities were evident as life expectancy at age 65 is 2.5 years longer for Latinx and white older adults (age 85) compared to African Americans (age 82.4). African American and Latinx older adults had higher rates of preventable hospitalizations per 10,000 (801.1 and 678.9, respectively) compared to white (492.4) and Asian (374.1) older adults. Findings from this report will spur

Chicago's continued progress as an Age-Friendly City for all its residents.

### THE INFLUENCE OF SOCIODEMOGRAPHIC FACTORS ON GENDER DISPARITIES IN DISABILITY TRENDS

Ya-Mei Chen,[1] Hsiao-Wei Yu,[2] Tung-Liang Chiang,[1] Duan-Rung Chen,[1] and Yu-Kang Tu[1], *1. National Taiwan University, Taipei, Taiwan, 2. Chang Gung University of Science and Technology, Taipei, Taiwan*

Objective. In Taiwan as well as in many other aging societies, decreasing disability is a key public health priority. Gender is known to be a significant factor for developing disability. Our study aimed to examine gender disparities in disability trends as well as how sociodemographic factors influence these disparities. Methods. We used multiple-group latent growth curve modeling (MG-LGM) to examine data drawn from the Taiwanese Longitudinal Study of Aging, a longitudinal and nationally representative survey database. Four waves of survey data and 3,429 older adults (mean ages = 50-96) were included for analysis. Disability trajectories among men and women were modeled separately using MG-LGM. Equality constraints were imposed on the six factors assessed: age, education, leisure activities, perceived (self-rated) health, health behaviors, and comorbidities. Results. Baseline disability levels were not significantly different between the two groups, but once disability began, the progression toward greater disability was almost 50% faster among older women. Greater age and more comorbidities added significantly more to baseline disability and speed of progression among older women than among older men ($p < 0.001$). However, having better health behaviors (e.g., no alcohol, more leisure activities) reduced baseline disability significantly more among women ($p < 0.05$). Particularly interesting findings include that perceived health reduced baseline disability only among men ($p < 0.05$), while having a better social network reduced baseline disability only among women ($p < 0.05$). Conclusion. For older women, disability prevention is crucial, and promoting positive health behaviors and strong social networks are promising strategies.

### ETHNIC DISPARITY IN NUTRITIONAL STATUS: EVIDENCE FROM ECUADOR'S NATIONAL SURVEY OF HEALTH, WELFARE AND AGING (SABE)

Takashi Amano,[1] and Carlos Andres Gallegos[1], *1. Brown School, Washington University in St. Louis, St. Louis, Missouri, United States*

Providing effective public services to improve the nutritional status among older adults is pivotal in countries experiencing population aging. Public investment and social policy in Ecuador have focused mainly on standard food-based interventions and cash transference programs. These efforts, however, may be not effective enough to reach those populations that need the most. This study aims to identify those populations that should be targeted by specific interventions. Data were drawn from Ecuador's Survey of Health, Welfare and Aging (SABE) – 2009, a probability sample of households with at least one person who were 60 years or older in Ecuador. The final sample consisted of 5,235 people who were 60 years or older. Ethnic identity was categorized into four categories: Indigenous People, Mestizo (Mixed of Spanish and Indigenous People), Afro-Ecuadorian/Mulato, and Other. Nutritional status was measured using Mini Nutritional Assessment (MNA). Ordered logistic regression analysis was utilized to assess the association between ethnic identity and nutritional status. Results revealed that the Indigenous ethnicity was significantly associated with worse nutritional status compared to Mestizo and Other even after controlling for a range of covariates including socio-economic status, health related factors, and social support. These findings suggest the existence of underlaying factors hindering the nutritional status of among indigenous older adults in Ecuador. Considering the information revealed by SABE, interventions and other strategies should be targeted and designed specifically accounting for the needs, preferences, and culture of the most vulnerable population.

### RACIAL BIAS IN STAFF OPTIMISM ABOUT ADL IMPROVEMENT AMONG NURSING HOME RESIDENTS

Kenneth J. Branco[1], *1. Stonehill College, Easton, United States*

Research on bias in health care has shown disparity in provision of care to and health outcomes of African Americans. Patient optimism was associated with improved physical and psychosocial outcomes, and nurse optimism was correlated with patient perceptions of care. We estimated effects of race using logistic regression, controlling for ADLs, cognitive impairment, and gender on both staff optimism and resident optimism about capacity for improvement in ADLs in a probability sample (n=2604) of nursing home residents who were evaluated with the resident assessment instrument (RAI). We found no difference between African American and White residents' optimism about their own capacity for improvement. Staff findings were quite different. Staff were most optimistic about the potential of residents who needed ADL assistance OR=1.82; 95% CI [1.42-2.32] over those who were ADL dependent or those who only needed oversight. Most importantly, it was in the oversight category of ADL impairment where the greatest indication of racial prejudice occurred. Staff were much less likely to be optimistic about African American residents (16%) compared to White residents (30%). With all control variables entered, staff were still less willing to be optimistic about African American resident improvement (AOR=0.65; 95% CI [0.44-0.96]. The implications of these findings are troubling. It is unlikely that staff would expend energy on improving the functioning of the African American residents whom they believe cannot improve. Further research is needed on the extent to which prejudice in nursing homes is accompanied by discrimination and how the bias can be overcome.

### HIGH-DEDUCTIBLE HEALTH PLANS AND COST OF HEARING AIDS AMONG ADULTS WITH HEARING LOSS: IMPLICATIONS FOR DISPARITIES

Elham Mahmoudi,[1] and Neil Kamdar[2], *1. University of Michigan, Department of Family Medicine, Ann Arbor, Michigan, United States, 2. University of Michigan, Ann Arbor, Michigan, United States*

High-deductible health plans (HDHPs) have shown potential to curb rising healthcare costs. We examined use and cost of hearing aids (HAs), comparing HDHPs with non-HDHPs. Using the 2009-2016 Truven Marketscan claims, we identified adults aged 50-64 who were diagnosed with hearing loss (HL) and whether they used HAs or not (n=1,247,113). We applied multivariable generalized linear models, adjusting for age, gender, hierarchical condition categories (HCCs). To control for potential selection bias, we applied an inverse propensity score weighting. Our outcomes of interest included: (1) utilization and (2) total and out-of-pocket costs of HAs (inflation adjusted to 2016 dollars), comparing HDHPs with non-HDHPs. Number of enrollees in HDHPs increased by 343%, from 1,717 in 2009 to 7,615, in 2016. The percentage of patients who used HA increased from 9.5% (95% CI:0.09-0.10) to 15% (95% CI:0.14-0.15) within non-HDHPs and from 5% (95% CI:0.04-0.06) to 16% (95% CI:0.15-0.17) within HDHPs. The average adjusted cost sharing and total cost of HAs increased by 74% ($85 to $148) and 52% ($589 to $894), respectively, among non-HDHPs; they increased by 80% ($173 to $312) and 91% ($589 to $1,126), respectively, among HDHPs. Average out-of-pocket costs for HAs in HDHPs were twice as much as in non-HDHPs (p <0.0001). Total and out-of-pocket costs for hearing aids were substantially higher among HDHPs compared with non-HDHPs. Many employers have started offering only HDHPs, leaving their employees with no other health insurance option. Higher cost sharing may worsen the existing socioeconomic disparities in access to HAs.

### OPPRESSIVE EXPERIENCES AMONG DEAF DIVERSE SENIOR CITIZENS

Audrey K. Frank[1], *1. Gallaudet University, Washington, District of Columbia, United States*

None of the limited literature on deaf seniors focuses on their experiences of oppression. There is an article that demonstrates that not many mental health professionals have the skills to work with deaf seniors. Obstacles have been noted in the literature among deaf people in general terms of being oppressed with employment, doctors, education, family discrimination, stereotyping, stigmas and cultural conflicts because they are deaf. The obstacles facing deaf seniors had not been specifically explored before this research. The shift attention to deaf seniors is needed in order to make their experiences known and at the same time their lives, values, and strengths need to be understood and recognized. Ninety-one deaf seniors from five states aged between 50 years and 93 years were interviewed to describe their recent experience on oppression. They shared their common experiences on oppression such as lack of communication with their doctors, hearing co-workers, and family members, struggling to get promoted at work, and being left out in the neighborhood. According to the deaf seniors, the community did not acknowledge or accommodate the special needs they had as deaf seniors. Their detailed descriptions provide consistent evidence that supports the importance of cultural awareness for medical and mental health professionals. The professionals are to enhance better understanding of experiences among deaf seniors. This lack of awareness highlights the need for research about deaf seniors' experiences of oppression and for research on what professionals should know about the special needs of this population.

### LIFE EXPECTANCY WITH CHRONIC DISEASE AMONG OLDER MEXICAN AMERICANS

Kerstin G. Emerson,[1] Anqi Pan,[1] and Hanwen Huang[1], *1. University of Georgia, Athens, Georgia, United States*

The Hispanic Paradox research consistently shows a survival advantage among Hispanics, where they have significantly longer life expectancy compared to non-Hispanic Whites. However, less is known about the quality of these additional years of life. Our goal was to calculate life expectancy for older Hispanics in the US, and to determine what proportion of their lives they could expect to live with chronic conditions. We used data from 2004-5 of the Hispanic Established Population for the Epidemiological Study of the Elderly (Hispanic-EPESE), linked to vital status data through 2016. To determine life expectancy with/without chronic conditions, we used Sullivan's method. Chronic conditions included: diabetes, stroke, heart attack, arthritis, hypertension, and cancer. Finally, we mapped life expectancy without chronic conditions across neighborhood characteristics. The sample consisted of 2,069 Mexican Americans aged 75 and older. Results showed that at age 75, Mexican Americans could expect to live a large portion of their lives with at least one chronic condition (88%). The largest proportion of life lived with disease was for hypertension (61%), arthritis (59%), and diabetes (33%). There was no pattern by neighborhood characteristics for disease-free life expectancy. Gender differences could not be examined because of small sample sizes. Our findings show that regardless of neighborhood characteristics, Mexican Americans can expect to live a high proportion of their life with at least one chronic condition. This is particularly high for hypertension, diabetes, and arthritis. It is important to consider quality as well as quantity of life when exploring the Hispanic Paradox.

### META-SYNTHESIS: OLDER ADULT SURVIVORS' RESILIENCE

Roberta R. Greene,[1] Harriet Cohen,[2]
Nancy A. Greene,[3] and Shira Hantman[4], *1. University of Texas at Austin, Austin, Texas, United States, 2. Jewish Family Service of Dallas, Dallas, Texas, United States, 3. Johnson C. Smith University, Charlotte, North Carolina, United States, 4. Tel Hai College, Upper galilee, Israel, Israel*

This paper presents the results of a meta synthesis of 8 qualitative studies that examined resilience among 270 older adult survivors following potentially traumatic adverse events (Bonanno, 2004). The primary data involved information about survivors' critical events during the Holocaust , the Cambodian genocide, and the discriminatory practices of the Jim Crow U.S. South. A narrative approach to gerontology was used to collect and analyze the original data. known as a constant comparison data analysis ( Glaser & Strauss, 2009; Miles & Huberman, 1994). This allowed for the understanding of risks such as such as discrimination, imprisonment, genocide, and possible loss of life and the development of themes of resilience at the personal, interpersonal, sociocultural, and societal levels (Kenyon & Randall, 2001) The meta synthesis of secondary data involved coding the original findings, grouping them into descriptive themes, and generating new overarching analytical themes (Thomas & Harden, 2008). The most prevailing theme at the personal-level (internal feelings) was overcoming the grief of losing a loved one. Interpersonal-level themes (relationships between people) were related to staying connected to family. Sociocultural-level themes (the beliefs and mores of the time) centered on making meaning of a critical event within its historical context Societal themes (government and institutions) themes were indicative of being able to contribute or influence one's current community. Implications for clinical practice and policy formation are provided.

### SESSION 1305 (POSTER)

### AGING IN PLACE | TRANSPORTATION AND HOUSING ISSUES

### IDENTIFYING HOUSING PREFERENCES IN AN AGE-FRIENDLY COMMUNITY

Natalie Pope,[1] and Allison Gibson[1], *1. University of Kentucky, Lexington, Kentucky, United States*

More than a decade ago, the World Health Organization's (WHO) age-friendly cities and communities program was established to guide communities in preparing for rapid population aging. WHO identified eight domains of livability that influence health and wellbeing of older adults—housing, one of the eight domains is the focus of this study. Partnering with our local Age Friendly initiative, we examined housing preferences and values for residents of a mid-sized urban community. A cross-sectional survey was administered online via Qualtrics and through face-to-face data collection across the community (e.g., public libraries, community farmers' markets). Participants aged 30+ completed a researcher-devised survey about housing-related preferences and values. Data were analyzed using descriptive and inferential statistics. Of our 518 respondents, half were 60+ and most were women (73.2%). Younger respondents (age 30-59) were more likely to be married or living with a partner (73.7%). There was little difference in housing preferences between older and younger respondents or among those with varying financial means. Analysis revealed the top five housing considerations were: "safety" (78.8%), "affordability" (73.2%), "privacy" (51.9%), "proximity to services I frequently use" (50.6%), and "accessibility" (42.4%). An unexpected finding was that almost 50% of older respondents expressed a willingness to share a home with a roommate. Data suggests that city planners and property developers should prioritize these preferences when planning for the housing related needs of older residents. Alternative housing models, such as cohousing, should be explored further. Future research should examine interpretation of such housing preferences (i.e., what's considered affordable?).

### DISTANCE FROM APARTMENT TO ACTIVITY AS A BARRIER TO PARTICIPATION IN GROUP ACTIVITIES FOR ASSISTED LIVING RESIDENTS

Rebecca L. Mauldin,[1] and Jason Fernandez[2], *1. University of Texas at Arlington, Arlington, Texas, United States, 2. University of Houston, Houston, Texas, United States*

Participating in group activities is potentially also beneficial for residents of assisted living facilities, who have decreased independence due to physical or cognitive limitations. However, their impaired abilities can make attending group activities difficult. One potential barrier is the physical distance from residents' apartments to the activity location. This study analyzed resident (N=30) attendance at group activities in an assisted living facility. Attendance records for 822 group activities over the course of six months and the distance between residents' apartments and activity location were analyzed. Exponential random graph models were used to estimate the effect of individual-level, activity-level, and social network factors on the likelihood that a resident attends an activity. On average, residents attended 128 events (SD = 151) during the six month study period. The average size of activities was 4.6 residents (SD = 2.9). The closest distance between a resident's apartment and an activity was 7.7 feet; however, some residents had to travel up to 382 feet to attend an activity (M=153 ft.; SD=52). After controlling for individual-, activity-, and social network-level factors, distance to the activity was significantly and negatively associated with the likelihood a resident attended the activity. In spite of the fact that assisted living facilities offer supports and services to help residents overcome their physical limitations, it appears that distances within the building can be barriers to participation in group activities. This should be taken into account in designing facilities, locating group activities, and devising strategies to increase participation in group activities.

### LIFE SPACE MOBILITY AND NEIGHBORHOODS: HOW HOME MODIFICATIONS IMPACT AGING IN PLACE

Alan DeLaTorre,[1] Ivis García,[2] Julianne Reno,[1] Ja Young Kim,[2] and Keith Diaz Moore[2], *1. Portland State University, Portland, Oregon, United States, 2. University of Utah, Salt Lake City, Utah, United States*

This presentation details a mixed methods study funded by the National Institute for Transportation and Communities that was conducted with 50 older adults aged 65 and older who lived in Portland, Oregon (n=25) and Salt Lake County, Utah (n=25). The purpose of the study was to improve understanding of how home modifications affect older adults' mobility in different life-spaces (e.g., one's bedroom, neighborhood), their understanding of aging in place and neighborhood, and their ability to age in place. During each home visit, a series of research protocols (i.e., surveys, interviews, mapping exercises) were carried out with each participant. The study found that home modifications (e.g., grab bars, replacing showers with bathtubs, and adding raised toilets) were reported to increase in-home mobility and, for some, their independence; however, for certain participants, those same modifications were less useful, especially to those with the need for caregiver supports. Life-space mobility outside the home was impacted by home modification such as ramps and railings on stairs; for some, those modifications bolstered social connections and access to services. Overall, home modification were seen as enabling both mobility and aging in place. Furthermore, respondents' understanding and description of their neighborhoods varied greatly and were influenced by mobility barriers (e.g., presence of sidewalks and crosswalks) and available amenities.

### BARRIERS AND FUTURE DIRECTIONS FOR SUFFICIENT ACADEMIC RESEARCH ON HOME MODIFICATION FOR OLDER ADULTS IN CHINA

Mengzhao Yan[1], *1. Leonard Davis School of Gerontology, University of Southern California, Los Angeles, California, United States*

Home modification has been perceived as an effective method to make current dwelling units more age-friendly and improve the well-being of older adults. In China, a country with an increasing number of older adults, home modification research is still in its infancy. By typing "适老化改造" (home modification for older adults) as a keyword for the theme of articles in China National Knowledge Infrastructure (CNKI), the most authoritative and comprehensive database for academic publication in China, only 158 related papers are found to be published until the end of 2017, among which the first was published in 2009. With an aim to determine the current state of home modification research in older adults and to determine what could be done to facilitate sufficient research on this topic, the present study conducted a systematical review of the 158 papers about home modification for older adults published in China between 2009 and 2017. Based on the systematical review, the present paper identifies four barriers to conducting this line of research and puts forward six practical suggestions for future research studies in this area so as to contribute to building age-friendly dwellings and promoting age-friendly China.

### MIND THE GAP: FINDINGS FROM THE AGE-FRIENDLY OXFORD COMMUNITY NEEDS ASSESSMENT

Meghan A. Young,[1] Usha Dhakal,[1]
Valerie L. Kessler,[1] and Suzanne R. Kunkel[2], *1. Scripps Gerontology Center - Miami University, Oxford, Ohio, United States, 2. Miami University Scripps Gerontology Center, Oxford, Ohio, United States*

In November 2017, Oxford, OH joined the AARP network of Age-Friendly Communities (AFCs). The first step in building an action plan through the AARP process is conducting a community needs assessment. Scripps Gerontology Center adapted the AARP Community Survey Questionnaire and mailed surveys to a random sample of 700 Oxford residents aged 50 years and older. The response rate was 46.8%. For seven of the eight domains of livability, individuals were asked how important is it to have particular services in the community (Likert scale) and whether the community provides the services (response options: yes, no, not sure). The responses to these questions were used to calculate a perceived gap score. The purpose of this project was to identify which domains had the largest perceived gaps, then further analyze individual item gaps. The three domains with the largest gaps were transportation (50.6%), housing (47.7%), and health (46.2%). Further analysis of the 60 individual domain items provided information about the type of gap. For example, 84% of respondents found the item "affordable public transportation" important. However, of those who said it is important, 73% perceived a gap in service provision, and 64% of the gap was due to not knowing if Oxford provides it. Communities may interpret a "not sure" gap as an opportunity to restructure how they promote services to older individuals. Implications of this research include proposing different ways of analyzing needs assessment data so AFCs can make efficient and effective changes for older adults to age in place.

### SILVER FRIEND: A SMART SPEAKER PROJECT TO ASSIST LOW-INCOME OLDER ADULTS LIVING ALONE IN SOUTH KOREA

Jongwoong Kim[1], *1. University of Cincinnati, Cincinnati, Ohio, United States*

Ownership rate of smart speakers in the U.S. reaches 21 percent in late 2018, and the growth of the global market is predicted to explode in the coming years. With this trend, there is an increasing expectation that smart speakers can assist older adults to stay independently in their homes as they age. However, there still is a shortage of empirical studies and evidence that these devices can actually be effective in helping older adults age in place. This paper describes a pilot project, Silver Friend, in South Korea, which utilizes a smart speaker, NUGU, to help low income older adults who live alone remain their homes as actively and safely as possible. The Silver Friend project has largely two goals: 1) reducing the older adults' loneliness and 2) improving emergency response via the NUGU's voice recognition and data monitoring system. Initially, the project served 90 older adults who live alone in government-purchased rental housing. This project was a result of a public-private partnership led by a South Korean tech/telecom giant, SK Corporation, practicing its corporate social responsibility. In South Korea, 15 percent of the population is age 65 and older, and 20 percent of them (approximately 1.5 million older adults) are living alone and/or in need of financial assistance. With the ever-expanding global market for smart speakers, this paper focusing on a case in South Korea describes evidence and a potential policy model in utilizing a smart speaker to help achieve the goal of aging in place.

### AGING IN COMMUNITY: A LEGAL MAPPING ANALYSIS OF MASSACHUSETTS MUNICIPAL ACCESSORY DWELLING UNIT ZONING BYLAWS

Travis M. Gagen[1], *1. Assumption College, Worcester, Massachusetts, United States*

Accessory-dwelling units (ADUs) are one alternative housing arrangement that enable older adults to remain in the home despite functional decline. Functional decline increases with age making older adults more susceptible to loosing independent housing. Involuntary relocation to institutional care can result in a decline of functional health, reduced life satisfaction, impairment of psychological well-being and increased mortality rate. The majority of older Americans (93%) wish to remain in their home for as long as possible. ADUs function to maintain, stimulate and support an older adult as a means to prevent relocation to an institution. The modified environment coupled with adaptable features maintains and supports activities of daily living (ADL) within a familiar place. Under Massachusetts law MGL c. 40A, the state gives authority to cities and towns to adopt ordinances and bylaws to regulate the use of land, buildings and structures. Restrictive zoning laws limit the ability to construct health-promoting built-environments to age-in-community. All 351 Massachusetts municipalities Accessory Dwelling Unit (ADU) zoning bylaws were coded using the ADU Friendliness Score. Once scored, the 351 municipalities were placed into four categories based off their ADU score; the four categories are poor (0-24), fair (25-49), good (50-74), and excellent (75-100). Eighty-nine municipalities (25%) are in the poor category; thirty municipalities (8.5%) are in the fair category; one hundred and eighty-five municipalities (53%) are in the good category; forty-seven municipalities (13.5%) are in the excellent category. These findings contributed to a model ADU bylaw specific for aging Americans for municipalities to adopt.

### HOW DRIVING STRESS, CONFIDENCE, AND THOUGHTS ABOUT DRIVING ALTERNATIVES AFFECT TRANSPORTATION PLANNING

Jonathon Vivoda,[1] Jiawei Cao,[1] and Annie Harmon[2], *1. Miami University, Oxford, Ohio, United States, 2. Washington University, St. Louis, Missouri, United States*

Although many older adults will eventually not be able to safely drive themselves, comprehensive planning for a non-driving future is infrequent. To better understand the factors that predict transportation planning, the role played by several different aspects of driving were assessed. We analyzed whether stress about driving, enjoyment of driving, driving confidence, recent experience with negative driving events, and thoughts about how well other transportation modes could meet one's future needs played a role in planning. Age, gender, race, and income were also controlled for in the models. More transportation planning was significantly associated with experiencing driving as more stressful and with reporting less confidence in one's driving skills. Level of driving enjoyment and the number of concerning driving events experienced during the past year (e.g., crash, near crash, driving conversations, health issues, etc.) were not significantly related to planning. Respondents were also asked about how well other transport modes (e.g., rides with other drivers, buses, light rail, taxis, etc.) could meet their needs when they can no longer drive. This factor explained more of the variance in the planning outcome than any of the other factors, with a larger number associated with more planning. This result suggests that engaging in more planning may be associated with an openness to non-driving options, but it is unclear if that is a result of more planning, or a precursor to this activity.

### FINDINGS FROM THE ENTOURAGE NOORD PROJECT: CO-CREATING NEW HOUSING MODELS FOR OLDER ADULTS IN BRUSSELS

An-Sofie Smetcoren,[1] and Liesbeth De Donder[1], *1. Vrije Universiteit Brussel, Brussels, Belgium*

In response to the challenge of an ageing population and the housing crisis in Brussels, the practice-based project 'Entour-Age Noord: Inspiring and innovative housing & work' was launched. A main objective was to develop various innovative and small-scaled housing models for older adults. The new housing models were designed to reinforce quality of life of older people who are ageing in the neighbourhood whilst allowing them to choose the models best suited to their needs and wishes. Given the complexity and multidimensionality, participatory-action research was used and the project was divided into six different 'work packages' (WP's). Within these WP's, different stakeholders and end users (older people, informal caregivers, neighbourhood residents, Community Land Trust, etc.) were involved during various activities (service design methodology, architectural workshops, inspiring visits, focus groups etc.) in order to co-create the answers. First, based on the results, eight personas (=conceptual models of targeted groups) were developed. Second, stemming from the participants needs and wishes (e.g. desire for more social interaction), architectural and spatial design characteristics were detected (e.g. provide common/shared spaces that stimulate encounter). Based on these personas and spatial characteristics, two prototypes of new housing models were conceptually elaborated on paper and two were operationalized and prepared in practice. The spatial and architectural characteristics are not limited to old age but can be of interest of any age group. Future housing developments could take these suggestions into consideration. Furthermore, experiences, opportunities and pitfalls of a co-creative decision-making process with older adults will be discussed.

### A STUDY ON CHANGES IN RESIDENTIAL LIFESTYLE AND AWARENESS OF RESIDENTS IN A HOUSING COMPLEX REGENERATION PROJECT

tomoya ashihara,[1] Akiko Nishino,[1] yonggeun Lee,[1] toshio otsuki,[1] Kazuhiro nishide,[1] and yuji matsuda[1], *1. The University of Tokyo, Tokyo, Japan*

In Japan, where the ratio of elderly citizens exceeds 27%, there are issues such as the aging of residents and an increase in the number of people living alone. The present study aims to identify the elements required for a community base, based on temporal changes in daily life activities of residents in Housing Complex T, where more than 40% of residents are elderly, and in the surrounding area. A questionnaire survey was conducted with residents of Housing Complex T and residents in the surrounding area in 2010 (856/4,940 responses) and in 2018 (1,194/5,049 responses). The survey results showed that half of the elderly people participated in group circle activities during the day, and there was no change between 2010 and 2018. More than half of the residents of the housing complex utilize the supermarket within the housing complex several times a week. However, only 20% of the residents in the surrounding area use the supermarket. Parks were being utilized by 29.9% of housing complex residents and 37.6% of the residents outside of the complex, while the library was being utilized by 37.1% of housing complex residents and 39.1% of the residents outside of the complex. Public facilities are being utilized. These results show that it is important to provide architectural functions with high public utility within the property of housing complexes; they should be well known by residents of the surrounding area in order to create a community.

### THE DUKE INTERAGENCY CARE TEAM: A BRIDGE TO GERIATRIC COMMUNITY RESOURCES

Geraldine E. Kanne,[1] Melissa Black,[2] Marilyn Disco,[3] Rhonda Mack-Minniefield,[3] David Halpern,[4] Gina Upchurch,[3] Heidi White,[5] and Mitchell T. Heflin[6], *1. Duke University Health System, Durham, North Carolina, United States, 2. Triangle J Area Agency on Aging, Durham, North Carolina, United States, 3. Senior PharmAssist, Durham, North Carolina, United States, 4. Durham Medical Center, Durham, North Carolina, United States, 5. Duke University Medical Center, Durham, North Carolina, United States, 6. Duke University Medical Center, Durham, North Carolina, United States*

The Duke Geriatric Workforce Enhancement Program aims to improve linkages between primary care practices (PCP's) and community-based organizations by developing an interdisciplinary, community-based team to consult with PCPs, identifying resources to help vulnerable older adults. The Inter-agency Care Team (ICT) includes a nurse practitioner, pharmacists, community resource specialists, geriatricians and geriatrics and advanced practice nursing fellows. PCP's refer older adults with complex care needs through the EHR for virtual consultation by the ICT. Team members review medical records and call participants and caregivers to obtain permission for the consult, gather information on function, social factors, medical problems, and their perceived needs. The ICT meets to review each case and sends written recommendations to the PCP and patient. To date, the ICT performed consultations for 73 older adults with a mean age of 76 years. 69% were female. 71% were black and 26% white. Frequently identified needs included personal/home safety (74%), medication management (64.3%), food security (63.0%), cognition (49.3%), transportation (38.4%) and advance care planning (31.5%). In the 90 days before consultation, 32.9% of patients had ED visits and 21.9% were hospitalized. In the 90 days after, 24.7% had ED visits and 13.7% were hospitalized. (Differences were not statistically significant.) ICT provided virtual consultation for complex older adults with prevalent social needs and high rates of ED visits and hospitalization. The team worked with PCP's to connect these patients more directly to community resources. Further study is needed to know rates of adherence with recommendations and true impact on health outcomes.

### THE SIGNIFICANT ROLE OF HOUSING COORDINATION IN CONNECTICUT'S MONEY FOLLOWS THE PERSON PROGRAM

Kathy G. Kellett,[1] Dawn Lambert,[2] Tamara Lopez,[2] and Julie Robison[3], *1. UConn Health, Farmington, Connecticut, United States, 2. Connecticut Department of Social Services, Hartford, Connecticut, United States, 3. University of Connecticut, Farmington, Connecticut, United States*

Housing coordination, a central component of the Money Follows the Person program, allows older adults and people with disabilities to experience greater independence and sense of well-being (Butler & Cabello, 2018; Koenig, 2015). This study was part of the 2017 Money Follows the Person (MFP) Process Evaluation involving 26 key informants (KIs) who completed telephone interviews sharing their experiences about program implementation. Of these, 13 KIs providing housing coordination services to the MFP transition team were asked about the housing coordination training they received and suggestions to improve the training. They were also asked about housing resources, how they develop their housing inventory, and recommendations for a "Housing Best Practices Report." Interviews were audiotaped and transcribed. Data were analyzed using ATLAS.ti. Results demonstrate a need for more training and assistance to enable housing coordinators to expand their knowledge of housing alternatives, increase their awareness of housing policy/process changes, and further inform consumers about housing choices. Suggestions to improve housing coordination included offering more creative solutions during monthly housing coordination phone calls. Housing inventory challenges mentioned by KIs included time constraints, limited staff, administrative delays, and lack of affordable housing. Suggestions for housing inventory development focused on improving the management/maintenance of housing inventories. Best housing practices underscored the importance of communicating, teaming, building relationships with landlords and management companies, and standardizing housing policies and procedures. Overall recommendations included strengthening collaboration among housing coordinators to identify and implement best practices and improving housing inventory development to widen housing options for consumers.

### PLANNING FOR AGING IN PLACE IN RURAL AREAS: PERCEPTIONS AND NEEDS OF OLDER ADULTS

Raven H. Weaver,[1] and Cory Bolkan[2], *1. Washington State University, Pullman, Washington, United States, 2. Washington State University, Vancouver, Washington, United States*

Most individuals prefer to live independently in their homes, but will need support to age-in-place safely. Rural-dwelling individuals historically have worse health, limited income, and restricted access to adequate services/supports compared to their urban counterparts. Community-based aging services organizations (i.e., Area Agency on Aging; AAA) offer in-home health, social support, and information/referral to community resources that support older adults in both urban and rural communities. A representative sample of adults aged 60+ (N=253, mean age=74) were surveyed via computer-assisted-telephone interviews about their health status, needs, and service utilization. Over half (54%) lived in rural counties, which was significantly associated with receiving insufficient health care services ($X2=9.227$, $p=.002$). Insufficient service access was also associated with experiencing a fall ($X2=7.315$, $p=.007$). While 53% reported having chronic conditions, most individuals still reported good health and their top reported needs included: yard work, interior/exterior house repairs, and housework. Content analysis of open-ended survey responses regarding future care needs revealed participants anticipate help from family/friends or neighbors; reliance on physicians for referrals; and expect insurance to cover their needs. Participants had varying awareness levels of available community resources and identified concerns about adequacy of services (e.g., mental health; transportation) and health insurance barriers (e.g., reimbursement; vision/dental coverage). Preparing for future needs and anticipating changing functional capacity is critical, especially among rural-dwelling older adults with chronic conditions. To improve ability for adults with diverse needs to age-in-place, preventive services/supports that span the continuum of care needs and that complement informal family care are necessary.

### INSTABILITY OVER THE LIFE COURSE AND POVERTY IN OLDER ADULTHOOD: A MIXED-METHODS STUDY

Sarah Dys,[1] Anna Steeves-Reece,[2] and Paula Carder[3], *1. Institute on Aging, Portland, Oregon, United States, 2. Oregon Health Science University and Portland State University School of Public Health, Portland, Oregon, United States, 3. Portland State University Institute on Aging, Portland, Oregon, United States*

Over half of older adults (age 65+) who rent their home are cost burdened, paying more than one-third of their income on rent (JCHS). Government programs are in short supply, and most who qualify for housing assistance will wait months or years to receive a voucher or unit. This mixed methods (QUANT to qual) study explored housing instability among older adults wait-listed for housing assistance. Analysis of surveys (n=268) and in-depth interviews (n=29) examined how financial instability influences participants' perceived risk of future homelessness. Perceived financial instability is associated with perceived risk of homelessness (p<.001). Compared to those whose current perceived financial status was now much worse from when they applied for assistance, those who were stable had lower perceived risk of homelessness. Thematic analysis of interview data provided insights into patterns of life-long experiences with various forms of instability, which are reproduced over the life course and met with little societal response or restrictive eligibility criteria. Major events (e.g., relationship changes, job loss, injury/illness) contributed to financial instability and housing instability among participants. Interviews wove together current status with individual histories, while the survey provided a cross-sectional perspective. An iterative analytic approach allowed us to conceptualize the relationships between health, employment, relationships, financial instability, food insecurity, and perceived risk of homelessness. Overall, this project describes how older Americans experience poverty and provides further evidence for how social determinants of health throughout the life course result in differential access to material resources, including income, food, health care, and housing.

### BUILDING SAFE ROUTES TO AGE IN PLACE THROUGH PARTNERSHIPS WITH AGE-FRIENDLY COMMUNITIES

Holly Dabelko-Schoeny,[1] Noelle L. Fields,[2] Ian Murphy,[1] Katie White,[1] Marisa Sheldon,[1] and Kristen E. Ravi[2], *1. The Ohio State University, Columbus, Ohio, United States, 2. University of Texas at Arlington, Arlington, Texas, United States*

Participation in active transportation by older persons has been associated with higher quality of life and longevity. Efforts across the United States to increase active transportation (walking, biking and fixed route transportation) by older adults have lacked meaningful input by older adults, are non-theoretical and have had inconclusive results. This paper describes the findings from Phase 1 of the Safe Routes to Age in Place project, a collaboration between Age-Friendly Franklin County, The Ohio State University (OSU) and the University of Texas-Arlington (UT-A), funded by the Ohio Department of Transportation. The aim of the study is to increase the options and safe utilization of active transportation by older persons in three pilot neighborhoods in Franklin County, Ohio utilizing community-based participatory action research strategies with a customized data collection app, MyAmble. The purpose of Phase 1 was to engage city and regional planning students, government officials and older adults to identify "hot spots" in the three pilot neighborhoods. Heat mapping of community data including locations of densely-populated vulnerable older adults, fixed transportation routes, bus stop signage and benches, shared bike stations, bike routes, paratransit routes and pedestrian crash data was completed. Maps were used to identify areas of focus and discussions with municipal leaders and older adult residents further refined target areas to be used in the second phase of the study. Older residents completed walk audits using the MyAmble app and identified issues with sidewalks, crosswalk timers and bus stops. Implications related to age-friendly communities and active transportation are offered.

### YOU CAN'T GET THERE FROM HERE! TRANSPORTATION ISSUES OF OLDER ADULTS AND PEOPLE WITH DISABILITIES

Angela M. Zell,[1] and Joan Ilardo[1], *1. Michigan State University College of Human Medicine, East Lansing, Michigan, United States*

Older adults and individuals with disabilities face transportation challenges on a daily basis that differ from the general population. Transportation is critical to all aspects of quality of life yet presents significant challenges. Lack of appropriate vehicles, reliable public transportation, and the high-cost of vehicle ownership lead to missed healthcare appointments, lack of access to proper nutrition, and social isolation. The purpose of the study was to provide information for the Commission on Services to the Aging. It was necessitated by the lack of existing data from the Department of Transportation because Michigan's transportation systems are locally controlled. An online questionnaire was emailed to public and private organizations serving older adults and people with disabilities to determine transportation services currently available in their geographic areas and innovative solutions employed to address barriers. The questionnaire was adapted from the National Center of Senior Transportation in 2009 that produced the report "Transportation: The Silent Need". The study analyzed data on current transportation services and innovative solutions piloted in Michigan. Respondents represented every Michigan county and included area agencies on aging, senior centers, councils on aging, healthcare agencies, transportation providers, community action agencies, and job training programs. Information includes: services provided by the agency; barriers to accessing transportation; access to transportation services information; transport services previously used, currently available, and being planned. Many of the 95 respondents commented on persistent lack of funding for viable, reliable transportation options and jurisdictional issues. Most pilots used fixed routes, volunteer drivers, demand response and expanded schedules.

### CHARACTERISTICS AND LIVING ARRANGEMENTS OF CHILDLESS ELDERLY AMERICANS

Xiao Xu,[1] Jersey Liang,[2] BoRin Kim,[3] Mary Beth Ofstedal,[4] and James Raymo[5], *1. Yale University, New Haven, Connecticut, United States, 2. University of Michigan, Ann Arbor, Michigan, United States, 3. University of New Hampshire, Durham, New Hampshire, United States, 4. University of Michigan, Ann Arbor, Michigan, United States, 5. University of Wisconsin-Madison, Madison, Wisconsin, United States*

Despite a growing number of Americans without children, information on characteristics of childless older adults and their living arrangements is sparse and often outdated. To address this knowledge gap, we examined data from the 2014 Health and Retirement Study (HRS) on childless Americans age ≥65 years (N=760) and compared with childless elders in 2004 HRS (N=830). All analyses accounted for complex sample design of HRS to generate nationally representative estimates. The proportion of elderly Americans without any living biological or step children increased from 8.1% in 2004 to 9.4% in 2014. Compared to childless elders in 2004, those in 2014 were younger (mean age=76.8 years versus 73.8 years, p<0.01) with a higher proportion completing college education (20.7% versus 37.5%, p<0.01) or were cognitively intact (64.6% versus 73.6%, p<0.01). However, childless elders in 2014 had more chronic conditions than those in 2004 (mean=2.5 versus 2.3, p<0.01). The proportion of childless elders living independently (alone or with a spouse) (81.6% in 2004 versus 82.6% in 2014) or living with others (10.0% in 2004 versus 13.5% in 2014) remained relatively stable, whereas the proportion living in nursing homes declined significantly from 8.4% in 2004 to 4.0% in 2014 (adjusted odds ratio [OR]=0.07, p<0.01). A sensitivity analysis limited to childless elders without biological children showed that they accounted for 9.9% of the elderly population in 2004 versus 11.9% in 2014, while other results were generally similar. These findings can help inform long term care needs of childless elders.

### AGE-FRIENDLY COMMUNITIES: ENHANCING COMMUNITY CONNECTIONS

Patricia A. Oh[1], *1. University of Massachusetts, Boston, Boston, Massachusetts, United States*

Age-friendly communities promote active, healthy, socially connected aging. Opportunities for social connections are key for older residents to enjoy the best possible health and well-being. Communities that join the AARP Network of Age-Friendly States and Communities (AARP NAFSC) include an aging lens in eight areas of community life—social participation, respect and social inclusion, civic participation and employment, communication and information, housing, transportation, community support and health services, and outdoor spaces and buildings. By addressing factors in these eight areas, communities encourage residents to enjoy formal participation in activities and groups and informal contacts with friends, neighbors and other residents. The purpose of this exploratory study was to find out if communities that join the AARP NAFSC plan and implement changes to enhance social connectedness. A review of 62 AARP-approved action plans nationwide, showed that social connectedness was included in 74% of the mission statements and was a goal in 92% of the plans. The lack of resources in rural communities creates special challenges; many age-friendly initiatives depend on community volunteers to implement changes on a shoe-string budget. To learn how rural age-friendly communities promote social connections, an email survey was distributed to 46 AARP NAFSC communities in rural Maine. All the communities responded. Fostering social connectedness was an explicit goal for 88% of the communities. Areas of implementation included services and activities (83%), communication (61%), transportation (30%), programming to include isolated residents (26%), accessible public spaces (22%), and intergenerational volunteering (17%). Implications will be discussed.

### IDENTIFYING SALIENT TRAINING AND SUPPORT NEEDS WITHIN A STATEWIDE LIFELONG COMMUNITIES NETWORK

Jennifer Crittenden,[1] Patricia A. Oh,[2] and Laura Lee[3], *1. UMaine Center on Aging, University of Maine, Bangor, Maine, United States, 2. University of Massachusetts, Boston, Boston, Massachusetts, United States, 3. Maine Community Foundation, Portland, Maine, United States*

As the older adult population grows in the United States, the need for community planning approaches that respond to the needs of older adults is of increasing importance. As a result, lifelong community movements, encompassing models such as Age-Friendly Communities, Livable Communities, and "Aging-in-Place" initiatives are proliferating. Maine, the oldest state by median age, currently hosts the largest number of AARP designated Age-Friendly Communities efforts. Given the size of this network, the purpose of this study was to collect descriptive information about the status of existing lifelong communities initiatives, their training and support needs, and the desired format and configuration of future training programming. An electronic survey was distributed to community representatives from 76 lifelong communities initiatives throughout Maine. A total of 38 communities responded to the survey representing a response rate of 50%. The majority of respondents (80.4%) reported having a committee or other coordinating group guiding their work. A large portion have completed planning phase activities including hosting focus groups (79.5%), carrying out a survey (66.7%), and identifying a list of local assets (59%). Fewer have completed the plan drafting phase (17.9%). A majority reported receiving assistance from AARP (66%) and a regional educational consortium (66%). The areas with highest self-reported training needs (based on mean ratings) are: Volunteer recruitment and retention, specialized trainings on Age-Friendly Community topics, Dementia-Friendly Communities topics, and outreach and community engagement strategies. Implications will be discussed including optimal configuration of training and support for similar such networks.

### GERIATRIC CONDITIONS AND HEALTH CARE UTILIZATION AMONG OLDER ADULTS LIVING IN SUBSIDIZED HOUSING

Sarah E. Kler,[1] Sun Young Jeon,[2] Kanan Patel,[2] Christine Ritchie,[2] Krista Harrison,[2] Kali S. Thomas,[3] and Rebecca Brown[4], *1. Warren Alpert Medical School of Brown University, Providence, Rhode Island, United States, 2. UCSF, San Francisco, California, United States, 3. Providence VA Medical Center, Providence, Rhode Island, United States, 4. University of Pennsylvania, Philadelphia, Pennsylvania, United States*

In the US, 1.7 million low income older adults live in subsidized housing. Previous research suggests that subsidized housing residents have poorer health status than older adults in the general community. However, little is known about the prevalence of geriatric conditions. To understand these factors we conducted a retrospective cohort analysis of 11,558 Medicare enrollees ages 65+ who were enrolled in the National Health and Aging Trends Study in 2011 or 2015, including 507 living in subsidized housing and 11,051 in the general community. We compared subsidized housing residents to general community residents across measures of sociodemographics, functional limitations, and geriatric syndromes. We also compared the prevalence of hospitalization, move to a higher level of care, and death within five years. Results suggest that compared to general community residents, subsidized housing residents were more likely to be women (66% vs. 55%, p<0.01), racial/ethnic minorities (50% vs. 18%, p<0.01), and to lack a high school diploma (50% vs. 20%, p<0.01). They also had poorer health status, including higher rates of self-reported functional impairment (difficulty with 2 or more ADLs; 16% vs. 10%, p<0.01), probable dementia (15% vs. 8%, p<0.01), and frailty using the three-level Fried frailty index (55% vs 26%, p<0.01). Subsidized housing residents also had higher rates of hospitalization (29% vs. 22%, p<0.01), move to a higher level of care (4% vs. 3%, p<0.01), and death (10% vs. 7%, p<0.01) compared to community-residing peers. These findings will help inform targeted interventions to improve aging in place for this vulnerable population.

### SESSION 1310 (POSTER)

### BIOLOGY OF AGING

### ANTI-AGING PROTEIN CD9 AFFECTS AGE-RELATED HEART FAILURE

Sriya T. Jonnakuti,[1] and Mujib Ullah[1], *1. Stanford University, Palo Alto, California, United States*

The CD9 is transmembrane protein that plays a critical role in many cellular processes including aging associated cardiac pathologies. The heart function declines in the aged population. Ageing is strongly associated with many age-related conditions such as increased risk of heart failure. If aging can be prevented slowed down or even reversed, heart failure and other signs of aging could be controlled or even cured. It is unknown whether CD9 is cardioprotective. The objective of this study is to investigate whether a decline CD9 levels contributes to aging-related heart failure. Our data shows that CD9-deficient aged mice develop cardiac abnormalities and pathological cardiac hypertrophy, Cardioprotection by CD9 in old mice is followed by the downregulation of SIRT6 in the heart, and CD9 overexpressed exosomes ameliorates cardiac pathologies in treated mice and improves their long-term survival. Additionally, the serum level of CD9 decreased significantly in aged mice. CD9 overexpressed exosomes are cardioprotective and improve cardiac function in aged mice. These exosomes mediate their paracrine effects by attenuating, blood pressure, heart beat, reactive oxygen species and fibrosis. Remarkably, CD9 overexpression reversed fibrosis associated brain natriuretic peptide (BNP), Sirt6, and galectin 3 (Gal-3). These results provide a new perspective on the pathogenesis of cardiomyopathies and open new avenues for treatment of the disease.

### THE ROLE OF POLYGENIC SCORE AND COGNITIVE ACTIVITIES ON COGNITIVE FUNCTIONING OF OLDER ADULTS

Su Hyun Shin,[1] Soohyun Park,[1] and Giyeon Kim[2], *1. The University of Alabama, Tuscaloosa, Alabama, United States, 2. Chung-Ang University, Seoul, Korea, Republic of*

Purpose of study: This study investigated whether and to what extent genetics for cognition and engagement in cognitive activities are related to trajectories of cognitive functioning in older adulthood. Furthermore, we explored whether engaging in cognitive activities could moderate the effect of genetic traits on cognitive functioning in general and across different dimensions: fluid and crystallized intelligence. Design and Methods: Growth curve models were estimated using the sample of 3,129 individuals aged 50 or older (10,000 observations) in the U.S. from 2000-2012 waves of the Health and Retirement Study. Polygenic score for general cognition (PGS) was used to measure genetic traits for cognition, and the number of hours spent per week on each of nine cognitive activities was used to measure individuals' level of the engagement in cognitive activities. Results: PGS for cognition, reading books, using a computer, and playing cards/games/solving puzzles had positive effects on cognitive functioning. The positive effect of PGS on cognitive functioning was reduced from excessive TV watching. The positive effect of PGS on cognitive functioning was strengthened by spending more hours reading papers/magazines. The measure of fluid, rather than crystallized intelligence, appeared to drive these results. Conclusion: Findings suggests that while genetic factors predict cognitive functioning, engaging in different types of cognitive activities could yield different cognitive functioning trajectories in later life. Practical implications are that older adults should be more selective when choosing their leisure activities to promote cognitive health.

### DETERIORATING HEALTH AMONG OLDER ADULTS AND CORTISOL: LONGITUDINAL EVIDENCE FROM THE MIDUS STUDY

Julie Ober Allen[1], *1. University of Michigan, Ann Arbor, Michigan, United States*

Various mental and physical health conditions common among older adults have been linked to cortisol dysregulation (i.e., blunting of daily cortisol patterns) in predominantly cross-sectional studies. Researchers have suggested that cortisol dysregulation interferes with regulatory functions throughout the body and brain, disrupting multiple biological systems, and contributing to the development or progression of negative health outcomes over time. Prospective studies are needed to investigate the causal direction of cortisol dysregulation and poor health outcomes. This study examined whether diurnal cortisol patterns predicted subsequent health deterioration using longitudinal data from the National Survey of Midlife in the US (MIDUS). Analysis was restricted to 1,336 participants who provided salivary cortisol (4 samples/day for 4 days) and health data in MIDUS II (2004-2009) and updated health data in MIDUS III (2013-2014) (mean age=56, 45% male, 94% White). We simultaneous modeled multiple measures of diurnal cortisol patterns and their relationships to changes in mental (depressive symptomology) and physical (self-rated physical health, functional limitations, and number of new chronic health conditions) health from MIDUS II to III. All indicators of physical health deterioration were associated with cortisol, though not all measures demonstrated relationships in the expected direction. Mental health change over time was unrelated to cortisol. Older age was also associated with increased functional limitations and more new chronic conditions but improvements in mental health over time. Findings suggest that diurnal cortisol patterns contribute to physical health deterioration over time, independent of age-related decline, but not mental health changes in later life.

### GLUTATHIONE, MITOCHONDRIAL DEFECTS, AND A UNIQUE METABOLIC CYCLE IN OLDER HUMANS: IMPLICATIONS FOR SARCOPENIA

Rajagopal V. Sekhar,[1] Jean Hsu,[2] Farook Jahoor,[1] Shaji Chacko,[2] Premranjan Kumar,[2] and Chun Liu[2], *1. Baylor College of Medicine, Houston, Texas, United States, 2. Baylor College of Medicine, Houston, United States*

Sarcopenia in aging leads to decreased muscle mass and physical-function (muscle strength and exercise capacity), but underlying mechanisms are not well understood and effective interventions are limited. We hypothesized that deficiency of the intracellular antioxidant protein Glutathione initiates a unique self-perpetuating metabolic cycle linking impaired fasted mitochondrial fuel-oxidation (fMFO) to protein catabolism and contributes to sarcopenia. We also hypothesized that supplementing the Glutathione precursor amino-acids glycine and N-acetylcysteine (GlyNAC) to correct Glutathione deficiency in older humans could reverse these defects. We tested our hypothesis in a 24-week open-label clinical-trial in 8 older-humans (74y) studied before and 24-weeks after GlyNAC supplementation, compared to 8 gender-matched unsupplemented young-controls (25y), and measured intracellular Glutathione concentrations, fMFO, physical-function, muscle-protein breakdown-rate (MPBR), gluconeogenesis, and urine nitrogen-excretion (UNE). GlyNAC supplementation in older humans corrected Glutathione deficiency and restored impaired fMFO (to levels in young controls), lowered MPBR and UNE, and increased physical-function, but did not affect gluconeogenesis or increase lean-mass, and suggest that muscle amino-acids are utilized for energy needs rather than glucose production. The absence of an increase in lean-mass suggests that GlyNAC should be combined with anabolic agents for potential benefits in combating sarcopenia. Overall, these results indicate the presence of a unique reversible metabolic cycle in older humans initiated by Glutathione deficiency which results in impaired mitochondrial fatty-acid and glucose oxidation, muscle-protein breakdown, UNE, and leads to deficiency of glycine and cysteine which re-initiate the cycle. These data have implications for improving physical-function and muscle mass in age-associated sarcopenia, and warrants further investigation.

### MTOR PROMOTES BBB BREAKDOWN IN A MODEL OF ALZHEIMER'S DISEASE

Stephen F. Hernandez,[1] Candice E. Van Skike,[2] Nick DeRosa,[1] and Veronica Galvan[3], *1. UT Health San Antonio, San Antonio, Texas, United States, 2. Barshop Institute - UT Health San Antonio, San Antonio, Texas, United States, 3. Barshop Institute for Longevity and Aging Studies, San Antonio, Texas, United States*

Cerebral amyloid angiopathy (CAA) is characterized by fibrillar amyloid β (Aβ) association with cerebrovasculature, which leads to impaired brain vascular function, and is present in 87% of people with Alzheimer's disease (AD). We previously showed that inhibition of mTOR by rapamycin prevented BBB breakdown and reduced vascular fibrillar Aβ in 18-19 month old Tg2576 mice that model AD-associated CAA. This finding suggests that mTOR attenuation restores integrity of the blood brain barrier (BBB) and concomitantly reduces vascular Aβ accumulation in this mouse model. Objective: To determine the mechanisms by which mTOR drives BBB breakdown we measured the abundance of tight junction proteins zonula occludens 1 (ZO-1), occludin, and claudin-5. Methods: We used immunofluorescent confocal microscopy on frozen brain tissue sections of the same Tg2576 mice used in the previous study. Results: We confirm BBB breakdown in Tg2576 mouse brains and showed that some, but not all tight junction proteins measured were decreased in cerebrovasculature of Tg2576 mice. Attenuation of mTOR by rapamycin preserved BBB integrity, decreased vascular Aβ accumulation, and increased levels of tight junction protein abundance in Tg2576 mice, which also showed a reduced numbers of cerebral microhemorrhages. Conclusions: Taken together, these data suggest that mTOR promotes brain vascular Aβ deposition, BBB breakdown and vascular damage in the Tg2576 mouse model. Thus, mTOR inhibitors such as rapamycin – an FDA approved drug - may have promise in the treatment of AD and other dementias with related cerebrovascular dysfunction.

### NOVEL STAIN SEPARATION METHOD FOR AUTOMATIC STEREOLOGY OF IMMUNOSTAINED TISSUE SECTIONS

Palak Dave,[1] Dmitry Goldgof,[1] Lawrence O. Hall,[1] SAEED Alahmari,[1] and Peter R. Mouton[2], *1. University of South Florida, Tampa, Florida, United States, 2. SRC Biosciences, Tampa, Florida, United States*

Many studies of brain aging and neurodegenerative disorders such as Alzheimer's and Parkinson's diseases require rapid counts of high signal: noise (S:N) stained brain cells such as neurons and neuroglial (microglia cells) on tissue sections. To increase throughput efficiency of this work, we have combined deep learned (DL) neural networks and computerized stereology (DL-stereology) for automatic cell counts with low error (<10%) compared to time-intensive manual counts. To date, however, this approach has been limited to sections with a single high S:N immunostain for neurons (NeuN) or microglial cells (Iba-1). The present study expands this approach to protocols that combine immunostains with counterstains, e.g., cresyl violet (CV). In our method, a stain separation technique called Sparse Non-negative Matrix Factorization (SNMF) converts a dual-stained color image to a single gray image showing only the principal immunostain. Validation testing was done using semi- and automatic stereology-based counts of sections immunostained for neurons or microglia with CV counterstaining from the neocortex of a transgenic mouse model of tauopathy (Tg4510 mouse) and controls. Cell count results with principal stain gray images show an average error rate of 16.78% and 28.47% for the semi-automatic approach and 8.51% and 9.36% for the fully-automatic DL-stereology approach for neurons and microglia, respectively, as compared to manual cell counts (ground truth). This work indicates that stain separation by SNMF can support high throughput, fully automatic DL-stereology based counts of neurons and microglia on counterstained tissue sections.

### SOLUBLE INTERCELLULAR ADHESION MOLECULE (SICAM-1) AS A BIOMARKER OF VASCULAR COGNITIVE IMPAIRMENT IN OLDER ADULTS

Rosalinda Sanchez Arenas,[1] Michael A. Gregory,[2] and Leticia Manuel-Apolinar[1], *1. IMSS, Mexico, Mexico City, Mexico, 2. McMaster University, Hamilton, Ontario, Canada*

Background: Endothelial dysfunction and subsequent inflammation contribute to the development of vascular cognitive impairment (VCI). Soluble intercellular adhesion molecule-1 (sICAM-1) is upregulated in endothelial dysfunction and promotes an inflammatory response; however, the relationship between sICAM-1 and VCI remains equivocal. Objective: To determine whether sICAM-1 contributes to the prediction of VCI. Methods: Community-dwelling older adults (n=172) from the "Cohort of Obesity, Sarcopenia and Frailty of Older Mexican Adults" (COSFOMA) study were identified as VCI or controls using standard neuropsychological evaluations and neuroimaging. sICAM-1 was quantified using ELISA, and multivariate logistic regression determined the association between sICAM-1 and VCI. Results: 31 VCI cases were identified. sICAM-1 was higher in VCI [VCI: 450.7 (241.6) ng/ml vs. Control: 296.9 (140.9) ng/ml]. sICAM-1 concentrations above the 90th percentile (464.1 ng/mL) was associated with VCI group membership in all models [OR = 6.9 (95% CI: 1.1- 42.2)]. The final saturated model explained 64% of the variance in VCI group membership. Conclusion: High concentrations of sICAM-1 are independently associated with VCI group membership. Efforts to further characterize the relationship between indices of endothelial dysfunction and pathological changes to the aging brain should be further pursued.

### SINGLE-CELL TRANSCRIPTOMICS OF AGING MOUSE ISLET REVEALS AGE-RELATED RECRUITMENT OF ISLET-RESIDENT MACROPHAGE

Jia Nie,[1] and Nicolas Musi[2], *1. University of Texas Health Science Center Barshop Aging Institute, San Antonio, Texas, United States, 2. Barshop Institute for Longevity and Aging Studies, University of Texas Health Science Center, San Antonio, Texas, United States*

Type 2 diabetes (T2D) prevalence increases with age. The notion of inevitable progression of T2D has been challenged by reports of remission in some human T2D cases; however, this remission is dependent on islet function reserve. To elucidate the molecular mechanisms driving islet cell dysfunction, it is necessary to understand islet cell composition, diversity, and function throughout the lifespan. We generated a single-cell transcriptomic atlas of healthy islets isolated from young (5 weeks old), middle-aged (12 months old), and older-aged (25 months old) mice. Cell clustering identified 13 initial cell clusters that were further sub-clustered. This single-cell RNAseq profile showed that each cell type/group has different markers and functional characteristics and that age causes a remarkable shift in islet cell composition, diversity, and number. By comparing macrophages from young and old mice, we also found that aged islets contain a higher number of islet-resident macrophages. Overall, this single-cell islet atlas covers nearly all cells in the normal islet and allows a comprehensive exploration of all transcriptional states throughout the lifespan.

### ASSESSING THE RELATIONSHIP BETWEEN SERUM IGF-1 AND ADIPOSITY BY AGE IN THE LONG LIFE FAMILY STUDY

Rehab A. Sherlala,[1] Candace M. Kammerer,[2] Allison L. Kuipers,[3] Mary K. Wojczynski,[4] Svetlana Ukraintseva,[5] Mary Feitosa,[6] Jonas Mengel-From,[7] and Ryan Minster[1], *1. University of Pittsburgh, Pittsburgh, Pennsylvania, United States, 2. Department of Human Genetics University of Pittsburgh; Pittsburgh, Pennsylvania, United States, 3. Department of Epidemiology University of Pittsburgh; Pittsburgh, Pennsylvania, United States, 4. Department of Genetics Washington University in St. Louis, Saint Louis, Missouri, United States, 5. Duke University, Durham, North Carolina, United States, 6. Washington University School of Medicine, St Louis, Missouri, United States, 7. University of Southern Denmark, Odense C, Denmark, Denmark*

Serum levels of insulin-like growth factor 1 (IGF-1) and measures of adiposity, such as body mass index (BMI), are associated with susceptibility to age-related diseases. Previous reports of the relationship between IGF-1 and BMI ranged from positive to negative to no relationship, perhaps because previous reports studied different age cohorts. Using data on 4270 participants (aged 24-110 years) from the Long Life Family Study, we investigated the relationship between IGF-1 and BMI overall and by age groups. IGF-1 and BMI were positively correlated in the total sample (β=0.161, r2= 0.0038, p=1.8-05). However, further analyses revealed that the relationship between IGF-1 and BMI varied by age quartile: in the 1st quartile (24-58yo) the relationship was negative (β=−0.204, r2= 0.011, p=0.0008); in the 2nd quartile (59-66yo) the relationship was negative but non-significant (β=−0.069, r2= 0.0012, p=0.28); in the 3rd quartile (67-86yo) the relationship was positive but non-significant (β=0.106, r2= 0.002, p=0.13); and in the 4th quartile (87-110yo) the relationship was positive (β=0.388, r2= 0.019, p=1.2−05). This pattern did not differ by sex. We also detected a similar age-related pattern between IGF-1 and BMI using an independent dataset (NHANES III), comprising 2550 men and women aged 20-90 years. Our results may clarify some of the inconsistency in previous literature about the relationship between IGF-1 and BMI. Additional studies of IGF-1 and adiposity measures are needed to better understand the underlying mechanisms involved.

### FASTING-MIMICKING DIET REDUCES RISK FACTORS FOR AGING-RELATED DISEASES IN PRECLINICAL AND CLINICAL STUDIES

Sebastian Brandhorst[1], *1. University of Southern California, Los Angeles, California, United States*

Prolonged fasting promotes stress resistance, but its effects on longevity are poorly understood. Calorie restriction or major dietary composition changes can have profound effects on healthy aging but the inability of many subjects to adhere to chronic and extreme diets together with the potential of adverse effects limit their application. Fasting-mimicking diets (FMDs) are effective in increasing health and lifespan, possibly by inducing stem cell-based regeneration, or as therapies in mouse models of a variety of diseases. FMDs reduce cancer incidence/progression, modulate the immune response, reduce immuno-senescence, ameliorate or reverse disease progression of multiple sclerosis, Type I and Type II diabetes, and reverse inflammatory bowel disease pathology. In a randomized clinical trial, markers/risk factors for metabolic syndrome and other age-related diseases were favorably impacted after completion of 3 FMD cycles. These effects were larger in participants at risk for age-related diseases.

Conclusions: We conclude that the FMD was safe and feasible in rodent and human studies. Larger studies on patients with diagnosed diseases are necessary to determine its impact on diabetes and cardiovascular disease treatment.

### SESSION 1315 (POSTER)

### CHRONIC DISEASE AND DISEASE MANAGEMENT

### MEDICAL CANNABIS USE: EXPLORING THE PERCEPTIONS AND EXPERIENCES OF OLDER ADULTS WITH CHRONIC CONDITIONS

Lydia K. Manning,[1] and Lauren M. Bouchard[2], *1. Concordia University Chicago, River Forest, Illinois, United States, 2. Concordia University, River Forest, Illinois, United States*

The past decade has witnessed an increased interest in the therapeutic properties of cannabis, and a growing body of research illustrates the varied uses of cannabis-based medicines for diverse symptoms, syndromes, disorder and both acute and chronic conditions, many of which are associated with advanced age (Lucas et. al, 2016). While the use of medical cannabis is on the rise in the older adult population (Kaskie et. al, 2017), more research is needed to advance the discourse on medical cannabis. With this study, we investigate older adult's perceptions and experiences of medical cannabis use to treat and/or manage chronic conditions, specifically as a substitute for prescription drugs. Our findings suggest that older adults are open to medical cannabis as an alternative to pharmaceutical. Additionally, narratives revealed that users are hopeful that medical cannabis will provide relief with regard to the management of symptoms and relief of pain. Participants discussed their awareness and ability to manage issues related to stigma both from their primary care providers as well as family and friends. Furthermore, older adults described the frustrations with a lack of education, awareness, and support with dosing. Findings are presented as an interpretation of the participants' perceptions of their medical cannabis use. Implications for putting medical cannabis use into everyday practice as well as policy implications are considered.

### EATING THE PAIN AWAY: THE USE OF FOOD AS A COPING MECHANISM IN OLDER ADULTS EXPERIENCING CHRONIC PAIN

Alexandra Felpeto,[1] Alexandra M. Ramos,[1] Diana Hincapie,[1] Madison B. Lenox,[1] and Rebecca Reinhardt[1], *1. Nova Southeastern University, Fort Lauderdale, Florida, United States*

Individuals experiencing pain rely on impulse to make decisions, including choices regarding food consumption (Darbor, Lench, & Carter-Sowell, 2016). This study examined whether older adults experiencing chronic pain report higher instances of emotional eating in comparison to a population of older adults not experiencing chronic pain. Data stemmed from the Midlife in the United States study was analyzed to investigate whether individuals used food as a coping mechanism for chronic pain symptoms (Ryff et al., 2017). The sample consisted of Americans aged 60 to 74 years of age. Pain conditions included: has chronic pain (n=686) and does not have chronic pain (n=1036). Results of the Independent Samples T-Test indicated that participants were found to be engaging in emotional eating when experiencing chronic pain symptoms, as hypothesized. Participants in the has chronic pain condition reported relying on food as a coping mechanism more (M= 3.66, SD= 1.87) than participants in the does not have chronic pain condition (M= 3.42, SD= 1.71); t(1370)= 2.71, p= .007, d= 0.13. Results suggest that older adults experiencing chronic pain report utilizing food as a coping mechanism more than older adults that do not experience chronic pain. These findings have health implications given the rising obesity rates associated with persistent pain. Future directions may include studies on the negative health outcomes that result from high instances of emotional eating in older adults experiencing chronic pain. Additionally, investigating alternative coping mechanisms for chronic pain would be beneficial to diminish the harmful health effects of emotional eating.

### EXPERIENCE OF STROKE RECOVERY FOR WOMEN 60 OR OLDER: USING AUTO-PHOTOGRAPHY TO ENHANCE THE NARRATIVE PROCESS

Iona Johnson[1], *1. Towson University, Towson, Maryland, United States*

Stroke is a common health concern in the U.S. with 795,000 new strokes each year. Women dominate these numbers, with 55,000 more strokes per year than men, yet they are underrepresented in stroke research. Some research indicates that women have worse physiological and psychosocial outcomes after stroke than men, yet little is known about how they experience recovery. This study used a qualitative phenomenological approach to address the question: "What is the experience of stroke recovery for community dwelling women age 60 or older?" The participants were 10 women, ages 60 – 78, with times post-stroke ranging from 4 months to 15 years. They participated in 2 semi-structured interviews, with auto-photography used to enhance sharing of information. Between the two interviews, they were provided with a digital camera and asked to take pictures that helped to explain their lives before and after stroke. During the 2nd interview, participants described their pictures, and answered additional questions about their recovery. Interviews were transcribed verbatim, and the narratives were coded and analyzed thematically to describe how this sample of individuals experienced stroke recovery. Four overarching themes emerged from the data: 1) the stroke event, 2) a new chapter, 3) meaning and process of recovery, and 4) self-identity. In general, narratives revealed that recovery is described as a complex, individualized, and subjective experience that extends beyond overt physical abilities. Participants in this study experienced changes in self-identity and described a "new normal" after stroke. Implications and recommendations for rehabilitation, research, and policy are discussed.

### MEASURING QUALITY OF LIFE IN OLDER ADULTS LIVING WITH T2DM: A COMPARISON WITH YOUNGER ADULTS USING 2015 BRFSS DATA

Brittany Smalls,[1] Myles Moody,[1] Matthew Rutledge,[1] and Amy Cowley[1], *1. University of Kentucky, Lexington, Kentucky, United States*

Challenges due to burden of disease can affect adherence to self-care behaviors and optimal health outcomes in those living with T2DM. This study utilized state- and national-level data from the 2015 BRFSS to compare QoL measured by the prevalence of physical and mental burden days among older adults (OAs) compared to younger adults living with T2DM. The results of our analysis showed that OAs living in the US were significantly less likely to experience at least one mental burden day when compared to their younger counterparts (OR =0.61, 95% CI: 0.58, 0.64), while gender, education, race, BMI, and depression, CVD, or another chronic condition were significantly associated with the odds of experiencing at least one mental burden day. Whereas, in Kentucky OAs were less likely to experience at least one mental burden day when compared to their younger counterparts (OR= 0.48, 95% CI: 0.35, 0.66). Gender, education, BMI, and depression were significantly associated with the odds of experiencing at least one mental burden day or one physical burden day. The findings of this study suggests that the questions used by BRFSS to measure QoL may not be the most suitable for OAs who likely have different criteria for self-reported mental or physical burden days. When assessing QoL or burden of disease among the aging at a population level, considerable thought should be given into the questions asked and if they appropriately examine patient-level QoL in this population.

### COGNITIVE FUNCTION IN COUPLES AND COLLABORATIVE INVOLVEMENT IN TYPE 1 DIABETES MANAGEMENT

Cynthia Berg,[1] Yana Suchy,[1] Nancy Allen,[1] Rob Kent de Grey,[2] MaryJane Campbell,[1] and Vicki Helgeson[3], *1. University of Utah, Salt Lake City, Utah, United States, 2. University of Utah, Salt Lake City, United States, 3. Carnegie Mellon University, Pittsburgh, Pennsylvania, United States*

Managing type 1 diabetes involves coordinating complex daily behaviors that benefit from higher cognitive function. One's spouse's cognitive function may also be beneficial as spouses may collaborate in daily adherence behaviors and may be especially beneficial for older adults who may be experiencing poorer cognitive function. We examined: 1) whether one's own and one's spouse's cognitive function predicted lower (better) HbA1c, 2) whether collaborating with a more cognitively capable spouse was especially beneficial, and 3) whether the benefit of partners' cognitive ability occurred through better adherence. 199 couples were recruited where one member was diagnosed with type 1 diabetes for at least one year (52% females, average age 46.8 years, range 25.9-74.9, average duration of diabetes 27 years). Both patients and spouses completed the information subtest from the Wechsler Adult Intelligence Scale-Fourth Addition as a measure of general intelligence. Patients rated the collaborative involvement of their spouse in their diabetes and their adherence to their medical regimen. Multiple regressions revealed that spouse' higher intelligence uniquely and solely predicted better HbA1c over patient's intelligence. Collaborating with a spouse of lower intelligence was associated with higher HbA1c for older adults; collaborating with a spouse of higher intelligence was associated with somewhat lower HbA1c. Mediational analyses indicated that spouse's intelligence was associated with higher HbA1c through better adherence behaviors. The results suggest that individuals with type 1 diabetes who have a spouse of lower cognitive function may benefit from support from others in their network to manage their diabetes.

### SOCIOEMOTIONAL SELECTIVITY THEORY AND THE PERCEPTION OF CHRONIC PAIN

Ruth A. Wagstaff,[1] Bob G. Knight,[1] and Liam Hendry[1], *1. University of Southern Queenslan, Toowoomba, Queensland, Australia*

The Fear Avoidance model of chronic pain (FAM) posits that pain perception is best understood in the context in which pain occurs. An aspect of context that has received little attention is the effect that future time perspective and its related goals may have on chronic pain perception. According the Socioemotional Selectivity Theory (SST), young adults have instrumental goals to prepare for the future and older adults have goals to establish and maintain wellbeing. These differences have profound effect on cognition including attentional bias. It was hypothesised that future time perspective would influence pain perception through attention to pain. A total of 306 participants, 18 – 88 year olds with chronic joint pain completed the Numeric Analogue Scale for pain, three scales to measure the FAM constructs: Pain Vigilance Awareness Questionnaire, Pain Catastrophization Scale, and the Pain Anxiety Symptoms Scales, and, two scales to measure the SST constructs: Future Time Perspective Scale, (measures the goals and opportunities anticipated in the remaining life span) and the Positive Affect Negative Affect Scale (measures mood). Structural equation modelling finding supported the hypothesis and the model of fit indices indicated a good fit ($\chi2$ (53) = 127.412, p = .000, TLI .958, CFI = .971, SRMSEA = .056, RMSEA = .068, CI 90% [.053, .083]). This is the first time that age, future time perspective and the positivity effect have been shown to contribute the pain experience through attention. It provides a possible framework for exploring age appropriate psychological treatments for chronic pain.

### HOW MULTIPLE CHRONIC DISEASE BURDEN INFLUENCES COMMUNITY-BASED BEHAVIORAL HEALTH PROGRAM PARTICIPANTS

Keith W. TURNER,[1] and CHENG YIN[1], *1. THE UNIVERSITY OF NORTH TEXAS, DENTON, Texas, United States*

Background: Studies have shown that participation in community-based self-management education programs can result in improved healthful behaviors (exercise, cognitive symptom management, coping, and communications with physicians), improved health status (self-reported health, fatigue, disability, social/role activities, and health distress), and decreased days in the hospital. Problem: One of the understudied factors thought to influence efficacy in community based self-management programs is the presence and impacts of multiple chronic conditions on participants within community based behavioral health program populations. Multiple chronic diseases when scaled collectively can be considered as a participant's individual disease burden to be included in other analyses. Methodology: This investigation explores possible ways disease burden associates with such important constructs as participant personal characteristics and participant confidence in controlling impacts of their disease symptoms and participant preferences for use of various methods of coping with disease impacts. Outcomes: Results indicate a complex pattern of relationships between such factors as personal characteristics of program participants and their perceived mastery over the impacts of their disease symptoms, and their preferred mechanisms for coping. Implications: program designers and managers can better understand the differential influences of disease burden on participants analyzed with their personal characteristics and their preferential uses for coping mechanisms and their perceived ability to withstand the added burdens of multiple chronic diseases.

### DOES ONSET OF CHRONIC CONDITIONS MODERATE THE IMPACT OF RELATIONAL LIFE EVENTS ON DEPRESSED MOOD?

Aaron M. Ogletree,[1] Rosemary Blieszner,[2] Rachel Pruchno,[3] Jyoti Savla,[2] and Laura P. Sands[2], *1. American Institutes for Research, Washington, District of Columbia, United States, 2. Virginia Tech, Blacksburg, Virginia, United States, 3. Rowan University, Stratford, New Jersey, United States*

More than 62% of adults aged 65+ have more than one chronic condition; this number increases to more than 82% for those 85+. Older adults simultaneously experience changes in their relationships due to negative relational life events, including illness, injury, or death of a loved one. Stressors occurring in tandem can overload psychological resources and increase risk for poor mental health. Informed by the stress process model, we assessed the influence of relational life events on depressive symptoms over time and evaluated the moderating effects of chronic condition onset. Self-reports of four stressful life events, five chronic conditions, and depressive symptoms as measured by the CES-D came from 2,948 older adults participating in the ORANJ BOWL panel. Using longitudinal multilevel mixed effect modeling, we examined trajectories of depressive symptoms across three waves. While depressive symptoms increased over time, they were greater for people who experienced more relational life events and the onset of more chronic conditions. Participants who reported experiencing all four relational life events but no chronic conditions had an average CES-D score of 5.28 ($p<.0001$); average CES-D score increased to 12.72 ($p<.0001$) for those who reported four life events and the onset of four or more new chronic conditions during the study period. In summary, chronic condition onset moderated the relationship between life events and depressive symptoms. Findings highlight the need for practitioner awareness of increased mental health risks for people experiencing stressors in multiple domains of life.

### SEX DIFFERENCES BETWEEN ATTITUDES TOWARD MEDICATIONS AND POOR ANTIHYPERTENSIVE MEDICATION ADHERENCE IN ELDERLY

Taylor Johnson,[1] Erin Peacock,[1] Julia Silver,[1] James Marsh,[2] Richard Petty,[3] and MA Krousel-Wood[1], *1. Tulane University School of Medicine, New Orleans, Louisiana, United States, 2. Tulane University, New Orleans, Louisiana, United States, 3. The Ohio State University, Columbus, Ohio, United States*

Despite its importance for blood pressure control, antihypertensive medication adherence remains a challenge in older adults. Explicit and implicit attitudinal ambivalence toward medications (holding both positive and negative explicit attitudes, and discrepant explicit and implicit attitudes, respectively) may underlie low adherence. We examined whether race, age, or sex affect the associations between attitudes, ambivalence, and adherence. A questionnaire and implicit association test captured medication attitudes from hypertensive adults aged ≥55 (n=199). Adherence was measured with the Krousel-Wood Medication Adherence Scale (K-Wood-MAS-4). Higher scores on the attitudes and adherence scales indicate more positive attitudes and worse adherence, respectively. Associations and effect modification by sex, race (white vs. nonwhite), and age (<65 vs. ≥65) were tested in separate ordinary least squares regressions. The sample was 51.0% female, 43.7% nonwhite, 35.5% aged ≥65, with mean K-Wood-MAS-4 0.64 (SD=0.88). Better adherence was associated with more positive net explicit attitudes ($\beta$=-0.18, 95% CI -0.30, -0.06, p=0.003), and worse adherence with higher explicit ambivalence ($\beta$=-0.05, 95% CI 0.01, 0.09, p=0.028). The associations with explicit attitudes and explicit ambivalence were significant for men ($\beta$=-0.30, 95% CI -0.48, -0.11, p=0.002 and $\beta$=0.09, 95% CI 0.03, 0.15, p=0.005, respectively) but not for women ($\beta$=-0.07, 95% CI -0.423, 0.09, p=0.378 and $\beta$=-0.00, 95% CI -0.06, 0.05, p=0.982, respectively) (p-values for interaction=0.062 and 0.031, respectively). No race or age differences were identified. Adherence was not associated with implicit attitudes or implicit ambivalence. In conclusion, explicit attitudes and explicit attitudinal ambivalence may underlie low adherence to antihypertensive medications, particularly for older men.

### ASSOCIATION OF MULTIMORBIDITY WITH INCIDENT SHINGLES AMONG OLDER AMERICANS

Hyewon Kang,[1] Eileen Crimmins,[2] and Jennifer A. Ailshire[1], *1. University of Southern California, Los Angeles, California, United States, 2. Davis School of Gerontology, University of Southern California, Los Angeles, California, United States*

Older adults with multimorbidity are susceptible to shingles due to deterioration of immune function related to coexisting chronic disorders and immunological change. Although the association between individual chronic diseases and incident shingles has been documented, little is known about the effect of having multiple conditions on the risk for developing a new case of the disease. Multimorbidity is a normal condition with advanced aging. This paper examines risk of shingles onset associated with multimorbidity defined as having one or more chronic diseases including hypertension, diabetes, cancer, heart disease, lung disease, arthritis, and stroke) among older American. Data for this study come from the 1992 -2016 Health and Retirement Study. The study finds that risk for onset of shingles linearly increases with number of chronic disorders when age, gender, and race/ethnicity were adjusted (adjusted OR: 1.58, 95% CI; 1.37, 1.80 for those with 1 chronic condition vs adjusted OR: 3.43, 95% CI: 2.21, 5.53 for those with 6 conditions). The risks for multimorbidity were little changed after additional adjustments for socioeconomic status and health behaviors. The effect of multimorbidity on developing shingles is greater among young-old adults in their 50s than among the older-old adults aged 80 and higher. Our finding challenges the single-disease framework that is often used in previous studies examining risk factors for shingles. Our finding highlights the need for primary prevention and treatment of multimorbidity for reduction of shingles cases among older adults, especially young old adults in 50s.

### RURAL-URBAN DISPARITY IN BIOMARKER RISKS FOR CARDIOVASCULAR DISEASE AMONG CHINESE MIDDLE-AGED AND OLDER ADULTS

Haowei Wang,[1] Jeffrey E. Stokes[1], *1. University of Massachusetts Boston, Boston, Massachusetts, United States*

Cardiovascular disease (CVD) is a leading cause of adult mortality in China, accounting for 45% of deaths from noncommunicable disease. Moreover, Chinese health status and health services are disproportionately divided between urban and rural areas. This study examined rural-urban differences in age trajectories of CVD risk, measured by C-reactive protein (CRP), high-density lipoprotein (HDL) cholesterol, body mass index (BMI), and waist circumference. This study also investigated whether community factors, including recreational amenities, infrastructure availability, physical environment, public facilities, and health services, may explain such rural-urban disparities. We used data from the baseline data of the China Health and Retirement Longitudinal Study (2011), including 11, 528 respondents from 440 communities, who were aged 45 and older and participated in the biomarker survey. Multilevel models revealed that rural adults had a higher level of HDL and lower levels of CRP, BMI, and waist circumference compared to their urban counterparts. Rural adults also had slower age-related increases in trajectories for CRP, HDL and BMI. Associations of physical environment and public facilities with CVD risks were largely explained by rural-urban disparity. However, the availability of infrastructure explained both between- and within- rural-urban differences in BMI and waist circumference. Models were controlled for previously diagnosed CVD conditions, individual demographic characteristics, self-rated health, activities of daily living, depressive symptoms, physical activity, smoking and drinking behaviors. Findings contribute to the understanding of prevalence and disparities in biomarker risks for CVD among Chinese middle-aged and older adults. Intervention implications are discussed to address the emerging health disparities.

### POORER DIET QUALITY OBSERVED IN OLDER ADULTS WITH A GREATER NUMBER OF CHRONIC DISEASES

Jessica L. Krok-Schoen,[1] Stephanie Fanelli,[1] Janell Pisegna,[1] Owen J. Kelly,[2] and Christopher A. Taylor[1], *1. School of Health and Rehabilitation Sciences, The Ohio State University, Columbus, Ohio, United States, 2. Abbott Nutrition, Columbus, Ohio, United States*

Unhealthy lifestyle behaviors, including poor diet over many years, contribute to the development of chronic diseases, especially overweight/obesity, hyperglycemia, hypercholesterolemia and hypertension. Because poor diet is common to the diseases, it supports the notion of concurrently managing comorbidities through improved diet. Therefore, the purpose of this study was to assess differences in diet quality and nutrient intakes, in adults aged 65 years and older, by the number of chronic conditions. Data from 7,169 adults, aged 65 years and older, from the 2005-2016 National Health and Nutrition Examination Survey were assessed for selected chronic diseases from laboratory data: overweight/obesity (body mass index >25); hyperglycemia (glycated hemoglobin >5.7%); hypercholesterolemia (total cholesterol >200 mg/dL); hypertension (blood pressure >120/80 mmHg). The number of chronic diseases was computed per participant. Dietary intakes and diet quality score were assessed using 24-hour dietary recalls. Few adults had none of the selected chronic disease (n=79;1.4%), with others presenting 1 indicator (n=677;9.8%), 2 indicators (n=1,762;25%), 3 indicators (n=2,741;38.9%) and all 4 indicators (n=1,910;24.9%). Diet quality was significantly lower in those with three or four chronic diseases (P<0.001). Adults without any of the selected chronic diseases consumed significantly more calories, carbohydrates, fiber and added sugars, as well as folate, vitamin C and calcium than those with chronic diseases (P<0.001). Overall, dietary intakes from the day of intake were different for those with or without chronic diseases. These findings strengthen the need to promote healthy eating in older adults with one or more chronic conditions to help improve outcomes.

### ASSOCIATION BETWEEN LEVEL OF PAIN AND DEPRESSION AMONG CHRONICALLY ILL OLDER ADULTS IN RURAL ALABAMA

Hyunjin Noh,[1] Anne Halli-Tierney,[2] Lewis Lee,[1] and Temilade Aladeokin[1], *1. The University of Alabama School of Social Work, Tuscaloosa, Alabama, United States, 2. The University of Alabama College of Community Health Sciences, Tuscaloosa, Alabama, United States*

Pain and depression, two of the common symptoms among chronically ill older adults, have been found to be related in various populations; however, further knowledge is needed about their relationships and moderating factors among community-dwelling, chronically ill older adults, particularly in lower-income, rural areas with limited healthcare resources. Therefore, this study aimed to examine the association between pain level and depression among chronically ill older adults in rural areas. A total of 100 residents of a rural county in West Alabama, who are 55+ and have chronic illnesses and pain, were recruited from four community senior centers and were interviewed using a structured questionnaire. Pain levels were assessed by the Philadelphia Geriatric Center (PGC) Pain Scale, and depression by an abbreviated version of the Center for Epidemiologic Studies Depression Scale (CES-D). Bivariate correlation and multivariate analysis were conducted. The correlation between pain and depression was significantly positive ($r = .35$, $p < .001$). The results of the model indicated that pain scores and other control variables explained approximately 18 percent of the variance in depression. The multivariate analysis results confirmed that those who had higher pain scores were significantly likely to have increased depression scores ($b = 4.97$, $SE = 1.52$, $p < .01$). Education marginally significantly moderated the relationship between pain and depression ($p = .059$). The previously reported positive pain-depression relationship exists among chronically ill older adults in rural areas, calling for tailored interventions to reduce their pain and its impact on depression.

### PAIN EXPERIENCES IN OLDER ADULTS WITH HEART FAILURE IN THE HOME HEALTHCARE SETTING AFTER HOSPITAL DISCHARGE

Youjeong Kang,[1] and Cary Reid[2], *1. University of Utah, Salt Lake City, Utah, United States, 2. Weill Cornell Medicine, New York, New York, United States*

Pain symptoms are underreported in older adults (i.e., those ages ≥65), because many consider it a part of aging. Pain is highly prevalent in patients with heart failure (HF). HF is increasing in incidence with the aging population. Pain is also an important risk factor for rehospitalization among home healthcare patients with HF. However, little is known about the pain experiences of older adults with HF after hospital discharge. Accordingly, this study sought to describe pain and other symptoms among older adults with HF in the home healthcare setting using a mixed-methods approach. We conducted semi-structured interviews. We used the Edmonton Symptom Assessment Scale and Brief Pain Instrument on older adults (N=17) with HF in the home healthcare setting within 10 days of hospital discharge. The mean age was 76±9.25 years, nearly 50% were female, and most (90%) were White. The majority of participants managed pain with medications. Loss of appetite had the highest mean severity score (3.88±2.50) of symptoms measured. The mean score for pain at its worst was 4.94±3.07. Pain interfering with sleep had the highest mean score (4.24±3.36). Using content analysis we identified 4 distinct themes regarding the pain experience: (1) differences in the pain experiences before and after being diagnosed of HF, (2) symptoms accompanied with pain, (3) health care providers' pain assessment, and (4) knowledge about pain management. These findings will contribute to the development and evaluation of a pain assessment and management protocol targeting older adults with HF in the home healthcare setting.

### RECIPROCITY AND INDIVIDUAL DIFFERENCES IN DIET-RELATED SPOUSAL INVOLVEMENT AMONG OLDER COUPLES MANAGING DIABETES

Kristin J. August,[1] Caitlin S. Kelly,[2]
Charlotte H. Markey,[1] and Shibin Yan[1], *1. Rutgers University, Camden, New Jersey, United States, 2. University of Utah, Salt Lake City, Utah, United States*

Spouses are commonly involved in supporting and regulating their partners' diabetic diet. Older spouses also may be managing their own condition that requires changes to their diet. Little is known about the extent to which this spousal involvement is reciprocal and if there are individual differences in the provision of diet-related support and control (regulation). This study sought to understand the reciprocity of diet-related support and both positive and negative forms of control, whether personality traits predicted the provision of diet-related spousal involvement, and whether gender moderated these associations. We used data from a cross-sectional survey of 150 couples (50+ years) in which one member had type 2 diabetes (Mage=65.94; Mmarital length=33.46; 50.7% male patients; 58.7% of spouses had a condition that required changes to their diet). The provision and receipt of social support and two forms of social control were moderately correlated within individuals (patient rs=.36-.45, ps<.001; spouse rs=.41-.73, ps<.001). Using Actor Partner Interdependence Models that controlled for age and responsibility for managing meals, results revealed that being more extraverted or having a partner who was more extraverted was associated with greater involvement, whereas being more neurotic or having a partner who was more neurotic or had more interpersonal problems was associated with less positive and greater negative involvement. Associations were particularly pronounced for men. Results suggest that there is moderate amount of reciprocity, as well as personality differences, in diet-related spousal involvement. These findings highlight the importance of targeting both members of the couple in diabetes intervention efforts.

### ASSOCIATION BETWEEN SEEKING HEALTH INFORMATION ON THE INTERNET AND PALLIATIVE CARE KNOWLEDGE

YuJun Zhu,[1] and Susan Enguidanos[1], *1. University of Southern California, Los Angeles, California, United States*

Background: National and state surveys have found that only 8% - 17% of people know what palliative care (PC) is. Anecdotal reports also suggest many of those knowledgeable have misconceptions about PC. This lack of knowledge or misconceptions about PC can serve as barriers to accessing palliative care for seriously ill patients. Additionally, individuals often use the internet to seek health information, but it is unclear if this improves palliative care health literacy. Methods: We used the Health Information National Trends Survey 5, Cycle 2 (2018), a nationally representative dataset (n=3,504) to compare self-rated PC knowledge level with actual knowledge. We conducted chi-square and logistic regression to examine the association between PC knowledge and seeking health information on the internet. Results: About 33% of participants self-reported having PC knowledge. When these individuals were queried about specific PC knowledge, 58% failed to answer three basic questions correctly. After controlling for demographic and socioeconomic status, using the Internet to seek health information was positively associated with being knowledge about PC (P=0.006, OR=1.64), while having less than high school education was associated with lower PC knowledge. Implications: We found that self-reported PC knowledge may not reflect actual PC knowledge and thus clinicians should be sure to define the service when introducing it to patients. In addition, since use of internet in seeking health information is significantly associated with PC knowledge, it is important to ensure that internet PC information is valid, reliable, easy to access, and presented in simple language.

### THE ASSOCIATION OF PAIN LOCUS OF CONTROL WITH PAIN OUTCOMES AMONG OLDER ADULTS

Shirley Musich,[1] Shaohung Wang,[1] Luke Slindee,[2]
Sandra Kraemer,[3] and Charlotte Yeh[4], *1. Optum, Ann Arbor, Michigan, United States, 2. Optum, Eden Prairie, Minnesota, United States, 3. UnitedHealthcare, Minneapolis, Minnesota, United States, 4. AARP Services, Inc., Washington, District of Columbia, United States*

Pain locus of control (LOC) refers to perceived control over health and pain. LOC has ramifications on pain severity, disability and treatment. Our objective was to determine prevalence of 3 subscales of LOC among older adults with pain; and examine associations of scales on pain severity, opioids and physical function. The sample was identified from adults age≥65 with back pain, osteoarthritis or rheumatoid arthritis and minimum of 12-months medical and drug plan enrollment. Members were mailed a survey in May 2018 assessing positive resources, negative attributes and pain outcomes of pain. Opioid and other medications were determined from drug claims. The population was propensity weighted to adjust for survey non-response bias; weighted to generalize to those with diagnosed pain. Multivariate logistic regression was used to determine protective associations of positive resources with negative attributes on outcomes. Among respondents, 30% were internal LOC; 34% powerful other; 36% chance. In multivariate models, demographics, socioeconomics, health status, pain medications, resilience and social network, internal LOC was associated with lower pain severity, reduced opioid use and increased physical function. Magnitude of impact was comparable to resilience and diverse social networks. Powerful other LOC was partially protective with improved psychosocial attributes but poorer pain outcomes; chance LOC was associated with poor psychosocial/pain outcomes. Despite positive resources, depression and poor sleep maintained associations with pain outcomes. Internal LOC is a strong positive resource associated with improved pain. Multidimensional programs should include enhancing positive resources, including LOC, and focus on improving negative attributes to maximize pain management.

### SESSION 1320 (POSTER)

### CHRONIC DISEASE MANAGEMENT 1

### SELF-GLUCOSE MONITORING AND HYPOGLYCEMIA RISK IN OLDER RURAL VETERANS WITH TYPE 2 DIABETES

Willy Marcos Valencia,[1] Kaicheng Wang,[2] Kiranmayee Muralidhar,[2] and Stuti Dang[3], *1. Miami Veterans Affairs Healthcare System- GRECC, Miami, Florida, United States, 2. Department of Public Health Sciences, University of Miami Miller School of Medicine, Miami, Florida, United States, 3. Miami VA GRECC, Miami, Florida, United States*

Hypoglycemia evaluation is expected in every encounter with diabetic patients. However, self-monitoring and self-management may not be complete at home, and limited by geriatric syndromes. Furthermore, hypoglycemia risk increases with age, and rurality may limit access to frequent monitoring. We identified 112 rural veterans with high hypoglycemia risk, using the local medication database (sulfonylureas and insulin), combined with age and glycated hemoglobin (HbA1c). Statistical analyses were conduct using SAS 9.4 (Cary, NC). We used Chi-square, Fisher's, One-way ANOVA for baseline variables, and a multivariate logistic regression model to assess the association of hypoglycemia and risk factors, including age, HbA1c%, self-monitoring, and knowledge. Hypoglycemia was reported in 30.4% of cases, of whom the majority were younger than those not reporting hypoglycemia (72.0±4.3 vs 75.0±6.5 years, p=.015). Baseline HbA1c% was higher in cases with hypoglycemia compared to those without (7.7±1.6% versus 7.3±1.2%, not statistically significant). There were no significant differences between pharmacologic regimens, self-monitoring, and general knowledge. Veterans who knew hypoglycemia symptoms were 6 times more likely to reported hypoglycemia, compared with veterans who did not know any symptoms. We contacted primary care teams (PCT) for whom medications were adjusted. Hypoglycemia risk is high in the older population, and telemedicine programs can support primary care teams to improve management of their patients. Poor symptom knowledge needs to be addressed, while considering special attention for hypoglycemia unawareness in the oldest age group. We are implementing a project using continuous glucose monitoring in this high-risk population.

### THE FL-REACH PROJECT: TRANSLATING AN EBP FOR AN OUTPATIENT CLINICAL SETTING TO REACH DIVERSE COMMUNITY MEMBERS

Tracy C. Wharton,[1] Daniel Paulson,[1] Rosemary Laird,[2] Judy Clark,[2] Gayle Shepherd,[2] and Felicia Bender[1], *1. University of Central Florida, Orlando, Florida, United States, 2. Advent Health Orlando, Orlando, Florida, United States*

With more than 500,000 patients with Alzheimer's disease, constituting nearly 10% of all cases in the US, the state of Florida spends an estimated $20 billion per year on care and treatment related to this disease. The Florida State Plan on Aging reported that 75% of informal caregivers felt that early education and training should be a high priority for the state, and that difficult behaviors and limited knowledge about dementias were among the most significant challenges that they faced. The REACH II intervention is the gold-standard for evidence-based practices that address burden, well-being, and skills training for dementia caregivers. This presentation describes the partnership of a Memory Disorder Clinic (MDC) team and two university-based researchers working to embed a modified REACH protocol into an outpatient clinic. Critical streamlined components and new material designed to innovations since the REACH trials. The FL-REACH protocol is significantly shorter, adds a structured assessment for both patient and family needs, expands to include advanced care planning and grief modules, and intentionally builds linkages to the medical care team, with a focus on outreach to diverse families. This manualized intervention is offered to families of patients diagnosed through the MDC, thus capitalizing on the rapport and trust that is built, and providing in-house opportunities to engage diverse populations with a program grounded in the evidence base. This intervention provides critical foundational training for families that will bridge to seamless team coordination in the future.

### SLEEP APNEA ASSOCIATED COMORBIDITIES AND MORTALITY IN AN OLDER VETERAN POPULATION

Denise M. Kresevic,[1] muralidahar pallaki,[2] Christopher J. Burant,[3] Clare Gideon,[1] Emily Schroeder,[1] and Peijun Chen[1], *1. Northeast Ohio VAMC, Cleveland, Ohio, United States, 2. Northeast Ohio va health system, Cleveland, Ohio, United States, 3. Case Western Reserve University, Cleveland, Ohio, United States*

Evidence continues to mount that sleep apnea (SA) occurs in 10-25% of Americans and is associated with significant morbidity and mortality (Schulman 2018). Among veterans,

SA has been reported four times more often as compared to other non-veteran cohorts. (Wong 2015). The risk of developing dementia is increased in older individuals with OSA (Shastri, Bangar, & Holmes, 2015). The prevalence and characteristics of older adults with dementia and sleep apnea is not well known and long-term population-based studies on mortality have been lacking. Recent studies have reported overall mortality rates of 19%, in those individuals with SA, an increased rate of 1.5-3 times the mortality rate as compared to those individuals those without SA. Current recommendations support SA screening of high risk individuals including those with symptoms of snoring, fatigue, memory and concentration problems and mood changes. (Krist 2018). Despite a large number of older adults with suspected SA and comorbidities, the majority are not screened, referred, diagnosed and treated. In this VA pilot study of outpatient older male veterans with dementia and SA, N=195, mean age 75.83 years, SD=9.1, 51.3% were white, 37.5% were black. Frequently found comorbidities were: hypertension 88%, congestive heart failure 41%, Diabetes. 62% and, stroke 21%. Of note, among those who died, SA was significantly related to congested heart failure (r=.32, p<.001) and COPD (r=.40, p<.001). The overall mortality rate of 27% was higher than previous reports. Further investigation is needed to better understand the relationship between comorbidities, and SA, screening, treatment and mortality.

### LIFITEGRAST 5% FOR DRY EYE DISEASE: COMBINED EFFICACY AND SAFETY FROM FIVE RANDOMIZED CONTROLLED TRIALS

Agustin L. Gonzalez,[1] Eric Donnenfeld,[2] Christophe Baudouin,[3] Edward Holland,[4] Kelly Nichols,[5] Paul Karpecki,[6] Mohamed Hamdani,[7] and Amir Shojaei[8], *1. Eye and Vision North Texas Center for Dry Eye and Ocular Surface Disease, Richardson, Texas, United States, 2. Ophthalmic Consultants of Long Island, Garden City, New York, United States, 3. Quinze-Vingts National Ophthalmology Hospital, Paris, France, 4. Cincinnati Eye Institute-Northern, Edgewood, Kentucky, United States, 5. University of Alabama, Birmingham, School of Optometry, Birmingham, Alabama, United States, 6. Kentucky Eye Institute, Lexington, Kentucky, United States, 7. Apellis Pharmaceuticals, Crestwood, Kentucky, United States, 8. Takeda, Vice President, Global Head - Ophthalmology, Lexington, Massachusetts, United States*

Five randomized, double-masked, placebo-controlled trials were conducted in the US in adults with DED, an eye dryness score (EDS, visual analogue scale [VAS], 0–100) ≥40, and inferior corneal staining score ([ICSS], 0-4) ≥2.0 at study entry: four 84-day efficacy trials (phase 2, lifitegrast n=58, placebo, n=58; phase 3: OPUS-1, n=293, n=295; OPUS-2, n=358, n=360; OPUS-3, n=355, n=356) and a 1-year safety study (SONATA, lifitegrast n=220, placebo n=111). The pooled population had a mean age of 59.4 years, and 76% were females. Lifitegrast treatment, versus placebo, significantly improved EDS from baseline to day 84 in three of the four trials: OPUS-1 (treatment effect [TE] 4.7; P=0.0311), OPUS-2 (TE 12.3; P<0.0001), and OPUS-3 (TE 7.5; P=0.0003). Lifitegrast significantly improved ICSS in the phase 2 (TE 0.25; P=0.0498) and OPUS-1 (TE 0.23; P=0.0007) trials, and nominally in OPUS-3 (TE 0.17; nominal P=0.0135). The responder analyses from OPUS-2 and OPUS-3 assessed the proportion of subjects with an EDS reduction from baseline (≥10, ≥15, ≥20 points), and percentage change from baseline (≥30, ≥50, ≥70%), to days 14, 42 and 84. More subjects achieved ≥30% EDS reduction with lifitegrast versus placebo in Opus-2, and Opus 3 at days 14, 42, and 84 (all nominal P<0.0001). A similar trend was seen at other response thresholds. Pooled safety data (lifitegrast n=1287; placebo, n=1177) indicated Lifitegrast was well tolerated with no serious ocular adverse events. Lifitegrast significantly improved signs and symptoms of DED in adult subjects, with EDS improvements observed starting at day 14 after treatment.

### PREDICTORS OF SHORTNESS OF BREATH DURING STAIR CLIMB IN COMMUNITY-DWELLING INDIVIDUALS WITH COPD

Shweta Gore[1], *1. MGH Institute of Health Professions, Boston, Massachusetts, United States*

Dyspnea is the primary and most disabling symptoms seen in chronic obstructive pulmonary disease (COPD). Primary pathophysiological changes such as dynamic hyperinflation have been associated with the etiology of dyspnea in chronic obstructive pulmonary disease (COPD). However, since the experience of dyspnea "derives from interactions among multiple physiological, psychosocial and environmental factors", a single correlate to accurately predict dyspnea has not been established. The purpose of this study was to identify factors that could predict shortness of breath during stair climb (SOB-SC) in community dwelling adults with COPD. We hypothesized that physical activity and muscle strength would significantly predict SOB-SC. Individuals with COPD who participated in the National Health and Nutrition Examination Survey (NHANES) between years 1999-2002 were selected for this study. Participants were excluded if they had significant mobility limitations. Socioeconomic, demographic variables, and clinical variables including BMI, physical activity, comorbidities, muscle strength, ankle brachial index, waist circumference and inflammatory markers were extracted. Logistic regression models were plotted with SOB-SC as the categorical dependent variable after assessing for collinearity using the forced-entry method. Individuals with COPD had a significantly greater proportion of SOB-SC ($\chi$ = 134.87, p < 0.001). Larger waist circumference (p = 0.002, CI = 0.04 -0.13), presence of cardiovascular disease (p = 0.001, CI = 0.76 -2.37) and Caucasian race were found to significantly predict SOB-SC after controlling for covariates. This study reinforces the importance of screening for cardiovascular disease and lifestyle modification in this population subgroup. Future studies examining differences in COPD severity are needed.

### SESSION 1325 (POSTER)

### CROSS CULTURAL STUDIES

### A QUALITATIVE REVIEW OF OLDER ADULT PERSPECTIVES ON HEALTHY AGING IN THE CIRCUMPOLAR NORTH

Britteny M. Howell,[1] and Jennifer R. Peterson[2], *1. University of Alaska Anchorage, Anchorage, Alaska, United*

*States, 2. University of Alaska Fairbanks, Fairbanks, Alaska, United States*

Cross-cultural research has shown marked variation in health outcomes across the world's older adult populations. Indeed, older adults in the Circumpolar North experience a variety of health disparities. Because aging is a biological process rooted in sociocultural context, there exists great variation in the ways older adults define and experience healthy, or "successful," aging in their communities. The aim of this analysis was to synthesize qualitative research among older residents (aged 50+ years) in the Circumpolar North to identify a definition of healthy aging common in the region. The Circumpolar North is defined as the Arctic and subarctic regions of Canada, Finland, Denmark, Greenland & the Faroe Islands, Iceland, Norway, Russia, Sweden, and the United States. A thorough review was conducted across a variety of academic search databases for peer-reviewed, qualitative studies conducted among community-dwelling older adults. The search strategy initially identified 194 articles; 22 articles met the inclusion criteria. Included studies were coded and analyzed using Grounded Theory to examine underlying themes of healthy aging in the Circumpolar North. The findings reveal the importance older adults place on incorporating social, environmental, and personal resilience factors into multidimensional models of healthy aging. This research also highlights the need for increased translational research with populations in the Circumpolar North that are under-represented in the gerontological literature.

### MEASURING SOCIOECONOMIC STATUS IN OLDER POPULATIONS: A SCOPING REVIEW AND INTERNATIONAL PERSPECTIVE

Gemma F. Spiers,[1] Barbara Hanratty,[1] Fiona E. Matthews,[1] E Moffatt,[1] and A Kingston[1], *1. Newcastle University, Newcastle upon Tyne, United Kingdom*

Socioeconomic status (SES) is often measured using indicators that are less relevant to older populations. Building on earlier debates about these issues, an up-to-date, critical review of contemporary evidence and approaches is needed. A key question is how these challenges might vary between countries and different socio-cultural contexts. An international systematic scoping review was undertaken to a) identify which measures of SES have been used in studies of older adults' health, healthcare utilization and social care utilization, and b) critically appraise the application and validity of these measures in older populations. Systematic searches were conducted in five databases (Medline, Scopus, EMBASE, PsychInfo, Web of Science and Health Management Information Consortium) in May 2018. Studies were eligible if they reported data about the relationship between a measure of SES and self-rated health, healthcare use or social care use for people aged 60+ years, and were published after 2000 in a high-income country (as defined by the Organisation for Economic Cooperation and Development). Sixty-two studies across seventeen countries were included. Measures used included: education (n=41), income (n=37), subjective SES (n=8), occupational or employment (n=10), area deprivation (n=10), combined wealth (n=7), home ownership (n=13), and housing conditions (n=2). A minority (n=7) used a range of proxy variables. The challenges of applying these measures to older populations will be considered. Attention is given to how these challenges may differ by country, whilst considering the added complexities of age, gender and socio-cultural context. Implications for future research on older adults' health inequalities are discussed.

### CHANGES IN DEPRESSIVE SYMPTOMS IN EAST ASIA: A COORDINATED ANALYSIS OF THREE LONGITUDINAL STUDIES

Takeshi Nakagawa,[1] Jinmyoung Cho,[2] and Dannii Yeung[3], *1. National Center for Geriatrics and Gerontology, Aichi, Japan, 2. Baylor Scott & White Health, Temple, Texas, United States, 3. City University of Hong Kong, Hong Kong, Hong Kong*

Evidence for changes in depressive symptoms is relatively sparse in Asian populations. We examined changes in depressive symptoms in China, Korea, and Japan. Data were derived from three longitudinal studies with three measurement waves: the China Health and Retirement Longitudinal Study (CHARLS between 2011—2015), the Korean Longitudinal Study of Aging (KLoSA between 2006—2010), and the Japanese Study of Aging and Retirement (JSTAR between 2007—2011). Participants aged 50—75 years were included in the analysis (CHARLS: n = 10,385; KLoSA: n = 6,683; JSTAR: n = 3,004). Multilevel analyses were conducted separately for each country to examine trajectories of depressive symptoms, controlling for age, age squared, gender, education, marital status, activities of daily living, and morbidity as covariates. Depressive symptoms were measured by the 10-item CES—D. The CES—D score was scaled to a T score metric (M = 50, SD =10) using the score at wave 1 in each country as a reference. Trends in depressive symptoms varied across countries, with stability in China but increase in Korea and Japan (Estimate = —0.05, 0.69, 0.40, respectively). Older Koreans reported higher levels of depressive symptoms than younger adults, whereas a reverse pattern was shown in China. Age differences were not found in Japan. Higher levels of education were associated with lower levels of depressive symptoms in China and Korea, whereas the opposite association emerged in Japan. These country differences will be discussed through the lens of societal and economic factors (e.g., welfare systems and economic recession).

### COGNITION, SELF-RATED COGNITIVE HEALTH, AND CONCERNS ABOUT ALZHEIMER'S DISEASE IN OLDER KOREAN AMERICANS

Yuri Jang,[1] Nan Sook Park,[2] David Chiriboga,[2] Hyunwoo Yoon,[3] and Min-Kyoung Rhee[1], *1. University of Southern California, Los Angeles, California, United States, 2. University of South Florida, Tampa, Florida, United States, 3. Texas State University, San Marcos, Texas, United States*

Responding to the dearth of research on cognitive health in older ethnic minorities, the present study explored the associations among cognitive performance, self-rated cognitive health, and concerns about Alzheimer's Disease (AD) in older Korean Americans. We hypothesized that (1) cognitive performance and self-rated cognitive health would be moderately associated; (2) both cognitive performance and self-rated cognitive health would be associated with concerns about AD; and (3) the effect of cognitive performance on concerns about AD would be mediated by self-rated cognitive health. Analyses of the survey data of 2,150 older Korean Americans (mean age = 73.2) residing in five states provided support for the hypotheses. Cognitive performance, measured with a Korean version of the Mini-Mental State Exam (MMSE), and a single item asking respondents to rate their overall cognitive status on a 5-point scale (excellent/very good/good/fair/poor) were moderately associated. Both poor performance in the MMSE and negative ratings of cognitive health status were associated with increased concerns about AD. Mediation analyses using the macro PROCESS showed that the indirect effect of cognitive performance on the concerns about AD through self-rated cognitive health (–.01 [.002]) was significant (bias corrected 95% confidence interval for the indirect effect = –.02, –.01). That is, the effect of cognitive performance on the concerns about AD was mediated by individuals' subjective evaluations of their cognitive health. Findings not only help better understand the psychological mechanisms that underlie cognitive health and AD concerns but also suggest avenues for interventions.

### LANGUAGE USE, LITERACY SKILLS, AND ONLINE HEALTH INFORMATION-SEEKING IN LATER LIFE AMONG HISPANICS IN THE U.S.

Roberto J. Millar,[1] Shalini Sahoo,[2] Takashi Yamashita,[1] and Phyllis Cummins[3], *1. University of Maryland Baltimore County, Baltimore, Maryland, United States, 2. University of Maryland, Baltimore, Baltimore, Maryland, United States, 3. Scripps Gerontology Center, Miami University, Oxford, Ohio, United States*

Online platforms, which are often in English, have become a common source of health information. Accordingly, language skills have been shown to be important for health information-seeking. In the United States, the use of online health information is generally low among Hispanics, particularly those with limited English proficiency. What is less clear, however, is how different measures of English proficiency may be linked to online health information seeking in later life. This study examines the associations between language spoken at home, literacy skills, and online health information seeking among middle age and older Hispanics in the U.S. Data of Hispanic adults aged 40 years and older (n = 315) come from the 2012/2014 Program for International Assessment of Adult Competencies (PIAAC). We used binary logistic regression models with complex sampling weights to examine online health information seeking as a function of primary language use at home (Spanish vs. English) and literacy skill assessment scores (low – high: 0 – 500 points). Results indicated that speaking Spanish at home (OR = 0.317, $p < 0.05$) is a negative predictor, and greater literacy skills (OR = 1.011, $p < 0.05$) is a positive predictor of online health information seeking. Findings from this study clarify possible health information disadvantages by limited English proficiency and lower literacy skills. We discuss how the impact of primary language use and literacy skills should be incorporated into future health communication and policy initiatives to address the barriers to health information among middle-aged and older Hispanic adults.

### AVOIDANT FILIAL PIETY IN KOREAN IMMIGRANT FAMILIES: WHEN DO WE TALK ABOUT MOM AND DAD?

Joonsik Yoon[1], *1. Syracuse University, Syracuse, New York, United States*

Parental caregiving for older adults is a challenge to most families, not only because of intangible factors such as role-reversals in family dynamics, but due to the many practical difficulties involved. At the same time, family members are important sources of support and care even in wealthy countries with established and relatively efficient social services for the older population. For migrant families from East Asia, where adult children have emigrated while their parents have remained in the country of origin, the distance and the transnational nature of the family ties present additional complications to the challenge of providing parental care within a highly filial culture. In this qualitative study, first-generation Korean Americans were interviewed in open-ended, in-depth interviews about their experiences and concerns surrounding transnational parental caregiving. The results show that none of the study participants or their families plan for parental care. Although most respondents expressed concerns over potential future care needs of their parents, none of them had taken steps to address those concerns or discussed the matter with other family members, who were equally reticent to discuss it. This was even though many stated that they were specifically worried about how to care for their parents in times of need given that they lived far away in another country. The discussion explores why migrant families consistently avoid what they themselves acknowledge to be an important, even necessary, discussion their families ought to have.

### AIR QUALITY AND WELL-BEING AMONG MIDDLE-AGED AND OLDER ADULTS IN EAST ASIA

Takashi Yamashita,[1] Giyeon Kim,[2] Darren Liu,[3] and Anthony R. Bardo[4], *1. University of Maryland, Baltimore County, Baltimore, Maryland, United States, 2. Chung-Ang University, Seoul, Korea, Republic of, 3. Des Moines University, Des Moines, Iowa, United States, 4. University of Kentucky, Lexington, Kentucky, United States*

There is a well-established link between air quality, an important component of one's local living environment, and well-being. However, the link between well-being and air quality is largely based on findings from western nations, and evidence from East Asia (where air pollution is a major challenge) is scant. Thus, the present study sheds much needed light on the association between well-being and air quality in four East Asian countries (i.e., China, Japan, South Korea, and Taiwan). Data for this study were drawn from the internationally representative 2010 East Asian Social Survey Health Module. The sample was limited to middle-age and older adults (i.e., 50 years and older) to account for differential exposure across the life course (N = 4,052). Linear regression models with robust standard error estimation and full information maximum likelihood were used to examine associations between four well-being indicators (self-rated health, SF-12 physical health and mental health, and happiness) and self-reported air quality. Results showed that air quality was negatively associated with well-being across East Asian nations --- with self-rated health in Japan (b = -0.09, p

< 0.05) and Taiwan (b = -0.14, p < 0.05); physical health in Japan (b = -0.96, p < 0.05); mental health in China (b = -1.05, p < 0.05) and Japan (b = -1.49 , p < 0.05); and happiness in China (b = -0.07, p < 0.05). Possible explanations underlying these distinct national patterns and strategies to enhance well-being through environmental and behavioral interventions are discussed.

### THE OLDER POPULATION LIVING WITH AND PROVIDING CARE TO GRANDCHILDREN, BY NATIVITY: 2013-2017

Bashiruddin Ahmed,[1] Andrew Roberts,[2] Matthew Spence,[3] Wan He,[2] and Laquitta Walker[3], *1. U.S. Census Bureau Population Division, Washington, District of Columbia, United States, 2. United States Census Bureau, Washington, District of Columbia, United States, 3. U.S. Census Bureau, Washington, District of Columbia, United States*

The rapid growth of the older population in the United States combined with changing living arrangements, marital status, and employment, increases the importance of multigenerational ties for the well-being of families. The U.S. Census Bureau's earlier reports on grandparents living with grandchildren mostly focused on the background characteristics of all grandparents without classifying them by nativity. This study expands on the research by presenting data for both native- and foreign-born grandparents aged 60 and older who live with and provide care to their grandchildren under 18. Data for this study come from the 2013-2017 American Community Survey 5-year estimates. Among native-born grandparents living with grandchildren, the majority were females, aged 60-69, White alone, non-Hispanic or Latino, married, high school graduate or higher, had no disability, lived in a household that was owned, uninsured, not in labor force, and not in poverty. The foreign-born grandparents were similar in most characteristics except for race component and educational attainment. Key findings include: • Among total older population, 14.3 percent of the foreign-born lived with grandchildren, compared with 4.1 percent of the native-born. • Among co-resident grandparents, the native-born (36 percent) were more likely to be caregivers, compared with the foreign-born (14 percent). • The proportions of co-resident grandparents widely vary by race and Hispanic origin. • Among grandparent caregivers, over 50 percent had been responsible for grandchildren for 5 years or more, while 14 percent for less than a year. • Both native- and foreign-born grandparents show declining patterns of care-giving by age.

### SESSION 1330 (POSTER)

### DEPRESSION, ANXIETY, AND MENTAL HEALTH

### INTRA-INDIVIDUAL PHYSICAL AND PSYCHOLOGICAL SYMPTOM VARIABILITY AND STRESS INFLUENCE ACTIVITIES OF DAILY LIVING

Scott E. Moore,[1] Kelly Wierenga,[2] and Heather K. Hardin[1], *1. Frances Payne Bolton School of Nursing, Case Western Reserve University, Cleveland, Ohio, United States, 2. Indiana University, Indianapolis, Indiana, United States*

Stress and symptomatology have been shown to have strong relationships to health outcomes in older adults. More specifically, perceived social stressors (PSS), whether related to disease, disability or demographics, is a contributor to health. Recently it was reported that intra-individual symptom variability (ISV) may predict poor health outcomes better than symptom severity in some chronic illnesses. Individual and combined influences of ISV and PSS on health behaviors are not fully described. Using a subset (n = 518, 46.5% men; mean age = 48.7) of MIDUS Refresher participants' 8 day daily diary data, we sought to determine the influences of physical and psychological ISV and PSS on independent and basic activities of daily living (iADLs, bADLs). The ISVs represent an average of day-to-day variation across each of the 22 physical and 27 psychological symptoms for each participant. Psychological ISV, physical ISV, PSS, and total number of chronic conditions were entered into two structural equation models as predictors for each ADL outcome (p<.01). The models depicted both direct and indirect influences of psychological ISV on ADLs (iADLs: B=-.43, P < .001; B = .51, P < .001 [through PSS]; bADLs: B=-.45, P < .001; B = .51, P < .001 [through PSS]). However, the influence of physical ISV on ADLs was indirect (B = .22, P = .001 [through PSS]). Individual-level influences of ISV and PSS on ADLs may better aide healthcare providers' identifying and intervening to disrupt poor health outcomes for those at risk.

### DETERMINANTS OF DEPRESSION AMONG AGING LGBT IN TEXAS

Ami Moore[1], *1. University of North Texas, Denton, Texas, United States*

Introduction: Aging LGBT experience significant health disparities compared to their heterosexual counterparts. As a sexual minority, they have a high likelihood of acquiring physical and mental illnesses. Methods: This study investigated factors that are associated with reports of depression among LGBT. Data came from Project Gray Pride, a study of aging gay, lesbian, bisexual, and transgender men and women living in a metropolitan area in Texas (N=126). Linear regression analysis was used. Results: Results show a negative association between respondents who self-identified as cisgender female and depression compared to those who self-identified as transgender. However, social vigilance and reports of health symptoms positively correlate with depression. Additionally, the interaction term of gender and race showed that respondents who were non-Whites and female reported higher levels of depression relative to their counterparts who were White males or White transgender. Other factors such as HIV status, age, education were not statistically significant. Conclusion: This study shows that being socially vigilant increases depression among aging LGBT. Also, aging female LGBT who are non-White may have an increased burden of negative social experiences leading to high levels of depression. As the population of older LGBT is increasing, intervention efforts must take into account these important findings in order to devise sound policies and programs to improve mental health among LGBT.

### SELF-REPORTED VISION IMPAIRMENT, VISION CORRECTION, AND DEPRESSION AMONG MIDDLE-AGED AND OLDER CHINESE

Beibei Xu,[1] Gui-Ying Cao,[2] Shan-Shan Yao,[2] zi-shuo Chen,[2] and Zi-Ting Huang[2], *1. Peking University Medical Informatics Center, Beijing, China, 2. Department of Epidemiology and bio-statistics School of Public Health, Peking University, Beijing, China*

This study utilized data from the China Health and Retirement Longitudinal Study (CHARLS) to investigate the associations between vision impairment (VI), vision correction (VC) and depression among middle-aged and older Chinese. Near or distance vision and VC were reported via questionnaires. For this study, participants were divided into four categories: no VI, distance vision impairment (DVI), near vision impairment (NVI), or both distance and near vision impairment (DNVI). Participants were also divided into four groups: no VI with VC (VI(-)/VC(+)), no VI without VC (VI(-)/VC(-)), VI with VC (VI(+)/VC(+)) and VI without VC (VI(+)/VC(-)). Depression was evaluated by Center for Epidemiological Studies Depression Scale (CES-D-10). Of 16,159 individuals aged 45 years and older included, the mean (SE) age was 58.5 (0.1) years and 52% of participants were women. Compared with no VI, DVI only (OR=1.92; 95% CI: 1.69, 2.19), NVI only (OR=1.34; 95% CI: 1.17, 1.53) and DNVI (OR=2.23; 95% CI: 1.86, 2.66) were associated with higher odds of depression at baseline after full adjustment with covariates. Only DNVI was significantly associated with higher odds of onset of depression two (OR=1.41; 95% CI: 1.12, 1.78) and four (OR=1.30; 95% CI: 1.02, 1.66) years later after full adjustment. Compared with VI(-)/VC(+), VI(-)/VC(-) (OR=1.36; 95% CI: 1.03, 1.81) and VI(+)/VC(-) (OR=1.55; 95% CI: 1.15, 2.10) were associated with higher odds of onset of depression four years later after full adjustment. VC might be a protective factor for preventing depression among those middle-aged and older individuals with VI or potential vision decline.

### MEANING IN LIFE AND REASONS FOR LIVING IN OLDER ADULTS WITH SUICIDAL IDEATION: AN INTEGRATIVE REVIEW

Atami Sagna[1], *1. The University of Texas at Austin, Austin, Texas, United States*

Suicide has ranked as the 10th leading cause of death for all ages in the United States. Although Healthy People 2020's target is to reduce suicide rates by 10.2 per 100,000 by 2020, it remains that suicide rates continue to increase, with suicide in older adults contributing substantially to this rise. Older adults have a higher risk for suicide, yet research in the area on positive psychological factors such as meaning in life and reasons for living is lacking. The purpose of this review is to investigate the associations among meaning in life, reasons for living and suicidal ideation in older adults (55+ years). Based on PRISMA guidelines, the PubMed, PsycINFO, PsycARTICLES, and CINAHL databases were systematically searched for relevant publications without date restrictions. Nine studies, qualitative and quantitative, are included in the review, showing a relationship among meaning in life, reasons for living and suicidal ideation in older adults. All the studies found that meaning in life and reasons for living were negatively associated with suicidal ideation in older adults. The findings of this review highlight the importance of including positive psychological factors in assessing suicide risk in older adults and in planning preventative measures and services for this high-risk group.

### SOCIAL TIES AND SUICIDAL IDEATION AMONG OLDER ADULTS REFERRED FOR A BEHAVIORAL HEALTH EVALUATION

Shahrzad Mavandadi,[1] Erin Ingram,[1] Johanna Klaus,[1] and David Oslin[1], *1. Corporal Michael J Crescenz VA Medical Center, Philadelphia, Pennsylvania, United States*

The association between the quality and nature of social relationships and suicidal outcomes across the lifespan is well established. However, social ties are rarely assessed in primary care and other clinical settings where older adults at high risk for suicide-related outcomes are seen. This study examined the unique associations between three indices of social ties (i.e., perceived social support, frequency of negative social exchanges, and degree of social integration) and death/suicidal ideation among 3,261 older veterans (aged 65+) who completed a clinical mental health/substance use (MH/SU) assessment upon referral to a Primary Care-Mental Health Integration (PCMHI) program. Data on sociodemographics, MH/SU conditions (e.g., depression, anxiety, and substance use), perceived health, the three indices of social ties, and death/suicidal ideation were extracted from clinical interviews. Veterans were on average 70.8 years old (+6.5 years) and primarily male. Approximately half were married and 60.3% were non-Hispanic white. Forty percent reported death ideation or suicidal ideation, as measured by the Paykel Suicide Scale. Logistic regression analyses revealed that, adjusting for covariates, while perceived social support was associated with a greater risk of reporting death ideation relative to no ideation, both social support and frequency of negative exchanges were uniquely associated with greater risk of reporting suicidal ideation relative to no ideation. Social integration was unrelated to odds of death or suicidal ideation. Findings underscore the value of integrating assessments of multiple aspects of social ties into routine PCMHI practice, as doing so has the potential to enhance suicide screening and intervention efforts.

### MESSAGE FRAMING AND ENGAGEMENT IN MENTAL HEALTH CARE: THE MODERATING ROLE OF AGE

Shahrzad Mavandadi,[1] Erin Wright,[1] Johanna Klaus,[1] and David Oslin[1], *1. Corporal Michael J Crescenz VA Medical Center, Philadelphia, Pennsylvania, United States*

Engagement rates in specialty mental health (MH) care for depression are suboptimal across the lifespan, prompting various efforts to promote treatment engagement. This study examined the extent to which age moderates the impact of message framing on appointment attendance among patients referred to specialty MH care. The study employed a randomized, prospective, experimental design. Patients meeting criteria for major depression and referred to specialty MH care at a Veterans Affairs Medical Center (n=360) were randomized to receive either a routine, neutrally worded patient reminder letter prior to their scheduled specialty MH care appointment or a letter that included one of two messages (gain-framed or loss-framed) that were added to the routine letter. Appointment/attendance data were extracted from the computerized patient record system. Logistic regression was used to determine the potential moderating effect of age on the association between message frame and appointment attendance. The sample (mean age = 51.5 (SD=13.5)) was primarily male (85%), non-white (62%), and reported an average depressive symptom score of 19.3 (SD=3.8, range=9-27). Age moderated the impact of message frame on the odds of attending the MH appointment: while younger adults were more likely to attend after receiving a gain-framed message, older adults' engagement rates did not differ significantly across conditions. Findings suggest that overtly highlighting the benefits of attending an initial specialty MH care appointment, even if in writing, can impact engagement rates among younger adults. Potential alternative, targeted approaches utilizing message framing that may be more effective among older adults should be explored.

### ANTIDEPRESSANT USE AND MORTALITY RISK IN OLDER ADULTS WITH HISTORY OF STROKE

Madison B. Lenox,[1] Victor Tran,[1] Grace Tworek,[1] Andrew Solow,[1] and Diana Hincapie[1], *1. Nova Southeastern University, Fort Lauderdale, Florida, United States*

The American Stroke Association reports stroke as the fourth leading cause of death in the United States, with 66% of hospitalized cases being older adults. Recovery from stroke is a public health issue, as post-stroke depression (PSD) is a significant concern. Approximately 20-23% of stroke survivors identified co-occurring diagnoses, which are associated with physical, functional, and cognitive limitations and increased mortality risk. Antidepressant use has exhibited its efficacy in treating PSD. This study explores the association between antidepressant use and mortality risk in older adults with history of stroke. Older adults aged 65 and older (N=3631, 55.4% female, 72.6% Caucasian, Mage=79.64 years, SDage=7.29 years, MEd=14.55 years, SDEd=8.269 years) with history of stroke were selected from the National Alzheimer's Coordinating Center database to explore the association between antidepressant use and mortality. A chi-squared test of independence was calculated comparing antidepressant use and mortality rates. A significant association was found ($\chi 2$ (1) = 15.933, $p < .001$) between current antidepressant use and mortality. Findings suggest antidepressant use is associated with lower mortality rates in subjects with a history of stroke. Implications include highlighting the role psychologists play in the early identification of PSD and early antidepressant intervention post-stroke to increase life longevity. Although findings only infer association, they demonstrate evidence for the link between PSD, antidepressant use, and lower mortality rates. Future directions include exploring other forms of depression treatment and mechanisms of antidepressant use. Limitations include examining potential moderators (e.g., gender, SES, type of stroke), and substance use within this population.

### EVALUATING THE ASSOCIATION BETWEEN SLEEP AND MEMORY IN OLDER VETERANS WITH PTSD

Katherine S. Hall,[1] Miriam C. Morey,[1] Hayden Bosworth,[1] Jean Beckham,[1] and Michelle Pebole[1], *1. Durham Veterans Affairs Health Care System, Durham, North Carolina, United States*

Sleep disturbances are core symptoms of posttraumatic stress disorder (PTSD), and recent studies also suggest a link between PTSD and cognitive impairment. There is some evidence of an association between sleep disturbances and cognitive abilities, such as memory, though few studies have focused on older adults and fewer still among those with mental health conditions. This study examined the association between subjective memory complaints and sleep (quality and quantity) in older veterans with PTSD. Fifty-four veterans with PTSD (M age=67.4, 85.2% African American, 90.7% men) participated in the study. Sleep was assessed using the Pittsburgh Sleep Quality Inventory (PSQI) and the PSQI Addendum for PTSD (PSQI-A). Memory was assessed using the Frequency of Forgetting Scale (FOF) derived from the Memory Functioning Questionnaire. The relationship between sleep quality parameters and FOF were examined using bivariate correlations and independent samples t test. Over 60% of participants met military-specific criteria for poor sleep (PSQI≥10; PSQI-A>5). Overall sleep quality on the PSQI-A was significantly associated with worse memory ($r=-0.38$, $p<.01$). Among specific sleep parameters (e.g., sleep latency, sleep duration), greater daytime dysfunction due to sleepiness was significantly associated with worse memory ($r=-0.44$, $p<.01$). Between-group analyses comparing memory complaints across participants classified as 'poor' versus 'good' sleepers on the PSQI-A approached significance ($t(52)=1.93$, $p=.06$). This study suggests that poor sleep may be a correlate of memory complaints among older adults with PTSD. Future studies are needed to determine whether poor sleep is an underlying factor in the link between PTSD and cognitive impairment.

### BACK TO BASICS: HOPELESSNESS AS THE MEDIATING FACTOR FOR COMPLETED SUICIDE AMONG OLDER ADULTS

Silvia C. Hernandez,[1] James C. Overholser,[1] James Lavacot,[1] Kristie L. Philips,[2] and Craig A. Stockmeier[3], *1. Case Western Reserve University, Cleveland, Ohio, United States, 2. Cleveland VA Medical Center, Cleveland, Ohio, United States, 3. University of Mississippi Medical Center, Jackson, Mississippi, United States*

Individuals 65 years and older are at high risk for completing suicide. Though risk factors have been established in the literature, the dominant atheoretical approach has left the field at an impasse. The present study aimed to integrate core risk factors of hopelessness, depression, physical illness, and social isolation by proposing a biopsychosocial framework of older adult suicide. A psychological autopsy was used to compare individuals 65 years and older who died either by suicide ($n = 32$) or natural causes ($n = 45$). Structural equation modeling results suggested that hopelessness was the only factor directly associated with suicide ($B = .01$, $\beta = 0.84$, $SE = 13.31$, $p \leq .001$), fully mediating the relationships between suicide and social isolation, negative attitudes about physical health, and depression. The proposed model adequately fit the data, explaining 71% of the variance in cause of death. Advanced age (75+ years) moderately increased social isolation, which weakly increased hopelessness, contributing to suicide in a smaller magnitude than expected. Though individuals in the advanced age group had a wider range of physical illnesses, this did not increase risk. Rather, negative perceptions of health increased risk for all individuals 65 years and older via depression and hopelessness, irrespective of the presence of impairing physical illness. Findings support the claim that hopelessness plays a pivotal role in the progression from suicidal ideation to completion among older adults. Directly targeting hopelessness could help prevent at-risk older adults from acting on their thoughts of suicide.

### MIND-BASED AND MOVEMENT-BASED MIND-BODY INTERVENTION FOR CHINESE OLDER PEOPLE WITH DEPRESSION

Sunny H.W. Chan,[1] Herman H.M. Lo,[1] and Chong Ho Yu[2], *1. The Hong Kong Polytechnic University, Hong Kong, Hong Kong, 2. Azusa Pacific University, Azusa, California, United States*

Mind-body intervention has been well established as an alternative psychosocial intervention for managing depression. Mindfulness-based intervention (MBI) and health qigong (HQ) are two common forms of mind-body intervention which share the common focus on breathing. However, they may represent two distinct approaches with different mechanisms. MBI focuses more on mind-based practices whereas HQ may focus predominantly on body-based movement practices. Thus, a large research gap in comparing the unique therapeutic effects of mind-based and movement-based health practices on alleviating depression among older people is worthy of further investigation. A total of 45 community-dwelling Chinese older adults aged 60 or above with symptoms of clinical depression were recruited. They were randomly assigned to three different groups, including an MBI group, a HQ group, and a waitlist control (WLC) group. Comparisons were made before and after 8-week interventions. Regarding the primary outcome, the effect sizes between the MBI and WLC groups, as well as between the HQ and WLC groups, were reasonably large (Hedges' g = 1.338 and 0.725, respectively), yet the effect size between the MBI and HQ groups was moderate (Hedges' g = 0.325). Specifically, participants in the MBI group showed more improvements on perceived stress, self-efficacy, and mental health, whereas participants in the HQ group showed relatively better performance regarding interoception and physical mobility. Findings from this research demonstrate the unique therapeutic effects of mind-based and movement-based interventions on alleviating depression among older people. The application of two distinct forms of mind-body intervention in a Chinese context is discussed.

### THE COURSE OF DEPRESSIVE SYMPTOMS IN OLDER ADULTS RECEIVING IN-HOME CARE

Anne-Sofie Helvik,[1] maria Barca,[2] Sverre Bergh,[3] Jūratė Šaltytė –Benth,[4] and Tom Borza[3], *1. Norwegian National Advisory Unit for Aging and Health, Trondheim, Norway, 2. Norwegian National Advisory Unit on aging and health, Tønsberg, Norway, 3. Innlandet Hospital Trust, Ottestad, Norway, 4. University of Oslo, Oslo, Norway*

The aim of the study was to describe the prevalence, incidence and persistence of depressive symptoms over a 36-month follow-up period among older people receiving in-home care, and to explore the association between cognitive function and the course of depressive symptoms. In all, 1001 older people (≥ 70 years) receiving in-home care were included in a longitudinal study over 36 months. Depressive symptoms, cognitive function, general medical health, activities of daily living, neuropsychiatric symptoms and use psychotropic drugs were assessed at three assessments. Dementia and mild cognitive impairment were diagnosed at all assessments. Baseline demographic characteristics and information on nursing home residency at follow-up were recorded. Linear mixed models were estimated. We found the prevalence and cumulative incidence of individual depressive symptoms to be higher in those with dementia at baseline than in those without. The persistence of depressive symptoms did not differ between those with or without dementia at baseline. The severity of cognitive decline and mean depressive symptom score assessed simultaneously were positively associated, but the strength of the association changed over time and was not significant at the last assessment. In conclusion: The differences in prevalence and cumulative incidence of depressive symptoms in those with and without dementia at baseline, and the association found between degree of cognitive decline and depressive symptoms over time shows that depression and dementia are interconnected. Nurses and clinicians should pay attention to cognitive status when observing or evaluating depression among older people receiving in-home care.

### BODY TYPES AND ASSOCIATION WITH DEPRESSIVE SYMPTOMS AMONG OLDER ADULTS: FINDINGS FROM NHANES 2013-2016

Queendaleen C. Chukwurah,[1] and Lydia Bazzano[2], *1. Tulane Center for Aging, School of Medicine, Tulane University, New Orleans, Louisiana, United States, 2. Department of Epidemiology, School of Public Health and Tropical Medicine, Tulane University, New Orleans, Louisiana, United States*

Older Americans are increasingly affected by overweight and obesity now as compared to previous decades. We examine the prevalence rates and association of Depressive Symptoms (DS) across body types created using the National Heart, Lung, Blood Institute recommended body mass index /waist circumference (WC) anthropometric cut off values among older Americans. 3,132 participants, 50 years and older from the National Health and Nutrition Examination Survey (NHANES) 2013-2016 was used for this analysis. Six body types were created using the anthropometric cut off values- normal weight with normal WC, overweight with normal WC, obese with normal WC, normal weight with high WC, overweight with high WC, and obese with high WC. The PHQ-9 score was used to create DS categories (1-4, 5-9, 10-14, 15-19, ≥20). The relationship of body types to DS categories was assessed using weighted multinomial logistic regression. The mean (SD) sample age was 63.4 (9.2). Approximately 12.9% of participants had a PHQ-9 score of at least 10. After adjusting for age, gender, race/ethnicity and poverty-income ratio, overweight with high WC (OR 7.61, 95% CI 2.37-24.48) had high odds of moderately severe DS. Obese with high WC had high odds of mild DS (OR 1.76, 95% CI 1.22-2.52), moderate DS (OR 2.14, 95% CI 1.09-4.20) and moderately severe DS (OR 5.59, 95% CI 2.75-11.39) compared to normal weight with normal WC. We demonstrate an association of body types with DS in an aging American population and these findings would not be identified if anthropometric measures were examined separately.

### STRESS AND PSYCHOLOGICAL CHARACTERISTICS PREDICT CLUSTERED HEALTH TRAJECTORIES IN OLDER ADULTS

Myriam V. Thoma,[1] Jan Höltge,[1] Shauna L. Mc Gee,[1] Andreas Maercker,[1] and Mareike Augsburger[1], *1. University of Zürich, Zürich, Switzerland*

The first aim of this study was the empirical identification of clustered health trajectories in older individuals, including an expected, more favorable or successful aging (SA) trajectory. The second aim was the identification and analysis of determinants useful for subgroup membership prediction. Particular focus was on early-life and chronic stress, as well as on a broad set of psychological characteristics, such as resilience, personality traits and general affect. A longitudinal survey study with two assessments one year apart has been conducted with older adults (N=224; mean age = 68 years; 72% women). The clustered health trajectories were identified using a longitudinal variant of k-means. For the prediction of subgroup allocation, random forests with conditional interferences were used. The applied machine learning-based approach revealed two latent clustered health trajectories: a 'constant high health' (66% of the sample) and a 'maintaining low health' trajectory (34%). Chronic stress and positive affect were found to be the most important predictors. Further predictors and their interactions were found to be important for predicting subgroup belonging, including resilience, self-esteem, social support, optimism, as well as negative affect and pessimism. Also, childhood adversities have to be included to predict subgroup belonging. With this study, we were able to show that individuals can be empirically allocated to two separate joint health trajectories in later life over the observation period of one year. In order to understand the current heterogeneity in health in older age, previous and current stress exposure and psychological characteristics have to be taken into account.

### EFFECTS OF ENVIRONMENTAL FACTORS ON THE INCIDENCE OF DEPRESSION IN OLDER ADULTS IN TAIWAN: A MULTI-LEVEL ANALYSIS

Chia Chun Li,[1] Der-Chiang Li,[1] Susan C. Hu[1], *1. National Cheng Kung University, Tainan City, Taiwan*

Depression is a common mental disorder worldwide, and also a leading cause of disability and global burden of disease. The risk factors of depression are multi-facets; however, past studies have focused more on individual factors than ecological factors. This study aims to use a health map framework with multi-level analysis to analyze the effects of environmental factors on the incidence of depression of older adults in Taiwan. We connected 3 national datasets, including the National Health Interview Survey (NHIS), Age-friendly Dataset at city-level, and the National Health Insurance Research Databases (NHRID), to examine the relationship between environmental factors and the incidence of depression in Taiwan. The dependent variable was the new cases of depression in older adults during 2010-2016. The study variables were environment factors including 5 build environments and 8 social environments. A total of 6494 valid participants aged 50 and over were recruited from the 2009 NHIS and were followed up to 2016. There were 292 (4.5%) new incident cases of depression during 2010-2016. Results of multi-level analysis showed that only one built environment (no. of heritage sites) was associated with the incidence of depression. No social factors was found significantly in the study. It seems that factors of individual-level appear more important than that of ecological-level as the determinants of depression. Results of this study could provide an exploratory information for future research regarding the relationships between depression and environments.

### ELDER MISTREATMENT AND ANXIETY SYMPTOMS AMONG U.S. CHINESE OLDER ADULTS

Ying-Yu Chao,[1] Yu-Ping Chang,[2] and XinQi Dong[3], *1. Rutgers School of Nursing, Newark, United States, 2. School of Nursing, The State University of New York, University at Buffalo, Buffalo, New York, United States, 3. Rutgers Institute for Health, Health Care Policy and Aging Research, New Brunswick, United States*

Purpose: Elder mistreatment is a critical public health issue across all cultural backgrounds. However, we have limited understanding of the mental health outcomes of elder mistreatment among older Chinese Americans. This study aimed to examine the association between different types of elder mistreatment and anxiety symptoms among U.S. Chinese older adults. Methods: Data were from the Population Study of Chinese Elderly in Chicago (PINE), a community-based participatory research study of 3,157 Chinese older adults in the Greater Chicago area. Elder abuse was assessed with the overall abuse screening test and Modified Conflict Tactic Scale. Anxiety symptoms were assessed by the Hospital Anxiety and Depression Scale (HADS-A). Multivariate logistic regression analyses were performed. Results: Participants had a mean age of 72.8 ± 8.3 years old (range 60-105). After controlling for potential covariates, participants with overall abuse (OR = 1.76, 95% CI: 1.57-1.98, $p < .001$), psychological abuse (OR = 1.76, 95% CI: 1.53-2.02, $p < .001$), physical abuse (OR = 1.56, 95% CI: 1.03-2.36, $p < .05$), and financial exploitation (OR = 1.24, 95% CI: 1.06 – 1.44, $p < .001$) were more likely to have anxiety symptoms. However, sexual abuse was not significantly associated with anxiety symptoms ($p = 0.83$). Conclusion & Implications: Our findings indicate that elder mistreatment may result in anxiety symptoms among Chinese older adults. Further studies can examine whether Chinese cultural traits (e.g., face, harmony) influence the relationships between elder mistreatment and anxiety symptoms.

### AGING WITH SCHIZOPHRENIA: RACIAL DISPARITIES IN COGNITIVE IMPAIRMENT

Sarah Dobbins,[1] Erin Hubbard,[2] and Heather C. Leutwyler[2], *1. UCSF SChool of Nursing, San Francisco, California, United States, 2. UCSF, San Francisco, California, United States*

Introduction: Cognitive impairment (CI) is a core feature of schizophrenia (SCZ). Comorbidities such as substances/smoking, metabolic syndrome, and medications contribute to CI. There are racial disparities in CI in the general US population. No study has evaluated CI racial disparities among people with schizophrenia (PWSCZ). The aims of this study were to describe the clinical/psychosocial correlates and racial disparities in CI. Methods: Cognitive performance in PWSCZ over 55 years old was measured using MATRICS Consensus

Cognitive Battery (N=66). We calculated age- and gender-corrected scores for global cognitive performance, which represent number of standard deviations away from the mean of the MCCB normative sample. Clinical and sociodemographic data were collected. A counterfactual approach was used to explore mediation of CI through education. Results: Our "all-comer" convenience sample represented 57.6% white, 25.8% black, and 16.6% other non-white groups. There was a black/non-black disparity in cognitive score (-2.33 v.s. -1.68, t=2.843, p<.01). This difference remained significant in a regression model adjusted for age, substance use, smoking, education, anti-psychotic medication, and positive/negative symptoms (-.6611, [95%CI:-1.12,-.20], overall F8, 57=3.690, p=.0016). In the mediation analysis, education accounted for 19% of the disparity in CI. In the counterfactual scenario in which education was distributed equally, education accounted for 48% of the disparity. Conclusion: There are significant racial disparities in cognitive performance among older PWSCZ, and educational attainment may account for a sizable portion of the disparity.

### ASSOCIATION OF WELL-BEING WITH ANXIETY, DEPRESSION, AND FUNCTIONAL IMPAIRMENT FOLLOWING REHABILITATION SERVICES

Adam Simning,[1] and Christopher L. Seplaki[1], *1. University of Rochester, Rochester, New York, United States*

Millions of older adults receive rehabilitation services yearly that aim to restore, sustain, or limit decline in functioning. Older adults who receive rehabilitation comprise a vulnerable population that is unfortunately at elevated risk for anxiety, depression, and functional impairment. We hypothesize that lower levels of wellbeing prior to rehabilitation services are associated with a greater risk of having clinically significant anxiety or depressive symptoms, or worsening impairments in self-care or household activities, following rehabilitation. This study uses data from 2015 and 2016 waves of the National Health and Aging Trends Study, and includes 853 participants with information on rehabilitation services, wellbeing, anxiety and depression, and functional impairment, as well as demographic characteristics, socioeconomic status, and health variables. In a series of multivariable logistic analyses with wellbeing serving as our primary independent variable, older adults in the lowest quartile of wellbeing (compared to those in the highest quartile of wellbeing) had greater odds for having anxiety symptoms (OR=3.04; 95% CI: 1.24-7.46), depressive symptoms (OR=6.54; 95% CI: 2.80-15.25), and worsening impairment in self-care (OR=2.15; 95% CI: 1.09-4.23), but not in household activities (OR=1.49; 95% CI: 0.67-3.32). This study's findings suggest that older adults with low levels of wellbeing at baseline may be more susceptible for having mental illness and functional impairment at follow-up. Conversely, the findings suggest that perhaps those with high levels of wellbeing may be able to experience significant health events with fewer residual consequences. The mechanism by which wellbeing may affect these outcomes is unclear and warrants further investigation.

### A TALE OF TWO CASE STUDIES: ACCELERATED RESOLUTION THERAPY FOR COMPLICATED GRIEF IN OLDER ADULTS

Harleah G. Buck,[1] Diego F. Hernandez,[2] Tina Mason,[3] Cindy Tofthagen,[4] and KEVIN E. KIP[1], *1. University of South Florida, Tampa, Florida, United States, 2. University of South Florida, Tampa, United States, 3. H.L. Moffitt Cancer Center, Tampa, Florida, United States, 4. Mayo Clinic, Jacksonville, Florida, United States*

Complicated grief (CG) is characterized by lengthy, intense, and functionally impairing grief which disproportionately affects older adults. Accelerated Resolution Therapy (ART) is a brief, protocol driven, exposure/imagery rescripting therapy which uses lateral left-right eye movements. ART, unlike traditional psychotherapy, directs the person to perform two tasks simultaneously (e.g. re-experiencing the grief experience and performing eye movements), taxing limited working memory capacity. Importantly, this may force memory traces representing events, emotions, and sensations to compete for permanence, as well as reduce the vividness and emotional intensity of the original grief. Two CG case studies are presented (expected; unexpected death) with their response to ART. Stake's instrumental case study methodology was used to identify and study cases which reflect a range of CG. Additionally, CG was measured by the Inventory of Complicated Grief (ICF). ICF's range is 0-76 with scores > 24 indicating CG. Case 1 was a spousal caregiver with a single, expected death where helplessness, guilt, shame, and a life alone had resulted in CG (baseline ICF 33). Her ICF at 8 weeks post-ART was 10. Case 2 was an adult child caregiver with multiple (parent, sibling), unexpected deaths in quick succession where loss, guilt, anger, and helplessness had resulted in CG (baseline ICF 25). Her ICF at 8 weeks post-ART was 9. Both participants were able to process the distressing sensations that emerged during the imaginal exposure component facilitated with the use of eye movements. This suggests that ART may be a powerful new mind-body treatment for CG.

### SOCIAL ISOLATION AND MENTAL HEALTH CHALLENGES AMONG HIGH-NEEDS VETERANS

Stuti Dang,[1] Kiranmayee Muralidhar,[2] Kaicheng Wang,[2] Diana Ruiz,[3] Carlos Gomez-Orozco,[4] and Willy Marcos Valencia[5], *1. Miami Veterans Affairs Healthcare System- GRECC, Miami, Florida, United States, 2. Department of Public Health Sciences, University of Miami Miller School of Medicine, Miami, Florida, United States, 3. Miami Veterans Affairs Healthcare System-Research Service, Miami, Florida, United States, 4. South Florida Veterans Affairs Foundation for Research and Education Miami, Miami, Florida, United States, 5. Miami Veterans Affairs Healthcare System- GRECC, Miami, Florida, United States*

Using predictive analytic modeling, the Veterans Affairs has identified vulnerable Veterans, labeled as High Need High Risk (HNHR), as those who need greater services and support. To better understand their need gaps, we assessed function, mobility, mood, and caregiver status using a mailed needs assessment questionnaire to 1112 HNHR Veterans. Among the 341(30.7%) respondents, they were primarily 274(80.4%) Non-Hispanics; 210(61.6%) Whites, and 119(34.9%) Black or African Americans; average age was 69.5±9.6 years old; 310(90.4%) had ≥high school education. The average Barthel ADL score was 81.5±22.8 and average Lawton IADL score was 5.8±2.2. Walking or balance issues were present among 260(75.8%), 227(66.2%) said they use an assistive device, and 167(48.7%) had suffered ≥1 fall, 43(12.5%). Regarding depression, 117(34.3%) screened positive (PHQ2 score≥3). These were significantly younger (66.7±9.1) than those who did not (70.8±9.3, p≤0.01). They were also significantly lower functioning (5.37±2.1 vs.6.38±2 Lawton IADL score, p≤0.01), more dependent (77.8±23.1 vs 86±19.2 Barthel ADL score, p≤0.01). We also observed significant differences in their telephone contact with family (never to once/week) [35(29.9%) vs. 27(13.4%), (p≤0.01)]; in meeting with friends or relatives ≥3times a week [12(10.3%) vs. 69(34.3%), (p≤0.01)]; and in likelihood of attending meetings with clubs or other organizations [94(80.3%) vs. 138(68.7%), p=0.040]. Detecting depression is a priority among HNHR Veterans. There is an urgent need to devise viable strategies to offer interventions that incorporate mental health needs and reduce social isolation, potentially addressing mobility, function, and transportation.

### SESSION 1335 (POSTER)

### EDUCATION AND TRAINING TO PREPARE AN INTERDISCIPLINARY WORKFORCE IN GERIATRICS AND GERONTOLOGY

### TEACHING ABOUT GERONTOLOGY AND AGING THROUGH INTERPROFESSIONAL EDUCATION AND COLLABORATION

Elaine T. Jurkowski,[1] Ruth Heitkamp,[2] Cherie Kelly,[3] and Sharon Smaga[4], *1. Southern Illinois University Carbondale, Carbondale, Illinois, United States, 2. Center for Rural Health and Social Service Development, SIU School of Medicine, Carbondale, Illinois, United States, 3. Physician Assistant Program, Southern Illinois University Carbondale, Carbondale, Illinois, United States, 4. Family Practice Residency Program, SIU School of Medicine, Carbondale, Illinois, United States*

Repeatedly as multidisciplinary professionals we are concerned about the individual patient we serve. Interprofessional collaboration across disciplines such as primary care medical practice, physician assistants, social workers and psychologists does not occur naturally, since educational programs are often taught independently of each other, but these disciplines are required to work collaboratively with each other. The objective was to promote communication across disciplines (Medicine, Social Work, Physician Assistant and Psychology) and help each discipline understand the roles played in promoting mental health and general health for older adults. An educational seminar was conducted using cases and guide questions focused on identifying strategies for care. The teams consisted of Medical Residents, a Social Work student, a Psychology student and a Physician Assistant student. A series of guide questions were provided, and teams were asked to discuss and identify a care plan. Debriefing followed to discuss the outcomes across all teams. Pre-post test results examined variables related to interdisciplinary collaboration. Findings suggest professionals were surprised at what they learned from the other disciplines they were collaborating with. They also learned about community based resources available as well as strategies to promote health outcomes. All participants felt that the opportunity to collaborate outside of their disciplines would strengthen their impact when working with older adults and their families. In conclusion, a problem based learning approach coupled with the opportunity to collaborate with other disciplines through (IPE) is a venue to improve overall collaboration across professionals and ultimately improve mental health outcomes of consumers.

### IMPROVING GERIATRIC CARE OF INTERPROFESSIONAL PRACTICING PROVIDERS

Becky Powers,[1] and Jeanette Ross[2], *1. South Texas Veterans Health Care System Geriatric Research Education and Clinical Center, San Antonio, Texas, United States, 2. South Texas Veterans Health Care System, San Antonio, Texas, United States*

Practicing providers often struggle with the care of older adults due to knowledge, skill, and attitude barriers. In an attempt to improve employee engagement in the care of older adults, the Geri-EMPOWER (Empowering Medical Providers and Older adults With strategies to Escape Readmission) program was initiated. In this program, case managers and visiting VA rural geriatric scholars participated in a two days of intensive educational sessions including lectures, shadowing inpatient teams and clinic providers, a dementia simulation learning exercise, and an Observed Structured Clinical Exam (OSCE) with standardized patient encounters. The 15 initial participating trainees came from a variety of medical backgrounds including physicians, nurse practitioners, nurses, social workers, and psychologists. A 10 item knowledge based pretest and posttest was constructed using the learning objectives of the course. Skills of attendees were directly observed during 4 OSCE stations. Attitudes towards older adults were measured before and after the intervention using the Caroline Opinions on Care of Older Adults (COCOA) scale. Geriatric and palliative care knowledge improved with average knowledge test scores improving from 63% to 86% before and after the course. Participants obtained all minimum competencies during their OSCE exam, and rated this session very highly in their course feedback. Attitudes towards older adults were also found to improve with an average COCOA score increase of 9 points before and after the educational sessions. This innovative course based in adult-learning theory demonstrates that employed interprofessional providers can quickly improve knowledge, skills, and attitudes towards older adults.

### FORMAL TRAINING IN TELEHEALTH UNIQUELY PREPARES AN INTERDISCIPLINARY WORKFORCE IN GERIATRICS AND GERONTOLOGY

Kathryn A. Nearing,[1] Hillary D. Lum,[1] Janna Hardland,[1] Skotti Church,[1] and Stephanie Hartz[2], *1. University of Colorado Anschutz Medical Campus, Division of Geriatrics, Aurora, Colorado, United States, 2. Rocky Mountain Regional VA Medical Center, Aurora, Colorado, United States*

Geriatric Research Education and Clinical Centers (GRECCs) are centers of excellence funded by the Veterans Administration for the advancement and integration of research, education and clinical activities in geriatrics and gerontology to improve the health, and health care of, older Veterans. The GRECC Connect program expands access to care for rural-residing older Veterans and caregivers. Eastern Colorado GRECC Connect integrates Associated Health Trainees (Audiology, Psychology, Social Work, Pharmacy) and Geriatric Medicine Fellows into interdisciplinary tele-geriatric and tele-palliative care consultations provided to outlying community-based outpatient clinics. The formal telehealth training includes: (1) an initial didactic orientation to introduce skills, common challenges, and important tips when working with older patients and caregivers via telehealth; (2) direct observation and modeling by preceptors, followed by a structured opportunity to debrief what the trainee observed and address questions; and (3) opportunities to provide Geriatric telehealth services, supported by the interprofessional team and feedback and reflection. A formal competency assessment, standardized observation protocol and debriefing guide support the development and assessment of telehealth competencies. During exit interviews, trainees indicated that these experiences offered unique opportunities to develop their clinical skills, particularly related to active listening and communication. They identified their involvement with GRECC Connect as a highly valued aspect of their Geriatrics training. Rigorous training in telehealth is an essential aspect of workforce development in Geriatrics and Gerontology given the concentration of older adults in rural areas. During this session, we will highlight the value of telehealth training for workforce development in Geriatrics and Gerontology.

### SESSION 1340 (POSTER)

### END OF LIFE | DEATH AND BEREAVEMENT

### WALKING THE TALK: ENGAGING PATIENTS AND CAREGIVERS AS COLLABORATORS IN PALLIATIVE CARE RESEARCH

Anna N. Rahman,[1] and Susan Enguidanos[2], *1. University of Southern California, Leonard Davis School of Gerontology, Los Angeles, California, United States, 2. University of Southern California, Los Angeles, California, United States*

Palliative care is inherently patient directed and family oriented. Given this, it is especially important that patients and caregivers participate in developing new services intended to meet their needs. Our experience with conducting a controlled trial of home-based palliative care demonstrates this point. This poster will describe the implementation and dissemination decisions that patient and caregiver stakeholders guided during a comparative effectiveness trial of palliative care. It also will present strategies for engaging stakeholders and identify the impact of these strategies on continuous study improvement. For the study, we convened an advisory committee (AC), which included eight patients and caregivers among its 34 members. These eight partners in particular helped us identify meaningful patient- and caregiver-reported outcome measures, Throughout the study's 2.5-year period, they offered suggestions that influenced the tone and topics addressed in quarterly AC meetings. They also helped refine the study's patient and caregiver assessment instruments by roleplaying with our research assistants. When we launched a Google group to promote discussion among AC members, patient and caregiver stakeholders were especially forthcoming. They identified gaps in care and consumer understanding of palliative care that we otherwise would have overlooked. Of note, our stakeholders provided input on how to best to introduce a new palliative care program to patients. These engagement strategies create feedback loops that open new opportunities for continuously improving research. Moreover, engagement of patients and caregivers in research planning and implementation provides important and otherwise overlooked perspectives while ensuring a patient-centered focus, consistent with palliative care goals.

### THE LAST MONTH OF LIFE: AN EXPLORATORY REVIEW OF THE NATIONAL HEALTH AND AGING TRENDS STUDY

Abigail Latimer,[1] Lauren Montemuro-Rode,[2] Brianna Garrison,[3] and Allison Gibson[4], *1. University of Kentucky College of Social Work, Lexington, Kentucky, United States, 2. Bryn Mawr College, Bryn Mawr, Pennsylvania, United States, 3. Baylor University, Houston, Texas, United States, 4. University of Kentucky, Lexington, Kentucky, United States*

Approximately 80% of Americans prefer to die at home. Hospice and palliative care services are associated with improved pain and symptom management, increasing capacity to meet preferences for end-of-life care at home. However, according to the NHPCO (2018) only 48% of Medicare beneficiaries were enrolled in hospice at the time of death. This poster presents trends in the last month of life for adult Medicare beneficiaries age 65 or older examining the influential factors contributing to the quality of end-of-life experiences. A cross-sectional survey design was utilized with the National Health and Aging Trends Study (NHATS). Descriptive and inferential statistics were generated to describe a sample of persons (n= 241) who died in 2017. The sample demographics are predominately white (77.6%) females (61.4%) over 90 years old (42.4%). 29.5% of individuals died at home, 29.5% at the hospital, and 27% at a nursing home. Only 32.2% had hospice care in the last month, with many experiencing pain (71.1%), shortness of breath (54.7%), and anxiety/sadness (56.9%). There were 33.6% of participants who lived alone at death and 70% did not receive hospice care. The majority of these individuals were widowed (70.4%) and 33.3% died in the hospital. The other 28.4% died at their home or someone else's and 25.9% died in a nursing home. Many older adults face multiple barriers to experiencing a quality end-of-life experience. Future research should examine the challenges facing those living alone at time of death.

### REGIONAL VARIATIONS IN ENGAGEMENT IN ADVANCE CARE PLANNING AMONG RACIALLY AND ETHNICALLY DIVERSE OLDER ADULTS

Kyeongmo Kim,[1] Michin Hong,[2] and Giyeon Kim[3], *1. Virginia Commonwealth University, Richmond, Virginia, United States, 2. Indiana University, Indianapolis, Illinois, United States, 3. Chung-Ang University, Seoul, Korea, Republic of*

Advance care planning (ACP) has positive effects on the quality of end-of-life of older adults. Given the influence of environmental factors on health and behavioral health behaviors among older adults, geographic variations may exist in engagement in ACP. This study examined whether 1) there was a regional variation in engagement in ACP and 2) there was a racial/ethnic difference in ACP across regions (Northeast, Midwest, South, and West). Drawn from the 2012 National Health and Aging Trends Study, 2,015 Medicare beneficiaries in the U.S. were included in analyses. Results from logistic regression analysis showed that older adults living in the West (OR=1.66, p=0.003) and the Midwest (OR=1.39, p=0.032) were more likely to be engaged in ACP compared to those living in the South. African Americans (OR=0.31, p<0.001), Hispanics (OR=0.30, p<0.001), and other races (OR=0.49, p=0.04) were less likely than their white counterparts to be engaged in ACP. We also conducted four separate logistic regression analyses by regions. In the Northeast, Midwest, and South, African Americans were less likely to be engaged in ACP compared to whites. In the West, Hispanics were less likely to be engaged in ACP compared to whites. Findings from this study provide a clear picture of racial/ethnic disparities in ACP across different regions in the U.S., suggesting where to target for future interventions to improve engagement in ACP among racial/ethnic minorities. Future research should be conducted at lower levels of geographic areas to find modifiable geographic factors to improve engagement in ACP among older adults.

### VARIATION IN ADVANCE DIRECTIVES TIMING BETWEEN END-STAGE RENAL DISEASE PATIENTS AND CANCER PATIENTS

Yu-Hsuan Wang,[1] and Susan Enguidanos[2], *1. Leonard Davis School of Gerontology, University of Southern California, Los Angeles, California, United States, 2. University of Southern California, Los Angeles, California, United States*

The United States has the third highest prevalence and the second highest incidence of End-Stage Renal Disease (ESRD). ESRD is associated with high mortality and lower quality of end-of-life experiences. Having an advance directive (AD) is associated with better care at the end of life. Although past ACP completion rates in ESRD patients has been studied, little is known about its timing differences between ESRD and cancer patients. This study investigates the timing difference of AD completion between ESRD and cancer patients We conducted logistic regression to analyze data from the Health and Retirement Study, a nationally representative longitudinal survey of older adults. The analytic sample included exit interviews from 2012 to 2016 among 971 proxies of deceased with ESRD or cancer. Among the sample, 47% of decedents completed an AD; 44% of cancer patients and 48% of ESRD patients. Being a racial minority (OR=0.38, p<0.001), and lower education (OR= 0.63, p=0.001) were associated with lower AD completion rates. No significant differences in AD completion rates were found between cancer patients and ESRD patients. Compared to cancer patients, ESRD patients were more likely to complete ADs more than one year before death (OR=3.15, p=0.001). However, there were no significant difference between cancer patients and ESRD patients in AD completion rates in the three months before death. Although both samples had comparable rates of AD completion, compared to cancer patients, ESRD patients tend to document care preferences earlier. Further studies are needed to investigate factors related to early documentation of ADs.

### LIFE RESTORATION AND CARE PREPARATION AMONG OLDER PARENTS WHO LOST THEIR ONLY ADULT CHILD IN SHANGHAI

Lin Chen,[1] Yajun Song,[2] and Yixin You[1], *1. Fudan University, Shanghai, China, 2. East China University of Science and Technology, Shanghai, China, China*

The combination of aging and losing an only adult child challenges an increasing number of older adults in China. Current literature primarily focuses on older parents' bereavement but seldom examines how they restore their lives. Guided by the Dual Process Model, this study explores how older parents who lost their only adult child restore their lives and prepare for future care in Shanghai. Twenty-four older adults were purposively sampled and participated in face-to-face, in-depth interviews. The findings suggest that participants tried to restore their lives by rationalizing grief and expanding their social networks. Despite their losses, participants remained in favor of family caregiving and reluctantly prepared for future care. They showed ambivalent attitudes toward current government support while proposing their preferred services. This study informs social work practice to incorporate caregiving plan services and emotional support for this vulnerable group.

### CHILDHOOD CONDITIONS AND ARTHRITIS AMONG MIDDLE-AGED AND OLDER ADULTS IN CHINA: THE MODERATING ROLES OF GENDER

Nan Lu,[1] Bei Wu,[2] Nan Jiang,[3] and Tingyue Dong[1], *1. Renmin University of China, Beijing, China, 2. New York University, New York, New York, United States, 3. Columbia University, New York, United States*

This study examined the moderating effect of gender on the relationship between childhood conditions and arthritis among middle-aged and older adults in China. The data were derived from the 2015 wave and the life history module of the China Health and Retirement Longitudinal study. The sample included 19800 respondents age 45 and over. Multiple imputation was used to handle the missing data. A multilevel logistic regression was used to test the proposed models. Childhood socioeconomic status, mother's education, subjective health, access to health care and medical catastrophic event were found to be significant factors associated with arthritis in later life, after controlling for adulthood and older age conditions. Furthermore, the effects of childhood socioeconomic status on arthritis were found to be higher among men than women. The findings highlight the important role of childhood conditions that affect the onset of arthritis in later life. Policy and intervention implications are discussed.

### DEATH NARRATIVE IN 19TH-CENTURY CHINA: HOW DID NEWSPAPERS FRAME DEATH AND DYING?

Yifan Lou[1], *1. Columbia University, New York, New York, United States*

This study explored the death narrative in the late Qin dynasty as expressed in Chinese newspapers in the 19th century. Using textual analysis to analyze the 646 pieces of news containing death-related topics, this study revealed the discourse regarding death and dying during this period can be understood at three levels: (a) euphemism of death: the language of death and its relationship with power and social hierarchy; (b)definition of "good death": including preferences for location, cause, and experiences of death and dying; and (c) Western influence on the death narrative: missionaries' efforts to incorporate Catholic and Chinese traditions to attract more believers. This paper argues that the current Chinese people's perception of death is inherited and evolved from those historical roots, which has practical implications for the systematic development of hospice care in China. Suggestions include changing the language used in the hospice policy, emphasizing the importance of confidentiality in home-based hospice programs, and building a hospice system based on public perceptions of so-called "good death" while advocating for individualized definitions of this concept.

### SESSION 1345 (POSTER)

### ENVIRONMENT AND AGING

### NAVIGATING DISASTERS: A CASE STUDY VA AND NON-VA HOME-BASED LONG-TERM CARE IN PUERTO RICO FOLLOWING HURRICANE MARIA

Leah M. Haverhals,[1] and Deisy Hernandez-Lujan[2], *1. Seattle-Denver Center of Innovation, Denver, Colorado, United States, 2. Seattle-Denver Center of Innovation, Aurora, Colorado, United States*

This research describes how Department of Veterans Affairs (VA) and non-VA home-based long-term care (LTC) environments prepared for and secured the safety and wellbeing of elderly and disabled persons in the wake of Hurricane Maria, which struck Puerto Rico on September 20, 2017. In-person interviews, home visits, and field observations were conducted in Puerto Rico from January-March 2019. Staff from three of the VA's Caribbean Healthcare System's home-based LTC programs were interviewed, as well as caregivers in non-VA LTC environments. Veterans, family members of Veterans, family members of VA caregivers, and community members with expertise in disaster recovery were also interviewed for a total of N = 58 interviews and N = 12 home visits. Preliminary results of qualitative content analysis show VA and non-VA LTC environments prepared residents and caregivers for Hurricane Maria through providing education and recommendations in advance, including having enough medications and food on hand. Participants described Hurricane Maria not simply as a disaster but a "crisis" and a storm unlike any they had ever experienced. The interconnected nature of the VA seemed to provide a stronger support network compared to non-VA environments that were often independently run. Health of Veterans and non-Veterans was reported to be mostly stable during recovery. Perspectives from VA and non-VA entities allowed for a fuller picture to emerge around how Hurricane Maria impacted home-based LTC environments in Puerto Rico. This research can inform policies and procedures for such environments caring for elderly and disabled populations in areas prone to disasters.

### HOUSING AFFORDABILITY AND INTER-REGIONAL MOVES AMONG OLDER ADULTS

Sarah Mawhorter,[1] and Jennifer A. Ailshire[1], *1. University of Southern California, Los Angeles, California, United States*

Housing prices have risen in urban areas across the US since 2000, with only a brief interruption after the housing crisis of 2008. At the same time, prosperous urban areas have pulled away from declining urban and rural areas. Older adults are more likely to be affected by both increases and divergence of housing prices: owners may not be able to afford rising property taxes (though they benefit from increasing home equity), and renters are especially vulnerable. Housing affordability constraints may also affect the places where older adults can afford to move. In this paper, we compare the residential mobility patterns of adults aged 50+ living in high-cost, mid-cost, and low-cost areas from 2000-2014, using data from the Health and Retirement Study with county-level US Census and American Community Survey contextual data, as well as the Zillow Home Value Index. We find that both homeowners and renters living in high-cost areas remain in place at higher rates compared with those living in mid-cost and low-cost areas. Among those who move, older adults living in high-cost regions move towards mid-cost and low-cost regions more often than the reverse. The differences are particularly pronounced for renters. The overall outcome is a net movement of older adults away from high-cost areas towards mid-cost and low-cost areas. These shifts have consequences for the well-being of older adults facing budget constraints that may limit the areas where they can afford to live or move, and broader implications for the future of urban areas.

### FLOURISHCARE: HELPING OLDER ADULTS FLOURISH IN RURAL COMMUNITIES

Anna Faul,[1] Joe D'Ambrosio,[1] Pamela Yankeelov,[1] Samantha G. Cotton,[1] Megan Austin,[1] Mona Huff,[1] and Darla Handy[1], *1. University of Louisville, Louisville, Kentucky, United States*

Flourishing represents living within an optimal range of human functioning. To foster older adults flourishing, our FlourishCare model of care coordination was developed as part of a HRSA Geriatric Workforce Enhancement Program (GWEP) to transform PC sites by delivering coordinated services in 6 rural KY counties. FlourishCare is unique in its integration of academic teams, community health teams and mental health specialists within age-friendly primary care health systems.FlourishCare recognizes that many of the largest drivers of health care costs fall outside the clinical care environment. One of the major components of Flourish is the involvement of community health navigators (CHNs), community education coordinators (CECs) and community coalitions working with the health teams to respond to the social determinants of health. For each patient that is referred and agrees to participation in the program, the CHN performs Flourish clinical and home assessments and obtains medical records. A repeated measure designed was used to assess the 60 patients who have baseline assessments results along with at least a 6-month assessment and in some cases a 12-month assessment (n= 20). Patients have demonstrated a significant increase in their flourishing across all determinants of health.

There was also a significant improvement the total FI from pre to post-test at 6-month. These outcomes illustrate that it is important to operationalize the outcomes for older adults by evaluating the success as the ability of the older adult to flourish when they can maintain current functioning, access to resources have sufficient support systems in place.

### NEIGHBORHOOD CONDITIONS AND SELF-NEGLECT IN LATER LIFE: LONGITUDINAL EVIDENCE FROM THE NSHAP

Laura Upenieks,[1] James Iveniuk,[2] and Markus H. Schafer[1], *1. University of Toronto, Toronto, Canada, Canada, 2. Wellesley Institute, Toronto, Ontario, Canada*

Self-neglect includes persistent inattention to personal hygiene and the conditions of one's immediate living environment and is known to be associated with an increased risk of mortality among older adults. Although previous studies have shown that many individual factors predict self-neglect, neighborhood characteristics have received much less attention. Extant research has yet to consider connections between the conditions of one's neighborhood and self care over time. Using nationally representative longitudinal data from the National Social Life, Health, and Aging Project (NSHAP), we consider several features of neighborhood context in later life, including self-reported perceptions of neighborhood cohesion and neighborhood danger, neighborhood disorder (measured by interviewer ratings), and concentrated neighborhood disadvantage (using census data). Adjusting for individual-level factors (including social connection, physical and cognitive health, and demographics), results from both lagged dependent variable and cross-lagged panel models find higher levels of neighborhood disorder to be associated with higher self-neglect scores (measured by interviewer ratings) over time. Social cohesion, perceived neighborhood danger, and collective efficacy were not associated with self-neglect when controlling for neighborhood disorder. These findings suggest that improving neighborhood disorder may be an effective approach for self-neglect prevention in later life

### THE PHYSICAL AND SOCIAL ENVIRONMENTS, SOCIAL ACTIVITIES, AND SUBJECTIVE WELL-BEING: FINDINGS FROM THE K2 STUDY

Midori Takayama,[1] Yoshiko Ishioka,[1] and Ikuko Sugawara[2], *1. Keio University, Tokyo, Japan, Japan, 2. University of Tokyo, Bunkyo, Tokyo, Japan*

It has been pointed that the environments effect subjective well-being(SWB). However, it is still not clear what aspects of environments effect SWB among older adults and if degree on physical condition of older adults cause the difference on relations between and environments and SWB. In this study, firstly, we examined the relationship between the physical and social environments, social activities, and SWB in a sample of older Japanese. Secondly, we examined the differences on the effects of environments on SWB between older adults with lower physical functions and those with higher physical functions. We used data from locally representative longitudinal study of older adults 75±1, 80±1, and 85±1 years of age (at baseline) , which was conducted in Japan (The Keio-Kawasaki Aging Study(K2 study) ; N = 1388). Concerning the environments, we assessed the physical environments (public spaces and buildings, and accessibility) and the social environments (culture and recreation programs, and inclusive social environment). Results from covariance structure analyses showed that the accessible physical environment and the social environments were significant predictors of SWB, and showed that accessibility and the social environments influenced SWB via participation of social activities, too. Moreover, results from multiple group structural equation modeling showed that accessibility was a stronger predictor of SWB among older adults with lower physical functions, while accessibility was not a predictor among older adults with higher physical functions. The potential benefits of this approach provide a basic developing compensation model of SWB for this population of older adults.

### COMPARISON OF NURSING HOME STAFF'S PERCEPTIONS OF NOISE TO MEASURED NOISE LEVELS

April Ames,[1] Stacie Dubin,[1] Michael Valigosky,[1] and Victoria Steiner[1], *1. University of Toledo, Toledo, Ohio, United States*

A noisy environment may affect the ability of healthcare staff in nursing facilities to effectively complete tasks and provide quality care to residents. Staff may also become irritable or annoyed due to their perception that noise levels are too loud. The purpose of this descriptive study was to examine the differences in nursing home staff's perceptions of noise levels compared to measured noise levels in four nursing home facilities in Ohio. A questionnaire was also distributed to examine the perceptions of noise levels by staff and the effects of noise on their health. The majority of the respondents (n=90) were white females. They described all facilities as being moderately noisy which was consistent with the measured noise levels. The loudest perceived noise sources included door/patients alarms and floor cleaners, which was confirmed by measured noise levels. The majority of facilities identified the nurses station as one of the noisiest locations; however, this was inconsistent with measured noise levels. Overall, respondents at all facilities felt neutral or disagreed that the noise levels impacted themselves or the residents. However, some respondents agreed that in a noisy environment it is easier to make job errors, difficult to concentrate on work, and they find themselves irritable or agitated. Perceptions of noise should be considered along with measured noise levels because tolerance levels differ among individuals and mental activities involving memory or complex analysis are sensitive to noise which may affect job performance.

### JOINING AND REMAINING: FACTORS THAT CONTRIBUTE TO MEMBERSHIP IN A VILLAGE

Ruth E. Dunkle,[1] Karen Harlow-Rosentraub,[1] Garrett T. Pace,[1] and Larry Coppard[1], *1. University Of Michigan, Ann Arbor, Michigan, United States*

Many older people want to age in place with one popular model of care being the Village Model. Understanding why people join and why they continue as members are important considerations for a Village to survive. Utilizing open-ended data from a representative sample of current members of a Village (N=100), we examined the reasons for people becoming members as well as continuing their membership. Twenty six percent of the sample were men, age ranged from 50-95, 30% lived alone and 58% believed that their health was very good. Data were coded by three researchers with discrepancies resolved by discussion to refine the codes. Three categories were identified: instrumental, social, and altruistic. The most frequent reason for joining was instrumental (35%) where the member wanted the services provided by the Village. It was also the most frequent reason for continuing membership (57%). An analysis was also conducted to examine predictors of reasons for joining and continuing membership in the Village. These included, age, gender, health, and living alone. Results indicate that men were less likely to join or continue their membership for instrumental reason compared to women, and members who live alone were more likely to become a member for social reasons. When age at entrance into the Village was examined, each increasing year of age was associated a .01 increase in the probability of continuing as a member for instrumental reasons. Findings provide guidance in issues related to sustaining membership in a Village.

### EFFECTS OF AGE ON CONNECTION TO NATURE AND POSITIVE AFFECT

Amy Knepple Carney,[1] and Julie Patrick[2], *1. University of Wisconsin-Oshkosh, Oshkosh, Wisconsin, United States, 2. West Virginia University, Morgantown, West Virginia, United States*

Socioemotional selectivity theory positis that when we feel our time as limited, when a person ages, emotion based goals become a priority (Carstensen, Isaacowitz, & Charles, 1999). Although previous studies have shown that all age groups benefit from a connection to nature (CN; Bisceglia, Perlman, Schaack, & Jenkins, 2009; Han, 2008; Mayer et al., 2009), there have been no studies conducted to determine if there are age differences in CN and how that relation contributes to positive affect. Analyses were conducted with a sample size of 152 participants with an average age of 37.55 years (SD = 15.64; Range 18 -89). Age was significantly positively associated with CN, $r(151)=.16$, $p<.05$. Additionally, an ANOVA showed that middle-aged to older adults reporting significantly higher CN than younger adults. The relation of positive affect to age and CN was then examined. In the analysis examining the effects of age and CN on positive affect, the model was significant, $F(3, 146)=8.48$, $p<.05$, R2 = .15. Both, CN, and age, uniquely contributed to the variance accounted for on positive affect, although, the interaction of CN and age did not uniquely contribute to the variance. These results may be indicative of socioemotional selectively theory, in that older adults were choosing connection to nature because it fulfilled more emotional activities/goals than the younger adults in the study. Because previous research has all but ignored the association of CN and age and their relation to positive affect, it should be considered in future research.

### DISASTER PERCEPTIONS AND PREPAREDNESS BEHAVIORS AMONG U.S. OLDER ADULTS

Melissa Krook,[1] and Peter Vitaliano[1], *1. University of Washington, Seattle, Washington, United States*

U.S. economic loss from natural disasters hit an all-time high in 2017 with 16 climate events totaling $306 billion. However, disasters' costliest effects may result from emotional and psychosocial health. Research suggests those who are: seniors, distressed, and/or experience early-life vulnerabilities have increased risk for negative health responses. This study addresses the need to reduce vulnerability/increase preparedness by evaluating how older adults (OA) perceive/prepare for disasters, including influential psychological factors. Literature review results indicate OA are: (1) among our most vulnerable populations for disasters, (2) underprepared, though resources are available, and (3) preparing friends/family before themselves. The Socioemotional Selectivity Theory (SST) posits: alongside aging, time perceptions become constrained, motivations shift, and we prefer positive over negative information. Therefore, I asked: (1) if OA are intuitively resistant to negative information, like impending disasters, how might we reframe it to align with their desire for positive information? (2) If we approach OA through positive experiences, will they be motivated to prepare? I employed a model: preparedness behavior (PB) is a function of vulnerability (V) and resilience (R). A survey was developed to assess how factors of V and R would interact/influence PB. I will pilot test this survey through evaluating community-living OA. PB is expected to be negatively related to V, positively related to R. This study extends disaster research by using psychological variables to predict preparedness and evaluating preparedness motivation using SST as a guiding framework. Results should increase knowledge about OA's disaster preparedness perceptions and factors to mitigate increased preparedness.

### THE ROLE OF SENSE OF COMMUNITY IN CHANGING THE HEALTH-PROMOTING EFFECT OF BUILT ENVIRONMENT: A COMMUNITY SURVEY

Jennifer Y.M. Tang,[1] Cheryl Chui,[2] Tuen Yi Chiu,[1] Rebecca Chiu,[3] Vivian W. Lou,[4] Michael Tse,[5] Angela YM Leung,[6] and Terry Lum[2], *1. Sau Po Centre on Ageing, The University of Hong Kong, Pok Fu Lam, Hong Kong, 2. Department of Social Work and Social Administration, Pok Fu Lam, Hong Kong, 3. Department of Urban Planning and Design, the University of Hong Kong, Pok Fu Lam, Hong Kong, 4. The University of Hong Kong, Hong Kong, P.R.C., Hong Kong, 5. Centre for Sports and Exercise, the University of Hong Kong, Pok Fu Lam, Hong Kong, 6. Centre for Gerontological Nursing, School of Nursing, The Hong Kong Polytechnic University, Hong Kong, Hong Kong*

Previous research that studies the impact of built environment on health often attribute the enabling effects of environment on physical activity participation and opportunities for social interaction. Few studies have explored how the role of subjective feeling, such as the feeling of connectedness with the community, affects the association between built environment and physical and mental health. We conducted a cross-sectional survey with 2,247 residents aged 50 years or above in five districts in Hong Kong. We tested the mediation effect of sense of community in the relationship between physical environment and health using the path analysis. We administered a questionnaire to assess the residents' perceived age-friendliness of outdoor spaces and buildings in the district. We used the Brief Sense of Community Scale and the 12-item Short-form Health Survey to measure sense of community and physical and mental health. We found that age-friendliness of outdoor spaces was modestly correlated with mental health (r = 0.10, P < 0.001) but not with physical health (r = 0.02, P = 0.4), whereas age-friendliness of buildings correlated with both (r = 0.05, P = 0.01; r = 0.06, P = 0.004). Sense of community mediated 25.9% of the total effect between outdoor space and physical health, 20.4% between outdoor space and mental health, and 42.5% between service and building on physical health. To conclude, sense of community was a partial mediator of the environment-health relationship. Future design of built environment should take into consideration its potential influence on sense of community and health.

### MEMORY FOR PICTURES AND WORDS AFTER A NATURAL DISASTER

Katie E. Cherry,[1] Katelyn McKneely,[1] Quyen Nguyen,[1] Shui Yu,[2] Laura Sampson,[3] Sandro Galea,[2]
Matthew R. Calamia,[1] and Emily M. Elliott[1], *1. Louisiana State University, Baton Rouge, Louisiana, United States, 2. Boston University, Boston, Massachusetts, United States, 3. Boston University School of Public Health, Boston, Massachusetts, United States*

The pictorial superiority effect (PSE) is the finding that memory for pictures exceeds that of memory for matching words for people of all ages (Cherry et al., 2012). We examined free recall of line drawings and matching words in adults enrolled in the LSU Flood Study, an interdisciplinary study of disaster stress and cognition. We tested the hypothesis that disaster stress would be associated with deficits in memory for pictures and words. Participants were sampled from a three-parish (county) region of Baton Rouge, LA that was severely devastated by the 2016 flood (N = 202, age range: 18-88 years). They received multiple tests, including the Montreal Cognitive Assessment (MoCA; Nasreddine et al., 2005), and self-report measures of executive function and functional impairment (Barkley, 2011). Three groups were compared: (1) non-flooded adults as controls, (2) once-flooded adults with structural damage to homes and property in 2016, and (3) twice-flooded adults who had relocated to Baton Rouge because of catastrophic losses in Hurricanes Katrina and Rita and flooded again in 2016. Results yielded a PSE in free recall for all disaster exposure groups (p < 0.001). Follow-up analyses by age group revealed that older adults showed the same memorial advantage of pictures relative to words as did their younger counterparts across all disaster exposure groups. These results imply that single and multiple disaster exposures do not appear to disrupt cognition assessed with traditional, laboratory-based measures. This research was supported by a grant from the National Science Foundation (Award Number 1708090).

### HAVE I GOT A JOB FOR YOU: OCCUPATION-RELATED ASTHMA THROUGHOUT LIFE IN THE UNITED STATES

Sarah B. Laditka,[1] James N. Laditka,[1]
Ahmed Arif,[1] and Jessica Hoyle[1], *1. University of Nortth Carolina at Charlotte, Charlotte, North Carolina, United States*

Work exposures to asthma triggers can cause or aggravate asthma, which affects twenty-five million Americans including many older workers, and retirees who want to work or need to do so for income. Asthma trigger exposures have particular risk for older workers. Older adults who develop asthma have poorer health outcomes than people who had childhood asthma, yet older workers with low incomes may have limited ability to leave a job despite health risks. We studied occupation-related asthma using the nationally representative Panel Study of Income Dynamics (PSID) (1968-2015, n=13,957, 205,498 person-years). We compared asthma outcomes in occupations with likely asthma trigger exposures to those in occupations with limited exposures. Methods included: prevalence ratios; incidence risk ratios (log-binomial regression adjusted for age, sex, race/ethnicity, education, atopy, current and past smoking, and survey design); attributable risk fractions; population attributable risks; and microsimulation. The adjusted prevalence ratio comparing high risk occupations to low was 4.1 (95% confidence interval, CI 3.5-4.8); adjusted risk ratio 2.6 (CI 1.8-3.9); attributable risk 16.7% (CI 8.5-23.6); population attributable risk 11.3% (CI 5.0-17.2). In microsimulations, 14.9% (CI 13.4-16.3) with low trigger exposures reported asthma during working life, compared with 23.9% (CI 22.3-26.0) with high exposures. Asthma triggers at work may cause or aggravate more than 10% of adult asthma, and increase asthma risk by 60%. Lung health contributes importantly to well-being, and the ability to work at older ages. Results highlight needs for policies and employer actions to reduce asthma trigger exposures, and for public education about lung health.

### NEIGHBORHOOD SOCIAL ENVIRONMENT AND PHYSICAL FUNCTION: EVIDENCE OF RACIAL AND ETHNIC DIFFERENCES

Roberto J. Millar[1], *1. University of Maryland, Baltimore County, Baltimore, Maryland, United States*

Empirical and theoretical scholarship suggest that as individuals age and their physical, cognitive, and social needs change, their neighborhood environment becomes increasingly important to their health and well-being. Despite recent advances in this area of research, a number of critical gaps remain. Namely, few studies examine the associations between neighborhood social environments and performance-based physical function. Furthermore, racial and ethnic differences are widely understudied. The objectives of this study are (1) to examine the association between neighborhood social cohesion and physical disorder on physical function in older adults, and (2) to identify potential racial/ethnic differences in these associations. Data come from round five (collected in 2015) of the National Health and Aging Trends Study (NHATS; N=5,619). A series of adjusted linear regression models were used to predict performance-based physical function based on characteristics of the neighborhood social environment (i.e., cohesion, disorder). Results showed that only neighborhood physical disorder was statistically significantly associated with poorer physical function (p < 0.05). Similarly, when stratified by race/ethnicity, only neighborhood physical disorder was associated with poorer physical function in Whites ($p < 0.05$). There was no significant association for either neighborhood social environment characteristic and physical function for Black or Hispanic older adults. Racial and ethnic differences warrant closer investigation in studies of neighborhood effects on health. Community-level interventions, policy makers, and researchers should consider the interactions between minority membership and neighborhood social environments when addressing issues of health and physical function.

### SESSION 1350 (POSTER)

### FAMILY & INTERGENERATIONAL RELATIONS I

### OLDER PARENTS' PERCEIVED SOCIAL SUPPORT AND STRAIN FROM ADULT CHILDREN AND INTERGENERATIONAL FINANCIAL TRANSFER

Dahee Kim,[1] and Peter Martin[1], *1. Iowa State University, Ames, Iowa, United States*

The gerontological literature indicates that both positive and negative relationships are dimensions of intergenerational relationships. Moreover, depending on intergenerational financial support, the association between older parents' perceived social support and strain from adult children can vary. The purpose of the current study is to investigate the association between intergenerational social support and strain. We also examined the impact of intergenerational financial transfers on intergenerational social support and strain. We analyzed data of 1,329 older adults aged 65 to 84 from the Health and Retirement Study collected at 2006(t1), 2010(t2), and 2014(t3). Cross-lagged panel models were performed to examine the reciprocal association between intergenerational social support and strain over time. Multiple group comparisons were conducted to estimate the impact of financial support exchange on social support and strain from adult children. The results demonstrated that social support and strain from adult children were stable over time. Furthermore, social strain had negative effects on the changes in social support from adult children. Multiple group comparisons suggested that in the parents' groups (financial support provision vs. no provision to adult children, and financial support receipt vs. no receipt groups from adult children) intergenerational social support and strain were stable over time. Additionally, the impact of social strain on subsequent social support from adult children differed depending on intergenerational financial support. These findings highlight the reciprocal association between intergenerational positive and negative relationships. Further, this research suggests the importance of intergenerational support in older parents' and adult children's positive and negative relationship quality.

### DEVELOPING A QUALITY-OF-RELATIONSHIP INTERVENTION FOR STROKE-SURVIVOR FAMILY CAREGIVER DYADS

Michael J. McCarthy,[1] Karen Lyons,[2] Dorothy Dunn,[1] Yolanda Garcia,[1] and Tamilyn Bakas[3], *1. Northern Arizona University, Flagsttaff, Arizona, United States, 2. Boston College William F. Connell School of Nursing, Chestnut Hill, Massachusetts, United States, 3. University of Cincinnati, Cincinnati, Ohio, United States*

A strong interpersonal relationship after stroke is important for the well-being of survivors and family caregivers. At present, few interventions are specifically designed to strengthen the relationship between members of the care dyad. The aim of this study is to develop, validate, and pilot test a quality of relationship intervention for stroke dyads. This poster presents findings from the content validity phase of the study and includes "tips" that dyads offered for maintaining a strong relationship. Semi-structured interviews were conducted with N=19 dyads to solicit information about relationship problems and tips. These data were used to develop a 17-item relationship assessment questionnaire with 17 "tip sheets" corresponding to each item. A Delphi process was used to obtain feedback from a 10-member expert panel about the degree to which the questionnaire and each tip sheet was (1) relevant; (2) clear; (3) accurate, and; (4) useful. Expert agreement ranged for 80% to 100%. The final materials included tips for dealing with issues that arise when one or both partners experience communication, mobility, cognitive, or emotional issues; reaching agreement about different aspects of recovery; working together to meet each partners' needs; adjusting to new roles after stroke; dealing with relationship problems that predated stroke, and; getting support from other family members, friends, peers, and professionals. Findings highlight areas to consider in promoting strong relationships in care dyads. Future research is needed to examine whether these materials can help to reduce stressful interactions for care dyads, promote dyadic coping, and improve overall relationship quality.

### TRANSITIONING OUT OF GRANDCHILDREN CAREGIVING: EFFECTS ON GRANDPARENTS' EMOTIONAL WELL-BEING

Rita X. Hu,[1] Lydia Li,[2] and Toni C. Antonucci[2], *1. University of Michigan, Ann Arbor, Ann Arbor, Michigan, United States, 2. University of Michigan, Ann Arbor, Michigan, United States*

Research has suggested that grandparents caring for grandchildren experience both psychological gains and loss. Less clear is what happens to these grandparents after they exit from the caregiving role. This study used the Health and Retirement Study (HRS) 2010 to 2014 data to examine the effects of transitioning out of caregiving on the psychological well-being of grandparents. Psychological well-being was measured by the Positive and Negative Affect Schedule. We defined caregiving grandparents as grandparents who provide 100+ hours of care per year to their grandchildren. In the first wave, 8,278 respondents in the HRS were identified as caregiving grandparents. Among them, 3,914 continued to be caregivers and 4,364 transitioned out of the caregiving role by indicating they are no longer providing care in the second wave. Grandparents who transitioned out of caregiving are more likely to be older in age, less educated and not married. Linear regression analysis was conducted to compare the two groups (continuing vs. exiting caregiving) on positive and negative affect, controlling for the first wave's measures of the dependent variable, sociodemographic characteristics and health status of respondents. Results show that grandparents who continued caregiving had less decline in positive affect than grandparents who transitioned out of caregiving (b = -0.05, SE = 0.02, p<0.01), adjusting for covariates. But they were not significantly different in negative affect. These findings indicate that older adults may have fewer sources of joy after exiting the role of caregivers of their grandchildren.

### SOCIAL STRATIFICATION AND TRAJECTORIES OF LIVING ARRANGEMENTS AMONG OLDER AMERICANS

Jersey Liang,[1] BoRin Kim,[2] Xiao Xu,[3] James Raymo,[4] Mary Beth Ofstedal,[1] and Qing Zheng[5], *1. University of Michigan, Ann Arbor, Michigan, United States, 2. University of New Hampshire, Durham, New Hampshire, United States, 3. Yale University, New Haven, Connecticut, United States, 4. University of Wisconsin-Madison, Madison, Wisconsin, United States, 5. Abt Associates, Durham, North Carolina, United States*

Living arrangements are critical to intra-family exchanges that affect older persons' health and well-being. The conventional conceptualization of living arrangements has emphasized coresidence with children, while overlooking proximate residence from children. Additionally, existing research often relied on cross-sectional data which confound intrapersonal differences with interpersonal variations. This study examined the dynamics of living arrangements in old age by depicting their trajectories as a function of social stratification (i.e., age, gender, race/ethnicity, education, income, and wealth). Data came from the Health and Retirement Study and included a national sample of 7,822 older Americans with at least one living child from 1998 to 2014. Multi-level mixed effects models were employed to analyze the trajectories of living arrangements and their key determinants for the young-old and the old-old separately. Among the young-old (age 65-74, N=4,917), the probability of coresidence increased slightly over time, whereas the probabilities of proximate residence and distant residence decreased slightly and remained stable respectively, and the risk for institutionalization increased moderately. Similar but more accelerated trajectories were observed among the old-old (age 75+, N=2,905). Age, gender, race/ethnicity, education, income, and asset were significantly associated with not only the levels of the probabilities of various living arrangements but also their slopes. For instance, among the old-old, Hispanics had a lower level of nursing home residence as well as a slower rate of increase in the risk of institutionalization than Whites. These findings may inform public policies to strengthen family-based support and long-term care for older people.

### AN INTERGENERATIONAL DYADIC APPROACH TO UNDERSTANDING GRANDPARENT DEATH WITHIN A FAMILY SYSTEM

Margaret M. Manoogian,[1] Heidi Igarashi,[2] and Maggie Leinenweber[1], *1. Western Oregon University, Monmouth, Oregon, United States, 2. Oregon State University, Corvallis, Oregon, United States*

Due to increased longevity and generational location, grandparent death creates new contexts for identity, family culture, and intergenerational relationships. To explore this loss from two perspectives, we conducted intensive interviews with young adults and their mothers (N = 16) who experienced a recent grandparent (parent) death. Guided by the life course perspective, we were interested to learn how grandparent death may shape identity, meaning, and behaviors among family members, and influence their shared parent-child relationship. Findings suggest that the relationship with the grandparent, the diverse expressions of grief, the navigation of family transitions during and after death, and the curation of grandparent memories influenced individual and family outcomes. Implications suggest the need for varied supports that are sensitive to how individual family members approach grief in distinct ways reflective of their developmental positions, past experiences, and relational expectations.

### IT MAKES ME FEEL LIKE I'M NOT ALONE: GRANDPARENT CAREGIVERS' DISCUSSIONS OF SOCIAL SUPPORT

Jessica Freeman,[1] and Jessica Elton[2], *1. University of Tennessee at Chattanooga, Chattanooga, Tennessee, United States, 2. Eastern Michigan University, Ypsilanti, Michigan, United States*

This study investigated skipped-generation "grandfamily" caregivers' sources of social support. Skipped-generation "grandfamilies" are defined as families in which grandparents co-reside with and take on parental responsibilities for grandchildren (Shakya, et al., 2012). Nearly 2.6 million U.S. grandparents are responsible for at least one grandchild (Generations United, 2017). How grandparent caregivers seek social support is an important topic of research because social support has the potential to influence health, personal relationships, and sense of self-worth (Burleson, 1990, 2003; Sarason & Sarason, 2009; Vangelisti, 2009). Thus, this exploratory study looked at if, why/why not, and how skipped-generation grandparents seek social support. The study also elaborated upon which types of social support grandparent caregivers described receiving. Interviews were conducted with grandparents (N = 21) who identified as primary caregiver for at least one grandchild. Two independent coders analyzed transcripts, applying Braun and Clarke's (2006) approach to thematic analysis. Results revealed that several grandparent caregivers report a sense of isolation and do not seek out formalized support structures due to lack of time, sense of connection, or interest. On the other hand, others receive social support formally and informally, via a number of channels including support groups (online and in-person), trained professionals, and friends/family. Following Cutrona and Suhr's (1992) social support categorization, the results indicate that grandparent caregivers most often seek support falling into categories ranging from informational/advice, emotional, tangible, and networking.

### A MICROLONGITUDINAL APPROACH TO STUDYING GRANDMOTHER-GRANDCHILD DAILY INTERACTIONS

Shelbie Turner,[1] Shannon T. Mejia,[2] Robert S. Stawski,[1] and Karen Hooker[1], *1. Oregon State University, Corvallis, Oregon, United States, 2. University of Illinois, Urbana-Champaign, Champaign, Illinois, United States*

Research suggests that grandparent-grandchild dyads shift in degree of solidarity over extended periods of time (e.g. Moorman & Stokes, 2016), but no work has considered grandparent-grandchild interactions microlongitudinally. This study utilized microlongitudinal data with an emphasis on intraindividual variability to examine the daily processes associated with relational aspects of grandparenting. Using data from 24 grandmothers in the Personal Understandings of Life and Social Experiences (PULSE) project, we explored how grandmother-reported satisfaction with grandchild interactions impacted grandmothers' same-day positive and negative affect over 100 days. We first justified the need for microlongitudinal analyses by assessing the degree to which there were within-person shifts in interaction satisfaction over time. Intra-class correlations indicated 86% of the variation in interaction satisfaction was within-persons, warranting an intraindividual variability approach. As such, we then employed multi-level models to examine the within-person and between-person effects of interaction satisfaction predicting same day positive and negative affect. At the within-person level, on days when grandmothers reported higher than their average interaction satisfaction, they reported more positive affect (Estimate = 0.09, SE = 0.03, p = 0.009) and lower negative affect overall that day (Estimate = -0.08, SE = 0.02, p = 0.005). At the between-person level, grandmothers who had, on average, higher interaction satisfaction had more positive affect (Estimate = 0.63, SE = 0.09, p<.0001) and lower negative affect on average (Estimate = -0.53, SE = 0.11, p<.0001).

### MENTAL HEALTH OF GRANDPARENTS RAISING GRANDCHILDREN: UNDERSTANDING PREDICTORS OF GRANDPARENTS' DEPRESSION

Youjung Lee,[1] and Kyeonghee Jang[2], *1. Binghamton University, Binghamton, New York, United States, 2. Abilene Christian University, Abilene, Texas, United States*

Grandparents raising grandchildren experience caregiving stress, negatively influencing their mental health. They experience limited social supports and suffer from a lack of respite care and community resources. The present study attempts to explore needs of grandparent-headed families and factors related to grandparents' depression. In 2015-19, surveys with 92 custodial grandparents were conducted in the northeastern U.S. The respondents were primarily white (77%) and 62 years old on average (ranged from 44 through 84) at the time of the interviews. The depressive symptoms ranged from 1 through 45, with the score of 16+ indicating clinical depression (41%). Sixty-three percent of grandparents reported a household income below $40,000 and 40% of them rated their health as poor or fair. Ninety-five percent reported at least one or more concerns in raising their grandchild (i.e., financial concerns, legal issues, and physical health). A multiple linear regression analysis was performed to examine the contributions of age, ethnicity, duration of care, factors related to multigenerational caregiving, social support from family members, social support from friends, and social support from significant others in accounting for grandparents' depressive symptoms. The model explained 29% of variance in the outcome (R2=.290; adjusted R2=.231). Among the predictors, only one factor was significant: social support from family members (beta=-.352, p=.006). Grandparents with increased social support from family members have lower rates of depression. This finding reinforces the importance of familial support for grandparents raising grandchildren, and recommends the development of family-centered programs to offer support for custodial grandparents to promote caregivers' well-being.

### BUILDING NETWORKS OF PROFESSIONALS TO SUPPORT KINSHIP CARE FAMILIES

Andrea B. Smith[1], *1. Western Michigan University, Kalamazoo, Michigan, United States*

Numbers of American children being raised by relatives continue to rise. Over 7.8 million children, representing 10.5% of American children under age 18, lived in a relative-headed home. Of these, 2.5 million have no parent present in their home (Generations United, 2016). Kinship families' needs are typically complex, needing prompt and sensitive responses. Varied professionals often serve kinship families but the majority report receiving little information or training related to kinship families. (Smith 2017 This pilot project surveyed professionals (n = 63) representing varied disciplines in a pre/post-test format to determine change in knowledge and strategies for working with kinship care family members. All respondents were enrolled in graduate courses specifically focused on kinship families. Respondents represented diverse fields including family therapists (n = 9), family service workers (n = 23), teachers (n = 16), school administrators (n = 4), child care providers (n = 2) and health care professionals (n = 7 ). Respondents completed a 17 question pre/post survey. Results demonstrated the majority (n = 59; 93%) had experience with kinship families but most (n = 47; 74%) had received little/no targeted professional training. Post-test results strongly indicated that completing any amount of kinship-related coursework positively impacted professionals' knowledge, confidence, and readiness to utilize learned strategies in their work with kinship families. Additionally, post-test results of students completing the entire series of classes (9 credits) revealed significantly greater changes, demonstrating the importance of providing comprehensive information to enhance professional practices for working with kinship families.

### SCAFFOLDING PARENTS TO ACCEPT ADULT CHILDREN'S INTERVENTION

Noriko Toyokawa,[1] Nancy Darling,[2] and Teru Toyokawa[1], *1. California State University San Marcos, San Marcos, California, United States, 2. Oberlin College, Oberlin, Ohio, United States*

Guided by social-cognitive domain specific theory (Smetana, 1997), this study explored the issue of role reversal in the aging parent-adult child relation when parents are experiencing age-related functional limitations. Data was collected from adult children (N=16, Mage=53.06, SD=6.08) with a living parent of 70 years old or older who participated in a focus group and were analyzed by a directed analysis (Potter & Levine-Donnerstein, 1999). Participants legitimated their intervention into parents' autonomy when they perceived a potential risk of parents' health and safety and involvement of those and of others. Eight types of intervention emerged: (1) monitoring and talking with potential risk with parent (2) convincing parents under the name of super power or an authority figure (3) scaffolding parent's task by teaching skills, (4) scaffolding by sharing role, (5) scaffolding by optimizing environment, (5) overriding parents' autonomy behind parents, (6) forcefully overriding, (7) giving up parents' behavioral modification by accepting parents' lifestyle, and (8) giving up because of discomfort of talking about the issue (i.e., potential risks of parent's sexual intercourse, parents' death preparation). Thus, adult children changed their strategies of intervention from monitoring their parents' behaviors to overriding parents' autonomy, depending on their appraisal of potential harms of parents' prudential and moral domains of life and of their own work/family conditions from monitoring to overriding. Adult children's possible ways of scaffolding in helping their parents accept their children's interventions as letting parents maintain their psychological autonomy, including communication skills to discuss uncomfortable topics is discussed.

### ADVERSE CHILDHOOD EXPERIENCES REPORTED BY CUSTODIAL GRANDMOTHERS AND THEIR ADOLESCENT GRANDCHILDREN

Gregory C. Smith,[1] Frank J. Infurna,[2] Britney A. Webster,[1] Megan L. Dolbin-MacNab,[3] Max Crowley,[4] and Carol M. Musil[5], *1. Kent State University, Kent, Ohio, United States, 2. Arizona State University, Tempe, Arizona, United States, 3. Virginia Tech, Blacksburg, Virginia, United States, 4. Pennsylvania State University, University Park, Pennsylvania, United States, 5. Case Western Reserve University, Cleveland, Ohio, United States*

The Risky Family Model postulates that adverse childhood experiences (ACE) are likely to be encountered across generations within custodial grandfamilies which, in turn, may adversely impact their overall well-being. The present study is a pioneering attempt to examine the patterns of ACEs self-reported by custodial grandmothers (CGM) and adolescent grandchildren (AGC) from the same families, and how their total ACE scores correlate with key physical and mental health outcomes. A total of 129 CGM-ACG dyads recruited for a nationwide RCT study completed separately at baseline the 10-item ACE-CDC and 4 items from the ACE-IQ, as well as various standardized measures of physical and emotional well-being. The most frequent ACEs reported by AGC were loss of a parent (60.5%), verbal abuse (58.1%), bullying by peers (46.5%), and living with someone jailed (45.0%). The predominant ACEs for CGM were bullying by peers (48.8%), verbal abuse (48.1%), living with a mentally ill person (34.1%), being touched sexually (29.5%), and loss of parent (29.5%). Only 10.1% of ACG and 15.5% of CGM reported 0 ACEs, whereas 65.1 % of ACG and 59% of CGM reported > 3 ACEs. For ACG, total ACE scores correlated significantly with externalizing (r=.32) and internalizing (r=.30) difficulties, self-esteem (r= -.28), loneliness (r=.27), school problems (r=.24), and physical health (r= -.26). For CGMs, anxiety (r=.23) and depression (r=.19) only were correlated significantly with total ACEs. We conclude that although both CGM and ACG reported alarmingly high levels of ACEs, different patterns and correlates exist between the generations. [Funded by R01AG054571]

### GRANDPARENTAL CHILDCARE AND ITS RELATIONSHIP WITH SOCIOECONOMIC INEQUALITIES AND HEALTH: EVIDENCE FROM ENGLAND

Giorgio Di Gessa,[1] and Karen Glaser[1], *1. King's College London, London, United Kingdom*

It is well recognised that grandparents play a vital economic and social role in providing grandchild care to families. In the UK, about two million grandparents provide care to their grandchildren for at least ten hours per week. However, to date, little is known about the nature and extent of care they provide and whether, and how, this relates to socio-economic and health inequalities. In this paper, we use wave 8 of the nationally representative English Longitudinal Study of Ageing. Using latent class analysis, we developed profiles of grandparent childcare combining newly collected information on the activities grandparents do with or for their grandchildren, as well as on the extent of (periodicity, frequency, and intensity), and motivations for such care. Using regressions, we then examined the extent to which grandparents' involvement in childcare is patterned according to current and lifetime socio-economic characteristics and whether such inequalities are an important modifier of the relationship between grandparenting and mental and physical health. Preliminary results suggest that more advantaged grandparents are the ones undertaking less arduous caring tasks and for shorter periods whereas those looking after grandchildren more intensively and for financial reasons are more likely to be of less advantageous backgrounds (not married, low-educated, not homeowners, and poorer). Moreover, undertaking more frequent and challenging grandchild care activities seems to be associated with poorer health but only among grandparents in the more disadvantaged groups. Our results contribute to better understand the nature of grandparental childcare and its effect on health.

### THE GREAT AND THE GRAND: THE ROLES OF GREAT-GRANDPARENTS AND GRANDPARENTS IN MULTIGENERATIONAL FAMILIES

Emily Schuler,[1] and Cristina Maria de Souza Brito Dias[1], *1. Universidade Católica de Pernambuco (UNICAP), Recife, Pernambuco, Brazil*

The increase of Human Aging has been observed rapidly in the whole world, as it has been in Brazil allowing the experience to live several roles within the family for a longer time. As a consequence, more multigenerational families emerge with a more vertical structure, formed by four or even five generations. While the oldest generation adds another generational role to their life, the one of great-grandparents, the youngest generation is born into an intergenerational network of relationships. There are various questions about the differences in the role of great-grandparents and grandparents, which motivated this present study. Thus, the objective of this study was to understand the roles of great-grandparents and grandparents in the family and their intergenerational repercussions. Four families with for generations, totaling 16 participants. One member of each generation was interviewed, using a specific script, which was afterwards analyzed by the Thematic Content Analysis. The results pointed out that both great-grandparents and grandparents have distinct roles that are constructed around the needs of the family; both figures provide emotional and material support to the family; both roles have transgenerational importance in the transmission of family legacies, which are related to faith, solidarity, education and order. It can also be said that the great-grandparents can be compared to the grandparents of the past, as the grandparents can be assimilated to the parents of older days. It is hoped that this research contributes to the visibility of these two generations and to sensitize professionals about this theme.

### THE IMPORTANCE OF EMOTIONAL CAPITAL ACROSS THE LIFESPAN: WHY THE LITTLE MOMENTS IN MARRIAGE MATTER

Lisa Neff,[1] Courtney Walsh,[1] and Jennifer Beer[1], *1. University of Texas at Austin, Austin, Texas, United States*

Throughout a marriage, couples will share countless ordinary moments together, such as laughing together or engaging in leisure activities. Although these moments may seem trivial in isolation, research suggests that accumulating small positive moments together helps couples build emotional capital, which serves as an essential resource for protecting marriages from the harmful consequences of relationship challenges. This study explored whether emotional capital may buffer couples not only from the negative effects of relational stressors, but also from the negative effects of life stressors encountered outside the relationship in a sample of younger (age 30-45) and older (age 60+) married couples. Drawing from theories of socioemotional expertise, we also examined whether the buffering effects of emotional capital may be stronger for older adults. One hundred forty-five couples completed a 21-day daily diary task assessing shared positive experiences with the partner, negative partner behaviors, marital satisfaction, life stress, and mood. Spouses who generally accrued more shared positive moments with their partner across the diary days maintained greater marital satisfaction on days of greater partner negativity compared to spouses who accrued fewer positive moments. Moreover, spouses who generally accrued more shared positive moments with their partner across the diary days also reported lower levels of negative mood on days in which they experienced more life stress compared to spouses who accrued fewer shared positive moments; in both cases, the buffering role of emotional capital was significantly stronger for older adults. All results held when adjusting for relationship length and general marital happiness.

### THE CONTEXTUAL EXPERIENCES OF SOCIAL CAPITAL AMONG OLDER GRANDPARENTS RAISING ADOLESCENT GRANDCHILDREN

Tina L. Peterson[1], *1. University of Oklahoma, Norman, Oklahoma, United States*

Older grandparents raising adolescent grandchildren are an understudied population. Greater understanding is needed of the social capital (e. g. information, emotional support, companionship, practical instrumental support, and influence, power, and control) harnessed by older grandparents raising adolescent grandchildren. This research applied a qualitative, phenomenological approach to explore social capital among older grandparents. Face-to-face interviews were conducted with 19 grandparent caregivers ranging in age from 55 to 88 years. Eligibility criteria were: primary caregiver for a grandchild 12 years or older; grandchild resides in home at least 3 days; grandparent 40 or older and resides in Oklahoma, Alabama, or Kentucky. Participants were recruited by word of mouth, newspapers, and flyers. Grandparents responded to a question prompt, "I am going to ask you some questions about your support system to assist with your concerns about your older grandchild." Interviews were conducted in public places, audiotaped, and transcribed verbatim. Data were analyzed using a question analysis approach to sort responses into matrices, develop memos, and identify themes. Most older grandparents were female (84.2%), Caucasian (52.6%), married (57.9%), and never attended a support group (68.4%). One overarching theme from these older grandparent caregivers is access to social capital exists on a continuum. Participants' experiences with social capital pertained to family proximity, evolving perception of friends, limited or no social engagement with neighbors, dynamics of church attendance and size, and familiarity with community resources. These older grandparents raising adolescents shared positive reactions to select social capital with some types emerging as less important or underdeveloped.

### BLACK AND WHITE YOUNG ADULTS' SUPPORT TO MIDLIFE PARENTS

Kristie A. Wood,[1] Meng Huo,[1] Yee To Ng,[1] and Karen Fingerman[1], *1. University of Texas, Austin, Austin, Texas, United States*

Researchers have observed racial differences in support to midlife parents. Black adults typically provide more support to parents and report greater rewards in doing so. We were interested in whether this differential trend can be observed in young adulthood. Furthermore, we aimed to understand cultural beliefs underlying any racial differences in support provided to parents. We examined support Black and White young adults provided to their parents, and beliefs associated with that support. Young adults (aged 18–35 years; 26%, n=184 Black and 74%, n=525 White) from the Family Exchanges Study II (2013) participated. They reported how often they provided 4 types of support (financial, technical, practical, emotional) to each parent (N =824) on a scale from 1 = once a year or less often to 8 = everyday. Multilevel models revealed Black young adults provided more frequent support to parents than White young adults, mediated by beliefs about familial obligation. Interestingly, we also found that Black young adults report significantly more negative relationship quality with parents and a stronger desire for support from parents when compared to White young adults. Findings suggest that Black young adults may espouse collective and interdependent values such as the ability to provide for a family and to receive support from family. Further, more frequent support may co-occur with conflicts that suggest that congruence between values and support do not necessarily inspire harmonious ties.

### SESSION 1355 (POSTER)

### FAMILY CAREGIVING I

### SPOUSE OR CAREGIVER? EXAMINING CORRELATES AND CONSEQUENCES OF SPOUSES BEING IDENTIFIED AS CANCER CAREGIVERS

Kristin Litzelman[1], *1. University of Wisconsin-Madison, Madison, Wisconsin, United States*

Spouses provide critical support to cancer survivors, but are not always identified as caregivers. This study sought to understand the differences between such spouses. Using the Medical Expenditures Panel Survey 2016 Experiences with Cancer Supplement, spousal dyads in which one spouse had cancer were identified (n=670). Cancer survivors reported which family members or friends, if any, provided care during or after their cancer treatment. Survivor and spouse sociodemographic characteristics and spouse psychosocial characteristics including depression (Patient Health Questionnaire-2), distress (Kessler-6), and self-rated health were self-reported in the survey. The proportion of spouses identified as caregivers was calculated and compared with those not identified as caregivers on dyadic characteristics. Multivariable logistic regressions compared spouses' depression, distress, and self-rated health by identification as a caregiver. All analyses employed survey weighting. Most married cancer survivors reported that their spouse was a caregiver (32%) or that they did not have a caregiver (65%); very few did not list their spouse as a caregiver (3%). Survivors who were white (non-Hispanic) or off treatment were less likely to report that their spouse was a caregiver (30% vs. 46%, p0.10; 9.6% vs 11.1%, p>0.10; and 46% vs 50%, p>0.10, respectively). The findings suggest that spouses' experiences may be similar regardless of whether they are considered a caregiver, with implications for research and service delivery.

### CAREGIVING EXPOSURE AND COGNITION: A PROPENSITY-MATCHED, 10-YEAR FOLLOW-UP

Victoria R. Marino,[1] Dylan J. Jester,[2] and William E. Haley[1], *1. University of South Florida, Tampa, Florida, United States, 2. School of Aging Studies, University of South Florida, Tampa, Florida, United States*

Evidence on the effect of caregiving on cognitive health is mixed. Literature suggests that highly-strained subgroups of CGs experience impairment especially related to depression and negative health behaviors. Conversely, others suggest that sustained higher amounts of caregiving is associated with better cognitive functioning over time. This study compared baseline level and 10-year change in executive function (EF) and episodic memory (EM) in middle aged adults with no caregiving exposure, short (less than one year), average (one to less than five years), and long (five or more years) caregiving exposure. Data came from waves 2 and 3 of the Midlife in the United States study. We statistically matched 265 CGs to 265 non-CGs on baseline sex, age, race, education, marital status, and physical health and controlled for depression and anxiety in analyses. Mixed models estimated fixed-effects of time, depression, and a time by caregiving exposure interaction relative to no caregiving exposure. For EF, CGs with short exposure had higher baseline scores relative to non-CGs ($\beta$=.294;p=.023). Though each group declined over time ($\beta$=.298;p<.001), caregiving exposure was not related to the rate of decline in EF. Baseline levels of EM were highest in CGs with average exposure ($\beta$ =.521;p=.011) and long exposure ($\beta$=.482;p =.017). However, not all participants declined in EM over 10 years; CGs with long exposure exhibited the only statistically significant decline ($\beta$ =-.262;p=.026). Results suggest that CG duration is intimately related to EF and EM at baseline, but perhaps only to rate of decline for EM.

### THE EFFECT OF CAREGIVING ON THE DEPRESSIVE SYMPTOMS OF OLDER SPOUSAL CAREGIVERS IN CHINA

Xinyi Zhao,[1] Huiying Liu,[2] Zhaoguang Wang,[3] Quan Zhang,[4] and Vivian W. Lou[5], *1. School of Health Humanities, Peking University, Beijing, China, 2. University of Hong Kong, Hong Kong, Hong Kong, 3. National School of Development, Peking University, Beijing, Beijing, China, 4. Peking University Health Science Center, Beijing, China, China, 5. The University of Hong Kong, Hong Kong, P.R.C., Hong Kong*

Objectives: This study investigated the influence of caregiving on the depressive symptoms of older spousal caregivers in China. Methods: Data came from the 2015 China Health and Retirement Longitudinal Study (CHARLS). The sample of this study included older adults aged 60 and above. The respondents were coded as spousal caregivers if they provided activities of daily life (ADL)/instrumental activities of daily life (IADL) assistance to their spouses and did not receive any ADL/IADL help. Ordinary Least Square (OLS) regression analyses were applied. Results: Results from the regression model indicated that spousal caregiving was significantly associated with depressive symptoms (p<0.01). The spousal caregivers showed more depressive symptoms than the non-caregivers. Among the spousal caregivers, the females and those who reported poorer health had more depressive symptoms than their counterparts (p<0.01). Conclusion: The results highlight the negative influence of caregiving on psychological health of spousal caregivers. Caregiver support should be targeted on the older spousal caregivers, especially the wife caregivers.

### LESSONS LEARNED FROM SAVVY EXPRESS: A FEASIBILITY STUDY OF A PSYCHODUCATIONAL INTERVENTION FOR CARE PARTNERS

Maria P. Aranda,[1] and Debra Cherry[2], *1. University of Southern California, Los Angeles, California, United States, 2. Alzheimer's Los Angeles, Los Angeles, California, United States*

An increasing number of families, funders, and community providers seek very brief psychosocial caregiver interventions, yet the evidence for such condensed interventions is not established. Based on the Savvy Caregiver Program, we explored the feasibility, acceptability, and outcome trends for a condensed 3-session version titled, Savvy Express. Based on a single-group, pre- and post-test intervention design, we examined post-intervention and 3-month data on 116 English-speaking racially and ethnically diverse care partners caring family members with Alzheimer's disease and related dementias. 41% of the sample was non-Latino white and comprised of Latinas, African Americans and Asian American/API. Most care partners were either adult children or spouses caring for someone with AD or other dementia. Over 80% were college educated. Two of three participants completed all 3 classes. Our findings indicate significant improvements in caregiver levels of depressive symptomatology and anxiety, competence, management of the situation, reduction of expectations, making positive comparisons, and reactivity to the family member's memory behavior. Upwards of 90% would recommend the program to other caregivers. Savvy Express is a brief caregiver intervention with high acceptability and feasibility. Improvements in care partner psychosocial outcomes signal a promising practice to reduce the burden of caregiving. A major focus of the paper focuses on barriers and facilitators to uptake of the study procedures and intervention with community-based partners. Future work is needed to establish the efficacy of Savvy Express across a longer observation period, and with less educated, low-income participants, and limited English-speaking families.

### TRANSITIONS OF FAMILY CAREGIVERS: A CONCEPTUAL FRAMEWORK FOR END-OF-LIFE CAREGIVING

Aimee Fox,[1] and Christine Fruhauf[1], *1. Colorado State University, Fort Collins, Colorado, United States*

Increased longevity and a growing aging population have led to rising numbers of informal, family caregivers. Compared to non-caregivers, caregivers are more stressed and depressed, and report lower levels of self-efficacy, physical health, and subjective well-being. Along the caregiving trajectory, end-of-life (EoL) care is emerging as a distinct phase that is not understood and warrants further research. This presentation will include an integrative literature review on EoL caregivers and propose a model for the transition to caregiving at the end-of-life. The review highlights the differences in strengths and needs of EoL caregivers when compared to non-EoL caregivers and age-related non-caregiving peers. For example, family members providing EoL care are more vulnerable to stress and psychological and physical strain, report low preparedness for EoL care, and often indicate they do not have the knowledge or skills needed for providing care at the EoL. The model for the transition to caregiving at the end-of-life presents new approaches to traditional stress and coping models and a conceptual framework for future EoL caregiving research and intervention efforts. The model highlights the need for expanding the theoretical foundations of EoL caregiving research and guides innovative methods of data collection and thoughtful approaches to the timing, recruitment, and utilization efforts of intervention programs. Ultimately, as researchers identify the needs of EoL caregivers, practitioners can improve caregivers health and well-being not only during this stage, but as they follow their own developmental path beyond loss and bereavement.

### SUPPORT AND EDUCATION NEED FULFILLMENT IN INFORMAL DEMENTIA CAREGIVERS

Nicholas James,[1] and Daniel Paulson[1], *1. University of Central Florida, Orlando, Florida, United States*

Caregivers vary widely in their need for and utilization of support services, and there are many reasons for this (e.g., time or financial cost, distance, perception of need). This study explored the disparity between services that are desired and those that are utilized, and examined the hypothesis that unmet needs are a determinant of caregiver burden. An online sample of informal caregivers (N=151) responded to a questionnaire containing a list of common caregiver support services: sharing duties, professional transportation, respite care, non-profit community organizations, financial counseling, caregiver education programs, support groups. Participants were presented with a list of support services and information and asked to indicate A) which they desired and B) which they had received while providing care. Respite care and financial counseling were identified as support that caregivers were unable to obtain, while caregiving education and family/friend support were most commonly fulfilled. A linear regression model controlling for demographic variables was constructed. Unfulfilled support needs accounted for 40.6% of variance in caregiver burden, however the final model included only total ratio of unfulfilled services, transportation services, stress management skills, professional treatment for the caregiver, and behavioral management skills. Results highlight the unique contribution of certain support services in burden reduction. These findings imply a need to improve accessibility to caregiver support, especially those which require payment (e.g., transportation aids and mental healthcare). Further implications and actionable changes related to caregiver support services are discussed.

### FACTORS ASSOCIATED WITH CAREGIVER REACTIONS TO PROBLEM BEHAVIORS OF PERSONS WITH ALZHEIMER'S DISEASE

Junrong Shi,[1] and Cathy Scott[1], *1. University of Tennessee at Chattanooga, Chattanooga, Tennessee, United States*

Problem behaviors among persons with Alzheimer's Disease (AD) can have a major impact on caregivers. However, caregiver's subjective reactions to the problem behaviors have a stronger impact on caregivers than the objective frequency of problem behaviors (Robinson et al., 2001). This study aims to examine the factors associated with caregiver's subjective reactions to problem behaviors. Data were collected from a sample recruited from community agencies served AD caregivers in the southeastern region of the country (N=109). The caregivers' reactions to problem behaviors were measured by the Revised Memory and Behavior Problem Checklist (Teri et al., 1992). Three subscale scores were used to measure the reactions to the behaviors related to memory loss, depression and disruption. Multivariate regression models were conducted including gender, race, employment, living arrangement, knowledge about the disease, resilience for caregivers; and ADL and IADL functioning, and frequency of problem behaviors for care recipients. Race ($B=-.162$; $p<.05$) and frequency of care-recipient problem behaviors ($B= 0.733$; $p<.001$) were significantly associated with caregiver's reaction to problem behaviors. Caregiver's knowledge about the disease ($p<.01$) only influence their reactions to memory loss problems but not for disruption and depression problems. African American caregivers had fewer reactions to disruption ($p<.01$) and more reactions to depression problems ($p=0.06$) than white caregivers, but no difference between the two groups in their reactions to memory loss problems. Personal resilience was not associated with reactions to any problem behaviors. Intervention should be tailored to the needs of caregivers to deal with behaviors of a person with AD.

### RELATIONSHIPS BETWEEN INFORMAL CAREGIVERS' MARITAL SATISFACTION, BURDEN, AND CAREGIVING STRESS

Hannah Ottmar,[1] Amy Houston,[2] Elisabeth Harfmann,[2] Amy Olzmann,[3] and Glenna Brewster[4], *1. Xavier University,*

*Cincinnati, Ohio, United States, 2. Milwaukee VAMC, Milwaukee, Wisconsin, United States, 3. Baltimore VAMC, Baltimore, Maryland, United States, 4. Emory University Nell Hodgson Woodruff School of Nursing, Atlanta, Georgia, United States*

Spouses often serve as the primary caregiver of individuals with dementia (Wright, 1991). As caregiving can be a stressful experience, marriage strains may occur. Researchers have found that caregiver spouses reporting low marital cohesion and satisfaction endorsed significant symptoms of depression (Rankin et al., 2001). Lower levels of marital intimacy have been found to be associated with higher levels of depression and strain among caregivers (Morris et al., 1998). The goal of the current study was to better understand the relationships between caregiver (N = 158) marital satisfaction, caregiving stress, and subjective and objective burden. Results indicated that marital satisfaction was a significant negative predictor of subjective burden as measured by the Zarit Burden Interview ($F(1,134) = 93.51$, $p < .001$, $R2 = .411$), subjective burden as measured by the Revised Memory and Behavior Problems Checklist (RMBPC), Reaction Scale ($F(1, 146) = 25.65$, $p < .001$, $R2 = .149$), and role captivity ($F(1, 139)$, $P < .001$, $R2 = .380$). Marital satisfaction was a significant positive predictor of caregiver competence ($F(1,140) = 32.45$, $p < .001$, $R2 = .188$). It was also found that marital satisfaction was not a significant predictor of objective burden as measured by the RMBPC Frequency Scale ($F(1, 146)$, $p = .065$, $R2 = .023$). The findings have implications for future interventions in that improving marital satisfaction of spouse caregivers may reduce subjective burden, decrease feeling trapped within the caregiving role, and increase caregivers' sense of competence within the caregiving role.

### IMPACT OF ELDER ABUSE ON SUBSEQUENT COGNITIVE DETERIORATION IN OLDER ADULTS WITH COGNITIVE IMPAIRMENT

Boye FANG,[1] and Elsie Yan[2], *1. Hong Kong Polytechnic University, Hong Kong Polytechnic University, Hong Kong, 2. The Hong Kong Polytechnic University, Hong Kong, Hong Kong*

Objectives: Considering the rapidly increasing older population, elder abuse has become a major public health problem. Using longitudinal data, this study examines the impact of elder abuse on subsequent cognitive deterioration. Methods: At baseline, one-thousand-and-two older adults (aged≥55 years) with a clinically valid diagnosis of mild-to-moderate cognitive impairment were consecutively recruited from the geriatric and neurological departments of three Grade-A hospitals in Guangdong Province of People's Republic of China from 2015 to 2016 and 958 of them have completed the present follow-up study after two years. The major independent variables were psychological abuse, physical abuse, caregiver neglect, and financial exploitation experienced at baseline. The outcome variable was cognitive deterioration defined by repeated measures using the Mini-Mental State Examination. Covariates included demographic characteristics, neuropsychiatric symptoms, depression, and IADL impairment. Results: According to the mixed-effect model, elder abuse at baseline did not have significant association with baseline cognitive impairment. However, experience of physical abuse (coefficient 0.11; 95% CI 0.06-0.15), psychological abuse (coefficient 0.28; 95% CI 0.09-0.47), and caregiver neglect (coefficient 0.03; 95% CI 0.01-0.05) at baseline predicted greater cognitive deterioration over the two-year observation period. Other contributing factors for greater cognitive deterioration included neuropsychiatric symptoms and depression. Discussion: Although no significant cross-sectional association between elder abuse and cognitive impairment was observed, physical abuse, psychological abuse, and caregiver neglect at baseline was found to have a long-term prominent effect on subsequent cognitive deterioration over the two-year observation period. The present results su

### FUTURE PLANNING AMONG OLDER CAREGIVERS OF FAMILY MEMBERS WITH INTELLECTUAL DISABILITY OR MENTAL ILLNESS

Fei Wang,[1] and Yangdi Han[2], *1. Jack, Joseph and Morton Mandel School of Applied Social Sciences, Case Western Reserve University, Cleveland, Ohio, United States, 2. School of Social Development and Public Policy, Fudan University, Shanghai, Shanghai, China*

Objectives: This study aims to examine future planning among older caregivers for family members with intellectual disability or mental illness, focusing on preferences, predictors and barriers. Method: Data were drawn from 260 caregivers (aged 50 or older) to a family member with intellectual disability or mental illness in Shanghai, China. Caregivers rated six types of future care arrangement under three circumstances: (1) the ideal situation, (2) unable to provide care due to age-related illnesses, and (3) caregivers are deceased. Socio-demographic factors associated with future planning were examined using multinomial logistic regression. Caregivers also rated twelve barriers to future planning. Results: Government-subsidized care facility is the most preferable care arrangement across the three circumstances. While continuing family care was still preferred if caregivers were to become sick or deceased, it was a less preferred option in the ideal situation. Common barriers were the cost of institutional care and the inadequate skills of the staff. Regarding the predictors of future planning, the older the caregivers were, the less likely they had no future plans. Caregivers were more likely to prefer family care over institutional care if their family members had mild impairment. Caregivers of a family member with mental illness were more likely to have no future planning than caregivers of a family member with intellectual disability. Conclusion: This study identified the needs of older caregivers for future planning specific to different circumstances. It also identified demographic profiles of future planning and the caregiver population at risk of no future planning.

### PAACC: A MINDFULNESS-BASED MULTICOMPONENT PROGRAM FOR FAMILY CAREGIVERS OF PERSONS WITH DEMENTIA

Lauren Hagemann,[1] Katherine Luci,[1] Mamta Sapra,[1] Jyoti Savla,[2] Lindsey Jacobs,[3] and Tonda Yates[1], *1. Salem VA Medical Center, Salem, Virginia, United States, 2. Virginia*

*Tech, Blacksburg, Virginia, United States, 3. VA Boston Healthcare System, Brockton, Massachusetts, United States*

Few interventions for family caregivers of persons with dementia (PwD) focus on both dementia care skill-building and the enhancement of acceptance and compassion towards oneself and the PwD. We designed a multicomponent, mindfulness-based 4-session caregiver intervention (Practice of Acceptance, Awareness, and Compassion in Caregiving, or "PAACC") to reduce burden in caregivers of family members with Alzheimer's disease and related dementias (AD/ADRD) and TBI-related AD. A prospective, randomized trial design is being implemented to compare the effectiveness of PAACC to the well-known, evidence-based REACH-VA intervention. Seventeen family caregivers (Mean Age = 68.71 years; 82% women; 30% had high school or less education) have participated in the trial thus far and provided qualitative responses to acceptability questions. High acceptability was noted for all intervention components of PAACC. Participants completed 95% of mindfulness homework during the study period. A majority also reported practicing spontaneous, informal mindfulness while engaged in daily activities (e.g., going for a walk, cooking). One participant noted incorporating mindfulness in her daily spiritual practice. The majority remarked that PAACC taught them to be more aware and accepting of the PwD's illness. Others mentioned becoming more aware of which stressors triggered them, and that they were able to avoid arguments with the PwD because of this increased awareness. Overall, our results suggest that this mindfulness-based multicomponent intervention is a promising method for promoting stress reduction for family caregivers of persons with dementia regardless of age, stage of dementia, education level, or rurality. Implications for research and practice will be discussed.

### ACTIONS TAKEN BY STROKE FAMILY CAREGIVERS TO POTENTIALLY PREVENT SURVIVORS' HOSPITAL READMISSIONS IN CHINA

Hairui Yu,[1] Jennifer Perion,[1] Victoria Steiner,[1] and Linda Pierce[1], *1. University of Toledo, Toledo, Ohio, United States*

Stroke is a leading cause of death in China; its level of burden on the Chinese population is greater than the global average. Family caregiving plays an essential role in prevention and management of stroke. The purpose of this qualitative descriptive study was to identify actions family caregivers of stroke survivors in China take to prevent hospital readmissions. Using purposive sampling, adult family caregivers (n=10) were enrolled from Huai'an city in Jiangsu province who provided care for a survivor in community settings for six months or longer. Caregivers were asked questions in a face-to-face, semi-structured interview with content validity established by experts in the field. Audiotaped interviews were transcribed/translated into English and the narrative data analyzed using Colaizzi's approach to content analysis. Seven female and three male caregivers with an average age of 55 years indicated actions that comprised six themes. These themes are: 1) Encouraging care recipients to be physically active, 2) Balancing a healthy diet with pleasurable foods, 3) Monitoring the physical health of care recipients and preventing injuries, 4) Developing personal and intimate strategies to motivate care recipients, 5) Providing emotional support and maintaining optimism, and 6) Gaining knowledge through relationships with doctors but desiring communication with other caregivers. Recognizing these actions taken by stroke family caregivers may improve education programs aimed at preventing hospital readmissions and be applicable throughout the world. Findings may also guide healthcare professionals who can advocate with Chinese local, provincial, and central health commissions on stroke survivors and their family caregivers' behalf.

### FEASIBILITY OF CARRES MODULES TO REDUCE POTENTIALLY AVOIDABLE HOSPITALIZATIONS IN PERSONS WITH COGNITIVE DEFICITS

Victoria Steiner,[1] Linda Pierce,[1] and Carol Bryan[1], *1. University of Toledo, Toledo, Ohio, United States*

Family caregiving is an essential, yet understudied, factor that can hasten, delay, or prevent hospital readmissions in individuals with cognitive deficits. This 3-month feasibility study examined 18 Internet-based educational CARREs (Communicate, Assist, Recognize & Report Events) Modules for family caregivers that address care recipients' potentially avoidable hospitalization (PAH) conditions, e.g. UTI. This study determined: 1) caregivers' perceptions about the use of the CARREs Modules, 2) caregivers' self-reported value of the Modules, and 3) potential outcomes for caregivers and care recipients. Community-dwelling family caregivers were recruited from local support/education programs and assigned 6-8 Modules based on their care recipients' needs. Links to online surveys were emailed at baseline, and 30 and 90 days post-enrollment. Descriptive statistics were performed on these data. Twenty potential subjects were screened but five were ineligible and three refused to participate. The remaining 12 subjects were primarily White females caring for a husband or parent. Subjects completed all the Modules they were assigned and did not experience any difficulties answering the survey questions. Subjects reported completing the Modules at least moderately increased their knowledge (67%), not being burdened by completing the Modules (67%), and very likely to participate again (58%). Many subjects stated the Modules taught them new things about preventing hospital readmissions in their care recipient (75%) and improved their well-being as a caregiver (83%). In collaboration with a home care agency, the investigators plan to implement and test a sustainable, "real-world" educational intervention incorporating the CARREs Modules that reaches a wide audience of family caregivers.

### RESULTS FROM RESEARCH ON A HEALTH-COACHED WALKING PROGRAM IN FAMILY CAREGIVERS OF PERSONS WITH DEMENTIA

Jane Flanagan,[1] Kathryn Post,[2] and Rebecca Hill[1], *1. Boston College, Chestnut Hill, Massachusetts, United States, 2. Massachusetts General Hospital, Boston, Massachusetts, United States*

Caregivers of persons with dementia demonstrate increased levels of stress, anxiety and depression, placing them at increased risk for poor health-related outcomes. Walking is widely supported as a cost-effective, accessible exercise and way to maintain physical fitness and mitigate stress levels. There is a critical gap in addressing health promotion strategies in caregivers. The specific aims of this health-coaching (HC) walking study using wireless pedometers in family caregivers of persons with dementia were to: 1) establish the feasibility of HC and wireless pedometer use, 2) examine outcomes of well-being, stress and activity level and 3) understand the experience of participation. This 2-group comparative study used a repeated measure design and mixed methods approach. We enrolled 27 females and 5 male caregivers (n=32), μ age 57 years: with both 16 in the control in the intervention arm. Pre-post measures of general health (body mass index [BMI], blood pressure, heart rate, cognition, well-being, stress and perceived activity level) were obtained from caregivers at baseline and again at 12 weeks. Results indicate that those who received HC had a statistically significant improvement in BMI (p = .01). There were no other statistically significant improvements in outcomes in either group. Qualitative findings suggest that participants reported many stresses that made self-care challenging. In summary, this was a feasible intervention that resulted in improved BMI in the HC group. Work is needed to understand the long-term impact of this outcome. Further exploration of other health-promoting interventions that may be beneficial for this population is essential.

### QUALITY OF LIFE OF GRANDPARENTS RAISING GRANDCHILDREN IN NIGERIA

Joshua O. Aransiola,[1] Funmi Togonu-Bickersteth,[1] Kolawole Aliyu,[2] Mojirayo Afolabi,[2] and Akanni Akinyemi[2], *1. Obafemi Awolowo University, Ile-Ife, Ile-Ife, Osun State, Nigeria, 2. Obafemi Awolowo University, Ile-Ife, Osun State, Nigeria*

This article examined the personal and household characteristics influencing the quality of life (QoL) of grandparents caring for grandchildren in Skipped Generation Households in Nigeria with a sample of 2, 144 grandparents in Imo, Lagos and Kano. Chi square and multinomial logistic regression were employed to understand the relationship between the dependent variable (QoL) and independent variables (personal and household characteristics). The level of the QoL of the grandparents almost spread evenly among low (34.3%), average (34.3%) and high (31.4%). Five domains of QoL were examined including level of independence (LI), psychological well-being (PW), social relation (SR), physical health (PH), environment (ENV) and engagement in income generating activities (IGA). Personal characteristics including; state of residence was significantly associated with all the domains except LI and PW, age was associated with IGA, LI and ENV, sex was associated with SR and ENV and level of education was associated with all the domains except IGA and ENV while religious affiliation was associated with IGA. Household characteristics including; sex of household head was significantly associated with PH, SR and ENV, age of household head was associated with IGA and LI and wealth index was associated with all the domains while the number of household members was significantly associated with ENV. The regression analysis shows that only state of residence and wealth index significantly influence the QoL of the grandparents (P≤0.05). The state of residence and wealth index are therefore important in any policy intervention for this category of elderly persons in Nigeria.

### SUPPORTING FAMILY CAREGIVERS: HOW DOES RELATIONSHIP STRAIN OCCUR IN CAREGIVING DYADS? A QUALITATIVE STUDY

Elizabeth S. Avent,[1] Laura Rath,[2] Kylie Meyer,[3] Donna Benton,[1] and Paul Nash[4], *1. University of Southern California, Los Angeles, California, United States, 2. Archstone Foundation, Long Beach, California, United States, 3. University of Texas Health Science Center San Antonio, San Antonio, Texas, United States, 4. University of Southern California, LA, California, United States*

Family members and spouses are usually the primary caregivers for older adults. Providing direct care can be stressful, strenuous, and time-consuming for caregivers, potentially leading to frustration and anger towards care recipients. This can be detrimental to the relationship quality of the caregiving dyad. Though caregiver strain and burden have been extensively studied, there is limited information on the development of relationship strain. To explore how relationship strain occurs between caregivers and care recipients, 8 focus groups (N=62) and 8 semi-structured telephone interviews were conducted with caregivers in Los Angeles, inquiring about relationship quality with their care recipients and when frustration and anger occurs. Inductive coding was used to create coding schemas. Findings showed that most caregivers reported relationship strain occurring after taking on the caregiving role, and frustration and anger arose when providing ADLs, especially during bathing and toileting. Although these caregivers had initially experienced strain in their relationships, a recurring theme that emerged was that they developed strategies to decrease frustration and anger and improve the quality of their relationships with their care recipients. Direct communication with caregivers is important in designing a structured and effective intervention. These findings help inform an intervention for new caregivers to help them identify what can lead to relationship strain, as well as teach them reliable strategies to manage frustration and anger towards their care recipients.

### SESSION 1360 (POSTER)

### FRAILTY, FALLS AND FALL PREVENTION

### THE EFFECT OF YOGA ON FALL PREVENTION OUTCOMES IN ADULTS 50 YEARS AND OLDER: A SYSTEMATIC REVIEW OF THE LITERATURE

Chinelo K. Nsobundu,[1] Margaret J. Foster,[2] and Yan Hong[1], *1. Texas A&M University - School of Public Health, College Station, Texas, United States, 2. Texas A&M University, College Station, Texas, United States*

Falls constitute a multitude of injuries irrespective of age. To combat these challenges, older adults are encouraged to engage in recreational activities. Yoga has been identified as an effective physical activity to promote mobility and balance for older adults. This study aims to systematically review the literature about yoga as a fall prevention intervention and synthesize the outcomes. Major databases (Ovid Medline & CINAHL) were searched for relevant articles. Studies were included if they met the criteria of 1) being a face to face yoga program, 2) aimed to recruit participants 50 years or older, and 3) reported at least one fall-related outcome (e.g., balance, mobility, fear of falling) as a result of the yoga program. 57 studies were identified: 32 from Ovid Medline and 25 from CINAHL. After removing the duplicates and applying a strict inclusion and exclusion criteria, 11 articles were included in the final analysis. A detailed synthesis of the results will be presented and quality assessment of included articles will be performed using the Modified Downs and Black checklist which appraises the methodological quality of both randomized and non-randomized studies. More research is needed to understand the impact of yoga in preventing falls among older adults at least 50 years of age. Additionally, research should establish a gold standard index that identifies which specific yoga programs ( based on type- individual vs. group; hatha, iyengar, kundalini, ashtanga, and etc.; frequency, and duration) have an enhanced effect on fall prevention.

### ASSOCIATION BETWEEN COGNITION AND FALL RISK BASED ON THE STEADI ALGORITHM: PROJECT VIBE

Tiffany F. Hughes,[1] Cara Carramusa,[1] and Daniel J. Van Dussen[1], *1. Youngstown State University, Youngstown, Ohio, United States*

Falls are a growing concern among older adults with estimates that one in four fall each year. Older adults who experience a fall are at higher risk for poor health outcomes that threaten independence and increase risk of death. Impairment in cognitive function is known to be associated with greater fall occurrence; however, cognitive testing is not an integral part of clinical fall risk assessment. The purpose of this study is to examine cognitive performance in relation to fall risk level and its components determined using the Stopping Elderly Accidents, Deaths, and Injuries (STEADI) algorithm. One hundred eight community dwelling older adults (mean age 79(SD 7.3) years, 76% women, and 56% college or higher education) were included. Cognition was assessed with the Montreal Cognitive Assessment (MoCA; >= 26 normal). The STEADI algorithm was used to assess fall risk (low vs. moderate/high) based on the Stay Independent screening (>= 4), impairment in gait (Timed Up and Go (TUG)), strength (30-second chair stand), and balance (4-stage balance), and number of falls (>= 2). Associations between cognition and fall risk and its components were assessed using logistic regression adjusting for age, gender, and education. Normal cognitive status was marginally associated with lower likelihood of moderate/high compared to low fall risk (OR 0.42, 95% CI 0.17-1.04), and with a lower likelihood of TUG impairment (OR 0.22, 95% CI 0.07-0.66). These results suggest cognitive status may contribute important information about older adults' fall risk and should be considered an integral part of fall risk assessment.

### FALLS AND MOTOR VEHICLE COLLISIONS: A LONGITUDINAL INVESTIGATION OF OLDER DRIVERS

Caitlin N. Pope,[1] Pariya Fazeli,[2] Tyler R. Bell,[3] Meghana Gaini,[2] Sylvie Mrug,[2] and Karlene Ball[2], *1. Center for Injury Research and Policy, The Research Institute at Nationwide Children's Hospital, Columbus, Ohio, United States, 2. University of Alabama at Birmingham, Birmingham, Alabama, United States, 3. Pennsylvania State University, State College, Pennsylvania, United States*

Longitudinal research is needed to better understand mobility and aging, as falls and motor vehicle collisions (MVCs) are the top two leading causes of unintentional injury-related deaths for adults 65 and older in the United States. Using a longitudinal sample of older adults, prior falls were assessed as a predictor and moderator of the rate of subsequent MVCs over a 15 year time period. Using a 15-year longitudinal sample of 1,911 older adults recruited from three Maryland State Motor Vehicle Administration (MVA) sites, we conducted group differences and Generalized Estimating Equation (GEE) Poisson regressions. Individuals who reported a fall at baseline were more likely to be female, older, have poorer physical functioning, and reported more situational driving avoidance at baseline compared to those who did not report a fall. Females who reported a fall at baseline had a 2x greater risk rate of subsequently reporting a MVC over the 15 year time period than males. Furthermore, individuals, irrespective of gender, with a prior fall at baseline who drove more days per week over the 15-year time span had a 23% higher risk rate of a subsequent MVC. The current findings further the discussion on aging and mobility as it offers a longitudinal perspective on the association between falls and MVCS. These findings promote the utility of investigating non-traditional driver screening methods to identify drivers who may be at an increased rate for further driving difficulties.

### FRAILTY IN REACH II CAREGIVERS: A SECONDARY DATA ANALYSIS

Jeananne Elkins,[1] and Patricia C. Griffiths[2], *1. Brenau University, Gainesville, Georgia, United States, 2. Emory University School of Medicine, Atlanta, Georgia, United States*

Frailty, a reduction in reserve capacity in people who are otherwise considered healthy, affects between 9 and 13% of adults who are older. Frailty is a poorly understood syndrome; however, frailty is correlated with negative CV procedure outcomes, falls and institutionalization. Little is known about frailty in caregivers. A secondary data analysis was conducted using the REACH II publicly available dataset and the Groningen Frailty Index (GFI). At consent two percent of REACH II caregivers had difficulty going to the toilet while 11% had difficulty walking outdoors. More than 1/3 had hearing and vision losses. 75% felt sad or dejected. 82% were taking more than 4 medications. Based on their calculated GFI, between 61% and 64% of the REACH II caregivers were frail. Frail caregivers and their care recipient were less likely to go to the emergency department (-0.110 coefficient; p = 0.004 95% CI -0.184 -0.035) and were less likely to be hospitalized overnight during the past 6 months (-0.121 coefficient; p=0.004; 95% CI -0.203 -0.040). Frailty is an under-recognized syndrome in caregivers. Little is known about the impact of frailty on the caregiving dyad; however, ED utilization and hospitalization was decreased in these caregivers and their care recipients. This decrease may imply a delay in seeking care; and, in fact, lead to worse health outcomes for the dyad. With the aging of Baby Boomers and the continued dependence for long term care delivered by unpaid caregivers, implementation of programs to prevent and treat frailty in caregivers is essential.

### LOWER BACK PAIN AFFECTS MOBILITY-RELATED INJURIES IN OLDER ADULTS

Tyler R. Bell,[1] Pariya Fazeli,[2] Caitlin N. Pope,[3] Michael Crowe,[2] and Karlene Ball[2], *1. Pennsylvania State University, State College, Pennsylvania, United States, 2. University of Alabama at Birmingham, Birmingham, Alabama, United States, 3. Center for Injury Research and Policy, The Research Institute at Nationwide Children's Hospital, Columbus, Ohio, United States*

Pain causes functional limitations and might elevate risk for mobility-related injuries in older adults. For this reason, we examined the longitudinal impact of lower back pain on the likelihood of MVCs and falls. Between 1998 and 1999, participants (ages >55 years) completed cognitive and physical measures at three Motor Vehicle departments. Participants then completed a telephone interview (n=1,248) assessing yearly health complications and injuries, which continued annually for 14 years. Separate longitudinal models examined the relationship between lower back pain and MVC and fall likelihood while controlling for demographics and mobility. Overall, those with lower back pain were twice as likely to have a fall than unafflicted peers (95%CI:1.69-2.47) and odds of MVC was just beyond statistical significance (95%CI: 0.97-1.94). In persons with lower back pain, problems in lower-limb function, divided attention, and task-switching were associated with MVCs whereas problems in lower-limb function were related to falls. Addressing limitations from pain might reduce mobility-related injury in older adults.

### THE FEASIBILITY OF USING COMMUNITY PHARMACISTS TO COUNSEL OLDER ADULTS ON FALL PREVENTION

Renae L. Smith-Ray,[1] Tanya Singh,[1] Michael W. Suwalski,[2] and Michael Taitel[3], *1. Walgreens Center for Health and Wellbeing Research, Deerfield, Illinois, United States, 2. Walgreen Co., Deerfield, Illinois, United States, 3. Walgreens Center for Health and Wellbeing Research, Deerfield, Illinois, United States*

Despite recognition as a serious public health problem, older adults falls increased 30% between 2007-2016. Numerous evidence-based fall prevention programs exist, but may have inadequate reach. Pharmacists are highly trained and accessible clinicians who have potential to counsel on fall prevention. This study describes the reach of a fall prevention outreach conducted by a large national pharmacy chain in partnership with local area agencies on aging (AAAs). On August 7, 2018, the pharmacy chain held an outreach during which older patients were incentivized to speak with pharmacists about their fall risk and prevention strategies. In Ohio, AAAs provided pharmacists additional support and availability of AAA fall prevention programs. A random sample of pharmacists was sent a follow-up survey to assess the program's reach, except in Ohio where all pharmacists received the survey. Response rates were 41% (N=111) and 59% (N=160) in Ohio and non-Ohio states, respectively. We estimate that pharmacists discussed fall prevention with an additional 57,642 on 8/7/2018. The difference in older patients counseled on fall prevention on 8/7/2018 vs. a typical day was significantly greater (p=0.03) for Ohio pharmacists ($\mu$=9.28) compared to non-Ohio pharmacists ($\mu$=5.94). The majority of pharmacists in Ohio and non-Ohio states were moderately or extremely confident in their ability to discuss fall prevention with older patients (69.82% vs. 72.72%) and play an important role in fall prevention (59.75% vs. 54.54%). This study demonstrates the feasibility of utilizing community pharmacists, in partnership with AAAs, to reach large numbers of older adults to counsel on fall prevention.

### FRAILTY MEDIATES SENILITY IN MEXICAN AMERICANS

Raymond F. Palmer,[1] Donald R. Royall,[2] Brian Downer,[3] and Kyriakos Markides[4], *1. Department of Family & Community Medicine, The University of Texas Health Science Center at San Antonio, San Antonio, Texas, United States, 2. Departments of Psychiatry, Medicine and Family & Community Medicine, The University of Texas Health Science Center at San Antonio, San Antonio, Texas, United States, 3. University of Texas Medical Branch, Galveston, TX, Galveston, Texas, United States, 4. University of Texas Medical Branch, Galveston, Texas, United States*

The latent construct "d" (for "dementia") offers a continuously distributed transdiagnostic dementia severity metric. Age is significantly associated with "d". We test whether frailty mediates age's effect on 6 year prospective change in dementia severity in Mexican-Americans (MA), using data from the Hispanic Established Population for Epidemiological Studies in the Elderly (HEPESE). Age was regressed onto the 6yr prospective slope of change in "d" in N = 880 [mean age = 77.4 (6.1) at wave 3]. Change in "d" was estimated by a latent growth curve (LGC) indicated by latent cognitive measures across three HEPESE waves (i.e., 3, 5 and 6). "Frailty" was assessed by a modified version of Fried et al.'s construct observed at wave 5, and was tested as a mediator of age's association with change in "d". The mediation effect was estimated by MacKinnon's method. "d" at each wave, and the LGC of change in "d" all had acceptable model fit (e.g. RMSEA <.05). Age was significantly associated with change in "d". 51% of their association was explained by frailty. Frailty mediates the majority of age's association with dementia severity. Not only does this support the existence of a cognitive "frailty" syndrome in MA, it also implicates an effect of frailty on intelligence (as "d" is derived from Spearman's general intelligence factor "g"). Their association may be mediated by blood-based serum biomarkers, including somatomedins, which may offer targets for the treatment and /or prevention of senility in frail elderly persons.

### MEDIATING EFFECT OF GRIP STRENGTH TRAJECTORY ON THE ASSOCIATION BETWEEN EXERCISE AND WEAKNESS: A DYADIC APPROACH

Susanna Joo,[1] Hye Won Chai,[2] and Hey Jung Jun[1], *1. Yonsei University, Seoul, Korea, Republic of, 2. The Pennsylvania State University, University Park, Pennsylvania, United States*

This study aimed to explore the mediating effect of grip strength trajectory on the longitudinal association between regular exercise and weakness in grip strength using a dyadic approach. We used six waves of the Korean Longitudinal Study of Aging (KLoSA) collected every two years from 2006 to 2016. The sample was middle and old-aged Korean couples who participated in all six waves of the survey (N=1,967). The outcome variables were husbands' and wives' grip strength at Wave 6, coded as a binary variable (0=clinically weak, 1=normal). The mediating variables were husbands' and wives' trajectories of grip strength across Waves 1 and 5. Independent variables were three dummy variables indicating couple's participation in regular exercise (both engaged in regular exercise, only husband did, only wife did). Reference group was both not doing regular exercise. Results showed several significant mediational pathways. For husbands, engagement in regular exercise of both spouses and only husband were associated with higher grip strength at Wave 1, and slower decline in grip across waves was related to lower likelihood of having a clinically weak grip strength at Wave 6. As for wives, engagement in exercise of both spouses was associated with higher grip strength at Wave 1 which in turn was related to lower likelihood of having a clinically weak grip strength at Wave 6. These results suggest longitudinal dyadic processes through which engagement in regular exercise affects weakness in grip strength among older Korean couples.

### MOTIVATIONAL INTERVIEWING TO ENGAGE OLDER INPATIENTS IN FALL PREVENTION: PILOT RANDOMIZED CONTROLLED TRIAL

Hiroko Kiyoshi-Teo,[1] Kathlynn Northrup-Snyder,[1] Elizabeth Eckstrom,[1] Debbie Cohen,[1] Nathan Dieckmann,[2] and Sydnee Stoyles[1], *1. Oregon Health & Science University, Portland, Oregon, United States, 2. Oregon Health & Science University School of Nursing, Portland, Oregon, United States*

Motivational Interviewing (MI) is an evidence-based approach for fostering behavior change and holds potential to engage patients in behavior change related to fall prevention. A two-arm, unblinded, pilot randomized controlled trial was conducted in a hospital setting to determine the feasibility (recruitment and retention), establish suitable procedures for the intervention (duration and quality of MI), and to test study measurements (fear of falling, importance and confidence related to fall prevention, patient activation, fall prevention behaviors, and fall rates). Participants were high fall risk older inpatients (age ≥ 65). The intervention arm received MI at one time during hospitalization in addition to routine hospital fall prevention intervention. The control arm received the routine hospital care for fall prevention only. Measures were collected at baseline, 2-days, 1-week, 1-month, and 3-month. A total of 120 inpatients were contacted by the study team and 67 were randomized: intervention arm (n=36) and control arm (n=31). Approximately 66% of participants completed the study at the 3-month data collection and MI intervention took an average of 21 minutes and was of adequate quality. The intervention group reported less fear of falling after the MI intervention and maintained fall prevention behaviors over time (p<.05). The study identified that MI for fall prevention at a hospital setting was feasible to deliver and provided insights into suitable study procedures and beginning evidence for a positive impact of MI.

### THE BUILT ENVIRONMENT INFLUENCED THE INCIDENCE OF FALLS IN OLDER ADULTS LIVING IN THE UNDERSERVED COMMUNITY

Rie Suzuki,[1] Jennifer Blackwood,[2] Shailee Shah,[1] Sabah Ganai,[1] and Kimberly Warden[3], *1. Department of Public Health and Health Sciences, University of Michigan-Flint, Flint, Michigan, United States, 2. Physical Therapy Department, University of Michigan- Flint, Flint, Michigan, United States, 3. Hamilton Community Health Network, Inc., Flint, Michigan, United States*

The built environment is commonly cited as a facilitator to local walking. Although health promotion programs targeting physical activity are available, few studies have investigated the associations of the perceived neighborhoods with the incidence of falls in the minority communities. Hence, the purpose of this preliminary study was to understand whether the perceived built environment influenced the fall experiences in older adults living in the underserved community. The preliminary cross-sectional survey was conducted at the regional health clinic in Flint, MI. Descriptive statistics and analysis of variance (ANOVA) were performed using SAS v8.4. The eligibility criteria included over 65 years old and Flint residents. Of 132 participants, the mean age was 69.75 (SD=5.00). The majority were female (68%), African Americans (80%), single, divorced or widowed (80%), and > GED (84%). The ANOVAs supported that "had fallen in the past year" was associated with "stores are within easy walking distance," "easy to walk to a transit stop" and "there is a dirt strip that separates the streets from the sidewalks." The fall experience was more likely to associate with the sedentary lifestyle and the comorbidities such as diabetes, fatigues, muscle spasms, and chronic pain. To summarize, the built environment increased the incidence of falls in the past year. Those who had fallen had poor health conditions. Further studies are needed for older adults to engage in physical activity. It is essential to develop the age-friendly support systems and accommodations to local walking in this community.

### EARLY FRAILTY PHENOTYPES AND PREDICTIONS OF COGNITIVE AGING: EVIDENCE FROM THE VICTORIA LONGITUDINAL STUDY

Linzy Bohn,[1] Yao Zheng,[1] G. Peggy McFall,[1] and Roger A. Dixon[1], *1. University of Alberta, Edmonton, Alberta, Canada*

Frailty is an aging condition that reflects multisystem decline. A prominent approach to frailty assessment is to create an index, whereby responses across multiple indicators of aging systems are summed to create a single score. These studies indicate that frailty is associated with adverse aging outcomes (e.g., mortality, dementia). We employ a data-driven approach to detecting and differentiating emerging frailty phenotypes and examine their associations with non-demented cognitive aging trajectories. Participants (n = 653; M age = 70.6, range 53-95) were community-dwelling older adults from the Victoria Longitudinal Study. Participants contributed (a) baseline data for 30 frailty-related items representing deficits across 7 domains (e.g., instrumental and cardiovascular health) and (b) longitudinal data for latent variables of executive function, speed, and memory. For each participant, we calculated the proportion of deficits present in each frailty-related domain and submitted these data to a latent profile analysis (LPA; Mplus 7.0). We used latent growth modeling (LGM) to test these frailty phenotypes for prediction of cognitive performance and decline. LPA results revealed three profiles, one large normal low-frailty profile and two emerging frailty phenotypes. Whereas the latter represented profiles of individuals with respiratory-type frailty (i.e., marked impairment in respiratory function; 7%) and mobility-type frailty (i.e., marked impairment in mobility function; 9%), the former featured limited impairment across frailty domains (83%). Findings from LGM indicated that these profiles were differentially related to cognitive performance and decline. Data-driven approaches can help detect early differentiation of frailty profiles and contribute to personalized intervention.

### SOCIAL ISOLATION AND FALLS RISK AMONG COMMUNITY DWELLING OLDER ADULTS: THE MEDIATING ROLE OF DEPRESSION

Jeffrey Burr,[1] and Lien Quach[2], *1. University of Massachusetts Boston, Boston, Massachusetts, United States, 2. Veterans Administration, Boston, Massachusetts, United States*

Relatively little is known about the relationship between social isolation and the risk of falls among older adults. Yet, a considerable amount of research demonstrates that lack of sufficient social relationships, broadly defined, represents a modifiable risk factor for many indicators of well-being in later life. This study examines the association between two types of social isolation and the risk of falls. The study also examines whether depression mediates the association between social isolation and risk of falls. Longitudinal data from the Health and Retirement Study (2006-2012) were collected from community-dwelling participants aged 65 and older (N=8,464). The outcome variable was number of falls self-reported over the observation period. Independent variables included perceived isolation (feeling lonely, perceptions of social support), social disconnectedness (e.g., having no friends or relatives living nearby, living alone), and number of depressive symptoms. Results from regression models indicated that social disconnectedness was associated with a 5% increase in the risk of falls (IRR=1.05, 95% CI=1.01-1.09). Perceived social support was associated with a 21% increase in the risk of falls; when examined together, perceived social support and loneliness were associated with a combined 37% increase in falls risk. Depression was associated with a 47% increase in falls. Depression mediated the association between perceived isolation and falls. Further, perceived isolation mediated the association between social disconnectedness and falls. Reducing perceived social isolation and social disconnectedness may be an avenue for designing interventions to reduce the risk of falls, especially for older adults with depression.

### FEASIBILITY OF A LOW-DOSE FRAILTY PREVENTION INTERVENTION AMONG OLDER AFRICAN AMERICANS

Heather A. Fritz,[1] Wassim Tarraf,[1] and Pragnesh Patel[1], *1. Wayne State University, Detroit, Michigan, United States*

Older African Americans (OAA) are at high risk for becoming frail in later life. Interventions can reverse or delay frailty, yet OAA have largely been excluded from frailty intervention research. Many interventions are also time and resource intensive, making them inaccessible to socially disadvantaged OAA. We present results of a feasibility trial of a low dose frailty prevention intervention among 60 community-dwelling, pre-frail OAA aged 55+ recruited from a primary care clinic between June 1st and October 31st 2018. Using a 2-arm RCT, participants were assigned to the intervention, which was delivered by an occupational therapist (OT) and comprised of four sessions over four months (an OT evaluation, and sessions on healthy dietary practices, increasing physical activity, and maintaining a healthy lifestyle), or enhanced usual care (publicly available information about healthy lifestyle, home safety, and local elder services). Feasibility criteria were set a priori at 75% for participant retention (including attrition due to death/hospitalization), 80% for session engagement, 2 participants/week for mean participant accrual, and 90% for program satisfaction. Participants were 65% female with an average age of 76.58 years, 51.67% of which lived alone and 51.67% lived off of less than 15K per year. Feasibility metrics were met. The study recruited 2.5 participants per week and retained 75% of participants who attended 95% of scheduled sessions. Mean satisfaction scores were 93%. The intervention was feasible to deliver. Qualitative findings from exit interviews suggested changes to the program dose, structure, and content that could improve it for future use.

### AM I FRAIL, LOVE? YES, I SUPPOSE I AM: WHAT 10 OLDER PEOPLE CAN TELL US ABOUT LIVING WITH FRAILTY

Oliver M. Todd,[1] Andrew Clegg,[1] and Mary Godfrey[1], *1. University of Leeds, Bradford, Yorkshire, United Kingdom*

We sought to explore what matters in later life with frailty from an older persons perspective. Between March and May 2018, we recruited ten people, purposively sampled from the CARE75+ ageing cohort study. Interviews took place at the participant's own home in two sittings, each 45 minutes long. Interviews were semi-structured, used narrative techniques based on a topic guide developed with a patient representative. We used systematic analysis of the narrative experience to identify meaning in the context of an individual's time, space and history. Participants had a mean age of 84 years (range 77 to 93), half were women, and three were interviewed with their care-givers. All had moderate or severe frailty: mean frailty index 0.36 (range 0.25 to 0.47); mean Fried score 4 (range 3 -5). Half knew hunger as children; most grew up in large families and left school early; two survived TB in early life; all lived through or were affected by war. The term frailty was: never voluntarily used; described negatively and in value laden terms; seen better in others than themselves. Decision making was best delegated to doctors who knew you and your family over time. Narratives focused on health events of a spouse; symptoms featured more than diagnoses. Survivorship, reciprocity and community were sustaining values. To engage elders in shared decision making we learnt to consider influences of cohort, of people closest to them; and to describe rather than declare someone to be frail, in terms that are real to them.

### SESSION 1365 (POSTER)

### GERONTOLOGY AND GERIATRICS EDUCATION

### INTERPROFESSIONAL SOCIALIZATION AND VALUING SCALE FOR HEALTH PROFESSIONAL STUDENTS WORKING WITH OLDER ADULTS

David Cicala,[1] Nicholas Ang,[1] Jennifer Mendez,[2] Shanique Brown,[1] and Martha Schiller[3], *1. Wayne State University, Detroit, Michigan, United States, 2. Wayne State University School of Medicine, Detroit, Michigan, United States, 3. Wayne State University, Detroit, United States*

Interprofessional education allows students from two or more professions the opportunity to collaboratively practice patient centered care. Given the importance of interprofessional education in helping individuals become effective team members and understanding their value to a healthcare team, there is a need to evaluate the experiences. The Interprofessional Socialization and Valuing Scale (ISVS), aims to measure self-perceived experiences with interprofessional collaborative teamwork, including the ability, value, and comfort in working with others. The Wayne State University Interprofessional Team Visit (IPTV) program is an older adult home visit program that places pharmacy, social work, occupational therapy, nursing, and medical students in teams. Students form teams of 3 different disciplines and interview the older adult to assess various aspects of health and wellbeing. In order to evaluate the interprofessional educational experiences of the students, they are given a pre- and post- survey utilizing the ISVS tool. 18 questions pertaining to perceptions of what students have learned about working with professionals from other disciplines. Students respond to each statement using a 7-point scale with 1 = "Not at All" and 7 = "To a Very Great Extent." Statistical analysis is conducted in order to compare pre- to post-surveys and also assess differences between groups. It is found that ISVS scores increase from pre- to post-survey, second year medical students and third year pharmacy students feel more comfortable working in teams, and teams consisting of these two have higher average scores.

### INTERDISCIPLINARY CURRICULUM FOR THE CARE OF OLDER ADULTS: CREATING NETWORKS OF COLLABORATIVE LEARNERS

Pamela Yankeelov,[1] Anna Faul,[1] Joe D'Ambrosio,[1] and Samantha G. Cotton[1], *1. University of Louisville, Louisville, Kentucky, United States*

Serving older adults with multiple chronic conditions and variable social, emotional, or physical support effectively within the primary care setting requires an interdisciplinary approach to care. Our GWEP program has developed an interprofessional education center that educates and prepares students and professionals from social work, medicine, nursing, dentistry, pharmacy, and community health partners, to function within a transformed integrated patient-centered geriatric primary care and community-based service delivery system. Learners from multiple disciplines attend a face-to-face Interdisciplinary Case Management Experience (ICME) session lasting 2.5 hours. Sessions include learners from each discipline and, if possible, at least one community practitioner in small groups of 6–8 learners at each table facilitated by 1 faculty member. Approximately 1,200 learners have received the curriculum. To evaluate the program, Kirkpatrick's Training Evaluation Model was used to determine if learners were satisfied with the content, skilled, and confident in their abilities to utilize the curriculum. Learners completed a satisfaction survey after taking each module, along with an interdisciplinary geriatric care knowledge test and self-efficacy test before and after taking each module to measure learning outcomes. Analysis showed that learners, irrespective of discipline, were satisfied with the program. All disciplines showed a significant increase from pre- to posttest for all 5 online modules achieving a mean post-knowledge score of 85% across all 5 online training modules. All disciplines experienced significant differences in their self-efficacy with working on interdisciplinary teams from pre to post ICME. Implications for future interprofessional curriculum will be discussed.

### PROVIDER AND SYSTEM IMPACTS OF THE UW ECHO IN RURAL AND FRONTIER CARE TRANSITIONS

Catherine Carrico,[1] Tonja Woods,[1] Robin A. Barry,[1] Kevin Franke,[2] and Christine McKibbin[1], *1. University of Wyoming, Laramie, Wyoming, United States, 2. Mountain Pacific Quality Health, Casper, Wyoming, United States*

Background: The Project ECHO model utilizes a hub and spoke approach through which a team of experts co-mentors local providers in the management of complex cases while disseminating information about best practices and evidence-based care. Project ECHO is a promising model for improving patient care through transformation of the care delivery system. The UW ECHO in Rural and Frontier Care Transitions created an online community of practice comprised of local care coalitions dedicated to improving care transitions in Wyoming and Montana. This ECHO network provided a unique opportunity to support system- and provider-level implementation of best practices in care transitions. Methods: Semi-structured interviews were conducted with thirty ECHO attendees following participation in an ECHO session as either a participant or case presenter. Thematic analysis was used to analyze interview data. Results: Two overarching themes emerged 1) impact of the ECHO on the provider or healthcare team and 2) impact on the system. Participants indicated that the impact on the provider/healthcare team included an increased sense of community, increased awareness of community resources, increased knowledge of care transition strategies, and increased confidence in implementing best practices. Additionally, providers indicated increased utilization of community resources. Systemic impacts included increased involvement of interprofessional team members in patient care and utilization of ECHO recommendations to present systemic interventions and changes to colleagues, administration, and leadership. Conclusions: This ECHO network had a particularly strong impact on the provider and healthcare team as participants increased their knowledge, confidence, and use of best practices in care transitions.

### A MIXED-METHODS EVALUATION OF A NURSE-LED INTERPROFESSIONAL INTERVENTION FOR COMMUNITY-DWELLING OLDER ADULTS

Anna-Rae L. Montano[1], *1. University of Connecticut, Storrs, Connecticut, United States*

This study aims to assess the relationship between an Interprofessional Collaborative Practice (IPCP) intervention for community-dwelling older adults, Geriatric Outreach and Training with Care! (GOT Care!), and the observed 26% reduction in Emergency Department (ED) visits for the 51 older adults who participated. This study utilized a convergent parallel mixed-methods design that included a historical prospective matched cohort study and semi-structured interview surveys. The 51 GOT Care! participants were retrospectively matched to 51 control participants on several variables and ED visits were assessed for each group at 6, 8 and 12 months. Mixed effects generalized linear modeling, with a Poisson response, a log link function, and a random effect for pair, was conducted to analyze the quantitative data. Stakeholders, including hospital administrators and faculty clinicians, of GOT Care! responded to electronic semi-structured interview surveys regarding the relationship between GOT Care! and the observed reduction in ED visits. Content analysis of the responses was completed. The themes from the stakeholder interviews were integrated into the historical prospective matched cohort study to further explore the relationship between GOT Care! and the reduction in ED visits. The results of this study are still pending. This study has implications regarding the utilization of an IPCP model within an academic-practice partnership in the aim of reducing ED visits for vulnerable community-dwelling older adults. Hospital administrators can also utilize the results of this study to gauge the value of IPCP models within their hospital systems.

### HOW MEDICAL EDUCATION ALLEVIATED ETHICAL DILEMMAS IN RESIDENTS DURING END-OF-LIFE FAMILY MEETINGS IN THE SOUTH TEXAS

Jessica G. Martin,[1] Andrya Rivera-Burciaga,[2] Cesar Gutierrez,[1] and Andrew Dentino[1], *1. University of Texas Rio Grande Valley, Edinburg, Texas, United States, 2. DHR Health, Edinburg, Texas, United States*

The UTRGV DHR Internal Medicine Program conducted a study addressing end of life (EOL) care focused on our Hispanic community in regards to communication and trust between patients, caregivers, and healthcare providers. Our residents train at a community hospital which cares for an 89% Hispanic population of 1.2 million, spanning over 4 counties of the Rio Grande Valley. Trainees are often involved in family meetings while treating hospitalized, terminally ill patients. Although family meetings are a standard approach in palliative care, Hispanic family meetings tend to occur more often and with a larger, extended family unit. Our intent was to educate our residents to initiate conversations about EOL care choices promoting delivery of patient-centered, family oriented care utilizing culturally appropriate information regarding EOL issues. Baseline surveys were provided to all 39 trainees which assessed anxiety, incompetence, and communication skills in delivering bad news during family meetings. An advanced care planning process was implemented over 3 months with a goal to engage patients in EOL conversations, initiation, and completion of advanced directives. Residents received weekly training on interactive methods and ethical concepts including group discussions, role-playing, and demonstrations which were culturally and linguistically appropriate. We found that physician competence in conducting Hispanic family meetings is vital. Residents completed a post-training survey resulting in 100% improved attitudes and behaviors such as confidence, satisfaction, caring and empathy. They felt more comfortable and prepared to speak to a larger family unit who was likely to ask a lot of questions and request multiple meetings.

### FOSTERING INTERPROFESSIONAL COLLABORATION IN HISPANIC GERIATRIC PATIENT CARE: NEW GME PROGRAM AND PALLIATIVE TEAM

Jessica G. Martin,[1] Andrya Rivera-Burciaga,[2] Cesar Gutierrez,[1] Andrew Dentino,[1] and Vianis Bravo[1], *1. University of Texas Rio Grande Valley, Edinburg, Texas, United States, 2. DHR Health, Edinburg, Texas, United States*

Hispanic minorities have a higher incidence of chronic disease which may result in increased hospitalizations and life-threatening illness. Our growing geriatric population led our community hospital to create a dedicated Palliative Care department, an interprofessional team of physicians, nursing, pharmacy, social work, counselors, and chaplains whose collaborative practice has improved outcomes thus strengthening healthcare delivery. When our new medical school established graduate programs, including Internal Medicine residency and Hospice/Palliative Medicine fellowship, our team embraced the opportunity to optimize our palliative service through enhanced interprofessional care. We created a geriatric rotation on July 1, 2018 in which 60% of residents worked with palliative care professionals as a consult team bringing together the inpatient resident service and palliative interdisciplinary team. This collaborative model allowed the palliative team to interface with our trainees and teach them to identify the range of needs of older adults early on in their care. Residents reported 100% satisfaction on evaluations, specifically on clinical training, goal fulfillment, and team support. Our learners valued the opportunity to learn with and from other healthcare professionals. Supervising providers also felt that working with residents was beneficial to their practice habits (i.e., providing evidence-based practices, application of guidelines), which offered them a more holistic approach in caring for patients and families. The interprofessional collaboration between a community hospital and medical school to educate and train clinicians who care for individuals with advanced illness has fostered confidence, trust, mutual respect, open communication and effective sharing of critical information for both clinicians and patients.

### ASSESSMENT OF PALLIATIVE CARE TRAINING NEEDS FOR CERTIFIED NURSING ASSISTANTS

Jinsook Kim,[1] and Jennifer A. Gray[1], *1. Northern Illinois University, DeKalb, Illinois, United States*

It is unknown whether certified nursing assistants (CNAs) receive up-to-date palliative care training through continuing education. Also unclear is whether existing trainings cover the issues that CNAs encounter at work or are tailored to CNAs' learning styles and preferences. This study aimed to assess the palliative care training needs of CNAs working at skilled nursing facilities (SNFs) in northern Illinois. CNAs (n=127) from 6 SNFs completed an online survey regarding palliative care training experience, perceived needs for palliative care training, and demographic and work-related information. The majority of the participants were female (88%) and White (58%) or African American (20%). On average, participants were 34 years old and worked for 8 years in the field. Four out of five preferred a training 90 minutes or shorter. Approximately one half preferred in-person training, and the rest preferred a hybrid (32%) or online delivery (19%). Discussions and videos were most preferred in training, while quizzes and mobile apps were least preferred. CNAs who worked longer in the field were less likely to have received training than their counterparts. The least-trained areas included utilizing advance directives and discussing death with patients. The most needed training areas were talking to a patient who wants to hasten death and addressing the complex needs of dying patients. The results indicate a relative deficiency of palliative care training among CNAs who have worked longer in the field. Training areas needing more attention include advance directives, discussing death with patients, and the complex needs of dying patients.

### EHEALTH PROGRAM BASED ON THE INFORMATION-MOTIVATION-BEHAVIORAL SKILLS MODEL FOR THE ELDERLY: A PILOT STUDY

Eunjin Yang,[1] Sun Ju Chang,[2] Kyoung-eun Lee,[1] and Hyunju Ryu[1], *1. College of Nursing, Seoul National University, Seoul, Korea, Republic of, 2. College of Nursing & Research Institute of Nursing Science, Seoul National University, Seoul, Korea, Republic of*

Although the internet is helpful and considered a preferred channel to get health information, some vulnerable populations such as the elderly have a digital divide. The purpose of this study was to test the development and intervention of the eHealth program based on the Information-Motivation-Behavioral Skills (IMB) model for improving internet health information seeking, understanding and utilization behaviors in the elderly. This study was a single group pretest-posttest design, and the eleven elderly aged 67-87 (mean74.6, SD 6.9) participated in 5 session program at a senior welfare center from 25 January to 22 February 2019. Each theory-based constructs of IMB model such as computer/web knowledge ($p<.01$), attitude toward the internet health information usage ($p<.01$), and eHealth literacy ($p<.01$) was significantly improved at posttest than pretest. More than half of the participants (6 of 11) gave up on searching internet health information on pretest; however, all of the participants searched the internet health information accurately on the posttest. Regarding health information understanding, a significant difference was found ($p=.03$), and participants reported positive behavioral change after the program (6.54 ± 2.42). This pilot study indicated that the theory-based eHealth program might be an effective way to decrease a digital divide for the elderly. Therefore, the preliminary findings show promise for the use of the IMB model-based eHealth program as an intervention to improve internet health information seeking, understanding, and utilization behaviors in the elderly.

### MOBILITY EDUCATION FOR THE DIVERSE AGING COMMUNITY, CAREGIVERS, AND THEIR HEALTH CARE PROVIDERS

Chrysanne Karnick,[1] Ruth Manna,[2] Natalie Gangai,[2] Rosario Costas Muniz,[2] and Beatriz Korc-Grodzicki[2], *1. Memorial Sloan Kettering Cancer Center, New York, New York, United States, 2. Memorial Sloan Kettering Cancer Center, New York, United States*

Older adults can reduce fall risk in their homes and the community. Health care professionals (HCP) have a role in preventing falls. An interprofessional team of HCPs at a comprehensive cancer center created and delivered educational workshops to increase knowledge about falls prevention. Educational workshops were provided in community centers, libraries, places of worship and at local hospitals to medically underserved, diverse community members, caregivers and HCP. An Occupational (OT) and Physical Therapist (PT) taught three workshops together and the OT taught nine workshops. Workshops included fall prevention, home modifications, safe patient handling (SPH), and the role of OT/ PT in geriatric oncology care. Practical and culturally competent steps were emphasized, with translation of written materials and live interpretation provided as appropriate. Knowledge increase was assessed, and post-session qualitative data was collected. The mean age of community members was 68 years, of nurses was 42, and of caregivers 63. A majority of participants were female. 220 older adults completed surveys, 40 caregivers, and 11 registered nurses. The Falls Prevention workshops with unmatched (n=79) and matched data (n=140) showed significant improvements in knowledge [$t(135)=-3.33$, $p<0.001$; $t(139)=-4.03$, $p<.001$; respectively). Caregivers who participated in the SPH workshop improved their learning for the unmatched (n=12) and matched data (n=28) after participating in the workshops [$t(22)=-3.50$, $p=.002$; $t(27)=-3.95$, $p<.001$] respectively. For nurses, the change in scores from pre (M=.56) to post scores (M=.73) were significant ($t=-2.76$, $df=10$, $p=.02$). Caregivers and HCPs benefit from continued education to promote safer, holistic care for family members and patients.

### EFFECTS OF A SHARED DECISION MAKING INTERVENTION FOR OLDER ADULTS WITH CHRONIC CONDITIONS: AN OBSERVATIONAL STUDY

Ruth E. Pel-Littel,[1] Bianca Buurman,[2] Marjolein van de Pol,[3] Linda Tulner,[4] Mirella Minkman,[5] Wilma Scholte op Reimer,[6] and Julia van Weert[7], *1. Vilans, Utrecht, Utrecht, Netherlands, 2. Department of Internal Medicine, Section of Geriatric Medicine, Academic Medical Centre, University of Amsterdam, Amsterdam, Netherlands, 3. Department of Primary and Community Care, Radboud University Medical Center, Nijmegen,*

*Netherlands, 4. Medisch Centrum Slotervaart, Amsterdam, Netherlands, 5. Vilans, Utrecht, Netherlands, 6. ACHIEVE, Centre of Applied Research, Faculty of Health, Amsterdam University of Applied Sciences, Amsterdam, Netherlands, 7. Amsterdam School of Communication Research/ASCoR, University of Amsterdam, Amsterdam, Netherlands*

Shared decision making (SDM) in older patients is more complex when multiple chronic conditions (MCC) have to be taken into account. The aim of this research is to explore the effect of the evidence based implementation intervention SDMMCC on (1) the preferred and perceived participation (2) decisional conflict and (3) actual SDM during consultations. 216 outpatients participated in a video observational study. The intervention existed of a SDM training for geriatricians and a preparatory tool for patients. Consultations were videotaped and coded with the OPTIONMCC. Pre- and post-consultation questionnaires were completed. Participation was measured by the Patients' perceived Involvement in Care Scale (PICS). Decisional conflict was measured by the Decisional Conflict Scale (DCS). The patients mean age was 77 years, 56% was female. The preparatory tool was completed by 56 older adults (52%), of which 64% rated the tool as positive. The preparatory tool was used in 12% of the consultations. The mean overall OPTIONMCC score showed no significant changes on the level of SDM(39.3 vs 39.3 P0.98), however there were significant improvements on discussing goals and options on sub-items of the scale. There were no significant differences found in the match on preferred and perceived participation (86.5% vs 85.0% P 0.595) or in decisional conflict (22.7 vs 22.9 P0.630). The limited use of the preparatory tool could have biased the effect of the intervention. In future research more attention must be paid towards the implementation of preparatory tools, not only among patients but also among geriatricians.

### THE DEVELOPMENT OF AN INTERVENTION TO IMPROVE SHARED DECISION MAKING IN GERIATRIC OUTPATIENTS

Ruth E. Pel-Littel,[1] Julia van Weert,[2] Mirella Minkman,[3] Wilma Scholte op Reimer,[4] Marjolein van de Pol,[5] and Bianca Buurman[6], *1. Vilans, Utrecht, Utrecht, Netherlands, 2. Amsterdam School of Communication Research/ASCoR, University of Amsterdam, Amsterdam, Netherlands, 3. Vilans, Utrecht, Netherlands, 4. ACHIEVE, Centre of Applied Research, Faculty of Health, Amsterdam University of Applied Sciences, Amsterdam, Netherlands, 5. Department of Primary and Community Care, Radboud University Medical Center, Nijmegen, Netherlands, 6. Department of Internal Medicine, Section of Geriatric Medicine, Academic Medical Centre, University of Amsterdam, Amsterdam, Netherlands*

Shared decision making (SDM) contributes to personalised decisions that fit the personal preferences of patients. However, older adults frequently face multiple chronic conditions (MCC). Therefore, implementing SDM requires special features. The aim of this paper is to describe the development of an intervention to improve SDM in older adults with MCC. Following the Medical Research Council framework for developing complex interventions, the SDMMCC intervention was developed step-wise. Based on a literature review and empirical research we developed in a co-creation process with the end-users a training for geriatricians and a preparatory tool for older patients with MCC and informal caregivers. After assessing feasibility the intervention was implemented at two outpatient geriatric clinics in a pilot study (N=108). Key elements of the training for geriatricians include: developing skills how to involve older adults with MCC and informal caregivers in SDM and learning how to explore personal goals related to quality of life. Key elements of the preparatory tool for patients include: an explicit invitation to participate in SDM, nomination that the patient's own knowledge is valuable, invitation to form a partnership with the geriatrician, encouragement to share information about daily and social functioning and exploration of possible goals. Furthermore, invitation of informal caregivers to share their concerns. Through a process of co-creation both a training for geriatricians and a preparatory tool for older adults and their informal caregivers were developed, tailored to the needs of the end-users and based on the 'Dynamic model of SDM with frail older adults'.

### BELIEFS REGARDING GERIATRICS PRIMARY CARE TOPICS AMONG MEDICAL STUDENTS AND INTERNAL MEDICINE RESIDENTS

Dylan J. Jester,[1] Kathryn Hyer,[2] and Ross Andel[2], *1. School of Aging Studies, University of South Florida, Tampa, Florida, United States, 2. University of South Florida, Tampa, Florida, United States*

Our study evaluated and contrasted responses to 25 content areas essential to the primary care of older adults by medical students and residents, and identified attitudes toward aging amongst students and residents. One hundred and thirty-six medical students and 61 Internal Medicine residents completed a survey including the 25-item Geriatrics Clinician-Educator Survey and 18-item Images of Aging Scale. Students and residents rated importance and knowledge for content areas from 1 (low) to 10 (high). Gap scores reflecting the difference in ratings between importance and knowledge were calculated. The Images of Aging scale ranges between 0 (furthest from what you think) and 6 (closest to what you think). Results indicated that students and residents reflected similar beliefs about the importance of content areas, but students provided lower ratings in knowledge. Students revealed larger gap scores in areas that reflected general primary care (e.g., assess chronic conditions, medications), whereas residents revealed larger gap scores in areas that reflected specialists' expertise (e.g., driving risk, cognition, psychiatric symptoms). Attitudes toward older adults did not differ appreciably between students and residents. In sum, primary care topics applicable for any age demographic were rated as most important by first-year medical students and Internal Medicine residents. Topics relevant to older populations – particularly those requiring specialists' knowledge of or requiring sensitive discussion with older adults – were rated as less important and were less well mastered.

### EFFECTIVENESS OF A SIMULATION-BASED EMPATHY ENHANCEMENT PROGRAM WITH MINDFULNESS ON SOCIAL WORKERS

Areum Han,[1] and Taehee Kim[2], *1. Yonsei University, Wonju, Gangwon, Korea, Republic of, 2. Yonsei University Wonju Severance Christian Hospital, Wonju, Gangwon, Korea, Republic of*

Empathy enhancement programs and mindfulness-based practices may reduce care practitioners' burnout and stress while increasing satisfaction, caring efficacy, and well-being. No study has been conducted to measure the effectiveness of a simulation-based empathy enhancement program combined with mindfulness practice on professionals working with older adults living alone. This study, therefore, assessed the effectiveness of a simulation-based empathy enhancement program with a brief mindfulness practice session on social workers working with older adults living alone. This study was a quasi-experimental study involving 105 social workers in South Korea. The experimental group received a simulation-based empathy enhancement program with mindfulness practice, and the attention control group watched a 30-minute-long educational video about empathy. Data were collected prior to the intervention and at two weeks after the intervention using self-reported questionnaires measuring empathy, caring efficacy, psychosocial stress, compassion satisfaction, burnout, and secondary traumatic stress. The experimental group had significantly lower levels of psychosocial stress compared to the attention control group. Both groups showed significant improvements in empathy but in different empathy measures. Also, the experimental group only showed significantly lower levels of burnout and secondary traumatic stress after the intervention while the attention control group only showed significant improvements in compassion satisfaction and caring efficacy. Although between-group differences were found in psychosocial stress only, pre-and post-test differences in different outcome measures from experimental and attention control groups indicate limited but possible effectiveness of each of the empathy enhancement programs on people in caring professionals.

### EMPOWERING OLDER ADULTS IN HEALTHCARE SETTINGS: COMMUNICATION SKILLS TRAINING FOR SURGICAL RESIDENTS

Linda Roberts,[1] Charles Cornell,[1] Mathias Bostrom,[1] Sandra Goldsmith,[1] Titilayo Ologhobo,[1] Timothy Roberts,[2] and Laura Robbins[1], *1. Hospital for Special Surgery, New York, New York, United States, 2. NYU Langone Medical Center, New York, New York, United States*

Older adults often perceive themselves as stigmatized and powerless in healthcare settings. Communication with them is complicated by age-related issues and negative stereotypes about older adults and aging. It is therefore vital for physicians and surgeons, who encounter the most vulnerable elderly, to communicate successfully with this population, who wish to maintain quality and dignity in their lives. Successful patient communication leads to better recall of information, compliance, adherence to medications, satisfaction, and overall better outcomes. We developed a two-part training program comprised of small group interactive didactic sessions on aging issues with third year surgical residents, and workshop demonstrations given by the residents to a group of older adults, followed by a question and answer session. Residents were assessed using a 22-item pre–post questionnaire covering medical knowledge of aging, attitudes toward older adults, and personal anxiety about aging. Since its inception, the program has reached 88 residents and 711 older adults. For residents, knowledge scores ($p \leq 0.001$), six of nine attitude items ($p \leq 0.01$) and one of four anxiety items ($p \leq 0.001$) improved significantly. This is notable as well since attitudes and anxiety levels are attributes that are deep-seated and hard to change. For older adults, post surveys showed that 96% strongly agreed/agreed that residents had demonstrated sensitivity toward them and 96% were very satisfied/satisfied with the program. Our replicable, low-cost program enables residents to learn and realistically practice universal underlying communication skills in order to maintain effective and sensitive communication with this vulnerable population.

### SESSION 1370 (POSTER)

### INNOVATIONS IN CLINICAL GERIATRICS EDUCATION

### HOW TO PREPARE PHYSICIAN ASSISTANTS FOR THE CARE OF OLDER ADULTS? PARTNER WITH GERONTOLOGY

Jacqueline Eaton,[1] and Trenton Honda[1], *1. University of Utah, Salt Lake City, Utah, United States*

Approximately 80% of older adults have chronic illness which requires complex care. Primary care providers require special training to improve the care they provide older adults. As primary care shortages increase, and the older adult population swells, physician assistants (PA) will increasingly be relied upon to provide care and advocacy for older adults. The purpose of this presentation is to describe the development of a dual degree program that facilitates enhanced gerontological training for students pursuing a Masters of PA Studies degree. The Gerontology Interdisciplinary Program and the PA Program at the University of Utah collaborated to assess program objectives, competencies, and coursework, while identifying student interest in this dual MS venture. Students were interested in increasing their skills to meet the growing need for geriatric care while also saving time and money. In addition, it was important that the combined program of study did not overburden students and accommodated participants off-site. Faculty and administrative buy-in was sought from within departments and colleges. Revisions to the proposed program of study included altering course offerings, changing program start dates, and removing course overlap. A 30-month dual MS was developed that incorporates 87 PA Program credits, 25 Gerontology Credits, and 6 credits shared through practicum, geriatric content, and evidence based practice. Students who graduate will complete a Gerontology focused Masters Project that combines their work from the two programs. This dual MS program prepares students to be competitive in the job market, while also targeting an area of need in primary care.

### VIRTUAL REALITY MEDICAL EDUCATION PROJECT ENHANCES EMPATHY

Marilyn R. Gugliucci[1], *1. University of New England College of Osteopathic Medicine, Biddeford, Maine, United States*

Introduction: It is particularly important that innovative learning modalities are utilized to augment medical students' learning about empathy in relation to older adult health care. As the older population increases and lives longer, their health care utilization is predicted to increase dramatically. Methods: 1st year osteopathic medical students (N=174) at the University of New England were required to complete the National Network of Libraries of Medicine (NN/LM) New England Region (NER) grant funded Embodied Labs' "We Are Alfred" Virtual Reality (VR) module (15 min) and a pre/post-test. The students assumed the role of Alfred, a 74 y/o African American male with macular degeneration and hearing loss. "We Are Alfred" utilizes a virtual reality headset, headphones, and a hand-tracking device to immerse students into Alfred's experiences as a patient. Descriptive statistics and t-tests were applied for data analyses. Results: Learning was broad and significant: 94% reported increased empathy; 92% reported increased learning about macular degeneration; and 90% reported increased learning about hearing loss. Qualitative data collected from the pre-tests and post-tests supported learning on empathy with 4 associated themes (Personal Experiences, Perceptions of Older Adults, Thoughts about Health, Descriptors of Aging).. Conclusion: Virtual reality was deemed a successful medical education learning tool for these medical students. Utilizing this technology to create an immersive case study taught these medical students about the aging experience from the first-person patient perspective.

### IS GHANA READY FOR GERIATRICS? MEDICAL STUDENTS' INTEREST AND INTENTION TOWARD GERIATRIC SPECIALIZATION IN GHANA

Grace Karikari,[1] Lesa L. Huber,[1] and
David K. Lohrmann[1], *1. Indiana University School of Public Health, Bloomington, Indiana, United States*

Aging is a global phenomenon, and the population of elderly persons continues to increase in many communities around the world. Ghana is not an exception. As the demographics are changing, it is vital that health care providers are trained to effectively diagnose, care for, and manage aging-related conditions to improve the quality of life of the elderly. The aim of this study is to explore the underlying reasons for medical students' interest in geriatrics and their intentions, likelihood of selecting geriatrics as a specialty for future practice. This exploratory study involved (n =12) fourth, fifth and sixth year medical students in a Ghanaian public university. Semi-structured interviews guided by the theory of planned behavior were conducted. The findings show a lack of geriatric education or training for students in this institution and region. Students' interest and preferences for geriatrics were mainly influenced by the lack of knowledge and exposure to the specialty, as well as what it entailed to be a geriatrician. Although some students expressed interest in working with older patients, none had the intention to pursue geriatrics as a career option. Creating positive exposure to geriatrics through educational and vocational opportunities are necessary. The findings from this study can be instrumental in developing a knowledge-base of aging and caring for the elderly. Findings can also serve as the foundation for a more comprehensive study, leading to effective geriatrics education interventions in Ghana and Africa at large.

### SESSION 1375 (POSTER)

### LATE-LIFE FINANCES AND ECONOMICS

### COST-BENEFIT ANALYSIS OF COMMUNITY-BASED SERVICES AS A MEANS OF PROMOTING ACTIVE AGING IN GALILEE

Shira Hantman[1], *1. Tel Hai College, Upper galilee, Israel, Israel*

Increase in life expectancy has benefits, but also costs from increased expenses related to morbidity and prevention. These costs may be reduced by adopting a healthier lifestyle. The goal of the study was to quantify the economic value of a variety of activities in which older adults partake: e.g. cultural, intellectual, physical and nutritional activities. Research questions: Are the monetary benefits of improved health different when measured on a subjective willingness-to-pay (WTP) approach or on an objective Cost-of-Illness (COI) approach? Is the monetary benefit of active aging larger than the cost associated in doing that? 300 older adults participating in various activities of the local senior center and a control group not participating filled out a life style survey. A choice modelling (CM) approach estimated a subjective monetary welfare and compared it to an objective measure of benefit associated with the occurrence of different health symptoms associated with more active aging. An association was found between the various activities explored and the objective and subjective perspective of health. Moreover, all older adult activities passed the cost benefit test albite the order was different between objective and subjective estimations. Nutrition related activities were found to be the most beneficial. Cultural activity ranked second objectively and subjectively. Intellectual activity ranked last objectively and physical activity ranked last subjectively. Participants will understand the need to provide optimal policy and efficient resource distribution between the various activities offered to older adults. This will result in better health lowering public health expenditures.

### NAVIGATING FINANCIAL CONVERSATIONS ABOUT STUDENT LOAN DEBT AMONG OLDER BORROWERS AND THEIR FAMILIES

Samantha Brady,[1] Julie Miller,[1] Alexa Balmuth,[1]
Lisa D'Ambrosio,[1] and Joseph Coughlin[1], *1. MIT AgeLab, Cambridge, Massachusetts, United States*

Currently, approximately 44 million people in the United States carry the weight of over 1.4 trillion dollars of student loan debt. As the cost of education continues to rise, the decision of taking on student loans is increasingly a family decision rather than an individual one. While the majority of research focuses on younger borrowers, little research has been done to understand the experiences of parents and grandparents taking on student loans for a loved one. In order to financially and emotionally manage this burden, borrowers may benefit from support from their social networks, including family and friends. For many, navigating these difficult conversations presents a challenge of its own. This presentation will spotlight an MIT AgeLab mixed methods study about how student loan borrowers between the ages of 51 and 75 experience and manage their student loans within family systems and how these loans may impact family dynamics. Data were collected through focus groups and a national survey. Preliminary findings suggest that older borrowers demonstrate several distinct communication typologies within their families in regards to finances, particularly regarding student loan accrual and repayment. Each of the four primary communication styles regarding loans impact borrowers' financial and emotional wellbeing throughout the life course, as well as perceived relationship dynamics. Moreover, older borrowers are more likely to report family conflict if student loans are less frequently discussed with family members. Findings also suggest strategies to help parents and grandparents facilitate conversations abound student loans based on their unique family communication styles.

### MEDICARE COVERAGE CHANGES: THE ROLE OF INDIVIDUAL CHARACTERISTICS AND INTERNET USE

Brian E. McGarry,[1] and David C. Grabowski[1], *1. Harvard Medical School, Boston, Massachusetts, United States*

An important challenge to the success of consumer choice within the Medicare program is older adults' proclivity to assess their coverage on an annual basis and make changes when appropriate. Every year, relatively few beneficiaries alter their coverage (e.g., by switching Part D plans, switching between MA and traditional Medicare coverage) despite annual coverage changes and new market entrants. Little is known about the factors that encourage re-evaluation of Medicare coverage choices or the role of technology in facilitating changes. The latter knowledge gap is particularly relevant as the internet is an increasingly important delivery mechanism for Medicare information and consumer support tools. This study uses a nationally-representative sample of Medicare beneficiaries to describe Medicare coverage changes, the individual factors that predict such changes, and the relationship between Medicare-related internet use and plan switching. On average, 12% of Medicare beneficiaries made changes to their coverage in a given year, with 25% of beneficiaries making a change at any point during the study period. Between 2011 and 2015, the self-reported rate of using the internet to handle Medicare/insurance matters increased from 5% to 11%. In adjusted models that included individual-level fixed effects and other time-varying characteristics (e.g., health status, prescription drug needs), Medicare-related internet use was associated with a 65% increase in the probability of making a coverage change. Although using the internet to handle insurance matters remains relatively rare among older adults, it may an important mechanism for obtaining information that encourages plan changes, facilitating such switches, or both.

### ASSESSING THE EFFECTS OF LONG-TERM CARE INSURANCE ON EQUITY AND EFFICIENCY: A CASE STUDY OF QINGDAO CITY IN CHINA

Wei Yang,[1] and Shuang Chang[2], *1. King's College London, London, United Kingdom, 2. Tokyo Institute of Technology, Tokyo, Japan*

The demand for equitable and efficient long-term care (LTC) has risen rapidly, and finding a suitable mechanism to finance LTC has become a pressing policy concern for many countries. A number of high income countries have chosen to use a social LTC insurance to fund the LTC system, but empirical assessments on such an insurance in low-and middle-income countries are limited. Using China as an example, this paper empirically assesses the performance of newly-piloted LTCNI by evaluating its impact on equity and efficiency in financing. We draw data from 47 in-depth interviews conducted with local government, care providers and family members of the LTCNI participants in Qingdao in 2016. We found that there remain sizable disparities in financial burden among LTCNI participants, despite of its emphasis on ensuring access to care based on people's needs; care providers are incentivised to provide care at the least cost even this care is deemed as insufficient or inadequate due to fixed payment for their services. Our paper offers critical insights into the potential and challenges in applying LTC insurance model to a LMIC, where critical lessons can be drawn for public LTC insurance in other LMICs.

### MEDICAID FINANCING IN ASSISTED LIVING AND CHARACTERISTICS OF MEDICARE DUAL-ELIGIBLE RESIDENTS

Chanee D. Fabius,[1] Portia Y. Cornell,[2] and Kali S. Thomas[3], *1. Johns Hopkins University, Baltimore, Maryland, United States, 2. Brown University, Providence, Rhode Island, United States, 3. Providence VA Medical Center, Providence, Rhode Island, United States*

An increasing number of assisted living (AL) residents rely on Medicaid waivers or state plans to pay for personal care and other supportive services. States may finance services for duals residing in AL through Medicaid waivers and state plans, but the availability of coverage varies – some states offer little to no Medicaid coverage for services in AL, and others offer multiple pathways to receiving assistance. Little is known about duals in AL, including how many have access to AL and the quality of care they receive there. The present study compares the characteristics of Medicare beneficiaries residing in large AL settings, by dual-eligibility status, and investigates the variability in the share of duals in AL among states. We identified 586,397 Medicare beneficiaries residing in AL in 2014. Medicare claims were used to measure health characteristics and health care utilization. Duals represented 16% of AL residents in our cohort. Compared to non-duals, duals were more often older adults of color (24 vs 4%), and more likely to qualify for Medicare due to disability (46 vs 7%). Duals had higher rates of hospitalizations (24 vs 21%) and skilled nursing facility use while in AL (11 vs 10%), and more chronic conditions than non-duals. States varied in the share of AL residents who are duals, ranging from 6% in New Hampshire to 41% in New York. State policies that may contribute to variation in the prevalence of duals in AL and implications of these findings for policy-makers and residents will be discussed.

### GENDER DIFFERENCE IN RETIREMENT TIMING

Hansol Kim,[1] Hyun Kang,[2] and David J. Ekerdt[2], *1. Gerontology, University of Kansas, Lawrence, Kansas, United States, 2. University of Kansas, Lawrence, Kansas, United States*

The aim of this research is to examine the retirement timing of older men and women in the United States and to find what factors impact such timings. This research used the 2014 Health and Retirement Study datasets. A total of 2,401 respondents were included in this research. All of the participants were over 60 years old, half were women, and the majority of participants were full-time workers (81.8%). The dependent variable was expected years until retirement which was measured as a continuous variable, asking when the respondent thinks he/she will stop work or retire. Controlling for age, race, marital status, education, health, full time, and a number of children, the results revealed that males expect to work 1.2 years longer than women. Yet women have reasons for working longer that are not found among men. Older age and poor health predict a sooner retirement for both men and women. Yet women differed from men in wanting longer work lives if they are African American, employed part-time, and have large families. Women are living longer than men, and the labor participation of women is increasing. Older women will have more challenge in preparing for retirement than men due to their greater need to extend work to secure income. Gender differences in expectation for retirement financial security and their effect on retirement timing. Deserves future research, to understand women's decision making at this life stage.

### HOME CARE EXECUTIVES SAY MEDICARE HOME HEALTH PAYMENT SYSTEM ENCOURAGES LESS-IS-BETTER PRACTICE

William D. Cabin[1], *1. Temple University, Philadelphia, Pennsylvania, United States*

There has been an increasing trend for Congress and the Centers for Medicare and Medicaid Services (CMS) to add non-skilled services to coverage under Medicare Advantage and Medicaid inpatient hospital. At the same time there has been a 75% decline in home health aide visits, the only Medicare home health non-skilled service, as a percentage of all Medicare home health visits from 2000-2016. A literature review indicates no studies addressing the potential factors accounting from these seemingly contradictory trends. The present study is based on interviews of five Chief Executive Officers (CEOs), five Chief Financial Officers (CFOs), and eight Chief Nursing Officers (CNOs) from Medicare-certified home health agencies between October 2017-July 2018. Results indicated agreement among interviewees on three themes: the Medicare home health relies on a medical model which focuses on intermittent skilled care; the Medicare home health prospective payment system (PPS) exacerbated the focus on skilled care by rewarding higher reimbursement for skilled care based episodes; and a synergy has evolved of "less is better" regarding utilization of home health aide services and reimbursement. Policymakers are urged to consider adding coverage of non-skilled services under Medicare home health, similar to Medicare Advantage, by funding demonstration projects with appropriate changes in reimbursement.

### SESSION 1380 (POSTER)

### LGBT+ AND SEXUALITY

### SEXUALLY TRANSMITTED INFECTION KNOWLEDGE AMONG OLDER ADULTS: PSYCHOMETRICS AND TEST-RETEST RELIABILITY

Matthew L. Smith,[1] Tammy Coffey,[2] Caroline D. Bergeron,[3] Enbal Shacham,[4] Heather H. Goltz,[2] and Ali Boolani[2], *1. Center for Population Health and Aging, Texas A&M University, College Station, Texas, United States, 2. Clarkson University, Potsdam, New York, United States, 3. Bexar County Community Health Collaborative, San Antonio, Texas, United States, 4. Saint Louis University, St. Louis, Missouri, United States*

Sexually transmitted infections (STI) among older adults have dramatically increased in recent years, especially among those who are widowed and divorced. The purposes of this study were to: (1) identify STI-related knowledge among older adults; (2) report the psychometric properties of a tool commonly used to assess STI-related knowledge among youth and young adults; and (3) determine the test-retest reliability of the tool. Data were analyzed from 43 adults between the ages of 65 and 94 using a 27-item Sexually Transmitted Disease Knowledge Questionnaire (STD-KQ). Participants completed identical instruments on two separate days with approximately two weeks between. After responses were coded for correctness, composite scores were created. Cronbach's reliability coefficients were calculated to determine response consistency, and Pearson's r coefficients were used to assess test-retest reliability. Of 27 possible correct answers, participants reported an average of 11.6 (±6.6) correct responses on Day 1 and 11.7 (±7.3) correct responses on Day 2. Cronbach's alpha coefficients for the 27-item composite scale were high for both days (0.89 and 0.92, respectively), which indicates strong response consistency. Pearson's r coefficients were high between responses for the 27-item composite scale on Days 1 and 2 ($r=0.88$, $P<0.01$), which indicates strong test-retest reliability. Pearson's r coefficients were high between responses for all but three of the 27 items when assessed separately. Findings suggest the utility of the STD-KQ to assess STI knowledge among older adults. However, the consistently low knowledge scores highlight the need for educational interventions among this population.

### OLDER LESBIAN HEALTH VIA RELATIONSHIP STATUS: HEALTH CONDITIONS AND HEALTH BEHAVIORS

Jie Yang,[1] and Paige Averett[2], *1. East Carolina University School of Social Work, Greenville, North Carolina, United States, 2. East Carolina University, Greenville, North Carolina, United States*

Existing research has demonstrated that those in committed relationships are healthier than those who are not. However, very little research on same-sex relationships and particularly older lesbian relationships exists. The current study fills the gap by providing a health profile of lesbians in different relationship status, including partnered, widowed, casual dating, single—not dating, and celibate. The outcomes include diagnosed health conditions (include arthritis, high blood pressure, diabetes, depression, heart problems, and stroke) and health behaviors (exercise, smoking, and drinking). Data are from an original sample of 456 older lesbians aged 55 and over. Convenience sampling was adopted for this hard-to-reach population. Bivariate analysis results showed that widowed and celibate lesbians are more likely to report depression than other relationship groups. No disparity was found regarding health behaviors among different relationship groups. To further understand the unique group identifying as celibate, multivariate logistic regression analysis was performed to examine celibacy's impact on lesbians' reported depression after controlling for demographics and reported physical health. Multiple imputations was performed to handle missing values. Results showed that being celibate was associated with higher odds of reporting depression compared with having a partner or casually dating. Being celibate was also associated with lower odds of reporting depression compared with widows and singles who are not dating. To our knowledge, this study is the first to examine in details the health consequence of different relationship status among older American lesbians. Findings have important implication for health promotion among older lesbians.

### REJECTION SENSITIVITY AS A MEDIATOR OF PERCEIVED SOCIAL ACCEPTANCE OF LGBTQ OLDER ADULTS

Kirenia Brunson,[1] Sarah Nanami Morehouse,[1] and Ashely M. Stripling[2], *1. Nova Southeastern University, Fort Lauderdale, Florida, United States, 2. Nova Southeastern University, Fort Lauderdale, United States*

As the US population becomes older and more diverse, examinations exploring intersections of identity become increasingly important. Specifically, lesbian, gay, bisexual and transgender (LGBT) adults face unique challenges as they move into late life including issues related to caregiving, social isolation, and access to competent care. As such, understanding the role of moderating psychological factors, such as rejection sensitivity, within this population becomes instrumental to providing appropriate healthcare. Thus the purpose of this review is to (a) increase understanding of role of rejection sensitivity in LGBT older adults (b) understand the effect of treatment setting on this relationship. Source documents for this literature review were identified through PsycInfo, PsyARTCILES and Google Scholar using the following keywords, and combinations: "Older Adult," "Gerontology," "LGBTQ," "gay," "sexual minority," "Rejection Sensitivity," and "Social Acceptance". Consistent with a thorough analysis, all English-Language abstracts were read, as were the full articles of those texts that appeared relevant to this literature review. After applying relevant exclusion criteria (i.e. young age), 6 texts warranted inclusion including: 2 books and 4 articles. A thorough review of the literature revealed that internalized homonegativity and rejection sensitivity mediate the degree of emotional dysregulation, internalization of symptoms and rejection-based proximal stress across multiple settings. However, the impact of these relationships in long-term care settings remains unknown. Implications of the current presentation include improving awareness of psychological health factors in LGBT older adults and provision of recommendations to reduce barriers to optimal care.

### LGBT WIDOWHOOD: THE ASSOCIATION BETWEEN PARTNER LOSS AND PSYCHOLOGICAL WELL-BEING

Jessica A. Noblitt,[1] and Anne Barrett[1], *1. Florida State University, Tallahassee, Florida, United States*

Death of a partner – one of the most stressful events many people will ever experience – has profound effects on psychological well-being. However, research on widowhood focuses almost exclusively on heterosexual couples, with little known about these studies' applicability to the LGBT population. Further, the few studies of partner loss among LGBT individuals are qualitative and many focus on gay men. As a result, little is known about the effects of partner loss in this population as a whole or among other sexual minorities, including lesbians, bisexuals, and transgender individuals. Addressing this issue, our study examines the association between partner loss and psychological well-being using data from the 2010 Aging with Pride: National Health, Aging, and Sexuality/Gender Study (NHAS) (n=2,322), the largest nationally representative sample of LGBT middle-aged and older adults. We use OLS regression to examine the association between partner loss and psychological well-being, measured as depressive symptoms, loneliness, and subjective mental health. Results reveal a significant association between partner loss and psychological well-being across all three measures. However, the association is significantly weaker in models controlling for current partner status. In models predicting depressive symptoms and loneliness, partner loss no longer reaches significance, though it remains significant in the model predicting subjective mental health. Results suggest that current partner status more strongly impacts psychological well-being than does partner loss, though further research is needed to identify how, for whom, and under what circumstances the negative effects of partner loss may linger.

### SEX AND THE BIOPSYCHOSOCIAL MODEL: SEXUAL ACTIVITY AND GENDER DISPARITY IN OLDER ADULTS

Rebecca Reinhardt,[1] Zaver D. Moore,[1] and Alexandria Nuccio[1], *1. Nova Southeastern University, Fort Lauderdale, Florida, United States*

The biopsychosocial model emphasizes relational factors such as quality and availability as key components to older adult sexual activity (Gillespie, 2017). Supporting these findings, a previous study found that older adults aged 65 and over reported having more sex in the past six months but fewer sexual partners in the past year than younger adults. The current study seeks to further explore sexual activity by gender specifically, number of sex partners in the last year, and frequency of sex over the past six months in older adults. To better understand sexual activity and gender differences, 499 (male=59.7%, female 40.3%) participants aged 65 to 93 were selected from the de-identified Survey of Midlife in the US database (MIDUS-3). A Weltch T-test was used examining sexual activity among older adults based on gender. Results indicated that the number of sex partners within the past year was similar for females (M=1.10, SD=.49) and males (M=1.04, SD=.28), t(477)=1.62, p=.10, d^=.07. Further, results indicated sex frequency within the last six months was similar between females (M=3.41, SD=1.55) and males (M=3.66, SD=1.48), t(415)=1.76, p=.08, d^=.14. Contrary to previous research, the present findings suggest there are no gender differences in number of sex partners or sex frequency for older adults. The current findings draw attention to potential discrepancies within this under-explored subject area. While implications of these findings can improve communication regarding sexual health, future research should focus on how aspects of the biopsychosocial model can be a protective factor for the sexual health of older adults.

### SEXUAL ATTITUDES IN MIDDLE-AGED AND OLDER ADULTS: DEVELOPING AN UPDATED AND AGE-RELATIVE MEASUREMENT SCALE

Allyson S. Graf[1] and Viviane Johnson[1], *1. Northern Kentucky University, Highland Heights, Kentucky, United States*

Sexual attitudes in later life contribute to sexual and psychological well-being. Existing scales of sexual attitudes specific to aging adults are narrowly focused on specific settings or population sub-groups and are not inclusive of the diverse range of sex-related activities and sexual identities represented in present and upcoming older cohorts. The aim of this study was to create and validate a multi-dimensional measure of sexual attitudes appropriate for a sexually-diverse aging population. Following focus groups and feedback, 51 survey items were constructed; other measures of sexual attitudes, knowledge, and behaviors were included for the purposes of validation. A sample of participants (N = 291; Mage = 56.69, SD = 8.82, range = 45-99; 63.2% female) was recruited through Amazon's Mechanical Turk and the local community to complete a survey on sexual attitudes, well-being, and health. Psychometric properties of the measure were assessed using exploratory factor analysis to reduce items and identify existing factor structures; construct validity was confirmed using correlations with existing measures of sexual attitudes; and internal consistency was assessed for each sub-scale. Five factors emerged: core personal values (8-items; $\alpha$ = .99), communication and expression (13-items; $\alpha$ = .92), sources of knowledge (9-items; $\alpha$ = .87), traditional taboos (9-items; $\alpha$ = .89), and consent within established relationships (4-items; $\alpha$ = .94). Results provide a relevant and multi-faceted measurement of sexual attitudes for use with middle-aged and older adult populations. Future studies may benefit from an alternative to dated, restrictive measures. Validation should be ongoing with more diverse samples.

### DOES SEXUAL ORIENTATION RELATE TO HEALTH AND WELL-BEING? A PROPENSITY-SCORE MATCHED ANALYSIS

Christi L. Nelson[1] and Ross Andel[1], *1. University of South Florida, Tampa, Florida, United States*

Around 2.7 million adults over the age of 50 self-identify as lesbian, gay, bisexual, and transgender (LGBT) in the United States. Past research suggests that additional stressors caused by being a socially stigmatized minority group can have a negative effect on health and well-being. The purpose of this study was to examine the relationships between sexual orientation and self-rated health, memory, and psychological well-being in a 1:3 propensity score-matched subsample from 2016 wave of Health and Retirement Study (HRS), a nationally representative study of older adults. Each lesbian/gay/bisexual (LGB) participant (n=140) was matched with three straight participants (n=420) on age, sex, and education. The average age was 53.8 years (SD=2.3 years), 54% were men, the average education was 14.3 years (SD=2.4 years). Logistic regression results indicated that LGB participants were almost twice as likely to report ever having depression (OR=1.85, 95% CI=1.23-2.80). Conversely, LGB participants were more likely to report having better health (OR=1.47, 95% CI= 1.04-2.07) than straight participants and the two groups did not differ significantly in memory (OR=1.16, 95% CI= 0.82-1.64) from their straight counterparts. In conclusion, it is possible that the stigma due to sexual orientation plays a role in psychological well-being but may also reflect in better physical health but not cognitive health. It is also possible that the better health in LGB participants reflects self-report bias.

### CONSTRUCTED FAMILY SYSTEMS FOR AGING LGBTQ ADULTS PLANNING FOR ELDER CARE

Lisa A. Hollis-Sawyer,[1] and Laura Tejada[1], *1. Northeastern Illinois University, Chicago, Illinois, United States*

Examining the intersectionality of sexuality, gender, and age, Fredriksen-Goldsen (2017) argued that the aging LGBTQ community is a marginalized aging group, and the lack of attention to their social and other support needs are creating negative aging trajectories for those who have experienced lifespan issues of disenfranchisement and unfair treatment affecting their health and care planning. The effect of a family can be a complex one and is further determined by social class, race, ethnicity, gender, and region. Allen and Roberto (2016) suggested that the LGBT adults may have three different types of family experiences, ranging from their "family of origin" (biological family) to "family of choice" (created kinship family). Oswald (2002) presented research on resilience in constructed family systems for aging gays and lesbians and identified two underlying processes toward establishing and maintaining family resilience: intentionality relates to the intentional actions of the older adult to establish and maintain the chosen family system while redefinition involves using language and symbols to reinforce their social support network. This idea of "doing family" is critical to comprehend from LGBTQ family members' perspectives for eldercare planning. Social support needs for changing family dynamics begins in middle adulthood among aging gays and lesbians (Barker et al., 2006). Hypothetical case studies (e.g., sibling dynamics) will be discussed with implications for support and intervention programs to assist in the eldercare decision making of this growing aging subpopulation. Resource tools will also be presented to help achieve positive eldercare outcomes in this process of "doing family."

### THE PROTECTIVE ROLE OF SOCIAL SUPPORT ON DEPRESSIVE SYMPTOMS AMONG AGING SEXUAL MINORITY CANADIANS

Arne Stinchcombe,[1] Nicole G. Hammond,[2] and Kimberley Wilson[3], *1. Saint Paul University (Ottawa), Ottawa, Ontario, Canada, 2. University of Ottawa, Ottawa, Ontario, Canada, 3. University of Guelph, Guelph, Ontario, Canada*

Sexual minority older adults face minority stressors that are associated with higher rates of mental illness. The stress buffering effects of social support within majority populations are well documented. Using a large population-based sample of aging Canadians, we sought to examine the relationship between sexual orientation and depressive symptoms, and determine whether this relationship is moderated by social support and sex. Baseline data from the Canadian Longitudinal Study on Aging (CLSA) were used (n = 46147). Participants were between the ages of 45-85 years at time of recruitment (mean age = 62.46, SD = 10.27), and self-reported their sexual orientation as heterosexual or lesbian, gay, or bisexual (LGB) (2.1%). Social support and depressive symptoms were measured using validated instruments. Four functional social support subscales were derived: tangible, positive social interaction, affectionate, and emotional/informational. Multiple linear regression models adjusted for relevant covariates were conducted. LGB identification was associated with greater depressive symptoms when compared to heterosexual participants (p = 0.032). As evidenced by a significant 3-way interaction (p = 0.030), increasing tangible social support was associated with a corresponding decrease in the risk of depressive symptoms; this relationship was most pronounced for lesbian and bisexual women. A significant 2-way interaction (p = 0.040) revealed that as emotional/informational social support increased, depressive symptoms decreased, with greater disparity between LGB and heterosexual participants at lower levels of social support. The results highlight the importance of social support in promoting mental health, especially among sexual minority older adults.

### SEXUAL ORIENTATION AS MULTIDIMENSIONAL: AGE AND GENDER DIFFERENCES IN SEXUAL MINORITY OLDER ADULTS

Eve Z. Root,[1] Grace Caskie,[1] Bethany Detwiler,[1] and Nicole L. Johnson[1], *1. Lehigh University, Bethlehem, Pennsylvania, United States*

Recommendations to conceptualize sexual orientation as a continuum and as multidimensional rather than one dichotomous variable (e.g., DeBlaere et al., 2010; Kinsey et al., 1948) have been largely unexplored in sexual minority older adults, including how these dimensions might differ by age and gender. In this study, participants indicated their sexuality using three continua representing (1) attraction in general, (2) emotional attraction, and (3) physical attraction. Possible responses ranged from 0=exclusively opposite sex to 7=exclusively same sex. The current sample included 187 participants (50-86 years; 73 men, 114 women) self-identifying their sexual attraction in general as not exclusively to the opposite sex. Age groups were 50-55 (n=56), 56-64 (n=84), and 65-86 (n=47) years. MANOVA results indicated a significant multivariate age group by gender interaction (p=.040) that was significant for all three attraction variables---attraction in general (p=.035), emotional attraction (p=.010), and physical attraction (p=.029). In the 50-55 age group, the average response for physical attraction was closer to exclusively same sex for men than for women. For the 56-64 age group, the average response for attraction in general and emotional attraction was closer to exclusively same sex for women than men. Among those 65+, women responded closer to exclusively same sex than men only for emotional attraction. Gender differences on all three sexual attraction continua were not consistent across age groups, which may reflect a more fluid and complex understanding of sexuality in older LGB adults. Future studies should consider using multidimensional and continuous variables when measuring sexual orientation.

### EXPLORING HOW AGE PREDICTS OUTNESS AND INTERNALIZED HOMOPHOBIA IN A LIFESPAN SAMPLE OF SEXUAL MINORITIES

Michael T. Vale,[1] Paige M. Pasta,[1] and Toni L. Bisconti[1], *1. University of Akron, Akron, Ohio, United States*

Minority Stress Theory posits that specific minority stressors, such as internalized homophobia and outness, predict negative health outcomes in sexual minorities. There has been substantial work in addressing these stressors in young adult samples; however, less in known about older adults. Older sexual minorities were socialized in a time in which same-sex relations were considered deviant and illegal, and therefore, have been exposed to a lifetime of marginalization. Although there is evidence that minority stressors negatively impact health in older adults, many studies exclude a complete lifespan sample. The goal of this study was to collect a sample of individuals in same-sex relationships ranging in age from 18-80 (N = 228, M = 40.93, SD = 15.87) and examine whether age correlates with outness and internalized homophobia. We found that older participants had higher degrees of outness (r = .21, p < .01) and less internalized homophobia (r = -.20, p < .01) resulting in less overall minority stress. We also analyzed different social resources that might explain the age-related decrease in minority stress and found that age was related to higher self-esteem (r = .24, p < .01). We tested whether self-esteem moderated the direct relationships between age and the minority stressors and found a significant interaction for internalized homophobia (B = .0175, p < .01), but not outness. Our findings provide support that older sexual minorities report less minority stress and more research is needed to explain what promotes these trends.

### FOR BETTER OR FOR WORSE: MARITAL TRANSITIONS AND SEXUAL LIFE IN MIDDLE AND OLDER AGE

Jeffrey E. Stokes,[1] Elizabeth A. Gallagher,[1] Remona Kanyat,[1] Cindy N. Bui,[1] and Celeste Beaulieu[1], *1. University of Massachusetts Boston, Boston, Massachusetts, United States*

Marital transitions have known implications for health and well-being. However, little research has examined the effects of such transitions on adults' sexual lives. This study uses longitudinal data from the National Study of Midlife Development in the United States (1995–2014) to compare different marital status and transition groups' sexual activity, satisfaction, control, and effort throughout mid-and-later life. Across all outcomes, effects of marital status/transitions were contingent upon baseline values of the outcome. Consistently married adults reported more frequent sexual activity, greater sexual satisfaction, and greater effort put into sexual life than other groups when baseline values of those outcomes were average or above-average; such group differences were reduced or reversed at below-average baseline values. Among the not-married, women reported significantly less sexual activity than men. The consistently divorced/separated, consistently widowed, newly divorced/separated, and newly widowed all reported greater control over sexual life at follow-up than the consistently married, when baseline sexual control was average and/or below-average. Lastly, women reported lesser effort put into sexual life at follow-up than men across all groups, accounting for baseline effort; these gender gaps were least pronounced among the consistently and newly married, and most pronounced among the newly widowed and newly divorced/separated. Overall, findings indicate that implications of marital transitions for midlife and older adults' sexual lives depend upon both gender and pre-transition context. Marriage is not always beneficial for sexual life; rather, poor quality sexual lives during marriage can reduce opportunities for improvement that may arise with marital transitions, including divorce and widowhood.

### THE IMPACT OF SEXUAL FLUIDITY AND SOCIAL SUPPORT ON EGO INTEGRITY IN OLDER ADULTS

Alicia C. Figueroa[1], *1. Northeastern Illinois University, Chicago, Illinois, United States*

The population of lesbian, gay, and bisexual (LGB) Americans is growing as the number of older adults "come out. " While we know that "coming out" later in life impacts the experience of aging, little research has examined ways in which social support and sexual fluidity influence ego integrity in older adults. The present study investigated ego integration, changed sexual behavior, and perceived social support in adults 45 years of age and older who had been in a long-term relationship with the opposite sex prior to "coming out" as LGB. The average of participants was 61 years, sixty-eight percent (N = 43) were currently married or in a domestic partnership, and eighty-six percent (N = 54) identified as Caucasian. Individuals were recruited to participate in the survey utilizing online social media. Results (N = 63) suggested that those whose behavior was more sexually fluid were least ego integrated. Timing of "coming out" LGB, Early (44 and younger) versus Late (after the age of 45) impacted the degree of sexual fluidity and perceived social support. Results indicated those who "came out" Late were more sexually fluid and were less fearful of "coming out. " The most significant result revealed social support from significant others to be most impactful compared to friends and family. This research expands on the challenges of those who are aging in a non-normative environment. The implications suggest that individuals who "come out" later have a more difficult time with accepting their sexual fluidity.

### SESSION 1385 (POSTER)

### LIFE REVIEW, REMINISCENCE, AND EMOTIONS

### EXPLORING AGE, GENDER, AND RELATIONSHIP CLOSENESS DIFFERENCES IN RESPONSES TO IGNORED DISRESPECT

Jenessa C. Steele,[1] Amanda Chappell,[1] and Rachel Scott[1], *1. Radford University, Radford, Virginia, United States*

Emotional responses to disrespect tend to be negative (Hawkins, 2015). Little is known about how responses to disrespect vary across age groups and relationship closeness. It is unknown whether older adults have more emotional protection against disrespectful experiences, or are more deeply affected due to relationship closeness. Overall, we might expect that older adults react less negatively to disrespect compared to young adults, as they are more-skilled emotion regulators (Carstensen, 1991; English & Carstensen, 2014). We aimed to explore if, and under which circumstances, older adults are more or less sensitive to disrespect compared to younger adults. Three hundred participants responded to six scenarios illustrating ignored disrespect. Participants were randomly assigned to close or distant relationship disrespect scenarios. Relationship closeness was first determined by requesting participants identify a person in each layer of Kahn and Antonucci's (1980) Social Convoy Model. Identified names were then automatically inserted into the six scenarios. Emotional responses and sensitivity to each scenario were recorded. Participants in the close condition reported more sensitivity to disrespect and negative emotions than participants in the distant condition. Females reported more sensitivity to disrespect and negative emotions than males. We did not find overwhelming support for age differences in responses to disrespect. A single scenario indicated younger participants more sensitive to disrespect than older participants. Findings suggest it is more hurtful to be disrespected by someone close to you and females may be more sensitive to disrespect than males. More research investigating the role of age in disrespect is needed.

### GENDER DIFFERENCES IN SOCIAL JUDGEMENTS RELATE TO SOCIAL EXPERIENCE ON THE JOB FOR OLDER ADULTS

Cassandra Richards,[1] and Jennifer T. Stanley[1], *1 The University of Akron, Akron, Ohio, United States*

Older adults (OA) are worse than young adults (YA) at recognizing emotional facial expressions (Ruffman, Henry, Livingstone, & Phillips, 2008). In particular, age differences in anger recognition remain even when other emotions improve via additional context (e.g., Richter, Dietzel, & Kunzmann, 2011; Stanley & Isaacowitz, 2015). We investigated whether job experiences with greater social components would relate to better anger recognition and interpersonal perception in OA. We expected OA who held jobs with more social requirements would be better at anger recognition and interpersonal perception, but that this would differ by gender. OA (N=194) reported their present job and completed an emotion perception task and the Interpersonal Perception Task-15 (IPT-15; Costanzo & Archer, 1989). Ratings from the O*Net database were used to determine the degree of social requirements (0-100) for reported jobs. For older females, more social experience in their present job was related to better anger recognition ($r = .45, p = .014$). More face-to-face experience in the job held the longest was related to better overall emotion perception in older females ($r = .20, p = .047$). For older males, more social experience in their present job was related to worse anger recognition ($r = -.45$, $p = .029$). More coordination and leadership experience in the job held the longest was related to better interpersonal perception in older males ($r = .28$, $p = .010$). These results suggest gender is important when examining the degree to which social experience in the workplace relates to social judgments. Future work should investigate whether gender differences in subordinate vs leadership roles can account for these findings.

### AGE MODERATES THE ASSOCIATION BETWEEN IMPLICIT AND EXPLICIT NEGATIVE EMOTIONAL REACTIVITY

Mai B. Mikkelsen,[1] Gitte Tramm,[1] and Mimi Mehlsen[1], *1. Department of Psychology and Behavioural Sciences, Aarhus University, Aarhus, Denmark*

Background: According to the notion of maturational dualism, the link between mind and body weakens with age and this weakening has important consequences for emotional experiences. Specifically, it is hypothesized that age-related decline in interoceptive awareness and physiological reactivity reduce the ability to use bodily states to guide judgments about emotions. If this hypothesis is valid, then age may moderate the association between explicit measures (based on consciously accessible mental representations of affect) and implicit measures (based on unconscious affective processes such physiological activation) of affective reactivity. Purpose: To investigate whether age moderates the association between explicit and implicit measures of negative affective reactivity. Methods: A sample of 275 participants (age range=20-78) viewed 25 pictures validated to induce negative emotions. Participants filled in the PANAS assessing explicit affect and the IPANAT assessing implicit affect before and after viewing the pictures. Emotional reactivity was operationalized as residualized gain scores derived from regressions of baseline affect on affect following picture viewing. Results: Age moderated the association between implicit negative affective reactivity and explicit negative affective reactivity (B=60). Discussion: The results showed a reduced association between explicit and implicit negative affective reactivity with age. This finding is consistent with the notion of maturational dualism and may indicate that older adults use affective processes that are not represented consciously (e.g., physiological activation) less than young adults when judging their emotional state.

### THE HIGHWAY OF LIFE: SOCIAL VIRTUAL REALITY AS A REMINISCENCE TOOL

Steven J. Baker,[1] Jenny Waycott,[1] Jeni Warburton,[2] and Frances Batchelor[1], *1. The University of Melbourne, Melbourne, Victoria, Australia, 2. La Trobe University, Wodonga, Victoria, Australia*

A large body of research demonstrates the positive impact that reminiscence activities can have on older adult wellbeing. Within this space, researchers have begun to explore how virtual reality (VR) technology might be used as a reminiscence tool. The immersive characteristics of VR could aid reminiscence by giving the sense of being fully present in a virtual environment that evokes the time being explored in the reminiscence session. However, to date, research into the use of VR as a reminiscence tool has overwhelmingly focussed on static environments that can only be viewed by a single user. This paper reports on a first-of-its-kind research project that used social VR (multiple users co-present in a single virtual environment), and 3D representations of personal artifacts (such as, photographs and recorded anecdotes), to allow a group of older adults to reminisce about their school experiences. Sixteen older adults aged 70-81 participated in a four-month user study, meeting in groups with a facilitator in a social virtual world called the Highway of Life. Results demonstrate how the social experience, tailored environment, and personal artifacts that were features of the social VR environment allowed the older adults to collaboratively reminisce about their school days. We conclude by considering the benefits and challenges associated with using social VR as a reminiscence tool with older adults.

### LEARNING FROM LIFE STORIES: RECRUITING NURSING HOME RESIDENTS FOR A LIFE STORY WORK PROGRAM

Brian K. Polk,[1] Farida Ejaz,[1] and Miriam Rose[1], *1. Benjamin Rose Institute on Aging, Cleveland, Ohio, United States*

Recruiting nursing home (NH) residents to participate in program evaluations is a consistent challenge. This was evident in a federally supported project to improve person-centered care of long-stay NH residents enrolled in Medicaid. Evaluators sought to examine the impact of a life story work intervention using a pre-post study design involving interviews of NH residents and surveys of their family members and staff. Other resident eligibility criteria included willingness to participate in both research and life story interviews, age 60+, a Brief Inventory Mental Status (BIMS) score of 8 or higher, English-speaking, and consent from a legal guardian, if applicable. A total of 16 NHs agreed to participate in the implementation and evaluation of the program, which developed complimentary, individualized life story booklets for residents and a companion summary for staff. Of the homes' combined population of 1,817 residents, 569 met eligibility criteria for the research study. Non-response from legal guardians excluded 37 residents, and 174 residents approached for recruitment declined to have their names released to the researchers. During baseline interviews, 20 residents failed the BIMS, 21 were unavailable, and 79 refused when approached by a research interviewer. Ultimately, 238 resident interviews were completed at baseline. Common themes for refusals included disinterest in participating in life story work, statements that theirs was not a good life worth talking about, and doubts that quality of care would improve. Strategies for addressing such challenges included displaying sample life story materials during recruitment and providing residents additional time to consider participation.

### PORTRAYAL OF A CENTENARIAN: EXPLORING ACTOR INSIGHTS ON AGING AND LONGEVITY

Alex J. Bishop,[1] Tanya Finchum,[2] and Julie Pearson-Littlethunder[1], *1. Oklahoma State University, Stillwater, Oklahoma, United States, 2. Oklahoma Oral History Research Program Oklahoma State University, Stillwater, Oklahoma, United States*

Data for this study originated the Oklahoma 100 Year Life Project. A total N = 7 volunteer actors (n = 5 women; n = 2 men) were recruited to participant as actors to play the role of centenarians in a living history play based on oral historical narratives. Actors participated in preliminary (prior to play performance) and post (after play performance) focus group sessions. Focus group questions addressed four key ideas: (1) Perceptions of aging and human longevity; (2) Loss and decline in aging; (3) Narrative storytelling and; (4) Personal life goal(s). Qualitative content analysis was performed to assess general thematic evidence stemming from actor perspectives. Four predominant themes emerged suggestive of adaptation in actor perceptions about human aging and longevity. These themes included: (1.) Purpose seeking (e.g. "It was interesting to see how much you can get out of life."); (2.) Age-embodiment (e.g., "I really wanted to focus on my facial movements and the way I talked"); (3.) Creative curiosity (e.g., "It takes a little bit of creativity. . . someone who has curiosity"); and (4.) Self-actualization

(e.g., "You just think it's not going to happen to me. . . and then you're like. . . I am probably going to live to 100 now."). Results have implications relative to how oral historical narratives can be used to enhance the personification of aging on stage, as well as demystify personal myths about longevity. Applications for use within educational and community settings for theatrical performances will be shared.

### LINKS BETWEEN POSITIVE AFFECT AND DISENGAGEMENT FROM NEGATIVE STIMULI IN YOUNGER AND OLDER ADULTS

Kathryn L. Ossenfort,[1] and Derek M. Isaacowitz[1], *1. Northeastern University, Boston, Massachusetts, United States*

Older adults attend to more positive than negative content compared to younger adults; this "age-related positivity" effect is often thought of as a way older adults may be regulating their moods. However, attentional disengagement abilities decline with age, which may make positive looking more challenging for older adults in some cases. To evaluate links between early attentional processes and affect, 48 younger adult and 49 older adult participants reported levels of positive and negative affect on the Positive and Negative Affect Scale (PANAS) and completed a spatial cueing task evaluating attentional orienting and disengagement from emotional stimuli. Participants were tasked with responding to the location of a spatial target after seeing a cue (emotional image) that either appeared on the same (orienting) or opposite (disengagement) side of the screen. Multilevel modeling analyses were conducted using age and self-reported affect from the PANAS as predictors at level-2, and trial characteristics as predictors at level-1. Positive affect (PA) was unrelated to task performance for younger adults. Older adults reporting higher PA responded more slowly overall, and higher PA scores predicted similar response times to positive and negative stimuli on both trial types. Older adults reporting lower PA oriented attention more quickly to positive stimuli, but took longer to disengage from negative. These results suggest that there may be a relationship between the ability to flexibly disengage from negative content and PA for older, but not younger adults, and also highlight the importance of teasing apart specific attentional processes when evaluating positivity effects.

### REMEMBER AND REMINISCE: CONNECTING OUR SCIENCE TO PEOPLE'S LIVES

Joseph E. Gaugler,[1] Robyn Birkeland,[1] Tamara L. Statz,[1] Rachel Zmora,[2] Colleen M. Peterson,[3] Hayley R. McCarron,[4] and Nancy Kazlauckas[5], *1. University of Minnesota - School of Public Health, Division of Health Policy and Management, Minneapolis, Minnesota, United States, 2. University of Minnesota, Minneapolis, Minnesota, United States, 3. University of Minnesota - School of Public Health, Division of Epidemiology and Community Health, Minneapolis, Minnesota, United States, 4. University of Minnesota School of Public Health, Minneapolis, Minnesota, United States, 5. Independent Quilting Artist, Sauk Centre, Minnesota, United States*

A driving paradigm of science, including much of the discipline of gerontology, remains post-positivism. However, the post-positivistic emphasis on experimental control, elimination of "bias," and internal validity may create a chasm between the science and scholarship we conduct and the lives of those who engage with us in such endeavors. The purpose of this presentation (a quilt provided to the authors by an individual who completed one of our studies evaluating the efficacy of a psychosocial support program for family members of relatives living in residential long-term care and with memory loss) is to offer an opportunity for scholars and others to reflect on the connections and partnerships we establish with those who volunteer to engage in our research initiatives. In particular, the aim of this presentation is to stimulate viewers to consider how we grow our relationships with those who volunteer in our research by creating true partnerships that outlast a single project. With the emergence of participatory research methodologies, person-centered care, and social media technologies, the importance of building relationships and partnerships with communities, families, and individuals in a more equitable manner is pressing. The use of the "Remember and Reminisce" quilt as well as other materials donated by individuals to dementia care researchers provide vivid, tactile reminders from those who engage with our science that our obligations to understanding their experiences must assume a broader, longer-term perspective than the boundaries that often dictate the life-cycle of a standard project/study.

### THE HISTORY OF BEAUTY AND THE AESTHETICS OF LONGEVITY

Naomi Woodspring[1], *1. University of the West of England, Bristol, United Kingdom*

A long standing cultural narrative is aging appearance is neither attractive nor acceptable. This has not always been the case; the aesthetics of older appearance has been appreciated other times in history. Significant numbers of older people in the public sphere, as a result of the longevity revolution, has created a sense of visibility of among older people, particularly women. The aim of this qualitative study was to explore current notions of beauty and age among the postwar generation. A diverse group of thirty-four women and men (born between 1945 -1955) from the US and the UK were interviewed with a focus on their own self-presentation and the acts of seeing and being seen. This paper explores the some of the findings from this study which asked the central question – can old people be beautiful and, if so, how is age and beauty defined? The majority of research participants answered in the affirmative and responded with clearly defined notions of age and beauty. The findings found significant gender differences; not within the central research question but in regard to their own appearance. There were also significant gender differences in terms of a 'competitive' or 'cooperative' gaze when observing other older people. This small study invites further research and points to a possible shift in the aesthetics of old age, in part, as a result of the longevity revolution. It provides an outline for further exploration of the importance of appearance, meaning, and a sense of self in old age.

### GENDER DIFFERENCES IN MEANINGFUL LIVED EXPERIENCES RECALLED BY CENTENARIANS

Jyoti Bhatta,[1] Alex J. Bishop,[1] Nadia Firdauysa,[1] and Tanya Finchum[2], *1. Oklahoma State University, Stillwater,*

*Oklahoma, United States, 2. Oklahoma Oral History Research Program Oklahoma State University, Stillwater, Oklahoma, United States*

The purpose of the investigation was to conduct a retrospective examination of meaningful live experiences reported by centenarians. Data for this study originated from N=111 centenarians (n = 43 men; n = 68 women) who participated in the Oklahoma 100-Year Life Project. Applying a hierarchical convoy mapping technique commonly used in social network evaluations, IBM/SPSS 23.0 was used to conduct a descriptive analysis of N = 654 recalled lived experiences. The Mini-Mental Status Examination-SF (MMSE-SF; M = 12.55; SD = 1.55) was used to screen all participants for cognitive orientation prior to participation to ensure capacity to consent and intact memory recall. Centenarian participants recalled a total of M = 6.90; SD= 2.61 lived experiences. Centenarian men recognized a significantly greater average number of meaningful experiences (F = (1, 653) = 30.53, p < .01) compared to centenarian women (M = 4.06 vs. M = 3.43). A good proportion of centenarians (40.50%) acknowledged meaningful events as occurring during young-adulthood. However, the timing of such events occurred significantly earlier (F = (1, 357) = 7.37, p < .01) on average for men compared to women (M = 27.60 yrs. vs. M = 34.11; 1.53). Further analysis revealed that over half of lived experiences considered meaningful among centenarians proportionally fit into three domain types: Family-oriented (19.5%); Work/employment related (18.7%); and Marriage (13.7%). Results have implications relative to understanding how variation in meaningful lived experiences among centenarians. Further evidence of a gendered life course in human longevity will be highlighted.

### PREDICTORS OF SELF-CONTINUITY IN LATER LIFE

Charikleia Lampraki,[1] Dario Spini,[1] and Daniela Jopp[2], *1. University of Lausanne, Swiss National Centre of Competence in Research LIVES, Lausanne, Vaud, Switzerland, 2. University of Lausanne, Lausanne, Switzerland, Switzerland*

Self-continuity is an identity mechanism that interconnects past and present experiences with future expectations, creating a coherent whole. However, research is limited regarding inter-individual differences and life course determinants of change in self-continuity. Using a life-course perspective on vulnerability, we investigate how the accumulation of resources (e.g., social, hopeful attitude) and the occurrence of critical life events (e.g., childhood adversity, divorce) across the life course may affect changes in self-continuity. Data derived from the LIVES Intimate Partner Loss Study conducted in Switzerland from 2012 to 2016 (3 waves). The sample consisted of individuals having experienced divorce (N = 403, Mage = 55.43) or bereavement (N = 295, Mage = 69.91) in the second half of life, using a continuously married group as a reference (N = 535, Mage = 65.60). Multilevel hierarchical models were used. Results indicated that as individuals grew older they experienced more self-continuity. More childhood adversity was negatively associated with inter-individual differences in self-continuity for all groups. Divorcees with more childhood adverse events felt significantly less self-continuity as they grew older than divorcees with less childhood adversity. In the bereaved group, more childhood adversity and less hope was linked to lower levels of self-continuity. More hopeful married individuals felt more self-continuity as they grew older than less hopeful ones. In sum, findings demonstrate that self-continuity changes as a function of age, but also differs with regard to the critical life events experienced across the life course and the availability of resources.

### EXPLORING THE EFFICACY AND EFFECTIVENESS OF REMINISCENCE USING THREE-DIMENSIONAL PRINTED OBJECTS

Keith A. Anderson,[1] Marla Berg-Weger,[2] Tom Plocher,[3] Anne Farina,[2] and Joseph E. Gaugler[4], *1. University of Montana, Missoula, Montana, United States, 2. Saint Louis University, St. Louis, Missouri, United States, 3. Moai Technologies, Maple Grove, Minnesota, United States, 4. University of Minnesota - School of Public Health, Division of Health Policy and Management, Minneapolis, Minnesota, United States*

Reminiscence therapy (RT) is a form of person-centered care that involves the exploration of past activities, events, and experiences with another person or in a small group. Research on RT has found an array of benefits for older adults, including improvements in mood, quality of life, social interaction, cognition, and memory. In this presentation, the researchers report on Phases I and II of an evaluation of a reminiscence using three-dimensional (3D) printed objects from older adults' pasts. Advances in 3D printing technology now allow researchers to create scale replicas of cherished items from peoples' pasts, such as toys, bicycles, pets, automobiles, boats, and houses. In Phase I, the researchers evaluated the efficacy of incorporating 3D objects in reminiscence using a parallel convergent mixed methods design. Participants agreed or strongly agreed that the 3D object reminiscence was well-received (88.9%), facilitated reminiscence (83.3%), increased engagement and alertness (72.3%). Qualitative data identified additional benefits of the use of 3D objects in reminiscence, including increases in social engagement and interactions with staff and family members. In Phase II, the researchers are evaluating the effectiveness of a formal 3D reminiscence intervention using a randomized control trial of 175 older adults. The researchers hypothesize that 3D object reminiscence will be more effective than reminiscence using verbal cues in stimulating memory and enhancing cognition, engagement, mood, and quality of life. Preliminary findings from Phase II will be reported along with the findings from Phase I.

### LINKS BETWEEN LONELINESS AND HAPPINESS IN JAPAN'S AGING POPULATION

Takashi Yamashita,[1] Anthony R. Bardo,[2] and Jack Lam[3], *1. University of Maryland, Baltimore County, Baltimore, Maryland, United States, 2. University of Kentucky, Lexington, Kentucky, United States, 3. The University of Queensland, Indooroopilly, Queensland, Australia*

Fertility rates in Japan have been historically low over several decades while life expectancy remains among the highest in the world. Consequently, traditional social networks consisting of immediate family and relatives have shrunk, and a growing number of older adults in contemporary Japanese society report feeling lonely. Thus, the well-being of Japans aging population is a major concern. While the negative effects of loneliness on perceived well-being (e.g., happiness) in later life have been well documented in western nations, relatively little is known from a Japanese context. Thus, we utilized a sample (n = 258) of urban community-dwelling Japanese adults age 65 years and older from the 2012 Survey of Mid-Life in Japan (MIDJA) to examine the association between happiness and loneliness. Consistent with findings from western nations, we identified strong links between happiness and loneliness in Japan. Results from ordinal logistic regression models showed that loneliness (OR = 0.80, $p < 0.05$) was negatively associated with happiness even after accounting for sociodemographic characteristics. Additionally, this study examined relevant demographic and cultural characteristics in order to contextualize the findings and identify possible explanations. For example, the cultural importance of family ties and gendered family roles was discussed in relation to the likely impact that increased levels of loneliness will have on the well-being of older Japanese adults. In sum, if the well-being of Japan's rapidly aging population is to be maintained (or possibly even enhanced), then the growing societal issue of loneliness must be addressed.

### LONGITUDINAL RELATIONSHIPS BETWEEN RESOURCES, MOTIVATION, AND ENGAGEMENT IN COGNITIVELY DEMANDING ACTIVITIES

Claire Growney,[1] and Thomas M. Hess[1], *1. North Carolina State University, Raleigh, North Carolina, United States*

Selective Engagement Theory (SET; Hess, 2014) suggests that decreases in personal resources and increases in the costs associated with activity engagement in old age negatively influence the motivation to engage in cognitively demanding activities. Here we explore these ideas longitudinally including a wide range of personal resources (cognitive ability, physical health, emotional health, and sensory functioning), with the expectation that emotional health might be a particularly important resource for older adults given its relative preservation with age. Young (n=125; age 19-42 at Time 1) and older adults (n=183; age 60-85 at Time 1) were tested from two to five times between years 2010 and 2016. Resources, motivation, and self-reported activity engagement (VLS Activity Questionnaire) were assessed at each time point. Using multilevel structural equation modeling, we found that changes in emotional health and sensory functioning predicted changes in motivation to engage in cognitively demanding activities. Additionally, increases in motivation predicted increases in engagement in cognitively demanding activities (e.g., technical, developmental), but decreases in less demanding activities (e.g., TV watching). Lastly, motivation partially mediated the relationships between emotional health and these activities, as well as between sensory functioning and engagement in technical activities. Results provide support for SET, demonstrating associations between changes in resources, motivation, and engagement in activities that are particularly demanding of cognitive resources, with the strength of these relationships being stronger in older than in young adults. Our results suggest that emotional resources may be particularly influential in determining the motivation for activity engagement in later life.

### THE INFLUENCE OF EMOTIONAL IQ ON THE LAGGED RELATIONSHIPS BETWEEN MOMENTARY PAIN AND AFFECT IN OLDER ADULTS

Shelley E. Condon,[1] Brian Cox,[2] Dylan M. Smith,[3] and Patricia A. Parmelee[1], *1. The University of Alabama, Tuscaloosa, Alabama, United States, 2. Alabama Research Institute of Aging, Tuscaloosa, Alabama, United States, 3. Stony Brook University, Stony Brook, New York, United States*

Emotion regulation is influenced by stage of life and time perspective (Carstensen, 1992), with older adults placing greater emphasis on optimizing positive moods and repairing negative ones. Recently, there has been growing interest in emotional intelligence (EI) as a broad indicator of these mood regulation processes. Multiple cross-sectional studies have examined EI, pain and affect in older adults with chronic pain; however, little research has addressed these relationships in temporal context. The current microlongitudinal study addressed this gap by examining (1) lagged relationships between momentary pain and affect, (2) main effects of emotional intelligence (mood attention, clarity and repair) on those relations, and (3) the moderating role of EI on lagged relationships between pain and affect. Three hundred twenty-five older adults (mean age = 63.9) with knee osteoarthritis completed in-person interviews and received four phone calls daily (random within 4-hour blocks) for one week. Multilevel models examined the predictive value of affect from the previous call on current pain, and vice versa, controlling for sociodemographic variables. Across all outcomes (positive affect, negative affect, pain), a significant main effect was found for mood clarity and repair, but not attention. However, EI did not moderate lagged associations between momentary pain and affect. Average pain (across the 28 calls) significantly predicted momentary negative affect, and vice versa. Thus, while emotional intelligence is significantly related to momentary mood states, it does not appear to be related to momentary pain. Implications and ideas for future research are discussed. (R01-AG041655, P. Parmelee & D. Smith, Co-PIs)

### RETIREES' MEMORIES OF WORK LIFE AND WELL-BEING AFTER RETIREMENT: A QUALITATIVE INQUIRY

Tina Hahnel,[1] Sabine Hommelhoff,[1] and Hsiao-Wen Liao[2], *1. Friedrich-Alexander University, Erlangen-Nürnberg, Germany, Germany, 2. Stanford University, Stanford, California, United States*

Reminiscence research has grown immensely in the past 30 years. Yet, research on personal memories of work lives is lacking. This is surprising because work is a crucial aspect of many people's lives and an important life story chapter (Thomsen, Pillemer, & Ivcevic, 2011). Part of a larger project, the present qualitative study aimed to understand (1) what retirees remember about their work lives and (2) whether and how retirees tie those memories to their current well-being. Six in-depth interviews on lives before and after retirement (4 women and 2 men with different careers, age range 65 to 87 years) were conducted and transcribed verbatim. Findings of a thematic analysis (Braun & Clarke, 2006) revealed that participants reported both big and small stories. They first narrated landmark events (e.g., job loss after the Fall of the

German Wall) and continued to recount many little incidents (e.g., a child asking an unretiring teacher if she is now "done with retirement"). Additionally, participants not only reminisced about work itself (i.e., what jobs were like) but equally about workplace relationships (e.g., particularly positive or negative relations with supervisors). Despite difficult times at work, participants reported that they were now at peace with how things went and generally satisfied with their current lives. We discuss how the type (i.e., big or small) and content (i.e., work- or relationship-focused) of retirees' memories and positive meaning-making (i.e., recounting work lives in a positive light) may contribute to well-being and propose a conceptual model for future research.

### EMOTIONS SURROUNDING THE 2018 MIDTERM ELECTIONS

Sydney Krueger,[1] and Kevin Ochsner[1], *1. Columbia University, New York, New York, United States*

Over the adult life-span there is a self-reported shift in daily life emotions towards feeling less negative and more positive. We hypothesized that variations in emotion regulation behavior over the life-span could explain why aging is associated with this "rosy glow". We collected survey data from 400 adults on Prolific (18-90, M = 47, SD = 16) at three time points: once before and two times following the 2018 Midterm Elections. We collected political engagement ratings, baseline emotion ratings, emotion ratings following the election, and self-reported emotion regulation behavior (e.g., situation modification, situation selection, reappraisal, seeking social-support). In our analyses we treated age as a continuous variable predicting differences in emotion ratings and emotion regulation reports. Consistent with past research, age predicted a decrease in negative and an increase in positive emotions at baseline (before the election). Controlling for political affiliation and we found that age predicted a lower likelihood of using social support regulation and situation modification. We also found that age was inversely predictive of the use of multiple strategies, such that younger adults are more likely to rely on a larger array of regulatory strategies than older adults. These results suggest that age-related differences in self-reported emotions in daily life may be attributed to a reduction in regulation-strategy usage over the life-span, and perhaps a reduced need to regulate negative emotion.

### SESSION 1390 (POSTER)

### MENTAL HEALTH

### RELATED FACTORS OF MENTAL HEALTH WELL-BEING IN THE PARTICIPANTS OF SENIOR CITIZENS' COLLEGE

Hayato Uchida,[1] and Shinro Matsuura[2], *1. University of Hyogo, Himeji, Hyogo, Japan, 2. Matsuura Clinic, Himeji, Hyogo, Japan*

The aim of this study was to clarify the mental health well-being and its related factors of participants in senior citizen's college. The participants were 364 persons (age 69.4+/-6.1) aged sixty years or over living in Hyogo Prefecture, Japan. We conducted a cross- sectional study that included age, family structure, employed status, self-related health, presence of chronic disorders, Instrumental ADL (TMIG index of competence score), dietary variety score (1-10), cognitive social capital, structural social capital, the scales of grandchild-grandparent relationships, Japanese version of the abbreviated Lubben Social Network Scale (LSNS-6), the frequency of going outdoors, and Mental health well-being was assessed using the Japanese version of the World Health Organization Mental Health Well-being Index-five items, WHO-5. We carried out the surveys in October in 2018. The total score of WHO-5 was 19. 6+/-4.7 among all participants. From the results of multivariate logistic regression analysis, "social isolation" (OR=4.001, 95%CI=1.584-10.043) was independently associated with low mental health well-being (WHO-5). These results suggest that, to advance the well-being of the elderly, it is necessary to develop and implement the projects which promote social functions including close relationship with others, neighbors or family members.

### MEASURING MINDFULNESS IN OLDER ADULTS: A LITERATURE REVIEW OF EXISTING MEASURES

M. Lindsey Jacobs,[1] Jacqueline Gurevitch,[2] Casey Catlin,[2] and Patricia M. Bamonti[2], *1. VA Boston Healthcare System, Brockton, Massachusetts, United States, 2. VA Boston Healthcare System, Boston, Massachusetts, United States*

Mindfulness is the process of non-judgmental, present-moment awareness and has increasingly been used in psychotherapy. Self-report measures that assess mindfulness are vital in clinical research and practice in order to determine the effectiveness of mindfulness-based psychotherapies. Many mindfulness measures have been developed, but it is unclear which measures have been validated with older adults. The purpose of this review was to identify measures that have been validated in adults age 50 and older. PubMed was searched through March 2019. Search terms were "mindfulness" and terms denoting measurement (e.g., "measure," "measurement"). Review articles, dissertations, and non-English publications were excluded. Articles were independently evaluated by three raters. Studies describing measures that did not exclusively evaluate mindfulness were excluded. Sixty-two articles were included and described 27 mindfulness measures. The most frequently studied measures were the Five Facet Mindfulness Questionnaire (n=13) and the Mindfulness Attention Awareness Scale (n=14). Only three psychometric studies had a participant sample with a mean age of 50 and over, with one study having a sample with a mean age of 71. The measures evaluated in these studies were the Five Facet Mindfulness Scale and Langer Mindfulness Scale. Eighteen studies included some older adults, though the numbers were not large enough to yield a mean age over 50. Validation of mindfulness measures in adults age 50 and over has been largely neglected. Additional psychometric research is needed to validate commonly used measures in this population.

### THE AGING JOURNEY RAILWAY, ILLICIT DRUG (DE) RAILS, AND CARE: OLDER USERS IN BRAZIL AND PORTUGAL

Maciane Lourenço,[1] Talita Bueno,[2] Priscila Muniz,[3] Robson Pavão,[4] Maria Gabriela França,[2]

Jaqueline Da Silva,[5] and Emiliane Ferreira[6], *1. Enfermeira do Hospital Federal Servidores do Estado, Federal University of Rio de Janeiro, Brazil, 2. Federal University of Rio de Janeiro, Rio de Janeiro - RJ, Brazil, 3. Federal University of RIo de Janeiro, Federal University of RIo de Janeiro, Brazil, 4. Federal University fo Rio de Janeiro, Rio de Janeiro, Brazil, 5. Hospital Federal Servidores do Estado, Petrópolis, RJ, Brazil, 6. Federal University of Rio de Janeiro / UFRJ - Psychiatry Institute / IPUB, Rio de Janeiro, Rio de Janeiro, Brazil*

The study object was motivation to sustain self-care of older drug users. Objective: Analyze older drug users´ motivation to sustain self-care practices. Specific objectives: (i) To characterize social-demographic profile, physical and mental health of older illicit drug users. (ii) Describe everyday practices classified as self-care by older drug users; (iii) Discuss what helps and hinders older drug users´ motivation to sustain self-care. Methodology: Qualitative, descriptive, study using Grounded Theory (GT) to guide the process of data collection, analysis and discussion. Sixty individuals 60+ who used or are current users of illicit drugs were contacted through the snowball technique and interviewed in Brazil and Portugal. Data collection instruments were: (i) a semi-structured question guide; (ii) a spreadsheet for quantitative data collection; (iii) field notes; and (iv) memos were filled out throughout the research process, with the purpose to contribute with and enlighten data analysis and discussion. Results: From data submitted to GT analytical processes, 152 codes emerged, were grouped in 10 subcategories and three categories: "Life trajectory"; "Drugs" and "Health tensions and balance practices". Conclusion: Data results on older drug users´ motivation to sustain self-care practices gave rise to the central category "the aging journey railway, drug (de) rails and care: ways in Brazil and Portugal" revealing self-care as key in the treatment, also highlighting significant individuals and therapeutic modalities as consubstantial influence for such maintenance.

### BURDENS OF HONOR: EXAMINING HONOR IDEOLOGY, SUICIDE RISK-FACTORS, AND AGEISM IN OLDER ADULTS

Jarrod Bock,[1] and Ryan P. Brown[2], *1. Oklahoma State University, Stillwater, Oklahoma, United States, 2. Rice University, Houston, Texas, United States*

Prior research has demonstrated that rates of suicide increase as men enter older adulthood and that these rates are even higher in honor-oriented regions of the U.S. (particularly among White men). Research into the honor-suicide link has suggested explanatory factors that coincide with the interpersonal theory of suicide, such as untreated depression, heightened risk-taking, and the use of firearms in suicide; however, factors related to aging (e.g., ageism, anxiety about aging) have yet to be examined. The present study examined ambivalent ageism, permissive attitudes toward suicide, and interpersonal risk-factors for suicide as explanations for the honor-suicide link among a sample of 201 older American men (Mage = 56.45, SD = 8.35, range = 44-77 years of age). After controlling for participant age and religiosity, participants with greater endorsement of honor ideology but lower levels of honor fulfillment expressed heightened levels of thwarted belongingness ($\beta = 0.35$, $p = .001$)—an established interpersonal risk-factor for suicide. Additionally, lower levels of honor fulfillment predicted greater anxiety about aging ($\beta = -0.41$, $p < .001$), greater perceived burdensomeness ($\beta = -0.39$, $p < .001$), and more positive implicit attitudes toward youth ($\beta = 0.27$, $p = .019$). Conversely, greater levels of honor fulfillment predicted more positive attitudes toward aging ($\beta = 0.20$, $p = .025$). Our results extend previous research on the honor-suicide relationship by demonstrating the utility of marrying the interpersonal theory of suicide with research on cultures of honor.

### STRENGTHENING AGE-FRIENDLY COMMUNITIES THROUGH CAPACITY BUILDING TO ADDRESS BEHAVIORAL HEALTH CONCERNS

Bronwyn Keefe,[1] and Rita Kostiuk[1], *1. Boston University, School of Social Work, Boston, Massachusetts, United States*

The Center for Aging and Disability Education and Research at Boston University in collaboration with the Age-Friendly New Bedford received funding from Tufts Health Plan Foundation to reduce the stigma of mental illness and increase awareness of the effects of social isolation in the community. In order for older adults to be fully engaged in community life, behavioral health concerns need to be addressed with a focus on social isolation, depression, and substance use. Many Age Friendly efforts don't address these issues even though significant numbers of older adults are impacted. Without a community-wide capacity building effort, behavioral health issues among older adults often fade into the background. We developed and implemented a 3-tiered approach to incorporating behavioral health into an Age Friendly initiative. In the first tier, we focused on increasing awareness of the impact of behavioral health concerns and stigma by creating an anti-stigma campaign in multiple languages. The second tier focused on holding workshops for older adults on behavioral health related issues. The third tier provided training to key stakeholders, including aging service providers, clergy, first responders, and resident coordinators focusing on the need to effectively identify and respond to older adults with behavioral health concerns. Using the Depression Stigma Scale, we measured perceived stigma among older adults pre and post workshop participation. We found statistically significant changes in how older adults perceive depression after participation in the workshop. Training results were also statistically significant with gains pre-post training in key competency areas.

### THE RELATIONSHIP BETWEEN MARITAL STATUS, COHORT, AND DEPRESSION SYMPTOMS IN TAIWAN

Fang-Yi Huang,[1] and Min Li[2], *1. Trinity Washington University, College Park, Maryland, United States, 2. University of Florida, GNV, Florida, United States*

Objectives: The relationship between marital status and depression symptoms is well documented. However, how the negative economic shock affect relationship differ by gender and cohort is still indecisive. The dataset "2011 wave of the Taiwan Longitudinal Study in Aging" and logistic regression models were used in the study. The results: Marital status is related to depression symptoms, but it differs by gendered cohort. With considering financial shock, there is no difference of depressive symptom between divorced and married female. The divorced and widowed have 4.81 and 2.47 times higher of getting depression symptom than the married for baby boom female. Being divorced is 3.67 times higher of getting depressive symptoms than being married for baby boom male. For WWII female, the widows are 1.78 times higher to have depressive symptoms than the married. being divorced, widowers, and single are 3.32, 2.21 and 2.90 times higher of getting depressive symptoms than being married for WWII male. Being divorced is 3.67 times higher of getting depressive symptoms than being married for baby boom male. In conclusions, people with unstable marital statuses are more depressed than the married. In particular, the effect of unstable marital statuses on depression could be account for by financial decline for women but not men. Given the policy emphasis on those with unstable marital status and economic decline, divorce female and single baby boom female may represent particular groups in whom interventions designed to financially support.

### RELATIONSHIPS BETWEEN ANXIETY AND PERSONALITY DISORDER FEATURES IN YOUNGER AND OLDER ADULTS

Olivia R. Noel,[1] Lisa E. Stone,[1] and Daniel L. Segal[1], *1. University of Colorado at Colorado Springs, Colorado Springs, Colorado, United States*

Introduction: Anxiety is a prevalent problem that has been found to be associated with multiple other mental disorders, functional impairments, and poor quality of life. Specifically, it appears that personality may play a major role in anxiety based on preferred dispositional coping methods and presence of normal and dysfunctional personality traits. The purpose of this study was to examine associations between anxiety and personality disorder (PD) features. It was hypothesized that anxiety would have positive associations with the avoidant, dependent, obsessive-compulsive, schizotypal, paranoid, and borderline PD scales. Method: Community-dwelling older adults (N = 130) and younger adults (N = 243) completed the Geriatric Anxiety Scale (GAS) and the Coolidge Axis Two Inventory (CATI) as part of a larger assessment battery. Correlations were computed between the GAS total score and the 14 PD scales from the CATI. Results: Results showed that anxiety was significantly and positively associated with all 14 PD scales. Specifically, as expected, the schizotypal (.52), paranoid (.55), avoidant (.56), obsessive-compulsive (.60), dependent (.62), and borderline (.69) PD scales were all significantly positively associated with anxiety. The remaining 8 PD scales also showed strong, positive correlations with anxiety: sadistic (.27), antisocial (.28), schizoid (.32), histrionic (.42), narcissistic (.44), passive-aggressive (.59), self-defeating (.64), and depressive (.69). Discussion: These results indicate that anxiety and abnormal personality traits are highly associated, showing a strong comorbidity. An implication is that PDs may play a role in the development of anxiety, or vice versa. Longitudinal studies are needed to clarify temporal and causative relationships.

### PERCEIVED NEIGHBORHOOD SOCIAL DISORDER, FRIENDS' SUPPORT, AND DEPRESSIVE SYMPTOMATOLOGY OF NON-MARRIED OLDER WOMEN

Seungjong Cho,[1] and Aloen Townsend[1], *1. Case Western Reserve University, Cleveland, Ohio, United States*

This study examined whether perceived neighborhood social disorder predicted depressive symptoms among non-married older women (N = 823) drawn from the 2016 Health and Retirement Study. It also tested the stress buffering effect of friends support. A negative binomial regression model showed that higher perceived neighborhood social disorder was associated with higher depressive symptoms. Presence of good friends in the same neighborhood and number of close friends were protective factors, but no stress buffering effect of friends support was identified. This study highlights the adverse effect of negative perceptions of the neighborhood social environment on non-married older women's depressive symptoms.

### MENTAL HEALTH POLICY OUTCOMES: AN EXAMINATION OF OLDER ADULTS' MENTAL HEALTH SERVICE USE, 2002-2012

Katy-Lauren Ford,[1] and Giyeon Kim[2], *1. UPMC, Pittsburgh, Pennsylvania, United States, 2. Chung-Ang University, Seoul, Korea, Republic of*

Objectives: Though several national-level mental health policies have been enacted and implemented over the past decade, older adults' rates of mental health service (MHS) utilization remain low. We aimed to examine individual- and community-level factors that have fostered the most successful implementations of national mental health policies in recent years. Methods: We conducted a multilevel growth curve analysis to examine older adults' MHS use using the Medical Expenditure Panel Survey – Household Component, or MEPS-HC. We considered MHS use in the MEPS-HC for the period of 2002-2012, during which members of MEPS Panels 6-17 provided responses. We identified 8,416 respondents aged 65+ with mental health need, and examined the rates of actual MHS utilization among this sample as it varied by insurance status, rural/urban location, and race/ethnicity. Results: Analyses revealed that rates of older adults' MHS use did not increase significantly over our examination period, regardless of race/ethnicity or rurality of location. Only insurance status was a significant predictor of change in MHS use rates over the years 2002-2012; $t=3.93$ (19), $p<0.001$. Conclusions: Findings suggest that rates of MHS use remained stagnant over the decade examined, revealing problems with implementation of relevant policies for older adults. Additionally, our analyses highlighted that although there were no disparities in rates of MHS use by geographic location or race/ethnicity, there may be significant disparities in identified need for services among older adults. We make suggestions for ensuring greater efficiency and efficacy of efforts to improve older adults' MHS use in the coming decade.

### DOES MUSICKING IMPROVE WELL-BEING IN LATER LIFE? USING BASIC PSYCHOLOGICAL NEEDS THEORY TO EXPLORE THE QUESTION

Anna Wong,[1] and Koji Matsunobu[2], *1. The Chinese University of Hong Kong, Shatin, New Territories, Hong Kong, 2. The Education University of Hong Kong, Tai Po, New Territories, Hong Kong*

Older adults are vulnerable to developing poor mental health as they experience significant life changes such as retirement, widowhood, living alone, institutionalisation, physical and/or cognitive deterioration in oneself or one's partner, which are associated with increased depression and loneliness. Understanding the mechanisms and identifying effective measures that strengthen their capacities to cope are therefore very important. Extensive research has suggested that active music-making with others has many psychosocial benefits for older adults. This study explored in detail the musical experiences drawn from different settings of Japanese and Hong Kong music communities. Semi-structured group interviews were conducted for members of music-making groups in Japan and Hong Kong who regularly practised, rehearsed, and performed their instruments together in community settings. A phenomenological approach was used to capture and analyse their lived experiences. A needs satisfaction theoretical framework was adopted to shed light on links between their musical engagement and wellbeing outcomes. Active musical engagement was found to be an important source of support for older musicians' psychological needs. It was central to their positive identity development and sense of purpose in old age. The presentation will further elaborate on underlying mechanisms that linked social, emotional, and artistic experiences of active musical engagement to wellbeing. This study identified active musical engagement as an effective agent in healthy ageing. Differential manifestations of need-supportive practices in musically and culturally distinct communities in Japan and Hong Kong were also described, giving evidence for the positive value of community music groups for promoting mental health and wellbeing.

### QUALITY OF LIFE IN OLDER ADULTS RECEIVING IMMUNE CHECKPOINT INHIBITOR THERAPY

Aasha I. Hoogland,[1] Sarah L. Eisel,[2]
Nathaly E. Irizarry-Arroyo,[2] Brian James,[3] Jori Mansfield,[4] Elizabeth A. Lafranchise,[4] and Heather S. Jim[2], *1. Cancer Center, Tampa, Florida, United States, 2. Moffitt Cancer Center, Tampa, Florida, United States, 3. Morsani College of Medicine, University of South Florida, Tampa, Florida, United States, 4. University of South Florida, Tampa, Florida, United States*

Immune checkpoint inhibitors (ICIs) have generated significant excitement for their ability to extend survival in patients with lung, head and neck, and other cancers. In older adults with cancer, emerging research suggests that ICIs improve overall and progression-free survival, but few studies have reported on quality of life (QOL). The goal of this study was to examine changes in QOL over time in older (65+ years) vs. younger (<65 years) lung and head and neck cancer patients. Eligible participants scheduled to begin ICI for lung or head and neck cancer completed the Functional Assessment of Cancer Therapy General (FACT-G) every 2-4 weeks until disease progression. Controlling for cancer site, age group differences in QOL over time were evaluated using linear mixed models. A total of 80 lung cancer (mean age=66.5, 55% female, 55% aged 65+) and 55 head and neck cancer patients (mean age=61.5, 15% female, 45% aged 65+) provided consent. At baseline, patients with head and neck cancer reported significantly lower overall QOL, physical well-being, and emotional well-being compared to patients with lung cancer (ps<.0001). Older patients had marginally higher baseline emotional well-being than younger patients (p=.07). Across groups, there were increases in social well-being (p=.04) and a trend toward decreasing physical well-being (p=.10) over time. Patients with head and neck cancer reported lower emotional well-being over time than patients with lung cancer (p<.01). There were no age differences in QOL over time. Larger longitudinal studies are needed to better understand QOL in older patients receiving ICIs.

### PSYCHOSOCIAL FACTORS INFLUENCING DEPRESSIVE SYMPTOMS AMONG OLDER ADULTS FROM NORTHERN INDIA

Kasturi Banerjee,[1] and Tamara A. Baker[1], *1. University of Kansas, Lawrence, Kansas, United States*

Global data show a significant increase in the number of adults 65+ years of age in India. Despite this increase, there is a dearth of available resources to adequately service their mental health needs. Data indicate that residents in Northern India, in particular, report poorer mental health outcomes than those in the South. The prevalence and impact of neuropsychiatric disorders and depression remain particularly significant, but largely unexplored. The aim of this study was to examine possible psychosocial and health factors affecting depressive symptoms in North India. Data were taken from the Longitudinal Ageing Study in India (LASI). Participants included adults aged 45 years and above (n=792), from the states of Rajasthan and Punjab. A multiple linear regression model was calculated to determine the influence of identified demographic and psychosocial factors (e.g., financial and social support, life satisfaction) on depressive symptoms. Data show that low life satisfaction ($\beta$= -0.19,p<0.001), poorer self-reported health ($\beta$=0.15,p<0.01), and being a care provider ($\beta$= -0.12,p<0.01) were significant predictors of depressive symptoms. These results indicate an increased need for care-giver mental health support along with policy aimed at awareness about caregiver burnout, health care access, and economic instrumental support services. A magnified view of the impact of life satisfaction on depression will be of immense value for understanding the unique needs and challenges of working with this population.

### THE IMPACT OF SUBJECTIVE WELL-BEING ON LONGEVITY AMONG OLDER ADULTS

Sylvia Y. Wang,[1] Giyeon Kim,[2] and Ansley Gilpin[1], *1. The University of Alabama, Tuscaloosa, Alabama, United States, 2. Chung-Ang University, Seoul, Korea, Republic of*

Objectives: People tend to believe happier people live longer. However, relatively few empirical studies have examined the influence of subjective well-being (SWB) on longevity among older adults. Thus, our study investigated the impact of SWB on longevity among older adult using national representative longitudinal data in the U.S. Methods: Drawn from the National Health and Aging Trends Study, 6,757 older adults aged 65 or older with completed information of SWB from 2011 were selected and followed until 2017 annually. The Kaplan-Meier estimator was used to estimate the survival time between different levels of SWB without covariates. In addition, the Cox Proportional Hazards Model was used to investigate the impact of SWB on longevity while adjusting the influences of covariates. Results: We found that a higher level of SWB predicted longer survival times among older adults. The impact of SWB on survival times remained to be significant, but weaker, after adjusting the influences of age, educational attainment, household income, gender, marital status, number of health insurances, self-rated health, chronical medical illness, and mental health. Conclusion: Findings suggest that happier older adults live longer. Recognizing the importance of SWB on longevity, healthcare providers should develop programs promoting higher SWB to prolong life for older adults.

### THE ASSOCIATION BETWEEN MULTIPLE CHRONIC CONDITIONS AND DEPRESSIVE SYMPTOMS: DISTINCTIONS BY RACE, GENDER, AND AGE

Christy L. Erving,[1] and Cleothia Frazier[1], *1. Vanderbilt University, Nashville, Tennessee, United States*

Multiple chronic conditions (MCC)—the co-occurrence of two or more chronic diseases—is a serious concern due to its high prevalence among middle-age and older-adults, and its association with increased disability, mortality risk, and healthcare costs. A growing body of work has shown that chronic physical conditions are associated with depressive symptoms. While MCC and depression affect a substantial proportion of older adults in general, there are important status variations in disease burden along the dimensions of race, gender, and age. This study employs an intersectional and multi-hierarchical approach to assess how these status characteristics (race, gender, and age) may condition the MCC-depression association. We use data from the 1994-2014 waves of the Health and Retirement Study (HRS), a nationally representative data source providing a longitudinal survey of U.S. adults over the age of 50 biennially. Results revealed that MCC was positively associated with depression in general. However, Black Americans, women, and younger adults were more likely to experience depression relative to their White, male, and older counterparts, respectively. The findings suggest that the lived experience of MCC differs by social status, and is perhaps due (in part) to status differences in access to social resources to counteract the potentially deleterious psychological effects of MCC. This research has also has practical implications: given the strong MCC-depression association, older adults with MCC should be offered psychological services to decrease the likelihood of developing mental health problems due to the stress associated with having multiple chronic conditions.

### THE EFFECT OF OLDER ADULTS' STRESS AND COPING ON LIFE SATISFACTION

Meeryoung Kim[1], *1. Daegu University, Daegu, Korea, Republic of*

Older adults experience stressors in everyday life, which can be acute or chronic stressors. When people are stressed, coping abilities and social support are important factors for increasing their life satisfaction. Using Pearlin et al.'s (1990) stress process model, this study compared whether acute or chronic stressors were more stressful. Additionally, the effectiveness of emotional, informational and instrumental support on life satisfaction were compared. This study used the 3rd and 5th addition wave of KReIS (Korean Retirement and Income Studies) which were collected in 2009 and 2014 respectively. For socio economic status, 3rd wave data was used. Independent and dependent variables were derived from 5th addition wave data. The sample size of this study was 4,072 older adults who were 65+. Daily hassles were used to indicate acute stressors, and physical and economic strain were used to indicate chronic stressors. For coping resources, social support and coping were used. Life satisfaction was used for dependent variable. Since the stress model is a process model, hierarchical multiple regression was used. Both acute and chronic stressors had a significant effect on reducing life satisfaction. Coping and social support both had significant positive effects on life satisfaction. In regard to social support, emotional and instrumental social support had significant effects on life satisfaction. These results implied that chronic stressors were more stressful than acute stressors. In addition, instrumental social support was found to be better than emotional social support for increasing life satisfaction for Korean older adults.

### HIGH RISK OF NEW PSYCHIATRIC DISORDERS AND SUICIDAL BEHAVIOR IN DEMENTIA

Amy Lai,[1] Yixia Li,[1] and Amy Byers[1], *1. SF Veterans Affairs Health Care System, San Francisco, California, United States*

Occurrence of new mental health (MH) disorders in patients with dementia is neglected, with next to nothing known. We examined association between dementia diagnosis and risk of new psychiatric disorders and suicide, and MH services use. We merged four national databases from US Department of Veterans Affairs. Sample included 2,529,181 patients (≥50 years) in fiscal years (FY) 2012-2013 with no MH disorders. Dementia, psychiatric disorders (mood, anxiety, substance), suicidal behavior (ideation, plan, attempt, death by suicide) were identified by ICD-9/10 codes and national suicide databases. Hazard ratios (HR) were estimated using Cox proportional hazard models, with time-to-event defined as age at first diagnosis of MH disorder during FY 2014-2016. Analyses adjusted for medical/sociodemographic factors. Compared to those without dementia, dementia patients showed roughly 2-fold increased risk of new mood (HR: 2.19, 95% Wald CI: 2.15-2.24, p<.001) or anxiety (HR: 1.56, 95% CI: 1.50-1.63, p<.001) disorders. Recent dementia diagnosis was associated with highest risk of these disorders than prior or no diagnosis; for example, patients with recent diagnosis showed 72% greater risk of anxiety disorders (HR: 1.72, 95% CI: 1.63-1.81, p<.001). Although patients with prior dementia diagnosis had lower risk of suicidal behavior, risk increased with recent dementia diagnosis. However, dementia patients with new MH disorders showed little MH services use (< 20%). Patients with dementia have increased risk of new MH disorders, especially recent dementia diagnosis. Furthermore, MH services are underutilized, highlighting critical need for integration of such services in caring for dementia patients.

### INCREMENTAL VALIDITY OF OPTIMISM AND PURPOSE FOR PREDICTING OLDER ADULTS' DEPRESSIVE SYMPTOMS

Lauren L. Mitchell,[1] Chris Erbes,[1] and Paul Arbisi[1], *1. Minneapolis VA Health Care System, Minneapolis, Minnesota, United States*

After age 60, depressive symptoms tend to increase slowly over time on average across the population. However, individual trajectories vary, with some increasing more steeply, and others remaining stable. A broad array of psychological constructs have been demonstrated to predict depressive symptoms, including neuroticism, extraversion, optimism, and sense of purpose in life. It is important for psychologists to understand which among these factors are the strongest and most robust predictors. A substantial body of research demonstrates that Big Five personality traits are strongly associated with depressive symptoms (e.g., Hakulinen et al., 2015). Optimism and purpose are also associated with well-being (Carver et al., 2009; Pinquart, 2002), but it is not clear whether such associations could be accounted for by Big Five traits, which are also correlated with optimism and purpose. Using data from the Health and Retirement Study (N = 14,021), we tested the incremental validity of optimism and purpose for predicting older adults' depressive symptoms, controlling for Big Five traits and demographics. A latent growth curve modeling approach allowed us to examine associations with trajectories of depressive symptoms over six waves (approximately 10 years). Results demonstrated that both optimism and purpose are significantly associated with baseline levels of depressive symptoms, over and above the Big Five. However, only Big Five traits were associated with linear and quadratic slope in depressive symptom trajectories. These findings suggest that optimism and purpose are not redundant with Big Five traits for predicting depressive symptoms, and may be valuable targets for intervention efforts.

### NEUROPSYCHIATRIC SYMPTOMS AND FUNCTIONAL ACTIVITIES AMONG OLDER ADULTS: THE ROLE OF ETHNICITY AND COGNITIVE STATUS

Shanna L. Burke,[1] Adrienne Grudzien,[1] Mitra Naseh,[1] and Tamara J. Cadet[2], *1. Florida International University, Miami, Florida, United States, 2. Simmons University, Boston, Massachusetts, United States*

Little is known about the likelihood of future functional deficits based on current neuropsychiatric symptoms (NPSs). This study seeks to examine the impact of NPSs on functional activities (FAs) by cognitive status and ethnicity. A secondary analysis of the National Alzheimer's Coordinating Center Uniform Data Set was conducted using ordered logistic regression to examine the effect of NPSs (based on the Neuropsychiatric Inventory Questionnaire [NPI-Q]) on FAs (based on Functional Assessment Questionnaire). Participants had a mean age of 74 (SD: 9.88) and were included if normal cognition was assessed at baseline (n= 13,470). Higher rates of NPSs were associated with higher dependency in almost all FAs. Among NPSs, apathy was the best predictor ($p<.05$) of FAs for participants in different cognitive groups and ethnicity subsamples. The impact of other NPSs varied. Anxiety and apathy were the best predictors of FAs among participants with cognitive impairment (but not MCI). Among those who eventually developed dementia (n= 6,818), delusions, hallucinations, agitation, depression, irritability, and motor disturbance were significantly associated ($p<.05$) with future deficits in FAs. Among Hispanics (n=1,095), hallucinations, agitation, apathy, and motor disturbance were significantly associated with dependency in FAs, while for non-Hispanics, all NPSs were associated with dependency in FAs, except elation and nighttime disturbance. Findings suggest as the severity of the NPSs increases, older adults experience higher levels of dependency in FAs. The nature and extent of NPSs' impact on FAs varied based on cognitive status and ethnicity, suggesting the importance of considering these factors in service provision.

### EVALUATION OF A TELEPHONE-BASED PROGRAM DESIGNED TO REDUCE LONELINESS IN OLDER VETERANS

Christine E. Gould,[1] Christine Juang,[2] Terri Huh,[3] and Sowmya Iyer[2], *1. VA Palo Alto Health Care System, Palo Alto, United States, 2. VA Palo Alto, Palo Alto, California, United States, 3. San Francisco VA Health Care System, San Francisco, California, United States*

We examined the benefits and challenges of implementing a program aiming to reduce older Veterans' loneliness. The program helped connect older Veterans with others through telephone-based group activities. Thirty-four Veterans were enrolled in the program, of which 14 attendees called in to one or more activities. Data were collected at baseline, 3-months, and 6-months. Differences in baseline characteristics between attendees and non-attendees were calculated. Changes in Veterans' perceived loneliness, depression, anxiety, and quality of life were evaluated. Client satisfaction were analyzed. Qualitative data analyses were conducted to identify perceived benefits and challenges of participating in this program. Attendees were more likely to be depressed and have limited environmental resources to promote quality of life than non-attendees. A significant decrease in loneliness was found among attendees from baseline to 3-months. Overall, Veterans were satisfied with this program and the services provided. Perceived benefits included a structured program with interesting topics to spend time on as well as the opportunity to socialize, exchange ideas, and connect with other Veterans. Perceived individual challenges (e.g., hard of hearing) and program-level challenges (e.g., complicated procedures) were observed. Results demonstrated that this telephone-based program is feasible and beneficial for older Veterans to reduce loneliness. Several barriers and challenges were noted, providing insight into ways to improve the implementation of this program.

### DISCRIMINATION AND DEPRESSION IN MID- TO LATE LIFE: THE ROLE OF OPTIMISM

Elliottnell Perez,[1] Joseph Dzierzewski,[1] and Scott Ravyts[1], *1. Virginia Commonwealth University, Richmond, Virginia, United States*

he positive association between discrimination and depression is well-supported throughout the literature. Previous evidence exploring potential mechanisms suggest discrimination is associated with depression via changes in social cognition. The goal of the current study was to investigate whether optimism explained the relationship between discrimination and depressive symptoms in mid-to-late life. Furthermore, the study assessed whether this mediated relationship was moderated by race or sex. This study included cross-sectional and longitudinal secondary data analysis of 2453 middle-aged and older adults (M age=68.30,SD=8.93) from the Midlife in the United States study. Discrimination was measured using an 11-item count of the number of discriminatory events experienced. Optimism was measured using the 6-item Life Orientation Test. Depressive symptoms were assessed using a 7-item count of the number of symptoms experienced. Optimism mediated the relationship between discrimination and depressive symptoms cross-sectionally, 95% CI:[.012, .034], and longitudinally, 95% CI:[.008, .024]. There was no evidence of moderated mediation; however, sex did moderate the direct relationship between discrimination and depressive symptoms cross-sectionally, b=.10, 95% CI:[.001, .194], and longitudinally, b=.03, 95% CI:[.01, .05]. The current study extends the literature by providing cross-sectional and longitudinal support for optimism as a mechanism through which discrimination leads to depressive symptoms in older adults. Evidence also suggests that women experience greater depressive symptoms than men in response to discrimination. Future research may wish to examine the developmental course of observed relationships and the impact of multiple marginalized identities on these relationships.

### SESSION 1395 (POSTER)

### MULTIMORBIDITY

### MUSCLE WEAKNESS IS ASSOCIATED WITH DISABILITY AND CHRONIC MULTIMORBIDITY IN MIDDLE-AGED AND OLDER AMERICANS

Mark Peterson,[1] and Jessica Faul[1], *1. University of Michigan, Ann Arbor, Michigan, United States*

Background: The objective of this study was to use nationally-representative data on Americans age 50+ to determine the association between grip strength and inflammation as independent predictors of incident disability, chronic multimorbidity and dementia. Methods: Older adults (n=12,618) from the 2006-2008 waves of the Health and Retirement Study with 8-years of follow-up were included. Longitudinal modeling was performed to examine the association between baseline grip strength (normalized to body mass: NGS) and high sensitivity c-reactive protein (hs-CRP) (≥3.0 mg/L) with incident physical disabilities (i.e., ≥2 limitations to activities of daily living), chronic multimorbidity (≥2 of chronic conditions), and dementia. Results: The odds of incident disability were 1.28 (95% CI: 1.19-1.37) and 1.27 (95% CI: 1.21-1.36) for men and women respectively, for each 0.05-unit lower NGS. The odds of incident chronic multimorbidity were 1.22 (95% CI: 1.06-1.18) and 1.12 (95% CI: 1.06-1.17) for men and women respectively for each 0.05-unit lower NGS. The odds of incident dementia were 1.10 for men (95% CI: 1.02-1.20) for each 0.05-unit lower NGS, but there was no significant effect for women. Elevated hs-CRP was only associated with chronic multimorbidity among women (OR=1.60; 95%CI: 1.26-2.02). Conclusions: Our findings indicate a robust inverse association between NGS and disability and chronic, multimorbidity in older men and women, and dementia in men. Elevated hs-CRP was only associated with chronic multimorbidity among women. Healthcare providers should implement measures of handgrip strength in routine health assessments and discuss the potential dangers of weakness and interventions to improve strength with their patients.

### MULTIPLE CHRONIC CONDITIONS AND RISK OF DEMENTIA AND COGNITIVE IMPAIRMENT

Yura Lee,[1] Youngjoo Cho,[1] and HYUNKYOUNG OH[2], *1. University of Wisconsin - Milwaukee, Milwaukee, Wisconsin, United States, 2. University of Wisconsin-Milwaukee College of Nursing, Milwaukee, Wisconsin, United States*

This study explores the relationship between the presence of multiple chronic condition and risk of dementia and cognitive impairment with no dementia (CIND) among older Americans. The study sample included 617 participants aged 70 years and older from the Aging, Demographics, and Memory Study (ADAMS). An expert consensus panel of the ADAMS data provided each participant a cognitive diagnosis into 1) no cognitive impairment, 2) CIND, or 3) dementia. The presence of multiple chronic condition was defined as having three or more chronic conditions in this study (e.g., heart attack, stroke, respiratory problems, cancer, hypertension, diabetes). Functional limitation, depression, cognitive activity engagement, apolipoprotein E (ApoE), and sociodemographic characteristics were included as covariates. A multinomial logistic regression analysis showed that individuals who have multiple chronic conditions have increased odds of being diagnosed with CIND versus no cognitive impairment controlling for other covariates. However, multiple chronic condition was not associated with increased risk of being diagnosed with dementia versus no cognitive impairment. Conclusion: The finding suggests that the presence of multiple chronic conditions may be a risk factor for cognitive impairment in later life. However, further investigation using a longitudinal design is needed to better understand the relationship between cognition and multiple chronic conditions.

### CHALLENGES IN SELF-MANAGEMENT AMONG OLDER ADULTS WITH HYPERTENSION AND DIABETES

HYUNKYOUNG OH,[1] YURA LEE,[2] Wonchan Choi,[3] and Zhi Zheng[4], *1. University of Wisconsin-Milwaukee College of Nursing, Milwaukee, Wisconsin, United States, 2. University of Wisconsin-Milwaukee Helen Bader School of Social Welfare, Milwaukee, Wisconsin, United States, 3. University of Wisconsin-Milwaukee School of Information Studies, Milwaukee, Wisconsin, United States, 4. University of Wisconsin-Milwaukee College of Engineering and Applied Science, Milwaukee, Wisconsin, United States*

This abstract introduces an ongoing research project that aimed to develop a patient-centered self-management program using health information and technologies for older adults with hypertension and diabetes. The purpose of the project in the first phase was to better understand challenges in self-management faced by older adults with both conditions. A semi-structured and face-to-face interview was conducted to explore the challenges in self-management of the target population living in Milwaukee areas, Wisconsin. Audio recordings were transcribed in verbatim; transcripts were analyzed; and themes were identified. A total of six individuals participated in this study by January 2019. Their age ranged from 56 to 75. Four of them were female; five of them were African Americans; and one was Caucasian. All participants reported more than two additional conditions that were arthritis, cardiovascular diseases, pain, kidney diseases, respiratory diseases, and depression. Most participants were self-managing their conditions mainly by taking prescribed medications. Several themes were emerged as challenges to self-management: monitoring blood pressure and glucose, engagement in physical activity, and healthy eating. Among these, participants reported healthy eating as the most difficult self-managing activity. Majority of participants expressed the need for physical activity support due to pain and/or vision problems known as one of diabetic complications. Understanding challenges and needs of a specific population is the first step for health care providers to support self-management of the patients appropriately. The results of this preliminary study will help health care providers develop effective self-management programs for older adults with both conditions.

### MULTIPLE CHRONIC CONDITIONS IN OLDER CANCER SURVIVORS WITH AND WITHOUT HIV

Siran Koroukian,[1] Nicholas Schiltz,[1] Johnie Rose,[1] Gregory Cooper,[1] Cynthia Owusu,[1] Abby Statler,[2] Scott E. Moore,[3] and Sarah Markt[1], *1. Case Western Reserve University, Cleveland, Ohio, United States, 2. Cleveland Clinic, Cleveland, Ohio, United States, 3. Frances Payne Bolton School of Nursing, Case Western Reserve University, Cleveland, Ohio, United States*

Background: With more effective treatment for both HIV and cancer, longevity among persons living with HIV (PLwHIV) has improved significantly. However, little is known about whether the comorbidity profile of cancer survivors differs between PLwHIV and their HIV-free counterparts. To address this critical gap in knowledge, we compared the occurrence and combination of multiple chronic conditions (MCCs) among older cancer survivors by HIV status. Methods: We used national data from the 2014 Chronic Conditions Data Warehouse (CCW) as part of the Medicare Beneficiary Summary File, which flags 66 conditions, including HIV/AIDS, and history of common cancers (colorectal, lung, prostate, and leukemias/lymphomas). We limited our study population to men age 65 years or older who were cancer survivors. In addition to descriptive analysis, we conducted association rule mining (ARM) analysis to compare the prevalence of the most common MCCs among cancer survivors, with and without HIV. Results: We identified 1.3 million individuals, of which 1,901 (0.15%) were PLwHIV. Compared to their HIV-free counterparts, PLwHIV were younger (mean of 72.5 and 77.0 years); more were non-White (41.8% vs. 13.5%); and more of them presented with anemia (44.8% vs. 35.3%), chronic kidney disease (CKD, 41.9% vs. 26.6%), depression (26.6% vs. 13.9%), viral hepatitis (20.4% vs. 0.70%), and/or liver disease (10.7% vs. 4.8%; $p < 0.001$ for all comparisons). ARM results showed a prominence of CKD in the most common top 5 combinations of conditions. Conclusion: Despite being younger than their HIV-free counterparts, PLwHIV with history of cancer carry a significantly greater comorbidity burden.

### COGNITION AND HISTORY OF MULTIPLE HEALTH CONDITIONS OVER 20 YEARS: EVIDENCE FROM THE HEALTH AND RETIREMENT STUDY

Rebecca Bendayan,[1] Alex D. Federman,[2] and Richard J Dobson[3], *1. Dept. Biostatistics and Health Informatics, Institute of Psychiatry, Psychology & Neuroscience (IoPPN), King's College London, London, United Kingdom, United Kingdom, 2. Division of General Internal Medicine, Department of Medicine, Icahn School of Medicine at Mount Sinai, New York, United States, 3. Dept. Biostatistics and Health Informatics, Institute of Psychiatry, Psychology & Neuroscience (IoPPN), King's College London, London, United Kingdom*

Research to date on multimorbidity and cognitive impairment is mainly cross-sectional or with limited history information of the health conditions. The present study explores the association between cognitive performance and previous history of health conditions over 24 years in a sample of 4858 respondents of the Health Retirement Study. Data from health conditions between 1998 and 2014 included self-reports for hypertension, diabetes, arthritis, stroke, cancer, lung and heart diseases and psychiatric problems. Duration of the health condition was categorized as more than 10 years, between 4 and 10 years, less than 4 years and no condition. Cognition was assessed using a summary index of cognitive performance including measures of memory, working memory, speed processing, knowledge, and language. ANOVA and post hoc tests were performed to explore the association between cognition and the duration of each health condition independently. Multiple linear regression analyses were performed to explore the association between multiple health conditions and cognitive performance. Results showed significant independent associations between cognitive performance in 2014 and each health condition, except for cancer [$F(1,4)=2.60$; $p =.51$]. When all the health conditions were considered in the regression models, we found that cognitive performance is negatively associated with high blood pressure and stroke (independently of the duration of the condition), long-term diabetes and lung diseases (i.e., for more than 10 years) and recent cancer (i.e., in the last 4 years). Our findings highlight that considering duration of co-existent health conditions is key for identifying individuals at greater risk of cognitive impairment.

### HEALTHCARE PROVIDERS' EXPERIENCES IN CARING FOR OLDER ADULTS WITH MULTIPLE CHRONIC CONDITIONS

Jenny Ploeg,[1] Marie-Lee Yous,[1] Kimberly Fraser,[2] Sinéad Dufour,[1] Sharon Kaasalainen,[1] Carrie McAiney,[3] Maureen Markle-Reid,[1] and Lisa Garland Baird[4], *1. McMaster University, Hamilton, Ontario, Canada, 2. University of Alberta, Edmonton, Alberta, Canada, 3. University of Waterloo, Waterloo, Ontario, Canada, 4. University of Prince Edward Island, Charlottetown, Prince Edward Island, Canada*

The management of multiple chronic conditions (MCC) in older adults living in the community is complex. Little is known about the experiences of interdisciplinary primary care and home providers who care for this vulnerable group. The aim of this study was to explore the experiences of healthcare providers in managing the care of community-living older adults with MCC and to highlight their recommendations for improving care delivery for this group. A qualitative interpretive description design was used. A total of 42 healthcare providers from two provinces in Canada participated in semi-structured interviews. Participants represented diverse disciplines (e.g., physicians, nurses, social workers, personal support workers) and settings (e.g., primary care and home care). Thematic analysis was used to analyze interview data. The experiences of healthcare providers managing care for older adults with MCC were organized into six major themes: (1) managing complexity associated with MCC, (2) implementing person-centred care, (3), involving and supporting family caregivers, (4) using a team approach for holistic care delivery, (5) encountering rewards and challenges in caring for older adults with MCC, and (6) recommending ways to address the challenges of the healthcare system. Healthcare providers highlighted the need for a more comprehensive integrated system of care to improve care management for older adults with MCC and their family caregivers. Specifically, they suggested increased care coordination, more comprehensive primary care visits with an interprofessional team, and increased home care support.

### CHRONIC CONDITION PATTERNS IN THE U.S. POPULATION AND THEIR RELATIONSHIP WITH MORTALITY

D. Diane Zheng,[1] Sharon L. Christ,[2] Byron Lam,[3] Kathryn McCollister,[1] Daniel J. Feaster,[1] and David J. Lee[1], *1. University of Miami, Miami, Florida, United States, 2. Purdue University, West Lafayette, Indiana, United States, 3. Bascom Palmer Eye Institute, Miami, Florida, United States*

Using data from 372,933 participants age >18 years from National Health Interview Survey 2002-2014, we employed latent class analysis to develop latent classes or subgroups of participants based on their combination of 13 self-reported chronic conditions. Mortality linkage with National Death Index was performed through December 31st, 2015. Survival analyses were conducted to assess how the derived latent class membership predicted all-cause mortality and cause specific mortalities. Five latent groups were identified with 70.5% of the participants belonging to the "healthy group". The other four groups represented various degrees and patterns of multi-morbidity and were labeled accordingly: "hypertensive group" 20%, "respiratory condition group" 3.9%, "heart condition group" 3.7%, and "severely impaired group" 1.9%. 32,609 deaths were identified with average follow-up time of 6.93 years. After controlling for survey design, age, gender, race, Hispanic origin, education, income, health insurance, BMI, smoking and alcohol drinking status, compared to the healthy group, participants in all four latent disease groups had elevated mortality risk: hypertensive group Hazard Ratio(HR) 1.57 (95% confidence interval [1.49, 1.65]); respiratory condition group (2.08 [1.93, 2.24]); heart disease group (2.27 [2.13, 2.42]); and severely impaired group (3.84 [3.55, 4.16]; all p-values <0.01). Patterns of the chronic condition classes were also strongly associated with the primary underline cause of death. Four multi-morbidity groups, comprising 29.5% of the US population were at significantly elevated risk of mortality. Assessing patterns of disease co-occurrence in the US population may be useful for identifying individuals in need of targeted interventions to reduce mortality risk.

### SELF-REGULATION STRATEGIES IN MANAGING MULTI-MORBIDITIES AMONG COMMUNITY-DWELLING PEOPLE AGING WITH ARTHRITIS

Wenhui Zhang,[1] Kavita Radhakrishnan,[1] Heather Becker,[1] Gayle Acton,[2] and Carole K. Holahan[2], *1. School of Nursing, the University of Texas at Austin, Austin, Texas, United States, 2. The University of Texas at Austin, Austin, Texas, United States*

Quantitative and qualitative evidence supported the self-regulation strategies of Selection, Optimization and Compensation (SR-SOC), used by people aging with single and multiple chronic conditions (MCCs) to adapt to chronic disabling symptoms and live well. This study investigated the SR-SOC Strategies in the self-management of community-dwelling people aging with arthritis and MCCs. 140 individuals aged > 50 completed the demographic questionnaire, Functional Comorbidity Index (FCI), Brief Health Literacy Screening, Lubben Social Network Scale, Patient-Healthcare Provider Communication Scale, Health Insurance Checklist, PROMIS Adult Self-Reported Health Measures, SOC Questionnaire, Arthritis Self-Efficacy Scale, Healthcare Service Utilization Questionnaire, and a visual analogue QOL scale. With the theoretical framework, multivariate hierarchical stepwise regression was used to predict SR-SOC Strategies, arthritis self-efficacy, healthcare utilizations and QOL. Majority of the sample were female (70%), with < Bachelor's degree (56%), White (34%) or African American (33%), with personal annual income < $25,000 (52%). Thirty-seven percent reported fairly and 26% poorly adequate income. Number of FCI count ranged from 2 to 14 (Mean =3.8). The top four comorbidities were obesity, diabetes, visual impairment and degenerative disc disease. QOL ranged from 0.5 to 10.0 (Mean=7.2, SD=2.2). Age, physical symptom cluster (pain, fatigue and cognitive abilities) and healthcare provider communication quality significantly predicted SR-SOC strategies. Income adequacy, physical symptom cluster and SR-SOC strategies significantly predicted arthritis self-efficacy. FCI significantly predicted healthcare utilization total, inpatient healthcare utilization, clinician visit and hospitalization. With income adequacy, FCI significantly predicted home health visit and emergency room visit. Being African American and FCI significantly predicted prescriptions filled.

### RACIAL-ETHNIC DIFFERENCES IN MULTIMORBIDITY PROGRESSION ACCORDING TO BODY-WEIGHT STATUS AMONG OLDER U.S. ADULTS

Anda Botoseneanu,[1] Sheila Markwardt,[2] Heather Allore,[3] Corey Nagel,[4] Jason T. Newsom,[5] David A. Dorr,[2] and Ana R. Quiñones[6], *1. University of Michigan, Dearborn, Michigan, United States, 2. Oregon Health & Science University, Portland, Oregon, United States, 3. Yale University, New Haven, Connecticut, United States, 4. University of Arkansas for Medical Sciences, Little Rock, Arkansas, United States, 5. Portland State University, Portland, Oregon, United States, 6. Family Medicine, Oregon Health & Science University, Portland, Oregon, United States*

Obesity and multimorbidity are more prevalent among underrepresented U.S. racial/ethnic minority groups. Evaluating whether racial/ethnic disparities in multimorbidity accumulation vary according to body-mass index (BMI) may guide interventions aimed at reducing multimorbidity burden in vulnerable racial/ethnic groups. We used 1998-2014 data from the Health & Retirement Study (N=8,635 participants, age 51-55 years old at baseline) and negative binomial models stratified by BMI category to evaluate differences in rates of accumulation of seven chronic conditions (arthritis, cancer, diabetes, heart disease, hypertension, lung disease, and stroke), focusing on differences between racial/ethnic groups [White (reference; 64.7%), Black (21.5%), Hispanic (13.8%)]. Overweight and obesity were more prevalent in Black (80.9%) and Hispanic (78.6%) than White (69.9%) participants at baseline; in all BMI categories, Black participants had higher rates of multimorbidity compared with White participants (normal BMI:$\beta$=0.304, p<0.001; overweight:$\beta$=0.243,p<0.001; and obese:$\beta$=0.135,p=0.013); initial burden of disease was similar between Whites and Hispanics in the normal and overweight categories, but significantly lower among Hispanics (vs. Whites) in the obese category ($\beta$= -0.180,p=0.017). We found no significant differences in rates of disease accumulation between the racial/ethnic groups in any of the BMI categories. There are substantial differences in initial disease burden between Black and White middle-aged/older adults, but not in the rate of accumulation of disease between the race/ethnic groups in the 3 main BMI categories. These findings suggest an opportunity to reduce racial disparities in multimorbidity by intervening early in the lifecourse to reduce the burden of chronic disease among vulnerable racial minorities prior to entering middle-age.

### CHRONIC ILLNESSES AND FATIGUE IN OLDER INDIVIDUALS: A LITERATURE REVIEW

Maral Torossian,[1] and Cynthia Jacelon[1], *1. University of Massachusetts Amherst, Amherst, Massachusetts, United States*

In the United states, 60% of adults have one chronic disease and 40% have at least two chronic diseases. Fatigue is a commonly reported symptom in individuals with chronic illnesses, the prevalence of which ranges between 40-74%. It is associated with multiple risk factors and has a tremendous impact on quality of life, social functioning, mood, motivation and cognition. Despite its high prevalence, the relationship between fatigue and chronic illness has not been well explored. Accordingly, the focus of this synthesis of literature is to explore fatigue-associated factors and their relation to chronic disease. The databases searched were CINAHL, PubMed, PsychInfo and Web of Science, where the following keywords were used: "Chronic disease" OR "Chronic illness" OR "Chronic conditions", "Fatigue", "Elderly" OR "Older adults" OR "Seniors" OR "Geriatrics". The synthesis resulted in four themes: understanding the concept of fatigue, factors related to fatigue, activity and fatigue, and self-management of fatigue. There were some inconsistencies in the findings among research studies which were addressed, in addition to the strengths and weaknesses of some of the fatigue measurement scales used. This literature review integrates findings about fatigue in chronic illnesses in various aspects, in the population of individuals who are of 65 age or older. The four emerged themes are of value to individuals with similar characteristics as the selected population, as well as to health care providers and researchers who may address the inconsistent findings and provide a strong evidence for best practice.

### SESSION 1400 (POSTER)

### SOCIAL DETERMINANTS OF HEALTH

### DIMENSIONS OF SOCIAL SUPPORT AND ASSOCIATIONS WITH HEALTH AMONG OLDER ADULTS

Samuel Asante,[1] and Eun-Jun Bang[1], *1. Northeastern State University, Broken Arrow, Oklahoma, United States*

Social support is fundamental to human survival, and is significantly involved in the attainment and maintenance of good health and wellbeing. Previous studies have often considered social support as a singular, non-dimensional construct. While this is important and enlightening, the method of adding up individual aspects to create a singular, non-dimensional construct has produced little understanding of these aspects/dimensions of social support and their implications for health. This study examined three dimensions or types of social support-affective, confidant, and instrumental support-and their associations with physical and mental health in older adults. Data for this study were obtain from Utah Fertility, Longevity, and Aging (FLAG) study. Participants involved 325 older adults, aged 50 years or older. Results showed a significant, strong positive correlation between affective support and physical and mental health, and weak association between confidant support and physical and mental health. The correlation between instrumental support and physical and mental health was moderate. After controlling for the influence of socio-demographic variables, affective and instrumental support significantly predicted physical and mental health. Confidant support was not a significant predictor of either physical or mental health. The findings suggest both affective and instrumental support might be relatively more important to the health and mental wellbeing of older adults than confidant support, underscoring the relative importance older adults attach to quality rather than confidant support, which essentially is quantity of social ties.

### GRANDPARENTAL CAREGIVING AND CO-RESIDENCE, SOCIOECONOMIC STATUS, AND MORTALITY IN RHODE ISLAND CITIES AND TOWNS

Julia Broccoli,[1] Steven A. Cohen,[1] and Mary L. Greaney[1], *1. University of Rhode Island, Kingston, Rhode Island, United States*

Grandparents co-residing with their grandchildren is becoming increasingly more common, with over 1.5 million grandchildren living with their grandchildren in the U.S. Furthermore, the number of grandparents who are primary caregivers for their grandchildren has also increased, which can negatively effects the grandparents' physical and mental health, and increase social isolation and financial burden. However, the associations between grandparental caregiving and health outcomes are not well understood on a population level. Therefore, the purpose of this study was to assess associations between grandparental caregiving, socioeconomic status, and population health outcomes. Using mortality data (2009-2011) from the Rhode Island (RI) Department of Health and life table methods for each RI city/town, life expectancy at age 65 (LE65) and age-standardized mortality rates (ASMR) were calculated and linked to data from the American Community Survey on grandparental caregiving responsibilities, grandparental living arrangements (co-residence), poverty status, and demographics. Correlations and multivariable linear regression modeling were used to evaluate associations among LE65, ASMR, grandparental caregiving and co-residence, demographics, and poverty. Both LE65 (rho=-0.382, p=0.016) and ASMR (rho=0.327, p=0.042) were associated with the percent of grandparents living with grandchildren. The percent of grandparents as primary caregivers to their grandchildren was not significantly associated with LE65 or ASMR. ASMR was associated with the percent of grandparents living in poverty (rho=0.401, p=0.013) and overall poverty (rho=0.363, p=0.023). These results highlight conditions of community-based living and role of primary caregivers at an older age that should be further explored to improve the health of grandparents, particularly in multi-generational homes.

### EARLY-LIFE MILITARY EXPOSURES AND HEALTH AMONG OLDER VETERANS: THE BUFFERING EFFECT OF PSYCHOLOGICAL RESILIENCE

Miles G. Taylor,[1] Stephanie Ureña,[2] Dawn Carr,[1] and Stella N. Min[1], *1. Florida State University, Tallahassee, Florida, United States, 2. Department of Sociology, Florida State University, Tallahassee, Florida, United States*

Objectives Drawing on the life course framework and theoretical concept of resilience, we examine the impact of early-life service-related exposures (SREs) on later-life functional impairment trajectories among older U.S. male veterans. We conceptualize resilience as a psychological resource potentially moderating the lasting negative consequences of traumatic military exposures. Method Using the 2013 Veterans Mail Survey linked to the Health and Retirement Study 2006–2014 Leave Behind Questionnaire and RAND Data File (v.N), we estimate latent growth curve models of functional impairment trajectories. Results SRE to death has a persistent positive effect on functional limitations and activities of daily living limitations. Psychological resilience significantly moderates this association, such that veterans maintaining higher levels of resilience in the face of adverse exposures have considerably less functional impairment over time compared to their counterparts with low levels of resilience. Discussion Our findings point to the importance of psychological resilience in later life, especially within the realm of traumas occurring in early life. We discuss implications for current military training programs, stressing the importance of research considering individual resources and processes that promote adaptation in the face of adverse life events.

### METABOLIC SYNDROME AND LATE-LIFE DEPRESSION AMONG MEXICAN AMERICANS: THE ROLE OF NATIVITY

Weihui Zhang[1], *1. University at Albany, Rensselaer, United States*

Metabolic syndrome (MS) has been reported to predict depression. However, studies evaluating if there are differences by nativity status among Mexican Americans are scarce. This study aims to examine the association between metabolic syndrome and depression among Mexican-American older adults. We also evaluated the role of nativity, sociodemographic and health risk factors. We use three waves (2006-2013) from the Hispanic Established Populations for the Epidemiologic Study of the Elderly (HEPESE; N=1,542, mean age =83.45 in 2006). MS was defined according to the National Cholesterol Education Programme (NCEP-ATP III) using abdominal obesity, use of antihypertensive medication, and insulin. Depression was ascertained by self-report of a CES-D score greater than 16. We applied random-effect logistic regression models which accounted for inter-individual correlation and adjusted for age, sex, education, smoking, alcohol use, physical performance, and self-esteem. We also tested for interaction between MS and nativity. Approximately 30% of foreign-born and 22% of US-born reported depression. The prevalence of MS was higher in the Foreign-born when compare to the US-born (5.89% vs. 5.35%). In the total sample, MS was associated with a higher risk of depression (OR=4.34, p=0.007). Foreign-born Mexican Americans were more likely to have depression (OR=1.70, p=0.002) when compared to US-born; however, foreign-born with MS reported lower depression (OR=0.26, p=0.052) after adjusting for potential confounders. Our finding adds to the concept of "metabolic depression," and further highlights the importance of evaluating nativity to explain the differences in physical and psychological health among a sample of the Hispanic population at old age.

### COHORT DIFFERENCES AND 4-YEAR CHANGE IN EVERYDAY DISCRIMINATION AMONG OLDER AMERICANS

Felicia V. Wheaton[1], *1. Bethune-Cookman University, Daytona Beach, Florida, United States*

Experiences of everyday discrimination, such as being treated with less courtesy or respect, are associated with poorer mental and physical health in later life. Yet not much is known about cohort differences in experiences of everyday discrimination, nor how subjective reports of discrimination change over time among older adults. This study assessed cohort differences and 4-year change in everyday discrimination using data from the 2006 and 2010 waves of the Health and Retirement Study (HRS), a nationally-representative study of Americans 50+. In both waves, participants were asked about 5 possible experiences of everyday discrimination: for example, "In your day-to-day life, how often have any of the following things happened to you? You receive poorer service than other people at restaurants and stores" and could respond from 0=never to 5=almost every day. Average everyday discrimination was lower for older cohorts compared with younger cohorts in both 2006 and 2010. Paired-samples t-tests showed that average everyday discrimination declined significantly over the 4-year period. However, it is unclear if changes are due to period or age effects. Implications of these findings will be discussed.

### MOVING AND GROOVING: ACTIVITY BENEFITS OF SOCIAL INTEGRATION MAY DEPEND ON RELATIONSHIP QUALITY

Colette J. Brown,[1] and Karen Rook[1], *1. University of California, Irvine, Irvine, California, United States*

New evidence indicates that being more socially integrated increases physical and leisure activities (Fingerman et al., 2019), thus reducing the health risks of being sedentary. Yet socially integrated individuals often have both positive (e.g., supportive) and ambivalent (e.g., supportive and conflictual) relationships. The current study investigated whether the two kinds of relationships exhibit comparable associations with activity levels, and in turn, health. In-person interviews conducted with a national sample of adults ages 65 to 91 (N = 916) assessed participants' social ties, frequency of vigorous physical activity and leisure activities, and functional health limitations. Hierarchical multiple regression analyses structured to test mediation were conducted. Having more social ties (both positive and ambivalent) was associated with more frequent physical and leisure activity. After adjusting for demographic characteristics, both kinds of social ties were related to health limitations, albeit in opposite directions, with positive ties related to fewer limitations and ambivalent ties related to more limitations. Once physical and leisure activities were included, the association between positive ties and health limitations became nonsignificant, whereas the adverse effect of ambivalent ties remained unchanged. These findings reveal that the health benefits of positive, but not ambivalent, social ties may be explained by physical and leisure activities. This ability to get people up and moving has, until recently, been an overlooked benefit of social integration. Further investigation of the quality of social ties will extend knowledge of the pathways by which they affect health.

### THE EFFECT OF FINANCIAL DIFFICULTIES ON COGNITIVE FUNCTION IN A JAPANESE ELDERLY POPULATION SAMPLE

Yumi Ishikawa[1], *1. Osaka School of International Public Policy, Osaka University, Osaka, Japan*

Background: Studies have been investigating the effect of financial difficulties on cognitive function especially in the US and Australia. However, there is still a discrepancy regarding the results. This study aims to estimate the effect of financial difficulties on cognitive function in a Japanese elderly population sample. It is rewarding to focus on the Japanese setting, which has the highest proportion of elderly in the world. Method: This study uses a longitudinal panel dataset which include randomly selected elderly Japanese citizens aged 60 and over; the National Survey of the Japanese Elderly. It is a panel dataset containing income and cognitive function. It is ideal dataset to capture the probability of onset of cognitive impairment as it focuses on the elderly. We estimate the effect of participants' equivalent income on the probability of onset of cognitive impairment at a following survey point using random-effect probit model. Result: The first main result is there is a significant negative effect from financial difficulties on cognitive function. The results indicate that when participants' equivalent income drops by 1%, they are 2.2% more likely to develop cognitive impairment. The second result is that this negative effect is heterogeneous, depending on their income level. Specifically, this negative effect is observed only at low income level, but not at high income level. That is, the deteriorating effect by impoverishment would be severe when the financially needy people faced income drops. Discussion: Income support plays an important role in improving recipients' cognitive function, especially among the poor.

### SOCIOECONOMIC STATUS AND THE GENDER GAPS IN LATE AGE SELF-REPORTED HEALTH IN TAIWAN

Robert White,[1] and Fang-Yi Huang[2], *1. University of Florida, Gainesville, Florida, United States, 2. Trinity Washington University, College Park, Maryland, United States*

The socio-economic status (SES) are the fundamental causes of gendered health disparity. However, how the gender gap in self-reported health (SRH) mediate by SES at old age over time is still unresolved. Some argue the SES measures play more and more important role to explain the gender gap in SRH at later age because the feminization of poverty and female's longer widowhood increased the gap over life course through cumulative disadvantage approach. But others SES-SRH gradients in gender gap keep convergent by age since the effects of SES on health for male has declined and make the gender gap in health disparity vanish over time. Our results show for every age, increasing SES is associated with declining risk of reporting poor health and the effects can explain much more for women than men, especially for the younger old below age 70. The effects of SES and marriage on the magnitude of the gender gap are substantial approximately 40 percent among seventy year olds to the full gender gap among 55-64 year olds in 2006.

### GENDER INFLUENCES ON LIFE SPACE TRAVEL AMONG THE OLD-OLD

Chengming Han,[1] Eva Kahana,[2] Boaz Kahana,[3] and Tirth Bhatta[4], *1. MA, Cleveland, Ohio, United States, 2. Case Western Reserve University, Cleveland, Ohio, United States, 3. Cleveland State University, Cleveland, Ohio, United States, 4. UNLV, Las Vegas, Nevada, United States*

This paper focuses on gender influences on life space mobility based on distance from home traveled by elderly retirees. Consideration of life space travel offers a window into environmental autonomy and complexity in late life. The gendered nature of time use and social networks have been primarily studied in younger age groups. Our sample included 437 older adults (mean age 83 for both men and women) living in a large Florida retirement community that offered no services. Mean age was 83 for both men and women. Fewer (37.6%) women than men (69.7%) were married and more men drove a car (83% vs 63%).Women reported poorer subjective health and had more IADL limitations. Compared to women, men were significantly more likely to travel long distances. Women's weekly and monthly travel tended to be local, limited to their neighborhood. On average, respondents of both genders visited their families and friends out of town every month. Better health, current driving and volunteering were related to longer driving distances for both genders, but these advantages were no longer significant after controlling for demographic characteristics. Our findings underscore the complex relationships between gender and life space travel in late life. Even among elderly men and women of the same age better health and driving resources contribute to larger life space for elderly men.

### EDUCATION, LITERACY, NUMERACY AND HEALTH INFORMATION SEEKING IN LATER LIFE

Takashi Yamashita,[1] Anthony R. Bardo,[2] Roberto J. Millar,[1] Shalini Sahoo,[3] Phyllis Cummins,[4] and Darren Liu[5], *1. University of Maryland, Baltimore County, Baltimore, Maryland, United States, 2. University of Kentucky, Lexington, Kentucky, United States, 3. University of Maryland, Baltimore, Baltimore, Maryland, United States, 4. Scripps Gerontology Center, Miami University, Oxford, Ohio, United States, 5. Des Moines University, Des Moines, Iowa, United States*

Health information plays a critical role for health promotion and maintenance in later life. While health information seeking is primarily driven by need (e.g., health), significantly less is known about the roles of education and health-literacy. Thus, we examine complex pathways that link health information seeking behaviors with education and health literacy (decomposed into general literacy and numeracy), and how these pathways differ by health status among a nationally representative sample of Americans age 50 and older (n = 2,750). Data come from the 2012/2014 Program for International Assessment of Adult Competencies. Multigroup structural equation models were used to examine the use of eight health information sources (newspapers, magazines, internet, radio, TV, books, friends/family, and health professionals) by health status (good vs. poor). Findings showed that literacy and numeracy are significant mediators of the relationship between education and health professional as an information source. Additionally, the mediation effects on health professionals by literacy status [indirect-effect (good vs. poor health) = 0.48 vs. 2.13, $p < 0.05$] and numeracy [indirect-effect (good vs. poor health) = -0.47 vs. -1.81, $p < 0.05$] were significantly moderated by health. At the same time, no moderated mediation effect was observed in the use of any other information sources. This study provides some of the first nationally representative evidence regarding how education functions through health literacy components to shape health information seeking behaviors by health status. Explanations and implications for differing effects of education, literacy, and numeracy on health information seeking in later life were evaluated.

### RACE, BIOLOGICAL AGE, AND COGNITION: THE SYSTEMATIC ASSESSMENT OF GERIATRIC ELEMENTS IN ATRIAL FIBRILLATION STUDY

Sarah N. Forrester,[1] David D. McManus,[1] Jane S. Saczynski,[2] and Catarina I. Kiefe[1], *1. University of Massachusetts Medical School, Worcester, Massachusetts, United States, 2. Northeastern University, Boston, Massachusetts, United States*

Atrial Fibrillation (AF) is associated with dementia and cognitive decline. AF is less prevalent among Blacks than Whites, although AF-related complications are more common in Blacks. In the general population, all-cause cognitive decline and dementia are more prevalent among Blacks than Whites. Thus, studying diverse populations with AF may advance our understanding of racial disparities in cognitive functioning. We created a measure of multisystem dysregulation (weathering), which includes but is more encompassing than aging, and examined its association with racial differences in cognition using data from the SAGE-AF study, a prospective cohort of >65-year olds with AF, at high stroke risk, and eligible for anticoagulation. Biological (as opposed to chronological) age among 974 participants was calculated using the Klemera and Doubal method using biomarkers representing physiological functioning, metabolism, and blood pressure. We defined weathering as the difference between biological and chronological age (weathering >0 indicates that biological age is higher than chronological age). We measured the association between weathering and the Montreal Cognitive Assessment (MoCA) score. Mean weathering (SD) was -0.7 (11.5) and 4.3 (12.6) for whites and non-whites, respectively. There was an interaction between race/ethnicity and weathering on cognition (P=0.004). In stratified analyses, higher weathering was associated with a lower MoCA score among both Whites and non-Whites but more so among non-whites (B = -0.09, 95% CI: -0.17, -0.02) for Whites (B = -0.03, 95% CI: -0.06, -0.01) for non-whites. Aging-related multisystem dysregulation is more strongly associated with worse cognition in non-whites than in whites.

### THE EDUCATION-HEALTH GRADIENT AND PATHWAYS TO MORTALITY

Justin J. Kiel,[1] Yenee Soh,[1] Muqi Guo,[1] and David Canning[1], *1. Harvard T.H. Chan School of Public Health, Boston, Massachusetts, United States*

Large and widening educational gaps in life expectancy exist in the United States, but the mechanisms behind the education-health gradient are not well understood. We aim to study the different pathways of mortality by education group and decompose the life expectancy gap at age 50 into three components: differences in initial health at age 50, differences in transition rates to poor health states, and differences in mortality rates, given the health state. We use 11 waves of the Health and Retirement Survey and model the evolution of mortality, and health states (measured by self-rated health, limitation on any activity of daily living, and ever diagnosed with a range of health conditions). We estimate Markov models of transitions between health states and mortality over two year periods and use these transition matrices to simulate how a cohort's health changes as they age. We find a 6-year gap in life expectancy between the highest and lowest education groups. Initial health states account for one-third of the gap in life expectancy, primarily from differences in objective health measures. The gap has widened over time, explained by comparatively better health status changes for the higher educated group, compared to the other education groups. The lower education groups have high rates of transition to poor health states, but not differential mortality rates conditional on their health state. These results suggest that educational gaps in life expectancy are due to the onset of ill health at earlier ages, and not different mortality outcomes after disease diagnoses.

### ASSOCIATIONS OF GENETICS AND LIFE COURSE CIRCUMSTANCES WITH A NOVEL AGING MEASURE THAT CAPTURES MORTALITY RISK

Zuyun Liu,[1] xi Chen,[2] Thomas M. Gill,[3] Chao Ma,[2] Eileen Crimmins,[4] and Morgan E. Levine[3], *1. Department*

*of Pathology, Yale School of Medicine, New Haven, Connecticut, United States, 2. Department of Health Policy and Management, Yale School of public Health Department of Economics Yale University, New Haven, Connecticut, United States, 3. Yale School of Medicine, New Haven, Connecticut, United States, 4. Davis School of Gerontology, University of Southern California, Los Angeles, California, United States*

We aimed to evaluate associations between a comprehensive set of factors, including genetics and childhood and adulthood circumstances, and a novel aging measure, Phenotypic Age (PhenoAge), which has been shown to capture mortality and morbidity risk in the U.S. population. Using data from 2339 adults (aged 51+) from the U.S. Health and Retirement Study, we found that together all 11 study domains (4 childhood and adulthood circumstances domains, 5 polygenic scores [PGSs] domains, and 1 demographics, and 1 behaviors domains) accounted for about 30% of variance in PhenoAge after accounting for chronological age. Among the 4 circumstances domains, adulthood adversity was the largest contributor (9%), while adulthood socioeconomic status (SES), childhood adversity, and childhood SES accounted for 2.8%, 2.1%, 0.7%, respectively. All PGSs contributed 3.8% of variance in PhenoAge (after accounting for chronological age). Further, using Hierarchical Clustering, we identified 6 distinct subpopulations/clusters based on the 4 circumstances domains, and 3 subpopulations/clusters of them that appear to represent disadvantaged circumstances were associated with higher PhenoAge. Finally, there was a significant gene-by-environment interaction between a previously validated PGS for coronary artery disease and the most apparently disadvantaged subpopulation/cluster, suggesting a multiplicative effect of adverse life course circumstances coupled with genetic risk on phenotypic aging. We concluded that socioenvironmental circumstances during childhood and adulthood account for a sizable proportion of differences in phenotypic aging among U.S. older adults. The disadvantaged subpopulations exhibited accelerated aging and the detrimental effects may be further exacerbated among persons with genetic predisposition to coronary artery disease.

### SOCIAL AND BEHAVIORAL FACTORS IN COGNITIVE AGING: APPLYING THE CAUSAL INFERENCE FRAMEWORK IN OBSERVATIONAL STUDIES

Anja K. Leist[1], *1. University of Luxembourg, Esch-sur-Alzette, Luxembourg*

Rationale: There is an urgent need to better understand how to maintain cognitive functioning at older ages with social and behavioral interventions, given that there is currently no medical cure available to prevent, halt or reverse the progression of cognitive decline and dementia. However, in current models, it is still not well established which factors (e.g. education, BMI, physical activity, sleep, depression) matter most at which ages, and which behavioral profiles are most protective against cognitive decline. In the last years, advances in the fields of causal inference and machine learning have equipped epidemiology and social sciences with methods and models to approach causal questions in observational studies. Method: The presentation will give an overview of the causal inference framework and different machine learning approaches to investigate cognitive aging. First, we will present relevant research questions on the role of social and behavioral factors in cognitive aging in observational studies. Second, we will introduce the causal inference framework and recent methods to visualize and compute the strength of causal paths. Third, promising machine learning approaches to arrive at robust predictions are presented. The 13-year follow-up from the European SHARE survey that employs well-established cognitive performance tests is used to demonstrate the usefulness of the approach. Discussion: The causal inference framework, combined with recent machine learning approaches and applied in observational studies, provides a robust alternative to intervention research. Advantages for investigations under the new framework, e.g., fewer ethical considerations compared to intervention research, as well as limitations are discussed.

### SUBJECTIVE APPRAISALS OF PHYSICAL AND EMOTIONAL HEALTH: EXAMINING AGE DIFFERENCES ACROSS RURAL URBAN COMMUNITIES

Hillary Dorman,[1] Rebecca S. Allen,[2]
Patricia A. Parmelee,[2] and Lisa C. McGuire[3], *1. University of Alabama, Tuscaloosa, Tuscaloosa, Alabama, United States, 2. The University of Alabama, Tuscaloosa, Alabama, United States, 3. CDC, Atlanta, Georgia, United States*

Using the Meikirch model as a theoretical underpinning, the present study aimed to examine population health disparities by discerning age variations within and across rural urban areas. Secondary data analysis was conducted using the CDC's 2017 Behavioral Risk Factor Surveillance System. The study's outcome variables included physical health and mental health burden (zero days, 1-13 days, 14+ days). A total sample of 96,568 adults were included with a mean age of 66.05 years (SD = 9.91). Individuals were classified in the following age groups: 43% middle-aged (45-64 years), 33% young-old (65-74 years), and 25% old-old adults (75+ years). The sample was largely female (61%), Non-Hispanic White (86%), and urban (67%). A series of chi-square tests of independence – post hoc tests when applicable – were completed. Overall, rural residents reported a higher prevalence of severe physical and mental health symptom burden. Regarding physical health burden, a significant difference was found within urban settings (X2(4) = 50.74, $p < .001$), where, unexpectedly, young-old adults reported the best physical health. Regarding mental health burden, a significant difference was found for both urban (X2(4) = 1661.72, $p < .001$)) and rural settings (X2(4) = 820.65, $p < .001$), with middle-aged adults reporting greater mental illness and the old-old adults reporting greater mental health resiliency. Findings suggest that a multidimensional framework of health usefully informs public health and clinical service interventions, identifying populations and locations in need (i.e., targeting rural physical health across age groups and mental health among the middle-age, regardless of location).

### OCCUPATIONAL MOBILITY AND CHRONIC ILLNESS IN LATE LIFE: A SYSTEMATIC REVIEW

Rong Fu,[1] Kathleen Abrahamson,[2] and Tara Campbell[1],
*1. Siena College, Loudonville, New York, United States,*
*2. Purdue University, West Lafayette, Indiana, United States*

Objectives: Occupational mobility at various stages in the life course may have a cumulative influence on health outcomes in later life. This study aims to (1) systematically review the association between occupational mobility and chronic illness in late life; and (2) identify potential mechanisms underlying this relationship. Methods: A systematic review of literature was carried out by searching two databases (PubMed and SocIndex) and reference lists. Eligible studies examined associations between occupational mobility and at least one measure of chronic illness among adults aged 50 years or above. Occupational disruptions (e.g., job loss) were reviewed as special cases of occupational mobility. Results: Downward occupational mobility and mid-life occupational disruptions have been consistently shown to predict higher risk of chronic illness in older adults. Several potential mechanisms were identified from the literature: (1) health behaviors, including dietary practices and alcohol consumption; (2) psychosocial factors, including stress, stigma, job control, job demands, and job satisfaction; (3) economic factors, including financial incentives or constraints; and (4) other individual characteristics, including personality traits and coping skills. There is also evidence that the timing of job mobility and the duration of (each) occupation modify the association between occupational mobility and health in late life. Discussion: These findings suggest that experiencing involuntary occupational mobility at various stages in the life course can increase the risk of chronic illness in late life. Health professionals and policymakers should target more resources to disadvantaged older adults who experience involuntary occupational transition.

### CORESIDENCE OF OLDER PARENTS AND ADULT CHILDREN BENEFITS OLDER ADULTS' PSYCHOLOGICAL WELL-BEING: PATH ANALYSIS

Soohyoung r. Lee[1], *1. Yeshiva University, New York, United States*

Even though the coresidence of older parents and their adult children is no longer a rare phenomenon in current society, a little is known about the benefit of living with adult children from older adults' perspectives compared to the risk of this living situation. Previous research suggests that older adults' psychological well-being is low when they live with their adult children, and this become more salient among single parents, such as widowed or divorced. The current paper utilizes the National Health Measurement Study with a sample of age 55 and over, and their SF-36 Mental Health Component score, and psychological well-being self-acceptance score was measured. Path analysis reveals while mental health and psychological well-being scores are lower among single older adults at the time of the survey (e.g., divorced, widowed) than non-single, coresidence of older adults and adult children completely mediates the negative relationship between being single and both mental health psychological well-being. A complete mediation effect of living with an adult child on older adults' mental health and psychological well-being is consistent with both white and non-white minority older adults. This suggests that living with adult child benefits older adults' mental health and psychological well-being. The current study seeks to stimulate ideas that might generate the next answer to community-based care in our current aging society.

### OLDER PEOPLE LIVING WITH HIV: THE RELATIONSHIP BETWEEN COMMUNITY-LEVEL FACTORS AND THEIR HIV HEALTH

Yookyong Lee,[1] Rick Walton,[2] Edward Jackson,[3] Lindsey Jackson,[4] and Scott Batey[3], *1. University of Alabama at Birmingham, Birmingham, Alabama, United States, 2. NHS CSAB, Birmingham, Alabama, United States, 3. University of Alabama at Birmingham Center for AIDS Research, Birmingham, Alabama, United States, 4. Birmingham AIDS Outreach/Magic City Wellness Center, Birmingham, Alabama, United States*

According to Centers for Disease Control and Prevention, older adults (aged 50 and older) accounted for 17% of new HIV diagnoses in 2016. The number of older people living with HIV (PLWH) is increasing because of antiretroviral therapy that allows a near normal life expectancy with HIV. Consequently, older PLWH are more likely to develop diseases associated with aging and be affected by polymedication. However, their lived experience and challenges that they face have been underexamined. In this study, we focused on community-level factors, which have potential health implications for older PLWH. We examined a subsample of participants who were at least 50 years-old (n=20; Mean=55; range 50–60) from a community-based participatory research study (n=40). Participants, who previously established HIV care, were recruited through word-of-mouth and flyers posted in the community. Interviews were recorded, transcribed, coded, and triangulated through an iterative process during team meetings. In this subsample (n=20), majority of participants were male (75%) and Black (80%). Older PLWH reported crime, social isolation (interacting with crime and stigma), and lack of access to resources (e.g., transportation, grocery store, pharmacy) as factors negatively associated with their physical and emotional health. A more holistic, complex approach to support older PLWH is essential. They should experience a greater quality of life along with longevity. Therefore, more attention to contextual and structural barriers associated with treatment and care is necessary to find a way for higher retention, (re)engagement, and adherence to care among older PLWH.

### THE ASSOCIATION OF RESILIENCE AND SOCIAL NETWORKS WITH PAIN OUTCOMES AMONG OLDER ADULTS

Shirley Musich,[1] Shaohung Wang,[1] Luke Slindee,[2] Sandra Kraemer,[3] and Charlotte Yeh[4], *1. Optum, Ann Arbor, Michigan, United States, 2. Optum, Eden Prairie, Minnesota, United States, 3. UnitedHealthcare, Minneapolis, Minnesota, United States, 4. AARP Services, Inc., Washington, District of Columbia, United States*

Depression, stress and poor sleep are associated with increased pain among older adults with chronic pain. Positive resources may help buffer the impacts of negative attributes on pain. Our primary objective was to determine effects on pain outcomes (severity; interference) of positive resources (resilience; social networks) on negative attributes among older adults with pain. The sample (N=15,000) came from older adults≥65 years with AARP®Medicare Supplement and AARP®MedicareRx plans (insured by UnitedHealthcare Insurance Company) with diagnosed back pain, osteoarthritis and/or rheumatoid arthritis. Members received a survey assessing positive resources, negative attributes and outcomes of pain. Depression, stress, sleep, resilience, social networks, pain severity and interference were measured. Opioid and other medications were determined from claims. The population was propensity weighted to adjust for survey non-response; weighted to be generalizable to members with diagnosed pain. Multinomial logistic regression was used to determine associations of positive/negative attributes on pain. Among respondents (N=4,161; 29%), prevalence of pain severity and interference for no/mild, moderate and severe categories was 61%, 21% and 18% for severity and 67%, 16% and 17% for interference. In bivariate models adjusted for demographics/health status, negative attributes of depression, stress and poor sleep had stronger associations with pain severity and interference than moderating effects. In full multivariate models, the strongest associations with moderate and severe severity and interference remained depression, stress and sleep. Based on results, multidimensional pain management strategies should include management of depression, stress and poor sleep along with enhancement of positive resources and analgesics as needed for pain management.

### HOME CARE SOCIAL WORKERS CLAIM MEDICARE IGNORES PATIENT NEEDS

William D. Cabin[1], *1. Temple University, Philadelphia, Pennsylvania, United States*

There is significant literature on the importance of addressing social determinants of health (SDOH) in order to improve health care outcomes. In response, the Centers for Medicare and Medicaid Services (CMS) has expanded Medicare Advantage plans ability to cover SDOH-related services. Medicare home health does not cover SDOH-related services. A literature review indicates no studies on the nature, significance, or impacts of the lack of SDOH coverage in Medicare home health. This article summarizes an initial, exploratory study to address the literature gap, based on interviews of a convenience sample of 29 home care social workers between January 2013 and May 2014 in the New York City metropolitan area. Results indicate social workers believe the lack of SDOH coverage in Medicare home health results in exacerbation of existing patient conditions; creation of new, additional patient conditions; increased home care readmissions and re-hospitalizations; increased caregiver burden; and exacerbation of patients' mental health and substance abuse needs. Policymakers are urged to consider adding coverage of SDOH to Medicare home health primarily through expanded social work coverage.

### SESSION 1401 (POSTER)

### TECHNOLOGY

### ELECTRONIC PERSONAL HEALTH RECORD USE AMONG ELDERLY CANCER SURVIVORS AND NON-CANCER SURVIVORS

Yan Luo[1], *1. University of Alabama, Tuscaloosa, Alabama, United States*

Background Electronic personal health records (ePHRs) are potential tools to improve clinical outcomes through increasing patients' self-management. Although elderly people, especially elderly cancer survivors, is a growing population who can benefit from ePHRs, little is known about its utilization among the elderly, particularly among those diagnosed with cancer. Objective By applying Anderson's Behavioral Model of Health Services Use, this study aims to examine and compare the associated factors with ePHRs use among elderly cancer survivors and non-cancer survivors. Methods The data collected from the 2018 Health Information National Trends Survey (HINTS) was analyzed. The level of access to ePHRs among the elderly were assessed. Predictors of ePHRs use among elderly cancer survivors and non-cancer survivors were compared by conducting multiple linear regression. According to Anderson's Model, predisposing factors, enabling factors, and need factors were included in the statistical model. Results The overall use of ePHRs remained low among 577 participants (mean = .87, SD = 1.72, range from 0 to 4). Non-Cancer survivors reported lower ePHRs use (mean = .83, SD = 1.77, range from 0 to 4). Race/ethnicity, education, regular health care providers, health insurance, social support, and medical conditions were associated with ePHRs use among non-cancer survivors, while age, gender, social support, and self-reported health status were related to ePHRs use among cancer survivors. Conclusion This study suggests additional efforts to increase ePHRs utilization among the elderly, especially the elderly cancer survivors. The predictive findings reported in this study will contribute valuable implications to enhance the ePHRs use.

### WHY DON'T ELDERS ADOPT TWO-FACTOR AUTHENTICATION? BECAUSE THEY ARE EXCLUDED BY DESIGN

Sanchari Das,[1] Joshua Streiff,[1] Lesa L. Huber,[2] and L Jean Camp[1], *1. Indiana University Bloomington, Bloomington, Indiana, United States, 2. Indiana University School of Public Health, Bloomington, Indiana, United States*

Two-Factor Authentication (2FA) provides effective protection for online accounts by providing efficient and highly robust access control. Adoption and usability, however, remain challenges for such technologies. Most research on 2FA focuses on students or employees in the tech sector. For example, our research with student populations found that lack of adoption was primarily due to a lack user risk concern matched with confidence in their 'strong' password strategies. The situation for older adults (> 60 years) was quite different, as we discovered through detailed interviews and think-aloud protocols targeted at understanding the registration, after installation, and their (un)willingness to use 2FA. We focused our research on USB security hardware tokens; additionally, we asked about other 2FA strategies which the participants adopted (if any). Their lack of adoption of the devices stemmed from its shortfall of inclusive design. Most available security tokens which were compliant with tablets have very small form factors; nearly invisible in a purse, and easy to slip through a pocket. The larger security keys are device and browser (Google Chrome) dependent. The organizations which would be most invested in protecting older people -- retirement management funds, the Social Security Administration, Medicare, and banking institutions

- reasonably do not adopt 2FA because of its lack of acceptability. Our negative result is that older adults are caught in a negative feedback loop where lack of adoption prevents availability, and vice versa. The positive result is that these concerns are straight-forward to overcome.

### HEALTH LITERACY, MEDICAL NON-ADHERENCE, AND SELF-REPORTED HEALTH PROBLEMS AMONG OLDER INTERNET USERS

Gul Seckin[1], *1. University of North Texas, Denton, Texas, United States*

The Internet presents new options for the elderly to gather information to support their health care. Health information gathering is among the major motivations for using the Internet among aging baby-boomers. However, insufficient e-health literacy presents challenges for the aging baby boomers. We examined the extent to which health-related internet use and e-health literacy are associated with non-adherence and self-reported negative health outcomes. Respondents were randomly sampled from the largest national online probability-based research panel (N = 710; M= 48.8, SD= 16.4). The age range in our research allowed us to examine the hypothesized associations across the full sample while focusing on older adults (age ≥ 60; N = 194). Older adults with greater e-health literacy reported higher averages for non-compliance because of information obtained from the Internet [(t (194) = 5.06, p ≤ .0001]. Ordinary least squares regression analyses showed that older adults who reported greater averages on health-related internet use reported higher averages on self-reported health problems (β = .292, p ≤ .01). However, women reported fewer health problems (β = -.217, p ≤ .01). Non-adherence with doctor recommendations is a significant positive predictor of self-reported health problem in the full sample (β = .244, p ≤ .0001) but not among older respondents (β = .032, p ≤ .061). Older individuals will make better utilization of the Internet if health professionals guide them to credible sources for health-related information. Empowerment of individuals to utilize the Internet in an informed manner requires addressing their needs for e-health literacy skills.

### INTERNET USE AND NEGATIVE HEALTH PERCEPTIONS: THE MODERATING ROLES OF EDUCATION AND HEALTH LITERACY

Gul Seckin,[1] Valarie Bell,[1] and Susan Hughes[1], *1. University of North Texas, Denton, Texas, United States*

There is a mixed support regarding the effect of Internet use on health and well-being. We estimated the extent to which e-health literacy predicted two domains of negative assessment of well-being: negative affect and self-reported experience of health problems. Respondents were randomly sampled from the largest national online probability-based research panel (N = 710). Hierarchical ordinary least squares regression analyses were employed for hypothesis testing. We computed interaction terms (e-health literacy x strain; e-health literacy x education; and education x strain) as determinants of negative subjective assessment of well-being. Older adults with higher levels of e-health literacy reported significantly more health information consumerism [(t (194) = 7.32, p ≤ .0001] but less strain in medical encounters [(t (194) = 2.92, p ≤ .004]. They reported less negative affect [(t (194) = 2.11, p ≤ .036] and more satisfaction with medical encounters [(t (194) = 4.70, p ≤ .0001]. The effect of perceived strain in medical encounters on negative affect was weaker among those with higher levels of education (β = -.314, p ≤ .01). Education had a significant moderating effect on the association between perceived strain in medical encounters and self-reported health problems, (β = -.550, p ≤ .05). Those who reported lower averages for e-health literacy but higher educational levels indicated lower averages on negative affect (β = -.597, p ≤ .05). Given that conventional methods of acquiring health-related information shift to the Internet, our study holds significant health and social implications for a rapidly growing Internet-connected older population.

### DOES CHANGE IN INTERNET USE PREDICT PSYCHOLOGICAL WELL-BEING AMONG OLDER ADULTS?

Ronald W. Berkowsky[1], *1. California State University Channel Islands, Camarillo, California, United States*

Previous work focusing on the relationship between Internet use and quality of life among older adults (aged 65+) has found evidence of various positive impacts. This project expands upon this work by examining the relationship between Internet use and measures of psychological well-being (PWB) including autonomy, environmental mastery, personal growth, positive relations with others, purpose in life, and self-acceptance. The analytic sample is derived from two waves of data (Time 1 = 2004, Time 2 = 2011) taken from the Wisconsin Longitudinal Study and includes a sample of older adults aged~65 at Time 1 (N = 4943). Participants were separated into four categories: those who did not use the Internet at Time 1 or 2, those who used the Internet at Time 1 only, those who used the Internet at Time 2 only, and those who used the Internet at both Time 1 and 2. Regression analyses were performed with the Time 2 PWB measures as the outcomes and the Internet use categories as the primary predictors. Results indicate that while continuous Internet users typically reported higher PWB scores compared to non-users, those who stopped use between Time 1 and 2 also reported higher scores and those who started use between Time 1 and 2 reported lower scores. These results generally held when introducing Time 1 PWB measures as controls, suggesting changes in Internet use may affect PWB but not necessarily in the predicted directions. Additional control variables, potential explanations, and implications for future research are discussed.

### A COMPREHENSIVE DIGITAL SELF-CARE SUPPORT SYSTEM FOR OLDER ADULTS: A MULTIDISCIPLINARY FRAMEWORK

Priya Nambisan,[1] Kalle Lyytinen,[2] Kurt Stange,[1] Eva Kahana,[2] and Gary L. Kreps[3], *1. University of Wisconsin - Milwaukee, Milwaukee, Wisconsin, United States, 2. Case Western Reserve University, Cleveland, Ohio, United States, 3. George Mason University, Fairfax, Virginia, United States*

This paper presents an innovative conceptual framework for designing a Comprehensive Digital Self-care Support System (CDSSS) to meet the health needs -physical, mental and social health needs of older adults and their caregivers. Older adults deal with multiple co-morbidities, medications and their side effects, fragmented care and often have poor understanding of their own health and treatments. These challenges call for solutions that lead to better empowerment and pro-active engagement and for support systems that focus on wellness and preventive care. The conceptual model we offer draws on diverse disciplines including health care management and medicine, information systems, communication, consumer behavior, and sociology to identify a set of key design principles for CDSSS. A review and analysis of the literature in the different fields led to the identification of 6 CDSSS design principles: (1) Systems approach; (2) User experience; (3) Ecosystem perspective for shared resources 4) Social and contextual learning; (5) Accessible design; (6) Designing for trust and empathy. The model clarifies how these design principles (or approaches) inform the development of the three main components of a CDSSS (data integration, communication, and resource integration) and enable the key CDSSS deliverables (learning, social & emotional support and care integration). The conceptual model also helps to lay out an agenda for future research on self-care support systems for older adults.

### LOOK WHO'S TALKING: TRADITIONAL AND ELECTRONIC MEDIUMS OF CONTACT LINKED WITH LATER-LIFE SIBLING RELATIONSHIPS

Melanie S. Hill,[1] Jeremy B. Yorgason,[1] Sarah Coyne,[1] and Alexander C. Jensen[1], *1. Brigham Young University, Provo, Utah, United States*

The sibling role is often the longest lasting relationship between individuals. As such, older adults may turn to siblings in later life as it is a relationship that is already familiar. Having a close and less conflictual relationship with a sibling may be especially important as older adults value siblings for emotional and practical support exhibited through contact. Minimal research has examined mediums of contact used between sibling dyads despite the increase use in technology among older adults. Using a sample of 491 Americans (Mage = 58.96) recruited via Amazon Mechanical Turk (Mturk), the current study examined five mediums of contact (i.e., in person, telephone, e-mail, texting, and social media) and how each type independently is related to sibling closeness and conflict. Further, using regression analyses in STATA, two and three-way interactions were examined to assess the role of sibling dyad composition affecting this relationship. Results indicated that contact through telephone was associated with higher sibling closeness for all sibling dyads, and that association was stronger for females with a sister compared to males with a brother. Further, in person and texting contact was especially beneficial for females with a brother. Main effects revealed contact in person, via social media, over the telephone, or through email, reported more sibling closeness, while those who engaged in more email contact reported less conflict. Thus, even in later life, siblings are keeping in contact with one another through both traditional and electronic mediums of communication, and this contact appears especially beneficial for sisters.

### CAREGIVER RESPONSES TO REMOTE ACTIVITY MONITOR ALERTS OF PERSONS WITH DEMENTIA

Rachel Zmora,[1] Jessica M. Finlay,[2] Lauren L. Mitchell,[3] and Joseph E. Gaugler[4], *1. University of Minnesota, Minneapolis, Minnesota, United States, 2. Social Environment and Health, Institute for Social Research, University of Michigan, Ann Arbor, Michigan, United States, 3. Minneapolis VA Health Care System, Minneapolis, Minnesota, United States, 4. University of Minnesota - School of Public Health, Division of Health Policy and Management, Minneapolis, Minnesota, United States*

The benefits of technology to alert family caregivers to the needs of persons with Alzheimer's disease or related dementias (ADRD) are unclear. Previous research indicates that remote activity monitoring (RAM) system alerts can be alternately reassuring and highly stressful for caregivers. We conducted a parallel convergent mixed-methods analysis of 62 primary caregivers of persons with ADRD to evaluate the association between the number of alerts and caregiver outcomes after 6 months. We assessed caregiver-reported usability of the system as well as self-efficacy, sense of competence, and distress as primary outcomes. Linear regression models tested the association between the number of alerts and caregiver-reported usability and primary outcomes. The number of alerts declined over the first 6 months of system use and was not associated with a change in system usability or primary outcomes. Thematic analysis of caregiver-reported perceptions of RAM use simultaneously probed for more in-depth understanding of caregiver experiences of and feelings towards RAM. Preliminary analyses reveal that 28% of caregivers comments were positive, noting benefits such as early warning of health concerns and peace of mind. 34% of comments were neutral or mixed, and 38% were negative. Concerns included false alarms and accidental triggers, losing sleep due to alarms, and difficulties using the system. These findings help characterize the adjustment period to use RAM technology. The mixed-method results inform future research studies and applications of RAM systems so that researchers and caregivers can better understand the initial adjustment period, address concerns, and avoid discontinuing RAM use prematurely.

### AGE-SPECIFIC VIEWS ON INVASIVE AND NON-INVASIVE HUMAN ENHANCEMENTS FOR COGNITIVE DECLINE

G. Rainville,[1] Laura Skufca,[1] and Madeline Eller[1], *1. AARP, Washington, District of Columbia, United States*

This research examines the degree to which younger and older Americans approve of addressing cognitive decline using either a pill-based or an implant-based intervention to restore prior functioning. Half of a probability-based online sample expressed concerns over side effects and levels of approval for a pill-based intervention whereas the remainder of the sample did so for a relatively invasive implant-based enhancement (data were interviews of 2,025 American adults gathered by NORC's AmeriSpeak panel as part of the AARP Human Enhancements study). We predicted and found that relative disapproval of the implant-based intervention was only significant among those with high concerns over side effects. However, when looking at two age groups for which cognitive decline differed in salience, relative disapproval of the implant-based enhancements were relatively stronger for those 50 and older even among those with few concerns over side effects. This age-based aversion to invasive forms of enhancements may have public health implications in that the subgroup who may most-immediately benefit from the enhancement and may be in the market for only non-invasive enhancements. It is not clear if such enhancements, however, could be delivered via a pill or other non-invasive forms.

### IDENTIFYING CARE ACTIVITIES THAT WERE SUPPORTED BY AMAZON ECHO FOR CARE PARTNERS AFTER ONSET OF COGNITIVE IMPAIRMENT

Galina Madjaroff[1], *1. University of Maryland Baltimore County, Baltimore, Maryland, United States*

There are several important challenges when addressing the needs of older adults with cognitive impairment and their care partners including the potential for diminishing emotional well-being and loss of autonomy, which could potentially lead to a lower overall quality of life for both care partners (CPs). The motivation of this study was to identify the care activities that were supported by home based technology for care partners after the onset of cognitive impairment. This work was done through gathering multiple sources of qualitative and quantitative data, including mobile application dialogue history logs, pre and post interviews, user feedback groups and home visits. The technology deployed in the home of the care partners was a Voice User Interface Intelligent Agent, specifically the Amazon Echo with its intelligent agent "Alexa." This technology was selected because it was not built from a traditional care model, yet embodies functions that could be used for all potential forms of care, including those that achieve a higher level of quality of life goals for care partners. From this study, we can further our understanding of how to deploy and design technology that shifts the perspective from "cure to care" with a focus on the older person and their lived experience, monitoring wellness, and not just addressing illness. Results and findings indicated that daily care activities of dyads that are seemingly fundamental are actually complex care activities that emerge from using the technology that support the care partners on multiple levels in satisfying multiple needs.

### PSYCHOMETRIC PROPERTIES OF THE OLDER ADULT TECHNOPHOBIA SCALE AND SMARTPHONE CHALLENGE TASK

Kelly J. Martin,[1] Sean A. Lauderdale,[1] Steven Thorpe,[2] and Jan Molhman[3], *1. Texas A&M University-Commerce, Commerce, Texas, United States, 2. California School of Professional Psychology (CSPP) Alliant, San Diego, California, United States, 3. William Paterson University, Wayne, New Jersey, United States*

Older adults are especially prone to anxiety if they are unable to keep pace with technological advances and are generally more technophobic than their younger counterparts. Older adults tend to limit their use of technology, if not avoid it altogether, such as using a smartphone for calls and text messages only, while eschewing more advanced functions. Currently, there is no measure of technophobia in older adults that captures fears and concerns about the use of these up-to-date technological tools. The purpose of this investigation was to evaluate the psychometric properties of a new scale of technophobia and corresponding smartphone challenge task in a sample of older adults. Community-dwelling older adults (N = 42, 81.0% female, Mage = 77.3) completed the following: the Older Adult Smartphone Challenge Task (OASCT), Older Adults' Technophobia Scale (OATS), Older Adult Social Anxiety Scale, Computer Anxiety Rating Scale, and the IPIP Five Factor Personality Domains. Preliminary data indicate good internal consistency for the OATS ($\alpha = .87$) and the OASCT ($\alpha = .86$). The OASCT was negatively correlated with age, computer anxiety, and OATS anxiety/avoidance scores, but positively correlated with education. The OATS scores were positively correlated with social anxiety, social avoidance, and computer anxiety, but negatively correlated with extraversion. To keep pace with the contemporary world, older adults must achieve a level of comfort with the use of technological devices. Administering the OASCT and OATS could be a valuable first step in identifying older adults with technology-related deficits and anxiety for individual and/or community-wide intervention.

### USE OF TECHNOLOGY BY FOUR DIVERSE COHORTS OF OLDER ADULTS: FINDINGS FROM THE CART STUDY

Katherine Wild,[1] Nora Mattek,[2] Nicole Sharma,[2] Jennifer Marcoe,[2] Rachel Wall,[2] and Jeffrey Kaye[2], *1. OHSU, Portland, Oregon, United States, 2. Oregon Health & Science University, Portland, Oregon, United States*

Early studies of technology adoption and computer use identified a "digital divide" between older adults and the general population. As that gap has narrowed, other demographic variables have been identified as continuing to foster disparities in access to and use of computers and related technologies. For example, gender, socioeconomic status, education, and ethnicity have been recognized as predictors of computer use among community living older adults. The ORCATECH Collaborative Aging (In Place) Research Using Technology (CART) initiative was designed to develop and validate an infrastructure for research utilizing technologies to facilitate healthy and independent aging. The CART program tests innovative technology applications in four diverse populations: residents in low income, section 202 housing in Portland; isolated, rural veterans in the Pacific Northwest; urban African American seniors in Chicago; and socially isolated, ethnically diverse low income seniors in Miami. As part of their participation in the CART project, older adults complete an annual survey of health and technology use. A total of 214 participants were enrolled and agreed to have their homes instrumented with the CART platform of monitoring technologies. Across all four cohorts 166 answered the technology survey thus far: 82 - 97% of participants own a cell phone; 64 - 78% perform some online banking activities. There were no differences among cohorts in computer use or cell phone ownership, or in other measures of technology use. Inclusion of ethnically and economically diverse populations in future technology research will be critical in the development of effective digital health interventions.

### SOCIAL MEDIA AND SOCIAL WELL-BEING IN LATER LIFE

Kelly Quinn[1], *1. University of Illinois at Chicago, Chicago, Illinois, United States*

Social well-being is important to health, but maintaining social relations often becomes difficult in later life due to retirement, chronic disease, and the death of spouses and friends. Social media platforms, such as Facebook and Twitter, present accessible and low cost communication technologies that are associated with enhanced feelings of social connection and reduced loneliness in younger age groups. This paper examines whether similar benefits might arise for adults at older ages. Using a four-week social media training workshop as a randomized, controlled wait-list intervention, this study examines whether social benefits are realized among a group of novice social media users, aged 65+. Measures of social well-being, including social capital, loneliness, social connectedness, and social provisions, were assessed at pre- and multiple post-test intervals for differences related to social media learning. Findings revealed only small differences between groups in one dimension of social connectedness, that of social integration. As these findings seemingly contradict studies conducted with younger persons, the contexts of social media use in older adulthood are discussed. These include the relevance of lower social media adoption rates, as well as influences that intersect with an older person's life stage, such as gaps in network coverage on technological platforms, perceptions of the value of weak connections, and a reduced digital skills base. These additional factors are relevant to understanding disparity in the benefits that can be obtained through the use of social media and highlight the differing needs that social media fulfill at varying life stages.

### USE OF INFORMATION AND COMMUNICATION TECHNOLOGY AND MEMORY PERFORMANCE IN OLDER ADULTS: WHICH COMES FIRST?

Eun Young Choi,[1] and Elizabeth M. Zelinski[1], *1. Davis School of Gerontology, University of Southern California, Los Angeles, California, United States*

The topic of older adults' information and communication technology (ICT) use looms large because of the beneficial effects of ICT use on physical health, emotional well-being, and social engagement. Previous research has shown that memory performance is also linked with ICT use, but the direction of influence is yet to be determined. Individuals with higher levels of memory function are more likely to use ICT devices, but ICT use may have protective effects on maintaining memory because using technologies includes mental exercises. The current study examined the temporal sequence of ICT use and memory performance, which can provide insight into the causation. Using three waves (2013, 2015, and 2017) from the National Health and Aging Trends Study (NHATS), a total of 4,048 community-dwelling older adults aged 65 and above were selected for the analysis. Memory performance was measured by summing scores of immediate and delayed word recall. Reciprocal 5-year lagged associations between ICT use and memory were examined, while controlling for age, gender, education, racial/ethnic minority status, and depressive symptoms. The final model showed adequate fit indices (CFI = .979 and RMSEA = .038). Word recall significantly predicted ICT use in later years. Reciprocally, greater use of ICT was significantly associated with better memory performance in following years. The effect of ICT use on memory performance was of greater magnitude in comparison with memory as a predictor for ICT use. These results suggest that ICT can have potential benefits for maintaining memory in old age.

### EXAMINING DISCREPANCIES IN SOCIAL ROBOT VERSUS HUMAN ASSESSMENTS OF GERIATRIC WELL-BEING

Erin Harrington,[1] Ha Do,[1] Alex J. Bishop,[1] Celinda Reese-Melancon,[1] and Weihua Sheng[1], *1. Oklahoma State University, Stillwater, Oklahoma, United States*

Socially assistive robotic (SAR) technologies represent a viable tool for monitoring the safety and health of older adults. However, it is unclear whether SARs can comprehensively screen geriatric well-being as effectively as trained human clinicians. The purpose of this study was to compare SAR versus human assessment of geriatric well-being. Participants included 30 older adults (Mage = 73.40, SD = 7.88) who completed a robot-administered well-being assessment session during which human-administered evaluation was simultaneously performed. Standardized clinical screening assessment tools common in geriatric care were administered (e.g., Short Blessed Test (SBT), UCLA Loneliness Scale, Geriatric Depression Scale, PHQ-4, Iowa Fatigue Scale, Fall Risk). Multiple dependent sample t-tests were used to explore variability in assessment scores between SAR and human evaluation. Assessment scores significantly differed on several measures, including the SBT ($t(29) = -9.33$, $p < .001$), UCLA Loneliness scale ($t(19) = 2.37$, $p < .05$), and fall risk assessment ($t(29) = 3.03$, $p < .01$). Specifically, the SAR indicated that older adults were significantly more cognitively impaired, less lonely, and more likely to fall compared to the human administrator. Other observed differences and hypothesized explanations will be discussed in greater detail. The current study indicates that there is a divergence in geriatric assessment outcomes based on human versus SAR administration. Findings have implications relative to further developing SAR technology to align with human-based evaluations to enhance cognitive well-being, social connectedness, and falls prevention.

### DEVELOPMENT AND EVALUATION OF EDUCATIONAL MATERIALS REGARDING MENTAL HEALTH MOBILE APPS AMONG OLDER VETERANS

Ashley Scales,[1] Julia Loup,[2] Christine Juang,[3] Erin Sakai,[3] Flora Ma,[4] and Christine E. Gould[5], *1. VA Palo Alto Health Care System, Palo Alto, California, United States, 2. University of Alabama, Department of Psychology, Tuscaloosa, Alabama, United States, 3. VA Palo Alto, Palo Alto, California, United States, 4. Palo Alto University, Palo Alto, California, United States, 5. VA Palo Alto Health Care System, Palo Alto, United States*

The number of older adults using mobile devices has doubled over recent years; however, many need assistance in learning how to use their device. To address this gap, we developed patient education materials teaching older Veterans how to download apps and the basics of mobile device and app use. For example, we developed step-by-step guides for three Veteran Affairs mobile apps that target mental health symptoms. Material development involved feedback from providers and older Veterans using a multi-step mixed methods evaluation process. Local technology and geriatric content experts provided initial feedback; all experts agreed the materials would be helpful to teach Veterans about mental health apps. We subsequently interviewed older Veterans (M = 78.5 years) who evaluated the materials. Over 50% of Veterans found the guides clear, articulate, and useful; 83.3% noted they would recommend to others. Lastly, providers who see older Veterans regularly rated the materials; 79% of providers rated the materials as helpful, with an average rating of 4.3 (1 = Strongly Disagree to 5 = Strongly Agree). Providers viewed the materials and apps as useful supplements to psychotherapy and especially useful for individuals who are unable to return to clinic. Overall, both providers and Veterans found the materials easy to understand and valuable for those new using mobile apps or devices. Findings from the evaluation process suggest the design of the materials may be vital to increasing the use of mental health mobile apps among older Veterans.

### THE INFLUENCE OF TECHNOLOGY ON QUALITY OF LIFE AND AGING IN PLACE

George Mois,[1] Tiffany R. Washington,[1] and Jenay M. Beer[1], *1. University of Georgia, Athens, Georgia, United States*

Many of the challenges that often accompany longevity can affect older adults' quality of life (QOL). Adoption of an assistive technology ecosystem presents the potential to alleviate these challenges and improve QOL. An assistive technology ecosystem refers to the use of multiple assistive technologies to address a set of challenges affecting single or multiple characteristics of older adults' QOL. However, little is known how technology can influence characteristics of older adult's QOL. The purpose of this study was to investigate how using technology can improve older adults QOL. Data from the 2016 wave of the National Health and Aging Trends Study (NHATS) were analyzed using four logistic regression models. The sample included are older adults age 65+ (N=5,488). The dependent variables used in this study were QOL indicators such as self-confidence, continue improving life, likes living arrangement, and self-determination. The variables used to measure technology included computer, cell phone, tablet, and internet use. Older adults who used the internet had significantly higher odds of reporting self-determination (OR=1.68), like living arrangement (OR= 1.97) and continue improving life. Tablet users had significantly higher odds of continuing to improve their life (OR= 1.249) and increased self-determination (OR= 1.174). Cellphone users had significantly higher odds of having self-confidence (OR= 2.814). These findings support the need for a network of resources accessed through an ecosystem of technologies to address the challenges encountered by older adults aging in place. This study's findings can inform technology education programs, interventions, and assist with the development of support networks.

### THE UNCANNY VALLEY REVISITED: AGE-RELATED DIFFERENCE AND THE EFFECT OF FUNCTION TYPE

Yun-Chen Tu,[1] Sung-En Chien,[1] Yueh-Yi Lai,[2] Jen-Chi Liu,[2] and Su-Ling Yeh[1], *1. Department of Psychology, National Taiwan University, Taipei City, Taiwan, 2. Intelligent Vision System Division, Electronics and Optoelectronics System Research Laboratories, Industrial Technology Research Institute, Hsin-Chu County, Taiwan*

Due to declined birthrate and the increased aging population, solving the problem of labor shortage has become important. Introducing robotic labors could effectively help older adults' daily lives. However, older adults' acceptance of robots was lower than younger adults. Robot's appearance might be one of the reasons. The Uncanny Valley (UV) refers to the phenomenon that people rate more positively as robots become more humanlike, but only up to a certain point; as it approaches near-perfect similarity of human appearance, likeability drops and forms an uncanny valley. Nonetheless, previous results supporting the UV were mainly from younger adults. We examined whether the UV is also applicable for older and middle-aged adults. We also examined whether the acceptance of function (companion vs. service) would change based on robot appearance, and whether robot-induced traits have any relation with the acceptance of robot function. We asked younger (N= 80, age 18-39), middle-aged (N= 87, age 40-59), and older (N= 88, age 60-87) adults to view each picture of 84 robots and evaluate their impression of each robot and intention of use regarding robot function. Contrary to the UV found for younger and middle-aged adults, older adults did not show UV–they preferred humanlike over non-humanlike robots, regardless of the robot function. Scores on each trait–except for authoritativeness–showed positive correlations with the acceptance of functions. These findings imply that the design of assistive robots should take UV into consideration by customizing robots' appearances and functions to different age groups.

### CROSS-COUNTRY COMPARISON OF INTERNET USE AND DEPRESSION BY GENDER: THE ROLE OF INTERGENERATIONAL FACTORS

Hyunju Shim,[1] Jennifer A. Ailshire,[2] and Eileen Crimmins[3], *1. University of Southern California, Los Angeles, United States, 2. University of Southern California, Los Angeles, California, United States, 3. Davis School of Gerontology, University of Southern California, Los Angeles, California, United States*

Technology may offer one approach to reducing depression as it provides medium to maintain connections (Cotton et al., 2014). Yet, depression, internet use, gender roles, and expectation of intergenerational interaction all differ across countries. Using nationally representative data from the U.S (Health and Retirement Study: HRS) and South Korea (Living Profiles of Older People Survey: LPOPS), the study examines 1) association between internet use and depressive symptoms by gender in two countries; 2) and whether intergenerational factors moderated this association. In the U.S., more than half of men and women aged 65+ used the internet, while approximately 30% of women and 47% of men used the internet in Korea. Using the internet was associated with lower depression for those living far from the closest child for women in the U.S., and for men in Korea. The findings indicate that the association of internet use on depressive symptoms can be influenced by intergenerational factors that may differentially affect men and women depending on the sociohistorical contexts.

### PROBLEM SOLVING SKILLS AND CANCER SCREENING BEHAVIORS IN MIDDLE-AGED AND OLDER MEN IN THE U.S.

Shalini Sahoo,[1] Roberto J. Millar,[1] Takashi Yamashita,[1] and Phyllis Cummins[2], *1. University of Maryland, Baltimore County, Baltimore, Maryland, United States, 2. Scripps Gerontology Center, Miami University, Oxford, Ohio, United States*

Routine cancer screening is widely recognized as an effective strategy for reducing cancer mortality – the second leading cause of death in the U.S. Research shows cancer screening rates need to be improved, and men are less likely to uptake recommended screening than women. Cancer screening requires an array of tasks such as seeking up-to-date guidelines, making appointments, planning a hospital visit, and communicating with health care professionals in the complex health care systems. Importantly, modern health care systems are rapidly adopting technology such as web-based applications for information dissemination and communication with patients. This current study is designed to better understand the roles of problem-solving skills in the technology-rich environment (PSTRE) in two selected cancer screening behaviors among middle-aged and older men. We obtained nationally representative data with a sophisticated PSTRE assessment from the 2012/2014 Program for the International Assessment of Adult Competencies (PIAAC). Binary logistic regression models with survey weights were used to estimate the association between PSTRE scores (1 – 500 points) and two cancer screening behaviors of men who meet the recommended guideline of age between 45 to 74 years old ($n = 1{,}168$). Results showed that greater PSTRE scores were positively associated with prostate cancer screening ($OR = 1.005$, $p < 0.05$). Improvement in PSTRE may promote the specific cancer screening behaviors. Our findings also inform future interventions that seek to improve cancer screening among a vulnerable section of older populations.

### CALL A TEENAGER . . . THAT'S WHAT I DO!: GRANDCHILDREN HELP OLDER ADULTS USE NEW TECHNOLOGIES

Jennifer D. Portz,[1] Christine Fruhauf,[2] Sheana Bull,[1] Rebecca S. Boxer,[3] David Bekelman,[1] Alejandra Casillas,[4] Kathy Gleason,[5] and Elizabeth Bayliss[6], *1. University of Colorado, Aurora, Colorado, United States, 2. Colorado State University, Fort Collins, Colorado, United States, 3. Kaiser Permanente Institute for Health Research, Aurora, Colorado, United States, 4. UCLA, LA, California, United States, 5. Kaiser Permanente, Aurora, Colorado, United States, 6. Kaiser Permanente Colorado, Aurora, Colorado, United States*

As older adults increasingly show interest in technology for their well-being, families will play an important role in promoting the adoption and use of beneficial health technologies. The purpose of this study was to conduct a sub-analysis of data collected from a large-scale qualitative project regarding older adults' experiences using health information technology. Specifically, the sub-analysis explored older adults' experiences with technology support from family members to inform strategies for promoting older adult engagement with new health technologies. While the primary analysis of the original study was theoretically driven, this paper reports results from the inductive, open-coding analysis. Twenty-four older patients (≥65 years) with multiple chronic conditions (Charlson Comorbidity Index > 2) participated in a focus group conducted at patients' primary clinic. While conducting the primary theoretically-driven analysis, coders also utilized an open-coding approach to ensure important ideas not reflected in the theoretical code-book were captured. Open-coding resulted in a primary theme, "family support", that was furthered categorized by who and how the tech-support was provided. Participants were not specifically asked about family support, yet family assistance and encouragement for technology emerged from every focus group. Participants repeatedly mentioned that they called their grandchildren and adult children for help with technology. Participants also reported that family members experienced difficulty when teaching technology use. Family members struggled to explain simple technology tasks and were frustrated by the slow teaching process. Family support, specifically via grandchildren, may have a key role in the successful adoption and use of emerging health technologies.

### IT'S MY BUDDY: EXPLORING THE PERCEPTIONS OF PEOPLE WITH DEMENTIA ABOUT THE SOCIAL ROBOT PARO IN A HOSPITAL SETTING

Lillian Hung,[1] and Habib Chaudhury[2], *1. Simon Fraser University, Vancouver, British Columbia, Canada, 2. Simon Fraser University, Vancouver, Canada*

New technology such as social robots opens up new opportunities in hospital settings. PARO, a robotic pet seal, was designed to provide emotional and social support for older people with dementia. This project aims to explore the perceptions of persons with dementia about PARO's role in a hospital setting. Video-ethnographic methods were applied. We had conversational interviews with and video observations of 10 older people with dementia in the geriatric unit of a large Canadian hospital. Also, semi-structured interviews and two focus groups were conducted with 10 staff members in the local unit to gain contextual information. Thematic analysis yielded three substantive themes: (a) "it's like a buddy"—the robot helps people with dementia uphold a sense of self in the world; (b) "it's a conversation piece"—the baby seal facilitates social connection; and (c) "it's all about love"—PARO transforms and humanizes the clinical setting. Our findings help provide a better understanding of the direct perspectives of patients with dementia on the use of social robots. Instead of substituting human contact, the social robot complements emotional care and supports our fundamental human need for love.

### TECHNOLOGY

### TRANSLATING THE RELATIONSHIP BETWEEN QUALITY OF LIFE AND MEMORY USING A NOVEL EEG TECHNOLOGY

Mitchell Roberts,[1] Orli Kehat,[2] Michaela Gross,[1] Nethanel Lasri,[2] Gil Issachar,[2] Erica Sappington VandeWeerd,[1] Ziv Peremen,[2] and Amir Geva[2], *1. University of South Florida, Tampa, Florida, United States, 2. elminda, Hertsliya, Israel*

Existing research has postulated a relationship between cognition and quality of life (QoL). Components of QoL such as satisfaction with social support may be particularly influential in memory for those with comorbidities. Additional research is needed to characterize the relationship between memory and QoL domains. Findings are presented from a clinical trial using BNA memory scores to assess brain health. BNA uses EEG technology and machine learning to map networks of brain functioning including working memory. Participants were older adults living in The Villages, an active lifestyle community in Florida, between the ages of 55-85, from 8/30/2017-3/11/2019. Participants were stratified into 2 groups: healthy (no CNS/psychiatric conditions; n=158) and multi-morbid (>1 CNS and/or psychiatric conditions; n=106) and compared across memory and QoL indicators. Subjective QoL was measured by the WHOQOL-BREF across 4 domains (physical, psychological, social, environmental). Scores on QoL domains were divided into 3 levels (high-medium-low) and tested for their relationship to BNA memory scores using ANOVA. Results indicate a relationship between health status, subjective QoL and BNA memory scores. Healthy subjects who scored high in the psychological QoL domain had significantly higher memory scores [$F(2,152)=4.30, p=.02$)]. In healthy subjects, satisfaction with social support ($p=.001$) had the strongest impact on memory for social QoL, while body image ($p=.06$) and concentration ($p=.06$) were the most salient predictors of psychological QoL and approached significance. Multi-morbid subjects who indicated high social ratings had higher memory scores ($F(2,100)=3.75, p=.03$) which relied heavily on satisfaction with social support ($p=.003$). Implications for policy and practice are discussed.

### AN INNOVATIVE DATA-DRIVEN FALL PREVENTION COMMUNICATION TOOL FOR ADMINISTRATORS, NURSE MANAGERS, AND STAFF NURSES

Ragnhildur Bjarnadottir,[1] and Robert Lucero[1], *1. University of Florida, Gainesville, Florida, United States*

Falls are the leading cause of injury among older adults, resulting in 3 million emergency department visits and 800,000 hospitalizations each year in the United States alone. In the hospital setting, falls are among the most common adverse events, causing longer stays and higher costs of care. Substantial efforts have been made to reduce falls in the past decade but with limited sustained effect. This may in part be due to limitations of existing tools for fall-risk assessment and evidence-based fall prevention. Therefore, there is a need to strengthen the evidence on risk factors for hospital-acquired falls and address barriers to translating the best available evidence into practice. To this end, we undertook the development of an innovative dissemination tool to implement fall-related evidence generated through state-of-the-art data science and information visualization approaches. Through a multidisciplinary academic-clinical partnership, we have developed an infographic to disseminate empirical evidence to nurses and administrators in the hospital setting. The infographic was developed based on principles of user-centered design and persuasive communication, and focuses on providing clear and accessible information about factors contributing to a patients' risk of falling in the hospital. This innovative dissemination approach is intended to foster dialogue between administrators, mid-level nursing management and staff nurses as well as evidence-based practice at the bedside. Future use and evaluation of this fall prevention tool will focus on adapting the infographic as an interactive digital tool for education and engagement of patients and families in the hospital setting.

### A MAGIC GATHERING: PILOTING AN AUGMENTED REALITY ENGINE TO PROMOTE MENTAL HEALTH AND WELL-BEING

Thomas Chan,[1] Daniel Matook,[1] Isaac Argueta,[1] Jonathan Kennedy,[1] Jeremy Argueta,[1] and Thomas Chan[1], *1. California State University Northridge, Northridge, California, United States*

Augmented reality (AR), superimposing digital assets onto the real-world (i.e., holograms)—offers a paradigm shift in delivering immersive interventions for aging adults. Reminiscence and life review are robust intervention techniques that have decades of empirical support to boost mood and meaning in older adults. In this presentation, we demonstrate the applied research and technology development process to create an AR reminiscence and life review engine for aging adults and their caretakers. We conducted 25 needs assessment interviews with community-dwelling older adults, and assisted living facility directors, nurses, and residents. Initial analyses revealed the need for: (1) finding scalable and cost-effective solutions to alleviate time burden for caregivers; (2) increasing variety of activities that do not need much instruction; (3) producing activities that grab and maintain attention; and, (4) generating more personalized activities that do not divert too much time from caretakers. We integrated the findings to develop a working prototype AR engine called "Project Phonado." From preliminary user testing, Project Phonado aids in boosting mental health and meaning in aging adults—at least temporarily (immediate and one day follow-up). We will show clips of some pilot user testing of the engine and discuss the next evaluation and development steps.

### WRIST-WORN TRI-AXIAL ACCELEROMETER VALIDATION IN YOUNG, MIDDLE-AGED, AND OLDER ADULTS

Todd M. Manini,[1] Santiago Saldana,[2] Duane Corbett,[1] Amal A. Wanigatunga,[3] Eduardo Navarro,[1] Mamoun M. Mardini,[1] and Ramon Casanova[2], *1. University of Florida, Gainesville, Florida, United States, 2. Wake Forest University, Winston-Salem, North Carolina, United States, 3. Johns Hopkins Bloomberg School of Public Health, Baltimore, Maryland, United States*

Purpose: This study evaluated wrist-worn accelerometers for estimating metabolic intensity and classifying activity types across a wide age spectrum. Methods: Participants (n=141, 67% women, aged 20-89 yrs) performed a battery of 31 common daily activities (e.g. washing dishes, walking) in a standardized laboratory setting. A tri-axial accelerometer was worn on the right wrist during each activity whiel a portable metabolic unit was used to measure oxygen consumption (ml/kg/min), which was converted into metabolic equivalents (METs). Random forest analyses estimated metabolic intensity and classified activity type based on seven data features.

Resulting estimates were cross-evaluated on a separate sample of 16 participants who performed a sub-set of activities in their home. Results: In the laboratory setting, mean differences between measured and predicted MET value for sedentary (0.36), lifestyle (0.02) and locomotor (0.30) activities were low, but the 95% limits of agreement ranges were relatively large (+/-1.0, +/-1.8, +/-3.1, respectively). Data features were 85%, 88%, and 71% accurate for identifying sedentary, lifestyle and locomotor activities. Prediction equations had an overall mean difference of 0.19 METs (95% limits of agreement = -1.3 to 1.7) when activities were performed at home. Conclusion: Data features extracted from a wrist worn tri-axial accelerometer provide a moderate-to-high group estimate of metabolic intensity and had modest accuracy in identifying activity types across a variety of daily activities. However, significant between person variations were evident. Additional work is needed to refine wrist-worn accelerometers for estimating physical activity type, intensity, duration and frequency across the age spectrum.

### DETECTION OF FORWARD PROPULSION USING A SINGLE ACCELEROMETER DURING WALKING IN OLDER POPULATION

Mohsen Zahiri,[1] Bijan Najafi,[1] Hung Nguyen,[1] and Gu Eon Kang[1], *1. Baylor College of Medicine, Houston, Texas, United States*

In the geriatric population, diminished ankle joint moment and weak plantar flexor can contribute to inadequate forward propulsion and negatively impact gait performance, which can lead to poor energetic efficiency. Detection of propulsion phase can help identify gait normality and guide rehabilitation therapy to improve functional performance. Current methods have limited application in daily life and unsuitable for continuous monitoring. In this study, we aim to develop algorithms based on a single sensor attached to the shin to accurately detect propulsion phase. Six elderly (age: 73 years, BMI: 30.4) were recruited. Participants walked at their normal pace while wearing a plantar pressure system and an accelerometer on the shin. The pressure data was used to define the beginning of the propulsion phase when the pressure switched from the heel to the forefoot. A wavelet algorithm was developed to automatically detect the start and end points of propulsion phase using an accelerometer. The Bland-Altman method was used to evaluate the agreement between these methods. Pearson's Coefficient was used to quantify the correlation. Based on the Bland-Altman analysis, A high agreement was obtained between the proposed method using accelerometer and pressure sensor (bias =9 ms, precision = 30 ms). Both algorithms are significantly correlated (r = 0.85, p<0.05). This study presents an innovative algorithm to automatically detect the propulsion phase for older adults during walking. Using wearable could facilitate the capture of propulsion phase during living activity, which might provide more insights into the mechanism of walking during rehabilitation therapy.

### LONG-TERM GAIT SPEED TELEMONITORING IN OLDER ADULTS WITH MILD COGNITIVE IMPAIRMENT OR MILD DEMENTIA. THE DECI STUDY

Lorenzo M. Donini,[1] Alberto Rainoldi,[2] Luca C. Feletti,[3] Gianluca Zia,[4] Eleonora Poggiogalle,[1] Susanna Del Signore,[4] and Monika Jurkeviciute on behalf of DECI Consortium[5], *1. Sapienza University, Rome, Italy, 2. University of Turin, Italy - Neuro Muscular Function Research group, School of Exercise and Sport Sciences, Department of Medical Sciences, Torino, Italy, 3. Caretek srl, Torino, Italy, 4. Bluecompanion LTD, London, England, United States, 5. Chalmers University of Technology, Gothenburg, Sweden*

Non-intrusive telemonitoring of physical activity in Older Adults suffering from Mild Cognitive Impairment (MCI), or Mild Dementia (MD), was implemented as part of a 6-month multicomponent digital intervention in the DECI study (EU Horizon2020 grant No 643588). Methods: To estimate gait speed long-term trajectory, a processing algorithm was applied on individual accelerometry data continuously recorded via the ADAMO wrist-watch accelerometer. Speed Trend Analysis was performed if patients wore the device ≥90 days. Only outdoor activity was analyzed to reflect patients' own natural gait speed. Only time spent in high or very-high-activity level is used, to eliminate rest periods (e.g. sitting on a bench, on a bus or driving). A raw mean walking speed was computed. Stride was computed from gender and height and walked distance from stride and step count. Mean walking speed was estimated by walking distance and duration. A rolling mean algorithm was applied to the computed mean 15-day baseline series, resulting in a new series representing normalized patient's gait speed trajectory during the study. Results: Baseline characteristics: F/M=21/19; MCI/MD=36/4; age=75.4±6.0 years; BMI= 24.6±5,2; MMSE=26.5±2.4; education=8.9±4.0 years. Monitoring days=147±29. Overall three main patterns of gait speed trajectory were identified: "relative stability", "improving trend" and "progressive decline": No evident correlation with cognitive status was observed in the sample. Examples of individual patterns are shown. Conclusions: Gait Speed Analysis can describe physical function trajectory over time and identify decliners from stable or improving older adults. Further analyses may clarify the relationship between physical function changes and cognitive status.

### AGE- AND ETHNICITY-RELATED DISPARITIES IN TECHNOLOGY USE AMONG HIGH-RISK VETERANS

Kiranmayee Muralidhar,[1] Willy Marcos Valencia,[2] Kaicheng Wang,[1] Diana Ruiz,[2] Carlos Gomez-Orozco,[3] and Stuti Dang[2], *1. Department of Public Health Sciences, University of Miami Miller School of Medicine, Miami, Florida, United States, 2. Miami Veterans Affairs Healthcare System- GRECC, Miami, Florida, United States, 3. South Florida Veterans Affairs Foundation for Research and Education Miami, Miami, Florida, United States*

Using predictive analytic modelling, the Veterans Affairs has identified Veterans considered to be High Need High Risk (HNHR) requiring increased support. This pilot study sent needs assessment questionnaires to 1112 HNHR Veterans to better understand gaps regarding technology use, access, physical function, and mobility. There were 341(30.7%) respondents: 270(80.4%) Non-Hispanic, 64(18.8%) Hispanic/Latino; 210(61.6%) White, 119(34.9%) Black/African Americans; and 310(90.4%)

had ≥high school education. Average Barthel(ADL) score was 81.5±22.8 and Lawton(IADL) score was 5.8±2.2. Younger Veterans (age<70) were more likely able to use Internet ((117(65%) vs 74(46%)),(p≤0.01) and email (106(58.9%) vs 67(41.6%),( p≤0.01). They were also more likely enrolled in MyHealtheVet (87(48.3%) vs 58(36%),(p=0.043). Secure messaging was used by 62(34.3%) younger and 37(23%) older Veterans,(p=0.026). More higher functioning Veterans (140(55.1%)) used email than lower functioning (33(37.9%)),(p=0.018). Among higher functioning Veterans, 148(58.3%) were willing to use videoconference for care coordination and 116(45.7%) owned a smartphone or computer with camera for this; more than lower functioning Veterans (33(37.9%) and 28(32.2%)), (p≤0.01 for both). Less dependent Veterans preferred to be contacted via cellphone (88(62.4%)) or Internet (10(7.1%)) compared to the more dependent (96(48%) and 6(3%)) respectively (p=0.01). Only 71(44.1%) of older Veterans were willing to use videoconference (p≤0.01) and 54(33.5%) owned a smartphone or computer with camera,(p≤0.01). There are significant variations in technology use by age and ethnicity. However, although there are differences by functional ability, a significant number of disabled veterans are willing and able to use technology, and this may provide a way to address access barriers in this population.

### WEBCAMERA-BASED VISUAL PAIRED COMPARISON AS A REMOTE COGNITIVE SCREENING METHOD

Michelle Gray, Joshua Gills, Spencer A. Smith, Emily Bates, Jordan M. Glenn,[2] Erica Madero,[2] and Nick Bott[2], *2. Neurotrack, Redwood City, California, United States*

Alzheimer's disease (AD) is a form of dementia impacting memory and cognitive function of 131 million individuals worldwide. Though early cognitive decline detection is important, cognitive screening is limited among older adults and many cases go undetected. As easy-to-use cognitive assessments are not readily available to the general population, the purpose of this investigation was to determine the ability of a 5-minute webcamera-based eye-tracking cognitive assessment to discriminate between cognitively intact adults and adults with mild cognitive impairment (MCI) or AD. This prospective study included 56 participants (age=55.9±26.8) divided into three groups: younger cognitively intact (ages 18-46 years, n=25), older cognitively intact (ages >60 years, n=20), and older cognitively impaired participants with MCI or AD (ages>60 years, n=13). All participants completed the Digit Symbol Substitution Test (DSST) and Visual Paired Comparison test (VPC) to assess cognition. One-way ANOVA detected differences in cognition between groups. A Pearson correlation determined the association between cognitive assessments. Additionally, multiple regression determined the ability of VPC and age to predict DSST scores. Results revealed significant differences between cognitively intact and cognitively impaired groups for VPC (p=.001) and DSST (p<.001). Follow-up analyses revealed significant differences between cognitively intact and cognitively impaired adults (p=.005) with no differences between younger and older cognitively intact adults (p=.34). There was a significant association between the VPC and DSST cognitive assessments (r=.54, p<.001), with VPC and age accounting for 69% of the variation in DSST. These results support the use of webcamera-based VPC as a viable option when screening tool MCI/AD.

### FEASIBILITY OF MOBILE HEALTH FOR LOW-INCOME MINORITY HISPANIC PATIENTS WITH A STROKE

Stuti Dang,[1] Kasra Sarhadi,[2] Sonjia Kenya,[2] Chuanhui Dong,[2] Natalie Ferras,[2] Jose Romano,[2] and Olveen Carrasquillo[2], *1. Miami Veterans Affairs Healthcare System- GRECC, Miami, Florida, United States, 2. University of Miami Miller School of Medicine, Miami, Florida, United States*

Stroke is a leading cause of death and functional impairment that disproportionately impacts Hispanics. Several studies have supported the feasibility of mobile health interventions (mHealth) to provide health monitoring and patient education for improving chronic disease management, but none have focused on Latino stroke patients. The Hispanic Secondary Stroke Prevention Initiative is a randomized study of 200 stroke patients designed to evaluate the impact of a 12-month multi-modal Community Health Worker (CHW) and mHealth intervention on blood pressure control. Eligible participants were Latinos who experienced a mild-moderate stroke within the last five years. The CHW component included home visits, telephone calls, and daily text messages to obtain home blood pressure readings and provide patient navigation and health education. Feasibility was defined as the proportion of patients that responded to at least half the messages. Pre-post paired t-tests assessed improvements in question accuracy while correlation coefficients highlighted improvements in response rates. Among the 65 participants randomized to the intervention, the response rate was as follows: 37% - >50% response, 21% - 25-50%, 19% - 10-25%, and 23% - <10%, This finding suggests that mHealth interventions may be challenging in this population. However, the proportion of questions answered correctly increased from 63% to 84% in the intervention period's last two months (p<0.05). There was a positive correlation between increased response rates and response accuracy to patient education assessments (r=0.82, p<0.05). These improvements in health knowledge suggest that a subset of patients may benefit from mHealth interventions, and the benefit correlates with use.

### NOVEL REMOTE ASSESSMENT OF THE STANDING POSTURAL CONTROL IN YOUNGER AND OLDER ADULTS USING SMARTPHONE APPLICATION

Junhong Zhou,[1] Wanting Yu,[1] Hao Zhu,[1] On-Yee Lo,[1] Thomas Travison,[1] Lewis Lipsitz,[2] and Brad Manor[1], *1. Hinda and Arthur Marcus Institute for Aging Research, Harvard Medical School, Roslindale, Massachusetts, United States, 2. Hebrew SeniorLife, Roslindale, Massachusetts, United States*

In older adults, assessment of standing postural control under various task and/or environmental conditions provides valuable insight into cognitive-motor function. To date, however, such assessments have been limited primarily to laboratory or clinical settings. We therefore created a smartphone App to enable remote assessments of postural control. This App provides users with standardized multi-media instructions and harnesses the phone's internal motion sensors to capture postural sway, with the phone placed in the user's pants pocket, during trials of standing with eyes open, eyes-closed, and while performing serial-subtractions (i.e., dual tasking). We then established the test-retest reliability of several metrics of postural sway derived from this assessment tool, as well as their sensitivity to the effect of age and standing condition. Fifteen healthy younger and 15 older adults completed multiple standing trials in two separate laboratory visits and on three separate days in their own homes. Postural sway metrics included the mean distance from the center of the trajectory and root mean square were derived from both transverse-plane acceleration and angular velocity time series. Each sway metric demonstrated excellent test-retest reliability, even when analyzed separately by group and standing condition (ICCs: 0.78-0.89). Moreover, each metric was sensitive to age group and standing condition, such that greater sway was observed in older adults as compared to younger adults ($p<0.03$), and in more challenging standing conditions ($p<0.0001$). These results suggest that sensitive metrics of standing postural control may be reliably obtained from remote smartphone-based assessments in both younger and older adults.

### AUTOMATIC QUANTIFICATION OF TANDEM WALKING USING A WEARABLE DEVICE: VALIDITY OF THE INSTRUMENTED TANDEM WALK

Jeffrey M. Hausdorff,[1] Natalie Ganz,[1] Eran Gazit,[1] Amit Hadad,[2] Aron S. Buchman,[3] and Anat Mirelman[1], *1. Center for the study of Movement, Cognition and Mobility, Neurological Institute, Tel Aviv Sourasky Medical Center, Israel, Tel Aviv, Israel, 2. Department of Physical Therapy, Sackler Faculty of Medicine, Tel Aviv University, Israel, Tel Aviv, Israel, 3. Rush Alzheimer's Disease Center, Rush University Medical Center, Chicago, USA, Chicago, Illinois, United States*

Tandem walk (TW) is typically assessed by the time to complete the task and the number of missteps, however, these measures suffer from limitations and may not fully capture the range of performance in this task. We developed metrics of TW by using a body-fixed, wearable sensor in young and older adults. Healthy young men (n=40) and older adult men (n=362) were studied. While wearing a 3D accelerometer on their lower back, subjects performed three different tasks: TW, usual-walking, and quiet standing. The extracted measures for TW were: High-to-Low frequency band ratio from the power spectral density from the ML axis [nu], signal vector magnitude[g], step duration[s], sample entropy from ML, AP axis[nu] and CV[%]. All of the TW metrics were significantly different in the young and older men ($p<0.001$). Older men completed the TW with higher CV, suggesting greater stride-to-stride variability and they walked more slowly, as seen by their step duration. Additionally, the frequency ratio measure suggests that the older adults displayed less complex corrective movements in the ML axis. TW measures were modestly correlated with usual-walking (e.g., average stride time with TW step time, $r=0.3$; $p<0.001$) and with quiet standing postural control (e.g., acceleration path length in the ML and AP axis with TW sample entropy in the ML axis, $r=0.13$; $p=0.014$). Metrics derived from a wearable device complement conventional TW measures and vary with age. Further work is needed to determine if TW, gait and posture metrics are differentially associated with distinct adverse health outcomes.

### WEARABLES REVEAL A GAP BETWEEN GAIT PERFORMANCE IN THE LAB AND DURING 24/7 MONITORING IN OLDER ADULTS

Inbar Hillel,[1] Laura Avanzino,[2] Andrea Cereatti,[3] Marcel Olde Rikkert,[4] Silvia Del Din,[5] Pieter Ginis,[6] Anat Mirelman,[1] and Jeffrey M. Hausdorff[1], *1. Center for the study of Movement, Cognition and Mobility, Neurological Institute, Tel Aviv Sourasky Medical Center, Israel, Tel Aviv, Israel, 2. IRCCS San Martino Teaching Hospital, Genoa, Italy., Genoa, Italy, 3. Department of Biomedical Sciences, Bioengineering unit, University of Sassari, Italy, Sassari, Italy, 4. Department of Geriatric Medicine, Donders Centre for Medical Neuroscience, Radboudumc Alzheimer Center, Radboud university medical center, Nijmegen, The Netherlands, Nijmegen, Netherlands, 5. Institute of Neuroscience, Newcastle University Institute for Ageing, Clinical Ageing Research Unit, Campus for Ageing and Vitality, Newcastle University, Newcastle upon Tyne, UK, Newcastle upon Tyne, United Kingdom, 6. Neuromotor Rehabilitation Research Group, Department of Rehabilitation Sciences, KU Leuven, Belgium, Leuven, Belgium*

We compared in-lab usual-walking (UW) and dual-task walking (DTW) to daily-living measures of gait obtained during 24/7 monitoring. In-lab gait features (e.g., gait speed, step and stride regularity) derived from UW and DTW were compared to the same gait features during daily-living in 150 elderly fallers (age: 76.5±6.3 years, 37.6% men). Features were extracted from a lower-back accelerometer. In daily-living setting, subjects wore the device for one week and pre-processing detected 30-second walking bouts. A histogram of all walking bouts was determined for each walking feature for each subject, then each subject's typical, worst and best values were determined. Statistics of reliability were assessed using ICC and Bland-Altman. As expected, in-lab gait speed, step regularity, and stride regularity were worse during DTW, compared to UW. Gait speed, step regularity, and stride regularity during UW were significantly higher (i.e., better) from the typical daily-living values ($p<0.0001$) and different ($p<0.000$) from the worst and best values. DTW values tended to be similar to typical daily-living values ($p=0.205$, $p=0.053$, $p=0.013$ respectively). ICC assessment and Bland-Altman plots indicated that in-lab values do not reliably reflect the daily-walking values. Gait values during relatively long daily-living walking bouts are more similar to the corresponding values obtained in the lab during DTW, as compared to UW. Still, gait performance during most daily-living walking bouts are worse than that measured in-lab and do not reliably reflect each other. That is, an older adult's typical daily-living gait cannot be estimated by simply measuring walking in a structured, laboratory setting.

### VIDEO RECORDING FOR CAPTURING INTERACTIONS BETWEEN RESIDENTS WITH DEMENTIA AND STAFF: A SYSTEMATIC REVIEW

Da Eun Kim,[1] Hae Sagong,[1] Eunjoo Kim,[1] Ah Ram Jang,[1] and Ju Young Yoon[1], *1. Seoul National University, Seoul, Korea, Republic of*

The use of video-recording offers important advantages in observing and assessing the relationships between specific behaviors in health care settings. The purpose of this systematic review is to investigate and synthesize the methodological characteristics of studies using video-technology for measuring interactions between the older persons with dementia and staff in long-term care facilities. We searched Medline, Embase and CINHAL databases for published articles in English using a video-recording method for both staff and persons with dementia. Quantitative research design studies (e.g., descriptive studies or an experimental studies) were included. Among 5,605 searched papers, a total of 20 studies were selected for this review. Situations of video-recording were providing personal care (n=12), mealtime (n=6), and conversation (n=3). Concepts of measuring by video-recording were classified into two groups: 1) Staff [care practice by staff (n=13) and communication by staff (n=11)] and 2) residents [communication by resident (n=4), activities of daily life function of resident (n=8), and behavioral and psychological symptoms of dementia (n=10)]. This review demonstrates that video technologies are actively used to evaluate the relationship between quality of care and health outcomes of the elderly with dementia in many international nursing studies. This study provides the foundation for a future research using video-recording technologies to examine the interactions and relationship between staff and the residents in long-term care settings.

### EXPLORING TECHNOLOGY-ENABLED ASSESSMENTS OF SYMPTOMS EXPERIENCE BY INFORMAL CAREGIVERS

Daniel J. Schulman,[1] Portia Singh,[1] Mladen Milosevic,[1] and Ali Samadani[2], *1. Philips Research North America, Cambridge, Massachusetts, United States, 2. Philips Research, Cambridge, Massachusetts, United States*

For community-dwelling older adults with chronic conditions, effective symptom management is a determinant of quality of life. Providers often have poor knowledge of an individual's symptoms experience, especially when contact is infrequent, leading to suboptimal symptom management. Many older adults receive frequent care and contact from family, friends, and other informal caregivers (ICGs). Subjective observation by ICGs is an underexplored information source, but faces barriers including ICG burden and lack of ICG knowledge. It is unclear what relevant information might be collected by ICG observations. We conducted a pilot evaluation of Philips CarePartners Mobile (CPM), a prototype smartphone application that provides communication and coordination support to a "circle" of ICGs assisting an older adult. CPM includes features enabling ICGs to share semi-structured observations. 19 caregivers (in 8 circles) used CPM for 12 weeks, contributing 397 observations and participating in interviews and other assessments. We performed a qualitative analysis of the observations, coding for presence of content relevant to dimensions in the UCSF Symptom Management Theory (perception of, evaluation of, and response to symptoms). Relevant content was found in 150 observations, with perception and assessment more common (141) than response (32). Common symptoms included mobility difficulty (31), fatigue (23), dizziness (21), pain (19), and confusion (18). Among observations without symptom-relevant content, many reported on overall mood (92), and reference to social activities was frequent. These results demonstrate that symptoms experience can be assessed using caregiver observations, although further work may be needed to enable caregivers to provide a comprehensive assessment.

### EHEALTH ON FRAILTY: FRAILSURVEY, A RELIABLE SMARTPHONE APPLICATION FOR SELF-ASSESSMENT OF FRAILTY

Luís Midão,[1] Carla Sá,[2] Elisa Marques,[2] Mafalda Duarte,[3] Constança Paúl,[4] João Viana,[2] and Elísio Costa[5], *1. ICBAS - Abel Salazar Institute of Biomedical Sciences, University of Porto, Porto, Portugal, Porto, Portugal, 2. CIDESD - Research Center in Sports Sciences, Health Sciences and Human Development, University Institute of Maia, Porto, Portugal, Porto, Portugal, 3. ISAVE – Superior Institute of Health, Amares, Braga, Portugal, ISAVE – Superior Institute of Health, Braga, Portugal, 4. Instituto de Ciências Biomédicas Abel Salazar, Universidade do Porto, Porto, Portugal, 5. UCIBIO/REQUIMTE, PORTO4AGEING - Competences Centre on Active and Healthy Ageing of the University of Porto, Faculty of Pharmacy of the University of Porto, Porto, Portugal, UCIBIO/REQUIMTE, Portugal*

Frailty is a clinical syndrome whose signs and symptoms are predictors of health complications, making this a major public health problem. FRAILSURVEY, which was considered a good practice by several entities, is a smartphone application that allows an easy assessment of frailty, available in Portuguese, English and Italian, on both iOS and Android stores. Smartphone-based assessment has been proven to be a useful diagnostic tool for patients, although few applications have demonstrated reliability. With this work we aimed to test the reliability of FRAILSURVEY as a tool to assess frailty, and to study people's preference on which way to assess frailty. FRAILSURVEY is a questionnaire that comprises two sets of questions: 19 about sociodemographic data, social resources, self-perception of health, nutrition, medication, psycho-social and cognitive status, and time occupation, plus a set of 15 used to assess frailty status, the Groningen Frailty Indicator (GFI). Including 427 older adults in this study, a randomized repeated measures crossover design was employed using the FRAILSURVEY questionnaire both in paper and in application, with a week of interval to reduce carryover effects. Reliability was assessed through the GFI scores obtained by the same person between the paper and application. There were no significant differences between the results on frailty assessment using the questionnaire on both paper and application. A significant correlation was noted in the total group (ICC=.794, $P<0.01$). Our work shows that FRAILSURVEY is a reliable mobile application for frailty assessment, that can be used by older adults, caregivers, and health professionals.

### DEVELOPMENT AND TESTING OF AN ACCELEROMETER-BASED POSITIONAL MONITORING SYSTEM

Leighanne Jarvis,[1] Sarah Moninger,[2] Chandra Throckmorton,[3] Juliessa M. Pavon,[1] and Kevin Caves[4], *1. Duke University, Durham, North Carolina, United States, 2. Duke University Department of Surgery, Durham, North Carolina, United States, 3. Signal Analysis Solutions, LLC, Bahama, North Carolina, United States, 4. Duke University, Durham, United States*

Health and fitness are contributing factors to physical resilience, or the ability to resist or recover from functional decline following health stressors. Accelerometer based activity monitors have been used in both the in-patient and outpatient setting to monitor mobility. While using sensors to track mobility is increasing, most clinical settings rely on patient reported outcomes. These measures often under or overestimate movement. The lack of a clinically meaningful way to measure mobility in the in-patient setting is a barrier to improving the mobility of hospitalized individuals. This is especially important when considering that over one-third of hospitalized older adults are discharged with a major new functional disability in performing activities of daily living. Our goal was to automatically determine if the subject is laying, reclining, sitting, standing, and walking to better reflect actual activity. Other platforms and studies indicate the ability to determine a difference in activity vs. inactivity or laying and reclining vs. standing and walking, but not all five phases of movement defined here. The aim of this study was to use accelerometer data to train a machine learning algorithm to automatically classify the postural changes (i.e. laying, reclining, sitting, standing, and walking). Preliminary results demonstrate that our trained algorithm is overall 95% accurate in determining each position from unlabeled data from the subject population. Additionally, this algorithm will be applied to in-patient hospitalized older adults for tracking of positions throughout the day.

### #GERIATRICS: AN ANALYSIS OF THE IMPACT OF THE GERIATRICS TWITTER NETWORK

Varun Ayyaswami,[1] Arpan Prabhu,[2] and Steven Gambert[1], *1. University of Maryland School of Medicine, Baltimore, Maryland, United States, 2. University of Pittsburgh School of Medicine, Pittsburgh, Pennsylvania, United States*

Twitter connects an international community of healthcare stakeholders, potentially augmenting access to information related to geriatric medicine. The purpose of this study is to analyze the geriatric medicine Twitter network, and we hypothesize this community has substantially grown in the last six years. We analyzed all publicly available tweets including the hashtag #geriatrics from January 1, 2013-January 1, 2019 using Symplur Signals, a health care social media analytics platform. We evaluated #geriatrics metrics over time related to activity, content analysis, user characteristics, engagement, and network analysis. A total of 159,008 tweets (containing 42.8% re-tweets) with the hashtag #geriatrics were written by 29,443 users, resulting in 393.6 million impressions. The number of tweets increased from 9,705 in 2013 to 39,151 in 2018 (32.2% compound annual growth); users increased from 3,366 in 2013 to 29,443 in 2018 (55.3% compound annual growth). Users were primarily found in the United States, United Kingdom, and Canada. The most commonly trending topic from 2013-2015 and from 2016-2018 was ‘older adults’. The top hashtags included in tweets with #geriatrics were #job, #aging, and #hpm (hospice and palliative medicine). Network analysis showed central hubs to be medical journals, provider organizations, individual physicians, and individual advocates. The top 150 influencers consisted primarily of physicians (49.1%), advocate/support organizations (13.8%), and media organizations (6.3%). The use of Twitter to promote geriatric medicine using #geriatrics has matured into an international digital community of interest. Future studies should examine hashtags related to age prevalent illnesses and hashtags likely to be used by patients.

### ALEXA, ASSESS MY MEMORY: THE FEASIBILITY OF EXTENDED HEALTH MONITORING IN AN OLDER-ADULT-LIVING COMMUNITY

Jensen Davis,[1] Shannon Howard,[1] Gregory King,[1] Phanidar Boddu,[1] Kiran Jyothi,[1] and Joan McDowd[1], *1. University of Missouri-Kansas City, Kansas City, Missouri, United States*

The goal of most older adults is to live independently in their own homes, for as long as possible. There are many advantages to aging in place for the individual, but also challenges as changes in cognitive and physical health can occur over time. Especially for older adults living alone, tracking these changes is critical for early intervention and prevention. The relatively easy availability of consumer technology may provide one mechanism for monitoring older adults in their homes. We designed a pilot study to test the feasibility and acceptability of using wearable sensors (Fitbit sensors), in conjunction with automated interactive voice recognition technology (Amazon Echo), to monitor older adults’ physical and cognitive health during daily activities. Participants (7 females, 2 males; 65-80 years of age) were recruited from a housing complex for older adults with low income. They were interviewed about health monitoring technology before and after a 2-week measurement period during which they were expected to wear the Fitbit daily and interact with the Amazon Echo for 8 consecutive days. Feasibility challenges included limited skill in Echo interactions, remembering to do the assessments, and charging/uploading Fitbit data. Qualitative analysis of interviews revealed generally positive attitudes about technology, but low comfort operating the devices. These preliminary findings suggest that with additional training for older adults, sensors and voice recognition technologies could have significant roles in maintaining older adult quality of life by contributing to early detection of decline and timely intervention.

### NURSING HOME TELEED INTERVENTION: ADVANCING NEW CARE MODELS

Charlene C. Quinn,[1] Anthony Roggio,[2] Barr Erik,[3] and Ann Gruber-Baldini[4], *1. University of Maryland School of Medicine, Baltimore, Maryland, United States, 2. University of Maryland Medical Center, Baltimore, Maryland, United States, 3. University of Maryland School of Medicine, Baltimore, Maryland, United States, 4. University of Maryland Baltimore School of Medicine, Baltimore, Maryland, United States*

New reimbursement and managed care models demonstrate the need to reduce avoidable Emergency Department (ED) use and limit preventable inpatient admissions for older adults in Skilled Nursing Facilities (SNF). The objective was to develop an ED telemedicine consultation intervention for SNF residents with acute medical problems. Secondary objectives including evaluation of health care utilization, provider satisfaction. Demonstration evaluation in three urban SNFs, telemedicine linked to university medical center ED. Mobile telemedicine cart equipment assessed SNF residents for any change in condition. ED physicians used tablets with secure access to conduct the resident assessment. Provider satisfaction measures imbedded in EMRs were completed at consultation visit end. 460 patients had changes in condition, 327 resulted in 911 calls, 85 deemed eligible for telemedicine consult. Conducted 57 telehealth consults. Forty (70%) telemedicine consult residents remained in the SNF. Fourteen residents were transferred to the ED. Average satisfaction scores were 5.8/7 for SNF nurses (n=49) and 5.6 for ED physicians (n=45). Lower-rated items related to technical equipment problems. ED physicians reported residents transferred to ED after telehealth visit had better continuity of care. The intervention was effective in preventing or delaying transfer of acutely ill, medically complex SNF residents. Implementation of the intervention identified need for SNF admission policy and procedure changes; weekly telemedicine training; SNF clinical advocates; on-site tracking and linkage of EMRs across providers; HIPAA shared medical record concerns. Future research plans include analyses of detailed SNF resident characteristics and business case assessment for reduction of transfers, ED and hospital utilization.

### DOES WEARING AUGMENTED-REALITY GOGGLES AFFECT OLDER ADULTS' KINEMATICS DURING GAIT?

Edgar R. Vieira,[1] Jansen Estrazulas,[2] Fernanda Civitella,[3] Jorge Carreno,[4] Newton D'Souza,[5] Ebru Ozer,[6] and Francisco Ortega[7], *1. Department of Physical Therapy, Florida International University, Miami, Florida, United States, 2. College of Health, Amazonas State University, Manaus, Amazonas, Brazil, 3. Department of Physical Therapy, Florida International University, Miami, Florida, United States, 4. Department of Physical Therapy, Florida International University, Miami, Florida, United States, 5. Department of Interior Architecture, Florida International University, Miami, Florida, United States, 6. Department of Landscape Architecture + Environmental and Urban Design, Florida International University, Miami, Florida, United States, 7. Department of Computer Science, Colorado State University, Fort Collins, Colorado, United States*

Virtual-reality (VR) testing can cause motion sickness and impair safety, especially for older adults, but augmented-reality (AR) may allow the testing of holograms embedded into a mixed-reality environment without the VR impediments. However, wearing AR googles may affect the way people walk, but this possibility has not being tested. The objective of this study was to evaluate if wearing AR googles during gait would affect the kinematics of older adults. Ten older adults (68±5 years), who could walk without assistive devices, participated in this study. The participants walked outdoors in a public park with and without the AR googles. The participants were instrumented inertial movement units to track their kinematics (MTw Awinda trackers, Xsens Technologies B.V., Enschede, the Netherlands). The goal of the study was to assess if simply wearing the googles would affect gait, therefore no holograms were displayed. Ten gait cycles were analyzed and the mean of each subject was used to compare the joint kinematics between the conditions (with vs without googles) using T-tests in SPSS 18. The foot, ankle, knee and hip angles were not different between the conditions (p>0.05), but there were significantly less trunk flexion at 44% of the gait cycle (p=0.035) and less forward head flexion throughout the gait cycle (p=0.023) when the participants were wearing the googles vs. when they were not. The findings indicate that wearing AR goggles changed the trunk and head posture cycle, but did not affect the lower limb kinematics during gait.

### HOME-BASED TRANSCRANIAL DIRECT-CURRENT STIMULATION AND EXPERIMENTAL PAIN SENSITIVITY

Hyochol Ahn,[1] Chengxue Zhong,[1] Setor Sorkpor,[1] and Hongyu Miao[1], *1. University of Texas Health Science Center at Houston, Houston, Texas, United States*

Osteoarthritis (OA) of the knee is one of the most common causes of pain in older adults. Clinic-based transcranial direct current stimulation (tDCS) is a noninvasive brain stimulation technique that has been shown to reduce pain, but no published studies have reported using home-based self-administered tDCS in older adults with knee OA. Thus, the purpose of this study was to examine the effect of home-based tDCS on experimental pain sensitivity in older adults with knee OA. Twenty community-dwelling participants aged 50–85 years with knee OA pain received ten daily sessions of 2 mA tDCS for 20 minutes at home. A multimodal quantitative sensory testing battery was completed, including heat pain tolerance, pressure pain threshold, and punctate mechanical pain. Participants (75% female) had a mean age of 61 years, and a mean body mass index in the sample was 28.33 kg/m2. All 20 participants completed all ten home-based tDCS sessions without serious adverse effects. The Wilcoxon Signed-Rank test showed that all the differences between the baseline measurements and experimental pain sensitivity measurements after 10 sessions were statistically significant. Effect sizes (Rosenthal's R) were R = 0.35 for heat pain tolerance (P = 0.02), R = 0.40 for pressure pain threshold (P < 0.01), and R = 0.32 for punctate mechanical pain (P = 0.02). We demonstrated that home-based self-administered tDCS was feasible and reduced experimental pain sensitivity in older adults with knee OA. Future studies with well-designed randomized controlled trials are needed to validate our findings.

### VETCONNECT: A VA TELEHEALTH PROGRAM PROVIDING TREATMENT, EDUCATION AND TRANSITIONS OF CARE TO NURSING HOME VETERANS

Cari Levy,[1] Anne Hale,[2] Emily Galenbeck,[3] and Leah M. Haverhals[4], *1. VA Rocky Mountain Regional VA Medical Center, Aurora, Colorado, United States, 2. VA Rocky Mountain Regional VA Medical Center, Aurora, Colorado, United States, 3. Seattle-Denver Center of Innovation, Denver, Colorado, United States, 4. Seattle-Denver Center of Innovation, Denver, Colorado, United States*

Transporting nursing home Veterans to hospitals for outpatient care can present many challenges, including lengthy time in transit, coordination difficulties between the hospital and nursing home, and travel burden on Veterans. In June 2017, the VetConnect program began offering Veterans in Colorado residing in Department of

Veterans Affairs (VA) contracted Community Nursing Homes (CNHs) and State Veteran Nursing Homes (SVHs) telehealth appointments in Geriatrics, Mental Health, Palliative Care, and Psychiatry via clinicians at the Rocky Mountain Regional VA Medical Center (VAMC). Then in June 2018, VetConnect expanded to offer Virtual Tours for Veterans transitioning out of inpatient care to CNHs the opportunity to virtually tour the CNH, connecting with CNH staff through video for a more stress-free and informed transition from hospital to CNH. In winter 2018, VetConnect expanded once more to include Dementia Educational Outreach Series for CNH and SVH staff, allowing them to connect virtually to VA staff with expertise in dementia care and other Veteran-specific topics such as treating post-traumatic stress disorder (PTSD). To date, VetConnect has provided 502 telehealth visits, 18 Virtual Tours, and 4 dementia ECHO trainings (with 58 nursing home staff participants). Evaluation measures to be presented include collection and analysis of telehealth nurse field notes (who are present with Veterans at CNHs/SVHs during telehealth visits), creation of process maps, conducting key stakeholder interviews, and identification of potential impacts the VetConnect program has had on VA to CNH and SVH relationships and Veteran health.

### ASSESSING THE IMPACT OF A GAME-CENTERED MOBILE APP ON COMMUNITY-DWELLING OLDER ADULTS' HEALTH ACTIVATION

Jason Crandall,[1] Matthew Shake,[2] and Uta Ziegler[3], *1. Western Kentucky University Center for Applied Science in Health and Aging, Bowling Green, Kentucky, United States, 2. Western Kentucky University Department of Psychological Sciences, Bowling Green, Kentucky, United States, 3. Western Kentucky University School of Engineering and Applied Science, Bowling Green, Kentucky, United States*

The objective of this investigation was to evaluate the impact of a game-centered mobile app (Bingocize®) on older adults' knowledge, skill, and confidence for managing aspects of their healthcare. Rural community-dwelling older adults (N=85) with mobility, not engaged in any structured exercise program, used the app in a group setting for 10 weeks, twice per week, for one hour. Participants were randomly assigned to (a) a version that included health education, or (b) health education and an exercise component. The Patient Activation Measure (PAM-10) was used to assess group changes in knowledge, skill, and confidence for managing aspects of their healthcare. Two (Group: Exercise + Health Education vs. Health Education-only) x Two (Time: Pre- vs. Post-intervention) analyses of variance, with significance $p<.05$, was used for statistical analysis. Results: PAM-10 values significantly increased from pre- to post-intervention for both groups, as did knowledge of the health topics (all $p < 0.05$). Attendance was >93% in both groups. Bingocize® engendered high attendance and improved health activation of older adults; however, additional research is needed to examine whether changes in activation result in long-term changes in health behavior. The Bingocize® mobile app is an enjoyable and effective way to increase health activation in community-dwelling older adults.

### SESSION 1405 (POSTER)

### SRPP SECTION STUDENT POSTER AWARD FINALISTS

### SOCIAL ISOLATION AMONG THE RECIPIENTS OF THE MINIMUM LIVELIHOOD GUARANTEE PROGRAM IN CHINA

Yalu Zhang,[1] and Ada Mui[1], *1. Columbia University, New York City, New York, United States*

The eligibility for the minimum livelihood guarantee program in China is generally based on means testing, including a complicated income test and a household registration test. Even though the fiscal budget for this program steadily increased in the last two decades, the number of beneficiaries has decreased in both rural and urban areas since 2011. The recipients were stigmatized because their names are reported in the public domain and are known to their neighbors. Some beneficiaries tended to reduce social participation for fear of losing the eligibilities due to the stricter criteria or the stigma associated with receiving benefits. This paper examined the adverse impact of this welfare program on social isolation among Chinese middle-aged and older adults (n=8,447) using the panel data from the China Health and Retirement Longitudinal Study and the propensity score weighted difference-in-differences method (PSM-DID). The results present disparities between urban and rural older adults—The older program recipients in urban areas significantly reduced social participation by 20.53%, while the adverse impact was not statistically significant among rural recipients. Compared to older adults (aged 60 and above), middle-aged adults (aged between 45 and 60) were not socially isolated due to program enrollment in both rural and urban areas at the 95% confidence level. This paper for the first time addresses the causal relationship between program enrollment and the adverse impact of social isolation. It also alerts policymakers and local program administrators to protect older program recipients from stigma, sense of shame, and reduced social connection.

### DO WELFARE RECEIPTS CROWD OUT PRIVATE TRANSFERS FROM FAMILIES AND FRIENDS? EVIDENCE FROM PANEL DATA IN CHINA

Yalu Zhang[1], *1. Columbia University, New York, New York, United States*

Many countries are undergoing an unprecedented challenge to provide financial support to the older generation and guarantee their livelihood and wellbeing. China is no exception. Rural older adults in China have been becoming even more vulnerable to lack of care and inadequate financial resources as the growth of urbanization and labor migration has intensified. Therefore, it becomes increasingly difficult to follow the traditional family support model for the aged. Using the panel data of the China Health and Retirement Longitudinal Study 2011, 2013, and 2015 and a system generalized methods of moment (GMM), this paper examined the dynamic relationship between welfare receipts and monetary transfers from families and friends among rural and urban older adults (n=9,496) in China. The results show that the welfare receipts do not induce any "crowd-out" or

"crowd-in" effects on rural older adults' private transfer receipts. The incidence and amount of private transfers that occurred among rural older adults are more likely to be determined by the private transfers they received in prior waves. The intensity of catastrophic health expenditure itself does not affect the occurrence and size of private transfers. This study, on the one hand, confirms that among rural recipients, public transfers do not substitute private transfers, which most of the policymakers have long been concerned about. However, on the other hand, it also reveals the shortcoming of current public transfer policies—the generosity of public transfers does not enable rural older adults to be financially independent of intra-family transfers.

### PROTECTIVE FACTORS IN THE CONTEXT OF SUCCESSFUL AGING IN URBAN-DWELLING ALASKA NATIVE ELDERS

Steffi M. Kim,[1] and Jordan P. Lewis[1], *1. University of Alaska Anchorage, Anchorage, Alaska, United States*

Recently, researchers have been exploring successful aging in rural communities of Alaska as it is experienced by Alaska Native Elders. Due to outmigration based on economic, medical, or familial influences, many Alaska Native elders leave their home communities to live in urban settings in Alaska, even though research suggests that most elders would like to remain in their home communities to grow old. Very little is known about the relocation process and how it impacts an elder's views on successful aging. While established protective factors in rural communities involve family and extended family members, community, subsistence, and traditional activities, there is little knowledge of which protective factors exist in urban settlements supporting successful aging. This exploratory, qualitative study presents the protective factors of successful aging in the context of relocation of Alaska Native Elders from rural to urban dwellings. A life course approach and discourse analysis were used to analyze individual interviews asking how Alaska Native Elders experienced relocation and how successful aging was experienced similarly and differently in rural and in urban settings. A community-based participatory approach allowed for collaboration between researchers and communities as equal partners at all phases of the process, both contributing their expertise to enhance understanding of successful aging and supportive factors. Access to informal supports and meaningful community engagement were more important to rural Elders, and access to health care services and family engagement were important to Elders in urban communities. Challenges remain for Elders in urban environments to establish a sense of community.

### HOW DOES MODE OF SERVICE DELIVERY IMPACT CAREGIVER STRAIN? A MIXED-METHODS STUDY OF CAREGIVERS IN LOS ANGELES

Haley B. Gallo,[1] Chia Ying Chen,[1] and Donna Benton[2], *1. Leonard Davis School of Gerontology, University of Southern California, Los Angeles, California, United States, 2. University of Southern California, Los Angeles, California, United States*

As the number of people living with Alzheimer's disease and related dementias increases, so too will the number of people who care for them. This growing population requires instrumental and emotional support as they fulfill their caregiving duties. CareJourney is an online platform that provides this support; it can be used either alone, or with traditional in-person and phone-mediated consultations with family care navigators (FCNs). The intent of CareJourney is to provide a greater opportunity for self-directed services, as well as flexibility for working caregivers and those who prefer using technology. Satisfaction surveys (N=222) were analyzed to evaluate caregivers' perceptions of CareJourney and the traditional service delivery modes. Additionally, five 30-minute interviews with caregivers were coded by two researchers (kappa=0.84) for a more in-depth understanding. Ten percent of survey respondents used CareJourney, and preliminary results from both the interviews and the survey suggest that caregiver burden, lack of time, and desire for a personal connection with FCNs influence mode of service delivery. Although a quarter of users relied on CareJourney to find caregiving resources and email their FCNs because it was more convenient than traditional modes, the interviews and surveys revealed that the caregivers most valued the personal touch that came from interacting with FCNs. Speaking with FCNs over the phone or in-person allowed caregivers to feel "reassured," "less isolated," and "comforted" as they discussed caregiving strains. Future interventions and services for caregivers should be offered using multiple modes to accommodate a range of time-demands and preferences for personal connection.

### BUILDING DISASTER RESILIENCE FOR OLDER ADULTS AGING IN PLACE: THE ROLE OF COMMUNITY-BASED ORGANIZATIONS

Claire Pendergrast,[1] Basia Belza,[1] Ann Bostrom,[1] and Nicole Errett[1], *1. University of Washington, Seattle, Washington, United States*

Older adults are more susceptible to adverse health outcomes during and after a disaster compared with their younger counterparts. Developing community resilience, or strengthening communities to reduce the negative impacts of disasters, has the potential support older adults' health and well-being. Community-based organizations (CBOs), such as senior centers and Villages, provide social services and programming that support aging in place and may support older adults' resilience to disasters. This study examines CBO leadership perspectives on the role of CBOs in building disaster resilience for older adults aging in place, as well as perceived barriers and facilitators to incorporating disaster resilience activities into organizational programming. In-depth interviews were conducted with a purposive sample of staff-members of CBOs serving older adults aging in place in King County, Washington. Participants included representatives from 14 organizations that varied in size, geographic setting, organizational structure, and ethnic, linguistic, and socio-economic backgrounds of organizational members. The sample included five government-run senior centers, seven non-profit senior centers, and two Villages. Interviews were audio-recorded and transcribed verbatim. We used a combined inductive and deductive approach to code and thematically analyze the data. Results indicate that local context, leadership risk perception, collaborations, and existing services and programming influence CBOs' willingness to engage in activities supporting disaster resilience for older adults aging in place. Findings suggest that CBOs supporting aging in place may support disaster resilience for older adults by serving as a trusted source of disaster preparedness information and tailoring disaster-related messages for an older adult audience.

### HOW AN INDIVIDUAL'S SENSE OF CONTROL INFLUENCES THEIR PERCEPTION OF SUCCESSFUL AGING

Alexandra P. Harris,[1] Alexandria Nuccio,[1] and Ashely M. Stripling[2], *1. Nova Southeastern University, Fort Lauderdale, Florida, United States, 2. Nova Southeastern University, Fort Lauderdale, United States*

Factors like physiology, mental health, personal resources, and social support have been identified to contribute to perceived successful aging (Cosco, Prina, Perales, Stephan & Brayne, 2015); however, sense of control's role in these relationships remains underexplored. Studying the impact of sense of control is crucial, given that many factors associated with well-being are correlated with later life success. The current study investigates associations among sense of control and constructs known to define successful aging. The data was derived from the Survey of Midlife in the US database (MIDUS3). Participants were primarily Caucasian (88.7%) and female (54.9%) with a mean age of 63.64 years (SD=11.35). A series of hierarchical multiple regressions revealed that sense of control impacts physical health ($F = 87.734$, $p<0.001$), depression ($F = 43.944$, $p<0.001$), anxiety ($F = 24.680$, $p<0.001$), social actualization ($F = 66.450$, $p<0.001$), and instrumental activities of daily living (IADL) ($F = 135.963$, $p<0.001$) over demographic correlates (i.e., age, sex, and race). The present findings suggest that higher levels of control results in increased comfort in social atmospheres, absence of mood symptoms, good health, and limited issues with IADL. Implications of the current findings include a deeper understanding of how psychological factors, such as sense of control, can impact physical and mental health in order to improve care and promote wellbeing in late life.

### THE IMPACT OF A PARO INTERVENTION ON DEPRESSION AND WELL-BEING IN OLDER ADULTS WITH DEPRESSION

Shu-Chuan Chen,[1] Wendy Moyle,[2] and Cindy Jones[3], *1. Griffith University, Brisbane, Queensland, Australia, 2. Griffith University, Queensland, Australia, 3. Bond University, Brisbane, Queensland, Australia*

Aim: This study aimed to explore the effect of a social robot Paro intervention on depression and well-being in older adults with depression living in long-term care facilities in Taiwan. Methods: This study was adopted a single group and quasi-experimental with repeated measures design. Each participant participated in two stages: observation and Paro intervention stages. Stage 1 was an 8-week observation stage in long-term care facilities where the purpose was to observe the normal mood, behaviour and activities of older adults with depression. In stage 2, each participant was given a Paro by the researcher to keep for 24 hours for 7 days in for 8 weeks. Outcome measurements were obtained 4 times: a week before the intervention (T1), immediately the end of 8-week observation (T2), mid-point of Paro intervention (T3), and immediately the end of 8-week Paro intervention (T4). Instruments included the Geriatric Depression Scale, the UCLA Loneliness Scale version 3, and the World Health Organization Quality of Life Questionnaire-OLD. Results: There were 20 participants completed the study. The mean age of participants was 81.1years (SD = 8.2). After 8-week Paro intervention, statistically significant differences in changes were found on depression, loneliness, and quality of life from pre-intervention to post-intervention. Conclusion: This study was found that Paro intervention has beneficial effects on depression and mental well-being for older people with depression in long-term care facilities. Paro Intervention might be a suitable psychosocial intervention for older people with depression and should be considered as a useful tool in clinical practice.

### ADMISSION AND CARE OF INDIVIDUALS WITH MENTAL ILLNESS IN MASSACHUSETTS NURSING HOMES: A PILOT STUDY

Adrita Barooah,[1] and Pamela Nadash[1], *1. University of Massachusetts Boston, Boston, Massachusetts, United States*

Due to the rising prevalence of mental illness in nursing homes (NHs), the US Congress passed the 1987 Pre-Admission Screening and Resident Review (PASRR) mandate, which aims both to limit inappropriate institutionalization of people with mental illness and to ensure that they are served appropriately when living in NHs. Although the PASRR is a federal mandate, states have considerable flexibility in implementing it, resulting in considerable variation across states. This study explores the Commonwealth of Massachusetts' policies on admission and care of individuals with mental illness in NHs, focusing on implementation of PASRR regulations. Semi-structured phone interviews were conducted with key informants identified through purposive snowball sampling (N=8). Key informants included representatives from NHs, the State Mental Health Authority, state Medicaid office, and independent contractors and an academic expert. Data were analyzed using qualitative content analysis. Participants agreed that the PASRR tools efficiently identified and screened people with mental illness -- thus achieving PASRR's first aim, but that the regulations did not successfully ensure appropriate services. Interviewees also identified a lack of services and options for diversion of people with mental illness into the community. Nursing home informants noticed a disconnect between the various supervising departments and felt instructions were unclear on the administration of the tool. This work builds a case for a national study to understand how PASRR implementation varies across states, resulting in variations in the proportion of people with mental illness admitted and served in NHs.

### VALIDATING THE PREFERENCES ASSESSMENT TOOL FOR NURSING HOME RESIDENTS USING ITEM RESPONSE THEORY

Yinfei Duan,[1] Weiwen Ng,[2] and Tetyana P. Shippee[3], *1. School of Nursing, University of Minnesota, Minneapolis, Minnesota, United States, 2. University of Minnesota, Minneapolis, Minnesota, United States, 3. University of Minnesota, Minneapolis, Minnesota, United States*

The Preference Assessment Tool (PAT), part of the Minimum Data Set (MDS), assesses residents' preferences to enable preference-based care in nursing homes (NHs). The two PAT sections including daily routine preferences and activity preferences are assumed to measure autonomy and meaningful activities as the underlying constructs associated with residents' psychosocial needs. Yet, the validity of this assumption has not been tested. This study examines PAT's construct validity using item response theory. We fitted graded response models to the two PAT sections using 2017 MDS annual assessments of 8,829 long-stay residents in 291 Minnesota NHs. Most items discriminated well between residents who had at a low versus high intensity of these two types of psychosocial needs (i.e. have discrimination parameters > 1). Two daily routine preference items (family's involvement in care planning, and having snacks), and three activity preference items (having pets, keeping up with news, and reading) had poor discrimination in measuring autonomy and meaningful activities respectively. Overall, the PAT appeared to be more sensitive in measuring the lower middle range of the two constructs. We estimated the correlation between the two constructs as 0.65. In conclusion, the PAT performs fairly well in measuring the two types of psychosocial needs for NH residents. Nevertheless, more items are needed to capture a broader range of psychosocial needs beyond autonomy or basic leisure activities. The findings of this study brought attention to the utility of the PAT in measuring residents' psychosocial needs and in guiding resident-centered care.

### KEEPING COOL IN FLORIDA ASSISTED LIVING COMMUNITIES: BARRIERS TO POWER RULE IMPLEMENTATION

Joseph June,[1] Debra J. Dobbs,[2] David Dosa,[3] Lindsay J. Peterson,[4] and Kathryn Hyer[5], *1. School of Aging Studies, University of South Florida, Tampa, Florida, United States, 2. University of South Florida, Tampa, Florida, United States, 3. Brown University School of Public Health, Providence, Rhode Island, United States, 4. University of South Florida School of Aging Studies, Tampa, Florida, United States, 5. University of South Florida, Tampa, Florida, United States*

In 2018, in response to deaths of nursing home residents after Hurricane Irma, Governor Scott and the Florida legislature enacted an emergency power rule for nursing homes and assisted living communities (ALCs). It mandates these settings to have a generator to regulate ambient air temperatures during loss of primary power by Jan. 1, 2019. However, due to cost and supply challenges the implementation of these plans has been difficult, particularly for small and independently owned ALCs. The purpose of this mixed methods study is to determine the characteristics of ALCs that were able to comply with the rule and concerns raised by ALC administrators. Using data from state regulatory agencies on all Florida ALCs (N=3082), we determine associations between ALC characteristics (size, specialty license, low-income residents) and non-compliance to the rule, using chi-square and t-tests. Additionally, we conducted interviews and focus groups with ALC administrators (N=60) about issues of implementing their emergency power plan. A content analysis approach was used and Atlas.ti v7 was used for initial and axial coding. Some prevalent themes were issues with time frames, coordination between local and state regulations, and financial burden. Themes varied by size and organizational structure of ALCs. Results will inform policy-makers on the barriers faced by ALCs to implement a new regulation that may cause financial difficulties and compromise quality care. This study has implications related to disaster preparedness regulations and their effects on independent ALCs with fewer financial resources.

### SESSION 1410 (POSTER)

### TRANSPORTATION/DRIVING IN OLDER ADULTHOOD

### DIFFERENCES IN SELF-REGULATION BETWEEN OLDER DRIVERS WITH AND WITHOUT DEMENTIA

Jennifer S. Zakrajsek,[1] Lisa J. Molnar,[1] and David W. Eby[2], *1. University of Michigan Transportation Research Institute, Ann Arbor, Michigan, United States, 2. University of Michigan Transportation Research Institute and Center for Advancing Transportation Leadership and Safety, Ann Arbor, Michigan, United States*

Given the high current prevalence of dementia and expected increases in the coming years, it is increasingly important to understand and address the process of transitioning from driving to non-driving among older drivers with dementia and the potential role of self-regulation (e.g., avoiding challenging driving situations). The purpose of this Alzheimer's Association-funded study was to examine differences in self-regulation between older drivers with and without dementia. Fifty-two active drivers age 65 or older (16 with dementia; 36 healthy controls), completed a validated self-regulation questionnaire and standardized assessment of cognition, vision, and psychomotor skills. The dementia group reported some awareness of cognitive challenges (lower driving-related abilities to remember things and concentrate on multiple things at a time than the comparison group). Similarly, the dementia group performed significantly worse on memory tasks and complex reaction time in the objective assessment. Both groups were confident they could drive safely where they needed to go. However, the dementia group reported being less comfortable driving in several situations (unprotected left turns, unfamiliar areas, alone, rush hour traffic, backing up). Despite lower driving comfort among the dementia group, there were few differences in self-regulation between the groups. The only statistically significant difference was that more of the dementia group had reduced their driving in the past year. There were no differences in self-regulation between groups for any of the specific driving situations for which the dementia group reported feeling less comfortable. Full study results will be presented along with implications for programs and future research.

### OLDER ADULTS' OPINIONS ON DIFFERENT VEHICLE TECHNOLOGIES

Sara A. Freed,[1] Lesley A. Ross,[1] and Despina Stavrinos[2], *1. The Pennsylvania State University, University Park, Pennsylvania, United States, 2. The University of Alabama at Birmingham, Birmingham, Alabama, United States*

Vehicle technologies have the potential to greatly improve road safety. Given normative changes in cognitive, sensory and physical functioning, older drivers may particularly benefit from such technologies. However, little research has examined older adults' opinions of vehicle technologies, descriptive differences of individuals more likely to have positive opinions of vehicle technologies, and how their opinions may differ depending on the type of vehicle technology. The current study examined older adults' opinions on vehicle technology in a sample of 72 adults between 65 and 85 years (M = 72.3, SD = 5.36, 48% women). Participants were asked, "How important is [parking assistance, crash avoidance systems, early collision warnings, built-in GPS] in choosing a new vehicle?" on a scale from 1 ("not at all") to 5 ("must have"). On average, participants rated built-in GPS as the most important (M = 3.81, SD=1.10) with parking assist as the least important (M=1.86, SD=1.13). We used correlational analyses to examine the association between demographic and personality and importance ratings. Women were more likely to rate greater importance for built-in GPS than men ($r=.35$, $p<.05$). Age, education, self-reported driving quality, and self-reported average weekly driving miles were not significantly associated with importance rating. In terms of personality, only higher levels of extraversion were associated with more positive ratings of early collision warning systems ($r=.24$, $p<.05$). Vehicle technology design and education should take older adults' preferences into account and consider individual differences, and future work should examine other predictors of vehicle technology preferences such as functional performance.

### BASELINE DRIVING MILEAGE PREDICTS STATE-REPORTED CRASHES ACROSS 5 YEARS

Junyan Tian,[1] Sara A. Freed,[1] and Lesley Ross[1], *1. The Pennsylvania State University, University Park, Pennsylvania, United States*

Although annual driving mileage has frequently been examined as a predictor of crashes among older adults, most research used cross-sectional design and relied on self-reported crash data. This study used multivariate regression to examine the number of state reported at-fault crashes between groups of low (14,000km) self-reported annual distance over five years. Additionally, key factors of interest including age, gender, and population density were examined as predictors. The sample included 519 healthy older adults aged 65-90 (M=73.17, SD=5.56) across five sites in the United States. 12% of participants experienced a crash across five years, and among those who crashed, the majority (87%) experienced one crash (range 0-3 crashes). After controlling for age, gender and testing site, people in the high annual mileage group had a greater number of crashes compared to the low mileage group ($\beta=.14$, $t(513)=2.37$, $p=.02$). There was not a significant difference in number of prospective crashes between the low and medium group. Also, people who drove in sites with low population density had more crashes than those who in high population density sites ($\beta=.10$, $t(513)=2.24$, $p=.03$). Higher age was associated with a greater number of prospective crashes ($\beta=.01$, $t(513)=3.67$, $p =.002$); however, gender was not a significant predictor of crashes. Our results highlight the importance of examining prospective crashes over time, and taking mileage and population density into consideration. Future research should examine trajectories of driving exposure in relation to prospective crashes using multilevel modeling.

### DRIVING CESSATION IS ASSOCIATED WITH POORER MENTAL HEALTH

Arne Stinchcombe,[1] Carlina Marchese,[2] Shauna Fossum,[2] Sylvain Gagnon,[3] Gary Naglie,[4] Mark Rapoport,[5] Bruce Weaver,[2] and Michel Bédard[2], *1. Saint Paul University (Ottawa), Ottawa, Ontario, Canada, 2. Lakehead University, Thunder Bay, Ontario, Canada, 3. University of Ottawa, Ottawa, Ontario, Canada, 4. Baycrest Health Sciences/University of Toronto, Toronto, Ontario, Canada, 5. Sunnybrook Health Sciences/University of Toronto, Toronto, Ontario, Canada*

The Canadian Longitudinal Study on Aging (CLSA) is a longitudinal health study that will follow individuals aged 45 to 85 for 20 years. At baseline, participants completed measures related to driving status and mental health outcomes (e.g., Center for Epidemiologic Studies Depression Scale; CES-D). In this study we examined the associations between driving status and mental health outcomes. In the baseline sample, 1,415 participants reported being former drivers and 44,694 reported being current drivers. A greater proportion of former drivers were female, older, and urban-dwelling. Compared to current drivers, former drivers had lower levels of social support, poorer self-rated physical health, and less community participation. After controlling for these covariates as well as age and sex, former drivers had greater odds than current drivers of being classified as depressed (OR=2.48, 95% CI=2.21-2.79), and of reporting psychological distress (OR=2.22, 95% CI=1.87-2.62). Using data from former drivers only, we also examined associations between variables that contributed to driving cessation and depression symptoms. Former drivers had greater odds of being depressed if they reported feeling nervous or intimidated behind the wheel (OR=1.77, 95% CI= 1.11 - 2.80), or if they experienced difficulties with the licensing process (OR=1.62, 95% CI=1.07 - 2.54), before they stopped driving. As a next step we will search for factors that may modify the relationship between driving status and mental health. The identification of factors that modify the impact of driving cessation on mental health is critical to the development of interventions that will support smoother transitions to non-driving.

### SCANNING FOR CRITICAL EVENTS DURING DRIVING: VISUAL SCANNING USING EYE TRACKING TECHNOLOGY ON A DRIVING SIMULATOR

Anne E. Dickerson,[1] Rachel Gartz,[2] Megan Eaker,[1] and Brittany Clark[1], *1. East Carolina University, Greenville, North Carolina, United States, 2. Vidant Medical Center, Greenville, North Carolina, United States*

This study explored age differences and scanning strategies for critical events using a driving simulator with eye-tracking technology. One critical skill in driving is to efficiently scan the environment. Ten young(M=26y) and 10 old(M=65y) healthy adults drove a wearing Tobii-Pro Glasses. The software analyzes areas of interest (AOI) for "percent time of fixation" as well as illustrative "heat maps" of duration of fixation. At a four-way-stop, the young fixated at the road ahead (25% fixation time), dashboard (29%), more often than left/right of their car (15%). The old fixed on the road ahead (21%) but spent more time outside the car (23%). At the hidden sign, older drivers fixated on the environment outside the road ahead more than younger drivers (26% versus 20%), but both had increased fixation to the right side of the vehicle (Y-23%, O-24%) with this event, likely looking for the sign. Heat maps of the hidden sign illustrate this, suggesting older drivers may more efficiently scan their environment. Other possibilities include the young trusting the simulator, young/old scan differently, or interference of previous simulator experience. Simulator outcomes showed age differences on gas-pedal-reaction times suggesting that older adults are more cautious, slowing down faster with critical events. Limitations include small sample and limited studies have used eye-tracking technology in driving. This study raises interesting questions, especially for medically-at-risk drivers with visual impairments. Using the eye-tracking may enhance targeting specific strategies for a variety of impairments as well as establishing a baseline of typical drivers' visual scanning habits.

### THE RELATIONSHIP BETWEEN OLDER DRIVERS' RESILIENCE AND SELF-REPORTED DRIVING MEASURES OVER 5 YEARS

Renée M. St. Louis,[1] Judith Charlton,[1] Sjaan Koppel,[1] Lisa J. Molnar,[2] Marilyn Di Stefano,[3] Peteris Darzins,[4] Michel Bédard,[5] and Shawn Marshall[6], *1. Monash University Accident Research Centre, Clayton, Victoria, Australia, 2. University of Michigan Transportation Research Institute, Ann Arbor, Michigan, United States, 3. VicRoads, Melbourne, Victoria, Australia, 4. Eastern Health Clinical School, Monash University, Forest Hill, Victoria, Australia, 5. Lakehead University, Thunder Bay, Ontario, Canada, 6. University of Ottawa/ Ottawa Hospital, Ottawa, Ontario, Canada*

As people age into older adulthood, they are more likely to experience events that impact their driving, such as age-related cognitive and functional declines, serious illness, or disability. The ability to demonstrate resilience following such adversity may influence one's decisions and feelings about driving. This study investigated whether resilience of older drivers changes over time, and if relationships between resilience, gender, and self-reported driving-related abilities, perceptions and practices remain stable or change. Participants were from the Candrive/Ozcandrive study, a prospective cohort study of older drivers from Canada, Australia and New Zealand. Analyses are presented from a subset of Ozcandrive participants (n=125) from Australia who completed a resilience scale at two time points approximately five years apart, as well as measures of driving comfort during the day and night, perceived driving abilities, and driving frequency. Participants were primarily male (67.2%) with a mean age of 81.6 years (SD=3.3, Range=76.0-90.0) at Time 1. Resilience increased significantly from Time 1 to Time 2 (Median=82.0/84.00, z=-2.9, p<.01). Although females had significantly higher resilience than males at both Time 1 (Median=84.0/81.0, U=2.3, p=.02) and Time 2 (Median=86.5/82.0, U=2.1, p=.03), there was a statistically significant increase in resilience of males over five years (p<.01) and no statistical change for females. Results show small but significant positive correlations, and increasingly stronger relationships over time between older drivers' resilience and driving comfort as well as perceived driving abilities. Future research will use modelling to examine the association of various factors on the change in resilience and driving-related measures.

### SESSION 1415 (SYMPOSIUM)

### INTEREST GROUP SESSION—RAINBOW RESEARCH GROUP: ADAPTING AN EVIDENCE-BASED INTERVENTION TO LGBT ADULTS WITH DEMENTIA AND CARE PARTNERS: MOBILIZING SUPPORT NETWORKS

Chair: Karen Fredriksen Goldsen, *University of Washington, Seattle, Washington, United States*
Discussant: Linda Teri, *University of Washington, Seattle, Washington, United States*

LGBT (lesbian, gay, bisexual, and transgender) older adults have been found to have elevated risks of cognitive impairment. Maintaining quality of life is a challenge for those experiencing cognitive decline and their caregivers. Whereas support networks are essential for quality of life, LGBT older adults with dementia may face unique risks, such as stigma, social isolation, lack of family support, and barriers to healthcare. Aging with Pride: IDEA (Innovations in Dementia Empowerment and Action), is the first federally funded clinical trial to test an intervention designed to improve quality of life of LGBT older adults with dementia and caregivers adapting a preexisting program teaching behavioral strategies and physical exercises. The intervention incorporated empirical findings from a longitudinal study, Aging with Pride: National Health, Aging, and Sexuality/ Gender Study (NHAS) and developed innovative and culturally responsive approaches. Kim and colleagues examine predictors of longitudinal changes in physical functioning among LGBT older adults with cognitive impairment focusing on physical, social, and recreational activities as well as stigma. Emlet and colleagues investigate caregiving experiences among LGBT older adults and identify factors that are associated with their physical and mental health. Lastly, Fredriksen Goldsen and colleagues introduce how the modifiable factors identified from the Aging with Pride: NHAS were incorporated in the IDEA intervention and evaluate the processes of the culturally-responsive approaches implemented in the study. The presentations in this symposium illustrate the importance of tailoring clinical trial studies for hard-to-reach and underserved populations with dementia responding to their unique health needs.

### MODIFIABLE DETERMINANTS OF PHYSICAL FUNCTIONING AMONG LGBT OLDER ADULTS WITH COGNITIVE IMPAIRMENT

Hyun-Jun Kim,[1] Hyun-Jun Kim,[1] Karen Fredriksen-Goldsen,[1] and Hyunzee Jung[1], *1. University of Washington, Seattle, Washington, United States*

Heightened risks of cognitive impairment are a critical health concern for lesbian, gay, bisexual, and transgender (LGBT) older adults. The physical and functional declines associated with cognitive impairment are known to increase risks of injury and decrease independent mobility. Guided by the Health Equity Promotion Model, this study analyzed longitudinal data (T0 to T2, N=646) to examine risk and protective factors predicting changes in physical functioning over time among LGBT older adults with cognitive impairment. According to the results of multilevel mixed models, social support, physical, recreational, and wellness activities, and nutrition were associated with improvement in physical functioning over two years; while stigma contributed to decline in physical functioning. LGBT older adults with cognitive impairment face unique challenges due to social stigma and isolation. The findings suggest that enhancing their access to community resources and their participations in physical, social, community, and recreational activities would improve their physical functioning.

### DETERMINANTS OF PHYSICAL AND MENTAL HEALTH AMONG LGBT OLDER ADULT CAREGIVERS

Charles Emlet,[1] Charles Emelt,[1] Hyunzee Jung,[2] Hyun-Jun Kim,[2] David La Fazia,[2] and Karen Fredriksen-Goldsen[2], *1. University of Washington, Tacoma, Washington, United States, 2. University of Washington, Seattle, Washington, United States*

Caregivers experience chronic stress that can negatively affect their health, resulting in lower quality of life. This study aims to understand the physical and mental health of caregivers by examining modifiable risk and protective factors. Life experiences of LGBT older adults are considered. In the longitudinal study, Aging with Pride: National Health Aging, Sexuality and Gender Study, approximately one third (31%, n=754) of LGBT older adults (50+ years) provide care: 38% to partner/spouse, 29% friends, 20% other relatives, and 8% ex-partner/ex-spouse. Linear regression models examined the effects of risk and protective factors at T0 on health outcomes at T2. Mastery, physical activity, and nutrition predicted better physical functioning, lower mental distress, and higher health-related quality of life (HRQOL). Social participation and support, community engagement, and lower identity stigma predicted better mental health. Microaggression predicted lower physical HRQOL. Interventions enhancing protective factors may promote physical and mental health of LGBT caregivers.

### LESSONS LEARNED FROM THE FIRST INTERVENTION STUDY ADDRESSING DEMENTIA IN THE LGBT COMMUNITY

Karen Fredriksen Goldsen,[1] Karen Fredriksen-Goldsen,[1] Linda Teri,[1] Hyun-Jun Kim,[1] Glenise McKenzie,[2] David La Fazia,[1] Charles Emlet,[3] and Ryan Petros[1], *1. University of Washington, Seattle, Washington, United States, 2. Oregon Health & Science University, Portland, Oregon, United States, 3. University of Washington, Tacoma, Washington, United States*

The cognitive health needs of LGBT older adults have not been adequately addressed in mainstream clinical trials. Aging with Pride: IDEA (Innovations in Dementia Empowerment and Action), is an intervention designed to improve physical functioning and quality of life of LGBT adults with dementia and caregivers. We evaluate the processes and effectiveness of culturally-responsive recruitment approaches implemented in this study. A strong research-community partnership was necessary for the development and implementation of the intervention. LGBT participants with dementia made the first contact to research team as often as caregivers did and showed a higher rate of living alone and having a friend-based care network. The most common reason for ineligibility was not having a caregiver. Participants learned about the study via multiple venues including community events, newsletters, and social media. This study illustrates important new ways to sustain collaborations with disadvantaged communities and conduct a clinical trial with hard-to-reach participants.

### SESSION 1420 (SYMPOSIUM)

### INTEREST GROUP SESSION—AGE-FRIENDLY UNIVERSITY (AFU): AGE-FRIENDLY UNIVERSITY CAMPUSES: PUTTING PRINCIPLES INTO PRACTICE

Chair: Joann M. Montepare, *Lasell College, Newton, Massachusetts, United States*
Co-Chair: Kimberly Farah, *Lasell College, Newton, Massachusetts, United States*
Discussant: Kenneth F. Ferraro, *Purdue University, West Lafayette, Indiana, United States*

The pioneering Age-Friendly University (AFU) initiative, endorsed in 2016 by GSA's Academy for Gerontology in Higher Education (AGHE), calls for institutions of higher education to respond to shifting demographics and the needs of our aging populations through more age-friendly programs, practices, and partnerships. Over 45 institutions in the United States, Canada, European countries, and beyond have joined the network and adopted the 10 AFU guiding principles. This symposium will feature leaders at AFU campuses who will discuss why their institution joined the initiative, their age-friendly campus vision, and how they are putting AFU principles into practice. AFU Washington University St. Louis leaders will describe efforts to increase age-diversity on their campus, especially through professional studies programs that support personal and career development in the second half of life. AFU University of Southern California leaders will discuss new age-friendly efforts entailing intergenerational exchange, best practices of age-friendly programming for retired employees and alumni, and emerging connections to local community aging initiatives. AFU Eastern Michigan University leaders will round out the presentation with an overview of accomplishments of their campus-wide steering committee and its age-friendly evaluation research that informed the needs and interests of older learners pursuing second careers, as well as how to actively engage emeritus faculty and staff as a retired community on campus. The discussant will provide integrating comments and age-friendly suggestions for putting AFU principles into practice.

### A CASE ARGUMENT FOR AGE DIVERSITY ON CAMPUS

Nancy Morrow-Howell,[1] Edward Lawlor,[1] Ed Macias,[1] Emma Swinford,[1] and Jeffrey Brandt[1], *1. Washington University, st. Louis, Missouri, United States*

Washington University for Life is an initiative to increase age-diversity on campus, especially in degree and certificate programs. We have developed a case argument for educating people across the longer life course; and we identify positive outcomes for society, younger students, the institution, and for older adults themselves. These arguments do not include the reality that the supply of young students will be constrained, as this demographic press is not viewed as threatening by many U.S. universities. This argument is being presented to administrators, admission and career service personnel, and classroom instructors, who do not universally accept the benefits, much less the necessity, of increased age-diversity; and they often express resistance to disrupting traditional practices with only the well-being of older adults in mind. A full discussion of reasons that Washington University should move purposively toward age inclusivity is important to build consensus and identify opportunities for new practices.

### MAKING CAMPUS GREAT AT ANY AGE: ONE UNIVERSITY'S APPROACH TO AFU PRINCIPLES

Cassandra Barragan,[1] Cassandra Barragan,[1] and Andrea Zakrajsek[1], *1. Eastern Michigan University, Ypslianti, Michigan, United States*

This presentation will discuss the focused approach to putting AFU principles into practice that we accomplished with our campus-wide steering committee. We performed surveys for students over the age of 40 to learn the perspectives of older learners and what resources they felt were available on campus. By doing this, we were able to understand more about learners pursuing second careers and through a variety of initiatives at the university level, we have improved visibility of older learners and the richness they bring to our campus. We also collected data from our emeritus faculty and staff to learn how they interact with campus in retirement to understand effective ways to actively engage with them as a retired community. Overall, we have been able to effectively use the AFU Initiative to enhance inclusion and diversity to include older learners on campus and make AFU efforts more visible across campus.

### BUILDING RECOGNITION ACROSS CAMPUS AND TOWN FOR USC'S AGE-FRIENDLY UNIVERSITY INITIATIVE

Paul Nash,[1] Paul Nash,[2] and Caroline Cicero[2], *1. University of Southern California, LA, California, United States, 2. University of Southern California, Leonard Davis School of Gerontology, Los Angeles, California, United States*

Details of efforts to highlight existing age-related programming on campus, create a new intergenerational experiential learning program, build a social media presence, and include 'age' in the university's diversity efforts will be discussed. Best practices in community engagement will be emphasized including successes with retired employees, alumni, activities programming, and local age-friendly efforts. Discussion includes the benefits of developing a multidisciplinary working group to ensure the Initiative is a university wide effort and the challenges of working in a large, multi-campus research university located in multilayered bureaucratic local jurisdictions. Future goals and collaboration with AFU Global Network partners and aspirations for addressing societal ageism will be addressed.

### SESSION 1425 (PAPER)

### BIOBEHAVIORAL HEALTH, HEALTH PROMOTION, AND BIOLOGICAL ASPECTS OF AGING

### A NOVEL METABOLITE COMPOSITE SCORE EXPLAINS THE HIGHER MORTALITY ASSOCIATED WITH FRAILTY AMONG OLDER BLACK MEN

Megan M. Marron,[1] Tamara B. Harris,[2] Robert M. Boudreau,[1] Steven C. Moore,[3] Jason L. Sanders,[4] Stacy G. Wendell,[1] Joseph M. Zmuda,7 and Anne B. Newman[1], *1. University of Pittsburgh, Pittsburgh, Pennsylvania, United States, 2. National Institute on Aging, Bethesda, Maryland, United States, 3. National Institutes of Health, Rockville, Maryland, United States, 4. Brigham and Women's Hospital, Boston, Massachusetts, United States, 5. Department of Epidemiology University of Pittsburgh; Pittsburgh, Pennsylvania, United States*

Frailty is more prevalent among black versus white older Americans. We previously sought to better characterize frailty among 287 black men ages 70-81 by identifying 37 plasma metabolites associated with vigor to frailty using the scale of aging vigor in epidemiology (SAVE). Using this information, we developed a metabolite score to determine if it explained the frailty-associated higher mortality. The Human Metabolome Database classified the metabolites as organic acids/derivatives (m=14), lipids/lipid-like molecules (m=12), organoheterocyclic compounds (m=4), benzenoids (m=3), organic nitrogen compounds (m=2), organic oxygen compounds (m=1), and nucleosides/nucleotides/analogues (m=1). Values for each were ranked into tertiles. The metabolite tertile associated with more vigorous SAVE scores was given a score of 0, the metabolite mid-tertile a score of 1, and the metabolite tertile associated with frailer SAVE scores a score of 2. The metabolite composite score was calculated as the sum of the metabolite tertile scores. One standard deviation frailer SAVE was associated with 30% higher mortality ($p=0.0002$), adjusting for age and study site. The association between frailty and mortality was attenuated by 56% after additionally adjusting for the metabolite score, where organic acids/derivatives and lipids/lipid-like molecules (mostly amino acids, glycerophospholipids, sphingolipids) accounted for most of the attenuation. In this model, one standard deviation higher metabolite score was associated with 46% higher mortality ($p<0.0001$). The metabolite score also predicted mortality among 48 community-dwelling (96% white) older men ($p=0.03$). These metabolites provide a deeper characterization of frailty that reproducibly explains a substantial portion of the vulnerability to death in these older men.

### GENE-ENVIRONMENT INTERPLAY BETWEEN SMOKING BEHAVIOR AND COGNITION AMONG OLDER ADULTS

Shandell Pahlen,[1] William Kremen,[2] and Chandra A. Reynolds[1], *1. University of California, Riverside, Riverside, California, United States, 2. University of California, San Diego, San Diego, California, United States*

Associations between smoking behavior and lower cognitive functioning have been observed but there is a paucity of evidence examining the etiological impact of smoking on cognition. The current study explored the moderation of genetic and environmental contributions to cognition across mid and late-adulthood by smoking behaviors in 8 twin studies from the international IGEMS consortium ($N=11{,}764$; $M_{age}=63.1$ years). Mixed effects regression models between smoking behavior and cognition found the strongest negative effects for smoking on Symbol Digit ($B_{packyears}=-1.42$, $p<.0001$) and Block Design ($B_{packyears}=-1.79$, $p=.0008$), while controlling for dependency between twin siblings, age, sex, and country. Although the negative effects tended to be more pronounced for males, we did not find significant sex moderation. Univariate biometric models considered smoking behavior (status and pack years) and age as moderators of genetic and environmental components contributing to cognitive performance. Results for both Symbol Digit and Block Design suggest that smoking (current and past) is associated with lower genetic, and higher environmental influences on cognition compared to non-smoking. For Block Design, but not for Symbol Digit, pack years moderated shared environmental contributions, with the highest contributions found for current smokers compared to former. Overall, results illustrate an increasing saliency of smoking related environmental influences for processing speed and spatial reasoning tasks. Cognitive tasks with speed components may be sensitive to age-related declines, and speed may also represent a factor vulnerable to smoking exposure, potentially implicating important health and neurobiological pathways. Supported by NIH Grant Nos. R56 AG037985, R01 AG060470.

### LINKAGES BETWEEN INDIGENOUS CULTURAL GENERATIVITY AND SOBRIETY TO PROMOTE ALASKA NATIVE SUCCESSFUL AGING

Jordan P. Lewis[1], *1. University of Alaska Anchorage, Anchorage, Alaska, United States*

The aim of this study was to explore motivating and maintenance factors for sobriety among older AN adult participants (age 50+) from across Alaska. Ten life history narratives of Alaska Native older adults, representing Alutiiq, Athabascan, Tlingit, Yup'ik/Cup'ik Eskimos, from the PA sample were explored using thematic analysis. AN older adults are motivated to abstain from, or to quit drinking alcohol through spirituality, family influence, role socialization and others' role modeling, and a desire to engage in indigenous cultural generative activities with their family and community. A desire to pass on their accumulated wisdom to a younger generation through engagement and sharing of culturally grounded activities and values, or indigenous cultural generativity, is a central unifying motivational and maintenance factor for sobriety. The implications of this research indicate that family, role expectations and socialization, desire for community and culture engagement, and spirituality are central features to both AN Elders' understanding of sobriety and more broadly, to their successful aging. Future research is needed to test these findings in population-based studies and to explore incorpo- ration of these findings into alcohol treatment programs to support older AN adults' desire to quit drinking and attain long-term sobriety. Sobriety can put older AN adults on a pathway to successful aging, in positions to serve as role models for their family and community, where they are provided opportunities to engage in meaningful indigenous cultural generative acts.

### NUTRITIONAL RISK PREDICTS HEALTH SERVICES UTILIZATION AND DEATH OVER 1 YEAR: RESULTS FROM THE UAB STUDY OF AGING II

David R. Buys,[1] Richard E. Kennedy,[2] Yue Zhang,[2] Julie Locher,[2] and Cynthia J. Brown[2], *1. Mississippi State University, Starkville, Mississippi, United States, 2. University of Alabama at Birmingham, Birmingham, Alabama, United States*

Nutritional risk has been demonstrated to be associated with poor health outcomes, increased risk of health services utilization (HSU), and mortality among older adults. The aim of this study was to assess the prospective relationship between nutritional risk; HSU focusing separately on emergency department visits, hospitalization, and nursing home admission; and mortality. Using the University of Alabama-Birmingham Study of Aging II, we examined this relationship among 419 community-dwelling older Alabamians (75+years). We used the Mini-Nutrition Assessment (MNA), a well-validated nutritional risk assessment, which classifies individuals as either well-nourished, at-risk or malnourished, collected at baseline. We assessed HSU by asking about healthcare encounters since the last monthly follow-up call for 12 months and verified death with family reports and official documents. We completed univariate, bivariate, and Cox proportional hazards regression analyses with one-year of follow-up data, adjusting for social support, social isolation, comorbidities, and demographic variables. Accounting for covariates, being either at-risk or malnourished, relative to well-nourished, was associated with emergency department visits (HR: 1.30, 95% CI:1.14,1.48), hospitalization (HR: 1.58, 95% CI:1.37,1.82), nursing home admission (HR: 8.94, 95% CI:3.99,20.02), and mortality (HR: 1.90, 95% CI:1.25,2.88). These findings underscore the growing awareness that nutritional risk, particularly for older adults, is a significant factor affecting their well-being and particularly their ability to continue living in the community. Nutrition assessment, interventions, and services for community-dwelling older adults may lead to a reduction in health care utilization, particularly nursing home placement, and ultimately to reduced healthcare costs to families and taxpayers.

### SENSE OF PURPOSE IN LIFE AND REDUCED LIKELIHOOD OF FUTURE DRUG MISUSE

Eric S. Kim,[1] Carol Ryff,[2] Afton Hassett,[3] Chad Brummett,[3] Charlotte Yeh,[4] and Victor Strecher[3], *1. Harvard T.H. Chan School of Public Health, Boston, United States, 2. University of Wisconsin, Madison, Wisconsin, United States, 3. University of Michigan, Ann Arbor, Michigan, United States, 4. AARP Services, Inc., Washington, District of Columbia, United States*

A stronger sense of purpose in life is hypothesized to reduce the likelihood of drug misuse because it has been linked with several protective factors including: increased ability to handle stress and pain tolerance, decreased impulsivity, and reduced risk of depression and chronic conditions. However, the association between purpose in life and drug misuse has been understudied. We tested whether people with a stronger sense of purpose in life had a decreased likelihood of incident drug misuse 9-10 years later. We also tested whether people with a stronger sense of purpose were less likely to cope with stress by misusing drugs. Participants were drawn from the Midlife in the United States Study (MIDUS; n=3,483) and from a stress coping module of the Health and Retirement Study (HRS; n=498). Among MIDUS respondents not misusing drugs at baseline, people in the highest quartile of purpose (compared to people in the lowest quartile) had 42% reduced odds (95% CI: 0.37-0.92) of incident drug misuse 9-10 years later in the fully-adjusted model (e.g., sociodemographic factors, depression, chronic conditions, and chronic pain). Among HRS respondents, people in the highest purpose quartile had 65% decreased odds (95% CI: 0.14-0.89) of misusing drugs to cope with stress in the fully-adjusted model. A growing number of intervention studies show that purpose in life can be raised. With additional research, these data suggest that sensitively tailored and administered purpose in life may reduce the likelihood of drug misuse and help stem the tide of our nation's growing drug epidemic.

### SESSION 1430 (SYMPOSIUM)

### INTEREST GROUP SESSION—EPIDEMIOLOGY OF AGING: BIOSOCIAL RESEARCH ON BRAIN AGING AND BIOLOGICAL AGING

Chair: Daniel W. Belsky, *Columbia University Mailman School of Public Health, New York, New York, United States*

Our aging global population presents a new set of challenges for public health. Individual-disease focused models are becoming outmoded as geriatricians recognize multimorbidity and frailty as the central challenges in preserving health for older adults. Evidence from research into the biology of aging suggests that a set of common cellular-level processes underpin decline in system integrity that induces vulnerability to disease across multiple organ systems, including the brain. In parallel, research in life-course gerontology indicates that the roots of aging-related decline in system integrity extend from early life and encompass histories of social, psychological, and biochemical exposures. The research presented in this symposium aims to integrate these emerging paradigms in aging research by mapping connections among measures of aging in the brain and body and social, psychological, and nutrition exposures. Our symposium focuses on (1) links between social-psychological determinants of health and biological aging in the brain and body; and (2) social and behavioral protective factors that may buffer emerging biological risk in aging. The overarching goal of this symposium is to introduce an approach to gerontology that integrates geroscience with life-course social and psychiatric epidemiology to advance understanding of cognitive aging and functional decline, and ultimately identify novel interventions to extend healthy lifespan.

### MENTAL AND PHYSICAL HEALTH SEQUELAE OF BEREAVEMENT IN OLDER ADULTS: U.S. HEATH AND RETIREMENT STUDY ANALYSIS

Benjamin Domingue,[1] Laramie Duncan,[2] Amal Harrati,[3] and Daniel Belsky[4], *1. Stanford University Graduate School of Education, Stanford, California, United States, 2. Psychiatry and Behavioral Sciences, Stanford, California, United States, 3. Primary Care & Population Health, Stanford, California, United States, 4. Epidemiology, New York, New York, United States*

Death of a spouse (bereavement) is associated with poor mental and physical health outcomes in older adults. But it is unknown how mental- and physical-health sequelae of bereavement are related and the clinical significance of bereavement-related depression has been questioned. We analyzed US Health and Retirement Study (HRS) data tracking mental and physical health of 36,034 older adults during 1992-2016. Post-bereavement data were available for N=4,985 participants with recorded date of spousal death. We analyzed longitudinal repeated-measures data on survivors' depression, disease, disability, and mortality. Bereavement effects on depression were immediate, but short-lived, attenuating within the year. In contrast, bereavement effects on physical health and mortality persisted over follow-up. Critically, the magnitude of short-lived effects on depression correlated with the magnitude of longer-lasting effects on disease, disability, and mortality. Results reveal connections between mental and physical health and aging and suggest bereavement-related depression as a biomarker of enduring health risk.

### PREDICTING TRANSDIAGNOSTIC PSYCHOPATHOLOGY FROM INDICES OF AGING IN THE HUMAN STRUCTURAL CONNECTOME

James Madole,[1] James W. Madole,[1] Simon R. Cox,[2] Colin R. Buchanan,[2] Stuart J. Ritchie,[3] Mark E. Bastin,[2] Ian J. Deary,[2] and Elliot M. Tucker-Drob[1], *1. The University of Texas at Austin, Austin, Texas, United States, 2. The University of Edinburgh, Edinburgh, Scotland, United Kingdom, 3. King's College London, London, England, United Kingdom*

Imaging-derived indices of brain structure and white-matter connectivity evince steep declines with adult age and are robustly linked to neurological disease and a wide range of psychopathologies. Risk for psychopathology may be related to rapid structural brain aging, but the specific patterns of relations are not well documented. Using structural and diffusion MRI data from UK Biobank, we estimated a structural connectome for each participant (N = 3155), and used empirically-driven machine-learning algorithms to identify features of the connectome most susceptible to brain aging. In an age-homogenous hold-out sample of older adults, we score participants' "connectome age" using the coefficients saved from the training sample. We examine associations between connectome age and both psychiatric symptom counts and polygenic risk scores for a range of psychiatric disease traits. This will be amongst the first and most comprehensive investigation of the extent to which psychopathology relates to signatures of structural connectome aging.

### INTEGRATIVE ANALYSIS OF ALZHEIMER'S DISEASE GWAS TO DEVELOP A NEW POLYGENIC PREDICTOR AND TEST BIOSOCIAL ETIOLOGY

Amal Harrati[1], *1. Stanford School of Medicine, Stanford, California, United States*

Alzheimer's disease (AD) has genetic and environmental causes and etiology is thought to reflect interplay among these factors. A barrier to integration of genetic and environmental etiologic factors in research to inform prevention and intervention is poor understanding of AD genetics beyond APOE4. We used the new Genomic SEM methodology to conduct integrative analysis of results from several AD genome-wide association studies (GWAS), including brain-imaging and autopsy AD GWAS, to derive a novel, polygenic genetic predictor of AD. We applied this polygenic predictor in the US Health and Retirement Study to test associations with (1) cognitive aging measured from repeated-measures longitudinal cognitive-test data; and (2) blood-chemistry-based biological age algorithms. In further exploratory analysis, we tested if risk measured by the novel AD polygenic predictor was correlated with and/or buffered by a known environmental factor influencing AD etiology, life-course socioeconomic position. Results map new directions for bio-social AD research.

### REVERSE TRANSLATION OF HUMAN BIO-AGING MEASURES TO CYNOMOLGUS MONKEYS TO TEST ASSOCIATIONS WITH DOMINANCE RANK

Noah Snyder-Mackler,[1] Carol Shively,[2] Tom Register,[2] and Daniel W. Belsky[3], *1. University of Washington, Seattle, Washington, United States, 2. Wake Forest University, Winston-Salem, North Carolina, United States, 3. Columbia University Mailman School of Public Health, New York, New York, United States*

Social status is a powerful correlate of aging-related health decline. Observational data in humans suggest that disadvantaged social status may be associated with accelerated biological aging. But establishing causality in this relationship poses challenges; experimental manipulation of human social status is not possible. In contrast, social status can be experimentally manipulated non-human primates (e.g. Snyder-Mackler 2016 Science). We conducted analysis to reverse-translate blood-chemistry of biological aging to cynomolgus monkeys using data from several hundred animals in the Wake Primate Center breeding colony. We are applying these measures in an independent sample of monkeys with ascertained dominance rank to test replication in the non-human primate model of the human social gradient in biological aging. Parallel analysis of DNA methylation-based measures of biological aging are ongoing and should be available to present by Fall 2019. Results will inform potential to use this non-human primate model to study social determinants of biological aging.

### IS SELF-RATED HEALTH A WINDOW ONTO THE BIOLOGY OF HEALTH AND AGING?

Daniel W. Belsky[1], *1. Columbia University Mailman School of Public Health, New York, New York, United States*

Self-ratings of health predict survival in older adults, suggesting that they capture important information about system integrity. We analyzed epigenetic clock, blood biochemistry, and functional test data alongside participant reports of disability, morbidity, and self-rated health in population-based cross-sectional and longitudinal datasets from the US and UK (total N>50,000) and in a randomized trial of caloric restriction (N=220). We (1) profiled cross-sectional biomarker correlates of self-rated health; (2) quantified residual biomarker associations with self-rated health after accounting for research-participant reports of morbidity and disability and evaluated variation in associations across strata of age, birth-cohort cohort, socioeconomic status, and cognitive functioning; and (3) tested coordinated change in biomarker indices of system integrity and self-ratings of health in response to caloric restriction. Results develop an understanding of self-rated health as a window onto biological processes of aging and highlight important design considerations for future research to illuminate the biological basis of health.

### SESSION 1435 (SYMPOSIUM)

### INTEREST GROUP SESSION—ORAL HEALTH: COGNITIVE FUNCTION, SOCIAL SUPPORT, AND ORAL HEALTH STATUS AMONG OLDER ADULTS IN THE U.S. AND ABROAD

Chair: Bei Wu, *New York University, New York, New York, United States*
Co-Chair: Stephen K. Shuman, *University of Minnesota School of Dentistry, Minneapolis, Minnesota, United States*
Discussant: Michele Saunders, *Div of Aging Research & Geriatric Psychiatry, UT Health-San Antonio, San Antonio, Texas, United States*

There is an increasing awareness of the importance of oral health and its associated risk factors among older adults. This symposium includes four papers that address cognitive function, social support and oral health problems and symptoms among older adults in the U.S. and China. Lu and his colleagues examined the reciprocal relationship between cognitive function and complete tooth loss Chinese adults age 50+ using the China Health and Retirement Longitudinal study. The results show that there is a reciprocal relationship between these two indicators. The second paper used the Population Study of Chinese Elderly in Chicago (PINE) and examined the associations between tooth/gums symptoms and changes in cognitive function in Chinese older immigrants. The results reveal that having teeth symptoms was associated with a decline in cognitive function. Using the same PINE data, the third paper examined the association between different characteristics of social relationships and the number of oral health problems among U.S. older Chinese adults. Wu and her colleagues conducted a partner-assisted pilot intervention to improve oral health for community-dwelling older adults with either mild cognitive impairment or mild dementia. The results of this 6-month intervention show that persons in the treatment group had more improvement in oral hygiene than those in the control group. Findings from these four papers illustrate that cognitive function, social support, and oral health are interrelated. This symposium highlights the importance of improving cognitive health, social support, and oral health for middle-aged and older adults.

### CARE PARTNER-ASSISTED PILOT ORAL HEALTH INTERVENTION FOR PERSONS WITH COGNITIVE IMPAIRMENT

Bei Wu,[1] Brenda L. Plassman,[2] Kathleen Nye,[2] Patricia Poole,[2] Melanie Bunn,[2] Hanzhang Xu,[2] Christine Downey,[3] and Ruth Anderson[3], *1. New York University, New York, New York, United States, 2. Duke University, Durham, North Carolina, United States, 3. University of North Carolina-Chapel Hill, Chapel Hill, North Carolina, United States*

This study pilot tested effectiveness of a care partner-assisted intervention on improving oral health among community-dwelling older adults with cognitive impairment. Twenty five participants (15 with mild cognitive impairment

[MCI] and 10 with mild dementia) and their care partners were enrolled. Eighteen participants were randomly assigned to the treatment and 7 to the control group. The treatment group received educational materials, an electronic toothbrush, coaching on communication and goal setting, and individualized instruction on oral hygiene technique. The control group received educational materials and an electronic toothbrush. There were 3-data collection points: baseline, the end of the 3-month intervention, and 3-month after the intervention. The intervention improved participants' oral hygiene (based on clinical measure of plaque index and gingival bleeding) for both groups; with more improvement in the treatment group. Improvement was greater for MCI than for mild dementia participants. This intervention showed promising results for a larger trial.

### RECIPROCAL RELATIONSHIP BETWEEN COGNITION AND EDENTULISM AMONG MIDDLE-AGED AND OLDER ADULTS IN CHINA

Nan Lu,[1] Bei Wu,[2] and Yaolin Pei[2], *1. Renmin University of China, Beijing, China, 2. New York University, New York, New York, United States*

While empirical evidence shows that cognitive function affects oral health and vice versa, there is a lack of empirical evidence to test the reciprocal relationship between these two indicators. This study aimed to examine this relationship among middle-aged and older adults in China. Data were derived from the 2011 and 2015 waves of the China Health and Retirement Longitudinal study. A two-wave cross-lag analysis was adopted to test the hypothesized model. Cognitive function in 2011 was found to be a significant predictor of complete tooth loss in 2015. Furthermore, complete tooth loss in 2011 was found to be a significant predictor of cognitive cognition in 2015. This finding demonstrates the reciprocal relationship between cognitive function and oral health. This study highlights the importance of improving both cognitive health and oral health for middle-aged and older adults. Policy and intervention implications are discussed.

### SOCIAL COHESION AND ORAL HEALTH PROBLEMS AMONG U.S. OLDER CHINESE ADULTS

Weiyu Mao,[1] Weiyu Mao,[1] Bei Wu,[2] Iris Chi,[3] Wei Yang,[1] and XinQi Dong[4], *1. University of Nevada, Reno, Reno, Nevada, United States, 2. New York University, New York, New York, United States, 3. University of Southern California, Los Angeles, California, United States, 4. Rutgers University, New Brunswick, New Jersey, United States*

This study examined the relationship between social cohesion (i.e., sense of community and neighborhood cohesion) and self-reported number of oral health problems and further investigated the potential moderating role of cognitive function in such a relationship among U.S. older Chinese adults. Data came from baseline of the Population Study of Chinese Elderly in Chicago between 2011 and 2013 (N = 3,157). Stepwise negative binomial regression models with interaction terms were used. Individuals with a stronger sense of community had 1% less risk of having oral health problems (RR = .99; 95% CI = .98, .99; $p < .001$). Individuals experiencing a stronger neighborhood cohesion had a 11% reduction in risk of having oral health problems (RR = .89; 95% CI = .86, .92; $p < .001$). To promote optimal oral health, interventions need to account for individuals' perception and actual integration with their neighborhood and communities.

### ORAL HEALTH AND COGNITION AMONG U.S. COMMUNITY-DWELLING CHINESE OLDER ADULTS

Darina V. Petrovsky,[1] Bei Wu,[2] Weiyu Mao,[3] and XinQi Dong[4], *1. University of Pennsylvania, Philadelphia, Pennsylvania, United States, 2. New York University Rory Meyers College of Nursing, New York, New York, United States, 3. University of Nevada, Reno, Reno, Nevada, United States, 4. Rutgers University Institute for Health, Health Care Policy and Aging Research, New Brunswick, New Jersey, United States*

The purpose of this study was to examine the associations between tooth/gums symptoms and changes in cognitive function. We used data from the Population Study of Chinese Elderly in Chicago, a two-wave epidemiological study of 2,713 U.S. Chinese older adults. We selected self-reported oral (tooth and gum) symptoms as independent variables. We measured global function and three cognitive domains: episodic memory, executive function and working memory. Adjusting for sociodemographic and health-related characteristics, participants who reported having teeth symptoms at baseline, experienced their global cognition and episodic memory decrease (both $p<0.05$). Participants who reported having teeth symptoms at baseline, experienced a faster rate of decline in global cognition for every additional year. However, this effect disappeared once we adjusted for all covariates. We found no significant relationship between baseline gum symptoms and change of cognitive function. Future research directions, clinical and policy implications will be discussed.

### SESSION 1440 (SYMPOSIUM)

### CUMULATIVE DISADVANTAGE OF EARLY-LIFE ADVERSITY AND HEALTH IN MIDLIFE AND LATER ADULTHOOD

Chair: Jooyoung Kong, *University of Wisconsin-Madison, Madison, United States*
Co-Chair: Agus Surachman, *The Pennsylvania State University, University Park, Pennsylvania, United States*
Discussant: Deborah Carr, *Boston University, Boston, Massachusetts, United States*

Cumulative dis/advantage (CDA) framework is one of the most influential theoretical frameworks in understanding how early adversity creates health disparities across adulthood. The CDA model posits that adverse experiences early in life may lead to subsequent adversities over time and accumulates across the life course. Various studies have shown that middle-aged and later adulthood are periods when accumulated disadvantages proliferate, resulting in heightened risks for an individual's health and well-being. This symposium includes four presentations that build on such existing knowledge, and its primary aim was to further examine the complexity of how various types of adverse childhood experiences may influence physical and psychological health in middle and later adulthood. This symposium addresses a wide range of early adversities, including low socioeconomic status, parental maltreatment, and household dysfunctions. The four presentations also focus on examining various aspects of physical and psychological health outcomes in later adulthood, including measures of body mass index, physical functional ability, somatic symptoms, and clinical risk for rapid declines in kidney function. Furthermore, these presentations will demonstrate the utilization of innovative and robust methodological approaches, including latent class analysis, multilevel structural equation modeling, and latent growth modeling on examining the association between early life adversity on the long-term trajectory of change in health status using large-scale longitudinal data. Lastly, this symposium consists of an outstanding group of multidisciplinary presenters with diverse backgrounds who aim to enhance the understanding of the processes and mechanisms of CDA and how they affect individuals' life courses.

### LATENT CLASS ANALYSIS OF RE-EXPERIENCING VIOLENCE ACROSS THE LIFE COURSE

Jooyoung Kong,[1] and Scott Easton[2], *1. University of Wisconsin-Madison, Madison, United States, 2. Boston College School of Social Work, Chestnut Hill, Massachusetts, United States*

Guided by the cumulative disadvantage hypothesis, the present study examines recurrent victimization experiences across the life course and their impact on psychological health in later life. Using data from the 2010-2011 Wisconsin Longitudinal Study, we explored the latent structure of histories of childhood maltreatment (i.e., neglect, emotional/physical abuse, witness of domestic violence) and elder abuse victimization among 5,968 older adults (average age of 71 years). We also investigated whether membership in specific latent classes, particularly experiencing both childhood and elder victimization, would be associated with psychological functioning in late life. We identified five latent classes: "Never victimized" (66% of respondents), "Abused as child" (16%), "Abused and neglected as child" (9%), "Abused as elder" (6%), and "Abused as child and elder" (2%). Also, the "abused as child and elder" class consistently was associated with negative psychological outcomes (i.e., distress and somatic symptom severity) and lower levels of psychological well-being.

### LIFE COURSE PATHWAYS OF CHILDHOOD SOCIOECONOMIC STATUS TOWARD CLINICAL RISK FACTORS FOR WORSE KIDNEY FUNCTION

Agus Surachman,[1] Alexis Santos,[1] Jonathan Rush,[2] Lacy Alexander,[1] Christopher Coe,[3] and David M. Almeida[1], *1. The Pennsylvania State University, University Park, Pennsylvania, United States, 2. University of Victoria, Victoria, British Columbia, Canada, 3. University of Wisconsin - Madison, Madison, Wisconsin, United States*

This study examines the roles of daily stress processes as a possible mediator of how life course socioeconomic inequality is reproduced in day-to-day experiences and creates disparities in clinical risk for rapid kidney function declines in adulthood. Data are from 1174 middle and older adults (56% female; ages 40–84, Mage = 56.2) who participated in the MIDUS study wave 2 and Refresher. We found significant pathways that childhood SES was associated with education and adult SES, adult SES was associated with exposure to daily stressors and daily stressor reactivity to negative affect, positive affect, and somatic symptom. In turn, higher report of daily somatic symptom and lower report of daily positive affect were associated with higher CKD risk factors. Finally, childhood SES was directly associated with CKD risk factors. Childhood SES is associated with clinical risks for rapid kidney function decline through direct association and life course accumulation.

### CHILDHOOD MISFORTUNE PREDICTS LIFESPAN HEALTH INTO LATE ADULTHOOD

Nicholas A. Turiano,[1] Nicholas A. Turiano,[1] Kate A. Leger,[2] and Patrick L. Hill[3], *1. West virginia University, Morgantown, West Virginia, United States, 2. University of kentucky, Lexington, Kentucky, United States, 3. Washington University in St. Louis, St. Louis, Missouri, United States*

Childhood misfortune encompasses a diverse set of negative early life experiences that have damaging effects on lifespan development. We extended this topic by examining how early life misfortunes predicted changes in measures of physical functioning (FUNC) and body mass index (BMI) in adulthood (ages 25-75). We used 3-wave data (N = 6,000) from the Midlife Development in the U.S. study across 20 years. Unconditional latent growth curve models (adjusting for age, sex, education) suggested significant ($p < .05$) mean-level change and variability in change for FUNC (Int = 1.47; Slope = 0.24) and BMI (Int = 26.71; Slope = 0.90). Higher levels of childhood misfortune (e.g., abuse, financial strain) significantly predicted worse FUNC (Int = 0.05; Slope = 0.02) and higher BMIs (Int = 0.24; Slope = 0.07) at baseline and steeper increases over time. Findings underscore the need to address adult health problems that emerge much earlier in life.

### EXAMINING CUMULATIVE INEQUALITY IN THE ASSOCIATION BETWEEN CHILDHOOD SES AND BMI FROM MIDLIFE TO OLD AGE

Chioun Lee,[1] Chioun Lee,[1] and Soojin Park[1], *1. University of California-Riverside, Riverside, California, United States*

Socioeconomic status (SES) is among the strongest determinants of body mass index (BMI). For older populations, selection bias is a large barrier to assessing cumulative disadvantages. We investigated the extent to which childhood SES affects BMI from midlife to old age and gender differences in the association. Data come from Midlife in the U.S. We used latent growth models to estimate BMI trajectory over a period of 20 years and examined results under different missing data patterns. Compared to individuals from higher childhood SES, those from lower childhood SES have higher BMI in midlife and experience a faster increase in BMI between midlife and old age. The observed associations remain significant even after controlling for midlife SES. After addressing nonrandom selection, the gap in BMI between high and low childhood SES widens from midlife to old age for women. The findings provide new evidence of cumulative inequality among older adults.

### SESSION 1445 (SYMPOSIUM)

### INTEREST GROUP SESSION—LIFELONG DISABILITIES: DEFINING HEALTH OUTCOMES FOR ADULTS AGING WITH DISABILITY

Chair: Kelly Munly, *Penn State Altoona, Altoona, Pennsylvania, United States*
Co-Chair: Caitlin E. Coyle, *Gerontology Institute, University of Massachusetts Boston, Boston, Massachusetts, United States*
Discussant: Caitlin E. Coyle, *Gerontology Institute, University of Massachusetts Boston, Boston, Massachusetts, United States*

The population of persons aging with disabilities is growing. Being able to segment aging with disability subpopulations within national data sets is becoming increasingly important in order to understand the relationship of aging with disability to a range of outcomes in later life including health and wellness, economic security, and health and long-term service and support need and use. This symposium includes four examples of how existing data can be used to draw conclusions about the experience of old age for persons with intellectual or developmental disabilities. In addition, the symposium offers insights into the limitations of these data and the presentations lend themselves to a discussion of how measurement across disability subpopulation can be ope rationalized. Two of the presentations focus on understanding mortality trends of adults with cerebral palsy and down syndrome--including an understanding of the health conditions facing these populations. A third presentation will focus on cardiovascular risk factors and co morbidity among adults with cerebral palsy. Finally, a fourth presentation will focus on pairing qualitative understanding with quantitative trends to offer a deeper understanding of the health management challenges for adults with disability as they age. Through a deeper understanding of the experience of health in later life for adults with disability, ideas about interventions and supports can be better aligned with the needs of these populations.

### COMPARISON OF AGE AND BIOLOGICAL SEX MORTALITY TRENDS BETWEEN ADULTS WITH AND WITHOUT DOWN SYNDROME

James D. Stevens,[1] James D. Stevens,[2] Scott D. Landes,[2] and Margaret A. Turk[3], *1. Syracuse University Department of Sociology, Syracuse, New York, United States, 2. Syracuse University, Syracuse, New York, United States, 3. SUNY Upstate, Syracuse, New York, United States*

Distinct mortality trends emerge from comparisons of mean and median age at death and specific causes of death between adults with and without cerebral palsy. We compare standardized mortality odds ratios (SMORs) for 20 leading causes of death for 11,895 adults with cerebral palsy and 13,047,988 without cerebral palsy in the US between 2012 and 2016. Male and female decadents with cerebral palsy died significantly younger than male and female decadents without cerebral palsy, and were more likely to die from respiratory diseases, choking, and unknown causes. Public health and preventive care efforts should account for respiratory, swallowing, and nutrition risks, as well as mortality trends' variation across age and biological sex. The CDC and WHO could better surveil this population's health and mortality by disallowing certifiers from using cerebral palsy as the underlying cause of death as the practice leads to high rates of unknown causes of death.

### INCREASED CARDIOVASCULAR DISEASE RISK FACTORS IN PERSONS WITH CEREBRAL PALSY

Patricia C. Heyn[1], *1. University of Colorado Denver Anschutz Medical Campus, Aurora, Colorado, United States*

Cardiovascular disease (CVD) remains the leading cause of global mortality. Individuals with disabilities are at increased risk of CVD in part due to their musculoskeletal and/or cognitive impairments that can challenge their participation in physical activities. Gait ability has been associated to several key health outcomes including morbidity. Unfortunately, there is little research done to understand how individuals with obstructed mobility (i.e. developmental or injury condition) grow older. Our research team (Heyn et al 2019), evaluated the prevalence of CVD risk factors and age-related health outcomes associated to aging on a cohort of adults with cerebral palsy with obstructed mobility. Metabolic syndrome was identified in 17.1% of the cohort, higher than the 10% in the NHANES sample. CVD risk factors were much higher in this cohort as compared to normative population data. There was a positive correlation between mobility level, waist circumference ($r=0.28$, $p=0.02$), and waist-to-hip ratio ($r=0.28$, $p=0.02$).

### COMPARISON OF AGE AND BIOLOGICAL SEX MORTALITY TRENDS BETWEEN ADULTS WITH AND WITHOUT DOWN SYNDROME

Scott D. Landes,[1] Scott D. Landes,[2] James D. Stevens,[2] and Margaret D. Turk[3], *1. Syracuse University, Aging Studies Institute, Syracuse, New York, United States, 2. Syracuse University, Syracuse, New York, United States, 3. Upstate University Hospital, Syracuse, New York, United States*

Age at death and cause of death comparisons between adults with and without Down syndrome reveal distinct mortality trends that can be utilized to inform preventive care efforts to reduce premature mortality in this population. We compare mean and median age at death, and standardized mortality odds ratios (SMORs) for 20 leading causes of death for 9,564 decedents with and 13,050,319 without Down syndrome in the U.S. between 2012 and 2016. Decedents with Down syndrome, on average, were substantially younger than those without Down syndrome, and were more likely to die from Alzheimer disease and dementia at younger ages. In addition, adults with Down syndrome also had higher risk of choking related deaths. Efforts to reduce premature mortality through public health and preventive care interventions for this population should be cognizant of these increased risk factors, as well as variation in age and biological sex mortality trends.

### UNDERSTANDING NEEDS AND CHALLENGES OF HEALTH SELF-MANAGEMENT ACTIVITIES FOR OLDER ADULTS WITH MOBILITY LIMITATIONS

Qiong Nie,[1] Qiong Nie,[2] and Wendy Rogers[2], *1. University of Illinois Urbana-Champaign, Illinois, United States, 2. University of Illinois, Champaign, Illinois, United States*

According to the U.S. Census, about 30% of older adults live with a mobility impairment, defined as difficulty walking and/or climbing stairs. Older people with mobility limitations are at risk for decreased access to medical services and negative health outcomes. However, few studies address the healthcare needs of these individuals. We used a combination of a population-based data set and an in-depth interview study to understand the challenges these older adults experience in managing medications, accessing health information, going to healthcare provider appointments, and getting help in case of emergency. The quantitative data were from 2,828 participants in the National Health & Aging Trends Study (NHATS). The qualitative data were from 60 participants in the Aging Concerns, Challenges, and Everyday Solution Strategies (ACCESS) interview study. These data provide insights about health self-management challenges for older adults with mobility impairments and guidance for support solutions and interventions.

### CHANGING NEEDS AND NEEDS ASSESSMENT IN THE TRANSITION BETWEEN DISABILITY AND OLD AGE

Tine Rostgaard,[1] and Lea Graff[2], *1. VIVE - The Danish Center for Social Science Research, Copenhagen, Denmark, 2. VIVE, Copenhagen, Hovedstaden, Denmark*

As our societies age, we see more people with disabilities living well into old age. However, there are different societal, systemic and individual assumptions about needs, rights and obligations associated with frail older people and people with disabilities. The paper presents quantitative results from a Danish study investigating what challenges ageing of society pose for the individual as well as for the welfare state in regards to meeting the needs of those who either age into disability or age with disability. Using panel data from the Danish Level of Living Survey from 1997-2017, we investigate how ADL related needs for care have changed for the 52+ year olds and we project how needs will change in the near future. Finally, we show how different systemic approaches to need assessment for those under or above 65, but with otherwise identical and socio-economic backgrounds, result in a very different service utility.

### SESSION 1450 (SYMPOSIUM)

### ESPO AND NIA BUTLER-WILLIAMS SCIENTIFIC SYMPOSIUM: INFLUENCE OF RACE, ETHNICITY, AND EXPERIENCE ON HEALTH IN AGING

Chair: Jamie N. Justice, *Wake Forest School of Medicine, Winston-Salem, North Carolina, United States*
Co-Chair: Carl V Hill, *NIH/National Institute on Aging, Bethesda, Maryland, United States*
Discussant: Roland J. Thorpe, *Johns Hopkins Bloomberg School of Public Health, Baltimore, Maryland, United States*

The NIA's Butler-Williams Scholars Program and GSA's Emerging Scholars and Professional Organization are united in providing career development opportunities for early career scholars in a manner that promotes leadership, diversity, and inclusivity. This provides a foundation to develop a network of next generation of scientists, clinicians, and policy makers capable of shaping health in aging. Among the chief concerns of our aging population are disparities in health associated with race/ethnicity, experience, environment, access to health care, and sociocultural and socioeconomic factors. GSA's early career professionals and alumni of the prestigious NIA Butler-Williams Scholars Program have tackled these issues directly and the scientific scholarship that results is astounding in its breadth and depth. Dr. Vicki Johnson-Lawrence, Ph.D. (Butler-Williams class of 2014), will present on the interacting effects of education and race/ethnicity on multi-morbidity, highlighting lessons learned from the National Health Interview Study. Dr. Lauren Parker (Butler-Williams class of 2018) will review efforts to develop culturally competent content for recruitment of Hispanic and black/African American persons to NIA-supported dementia-caregiving studies. Dr. Ryon Cobb (Butler-Williams class of 2016) will discuss the impact of race/ethnicity on kidney function among older adults, with evidence from the Health and Retirement Study. The final speaker, Dr. Ana Quiñones (Butler-Williams class of 2012), will present on longitudinal tracking of multi-morbidity in racially/ethnically diverse older adults. In sum, the featured talks by rising stars in aging research deepen our understanding of the influence of race, ethnicity, and experience on health and chronic disease in diverse aging populations.

### EDUCATION, RACE-ETHNICITY, AND MULTI-MORBIDITY AMONG ADULTS AGES 30-64 IN THE NATIONAL HEALTH INTERVIEW STUDY

Vicki Johnson-Lawrence,[1] Anna Zajacova,[2] and Rodlescia Sneed[1], *1. Michigan State University, Flint, Michigan, United States, 2. University of Western Ontario, London, Ontario, Canada*

Demographic risk factors for multimorbidity (living with 2+ chronic conditions) have been identified in numerous population-based studies of older adults; however, there is less data on younger populations, despite the fact that approximately 24% of US adults age 18+ have multimorbidity. To examine the associations of education and race/ethnicity with mutimorbidity among adults aged 30-64 using cross-sectional data from the 2002-2014 National Health Interview Surveys. Compared to having a bachelor's degree or higher, completing less than HS (OR=1.58, 95% CI = 1.50-1.66) or HS/some college (OR=1.32, 95% CI = 1.27-1.37) were both associated with increased odds of multimorbidity. Non-Hispanic Blacks had greater odds of multimorbidity (OR=1.07, 95% CI = 1.02-1.11) compared to Non-Hispanic Whites with comparable characteristics. Reducing multimorbidity through health promotion efforts across the socioeconomic spectrum and earlier in the life course will be a requirement to age successfully and support overall well-being in the aging US population.

### RECRUITMENT AND RETENTION OF DEMENTIA CAREGIVERS: A REVIEW OF TWO STUDIES

Lauren Parker,[1] and Laura N. Gitlin[2], *1. Johns Hopkins Bloomberg School of Public Health, Baltimore, Maryland, United States, 2. College of Nursing and Health Professions, Drexel University, Philadelphia, Pennsylvania, United States*

Two of the goals of the National Strategy for Recruitment and Participation in Alzheimer's Disease and Related Dementias Clinical Research are to "increase awareness and engagement (Goal 1)" and to "engage local communities and support participants (Goal 3)". Leveraging the findings from two National Institute on Aging funded dementia-caregiving related research studies, ADS-Plus and The Providing Evidence-Based Approaches to Caregiver Stress, the goal of this presentation is to discuss methodologies and approaches to develop culturally competent content for outreach. Recruitment strategies from the two studies will explore the impact of race/ethnicity on recruiting Hispanic (ADS-Plus), and Black/African American populations. Further, the effectiveness of recruitment strategies from both studies will be discussed, as an effort to (1) conceptualize best practices necessary to develop and sustain equitable and sustainable community partnership, and (2) create and improve evidence-based recruitment resources.

### RACE BY AGE PATTERNS IN KIDNEY FUNCTIONING AMONG OLDER ADULTS: EVIDENCE FROM THE HEALTH AND RETIREMENT STUDY

Ryon J. Cobb[1], *1. University of Texas at Arlington, Arlington, Texas, United States*

The present study considers how race combines with chronological age to shape kidney function among older adults. We analyzed cross-sectional data from a nationally representative study of older adults. Our measure of kidney function derived from the cystatin C-based estimated glomerular filtration rate. We use a pattern variable to divide White and Black respondents into four groups based on their age group membership: early midlife (age 52–59), late midlife (age 60–69), young old (age 70–79), and oldest old (80s+ years). Results from our ordinary least squares models reveal that Blacks and Whites in late midlife, young old, and oldest old exhibited poorer kidney function than Whites in early midlife. Our study uncovers evidence of race by age disparities in kidney function among older adults. Future longitudinal studies will provide further insight into how and why race combines with age to pattern kidney function over time.

### TRACKING CHANGES IN MULTIMORBIDITY AMONG RACIALLY AND ETHNICALLY DIVERSE POPULATIONS

Ana R. Quiñones,[1] Anda Botoseneanu,[2] Sheila Markwardt,[3] Corey Nagel,[4] Jason T. Newsom,[5] David A. Dorr,[3] and Heather G. Allore[6], *1. Family Medicine, Oregon Health & Science University, Portland, Oregon, United States, 2. University of Michigan, Dearborn, Michigan, United States, 3. Oregon Health & Science University, Portland, Oregon, United States, 4. University of Arkansas for Medical Sciences, Little Rock, Arkansas, United States, 5. Portland State University, Portland, Oregon, United States, 6. Yale University, New Haven, Connecticut, United States*

Multimorbidity is widely recognized as having adverse effects on health and wellbeing above and beyond the risk attributable to individual chronic disease. Much of what is known about multimorbidity rests on research that has largely focused on one point-in-time, or from a static perspective, with little consideration to issues involved in assessing longitudinal changes in multimorbidity. In addition, less focus has been placed on assessing racial and ethnic variations in longitudinal changes of multimorbidity. Addressing this knowledge gap, we highlight important issues and considerations in addressing multimorbidity research from a longitudinal perspective and present findings from longitudinal models that examine differences in the rate of chronic disease accumulation and multimorbidity onset between non-Hispanic white (white), non-Hispanic black (black), and Hispanic study participants in the Health and Retirement Study starting in middle-age and followed for up to 16 years.

### SESSION 1455 (SYMPOSIUM)

### INTEREST GROUP SESSION—WOMEN'S ISSUES: FROM MENOPAUSE TO MEDICARE: CONTRIBUTIONS FROM COHORT STUDIES OF AGING WOMEN'S HEALTH

Chair: Nancy F. Woods, *University of Washington, Department of Biobehavioral Nursing and Health Informatics, Seattle, Washington, United States*
Co-Chair: Barbara B. Cochrane, *University of Washington School of Nursing, Seattle, Washington, United States*
Discussant: Barbara B. Cochrane, *University of Washington School of Nursing, Seattle, Washington, United States*

Although some countries have well-established birth cohorts for research on aging, few US projects have followed the evolution of women's health from midlife through older age, leaving gaps in understanding their health and ignoring a window of time spanning age groups during which women may benefit from health promotion and prevention efforts. Cohort studies of midlife and older women's health have begun to address this gap, providing opportunities to advance our understanding of both reproductive and healthy aging, including multiple racial/ethnic groups and US regions. Studying existing cohorts of aging women provides investigators with opportunities to: track changes over time, identifying trajectories of health along with aging; clarify antecedent-consequent relationships; identify how historical events may be related to emergent patterns of health; incorporate common data elements that allow comparisons within and across cohorts over time; and introduce new measures/indicators, such as genomic markers. This symposium will provide a foundation for future research using existing databases from studies providing longitudinal health-related measures of women as they age. Opportunities for conducting new analyses on midlife and older women's health will be described for four large cohort studies: two studies of women exclusively -- the Study of Women's Health Across the Nation and the Women's Health Initiative – and two studies with large numbers of women participants – the Baltimore Longitudinal Study of Aging and the Rancho Bernardo Study of Healthy Aging. Unique and common considerations for answering future questions about women's health from menopause to Medicare in these studies will be discussed.

### THE STUDY OF WOMEN'S HEALTH ACROSS THE NATION (SWAN): FROM MIDLIFE ONWARD

Nancy E. Avis,[1] Nancy E. Avis,[2] Sybil Crawford,[3] Alicia Colvin,[4] Carol A. Derby,[5] Carrie Karvonen-Gutierrez,[6] and Maria M. Brooks[7], *1. Wake Forest School of Medicine, Winston-Salem, North*

*Carolina, United States, 2. Department of Social Sciences and Health Policy, Wake Forest School of Medicine, Winston-Salem, North Carolina, United States, 3. Graduate School of Nursing, University of Massachusetts Medical School, Worcester, Massachusetts, United States, 4. Department of Epidemiology, Graduate School of Public Health, University of Pittsburgh, Pittsburgh, Pennsylvania, United States, 5. Department of Epidemiology and Population Health, Albert Einstein College of Medicine, Bronx, New York, United States, 6. Department of Epidemiology, University of Michigan School of Public Health, Ann Arbor, Michigan, United States, 7. Department of Epidemiology, Graduate School of Public Health, University of Pittsburgh, Pittsburgh, Pennsylvania, United States*

The Study of Women's Health Across the Nation (SWAN) is a multisite, multiracial/ethnic longitudinal study of women initially aged 42-52 (N=3302) designed to characterize the physiological and psychosocial changes that occur during the menopause transition and to assess their relations to subsequent health and age-related diseases. Each of seven clinical sites recruited non-Hispanic white women and women belonging to a predetermined racial/ethnic minority (African American, Hispanic, Chinese, or Japanese). Cohort eligibility was determined from a cross-sectional survey of 16,065 women in 1996-97 aged 40-55 who were aged 42-52, had an intact uterus and at least one ovary, and not using hormone therapy. Cohort participants have been assessed in-person approximately annually through follow-up visit 15 in 2017 using a standardized protocol of detailed questions about medical, reproductive and menstrual history; lifestyle and psychosocial factors; physical and psychological symptoms; and anthropometric measurements, reproductive hormones, bone and body composition, and cardiovascular health.

### THE WOMEN'S HEALTH INITIATIVE (WHI): STILL LEARNING FROM 161,808 POSTMENOPAUSAL WOMEN

Andrea Z. LaCroix,[1] and Andrea Z. LaCroix[2], *1. University of California San Diego, La Jolla, California, United States, 2. Family Medicine and Public Health, University of California, San Diego, La Jolla, California, United States*

The WHI enrolled 161,808 women ages 50-79 into 1-3 Clinical Trials (n=68,132) or the Observational Study (n=93,676) from 1993-1998. As of March 31, 2018, 70,812 women are alive and continue to be enrolled including 10,179 over the age of 90, 4588 Black/African American, and 1998 Hispanic/Latina women. 54,877 women have died, including 17,010 from cardiovascular disease and 14,553 from cancer. 64,344 reported a fracture during follow-up. Between 2012-2014, 7875 women completed a brief in-person visit as part of The Long Life Study. This examination included the Short Physical Performance Battery, grip strength, height, weight, blood pressure, heart rate and a blood draw. Genetic data of various types have been collected for over 30,000 WHI participants. Whole genome sequencing data is available for over 11,000 WHI participants. Over 1700 manuscripts have been published. The WHI biospecimen repository contains millions of stored biospecimens. CMS (Medicare) data is available and regularly updated.

### THE RANCHO BERNARDO STUDY (RBS) OF HEALTHY AGING: A RICH RESOURCE FOR STUDYING AGING IN WOMEN

Linda McEvoy,[1] Linda K. McEvoy,[2] Gail A. Laughlin,[3] Donna Kritz-Silverstein,[3] Richelle Bettencourt,[3] and Jaclyn Bergstrom[3], *1. University of California San Diego, San Diego, California, United States, 2. Department of Radiology, Department of Family Medicine and Public Health, University of California, San Diego, La Jolla, California, United States, 3. Department of Family Medicine and Public Health, University of California, San Diego, La Jolla, California, United States*

RBS is an ideal cohort to study healthy aging in women and to examine sex differences in aging. Initiated in 1972, RBS enrolled 82% of adult residents of Rancho Bernardo, a suburb of San Diego. Residents were white, and middle to upper-middle class. Participants have been followed via 12 research clinic visits at ~4 year intervals and 32 mailed surveys. RBS contains detailed assessment of cardiometabolic disease risk factors, bone health, biomarkers, physical ability, cognitive function, health and reproductive history, medications, behaviors (smoking, drinking, diet, exercise) and psychosocial measures. Of the 6726 participants, 54% are women and 65% were aged ≥50 at enrollment. Vital status is known for 91% of the cohort; overall mortality is 71%. Death certificates have been coded for cause of death for 91% of decedents. We will discuss contributors to healthy longevity in RBS women (average age of death 86.4 yrs; 29% lived to age ≥90).

### THE BALTIMORE LONGITUDINAL STUDY OF AGING: OPPORTUNITIES FOR RESEARCH ON WOMEN'S AGING AND HEALTH

Eleanor M. Simonsick,[1] Eleanor Simonsick,[2] Ann Z. Moore,[3] Michelle Shardell,[3] Nancy C. Shaffer,[3] and Toshiko Tanaka[3], *1. Longitudinal Studies Section, Intramural Research Program, National Institute on Aging, Baltimore, Maryland, United States, 2. Intramural Research Program, National Institute on Aging, Baltimore, Maryland, United States, 3. National Institute on Aging, Baltimore, Maryland, United States*

The Baltimore Longitudinal Study of Aging (BLSA), an ongoing continuous enrollment cohort study of normative aging established in 1958 currently conducted by the NIA Intramural Research Program, began including women in 1978. To date, nearly 1200 women aged 17-94 at enrollment (median=53 Q1-Q3=40-70) have been followed for up to 21 visits spanning 40 years (median visits=6; Q1-Q3=3-9). Over 3 days, participants receive comprehensive examinations, interviews, imaging and functional and cognitive evaluations; repeat visits occur every 1-4 years depending on age. The BLSA offers opportunities to examine distributions of, change in and interrelationships among several rarely concurrently ascertained parameters (e.g., cardiovascular fitness, resting metabolic rate, glucose challenge response, five-factor personality, brain volumes, and diet) over the life course and across birth cohorts. The BLSA also maintains an extensive biorepository. This talk will summarize the extensive measurement catalogue and timeline and provide illustrative examples from ongoing research on women's aging and health.

### SESSION 1460 (SYMPOSIUM)

### INTEREST GROUP SESSION—GRANDPARENTS AS CAREGIVERS: GERONTOLOGICAL INTERVENTION SCIENCE: FOUR DIFFERENT APPROACHES TO GRANDFAMILY RESEARCH

Chair: Christine Fruhauf, *Colorado State University, Fort Collins, Colorado, United States*
Co-Chair: Loriena Yancura, *University of Hawaii at Manoa, Honolulu, Hawaii, United States*
Discussant: Bert Hayslip, *University of North Texas, Denton, Texas, United States*

For nearly three decades, the interest in custodial grandparents and their grandchildren has resulted in empirical studies from scholars addressing both the challenges and strengths often experienced by grandfamilies (i.e., grandparents and the grandchildren in their home). As the scholarship of intervention science increases in interest and as scholars continue to advance the field of knowledge with grandfamilies, it is important to share best-practices as we plan the future of gerontological intervention science. In this symposium, investigators with extensive experience researching custodial grandparents and in creating, testing, and refining interventions with grandfamilies will present theoretical, methodological, and practical lessons learned and empirical findings from their interventions. The first paper, by Musil and colleagues, will address a current online training program addressing resourcefulness with grandmothers. Second, Montoro-Rodriguez and Hayslip will discuss the use of the selective optimization with compensation model as applied to a goal-setting intervention with grandparents. The third paper by Fruhauf, Yancura and colleagues will address the progress and impact from the first two years of their intervention with grandparents, grandchildren, and service providers. The final paper, presented by Webster, Smith, and Infurna will focus on a dyadic intervention with grandparents and adolescent grandchildren as they seek to build social intelligence. Hayslip, the discussant, will integrate key points from these interventions while addressing considerations for future research as scholars embark on refining and testing their interventions related to grandparents raising grandchildren.

### IMPROVING HEALTH AND WELL-BEING OF GRANDPARENTS RAISING GRANDCHILDREN: A STRENGTHS-BASED INTERVENTION

Christine Fruhauf,[1] Loriena Yancura,[2] Aimee Fox,[1] Nathaniel Riggs,[1] Heather Greenwood-Junkiermeyer,[2] and A. N. Mendoza[3], *1. Colorado State University, Fort Collins, Colorado, United States, 2. University of Hawaii at Manoa, Manoa, Hawaii, United States, 3. The Ohio State University, Columbus, Ohio, United States*

Many grandparents raising grandchildren experience depression. Few interventions take a strengths-based approach to improve their mental health. To address this gap, this study utilized an adapted version of Powerful Tools for Caregivers (PTC) for grandparents (PTC-G) to improve their self-care, communication, and self-efficacy. Grandparents completed self-assessments including the CES-D short form prior to the intervention, immediately after the 6-week program, and at 6-months. Focus groups were also conducted during the 6-month follow-up to further explore positive behavior change. Data from all sources were analyzed to show that the PTC-G program significantly lowered depressive symptoms of grandparents raising grandchildren. Qualitative data shows that grandparents report increased awareness and use of self-care practices and community services. By improving the health and well-being of grandparents raising grandchildren, the PTC-G intervention shows promise in reducing depression and improving long-term mental health outcomes in vulnerable grandfamilies.

### GIFT: GRANDMOTHER INITIATIVES IN FAMILY TRANSFORMATION

Carol M. Musil,[1] Jaclene Zauszniewski,[1] Alexandra Jeanblanc,[1] McKenzie Wallace,[1] Christina Henrich,[1] Chris Burant,[1] and Elizabeth Tracy[1], *1. Case Western Reserve University, Cleveland, Ohio, United States*

Little research has been conducted on the development and testing of interventions to support grandmothers during their caregiving experience. Our descriptive and pilot intervention studies provide the foundation for converting a face-to-face resourcefulness intervention into an NIH-funded self-administered, online intervention for grandmother caregivers, to determine its impact on individual health and family well-being. The online intervention provides personal and social resourcefulness training using online video content and structured reflective journaling to reinforce resourcefulness skills compared to reflective journaling. We are testing the resourcefulness intervention in a randomized clinical trial (RCT) with a national sample of 334 grandmothers living with/raising grandchildren, comparing the resourcefulness training protocol vs. unstructured journaling alone. Given the limited interventions available to grandmother caregivers, an intervention that transcends time and place and is available 24 hours a day is expected to bolster the personal and social resourcefulness, which will in turn affect individual and family outcomes.

### THE USE OF SOLUTION-ORIENTED STRATEGIES TO SUPPORT GRANDPARENTS RAISING GRANDCHILDREN

Julian Montoro-Rodriguez,[1] Bert Hayslip Jr.,[2] Jennifer Ramsey,[3] and Jane l. Jooste[4], *1. University of North Carolina at Charlotte, Charlotte, North Carolina, United States, 2. University of North Texas, Denton, Texas, United States, 3. University of North Carolina - Charlotte, Charlotte, North Carolina, United States, 4. Lewisville ISD Special Education, Purnell Support Center, Lewisville, Texas, United States*

There is consistent evidence that caregiving for grandchildren is psychologically, emotionally and physically challenging for grandparents. However, many of them may not be able to receive the support they need from their own families or public services. Using the Selective Optimization with Compensation framework and principles of solution-oriented cognitive models, we implemented a six-week session pilot program to increase grandparents' effective coping strategies, reduce stress, and improve parenting and communication with grandchildren. Preliminary data from 22

grandparents suggest that participants improved their use of effective selection strategies to set priorities and pursue goals related to their wellbeing and the quality of their relationship with grandchildren. Their pre-post test scores also indicated a higher level of parental efficacy, social relations and confidence in organizing their time and priorities. Results indicate that focusing on solution-oriented strategies may empower grandparents to improve their psychological adjustment and take care of their needs.

### SOCIAL INTELLIGENCE TRAINING FOR CUSTODIAL GRANDMOTHER-ADOLESCENT GRANDCHILDREN DYADS

Britney A. Webster,[1] Greg Smith,[1] and Frank Infurna[2], *1. Kent State University, Kent, Ohio, United States, 2. Arizona State University, Tempe, Arizona, United States*

Custodial grandmothers (CGMs) and adolescent custodial grandchildren (ACG) face risk of poorer social skills and competencies due to early life adversities which have downstream negative consequences for mental and physical health. We describe an RCT examining the efficacy of an online social intelligence intervention (SII) at improving the emotional, interpersonal, and physical well-being of CGM-ACG dyads through mutual enhancement of their social competencies. Our SII is particularly valuable for these dyads because it enhances their social competencies and relationships, thereby leading to positive outcomes. Additionally, adolescence is a critical period for developing social competencies, largely through interactions with female caregivers. Our longitudinal mixed-methods approach addresses four aims: (1) Investigating if SII improves social competencies and overall well-being through both actor and partner effects; (2) Exploring moderators of SII efficacy; (3) Studying qualitatively how dyads view SII as changing their lives; and (4) Conducting a SII cost-benefit analysis. [Funded by R01AG054571]

### SESSION 1465 (SYMPOSIUM)

### HAPPY WHERE I AM: HOME AND COMMUNITY PREFERENCES, TELEHEALTH, SOCIAL ISOLATION, AND WELLNESS OF OLDER RURAL RESIDENTS

Chair: Cassandra Cantave Burton, *AARP, Washington, D.C, United States*

About 16 percent of adults 50-plus and 25 percent of 65-plus adults reside in rural areas or small towns in the United States . The percentage increases to rural communities could mean a higher prevalence of chronic disease, a higher disability rate, a lower prevalence of healthy behaviors, and a widening gap in life expectancy relative to the nation as a whole. Moreover, rural areas face additional obstacles and challenges such: Difficulty forming community partnerships because of proximity challenges; migration of younger individuals to cities for career and social opportunities, resulting in a smaller pool of potential caregivers; an aging housing stock that also may be unsafe and in need of repair; and inadequate resources available to meet the broad range of needs among older adults. AARP has been engaged with policy makers and community members to ensure that older residents who live in rural areas have access to community supports so they can remain in their homes and communities and have the services that they need as they get older. Presenters in this symposium will present data supporting AARP's work to better the lives of older rural residents. Findings from AARP studies on home and community preferences, social isolation, telehealth and broadband access, and brain health will be presented.

### FACTORS CONTRIBUTING TO SOCIAL ISOLATION AMONG OLDER RURAL RESIDENTS

Cassandra Cantave Burton[1], *1. AARP, Washington, D.C, United States*

AARP research finds one-third of adults age 45 and older consider themselves lonely. Analysis from the National Survey of Adults Age 45-plus on Loneliness and Social Connections will be presented. Findings indicate that sixty-one percent of respondents who have never spoken to a neighbor are lonely, compared with 33 percent who have spoken to a neighbor. Individuals earning less than $25,000, caregivers and LGBTQ people are more likely to be lonely. Moreover, the structure of one's community also plays an important role in predicting loneliness and was significantly related to a person feeling lonely.

### HAPPY IN MY HOME AND COMMUNITY: PREFERENCES OF OLDER RURAL RESIDENTS

Joanne V. Binette[1], *1. AARP, Washington, District of Columbia, United States*

AARP will introduce research on rural residents from the 2018 Home and Community Preferences Survey of Adults Age 18 and Older that shows the importance of making communities places where rural residents can successfully age throughout all stages of life. This data will provide useful insights regarding what adults who live in rural areas want and need in their communities to positively contribute to their overall health and well-being and keep them actively engaged and involved in their community. The survey data can serve as a key tool for communities to understand what rural residents view as important for successfully aging in place and strategies that communities might employ to create community supports and services conducive for older rural residents.

### WHO, ME, WORRIED?: A LOOK AT BRAIN HEALTH AND STRESS AMONG RESIDENTS IN GEOGRAPHICALLY DIFFERENT AREAS

Katherine Bridges[1], *1. AARP, Washington, District of Columbia, United States*

The 2018 AARP Brain Health and Mental Well-Being Survey reveals Millennials (age 22 to 37) have the highest level of stress while those in the Silent/Greatest Generation (over 73) have the lowest. Adults in their 50s and beyond have higher average mental well-being scores compared to younger adults. On a scale of mental well-being with an average score of 52, the average well-being for those age 18-39 is about 50, compared to about 54 for those 60 and older. This presentation will highlight generational difference in mental well-being and will examine community difference for older adults particularly those who reside in metropolitan areas compared to non-metropolitan areas.

### THE DIAL-UP BLUES: OPINIONS ON TELE-HEALTH AND BROADBAND AMONG RURAL RESIDENTS

Aisha Bonner Cozad[1], *1. AARP, Washington, District of Columbia, United States*

Findings from several AARP telephone studies on Caregiving and telehealth will be presented. Starting in 2017, AARP began collecting data on telehealth issues in the caregiving studies. As the amount of data began to grow, patterns of telehealth use began to emerge. Using a sample of 1,000 respondent's age 45-plus, the data shows respondents are generally supportive of the telehealth. Telehealth activities are often billed as a way to reduce healthcare cost and to provide much needed healthcare to geographically isolated populations particularly those residing in rural areas. A key component to accessing telehealth services is having access to adequate broadband which can be challenging in rural communities. Data shows that older adults (65+) are less likely to be very interested in telehealth services. Research finds that those employed, women and AARP members are all more likely to be very interested in using the telehealth services.

### SESSION 1470 (SYMPOSIUM)

### JOSEPH T. FREEMAN AND EXCELLENCE IN REHABILITATION OF AGING PERSONS AWARD LECTURES

Chair: Christine Mueller, *University of Minnesota, Minneapolis, Minnesota, United States*

The Joseph T. Freeman Award is a lectureship in geriatrics awarded to a prominent physician in the field of aging, both in research and practice. The award was established in 1977 through a bequest from a patient's estate as a tribute to Dr. Joseph T. Freeman. The Joseph T. Freeman Award lecture will feature an address by the 2019 Freeman Award recipient, Anne Newman, MD, MPH, of the University of Pittsburgh. The Excellence in Rehabilitation of Aging Persons Award is designed to acknowledge outstanding contributions in the field of the rehabilitation of aging individuals. The Excellence in Rehabilitation of Aging Persons Award lecture will feature an address by the 2019 Excellence in Rehabilitation Award recipient, Neil Alexander, MD, of the University of Michigan.

### SESSION 1475 (SYMPOSIUM)

### INTEREST GROUP SESSION—RELIGION, SPIRITUALITY AND AGING: KEEPING FAITH ALIVE: ADVANCING THE NEXT GENERATION OF RELIGIOUS AND SPIRITUAL INQUIRY IN GERONTOLOGY

Chair: Alex J. Bishop, *Oklahoma State University, Stillwater, Oklahoma, United States*
Discussant: James W. Ellor, *Baylor University Garland School of Social Work, Texas, United States*

A compelling body of evidence suggests that church attendance and spiritual practices increase as persons approach the end life. In fact, a growing number of baby boomers in the United States are returning to church and reconnecting with their faith communities. A primary reason for this surge in religious engagement involves a personal desire to turn away from the material pursuits of life and achieve a sense of meaning and fulfillment amid increasing frailty and vulnerability. However, most religious institutions do not have the necessary resources to support an ever increasing older membership. The graying of religion will require gerontologists to develop innovative approaches to advance theoretical thinking, measurement and assessment practices, and program evaluation. Therefore, the purpose of this symposium is to advocate and synergize a new generation of religious and spiritual inquiry in gerontology. Of particular interest is exposing theoretical models, measurement strategies, mixed-methodologies, and evidence-based findings to promote and sustain positive faith connections as persons grow old. Empirical advancements surrounding commitment and practice of one's religious faith in old age, evolving feelings of religious belief versus doubt, disposition to seek and engage in acts of forgiveness, and faith-community responses to dementia care will be shared. Implications relevant for building interdisciplinary and transdisciplinary connections across behavioral and social science research, social work, clinical counseling, and pastoral ministry will be highlighted.

### EXAMINING RELIGIOUS DOUBT AND WELL-BEING OVER TIME

Julie Hicks Patrick,[1] Laura Bernstein,[1]
Madeline M. Marello,[1] and Heather R. Moore[1], *1. West Virginia University, Morgantown, West Virginia, United States*

Drawing on seminal pieces in the field (e.g., Krause et al., 1999; Krause & Ellison, 2009) and our own work (e.g., Henrie & Patrick, 2014; Patrick & Henrie, 2015; 2016), we examine several aspects of religious doubt, including: 1) identifying a multidimensional measure, 2) replicating cotemporaneous associations among doubt and key well-being outcomes, 3) demonstrating the longitudinal stability of doubt over a 2-year period, and 4) examining whether doubt influences the stability or levels of well-being over a two-year period. Our factor analysis supports a 4-factor model of religious doubt, with stability over the 2-year period. Associations among religious doubt and well-being were consistent across multiple samples. Latent growth curve models showed that religious doubt and age significantly influence mean levels of well-being, although neither age nor doubt influenced the trajectory of change in well-being over the 2-year period. Future directions for design and analyses are discussed.

### SPIRITUAL AND RELIGIOUS CHANGE IN LATER LIFE

Vern Bengtson,[1] Merril Silverstein,[2]
Samantha Copping,[3] and Camille Endacott[3], *1. University of Southern California, Los Angeles, California, United States, 2. Syracuse University, Syracuse, New York, United States, 3. University of California-Santa Barbara, Santa Barbara, California, United States*

How does religious and or spiritual engagement change with aging? Is there a "return to religion" among aging baby boomers these days? Some theories of aging posit an increase in spirituality toward the end of life, such as the psycho-developmental theories of James, Jung, Erickson, and Tornstam, while others emphasize continuity (no change). We explore this issue in a mixed-methods longitudinal study of older adults' spiritual and religious trajectories. Data are from surveys with 981 baby boomer participants of the 45-year Longitudinal Study of Generations panel, and intensive interviews with 70 individuals 65-95 from a variety of religious and non-religious backgrounds. Results indicate complex trajectories of increase, decrease, and continuity as individuals move into their later years. We also detect a "return to religion" among baby boomers. We discuss these findings in terms of both life-course personality theories and social integration/support

### A TEST OF THE OKLAHOMA INMATE FORGIVENESS AND WELL-BEING MODEL

George K. Randall[1] and Alex Bishop[2], *1. Sam Houston State University, Huntsville, Texas, United States, 2. Oklahoma State University, Stillwater, Oklahoma, United States*

Data was collected from older male offenders (N = 86 non-violent; N = 163 violent) incarcerated in Oklahoma. Testing a forgiveness model, positive evaluation of life PVOL was regressed on religiosity (REL) and forgiveness of self (FSelf), others (FOthers), and situation (FSit) using hierarchical OLS regression. Blocks of predictors included: a) age and education; b) religiosity; and c) FSelf, FOthers, and FSit. For the non-violent model of PVOL significant predictors included REL ($\beta = .26, p \leq .01$) and FSelf ($\beta = .40, p \leq .01$). For the violent offender model of PVOL significant predictors included REL ($\beta = .31, p \leq .001$), FS ($\beta = .21, p \leq .01$) and FSit ($\beta = .33, p \leq .001$). Result indicate effects of REL and FSelf for both non-violent and violent offenders but a unique association of FSit for violent offenders. Implications for gerontological inquiry, practice, and policy are discussed.

### SHARING ONE ANOTHER'S BURDENS: CONGREGATIONAL SUPPORT OF CAREGIVERS FOR PERSONS LIVING WITH DEMENTIA

Brianna Garrison[1], *1. Baylor University, Houston, Texas, United States*

This presentation will discuss the findings and implications from a mixed-methods study examining the impact of support services for caregivers of persons living with Dementia in their faith community. Caregivers and persons living with dementia participating in religious activities report numerous psychosocial benefits. Faith communities are the primary social network for older adults, with 48% of older adults attending religious services at least once weekly. Results will highlight specific opportunities for local congregations to foster spiritual connection and meaningful engagement with caregivers of persons living with Dementia. Findings will also describe key considerations and pathways for social work practitioners, researchers, and religious leaders to better serve older adults in their communities by providing education and strengths-based interventions in the context of local congregations. These findings have the potential to increase the reach of such programs to diverse, underserved populations.

### SESSION 1480 (SYMPOSIUM)

### INTEREST GROUP SESSION—RESEARCH IN QUALITY OF CARE: LIVING IN AND LEAVING NURSING HOMES: THE FACTORS THAT CONTRIBUTE TO QUALITY OF LIFE, HEALTH, AND SAFETY OUTCOMES

Chair: Nancy Kusmaul, *University of Maryland, Baltimore County, Baltimore, Maryland, United States*
Discussant: Mercedes Bern-Klug, *University of Iowa, Iowa City, Iowa, Andorra*

Nursing homes house some of the most vulnerable older adults. They often have complex medical conditions and/or cognitive impairments that put them at risk for negative outcomes and poor quality of life. These outcomes can be altered through incorporating evidence-based practices aimed to improve care and residents' life experiences. In this symposium we will explore factors that are shown to influence outcomes and quality of life for people that live in and are discharged from, long term care settings. Amy Roberts and colleagues will explore the influences of nursing home social service staff qualifications on residents' discharge outcomes. Colleen Galambos and colleagues will present findings on advance directives and their impact on reducing potentially avoidable hospitalizations. Kelsey Simons and colleagues will discuss the potential for unmet needs for mental health services as part of nursing home care transitions, and will discuss a model of quality improvement that addresses this gap in care. Vivian Miller will present findings on the impact transportation access has on the ability of community-dwelling family members to visit and provide social support to their family member residents in long-term care. Finally, Nancy Kusmaul and Gretchen Tucker report the findings of their study comparing perceptions of nursing home residents, direct care staff, management, and families on the care practices that influence resident health and quality of life while they live in a long term care setting.

### PERSON-CENTERED CARE: DEFINITIONS AND PERCEPTIONS OF VARIOUS STAKEHOLDERS

Nancy Kusmaul,[1] and Gretchen Tucker[1], *1. University of Maryland Baltimore County, Baltimore, Maryland, United States*

Implementation of culture change in nursing homes shifts the care model from a traditional, more medically focused approach to person-directed care. Person-directed care promotes resident autonomy and decision making and the empowerment of direct care staff. In this paper, we examine how different stakeholders in nursing homes (residents, family members, direct care staff, administrative staff) conceptualize and experience a selection of person-centered care concepts (consistent assignment, meal choice, waking/bedtime practices, and bathing). We describe the commonalities and differences in the ways different groups of stakeholders operationalize these core person centered care practices and describe areas of potential conflict of views. Lastly, we consider how the well-being and quality of life for residents is affected by the use of these practices.

### THE IMPACT OF SOCIAL WORKERS ON POST-ACUTE CARE DISCHARGE OUTCOMES

Amy Restorick Roberts,[1] Amy Restorick Roberts,[1] John R. Bowblis,[1] and Austin C. Smith[1], *1. Miami University, Oxford, Ohio, United States*

Background: Social service staff may play a key role in helping post-acute care patients in skilled nursing facilities return home, yet few studies quantify how social service staff contribute to better patient outcomes. Method: A quasi-experimental statistical approach, regression discontinuity, was used among newly-admitted, Medicare post-acute care patients (65+) to examine the relationship between higher qualifications of social service workers and various discharge outcomes. National data (2011-2015) were drawn from the Online Survey Certification and Reporting system, the Certification and Survey Provider Enhanced Reports, and the Minimum Data Set. Findings: Patients in facilities with a greater proportion of more qualified social service staff (qualified social workers vs. paraprofessionals) had better discharge outcomes. Post-acute care patients were more likely to be discharged home within 30 days, compared to being re-hospitalized or remaining in the facility. Conclusion: Policymakers and providers should support efforts to increase the qualifications of social service staff.

### THE RELATIONSHIP BETWEEN ADVANCE DIRECTIVE COMPLETION AND POTENTIALLY AVOIDABLE HOSPITALIZATIONS

Colleen M. Galambos,[1] Marilyn Rantz,[2] Lori Popejoy,[2] Pritchett Angelita,[2] and Greg Petroski[3], *1. University of Wisconsin Milwaukee, Milwaukee, Wisconsin, United States, 2. University of Missouri Sinclair School of Nursing, Columbia, Missouri, United States, 3. Office of Medical research, Columbia, Missouri, United States*

Advance directive (AD) completion can improve transitions between hospitals and skilled nursing facilities (SNF's). One CMS Innovations Demonstration Project, The Missouri Quality Initiative (MOQI), focused on improving advance directive documentation and use in sixteen SNF's. An analysis was conducted of data collected from annual chart inventories occurring over four years. Using a logistic mixed model, results indicated statistical significance ($p<0.001$) for increased AD documentation. Greatest gains occurred at project mid-point. The relationship between having an advance directive and occurrence of transfer to a hospital was tested on a sample of 1563 residents with length of stays more than 30 days. Residents who did not have an advance directive were more likely to be transferred. A logistic regression was conducted and the results were statistically significant ($p<0.02$). The MOQI model and initiatives will be explained followed by a discussion of research methodology, data collection, and analyses. Practice implications will be discussed.

### ADDRESSING AN UNMET NEED FOR MENTAL HEALTH SUPPORT DURING CARE TRANSITIONS FROM NURSING FACILITIES

Kelsey Simons,[1] Katherine Luci,[2] Lauren Hagemann,[2] M. Lindsey Jacobs,[4] Emily Bower,[5] Morgan Eichorst,[6] and Michelle Hilgeman[7], *1. VISN Center of Excellence for Suicide Prevention, Canandaigua, New York, United States, 2. Salem VA Medical Center, Salem, Virginia, United States, 4. VA Boston Healthcare System, Brockton, Massachusetts, United States, 5. VISN Center of Excellence for Suicide Prevention, Canandaigua, New York, United States, 6. VA Northern Indiana Health Care System, Mishawaka, Indiana, United States, 7. Research & Development Service, Tuscaloosa, Alabama, United States*

Mental health (MH) disorders are common among skilled nursing facility (SNF) residents and may inhibit rehabilitation goals. Moreover, discharges to the community from SNFs are periods of heightened suicide risk within the Veterans Health Administration (VHA), suggesting an urgent need for improved continuity of MH care. This paper presents results of medical records reviews indicating a potential gap in MH services at discharge from VHA SNFs. A quality improvement project ("Suicide Awareness for Veterans Exiting Community Living Centers" – SAVE-CLC), designed to address this gap, will also be discussed. Piloted in 3 sites (N = 66) in 2018, SAVE-CLC clinicians administered depression screens by phone to 47 Veterans (71%) after SNF discharge and helped connect Veterans to MH services. 24 Veterans (26%) received a second such call. Patients and caregivers expressed high satisfaction with SAVE-CLC (n = 35, 97%). Implications for quality improvements in SNF care transitions will be discussed.

### TRANSPORTATION IS THE DRIVER TO VISIT: DOES COMMUNITY CONNECTEDNESS AFFECT DEPRESSION SYMPTOMS OF RESIDENTS?

Vivian J. Miller[1], *1. University of Texas at Arlington, Arlington, Texas, United States*

A recent study found that lack of transportation access (or, transportation disadvantage), together with travel time, is a major barrier for community members to visit their loved ones residing in long-term care nursing homes (Miller, 2018). This transportation disadvantage not only may contribute to decreased visitation but also prohibits family from providing social support to residents, which is imperative for residents to maintain decreased symptoms of depression, a sense of belonging, and highest well-being. A sequential mixed-methodological study was conducted across 11 CMS-certifiable nursing homes in North Central Texas to examine this effect of transportation disadvantage of community members on depressive symptoms of their family in long term care (N=89 dyads). Findings from this study will be presented. Additionally, implications for social work, transportation planning, policymakers, and other key professions will be discussed.

### SESSION 1485 (SYMPOSIUM)

### MIGC 2019 SYMPOSIUM: STRENGTH IN CREATING DIVERSIFIED SPACES IN EDUCATION AND RESEARCH ON AGING

Chair: Adrienne T. Aiken Morgan, *North Carolina A&T State University, Greensboro, North Carolina, United States*
Co-Chair: Candace Brown, *Duke University, Durham, North Carolina, United States*
Discussant: Gregory R. Samanez-Larkin, *Duke University, Durham, North Carolina, United States*

Populations of minority older adults will continue to increase at an accelerated pace in the coming decades. As such, it is increasingly important to disseminate minority aging education and research topics in spaces that will prepare gerontology scholars to address the needs of diverse elders. This symposium will highlight efforts to diversify academic spaces by scholars engaged in minority aging education and research. The first presentation describes a service-learning pedagogical approach to teaching minority aging topics to graduate students. It will discuss how a gerontological social work course seeks to offer real-world learning experiences through community partnership. The second presentation discusses an experiential learning pedagogical approach to teaching social determinants of health to graduate students at a historically Black university and highlights how to apply theoretical concepts to creating community needs assessments and health promotion programming for the local community. The third presentation discusses efforts to teach undergraduate students about older LGBT individuals, who represent a growing group of minority elders. This presentation advocates for the use of various strategies for integrating both research and pedagogical approaches to increase knowledge and awareness of LGBT aging topics. The last presentation focuses on the promotion and dissemination of scholarship produced at minority-serving institutions (MSI) through the creation of a new open-source journal. This presentation describes publication challenges for tenure-track MSI faculty and developed opportunities for inclusiveness of such scholarship. The symposium discussant will summarize these challenges, opportunities, and implications to promote minority-focused gerontological topics in academia.

### MINORITY AGING BEYOND THE CLASSROOM

Tiffany R. Washington[1], *1. University of Georgia, Athens, Georgia, United States*

Service-learning is the pedagogical approach of integrating classroom learning objectives with community-based experiences. In gerontology education, service-learning is one way educators can collaborate with their respective communities to expose students to topics in minority aging. This paper presentation describes students' experiences in a service-learning gerontological social work course embedded in a university-community partnership that took students beyond the classroom and into the community to learn about minority aging. In-classroom topics included social injustices in aging, caregiving in African American families, culturally-tailored caregiving interventions, and health disparities. In the community, students conducted in-home visits engaging persons with dementia in a tailored activities, thus freeing caregivers to engage in self-care activities. Classroom and community experiences were connected through ongoing reflection, critical thinking, and problem solving activities. Survey data revealed students experienced increased gerontological self-efficacy and increased knowledge and attitudes about dementia. This study has implications for future course design around minority aging.

### DIVERSITY IN ACTION: PRACTICE-BASED LEARNING FOR IMPROVING COMMUNITY HEALTH

Amy Thierry[1], *1. Xavier University of Louisiana, New Orleans, Louisiana, United States*

Non-traditional learning methods in diverse classrooms, particularly at HBCUs, can prepare students for successful careers in aging. In a graduate-level public health course on the social determinants of health, students engaged in an experiential learning opportunity to address health disparities within a historic Black neighborhood in New Orleans. Through a combination of classroom lectures, community integration activities, and skills-building training with faculty and public health professionals, students applied a health equity life course framework in developing, implementing, and evaluating a multi-faceted community needs assessment used to guide health promotion programming for neighborhood residents. In this example, student mastery of complex concepts was achieved through hands-on experiences that had a direct impact on the community. Moreover, a community-engaged educational environment allowed students to take ownership of their learning, develop their professional identity, as well as initiate a sustainable partnership with the community through which improvements in health can be made.

### CREATING DIVERSE SPACES IN UNDERGRADUATE EDUCATION: OLDER LGBT RESEARCH AND TEACHING

Maggie Syme[1], *1. Kansas State University, Manhattan, Kansas, United States*

According to SAGE (Services and Advocacy for GLBT Elders), an estimated 3 million older adults currently identity as lesbian, gay, bisexual, or transgendered (LGBT), which is expected to double in the next decade, making them one of the fastest growing minority aging groups. Yet, there is very little attention being paid to older LGBT individuals, a dearth of information on their experiences, and multiple stigmas that compound to make this a challenging space for education and research. This presentation will discuss several different strategies to integrate research and teaching in order to make LGBT elders a relevant and impactful topic in undergraduate education. These include students undertaking field research on dating ads, creating infographics for health challenges, and evaluating sexual wellness research to illustrate similarities and differences among LGBT older adults and their heterosexual counterparts. Specific considerations for approaching topics in undergraduate contexts will be discussed.

### USING OSJ PUBLISHING AND TEAM PEER REVIEWS TO EXPAND SCHOLARLY WORKS FROM A DIVERSIFIED AND INCLUSIVE PERSPECTIVE

Pamela P. Brown[1], *1. Albany State University, Albany, Georgia, United States*

While Minority Serving Institutions (MSI) are often more teaching than research focused, promotion and tenure heavily rest on the success of being published. It is challenging to publish with high teaching loads and limited research time. A viable alternative is to publish the scholarship of teaching/learning work conducted within MSI classrooms. Open Source Journals (OSJ) assists with the publication needs of MSI faculty and can bring a more diversified andragogic practice out to the public sphere. It also allows for the publishing of more peer-reviewed journals, thus allowing for a greater expansion of diversity in the editorial process. This presentation will focus on a newly created OSJ, which focuses on MSI faculty and innovative teaching. A discussion of a team peer review process is included which focuses on the ability to be inclusive in publishing scholarly works from MSI faculty and those who work with historically underserved student populations.

### SESSION 1490 (SYMPOSIUM)

### PRESIDENTIAL SYMPOSIUM: MUSEUMS AND AGING: NOVEL NETWORK OPPORTUNITIES TO SUPPORT OPTIMAL AGING

Chair: Desmond O'Neill, *Trinity College Dublin, Dublin, Ireland*

Co-Chair: Robert Roush, *Huffington Center on Aging, Baylor College of Medicine, Houston, Texas, United States*
Discussant: Robert Roush, *Huffington Center on Aging, Baylor College of Medicine, Houston, Texas, United States*

Museums represent an evolving and under-recognized network of opportunity for examining aging while supporting optimal aging across the lifespan. Museums bind communities together in a civic body by "…identifying its highest values, its proudest memories, and its truest truths."(Duncan, 1991). They represent a secular ritual of the modern state in which the spiritual heritage of the nation is offered as a public reinforcement of political values. Art museums are also sites which enable individuals to achieve liminal experience - to move beyond the psychic constraints of mundane existence, step out of time, and attain new, larger perspectives. The interaction and potential synergies between museums and aging have been insufficiently explored in gerontological scholarship, with the existing emphasis largely focussing on facilitating access to older people and those with age-related health conditions. This symposium reflects and magnifies the networking of GSA with a major art museum through an Educational Site Visit during GSA 2019 to the Blanton Art Museum. It proposes to review museums and ageing in a broader context, exploring the context within which aging is represented in the discourse of heritage and museums, museums networking to provide a repository of late-life creativity, networks of older people as a key resource and client group for museums, life-course and inter-generational engagement with museums. Finally, the insights that the ageing of art works provide for curating the longevity dividend through developing scholarly networks between gerontologists and curators.

### MUSEUMS AND AGING: VISTAS, SYNERGIES, AND OPPORTUNITIES

Desmond O'Neill[1], *1. Trinity College Dublin, Dublin, Ireland*

Against a background of an overall decrease in attendance at museums, older Americans represent a contrast with a marked increase in attendance of those aged over 75. Drawing on theorists of museums and the narratives on ageing in the literature of museums, this introductory presentation gives an overview of the socio-political context of museums, the activities and roles of older people as currently presented through networks such as the Network of European Museums Organizations, and propose a fresh vision of the networks and synergies between older people, museums and gerontology.

### MUSEUMS AS A NETWORK REPOSITORY OF LATE-LIFE CREATIVITY

Manfred Gogol[1], *1. Heidelberg University, Heidelberg, Germany*

Through collaboration and exchange for exhibitions, art museums represent an under-explored network for displaying late-life creativity and the longevity dividend. Drawing on the collection from many galleries brought together for the Letzte Bilder (Last Pictures) exhibition by the Schirm Gallery Frankfurt, this talk will illustrate the possibility of creating educational programs, Late Life Creativity trails both virtual through podcasts and structural, as well as specific exhibitions of late-life creativity as a catalyst for reimagining and reframing ageing. Further approaches are the focus on the life course of works of artist like Cy Twombly at the Museum Brandhorst Munich or Pablo Picasso at the Museum Barberini Potsdam, as well as studying aging in artists self-portraits. The interaction of creativity with age, diseases, and functional decline in the museum context can promote the changing of role models and our understanding of productivity and creativity by ourselves as well as in the society.

### EDUCATION, GERONTOLOGY, AND MUSEUMS

Ray Williams[1], *1. Blanton Museum of Art, Austin, Texas, United States*

Many adult museum visitors welcome the invitation to make personal connections to works of art. Time for individual reflection and sharing with others may enable relationships to deepen and new insights to emerge. This presentation describes an approach to gallery teaching that honors the memories, associations, and emotions that visitors bring to their encounters with works of art. The approach has been particularly effective with groups of health care professionals, and as a reminder to docents of the powerful affective experiences that will naturally occur for some members of the public. Drawing on the experiences of collaborative networks of museum and medical educators, we outline the basis of rich opportunities for developing lifespan and gerontological educational projects in museums.

### NETWORKING MUSEUMS, OLDER PEOPLE, AND COMMUNITIES: UNCOVERING AND SUSTAINING STRENGTHS IN AGING

Teresa Bonner[1], *1. Aroha Philanthropies, Minneapolis, Minnesota, United States*

Museums, like other cultural institutions, are beginning to embrace a new role: facilitating creativity of older adults through education programs. A cohort of 20 American museums from Alaska to Puerto Rico are embarking on an ambitious two-year program, funded by Aroha Philanthropies, to develop successful creative aging programs in their communities. The cohort includes art museums, history centers, botanical gardens and a science museum. The group has received extensive training and technical assistance to build their capacity and awareness of needs and desires of older adults. The American Alliance of Museums (AAM), a partner in this initiative, is leading a deep dive into the potential of museums to enable older adults to learn, make and share the arts. With Aroha's support, AAM has created a two-year position, the Aroha Senior Fellowship in Museums and Creative Aging to lead an exploration of how museums can deepen their engagement with creative aging.

### KEEPING THINGS, BUT ONLY FOR A WHILE

David J. Ekerdt[1], *1. University of Kansas, Lawrence, Kansas, United States*

The life course is accomplished by material culture held as a convoy of possessions, but also sustained by public affordances and amenities that include the artifacts and artworks to be found in museums. In both places—household and museum—objects come and go, but there is mainly keeping. The difference lies in the capacity to keep things indefinitely: it is virtue for museums but a predicament for households of aging adults. Museums model ideals of permanence and responsibility toward things, ideals that, in the long run, households can only faintly attain. For older adults and for gerontologists, preservation is the wrong lesson to take away from the galleries. Rather, what we can learn there is how single, selected things can show, in a thoughtful way, an entire world of ideas and universe of meaning. No need to keep it all—and forever—but we can honor things while we can.

### SESSION 1495 (SYMPOSIUM)

### INTEREST GROUP SESSION—ALZHEIMER'S DISEASE AND RELATED DIMENTIAS: OPPORTUNITIES AND CHALLENGES TO DEVELOPING AND TESTING PRAGMATIC ADRD INTERVENTIONS

Chair: Abraham A. Brody, *NYU Rory Meyers College of Nursing, New York, United States*
Discussant: Laura N. Gitlin, *Drexel University, Philadephia, Pennsylvania, United States*

Many clinical trials have been performed to develop the evidence for caring for persons with Alzheimer's Disease and Related Disorders (ADRD) in tightly controlled settings. These trials have found efficacy of a wide spectrum of interventions to address issues from advanced care planning to behavioral and psychological symptoms of dementia (BPSD). However, few ADRD interventions have been tested in wide-scale pragmatic fashion in long term supportive settings (LTSS) such as nursing homes, primary care clinics, hospices, or community based organizations. This is due to a variety factors, principle amongst them are the difficulty in implementing pragmatic trials, and that many of the interventions developed in tightly controlled settings are not directly translatable to real-world settings. Without translating and testing interventions in real world settings, the evidence base remains largely inaccessible to the end user, the persons with ADRD and their caregivers. Moreover, effectiveness remains unclear. The lack of pragmatic trials in ADRD exists despite significant recent investment from the NIH Office of the Director in a health systems collaboratory to support pragmatic clinical trials. In 2018, NIA therefore released a call for 2-phase intervention development and pragmatic trial testing via an R61-R33 mechanism (PAR-18-585). Four proposals were funded in September 2018 from this PAR. This symposium will explore the opportunities and challenges present in developing and testing pragmatic interventions in ADRD in LTSS. The speakers will also share specific scientific methodological and implementation questions that need to be addressed in applying for pragmatic trial awards.

### DEVELOPING AND IMPLEMENTING ALIVIADO DEMENTIA CARE IN HOSPICES: CHALLENGES ENCOUNTERED AND LESSONS LEARNED

Abraham A. Brody,[1] Shih-Yin Lin,[2] Catherine E. Schneider,[2] Alycia A. Bristol,[2] Kimberly E. Convery,[2] and Victor Sotelo[2], *1. NYU Rory Meyers College of Nursing, New York, United States, 2. NYU Rory Meyers College of Nursing, New York, New York, United States*

Hospice was originally developed to care for individuals with metastatic, solid-tumor cancers. While advanced ADRD is now the primary illness in approximately 19% of the hospice population and presents as a co-morbid condition in many more, little evidence-based work has been performed to retool hospice to care for persons with ADRD and their caregivers. Aliviado Dementia Care-Hospice Edition is a systems level change program consisting of hospice workforce training, an implementation toolbox, and agency-wide workflow changes. Aliviado seeks to improve the quality of life for persons with ADRD and their caregivers receiving hospice, focused specifically on BPSD and pain assessment and management. In developing a coalition of hospice agencies and implementing this pragmatic intervention, we discuss our solutions to overcoming a number of barriers, including varying electronic health records, performing culture change with a disseminated workforce, scaling to 25 hospices, and working with some hospices who lack experience performing research.

### THE PORCHLIGHT PROJECT: COLLABORATING TO ENHANCE THE DEMENTIA CAPABILITY OF COMMUNITY-BASED VOLUNTEERS

Joseph E. Gaugler,[1] Gabriela Bustamante,[2] Christina Rosebush,[2] Jeri Schoonover,[3] Roxanne Jenkins,[3] Nicole Bauer,[3] Lisa Beardsley,[3] and Laura Rowe[3], *1. University of Minnesota - School of Public Health, Division of Health Policy and Management, Minneapolis, Minnesota, United States, 2. University of Minnesota, Minneapolis, Minnesota, United States, 3. Lutheran Social Service of Minnesota, Saint Paul, Minnesota, United States*

Public health efforts to address Alzheimer' disease and related dementias (ADRD) are limited. Utilization of lay/peer intervention providers in the community to reach older persons and their families may offer a novel method to reach those in need. Such an approach may also serve as a fulcrum around which formal healthcare delivery and community-based LTSS are better integrated. This pragmatic trial, the Porchlight Project, aims to refine a multicomponent training approach for lay volunteers in Minnesota (i.e., Senior Companions) that enhances their capability to deliver dementia care and support to underserved older persons in need. This presentation will highlight the development and collaboration with Lutheran Social Services of Minnesota to refine and deliver a useful and feasible training program to enhance Senior Companions (n = 20) dementia care capability, as well as the potential and challenges of delivering a pragmatic trial of this type across the state of Minnesota.

### CHALLENGES FOR IMPLEMENTING PRAGMATIC COMMUNICATION INTERVENTIONS IN NURSING HOME SETTINGS

Kristine N. Williams,[1] Clarissa Shaw,[2] and Carissa K. Coleman[3], *1. University of Kansas Medical Center, Kansas City, Kansas, United States, 2. University of Iowa College of Nursing, Iowa City, Iowa, United States, 3. University of Kansas School of Nursing, Kansas City, Kansas, United States*

Interventions to improve nursing home care have been developed and tested. However readily disseminated interventions are lacking. Barriers include low staffing levels contributing to limited time for education and high turnover of direct care and administrative staff. Educational interventions must be accessible to accommodate busy staff. Meaningful outcome measures are needed and interventions must fit varied nursing home sizes, ownership, resident population, and regions. Changing Talk Online (CHATO) was adapted from the effective, yet time-intensive, Changing Talk program addressing nursing home staff communication. The original classroom-based program significantly improved staff communication with residents and resulted in subsequent reductions in resident behavioral and psychological symptoms of dementia. Strategies for marketing and recruiting nursing homes and to engage administrators and staff will be discussed as implemented in the Changing Talk Online (CHATO) R61 trial. Approaches addressing unique nursing home challenges to implementation are essential for successful dissemination to improve care.

### INVOLVING FAMILY TO IMPROVE COMMUNICATION IN PRIMARY CARE FOR PRIMARY CARE PATIENTS WITH DEMENTIA

Jennifer L. Wolff,[1] Sydney Dy,[2] Jennifer Aufill,[2] Diane Echavarria,[2] Cynthia Boyd,[3] David Roth,[4] Jessica Colburn,[4] and Danetta Sloan[2], *1. Johns Hopkins University, Maryland, United States, 2. Johns Hopkins Bloomberg School of Public Health, Baltimore, Maryland, United States, 3. Johns Hopkins School of Medicine, Baltimore, Maryland, United States, 4. Johns Hopkins University, Baltimore, Maryland, United States*

Family caregivers are at the forefront of managing dementia but are not routinely included in discussions about prognosis and are often poorly prepared to engage in surrogate decision-making. Few interventions target advance care planning in primary care, which is where most persons with dementia are initially diagnosed and medically managed. SHARING CHOICES proactively engages family and support advance care planning in primary care by normalizing advance care planning discussions, clarifying the role of the family during interactions with primary care clinicians, providing ongoing access to a non-clinician trained to lead advance care planning conversations, facilitating registration for the patient portal enable and extending electronic interactions and information access to family caregivers, and providing education and resources about dementia for clinic staff. This presentation will discuss refinement of the SHARING CHOICES protocol and facilitators and challenges of executing a pragmatic trial of this type across two large primary care systems.

### SESSION 1500 (SYMPOSIUM)

### INTEREST GROUP SESSION—AGING AMONG ASIANS: RESEARCH METHODS IN AGING AMONG ASIANS

Chair: Wenjun Li, *University of Massachusetts Medical School, Worcester, Massachusetts, United States*
Co-Chair: Shantha Balaswamy, *The Ohio State University, Columbus, Ohio, United States*
Discussant: Allen Glicksman, *Philadelphia Corporation for Aging, Philadelphia, Pennsylvania, United States*

Asians are the largest and the fastest growing segment of the world population. Asian immigrants are the second largest immigrant population in the U.S. However, age-related social and health issues are understudied among late-life immigrant and the oldest old Asians. Little data exist to support public health promotion, policy studies and clinical practice in this population. To advance research into aging among Asians living in the U.S. and elsewhere in the world, sound methodologies can be adopted from those well-developed in other settings while novel methodologies are to be developed to meet the unique needs of Asian studies. This symposium brings together four abstracts that address a variety of common methodological issues in social and health studies among Asian older adults. The topics range from culturally and linguistically appropriate strategies for recruiting non-English speaking research participants, assessment of social isolation and transportation barriers using an ethnographical approach, development of a new culturally appropriate measure for successful aging among the oldest old Chinese in China, and evaluation of preventive healthcare use among faith-based first-generation Chinese immigrants using self-administered surveys in the U.S. These studies involve qualitative ethnographical analysis, mixed methods for instrument development, quantitative data analysis, use of geographic information systems and demography to plan participant recruitment, and use of staged community engagement to increase efficiency and representativeness of participant recruitment. Lessons learned from these studies are valuable to future studies on aging among Asians. This symposium is a collaborative effort of the GSA Aging Among Asians Interest Group.

### APPROACHES TO RECRUITING NON-ENGLISH-SPEAKING LATE-LIFE CHINESE IMMIGRANTS FOR HEALTHY AGING STUDIES

Wenjun Li,[1] Linda Churchill,[1] Jie Cheng,[1] Rachel Siden,[1] and Annabella Aguirre[1], *1. University of Massachusetts Medical School, Worcester, Massachusetts, United States*

Non-English Speaking late-life Chinese immigrants are hard to reach. We developed a staged, multi-facet, community-engaged approach to recruiting participants for aging research. We first used a direct mail campaign targeting neighborhoods with high concentrations of racial minorities, and sent mails to households with a possible Chinese family name. Invitational letter, interest survey and flyers are printed in traditional and simplified Chinese using large font. Flyers include a colorful graphic portraying diverse racial background. Prior to the mailing, we presented the study at senior centers, faith-based organizations, community centers and bingos that hosted higher rates of minority older adults. We posted study materials in Chinese language schools and Chinese "WeChat" groups. We also encouraged current participants to "tell a friend". Chinese-English bilingual staff are trained and certified as recruiters. Within two months, over 50 participants from diverse neighborhoods are recruited.

Our community-engaged, linguistically and culturally appropriate approach has been highly effective.

### SUCCESSFUL AGING SCALE DEVELOPMENT FOR COMMUNITY-DWELLING OLD-OLD (75+) IN CHINA: A PRELIMINARY ANALYSIS

Minzhi Ye,[1] Lin Chen,[2] and Lin Gu[3], *1. Benjamin Rose Institute on Aging, Cleveland, Ohio, United States, 2. Department of Social Work, Fudan University, Shanghai, Shanghai, China (People's Republic), 3. Shanghai Volunteer Guidance Center for Longevity, Shanghai, Shanghai, China (People's Republic)*

Thousands of articles have discussed the concept of successful aging, yet no existed scale to measure its components among old-old (age 75+) in urban China. This study used a mixed method to develop a valid scale in Shanghai from July 2016 to Nov 2018. We first conducted 97 semi-structured, in-depth interviews on community-dwelling old-old and listed 70 items based on the findings and previous studies (e.g., Lubben, 1988). Ten multidisciplinary professionals and eight old-old had focus groups to evaluate the content validity of the scale. We used the final 22 items to survey 208 community-dwelling old-old in twelve neighborhoods. The mean age is 81, with 40% females. The principal component analysis showed a 13-item scale representing four domains (self-reliant, aging attitude, self-value expression, and social support), with 0.67 total reliability (Cronbach's Alpha). This study provided a set of quality indicators to evaluate old-old's life in urban China.

### BARRIERS TO TRANSIT MOBILITY AND WELL-BEING AMONG LATE-LIFE ASIAN INDIAN IMMIGRANTS

Rupal Parekh[1], *1. University of Connecticut, Hartford, Connecticut, United States*

Using qualitative interpretative phenomenological analysis and semi-structured interviews, we explored transit mobility needs and its impact on well-being and quality of life among late-life Asian Indian immigrants. Using inductive and deductive methods, we analyzed qualitative data collected from 18 participants. Four themes emerged specific to the influence of contextual factors on transportation mobility barriers among participants. Findings suggest that cultural and individual attitudes combined with the 'built environment' hinder participation at the temple and other cultural and religious activities and increase dependence on adult children. The reciprocal fit between transit mobility needs, access to culturally familiar environments and the built environment is critical to freedom, independence, and healthy aging for diverse older adults. Research at the intersection of global aging and transportation mobility requires equal grounding in a range of justice principles (environmental, social, and economic) in the pursuit of sustainable transportation options for diverse older adult populations.

### LENGTH OF STAY IN U.S. AN ANOMALY: PREVENTIVE HEALTHCARE UTILIZATION AMONG FAITH-BASED FIRST-GENERATION CHINESE

Su-I Hou[1], *1. The University of Central Florida, Orlando, Florida, United States*

Limited knowledge exists on preventive healthcare services utilization (PHSU) among Chinese immigrants. This study examined factors related to PHSU among faith-based first-generation Chinese. A self-administered survey was conducted in five Chinese churches in southeastern US.(n=184). Mean age was 43 (SD=14) and 50% were males. Comparing with recent immigrants (<10 years), those who lived in the US for more than 10 years were more likely to be married (94.6% vs. 50.0%), have annual exams (74.5% vs. 39.6%), report doctors recommending cancer screenings (35.3% vs. 12.5%), have talked to doctors about screenings (25.5% vs. 4.2%), and perceive higher cancer knowledge (20.0% vs. 2.1%) (all p<.05). Considering all factors together, regressions showed perceived knowledge was the only significant predictor on having talked to doctors (OR=3.11) and doctor recommending screening (OR=2.15). Married status was the only strong predictor on annual exam (OR=29.13). Our findings highlight the urgent need for promoting PHSU in this population.

### SESSION 1505 (SYMPOSIUM)

### INTEREST GROUP SESSION—INDIGENOUS PEOPLES: REVEALING THE HEALTHY BRAIN INITIATIVE'S ROAD MAP FOR INDIAN COUNTRY: OPPORTUNITIES FOR ENGAGEMENT

Chair: Heidi Holt, *Centers for Disease Control and Prevention, Atlanta, Georgia, United States*
Discussant: Blythe S. Winchester, *Cherokee Indian Hospital, Cherokee, North Carolina, United States*

This landmark document, The Healthy Brain Initiative: Road Map for Indian Country, is the first-ever public health guide focused on dementia in American Indian/Alaska Native (AI/AN) communities. It is intended as a tool for leaders of the 573 federally recognized Indian tribes, as well as state-recognized tribes, to engage their communities in this important issue. Early in the development of the HBI Public Health Road Map for Dementia, CDC recognized strategies that may work for state and local public health agencies likely would need to be tailored by native communities due to culture and unique contexts. This Road Map for Indian Country (Road Map) can be used to support a dialogue within a Native community about how to promote wellness across the lifespan and best support older adults with dementia, their families, and caregivers. The panel will consist of 5 presenters and 1 discussant. Bill Benson, International Association of Indigenous Aging, will discuss the background and need for the project. Molly French, the Alzheimer's Association, will describe the process used to create the Road Map. Heidi Holt, CDC, will describe the content and goals of the Road Map. Kelsey Donnellan, Association for State and Territorial Health Officials (ASTHO), will present key Indian Country products and Messages that accompany the Road Map. Lisa McGuire will present relevant Behavioral Risk Factor Data, including data specific to the AI/AN population. The discussant will wrap up the panel and provide unique insights as to the use and future for this important document.

### WHAT'S IN IT? AN IN-DEPTH DESCRIPTION OF THE INDIAN COUNTRY ROAD MAP

Heidi Holt[1], *1. Centers for Disease Control and Prevention, Atlanta, Georgia, United States*

As tribal health and aging leaders become concerned about the growing problem of dementia, they can build on strengths in their cultures and traditions, which provide unique opportunities to improve the lives of older adults living with dementia, their families, and their communities. To offer a tool for tribal leaders, CDC, together with key partners, created The Healthy Brain Initiative: The Road Map for Indian Country. Designed to support discussion about dementia and caregiving within tribal communities, this Road Map encourages a public health approach as part of a holistic response. During this presentation, an in-depth review of the themes that shaped this Road Map will be provides, gaps in knowledge and practice will be described, and explain the 8 recommended actions in the Road Map. Discussion will wrap up with recommended actions for moving forward, as well as CDC's plans for supporting implementation of the Road Map.

### ALZHEIMER'S DISEASE IN AI/AN COMMUNITIES: THE NEED FOR A CULTURALLY TAILORED ROAD MAP FOR ACTION

William F. Benson[1], *1. International Association for Indigenous Aging, Silver Spring, Maryland, United States*

Although little is known about Alzheimer's disease and other dementias among our American Indian and Alaska Native (AIAN) population, the numbers of AIAN elders is growing rapidly as is the overall AIAN population. As noted in The Gerontologist, the number of AIAN elders 65 and older will triple by 2030. Between 2014–2060, the number of AIAN people aged 65 and older living with dementia is projected to grow over 475%. This poses challenges for tribal officials, health/social service providers and families. Among these are limited awareness/knowledge about Alzheimer's disease, inadequate services and resources, and limited experience in providing proven responses to the needs of those with Alzheimer's and their families. To help tribes and others serving the AIAN population to respond to such challenges, it was important to develop a menu of options for tribal communities and leaders to consider appropriate to their needs, circumstances and values.

### LISTENING AND LEARNING TO FORM THE HBI ROAD MAP FOR INDIAN COUNTRY

Molly French,[1] Michael Splaine,[2] John Shean,[3] and Heidi Holt[4], *1. Alzheimer's Association, Washington, District of Columbia, United States, 2. Splaine Consulting, Columbia, Maryland, United States, 3. Alzheimer's Association, Washington DC, District of Columbia, United States, 4. CDC, Atlanta, Georgia, United States*

American Indian and Alaska Native (AI/AN) communities are establishing new paths as more older adults develop Alzheimer's and other dementias along with other co-morbidities. To offer a flexible framework of public health strategies that proactively address the growing issue of dementia among AI/ANs, Alzheimer's Association and Centers for Disease Control and Prevention (CDC) developed the first-ever Healthy Brain Initiative Road Map for Indian Country. Partnering with International Association for Indigenous Aging supported Road Map development through virtual listening sessions and written comments from regional Native health experts, tribal aging service leaders, and tribal government officials. Many additional discussions, engagement of a cultural guide, and an additional partnership with National Indian Health Board further informed Road Map contents, graphic design, and marketing. Presenter will describe rationale for the process, themes from the consultations, and lessons learned by the Association and CDC that can apply to similar initiatives.

### CONNECTING HEALTHY BRAINS AND HEALTHY HEARTS IN INDIAN COUNTRY

Kelsey M. Donnellan[1], *1. Association of State and Territorial Health Officials (ASTHO), Washington, D.C., United States*

The release of the Healthy Brain Initiative Road Map for Indian Country inspired the International Association for Indigenous Aging (IA2), Association of State and Territorial Health Officials (ASTHO), and the Centers for Disease Control and Prevention (CDC) to develop health communication materials to promote heart health and brain health among states with American Indian and Alaska Native (AI/AN) communities. IA2 engaged public health, tribal health, and brain health experts to inform the key messages and intended audiences. The final package includes two posters, one flyer, one provider guide, four radio public service announcements, and two short videos. Tribal health officials and state health are encouraged to distribute the resources through senior centers and inter-tribal organizations, healthcare facilities, administrative offices, tribal newspapers/radio stations, and as mailers to tribal members. The session will focus on how the resources raise awareness of and promote action on heart and brain health among AI/AN communities.

### DATA FOR ACTION: CDC'S BEHAVIORAL RISK FACTOR SURVEILLANCE SYSTEM (BRFSS) COGNITIVE DECLINE AND CAREGIVING DATA

Lisa C. McGuire[1], *1. CDC, Atlanta, Georgia, United States*

The Centers for Disease Control and Prevention's (CDC) Behavioral Risk Factor Surveillance System (BRFSS) is the world's largest ongoing health survey, administered in all 50 U.S. states, as well as the District of Columbia and the three U.S. territories, with data collected from more than 400,000 respondents. The BRFSS collects data on cognitive decline and caregiving as well as many health behaviors, annually. In 2015-2017, the 6-item cognitive decline module has been administered in 49 states, DC, and Puerto Rico, while the 9-item caregiving has been administered in 44 states, DC, and Puerto Rico. CDC's Alzheimer's Disease and Healthy Aging Program has developed many data for action resources for use by states and other partners to help identify populations and communities most at need. This presentation will describe the series of resources to facilitate data utilization among Tribal Communities, including state and race/ethnicity-specific infographics, topic-specific briefs, and the online portal.

### SESSION 1510 (PAPER)

### SLEEP DISORDERS

### BIDIRECTIONAL ASSOCIATIONS BETWEEN SLEEP COMPLAINTS AND DEPRESSION: FINDINGS FROM THE NHATS STUDY

Minhui Liu,[1] Sarah L. Szanton,[2] and Michael V. Vitiello[3], *1. Johns Hopkins University School of Nursing, Baltimore, Maryland, United States, 2. Johns Hopkins School of Nursing, Baltimore, Maryland, United States, 3. University of Washington, Seattle, Washington, United States*

Depression and insomnia are prevalent in older adults and show bidirectional relationships. Sleep initiating and maintenance difficulties are the two frequently seen complaints of insomnia diagnostic criteria. Whether these two sleep complaints differ in their associations with depression is unknown. Using the National Health and Aging Trends Study (NHATS), we examined whether sleep initiating and maintenance difficulties at baseline (T1) predicted depression onset at 12 months (T2) and 24 months (T3) in 4,048 T1 non-depressed participants and whether depression at T1 predicted these two sleep complaints at T2 and T3 in 3,581 T1 non-insomnia participants. Participants who developed depression at T2 tended to be Hispanic, non-Hispanic black, less educated, live alone, physically inactive, and have more painful locations and chronic conditions. Participants with sleep complaints at T2 tended to be less educated, live alone, physically inactive, and have more painful locations and chronic conditions. Sleep initiating difficulty persistently predicted depression onset at T2 (OR: 1.62, 95% CI: 1.14, 2.31) and T3 (OR: 1.84, 95% CI: 1.21, 2.81) after adjusting demographics, lifestyles and health condition-related covariates. Depression at T1 persistently predicted sleep initiating difficulty at T2 (RRR: 2.19, 95% CI: 1.44, 3.34) and T3 (RRR: 1.70, 95% CI: 1.07, 2.70) after adjustment. Sleep maintenance difficulty at T1 did not predict depression onset at either time point and vice versa. This study suggests a bidirectional association of depression with sleep initiating difficulty but not sleep maintenance difficulty in older adults. Interventions targeting difficulty initiating sleep may moderate depression onset in older adults.

### DYADIC FACTORS THAT ASSOCIATE WITH INSOMNIA IN CAREGIVERS OF PERSONS LIVING WITH DEMENTIA

Glenna S. Brewster,[1] Donald Bliwise,[2] Fayron Epps,[3] Kate Yeager,[1] and Ken Hepburn[4], *1. Emory University, Nell Hodgson Woodruff School of Nursing, Atlanta, Georgia, United States, 2. Emory University School of Medicine, Atlanta, Georgia, United States, 3. Georgia State University, Atlanta, Georgia, United States, 4. Emory University, Atlanta, Georgia, United States*

Insomnia is prevalent in caregivers of persons living with dementia (PLWD); however, more research is needed to identify which dyadic factors most impact caregiver sleep. This study aimed to identify the factors associated with caregiver insomnia in the baseline component of a randomized clinical trial. A linear regression was conducted with caregiver variables (e.g., depression), and PLWD variables (e.g., disruptive nighttime behaviors) as independent variables in relation to insomnia, as assessed with Insomnia Severity Index (ISI). Caregivers (n=49) were on average 63 years, mostly female (65.3%), White (69.4%), and spouses (65.3%). Mean ISI was 6.8, indicating mild-to-moderately disturbed sleep. Multiple linear regression ($F(11,32) = 13.4$, $p<.001$) showed that both caregiver-based measures (depression, $p<.023$) and PLWD-based measures (disruptive nighttime behaviors, $p<.001$) were independently associated with ISI. Ideas about needed multicomponent dyadic interventions which target both PLWD nighttime behaviors, caregiver depression, and sleep disturbances will be discussed.

### MULTIMODAL PERSONALIZED CHRONOTHERAPY IMPROVES SLEEP IN ADULTS WITH MILD COGNITIVE IMPAIRMENT: A RANDOMIZED TRIAL

Ryan S. Falck,[1] John R. Best,[1] Jennifer C. Davis,[2] Patrick Chan,[1] Daniel Backhouse,[1] Glenn J. Landry,[1] and Teresa Liu-Ambrose[1], *1. University of British Columbia, Vancouver, British Columbia, Canada*
*2. University of British Columbia - Okanagan Campus, Kelowna, British Columbia, Canada*

Poor sleep is common among older adults with Mild Cognitive Impairment (MCI) and may contribute to their increased risk for dementia. Chronotherapy is a set of intervention strategies which can improve sleep quality by strengthening the entrainment of the biological clock to the solar light-dark cycle, and includes strategies such as: 1) bright light therapy (BLT); 2) physical activity (PA); and 3) good sleep hygiene. Thus, in this 24-week randomized controlled trial (RCT; NCT02926157), we aimed to examine the efficacy of a multimodal, personalized chronotherapy intervention to improve sleep quality among older adults with MCI. Ninety-six older adults (65+ years) with MCI were randomized to either: 1) a multimodal personalized chronotherapy group (INT); or 2) a waitlist-plus-education control group (CON). Participants allocated to the INT received four once-weekly, general sleep hygiene education classes, followed by 20 weeks of 1) individually-timed BLT; and 2) bi-weekly, individually-tailored PA counselling in conjunction with receiving a consumer-available PA tracker (Fitbit® FlexTM). We found a significant group x time interaction for objectively measured sleep fragmentation (5.01; $p< 0.01$) and also for Pittsburgh Sleep Quality Index (PSQI) score ($p= 0.03$), such that the INT: 1) maintained sleep fragmentation while CON worsened at 12 weeks ($p< 0.01$); and 2) had improved PSQI score compared to CON at both 12 weeks ($p< 0.01$) and 24 weeks ($p= 0.04$). Our results provide novel evidence that a multimodal personalized chronotherapy approach may promote both objective and subjective aspects of sleep quality in older adults with MCI.

### POOR SLEEP QUALITY IS RELATED TO DECREASED WHITE MATTER INTEGRITY IN BRAIN NOCICEPTIVE PATHWAYS IN OLDER ADULTS

Anna R. Egbert,[1] Ryan S. Falck,[1] John R. Best,[1] Linda Li,[1] Lynne Feehan,[1] and Teresa Liu-Ambrose[1], *1. University of British Columbia, Vancouver, British Columbia, Canada*

Poor sleep quality, decreased physical activity (PA) and increased sedentary behavior (SB) are common characteristics of older adults. Notably, these factors play an important role in brain health. We examined the relationship between sleep quality, PA, SB and brain white matter integrity (WM) in older adults with osteoarthritis (OA). We retained data on 16 participants (mean age 60, SD=7.7) from a larger Monitor-OA cohort recruited from Metro Vancouver, BC, Canada. Sleep efficiency and duration, amount of time spent on PA and SB daily over a period of one week was acquired with an objective measure – the multi-sensor monitor SenseWear Mini which integrates tri-axial accelerometer data, physiological sensor data and personal demographic information. Brain WM tractography was calculated from fractional anisotropy data obtained with diffusion weighted magnetic resonance imaging. Voxelwise group-level statistics examined the effects of our variables of interest on the integrity of brain WM tracts while controlling for participants age. We found that lower sleep efficiency was related to decreased integrity in WM tracts of frontal, temporal lobes, precuneus and thalamus (Bonferroni corrected $p<0.05$). Shorter sleep was related to lower WM integrity in frontal regions, posterior cingulate and insula radiations (Bonferroni corrected $p<0.05$). No significant effects were noted for PA or SB. The identified brain regions are involved in sleep processes but further overlap with the nociceptive brain network. Our findings suggest that neural mechanisms related to sleep disturbance may also involve pain-related processing in older adults.

### SESSION 1515 (PAPER)

### SOCIAL ISOLATION AND NETWORKS

### DO DENSE URBAN CENTERS PROVIDE LARGE SUPPORTIVE SOCIAL NETWORK TO OLDER ADULTS? A CROSS-COUNTRY COMPARATIVE STUDY

Nicola Palmarini,[1] Monica Cominato,[2] Francesca Luppi,[3] Margherita Ruggeri,[4] Ivana Pais,[5] Rosalba Schino,[6] and Mark Soto[6], *1. IBM Research, Cambridge, Massachusetts, United States, 2. Province of Vicenza (Public Administration), department of statistics, Vicenza, Italy, Italy, 3. Catholic University of the Sacred Hearth of Milan, Milan, Italy, Italy, 4. University of Padua, Department of Statistical Sciences, Padua, Italy, Italy, 5. Università Cattolica del Sacro Cuore, Milan, Italy, Italy, 6. University of Massachusetts Boston, Boston, Massachusetts, United States*

The urban-rural dichotomy underpins the common approach in studying environmental conditions influencing older adults' lives characterized by post-Second World War urban migration in both Italy and the United States (US). However, the traditional opposition urban-rural dichotomy is inadequate to study how the environmental characteristics of a geographical area can account for the heterogeneous profile of its populations and its age distribution. This study aims to overcome the traditional mobility theories as an explanatory dichotomy for understanding the distribution of the age structure of a given population. The extent of a supportive network, and the connection between the place of residence to the proximity of other residential centres can be seen as potential resources for understanding the attractiveness of certain areas for older adults. A large harmonized set of demographic and socio-economic data were collected from the Italian National Institute of Statistics (ISTAT) and the American Community Survey (ACS). An analysis at the Italian municipality- and US county-level finds the population over 75 years old are overrepresented in rural areas of both countries as would be expected by available employment opportunities, but considerable heterogeneity among both urban and rural areas exist. In particular, rural more than urban settings are based on an informal support network that is argued to rely on human proximity to produce successful aging in the community. The variation in population of older adults in rural areas, therefore, might have implications on how to achieve age-friendly communities, aside from population's traditional mobility theories and formal support network.

### PREDICTORS OF LONELINESS INCIDENCE IN CHINESE OLDER ADULTS FROM A LIFE COURSE PERSPECTIVE

Fang Yang[1] and Danan Gu[2], *1. Shanghai University, Shanghai, China, 2. United Nations, New York, United States*

Loneliness in older adults is an increasing public health issue. Research on predictors of loneliness incidence using longitudinal data in China is limited. We aim to examine what factors are predictive of loneliness incidence from a life course perspective and whether predictors differ between women and men based on a nationally representative longitudinal dataset in China. A total of 5,043 older adults aged 65 or above from the Chinese Longitudinal Healthy Longevity Survey who were not lonely in the 2008 wave were included in the analysis. Logistic regression models were applied to examine what factors in the 2008 wave predicted loneliness incidence in the 2011 wave. Analyses were also stratified by gender to examine gender differences. Older ages and self-rated poor health increased the odds of loneliness incidence, whereas receiving one or more years of schooling, rural-urban migration, living with family members, having a white-collar job, having a good family economic status, being currently married, having a higher resilience and social support decreased the odds of loneliness incidence. We also found gender differences: socioeconomic factors were significant only for older men, whereas self-rated health, resilience, and social support were significant only for older women. This study offers insights into disentangling the complexity of factors associated with loneliness incidence in Chinese older adults from a life course perspective and from a gendered perspective.

### SOCIAL ISOLATION AND OLDER ADULTS: UNDERSTANDING THEIR EXPERIENCE

Bonnie Jeffery,[1] Tom McIntosh,[1] and Nuelle Novik[1], *1. University of Regina, Regina, Saskatchewan, Canada*

This presentation will focus on a unique undertaking where three provincial organizations in Saskatchewan, Canada implemented nine projects to address social isolation for rural and urban older adults across a geography that encompasses one-half of the province. A survey of older adults was conducted to assess their level of social isolation in order to gain a more thorough understanding of the experiences of social isolation among community dwelling older adults. Key variables of interest included: older adult access to services and supports, participation in activities, feelings of being valued by others, barriers to supports and services, and the overall isolation experienced by older adults. The responses from 1,719 urban and rural older adults indicate that 24.1% of respondents felt that they lack support, 17.2% feel less connected to family and friends, and 16.8% of respondents do not feel valued by their friends and family. Overall, almost one-quarter (23.9%) of the survey respondents score 'high' or 'medium' on a Social Isolation Index. One-third of respondents report they experience barriers to participation in activities outside the home. Several key categories of barriers were identified: health, personal, environmental, social, transportation and systemic. Respondents identified accommodation, services, practices, and activities as areas where their community could assist in participation of community activities outside of the home. Social isolation can have serious health consequences for older adults. The results of this survey highlight several key areas that older adults identify as important for reducing their feelings of isolation and enhancing their overall health and well-being.

### THE EFFECTIVENESS OF A PEER-BASED INTERVENTION ON SOCIALLY ISOLATED OLDER CHINESE IMMIGRANTS IN CANADA

Daniel, W. L. Lai,[1] Jia Li,[1] Liza Chan,[2] Celia Li,[3] and Alison X. Ou[1], *1. The Hong Kong Polytechnic University, Hong Kong, Hong Kong, 2. Calgary Chinese Elderly Citizens' Association, Calgary, Canada, 3. Calgary Chinese Elderly Citizens' Association, Calgary, Alberta, Canada*

Objectives: This study aimed to examine the effectiveness of a peer-based intervention in reducing older Chinese immigrants' loneliness and social isolation to improve their psychosocial well-being. Method: A randomized controlled trial design was adopted. A sample of 60 community-dwelling older Chinese immigrants aged 65 and above were randomly assigned to the intervention group (n=30) and the control group (n=30). Intervention group participants received an eight-week peer support intervention. 25 volunteers aged 48 to 76 were recruited and trained to provide one-to-one peer support services through home visits and telephone. The services included multiple activities such as providing emotional support, assisting in problem-solving, and community resources sharing. Different types of activities were consecutively executed throughout the eight weeks in accordance with the service protocol. Standardized assessments including loneliness, social support, social participation, and other psychosocial outcomes such as life satisfaction, happiness, depression, and resilience at baseline and after intervention were measured. Results: After the intervention, as compared to control group participants, intervention group participants reported a significant decrease in loneliness, fewer barriers in social participation, and a significant increase in resilience. They also reported fewer depressive symptoms, increased life satisfaction, and happiness, but no such improvements were observed in the control group. Discussion: The study findings illustrate the need to further examine the use of peer-based interventions for both program effectiveness and delivery efficiency. In the era of population aging, baby boomers can be trained to take up more volunteer roles to serve older adults in distress via peer-based intervention approaches.

### WEALTH AND HEALTH IN PREDICTING ELDERS' SOCIAL CAPABILITIES IN CHINA: MEDIATING ROLE OF SOCIAL NETWORK

Yalu Zhang,[1] Qin Gao,[1] Fuhua Zhai,[2] and Paul Anand[3], *1. Columbia University, New York, New York, United States, 2. Fordham University, New York, New York, United States, 3. The Open University, Milton Keynes, United Kingdom*

Despite an established positive link between social wealth, health, and social capability among older adults, the effect and mechanisms among these factors are understudied. This paper uses the WHO Study on Global Ageing and Adult Health (SAGE) data and a mediation analysis method, combing social capital theory and a social capabilities approach, to provide new evidence on the effects of financial resource, physical function, and cognitive function on the social capabilities of older adults (aged 55 and above) in China and the possible mediating role of social network in this relationship. The descriptive analysis results show that urban older adults (n=5,274), on average, had lower freedom of expression, lower sense of living safety, and less frequent community participation, while having better self-perceived health, higher physical and cognitive functions, more household income, and higher educational background than their rural peers (n=5,270). The Baron and Kenny's mediation analysis results show that social networks accounted for a substantial proportion of the effects of wealth and health on social capabilities, but wealth and health still had strong, positive direct effects of its own. Higher mediating effects of social networks were found in the association between functions and social capabilities of freedom of expression (9.46%) and sense of safety (36.33%) among rural older adults. Results of this study urge for further social policies and intervention programs to enhance older adults' social capabilities, including social cohesion, sense of trust and safety, physical and mental functioning, and subjective well-being.

### SESSION 1520 (SYMPOSIUM)

### TARGETING THE MTOR NETWORK TO MAXIMIZE HEALTHSPAN

Chair: Viviana I. Perez, *Linus Pauling Institute, Oregon State University, Corvallis, Oregon, United States*

The mechanistic target of rapamycin (mTOR) is a key nutrient and growth factor-responsive pathway that has emerged as a central regulator of aging. Inhibition of mTOR complex I in particular has been found to increase lifespan and improve functional declines during aging in yeast, worms, flies, and mice. Recently clinical studies have provided the first evidence that similar effects may be achievable in pet dogs and in elderly people. This session will focus on new discoveries related to mTOR signaling and aging.

### MECHANISMS OF NEUROPROTECTION BY MTOR INHIBITORS

Veronica Galvan[1], *1. Barshop Institute for Longevity and Aging Studies, San Antonio, Texas, United States*

The mammalian/mechanistic target-of-rapamycin (mTOR) inhibitor rapamycin, that delays aging in mice, halts and even reverses memory deficits, and restores cerebral blood flow (CBF), neuronal activation, and neurovascular coupling in models of Alzheimer's disease (AD), cognitive dysfunction of atherosclerosis, and normative aging. Genetic reduction of TORC1 in neurons to levels similar to those achieved by rapamycin, promoted synaptic bouton remodeling, enhanced memory, and increased brain glucose metabolism. In AD mice, the restoration of CBF and neurovascular coupling by mTOR attenuation was dependent on the activation of both constitutive nitric oxide synthase (NOS) isoforms, possibly due to stabilization of their mRNAs. The mechanisms by which mTOR attenuation preserves brain healthspan may be common to different models of age-associated neurological disease. We singled out (a) ablation of NOS activity, and (b) synaptic bouton loss as key mechanisms by which TOR drives brain aging and contributes to the pathogenesis of dementias modeled in mice.

### SEX AND TISSUE SPECIFIC ROLES OF MTORC2 IN HEALTHSPAN, METABOLISM, AND SURVIVAL

Dudley W. Lamming[1], *1. University of Wisconsin-Madison, Madison, Wisconsin, United States*

Genetic and pharmacological inhibition of the mechanistic Target Of Rapamycin Complex 1 (mTORC1) promotes health and longevity in organisms ranging from yeast to mice, and may also have rejuvenative effects in dogs and humans. A potential barrier to the translation of rapamycin-based therapies to the clinic are the side effects of rapamycin, which include metabolic disruption. We and others have demonstrated that many of these side effects are mediated not by inhibition of mTORC1, but by "off-target" inhibition of a second mTOR complex, mTORC2. However, the effect of inhibiting mTORC2 on the healthspan and longevity of mammals has been largely unaddressed. Here, we will discuss our research exploring the contribution of mTORC2 signaling in three different tissues to the healthspan, metabolism, and longevity of mice.

### ANTI-CELL SENESCENT EFFECTS OF RAPAMYCIN AND THEIR ROLE IN DISEASES, INCLUDING ALZHEIMER'S

Viviana I. Perez[1], *1. Linus Pauling Institute, Oregon State University, Corvallis, Oregon, United States*

Senescent cells contribute to age-related pathology and loss of function, and their selective removal improves physiology and extends longevity. We have shown that deficiency in Nrf2 in young Nrf2KO mice leads to an increase in senescent cells, with increased levels of pro-inflammatory cytokines in various tissues, including the brain. Both the cellular senescence and SASP decrease significantly when these mice are treated with rapamycin. Our current work focuses on determining whether cellular senescence contributes to premature AD-like pathogenesis in mouse models of AD. Indeed, it has been shown that Nrf2 deficiency in mouse models for AD leads to exacerbated pathology, suggesting that increased inflammation, due in part to the increased SASP-producing senescent cells, might be contribute to this phenotype. We are testing whether the burden of senescent cells in the brain on mouse models of AD can be reverted by rapamycin.

### LOW TRANSLATION DOWNSTREAM OF TOR ACTIVATES MYOGENE EXPRESSION AND ENHANCES HEALTH OF BODY MUSCLE IN C. ELEGANS

Juyoung K. Shim,[1] Santina Snow,[2] Amber Howard,[2] Jordan Horrocks,[2] Joshua Kelley,[3] Hussein Sayed,[2] and Aric Rogers[2], *1. MDI Biological Laboratory, Bar Harbor, Maine, United States, 2. MDI Biological laboratory, Salisbury Cove, Maine, United States, 3. University of Maine, Orono, Maine, United States*

Previous studies reported that DR or low nutrient signaling reduces translation and extends lifespan, while attenuating growth and reproduction. In this study using C. elegans as a model organism, we found that reduced translation in neurons or germ tissues increases survival and primes the HSR against unfolded protein stress in adult animals. Surprisingly, lowering translation in these tissues significantly upregulates transcription of several key muscle maintenance genes, including those encoding the myogenic response factor HLH-1 and heavy myosin chain factor UNC-54 and MYO-3. It also improves muscle maintenance according to motility assays. However, reduced translation in muscle tissue does not activate the same level of myogenic gene expression. Instead, lowering translation in striated body muscle results in increased reproduction and accelerated development. To further investigate, we examined maintenance of muscle health via deconvolution fluorescent microscopy and immunostaining. Using computational analyses involving Fiji ImageJ and MATLAB program, we discovered that lowering translation delays decrepitude in body muscle structure, resulting in improved mitochondrial morphology and increased thermotolerance. Here we report that, combining physiological, molecular, and microscopic technique, muscle is protected during low translation, low TOR signaling, or DR. We hypothesize that results reflect evolutionary responses that preserve function required for foraging during nutrient scarcity and accelerate development during times low energy usage and high nutrient abundance.

### SESSION 1525 (SYMPOSIUM)

### TECHNOLOGY-BASED HOME MONITORING TO ASSESS AND IMPROVE THE HEALTH AND WELL-BEING OF OLDER ADULTS

Chair: Walter R. Boot, *Florida State University, Tallahassee, Florida, United States*

Technology, including in-home sensors, monitoring systems, and wearables, holds great promise with respect to being able to help manage chronic conditions, improve older adults' health, support their safety, prolong independence, and collect data for healthcare and research purposes. This symposium will introduce novel ways in which these systems are being deployed, and also important challenges to their implementation that could decrease their potential benefits. This technology will only be successful if it addresses key issues of adherence and privacy, and considers users' preferences. The first talk in this session will explore the novel use of GPS-based wearable sensors to capture the concept of life-space mobility, a measure critically related to quality of life and independence. The next talk focuses on a mobile health cardiovascular system and smartphone application, and finds that such systems can successfully transmit meaningful mHealth data while maintaining reasonable protocol adherence. Next, issues of user preference are discussed, including the types of data older adults and their children are willing to share, and the tradeoffs between values of privacy and independence. The session will conclude with a talk on older adults' attitudes toward privacy related to in-home monitoring, and individual difference predictors of privacy concerns. In sum, this session will provide an overview of the potential and pitfalls related to in-home sensors and monitoring systems for older adults, their formal and informal caregivers, and aging researchers.

### USING GPS-BASED WEARABLE SENSORS TO CAPTURE LIFE-SPACE MOBILITY AMONG COMMUNITY-DWELLING OLDER ADULTS

Jane Chung[1] and Orrin Myers[2], *1. Virginia Commonwealth University School of Nursing, Richmond, Virginia, United States, 2. University of New Mexico School of Medicine, Albuquerque, New Mexico, United States*

Life-space mobility (LSM) is critical to quality of life among older adults. Due to the limitations of current mobility measures, GPS-based sensors are suggested as a potential tool that can collect high-resolution spatial and temporal data on mobility. We aimed to examine the feasibility of using a GPS watch to measure LSM among older adults. Participants were asked to use the device for 8 hours a day for three days. GPS data were analyzed with QGIS and SAS. GIS measures were used to characterize LSM. Participants walked 3.2km/day and moved 67km on average. Nearly all movements at home were < 0.8 m/s, indicating slower gait speed. GPS data suggest that community-dwelling older adults made active trips outside the home, but they were tightly tethered to their residential environments and spent most of their time at home. GPS-based sensors may be particularly beneficial to continuously monitor any changes in elderly mobility.

### FRAMINGHAM HEART STUDY NOVEL EXAMINATION USING TECHNOLOGY IN COMMUNITY-DWELLING ADULTS

Joanne Murabito,[1] Joanne M. Murabito,[2] Ludovic Trinquart,[3] Emelia J. Benjamin,[4] Honghuang Lin,[5] Nicole Spartano,[4] Mayank Sardana,[6] and David D. McManus[7], *1. Boston University, Boston, Massachusetts, United States, 2. Boston University School of Medicine, Boston, Massachusetts, United States, 3. Boston University School of Public Health, Boston, Massachusetts, United States, 4. Boston University School of Medicine, Framingham, Massachusetts, United States, 5. Boston University, Framingham, Massachusetts, United States, 6. University of Calfornia San Francisco, San Francisco, California, United States, 7. University of Massachusetts Medical School, Worcester, Massachusetts, United States*

The electronic Framingham Heart Study (eFHS) is a mobile health cardiovascular monitoring system deployed at the current in-person exam. We developed a smartphone app which includes an electronic consent, health questionnaires, and integrates with a wireless blood pressure (BP) cuff and Apple Watch. We have deployed the app in 1927 FHS participants (mean age 52.8, range 32-80 years, 57% women, 7% multi-ethnic) who owned a compatible smartphone (iPhone or Android). We have observed high rates of initial survey adherence (85% completed baseline surveys). Preliminary results demonstrate about 76% of participants who transmit data from the Apple Watch continued to transmit data 12 weeks following enrollment. Our experience highlights the capacity of FHS participants to transmit meaningful mHealth data. We plan to identify factors associated with long-term use and to compare digital data to data collected in the Research Center to understand the value of this novel means of data collection.

### DO OLDER ADULTS AND THEIR CHILDREN AGREE ABOUT THE USE OF LOCATION TRACKING, IN-HOME SENSORS, AND WEB-CAMERAS?

Clara Berridge[1], *1. University of Washington, Seattle, Washington, United States*

On the dyad and aggregate level, we compare preferences of older adult women and their adult children for three remote monitoring technologies: location tracking, in-home sensors, and Web-cameras. Their assessments of each technology's impact on privacy, safety, independence, freedom, relationship with family member, social life, and identity are also compared. Twenty-eight individual, in-depth structured interviews were conducted with 18 women who are Meals on Wheels clients and 10 of their adult children. Adult children preferred each technology more than their mothers did and underestimated both their mothers' ability to comprehend the functions of the technologies and the importance of engaging them fully in decision making. For both groups, privacy was the most-cited concern, and participants perceived significant overlap between values of privacy, independence, identity, and freedom. Shared decision-making tools are needed to promote remote monitoring use consistent with older adults' values and to prevent conflict and caregiver overreach.

### IN-HOME MONITORING AND PRIVACY CONCERNS AMONG OLDER ADULTS

Moon Choi[1], *1. KAIST Graduate School of Science and Technology Policy, Daejeon, Korea, Republic of*

This study aimed to contribute to understanding older adults' perspectives on privacy related to in-home monitoring by analyzing factors influencing the willingness to share their information collected by in-home monitoring systems. Study participants were interviewed at local senior centers in South Korea (N=170; mean age=77; 45.9% women). One out of eight (13.5%) participants did not want to share their information with anyone. Participants were more inclined to share their information with family and hospitals than with researchers, government agencies or insurance companies. The logistic regression results showed that participants living with others were more likely to share their information of vital signs, medication, and toileting compared to those living alone (ORs=20.5, 5.3, 4.5, respectively). Younger age and advanced ability to use the Internet were negatively associated with the willingness to share, overall. Information granularity and socioeconomic characteristics are important factors to consider when developing in-home monitoring systems for older adults.

### SESSION 1530 (SYMPOSIUM)

### POLICY SERIES: THE HRSA GERIATRICS EDUCATION INITIATIVES: A POLICY AND PROGRAM UPDATE

Chair: Catherine Carrico, *University of Wyoming, Laramie, Wyoming, United States*

Co-Chair: Kathryn Hyer, *University of South Florida, Tampa, Florida, United States*
Discussant: Brian W. Lindberg, *The Gerontological Society of America, Washington, District of Columbia, United States*

This session will address the evolving work of Geriatric Workforce Enhancement Programs (GWEP) to educate and train the primary care workforce and partner with community based organizations to address gaps in healthcare. The inaugural GWEP awardees were announced at the 2015 White House Conference on Aging. In November 2018 the Health Resources and Services Administration (HRSA), released the second GWEP Notice of Funding Opportunity (NoFO). The new funding opportunity reflects current policy priorities. In addition, for the first time in 9 years a Notice of Funding Opportunity was released for the Geriatric Academic Career Award (GACA) program. The return of the GACA program indicates the success of advocacy efforts to increase programs to support geriatric training and the development of geriatric academic professionals. Individual symposiums will explore the policy priorities reflected in the GWEP NoFO including the use of technology for training and care delivery, the age-friendly healthcare and dementia-friendly community initiatives, and value-based care including improving partnership with community based organizations. Additionally, an individual symposium will present the GACA program and discuss the advocacy process leading to the reintroduction of this program. During the last congress advocates of the GWEP and GACA programs supported authorization and funding of geriatric programs. Geriatrics programs describing the work of the GWEPs and GACAs were reinserted into the legislative process, although this reauthorization was not completed. This advocacy process garnered strong bipartisan and agency support. The session will describe these efforts and will conclude with an update on current authorization and appropriation status.

### AN UPDATE ON THE RETURN OF THE GERIATRICS ACADEMIC CAREER AWARDS PROGRAM

Leland Waters[1] and Maura Brennan[2], *1. Virginia Commonwealth University, Richmond, Virginia, United States, 2. University of Massachusetts Medical School-Baystate, Springfield, Massachusetts, United States*

The Health Resources and Services Administration recently announced a notice of funding opportunity (NOFO) for the Geriatrics Academic Career Award Program (GACA). The purpose of the GACA program is to support the career development of individual junior faculty in geriatrics as academic geriatrics specialists and to provide clinical training in geriatrics, including the training of interprofessional teams of health care professionals. The GACA program is a mentorship model where the awardee is guided through a faculty career development plan, with required project deliverables, over the course of four years. The GACA supported 222 junior faculty members between 1998 and 2006, when it was discontinued. This initial NOFO provides funding for up to 26 awardees in the schools of allopathic medicine, osteopathic medicine, nursing, social work, psychology, dentistry, pharmacy, and allied health. This presentation will provide an up-to-date overview of the program with awardee demographics and project deliverables.

### INTEGRATING PRINCIPLES OF VALUE-BASED CARE THROUGH COMMUNITY-BASED ORGANIZATION PARTNERSHIPS

Marla Berg-Weger[1] and Catherine Taylor[2], *1. Saint Louis University, St. Louis, Missouri, United States, 2. The George and Anne Ryan Institute for Neuroscience The Rhode Island Geriatric Education Center The University of Rhode Island, Providence, Rhode Island, United States*

To develop programming consistent with value-based care principles, two GWEPs share strategies and policy implications when partnering with community-based organizations (CBO). Because the aims of value-based care – better patient outcomes and population health, reduced health care costs – cannot be achieved by changing medical practice alone, RI Geriatric Education Center (RIGEC) partners with multiple CBOs to deliver evidence-based patient education programming to empower patients/caregivers to take charge of their health. RIGEC will work directly with physician practices to bring education directly to their high-risk patient census. Saint Louis University CBO partners integrated geriatric assessment into routine care to identify geriatric syndromes. Introduction of protocols for Medicare Annual Wellness Visits, Cognitive Stimulation Therapy and exercise/strengthening enables CBOs to offer interventions. Value-based care is achieved by reducing costs as geriatric syndromes are identified earlier, enabling diagnosis, treatment, and management; improving patient outcomes and satisfaction; and strengthening provider competence in geriatric care.

### GWEPS WORKING TO SUPPORT THE AGE-FRIENDLY HEALTHCARE SYSTEM MOVEMENT

Tara A. Cortes,[1] Cinnamon St.John,[2] and Jeff Lucas[3], *1. Hartford Institute for Geriatric Nursing at NYU Rory Meyers College of Nursing, New York, New York, United States, 2. Hartford Institute for Geriatric Nursing, United States of America, New York, United States, 3. RAIN, Bronx, New York, United States*

Age friendly health systems aim to help people age and die with dignity. As this social movement progresses it is important to remember the community as a critical stakeholder. To ensure their engagement requires input from the community to understand their needs and education of the community to empower people to know what standard should be expected from an age friendly health system. The NYU GWEP has trained 140 community volunteers who have educated >4,500 older adults in the Bronx on healthy behaviors and chronic disease management. 85% responded that they have changed their behaviors as a result of the teaching. Qualitative data reveals that people feel they can now talk to their primary care provider in a meaningful way about what matters to them, the medications they take, their mental state, and mobility.

### FOSTERING DEMENTIA-FRIENDLY COMMUNITIES AND SUPPORTING CAREGIVERS THROUGH THE GWEP PROGRAMS

Jennifer J. Severance,[1] Lisa O'Neil,[2] and Mindy Fain[3], *1. University of North Texas Health Science Center, Fort Worth, Texas, United States, 2. University of Arizona,*

*Tuscon, Arizona, United States, 3. University of Arizona, Tucson, Arizona, United States*

As the number of people affected by dementia increases, it is essential that caregivers are provided resources and communities educated on how to engage and support people living with dementia. Supporting caregivers and providing education about dementia-friendly communities are key components of the Geriatric Workforce Enhancement Programs. This symposium will provide examples of GWEP projects which foster dementia-friendly communities and support caregivers. Additionally, recent and current legislation targeting dementia and caregivers will be presented.

### HUBS OF INNOVATION: GWEP UTILIZATION OF TECHNOLOGY AND INNOVATION FOR TRAINING AND SERVICE DELIVERY

Thomas V. Caprio,[1] Katherine A. Bennett,[2] Greg O'Barr,[3] Catherine P. Carrico,[4] and Christine McKibbin[5], *1. University of Rochester, Rochester, New York, United States, 2. University of Washington, Seattle, Washington, United States, 3. Cheyenne Regional Medical Center, Cheyenne, Wyoming, United States, 4. University of Wyoming, Laramie, Wyoming, United States, 5. University of Wyoming, Laramie, Wyoming, United States*

Technology offers powerful tools for meeting the increasing needs of older adults and caregivers. The Geriatric Workforce Enhancement Program recognizes the important role technology will play in service delivery and training by fostering innovation to meet local training and healthcare needs. This symposium will review the innovative models and strategies GWEPs are utilizing to improve care including Project ECHO, remote monitoring, and telehealth. The policy implications of these models will be presented, including current legislative action to support technology and innovation in the care of older adults.

### SESSION 1535 (SYMPOSIUM)

### INTEREST GROUP SESSION—LONELINESS AND SOCIAL ISOLATION: THE LANGUAGE(S) OF LONELINESS

Chair: Christina Victor, *Brunel University London, Uxbridge, Middlesex, United Kingdom*
Discussant: Kimberley Smith, *University of Surrey, Guildford, Surrey, Canada*

We offer a novel perspective on the burgeoning literature focused on loneliness later life by examining the language(s) used to describe, define and depict loneliness. We have an extensive body of work describing the prevalence of , 'vulnerability factors' and consequences of loneliness in later life. These activities start with pre-defined concepts of what loneliness is and often use scales and questions which may/may not use the term loneliness. How well does the contemporary language of loneliness used in research, policy, practice and the media really capture the depth and complexity of what people are experiencing? Do the terms and words use in our measurement scales and quantitative research resonate with this vocabulary? In qualitative research interviews how do older adults talk (or avoid talking) about loneliness? How does the media talk about loneliness and what images does this convey about later life? We will address these three issues in our seminar. Using data from qualitative interviews undertaken as part of a mixed methods study of temporal variations in loneliness, Thomas uncovers the strategies participants used to talk or avoid talking about loneliness. Victor uses qualitative data from 12,000 adults aged 60+ collected as part of the BBC loneliness experiment to examine the terms used to describe loneliness and to identify both the opposite of loneliness and the positive aspects of loneliness. Sullivan exposes how loneliness is constructed in print and digital media over a 10-year period in the UK and Canada and its role in framing the loneliness problem.

### WHAT IS LONELINESS: INSIGHTS FROM THE BBC LONELINESS EXPERIMENT

Christina Victor,[1] Pamela Qualter,[2] and Manuela Barreto[3], *1. Brunel University London, Uxbridge, Middlesex, United Kingdom, 2. University of Manchester, Manchester, England, United Kingdom, 3. University of Exeter, Exeter, England, United Kingdom*

Older peoples' views on what defines loneliness are conspicuous by their absence. The BBC Loneliness Experiment included 3 free-text questions which aimed to address this gap in our knowledge. Participants were asked to define what loneliness meant to them; their understanding of the opposite of loneliness and if loneliness could be positive and why. There were 55,000 survey responses:12,000 aged 60+. The 'top five' loneliness definition were: having no one to talk to; feeling disconnected from the world; feeling left out; sadness and feeling misunderstood. The most common terms used to describe the opposite of loneliness were: being connected; contentment with social relationships; happiness; friendship and availability of people. Almost 50% reported that loneliness could be positive as did 16% of those who were often/always lonely with the reasons given for this including opportunities for personal growth, the enjoyment of being alone and knowledge that the feeling would pass

### "BUT WE ARE NOT POWERLESS AGAINST THIS PROBLEM": OLDER PEOPLE, LONELINESS AND THE MEDIA

Mary Pat Sullivan[1], *1. Nipissing University, North Bay, Ontario, Canada*

Media campaigns play a critical role in framing public perceptions or 'public talk' around social issues. The media's role in characterizing the loneliness 'problem' is, however, an under explored area. This paper presents the language of loneliness and loneliness representations in the media in Canada and England over a 10-year period (2009-2018) and their relationship with key policy initiatives specific to an ageing population. Using qualitative content analysis, the findings illustrate the use of skilled marketing techniques and highly stigmatizing discourse. These media approaches act to: (1) reinforce the threat of an ageing population; (2) endorse responsibilization and governmentality of the body; and (3) promote individual and/or family shame and morally responsible actions by charities and volunteers. We conclude that there is a need for a critical analysis of loneliness from the perspective of social and cultural constructions of ageing, the positioning of older people in society, and neo-liberalist ideology

### TALKING ABOUT LONELINESS

Michael Thomas[1], *1. Brunel University London, Uxbridge, United Kingdom*

his paper draws on insights from a mixed methods study on temporal variations in loneliness among older people (aged 50 years plus) in England. The paper highlights alternative strategies available to participants in research interviews, including engaging with personal experience of loneliness or avoidant tactics such as digression, projection onto others or claiming ignorance of the topic. The data point towards complicating factors including a reluctance to disclose loneliness as a socially undesirable phenomenon, as well as difficulties in articulating the complexities of this topic. These considerations remind researchers of the need for reflection on the use of qualitative interviews when investigating loneliness and other difficult aspects of personal experience.

### SESSION 1540 (SYMPOSIUM)

### INTEREST GROUP SESSION—AGING WORKFORCE: WORK AND PRODUCTIVE AGING: OPPORTUNITIES AND CHALLENGES

Chair: Jim Emerman, *Encore.org, San Francisco, California, United States*
Co-Chair: Cal J. Halvorsen, *Boston College School of Social Work, Chestnut Hill, Massachusetts, United States*
Discussant: Jim Emerman, *Encore.org, San Francisco, California, United States*

With much of the world experiencing population aging and a strong need—and desire—among many approaching later life to work longer than past norms, individuals and institutions are experimenting with new ways of working. Yet given the complexities of navigating the work environment in later life, including aspects of cumulative (dis)advantage that help or hinder one's work prospects, the pull to socially impactful work in the nonprofit sector, and the day-to-day experience of such work in later life, outcomes from this work can vary. Consequently, this symposium will focus on the challenges and opportunities of working longer and their relevance to a productive aging model. The first paper will provide a framework for engaging in the conversation on productive engagement in later life. It will give particular consideration to older workers with lower levels of socioeconomic status in OECD countries. The second paper will discuss results from more than 1,400 surveys of fellows and organizational hosts that have participated in the Encore Fellowships Network, which matches mid- and late-career workers (typically corporate retirees) to non-profit organizations seeking their skills and experience. The third and final paper will reveal findings from an experience sampling methods study of two groups of older adults over the age of 60: founders or leaders of social purpose organizations, and older volunteers. We will conclude by facilitating a discussion on ideas for future scholarship on longer working lives, with particular emphasis on individuals with less advantage as well as those pursuing social purpose work.

### CAN PRODUCTIVE AGING BE MADE TO WORK FOR ALL WORKERS?

Philip Taylor[1], *1. Federation University Australia, Melbourne, Victoria, Australia*

This paper considers the changing status of older workers in Organisation for Economic Co-operation and Development (OECD) countries and addresses the questions of if and how they can be supported to age productively. This paper questions the utility of the dominant pro-work policy framework which, while ostensibly aimed at all older workers, mostly benefits those for whom greater choice around work and retirement was always available. Analysis of OECD data concerned with employment and unemployment demonstrates that, for a significant proportion of older workers, choice in terms of labor force participation is severely constrained, with potentially adverse consequences for the transition to old age. The soundness of the pro-work agenda is challenged and it is argued that older people's advocacy, in particular, has an important role to play in offering a vision of what it means to grow old successfully that is not limited by narrow conceptions of productivity.

### THE ENCORE FELLOWSHIPS PROGRAM: FINDINGS FROM HOST AND FELLOW FOLLOW-UP EVALUATIONS

Cal J. Halvorsen[1] and Jim Emerman[2], *1. Boston College School of Social Work, Chestnut Hill, Massachusetts, United States, 2. Encore.org, San Francisco, California, United States*

The Encore Fellowships Network (EFN) began as a pilot program in 2009 to match mid- and late-career for-profit employees in the San Francisco Bay Area to non-profit organizations seeking their skills and experience. After a successful pilot, the EFN expanded nationally, reaching 26 states and placing close to 2,000 Fellows in 10 years. This presentation will reveal analysis from more than 1,400 linked surveys of fellows and their organizational hosts both pre- and post-fellowship, totaling more than 350 observations. Results indicate a high sense of program satisfaction and enduring impact on the work of the non-profits. Further, the efficiency of the matching process was positively associated with fellows' recommendation of the program to friends or colleagues and a higher sense of enduring impact among fellows was positively associated with the likelihood of pursuing post-fellowship non-profit work. Implications for the EFN and the broader nonprofit sector will be discussed.

### THE DAY-TO-DAY EXPERIENCES OF LATER-LIFE PROSOCIAL WORK: AN EXPERIENCE SAMPLING METHOD STUDY

Jeanne Nakamura,[1] Dwight C.K. Tse,[1] Ajit Mann,[1] Laura Graham,[1] Jordan Boeder,[1] and Kelsey Finley[1], *1. Claremont Graduate University, Claremont, California, United States*

As one form of productive aging, many older adults undertake significant prosocial activity. Alongside its contribution to the welfare of others, prosocial activity has been linked to a variety of positive outcomes for those undertaking it (e.g., higher life satisfaction). However, little is known about the impact of this activity on older adults' day-to-day lives. We studied a national sample of about 150 older adults who give back to their communities on a regular basis in one of two ways: either by playing leadership roles in social-purpose organizations or as more traditional volunteer workers in such organizations. We employed the experience sampling method to describe and compare the experience of prosocial activity during a typical week for these two groups of highly engaged adults. While prosocial activity carries both costs and rewards, these show differences as well as similarities for the two groups. Implications for research and practice are discussed.

### SESSION 2000 (SYMPOSIUM)

### INTEREST GROUP SESSION—INTERNATIONAL AGING AND MIGRATION: AGING SOCIETY: INDIVIDUAL, FAMILY, AND COMMUNITY COLLABORATION FROM A GLOBAL PERSPECTIVE

Chair: Noriko Toyokawa, *California State University San Marcos, San Marcos, California, United States*
Discussant: Vivian W. Lou, *The University of Hong Kong, Hong Kong, P.R.C., Hong Kong*

The purpose of this symposium is two-fold: (1) to promote mutual understanding and communication regarding the long-term caregiving plans for older immigrants/refugees between policymakers and researchers in the fields of behavioral and social sciences and (2) to discuss the needs for connecting local needs of older immigrants/refugees and their families with a global plan for aging society. The symposium is structured by three empirical studies on older adults and their caregivers by behavioral and social sciences researchers, followed by a presentation of the needs for an international convention on the rights of older people by an advocate in the global network to promote older people's health. Miyawaki and colleagues focus on the residential status quo, family relations, and prevalence of chronic diseases among older Vietnamese refugees in Houston, TX, U.S.A. Liu's qualitative study on Taiwanese professional caregivers' perceptions of clients in adult daycare services reveals the relation between staff's negative image of aging and their practice. Toyokawa conceptualizes middle-aged Mexican immigrants' sense of family obligation, as their obligation for reducing children's caregiving burden and the endorsement predicts their well-being. Three presenters point out the need for standards for basic needs of refugees/immigrants, staff training, and the quality of long-term care, and discuss the meaning of culturally sensitive support based on their studies. Finally, Marumoto advocates the need for an international convention of the rights of older people and standardization of the quality of long-term care. Specific approaches to 'harness the network' between local and global efforts are discussed.

### I WILL KEEP CONTRIBUTING TO MY FAMILY: FAMILY OBLIGATION AMONG OLDER ADULTS WITH MEXICAN IMMIGRANT BACKGROUNDS

Noriko Toyokawa[1], *1. California State University San Marcos, San Marcos, California, United States*

Providing care of older parents is a family obligation for children with Mexican cultural contexts (Knight et al., 2010). Nevertheless, little is known about how parents with Mexican cultural backgrounds believe about their family obligations. The current study conceptualized Mexican American older adults' sense of family obligation. Data was collected from 307 Mexican Americans (Mage=54, SD=8, range 45-77 years old, females=56%) through an online survey. A 2-factor model: Expectation on children's caregiving (3-item) and Efforts to reduce children's burden (7-item) were identified as the best-fit model through EFA and CFA analyses (CFI=.96, SRMSA=.4). The component of efforts to reduce children's burden predicted participants' generativity assessed by the scale of McAdams and Aubin (1992). The findings suggest that Mexican American older adults expect their children to take care of them, as they feel obligated to reduce their children's caregiving burden. The function of the cultural value in intergenerational relations is discussed.

### WHY DO WE NEED AN INTERNATIONAL CONVENTION ON THE RIGHTS OF OLDER PEOPLE?

Mika Marumoto[1], *1. HelpAge International, London, United Kingdom*

This presentation discusses the increasing need for an international convention on the rights of older people. Such a convention would contextualize global, regional and national demographic shifts and identify gaps in existing international human rights laws, so as to better protect older persons' rights to health and well-being. Persons aged 60 or above are expected to more than double from 2015 to reach 2 billion in 2050, with their proportion of the world population rising from 12% to 21%. By 2050, 80% of older persons are expected to live in societies that are currently labeled developing countries. Existing international human rights instruments fall short regarding pensions and protection from poverty. The presentation demonstrates ongoing global efforts, specifically through the UN Open-ended Working Group on Ageing, to set global rights-based standards, and the roles played by civil society organizations that use network approaches in advocating for the rights of older people.

### HEALTH STATUS OF OLDER VIETNAMESE REFUGEES: RESULTS FROM THE VIETNAMESE AGING AND CARE SURVEY (VACS)

Christina E. Miyawaki,[1] Nai-Wei Chen,[2] Oanh L. Meyer,[3] Mindy Thy Tran,[4] and Kyriakos S. Markides[5], *1. Graduate College of Social Work, University of Houston, Houston, Texas, United States, 2. Beaumont Research Institute, Beaumont Health, Royal Oak, Michigan, United States, 3. University of California, Davis, Sacramento, California, United States, 4. University of Houston, Houston, Texas, United States, 5. University of Texas Medical Branch, Galveston, Texas, United States*

Over 1.3 million Vietnamese including refugees migrated to the U.S., after the Vietnam War. Vietnamese are the 4th largest Asian ethnic group in the U.S. Despite the number, little is known about their health conditions. To fill this gap, the Vietnamese Aging and Care Survey (VACS) was developed, and sociodemographic and health data on 132 refugees (≥65 years) were collected in Houston, Texas. They were on average 75.4 years-old, retired (77%), married (58%), female (55%) with less than high school education (86%) in poor/fair health (76%). They immigrated around age 49 years-old, and have hypertension (74%), arthritis (48%), and diabetes (41%). They manage their lives by living in a multi-generation tightly-knit enclaves, and show resilience to their low sociodemographic status (≤25K, 94%).

Findings suggest healthcare professionals to introduce more social services such as adult daycare programs in culturally-sensitive ways to ease their transition to new lives in the U.S.

### PROFESSIONAL CAREGIVERS' PERCEPTIONS OF PEOPLE WITH DEMENTIA AT ADULT DAY SERVICES IN TAIWAN: THE EFFECTS ON CLIENTS

Chih-ling Liou[1], *1. Kent State University, North Canton, Ohio, United States*

This study aims to explore staff perception of their clients with dementia in five ADS centers in Taiwan. Focused ethnography was used to collect data from 45 individual interviews with nurse's aides, nurses, social workers, housekeepers, volunteers, bus drivers and centers' directors and 600 hours of field observations. The findings from the content analysis revealed two themes reflecting ADS staff's attitudes towards clients: (1) labeling the clients as "old" or "sick" to distance themselves from the clients and (2) viewing the clients as their aging relatives and over-helping them. Both attitudes not only affected staff-client relationships but also influenced how clients viewed themselves by inducing self-labeling of incompetent and dependent. More dementia-specific and communication training is needed for staff at ADS centers in Taiwan to change their attitudes and behavior toward clients with dementia. With a more positive attitude of people with dementia, the prerequisites for person-centered care will improve.

### SESSION 2005 (SYMPOSIUM)

### INTEREST GROUP SESSION—MEASUREMENT, STATISTICS, AND RESEARCH DESIGN: ANALYSIS OF DEVELOPMENTAL AND COMPLEX SYSTEMS: UNDERSTANDING THE DYNAMICS OF AGING

Co-Chair: Xiao Yang, *Pennsylvania State University, University Park, Pennsylvania, United States*
Co-Chair: Nilam Ram, *Pennsylvania State University, University Park, Pennsylvania, United States*
Discussant: Nilam Ram, *Pennsylvania State University, University Park, Pennsylvania, United States*

Aging is the product of numerous dynamic processes that span multiple domains of functioning (e.g., biological, psychological, social), multiple levels of analysis, and multiple time-scales. Scientific inquiry in many fields has benefited from articulation and analysis of complex systems. This symposium brings together a collection of papers that illustrate how dynamical systems modeling is contributing to both theory and understanding of aging. Yang and colleagues apply Boolean network approach to intensive longitudinal data to identify sequences of emotion and behavior that lead to a stable equilibrium, and suggest how that information can be used to design interventions that push individuals toward a healthier equilibrium. Rector and colleagues illustrate use of dynamic indicators and multiscale entropy measures as indicators of resilience and explain how those measures may be used in prediction of physical recovery. Brick highlights how sequence mining methods can be used to identify commonalities and differences in dynamic change, and how those patterns characterize and distinguish groups with respect to aging trajectories. Moulder and colleagues demonstrate how latent maximum Lyapunov exponents can be used to study sensitivity of individuals' developmental trajectories to initial conditions. Boker and colleagues provide a general overview of how dynamic models, including an adaptive equilibrium regulation model, distinguish resilience to acute versus chronic stressors and patterns of regulation. Together these papers highlight the value complex system thinking can add to our understanding and optimization of aging.

### OPTIMIZING INDIVIDUALS' DAILY FUNCTION: A BOOLEAN NETWORK APPROACH FOR IDENTIFYING INTERVENTION SEQUENCES

Xiao Yang,[1] Xiao Yang,[1] Nilam Ram,[1] David Conroy,[1] Aaron Pincus,[1] and Denis Gerstorf[2], *1. Pennsylvania State University, University Park, Pennsylvania, United States, 2. Humboldt-Universität zu Berlin, Berlin, Berlin, Germany*

Development and aging are the product of a process wherein an individuals' functional components co-act to produce change. System dynamics can be described using a variety of methods. In this paper we illustrate how Boolean network methods may be used to describe the sequences of emotion and behavior states that lead to a stable equilibrium – e.g., healthy function; and the interventions needed to push an individual toward healthier equilibria. We applied Boolean network models to intensive longitudinal data obtained from 150 participants (age 18-89 years) to describe individuals' on-going psychosocial dynamics and identify the specific social behaviors that may be driving them toward undesirable and/or desirable equilibria (e.g., high and low negative emotions). Results are discussed with respect to how they inform theory about developmental systems, and construction of interventions meant to guide individuals toward healthy aging.

### RESILIENCE AND RECOVERY IN OLDER ADULTS: A COMPLEX SYSTEMS APPROACH

Jerrald Rector,[1] Jerrald Rector,[2]
Marcel G. Olde Rikkert,[2] and René Melis[2], *1. Radboud University medical center, Nijmegen, Netherlands, 2. Radboud university medical center, Nijmegen, Gelderland, Netherlands*

In medicine, we still cannot objectively assess who will recover from health stressors imposed by disease or its treatment. If resilience, the dynamic ability to respond to and recover from health stressors, is considered as an emergent feature of a complex system, then methodology from complexity science may help us quantify the health-promoting features that support the recovery process. This presentation describes ongoing work aimed at empirically testing the concept and predictive value of resilience by examining the extent to which small-scale responses of bodily systems (i.e. heart rate, activity) to natural (micro)perturbations and indicators of loss of complexity are related to physical functioning and recovery throughout the journey of 120 geriatric inpatients. Dynamic indicators of resilience (variance, autocorrelation) and multiscale entropy measures were estimated from continuous heart rate and accelerometer data and compared to measures of patients' physical functioning (e.g., ADLs, frailty) at admission, discharge and 3 months later.

### AN ADAPTIVE EQUILIBRIUM REGULATION MODEL OF RESILIENCE

Steve Boker,[1] Steve Boker,[1] and Cindy Bergeman, *1. University of Virginia, Charlottesville, Virginia, United States, 2. University of Notre Dame, Notre Dame, Indiana, United States*

Adaptive equilibrium regulation (AER) models distinguish between the effects of acute versus chronic stressors as a system responds to changes in the environment. Acute stressors have a short time interval during which the stressor is present. Chronic stressors have an onset and may also have an offset, but the stress persists over a period of weeks, months or years. Resilience to an acute stressors may involve rapid self-regulation back to equilibrium without affecting the regulation process itself. Resilience to a chronic stressor may require the system to readapt itself so that regulation of the chronic stressor becomes more effective over time. We present a differential equation model that allows for adaptation of regulation in response to chronic stress and illustrate its use in intensive longitudinal burst data from the Notre Dame Study of Health and Wellbeing.

### QUANTIFYING SENSITIVE DEPENDENCE OF INITIAL CONDITION USING STRUCTURAL EQUATION MODELING

Robert Moulder[1] and Steve Boker[1], *1. University of Virginia, Charlottesville, Virginia, United States*

Human systems display sensitive dependence of initial condition. That is, even though two individuals may be similar in most regards, small differences between these individuals may have far reaching consequences later in life. In dynamical systems analysis, this sort of behavior is quantified with maximum Lyapunov exponents. These exponents quantify the degree to which small differences in initial condition between two systems affect trajectories of these systems later in time. Current methods for estimating maximum Lyapunov exponents are sensitive to noise and this sensitivity leads to estimation errors when researchers attempt to estimate these exponents on data obtained from human participants. Additionally, most current methods only allow for maximum Lyapunov exponent estimation using univariate time series. In this presentation, we present a method for using structural equation modeling for estimating latent maximum Lyapunov exponents from noisy multivariate time series and discuss applications of this method for analyzing human generated data.

### SEQUENCE MINING FOR COMPLEX PATTERN FINDING

Tim Brick[1], *1. Pennsylvania State University, University Park, Pennsylvania, United States*

The processes of aging play out across multiple variables and multiple timescales, with patterns of daily, and weekly behavior that may be influenced by each other and by changes across the aging process. Further, many of these patterns do not fit neatly into the linear modeling approaches common in the field. Sequence mining, an approach from the data mining literature, provides a means of identifying commonalities and differences in these sequences in ways that can begin to handle the multivariate and multi-timescale nature of behaviors in aging. In this talk, I present an example of sequence mining to illustrate its ability to find arbitrarily complex patterns of behavior that characterize and distinguish groups and individuals.

### SESSION 2010 (PAPER)

### COGNITIVE FUNCTIONING AND IMPAIRMENT

### ASSOCIATION BETWEEN FAMILY TYPOLOGY AND COGNITIVE FUNCTION AMONG OLDER ADULTS IN THE US? FINDINGS FROM THE PINE STUDY

Mengting Li,[1] Man Guo,[2] Meredith Stensland,[3] and XinQi Dong[4], *1. Rutgers, The State University of New Jersey, New Brunswick, New Jersey, United States, 2. University of Iowa, Iowa, United States, 3. The University of Texas at Austin, Austin, Texas, United States, 4. Rutgers Institute for Health, Health Care Policy and Aging Research, New Brunswick, United States*

A broad literature has explored racial and ethnic disadvantages in cognitive aging. Migration and acculturation created additional challenges on cognitive aging of minority older immigrants. Asian Americans are the fastest growing minority group in the United States. Chinese Americans constitute the largest segment of Asian Americans. Family is a core social value in Chinese culture. Less is known regarding the impact of family relationship on cognitive function for US Chinese older immigrants. Data were derived from the Population Study of Chinese Elderly (PINE), a community-engaged, population-based epidemiological study of 3,157 US Chinese older adults aged 60 and above in the greater Chicago area from 2011-2013. A typology approach is a useful tool to operationalize multifaceted family relationships. Our prior study used Latent Class Analysis to cluster family typologies, evaluating structural, associational, affectual, functional and normative aspects of family relationship. Cognitive function was evaluated by global cognition, episodic memory, executive function, working memory, and Chinese Mini-Mental State Examination (C-MMSE). Linear regression and quantile regression were used. The findings showed detached and commanding conflicted typologies were associated with lower global cognitive function compared with unobligated ambivalent typology. Wish respect to cognitive domains, detached, commanding conflicted, and tight-knit typologies were associated with lower episodic memory, working memory, and C-MMSE than unobligated ambivalent typology, respectively. Commanding conflicted typology, featured by high intergenerational conflicts, was associated with lowest cognitive function among all typologies. Health care professionals and social service providers should focus on older adults with commanding conflicted typology and prevent them from cognitive impairment.

### CAN HEARING AIDS DELAY THE ONSET OF ALZHEIMER'S AND OTHER AGE-RELATED CONDITIONS AMONG ADULTS WITH HEARING LOSS?

Elham Mahmoudi,[1] Tanima Basu,[2] Kenneth Langa,[2] Michael McKee,[2] Phillip zazove,[2] and Neil Kamdar[2], *1.*

University of Michigan, Department of Family Medicine, Ann Arbor, Michigan, United States, 2. University of Michigan, Ann Arbor, Michigan, United States

In this study, we examined the association between hearing aids (HAs) and the onset of Alzheimer's disease or dementia; depression or anxiety; drug or alcohol disorders; and falls among adults aged 50 and older with hearing loss (HL). We performed a retrospective study of 176,716 adults (50+) with HL diagnoses using a national, insurance claims data (2008-2016). We used Kaplan Meier curves to examine disease-free survival and Cox regression models to examine the risk-adjusted association between HAs and time to diagnosis of 4 age-related/HL-associated conditions within 3 years of HL diagnosis. Large gender and racial/ethnic differences exist in HAs use. Approximately 11.3% of women vs. 14.5% of men used HAs (95% CI Difference: -0.04, -0.03). About 14.1% of Whites (95% CI: 0.14, 0.14) vs. 9.5% of Blacks (95% CI: 0.09, 0.10) and 7.8% of Hispanics (95% CI: 0.07, 0.08) used HAs. The risk-adjusted hazard ratios of being diagnosed with Alzheimer's disease or dementia, depression or anxiety, drug/alcohol disorders, and injurious falls within 3 years after HL diagnosis, for those who used HA vs. those who did not, were lower by 0.82 (95% CI: 0.76-0.88), 0.92 (95% CI: 0.89-0.95), 0.91 (95% CI:0.80-1.04), and 0.86 (95% CI: 0.81-0.92), respectively. Use of HAs is associated with delayed onset of Alzheimer's, dementia, depression, anxiety, and injurious falls among adults 50 years of age and older with HL. This is important because HL are increasingly common among older adults and early HL diagnosis and use of HAs may prevent or delay physical and mental decline.

### INTRODUCING ARTIFICIAL COMPANIONS TO USERS WITH DEMENTIA IN UNREGULATED MARKETS: OPPORTUNITIES VS. ETHICAL ISSUES

Elena Portacolone,[1] Jodi halpern,[2] Jay Luxenberg,[3] Krista Harrison,[4] and Kenneth Covinsky[1], *1. University of California San Francisco, San Francisco, California, United States, 2. University of California Berkeley, Berkeley, California, United States, 3. On Lok, San Francisco, California, United States, 4. UCSF, San Francisco, California, United States*

Because of the high costs of providing long-term care, artificial companions are increasingly considered an opportunity to provide support to older adults with cognitive impairment while saving costs. Artificial companion can comfort and inform, thus inducing a sense of being in a relationship. Sensors and algorithms usually allow these applications to exude a life-like feel. The explosion of these technologies has created a "cultural lag" between their rapid commercial introduction and the slower evolution of regulations. An outcome of this cultural lag is a tension between the potential of artificial companions to support users and a series of unresolved ethical issues related to the fact that users might lack the capacity to fully understand the implications of using these technologies. Specific challenges of deception, surveillance, consent and social isolation are raised by the introduction of these technologies in users with cognitive impairment. The case study of a sophisticated artificial companion commercially available in the United States lends the opportunity to examine the tension between the potential of this technologies vs. unresolved ethical issues. This companion is an avatar on an electronic tablet that is displayed as a dog or a cat. Whereas artificial intelligence guides most artificial companions, this application is a hybrid of robots and human beings because it also relies on technicians "behind" the on-screen avatar, who via surveillance, interact with users. We conclude with a call to develop regulations promoting artificial companions as "human-driven technologies," i.e. technologies focused on truly empowering users according to their cognitive abilities.

### LONELINESS, MARITAL STATUS, AND COGNITIVE IMPAIRMENT AMONG OLDER AMERICANS

Jack Lam,[1] Anthony R. Bardo,[2] and Takashi Yamashita[3], *1. University of Queensland, Brisbane, Queensland, Australia, 2. University of Kentucky, Lexington, Kentucky, United States, 3. University of Maryland, Baltimore County, Baltimore, Maryland, United States*

Loneliness has been linked to increased risk of mortality and morbidity, and emergent research has identified a negative association between loneliness and cognitive functioning. While the determinants of loneliness are wide in scope, loneliness is closely tied to marital status in later life. At the same time, research has shown that those who are married have lower risk of cognitive impairment. The aim of this study was to determine the association between loneliness and cognitive impairment, and examine whether it is moderated by marital status. Data come from 9 waves of the RAND version of the HRS (1998 - 2014). Consistent with previous research, results from random effects logit models showed that loneliness is associated with greater risk of cognitive impairment [Odds-ratio (OR) = 1.41, $p < 0.01$]. Additionally, those who are widowed (OR = 1.29, $p < 0.01$), separated/divorced (OR = 1.33, $p < 0.01$), or never married (OR = 1.70, $p < 0.01$) are also more likely to have a cognitive impairment, compared to those who are married. However, the association between loneliness and cognitive function was found to only differ among those who are widowed. Contrary to expectations, widows who report feeling lonely are 29% ($p < 0.01$) less likely to have a cognitive impairment. In sum, while loneliness and marital status are closely linked with one another, they are both independent determinants of cognitive impairment. The distinct theoretical mechanisms linking loneliness and marital status to cognitive function in later life are discussed.

### PHYSICAL ACTIVITY MEDIATES THE EFFECTS OF DEPRESSIVE SYMPTOMS ON THE COGNITIVE FUNCTION OF OLDER ADULTS

Yit Mui Khoo,[1] and Hisako Matsuo[1], *1. Saint Louis University, St. Louis, Missouri, United States*

Evidence suggests that depressive symptoms among older adults were associated with cognitive impairment and affect cognitive decline over time, while physical activity was associated with lower risk of cognitive decline or have positive effect on cognitive function. The purpose of this study is to examine whether physical activity could mediate the effects of depressive symptoms on the cognitive function of older adults. Data from the 2014 Health and Retirement Survey (HRS) of older adults $\geq$ 60 years (N=9,753) were used. Hierarchical regression was conducted to examine the relationship between depressive symptoms, physical activity, and cognitive function. Mediation analysis was used to examine whether physical activity could mediate the effects of depressive symptoms on cognitive function. Regression results indicated that increased depressive symptoms was associated with poorer cognitive function, while increased moderate and mild physical activity were associated with better cognitive function. Mediation analysis indicated that the direct effect of depressive symptoms on cognitive function was significant. The indirect effect of depressive symptoms on cognitive function mediated by moderate and mild physical activity were also significant. Findings suggest that physical activity could potentially improve the cognitive function of older adults who have depressive symptoms. Moderate and mild physical activity could benefit older adults with depressive symptoms and reduce the risk of cognitive decline. Frail, disabled or chronically ill older adults are less likely to participate in vigorous physical activity, but they could benefit from moderate or mild physical activity and have better cognitive health.

### SESSION 2015 (SYMPOSIUM)

### DEMENTIA CARE AND BEHAVIORAL HEALTH WORKFORCE DEVELOPMENT: INNOVATIONS IN TRAINING ACROSS SETTINGS

Chair: Mary F. Wyman, *WS MIDDLETON MEMORIAL VETERANS HOSPITAL, Madison, Wisconsin, United States*
Co-Chair: Verena R. Cimarolli, *LeadingAge LTSS Center @ UMass Boston, Washington, District of Columbia, United States*
Discussant: Robyn Stone, *LeadingAge, Washington, District of Columbia, United States*

It is well-established that there is a critical shortage of trained health care staff who can deliver high-quality behavioral health services to persons with dementia. The development of innovative professional training curricula, and effectively implementing and sustaining such programs, is a key element in addressing this workforce crisis. This symposium highlights cutting-edge efforts being conducted across the continuum of care, to train health care professionals to support optimal behavioral health for persons with dementia. In the outpatient setting, Wyman et al. present data from a survey of mental health clinicians on their perspectives about working with persons with dementia and caregivers, revealing critical gaps in knowledge and skills to target in continuing education programming. Wray and colleagues focus on integrated behavioral health in primary care, discussing how geriatric experts can most effectively contribute to improved assessment and treatment within this setting. Koepp presents an innovative program to transform outpatient mental health care for persons with dementia through provider training and consultation. In the residential care setting, Reinhardt and colleagues report on the implementation of a comprehensive approach to the alleviation of behavioral health problems through training direct care staff in person-directed care in nursing homes. Finally, Karel et al share how VA interprofessional nursing home teams learn and sustain an evidence-based program to address behavioral concerns among residents with dementia. The discussant will lend a deep expertise in research and policy related to the geriatric workforce to her remarks on the presentations.

### DEVELOPING DEMENTIA-SPECIFIC PROVIDER TRAINING IN MENTAL HEALTH: WHAT IS THE TARGET?

Mary F. Wyman,[1] Corrine I. Voils,[2] Ranak B. Trivedi,[3] and Carey E. Gleason[4], *1. WS MIDDLETON MEMORIAL VETERANS HOSPITAL, Madison, Wisconsin, United States, 2. WS Middleton Memorial Veterans Hospital, Madison, Wisconsin, United States, 3. Stanford University/ Palo Alto VA, Menlo Park, California, United States, 4. W.S. Middleton Memorial Veterans Hospital, Madison, Wisconsin, United States*

Most persons with dementia (PwD) live in the community and receive mental health care in the outpatient setting, making these providers an important target for education to improve dementia care. To inform the development of training curricula, we surveyed 65 mental health providers in a Veterans Affairs outpatient clinic on perceived barriers and training needs related to service delivery to PwD and caregivers. We used an adapted version of the Sense of Competence in Dementia Care Staff scale to assess domain-specific competencies. Respondents rated this work as highly important and wanted dementia-related training. They reported low competency in person-centered care approaches and challenging clinical situations, e.g., managing risk of harm. System-level barriers affecting services for PwD were noted. Findings suggest that outpatient mental health professionals are an underutilized resource in dementia care. This work can inform the development of provider training and identification of system-level barriers in this setting.

### BEHAVIORAL HEALTH WORKFORCE DEVELOPMENT TO IMPROVE DEMENTIA CARE IN INTEGRATED PRIMARY CARE

Laura O. Wray,[1] Bonnie M. Vest,[2]
Laura Levon Brady,[2] and Paul R. King[3], *1. VA Center for Integrated Healthcare, Buffalo NY, United States, 2. University at Buffalo, jacobs School of medicine and biomedical sciences, Buffalo, New York, United States, 3. VA Center for Integrated Healthcare, Buffalo, New York, United States*

People with dementia (PwD) receive most of their health care in primary care, yet timely recognition and optimal management of dementia in that setting continues to be challenging. Implementation of primary care medical home models in the Veterans Health Administration (VHA) holds promise for improving quality and coordination of dementia care through interprofessional collaboration. Integrating behavioral health providers (BHPs) into primary care may help to support the care of people with dementia and their families. However, most integrated BHPs have a generalist training background and likely require professional education to address the unique needs of patients with dementia. We will describe findings from a national VHA education needs survey of integrated BHPs and an in-depth qualitative study examining primary care for PwD in two large VHA healthcare systems. We will discuss how geriatric experts can serve as trainers to address current gaps in primary care of PwD.

### TRANSFORMING DEMENTIA SERVICE DELIVERY: THE MENTAL HEALTH GERO-CHAMPIONS PROGRAM

Regina M. Koepp[1], *1. Atlanta VA Health Care System, Decatur, Georgia, United States*

In order to provide effective mental health care to older adults with major neurocognitive disorders (e.g., Alzheimer's disease and related dementias) within outpatient mental health clinics, mental health practitioners must possess a basic understanding of these disorders, the needs of and challenges faced by people living with dementia and their families, and effective treatment approaches for this population. The Mental Health Gero-Champions Program was established in 2015 at a large Veterans Affairs medical center with the aim of providing clinical support and opportunities for training to multidisciplinary mental health providers to enhance skills in assessing and treating older adults with neurocognitive disorders. This presentation will provide an overview of the Mental Health Gero-Champions Program, describe the development and implementation of this program, and discuss challenges and successes in sustaining this transformative initiative over time.

### CHALLENGES AND STRATEGIES FOR SUSTAINING A NEW DEMENTIA BEHAVIORAL CARE APPROACH IN NURSING HOMES

Michele J. Karel,[1] Karen M. Benson,[2] Youliana Piscopo,[3] Susan Maataoui,[4] and Kimberly Curyto[5], *1. Office of Mental Health and Suicide Prevention, Veterans Health Administration, Washington, District of Columbia, United States, 2. Michael E. DeBakey VA Medical Center, Houston, Texas, United States, 3. John D Dingell VA Medical Center, Detroit, Michigan, United States, 4. VA Maine Healthcare System, Augusta, Maine, United States, 5. Batavia VA Medical Center, Batavia, New York, United States*

The STAR-VA training program in Veterans Health Administration Community Living Centers (CLCs) has been helping interdisciplinary care teams understand and manage dementia-related behaviors in the nursing home setting, with promising clinical outcomes. However, sustaining a new care approach in a health care system poses multiple challenges. This presentation will discuss facilitators and barriers to STAR-VA sustainability based on CLC team and nurse leader feedback. Findings are informing development of a new site coaching program and a sustainability toolkit. Feedback to date suggests that critical STAR-VA implementation and sustainability strategies include: regularly scheduled team meetings to discuss behavioral assessment and care plans; ongoing staff training (e.g., new staff orientation); communicating care plans across shifts and in the health record; multiple nurse/shift champions; impromptu huddles; acknowledging staff successes; leadership engagement. The coaching program engages teams in setting and tracking site sustainability goals. Lessons learned will be discussed.

### DEMENTIA-FOCUSED PERSON-DIRECTED CARE TRAINING IN THE NURSING HOME: FIDELITY AND OUTCOMES

Joann P. Reinhardt,[1] Orah Burack,[1] Verena Cimarolli,[2] and Audrey Weiner[1], *1. The New Jewish Home, New York, New York, United States, 2. Leading Age, Washington, District of Columbia, United States*

To address the behavioral health of nursing home (NH) residents living with dementia, training direct care staff (DCS) is essential, for the well-being of both residents and staff. We evaluated a training for DCS focused on providing care for persons with advanced dementia who are at-risk of not having care needs met, largely due to communication deficits. Staff were trained in promoting comfort and reducing distress through person-directed care (PDC), deeply knowing each resident, and anticipating needs. Subsequent fidelity interviews with staff showed a higher number of PDC practices utilized by staff in the intervention communities compared to usual care. We also compared the impact of the PDC model versus a traditional model of NH care on resident clinical outcomes, finding a significant interaction where those in the intervention group had fewer clinical symptoms over a 6-month period. Implications for training in the NH setting will be discussed.

### SESSION 2020 (SYMPOSIUM)

### INTEREST GROUP SESSION—SYSTEMS RESEARCH IN LONG TERM CARE: DEVELOPING AN EVIDENCE BASE FOR NURSING HOME STAFFING IN EUROPE AND BEYOND: MANY PATHS TO ONE GOAL?

Chair: Jan Hamers, *Maastricht University, Maastricht, Netherlands*
Co-Chair: Ramona Backhaus, *Maastricht University, Maastricht, Netherlands*
Discussant: Kirsten Corazzini, *Duke University, Durham, North Carolina, United States*

Despite heterogeneity across countries, nursing homes worldwide have to ensure the delivery of high quality care. At the same time, adequately staffing the homes remains a major concern in most countries. It is a significant challenge to determine the numbers and type of staff as well as staff's competencies that are necessary to meet the complex needs of nursing home residents. While, especially in the US, research on the relationship between staffing and quality in nursing homes has received considerable attention, the research literature is contradictory. Evidence shows that employing more staff or more registered nurses instead of nurse assistants will not automatically lead to better nursing home quality. This inconsistency of evidence might be explained by a myriad of methodological and theoretical challenges. This symposium aims to provide an international perspective on how researchers from different European countries contribute to the development of an evidence base for nursing home staffing. The first presenter will draw on findings from a realist review conducted in the UK, focusing on the mechanisms (how, why and in what circumstances) under which staff influence care home quality. The second presenter will offer insights into a Swiss study, taking a broader perspective on measuring staffing and quality in nursing homes. The third presenter will present a new instrument to identify staffing ratios in German nursing homes and provide insight into its development process. The fourth presenter will discuss the results of a critical review on the evidence base of a Dutch nursing home staffing guideline.

### THE RELATIONSHIP BETWEEN STAFFING AND QUALITY FOR CARE HOME RESIDENTS: A REALIST REVIEW

Karen Spilsbury[1] and Kirsty Haunch[2], *1. University of Leeds, Leeds, United Kingdom, 2. University of Leeds, Leeds, England, United Kingdom*

In recent years, quality of care in the sector has come under increasing scrutiny, and practice and policy concerns have been raised about staffing levels, recruitment and retention. Beyond broad recognition that 'staff influence quality', little is known about the care home workforce and its relationship to quality. We are undertaking the first study in the UK to address this important question, considering quality from a range of perspectives. One work package comprises a realist review to develop theoretical explanations of how, why and in what circumstances staffing promotes quality for residents and relatives. We will present three mechanisms that promote quality - individual commitment to resident focused care, reciprocity in the team, and a mandate to be creative and flexible to meet residents' needs. We will consider the contexts required to trigger these mechanisms – staffing numbers, stability of the team, size of the team, and clear leadership.

### ORGANIZATIONAL FACTORS RELATED TO RESIDENT-REPORTED QUALITY OF LIFE IN NURSING HOMES

Franziska Zúñiga[1] and Sabine Hahn[2], *1. University of Basel, Basel, Switzerland, 2. Bern University of Applied Sciences, Health Division, Applied Research & Development in Nursing, Bern, Bern, Switzerland*

Few studies so far take a broader perspective at staffing in nursing homes (NH) including e.g. the impact of activity staff on quality outcomes. Moreover, few assess resident-reported quality of life (QoL). Examining the relationship of organizational and resident factors with QoL, we report the results from a questionnaire survey of organizational characteristics from 51 Swiss nursing homes and of structured interviews with 863 residents. Residents rated their quality of life with a single item. A logistic regression model was applied. At the organizational level, a higher number of activity staff was significantly related to QoL, while at resident level, both the possibility to select their NH and less care dependency were significant predictors. Meaningful activities as well as autonomous decision-making concerning one's living place seem of paramount importance for residents' better perception of their QoL. NH staff mix needs to address the possibility to offer enjoyable activities.

### A NEW INSTRUMENT TO DETERMINE ADEQUATE STAFFING RATIOS IN GERMAN NURSING HOMES

Karin Wolf-Ostermann,[1] Heinz Rothgang,[1] Ingrid Darmann-Fink,[1] Thomas Kalwitzki,[1] and Mathias Fünfstück[1], *1. University of Bremen, Bremen, Bremen, Germany*

Ever since mandatory long-term care insurance was introduced in Germany there has been concern about staffing of nursing homes. As attempts to introduce the Canadian PLAISIR system failed today staffing ratios between federal states differ by more than 20 % and perceived understaffing is a major reason for the lack of nurses. Against this background the reform act from December 2015 commissioned the development of a new instrument to identify necessary staffing ratios. The University of Bremen was mandated to develop this instrument, and will present the final product in August 2019 to the Commissioner. The contribution will describe the methods applied in developing the instrument and the resulting instrument itself which translates the number and characteristics of any given nursing home into staffing requirements according to certain degrees of qualification, thus replacing general quotas of registered nurses to auxiliary staff by individual ratios for each nursing home.

### EVALUATING THE EVIDENCE BASE OF A STAFFING GUIDELINE FOR DUTCH NURSING HOMES

Ramona Backhaus,[1] Hilde Verbeek,[2] Bram De Boer,[3] Erik Van Rossum,[4] Jos Schols,[5] and Jan Hamers[6], *1. Maastricht University, Maastricht, Netherlands, 2. Maastricht University, Maastricht, Limburg, Netherlands, 3. Maastricht University, Maastricht, Limburg, Netherlands, 4. Zuyd Hogeschool, Heerlen, Limburg, Netherlands, 5. Maastricht University, Maastricht, Limburg, Netherlands, 6. Maastricht University, Maastricht, Limburg, Netherlands*

Related to the Dutch nursing home quality framework implemented in 2017, a staffing guideline was developed, aimed at assisting nursing homes to adequately staff their wards. For the Dutch Ministry of Health, we investigated the evidence base of this guideline. We critically reviewed scientific literature (n=65) and interviewed (inter)national experts (n=8) and potential guideline users (n=5). We found that departing a quality improvement dialogue directly from teams, clients and their families is positive. However, weaknesses were identified as well. Several risks exist for employees to adequately assess resident needs. Furthermore, buy-in is needed from board level to develop a vision on which competencies and amount of staff are needed to fulfill these needs. Examples of guideline improvement recommendations were assisting teams in how to assess resident needs, critically reflect on care provision and considering a role for (top)management. Overall, it was concluded that the value of the guideline was limited.

### SESSION 2025 (SYMPOSIUM)

### DISTINGUISHED FACULTY AND RISING STAR EARLY-CAREER FACULTY AWARD LECTURES

Chair: Cynthia Hancock, *University of North Carolina, Charlotte, Charlotte, North Carolina, United States*

The Distinguished Faculty Award acknowledges individuals whose teaching stands out as exemplary, innovative, or influential—or any combination thereof. The Distinguished Faculty Award lecture will feature an address by 2019 recipient Gayle Doll, PhD, of Kansas State University. The Rising Star Early-Career Faculty Award acknowledges new faculty whose teaching and leadership stand out as influential and innovative. The Rising Star Early-Career Faculty Award lecture will feature an address by 2019 recipient Katarina Felstedof, PhD, of the University of Utah.

### SESSION 2030 (PAPER)

### ECONOMICS OF LATER LIFE AND RETIREMENT

### CAREGIVING AND PREPARATION FOR RETIREMENT

Shirley L. Porterfield,[1] Eunsun Kwon[2], *1. University of Missouri-St Louis, St. Louis, Missouri, United States, 2. St. Cloud State University, St Cloud, Minnesota, United States*

Saving for retirement should begin with the first job, but preparation with respect to determining a specific retirement age and plans for post-retirement life, generally occurs closer to the retirement date. However, among those who provide care for family or close friends who are elderly and/or have disabilities, retirement preparation may take a back seat to more pressing current concerns. While we know quite a lot about patterns of saving for retirement and the factors that influence those patterns, we know little about retirement expectations and patterns of thinking about and planning for the broader retirement experience, particularly among caregivers. This paper uses data from the 2008-2016 rounds of the nationally-representative 1979 National Longitudinal Survey of Youth to examine retirement expectations and five areas of retirement preparation (reading, using a computer app, consulting a financial planner, calculating income, or attending meetings) among employed adults (ages 51-59 in 2016) who are or are not providing care for someone in or out of their household. Longitudinal analysis finds significantly lower retirement preparation among adults caring for someone inside versus outside the household, as well as significantly lower preparation activities among female versus male caregivers. Caregiving influences employment and, in turn, the types of retirement accounts held by men and women. Although caregiving is associated with decreased retirement savings among both men and women who have pension accounts, retirement preparation activities in 2008 and 2012 are associated with higher retirement savings in 2016.

### LONG-TERM POVERTY, SPATIAL DISADVANTAGE, AND MULTIPLE EXCLUSIONS IN LATER LIFE: A CASE IN SHANGHAI

Hongmei Tong,[1] Daniel, W. L. Lai,[2] and Lun Li[3], *1. MacEwan University, Edmonton, Alberta, Canada, 2. The Hong Kong Polytechnic University, Hong Kong, Hong Kong, 3. University of Calgary, Calgary, Canada*

Objectives: This study aimed to examine the associations among three types of cumulative disadvantages: long term poverty, spatial disadvantage, and multiple exclusions using a Cumulative dis/advantage (CDA) and life course perspective. Method: A sample of 419 Chinese adults aged 60 and older from three communities in Shanghai completed a structured questionnaire. Multiple exclusions were measured by variables related to material resources, housing conditions, social relations, civic activities, basic services, and neighbourhood factors. Hierarchical regression was implemented by SPSS 25 and moderation analysis was performed with the SPSS macro PROCESS from Hayes (2013). Results: 39% of respondents reported that they experienced multiple exclusions and one in five respondents report often or most time living in poverty. Regression analysis indicated that experience long-term poverty and length of living in the same neighbourhood is positively associated with multiple exclusions in later life and these associations are not attenuated by demographics, and health factors. But, moderation analysis showed the length of living in the same neighbourhood has significant moderating effect on the relationship between long term poverty and multiple exclusions, particularly for older adults living in the same neighbourhood for more than 30 years. Discussion: The study findings illustrate the need to consider not only life course risks such as long-term poverty but also spatial disadvantages in addressing multiple social exclusions among older Chinese adults.

### PATTERNS OF WEALTH TRAJECTORY IN LATER LIFE: CRITICAL PERIOD, ACCUMULATION, AND SOCIAL MOBILITY MODELS

Yu-Chih Chen,[1] Sojung Park,[2] and Nancy Morrow-Howell[3], *1. Washington University in St. Louis, St. Louis, Missouri, United States, 2. Brown School, Washington University in St. Louis, St. Louis, Missouri, United States, 3. Washington University, St. Louis, Missouri, United States*

Wealth, an important financial cushion for older adults to buffer economic stress, requires a longer time to accumulate and develop in one's course of life. However, little is known about the trajectories of wealth in later life, and how the life course socioeconomic status (SES) may contribute to the development of wealth at old-age. This study investigated longitudinal patterns of wealth trajectory and whether SES across the life course affects these trajectories using critical period, accumulation, and social mobility models. Using data from 16,189 adults aged 51 and older from the 2004-2014 Health and Retirement Study, a growth mixture model was used to explore distinct wealth trajectories. Impacts of life course models were studied using multinomial logistic regression. Results showed that four heterogeneous latent classes of wealth were identified: Stable high (reference group), Low and increasing, Stable low, and High but decline. Disadvantaged adulthood SES, accumulated exposure to socioeconomic risks, and downward or persistent socioeconomic disadvantage over the life course were associated with Stable low, Low and increasing, and High but decline, supporting all three life course mechanisms on wealth development in later life. Evidence suggests that wealth development is heterogeneous across individuals, and a strong gradient effect of life-course SES on wealth trajectories are clearly observed. Programs and policies should address the effects of life course on wealth development to strengthen the economic well-being in later life.

### THE ROLE OF SECURED AND UNSECURED DEBT IN RETIREMENT PLANNING AMONG PRE-RETIREES

Zibei Chen[1] and Karen Zurlo[2], *1. University of Michigan, Ann Arbor, Michigan, United States, 2. Rutgers, New Brunswick, New Jersey, United States*

The retirement landscape is transformed by the shifting of risk and responsibility to individuals, who are increasingly responsible for their retirement security. Many factors lead to indebted and overleveraged American households. Specifically, nearly 40% of Americans approaching retirement are heavily indebted. Understanding the role of secured and unsecured debt in retirement planning becomes an urgent concern because debt is highly related to well-being in retirement among a growing number of older Americans. We focus on pre-retirees because these individuals have time to earn an income and plan ahead before they commit to a fully retired lifestyle. Utilizing data from the 2015 National Financial Capability Study, we identified the secured and unsecured debt that influences retirement planning among a national sample of pre-retirees, aged 51 to 61 years. Regression and mediation analyses were used to examine the relationship between debt and retirement planning and to identify the mediating effect of having a retirement account on the relationship between unsecured and secured debt and retirement planning. Our results indicated that mortgage debt and credit card debt were negatively associated with retirement planning. Having a retirement account is positively associated with retirement planning and it also mediates the relationship between credit card debt, specifically, and retirement planning. In conclusion, we urge individuals and financial planning executives to take time during the pre-retirement years to assess various forms of debt and determine how it is affecting their retirement planning objectives. And policy-makers should address the challenges faced by indebted pre-retirees.

### THE SOCIAL CONSTRUCTION OF RETIREMENT AND GENDER DIFFERENCES IN RETIREMENT TIMING

Michelle Silver[1], *1. University of Toronto, Toronto, Ontario, Canada*

Retirement is an ever-evolving, dynamic, and complex social construct we associate with the end of one's career. For some the term is a bad word and a term that needs to be retired, while others can't wait to retire and enjoy the good life. This paper examines a brief history of retirement and theoretical work from feminist gerontology, while focusing on gender differences in the social construction of retirement and policy implications of 10 different government pension plans. In doing so, it looks at policy implications associated with the standard retirement age tied to public pension plans in the United States, Canada, and the European Union. Findings indicate that women live longer than men in each country, yet women retire earlier and receive lower pensions than men. As the landscape surrounding women's work experiences changes and concerns about gender equity in salaries and workplace compensation continue to be raised, this paper extends the concerns to raise important questions about inequities in retirement.

### SESSION 2035 (SYMPOSIUM)

### ELDER FINANCIAL ABUSE IN FAMILIES: EXPANDING THEORY AND RESEARCH

Chair: Cory Bolkan, *Washington State University, Vancouver, Washington, United States*
Co-Chair: Marlene Stum, *University of Minnesota, St. Paul, Minnesota, United States*
Discussant: Pamela B. Teaster, *Virginia Tech, Blacksburg, Virginia, United States*

Elder family financial exploitation (EFFE) is widespread and increasing. The effect is devastating, causing significant financial losses, reducing health and well-being of elders, and disrupting family systems. Research reveals that most (90%) perpetrators are family members or trusted others and researchers typically focus on identification of the problem, rather than understanding how and why exploitation occurs within the family unit. Furthermore, limited consensus exists regarding a theoretical understanding of the complexities of EFFE. Theory-driven, empirical explanations of how and why EFFE transpires are urgently needed to enhance and deepen intervention and prevention efforts. In this symposium, we extend both theory and research by using a common theoretical lens to present research findings from three distinct EFFE studies. The first paper reviews the current literature on EFFE and theory and introduces Bronfennbrenner's bioecological Process-Person-Context-Time (PPCT) model as an under-utilized, but useful framework for understanding EFFE. The second paper reports on findings from in-depth interviews with non-perpetrator family members who experienced EFFE and will highlight complex intergenerational family systems processes in PPCT. The third paper highlights findings from a national study of substantiated and investigated cases of EFFE in which family member perpetrators were designated as surrogate decision makers. The fourth paper shares results from a qualitative study of family member POAs and how components of the PPCT model can be interpreted for use by helping professionals assisting families. We will also focus on the opportunities and challenges of developing theoretically sound EFFE research and the implications for improving practice and policy.

### PRELIMINARY FINDINGS ON FAMILY PERPETRATORS AS DESIGNATED SURROGATE DECISION MAKERS

Cory Bolkan,[1] Pamela Teaster,[2]
Holly Ramsey-Klawsnik,[3] and Kenneth Gerow[4], *1. Washington State University, Vancouver, Washington, United States, 2. Virginia Tech, Blacksburg, Vermont, United States, 3. National Adult Protective Services Association, Washington, District of Columbia, United States, 4. University of Wyoming, Laramie, Wyoming, United States*

Vulnerable older adults needing surrogate decision makers typically rely upon others for care and are unable to advocate for themselves. The issue of EFFE perpetrated by family members designated as surrogates has become highly visible nationally, yet no reliable, empirical documentation exists on the nature or extent of exploitation by surrogate perpetrators. In collaboration with the National Adult Protective Services Association (NAPSA), we prospectively gathered APS data from six geographically diverse counties on 450 substantiated cases of abuse by POAs, representative payees, and guardians of vulnerable adults 65+ living in community settings. This presentation will highlight how family member surrogates perpetuated abuse and the outcomes on elder victims. These findings elucidate person and process-level factors (e.g., characteristics of victims, perpetrators, and their relationships) within the context of the APS system and can inform practice and policy recommendations for better prevention, detection, investigation, and intervention in these challenging cases.

### EXAMINING THE NATURE AND ROLE OF FAMILY SYSTEM INTERACTIONS AND ELDER FAMILY FINANCIAL EXPLOITATION

Marlene Stum[1], *1. University of Minnesota, St. Paul, Minnesota, United States*

Guided by Bronfenbrenner's Bioecological Theory, this paper contributes insight into family processes associated with elder family financial exploitation (EFFE). Processes involve "everyday" reciprocal interactions in the family microsystem between and among elder victims, perpetrator family members (i.e. adult children), and involved non-perpetrator family members (i.e. adult children, in-laws). Qualitative data from a purposeful sample of 28 non-perpetrator/non-victim family members who had experienced EFFE were thematically coded and analyzed. The findings suggest eight intergenerational family processes are relevant for understanding EFFE: a) parent/child resource exchange patterns, b) negotiating a "fair" use of resources, c) quality of parent/child and sibling relationships, d) family of origin functioning and dynamics (e.g. trust, respect, closeness), e) communication patterns, f) alliances and taking sides, g) role negotiation, and h) physical interactions. Consistent definitions and quality measures relevant for parent/adult child relationships over the life course and in later life developmental stages are needed next steps.

### ADDRESSING THE THEORETICAL GAPS IN ELDER FAMILY FINANCIAL EXPLOITATION

Megan Gilligan,[1] Axton Betz-Hamilton,[2] and Ashton Chapman[3], *1. Iowa State, Ames, Iowa, United States, 2. South Dakota State, Brookings, South Dakota, United States, 3. Robert Wood Johnson Foundation, Princeton, New Jersey, United States*

A systematic review of elder family financial exploitation (EFFE) literature from the past five years reflects limited use or consensus of theoretical perspectives. In this paper, we propose using Bronfenbrenner's Bioecological Theory to frame the dynamic, interrelated factors associated with EFFE. Bronfenbrenner's Bioecological Theory, specifically the PPCT model includes Proximal processes, Person characteristics, Contextual systems, and Time. Proximal processes are increasingly complex interactions between individuals (e.g., family communication). Person characteristics include demand (e.g., gender, age), resource (e.g., education), and force (e.g., temperament) characteristics affecting interactions. Contextual levels drawn from Bronfenbrenner's original model (e.g., micro, meso, exo, and macro) emphasize the effect of interrelated systems on development. T refers to Time, including changes occurring in time (e.g., longitudinal) and over time (e.g., historical). Collectively, the PPCT model provides a framework for understanding the iterative, complex factors linked to EFFE.

### ELDER FAMILY FINANCIAL EXPLOITATION BY POWER-OF-ATTORNEY AGENTS THROUGH THE LENS OF BRONFENBRENNER'S PPCT MODEL

Virginia B. Vincenti[1] and Ashton Chapman[2], *1. University of Wyoming, Laramie, Wyoming, United States, 2. Social Grove, Inc., Joplin, Missouri, United States*

This paper presents a subset of qualitative data from a phenomenological study of 3 men and 17 women (N=20) from families with designated power of attorney (POA) agents who allegedly perpetrated elder family financial exploitation (EFFE). Participants were aged 22 to 63 (M = 43.5) with varying educational and income levels. The study explored EFFE by POA within Bronfenbrenner's PPCT model. Data were analyzed using a thematic, inductive approach. Person characteristics (e.g. perpetrators' personality and victims' cognitive functioning), proximal processes (e.g. family patterns of communication and resource sharing), context (e.g. geographic location), and time (e.g. prevailing legal, economic, and cohort factors) emerged as relevant for EFFE experiences. Given that EFFE helping professionals (e.g., attorneys, practitioners) often lack training in family- or systems-focused dynamics and interventions, implications and applications of the PPCT model will be discussed with the goal of raising awareness of factors related to EFFE identification and prevention.

### SESSION 2040 (SYMPOSIUM)

### EXAMINING THE DETERMINANTS AND OUTCOMES OF SUBJECTIVE AGING

Chair: Allyson F. Brothers, *Colorado State University, Fort Collins, Colorado, United States*
Co-Chair: Serena Sabatini, *Centre for Research in Ageing and Cognitive Health, University of Exeter, Exeter, United Kingdom, University of Exeter, United Kingdom*
Discussant: Shevaun D. Neupert, *North Carolina State University, Raleigh, North Carolina, United States*

Given a growing body of evidence for the developmental relevance of the perceived experience of aging and for the presence of interindividual variability in the way people experience aging, this symposium examines the determinants and outcomes of various subjective aging constructs. This session will explore the role of various psychological variables in explaining variability in subjective aging experiences. Consequences of various subjective aging concepts on cognitive functioning, emotional and physical well-being will also be discussed. The first two presentations examine Attitudes Toward Own Aging (ATOA). Kornadt, Siebert and Wahl will address the developmental co-dynamics of personality and ATOA across the second half of life. Siebert and Wahl will examine the associations of ATOA with subjective and objective cognitive functioning. The last two presentations focus on awareness of age-related change (AARC). Sabatini, Silarova, Collins, Martyr, Ballard, Anstey, Kim & Clare will present findings from a systematic-review and meta-analysis synthesizing and quantifying associations of awareness of age-related change (AARC) with emotional and physical well-being and cognitive functioning. Finally, Rothermund and de Paula Couto will show how both the experience of positive and/or negative changes (gains and losses) and the presence of positive and/or negative age stereotypes predict individual's attributions of change to age. This last presentation will also examine how together the presence of change and attribution of change to age predict developmental outcomes. The symposium will conclude with summarizing remarks from the discussant who suggests possible directions for future research on determinants and outcomes of perceived experience of aging.

### META-ANALYSIS OF AARC AND EMOTIONAL AND PHYSICAL WELL-BEING

Serena Sabatini,[1] Barbora Silarova,[2] Anthony Martyr,[2] Rachel Collins,[2] Clive Ballard,[2] Kaarin J. Anstey,[3]

Sarang Kim,[4] and Linda Clare[2], *1. Centre for Research in Ageing and Cognitive Health, University of Exeter, Exeter, United Kingdom, University of Exeter, United Kingdom, 2. University of Exeter, Exeter, England, United Kingdom, 3. University of New South Wales, Sidney, New South Wales, Australia, 4. University of Tasmania, Hobart, Tasmania, Australia*

Associations of awareness of age-related change (AARC) with emotional and physical well-being and cognitive functioning were synthesised in a systematic review with a correlational random-effects meta-analysis. Twelve studies were included in the review, nine exploring the association between AARC and emotional well-being and eleven exploring the association between AARC and physical well-being. No study explored the association between AARC and cognition. There is evidence of weak associations between higher level of AARC gains and better emotional well-being and between higher level of AARC losses and both poorer emotional well-being and poorer physical well-being. There was no association between AARC gains and physical well-being. There is some indication that AARC gains and losses can play a role in emotional well-being and that AARC losses are associated with physical well-being but these associations are weak. Due to the limited number of studies and their high heterogeneity, interpretation of these results remains unclear.

### ATTITUDES TOWARD OWN AGING AND PERSONALITY IN LATER LIFE: EXAMINATION OF BIDIRECTIONALITY OVER 20 YEARS

Anna E. Kornadt,[1] Jelena S. Siebert,[2] and Hans-Werner Wahl[2], *1. Bielefeld University, Bielefeld, Nordrhein-Westfalen, Germany, 2. Heidelberg University, Heidelberg, Baden-Wurttemberg, Germany*

Big Five personality traits are assumed to be linked with attitudes toward own aging (ATOA). Both constructs have central importance for the aging process, it is thus important to comprehensively address their mutual connection over time. We used data from the ILSE study, a longitudinal study with four measurement occasions, spanning 20 years and including two participant cohorts (n = 501; born 1950-52 and n = 500; born 1930-32). Dual latent change score models showed that personality was longitudinally related to change in ATOA: Lower Neuroticism, higher Conscientiousness, and higher Openness predicted more positive attitudes; the effect for Extraversion varied by time. Furthermore, the role of personality seems to be confined to certain sensitive periods in midlife and early old age. ATOA had only marginal longitudinal impact on personality. Our results shed light on the developmental co-dynamics of personality and subjective perceptions of aging across the second half of life.

### COGNITIVE PERFORMANCE AND COGNITIVE COMPLAINTS WITH ATTITUDES TOWARD AGING IN MIDLIFE AND OLD AGE

Jelena Siebert,[1] Jelena S. Siebert,[2] Tina Braun,[3] and Hans-Werner Wahl[2], *1. Heidelberg University, Heidelberg, Germany, 2. Heidelberg University, Heidelberg, Baden-Wurttemberg, Germany, 3. Universität der Bundeswehr, Neubibberg, Bayern, Germany.*

Converging longitudinal research suggests that more negative views on aging go along with higher cognitive impairment. In some contrast, although conceptually suggested, possible reciprocal relationships remain less clear empirically. Using 20-year data from the Interdisciplinary Longitudinal Study of Adult Development (ILSE), we aim to better understand developmental co-dynamics between cognitive factors and attitude toward own aging (ATOA). Drawing on 1002 baseline participants (445 at T4) from two age cohorts (midlife: 40 years at baseline; old age: 60 years), longitudinal trajectories between ATOA, performance-based cognitive measures and subjective cognitive complaints are examined. Findings based on multi-group latent growth curve models (a) reveal substantial associations between ATOA and subjective complaints as well as objective cognition in both age groups; (b) confirm previous findings that ATOA predicts cognitive change over 20 years; and (c) find cognitive complaints but not cognitive performance able to predict change in ATOA in later life.

### AGE-RELATED ATTRIBUTIONS OF CHANGE: DETERMINANTS AND CONSEQUENCES

Klaus Rothermund[1] and Clara de Paula Couto[2], *1. FSU Jena, Jena, Thuringen, Germany, 2. FSU Jena, Dept. of Psychology, Jena, Thuringen, Germany*

Diehl and colleagues recently introduced the concept of "Awareness of Age-related Change" (AARC; Diehl & Wahl, 2010), emphasizing the need to investigate the subjective experience of one's own aging. In the Aging-as-Future project (n=1,300 participants, age range 35-85), we separately assessed the experience of changes and attributions of these changes to age. Attributions of changes were driven by a correspondence between the direction of change (gain vs. loss) and negative vs. positive age stereotypes. Importantly, our data also support the assumption that changes interact with age-related attributions in predicting life satisfaction. Specifically, age-related attributions were shown to exacerbate negative effects of losses on well-being.

### SESSION 2045 (PAPER)

### FAMILY CAREGIVING: INTERVENTIONS AND UNMET NEEDS

### CAREGIVER DEPRESSIVE SYMPTOM SEVERITY AMONG OLDER ADULTS WITH COGNITIVE VULNERABILITY

Richard H. Fortinsky[1] and Dorothy Wakefield[2], *1. University of Connecticut School of Medicine, Farmington, Connecticut, United States, 2. UCONN Center on Aging, Farmington, Connecticut, United States*

While caregivers of older adults with dementia often report considerable levels of depressive symptoms, much less is known about depressive symptoms among family members of older adults with depression or recent delirium. As part of an ongoing randomized clinical trial testing an in-home multidisciplinary team intervention for older adults with cognitive vulnerability due to dementia, depression, and/or delirium (care recipients, or CR) and their caregivers, in this presentation we report baseline data from the first 211 dyads enrolled in the trial to determine how caregiver depressive symptom severity is related to: CR diagnoses; CR cognitive impairment severity; and CR depressive symptom severity. CR diagnostic groups: Depression Only (n=49); Dementia Only (n=61); Depression and Dementia Only (n=47); Delirium Plus (n=54). Depressive symptom severity was measured using the Center for Epidemiologic Studies Depression Scale; CR cognitive symptom severity was measured using the Telephone Interview for Cognitive Status. Among CR, 57% were female, mean/sd age=77/6.9, 93% White; among caregivers, 64% were female, mean/sd age=66/13.7, 91% White, 55% spouses, 25% daughters, 9% sons. In multivariate linear regression models, which included covariates caregiver gender, relationship to CR, and number of hours/week providing care, we found that caregiver depressive symptom severity was less severe among caregivers of CR with Dementia Only compared to CR with Depression Only (b=-3.32; p=0.06); not associated with CR cognitive symptom severity; and significantly associated with CR depressive symptom severity (b=0.14; p<0.01). We conclude that family members of older adults with depression deserve greater attention to address their own depressive symptoms.

### FACTORS ASSOCIATED WITH UNMET NEED AMONG FAMILY CAREGIVERS

Joanne R. Campione[1] and Katarzyna Zebrak[2], *1. Westat, Durham, North Carolina, United States, 2. Westat, Rockville, Maryland, United States*

This study investigates the relationship between caregiver demographics, caregiving intensity, caregiver support use, and aspects of the caregiving situation with perceived unmet need. The data source was survey response data from 1,558 informal caregivers caring for older adults (age 60+) or caring for any adult with Alzheimer's disease or related dementia (ADRD). Participants were randomly recruited through U.S. Area Agencies on Aging client lists of any service and interviewed in December 2016. Caregivers were asked, "Are you receiving all the help you need?" Twenty-two percent (n=345) said "Definitely No" and were classified as having unmet need. We placed caregivers in tertiles based on their Zarit burden score; unmet need was 14% among low burden, 20% among medium, and 34% among high. The mean age of the care recipient (CR) was 81 years. Caregivers were 70% non-Hispanic White, 52% caring for CR with ADRD, and 43% spouse of CR. A multivariable logistic regression found two predictive factors (odds ratio p-value < 0.01) that increased the likelihood of reporting unmet need: daily intensity of caregiving and not feeling appreciated by CR. Usage of caregiver education, counseling or support group services in the past 6 months decreased the likelihood of reporting unmet need. In the high burden subgroup, Black caregivers were more likely to report unmet need than White caregivers. Understanding the relationship between caregiver demographics, self-reported burden level, service use, and the caregiver's need for more help can assist caregiver support programs in assessing, measuring, and addressing the ongoing needs of caregivers.

### MOVING TOWARD BEST PRACTICES IN STATE APPROACHES TO SUPPORTING FAMILY CAREGIVERS OF OLDER ADULTS

Julia Burgdorf,[1] Jennifer Aufill,[2] and Jennifer L. Wolff[3], *1. Johns Hopkins Bloomberg School of Public Health, Baltimore, Maryland, United States, 2. Johns Hopkins University Bloomberg School of Public Health, Baltimore, Maryland, United States, 3. Johns Hopkins University, Maryland, United States*

In January 2018, Congress passed the Recognize, Assist, Include, Support, and Engage (RAISE) Family Caregivers Act to establish a national strategy to acknowledge and assist family caregivers. The success of such an effort will be contingent on knowledge and dissemination of best practices. We conducted a policy project funded by the Milbank Memorial Fund that profiles five geographically and politically diverse states that have pursued novel approaches to supporting family and unpaid caregivers of older adults. We discuss findings from national and state reports, state aging plans, and interviews with 26 key informants. We find that each state (Hawaii, Maine, Minnesota, Tennessee, Washington) sought to strengthen supports for family caregivers as an element of broader community-based long-term services and supports strategies. Supports for family caregivers included financial support for working caregivers, caregiver assessment and care planning, and expanded access to respite care: several states included these services within programs targeted to persons at high risk of institutionalization or Medicaid-entry. Key informants noted the importance of tailoring programs to suit each state's unique demographic, geographic, and service delivery context, and reported that individual stories, advocacy, and data were critical to placing family caregiving on legislative policy agendas. State leaders highlighted the pivotal role of state aging networks in efforts to supporting caregivers, given these entities' deep knowledge of local community needs and challenges and established relationships with local service providers. Meaningful change often required a long-term commitment and sustained incrementalism, typically by innovating on a smaller scale before expanding statewide.

### NOTHING FOR ME WITHOUT ME: CAREGIVER TRAINING NEEDS

Jeananne Elkins,[1] and Phillip Rustin[1], *1. Brenau University, Gainesville, Georgia, United States*

Over 34 million informal caregivers provide care to an adult aged 50 or older with over 15 million caregivers caring for a person with dementia. People often become caregivers unexpectedly. For most caregivers little training is available, and most skills are learned "on the job". The objective of this study is to determine the training needs of caregivers. An anonymous survey was conducted at two caregiver conferences. Caregivers were asked to name up to 5 training needs. For this abstract we report the percent of responses by caregivers. Seventy-nine caregivers completed the survey. Seventy-seven percent (77%) of the caregivers cared for a person over age 50. Increased knowledge of health issues (17%) and resources (17%) were the highest responses. Training in physical caregiving skills i.e. bathing (10%), transfers (8%) and diaper changes (4%) were voiced as important. Training for improving personal interactions with the care recipient was important (9%) as well as how to practice patience, kindness and compassion (7%). Two areas where training is available but perhaps not accessible – caregiver support (9%) and care recipient behaviors (8%) were all important to caregivers. Healthy eating (9%) and stopping or limiting driving by the care recipient (2%) were enunciated as areas requiring training. Many of the training needs should be addressed by healthcare professionals in their interactions with caregivers and care recipients. Programs are available online, but caregivers are not accessing this training. To improve outcomes for both caregivers and care recipients targeted training for caregivers is needed.

### PUBLIC HEALTH AND SOCIAL SERVICES SYSTEM: THE DISENCHANTMENT OF ISOLATED SENIOR CAREGIVERS

Melanie Couture,[1] Pam Orzeck,[2] and Apostolia Petropoulos[3], *1. Centre for research and expertise in social gerontology, CIUSSS West-Central Montreal, Montreal, Quebec, Canada, 2. McGill University, Montreal, Quebec, Canada, 3. Jewish Eldercare Centre, Montreal, Quebec, Canada*

Social isolation is one of the negative consequences associated with caregiving and is experienced by approximately 20% of Canadian family caregivers. Being in a public health and social services system, Canadian caregivers should normally turn to their local community service centres (CLSC) to access formal services and feel less isolated. However, studies have shown that satisfaction is low regarding accessibility and continuity of formal support services. In an effort to develop interventions that meet the needs of isolated senior caregivers, the purpose of this exploratory descriptive qualitative study was to identify challenges encountered in accessing and utilizing formal supports within the public health and social services system in Canada. Nineteen isolated senior caregivers participated in seven focus groups. Data analysis was performed using the Miles, Huberman, and Saldana (2014) approach. Results showed that isolated caregivers do not know where to get information about existing services within the formal system. Once services are found, waiting lists are linked to unbearable delays. Some caregivers are actually redirected to private services, if they can afford it. Isolated caregivers also criticize the unpredictability of the system as they face relentless changes of care providers, inadequate services and sometimes unwarranted cancellations or terminations. In addition, they find formal services lacking human sensitivity. Many of them come to the conclusion that formal services are not worthwhile and exclude themselves from the formal system. This research demonstrated that the health and social services system can actually contribute to the social isolation of senior caregivers longing for support.

### SESSION 2050 (SYMPOSIUM)

### FROM BIG DATA TO COMMUNITY SETTINGS: HOW CAN EXERCISE IMPROVE HEALTH OUTCOMES IN OLDER CANCER SURVIVORS?

Chair: Shirley M. Bluethmann, *The Pennsylvania State University College of Medicine, Hershey, Pennsylvania, United States*
Discussant: Corinne Leach, *American Cancer Society, Atlanta, Georgia, United States*

Current guidelines recommend that survivors achieve regular physical activity and reduced inactivity to attenuate cancer and mortality risk and to promote quality of life. Optimal ways of obtaining physical activity or measuring how much exercise has been achieved continues to be a challenge, especially among older adults and cancer survivors. Our symposium will provide insights using big data to understand population level patterns of activity and potential benefits for survivors, examining implications for survival and key health outcomes, including multimorbidity and functional limitations. We will also discuss social and environmental determinants that may be important for older survivors in designing community interventions, especially in rural communities. These include considerations related to the built environment and social support to promote leisure-time physical activity in older survivors.

### PHYSICAL ACTIVITY AND SEDENTARY BEHAVIORS: IMPACT ON SELF-RATED HEALTH AND PHYSICAL OUTCOMES IN CANCER SURVIVORS

Shirley M. Bluethmann,[1] Eileen Flores,[2] Charles Matthews,[3] and Frank Perna[3], *1. The Pennsylvania State University College of Medicine, Hershey, Pennsylvania, United States, 2. Penn State College of Medicine, Hershey, Pennsylvania, United States, 3. National Cancer Institute, Rockville, Maryland, United States*

Physical activity (PA) and avoidance of inactivity are recommended in cancer survivorship. But survivors are not meeting these recommendations. We used national data (NHANES) collected 2011-2014 (n=9620) to estimate associations of PA and TV viewing with 3 health outcomes: self-rated health, functional limitations and multimorbidity in older cancer survivors and adults without cancer. Greater PA was associated with reporting excellent health in survivors. Survivors that obtained 22.5+ MET-hours/week were 5.5 times more likely to report excellent health than those that did no exercise (OR=5.5, p<.001). We observed a decrease in likelihood of multimorbidity and functional limitations with increasing PA (both significant at p<.001). We noted survivors that abstained from watching TV were 3x more likely to report excellent health and between 60-80% less likely to report functional limitations and multimorbidity than TV watchers (p<001). Findings with non-cancer adults were similar. Survivors need PA and reduced TV to maximize health outcomes.

### SELF-REPORTED WALKING PACE AND ALL-CAUSE MORTALITY AMONG CANCER SURVIVORS IN THE NIH-AARP DIET AND HEALTH STUDY

Elizabeth Salerno,[1] Pedro Saint Maurice,[1] Erik Willis,[1] Loretta DiPietro,[2] and Charles Matthews[1], *1. National Cancer Institute, Rockville, Maryland, United States, 2. George Washington University, Washington, District of Columbia, United States*

We examined the association between self-reported walking pace and all-cause mortality among cancer survivors in the NIH-AARP Diet and Health Study. Participants included 30,110 adults (Mage=62.4+/-5.14 years) diagnosed with cancer between study enrollment and follow-up, when they self-reported walking pace. Individuals were followed until death or administrative censoring in 2011. We estimated the hazards ratios (HR) and 95% confidence intervals (CI) for walking pace and all-cause mortality adjusting for age, sex, race, BMI, health status, physical activity and cancer type. Cancer survivors reporting faster walking paces had significantly reduced mortality risk. Relative to those reporting an 'easy' walking pace, walking at a 'normal,' 'brisk,' or 'very brisk' pace was associated with significantly lower risk: [HR=0.74 (0.70,0.78)], [HR=0.66 (0.61,0.71)], and [HR=0.73 (0.60,0.89)], respectively. Being 'unable to walk' was associated with 30% increased mortality [HR=1.30 (1.15,1.46)]. These findings provide novel support for the association between self-reported walking pace and survival after cancer.

### DETERMINANTS OF LEISURE-TIME PHYSICAL ACTIVITY IN OLDER, RURAL CANCER SURVIVORS IN CENTRAL PENNSYLVANIA

Scherezade K. Mama,[1] Nishat Bhuiyan,[1] Eugene Lengerich,[2] and Kathryn Schmitz[3], *1. The Pennsylvania State University, University Park, Pennsylvania, United States, 2. Pennsylvania State University, Hershey, Pennsylvania, United States, 3. The Pennsylvania State University College of Medicine, Hershey, Pennsylvania, United States*

This study explored social and environmental determinants of leisure-time physical activity (LTPA) in cancer survivors (CS) residing in Central Pennsylvania, a largely rural region. Rural CS completed questionnaires assessing LTPA, social support (SS) for LTPA, home and neighborhood environments for LTPA. Logistic regression models were used to assess associations with being active/inactive. Participants (n=219) were categorized as mature survivors (<75 years, 80.7%) or elderly survivors (>=75 years, 19.3%). Only 28.2% of elderly survivors reported meeting LTPA guidelines compared to 45.6% of mature survivors. Survivors reporting SS for LTPA were 10% more likely to active than those who did not have SS (OR=1.1, CI 1-1.1). Mature survivors that reported environmental support (home: OR=1.2: CI 1-1.3; neighborhood: OR=1.8, CI: 1-3.2) were more likely to be active than those without strong environmental support. Creating more supportive environments to foster LTPA in elderly survivors in rural areas is a key priority for future research.

### SESSION 2055 (SYMPOSIUM)

### GAPS AND OPPORTUNITIES TO IMPROVE ACCESS TO HEALTHCARE FOR OLDER RURAL VETERANS

Chair: Bret Hicken, *Veterans Rural Health Resource Center-SLC, Salt Lake City, Utah, United States*
Co-Chair: Aaron T. Seaman, *Iowa City VA Healthcare System, Iowa City, Iowa, United States*
Discussant: Lauren Moo, *Geriatric Research Education & Clinical Center, Bedford, Massachusetts, United States*

Almost 25% of US military veterans live in rural areas and over 70% of these are age 55 years or older. The physical, cognitive, and functional declines common to aging coupled with accumulated physical and psychological traumas often incurred through military service make caring for older Veterans an especially difficult challenge. Furthermore, the geographic distances associated with rural living pose a significant barrier to timely access to care for aging Veterans and caregivers living in remote communities. Significant gaps remain in VA's understanding of the needs of this population, which is essential for developing adequate models of care. This symposium highlights various aspects of the rural older Veteran experience and will suggest opportunities for improving access to care for this population. The first presentation estimates the differential impact of rural status on mortality rates among older Veterans using mortality data from the General Social Survey (Wilmoth et al). A second study is an analysis of urban/rural differences in PTSD symptoms as function of multiple co-factors and accounting for geographic location (Kurth et al). Seaman et al present qualitative data regarding the experience of Veterans with head and neck cancer to understand how rurality impacts cancer survival (Seaman et al). The final presentation will highlight a model to address mental health problems in older Veterans in primary care (Geri-PACT [Kube et al]) and will specifically highlight how this VA program may be adapted to address such needs the needs of older Veterans in rural areas.

### VETERAN MORTALITY DISADVANTAGE AMONG RURAL, SUBURBAN, AND URBAN RESIDENTS

Janet M. Wilmoth,[1] Scott D. Landes,[1] and Andrew S. London[1], *1. Syracuse University, Syracuse, New York, United States*

Although veterans tend to have higher mortality rates than non-veterans, recent research suggests there is substantial heterogeneity in veteran mortality on the basis of various characteristics such as race, period of service, type of health insurance coverage, and service-connected disability status. This analysis extends the extant literature by using the 1978-2014 General Social Survey linked to the National Death Index (GSS-NDI) to examine veteran status differences in mortality by geographic location. We estimate a series of Cox regression models predicting death for male veterans and nonveterans, controlling for age, race/ethnicity, education, income, and work status. Separate models are presented for rural, suburban, and urban residents. All models are weighted and include robust standard errors. The results indicate that rural veterans have higher mortality risk than rural non-veterans, particularly among older adults. There are no significant differences in mortality risk between veteran and non-veterans living in suburban and urban areas.

### PTSD SYMPTOMS AMONG VIETNAM, PERSIAN GULF, AND OEF/OIF/OND VETERANS: A RURAL/URBAN COMPARISON

Maria Kurth,[1] Soyoung Choun,[2] Dylan Lee,[2] David Rothwell,[2] and Carolyn aldwin[2], *1. OSU Oregon State University, Corvallis, Oregon, United States, 2. Oregon State University, Corvallis, Oregon, United States*

There are mixed results in studies examining rural/urban differences in PTSD symptoms among veterans; however, many of these studies failed to consider possible confounds with geographic location. This study examined rural/urban differences in PTSD symptoms by combat exposure, war cohort, and gender. The VALOR (Veterans Aging: Longitudinal studies in Oregon) pilot study sampled Vietnam, Persian Gulf, and OEF/OIF/OND war cohorts using an online survey. The sample (N=237, Mage=57.84, SD=12.68) was mainly male (65%), White (85%), and urban (75.95%); most reported combat exposure (71%). Participants completed measures of PTSD, combat exposure, and demographics. Results indicate no effect of cohort or rural/urban status on PTSD symptoms. There was a significant effect of combat exposure, $F(1,224)=4.58$, $p=.03$, and gender, $F(1,224)=4.13$, $p=.04$, with males reporting higher levels of PTSD symptoms and combat exposure. Contrary to our expectations, there were no effects of cohort or geographic location on PTSD symptoms.

### THE GRAYING OF PRIMARY CARE: THE ROLE OF PSYCHOLOGY IN GERIATRIC PRIMARY CARE

Erin Kube,[1] Grant Harris,[2] and Bret Hicken[3], *1. Hines VAMC, Hines, Illinois, United States, 2. St. Louis VAMC, St. Louis, Missouri, United States, 3. SLC VAMC, Salt Lake City, Utah, United States*

As of 2012, more than half of Veterans receiving care within a VA medical facility were age 65 or older. They have complex co-occurring medical and mental health needs, cognitive impairments, functional deficits, and psychosocial complexity. In 2015, GeriPACT emerged as a specialized geriatric primary care clinic model to serve this vulnerable population. The presence of psychologists in geriatrics has significant implications for treatment of cognitive, behavioral, and psychosocial needs. This mixed methods project aimed to describe the current scope and functions of GeriPACT psychologists and differentiate their services from other PACT clinics. Twenty total GeriPACT psychologists participated. The results suggest that mental health services within GeriPACT are multifaceted and need-driven. Significant themes highlight role specific characteristics of psychologists, clinician backgrounds, team education, and referral processes to improve access to care. Recommendations for implementation, clinician training, and future policy planning will also be presented.

### SESSION 2060 (SYMPOSIUM)

### HARNESSING TECHNOLOGY TO SUPPORT PERSONS WITH DEMENTIA AND THEIR CAREGIVERS

Chair: Stacy L. Andersen, *Boston University School of Medicine, Boston, Massachusetts, United States*
Co-Chair: Walter Boot, *Florida State University, Tallahassee, Florida, United States*
Discussant: Jeffrey Kaye, *Oregon Health & Science University, Portland, Oregon, United States*

One in eight older adults in the US has Alzheimer's disease or a related dementia, which are characterized by progressive cognitive and physical declines. The impact of dementia also goes beyond the individual since 92% of persons with dementia receive functional and emotional support from family members and other informal caregivers. The time demands, financial strain, and emotional toll of caregiving are known to cause increased stress and health problems. Therefore, there is a wealth of opportunities to develop new ways to intervene in the progressive loss of function among persons with dementia and ways to support them and their caregivers. Co-sponsored by the Alzheimer's Disease and Related Dementias and Technology and Aging Interest Groups, this symposium addresses innovations in the implementation of new and existing technologies in the dementia care continuum. We will discuss the development and testing of a new mobile application designed to integrate both physical activity and cognitive training. Then we will discuss results from a virtual support group intervention to provide disease education, care planning, and emotional and social support among persons newly diagnosed with Alzheimer's disease and living alone. Next we will share results from a study using customized voice-assisted technologies to enable individuals with memory impairment to maintain independence and quality of life and reduce caregiver burden. Finally, we will present findings regarding the validity and accuracy of a wearable sensor-based device that measures skin conductivity and heart rate variability to monitor stress level among caregivers of persons with dementia.

### DEVELOPMENT AND TESTING OF A MOBILE APPLICATION INTEGRATING PHYSICAL ACTIVITY AND COGNITIVE TRAINING

Lenora Smith[1], *1. University of Alabama in Huntsville, Huntsville, Alabama, United States*

Dementia is progressive, which causes a debility in activities of daily living, leading to bedridden and uncommunicative individuals. Evidence has shown that physical activity may protect the brain from memory impairment. Based on a previous study we conducted using consumer-based applications with people with mild cognitive impairment, we developed a mobile application called the mPACT app (mobile physical activity and cognitive training). The System Usability Scale and Usability Test Observation Coding Form were used to capture usability and verbal/nonverbal behaviors respectfully of 5 participants. Results noted an above average score for usability, with a mean score of 72.5. Observations noted that almost all participants were smiling and enjoying most of the games within the app; some had difficulty with a couple of the games. Overall, there was a positive response to the mPACT app, revisions have been made, and next steps include another beta test with people with MCI.

### A VIRTUAL SUPPORT GROUP FOR PERSONS LIVING ALONE WITH ALZHEIMER'S DISEASE

Allison Gibson[1], *1. University of Kentucky, Lexington, Kentucky, United States*

To date, early stage programming for the persons with the disease has been limited. The purpose of this study was to explore the challenges and opportunities of a virtual support group intervention for persons living alone with Alzheimer's disease (AD). Following recruitment, participants who were newly diagnosed (within 2 years of diagnosis) and residing alone in their own residence were included in a pilot study of a virtual support group intervention for a 3-month period. Data were collected before and after the intervention through the use of surveys and one-on-one interviews with all 12 participants (n=12). Data were analyzed using a mixed methods approach including thematic analysis. Results indicated that the virtual support group intervention increased group members' education of the disease, knowledge regarding care planning, feelings of empowerment towards the diagnosis of the disease, and increased feelings of social support. Implications for such interventions will also be discussed.

### VOICE FIRST TECHNOLOGY: SIMPLIFYING LIFE FOR COMMUNITY-DWELLING OLDER ADULTS LIVING WITH DEMENTIA

Debra J. Sheets,[1] Marilyn Malone,[2] Stuart W. MacDonald,[1] Carl Asche,[3] and Andre Smith[1], *1. University of Victoria, Victoria, British Columbia, Canada, 2. Island Health, Victoria, British Columbia, Canada, 3. University of Illinois-Peoria, Peoria, Illinois, United States*

Voice first technology offers older adults with dementia support that may maintain independence, reduce social isolation and improve quality of life (QoL). This study investigates the impact of a voice-controlled technology customized to the needs of participants living with dementia and their caregivers. A mixed methods design focused on psychosocial factors and usability characteristics. The purposive sample consisted of older adults with dementia (n=12) and their care partners (n=12)) living independently in the community. Validated measures for cognition, depression, caregiver burden, quality of life and usability were included. Qualitative in-home interviews were conducted to assess impact on social connections and independence. Results indicate that voice first technology can reduce caregiver burden and can support the independence and QoL of older adults with dementia. The discussion considers the value of low cost voice first technology as a way to support older adults with dementia and their caregivers.

### DETECTING STRESS INCIDENCE WITH WRISTBAND SENSOR-BASED DEVICE: A WAY TO SUPPORT CAREGIVERS OF PERSONS WITH DEMENTIA

Angela YM Leung[1], *1. Centre for Gerontological Nursing, School of Nursing, The Hong Kong Polytechnic University, Hong Kong, Hong Kong*

This experimental study aimed to investigate the sensitivity of detecting stress level with a wearable device. A total of 10 caregivers underwent a psychological stress test with a wearable sensor-based device that measured skin conductivity (Electrodermal Activity, EDA) and heart rate variability (Interbeat Interval, IBI). Two sample t-tests were used to compare the mean scores of EDA and IBI at baseline and during stress. The accuracy for classifying stress test epochs was 70%. There was a significant increase in EDA at the time when the participants were under stress [mean score (SD) at baseline vs at stress: 0.089 (SD 0.150) vs 0.427 (SD 0.430), $p < 0.001$]. However, insignificant changes in IBI were observed. In conclusion, this wearable sensor-based device was able to recognize stress incidence and accurately capture physiological changes. It has a potential to be part of the sensor-based stress monitoring and alleviating system (SbSMA).

### SESSION 2065 (SYMPOSIUM)

### INTEREST GROUP SESSION—DISASTERS AND OLDER ADULTS: HARNESSING THE POWER OF SOCIAL NETWORKS DURING DISASTERS TO FACILITATE PREPARATION AND RECOVERY

Chair: Judith Robertson R. Phillips, *California State University San Marcos, San Marcos, California, United States*
Discussant: Katie E. Cherry, *Louisiana State University, Baton Rouge, Louisiana, United States*

With the increasing occurrences of disasters throughout the world, researchers, communities, and organizations have become interested in how the use of social networks during and after a disaster can ease the psychological recovery of older adults who are affected by traumatic disaster events such as hurricanes and wildfires. This symposium will focus on the power of social networks and highlight the importance of preparedness, informal and formal groups, and interventions to assist recovery of older adults. First, Dr. Judith Phillips will present data on how informal and formal social network groups affected the psychological well-being of older adults who experienced exposure to multiple wildfires. Second, Dr. Denise Eldemire-Shearer will address how both formal and informal social network groups in Jamaica are informed and mobilized to provide support to older adults on the island during hurricanes and other water-related disasters. Third, Dr. Lisa Brown will introduce the Skills for Psychological Recovery intervention and provide an overview and modifications needed when using with older adults; she will also demonstrate an exercise. Fourth, Dr. Debra Dobbs will present themes examining the role of community engagement in hurricane preparedness which were gathered from focus groups and interviews with assisted living administrators in Florida. Lastly, Dr. Mary Helen McSweeney-Feld will address how voluntary organizations active during disasters aid older adults with disaster relief efforts. Together these presenters will provide evidence of the power of various social networks that will lessen the vulnerability of older adults after disasters.

### IMPACT OF INFORMAL AND FORMAL SOCIAL NETWORKS DURING A WILDFIRE ON PSYCHOLOGICAL RECOVERY

Judith Robertson R. Phillips[1], *1. California State University San Marcos, San Marcos, California, United States*

Having the support of a social network can play an important role towards psychological well-being for those impacted by a disaster. The purpose of this study was to investigate how type (emotional, tangible, informational) and source (family, friends, others) of social support during three Southern California wildfires impacted the psychological well-being of three victim-groups (based on severity of exposure) of community-residing adults (N= 203; meanage = 63.4 years; range 50-94 years) who responded to surveys about their experiences during the 2007, 2014, and 2017 northern San Diego County, CA wildfires. Analyses revealed that emotional social support from friends and neighbors was the most frequently received type and source of support, especially for the primary victim-group, those with residential loss. All victim-groups exhibited healthy psychological well-being. Discussion will focus on how informal groups differed and were similar to formal groups in providing social support during a disaster to encourage psychological recovery.

### PERSPECTIVE FROM A CARIBBEAN ISLAND, JAMAICA

Denise Eldemire-Shearer,[1] Kayon M. Donaldson-Davis,[2] Julian McKoy Davis,[3] and Douladel M. Willie-Tyndale[3], *1. Mona Ageing & Wellness Centre, Kingston, Jamaica, 2. Mona Ageing & Wellness Centre, Kingston 7, Saint Andrew,*

*Jamaica, 3. Mona Ageing & Wellness Centre, Kingston 7, Kingston, Jamaica*

Jamaica is prone to hurricanes and associated flooding. The older adult population is vulnerable given the increasing ageing population (12% over age 60), high rate of disability (20% - 30%) low literacy levels, increased feminization, and high numbers of old-old. This paper describes strategies employed in disaster management with a special focus on older persons as a vulnerable group. The significant contribution of social networks to disaster mitigation will also be discussed. Age-friendly strategies include older person representation on local disaster committees; mainstreaming the needs of older persons in community disaster plans; training of disaster workers on ageing and the special needs of older persons; maintaining community lists of frail, single and house-bound older persons. Social networks especially faith-based organisations are integral in disaster preparedness and recovery activities.

### SKILLS FOR PSYCHOLOGICAL RECOVERY DURING AND AFTER DISASTERS TO STRENGTHEN SOCIAL SUPPORT

Lisa M. Brown[1], *1. Palo Alto University, Plo Alto, California, United States*

The combination of an aging population and a limited number of disaster responders reveals a need for trained laypeople who can implement interventions that facilitate recovery after traumatic events. Skills for Psychological Recovery (SPR) is an intervention that is designed to be implemented after use of Psychological First Aid with people who were exposed to a traumatic stressor. SPR uses a skills-building approach to promote self-efficacy, support resilience, strengthen social networks, and ameliorate the negative impact of traumatic life events. Strengthening social support is beneficial as research clearly demonstrates that destruction of communities and loss of informal social support networks produces long-term psychological distress for older adults. This presentation will demonstrate a SPR social support exercise with attendees, provide an overview of the other modules, and describe modifications for using SPR with older adults. During times of crisis, social support systems are critical to the psychological well-being of older adults.

### THE ROLE OF COMMUNITY ENGAGEMENT IN HURRICANE PREPAREDNESS AS PERCEIVED BY ASSISTED LIVING ADMINISTRATORS

Debra J. Dobbs,[1] Joseph June,[1] Lindsay J. Peterson,[1] David Dosa,[2] Dylan Jester,[1] and Kathyrn Hyer[1], *1. University of South Florida, Tampa, Florida, United States, 2. Brown University, Providence, Rhode Island, United States*

The importance of communities in disasters has been well established since Hurricane Katrina. Smit and Wandel's bottom-up approach to assess risks during a disaster involves community stakeholders. Administrators of assisted living (AL) environments increasingly have to assess the risks of hurricane evacuation for vulnerable older adults. The current study examines intersections between social networks, communication and preparedness during a hurricane for AL administrators. We conducted focus groups and interviews with AL administrators (N=60) in Florida about communication patterns with community associations, emergency management officials, AL staff, residents and their families during Hurricane Irma (2017) and about their perceptions of preparedness. A content analysis approach was used. Atlas.ti v7 was used for initial and axial coding. Co-occurrences were found among communication and preparedness themes. Some prevalent themes included "social capital", "high versus low tech strategies" and "leadership effectiveness". Themes intersected with individual administrator and AL organizational characteristics.

### VOADS AND OLDER ADULTS IN DISASTERS

Mary H. mcsweeney-feld[1], *1. towson university, Towson, Maryland, United States*

Voluntary Organizations Active in Disasters (VOADs) are a key mechanism for sharing knowledge and resources throughout a disaster cycle. VOADs work as a coalition of not-for-profit organizations that can respond to disasters on a local level in a coordinated response. Given the changes in FEMA's Strategic Plan for 2018-2022, the role of VOADs will become more important over time. It is unclear whether the needs of older adults in disasters will be addressed by VOAD coalitions. Organizations such as public health departments and emergency management services are typically involved in the activities of VOAD organizations, but it is unclear whether organizations serving older adults know about VOAD coalitions or have considered becoming members of these organizations. A case study of the state of Maryland's VOAD coalition will be used as an example of how one state VOAD is able to provide services and supports for older adults in disasters.

### SESSION 2070 (SYMPOSIUM)

### INTEREST GROUP SESSION—NUTRITION: IMPLEMENTATION OF NUTRITION, BODY COMPOSITION, AND FUNCTIONAL ASSESSMENT IN VULNERABLE OLDER ADULTS

Chair: Rose Ann DiMaria-Ghalili, *Drexel University, Philadelphia, Pennsylvania, United States*
Discussant: Julie Locher, *University of Alabama at Birmingham, Birmingham, Alabama, United States*

This symposium will highlight the implementation of nutrition, body composition and functional assessment in vulnerable older adults. Understanding the barriers and enablers of implementing traditional nutrition screening and assessment tools, nutrition focused physical examination, body composition analysis, functional assessment and biomarkers can aid researchers in the design of pragmatic nutrition studies in vulnerable older adults. DiMaria-Ghalili will provide an introduction to overall nutrition assessment in community-dwelling older adults and present lessons learned in regards to nutrition assessment protocols (Mini-Nutritional Assessment, hand-grip strength, and inflammatory biomarkers) from an ongoing clinical trial. Starr will discuss the issues surrounding nutrition screening and assessment in high-risk older adults undergoing abdominal surgery. A comparison of data on different nutrition screening and assessment tools (NRS-2002; PG-SGA, Nutrition Focused Physical Examination) in older adults undergoing abdominal surgery will be presented. Batsis will discuss the controversies and challenges in body composition assessment focusing on sarcopenic obesity. Data highlighting the different diagnostic criteria of sarcopenic obesity and diagnostic accuracy will be presented. The link between overall nutrition status and physical function on health outcomes is emerging in older adults. Bales will discuss the types of physical function assessment tools used in studies of older adults and discuss her findings on the impact of higher protein intake on functional outcomes. The symposium will conclude with a discussion led by Locher on practical implications of integrating best assessment techniques into clinical practice.

### NUTRITION ASSESSMENT PROTOCOLS IN COMMUNITY-DWELLING OLDER ADULTS WITH CHRONIC WOUNDS

Rose Ann DiMaria-Ghalili,[1] Sarah Charbonneau,[1] Keyanna Bynum,[1] Michael Neidrauer,[1] Michael S. Weingarten,[1] and Peter A. Lewin[1], *1. Drexel University, Philadelphia, Pennsylvania, United States*

Older adults are at risk for altered nutritional status and functional impairment due to physiological (e.g., age-related changes, acute and chronic co-morbid conditions) and psychosocial factors (e.g., depression, loneliness, cognitive impairment). Those with alterations in nutritional and/or functional status are at risk for poor health outcomes-including delayed healing of chronic wounds. We will discuss our lessons learned when devising nutrition assessment protocols from our ongoing double-blind randomized control clinical trial testing the effectiveness of ultrasound treatment on healing chronic leg wounds. The discussion will focus on the following measures: the Mini-Nutritional Assessment, handgrip strength assessment, and inflammatory biomarkers.

### NUTRITION ASSESSMENT IN HIGH-RISK PATIENTS: NRS-2002, PG-SGA, AND NFPE IN OLDER ADULTS PREPARING FOR ELECTIVE SURGERY

Kathryn N. Porter Starr,[1] Kenlyn Young,[1] Shelley R. McDonald,[1] Nancy Loyack,[2] Sandhya Sandhya Lagoo-Deenadayalan,[1] Mitchell T. Heflin,[1] Carl F. Pieper,[3] and Connie W. Bales[3], *1. Duke University School of Medicine, Durham, North Carolina, United States, 2. Durham VA Medical Center, Durham, North Carolina, United States, 3. Duke University Medical Center, Durham, North Carolina, United States*

Post-surgical complications are most common in older adults. While a number of factors contribute, one key determinant is malnutrition. Malnutrition is seen in up to 86% of older adults at hospital admission. Malnutrition and post-surgical complications are linked through two critical observations: 1) malnutrition dramatically reduces the ability of older adults to overcome postsurgical health stressors, and 2) nutritional status is likely to deteriorate further during hospitalization and after discharge. Despite convincing evidence that perioperative nutrition intervention can improve surgical outcomes, nutrition screening and assessment in the preoperative period is not required or standardized. We will review issues surrounding screening and assessment of malnutrition in older adults preparing for elective surgery and present data on screening (NRS-2002) and assessment tools (Nutrition Focused Physical Exam and PG-SGA) used in this high-risk population. Finally, we will discuss best practices for identifying and intervening with malnourished older adults in the preoperative setting.

### PHYSICAL FUNCTION AND BODY COMPOSITION AS NUTRITIONAL OUTCOMES: ASSESSMENT APPROACHES

Connie W. Bales,[1] Kathryn N. Porter Starr,[1] and Marshall Miller[1], *1. Duke University, Durham, North Carolina, United States*

Nutritional status is a strong determinant of both body composition and physical function (PF), parameters that are closely interrelated but rarely evaluated in the clinical setting due to cost, access, and lack of agreement on best approaches in older adults. Recent evidence that changes in muscle mass do not closely correspond to changes in muscle function will be reviewed in the context of our studies of higher protein obesity interventions. PF assessments, including indices for older adults (Short Physical Performance Battery and Physical Performance Test), as well as specific tests like gait speed and handgrip strength, will be explained as nutrition outcomes and in relation to body composition from air displacement (BodPod) and dual energy x-ray absorptiometry (DXA). These results, along with new studies of muscle quality, will bring a better understanding of the complexity of responses to nutritional interventions designed to optimize body mass and composition in older adults.

### CONTROVERSIES AND CHALLENGES IN DEFINING SARCOPENIC OBESITY

John A. Batsis[1], *1. Geisel School of Medicine at Dartmouth, Lebanon, New Hampshire, United States*

Sarcopenia is defined as the loss of muscle mass, strength and physical function with aging and in conjunction with obesity, leads to incrementally adverse outcomes. There is no current consensus for defining this disease entity, making an accurate evaluation challenging in both the research and clinical settings. We will review the definitions put forth by the Sarcopenia Definition and Outcomes Consortium and the European Working Group for the Study of Sarcopenia. We will present data highlighting the different diagnostic criteria of sarcopenic obesity and the diagnostic accuracy of common anthropometric measures in measuring adiposity using data from the National Health and Nutrition Examination surveys. The advantages and disadvantages of the different modalities of assessing body composition will be discussed, including body impedance analysis, dual energy x-ray absorptiometry, computer tomography and magnetic resonance imaging in addition to simple, novel clinical screening tools for obesity sarcopenia will be presented.

### SESSION 2075 (SYMPOSIUM)

### IMPLEMENTING THE MEDICARE ANNUAL WELLNESS VISIT: A GWEP COLLABORATION

Chair: Ellen Flaherty, *Dartmouth-Hitchcock Medical Center, Lebanon, New Hampshire, United States*
Discussant: Nina Tumosa, *Health Resources and Services Administration, Rockville, Maryland, United States*

The Geriatric Interprofessional Team Transformation in Primary Care (GITT-PC) model improves delivery of healthcare to older adults in primary care by training healthcare professionals in team functioning, rapid cycle QI, and evidence based geriatric practice. The program capitalizes on the role of nursing and other healthcare disciplines. To maximize sustainability, it focuses on Medicare-reimbursable visits. This program will focus on the implementation of the Annual Wellness Visit (AWV) through a collaboration between Dartmouth and 3 GWEPs: University of Florida, University of Louisville and the University of Wyoming. The model's standardized approach to implementation begins with practice assessments and two trainings. The first training focuses on team functioning & rapid cycle QI and the second is a deep dive training focused on the implementation of the AWV. After the training, practices participate in a data-driven, virtual learning collaborative with monthly data collection and learning sessions. Since 2015, the AWV, through the GITT-PC model has been implemented in 14 sites in northern New England, 10 sites in upstate New York, and nationally through five other GWEP awardees across the country.

### A GWEP COLLABORATION: IMPLEMENTING THE AWV IN A RURAL PRIMARY CARE CLINIC

Christine McKibbin[1], *1. University of Wyoming, Laramie, Wyoming, United States*

This presentation will focus on the collaboration with the Dartmouth GWEP to implement the AWV in a rural primary care clinic. The challenges of practice transformation in busy primary care clinics will be discussed along with lessons learned on a successful GWEP partnership to achieve improved patient outcomes in primary care.

### A TEAM APPROACH TO THE IMPLEMENTATION OF THE ANNUAL WELLNESS VISIT

Lucy Guerra[1], *1. University of Florida, Tampa, Florida, United States*

The success of any practice change initiative is dependent upon a highly effective team. This presentation will focus on the "secret sauce" of our implementation of the AWV in collaboration with the Dartmouth GWEP. Participating in both asynchronous and live virtual training enabled our team to come together to successfully implement the AWV using a team based model. Using the principles of highly effective teaming and a rapid cycle QI approach we have been able to improve patient outcomes in primary care.

### A UNIQUE APPROACH TO INTERPROFESSIONAL TRAINING USING THE ANNUAL WELLNESS VISIT HEALTH RISK ASSESSMENT

Anna Faul[1], *1. University of Louisville, Louisville, Kentucky, United States*

The Kentucky (KY) Rural & Underserved Geriatric Interprofessional Education Program (KRUGIEP) participated in a unique innovative approach to the implementation of the Medicare Annual Wellness visit in collaboration with the Dartmouth GWEP. The model focused on the integration of students into the process of conducting the Health Risk Assessment through community based home visits. This talk will focus on this unique program and the participation in a multi-GWEP learning collaborative.

### SESSION 2080 (SYMPOSIUM)

### INTEREST GROUP SESSION—MENTAL HEALTH PRACTICE AND AGING: INTEGRATION OF SOCIAL DETERMINANTS OF HEALTH IN DEFINING HEALTH BEHAVIORS AND OUTCOMES AMONG DIVERSE OLDER ADULTS

Chair: Jacquelyn Minahan, *University of Kansas, Lawrence, Kansas, United States*
Discussant: Tamara A. Baker, *University of Kansas, Lawrence, Kansas, United States*

Social determinants of health (SDoH) are conditions in which individuals live, learn, work, and play. Specifically, they are influenced by the distribution of resources, money, and power, and have significant implications on health behaviors and outcomes across the life span. Existent data show the influence these indictors may have in the onset and progression of chronic illnesses. However, much of these data focus on the effect of race and health, as social determinants, but fail to adequately address the myriad other factors (e.g., health care, social and community context) that influence the social patterning across the life course. This symposium presents findings from several studies highlighting the nuanced role of SDoH across diverse populations of older adults. Scholars will present findings on the influence that identified determinants, such as social networks, lifestyle behaviors, and gender, have in defining health outcomes across the life course. Minahan presents the relationship between chronic illnesses and depression and compares depressive symptomatology according to disease cluster in a nationally-representative sample of older adults. Atakere discusses determinants of well-being among African American males with chronic illnesses and the challenges associated with this marginalized population. Booker examines spirituality as a mechanism for pain management among older African Americans and presents this as a crucial determinant of health. This symposium will expand on the existing body of literature by emphasizing social and cultural determinants, aside from race, that influence health behaviors and outcomes across the life span.

### MULTIMORBIDITY IN OLDER ADULTS: CAN DISEASE CLUSTER PREDICT DEPRESSION SEVERITY?

Jacquelyn Minahan[1], *1. University of Kansas, Lawrence, Kansas, United States*

Multimorbidity, defined as the co-occurrence of two or more chronic conditions, is positively correlated with depression severity among older adults. However, few studies have compared depression outcomes by disease cluster. To address this gap, secondary data analyses were performed using data from the National Social Life, Health, and Aging Project (NSHAP), Wave 2. For the purpose of this study, disease clusters are composed of conditions that implicate similar body systems (e.g., musculoskeletal system, cardiovascular system). Participants reported an average of 2.69 (+/- 1.97) chronic conditions. Multimorbidity and depressive symptom severity, as measured by the Center for Epidemiological Studies – Depression, Iowa Form (CES-D) were positively associated ($p<0.001$). Individual disease clusters, age, self-identifying as female, and lower educational attainment were predictive of depressive symptom severity ($p<0.001$). Findings support the necessary inclusion of social determinants (health status, gender, education, age) in the conceptualization of health and health outcomes within an aging population.

### DETERMINANTS OF GENERAL WELL-BEING IN BLACK MALES WITH CHRONIC ILLNESS

Darlingtina Atakere[1], *1. University of Kansas, Lawrence, Kansas, United States*

Over the last decades, considerable attention has been directed towards examining the well-being of people living with chronic illness. The presence of one or more chronic illnesses challenges their quality of life and general well-being, thus, impacting their abilities to function physically, psychologically, and socially. I investigated reports of general well-being in Black males with chronic illness(es) in a sample of N=242 participants. The males were aged 35–63 and identified as Black/African American males. The participants responded to items assessing general well-being; ethnic identity; self-esteem; active coping; the presence of chronic illness(es); and additional demographic, social and ecological characteristics. Analyses of responses indicated that marital status, ethnic identity, self-esteem are significant determinants of general well-being in Black males with chronic illness(es). Data further showed active coping to be negatively correlated with well-being. I discuss the implications of results for the understanding of health outcomes among this marginalized population.

### SPIRITUALITY IN CHRONIC PAIN SELF-MANAGEMENT: LOWER USE BUT EQUAL IMPORTANCE IN OLDER AFRICAN AMERICANS

Staja Booker[1], *1. The University of Florida, Gainesville, Florida, United States*

Spirituality is a key social determinant of health for African Americans (AAs) and strongly impacts management of chronic pain. Older AAs (average age 68± 12.37) from urban and rural communities completed questionnaires (N= 110) and audio-recorded, semi-structured individual interviews (N= 18) describing osteoarthritis pain self-management. Prayer was used by 42% of AAs, with substantially fewer attending church (23.6%), watching religious television or reading the Bible/Christian literature (20.9%), listening to gospel music (18.2%), and laying of hands (8.2%). Interestingly, prayer and church attendance were the only pain strategies rated by more participants as very helpful. Regardless of religiosity, most AAs believed that spirituality was "an important aspect, whether we realize it always or not". Specifically, prayer was considered "number one… 'cause I know it's gonna be all right once I do pray…prayer help heal the pain". Spiritual strategies remain integral for chronic pain self-management despite lower than expected use among AAs.

### SESSION 2085 (SYMPOSIUM)

### POLICY SERIES: INTERDISCIPLINARY PUBLIC POLICY DISCUSSION SESSION

Chair: Linda K. Harootyan, *Wilmington, North Carolina, United States*
Discussant: Brian W. Lindberg, *The Gerontological Society of America, Washington, District of Columbia, United States*

This session, organized by the GSA Public Policy Committee, will provide both GSA section leadership and attendees an opportunity to have an open dialogue on important public policy issues of significance to the aging population. Section leaders will discuss key policy issues of particular relevance to their section's mission and purpose. They also will comment on improving physical and mental health to illustrate how their different disciplines and perspectives inform and apply to public policy on that issue. This will be an interactive session with plenty of opportunity for audience feedback and input.

### PUBLIC POLICY ISSUES OF SIGNIFICANCE TO THE BIOLOGICAL SCIENCES

Scott Leiser[1], *1. University of Michigan, Ann Arbor, Michigan, United States*

This presentation will cover public policy issues of significance to the aging population, focusing on the perspective of the biological sciences and on policies that may improve physical and mental health.

### PUBLIC POLICY ISSUES OF SIGNIFICANCE IN SOCIAL RESEARCH, POLICY, AND PRACTICE

Robert Harootyan[1], *1. Senior Service America Inc., Silver Spring, Maryland, United States*

This presentation will cover public policy issues of significance to the aging population, focusing on the perspective of the social research, policy and practice community and on policies that may improve physical and mental health.

### POLICY PRIORITIES: PERSPECTIVES FROM THE BEHAVIORAL AND SOCIAL SCIENCES

Karl Pillemer[1], *1. Cornell University, Ithaca, New York, United States*

This presentation will cover public policy issues of significance to the aging population, focusing on the perspective of the behavioral and social sciences and on policies that may improve physical and mental health.

### SIGNIFICANT PUBLIC POLICY ISSUES IN THE HEALTH SCIENCES

Stephen Kritchevsky[1], *1. Wake Forest School of Medicine, Winston-Salem, North Carolina, United States*

This presentation will cover public policy issues of significance to the aging population, focusing on the perspective of the health sciences and on policies that may improve physical and mental health.

### SESSION 2090 (SYMPOSIUM)

### IS AGING AN ACQUIRED MITOCHONDRIAL DISEASE?

Chair: Alessandro Bitto, *University of Washington, Seattle, Washington, United States*

Mitochondrial dysfunction is a hallmark of aging, but severe mitochondrial dysfunction leads to rare childhood disorders such as Leigh Syndrome. This session explores the similarities and differences between normative aging and mitochondrial disease and the potential for interventions to positively impact both conditions.

### AGING AND MITOCHONDRIAL DISEASE: SHARED MECHANISMS AND THERAPIES?

Alessandro Bitto,[1] Herman Tung,[2] Kejun Ying,[3] Daniel L. Smith,[4] Ernst-Bernhard Kayser,[5] Philip G. Morgan,[5] Margaret M. Sedensky,[5] and Matt Kaeberlein[6], *1. University of Washington, Seattle, Washington, United States, 2. Allen Institute for brain science, Seattle, Washington, United States, 3. School of Life Sciences, Sun Yat-sen University, Guangzhou Shi, Guangdong, China (People's Republic), 4. Department of Nutrition Science, University of Alabama, Birmingham, Alabama, United States, 5. Seattle Children's Research Institute, Seattle, Washington, United States, 6. Department of Pathology, University of Washington, Seattle, Washington, United States*

Mitochondrial disease describes multiple pathologies characterized by a wide array of disease symptoms and severity, caused by mitochondrial dysfunction in one or multiple organs. Aging organisms display a similar variety of disease phenotypes, which are often characterized by mitochondrial impairment. Despite the heterogeneity of aging phenotypes, several interventions have been identified which can increase lifespan and delay the onset of age-related diseases in multiple organisms. Two age-delaying interventions, rapamycin and acarbose, dramatically suppress pathology in a mouse model of mitochondrial disease caused by depletion of the NADH-Ubiquinone Oxidoreductase Complex (Ndufs4-/-). This model recapitulates human Leigh syndrome, a childhood mitochondrial disease. Upon treatment with either drug, disease suppression is accompanied by a remodeling of nutrient metabolism and restoration of the NAD+/NADH ratio in the brain without affecting the electron transport chain. Thus, we propose that metabolic derangements induced by mitochondrial dysfunction may be a shared mechanism of aging and mitochondrial disease.

### TURNING THE OXYGEN DIAL AS A THERAPY: HYPOXIA TREATMENT FOR MITOCHONDRIAL DYSFUNCTION

Isha Jain[1], *1. University of California, San Francisco, San Francisco, California, United States*

Mitochondrial disease affects 1 in 4800 live births, with little in the way of therapies. We found that chronic hypoxia extends the life of a Complex 1 disease model by 5-fold. Starting hypoxia therapy at a late-stage of disease can even reverse the MRI-detectable lesions. At the other extreme, mild hyperoxia greatly exacerbates disease and leads to death within several days. These findings have now led to a phase 1 clinical trial in healthy volunteers, with the ultimate goal of human translation. We believe we have identified a new mode of treatment that will be broadly applicable to different forms of mitochondrial dysfunction, ranging from rare inborn errors of metabolism to more common, age-associated pathologies. We believe that "turning the oxygen dial" to low or high oxygen will serve as a novel therapeutic for a range of conditions in the coming years.

### MITOCHONDRIA AS REVERSIBLE REGULATORS OF AGING ASSOCIATED SKIN WRINKLES AND HAIR LOSS IN MICE

Keshav K. Singh[1], *1. University of Alabama at Birmingham, Birmingham, Alabama, United States*

To evaluate the consequences of the decline in mtDNA content associated with aging we have created an inducible mouse model expressing, in the polymerase domain of POLG1, a dominant-negative mutation that induces depletion of mtDNA. We utilized this inducible mouse model to modulate mitochondrial function by depleting and repleting the mtDNA content. We demonstrate that, in mice, ubiquitous expression of dominant-negative mutant POLG1 leads to 1) reduction of mtDNA content in skin, 2) skin wrinkles, and 3) hair loss. By turning off the mutant POLG1 transgene expression in the whole animal, the skin and hair phenotypes revert to normal after repletion of mtDNA. Thus, we have developed whole-animal mtDNA depleter-repleter mice. These mice present evidence that mtDNA homeostasis is involved in skin aging phenotype and loss of hair and provide an unprecedented opportunity to create tissue-specific mitochondrial modulation to determine the role of the mitochondria in a particular tissue.

### DOES AUGMENTATION OF MITOCHONDRIAL THIOREDOXIN REDUCTASE 2 IMPROVE METABOLIC FITNESS?

Sandra Chocron,[1] and Andrew Pickering[2], *1. Barshop Institute for aging and longevity studies, University of Texas Health Science Center, San Antonio, Texas, United States, 2. Barshop Institute for aging and Longevity studies, UTHSCSA, San Antonio, Texas, United States*

Mitochondrial Thioredoxin Reductase (TrxR2) is a rate limiting enzyme in the mitochondrial thioredoxin system which serves as one of the major mitochondrial ROS scavenging pathways. Txnrd2 is also a repressor of the ASK-1 oxidative stress induced apoptotic pathway. Our group previously identified a correlation with the expression of this protein and long-lived species and its overexpression prolonged lifespan in Drosophila. We have generated a TrxR2 transgenic (T-tg) mouse which has ubiquitously heighted (two-fold) TrxR2 expression. We found that overexpression of TrxR2 leads to enhanced mitochondrial metabolism and increased resistance to mitochondrial oxidative damage in MEFs (data not shown). We also found that female T-tg mice showed a leaner trend and reduced food consumption, with improved glucose tolerance but no difference in insulin sensitivity. These mice showed a lower Oxygen consumption and CO2 production with lower energy expenditure in individual metabolic cages. We further tested their exercise capacity where T-tg mice had a similar performance to control mice. These results suggest that TrxR2 overexpression can lead to some beneficial metabolic changes that need to be further understood. (Acknowledgments to Nathan Shock Lifespan assessment Core for the help developing all these assays in mice).

### SESSION 2095 (SYMPOSIUM)

### LATEST ADVANCES IN UNDERSTANDING THE RELATIONSHIPS BETWEEN FRAILTY AND COGNITIVE IMPAIRMENT

Chair: Qianli Xue, *Department of Medicine, School of Medicine, Johns Hopkins University, Baltimore, Maryland, United States*
Discussant: Michelle C. Carlson, *Johns Hopkins Bloomberg School of Public Health, Baltimore, Maryland, United States*

As two of the most common geriatric conditions, frailty and cognitive impairment often coexist, and are known to predict poor health outcomes separately and jointly. The link between frailty and cognitive impairment may result from the fact that many of the aging processes underlying frailty may also be responsible for brain aging and cognitive decline. What is unknown is: (1) whether frailty as a measure of physiological resilience is predictive of dementia above and beyond neuropathology and cognitive decline; (2) whether there are individual characteristics that uniquely identify separate vs. joint presence of physical frailty and cognitive impairment. To begin to address these questions, Talk 1 reviews concept of frailty in relationship to reserve and resilience and discusses theoretical underpinnings of three integrated phenotypes of physical and cognitive impairment; Talk 2 uses data from two epidemiological cohorts to study the relationship between frailty and dementia after accounting for neuropathology. Talk 3 uses data from the Gait & Brain Study to compare the strength of associations of cognitive impairment alone vs. cognitive impairment plus physical frailty (or gait performance) with incident dementia. Talk 4 uses data from the National Health and Aging Trends Study to develop a U.S. national profile on the intersection between physical frailty and cognitive impairment in community-dwelling older adults. Together, findings from this study help elucidate the interconnection between physical frailty and cognitive impairment, as well as clinical utility of joint consideration of cognitive decline and physical frailty for predicting risk of dementia.

### INTEGRATING FRAILTY AND COGNITIVE PHENOTYPES: THEORY, MEASUREMENT, APPLICATIONS

Qianli Xue,[1] Brian Buta,[2] Lina Ma,[3] Meiling Ge,[4] and Michelle carlson[5], *1. Department of Medicine, School of Medicine, Johns Hopkins University, Baltimore, Maryland, United States, 2. Department of Medicine Division of Geriatric Medicine and Gerontology, School of Medicine, Johns Hopkins University, Baltimore, Maryland, United States, 3. Department of Geriatrics, Xuanwu Hospital, Capital Medical University, Beijing, Beijing, China (People's Republic), 4. The Center of Gerontology and Geriatrics (National Clinical Research Center for Geriatrics), West China Hospital, Sichuan University, Chengdu, Sichuan, China (People's Republic), 5. Department of Mental Health, Bloomberg School of Public Health, Johns Hopkins University, Baltimore, Maryland, United States*

The fact that frailty and cognitive impairment are associated and often coexist in older adults has led to the popular view of expanding the definition of frailty to include cognitive impairment. However, there is great variability in approaches to and assumptions regarding the integrated phenotypes of physical frailty and cognitive impairment. By reviewing the theoretical underpinnings of three integrated phenotypes of physical and cognitive impairments, this talk advocates the incorporation of biological theories in phenotype development that helps determine shared and distinct pathways in the progression to physical and cognitive impairments.

### THE ROLE OF FRAILTY IN DEMENTIA EXPRESSION: EVIDENCE FROM CLINICAL-PATHOLOGIC COHORT STUDIES

Lindsay M. Wallace,[1] Olga Theou,[1] Melissa K. Andrew,[1] David Bennett,[2] Carol Brayne,[3] and Kenneth Rockwood[1], *1. Dalhousie University, Halifax, Nova Scotia, Canada, 2. Rush University Medical Centre, Chicago, Illinois, United States, 3. University of cambridge, Cambridge, England, United Kingdom*

A growing body of evidence suggests that pathological features of dementia are diverse and don't wholly explain variability in dementia incidence. Objective: discuss the role of frailty in dementia expression using evidence from clinical-pathologic cohort studies. Methods: Cross-sectional relationships between neuropathology (Braak, CERAD staging), frailty (frailty index at last study visit before death), and dementia diagnosis at death were performed using data from the Rush Memory and Aging Project (MAP) and the Cambridge City Over 75s Cohort study (CC75C). Results: Participants were 89.7±6.2 and 92.2±4.5 years in MAP (n=451) and CC75C (n=177) and mostly female (69.4-70.1%). Most had dementia (52.8%-59.3%). Frailty was normally distributed (mean FI0.42±0.18 in MAP and 0.34±0.16 in CC75C). Frailty was associated with dementia in MAP (OR=1.88, p<0.001) and CC75C (OR=1.30, p=0.03) after controlling for age, sex, time to death, and neuropathology. Longitudinal analysis is in progress. Frailty appears to play a meaningful role in dementia expression.

### PHYSICAL FRAILTY, COGNITIVE FRAILTY, AND THE RISK OF DEMENTIA IN THE GAIT AND BRAIN STUDY

Manuel Montero-Odasso,[1] Yanina S. Adamson,[2] Susan Muir-Hunter,[1] Tim Doherty,[3] Alvaro Casas-Herrero,[4] Richard Camicioli,[5] Jennie Wells,[3] and Mark Speechley[1], *1. University of Western Ontario, London, Ontario, Canada, 2. Parkwood Institute, University of Western Ontario, London, Ontario, Canada, 3. Parkwood Hospital, London, Ontario, Canada, 4. F.E.A Geriatría Complejo Hospitalario, Pamplona, Navarra, Spain, 5. WC Mackenzie Health Sciences Centre, Edmonton, Alberta, Canada*

Cognitive-frailty has been proposed as a distinctive entity which preludes dementia. We examined the relationship between physical frailty, cognitive status, and gait performance as predictors of cognitive decline and incident dementia. Using a cohort study of 252 community older adults free of dementia at baseline, we found that participants with frailty had a higher prevalence of cognitive impairment (77%) compared to those without (54%, p=0.02) but the risk of progression to dementia was not significant. Adding cognitive impairment to the frailty phenotype (cognitive-frailty)

predicted further cognitive impairment and progression to dementia. However, when the slow gait component of frailty was combined with baseline cognitive impairment, it showed the highest risk of progression to dementia (HR: 35.9; 95%CI: 4.0–319.2; p= 0.001). Frailty and Cognitive impairment are common and co-exist in the same individuals. However, slowing gait seems to be the frailty component driving the association with future dementia.

### A U.S. NATIONAL PROFILE OF OLD ADULTS WITH COGNITIVE IMPAIRMENT ALONE, PHYSICAL FRAILTY ALONE, AND BOTH

Meiling Ge,[1] and QIANLI XUE[2], *1. Center on Aging and Health, Johns Hopkins Medical Institutions, Baltimore, Maryland, United States, 2. Department of Medicine Division of Geriatric Medicine and Gerontology, School of Medicine, Johns Hopkins University, Baltimore, Maryland, United States*

Using data from NHATS, we aimed to identify characteristics (demographics, health conditions/events, self-care behaviors, psychological wellbeing) that distinguish joint vs. separate presence of physical frailty (by the Fried's) and cognitive impairment (CI: bottom quintile of test performance in executive function and memory; or proxy-report of dementia diagnosis or AD8 score >=2). Of the 7,497 older adults, 25.5%, 5.6%, and 8.7% had CI only, frailty only, and both, respectively. After adjusting for demographic characteristics, current smoker, single disease, and knee surgery history uniquely identified "frailty only". Although none was found to uniquely identify "CI only" or "both", surgery history and comorbidity were strongly associated with "frailty only" and, to a lesser degree, "both", but not "CI only". The findings advocate for treating physical frailty and CI as overlapping yet distinct conditions, and prioritizing comorbidity, surgery history, and smoking status in clinical screening of frailty and CI before formal diagnostic assessments.

### SESSION 2100 (SYMPOSIUM)

### LESSONS LEARNED FROM ACCREDITING GERONTOLOGY PROGRAMS: GETTING READY!

Chair: Harvey L. Sterns, *The University of Akron, Akron, Ohio, United States*
Co-Chair: Janet S. Hahn, *Western Michigan University, Kalamazoo, Michigan, United States*

Accreditation for Gerontology Education Council (AGEC) is an organization that collaborates with, but is independent from the Gerontological Society of America and the Academy for Gerontology in Higher Education. It is directed by a Board of Governors consisting of nine members representing higher education gerontology programs and entities associated with the field of aging. The organizational structure also includes review teams, site visitors, and staff support. Higher education degree granting programs in gerontology, specifically associate arts degree, baccalaureate degree, and master's degree programs, are eligible to apply to AGEC for accreditation. This symposium will have presentations that focus on Overview and Experiences to Date that will describe the accreditation process and what has been learned by the accreditation of the first three degree programs. The second presentation will focus on How and Why to Apply for Accreditation and will provide background information on the steps and processes necessary to submit for the accreditation review with clarification updates. The third presentation will provide lessons learned from our first reviews with suggestions on Preparing the Self- Study and will include guidance on approaches to be taken. The fourth presentation is also lessons learned with a focus on Mapping the Competencies as part of the Self Study. Symposium presenters share important information to encourage gerontology degree programs to apply for AGEC accreditation.

### ACCREDITATION FOR GERONTOLOGY EDUCATION COUNCIL: OVERVIEW AND EXPERIENCES TO DATE

Harvey L. Sterns[1], *1. The University of Akron, Akron, Ohio, United States*

This presentation will describe the accreditation process that was developed by a special committee under the sponsorship of the Association for Gerontology in Higher Education. With support from The Russell & Josephine Kott Memorial Charitable Trust, the committee developed materials to guide the evaluation process and an operations manual, formed a Board of Governors, registered as an independent non-profit and gained approval as a 501C3 entity. This resulted in the official formation of the Accreditation for Gerontology Education Council. Three schools have gone through the accreditation process. Results of the process will be described and lessons learned will be discussed. Refinement of the process is ongoing. There is a marketing committee that has reached out to additional schools, and new applications are being accepted.

### HOW AND WHY TO APPLY FOR GERONTOLOGY ACCREDITATION

Janet S. Hahn,[1] and Donna E. Schafer[2], *1. Western Michigan University, Kalamazoo, Michigan, United States, 2. National Association for Professional Gerontologists, Healdsburg, California, United States*

This presentation covers the steps to achieve accreditation of a gerontology program as well as the costs and benefits of accreditation. The overall timeline for a typical accreditation process is presented as well as the organization of required standards. Advance planning and a close review of Accreditation for Gerontology Education Council standards will allow time for a program to comply and document compliance with standards. Communication and cooperation within an institution are needed for a program to successfully seek accreditation. Key individuals and important conversations will be identified to assist those who are considering accreditation for their gerontology program.

### PREPARING YOUR AGEC SELF-STUDY

Donna Weinreich,[1] and Donna Schafer[2], *1. Western Michigan University, Kalamazoo, Michigan, United States, 2. National Association for Professional Gerontologists, Healdsburg, California, United States*

Section VI of the AGEC Handbook provides guidelines for writing the self-study. This Handbook section is central to the accreditation review process because it provides information about how your program demonstrates that accreditation standards have been met/exceeded. It also specifies preparatory work that programs should undertake prior to applying for accreditation. Based on insights gained from the first round of AGEC accreditation reviews at the master's, baccalaureate and associate levels, Section VI of the Handbook has been revised to clarify expectations about the self-study. Specifically, greater emphasis has been placed on 1) following the Standards outline in Section V of the Handbook, 2) insuring that all relevant information is contained in the self-study and, 3) requiring that the complete document, including appendices, is submitted well in advance of the site visit. This presentation describes the content and revisions in Section VI, as well as the process for submitting the self-study.

### ASSESSMENT OF THE AGEC STANDARD DEMONSTRATING STUDENT LEARNING

Robert J. Maiden,[1] and Jan Abushakrah[2], *1. Alfred University, Alfred, New York, United States, 2. Portland Community College, Portland, Oregon, United States*

Addressing the gerontology program's experience in measuring and integrating the competencies in their curricula is a fundamental challenge in program evaluation. Using the AGHE Gerontology Competencies for Undergraduate and Graduate Education is the key. We will demonstrate how one identifies the learning outcome measures across the curriculum based on the competencies by adumbrating a four-step process. First, it entails developing a written statement of the key learning outcomes, expressing them in objective, measurable terms. Second, the learning outcomes are assessed. Third, the results of the assessments are posted in a matrix format across a four or five year period. Fourth, the results of the learning outcome assessments are discussed, evaluated, and implemented in a formative process to improve teaching and learning. In addition, the results can be applied in a summative way to evaluate and improve the gerontology program.

### SESSION 2105 (SYMPOSIUM)

### LIVING IN RURAL CONTEXTS: TOWARD A CRITICAL INTERDISCIPLINARY PERSPECTIVE ON RURAL AGING

Chair: Kieran Walsh, *NUI Galway, Galway, Ireland, Ireland*
Co-Chair: Mark Skinner, *Trent University, Peterborough, Ontario, Canada*

Despite a growing focus on rural ageing, international literature in this field remains underdeveloped in critical and interdisciplinary perspectives. Reflecting traditional divisions across geographic, gerontological and health literatures, how we understand experiences of growing older in rural settings can still be characterised by a narrow, applied approach. This has implications for our capacity to disentangle multifaceted lived realities from rural contexts, and macro socio-economic and structural environments. There then remains questions about the ways in which the study of rural ageing needs to develop to direct policy, research and practice agendas to be a more critical reflection of these complexities. This symposium aims to draw together interdisciplinary critical perspectives on ageing and rurality as a means to advance this development. It will consider different theoretical approaches and major cross cutting challenges in relation to rural ageing. Burholt and Scharf will examine how critical gerontology has raised awareness of the heterogeneity of rural ageing across social justice elements of demography, resources, recognition and representation. Keogh and Walsh address these same elements in relation to the empirical intersection of exclusion and change in the production of a new rurality for older people. Cutchin and Rowles present a pragmatist theoretical perspective to encapsulate the essence of rural integration within an ever-changing milieu. Poulin et al. offer a critical approach to rural gerontological health that emphasizes intersectionality in the formation and development of older adult health. Herron and Skinner explore the intersectional construction of dementia and mental health in rural settings for older adults.

### CRITICAL SOCIAL GERONTOLOGY AND RURAL AGING

Vanessa Burholt,[1] and thomas Scharf[2], *1. Swansea University, Swansea, United Kingdom, 2. Newcastle University, Newcastle, England, United Kingdom*

This paper examines the extent to which critical gerontology has raised awareness of the heterogeneity of rural ageing in High Income Countries (HICs) and compare this to our knowledge of the issues that are associated with rural ageing in Low to Middle Income Countries (LMICS). We will draw on Nancy Fraser's social justice framework to summarize key issues around: (1) Demography (such as globalization, urbanization, counter-urbanization and rural population ageing); (2) Resources (individual material and social resources; community resources such as access to services); (3) Recognition (social status, cultural visibility through social participation and cultural worth through valued social roles); (4) Representation (in social, health and rural development policies; and in private sector and NGO approaches). We argue that an intersectional approach that takes into account location and context (structural/economic/political) alongside other dimensions of oppression and/or privilege can provide a better understanding of the experience of ageing in rural areas.

### PROCESSES OF EXCLUSION AND NEW RURALITY: COMMUNITY CHANGE IN THE LIVES OF RURAL OLDER PEOPLE

Sinead Keogh,[1] and Kieran Walsh[2], *1. National University of Ireland Galway, Galway, Ireland, Ireland, 2. Irish Centre for Social Gerontology, Galway, Galway, Ireland*

Rural settings are sites of rapid change. Now sharing many of the processes that characterise their urban neighbourhood counterparts, older people's rural communities, even those in remote locations, are being altered by forces driven by gentrification and population churn. While the potential for displacement is apparent, the extent to which older people respond to these processes is not well understood. The degree to which these shifting contexts produce new exclusionary mechanisms for older people to contend with and new opportunities for them to exploit has yet to be sufficiently explored. This paper aims to address the intersection of exclusion and community change in the production of a new rurality for older people. The analysis will 1) present an overview of the relevant international literature, and 2) highlight the current and emerging exclusionary processes that are impacting on the lives of older people using data from individual narratives and time-use diaries.

### RETHINKING RURAL GERONTOLOGY THROUGH A DEWEYAN PRAGMATIST PERSPECTIVE

Malcolm Cutchin,[1] Malcolm P. Cutchin,[1] and Graham D. Rowles[2], *1. Wayne State University, Detroit, Michigan, United States, 2. University of Kentucky, Lexington, Kentucky, United States*

Rural aging as we have conceived of it in the gerontological literature of the past 50 years no longer exists, if it ever did. In this presentation, we contribute toward a reframing of the discourse on rural aging through a critique of established views of rural aging as an ecological, cultural, and phenomenological experience. We argue that each view is limited in its ability to encapsulate the essence of rural living and community. Our critique provides a context for a dynamic perspective on rural aging that embraces the situational uniqueness of each rural environment. We introduce that perspective, based in John Dewey's philosophy, and grounded in the idea of situationally defined manifestations of place integration within an ever-changing milieu. We conclude with a discussion of key implications, including how this perspective reshapes the roles of researchers and older rural residents in the process of ongoing rural gerontological inquiry.

### LEVERAGING CRITICAL RURAL GERONTOLOGY TO IMPROVE RURAL GERONTOLOGICAL HEALTH

Laura Poulin,[1] and Neil Hanlon[2], *1. Trent University, Ontario, Peterborough, Canada, 2. University of Northern British Columbia, Prince George, British Columbia, Canada*

A critical approach in rural gerontology has led to a better understanding of the complex interplay between older adults unique aging experiences and the multidimensional and dynamic communities in which they live. The evolution of critical rural gerontology will be explored, outlining why a similar approach is needed in rural gerontological health. In particular, rural gerontological health literature must expand beyond a deficit focus that homogenizes older adult health experiences and recognize the complexities of negotiating older adult health within multidimensional rural spaces. Inherent in this approach is recognizing the intersectionality of older adult health as well as the need to study rural gerontological health as an experience enhanced and inhibited by interactions within and across formal health services, informal social services and informal care. This approach will contribute to innovations in policy and practice addressing the burgeoning interest of how to effectively care for older adults in rural settings.

### EXPLORING THE CRITICAL CONTRIBUTIONS OF ARTS-BASED APPROACHES IN RURAL DEMENTIA CARE

Rachel Herron[1], *1. Brandon University, Manitoba, Canada*

People living with dementia can experience significant barriers to meaningful participation in their communities, particularly in underserviced rural and small-town settings. Drawing on a multi-method pilot study employing observations, diaries, focus groups and interviews in rural Canada, we examine the potential of an innovative dance program developed by Baycrest Health Sciences and Canada's National Ballet School, to transform the experiences of people living with dementia and the rural places in which they live. Our findings identify moments, processes, and places of transformation throughout the program including moments of individual self-expression; changing interactions with staff, volunteers, and carers; and changing relationships with home and community. We argue that art-based programs can challenge dominant assumptions about people living with dementia and contribute to the creation of more just health and social care in rural places. In doing so, we illustrate the value of critical arts-based approaches to aging in rural places.

### SESSION 2110 (SYMPOSIUM)

### OPEN SCIENCE AND TRANSPARENT RESEARCH PRACTICES: IMPLICATIONS FOR GERONTOLOGY

Chair: Derek M. Isaacowitz, *Northeastern University, Boston, Massachusetts, United States*
Discussant: Jonathan W. King, *National Institute on Aging, Bethesda, Maryland, United States*

Scientists from many disciplines have recently suggested changes in research practices, with the goal of ensuring greater scientific integrity. Some suggestions have focused on reducing researcher degrees of freedom to extract significant findings from exploratory analyses, whereas others concern how best to power studies and analyze results. Yet others involve ensuring that other interested researchers can easily access study materials, code, and data, to help with re-analysis and/or replication. These changes are moving targets, with discussions and suggested practices ongoing. However, aging researchers have not yet been major participants in these discussions, and aging journals are just starting to consider open science policies. This symposium, sponsored by the GSA Publications Committee, will highlight transparency and open science practices that seem most relevant to aging researchers, discuss potential challenges to implementing them as well as reasons for doing so, and will consider how aging journals may implement these practices. Open science practices to be considered include: preregistration, open data, open materials and code, sample size justification and analytic tools for considering null effects. Presenters from a range of areas of aging research (lab, secondary data, qualitative) will show examples of open science practices in their work and will discuss concerns about, and challenges of, implementing them. Then, editorial team members will discuss the implications of these changes for aging journals. Finally, discussant Jon King will give NIA's perspective on the importance of encouraging open science practices in the aging field.

### OPEN SCIENCE IN GERONTOLOGY: IMPLICATIONS FOR PUBLISHING

Derek M. Isaacowitz[1], *1. Northeastern University, Boston, Massachusetts, United States*

One big push in open science is to change journal practices to encourage a more transparent and replicable scientific record. I will start by considering why these issues are important from the perspective of a journal editor. The Transparency and Openness Promotion Guidelines were developed as a modular way for journals to encourage and/or require certain practices by authors before submitting. I will describe the TOP guidelines and will recount my experience in working to bring the Journals of Gerontology: Psychological Sciences to Level 1 of the TOP guidelines. Despite the challenges involved in changing journal practices to encourage and/or mandate greater use of open science practices, these changes will likely be coming to more journals in gerontology and beyond in the coming years, making it important for authors to be aware of changes in expectations and practices.

### PREREGISTRATION TO FACILITATE A REPLICABLE SCIENCE OF AGING

Walter R. Boot[1], *1. Florida State University, Tallahassee, Florida, United States*

Psychology and other sciences have been in the midst of a replication crisis. One proposal to address this crisis is the preregistration of studies, including study hypotheses, methods, measures, and analysis approaches to reduce false positive findings resulting from "experimenter degrees of freedom." This talk will explore the benefits, and also the challenges, of preregistration, along with common misconceptions about preregistration. A preregistration case study will be presented involving a series of experiments exploring different hypotheses regarding the mechanism behind changes in attentional processing associated with aging (http://doi.org/10.1525/collabra.26). This talk will present a brief tutorial of how to preregister studies and where to preregister them. The importance of preregistration for intervention studies will be emphasized.

### CHALLENGES IN PRE-REGISTRATION AND APPLYING OPEN SCIENCE PRINCIPLES WHEN USING SECONDARY OR LONGITUDINAL DATA

Daniel K. Mroczek[1], *1. Northwestern University, Chicago, Illinois, United States*

The application of open science, preregistration, and transparency principle is challenging when using existing data, including ongoing long-term longitudinal data. The goal is to distinguish clearly between exploratory and confirmatory research, but in the context of archival or longitudinal work there are risks associated with prior knowledge that has been obtained from these secondary sources. That said, new principles are being developed, including specialized pre-registration templates, that can guide the application of open science and transparency ideas to longitudinal and other secondary data, thereby increasing credibility of such work. These include: 1) disclosure of prior knowledge about a given dataset, ranging from "never worked with these data" to having multiple publications, in the pre-registration, 2) use of hold-out subsamples that can be used for validation or confirmatory purposes, and 3) making more clear what research questions are exploratory and confirmatory.

### PRE-REGISTERING QUALITATIVE RESEARCH: BENEFITS, UNINTENDED CONSEQUENCES, AND UNANSWERED QUESTIONS

Barbara Bowers[1], *1. University of Wisconsin-Madison, School of Nursing, Madison, Wisconsin, United States*

While preregistration has gained increasing acceptance for quantitative, particularly experimental, studies, its relevance and implementation for qualitative research has only recently been proposed. This presentation provides an overview of the very recent and ongoing debate on the potential benefits and costs of implementing preregistration for qualitative research. The presentation summarizes the debates about whether and how preregistration will lead to greater transparency in qualitative research, explores the implications of preregistration for qualitative research, identifies some of the costs incurred in preregistering qualitative studies, describes the inaccurate assumptions about qualitative research that are repeatedly embedded in calls for preregistration, and identifies some likely, unintended consequences of adopting the same approaches employed or proposed for quantitative studies. Acknowledging the importance of greater transparency and reduced publication bias for all research, including qualitative studies, questions about transparency that qualitative researchers must urgently address are also suggested.

### SESSION 2115 (SYMPOSIUM)

### PRESIDENTIAL SYMPOSIUM: OPTIMIZING SURGICAL CARE FOR ALL OLDER ADULTS

Chair: Thomas Robinson, *University of Colorado, Aurora, Colorado, United States*
Co-Chair: Ronnie Rosenthal, *Yale School of Medicine, New Haven, Connecticut, United States*

Our program will provide a detailed overview with an emphasis on the research aspects of the new Coalition for Quality in Geriatric Surgery, a project supported by the American College of Surgeons and the John A. Hartford Foundation. This project is a national endeavor which aims to systematically improve the surgical care of older adults by establishing a verifiable quality improvement program with standards based on best evidence focused on what matters most to the individual patient. We believe there is a critical need for safe, high-quality, patient-centered surgical care for older adults. Aging surgical patients have unique physiological needs, social needs and unique goals of care. We formed the Coalition to help hospitals meet these rising needs by setting and verifying interdisciplinary standards and developing outcome measures that matter to older patients, families and caregivers. In collaboration with our 50+ stakeholder organizations, we have set the standards, developed measures that matter, educated providers and patients, and created awareness about the surgical needs of older adults at all hospitals through the program. The geriatric surgery program, set to launch in the Summer of 2019, will use the four principles of continuous quality improvement: set standards, define the right infrastructure, collect rigorous data, and verify. The program not only improves perioperative care, but also impacts the full cycle of care for older adults. Our group has harnessed the power of networks through partnership and collaboration of all disciplines involved in the peri-operative care of older adults.

### CREATION OF EVIDENCE-BASED GERIATRIC SURGERY STANDARDS

Marcia McGory Russell[1], *1. UCLA Medical Center, Los Angeles, California, United States*

The aim of this abstract is to describe how to establish high-quality, valid standards to improve surgical care of the older adult. The older adult population has high demand for high-quality surgical care. Building upon prior guidelines, quality indicators, and pilot projects, the Coalition for Quality in Geriatric Surgery (CQGS) included 58 diverse stakeholder organizations committed to improving surgery for older adults. Using a modified RAND-UCLA Appropriateness Methodology, 44 of 58 CQGS Stakeholders twice rated validity (primary outcome) and feasibility for 308 standards, ranging from goals and decision-making, pre-operative assessment and optimization, perioperative and postoperative care, to transitions of care beyond the acute care hospital. Stakeholders rated the vast majority of standards of care as highly valid (99%) and feasible (94%) for improving the quality of surgical care provided to older adults.

### MEASUREMENT OF GERIATRIC SURGERY OUTCOMES IN A NATIONAL REGISTRY

Emily Finlayson[1], *1. University of California San Francisco, San Francisco, California, United States*

The American College of Surgeons National Quality Improvement Program started a “Geriatric Pilot” in January 2014. This project has already collected 19 additional older adult specialty variables in more than 60,000 patients undergoing operations. Twenty-six medical centers participate from across North America. The variables collect information in the domains of cognition, function, mobility and decision-making. Variables are collected in both the pre- and post-operative settings. It is clear that the quality of surgical care cannot be limited to the immediate hospitalization. The pilot has recently expanded its use of longer-term outcomes and has begun collecting 30-day outcomes of functional status and living location.

### EFFECTIVENESS VS. EFFICACY: CREATING EVIDENCE FROM GERIATRIC SURGERY QUALITY PROGRAMS

Meixi Ma[1], *1. American College of Surgeons, Chicago, Illinois, United States*

The American College of Surgeons National Quality Improvement Program started a “Geriatric Pilot” in January 2014. This initiative has collecting specialty variable related to older adults in more than 60,000 patients 65 years and older undergoing operations. Multiple research publications have been generated from this pioneering national quality program focused on surgical quality in older adults. The purpose of this presentation will be to review the major findings of this new body of research. Studies have focused on functional trajectory following operations, postoperative delirium, and peri-operative decision-making.

### LAUNCHING STANDARDIZED GERIATRIC SURGERY PROGRAMS: THE ALPHA AND BETA PILOTS

Lindsey Zhang[1], *1. American College of Surgeons, Chicago IL, United States*

Then Coalition for Quality in Geriatric Surgery has completed both alpha and beta pilots which represent the initial efforts to launch this national quality initiative to improve the care of all older adults undergoing operations. The alpha phase included hospital stakeholders rating the feasibility of implementing standards related to evidence-based high quality surgical care for older adults on the topics of eliciting patient goals, completing a preoperative frailty risk assessment, educating healthcare professional about care specific to older adults, and implementing postoperative age-friendly care models. The beta pilot phase required 9 medical centers nationally (including an academic hospital, an urban medical center, a Kaiser hospital, a VA hospital and a rural hospital) to implement the evidenced based standards for high quality surgical care of older adults. Site verification visits were completed in the Summer 2018 which evaluated the effectiveness of each medical center’s ability to implement each of the standards.

### FUTURE DIRECTIONS: DISSEMINATION OF STANDARDIZED GERIATRIC SURGERY CARE NATIONALLY

Mark Katlic[1], *1. Sinai Medical Center, Baltimore, Maryland, United States*

The American College of Surgeon’s Coalition for Quality in Geriatric Surgery will formally launch a national initiative aimed to improve the quality of surgical care for all older adults in July 2019. The first-year goal will be to recruit and successfully verify 100+ medical centers. This presentation will provide an overview of dissemination efforts for the standards set for providing high quality surgical care for older adults as well as processes to measure the quality of care provided to older adults at these medical centers. It our vision that this national initiative will lead the effort to the improvement of surgical care of all older adults.

### SESSION 2120 (SYMPOSIUM)

### SAFE AND EFFECTIVE PAIN CONTROL FOR GERIATRIC PATIENTS: A MULTIDISCIPLINARY APPROACH DURING THE OPIOID EPIDEMIC

Chair: Tatyana Gurvich, *University of Southern California, Los Angeles, California, United States*

Opioid use is at a crisis level. According to the CDC, an estimated 20 % of patients presenting to physician offices with non-cancer pain receive an opioid prescription (1). According to the Administration on Aging and Substance Abuse and Mental Health Services Administration, the population of older adults who misuse opioids is expected to double by 2020. Today’s mandate to reduce opioid use and to manage patients safely with adjuvant medications comes with many challenges in geriatrics. Many patients have comorbidities which limit the use of adjuvant pain medications. A careful balance must be achieved, in order to provide good pain management and improve quality of life in this patient population. This symposium will explore multidisciplinary approaches to managing pain in geriatrics to reduce opioid use and manage safe opioid use where necessary. Pharmacological strategies for adjusting dosing and managing compliance will be discussed. Cooperative education to improve prescribing practices along with patient education to improve safe use, are important elements. Adjunct use of physical therapy and integrative medicine are also discussed as viable and effective adjuncts or alternatives to traditional pain management. You will learn how to use medications safely, utilize physical therapy to its maximum potential and learn more about innovative integrative medicine techniques, all of which decrease pain and improve function and most importantly quality of life. (1) Daubresse M, Chang HY, Yu Y, et al. Ambulatory diagnosis and treatment of nonmalignant pain in the United States, 200-2010. Med Care 2013; 51:870-8

### SAFE PHARMACOLOGIC APPROACHES TO PAIN MANAGEMENT IN GERIATRIC PATIENTS

Tatyana Gurvich[1], *1. University of Southern California, Los Angeles, California, United States*

We are in the midst of an opioid crisis, but have more patients in pain than ever before. Seniors have painful conditions such as chronic back pain, arthritis, neuropathy and fibromyalgia. As many as 50% of older Americans have pain daily from more advanced disease. CDC guidelines mandate that we use opioids less and adjuvant medications more. But using adjuvants in this patient population adds an extra layer of complexity. Some are not well tolerated contributing to cognitive decline, some have to be dose adjusted for declining kidney or liver function. Some medications are safe but underutilized by this patient population. Strategies for safe opioid use will be discussed from both provider and patient perspectives. A review of adjuvant medication will be provided focusing on safe medication use in seniors. Prescription and over-the-counter and alternative medications will be reviewed.

### MEDICAL MANAGEMENT OF PAIN IN GERIATRIC PATIENTS

Sonia Sehgal[1], *1. University of California Irvine, Orange, California, United States*

Pain and pain management is a growing concern for older adult patients and their health care providers. Upwards of 50% of older adults report having bothersome pain and 75% of these individuals report having pain in more than one location. Persistent pain is associated with decreased physical function, depression, isolation, and a decreased quality of life. Managing chronic pain in the setting of an opioid crisis requires special knowledge about the aging body so that medications can be used safely and effectively; and adjuvant therapies such as PT or acupuncture can be maximized. This segment of the symposium will review common challenges faced by primary care providers when managing chronic pain in older adults: the natural physiologic changes that occur in the aging body, co-morbid chronic diseases which can complicate pain syndromes, and assessing pain in cognitively impaired patients.

### INTEGRATIVE APPROACHES TO MANAGING PAIN IN GERIATRIC PATIENTS

David Kilgore[1], *1. University of California Irvine, orange, California, United States*

In the United States, over 100 million people have chronic pain, which is associated not only with high care costs, disability and suffering, but more recently, with growing concerns about potential side effects and complications from medications commonly used to treat pain, in particular NSAIDs and opioid therapies. Pain is a multifaceted issue involving nutritional, psychosocial, biochemical, neurological and physical components. Our current medical model typically addresses pain in a unidimensional mode that often only focuses on medications. The last decade has seen advancement in the evidence base behind a number of Integrative therapies used to treat pain, most of which offer a safe side effect profile and can help expand the toolkit of available options to treat this challenging problem in the older patient. This session will briefly review several examples of Integrative therapies for pain, including Acupuncture, Mind Body treatments, Nutrition and selected supplements.

### EXERCISE THERAPY: AN ALTERNATIVE APPROACH TO MANAGING PAIN IN GERIATRIC PATIENTS

Lydia In[1], *1. University of Southern California, Los Angeles, California, United States*

Physical therapy improves the physical function and strength of older adults. Exercise decreases pain associated with osteoarthritis of the knee and hip, while improving well-being and physical function in patients with fibromyalgia. Current CDC guidelines for chronic pain management recommend exercise therapy as one of the non-pharmacologic strategies to employ. Based on a patient's personal goals, physical therapists, experts in human movement, design individualized care plans, provide appropriate interventions, and modify treatment as necessary to optimize or maintain function, improve quality of life, and delay dependency and need of care. When PTs work with patients in pain, they focus on the movement patterns that may contribute to the pain. PTs also evaluate risk factors for pain to help prevent future pain issues. Strategies employed by physical therapists will be discussed, and well as ways to integrate exercise into a holistic and multi-disciplinary pain management approach.

### SESSION 2125 (SYMPOSIUM)

### INTEREST GROUP SESSION—SLEEP, CIRCADIAN RHYTHMS AND AGING INTEREST GROUP: SLEEP, PHYSICAL ACTIVITY, AND BRAIN AGING

Chair: Christopher N. Kaufmann, *Division of Geriatrics and Gerontology, Department of Medicine, University of California San Diego, La Jolla, California, United States*
Discussant: Katie L. Stone, *California Pacific Medical Center Research Institute, San Francisco, California, United States*

As the US population ages, the prevalence of Alzheimer's Disease (AD) and related dementias is expected to increase, making dementia prevention efforts a major public health priority. Impaired sleep and circadian rhythms, along with other lifestyle factors, have emerged as important modifiable disease risk factors—recent studies demonstrate the importance of sleep in preventing the development of key biomarkers for AD/dementia pathology. In this symposium, we will highlight findings on the associations of sleep, circadian rhythm disruptions, and daytime activity patterns on development of cognitive decline and dementia, exploring not only the mechanisms driving these associations, but the potential impact of sleep and lifestyle interventions in promoting healthy brain aging. The symposium consists of four presentations which use data from large national cohort studies. First, we will present analyses on patterns of 24-hour (circadian) activity rhythms (e.g., usual time of day for peak activity, regularity of circadian patterns) and incident dementia risk. The second presentation will present findings pertaining to understanding the link between sleep disturbance and inflammation (a substantial contributor to cognitive aging). The third will examine whether detailed daytime activity patterns associate with imaging-based brain volumes, independent of sleep disturbance. The final presentation will explore whether initiation of sleep disorder treatments may have the potential to change trajectories of cognitive performance as individuals age. Overall, the symposium will highlight the importance of sleep and activity patterns to brain health and stimulate discussion about improving sleep and circadian disruption as a target for dementia prevention efforts.

### ANALYSIS OF REST-ACTIVITY RHYTHMS AND RISK OF INCIDENT MILD COGNITIVE IMPAIRMENT AND DEMENTIA IN OLDER WOMEN

Katie L. Stone,[1] Katie L. Stone,[2] Terri Blackwell,[2] Jamie Zeitzer,[3] Kristine Yaffe,[4] Sonia Ancoli-Israel,[5] Susan Redline,[6] and Gregory J. Tranah[7], *1. California Pacific Medical Center Research Institute, San Francisco, California, United States, 2. California Pacific Medical Center, San Francisco, California, United States, 3. Stanford University, Palo Alto, California, United States, 4. University of California, San Francisco, San Francisco, California, United States, 5. University of California, San Diego, San Diego, California, United States, 6. Brigham and Women's Hospital, Boston, Massachusetts, United States, 7. Sutter Health, San Francisco, California, United States*

In older adults, desynchronized circadian rhythms have been associated with medical illness, including Alzheimer Disease. Activity, which can be easily measured using actigraphy over consecutive 24-hour periods, is a valid marker of entrained sleep phase and correlates with entrained endogenous circadian phase. We compare results of both parametric and non-parametric analyses to test the association of rest-activity patterns with incident MCI and dementia in 2132 older women who had 2 or more 24-hrs periods of actigraphy data collected at baseline. Follow-up neuropsychological testing approximately 5 years later is used to classify women as normal, MCI, or dementia. Logistic regression models are adjusted for age, clinic site, race, education, body mass index, functional status, comorbidities, medication use, and health habits. Results suggest the importance of overall amplitude and rhythmicity, as well as timing of activity patterns over the 24-hour day as risk factors for incident MCI/dementia.

### CROSS-SECTIONAL AND LONGITUDINAL RELATIONSHIPS BETWEEN REST-ACTIVITY RHYTHMS AND INFLAMMATION IN OLDER MEN

Xiao Qian,[1] Qian Xiao,[1] Daniel S. Evans,[2] Susan Redline,[3] Nancy Lane,[4] Sonia Ancoli-Israel,[5] and Katie S. Stone[6], *1. University of Iowa, Iowa City, Iowa, United States, 2. California Pacific Medical Center Research Institute, San Francisco, California, United States, 3. Brigham and Women's Hospital, Boston, Massachusetts, United States, 4. UC Davis, Davis, California, United States, 5. University of California San Diego, La Jolla, California, United States, 6. California Pacific Medical Center Research Institute, San Francisco, California, United States*

Sleep disturbances and physical inactivity have been associated with chronic inflammation, an important risk factor for cognitive decline in the aging population. However most previous studies focused on the cross-sectional relationships between sleep and physical activity and inflammation. In the Outcomes of Sleep Disorders in Older Men (MrOS Sleep) study, we studied both the cross-sectional and prospective associations between characteristics of 24-hour rest-activity rhythms measured by actigraphy and inflammation index measured by multiple circulating markers. In cross-sectional analysis, a lower amplitude is associated with elevated inflammation (Odds ratio Q4 vs Q1 (95% Confidence interval): 1.65 (1.22, 2.24)). In prospective analysis, an earlier acrophase (<12:30) is associated with a two-fold increase in the risk of developing elevated inflammation over four years of follow up (2.08 (1.02, 4.23)). No individual inflammatory markers are associated with rest-activity rhythms. Our findings suggest that rest-activity rhythm characteristics predicts elevated inflammation.

### ASSOCIATION BETWEEN BRAIN VOLUMES AND PATTERNS OF COMMUNITY-DWELLING PHYSICAL ACTIVITY

Amal A. Wanigatunga,[1] Yang An,[2] Christos Davatzikos,[3] Jacek K. Urbanek,[4] Adam P. Spira,[1] Eleanor M. Simonsick,[2] Susan M. Resnick,[2] and Jennifer A. Schrack[1], *1. Johns Hopkins Bloomberg School of Public Health, Baltimore, Maryland, United States, 2. National Institute on Aging, Baltimore, Maryland, United States, 3. University of Pennsylvania, Philadelphia, Pennsylvania, United States, 4. Johns Hopkins University and Medical Institutions, Baltimore, Maryland, United States*

With aging, brain structural integrity may influence patterns of physical activity (PA) performed in community-dwelling settings. In 281 cognitively-intact adults aged ≥65 years, linear regression models were fitted to examine whether MRI brain volumes (cc), assessed using an automated multi-atlas approach, were cross-sectionally associated with accelerometer-derived: 1) daily PA minutes and 2) activity fragmentation defined as the ratio of # of contiguous PA minutes over total PA minutes x 100. Higher white matter in the parietal and temporal lobes were associated with more daily active minutes (2.8 (SE=1.0) and 3.1 (0.9) min/day, respectively; p<0.005 for both) after adjusting for demographics, behavioral factors, medical conditions, and intracranial volume. Higher white matter in the temporal region was associated with lower fragmentation (-0.15 (0.05) %, p=0.004). Our results suggest sensorimotor-related brain morphometry is connected with both the amount and manner in which PA is performed throughout the day in well-functioning older adults.

### COGNITIVE TRAJECTORIES BEFORE AND AFTER SLEEP TREATMENT INITIATION IN U.S. OLDER ADULTS WITH SLEEP DISTURBANCE

Christopher N. Kaufmann,[1] Mark W. Bondi,[2] James D. Murphy,[3] Xin Tu,[4] and Alison A. Moore[1], *1. Division of Geriatrics and Gerontology, Department of Medicine, University of California San Diego, La Jolla, California, United States, 2. Department of Psychiatry, University of California San Diego, La Jolla, California,*

*United States, 3. Department of Radiation Medicine and Applied Sciences, University of California San Diego, La Jolla, California, United States, 4. Division of Biostatistics, Department of Family Medicine and Public Health, University of California San Diego, La Jolla, California, United States*

Sleep disturbances are associated with cognitive decline but it is not clear if initiation of sleep treatments mitigates decline. We used the 2006-2014 Health and Retirement Study. At each wave, participants were administered cognitive assessments and scores were summed (values=0-35; higher=better cognition). All participants also reported if, in the past two weeks, they had taken medications or used other treatments to improve sleep. Our sample (N=4,650) included individuals who at baseline were cognitively normal and untreated for sleep, and at any wave reported some sleep disturbance. We characterized cognitive performance over study period with comparisons before and after sleep treatment initiation. Between 2006-2014, participants exhibited declines in cognitive performance (B=-2.40; 95% CI=-2.73, -2.06; p<0.001) after controlling for confounders. Following sleep treatment, cognitive decline became less pronounced (interaction B=0.94; 95% CI=0.21, 1.67; p=0.013). Results suggest that in older adults with sleep disturbance, initiation of sleep treatment may slow cognitive decline.

### SESSION 2130 (SYMPOSIUM)

### INTEREST GROUP SESSION—PATIENT/PERSON ENGAGEMENT IN RESEARCH INTEREST GROUP (PPER-IG): STRENGTH IN AGE: ENGAGING OLDER ADULTS AS HEALTH RESEARCHERS THROUGH COMMUNITY-BASED PARTNERSHIPS

Chair: Silvia Sörensen, *University of Rochester Warner School for Education and Human Development, Rochester NY, United States*
Co-Chair: Rebecca S. Allen, *The University of Alabama, Tuscaloosa, Alabama, United States*
Discussant: Reza Yousefi Nooraie, *URMC, University of Rochester School of Medicine and Dentistry, New York, United States*

The lack of clear translation of health research to improving older under-served patients' lives presents a serious problem. Studies of aging rarely include the older adults themselves in the process of conceptualizing questions, implementing the research, and applying and evaluating the results. Lack of input particularly from marginalized and minority older adults may compromise the relevance and accuracy of health research findings. In this symposium, we present the design and evaluation of two projects funded by the Patient-Centered Outcomes Research Institute (PCORI), in which older adults are trained to understand research language, culture, and methods, and are subsequently incorporated into research projects in a variety of roles. Silvia Sörensen will describe the "Engaging Older Adult Learners as Health Researchers" (ENGOAL) in Rochester, NY. This program provides six months of weekly classes and 4-6 months of research apprenticeships for older adults. Dorine Otieno and Kate Kondolf will describe evaluation results from both quantitative and qualitative analyses. Rebecca Allen will describe the design and implementation of "Sharing Opinions and Advice about Research (SOAR) in the Deep South," a partnership of The University of Alabama with community stakeholders from Sumter and Holt County to recruit and train community members to assist in the formulation of research questions based on the needs of their communities. Allen and Dragan will present the evaluation results from this project with regard to implementation and graduate education. Reza Yousefi-Nooraie will synthesize the insights from these projects and add the perspective of a social network analyst.

### ENGAGING OLDER ADULT LEARNERS AS HEALTH RESEARCHERS: PROGRAM OVERVIEW

Silvia Sörensen,[1] Sandhya R. Seshadri,[2] and Joyce Duckles[3], *1. University of Rochester Warner School for Education and Human Development, Rochester NY, United States, 2. University of Rochester School of Nursing, rochester, New York, United States, 3. Warner School for Education and Human Development, Rochester, New York, United States*

Studies of aging rarely include the older adults themselves in the process of conceptualizing questions, implementing the research, and evaluating the results. To provide opportunities for community members to become engaged in research, researchers and community stakeholders developed "Engaging Older Adult Learners as Health Researchers (ENGOAL)." This program educates older adults from underserved and underresourced communities about geriatric health and research methods, enabling them to become Research Partners. Two cohorts of African-American seniors (N=21) aged 53-79 have participated or are currently participating in six months of weekly classes followed by 4-6 months of research apprenticeships. Content and structure of classes (covering qualitative and quantitative research approaches and language, salient health themes, and developing a research proposal) will be described. Challenges and successes in providing research apprenticeships (interviewing for a study on vision, reviewing recruitment materials, evaluating a mentorship program, and testing a diabetes management program) will be discussed.

### PROJECT SOAR PARTNERSHIPS: EXPLORING ADVANCE CARE PLANNING AMONG RURAL AFRICAN AMERICANS

Rebecca S. Allen,[1] Pamela Payne-Foster,[1] JoAnn S. Oliver,[2] Christopher H. Spencer,[1] and Deanne M. Dragan[1], *1. The University of Alabama, Tuscaloosa, Alabama, United States, 2. The University of Alabama College of Nursing, Tuscaloosa, Alabama, United States*

Sharing Opinions and Advice about Research (Project SOAR), funded by PCORI, trained individuals living in under-resourced and underserved communities how to evaluate and provide advice to scientists about recruitment procedures, survey items, and intervention components for implementation in their communities. In partnership with the HELLO Project (Van Scoy, Green, & Volpe, 2019), Project SOAR community partners recruited 50 rural African American adults to consider their values, plans, and treatment preferences near the end of life while playing the HELLO game. Community and research partners along with a HELLO Project representative facilitated questionnaire completion and the process of the HELLO game to promote advance care planning. Tables of 10 participants held facilitated discussions for each HELLO game item and recorded their individual wishes and plans in HELLO booklets. Community participants had difficulty completing questionnaires without assistance; however, all were engaged and accepting of game-like discussions regarding advance care planning.

### EVALUATING THE ENGAGING OLDER ADULT LEARNERS AS HEALTH RESEARCHERS PROJECT I

Kate Kondolf,[1] Joyce Duckles,[2] sandhya Seshadri,[3] Phyllis Jackson,[4] Craig Sellers,[3] Doreen Young,[5] George Moses,[6] and Silvia Sörensen[2], *1. Warner School for Education and Human Development, University of Rochester, Rochester, New York, United States, 2. Warner School for Education and Human Development, Rochester, New York, United States, 3. University of Rochester School of nursing, Rochester, New York, United States, 4. Common Ground Health, Rochester, New York, United States, 5. Beechwood Neighborhood Center, Rochester, New York, United States, 6. North East Development Corporation, Rochester, New York, United States*

We discuss the evaluation of the Engaging Older Adult Learners as Health Researchers (ENGOAL), a program designed to educate older adults from underserved and underresourced communities about geriatric health and research methods, enabling them to become Research Partners. Quantitative and qualitative data were collected for evaluation from 21 participants aged 53-79. We used All Aspects of Health Literacy Scale (AAHLS, King's College, London) and an adaptation of Stanford Patient Education Research Center Chronic Disease Self-Efficacy Scale to assess pre-post and follow-up changes, using repeated measures analyses. Results suggest notable increases in self-efficacy, but only small improvements in subjective health literacy. Themes identified through qualitative analysis of interviews with participants included: (1) Finding our Voices (2) Race and Health (3) Faith and Health (4) Communicating with Providers and (5) Sharing and Advocacy. Emerging community leadership of participants are further evidence of confidence gains in our participants.

### EVALUATING THE ENGAGING OLDER ADULT LEARNERS AS HEALTH RESEARCHERS PROJECT II

Dorine Otieno,[1] Kate Kondolf,[2] Phyllis Jackson,[3] Joyce Duckles,[2] Sandhya Seshadri,[4] George Moses,[5] Doreen Young,[6] and Silvia Sörensen[2], *1. Warner School for Education and Human Development, University of Rochester, Rochester, New York, United States, 2. Warner School for Education and Human Development, Rochester, New York, United States, 3. Common Ground Health, Rochester, New York, United States, 4. University of Rochester School of nursing, rochester, New York, United States, 5. North East Development Corporation, Rochester, New York, United States, 6. Beechwood Neighborhood Center, Rochester, New York, United States*

We discuss the evaluation of the Engaging Older Adult Learners as Health Researchers (ENGOAL), a program designed to educate older adults from underserved and underresourced communities about geriatric health and research methods, enabling them to become Research Partners. Quantitative and qualitative data were collected for evaluation from 21 participants aged 53-79. We used All Aspects of Health Literacy Scale (AAHLS, King's College, London) and an adaptation of Stanford Patient Education Research Center Chronic Disease Self-Efficacy Scale to assess pre-post and follow-up changes, using repeated measures analyses. Results suggest notable increases in self-efficacy, but only small improvements in subjective health literacy. Themes identified through qualitative analysis of interviews with participants included: (1) Finding our Voices (2) Race and Health (3) Faith and Health (4) Communicating with Providers and (5) Sharing and Advocacy. Emerging community leadership of participants are further evidence of confidence gains in our participants.

### STUDENTS' LEARNING IN COMMUNITY-BASED PARTICIPATORY RESEARCH: THE POTTED PLANT PROJECT IN PROJECT SOAR

Deanna Dragan,[1] Jermaine Mitchell,[1] Rebecca S. Allen,[1] Pamela Payne-Foster,[1] JoAnn Oliver,[2] and Christopher Spencer[1], *1. The University of Alabama, Tuscaloosa, Alabama, United States, 2. The University of Alabama School of Nursing, Tuscaloosa, Alabama, United States*

Sharing Opinions and Advice about Research (Project SOAR), funded by PCORI, trained individuals living in under-resourced and underserved communities how to evaluate and provide advice to scientists about recruitment procedures, survey items, and intervention components for implementation in their communities. Moreover, graduate students learned community-based participatory research (CBPR) procedures and interacted with communities in implementing their own research projects. Students worked with the urban Holt community in western Alabama on issues of food insecurity due to pollution and concerns about growing vegetables and herbs in the soil. Students participated in the Potted Plant Project and plant give away, collecting questionnaire and health data. Students reported finding the fluid nature of research during this community event both stressful and rewarding. Students also identified how familiarity with CBPR procedures enhanced their clinical service provision in the community. Discussion will focus on future graduate training needs in implementation of CBPR.

### SESSION 2135 (SYMPOSIUM)

### STRENGTH IN AGE: HARNESSING THE POWER OF THE NATIONAL INSTITUTE ON AGING FOR EARLY-CAREER SCIENTISTS

Chair: Marie A. Bernard, *National Institute on Aging, National Institutes of Health, Bethesda, Maryland, United States*

The National Institute on Aging (NIA) at the National Institutes of Health, Department of Health and Human Services, supports biomedical and behavioral research with a life-span focus. There is attention to understanding basic processes of aging, improving prevention and treatment of diseases and conditions common in later years, improving the health of older persons, as well as a focus on Alzheimer's disease and related dementias. The NIA also supports the training and career development of scientists focusing on aging research and the development of research resources. The symposium, meant for junior faculty and emerging scholars, will provide an update on the latest research findings from the NIA followed by a brief update on funding mechanisms. An opportunity is provided to meet and consult with NIA extramural staff.

### OVERVIEW OF NIA RESEARCH

Richard Hodes[1], *1. National Institute on Aging, National Institutes of Health, Bethesda, Maryland, United States*

Dr. Hodes will provide an overview of the latest research in basic biology, neuroscience, behavioral and social science, and geriatrics and clinical gerontology supported by the NIA.

### HOW TO GET AN NIA GRANT

Robin A. Barr[1], *1. National Institute on Aging, National Institutes of Health, Bethesda, Maryland, United States*

Dr. Barr will provide an overview of the various funding mechanisms supported by NIA, with emphasis on mechanisms that are most appropriate for early career scientists.

### SESSION 2140 (SYMPOSIUM)

### INTEREST GROUP SESSION—KOREAN AND KOREAN AMERICAN AND AGING: UNDERSTANDING THE HEALTH AND WELL-BEING OF OLDER KOREAN AMERICANS FROM CULTURAL PERSPECTIVES

Chair: Miyong T. Kim, *University of Texas at Austin, Austin, Texas, United States*
Co-Chair: David Chiriboga, *University of South Florida, Tampa, Florida, United States*
Discussant: Barbara Yee, *University of Hawaii at Mānoa, Honolulu, Hawaii, United States*

Despite tremendous progress in improving health in the U.S. in recent years, many ethnic minority populations still experience significant health disparity gaps that stem from a lack of valid research and culturally sensitive service infrastructures for those populations. Our previous research indicates that overwhelming numbers of older Korean Americans, a first-generation immigrant group, suffer not only from health issues but also from a lack of self-confidence and a feeling of social isolation because of language and cultural barriers. In addition, many older Korean Americans lack personal resources and health literacy to overcome those barriers when they attempt to access the complicated U.S. health care system. The purpose of this symposium is to promote the understanding of the health and well-being of older Korean Americans and identify their vulnerabilities and resilience. A series of five community-based studies of older Korean Americans conducted in multiple locations (e.g., FL, HI, TX, NY, CA, MD, VA, DC, MN, and IL) that covers diverse topics on health and well-being (e.g., health literacy, chronic disease management intervention, physical/mental/oral/cognitive health, end-of-life issues, diabetes, cancer, and dementia), using various methodologies (e.g., quantitative surveys, qualitative interviews, focus groups, and randomized controlled trials) will be presented. The issues of diversities and disparities will be discussed from the cultural perspectives, as well as implications for future research and practice.

### PHYSICAL-MENTAL-ORAL-COGNITIVE HEALTH IN OLDER KOREAN AMERICANS: A MULTISITE STUDY

Yuri Jang,[1] Nan Sook Park,[2] Min-Kyoung Rhee,[1] Hyunwoo Yoon,[3] Yong Ju Cho,[4] miyong T. Kim,[4] and David A. Chiriboga[2], *1. University of Southern California, Los Angeles, California, United States, 2. University of South Florida, Tampa, Florida, United States, 3. Texas State University, san Marcos, Texas, United States, 4. University of Texas at Austin, Austin, Texas, United States*

Using data from surveys with older Korean Americans (n = 2,150) conducted at five sites in the U.S. (California, New York, Texas, Hawaii, and Florida), the present study explored the status of physical/mental/oral/cognitive health and its determinants. For each health domain, we examined how self-rating (excellent/very good/good/fair/poor) of health was associated with other domain-relevant indicators (e.g., the number of chronic diseases, symptoms of depression, problems with teeth or gums, or cognitive performance) and sociocultural factors (e.g., socioeconomic status, acculturation, social network, and social cohesion). Geographic variation was also considered. The correlations between self-ratings and domain-relevant indicators in all health domains were significant but moderate. A series of multivariate regression models of self-ratings of physical/mental/oral/cognitive health not only confirmed the effect of the domain-relevant health indicators but also demonstrated a critical contribution of sociocultural determinants. Implications for older immigrants were discussed in terms of place, culture, and context.

### THE ROLE OF HEALTH LITERACY ON PROCESS AND OUTCOMES OF A DIABETES SELF-HELP INTERVENTION WITH KOREAN AMERICANS

Miyong T. Kim[1], *1. University of Texas at Austin, Austin, Texas, United States*

The purpose of this study was to explicate the underlying mechanisms of the role of health literacy (HL) in diabetes management process involving a group of Korean Americans with type 2 diabetes mellitus (DM). We used data from a randomized clinical trial of an HL-focused diabetes self-management intervention (n = 250). A series of path analyses identified the level of self-efficacy and self-care skills as a significant mediator between HL and glucose control (HbA1C) and quality of life for the target population. In addition, education and acculturation were revealed as the most significant correlates of HL for this new immigrant group. Despite inconsistent empirical findings regarding the statistically significant effect of HL on glucose control, this study confirmed the apriority hypothesis that HL indirectly influences health outcome through mediators such self-care skills as self-efficacy. This study highlighted the importance of HL in chronic disease management for people with limited HL.

### MOBILE APP INTERVENTION TO PROMOTE BREAST CANCER SCREENING IN OLDER KOREAN AMERICAN WOMEN

Hee Y. Lee[1], *1. The University of Alabama, Tuscaloosa, Tuscaloosa, Alabama, United States*

To promote breast cancer screening behavior in older Korean American women, a mobile phone multimedia messaging intervention (mMammogram) was developed.

The current study explored how the mMammogram intervention can be applied and improved for wide dissemination and implementation in other older racial/ethnic immigrant populations. Three focus groups were conducted with 14 older Korean American women who completed the mMammogram. Three themes emerged: (1) emphasis on knowledge improvement (understanding of breast cancer screening); (2) assistance of bilingual health navigators to promote mammography (e.g., setting up appointment and transportation); and (3) consideration of technology literacy (e.g., training for use of smartphone and connectivity issues). Mobile app intervention combined with bilingual health navigation services can be an acceptable and effective intervention medium to promote mammogram, which will bring about a positive change in women's health screening behavior, especially for newly arrived immigrant populations.

### ATTITUDES TOWARD AND PREFERENCE FOR ADVANCE CARE PLANNING AMONG OLDER KOREAN AMERICANS

Michin Hong,[1] Hyunjun Noh,[2] and Kyeongmo Kim[3], *1. Indiana University, Indianapolis, Illinois, United States, 2. the university of alabama, Tuscaloosa, Alabama, United States, 3. Virginia Commonwealth University, Richmond, Virginia, United States*

Advance care planning (ACP), referring to a decision-making process for end-of-life (EOL) care in advance, is critical in ensuring satisfaction with care and quality of life among patients and caregivers at the EOL. However, Korean Americans consistently report lower levels of ACP engagement compared to whites, indicating their potential vulnerability at the EOL. To gain insight into strategies to address the disparity, this qualitative study explored attitudes toward and preference for ACP among older Korean Americans. We conducted three focus group interviews with 31 older Korean Americans. Through iterative data analysis process, four themes emerged: 1) moderate levels of awareness of and prior experiences with ACP: 2) favorable attitudes toward ACP, while concerned about non-compliance by children: 3) preference for natural dying without life prolonging treatments and pain: and 4) preference for physician-initiated ACP. Our findings suggest the necessity of culturally tailored approaches to promote ACP for this population.

### PREVALENCE AND CORRELATES OF MILD COGNITIVE IMPAIRMENT AND DEMENTIA IN OLDER KOREAN AMERICANS

Hochang Lee,[1] Miyong Kim,[2] Peter Walsh,[3]
Kim B. Kim,[4] and Haera Han[5], *1. University of Rochester Medical Center, Rochester, New York, United States, 2. University of Texas at Austin, Austin, Texas, United States, 3. University of Rochester, Rochester, New York, United States, 4. Korean Resource Center, Ellicott City, Maryland, United States, 5. Johns Hopkins University, Baltimore, Maryland, United States*

With a high prevalence of well-established risk dementia risk factors (e.g. tobacco, alcohol use, hypertension, and diabetes), there is a dire need for data on prevalence and correlates of cognitive impairment and related service utilization among older Korean Americans. Memory and Aging study of Koreans (MASK) is a two-stage epidemiological survey with two-step sampling strategy to obtain a representative sample. We screened 1,118 older Korean Americans (mean age: 70.7, female: 67.2%) with a Korean version of the Mini-Mental State Exam, and 118 persons sampled from two levels of performance on the MMSE underwent the second stage clinical evaluation that included the Consortium to Establish a Registry for Alzheimer's Disease (CERAD). Preliminary analysis estimated that community prevalence of mild cognitive impairment in this population was 28.4% (CI: 18.2%-41.5%) and dementia was 7.2% (3.8% - 13.2%). Correlates of cognitive impairment, and potential barriers to the services will be discussed during this presentation.

### SESSION 2145 (SYMPOSIUM)

### WHAT STORIES DO FILMS TELL ABOUT AGING?

Chair: Helen Q. Kivnick, *University of Minnesota School of Social Work, Saint Paul, Minnesota, United States*
Discussant: Jim Vandenbosch, *Terra Nova Films, Chicago, Illinois, United States*

With the aging of the worldwide population, films inevitably include an increasing number of stories about aging and its vicissitudes. Gerontologists acknowledge that aging (a process that is at once biological, social, and psychological) includes elements of decline, loss, and fear– along with opportunities for new learning and creativity, contributions, love, and satisfactions. However given mainstream society's predominant demand for the status quo, we should not be surprised that films made for popular, commercial audiences tend to reflect self-reinforcing, negative stereotypes and defenses against them (e.g., denial; fantasy; melodrama). This symposium seeks to: 1) Name and illustrate this filmic process of reinforcing negative stereotypes and misconceptions; 2) Describe and illustrate two existing theories that. explain positive processes associated with healthy aging; 3) Identify and illustrate films that constitute positive alternatives to the status quo and, in so doing, open the door to films presenting realistic stories of the positive balancing of later-life Integrity with Despair. The first presenter will articulate and illustrate this filmic process of reinforcing negative stereotypes and misconceptions. Presenters #2 and 3 will describe and illustrate two existing theories that explain positive processes associated with healthy aging (Baltes & Baltes' Selective Optimization and Compensation; Erikson's Integrity & Despair). Presenter #1 will return to identify and illustrate films that constitute positive alternatives to the status quo and, in so doing, open the door to discussing films that present realistic stories of older adulthood's complexities.

### VITAL INVOLVEMENT: A SOURCE OF REALISTIC OPTIMISM FOR OLDER ADULTHOOD

Helen Q. Kivnick[1], *1. University of Minnesota School of Social Work, Saint Paul, Minnesota, United States*

Vital Involvement (VI) was initially proposed (Erikson et al., 1986) as one of three principles around which lifelong healthy psychosocial development takes place. As more recently elaborated, VI has come to describe a person's meaningful, reciprocal engagement with the world outside the integrating "self." It is through VI that the person engages in healthy psychosocial development throughout life, including balancing Older Adulthood's focal tension between Integrity and Despair. This life stage is widely associated with the physical, cognitive, and social losses, and societal constraints that give rise to later-life despair. However, VI functions as a lifelong psychosocial model for the meaningful environmental engagement that supports later life's wisdom and integrity. Notably few films present an integrated view of older adulthood's losses along with opportunities. But those few can be a source of optimism to elders for whom VI may not be intuitive, but who can learn its practice.

### THE COURAGE TO EMBRACE OLD AGE

Jim Vandenbosch[1], *1. Terra Nova Films, Chicago, Illinois, United States*

Most popular films and television programs reflect, and thereby reinforce, the common and entrenched cultural perception of aging as the "enemy" of vital living. Aging used to be kept at bay in popular films and TV programs through a stereotyping that allowed ridicule and avoidance. Today, such overt negative portrayals have begun to fall out of favor but are being replaced by a subtler form of ageism—that of "super-aging" where older adults who are seen as successfully holding onto their youthful ways are celebrated and held up as models of "successful" aging. This presentation will give an overview of how most popular films and television programs frame the experience of older adulthood, and will illustrate this with clips from such films. Then, in contrast, clips will be presented from films that present a more holistic and balanced view of elderhood.

### THE S.O.C. MODEL OF AGING IN DOCUMENTARY FILMS: POSITIVE ADAPTATION TO AGE-RELATED LOSS IN THEORY AND EVERYDAY LIFE

Rick Scheidt[1], *1. Kansas State University, Manhattan, Kansas, United States*

There is overwhelming evidence that "pop culture" depictions of age-related losses are primarily negative, ignoring positive adaptive experiences associated with the second half of life. Unfortunately, film as an entertainment medium often creates and reinforces this negative status quo. This presentation describes the usefulness of the Baltes and Baltes S.O.C. Model for offsetting losses – via narrowing and revision of goal priorities (Selection), locating and enhancing resources to achieve positive outcomes (Optimization), and using these to increase one's personal limits and reserve capacities (Compensation). In addition, positive "S.O.C. Solutions" (Spiehs, 2018) are illustrated for everyday loss scenarios within four new documentary films. These include positive adaptations to four loss domains – personal autonomy (driving), physical capacity (sexual responsiveness), psychological well-being (loneliness and belonging), and environmental destruction (place dependency). Annotated sources will be made available.

### SESSION 2150 (SYMPOSIUM)

### AGING-IN-COMMUNITY MODELS AND PROGRAMS: PROGRAM AND POLICY PLANNING STRATEGIES

Chair: Su-I Hou, *The University of Central Florida, Orlando, Florida, United States*
Co-Chair: Carrie Graham, *University of California, Berkeley, California, United States*
Discussant: Emily Greenfield, *Rutgers University, New Brunswick, New Jersey, United States*

This symposium introduces key aging-in-community models and programs, with a particular focus on program and policy planning strategies. Villages are a new, consumer-directed model that aim to promote aging -in-community through a combination of facilitated social engagement, member-to-member support, volunteer opportunities, and collective bargaining for services. Dr. Graham from the University of California will share results from both a national survey of Village directors and a survey of village members, summarizing Village organizational development trends and members' perceived impacts. Dr. Gilcksman from Philadelphia Corporation for Aging will share how older adults who do not participate in a Village create their own informal social and service network to maintain themselves and to accomplish the same goals as a Village, building community at the neighborhood level. Additionally, Dr. Hou from the University of Central Florida will discuss lessons learned on program planning strategies among older adults participating in three programs promoting aging-in-community: a university-based lifelong learning program (LLP), a county neighborhood lunch program (NLP), and a Florida Village program as a comparative case study. Finally, Dr. Glass from the University of North Carolina Wilmington will share the current trend of the new senior cohousing model, promises and challenges for older adults providing mutual support to each other as they age together. This symposium will further discuss strengths and weakness, and planning strategies of the various AIC models and programs.

### ELDER COHOUSING: THE EMBODIMENT OF AGING IN COMMUNITY

Anne P. Glass[1], *1. University of North Carolina Wilmington, Wilmington, North Carolina, United States*

A new alternative living arrangement has emerged in the U.S., in which older adults proactively choose how, where, and with whom they want to live, in a close-knit community where neighbors look out for each other. Adopting the cohousing model originally established in northern Europe, these elder intentional communities are distinctive, as they are run by the residents themselves, and there is a focus on neighbors helping each other. Drawing from over a decade of research incorporating data from six communities, the challenges and benefits of establishing such a community will be addressed and the requirements necessary for mutual support to thrive will be identified. Finally, the model of aging better together intentionally, developed from the senior cohousing experience, will be shared, as well as the implications for how the model can be used for planning and policy in other settings.

### PROGRAM PLANNING STRATEGIES AMONG OLDER ADULTS PARTICIPATING IN THREE PROGRAMS PROMOTING AGING-IN-COMMUNITY

Su-I Hou[1], *1. The University of Central Florida, Orlando, Florida, United States*

This study examines program planning strategies among older adults participating in programs promoting aging-in-community (AIC) programs. Older adults from three programs were recruited (n=290): a university-based lifelong learning program (LLP; n=110), a county neighborhood lunch program (NLP; n=84), and a village program (n=96). Mean age was 72.4 (SD=8.68) years and 78% female. Findings showed NLP participants were more likely to obtain health information from TV (p=.030), friends and neighbors (p=.016), family members (p<.001), or mailed advertisement (p<.001); while less likely to obtain health information online (p<.001). Village members preferred afternoon while NLP participants preferred morning programs (p=.025). Most desired frequency was weekly (45%) and delivered in small group modes (68%). NLP participants were more likely to report self as risk takers (29% vs. 17%) or old tradition (23% vs. 3-8%) towards new technology adoption (p<.001). Results have implications on tailored program planning for older adults in different AIC programs.

### THE DO IT MYSELF VILLAGE: BUILDING A VILLAGE-LIKE SUPPORT SYSTEM WITHOUT A VILLAGE

Allen Glicksman,[1] Lauren Ring,[1] and Carrie Graham[2],
*1. Philadelphia Corporation for Aging, Philadelphia, Pennsylvania, United States, 2. University of California Berkeley, Berkeley, California, United States*

Villages provide members with a wide range of support including socialization, vetted vendors and other services that assist the elder to age in place. While not every Village offers the same types of support many older adults join Villages to gain benefits they may have lost (such as an informal support network) or ones they cannot find (such as identifying reliable providers of home repair). However, Villages are not available everywhere and there are barriers to Village membership, especially cost. Do older adults without access to a Village simply "do without" or do some of them create the same type of support system on their own? This presentation, using data collected in focus groups and individual interviews for a study of aging in community will describe the ways in which older adults have fashioned their own set of services and socialization opportunities to achieve the same goals as Village membership.

### THE VILLAGE MODEL, WHAT'S NEXT?

Carrie Graham,[1] and Andrew Scharlach[1], *1. University of California, Berkeley, California, United States*

Researchers at UC Berkeley will present some key findings of their research on Villages spanning the last decade. First, results of a longitudinal study of operational Village organizations in the US (conducted in 2009, 2012, and 2016) reveals that the Village model has expanded and developed over time, with some changes in organizational structure. A national survey of Village members (N=2000) shows that Village remain homogeneous, and impact different types of members in different ways, with older, more frail members perceiving more quality of life benefit, while younger, healthier members perceive more benefits in the areas of social engagement. Finally, two studies looking at Village retention/participation show that issues such as lack of diversity, focus on social engagement can be barriers to inclusion, retention and ultimately, scalability of the model.

### SESSION 2155 (PAPER)

### ALZHEIMER'S DISEASE AND RELATED DEMENTIAS

### MY PARENT'S BODY IS SACRED: LATINO PERSPECTIVES ON BRAIN DONATION FOR ALZHEIMER'S DISEASE RESEARCH

Yadira Montoya,[1] Guilherme M. Balbim,[2]
Crystal M. Glover,[3] and David X. Marquez[2], *1. NORC at the University of Chicago, Chicago, Illinois, United States, 2. University of Illinois at Chicago, Chicago, Illinois, United States, 3. Rush Alzheimer's Disease Center, Chicago, Illinois, United States*

Brain donation is a critical part of advancing research addressing Alzheimer's disease and related dementias (ADRD). Latinos are at a higher risk of developing ADRD compared to non-Latino Whites. However, there is limited knowledge regarding causes and mechanisms related to ADRD health disparities among Latinos partially due to lower research participation and brain donation rates. Family members play a pivotal role in increasing brain donation rates, particularly, among underrepresented groups. In this study, we examine the perceptions of brain donation among adult children of older Latinos. We invited Latino men and women (N=15) with a parental-figure who was 65 years and over to participate in one of three focus groups. During the focus groups, participants discussed the meaning of brain donation for research, reasons to donate or not, and their reactions to the possibility of their parental-figure being a brain donor. All focus groups were audio-recorded and transcribed with transcripts used for data analysis. We used a Grounded Theory Approach to analyze focus group data. Results yielded three themes: (1) social and cultural factors influencing a family's willingness to support organ donation; (2) lack of knowledge about the brain donation process; and (3) recommendations for engaging more Latinos in ADRD research and brain donation. Findings provide insight into how family participation may facilitate increased brain donation rates in ADRD studies among older Latinos. A main recommendation for researchers is to adopt a family-centered approach throughout the research process with a focus on addressing information gaps - from recruitment to dissemination.

### PERCEPTIONS OF BRAIN HEALTH AND INTEREST IN PARTICIPATING IN BRAIN HEALTH RESEARCH AMONG ADULTS AGE 50 TO 64

Erica Solway,[1] Donovan Maust,[2] Matthias Kirch,[3]
Dianne Singer,[2] Jeffrey Kullgren,[2] and Preeti Malani[3], *1. Institute for Healthcare Policy and Innovation, University of Michigan, Ann Arbor, Michigan, United States, 2. University of Michigan, Ann Arbor, Michigan, United States, 3. University of Michigan Medical School, Ann Arbor, Michigan, United States*

Evidence suggests it may be possible to reduce the risk of developing dementia during midlife. The University of Michigan National Poll on Healthy Aging (NPHA), a nationally representative online survey, sought to determine to what extent adults age 50 to 64 anticipate and worry about developing dementia, are taking steps to prevent dementia, and are likely to participate in dementia-related research.

Nearly 50% of poll respondents (n=1,025) perceived themselves as being somewhat or very likely to develop dementia. Worry about developing dementia was higher among respondents who had a family member with dementia (66.3% vs. 31.8%; Pearson's Chi squared, p<0.001) and those who had been a caregiver of a person with dementia (65.2% v. 38.9%; Pearson's Chi squared p<0.001). Only 5% of respondents had discussed preventing dementia with their doctor. In contrast, many respondents endorsed pursuing a variety of strategies to help maintain their memory. For example, 55% did crossword puzzles or other brain games; more than 30% reported taking fish oil or omega-3 supplements. Finally, 44% of respondents said they were likely to participate in studies to test a new medicine to prevent dementia and to test a new treatment for dementia. According to this NPHA, while many adults age 50 to 64 in the U.S. are worried about developing dementia, fewer are willing to participate in research to prevent or treat dementia. The low percentage who discussed dementia prevention with their doctor is concerning, particularly because many report using non-evidence-based prevention strategies such as dietary supplements.

### PERMANENT TRANSITION OF HOMECARE RECIPIENTS WITH DEMENTIA TO NURSING HOMES IN NYS: RISK FACTORS

Yuchi Young,[1] Maksim Papenkov,[1] Wan-Hsaing Hsu,[2] Faryal Shahid,[1] and Yen-Hong Kuo[3], *1. SUNY at Albany, Albany, New York, United States, 2. New York State Department of Health, Albany, New York, United States, 3. Hackensack Meridian Health, Neptune, New Jersey, United States*

Objective. To determine predictors associated with the permanent transition to nursing homes among home care recipients with dementia. Methods. Retrospective cohort study (01/2007-12/2012). Study participants (n=48,338) include older adults age 65+ with dementia who received home health services in NYS for at least 2 months prior to permanent transition to nursing homes. Permanent transition is defined as discharge from a home care agency to a nursing home where participants must reside for 3+ months continuously. Two data sets were used—the Minimum Data Set (MDS) and Outcome and Assessment Information Set (OASIS). Multivariate logistic regression was used to quantify the association between predictors and permanent transition to nursing homes. Results: 29% of home care recipients with dementia made permanent transitions to nursing homes. The mean age was 83 years old ranging from 65-111. Among the permanent transition group, 47% were age 85+, 68% female, 75% white, 13% black, 9% Hispanic, 29% with both urinary & bowel incontinence, and 15% with depression. Risk factors associated with permanent transition included increasing age (OR=1.1; 95% CI 1.03-1.18); being white (OR =1.25; 95%CI 0.83-0.94) compared to black, urinary and bowel incontinence vs. continence (OR=1.46; 95% CI 1.37-1.56); depression vs. no depression (OR=1.2; 95% CI 1.11-1.25); hip fracture vs. no hip fracture (OR=2.63; 95% CI 2.27-3.05), and 3+ hospitalizations vs. no hospitalization (OR=3.02; 95% CI 2.77-3.29). Conclusion: Two potential modifiable risk factors related to permanent transition to nursing homes are depression and incontinence. Early diagnosis and treatment may delay or avert nursing home entry.

### RACIAL-ETHNIC DIFFERENCES IN NURSING HOME QUALITY OF LIFE FOR ALZHEIMER'S DISEASE AND DEMENTIA RESIDENTS

Tetyana P. Shippee,[1] Stephanie Jarosek,[1] Xuanzi Qin,[2] and Mark Woodhouse[3], *1. University of Minnesota, Minneapolis, Minnesota, United States, 2. U of MN, Minneapolis, Minnesota, United States, 3. School of Public Health, University of Minnesota, Minneapolis, Minnesota, United States*

Nursing homes (NHs) are often racially segregated, and minority residents admitted to NHs usually have more advanced stages of dementia at the time of admission than their white counterparts, with different care needs. Previous work has shown that racial disparities in NH quality of life (QoL) were partially due to different case mix of white and minority residents; it is unclear if disparities persist when comparing residents with similar ADRD diagnoses. The 2011-2015 Minnesota Resident Quality of Life and Satisfaction with Care Survey data contain in-person resident responses from a random sample of residents of all Medicare/Medicaid certified NHs in the state, about 40% of whom have AD/ADRD. These data were linked to the Minimum Data Set (MDS) and facility characteristics data. The population consists of 25,039 White, 580 Black, 94 Hispanic, 229 Native Americans, and 99 Asian/Pacific Islander NH residents with ADRD residing in 376 NHs. Racial/ethnic minority residents reported significantly lower QoL scores compared to their white counterparts, with the largest disparities in the food and relationships domains. We adjusted for resident (age, marital status, education, sex, length of stay, anxiety/mood disorder, activities of daily living scores) and facility characteristics (proportion of minority residents, ownership, urban vs rural, size, and occupancy ratio) using a multivariate random intercept model. After adjustment, significant differences remained in total QoL score and several QoL domains for Black, Asian and Hispanic residents (no significant differences for Native American residents). Practice guidelines should consider different care needs of racial/ethnic minority NH residents with ADRD.

### THE BUDDY PROGRAM: IMPACT ON PERSONS LIVING WITH DEMENTIA AND FAMILY CAREGIVERS

Darby Morhardt,[1] Mary Mittelman,[2] Ann Burgunder,[2] and Thea Micoli[2], *1. Northwestern University, Chicago, Illinois, United States, 2. New York University, New York, New York, United States*

Psychosocial interventions have the potential to offer substantial benefit to people with dementia and their family caregivers. The Buddy Program is an experiential learning program that pairs students with persons with dementia for activities and relationship-building. Previous studies have demonstrated the program's positive impact on student knowledge and attitudes. New York University's (NYU) Alzheimer's Family Support Program began replicating the Buddy Program in 2017 and has enrolled 80 students. Northwestern University's (NU) Buddy Program, in its 22nd year, has enrolled 260. This presentation describes the impact of the program on the mentors (NU) and the caregivers (NYU). Post program focus groups with mentors and student journals describing interactions with the caregiver were thematically analyzed. Mentors describe feelings of pride in the mentorship role, enjoyment being with student, and the opportunity to develop a new friendship. Caregivers enjoyed the respite provided by the buddies, knowing that their relatives with dementia were enjoying themselves and seeing their relatives with dementia in the role of valued companion. Qualitative data from caregivers and mentors participating in the buddy programs at NU and NYU underscore the possibility that people with dementia can still make contributions to society. The buddy program has a positive impact on quality of life for persons living with dementia and their family members, while providing a valuable educational experience for students. Further research on the buddy program's benefits is warranted.

### SESSION 2160 (SYMPOSIUM)

### APPLYING MIXED-METHODS APPROACHES TO THE STUDY OF SOCIAL TIES IN LATER LIFE

Chair: J Jill Suitor, *Purdue University, West Lafayette, Indiana, United States*
Co-Chair: Megan Gilligan, *Iowa State, Ames, Iowa, United States*

This symposium brings together a diverse set of studies applying mixed-methods approaches, with an emphasis on illustrating the ways in which such designs can provide greater understanding of interpersonal processes in the middle and later years. The papers span a wide range of relational contexts, including ties between parents and adult children, grandparents and grandchildren, and couples in gay, lesbian, heterosexual partnerships. They illustrate a variety of ways to combine quantitative and qualitative data collecting using surveys, in-depth interviews, timeline data, and technological devices. In the first paper, Silverstein and Bengtson present a study of continuity, change, and conflict across the generations regarding religion. The next two papers explore the impact of social relations on well-being. Fingerman and colleagues report findings from a study of social engagement and sedentary activities; and Suitor and colleagues investigate gender differences in the effects of mothers' favoritism on adult children's depressive symptoms. The final two papers focus on couples' experiences when facing potentially demanding and/or challenging life circumstances. Thomeer and colleagues present findings from a study of same-sex couples in the context of marriage and parenthood; and Umberson and colleagues shed light on marital dynamics in same-sex and different-sex couples when one partner experiences psychological distress. This diverse set of studies provides a rich overview of the ways in which mixed-methods approaches can shed more light on patterns and consequences of relationships in adulthood than could be learned using single-method designs.

### CHANGES IN THE PSYCHOLOGICAL COSTS OF MATERNAL FAVORITISM FOR DAUGHTERS AND SONS AS MOTHERS AGE

J Jill Suitor,[1] Megan Gilligan,[2] Marissa Rurka,[1]
Yifei Hou,[1] and Catherine Stepniak[1], *1. Purdue University, West Lafayette, Indiana, United States, 2. HDFS, Iowa State University, Ames, Iowa, United States*

Life course perspectives suggest that the consequences of being mothers' favorite children will vary, depending on the expectations associated with that status at different points in mothers' lives. We propose that maternal favoritism predicts depressive symptoms only when mothers are older and at greater risk of facing losses for which favored children perceive they should provide additional emotional support. To address this question we used mixed-methods panel data collected from 479 adult children as part of the Within-Family Differences Study. Multi-level regression analyses revealed that perceiving oneself as the child most emotionally close to the mother did not predict depressive symptoms for daughters or sons at T1, but was a predictor of daughters' depressive symptoms at T2. Qualitative analyses revealed that by T2, favored daughters had begun perceiving themselves as emotional caregivers when mothers faced age-related losses, whereas favored sons did not hold these role perceptions at either wave.

### DEALING WITH A DISTRESSED SPOUSE: MARITAL DYNAMICS IN SAME-SEX AND DIFFERENT-SEX COUPLES

Debra Umberson[1], *1. University of Texas, Austin, Texas, United States*

We use a mixed-methods strategy to generate insights into gendered marital dynamics when one partner is experiencing high levels of psychological distress/depression. Our data are unique in their dyadic design and in the inclusion of same-sex and well as different-sex marital dyads. The data are from closed- and open-ended survey responses (from 808 gay, lesbian, and heterosexual spouses in 404 unions) as well as in-depth interview data (with a subsample of 45 gay, lesbian, and heterosexual couples in 90 unions). Respondents were asked about their most significant period of emotional distress during the marriage, how their spouse reacted to their distress (e.g., providing emotional or instrumental support, withdrawing), and how much they worried about burdening their spouse. Respondents were also asked how they reacted to their spouse's periods of emotional distress. Preliminary results point to gendered experiences of distress within marriage that sometimes differ for same-sex compared to different-sex couples.

### MULTIPLE METHODS OF ASSESSING DAILY MEDIA USE IN LATE LIFE

Karen Fingerman,[1] Crystal L. Ng,[2] Meng L. Huo,[2] and Shiyang L. Zhang[2], *1. University of Texas, Austin, Austin, Texas, United States, 2. The University of Texas at Austin, Austin, Texas, United States*

Television viewing is a risk factor for obesity and poor physical health. By contrast, close ties to family and friends in late life are often beneficial. This study examined associations between social engagement and television viewing. Participants (N = 313) from the Daily Experiences and Well-being Study completed an initial interview about their social partners and participated in a 5 to 6 day intensive data collection including Ecological Momentary Assessments about their social contact and activities every 3 hours. Participants also wore Electronically Activated Recorders (EAR) which captured snippets of sound in the environment. Multilevel models using self report and EAR data revealed that participants were more likely to watch TV when they were with close family members (e.g., spouse, grown children) than with friends or acquaintances. Findings from these multiple methods suggest that close family may encourage risks (e.g., sedentary behaviors) as well as benefits in late life.

### OF VALUES AND P-VALUES: USING MIXED METHODS TO STUDY FAMILIES AND RELIGION IN THE LONGITUDINAL STUDY OF GENERATIONS

Merril D. Silverstein[1], *1. Syracuse University, Syracuse, New York, United States*

In this presentation we discuss challenges and opportunities presented by the use of mixed methods to study continuity, change, and conflict related to religion—and the absence of religion—in multigenerational families. Discussion centers on the complementarity of quantitative and qualitative approaches, strategic decisions in tone and emphasis of the manuscript, and uncertainties in navigating the peer review process. An empirical example is provided to illustrates these points. We present an analysis that tracked patterns of religious change across three generations using survey data and in depth interviews emphasizing epistemological issues in knowledge generation.

### PARENTHOOD AND MARRIAGE WITHIN SAME-SEX COUPLES: AN ANALYSIS USING SURVEY, TIMELINE, AND IN-DEPTH INTERVIEW DATA

Mieke Thomeer,[1] Allen J. LeBlanc,[2] and David M. Frost[3], *1. University of Alabama-Birmingham, Birmingham, Alabama, United States, 2. San Francisco State University, San Francisco, California, United States, 3. University College London, London, England, United Kingdom*

In this study, the authors analyze data from Project SHARe, a mixed-methods study of same-sex couples in two cities, Atlanta and San Francisco. This study considers both past experiences of parenthood and marriage among same-sex couples, as well as what same-sex couples anticipate will occur regarding their own future experiences of parenthood and marriage, based on analysis of survey responses, timeline data, and in-depth interviews.

### SESSION 2165 (SYMPOSIUM)

### ASIAN OLDER ADULTS' ONLINE AND OFFLINE HEALTH INFORMATION PREFERENCES AND BEHAVIORS

Chair: Bo Xie, *The University of Texas at Austin, Austin, Texas, United States*
Co-Chair: Kristina Shiroma, *The University of Texas at Austin, Austin, Texas, United States*

Older adults living in Asia or of Asian origin have unique preferences for information that require special attention. This symposium focuses on the health information preferences and behaviors of Asian older adults. Song et al. investigated the relationship between Internet use and perceived loneliness among Older Chinese using from survey data collected in the 2015 wave of the China Health and Retirement Longitudinal Study (CHARLS), a national study involving 12,400 households in Mainland China. Multiple regression results suggest that older Chinese Internet users perceived significantly less loneliness compared with their age peers who were non-Internet users. Zhang et al. investigated the role of information and communication technologies in supporting antiretroviral therapy (ART)-related knowledge seeking among older Chinese with HIV. Their cross-sectional survey data were collected from 2012 to 2013 in Guangxi, China. The results suggest that less than 5% of the participants sought HIV-related information via computers. Patients less knowledgeable about ART were more likely than those more knowledgeable to consult medical professionals about the disease via cell phones. Shiroma et al. report findings of a systematic literature review conducted in spring 2019 that examined Asian ethnic minority older adults' preferences for end-of-Life (EOL) information seeking and decision making. The results suggest Asian ethnic minority older adults are understudied in the literature on EOL information and decision making, especially in terms of their unique cultural contexts. Du et al. examined how health information obtained from different types of social networks affect osteoporosis self-management behaviors among older White and Asian women.

### END-OF-LIFE INFORMATION PREFERENCES OF ASIAN OLDER ADULTS: A SYSTEMATIC REVIEW OF THE LITERATURE

Kristina Shiroma,[1] Nathan Davis,[1] and Bo Xie[1], *1. The University of Texas at Austin, Austin, Texas, United States*

Older adults of Asian ethnic minority groups are often underrepresented in the literature on cultural aspects of end-of-life (EOL) decision making. This literature review aimed to systematically investigate the cultural aspects of EOL decision making for aging adults of Asian ethnic minority groups. In February 2019, systematic searches were conducted in PubMed using MeSH terms "end-of-life", "decision-making", and "culture OR cultural". Articles with human subjects, full text in English, published in the past 10 years, with original, empirical findings were included. After multiple rounds of screening, the final sample included 22 results, with sample sizes ranging from 11 to over 9 millions representing South Asian, Chinese, Korean, Taiwanese, Singaporean, Asian and Asian/Pacific adults. The findings suggest the literature on older Asian adults is present, but limited. Future research is needed to explore cultural aspects of Asian ethnic minority groups in respect to older adult's information preferences in EOL decision-making.

### HEALTH INFORMATION'S IMPACT ON OSTEOPOROSIS SELF-MANAGEMENT BEHAVIORS IN OLDER WHITE AND ASIAN WOMEN

Yan Du,[1] Jing Wang,[1] and Qingwen Xu[2], *1. The University of Texas at San Antonio, School of Nursing, San Antonio, Texas, United States, 2. School of Social Work, NY, New York, United States*

Little is known about how health information obtained from different types of social networks affect health behaviors. This study aimed to explore the effect of health information on osteoporosis management behaviors among White and Asian women from a social capital (SC) perspective using a variety of SC measures (e.g. bonding: family, friends, coworkers; bridging: churches, clubs; linking: health providers). Semi-structured interviews were conducted with

10 White and 10 Asian women aged 50 and over in 2016. Through content analysis, we found that SC possession was different between older White and Asian women, and SC utilization to obtain health information corresponded with their possession of SC. Comparing to other diseases, health information relevant to osteoporosis was less frequently communicated. Health information from different types of SC interactively shaped participants' behaviors. The findings suggest that culturally appropriate health interventions might improve older White and Asian women's self-management behaviors of osteoporosis.

### INVESTIGATING THE RELATIONSHIP BETWEEN INTERNET USE AND PERCEIVED LONELINESS AMONG OLDER CHINESE

Shijie Song,[1] Shijie Song,[2] Yuxiang (Chris) Zhao,[3] and Qinghua Zhu[1], *1. Institute of Health Informatics, School of Information Management, Nanjing University, Nanjing, Jiangsu, China, 2. Nanjing University, Nanjing, Jiangsu, China (People's Republic), 3. Nanjing University of Science and Technology, Nanjing, Jiangsu, China (People's Republic)*

There is a long debate on the impact of Internet use on individuals' perceived loneliness. This study aimed to investigate the relationship between older Chinese people's Internet use and their perceived loneliness. We employed cross-sectional data from the 2015 China Health and Retirement Longitudinal Study (N = 7,343; age range: 50-80 years old; Mean = 62.48; SD=8.00). Loneliness was measured by a 4-point Likert scale (Mean = 1.60, SD = 1.05) on the frequency of loneliness perception (higher score indicates higher loneliness). Internet use was measured by a dummy variable (Mean = 0.05, SD = 0.23). The results of multiple regression suggest that older Internet users reported significantly lower loneliness (B = -0.127, SD = 0.045, $p = 0.005$) compared with non-Internet users, suggesting a mitigating effect of Internet use on loneliness. Thus, the Internet might be implemented as an intervention to reduce loneliness among older adults in China.

### THE ROLE OF ICT IN ANTIRETROVIRAL THERAPY-RELATED KNOWLEDGE SEEKING AMONG OLDER CHINESE LIVING WITH HIV

Yao Zhang,[1] Xiaoming Li,[2] Shan Qiao,[2] Yuejiao Zhou,[3] and Zhiyong Shen[3], *1. Kent State University, School of Information, Kent, Ohio, United States, 2. University of South Carolina, Arnold School of Public Health, Columbia, South Carolina, United States, 3. Guangxi Center for Disease Control and Prevention, Nanning, Guangxi, China (People's Republic)*

Antiretroviral therapy (ART) enables HIV patients to reduce disease progression. ART adherence is closely related to patients' knowledge about the medical treatment. This study investigated the role of information and communication technologies (ICTs) in supporting ART-related knowledge seeking among older Chinese with HIV, using cross-sectional data collected from 2012 to 2013 in Guangxi, China. Of the 2987 HIV patients, 688 were 45 years or older and going through ART. We used an 11-item scale ($\alpha$=0.69), which was developed based on existing literature, to assess ART-related knowledge to obtain a composite score (0-11). Less than 5% of the participants sought HIV-related information via computers. Patients with lower ART-related knowledge were more likely to consult medical professionals about the disease via cell phones than those with higher scores (8.28 vs. 9.45, $p < 0.001$). The results suggest that future interventions should integrate cell phones to promote ART-related knowledge dissemination among older patients.

### SESSION 2170 (SYMPOSIUM)

### AT THE INTERFACE OF THE BRAIN AND ENVIRONMENT: DETERMINANTS OF COGNITIVE FUNCTION AND MOBILITY

Chair: Andrea Rosso, *University of Pittsburgh, Department of Epidemiology, Pittsburgh, Pennsylvania, United States*
Discussant: Michelle C. Carlson, *Johns Hopkins Bloomberg School of Public Health, Baltimore, Maryland, United States*

It is hypothesized that older adults are more influenced by environmental factors due to reduced tolerance to stressors and greater cumulative exposure. The role of environmental factors in brain and cognitive aging, specifically, has not been well studied. In this symposium, we highlight novel work at the interface of the environment and the brain in aging. The presentations assess varying aspects of the environment/brain interface within three cohorts. Two of the presentations focus on the environment as a risk factor for brain or cognitive aging. Sara Godina will present analyses of neighborhood socioeconomic status and neuroimaging markers of brain atrophy within the Health ABC Study, with the aim of understanding documented associations of neighborhood socioeconomic status with cognitive function. Jessica Finlay will present data from the REGARDS study linking weather conditions with cognitive changes. Climate influences social and physical activities and could thereby, impact cognitive aging. The brain also serves to process and integrate environmental data in order to direct behaviors in an adaptive manner. Two of the presentations assess how cognitive aging may impact individuals' interactions with the environment. Using residential histories of participants in the Cardiovascular Health Study, Jonathan Platt will explore how cognitive trajectories change in relation to changes in residence. These analyses will demonstrate how residential changes may be either supportive or detrimental to cognitive aging. Finally, Andrea Rosso will present data from the Health ABC that demonstrates how cognitive aging may determine the vulnerability of older individuals to environmental influences on mobility declines.

### COGNITIVE DECLINE ENHANCES VULNERABILITY TO ENVIRONMENTAL DETERMINANTS OF MOBILITY

Andrea Rosso,[1] Philippa Clarke,[2] Stephanie L. Studenski,[3] and Caterina L. Rosano[3], *1. University of Pittsburgh, Department of Epidemiology, Pittsburgh, Pennsylvania, United States, 2. university of michigan, Ann Arbor, Michigan, United States, 3. University of Pittsburgh, Pittsburgh, Pennsylvania, United States*

Neighborhood and residential characteristics can potentially influence mobility of older adults but associations may differ based on cognitive function. We tested whether neighborhood and residential characteristics derived from audits of Google Streetview images were related to 4-year incident mobility disability and whether these associations differed by cognitive trajectories (maintainer vs decliner over 14 years). In 260 participants from the Health ABC (mean age=81.8 years, 57% female, 39% black), Cox proportional hazard models tested associations stratified by cognitive trajectory, adjusted for demographics. Mixed compared to residential land use was associated with greater risk of mobility disability among cognitive decliners (HR=1.27, 95% CI: 1.05-1.54) but not cognitive maintainers (HR=1.07, 95% CI: 0.90-1.28). Presence of slopes near the home and having a ramp at the home entrance were associated with lower mobility disability risk, again in decliners only. Lower cognitive function may increase vulnerability to poorer neighborhood and residential characteristics for mobility outcomes.

### NEIGHBORHOOD SOCIOECONOMIC STATUS AND GRAY MATTER VOLUME IN OLDER ADULTS

Sara L. Godina,[1] Caterina Rosano,[1] Peter J. Gianaros,[1] Howard J. Aizenstein,[1] Michelle C. Carlson,[2] Philippa Clarke,[3] and Andrea L. Rosso[1], *1. University of Pittsburgh, Pittsburgh, Pennsylvania, United States, 2. Johns Hopkins University, Baltimore, Maryland, United States, 3. University of Michigan, Ann Arbor, Michigan, United States*

Lower neighborhood socioeconomic status (nSES) is associated with poorer cognitive function; underlying neural correlates are unknown. Cross-sectional associations of nSES (six census-derived measures of income, education, and occupation) and gray matter volume (GMV) of eight memory-related regions (hippocampus, middle frontal gyrus, amygdala, insula, parahippocampal gyrus, anterior, middle, and posterior cingulum) were examined in 264 community-dwelling older adults (mean age=83, 56.82% female, 39.02% black). In linear mixed effects models adjusted for total brain atrophy and accounting for geographic clustering, higher nSES was associated with greater GMV of the left hippocampus, left posterior cingulum, and bilateral insula, middle frontal, and parahippocampal gyri. nSES remained associated with GMV of the right insula (β= -32.26, p=0.026, 95%CI: -60.66, -3.86) after adjusting for individual level age, gender, race, income, and education. The nSES and cognitive function association may not be due to gray matter volume differences; other behavioral and biological mediators should be explored.

### THE BIDIRECTIONAL RELATIONSHIP BETWEEN AGING IN PLACE AND COGNITIVE FUNCTIONING OVER TIME

Jonathan Platt,[1] Yvonne Michael,[2] Gina Lovasi,[2] and Andrea Rosso[3], *1. Columbia University, New York, New York, United States, 2. Drexel University, Philadelphia, Pennsylvania, United States, 3. Pittsburgh School of public health, pittsburgh, Pennsylvania, United States*

Residential stability (aging in place) in older adults may be either supportive or detrimental to cognitive aging, and may be dynamic over time. Using residential histories of 3608 older adults in the Cardiovascular Health Study, this study seeks to estimate the potentially bidirectional relationship between residential change and cognitive functioning. Residential data were recorded and georeferenced annually, and the Modified Mini-Mental State Examination assessed global cognitive functioning. Marginal structural models will be used to assess the effect of residential and cognitive exposures over time, in the presence of time-varying covariates that may act as confounders and mediators at different time points. We hypothesize that residential stability will have a bidirectional relationship with cognitive functioning over time. Aging in place will be associated with higher cognitive function during follow-up, and predict longer dementia-free survival. In turn, time to residential relocation during follow-up will be shorter among those with lower cognitive function.

### WINTER WOES? IMPACT OF SNOW, SLEET, AND RAIN ON AGE-RELATED COGNITIVE DECLINE

Jessica M. Finlay,[1] Philippa Clarke,[2] Anam Khan,[2] Carina Gronlund,[2] Robert Melendez,[2] Suzanne Judd,[3] and Virginia Wadley[3], *1. Social Environment and Health, Institute for Social Research, University of Michigan, Ann Arbor, Michigan, United States, 2. Institute for Social Research, University of Michigan, Ann Arbor, Michigan, United States, 3. The University of Alabama at Birmingham, Birmingham, Alabama, United States*

Snowfall, sleet and rain can adversely affect the mobility of older adults, with negative consequences for engagement in daily activities and socializing. This can lead to isolation and loneliness, which can negatively impact cognitive functioning. We tested whether long-term exposure to precipitation – particularly snow and cold rain (precipitation at ambient temperatures between 0°C and 10°C) – negatively impacts age-related cognitive function trajectories among a national sample of over 30,000 Americans (aged 45+) in the Reasons for Geographic and Racial Differences in Stroke study followed since 2003. Linear growth mixture models showed that living in an area with a 25% greater proportion of days with snow/rain in the past year was associated with a 0.6 unit decrease in cognitive function score (p<.001). Effects were stronger among those aged 75+, who experienced faster rates of cognitive decline. The findings motivate further research on the role of cold-season precipitation for cognitive decline.

### SESSION 2185 (SYMPOSIUM)

### ESPO/SOCIAL RESEARCH, POLICY, AND PRACTICE: CHOT: AN INDUSTRY-ACADEMIC MODEL TO IMPROVE HEALTH

Chair: Justin C. Lord, *Louisiana State University at Shreveport, Shreveport, United States*
Co-Chair: Danielle Waldron, *Department of Gerontology, University of Massachusetts Boston, Boston, Massachusetts, United States*

This proposed symposium will bring together a diverse panel of emerging and established academics, as well as, industry leaders to speak about the power of collaboration. As the delivery of health care in the field of gerontology becomes increasingly complex – researchers and practitioners will have to find new ways to address these challenges. One potential solution is to become more interdisciplinary and collaborative in our approach. This symposium will bring together academic and industry leaders from the National Science Foundation's Centers for Health Organization Transformation (CHOT) to share with our membership an industry-academic partnership model that has benefited industry-focused research across more than 50 disciplines. As the ESPO representatives for the SRPP division, we believe that our membership would benefit from a symposium of this nature. This symposium would also reflect this year's conference is "Harnessing the Power of Networks." The SRPP section has the strongest commitment to practice and research on practice such as ways to improve the delivery of services to an increasingly diverse older population. A model of industry partnership holds tremendous value for all those involved; however, it is incredibly valuable for junior researchers who are just beginning their careers. The CHOT model provides opportunities and funding for researchers to develop collaborative relationships; engage in research; access new sources of data; disseminate findings directly to a broader audience, and have the ability to address some of health care's wicked problems.

### CHOT LEADERSHIP

Thomas Ferris[1], *1. Texas A&M University, Texas A&M, Texas, United States*

The first speaker is Dr. Thomas Ferris the Center Director of the NSF Center for Health Organization Transformation (CHOT). Dr. Ferris will provide a high-level overview of CHOT and provide the audience with a greater understanding of its history, funding, partners, and accomplishments. In this section, the mission, annual research model, and the I/UCRC planning integration framework will be reviewed and discussed. The NSF CHOT is the only Industry-University Cooperative Research Center (I/UCRC) focused on innovations in healthcare delivery in the nation. CHOT researchers work alongside the Industry Advisory Board (IAB) to conduct research that supports the implementation of evidence-based transformational strategies within the healthcare sector. During this symposium, Dr. Ferris will discuss some of the challenges and opportunities that can arise from these partnerships. More importantly, in this section will speak to how value is created through these collaborative efforts and how ultimately this can improve health outcomes.

### CHOT ACADEMIC PARTNER

Robert Weech-Maldonado[1], *1. University of Alabama at Birmingham, Birmingham, Alabama, United States*

The second speaker will be Dr. Robert Weech-Maldonado, an academic representative and CHOT Site Director at the University of Alabama at Birmingham. Dr. Weech-Maldonado will share what got UAB involved in CHOT; the process to get involved; what it means to be a CHOT partner, and the pros/cons of this program from the academic side. During this symposium, we will explore why this model needs to be expanded to the field of gerontology and the impact it can have on the aging population. Relating to the academic experience, Dr. Maldonado will speak to how CHOT can result in financial support to the academic institution; provide access to unique data sets / populations; outcomes, such as, publications, and how this program can further academics careers. We will also explore some of the struggles related to working with an industry partner as it relates to deliverables and expectations.

### CHOT STUDENT REPRESENTATIVE

Neeraj Puro[1], *1. University of Alabama at Birmingham, UAB, Alabama, United States*

As the Senior Student Representative for the Social Research, Policy, and Practice Section for GSA one of the goals is to organize a symposium that would be beneficial for students and junior researchers. In this section, we will have a student who was involved in CHOT to speak about his experience. Neeraj Puro is a doctoral candidate who has been involved in several CHOT projects during his academic career. The CHOT has helped fund his doctoral work and allowed him to travel and present at more conferences. The projects that he was involved in helped him with early publications and assisted when he was out in the career market. He will speak about his experience; what he learned, and his opinion about the process. He will reflect on this program from a student/junior researcher's perspective and how it has influenced his doctoral journey and the beginning of his career.

### CHOT INDUSTRY PARTNER

Kenneth Cochran[1], *1. University of Alabama at Birmingham, UAB, Alabama, United States*

The third speaker will be the industry partner representative, Kenneth Cochran, President and CEO of Opelousas General Health System. Here we will hear from industry partners about what drew them to this program of inter-collaboration; the benefits they have received, and what was required of them to participate. This will be helpful as it will provide an opportunity to have a first-hand account about the value that practitioners find in this program as it relates to testing interventions, developing new systems, and improving financial performance and/or quality. Kenneth will speak to a particular project at Opelousas and describe entire process. Kenneth will highlight the challenges and opportunities that come from partnering with an academic institution. Also, how CHOT brings together industry partners who are facing the same challenges and how they learn and adopt best practices from each other. This should further illustrate the value that academic/industry collaboration can bring.

### SESSION 2190 (SYMPOSIUM)

### CORRECTING GLUTATHIONE DEFICIENCY IN AGING: IMPACT ON MITOCHONDRIA, STRENGTH, INFLAMMATION AND METABOLIC DEFECTS

Chair: Rajagopal Sekhar, *Baylor College of Medicine, Houston, Texas, United States*
Co-Chair: George E. Taffet, *Baylor College of Medicine, Houston, Texas, United States*
Discussant: Roger Fielding, *Tufts University, Boston, Massachusetts, United States*

Aging is associated with deficiency of Glutathione, the most abundant, intracellular, antioxidant protein, but underlying mechanisms are unknown and interventions limited. This symposium is primarily focused on the results of placebo-controlled, double-blind randomized clinical-trial (RCT) on the impact of correcting Glutathione deficiency in older humans on mitochondrial impairment, oxidative stress, strength, inflammation, and insulin resistance. Dr. Jahoor's presentation will serve as an introduction by discussing mechanisms underlying Glutathione deficiency and validation of a novel nutritional intervention based on supplementing glycine and N-acetylcysteine (GlyNAC) to correct Glutathione deficiency in older-humans. Dr. Sekhar will present the results of a pilot 16-week pilot randomized, placebo-controlled, double-blind clinical trial in older humans investigating the effect of supplementing GlyNAC (vs. placebo) to improve Glutathione levels and oxidative-stress in 24 older-humans and 12 young-humans on impaired mitochondrial fuel-oxidation (MFO) and other defects. The trial met its primary objective that that GlyNAC supplementation (and not placebo) significantly improved Glutathione deficiency and corrected impaired MFO (and defects in its molecular regulation), and also significantly improved gait-speed (increased 19% increase to match young-humans), muscle-strength, exercise-capacity, and lowered oxidative-stress (80%) inflammation (IL-6 83%, TNF-alpha 58%), and insulin-resistance (68%). Dr. Taffet will discuss age-induced diastolic heart failure, and the effect of supplementing GlyNAC (vs. NAC alone) in aged 24-month old mice with diastolic heart-failure, impaired myocardial MFO and cardiac-inflammation. Collectively this symposium on Glutathione and Aging will highlight the discovery that supplementing GlyNAC to correct Glutathione deficiency in older-humans has significant health benefits, and could be a novel nutritional-intervention in aging.

### GLUTATHIONE DEFICIENCY AND OXIDATIVE STRESS IN AGING: METABOLIC MECHANISM AND TARGETED INTERVENTION

Farook Jahoor,[1] George E. Taffet,[1] and Rajagopal V. Sekhar[1], *1. Baylor College of Medicine, Houston, Texas, United States*

The free-radical theory of aging suggests that age-related functional decline is mediated by increases in free-radical induced oxidative-stress. Cells normally depend on antioxidants for protection against oxidative-stress. Glutathione is the most abundant endogenous intracellular antioxidant protein composed of 3 amino-acids, cysteine, glycine and glutamic-acid, and is known to be deficient in older-humans. We investigated Glutathione kinetics in older humans using a stable-isotope tracer-based approach, and found that compared to younger humans, older-humans had severe Glutathione deficiency as a result of decreased synthesis caused by limited availability of glycine and cysteine, and associated with elevated oxidative-stress. Orally supplementing glycine and cysteine (provided as N-acetylcysteine) at doses of 1.33mmol/kg/d and 0.81mmol/kg/d respectively for 2-weeks corrected their intracellular deficiency, normalized Glutathione synthesis rates and lowered oxidative-stress to levels in younger controls. These results suggest that short-term supplementation of GlyNAC at these doses can successfully correct intracellular Glutathione deficiency in older-humans.

### CORRECTING GLUTATHIONE DEFICIENCY AND MITOCHONDRIAL DYSFUNCTION IN OLDER HUMANS: A RANDOMIZED CLINICAL TRIAL

Rajagopal V. Sekhar,[1] Premranjan Kumar,[1] Jean W. Hsu,[1] James Suliburk,[1] George E. Taffet,[1] Charles G. Minard,[1] Farook Jahoor,[1] and Chun Liu[1], *1. Baylor College of Medicine, Houston, Texas, United States*

Aging is associated with impaired mitochondrial fatty-acid oxidation (MFO) due to unknown mechanisms, and interventions are lacking. We hypothesized that impaired MFO in aging occurs due to Glutathione-deficiency and tested this in a randomized, placebo-controlled double-blind clinical-trial in 24 older-humans (71.1y) and 12 young-controls (25.5y) using calorimetry, muscle-biopsy and tracer-protocols. Older-humans received either GlyNAC (Glycine 1.33mmol/kg/d and N-acetylcysteine 0.83mmol/kg/d as Glutathione precursors) or isonitrogenous-placebo for 16-weeks; young-controls received GlyNAC for 2-weeks. Compared to young-controls, older humans had significantly lower Glutathione, impaired MFO, lower gait-speed and physical-function, and higher oxidative-stress, inflammation and insulin-resistance. GlyNAC supplementation in older-humans significantly improved and restored MFO; increased gait-speed (19%,) and physical-function; and decreased oxidative-stress (TBARS 80%), inflammation (IL-6 83%; TNF-alpha 58%), and insulin-resistance (HOMA-IR 68%), but young-controls were unaffected. These data provide proof-of-concept that GlyNAC supplementation could improve the health of older-humans by correcting Glutathione-deficiency and mitochondrial-defects to improve gait-speed, oxidative-stress, inflammation and insulin-resistance.

### GLUTATHIONE, INFLAMMATION, MITOCHONDRIAL FAT OXIDATION AND DIASTOLIC HEART FUNCTION IN OLD MICE

George E. Taffet,[1] K A. Cieslik,[1] R V. Sekhar,[1] Celia A. Pena Heredia,[2] Dale J. Hamilton,[2] and Mark L. Entman[1], *1. Baylor College of Medicine, Houston, Texas, United States, 2. Houston Methodist Hospital, Houston, Texas, United States*

Impaired diastolic function is a risk factor for diastolic heart failure, may limit exercise performance, and is common in aging in both people and animals. This diastolic dysfunction seems to be associated with cardiac inflammation, fibrosis and impaired mitochondrial fatty acid metabolism. Old (24-28 m) mice fed a GlyNAC supplemented diet for 8 weeks were compared to those on control diet, and had dramatic improvement in all these parameters. For example, ATP generation from fatty acids with five-fold higher in the GlyNAC supplemented mice. In vitro studies compared NAC with GlyNAC and demonstrated the benefits only with supplementing both amino acids as compared to NAC alone. These data suggest that GlyNAC may have a role in improving cardiac function thus improving exercise tolerance and quality of life for older people.

### SESSION 2200 (SYMPOSIUM)

### CREATIVITY IN LATER LIFE: PERSPECTIVES ON ITS FORMS AND MEANINGS

Chair: Carolyn E. Adams-Price, *Department of Psychology Mississippi State University, Starkville, Mississippi, United States*
Discussant: Danielle K. Nadorff, *Mississippi State University, Starkville, Mississippi, United States*

Over the past 15 years, gerontologists have become increasingly interested in identifying activities that increase meaning and well-being in older adults' lives. One such activity is creative activity. New research suggests that creative activity can improve social connections, well-being and self-esteem, and sometimes cognitive processing. Yet some culturally-specific creative activities in which older people participate are not considered creative using standard psychological paradigms of creativity. This symposium will examine new perspectives on creativity in older adults with emphases on creative meaning across the life-span, and the benefits of new and long-term creative hobbies in healthy and frail older adults. Age differences in artists' perceptions of their creativity will also be discussed. The first presentation by Helen Kivnick will discuss the overall theme of vital involvement in late life from an Eriksonian perspective and its link to the benefits of creative activities. The second presentation by Kate de Medeiros will discuss the creative benefits of a poetry intervention for older adults with dementia. The third presentation by Monika Ardelt will examine young and older nominated visual artists' perceptions of their own creativity and the creative process, in the context of creative careers. Finally, Carolyn Adams-Price will describe Glaveanu's sociocultural model of creativity, which can be used to explain the significance of different levels of creativity in a cultural context, from everyday creativity to genius creativity. Glaveanu's model has great promise for helping us understand the significance of various creative activities for older adults. Danielle Nadorff will be our discussant.

### THE SOCIOCULTURAL MODEL OF CREATIVITY: HOW IT CAN EXPLAIN THE SIGNIFICANCE OF MULTIPLE FORMS OF LATE-LIFE CREATIVITY

Carolyn E. Adams-Price[1], *1. Department of Psychology Mississippi State University, Starkville, Mississippi, United States*

Creativity in later life comes in many forms, ranging from everyday creativity to genius-level creativity, and including both newly learned creative activities and life-long creative hobbies. Previous psychosocial models of creativity have had limited utility in explaining the significance of late life creativity. Glaveanu's sociocultural model has not been previously applied to older adults, but its inclusiveness makes it supremely useful for describing the range of creative activities and products in context. Creativity, according to Glaveanu, involves five interconnected components: actors, actions, artifacts, audiences, and affordances, which can be used to describe many different points along continuum of creative activities. The sociocultural model recognizes the value of different levels of creativity, including culturally-specific crafts, practice and learning new skills, the role of large and small audiences for creativity, and the connection of creativity and community. Glaveanu's model will be discussed in the context of Erikson's theory of late life development.

### VITAL INVOLVEMENT: LINKING CREATIVITY TO LATER-LIFE WELL-BEING

Helen Q. Kivnick[1], *1. University of Minnesota School of Social Work, Saint Paul, Minnesota, United States*

Erikson's principle of Vital Involvement (VI) holds that psychosocial health in older adulthood rests on elders' meaningful, reciprocal engagement with the world outside the self. Older adulthood's focal tension between Integrity and Despair is fundamentally grounded in elders' "…vital involvement, with life's people, materials, activities, ideas, institutions, and so forth. [This engagement is] … every bit as important as …[the] reminiscence" (Kivnick &Wells, 2014) we have long identified as a path to wisdom. Through several empirical projects, our team has identified five dimensions of VI in older adulthood. Each is clearly invoked as an elder engages such arts media as clay, paint, paper, fiber, wood, words, music, movement, and more. These VI dimensions are also invoked in an elder's creative engagement with the people, materials, activities, and institutions of everyday life. This presentation illustrates ways that VI, through these dimensions, both facilitates and also expresses psychosocial well-being in older adults.

### CREATIVITY, HOPE, AND EXPECTATION IN A POETRY PROGRAM: RETHINKING WHAT COUNTS AS SUCCESS IN DEMENTIA CARE

Kate de Medeiros[1], *1. Miami University, Oxford, Ohio, United States*

Creativity offers liberation from the framework of decline in later life in general, and within the context of dementia specifically. Cohen described the synergy of hope and expectation as ways through which people access their creative potential, especially in the face of loss. This paper explores three case drawn from a qualitative study. Eight residents living in a secure dementia care facility participated in six 30-minute interactive poetry sessions using guidelines from the Alzheimer's Poetry Project (APP). Observations were conducted at baseline, during the intervention, and one week afterwards. All sessions were audio recorded, transcribed and analysed. Important findings included the positive potential of imagined futures, creative engagement as a tool for building social bonds, and the importance of language play. Overall, these findings point to new ways to consider success in dementia interventions by focusing on the potential to create meaningful and creative engagement opportunities.

### DOES AGE AFFECT CREATIVE PROCESS AND STYLE? A COMPARISON OF OLDER AND YOUNGER VISUAL ARTISTS

Monika Ardelt,[1] and Lucinda Orwoll[2], *1. University of Florida, Gainesville, Florida, United States, 2. Private Practice, Ann Arbor, Michigan, United States*

This study investigated differences in the creative process and style between 85 older (age 60-89 years, M=72.39) and 63 younger (age 27-58 years, M=41.95) visual artists who were nominated as artistically creative exemplars. Answers to open-ended survey questions were coded and compared by age group. Results of t-tests showed that the described creative process and style were similar for older and younger artists, with many being inspired by their environment or ideas and engaging in an intuitive and visual style. However, younger artists were more likely than older artists to be inspired by ideas, words, life, and the work process and to use an intuitive, expressionistic, eclectic, spiritual, and textured style. Interestingly, younger artists were more likely to believe that older artists have greater artistic experience, maturity, willingness to take risks, and understanding of the art world, whereas older artists tended to think that young artists are more original.

### SESSION 2205 (SYMPOSIUM)

### CUMULATIVE ADVANTAGE IN CHANGING ECONOMIC TIMES: STRESS, DISTRESS, AND FUTURE PROSPECTS FOR AGING COHORTS

Chair: Dale Dannefer, *Department of Sociology, Case Western Reserve University, Cleveland, Ohio, United States*
Co-Chair: Stephen Crystal, *Rutgers University, New Brunswick, New Jersey, United States*
Discussant: Angela O'Rand, *Departnbet of Sociology, Duke University, Durhan, North Carolina, United States*

Processes of cumulative dis/advantage operate within cohorts and across historical time. In the ongoing dance of age, cohort and period, each cohort encounters distinctive social and economic environments at particular ages that may ameliorate or exacerbate the cumulative and systemic processes of inequality production that operate over its collective life course. We explore issues of current and future late-life inequality and its consequences. As overall income inequality has grown, what are the likely consequences for late-life outcomes? How have cohorts currently in midlife been affected by the Great Recession of 2008 and subsequent recovery? What are the mental and physical consequences of these developments, and to what extent can they be ameliorated by interventions in middle and later adulthood? This symposium addresses how variation in economic circumstances and social and psychological stresses may affect outcomes over the life course, and how these complex, interacting processes can be best conceptualized and examined. One paper examines the impact of the Great Recession and subsequent events on the intracohort distribution of income, suggesting inordinate setbacks during the Recession with likely long-term effects for economically vulnerable subpopulations. Another explores the role of psychosocial stressors in the process of cumulative dis/advantage, focusing on linkages between functional limitations and psychological well-being in later life, and how these linkages are amplified by diverse dimensions of disadvantage (e.g., education, employment; coping strategies; caregiving). A third paper examines the intergenerational dimensions of cumulative advantage processes. Finally, contrasting theoretical frameworks for apprehending life-course processes and historical change will be explored.

### LATENCY/PATHWAYS, FUNCTIONALISM/MORPHOGENESIS: CONTRASTING PARADIGMS OF LIFE-COURSE INEQUALITY AND CHANGE

Dale Dannefer[1], *1. Department of Sociology, Case Western Reserve University, Cleveland, Ohio, United States*

Attention to dis/advantage during childhood has become a major interest of life-course studies. It has been a force in advancing attention to inequality over the undifferentiated "normal aging" versions of life-course and gerontological research, making clear the irreducible importance of the presence/ absence of key resources in accounting for life-course outcomes, from early onward. Explanatory strategies set forth within this work often contrast "latency/early origins" models (with explanation anchored in the early years) with "pathways" models (which examine the independent effects of adult life-course circumstances). This paper argues that these two types of models actually are aligned with distinct conceptual paradigms that imply fundamentally different understandings of aging in society ("functionalist/organismic" and "systemic/morphogenetic"). The differential implications of these two models for the relation of cumulative dis/advantage and social change is explored.

### CUMULATIVE DIS/ADVANTAGE, COHORTS, AND ECONOMIC SHOCKS BEFORE AND AFTER: LIFE COURSE INEQUALITY AND THE RECESSION

Stephen Crystal,[1] and Naomi Zewde[2], *1. Rutgers University, New Brunswick, New Jersey, United States, 2. School of Social Work, Columbia University, New York, New York, United States*

We examined age/cohort profiles of income inequality in the Survey of Consumer Finances for 2007, 2010, and 2016. Overall, the Gini coefficient decreased somewhat following the 2008 onset of the Recession, but rebounded by 2016 to a higher level than the 2007 baseline. Within-cohort inequality trends from 2007-2016 differed considerably across cohorts. For late baby boomers and millennials, inequality increased sharply – from .56 to .67 for the 1963-1972 birth cohort and from .49 to .60 for the 1973-1982 birth cohort. Inequality was higher at baseline for early baby boomers than later cohorts but did not increase as they moved into midlife and early late life. The Great Recession, with its large drops in asset values, initially led to slight reductions in inequality, but this effect was temporary. Late boomers and millennials experienced sharp increases in inequality following the Recession, presaging a very high-inequality late-life experience for these cohorts.

### UNDERSTANDING THE ROLE OF STRESS PROCESSES IN CUMULATIVE DIS/ADVANTAGE PROCESSES

Deborah Carr[1], *1. Boston University, Boston, Massachusetts, United States*

In my book Golden Years (2019), I argue that the psychosocial consequences of normal biological processes of aging are intensified for those who have had lives of disadvantage, just as the harmful consequences of life-course disadvantages are particularly acute for those experiencing age-related physical health declines. In this paper, I discuss the role of stress processes, including stress proliferation and amplification, as possible mechanisms contributing to cumulative dis/advantage. I evaluate these ideas empirically by focusing on the linkages between functional limitations (an indicator of normal biological aging) and psychological well-being in later life, and explore the extent to which these linkages are amplified diverse indicators of life course disadvantage including low education; poor-quality employment; avoidant coping strategies; and family-related strains including intensive caregiving. Analyses are based on data from the Midlife in the United States (MIDUS) study. I discuss the implications of incorporating stress process models in cumulative dis/advantage research.

### CUMULATIVE DIS/ADVANTAGE IN HEALTH AS AN INTERGENERATIONAL PROCESS

Kim Shuey[1], *1. Department of Sociology, :University of Western Ontario, London, Ontario, Canada*

Empirical applications of cumulative dis/advantage primarily focus on accumulative processes within an individual life course as a mechanism generating growing intracohort inequality with age. However, adult health reflects the accumulation of exposures to advantages and disadvantages across the individual life course as well as the transmission of resources and practices across generations within one's family of origin, which forms the foundation from which children are launched and inequality is reproduced. In analyses using two panel studies (PSID and Add Health), we integrate literature on the early-life origins of health with the concept of linked lives and the intergenerational transmission of resources to examine the relationship between parents' health and the health of their children in adulthood. Results indicate an intergenerational persistence in health and demonstrate the importance of looking beyond the individual life course to better understand cumulative dis/advantage in health as a process operating across generations within families.

### SESSION 2210 (SYMPOSIUM)

### CURRENT EFFORTS TO UNDERSTAND AND IMPROVE OLDER ADULTS' FUNCTIONAL RECOVERY AFTER HIP FRACTURE

Chair: Richard H. Fortinsky, *University of Connecticut School of Medicine, Farmington, Connecticut, United States*
Discussant: Nancy Latham, *Brigham and Women's Hospital, Boston, Massachusetts, United States*

The annual number of hip fractures in the US is projected to increase from 258,000 in 2010 to 458,000 by 2050. Globally, annual hip fractures are projected to reach 4.5 million by 2050. Yet the majority of older adults experience long-term mobility disability following hip fracture and do not return to pre-fracture functional capacity. Published reviews have concluded there is insufficient evidence regarding effectiveness of interventions designed to reduce residual disability and enhance mobility post-hip fracture. This Symposium features current efforts to understand and improve functional recovery post-hip fracture. Dr. Magaziner will present results from the recently-completed Community Ambulation Project (CAP), a multi-site randomized trial in which two in-home physical therapy interventions were compared: PUSH, which included aerobic conditioning, strengthening, balance and functional training; and PULSE, which included transcutaneous electrical nerve stimulation, flexibility and active range of motion exercises. Dr. Fortinsky will present CAP data examining the role of psychological resilience, optimism, depression, and balance confidence on mobility measures. Dr. Gruber-Baldini will present CAP data examining differences between PUSH and PULSE on study participants' cognition and the impact of cognition on community ambulation. Dr. Binder will present the study design and early results from the STEP-HI study, a multi-site randomized trial evaluating whether structured exercise and topical testosterone therapy can improve function post-hip fracture among older women. Discussant Dr. Latham will comment on design, results, and implications of these two studies for research, policy, and practice intended to improve older adults' recovery after hip fracture.

### A MULTI-COMPONENT HOME-BASED PHYSICAL THERAPY INTERVENTION FOR IMPROVING COMMUNITY AMBULATION AFTER HIP FRACTURE

Jay S. Magaziner[1], *1. University of Maryland Baltimore School of Medicine, Baltimore, Maryland, United States*

Presented is a two-group RCT evaluating a multi-component exercise program for hip fracture patients to determine if it is effective in improving the ability to walk independently in the community. Hip fracture patients age ≥60 years (N=210) were assessed and randomized within 26 weeks of hospitalization, and reassessed 16 and 40 weeks later. The primary outcome was ability to walk 300m in six minutes. PUSH (active treatment) included aerobic conditioning, strengthening, balance and functional training. PULSE (attention control) included transcutaneous electrical nerve stimulation, flexibility and active range of motion exercises. Both groups received 2-3 visits per week for 16 weeks in their residences from a physical therapist. 22/96 in PUSH (22.9%) and 18/101 in PULSE (17.8%) (difference 5.1%; 95% CI: -6.1%, 16.3%; P=.37) became community ambulators. We conclude that advancing substantial proportions of hip fracture patients to community ambulation will require more than the intervention evaluated in this study.

### PSYCHOLOGICAL FACTORS ASSOCIATED WITH AMBULATION PERFORMANCE AFTER HIP FRACTURE AMONG HOME-DWELLING OLDER ADULTS

Richard H. Fortinsky[1], *1. University of Connecticut School of Medicine, Farmington, Connecticut, United States*

Many older adults fail to resume optimal community living after hip fracture due to sustained limitations in ambulation capacity, yet reasons remain poorly understood. Roles of psychological factors in affecting ambulation performance post-hip fracture remain particularly understudied; depression has been associated with poorer self-reported functional status, and little is known about self-perceived balance confidence, resilience, and optimism. This presentation reports associations between each psychological factor, measured at CAP baseline, and gait speed and walking endurance, measured at baseline and 16 weeks later. In the CAP cohort (N=210), baseline mean/sd 4-meter gait speed (gs), 50-foot walk gs, and 6-minute walk distance were: 0.60/0.19 meters per second (mps); 0.67/0.20 mps; and 186.9/55.4 meters, respectively. In multivariate models, balance confidence was positively associated with all baseline ambulation measures ($p<0.001$ in all models), and resilience was positively associated with all 16-week follow-up ambulation measures ($p>0.05$ in all models). Implications of results will be discussed.

### IMPACT OF THE MULTI-COMPONENT HOME-BASED PHYSICAL THERAPY INTERVENTION ON COGNITIVE OUTCOMES IN THE CAP TRIAL

Ann Gruber-Baldini[1], *1. University of Maryland Baltimore School of Medicine, Baltimore, Maryland, United States*

Cognitive impairment after hip fracture influences recovery and some RCTs suggest aerobic and resistance exercise may improve cognition. This presentation examines differences in PUSH versus PULSE on cognition and the impact of cognition on community ambulation. In CAP, the

Modified Mini-Mental State Examination (3MS) was pre-specified for subgroup (3MS<91 versus 3MS≥91) evaluation and as a tertiary outcome. The CAP Mechanistic Pathways ancillary study (subgroup n=40) included Hooper Visual Organization Test and Trails A&B as additional outcomes. At baseline, PUSH did not differ from PULSE on cognitive measures. Over 16 weeks, PUSH became faster on Trails A (Δ= 6.7(29.9)) while PULSE became slower (Δ=9.4(24.2)) (p<.05). Those with higher baseline cognition were more likely to become community ambulators (28.3% 3MS≥91 versus 8.8% 3MS<91), but PUSH versus PULSE effects didn't differ by 3MS (no effect modification). Results suggest potential differences in recovery by cognition, but no treatment effect differences by level of cognition.

### STARTING A TESTOSTERONE AND EXERCISE PROGRAM AFTER HIP INJURY (STEP-HI) TRIAL: RATIONALE, METHODS, AND FIDELITY

Ellen Binder[1], *1. Washington University School of Medicine, St. Louis, Missouri, United States*

The STEP-HI study is an ongoing multi-center, randomized, controlled, double-blinded clinical trial that is evaluating whether six months of topical testosterone therapy combined with a supervised center-based exercise-training program can improve mobility, functional performance, and quality of life after hip fracture, compared to exercise training alone or Enhanced Usual Care. Female hip fracture patients ≥ 65 yrs. old who are living in the community or assisted living are being randomized within 16 weeks of surgical repair for the fracture, and re-evaluated 24 weeks later. Three hundred patients will be recruited from six clinical centers in the USA. This presentation discusses the rationale and study design, including innovative methods for fidelity monitoring of exercise procedures across the clinical sites. Strategies for recruitment and related challenges will be discussed. Preliminary enrollment and fidelity data will be presented.

### SESSION 2215 (PAPER)

### DEMENTIA AND COGNITIVE IMPAIRMENT

### BRAIN FUNCTIONAL CONNECTIVITY IN OLDER ADULTS WITH MCI: DO FALLS MATTER?

Anna R. Egbert,[1] Chun Liang Hsu,[1] Rachel Crockett,[1] and Teresa Liu-Ambrose[1], *1. University of British Columbia, Vancouver, British Columbia, Canada*

Older adults with mild cognitive impairment (MCI) are at an elevated risk of falls. We conducted a pilot longitudinal observational study to examine the natural course of brain intrinsic functional connectivity (FC) and cognitive function changes in association to falling history in older adults with MCI. 15 MCI participants (mean age 75.9, range 67-86) included 10 non-fallers and 5 fallers (minimum two falls in the previous 12 months with one in the last 6 months) from Metro Vancouver, BC, Canada. At study entry and 1-year follow-up, participants completed brain scanning session of structural MRI and resting state (RS) functional MRI, the Montreal Cognitive Assessment (MoCA), and the Mini Mental State Examination (MMSE). Results indicated an interaction between time (baseline vs. follow-up) and falls history on RS-FC in individuals with MCI (p<0.001). At 1-year follow-up, MCI non-fallers showed increased FC between frontal, parietal and occipital cortex (from baseline R=0.141 to follow-up R=0.321) and lack of decline on cognitive measures. Meanwhile, MCI fallers showed weakening of FC between those brain regions (from baseline R=0.314 to follow-up R=0.201) with simultaneous cognitive deterioration. Significant relationships between FC strength and cognitive status existed only at follow-up (R=0.525, p<0.05), suggesting that the triggered functional compensatory brain mechanisms in MCI non-fallers are not successfully executed in MCI fallers. Together, our pilot data suggest that older adults with MCI who fall show more advanced brain functional degradation with adjacent cognitive decline as compared to MCI individuals who do not fall.

### EVENT-SPECIFIC EMOTIONAL EXPRESSION OF PERSONS WITH DEMENTIA IN LONG-TERM CARE: PRELIMINARY RESULTS

Kyung Hee Lee,[1] Ji Yeon Lee,[2] Bora Kim,[1] and Marie Boltz[3], *1. Yonsei University College of Nursing, Seoul, Korea, Republic of, 2. Yonsei University, Seoul, Korea, Republic of, 3. Pennsylvania State University, University Park, Pennsylvania, United States*

Although dementia-related language, comprehension, and memory deficits occur fairly early stage in dementia, persons with dementia retain the ability to express their emotion even in the late stage of disease. However, health care providers do not know how to interpret emotional expressions that could be utilized as important signals of underlying needs and care preferences in persons with dementia. The purpose of this study was to explore the event-specific emotional expressions of persons with dementia in long-term care over a 6-month period. This was a longitudinal study using repeated observations. Emotional expressions were videotaped when three specific events (personal care, meal time, and activity) occurred at baseline, month 3 and month 6. A total of nine observations was made for each participant. We enrolled 13 participants so far; ten participants were completed 6 month follow up. The mean score of MMSE at baseline was 4.38; that of ADL was 16.62. On average, persons with dementia showed 9.93 episodes of positive emotional expression (PEE) per minute and 1.81 episodes of negative emotional expression (NEE) per minute. We found between person variations for both PEE and NEE. PEE and NEE were different by three types of events. Specifically, persons with dementia showed more PEE with activity than personal care and meal time and more NEE with personal care than the other two events. This study will provide better understanding of event-specific emotional expressions, and inform the development of emotion-oriented interventions programs to improve the psychological well-being of persons with dementia.

### SATISFACTION WITH OUTDOOR ACTIVITIES AMONG LONG-TERM SERVICES AND SUPPORTS RECIPIENTS

Justine S. Sefcik,[1] Karen Hirschman,[2] Darina V. Petrovsky,[2] Nancy Hodgson,[2] and Mary Naylor[2], *1. University of Pennsylvania School of Nursing, Philadelphia, Pennsylvania, United States, 2. University of Pennsylvania, Philadelphia, Pennsylvania, United States*

Being outdoors in nature has been associated with improved mental and physical health. There are no known studies exploring older adults' satisfaction with outdoor activities at the start of long-term services and supports (LTSS; in nursing homes, assisted living, or at home). We examined characteristics of older adults receiving LTSS and factors associated with outdoor activities satisfaction. A secondary analysis was conducted of baseline data involving structured interviews with older adults new to LTSS (Health-Related Quality of Life: Elders in Long-Term Care; R01AG025524-05). Primary outcome was a single item on the satisfaction with outdoor activities (not at all satisfied to extremely satisfied). We conducted multivariable linear regression models controlling for the influence of the characteristics important to health-related quality of life (LTSS setting, gender, age, number of comorbidities, and sensory impairment [vision/hearing].) Among 356 people, the majority (59%) were satisfied with their outdoor activities. Of 339 participants with complete data, more depressive symptoms (higher Geriatric Depression Score; p<.001) and higher cognitive functioning (higher MMSE score; p=.038) were associated with lower ratings of satisfaction with outdoor activities. Higher self-rated physical health (p=.038) and more independence with activities of daily living (p=.017) were associated with greater satisfaction with outdoor activities. Due to the cross-sectional nature of this study it is difficult to determine causality; however, outdoor activity is important to people receiving LTSS. Interdisciplinary teams can work with older adults receiving LTSS to assess interest level with outdoor activities and create a person-centered plan to increase outdoor activity and satisfaction levels.

### THE MEANINGS OF TRUST IN AFRICAN AMERICAN COMMUNITIES AND THEIR ASSOCIATION WITH PARTICIPATION IN DEMENTIA RESEARCH

Elena Portacolone,[1] Peter Lichtenberg,[2] Sahru Keiser,[3] Leah Vest,[4] Marsha Maloof,[5] and Julene Johnson[1], *1. University of California San Francisco, San Francisco, California, United States, 2. Wayne State University, Detroit, Michigan, United States, 3. UCSF, San Francisco, California, United States, 4. WSU, Detroit, Michigan, United States, 5. Pendergrass Smith, San Francisco, California, United States*

African American /Black American older adults' low participation in research reduces the generalizability of research findings and hinders understanding of dementia mechanisms, further widening health disparities. Both the Alzheimer's Association and the National Institutes of Health have identified recruitment of African Americans with cognitive impairment into dementia research as an area of high priority. Distrust of research and medical institutions is often cited as a barrier to participation of African Americans in dementia research. Therefore, the goal of this study is to better understand African American community members' expectations associated with trust. We used focus groups and semi-structured interviews to examine the expectations associated with overall trust. We conducted 6 focus groups: 4 with African American older adults and 2 with caregivers of African American older adults with cognitive impairment. We also interviewed 5 African American older adults with cognitive impairment (total n=59). Data were analyzed with content analysis. Five themes emerged: 1) Importance of providing truthful help/information leading to trust; 2) Long relationships leading to trust; 3) Acting efficiently and consistently (e.g., "not fooling around") leading to trust; 4) Transference of trust (e.g., I can likely trust someone trusted by a trusted person); 5) Difficult to trust because of a harsh social environment. To conclude, trust is a complex belief associated with multiple expectations and relationships. It is critical that researchers understand these expectations related to trust in order to increase recruitment of African American older adults into dementia research.

### TREATMENT WITH ANTIPSYCHOTICS AND RISK OF DEATH IN 58,000 PATIENTS FROM SWEDISH DEMENTIA REGISTRY

Dorota Religa,[1] Emilia Schwertner,[1] sara Garcia-ptacek,[1] Bjorn Johansson,[1] Katarina Nägga,[2] Bengt Winblad,[1] Maria Eriksdotter,[1] and Juraj Secnik[1], *1. Karolinska Institutet, Stockholm, Sweden, 2. Clinical Memory Research Unit, Department of Clinical Sciences Malmö, Lund University, Malmö, Malmö, Sweden*

Background: Behavioral and Psychological symptoms of Dementia (BPSD) are a heterogeneous group of clinical phenomena that is subjectively experienced by the patient and/or observable by an examiner (e.g., caregiver, physician) consisting in disturbed emotions, perception, thought, motor activity, and altered personality. There are recommendations to limit antipsychotic use in patients with dementia, and the big educational effort is made to follow them. In Sweden antipsychotics use changed from 10.1% in 2007-2008 to 5.2% in 2014-2015. Aims: The aim of the study is to analyze the actors associated with treatment and mortality of dementia patients, particularly those suffering from BPSD. Particularly we focus on assessing all-cause mortality patients with dementia treated with antipsychotic drugs (APDs). Methods: We have analyzed 58,412 patients newly diagnosed with dementia. We have found that 2526 of the patients were prescribed APDs (602 typical APDs and 1833 atypical APDs)- Results In the adjusted models, use of APDs at the time of dementia diagnosis was associated with increased mortality risk in the total cohort (hazard ratio = 1.4; 95% confidence interval 1.3-1.5). We have also stratified the results. Conclusions: The risk of death in patients with dementia was increased in group that used atypical and typical APDs. Our study gives more evidence to advice caution in APD prescription for patients with dementia.

### SESSION 2220 (SYMPOSIUM)

### PRESIDENTIAL SYMPOSIUM: EDUCATION NETWORKS: STRENGTHENING GERONTOLOGY AND GERIATRICS THROUGH CONNECTIVITY

Chair: Judith L. Howe, *James J Peters VA Medical Center, GRECC, Bronx, New York, United States*
Discussant: Kathryn Hyer, *University of South Florida, Tampa, Florida, United States*

The AGHE Presidential Symposium, related to the theme of the annual scientific meeting, underscores the importance of networks, collaborations and partnerships in advancing education in gerontology and geriatrics. AGHE has been at the forefront of many innovative programs since it was founded in 1974, contributing to the growth of the field and the recognition of education as one pillar of the field of gerontology and geriatrics, along with research, policy and practice. This symposium highlights three ongoing initiatives that promote connections and collaborations. The first paper discusses the Age-Friendly University (AFU) network which is made of institutions around the globe who have committed themselves to becoming more age-friendly in their programs and policies. AGHE endorses the AFU principles and invites its members and affiliates to call upon their institutions become part of this pioneering initiative. The AFU initiative is one of several international activities that AGHE, global leaders in education on aging, has engaged in. The second paper describes international networking activities such as collaborations with international organizations including the World Health Organization and connecting international and US students. In the third paper, initiatives to connect disciplines and professions through competency-based education and curricula are discussed. For instance, the Gerontology Competencies for Undergraduate and Graduate Education and the Program of Merit promote competency-based gerontology education across disciplines and professions.

### PERILS, PITFALLS, AND POTENTIALS OF NETWORKING IN INTERPROFESSIONAL EDUCATION: TAKING ON THE CHALLENGES

Phillip Clark[1], *1. University of Rhode Island, Kingston, Rhode Island, United States*

Gerontology and geriatrics are conceptualized as quintessential interdisciplinary fields. To understand aging and provide quality care to older adults, you need an interdisciplinary perspective and an interprofessional team. However, academic and clinical settings—with their vertical structures—require bridging strategies to connect the disciplinary dots. Implicit in this approach is the need for creating networks to support interdisciplinary education in both classroom and experiential settings. Taking on these challenges requires emphasizing the importance of key competencies for both gerontology and interprofessional practice with older adults, including foundational, interactional, and contextual dimensions. These competencies recognize the unique perspectives, contributions, and roles of different disciplines, and create the connections critical for promoting and sustaining interprofessional education. Strategies for developing and maintaining interprofessional networks include: (1) identifying forces driving and restraining change, (2) matching strategies for promoting networks to readiness for system change, and (3) enlisting external forces to make and maintain changes.

### AGE-FRIENDLY UNIVERSITIES: POSSIBILITIES AND POWER IN CAMPUS CONNECTIONS

Joann M. Montepare[1], *1. Lasell College, Newton, Massachusetts, United States*

Populations are aging locally, nationally, and globally – and challenging institutions of higher education to consider how they can respond to these changing demographics through new approaches to teaching, research, and community engagement. The Age-Friendly University (AFU) initiative was recently launched by an international team convened by Dublin City University, and endorsed by the Academy for Gerontology in Higher Education (AGHE). The AFU concept and 10 guiding principles provide a guiding campus-wide framework that colleges and universities can use for distinguishing and evaluating age-friendly programs and policies, as well as identifying institutional gaps and opportunities for growth. To date, over 45 institutions have joined the AFU global network. This presentation will describe how collaborations across aging-focused programs and campus units devoted to diversity, community engagement, professional studies, and related educational efforts offer prime opportunities to build and sustain an AFU vision.

### THE EVOLUTION OF AGHE AS A GLOBAL LEADER IN EDUCATION ON AGING: HOW, WHY, AND WHAT'S NEXT

Margaret A. Perkinson[1], *1. Univ. of Hawai'i at Manoa, Honolulu, Hawaii, United States*

The scope of AGHE's responsibility to gerontology and geriatrics extends worldwide, as reflected in its tag line, "Global Leaders in Education on Aging." Optimal responses to worldwide demographic transitions can only come from persons well-versed in the dimensions of aging and trained and globally situated to translate that knowledge into effective and culturally-appropriate solutions. This presentation reviews the evolution of AGHE's role in initiating and fostering global networks of educators in gerontology and geriatrics, including collaborative efforts with major international organizations (e.g., WHO, UN, IAGG) to increase the visibility and appreciation of aging-related issues among world leaders; sponsoring national and international meetings to promote exchange of ideas and refinement of teaching methodologies; initiating and adapting new models of gerontological training enhanced by advances in information and communication technology; and supporting world-wide cohorts of emerging scholars to assume leadership roles within the organization. Recommendations for next steps are considered.

### SESSION 2225 (PAPER)

### ELDER ABUSE AND OBSTACLES TO SUCCESSFUL AGING

### BARRIERS AND OPPORTUNITIES TO HEALTHY AGING IN ANCHORAGE, ALASKA, USING CONCEPT MAPPING

Britteny M. Howell,[1] and Daniel McLinden[2], *1. University of Alaska Anchorage, Anchorage, Alaska, United States, 2. Idea Networks, LLC, Cincinnati, Ohio, United States*

Alaska currently has the fastest growing proportion of older adults than any state in the country, and seniors are choosing to age-in-place in Anchorage in record numbers. Research shows that including older adults with community-based professionals (aging advocates, researchers, service providers) in focus group activities can provide a rich and holistic model of aging that demonstrates a robust foundation for supporting aging and addressing health disparities. This paper presents the results of a project conducted with older adults (50+ years), advocates, and other stakeholders in Anchorage using Concept Mapping (CM) methodology, a technique not often used in the gerontology literature. CM is a mixed-method, participatory approach that uses brainstorming and unstructured card-sorting combined with multivariate statistics (multi-dimensional scaling, hierarchical cluster analysis) to create a data-driven visual representation of thoughts or ideas of a community. CM is well suited to integrating perspectives from multiple points of view. Participants were prompted to address the research question: how do we think about aging in Anchorage & what are the barriers and facilitators to aging well? Results indicate services for seniors should include culturally responsive health programming, low-cost opportunities for social engagement, inclusion of older adults with intellectual/developmental disabilities, transportation considerations, navigators to locate services in Anchorage, and more. CM allowed the researchers to identify how residents view healthy aging in this urban subarctic location and brainstorm practical solutions with stakeholders and local policy-makers. This presentation will also share lessons-learned regarding the use of this participatory approach with older adults.

### EXISTING STRATEGIES FOR ELECTRONIC DATA COLLECTION BY ELDER ABUSE MULTI-DISCIPLINARY TEAMS

Tony Rosen,[1] David Burnes,[2] Darin Kirchin,[2] Alyssa Elman,[3] Risa Breckman,[3] and Mark Lachs[1], *1. Weill Cornell Medical College / NewYork-Presbyterian Hospital, New York, New York, United States, 2. University of Toronto, Toronto, Ontario, Canada, 3. Weill Cornell Medical College, New York, New York, United States*

Elder abuse cases often require integrated responses from social services, medicine, civil legal, and criminal justice. Multi-disciplinary teams (MDTs), which meet periodically to discuss and coordinate interventions for complex cases, have developed in many communities. Little is known about how these MDTs collect case-level data. Our objective was to describe existing strategies of case-level electronic data collection conducted by MDTs across the United States as a preliminary step in developing a comprehensive database strategy. To identify MDTs currently collecting data electronically, we used a snowball sampling approach discussing with national leaders. We also sent an e-mail to the National Center for Elder Abuse listserv inviting participation. We identified and reviewed 11 databases from MDTs. Strategies for and comprehensiveness of data collection varied widely. Databases used ranged from a simple spreadsheet to a customized Microsoft Access database to large databases designed and managed by a third-party vendor. Total data fields collected ranged from 12-338. Types of data included intake/baseline case/client information, case tracking/follow-up, and case closure/outcomes. Information tracked by many MDTs, such as type of mistreatment, was not captured in a single standard fashion. Documentation about data entry processes varied from absent to detailed. We concluded that MDTs currently use widely varied strategies to track data electronically and are not capturing data in a standardized fashion. Many MDTs collect only minimal data. Based on this, we have developed recommendations for a minimum data set and optimal data structure. If widely adopted, this would potentially improve ability to conduct large-scale comparative research.

### PROTECTIVE EFFECTS OF NEIGHBORHOOD SOCIAL COHESION ON ELDER ABUSE IN INDIAN OLDER ADULTS

E-Shien Chang,[1] and Becca Levy[1], *1. Yale School of Public Health, New Haven, Connecticut, United States*

It is estimated that elder abuse impacts 16% of older persons globally. There is a need to understand factors that protect older persons. In this study, we examined whether neighborhood social cohesion, or the mutual support, trust, and interaction among neighbors, could be such a factor. As it has been found to be protective of child abuse and domestic violence, we hypothesized that higher neighborhood social cohesion would extend to reduce the risks of elder abuse. Our cohort consisted of participants aged 60 and over in the Longitudinal Aging Study in India (LASI) pilot survey. Elder abuse was measured by asking participants if they experienced ill-treatment by family members. Neighborhood social cohesion was measured by a five-item instrument that captured perceived support and trust among neighbors. The final sample consisted of 541 participants with a mean age of 69 who largely (72.9%) resided in rural area. The hypothesis was supported. Compared to older persons with low neighborhood social cohesion, older persons with high neighborhood social cohesion were significantly less likely to experience elder abuse (OR= 0.57, 95% CI=0.35-0.92), after controlling for socio-demographics, health, and neighborhood contextual covariates. This study, for the first time, suggests that neighborhood social environment may exert a protective effect on risks of elder abuse. Neighborhood resources may both prevent family members from being abusive and may help older persons stop abuse. Incorporating structural factors such as neighborhood cohesion may be critical in devising elder abuse preventions that increase the safety and well-being of older persons.

### RESILIENCE THROUGH CONNECTION: SOCIAL SUPPORT AND ELDER ABUSE, DISASTER, BEREAVEMENT, AND COMBAT

Ron Acierno,[1] and Wendy Muzzy[1], *1. Medical University of South Carolina, Charleston, South Carolina, United States*

Elder abuse prevalence among community residing adults is 10%, but this prevalence is cut by more than half among those who report high levels of social connection. Relatedly, elder abuse outcomes are significant, producing increased prevalence of anxiety and depressive disorders, the prevalences of which are, again, halved when one experiences abuse in the context of high social support. Similarly, mental health effects of natural disaster on older adults are virtually eliminated in the presence of high social support. Moreover, treatment for anxiety and depressive disorders is improved when high social support is present. We will present findings from five of our major studies in the aforementioned areas that underscore this point.

### UNCIVILIZED CHILDREN OR VICTIMS OF DEMENTIA: INTERPRETATIONS OF AGGRESSION IN ASSISTED LIVING

Laura M. Funk,[1] Rachel Herron,[2] and Dale Spencer[3], *1. University of Manitoba, Winnipeg, Manitoba, Canada, 2. Brandon University, Manitoba, Canada, 3. Carleton University, Ottawa, Ontario, Canada*

There is increasing interest in examining aggression in older adults and persons living with dementia (PLWD) in nursing homes. Relational or indirect aggression between tenants is also of concern in assisted living (A/L). However, there is a dearth of knowledge about how people interpret the meaning of aggression in older adults in A/L. Such interpretations inform responses within interactions as well as in management, practice and policy. This study explored interpretations of aggression in older tenants of A/L through thematic and narrative analyses of qualitative interview data from 32 participants: 13 tenants and 19 staff. Tenants downplayed the existence of 'bullies' but spoke of (or themselves enacted, in the interview) relational aggression in tenant conflicts with 'troublemakers,' complainers, slackers and PLWD. Dementia was not universally interpreted as excusing inappropriate or aggressive behaviours, and tenants expressed discomfort interacting with PLWD. The narratives of both groups (tenants and workers) drew on and reproduced stigmatizing and/or patronizing views of dementia and aging. Workers commonly positioned dementia (or other conditions such as physical illness, changes in routine, isolation, and loss of independence) as mitigating culpability for aggression. They did so in part to avoid taking aggression personally; some were also concerned about protecting tenants from disciplinary measures. Narratives about aggression reflect and further reinforce relations of power within A/L, with implications for everyday interactions of life and work in these settings. A/L facilities can address relational aggression in part through addressing talk and actions that perpetuate ageism and dementiaism.

### SESSION 2230 (PAPER)

### ESPO/ BIOLOGICAL SCIENCES SECTION SYMPOSIUM: EMERGING INSIGHTS IN INTERACTIONS AND NETWORKS IN THE BIOLOGY OF AGING

### DEVELOPING C. ELEGANS TO STUDY AGE-RELATED EXTRACELLULAR VESICLE SIGNALS

Joshua Russell,[1] and Matt Kaeberlein[1], *1. University of Washington, Seattle, Washington, United States*

All cells release vesicles into their extracellular environment. These extracellular vesicles (EVs) contain multiple classes of molecules, including nucleic acids, proteins, and lipids. EV-signaling has been shown to be impacted by many age-related physiological processes such as inflammation, mitochondrial stress, and autophagy as well as directly mediate critical functions in cellular senescence and aging. The isolation and analysis of EV cargos from mammalian cell culture and liquid biopsy samples has become a powerful approach for uncovering the messages that are packaged into these organelles. Caenorhabditis elegans is a premier model for dissecting the genetics of aging however, EV analysis has not been tenable in invertebrate model systems due to lack of methods for obtaining sufficient amounts of pure EVs. We developed a method for isolating pure EVs from C. elegans with yields sufficient for mass spectrometry and RNAseq. Here we present the analysis of the genetic and protein cargos of EVs collected from wild type and long-lived mutants collected at different time points across their lifespans. As the first investigation of age-related EV signals in an invertebrate model system we believe these results will provide insights into cell non-autonomous mechanisms of aging.

### EPIGENETIC PROFILES OF BIOLOGICAL AGING HALLMARKS

Diana L. Leung,[1] Zuyun Liu,[2] and Morgan E. Levine[3], *1. Yale University, New Haven, Connecticut, United States, 2. Department of Pathology, Yale School of Medicine, New Haven, Connecticut, United States, 3. Yale University School of Medicine, New Haven, Connecticut, United States*

Investigation into the hallmarks of aging point to the existence of shared mechanisms that underlie the biological aging process. While there is a general consensus that hallmarks of aging rarely occur in isolation, little is known in regards to their overlapping networks or how interactions contribute to manifestations at the clinical level. Here, we examine whether shared epigenetic alterations—one of the proposed hallmark of aging—underlies diverse conditions characterized by other hallmarks, including cellular senescence, loss of proteostasis, genomic instability, mitochondrial dysfunction, and inflammation. Using weighted network analysis, we identified consistent overlaps in the methylation profiles across the different traits. For instance, epigenetic modules that were distinct in senescence were also affected in progeroid syndromes (Hutchinson-Gilford Progeria Syndrome and Werner's Syndrome) and smokers. These CpGs tended to be located in CpG islands, which are notable for their strong association with transcriptional regulation. Overall, our results suggest that epigenetic alterations intersect with various hallmarks of aging. In moving forward, incorporation of this understanding may lead to the development of biomarkers that better capture the biological (rather than chronological) aging process.

### THE EFFECTS OF AGE AND SIZE ON THE COMPANION DOG METABOLOME: ROLES OF TRYPTOPHAN AND FATTY ACID METABOLISM

Jessica M. Hoffman,[1] and Steven Austad[1], *1. Department of Biology, University of Alabama at Birmingham, Birmingham, Alabama, United States*

Recently, the companion dog has been promoted as an ideal animal model for human aging. Dogs show an interesting phenomenon where smaller individuals are longer lived than their larger counterparts. However, many of the underlying molecular mechanisms that influence aging and longevity in the dog are unknown. To begin to uncover these physiological changes, we completed the largest metabolomics study to date in the companion dog. Here, we collected blood plasma samples from companion dogs in three in the United States for metabolomics analysis. We then looked at the effects of age and size on the metabolome to develop new hypotheses about healthy canine aging. Our most striking differences were found with regards to geographic location in the canine metabolome, in which metabolic profiles were more similar between dogs in the same city than across cities. After controlling for this location effect, we found a strong signal of amino acid metabolism, specifically tryptophan metabolism, associated with weight in the dog where metabolites in the tryptophan metabolism pathway were always higher in small, long-lived dogs. Future studies will directly investigate the role of tryptophan metabolism in model organisms.

### SESSION 2235 (SYMPOSIUM)

### FUTURE CARE PLANNING AND END-OF-LIFE CARE DECISION MAKING: INDIVIDUAL AND SOCIAL INFLUENCES

Chair: Jeong Eun Lee, *Iowa State University, Ames, Iowa, United States*
Discussant: Silvia Sörensen, *University of Rochester Warner School for Education and Human Development, Rochester NY, United States*

As a growing number of older adults reach very old age, future care planning and end-of-life care decision making becomes increasingly important. Previous studies have shown that concrete future care planning steps are related to improved ability to manage illness and to better mental and physical health outcomes among older adults. Yet, relatively few older adults sufficiently plan for their future care. The purpose of this symposium is to highlight a collection of studies that each brings a unique perspective to the issue, reporting on individual and social factors that influence future care planning, end-of-life care decision making, and strategies to enhance future care planning among older adults. First, Chen and Siconolfi address common barriers and facilitators across diverse domains of age-related planning using content analysis. Second, Boerner and colleagues focus on the completion of formal planning without discussing the contents and factors associated with formal planning completion. Third, Strum investigates the complexities of navigating "fair" later life decisions involving family resources. Fourth, Moorman examines the racial differences in decisions of euthanasia and physician assisted suicide. Finally, Lee and colleagues report the findings from a future care planning intervention with older adults in rural area. The discussion by Sörensen will integrate the five papers with the goal of connecting the current evidence for meaningful steps in research and practice related to future care planning in older populations.

### FUTURE CARE PLANNING INTERVENTION WITH RURAL OLDER ADULTS

Jeong Eun Lee,[1] Dahee L. Kim,[1] Louise Peitz,[1] Eva Kahana,[2] and Boaz Kahana[3], *1. Iowa State University, Ames, Iowa, United States, 2. Case Western Reserve University, Cleveland, Ohio, United States, 3. Cleveland State University, Cleveland, Ohio, United States*

To address their needs for proactive self-care and end of life planning, we implemented a community based educational program that promotes future care planning (FCP) for community residing older adults. Extension specialists from Iowa State University implemented two brief FCP program sessions with older adults. Topics included both short-term and long-term future care planning activities and strategies. Baseline and post program surveys were completed by 216 community dwelling older adults (M=78.21). The program was successful in getting older Iowans in the rural area start end of life care preparation as well as helping them to make changes in their health care decisions. The majority of participants (89.6 %) reported high satisfaction with the programs. We also found a high rate of change (62%) in opinion regarding future care. The implication of future care planning for is discussed with recommendations for future research.

### COMMON BARRIERS AND FACILITATORS ACROSS DIVERSE DOMAINS OF AGE-RELATED PLANNING

Emily K. Chen,[1] Emily K. Chen,[2] and Daniel Siconolfi[3], *1. RAND Corporation, Arlington VA, Virginia, United States, 2. RAND Corporation, Arlington, Virginia, United States, 3. RAND Corporation, Pittsburgh, Pennsylvania, United States*

Advance care planning (ACP) – plans that express wishes for healthcare for when a person is unable to communicate – is often studied independently of other age-related planning activities. This study explored a broad set of age-related planning activities, such as retirement, finances, aging in place, and healthcare, including ACP. We used directed content analysis to identify barriers and facilitators to age-related planning from semi-structured interviews with 38 respondents (ages 55-74). Surprisingly, a common set of structural, interpersonal, and individual barriers and facilitators emerged across domains. Barriers included competing demands, resistance from family members, and aversion to planning in general. Facilitators included exposure to planning behaviors through professional and social networks, having witnessed negative outcomes from others' failure to plan, and a belief that planning would spare others future distress. These results reinforce the idea that ACP exists within of a set of age-related planning behaviors that share common characteristics.

### INCOMPLETE ADVANCE CARE PLANNING? CORRELATES OF PLANNING WITHOUT PERSONAL CONVERSATIONS

Kathrin Boerner,[1] Deborah Carr,[2] Katherine M. Ornstein,[3] and Sara Moorman[4], *1. University of Massachusetts Boston, Boston, Massachusetts, United States, 2. Boston University, Boston, Massachusetts, United States, 3. Icahn School of Medicine at Mount Sinai, New York, New York, United States, 4. Boston College, Chestnut Hill, Massachusetts, United States*

In the course of advance care planning (ACP), people may elect any of the following: a living will, a durable power of attorney for health care, and discussions with family members and health care providers. A small proportion of planners complete legal documents without discussing them with others (formal planning only, FPO). If people who have done FPO become incapacitated, their family and health care professionals may lack guidance on how to direct their care. To better understand this group, we drew on four large surveys of community-dwelling adults. Social isolation, measured by living alone and lack of a confidante, increased the odds of FPO across all studies. We also found some evidence that economic disadvantage and depressive symptoms were linked with FPO. We discuss implications for policy and practice, underscoring that ACP is yet another important domain affected by the crisis of social isolation in old age.

### RACIAL-ETHNIC DISPARITIES IN ATTITUDES TOWARD PASSIVE AND ACTIVE EUTHANASIA

Sara M. Moorman[1], *1. Boston College, Chestnut Hill, Massachusetts, United States*

This paper examined racial/ethnic differences in opinions about passive euthanasia (withdrawing or withholding treatment), suicide, and physician-assisted suicide. Data came from 1,832 participants in the 2013 Pew Religion and Public Life Project. Respondents from all racial/ethnic backgrounds were most likely to favor multiple forms of euthanasia. However, persons of color had a wider variety of opinions about euthanasia than did non-Hispanic whites. In multivariate multinomial logistic regressions, non-Hispanic whites had a 63% chance of approving broadly of euthanasia, while non-Hispanic blacks had a 40% chance, and Hispanics, a 49% chance. Opposition to euthanasia was most common among people with multiple disadvantages (e.g., educational attainment, immigrant status). Neither trust in health care providers nor recent experience with the death of a loved one explained these group differences. Results highlight large differences of opinion between the people who set policy and practice guidelines and those who lack this power and access.

### A FAIRNESS LENS TO LATER-LIFE PLANNING

Marlene Stum[1], *1. University of Minnesota, St. Paul, Minnesota, United States*

Being "fair" is often a desired goal when individuals are planning for financing future care, leaving an inheritance, and selecting an attorney-in-fact for financial and/or health care. However, differing perceptions about the fair use of an older parent's resources (paying for formal care, leaving an inheritance, compensating family caregivers, rewarding a sense of entitlement), and issues of who can or should be involved in decision processes, can lead to avoiding planning and be a source of family conflict. The complexities of fair decision rules for distributing resources, and important criteria for determining fair decision processes will be discussed, guided by interpersonal social justice theories, and findings from a qualitative study of inheritance involving older adults and adult children from the same family system (N= 18). Implications for helping family members, and professionals working with them, navigate "being fair" and increase later life planning will be shared.

### SESSION 2240 (SYMPOSIUM)

### LEVERAGING ANALYTIC METHODS TO EXPAND OPPORTUNITIES IN AGING-RELATED HEALTH DISPARITIES RESEARCH

Chair: Igor Akushevich, *Duke University, Durham, North Carolina, United States*
Co-Chair: Carl V Hill, *NIH/National Institute on Aging, Bethesda, Maryland, United States*
Discussant: Heather E. Whitson, *Duke University Medical Center, Durham, North Carolina, United States*

The objective of the Symposium is to improve the understanding of how existing analytic methods and data can be leveraged to make progress in understanding the causes and mechanisms of health-related disparities in Alzheimer's disease, related dementias and other prominent age-related diseases. Topics will cover a range of academic and administrative topics including: i) advanced analytic methods and modeling of health disparities with application to racial and geographic disparities in AD/ADRD; ii) the role of repeated anesthetic and surgical exposure in generation of disparities in AD/ADRD risk; iii) the nature of health disparities in cognitive aging as parallel to or distinct from health disparities in patterns of aging in other systems in the body; iv) recent advances in machine learning applied to large claims databases involving medical disparities; and v) geographic-related disparities in life expectancy across the U.S. A focus will be made on demonstrating how studies using established administrative data resources such as Medicare claims databases combined with innovative analytic approaches such as partitioning analyses, time-series based methods of projection and forecasting, and stochastic process models can be used to uncover previously overlooked or understudied aspects in this area of research. Analyses of such increasingly available large health datasets provides an opportunity to obtain nationally representative multiethnic results based on individual-level measures that reflect the real care-related and epidemiological processes ongoing in the U.S. healthcare system and allows the targetting of relatively rare diseases in relatively small population subgroups.

### USE OF ANALYTIC METHODS AND MEDICARE DATA IN THE ANALYSES OF DISPARITIES IN AD/ADRD HEALTH OUTCOMES

Igor Akushevich,[1] Arseniy Yashkin,[2] Svetlana Ukraintseva,[1] and Anatoliy Yashin[1], *1. Duke University, Durham, North Carolina, United States, 2. Duke University, Durham, NC, North Carolina, United States*

We demonstrate how application of analytic approaches developed in demography, biodemography, epidemiology, and population studies to Medicare and Medicare-linked data allows to identify causes and mechanisms of disparities in AD/ADRD. Our studies i) confirmed geographic disparities in AD mortality (e.g., existence of hot spots, West-East gradient) but did not detect them in Medicare data, ii) confirmed racial disparities in AD/ADRD incidence and survival (e.g., higher incidence and better survival in Black population) and demonstrated that they can be partly explained by heterogeneity in diagnosis severity and partly by genetic factors, iii) detected unexpectedly strong effects of systemic hypotension, chronic kidney and liver diseases on the risk of AD/ADRD. We concluded that further progress in understanding of mechanisms and causes of the disparities in AD/ADRD is possible by clarifying the role of cause-of-death coding, the effects of comorbidity and their treatment, dynamics in cognition scores before AD/ADRD diagnosis, and genetic factors.

### DISPARITIES IN ALZHEIMER'S DISEASE: THE ROLE OF REPEATED ANESTHETIC AND SURGICAL EXPOSURE

Miklos D. Kertai[1], *1. Vanderbilt University Medical Center, Nashville, Tennessee, United States*

Annually, there are 7 million patients > 65 years who undergo noncardiac surgery in the US. This number is expected to increase by 30% over the next 3 decades, and given the prevalence of surgically correctable comorbidities, the elderly will continue to undergo multiple surgical procedures. Currently, it is unclear what factors initiate or promote the development of Alzheimer's disease (AD). Preclinical studies suggest that exposure to anesthesia and/or surgery could be one of the environmental exposures that increase AD risk through neuroinflammation and neuroapoptosis. However, previous studies indicated substantial disparities in AD risk by gender, ethnicity, and race, but failed to explore the role of anesthesia and/or surgery exposure in the risk for AD. This presentation will review the role of disparities and anesthesia and/or surgery exposure in the risk for AD in elderly surgical patients.

### TESTING HEALTH DISPARITIES IN COGNITIVE AND BIOLOGICAL AGING IN OLDER ADULTS IN THE UNITED STATES

Daniel Belsky[1], *1. Columbia University Mailman School of Public Health, New York, New York, United States*

We conducted analysis to test if health disparities in cognitive aging were parallel to or different from health disparities in patterns of aging in other systems in the body, and if race/ethnicity-related disparities could be accounted for by differences in socioeconomic circumstances across the life-course. We analyzed data from more than 10,000 adults participating in the US NHANES and US Health and Retirement Study. We measured cognitive aging using neuropsychological tests of processing speed and memory. We measured aging in other systems using composite indices of biological aging based on organ-system function tests and blood chemistries. We conducted analysis to (i) quantify and compare health disparities in cognitive aging and biological aging; (ii) test if individuals exhibiting accelerated cognitive aging were also exhibiting accelerated biological aging; and (iii) test if race/ethnic disparities in cognitive and biological aging could be explained by measured socioeconomic resource differences in childhood and later life.

### MACHINE LEARNING APPROACHES TO ENHANCE CLAIMS DATA ANALYSES

Ricardo Pietrobon,[1] and David Marcozzi[2], *1. SporeData Inc, Durham, North Carolina, United States, 2. University of Maryland, Baltimore, Maryland, United States*

This presentation will cover recent advances in machine learning applied to large claims databases involving medical disparities. First, we will describe methods involving the enrichment of existing claims data with social determinants of health from census data, where variables are imputed from one dataset to another, ultimately resulting in clinical models with enhanced predictive performance. Second, we will discuss the inclusion of variables representing imaging signs from MRI and CT exams, presenting large scalability and interobserver reliability, representing a method that can be used to enrich large state and national registries through the use of image recognition. Finally, we will discuss novel protocols for Natural Language Processing involving a combination of rule-based creation of corpora for radiology and discharge reports, with highly accurate deep learning methods for concept extraction and classification.

### GEOGRAPHIC DISPARITIES IN LIFE EXPECTANCY AND MORTALITY IN THE U.S.

Julia Kravchenko,[1] and H Kim Lyerly[2], *1. Duke University School of Medicine, Durham, North Carolina, United States, 2. Duke University Medical Center, Durham, North Carolina, United States*

Although the US has one of the highest per-capita health expenditures in the world, it noticeably lags behind a number of other industrialized countries in terms of life expectancy (LE). These disparities remain unexplained by individual demographic, socioeconomic, and healthcare factors. Analysis of death certificates for 1999-2016 revealed that diseases contributed most to LE variability are myocardial infarction (explained 12.9% of the difference in mortality), heart failure (10.6%), stroke (8.2%), lung cancer (7.5%) and COPD (7.2%). Analysis of histories of diseased patients in Medicare records showed that septicemia (15.7%), low weight (13.8%), renal disease (13.3%), disorders of electrolyte and fluid balance (9.0%) and heart failure (7.3%) contributed most to the disparities. Diseases that substantially contribute to disparities in LE in the US include both common and less-often-discussed diseases. Future studies of variations in treatment patterns, access-to and quality-of medical care for these diseases could provide important insight in observed patterns.

### SESSION 2245 (SYMPOSIUM)

### M. POWELL LAWTON AWARD LECTURE

Chair: Sharon Inouye, *Harvard Medical School Aging Brain Center, Boston, Massachusetts, United States*

The M. Powell Lawton Award is presented annually to an individual who has made outstanding contributions from applied research that has benefited older people and their care. The lecture will be given by the 2018 recipient, Carol Whitlatch, PhD, Benjamin Rose Institute on Aging. The session will also include the presentation of the 2019 Lawton Award. The 2019 Lawton Award recipient is Barbara Resnick, PhD, CRNP, FGSA, of the University of Maryland. Supported by the Polisher Research Institute of the Madlyn and Leonard Abramson Center for Jewish Life.

### FOLLOWING IN THE FOOTSTEPS OF A GREAT APPLIED GERONTOLOGIST (WHILE SIMULTANEOUSLY FORGING YOUR OWN PATH)

Carol Whitlatch[1], *1. Benjamin Rose Institute on Aging, Cleveland, Ohio, United States*

Dr. M. Powell Lawton was an inspirational and productive scholar whose work had application for improving personal care and well-being for older adults and their families. He held strong to the principles of Person- and Family-Centered Care long before this terminology and model of care was commonly practiced. He was a living example of PFCC always wearing comfortable clothes and shoes to ensure his unique creativity was not obstructed by physical discomfort. Dr. Lawton's work has inspired countless gerontologists who have taken the next steps towards ensuring quality of care by understanding personal preferences, activities, and care values. Dr. Whitlatch will discuss Dr. Lawton's ongoing influence on care and best practices focusing on her own research that gives voice to the care values, preferences, and activities of families facing early-stage dementia. Dr. Whitlatch encourages attendees to wear their most comfortable shoes to the lecture in honor of Dr. Lawton.

### SESSION 2250 (PAPER)

### MINORITY AND DIVERSE POPULATIONS III

### 2009-2017 RACIAL AND ETHNIC DISPARITIES IN MEDICARE PATIENT EXPERIENCE AND INFLUENZA IMMUNIZATION

Marc N. Elliott,[1] Steven Martino,[2] Katrin Hambarsoomian,[1] Shondelle Wilson-Frederick,[3] Jacob Dembosky,[2] Sarah Gaillot,[3] Robert Weech-Maldonado,[4] and Amelia Haviland[5], *5. Carnegie Mellon University, Pittsburgh, Pennsylvania, United States, 2. RAND Corporation, Pittsburgh, Pennsylvania, United States, 3. Centers for Medicare & Medicaid Services, Baltimore, Maryland, United States, 4. University of Alabama at Birmingham, Birmingham, Alabama, United States, 1. RAND Corporation, Santa Monica, California, United States*

We sought to understand the extent to which racial and ethnic disparities in the immunization rates and case-mix adjusted patient experiences of access (getting needed care and getting care quickly) of Black, Hispanic, and non-Hispanic White Medicare beneficiaries have changed over time. Accordingly, we analyzed 2009-2017 CAHPS data from 2,725,614 Medicare beneficiaries. In 2009, flu immunization rates for Black and Hispanic beneficiaries were lower than non-Hispanic White beneficiaries by 17 and 14 percentage points, respectively. Over 9 years, these gaps were reduced to 12 and 8 points, respectively ($p<.01$ for all comparisons). In 2009, Black beneficiaries had 2-point and 5-point disparities on getting needed care and getting care quickly (on a 0-100 scale) respectively, relative to non-Hispanic Whites. For getting needed care, there was no significant change over time in the gap between Blacks and non-Hispanic Whites. For getting care quickly, the gap between Blacks and non-Hispanic Whites narrowed to 3 points in 2017. In 2009, Hispanic beneficiaries had 2-point and 5-point disparities on getting needed care and getting care quickly, respectively, compared to non-Hispanic Whites. The gap on getting needed care widened by 1 point to a 2017 disparity of 3 points. For getting care quickly, there was no significant change over time in the gap between Hispanics and non-Hispanic Whites. These findings suggest that flu immunization rates for Black and Hispanic Medicare beneficiaries have improved significantly relative to non-Hispanic Whites; however, substantial disparities remain. For the patient experience measures, the findings are more mixed.

### DOES DEMENTIA POLICY ACCOUNT FOR DIVERSITY? AN ANALYSIS OF CANADIAN DEMENTIA POLICIES

Kimberley Wilson[1], *1. University of Guelph, Guelph, Ontario, Canada*

Background: The World Report on Ageing and Health outlines key policy challenges that need to be addressed to have successful public health response to population aging, including 'dealing with diversity' and 'reducing inequity.' Dementia has been framed as a global health challenge affecting approximately 46 million people worldwide. To reduce inequality, dementia policies must account for and respond to diversity. The purpose of this research was to conduct an analysis of current Canadian federal, provincial, and territorial dementia strategies to examine their inclusion of dimensions of diversity. Method: We conducted an internet-based search and identified 13 unique Canadian federal, provincial, and territorial dementia documents. We completed a deductive content analysis to review each policy for content on: age and sex; racial and ethnic identity; sociocultural identity; religion; socioeconomic status; gender identity and sexual orientation; geographical location; and language fluency and communicative ability. Results: Within Canadian dementia policies there is minimal focus on diversity. When diverse identities were acknowledged in policies, very little guidance was provided to local policymakers, healthcare administrators, or service providers in how to acknowledge and accommodate different group' needs with services. Further, none of the policies adopted an intersectional approach; that is, they failed to recognize that older adults have several overlapping and interrelating identities. Conclusion: As Canada and other countries move towards developing and revising dementia policies it is imperative that they account for diversity within aging populations.

### HARNESSING THE HEALING POWER OF WESTERN AND NON-WESTERN HEALERS: IMPLICATIONS FOR SOCIAL WORK PRACTICE

Rashmi Gupta,[1] and Vijayan Pillai[2], *1. san francisco state university, san francisco, California, United States, 2. University of Texas at Arlington, Texas, United States*

Non Western therapies have played a vital role in dealing with a variety of crisis such as physical, emotional and existential over the last few centuries (Ejaz, 2000). Since the introduction of Western medicine in India as early as the eighteenth century, the two systems of medicine have coexisted and influenced each other. Mutual recognition of the therapeutic values of the two systems was further bolstered when American medicine recognized the role of alternative therapies as viable option for healing in the early eighties. The purpose of this paper is to examine the roles two kinds of healers (western and non- western) play in the Asian Indian Hindu context. For this qualitative study, 30 physicians (trained in Western medicine) and 5 sadhus (holy men) were interviewed in the city of Allahabad, during the Kumbh Mela. Open ended questions ranged from: a) field of practice (physicians), sadhus (mystics), b) years of practice, c) number of patients/followers, d) treatment offered (physician –surgery or medications), sadhus (prayers, fasts, charitable donations), e) adverse reactions. In-depth interviews were recorded and transcribed by two researchers from Hindi to English and coded into themes. Results indicate that about a quarter of Western trained physicians not only sought assistance from the sadhus for their emotional/spiritual issues, but also referred patients with terminal disease. Besides individual consults, the mystics conduct lectures on climate change, on holistic diet, meditation, yoga practices, and healing. Social workers need to assess the value Hindu immigrant families in United States attach to Non-Western therapies.

### HEALTHCARE EXPERIENCES OF NHOPI AND OTHER U.S. ASIAN MEDICARE BENEFICIARIES

Megan K. Beckett,[1] Marc N. Elliott,[1] Katrin Hambarsoomian,[1] Jacob Dembosky,[2] Shondelle Wilson-Frederick,[3] Sarah Gaillot,[3] and

Steven Martino[2], *1. RAND Corporation, Santa Monica, California, United States, 2. RAND Corporation, Pittsburgh, Pennsylvania, United States, 3. Centers for Medicare & Medicaid Services, Baltimore, Maryland, United States*

Native Hawaiians and Other Pacific Islanders (NHOPI) and Asians are the fastest-growing US racial and ethnic group. Differences in healthcare experiences may contribute to the better health outcomes of Asian-Only adults than NHOPIs, but little is known about NHOPI healthcare experiences. Using data from 1067 NHOPI and 9361 Asian-Only Medicare beneficiaries responding to the 2017 Medicare Consumer Assessment of Healthcare Providers and Systems (CAHPS) survey, we investigated NHOPI and Asian-Only differences in flu vaccination and six case-mix adjusted composite measures of healthcare experiences (scaled 0-100), with and without adjustment for Medicare coverage type and state of residence. Compared with Asian-Only beneficiaries, NHOPI beneficiaries reported better experiences getting needed care (adjusted-difference of +7 points), customer service (+5 points), doctor communication (+5 points), getting care quickly (+4 points), care coordination (+4 points), and getting needed prescription drugs (+3 points). In contrast, NHOPI beneficiaries reported worse flu immunization (-7 points; $p<0.05$ for all reported differences). Adjustment for Medicare Advantage (MA) coverage and state of residence accounted for <25% of differences. NHOPI vs. Asian-Only care experience differences were similar for MA and Fee-for-Service beneficiaries. NHOPI were immunized for influenza less often than Asian-Only beneficiaries in MA (-15 points), but not Fee-for-Service; this did not differ by gender. NHOPI men reported better experiences than Asian-Only men for customer service (+11 points) and getting care quickly (+6 points). Patient experience does not explain worse health outcomes found elsewhere for NHOPI than Asian-Only. Further study may suggest means of improving Asian-Only healthcare experiences and NHOPI immunization.

### RATES OF DISENROLLMENT FROM MEDICARE MANAGED CARE PLANS ARE HIGHER AMONG RACIAL, ETHNIC, AND LINGUISTIC MINORITIES

Steven Martino,[1] Megan Mathews,[2] Cheryl Damberg,[2] Judy Ng,[3] Denis Agniel,[2] Loida Tamayo,[4] Shondelle Wilson-Frederick,[4] and Marc N. Elliott[2], *1. RAND Corporation, Pittsburgh, Pennsylvania, United States, 2. RAND Corporation, Santa Monica, California, United States, 3. National Committee for Quality Assurance, Washington, District of Columbia, United States, 4. Centers for Medicare & Medicaid Services, Baltimore, Maryland, United States*

Voluntary disenrollment from Medicare managed care (Medicare Advantage; MA) plans is related to beneficiaries' negative experiences with their plan, disrupts continuity of care, and conflicts with goals to reduce Medicare costs. Information on associated factors may help illuminate the dynamics that drive decisions to disenroll. We used data from 17,517,852 beneficiaries enrolled in 736 MA plans in 2015 to investigate differences in rates of disenrollment by race, ethnicity, and preferred language. Disenrollment data came from Medicare's enrollment system. Social Security Administration data on race and ethnicity were augmented with surname, address, and other Medicare administrative data to calculate probabilities of membership in seven race/ethnicity/language-preference groups: White, Black, English-preferring Hispanic, Spanish-preferring Hispanic, Asian or Pacific Islander (API), American Indian or Alaska Native, and multiracial. We summarized disparities across groups using regression models with and without plan intercepts, controlling for gender, disability, and Medicaid eligibility. Adjusted rates of disenrollment were significantly higher for Spanish-preferring Hispanics (19.1%), Blacks (10.2%), and APIs (9.4%) than for Whites (7.7%), and significantly lower for English-preferring Hispanics (7.4%, p's<0.001). Within-plan disparities accounted for only a small fraction of overall disparities, indicating that Spanish-preferring Hispanics, Blacks, and APIs tended to be enrolled in plans with higher disenrollment than plans in which Whites were enrolled, whereas English-preferring Hispanics tended to be enrolled in plans with lower disenrollment. These between-plan differences may indicate that high-minority-enrollment plans less effectively inform beneficiaries about the cost and coverage of care or that racial/ethnic/linguistic minorities more often select plans that raise rates or restrict coverage.

### SESSION 2255 (SYMPOSIUM)

### MOBILITY AND PHYSICAL ACTIVITY IN OLDER ADULTS: OBSERVATIONAL AND INTERVENTION RESEARCH

Chair: Patricia M. Bamonti, *VA Boston Healthcare System, Boston, Massachusetts, United States*
Discussant: Jonathan Bean, *New England GRECC, VA Boston Healthcare System, Boston, Massachusetts, United States*

Mobility disability is associated with considerable morbidity and mortality in late life. Physical activity [PA] is a modifiable behavior that can reduce mobility disability, as well as improve physical and mental health outcomes in older adults. However, only a minority of older adults meet the minimum PA requirements based on national guidelines. Research examining factors impacting PA and mobility in late life, as well as novel interventions to increase PA and improve mobility is essential to enhancing health and wellbeing. This symposium will provide an overview of observational and intervention research focused on understanding factors associated with PA and mobility, as well as intervention research designed to increase PA and improve mobility in older adults. First, Dr. Lien Quach will present research examining the impact of social engagement in reducing the risk of mobility decline among older adults with mild cognitive impairment. Second, Dr. Patricia Bamonti will examine psychological factors related to uptake and adherence of pulmonary rehabilitation in older Veterans. Third, Dr. Stephanie Robinson will explore engagement, feasibility, acceptability, and change in PA following a web-based intervention in middle-aged and older adult patients with COPD. Fourth, Dr. Elisa Ogawa will present research examining the feasibility and acceptability of an exergaming intervention compared to an exercise control condition among older adults at risk for falls. The symposium will conclude with discussion led by Jonathan Bean, MD, MPH, who will highlight implications of findings across studies and identify areas for future research.

### HEALTH-RELATED QOL IS ASSOCIATED WITH COMPLETION OF PULMONARY REHABILITATION IN OLDER VETERANS WITH COPD

Patricia M. Bamonti,[1] Marilyn L. Moy,[2]
Christina Goodwin,[3] Kdee Elsen,[1] and Amy Silberbogen[1], *1. VA Boston Healthcare System, Boston, Massachusetts, United States, 2. VA Boston Healthcare System Harvard Medical School; Boston, Massachusetts, United States, 3. Cooper University Hospital, camden, Massachusetts, United States*

Pulmonary rehabilitation [PR] is the standard of care for Veterans with COPD. Psychosocial factors may play a role in PR participation. This study examined psychosocial factors among 253 Veterans with COPD (M age = 70.08, SD = 8.14) who completed (n = 110), did not complete (n = 75; "drop outs"), or never started (n = 68) PR. Measures completed at baseline were health-related quality of life (HRQL; Chronic Respiratory Questionnaire), depression (Beck Depression Inventory II), and exercise self-efficacy (Exercise Self-Regulatory Efficacy). One-way ANOVAs produced no significant differences in self-efficacy or depression. Significant differences were observed for HRQL, $F(2,170)=10.58$, $p<0.001$. Post hoc analysis revealed significant differences between never starters (M=69.72, SD=16.65) and completers (M=80.22, SD=19.29), p = 0.007 and differences between drop outs (M=66.69, SD=15.24) compared to completers, $p < 0.001$, such that better HQRL predicted completion. These findings have important clinical implications for engaging Veterans with COPD in PR.

### SOCIAL ENGAGEMENT, COGNITIVE IMPAIRMENT, AND MOBILITY IN THE BOSTON REHABILITATIVE IMPAIRMENT STUDY OF THE ELDERLY

Lien Quach,[1] David Gagnon,[2] Jennifer Moye,[2]
Kelly Cho,[2] and Jonathan Bean[2], *1. Veterans Administration, Boston, Massachusetts, United States, 2. VA Boston Healthcare System, Boston, Massachusetts, United States*

This study examined how social engagement (SE) and mild cognitive impairment (MCI) influence changes in mobility over three years of follow-up. We performed a secondary analysis of longitudinal data among primary care patients aged >64 years (N=430). Mobility outcomes include performance-based function via the Short Physical Performance Battery (SPPB) and patient reported function via the Late-Life Function Instrument (LLFI). Independent variables include: 1) MCI determined by a comprehensive cognitive battery and scores 1.5 SD < age-adjusted mean; 2) SE measured by standardized self-report of social activities. Multivariate linear mixed regression models demonstrate that MCI is associated with lower scores on SPPB and LLFI ($\beta= -0.76$, $p<0.0001$; $\beta= -1.47$, $p=0.005$ respectively). SE is associated with higher scores on SPPB and LLFI ($\beta=0.56$, $p<0.0001$; $\beta= 1.95$, $p<0.0001$ respectively), and partially mediates the association between MCI and on each outcome. SE is linked to mobility decline especially among participants with MCI.

### WEB-BASED PHYSICAL ACTIVITY INTERVENTION IN MIDDLE-AGED AND OLDER ADULT VETERANS WITH COPD

Stephanie A. Robinson,[1] Stephanie A. Robinson,[1]
Deanna L. Mori,[2] Caroline R. Richardson,[3] and
Marilyn L. Moy[4], *1. Edith Nourse Rogers Memorial Veterans Hospital, Bedford, Massachusetts, United States, 2. VA Boston Healthcare System, Boston, Massachusetts, United States, 3. University of Michigan, Ann Arbor, Michigan, United States, 4. VA Boston Healthcare System, West Roxbury, Massachusetts, United States*

Physical activity (PA) is recommended in all patients with chronic obstructive pulmonary disease (COPD). Technology-based interventions can deliver effective, scalable behavior-change techniques; though feasibility and acceptability among older adults is not established. Veterans with COPD (N=112, aged 49-89 years, median=68) were randomized to a 12-week web-based and pedometer intervention or a pedometer alone (control). Across groups, there was no significant difference between middle-aged (<68 years) and older (≥68 years) adults in percentage of pedometer wear-days over the study period (83.6% vs. 89.9%). In the intervention, there were no significant differences between middle-aged and older adults in total number of website logons (15.04 vs. 16.31), or proportion who reported they recommended the intervention (96.4% vs. 96.7%), found it easy to use (93.1% vs. 90.0%), and would continue to walk (93.1% vs. 89.7%). We conclude that a web-based PA intervention with a pedometer is feasible and acceptable in an older COPD population.

### FEASIBILITY AND ACCEPTABILITY OF AN EXERGAMING INTERVENTION FOR OLDER ADULTS AT RISK FOR FALLS

Elisa F. Ogawa,[1] Haikun Huang,[2] Lap-Fai Yu,[3]
Philimon N. Gona,[2] Richard K. Fleming,[2]
Jonathan F. Bean,[1] Suzanne G. Leveille,[2] and Tongjian You[2], *1. New England GRECC, VA Boston Healthcare System, Boston, Massachusetts, United States, 2. University of massachusetts Boston, Boston, Massachusetts, United States, 3. George Mason University, Fairfax, Virginia, United States*

This study examined the feasibility and acceptability of an exergaming program that utilized custom exergames, and compared it to a traditional physical exercise (control) program in older adults at risk for falls. A quasi-experimental study was conducted in older adults who lived in senior living communities. Participants enrolled in either program offered twice weekly for 8 weeks based on their residential site. Thirty-five participants enrolled in the study (mean age 77±7y) and 29 (82%) completed the follow-up assessment (exergaming: 93%; control: 73%). Overall attendance was 73% (exergaming: 79%; control: 68%), and 22 participants returned their program satisfaction form. There were no significant between-group differences in ratings of overall quality, enjoyment, instructors, peers, and facility of their assigned exercise programs. The exergaming intervention was well received and perceived as enjoyable. This study demonstrates that an 8-week exergaming intervention is feasible and acceptable for older adults at risk for falls.

### SESSION 2260 (SYMPOSIUM)

### NOVEL BIOMARKERS OF BIOLOGICAL AGE IN THE HEALTH AND RETIREMENT STUDY

Chair: Bharat Thyagarajan, *Department of Laboratory Medicine and Pathology University of Minnesota; Minneapolis, Minnesota, United States*

Discussant: Morgan E. Levine, *Yale University School of Medicine, New Haven, Connecticut, United States*

This symposium presents early results on epigenetic, transcriptomic and other aging biomarkers such as telomere length and mitochondrial DNA copy number from the Health and Retirement Study that allows a detailed examination of the biological pathways through which socioeconomic conditions influence the human aging process. In 2016 HRS collected 6 tubes of blood from people who completed the 2016 interview, had been in the sample at the prior wave and were not in a nursing home (n=9,973) to maintain a nationally-representative sample. These blood samples were analyzed for novel biomarkers of aging that included global methylation arrays, whole transcriptome sequencing, telomere length and mitochondrial DNA copy number among other biomarkers that were shown to be related to both social and economic circumstances and health outcomes at older ages. This level of integration of biological data to address social disparities hasn't been accomplished before on a large nationally-representative sample of Americans and will provide a unique opportunity to understand the biological mechanisms through which social disparities affect human health. The symposium will describe the utility of measuring novel age related biomarkers in a nationally representative population study such as HRS and the potential research opportunities that can be pursued using this publicly available resource. It will provide an overview of the measurement and distribution of epigenetic, transcriptomic and telomere length and mitochondrial DNA copy number as novel aging biomarkers. It will also describe the utility of these biomarkers in further understanding the biological underpinnings of socioeconomic differences in health and mortality.

### NOVEL AGING BIOMARKERS IN THE HRS

Eileen Crimmins[1], *1. Davis School of Gerontology, University of Southern California, Los Angeles, California, United States*

In addition to the broad panel of aging related biomarkers available in HRS, we will describe measurement of novel aging biomarkers such as telomere length and mitochondrial DNA copy number in 4000 HRS participants. Both these biomarkers were measured in DNA obtained from whole unsorted blood using quantitative real time polymerase chain reaction (PCR) and were adjusted for individual cell composition measured from flow cytometry. We will describe the relationship between these two biomarkers and other measures of biological age available in HRS. Differences in these two novel aging biomarker by socioeconomic status, race/ethnicity, and exposure to early life hardships will be presented to clarify the value of the data to further unravel how social factors get under the skin to affect the process of aging.

### TRANSCRIPTOMIC AGING IN THE HRS

Bharat Thyagarajan[1], *1. Department of Laboratory Medicine and Pathology University of Minnesota; Minneapolis, Minnesota, United States*

Since age related perturbations in gene expression profiles have been described and transcriptomic changes in specific biological pathways have been implicated in the aging process, we performed whole transcriptome sequencing on 4000 HRS participants using RNA obtained from Paxgene tubes collected during the 2016 interview. We will describe design and implementation of innovative quality control procedures to minimize technical variability in transcriptomic measurements and monitor analytical variation in large population studies such as HRS. We will also report the distribution of transcriptomic profiles according to various demographic characteristics (age, sex, racial/ethnic and socioeconomic differences) and describe the prevalence of previously reported aging related transcriptomic signatures in HRS. We will describe the associations between transcriptomic profiles and other measures of biological aging in HRS and report how changes in cell composition can affect transcriptomic profiles observed in population studies such as HRS.

### EPIGENETIC AGING IN THE HRS

Jessica Faul[1], *1. University of Michigan, Ann Arbor, Michigan, United States*

Biological aging can be characterized by molecular, cellular, and epigenetic changes that in addition to being related to chronologic age, are also associated with social disadvantage and associated morbidity and mortality. These biological markers can help explain at a biological level why socially disadvantaged individuals are at greater risk of aging-related disease and premature death. From DNA extracted from venous blood collected from over 4,000 HRS participants we measured array based DNA methylation. These assessments were made from unsorted cells, but are adjusted for individual cell composition measured from flow cytometry. We present genome-wide methylation differentials by age, race/ethnicity and SES using the largest, nationally representative sample with these data available to date. Understanding basic biological changes related to age and social disadvantage is essential for identifying translational opportunities to improve health.

### SESSION 2265 (SYMPOSIUM)

### NOVEL FINDINGS TWO DECADES FOLLOWING COGNITIVE TRAINING: FINDINGS FROM THE ACTIVE TRIAL

Chair: Alden L. Gross, *Johns Hopkins Bloomberg School of Public Health, Baltimore, Maryland, United States*
Co-Chair: George W. Rebok, *Johns Hopkins Bloomberg School of Public Health, Baltimore, Maryland, United States*
Discussant: Walter Boot, *Florida State University, Tallahassee, Florida, United States*

Although the only demonstrated panacea against cognitive decline, behavioral cognitive training usually fails to demonstrate transfer either to untrained cognitive abilities or to distal outcomes like everyday functioning. No such trials, however, have leveraged more than a decade of follow-up to adapt life-course perspectives. The Advanced Cognitive Training for Independent and Vital Elderly (ACTIVE) study remains the largest NIA-funded clinical trial of cognitively normal older adults. Efforts to re-invigorate the cohort after 20 years with external data linkages have coalesced around renewed interests in how training-related cognitive improvements affect long-term dementia risk, health care utilization and costs, credit scores, and active years in later life. The first presentation by Rebok overviews ACTIVE and its 20-year follow-up plans. Next, Gross and colleagues tested whether cognitive training attenuates the relationship between IADL difficulty and mortality; negative findings suggest proposed relationships between cognition and IADL difficulty are correlational, not causal, which has implications for transfer and trial outcomes. Third, Thomas tests a novel algorithm for classifying MCI, ushering opportunities to examine training effects on incidence of MCI after up to 20 years post-training. Marsiske then evaluates temporal transfer of training in reasoning ability, concluding that reasoning training and age, but not other baseline demographics, predict maintained alterations in reasoning abilities. Finally, Felix evaluates the stifling role of depressive symptoms on ability to benefit from training. Dr. Wally Boot, a recognized thought leader in transfer of cognitive training, will provide an illuminating discussion of promises and pitfalls of these lines of research.

### IADL DIFFICULTY AND MORTALITY IN ACTIVE: IMPLICATIONS FOR TRANSFER OF COGNITIVE TRAINING

Alden L. Gross,[1] Olivio Clay,[2] Cynthia Felix,[1] Fred Unverzagt,[3] Michael Marsiske,[4] and Norma Coe[5], *1. Johns Hopkins Bloomberg School of Public Health, Baltimore, Maryland, United States, 2. University of Alabama, Birmingham, Alabama, United States, 3. Indiana University, Indianapolis, Indiana, United States, 4. University of Florida, Gainesville, Florida, United States, 5. University of Pennsylvania, Pennsylvania, Pennsylvania, United States*

Objectives: Our goals were to externally scale an IADL difficulty scale to the more recognizable Functional Activities Questionnaire (FAQ), and test whether cognitive training attenuates the relationship between IADL difficulty and mortality. Method: We leveraged externally available FAQ data from NACC to scale questions about IADL activities administered in ACTIVE (N=2,802) using item response theory. We modeled time to death as a function of IADL difficulty in ACTIVE using survival analysis, testing whether ACTIVE intervention group status modified the association between FAQ and mortality. Results: IADL difficulty in ACTIVE, scaled to the FAQ, was associated with a higher risk of death (Hazard Ratio, HR, 1.01, 95% Confidence Interval, CI: 1.001, 1.02). The relationship did not differ by ACTIVE intervention status. Discussion: Cognitive training does not modify the relationship between IADL difficulty and mortality, consistent with a hypothesis that proposed relationships between cognitive ability and IADL difficulty are correlational, not causal.

### OVERVIEW OF THE ACTIVE STUDY AT 20 YEARS

Sherry Willis,[1] and George Rebok[2], *1. University of Washington, Seattle, Washington, United States, 2. Johns Hopkins Bloomberg School of Public Health, Baltimore, Maryland, United States*

ACTIVE (1998-2019), the largest NIA-funded clinical trial of cognitively normal elderly, was designed to test if cognitive intervention maintains functional independence in older adults (N = 2,802, aged 65-94) by improving basic mental abilities. In this paper we overview major aims of ACTIVE to investigate 1) effectiveness and durability (through 1,2,3,5, and 10 years of follow-up) of three cognitive interventions (memory, reasoning, processing speed) in improving basic measures of cognition; 2) if training in specific cognitive abilities improves or maintains cognitively demanding daily living skills (e.g., medication use, driving); and 3) impact of intervention on everyday mobility, health-related quality of life, and health service utilization. We also review aims of a recent NIA-funded 20-year follow-up of ACTIVE to examine whether improved cognition and daily function results in long-term reduction in dementia risk, years of disability, health care utilization and costs, and increased active years of life in advanced old age.

### ACTUARIAL CRITERIA FOR MCI DIAGNOSIS IN ACTIVE: IMPLICATIONS OF ADJUSTMENT FOR RACE

Kelsey Thomas,[1] Sarah Cook,[2] Fred Unverzagt,[3] Mark W. Bondi,[4] and Michael Marsiske[5], *1. University of California, San Diego School of Medicine, San Diego, California, United States, 2. Duke University, Durham, North Carolina, United States, 3. Indiana University, Indianapolis, Indiana, United States, 4. University of California, San Diego, San Diego, California, United States, 5. University of Florida, Gainesville, Florida, United States*

This study examined the baseline prevalence of mild cognitive impairment (MCI) in the ACTIVE study using actuarial criteria for MCI. Participants (n=2763; 26% Black) were classified as probable MCI cases if they had two observed test scores within the same cognitive domain (memory, reasoning, speed) that were >1SD below a demographically-adjusted expected score (based on the regression weights of a "robust" normal control group). Each score was adjusted using two approaches: Method 1 adjusted for age, sex, and education; Method 2 also adjusted for race. The estimated prevalence of MCI was 33.5% (n=925) in Method 1 and 32.1% (n=887) in Method 2. Adjusting for race reduced the proportion of Black participants classified as probable MCI from 42.3% to 34.9%. Future work will examine whether adjustment for social determinants of health (e.g., education quality, neighborhood/healthcare access) might further improve the utility of this classification method in diverse samples.

### LONG-TERM BENEFITS OF REASONING TRAINING: A PREDICTED DIFFERENCE APPROACH

Michael Marsiske[1], *1. University of Florida, Gainesville, Florida, United States*

In 928 ACTIVE participants, we investigated predictors of exceptional reasoning performance ten years post-enrollment. Participants had been randomized into a training arm (memory, reasoning, or speed of processing) or a no-contact control group. Each participant received an age- and education adjusted expected normative trajectory on a reasoning composite score, derived from the untrained control group. They were then classified as within- (n=467, 50%), above- (n=285, 31%), or below-normative expectation (n=176, 19%) ten years post-training. At a p<.001 significance criterion, reasoning training (b=, 0.632, OR =1.88) and younger age (b=-0.048/year, OR = 1.05) were associated with 10-year above-normative expectations. No other baseline factors considered (other training arms, education, cardiovascular risk, life space, mobility, locus of control, morale, motivation) predicted ten year status, nor did they interact with training arm. Reasoning training appears to have produced long term alterations in reasoning trajectory for many participants.

### DEPRESSIVE SYMPTOMS: HAMPERING MAINTENANCE OF COGNITIVE TRAINING GAINS

Cynthia Felix,[1] Simo Du,[1] Brad Taylor,[2] and George Rebok[1], *1. Johns Hopkins Bloomberg School of Public Health, Baltimore, Maryland, United States, 2. University of Florida, Gainesville, Florida, United States*

To study the impact of depressive symptoms on the effectiveness of cognitive training interventions, the ACTIVE 10-year follow up data was analyzed for cognition and difficulty in everyday functioning outcomes. Because depression is treatable, this topic is of high public health significance. Analyses showed that females had greater prevalence of depressive symptoms. Reasoning, speed of processing and daily function change scores were significantly worse in the depressive symptom group, while memory change score was non-significantly worse, showing poor proximal and downstream primary (functional) outcomes. Depressive symptoms may lead to lower retention of cognitive training benefits.

### SESSION 2270 (PAPER)

### PERSONALITY, CULTURAL ELEMENTS, AND COGNITIVE FUNCTIONING

### ADDRESSING THE NEEDS OF INCARCERATED INMATES WITH DEMENTIA: CREATING DEMENTIA-FRIENDLY PRISONS

Valerie Gruss,[1] and Memoona Hasnain[1], *1. University of Illinois at Chicago, Chicago, Illinois, United States*

U.S. prisons are experiencing a graying of their population, with many older inmates experiencing chronic conditions, including dementia. Older prisoners now represent 10% of the U.S. prison population and 18% of Illinois' prison population. Aging inmates cost more to incarcerate due to their medical needs. Bureau of Prisons data estimate $881 million (19%) of its budget was spent to incarcerate aging inmates. Prisons are seeking solutions to address the unmet needs of older inmates, especially those with dementia. These older inmates with dementia face discrimination and exploitation within the prison population, and Correction Officers and clinicians lack training to understand and address their complex needs. Utilizing the Alzheimer's Association's, ACT on Alzheimer's Toolkit we implemented four phases guiding communities' adoption of dementia-friendly practices: we convened meetings with Illinois Department of Correction leaders, assessed community strengths and gaps by surveying prison wardens, analyzed findings and created an action plan to provide dementia training of prison staff to create a dementia friendly community. Our "Dementia-Friendly Prisons" program trains prison staff on understanding and providing supportive care and management to inmates with dementia, enabling staff to meet inmates' needs, thereby creating an environment where inmates with dementia are safe and treated respectfully. Our program used an Appreciative Inquiry Four-D cycle approach (Discover-Dream-Design-Destiny) to engage and empower learners from a highly diverse workforce to develop into collaborative teams. Our program remedies a problem in the delivery of prison healthcare, serving a particularly vulnerable population and creates an adaptable model for other prisons and communities.

### CARING FOR OLDER INDIGENOUS PEOPLE WITH DEMENTIA: ATTENDING TO THE PERSONALITY

Kathleen R. Mason,[1] Tess H. Moeke-Maxwell,[1] and Merryn Gott[1], *1. University of Auckland, Auckland, New Zealand*

Māori, the indigenous people of New Zealand, are living longer and dying older. The prevalence of conditions associated with older people, such as Dementia are expected to increase amongst the Māori population. Pae Herenga, a qualitative research project investigating traditional Māori end-of-life care customs, identified an indigenous narrative of Dementia care, as carried out by their families. Sixty participants took part in face-to-face interviews to systematically record the traditional care customs employed by Māori families. Of these families, five experienced caring for someone with dementia. A traditional Māori family values approach based on biological connections, relationships, empathy, love, patience and inclusiveness aimed to care for the individual with Dementia as an important member of the family, and sought to maintain as much of the person's autonomy as possible, for as long as possible. Sharing care roles between family members and maintaining connections to Māori communities helped to prevent isolation of the person with Dementia and their family members caring for them. Involvement in family and community activities, and attending to the individual's personality and their spiritual needs were just as important as tending to their physical care needs. These findings emphasize the importance of a holistic approach to caring for indigenous people with Dementia.

### INSIGHTS FROM AFRICAN AMERICAN OLDER ADULTS ON BRAIN HEALTH RESEARCH ENGAGEMENT: NEED TO SEE THE NEED

Shani H. Bardach,[1] Markeda Yarbrough,[1] Charlene Walker,[2] Doris L. Alfred,[3] Eseosa Ighodaro,[1] and Gregory A. Jicha[1], *1. University of Kentucky, Lexington, Kentucky, United States, 2. Bluegrass Community and Technical College, Lexington, Kentucky, United States, 3. Love's Angels Early Childhood Development Center, Paris, Kentucky, United States*

Recruitment of African American (AA) participants into clinical research trials in the area of aging and dementia is a major problem facing the field. Although AAs are at a significantly elevated risk of developing Alzheimer's disease (AD), they are underrepresented in clinical trials and research studies. While previous research has identified a number of barriers to participation, relatively little is known about how to overcome these barriers and engage AA individuals in research. Photovoice may provide a novel approach to advance our understanding of AA perceptions in regards to barriers and strategies to increase AA engagement in brain aging research. The purpose of this project is to add to existing understanding of barriers and facilitators and identify strategies to enhance engagement. Three AA research advocates served as community facilitators to identify and guide groups of AA adults though an 8-10 session photovoice project. Group sessions involved discussions and sharing of images pertaining to various prompts in the area of brain health and research participation. Sessions were audiotaped and transcribed verbatim and photos were uploaded. Participants identified four categories of barriers to AA research participation: Mistrust, the belief that all research involves medication, avoidance and fear of acknowledging problems, and seeing the risks of research but not the need. Participants had various suggestions and approaches for ameliorating each of these barriers. This photovoice community engagement process revealed unique insights into barriers and opportunities for increasing AA engagement in brain aging research.

### REDUCING ANTIPSYCHOTIC USE IN LONG-TERM CARE: CONSIDERING THE ROLE OF CULTURE OF CARE AND INFORMAL COMMUNICATION

Andre Smith,[1] Sue Kurucz,[2] Tara Erb,[1] and Ruth Kampen[1], *1. Department of Sociology, University of Victoria, Victoria, British Columbia, Canada, 2. Island Health, Victoria, British Columbia, Canada*

Persons living with dementia-related disorders (PwD) can experience challenging behavioural and psychological symptoms (BPSD) as their illness progresses. There is a continued reliance on antipsychotic drugs (APD) in long-term care to manage this issue despite the well-documented risks of adverse events and increased morbidity and mortality. This study examines the role of culture of care in relation to efforts at reducing inappropriate APD use in managing BPSD within long-term care. Culture of care consists of shared norms, beliefs, and cognitive frames which guide clinical practice and inform the development and implementation of care strategies. Findings were obtained from three Canadian long-term care facilities working on reducing inappropriate use of APD. Data came from interviews with 6 nurses, 18 licenced practical nurses, 14 health care assistants, 4 activity leaders, 4 directors of care, 1 chaplain, and 10 physicians. We found that direct care providers initially varied in their perceived ability to develop and use alternate care strategies with health care assistants being most concerned about safety and exposure to violence. Change involved detective work and innovative thinking in assessing possible causes of BPSD beyond psychosis, including pain and feelings of confusion. Informal reciprocal patterns of communication emerged among health care assistants to identify effective non-pharmaceutical strategies to manage BPSD. Overall, the study shows how shared beliefs in the need for and value of alternate care practices among direct care providers along with the existence of effective informal communication can contribute to successful reduction in APD use when managing BPSD in PwD.

### SYSTEMATIC REVIEW AND META-ANALYSIS OF RACIAL AND ETHNIC DIFFERENCES IN DEMENTIA CAREGIVER WELL-BEING

Chelsea Liu,[1] Adrian Badana,[2] Julia Burgdorf,[1] Chanee D. Fabius,[3] William E. Haley,[4] and David L. Roth[5], *1. Johns Hopkins Bloomberg School of Public Health, Baltimore, Maryland, United States, 2. School of Aging Studies, University of South Florida, Tampa, Florida, United States, 3. Johns Hopkins University, Baltimore, Maryland, United States, 4. University of South Florida, Tampa, Florida, United States, 5. Johns Hopkins University School of Medicine, Baltimore, Maryland, United States*

Studies comparing racial/ethnic differences on psychological and physical outcomes of dementia caregivers have often reported differences in well-being for minority groups compared to Whites. However, due to issues with enrolling minorities into studies, recruitment methods often differ for minority and White participants and may lead to biased comparisons. We conducted a systematic review and meta-analysis to examine racial/ethnic differences in dementia caregiver outcomes and to determine whether any differences vary among studies with population-based samples compared to convenience samples. We systematically reviewed articles with primary data from PubMed, Google Scholar and PsycINFO, and included studies comparing either African American (AA) or Hispanic/Latino dementia caregivers to White caregivers on measures of psychological health (e.g. depression, anxiety, burden) and physical health (e.g. self-rated health, cardiovascular measures, stress biomarkers). Reviewers screened titles and abstracts, reviewed full texts and conducted risk-of-bias assessments. A total of 207 effects were extracted from 40 studies. Random-effects models showed that Hispanics/Latinos reported significantly lower levels of well-being than Whites ($ps < .05$) for both psychological outcomes (37 effects) and physical outcomes (15 effects), while AAs were not significantly different from Whites in either domain. No differences were observed for population-based studies (N=3; 23 effects) or convenience-sample studies (N=37; 184 effects). Although some previous studies with convenience samples found better psychological well-being in AA caregivers, that pattern was not confirmed in our meta-analysis. Additional analyses for the different indicators of well-being and the relationship of quality ratings to effect sizes will be discussed along with implications for future research.

### SESSION 2275 (SYMPOSIUM)

### PHYSIOLOGY OF SUCCESSFUL AGING: IMPLICATIONS FOR HEALTH AND WELL-BEING

Chair: Konstantinos Mantantzis, *Humboldt University of Berlin, Berlin, Germany*
Co-Chair: Denis Gerstorf, *Humboldt University Berlin, Berlin, Germany*
Discussant: Thomas M. Hess, *North Carolina State University, Raleigh, North Carolina, United States*

Research into peripheral physiology and its association with cognition, emotionality, and social/physical functioning has received considerable attention over the years. However, many of the underlying mechanisms are not well understood. In this symposium, we have compiled a set of four empirical projects that showcase current and future endeavors to address some of the long-standing questions about when, how, and why physiology shapes and is shaped by key psychosocial resources. Hawkley et al. make use of data from the NSHAP and HRS longitudinal studies to investigate whether social relationships such as number of friends predicts risk of diabetes among older adults. Wilson et al. use dyadic data from young and middle-aged couples to examine cardiometabolic similarity among spouses, and how such concordance is shaped by key relationship factors such as emotional closeness. Pauly et al. use data from two daily-life studies of older couples to investigate how physiological synchrony in cortisol is modulated by partner interactions, empathy, and empathic accuracy. Finally, Mantantzis et al. make use of multi-year longitudinal data from the Berlin Aging Study II to examine the role of glucose regulation capacity for trajectories of subjective well-being among older adults. Thomas Hess will discuss the importance of these papers, discuss strengths and weaknesses of the approaches chosen, and consider implications for future research.

### POOR GLUCOSE REGULATION IS ASSOCIATED WITH LOWER WELL-BEING AMONG OLDER MEN, BUT NOT WOMEN

Konstantinos Mantantzis,[1] Johanna Drewelies,[2] Gert G. Wagner,[3] Ilja Demuth,[4] Elizabeth Steinhagen-Thiessen,[4] Ulman Lindenberger,[5] Sandra Düzel,[5] and Denis Gerstorf[2], *1. Humboldt University of Berlin, Berlin, Germany, 2. Humboldt University of Berlin, Berlin, Berlin, Germany, 3. German Institute for Economic Research, Berlin, Berlin, Germany, 4. Charité University Hospital, berlin, Berlin, Germany, 5. Max Planck Institute for Human Development, berlin, Berlin, Germany*

Glucose regulation is a key aspect of healthy aging, but little is known about gluco-regulatory capacity and older adults' well-being. In this study, we examine whether gluco-regulatory capacity is predictive of within-person age-related trajectories of three major well-being indicators. We applied growth models to multi-year longitudinal data obtained in the Berlin Aging Study II (N = 1437; age 60-89; 53% women) and used insulin resistance as an index of glucose regulation capacity. Poor glucose regulation was associated with lower levels of well-being in men, but not women. These associations among men emerged for two of the three well-being indicators, were maintained across old age, and were independent of the other cognitive and physical factors examined. We discuss how sexual dimorphism may have contributed to our findings, and conclude that our results provide initial evidence for the relevance of glucose regulation for quality of life among older men.

### NUMBER OF FRIENDS AND THE RISK OF DEVELOPING DIABETES: A REPLICATION USING NSHAP AND HRS

Louise Hawkley,[1] Phil Schumm,[2] Elbert Huang,[3] and Martha McClintock[4], *1. NORC, Chicago, Illinois, United States, 2. Biostat Consulting Lab, University of Chicago, Chicago, Illinois, United States, 3. General Internal Medicine, University of Chicago, Chicago, Illinois, United States, 4. Institute for Mind & Biology, University of Chicago, Chicago, Illinois, United States*

The epidemic increase in diabetes prevalence (primarily type 2) is a public health crisis. We hypothesized that the rates of movement among diabetic states depend in part on one's social relationships and environment. Using population-based samples from both NSHAP and HRS, collected in 2005–15, we found that having more friends was associated with a lower risk for acquiring diabetes over the next 4-5 years. As an independent replication, separate logistic models for NSHAP and HRS data yielded similar odds-ratios for the protective effect of having friends (OR = 0.82 and 0.92 respectively), adjusting for gender, age, race/ethnicity, and BMI. This effect was concentrated entirely between 0–4 friends; differences in the number of friends above 4 were not associated with differences in diabetes risk.

### FOR BETTER AND WORSE? THE IMPORTANCE OF CLOSENESS AND AGE FOR SPOUSES' CARDIOMETABOLIC SIMILARITY

Stephanie J. Wilson,[1] Juan Peng,[2] Rebecca Andridge,[2] Lisa M. Jaremka,[3] Christopher P. Fagundes,[4] William B. Malarkey,[5] Martha A. Belury,[2] and Janice K. Kiecolt-Glaser[1], *1. The Ohio State University College of Medicine, Columbus, Ohio, United States, 2. The Ohio state University, Columbus, Ohio, United States, 3. University of Delaware, Newark, Delaware, United States, 4. Rice University, Houston, Texas, United States, 5. The Ohio State College of Medicine, Columbus, Ohio, United States*

Spouses share age-related disease risks: a person's diabetes or hypertension raises the partner's odds for the same condition. To probe the importance of partners' closeness, marital satisfaction, and age for spouses' similarity in cardiometabolic health, 43 disease-free couples ages 24-61 provided fasting glucose, fat and carbohydrate oxidation, and blood pressure at two study visits. Couples who felt closer had more similar rates of carbohydrate oxidation compared to those who felt less close. Likewise, happier couples had more similar carbohydrate and fat oxidation. Fasting glucose and blood pressure were more similar within middle-aged couples compared to younger pairs. In follow-up analyses, partners' health behavior concordance did not explain these effects. In sum, closer, happier, and older couples shared more similar cardiometabolic profiles, perhaps driven by joint stress and emotional spillover. Findings suggest that closer, happier relationships may confer both larger health risks and benefits, and increasing age may raise the stakes.

### LINKS BETWEEN PARTNER INTERACTIONS, EMPATHY, AND EVERYDAY PHYSIOLOGICAL SYNCHRONY IN OLDER COUPLES

Theresa Pauly,[1] Victoria I. Michalowski,[2] Johanna Drewelies,[3] Denis Gerstorf,[3] Maureen C. Ashe,[2] Kenneth M. Madden,[2] and Christiane A. Hoppmann[2], *1. University of British Columbia, Vancouver, Canada, 2. University of British Columbia, Vancouver, British Columbia, Canada, 3. Humboldt-Universität Zu Berlin, Berlin, Berlin, Germany*

Romantic partners exhibit dyadic covariation (synchrony) in physiological parameters. This study aims to link everyday cortisol synchrony to daily partner interactions and empathy. We conducted coordinated multilevel analysis using data from two independently collected samples of older couples (Study 1: N = 85 couples, aged 60-87 years; Study 2: N = 77 couples, aged 66-85 years) who completed questionnaires and provided salivary cortisol samples 5 to 7 times daily for 7 days. Cortisol levels were significantly correlated among partners in both studies. Cortisol synchrony was higher when partners were present (Study 1), and when partner interactions involved feeling understood and valued (Study 1) and seeking help or closeness (Study 2). Higher cortisol synchrony was further related to greater empathic accuracy (Study 1) and greater empathy (Study 2). Thus, social bonding processes and the ability to consider other's thoughts and feelings may be intertwined with physiological synchrony in everyday life.

### SESSION 2280 (PAPER)

### RACE, SOCIETY, AND HEALTH OUTCOMES

### DEATH OF A CHILD AND MORTALITY RISK OVER THE LIFE COURSE: RACIAL DISADVANTAGE IN THE U.S.

Rachel Donnelly,[1] Debra Umberson,[2] Robert Hummer,[3] and Michael Garcia[4], *1. Vanderbilt University, Nashville, Tennessee, United States, 2. University of Texas, Austin, Texas, United States, 3. University of North Carolina, Chapel Hill, North Carolina, United States, 4. Population Research Center, Austin, Texas, United States*

Numerous studies show that bereavement, including bereavement following the death of a minor child, increases mortality risk in white populations. The death of a child prior to midlife has received much less attention. Moreover, recent research shows that black Americans are substantially more likely to lose a child compared to white Americans, but this racial disadvantage is largely unexplored. Losing a child is a traumatic event that may activate biopsychosocial and behavioral risk factors that add to mortality risk. We analyze longitudinal data from the Health and Retirement Study (1992-2014) to assess the association of child loss prior to midlife with mortality risk in mid to later life, and the possible biopsychosocial and behavioral covariates linking child death to mortality. The analytic sample includes 20,489 non-Hispanic white respondents and 5,328 non-Hispanic black respondents who have ever given birth to or fathered at least one child. Findings suggest that that the death of a child prior to midlife is associated with increased mortality risk, net of sociodemographic controls. Psychological (e.g., depressive symptoms), behavioral (e.g., alcohol use, smoking), and social (e.g., income, marital status) factors explain this heightened mortality risk. Although the heightened mortality risk for child loss is similar for black and white parents, black parents experience a greater disadvantage as they are almost twice as likely as white parents to lose a child prior to midlife. Child loss and the resulting health risks disproportionately burden black families, functioning as a unique source of disadvantage for black Americans.

### RACE-ETHNICITY AND HOPELESSNESS IN OLDER AMERICANS: WHO'S AT GREATEST RISK AND WHY?

Uchechi Mitchell[1], *1. University of Illinois at Chicago, Chicago, Illinois, United States*

Racial and ethnic minorities are more likely to experience adversity throughout their lives, which puts them at greater risk feelings of despair and powerlessness. This study uses data from 5,500 respondents from the Health and Retirement Study to assess racial/ethnic differences in hopelessness and test whether older blacks and Hispanics experience greater increases in hopelessness as they age. Hopelessness was assessed using 4-items that capture the extent to which a person has a negative outlook towards the future and believes they are powerlessness to overcome the obstacles they face; it is measured at three time points: 2006/2008, 2010/2012 and 2014/2016. Linear regression models were used to assess differences in hopelessness by race/ethnicity and linear mixed models were used to assess racial/ethnic differences in trajectories of hopelessness over time. Older blacks and Hispanics were more likely to report feelings of hopelessness at each timepoint of the study. Differences between blacks and whites were completely explained by differences in education and poverty status, while differences between Hispanics and whites remained. Although minority elders had higher levels of hopelessness at each time point, older whites experienced steeper increases in hopelessness over time. These findings suggest that structural factors influence feelings of hopelessness among minority elders. However, older blacks and Hispanics may develop resilience to hopelessness as they age.

### RACIAL DISPARITIES IN HOSPICE USE AT THE END OF LIFE: FINDINGS FROM THE REGARDS STUDY

Katherine Ornstein,[1] Orla C. Sheehan,[2] Jin Huang,[3] J. David Rhodes,[4] and David L. Roth[3], *1. Icahn School of Medicine at Mount Sinai, New York, New York, United States, 2. Center on Aging and Health, Johns Hopkins University School of Medicine, Baltimore, Maryland, United States, 3. Johns Hopkins University School of Medicine, Baltimore, Maryland, United States, 4. School of Public Health, Department of Biostatistics, Birmingham, Alabama, United States*

Hospice supports patients and families through interdisciplinary care focused on symptom management and maximization of quality of life. Although hospice care confers well-documented benefits, it remains underutilized: many patients do not use it at all or enter care too late to receive any benefit. While racial disparities in hospice use have been documented, hospice utilization among non-white decedents remains understudied, particularly among those with non-cancer diagnoses. Therefore, we used the REasons for Geographic and Racial Differences in Stroke (REGARDS) study, a population-based investigation of stroke incidence with oversampling of Blacks and cause of death adjudication by expert panel review, linked to Medicare claims data to examine racial disparities in end-of-life care. We identified 1221 participants who died between 2013-2015 due to natural causes excluding sudden death. More than half (52.8%) used hospice during the last 6 months of life (median =15 days), with use among cancer decedents over 70%. Overall, Blacks were significantly less likely to use hospice (OR=0.570) compared to Whites in adjusted analyses. Among hospice users, Blacks did not significantly differ from Whites in length of stay. In analyses stratified by cause of death (dementia, cancer, CVD and other), Blacks were significantly less likely than Whites to use hospice for all causes of death other than dementia. Despite tremendous growth of hospice in recent decades, our findings suggest that this effective service remains highly underutilized among Blacks dying from cancer, CVD and other serious illnesses, suggesting a need for targeted intervention to eliminate disparities in quality end-of-life care.

### RACIAL SIMILARITIES AND DIFFERENCES IN FUTURE CARE PLANNING

Natasha L. Peterson,[1] and Jeong Eun Lee[1], *1. Iowa State University, Ames, Iowa, United States*

Recent population trends for the United States show substantial increases in racial diversity as well as an increase in the older adult demographic. Accordingly, the expansion in minority older adults suggests the importance of recognizing how race affects future care planning (FCP) in late life. The aim of this paper is to investigate the racial similarities and differences in planning behavior and intentions related to end-of-life care planning. This study utilized data from the Future Care Planning intervention based on the initial assessment with 237 older adults in rural Iowa (Mage= 75.93, SD=11.14) with 73 non-White participants. Results indicated that both Whites and non-Whites had similar levels of intentions in terms of future care. However, non-Whites were less likely than Whites to engage in multiple areas of FCP including planning behavior and preparing for end-of-life (F=23.957, p<.000). These findings suggest a need for more targeted FCP assessments and interventions across race groups in rural contexts.

### RACIAL VARIATION IN THE RELATIONSHIP BETWEEN LIFE-COURSE TURNING POINTS AND LIFE SATISFACTION

Dawn C. Carr,[1] Ben L. Kail,[2] and Rocio Calvo[3], *1. Florida State University, Tallahassee, Florida, United States, 2. Georgia State University, Atlanta, Georgia, United States, 3. Boston College, Boston, Massachusetts, United States*

According to many social gerontologist and life course scholars, major life course transitions, referred to as "turning points," have a significant impact on well-being. Although the relationship between major later life turning points and general well-being is fairly well understood, it is unclear whether there is systematic racial/ethnic variation in response to turning points in general. Moreover, much of sparse research on racial/ethnic variation that does exits overlooks how Hispanic Americans may respond differently to turning points than do either white Americans or African Americans. To that end, in this paper, we draw on life course theory to assess whether the relationship between retirement and the death of a spouse (i.e. turning points) and life satisfaction (a measure of well-being) vary by race/ethnicity. We focus on differences between whites, Hispanics, and African Americans. Moreover, we draw on stress process theory to identify mechanisms that may explain any observed racial/ethnic variation in these relationships. Using the Health and Retirement Study, in preliminary results we find: 1) before adjusting for turning points, Hispanics appear to have higher life satisfaction than whites, and African Americans do not differ significantly from whites; 2) however, after adjusting for turning points, only Hispanics who make life course transitions have significantly higher life satisfaction than whites; and 3) this higher life satisfaction observed among Hispanics who experience turning points is largely not accounted for by several factors derived from stress process theory.

### SESSION 2285 (SYMPOSIUM)

### POLICY SERIES: RESEARCH, POLICY, AND PRACTICE: IMPACTS OF DISCRIMINATION

Chair: Patricia M. D'Antonio, *The Gerontological Society of America, Washington, District of Columbia, United States*

Discussant: John Feather Aging crosses all domestic and international borders and affects everyone regardless of religion, race, nationality, ethnicity, sexual orientation, or gender identity. The main purpose of The Gerontological Society of America is "to advance the scientific and scholarly study of aging and to promote human welfare by the encouragement of gerontology in all its areas." Yet in 2019, policies remain in effect that impact individuals in a discriminatory manner. The program will highlight research in several areas that demonstrate the effects of these discriminatory practices.

### DEFINING DIFFERENT FORMS OF DISCRIMINATION IMPACTING SOCIETY

Patricia M. D'Antonio, *The Gerontological Society of America, Washington, District of Columbia, United States*

Aging crosses all domestic and international borders. It's an issue that affects everyone regardless of religion, race, nationality, ethnicity, sexual orientation, or gender identity. The main purpose of The Gerontological Society of America (GSA) is "to advance the scientific and scholarly study of aging and to promote human welfare by the encouragement of gerontology in all its areas." Yet in 2019, policies remain in effect that impact individuals in a discriminatory manner. The program will highlight research in several areas that demonstrate the effects of these discriminatory practices.

### THE IMPACT OF DISCRIMINATORY POLICIES AND PRACTICES ACROSS THE AGING CONTINUUM

Brian W. Lindberg[1], *1. The Gerontological Society of America, Washington, District of Columbia, United States*

Aging crosses all domestic and international borders. It's an issue that affects everyone regardless of religion, race, nationality, ethnicity, sexual orientation, or gender identity. The main purpose of The Gerontological Society of America (GSA) is "to advance the scientific and scholarly study of aging and to promote human welfare by the encouragement of gerontology in all its areas." Yet in 2019, policies remain in effect that impact individuals in a discriminatory manner. The program will highlight research in several areas that demonstrate the effects of these discriminatory practices.

### SESSION 2290 (SYMPOSIUM)

### STRENGTH IN AGE: HARNESSING THE BENEFITS OF NIA'S 45 YEARS

Chair: Marie A. Bernard, *National Institute on Aging, National Institutes of Health, Bethesda, Maryland, United States*
Co-Chair: Richard Hodes, *National Institute on Aging, National Institutes of Health, Bethesda, Maryland, United States*
Discussant: Robin A. Barr, *National Institute on Aging, National Institutes of Health, Bethesda, Maryland, United States*

The National Institute on Aging at the National Institutes of Health was established in 1974 with a mission to support and conduct research on aging processes, age-related diseases, and special problems and needs of the aged. Shortly thereafter, the Institute was designated by Congress as the lead research agency in Alzheimer's disease. During this symposium, NIA's scientific leadership will reflect on major accomplishments to improve the health of older individuals over the past 45 years and look to the future of the NIA and aging research. This symposium will describe progress led by NIA's extramural divisions, the intramural program, and the Office of Special Populations. A brief panel discussion will ensue, followed by an informal reception and opportunities for networking in celebration of NIA's 45 years

### VIEWPOINT FROM THE DIVISION OF AGING BIOLOGY

Felipe Sierra[1], *1. National Institute on Aging, National Institutes of Health, Bethesda, Maryland, United States*

Dr. Sierra will review accomplishments of the Division of Aging Biology and make projections of future growth in the field.

### VIEWPOINT FROM THE DIVISION OF NEUROSCIENCE

Eliezer Masliah[1], *1. National Institute on Aging, National Institutes of Health, Bethesda, Maryland, United States*

Dr. Masliah will review accomplishments of the Division of Neurosciences and make projections for the future.

### VIEWPOINT FROM THE DIVISION OF BEHAVIORAL AND SOCIAL RESEARCH

John Haaga[1], *1. National Institute on Aging, National Institutes of Health, Bethesda, Maryland, United States*

Dr. Haaga will review accomplishments of the Division of Behavioral and Social Research and make projections for the future.

### VIEWPOINT FROM THE DIVISION OF GERIATRICS AND CLINICAL GERONTOLOGY

Evan Hadley[1], *1. National Institute on Aging, National Institutes of Health, Bethesda, Maryland, United States*

Dr. Hadley will review accomplishments of the Division of Geriatrics and Clinical Gerontology and make projections for the future.

### VIEWPOINT FROM THE INTRAMURAL RESEARCH PROGRAM

Luigi Ferrucci[1], *1. Translational Gerontology Branch, Intramural Research Program, National Institute on Aging, National Institutes of Health, Baltimore, Maryland, United States*

Dr. Ferrucci will review accomplishments of the Intramural Research Program and make projections for the future

### SESSION 2295 (SYMPOSIUM)

### STRENGTH IN AGE: INCREASING THE POWER OF FAMILY CAREGIVERS

Chair: Heather Young, *Betty Irene Moore School of Nursing - UC Davis, Sacramento, California, United States*
Co-Chair: Theresa Harvath, *Betty Irene Moore School of Nursing at University of California Davis, Sacramento, California, United States*
Discussant: Fawn Cothran, *Betty Irene Moore School of Nursing at University of California Davis, Sacramento, California, United States*

This session addresses approaches to strengthening the capacity of family caregivers in the context of intense and complex care. Recognizing the increasing role that families play in delivering complex care at home to individuals with multiple conditions, this symposium highlights approaches to enhancing support and increasing the power of family caregivers. In the first half of the symposium, the papers elucidate characteristics of the caregiving situation that put caregivers at risk and suggest potential areas for intervention by health systems. The second half explores system level approaches to enhancing capacity for family caregivers. On the demand side, the first paper will examine the social network of family caregivers, highlighting effects of social isolation on caregiver health. The second paper uses national data to understand the relationship between higher demand caregiving situations and the strain and challenges that caregivers experience. On the potential solutions side, the third paper addresses a collaborative design of an intervention to enhance supports for family caregivers of persons with dementia at a critical time, during hospitalization. The last paper provides an overview of evidence-based technological solutions to support family caregiving. Taken together, these papers establish some of the demand characteristics of the caregiving situation and provide potential health system solutions to improve capacity of family caregivers. In the final segment, three discussants will reflect on the implications of these papers for clinical practice, education, research and policy.

### FAMILY CAREGIVER SOCIAL ISOLATION AND HEALTH: FINDINGS IN THE NATIONAL SURVEY OF CAREGIVING

Janet S. Pohl,[1] Janice F. Bell,[2] Nancy Woods,[3] and Daniel J. Tancredi[4], *1. Betty Irene Moore School of Nursing at University of California Davis, Sacramento, California, United States, 2. Betty Irene Moore School of Nursing, University of California Davis, Sacramento, California, United States, 3. University of Washington School of Nursing, Seattle, Washington, United States, 4. University Of California Davis, School Of Medicine, Sacramento, California, United States*

Social relationships are important for family/informal caregiver health. Due to caregiving commitments caregivers are at risk for social isolation. Social isolation is associated with adverse health outcomes in the general population, but few studies have examined this association among caregivers. Employing the Convoy Model of social relations, we examined associations between caregiver self-reported health and social isolation—operationalized as a measure that included multiple domains and as specific domains modeled independently. Social isolation prevalence was 24.74% (n=2,175); mean health was 3.46 (SE=0.02) on a scale of 1 (poor) to 5 (excellent). In adjusted models, domain-inclusive social isolation was inversely associated with health ($\beta$=-.07; CI=-0.14,-0.02, p=0.01). In independent domain adjusted models, only participation in club activities was associated with health ($\beta$=-.22; CI=-0.35,-0.10, p<0.01). Social isolation predicted caregiver health, with club participation explaining much of the variance. Domain inclusive social isolation measures can identify targets for intervention studies to improve caregiver health.

### FAMILY CAREGIVING IN THE CONTEXT OF INTENSE AND COMPLEX CARE

Janice Bell,[1] Robin L. Whitney,[2] and Heather M. Young[1], *1. Betty Irene Moore School of Nursing at University of California Davis, Sacramento, California, United States, 2. Hillblom Center on Aging UCSF Fresno Medical Education Program, University of California San Francisco, Fresno, California, United States*

Approximately four in ten family caregivers experience high intensity care, based on the number of caregiving hours, activities of daily living (ADLs) and instrumental ADLs supported for the care recipient. Using the 2015 Caregiving in the U.S. Survey, we examined outcomes associated with high (compared to low or medium) intensity of care. High intensity was positively associated with emotional stress (OR=2.10; 95%CI: 1.52-2.91); financial strain (OR=1.69; 95% CI: 1.210-2.36); physical strain (OR=3.09; 95% CI: 2.21-4.34); and declines in caregiver health (OR=2.14; 95% CI: 1.56-2.93). High intensity was also associated with greater difficulty coordinating recipient care (OR=1.96; 95%CI: 1.42-2.71), higher odds of performing complex medical/nursing tasks (OR=6.85; 95% CI: 5.27-8.90) and, among task performers, greater difficulty performing tasks (OR=2.10; 95% CI: 1.43-3.08). High intensity of care impacts caregiver health and the caregiving role in multiple domains; new clinical and policy approaches are needed to mitigate risks and ensure adequate support.

### AN ACADEMIC-HEALTH SYSTEM COLLABORATION TO DEVELOP A PROGRAM FOR FAMILY CAREGIVERS OF PERSONS WITH DEMENTIA

Elena Siegel,[1] Ladson Hinton,[2] Elizabeth Rice,[1] Nilpa Shah,[1] Vanessa McElroy,[2] Jasmine DeGuzman Lacsamana,[3] Heather M. Young,[1] and Gregory A. Maynard[2], *1. Betty Irene Moore School Of Nursing, University Of California Davis, Sacramento, California, United States, 2. University of California Davis Health, Sacramento, California, United States, 3. Archstone Foundation, Long Beach, California, United States*

Hospitalization of persons with dementia can pose specific challenges for family caregivers, with post-discharge issues in managing acute clinical care needs coupled with dementia-related care that can exacerbate caregiver fatigue and capacity. We established an academic-practice collaboration to develop an evidence-based and innovative multi-component health system-level program to support family caregivers of persons with dementia in transition from hospital to community. Using an implementation science approach aimed to decrease the gap in translation of caregiver research into practice, we co-designed the program/implementation plan as a quality improvement initiative reflecting an integration of evidence from family caregiving literature and the health system's unique context, workflows, stakeholder perspectives, resources, and values/priorities. This paper highlights insights gained and lessons learned in establishing a successful academic-practice collaboration, including time/investment to establish a shared project vision and identify/leverage existing organizational capacity to successfully deliver a program to improve the health and wellbeing of family caregivers.

### STRENGTHENING FAMILY CAREGIVING THROUGH INNOVATIVE TECHNOLOGY SOLUTIONS

David Lindeman,[1] and Katherine K. Kim[2], *1. University of California Berkeley, Berkeley, California, United States, 2. Betty Irene Moore School Of Nursing and School Of Medicine Public Health Sciences, University Of California Davis, Sacramento, California, United States*

Technology has the potential to enhance the repertoire of tools for family caregiving to address the complexities of caring for older adults. There are examples of technology-enabled interventions helping older adults remain independent and safe in their home; easing the financial, physical, and psychological challenges of family caregiving; assisting in the management of chronic illness; improving socialization and support; offering information and resources on a "just in time" basis; and improving the quality of care and quality of life for both older adults and their family caregivers. This session will review eight evidence-based, technology-enabled solutions for family caregivers, including technology solutions that address medication adherence, falls prevention, personal emergency response, remote monitoring, telehealth, dementia tracking, social engagement, and care training. Key drivers for successful application of these interventions (e.g., technology, analytics, user experience design) as well as barriers to scaling (e.g., accessibility, affordability, regulation) will be reviewed.

### SESSION 2300 (SYMPOSIUM)

### SUCCESSES IN THE TRANSLATION OF CAREGIVER INTERVENTIONS IN COMMUNITY-BASED ORGANIZATIONS AND HEALTH CARE SYSTEMS

Chair: Jinmyoung Cho, *Baylor Scott & White Health, Temple, Texas, United States*
Discussant: Alan B. Stevens, *Baylor Scott & White Health, Temple, Texas, United States*

Family caregiving of an older adult has become an essential element of the U.S. health care system, with 83 percent of long-term care provided to older adults coming from family members or other unpaid helpers. With the amount and type of care provided by families expected to increase, caregiving demands should be coupled with community and health care systems-based supports. While scientific research has demonstrated the value of providing education, skills training and support to family caregivers, health care and social service providers do not systematically include these interventions in their services. Thus, for the vast majority of family caregivers, caregiving support services remain extremely fragmented, if not elusive. This symposium provides four examples of how health care systems that frequently see patients with dementia and community-based organizations who provide ongoing supportive services to family caregivers, have adapted evidence-based caregiver interventions into branded service programs. Dr. Jinmyoung Cho will present racial/ethnic comparisons on the impact of community-based implementation of a caregiver education program, REACH-TX. Dr. Leah Hanson will introduce the implementation of Mindfulness-Based Dementia-Care (MBDC) within a health care system. Dr. Christine Jensen will address how caregivers can benefit from evidence-driven programs in health care settings. Lastly, Dr. David Coon will present two different approaches to translation of evidence-based programs through community-based organizations, with CarePRO embedded after completion of a clinical trial and EPIC embedded from the program's initial pilot phase. The discussant, Dr. Alan Stevens, will highlight the needs of caregivers and support services recognized by all key stakeholders.

### COMMUNITY-BASED IMPLEMENTATION OF A CAREGIVER EDUCATION PROGRAM, REACH-TX: RACIAL-ETHNIC GROUP COMPARISON

Jinmyoung Cho,[1] Donald R. Smith,[2] and Alan B. Stevens[3], *1. Baylor Scott & White Health, Temple, Texas, United States, 2. Area Agency on Aging/Community Investment United Way of Tarrant County, Fort Worth, Texas, United States, 3. Baylor Scott & White Health, Temple, Texas, United States*

We present the effect of racial/ethnic group difference on the impact of REACH TX on measures of quality of life as implemented by the Alzheimer's Association North Central Texas Chapter. Five dimensions of quality of life (burden, depression, social support, self-care, and problem behaviors) were assessed at baseline and 6-month follow-up among three racial/ethnic groups of caregivers (White: 1,050; African American: 269; Hispanic: 176). Generalized estimating equations (GEEs) were used to assess racial/ethnic differences in the changes of quality of life after adjusting covariates. Significant interaction effects between racial/ethnic group and time (from baseline to follow-up) were found in burden, depression, and social support. White and Hispanic caregivers showed significant improvements, while the improvement among African American Caregivers was not statistically significant. The disparity in outcomes among diverse racial/ethnic groups in the program suggests the REACH TX intervention would benefit from tailoring interventions for African American caregivers.

### EVIDENCE-DRIVEN PROGRAMS IN THE HEALTHCARE SETTING: HOW CAREGIVERS CAN BENEFIT

Christine J. Jensen,[1] and Christine J. Jensen[1], *1. Riverside Center for Excellence in Aging and Lifelong Health, Williamsburg, Virginia, United States*

How do family caregivers who interface with the healthcare system benefit from evidence-driven programs designed to support them? Education, training, and care coordination programs have all been found to significantly improve the well- being of caregivers. These programs include Operation Family Caregiver; Caring For You, Caring For Me; BRI Care Consultation; the New York University Caregiver Intervention; and patient and family advisory councils. Healthcare systems that host, translate and scale these programs make them more readily accessible for patients and their families, staff, and the larger community. Further, there is legislation, including the CARE Act, which recognizes the key role a family caregiver provides as a partner in their love one's care. The CARE Act, now enacted in 36 states, states that family caregivers must be identified in the medical record and provided with the necessary resources to be able to care for their loved one after a hospital stay.

### IMPLEMENTATION OF A MINDFULNESS BASED DEMENTIA-CARE PROGRAM WITHIN A HEALTHCARE SYSTEM

Leah Hanson,[1] Bhavani Kashyap,[1] and Michelle P. Barclay[2], *1. HealthPartners Institute, St. Paul, Minnesota, United States, 2. The Barclay Group, LLC, Minneapolis, Minnesota, United States*

The stress associated with caring for a loved one with Alzheimer's disease or a related dementia can negatively affect mental and physical health. Mindfulness-based stress reduction (MBSR), teaching caregivers to focus on the present moment with non-judgmental awareness, has been shown to improve overall mental health, reduce perceived stress, and decrease depression. At HealthPartners, we implemented the Mindfulness Based Dementia-Care (MBDC) program, an 8-week program designed for family caregivers for those living with dementia. MBDC combines teaching of MBSR skills along with essential education for this chronic disease. Participants attend a 1-hour orientation session, 2-hour classes each week for 8 weeks, at-home practice between classes, and a 6-hour Saturday retreat. Secure electronic surveys are administered at baseline, program completion and follow-up for evaluation of the program. We will discuss barriers and learnings of implementation as well as preliminary results from the evaluation.

### TWO TALES OF TRANSLATION: EVIDENCE-BASED CAREGIVER PROGRAMS THROUGH COMMUNITY-BASED ORGANIZATIONS

DAVID Coon,[1] and Carol Whitlatch[2], *1. Arizona State University, Phoenix, Arizona, United States, 2. Benjamin Rose Institute, Cleveland, Ohio, United States*

This presentation reports findings from on different translation approaches that embedded into the community two evidence-based interventions for family caregivers of people with dementia. CarePRO was embedded into the community after completion of a clinical trial, whereas EPIC was embedded from the program's inception. CarePRO is a group and telephone coach call intervention targeting family caregivers and EPIC is a group dyadic intervention for both early-stage people and their care partners. Available in English and Spanish, these programs are still being delivered across two states through community-based organizations. Findings from both CarePRO and EPIC demonstrate significant levels of participant benefit (e.g., reduced negative mood states, negative coping strategies, and negative network interactions as well as increased positive mood states, self-efficacy, care preparedness). Presenters will share lessons learned from the translation process regarding design modifications, training and supervision of interventionists, participant recruitment and retention, and adaptation of outcome assessments.

### SESSION 2305 (SYMPOSIUM)

### THE IMPORTANCE OF THE LONG-TERM CARE WORKFORCE ON QUALITY OUTCOMES FOR INDIVIDUALS RECEIVING SERVICES

Chair: Lindsay Schwartz, *American Health Care Association/National Center for Assisted Living, Washington, District of Columbia, United States*

Co-Chair: Howard Degenholtz, *University of Pittsburgh, Pittsburgh, Pennsylvania, United States*
Discussant: Amy M. York, *Eldercare Workforce Alliance, Washington, District of Columbia, United States*

A strong, supported long-term care (LTC) workforce is vital to quality outcomes of individuals receiving LTC. With the sector facing issues around recruitment and retention, it is important to understand factors impacting the workforce. This symposium includes four presentations, a mix of both quantitative and qualitative research. First, Scales and colleagues will provide an overview of the workforce crisis using extensive policy analysis of home and community-based services (HCBS) in the US. Factors impacting the HCBS direct care workforce (DCW), including training, supply and demand, models of care and compensation, will be addressed. Next, Carder et al. will compare workforce recommendations from the 2003 Assisted Living Workgroup (ALW) report to current regulations. Many states have incorporated recommendations including criminal background checks and training while few have required staff performance evaluations and policies to improve retention. Morgan et al. examine AL residents' care convoys' impact on resident outcomes utilizing data from interviews with AL staff, external health care professionals, residents and family members (n=219). Policies, practices, work overload, time constraints, lack of training and turnover impacted DCW involvement in care convoys. Bender et al. analyze data from 14 DCWs and 16 executive directors from 4 ALs to examine how staff implement and understands end-of-life care policies and procedures. Limited training and communication around death present opportunities for improvement to support DCWs experiencing grief and bereavement. The discussant will address the importance of workforce as part of the network providing quality of care and improving quality of life of individuals receiving LTC.

### HOW STATES CHANGED REGULATORY REQUIREMENTS FOR ASSISTED LIVING STAFF SINCE 2003

Paula Carder,[1] and Sarah Dys[2], *1. Portland State University Institute on Aging, Portland, Oregon, United States, 2. Portland State University, Portland, Oregon, United States*

In 2003, the Assisted Living Workgroup (ALW) published quality improvement recommendations for states' regulations, including 26 regarding staffing/workforce. We reviewed states' 2003 and current regulations to identify the presence of ALW standards. Over half of states' regulations reflect 7 of the 26 staffing/workforce recommendations. Those most often added after 2003 concern criminal background checks, with a 58.8 percent increase in states that added federal background checks and use of criminal background checks to inform hiring. At least 40 states' regulations reflect the ALW recommendations for administrator and direct care staff training. Very few states require staff performance evaluations (n=13), human resource policies to improve retention (n=1), or management practices to improve retention (0). The 10 ALW recommendations concerning staff who administer medications have been adopted by fewer than 23 states. These findings can inform future policy analysis and research on staffing/workforce in assisted living communities.

### HOME AND COMMUNITY-BASED CARE FOR A DIVERSE AND GROWING POPULATION: WORKFORCE POLICY AND PRACTICE IMPLICATIONS

Kezia Scales,[1] Jodi M. Sturgeon,[1] Lisa I. Iezzoni,[2] Robert Espinoza,[1] Stephen Campbell,[3] Allison Cook,[1] and Naomi I. Gallopyn[2], *1. PHI, Bronx, New York, United States, 2. Health Policy Research Center, Mongan Institute, Massachusetts General Hospital, Boston, Massachusetts, United States, 3. PHI (Paraprofessional Healthcare Institute), Bronx, New York, United States*

Most Americans would prefer to continue living in their homes and communities as they age, even when they require support with daily activities due to illness or disability. Much of this support is provided by unpaid caregivers, but the paid home care workforce also plays an essential role. Due to demographic changes and poor job quality, however, the home and community-based services (HCBS) sector is struggling to attract and retain enough workers to meet demand. Drawing from an extensive analysis of HCBS in the United States, this paper examines key factors impacting the home care workforce, including: supply and demand trends; financing policies; service-delivery models; and policies and practices defining workers' compensation, training, and career development. From these findings, we provide recommendations for addressing the home care workforce crisis and maximizing home care workers' contribution to the delivery of high-quality supports for a growing and evolving population of HCBS consumers.

### WE'RE NEVER TALKING ABOUT IT: POLICY AND PRACTICE ABOUT END OF LIFE IN ASSISTED LIVING

Alexis A. Bender,[1] Ann E. Vandenberg,[1] Candace L. Kemp,[2] Elisabeth O. Burgess,[2] and Molly M. Perkins[1], *1. Emory University, Atlanta, Georgia, United States, 2. Georgia State University, Atlanta, Georgia, United States*

Increasing numbers of people are aging in place and dying in assisted living (AL) and AL is increasingly becoming a site of end-of-life care in the US. Consequently, AL staff frequently experience death and decline of the residents they care for. Using data from ethnographic observations and in-depth qualitative interviews with 14 direct care workers and 16 administrators participating in a 5-year NIA-funded study (R01AG047048) examining end-of-life care in four diverse ALs, we used thematic analysis to examine how AL administrators and staff implement and understand policies and norms around death and dying (e.g., training or use of advance care directives). We found there is limited training regarding death and bereavement and limited formal grief support for staff. These findings identify gaps in communication between administrators and direct care workers with implications for increasing communication about death and dying and improving support for direct care workers experiencing grief and bereavement.

### BUILDING COHESIVE CONVOYS: IMPROVING STABILITY AND PREPAREDNESS OF DIRECT CARE WORKERS IN ASSISTED LIVING

Jennifer C. Morgan,[1] Elisabeth Burgess,[1] Christina Barmon,[2] and Candace L. Kemp[1], *1. Georgia State University, Atlanta, Georgia, United States, 2. Central Connecticut State University, New Britain, Connecticut, United States*

Person-centered collaborative care arrangements that empower residents, families, and care partners require supports for resident and their "care convoys"—the evolving collection of individuals who provide formal and informal care. Direct care workers (DCWs) are essential in supporting resident needs within the complex and dynamic environment of assisted living. The stability and preparedness of this workforce is central to improving quality of life for residents. This paper identifies key factors influencing the integration of DCWs in the convoys and explore supportive employment practices to strengthen the convoy. This analysis uses data from a 5-year mixed-method qualitative study of eight assisted living communities. Time pressures, AL policies and practices, work overload, lack of training, and turnover impact whether direct care workers are empowered as full members of the care convoy. Supportive employment practices (e.g. training, onboarding, career opportunities, rewards and recognition) are discussed as potential solutions for building consensus and collaboration.

### SESSION 2310 (PAPER)

### USE OF TECHNOLOGY AND INTERVENTIONS AS RELATED TO HEALTH BEHAVIORS

#### A VOICE-INTERACTION-MEDIATED SMART HOME TO SUPPORT AGING IN PLACE: A NEW PARADIGM TO ENGAGE WITH HEALTH-RELATED DATA

Yong K. Choi,[1] Hilaire J. Thompson,[2] and George Demiris[3], *1. University of California Davis, Sacramento, California, United States, 2. University of Washington, Seattle, Washington, United States, 3. University of Pennsylvania, Philadelphia, Pennsylvania, United States*

Current tools for health management such as personal health records (PHR), mobile applications, and wearable devices rely on conventional interfaces such as keyboard and mouse with screens for reading information. More recently, Internet-of-things (IoT) voice-activated smart speakers and embedded Artificial Intelligence (AI) assistants have emerged that may provide an effective user interaction platform to support healthy aging, creating a hands-free, conversational way to access and share health related data. We conducted an exploratory study with nineteen older adults (65+) who chose to evaluate a smart speaker for a 2-month as part of a larger IoT feasibility study. Three interview sessions were conducted to gather attitudes towards this technology. Participants provided feedback on future improvements or desirable features for health maintenance. A content analysis was performed to extract common themes. In general, participants expressed a positive experience with the voice interface and discussed its potential as an integral tool for their home health management. Based on these findings, we propose a voice interaction smart home platform to support healthy aging. This platform provides an intuitive voice user interface to execute commands to access, record, and query data from IoT sensors, a PHR and a hospital EHR. For example, the voice interaction platform will help individuals to dynamically explore their own health data thus eliminating manual and tedious searching of PHR data. We demonstrate how this platform can potentially improve usability issues including difficulties navigating charts and entering or retrieving health data using conventional interfaces. Finally, we highlight ethical and technical considerations.

#### BETTER LIVING THROUGH TECH: TECHNOLOGY-MEDIATED RECREATION AND LONG-TERM CARE FACILITY RESIDENTS' QUALITY OF LIFE

Ethan A. McMahan,[1] Marion Godoy,[2] Abiola Awosanya,[3] Robert Winningham,[1] Charles De Vilmorin,[4] and Meaghan McMahon[5], *1. Western Oregon University, Monmouth, Oregon, United States, 2. Responsive Health Management Inc., Toronto, Ontario, Canada, 3. Cedarvale Long Term Care Home, Toronto, Ontario, Canada, 4. Linked Senior, Washington, D.C., United States, 5. MBM Consulting, Washington, D.C., United States*

Empirical research on long-term care facility resident engagement has consistently indicated that increased engagement is associated with more positive clinical outcomes and increased quality of life. The current study adds to this existing literature by documenting the positive effects of technologically-mediated recreational programing on quality of life and medication usage in aged residents living in long-term care facilities. Technologically-mediated recreational programming was defined as recreational programming that was developed, implemented, and /or monitored using software platforms dedicated specifically for these types of activities. This study utilized a longitudinal design and was part of a larger project examining quality of life in older adults. A sample of 272 residents from three long-term care facilities in Toronto, Ontario participated in this project. Resident quality of life was assessed at multiple time points across a span of approximately 12 months, and resident engagement in recreational programming was monitored continuously during this twelve-month period. Quality of life was measured using the Resident Assessment Instrument Minimum Data Set Version 2.0. Number of pharmacological medication prescriptions received during the twelve-month study period was also assessed. Descriptive analyses indicated that, in general, resident functioning tended to decrease over time. However, when controlling for age, gender, and baseline measures of resident functioning, engagement in technologically-mediated recreational programming was positively associated with several indicators of quality of life. The current findings thus indicate that engagement in technology-mediated recreational programming is associated with increased quality of life of residents in long-term care facilities.

#### DIGITAL INDEPENDENCE: HEALTH-RELATED DIFFICULTY IN INTERNET USE AND HOW IT AFFECTS OLDER ADULTS' QUALITY OF LIFE

Shannon Ang,[1] Emily Lim,[2] and Rahul Malhotra[3], *1. University of Michigan, Michigan, United States, 2. University of Massachusetts Boston, Boston, Massachusetts, United States, 3. Duke-NUS Medical School, Singapore, Singapore, Singapore*

Using the internet is increasingly a necessity. However, older adults may not do so due to either non-health reasons (e.g., lack of digital literacy or internet access) or health-related reasons. While researchers have studied internet use among older adults, most do not discriminate whether non-use is due to health reasons or otherwise. Recent studies also reveal that older adults use the internet to keep in touch with family and friends, highlighting that limitations in internet use may be detrimental for their well-being. We therefore, examine the key correlates of health-related difficulty in internet use, and how it may affect quality of life by reducing the size of their social support networks. Data were from a national survey of older Singaporeans (n=3966) conducted in 2016-17. Multinomial logistic regression and mediation analysis were used to identify older adult subgroups more likely to experience health-related difficulty in internet use, and whether such difficulty affected older adults' quality of life through their social support networks. Results showed that males, those of Malay ethnicity, those with less education, and those with more instrumental activity of daily living (IADL) limitations were more likely to experience health-related difficulty in internet use. Social support networks mediated the relationship between health-related difficulty in internet use and quality of life. These findings suggest that other than managing the health conditions of older adults who face health-related difficulty in internet use, offline modes of keeping them socially connected may promote their quality of life.

### EXPLORING THE POWER OF COMBINATORIAL HEALTH TECHNOLOGIES TO SUPPORT SELF-MANAGEMENT OF COPD AMONG OLDER PEOPLE

Sandra Varey,[1] Mandy Dixon,[1] Alejandra Hernandez,[1] Ceu Mateus,[1] Tom Palmer,[1] and Christine Milligan[1], *1. Lancaster University, Lancaster, United Kingdom*

Ways to address the increasing healthcare needs of older people are a priority for the National Health Service (NHS) in England. The NHS England Test Bed programme was designed to trial new models of care that are supported by digital health technologies. This paper reports on findings from one Test Bed programme, the Lancashire and Cumbria Innovation Alliance (LCIA) – a partnership between NHS England, industry and Lancaster University, which ran from 2016 to 2018. A key aim of the LCIA Test Bed was to explore the extent to which supported self-care telehealth technology helped older people with long-term conditions to better self-manage their own care, promoting independence and enabling them to remain at home for longer. Each patient received a combination of health technologies over a six-month period. This paper presents results from the qualitative data that formed part of a large-scale mixed-methods evaluation. Specifically it draws on the analysis of 34 observational interviews with 17 participants with chronic obstructive pulmonary disease (COPD) to understand the role of these technologies in the self-management of their care. The data revealed that the majority of participants felt more confident about self-managing COPD as a result of their participation in the programme. These increases in confidence were the result of participants' increased knowledge and skills in managing their COPD. The paper demonstrates how patients learned to better manage their respiratory condition, the impact of this learning on their daily lives and that of their family carers, and the implications for healthcare practice.

### INTERVENTION DEVELOPMENT: PARTICIPANT EXPERIENCES WITH NOVEL TECHNOLOGICAL DEPRESSION TREATMENTS

Avani A. Shah,[1] Martin Morthland,[1] Forrest Scogin,[1] and Andrew Presnell[1], *1. University of Alabama, Tuscaloosa, Alabama, United States*

The purpose of this presentation is to discuss the development process of two novel technology-based interventions for depression in older adults while comparing older adults' preferences for audio-based and computer-based cognitive behavioral therapy for depressive symptoms. The audio program consisted of eight compact discs and a workbook while the computer program consisted of 11 modules of similar duration provided on a tablet PC. Fifty-one older adults were recruited from medical settings and rural communities and randomly assigned to an immediate treatment group (computer or audio) with minimal contact or a four-week minimal contact delayed treatment control condition. Participants rated computer-based and audio-based cognitive behavioral therapy fairly equally, with 75% of those who received audio treatment and 85% of those who received computer-based treatment indicating benefits to their mood. Qualitative experiences will be presented by themes: challenges, benefits, recommended improvements, and technological issues. The goal of the presentation is to share information that may help clinicians and researchers develop other mental health technological interventions tailored for older adults needs.

### SESSION 2313 (SYMPOSIUM)

### EAST MEETS WEST: BUILDING DEMENTIA-FRIENDLY COMMUNITIES: REFLECTIONS ON GLOBAL EXPERIENCES

Chair: Fei Sun, *Michigan State University, East Lansing, Michigan, United States*
Discussant: Nancy Hooyman, *University of Washington, Seattle, United States*

The emergence and rapid development of dementia friendly initiatives (DFIs) represents growing global awareness of needs of persons living with dementia (PWD). Policy and practice efforts across regions and countries made to make physical and social environments more friendly, inclusive, and capable for PWD vary. When designing, implementing and evaluating DFIs, countries at different socioeconomic development stages need to set priorities based upon their local needs and cultural norms. This East Meets West symposium aims to understand the variability and progress of the DFI in the global context, highlighting experiences from two countries: U.S.A. and China, where about one third of the world's total estimated 47 million PWD live. The first study from Shanghai emphasizes efforts by local health care professionals to promote dementia screening and improve diagnosis, with the ultimate goal to improve dementia literacy and build a dementia friendly city. The second paper describes a community-based participatory approach to develop dementia friendly communities in Beijing, highlighting the significance of non-governmental and governmental collaboration. The DFI in Florida underscores the partnership among multiple sectors with an emphasis of the involvement of PWD, while the DFI in rural Michigan stresses care system coordination. The last paper based upon an online survey of DFI stakeholders from six WHO regions echoes successful features of DFIs (i.e., involvement of PWD, multi-sectorial partnership) identified in Chinese and U.S. examples, and identifies variations in strategies used to modify physical and social environments across countries. Invited panelists from different sectors will share comments in the end.

### BUILDING A CONTINUOUS DEMENTIA MANAGEMENT MODEL IN COMMUNITIES OF SHANGHAI

Xia Li,[1] Qi Qiu,[2] Yinghua Yang,[2] Ling Sun,[2] MinJun Jiang,[2] chunling Gu,[2] Ming Cui,[2] and Xiang Lin[2], *1. Shanghai Mental Health Center, Shanghai, Shanghai, China, 2. Shanghai Mental Health Center, Shanghai, Shanghai, China (People's Republic)*

Over 10 million people with Alzheimer's disease or related dementias (ADRD) live in China. In Shanghai, the prevalence of ADRD is about 3-4% among aged 60 or older, and approximately 70-85% have never been diagnosed. This study reported the pilot testing results of a dementia management model launched by Shanghai Mental Health Center to build dementia friendly communities. The dementia management model links screening, diagnosis, care planning, treatment, and services, to improve dementia literacy and standard diagnosis rate. About 3,786 senior residents were screened using the AD 8 and MoCA scales. The cognitively intact group was suggested for annual check-up, while at -risk groups were referred to formal diagnosis and intervention. About 125 older adults with a mild cognitive impairment diagnosis were provided referrals for cognitive training, and 109 diagnosed with dementia were provided medical and social interventions. This management model adds to dementia awareness and education.

### INVOLVING COMMUNITY GOVERNMENTAL ORGANIZATIONS IN THE ESTABLISHMENT OF DEMENTIA-FRIENDLY COMMUNITIES IN BEIJING

Yu Liu,[1] Hexin Zhao,[2] and Hong Guo[3], *1. China Medical University, Shenyang, Liaoning, China, 2. Beijing Hospital, beijing, Beijing, China (People's Republic), 3. Beijing Traditional Chinese Medicine University School of Nursing, Beijing, Beijing, China (People's Republic)*

China has about 20% of the world's total dementia population. Since most elders with dementia are living at home with care by family members, communities are fundamental support resources for families as well as patients. Dementia-friendly community initiatives aim to empower families with dementia and increase their social inclusion. Within the Chinese political context, the community level governmental organization called Ju-wei-hui has played a key role in community engagement. Within this context, a Community Based Participatory Action Research (CBPAR) process is utilized to increase public awareness on dementia and caring strategies. Our team collaborated with 15 Ju-wei-hui offices across Beijing to design a series of courses and teaching modules together. Five hundred community residents participated and positively evaluated the project. A major finding is that CBPAR could be an effective strategy to develop dementia-friendly communities across China.

### CREATING DEMENTIA-FRIENDLY COMMUNITIES IN CENTRAL FLORIDA IN THE UNITED STATES

Tracy Wharton,[1] Daniel Paulson,[2] and Courtney Wagner[2], *1. Central Florida University, Orlando, Florida, United States, 2. University of Central Florida, Orlando, Florida, United States*

The Dementia Care & Cure Initiative in Florida is a statewide movement to advance dementia friendly communities. With 25% of the state over the age of 65, Florida has one of the highest rates of dementia in the nation. The taskforce based in Orlando involves a partnership of representatives from social service agencies, law enforcement, healthcare providers, and research partners, as well as consumers. The task force commissioned a series of five focus groups with 43 consumers. These focus groups produced short and long-term recommendations, identifying such issues as needed training for emergency personnel and law enforcement, improving inter-provider communication, and providing culturally competent programming for a diverse region. The taskforce has been planning with the Mayor's office and law enforcement to initiate training and support for community engagement, and planning for implementation of these goals. Recommendations from the groups and from the taskforce to community leaders will be discussed.

### BUILDING DEMENTIA-FRIENDLY COMMUNITY IN MICHIGAN: THE EXPERIENCES OF BATTLE CREEK

Karla Fales[1], *1. Region 3B Area Agency on Aging, Battle Creek, Michigan, United States*

Alzheimer's disease is now the 6th leading cause of death in Calhoun County – a mostly rural county of 156,000 people in southwest Michigan. In 2017, a cross-sector collaborative launched Dementia Friendly Battle Creek led by CareWell Services Area Agency on Aging. This study focuses on evaluation of the progress and outcomes of this initiative. The goal is to create a community that features high awareness of dementia, coordinated systems of care, and an environment that promotes dignity and engagement of PWD. Using participatory community planning, three Pillars of Impact were developed: Awareness, Coordination, and Environment. More than 500 people have engaged in the effort through trainings, educational programs, expanded supportive services, and the creation of integrated pathways of care for PWD. Additionally, more than $100,000 has been leveraged to support the effort. Challenges included sustaining momentum & gaining buy-in from local municipalities and health care providers.

### DEVELOPING A DEMENTIA-FRIENDLY COMMUNITY GLOBAL TOOLKIT: INSIGHTS FROM STAKEHOLDERS IN WHO MEMBER COUNTRIES

Fei Sun,[1] Emmanuel Chima,[1] Katrin Seeher,[2] Tarun Dua,[2] and Devora Kestal[2], *1. Michigan State University, East Lansing, Michigan, United States, 2. World Health Organization, Genève, Geneve, Switzerland*

Drawing on perspectives from stakeholders involved in dementia friendly initiatives (DFIs) in WHO member countries, this paper describes the characteristics of DFIs around the world and summarizes success factors and barriers to implementation. Data were collected through an online consultation survey of 129 stakeholders from 46 countries in all six regions of WHO. Most DFIs present three essential features of WHO's definition for DFI, that is, centering on the needs of persons living with dementia (PWD), multi-sector collaboration, and physical and social environmental changes. Over 70% participants reported their DFIs targeted PWD and included PWD as important partners. High-income countries tend to focus on enhancing professional capacity and environmental adaptation, while low-middle income countries prioritize dementia awareness campaigns. This corresponds to the reported disparities in levels of inclusion of PWD in societies, support to PWD, and service access for PWD found between low-middle income countries and high-income countries.

### SESSION 2315 (POSTER)

### ACUTE CARE AND HOSPITALIZATION

### GERIATRIC MEASURES AS PREDICTORS OF 1-YEAR MORTALITY IN MAJOR SURGERY PATIENTS

Victoria L. Tang,[1] Kenneth Covinsky,[1] Emily Finlayson,[1] Bocheng Jing,[1] John Boscardin,[1] and Sarah Ngo[1], *1. University of California San Francisco, San Francisco, California, United States*

A growing proportion of older adults are undergoing major surgery despite the higher risk of post-operative mortality. Geriatric measures (i.e. physical, cognitive, and psychosocial function) are often not included in studies evaluating post-operative outcomes in older adults. Our goal was to determine the association of geriatric measures and 1-year mortality in older adults after major surgery. We analyzed longitudinal data from the Health and Retirement Study linked to Medicare claims (N=1364 participants), age ≥ 65 and who underwent abdominal aortic aneurysm [AAA] repair, coronary artery bypass graft [CABG], or colectomy. Our outcome was mortality within 1 year of the major operation. Predictors included the following geriatric measures: dependence in activities of daily living (ADL), dependence in independent activities of daily living (IADL), mobility ability, and dementia, and depression. We analyzed using multivariate cox proportional hazard models. Mean participant age was 76±6 years, 56% were women, 11% underwent a AAA repair, 50% CABG, 40% colectomy; 18% died within 1 year of their major operation. After adjusting for age, comorbidity burden, surgical type, gender, race, wealth, income, and education, the following measures were significantly associated with 1-year mortality: depression (adjusted HR (aHR): 1.53, p=0.03), dementia (aHR: 1.90, p=0.03), >1 ADL dependence (aHR: 2.35, p<0.01), >1 IADL dependence (aHR: 1.95, p<0.01), and inability to walk several blocks (aHR: 1.69, p<0.01). In this cohort, 18% of participants who underwent major surgery died within 1 year and function, cognition, and psychological well-being were significantly associated with mortality. These measures should be incorporated into pre-operative assessment.

### WORKING BACK TO NORMAL FUNCTION FOLLOWING HOSPITALIZATION: A GROUNDED THEORY STUDY

Daniel Liebzeit,[1] Lisa Bratzke,[1] and BARBARA KING[2], *1. University of Wisconsin-Madison School of Nursing, Madison, Wisconsin, United States, 2. University of Wisconsin-Madison, Madison, Wisconsin, United States*

Transitions older adults experience post hospital discharge have primarily focused on the process of moving care from one setting to another (e.g. hospital to home). Older adults often experience a significant transition in terms of losing functional status after a hospital stay. Little is known about how older adults regain their functional ability, the type of work they engage in to recover, and conditions that influence their ability to work after a hospital stay. The objective of this Grounded Theory study was to understand strategies older adults use post discharge as they work to regain their functional status and what conditions facilitate or limit their ability to work toward returning to normal. A qualitative study was conducted. Adults aged 65 and older discharged from a large Midwestern teaching hospital (N = 14) were interviewed using in-depth one-on-one interviews. Data were analyzed using open, axial, and selective coding. Participants described key strategies they employed to regain their normal function following hospitalization and illness: doing exercises, expanding physical space, resuming prior activities and daily cares, and tracking improvement with benchmarks. Several conditions such as, presence of informal (family, friends) and formal (healthcare providers) support, perceived threats (relocation), and having poor physical or physiologic function, acted as barriers and facilitators to participants ability to work back to normal function. This study provides empirical data on work older adults engage in to transition back to normal function during the post discharge period. It presents opportunities for better supporting their work of regaining function.

### PAIN, PAIN MANAGEMENT, AND CONSEQUENCES OF PAIN AMONG HOSPITALIZED PERSONS WITH DEMENTIA

Rachel Arendacs,[1] Marie Boltz,[2] Ashley Kuzmik,[3] and Barbara Resnick[4], *1. Penn State University College of Nursing, State College, Pennsylvania, United States, 2. Pennsylvania State University, University Park, Pennsylvania, United States, 3. Penn State College of Nursing, State College, Pennsylvania, United States, 4. University of Maryland School of Nursing, Baltimore, Maryland, United States*

The purpose of this study was to describe the incidence, management and impact of pain on behavior and delirium in hospitalized older adults living with dementia. This was a descriptive study using baseline data from patients in the first cohort of the Fam-FFC study which evaluates the impact of a family-engaged function-focused care intervention in hospitalized patients with dementia. The majority of the sample was female (70%) and black (80%) with a mean age of 82.5 (SD=8.9). Pain (PAINAD) was reported by 36% of the patients; 42% of those demonstrating pain were prescribed analgesics. Controlling for age, gender and baseline cognition, pain was significantly associated with behavioral and psychiatric symptoms of dementia ( t =2.1, p= .034) and delirium severity (t = 4.9, p<.0001). Results suggest the need for pain assessment and individualized treatment plans to promote comfort and decrease behavioral and delirium symptoms in hospitalized persons with dementia.

### AGE-FRIENDLY CARE AND MOBILITY IN HOSPITALIZED OLDER ADULTS WITH COGNITIVE IMPAIRMENT

Jasmine K. Vickers,[1] Richard E. Kennedy,[1] Shari Biswal,[2] David James,[2] Katrina Booth,[1] Emily Simmons,[2] and Kellie Flood[1], *1. University of Alabama at Birmingham, Birmingham, Alabama, United States, 2. University of Alabama at Birmingham (UAB) Hospital, Birmingham, Alabama, United States*

Hospitalization of older adults with cognitive impairment (CI) has been associated with higher risk for adverse outcomes. Acute Care for Elders (ACE) Units were developed to meet the unique hospital care needs of older adults and have been associated with reductions in functional decline and readmissions. The Virtual ACE intervention was developed to disseminate ACE principles across hospital units. Virtual ACE included training interprofessional providers to utilize screens and care protocols to optimize care for older adults on eight units at a large academic medical center. We conducted a preliminary analysis of mobility and patient outcomes before and after Virtual ACE among 192 older adults with CI on hospital admission. Chi-Square tests were used to examine the associations between Virtual ACE and patient outcomes. There were statistically significant pre vs. post improvements in patients' mobility from bed to chair (30% vs. 51%, p=0.011) and on the unit hallway (12% vs. 27%, p=0.046). Although not statistically significant, there were also improvements in hospital room mobility (39% vs. 50%, p=0.214) and documentation of activities of daily living (ADL) screens (70% vs. 80%, p=0.196). There were non-significant reductions in pressure ulcer prevalence (26% vs. 22%) and restraint use (5% vs. 0%) during the hospital stay. Pain was similar before and after Virtual ACE. Virtual ACE was associated with increased mobility and slight reductions in adverse outcomes. As increased hospital mobility improves patient functioning post-discharge, Virtual ACE has the potential to maintain function and enhance outcomes in hospitalized older adults with CI.

### MULTISENSORY STIMULATION FOR HOSPITALIZED PATIENTS WITH DEMENTIA: A PILOT STUDY

Sheila L. Molony,[1] and Christine Waszynski[2], *1. Quinnipiac University, Hamden, Connecticut, United States, 2. Hartford Hospital, Hartford, Connecticut, United States*

Introduction: Delirium occurs in up to 50% of hospitalized patients and the risk is higher in persons with dementia. Multi-sensory stimulation environments (MSSE), including trademarked "Snoezelen" rooms, have been effective in achieving positive outcomes in persons with dementia, but there have been no studies in the acute-care setting. Purpose: This pilot study tested the effect of a therapeutic Multisensory Stimulation Environment known as "the Hub" in an acute-care hospital. Methods: A sample of 56 patients were randomized to receive usual care or the Therapeutic Hub intervention during hospital days 2-4. Hub activities were multi-sensory and tailored based on preferences and abilities. We will describe techniques to address methodological challenges in the study with acutely ill, cognitively vulnerable participants. We will also present qualitative data describing the experience of participants receiving the Hub intervention, and will present preliminary findings regarding between group-differences in function (Functional Independence Measure), mobility, falls, wellbeing (Warwick-Edinburgh Mental Wellbeing Scale)– and person-environment relationship conceptualized as situational at-homeness (S-EOH). Conclusion: The results of this study will inform future trials on the effects of unique therapeutic environments for hospitalized persons at highest risk for delirium.

### VISIBILITY OF PROGRAMS AND SERVICES ON HOSPITAL WEBSITES: GERIATRICS COMPARED WITH OBSTETRICS

Brenda P. Johnson,[1] MyoungJin Kim,[1] Emily E. McMahon,[1] and Ali Mojadam[1], *1. Illinois State University, Normal, Illinois, United States*

Hospitals increasingly use a digital platform to market their services as well as reflect the values and mission of the organization. As payment incentives and national initiatives grow for making hospitals more "age friendly" in both the quality and extent of specialized services for older adults, the visibility of these services on a digital platform is of interest. This exploratory, descriptive analysis of hospital websites was to illuminate the ease with which a consumer could identify that a hospital has specialized services in geriatrics as compared with obstetrics. A proportionate stratified random sampling based on the five geographical regions of the country was used to select 220 hospitals with 5% margin of error, 95% confidence interval and 50% sample proportion with no prior information from a population of over 600 hospitals known to have implemented at least one quality initiative specific to older adults (NICHE). A binary matrix was developed for collecting data related to the visibility of services in three main areas of hospital websites: 1) images and terms on the homepage 2) identification of programs in the "Services" function and 3) availability of the specialty in the "Search for Doctor" function. Data show that the frequency of all key words used to identify obstetrical services was greater than those used to describe geriatric services across all functional areas explored in this study. Implications are that hospital websites may be an underutilized resource for marketing services as well as accurately reflecting specialized services for the older adult population.

### SYMPTOM EXPERIENCES OF HOSPITALIZED OLDER ADULTS ON ADMISSION AND FUNCTIONAL OUTCOMES 1 MONTH POST-DISCHARGE

Anna Zisberg,[1] Ksenya Shulyaev,[2] Anat Liberty,[3] Nurit Gur-Yash,[4] Maayan Agmon,[2] and Dorit Pud[2], *1. The Cheryl Spencer Department of Nursing Faculty of Social Welfare and Health Science, University of Haifa, Israel, Haifa, Isreal, Israel, 2. The Cheryl Spencer Department of Nursing Faculty of Social Welfare and Health Science, University of Haifa, Mount Carmel, Israel, Haifa, Israel, Israel, 3. HaEmek Medical Center, Clalit Health Services, Afula, Israel, Afula, Israel, Israel, 4. Oranim, College of Educational, Israel., Oranim, Israel*

The aims of this study were to identify subgroups of older adults admitted to hospital based on their experience with multiple symptoms and to explore if these subgroups differed on physical and cognitive characteristics at time of admission, and on functional outcomes one-month post-discharge. The study included 331 older adults (mean age 75.5±7.1), admitted to hospital and were hospitalized in internal-medicine units. Demographic, functional, cognitive, psychological and mobility characteristics and symptoms' disturbance were assessed within the first 24 hours of admission and one moth following discharge. Cluster analysis identified three distinct subgroups based on patients' experiences with five highly prevalent symptoms (tiredness, dyspnea, dizziness, sleep disturbance and pain): Low or high levels of all five symptoms (70%, 14%, respectively), and moderate levels of four symptoms with high dyspnea (14%). "All high" cluster was characterized by the worst cognitive and instrumental function, and highest anxiety and depression levels. The "moderate with high dyspnea" subgroup expressed the highest comorbidity score. Multivariate Logistic regression showed that the odds of decline in instrumental ADL one month post-discharge was 3.28 (95% CI 3.21-3.25, p=.021) for "all high" and 2.35 (95% CI 2.33-2.36, p=.043) for "all low" symptom-subgroups compared to "medium with high dyspnea" subgroup adjusted for pre-morbid function, health conditions and demographic characteristics. Belonging to certain symptom-subgroups is an important risk factor in predicting negative consequences of hospitalization. These findings emphasize the importance of evaluating and subgrouping broad range of symptoms among hospitalized older adults.

### CHARACTERISTICS OF HOSPITALIZED OLDER ADULTS WITH RECURRENT CLOSTRIDIUM DIFFICILE: INTERVENTION OPPORTUNITIES

Anna Boone,[1] Laurie Kennedy-Malone,[2] Thomas McCoy,[2] Deborah A. Lekan,[3] Debra Wallace,[2] and Robert Rourk[1], *1. Cone Health, Reidsville, North Carolina, United States, 2. University of North Carolina at Greensboro, Greensboro, North Carolina, United States, 3. UNC-Greensboro School of Nursing, Greensborol, North Carolina, United States*

Clostridium difficile infection (CDI) is the leading cause for gastroenteritis-associated deaths (Hall et al., 2012). Risk factors include advanced age, antibiotic use, and hospital admission, yet multiple others are not widely known. CDI recurrence risk may be as high as 40% after initial treatment (Garey et al., 2008; Kelly & Lamont, 2008). Older adults may present atypically, and treatment guidelines for initial and recurrent CDI have evolved from older standards. A retrospective cohort research study explored characteristics of hospitalized adults ages 55 and older with CDI between December 31, 2013 through December 31, 2015, identified by ICD diagnosis codes. Recurrence within one year, laboratory measurements, chronic diseases, and psychosocial data were captured from the electronic health record (EHR). Over the study period, 871 patients had a recurrence rate of 23.9% (n=208). Caucasian females comprised over half the sample, and 9.1% expired during initial hospitalization. Almost two-thirds (n=576, 66.1%) lived in private residences prior to admission. CDI recurrence was more prevalent if discharged to skilled nursing facility and home health care services. Hypertension, heart failure, and chronic kidney disease were most prevalent in the recurrent CDI group. Polypharmacy was noted in over two-thirds of sample. A large portion of the sample displayed hypoalbuminemia on admission.Utilizing the EHR to aggregate data promotes interventions to reduce recurrence, prolonged stay, and aggressive treatment per current guidelines. Multidisciplinary approaches include deprescribing, nutritional support, and chronic disease management. Person-centered care and individualized interventions should begin on admission with close outpatient follow-up and hopeful reduced readmissions.

### PROLONGED MECHANICAL VENTILATION: CHARACTERISTICS OF PATIENTS TREATED AT HOME COMPARED TO HOSPITAL LONG-TERM CARE

Jeremy M. Jacobs,[1] Esther-Lee Marcus,[2] and Jochanan Stessman[1], *1. Dept of Geriatrics and Rehabilitation, Jerusalem Institute for Aging Research, Hadassah Hebrew University Hospital, and Clalit Health services, Jerusalem, Israel, 2. Head, Chronic Ventilator-dependent Division, Herzog Hospital, Jerusalem, Israel*

Rising numbers of patients receiving Prolonged Mechanical Ventilation (PMV) pose a challenge, and advancing technology supports ventilators appropriate for either Home or Hospital Long Term Care (HLTC).Data guiding decisions concerning place of care are lacking. This study describes the characteristics of the majority (120/123) of all PMV patients aged ≥18 (and their caregivers) in Jerusalem, covered by the Clalit Health Service, treated either with Home Hospital or HLTC. Patients were more alert and communicative at Home vs. HLTC (40/46 vs. 22/74), younger (54 vs.73 years, p12 years (36% vs.21%, p=0.1), and without legal guardian (59% vs. 12%, p<0.01). Primary reason for PMV at home was degenerative neuromuscular disease (59% vs. 28%), compared to post resuscitation/sepsis/CVA in HLTC patients (17% vs. 62%), who suffered more comorbidity, functional decline post-PMV, and pressure sores (0% vs. 42%). Ventilation was more likely to be planned at home vs HLTC (33% vs. 8%), and yet 119/120 were without Advanced Directives prior to PMV. Caregivers at home tended to be spouses (48% vs. 31%) and offspring at HLTC (17% vs. 47%), with reduced Modified Caregiver Strain Index at home (10.5 vs. 12.9, p=0.12). Mortality during follow-up was lower at home (15.2% vs. 27%). Costs to the health fund for home versus HLTC were approximately 1:3. Our findings suggest that with appropriate targeting of eligible PMV patients, Home Hospital may be the preferred model of care for patients, caregivers and healthcare providers.

### WELL-BEING AND ADVANCE DIRECTIVES AMONG PROLONGED MECHANICAL VENTILATION PATIENTS AT HOME VERSUS HOSPITAL

Jochanan Stessman,[1] Esther-Lee Marcus,[2] and Jeremy M. Jacobs[1], *1. Dept of Geriatrics and Rehabilitation, Jerusalem Institute for Aging Research, Hadassah Hebrew University Hospital, and Clalit Health services, Jerusalem, Israel, 2. Head, Chronic Ventilator-dependent Division, Herzog Hospital, Jerusalem, Israel*

Despite increasing numbers of older patients requiring Prolonged Mechanical Ventilation (PMV), little is known concerning their mood, well-being, distressing symptoms and attitudes towards ventilation. Furthermore differences may exist according to place of care- whether Home Hospital or Hospital Long Term Care (HLTC). These issues were addressed using the revised Edmonton Symptom Assessment System (r-ESAS)(10 items, max severity score=100), and Short Geriatric Depression Scale, in a study of the majority of PMV patients (n=120/123) aged ≥18 (range 18-96 years) all Clalit

Health Service insurees, in Jerusalem. Communicative patients were interviewed (40/46 at home, 22/74 in HLTC, average age 54 vs.73 years, p<0.01). The following symptoms (dichotomized to "not-a-problem"/"problematic") were frequently reported as "not-a-problem" among patients (Home,HLTC): tiredness (59%,58%), poor appetite (95%,90%), pain (69%,84%), drowsiness (77%,90%), nausea (85%,84%), and shortness of breath (82%,90%). General well-being (categorized to good/mild/moderate/severe impairment) was reported more frequently as good or mildly impaired at Home vs. HLTC (54% vs.26%), as was lower frequency of depression (34.4% vs. 44.4%, p=0.049). The total r-ESAS score was similar irrespective of setting: Home-24.9/100 (inter quartile range {IQR} 13.5-32.5) vs. HLTC-23.7/100 (IQR 17.5-32),p=0.74. The majority (119/120) of patients were without advanced directives prior to initiation of PMV. When asked if they had to choose again, 82% and 91% of communicative patients at home vs. HLTC would opt again for ventilation, as would 75% of caregivers of uncommunicative patients. Our findings emphasize the resilience and low levels of distressing symptoms among PMV patients, and may contribute to the decision-making process concerning advanced directives.

### INNOVATIONS IN EMERGENCY MEDICAL CARE: EARLY ADOPTERS OF GERIATRIC EMERGENCY DEPARTMENTS

John G. Schumacher[1], *1. University of Maryland, Baltimore County (UMBC), Baltimore, Maryland, United States*

Hospital emergency departments (EDs) treat more than 20 million older adults each year making it a remarkably significant site of healthcare delivery. To date, the nation's nearly 5,000 EDs have been slow to modify their staffing, ED training, procedures, or physical environment to better serve the unique needs our heterogeneous older adult population. Nonetheless, nationwide a small set of innovative EDs have redesigned their care and now promote themselves as Geriatric Emergency Departments (GEDs) which specifically tailor care to older adults and their families. Using a systematic, nationwide search process of U.S. hospitals, this research identified n=83 EDs which clearly self-identified as GEDs. All eligible GEDs were contacted with n=54 (65%) responding to our self-administered survey regarding their organization, delivery of care, and adherence to national guidelines on the emergency medical care of older adults. Results document a wide variety of care models, staffing patterns, screening procedures, clinical care modifications, quality improvement efforts, physical environment enhancements, referral patterns, and tracking of older patient outcomes. Analysis of open-ended responses demonstrated widely divergent interpretations of the national guidelines on emergency care for older adults including the definition of a GED. Based on the findings, research recommendations are made to researchers regarding the conceptualization and specific wording of future survey items in order to increase the reliability and validity of research into GEDs.

### GERIATRICS MICU CO-MANAGEMENT: INTEGRATING GERIATRIC ASSESSMENTS FOR CRITICALLY ILL PATIENTS

Gowrishankar Gnanasekaran,[1] and Eduardo Mireles-Cabodevila[1], *1. Cleveland Clinic Foundation, Cleveland, Ohio, United States*

Programs like orthogeriatrics, geriatric cardiology have shown to improve outcomes in hospitalized geriatric patients. Our Geriatrics MICU Co-management program is a quality improvement initiative that instigates a partnership approach with critical care medicine in integrating geriatric assessments and build foundation for interdisciplinary care of critically ill patients. MICU (Medical Intensive Care Unit) protocols do not have standard geriatrics assessments integrated in clinical care. An electronic dash-board identifies high risk elderly (HRE) patients admitted at a MICU in a large teaching hospital in Northeast Ohio based on nursing specific screening triggers. A geriatrics co-management team engages in a comprehensive geriatric assessments and care transition. 386 patient were identified using HRE screening triggers in a period of 100 days. 33 % (n=131) were generated as consults for co-management. A pilot review on 131 HRE patients was conducted. 70% (n=93) patients had incident frailty. 93% (n=87) of patients with frailty were diagnosed with incident delirium. 56% (n=74) of patients were newly diagnosed with cognitive impairment. 56 % (N=74) of patients had a medication reduction. An average of 1.23 medication was changed. 85% (n =112) of patients had a warm hand off to the next level of provider on discharge. 90% (n=119) of patients notified improved self-management skills and better understanding of discharge process. The Geri-MICU program demonstrates a patient -centered approach in integrating geriatric assessments for critically ill patients and build foundation of a geriatrics-critical care task force. The program would be a mile stone in optimizing elderly care in critical care units.

### GENERALIZABILITY OF CLINICAL TRIALS OF DELIRIUM INTERVENTIONS

Christina DiBlasio,[1] Mackenzie E. Fowler,[1] Roy Martin,[1] Yue Zhang,[1] and Richard E. Kennedy[1], *1. University of Alabama at Birmingham, Birmingham, Alabama, United States*

Delirium, or acute confusional state, affects up to 7 million hospitalized older adults annually, and is associated with increased risk of mortality, institutionalization, and cognitive and functional impairment. There has been a proliferation of both pharmacological and nonpharmacological clinical trials to reduce the incidence and sequelae of delirium. In other neuropsychiatric disorders, exclusion criteria prevent up to 75% of individuals with the condition under study from participating. It is unclear how well these trial samples represent the population of older adults with delirium. We selected all intervention trials registered at ClinicalTrials.gov containing the keyword "delirium" (N=131), regardless of type of intervention. We manually examined study descriptions to restrict analysis to studies with delirium as a primary or secondary outcome (N=92). Of these 92 studies, 76% enrolled only adults, with 45% enrolling only older adults. 38% of studies were restricted to surgical units, 27% to intensive care units, and 7.6% to medical units. Only 1 study examined nursing homes and 4 studies examined palliative care. 50% of studies excluded individuals with pre-existing dementia, 28% excluded individuals with psychiatric disorders, and 30% excluded individuals with neurological disorders. 34% of studies excluded individuals with alcohol or drug abuse. Overall, many intervention studies for delirium are limited to the surgical and ICU populations, and they exclude individuals with common comorbidities associated with an increased risk of delirium. Similar to other neuropsychiatric disorders, these findings raise significant concerns about the generalizability of clinical trials in delirium to the hospitalized older adult population.

### PREDICTORS OF PRESSURE ULCER DEVELOPMENT AMONG SENIORS DURING EMERGENCY TRANSFERS TO HOSPITAL EMERGENCY DEPARTMENT

Colin Reid,[1] Hannah Jiwani,[1] Kaitlyn C. Tate,[2] and Greta G. Cummings[2], *1. University of British Columbia, Kelowna, British Columbia, Canada, 2. University of Alberta, Edmonton, Alberta, Canada*

Emergency transfers of seniors in long-term care facilities (LTCF) aged >65 to hospital emergency departments (ED) are common and carry with them risks that can lead to less-than-optimal quality of care and quality of life. Pressure ulcers are one such risk. We used data from the Older Persons Transitions in Care (OPTIC; N=637) study, conducted in two Canadian provinces in 2011 and 2012, to assess potential predictors of pressure ulcer development between the time that a resident is transported to the ED until the time they return to their original nursing home. Step-wise binary logistic regression was employed to identify predictors of pressure ulcer development during the transition. Potential predictors included length of transition, inpatient status, demographic, health variables (including incontinence). Among the 335 residents for whom we were able to gather new pressure ulcer data, 56 (16.7%) were identified as having developed new skin wounds upon return to the LTCF. Transitions from ED admission to return to LTCF averaged 106.7 hours (sd=143.6) with a median of 50.0 hours. Length of transition and whether the resident spent time as an inpatient emerged as the only predictors: longer transition times and spending time as an inpatient predict development of bed sores. These results speak to the need for improved monitoring and treatment of skin wounds during emergency transitions of older adults from LTCF.

### SESSION 2320 (POSTER)

### ADULT PROTECTION | ELDER ABUSE

### IMPROVEMENTS NEEDED FOR JAPANESE ELDER ABUSE PREVENTION LAW: COMPARISONS WITH OTHER ABUSE PREVENTION LAWS

Asako Katsumata,[1] and Noriko Tsukada[2],
*1. Higashigaokaso rehabilitation institution, Japan,*
*2. Nihon University, Tokyo, Japan*

Japan has four stand alone abuse prevention laws, including child abuse (enforced in 2000), domestic abuse (2001), elder abuse (2006) and abuse for people with disabilities (2011). These laws are distinctive in that they aim to prevent abuse, not merely to address abuse after it occurs. This paper compares components of these four abuse prevention laws, delineating major strengths and weaknesses of the elder abuse prevention law in comparison to the other three. The analysis considers both institutional and domestic settings and suggests possible improvements of elder abuse prevention law that need to be made. Evidence for this analysis is supplied through examination of trends abuse using longitudinal data (2012-2017) collected by Japan's Ministry of Health, Labour and Welfare. The analysis shows commonalities in trends in the four abuse categories. For example, the number of abuse cases has risen over time despite the dissemination and implementation of abuse prevention training, program implementation, and public outreach. Differences include reporting – police more often report abuse cases of children and people with disabilities, while elder abuse cases are more often reported by professional staff members responsible for dealing with elder abuse cases. Although some amendments have been made to the child abuse and domestic abuse prevention laws, no amendments have been made to the elder abuse prevention law despite the requirement to review its success. Needed revisions include provisions of protection orders and temporary shelters to protect elder victims from abusers as soon as possible.

### ELDER ABUSE BY FAMILY CAREGIVER: DOES COGNITIVE IMPAIRMENT REALLY MATTER?

Elsie Yan[1], *1. The Hong Kong Polytechnic University, Hong Kong, Hong Kong*

Previous research suggested that cognitive impairment is a risk factor for elder abuse. Persons with dementia experience elevated risk of abuse as compared to the general aging population. The present study compared rates of abuse reported by family caregivers of older persons with and without dementia. A total of 693 family caregivers participated, among which 592 were providing care older persons with dementia and 101 were providing care to older persons without. Participants provided information on their demographic characteristics, care recipient physical functioning (Instrumental Activities of Daily Living), behavioral problems (Cohen Mansfield Agitation Inventory), caregiver stress (Zarit Burden Interview), emotional and instrumental social support, and abusive behaviors directed at the care recipients (Conflict Tactics Scale and Potentially Harmful Behaviors). Abuse is common in this sample: 46.8% reported potentially harmful behaviors, 52.7% reported psychological aggression, 11% physical assault, and 1.3% injury. No significant difference was observed between caregivers providing care to older persons with or without dementia (p>.05). A series of logistic regression was conducted to determine factors associated with abuse. Care recipient behavioral problems and caregiver burden were two prominent factors associated with potentially harmful behaviors and all forms of abuse. Behavioral problems are common in persons with cognitive impairment and many caregivers feel stressful managing them. It is plausible that cognitive impairments per sec do not increases risk of abuse, but the associated characteristics do. Helping family caregivers manage the caregiving situation and their expectation, positive appraisal and cognitive restructuring may help prevent elder abuse.

### CRIME-RELATED PHYSICAL INJURIES AMONG OLDER ADULTS IN THE UNITED STATES: FINDINGS FROM 2015 NIBRS DATA

Gerson Galdamez,[1] and Zach Gassoumis[2], *1. University of Southern California, Los Angeles, California, United States, 2. Leonard Davis School of Gerontology, University of Southern California, Los Angeles, California, United States*

Approaches to addressing crimes within the justice system typically do not differentiate between younger and older adults. However, approaches to addressing elder abuse cases—which involve older adult victims exclusively—often rely on the preconception that abuse can only occur if a visible physical injury exists. This preconception is driven in part by perceptions of frailty and ease-of-injury in older, vulnerable victims. We aimed to quantify the prevalence of physical injuries among older victims of violent crimes. We used data reported by the U.S. National Incidence-Based Reporting System (NIBRS) in 2015 to quantify the frequency of reported crimes which resulted in visible, physical injuries to victims aged 60+. Logistic regression was used to determine effects of age, race/ethnicity, type of crime, relationship to offender, and victim locality on physical injury from crime. Among 1,373,417 crime victims, 80.63% of older adults (60+) sustained an injury, as opposed to 65.17% of younger adults (<60). The proportion of individuals who showed physical injuries consistently increased with age until age 90. Knowing the offender was associated with higher odds of sustaining a visible injury (OR 1.47, 95% CI 1.38-1.58). Although older adults show higher risk of visible physical injuries from violent crimes than younger adults, it is possible for a crime against an older adult to have occurred without a visible injury. Medical and criminal justice practitioners should utilize this evidence on elder crime victimization to aid in expert witness testimony and other activities related to justice in crimes against older adults.

### ELDER SELF-NEGLECT: REPORTING AND RESPONSE WITHOUT APS OVERSIGHT

Kelly A. Melekis[1], *1. Skidmore College, Saratoga Springs, New York, United States*

Self-neglect is generally considered a geriatric phenomenon and subsumed within the category of elder abuse and neglect. In the U.S., self-neglect is the most commonly reported form of elder abuse and neglect and has been linked to decreased physical and psychological well-being, higher mortality rate, and increase health care services utilization. While self-neglect typically falls under the purview of a state's adult protective service (APS) system, there are several states where this is not codified and/or practiced. This study examined the state of existing laws, policies and programs that address elder self-neglect in the U.S. in an effort to better understand how self-neglect reporting and response is handled, particularly in states where APS does not have oversight. A 50-state review of existing policies and laws addressing elder self-neglect was followed by key stakeholder interviews with those who oversee self-neglect reporting and response in states where APS does not have oversight. Findings indicate that a range of rationales explain variation in responsibility for self-neglect cases, including a lack of specificity in statutes, concerns that self-neglect does not fit into legal/criminal models due to the lack of perpetrator/victim relationship, and strong sentiments that those engaging in self-neglect need human services and support rather than investigatory or legal approaches. While the inclusion of self-neglect into the broad category of elder abuse and neglect is often perceived as vital for the provision of services, findings indicate it may exacerbate the complex challenges inherent in defining and conceptualizing both the term and its response.

### SESSION 2325 (POSTER)

### ALZHEIMER'S DISEASE AND RELATED DEMENTIAS: POLICIES, PRACTICES, AND RESEARCH

### LONGITUDINAL CHARACTERISTICS OF INFORMAL CAREGIVERS OF COMMUNITY-DWELLING PERSONS WITH DEMENTIA

Eric Jutkowitz,[1] Joseph E. Gaugler,[2] Amal Trivedi,[1] Lauren L. Mitchell,[3] and Pedro L. Gozalo[1], *1. Brown University, Providence, Rhode Island, United States, 2. University of Minnesota - School of Public Health, Division of Health Policy and Management, Minneapolis, Minnesota, United States, 3. Minneapolis VA Health Care System, Minneapolis, Minnesota, United States*

Alzheimer's disease and related dementias (ADRD) affect >5 million Americans. Persons with ADRD experience functional limitations and often require support from informal caregivers (e.g., help with feeding). Little is known about how caregiving evolves over the full course of the disease. We used data from the Health and Retirement Study to identify incident predicted community-dwelling persons with ADRD (n=565), their informal caregivers, and the total hours of care they received in a month from predicted incidence up to 8-years post incidence. We estimated linear mixed-effects models to determine the characteristics of the person with ADRD that are associated with trajectories of caregiving hours. At predicted incidence, persons with ADRD received 120 hours of care in a month of which spouses provided 30% of care hours, adult children provided 32% of care hours, other relatives provided 12% of care hours, and non-relatives (including paid support) provided 25% of care hours. By 8-years post incidence, persons with ADRD still in the community (n=23) received 303 hours of care in a month of which spouses provided 28% of care hours, adult children provided 41% of care hours, other relatives provided 3% of care hours, and non-relatives provided 28% of care hours. Having great grandchildren and more functional limitations were associated with receiving more hours of informal care. Attrition (mortality and residing in a nursing home) was influenced by hours of care received in the previous interview and resulted in those that remained in the community being persons that required less care.

### THE ROLE OF DEMENTIA FRIENDS IN CREATING STRONGER NETWORKS WITHIN FAITH-BASED COMMUNITIES

Christy M. Nishita,[1] and Heather Chun[2], *1. University of Hawaii, Center on Aging, Honolulu, Hawaii, United States, 2. National Asian Pacific Center on Aging, Seattle, Washington, United States*

Faith-based communities are uniquely positioned to support members of their congregation with dementia and their caregivers. Aligned with their ministry and service work, congregations can identify individuals who need help and provide needed instrumental and emotional support. This poster will present preliminary findings from a pilot study with a Chinese, Mandarin-speaking church and a Samoan church in Honolulu, Hawaii. Using the Dementia Friends USA curriculum, which was translated into Mandarin and Samoan, the pilot study examined the potential for the Dementia Friends approach to mobilize a congregation and support caregivers. Findings indicate that there was great interest and need for information and dementia within the church community. In addition, a strong ministry and high proportions of older congregation members were keys to the success of the pilot. Finally, misperceptions of dementia within the Chinese and Mandarin communities were common and needed to be addressed in order for church members to fully embrace Dementia Friends principles (i.e., it is possible to have a good quality of life with dementia.) Future work will further explore faith-based community characteristics and other program components that lead to improved support for persons with dementia and their caregivers, as well as stronger dementia-friendly, faith based communities.

### THE PRICE OF DEMENTIA: POLICY STRATEGIES TO NARROW HEALTH DISPARITY GAPS, REDUCE COSTS, AND IMPROVE QUALITY OF LIFE

Nora Super,[1] Rajiv Ahuja,[2] and Kevin Proff[3], *1. Milken Institute Center for the Future of Aging, Washington, District of Columbia, United States, 2. Milken Institute, Washington, District of Columbia, United States, 3. Milken Institute, Los Angeles, California, United States*

Historically, a diagnosis of Alzheimer's disease and related dementias (ADRDs) created a sense of hopelessness among those diagnosed, their families, and physicians. The misperception that there is no available medical treatment or interventions inhibited patients from receiving cognitive assessments or exploring home- and community-based interventions that could improve quality of life. Today, researchers have developed evidence-based pathways to improve brain health, reduce dementia risk, and enhance lifelong cognitive function. This report highlights recent scientific advancements focused on medical and lifestyle interventions that reduce the risk of ADRDs and slow the disease progression. To improve the current system and reduce the risk of dementia and improve brain health, the following policy areas are recommended: 1) promote brain health strategies to delay onset and reduce the stigma associated with cognitive decline; 2) increase early detection and cognitive screening efforts; 3) build a dementia-capable workforce; 4) create seamless transitions from health systems to community-based services to better support those with dementia; 5) improve caregiver training and support; and 6) increase funding for research related to ADRDs. Understanding that ADRDs disproportionately impact certain populations, these policy recommendations include strategies to narrow health disparities. Previous Milken Institute research highlighted the disproportionate health impacts of dementia on women, which costs the economy trillions of dollars. This report expands on that analyses and stratifies the data by race and income as well. Ensuring that these policy recommendations address disproportionately impacted groups will benefit the broader healthcare system and alleviate the burden felt by all patients and families.

### A PILOT STUDY TO TEST METHODS FOR DATA COLLECTION ON NURSES CARING FOR RESIDENTS WITH DEMENTIA IN THE NURSING HOME

Rebekah Perkins,[1] Elizabeth Cashdan,[1] and Katherine Supiano[1], *1. University of Utah, Salt Lake City, Utah, United States*

Nurses draw from their experiences and intuition to detect changes in patient condition, patterns of patient behaviors, and evidence of distress. In the nursing home setting, nurses care for residents with dementia and manage challenging behavioral and psychological symptoms of dementia (BPSD), and may rely on informed intuition to assess and capably respond to such behaviors. To date, no observational method has been developed to discern nurse-resident interactions that identify expert nurses who effectively address BPSD. It is not known if or how nurses in this setting use intuition to make clinical judgments and decisions to manage BPSD events. Using an ethogram approach, we developed an observational tool and spot interview method to discern BPSD events, background and proximal factors and nurse responses to BPSD. Pilot observations took place over three nursing shifts to identify nurse-resident interactions during BPSD events using the observation tool. Nurse-resident interactions were followed by spot interviews with each nurse to clarify their responses to BPSD. Semi-structured interviews were conducted with nurse participants to further develop an interview guide and identify elements of nurse intuition. The pilot study affirmed the feasibility of gaining access to facilities with residents with BPSD, of nurse comfort with field observation and interviews, and established preliminary construct validity of the "expert nurse." Verification of the utility of this observation and interview method permit further examination of effective nurse engagement with nursing home residents with BPSD, informs our understanding of nurse intuition and permits further exploration of the broader context of BPSD.

### STRENGTHENING DEMENTIA CAREGIVING NETWORKS WITH TECHNOLOGIES: INTERSECTIONS OF POLICY, RESEARCH, AND PRACTICE

Nicole Ruggiano,[1] Lauren R. Bangerter,[2] Melinda Gruber,[3] Hannah Luetke-Stahlman,[4] Kelly A. O Malley,[5] and Jennifer L. Wolff[6], *1. University of Alabama, Tuscaloosa, Alabama, United States, 2. Mayo Clinic, Rochester, Minnesota, United States, 3. Lakeland Health, Joseph, Michigan, United States, 4. Cerner Corporation, Kansas City, Missouri, United States, 5. New England GRECC, Boston VA Healthcare System, Boston, Massachusetts, United States, 6. Johns Hopkins University, Maryland, United States*

Both family caregivers and providers report numerous challenges to providing care for of adults with Alzheimer's disease and related dementias (AD/RD). Many of these challenges stem from ineffective communication and coordination within the health and support service networks, including: (1) communication challenges posed by AD/RD patients; (2) communication barriers between caregivers and providers; and (3) communication barriers among providers across institutions. Caregivers increasingly rely on technology to fill gaps in their knowledge, identify social support, and learn how to execute more complex care demands. While technology has been suggested as an effective strategy for improving the quality of AD/RD care and support caregivers, the role that public policy and research can and should play in advancing the goal of such tech-based solutions is still emerging. This is especially true in respect to how technology can strengthen care networks for patients and caregivers who are racial/ethnic minorities and/or living in rural communities. This presentation will identify the role that past legislation (e.g. the Affordable Care Act, HITECH, Medicaid) has played, as well as the role that recent and proposed legislation (e.g. RAISE Caregiver Act, CARE Act, 21st Century Cures Act, BOLD Infrastructure for Alzheimer's Act, Older American's Act reauthorization) should play in strengthening the health, support networks that caregivers interact with to provide ongoing care. This presentation will also summarize the current state of research on caregiver technologies and discusses how future research and policy initiatives can promote the translation of tech-based interventions into everyday care settings.

### MOVING PICTURES: RAISING AWARENESS OF DEMENTIA IN CALD COMMUNITIES THROUGH MULTIMEDIA

Bianca Brijnath,[1] Josefine Antoniades,[2] Jon Adams,[3] Collette Browning,[4] Dianne Goeman,[5] Katie Ellis,[6] and Mike Kent[6], *1. National Ageing Research Institution (NARI), Parkville, Victoria, Australia, 2. National Ageing Research Institute, Parkville, Victoria, Australia, 3. University of Technology Sydney, Ultimo, New South Wales, Australia, 4. Australian National University, Canberra, Australian Capital Territory, Australia, 5. University of Newcastle, Callaghan, New South Wales, Australia, 6. Curtin University, Bentley, Western Australia, Australia*

Limited awareness of dementia in people from culturally and linguistically diverse (CALD) backgrounds often results in delayed diagnosis, poorer prognosis, and a higher burden of care on families and health systems. Given the rapidly ageing and multicultural populations in migrant-receiving countries such as Australia and the United States, this disparity needs to be addressed urgently. This project aimed to inform and educate people from five linguistically diverse backgrounds – Hindi, Tamil, Mandarin, Cantonese, and Arabic – about dementia. A mixed methods, multimedia design comprising video-interviews with 76 participants including carers from the five language groups and key service providers was employed. Data were gathered nationally across Australia in 2018 and thematically analysed. Data were used to co-produce 15 short films, comics, and a mobile-optimised website from which data analytics were measured. The films and comics focused on dementia detection and timely diagnosis, how to navigate the aged care system, and the importance of self-care. Analytics data is currently being collected online and via community forums. In conclusion, co-production methods in tandem with digital multimedia are fundamental to developing culturally salient interventions to address dementia disparities in CALD populations in Australia and internationally.

### DEFINING DEMENTIA-FRIENDLY COMMUNITIES FROM THE PERSPECTIVE OF THOSE AFFECTED: A QUALITATIVE APPROACH

Iris A. Wernher[1], *1. Portland State University, Institute on Aging, Portland, Oregon, United States*

Cities and communities across the globe are pledging to become more "dementia friendly," yet many communities lack direction as to what this pledge might entail. This poster describes findings from a qualitative study conducted in and around Portland, Oregon. The goal was to better understand how communities can become more dementia friendly – from the perspective of those directly affected by the disease. The study further aimed to clarify how a city or community's age- and dementia-friendly efforts can be integrated. Fifty community-dwelling participants – 25 individuals living with dementia and their 25 informal care partners – were interviewed separately. The questions centered on the participants' daily lives, barriers to and opportunities for realizing desired activities, and the participants' thoughts on how communities can become better and more inclusive places for people living with dementia. The analysis of the interviews yielded common themes, such as social inclusion, public awareness, and transportation, which served to develop a framework of dementia friendliness. This framework was compared to the World Health Organization's framework of age friendliness to identify areas of overlap and divergence, providing the foundation for a synergistic integration of dementia-friendly initiatives into the greater context of age friendliness. Finally, the answers of individuals living with dementia and those of their care partners were compared to identify similarities and differences in their perspectives. The study was funded, in part, by Oregon citizens through the Alzheimer's Disease Research Fund of the Oregon Charitable Checkoff Program, administered by the Oregon Partnership for Alzheimer's Research.

### DEMENTIA KNOWLEDGE, ATTITUDES, AND BELIEFS: A 3-YEAR UPDATE

Pedro M. Santos,[1] Constança Paúl,[1] Catarina Alvarez,[2] Ana Margarida Cavaleiro,[2] Tatiana Nunes,[2] and Silvana Paules[2], *1. CINTESIS – Center for Health Technology and Services Research, Oporto, Portugal, 2. Alzheimer Portugal, Lisbon, Portugal*

Objective: To characterise and analyse beliefs, knowledge, and attitudes regarding dementia three years after the first Portuguese survey (2015), in order to inform mental health research and policy-making, and establish the baseline to assess "Dementia Friends" campaign impact. Method: An advisory group, including academics and representatives of Alzheimer Portugal and Alzheimer Society UK, drafted the survey based on previous international research and the first national survey. The survey was uploaded to the Directorate-General of Health website and disseminated through relevant stakeholders and social networks. The survey was cascaded by inviting recipients to further share it. Data were collected during July 2018. Results: 1716 individuals completed the survey: median age 43y, 83.4% female, 25.1% professionals working in the field of dementia. 31.7% of respondents know someone close with dementia, and 42.5% have/had relatives with dementia. 14.7% consider people with dementia must leave/stop to attend ceremonies and social events, but this percentage increased to over one-third (37.3%) when asked about the opinion of most people living in their community. Regarding the key messages of "Dementia Friends" campaign, results reveal that 25.6% of respondents consider dementia is a natural part of ageing, 21.3% that it is not caused by diseases of the brain and 17.4% that it is just about losing memory. Conclusions: The second survey results reinforce the need for initiatives to change people's perceptions of dementia, that build up on evidence-based decision-making and international learning and cooperation as part of "Dementia Friends" global movement.

### COGNITIVE IMPAIRMENT AND SLEEP DIFFICULTIES OVER 10 YEARS IN A NATIONAL SAMPLE OF OLDER ADULTS

Rebecca Robbins,[1] Amanda Sonnega,[2] Robert W. Turner II,[3] Girardin Jean-Louis,[1] and Kenneth Langa[4], *1. NYU School of Medicine, New York, United States, 2. University of Michigan, Michigan, United States, 3. The George Washington University, Washington, District of Columbia, United States, 4. University of Michigan, Ann Arbor, Michigan, United States*

Prior studies suggest that sleep difficulties (e.g., trouble falling asleep) may be associated with cognitive impairment. We used a large, nationally representative longitudinal survey of adults over the age of 50 in the US to examine the relationship between sleep difficulties and cognitive functioning. Generalized estimation equation (GEE) linear regression models were used to analyze data from the 2004-2014 waves of the Health and Retirement Study. We examined sleep difficulties and cognitive functioning within participants and across time (n=17,642). Sleep difficulty was measured as trouble falling asleep, nocturnal awakenings, and waking too early scored as 1= rarely/never, 2=sometimes, and 3=most of the time. A summary score indicated cognitive functioning (range 0-27). Models controlled for age, gender, race/ethnicity, marital status, education, chronic medical conditions, depressive symptoms, and body mass index (BMI). Compared to those with no sleep difficulties, those who reported difficulty falling asleep ["sometimes" OR=0.83,95%CI:0.71-0.96 and "most of the time" OR=0.79,95%CI: 0.64-0.98] and waking too early ["most of the time" OR=0.79,95%CI: 0.63-0.98] had worse cognitive functioning. Compared to those with no sleep difficulties, those who reported nocturnal awakenings ["most of the time" OR=1.29,95%CI:1.08-1.54] had higher cognitive functioning. Over time, lower cognitive function was more likely among those reporting difficulty falling asleep (OR=0.73,95%CI:0.54-0.97), nocturnal awakenings (OR=0.77,95%CI:0.61-0.97) and waking too early (OR=0.65,95%CI: 0.47-0.88). In this nationally representative, longitudinal sample of older US adults, we found that over time lower cognitive function was more likely among those who reported difficulty falling asleep, nocturnal awakenings, and waking too early.

### SKILLS AND STRATEGIES OF FACILITATORS WHO CO-CREATE DIGITAL STORIES WITH PERSONS WITH DEMENTIA

Christine H. Daum,[1] Lili Liu,[1] Kara Hollinda,[2] David Kaufman,[3] and Arlene Astell[4], *1. University of Alberta, Edmonton, Alberta, Canada, 2. Independent, Saskatoon, Saskatchewan, Canada, 3. Simon Fraser University, Surrey, British Columbia, Canada, 4. University of Toronto, Toronto, Ontario, Canada*

Digital storytelling combines storytelling and digital tools to create brief video clips in which narrative, images, and music are embedded, in order to share personal stories. Digital storytelling facilitators can be health and social care providers as well as care partners who collaborate with persons living with dementia to co-create their stories. These facilitators elicit people's stories and use the technology to create the digital story. Despite their important role, there is a paucity of information on facilitators' specific skills and strategies used in working with persons living with dementia. The purpose of this project was to examine skills and strategies used by facilitators who co-create digital stories with persons living with mild dementia. Audio recordings of 70 digital storytelling co-creation sessions conducted in three Canadian cities (Edmonton, Vancouver, Toronto) were transcribed and subjected to qualitative content analysis. Regardless of their disciplinary background, facilitators acted as weavers, bringing together narrative threads to co-construct a digital story with participants. Essential communication skills and strategies included active listening, strategic questioning, being comfortable with silence, and therapeutic responding. Building relationships and collaboration were achieved through flexibility, empathy, and encouraging autonomy. To be an effective facilitator of the digital storytelling process with older adults living with dementia, facilitators must adapt their communication strategies and relational skills to the strengths and needs of the older adults with whom they are collaborating.

### ACCURACY AND USABILITY OF A MOBILE ALERT SYSTEM FOR COMMUNITY CITIZENS TO LOCATE PERSONS WITH DEMENTIA WHO GET LOST

Christine H. Daum,[1] Noelannah Neubauer,[1] Carlo Oliva,[1] Ron Beleno,[2] and Lili Liu[1], *1. University of Alberta, Edmonton, Alberta, Canada, 2. Independent/Community ASAP, Toronto, Ontario, Canada*

Three out of five persons with dementia wander. While the literature supports community engagement in the location of lost older adults, publically funded Silver Alert programs are associated with jurisdictional issues and alert fatigue. In collaboration and consultation with key stakeholders (older adults living with dementia, care partners, service providers, advocates, police organizations), we developed Community ASAP, an alert system (mobile website and app) that mobilizes community citizens who volunteer to keep watch for persons with dementia reported to be lost. The purpose of this study was to evaluate usability and functionality of the Community ASAP app. Thirteen participants from six regions in Canada received a total of 130 missing person notifications. They recorded the time and content of these notifications, completed the Website Usability Questionnaire, and provided written feedback or participated in a group interview about their experiences using the app and suggestions for improvements. The Community ASAP app delivered notifications with 100% accuracy and received messages from participants with 98% accuracy. Overall, participants thought the interface was easy to navigate, graphics were pleasing, easy to use, and was clearly organized. Suggestions for improvements to increase usability included: 1) Multisensory alerts to make them more noticeable and increase readability; 2) Clearer navigation within the home screen and preferences; 3) User support (instructions, demo video, technical support). Evaluation results for this innovative app were favourable; suggestions will be used to further improve usability, particularly for end users who are novices at using mobile applications.

### TOTAL AND POST-DISCHARGE 30-DAY EPISODE PAYMENTS FOR BENEFICIARIES WITH ALZHEIMER'S AND DEMENTIA

Neil Kamdar,[1] and Elham Mahmoudi[2], *1. University of Michigan, Ann Arbor, Michigan, United States, 2. University of Michigan, Department of Family Medicine, Ann Arbor, Michigan, United States*

There exists lack of evidence regarding incremental post-discharge cost and utilization of healthcare services for older adults with Alzheimer's and dementia (AD). We quantified episode payments associated with AD vs. non-AD 30 days after medical or surgical procedures. We utilized administrative claims between January 2012 and June 2017 from the Michigan Value Collaborative (MVC) across 31 different medical and surgical services. We identified all patients with any AD diagnosis code throughout their enrollment using ICD-9-CM, ICD-10-CM codes. We price standardized 30-day episode payments and split them based on patient setting. Payments were risk adjusted and winsorized at the 99th /1st percentile. Propensity score matching using calipers without replacement adjusted for clinically relevant surgical and medical procedures, HCCs, insurance type, and age to control for selection bias. We identified 66,676 AD episodes and 656,235 non-AD episodes. After propensity score matching, there were 58,485 AD and non-AD episodes with significant differences in total episode payments of ($22,378 vs. $19,595, 95% CI Diff: ($2,658, $2,910)). Post-acute care and readmission payments were significant ($4,561 vs. $3,272, 95% CI Diff: ($1,235, $1,342)) and ($1,807 vs. $1,165, 95% CI Diff: ($595, $691)), respectively. AD episodes had a higher readmission rate (21.6% vs. 14.8%, $p<0.0001$). County variation in payments for AD episodes was substantial (Median: $4,370, Range: $3,881). AD patients are at higher risk of readmission and more resource intensive to hospitals and health systems. Examining drivers of post-discharge cost variation can influence practice pattern changes in management of AD patients.

### ATTITUDES TOWARDS AGING

### PERCEPTIONS OF AGING AND HEALTH OF OLDER ADULT SPOUSES

Jennifer L. Smith,[1] Joseph G. Bihary,[1] Dugan O'Connor,[1] Ajla Basic,[1] and Cate O'Brien[1], *1. Mather LifeWays Institute on Aging, Evanston, Illinois, United States*

Evidence suggests that negative perceptions of aging are associated with worse health outcomes. Research also shows that marital partners can mutually influence each other's health; however, limited research has explored perceptions of aging within couples. The purpose of the current study was to examine the association between self and partner perceptions of aging and health among married older adult couples. Both members of 452 couples (average age: wives = 80.6 years, husbands = 82.7 years) completed a survey that measured perceptions of aging and health (i.e., self-reported health, healthy diet, physical functioning, stress, and physical activity). Data were analyzed using actor-partner interdependence models, and analyses were conducted separately for positive and negative perceptions of aging, controlling for self and partner's age, depression, and chronic health conditions as well as gender and income. Consistent with previous research, analyses revealed that one's own positive perceptions of aging were related to better health across all measures, and the opposite pattern was found for negative perceptions of aging. An examination of partner effects revealed that people reported better physical functioning and greater physical activity when their spouses had more positive perceptions of aging. Similarly, husbands reported less stress when wives had more positive perceptions of aging, but there was not a partner effect for wives. In contrast, people reported lower subjective health and less healthy diets when their spouses had more negative perceptions of aging. These findings suggest that health-promotion efforts should consider partners' perceptions of aging as a potential resource or risk factor.

### CHANGES IN PERSONAL RESOURCES AND SUBJECTIVE AGE ACROSS 8 YEARS

Allura F. Lothary,[1] and Thomas M. Hess[1], *1. North Carolina State University, Raleigh, North Carolina, United States*

Subjective age has been extensively researched as a predictor of physical and mental health outcomes in older adulthood. However, the mechanisms behind subjective age have not yet been established and reasons for longitudinal changes are unclear. Selective engagement theory (SET; Hess, 2014) proposes a connection between increases in objective and subjective cognitive costs and motivation to engage future resources. Costs are influenced by personal resources, mainly physical and mental health. These changes in costs may also influence subjective experiences of the aging process. For example, aging attitudes have been shown to partially mediate the associations between resources and both motivation and activity engagement (Hess et al., 2018). Using the Health and Retirement Study (HRS) data, changes in resources (physical and mental health) were hypothesized to predict changes in subjective age across 8 years. More specifically, reductions in resources were predicted to be associated with older subjective ages over time. This relationship was expected to be mediated through aging attitudes. Results indicate an association between changes in resources and changes in subjective age over time in the expected direction. Changes in resources were partially mediated by aging attitudes. However, resources function differently in predicting changes in subjective age based on the resource (physical and mental health) and chronological age of the participant. These findings provide more guidance as to the mechanisms behind subjective age and may offer theoretical guidance for future work looking at understanding changes in subjective age.

### LIFE AS A JOURNEY: A VIEW OF LIFE AS A JOURNEY MODERATES THE RELATIONSHIP BETWEEN SUBJECTIVE AGE AND SUBJECTIVE HEALTH

Ga-Eun (Grace) Oh[1], *1. Open University of Hong Kong, Kowloon, Hong Kong*

Previous research has shown the relationship of subjective age and health status: feeling younger than one's age is correlated with better health outcomes including both subjective and objective measures. This research investigates how the view of life as a journey might moderate the relationship between subjective age and subjective health. A view to look at life as a journey is a common metaphor to view life as an ongoing process. Prior work has suggested that people who went through difficult situations successfully tend to construe their life experience as a journey. This suggests that thinking of life as a journey might help people cope better with their negative experiences in general such as feeling older. Thus, we investigate to see if believing 'life as a journey' can buffer against the negative effect of feeling older on subjective health perception. To test this, we collected the data from American participants (N = 724) of various ages. The results showed that more life was viewed as a journey, smaller the detrimental effect of subjective age on subjective health. Although feeling older generally reduced subjective health, this negative effect of feeling older was smaller among those who thought life as a journey. This research suggests that thinking life as a journey might be used to reduce the negative impact of older subjective age on health perceptions.

### DISENTANGLING KNOWLEDGE AND BIAS: COMBATING AGEISM WITH THE REVISED FACTS ON AGING QUIZ

Jensen Davis,[1] Linda Breytspraak,[1] Jacob Marszalek,[1] and Joan McDowd[1], *1. University of Missouri-Kansas City, Kansas City, Missouri, United States*

The Facts on Aging Quiz (FoAQ) was developed in 1977 as a 25-item True/False test of knowledge about older adults. Since that time, it has been utilized in hundreds of studies involving clarifying misconceptions, measuring factual knowledge across different groups, and assessing bias toward older adults. The current study examines the psychometric properties of a revision to the FoAQ created in 2015 that modified the original items and added 25 more to better reflect contemporary aging research. Participants were sampled using Qualtrics and MTurk platforms and targeted to equally represent the following four age groups: 18-34, 35-49, 50-64, and 65 and older. Exploratory factor analysis (n=956) did not support a multi-factor structure, contrary to previous theories of it having cognitive, physical, societal, and psychological health factors. A single factor model was forced which contained 28 items that only accounted for 26% of the variance in scores. The reliability reached satisfactory levels in the younger three age groups with the 28-item version but remained inadequate among those 65 and older. Small associations with the Expectations Regarding Aging-12 and Aging Semantic Differential scales were observed. In its present format, the FoAQ is not sufficient for research use but remains a useful tool in provoking discussion about age bias and areas in which people of all ages lack factual information. Researchers suggest an expansion in response options and further clarifying the use of this instrument as a measure of knowledge or bias.

### BOSTON AGING TOGETHER STUDY: ATTITUDES TOWARD OWN AGING AMONG OLDEST-OLD PARENTS AND CHILDREN

Yijung K. Kim,[1] Kathrin Boerner,[1] Kyungmin Kim,[1] and Daniela Jopp[2], *1. University of Massachusetts Boston, Boston, Massachusetts, United States, 2. University of Lausanne, Lausanne, Switzerland, Switzerland*

One consequence of modern longevity is the growing number of older adults with very old parents. While family members are often interdependent in their development and aging, less is known about how intergenerational relationships may influence individuals' attitudes toward their own aging in later life. Using 70 dyads of oldest-old parents (Mage = 93) and their children (Mage = 67) from the Boston Aging Together Study, we examined the dyadic concordance in positive attitudes toward own aging, and how perceptions of giving and receiving care are associated with attitudes toward own aging for parents and children. On average, parents reported more negative attitudes toward own aging than did children. In less than half of all dyads (46%), both parents and children reported positive (i.e., score three or higher on a scale that ranged from one to five) attitudes toward own aging. T-test results showed that the dyads with positive attitudes toward own aging had more within-dyad age difference, better average self-rated health, fewer depressive symptoms and less loneliness than others. For children, higher level of caregiver's burden was associated with more negative attitudes toward own aging. For parents, perception of received support was not associated with their attitudes toward own aging. This study sheds light on how both individual and family characteristics may influence individuals' aging perceptions. Findings suggest the context of parent-child ties may particularly be relevant to those older adults who may have to deal with their own aging- related challenges as well as those of their parents.

### AGE-BASED STEREOTYPE THREAT: A SYSTEMATIC REVIEW OF PRIMING TECHNIQUES AND THEIR EFFECTS

Francesco Vailati Riboni,[1] and Francesco Pagnini[1], *1. Università Cattolica del Sacro Cuore di Milano, Milan, Italy, Italy*

Age-based stereotype threat (ABST) occurs when older adults are influenced by negative stereotypes about age-related decline and functional losses and ironically behave in disengaging and self-defeating ways that confirm the stereotype (Steele & Aronson, 1995). Aging stereotypes are found to be strong predictors of health and illness outcomes in later life, and are associated with performance in specific areas, mainly in cognitive and physical domains. The current study reviewed the experimental methods and their reported effects previously published in the literature to determine if there were different ABST methods were associated with different types of age-related outcomes. We conducted a systematic review, screening the scientific literature for papers that included experimental manipulation of age-related stereotypes as an independent variable, focusing on samples of older adults (1113 articles, most published after 2003). Through a classification of the common and distinctive characteristics of the different stereotype manipulation techniques, we were able to identify three specific types of experimental methods: by instruction, tests, and interpersonal exposure. Although the mechanism by which stereotypes are associated with functional outcomes in older adults remains unclear, our review suggests it is possible to experimentally control the activation of the stereotype by manipulating its specific characteristics and the way older participants are exposed to it. Findings also highlight the possibility that specific experimental methods used to induce ABST in older individuals may lead to unique and different consequences on functional performance variation.

### VIEWS ON AGING ALTER THE IMPACT OF DEATH FEARS ON GENERALIZED ANXIETY

G. Rainville,[1] and Laura Mehegan[1], *1. AARP, Washington, District of Columbia, United States*

This study looks at the relationship between fear of death, views on aging, and generalized anxiety. We theorize that positive views on aging (such as disagreeing that old age is a time of loss and decline) will buffer anxieties related to the unavoidable event of death. To test an implication of this theory, we ran a model looking at whether scores from a five-item views of aging scale altered the relationship between a fear of death measure (i.e., Collet-Lester's "Your death" subscale) and generalized anxiety as measured on the GAD-7. Covariates related to religious beliefs, history with depression and anxiety, stress, social desirability reporting, and routine behaviors were included. An online probability sample of American adults age 18 and older (fielded January 2019) yielded results consistent with a "views-on-aging-as-an-anxiety-buffer" theory. Specifically, visualizations indicate that in a high fear of death condition, those with positive views of aging have significantly lower anxiety levels than those who do not. As such, this research suggests that positive views on aging may console those harboring fears about death.

### PLANNING FOR FUTURE CARE NEEDS: THE IMPORTANCE OF PERCEIVED NEED

Julie A. Gorenko,[1] Calandra Speirs,[1] Candace Konnert,[1] Claire McGuinness,[1] and Camille Mori[1], *1. University of Calgary, Calgary, Alberta, Canada*

Despite the demonstrated need to plan for future care needs, many individuals fail to engage in planning, often with negative consequences for their future health and well-being (Lee, Mason, & Cotlear, 2010). Theoretically, the propensity to utilize planning resources may be related to the perceived need for care in the future, a demonstrated predictor of the utilization of health and mental health services (Andersen, 1995; Karlin, Duffy, & Greaves, 2008). The purpose of this study was to examine perceptions of need for future care in combination with predisposing (age, financial security, attitudes towards planning) and enabling (anticipated support, satisfaction with family discussions about future care needs) variables in predicting planning behavior. The sample was comprised of 385 adults, aged 50 years and older (M=66.5, SD=9.3, range=50-92). Hierarchical regression analyses entered two well-established predictors, age and financial security in step 1, and attitudes towards planning, anticipated support, satisfaction with family discussions, and perception of need in step 2. Age and financial security explained 17% of the variance in planning; the addition of step 2 variables explained 33% of the variance and R-squared was significant ($p<.001$). All predictors were significant at $p<.001$, with the exception of anticipatory support ($p<.05$). These results support both the individual (i.e. positive attitudes, perceived need) and contextual nature of planning, in particular the belief that support will be available when you need it and the benefits of family discussions in facilitating planning. Recommendations for enhancing successful planning among individuals and their families will be presented.

### COGNITIVE HEALTH STATUS AND GENDER DIFFERENCES IN ATTITUDES AND EMOTIONS TOWARD OLDER ADULTS

Grace Caskie,[1] Abigail R. Voelkner,[1] and Hannah M. Bashian[1], *1. Lehigh University, Bethlehem, Pennsylvania, United States*

The health and gender of older adults can elicit differing attitudes and emotions within young and middle-aged adults (Bergman & Bodner, 2015); one's own gender may also influence these differences (Bergman & Cohen-Fridel, 2012). In this study, 287 participants (173 males, 114 females), aged 19-55 years (M=32.8), were randomly assigned to read one description of an older adult that varied cognitive health status (healthy/Alzheimer's) and gender (male/female). Factorial MANOVAs examined differences by gender, health, and participant gender for participants' (a) emotions about the older adult (compassion and emotional distance) and (b) negative perceptions about aging (ageist attitudes and aging anxiety). The first MANOVA found a significant main effect for health status; participants expressed more compassion ($p=.013$) and less emotional distance ($p<.001$) for the older adult with Alzheimer's than for the healthy older adult. Also, the Target Gender X Participant Gender interaction was significant for emotional distance ($p=.032$), but not for compassion ($p=.616$); men reported more emotional distance than women for the female older adult, regardless of target health status, but men and women's emotional distance were very similar for the male older adult. The second MANOVA showed only a significant health status main effect; ageist attitudes ($p=.021$), but not aging anxiety ($p=.062$), differed by health status of the older adult, with more ageist attitudes expressed for the healthy older adult than the older adult with Alzheimer's. Overall, these results show that individual factors can influence young and middle-aged adults' negative attitudes and emotions towards older adults. Implications will be discussed.

### INTERRELATIONSHIPS AMONG COLLECTIVE PERSONALITY, ATTITUDES TOWARD INTERGENERATIONAL SUPPORT, AND CO-RESIDENCE

Yooumi Lee[1], *1. Syracuse University, Syracuse, New York, United States*

This study investigates if collective personality influences positive attitude towards family caregiving and support for elderly parents, which, in turn, engenders positive attitude toward intergenerational coresidence. To test the hypothesis, the author examines the interconnections among collective personality; attitude towards family caregiving, and attitude towards support for aged parents; and attitude towards intergenerational coresidence in the U.S. The survey data is collected from total 67 undergraduate students in classroom environment. Particular attention is given to their attitude towards family caregiving and aged parents support. Results show that those three predictors are positively correlated with the attitude towards intergenerational coresidence i.e. respondents with more positive attitude towards family caregiving and aged parents support and with collective personality are more likely to have positive attitude towards intergenerational coresidence. Among the three predictors, collective personality is positively connected to the attitudes towards family caregiving and support for aged parents. A positive correlation is also observed between two attitude predictors i.e. respondents with more positive attitudes towards the family caregiving are likely to have positive attitude towards the support for aged parents and vice versa. The results highlights the important influence of collective personality, and attitude towards family caregiving and support for older parents on the intergenerational coresidence, even in the individualistic society.

### WORRIES OF OLD-AGE SUPPORT FOR PARENTS AND THEMSELVES: VIEWS FROM OLDER BACHELORS IN RURAL CHINA

Ying Wang,[1] Huijun Liu,[1] Bei Wu,[2] and Yaolin Pei[2], *1. Xi'an Jiaotong University, Xi'an Shaanxi, China, 2. New York University, New York, New York, United States*

Due to China's gender imbalance, it is estimated that more than 30 million adult males were unable to get married. The old-age support for older unmarried sons (so-called forced bachelors) and their parents faces a significant challenge. Using data from a survey in Central and Western rural China, the present study examined the impact of family structure and health status on the worries about old-age support for themselves and their parents from the perspective of older unmarried sons. The sample included 359 older unmarried sons with rural Hukou (housing registration) status. The age of he sample ranges from 28 to 51. The results showed that 52.64% and 54.8% of respondents were worried about their own and their parents' old-age support, respectively. Ordered logistic regression showed that having a sister(s) was negatively related to worries about their own and parents' old-age support. Those with living mothers had less worries than their counterparts, and those who had a brother(s) had less worries about their parents' old age support. Moreover, having any brothers who were also older unmarried sons was positively related to worries about their own and parents' old-age support. Older unmarried sons who had two frail old parents had more worries for their parents' old-age support than those whose parents were physically independent. The study highlights the importance of family structure and parental health status as important factors in worries over old-age support in China.

### OPTIMISM AND EXPECTATIONS REGARDING AGING AMONG YOUNG ADULTS AND OLDER ADULTS

Samuel C. Van Vleet,[1] Jenna M. Moore,[2] Sarah Edzards,[2] Leah N. Smith,[2] Jennifer Sawyer,[2] Allyson M. Coldiron,[2] Allie M. Sandlin,[2] and Michael D. Barnett[2], *1. University of North Texas, Denton, United States, 2. The University of Texas at Tyler, Tyler, Texas, United States*

A large body of research has found that individuals' attitudes toward aging may influence their future health. Previous research has found that age is associated with more negative expectations toward aging. However, it is possible that optimism, or generalized positive expectancies regarding future outcomes may play a role in expectations regarding aging. Optimism has been identified as a key component of successful aging. The purpose of this study was to compare expectations regarding aging among young adult and older adult age cohorts, controlling for optimism, and to investigate the differential relationships between optimism and expectations regarding aging by age cohort. Young adults (n = 130) and older adults (n = 335) completed a survey containing the Expectations Regarding Aging – 12 and the Optimism-Pessimism Scale. Results found that, after controlling for optimism, older adults endorsed more negative expectations regarding aging. Comparison of the correlation coefficients between optimism and expectations regarding aging among age cohorts found that optimism was significantly associated with expectations regarding aging among older adults but not young adults and that this difference was significant. Taken together, the results suggest that older adults have more negative expectations for aging and that optimism may play a key role in older adults' expectations regarding aging.

### ADOLESCENTS' ATTITUDES ON GRANDPARENT INVOLVEMENT IN CHILD CARE

Katherine E. del Rosario,[1] and Raphael Ferrer[2], *1. University of the Philippines, Los Banos, Laguna, Manila, Philippines, 2. University of the Philippines Los Banos, Laguna, Philippines*

This descriptive associative study focused on the attitudes of selected adolescents toward the involvement of grandparents (maternal grandmother, maternal grandfather, paternal grandmother, paternal grandfather) in childcare. Specifically, it aimed at determining the rate of closeness of these adolescents toward their own grandparents; their attitudes toward the involvement of their grandparents; and the relationship between the students' closeness with their grandparents and their attitudes toward grandparents' involvement. A survey was administered to 185 adolescents aged 16 to 22 from the University of the Philippines. Results showed that most of the respondents (43.38%) are either very close or close to their grandparents, especially their maternal grandmother (n = 126). The attitudes of the respondents toward grandparents' involvement were measured by looking at their rate of agreeableness on 17 statements. Results showed that most of the respondents either agree or neither agree nor disagree (M = 2.60) on grandparents' involvement. The correlation analysis showed a moderate to high positive linear association between MGM closeness and MGM involvement (r=.72); MGF closeness and MGF involvement (r=.74); PGM closeness and PGM involvement (r=.63); and PGF closeness and PGF involvement. This implies that perceived closeness increases with perceived involvement. The findings of this study emphasized on the role grandparents play in the lives of adolescent grandchildren. Some implications of this study with regards to future research in this field are described.

### RECEIVING ELDERLY PRIVILEGE AND POSITIVE ATTITUDES ABOUT AGING: THE MEDIATING ROLE OF SUPPORT FROM CHILDREN

Lingya Zhao,[1] and Ling Xu[2], *1. Wuhan University, School of Political Science and Public Administration, Wuhan, Wubei province, China, 2. University of Texas at Arlington, Arlington, Texas, United States*

Objectives. This study investigated the influence of receiving elderly privilege on positive attitudes about aging, and examined the mediating roles of support from children in such associations among Chinese older adults. Methods. Data were derived from China Longitudinal Aging Social Survey (CLASS), a nationwide social survey project of Chinese older adults aged 60 or older (N = 7,184) conducted in 2014. The positive attitudes about aging were measured by Attitudes to Aging Questionnaire. Sum scores were calculated with higher scores indicating more positive attitudes about aging. Analyses were conducted by PROCESS v3.3 for SPSS 20.0. Results. Receiving elderly privilege was significantly associated with more financial support ($b = 0.303$, $p < .001$) and instrumental support ($b = 0.225$, $p < .001$) from children. Financial support ($b = 0.100$, $p < .05$), instrumental support ($b = 0.090$, $p < .05$), and emotional support ($b = 0.405$, $p < .001$) received from children were significantly correlated with more positive attitudes about aging. Financial support ($b = 0.030$, BCa CI [0.010, 0.057]) and instrumental support ($b = 0.020$, BCa CI [0.000, 0.042]) from children mediated the association between receiving elderly privilege and positive attitudes about aging. Conclusion. With the rapid increase of aging population in China, it's important to help Chinese older adults maintain high quality of life by encouraging society and family to work together. To maintain higher levels of positive attitudes about aging among Chinese older adults, social program of providing elderly privilege and support from children are both needed.

### DIMINISHED COMPLEXITY OF RELIGIOUS BELIEF AMONG DROPOUTS FROM A LONGITUDINAL STUDY OF COPING

Jordan A. Anderson,[1] Shaelyn M. Harris,[1] Erica L. Nelson,[1] Ingrid Teuber,[1] and Andrew Futterman[1], *1. Department of Psychology, Loyola University Maryland, Baltimore, Maryland, United States*

Selective attrition is a common problem in longitudinal studies of older adults. Dropout is due to many factors, but frequently health concerns figure prominently as a reason for attrition. In light of previous work that suggests health problems reduce complexity of religious and other social involvements, the current study examines complexity of religiousness among dropouts and continuers in a longitudinal study of religion and health in later life. A random sample of 287 older adults living in Worcester, MA was assessed at two times of measurement 12 months apart using interview-based measures of religious orientation (Batson, Schoenrade, and Ventis, 1993) and health (OARS). Of the 287 who began the study, 72 dropped out and were not available to be assessed at 12 month assessment. Using Mplus, a three-factor model of Ends, Means, and Quest orientations demonstrated a good fit to the data in both dropout and continuer subsamples (e.g., CFI's equaled .959 and .966, respectively). Diminished correlations between Ends, Means, and Quest orientations in the dropout vs. continuer subsample suggests diminished complexity of religious orientation among dropouts. Dropouts were more seriously ill, had higher levels of functional impairment, and demonstrated lower SES, suggesting increased vulnerability and fewer resources may have been the reason for dropping from the study as well as one possible cause of their diminished religious complexity.

### AGING BODIES IN PARADISE? AN ETHNOGRAPHIC STUDY OF FANTASY FEST IN KEY WEST, FLORIDA

Rachel A. Douglas,[1] and Anne Barrett[1], *1. Florida State University, Tallahassee, Florida, United States*

Dominant cultural constructions of aging bodies, particularly those of women, as unattractive and asexual may be challenged within politically and socially progressive leisure environments, like Key West, Florida, that promote out-group acceptance, collectivity, and cultural diversity. However, this possibility receives limited scholarly attention. Addressing this gap, our study employs observational and interview data (n=60) collected in 2017 and 2018 at Key West's Fantasy Fest – an annual event marketed as a "10-day party in paradise for grown-ups." The festival, drawing as many as 100,000 people, cultivates a relaxed atmosphere permissive of nudity and theatrical body adornment, including body paint and costume. This feature makes it an ideal site for examining the effect of inequalities, including age and gender, on body displays and social reactions to them. Data analysis revealed four themes centering on aging bodies – Judging Bodies, Limiting Body Displays, Displaying Bodily Difference, and Liberating Bodies. Age and gender inequality strongly influenced judgments of attractiveness and sexual appeal, contributing to older participants' more limited body displays. Nevertheless, both young and old participants collectively contributed to creating a liberating environment that celebrates bodily difference and encourages cross-age interaction. While limited to one site with a unique political and social climate, our study suggests the potential of progressive leisure environments to broaden notions of aging bodies and encourage cross-age connections.

### DYADIC PREDICTORS OF AGING SATISFACTION: RELATIONSHIP QUALITY MODULATES THE IMPACT OF HEALTH RESTRICTIONS

Daniela Jopp,[1] Charikleia Lampraki,[2] Claudia Meystre,[3] Kyungmin Kim,[4] Yijung K. Kim,[4] and Kathrin Boerner[4], *1. University of Lausanne, Lausanne, Switzerland, Switzerland, 2. University of Lausanne, Institute of Psychology, National Pole of Research LIVES, Lausanne, Switzerland, 3. University of Lausanne, Institute of Psychology, Lausanne, Vaud, Switzerland, 4. University of Massachusetts Boston, Boston, Massachusetts, United States*

How individuals develop perceptions of their own aging process is receiving increasing attention. While own age-related experiences are important, the aging of close others, such as parents, has been also found to play a role. Of particular interest may be parental health, yet relationship quality aspects may influence the extent to which health restrictions affect the children's aging perceptions. Dyadic data from the Swiss "Aging Together" study were analyzed (dyad N = 98, Parent Mage = 83.85; Child Mage = 56.43). For parents, actor-partner interdependence models indicated a negative relationship between health restrictions and aging satisfaction; the more health restrictions they experienced, the less happy they were with their aging process. Parental health restrictions also played a role for children's aging satisfaction, but the effect was dependent on how the relationship with the parent was perceived by the child; parents' health restrictions were negatively associated with aging satisfaction of children who reported better relationship quality (i.e., less conflictual), but not with aging satisfaction of children who reported worse relationship quality (i.e., more conflictual). In addition, when the parent was more restricted, children who perceived the parent as more supportive showed lower levels of aging satisfaction, compared to children who perceived the parent as less supportive. Lastly, children's own health restrictions had no negative impact on their aging satisfaction when they perceived the parent as more supportive. In sum, health restrictions may influence both dyadic partners' aging satisfaction, but relationship quality modulates their impact.

### ATTITUDES OF OLDER ADULTS TOWARD PHARMACEUTICAL DIRECT-TO-CONSUMER ADVERTISING

Ann M. O'Hanlon,[1] and Carlen L. McLin[1], *1. University of New Orleans, New Orleans, Louisiana, United States*

According to national data, older adults view more television than any other age group. However, further research is needed about how older adults respond to programming. In particular, this study focused on the attitudes of older viewers toward pharmaceutical direct-to-consumer advertising (DTCA). Prior research has shown DTCA increases consumers' demand for drugs, but less research has addressed how older consumers perceive such advertising. Researchers surveyed 293 older adults who attended Senior Centers throughout New Orleans about their television viewing and response to direct-to-consumer drug advertising. The sample was predominantly female (96%) and African American (87%) with a mean age of 76 years. The primary motivations selected for viewing television were to watch a favorite show, to be entertained, or learn information; older adults did not often endorse reasons of boredom, escapism, or relaxation. A majority of the sample (62%) perceived commercials as an interruption or annoying, and indicated they were uninfluenced by advertising (74%). When asked about direct-to-consumer advertising, 54 percent disapproved of such advertising, but 50% reported attending to such commercials when aired. Some older adults reported that advertisements on medicines could provide valuable information (46%), but also perceived the ads as confusing (57%) or alarming (61%). After viewing DTCA, older adults most often reported speaking to a doctor (46%) or seeking information (27%) about a drug, but some reported that they decreased (11%), increased (3%) or stopped (3%) taking medicine. The results provide additional information about how direct-to-consumer advertising is perceived by older adults and affects compliance behavior.

### IMPACT OF FAMILY FUNCTION ON THE RELATIONSHIP BETWEEN SELF-PERCEPTION OF AGING AND STRESS IN MIDLIFE

Hyun-E Yeom,[1] Eunyoung Park,[1] and Misook Jung[1], *1. Chungnam National University, Daejeon, Korea, Republic of*

Midlife is a transitional period with considerable stress related to health changes and interpersonal responsibilities. Understanding how self-perception of aging and family function affect stress is essential to improve quality of life in midlife and beyond. The purpose of this study was to examine the association among self-perception of aging, family function, and stress with a specific focus on the interaction between self-perception of aging and family function, which affect stress in midlife Koreans. This is a cross-sectional study. Data on a convenience sample of 249 midlife Koreans (age mean= 50.6, 50.1% male) were collected through a self-administered survey and analyzed using the PROCESS macro for SPSS. Self-perception of aging was significantly related to family function ($r= -.121$, $p=.045$), and family function was related to stress ($r= -.402$, $p<.000$). Self-perception of aging was a significant predictor for stress ($\beta= -.130$, $p=.008$) after adjusting for age, gender, subjective health status, and chronic health problems. A significant interaction between family function and self-perception of aging on stress was found ($\beta= -.261$, $p=.006$), indicating that the influence of self-perception of aging on stress was different depending on family function. Self-perception of aging was a strong predictor of stress in individuals who reported poorer support from family members, but not in those who reported better support. Our findings emphasize the importance of supportive family function, which could regulate the impact of self-perception of aging on stress in midlife. Developing psycho-cognitive interventions to improve self-perception of aging and supportive interaction between family members is warranted.

### IMPACT OF SELF-PERCEPTION OF AGING ON THE RELATIONSHIP BETWEEN HEALTHY BEHAVIOR, STRESS, AND HEALTH IN MIDLIFE

Hyun-E Yeom,[1] Eunyoung Park,[1] and Misook Jung[1], *1. Chungnam National University, Daejeon, Korea, Republic of*

Self-perception of aging may affect the interpretation of health-related changes that influence behaviors. Understanding how self-perception of aging is associated with healthy behavior, stress, and health is essential to prepare healthy aging. The purposes of this study are to examine the influence of healthy behavior on stress and subjective health status and to investigate whether self-perception of aging affects the association in midlife Koreans. This is a cross-sectional study. Data were collected through a self-administered survey using a convenience sampling of 466 midlife Koreans (mean age = 50.7, 52.4% male). The PROCESS macro for SPSS was applied for data analysis. Self-perception of aging was significantly related to stress ($r= .20$, $p<.00$), and subjective health was related to healthy behavior ($r= .22$, $p<.00$) and stress ($r= -.38$, $p<.00$). Healthy behavior predicted stress, which, in turn, affected subjective health status (index=.05, 95% CI [.01, .10]). A significant interaction between healthy behavior and self-perception of aging was found ($\beta= -.22$, $p=.002$), indicating that the influence of healthy behavior on stress was different depending on the individual's self-perception of aging. It means that the impact of healthy behavior on stress was stronger in individuals with a more negative self-perception of aging. The findings demonstrate the critical role of self-perception of aging, which affects the benefits of healthy behavior on stress and subjective health status. This study highlights the importance of assessing self-perception of aging and developing cognitive behavioral interventions that contribute to modifying stereotyped beliefs about aging for better quality of life in midlife.

### SESSION 2345 (POSTER)

### CANCER

### ASSESSING RISK FOR ENDOMETRIAL CANCER AMONG HISPANIC FEMALES AGE 50 YEARS AND OLDER

Ana Rodriguez,[1] Yong-Fang Kuo,[2] and Enshuo Hsu[3], *1. The University of Texas Medical Branch at Galveston, Galveston, Texas, United States, 2. University of Texas Medical Branch, Galveston, Texas, United States, 3. UTMB, Gaveston, Texas, United States*

Endometrial cancer is the most common gynecological cancer in the US, with most women diagnosed between 55 and 64 years old. Seventy-five percent of women with endometrial cancer are postmenopausal, and the most common symptom is postmenopausal bleeding. Only a few studies have addressed the lack of knowledge and awareness of risk factors and/or health care utilization for early signs and symptoms of endometrial cancer. The objective of this study was to evaluate health care utilization among Hispanic women aged ≥50 years who are at risk for endometrial cancer. This retrospective cohort study used a combination of diagnosis and procedure codes from UTMB's electronic health records to identify Texas Hispanic females who had a health encounter at ≥50 years of age between 2012 and 2016. Risk factors included conditions/treatments affecting hormone levels, age, body mass index, diabetes, gravidity, parity, family history of endometrial or colorectal cancer, previous diagnosis of breast or ovarian cancer or endometrial hyperplasia, smoking or alcohol use, and treatment with radiation therapy in the pelvis area. Multivariate logistic regression models evaluated for predictors of endometrial cancer. The study included 11,563 Hispanic females aged ≥50 years (median age=57). Most women were overweight. Currently, we identified 705 Hispanic females (6.1%) with possible endometrial cancer with validation underway. Females who have a history of vaginal spotting/bleeding, pelvic bleeding, and pelvic pain are at higher risk for endometrial cancer. It is important for physicians to educate patients on recognizing the signs and symptoms of endometrial cancer.

### CANCER PREVALENCE AND THE ROLE OF MULTIMORBIDITY AMONG OLDER ADULTS: USING NHANES 2011-2016

Fei Tang,[1] and Elizabeth Vasquez[2], *1. University at Albany - State University of New York, Albany, New York, United States, 2. University at Albany, SUNY, Albany, New York, United States*

Cancer risk increases as age, understanding the potential risk factors of cancer is essential for cancer prevention. Biological and epidemiologic studies suggest relationships between individual chronic conditions and increased cancer risk. However, limited researches have analyzed the association between multimorbidity (simultaneous presentation of two or more chronic diseases) and cancer. The current study is aimed to evaluate whether having multimorbidity is associated with increased all-site and site-specific cancer prevalence among older adults. Data of 5,200 older adults aged 55 years and older who participated in the 2011-2016 National Health and Nutrition Examination Survey (NHANES) were included in the study. Single and multiple logistic regression models were used to evaluate the associations between multimorbidity and cancer. 3,623 (70%) individuals in our study were identified as having multimorbidity and 992 (19%) individuals were diagnosed with cancer. After adjusting for demographic covariates and smoking status, having multimorbidity was significantly associated with having all-site cancer (AOR: 1.57; 95% CI: 1.25 – 1.98) and lung cancer (AOR: 8.91; 95% CI: 1.51 - 52.73). Multimorbidity was associated with increased odds of having cancers among older adults. Our findings add to the evidence suggesting the potential relationships between multimorbidity and cancer. Future longitudinal studies are needed to examine the biological mechanisms and temporality of the association. If the association between multimorbidity and cancer is affirmed, it could have substantial implications in public health, as management of multiple chronic conditions could also advantage cancer prevention among older adults.

### LONG-LIVED INDIVIDUALS PRESENTING WITH LARGE BREAST AND COLON TUMORS HAVE A LOWER RISK OF CONCURRENT METASTASIS

Benjamin Sweigart,[1] Michelle R. Johnson,[2] Michael Voisine,[3] Zeyuan Song,[1] Kimberly Bertrand,[4] Stacy L. Andersen,[5] Paola Sebastiani,[1] and Thomas T. Perls[5], *1. Boston University, Department of Biostatistics, Boston, Massachusetts, United States, 2. Cambridge Health Alliance, Cambridge, Massachusetts, United States, 3. Boston University Medical Center, Boston, Massachusetts, United States, 4. Slone Epidemiology Center at Boston University, Boston, Massachusetts, United States, 5. Boston University School of Medicine, Boston, Massachusetts, United States*

We hypothesized large tumors (stage T3 or T4) are less likely to metastasize in centenarians compared to younger patients. We analyzed 2004 to 2015 Surveillance, Epidemiology, and End Results (SEER) data for the most common cancer types (breast, colon, lung, and prostate) among patients with T3 or T4 tumors and compared rates of M1 (presence of metastases) at time of diagnosis according to ages 30-110 years. Among 44,066 breast cancer patients, metastasis rates fell after age 80 for T3 and after age 74 for T4 tumors. The relative risk of metastasis [RR] for T3 patients ages 90-110 years compared to ages 50-89 years was 0.73, 95% CI 0.57;0.94, and the RR for T4 patients was 0.48, 95% CI 0.42;0.55. Among 296,041 colon cancer patients, metastasis rates for T3 and T4 tumors steadily declined after age 60; RR for T3 patients was 0.66, 95% CI 0.62;0.71 and for T4 was 0.73, 95% CI 0.69;0.78 for the older and younger age groups. No difference in metastasis rates at diagnosis was observed for ages 90-110 with small cell and non-small cell lung cancers. Among 52,738 men presenting with stage T3 prostate cancer, the rate of metastasis steadily increased after age 70 (RR = 6.00, 95% CI 4.72;7.63) while there was no substantial difference in metastasis rate according to age for T4 patients. More work is needed to determine whether these findings are related to differences in screening and detection among those at older ages or whether they have a greater resilience to metastasis.

### GERIATRIC ASSESSMENT BEFORE HEMATOPOIETIC STEM CELL TRANSPLANT IDENTIFIES DEFICITS ACROSS ALL AGES

yen p. lowder,[1] Kristi Romero,[1] Yi Ren,[1] Amy M. Pastva,[1] Miriam C. Morey,[2] Harvey J. Cohen,[3] Nelson J. Chao,[1] and Anthony D. Sung[1], *1. Duke University Hospital, Durham, North Carolina, United States, 2. Durham Veterans Affairs Health Care system, Durham, North Carolina, United States, 3. Duke University Medical Center, Durham, North Carolina, United States*

Allogeneic hematopoietic stem cell transplant (HCT) is a lifesaving procedure; however, it is associated with significant morbidity, and treatment-related mortality ranges from 10-30%. Morbidity and mortality have been associated with poor functional status. The geriatric assessment (GA) may allow identification of deficits pre-HCT, allowing intervention and improvement. While focused on older adults, we hypothesize that GA may also identify deficits in younger patients who may be debilitated by chemotherapy or cancer before HCT. We performed a GA in all adult patients at the time of initial evaluation for HCT (between 10/1/17-1/31/19) and again immediately before HCT. Deficits were identified and patients referred to specialists (physical therapy, neuropsychology, etc.) prior to HCT. Among 83 patients, the median age was 58 years (age range: 19-75), 59 (71%) had ≥1 deficits, including 41 (49%) had ≥2 deficits that required referral. The most common deficit was physical function (45, 54%), followed by cognitive function (29, 35%), nutrition (26, 31%), and mental health (7, 8%). Deficits were common across all age groups: 9/16 (56%) 60 years old. To date, 40 patients have undergone HCT; of the 24 with deficits at initial evaluation, 10 (42%) improved at least one deficit, 5 (21%) were unchanged, and 9 (38%) not evaluated. Physical and nutrition deficits were most responsive to intervention. These results suggest that there is a high degree of impairment prior to HCT among both older and younger patients; however, these deficits are amenable to improvement prior to HCT.

### COMBINED EFFECT OF CMV SEROPOSITIVITY AND SYSTEMIC INFLAMMATION ON DEMENTIA PREVALENCE IN CANCER SURVIVORS

Sithara Vivek,[1] Bharat Thyagarajan,[2] Heather Nelson,[1] Anna Prizment,[1] Eileen Crimmins,[3] and Jessica Faul[4], *1. University of Minnesota, Minneapolis, Minnesota, United States, 2. Department of Laboratory Medicine and Pathology University of Minnesota; Minneapolis, Minnesota, United States, 3. Davis School of Gerontology, University of Southern California, Los Angeles, California, United States, 4. University of Michigan, Ann Arbor, Michigan, United States*

Though cancer patients treated with multi-modal therapies demonstrate higher levels of systemic inflammation, which is associated with dementia, cancer survivors have not shown a consistent association with dementia. Since several studies reported an independent association between cytomegalovirus (CMV) infection, inflammation and dementia in non-cancer populations, we have evaluated whether CMV infection and systemic inflammation were associated with increased prevalence of dementia in cancer survivors in Health and Retirement Study (HRS). We evaluated prevalence of dementia (using score ≤7 on the 27-point scale) among 1607 cancer survivors, in whom we measured CMV seropositivity and two biomarkers of systemic inflammation: C-reactive protein (CRP) and neutrophil-lymphocyte ratio (NLR). The prevalence of CMV seropositivity was 68.26% (n=1097), while prevalence of increased systemic inflammation [CRP >5mg/L and NLR >4] was 4.23% (n=68). Using survey logistic regression, adjusted for age, race, gender, BMI (Body Mass Index) and sampling design, cancer survivors who were both CMV seropositive and had increased systemic inflammation had the highest odds of dementia compared to those who were CMV seronegative and had low levels of systemic inflammation (OR=6.59; 95% CI [2.81, 15.44]; p<.0001). Cancer survivors who were CMV seropositive without evidence of systemic inflammation had a lower but increased odds of dementia (OR=2.02; 95% CI [1.17, 3.47]; p=0.01). Odds of dementia among those who were CMV seronegative with elevated systemic inflammation was not significant (p=0.09). Our study demonstrates a possible role for ongoing CMV induced inflammation in determining dementia prevalence among cancer survivors that needs further confirmation.

### CHARACTERIZING CONCURRENT ALZHEIMER'S DISEASE AND CANCER IN U.S. ADULTS OVER 65

Melody K. Schiaffino,[1] and James Murphy[2], *1. SDSU, San Diego, California, United States, 2. UCSD Moores Cancer Center, La Jolla, California, United States*

Cancer (CA) care delivery fragmentation persists for patients across the cancer continuum. Racial and ethnic disparities are one of the primary factors attributable for variation in treatment outcomes, in addition to language and patient-provider communication barriers. Latino and African-American communities also bear a greater burden of Alzheimer's Disease (AD) risk than White making patients experiencing AD+CA at risk for poor quality and treatment disparities. This study aims to characterize AD+CA in a population-based sample. Using 2004-2013 SEER-Medicare data we identified multiple cancers and the prevalence of concurrent AD+CA in the database (N=273,349). Patients selected for a first primary, histologically confirmed, any stage, not diagnosed in death certificate or autopsy and had at least 24 months of data prior to diagnosis to calculate a comorbidity index. All analyses were conducted in SAS 9.4 (Cary, N.C.). Across lung (LC), colorectal, head and neck (HNC), prostate (PC), and cervical cancer (CC) we found 5890 cases of AD+CA or 2.15%. While lung represented the largest sample, colorectal (CRC) cancer was responsible for the largest proportion of concurrent AD+CA cases at 3.52% of all CRC. Black and Latino CRC, HNC patients had higher than overall prevalence of AD+CC. Black CRC patients had 6.13% AD+CA vs White 3.27 and Latino HNC patients reported 5.06% vs 3.25 White. Earlier stage patients had higher AD+CA vs later stages for CRC, HNC, and CC. The opposite was true for LC. Finally, women had slightly higher prevalence of AD+LC but significantly higher AD+CRC and AD+HNC compared to men.

### SESSION 2350 (POSTER)

### CHRONIC DISEASE MANAGEMENT 2

### GREATER HYPOGLYCEMIA UNAWARENESS IN OLDER COMPARED TO YOUNGER RURAL VETERANS WITH TYPE 2 DIABETES

Willy Marcos Valencia,[1] Kaicheng Wang,[2] Kiranmayee Muralidhar,[2] and Stuti Dang[3], *1. Miami Veterans Affairs Healthcare System- GRECC, Miami, Florida, United States, 2. Department of Public Health Sciences, University of Miami Miller School of Medicine, Miami, Florida, United States, 3. Miami VA GRECC, Miami, Florida, United States*

Hypoglycemia is of great concern in older patients, especially when complicated with multimorbidity and geriatric syndromes. We implemented a telemedicine model to address hypoglycemia knowledge, risk factors, incidence and comanagement with their primary care teams (PCT). We identified 166 consecutive rural veterans with high hypoglycemia risk, based on a local medication database (sulfonylureas and insulin), age, and recent glycated hemoglobin A1c (HbA1c). We conducted a telephone medication reconciliation and survey assessing glucose self-monitoring (GSM), hypoglycemia knowledge and symptoms. Variables were tested using chi-square, Fisher's, and one-way ANOVA. Multivariable logistic regression model was built to assess the association of hypoglycemia and age group, adjusted with treatment, HbA1c%, self-monitoring, and knowledge. There were 54 veterans aged <65 (younger), and 112 veterans aged ≥65 years (older). Average HbA1c was higher in younger than older (8.20±1.96 vs 7.43±1.34%, p=.003). There was no difference in treatment regimens, but the older had greater GSM (p=.028) and lower hypoglycemia symptom knowledge (p=.026). Symptomatic hypoglycemia was greater in younger versus older (50.0% vs 30.4%, p=0.014). Recent (past-2-weeks) hypoglycemic events were more frequent in younger than older (24.1 vs 1.79%, p<.001). Regression analyses showed that younger veterans were more likely to have hypoglycemia (OD=2.37, 95% CI 1.11-5.04). Our results indicate a great need to evaluate older adults with high hypoglycemia risk, in whom we observed less reports of hypoglycemia albeit with similar regimens and lower HbA1c. We suspect greater hypoglycemia unawareness, thus we are implementing a project using continuous glucose monitoring in this high-risk population.

### SESSION 2355 (POSTER)

### DEMENTIA AND DEMENTIA CARE

### SOCIAL NETWORK BY ENVIRONMENTAL DESIGN: FACILITATING THERAPEUTIC EFFECT IN LTCF FOR PEOPLE EXPERIENCING DEMENTIA

Farhana Ferdous[1], *1. Assistant Professor, Department of Architecture, College of Engineering and Architecture Howard University, Washington, D.C., United States*

The relationship between the physical environment and the prevalence of social interaction have been a core topic of inquiry in environmental gerontology. It has been estimated that around 25 million people worldwide have dementia, and the number will exceed 80 million by 2040. A growing body of literature in the areas of environment-behavior studies shows that the physical environment affects positive behavioral changes, in turn, affecting individual, group and organizational outcomes, but little research has focused on older adults especially those with cognitive impairment by targeting the Dementia Enabling Environment of care facilities. By using non-pharmacological interventions, the purpose of this study is to initiate positive social network among dementia residents and staff by analyzing the spatial configuration of the physical environment and layout in long-term care facilities (LTCF). The findings may give evidence-based design guidelines for future research and design of memory care facilities to promote therapeutic experience for older people experiencing early to moderate stage dementia. Using a 3-stage, multi-method research design such as space syntax, behavior mapping techniques and direct observations, this study objectively measured the spatial configuration of LTCF (physical environment) to evaluate the provision of social interactions (among dementia residents and staff), promote positive health outcomes and healthy living for people experiencing early to moderate stage dementia. This study was able to establish that the architectural layout and environmental design could have a positive and protective effect against dementia in environmental gerontology and geriatrics.

### DESIGN AND EVALUATION OF AN ECOLOGICAL MOMENTARY ASSESSMENT TOOL FOR ASSESSING DEPRESSION IN DEMENTIA

Iulia Niculescu,[1] and Andrea Iaboni[1], *1. University Health Network, Toronto, Ontario, Canada*

Background. Assessing depression in people with dementia is challenging due to limitations of retrospectivity. Mobile Ecological Momentary Assessment (EMA) provides a novel approach in assessing depressive symptoms by collecting informant measures in intervals throughout the day, decreasing recall bias and increasing representativeness. Objective. The objective of this study is to design an EMA application for assessing depression in individuals with dementia. Methods. A literature review was conducted to determine commonly used and validated assessments for depression in dementia. Assessments were analyzed for common items, where items less commonly used (<50% of assessments sharing the item) or not relevant to be collected using EMA were excluded. Wording of items were also analyzed to develop the specific structure of questions for the EMA assessment. Results: Six assessments were found and demonstrated adequate performance outcomes. Items fell into either mood-related, dementia-related, vegetative, psychotic or positive mood symptom groups. The mood-related group was analyzed separately for prominent items, which were sadness, anxiety, pessimism, loss of interest and irritability. Wording of items were modified to be consistent with being collected throughout the day, rather than retrospectively. These items were incorporated as core observational domains in the application to be tested. Sadness and anxiety were additionally included as self-report items as studies have shown these to be most discordant between individuals with dementia and informants. Conclusions: This research is a first step towards an innovative approach to assessing depression in dementia. Next steps involve evaluation of the application's feasibility and reliability for assessing depression in dementia.

### INTERVENTIONS TO REDUCE STIGMA OF DEMENTIA: FINDINGS FROM A SCOPING REVIEW

Juanita R. Bacsu,[1] Marc Viger,[2] Shanthi Johnson,[3] Tom McIntosh,[1] Bonnie Jeffery,[1] Nuelle Novik,[1] and Paul Hackett[2], *1. University of Regina, Regina, Saskatchewan, Canada, 2. University of Saskatchewan, Saskatoon, Saskatchewan, Canada, 3. University of Alberta, Edmonton, Alberta, Canada*

Although there is significant stigma attached to dementia, there is a paucity of knowledge on stigma reduction interventions. Guided by a strength-based approach, this presentation consists of two objectives: 1) to identify the literature on interventions to reduce dementia-related stigma; and 2) to recognize the strength-based components of existing anti-stigma interventions. A five-stage scoping review process was used to examine peer-reviewed literature of anti-stigma interventions of dementia from 2008 to 2018. From 744 initial records, 21 articles matched our inclusion criteria and were reviewed. A stigma reduction framework was used for classifying interventions: education (to dispel myths with accurate information), contact (to provide interaction with people with dementia), mixed (education and contact interventions), and protest (to challenge negative attitudes of dementia). A range of education, contact, and mixed interventions were identified. Strength-based components of education interventions included using: facts to dispel myths, multiple mediums to support dementia information, and culturally-informed strategies for specific audiences. Key components of contact and mixed interventions included: showcasing the achievements of people with dementia, relationship-building, and engaging in purposeful learning. Findings from this study can help to inform future interventions to reduce stigma and improve the quality of life for people affected by dementia.

### DECIDE, DISCOVER, AND DO: AN INTERVENTION FOR COMMUNITY-DWELLING INDIVIDUALS WITH DEMENTIA

Gregg Gorzelle,[1] Michael Skrajner,[1] John Zeisel,[1] Cassie Best,[2] and Drew Walker[3], *1. Hearthstone Alzheimer Care, Woburn, Massachusetts, United States, 2. Hearthstone Alzheimer Care, Mass, United States, 3. Hearthstone Alzheimer Care, MA, United States*

Decide, Discover, and Do!TM (D3) is a mobile application being developed and evaluated in an NIA-funded Phase 1 SBIR project. The goal of D3 is to enhance the quality of life and care for community-dwelling individuals with dementia (IWD) whose primary care partners (CPs) are their family members. D3 consists of (1) video-based, interactive training for family CPs on best practices in dementia care and (2) evidence-based activities for CPs to facilitate with their loved ones. The study examines the impact of D3 training modules on knowledge transfer and the effects of D3 activities on engagement/affect. The activities are unique in that they create an overarching narrative for daily activities that creates a consistent routine, thereby capitalizing on procedural memory. The activities build upon one another, starting with the IWD choosing a topic (e.g., nature) early in the day, followed by the dyad engaging in a tablet-based activity related to the topic (e.g., reading an article about rainforests), and culminating in an experiential activity (e.g., tasting various foods found in rainforests ). In pilot work, when training was implemented with homecare workers, there was a significant increase in knowledge from pre- to post-test (+21.0, $p<.01$). In addition, similar activities were shown to be highly engaging, with IWD exhibiting significantly higher levels of constructive engagement (+0.40, $p<.01$), and significant lower levels of other engagement (-0.13, $p<.05$), when compared to standard programming. This Phase 1 study will be complete by the conference; outcome data will be presented.

### THE EPIDEMIOLOGY OF ALZHEIMER'S DISEASE AND RELATED DEMENTIAS AMONG ARAB AMERICANS

Florence Dallo,[1] and Tiffany Kindratt[2], *1. Oakland University, Rochester, Michigan, United States, 2. University of Texas Southwestern Medical Center, Dallas, Texas, United States*

In the United States (U.S.), Alzheimer's Disease and Related Dementias (ADRD) afflict over 4.7 million individuals ages 65 or older. Most studies compare the prevalence of ADRD between minorities and whites. Arab Americans are a subgroup of whites, and ADRD is not understood among Arab Americans. The overall goal of this study is to estimate the prevalence of and risk factors for ADRD among Arab Americans ages 45 or older compared to non-Hispanic whites, non-Hispanic blacks, Hispanics and Asian Americans. Data for 2000-2017 from the National Health Interview Survey (NHIS) using the region of birth question was be used (N=222,219). Percents, chi-square and logistic regression will be estimated. Age- and sex-adjusted prevalence of ADRD was 10.3% for foreign-born Arab Americans compared to approximately 7.5% for US-born non-Hispanic whites (NHW), blacks and Asians. The prevalence of ADRD was 8.6% for Hispanics (all p-values <.0001). When controlling for age and sex, Arab Americans were 1.4 times (OR=1.02,1.93) more likely to have ADRD compared to US-born NHW. This is the first study to focus on ADRD among Arab Americans and the findings suggest ADRD is a burden in this population. Future studies should capture other generations of Arab Americans to better understand the trend of ADRD among this understudied, often invisible population.

### OLDER ADULTS' PERSPECTIVES OF COGNITIVE HEALTH PROMOTION AND ACTIVITIES TO AMELIORATE COGNITIVE DECLINE

Juanita R. Bacsu,[1] Shanthi Johnson,[2] and Marc Viger[3], *1. University of Regina, Regina, Saskatchewan, Canada, 2. University of Alberta, Edmonton, Alberta, Canada, 3. University of Saskatchewan, Saskatoon, Saskatchewan, Canada*

There is a paucity of research on cognitive health promotion from the perspective of older adults, especially within a rural context. However, dementia and cognitive impairment are more prevalent among rural older adults than urban older adults. This presentation has two objectives: to examine cognitive health promotion from the perspective of rural older adults; and 2) to identify key activities associated with ameliorating cognitive decline in rural communities. Drawing on a community-based participatory research approach, data was collected through two waves of semi-structured interviews with the same group of 42 older adults in rural Saskatchewan, Canada. Guided by lay theory and cultural schema theory, data was coded using thematic analysis. In describing cognitive health promotion, four key areas emerged including emotional health, intellectual health, social health, and functional health. In discussing activities to support cognitive health promotion, participants emphasized the importance of thinking positively, keeping your brain active, mingling with others, and managing your daily affairs. Focusing on older adults' perceptions of cognitive health promotion provides valuable information to advance knowledge of key strategies and activities to support cognitive health. In developing effective strategies to promote cognitive health, it is essential to engage in collaborative research and partnerships with older adults.

### PREDICTORS OF ATTRITION IN THE MULTI-ETHNIC 1FLORIDA ADRC CLINICAL CORE

Shanna L. Burke,[1] Warren Barker,[2] Monica Rosselli,[3] Miriam Rodriguez,[4] Carolina Robayo,[2] Cesar L. Chirinos,[2] Raquel Behar,[2] and Maria T. Greig-Custo[5], *1. Florida International University, Miami, Florida, United States, 2. Mount Sinai Medical Center, Miami Beach, Florida, United States, 3. Florida Atlantic University, Department of Psychology, Florida Atlantic University, Department of Psychology, Florida, United States, 4. Albizu University, Miami, Florida, United States, 5. Wien Center for Alzheimer's Disease and Memory Disorders, Miami Beach, Florida, United States*

Understanding predictors of attrition can position researchers to increase retention efforts and focus on preventing attrition. Attrition, or dropout of participants during a study prior to completion, can threaten the internal and external validity of a study's findings. Data from the 1FloridaADRC Clinical Core was analyzed, and included 271 participants within a two-year follow-up window, of which 216 (79.7%) were retained. T-tests and chi-square analyses were used to determine if a number of demographic, clinical, acculturation, and neuroimaging predictors were associated with attrition. The participant cohort included: 85% with cognitive impairment; 60% Hispanic; 42% over the age of 75; and 62% female. Predictors of greater attrition included: age over 75 years ($p< .003$); cognitive diagnosis of MCI or dementia ($p< .01$); and lower scores on the Mini-Mental Status Exam ($p<.04$), the Hopkins Verbal Learning Test (HVLT) immediate ($p< .02$), and delayed ($p<.002$) Higher total score on the Neuropsychiatric Inventory Questionnaire ($p<.06$), endorsement of night time behaviors ($p<.05$) and greater hippocampal atrophy ($p<.02$) were also predictive of attrition. Hispanic ethnicity was not a predictor of attrition, as retention was 81% for Hispanics versus 79% for non-Hispanics. However, among Hispanic participants, English acculturation measured by the Bidimensional Acculturation Scale for Hispanics was lower for those who dropped out ($t=2.8$; $p=.006$).

### PREDICTORS OF INSOMNIA IN INDIVIDUALS WITH MILD COGNITIVE IMPAIRMENT

Meghan Mattos,[1] Eric M. Davis,[1] and Carol Manning[1], *1. University of Virginia, Charlottesville, Virginia, United States*

Insomnia is a common disorder that affects up to 40% of people age 65 and older. Untreated insomnia can decrease quality of life, increase healthcare use, and exacerbate cognitive problems. Individuals with cognitive impairment experience more sleep disorders than those without cognitive concerns, yet little is known about insomnia and mild cognitive impairment (MCI). Our objective was to examine predictors of insomnia in persons with MCI (PwMCI). Using data from the National Alzheimer's Coordinating Center Uniform Data Set, a cross-sectional study of older PwMCI was conducted. Independent sample t-tests and contingency tables with chi-square tests of independence were used to examine differences between PwMCI with and without insomnia. Multivariate binary logistic modeling was performed. The total sample (N=1543) was comprised of 234 (15.1%) with clinician-reported insomnia and 1309 (84.9%) without insomnia. PwMCI and insomnia were more likely to be younger, take more medications, and smoke cigarettes (p.05). Three variables significantly predicted insomnia in PwMCI subjects in a multivariate model: active depression (OR 1.66, 95%CI 1.21, 2.27), active anxiety (OR 2.16, 95%CI 1.57, 2.99) and arthritis (OR 1.78, 95%CI 1.33, 2.39). Differences in predictors of insomnia in PwMCI highlight the need for geriatric and mental health specialists to provide specialized care to this population. Future studies should examine conversion of PwMCI with insomnia to dementia and the compounding effects of insomnia on cognition.

### AGE AND ADVANCE CARE PLANNING PREDICTS SELF-PERCEIVED RISK FOR DEMENTIA

Stacy W. Yun,[1] and Molly Maxfield[1], *1. University of Colorado Colorado Springs, Colorado Springs, Colorado, United States*

Self-perceived Alzheimer's disease and related disorders (ADRD) risk may be highly influenced by the sense of control which one has over the prevention and negative impact of such diagnoses (Kessler et al., 2012). This study examined whether age, advance care planning (ACP), and experimentally manipulated dementia salience (DS) are related to self-perceived risk of dementia. Participants ($N = 122$, 40 to 88 years old, $M = 65.66$, $SD = 9.71$) completed the computerized study using Qualtrics software. Participants completed questionnaires assessing individual differences, self-perceived risk, and demographics. Multiple regression was calculated to predict self-perceived ADRD risk based on age, ACP, and DS induction. The set of predictors explained 15.3% of variance in participants' self-perceived risk of dementia, $F(3, 118) = 7.08$, $p < 0.001$. Specifically, being older ($\beta = -0.40$, $p < 0.001$) and having less ACP items completed ($\beta = 0.26$, $p < 0.01$) uniquely predicted lower perceived risk. DS condition ($\beta = -0.01$) did not attain significance in the model. Younger age might be associated with less ADRD exposure and understanding, which may influence perceived risk. It is possible that older adults are less worried about developing dementia as they may (erroneously) believe they have "passed" the time to develop ADRD. Additionally, high levels of self-perceived ADRD risk may have resulted in motivation for greater levels of planning for the future among those with greater ACP. Future studies will investigate the level or point in which perceived risk changes from being beneficial and health-promoting to problematic and anxiety-provoking.

### PREDICTORS OF DEMENTIA IN OLDER ADULTS: VARIATION BY NATIVITY STATUS AND RACE-ETHNICITY

Heehyul Moon,[1] Adrian Badana,[2] So-Yeon Hwang,[3] Jeanelle Sears,[1] and William E. Haley[4], *1. University of Louisville, Louisville, Kentucky, United States, 2. School of Aging Studies, University of South Florida, Tampa, Florida, United States, 3. NONE, Cincinnati, Ohio, United States, 4. University of South Florida, Tampa, Florida, United States*

Among many possible factors that are associated with increased risk for dementia, one important topic is the role of race and ethnicity. However, previous studies did not examine rates separately by racial/ethnic groups and by nativity status, and little of this research comes from nationally representative data. The current study investigated different predictors of dementia by race/ethnicity and nativity status using Round 1 of the National Health and Aging Trends Study (NHATS, N=7,473). Our stratified log-binominal regression analyses by race/ethnicity (Non-Hispanic White (NHW), non-Hispanic Black (NHB), Hispanic, and Other) show that the relationship of nativity status to dementia (Probable dementia vs Possible and no dementia) is likely determined by multiple factors (e.g., education, # of cardiovascular conditions/risk factors, # in household) that may vary across race and ethnicity. The relative risks of Probable dementia were higher among immigrants in the Hispanic, and other groups, but lower among immigrants in NHBs, although the relative risk of dementia among NHBs was marginally significant. Gender was not a significant factor of Probable dementia across the four groups after adjustment for other variables including nativity status. Older age was significantly associated with the higher prevalence of Probable dementia across the four groups. However, other risk factors for dementia varied considerably between the four racial/ethnic groups after controlling for nativity status. Healthcare providers systems must be prepared to deliver culturally competent supports and services to diverse groups of older Americans and their family members.

### USE OF FDA-APPROVED ALZHEIMER'S DISEASE MEDICATIONS AMONG NON-INSTITUTIONALIZED U.S. ADULTS AGE 60 YEARS AND OLDER

Steven Frenk,[1] and Jessica M. Sautter[2], *1. CDC/National Center for Health Statistics, Hyattsville, Maryland, United States, 2. University of the Sciences, Philadelphia, Pennsylvania, United States*

This study examined the use of Alzheimer's Disease medications (ADMs) among non-institutionalized U.S. adults aged 60 years and older. It described the characteristics of ADM users, compared them to adults who were not currently using ADMs, and examined how demographic characteristics, living arrangements, healthcare utilization, and self-perceived cognition are associated with use of ADMs. It also identified the duration of ADM use and the most commonly used ADMs. Data come from the National Health and Nutrition Examination Survey, a nationally representative study of the non-institutionalized U.S. population (2009-2014). Respondents were classified as ADM users if they reported using one or more of the following prescription ADMs in the past 30 days: donepezil, rivastigmine, memantine, galantamine, or a drug combination of donepezil and memantine. During the study period, 1.9% of non-institutionalized U.S. adults aged 60 years and older reported using an ADM in the past 30 days. Use of ADMs was associated with concurrent use of antidepressant medications and also frequent or worsening memory loss and confusion. Most ADM users (82.3%) reported using ADMs for more than one year and the most frequently used ADM was donepezil. In 2009-2014, about 1.1 million non-institutionalized adults aged 60 years and older used an ADM in the past 30 days. They reported relatively poor cognitive abilities, frequent healthcare utilization, and concurrent use of antidepressant medications compared to non-users. Newly released public survey data will be useful to provide updated estimates of ADM use in the community.

### A WEB-BASED MOBILE APPLICATION TO SUPPORT SOCIAL ENGAGEMENT IN PERSONS WITH DEMENTIA

Hayley R. McCarron,[1] Rachel Zmora,[2] and Joseph E. Gaugler[3], *1. University of Minnesota School of Public Health, Minneapolis, Minnesota, United States, 2. University of Minnesota, Minneapolis, Minnesota, United States, 3. University of Minnesota - School of Public Health, Division of Health Policy and Management, Minneapolis, Minnesota, United States*

Assistive technology has been recognized as a promising tool to improve the lives of persons living with dementia and their caregivers. The use of assistive technology in dementia care is expanding, although it is most often applied to manage care and promote safety. There is a lack of assistive technology designed to aid persons with dementia to participate in meaningful activities. The present study utilizes a pilot randomized controlled trial (RCT) design to evaluate the feasibility, acceptability, and effects of an assistive technology device, the Social Support Aid (SSA), designed to assist persons with dementia engage in social interaction. Quantitative data were collected initially and three and six months later, and participants in the technology group participated in qualitative interviews. Feasibility and utility scores indicated that participants felt neutral about the technology. Use of the SSA was not significantly associated with changes in quality of social interactions or quality of life measures over the six months of follow-up ($P > .05$). The qualitative analysis revealed three themes that described how and why the SSA worked or did not: (1) outcomes (2) reasons why (not) useful and (3) recommendations. There is a need to develop effective assistive technology that improves the quality of life of persons with dementia. Assistive technology that allows persons living with dementia to maintain some level of autonomy should be a priority for future research. Results provide recommendations for future assistive technology development and evaluation.

### MILD BEHAVIORAL IMPAIRMENT AS A PREDICTOR OF COGNITIVE FUNCTIONING IN OLDER ADULTS

Hillary J. Rouse,[1] Brent J. Small,[1] and John Schinka[1], *1. University of South Florida, Tampa, Florida, United States*

Background: Mild behavioral impairment (MBI) is considered to be a late life transitional state between normal aging and dementia that describes individuals who have persistent behavioral changes and/or psychiatric symptoms. Individuals with MBI are found to be at greater risk of dementia compared to those without these symptoms. Identifying how MBI might relate to different domains of cognition is of key importance, as it could be an early indicator of a future dementia diagnoses. Method: Secondary data analysis of a sample (n=512) of older adults from the Florida Alzheimer's Disease Research Center who were either cognitively healthy or presenting with mild cognitive impairment (MCI). Some individuals presented with MBI, as defined by decreased motivation, affective dysregulation, impulse dyscontrol, social inappropriateness, or abnormal perception/thought content. Executive function, attention, short-term memory, and episodic memory, were compared using a battery of neuropsychological assessments. Results: Individuals with MCI performed worse on all tasks across all cognitive domains, where individuals with MBI performed worse on several tasks associated with executive function, attention, and episodic memory. Compared to individuals with only MCI, individuals with MCI and MBI performed significantly worse on tasks associated with executive function and episodic memory. Conclusion: The present study found evidence that individuals with MBI will perform worse on tasks of executive function, attention, and episodic memory. Further, those with MCI and MBI will perform significantly worse on executive function and episodic memory tasks. Future research should explore if these findings can help to predict specific dementia diagnoses.

### TASKS AND EDUCATION RESOURCES OF ADOLESCENTS WHO ASSIST WITH CAREGIVING FOR A FAMILY MEMBER WITH DEMENTIA

Jennifer Perion,[1] April Ames,[1] and Victoria Steiner[1], *1. University of Toledo, Toledo, Ohio, United States*

An abundance of research involving adults who care for family members with dementia has guided the creation of supportive programs/services. Much less is known about adolescents who are dementia caregivers. This descriptive secondary analysis utilized data collected during a qualitative examination into the psychological well-being of adolescent dementia caregivers. Eleven adolescent/adult dyads who provided dementia care for a family member completed surveys prior to the adolescents' participation in focus groups. Five male and six female adolescents ages 12 to 17 and eleven female adults were asked similar questions about caregiving tasks, education resources, and demographic information. Using descriptive statistics, the results of the surveys provide a snapshot of caregiving among a group of adolescents living in northwest Ohio and highlight differences reported by the dyads. Adult accounts of adolescent preparatory education were incongruent with the adolescents' and did not report their use of books or online caregiving resources. Conversely, three adults (27%), but no adolescents, identified hands-on and observational opportunities as education resources. Adults reported greater adolescent involvement in ten activities of daily living (71%), especially related to bathing, shopping, transportation, and managing medication and finances. Adolescents reported helping with tasks such as eating and laundry more often than adults. While the sample size was small, these findings suggest a need for triangulation when seeking knowledge about adolescent caregiving. These results may inform researchers wishing to investigate the role of adolescent caregivers, as well as guide supportive agencies who provide education materials to families caring for individuals with dementia.

### DEVELOPMENT, RELIABILITY, AND VALIDITY OF THE CAREGIVER MEALTIME ENGAGEMENT SCALE IN NURSING HOMES

WEN LIU,[1] Melissa Bachelar-Murphy,[2] and Kristine N. Williams[3], *1. The University of Iowa, Iowa City, Iowa, United States, 2. George Washington University, Washington, D.C., United States, 3. University of Kansas Medical Center, Kansas City, Kansas, United States*

Persons with dementia commonly experience mealtime challenging behaviors resulting in negative outcomes. Appropriate caregiver engagement is critical in engaging residents in eating. Current caregiver behavior measures are neither validated nor specific for mealtime care. A feasible and reliable measure to evaluate caregiver engagement during mealtimes is needed. Our team developed the Caregiver Mealtime Engagement Scale (CMES), a 29-item observational measure with good content validity (Content Validity Index = 1.00). The CMES includes 24 positive behaviors (e.g., position resident upright) and 5 negative behaviors (e.g., interrupt resident). Each item is scored by frequency on a 0 (never) – 3 (always) scale. Total score ranges from 0-87; higher score indicates better engagement. This study aimed to test the CMES' reliability and validity through a secondary analysis of 87 mealtime video-recorded observations from a hand feeding trial (P30). The sample included 7 residents and 25 staff from 2 nursing homes. The CMES has good internal consistency (Cronbach's $\alpha$ =.775). Inter-rater reliability was good ($r = .861$, $p<.001$) based on ratings of 20 videos by two independent trained coders. Intra-rater reliability was excellent ($r = .905$, $p<.001$) based on ratings of 20 videos by one trained coder at two times (2-3 weeks apart). The CMES demonstrated good convergent validity based on association with the Relational Behavior Scale ($r = .822$, $p<.001$) and Mealtime Relational Care Checklist ($r = .324$, $p=.002$). Findings support the CMES' reliability and validity. Future research is needed to test CMES among a larger diverse sample of caregivers in different settings.

### EMPOWERMENT AMONG FORMAL CAREGIVERS WORKING WITH PERSONS WITH DEMENTIA IN HOME CARE

Heather A. McIlveen,[1] Marie Y. Savundranayagam,[1] JB Orange,[1] and Marita Kloseck[1], *1. Western University, London, Ontario, Canada*

There is significant literature on workplace empowerment that focuses on individuals in positions of power rather than those who lack it. However, there is limited research on empowerment of home care workers, such as personal support workers (PSW) who work in dementia care. Empowerment is an active process based on a multifaceted model consisting of four components: meaning, self-determination, impact and competence. This study explored the roles of education and employer support in empowering PSWs to care for persons with dementia who live at home. Empowerment was investigated using semi-structured interviews with PSWs (N=15). A phenomenological approach was to understand the lived experiences of home-care based PSWs who work with persons with dementia. Components of empowerment were reflected through five emerging themes: "providing best care", "autonomy", "employer support", "career long learning", and "experiential learning". The theme "providing best care possible" support the component of meaning, which included the motivation for training among PSWs and their value of aging in place. The theme "autonomy" supported the component of self-determination, which focused on PSW workload and feelings regarding their control working in home care versus long term care. The theme "employer support" supported the component impact, which included both PSW compensation and their perceived lack of emotional support. Finally, the themes "career-long learning" and "experiential learning", were linked with impact and competence components, respectively. Overall, these findings support relationships between education and employer support in empowering PSWs who care for persons with dementia who live at home.

### FOSTERING COMPASSIONATE CARE FOR PERSONS WITH ALZHEIMER'S DISEASE AND RELATED DEMENTIAS

Samantha G. Cotton,[1] Anna Faul,[1] Joe D'Ambrosio,[1] and Pamela Yankeelov[1], *1. University of Louisville, Louisville, Kentucky, United States*

The aim of this study was to examine the impact of the implementation of a new Compassionate Care (CC) curriculum, designed by social workers, on the quality of care provided by Certified Nursing Assistants (CNAs) to residents with Alzheimer's disease (AD). Additionally, the purpose was to create a collaborative network of CNAs that supported each other. The sample included residents and CNAs from an experimental nursing facility and a control nursing facility. At baseline and 12-weeks, data were collected on AD knowledge, self-efficacy, caregiving satisfaction, and affiliate stigma. CNA changes in terms of their knowledge of AD, self-efficacy, caregiving satisfaction and affiliate stigma were analyzed using a two-way mixed method MANOVA. The stress levels of the residents, specifically agitation and salivary cortisol levels, was examined by testing a hybrid multilevel growth model. The final models were able to show how the changes in the CNAs specifically affected these positive outcomes. CNA knowledge and self-efficacy had the most impact on changing agitation levels, and CNA knowledge and agitation levels had the most impact on salivary cortisol levels. The results of this study showed that integrating a compassionate care curriculum into the work that CNAs perform can lead to positive outcomes on knowledge, self-efficacy, caregiving satisfaction, affiliate stigma and a reduction of agitation and cortisol levels in persons with AD.

### DEVELOPING A CONCEPTUAL MODEL OF FAMILY PREPAREDNESS FOR FUTURE DEMENTIA CAREGIVING IN CHINESE FAMILIES

Jacky CP Choy[1], *1. Department of Social Work and Social Administration, The University of Hong Kong, Hong Kong, Hong Kong*

Dementia is a growing health challenge that demands better public preparedness. Persons with dementia often lack the capacity to make and execute plans such that family involvement in care preparation becomes necessary. It is commonly observed in Chinese societies that there are more than one family members involved in the taking care of the person with dementia. The current qualitative study aims to understand preparedness for dementia caregiving of a family as unit in a Chinese society. In-depth interviews with 10 family units of dementia caregivers were conducted. Participants (4 spousal caregivers; 44 to 80 years old; mean years of caregiving: 3.3) reflected on how prepared their families were before the caregiving began. Thematic analysis was applied to examine the family preparedness and the family dynamics throughout the caregiving journey. As opposed to a crisis-driven involvement, involvement of more family members before crisis was helpful for reducing the damage brought to the family. Furthermore, families that could align their expectation and understanding of the situation, share knowledge and resources, negotiate the allocation of caregiving duties, and provide emotional support among family members were more likely to provide proper care with minimal sacrifice in family wellbeing. Chinese families often worked as a caregiving team, yet, with uneven distribution of caregiving duties and a lack of proper communication to sustain their caregiving role healthily. Timing and quality of family involvement were more influential factors than family resources to successful adaptation to caregiving.

### PERSONHOOD-BASED KNOWLEDGE: A NEW CONSTRUCT PREDICTING PERSONAL DEMENTIA FEAR

Alexandria R. Ebert,[1] Susan McFadden,[2] and Danica Kulibert[3], *1. West Virginia University, Morgantown, United States, 2. University of Wisconsin Oshkosh, Oshkosh, United States, 3. Tulane University, New Orleans, United States*

Perhaps because the public is not well-versed on the biological and medical facts of dementia (biomedical knowledge; BK), or the life experiences and capabilities of persons living with dementia (personhood-based knowledge; PBK), dementia is one the most feared and stigmatized terminal illnesses (Alzheimer's Society, 2007), typically resulting in social isolation (George, 2010). Similar to personal dementia fear (fear of developing dementia; PDF), dementia worry has been associated with suicide ideation (Cui et al., under review), which is a predictor of accepting attitudes toward physician-assisted suicide (Wolfgag, 2017). Findings from the development and testing of a conceptual model of social comfort indicated that people with higher levels of PBK had higher levels of social comfort towards persons with dementia (Ebert, Kulibert, & McFadden, 2019). The present study is a secondary analysis of data obtained from Wisconsin residents through an online platform and community outreach efforts (Ebert, Kulibert, and McFadden, 2019; N = 645) to examine whether individuals with higher levels of PBK have lower levels of PDF. A hierarchical linear regression revealed that PBK and BK were significant predictors of PDF ($\beta = -.13$, $p < .05$; $\beta = .108$, $p < .05$, respectively). However, when controlling for age ($\beta = .14$, $p < .01$) and knowing a friend or family member with dementia ($\beta = -.19$, $p < .001$), only PBK remained a significant predictor ($\beta = -.16$, $p < .01$). Results suggest that enhancing PBK through interactions with people living as well as possible with dementia could reduce PDF.

### REJECTION OF CARE, AGGRESSION, AND AGITATION IN PERSONS WITH DEMENTIA: A REVIEW OF DEFINITIONS IN DEMENTIA MEASURES

Scott Choi,[1] Maan Cajita,[2] and Laura N. Gitlin[3], *1. Towson University, Towson, Maryland, United States, 2. University of Pittsburgh, Pittsburgh, Pennsylvania, United States, 3. Drexel University, Philadephia, Pennsylvania, United States*

Objective: To provide a systematic review of how rejection of care, aggression, and agitation are described and operationalized in existing measures of dementia-related behaviors with a particular focus on whether these behaviors are conceptualized as separate phenomena in rating scales. Methods: We reviewed two systematic reviews of behavioral measures in dementia to evaluate their definitions and operationalization of rejection, aggression, and agitation. Additionally, we conducted a systematic review of English-language peer-reviewed articles published from 1980 to 2017 to update the previous list instruments and identify additional measures that were not captured in previous reviews. Results: 43 instruments (23 general behavior measures, 20 symptom-specific measures) developed to measure behavioral symptoms were included. Of these, 25 (58.1%) included items related to rejection of care; 32 (74.4%) included aggression items; and 35 (81.4%) had agitation items. Descriptions and definitions of the behaviors were highly variable across instruments. 13 of 23 general measures and 3 of 20 symptom specific measures included items separately representing all three behaviors while the rest of the measures lacked items measuring one or two behaviors of our interest. Conclusions: The review demonstrated that rejection, aggression, and agitation are measured in most scales yet their measurement is highly variable and they are often not distinguished from each other. Researchers and clinicians need to consider each symptom in its own right and revise existing instruments to address possible misnomers to improve measurement of dementia behaviors.

### THE RELATIONSHIP BETWEEN HAIR CORTISOL, CHRONIC STRESS, AND WELL-BEING AMONG OLDER ADULTS WITH DEMENTIA

Eunsaem Kim,[1] Cory Bolkan,[1] Erica Crespi,[2] and Jennifer Madigan[2], *1. Washington State University, Vancouver, Washington, United States, 2. Washington State University, Pullman, Washington, United States*

Hair cortisol concentration (HCC) is a new method of measuring chronic stress by analyzing a small sample of hair. Given the strong relationship between chronic stress and quality of life (QOL) in people with dementia, HCC may enhance our understanding of their well-being. Our primary goal was to establish feasibility of HCC testing in people with dementia as a biomarker of chronic stress. To do so, we examined the HCC levels of chronic stress during the transition to memory care. Newly admitted memory care residents (N = 13, mean age = 82) were followed over nine months. Residents' hair samples and health information were collected at three-month intervals. HCC in individuals with dementia (16.29 pg/mg) were considerably lower than older adults without dementia (30.48-57.8 pg/mg). Transition to memory care was associated with significantly decreased HCC levels ($\beta = -.70$, $p < .001$). Depression ($\beta = -.32$, $p < .05$) and psychotropic medication use ($\beta = -.20$, $p < .01$) facilitated the rate of HCC decline. Additionally, greater fluctuations in HCC over time were associated with higher HCC levels ($\beta = .048$, $p < .05$). Elevated HCC levels were related to poor functional capabilities ($\beta = 8.31$, $p < .05$), low social engagement ($\beta = -6.01$, $p < 05$), and poor overall QOL ($\beta = -3.61$, $p < .01$). These results underscore the significance of positive care environments to minimize chronic stress in individuals with dementia and indicate that HCC may be a useful stress measure in this population.

### LIFESTYLE BEHAVIORS AND RACIAL DIFFERENCES IN ALZHEIMER'S DISEASE RISK

Roger Wong[1], *1. Washington University in St. Louis, Saint Louis, Missouri, United States*

There is substantial evidence indicating that among all racial groups, Blacks have the highest Alzheimer's disease (AD) risk. Despite the wide support for this disparity, the reasons for these racial differences in AD risk remain unclear. The purpose of this study was to examine how lifestyle behaviors (physical activity, smoking, and social contacts) mediate and moderate the relationship between race and AD risk among Black and White older adults. This study used seven annual waves (2011-2017) of prospective data from the National Health and Aging Trends Study (NHATS), a large nationally representative U.S. sample of older adults. At each wave, physical activity was measured as whether they engaged in vigorous physical activities; smoking was measured as whether they were cigarette smokers; and social contacts was measured as whether they visited friends/family outside of their home. The dependent variable was age of AD diagnosis. Multivariate analyses were conducted using the Cox proportional hazards model. Blacks had a 1.3 times significantly higher risk for AD compared to Whites (Hazard Ratio [HR]=1.31, p=.03). After controlling for lifestyle behaviors as a mediator, racial differences in AD risk were attenuated and no longer significantly different between Blacks and Whites (HR=1.05, p=.74). For the moderation model, interactions between race and each lifestyle behavior generated no statistically significant results. Our findings indicate lifestyle behaviors contribute to racial differences in AD risk between Blacks and Whites. Future research is needed among Black populations to identify specific lifestyle behaviors that are especially protective against AD as targets of intervention.

### SESSION 2360 (POSTER)

### DISABILITY, FALLS, AND MOBILITY

### PROXIMAL DETERMINANTS OF FALLS IN OLDER ADULTS: THE MOBILIZE BOSTON STUDY

Hyun Gu Kang,[1] and Jonathan Hsu[1], *1. California State University San Marcos, San Marcos, California, United States*

Objectives: To study risk factors for falling, we examined risk factors for falls among older people according to the proximal determinants present at the time of the fall. Methods: Data came from MOBILIZE Boston, a prospective cohort study of 765 community-dwelling women and men, mainly aged 70 years or older. Over 4.3 years, 1737 falls were recorded, along with narrative reports describing proximal determinants at the time of the fall. Proximal determinants were identified from narrative reports and falls were classified into categories. Categorization was verified using three raters. Results: 14 categories of proximal determinants were identified. Of these, environmental determinants were the largest contributor to falls (74%). Participants with poor mobility and executive function were more likely to fall while performing activities of daily living, specifically while trying to stand and bending over. However, participants with poor mobility also had lower likelihood of falling to environmental hazards and dual-tasking cognition. In contrast, high-functioning older adults with naturally fast movement speed tend to fall to environmental factors while engaging in complex motor activities. Conclusions: Our results suggest there may be two populations of fallers, the healthy and the disabled, each with their own set of distinct risk factors and triggers. Cognitively functional older adults who choose to engage in vigorous activities in hazardous environments may increase their chances of falling to dual-task cognition. Community fall prevention efforts may benefit from examining the needs of specific subpopulations.

### RACIAL DISPARITIES IN HIP FRACTURE CARE

Iman Ali,[1] Jessica Jang,[1] Sanjana Vattigunta,[1] Ankit Bansal,[1] and Uma Srikumaran[2], *1. Johns Hopkins University, Baltimore, Maryland, United States, 2. Johns Hopkins, Baltimore, Maryland, United States*

Hip fractures are associated with significant morbidity and mortality. Delaying surgery for more than 24 hours after presentation results in more complications, higher 30-day mortality rate, and longer stays in the hospital. As such, high-quality care should be provided consistently to an increasingly diverse patient population. We determined if race characteristics influence the quality of care provided to patients with hip fractures. We conducted a retrospective analysis on patients at our institution between January 2015 and December 2017. Patients were categorized as white, Black, Asian, and other. The primary outcome variable was the time between presentation to surgery. Other outcomes included length of hospital stay and narcotic pain medication consumption in the first 24 hours postoperatively. Adjusted analysis was performed, controlling for sex, age, body mass index (BMI), American Society of Anesthesiologists' (ASA) classification of health, and Charlson Comorbidity Index (CCI). There were 1544 hip fracture patients included in the study. The majority of patients were white (84.1%) followed by Black (7.6%), Asian (4.5%), and other (3.7%). Most patients were female (69.6%). After adjusting for patient characteristics, Black patients experienced a significantly greater delay to surgery after presentation than white patients (42.1 vs. 34.9 hours). In addition, Black patients experienced significantly longer length of hospital stays compared to their white counterparts (6.9 vs. 5.8 days). Racial disparities in the quality of care provided to hip fracture patients persist even after adjusting for patient characteristics. Addressing these disparities can possibly enhance outcomes for minority patients.

### LINKING VISUAL-SOMATOSENSORY INTEGRATION TO COGNITIVE AND MOTOR OUTCOMES IN AGING

Jeannette R. Mahoney[1], *1. Albert Einstein College of Medicine, Bronx, New York, United States*

Ability to successfully integrate simultaneous information relayed across multiple sensory systems is an integral aspect of daily functioning. Unisensory impairments have been individually linked to slower gait, functional decline, and increased risks for falls in aging. Yet, research investigating age-related changes in multisensory integration (MSI) processes still remains relatively scarce. To date, there has been converging evidence for larger behavioral multisensory effects in older compared to younger adults; however, the question of whether larger effects are actually beneficial remains largely unanswered. Findings from our studies provide support for differential multisensory processing in aging, where decreased magnitude of visual-somatosensory integration was associated with worse balance, increased falls, and slower gait. Furthermore, we established a link between visual-somatosensory integration and cognition in aging. That is, magnitude of visual-somatosensory integration was largest in older adults with normal cognitive functioning, and presence of MCI/dementia significantly decreased magnitude of visual-somatosensory integration which in turn adversely impacted balance and gait performance. While the effect of MSI has been attributed to basic degenerative changes in neuronal architecture during the aging process, this speculative interpretation has yet to be formally tested. Future studies are clearly needed to establish the structural and functional correlates of MSI in aging, specifically visual-somatosensory integration, in order to further establish the link between differential multisensory effects with other important age-related clinical outcomes. Nevertheless, these studies stress the importance of successful MSI in aging, and highlight the need for multisensory based interventions that could potentially ameliorate disability.

### RISK FACTORS FOR FALLS IN OLDER ADULTS WITH TYPE 2 DIABETES

Edgar R. Vieira,[1] FABIELI FONTES,[2] BARTOLOMEU FILHO,[2] FABRICIA CAVALCANTI,[3] and JULIANA GAZZOLA[4], *1. Department of Physical Therapy, Florida International University, Miami, Florida, United States, 2. Graduate Program in Physical Therapy, Federal University of Rio Grande do Norte, Natal, RN, Brazil, 3. Graduate Program in Physical Therapy, Federal University of Rio Grande do Norte, Natal, RN, Brazil, NATAL, Brazil, 4. Graduate Program in Physical Therapy, Federal University of Rio Grande do Norte, Natal, RN, Brazil, NATAL, Rio Grande do Norte, Brazil*

The objective of this study was to evaluate the probability of the risk of falls in the older adults with type 2 diabetes.

One-hundred and eleven older adults (age: 69±7 years) with type 2 diabetes participated in this cross-sectional observational study. The participants sociodemographics, physical function, cognitive status (Mini Mental State Exam – MEEM and Geriatric Depression Scale – GDS), balance (Mini BEST test), functional performance (WHODAS 2.0) and falls risk (Quick Screen Clinical Falls Risk Assessment – QuickScreen) were evaluated. The data was analyzed using the Kruskall-Wallis, Chi-square, and Fisher's exact tests (p<0.05). Thirty percent of the participants had fallen during the previous 12 months, and 80% of the participants reported fear of falling. The average number of falls risks was 3.5±2. Increased number of falls risks were associated with lower educational level (p=0.005), poorer general health (p=0.001), vision impairment (p=0.017), higher number of diseases (p<0.0001), higher number of medications (p<0.0001), longer diabetes duration (p<0.0001), lower limb pain (p<0.0001), depression (p<0.001), worse functional performance (p<0.0001), and worse balance (p<0.0001). Older adults with type 2 diabetes with lower education, worse health and vision, greater number of diseases and medications, longer diagnosis of diabetes, lower limb pain, depressive symptoms, worse functional performance and balance presented more risks for falls.

### SERUM 25-HYDROXYVITAMIN D LEVELS IMPACT RISK OF EXTENSIVE ASSISTANCE NEEDED BY OLDER ADULTS TO WALK

Krista N. Jenney,[1] Ronna Robbins,[1] Sara Sweitzer,[1] Nalini Ranjit,[2] and Margaret Briley[1], *1. The University of Texas at Austin, Austin, Texas, United States, 2. The University of Texas Health Science Center at Houston School of Public Health in Austin, Austin, Texas, United States*

Study objective is to determine the association between deficient 25-hydroxyvitamin D [25(OH)D] serum levels and the amount of assistance needed to walk in a room by older adults living in long-term care (LTC) communities. Participants (age ≥ 65) from five LTC communities in Central Texas were recruited for a multi-site, cross-sectional study (n=169). Double-blinded data abstraction protocols were used to collect a one-year medical history. Laboratory blood draws measured serum 25(OH)D levels. Level of assistance was measured by the activities of daily living score for walking in room from section G of the Minimum Data Set (MDS). To determine the association between deficient 25(OH)D serum levels (≤20 ng/ml) and assistance with walking, adjusted logistic regression was used. Total vitamin D per day (supplementation and meals), therapy and/or restorative programs, body mass index, race, gender, age, and years living in the community were used as confounders. Of the 169 participants (mean age=83) 27.17% had deficient serum 25(OH)D and 9.25% required extensive assistance to walk in a room. The mean serum level and supplementation rate of participants was 32.61 ng/ml and 1,160.64 IU per/d, respectively. Participants with deficient 25(OH) D serum levels had significantly elevated odds (OR=8.73; CL: 1.28, 8.54; p=0.027) of requiring extensive assistance to walk in a room compared to those with adequate serum levels (>20 ng/ml). Deficient 25(OH)D serum levels are associated with increased assistance to walk in a room indicating that adequate serum levels in LTC residents could potentially decrease burden on staff.

### ONE-LEGGED STANCE BALANCE OF OLDER ADULTS WITH AND WITHOUT FALLS

Edgar R. Vieira,[1] Márcio Oliveira,[2] Andre Gil,[3] Karen Fernandes,[4] Denilson Teixeira,[5] Cesar F. Amorim,[6] and Rubens da Silva[7], *1. Department of Physical Therapy, Florida International University, Miami, Florida, United States, 2. Clinical Master's Program in Physical Exercise in Health Promotion, Pitagoras Unopar, Londrina, Parana, Brazil, 3. Department of Physical Therapy, Pitagoras Unopar, Londrina, Parana, Brazil, 4. Graduate Programs in Rehabilitation Sciences UEL/UNOPAR, Londrina, Parana, Brazil, 5. Department of Physical Education, Universidade Estadual de Londrina, Londrina, Parana, Brazil, 6. Department of Physical Therapy, Florida International University, Miami, Florida, United States, 7. Departement des Sciences de la Sante, Universite du Québec à Chicoutimi (UQAC), Saguenay, Quebec, Canada*

Balance impairment is a common problem among older adults. Poor balance in older adults is often associated with mobility impairments, activity limitations and fear of falling in older adults. Thus, balance assessment is useful for early detection of postural control deficits to prevent mobility impairments and falls in older adults. The aim of this study was to assess if balance measures based in center of pressure (COP) parameters during one-legged stance could differentiate between older adults with and without falls in the past 12 months. One-hundred and seventy older adults (50 fallers and 120 non-fallers, age range: 63-72 years) performed three 30s one-legged stance trials with eyes open on a force platform with 30s of rest between each trial. The following variables were evaluated: COP 95% elliptical area, COP velocity in the anterior-posterior and medio-lateral directions, and test duration (how long the participant was able to stay in one-legged stance, up to 30s). Fallers had poorer balance than non-fallers (P ≤0.004). The COP parameters presented an area under the curve between 0.65-0.72, with sensitivity varying from 66 to 78% and specificity from 54 to 68%. There were no significant differences between fallers and non-fallers on test duration (17 vs. 18s, respectively). The findings showed that the fallers had similar duration time, but poorer balance than the non-fallers during one-legged stance. The COP parameters were able to differentiate the balance between fallers and non-fallers with acceptable area under curve, sensitivity and specificity.

### CHARACTERISTICS OF MALADAPTIVE FALL RISK APPRAISAL AMONG OLDER ADULTS AGES 60 YEARS AND OLDER

Ladda Thiamwong,[1] and Boon Peng Ng[1], *1. College of Nursing, University of Central Florida, Orlando, Florida, United States*

Even though one-third of older adults have maladaptive fall risk appraisal (FRA), no studies has examined this discrepancy between perceived fall risk and physical fall risk among older adults in Thailand. We examined the characteristics of fall risk appraisal (FRA). 433 community-dwelling older adults were randomly selected from two provinces in

Southern Thailand. Physical fall risk was assessed by a full tandem stand test and perceived fall risk was assessed by the Fall-Efficacy Scale International. We classified FRA into: 1) Rational FRA means low physical fall risk and low perceived fall risk; 2) Irrational FRA means low physical fall risk but high-perceived fall risk; 3) Congruent FRA means high physical fall risk and high-perceived fall risk; and 4) Incongruent FRA means high physical fall risk but low perceived fall risk. Irrational FRA and Incongruent FRA are a maladaptive FRA. About 60% of the participants had maladaptive FRA, which consisted of irrational FRA (57.3%) and incongruent FRA (2.3%). 20.8% were in rational FRA and 19.6% in congruent FRA. Among those with rational FRA, incongruent FRA, irrational FRA, and congruent FRA, 27.8%, 60%, 41.1%, and 74.1% reported having at least one fall in the past year, respectively. After covariate adjustment, participants in the congruent FRA group were 3.29 times more likely (p=0.006) to fall than those in rational FRA. High proportion of participants had maladaptive FRA so screening individuals with maladaptive FRA and prevent them to transition into the congruent FRA group is important efforts to mitigate health and economic burdens.

### FALLS AND NUTRITIONAL RISK AMONG RURAL STATE RESIDENTS

Mariana Wingood,[1] Nancy M. Gell,[1] and Emily Tarleton[1], *1. University of Vermont, Burlington, Vermont, United States*

Vermont continues to have one of the nation's highest fall rates and its rurality may be a contributing factor. The purpose of our study was to compare fall history and nutritional risk (a fall risk factor also associated with rurality) in participants from rural and metropolitan areas. We collected data at statewide community-based fall risk screenings. During the events, nutritional data was collected using the DETERMINE Your Nutritional Health Screening Tool Questionnaire. We used descriptive statistics (chi2) to examine the relationship between fall history, nutritional risk, and rurality. From 123 subjects, 67% were classified as rural residents. There was no relationship between fall history and nutritional risk (p=0.6). Compared to rural residents, a significantly higher percentage of those living in metropolitan areas reported falls (54% versus 35% p=0.05). However, metropolitan residents were not at higher nutritional risk (49% versus 54%, p=0.61). National nutritional risk rates are lacking, but food insecurity is associated with nutritional risk. Our overall reported high nutritional risk (20%) is higher than the prevalence of food insecurity, both nationally (11%) and in Vermont (9%). In conclusion, we did not identify a relationship between fall history and nutritional risk. We did find a higher percentage of metropolitan residents reporting falls. Furthermore, we identified that DETERMINE is a feasible nutritional screening tool to use at fall risk screenings. It can be used to identify community-dwelling older adults at nutritional risk, but it may not have the sensitivity to identify an association between nutritional risk and falls.

### ABILITY OF THE PHYSIOLOGICAL PROFILE ASSESSMENT TO CLASSIFY FALLER TYPE: A PROSPECTIVE COHORT STUDY

Deborah A. Jehu,[1] Jennifer C. Davis,[2] Kristin Velsey,[3] Winnie Cheung,[1] and Teresa Liu-Ambrose[1], *1. University of British Columbia, Vancouver, British Columbia, Canada, 2. University of British Columbia - Okanagan Campus, Kelowna, British Columbia, Canada, 3. University of British Columbia, Vancouver, Canada, Canada*

Accurately identifying older adults who will experience subsequent falls is important for the provision of secondary fall prevention. The purpose of this study was to determine the accuracy of the Physiological Profile Assessment (PPA) – a valid and reliable fall-risk assessment [1] – in predicting subsequent falls over a 12-month period in older adults who sought for medical attention after an index fall. Seven hundred thirty-seven community-dwelling adults, aged 70 years and older, who were seen at the Vancouver General Hospital Fall Prevention Clinic, completed the PPA at their initial visit. Falls over the subsequent 12 months were tracked prospectively via monthly falls calendars. All individuals received geriatric care at baseline. Binary logistic regressions were performed to determine the accuracy of classifying two prospective faller types: 1) no additional falls; 2) one or more additional fall(s). Baseline PPA, age, and sex were entered as independent variables. During the 12 month observation period, 345 participants had no additional falls (Age:81.3±6.6yrs;Female=251) and 392 fell one or more times (Age:82.3±6.5yrs;Female=230). The classification accuracy was 51.3% for those who had no additional falls and 64.8% for those with one or more additional fall(s) (Overall:58.5%;$\chi2$=29.0;PPA:$\beta$=-0.21;Age:$\beta$=-0.01;Sex:$\beta$=-60). The PPA was not able to accurately differentiate between those who did and did not subsequently fall. Fall-risk assessment sensitivity and specificity should be improved in older adults seeking medical attention following an index fall to inform secondary fall prevention. [1] Lord SR, et al., 2003. Phys Ther.

### FALL RISK BEHAVIORS AND INTRINSIC RISK FACTORS FOR FALLS IN INDIGENOUS AND NON-INDIGENOUS RURAL OLDER ADULTS

Andre G. Bouweraerts,[1] and Justus Ortega[1], *1. Humboldt State University, Arcata, California, United States*

Within California, older adults living in rural counties have reported higher rates of falls than urban dwelling older adults. Although many Indigenous people live in rural areas, it is unclear whether the rate of falls among Indigenous older adults is similar to that of non-indigenous older adults living in rural areas. Thus, the purpose of this study was to examine fall risk behaviors and intrinsic risk factors for falls in rural dwelling Indigenous (N = 89), and non-Indigenous (N = 68) older adults 60-95 years of age living in California. Results showed that both Indigenous and non-Indigenous older adults share similarly high fall rates, but there are a much greater number of Indigenous older adults falling multiple times a year. Moreover, fall risk behaviors and intrinsic fall risk factors were significantly different between Indigenous and non-Indigenous rural-dwelling older adults. Future studies should investigate falls and fall risk factors in different tribes/locations of Indigenous older adults to better understand whether these risk factors differ among tribes. Moreover, it would be beneficial for future studies to assess the effectiveness of fall prevention exercises on fall risk in these communities. Information gained from this study helps to inform clinicians and researchers alike about the prevalence of falls and factors contributing to falls among Indigenous older adults living in rural communities; and helps to influence decisions in the future of programs for reducing fall risk in this often neglected population.

### CRITICAL ASPECTS OF MOBILITY FOR IMPROVING PATIENT OUTCOMES AFTER CARDIAC SURGERY

Tiffany Tsay,[1] Jennifer A. Schrack,[2] Allyse Depenbrock,[1] Amal A. Wanigatunga,[1] Jacek Urbanek,[3] and Charles H. Brown[4], *1. Johns Hopkins Bloomberg School of Public Health, Baltimore, Maryland, United States, 2. Johns Hopkins University, Baltimore, Maryland, United States, 3. John Hopkins University School of Medicine, Department of Medicine, Division of Geriatric Medicine and Gerontology, Center on Aging and Health, Baltimore, Maryland, United States, 4. Johns Hopkins University School of Medicine, Baltimore, Maryland, United States*

Physical activity after major cardiac surgery has been associated with length of stay, discharge location, risk of readmission, and functional change. However, in-hospital mobility is not currently assessed in a standardized way with nurse reports being the primary mechanism of tracking patient activity. Furthermore, it is unclear whether it is the total amount, frequency, or type of activity that is most important for improving patient outcomes post-surgery. To better understand the duration, frequency, and intensity of patient activity post-cardiac surgery, we conducted an observational study of 206 patients using a wrist-worn accelerometer and ankle-worn pedometer. Patients with lower levels of average daily pedometer-based ambulation in the first four days post-surgery, when compared to counterparts who ambulated more, had higher odds of a longer length of stay (OR=4.55, p<0.0001) or being discharged to rehab vs. home (OR=7.7 p=0.012), independent of age, race, bypass time, and EuroSCORE (cardiac surgery risk score). Engaging in an average of less than two bouts of accelerometer-derived activity lasting 5 minutes or more each day was associated with higher odds of having a longer length of stay (OR=2.69, p=0.008) or being discharged to rehab vs. home (OR=20.9, p=0.019). A slower speed of recovery during the first four postoperative days, characterized by a smaller increase in pedometer-based ambulation with each successive day, was also associated with higher odds of being discharged to rehab vs. home (OR=6.62, p=0.008). Further research is needed to develop appropriate frequency and activity thresholds for use as intervention tools to improve patient outcomes post-surgery.

### NEURAL CORRELATES OF COMPLEX WALKING TASKS IN OLDER ADULTS

Nemin Chen,[1] Caterina Rosano,[1] Helmet Karim,[1] Stephanie Studenski,[1] Howard Aizenstein,[1] and Andrea Rosso[2], *1. University of Pittsburgh, Pittsburgh, Pennsylvania, United States, 2. University of Pittsburgh, Department of Epidemiology, Pittsburgh, Pennsylvania, United States*

Walking on a narrow path challenges attention and balance control but its neural correlates are unknown. We assessed the association between gray matter microstructural integrity and gait speed along a 6 m long and 20 cm wide path in participants from the Health, Aging, and Body Composition Study (n=155; mean age=83, 53% women, 35% black). Micro-structural integrity was measured by mean diffusivity (MD) in gray matter computed from diffusion weighted imaging (DWI); higher MD indicates lower integrity. We conducted general linear models to assess this association with gray matter microstructural integrity of regions of interest (based on known associations of usual pace gait speed): middle frontal gyrus; caudate; putamen; anterior, middle, and posterior cingulate; hippocampus; precentral gyrus; and supplementary motor area. We adjusted for total brain atrophy, usual pace gait speed, age, sex, race, and education. The average narrow-path gait speed was 0.97 m/s (standard deviation: 0.21). Average usual pace gait speed was 1.1 m/s (standard deviation: 0.21). After adjusting for covariates, we identified significant negative associations between narrow-path gait speed and gray matter MD of left posterior cingulate, left and right hippocampus, and left precentral gyrus (p<0.05). Narrow-path gait speed is associated with lower microstructural integrity in gray matter related to network connectivity (posterior cingulate), spatial cognition (hippocampus), and motor function (precentral gyrus).

### INDOOR AND REAL-LIFE OUTDOOR GAIT SPEED AND ITS ASSOCIATION ON GEOGRAPHIC LIFE-SPACE AND OUTDOOR MOBILITY

Chek Hooi Wong,[1] Pey June Tan,[1] Mei Sian Chong,[1] Jagadish U. Mallya,[2] Mimaika L. Ginting,[1] Isabel H. Ng,[1] Soon Hoe Ho,[1] and Belinda Yuen[3], *1. Geriatric Education and Research Institute, Singapore, Singapore, 2. Department of Geriatric Medicine, Khoo Teck Puat Hospital, Singapore, Singapore, 3. Singapore University of Technology and Design, Singapore, Singapore*

Independent outdoor mobility is important to community-dwelling older adults as it enables reach and access to resources for everyday activities, but this becomes increasingly challenging with the progressive decline in physical performance in ageing. We aim to understand the relationship between Indoor (IGS) and Real-Life Outdoor Gait Speed (OGS) with objectively-measured geographic life-space extent and outdoor mobility among community-ambulant older adults in Singapore. Thirty-three participants aged ≥55 years living in three neighbourhoods wore hybrid mobility trackers continuously for 7 days. Baseline 6-metre IGS was measured with a stopwatch, while OGS was from outdoor accelerometer data. Nodes were defined as significant places visited for ≥5 minutes. Multiple linear regressions examined each association between IGS and OGS on geographic life-space extent and outdoor mobility measures adjusting for confounders. Participants' mean age was 69.2±7.1 years with mean IGS and OGS of 1.11m/s and 0.85m/s respectively. They spent on average 4.7 hours/day out-of-home with the majority (57%) of nodes located within 500m from home. There were no significant associations between gait speeds and geographic life-space measures. Higher OGS was associated with higher total number of nodes/week while higher IGS was significantly associated with lower percentage of nodes within 500m from home. Our findings highlighted the complexity and multi-dimensionality of independent outdoor mobility as both gait speed performances were not significantly associated with geographic life-space extent, but rather with spatiotemporal interaction and opportunities to access locations for older adults' daily activities.

### COGNITIVE APPRAISAL AND KNOWLEDGE AS RISK FACTORS FOR HIGH FEAR OF FALLING IN OLDER AND MIDDLE-AGED ADULTS IN SINGAPORE

Pey June Tan,[1] Reuben Ng,[1] Angelique Chan,[2] Jagadish U. Mallya,[3] Noor Hafizah Ismail,[4] Chek Hooi Wong,[1] and Peter K. Tay[5], *1. Geriatric Education and Research Institute, Singapore, Singapore, 2. Centre for Ageing Research and Education, Duke-NUS Medical School, Singapore, Singapore, 3. Department of Geriatric Medicine, Khoo Teck Puat Hospital, Singapore, Singapore, 4. Department of Continuing and Community Care, Tan Tock Seng Hospital, Singapore, Singapore, 5. Centre for Ageing Research and Education, Singapore, Singapore*

Fear-of-falling (FOF) can be adaptive or maladaptive depending on one's appraisal of knowledge and beliefs, but few have elucidated this cognitive process in older adults surrounding falls. We aim to identify risk factors for high FOF amongst community-dwelling older adults (OA) and middle-aged adults (MA) in Singapore. This was a cross-sectional survey of a nationally-representative sample of OA (≥60 years) and MA (40-59 years) identified by stratified random sampling. Primary outcome was high FOF measured by a single-item (4-point scale). Independent variables were history-of-falls, quality-of-life, fall-related cognitive appraisal (balance problems, importance to restrict activities to prevent falls) and knowledge indicators (knowledge of other OA who fell, ability to identify out of 13 fall risk factors). MA were also asked if they're caregivers. Multiple logistic regressions identified risk factors for high FOF separately by age-groups, adjusting for socio-demographics and comorbidities. The final analysis included 549 OA (70.6±6.88 years) and 309 MA (49.7±5.89 years). No differences in high FOF was found among OA and MA (37% vs. 38%, p=0.305), but there were more falls among OA (19% vs 12%, p=0.010). Higher knowledge of fall risk factors and self-reported balance problems were significant risk factors for high FOF among OA only, while a history-of-falls and being a caregiver were significant among MA only. Perceived importance to restrict activities was associated with high FOF in both age-groups. Although findings suggest differences in the mechanism of high FOF between OA and MA, both age-groups have maladaptive appraisal tendencies related to restrict activities to prevent falls.

### ABERRANT FUNCTIONAL CONNECTIVITY UNDERPINNING CHANGES IN LIFE-SPACE MOBILITY IN OLDER ADULTS

Chun Liang Hsu,[1] John R. Best,[1] Rachel A. Crockett,[1] Patrick Chan,[1] and Teresa Liu-Ambrose[1], *1. University of British Columbia, Vancouver, British Columbia, Canada*

Subtle, but observable, changes in mobility often exist among older adults. Life-space mobility defines the frequency and extent of movements in the environment, and lower life-space mobility is associated with adverse health outcomes and mild cognitive impairment (MCI). Currently, the underlying mechanism of this association is not well understood. The aim of this study was to examine the functional neural correlates of reduced life-space mobility over 12 months. Thirty-five older adults over the age of 65 years with MCI were recruited and enrolled into this 12-month prospective study. Resting-state functional magnetic resonance imaging was completed at study baseline. Clinical assessment of anthropometric, behavioural measurements, and life-space mobility was conducted at study baseline and at the 12-month period. Over the 12-month study, the 35 participants demonstrated a significant reduction in LSA scores (paired sample t-test mean change=-6.53, p=0.01); greater baseline connectivity between the default mode network and the sensorimotor network was significantly associated with lower life-space mobility ($R2=0.44$, p=0.04). These findings suggest reduced life-space mobility in older adults may be partially due to altered inter-network connectivity in the brain such that normal neuro-cognitive motor behaviours is disrupted. Therefore, the maintenance of functional architecture of the brain may be essential in preserving the extent and frequency of older individuals' movement in their environment.

### SYSTEMATIC REVIEW OF THE EFFECTS OF NON-VR EXERGAMING ON MOBILITY OUTCOMES OF OLDER ADULTS LIVING IN LONG-TERM CARE

Charlene H. Chu,[1] Renee Biss,[2] Afroza Sultana,[3] Amelie Gauthier-Beaupré,[4] and Arezoo Talebzadeh[5], *1. Lawrence S. Bloomberg Faculty of Nursing, University of Toronto, Toronto, Ontario, Canada, 2. York University, Toronto, Ontario, Canada, 3. McGill University, Montreal, Quebec, Canada, 4. University of Ottawa, Ottawa, Ontario, Canada, 5. OCAD, Toronto, Ontario, Canada*

Introduction: Institutionalized older adults have high-rates of mobility decline resulting in reduced quality of life and increased dependency. Given the ageing population, there has been a proliferation of exergaming technology targeting older adults to maintain their physical activity (PA) levels and prevent decline. However, it is unclear if exergaming is effective to maintain or improve the PA of institutionalized older adults. Method: Four databases (MEDLINE/CINAHL/PsycINFO/Compendex) were systematically searched (key terms like "nursing homes", exergaming"). Quantitative manuscripts examining the effects of exergaming on PA measures of institutionalized older adults published in English between 2006-present were eligible. Virtual reality was excluded from the search. No meta-analysis was conducted due to hetereogeneity of the results. Results: 11 studies were included from a search that yielded 208 results. The exergaming platforms that were used the most were the Kinect and Wii. The most commonly used PA measures were the Berg Balance Scale and the Timed-up-and-Go (n=4 studies) with no other measures being used in more than one study. Interventions ranged in exercise (e.g. cognitive-motor training, strength training, balance, etc), frequency, duration, and modality. Study designs were also heterogeneous. Articles were of very poor to poor quality. There was minimal reporting on adverse events. Older adults with cognitive impairment were commonly excluded. Challenges in current technology and studying this group are highlighted. Conclusion: Exergaming may be promising to maintain PA but more robust research is needed. More exergaming technology designed for long-term care to meet the specific complex needs of this population is warranted.

### CHARACTERISTICS OF THE GAIT INITIATION PHASE IN OLDER ADULTS WITH DIABETIC PERIPHERAL NEUROPATHY

Gu Eon Kang,[1] Hung Nguyen,[1] Mohsen Zahiri,[1] He Zhou,[1] Changhong Wang,[1] Rahul Goel,[1] and Bijan Najafi[1], *1. Baylor College of Medicine, Houston, Texas, United States*

Impairment in steady-state gait in older adults with diabetic peripheral neuropathy (OADPN) is well-known, however little attention has been paid to the gait initiation phase in which postural transitions occur from upright standing to steady-state gait. Given the risk of falls in the gait initiation phase in older adults, knowing its characteristics may be as important as steady-state gait. The aim of this study was to investigate kinematic characteristics of the gait initiation phase in OADPN compared to healthy older adults (HOA). Thirteen OADPN (72.9±6.1 years; 33.0±4.8 kg/m2), and 11 HOA (71.8±2.7 years; 26.5±4.3 kg/m2; no cardiovascular, neurological or orthopedic condition, no history of falling) performed gait on level ground for minimum 10 meters at self-selected comfortable speed. We collected kinematic data using five wearable sensors (LEGSysTM, BioSensics LLC, Watertown, MA) attached on the shanks, thighs and lower back. We used previously validated algorithm to analyze kinematic parameters for the gait initiation phase. Our statistical model showed that the number of steps, stride velocity, gait cycle time, double limb support and mediolateral center-of-mass sway during the gait initiation phase is significantly different between HOA (2.4±0.7 steps; 1.16±0.15 m/s; 1.12±0.10 seconds; 20.3±4.8%; 4.0±1.5°, respectively) and OADPN (4.0±2.1 steps; 0.92±0.29 m/s; 1.23±0.12 seconds; 29.2±10.3%; 7.0±2.9°, respectively) (all $p<0.05$). The results suggest that OADPN take more, slower and more unstable steps to reach steady-state gait from upright standing compared to HOA. The results also provide implications for needs to develop new interventions targeting the gait initiation phase in OADPN.

### FRESH AND LEAN BEEF INTAKE IN RELATION TO FUNCTIONAL LIMITATIONS AMONG U.S. OLDER ADULTS, 2005-2016

Ruopeng An,[1] Sharon Nickols-Richardson,[2] Reginald Alston,[2] Sa Shen,[2] and Caitlin Clarke[2], *1. University of Illinois at Urban-Champaign, Champaign, Illinois, United States, 2. University of Illinois at Urbana-Champaign, Champaign, United States*

Beef is a key component in the American diet. This study assessed fresh and fresh lean beef intake in relation to functional limitations among U.S. older adults 65 years and older. Logistic regressions were performed on individual-level 24-hour dietary recall and health indicator data (N=6,135) retrieved from 2005–2016 National Health and Nutrition Examination Survey. Approximately 51%, 14%, and 9% of older adults consumed beef, fresh beef, and fresh lean beef, respectively. Daily increase in fresh beef consumption by 1 ounce-equivalent was associated with a reduction in the odds of lower extremity mobility limitation (LEM) by 16% (95% confidence interval=4%–27%), general physical activities limitation by 13% (1%–24%), and any functional limitation by 14% (2%–24%). Daily increase in fresh lean beef consumption by 1 ounce-equivalent was associated with a reduction in the odds of LEM by 22% (7%–34%) and any functional limitation by 15% (1%–28%). No association with activities of daily living, instrumental activities of daily living, or leisure and social activities limitations was identified. In conclusion, preliminary evidence links fresh and fresh lean beef consumption to reduced functional limitation risk. Older beef consumers are encouraged to modestly increase their intakes of fresh and lean beef, rather than total beef, to maximize attributes of functional health associated with beef consumption while concurrently avoiding additional saturated fat and sodium intake. Limitations of this study include measurement errors and cross-sectional study design. Future studies with longitudinal/experimental design are warranted to examine the influence of fresh/lean beef consumption on functional limitations among older adults.

### ACADEMIC PARTNERING IN A COMMUNITY-BASED FALL PREVENTION PROGRAM

Dennis W. Klima[1], Emily Wehlandl,[2] Aspen Holmes,[2] Jessica Weimert,[2] Nicholas Rhoten,[2] Jestine Wolfe,[2] Katherine Avila,[2] and Nate Austin[2], *1. UMES, Princess Anne, Maryland, United States, 2. UMES, Princess Anne, Maryland, United States*

Falls are a major cause of disability nationally are and linked to both fractures and fear of falling. The purpose of this study was to assess the effectiveness of a rural community-based fall prevention program using an academic partnership model with seven physical therapy students rotating through six senior centers. One hundred and fifty-four older adults (Mean age: 76.1+/- 8.5) completed Stepping On at six locations. A mixed-methods design was utilized. Physical therapy students partnered with older adult participants to teach exercises, strategize floor recovery techniques, and identify community safety barriers. A descriptive survey tool assessed demographic profiles, falls efficacy, and program effectiveness among participants. Students participated in a follow-up focus group to discuss perspectives on their role in the fall prevention program. Most participants were female (86.4%), lived alone (50.0%) and taking four or more medications (74%). Thirty-eight participants (24.7%) had fallen over the past year. Eighty-eight (57.1%) subjects noted they had less fear of falling following the community-based intervention; moreover, most subjects (74.7%) reported having an improved plan for floor rise after a fall. Major focus group themes underscored students' enhanced ability to teach exercise and mobility activities with an increased awareness of interdisciplinary fall prevention. Following a collaborative community-based fall prevention program, seniors have a better understanding of fall causes and plan to seek floor recovery assistance. In turn, student teaching and communication skills are reinforced. Student partnering with seniors promotes fall prevention strategies and affords benefits to both students and participants.

### RISING FROM THE FLOOR IN PERSONS WITH PARKINSON'S DISEASE

Dennis W. Klima,[1] Jeremy Stewart,[1] Frank Freijomil,[1] and Mary DiBartolo[2], *1. UMES, Princess Anne, Maryland, United States, 2. Salisbury University, Salisbury University, United States*

While considerable research has targeted gait, balance and preventing falls in individuals with Parkinson's disease (PD), less in known about the ability to rise from the floor in this population. The aims of this study were to 1) Examine the relationship between locomotion and physical performance tests and the timed supine to stand performance measure and to 2) Identify both the time required and predominant motor patterns utilized by persons with PD to complete to floor rise transition. A cross-sectional design was utilized. Twenty community-dwelling older adults with PD (mean age 74.8+/-9.5 years; 13 men) performed a standardized floor rise test and locomotion tests in a structured task circuit. Subject demographic and anthropometric data were also collected. Statistical analyses included descriptive statistics and Pearson Product Moment correlations. Fifteen subjects (75%) demonstrated the crouch kneel pattern and fourteen (70%) used an all-4's strategy to rise to stand. The mean time to rise from the floor was 14.9 (+/- 7.6) seconds and slower than published norms for persons without PD. Nine subjects required the use of a chair to perform floor recovery. Supine to stand performance time was significantly correlated with the: Dynamic Gait Index (r= - 0.66; p<0.002), Five Times Sit to Stand Test (r=0.78; p<0.001), Timed Up and Go Test (r=0.74; p<0.001), and gait velocity (r= -0.77; p<0.001). Rising from the floor demonstrates concurrent validity with locomotion and physical performance tests. Floor recovery techniques can be incorporated in fall prevention initiatives in conjunction with PD symptom management.

### ACHIEVING THE TRIPLE AIM THROUGH A FALLS PREVENTION INNOVATION IN LONG-TERM CARE

Deanna L. Gray-Miceli,[1] Sarah J. Ratcliffe,[2] Matthew L. Smith,[3] and Jeannette Rogowski[4], *1. Florida Atlantic University, Boca Raton, Florida, United States, 2. University of Virginia, Charlottesville, Virginia, United States, 3. Center for Population Health and Aging, Texas A&M University, College Station, Texas, United States, 4. The Pennsylvania State University, University Park, Pennsylvania, United States*

Falls disproportionately burden frail older adults in long-term care, which is evidenced by the 60% fall rate annually in this setting. Our health systems improvement model, framed by the Health Outcomes Model for falls prevention, is targeted to achieve the triple aim of better health, better care, and better value. Highlighting evidence from three inter-related studies, this presentation describes the impact of a practice change model and its impact on patient, unit/staff, and organizational level factors as related to facility-wide falls prevention. In study one, we used an evidenced-based practice approach and assessment tool to achieve total and recurrent falls reduction over one year by 32% and 25%, respectively (p<0.001). In study two, the annual savings of using this approach was assessed and showed a total savings of $53,531.00, which equates to $700.00 per falling person. In pilot study three, a convenience sample of 15 older adults who were interviewed about their fall by nurses using this approach stated they felt valued and that the nurse cared about their story (n= 6; 40%). Purposive efforts to embed the model and effective strategies for adoption and implementation are presented.

### PATTERNS OF DISABILITY ACCORDING TO ENROLLMENT TO HEALTH INSTITUTIONS AMONG ADULTS AGE 50 AND OLDER IN MEXICO

Eduardo Cabrero Castro[1], *1. Universidad Nacional Autónoma de México, Ciudad de México, Mexico*

The aim is to describe and analyze the progression of the limitation in the Basic Activities of Daily Living (ADL) in the period 2012 - 2015, in adults over 50, grouped by their enrollment to public health services and private health institutions in Mexico (6 groups). The sociodemographic and health characteristics of each set of adults are included in this study. Disability is measured using the ADL scale. Data come from the longitudinal Mexican Health and Aging Study in the years 2012 and 2015. Multinomial logistic regressions and multi-state life tables were carried out for the analysis. Between 35% and 50% of the participants with disabilities at the beginning of the study, recovered their functionality regardless of which health institution they were enrolled to. Those individuals enrolled in two or more institutions are .33 times more likely to have no disability, compared to those who are not enrolled in any institution. Transition probabilities from active life to ADL disability rise sharply with age until 86 years old (21%), from which it begins to descend. Conditional life expectancy estimates for those initially healthy at the age 60 is 30.07 years. Of the above, 4.74 years are lived with disabilities. The disability process follows different trajectories in each group of enrollees. These trajectories were associated with socioeconomic and health factors (age, sex, diabetes and cognition). Being enrolled in 2 or more institutions is related to the prevention of disability. This is probably due to the increased access to health services.

### NOVEL INSIGHTS ON THE RELATIVE IMPORTANCE OF CLINICAL AND GAIT MEASURES FOR DETECTING FALL RISK IN OLDER ADULTS

Sandra Hundza,[1] Stuart MacDonald,[1] Drew T. Commandeur,[2] and Mark Klimstra[1], *1. University of Victoria, Victoria, British Columbia, Canada, 2. University of Victoria, Victoria, Canada*

We sought to extend recent research that explored model-based approaches for combining clinical and gait measures to determine the most sensitive grouping for retrospectively classifying fallers from non-fallers which resulted in a model with 92% sensitivity and 66% specificity and an overall model of 83%. In the present study, the clinical assessment battery was augmented by incorporating more challenging balance items while removing clinical measures characterized by ceiling effects and restricted range. Thirty-two community-dwelling older adults (>70yrs, 16 fallers, 16 non-fallers) completed a battery comprising 76 measures of more challenging clinical measures of mobility and balance, and retained gait (GaitRITE), postural sway and physiological measures. Within each domain, highly collinear and theoretically-redundant measures were removed. Next, a Principal Component Analysis (PCA) identified those clinical and gait variables that accounted for the most unique variance. Finally, a backward stepwise logistic regression was performed on the reduced set of variables from the PCA to develop predictive equations. The current analysis yielded improved specificity of 75%, but slightly lower sensitivity 81%.

Interestingly, when the results for the PCA from the previous study were used with the current data, the model classified fallers with 87% sensitivity and 86% specificity and an overall model of 86%. Notably, in all analyses, gait variables were central in identifying fall risk, with single- vs. dual-task difference scores of particular predictive importance. The differences observed between the best-fitting models across the two cohorts implies that modelling methods should accommodate and harness individual differences (e.g., machine learning techniques).

### SESSION 2365 (POSTER)

### ELDER ABUSE

### NATIONAL IMPLEMENTATION OF A VHA DIFFUSION OF EXCELLENCE GOLD STATUS PRACTICE

Kimberly K. Garner,[1] Jamie Jensen,[1] Lisa Nabholz,[1] Melissa Harding,[1] and Laura Taylor[2], *1. VISN 16/Little Rock Geriatric Research Education Clinical Center - Department of Veteran Affairs, Little Rock, Arkansas, United States, 2. National Social Work Program Office - Department of Veteran Affairs, Washington, D.C., United States*

Advance care planning (ACP) is a health behavior that requires in-depth discussions support by trained professionals and motivational strategies to promote goal-setting and actions. Group visits in the healthcare setting can effectively increase an individual's motivation and self-efficacy for ACP. The Department of Veterans Affairs (VA) has developed the Diffusion of Excellence Initiative to identify and spread innovative practices such as Advance Care Planning via Group Visits (ACP-GV), which uses an interactive group session to engage Veterans in thinking about and planning for their future medical decisions. In these sessions, social workers, nurses, psychologists, and chaplains, facilitate group discussions to increase the chance that a Veteran's care preferences are known and reflect with their wishes. This also can relieve trusted others of having to make these tough decisions without much guidance. In addition, ACP-GV increases the effectiveness of advance care planning through allowing Veterans to discuss and process this complex topic with their peers. To date, 36 VA Medical Centers (VAMCs) are currently adopting, implementing or sustaining this practice and more than 15,250 Veterans have attended ACP-GV sessions. In addition, another 40 VAMCs are exploring participating this practice. Of those participants, approximately 18-20% develop a new advance directive and 86% set a smart goal to take steps toward advance care planning. Continued dissemination and implementation of this innovative practice is ongoing. After the session, attendees will have practical guidance for implementation of ACP-GV discussions in integrated (VA) or fee-for-service (Medicare) settings.

### THE NATIONAL ELDER MISTREATMENT STUDY 8 YEARS LATER: VICTIM MENTAL HEALTH OUTCOMES AND PROTECTIVE FACTORS

Melba Hernandez-Tejada,[1] Ron Acierno,[1] Georgia Anetzberger,[2] Daniel Loew,[3] and Wendy Muzzy[1], *1. Medical University of South Carolina, Charleston, South Carolina, United States, 2. Case Western Reserve University, Cleveland, Ohio, United States, 3. Abt SRBI, New York, New York, United States*

The purpose of this study was to conduct an 8-year follow-up of the National Elder Mistreatment Study (NEMS) and specify risk ratios for negative outcomes of elder abuse, including DSM-5 defined depression, generalized anxiety disorder (GAD), post-traumatic stress disorder (PTSD), and poor self-reported health. Methodology: Attempts were made to re-contact, via Computer Assisted Telephone Interview, all 752 NEMS participants who reported mistreatment since age 60 at Wave I, as well as a randomly selected sample of non-mistreated NEMS participants Results: 183 NEMS Wave I elder abuse victims and 591 non-victims provided data. In bivariate analyses, elder mistreatment 8 years earlier increased risk of negative outcomes by 200-700%. However, multivariate analyses revealed that Current (Wave II) social support was highly protective against most negative outcomes (excepting PTSD), and even appeared to nullify effects of mistreatment on GAD and poor self-reported health. Conclusions: Outcomes of elder mistreatment have not been studied prospectively in a national sample. The NEMS 8-year follow-up findings indicate a strong relationship between elder mistreatment at Wave I and negative emotional and physical health 8 years later. Fortunately, current (Wave II) social support appears to be both consistently and powerfully protective against most negative outcomes.

### FACILITATORS AND BARRIERS FOR ELDER ABUSE VICTIMS SEEKING HELP: FINDINGS FROM THE NATIONAL ELDER MISTREATMENT STUDY

David Burnes,[1] Ron Acierno,[2] and Melba Hernandez-Tejada[2], *1. University of Toronto, Toronto, Ontario, Canada, 2. Medical University of South Carolina, Charleston, South Carolina, United States*

Understanding help-seeking among victims of elder abuse is a critical challenge in the field. The vast majority of elder abuse victims remain hidden from formal support/protective response systems, such as adult protective services, legal/justice, law enforcement, or other agencies responsible for addressing this issue in the community. Guided by the Behavioral Model of Health Services Use, this study examined factors that facilitate or impede formal help-seeking among victims of elder emotional, physical and sexual abuse, represented by a call for help in the form of a report to police or other authorities. Data came from a national, population-based elder abuse study in the U.S. with a representative sample (n=304) of victims reporting abuse in the past year. Gold-standard measurement strategies were used to assess each elder abuse subtype. Multivariable logistic regression was conducted to identify help-seeking facilitators/barriers. Help-seeking through reporting to police or other authorities occurred among only 15.4% of elder abuse victims nationwide. Help-seeking was predicted by factors attached to the victim (abuse type, poly-victimization), perpetrator (prior police trouble, social network size), and victim-perpetrator relationship (victim dependence on perpetrator). This study highlights the extremely hidden nature of elder abuse in our society, as well as the need to develop strategies that incorporate victim, perpetrator, and victim-perpetrator relationship factors to promote greater help-seeking among victims.

### A SYSTEMATIC REVIEW AND META-ANALYSIS OF AGE-RELATED DIFFERENCES IN TRUST

Phoebe E. Bailey,[1] and Tarren Leon[1], *1. Western Sydney University, NSW, Australia*

This systematic review and meta-analysis quantifies the magnitude and breadth of age-related differences in trust. Thirty-eight independent data sets met criteria for inclusion. Overall, there was a moderate effect of age group on trust (g = 0.22), whereby older adults were more trusting than young adults. Three additional meta-analyses assessed age-related differences in trust in response to varying degrees of trustworthiness. This revealed that older adults were more trusting than young adults in response to neutral (g = 0.31) and negative (g = 0.33), but not positive (g = 0.15), indicators of trustworthiness. The effect of age group on trust in response to positive and neutral cues was moderated by type of trust (financial vs. non-financial) and type of responding (self-report vs. behavioral). Older adults were more trusting than young adults in response to positive and neutral indicators of trustworthiness when trust was expressed non-financially, but not financially. There was also an age-related increase in self-reported, but not behavioral, trust in response to neutral cues. Older adults were more trusting than young adults in response to negative indicators of trustworthiness regardless of the type of trust or type of responding. The reliability of information about trustworthiness (superficial vs. genuine cues) did not moderate any effects of age on trust. Implications of these findings and directions for future research are discussed.

### CHALLENGES AND BARRIERS FOR SERVICE PROVIDERS IN RURAL SETTINGS: AN ELDER-ABUSE NEEDS ASSESSMENT

Louise Peitz,[1] Jeong Eun Lee,[1] Megan Gilligan,[2] and Naomi R. Meinertz[1], *1. Iowa State University, Ames, Iowa, United States, 2. Iowa State, Ames, Iowa, United States*

There has been concern about the increasing prevalence of elder abuse in the rural settings. This trend has called for more coordinated efforts to address elder abuse in the rural community. The current literature regarding elder abuse suggest there is more to learn about systematic obstacles service providers face. The framework applied in this study include elements of Bronfenbrenner's Bioecological model, showing a multilevel application to the obstacles agencies encounter when supporting else abuse victims. Little research has been conducted on the providers who work with elders in the rural context. In this study, based on the needs assessment of Iowa, we have examined the perceived barriers service providers face. Our sample included 222 providers across Iowa who took an online survey. The survey was initially sent to members of the Coordinated Community Response team of Iowa. Our findings indicated that service providers revealed multilevel challenges (i.e., individual vs. organization level) in working with elder abuse cases. Service providers' perceived effectiveness training was negatively associated with the quality of training they evaluated ($\beta$=.24, p<.05). The data also showed us that a large portion of agencies offered no training and education on elder abuse. Common themes described by the providers as barriers to addressing abuse was lack of education, social isolation, low awareness and ageism. These results highlight that training for elder abuse in the rural setting needs multi-level systematic efforts.

### ABUSE, NEGLECT, AND EXPLOITATION IN ASSISTED LIVING: AN EXAMINATION OF LONG-TERM CARE OMBUDSMAN COMPLAINT DATA

Karen Magruder,[1] Ling Xu,[2] and Noelle L. Fields[2], *1. Senior Source at Dallas, Senior Source in Dallas, Texas, United States, 2. University of Texas at Arlington, Arlington, Texas, United States*

Adult Protective Services (APS) has seen cases of elder abuse increase recently and older adults living in long-term care facilities are subject to abuse, neglect and exploitation (ANE) at higher rates than community-dwelling seniors. However, there is scant empirical literature about ANE in assisted living (AL) settings and skilled nursing facilities (SNFs). The purpose of this study is to examine ANE complaints in AL and SNFs as recorded by Long-term Care Ombudsman Programs (LTCOPs) utilizing secondary data from a statewide database as well as an agency database used by staff ombudsmen who work in a large Metropolitan city in Texas. The sample included 140,497 complaints made at 1,940 licensed ALs with approximately 45,107 residents and 1,231 SNFs with 93,932 residents in Texas from 2010-2017. The percent of total complaints coded as ANE was higher in AL (2.01%) than in SNFs (1.46%) (p < .001). However, after controlling for number of residents, the rate of total ANE complaints per resident was higher (0.019) in SNF compared to AL (0.007) (z = -17.221, p <. 001). The incidence of financial exploitation was significantly higher in AL (23.46%) than in SNF (11.90%) (z = 5.582, p < .001). The percentage of verbal/psychological abuse in SNF (34.78%) was significantly higher than that in AL (23.4%) (z = -2.238, p < .05). Study findings contribute to the knowledge about the prevalence and nature of ANE in long-term care communities and bolster support for increased AL ombudsman presence, staff training, resident-centered care models, and AL oversight.

### FINANCIAL DECISION MAKING SELF-EFFICACY IN COGNITIVELY AND FINANCIALLY VULNERABLE OLDER ADULTS

Evan Z. Gross,[1] Rebecca J. Campbell,[1] LaToya Hall,[1] and Peter Lichtenberg[1], *1. Wayne State University, Detroit, Michigan, United States*

Financial decision making self-efficacy (FDMSE) is a novel construct that may influence how older adults make financial decisions. Our previous research with a community sample of older adults demonstrated that cognitive functioning and suspected history of financial exploitation were both associated with low FDMSE. We sought to replicate these findings in two clinical samples of older adults: people with mild cognitive impairment (MCI) or probable Alzheimer's disease (PAD) and current victims of scams or exploitation as determined by a financial coach. Samples were obtained from the Michigan Alzheimer's Disease Center and a financial coaching intervention study. All participants completed a 4-item FDMSE measure. One-way ANOVAs, t-tests and chi-square tests were conducted to test for group differences with controls on demographics. There was a main effect of cognitive status on FDMSE, F(2,138) = 8.10, p < .001, which was driven by higher FDMSE in the healthy group (N = 63)

than the MCI (N = 76) or PAD (N = 28) groups. Similarly, scam victims (N = 25) had significantly lower FDMSE than non-exploited (N = 25) peers, $t(48)=2.33$, $p < 05$. Cognitive impairment and current financial scams are both associated with low FDMSE levels. Low FDMSE may exacerbate cognitive and psychosocial vulnerabilities that contribute to risk for poor financial decisions among older adults. Future interventions to enhance FDMSE may help older adults make better decisions despite changes in thinking abilities or previous negative financial experiences.

### TECHNOLOGICAL AND FINANCIAL PREDICTORS OF FEAR OF FINANCIAL ABUSE AMONG OLDER ADULTS

Shaina Alves,[1] and Erin Grinshteyn[1], *1. University of San Francisco, San Francisco, California, United States*

Much research has focused on elder abuse. Less research focuses on fear of abuse. This analysis examines the associations between feelings of technological competence and variables assessing financial confidence with fear of financial victimization. Data were collected among community dwelling older adults in Nevada (n=467). Questions were asked regarding technological competence, confidence in navigating the financial system, asset protection, trust in financial institutions, and previous financial abuse victimization. The outcome was assessed by asking how afraid the respondent was of becoming a victim of financial abuse. Multivariate logistic regression models were run controlling for confounding. Controlling for all covariates, those who reported feeling unconfident in their technological competence had 2.5 times the odds of being afraid of financial abuse compared with those who felt confident ($p<0.02$). Those who reported feeling like their assets were at risk had 4.12 times the odds of being afraid of financial abuse ($p<0.0001$). Older adults who reported feeling vulnerable to financial victimization had 9.4 times the odds of being afraid of financial abuse compared with those who felt invulnerable ($p<0.0001$). Those who were previously victims of financial abuse had 4.33 times odds of being afraid of financial abuse compared with those who had no history of financial abuse ($p<0.0001$). Feeling confident in the financial system, asset protection, fear of credit card use, and trust in financial institutions were not associated with fear of financial abuse. These data provide a better understanding of fear of financial abuse, which will allow for better prevention of this issue.

### USING DATA SCIENCE TO GENERATE PSYCHOSOCIAL PROFILES OF FINANCIAL EXPLOITATION IN SENIORS

Cassandra J. Enzler,[1] Robert Suchting,[1] Charles Green,[1] and Jason Burnett[1], *1. University of Texas Health McGovern Medical School, Houston, Texas, United States*

Financial exploitation (FE) in older adults is a significant public health problem linked to outcomes including depression, financial ruin and early mortality. This study applied exploratory data science techniques to a multi-year statewide protective services dataset of over 8,000 elder abuse cases. The goal was to derive data-driven psychosocial profiles of abuse with an emphasis on determining which factors, commonly shared across abuse cases, were most important for determining when elder FE was occurring and whether it was occurring alone or in conjunction with other types of abuse. We found that pronounced psychological distress (i.e. verbalizing suicide, homicide, self-harm) was most important for indicating when abuse had occurred and predicted non-FE related abuse. Drug paraphernalia in the home and perpetrator drug/alcohol use were important predictors of FE-related abuse. When differentiating pure FE from hybrid FE, factors indicative of long-term FE occurrence and substantial financial loss were most important (i.e. facing foreclosure, lack of food, medications, and utilities). The findings parallel some existing work characterizing pure and hybrid FE, but also highlight new profile factors that may help determine when FE is occurring and when it is less likely. Applying data science approaches to other large protective service datasets and national datasets such as the National Adult Maltreatment Registry could help improve characterization of abuse types such as pure and hybrid FE resulting in better detection, response and prevention.

### PERPETRATOR DEMOGRAPHICS ASSOCIATED WITH ADULT PROTECTIVE SERVICES—CONFIRMED FINANCIAL EXPLOITATION CASES

Cassandra J. Enzler,[1] Carlos Reyes-Ortiz,[1] and Jason Burnett[1], *1. University of Texas Health McGovern Medical School, Houston, Texas, United States*

Financial exploitation (FE) in older adults is a significant public health problem linked to outcomes including depression, financial ruin and early mortality. Studies have demonstrated risk factors associated with FE, but less is known about perpetrator characteristics. We performed a secondary data analysis of over 16,000 reported cases of FE utilizing a cross sectional design. Using multivariate logistic regression, confirmed and unconfirmed cases of FE were predicted from the following perpetrator demographics: age, gender, marital status, ethnicity, relationship to the victim, living status, and histories of drug abuse, alcohol abuse, and mental illness. Significant perpetrator demographics predicting confirmed FE were separation/divorce (OR=1.48), identifying as White (OR=1.33) or Black (OR=1.44), being a daughter (OR=1.61), son (OR=1.75), grandchild (OR=2.72), or other family member (OR=1.41), not residing with the victim (OR=2.32), and having a history of drug abuse (OR=2.56), alcohol abuse (OR=1.80), or mental illness (OR=1.91). These findings are based on a large statewide dataset and describe important perpetrator characteristics that could potentially be targeted for both intervention and prevention programs. This is especially important as many victims are reluctant to seek criminal action against a family member or trusted individual. This information is valuable as it may help APS, who has limited funding and staff, investigate and intervene in more difficult elder abuse FE cases.

### POLICIES AND PREVENTION OF U.S. WOMEN'S VIOLENT DEATH ACROSS AGES

Sonia L. Salari,[1] and Carrie Sillito[1], *1. University of Utah, Salt Lake City, Utah, United States*

U.S. violent death rates (homicide and suicide) are the highest in the developed world. Of all female murders (femicide), the majority are male perpetrated, intimate partner violence (IPV- 55-63%). Men are more often killed and by other male acquaintances, with only 2.8% IPV. Proportionally, older women (50+) have the top homicide victim rate (26%) among women. The baby boom cohort has been suicidal and aging has exacerbated the problem. Women are less likely to kill themselves, and the methods differ. We ask are mid and later life women's lethal victimization similar to younger women? What are policy implications for prevention? Our research uses national level data from news surveillance of 728 intimate partner homicide suicide (IPHS) events and the State Firearm Law Database (SFLD) to improve our understanding of violent cause mortality by sex, age, method and location. IPHS patterns show 90% of events used firearm and 90% were male perpetrated. Results of multivariate analyses show young women had greater awareness and fear before IPHS. Evidence finds older men sometimes decided to kill their IP as part of their own suicide, without a history of known domestic violence. Older women have disproportionately low use of shelters, police and protective orders. SFLD shows population adjusted states with more DV firearms laws have significantly fewer IPHS events. Firearm culture has restricted research, blocked law enforcement and has done little to reduce gun access in households with vulnerable populations (e.g., suicidal husbands). Lethality Assessment Protocols could be modified for elder women's unique situation.

### SESSION 2370 (POSTER)

### EPIDEMIOLOGY

### COMPARABILITY OF BIOLOGICAL AGING MEASURES IN THE NATIONAL HEALTH AND NUTRITION EXAMINATION STUDY, 1999-2002

Waylon J. Hastings,[1] Daniel Belsky,[2] and Idan Shalev[1], *1. Pennsylvania State University, State College, Pennsylvania, United States, 2. Columbia University Mailman School of Public Health, New York, New York, United States*

Biological processes of aging are thought to be modifiable causes of many chronic diseases. Measures of biological aging could provide sensitive endpoints for studies of risk factors hypothesized to shorten healthy lifespan and/or interventions that extend it. However, uncertainty remains about how to measure biological aging and if proposed measures assess the same thing. We tested four proposed measures of biological aging with available data from NHANES 1999-2002: Klemera-Doubal method (KDM) Biological Age, homeostatic dysregulation, Levine Method (LM) Biological Age, and leukocyte telomere length. All measures of biological aging were correlated with chronological age. KDM Biological Age, homeostatic dysregulation, and LM Biological Age were all significantly associated with each other, but were each not associated with telomere length. NHANES participants with older biological ages performed worse on tests of physical, cognitive, perceptual, and subjective functions known to decline with advancing chronological age and thought to mediate age-related disability. Further, NHANES participants with higher levels of exposure to life-course risk factors were measured as having older biological ages. In both sets of analyses, effect-sizes tended to be larger for KDM Biological Age, homeostatic dysregulation, and LM Biological Age as compared to telomere length. Composite measures combining cellular- and patient-level information tended to have the largest effect-sizes. The cellular-level aging biomarker telomere length may measure different aspects of the aging process relative to the patient-level physiological measures. Studies aiming to test if risk factors accelerate aging or if interventions may slow aging should not treat proposed measures of biological aging as interchangeable.

### TRENDS IN GERIATRIC PHYSICAL ASSAULT INJURIES TREATED IN U.S. EMERGENCY DEPARTMENTS, 2006-2015

Tony Rosen,[1] Sunday Clark,[2] Zach Gassoumis,[3] Jeanine Yonashiro-Cho,[3] Alyssa Elman,[4] David Lachs,[1] Elizabeth Bloemen,[5] and Jeffrey Hall[6], *1. Weill Cornell Medical College / NewYork-Presbyterian Hospital, New York, New York, United States, 2. Department of Emergency Medicine, Weill Cornell Medical College / NewYork-Presbyterian Hospital, New York, New York, United States, 3. Leonard Davis School of Gerontology, University of Southern California, Los Angeles, California, United States, 4. Weill Cornell Medical College, New York, New York, United States, 5. University of Colorado School of Medicine, Aurora, Colorado, United States, 6. Centers for Disease Control and Prevention, Atlanta, Georgia, United States*

Trends in Geriatric Physical Assault Injuries Treated in US Emergency Departments, 2006-2015 Older adults are common victims of assault, many of which may result in severe injuries. Our objective was to understand temporal and demographic trends in geriatric assault injuries treated at U.S. Emergency Departments (EDs) and to compare these trends to assault injuries in younger adults. We conducted an analysis of assault injuries in patients aged ≥60 compared to patients aged 18-59 treated in EDs during 2006-2015 using the National Electronic Injury Surveillance System-All Injury Program Special Study of Assaults, which collects data from a nationally representative stratified probability sample of U.S. hospitals. Total geriatric assaults seen in EDs increased from 35,135 in 2006 to 69,657 in 2015, a 98% increase. These injuries increased as a percentage of all geriatric injuries treated from 0.9% to 1.1%. Assaults in older men increased 119%, while assaults in older women increased 68%. Among age groups, the biggest percentage increases were among adults aged 60-64 (138%) and aged 65-74 (89%). ED visits for injuries associated with physical elder abuse increased from 13,241 in 2006 to 27,406 in 2015, a 107% increase. During this period, number of younger adults treated for assault did not significantly change. We concluded that geriatric assault injuries, particularly in older men in younger age groups, are dramatically increasing. Further research is needed to better understand these assaults to develop prevention strategies.

### USE OF EXPOSURE CROSSOVER DESIGN TO CONTROL FOR UNMEASURED BASELINE CONFOUNDING IN OBSERVATIONAL STUDIES

Ling Han,[1] Robert D. Kerns,[2] Melissa Skanderson,[3] Joseph L. Goulet,[4] Stephen Luther,[5] and Cynthia Brandt[1], *1. Yale School of Medicine, New Haven, Connecticut, United States, 2. Yale University, New Haven, Connecticut, United States, 3. VA Connecticut, West Haven, Connecticut, United States, 4. VA Connecticut Healthcare System, West Haven, Connecticut, United States, 5. James A. Haley Veterans Hospital, Tampa, Florida, United States*

Observational comparative effectiveness studies face the challenge of selection bias. Due to lack of randomization, an alleged treatment effect may reflect inherent differences in baseline characteristics between comparison groups, rather than the outcome of treatment. Propensity score methods were devised to "resample" a most comparable comparison group, under a strong yet untestable assumption of no unmeasured confounding. We present an "exposure crossover" study evaluating complementary and integrative health approaches (CIH) among 6,379 US veterans who received acupuncture, massage or chiropractic therapies between 10/1/2011-9/30/2013. Their average pain intensity ratings (PIRs) during the 12-months after CIH initiation (effect period, EP) were compared with the 12-months before (baseline period, BP). Through this built-in self-matching, veterans' characteristics and other stable baseline confounding, measured and unmeasured, were presumably eliminated. After accounting for time-varying opioid use and within-subject correlations using a generalized estimating equation, we found that in comparison to the BP, the adjusted mean PIR during the EP was -0.40 (95% Confidence Interval (CI): -0.51, -0.29) points lower; while the adjusted rate ratio of moderate to severe pain (PIRs $\geq 4$) was 34% lower [0.66 (95% CI: 0.62, 0.70)]. The effect sizes were greater among veterans older than 65 years, yet diminished to null after 6-9 months. Assuming a 3-month induction period, using alternative random-intercept model, and examining post-CIH opioid use as an alternative outcome, derived similar results. These observations echo some randomized trials suggesting a modest, short-term CIH benefit, and highlight the merits and usefulness of exposure-crossover design to observational studies of medical interventions.

### ADVANCING AN INTERDISCIPLINARY SCIENCE OF AGING THROUGH A PRACTICE-BASED DATA SCIENCE APPROACH

Robert Lucero,[1] and Ragnhildur Bjarnadottir[1], *1. University of Florida, Gainesville, Florida, United States*

Two hundred and fifty thousand older adults die annually in United States hospitals because of iatrogenic conditions (ICs). Clinicians, aging experts, patient advocates and federal policy makers agree that there is a need to enhance the safety of hospitalized older adults through improved identification and prevention of ICs. To this end, we are building a research program with the goal of enhancing the safety of hospitalized older adults by reducing ICs through an effective learning health system. Leveraging unique electronic data and healthcare system and human resources at the University of Florida, we are applying a state-of-the-art practice-based data science approach to identify risk factors of ICs (e.g., falls) from structured (i.e., nursing, clinical, administrative) and unstructured or text (i.e., registered nurse's progress notes) data. Our interdisciplinary academic-clinical partnership includes scientific and clinical experts in patient safety, care quality, health outcomes, nursing and health informatics, natural language processing, data science, aging, standardized terminology, clinical decision support, statistics, machine learning, and hospital operations. Results to date have uncovered previously unknown fall risk factors within nursing (i.e., physical therapy initiation), clinical (i.e., number of fall risk increasing drugs, hemoglobin level), and administrative (i.e., Charlson Comorbidity Index, nurse skill mix, and registered nurse staffing ratio) structured data as well as patient cognitive, environmental, workflow, and communication factors in text data. The application of data science methods (i.e., machine learning and text-mining) and findings from this research will be used to develop text-mining pipelines to support sustained data-driven interdisciplinary aging studies to reduce ICs.

### MEDICAL COMPLICATIONS AND INJURY LEADING TO EMERGENCY DEPARTMENT USE AMONG OLDER ADULTS

Mary W. Carter,[1] Bo Kyum Yang,[1] and Cyrus Y. Engineer[1], *1. Towson University, Towson, Maryland, United States*

Medical injury consistently ranks among the most expensive hospital stay diagnoses and represents a frequent cause of hospital readmission. Although older adults are at greater risk of medical injury, in part, because of greater incidence of comorbidity and increased medical complexity, little is known about the burden of medical injury leading to ED use or the costs and outcomes associated with these events among older adults. In response, this study used nationally representative data from the 2014 Nationwide Emergency Department Survey to examine the epidemiology of older adult ED-visits for medical injury. Principal diagnosis codes were grouped using AHRQ's Clinical Classification Software to identify medical injury-related visits. Results indicated that in 2014, 506,466 ED-visits for medical injuries occurred, comprising 2% of all older adult ED-visits. Leading causes of medical injury included malfunction of device, implant and grafts (24%); infection and inflammation of internal prosthetic device, implant, and graft (16%), and other complications of surgical and medical procedures (15%). Risk factors for medical injury included being male, Medicaid as primary payor, and number of chronic conditions. Multinominal logistic regression and multivariate regression results indicate that Medical injury-related ED visits were associated with higher hospitalization risk (RRR=2.08, $p<0.000$), 27% longer hospital stays, and 24% higher total charges relative to non-medical injury related visits. However, medical injury was not associated with risk of death after adjustment. Study findings suggest that ED-visits for medical injury occur frequently among older adults and are associated with significant burden and cost.

### IN HIP FRACTURE, GENDER CONFOUNDS COGNITION ASSESSMENT, TIME TO DEATH, AND COGNITION-RELATED MORTALITY

Heather L. Mutchie,[1] Denise Orwig,[1] and Ann Gruber-Baldini[1], *1. University of Maryland Baltimore School of Medicine, Baltimore, Maryland, United States*

Of 300,000 annual hip fractures in the US, about 30% occur in men, over 30% experience cognitive impairment or dementia, and 30% die within one year. This study compares time-to-death and cognition-related cause of death (CR-COD) by gender after hip fracture using different methods of cognitive impairment ascertainment. Baseline hospital charts and Modified Mini-Mental State Examination (3MS) were from the Baltimore Hip Studies 7th cohort (2006-2011) (171 women, 168 men). National Death Index was obtained up to December 31, 2014. Cox models assessed dementia diagnosis in chart and 3MS<78 as predictors of mortality and CR-COD (died n=204). Men were more likely to fail the 3MS (Men:26.2%, Women:16.2%; p=0.025), die (Men:73.8%, Women:46.8%; p<.0001), and die sooner (Men:μ=30.8months, Women:μ=39.2months; p=0.0159). Significantly more women died with CR-COD (Men:17.7%, Women:31.2%, p=0.0253). Among men, those with any cognitive impairment (3MS:μ=21.5months, chart:μ=21.3months; both:μ=22.0months) died sooner than non-impaired (μ=22.0months). Among women only those with both chart and 3MS (μ=16.7months) died sooner than all other groups (3MS:μ=47.8months, chart:μ=35.1months, none:μ=42.9months). With respect to CR-COD, men with cognitive impairment from any source (3MS:μ=5.0months, chart:μ=43.4months, both:μ=30.5months) differed from non-impaired (μ=91.6months). While women with hospital chart alone (μ=14.0months) differed from non-impaired (μ=3.6months), 3MS alone (μ=8.5months) and both (μ=9.5months). Men had worse 3MS scores but similar chart diagnosis, indicating a potential underdiagnosis of men's cognitive impairment. Gender confounds the relationship between cognition and death or CR-COD. In men, any impairment predicts death overall and CR-COD. In women, chart with 3MS impairment predicted overall death, but chart alone was more predictive for CR-COD.

### OSTEOSARCOPENIC OBESITY AND VITAMIN D IN U.S. ADULTS AGES 50 AND OLDER: A CROSS-SECTIONAL STUDY

Barbara S. Saltzman,[1] and Kevin C. Kenney[1], *1. University of Toledo, Toledo, Ohio, United States*

Osteosarcopenic Obesity (OSO) is the concurrent presence of obesity (excess body fat), sarcopenia (low muscle mass) and osteoporosis (low bone mineral density). Low levels of serum vitamin D (vitD) have been associated with each disease and their simultaneous presence. We examined this association in adults aged ≥50 years from the 2001-2006 survey cycles of the National Health and Nutrition Examination Survey (NHANES) (N=3791). SAS v9.4 complex survey procedures were used. Demographics and vitD status were compared across OSO components (0,1,2,3) by chi-squared tests and ANOVAs. Odds ratios and 95% confidence intervals for the association between vitD status and increasing number of OSO components were calculated using multinomial logistic regression (alpha=0.05). The mean age was 65.0 years (30% were older adults ≥ 65), 45.6% were female, and prevalences for obesity, osteoporosis, sarcopenia and OSO were 84.7%, 8.0%, 16.3%, and 1.8%, respectively. Additionally, 30.9% of the sample was vitD deficient. After adjusting for supplement use, and race, women with 1 or 2, and men with 1,2 or 3 OSO components were more likely to be vitD deficient than sufficient, compared to those with none [women: 2.8(1.4-5.7), 2.1(1.4-6.8), and men: 2.3 (1.5-3.6), 3.5(2.1-5.9) and 6.3(1.0-38.5), respectively]. Vitamin D status was associated with having one or two OSO components in women, and OSO and components in men, after adjusting for supplement use, sex, and race. These results suggest it is important to consider obesity, osteoporosis, and sarcopenia jointly with respect to vitamin D status in the chronic disease management of aging and older adults.

### NATIVITY AND DISABILITY TRAJECTORIES AMONG TWO COHORTS

Elizabeth Vasquez,[1] Weihui Zhang,[2] and Anda Botoseneanu[3], *1. University at Albany, SUNY, Albany, New York, United States, 2. University at Albany, Rensselaer, United States, 3. University of Michigan, Dearborn, Michigan, United States*

Nativity is an important characteristic in the context of disability in older adults, as it may influence attitudes and behaviors that can delay or accelerate the disability process. This study aims to assess trajectories of disability (defined as lower-body functional limitations, limitations in daily and instrumental activities of daily living, and gross mobility) in foreign-born and US-born Mexican Americans between 1993 and 2013. We used eight waves (1993 -2013) from the Hispanic Established Populations for the Epidemiologic Study of the Elderly (HEPESE; N=3050, mean age at baseline=73.6 (±6.8). Disability was assessed using self-reported limitations in activities of daily living (ADL /IADL). Nativity and age-at-migration were collected by self-report. We used linear and quadratic growth curve models to evaluate the trajectory of ADL/IADL disability over a period of 20 years and assessed variations by nativity status while adjusting for potential confounders. Approximately 19% of foreign-born and 17% of US-born reported having at least one ADL, while 51.5% natives and 58.7% of foreign-born had at least one IADL. Our results showed that after controlling for age at baseline, sex, marital status, self-reported health, chronic conditions, and education, foreign-born older adults were less likely to have ADL/IADL disability at baseline, but exhibited a faster rate of increase in disability over time (β =0.31 for ADL, β=0.07 for IADL). Our findings show that foreign-born older adults are accumulating disability at faster compared with their native peers, highlighting the importance of evaluating nativity differences in the health outcomes of Hispanic population at old age.

### PARADOXICAL ASSOCIATION OF ADIPONECTIN WITH MORTALITY AMONG LONG-LIVED INDIVIDUALS AND THEIR OFFSPRING

Iva Miljkovic,[1] Robert M. Boudreau,[1] Ping An,[2] Bharat Thyagarajan,[3] Kaare Christensen,[4] Nicole Schupf,[5] Alexander Kulminski,[6] and Joseph M. Zmuda[7], *1. University of Pittsburgh, Pittsburgh, Pennsylvania, United States, 2. St. Louis, Washington University, Missouri, United States, 3. Department of Laboratory Medicine and Pathology University of Minnesota; Minneapolis, Minnesota, United States, 4. Danish Aging Research Center University of Southern Denmark, Odense C, Denmark, 5. Columbia University, Columbia University, United States, 6. Duke University, Durham, North Carolina, United States, 7. Department of Epidemiology, University of Pittsburgh; Pittsburgh, Pennsylvania, United States*

Adiponectin is an adipokine with well-recognized roles as an insulin-sensitizing, anti-inflammatory, and cardioprotective factor. Despite these biological properties, higher circulating adiponectin levels have recently emerged as a potential risk factor for mortality among older adults. We evaluated the relationship between adiponectin and all-cause mortality further using data collected on 3525 men and women who were participants in the Long Life Family Study (LLFS), a multicenter cohort study of two-generation families with a clustering of healthy aging and exceptional survival. Fasting serum adiponectin levels were measured at study entry. Date of death was abstracted from death certificates and proxy. Cox proportional hazards models were used to estimate the risk of mortality per one SD increase in adiponectin. We adjusted for age, sex, field center, education, lifestyle factors, BMI, comorbid conditions (diabetes, hypertension, lung diseases, heart disease, stroke, and cancer) and intra-familial correlation structure. The mean age of participants at study entry was 71.7 ± 16.3 years (range, 32-110 years; 44.4% male). A total of 1147 deaths occurred during an average 11 years of follow-up (25295 person-years). Higher serum adiponectin was associated with increased mortality even after adjusting for several potential confounding factors (Hazard ratio [95% CI]: 1.19 [1.12, 1.27]; P<0.0001). Consistent with prior studies and contrary to its beneficial metabolic properties, higher adiponectin level was associated with greater all-cause mortality among long-lived individuals and their offspring. Further studies are needed to assess the potential mechanisms underlying this paradoxical association.

### DETERMINANTS OF QUALITY OF LIFE IN COMMUNITY-DWELLING OLD ADULTS

Alfons Ramel[1], *1. University of Iceland, Reykjavik, Capital area, Iceland*

Background: Quality of life (QoL) has been regarded as a critical predictor of successful aging in gerontological research. Aim: The aim of this study was to examine the associations between physical activity, muscle strength, body composition, physical-/cognitive function and disease with quality of life community-dwelling older adults. Methods: Participants (N=225, 73.7±5.7yrs, 58.2% female) from the Reykjavik capital area in Iceland took part in this cross-sectional study. Socioeconomics, QoL, body composition, muscular strength, timed up and go test (TUG), six minute walk for distance (6MWD) and disease related information were measured. Fasting blood samples were analyzed for routine clinical measures. Results: In our subjects, only 19.1% had QoL below the age and gender corrected norm score of 50. A simple comparison between subjects with QoL below 50 vs subjects with a score above 50 indicated that participants with higher QoL had higher physical and cognitive function, higher muscular strength, lower blood glucose, exercised more and used a lower number of medicines. Differences in education, smoking, alcohol consumption, dietary intake and gender distribution were not significant. According to age and gender corrected linear models, TUG (B=-0.54,P=0.022), number of drugs (B=-0.67,P=0.018) and fasting glucose (B=-0.96,P=0.025) were the strongest independent correlates of QoL. In the models insulin/glucose and TUG/6MWD were interchangeable. Conclusion: Physical function, number of drugs and glucose metabolism are independently related to QoL and represent therefore potentially modifyable targets for future interventions in order to improve QoL in community dwelling old adults.

### LIVING ALONE IS ASSOCIATED WITH POORER PHYSICAL FUNCTION AND BONE MINERAL DENSITY IN ICELANDIC OLD ADULTS

Alfons Ramel[1], *1. University of Iceland, Reykjavik, Capital area, Iceland*

Background: Loneliness and living alone have been significant public health concerns among older adults given their association with a wide range of adverse health outcomes. Aim: The aim of this study was to examine whether living alone is associated with physical function and bone health in community-dwelling older adults. Methods: This was a secondary analysis of existing cross-sectional data of old adults (N=182, 73.7±5.7yrs, 58.2% female) from the Reykjavik capital area in Iceland. Information on socioeconomics, health, dietary intake and physical function was collected. 25-hydroxy-vitamin D (25OHD) and bone mineral density (BM were grouped retrospectively into "living alone" and into "in cohabitation". Results: Of our subjects, 76.4% were in cohabitation and and 23.6% lived alone. Participants who lived alone were older (74.5±5.6 vs. 72.1±5.0,P=0.008) and more often female (74.4 vs. 53.2%,P=0.014), but there were no differences in education, smoking, number of medications, physical activity (PA) or body mass index (BMI). According to age and gender corrected analyses, participants in cohabitation had higher grip strength (6.2±2.4lb,P=0.011), higher 25OHD (13.1±6.3nmol/L,P=0.037) and higher BMD (z-score lumbal: 1.195±0.417,P=0.005; z-score femur: 0.421±0.219,P=0.054; z-score total: 0.846±0.290,P=0.004). Statistical correction for PA, BMI, education and fish oil intake did not change the results. Conclusion: In comparison to old adults who live in cohabitation, Icelandic old adults who live alone have poorer physical function, lower 25OHD and lower BMD, which increases their risk for wrist or hip fracture. These differences between groups were not explained by physical, dietary or social confounding variables.

### POLYPHARMACY IN DIABETIC PEOPLE: EVIDENCE FROM THE ENGLISH LONGITUDINAL STUDY OF AGEING (ELSA)

Yun-Ting Huang,[1] Paola Zaninotto,[1] Andrew Steptoe,[1] and Li Wei[1], *1. University College London, London, United Kingdom*

Diabetes among older people is becoming more common worldwide, and usually accompanied by polypharmacy. However, the role of polypharmacy in older people with diabetes remains uncertain. A nationally representative cross-sectional study, ELSA 2012/2013, was used and 7729 participants aged 50-109 were investigated. Polypharmacy was defined as taking five to nine long-term used medications daily for chronic diseases or chronic symptoms, while using ten or more medications was excessive polypharmacy. The presence of illness was defined as either self-reported diagnosis or being prescribed specific medications for the condition. Data showed the prevalence of polypharmacy was 21.4%, and only 3% was excessive polypharmacy. 51.6% of diabetic people reported polypharmacy and 10.2% excessive polypharmacy. These rates were significantly higher than the 16.4% polypharmacy and 1.8% excessive polypharmacy among people without diabetes ($p < 0.001$). Among people with three or more comorbidities, polypharmacy was present in 61.5% of people with diabetes, compared with 36.0% in people without diabetes. Significant risk factors for polypharmacy were diabetes (Relative-risk ratios/RRR=4.06, 95% CI 3.38, 4.86), older age (RRR=1.02, 95% CI 1.01, 1.03), male (RRR=0.64, 95% CI 0.55, 0.75), more comorbidity (RRR=2.46, 95% CI 2.30, 2.62), living with a partner (RRR=1.20, 95% CI 1.01, 1.42), and less wealth (RRR=0.93, 95% CI 0.87, 0.98). However, age, cohabitation, and wealth were not significantly related to excessive polypharmacy. Diabetes and the number of comorbidities were predominant risk factors for excessive polypharmacy. Current evidences confirmed both health condition and socioeconomic status were associated with medication use in older adults.

### LONGITUDINAL RELATIONSHIP BETWEEN ENERGY UTILIZATION AND PHYSICAL AND COGNITIVE PERFORMANCE AS A FUNCTION OF AGE

Pei-Lun Kuo,[1] Yang An,[2] Alden Gross,[1] Eleanor M. Simonsick,[3] Susan M. Resnick,[2] and Jennifer A. Schrack[4], *1. Johns Hopkins University Bloomberg School of Public Health, Baltimore, Maryland, United States, 2. Laboratory Of Behavioral Neuroscience, National Institute On Aging, NIH, Baltimore, Maryland, United States, 3. Longitudinal Studies Section, Translational Gerontology Branch, National Institute On Aging, NIH, Baltimore, Maryland, United States, 4. Johns Hopkins University, Baltimore, Maryland, United States*

Energy utilization, which becomes more inefficient with age and is measured by a ratio of energy-cost-to-energy-capacity ("cost-ratio"), has been associated with functional decline. However, the interplay between longitudinal changes in energy efficiency and physical/cognitive functioning remains unclear. We investigated this relationship in 1020 participants of the Baltimore Longitudinal Study of Aging (baseline age: 68.9 (IQR: 59.8, 80.5), male: 44.7%). In linear mixed effects models adjusted for baseline age, sex, and height, an increasing cost-ratio was associated with faster decline in usual gait speed among those aged 50-64 years (beta = -0.20 m/s, p = 0.003), and >=65 years (beta = -0.16 m/s, p less than 0.001), but not those less than 50 years (beta = -0.22 m/s, p = 0.178). In models adjusted for baseline age, sex, race, and years of education, higher cost-ratio was associated with faster declines in executive function, as measured by time to finish Trail B, among those aged >=65 years (beta = 22.96 seconds, p = 0.020), but not <50 years (beta = -13.65 seconds, p = 0.557) or 50-64 years (beta = -15.61 seconds, p = 0.353). Together, these results suggest that energy utilization becomes more inefficient in the two to three decades prior to change in physical and cognitive functioning, implying it may act as an early marker of physiological aging. Further research is needed to understand the drivers of energy inefficiency, which may shed light on the biological mechanisms contributing to these declines.

### SENSITIVITY OF SELF-REPORTED COMORBIDITIES COMPARED TO MEDICARE CLAIMS IN OLDER ADULTS WITH TRAUMATIC BRAIN INJURY

Aparna Vadlamani,[1] and Jennifer Albrecht[1], *1. University of Maryland Baltimore, Baltimore, Maryland, United States*

Patient reported history of comorbid illness may be the only information available to the treatment team during an acute injury admission. Nevertheless, acute injury, particularly traumatic brain injury (TBI) which affects cognition, may decrease the patient's ability to accurately report medical history. Thus, the objective of this study was to evaluate the accuracy of patient-reported comorbid illness burden compared to the patient's Medicare administrative claims. Records of older adults treated for TBI at an urban level 1 trauma center 2006-2010 were linked to their Medicare administrative. Comorbidities were recorded in Medicare claims based on ICD9 codes and were reported in the trauma registry (TR) based on patient medical history recorded by a physician or nurse. Prevalence of each of the following comorbidities was calculated using information from the TR and claims: Alzheimer's disease and related dementias, chronic kidney disease, COPD, heart failure, diabetes, depression, stroke, and hypertension. Sensitivity of each patient-reported comorbidity was calculated using Medicare claims as the gold standard. We identified patient factors associated with accurate self-report using logistic regression. Among 408 older adults with TBI that linked to their Medicare claims, prevalence of each comorbidity was higher in Medicare claims compared to the TR, except for hypertension. Sensitivity for detecting these comorbidities using the TR ranged from 2% to 68%, with the highest sensitivity observed for hypertension. Older age and race were predictors of less accurate reported medical history. Reconciling self-reported patient history of these comorbidities with those reported in claims can better inform decisions regarding treatment.

### ASSOCIATION BETWEEN GRIP STRENGTH AND COGNITIVE FUNCTION AMONG COMMUNITY-DWELLING OLDER ADULTS

Milan Chang,[1] Alfons Ramel,[2] Palmi V. Jonsson,[3] Inga Thorsdottir,[4] and Olof Geirsdottir[4], *1. University of Iceland, School of Education, Faculty of Health Promotion, Sports and Leisure Studies, Reykjavik, Iceland, 2. University of Iceland, Reykjavik, Capital area, Iceland, 3. Landspitali University Hospital, Reykjavik, Iceland, 4. University of Iceland, Reykjavik, Iceland*

Background: Decline in both physical function and cognition among older adults has been associated with increased risk of dementia. Physical activity (PA) is beneficial for the improvement of both physical and cognitive function. The purpose of the study was to investigate the association between baseline physical function and cognitive function after 12 weeks of resistance training among older adults. Methods: Two hundred and thirty-seven community-dwelling older adults (N=237, 73.7±5.7 years, 58.2% female) participated in a 12-week resistance exercise program (3 times/week; 3 sets, 6-8 repetitions at 75-80% of the 1-repetition maximum), designed to increase strength and muscle mass of major muscle groups. Body composition, physical activity status, grip strength, cardiovascular risk factors, 6 minutes walking distance (6MWD), and Mini-Mental State Examination (MMSE) were measured at baseline and endpoint. The linear regression model was used to examine the association. Results:

Mean MMSE score was 27.5±2.1 at baseline and 28.1±2.2 after the exercise intervention. After the intervention, 57 declined, 55 remained the same, and 120 have improved in MMSE scores. We found that the MMSE score after the intervention was significantly associated with baseline grip strength (beta=.03, P<.05) among healthy older adults, after adjusting basic characteristics, cardiovascular risk factors and mobility at baseline. Conclusion: Our study found that baseline grip strength was strongly associated with cognitive function after the 12 weeks of resistance training. Muscle power, such as grip strength may play an important role in the effect of exercise intervention on cognition even among healthy independent older adults.

### EFFECTS OF MEDICARE COMORBIDITIES, SELF-REPORTED FACTORS, AND POLYGENIC RISK SCORES IN RISKS OF AD/ADRD

Igor Akushevich,[1] Arseniy Yashkin,[1] Julia Kravchenko,[2] Svetlana Ukraintseva,[1] and Anatoliy Yashin[1], *1. Duke University, Durham, North Carolina, United States, 2. Duke University School of Medicine, Durham, North Carolina, United States*

At this time there is no consensus on the origin, development, and progression of Alzheimer's Disease and related dementias (AD/ADRD) and the extent to which variation in the effects of potential risk factors affects the risk for this disorder is underexplored. In this paper we used HRS-Medicare-genetics data to evaluate the effects of risk factors from three groups: i) Medicare-based indicators of chronic diseases that have shown an association with AD/ADRD in the literature, ii) individual heath state, behavior, functional status, education and socioeconomic status, and iii) polygenic risk scores that incorporate known-to-date genetic risk factors for AD/ADRD. We found that: i) the effects of Medicare disease indicators are higher than the effects of self-reported diseases; ii) heart diseases, cerebrovascular diseases, and depression had a strong impact on AD/ADRD, while the presence of cancers sometimes decreases the risk of AD/ADRD; iii) systemic hypotension, chronic kidney disease, and chronic liver disease showed unexpectedly strong effects; iv) compared to females, males are affected by a lower number of risk factors albeit at higher magnitudes; v) BMI, alcohol, drinking, income, and number of education years are protective, vi) genetic scores associated with neurotransmitters (synapse functioning and loss) and neuroinflammation demonstrated strong significant effects, and vii) Blinder-Oaxaca decomposition demonstrated the important role of genetic factors in racial disparities in AD risk. The analyses show the extent to which links between the distinct differences in comorbidities, behavioral and socioeconomic factors can predict the risk for AD/ADRD.

### LONGITUDINAL ASSOCIATION BETWEEN SERUM 25 HYDROXY VITAMIN D AND COGNITIVE FUNCTION AMONG ICELANDIC OLDER ADULTS

Hrafnhildur Eymundsdottir,[1] Milan Chang,[2] Olof Geirsdottir,[3] Maria Jonsdottir,[3] Palmi V. Jonsson,[4] Vilmundur Gudnason,[5] and Alfons Ramel[6], *1. The Icelandic Gerontological Research Institute, Reykjavik, Iceland, 2. University of Iceland, School of Education, Faculty of Health Promotion, Sports and Leisure Studies, Reykjavik, Iceland, 3. Reykjavik University, Reykjavik, Iceland, 4. Landspitali University Hospital, Reykjavik, Iceland, 5. Icelandic Heart Association, Kopavogur, Iceland, 6. University of Iceland, Reykjavik, Capital area, Iceland*

Studies have indicated that low levels of serum 25 hydroxy vitamin D (25OHD) are associated with lower cognitive function among older adults while longitudinal studies have revealed controversial results. The aim was to investigate the longitudinal associations between 25OHD and cognitive function among older adults with 5-years follow up. The Age, Gene/Environment Susceptibility (AGES)-Reykjavik Study (N=3411) assessed cognitive function measuring memory function, speed of processing and executive function. 25OHD was measured using the Liaison chemiluminescence immunoassay and used as a continuous variable. Multivariate linear analysis, adjusting for numerous confounding factors, was used to calculate the longitudinal associations. All analyses were performed separated by gender. There was a high tendency for low levels of 25OHD i.e. 29.6% men and 37.7% women had hypovitaminosis D (<50 nmol/l). Both men and women had significantly lower scores in all aspects of cognitive function at the follow-up time period. Unadjusted correlations between 25OHD and cognitive functions showed a stronger correlation for women, whereas women had lower scores in all aspects of cognitive function associated with low 25OHD. After adjusting for potential confounders, e.g. age, education, lifestyle and health-related factors, 25OHD and cognitive function were not significantly associated. Observational studies indicate that lower levels of vitamin D are associated with lower cognitive function. Intervention studies are yet to show a clear benefit from vitamin D supplementation. More longitudinal- and interventional studies, with longer follow-up duration, are needed.

### TOWARD AN OPERATIONAL DEFINITION OF COGNITIVE LIFESTYLE: MODELING RESERVE ACROSS THE LIFESPAN

Lisa Swann,[1] and Fiona E. Matthews[1], *1. Newcastle University, Newcastle upon Tyne, United Kingdom*

A recent systematic review of current definitions of cognitive reserve across the lifespan was undertaken by our group and five reserve constructs were identified: Educational attainment, sociological position, occupational complexity, cognitive ability, and engagement in leisure activities. The aim of this study was to test whether the different constructs are predictive of cognitive performance in older adults. A theoretical model of cognitive lifestyle was designed to assess reserve across the lifespan and the different measures were mapped in individuals aged >=65 years (N=7,762; 54.47% women) from the Cognitive Function and Ageing Study (CFAS II). Multivariable logistic regression analyses, controlling for age and sex, were used to determine the relationship between later life cognitive function and the five identified measures of reserve. In support of previous findings, the results show that risk of cognitive decline decreases with additional education (OR=0.89; 95% CI= 0.86, 0.94), increasing fluid intelligence (OR=1.00; 95% CI= 0.99, 1.00), and participation in leisure activities (OR= 0.27; 95% CI=0.17, 0.42), and that the lower the hierarchical position of a person's social strata, the greater their odds of cognitive impairment in later life (OR=1.02; 95% CI=1.00, 1.05). However, though greater occupational complexity with data (OR=1.11; 95% CI=1.03, 1.19) and with people (OR=1.10; 95% CI= 1.03, 1.19) was found to be protective of cognitive decline as expected, greater occupational complexity with things was associated with greater decline (OR=0.98; 95% CI=0.93, 1.04). Given the cross-sectional nature of the present study, further longitudinal work is needed to ascertain what is driving these results.

### HOW NETWORKS OF MEDICARE BENEFICIARY PATIENT TRANSFERS AFFECT HOSPITAL C. DIFFICILE OUTBREAK RISK

Catherine C. Cohen,[1] and Timothy Luke Muggy[1], *1. RAND Corporation, Santa Monica, California, United States*

Clostridium difficile (CDI) is the most common healthcare associated infection (HAI), and it causes particularly high morbidity and mortality among older adults. While risk factors for this deadly HAI have been explored at the patient and facility levels, less is known about transmission between facilities. Recent literature suggests that more patient transfers to a hospital are correlated with elevated CDI levels. However, this work excludes skilled nursing facilities (SNFs), although SNFs are a major contributor of older adult patients to hospitals. We use multiple large data sets to make progress in filling several existing knowledge gaps. First, we construct and analyze healthcare facility networks using transfers of Medicare beneficiaries in the Minimum Data Set 3.0 and Medicare claims data from New Mexico, New York, Connecticut, and Colorado. We evaluate the role of SNFs in networks through the volume and frequency of patient flows in and out of individual hospitals. We also assess the level of interaction (i.e., transfers) between facilities of different sizes, locations, and types. Second, using the Healthcare Cost and Utilization Project State Inpatient Databases, we examine whether a hospital CDI outbreak is predictive of CDI incidence across a network of hospitals, using multiple existing metrics of CDI incidence, noting strengths and weaknesses for each. These two aims lay the foundation for future work to examine the relationship between patient transfers and the distribution of outbreaks. Such work may be able to identify facilities that present the greatest CDI risk to older adults across a facility network.

### BODY COMPOSITION ACROSS THE LIFESPAN IN AFRICAN CARIBBEAN MEN: FINDINGS FROM THE TOBAGO LONGITUDINAL STUDY OF AGING

Adam J. Santanasto,[1] Iva Miljkovic,[1] Ryan K. Cvejkus,[2] Allison L. Kuipers,[1] Victor Wheeler,[3] and Joseph M. Zmuda[2], *1. University of Pittsburgh, Pittsburgh, Pennsylvania, United States, 2. Department of Epidemiology, University of Pittsburgh; Pittsburgh, Pennsylvania, United States, 3. Tobago Health Studies Office, Scarborough, Tobago, Trinidad & Tobago, Tobago, Trinidad and Tobago*

Body composition changes vary by age and ethnicity and have a major impact on health and physical function. However, little is known about the magnitude, tempo and patterns of these changes in African-ancestry populations, particularly outside the U.S. Thus, we examined age-specific rates-of-change in lean and fat mass in a unique population-based, longitudinal cohort study of 2621 African-ancestry men on the Caribbean island of Tobago (age: 62.0±11.8 years, range: 32-99 years). Body composition was measured with DXA at study entry and after an average of 4 and 9 years. Annualized rates of change and 95% confidence intervals were calculated using all 3 time-points with Generalized Estimating Equations stratified by 5-year baseline age-groups. Lean mass declined at a fairly constant rate in age-strata up through age ≤64 years (-0.72; -0.76, -0.67%/yr), but accelerated to -0.92 %/yr (-1.02, -0.82 %/yr) among those aged 65-69, and to -1.16 %/yr (-1.30, -1.03 %/yr) among those aged 70-74 years – plateauing in those aged 75+. This pattern of age acceleration was observed in arm but not leg lean mass. The age-specific rates of decline in lean mass in this cohort of African Caribbean men appear to be lower than those reported in older African American men. In contrast to lean mas, fat mass increased by 2.93 %/yr (2.72, 3.15 %/yr) and this rate of increase was fairly uniform across the lifespan. Additional research is needed to better define the lifestyle, medical and biological factors contributing to body composition changes across the lifespan in African-ancestry populations.

### TEMPORAL TRENDS IN INCIDENCE RATES OF VISION IMPAIRMENT AMONG MIDDLE-AGED AND OLDER CHINESE

Gui-Ying Cao,[1] Shan-Shan Yao,[1] zi-shuo Chen,[1] Zi-Ting Huang,[1] and Beibei Xu[2], *1. Department of Epidemiology and bio-statistics School of Public Health, Peking University, Beijing, China, 2. Peking University Medical Informatics Center, Beijing, China*

This study aimed to investigate temporal trends in the incidence rates (IRs) of vision impairment (VI) and explore its associated factors among middle-aged and older Chinese. Data were obtained from China Health and Retirement Longitudinal Study (CHARLS, 2011-2015) and Chinese Longitudinal Healthy Longevity Survey (CLHLS, 1998-2014). There were 20,467 participants with a mean age of 57.4 years from CHARLS and 21,210 participants with a mean age of 90.0 years from CLHLS. Distance and near vision were self-reported in CHARLS and performance-based vision was assessed in CLHLS. Crude and age-adjusted IRs per 1,000 person years (PY) of distance vision impairment (DVI), near vision impairment (NVI) and both distance and near vision impairment (DNVI) in CHARLS and VI in CLHLS were calculated for each wave of surveys. Risk factor adjusted temporal trends in the incidence were examined using regression models. Age-standardised IRs for DVI, NVI and DNVI significantly decreased from 79.4 to 41.2, 95.1 to 48.0 and 40.0 to 21.3 (P < 0.001) from 2013 to 2015, respectively. Male gender, higher education, no ADL disability, no depression, no multimorbidity were associated with lower IRs of DVI, NVI or DNVI. Younger age was associated with lower IRs of DVI and DNVI but higher IRs of NVI. Age-standardised IR of VI significantly decreased from 131.6 to 71.9 (P = 0.010) from 2000 to 2014, and higher education and living in the South were associated with lower IR of VI. Future studies may further investigate the causality of such phenomenon.

### SESSION 2375 (POSTER)

### FAMILY CAREGIVING II

### GRANDPARENTS RAISING GRANDCHILDREN IN KOREA: CULTURAL UNDERSTANDING OF MULTIGENERATIONAL CAREGIVING

Youjung Lee,[1] and Sok An[2], *1. Binghamton University, Binghamton, New York, United States, 2. Korea Rural Economic Institute (KREI), Naju-si, Korea, Republic of*

Korean grandparents raising their grandchildren play significant roles as social and family resources in Korea. They experience caregiving stress that negatively influences their mental health while struggling with limited social support from peers, and minimal respite care and community resources. Despite a need for resources and information concerning this growing population, there is limited knowledge on Korean custodial grandparents, especially the distinctive cultural factors influencing their multigenerational caregiving experiences. This qualitative study explored Korean custodial grandparents' experiences of raising grandchildren and cultural meanings of multigenerational caregiving. Using a phenomenological approach, semi-structured interviews with 23 custodial grandparents were conducted from December 2018- January 2019 in two urban and three rural places in Korea. Among the grandparents, five were grandfathers. The mean age of the grandparents was 72 years old (range: 61-82), and the mean age of their grandchildren was 13 years old (range: 1-21). On average, the grandparents had raised 1.5 grandchildren (range: 1-3) for 11 years (range: 1.5-20). Four key themes emerged from the general structure of the lived experiences of Korean grandparents: a) intensive child care support from the grandchildren's uncle and aunt; (b) stigma of a child's divorce and custodial grandparenting; (c) different social expectations toward maternal grandparents; and (d) increased needs on financial educational support for grandchildren. The finding reinforces the significant function of collectivistic and family-based Asian culture in understanding experiences of Korean custodial grandparents. It also highlights society's view of only men carrying forward family lineage and its implications on grandparents' perceptions about multigenerational caregiving.

### CARING FOR CHILDREN WITH DEVELOPMENTAL DISABILITIES: WHAT ARE THE OUTCOMES FOR HEALTH IN AGING?

Jessica Hoyle,[1] James N. Laditka,[1] and Sarah B. Laditka[1], *1. University of North Carolina at Charlotte, Charlotte, North Carolina, United States*

Eight to ten percent of children have a developmental disability (DD). Caring for a child with DD can be rewarding. It can also be difficult, particularly for older parents or other caregivers (hereafter parents). Few studies have examined their experiences. We followed parents for 18 years using the nationally representative Panel Study of Income Dynamics (PSID). We defined DD with criteria used in research and education law, as: (1) autism, intellectual disability, learning disorder, epilepsy with seizures, attention deficit disorder/attention deficit hyperactivity disorder, cerebral palsy, or traumatic brain injury; and (2) qualification for professional services. We used three waves of the PSID's Childhood Development Study, 1997-2007, linking children's and parents' PSID data (n=4,070) to study three outcomes: parents "not very" or "not at all" satisfied with "life as a whole"; nonspecific psychological distress; and reports that depressive feelings interfered with life or activities "a lot." Discrete-time hazard models controlled for: child disability; children's and parents' age, sex, and education; survey design; and repeated measures. Among parents ages 47-61 at baseline (65-79 in 2015), those who cared for a child with DD were significantly more likely to report low satisfaction with life (hazard ratio, HR 4.6, 95% confidence interval, CI 1.6-13.2), distress (HR 2.8, CI 1.1-6.9) and, among women, depressive feelings (HR 1.3 CI 1.0-1.8). We found no such differences for parents less than age 65 in 2015. Results highlight the need to provide emotional support, respite, or other services for older adults caring for children with disabilities.

### IDENTIFYING PREDICTORS OF GUILT FOLLOWING PLACEMENT OF FAMILY MEMBER WITH DEMENTIA INTO RESIDENTIAL LONG-TERM CARE

Tamara L. Statz,[1] Colleen M. Peterson,[1] Robyn Birkeland,[1] Aneri Shah,[1] Kobe Perez,[1] and Joseph E. Gaugler[1], *1. University of Minnesota - School of Public Health, Division of Health Policy and Management, Minneapolis, Minnesota, United States*

Family caregivers of persons with dementia experience guilt for various reasons when placing their family member into residential long-term care (RLTC). Research has shown a relationship between guilt and overall caregiver burden; however, literature on predictors of guilt related to caregiving following RLTC placement is limited. The Residential Care Transition Module (RCTM) provides counseling and psychoeducation to family caregivers who have recently moved their family member with Alzheimer's disease or a related dementias into RLTC. This semi-structured intervention provides counseling on various topics including guilt, grief, and family dynamics. Using treatment group data (N=87) from the parent RCTM randomized controlled trial, we identified the impact of caregiver status (i.e., adult child vs spousal caregivers), sense of caregiver competence, and relationship closeness on baseline measures of guilt status and magnitude. Preliminary analyses showed that adult child status was associated with greater prevalence of guilt (37.5%) compared to spousal caregivers (26.7%). In addition, qualitative case notes were coded to identify common themes of experiences of guilt to inform practice. Examples include: Guilt related to no longer being able to care for the family member at home; not meeting self-dictated expectations (e.g., not visiting or staying long enough); and feeling guilted into being a caregiver in the first place. The current analyses aim to help practitioners better predict risk for placement-related guilt and highlight specific issues practitioners should consider to help mitigate such feelings. Specific opportunities for intervention are discussed.

### USING A STRESS PROCESS MODEL TO EXAMINE RACIAL DIFFERENCES IN CAREGIVER WELL-BEING AND HEALTH

Adrian Badana,[1] and William E. Haley[2], *1. School of Aging Studies, University of South Florida, Tampa, Florida, United States, 2. University of South Florida, Tampa, Florida, United States*

Current research must utilize nationally-representative samples of older adults and their family caregivers to accurately reflect the growing diversity of the United States. This study aims to use a stress process model to examine potential racial differences in caregiving in a population-based sample of 844 White and 389 Black family caregivers in the United States. We conducted 3 x 2 x 2 (relationship type x race x dementia care status) factorial ANOVAs to examine potential differences in caregiving stressors, appraisals, resources, and mental and physical health outcomes among primary family caregivers. Results indicated significant racial differences in caregiving on several stress process measures. Although Black caregivers reported more caregiving stressors, compared to White caregivers, they tended to report more positive appraisals of caregiving and more caregiving resources. Dementia caregivers tended to report greater caregiving stressors and worse measures of appraisal compared to non-dementia caregivers. There was a significant two-way interaction among relationship type and dementia care status for the caregiving stressor, hours of care. A stress process model can allow researchers to investigate various factors associated with racial differences in caregiving.

### DEPRESSION IN CAREGIVERS OF VETERANS: THE INTERACTION OF PERCEIVED SOCIAL SUPPORT AND AGE

Monica Scicolone,[1] and Patricia A. Parmelee[1], *1. The University of Alabama, Tuscaloosa, Alabama, United States*

Caregiving is a risk factor for increased psychological stress and depression (Pinquart & Sörensen, 2003). Perceived social support (PSS) is strongly associated with emotional well-being and, for informal family caregivers, may be an important predictor of caregiver psychological outcomes. Although much is known about the effects of global PSS, there is a gap in research regarding numerous identified functional dimensions of support, particularly among family caregivers. Thus, this secondary data analysis examined how dimensions of PSS predict depression, and the moderating effects of age on this relationship, utilizing data from 240 family caregivers of elderly veterans receiving outpatient care at the Atlanta VA. The analysis utilizes a multidimensional measure of PSS (Sherbourne & Stewart, 1991) with four sub scales: emotional/informational, tangible, affectionate, and positive social interaction. Preliminary OLS regression analyses, regressing depression on relevant demographic variables and PSS domains, revealed a significant overall model ($p < .001$). All domains besides tangible support significantly predicted depression. Because age was the only demographic variable associated with depression, we tested hypotheses of an interaction of PSS and age with PROCESS macro (Hayes, 2017). Moderation analyses revealed a significant interaction of support and age on caregiver depression ($p = .0145$). Of unique social support dimensions, only emotional/informational social support interacted with age ($p = .0075$), demonstrating decreased depression at high levels of emotional/informational support, but a weaker effect for those of increased age. The original study was supported by the U.S. Department of Veterans Affairs Rehabilitation R&D Service.

### CHARACTERISTICS OF CAREGIVERS WITH FAMILY CONFLICT

JoAnna Dieker,[1] Kelsey Bacharz,[1] Kendall Weber,[1] and Sara H. Qualls[1], *1. University of Colorado Colorado Springs, Colorado Springs, Colorado, United States*

The family environment is often overlooked in caregiver research and assessment, despite having implications for caregiver health and well-being (Zarit et al., 2019). The purpose of the present study was to examine differences on two types of family conflict (beliefs and support) among a diverse sample of caregivers. The present sample consisted of help-seeking ($n = 375$) and non-help-seeking ($n = 415$) caregivers (total $n = 790$). Caregivers filled out the Caregiver Reaction Scale (O'Malley & Qualls, 2017), a multidimensional assessment of the caregiver experience. Results of a 2 (adult children, spouse) x 2 (help-seeking, non-help-seeking) ANOVA indicated that help-seeking caregivers reported significantly more conflict over family beliefs than did non-help-seeking caregivers ($M = 1.93$ and $1.58$, respectively), $F(3,606) = 21.10$ $p < .001$. Adult children caregivers reported significantly greater conflict over family beliefs ($M = 1.91$) than did spouse caregivers ($M = 1.60$), $F(3,606) = 10.66$, $p < .001$. Adult children caregivers also reported significantly greater conflict over family support ($M = 1.87$) than did spouse caregivers ($M = 1.57$), $F(3,600) = 16.23$, $p < .001$. Results highlight that certain caregiving contexts (e.g., adult children caring for a parent) potentially increase family conflict, which has implications for caregiver burden. Family conflict over beliefs is also related to help-seeking in caregivers. Findings inform appropriate assessment and intervention regarding the family environment in caregiving.

### STRESS, DEPRESSION, AND COGNITIVE FUNCTION IN ADRD FAMILY CAREGIVERS

Frank Puga,[1] Kylie Meyer,[2] and Carolyn E. Pickering[2], *1. University of Texas Health San Antonio, San Antonio, Texas, United States, 2. University of Texas Health Science Center San Antonio, San Antonio, Texas, United States*

The rapidly growing number of individuals over the age of 55 stresses the need to identify unique factors to decrease older adults' vulnerability to psychiatric and neurodegenerative disorders, especially among high-risk groups such as family caregivers to persons with Alzheimer's disease or related dementias (ADRD). In this project, we investigated physiological stress responses, depression, and cognitive functioning. Using data from the Midlife in the United States database, we examined differences in physiological stress responses, depressive symptoms, and cognitive function (including working memory, episodic memory, verbal ability, processing speed, and executive functioning) between ADRD and non-ADRD caregivers. Results from the secondary analysis revealed a decrease in cognitive function, an increase in depressive symptoms and disrupted physiological responses in ADRD caregivers. These data suggest that the unique aspects of ADRD caregiving are a risk factor for the onset of psychiatric and neurodegenerative disorders. The results of this study support the development of targeted interventions to reduce the risk of depression and cognitive decline among ADRD caregivers. The specific focus on older adult ADRD caregivers, physiological processes, mental health, and dementia is unique in the field of aging and provides a critically needed new paradigm for identifying strategies to support ADRD caregivers and understanding the development of ADRD.

### SOCIOECONOMIC DETERMINANTS OF INFORMAL CAREGIVING IN INDIA

Kent Jason G. Cheng,[1] and Janet M. Wilmoth[1], *1. Syracuse University, Syracuse, New York, United States*

This study investigates the socio-economic determinants of informal caregiving for elderly parents in urban India, with a focus on caste differences. Bivariate and multivariate logistic regression models of caregiving are estimated with data from 2011 data of Osaka University's Preference Parameters Study. Three types of caregiving are examined: helping with housework, financial assistance, and providing care. The control variables include: age, sex, marital status, wealth, religiosity, self-rated health, parents requiring care, number of siblings, and number of co-resident children. The bivariate analysis indicates that the highest caste is significantly less likely than the lowest caste to help with housework (OR=.734, SE=.127). In the fully specified models, there is not a significant difference between caste groups in the likelihood of helping with housework or providing financial assistance, but the highest caste is more likely than the lowest caste to provide care (OR=1.443, SE=.309). Being female and married significantly lowers the odds of each type of caregiving. Wealth increases the likelihood of providing help with housework and financial assistance. When both parents require care, children are more likely to provide financial assistance and help with housework, but when one parent requires care, children are more likely to provide care. Overall, sex, marital status, and wealth are the strongest predictors of helping with housework and financial assistance, whereas sex, marital status, and caste are the most important predictors of providing care. The implications of these findings for aging parents and adult children in urban India are discussed.

### COGNITIVE ENHANCEMENT FOR GRANDPARENTING: REDUCED LONELINESS AS A MEDIATOR

Yuanqing Chang,[1] Yin Li,[2] and Xin Zhang[2], *1. School of Psychological and Cognitive Sciences,Peking University, Beijing, China, 2. Peking University, Beijing, China*

Grand-parental care, an important form of family care in China, However, its health effect on older adults, including their physical and mental health and cognitive function, is often not well understood. In the present study, we investigated the differences in terms of physical and mental health (measured by SF-36 Quality of Life Scale), self-aging attitudes (measured by the Philadelphia Senior Center Morale Scale), and cognitive functions (measured by WAIS, the subscales of Wechsler Adult Intelligence Scale) between those who provide grand-parental care and those who do not. Two studies were conducted. The first study examined 150 older adults who provided grandparental care and another 150 older adults who did not. Six months later, we randomly selected 103 older adults to conduct a longitudinal follow-up, of which 53 were older adults who provided grandparental care, and another 50 older adults were those who didn't. The results of both cross-sectional and longitudinal studies showed that, compared with older adults who did not provide grandparental care, those providing grandparental care has significantly better physical and mental health, more positive self-aging attitudes and even enhanced cognitive functions. Further path analysis showed that loneliness mediated the association between providing grandparental care and enhancement in functions, such that providing grandparental care can reduce loneliness of the older adults, which in turn can improve their physical and mental health and even enhance their cognitive functions. These results shed light on the practical implications of grandparenting in China for the society.

### RESILIENCE AND GRANDPARENT CAREGIVERS: A LONGITUDINAL ANALYSIS

Bert Hayslip[1], *1. university of north texas, Denton, Texas, United States*

Previous work reflecting a shift in views about custodial grandparents has emphasized such persons' strengths. Such research has specifically indicated that resilience cross sectionally mediates the relationship between the stressfulness of the demands of raising a grandchild and both adjustment-related and parentally relevant outcomes. To explore the dynamics of such relationships in a one-year longitudinal framework, 86 grandparent caregivers completed a variety of measures targeting personal and parental functioning as well as resilience at initial and one-year follow-up occasions. Findings indicated that resilience at T1 predicated ($p < .05$) the following at T2: parental stress, parental efficacy, health, well-being, depression, grandparent role satisfaction, self-rated life disruption, and grandchild and grandchild behavioral/emotional difficulties. T1 resilience also predicted a T2 index of overall personal resources (better health/social support, less life disruption). At the same time, the following at T1 emerged as antecedents ($p < .05$) of T2 resilience: parental stress, parental efficacy, grandparent role satisfaction, depression, health, grandchild attachment, and well-being. In addition, overall personal adjustment (higher grandparent role satisfaction, more positive caregiver role appraisal, greater well-being, higher grandchild attachment, less depression) as well as greater overall personal resources each predicted ($p < .05$) greater T2 resilience. These findings not only extend previous cross-sectional research reinforcing the value of resilience in understanding grandparent caregivers, but also indicate that numerous parental and personal variables may lay the groundwork for the development of resilience, wherein these data also suggest that the relationship between resilience and grandparent caregiver functioning may be bidirectional in nature.

### LONGITUDINAL CHANGES IN WELL-BEING OF PARENTS OF INDIVIDUALS WITH DEVELOPMENTAL OR MENTAL HEALTH PROBLEMS

Carlie J. Sloan,[1] Marsha R. Mailick,[2] Jinkuk Hong,[2] Jung-Hwa Ha,[3] Jan S. Greenberg,[2] and David M. Almeida[1], *1. The Pennsylvania State University, University Park, Pennsylvania, United States, 2. University of Wisconsin - Madison, Madison, Wisconsin, United States, 3. Seoul National University, Seoul, Korea, Republic of*

The negative impact of having a child with special needs on parental well-being is well documented. Previous research has suggested age attenuation of these impacts. However, this has not yet been examined longitudinally in late life. Therefore, it is unclear how the effect of having a child with a developmental disability or mental health problem changes as parents age and children become less likely to live at home. Using responses from the Study of Midlife in the United States (MIDUS), this study investigates: (1) longitudinal changes in the effect of having a child with a developmental or mental health problem on parental well-being, (2) age and gender moderations on these effects, and (3) the unique impact of factors directly related to the child's condition. Multiple linear regressions revealed that having a child with a developmental disability was predictive of higher negative affect, more somatic symptoms, and lower psychological well-being longitudinally. Additionally, there was a main effect of having a child with a mental health problem in predicting higher negative affect. However, age moderations were revealed such that the effect of having a child with a developmental disability or mental health problem was diminished for older parents. Additionally, within-group analyses revealed that longer duration of developmental disabilities and later parental age of onset of mental health problems were predictive of better outcomes. Overall, results suggest that although having a child with special needs is related to poorer well-being, these effects can attenuate as parents age and adapt.

### EXPERIENTIAL AVOIDANCE IN A SAMPLE OF DEMENTIA CAREGIVERS

Amy M. Houston,[1] Elisabeth Harfmann,[2] Amy Olzmann,[3] Glenna Brewster,[4] and Hannah Ottmar[5], *1. Milwaukee VA, Milwaukee, Wisconsin, United States, 2. Milwaukee VA, Milwaukee, Virginia, United States, 3. Baltimore VA, Baltimore VA, United States, 4. Emory University Nell Hodgson Woodruff School of Nursing, Atlanta, Georgia, United States, 5. Xavier University, Cincinnati, Ohio, United States*

Given the rapidly changing demographics, there will be an increasing number of individuals with dementia who will need significant support from informal caregivers. Providing care for an individual with dementia has been associated with negative outcomes in a number of domains including physical health, mental health, financial status and social functioning. There is a small but growing base of literature suggesting that fostering psychological flexibility, including acceptance, with dementia caregivers may be a helpful intervention. Experiential avoidance, which is the less adaptive alternative to acceptance, is the aversion from negative internal experiences including thoughts, feeling and physical sensations. Experiential avoidance has been found to be significantly related to depression and negative affect. The present study utilized online dementia caregiver support group samples ($n = 158$) to evaluate the relationship between experiential avoidance and general demographics, other aspects of psychological flexibility, and caregiver distress. Experiential avoidance was positively correlated with cognitive fusion ($r(134) = .231$, $p < .01$), caregiver burden ($r(127) = .258$, $p < .01$), and distress associated with dementia related behaviors ($r(140) = .225$, $p < .01$). Experiential avoidance was negatively correlated with engaged living ($r(133) = -.244$, $p < .01$), mindfulness ($r(123) = -.187$, $p < .05$), and self-rated health ($r(138) = -.193$, $p < .05$). Additionally, experiential avoidance was significantly higher for male caregivers ($t(136)=2.462$, $p=.015$) and those age 65 and over ($t(134)=-2.421$, $p=.017$). These findings support previous research that suggests experiential avoidance may be an important construct to target in future interventions with dementia caregivers.

### SUPPORTING FAMILY CAREGIVERS: HOW DO CAREGIVERS OF OLDER ADULTS COPE WITH ROLE STRAIN? A QUALITATIVE STUDY

Laura Rath,[1] Kylie Meyer,[2] Elizabeth S. Avent,[3] Paul Nash,[4] Donna Benton,[3] Zach Gassoumis,[5] and Kathleen Wilber[5], *1. Archstone Foundation, Long Beach, California, United States, 2. University of Texas Health Science Center San Antonio, San Antonio, Texas, United States, 3. University of Southern California, Los Angeles, California, United States, 4. University of Southern California, LA, California, United States, 5. Leonard Davis School of Gerontology, University of Southern California, Los Angeles, California, United States*

Qualitative research on positive coping approaches actually used by caregivers can inform interventions that can be feasibly implemented. Absent from previous qualitative research is how caregivers respond to strain in the relationship, specifically. Eight focus groups were conducted with a purposeful sample of racially and ethnically diverse family caregivers in Los Angeles (n=75). An additional 8 in-depth follow-up interviews were conducted. Content analysis was used to understand the mechanisms employed by caregivers to cope with strain and tension in the caregiving relationship. Preliminary results revealed twenty-two individual themes, which were subsequently grouped into four main superordinate themes: 1) Self-care; 2) Adaptation of behaviors and feelings; 3) Seeking and utilizing assistance and respite; and 4) Education and support groups. This work can help inform the design of programs to support caregivers and prevent potentially harmful behaviors, through understanding the experiences of caregivers in their own words.

### NONTRADITIONAL VIEWS? HOW SIBLINGS MATTER FOR PERCEIVED PARENTAL CARE RESPONSIBILITY IN JAPAN

Ryo Hirayama,[1] and Tomoko Wakui[1], *1. Tokyo Metropolitan Institute of Gerontology, Tokyo, Japan*

Our aim in this study was to explore whether and how siblings' marital and work status influence Japanese adult children's perceived responsibility for parental care. Within traditional familial institutions in Japan, married sons were expected to assume parental care responsibility. At the same time, such care arrangements built on gendered division of labor; sons served as family breadwinners, and their wives cared for their parents-in-law while out of the paid labor force. Yet, because of sociodemographic shifts such as a greater percentage of unmarried persons and a growing number of women who seek to maintain their job, it has been increasingly unclear which adult children can and should assume the role of parental caregiver. Using online survey data from 989 Japanese adult children who were all employees with no parental care experiences ever, we sought to clarify the influences of siblings' circumstances on whether these children anticipated assuming responsibility for conducting different care tasks for their parents. In doing so, we focused on how siblings' gender and work and marital status might combine to affect adult children's anticipation of parental care responsibility. A series of logistic regression analyses revealed that having a married brother made it less likely for adult daughters to anticipate assuming responsibility for conducting typical care tasks (e.g., ADL assistance) whereas for adult sons, having a single sister declined such anticipation. We discuss our findings in terms of how traditional familial institutions still impinge on Japanese adult children's views of parental care responsibility.

### ASSOCIATION BETWEEN ELDER ABUSE AND TELOMERE LENGTH IN OLDER ADULTS WITH COGNITIVE IMPAIRMENT

Boye FANG,[1] Elsie Yan,[2] Keith Tung,[3] and Gengzhen Chen[4], *1. Hong Kong Polytechnic University, Hong Kong Polytechnic University, Hong Kong, 2. The Hong Kong Polytechnic University, Hong Kong, Hong Kong, 3. University of Hong Kong, Hong Kong, Hong Kong, 4. Shantou University Medical College, Shantou, China*

Objectives: Elder abuse is a stressful event that can lead to compromised psychological and physical health consequences. This study examines the association between elder abuse and telomere length (TL), a biomarker reflecting cellular aging and disease pathogenesis. Methods: Between 2015 and 2016, 1,002 older adults (aged≥55 years) with cognitive impairment were consecutively recruited from the geriatric and neurological departments of three Grade-A hospitals in Guangdong Province of People's Republic of China. At two-year follow-up, 958 of these participants were reassessed and 600 of them were randomly selected for this study. The outcome variable is TL (measured in blood cells using a multiplex quantitative polymerase chain reaction) and the major independent variables were current experience of overall abuse, psychological abuse, physical abuse, caregiver neglect, and previous experience of domestic abuse in the past two years. Potential confounding variables include demographic variables and increased severity of cognitive impairment, neuropsychiatric symptoms, sleep disorders, and depressive symptoms. Results: Multivariate regression models show that current experience of overall domestic abuse ($t= -5.116$, $\beta= -0.376$, confidence interval[CI] -20.231--9.006), psychological abuse ($t= -4.431$, $\beta= -0.231$, [CI] -13.023--5.023), physical abuse ($t= -2.474$, $\beta= -0.151$, CI -14.116--1.621), and caregiver neglect were associated with shorter TL ($t= -4.470$, $\beta= -0.185$, CI -10.457--4.072). Other predictors of shorter TL were previous experience of domestic abuse and increased severity of depression. Discussion: Both current and previous experiences of elder abuse are associated with shorter TL. Multidisciplinary efforts were needed to prevent and intervene elder abuse.

### IT'S BETTER TO GIVE THAN RECEIVE, OR IS IT? THE EFFECT OF SOCIAL SUPPORT ON THE PSYCHOLOGICAL WELL-BEING OF OLDER PARENTS

Erik Blanco[1], *1. California State University Los Angeles, Los Angeles, California, United States*

The longevity revolution has led to more years of shared lives between older parents and adult children. Having these extra years together can be offset by the stressful life transitions of widowhood, health declines, and increased level of disability experienced by older parents. These transitions can lower older parents' psychological well-being. Although social support to/from adult children has the potential to buffer these effects, most older parents wish to remain independent, even in later life, making them reluctant to accept social support from their adult children. The purpose of this paper is to examine whether the provision or receipt of social support between older parents and adult children, influences positive mood and negative mood. Secondary data on older adults ($n = 461$) with adult children who participated in the 2004 wave of the LSOG were used. The results revealed that the provision of social support by older parents to adult children significantly increased parents' positive mood showing that it is better to give than receive. The results for the receipt of social support were more complex. Results suggest that when someone has a higher level of disability and does not receive social support their negative mood increases, but when someone has a high level of disability and does receive social support there is no effect on negative mood. This proposes that the receipt of social support is particularly important when the parent is in need of support and it is better to receive than give when parents are in need.

### SESSION 2380 (POSTER)

### HEALTH CARE

### THE ROLE OF COLLECTIVISM IN EXPLAINING LIFE COURSE HEALTH DISPARITIES IN OLDER AGES

Joelle Saad-Lessler,[1] and Karen Richman[2], *1. Stevens Institute of Technology, School of Business, Hoboken, New Jersey, United States, 2. University of Notre Dame, Notre Dame, Indiana, United States*

Collectivism refers to the social practice of investing in and relying on one's social network, rather than formal institutions, to ensure personal security. Using the re-engineered 2014 Survey of Income and Pension Participation (SIPP), we investigate how collectivist practices affect life course health disparities at older ages in the US. Indicators of Collectivism include measures of caregiving, inter and intrahousehold financial and material support and help from charities, friends and family members. Regression results indicate that increased collectivist interactions are associated with improved self-reported health status outcomes. Government support for collectivist behaviors can thus yield a low cost means of improving health outcomes among the elderly.

### LOWER URINARY TRACT AND BOWEL SYMPTOMS PREDICT QUALITY OF LIFE IN GAY AND BISEXUAL PROSTATE CANCER SURVIVORS

Kristine M. Talley,[1] Elizabeth Polter,[1] Benjamin D. Capistrant,[2] William G. West,[1] Badrinath Konety,[1] and B.R. Simon Rosser[1], *1. University of*

*Minnesota, Minneapolis, Minnesota, United States, 2. Smith College, Northampton, Massachusetts, United States*

Little is known about disparities in prostate cancer survivorship experienced by gay and bisexual men (GBM). However, early evidence suggests GBM may experience worse urinary and bowel symptoms than heterosexual men. This cross-sectional Internet-based survey describes the prevalence of lower urinary tract (LUTS) and bowel symptoms and their associations with physical and mental health related quality of life (QOL) in GBM treated for prostate cancer. This study enrolled 193 men who identified as gay or bisexual and had received prostate cancer treatment. The Expanded Prostate Cancer Index Composite instrument measured LUTS and bowel symptoms. The MOS SF-12 measured physical and mental QOL. Participants had a mean age of 63.4 years, were 5.6 years past treatment, and were treated with prostatectomy (52%), radiation (19%), or combined or systemic treatment (29%). The most common symptoms were nocturia (77%), urinary frequency (67%), urinary leakage (59%), bowel urgency (45%), bowel frequency (35%), and watery bowel movements (34%). Mean scores were 81.4±19.2 for urinary function, 74.5±20.7 for urinary bother, 88.9±12.1 for bowel function, 84.5±16.3 for bowel bother, 52.5±8.8 for physical QOL, and 46.0±11.4 for mental QOL. In multivariable models adjusted for age, race, treatment type, and time since diagnosis, urinary bother was associated with worse physical QOL (Adjusted Mean Difference (AMD): 0.11, 95%CI: 0.02-0.21), and bowel bother was associated with worse mental QOL (AMD: 0.23, 95%CI: 0.05-0.42). LUTS and bowel symptoms were common. Symptom bother rather than function predicted QOL. Understanding these disparities will help tailor treatments for this underserved population.

### THE ATTITUDE OF MEDICAL PRACTICES TOWARD LGBTQ OLDER ADULTS BEFORE AND AFTER INTERVENTION

Mackenzi Kim,[1] Lynn M. Wilson,[1] Nyann Biery,[1] and Brenda Frutos[1], *1. Lehigh Valley Health Network, Allentown, Pennsylvania, United States*

Individuals who identify as lesbian, gay, bisexual, transgender or other non-heterosexual or binary gender identifiers (LGBTQ) face tremendous obstacles in search of quality healthcare. Older LGBTQ adults face these obstacles in the setting of more complex health problems with few social services and support. Negative treatment from healthcare professionals has proven to be one of the most pervasive barriers to care faced by older LGBTQ adults. Sensitization training with the film, Gen Silent, is one way knowledge gaps and biases of healthcare professionals has been addressed. By utilizing the survey previously validated by Porter et al., health professionals' knowledge, perceptions, and attitudes toward LGBTQ older adults before and after viewing Gen Silent were assessed in Lehigh Valley Health Network (LVHN)-affiliated primary care practices. The principle outcome of this study was a statistically significant change in responses. Primary care practices were recruited for 45-minute sessions that included the showing of an educational, abbreviated version of Gen Silent to available staff. It was preceded by administration of a pretest survey and followed by a posttest survey and discussion. A paired t-test was conducted to determine significance of differences between pre- and posttest responses. Seventeen individuals (N=17) viewed the film and finished pre- and posttest surveys. Nearly all questions exhibited changes between pre- and posttests. Significantly, respondents indicated increased awareness of additional barriers to care faced by LGBTQ older adults compared to heterosexual peers. While limited, these results indicate that primary care professionals would benefit from training specific to the aging LGBT population.

### CONNECTING PROVIDER TO HOME: BRIDGING GAPS IN CARE

Melanie Weir,[1] Gerardo Moreno,[2] Rosaneli Loza,[1] Lisa Desai,[1] and Chi Hong Tseng[2], *1. SCAN Health Plan, Long Beach, California, United States, 2. University of California, Los Angeles, Los Angeles, California, United States*

When the physician has limited knowledge of the patient's condition and functioning at home it may result in nonadherence to treatment plans, goals of care not being met and avoidable utilization. Connecting Provider to Home (CP2H) deployed teams of a social worker and community health worker to act as the eyes and ears of the doctor in patients' homes and close the information gap in primary care. Study objectives were to 1) reduce unnecessary utilization, 2) increase provider and patient satisfaction, and 3) Improve communication between patient/caregiver and the healthcare team. A total of 416 adult patients were enrolled with a mean age of 76 years, and 58% were female. CP2H participants demonstrated statistically significant reductions in acute hospitalizations and ER use when compared to 700 controls. Acute hospitalizations were reduced by 216 and ER visits by 531 in the intervention group. The average per patient per year reduction in acute hospitalizations was 0.67. The average per patient reduction in ER use was 0.58. CP2H patients reported high levels of satisfaction and rated the program favorably. Stakeholder interviews found that physicians and staff believed the program improved clinical outcomes, provided valuable insight about patients' social barriers to self-care and added value. CP2H study results provide evidence that social workers and community health workers can be successfully and cost-effectively incorporated into the primary care team to address patient needs and priorities, observe the patient in the home environment and assist the physician in adapting treatment plans to optimize patient care.

### NURSES' OPINIONS AND BELIEFS ABOUT MALNUTRITION IN OLDER ADULTS: A CROSS-SECTIONAL STUDY

Debbie Ten Cate,[1] Roelof Ettema,[1] Marieke J. Schuurmans,[2] and Lisette Schoonhoven[2], *1. University of Applied Sciences Utrecht, Utrecht, Netherlands, 2. University Medical Center Utrecht, Utrecht, Netherlands*

Malnutrition in older adults is a frequent and major problem. Despite the fact that nurses have an essential role in nutritional care, they fail to ensure appropriate delivery in preventing and treating malnutrition. For improvement, it is necessary to understand the perspective of nurses about malnutrition. The aim of this study was to gain insight into nurses' opinions and beliefs about malnutrition in older adults. A cross-sectional study was conducted where nurses working in different health care settings were asked to fill in a survey with twelve questions regarding different aspects of malnutrition. Nurses (n = 557) frequently observe malnutrition in older care recipients, and they consider this as a serious health problem. They believe that prevention and treatment of malnutrition is important and they see screening of malnutrition as a relevant nursing activity. They also consider nutritional care as multidisciplinary. Nurses state their need for education to give adequate nutritional care. Nurses' opinions and beliefs about malnutrition in older adults is positive, which enhances nurses' behavior to give sufficient nutritional care to older adults. To gain more benefit in improving nursing activities within nutritional care for older adults, more education is needed targeting nurse professionals and nurse students.

### DOMICILIARY SERVICES IMPROVE ACCESS TO DENTAL CARE FOR FUNCTIONALLY DEPENDENT OLDER ADULTS

Mathew A. Lim,[1] and Gelsomina L. Borromeo[1],
*1. Melbourne Dental School, University of Melbourne, Melbourne, Victoria, Australia*

There is growing evidence demonstrating links between oral diseases and general health. The increased retention of teeth among functionally-dependent older adults presents a unique challenge in maintaining the oral health of these individuals from basic oral hygiene to accessing dental services. The results of our cross-sectional study demonstrate the important role domiciliary dental services play in reducing the barriers to accessing oral health care in this cohort. In our study, most individuals treated by domiciliary services lived in residential aged care facilities and were significantly older than those treated by hospital and community-based dental services dedicated to the specialized care of individuals with additional health care needs. A significantly higher number of those receiving domiciliary care were unable to self-consent for treatment compared to those managed in other settings. 27.4% of these patients had a diagnosis of dementia. More than half (56.9%) of patients treated by domiciliary services received some form of treatment with almost half (48.1%) of these requiring a dental extraction. Only two of these patients were not diagnosed with a chronic condition known to affect oral health (dementia, Parkinson's disease, diabetes mellitus, arthritis, stroke, osteoporosis). 23.7% of domiciliary appointments were used for denture fabrication. The results depict the worrying level of unmet treatment need in residents of aged care facilities. However, they also demonstrate the potential for domiciliary dental services to play a role in developing partnerships between carers and oral health professionals to improve the oral health of functionally-dependent older adults.

### HEALTH CARE AND SOCIAL CARE AMONG FRAIL OLDER ADULTS IN SWEDEN: A REGISTRY-BASED STUDY

Marie Ernsth Bravell,[1] Deborah G. Finkel,[2] and Linda Johansson[1], *1. Institute of gerontology, School of Health and Welfare, Jönköping University, Jönköping, Sweden, 2. Indiana University Southeast, New Albany, Indiana, United States*

In 2010 the Swedish government made a large investment to improve the quality of care for the "frailest older adults" but there have been few follow-ups of the care of this group. The aim of this study is to therefore describe use of care over the time in 2010-2014. Methods: In 2014, 9 National Quality Registries, 3 Care Registries (drug-, patient and death-registers) and the registry for care and social services for older persons (SOL) were individually matched to an older population in the Swedish Twin Registry (n≈45000). Identification of the "frailest older adults" was achieved via SOL and the Patient Registry. Results: 280 persons were identified as "frailest" in 2010 these were followed over time. About two thirds (60,7%) were women, mean age: 81.2±7.7, about one third (35%) lived in a nursing home, one third (34%) had more than three hospitals stays, almost three quarters (72.5%) had more than 19 hospital days and almost one third (28%) had more than seven outpatient care visits. By the end of 2012, 119 persons (42.5%) were deceased. Among those alive (n=161) 42 persons did not receive any inpatient care between 2010-2012, but more than 85% received outpatient care and 44.1% were living in nursing homes. In 2014, 90 persons (32.1%) were still alive and half of them lived in a nursing home. In conclusion, over time the use of care as well as the risk of mortality was high confirming the need of further improvement of the care for these persons.

### THE SAFEST REVIEW: THE SHOCK-ABSORBING FLOORING EFFECTIVENESS SYSTEMATIC REVIEW IN CARE SETTINGS

Amy K. Drahota,[1] Bethany E. Keenan,[2]
Chantelle Lachance,[3] Lambert Felix,[1] James P. Raftery,[4]
Kirsten R. Farrell-Savage,[1] and Dawn Mackey[5],
*1. University of Portsmouth, Portsmouth, Hampshire, United Kingdom, 2. Cardiff University, Cardiff, Wales, United Kingdom, 3. St. Michael's Hospital, Toronto, Ontario, Canada, 4. University of Southampton, Southampton, Hampshire, United Kingdom, 5. Simon Fraser University, Burnaby, British Columbia, Canada*

Falls in hospitals and care homes are a major issue of international concern. Falls cost the US $34 billion a year, with injurious falls being particularly life-limiting and costly. Shock-absorbing flooring decreases the stiffness of the ground surface to reduce the impact of a fall. There is a growing body of evidence on flooring for fall-related injury prevention, however no systematic review exists to inform practice. We systematically reviewed the evidence on the clinical and cost-effectiveness of shock-absorbing flooring use for fall-related injury prevention in care settings. We searched six databases, clinical trial registries, conference proceedings, theses/dissertations, websites, reference lists, conducted forward citation searches, and liaised with experts in the field. We conducted study selection, data collection, and critical appraisal independently in duplicate. We evaluated the influence of shock-absorbing flooring on fall-related injuries, falls, and staff work-related injuries. We adopted a mixed methods approach considering evidence from randomised, non-randomised, economic, qualitative, and implementation studies. We assessed and reported the quality of outcomes using the GRADE approach and Summary of Findings Tables. This review, conducted over the course of 2019, summarises the certainty of the evidence on whether and which shock-absorbing floors influence injuries from falls, the chance of someone falling over, and work-related injuries in staff (e.g. from manoeuvring equipment across softer floors). Our findings are applicable to health and social care professionals, buildings and facilities managers, carers, older adults, architects, and designers. Funded by National Institute for Health Research, Health Technology Assessment (ref 17/148/11); registered in PROSPERO (CRD42019118834).

### ASSISTED LIVING IMMUNIZATION PROJECT

Maureen S. Beck[1], *1. McGovern Medical School at UTHealth, Houston, Texas, United States*

Influenza immunization rates in the United States hover around 60% for people 65 years of age and older. Healthy People 2020 set a target rate of 90% for flu immunization. The influenza vaccine can reduce disease and expense with approximately 7 million flu related diseases, 109,000 hospital admissions, and 8,000 deaths prevented. A local assisted living had an unusually low influenza immunization rate last year of 40%. The goal of the quality improvement project was to increase the rate of influenza immunizations in this facility. The rational was that by providing education, promoting the event, and increasing direct communication with families and residents through phone call and emails the rate would improve. A collaborative project was begun in the summer of 2018. Approval was received by IRB and the facility administrators. Plans were made for two presentations to family and residents, promotional advertisement, family communication, and a scheduled flu day with the pharmacy. The presentation provided flu history and facts and myths about the immunization. Correct answers were rewarded with bookmarks with flu information. The assisted living director provided follow-up immunization data. Compared to the previous year, the immunization rate increased to 90%, counting immunizations on flu shot day and records of outside immunizations. Data concerning flu incidence from the 2017-2018 and 2018-2019 seasons will be requested to determine project impact. A partnership between primary care providers and long term care facilities focused on improving standards or meeting metrics can enhance care for your patient and the community.

### ILLNESS PERCEPTION AS A DETERMINANT OF SELF-RATED HEALTH AMONG OLDER CHINESE AMERICANS WITH TYPE 2 DIABETES

Ya-Ching A. Huang,[1] and Alexandra Garcia[2], *1. Texas State University, Round Rock, Texas, United States, 2. University of Texas at Austin, Austin, Texas, United States*

Self-Rated Health (SRH) has been used as a proxy to evaluate individuals' quality of life, overall well-being, and mortality. However, little is known about how illness perception influences SRH in Chinese American patients with type 2 diabetes (T2DM). This study, guided by Leventhal's Self-Regulatory Model, explored the association between illness perception and SRH beyond socioeconomic and health factors. A cross-sectional survey from 109 community-dwelling foreign-born Chinese Americans with T2DM (60-95 years old; 51.4% females; Mean age= 74.17, SD=6.83). In addition to descriptive and correlation analysis, hierarchical regression models of SRH was estimated by subsequently entering the following set of predictors: (1) demographics (age, gender, marital status, education, financial status, and acculturation), (2) health factors (insulin usage, length of diabetes, number of chronic condition, depression, and diabetes distress) and (3) illness perception (timeline, disease consequence, personal and treatment control). SRH was measured by asking "How would you rate your overall health?" with a likert response scale: 1 (poor) to 4 (very good). Only 50.5 % of participants rated their health as good or very good. Participants with lower acculturation, higher number of chronic health conditions, higher diabetes distress, and a belief that illness will have more negative consequences were highly correlated with worse SRH. The total amount of variance explained by the entire model was 56% ($F = 6.07$, $p < .001$). These findings suggest that the modifiable factors such as acculturation, diabetes distress, and the belief of diabetes consequences should be incorporated into integrative health promotion efforts for this population.

### CHALLENGES IN RECEIVING, ORGANIZING, AND PROVIDING CARE IN ACTIVITIES OF DAILY LIVING: A WORLD-CAFé DIALOGUE

Svenja Cremer,[1] Michel Bleijlevens,[2] Silke F. Metzelthin,[3] Janneke M. de Man-van Ginkel,[4] and Sandra M. Zwakhalen[5], *1. Department of Health Services Research, Care and Public Health Research Institute (CAPHRI), Maastricht University, Maastricht, The Netherlands, Netherlands, 2. Department of Health Services Research, Care and Public Health Research Institute (CAPHRI), Maastricht University., Limburg, Netherlands, 3. Maastricht University, Department of Health Services Research, Care and Public Health Research Institute (CAPHRI), Maastricht, Netherlands, 4. University Medical Center Utrecht, Utrecht, Netherlands, 5. Maastricht University, School for Public Health and Primary Care (CAPHRI), Department of Health Services Research, Living Lab in Ageing and Long-term Care, Maastricht, Limburg, Netherlands*

Activities of Daily Living are a series of basic activities performed by individuals on a daily basis necessary for independent living at home or in the community. ADL are part of basic nursing care which describe aspects of care that are fundamental to all patients' health and well-being, regardless of diagnosis, cultural background or healthcare setting. In daily practice, nursing staff is confronted with challenges when providing ADL-care. In order to provide nursing professionals with practical tools to tackle daily challenges, a deeper insight into the perceived challenges by nursing staff, care receivers and informal caregivers is necessary. Therefore, two 'world-café' sessions were organized to explore daily challenges in ADL-care. In a World Café, participants are regarded as experts of their own lived experience and experiential knowledge. By creating a hospitable environment (i.e. a café-style ambience) individual and collective knowledge and ideas can be shared. Care receivers, informal caregivers and professionals including nurses, nursing-assistants, physical therapists, occupational therapist, and other stakeholders (N=53) participated in these 'world-cafés'. The session's output comprised 'graphic recordings' (paper tablecloths and post its) of the conversations that have been analyzed using content analysis. Findings showed reported challenges regarding receiving, providing and organizing ADL-care. Challenges on organizational level comprised for example both bilateral agreements and communication. Whereas reported care related issues related to challenges such as the assessment of care receivers' needs and the use of assistive devices in the provision of ADL-care. The inventoried challenges will be addressed in an ADL-quality standard for nursing staff.

### PATTERNS OF PERFORMANCE ON QUALITY OF CARE AMONG U.S. HOME HEALTH AGENCIES, 2015-2017

Chenjuan Ma[1], *1. New York University, New York, New York, United States*

Home healthcare is a critical care source for community-dwelling older adults. As the fastest growing healthcare sector in the US, quality of home healthcare is under increasing scrutiny. The purpose of this study is to examine patterns of performance on quality of care among US home health agencies. This is a 3-year cohort study using 2015-2017 Home Health Compare data and Provider of Services (POS) Files. In the dataset, each HHA was assigned a star rating (1-5) to reflect the overall quality of care. This indicator was calculated based on two process measures (timely initiation of care and drug education) and six outcome measures (e.g., hospitalization). We examined 8,020 HHAs in the US. Over the 3-year period, the number of HHAs receiving a star rating of 4 or 5 increased from 27% in 2015, 31% in 2016, to 32% in 2017. Roughly, 32% of the HHAs received a lower star rating and another 32% received a higher star rating from 2015 to 2016. Similarly, 30% of the HHAs received a lower star rating and 29% of the HHAs received a higher star rating from 2016 to 2017. Hospital-based HHAs were less likely to receive a star rating of 4 or 5. Larger HHAs (OR 1.34; 95% CI, 1.13-1.59) and HHAs with ownership changes (OR, 1.38; 95% CI 1.20-1.59) were more likely to improve their star ratings overtime. Our finding indicates dynamic changes in the quality of care within the US home healthcare sector.

### TRENDS IN HEALTHCARE COSTS AND UTILIZATION ASSOCIATED WITH HEARING LOSS DIAGNOSIS OVER 10 YEARS

Nicholas Reed,[1] Jennifer A. Deal,[2] Frank Lin,[1] and Charlotte Yeh[3], *1. Johns Hopkins University, Baltimore, Maryland, United States, 2. Johns Hopkins University Bloomberg School of Public Health, Baltimore, Maryland, United States, 3. AARP Services, Inc., Washington, District of Columbia, United States*

Hearing loss affects 38 million Americans and is associated with cognitive and physical decline. Moreover, hearing loss limits communication between patients and providers. In this study, we aimed to determine whether hearing loss diagnosis impacts healthcare cost and utilization. In the OptumLabs Data Warehouse insurance claims database (January 1, 1999 to December 31, 2016) we identified cases of age-related hearing loss (i.e. excluding conductive, ototoxic-induced, surgical-related hearing losses) in adults over the age of 50 years. Hearing loss cases were propensity-matched (nearest neighbor) to persons without hearing loss on multiple socioeconomic (income, region), demographic (age, sex, race, education), and health status (comorbidity count, cancer, dementia, baseline resource utilization) indices. There were 154,414, 44,852, and 4,728 subjects, respectively, at the 2-, 5-, and 10-year follow-up time points. We used regression modeling to examine healthcare cost (total expenditures), inpatient hospitalization, length of inpatient stay, outpatient visits, 30-day readmissions, and emergency department visits. Among 4,728 matched adults, hearing loss was associated with $22,434 (95% CI $18,219-$26,648) or 46% higher total healthcare costs over a 10-year period compared to those without hearing loss. Persons with hearing loss experienced more inpatient stays (Incident Rate Ratio, 1.47; 95% CI 1.29-1.68) and were at-risk (44% higher risk) for greater 30-day hospital readmission (Relative Risk, 1.44; 95% CI 1.14-1.81) at 10-years post index date. Similar trends were observed at 2- and 5-year time points across all measures. Importantly, these pathways may be amendable to hearing treatment via amplification which warrants future research preventative strategies.

### EXPLORING THE IMPACT OF RACE-ETHNICITY ON RESPONSE TO WEIGHT-LOSS TREATMENT: RESULTS FROM THE POWR-UP STUDY

Marshall G. Miller,[1] Cassandra M. Germain,[2] Kathryn N. Porter Starr,[3] Martha E. Payne,[1] Richard Sloane,[4] and Connie W. Bales[1], *1. Duke University, Durham, North Carolina, United States, 2. North Carolina A&T State University, Greensboro, North Carolina, United States, 3. Duke University School of Medicine, Durham, North Carolina, United States, 4. Duke University Medical Center, Durham, North Carolina, United States*

Racial/ethnic differences in obesity prevalence and in responses to weight-loss treatment between Black and White women are well documented. Whether these differences influence responses to weight-loss treatment among older women is unknown. Therefore, we evaluated racial/ethnic differences among participants in a 6-month weight-loss study with traditional versus higher protein intake. Participants were obese (BMI ≥ 30 kg/m2) community-dwelling women, age 45 years or older, who self-identified as either Black or White. Change in body-weight, 6 minute walk test (6MWT), general health (SF-36), and satisfaction with life (SWL) were evaluated at 0, 4 and 6 months. Both racial groups reduced ($ps < 0.01$) body weight at 4 and 6 months, with a trend toward more weight loss among White women ($p = 0.07$), relative to Black women. Other racial/ethnic differences included greater improvements in general health ($p = 0.05$) and 6MWT ($p < 0.05$) for White versus Black women at 6 months; these differences persisted after adjusting for treatment group, age/education, and comorbidity. Although racial/ethnic differences in SWL were not observed, significant improvement was observed only among White women ($p < 0.01$). Interestingly, weight loss was associated with improved 6MWT only among Black women ($r = -0.66$, $p < 0.05$) and with general health only among White women ($r = -0.44$, $p < 0.05$). Overall, White women experienced greater improvements in health and physical function as a result of weight-loss than did Black women. Further research is needed to identify equitable intervention strategies for the treatment of sarcopenic obesity.

### FUNCTIONAL INDEPENDENCE, ACCESS TO KIDNEY TRANSPLANTATION, AND WAITLIST MORTALITY

Nadia M Chu,[1] Stephanie Sison,[2] Abimereki Muzaale,[3] Christine Haugen,[4] Jacqueline Garonzik Wang,[4] Silas Norman,[5] Dorry Segev,[3] and

Mara McAdams-DeMarco[6], *1. Department of Surgery, Johns Hopkins School of Medicine, Baltimore, Maryland, United States, 2. Department of Medicine, Johns Hopkins University School of Medicine, Baltimore, Maryland, United States, 3. Department of Surgery, Johns Hopkins School of Medicine, Maryland, Baltimore, United States, 4. Department of Surgery, Johns Hopkins School of Medicine, Baltimore, Maryland, United States, 5. Department of Medicine, Division of Nephrology, University of Michigan, Ann Arbor, Michigan, United States, 6. Department of Epidemiology, Johns Hopkins Bloomberg School of Public Health, Baltimore, Maryland, United States*

Although functional independence is a health priority for patients with advanced CKD, 50% of those who progress to end-stage kidney disease (ESKD) develop difficulties carrying-out essential day-to-day activities. Functional independence is not routinely assessed at kidney transplant (KT) evaluation; therefore, it is unclear what percentage of candidates are functionally independent and whether independence is associated with access to KT and waitlist mortality. We studied a prospective cohort of 3,168 ESKD participants (1/2009-6/2018) who self-reported functional independence in basic Activities of Daily Living (ADL) and more complex Instrumental Activities of Daily Living (IADL). We estimated adjusted associations between functional independence (separately) and listing (Cox), waitlist mortality (competing risks), and transplant rates (Poisson). At evaluation, 92.4% were independent in ADLs, but only 68.5% were independent in IADLs. Functionally independent participants had a higher chance of listing for KT (ADL:aHR=1.55,95%CI:1.30-1.87; IADL:aHR=1.39,95%CI 1.26-1.52). Among KT candidates, ADL independence was associated with lower waitlist mortality risk (SHR=0.66,95%CI:0.44-0.98) and higher rate of KT (IRR=1.58,95%CI:1.12-2.22); the same was not observed for IADL independence (SHR=0.86,95%CI:0.65-1.12; IRR=1.01,95%CI:0.97-1.19). ADL independence was associated with better KT access and lower waitlist mortality; clinicians should screen KT candidates for ADL independence, and identify interventions to maintain independence to improve waitlist outcomes.

### HEALTH, WORRY, AND FOOD INSECURITY IN LOW-INCOME U.S. ADULTS: AN EXPLORATION OF MIDLIFE VULNERABILITY

Lisa Miller[1], *1. University of California, Davis, Davis, California, United States*

Food insecurity, defined as the inability to afford and access nutritious foods to eat, is associated with poor health, higher healthcare costs, and increased risk of mortality (Gundersen et al., 2018). Moreover, food insecurity appears to accelerate aging processes. For example, food insecurity is associated with inadequate nutrition, which expedites the loss of muscle mass, increases mobility problems, increases financial worry, and increases the risk of frailty. While a good deal is known about food insecurity in later life, far less is known about midlife, which may be a time of unexplored vulnerability. We examined food insecurity in a sample (n=17,866; 2014 NHIS) of low-income (PIR<3) young, early-middle, late-middle, and older adults (18-84), focusing on health challenges (chronic conditions, functional limitations) and financial worry as predictors and whether their effects varied with age. Multinomial logistic regression was used to assess the association of predictors with food insecurity and determine whether associations differed by age group, adjusted for covariates (e.g., sex, race/ethnicity, education, social security). Food insecurity rates were highest in late- (37.5%) and early- (36.0%) midlife followed by young (33.7%) and older (20.2%) adults. Age moderated the relationship between food insecurity and risk factors (p < .05 for both) such that health was stronger- but financial worry was weaker- in midlife (due to higher food insecurity in low- and high- worry groups). Midlife is a period of increased vulnerability to food insecurity, particularly for those with health challenges. Research is needed to inform prevention strategies to help ensure optimal aging.

### SESSION 2385 (POSTER)

### HEALTH, MEDICAL AND SOCIAL SERVICES

### FALL INJURY CHARACTERISTICS ASSOCIATED WITH EMERGENCY DEPARTMENT VISITS AND HOSPITALIZATIONS AMONG OLDER ADULTS

Namkee G. Choi,[1] Diana M. DiNitto,[1] and Mark E. Kunik[2], *1. University of Texas at Austin, Austin, Texas, United States, 2. Baylor College of Medicine, Houston, Texas, United States*

Fall injuries and related healthcare use among older adults are increasing in the US. Based on the 2013-2017 US National Health Interview Survey public use data, this study examined fall injury characteristics that are associated with emergency department (ED) visits and hospitalizations among those aged ≥60 years who received medical attention for their fall injuries within a 91-day reference period (N=1,840). Our findings show that nearly a third of these older adults received care from emergency medical services (EMS), presumably for a “lift assist” to get off the floor and/or for ED or hospital transport; a little more than one-third had an ED visit only; and a little less than a fifth had an overnight hospital stay. Multivariable analysis showed that hip and head injuries, face injuries, and broken bones/fractures (from any type of injury) were likelier causes of hospitalization than injuries to other parts of the body. Fall injuries sustained inside the home, falls from loss of balance/dizziness, and living alone were also more likely to result in hospitalization, while fall injuries that occurred away from home and those with lung disease and memory problems were associated with higher risk of ED use only. These healthcare use data indicate the significant toll that fall injuries exact upon older adults and healthcare system. Fall prevention programs should target risk factors that are specific to serious injuries and be made more accessible. Strategies for implementing scalable, adaptable, and measurable fall prevention models by EMS providers and ED staff are also needed.

### SEX DIFFERENCE IN EARLY FRAILTY TRANSITIONS, HEALTHCARE USE, AND MEDICARE PAYMENT IN OLDER MEXICAN AMERICANS

Chih-Ying Cynthia Li,[1] Amol Karmarkar,[1] Lin-Na Chou,[1] Soham Al Snih,[2] Yong-Fang Kuo,[1] and Kenneth Ottenbacher[1], *1. University of Texas Medical Branch, Galveston, Texas, United States, 2. University of*

*Texas Medical Branch at Galveston, Galveston, Texas, United States*

This study investigated sex difference in early frailty transitions on one-year follow-up healthcare utilization and Medicare payment. We used the linked Medicare claims data and the Hispanic Established Populations for the Epidemiological Study of the Elderly (Hispanic-EPESE) survey, using longitudinal analyses for 789 older Mexican Americans ≥70 years old in 1998/99. Participants were divided into five transition groups: 1) remain non-frail, 2) improve (pre-frail to non-frail, frail to non-frail, frail to pre-frail), 3) remained pre-frail, 4) remained frail, 5) worse (non-frail to pre-frail, non-frail to frail, pre-frail to frail) based on their frailty status between Wave 3 (1998/99) and Wave 4 (2000/01). Main outcomes were: (a) healthcare utilization (hospitalization, emergency room admission, physician visit) and (b) Medicare payment (total and outpatient payments) from 2000/01 to 12 months after. Mean age was 78.8 (SD=5.1) and 60.3% were female in 1998/99. We found sex had significant interaction effects on one-year follow-up hospitalization and Medicare outpatient payment. Compared to the remained no-frail group, males who remained pre-frail (Odds Ratio [OR]= 3.62, 95% CI=1.18-11.2), remained frail (OR= 7.59. 95% CI= 1.74-33.1) and worse (OR=4.54, CI=1.74-11.8) had higher risk for hospitalization. Males in the worse group also had significantly higher Medicare outpatient payment (OR=2.58, CI=1.46-4.56). Same associations were not observed in females. However, both genders used similar frequency and type of outpatient services, as the top services were evaluation and management services. Our results suggested research is needed to examine balance between sex differences, frailty improvements, resources needed and total care expenditure in this population.

### OLDER ADULTS' IMMIGRANT STATUS AND SELF-REPORTED ABILITY TO NAVIGATE THROUGH THE HEALTHCARE SYSTEM

Huey-Ming Tzeng[1], *1. University of Saskatchewan, Saskatoon, Saskatchewan, Canada*

This exploratory study examined the association between older adults' immigrant status and their self-reported ability to perform each of the 51 self-care behaviors that are needed for them to navigate through the healthcare system. Secondary data analysis was conducted based on a 2018 telephone survey of community-dwelling adults 65 y/o or older, living in a western Canada province (N = 1,000). A previously validated survey tool, Patient Involvement Behaviors in Health Care (e.g., indicating Yes=1 or No=0 regarding their ability to perform each self-care behavior), and a demographic data form (e.g., are you an immigrant? Yes=1 or No=0) were used. Descriptive analyses and chi-square tests for independence (alpha= 0.05) were conducted. Among the 993 adults who indicated their immigrant status, 51 (5.1%) self-declared as immigrants. 32 (62.7%) of the immigrant participants and 457 (48.5%) of the non-immigrant participants resided in the urban areas. 88.2% of these immigrant participants was white, 7.8% was Asian, and 2% was black; 72.5% indicated that English is their first language. Immigrant participants were less likely to report being able to perform 5 self-care behaviors than non-immigrant participants. These 5 behaviors were: bringing someone to help you move around when needed; asking your providers to share your medical record with each other; finding insurance that best matches your needs; changing health insurance coverage as needed; and knowing of any interactions with old and new treatments. Clinicians should co-create approaches with older adult immigrants to improve their self-care capacity (e.g., connecting with relevant peer support networks).

### ACCESS TO CARE ROUNDS: A UNIQUE FORUM FOSTERING HEALTH SYSTEM AND SOCIAL SERVICE PARTNERSHIPS FOR OLDER VETERANS

Kathryn A. Nearing,[1] Hillary D. Lum,[1] Heather Kamper,[2] Kaily A. Cannizzaro,[3] Courtney Bauers,[4] and Joleen C. Sussman[5], *1. University of Colorado Anschutz Medical Campus, Division of Geriatrics, Aurora, Colorado, United States, 2. Area Agency on Aging, Denver Regional Council of Governments, Denver, Colorado, United States, 3. Rocky Mountain Mental Illness Research Education Clinical Center for Suicide Prevention, Aurora, Colorado, United States, 4. Denver-Seattle Center of Innovation for Veteran-Centered and Value-Driven Care, Aurora, Colorado, United States, 5. Rocky Mountain Regional VA Medical Center, Aurora, Colorado, United States*

Colorado Veteran Community Partnership (VCP) aims to connect Rocky Mountain Regional VA Medical Center front-line teams with diverse community partners to create integrated networks of support for older Veterans with complex needs and their family members and caregivers. To accomplish this goal, VCP launched Access to Care Rounds in January 2018 to build bridges between the healthcare system and community-based organizations. Each Access to Care Rounds features a cross-sector panel that discusses specific efforts to link a medically-complex, older Veteran to resources. This model was developed with stakeholder input and has highlighted topics related to chronic pain management, suicide prevention, homelessness, adult protective services, transportation, home-based primary care, hospice care, and firearm safety. Each Access to Care Rounds focuses on connecting VCP members, sharing expertise and resources, and highlights lessons learned related to care coordination, communication, and key processes that others can adopt/adapt to better serve older Veterans. On average, 30 individuals attend each session. Access to Care Rounds draw diverse audiences representing social services, mental health and other healthcare specialties. The latter include Social Workers (47%), Physicians (11%), Psychologists (8%), Registered Nurses (6%), and students/trainees (6%). Participants receive a description of the Veteran situation; the names, credentials, organizational affiliations and roles/expertise of each panelist; and, a resource list relevant to the constellation of issues addressed to enhance access to information and resources. Over 38% of respondents to session evaluations reported intentions to change their professional practice as a result of what they learned during an Access to Care Rounds.

### INTEGRATING SOCIAL NEEDS CARE INTO THE DELIVERY OF HEALTH CARE TO IMPROVE THE NATION'S HEALTH FOR OLDER ADULTS

Tamara J. Cadet,[1] Robyn Golden,[2] and Keegan Warren-Clem[3], *1. Simmons University, Boston,*

*Massachusetts, United States, 2. Rush University Medical Center, Chicago, Illinois, United States, 3. The University of Texas at Austin School of Law and McCombs School of Business, Austin, Texas, United States*

An ad hoc committee of the National Academies of Sciences, Engineering, and Medicine examined the potential for integrating services addressing social needs and the social determinants of health into the delivery of health care to achieve better health outcomes and to address major challenges facing the U.S. health care system. These challenges, include persisting disparities in health outcomes among vulnerable subpopulations, often defined by a number of factors including age. Presenters will discuss and provide recommendations in the following areas: 1. evidence of impact of social needs care on patient and caregiver/family health and wellbeing, patient activation, health care utilization, cost savings, and patient and provider satisfaction; 2. opportunities and barriers to expanding historical roles and leadership of social workers in providing health-related social needs care and evidence-based care models that incorporate social workers and/or other social needs care providers in interprofessional care teams across the care continuum (e.g., acute, ambulatory, community-based, long-term care, hospice care, public health, care planning) and in delivery system reform efforts (e.g., enhancing prevention and functional status, care management, and transitional care; improving end-of-life care; integration of behavioral, mental, and physical health services); and 3. realized and potential contributions of social needs care to make health care delivery systems more community based, person- and family/caregiver-centered, and responsive to social and structural determinants of health, particularly for vulnerable populations and communities, such as older adults and low-income families. Examples for each of the three areas will also be presented.

### DEPRESSION AND MEDICAL COST OF CARDIOVASCULAR DISEASES

Guijing Wang,[1] Chanhyun Park,[2] Heesoo Joo,[1] Nikki Hawkins,[1] and Jing Fang[1], *1. CDC, Atlanta, Georgia, United States, 2. Northeastern University, Boston, Massachusetts, United States*

Prevalence of cardiovascular disease (CVD), the leading cause of death worldwide, increases with age. Depression is a prevalent comorbidity with CVD. This study investigates the medical costs of CVD associated with depression using a nationally representative data, 2015 Medical Expenditure Panel Survey. Patients aged ≥18 were identified by using the International Classification of Disease, 9th Revision codes of 390-459 for CVD and 296 or 311 for depression (N=23,755). Medical costs were actual payments received by providers and classified by service types and payment sources. We estimated the medical costs for each service type and payment source using economic modelling techniques controlling for various potential confounders. Overall prevalence of depression was 11.4%; 17.0% in persons with CVD and 8.7% in persons without CVD ($p<0.001$). Medical cost with depression was estimated at \$6900 ($p<0.001$) for persons with CVD and \$2211 ($p<0.001$) for those without. Costs on depression-related prescription medicines accounted for the largest portion of medical costs among persons with CVD (\$3095, $p<0.001$). For persons with depression but without CVD, costs on outpatient visits accounted for the largest proportion (\$1179, $p<0.001$). Medicare payments accounted for the largest portion of the depression-associated costs at \$3338 ($p=0.014$) for persons with CVD. Compared with persons without CVD, those with CVD demonstrated doubled rates of depression. Depression-associated medical costs among individuals with CVD were tripled what they were for persons without CVD. Increased costs associated with depression were mainly for prescribed medicines and were financed by Medicare programs for persons with CVD.

### AGE AND USE OF COMPLEMENTARY AND ALTERNATIVE MEDICINE

Sean N. Halpin,[1] Nivedita R. Potapragaa,[2] Sharon H. Bergquist,[2] and Thomas Jarrett[2], *1. University of Georgia, Athens, Georgia, United States, 2. Emory University, Atlanta, Georgia, United States*

Complementary and alternative medicines (CAM) are often obtained over the counter and not disclosed to health care practitioners—leading to possible unforeseen, harmful drug interactions. These concerns are especially true for older adults who have a high likelihood of experiencing multiple comorbidities. Yet few studies examine the patterns of CAM use and disclosure across a wide age range. We used a mixed-methods in a study on patient attitudes toward CAM in a large primary care setting. Participants (n=279) ranged in age from 21-85 (mean=58), were mostly white (75%), and had a bachelor's degree or higher (83%). Most rated their physical health as good or very good (90%) and had a score of zero on the Charlson Comorbidity Index (76%). Use and disclosure of twelve types of CAM were assessed across three modalities including ingestible (e.g., herbs), psychological/mind-body (e.g., meditation), and physical (e.g., acupuncture). Age was not predictive of disclosure across the larger sample, but within respondents aged 65-85 (n=90), linear regression analyses showed likelihood of disclosure was associated with younger age, positive attitudes toward CAM, and expectation that their physician had positive attitudes about CAM. Semi-structured phone interviews (n=32) revealed older adults were more likely to have long-term CAM use, particularly for pain, and not feel it necessary to disclose to their physician. Meanwhile younger individuals reported trying CAM episodically for preventative health purposes. Understanding patterns of CAM use can help guide age-appropriate conversations and limit possible adverse outcomes from non-disclosure.

### CUSTODIAL AND CO-RESIDENT GRANDMOTHERS SERVICE USE AND NEEDS: 10 YEARS APART

Alexandra B. Jeanblanc,[1] McKenzie Wallace,[1] and Carol M. Musil[1], *1. Case Western Reserve University, Cleveland, Ohio, United States*

The period between 2008 and 2017 spans a period of economic and societal flux in America, including the 2008 recession, the Affordable Care Act, and the emergence of the opioid crisis. These changes have had profound effects on families, including the rise in grandparent-headed and multigenerational households and their financial, resource, and service needs. We compared the service needs and utilization patterns of 334 co-resident grandmothers who were participants in two independent NIH-funded studies of grandmothers raising grandchildren, conducted 10 years apart (2008-2009:n=145 and 2018-2019:n=189) Researchers asked both samples whether they received or had an unmet need for 16 types of services (ex: financial, legal, substance abuse, respite care). There were statistically significant differences between the samples on 8 of 16 services received, and 15 of 16 unmet, but recognized, service needs, with our current sample reporting higher usage and need. The largest percentage increases in need were financial assistance (31.5%), respite care (29.9%), exercise/fitness (26.4%), and family therapy (25%). Although there is a 7% increase in legal services received between the samples, 48.1% of the 2018-19 sample still report unmet legal needs, compared to 16.6% of the 2008-09 sample. We observed a significant difference in an unmet need for help to families dealing with drug and alcohol abuse, with 23% of current participants reporting unmet need, compared to 9% in the prior study. Our results highlight a gap in resources available to grandfamilies and highlight the need for intervention and evaluation research targeting service needs to inform policy change.

### EVALUATING A CONSUMER-DIRECTED HEALTH CARE PILOT FOR OLDER PEOPLE IN THE COMMUNITY

Amanda A. Phelan,[1] Alice Duggan,[1] Deirdre O'Donnell,[1] and Fealy Gerard[1], *1. University College Dublin, Dublin, Ireland, Ireland*

In an ageing society with a policy focus on community (Oireachtas 2017), providing choice and self-determination in home care provision for older people is fundamental. Consumer directed care has increased in popularity in many countries (Low, Yap & Brodaty, 2011). In 2016, planning began in Community Healthcare Organisation 3 to pilot a CDHC pilot for community based older people. This study presents the findings of this pilot project. The study used the Moore et al.'s (2015) evaluation approach. Data collection methods were via descriptive questionnaires with qualitative investigation of stakeholder experiences as well as an economic review. Findings demonstrated the need for additional clarity in understandings of the CDHC. Care recipients and caregivers did report satisfaction with care, however, it was the accessibility of care rather than choice that was considered important. There were also issues related to capacity to manage the CDHC as well as capacity to give a choice of providers, particularly in rural areas. Although, broadly cost-neutral, processes and structures also require additional alignment to enhance both effectiveness and efficiency. Empowering care recipients and enabling greater choice, flexibility and autonomy in care organisation and delivery was a central focus of the CDHC pilot. However, additional consumer awareness and programme enhancement is needed to fully realize the potential of the CDHC. Study recommendations focus on areas of increased clarity about the CDHC, implementation enhancement, complementarity and incorporation of CDHC with existing home support service provision arrangements

### WHY DON'T OLDER ADULTS USE SENIOR CENTERS? EVIDENCE FROM A SAMPLE OF MASSACHUSETTS ADULTS AGE 50 AND OLDER

Ceara Somerville,[1] Nidya Velasco Roldan,[1] Cindy N. Bui,[1] and Caitlin E. Coyle[2], *1. University of Massachusetts Boston, Boston, Massachusetts, United States, 2. Gerontology Institute, University of Massachusetts Boston, Boston, Massachusetts, United States*

Senior centers are an integral community resource, providing programs and services intended to meet the vast range of needs and interests of older adults. There is a growing literature describing senior center participants and benefits to participation, but little is known about those who choose not to participate at a local senior center. This presentation uniquely characterizes non-users of senior centers, based on a sample of community-dwelling adults aged 50+ from seven communities in Massachusetts (N = 9,462). To date, this is the largest data set that describes senior center usage. Most of the sample were women (60%) and in the 60-69 age group (36%). More than three quarters of the sample do not use the local senior center (77%). The most common reasons for non-usage were lack of interest (27%) and not feeling old enough (26%). There are significant differences in reasons of non-usage among age groups and gender ($p < .001$). Younger age groups' (50-69) most popular reasons for non-usage were not feeling old enough, not having time, inconvenient senior center hours, and not knowing what is offered. In contrast, older age groups (80+) more frequently reported having no interest or using programs elsewhere. Men were more likely to report not being interested and not being familiar with what is offered. Women were more likely to report not having time, inconvenient hours of programming, and using programs elsewhere. Based on results from this study, this presentation will outline implications for the future of senior centers and their programming.

### MANAGING MANAGED CARE: PERSPECTIVES FROM KEY STAKEHOLDERS IN SKILLED NURSING FACILITIES

Andrea E. Daddato,[1] Cynthia Drake,[2] Edward A. Miller,[3] Pamela Nadash,[3] Denise Tyler,[4] and Rebecca S. Boxer[5], *1. University of Colorado School of Medicine, Aurora, Colorado, United States, 2. Colorado School of Public Health, Aurora, Colorado, United States, 3. University of Massachusetts Boston, Boston, Massachusetts, United States, 4. RTI International, Waltham, Massachusetts, United States, 5. Kaiser Permanente Institute for Health Research, Aurora, Colorado, United States*

In recent years, Medicare Advantage (MA) plan enrollment has increased, a trend that is expected to continue. Many skilled nursing facilities (SNFs) rely on MA managed care insurer referrals to maintain their census in a market with high competition for post-acute care patients. This study used semi-structured interviews to describe the relationship between MA plans and SNFs from the perspective of key decision-makers in SNFs. Twenty-three interviews were conducted with key stakeholders from 11 Denver Metropolitan area SNFs. A combined purposive-snowball sampling approach was used to identify and recruit select staff from the participating facilities. Interviews focused on the relationship between MA plans and SNFs, including mechanisms of control, power dynamics, and preferences for MA versus Fee-for-Service (FFS) Medicare patients. Key findings included: 1) challenges SNF staff had navigating MA plans' case management processes, a key mechanism used by MA plans to influence the behavior of SNF decision-makers; 2) MA plans exercising power over beneficiaries' length of stay, potentially leading to early discharge and heightened risk for rehospitalization; 3) SNF preference for admitting Medicare FFS over MA patients due to higher rates of Medicare FFS reimbursement and greater control over patient care. SNFs are increasingly reliant on MA plans for patient referrals and revenue. The themes suggest that this growing reliance may place SNFs at odds with MA plans on how best to manage overall patient care. It is therefore important that future research investigate how MA plans' influence over care affects patient outcomes in SNFs and other post-acute settings.

### MANAGING DEATH IN THE FIELD: HOW EMERGENCY MEDICAL SERVICES TEAMS PROVIDE END-OF-LIFE CARE

Deborah P. Waldrop,[1] Jacqueline M. McGinley,[2] and Brian M. Clemency[3], *1. University at Buffalo School of Social Work, Buffalo, New York, United States, 2. Buffalo State College, Buffalo, New York, United States, 3. Erie County Medical Center, Buffalo, New York, United States*

Emergency medical services (EMS) providers respond more frequently to calls for older adults with serious illness than for people in other age groups. Recent legislation that makes it possible to document healthcare decisions has facilitated an era of choice in end-of-life care. EMS teams make time-sensitive decisions about care, resuscitation and hospital transport that influence how and where a seriously ill older adult will die and how his/her family will experience the death. Yet, EMS providers' perspectives on urgent decision-making and how they work with families are unknown. The purpose of this study was to explore the decision-making process that occurs how EMS teams respond when someone is dying from a serious illness (vs. an injury). In-depth in-person interviews were conducted with 50 EMS providers (24 emergency medical technicians [EMTs] and 26 Paramedics) from four ambulance services. Participants' ages ranged from 21-57 (M=37.9) and 70% were male. Qualitative data was coded using Atlas.ti software. Three themes illuminated participants' experiences with end-of-life calls: (1) How legally binding documents (e.g. Do Not Resuscitate [DNR] orders, Medical Orders for Life Sustaining Treatment [MOLST]) inform care; (2) Decision-making about foregoing or halting resuscitation (e.g. no hospitalization, death at home); and (3) Family care, support and education. The results suggest that EMS providers have critically important roles in upholding the wishes of seriously ill older adults and helping caregiving families through the end-of-life transition. Implications: Discussions about the meaning of legally binding documents (e.g. DNR, MOLST) and EMS calls are important in advance care planning.

### PRELIMINARY YEAR-ONE RESULTS OF A 5-YEAR PROJECT TO DEVELOP AND IMPLEMENT PATIENT- AND FAMILY-CENTERED CARE MODEL

Ya-Mei Chen,[1] Yuchi Young,[2] Dulmaa Munkhtogoo,[3] Ming-Ting Yang,[3] Hsin-Yun Tsai,[3] Nien-Chen Kuo,[3] Tsung-Hsien Yu,[4] and Kuo-Piao Chung[3], *1. National Taiwan University, Taipei, Taiwan, 2. SUNY at Albany, Albany, New York, United States, 3. Institute of Health Policy and Management, National Taiwan University, Taipei, Taiwan, 4. Department of Health Care Management, National Taipei University of Nursing and Health Sciences, Taipei, Taiwan*

Objective. The overarching goal of this 5-year study is to develop and evaluate an innovative patient- and family-centered care (PFCC) model to address the challenges of quality care and high cost care in Changhua County, Taiwan. The year-1 study examines the baseline differences on selected outcomes between Changhua County and the comparison group (nationally). Methods. Five-year longitudinal study. Participants: stroke patients (n=2,931) from Changhua County Hospitals. Year-1 baseline discharge registry data (2018) for stroke patients obtained from Changhua Health Bureau. Summary statistics and bivariate analysis performed. Providers: care provider [n=28] were interviewed through five focus groups for the support needed to begin implementing PFCC. Results. The year-1 results show the stroke incidence rate was 3.8%. Sixty-three percent of stroke patients were 65+ with an average age of 69, and 60% were male. Compared to the Taiwan national average, the stroke patients in Changhua County had a longer length of stay in acute and postacute care settings (41.1days vs. 29.3 days; $p < 0.001$), and higher hospitalization cost (US7,815 vs. US$5,905; $p<0.001$). The focus group data found lack of effective tools and platforms to facilitate the provider-to-provider communication necessary for PFCC. Conclusion. These findings suggest that stroke patients in Changhua County had a longer average length of stay and higher healthcare costs compared to stroke patients nationally. To address these discrepancies, our year-2 program will focus on PFCC intervention program development and pilot testing, which include a Taiwan-specific PFCC tool and platform, followed by three years of intervention implementation and evaluation.

### MEETING SOCIAL NEEDS TO IMPROVE HEALTH OUTCOMES: PARTNERSHIPS BETWEEN COMMUNITY-BASED ORGS AND HEALTH CARE

Traci L. Wilson,[1] Suzanne R. Kunkel,[1] Jane Straker,[1] Marisa Scala-Foley,[2] and Elizabeth Blair[2], *1. Miami University Scripps Gerontology Center, Oxford, Ohio, United States, 2. National Association for Area Agencies on Aging (n4a), Washington, D.C., United States*

Unmet social needs negatively affect individual and population health, and better integration of community-based supports and health systems is a promising approach to improve health outcomes and avoid unnecessary health care use. Community-based organizations (CBOs) such as Area Agencies on Aging (AAAs) and Centers for Independent Living (CILs), as providers and coordinators of social services, are well-positioned within their communities to coordinate care and provide for unmet social needs. Partnerships between CBOs and health care entities have clear potential to improve health care outcomes while also reducing expenditures. This paper will present a cross-sectional analysis of a national survey of AAAs, CILS, and other CBOs at two time points (2017: n=593; 2018: n=763) to understand the extent, type, and evolution of CBO engagement with health care providers. In addition, longitudinal analysis (n=374) shows movement at the organization level: 33% of organizations who did not have a contract at T1 but were pursuing one had achieved a contract by T2. This presentation will: describe details of the services delivered, contracting arrangements, and populations served under CBO/health care contracts, as well as challenges experienced by CBOs; examine differences by state and organizational structure; and discuss the implications of state policy on integrated care and contracting.

### HOSPICE SERVICES IN THE HOME SETTING AND CHARACTERISTICS OF MEDICARE DECEDENTS

Pauline Karikari-Martin,[1] Lirong Zhao,[2] and Lynn Miescier[2], *1. U.S. Public Health Service Commissioned Corps, Rockville, Maryland, United States, 2. Centers for Medicare & Medicaid Services, Baltimore, Maryland, United States*

There is little empirical work documenting the characteristics of Medicare beneficiaries receiving hospice services in the home setting using the Medicare place of service codes. The objective of this study is to examine differences in Medicare decedents who received hospice services in traditional and non-traditional homes (assisted living, and nursing home settings) defined by these codes. We conducted a secondary analysis of 675,782 Medicare decedents who received hospice services in 2015. Chi-squared and ANOVA tests were used to describe the socio-demographics, health conditions, utilization, and hospice payments of the decedents. Most of the decedents received hospice care in a traditional home (64.9%), but beneficiaries, aged 85 years and over, received hospice services in assisted living (72.1%) or nursing homes (59.8%). Overall, decedents who received Medicare hospice benefits in assisted living had the highest number of hospice days (mean=149.7 lifetime days; median = 30; standard deviation (S.D.) =245), and decedents in traditional homes had the fewest number of hospice days (mean=86.7 lifetime days; median = 24; S.D. =179). Among Medicare–Medicaid (duals) decedents who used hospice care, 49% received hospice care in nursing homes, and infrequently, 7% at assisted living. Medicare hospice payments were highest ($20,439 per beneficiary) for decedents in assisted living, but least for those in traditional homes ($11,830). Hospice services offered to Medicare beneficiaries who are 85 years and older, duals, or have a diagnosis of dementia may require more oversight and coordination of resources to ensure that they receive appropriate hospice services in non-traditional homes.

### PHYSICAL ACTIVITY PREFERENCES OF OLDER ADULT MEDICAID HOME AND COMMUNITY-BASED SERVICES WAIVER CLIENTS

Victoria S. Davila,[1] Lauren Pietzak,[1] Danielle Rossi,[1] Karen Phipps,[1] and Margaret Danilovich[1], *1. Northwestern University, Chicago, Illinois, United States*

Despite the known effectiveness, physical activity (PA) is not currently offered to older adult clients receiving Medicaid Home and Community Based Services (HCBS). To optimize PA implementation within Medicaid HCBS, understanding client preferences for PA programming is needed. Thus, the objective of this exploratory qualitative study was to identify the PA preferences of HCBS clients including mode, duration, implementation strategy, and frequency, as well as barriers and motivators to PA. We recruited participants from the Illinois' Department on Aging Community Care Program. We conducted semi-structured interviews in participants' homes which were audio recorded, transcribed, and analyzed using Dedoose (version 7.0.23). We derived semi-structured interview questions from the Health Belief, Social Cognitive, and Health Action Process Approach framework. We used a structured coding approach using conventional content analysis to derive codes from the text, then applied these codes to each interview and examined the frequency to determine themes. The most frequently referenced theme was barriers to PA, primarily co-morbidities. The primary motivator was social support by a peer or instructor. The preferred PA program components were walking 2-3 days per week with duration varying from 20 minutes to 2 hours. Clients also preferred individualized PA instruction versus a passive strategy such as pamphlets or videotapes. Our findings show that individual-level factors most significantly influence PA participation and should be addressed among Medicaid HCBS clients. We recommend Medicaid HCBS consider a personalized approach of PA implementation with their clients.

### WHAT CAN COUNTY-LEVEL EMERGENCY MEDICAL SERVICES DATA TEACH PUBLIC HEALTH ABOUT OLDER ADULT FALLS

Carolyn R. Ham,[1] Xinyao deGrauw,[1] and Scott Dorsey[2], *1. Washington State Department of Health, Washington, United States, 2. Snohomish County Fire District 7, Snohomish County, Washington, United States*

Fall-related injuries in older adults contribute to an increasing number of deaths, hospitalizations and Emergency Medical Services (EMS) responses. In Snohomish County, the third largest county in Washington State, EMS agencies with electronic health records cover 96% of the county's population. This EMS data contains unique information on falls, which can estimate costs and clarify intervention priorities. We analyzed 2018 data from EMS in Snohomish County. Fall incidents were summarized by count, frequency, and rate per 1,000 population. Costs for transferring patients to emergency departments (ED) were estimated using 2015 Snohomish Community Paramedic Analysis, and direct medical costs averted by implementing a single intervention were estimated based on prior research by Stevens and Lee (2018). There were total 38,910 incidents in older adults, of those 4,777 incidents were caused by falls (1606 in males and 2906 in females). The mean age (SD) was 81.0 (±8.9). The incidence rate was 45.6 per 1000 (55.8 in females and 30.6 in males). There were 573 repeated falls (12%). Most of the falls happened at home (54.85%), followed by assisted living and nursing homes (27.84%). 85.53% of the falls were transferred to ED, at an estimated cost of 3.15 million dollars. We calculated that one million dollars in medical cost could be averted by implementing home modifications delivered by an occupational therapist (OT). This research demonstrates the utility of EMS data for describing fall injury and determining interventions. Fall prevention programs should focus on preventing repeated falls and addressing home safety risks.

### VALIDATION OF A NEW FUNCTIONAL COGNITION SCREENING MEASURE

Dorothy F. Edwards,[1] Muhammad O. Al- heizan,[1] Timothy Marks,[2] and Gordon M. Giles[3], *1. University of Wisconsin Madison, Madison, Wisconsin, United States, 2. University of Wisconsin Madison, Madison Wisconsin, Wisconsin, United States, 3. Samuel Merritt University, Oakland, California, United States*

Identification of cognitive deficits that contribute to functional dependence in acute and post-acute care settings is important. The Menu Task (MT) is a brief cognitive 12 item screening measure designed to identify older adults at risk for IADL impairment in community settings. The objective was to compare the psychometric properties of the Menu Task a series of neurocognitive and functional cognitive performance measures including the Brief Interview of Mental Status (BIMS), the Montreal Cognitive Assessment (MoCA) the Weekly Calendar Planning Activity (WCPA) and the Performance Assessment of Self-Care Skills (PASS) in a sample of 200 community dwelling older adults. Participants were administered the MT, BIMS, MoCA, WCPA, and the PASS checkbook and shopping tasks. ROC analysis identified an MT cut score, sensitivity and specificity. We computed Cronbach's alpha, correlations among study measures and t-tests between groups impaired or unimpaired on the MT. Mean age of participants was 70.44(SD 8.3), the sample was predominately female ((76%), and white (81%). Mean MT score was 8.22 (SD 2.01) and the mean completion time was 185.5 sec (SD 108.11). The Menu Task has moderate internal consistency ($\alpha$= 0.65). The AUC statistic was 0.83 with an optimal MT cut score of "7" and sensitivity of .90 and specificity of .70. Significant differences ($p < 0.01$) were observed between impaired and not impaired MT groups on BIMS, MoCA, WCPA, and PASS. The Menu Task has moderate to strong evidence supporting its psychometric properties and the value of screening for functional cognitive deficits.

### THE INTERVIEW FOR DECISIONAL ABILITIES: DEVELOPING THE EVIDENCE BASE FOR ADULT PROTECTIVE SERVICES

Theresa Sivers-Teixeira,[1] Gregory Stevens,[1] Kelly Sadamitsu,[1] Christina Penate,[1] and Bonnie Olsen[1], *1. USC Keck School of Medicine, Los Angeles, California, United States*

Adult Protective Services (APS) workers assess clients for abuse and neglect and are asked to determine the client's understanding of risks they face. Yet, APS workers have little structured training in how to make such judgements. The Interview for Decisional Abilities (IDA 3.0-CA) is a tool designed for use by APS workers to assess the ability of suspected victims of elder mistreatment to make decisions about the risks they face. This study evaluates the impact of training and use of this tool on the knowledge, experiences and ability of APS workers to determine decisional ability. APS workers and supervisors were recruited from central and northern California APS programs and randomized into either control (n=94) or IDA 3.0-CA training groups (n= 95). Baseline surveys measure knowledge of, and experiences with, assessing decisional ability and determining next steps for case management. Additionally, respondents determine the decisional ability of three case scenarios. Three months post-training, controls and trained subjects complete the same survey with a new set of cases. Preliminary results at baseline indicate there were no statistically-significant differences between trained subjects (n=42) and controls (n=50) in their knowledge scores (78.6% correct vs. 81.0%, p=0.6641) or performance assessing decisional ability in the case scenarios (60.1% correct vs. 63.3%, p=0.3497). Reported experiences assessing decisional ability and determining next steps in case management were also similar for trained subjects and controls. Complete results will be presented regarding change in knowledge scores, experiences, and assessing decisional ability in case scenarios compared across trained subjects and controls.

### IMPLEMENTATION CHALLENGES AND OPPORTUNITIES IN A COMMUNITY-BASED TRANSITIONAL CARE SERVICES PROGRAM

Raven H. Weaver,[1] and Cory Bolkan[2], *1. Washington State University, Pullman, Washington, United States, 2. Washington State University, Vancouver, Washington, United States*

High-risk older adults (i.e., low-income, chronically ill) often have complex, costly healthcare needs and are at risk of re-hospitalization. Hospitals traditionally lead efforts to reduce readmissions, while community-based aging services organizations (e.g., Area Agency on Aging AAA) offer older adults in-home health, social support, and information/referral to community resources. Thus, creating, sustaining, and scaling up hospital-community partnerships can better meet older adults' comprehensive needs. We evaluated efforts of a hospital-AAA project to develop and implement a local transitional care services program (TCSP) that provided in-home/phone support post-discharge for high-needs older adults. Over a four-year period, 1,921 individuals (mean= 75 years; 57% women) were referred from hospital as eligible for TCSP. After referral, however, only 22.8% were successfully connected to community-based services and men were more likely than women to complete TCSP ($X2= 6.92$; $p= .009$). Of those referred, only 4% were re-hospitalized, indicating potential success of TCSP. Data revealed most were unable to be contacted (27.9%), refused the program (21.6%) or utilized alternative services, including SNFs (20.3%); inconsistent data collection and procedures yielded problematic missing data and inability to assess reasons for low engagement. We also surveyed and interviewed AAA staff (n=16) and found most staff exhibited high readiness for evidence-informed practices, supported proactive data use to improve planning, advocating, and serving clients, and identified significance of multidisciplinary community partnerships. Our findings generated recommendations to enhance staff engagement in TCSP, improve data collection for transforming data utility beyond enrollment purposes, and consider programmatic modifications to reach vulnerable elders.

### THE EFFECT OF DUAL ELIGIBILITY ON IMPROVEMENT IN MOBILITY: VARIATION BY PATIENTS' PRIMARY DIAGNOSIS

Molly W. Vaughan,[1] Ila H. Broyles,[2] Melvin J. Ingber,[3] Lauren A. Palmer,[1] Tara McMullen,[4] Karyn K. Anderson,[4] and Anne Deutsch[5], *1. RTI*

*International, Waltham, Massachusetts, United States, 2. RTI International, Research Triangle Park, North Carolina, United States, 3. RTI International, Rockville, Maryland, United States, 4. Centers for Medicare & Medicaid Services, Baltimore, Maryland, United States, 5. RTI International, Chicago, Illinois, United States*

Dually eligible individuals (i.e., eligible for Medicare and Medicaid) often have worse health and greater functional limitations than patients eligible for Medicare only. For dually eligible patients receiving inpatient rehabilitation services following a major illness or injury, improvement in function may be lower than for Medicare-only patients. To our knowledge, this is the first study to examine the relationship of dual eligibility on improvement in mobility for inpatient rehabilitation facility (IRF) patients by 13 primary diagnosis groups (e.g., Stroke, Amputation). Data was collected on the IRF-Patient Assessment Instrument at admission and discharge for all IRF patients discharged during 2017 (N = 428,631). A generalized linear model was run for each primary diagnosis group to examine the effect of dual eligibility on change in mobility during an IRF stay, adjusting for sociodemographic factors, clinical factors, and comorbidities. The proportion of patients who were dually eligible varied among primary diagnosis groups (9.6% for Hip/Knee Replacements, Fractures and Multiple Trauma to 21.7% for Amputation). Compared to patients who were non-dually eligible, dually eligible patients had lower improvement in mobility across all 13 diagnostic groups. The strongest effect of dual eligibility on lower improvement in mobility was among patients with hip and/or knee replacements ($\beta$: -1.99, $p<0.001$) and patients with non-traumatic spinal cord dysfunction ($\beta$: -1.83, $p<0.001$). This research indicates that dually eligible patients may have worse functional mobility outcomes than non-dually eligible patients for some IRF primary diagnosis groups, and these patients may need additional support after discharge.

### ELDER CARE COMPLEXITIES AND OUTCOMES: A MANDATE FOR INTERDISCIPLINARY GERIATRIC CLINICAL ASSESSMENT

Peter S. Reed,[1] and Zebbedia Gibb[1], *1. Sanford Center for Aging, University of Nevada Reno School of Medicine, Reno, Nevada, United States*

Interdisciplinary geriatric assessment has long been considered best practice to identify the full range of elder care needs. In 2015, the Sanford Center for Aging launched an interdisciplinary comprehensive geriatric assessment clinic. This team-based assessment includes a geriatrician, social worker and pharmacist meeting together with each client to review all aspects of their health and well-being, resulting in a comprehensive care plan to coordinate care with the client's primary care provider. To assess this approach, a survey was conducted with 415 randomly-selected clients prior to the clinical visit, with a 6-month follow-up survey completed for 170 clients (41%), gathering data on hospitalizations, long-term care utilization and quality of life. Combining these data with clinical assessment data provides a picture of clinical complexities of elder clients and offers a mandate for comprehensive interdisciplinary care. Baseline data showed 44% hospitalized in the prior year; an average of 4 chronic conditions and 10 medications; 44% with dementia or MCI and 29% with frailty. At 6-months post-assessment, 29% reported being hospitalized, 3.5% reported moving into long-term care, and there was a slight, non-significant decrease in quality of life. These data demonstrate the profound complexities that can be identified and addressed through comprehensive assessment and care, as well as the potential to reduce hospitalizations, enable people to age-in-place and maintain quality of life. Despite the well-documented value of these approaches, Medicare and other payers have not fully embraced the opportunity to achieve these positive outcomes and remain hesitant to adequately fund comprehensive approaches to care.

### PSYCHOMETRIC APPRAISAL OF THE GERIATRIC SOCIAL WORK COMPETENCY SCALE II WITH LIFELONG LEADERSHIP SKILLS

Scott E. Wilks,[1] Sarah Choate,[1] Danielle Eugene,[1] and Xi Du[1], *1. Louisiana State University, Baton Rouge, Louisiana, United States*

The Council on Social Work Education (CSWE) Gero-Ed Center and Hartford Partnership Program in Aging Education (HPPAE) emphasize five competency areas specific to social work practice with older adult clientele (i.e., gero social work), namely knowledge and skills applicable to (a) values, ethics and theories; (b) assessment; (c) intervention; (d) aging programs, services and policies; and (e) leadership in aging environments. Accordingly, CSWE/HPPAE created a standardized measure – Geriatric Social Work Competency Scale II with Life-long Leadership Skills (GSWCS II) – to assess empirically these practice competencies among social work students and gero social work practitioners. A scant amount of literature exists that reports properties of this measure. Consequently, the purpose of this study was to conduct a psychometric examination of the GSWCS II, namely its factor structure and reliability among the competency areas. The sample consisted of three, advanced year MSW cohorts (N=170) from a state flagship university in the southern United States. Almost one-third of the sample were enrolled in a gerontology specialization during their advanced year. The typical participant was a 27-year-old female enrolled full time, completing the 60-credit hour MSW program. Principal axis factor results indicated unidimensionality, using the traditional 1.0 eigenvalue threshold, for each competency scale. All items loaded moderately-to-strongly on their respective competency scales; loadings ranged from 0.569 to 0.906. Regarding internal consistency for each of the competency scales, Cronbach's alphas ranged from 0.932 to 0.959; Guttman split-half coefficients (lambda-4) ranged from 0.896 to .941. Implications for gero practice competency assessment are discussed.

### A LOGIC MODEL TO DESCRIBE ADULT PROTECTIVE SERVICES IN THE U.S

Zach Gassoumis,[1] Karl Urban,[2] Gila Shusterman,[2] and Stephanie Whittier Eliason[3], *1. Leonard Davis School of Gerontology, University of Southern California, Los Angeles, California, United States, 2. WRMA, Inc, Rockville, Maryland, United States, 3. Administration for Community Living, Washington, D.C., United States*

The adult protective services (APS) system in the U.S. serves as an investigative and service delivery system, targeting cases of reported abuse, neglect, and exploitation of older adults and adults with disabilities. APS developed from a piecemeal investigatory system, driven by state and local processes.

This system has persisted, with little federal oversight or uniformity. In turn, little systematic information exists about the structure and process of the APS system nationwide. This poster presents an effort to construct a logic model for APS in the U.S., compiled jointly by researchers and practitioners under the auspices of the National APS Technical Assistance Resource Center. The process included initial drafting by six experts from various backgrounds and was reviewed by diverse stakeholders in three iterative rounds of review. Because of the unique nature of APS, the product has a partially non-traditional structure for a logic model, plotting context, inputs/resources, activities, activity metrics, and results. Inputs/resources are plotted across various sources: APS staff, consultative experts, community partners, operational supports, funding for services, and legal and ethical processes. Activities, activity metrics, and results are mapped across three stages of service provision—intake, investigation, and post-investigation services—and at the quality assurance level. The resultant logic model can be used at the national level to drive system change and evaluation; alternatively, it can be customized to specific state/local contexts to enable quality improvement and evaluation efforts. The pursuit of these efforts, coupled with existing national strategies, can contribute to continued system change and evolution within APS.

### SESSION 2390 (POSTER)

### LONG TERM CARE

### FACTORS INFLUENCING STAFF-RESIDENT INTERACTIONS IN NURSING HOMES

Anju Paudel,[1] Elizabeth Galik,[2] and Barbara Resnick[2], *1. University of Maryland School of Nursing, Maryland, United States, 2. University of Maryland School of Nursing, Baltimore, Maryland, United States*

Staff-resident interaction is an integral part of daily life of nursing home residents and has an influence on residents' well-being. However, less is known about the factors that influence these interactions. The purpose of this study was to describe the quality of interaction between staff and residents with dementia in nursing homes, and explore the factors associated with 'positive' and 'negative/neutral' interactions. This cross-sectional analysis utilized baseline data from the first two cohorts in a randomized clinical trial, EIT-4-BPSD, including 338 residents from 35 nursing homes. Generalized linear mixed model (GLMM) was used to explore the factors associated with interactions. It was hypothesized that the resident factors (age, gender, race, marital status, cognition, comorbidities, depressive symptoms, agitation, functional status) and facility factors (facility ownership, facility size, RN hours, LPN hours, CNA hours, and star rating) would be associated with staff-resident interactions. The staff-resident interactions were mostly positive. Overall, the models for 'positive interactions' and 'negative/neutral interactions' correctly classified 82.8% and 85.3% of the cases respectively. Both 'positive' and 'negative/neutral' interactions were significantly associated with marital status, and profit status of the facility. Being married and living in a not for profit facility was associated with lower odds of positive interaction and higher odds of negative/neutral interaction. There is some evidence that marital status influences staff-resident interactions and that profit status of facilities are associated with staff resident interactions. Future studies could explore staff factors such as consistent assignment, job satisfaction, staff characteristics, and training.

### EXPLORING RELATIONSHIPS BETWEEN RELIGIOUS COPING AND GENERAL COPING STRATEGIES IN OLDER LONG-TERM CARE RESIDENTS

Nathaniel Andrew,[1] and Suzanne Meeks[1], *1. University of Louisville, Louisville, Kentucky, United States*

Aging long-term care residents utilize a variety of coping strategies to manage stressors. While many older adults report religious faith is important in the coping process, it is unclear how religious coping appraisals and religious coping styles fit within a broader coping framework. This poster explores relationships between religious coping and general coping strategies in a convenience sample of older nursing home and assisted living residents (median age = 71.5). In this cross-sectional study, we interviewed residents (N = 102) from long-term care facilities (N = 11) in the Louisville metropolitan area and southern Indiana. Participants responded to questions about religious practices, religious coping, general coping, stress, life satisfaction, psychological distress, and health. The present analyses examined correlations between religious coping appraisals/styles and general coping strategies. We found: 1) small to moderate associations between theoretically adaptive religious coping appraisals/styles (e.g., positive appraisals, collaborative/deferring styles) and theoretically adaptive general coping strategies (e.g., positive reframing, instrumental support), and 2) small to moderate associations between theoretically maladaptive religious coping appraisals/styles (e.g., negative appraisals, self-directing styles) and theoretically maladaptive general coping strategies (e.g., denial, behavioral disengagement). Our results identify interesting conceptual relationships suggesting residents who report positive religious coping appraisals and less independent religious coping styles use adaptive coping strategies more frequently. These constructs may be explored in future research through examining their theoretical uniqueness and whether they independently account for variance in clinically-relevant outcomes. Further study of religious coping in these settings may help promote resilience and optimal aging for long-term care residents.

### TECHNOLOGY REDUCES DISTRESS IN A GERIATRIC COHORT WITH DEPRESSION AND NEUROCOGNITIVE IMPAIRMENTS

James A. Mazzone,[1] Jonathan Sills,[2] Flora Ma,[3] Peter Louras,[3] and Erickson Alexander[3], *1. Veterans Affairs Palo Alto Health Care System, Menlo Park, California, United States, 2. Veterans Affairs Palo Alto Health Care System, Palo Alto, California, United States, 3. Palo Alto University, Palo Alto, California, United States*

Many older adult Veterans with Depression and superimposed Neurocognitive Impairments may demonstrate behavioral agitation. To buffer patient agitation seen within a Veteran's Affairs residential extended care facility, psychological services were expanded to include the use of mobile technologies. To evaluate the effectiveness of adding technology supported psychological services, outcomes were gathered as part of continuous process improvement efforts. 28

Veterans with Depression and NCI who received technology enhanced services were rated by staff on observed agitation behaviors prior and following clinical encounters. In addition, a subset of 17 Veterans also provided subjective unit of distress (SUD's) ratings based on the Brief Interview for Mental Status inclusion criteria. Paired sample T-Tests were completed to assess if technology-enhanced interventions resulted in average reductions of clinician rated observations of Veteran agitation behaviors. Significant differences were found in observations of Veteran facial tension (t(27)=3.722, p=.001), agitated body movement (t(14)=2.020, p=.053), and threatening posture (t(27)=2.243, p=.044). Evaluation of a subset of those residents who also provided SUD's ratings show a significant change in self-reported distress after intervention (t(16)=4.3, p=.001). Effect size for this difference was large (d=1.042). These results suggest that mobile technologies can help reduce agitation and Veteran self-reported distress among older Veterans with Depression and superimposed Neurocognitive Impairments.

### TECHNOLOGY-ENHANCED PSYCHOTHERAPY IMPROVES LIFE SATISFACTION AMONG OLDER ADULT VETERANS IN LONG-TERM CARE

Jonathan Sills,[1] James A. Mazzone,[2] Flora Ma,[3] Peter Louras,[3] and Erickson Alexander[3], *1. Veterans Affairs Palo Alto Health Care System, Palo Alto, California, United States, 2. Veterans Affairs Palo Alto Health Care System, Menlo Park, California, United States, 3. Palo Alto University, Palo Alto, California, United States*

To buffer the risk of declining life satisfaction among a Veteran cohort residing within a Veteran's Affairs long term care facility, a new model of care called Individualized Non-Pharmacological Services Integrating Geriatric Health and Technology (INSIGHT) therapy was developed and evaluated. Consistent with the INSIGHT therapy model, traditional psychotherapy interventions including reminiscence, behavioral activation, and relaxation exercises were modified such that they could be delivered on a digital platform. A paired sample T-test was performed to identify the effects INSIGHT Therapy had on Veteran satisfaction with life. Findings indicated that Veteran life satisfaction ratings the month prior (M= 19.6522) to the initiation of INSIGHT intervention and the month following three months of INSIGHT intervention (M=22.4783) show that the satisfaction with life increased among residents (t(22)=-2.334, p=.028). Effect size = 0.489. These results suggest that INSIGHT therapy interventions help to contribute to an increase in life satisfaction among an older adult Veteran cohort residing within a Veteran's Affairs long term care facility.

### NURSING HOME ADMINISTRATOR STRESS, SATISFACTION, AND INTENTIONS TO LEAVE

H. Wayne Nelson,[1] Bo Kyum Yang,[1] Cyrus Y. Engineer,[1] and Mary W. Carter[1], *1. Towson University, Towson, Maryland, United States*

Previous studies reported that high Nursing Home Administrator (NHA) turnover correlates with low staff morale and poorer care outcomes. The purpose of this study was to assess how NHA job satisfaction (JS) (in five subscales: job content, coworkers, work demands, work load, work skills, and rewards) interacts with role conflict and ambiguity, autonomy, work conflict, and influence and to estimate the odds of having NHA's intent to quit by degree of job satisfaction. A total 208 responses were collected from the online survey in 2017 among NHAs currently working in nursing homes in 5 states. We analyzed the data using descriptive statistics and ordinal logistic regression models. The findings suggested that NHAs were generally satisfied in all JS subscales and expressed moderately high levels of autonomy, neutral levels of work conflict, role conflict and role ambiguity. NHAs with good coworker relations (adjusted odds ratio [AOR]=0.67), fair job demands (AOR=0.68) and rewards (A0R=0.8), were less likely to harbor quitting intents. Interestingly, NHAs reporting higher job skills were more likely to consider leaving nursing homes (AOR=1.46). Overall, study findings are consistent with previous JS research with the exception that higher perceived skill efficacy was found to be associated with greater likelihood of quitting in the near future. This suggests perhaps that more highly skilled NHAs may now have less tolerance for work discomfort. These findings are presented in the context of earlier studies on NHA turnover as well as likely implications of changing market conditions.

### THE STRUCTURE OF CARE MANAGERS' PRACTICE RESPECTING THE AUTONOMY OF THE FRAIL ELDERLY IN JAPAN

Chiemi Hata,[1] and Sachiko Kasahara[1], *1. Shitennoji University, Habikino, Osaka, Japan*

The objectives of current study are to clarify the structure of practice respecting the autonomy of the frail elderly under the Long-Term Care Insurance system in Japan and to discuss the related factors to the practice. The mailed self-administered questionnaire survey was conducted on 1398 care managers who working in In-Home Long-Term Care Support Providers in A City in Osaka with the condition that "office with multiple care managers engaged and one care manager with more than 5 years' experience". The response rate was 51.0% (713persons) and no missing data 615 (44.0%) was analyzed. Analysis was carried out using Mplus.ver8. The structure of practice respecting the autonomy of the frail elderly and the rerated factors were examined as a causal model using structural equation modeling. As the result, it was confirmed the goodness of fit to the data (RMSEA=0.049, CFI=0.927). By the confirmatory factor analysis, the care manager's practice respecting the autonomy of the elderly was confirmed associating with three-factors structure; (1)data collection and assessment, (2) strength perspective and (3) professional relationship. Furthermore the practice was significantly affected by self-esteem of care-managers performance (β=0.494) and self-reflection to own work (β=0.269). In conclusion, the current study supported the hypothetical consideration in which self-esteem and self-reflection in care manager's practice significantly affected the practice respecting the autonomy of the frail elderly.

### GENDER DIFFERENCES IN NURSING HOME RESIDENT QUALITY OF LIFE: WHY WOMEN DO BETTER

Heather Davila,[1] Tetyana P. Shippee,[2] Weiwen Ng,[2] Odichinma C. Akosionu,[2] and Beth Virnig[2], *1. VA Boston Healthcare System, Boston, Massachusetts, United States, 2. University of Minnesota, Minneapolis, Minnesota, United States*

Despite research documenting gender differences in numerous outcomes in later life, we know little about gender differences in quality of life (QoL) for older adults who receive institutional long-term care. To address this gap, this study examines the relationship between gender and nursing home (NH) residents' QoL, including possible reasons for differences observed. We used a mixed methods design including surveys with a random sample of Minnesota NH residents using a multidimensional measure of QoL (n=8,870), resident clinical data, facility-level characteristics, and qualitative interviews with NH residents (n=64). We used mixed models and thematic analysis of resident interviews to examine possible differences in resident QoL based on gender. After controlling for individual and facility characteristics, women reported higher overall QoL than men, with men reporting significantly lower QoL in 4 of 8 QoL domains. In interviews, men noted being especially dissatisfied with facility activities, whereas women more frequently described having friends in the facility and relying on family for support. Some women viewed the NH as a place of respite and described wanting to stay long-term, even though their families asked them to return home. In contrast, men more often described the NH as necessary due to physical needs, but undesirable for long-term living. Our findings provide preliminary evidence that men and women experience QoL differently, with men reporting lower QoL in several domains. Tailoring more activities for men and finding ways to strengthen relationships for men within the facility may help reduce the gender disparities in QoL we observed.

### INDIVIDUAL AND INTERPERSONAL INFLUENCERS OF MEALTIME EXPERIENCES: PERSPECTIVES OF NURSING ASSISTANTS

Joy W. Douglas,[1] Seung Eun Jung,[1] Hyunjin Noh,[2] Amy Ellis,[1] and Christine Ferguson[1], *1. University of Alabama, Tuscaloosa, Alabama, United States, 2. The University of Alabama School of Social Work, Tuscaloosa, Alabama, United States*

In the United States, long-term care Certified Nursing Assistants (CNAs)'s central role is to provide direct care to residents, including mealtime assistance. It has been reported that employee turnover among CNAs is nearly 75% annually. High turnover rates of CNAs can increase the workload for remaining CNAs, interrupt quality of care for residents, and require extra resources for recruiting new staff. The aim of this qualitative study was to explore the individual and interpersonal barriers and facilitators CNAs experience when providing mealtime assistance to residents with dementia. Using purposive sampling, nine focus groups were conducted with a total of 53 CNAs who had at least one year of experience as a CNA working with older adults. Focus group questions were developed using the Social Ecological Model. All focus groups were audio recorded and transcribed verbatim. Data were analyzed using the directed content analysis approach. At the individual level, CNAs identified that communication skills with residents and coworkers, and the ability to accurately interpret resident behavior positively affected their ability to provide mealtime assistance. At the interpersonal level, interdisciplinary collaboration was identified as a significant facilitator. Reported barriers included negative interference from residents' family members, unpredictable resident behaviors, and lack of support from coworkers. CNAs reported individual and interpersonal factors that may influence their ability to effectively feed residents with dementia. Our findings will inform future investigations regarding job turnover. Equally important, providing CNAs with the training and opportunity to perform their duties efficiently can ultimately benefit the residents' mealtime experiences.

### PERSON-CENTERED CARE, BURNOUT, AND BARRIERS AMONG DIRECT CARE PROFESSIONALS: TARGETED TRAINING INTERVENTION 360

Lauren Stratton,[1] Hannah Dannewitz,[1] Jennifer Margrett,[1] Mack Shelley,[1] Linda Brown,[1] and Ann C. Drobot[2], *1. Iowa State University, Ames, Iowa, United States, 2. Alzheimer's Association, Iowa Chapter, Cedar Rapids, Iowa, United States*

Long-term care staff outcomes, such as job satisfaction and providing personalized care, are positively influenced by person-centered interventions. Implemented in eight facilities across Iowa, the Targeted Training Intervention 360 (TTI) program aimed to increase person-centered care among direct care professionals (DCP). Throughout the course of TTI, three waves of data were collected from DCPs regarding person-centered care (Person-Centered Care Assessment Tool; P-CAT) and feelings of burnout (Maslach Burnout Inventory; MBI). Analysis of variance tests were employed to identify significant differences in subscale scores across the three waves. Between waves one and two, results revealed significant increases in the P-CAT Extent of Personalizing Care (p=0.03) and Amount of Organizational Support subscales (p=0.001). Additionally, significant decreases from waves one and two were found in the MBI Emotional Exhaustion subscale (p=0.04). Between waves two and three, there were no significant changes in the P-CAT subscales; however, there was a significant increase in the MBI Emotional Exhaustion subscale (p=0.04). To supplement these findings, in wave three DCPs indicated barriers to implementing person-centered care, which included lack of time (49.0%), lack of experience (29.4%), and lack of administrative support (21.6%). Though there were no significant changes in P-CAT scores between the last two waves as well as barriers that must be addressed, DCPs described positive organizational and personal changes regarding person-centered care in the facility, including consistent staffing, using person-centered techniques in care, and individualized activities. Discussion focuses on ways to address barriers to person-centered care and sustain efforts in implementing change.

### SERVICE PROVISION IN RESIDENTIAL CARE COMMUNITIES FOR RESIDENTS WITH DEMENTIA AND DEPRESSION

Manisha Sengupta,[1] and Christine Caffrey[2], *1. CDC/National Center for Health Statistics, Hyattsville, Maryland, United States, 2. Centers for Disease Control and Prevention/National Center for Health Statistics, Hyattsville, Maryland, United States*

Assisted living and similar residential care communities (RCCs) are an important source of care for older adults, many of whom have dementia or depression. In 2016, 42% of residents in RCCs were diagnosed with dementia and 31% were diagnosed with depression. About two-thirds of RCCs (63%) provided social work and mental health services and 37% provided neither service. Using the 2016 National Study of Long-Term Care Providers, this study includes bivariate and ANOVA modeling to (1.) examine the variation in dementia and depression between RCCs that provide social work and mental health services and those that do not, and (2.) assess if there is an interaction between provision of services and RCC bed size in their association with dementia and depression. Bivariate results show that the prevalence of dementia does not vary by service provision, but the prevalence of depression does. The percent of residents with depression varied from 34% in RCCs that provided both social work and mental health services to 31% in RCCs that provided only one service to 28% in RCCs that provided neither service. The prevalence of dementia was around 4 in 10 residents, regardless of service provision. Fitting a two-way ANOVA model for dementia indicated that the effect was 4 percentage points higher for RCCs that provided services than those that did not, but only among small RCCs. Findings from this study can inform strategies to care for the needs of residents with dementia and depression across the various sizes of RCCs.

### BEST INTENTIONS OR BEST PRACTICE? HOW DO STAFF PERCEIVE BEST CARE FOR PEOPLE WITH DEMENTIA IN TAIWAN?

Chih-ling Liou[1], *1. Kent State University, North Canton, Ohio, United States*

Care of people with dementia is a global issue, and the quality of dementia care in the long-term care facility is of concern internationally. The Taiwanese government seeks to expand the availability of adult day services (ADS) to meet the needs of an aging population with dementia; however, research about care in ADS in Taiwan is limited. This study aims to investigate how ADS staff perceptions of best care affected care delivery and clients' quality of life. A focused ethnographic method was employed to collect data through 480 hours of participant observation and 31 staff interviews at three centers. Content analysis was used to analyze the data. The findings revealed a contradiction on how staff perceived best care and what they really performed at center. Thirteen staff members who were interviewed identified that in their view the best care is to "put yourself in the shoes of the client," and another nine staff members chose "keep clients happy" as the best care. From the observation data, however, the staff performed the "good care" by forcing the clients to follow their orders absolutely unless the clients refused violently. Staff-led activities were structured as tasks to be completed rather than activities to be enjoyed. The clients complained about the bureaucratic management that shaped their lives at centers but could only endure or accept. To support clients' quality of life and improve the care quality, ADS staff need to be educated more with the evidence-based best care for people with dementia.

### QUALITY OF CARE IN A LONG-TERM CARE COMMUNITY

Laura A. Murray,[1] and Melinda Heinz[1], *1. Upper Iowa University, Fayette, Iowa, United States*

Older adults may need to reside in long-term care facilities for additional assistance. However, research indicates differences in the quality of care. The purpose of this study was to conduct a naturalistic observation, recording factors affecting the quality of care residents received in a long-term care community. Over a three-week period, observations took place in the nursing home, assisted living, and memory care portions of the community. We predicted that there would be more issues negatively impacting quality of care in the nursing home area due to its medical model philosophy. Open-observations were recorded, coded, and analyzed for themes. Results indicated that the most significant issue influencing quality of care in all areas of the long-term care facility was communication (N = 57 recorded instances). Approximately 66% of recorded communication issues in the nursing home were negative compared to positive (25%) or neutral (8%) instances. Elderspeak was prevalent with staff using high pitched voices or saying "hun" to residents. At times, staff spoke too loudly to residents who did not have hearing impairment or would talk about residents in front of other residents, not taking into consideration privacy. In the memory care environment, positive examples were noted. Staff was friendly and worked together as a team, creating a positive work environment. Overall, results indicated staff members may need professional development in the area of communication, particularly staff working in the nursing home. In addition, reminding staff while it is their workplace it is also the resident's home would be beneficial.

### FROM BEING ACTIVATED TO SELF-ORGANIZATION IN ELDER CARE: CONCEPTS OF AGING AND THEIR IMPACT ON RESIDENTS' AGENCY

Irene Strasser,[1] Ines Hopfgartner,[2] and Carmen Payer[2], *1. The American University of Paris, Paris, France, 2. University of Klagenfurt, Klagenfurt, Austria*

In a research project together with an elder care facility in Austria, we were following a participatory approach to investigate important factors and processes to increase quality of life for residents with dementia. The aim was to better understand how we can foster participation, agency and freedom of choice within a care setting. Together with care workers, residents and relatives we were working on how to identify overall strategies and aims, as well as particular processes and procedures that allow for a higher involvement of all these groups of individuals. Doing research explicitly together with care workers we also aimed to trigger reflection of individual and organizational concepts of aging. We interviewed residents, relatives and care workers, conducted research workshops, and engaged in participant observation. In the talk the focus will be on care workers' perspectives. We wanted to better understand the multiple stressors within daily routines in care work. Particularly, we wanted to find out more about the multifaceted processes of integrating one's experiences into everyday care work, and integrating care work into one's life story, to make meaning of the important work they are providing under constantly stressful working conditions. Finally, together with care workers, we developed a model for participation, that is explaining a wide range of residents' possibilities for involvement: From activity orientation to actual participation and the realization of individually meaningful activities. What helps to initialize participation, and what we identified as obstacles in supporting residents' autonomy will also be discussed in the talk.

### LONG TERM CARE

### FEASIBILITY OF NURSING HOME LVNS USING AN ALGORITHM-GUIDED ASSESSMENT SUPPORT TOOL

Alyce S. Ashcraft,[1] Donna C. Owen,[1] Kyle Johnson,[1] and Huaxin Song[1], *1. Texas Tech University Health Sciences Center, Lubbock, Texas, United States*

Licensed Vocational Nurses (LVNs) are the frontline staff communicating with off-site physicians to manage new or unexpected symptoms in nursing home (NH) residents. Care decisions following physician-LVN conversation impact Alzheimer's disease (AD) resident quality of care. LVN difficulties using standardized communication forms (e.g. SBAR) spurred creation of an alternative not requiring high levels of RN supervision. The aim of this study was to examine the feasibility of an algorithm guided assessment support tool (AGAST) to help LVNs collect and cluster information using a simulated clinical scenario. A descriptive, participant observation design (video-taped) was used with ten practicing LVNs. AGAST was implemented in the Simulation Center in a clinical scenario of a NH resident with AD experiencing a urinary tract infection. The algorithm was placed within an online survey and participants were directed through a series of sequenced question and answer choices to identify and collect information pertinent to describing a change in resident condition. Duration of videos, description of AGAST-linked assessment behaviors, and content analysis of post-intervention transcripts were reported. AGAST prompts were seen with LVN physical assessment behavior and followed by questions about relevant day-to-day activity/level of performance. AGAST mean completion duration was 11.20 (SD=4.67) minutes. AGAST was rated as easy or very easy (90%). Major concerns included small font size on lab screens, insufficient open text space to add more information, and direct computer input. AGAST was a feasible alternative for assessment by LVNs. Further testing is needed in a variety of conditions.

### MANAGEMENT OF NONCOGNITIVE NEUROPSYCHIATRIC SYMPTOMS OF DEMENTIA IN NURSING HOME RESIDENTS

Prasanna Basnet,[1] Gayle Acton,[1] and Jane D. Champion[1], *1. The University of Texas at Austin, Austin, Texas, United States*

Background: It is challenging for nursing home (NH) staff to manage non-cognitive neuropsychiatric symptoms (NPS) of dementia. There is a need for an assessment of staff knowledge regarding non-pharmacological approaches to manage NPS of dementia. This assessment will inform development of policies/procedures to assist NH staff for management of problematic behaviors in residents with dementia (RWD) using non-pharmacological approaches thereby complying with CMS directives to reduce psychotropic medication use. Methods: NH staff members were interviewed using semi-structured interview methods. Interviews continued until thematic saturation was reached. A total of 17 interviews were completed. Findings: Forgetfulness, hallucinations, anger, agitation, combativeness are the most common problematic dementia behaviors. Participants reported that these behaviors make activities of daily living (ADL) care challenging and time-consuming. Redirection, distraction, and recreational activities are the most common approaches identified by participants to manage non-cognitive NPS using non-pharmacological techniques. Participants reported rushing residents to get things done hinders cooperation and escalate problematic behavior. Some participants believed the use of a low dose PRN benzodiazepine is effective as this calms the resident with dementia and reduces NH staff time requirements for assistance with ADL care. Participants described adverse reactions such as weight loss, and general decline as an outcome of somnolence secondary to psychotropic medications use. Implications: NH staff who participated in this project had not received any formal education or instruction concerning non-pharmacological approaches for the management of non-cognitive NPS. NH protocols are indicated for non-pharmacologic behavior management techniques prior to the administration of psychotropic medications.

### MEASURING CLIENT EXPERIENCE IN RESIDENTIAL AGED CARE TO INFORM AND SUPPORT CONSUMER CHOICE

Deirdre M. Fetherstonhaugh,[1] Yvonne Wells,[1] and Angela Herd[1], *1. La Trobe University, Victoria, Australia*

The Australian Government Aged Care Quality and Safety Commission (prior to January 2019 known as the Australian Aged Care Quality Agency) is responsible for accreditation of Australian aged care services which are audited against the Australian Accreditation Standards. Accreditation reports are publicly available. Prior to 2017, some clients were interviewed about their experiences, but the resulting information could not represent the client experience within a service due to low numbers, biased sampling, and an unsystematic approach to asking questions. La Trobe University was engaged to develop and pilot an interview tool to measure client experience for use in accreditation. Potential questions were identified through a literature review, mapped against the Accreditation Standards, and workshopped with an expert reference group. Twenty-four questions and a visual analogue were then piloted. Consumer groups and groups of Indigenous and culturally diverse clients in residential aged care homes were consulted. The perspective of Quality Agency surveyors was sought on the questions' usability. Statistical analyses sought to identify questions that minimised missing data, were responded to similarly by residents and their representatives, and elicited stable responses on retest. Twelve questions were identified as optimal. The 10 quantitative questions proved to reflect a single underlying dimension (consumer experience) and, when summed and explored through regression analyses, differentiated services significantly. The consumer experience interview tool is now used in all accreditation audits in Australia. Results are then used to generate consumer experience reports, which are published online and can support consumer choice of a residential aged care home.

### NURSING ASSISTANTS' OPINIONS ON CREATING THE PERFECT DINING EXPERIENCE FOR RESIDENTS WITH DEMENTIA

Christine Ferguson,[1] Joy W. Douglas,[1] Seung Eun Jung,[1] Hyunjin Noh,[2] and Amy Ellis[1], *1. University of Alabama, Tuscaloosa, Alabama, United States, 2. The University of Alabama School of Social Work, Tuscaloosa, Alabama, United States*

Feeding residents with dementia in long-term care settings can be challenging, partly related to environmental factors. Certified Nursing Assistants (CNAs) are primarily responsible for feeding residents with dementia who need assistance. Given that older adults with dementia have an increased risk of developing malnutrition, there is a need to develop standards in place for constructing an ideal dining environment to optimize residents' dietary intake. This qualitative study was conducted to explore CNA's perspectives of how dining areas could be enhanced to improve food intake of residents with dementia. Nine focus groups were conducted with a total of 53 CNAs who had at least one year of experience feeding residents. Focus groups were audio recorded and transcribed verbatim. Data were analyzed using directed content analysis guided by the Social Ecological Model. CNAs reported that distractions can significantly inhibit residents' food intake; therefore, limiting distractions such as noise and crowding is important. CNAs also reported the benefit of playing music in the dining area depended on the individual resident. Additionally, CNAs emphasized the importance of offering a variety of appetizing menu choices tailored to residents' preferences. CNAs have firsthand experience with residents with dementia and can provide valuable insights. Long-term care administration should consider interdisciplinary support to improve the mealtime experience of residents with dementia in an effort to enhance their dietary intake. In particular, providing a variety of menu choices in a well-lit, calm, spacious, and homelike dining environment can be beneficial.

### EVALUATING AN INNOVATIVE USE OF CIVIL MONETARY PENALTY FUNDS: THE MA SUPPORTIVE PLANNING AND OPERATIONS TEAM (SPOT)

Rosanna M. Bertrand,[1] Gabrielle R. Katz,[1] Teresa M. Mota,[1] Terry Moore,[1] Jennifer Pettis,[2] Katherine T. Fillo,[3] Katherine C. Saunders,[3] and Chiara S. Moore[3], *1. Abt Associates, Inc., Cambridge, Massachusetts, United States, 2. New York University Rory Meyers College of Nursing, Nurses Improving Care for Healthsystem Elders (NICHE), New York, New York, United States, 3. Massachusetts Department of Public Health, Bureau of Health Care Safety & Quality, Boston, Massachusetts, United States*

The Office of the Inspector General reported in 2014, that one in three NH residents experienced an adverse event within 35 days of admissions causing lasting or temporary harm. Thus, state departments of public health (DPH) were implored to invest in improving NH quality and safety. Using Civil Monetary Penalty funds, the Massachusetts DPH, developed the SPOT Initiative to innovatively provide NH teams with technical assistance and training to enhance their federally required Quality Assurance & Performance Improvement (QAPI) programs. Selection criteria included NH Compare 5-Star and MA scorecard ratings and geographic spread. To assess program effectiveness, the SPOT Team collected a range of data in each of the three SPOT years (e.g., QAPI assessments, leadership interviews and surveys, and training evaluations). Results demonstrated the success of the Initiative. Assessment data indicated an increase in QAPI readiness in each subsequent year overall and within of the each QAPI assessment domains (Design and Scope; Governance and Leadership; Feedback, Data Systems, and Monitoring/Systematic Analysis; Performance Improvement Projects and Systematic Analysis/ Systemic Action). In Year 1, the overall data collected from the assessments demonstrated that 78% of the NHs that engaged with SPOT had "Not Started" or "Just Started" (1.8/5) implementation of the key QAPI measures. By Year 3, only 13% of NH teams rated themselves in these initial categories, whereas, 57% rated themselves as "Almost There" or "Doing Great" (3.92/5). Further, feedback from most SPOT NH teams was extremely positive as evidenced by high evaluation rankings following initiative learning sessions.

### ARE TRACK-AND-TRIGGER SYSTEMS GOOD NEWS FOR RESIDENTS IN LONG-TERM CARE FACILITIES? A MULTI-METHOD EVALUATION

Robert O. Barker,[1] Siân Russell,[1] Rachel Stocker,[1] Jennifer Liddle,[1] Joy Adamson,[1] and Barbara Hanratty[1], *1. Newcastle University, Newcastle upon Tyne, United Kingdom*

Changes in physiological measurements such as blood pressure or temperature may signal deteriorating health before it is apparent. Early warning (or track and trigger) systems provide a framework to use such measurements to identify acute illness and prompt a timely response. They are in widespread use in acute hospitals across North America and Europe, but few have been validated in community settings. Hospitals in the UK have adopted the National Early Warning Score (NEWS) which measures temperature, respiratory rate, pulse, blood pressure, oxygen saturation and conscious level. This presentation describes a multi-method evaluation of the introduction of NEWS into 47 long-term care facilities. Staff with little or no healthcare training were tasked with digital recording of the NEWS. This multi-method evaluation consisted of a survey to explore staff views (n=42), a quantitative analysis of approximately 17,000 NEWS readings, and 21 semi-structured qualitative interviews with stakeholders. Survey and interview findings suggested that use of the score increased staff confidence in communication and care. There were many challenges to implementation, including practical difficulties in measuring vital signs, competing priorities for staff and a persistent lack of shared understanding across professional boundaries. Quantitative analysis of recorded scores described an increase in use of the NEWS over time, but wide variation in uptake between different facilities. Early warning systems may enhance management of acute illness in long-term care facilities but implementation is not straightforward. This presentation will discuss will discuss findings in depth - what worked, lessons learned and implications for the future.

### FACTORS ASSOCIATED WITH RESIDENTIAL CARE COMMUNITIES THAT HAVE RESIDENTS VISITING EMERGENCY DEPARTMENTS

Amanuel Melekin,[1] Christine Caffrey,[1] and Vincent Rome[1], *1. Centers for Disease Control and Prevention/National Center for Health Statistics, Hyattsville, Maryland, United States*

Emergency department (ED) visits are an important part of healthcare utilization. However, ED visits can be costly, lead to hospitalizations, and are sometimes unnecessary. Studies characterizing ED visits among long-term care settings have been largely focused on nursing homes where the unit of analysis is typically the resident. Facility- or community-level analyses describing residential care communities (RCCs) with ED visits are limited. Using RCCs as the unit of analysis, this study examines community-level factors associated with RCCs that have residents with ED visits. Community-level factors include ownership and chain affiliation, Medicaid participation, electronic health records use, service provision, nurse staffing, U.S. census region and metropolitan status. The study uses data from the 2016 National Study of Long-Term Care Providers conducted by the National Center for Health Statistics. In 2016, about 81% of RCCs had at least one resident visiting the ED in the past 90 days and around 19% of RCCs had no residents with ED visits in the past 90 days. Bivariate analyses indicated that ED visits varied by chain affiliation, ownership status, electronic health records use, and Medicaid participation. Logistic regression modeling to examine factors associated with whether or not RCCs had any residents with ED visits in the past 90 days will also be presented. Results may benefit efforts focused on implementing practices to reduce ED visits in RCCs.

### IMPROVING SLEEP USING MENTORED BEHAVIORAL AND ENVIRONMENTAL RESTRUCTURING (SLUMBER)

Joshua Chodosh,[1] Diana Hernandez,[2] Michael Mitchell,[3] Mary Cadogan,[4] Abraham A. Brody,[5] Cathy Alessi,[6] Jessica Smilowitz,[2] and Jennifer Martin[7], *1. New York University Langone Health, New York, New York, United States, 2. NYU Langone Health, New York, New York, United States, 3. VA Greater Los Angeles Healthcare System, North Hills, California, United States, 4. UCLA School of Nursing, Los Angeles, California, United States, 5. NYU Rory Meyers College of Nursing, New York, United States, 6. VA Greater Los Angeles Healthcare System, Los Angeles, California, United States, 7. VA Greater Los Angeles Healthcare System, North Hills, California, United States*

Sleep disturbances are common in skilled nursing facilities (SNF) affecting up to 70% of residents. Poor sleep is linked to depressed mood, cognitive impairment, increased pain, and functional disability. SNF residents depend on staff for basic day-to-day needs making it essential that staff be empowered in sleep improvement efforts. In SLUMBER, we are using a multi-site stepped-wedge design to implement a program for SNF staff to improve common sleep-disruptive factors. This three-month program includes four in-person staff meetings and three didactic webinars covering three content areas: 1) improving the nighttime sleep environment, 2) increasing daytime activities, light exposure, reducing daytime sleeping, and 3) helping individual residents having difficulty with sleep. In addition to mentoring staff on sleep improvement strategies, technology provides feedback on noise levels from decibel meters throughout the unit and weekly “sleep pearls” text messages sent to staff to reinforce teachings. We measured noise readings (in decibels) in one second increments. For sleeping hours, 10pm to 6am, we calculated the percentage of observations exceeding 60 decibels. Post intervention in the first of six study units, 78% of noise readings exceeded 60db during sleeping hours compared to three months later where 50.3% of noise readings exceeded 60db, suggesting benefits of noise-reduction efforts. SNF staff reported several instances of improving sleep among chronically poor sleepers and an improved work environment. This mentoring program can achieve important environmental improvements with perceived benefit to residents and staff. Whether this leads to objective symptom and physiological improvements awaits conclusion of this four-year trial.

### AN IOT TROLLEY FOR LONG-TERM CARE FACILITIES TO PROVIDE EFFICIENCY AND REDUCE RISKS

Chia-Ching Chou,[1] Ting-Ju Liu,[1] and Shih-Chung Kang[1], *1. Smart Aging Tech, New Taipei City, Taiwan, Taiwan*

One of challenges facing the long-term care facilities in Taiwan is the burden of the paperwork affecting nurses, which limits their time to look after the residents. Nurses usually estimate spending one quarter of their shifts with paperwork. The aim of this study is to develop a mobile care information system - Jubo IoT Trolley: a trolley with IoT vital-sign devices collecting and delivering timely care information to care professionals. Based on user-centered design (i.e., discover-define-develop-delivery), we conducted stakeholder interviews and rapid prototypes to zero in on the communication problem, and designed the IoT Trolley to support nurses in their daily workflow, facilitate vital-sign measurements at the bedsides, and collect the measured values to the cloud database automatically. Through design iterations, we have validated usability of the system in multiple care facilities. The result shows, with the IoT Trolley, the nurses can receive the senior’s critical vital status from the caregivers more promptly, provide instructions remotely and therefore, reduce potential care risks. Furthermore, the cloud analyzes the collected residents’ health data, the vital sign alerts can be sent to the nursing directors, so they can coordinate and intervene instantly. At last, this work demonstrates that through the technology, care qualities are improved, and care professionals can spend more valuable time with residents in the long-term care facilities.

### ADVANCED ILLNESS AMONG ELDERLY NURSING HOME RESIDENTS WITH ALZHEIMER’S DISEASE AND RELATED DEMENTIA

Tadeja Gracner,[1] Mark Sorbero,[2] Patricia W. Stone,[3] Mansi Agarwal,[3] and Andrew W. Dick[4], *1. RAND Corporation, Washington, District of Columbia, United States, 2. RAND Corporation, Pittsburgh, Pennsylvania, United States, 3. Columbia University School of Nursing, New York, New York, United States, 4. RAND Corporation, Boston, Massachusetts, United States*

Alzheimer’s disease and related dementia (AD/ADRD) are leading causes of mortality in the United States. Identifying advanced illness (AI) in NH residents is key for developing therapeutic and palliative care plans for end of life. We refined and extended existing measures of AI in NH residents with AD/ADRD and described patterns of survival for each measure. Using the Minimum Data Set (MDS; 2011 to 2013) linked to vital status (through 2016), we defined categories of AD/ADRD residents at AI onset: (1) those with ADRD, (2) and those with both, AD and ADRD. We estimated survival functions and multivariable duration models to describe patterns of survival from AI onset until death, stratified by AD/ADRD classifications, sex and functional status at AI onset, conditional on socio-demographics and co-morbidities. We limited our sample to adults ages >64 for whom we observed the incident AI assessment in the MDS. Median survival was 229 days for all classifications of AI, but higher for those with only ADRD (300 days). Survival declined substantially for residents with eating difficulties; to 122 days for residents with AD and ADRD. A stark survival decline (40 days) occurred among residents with shortness of breath. Across all AI classifications, survival was negatively associated with male sex, age, diabetes, substantial weight-loss and events such as heart failure. Depression, hypertension, and UTI were associated with small or insignificant increases in mortality risk. AI can be defined using MDS data, allowing for examination of policies designed to improve end of life care.

### ORGANIZATIONAL AND COMMUNITY FACTORS ASSOCIATED WITH NURSING HOME ORGANIZATIONAL CULTURE

Akbar Ghiasi,[1] Ganisher K. Davlyatov,[1]
Justin C. Lord,[2] and Robert Weech-Maldonado[1],
*1. University of Alabama at Birmingham, Birmingham, Alabama, United States, 2. Louisiana State University at Shreveport, Shreveport, United States*

This study examines how organizational and community factors are associated with organizational culture among high Medicaid nursing homes (70% or higher Medicaid census). According to the Competing Values, Framework, there are four types of organizational culture: clan culture (friendly working environment); adhocracy culture (dynamic/creative working environment); market culture (results-based organization); and hierarchy culture (formalized/structured work environment). Survey data from 324 nursing home administrators (30% response rate) from 2017- 2018 were merged with secondary data from LTCFocus, Area Health Resource File, and Medicare Cost Reports. The dependent variable consisted of organizational culture type. The independent variables comprised organizational factors (facility has nurse practitioner/physician assistant (NP/PA), RN/LPN/CNA hours per resident day, RN skill mix, ownership, chain affiliation, size, occupancy rate, and Medicare and Medicaid payer mix) and community factors (Medicare Advantage penetration, per capita income, educational level, unemployment rate, poverty level and competition). Multinomial regression results showed that, compared to facilities with hierarchical cultures, those with a market culture have greater odds of having an NP/PA and higher RN skill mix and LPN intensity, but lower odds for RN intensity, Medicaid payer mix, occupancy rate, and Black residents. Also compared to facilities with hierarchical cultures, those with a clan culture have lower odds for having an NP/PA, beds and occupancy rate, but higher odds of being located in communities with lower unemployment and higher Medicare managed care. In conclusion, different organizational cultures are associated with different staffing patterns, as well as organizational and community factors.

### CONSISTENCY IN DOCUMENTATION OF TRIGGER EVENTS AND RELATED DIAGNOSES DURING 911 TRANSFERS TO ED NH RESIDENTS

Colin Reid, University of British Columbia, Kelowna, Britis h Columbia, Canada Chloe Sollid-Gagner,[1] Rachel Ma,[1] Lee Morgan,[1] Kaitlyn C. Tate,[2] and Greta G. Cummings[2],
*1. University of British Columbia, Kelowna, British Columbia, Canada, 2. University of Alberta, Edmonton, Alberta, Canada*

Previous research has identified that documentation practices during resident transitions from long-term care (LTC) to the emergency department (ED) can be inconsistent or nonspecific, leading to the receipt of inappropriate or insufficient care. When many older adults (>65 years) in LTC have cognitive impairments that make communication difficult and changes in resident health can present ambiguously, consistent documentation becomes particularly critical for the provision of safe, timely and appropriate care. The purpose of this study was to examine documentation practices across care settings related to reason for transfer during transitions of older adults from LTC to the ED. We tracked every resident transfer from 38 participating LTC facilities to two included EDs in two Western Canadian provinces from July 2011 to July 2012. Using case-related data gathered from 637 transitions, we employed qualitative content analysis to categorize whether documentation practices from LTC to the ED were sufficiently reported and clinically consistent. Transitions were defined as consistent when symptomatology, trigger events and diagnoses aligned in a medically-intuitive manner. Inconsistency patterns were further categorized as minor (indicating one outlying symptom/trigger) and major (indicating more than two inconsistencies). Of the total 637 transitions, 2.67% contained too little data to be accurately categorized. The majority of cases (75.82%) of cases had consistent documentation, 13.19% had minor inconsistencies, and 8.32% had major inconsistencies. These results support that shared continuing education for documentation practices should occur across care settings to ensure that documentation practices are sufficient, support a geriatric focus and consider differing clinician perspectives.

### SESSION 2400 (POSTER)

### MENTAL HEALTH | DEPRESSION

### HIGH ON SMI: QUALITY CONCERNS ABOUT NURSING HOMES SERVING HIGH PROPORTIONS OF SERIOUSLY MENTALLY ILL RESIDENTS

Dylan J. Jester,[1] Kathryn Hyer,[2] and John R. Bowblis[3],
*1. School of Aging Studies, University of South Florida, Tampa, Florida, United States, 2. University of South Florida, Tampa, Florida, United States, 3. Department of Economics, Miami University, Oxford, Ohio, United States*

The proportion of nursing home (NH) residents that have serious mental illness (SMI) has increased over the least two decades. Residents with SMI tend be younger and have different medical needs than traditional residents. To better understand this population, our study examined the facility, staffing, and resident characteristics of NHs that were more likely to specialize in SMI. Utilizing the Certification and Survey Provider Enhanced Reports, low-SMI (N = 3,616) and high-SMI (N = 3,615) NHs were defined as the first and fourth quartile of the distribution of the proportion of SMI residents, respectively. We performed bivariate tests and multivariate logistic regression to compare facility, staffing, resident, and star-ratings characteristics between NHs. High-SMI NHs were less likely to be Not-For-Profit, have fewer beds, have more Medicaid-paying residents, lower registered nurse staffing, and lower certified nurse aide staffing levels (p's<.001). Residents in high-SMI NHs were more likely to require behavioral healthcare (p<.001) and be treated with psychoactive medications (any psychoactive, antidepressants, antipsychotics, anxiolytics (p's<.001), hypnotics (p<.01)). Finally, high-SMI facilities had lower overall quality, health inspection, quality measure, staffing, and registered nurse staffing star-ratings (p's<.001). High-SMI NHs have characteristics that are associated with lower quality-of-care (e.g., For-Profit, more Medicaid), lower staffing, prescribe more psychoactive medications, and have lower star-ratings. As the SMI population grows, large numbers of SMI residents will concentrate in a few NHs. While further research is needed to understand the implications of these trends, policy-makers must be aware of this population when affecting the resources and staffing of NHs.

### ADVERSE CHILDHOOD EXPERIENCES PREDICTING INMATE STATUS AMONG OLDER COMMUNITY DWELLERS

Mai S. Yang,[1] and Donald Hedeker[1], *1. University of Chicago, Chicago, Illinois, United States*

The goal of this study was to see if whether having exposure to early child adversity predicted inmate status among older adults. Very little research has focused on the relationship between inmate status and older adults, and factors that could predict someone being imprisoned. This study used data from the Health and Retirement Study (2006 – 2014) (N = 1,070) to examine this association. We used logit models to test if specific adverse childhood experience and race groups predicted inmate status among community dwellers age 55 and older (M = 72.2, SD = 8.5). More than half of the respondents were female (59%). About 1.9% respondent identified as non-Hispanic other, 7.2 % as Hispanics, 14.2% as non-Hispanic black, and 76.7% as non-Hispanic Whites. In terms of former inmate status 14% of the respondent indicated having been an inmate in jail or other correctional facility. Preliminary findings suggest predictors of inmate status were those who had trouble with the law in early adolescent years and those who self-reported as black. Findings from this study could provide insight into how early childhood experiences could predict inmate status in adulthood among older adults.

### LIVING LIFE TO THE FULL 55-PLUS: A COMMUNITY-BASED INTERVENTION FOR REDUCING DEPRESSION AND ANXIETY

Nasreen Khatri,[1] Stephen Perkovic,[2] and Tara Faghani Hamadani[3], *1. Rotman Research Institute, Baycrest, Toronto, Ontario, Canada, 2. York University, Toronto, Ontario, Canada, 3. Dalla Lana School of Public Health, University of Toronto, Toronto, Ontario, Canada*

Living Life to the Full 55+ (LLTTF) was an 8-week, 12-hour, community educational program based on CBT principles. The program aim was to teach participant skills and techniques to cope effectively with life stress. Participants self-referred to the program by responding to advertisements or were otherwise referred to the program by local community centres. Following the promising results of the pilot study, a follow-up empirical study was conducted to assess the impact of the program on clinical measures and other notable measures over time. Study participants (N = 514) were recruited at partner sites hosting the program. Demographic and other data was collected to assess the impact of the program. Measures collected to assess the impact of the program were the Beck Depression Inventory (BDI-II), Beck Anxiety Inventory (BAI), Warwick-Edinburgh Mental Well-being Scale (WEMWBS), and a brief measure of loneliness/social connectedness. Participant data was collected pre/post-intervention, and at post-course follow-up periods (3/6/9-months). Preliminary repeated-measures ANOVA analyses found statistically significant changes in depression, anxiety, mental well-being, and loneliness scores, from pre- to post-intervention. Participant' depression, anxiety, and loneliness scores significantly reduced from pre- to post-course. Participants also experienced a significant improvement in mental well-being from pre- to post-course. Observed decreases in loneliness and depression did not significantly change from post-course to any of the follow-up periods. Results suggest the viability of a nonclinical program for addressing depression and loneliness in older individuals, and have implications for community-based strategies attempting to address mental health issues in older Canadians

### PSYCHOSOCIAL STATUS, RESILIENCE, AND DEPRESSION OF OLDER CAREGIVERS IN TAIWAN

Tsuann Kuo,[1] Hei-Yin Ng,[2] and Der-Yan Han[3], *1. Chung Shan Medical University, Taichung City, Taiwan, 2. Taipei Medical University, Taipei, Taiwan, Taiwan, 3. Taipei Medical University, Taipei City, Taiwan*

A rapidly increasing older population and changing family structures in Taiwan have resulted in a significant shift in caregiving resources; there are now more older caregivers, and their psychosocial well-being and quality of life may be affected as a result of their new responsibilities. This study used secondary data from the 2018 Survey of Elderly Mental Health (N=1,113) to examine the psychosocial status, resilience, and depression of caregivers who were 65 years old and older. The results showed that the typical older caregiver was 74.15 years old, likely to be married and living with a spouse (63.6%), retired (57.4%), and had a monthly income less than 30,000 NT (30.7%). Compared with caregivers under 65 years of age, older caregivers had higher co-morbidity and lower quality of life as well as greater resilience and higher levels of depression. Both positive emotions ($p<0.05$) and self-assessed health ($p<0.001$) were found to be significant variables affecting depression and resilience. And negative emotions ($p<0.001$) also influenced depression. This study was the first in the nation to tackle issues related to aging caregivers that can identify policy, research, and practice implications to improve the general health and lives of older caregivers. Future research can use the results to explore opportunities for older caregivers that will minimize negative emotions in order to build resilience and combat depression.

### TRADITIONAL NETWORK ON MENTAL HEALTH: SUPPORT FROM CHILDREN AND DEPRESSIVE SYMPTOMS FOR OLDER ADULTS IN RURAL CHINA

Lei Chen,[1] and Fernando Torres-Gil[2], *1. Department of Social Welfare, Luskin School of Public Affairs, University of California, Los Angeles (UCLA), Los Angeles, California, United States, 2. Department of Social welfare and Public Policy, Luskin School of Public Affairs, University of California, Los Angeles, Los Angeles, California, United States*

In rural China, support from children is the traditional network for older adults. Their mental health problem is poorly understood and remains unsolved. The study aims to explore the relationships between older adults' depressive symptoms and characteristics of support from children under the current social-structural conditions in rural China. The study is informed by the intergenerational solidarity theory and the theoretical framework of social relationships and their influence on health. The quantitative study is based on the recent wave of Harmonized China Health and Retirement Longitudinal Study (Harmonized CHARLS) in 2015, which is a high-quality national-level dataset. The study applies conditional process analysis to do the data analysis. The key findings include: the number of children has a negative association with older adults' depressive symptoms (c= -.2390, p=.0002<0.05); the number of children influenced older adults' depressive symptoms indirectly through financial support from children (If children do not live with older parents, ab=-.0141, CI= -.393 to -.0038; If children live with older parents, ab=-.0153, CI= -.369 to -.0056;). However, both theses direct and indirect relationships do not depend on the co-residence situation between older adults and their children. The controlling variables include age, gender, and self-rated health. Under China's current transition period of population policy, this study provides policy implications regards to the characteristics of children support and their influence on older adults' mental health, especially in rural China. This study also tests the two theories to some extent under the Chinese context, which were initially developed in Western countries.

### FACTORS ASSOCIATED WITH SEVERITY OF DEPRESSION: EVIDENCE FROM THE CHINA HEALTH AND RETIREMENT LONGITUDINAL STUDY

Mingyang Zheng,[1] and Xian Liu[2], *1. University of Minnesota - Twin Cities, Minneapolis, Minnesota, United States, 2. Adelphi University, Garden City, New York, United States*

Depression among older adults in China is widespread. To explore factors associated with depression among older adults, most studies focus on the individual as the unit of analysis. However, since individuals are nested in families, it is important to understand depression within a family context. To address this gap, the current study examines the degree to which individual and dyad-level characteristics were associated with the severity of depression among older couples in China. Data for the study were drawn from wave 4 of the China Health and Retirement Longitudinal Study. The total sample size in the study was 2560 older couples aged 60 and above. Multilevel modeling was used to analyze the dyadic data. Our preliminary findings suggested the partial intraclass correlation between a dyad's depression scores was 0.32, which shows that a couple's scores were similar to one another. Those who were female, were younger, lived in rural areas, had lower cognition, and those whose spouses had lower cognitive ability were associated with more severe depression. The findings provided empirical evidence to support the argument that more community mental health resources should be allocated to rural areas in China. Moreover, given that female older adults are more vulnerable to depression compared to male older adults, it is imperative to develop tailored services to support women to enhance their psychological wellbeing. In addition, as spousal cognition was negatively correlated with depression, services to support older couples where one has dementia are needed.

### CAN THE BRIEF INTERVIEW OF MENTAL STATUS (BIMS) IDENTIFY RISK FOR IMPAIRED IADLS DUE TO FUNCTIONAL COGNITIVE DEFICITS

Timothy Marks,[1] Dorothy F. Edwards,[2] Muhammad O. Al- heizan,[2] and Gordon M. Giles[3], *1. University of Wisconsin Madison, Madison Wisconsin, Wisconsin, United States, 2. University of Wisconsin Madison, Madison, Wisconsin, United States, 3. Samuel Merritt University, Oakland, California, United States*

The Brief Interview of Mental Status (BIMS) was introduced to the Minimum Data Set (MDS) 3.0 as a cognitive screening tool. It includes temporal orientation and word recall (Saliba et al., 2012). This study examined the ability of the BIMS to identify impairment on performance-based functional cognitive screening tests that assess instrumental activities of daily living (IADLs). We recruited a cross-sectional sample of 200 participants who met the following inclusion criteria: age 55 and older, living independently in the community, and able to read and communicate in English.. Participants ranged in age from 55 to 92 years (Mean 70.96:SD = 8.56),were predominantly White (68%) and female (65%). Participants were administered the BIMS and a battery of performance-based tests of functional cognition -the Performance Assessment of Self Care Skills and the Weekly Calendar Planning Activity (WCPA). There was a mismatch in screening results: Among Individuals identified as cognitively intact on the BIMS 22-45% were found to be impaired on the PASS and/or the WCPA. Sensitivity of the BIMS to identify impaired IADL function did not exceed .06, although specificities were high (< .95). These findings suggest that individuals categorized as cognitively normal by the BIMS may be impaired on more complex IADL tasks. Individuals classified as unimpaired on the BIMS, may benefit from more complex functional cognitive screening to further assess IADLs function to better estimate ability to live independently in the community. Performance based assessments can improve discharge planning by identifying elders at risk after hospitalization.

### EFFECTIVENESS OF A COLLABORATIVE STEPPED-CARE MODEL FOR OLDER ADULTS WITH DEPRESSION

Terry Lum,[1] Gloria Wong,[2] Tianyin Liu,[3] Shiyu Lu,[3] Dara Leung,[3] Lesley Sze,[3] Joyce Sing,[3] and Weiwei Kwok[2], *1. Department of Social Work and Social Administration, PokFuLam, Hong Kong, 2. The University of Hong Kong, Pofulam, Hong Kong, 3. The University of Hong Kong, Hong Kong, Hong Kong*

Background: Depression is common among older adults and creates a substantial burden on individuals, caregivers, and healthcare system. This paper presents an innovative collaborative stepped care intervention that promotes the coordination between elderly center and community mental health center to provide nonpharmacological intervention to elders with mild to moderate level of depression. Methods: The stepped care model were implemented in four districts in Hong Kong between September 2017 and February 2019. In each district, one community mental health center and one elderly center worked together to implement this stepped care model. A quasi-experimental design was used to study the effectiveness of this intervention. Findings: A total of 853

older adults completed the intervention and additional 500 elders were recruited as control. The average age of intervention participants was 76.3 years. Their levels of depression and anxiety were measured by Patient Health Questionnaire (PHQ-9) and Generalized Anxiety Disorder 7-item (GAD-7) respectively. The average intervention lasted for 10 months. Their average PhQ9 score reduced from 7.2 before intervention to 2.7 after intervention (t= 34.7, p < .001). Their level of anxiety was lowered from 4.9 to 2.0 (t=16.9, p < .001). The different between the intervention and control groups were statistically significant. Conclusion: The stepped care model was effective in reducing the levels of depression and anxiety among Chinese older people. This paper will give detailed information about the stepped care model and its implementation.

### PROTOTYPE OF A VIRTUAL REALITY APPROACH TO INTEGRATE MENTAL HEALTH ACROSS GERONTOLOGICAL ENVIRONMENTS

Daniel Velez Ortiz[1], *1. Michigan State University, East Lansing, Michigan, United States*

Background: Puerto Ricans have the highest likelihood of psychiatric disorders among Latinos. This study developed and evaluated a prototype depression literacy curriculum; culturally grounded with perspectives and narratives of Puerto Rican older adults. The way a person determines need for services and decides to seek help has been found to be influenced by their perceptions of services and providers. McGuire (1989) presents the Communication Persuasion Model (CPM) that takes into account how persuasive communication changes attitudes and behaviors of consumers. Using the CPM as a theoretical foundation, this study presented a culturally grounded story through a Virtual Reality (VR) platform. Methods: A script was developed based on narratives of Puerto Rican older adults about depression. Filmed in 360° format and enhanced with supporting imagery, participants were presented two versions of the video, one with a VR headset and the other with a smartphone. Two focus group interviews were conducted with community-dwelling Puerto Rican older adults (n=14) in Orlando, FL. Results: Participants preferred the VR headset and found it was beneficial to educate about depression because it felt more immersive and encouraged an environment conducive to identifying their own experiences about depression. They noted that presenting the material with a case narrative was more culturally sensitive for the population. All participants needed minor assistance with operating technology. Conclusions and Implications: A narrative approach to depression literacy may be effective in personalizing messages. Assisted VR technology with supporting imagery may be efficacious and standardize positive messages to underrepresented and low resource populations.

### DEPRESSION AND HYPERTENSION AS RISK FACTORS OF CARDIOVASCULAR EVENTS AMONG MIDDLE-AGED AND OLDER ADULTS IN CHINA

Lydia Li,[1] Zhenmei Zhang,[2] Hongwei Xu,[3] and Jinyu Liu[4], *1. University of Michigan, Ann Arbor, Michigan, United States, 2. Michigan State University, Michigan, United States, 3. Queens College in the City University of New York, New York, United States, 4. Columbia University, New York City, New York, United States*

Objectives: 1) Examine effects of depression and hypertension on cardiovascular events (CV) in a two-year period. 2) Explore urban and rural differences Methods: Data from the first two waves of Chinese Health and Retirement Longitudinal Study, with a national sample of 14,560 adults age 45+, were used. The dependent variable is whether a CV (defined as heart attack or stroke) occurred between baseline and W2 (1=Yes, 0=No), based on respondents' report at W2. Depression was dichotomized using a score of 12 on the 10-item CES-D. Hypertension was based on self-report. Logistic regression was conducted. Covariates included sociodemographic characteristics and nine other chronic conditions. All independent variables were measured at baseline. Results: About 5.3% (n=768) of the sample had a CV between baseline and W2. Depression increases the risks of CV by 67% for rural (OR=1.67, 95% CI=1.3, 2.12) and 42% for urban respondents (OR=1.42, 95% CI=1.05, 1.91). Hypertension increases the risk by 51% for rural (OR=1.51, 95%CI=1.18, 1.94) but is not statistically significant among urban respondents. Interaction effects of hypertension and residential areas are statistically significant ($\chi 2$ (1) = 6.44, p = .01) Conclusion and Discussion: Given the high cost associated with heart attack and stroke, treating depression is an effective approach to reduce health care cost. Hypertension increases the risk of CV for rural but not urban respondents. It may be that hypertension is not as well managed among rural residents as in their urban counterparts. Improving hypertension management among rural residents should be a priority in China.

### A SYSTEMATIC REVIEW OF DIGITAL INTERVENTIONS ADDRESSING MENTAL DISORDERS AMONG THE ELDERLY POPULATION

Md Mahbub Hossain,[1] Samia Tasnim,[1] Abida Sultana,[2] Nishat Tasnim Hasan,[3] Hoimonty Mazumder,[2] Nusrat Khan,[4] and Farah Faizah[5], *1. Texas A&M School of Public Health, College Station, Texas, United States, 2. Nature Study Society of Bangladesh, Khulna, Bangladesh, 3. Independent Researcher, College Station, Texas, United States, 4. University of Cambridge, Cambridge, United Kingdom, 5. United Nations Population Fund (UNFPA), Dhaka, Bangladesh*

Background: Many studies have reported the widespread application of digital technologies in improving mental health. However, little is known about how these technological advancements can help the geriatric population who suffer from a wide range of mental disorders. There is no extensive review of evidence which can guide effective policy-making and implementation of such interventions. Objectives: To identify digital interventions addressing mental disorders among elderly people and evaluate the outcomes of these interventions. Methodology: According to the PRISMA guidelines, we searched six major health databases and screened the literature using these criteria: 1) journal articles reporting an intervention delivered using any of the digital platforms, 2) the interventions aimed to improve at least one mental disorder among geriatric population, 3) articles published in English language, 4) studies conducted in in any settings and time frame reporting any of the mental health-related outcomes. Results: Among 4870 articles found in the preliminary literature search, only 19 studies met our criteria. Most of the studies (n=14) described digital interventions addressing depressive illness among the elderly population. However, many interventions targeted multiple mental conditions including dementia, stress, anxiety, mood disorders, phobia, and functional disabilities. These interventions used internet-based therapies, mindfulness, digital assistants, and applications improving mental health behavior and practices. Most of the interventions (n=12) were evaluated using randomized study designs. Reported outcomes included improved symptoms, better quality of living, emotional and functional advancements, and decreased cost of treatment. This evidence necessitates further research and application of such technologies to improve geriatric mental health.

### MINORITY & DIVERSE POPULATIONS I

### UNWAVERING STRENGTH IN THE FACE OF ADVERSITY: THE STRONG BLACK WOMAN, DEPRESSION, AND BEREAVEMENT

Danielle L. McDuffie,[1] Rebecca S. Allen,[1] Sheila Black,[1] Martha R. Crowther,[1] Ryan Whitlow,[1] and Laura Acker[1], *1. The University of Alabama, Tuscaloosa, Alabama, United States*

This study sought to investigate grief outcomes among older African American (AA) women following the recent loss of a loved one. Whether or not the "strong Black woman" (SBW) schema of presenting unwavering strength despite adversity was present in recently bereaved older AA women, specifically related to depressive outcomes, also was explored. Eleven AA women aged 46 years and older (M=64.2), completed one time, in-person semi-structured interviews detailing their grief experiences. Interview transcripts were then coded by a team using an inductive qualitative approach. Four themes emerged throughout the women's bereavement experience: 1) Acceptance of Loss and Preparation, 2) Coping as a Gradual Passing of Time, 3) Engaging in Other Activities to Cope with Loss, and 4) Helping Others Cope. About a third of the women in the sample reported being clinically depressed in accordance with the specifications for Major Depressive Disorder reported in the DSM 5. AA women in the sample were found to portray not only the stoicism consistent with the SBW schema, but also themes consistent with embodying the schema during their bereavement experience. More research and attention should be paid to AA women's manifestations of depression, under the knowledge that AA women may have nontraditional depressive symptom presentations. Further, the tendency for the SBW schema to emerge during bereavement should be addressed with AA women in clinical practice, as lack of awareness of the use of this mechanism could lead to exacerbated, adverse adjustment.

### CORRELATES OF FAMILY CAREGIVING FOR OLDER ADULTS AND EMOTIONAL STRAIN AMONG AFRICAN AMERICANS

Fei Wang,[1] and Ann W. Nguyen[1], *1. Jack, Joseph and Morton Mandel School of Applied Social Sciences, Case Western Reserve University, Cleveland, Ohio, United States*

Objectives: Despite the growing older African American population and its increasing needs for informal care, few caregiving studies have focused specifically on African Americans. This study aims to 1) identify demographic correlates of caregiving for older family members among African Americans and 2) identify caregiving and demographic correlates of emotional strain among African American caregivers. Method: Logistic regression and linear regression were based on the African American sub-sample of the 2015 Caregiving in the U.S. Survey (N=260). Demographic characteristics included age, gender, education, income, marital status, co-residence of care recipient in the caregiver's home, relationship of care recipients to caregivers, and household size. Caregiving characteristics included hours of caregiving and whether respondents provide care for an older adult. Results: With respect to demographic correlates of family caregiving, older respondents were more likely to provide care for an older family member, and respondents were more likely to provide care to a parent/parent-in-law than to other relatives. Regarding emotional strain, age and household size were negatively associated with emotional strain, and hours of caregiving was positively associated with emotional strain. Discussion: This study identified demographic profiles of family caregiving and emotional strain. It also suggested the presence of unique risk and protective factors among older African American caregivers. Future research should test the underlying mechanisms between these factors and mental health outcomes for a better understanding of how caregiving strain can be attenuated.

### LIFETIME EXPERIENCES OF GRIEF AMONG RECENTLY BEREAVED AFRICAN AMERICANS

Danielle L. McDuffie,[1] Rebecca S. Allen,[1] Sheila Black,[1] Martha R. Crowther,[1] Ryan Whitlow,[1] and Laura Acker[1], *1. The University of Alabama, Tuscaloosa, Alabama, United States*

This study sought to investigate the ways recently bereaved African American middle to older aged adults conceptualized both prior and present loss. Fourteen African American men and women aged 46 years and older (M=62.6) completed one time, in-person semi-structured interviews detailing their grief experiences. Interview transcripts were then coded using a content analysis. Four themes were reported during prior loss (Continuing on with Normal Life/ Time, Faith/ Religion, Reminiscing/ Reminiscence, Social Support) along with present loss (Faith/ Religion, Keeping Busy, Reminiscence, Social Support). Men and women in the sample were found to cope in relatively consistent manners despite the timing of the loss, and in manners consistent with literature detailing African American grief outcomes. This information could help inform both bereaved African Americans and those seeking to aid African Americans during times of bereavement in proactively having knowledge of coping mechanisms that have been used historically and found to be beneficial.

### GENDER DIFFERENCES IN RECEIVING ASSISTANCE WITH DAILY CARE NEEDS AMONG OLDER BLACK AMERICANS LIVING WITH DIABETES

Ledric D. Sherman,[1] Chanee D. Fabius,[2] Timethia Bonner,[3] and Erica Spears[4], *1. Texas A&M University, College Station, Texas, United States, 2. Johns*

*Hopkins University, Baltimore, Maryland, United States, 3. Centers for Disease Control and Prevention, Atlanta, Georgia, United States, 4. Auburn University, Auburn, Alabama, United States*

Older Black Americans living with diabetes often receive social support critical to their self-care management practices and quality of life. Studies have reported positive relationships between support and diabetes care among this population, although gender differences exist, with men reporting better quality of life outcomes associated with diabetes self-management than women. More information is needed to to assist healthcare providers indeveloping gender-tailored interventions to improve diabetes self-management. Using data from the 2015 National Health and Aging Trends Study (NHATS; Round 5), a nationally representative sample of Medicare beneficiaries aged 65 and older, our cross-sectional study describes gender differences in receiving assistance with self-care, mobility, and household needs among older Black adults with diabetes (N=621). Participants were majority female (59% Females; 41%Males). Bivariate analyses showed women were often older with fewer years of education, lower incomes, and were more likely to live with others than men. A larger share of respondents reported receiving assistance with household activities (34%; e.g. shopping, medication administration), followed by self-care (21%; e.g. bathing, dressing), and mobility tasks (17%; e.g. getting around inside and outside of the house). Binary logistic regression showed that women were more likely to report receiving assistance with all three tasks after adjusting for age, education, income, living arrangements, number of health conditions, and self-rated health. Future research should identify the relationships between caregivers and care recipients (e.g. spouse/partner, children), as receiving support with daily needs has the potential to impact both the health and quality of life of both caregivers and care-recipients.

### POSITIVE AND NEGATIVE SOCIAL INTERACTIONS ON MENTAL HEALTH IN OLDER KOREAN AMERICANS: GENDER DIFFERENCES

Nan Sook Park,[1] Yuri Jang,[2] Min-Kyoung Rhee,[2] David Chiriboga,[1] and Soondool Chung[3], *1. University of South Florida, Tampa, Florida, United States, 2. University of Southern California, Los Angeles, California, United States, 3. Ewha Womans University, Seoul, Korea, Republic of*

While there is substantial documentation of a positive relationship between objective social engagement and mental health, relatively little is known about how perceived quality of social interactions affects mental health and how men and women differ. Considering the gap, the purpose of this study was to investigate gender difference in how social interactions associate with self-rated mental health in older Korean Americans. Data came from a survey with older Korean Americans aged 60 or over that included 713 men and 1437 women living in five sites (California, New York, Texas, Hawaii, and Florida), conducted during 2017–2018. In multiple regression models run separately for men and women, self-rated mental health on a five-point scale (excellent/very good/good/fair/poor) was regressed on four blocks of variables: socio-demographic characteristics (age, marital status, education, financial status, self-rated health, and region), immigration-related variables (length of stay in the U.S. and acculturation), social engagement (family network, friend network, and activity participation), and perceived quality of social interactions (positive or negative family interactions and negative community interactions). In the final models with all covariates, younger age, more years in education, better physical health, higher levels of acculturation, and more positive family interactions were commonly associated with more positively rated mental health for both men and women. For women, stronger family network and fewer negative family interactions were additional contributors. Results suggest that negative and positive indicators of family interactions differentially affect self-rated mental health for older Korean American men and women.

### ADAPTIVE GARDENING PRACTICES AMONG OLDER AFRICAN AMERICANS IN DETROIT

Jessica C. Robbins,[1] and Kimberly Seibel[1], *1. Wayne State University, Detroit, Michigan, United States*

It is well established that gardening can promote physical, social, and emotional wellbeing for many older adults in varied circumstances (Milligan, Gatrell, and Bingley 2004; Nicklett, Anderson, and Yen 2016; Wang and MacMillan 2013). In post-industrial cities formed by historical and ongoing processes of structural inequality such as Detroit, Michigan, gardening is beneficial for residents in terms of health, economic activity, community-building, and city beautification (Lawson 2005; Pitt 2014; Pothukuchi 2015; White 2011). However, research has less frequently investigated how gardening can promote wellbeing for older adults living in contexts of urban structural inequality. This poster addresses this gap by exploring how older African American gardeners in Detroit adapt their gardening practices to changing physical abilities and capacities. Drawing on ethnographic research conducted during one gardening season (March-October 2017) with older African Americans in Detroit (n= 27), we employ a selective-optimization-with-compensation framework (Baltes and Baltes 1990) to understand the modifications that older Detroiters make in their gardening practices as they age. Findings demonstrate that older African Americans in Detroit engage in gardening in flexible, creative ways that accommodate new physical limitations, while also connecting to changes occurring in the city of Detroit. This study thus has implications for further understanding how gardening can benefit older adults, and how older adults can contribute vitality to contexts of structural inequality.

### HEALTH-RELATED QUALITY OF LIFE AND CARDIOVASCULAR DISEASE IN ASIAN AMERICAN AND PACIFIC ISLANDER OLDER ADULTS

Lan Doan,[1] Yumie Takata,[1] Karen Hooker,[1] Carolyn Mendez-Luck,[1] and Veronica L. Irvin[1], *1. Oregon State University, Corvallis, Oregon, United States*

Cardiovascular disease (CVD) is the leading cause of death for Asian American (AA), Native Hawaiian, and Pacific Islander (NHPI) older adults, and AAs/NHPIs have not enjoyed decreases in CVD mortality rates, as have non-Hispanic whites (NHWs). Heterogeneity exists in the prevalence of traditional CVD risk factors for AAs/NHPIs. Health-related quality of life (HRQOL) reflect physical and mental burdens beyond clinical burdens, which may help explain discrepant CVD rates and risk factors in AAs/NHPIs. We examined HRQOL among NHW and AA/NHPI Medicare Advantage enrollees with and without a CVD (i.e., coronary artery disease, congestive heart failure, myocardial infarction, and stroke) using the Medicare Health Outcomes Survey. The sample included 655,914 older adults who were 65 years or older, self-reported as AA/NHPI or NHW, and were enrolled in Medicare Advantage plans in 2011-2015. HRQOL was measured using the Veterans RAND 12-item survey and is composed of a physical component score (PCS) and mental component score (MCS), where higher scores reflect better physical and mental health, respectively. Multivariable linear regression was used to explore HRQOL and CVD prevalence. Asian Indian, Filipino, Vietnamese, Other Asian, and NHPI subgroups had lower overall PCS, and all AA/NHPI subgroups had lower overall MCS, compared to NHWs. Among those reporting having any CVD, PCS varied by CVD outcomes and subgroups, whereas MCS was lower for all CVD outcomes and for all but one AA/NHPI subgroups (Japanese), compared to NHWs. Attention to mental health for AA/NHPI older adults could be important for the equitable realization of healthy aging.

### EXAMINATION OF TRUST IN THE HEALTH CARE SYSTEM AMONG OLDER IMMIGRANTS RESIDING IN THE MIDWEST

Robyn Husa,[1] Hisako Matsuo,[1]
Jennifer Hale Gallardo,[2] and Lisa Willoughby[1], *1. Saint Louis University, St. Louis, Missouri, United States, 2. CINDRR-Gainsville, Gainesville, Florida, United States*

Ethnic minority populations, such as immigrants, have demonstrated lower levels of trust in the health care system and their health care providers compared with non-migrant populations (Navaza et al., 2012; Renzaho, Polonsky, McQuilten, & Waters, 2013). This medical mistrust may adversely influence older adult immigrants' use of and satisfaction with health services (Hong et al., 2018a; Jang, Kim, & Chiriboga, 2005). Thus, the current project aimed to characterize influences of medical mistrust (healthcare system and healthcare providers) in older adult immigrant populations living in the United States of America (U.S.). We interviewed 142 older adult immigrants and refugees (aged 60+ years) who identified as Bosnian, Chinese, Indian, Korean, Latino, and Vietnamese about their perceptions on living in the U.S., of the healthcare system, and healthcare utilization as a part of the Successful Aging among Immigrants in Midlife (SAIM) project. Linear regression models predicting trust in the healthcare system and trust in healthcare providers were tested with healthcare knowledge (measured with a single item about flu shots) , acculturation, length of time in the U.S. , and age as predictors. We found that older age and healthcare knowledge was predictive of higher levels of trust in healthcare providers for Chinese. Although healthcare knowledge was predictive of trust in the health care system for the Chinese participants, greater length of time and higher acculturation were associated with higher trust in the healthcare system among Indian participants. The implications of the different predictive variables in each of the hypothesized models will be discussed.

### THE SOCIAL DETERMINANTS OF LATE-LIFE MIGRATION IN THE U.S.: FINDINGS FROM THE NEW AMERICANS PROJECT

Arati Maleku,[1] Megan Espana,[1] Sharvari Karandikar,[1]
Njeri Kagotho,[1] Rupal Parekh,[2] and Shannon Jarrott[1], *1. The Ohio State University, Columbus, Ohio, United States, 2. University of Connecticut, Hartford, Connecticut, United States*

Globally, late-life migration has been a growing phenomenon. Literature on aging and migration however, has primarily focused on immigrant populations who migrated early in life. To expand our conceptualization of aging and to plan for the care of growing older immigrant populations, it is crucial to understand the compounding effects of late-life migration and aging in new spaces. Drawing on the qualitative data (N=71) from a large-scale community-based participatory research project in a mid-western U.S. region, we examined the social determinants of late-life migration on the health and well-being of older immigrants by exploring: (a) barriers and facilitators of socio-cultural adaptation, (b) patterns of human service provision in a local context, and (c) societal patterns of caring for older immigrants in places of relocation. Life course and social convoy perspectives formed the conceptual basis of the study. Using Respondent Driven Sampling method, data collection included six focus group discussions (n=48) with immigrant communities and in-depth interviews with human service providers (n=23). Data analysis followed the Rapid and Rigorous Qualitative Data Analysis technique that generated six salient themes: cultural context of aging; challenges of late-life migration; broken convoy and social isolation; gender and age intersections; human services, and community efforts and solutions. Findings suggest that late-life migration is a conglomeration of losses and gains, contingent on complex determinants such as living arrangements, language, transportation, the built environment, inter-generational relationships, socio-economic status, and social convoys. We conclude with a call to develop age-friendly, culturally responsive human services and health policies.

### FACTORS ASSOCIATED WITH MEANINGFUL ACTIVITIES AMONG ETHNICALLY DIVERSE OLDER ADULTS

Dolapo O. Adeniji,[1] and Michin Hong[2], *1. School of Social work, Indiana Purdue University Indianapolis, Indianapolis, Indiana, United States, 2. Indiana University, Indianapolis, Illinois, United States*

Objectives: With aging, there is an increased chance for older adults to experience negative health outcome and lose independence. Previous studies have shown the positive influence of engagement in physical, religious, recreational and social activities on healthy aging. This study aims to examine the factors contributing to the frequency of activity participation that helps older adults achieve the goal of staying healthy. Method: A sample (n=480) aged 60-90 with M=74.31, SD=7.65, and female 55.6 % from Well Elderly II were surveyed for the study. Hierarchical regression analysis was performed to examine factors associated with the frequency of activity participation. Age, ethnicity, education and gender controlling for meaning ascribed to activity were entered in model I. While the independent variables: social support, perceived mental and physical health and meaning ascribed for activity participation were in model II. Model II (R2=57.8%) significantly improved the prediction of the outcome variable greater than model I (R2=13.4%). Results: Results showed that age, education, ethnicity, perceived mental health, physical health, social support were significant. Of all variables the meaning attributed to activity participation (β =59.2%, <.001) is the most predictor of meaningful frequency of activity participation in older adults. This implies that the meaning individuals attribute to activity participation increases their frequency of activity participation. Also, age, education, and ethnicity play important role in understanding the importance of consistency in activity participation. Implications: Meaningful activity participation should be encouraged to enable older adults increase their participation in activities that promote healthy living.

### PAIRING STORIES AND EDUCATIONAL CONTENT FOR A CULTURALLY CELEBRATORY HEALTHY AGING COMMUNITY RESOURCE

Raina L. Croff,[1] Edline Francois,[1] Caroline Lawrence,[2] Zoe Rothberg,[2] Juell Towns,[3] Patrice Fuller,[1] Andre Pruitt,[1] and Jeffrey Kaye[1], *1. Oregon Health & Science University, Portland, Oregon, United States, 2. Lewis and Clark College, Portland, Oregon, United States, 3. Portland State University, Portland, Oregon, United States*

Culturally celebratory programming exceeds cultural relevancy, engaging participants in celebration-making and culture-creating. African Americans aged 55+ in the Sharing History through Active Reminiscence and Photo-imagery (SHARP) study celebrate their heritage in gentrifying neighborhoods through walking-reminiscence sessions; they create culture through discussing ideas, beliefs, norms, values, and shared experiences of the past while considering these aspects within presently changing cultural dynamics. The SHARP study's narrative approach supports cognitively healthy behaviors and community priorities of cultural preservation in response to marginalization. The SHARP smartphone application houses 72 themed walking routes in Portland, Oregon's historically Black neighborhoods. One-mile routes with GPS-triggered historical neighborhood images prompt conversational reminiscence among walking triads. Recorded narratives are organized in a process called storytabling and thematically coded. Selections referencing cognitively healthy behaviors are flagged for pairing with online brain health content tested by 12 African Americans aged 55+. Historical images and narratives anchor educational content to relatable life experiences, framing healthy aging in a culturally celebratory, neighborhood context to improve applicability. The online resource, routes, and recorded narratives are community deliverables. Currently, 254 walking narratives from 2016-2018 walkers (n=40; 8 with mild cognitive impairment) have been transcribed and 60 analyzed. Walkers found image prompts and walking within triads of similar sociocultural backgrounds as highly motivating, healthy ways of addressing change. Content testers found narratives lent depth, meaning, and a sense of cultural resilience to educational content. Narrative approaches situate cognitive health in participant-driven terms and experiences, informing brain health best practices for marginalized and minority populations.

### WALKING AND TALKING ABOUT WHAT USED TO BE: THE SHARP NEIGHBORHOOD WALKING PROGRAM FOR OLDER AFRICAN AMERICANS

Juell Towns,[1] Patrice Fuller,[2] Edline Francois,[2] Raina L. Croff,[2] and Jeffrey Kaye[2], *1. Portland State University, Portland, Oregon, United States, 2. Oregon Health & Science University, Portland, Oregon, United States*

The Sharing History through Active Reminiscence and Photo-Imagery (SHARP) study aims to preserve African American cognitive health through neighborhood walking and social engagement in a way that celebrates Black culture. For 6 months, African Americans aged 55+ (2016 n=19; 2017 n=21) grouped in triads walked 1-mile routes accessible via the SHARP application. Routes included historical image prompts about Portland, Oregon's historically Black neighborhoods. Participant focus groups at months 1, 3, and 6 drove program development and refinements, and provided valuable insight into the program's meaning for participants. Discussions were thematically coded. Emergent themes included "suggested improvements," "technology," "mental health impact," "cultural incongruence," and "cultural significance." Participants suggested improvements to the application's navigational aspects and expressed willingness to engage technology despite initial apprehension. The triadic structure and place-based memory prompts aided reminiscence, allowing participants to make meaningful links between their own life experiences and their walking partners'. Neighborhood walking brought to the surface participant concerns about a lack of understanding between African American generations, and between long-time residents and whiter, wealthier demographics moving in. Some participants found it emotionally taxing to walk in the now gentrified historically Black neighborhoods, but still saw the program overall as useful, interesting, and necessary--to their physical and cognitive health, to their mental health as they processed neighborhood changes and community loss, and as an important contribution to preserving community history. Addressing individual health alongside pressing issues affecting older African Americans' sense of well-being and community may make cognitive health programs more meaningful and applicable.

### GEROTRANSCENDENCE OF ALASKA NATIVE ELDERS AGING SUCCESSFULLY IN THE ALEUTIAN AND PRIBILOF ISLANDS

Jordan P. Lewis,[1] and Eric Wortman[2], *1. University of Alaska Anchorage, Anchorage, Alaska, United States, 2. UW School of Medicine, Anchorage, Alaska, United States*

Meeting the healthcare needs of Alaska Native (AN) Elders in remote communities is critical to support successful aging and this study allows AN Elders from the Aleutian region to share their experiences and define successful aging, supporting the limited research on AN successful aging. This study interviewed 19 Elders in two communities from the Aleutian region of Alaska. Using a 20-item questionnaire based on Kleinman's explanatory model to explore successful aging and experiences of being an Elder. Thematic analysis was employed to identify the characteristics and activities of Elders coping and adapting to aging-related changes. This study identified 5 core elements of successful aging, 4 of which formed Lewis's AN model of successful aging (2011): Mental and Emotional Wellbeing, Spirituality, Purposefulness, and Physical Health and Mobility, and the new element of Gerotranscendence. The unique finding of this study that expands Lewis's model is the change in mindset Elders experience as they self-reflect. Elders describe being more intentional in their relationships and a stronger connection to traditional cultural and spiritual activities, described by Tornstam (2005) as gerotranscendence. This research will be used locally to develop community specific health promotion and prevention programs to improve Elder services. These findings can also be used by health care providers to help Elders find meaningful activities that promote health and teach individuals to cope with aging-related changes.

### THE MEDIATING ROLE OF ADVANCE CARE PLANNING IN ACCULTURATION AND USE OF NURSING HOME AMONG KOREAN AMERICANS

Soyeon Cho[1], *1. City University of New York-New York City College of Technology, Brooklyn, New York, United States*

The study examined attitudes towards Advance Care Planning (ACP) as a potential mediator in the association between acculturation and willingness to use nursing home in Korean American older adults (aged 60 and older). Data were driven from a cross-sectional study of 235 community-dwelling Korean American older adults (aged 60 and older) in 2013. Multivariate regression models of willingness to use nursing home were entered in the following order: (1) demographics, (2) health, (3) acculturation, and (4) attitudes towards advance care planning. The mediation effect of attitudes towards ACP in the relationship between acculturation and willingness to use nursing home was separately examined using the bootstrapping method. Higher acculturation was associated with positive attitudes towards ACP and more likelihood of using nursing home. The proposed mediation model was fully supported: positive attitudes towards ACP served as an intervening step between acculturation and willingness to use nursing home. The mediating role of attitudes towards ACP yields implications for developing culturally sensitive advance care planning education program targeting older individuals.

### ROLE OF RELIGIOUSNESS AND SPIRITUALITY AND SOCIAL NETWORKS IN DETERMINING DEPRESSION AMONG OLDER KOREAN AMERICANS

Soonhee Roh,[1] Yeon-Shim Lee,[2] and So-Young Park[3], *1. University of South Dakota, Sioux Falls, South Dakota, United States, 2. San Francisco State University, San Francisco, California, United States, 3. New York University, New York, New York, United States*

Korean Americans (KAs) are one of the fastest growing minority populations in the U.S. Depression is the most common psychological problem among older KAs. While the relationship between religiousness/spirituality (R/S) and well-being in later life is an important health concern, older KAs are often affiliated with a protestant church and have the highest church participation. This study assessed the role of R/S and social networks in determining depressive symptoms and identified the best predictors of depressive symptoms. Data were drawn from a cross-sectional survey with 200 older KAs residing in New York. Best-subsets regressions were used to evaluate the best predictors of depression. Findings indicated that nearly 30% of older KAs reported experiencing mild or severe depressive symptoms. The best model fit for depression involved physical health, R/S coping skills, social networks, and annual household income. Social networks and R/S coping skills were found to be a protective factor against depressive symptoms and may be an effective tool for health care strategies in the management of depression and health-promoting behaviors. Careful assessment of R/S and social networks among older KAs may provide a more comprehensive approach to traditional, biomedically derived clinical practices by focusing on the whole person in early identification of risk factors, prognosis, and intervention for depression. Health education and intervention could be framed in ways that strengthen such psychosocial coping resources for older KAs. Facilitating social participation and mobilizing R/S resources in a wide range of personally meaningful activities may mitigate psychological distress and enhance life satisfaction.

### SESSION 2410 (POSTER)

### MOBILITY, DISABILITY, AND PHYSICAL ACTIVITY

### FEELING AUTHENTIC DURING PLAYING PICKLEBALL IN LATER LIFE: PREDICTING POSITIVE PSYCHOLOGICAL FUNCTIONING

Jungsu Ryu,[1] Jinmoo Heo,[2] Chungsup Lee,[3] Amy Chan Hyung Kim,[4] Kyung Min Kim,[5] and Hyunmin Yang[6], *1. Marshall University, Huntington, West Virginia, United States, 2. Yonsei University, Seoul, Korea, Republic of, 3. California State University, Long Beach, Long Beach, California, United States, 4. Florida State University, Tallahassee, Florida, United States, 5. University of Miami, Coral Gables, Florida, United States, 6. Texas A&M University, College Station, Texas, United States*

Authenticity, being trustful with oneself, is regarded as a principle predictor of healthy functioning. However, the association between authenticity and psychological functioning has not been examined, even though leisure is an ideal context within which to experience authenticity. Therefore, this study examined the association between authenticity and psychological functioning in older adults playing pickleball. A convenience sample of 112 males and 96 females (64.11±6.56, 50 to 82yrs) was recruited from the 2017 U.S. Open Pickleball Championship which is an annual international pickleball tournament. The participants were asked to fill out a questionnaire primarily asking about their levels of authenticity (4-items) and psychological functioning measured by both perceived stress (4-items) and happiness (single item). The pearson correlation tests found higher levels of authenticity were significantly correlated with being less stressed ($r = -.373$, $p < .01$) and happier ($r = .203$, $p < .01$). A two-step hierarchical regression was used to determine the unique contribution of authenticity to either perceived stress or happiness, and found that authenticity uniquely contributed to 10% of the variance in perceived stress ($F= 4.678$, $p <.001$) and 2.3% of the variance in happiness ($F= 3.046$, $p <.01$). These results suggest that feeling authentic in older adults playing pickleball may play an important role in positive psychological functioning.

### PREDICTING FUNCTIONAL DECLINE IN OLDER ADULTS: MORE ACTIVITY NOW EQUALS LESS DECLINE LATER

Brenda Whitehead[1], *1. University of Michigan-Dearborn, Dearborn, Michigan, United States*

According to the Function Spiral Model (Whitehead, 2017), aging attitudes influence activity engagement, which impacts functional ability via physical conditioning (or deconditioning). This study tests the activity ◊ conditioning ◊ function segment of the model using 59 older adults aged 61-92 (Mage = 76 at Time 1) who participated in 2 in-person assessments of physical health, gait, and function, spaced 3 years apart. Participants also completed mail-in questionnaires, reporting engagement in activities (walking, gardening, household chores, social clubs, etc.) at each time point. Hypotheses were 1) a lower activity level at Time 1 would predict greater decline in physical function across the 3-year span, and 2) that this effect would be mediated by changes in physical conditioning. Dependent t-tests revealed that both physical function—as indicated by the timed Get Up and Go test—and physical conditioning—as indicated by peak respiratory flow—declined during the period. The regression model testing the effect of activity engagement at Time 1 on decline in physical function (controlling for age, baseline function, and activity change) supported hypothesis 1 (-0.43, p = .003): more activity at Time 1 predicted less decline in physical function over time. Instead of supporting the mediation hypothesis, the model including both activity and conditioning demonstrated the strength of the activity at Time 1 effect, which actually increased in magnitude (-0.48, p = .001). Although the hypothesized mediation was not supported, the findings highlight current activity engagement as an important mechanism for slowing the progression of future age-related functional decline.

### A PRESCRIPTION FOR WELLNESS: EXERCISE REFERRALS FOR PATIENTS AT A FEDERALLY QUALIFIED HEALTH CENTER

Gabriel Benavidez,[1] and Kelly Ylitalo[1], *1. Baylor University, Waco, Texas, United States*

Physical activity improves quality of life and prevents or delays chronic disease, but most adults in the United States are inactive. Consultation and planning with a health care provider, specifically with an exercise “prescription,” may increase physical activity, but utilization patterns and success of such programs are not well understood. This study assessed the initial 6 months of an exercise prescription program at a large, federally-qualified health center during 2018 whereby adult patients were referred via prescription to personalized health coaching by a fitness advisor. A census of all adults (n=512) who received an exercise prescription was combined with attendance data from the on-site exercise facility to classify patients as never attended, 1 to 3 visits, and ≥4 visits. Ordinal logistic regression was used to examine patient characteristics from the electronic health record that influenced exercise facility attendance. Only 30.2% of adults (mean age 44.7 years (SD 14.4)) completed ≥1 visit and 21.7% completed ≥4 visits. We identified no significant utilization differences by sex, race/ethnicity, body mass index, diabetes, hypertension, or coronary artery disease, but adults aged ≥60 years had almost twice the odds of ≥4 visits (OR=1.97; 95% CI: 1.18, 3.33; p=0.01) compared to younger patients. Many adult patients did not participate in the exercise prescription program, but older adults were more likely to participate. Exercise prescription programs with personalized health coaching may be useful for older adult patients receiving care at a federally-qualified health center. Future work will examine if or how exercise prescriptions impact chronic disease self-management.

### EXPLORING FUNCTION OVER TIME IN OLDER ADULTS WITH ADVANCED HEART FAILURE

Theresa Floegel,[1] Alexander Schoemann,[1] and Catherine Taylor[1], *1. East Carolina Unviersity, Greenville, North Carolina, United States*

Heart failure contributes to functional limitation in older adults, which is associated with rehospitalization and poorer health. However little is known about the trajectory of function after hospitalization in this population or its' association with readmission. Objective measurement over time to assess physical function trajectory in older adults with heart failure is needed. Purpose: To explore trajectory of function in older adults with heart failure who have history of hospitalization. Methods: Exploratory longitudinal design. Bi-weekly visits were conducted over 4 months with home-dwelling, ambulatory adults with heart failure (60+ years, NYHA classification III) who had a recent acute hospitalization. Function was assessed by Short Physical Performance Battery (SPPB) test and hand-grip strength (9 time-points). Descriptive analyses across time and groups comparison were performed. Results: Participants (N=10) were 75.3±4.6 years (2 female, 5 African American), with EF(%) 39±10 and 8.2±2.9 comorbidities. Half (n=5) were re-hospitalized during the study period. Those re-hospitalized scored an average 1.3 points lower on SPPB and had 2.03 kg less hand strength over time than those not re-hospitalized. An SPPB score <6 was observed across time in 51% of those re-hospitalized, compared to 31% of those remaining at home. Intra-individual function varied but did not decline over this time period. Conclusions: In a sample of older adults with heart failure, physical function ranged from some level of functional disability to functional dependence, with higher levels of disability observed in those re-hospitalized. A longer study period and larger sample may be needed to adequately assess physical function trajectory.

### EXAMINING SEX DIFFERENCES WITHIN THE RELATIONSHIP BETWEEN PHYSICAL ACTIVITY AND EXECUTIVE FUNCTION IN OLDER ADULTS

Genna Losinski,[1] Hilary J. Hicks,[1] Alex Laffer,[1] and Amber Watts[1], *1. University of Kansas, Lawrence, Kansas, United States*

Research has demonstrated sex-associated differences in physical activity and its benefits on cognition in older adults. The present study explored differential associations between moderate-to-vigorous physical activity (MVPA) and executive function, which is known to decline with aging. N = 53 older adults without cognitive impairment (M = 73.19 years,

SD = 6.53) wore accelerometers (Actigraph GT3X+) during 7 consecutive days. Activity intensity was categorized as light, moderate, or vigorous based on Freedson Adult Vector Magnitude cutpoints. Participants completed a battery of executive function tests: Digit Symbol Substitution Test, Verbal Fluency, Trail Making Test, and Stroop Color-Word Test. A cognitive composite score was created using confirmatory factor analysis. Women had a higher mean MVPA (4.57%) than men (2.64%, t (19.04) = -2.49, p = .022). However, executive function performance did not differ by sex (t (26.20) = 1.67, p =.107). The interaction between sex and time in MVPA did not predict performance on executive function, adjusting for age and education. Older age was the only significant predictor of poorer executive function (β = -0.038, p = .003). The current sample had limited engagement in MVPA (range 0.18-10.87%). These findings suggest that the amount of engagement in MVPA in a free-living environment may not be sufficient to demonstrate sex-associated differences in executive function performance. Future studies should explore executive function performance with other intensity levels and examine other areas of cognition.

### ACTIVITIES OF DAILY LIVING DIFFICULTIES AND TOILETING AMONG OLDER GHANAIANS: AN APPLICATION OF WHO-ICF FRAMEWORK

Kofi Awuviry-Newton,[1] Meredith Tavener,[2] Kylie Wales,[3] Paul Kowal,[4] and Julie Byles[5], *1. Priority Research Centre for Generational Health and Ageing, Faculty of Health and Medicine, The University of Newcastle, Newcastle, Australia, Australia, 2. Priority Research Centre for Generational Health and Ageing, Faculty of Health and Medicine, The University of Newcastle, Newcastle, NSW, Australia, 3. School of Health Sciences, The University of Newcastle, Newcastle, NSW, Australia, 4. The University of Newcastle, School of Medicine and Public Health, Faculty of Health and Medicine, Australia, 5. Priority Research Center for generational Health and Ageing, Newcastle, NSW, Australia*

The aim of the study was to analyze the prevalence of activities of daily living (ADL) difficulties among older Ghanaians and specifically how one ADL, toileting difficulty, predicts care and supports needs using the World Health Organization International Classification of Disability and Health framework (WHO-ICF). Toileting difficulty requiring upper extremity strength is among ADLs that can lead to functional loss of independence among older people globally. A sample of n=5,096 adults aged 50 years and older from the WHO Study on global AGEing and adult health (SAGE) Ghana Wave 1 was used to analyze difficulties with ADLs and toileting. Level of difficulty was assessed against 22 other functioning items from the interview. Out of the 22 functioning items, climbing one flight of stairs without resting was the most difficult activity to be completed by older Ghanaians, and difficulty eating being the least endorsed item. Toileting was ranked the 16th in terms of reported difficulty and was related to other ADLs. Logistics multivariate regression was used to analyze data. Including significant variables from the univariate analysis in parsimonious model based on WHO-ICF framework, age, self-report health, memory, bodily pain, short distance vision, stroke, neighborhood trust, toilet facility type, and religious meeting attendance, were significantly independently associated with toileting difficulty. Gender was significant at the univariate level but became insignificant after adjusting for body function and structural variables. Toileting difficulty was associated with factors across different components in the WHO-ICF making the WHO-ICF an appropriate tool for understanding health and disability.

### FACTORS ASSOCIATED WITH LIFE-SPACE CONSTRICTION IN LATER LIFE: EVIDENCE FROM THE HEALTH AND RETIREMENT STUDY

Moon Choi,[1] and Briana Mezuk[2], *1. KAIST Graduate School of Science and Technology Policy, Daejeon, Korea, Republic of, 2. University of Michigan School of Public Health, Ann Arbor, Michigan, United States*

This study aimed to examine factors associated with life-space constriction, using the data from the Health and Retirement Study, a nationally representative sample. We limited our analysis to those who were 65 years and older and answered to the 2012 experimental module on life-space (N=895; mean age=75.3; 59.4% women). Life-space was assessed with the modified version of the UAB Study of Aging Life-Space Assessment, ranging nine zones: room, home, own property, immediate neighborhood, town, community, county, state, and region. A series of logistic regression models were used to estimate odds ratios for life-space constriction by sociodemographic and health characteristics. The results showed that 3.0% and 6.7% of older adults reported that they had never been to places beyond their home and own property/apartment building for the past four weeks, i.e. the critical boundaries in terms of social isolation. The significant factor associated with the life-space constriction within home, immediate neighborhood, and town was physical mobility limitation (OR: 1.18, 1.09, 1.11, respectively), while the constriction within county was associated with education level (OR: 0.91). Driving a car was negatively associated with the life-space constriction within own property/apartment building and home (OR: 0.48 and 0.22, respectively). Policy makers need to pay more attention to social and environmental factors influencing social isolation among older adults such as transportation options and social class disparity.

### ACTIGRAPH'S LOW-FREQUENCY EXTENSION FILTER FOR ESTIMATING WRIST-WORN PHYSICAL ACTIVITY IN OLDER ADULTS

Hilary J. Hicks,[1] Alex Laffer,[1] Genna Losinski,[1] and Amber Watts[1], *1. University of Kansas, Lawrence, Kansas, United States*

Advancements in body-worn activity devices make them valuable for objective physical activity measurement. Research-grade monitors utilize software algorithms developed with younger populations using waist-worn devices. ActiGraph offers the low frequency extension (LFE) filter which reduces the movement threshold to capture low acceleration activity that is more common in older adults. It is unclear how this filter changes activity variable calculations in older adults. We investigated the effects of the LFE filter on wrist-worn activity estimates in this population. Participants were 21 older adults who wore the GT9X on their non-dominant wrist for 7 days in a free-living environment. Activity counts were estimated both with and without the LFE filter. Paired samples t-tests revealed that the LFE estimated significantly higher number of counts than non-LFE calculated counts per minute on all three axes ($p < .001$). Step count estimates were higher with ($M = 20{,}780.09$, $SD = 5300.85$) vs. without ($M = 10{,}896.54$, $SD = 3489.45$) the LFE filter, ($t(20) = -22.21$, $p < .001$). These differences have implications for calculations based on axis counts (e.g., Axis-1 calculated steps, intensity level classifications) that rely on waist-worn standards. For example, even without the filter, the GT9X calculated an average of 10,897 steps, which is likely an overestimate in this population. This suggests that axes-based variables should be interpreted with caution when generated with wrist-worn data, and future studies should aim to develop separate wrist and waist-worn standard estimates of these variables in older adult populations.

### INCREASING PHYSICAL ACTIVITY POST-KIDNEY TRANSPLANT: A PILOT RANDOMIZED CONTROLLED TRIAL

Tara O'Brien,[1] Cynthia Russell,[2] and Donna Hathaway[3], *1. The Ohio State University, Columbus, United States, 2. University of Missouri-Kansas City, Missouri-Kansas City, United States, 3. University of Tennessee Health Science Center, Memphis, Tennessee, United States*

Older kidney transplant recipients are at risk for graft failure and death due to lack of physical activity. Physical activity after transplant is the most modifiable non-pharmacological factor for improving physical function. One personal system intervention called, SystemCHANGE™ in combination with activity trackers, holds promise for increasing physical activity among this population. The purpose of this pilot randomized controlled trial was to evaluate the efficacy of SystemCHANGE™ on increasing average daily steps in older (age 60 and over) kidney transplant recipients from baseline to 6 months. The intervention group met monthly to implement a successful personal system solution based on their daily routines and step-data collected from the activity tracker. The control group received monthly educational information on healthy living with a transplant. Participants were randomized 1:1 to the intervention or control group. The sample consisted of 31 participants ($n = 15$ intervention, and $n = 16$ control). No significant differences were found at baseline among the groups for demographics, self-efficacy and health outcomes (blood pressure, weight, waist circumference, 6 minute Walk Test). However, the intervention group had greater increase in the average daily steps from baseline to 6 months (mean ± SD: $1511 \pm 2320$) as compared to the control group ($181 \pm 2419$). The between-group difference was of medium effect size ($d = .56$).The data suggests SystemCHANGE™ in combination with activity trackers may be feasible for older kidney transplant recipients to enhance daily steps.

### INDEX OF RELATIVE RURALITY AS A PREDICTOR OF PHYSICAL ACTIVITY CORRELATES FOR OLDER ADULTS

Mary K. Huffman,[1] and Steve Amireault[1], *1. Purdue University, West Lafayette, Indiana, United States*

According to the ecological model, physical activity (PA) results from interactions between personal (e.g., intentions), social (e.g., autonomy support), and environmental factors (e.g., walkability, rurality). However, past research has commonly used dichotomous measures of rurality (i.e., rural versus urban), which has limited our understanding of the relationship between rurality and other PA correlates. Therefore, the purpose of this study was to investigate the associations between rurality and known correlates of PA among older adults. Ninety-one older adults aged ≥ 60 years, without severe cognitive impairment, completed a questionnaire assessing PA intentions ($\alpha = 0.89$), autonomy support for PA ($\alpha = 0.91$), and walkability ($\alpha = 0.76$). The Index of Relative Rurality, a continuous, multidimensional measure of rurality, was used to evaluate the degree of rurality based on residential zip-code. Regression analyses revealed that the rurality-autonomy support association followed an inverted-U shape function ($p = 0.01$), whereas rurality was negatively associated with walkability ($p = 0.02$). Rurality was not associated with PA intentions; however, autonomy support was positively associated with PA intentions ($p = 0.01$). The use of non-dichotomous measures of rurality appears essential in our understanding of its association with other PA correlates. Failure to use such measures may result in incomplete portrayals of relationships.

### EFFECTS OF A SITTING REDUCTION INTERVENTION FOR OBESE OLDER ADULTS WITH DEPRESSION

Sarita A. Hemmady,[1] Dori Rosenberg,[2] Peggy Hannon,[1] and Jing Zhou[2], *1. University of Washington, Seattle, Washington, United States, 2. Kaiser Permanente Washington Health Research Institute, Seattle, Washington, United States*

Background: Little is known about the impact of sedentary behavior (SB) reduction interventions on older adults with obesity and depressed mood. An exploratory analysis examined behavioral and mental health effects of a SB reduction among participants with depressed moods. Methods: Participants were obese older adults (n=30, mean age=66, 77% female, 23% male, mean PHQ-8-Score=13.67) that were randomized to receive a sitting reduction intervention ( I-STAND); N=16) or a control condition (N=14) as part of a larger trial. Participants wore activPAL devices to assess sitting time at baseline and 12-weeks; they also completed the Patient-Health Questionnaire-8 (PHQ-8) to assess depressive symptoms. Linear regression models compared baseline and 12-week measures between groups adjusting for baseline values. A post-hoc qualitative analysis assessed ISTAND participant interview data. Results: I-STAND participants had greater reductions in sitting time than control participants by 57-minutes (p=0.04), as well as greater reductions in percent sitting time by 5.89-percent (p=0.03). Mean PHQ-8 scores were decreased by 0.14-points among the I-STAND group compared to the control (P=0.90). Qualitative themes included physical and social barriers to standing; varying perceptions of the presence of depression; physical health improvements (i.e. mood improvement) and perceptions of physical activity (i.e. feasibility to exercise). Conclusion: We found significant associations between sitting reduction and a SB intervention among older adults with obesity and depression, however this did not impact depressive symptoms. Further research should examine whether sitting reduction can improve mood or standing time among older adults with obesity and depressed mood.

### THE RELATIONSHIP OF DAILY STEPS TO MOOD AND ENERGY: APPLICATION OF STEPMATE (MOBILE APP FOR TRACKING EXERCISE)

Alycia N. Bisson,[1] Victoria A. Sorrentino,[1] and Margie E. Lachman[1], *1. Brandeis University, Waltham, Massachusetts, United States*

Physical activity is one of the most promising and accessible strategies to promote healthy aging. Yet, the majority of middle-aged and older adults do not engage in the recommended amount of exercise despite awareness of its widespread benefits. Smartphone apps have the potential to be valuable tools in tracking and encouraging physical activity; however, few apps incorporate successful behavior change strategies. Drawing from interviews with older adults, we created a new smartphone app to encourage and track daily walking, StepMATE (Mobile App for Tracking Exercise). StepMATE uses behavior change strategies including action planning and social support to help users determine where, when, and with whom they will walk. The app records steps and also uses experience sampling to assess mood and energy levels twice a day. Adults ages 50 and over (N=58) participated in a one-month study where they used the app to set their own walking goals and track their daily walking. Using multilevel modeling, we found that on days in which adults take more steps than their average, they report higher mood and energy. We also found that on days in which participants achieve their step goal, they report higher mood and energy than on days when they do not achieve their goal. Discussion will center on motivational approaches to behavior change among sedentary older adults.

### RELATIONSHIP OF AGING EXPECTATIONS, GOAL CONGRUENCE, AND ACTIVITY TO MUSCLE FUNCTION IN OLDER ADULTS

Murad H. Taani,[1] Christina Sima,[1] Immaculate Apchemengich,[1] Andrew Kaplan,[1] Michael Fendrich,[2] Rachel Schiffman,[1] and Scott Strath[1], *1. University of Wisconsin Milwaukee, Milwaukee, Wisconsin, United States, 2. University of Connecticut, Hartford, Connecticut, United States*

Poor muscle function is a major source of disability among older adults and leads to negative health outcomes including falls and fractures, exacerbating healthcare cost. This study was undertaken to understand: a) the characteristics of muscle function; and b) the relationship of self-management process variables (expectations regarding aging, goal congruence, and self-efficacy for physical activity) and physical activity self-management behavior to muscle function in a sample of older adults (N = 65) 75-93 years of age living in Continuing Care Retirement Communities. Using a descriptive correlational design, muscle function was measured with the Short Physical Performance Battery (SPPB) test and physical activity level with ActiGraph GT3X. Questionnaires included Expectations Regarding Aging and goal congruence scales and Physical Activity Assessment Inventory to assess self-efficacy. Pain was assessed by the PROMIS Pain Intensity 3a. Most participants (77%) performed poorly on the SPPB test. Controlling for pain, expectations regarding aging, goal congruence, self-efficacy and physical activity explained 46% of the variance in SPPB score. The model demonstrated that self-efficacy and light-intensity physical activity significantly explained 24.6 % of the variance in SPPB score; suggesting that low self-efficacy and decreased levels of light-intensity physical activity were significant predictors of low SPPB score. The findings demonstrate the need for more research documenting the underlying processes and risk factors for reduced muscle function. The potential benefits of this approach provide a basis for designing interventions to improve muscle function and delay the transfer to more restrictive living environments.

### INCIDENT DIFFICULTY IN INSTRUMENTAL ACTIVITIES OF DAILY LIVING: WHICH COMES FIRST?

Danielle L. Feger,[1] George W. Rebok,[1] Sherry Willis,[2] and Alden L. Gross[1], *1. Johns Hopkins Bloomberg School of Public Health, Baltimore, Maryland, United States, 2. University of Washington, Seattle, Washington, United States*

Background: Instrumental activities of daily living (IADLs) are necessary for successful independent living. Older adults may develop difficulty completing IADLs as they become physically and/or cognitively frail. The relative ordering in which IADLs deteriorate, and the importance of this ordering, is not well understood. Methods: Participants from the Advanced Cognitive Training for Independent and Vital Elderly (ACTIVE) study who reported no difficulty with IADLs at baseline were included. Individuals were followed up to 10 years for incidence of self-reported difficulty in 19 specific IADLs. The outcome of interest was time to any incident difficulty. We used Cox proportional hazards regression to estimate the hazard ratio (HR) of incident IADL difficulty for each IADL. Results: Of N=1,273 participants who contributed 6,144 person-years to the analysis, 887 developed difficulty with at least 1 IADL during the study period. The tasks in which participants reported difficulty earliest included giving self-injections (HR=5.69, [4.77, 6.79]), balancing checkbooks (HR=5.56, [4.32-7.16]), remembering often called numbers without having to look them up (HR=5.47, [4.55-6.59]), and household chores (HR=4.18, [3.43-5.11]). The last tasks to become difficult included keeping household expenses balanced (HR=0.07, [0.04-0.14]) and hanging up at the end of a phone call (HR=0.23, [0.09-0.56]). Conclusion: Independent older adults reported earlier difficulty with balancing checkbooks, remembering often called phone numbers, and doing household cleaning. Recognizing these early difficult tasks may facilitate early planning for family members and adoption of compensatory strategies.

### ASSOCIATION OF RESTRICTED LIFE SPACE MOBILITY WITH COGNITIVE FUNCTION WITHIN OLDER BLACKS AND WHITES WITH DIABETES

Olivio J. Clay,[1] Pamela Bowen,[1] Loretta Lee,[1] Gina McCaskill,[1] Olivia Affuso,[1] and Michael Crowe[1], *1. University of Alabama at Birmingham, Birmingham, Alabama, United States*

The Centers for Disease Control and Prevention have reported that approximately one of every eight older adults self-report experiencing confusion or memory loss that is becoming more frequent or getting worse. Thus, identifying individuals who are at-risk for cognitive problems is essential. The purpose of this investigation was to assess the relationship between life space mobility and cognition within older Blacks and Whites with diabetes. Baseline data from the University of Alabama at Birmingham (UAB) Diabetes and Aging Study of Health (DASH) were utilized. Multiple regression models adjusted for age, education, income, gender, and race were utilized to assess the association between restricted life space (a score of less than 60 on the UAB Life Space Assessment) and cognitive function as assessed by the Telephone Interview for Cognitive Status (TICS-M). The analytic sample consisted of 224 older adults with diabetes (mean age = 73.52) with 54% being female and 53% White. Of the participants, 75 (32%) had a restricted life space and individuals with restricted life space on average had cognition scores that were over 2 points lower than participants categorized as not having restricted life space (B = -0.18, $p < .01$). Additionally, Black participants had lower levels of cognition when compared to Whites in the covariate-adjusted models (B = -0.23, $p < .01$). Results of this investigation provide additional evidence to support the relationship between mobility and cognition. Longitudinal investigations assessing the association between mobility and cognition within older adults with diabetes are needed.

### CHRONIC KIDNEY DISEASE, MUSCLE WEAKNESS, AND MOBILITY LIMITATION

Kenzie Latham-Mintus,[1] Simit Doshi,[2] and Ranjani Moorthi[3], *1. IUPUI, Indianapolis, Indiana, United States, 2. Indiana University, Indianapolis, Indiana, United States, 3. Indiana University Purdue University Indianapolis, Indianapolis, Indiana, United States*

Objectives: Chronic kidney disease (CKD) is associated with increased mobility limitation. Prior research has documented that peripheral nerve abnormalities occur early in CKD and progressively worsen. Loss of balance, impaired muscle strength, and slow gait predispose older adults to falls and frailty. However, the current literature is limited by a lack of nationally representative data that includes objective measures of kidney disease and physical functioning. Thus, this research examines whether CKD is associated with muscle strength, balance, gait, and self-reported mobility limitations. Methods: Data come from the 2016 Health and Retirement Study (HRS). Estimated GFR, a measure of kidney functioning derived from creatinine levels in the blood, was used to classify CKD (i.e, eGFR<45 or Stage 3b CKD). Logistic and linear regression models were generated to examine the association of CKD with physical functioning, net of demographic characteristics (i.e., age, sex, race, and education) and comorbidities (i.e., obesity, pain, and number of diagnosed medical conditions). Results: In unadjusted models, CKD was significantly associated ($p<0.05$) with more mobility limitations, slower walking speeds, stronger grip strengths, and non-participation in balance tests. After adjusting for covariates, CKD ($\beta=-1.43$, $p=0.01$) was negatively associated with grip strength. In sex-stratified models, CKD was associated with slower walking speeds among men, whereas CKD was associated with more mobility limitations among women. Discussion: In a nationally representative sample of older adults, CKD was associated with poorer physical functioning on multiple measures. After adjusting for demographic characteristics and comorbidities, CKD was associated with increased muscle weakness.

### PERCEIVED PHYSICAL LITERACY FOR CHINESE ELDERLY QUESTIONNAIRE DEVELOPMENT: PRELIMINARY VALIDITY AND RELIABILITY

HAOCEN WANG,[1] and Barbara King[1], *1. University of Wisconsin-Madison, Madison, Wisconsin, United States*

Physical activity (PA) is an essential health prompting behavior. Unfortunately, in China, only about 12% to 40% of older adults met the PA recommendations. To guide the design of future PA interventions and policy-making, innovative solutions are needed to be introduced. Physical literacy, a relatively novel concept, has been recently introduced in the field of older adults' PA. This concept takes a holistic view of PA behavior, which proposed that a person need to be motivationally, physically, strategically, effectively, socially, and knowledgeably prepared to be and stay physically active. The aim of this study was to develop the Perceived Physical literacy for Chinese Elderly (PPLCE) questionnaire and to establish its reliability and validity. An item pool for the PPLCE was generated from literature and interviews with Chinese older adults. Expert panel reviews and cognitive interviews were applied to establish the face and content validity of the questionnaire. A convenient sample of 388 Chinese older adults was recruited to assess the psychometric properties of the PPLCE. The item-level analysis and exploratory factor analysis resulted in a 46-item self-report measure, consisting with four factors: motivation, physical competence, interaction with environment, sense of self, interaction with others, and knowledge and understanding. The Cronbach's alpha coefficient and test-retest reliability of the PPLCE were 0.88 and 0.79 respectively. A positive correlation between PPLCE and leisure-time PA was found ($r=0.43$). The PPLCE has the potential to be used as a valid and reliable measure to assess Chinese older adults' perceived physical literacy.

### MOBILITY TRAJECTORIES, HEALTHCARE SATISFACTION, AND PERCEIVED DISABILITY DISCRIMINATION AMONG OLDER ADULTS

Collin Mueller,[1] and Jessica S. West[1], *1. Duke University, Durham, North Carolina, United States*

Although functional mobility limitations are associated with increased healthcare needs in later life, little research explores how older adults with varying functional mobility trajectories experience healthcare quality. To this end, we explore the effects of functional mobility trajectories on differences in healthcare treatment satisfaction, perceived disability discrimination in healthcare settings, and perceived everyday disability discrimination. We analyzed 9 waves of the Health and Retirement Study (n=29,284, 1998-2014, ages 50-84). First, we estimate age-specific group-based trajectories of functional mobility across age using finite mixture models. Second, we use multinomial logistic regression to identify sociodemographic factors that place individuals at elevated risk of membership in each group. Third, we explore how membership in one disability trajectory group over another affects healthcare satisfaction, perceptions of everyday discrimination in the context of healthcare treatment settings, and perceived discrimination in everyday life. Regression models include clustered standard errors to account for heteroscedasticity across repeated observations of individuals over time. We identify six group-based trajectories of functional mobility limitation among aging Americans. Black, female, and less-educated Americans are at higher risk of membership in disadvantaged trajectories, characterized by more rapidly increasing counts of functional mobility limitations, than their counterparts. Disadvantaged functional limitation trajectories are associated with lower levels of healthcare satisfaction, higher levels of perceived physical disability discrimination in healthcare treatment settings, and higher levels of perceived physical discrimination in other contexts of everyday life. The present study advances our knowledge of how older adults experience healthcare settings and discrimination across functional mobility status trajectories.

### EXCURSION FROM HOME AND ECOLOGICAL PAIN IN OLDER ADULTS WITH KNEE PAIN

Mamoun M. Mardini,[1] Parisa Rashidi,[1] Subhash Nerella,[1] Dottington Fullwood,[1] Duane Corbett,[1]
Sanjay Ranka,[1] and Todd M. Manini[1], *1. University of Florida, Gainesville, Florida, United States*

Background: Pain is an essential factor in limiting life-space mobility. Ecological momentary assessment (EMA) is crucial to understanding pain intensity and frequency. This study evaluated a customized smartwatch app for daily ecological pain reporting and GPS (Global Positioning System) coordinates collection to understand the impact of pain on daily excursion from home in older adults who report knee pain. Methods: Participants (n=14, 73.2 +/- 5.4 yrs, 64% female) wore a smartwatch with a customized app called Patient Reported Outcome of Mood, Pain, and fatigue (PROMPT) for 6.5 (4.0) days. Participants were prompted in their free-living environment about their pain intensity (range 0-10) in the morning, afternoon and evening. Additionally, GPS data were collected at 15 min intervals throughout the day. Geodesic distance was used to calculate the distance from the home address. Daily pain values were binned into high and low levels to compare to maximum daily excursions. Results: Individuals with average daily pain > 2 traveled 4.1 fewer miles than those individuals reporting pain 2 exceeded a distance of 5 miles compared to 17.9% of those individuals reporting pain <= 2 (X2=6.89, p < 0.05). Conclusion: In older adults, higher level of knee pain is associated with a decline in life-space mobility. Using custom designed smartwatch applications provides new opportunity to investigate how pain impacts community mobility.

### SESSION 2415 (POSTER)

### RELIGION AND SPIRITUALITY

### THE RELATIONSHIP BETWEEN GLOBAL AND DAILY LEVELS OF PAIN AND RELIGIOUS COPING

Katherine L. Cheesman,[1] Brian Cox,[2]
Dylan M. Smith,[3] and Patricia A. Parmelee[1], *1. The University of Alabama, Tuscaloosa, Alabama, United States, 2. Alabama Research Institute of Aging, Tuscaloosa, Alabama, United States, 3. Stony Brook University, Stony Brook, New York, United States*

Objective: This research examines associations between global and daily levels of pain and the use of religious coping strategies among African American (AA) and non-Hispanic White (NHW) older adults with physician-confirmed knee osteoarthritis (OA). Methods: As part of a larger study of racial/ethnic differences in everyday quality of life with OA, 125 persons over the age of 50 completed a global measure of religious coping using the Brief RCOPE. Daily variability in pain and coping was assessed using a daily diary methodology consisting of 4 daily phone calls over 7 days. Hypotheses: Demographic characteristics (sex, race) were expected to predict religious coping at both the global and daily levels. Specifically, we expected women and AAs would use more religious coping than their male and NHW counterparts. Further, daily levels of pain were predicted to correlate with daily levels of coping. Results: AAs were found to engage in significantly more religious coping behaviors than NHWs at the global, but not daily, level. An intraclass correlation coefficient of .316 indicated sufficient within-person variability for the use of multi-level modeling to determine the association of daily pain and religious coping. Within individuals, pain was significantly lower on days when religious coping was not used. Implications: Results contribute to a growing understanding of how individuals use their religious beliefs to cope with daily pain and perhaps, to the formulation of more targeted therapies for individuals living with chronic illness. (Supported by R01-AG041655 D. Smith and P. Parmelee, PIs.)

### LIFE AFTER 100: IS GOD THE SECRET TO A SATISFYING AND PURPOSEFUL LIFE?

Nadia Firdauysa,[1] Jyoti Bhatta,[1] Alex J. Bishop,[1]
Tanya Finchum,[2] and James Grice[1], *1. Oklahoma State University, Stillwater, Oklahoma, United States, 2. Oklahoma Oral History Research Program, Oklahoma State University; Stillwater, Oklahoma, United States*

Data from N = 111 centenarians (M = 100.88; SD = 1.48) residing in Oklahoma was used to examine patterns in the relationship between the God oriented vs. non-God oriented longevity secrets and subjective well-being. Observational Oriented Modeling (OOM) was then used to conduct an ordinal analysis using concatenated ordering to produce degree of fitness between data and underlying patterns in life satisfaction and purpose-in-life across three time points. OOM is a data analysis method used to evaluate fitness of proposed patterns to data called PCC. Results indicated that centenarians maintaining a God-oriented longevity secret fit a decreased pattern in life satisfaction (PCC = 25.00, c-value = .09); whereas centenarians not maintaining a God-oriented longevity secret fit the same pattern (PCC = 49.18, c-value = .06). Meanwhile, centenarians having a God-oriented longevity secret fit a decreased pattern of purpose-in-life (PCC = 71.43, c-value =.12); whereas centenarians having a non-God oriented longevity secret fit the same pattern (PCC = 53.45, c-value = .28). In comparison to centenarians who acknowledged something other than God as the secret to their longevity, those who cite God as the reason for longevity tend to proportionately maintain a more satisfying view of life, yet experience a deteriorating sense of purpose over time. Results indicate that longevity secrets reflect divergent patterns in subjective well-being among persons living beyond 100 years. This has implications relative to how geriatric practitioners design interventions, services, or programs to enhance quality-of-life for long-lived adults.

### RELIGIOSITY AND HEALTH AMONG CHINESE OLDER ADULTS IN MAINLAND CHINA, HONG KONG, AND TAIWAN: A META-ANALYTIC REVIEW

Jia Li,[1] and Qi Wang[2], *1. The Hong Kong Polytechnic University, Hong Kong, Hong Kong, 2. University of Hong Kong, Hong Kong, Hong Kong*

Abstract: Religion plays an important role in people's later life. However, most existing studies on health and religiosity focused on Western settings. China has the largest aging population in the world and distinct contexts of religion. This study aims to examine the relationship between religiosity and health of Chinese older adults through a meta-analysis. We conducted a comprehensive database (English and Chinese) and gray literature searching. Two researchers independently extracted the studies and evaluated the quality of the eligible ones. A random-effect model was adopted to combine the results. Hedges' g was computed as a standardized measure of the effect size. Subgroup analysis was conducted to examine the potential moderators. From the 3776 potentially eligible papers, 74 were eventually included. The results showed that, having a religious belief or ever attending religious activities was significantly related to a higher level of anxiety (Hedges' g= 0.392, 95% CI[0.230, 0.556]), escape acceptance of death (0.477[ 0.154, 0.801]), death avoidance (0.498 [0.127, 0.870]), death anxiety (0.448[0.122, 0.774]), more positive coping practices (0.581[0.073, 1.094]), and subjective social support (0.313[0.143, 0.483]). However, the subgroup analysis did not conclude any significant moderators. Religiosity is significantly related to a variety of psychosocial characteristics of older adults, including both negative and positive traits. It calls for more future studies to investigate the competing mechanisms regarding how religiosity can influence older adults' health and vice versa.

### EXAMINING GENDER DIFFERENCES IN RELIGIOUS INVOLVEMENT AND DEPRESSION IN LATER LIFE

Hailey J. Santiago,[1] Caitlin Curtin,[1] Julia Stengel,[1] Edward H. Thompson,[2] and Andrew Futterman[3], *1. Loyola University Maryland, Baltimore, Maryland, United States, 2. College of the Holy Cross, Worcester, Massachusetts, United States, 3. Department of Psychology, Loyola University Maryland, Baltimore, Maryland, United States*

This study examines gender differences in a causal model of religious motivation, religious participation and depression. Using a random sample of 287 community-dwelling older adults living in Worcester, MA, the model hypothesizes that motivations for religious involvement (intrinsic vs. extrinsic) differentially predict religious participation (organizational and non-organizational) as well as depression at both initial and 12-month assessments. In this model, participation also mediates direct relationships between religious motivation and depression. Religious motivation and participation are assessed using standard measures (e.g., Allport & Ross, 1967; Ainlay & Smith, 1982), and depression is assessed both by self-report (CESD and by interview (Hamilton Rating Scale for Depression derived from the Schedule for Affective Disorders and Schizophrenia, SADS). Using MPlus, confirmatory analyses of the model were conducted separately in male and female samples. The model which includes both direct effects of religious motivation and participation on depression and with religious participation as mediating variable demonstrated reasonably good fit to the data in both male and female samples (e.g., CFI=.956 and .943, respectively). Consistent with previous research (e.g., McFarland, 2009), gender differences in the models emerge. For example, men report higher levels of religious participation and less depression than women. In addition, older men demonstrate stronger positive associations between extrinsic religiousness and organizational participation and a more negative association between extrinsic religiousness and depression, than older women. Elucidating the structural relationships among religious orientation, religious participation, and depression in older adults benefits our understanding of vulnerability and treatment of depression in this population.

### RELIGIOUS EXPERIENCE AS A KEY FACTOR IN THE DEVELOPMENT OF LATE-LIFE TRUE BELIEF

Austin Prusak,[1] Margaret Iapalucci,[1] Eoin Walsh,[1] Ann-Kathrin Wolfram,[1] and Andrew Futterman[2], *1. Loyola University Maryland, Baltimore, Maryland, United States, 2. Department of Psychology, Loyola University Maryland, Baltimore, Maryland, United States*

Hoffer (1951) argued that "true believers" are individuals who cannot tolerate questions about their views. In terms of "true" religious believers, such individuals would not be able to tolerate questions about religious doctrine. In this study, we examine potential causes of religious "true" belief in later life. Using a model of religious orientation developed by Allport (1950) and Batson (1991), we defined "true" religious believers as those individuals who describe deep and broad religious commitments (score high on measures of intrinsic religious orientation) but refuse to question or doubt their commitments (score low on quest religious orientations). Using logistic regression analyses, we examined predictors of religious true belief in a sample of 357 denominationally diverse older adults living in New England. Predictors in the model included denominational affiliation, religious knowledge, organizational and non-organizational religious participation, and religious experiences, as well as non-religious factors such as personality traits (NEO), severity of illness and self-rated health, overall life stress, and other background factors (SES, gender, age) Of these 14 predictors, one factor in particular stood out as a particularly robust predictor of membership in the "true" believer group (n=47) – high scores on self-reported religious experience. Religious life experiences may be influenced by cohort and personality, resulting in extremely high scores of the individual on the intrinsic orientation scale and extremely low scores on the quest scale; together these embody the "true believer".

### SESSION 2420 (POSTER)

### SLEEP

### PSYCHOSOCIAL FACTORS ASSOCIATED WITH SLEEP QUALITY AND DURATION AMONG OLDER ADULTS WITH CHRONIC PAIN

Catherine Zaidel,[1] Shirley Musich,[1] Jaycee Karl,[2] Shaohung Wang,[1] Sandra Kraemer,[3] and Charlotte Yeh[4], *1. Optum, Ann Arbor, Michigan, United States, 2. Optum, Minneapolis, Minnesota, United States, 3. UnitedHealthcare, Minneapolis, Minnesota, United States, 4. AARP Services, Inc., Washington, District of Columbia, United States*

Sleep complaints are common among older adults with pain. Due to the risk of side effects, sleep medications are not recommended. Little is known about the association between psychosocial factors and sleep, but further awareness could support non-drug strategies for poor sleep. Our objective was to determine prevalence of self-reported poor sleep and duration among older adults with pain; and examine associations of positive psychosocial characteristics on sleep. This study analyzed surveys and claims from older adults with AARP® Medicare Supplement plans insured by UnitedHealthcare. Participants were 65+ with diagnosed back pain, osteoarthritis and/or rheumatoid arthritis; 12-months plan enrollment. All participants responded to a survey in May 2018 assessing sleep quality. Prescriptions were determined from claims. Propensity weighting was used to adjust for non-response bias. Results were weighted to generalize to those with pain. Multivariate logistic regression was used to evaluate associations. Short sleep duration was most common (39%), followed by poor quality (22%), and long duration (9%). Higher resilience and diverse social networks were associated with good quality and duration. Strongest associations with bad quality and short duration were stress, depression and sleep medications. Psychosocial factors were strongly associated with sleep quality and duration among older adults with pain. Results underscore the importance of social factors on sleep and need for non-drug sleep strategies.

### ELECTRONIC USE AND SLEEP, SURPRISING BEDFELLOWS: RESTFULNESS EFFECTS OF ELECTRONICS USE PRIOR TO BEDTIME FOR THE 40-PLUS

G. Rainville,[1] and Cheryl Lampkin[1], *1. AARP, Washington, District of Columbia, United States*

Getting restful sleep is essential to well-being but stress and poor sleep habits may make sleeping through the night challenging. This research explored life event stressors and pre-sleep activities among 2,464 randomly selected Americans age 40 and older (using Ipsos' KnowledgeNetwork panel) to determine their joint effects on mental well-being. Respondents reported how often they engaged in twelve individual behaviors within an hour of going to sleep. These behaviors (found to be inter-correlated) were combined using EFA into four factors representing levels of engagement in each of four classes of pre-sleep activities: pre-sleep electronics use (e.g. texting/e-mail before bed), deep relaxation activities, reliance on sleep-aids, and "nightowl" behaviors (i.e., snacking). Counter to expectations, only electronics use had significant conditional effects on the path between a life events stressor index (a count of current, potentially stressful life events) and scores on the positively-framed Warwick Edinburgh well-being scale (WEMWBS). How often one sleeps through the night also had unexpected effects in a conditional path analysis. A somewhat-involved relationship emerges between each of the theoretically-relevant measures. First, the negative impact of stress is moderated by sleeping through the night. Sleeping through the night is, counter to previous studies on electronics use and sleep, mediated by the use of electronics prior to sleep. We propose that mechanisms (such as the nature of backlighting used in electronics) that hamper restfulness may be offset by relaxation effects or by setting one's ducks in a row by texting/emailing before going to sleep.

### VALIDATION AND CLINICAL USEFULNESS OF A SLEEP HEALTH SCALE IN LATE LIFE

Scott Ravyts,[1] Joseph Dzierzewski,[1] Elliottnell Perez,[1] and Emily Donovan[1], *1. Virginia Commonwealth University, Richmond, Virginia, United States*

Sleep health is a multidimensional construct of sleep and wakefulness which can be conceptualized as the opposite of sleep dysfunction. Assessing sleep health is particularly relevant among older adults who disproportionally experience sleep-related adverse outcomes. Yet, empirically-validated sleep health scales are lacking. The objectives of the present study were to assess the psychometric properties of a newly designed measure of sleep health (RU-SATED) among older adults and examine the association between sleep health and well-being in late-life. Data included 773 older adults (M=67.68, 52% female) who completed an online survey of their sleep and health. Respondents completed the six-item RU-SATED scale, the Insomnia Severity Index (ISI), and the Satisfaction with Life Scale (SWLS). Sleep health scores ranged from 1 to 12, (M=8.13, SD=2.68), with higher scores indicating better sleep health. Exploratory factor analysis revealed a one-factor model. Confirmatory factor analysis showed that a one-factor model was associated with model fit indices in the adequate range. Additionally, a hierarchical linear regression indicated that sleep health was positively associated with life satisfaction ($\beta$=.25, p<.001) and accounted for significant variance in life satisfaction above and beyond insomnia severity ($\Delta R2$=.04, p<.001). In conclusion, RU-SATED appears to be a valid measure of sleep health among older adults with potentially useful clinical applications. Future research would benefit from examining the association between sleep health and other relevant health outcomes, as well as assessing the prospective ability of sleep health to predict relevant outcomes above and beyond traditional measures of sleep quality or insomnia.

### REDUCING DYSFUNCTIONAL BELIEFS ABOUT SLEEP PROVIDES LONG-TERM BENEFIT IN OLDER ADULTS WITH INSOMNIA

Yeonsu Song,[1] Constance Fung,[2] Joseph Dzierzewski,[3] Michael Mitchell,[4] Karen Josephson,[2] Lavinia Fiorentino,[2] Jennifer Martin,[4] and Cathy Alessi[2], *1. School of Nursing,*

*UCLA, Los Angeles, California, United States, 2. VA Greater Los Angeles Healthcare System, Los Angeles, California, United States, 3. Virginia Commonwealth University, Richmond, Virginia, United States, 4. VA Greater Los Angeles Healthcare System, North Hills, California, United States*

Cognitive behavioral therapy for insomnia (CBTI) is recommended as first-line treatment in older adults. Changing dysfunctional beliefs and attitudes about sleep is an important component of CBTI, but the long-term impact of these changes are unknown, particularly in older adults. Methods involved secondary analyses of data from a large randomized controlled trial comparing CBTI (provided in 5 weekly sessions) to sleep education control, among older veterans with insomnia (N=159, mean age 72.2 years, 97% male, 79% non-Hispanic white). The purpose was to examine whether changes in a validated scale of Dysfunctional Beliefs and Attitudes about Sleep (DBAS) with CBTI treatment (baseline to post-treatment) was associated with later changes in self-reported sleep (post-treatment to 6 months follow-up). Sleep measures included Pittsburgh Sleep Quality Index (PSQI), Insomnia Severity Index (ISI), Epworth Sleepiness Scale (ESS) and 7-day sleep diary measures. Analyses compared the slope of change in DBAS (baseline to post-treatment) between CBTI and control with respect to the slope of change in sleep outcomes (post-treatment to 6-months). Compared to controls, the CBTI group had stronger associations between DBAS improvement (baseline to post-treatment) and subsequent PSQI improvement (post-treatment to 6-months) (difference in slopes=1.3, 95% CI=[.52,2.1], p=0.001). This pattern of significant results was also found for ISI (difference in slopes=1.8, 95% CI=[.58,3.0], p=0.004) and ESS (difference in slopes=1.0, 95% CI=[.25,1.7], p=0.009). Slopes were not different for sleep diary measures. These findings suggest that changing dysfunctional beliefs and attitudes may continue to confer sleep benefits well after completion of CBT-I in older adults.

### RELIGIOUS ATTENDANCE AND SLEEP DISTURBANCE IN OLDER MEXICAN AMERICANS

Terrence D. Hill,[1] Christopher Ellison,[2] and Lauren Hale[3], *1. University of Arizona, Tucson, Arizona, United States, 2. University of Texas at San Antonio, San Antonio, Texas, United States, 3. Stony Brook University, Stony Brook, New York, United States*

Although numerous studies have shown that religious involvement is associated with better health across the life course, researchers have virtually ignored possible links between religious involvement and sleep-related outcomes. Building on previous work, we tested whether religious attendance was inversely associated with sleep disturbance among older Mexican Americans. We also assessed whether depressive symptoms could mediate or explain any of the inverse association between religious attendance and sleep disturbance. Relevant hypotheses were tested using ordinary least squares regression and conditional process mediation analysis of cross-sectional data collected from the original cohort of the Hispanic Established Population for the Epidemiologic Study of the Elderly (H-EPESE). The baseline H-EPESE (1993-1994) included a probability sample of 3,050 Mexican Americans ages 65 and older. Due to missing data on our focal variables, our final analytic sample included 2,323 respondents. Regression models show that religious attendance is inversely associated with depressive symptoms and sleep disturbance, even with adjustments for smoking, drinking, body mass, chronic disease, mobility, marital status, living arrangements, family engagement, secular group participation, social support, age, gender, immigrant status, language proficiency, education, household income, and religious affiliation. Mediation analyses also indicate that depressive symptoms fully mediate the association between religious attendance and sleep disturbance. These findings contribute to previous work by showing that regular religious attendance may protect against sleep disturbance by promoting mental health in an understudied population of older Mexican Americans. The importance of religious involvement is supported by the fact that secular group participation was unrelated to sleep disturbance.

### RUMINATION, DEPRESSIVE SYMPTOMS, AND SLEEP QUALITY: SOCIAL SUPPORT AS A BUFFER

Christina M. Marini,[1] Stephanie J. Wilson,[2] and Lynn Martire[3], *1. The Center for Healthy Aging at The Pennsylvania State University, University Park, Pennsylvania, United States, 2. The Ohio State University College of Medicine, Columbus, Ohio, United States, 3. The Pennsylvania State University, University Park, Pennsylvania, United States*

Rumination, the act of dwelling on negative, unwanted thoughts, can stoke depression and disrupt sleep, both of which may threaten older adults' well-being. In line with a support buffering hypothesis, a previous study of younger and middle-aged adults found that social support mitigated the positive association between rumination and negative mood. To extend this research, we distinguished between spousal and family/friend support as moderators of rumination's links both to depressive symptoms and sleep quality among older adults. Data came from a sample of 128 adults who were, on average, 77 years old at study onset. Rumination was measured via the Rumination-Reflection Questionnaire. Perceived support was measured by items utilized in multiple nationally representative studies of older adults. Depressive symptoms were measured via the NIH PROMIS measure, and sleep quality was measured via items from the Pittsburgh Sleep Quality Index. Results indicated that support from family/friends (but not spouses) buffered the positive association between rumination and depressive symptoms, even after controlling for depressive symptoms six months prior. Conversely, when sleep quality served as the outcome, support from spouses (but not family/friends) buffered the negative association between rumination and sleep quality, even after controlling for sleep quality six months prior. Findings highlight the potential for specific sources of social support to buffer different consequences of rumination on older adults' health and well-being.

### OBJECTIVE AND SUBJECTIVE SLEEP DURATION AND ACTIVITY LEVEL IN OLDER ADULTS WITH MILD DEMENTIA

Miranda V. McPhillips,[1] Jinyoung Kim,[1] Darina V. Petrovsky,[1] Junxin Li,[2] Sonia Talwar,[1] Laurel Caffeé,[3] and Nancy Hodgson[1], *1. University of*

*Pennsylvania, Philadelphia, Pennsylvania, United States, 2. John Hopkins University, Baltimore, Maryland, United States, 3. University of Pennsylvania, Philadelphia, United States*

Little is known about the relationship between sleep duration and activities of daily living (ADLs) in those with mild dementia. We sought to examine the independent relationship between objective and subjective sleep duration and ADLs in community-dwelling older adults with mild dementia. Analyses were conducted on baseline data from participants enrolled in the Healthy Patterns Clinical Trial (Hodgson; R01NR015226). Measures included 24-hour wrist actigraphy for objective sleep duration, proxy-reported Pittsburgh Sleep Quality Index (sleep duration subscale) for subjective sleep duration and the Barthel Index for performance of ADLs. We used Spearman's correlation and multivariate linear regression. A total of 30 individuals (56.7% male) aged 74.6 (SD 7.4) with mean Clinical Dementia Rating (CDR) scores of 1 (SD 0.5) were enrolled. Objective sleep duration ranged from 2.7 to 11.5 with mean 6.7 (SD 2.4) hours; subjective sleep duration ranged from 4 to 13.5 with mean 7.9 (SD 2.4) hours. Longer objective and subjective sleep duration were significantly associated with worse ADL scores ($r = -0.48$, $p = 0.03$; $r = -0.59$, $p=0.007$, respectively) in bivariate analyses. After controlling for age, CDR, and depression, subjective sleep duration was independently associated with ADLs ($\beta = -1.90$, $p = 0.03$) and objective sleep duration trended toward significance ($\beta = -1.47$, $p = 0.10$). These preliminary results suggest self-reported longer sleep could be indicative of declines in ADLs in older adults with mild dementia. Further prospective studies are necessary to determine the independent association between objectively assessed sleep duration and ADLs in patients with mild dementia.

### COHORT DIFFERENCES IN SLEEP ENVIRONMENT, BEHAVIORS, AND CONCERNS: DESCRIPTIVE ANALYSES USING MY SLEEP SCRIPT APP

Christina Pierpaoli Parker,[1] Rachel Salas,[2] Charlene Gamaldo,[3] Alyssa Gamaldo,[4] and Peter Dziedzic[5], *1. University of Alabama, Birmingham, Alabama, United States, 2. Johns Hopkins Medicine, Baltimore, Maryland, United States, 3. Johns Hopkins Medicine, Johns Hopkins Sleep Disorders Center, Maryland, United States, 4. Penn State College of Health & Human Development, Assistant Professor, Human Development and Family Studies, Penn State, Pennsylvania, United States, 5. Johns Hopkins University School of Medicine, Director of The Center of mHealth and Innovations, United States*

Behavioral and environmental factors influence sleep outcomes. However, we understand little about how enviro-behavioral sleep hygiene practices and related sleep concerns vary across age cohorts. Using data from My Sleep Script, an app-based diagnostic checklist for identifying at risk patients, we described cohort differences in sleep hygiene and new sleep disturbances. 323 adults (46.6% female, 63.9% Caucasian) reported basic demographic, health, sleep, as well as enviro-behavioral data using the Epworth Sleepiness Scale (ESS), Pittsburg Sleep Quality Index (PSQI), and the Johns Hopkins Sleep Environment Instrument. We partitioned participants into four cohorts corresponding to birth year: The Silent Generation (N = 48, 14.9%), Baby Boomers (N = 124, 38.4%), Generation Xers (n = 109, 33.7%), and Millennials (N = 42, 13.0%). Spearman correlations described linkages among environment, behaviors, and sleep outcomes; a chi-square analysis, cohort differences in new sleep concerns. Having weapons, music players, lights, pets, and a disruptive sleep surface in the environment correlated with worse sleep quality. Eating, exercising, working, and sexual activity one hour before bed also correlated with worse sleep quality. Sleeping with pets, electronics, and on a disruptive surface correlated with lower sleep duration. Regarding cohort, we observed significant generational differences in new snoring and sleepiness complaints. Results confirm associations of suboptimal sleep hygiene with poor sleep outcomes and provide insights into their generational differences, warranting additional investigation.

### SESSION 2425 (POSTER)

### SOCIAL ISOLATION AND LONELINESS

### THE ISOLATION OF OLDER ADULTS: A COMPARISON OF JAPANESE GENDERS AND HOUSEHOLDS

Keiko Katagiri[1], *1. Kobe University, Kobe, Hyogo, Japan*

As the aging population increases, large changes have occurred among household structures in Japan. Half of older adults lived with their children's families in 1980. Now around 60 percent of them live by themselves: 27% in single households and 31% as older couples alone. Older adults in single households are said to be at higher risk of social isolation. A Japanese white paper reported that they had scanty of social interactions compared to other types of households. This study examines differences in the social relationships and health statuses among household types by gender and explores the risk factors of social isolation. Nationally representative 2012 Japanese Social Survey data were used for analyses; a subsample comprised participants aged 60 to 74 years. A series of ANCOVAs were conducted. The distribution of the gender and household types were single male 105 (10.0%), married male 387 (36.8%), single female 180 (17.1%), and married female 381 (36.2%); the main effects were being female and married. An interaction effect between them (single males were less happy than married males) was observed. Neighborhood relationships were better among females and married participants. Married participants were more active in community meetings, social participation, and volunteering. However, no difference was observed in social network size. Thus, network size alone was not related to social isolation, but being active in social relationships and the quality of relationships influenced social isolation and well-being. Being married and female may facilitate higher quality relationships and may lead to activity and buffer social isolation.

### IMPACT OF SOCIALIZATION IN LOW-INCOME URBAN ELDERLY COMMUNITIES

Taylor Wilkerson,[1] Schanea Ward,[2] Amy Popovich,[2] Pamela Parsons,[1] and Faika A. Zanjani[1], *1. Virginia*

*Commonwealth, Richmond, Virginia, United States, 2. Richmond City Health District, Richmond, Virginia, United States*

Older adults who experience social isolation have higher rates of mortality relative to their counterparts. Social interactions are an important way to combat this isolation. This research aims to better understand how social isolation in older adults living in low-income households in Richmond, Virginia (RVA) is related to their economic, physical, and psychological health status. As part of the VCU iCubed Health and Wellness Aging Core and in collaboration with the Richmond Memorial: East End Housing Coalition for Older Adults, older adults from a selected public housing unit (n=28) self-reported their financial status, experiences with physical and psycho-social health, and feelings of social isolation. Survey participants were 71.4% female, the mean age was 69.75 years, and 25% were high school graduates. Participants averaged 34 years living in the area and reported an average of $300 to spend on rent monthly. Overall, 55% (n=20) reported having two or more supports and 61% (n=22) reported hardly ever feeling isolated. However, a small subset of the sample reported having either no supports (5.6%, n=2) and 41.7% (n=15) lacked companionship some of the time or often. A one-way ANOVA was conducted and it was determined that participants who reported feeling left out more often were significantly more likely to report stress, anxiety, and depression (F[2, 25] = 6.998). Findings support the existence of supportive communities formed in low-income areas. Findings also indicate some older individuals residing in public housing in RVA experience social isolation, linking them to poorer psycho-social health.

### IMPACT OF OFFLINE AND ONLINE SOCIAL NETWORKS ON LONELINESS AND MENTAL HEALTH AMONG ITALIAN OLDER PEOPLE

Daniele Zaccaria,[1] Georgia Casanova,[1] and Antonio Guaita[1], *1. Golgi Cenci Foundation, Abbiategrasso, Italy*

In the last decades the study of older people and social networks has been at the core of gerontology research. The literature underlines the positive health effects of traditional and online social connections and also the social networks's positive impact on cognitive performance, mental health and quality of life. Aging in a Networked Society is a randomized controlled study aimed at investigating causal impact of traditional face-to-face social networks and online social networks (e.g. Social Network Sites) on older people' health, cognitive functions and well-being. A social experiment, based on a pre-existing longitudinal study (InveCe - Brain Aging in Abbiategrasso) has involved 180 older people born from 1935 to 1939 living in Abbiategrasso, a municipality near Milan. We analyse effects on health and well-being of smartphones and Facebook use (compared to engagement in a more traditional face-to-face activity), exploiting the research potential of past waves of InveCe study, which collected information concerning physical, cognitive and mental health using international validate scale, blood samples, genetic markers and information on social networks and socio-demographic characteristics of all participants. Results of statistical analysis show that poor social relations and high level of perceived loneliness (measured by Lubben Scale and UCLA Loneliness scale) affect negatively physical and mental outcomes. We also found that gender and marital status mediate the relationship between loneliness and mental wellbeing, while education has not significant effect. Moreover, trial results underline the causal impact of ICT use (smartphones, internet, social network sites) on self-perceived loneliness and cognitive and physical health.

### LONELINESS PREDICTS REDUCTIONS IN SLEEP QUALITY IN OLDER AMERICANS

Sarah Griffin,[1] Baylor Williams,[1] Scott Ravyts,[1] Joseph Dzierzewski,[1] and Bruce Rybarczyk[1], *1. Virginia Commonwealth University, Richmond, Virginia, United States*

Research documenting loneliness as a factor predicting health decline accumulates, yet the mechanisms underlying this relationship remain obscure. A potential mechanism is sleep disturbance, which is associated with loneliness. However, it remains unclear whether loneliness is a risk factor for subsequent sleep disturbance. The present study aimed to examine loneliness (measured via the Hughes Loneliness Scale) as a risk factor for sleep disturbance in a nationally representative sample of older (>65) Americans. Weighted linear regressions (accounting for complex sampling) were conducted on data from the Health and Retirement Study (n=3,042; 2006 & 2012 waves). Higher levels of loneliness in 2006 predicted sleep disturbance in 2014 when controlling for baseline sleep (B=.08, 95% confidence interval [CI]=[.04, .13]). This association remained after controlling for age, gender, race, ethnicity, education, net worth, and depressive symptoms (B=.07, 95% CI=[.04, .11]). These results identify loneliness as a risk factor for sleep disturbance over an eight-year span in older Americans. Further research is necessary to tease apart this relationship: specifically, to assess reciprocal effects over multiple timepoints, investigate the role of depression in loneliness and sleep disturbance, and employ experimental methods to address causality.

### THE INFLUENCE OF SOCIAL ISOLATION AND NEIGHBORHOOD DANGER ON OLDER ADULTS' FUNCTIONAL STATUS

Ilana J. Engel,[1] and Tamara A. Baker[1], *1. University of Kansas, Lawrence, Kansas, United States*

Social isolation is often associated with smaller social networks, bereavement, and chronic health problems. In addition, underserved neighborhoods, without the resources and social support of other areas, may further promote social isolation among older adults. This study utilized data from the 2nd wave of the nationally representative National Social Life, Health, and Aging Project (NSHAP) to examine if perceived neighborhood danger mediates the relationship between social isolation and functional impairment. We hypothesized that those who are less socially connected and feel less safe in their communities may experience worse health outcomes. Data for the total sample (N=1,804; 62-91 years of age) showed that partial mediation was supported, (F 2, 1801 = 22.91, p<0.01). Similar statistics were found by gender (men, F 2, 985 = 8.20, p<0.01; women, F 2, 813 = 14.79, p<0.01). This relationship, however, showed a stronger association among women ($\beta$ = -.39, p<.01) than men ($\beta$ = -.26, p<.05). Findings indicate that the relationship between perceived social isolation and impaired functional status may be partially explained by perceived neighborhood danger. These findings suggest that older adults who perceive their neighborhoods as dangerous, may be more socially isolated, and at risk for functional decline. These results support the Reserve Capacity Model, which posits that social resources are of increased importance for socioeconomically disadvantaged individuals. Additional research is needed to examine how such factors as stress, environment, and access to care contribute to our understanding of health outcomes among this population of adults.

### SOCIAL CAPITAL AND THE PET EFFECT

Judith Robertson R. Phillips,[1] Edith Jimenez,[1] and Heather Nicholson[1], *1. California State University San Marcos, San Marcos, California, United States*

Social capital such as positive relationships and social support play an important role in older adults' well-being. Typically, researchers have investigated family and friends as providers of interpersonal resources to older adults but there has been an increasing trend to explore companion pets as providers of social capital and to investigate the impact of pet companionship on older adults' psychological well-being. Inconsistencies have appeared in the literature though as to whether there is a "pet effect," the positive benefit of companion pets on older adults' psychological health. The purpose of this study was to investigate whether having a companion pet would provide greater social connection and better psychological well-being among 83 community-residing older adults (meanage = 62.87 years; males = 38; females = 45) who owned a companion pet, a dog (n= 53) , cat, (n= 21) or both (n= 9), versus 42 community-residing older adults (meanage = 65.69 years; males = 25; females = 17) who didn't own companion pets. Analyses revealed that no "pet effect" was found for any measure of psychological well-being: self-reported loneliness, happiness, life satisfaction, or mental health. This was especially true for cat owners in that the more one viewed a cat as a family member, the lower one's life satisfaction and happiness. In addition, as the number of cats in the household increased, the perceived social support from a significant other, family, and friends lowered. Discussion will focus on the implications of these results for pets as providers of social capital.

### IMPACT OF ICT AND SOCIAL NETWORKS SITES UTILIZATION ON OLDER PEOPLE'S WELL-BEING AND LONELINESS: A SCOPING REVIEW

Daniele Zaccaria,[1] Georgia Casanova,[1] and Antonio Guaita[1], *1. Golgi Cenci Foundation, Abbiategrasso, Italy*

In the last decade the debate on ageing issues has been powered by the relationship between older people wellbeing, loneliness, Information Communication Technology (ICT) and Social Networks Sites (SNSs). A scoping review on social experiment studies, analysing the casual effect of technologies use on older adults' wellbeing and loneliness, has been realized to support the randomized controlled trial included in "Aging in a Networked Society" project. The study aims to review the social experiments on the relationship between technology-use, older people wellbeing and loneliness, to provide a critical analysis of studies, to underline drivers and barriers in existing literature and to provide recommendations for future study and policy. 133 papers have been selected using interdisciplinary search engines (Scopus, Pubmed, Web of sciences, Google Scholar), taking into account contents and methods used. An in-depth examination of 9 experiences of social experiment have been provided, focused on six dimensions: "aims", "design", "sampling", "intervention", "findings" and "limitations". The literature show the positive effects of ICT, internet and SNSs use on older people wellbeing and quality of life. Our review underlines how the older people shown interest in ICT use to support their social relations, and how it should become a social driver. The low widespread of experimental studies impacts on the literature evidences. The main limits are related to the recruitment and sampling. Social experiment practices, based on controlled randomized trial, should be widespread to better support the evidences in ageing issue.

### LONELINESS SHAPES THE RELATIONSHIP BETWEEN ICT USE AND PSYCHOLOGICAL ADJUSTMENT AMONG OLDER ADULTS

Yang Fang,[1] Anson Chau,[2] Helene H. Fung,[2] and Jean Woo[3], *1. Nanyang Technological University, Singapore, Singapore, 2. Chinese University of Hong Kong, Hong Kong, Hong Kong, 3. The Chinese University of Hong Kong, New Territories, Hong Kong*

Background: Given findings that generally support the benefits of information and communication technology (ICT) for older adults' psychosocial adjustment, one might surmise that lonely older adults, who have a stronger need for psychological support, would reap more psychosocial benefits from ICT use. However, scant research has examined this view, much less the likelihood that ICT use might worsen the psychological well-being of lonely older adults, as has been shown to be the case in younger adults. Objective: To examine whether the association between ICT use and psychological adjustment (i.e., psychological distress and sense of community [SOC]) among older adults depends on their loneliness levels. Methods: A representative sample of 738 Hong Kong SAR Chinese older adults aged 60 years or older (56% female) was interviewed in 2017 on loneliness, frequency of ICT use (i.e., Internet and smart devices), psychological distress (6-item Kessler scale; K6), and SOC. Results: Regression analyses showed that loneliness significantly moderated the relationship between ICT use frequency and psychological adjustment (psychological distress and SOC); more frequent ICT use was associated with more psychological distress and less SOC, with higher levels of loneliness. Conclusion: These findings suggest that ICT use may be a mixed blessing for older adults, i.e., using more ICT might predict worse psychological adjustment among lonelier older adults. Efforts that promote ICT use among older adults should take their loneliness levels into account.

### DETECTION OF DEPRESSION USING THE UCLA THREE-ITEM LONELINESS SCALE IN OLDER CHINESE

Tianyin Liu,[1] Gloria H.Y. Wong,[1] Jennifer Y.M. Tang,[2] Shiyu Lu,[1] Dara Leung,[1] Lesley Sze,[1] Joyce Sing,[1] and Terry Lum[3], *1. The University of Hong Kong, Hong Kong, Hong Kong, 2. Sau Po Centre on Ageing, The University of*

*Hong Kong, Pok Fu Lam, Hong Kong, 3. Department of Social Work and Social Administration, PokFuLam, Hong Kong*

Loneliness is a significant and independent risk factor for later life depression. This study aimed to validate the Chinese version of the UCLA 3-item Loneliness Scale and to ascertain the cut-off point in detecting depression in community-dwelling older adults. The English version of the UCLA was translated into Chinese by six experienced social workers, clinical psychologists and researchers, and content validity was established by consultation and revision with 10 older adults. 1,919 older adults aged 60 years and over (average age = 76.3±8.0) were recruited from local NGOs, they were interviewed for demographic information, and assessed using the 3-item loneliness scale and the Patient Health Questionnaire (PHQ-9). Cronbach's α of the Chinese loneliness scale was 0.87; the average score was 4.0 ± 3.0 out of 9, and it significantly correlated with living alone ($r = 0.18$, $p < 0.001$), unmarried ($r = 0.12$, $p < 0.001$), no emotional support ($r = 0.14$, $p < 0.001$), and depression ($r = 0.41$, $p < 0.001$). Using PHQ-9 cut-off score of 5 for mild and above depression, the area under the curve (AUC) was 0.73 ± 0.1 ($p < .001$, 95% CI 0.71-0.76) with 77% sensitivity and 61% specificity. We determined a cut-off point of 3 for loneliness using the Youden index, which revealed better sensitivity over alternative definitions of loneliness. A cut-off point of 3 in the Chinese UCLA 3-item loneliness scale can reliably identify possible depression in community-dwelling older Chinese.

### RAMBLE ON: LONELINESS AND OFF-TOPIC VERBOSITY IN OLDER ADULTS

Leah N. Smith,[1] Jessica H. Helphrey,[1] Jennifer Sawyer,[1] Leigh A. Fierro,[2] Ben K. Mokhtari,[1] Jenna M. Moore,[1] Daniel Rodriguez,[1] and Michael D. Barnett[1], *1. The University of Texas at Tyler, Tyler, Texas, United States, 2. The University of Texas at Tyler, Texas, United States*

Numerous studies have found off-topic verbosity occurs more frequently in older adults than younger adults. Previous theories have attributed this to age-related decline, emotion recognition, and communication style. Previous research has linked lower loneliness with more off-topic verbosity; however, the precise nature of this relationship remains unclear. Loneliness has been defined as an inconsistency between an individual's actual and desired social relationships, and previous research has found that loneliness is associated with lower cognitive and social outcomes among diverse populations including older adults. The purpose of this study was to investigate the relationship between loneliness and off-topic verbosity among older adults. Healthy, community dwelling older adult participants ($N = 82$; age 60-99, $M = 76.66$, $SD = 8.52$) completed the Three-Item Loneliness Scale and provided a verbal sample in which they recounted an autobiographical memory (a vacation) and a procedural memory (how to make a breakfast); the verbal samples were transcribed and rated by three independent judges. In contrast with previous research, results found that loneliness was associated with a greater tendency to engage in tangential verbal topics. This suggests that social factors such as loneliness may impact the way some older adults express themselves verbally.

### HOPE AND RESILIENCE AMONG VULNERABLE, COMMUNITY-DWELLING OLDER PERSONS

Dennis R. Myers,[1] Clay Polson,[1] Jocelyn S. McGee,[2] and Rachel Gillespie[1], *1. Garland School of Social Work-Baylor University, Waco, Texas, United States, 2. Baylor University, Waco, Texas, United States*

Community-dwelling older adults in the U.S. are at risk for experiencing a number of physical, emotional, and social issues including poverty, social isolation, and deteriorating health and daily functioning. Despite such challenges, research indicates that many older adults remain resilient and that factors such as social support, spirituality, and self-esteem contribute to resilience and improved outcomes. One factor that has been found to be particularly important for resilience among older adults is a sense of hopefulness. However, research has not looked specifically at the effects of hope on older adults experiencing severe economic and psychosocial challenges. Utilizing survey data drawn from a unique sample ($n = 64$) randomly drawn from 224 clients of a Meals on Wheels program, we explore the relationship between hope and resilience among a group of at-risk, community-dwelling older adults in one central Texas community. We find that hope, after accounting for the effects of social support, spiritual experience, health (ADL), and ethnicity, is a strong and significant predictor of resilience among at-risk older adults and that hope tends to mediate the effect of spiritual experience on resilience. Drawing on these findings, we discuss potential implications for social workers and congregational leaders working with older adults and for future scholarship on hope and resilience.

### LONELINESS AND HYPOCHONDRIASIS AMONG OLDER ADULTS: THE MEDIATING ROLE OF INTOLERANCE OF UNCERTAINTY AND ANXIETY

Jenna M. Moore,[1] William P. Archuleta,[2] Jessica H. Helphrey,[1] Leah N. Smith,[1] Jennifer Sawyer,[1] David W. Rose III,[1] Christopher Reed,[1] and Michael D. Barnett[1], *1. The University of Texas at Tyler, Tyler, Texas, United States, 2. University of North Texas, Denton, Texas, United States*

Loneliness is prevalent among older adults and is associated with adverse outcomes for health and mortality. Additionally, researchers have suggested that loneliness may cause a person to direct attention inward and become preoccupied with bodily symptoms which may subsequently lead to health anxiety. However, little extant research has examined the association among older adults. In this study, we proposed a loneliness model of hypochondriasis in which loneliness contributes to hypochondriasis through intolerance of uncertainty and anxiety. Healthy, community-dwelling older adults ($N = 280$; 64.4% female; age range: 65-95; $M = 76.08$, $SD = 7.59$) completed an interview survey. Loneliness was associated with higher hypochondriasis and had an indirect effect on hypochondriasis through intolerance of uncertainty and anxiety. Lonelier older adults may have an activated threat system which prompts greater intolerance of uncertainty and anxiety and thereby results in greater hypochondriasis.

### SUBJECTIVE HEARING IMPAIRMENT AND LONELINESS IN COMMUNITY-DWELLING OLDER ADULTS

Alison Huang,[1] Jennifer A. Deal,[2] George W. Rebok,[3] Jay Pinto,[4] Linda J. Waite,[4] and Frank Lin[5], *1. Department of Mental Health, Johns Hopkins Bloomberg School of Public Health, Baltimore, Maryland, United States, 2. Johns Hopkins University Bloomberg School of Public Health, Baltimore, Maryland, United States, 3. Johns Hopkins Bloomberg School of Public Health, Baltimore, Maryland, United States, 4. The University of Chicago, Chicago, Illinois, United States, 5. Johns Hopkins University, Baltimore, Maryland, United States*

Loneliness in older adults is most often attributed to marital and living status, social life factors, and physical health. Hearing impairment, however, is an understudied, potentially modifiable risk factor for loneliness. Older adults with hearing impairment experience difficulties with communication and social functioning, which also could contribute to loneliness. For this analysis, we used data from Wave 2 of the National Social Life, Health, and Aging Project. Participants (N=3,174) were a nationally representative sample of community dwelling older adults aged 62 - 91 years. Poisson regression models with robust variance were used to model the cross-sectional relationship between self-reported hearing impairment and loneliness. We found a dose-response relationship such that individuals reporting very good/good and fair/poor hearing had a 9% [95% CI: 0.93 - 1.28] and 26% [95% CI: 1.10-1.46], respectively, higher prevalence of loneliness compared to individuals reporting excellent hearing, adjusting for chronic conditions, functional and cognitive ability, and demographic factors. Results were robust to exclusion of participants who reported hearing aid use. These findings suggest that self-reported hearing impairment is a strong factor associated with loneliness in older adults. Given the negative implications of loneliness on multiple facets of mental and physical health, functional ability, and premature mortality, efforts to further explore hearing impairment as a causal and modifiable risk factor for loneliness should be undertaken.

### SESSION 2430 (POSTER)

### SOCIAL ISOLATION, LONELINESS AND SOCIAL NETWORKS

### GENDER DIFFERENCES IN CARE USE IN THE NETHERLANDS: CHANGES BETWEEN 1995 AND 2016

Mari Aaltonen,[1] Dorly Deeg,[2] and Marjolein Broese van Groenou[3], *1. Tampere University, Faculty of Social Sciences, Tampere, Finland, 2. Amsterdam University Medical Centres, location VU University Medical Center, Department of Epidemiology and Biostatistics, Amsterdam, Netherlands, 3. VU University Amsterdam, Faculty of Social Sciences, Amsterdam, Netherlands*

In recent decades, care policy in the Netherlands reduced budgets for residential care and formal home care, which increased the demand for informal care. Women use formal care more often than men, but we lack information on the extent to which the gender gap in care use is explained by differences in individual chracteristics and changes in care policy. Data from the Longitudinal Aging Study Amsterdam (LASA) were employed to explore the gender gap in the use of informal, formal and private home care, community services, and residential care in the years 1996-2016, analyzed using Generalized Estimating Equations (GEE). The data consisted of 9,497 observations, gathered from 3,369 respondents aged 65-85. Women used all types of formal care more than men. The gender differences persisted even when individual characteristics were taken into account; however, only in residential care the differences diminished after care preferences were included in the analysis. During the study years, the gender gap increased in formal home care and in non-use of care, as women increasingly used formal home care and the proportion of men without care expanded. The gender gap in informal care use reversed, with men using more informal care during the earlier years and women using more in the later years. The persistent and even increasing gender differences in care use deserve further exploration of the role of gender in current care culture. The growing gender gab in non-use of care raises concern for older men and their possible increase in unmet care needs.

### THE EXTREME FACE OF SOCIAL ISOLATION: A COHORT STUDY OF UNBEFRIENDED INDIVIDUALS IN LONG-TERM CARE

Stephanie Chamberlain,[1] Wendy Duggleby,[1] Pamela B. Teaster,[2] Janet Fast,[1] and Carole Estabrooks[1], *1. University of Alberta, Edmonton, Alberta, Canada, 2. Virginia Tech, Blacksburg, Virginia, United States*

Even though social isolation is a significant predictor of poor health and mortality in older adults, very little is known about social isolation in long-term care (LTC) settings. The aim of this study was to describe the prevalence, demographic characteristics, health outcomes, and disease diagnoses of residents without family contact in Alberta LTC homes. Using data collected between April 2008 and March 2018, we conducted a retrospective cohort study using the Resident Assessment Instrument, Minimum Data Set, (RAI-MDS 2.0) data from 34 LTC facilities in Alberta. We identified individuals who had no contact with family or friends. Using descriptive statistics and binary logistic regression, we compared the characteristics, disease diagnoses, and functional status of individuals who had no contact with family and individuals who did have contact with family. We identified a cohort of 25,330 individuals, of whom 945 had no contact with family or friends. Different from residents who had family, the cohort with no contact was younger (81.47 years, SD=11.79), and had a longer length of stay (2.71 years, SD=3.63). For residents who had contact with family, residents with no contact had a greater number of mental health diagnoses, including depression (OR: 1.21, [95% CI: 1.06-1.39]), bipolar disorder (OR: 1.80, [95% CI: 1.22-2.68]), and schizophrenia (OR: 3.9, [95% CI: 2.96-5.14]). Interpretation: Residents without family contact had a number of unique care concerns, including mental health issues and poor health outcomes. These findings have implications for the training of staff and LTC services available to these vulnerable residents.

### GENDER DIFFERENCES IN THE MECHANISM OF INVOLUNTARY RETIREMENT ON LONELINESS

Oejin Shin,[1] hyunjoo Lee,[2] Sojung Park,[3] and Ji Young Kang[4], *1. University of Illinois at Urbana Champaign, Urbana Champaign, Illinois, United States, 2. Daegu University, Gyeongsan-si, Gyeongsangbuk-do, Korea, Republic of, 3. Brown School, Washington University in St. Louis, St. Louis, Missouri, United States, 4. Hannam University, Daejeon, South Korea, Korea, Republic of*

Involuntary retirement is known to be associated with long-lasting negative effects on well-being compared to voluntary retirement. However, little is known about complex mechanism connecting the path from social contexts and psychological factors of retirees, especially involuntary retirees to later year well-being. Also, despite the well-known gendered pattern of preretirement employment histories over the life course in general, gender differences in the pathway on well-being after involuntary retirement is still unclear. Drawing on the stress process theory, this study examined gender difference on the pathway linking involuntary retirement (primary stressor) to loneliness through material/physical vulnerability (secondary stressor) and social support/self-efficacy (coping resources). Data are from the 2014 HRS with 2,087 retirees aged 65+. Two-step structural equation modeling (SEM) was utilized to examine the significance of the specific effects of multiple mediators (material/physical vulnerability, coping resources). For male retirees, involuntary retirement was associated with a higher level of loneliness mediated through physical vulnerability and social-efficacy. For female retirees, involuntary retirement was directly associated with loneliness as well as indirectly associated through 1) material vulnerability connected to low social support, and 2) physical vulnerability related to low social support and low social-efficacy. The different impact of involuntary retirement may be due to differences in work history, previous work quality, and accumulated financial condition across gender. The results suggest important gender specified implications for social policy and practice for involuntary retirees.

### MAKING THE AGING EXPERIENCE WORTHWHILE: HOW SOCIAL INTERACTION CONTRIBUTES TO ACTIVE AGING

Daniel Doh,[1] Kwadwo Adusei-Asante,[2] and Vicki Banham[3], *1. Western Sydney University, Sydney, Australia, 2. Edith Cowan University, Joondalup, Perth, Australia, 3. Edith Cowan University, WA, Joondalup, Perth, Australia*

In most parts of the world, people are now living longer lives, which presents both opportunities and concerns over how to make the ageing process a worthwhile experience. The World Health Organisation's Active Ageing model became a prominent global policy response since 2002 and has evolved into different country-level ageing policies. While a considerable volume of literature exists on active ageing – testing the validity of its various components, there is limited empirical evidence of how social interaction contributes to active ageing for older people and how it can be promoted through policy. In this paper, we examine social interaction and how it contributes to lived experiences of active ageing among a sample of 30 older Ghanaians living in Australia and Ghana. Our findings confirm the significance of social interaction for active ageing, and shows that social interaction creates a sense of purpose for living, which leads to the ability of the individual to build resilience, which mitigates anxieties and pains associated with ill health (especially for frail older people); enhances self-motivation for play and fun; empowers the individual to explore opportunities for continuous activity including leisure, and improves the general feeling of happiness resulting in active ageing – quality of life. The paper's main argument is that social interaction presents potentials for improving the quality of life (active ageing) for older people and needs to be carefully considered in policy, research and practice.

### ADDRESSING SOCIAL ISOLATION OF OLDER ADULTS: THE COMMUNITY PERSPECTIVE AND CONTRIBUTION

Bonnie Jeffery,[1] Tom McIntosh,[1] and Nuelle Novik[1], *1. University of Regina, Regina, Saskatchewan, Canada*

This presentation will discuss the Reducing Isolation of Seniors Collective (RISC), a collaboration of three organizations that have implemented nine projects to address social isolation for rural and urban older adults in Saskatchewan, Canada. A survey was completed with the purpose of identifying community-level awareness, knowledge and perspectives on the extent of social isolation of older adults in their communities. Key variables of interest included contributors to social isolation of older adults, barriers to overcoming social isolation, and community efforts and promising assets for addressing social isolation of older adults in their own communities. To develop an understanding of the extent to which respondents are involved with older adults, the survey asked respondents to report how often they supported, observed, advocated for, and/or interacted with seniors. The 271 respondents identified their roles in the community as human service professionals, healthcare professionals, program facilitators, community leaders, organization members, and community volunteers. Three-quarters of respondents reported that they were involved with seniors at least daily or weekly. While 75.3% believed that social isolation of older adults was "somewhat" or "very" common, almost one-half (41.2%) of respondents believed there was not general awareness of social isolation of older adults by other members in their community. Over one-half of the respondents mentioned community programming (55.7%), friends and neighbours (63.1%), and volunteers (57.2%) as community assets that can reduce isolation of older adults. Respondents reported examples of promising interventions in their communities: church support, library programs, transportation service, visiting programs, advocacy groups, and information sessions.

### EXPLORING THE EFFECTS OF RURAL AND URBAN LIVING ON LONELINESS AND REINSTITUTIONALIZATION

Kaleigh Ligus,[1] Alexandra Grimaldi,[2] and Julie Robison[3], *1. University of Connecticut, Storrs, Connecticut, United States, 2. Yale University, New Haven, Connecticut, United States, 3. University of Connecticut, Farmington, Connecticut, United States*

The following study employs secondary data from the Money Follows the Person Rebalancing Demonstration (MFP) in Connecticut (CT) to assess relationships between rural and urban living on loneliness and reinstitutionalization among an older adult (65+) sample. MFP is a federal initiative to help states transition people from institutional settings to the community. Older adults (n=1,301) who transitioned from institutional care to the community between 2009 and 2015 were surveyed 6, 12 and 24 months after transition. Rurality was determined according to the CT State Office of Rural Health and US Census Bureau definitions: urban area (UA), urban cluster (UC) and rural, utilizing 2017 CT Population data. SPSS was used to conduct chi-square tests and one-way ANOVAs to examine relationships. Almost half of participants (48%) resided in UAs, another 43% lived in UCs and 8% lived in rural towns. A statistically significant relationship was found between rural and UC groups and loneliness, indicated by a three-item modified version of the R-UCLA loneliness scale. Rural residents reported lower rates of loneliness (3.84 out of 9) than did UC (4.61) or UA (4.64) residents. However, a significantly higher percentage of rural residents (44%) reported at least one instance of reinstitutionalization at 24 months compared to UC (36%) or UA (30%) residents. Multivariate analyses seek to clarify these contradictory results. The findings of this study have the potential to further inform the literature regarding loneliness and connections between reinstitutionalization among older adults living in rural and urban environments.

### COASTAL RETIREMENT: IT'S ALL FUN AND GAMES UNTIL THE HURRICANE HITS

Anne P. Glass,[1] Jenni Blair,[1] and Judith Nichols[1], *1. University of North Carolina Wilmington, Wilmington, North Carolina, United States*

Many people dream of retiring to the beach. Twenty-four individuals who retired from out-of-state to a beach area in southern North Carolina had previously been interviewed regarding their retirement process and decision to move to this destination. Hurricane Florence brought major flooding and devastation to the area in September 2018. Shortly after, 10 participants agreed to complete a second interview and data collection about their hurricane-related experiences. This sample consisted of 8 women and 2 men, average age of 74.4 (range=68-88), and all were white. Nine evacuated, including one who went to a shelter. This project provided a unique opportunity to compare answers about stress levels and how they felt about their choice to move to the area, before and after the storm. Six and five rated their stress high/very high just prior to, and during Florence, respectively, but stress levels returned to low/very low for 90%. Two stated the storm caused them to rethink their decision to move; one now says she feels ambiguous about her move and would probably not choose it again. Stress caused by uncertainty was a thread across all interviews. Anxiety and concern were experienced, but no one reported fear. Neighbors played an important role pre-, during, and post-storm. No participants had significant damages, although one had a break-in; all expressed gratitude. They reported some lessons learned to apply the next time. These findings will be of interest to planners and others. They also demonstrate the resilience of older adults in dealing with natural disasters.

### DOES A PERCEIVED CONNECTION TO A NEIGHBORHOOD REDUCE LONELINESS?

Kimberly J. Johnson,[1] and Dolapo O. Adeniji[2], *1. Indiana University-Purdue University Indianapolis, Indianapolis, Indiana, United States, 2. School of Social Work, Indiana Purdue University Indianapolis, Indianapolis, Indiana, United States*

This study investigated whether perceived neighborhood quality was associated with chronic loneliness for adults 60 and older in the United States. Although loneliness can be episodic and overcome, chronic loneliness has been identified as a social determinant of health. Utilizing ecological systems theory we hypothesized that higher levels of neighborhood social cohesiveness would be associated with lower odds of chronic loneliness. We postulated that the networks available to people in the proximal area where they live could provide social opportunities for reducing loneliness. This idea was consistent with prior findings indicating the salience of neighborhoods for retirees, but inconsistent with research indicating the importance of a confidant in reducing loneliness. Data from the 2008 and 2012 Health and Retirement Study Psychosocial Surveys were used (n = 3530). Loneliness was measured using the 3-item scale developed by Hughes and colleagues in 2004. Findings from unadjusted logistic regression indicated that loneliness was inversely related to neighborhood cohesion as measured by an index of the trustworthiness, friendliness and helpfulness of neighbors and cleanliness, occupancy, lack of graffiti, and sense of belonging in the area ($OR = .73, p < .001$). When demographic and health-related factors were entered into the model the odds of being lonely were significantly lower for those with higher ratings of social cohesion ($OR = .83, p < .001$). These findings were consistent with the idea that neighborhoods are an important social place for older persons and interventions at the neighborhood level may be more effective than individualized treatment plans.

### COUPLES LIVING WITH ADVANCED CANCER: RAMIFICATIONS OF SOCIAL ISOLATION AND BENEFITS OF AN EMBEDDED SOCIAL NETWORK

Victoria H. Raveis,[1] Sheindy Pretter,[2] Monique Carrero-Tagle,[1] Daniel G. Karus,[1] and Avani Shah[3], *1. New York University, New York, New York, United States, 2. Touro College, New York, New York, United States, 3. University of Alabama, School of Social Work, Tuscaloosa, Alabama, United States*

Cancer remains a leading cause of death, especially among older adults. While spouses are commonly involved in the provision of emotional and practical assistance to their ill spouse, their caregiving is not without cost. Although knowledge of an impending death permits preparation for the loss, a long and protracted illness, or one marked by intense caregiving demands, can deplete the well spouse's personal resources, increasing the risk of morbid bereavement outcomes. Well spouses (n=138), aged 50 and older (mean age 63.6), 41% male, providing 8+ hours of caregiving to a spouse with advanced cancer and a life expectancy of 6 months or less were followed over the terminal illness period. Caregiving spouses' anticipatory grief, depression and anxiety were all significantly, inversely correlated with sufficiency of social support, specifically tangible, informational and emotional support ($p<.05$). At 2-3 months post-death (widowed subsample, n=82), the surviving spouses' grief was inversely correlated with informational support ($p<.01$) and their social re-engagement post-death was directly correlated with sufficient tangible support ($p<.05$). Their narrative accounts about the illness and post-death period reveal various demands and negative social encounters they experienced with family members and others during this period, as well as, documenting support received. Spousal death presents a significant adaptive challenge, particularly to older adults who must adjust to the dissolution of longstanding bonds and altered life circumstances. Awareness of these complex relationship issues, i.e. problematic network relationships, will enable clinicians to attend to the needs and preferences of bereaved spouses during this period of heightened vulnerability.

### A LIFE MORE ORDINARY: CREATING DEMENTIA-FRIENDLY SPACES THROUGH THE PERFORMATIVE ARTS

Christine Milligan[1], *1. Lancaster University, Lancaster, United Kingdom*

The concept of dementia friendly communities emerged from the 'age-friendly' movement that has been supported by the WHO for some time. This recognizes that like most older people, those with dementia desire to remain in their own homes, and their own communities, for as long as possible. But it also recognizes the significant socio-environmental challenges this can present. Transforming attitudes to dementia, supporting family and friend caregivers, and promoting meaningful participation for all in the community are essential to the success of any such movement. This paper draws on a qualitative evaluation of one such programme that has sought to develop innovative dementia friendly spaces through the arts and arts performance. Focusing on the Dukes Theatre in Lancaster, UK and its partner theatres and cinemas, I discuss how these venues have, over a three year period, developed spaces in which both people with dementia and their family carers can continue to meaningfully participate in ordinary everyday activities that can be crucial to maintaining the sense of belonging and partnership that is often lost as the dementia journey progresses. Whilst efforts to implement change at the city and community level are laudable, I suggest that it is perhaps at the micro-scale of individual places that we are most likely to successfully stimulate change.

### HARNESSING THE POWER OF NETWORKS TO ADDRESS SOCIAL ISOLATION, LONELINESS, DEPRESSION, AND ELDER ABUSE

Tobi A. Abramson,[1] Berman Jacquelin,[1] and Jo Anne Sirey[2], *1. New York City Department for the Aging, New York, New York, United States, 2. Weill Cornell Medicine, White Plains, New York, United States*

Challenges to aging and risks to physical health and mortality arise when older adults have untreated mental health needs, experiences social isolation, loneliness, or is a victim of elder abuse. This symposium presents three innovative models that harness the power of networks and collaboration between aging services and an academic medical center to meet the needs of ethnically diverse community seniors. These model programs include: embedded mental health services on-site in community senior centers; Friendly Visiting to homebound seniors; and PROTECT intervention to treat elder abuse victims' mental health needs. Research from these innovative, collaborative programs indicate that over 50% of senior center members screened positive for depression and anxiety (higher than the national average of 3-20%), social isolation, loneliness, and elder abuse. Of the 75% who engage in treatment, 37.3% and 41% showed a 3-month improvement of depression and anxiety, respectively. For seniors who have a friendly visitor, one-third also suffer from depression and/or anxiety. Three months after being visited by a friendly visitor, 42% and 53% see improvement in loneliness and social isolation, respectively. Among victims of elder mistreatment, 33% screened positive for depression or anxiety and 16% reported suicidal ideation. Clients receiving the PROTECT intervention had a greater decrease in depression, felt services were more useful, and reported greater improvement in the abuse. To find and build strength in age, it is essential that programs and policy be developed to support collaboration and provide the opportunities for building and utilizing networks across different domains of aging.

### SOCIAL ISOLATION AND LONELINESS: A LATENT CLASS APPROACH TO COSTING HEALTH SERVICE ENGAGEMENT IN AGING POPULATIONS

Elaine Douglas,[1] and David Bell[2], *1. University of Stirling, Stirling, Scotland, United Kingdom, 2. University of Stirling, Stirling, United Kingdom*

Social isolation and loneliness are associated with poorer health status and poorer health outcomes. Little is known the impact on health service usage, and its inherent cost, although it is considered to be higher. Latent class analysis (LCA) was used to determine profiles (population groups) of loneliness and social isolation in older people (aged 50+, n=1,057) using model-fit criteria. Loneliness was measured using the UCLA Loneliness Scale and social isolation used a measure of social networks and social contact. We then analysed the socio-demographic, perceived health, and health behaviour of these profiles using descriptive statistics and logistic regression. The survey data (HAGIS, 2016/17) were linked to retrospective administrative health data to investigate patterns of repeat prescription use (from 2009) and health service usage (from 2005) and their associated costs. Our results highlight the distinction and inter-relation between social isolation and loneliness (including associations with socio-demographic and health characteristics), and the variation in health service usage and costs between the population groups. LCA profiles may help focussed targeting of these groups for health interventions. Further, the data-driven approach of LCA may overcome some of the limitations of indices of social isolation and loneliness. As such, this will extend the existing methodological approaches to quantitative analyses of social isolation and loneliness and demonstrate the benefits of using linked administrative health data. Significantly, this study incorporates the social and financial cost of social isolation and loneliness on health and its implications for health services.

### PARTICIPATION AND SOCIAL CONNECTIONS IN A RECREATIONAL SENIOR SOFTBALL LEAGUE

Dawn S. Tarabochia[1], *1. Montana State University, Bozeman, Montana, United States*

Senior only communities have long been an option for adults over a certain age. A variety of activities and clubs are often available to residents of these communities. The purpose of this research project was to understand the lived experience of recreational softball players regarding players decision to play senior softball and to determine what social opportunities were associated with recreational senior softball leagues. A phenomenological research study was constructed to seek further inquiry into two research questions associated with this project. Participants were members of a senior living community and members of a recreational senior softball league. Convenience and snowball sampling techniques were utilized, and 25 interviews were conducted. The interview transcripts were analyzed for phenomenological themes by the research team. The researchers used Van Manen's (1990) hermeneutical approach to analyze data. Trustworthiness was established by the use of a peer reviewer to assess the themes for accuracy. Themes associated with the first research question indicate that interviewees participated in softball for a variety of reasons, including having played softball as a younger adult, wanting to maintain a level of physical fitness, and for the social connections that participation in a softball league provided. Themes associated with the second research question found that participants enjoyed many social benefits from playing softball, including informal and formal social opportunities. In conclusion, the willingness and opportunity to play senior softball provided older adults the ability to maintain a certain level of physical activity and to engage in meaningful social interactions.

### SESSION 2435 (POSTER)

### UPDATE ON TEACHING STRATEGIES IN GERONTOLOGY AND GERIATRICS

### SERVICE LEARNING AS A MEANS TO ENHANCE CONNECTIONS TO OLDER ADULTS

Marjorie A. Getz[1], *1. Methodist College, Peoria, Illinois, United States*

Aging is a distinct part of the life cycle. College students enrolled in courses in gerontology often have difficulty relating to aging, that part of life not yet experienced. They may not fully appreciate that adults become more unique, not more similar, as they age. We describe courses in an undergraduate gerontology certificate program that incorporate experiential learning activities with older adults across a hierarchical sequence of courses. These courses feature service learning opportunities focused on increased understanding of course content, broader appreciation of the discipline and improved sense of civic responsibility. Much like the course content of the curriculum, the incorporated experiential learning opportunities for each course level fit a hierarchy leading to student competence and skills development needed for success in the final independent practicum. For the described courses, students provided community service, experienced direct contact with older adults and used reflective practices to integrate course content into service learning activities. We report on qualitative data obtained from students enrolled in the foundational course, Biophysical Aspects of Aging and the third level course, Aging and Mental Health. Content analyses of reflective essays identified five themes: (a) insights about the realities of aging in America (b) perceptions concerning personal negative stereotypes about older adults; (c) feelings of accomplishment/awareness of new skills in providing community services; (d) understandings related to the importance/value of community service; and (e) successes in integrating the course work on aging into service-learning experiences. Other experiential learning activities incorporated into this gerontology certificate program are highlighted.

### UNDERGRADUATE EXERCISE AND AGING STUDENTS KNOWLEDGE AND PERCEPTIONS REGARDING OLDER ADULTS

Tracy Davis,[1] and Michelle Zechner[2], *1. Rutgers University, Blackwood, New Jersey, United States, 2. Rutgers University, Scotch Plains, New Jersey, United States*

The aging population presents far ranging implications and opportunities for individuals, families, policy makers, and healthcare providers. The U.S. Census Bureau estimates an increase in the population aged 65+, which is projected to reach 83.7 million by 2050. With this changing demographic environment, human services and health care professionals with specialized training in aging are needed, regardless of career goals all students deserve exposure to aging education. Undergraduate students have varying knowledge and attitudes towards older adults. Many times undergraduate students have had limited interactions with older adults and their attitudes and perceptions are based on interactions with grandparents and other relatives. In order to better understand undergraduate student's knowledge and attitudes regarding older adults we surveyed a group of 50 undergraduate students enrolled in an exercise and aging course offered through the Department of Kinesiology and Health. Students were surveyed using the Facts on Aging Quiz (Breytspraak & Bandura, 2015) and the Aging Semantic Differential (ASD) (Rosencranz & McNevin, 1969). Preliminary findings suggest that students have relatively low knowledge about older adults, as the average score of the Facts on Aging Quiz was 30.4 out of 50 (SD= 3.86). However, scores on the ASD indicate that the student have generally more positive attitudes towards older adults (M=74.29; SD=20.9). At the end of the semester the same students will be surveyed again to evaluate the impact of the course. Findings from this study will be used to augment course content to increase student knowledge and attitudes about older adults.

### CREATING THE CT GERONTOLOGY CONSORTIUM TO BRING FACULTY AND STUDENTS TOGETHER TO PROMOTE GERONTOLOGICAL EDUCATION

Christina Barmon,[1] Claudia E. Oakes,[2] Donna Fedus,[3] Erica Michalowski,[4] Andrea June,[1] and Carrie Andreoletti[1], *1. Central Connecticut State University, New Britain, Connecticut, United States, 2. University of Hartford, West Hartford, Connecticut, United States, 3. Borrow My Glasses, Madison, Connecticut, United States, 4. AARP CT, Hartford, Connecticut, United States*

Although commonplace for faculty across universities to collaborate on research, it is less common to bring faculty and students from different institutions together for pedagogical purposes. In this poster, we describe the development of the Connecticut Gerontology Consortium, comprised of gerontology instructors at several academic institutions. We created this consortium in order to 1) develop a support network for faculty interested in teaching about aging, and 2) to support collaboration on efforts to promote creative, interdisciplinary student experiences that foster a passion for gerontology. In addition, we describe one endeavor, a student project, from the planning stages to post-project reflections. The intercollegiate and interdisciplinary "Careers in Aging" project brought together 62 students from four academic institutions with a broad range of experiences and interests. Students were assigned to groups from different schools to investigate careers in the aging network in Connecticut. Each student group interviewed a professional, delivered a presentation about their career path and day-to-day experiences, and created an informational brochure about the profession. Students indicated that they valued the experience of learning about the aging network and engaging with other students who shared an interest in gerontology. However, they cited challenges in communicating with students from other classes and at other institutions. This project has the potential to build networking opportunities for students as well as strengthening the community of gerontology professionals. The presentation will include lessons learned and how the consortium plans to move forward.

### SUITABLE? THE RHETORICAL AND LIFE COURSE IMPLICATIONS OF SERENA WILLIAMS AND THE CATSUIT

Brianne M. Stanback[1], *1. University of South Florida, Tampa, Florida, United States*

Rhetorical inquires have shown connections between representation and power, workplace fashion and development of ethos, and the rhetoric of glamour through women's fashion and dress. One element absent from that conversation is how the life course, which typically differs for women because of existing power structures advantaging men, may impact the experience of women as they age, their choice of dress, and the rhetorical implications of those decisions. To explore dress and rhetoric from a life course perspective, this project traces the evolution of Serena Williams' work apparel across her professional tennis career to the catsuit worn at the 2018 French Open, which is the focus of the project. Press reports on the 2018 catsuit by Nike, New York Times, Sports Illustrated, Business Insider, BBC Sport, Washington Post, and Los Angeles Times, interviews given by Williams, and the television documentary, Becoming Serena, will be analyzed for their treatment of Williams' work attire and the life course. Responses to the catsuit emphasize attitudes about gender, race, and class, either discounting or ignoring the life course implications such as motherhood and changes in health status. Despite professional success, responses about the catsuit may reflect that Williams faces the same jeopardies, and invisibility, common to many women as they age, and the rhetorical perspective provides new methodological and pedagogical possibilities for instruction in aging.

### MAKING IT WORK: SECURING A GERONTOLOGY INTERNSHIP FOR THE NONTRADITIONAL DISTANCE EDUCATION LEARNER

Katherine Im[1], *1. UMUC, Adelphi, Maryland, United States*

Some gerontology programs have required students to complete internships in order to satisfy the "interactional" Category II Competencies for gerontology as outlined by AGHE in 2014. For many non-traditional learners, a required internship in gerontology poses major challenges that may contribute to declines in enrollment and retention. Non-traditional learners are often older, employed, and have multiple responsibilities that compete for their time. This is especially true for students who attend university from a distance. This presentation reports the challenges faced by non-traditional, online students at a large state university who have an interest in gerontology. For these learners, the internship requirement is a deterrent to becoming a gerontology major. Challenges include student constraints, identifying potential placement sites in remote locations, and negotiating a complex administrative process. I review the programmatic measures taken to address these challenges and discuss the ways in which the internship process can be facilitated for non-traditional students. By developing a better process for securing internships, students have more freedom to focus on workplace learning. In addition, prospective gerontology students may come to regard the internship as an excellent opportunity for professional growth rather than a burden , thereby improving retention and interest in the major.

### UTILIZING UNDERGRADUATE RESEARCH TO INFORM COMMUNITY RESOURCES FOR OLDER ADULTS: A SERVICE-LEARNING PROJECT

Amanda E. Barnett,[1] and Sara Olinger[1], *1. Dunn County Human Services, Menomonie, Wisconsin, United States*

Engaging undergraduate students with aging-focused community resources is critical for preparing students to work with older adults and make positive contributions to aging societies. During the fall, 2018 semester, undergraduate students in a human development course on middle and late adulthood partnered with a county aging and disability resource center (ADRC) in Wisconsin to evaluate and update several of their existing programs and resources using empirical research. Upon completion of this project, students synthesized course material to meet all course learning objectives such as: (1) critically analyze physical, psychological, and sociological processes of aging across categories of difference (e.g. cultural, ethnic, class); (2) evaluate social policies and their multigenerational implications for midlife and older adults; and (3) construct a personal position on aging that integrates theory, research, and policy to demonstrate a sensitive and competent approach to working with midlife and older adults. Students researched, wrote reports, and presented to ADRC staff on the impact of social isolation on older adults, best practices for home visitor and transportation programs serving older adults, cognitive competency tools and best practices for utilizing memory assessments, grief supports and groups for family caregivers, and best practices for supporting veterans as they age. The outcome of these projects are research-based recommendations for any ADRC to consider when developing and implementing related programs. All stakeholders (students, professor, and ADRC staff) were satisfied with the process and outcomes of the project. Strengths and challenges of carrying out such a collaborative project will be reviewed.

### ADVANCE CARE PLANNING ASSIGNMENTS IN UNDERGRADUATE COURSES: EXAMINING STRESS APPRAISALS, COPING, AND RESPONSES

Maureen Templeman,[1] Victoria R. Marino,[1] Debra J. Dobbs,[1] and William E. Haley[1], *1. University of South Florida, Tampa, Florida, United States*

A common assignment in undergraduate aging courses requires students to consider or complete advance care plans for themselves. Due to the sensitive nature of this topic, it is important to consider student appraisals of such assignments. Using the transactional model of stress and coping, the current study used content analysis to examine student reactions to considering their own advance care planning. Participants included 151 students, diverse in race/ethnicity, in a Psychology of Aging undergraduate course at a large public university in the United States. Students were first exposed to course material related to death, dying, and bereavement, in addition to detailed advance directive forms, and were subsequently asked to discuss the importance of, and their preparedness for, their own advance care planning via a written assignment. Codes represented primary and secondary appraisals, coping efforts, and responses, and data was analyzed using ATLAS.ti software. Findings suggest that primary appraisals (perception of effect on well-being) ranged from positive to threatening, and secondary appraisals (perception of coping resources) focused on availability of internal and/or external resources. Themes such as traumatic experiences with death, feeling ill-prepared due to lack of knowledge or experience, and welcoming the opportunity to consider completing advance care plans emerged. Overall, results suggest that when designing assignments that require completion of advance care plans among undergraduate students, students should have the opportunity to discuss personal concerns first, and that some initial class activities should focus on preparatory tasks rather than just completion of advance care plans.

### SERVICE LEARNING TO INCREASE EXERCISE IN OLDER ADULTS: TESTING A GROWTH MINDSET INTERVENTION

Abby L. Heckman Coats,[1] Zhaoming Wang,[1] and Therese Miller[1], *1. Westminster College, Fulton, Missouri, United States*

Exercise is one of the best ways to maintain psychological and physical health in late life (Kohl et al., 2012). However, many older adults experience barriers to developing an exercise routine, such as fear/anxiety, limited perceived control, and lack of self-efficacy (Bock et al., 2014). Recently, successful psychological interventions to encourage exercise have been demonstrated (Brothers & Diehl, 2017; Lachman et al., 2018). Inspired by this success, we developed a service learning activity to address this community-based problem. Undergraduate psychology students visited local senior centers. During the visits, the students implemented an intervention to increase physical activity in older adults. Traditionally-aged undergraduate students ate lunch with the older adults and engaged in informal discussions. Then, after establishing rapport, they implemented an exercise-boosting intervention to the experimental group. (A control group did not receive the intervention.) The older adults in the experimental group heard quotes from peers explaining how they had overcome barriers to exercise. The peers' stories included growth-mindset language (Yeager, Paunesku, Walton, & Dweck, 2013), such as an initial reluctance to exercise, followed by hard work and finally implementing a successful exercise regimen. Then the older adults wrote their own letters to a peer, explaining how they have overcome barriers in the past and how they could continue to do in the domain of exercise. This activity was helpful for both the older adults and the undergraduate students, as documented by qualitative interviews and questionnaire responses. The students reported more positive attitudes about aging after the activity.

### TEACHING SEX AND AGING TO PRE-PROFESSIONALS: EXPERIENTIAL LEARNING OF A TABOO TOPIC

Jill Juris Naar,[1] Raven H. Weaver,[2] and Libbie Sonnier-Netto[3], *1. Appalachian State University, Boone, North Carolina, United States, 2. Washington State University, Pullman, Washington, United States, 3. Center for Development and Learning/Virginia Tech, New Orleans, Louisiana, United States*

Myths and stereotypes of sex in late life prevail (e.g., sex becomes unimportant or irrelevant; sex gets worse as individuals age; older adults are asexual). Unless the workforce is adequately prepared and knowledgeable to address the sexual health needs of older adults, it is difficult to disrupt ageism that contribute to discrimination against older adults. Methods: We evaluated a two-day experiential workshop designed for students to develop factual community resources and educational materials related to sex and aging. Results: Using content analysis, we examined open-ended responses from 41 students. We identified three themes that depicted the value of experiential learning, specifically for this taboo topic. Students (1) increased awareness of late life sexual health and behaviors, (2) demonstrated comfort and creative strategies to discuss sex, and (3) appreciated the opportunity for transferable pre-professional skill development. Discussion: Providing pre-professional training helped close the knowledge gap about basic gerontological issues and issues specific to sexual health and sexual behaviors in late life. Students perceived the flipped classroom and collaborative structure of this experiential workshop as beneficial and practical for their learning and professional preparation. They developed skills for translating knowledge into practical resources that likely will transcend professions. Emphasizing the potentially uncomfortable topic of sex and aging provided students an opportunity to increase their comfort when addressing issues they will experience in their various professions. It is critical for gerontology educators to identify strategies to deliver sex and aging education among professionals to enhance interactions with older adults.

### SESSION 2440 (SYMPOSIUM)

### 2019 GSA FELLOWS SYMPOSIUM: STRENGTH IN AGE: HARNESSING THE POWER OF NETWORKS

Chair: Peter Martin, *Iowa State University, Ames, Iowa, United States*

The 2019 GSA Fellows Symposium includes GSA members who were recently granted GSA Fellow status. They represent each of the GSA sections: Biological Sciences (BS), Health Sciences (HS), Behavioral & Social Sciences (BSS), Social Research, Policy & Practice (SRPP), and the Academy for Gerontology in Higher Education (AGHE). The theme of the 2019 GSA Fellows Symposium is focusing on the power of networks and will highlight the importance of neuroendocrine networks (Christian Sell), the role of social support for refugees in Canada (Esme Fuller-Thomson), the importance of professional partnerships to promote health (Heather Young), aging-friendly communities (Emily Greenfield), and the role of networks in teaching gerontology (Tina Kruger). The importance of networks across disciplinary boundaries will be discussed.

### AGING-FRIENDLY COMMUNITY INITIATIVES: HARNESSING NETWORKS FOR CHANGE FROM THE LOCAL TO THE GLOBAL

Emily Greenfield,[1] and Laurent Reyes[2], *1. Rutgers University, New Brunswick, New Jersey, United States, 2. Rutgers, The State University Of New Jersey, New Brunswick, New Jersey, United States*

Aging-friendly community initiatives (AFCIs) represent multi-sectorial efforts to systematically make localities better for long lives. AFCIs typically work toward progress in multiple domains of community life, focusing both on social and built environments. Despite tremendous growth in AFCIs worldwide over the past decade, there has been little empirical research on how they develop and work toward their aspirational goals. This paper presents findings from a community-partnered study on the development of nine philanthropically supported AFCIs in northern New Jersey. Drawing on fives waves of in-depth interview data collected with leaders over three years, the paper addresses the role of the leaders' interpersonal and interorganizational networks as they work toward aging-friendly community change. Findings on the centrality of networks across various levels of geographic scale for the development of AFCIs has implications for program theory, as well as broader issues concerning inclusion and equity within community-level responses to population aging.

### DEPRESSION AMONG OLDER CANADIAN REFUGEES: THE PROTECTIVE ROLE OF SOCIAL SUPPORT

Esme Fuller-Thomson,[1] Shen (Lamson) Lin,[1] Karen Kobayashi,[2] Simran R. Arora,[1] Hongmei Tong,[3] and Karen Davison[4], *1. University of Toronto, TORONTO, Ontario, Canada, 2. University of Victoria, Victoria, British Columbia, Canada, 3. MacEwan University, Edmonton, Alberta, Canada, 4. Kwantlen Polytechnic University, Surrey, British Columbia, Canada*

This study's objective was to identify which factors attenuate refugees' higher odds of depression. A secondary analysis of 272 refugees and 29,398 non-refugees in the Canadian Longitudinal Study on Aging, a 2012 study of Canadians aged 45 to 85, was conducted. The prevalence of depression was higher among refugees than non-refugees (22.1% vs 15.2%, p<.001). The age-sex adjusted odds of depression for refugees (OR=1.70, p<.001) was only modestly attenuated when sociodemographic characteristics, physical health conditions, chronic pain, binge drinking and level of physical activity were taken into account (ORs ranged from 1.61 to 1.70, all p<.05). However, in the model adjusting for social support, the odds of depression for refugees was reduced to non-significance (OR=1.30, p=0.92). Refugees have higher odds of depression than non-refugees, and this excess vulnerability is associated with lower levels of social support. Targeted interventions to decrease isolation and improve refugees' social support warrant greater attention

### HARNESSING THE POWER OF NETWORKS: PARTNERSHIPS TO PROMOTE HEALTH AND WELL-BEING FOR FAMILY CAREGIVERS

Heather Young[1], *1. Betty Irene Moore School of Nursing - UC Davis, Sacramento, California, United States*

This paper will address social and professional networks that support family caregivers who are providing care to older adults. Family caregiving is commonly a long-term commitment that can include functional, health care, social, financial and emotional support. This role can have a major impact on the health, well-being and economic security of the family caregiver, particularly for those who are socially isolated. This paper will highlight the nature of caregiving demands on individuals and families, particularly those providing intense and complex care, with a focus on cultural diversity. Health care professionals encounter family caregivers during routine care and at times of crisis and have an opportunity to enhance support and preparation. This paper will discuss effective community-level and health system partnerships and strategies that promote health and well-being for family caregivers, examine the potential for scaling these approaches, and suggest priorities for education, practice and future research.

### NEUROENDOCRINE NETWORKS IN AGING

Christian Sell[1], *1. Drexel Uiversity College of Medicine, Philadelphia, Pennsylvania, United States*

It has become clear that nutrient regulatory pathways are the major determinants of lifespan. From early studies on caloric restriction to genetic ablation studies in yeast, worms, flies, and more recently mice, a unifying theme has emerged that suppressing nutrient sensing pathways and neuroendocrine networks results in lifespan extension. This work has laid the foundation for interventions designed to ameliorate late-life dysfunction. Inhibition of the mechanistic target of rapamycin (mTOR) pathway using rapamycin, an FDA-approved drug used clinically to inhibit solid organ rejection, is one of the most promising of these interventions. It has been demonstrated that rapamycin enhances longevity in mice, even when initiated in relatively old animals. We will discuss the development of anti-aging therapies, the potential for emerging therapies, and pitfalls associated with clinical trials designed to test these therapies.

### THE ROLE OF NETWORKS IN TEACHING GERONTOLOGY THROUGH COMMUNITY ENGAGEMENT AND SERVICE-LEARNING PROJECTS

Tina M. Kruger[1], *1. Indiana State University, Terre Haute, Indiana, United States*

As we seek to prepare the next generation of researchers, teachers, and service providers in the field of aging, it is vital that students garner experience interacting directly and appropriately with older adults. Community engagement and service-learning (CE/SL) are high impact pedagogical practices associated with enhanced learning, retention, and student success and can facilitate positive interactions across generations. Creating CE/SL opportunities requires authentic relationships with community members and organizations, allowing faculty, staff, and students the opportunity to network with a variety of individuals and agencies. In this presentation, we define CE/SL, examine the role of networks in creating CE/SL opportunities, and discuss challenges and threats to effective CE/SL projects. Tools and resources will be highlighted to provide guidance to those wishing to harness the power of networks to teach gerontology content while also working to have positive, lasting, and measurable impact on the communities in which they serve.

### SESSION 2445 (SYMPOSIUM)

### A LIFE COURSE PERSPECTIVE OF LATE-LIFE FUNCTIONING: THE STUDY OF WOMEN'S HEALTH ACROSS THE NATION

Chair: Carrie Karvonen-Gutierrez, *University of Michigan, Ann Arbor, Michigan, United States*
Co-Chair: Elsa Strotmeyer, *University of Pittsburgh, Pittsburgh, Pennsylvania, United States*
Discussant: Carol Derby, *Albert Einstein College of Medicine, Bronx, New York, United States*

Early late-life deficits in physical functioning are highly relevant among women, who experience a more rapid decline in physical functioning vs. age-matched men. Trends showing an increasing prevalence of disability among mid-life adults, with an evolving understanding that late life health and functioning is the product of exposures accumulated across ones life, suggests an urgent need to understand the causes and consequences of functional limitations at the transition between mid- to late-life. The Study of Women's Health Across the Nation (SWAN) is an observational multi-racial/ethnic (White, Black, Chinese, Japanese, Hispanic) longitudinal study of 3,302 women recruited in 1996 from 7 U.S. clinical sites; Boston, Chicago, Detroit-area, Los Angeles, Newark (NJ), Oakland (CA), and Pittsburgh. Over the past 23 years, women have participated in up to 16 near-annual study visits, spanning from mid-life (age 42-52 years at baseline) to late life (age 65-73 years at follow-up visit 15). Retention at the most recently completed in-person visit (2015) was 74%. This symposium identifies mid-life risk factors for poor late-life performance-based physical functioning among women, including race/ethnicity (Sternfeld) and chronic health conditions (Lange-Maia). Further, early late-life decrements in stair climb time and muscle power (Strotmeyer) and falls and fall injuries (Ylitalo) were observed with key factors identified. The Discussant will examine implications for life course epidemiology in advancing our understanding of predictors of late-life physical functioning. Consideration of functioning during the transition from mid-life to late life is critical to target interventions that are most efficacious in promoting late life health and function.

### IMPACT OF MULTIPLE CHRONIC CONDITIONS ON CHANGE IN PHYSICAL FUNCTION FROM MID- TO EARLY LATE LIFE

Brittney S. Lange Maia,[1] Kelly Karavolos,[1] Elsa Strotmeyer,[2] Carrie Karvonen-Gutierrez,[3] Elizabeth Avery,[1] Imke Janssen,[1] Sheila Dugan,[1] and Howard Kravitz[1], *1. Rush University Medical Center, Chicago, Illinois, United States, 2. University of Pittsburgh, Pittsburgh, Pennsylvania, United States, 3. University of Michigan, Ann Arbor, Michigan, United States*

Chronic conditions emerging in midlife may be modifiable to prevent progression ultimately preserve physical function (PF) in late life. We quantified change in perceived PF in relation to several common chronic conditions known to impact PF in late life (osteoarthritis, diabetes, stroke, hypertension, heart disease, cancer, osteoporosis, and depression). Physical function (PF) was assessed using the Physical Functioning Scale of the SF-36 among 2,283 women in SWAN from an average age of 50.0±2.7 to 64.0±3.7 years. In covariate-adjusted Poisson models, each additional condition was associated with 3% worse PF ($p<0.001$), and an additional 0.4% annual worsening ($p<0.001$). Thus, holding demographic, lifestyle, socioeconomic, and other health factors constant, a woman a decade later entering old age with no chronic conditions would have 8.1%, 15.5%, and 17.0% better PF vs. having one, two, or three conditions, respectively. Preventing or delaying chronic disease progression in midlife may improve PF trajectories into late life.

### PHYSICAL ACTIVITY, FALLS, AND FALL INJURIES: THE STUDY OF WOMEN'S HEALTH ACROSS THE NATION

Kelly Ylitalo,[1] and Kelley Gabriel[2], *1. Baylor University, Waco, Texas, United States, 2. University of Texas Health Science Center-Houston, School of Public Health-Austin Campus, Austin, Texas, United States*

Physical activity (PA) is positively associated with physical functioning, but relations of domain-specific PA with falls is less clear. In 2015, women (n=1,962) self-reported fall history and PA. The Kaiser Physical Activity Survey (KPAS) assessed sports, non-sports leisure, household/caregiving, and total activity. Risk ratios (RR) were generated from poisson regression. One-third (29.7%) of women (mean age=65.1 years [SD 2.7]) reported ≥1 fall and 18.3% reported ≥1 fall injury in the previous 12 months. Women in the highest quartile of sports activity had an increased risk of ≥1 fall (RR=1.19;95%CI:1.00,1.41;p=0.049). Women in the highest quartile of total activity had an increased risk of injurious falls (RR=1.31;95%CI:1.04,1.65;p=0.021), but there was no association for domain-specific indices. KPAS may identify women and activity domains, such as sports, at high risk for falls and injuries. Future work should incorporate device-based PA assessments within domains to understand fall patterns among community-dwelling older adult women.

### RACIAL-ETHNIC DISPARITIES IN PHYSICAL PERFORMANCE: THE STUDY OF WOMEN'S HEALTH ACROSS THE NATION

Carrie Karvonen-Gutierrez,[1] Alicia Colvin,[2] Andrea Stewart,[2] Bradley Appelhans,[3] Jane Cauley,[2] Sheila Dugan,[3] Samar El Khoudary,[4] and Barbara Sternfeld[5], *1. University of Michigan, Ann Arbor, Michigan, United States, 2. Department of Epidemiology, Graduate School of Public Health, University of Pittsburgh, Pittsburgh, Pennsylvania, United States, 3. Rush University Medical Center, Chicago, Illinois, United States, 4. Department*

*of Epidemiology, Graduate School of Public Health, Pittsburgh, Pennsylvania, United States, 5. Division of Research, Kaiser Permanente, Kensington, California, United States*

Racial/ethnic disparities exist in physical performance among older adults, but little is known about the factors leading to these differences. We evaluated how sociodemographic, health, behavioral, and psychosocial factors mediated the relationship between race/ethnicity and physical functioning performance among 1,855 Black, Chinese, Hispanic, Japanese and White postmenopausal women from the Study of Women's Health Across the Nation. White women had better mean physical performance scores (decile-based score including relative performance on grip strength, timed 4-meter walk, and timed repeat chair stand tasks) as compared to Black, Hispanic and Chinese women but slightly poorer scores vs. Japanese women. In Blacks and Hispanics, 75% and 95% of that disparity, respectively, was through mediators, particularly education, financial strain, BMI, physical activity and pain. Addressing issues of poverty, racial inequality, bodily pain and obesity could reduce some racial/ethnic disparity in functional limitations as women age.

### STAIR CLIMB TIME AND FUNCTIONAL POWER ASSOCIATIONS IN EARLY OLD AGE: SWAN

Elsa Strotmeyer,[1] Brittney S. Lange-Maia,[2] Jane A. Cauley,[1] Sheila A. Dugan,[2] Samar R. El Khoudary,[1] Kelley Pettee Gabriel,[3] Nancy W. Glynn,[1] and Carrie A. Karvonen-Gutierrez[4], *1. University of Pittsburgh, Pittsburgh, Pennsylvania, United States, 2. Rush University Medical Center, Chicago, Illinois, United States, 3. University of Texas, Austin, Texas, United States, 4. University of Michigan, Ann Arbor, Michigan, United States*

Stair climbing assesses neuromuscular components of movement, including muscle power (force*velocity) which may decline earlier in aging vs. strength. We hypothesized age and age-related factor (N=1370; 65.5±2.7 years) associations to stair climb total time (sec), ascend lap time degradation (lap 1 minus 3), power (W/kg body weight) and power degradation (lap 1 minus 3). Adjusting for demographic, lifestyle and age-related comorbidity factors using multivariate linear regression, older age independently related to slower total time and lower power. Non-white ethnicity had slower total time (Black, Hispanic), higher ascend time degradation (Hispanic), and lower power (Hispanic, Chinese, Japanese) vs. Whites. Higher 36-Item Short Form Health Survey (SF-36) and Modified Baecke physical activity scores indicated better performance: lower total time, higher power (SF-36 only), and less degradation in ascend time and power. Stair climb time and power in early old age may capture initial functional loss targets for interventions to prevent late-life disability.

### SESSION 2450 (SYMPOSIUM)

### AGING, DISASTERS, AND THE ENVIRONMENT: ROLE OF CLIMATE CHANGE IN DISASTER PREPAREDNESS AND RESPONSE

Chair: Judith Robertson R. Phillips, *California State University San Marcos, San Marcos, California, United States*
Co-Chair: Melissa L. Cannon, *Western Oregon University, Monmouth, Oregon, United States*
Discussant: Zhen Cong, *School of Social Work, University of Texas at Arlington, Arlington, Texas, United States*

Continuing changes in the world's climate put the older adult population more at risk than other age groups. Multiple circumstances such as disasters and environmental events brought about by climate change impair the ability of older adults to maintain healthy physical and psychological well-being. Unfortunately, during these times, public and individual emergency preparation plans often fall short of having the needed resources to reach out and offer assistance to older adults. Co-sponsored by the Environmental Gerontology and Disasters and Older Adults Interest Groups, four presenters will highlight multiple circumstances where older adults face serious physical and psychological consequences as a result of climate change issues: 1) the loss of electrical power and its impact on hypothermia and hyperthermia in older adults, 2) the loss of basic public health services after Hurricane Maria and its contribution to older adults' reduced psychological well-being including increased levels of suicide, 3) homeless older adults experiencing a disaster and their resulting increased need for resources, and 4) older adults in the path of violent tornadoes and their ability to cope on their own. These presenters will discuss actions, interventions, and development of emergency disaster preparation plans that would be beneficial for older adult response and recovery. The fifth paper will review the theoretical backgrounds of and previous studies on Anthropocene with a focus on climate change policy and practice. Collectively, these papers will address various means by which older adults are affected by climate change and optimal practices to speed recovery.

### ACTIONS TO REDUCE RISK OF OLDER ADULTS DURING EXTREME WEATHER EVENTS THAT THREATEN HEALTH AND LIFE

Lisa M. Brown[1], *1. Palo Alto University, Plo Alto, California, United States*

Climate change places many older adults at unique risk for adverse, potentially life-threatening consequences. Compared to younger adults, older adults are more susceptible to the effects of extreme weather events and at greater risk for heat stroke and hypothermia. With advanced age, some people have vulnerabilities, such as social isolation and physical and mental health conditions that predispose them to a greater level of danger. Loss of power, that leaves people without functioning cooling or heating systems, can cause death because it exacerbates preexisting medical conditions and impairs thermoregulatory function for those with medical illnesses or taking certain medications. Many older adults who function independently may not self-identify as being vulnerable and take necessary precautions to remain safe. This presentation will describe recent research that found that many older adults are unaware of hypothermia and hyperthermia symptoms and the actions needed to maintain health and life.

### CLIMATE CHANGE FOR OLDER INDIVIDUALS EXPERIENCING HOMELESSNESS: CONSIDERATIONS FOR DISASTER EVENTS

Allison Gibson[1], *1. University of Kentucky, Lexington, Kentucky, United States*

There is a growing field of evidence that individuals experiencing homelessness are disproportionately impacted by climate change due to factors like exposure to the elements, lack of resources and services, as well as disenfranchisement and stigma; all while experiencing greater occurrences of environmental injustice. Given that there are distinct needs for older individuals experiencing homelessness when affected by disasters, this study will report salient themes identified from qualitative interviews with five residential shelters on considerations they incorporate when disaster planning for their older residents. Thematic analysis revealed older age and homelessness have serious implications for planning, responding and in the recovery of disasters. Challenges to finding accessible transitional and permanent housing, limitations to workforce re-entry, increasing income inequality between classes, limitations to mental health services and policies, and cultural justifications for criminalizing poverty and homelessness contribute to our collective understanding of disaster vulnerability when older adults experience homelessness.

### DEVELOPMENT AND REVISION OF THE FAMILY EMERGENCY PREPARATION PLAN AMONG OLDER ADULTS

Zhen Cong,[1] Zhen Cong,[1] James Craig Keaton,[1] and Daan Liang[2], *1. School of Social Work, University of Texas at Arlington, Arlington, Texas, United States, 2. Department Of Civil, Environmental, And Construction Engineering, Texas Tech University, Lubbock, Texas, United States*

This study examines factors related to holding, revising, and developing an individual emergency preparation plan among older adults who experienced violent tornadoes. A telephone survey was conducted with 543 respondents approximately one year after two violent tornadoes in 2013 with 276 respondents aged 65 or above. Logistic and multinomial logistic regression showed that education was positively associated with a higher likelihood of having a plan among younger but not older adults. Among those who had a plan before the tornado, older, but not younger, adults who experienced more stress were more likely to report the plan as helpful. More stress and having someone in the household with disability increased the likelihood of revising plans afterward among older adults but not among younger adults. Older adults were less likely to develop a new plan and older, but not younger, adults who reported more stress were more likely to develop a plan.

### FEASIBILITY OF AN EVIDENCE-BASED MENTAL HEALTH INTERVENTION WITH OLDER ADULTS IN POST-HURRICANE MARIA PUERTO RICO

Denise Burnette[1], *1. Virginia Commonwealth University, Richmond, Virginia, United States*

Owing to out-migration, decreased fertility and longer lifespans, 18.5% of Puerto Rico's population is aged 60+ -- 36% live alone and 40% are below poverty. Out-migration after Hurricane Maria may well raise the proportion of older adults to 30%. Mental health problems are among the most widespread and enduring effects of disasters. Common Mental Disorders (CMD) (anxiety, depression, traumatic stress) are most common. Before Maria, CDC data showed 38 % of persons age 65+ reported fair or poor health and 20% had been told they had a depressive disorder. In the months after Maria, the overall suicide rate rose 29%, while it more than doubled for people aged 65-69 and tripled for those aged 75- 79. This pilot study assesses the feasibility and acceptability of adapting the Friendship Bench, an evidence-based intervention developed in Zimbabwe for CMD, for use with older adults in primary care in Puerto Rico.

### THE ANTHROPOCENE AND THE LONGEVITY REVOLUTION

Moon Choi[1], *1. KAIST Graduate School of Science and Technology Policy, Daejeon, Korea, Republic of*

The Anthropocene, a term popularized in 2010 by Nobel Prize-winning chemist Paul Crutzen, refers to the current epoch during which human beings have begun to have a significant impact on the earth, e.g., the environment and climate change. Global population has grown approximately seven-fold over the past 200 years, while average life expectancy at birth has dramatically increased due to improvements in nutrition, medicine, and technology. The human Longevity Revolution thus provides important evidence of the Anthropocene. Yet, in the face of the Anthropocene, contemporary lifestyles rooted in capitalism–continually seeking more and bigger–are not sustainable; changes are needed for humanity to "live long on the damaged planet." This presentation will discuss the Longevity Revolution in the context of the theory and previous research on the Anthropocene, then suggest an agenda for future research related to the intersection between the Anthropocene and the Longevity Revolution.

### SESSION 2455 (SYMPOSIUM)

### COGNITIVE HEALTH AND ADRD: GENDER, RACE, AND ETHNIC ISSUES

Co-Chair: Toni C. Antonucci, *University of Michigan, Ann Arbor, Michigan, United States*
Chair: Kristine Ajrouch, *Eastern Michigan University, Ypsilanti, Michigan, United States*
Discussant: Jonathan W. King, *National Institute on Aging, Bethesda, Maryland, United States*

This symposium addresses minority issues in aging, specifically issues of gender, race and ethnicity, in the study of cognitive health and Alzheimer's Disease and Related Dementia (ADRD). Cabrera examines ADRD in the Latino community noting that there are or may be important differences among subgroups of Latinos, e.g. Mexicans and Puerto Ricans, who are too often considered a homogenous group. Using qualitative methods, specifically focus groups, she explores whether these two groups have a different understanding of or different concerns about ADRD. Dallo considers the epidemiology of ADRD among Arab Americans. Noting the dearth of evidence on this ethnic group, she uses the National Health Interview Survey from 2000-2017 to examine the prevalence of ADRD among foreign-born Arab American compared to Whites, Blacks and Asians. Indiro et al., consider the long reach of childhood SES on age-related brain changes in different racial and ethnic older adults. Finally, Byrd considers gender differences in cognitive health in the Baltimore Study of Black Aging and finds that women actually report better cognitive health than men, controlling for age, education and health status despite previous literature suggesting that women experience more dementia than men. In total, this symposium highlights the need to consider contexts such as gender, race and ethnicity in order to fully understand factors influencing cognitive health and ADRD

### LATINOS' PERCEPTIONS AND CONCERNS ABOUT ALZHEIMER'S DISEASE

Laura Y. Cabrera[1], *1. Michigan State University, East Lansing, Michigan, United States*

Several studies indicate that Latinos are at higher risk of developing Alzheimer Disease (AD). While research has centered on African-American/White or Latino/non-Latino differences, there exists heterogeneity within those groups. Clustering Latinos under a single group in AD resources, neglects cultural, biological and environmental differences. To address this complexity we examine perceptions and concerns about AD symptoms, diagnosis, and care among Mexicans and Puerto Ricans via six focus groups. A priori variables for thematic exploration include familiarity, cultural beliefs, trust, privacy, notions of identity and personhood. We use a pragmatic neuroethics framework as a lens to discuss and assess our findings and related implications. This will help address the multidimensional and multidirectional nature of knowledge and communication about diagnosis, treatments and nature of AD. These findings will help to identify differences and similarities among two distinct Latino groups, thereby contributing to scholarship in the fields of Latino's health, aging, and neuroethics.

### PREVALENCE AND RISK FACTORS FOR ADRD AMONG ARAB AMERICANS

Florence Dallo[1], *1. Oakland University, Rochester, Michigan, United States*

In the United States (U.S.), Alzheimer's Disease and Related Dementias (ADRD) afflict over 4.7 million individuals ages 65 or older. Arab Americans are a subgroup of whites in which ADRD is not well understood. This study estimates prevalence and risk factors for ADRD among Arab Americans ages 45 or older. Data for 2000-2017 from the National Health Interview Survey (NHIS) using the region of birth question was used (N=222,219). The age- and sex-adjusted prevalence of ADRD was 10.3% for foreign-born Arab Americans compared to approximately 7.5% for US-born non-Hispanic whites (NHW), blacks and Asians. The prevalence of ADRD was 8.6% for Hispanics (all p-values <.0001). When controlling for age and sex, Arab Americans were 1.4 times (OR=1.02,1.93) more likely to have ADRD compared to US-born NHW. Future studies should capture other generations of Arab Americans to better understand the trend of ADRD among this understudied, often invisible population.

### CHILDHOOD SES AND AGE-RELATED BRAIN CHANGES RACIALLY-ETHNICALLY DIVERSE OLDER ADULTS

Indira C. Turney,[1] Miguel Arce Rentería,[1] Anthony G. Chesebro,[1] Juliet M. Colon,[1] Nicole Schupf,[1] Richard P. Mayeux,[1] Adam M. Brickman,[1] and Jennifer J. Manly[1], *1. Columbia University Medical Center, New York, New York, United States*

Socioeconomic disadvantages in childhood has been linked to dementia in late life. However, the underlying pathways through which childhood socioeconomic status (CSES) affects health in old age is unclear. CSES has been linked to age-related differences in regions affected by Alzheimer's disease (AD; e.g., hippocampus). CSES varies across race/ethnicity; It is critical to examine the relationship between CSES and age-related brain structural changes across diverse aging populations. We used an established proxy for CSES, number of siblings (i.e., sibship size), to examine whether CSES buffered age-related changes in hippocampal volume in a community-based sample of racially/ethnically diverse older adults. Sibship size moderated age-related differences in hippocampal volume in Whites (β=-5.61[-11.09,-0.12]), but not in Blacks and Hispanics. Results indicate that Whites with no sibling (vs. Whites with siblings) show less age-related difference in hippocampal volume. Future analyses will examine other CSES factors (i.e., parental education/occupation) on age-related structural changes across race/ethnicity.

### EXPLORING SEX DIFFERENCES IN COGNITION IN OLDER BLACKS

DeAnnah R. Byrd,[1] and Roland J. Thorpe[2], *1. Wayne State University, Detroit, Michigan, United States, 2. Johns Hopkins, Baltimore, Maryland, United States*
Keith E. Whitfield[1]

Previous literature suggest that women experience more dementia than men. However, it is unclear what accounts for these differences and whether sex differences exist among Blacks over time. We hypothesize that Black women will have worse cognitive outcomes than men and smoking may potentially explain these differences. Longitudinal data from the Baltimore Study of Black Aging-Patterns of Cognitive Aging was used to assess cognitive change over 33 months in five domains. The sample consisted of 602 community-dwelling Blacks, aged 48-92 years at baseline and 450 at follow-up. Findings indicated that Black women reported better vocabulary, working and verbal memory than Black men, controlling for age, education, smoking, and health status. These findings suggest that Black women may have some cognitive advantages in mid to later life compared to Black men. Future research should continue exploring longitudinal sex differences in cognitive domains among Blacks and the underlying drivers of these differences.

### SESSION 2460 (SYMPOSIUM)

### POLICY SERIES: CONGRESSIONAL UPDATE

Chair: Brian W. Lindberg, *The Gerontological Society of America, Washington, District of Columbia, United States*

This popular annual session will provide cutting-edge information on what the 116th Congress has and has not accomplished to date, and what may be left for this year. Speakers will discuss key issues such as Social Security, Medicare, Medicaid, and the Older Americans Act, caregiving, the National Institutes of Health. Hill staffers, advocates, and lobbyists will present.

### SESSION 2465 (SYMPOSIUM)

### INTEREST GROUP SESSION—ABUSE, NEGLECT AND EXPLOITATION OF ELDERLY PEOPLE: DAILY REFLECTIONS OF DEMENTIA CAREGIVERS: MICRO-LONGITUDINAL APPROACHES TO UNDERSTANDING ELDER ABUSE AND NEGLECT

Chair: Carolyn E. Pickering, *University of Texas Health Science Center San Antonio, San Antonio, Texas, United States*
Discussant: Zach Gassoumis, *Leonard Davis School of Gerontology, University of Southern California, Los Angeles, California, United States*

Studies with clinic samples have found approximately 50% of family caregivers self-report engaging in abusive and neglectful acts towards the person with dementia whom they assist. Despite this, interventions to reduce and prevent elder abuse and neglect in dementia caregiving are lacking. To develop targeted interventions, the field still has much to learn about what happens during single incidents of elder abuse and neglect including (1) the types of tactics used (2) contextually-based risk/protective factors and (3) circumstances surrounding acts. This symposium will advance discussions on these topics through presentation of pilot data from a micro-longitudinal study on abuse and neglect within dementia family caregiving. Micro-longitudinal methods, such as daily diary studies, rely on intensive longitudinal measures over shorter periods of time. This approach can better ascertain ecologically-valid factors and identify temporal patterning between variables than traditional longitudinal and cross-sectional methods. First, we will provide an overview of the pilot project with family caregivers (N=50) completing diaries for 21 days. The second presenter will discuss data on the co-occurrence of different types of elder abuse and neglect as they manifest in daily lives of family caregivers, and lead discussion on measurement considerations. The next presenter will discuss data on contextually-based risk and protective factors for abuse and neglect that occur during daily caregiving activities. The final presentation will discuss findings on caregivers' perceptions of the circumstances surrounding abusive and neglectful behaviors. Discussion will focus on how these findings, and methods, can be used to advance intervention development for the field.

### CO-OCCURRENCE OF ABUSE AND NEGLECT AS REPORTED BY DEMENTIA FAMILY CAREGIVERS

Carolyn E. Pickering,[1] Maria Yefimova,[2] and Christopher Maxwell[3], *1. University of Texas Health Science Center San Antonio, San Antonio, Texas, United States, 2. Stanford Healthcare & VA Palo Alto, Menlo Park, California, United States, 3. Michigan State University, East Lansing, Michigan, United States*

Understanding the co-occurrence or overlap among multiple forms of elder abuse and neglect (EAN) is important for designing effective interventions. This paper reports patterns of family caregiver's daily behaviors related to physical assault, psychological mistreatment, and neglect. Majority of participants self-reported at least one EAN behavior (74%), with most reporting using multiple forms of EAN over the 21-day period (52%). On a given day, psychological mistreatment and neglect were more likely to happen in isolation, while physical assault was more likely to co-occur with psychological mistreatment. The mixed model's intra-class coefficient suggests the daily context, rather than personal characteristics, explain the variance in the use of EAN. These findings highlight the importance of never minimizing a single event of EAN reported in clinical practice, give the high rate of polyvictimization, and reinforces the need to understand why caregivers use one form of EAN over another on a given day.

### RISK AND PROTECTIVE FACTORS FOR ABUSE AND NEGLECT IN DAILY CAREGIVING

Maria Yefimova,[1] Carolyn Pickering,[2] Christopher Maxwell,[3] Frank Puga,[2] and Tami Sullivan[4], *1. Stanford Healthcare, Palo Alto, California, United States, 2. School of Nursing, University Of Texas Health Science Center At San Antonio, San Antonio, Texas, United States, 3. School of Criminal Justice, Michigan State University, East Lancing, Michigan, United States, 4. Department of Psychiatry, School of Medicine, Yale University, New Haven, Connecticut, United States*

The stress-process model suggests a variety of factors related to the stress-experience as important in the formation of outcomes including elder abuse and neglect (EAN). Multi-level modeling with days (n=831) nested within caregivers (N=50) was used to evaluate relationships between theoretically-based risk and protective factors and odds of EAN. Disruptions in the daily routine are a significant risk factor for abuse and neglect. Participating in a meaningful activity at least twice a day with the care recipient is a significant protective factor for neglect (OR=0.19; CI=0.06-0.64; p=0.01), but not abuse. Hypotheses that spending the full day together would increase the risk of EAN, and receipt of instrumental support and caregiver participation in self-care would decrease risk, were not supported. Findings demonstrate that the risk of EAN varies from day-to-day in the presence and absence of contextual factors. Moreover, abuse and neglect may have different etiologic pathways.

### CONTEXTS SURROUNDING PSYCHOLOGICAL MISTREATMENT AND NEGLECT BY CAREGIVERS TO PEOPLE WITH DEMENTIA

Kylie Meyer,[1] Kyungmi Lee,[2] Maria Yefimova,[3] and Carolyn Pickering[2], *1. University of Texas Health Science Center San Antonio, San Antonio, Texas, United States, 2. UT Health Sciences at San Antonio, San Antonio, Texas, United States, 3. Stanford Healthcare, Palo Alto, California, United States*

This presentation examines the individual experiences of caregivers using psychological mistreatment and neglect. When a caregiver indicated psychological mistreatment (e.g., yelling) or neglect (e.g., skipping necessary care) occurred on their daily diary, they were asked what they were doing and how they felt when the event occurred. Text responses were analyzed using content analysis. When psychological mistreatment occurred, there was often a triggering event. For example, 43.8% of caregivers reported they were responding to a behavioral symptom, and 28% indicated something inconvenient occurred. Caregivers were mostly frustrated/angry (68.8%) and annoyed (21.9%) when they used psychological mistreatment. When the caregiver neglected the recipient, 43.5% of caregivers reported the recipient refused to receive care and 30% reported prioritizing other care activities. In cases of neglect, caregivers were frustrated/angry (39.1%) and worried/anxious (30.4%). Findings indicate psychological mistreatment and neglect occur in unique contexts; prevention of these behaviors likely will require distinct intervention strategies.

### SESSION 2470 (SYMPOSIUM)

### INTEREST GROUP SESSION—ASSISTED LIVING: DEMENTIA IN ASSISTED LIVING: STATE VARIABILITY IN REGULATIONS, OVERSIGHT, AND RESIDENT OUTCOMES

Chair: Kali S. Thomas, *Providence VA Medical Center, Providence, Rhode Island, United States*
Discussant: Lindsay Schwartz, *American Health Care Association/National Center for Assisted Living, Washington, District of Columbia, United States*

Approximately one million individuals, an estimated 40% with a diagnosis of Alzheimer's disease-related dementias (ADRD), reside in assisted living (AL); yet, little is known about their experience or the quality of care provided in AL. Unlike other forms of long-term care (LTC), the licensing, operating, and enforcement requirements for AL falls to the states, which vary dramatically in their regulatory approaches. The overall objective of this symposium is to examine states' AL regulatory environments and understand if and how the health outcomes of AL residents with ADRD are impacted by states' regulatory decisions. Presenters will highlight the state variability in the regulation, oversight, resident composition, and outcomes of AL residents with ADRD. The first presentation will describe states' different regulatory requirements for staffing and admission/discharge criteria as it relates to residents with ADRD and how those have changed over the last decade. The second presentation will report results from a national survey of state agents regarding their oversight and enforcement activities in AL. The third presentation will characterize differences in the resident composition and healthcare utilization among residents with ADRD across states. The fourth presenter will report on the effect of residing in an AL licensed to provide specialized dementia care versus a standard-licensed AL on ADRD residents' outcomes. The discussant will contextualize findings as they relate to the current state of the AL industry. Results will ultimately inform policy-makers, organizational leaders, and clinicians as they seek the most effective ways to ensure optimal outcomes vulnerable residents with ADRD.

### TWELVE YEARS OF CHANGES IN STATES' ASSISTED LIVING REQUIREMENTS FOR DEMENTIA-SPECIFIC STAFF TRAINING

Paula Carder,[1] Lindsey Smith,[1] Seamus Taylor,[2] Brian Kaskie,[2] and Kali S. Thomas[3], *1. OHSU_PSU School of Public Health, Portland, Oregon, United States, 2. University of Iowa, Iowa City, Iowa, United States, 3. Brown University, Providence, Rhode Island, United States*

We describe two categories of dementia-specific AL requirements: staff training and admission/discharge criteria. We reviewed current requirements for all states and the District of Columbia, and amendments made over 12 years. Current and historic regulations were collected and analyzed using policy surveillance and qualitative coding. Twenty-three states currently require dementia-specific training, and 22 require continuing education. Nearly all states (49) require administrators to complete dementia-specific training. Of these, 13 states specified 7 to 120 hours of dementia care training. Some states added pre-admission screening for cognitive impairment; a few require a dementia diagnosis for admission. We describe state variation longitudinally in direct care staff training requirements, including: number of training hours, training content, and use of examinations or other tests of knowledge, skills and abilities. In addition, we categorize changes in admission/discharge criteria over time, including the use of medical versus behavioral health symptoms.

### RESULTS FROM A 2019 SURVEY OF STATE AGENTS ON ASSISTED LIVING REGULATIONS AND TRENDS

Brian P. Kaskie,[1] Portia Cornell,[2] Paula Carder,[3] and Kali Thomas[2], *1. University of Iowa, College of Public Health, Iowa City, Iowa, United States, 2. Brown University, Providence, Rhode Island, United States, 3. OHSU-PSU School of Public Health, Porland, Oregon, United States*

AL is regulated at the state level. Yet, little is known about the structure and function of state agencies that license and monitor AL. We fielded a 21-question survey among state agents with responsibility for AL in all 50 states. While licensure definitions of AL vary, state efforts appear uniform in regard to administrative alignment with departments of health as well as roles with facility licensing, renewal, and monitoring. However, we observed variability in the approaches used to monitor AL. While 80% of agents reported being able to issue fines for failures to meet regulatory standards, only 40% of states collected information concerning individual resident status. Only 20% issue separate licenses for providing care to persons with dementia, whereas 30% of state agents affirmed that non-licensed AL facilities were operating within their state. We consider how these varied regulatory approaches may shape facility operations and impact resident outcomes.

### DOES MEMORY CARE MATTER? EXAMINING THE EFFECT OF DEMENTIA-LICENSED CARE ON RESIDENTS' OUTCOMES

Portia Y. Cornell,[1] Momotazur Rahman,[1] Wenhan Zhang,[1] and Kali Thomas[1], *1. Brown University, Providence, Rhode Island, United States*

The objective of this study is to estimate the effect of receiving care in a dementia-care licensed (DCL) assisted living community, versus a standard AL, on outcomes of residents with ADRD. In four states that issue a license for specialized dementia care (AL, CO, MS, and NY), we identify a cohort of 5,720 Medicare fee-for-services beneficiaries with ADRD who moved to an AL in 2014. To control for unobserved factors that contribute to a patient's selection of AL type, we use the difference in the log-distances from an individual's home address to the nearest DCL and standard AL as an instrumental variable. We will report the effect of residence in a DCL AL on mortality, inpatient hospital days, emergency department utilization, and hospice use, showing how the use the distance instrument offers differing estimates from unadjusted or multiple-regression methods.

### STATE VARIABILITY IN HEALTHCARE UTILIZATION AMONG ASSISTED LIVING RESIDENTS WITH DEMENTIA

Kali S. Thomas,[1] Portia Cornell,[2] Wenhan Zhang,[2] Momotazur Rahman,[2] David Dosa,[2] Brian Kaskie,[3] and Paula Carder[4], *1. Providence VA Medical Center, Providence, Rhode Island, United States, 2. Brown University, Providence, Rhode Island, United States, 3. Iowa State University, Aimes, Iowa, United States, 4. OHSU-PSU School of Public Health, Porland, Oregon, United States*

The objectives of this study are to describe the state variability in the prevalence of ADRD among AL residents and in this group's healthcare utilization. Using 2014 Medicare enrollment data, Medicare claims, and the Minimum Data Set for a sample of 493,867 Medicare fee-for-service beneficiaries in AL (178,787 with ADRD), we present their rates of hospital and emergency department (ED) use, by state, adjusting for age, sex, race, and dual-eligibility status. States varied in their unadjusted percent of residents with ADRD from 25% in MN to 48% in WV. In 2014, 40% of AL residents with an ADRD diagnosis were hospitalized during the year, ranging from 30% in UT to 47% in FL. Forty-two percent of residents with ADRD used an ED in 2014, ranging from 36% in MD to 56% in NC. The potential influence of state regulations, enforcement, and market factors will be discussed.

### SESSION 2475 (SYMPOSIUM)

### INTEREST GROUP SESSION—CHINESE GERONTOLOGY STUDIES: DEVELOPING A BODY-MIND-SPIRIT CAREGIVER SELF-MANAGEMENT PROGRAM AND A MOBILE APPLICATION FOR CHINESE AMERICANS

Chair: Iris Chi, *University of Southern California, Los Angeles, California, United States*
Discussant: Walter Boot, *Florida State University, Tallahassee, Florida, United States*

Caregivers of older adults are at risk for poor physical and mental health outcomes. Chinese American caregivers, especially those who are recent immigrants, are at risks for developing health symptoms while less likely to seek outside help and utilize caregiver resources due to cultural barriers, low health literacy, and limited-English proficiency. This symposium will introduce the Caregiver Self-Management Program (CSMP), a training program based on the Body-Mind-Spirit model to equip Chinese immigrant caregivers with self-care knowledge, skills, and self-efficacy. We designed the in-person training to be four 3-hour sessions and pilot tested it among 11 Chinese caregivers. The training was well-received by participants; however, the caregivers' duties and their working status, compounded with transportation barriers, leave them little time to participate in the intervention. To address these challenges, we further employed a user participatory approach to design, prototype, and pilot-test a CSMP mobile application (app) to meet the needs of Chinese immigrant caregivers. App functions included interactive multi-media lessons and practice, scheduling of coaching sessions for problem solving, extended readings, community resources, etc. We co-designed the app with seven caregiver co-designers, alpha-tested the app prototype with 12 caregivers, and beta-tested the app in real-life settings with 20 caregivers. Four presentations in the symposium will discuss the CSMP in-person training design and evaluation, influence of Chinese culture on caregiving and impacts of CSMP, user-participatory approach used in co-design and alpha-testing of the app prototype, and preliminary results from the beta-testing to assess external user acceptance and feasibility of the CSMP app.

### A BODY-MIND-SPIRIT CAREGIVER SELF-MANAGEMENT PROGRAM FOR CHINESE AMERICANS: PROGRAM DESIGN

Mandong Liu,[1] Jinli Wu,[1] Geng Zhang,[1] and Iris Chi[1], *1. University of Southern California, Los Angeles, California, United States*

The aging society presents a growing demand for formal and informal caregivers. Literature review reveals that cultural practices and immigration challenges compound the caregiving burden in Chinese American caregivers. The team designed a culturally sensitive and linguistically appropriate training program targeting the overall well-being of Chinese caregivers. The intervention pivots on the Body-Mind-Sprit model, a health model rooted in Chinese traditional philosophies and integrating three components into an inseparable unity. We designed the in-person training to be four 3-hour sessions and incorporated modalities, which include mini lectures, skill training, discussion, exercise videos, homework activities, and a website. We pilot tested the intervention among 11 Chinese caregivers, followed by two focus group discussions and formative evaluation. Participants provided positive feedback in program evaluation survey, while analysis of focus group revealed three major topics: program benefits, barriers to receiving training and applying its content, and suggestions.

### A MIXED-METHODS STUDY ON THE INFLUENCE OF CHINESE CULTURE ON CAREGIVING AND IMPACTS OF CSMP

Jinli Wu,[1] Mandong Liu,[1] Geng Zhang,[1] and Iris Chi[1], *1. University of Southern California, Los Angeles, California, United States*

As a pilot study, the CSMP utilizes a mixed-methods design to tackle a lack of previous evidence and form a well-rounded perspective, using a randomized controlled trial with pre, post and 3-month follow-up assessments and focus group discussions. The CSMP not only targets cultural practices that exacerbates caregiving burden but also forms its basis upon Chinese culture and philosophies. New cultural factors together with previously-identified ones surfaced in discussions, including guanxi (Chinese relationship), renqing (favor), mianzi (self-esteem), social status, respect of authority, communication pattern, age respect, and more. The implications of cultural factors are discussed, together with corresponding areas for future improvement in CSMP and suggestions for professions working with this population. On the other side of the coin, participants who finished the CSMP achieved statistically significantly lower depression level, higher self-efficacy, and lower caregiver burden, compared to the control group.

### REFLECTIVE THINKING OF THE CSMP APP CO-DESIGN AND ALPHA-TESTING PROCESS

Kexin Yu,[1] Shinyi Wu,[1] Mandong Liu,[1] Haojun Jiang,[2] and Iris Chi[1], *1. University of Southern California, Los Angeles, California, United States, 2. Nanjing Agricultural University, Nanjing, Jiangsu, China (People's Republic)*

Time and transportation barriers prevented caregivers with busy schedules to attend the CSMP. Developing a CSMP mobile application (app) and testing its feasibility was hypothesized to increase the accessibility of the CSMP to caregivers. Applying a user-participatory approach, seven caregivers who attended the CSMP in-person intervention participated in a 2-hour co-design session. A CSMP app prototype was developed based on the co-design interview results. The same seven caregivers and another five caregivers who were naïve to the CSMP intervention participated in a 2-hour alpha testing interview to provide feedback to the app prototype. Compared to co-design, participants were able to provide more constructive suggestions regarding app design when the app prototype was shown. The user participatory approach is a dialogue between researchers' knowledge framework and users' lived experience. Both agreement and disagreement regarding app design were identified, yet how to address the discrepancy has not been discussed in the literature.

### BETA-TESTING OF A CSMP APP: AN ASSESSMENT OF USABILITY, UTILITIES, AND CHALLENGES

Shinyi Wu,[1] Kexin Yu,[1] Mandong Liu,[1] Haojun Jiang,[2] and Iris Chi[1], *1. University of Southern California, Los Angeles, California, United States, 2. Nanjing Agricultural University, Nanjing, Jiangsu, China (People's Republic)*

A beta-version of a CSMP app was designed to empower Chinese caregivers with the knowledge, skills, and self-efficacy needed for holistic health self-care at the convenience of using their mobile phone. The key app functions included interactive multi-media lessons and practice, scheduling of coaching sessions for problem solving, and extended readings. Four groups of 5 caregivers (totaling 20) sequentially participated in one-hour lab and one-week in-home testing of the app to identify usability, utilities and challenges of using the app. They also were invited to join a social media group chat to enable peer-to-peer communication for support and sharing. Using post-lab interviews and exit survey, the beta-testing allowed the research team to assess external user acceptance and feasibility of using the CSMP app in real-world settings, and helped the team explore the perceived training requirements, values, challenges, and improvement priorities to materialize learning CSMP curriculum in a mobile environment.

### SESSION 2480 (SYMPOSIUM)

### INTEREST GROUP SESSION—ECONOMICS OF AGING: EVOLVING CARE SYSTEMS FOR OLDER ADULTS: UTILIZATION IMPACTS OF MEDICARE AND MEDICAID PAYMENT TRENDS

Chair: Christine E. Bishop, *Brandeis University, Waltham, Massachusetts, United States*

With the promise of better care coordination, better member outcomes, and lower costs, Medicare and state Medicaid programs are implementing population-based payment systems for older adults. Medicare Advantage (MA) plans are responsible for Medicare services for their members, Medicaid managed long-term services and supports (MLTSS) programs cover a broad span of Medicaid benefits, and some state initiatives enroll beneficiaries dually eligible for both Medicaid and Medicare and integrate benefits from the two programs. Simultaneously, Medicaid programs are attempting to shift LTSS utilization away from nursing homes and toward home and community based services (HCBS). The presentations for this symposium address aspects of this changing landscape using Medicare and Medicaid claims and other data and causal econometric models. The first paper considers the effect of MA utilization on SNF staffing, quality, and financial health. The second paper compares medical care utilization outcomes, specifically risk of hospitalization, for Medicaid nursing home residents to outcomes for similar Medicaid members receiving HCBS. The third paper presents an MLTSS initiative in one state in the context of national developments and considers the challenges of evaluating its impact. The fourth paper compares hospitalization rates for MLTSS populations to rates for dually eligible-beneficiaries not enrolled in MLTSS. The discussion will bring findings together to assess early gains and costs as these systems of care evolve.

### THE IMPACT OF MEDICARE ADVANTAGE ON SKILLED NURSING FACILITY FINANCIAL PERFORMANCE, STAFFING, AND QUALITY

John R. Bowblis,[1] and Sean Huang[2], *1. Department of Economics, Miami University, Oxford, Ohio, United States, 2. Georgetown University, Washington, District of Columbia, United States*

Since the mid-2000s, skilled nursing facilities (SNFs) face an increasing percentage of post-acute care patients enrolled in Medicare Advantage (MA), yet our understanding the of how this affects SNFs is limited. Managed care may provide better coordination and continuous care that enhances SNF quality, but MA plans can also negotiate lower payments, providing SNFs with fewer financial resources to invest in staffing and quality. We use data from 2011-2015 from Medicare Beneficiary Summary File, Minimum Data Set, Certification and Survey Provider Enhanced Reporting, and Medicare Cost Reports to estimate linear fixed effect panel regressions with instrumental variables. We find that SNFs with greater MA share of post-acute care admissions have worse financial performance, lower nursing staff levels, and worse quality as measured by deficiency scores. Our finding favors the hypothesis that MA creates downward financial pressure and strong MA presence in local markets can potentially to spillover to non-MA residents.

### IS BEING HOME GOOD FOR YOUR HEALTH? OUTCOMES OF MEDICAID HOME- AND COMMUNITY-BASED SERVICES

R. Tamara Konetzka,[1] Rebecca J. Gorges,[1] Daniel H. Jung,[1] and Prachi H. Sanghavi[1], *1. University of Chicago, Chicago, Illinois, United States*

Expanding home- and community-based services (HCBS) alternatives to nursing homes has become a priority for many state Medicaid programs. Dramatic policy changes affecting diverse patient and family populations are being made on the basis of surprisingly little evidence about the outcomes of HCBS relative to the alternatives. Our study is designed to address that gap, providing the first national estimates of plausibly causal health outcomes for recipients of Medicaid HCBS relative to nursing home care. We also examine heterogeneity of these effects by several important subgroups. Results indicate that HCBS users have rates of hospitalization similar to their nursing home counterparts, and rates are higher for those with dementia. Furthermore, disparities by race and ethnicity that have been well-documented in nursing homes also appear to extend to the HCBS setting. The costs of potentially poor outcomes need to be part of the policy equation.

### MEDICAID MANAGED LONG-TERM SERVICES AND SUPPORTS: POLICY INNOVATION AND EVIDENCE GENERATION

Howard Degenholtz[1], *1. University of Pittsburgh, Pittsburgh, Pennsylvania, United States*

Twenty-two states have turned to managed care organizations to finance and deliver long-term services and supports (LTSS) as a way to control costs, improve quality and shift the locus of care away from institutional settings. These programs, referred to as Managed Long-Term Services and Supports (MLTSS) have been undertaken under several CMS authorities, and vary in terms of the program design and populations covered. Some programs such as the financial alignment demonstration, have integrated Medicaid and Medicare financing, while others have used waiver authority and three-party agreements to achieve coordination. However, all efforts share the same goal of improving the linkage between LTSS and both physical and behavioral health care. This presentation will provide conceptual framework for understanding and assessing program impact using the example of the multi-method evaluation of the $4 billion Pennsylvania Community HealthChoices program, one of the largest transitions in Medicaid policy to date.

### EFFECTS OF MEDICAID MANAGED CARE ENROLLMENT ON HOSPITALIZATION RISK AMONG DUAL-ELIGIBLES

Rebecca Gorges[1], *1. University of Chicago, Chicago, Illinois, United States*

Many state Medicaid agencies have recently expanded Medicaid managed care (MMC) to include low-income older adults that are Medicare-Medicaid dually enrolled (duals). While little evidence exists regarding the effects of these expansions on health outcomes, duals may be especially vulnerable to low-quality or low-intensity care delivered by MMC due to their high use of services and the fragmentation inherent in the two-payer system. Using difference-in-differences (DID) and instrumental variables estimation, this study provides the first national examination of the impact of MMC on hospitalization for duals using claims data. DID results indicate that managed long-term services and supports (LTSS) expansions among duals are associated with increased rates of hospitalization while comprehensive managed care results in no change in hospital use. Further analyses exploring heterogeneity shed light on how these managed care payment models affect older adults and individuals using LTSS.

### SESSION 2485 (SYMPOSIUM)

### INTEREST GROUP SESSION—HIV, AIDS AND OLDER ADULTS: HIV AND AGING: IMPROVING BIOPSYCHOSOCIAL HEALTH AND FUNCTIONAL WELLNESS

Chair: Monique J. Brown, *University of South Carolina, Columbia, South Carolina, United States*
Discussant: Molly M. Perkins, *Emory University School of Medicine, Atlanta, Georgia, United States*

People living with HIV/AIDS (PLWH) are living longer due to the success of antiretroviral therapy. In 2016, approximately 50% of PLWH in the U.S. were age 50 and older and 17% of new infections were among this age group. One key factor that contributes to the HIV prevalence and incidence among older adults is the lack of sexual health discussion during healthcare visits. Among older (50+) PLWH, most are exhibiting signs of accelerated aging and declines in functional (physical, cognitive and psychosocial) wellness and little is known about the lasting impact of childhood sexual abuse (CSA). In this symposium, we present new research assessing the biopsychosocial challenges faced by older PLWH and older adults at risk for HIV. Dr. Robinson will present findings on the frequency, content and barriers and facilitators in provider communication with older patients on sexual health and HIV. Dr. Brennan-Ing will examine the association between CSA and psychosocial health among older PLWH. Dr. Schrack will assess the impact of energy utilization on physical activity and function among older HIV+ and HIV- men. Dr. Taylor will assess predictors of physical, cognitive and psychosocial wellness among older men with and without HIV. Dr. Sekhar will present findings from a trial on the impact of glycine and N-acetylcysteine supplementation on accelerated aging among older PLWH. Dr. Perkins, will discuss the public health implications of the presented research for HIV prevention and wellness interventions among older adults, and suggest future areas of inquiry to improve clinical outcomes for this population.

### EXPLORING SEXUAL HEALTH AND HIV COMMUNICATION BETWEEN HEALTHCARE PROVIDERS AND OLDER PATIENTS

Erin L. Robinson,[1] Karla Washington,[2] Geuhye Park,[3] Laneshia Conner,[4] and Tara Lusby[1], *1. University of Missouri, School of Social Work, Columbia, Missouri, United States, 2. University of Missouri School of Medicine, Department of Family and Community Medicine, Columbia, Missouri, United States, 3. University of Missouri School of Social Work, Colu, Missouri, United States, 4. Spalding University School of Social Work, Louisville, Kentucky, United States*

While HIV and aging is a complex issue, healthcare providers can play a vital role in education and screening among older patients. Therefore, this study aimed to better understand the frequency, content, and barriers/facilitators of provider communication with older patients around sexual health and HIV. One-on-one interviews were conducted with healthcare providers (N=18) and analyzed using Nvivo12. Providers reported that approximately 1-30% of their older patients show risk factors for STI's. The majority of providers tailor their communication to older patients regarding sex/HIV, however they were divided in who initiates such conversations (themselves or the patient). Providers stated that standardized/routine health assessments help facilitate those conversations. Results indicate several barriers to conversations, such as age/gender differentials and time. Additional results will be discussed. Study findings can help guide provider education and screening of older adults regarding sexual health/HIV.

### LONG-TERM EFFECTS OF CHILDHOOD SEXUAL ASSAULT AMONG OLDER PEOPLE WITH HIV

Mark Brennan-Ing,[1] Liz Seidel,[2] Rebecca Erenrich,[3] and Stephen E. Karpiak[2], *1. Brookdale Center for Healthy Aging, Hunter College, The City University of New York, New York, New York, United States, 2. ACRIA Centers at GMHC, new York, New York, United States, 3. ACRIA Centers at GMHC, San Francisco, California, United States*

Research finds high rates of childhood sexual assault (CSA) among people with HIV (PWH). CSA is related to depression, post-traumatic stress disorder, substance abuse, and poor health. PWH age 50 and older account for the majority of this population in the U.S., but we have little information on the impact of CSA on these older adults. Data were obtained from the San Francisco arm of the Research on Older Adults with HIV 2.0 study (n=197). Fifty percent reported CSA. Cisgender women and transgender people were more likely to report CSA compared to other groups. PWH reporting CSA were more likely to meet the diagnostic criteria for PTSD (42% vs. 27%), and had higher mean PHQ-9 depression scores (9.3 vs. 6.8). Those reporting CSA had significantly more comorbid health conditions compared to their peers. Implications for using a trauma-informed care model with older adults living with HIV will be discussed.

### ENERGY EXPENDITURE AND PHYSICAL ACTIVITY AMONG OLDER PARTICIPANTS FROM THE MULTICENTER AIDS COHORT STUDY (MACS)

Jennifer A. Schrack,[1] Todd T. Brown,[2] and Joseph B. Margolick[3], *1. Johns Hopkins University, Baltimore, Maryland, United States, 2. Johns Hopkins Medical Institutions, Baltimore, Maryland, United States, 3. Johns Hopkins Bloomberg School of Public Health, Baltimore, Maryland, United States*

Energy utilization becomes more inefficient with age and is linked to low physical activity and functional decline. Persons aging with HIV exhibit accelerated functional decline, but the effect of chronic HIV infection on energy utilization and free-living physical activity remains unclear. We investigated cross-sectional associations between age and: resting metabolic rate, peak walking energy (VO2), and 7-day physical activity by accelerometry in 100 men in the MACS (age: 60.8+/-6.8 years, 35% black, 46.1% HIV+, 94% virally suppressed). In multivariable regression models adjusted for age, BMI, race, chronic conditions, and HIV viral load, HIV+ men had a higher resting metabolic rate ($\beta$=103.2 kcals/day, p=0.03) and lower peak walking VO2 ($\beta$=-1.8 ml/kg/min, p<0.02) than HIV- men. Moreover, HIV+ men demonstrated lower physical activity, overall and by time of day (p<0.05). These results suggest that energy utilization differs by HIV serostatus, which may contribute to lower physical activity and function with aging.

### FUNCTIONAL WELLNESS AND OLDER MEN WITH HIV

Tonya N. Taylor,[1] Jeremy Weedon,[1] and Tarik Silk[1], *1. SUNY Downstate Medical Center, Brooklyn, New York, United States*

Aging-related stressors, such as changing physical function, poorly managed multimorbidity, increasing pill burden and social losses can diminish for some older adults with HIV the capacity for self-care. These challenges, however, can be improved by maintaining physical, cognitive and social function, or functional wellness. Using cross-sectional data from a men's health study with younger and older men with and without HIV, we conducted general linear models to identify individual and clinical predictors for physical, cognitive, social and role function, as measured by the Medical Outcomes Study HIV Health Survey. We found that older HIV+ men had lower burdens of functional deficits compared to older HIV- men and younger HIV+ men and that across all models, depression, followed by diabetes, housing, and employment were predictive of functional wellness. Functional wellness for older HIV+ men is a multidimensional construct that includes optimizing internal and external resources to maintain healthy living and wellness.

### CORRECTING GLUTATHIONE DEFICIENCY REVERSES MITOCHONDRIAL DYSFUNCTION AND ACCELERATED AGING IN PATIENTS WITH HIV

Rajagopal V. Sekhar,[1] Premranjan Kumar,[1] James Suliburk,[1] Charles G. Minard,[1] and Chun Liu[1], *1. Baylor College of Medicine, Houston, Texas, United States*

Patients with HIV (PWH) have 'accelerated' aging based on early manifestation of geriatric comorbidities of declining physical-function, elevated inflammation, insulin-resistance, cognitive-impairment and abdominal-obesity, but contributing mechanisms are not well understood and interventions are lacking. We hypothesized that deficiency of the intracellular-antioxidant Glutathione results in impaired mitochondrial fuel-oxidation (MFO) and contributes to these defects, and that supplementing Glutathione precursors glycine and N-acetylcysteine (GlyNAC) could improve these defects. In an open-label trial, 8 PWH were studied before and after 12-weeks of GlyNAC supplementation (and 8-weeks after stopping GlyNAC), and compared to 8 matched, unsupplemented, uninfected controls. PWH had significantly impaired MFO, abnormal molecular regulation of MFO, muscle Glutathione deficiency, physical decline, cognitive-impairment, and higher oxidative-stress, inflammation, insulin-resistance and total body fat. GlyNAC supplementation significantly improved these defects, but benefits receded on stopping GlyNAC. These data suggest that GlyNAC supplementation could reverse 'accelerated aging' in PWH by improving defects linked to impaired MFO.

### SESSION 2490 (SYMPOSIUM)

### HOME IS WHERE THE HEART IS: OPTIMIZING AND TAILORING HOME AND COMMUNITY-BASED SUPPORT FOR DEMENTIA CAREGIVERS

Chair: Quincy M. Samus, *The Johns Hopkins University, Baltimore, Maryland, United States*

Co-Chair: Nancy Hodgson, *University of Pennsylvania, Philadelphia, Pennsylvania, United States*
Discussant: Joseph E. Gaugler, *University of Minnesota - School of Public Health, Division of Health Policy and Management, Minneapolis, Minnesota, United States*

Family caregivers, often "de facto" members of the care team for persons with dementia, play a central role in ensuring safety, support, quality of life, and continuity of care. Most often, they provide this care for loved ones at home and over a long period of time, as the illness progresses and care need intensifies. This session will provide a unique understanding of potential ways to optimize support for family caregivers in provision of day-to-day care in the home by examining often-overlooked factors influential in the health and wellbeing for both caregiver and persons with dementia. Presentations will draw from three large community-based trials testing interventions to support dementia caregivers. Drs. Samus and Sloan will present on common unmet needs identified by family caregivers and explore how needs differ by disease stage and race. Dr. Fortinsky will present baseline caregiver care-related challenges in a diverse cohort of caregivers and the effects of a caregiver intervention designed to mitigate these challenges. Dr. Hodgson will present the common symptoms clusters among home-dwelling persons with dementia patients and how these are associated with caregiver distress. Dr. Jutkowitz will discuss factors influencing dementia caregiver's willingness to pay for help at home. Findings from this session will help elucidate care needs that matter most to family caregivers in diverse community-living cohorts and how we might optimize and tailor supportive home-based interventions to target these needs and challenges.

### COMMON UNMET NEEDS OF DEMENTIA CAREGIVERS AND HOW THESE DIFFER BY DISEASE STAGE AND RACE

Quincy M. Samus,[1] Danetta Sloan,[1] Jeannie-Marie Leoutsakos,[1] Betty Black,[1] and Deirdre Johnston[1], *1. The Johns Hopkins University, Baltimore, Maryland, United States*

This presentation (co-presentation Samus and Sloan) will use combined cross-sectional, baseline data from two intervention studies (n=-642) conducted in Maryland evaluating the impact of dementia care coordination model (MIND at home) to provide a detailed description of common modifiable unmet care needs of family caregivers of community-living persons with dementia (PWD), explore how care needs may differ by the disease stage of the PWD, and presence of racial disparities in care needs. Unmet caregiver needs were identified based on comprehensive in-home assessments using a standardized tool (JHDCNA 2.0) with 6 care domains and 18 items covering caregiver needs. Family caregivers were 77% women; 63 years old (mean); 60% White; and 52% adult children. The most prevalent needs were for education/resources (98%), legal (74%), mental health (44%), and informal support (43%). Needs varied based on dementia severity. African American caregivers (vs. white) had significantly more unmet caregiver needs at baseline (p<.001).

### FACTOR STRUCTURE OF NEUROPSYCHIATRIC SYMPTOM FREQUENCY IN HOME-RESIDING PERSONS WITH DEMENTIA

Nancy Hodgson,[1] Natalie Regier,[2] and Laura Gitlin[3], *1. University of Pennsylvania, Philadelphia, Pennsylvania, United States, 2. The Johns Hopkins University, Baltimore, Maryland, United States, 3. Drexel, philadelphia, Pennsylvania, United States*

This study evaluated neuropsychiatric symptom clusters in a sample of home residing persons with dementia, and the impact of distinct clusters on family caregiver outcomes. The expanded Neuropsychiatric Inventory (NPI-C), including measures of frequency, was collected at baseline from 250 caregivers enrolled in Reducing Agitation in People With Dementia: the Customized Activity Trial [NCT01892579]. Principle component analyses were conducted resulting in an eight behavioral clusters, accounting for 44% of total variance: 1=agitation/aggression; 2=anxiety; 3=apathy/withdrawl; 4=impulsivity; 5=psychosis; 6=restlessness; 7=circadian disturbance; 8=depression. In multiple linear regressions caregiver burden was significantly influenced by the anxiety cluster. Caregiver depression was significantly influenced by apathy/withdrawal cluster, and quality of life was significantly associated with anxiety and circadian disturbance clusters. Dimensional representation of neuropsychiatric symptom clusters can be useful in assessing the effect of multiple co-occurring symptoms (versus discrete individual symptoms) on caregiver outcomes and for planning personalized intervention strategies for persons with dementia.

### INTERVENTION EFFECTS ON TARGET PROBLEMS EXPRESSED BY CAREGIVERS OF HOME-DWELLING PERSONS WITH DEMENTIA

Richard H. Fortinsky,[1] Laura N. Gitlin,[2] and catherine V. Piersol[3], *1. University of Connecticut School of Medicine, Farmington, Connecticut, United States, 2. Drexel University, Philadelphia, Pennsylvania, United States, 3. Thomas Jefferson university, Philadelphia, Pennsylvania, United States*

Care of Persons with Dementia in their Environments (COPE) is an evidence-based in-home intervention designed to optimize function and activity engagement in persons with dementia (PWD), and teach family caregivers (CG) how to manage dementia care-related problems. In this presentation, we report problems expressed by CGs, and intervention effects on these problems and CG outcomes, in the COPE CT translational study. CGs randomized to COPE who completed the assessment phase (N=134) expressed a total of 409 target problems, grouped as managing PWD behavioral problems (32%), caring for themselves (30%), managing PWD daily activities (24%) and engaging PWD in meaningful activities (14%). Most problems were reduced (75%) or eliminated (21%) among CG completing the intervention. In preliminary outcome analyses, compared to CG not receiving COPE, CG receiving COPE were more likely to report improved ability to manage dementia-related cognitive and behavioral symptoms (p<0.001). Implications for scaling COPE will be discussed.

### CHANGE IN DEMENTIA FAMILY CAREGIVERS' WILLINGNESS TO PAY FOR A NONPHARMACOLOGIC INTERVENTION

Eric Jutkowitz,[1] Daniel Scerpella,[2] Katherine Prioli,[3] Katherine Marx,[2] Laura N. Gitlin,[4] Laura Pizzi,[3] and Jonah Popp[1], *1. Brown University, Providence, Rhode Island, United States, 2. Johns Hopkins University, Baltimore, Maryland, United States, 3. Rutgers University, Piscataway, Maryland, United States, 4. Drexel University, Philadelphia, Pennsylvania, United States*

Family caregivers provide a majority of care for persons with dementia (PwD); however, little is known about caregiver's willingness to pay (WTP) for an intervention to help them manage dementia symptoms. To fill this gap, caregiver/PwD dyads (n=223) were recruited to participate in a randomized trial evaluating tailored activities to minimize behavioral symptoms and functional decline. At baseline and 6-months caregivers were asked their WTP per session for the 8-session 3-month program compared to caregiver education/support only. At baseline, treatment caregivers were WTP $26.20, which was $11.50 (95%CI:-$12.70, -$10.3) less per session compared to control group caregivers WTP $37.30. At 6-months, treatment caregivers were WTP $22.90 and control caregivers $27.30. From baseline to 6-months, a change in WTP was $7.00 (95%CI:$5.80, $8.30) greater than the change in WTP for control group caregivers. Caregivers WTP slightly decreases over time in both groups but decrease is less for TAP following program participation.

### SESSION 2495 (PAPER)

### IMPACT OF VISION AND HEARING IMPAIRMENTS

### A MODEL OF CARE FOR OLDER ADULTS WITH IMPAIRED VISION OR BLINDNESS AT THE END OF LIFE

Ximena Morales,[1] Ricardo Blondet,[2] Marcos Milanez,[2] Deepak Mandi,[2] Gabino Lares,[2] and Michael A. Silverman[2], *1. Trustbridge Hospice, West Palm Beach, Florida, United States, 2. West Palm Beach VA Medical Center, West Palm Beach, Florida, United States*

Our objectives were to describe unique challenges of visually-impaired patients receiving end-of-life institutional care and to propose a model for the comprehensive care of the vision-impaired patients incorporating bedside techniques and advanced assistive technology. The prevalence of visual impairment in long-term care is increasing. Collaborating with our Blind Rehabilitation Center, we have summarized a care model including the identification of patients who have impaired vision and adjustments of daily routine. Care was consistently provided by staff with voices familiar to patients. Staff is trained to introduced themselves clearly by voice when entering the room. Patients engage in hobbies that are less dependent on vision, such as music therapy. Safety measures are taken to facilitate mobility. We describe the case of a 90-year-old WWII Veteran with dementia and dysphagia who was legally blind and required extensive assistance with his ADLs. Although initially calm, the patient eventually became delirious, reliving his time as a gunner in the Navy and believing he was firing on Japanese Kamikaze planes. After his visual impairment was addressed using the approach described above, the patient became calmer. Listening to his wife's voice and enjoying his favorite gospel music helped him cope better with the situation until he died peacefully on hospice care. In conclusion, a model of care considering visual impairment was effective at alleviating distress. More emphasis needs to be placed on evaluating and managing sensory impairment when providing care for older adults approaching the end of life.

### HEARING IMPAIRMENT, COGNITIVE PERFORMANCE, AND BETA-AMYLOID DEPOSITION IN THE ARIC-PET AMYLOID IMAGING STUDY

Jennifer A. Deal,[1] Andreea Rawlings,[2] A. Richey Sharrett,[3] Nicholas Reed,[3] Joshua Betz,[3] Thomas Mosley,[4] Frank Lin,[3] and Rebecca Gottesman[3], *1. Johns Hopkins University Bloomberg School of Public Health, Baltimore, Maryland, United States, 2. Kaiser Permanente Center for Health Research, Portland, Oregon, United States, 3. Johns Hopkins University, Baltimore, Maryland, United States, 4. University of Mississippi Medical Center MIND Center, Jackson, Mississippi, United States*

Hearing impairment is a risk factor for dementia but the mechanism underlying this association is unknown. We investigated the relationship between hearing and cognitive performance and brain β-amyloid deposition in 252 adults aged 67-88 years (37% black race) without dementia from three U.S. communities. Global cortical standardized uptake value ratios (SUVRs) were calculated from florbetapir-positron emission tomography scans, with elevated SUVR defined as >1.2 (the median value). Air conduction hearing threshold levels for the frequencies of 0.5, 1, 2 and 4 kHz were obtained by pure tone audiometry and averaged for the better-hearing ear to yield a pure tone average (PTA) in decibels hearing level (dB). A composite cognitive score was created from ten neuropsychological tests summarized using latent variable methods. Multivariable-adjusted linear and Poisson regression with robust standard errors were used to estimate the average difference in cognitive scores and prevalence of elevated SUVR, respectively, by hearing impairment status. In analyses adjusted for age, sex, education and APOE ε4 status, hearing was not associated with elevated SUVR [prevalence ratio per 10 db increase (worse hearing) = 0.94 (95% CI: 0.84, 1.04)]. Results did not differ by race. In contrast, each 10 db increase in hearing impairment was associated with 0.08 standard deviation (95% CI: 0.02, 0.15) lower cognitive score, after adjustment for demographic and cardiovascular factors. Poorer hearing is associated with poorer cognitive performance but not with amyloid deposition, suggesting that the mechanism underlying the relationship between hearing and cognition may be independent of Alzheimer's-related pathologic brain changes.

### SELF-REPORTED VISION IMPAIRMENT AND SUBJECTIVE WELL-BEING IN OLDER ADULTS: A LONGITUDINAL MEDIATION ANALYSIS

Joshua R. Ehrlich,[1] Xiaoling Xiang,[1] Khushali Shah,[2] Rita X. Hu,[3] Brian C. Stagg,[4] and Vicki A. Freedman[1], *1. University of Michigan, Ann Arbor, Michigan, United States, 2. University of Miami, Miami, Florida, United States, 3. University of Michigan, Ann Arbor, Ann Arbor, Michigan, United States, 4. Duke University, Durham, North Carolina, United States*

Vision impairment (VI) in older adults is associated with declines in well-being. However, the pathways through which poor vision leads to declines in well-being have not been well described. The purpose of this study was to determine whether activity limitations and social participation restrictions mediate the impact of self-reported VI on subjective well-being. This study used data from the National Health and Aging Trends Study (NHATS), a nationally-representative longitudinal study of Medicare beneficiaries 65 and older that includes detailed measures of the disablement process. We conceptualized a longitudinal mediation model linking self-reported VI and subjective well-being. Structural equation modeling was used to test the mediating effects of activity limitations and social participation restrictions while adjusting for covariates. The final sample included 5,431 respondents. At baseline, 8.0% of Medicare beneficiaries had self-reported VI. Subjective well-being scores were significantly lower among respondents with self-reported VI (15.7, 95% CI=15.2, 16.2) compared to those without VI (17.6, 95% CI=17.5, 17.7). Self-reported VI had a significant indirect effect on subjective well-being through limiting mobility (β=-.04, 95% CI=-.07, -.03) and household activities (β=-.05, 95% CI=-.08, -.03), but not self-care limitations (β=0.0, 95% CI=0.0, 0.0) or participation restrictions (β=0.0, 95% CI=-0.01, 0.00). Total indirect effects from all mediation paths accounted for 42% of the effect of VI on well-being. In conclusion, mobility and household activity limitations are significant mediators that explain a considerable portion of the impact of poor vision on well-being. Interventions to promote successful accommodation may result in greater well-being for visually impaired older adults.

### SUBCLINICAL AGE-RELATED HEARING LOSS IS INVERSELY ASSOCIATED WITH DEPRESSIVE SYMPTOMS

Justin S. Golub,[1] Katharine K. Brewster,[2] Adam Brickman,[1] Adam Ciarleglio,[3] José Luchsinger,[1] and Bret Rutherford[1], *1. Columbia University, New York, New York, United States, 2. Columbia University Medical Center, New York, New York, United States, 3. George Washington University, Washington, District of Columbia, United States*

Age-related hearing loss (HL), defined by a pure-tone average (PTA) >25 decibels (dB) has been associated with depressive symptoms. We aimed to assess whether this association is present when hearing is better than the arbitrary, but widely-used, 25 dB threshold. The sampled population was the multicentered Hispanic Community Health Study (n=5,165). Cross-sectional data from 2008-2011 were available. Hearing was measured with pure tone audiometry. Clinically-significant depressive symptoms (CSDS) were defined by a score ≥10 on the 10-item Center for Epidemiologic Studies Depression Scale (CESD-10). Participants' mean age was 58.3 years (SD=6.2, range=50-76). Among those with classically-defined normal hearing (PTA ≤25 dB), a 10 dB increase in HL was associated with 1.26 times the odds (95% CI=1.11, 1.42) of CSDS, adjusting for age, gender, education, vascular disease, and hearing aid use (p25 dB; p<0.001). Results held even for a stricter HL cutpoint of 15 dB. Among subjects with strictly normal hearing (PTA ≤15 dB), a 10 dB increase in HL was associated with 1.47 (1.14, 1.90) times the odds of CSDS, adjusting for confounders (p<0.01). Results also held when defining CSDS by an alternative CESD-10 score ≥16. In conclusion, increasing hearing thresholds were independently associated with CSDS among adults with subclinical HL (PTA ≤25 dB). Studies investigating whether treating HL can prevent late life depression should consider a lower threshold for defining HL.

### SESSION 2500 (SYMPOSIUM)

### INTEREST GROUP SESSION—RURAL AGING: INNOVATIONS THAT SUPPORT RURAL OLDER ADULTS' HEALTH AND WELL-BEING: MODELS, NETWORKS, CASE STUDIES, REFLECTIONS

Chair: Roger O'Sullivan, *Institute of Public Health in Ireland, Belfast & Dublin, Ireland*
Co-Chair: Lyn Holley, *University of Nebraska, Omaha, United States*
Discussant: Marc A. Guest, *University of Kentucky, Lexington, Kentucky, United States*

Access of rural older people to health and wellness services is limited and becoming progressively more limited as trends toward increasing centralization of Government and private services continue. "Top-down" or urban centric models for rural service delivery often miss context essential to effectiveness and sustainability. In this symposium, each presenter in this multidisciplinary group of researchers presents innovative, community-based interventions that address these challenges using different methodologies and in respect to different needs Maiden (Psychology) compares the utilization of mental health services by rural older adults over time with their need for such services. Through the lens of social gerontology Holley examines networks of support that have intersected successfully to generate local solutions to unmet needs of rural-dwelling older adults. Crowther and Ford within a nursing and care context explore community-based models that draw upon the role of culture to integrate care for rural older adults. Katz, from an adult development perspective, reports on an educational game-intervention developed to reduce cognitive decline which is tailored specifically for older adults in rural areas. Wiese presents evidence from a pilot home-based approach that demonstrates a model for increasing rates of AD detection and treatment in a rural retired farm worker community in Florida. Our discussant, an emerging scholar in the field of rural gerontology, will reflect on the major themes that emerge from these multidisciplinary perspectives, especially the role of intersecting networks in community-based innovations and rural aging.

### CASES OF OPTIMALLY LOCAL SOLUTIONS TO UNMET NEEDS OF RURAL-DWELLING OLDER ADULTS: ROLES OF NESTED NETWORKS

Lyn Holley,[1] Kara l. Kohel,[2] Holly Hatton-Bowers,[2] and Susan Harris-Broomfield[2], *1. University of Nebraska, Omaha, United States, 2. University of Nebraska–Lincoln, Lincoln, Nebraska, United States*

Solutions developed top-down frequently make sub-optimal use of resources. Programs (e.g., caregiver respite) are studied extensively; study focused on the roles of nested networks (family/locality/state/nation) that intersect in care is lacking. To identify and assess potential for improving solutions, this study examines cases acknowledged to provide optimal support. It identifies and describes network roles and intersections critical to success, with particular attention to timing and intentionality of family and community interfaces. Findings may suggest improved design and operation of programs through targeted empowerment of networks Cases were identified in cooperation with the Nebraska Extension service, and analyzed by a multidisciplinary team that included Family Science and Gerontology. Rural-dwelling older adults who benefited from the solution, expert practitioners, officials and local champions were interviewed. Analysis included private and public actors, and explains outcomes within a cultural (e.g., individualist, independent) and opportunity (e.g. information, financial and human resources) framework (Gelfand, 2003).

### IDENTIFYING BARRIERS TO MENTAL HEALTH SERVICES IN A RURAL COMMUNITY

Robert J. Maiden,[1] Danielle Gagne,[2] Daniel l. Segal,[3] and Bert Hayslip Jr.[4], *1. Alfred University, Alfred, New York, United States, 2. alfred University, New York, New York, United States, 3. University Of Colorado, Colorado, Colorado, United States, 4. University of North Texas, Denton, Texas, United States*

Unmet mental health care needs of older people living in rural areas have been identified as a fundamental problem. This project engaged a rural consortium of service agencies to support recruitment through advertising, word of mouth, social media. So far, 100 rural participants aged 50 and older have completed our survey which includes the revised Barriers to Mental Health Services Scale, (BMHSS-R) which measures intrinsic barriers attributed to internal characteristics and beliefs, e.g. stigma, and extrinsic barriers, e.g. insurance costs, and lack of transportation. Preliminary results revealed increased services utilization compared to past research. However, several serious barriers remained, e.g. as lack of insurance/costs, distance/location, stigma, and lack of knowledge. The BMHSS-R results e demonstrate how the two types of barriers are related and interact within individuals. Implications are that internal barriers (e.g., stigma) and external ones (location, costs) can be reduced or eliminated through integrated medical/behavioral services.

### USING COMMUNITY-BASED MODELS TO PROVIDE INTEGRATED CARE FOR RURAL OLDER ADULTS

Martha R. Crowther,[1] and Cassandra D. Ford[2], *1. The University of Alabama, Tuscaloosa, Alabama, United States, 2. The University of Alabama, Capstone College of Nursing, Tuscaloosa, Alabama, United States*

Rural elders are one of the most at-risk populations for experiencing physical and mental health problems. In many rural communities, there are no psychosocial services available to meet the needs of the rural elderly. To provide rural older adults with integrated healthcare, we build upon our existing community-based infrastructure that has fostered community capacity for active engagement in clinical activities and has served as a catalyst to increase participation of rural older adults in clinical services. Our rural community model draws upon the role of culture in promoting health among rural older adults to provide rural service delivery. This model is built upon our network of partnerships with surrounding communities, including potential research participants, community-based organizations, community leaders, and community health-care systems and providers. By engaging the community we can create a sustainable system that will encourage rural older adults to utilize the health care system at a higher rate.

### A COMMUNITY-BASED INTERVENTION TO IMPROVE COGNITIVE FUNCTION IN RURAL APPALACHIAN OLDER ADULTS

Benjamin Katz[1], *1. Virginia Tech, Blacksburg, Virginia, United States*

Research suggests that social isolation in Appalachian older adults may be associated with reduced cognitive function. Despite this, few interventions for these individuals incorporate both social and cognitive components in a community-based setting. The “Memory Masterclass” program was developed to address this care challenge, and implemented through an Adult Day Services Center. With 29 community-dwelling older adult participants from western Virginia, the six-week pilot program focused on strategies and practices associated with improving long-term memory or executive function. In addition to the lifestyle-focused curricula, the course included group activities focused on connecting participants to community networks of resources that might enable them to successfully implement lifestyle changes. Findings support the feasibility of implementation with a wider group of rural Appalachian older adults and suggest that individual differences in self-reported memory may be closely tied to improvements following the program.

### LEARNING FROM IN-DEPTH COGNITIVE ASSESSMENTS CONDUCTED IN RURAL INDEPENDENT LIVING FACILITIES

Lisa K. Wiese,[1] Christine Williams,[2] James E. Galvin,[2] Debra Hain,[2] and David Newman[2], *1. C.E. Lynn College of Nursing, Florida Atlantic University, Boca Raton, United States, 2. Florida Atlantic University, Boca Raton, Florida, United States*

Despite benefits to earlier Alzheimer’s disease interventions, efforts to increase dementia detection and treatment in high-risk rural areas are lacking. Barriers include lack of resources, including limited time for providers to conduct in-depth cognitive assessments. A pilot program tested the effectiveness of a home-based approach for increasing rates of AD detection and treatment in a rural retired farmworker community. Depression and cognitive screenings of 139 residents conducted by community health workers were followed up with in-depth geriatric-focused assessments, including the Moca-B, by experienced, culturally diverse adult gerontological nurse practitioners (AGNPs). Their findings were forwarded to primary providers. This approach was evaluated for effectiveness using correlations, regressions, and Chi-square analyses of variables on rates of ADRD screening, referrals, and ADRD diagnosis.

### SESSION 2505 (SYMPOSIUM)

### INTEREST GROUP SESSION—QUALITATIVE RESEARCH: INNOVATIVE QUALITATIVE AND MIXED-METHODS STUDIES OF OLDER ADULTS' LIVED EXPERIENCES IN NETWORKS OF CARE AND CARING

Chair: Allyson M. Washburn, *National University, La Jolla, California, United States*
Discussant: Abby Schwartz, *East Carolina University, School of Social Work, Greenville, North Carolina, United States*

Researchers are challenged both when querying older adults about their lived experience and later when analyzing these rich interview data to characterize and preserve their truths. This symposium presents innovative strategies to meet these challenges in ways that deepen our understanding of older adults' lives within their networks of care and caring. Participants in two interpretive phenomenological studies were family caregivers. One examined the impact of the Virtual Dementia Tour® on participants' perceptions of their family member's lived experience of dementia, identifying changed realities and approaches to caregiving following their experience. The second analyzed in-depth interviews of aging caregivers about future care for their adult children diagnosed with autism spectrum disorders, finding that they had difficulty identifying caregiving support for the future while feeling the need to make plans and decisions now. Two mixed methods studies explored older adults' experience of their current environment. In the first, measures of social cognition and engagement informed the interpretation of combined phenomenological-hermeneutic and conventional content analyses of nursing home residents' responses to questions about their relationships, finding that they valued day-to-day social interactions as connections with longtime friends were maintained. The second study combined focus groups, in-depth interviews, and web-based surveys during the psychometric testing of the Person-Place Fit Measure for Older Adults to assess older adults' experience of aging in a particular place with people they find important. The strategies detailed in these four papers transformed more conventional approaches to deepen our understanding of older adults' experiences of their lives with others.

### FAMILY DEMENTIA CAREGIVERS' EPIPHANIES AND CHANGES IN THEIR APPROACH TO CARING FOLLOWING THE VIRTUAL DEMENTIA TOUR

Candace C. Harrington,[1] and Candace C. Harrington[2], *1. East Carolina University, Greenville, United States, 2. East Carolina University, Zebulon, North Carolina, United States*

Previous interventional studies have failed to show long-term improvements in caregiver stress, health indices, burden, or delay in long-term care placement. The Virtual Dementia Tour® (VDT) provides a vicarious first-person perspective of symptoms related to dementia. This interpretative phenomenological study revealed family dementia caregivers' perceptions of the VDT® and its impact on their perception of a person living with dementia. In-depth open semi-structured interviews were conducted with ten VDT® participants following a community event. Participants' statements described a life-changing process with eye-opening epiphanies about the lived experience of dementia and served as a "call to action" to change their approach to caring. Innovative advances in family caregiving research are critical to support this valuable geriatric workforce. This original study provided new knowledge about the value of the VDT® to inform interventions that harness the unrecognized power of vicarious experiences like the VDT® for family dementia caregivers to improve long-term outcomes.

### THE USE OF QUALITATIVE METHODS IN UNDERSTANDING FEARS FOR THE FUTURE AMONG AGING CAREGIVERS OF ADULTS WITH ASD

Christina N. Marsack-Topolewski,[1]
Christina Topolewski,[1] and Jillian Graves[1], *1. Eastern Michigan University, Ypsilanti, Michigan, United States*

Understanding the realities of enduring caregivers as they age is essential to providing adequate supports and services to support the both caregiver and care recipient. An analysis of 51 one-on-one interviews was used to examine the lived experiences of aging caregivers, specifically regarding future care plans and barriers for their adult children diagnosed with autism spectrum disorders (ASD). Using a phenomenological research design, our qualitative analysis included line-by-line analysis, followed by PI and co-investigator collaboration to discuss findings. As a collaborative process, the codes and themes were refined by collapsing and consolidating codes. Intensive interviewing, coupled with systematic qualitative analysis, yielded substantive responses around aging caregivers' experiences. Results highlighted four main themes that emerged from the interview responses, including (1) difficulty identifying caregiving support for the future, (2) barriers to making plans/decisions, (3) fear of the unknown, and (4) feeling the need to make plans and decisions now.

### THE SYNERGY OF QUALITATIVE AND MIXED METHODS TO EXPLORE THE RELATIONSHIP BETWEEN OLDER ADULTS AND THEIR SENSE OF PLACE

Joyce Weil[1], *1. University of Northern Colorado, Colorado, United States*

Understanding person-environment fit, or how well older adults' environment suits their needs, is complex. While all methodological approaches to gain deeper knowledge about fit have great value, the combination of methodologies provides a layered account of the experience of aging in a place, including the people older adults find important. This research found a clear benefit of combining results of 2 focus groups, 85 in-depth qualitative interviews, and 100 web-based surveys to assess how older adults discuss who and what is meaningful to them. Results show the combined benefit of using a qualitative and mixed-method process during the psychometric testing of the Person-Place Fit Measure for Older Adults. Qualitative focus groups and interviews offered additional domains and exact language older adults used to discuss place. Early qualitative phases improved later stages of this research, namely, online testing, reduction of survey items, and psychometric testing of the final quantitative instrument.

### A MIXED-METHODS STUDY OF NURSING HOME RESIDENTS' EXPERIENCES OF RELATIONSHIPS AND DAY-TO-DAY SOCIAL INTERACTIONS

Allyson M. Washburn,[1] and Susan Williams[2], *1. National University, La Jolla, California, United States, 2. National University, Stockton, California, United States*

Nursing home residents with and without cognitive impairment (N=38) answered open-ended questions about their day-to-day social interactions and ongoing relationships with family and friends. One author (SW) completed a conventional content analysis of the transcripts and the other (AW), a phenomenological-hermeneutic analysis. Findings from these analyses were combined and examined further using data from measures of social cognition and staff ratings of social behavior. Participants' social experiences appeared to be determined not only by long-established habits and preferences and length of nursing home stay but also by their cognitive status and social cognition competencies. A central theme was the importance of managing ongoing relationships and day-to-day interactions so as to reduce one's own stress as well as the burden on others. This presentation details how findings from distinct analytic strategies were combined to characterize the researchers' understanding of participants' lives in their networks of care from their own perspective.

### SESSION 2510 (SYMPOSIUM)

### IRVING S. WRIGHT AND VINCENT CRISTOFALO AWARD LECTURE

Chair: Stephanie Lederman, *American Federation for Aging Research, New York, New York, United States*
Co-Chair: Hattie Herman, *American Federation for Aging Research, New York, New York, United States*

The Vincent Cristofalo Rising Star Award in Aging Research lecture will feature an address by the 2018 recipient, Nathan K. LeBrasseur, PT, PhD, of the Robert and Arlene Kogod Center on Aging, titled "Biomarkers of Senescent Cell Burden." The Irving S. Wright Award of Distinction Lecture will feature an address by the 2018 recipient Pinchas Cohen, MD, of the USC Leonard Davis School of Gerontology, titled "Mitochondrial System Biology as a Window Into Diseases of Aging." These awards are given by the American Federation for Aging Research, Inc.

### MITOCHONDRIAL SYSTEM BIOLOGY AS A WINDOW INTO DISEASES OF AGING

Pinchas Cohen[1], *1. Leonard Davis School of Gerontology, Los Angeles, California, United States*

We identified multiple open-reading-frames (ORFs) within the mitochondrial genome. These ORFs encode putative peptides that we call Mitochondrial-Derived-Peptides (MDPs) which represent a sub-class of a growing group of novel micropeptides (from both mtDNA and nuclear chromosomes) that serve as signals related to cell and organismal protection and energy expenditure. We described multiple peptides including humanin, SHLPs, and MOTS-c. Exploring mtDNA methylation patterns as well as mito-transcriptomics demonstrated changes in specific ORFs/MDPs in certain diseases. We developed a modified GWAS bioinformatic technique (MiWAS) that identifies SNPs within MDPs that associate with diseases of aging. MOTS-c, and MENTSH, novel anti-obesity/diabetes MDPs, harbors mutation in Asians and Native-Americans, associated with diabetes risk. Thus, MDPs are expressed in an ethno-specific fashion and may contribute to health disparities in a manner related to relevant mitochondrial DNA SNPs. In summary, MDPs are a new class of mitochondrial-hormones that have diagnostic and therapeutic potential in human disease.

### BIOMARKERS OF SENESCENT CELL BURDEN

Nathan LeBrasseur[1], *1. Robert and Arlene Kogod Center on Aging, Mayo Clinic, Rochester, Minnesota, United States*

Senescent cells drive aging. Preclinical studies suggest that targeted elimination of senescent cells offers a unique therapeutic approach to counter numerous chronic diseases and geriatric syndromes. To foster the translation of basic science discoveries to clinical application, we have sought to identify circulating biomarkers that reflect systemic senescent cell burden. We first analyzed the secretome of multiple senescent human cell-types and developed a candidate panel of proteins that could be reliably measured in human blood. Multiple proteins demonstrated significant associations with chronological age in a community-based cohort of adults aged 20-to-90 years. Impressively, in two distinct surgical cohorts (severe aortic stenosis and ovarian cancer), candidate protein concentrations were associated with biological age indices, including frailty and adverse outcomes. Our data suggest senescence biomarkers may have utility for clinical practice as indicators of risk, and for clinical research as surrogate endpoints in trials of interventions targeting senescent cells.

### SESSION 2515 (SYMPOSIUM)

### KANSAS'S PEAK 2.0: AN ACADEMIC-STATE PARTNERSHIP IMPROVING THE LIVES OF NURSING HOME RESIDENTS

Chair: Gayle Doll, *Kansas State University, Manhattan, Kansas, United States*
Co-Chair: Laci Cornelison, *Kansas State University, Manhattan, Kansas, United States*
Discussant: Robyn Stone, *LeadingAge, Washington, District of Columbia, United States*

Most academic institutions welcome partnerships with industry and state government. These collaborations can lead to interventions to create social and environmental changes on a broad scale. Along with the opportunities, some challenges are inherent with these working relationships. The Kansas State University Center on Aging and the Kansas Department for Aging and Disability Services has been working together for more than 15 years on the Promoting Excellent Alternatives for Kansas nursing homes (PEAK) program. This collaboration has led to beneficial changes for nursing home residents and provided fertile ground for researchers wanting to examine these environments. This symposium will offer researcher insights as well as to elucidate process and procedures related to developing and maintaining collaborations with a state agency.

### PEAK 2.0: OPERATIONALIZING PERSON-CENTERED CARE AIDS NURSING HOMES IMPLEMENT AND SUSTAIN PRACTICES

Laci Cornelison,[1] Gayle Doll,[1] Maggie Syme,[1] and Migette Kaup[1], *1. Kansas State University, Manhattan, Kansas, United States*

Pay-for-performance programs to incentivize quality are on the rise nationally (Werner, Konetzka, & Polsky, 2013 & Arling, Job, & Cooke, 2009). Kansas initiated a Medicaid P4P program to incentivize person-centered care (PCC) beginning in 2012 called PEAK 2.0. This program created an operationalized definition of PCC through stakeholder collaboration and research outcomes (Harris, Poulsen, & Vlangas, 2006). Homes enrolled in the program undergo both self-evaluation and objective external evaluation based on the operationalized definition. These key features inherent in the PEAK 2.0 program make up has aided homes to implement PCC as well as, the ability to research homes that have implemented PCC in a new a different way than ever before.

### PEAK RESIDENT SATISFACTION OUTCOMES AND RESEARCH

Judy Poey,[1] and Laci Cornelison[2], *1. United Way of Central Maryland, Baltimore, Maryland, United States, 2. Kansas State University, Manhattan, Kansas, United States*

Outcomes related to person-centered care in nursing homes have been difficult to ascertain. Much of the extant literature has suffered from differing definitions of what it means to be person-centered, variation in the levels of implementation of person-centered care that an organization has achieved, and small sample sizes. The PEAK program provides a unique opportunity to control for these variables across a large sample of nursing homes throughout the state of Kansas. This presentation will discuss the methodological advantages of evaluating the PEAK program and the findings from an evaluation of resident satisfaction in nursing homes at varying levels of implementation of person-centeredness.

### MAKING IT WORK: PEAK 2.0 PROGRAM EVALUATION RESEARCH

Maggie Syme,[1] Gayle Doll,[1] Laci Cornelison,[1] and Migette Kaup[1], *1. Kansas State University, Manhattan, Kansas, United States*

Research opportunities from our organizational partnerships allow us to pursue answers to questions about PCC outcomes that have largely been out of reach for the field. Our program evaluation research agenda includes groundbreaking, short-term clinical outcomes from older Kansans living in nursing facilities implementing increasingly levels of PCC, including a 49% lower prevalence of depression for residents in strong PCC adopting homes (Hermer et al., 2017; Hermer et al., 2018). Yet there have been several challenges to achieving these goals that are atypical to the common academic research process. This presentation will further highlight the clinical outcomes association with PEAK 2.0, our continued research partnership with the state, and highlight some of the unique opportunities and challenges large-scale research with a state organization presents.

### APPLICATIONS OF QUALITATIVE RESEARCH IN DESCRIBING PCC ENVIRONMENTS: THE STORY BEHIND THE STATISTIC

Migette Kaup,[1] Judith Poey,[2] Gayle Doll,[1] and Laci Cornelison[1], *1. Kansas State University, Manhattan, Kansas, United States, 2. United Way of Central Maryland, Baltimore, Maryland, United States*

The goal of PCC is to enhance quality and bring meaning to the lived experience in long-term care. This requires attention to patterns of life, and the creation of residential experiences within the environment. Ten case studies of homes participating in a pay-for-performance PCC program in the Midwest reveals how environmental affordances may be critical in the implementation and sustainability of PCC. Data collected through an in-depth environmental assessment revealed multiple strategies and attributes related to organizational, operational, and environmental practices. This presentation will demonstrate a diagrammatic and empirical comparison of the environments of those early in the process and those who have fully implemented and sustained PCC practices. Specific features will be highlighted and patterns discussed.

### PARTNERSHIPS THAT WORK: AN ACADEMIC AND STATE COLLABORATION IN KANSAS

Gayle Doll,[1] Migette Kaup,[1] and Laci Cornelison[1], *1. Kansas State University, Manhattan, Kansas, United States*

Academic partnership with state government may be a researcher's dream or a tremendous burden. This presentation demonstrates the perspectives of university personnel as well as government leaders when contracts and grants are issued for the provision of research and services. Opportunities and barriers for researchers will be discussed.

### SESSION 2520 (SYMPOSIUM)

### LONG-DISTANCE CAREGIVING: MENTAL HEALTH CONSEQUENCES AND USE OF RESOURCES

Chair: Verena R. Cimarolli, *LeadingAge LTSS Center @ UMass Boston, Washington, District of Columbia, United States*
Co-Chair: Amy Horowitz, *Fordham University, New York, New York, United States*
Discussant: Rachel Pruchno, *Rowan University, Stratford, New Jersey, United States*

Long-distance caregiving (LDC) is a growing phenomenon and common experience for caregivers of frail older adults. In fact, 11% of family caregivers in the US live more than two hours distance from the care recipient (CR). Unfortunately, there is a paucity of research on unique experiences of LDCs and the impact of LDC on the mental health of LDCs. This symposium presents findings from the NIA funded Fordham Long-Distance Caregiving Study (R21AG050018) analyzing data of 304 long-distance caregivers (LDCs). The overall study goal was to better understand how LDCs deal with the structural constraint of distance, and to examine LDC consequences and resources. First, Horowitz presents the study background, characteristics of the sample, and provides a description of the unique experiences of LDCs. Next, Cimarolli concentrates on the Sociocultural Stress Process Model applied to LDC. Her study tested the impact of LDC on mental health and investigated resources (e.g., coping skills) which could mediate the association between caregiving stressors and mental health outcomes. The third paper (Falzarano) presents data related to satisfaction with formal service providers for four subgroups of LDCs based on CR residence and dementia status. Finally, Jimenez focuses on the characteristics of LDCs' network of other informal caregivers

(IC) providing assistance to the CR and factors are associated with more help received from other ICs. Dr. Pruchno, an expert in caregiving research, will discuss study findings. The symposium provides insights into unique experiences of LDCs, the impact of LDC on mental health, and resource use among LDCs.

### WHAT DOES LONG-DISTANCE CAREGIVING LOOK LIKE?

Amy Horowitz,[1] Danielle Jimenez,[1] Verena Cimarolli,[2] Francesca Falzarano,[1] and Jillian Minahan[1], *1. Fordham University, New York, New York, United States, 2. LeadingAge LTSS Center @UMass Boston, Washington, District of Columbia, United States*

Long-distance caregivers (LDCs) are defined by geography, with little known about what they actually do when visiting and from afar. Both quantitative and qualitative data were collected from 304 LDCs. Half of LDCs lived more than 500 miles away from the care receiver (CR); 38% visited at least 1x a month, another 53% visited several times a year. Visit length varied extensively, ranging from one to 90 days at a time, with a median of 3 days. A wide range of care management tasks were common both when visiting and from afar; and targeted both formal providers and other informal caregivers. Emotional support and help with ADLs and IADLs were common during in-person visits. Other examples of emerging themes include: building relationships with formal care providers; personalizing care through, for example, special foods and/or activities; and the role of resources in determining visit length and help provided.

### THE SOCIOCULTURAL STRESS PROCESS MODEL APPLIED TO LONG-DISTANCE CAREGIVING

Verena R. Cimarolli,[1] Amy Horowitz,[2] Danielle Jimenez,[2] Xiaomei Shi,[3] Francesca Falzarano,[2] and Jillian Minahan[2], *1. LeadingAge LTSS Center @UMass Boston, Washington, District of Columbia, United States, 2. Fordham University, New York, New York, United States, 3. LeadingAge LTSS Center @UMass Boston, Boston, Massachusetts, United States*

This study investigated the impact of LDC on mental health utilizing the Sociocultural Stress Process Model as a conceptual framework. A path analytic model tested the impact of caregiving stressors (i.e. distance, frequencies of visits, hours spent helping, burden) and sociocultural values (i.e. familialism) on LDCs' mental health outcomes (i.e. depression, anxiety), and resources (i.e. coping strategies, social support) which can mediate the association between stressors and mental health outcomes while controlling for socio-demographics. Results show that resources did not mediate the effects of stressors on the mental health outcomes. However, both higher depression and anxiety were associated with living closer to the care recipient (CR), less frequent visits, higher burden, being younger, being female, and less optimal income adequacy. In addition, higher depression was associated with lower use of coping strategies and higher education. Higher anxiety was also associated with lower levels of social support and higher familialism.

### LONG-DISTANCE CAREGIVERS' SATISFACTION AND CHALLENGES WITH FORMAL CARE PROVIDERS

Francesca Falzarano,[1] Jillian Minahan,[1] Verena Cimarolli,[2] Amy Horowitz,[3] and Danielle Jimenez[3], *1. Fordham University, Bronx, New York, United States, 2. LeadingAge LTSS Center @UMass Boston, Washington, District of Columbia, United States, 3. Fordham University, New York, New York, United States*

The purpose of this study (N=304) was to identify differences in LDCs' experiences with their care recipient's (CR) formal care providers (FCPs) among four LDC groups based on CR dementia status and residential setting (community/nursing home [NH]). Results show that LDCs of CRs without dementia living in a NH are less likely to be satisfied with information/communication provided by FCPs compared to LDCs of CRs with dementia in the community. FCP-related challenges were significantly greater among LDCs of CRs in a NH, with or without dementia, compared to LDCs of CRs without dementia living in the community. A significantly greater proportion of LDCs of CRs living in a NH, with or without dementia, reported dealing with inadequate care as a challenge compared to LDCs of CRs with dementia living in the community. This highlights LDCs' unique experiences related to FCPs based on differences in CR dementia status and residential setting.

### LONG-DISTANCE CAREGIVERS' INFORMAL CARE NETWORKS

Danielle Jimenez,[1] Francesca Falzarano,[2] Amy Horowitz,[1] Verena Cimarolli,[3] and Jillian Minahan[2], *1. Fordham University, New York, New York, United States, 2. Fordham University, Bronx, New York, United States, 3. LeadingAge LTSS Center @UMass Boston, Washington, District of Columbia, United States*

The purpose of this study (N=304) was to examine the characteristics of LDCs' informal caregiver (IC) network (Co-caregivers [Co-CG], other informal helpers) providing assistance to the care recipient (CR), and factors associated with more help received from ICs. The majority of LDCs in the sample reported working with at least one IC (81.9%) indicating the existence of a secondary care network. LDCs and Co-CGs were often siblings in comparison to other informal helpers that were more likely to be the CR's friend. Results also show that CRs with children, living in the community, receiving no formal services, and lower levels of cognitive impairment receive more hours of help from ICs. In addition, more hours of help by ICs were associated with LDCs' having higher scores of depression and anxiety, spending more hours per month helping the CR, and more frequent contact with CR. These seemingly discrepant findings are discussed.

### SESSION 2525 (SYMPOSIUM)

### INTEREST GROUP SESSION—NURSING CARE OF OLDER ADULTS: MAKING IT HAPPEN: PERSON-CENTERED DEMENTIA CARE FOR BEHAVIORAL AND PSYCHOLOGICAL SYMPTOMS

Chair: Ann M. Kolanowski, *Penn State, University Park, Pa., United States*

Discussant: Barbara Bowers, *University of Wisconsin-Madison, School of Nursing, Madison, Wisconsin, United States*

Person-centered care is the standard of practice in communal living sites, yet many facilities struggle to make this a reality. Both direct care staff and administrators find residents' behavioral and psychological symptoms of dementia (BPSD) particularly challenging. Our research team is conducting a pragmatic trial, the goal of which is to help staff use person-centered, non-pharmacological approaches for these symptoms. During the past three years we have gained insights into what may affect the ability of staff to deliver high quality person-centered care. We share these insights in this symposium. In the first paper, the investigators present data indicating that a high rate of psychotropic drug use among residents with dementia persists despite little association to BPSD, and bring into question the need for education around de-prescribing practices. In the second paper, the investigators discuss the conceptual basis and empirical evidence for using affect balance, in addition to symptom reduction, as an important and meaningful outcome for residents. The third paper examines gender differences in the expression of BPSD and how these differences may work to limit staff ability to identify treatment approaches for women who, nonetheless, have significant symptoms. In the final paper the psychometric properties and results of a new Knowledge of Person-centered Approaches for BPSD Test, that was developed by the team and given to staff, are examined and the implications of the findings for the delivery of person-centered care are considered. The discussant will reflect on these findings and provide direction for future research and practice.

### RESIDENT, STAFF, AND FACILITY FACTORS ASSOCIATED WITH AFFECT BALANCE

Ann M. Kolanowski,[1] Liza Behrens,[2] Kimberly Van Haitsma,[2] and Barbara Resnick[3], *1. Penn State, University Park, Pa., United States, 2. Penn State, University Park, Pennsylvania, United States, 3. University of Maryland, University Park, Maryland, United States*

Affect balance is a concept that is measured by comparing the relative frequency of experiencing positive affect versus negative affect. A higher ratio of positive to negative affect has been associated with greater well-being, improved function and resilience. Baseline data from an ongoing pragmatic trial were used to determine the resident, staff and facility factors associated with affect balance in 325 nursing home residents with moderate to severe cognitive impairments. Measures of resident demographics, cognitive status (BIMS), function (Barthel Index), staff hours, staff knowledge of person-centered care approaches, facility policies and the facility environment were taken. Affect balance was measured using items from the Quality of Life in AD scale. Initial correlational analyses indicate that affect balance is associated with being male (p=0.03), having better function (P=0.04) and receiving a greater number of RN and CNA hours of care (p=0.003 and P=0.02 respectively).

### FACTORS THAT INFLUENCE PSYCHOTROPIC MEDICATION USE

Barbara Resnick,[1] and Elizabeth Galik[2], *1. University of Maryland School of Nursing, Baltimore, Maryland, United States, 2. University of Maryland, Baltimore, Maryland, United States*

Understanding the factors that influence psychotropic can guide reduction in use of these medications. This study described predictors of psychotropics use among residents with moderate to severe cognitive impairment. This was a secondary data analysis using baseline data from the first 341 residents in the EIT-4-BPSD trial. Predictive measures included demographics, agitation, resistiveness to care, depression, cognition, pain, facility factors and state. Overall 63% (n=211) received at least one psychotropic medication, 16% (n=52) an anti-seizure medication, 23% (n=77) an anxiolytic, 30% (n=99) an antidepressant, 2% (n=8) a sedative hypnotic, 28% (n=93) an antipsychotic medication, and 9% (n=29) an opioid. Model testing explained 9 to 15% of psychotropic medication use. There were high rates of psychotropic medication use and a limited association between demographic factors, behavioral symptoms, and psychotropic medication use. Continued research is needed to explore additional factors associated with psychotropic medication use such as beliefs of providers.

### BEHAVIORAL AND PSYCHOLOGICAL SYMPTOMS OF DEMENTIA: DOES GENDER MATTER?

Elizabeth Galik,[1] Marie Boltz,[2] Barbara Resnick,[1] Ann Kolanowski,[2] and Kimberly Van Haitsma[2], *1. University of Maryland School of Nursing, Baltimore, Maryland, United States, 2. Pennsylvania State University, State College, Pennsylvania, United States*

Regardless of presenting symptoms, there are concerns that BPSD is more often identified in males versus females and males are more likely to be treated with pharmacologic and non-pharmacologic interventions than females. In part this is due to the behaviors in men, specifically aggression, being more distressing for staff and more difficult to manage. The purpose of this study was to test for gender differences in identification and management of BPSD. This was a secondary data analysis using data from the EIT-4-BPSD study including 357 residents, 114 males and 243 females. Men had more aggressive behavior (p=.03) and women more refusal of care (p=.05) and repetitive verbal behavior (p=.03). Men received more mood stabilizers (p=.02) than women. Ongoing research is needed to evaluate if aggression in females may not be recognized or treated as aggressive women are less distressing for staff than these same behaviors in males.

### RELIABILITY AND VALIDITY OF THE KNOWLEDGE OF PERSON-CENTERED BEHAVIORAL APPROACHES FOR BPSD

Kimberly Van Haitsma,[1] Caroline Madrigal,[1] Ann M. Kolanowski,[1] Barb Resnick,[2] Beth Galik,[2] Jeanette Ellis,[2] and Karen Eshraghi[3], *1. penn State, University Park, Pennsylvania, United States, 2. University of Maryland, Baltimore, Maryland, United States, 3. Penn State, Baltimore, Maryland, United States*

How behavioral and psychological symptoms of dementia (BPSD) are understood and managed is important to person-centered care. No knowledge tests associated with dementia specifically address staff knowledge of person-centered behavioral approaches to BPSD. The Knowledge of Person-Centered Behavioral Approaches for BPSD Test was developed by our team to fill that gap. We tested the reliability and validity of this measure using a Rasch analysis and additional psychometric testing. 1,071 nurses from 35 nursing homes participated in the study. Reliability was evident based on an item separation of 11.00 and item reliability of 0.99. Construct validity was evident in that all of the items fit the model with INFIT and OUTFIT statistics (0.6-1.4). Associations between test scores and observed positive and negative care interactions (r=.38, p=.03; r=-.26, p=.12), person-centered care approaches (r=-.25, p=.15), and resistance to care (r=-.31, p=.07) will be examined and implications for person-centered care discussed.

### SESSION 2530 (SYMPOSIUM)

### PRESIDENTIAL SYMPOSIUM: MULTIDISCIPLINARY EMERGING PERSPECTIVES ON BUILDING AND MAINTAINING NETWORKS IN AGING.

Chair: Darina V. Petrovsky, *University of Pennsylvania, Philadelphia, Pennsylvania, United States*
Co-Chair: Jamie N. Justice, *Wake Forest School of Medicine, Winston-Salem, North Carolina, United States*

This ESPO Presidential Symposium features a multidisciplinary perspective and recent scientific advances made by early career researchers from each of the GSA scientific sections. They will provide examples of how their work is addressing ways to build and maintain networks in aging and gerontological workforce. These talks will span research on the age-associated transcriptional networks (Biological Sciences, Kulkarni), enhancing care for persons with dementia using a professional healthcare network (Health Sciences, Kovaleva), ways to maintain care networks in nursing home residents (Behavioral and Social Sciences, Kennedy), exploring the impact of social isolation in older adults on the Autism Spectrum (Social Research, Policy, and Practice, Waldron) and reflections on a project that linked aging education and student involvement within the aging network at the state level (Academy for Gerontology in Higher Education, Stephenson). These talks will demonstrate the diversity of aims, strategies, methodologies, and tools employed across disciplines. In addition, these early career researchers will share how they use networks in their own disciplines to advance their science with the goal of building an independent program of research. We will conclude with a discussion on ways to identify synergies across different fields and promote strategies for successful cross-discipline collaboration.

### INVESTIGATING DYNAMICS OF AGE-ASSOCIATED TRANSCRIPTIONAL NETWORKS WITH INTERVENTIONS TARGETING AGING

Ameya S. Kulkarni,[1] Jessica C. Mar,[2] and Nir Barzilai[1], *1. Albert Einstein College of Medicine, Bronx, New York, United States, 2. Australian Institute for Bioengineering and Nanotechnology, University of Queensland, Brisbane, Brisbane, Queensland, Australia*

Biological aging is characterized by a progressive decline in physiological function from molecular to organismal levels, manifesting through adaptive transcriptional networks. We present an overview of the complex regulation of transcriptional networks in species- and tissue-specific aging. We aimed to: 1) capture the age-associated changes in gene-gene connectivity, and 2) evaluate the effect of two interventions targeting biological aging (metformin, acarbose) on the regulation of gene networks. Aim 1) Using RNA-Seq we modeled co-expression networks and identified differentially co-expressed gene-pairs between young, middle-aged and older-aged groups. Aim 2) Using short-term clinical studies in older humans (metformin: MILES-trial; acarbose: SAIL-trial), and complementary mouse studies, we revealed the genes and novel pathways underlying the drugs' effects on biological aging in muscle and adipose. Importantly, these interventions shifted transcripts to a more youthful expression. Overall, we provide evidence of age-associated gene-network topology changes and identify upstream transcriptional factors affected by age-targeting drugs.

### THE INTEGRATED MEMORY CARE CLINIC AS A HEALTHCARE NETWORK

Mariya A. Kovaleva[1], *1. Vanderbilt University School of Nursing, Nashville, Tennessee, United States*
Melinda Higgins,[2] Bonnie Mowinski Jennings,[2]
Mi-Kyung Song,[2] and Carolyn K. Clevenger[2], *2. Nell Hodgson Woodruff School of Nursing Emory University, Atlanta, Georgia, United States*
Patricia C. Griffiths[3], *3. Atlanta VA Medical Center, Center for Visual and Neurocognitive Rehabilitation, Decatur, Georgia, United States*
Kenneth W. Hepburn[2]

The Integrated Memory Care Clinic (IMCC) at Emory Healthcare is a patient-centered medical home led by advanced practice registered nurses (APRNs) who provide dementia and primary care. This presentation describes the experiences of persons living with dementia and their caregivers during their first year at the IMCC, through the lens of the IMCC as a healthcare network. Forty-two caregivers were evaluated in three survey-based assessments over nine months. Twelve caregivers completed qualitative interviews about their experience at the IMCC. Severity of depression and delusions and total symptom severity improved significantly for persons living with dementia. Caregivers described their sense of belonging to the IMCC healthcare team and valued direct telephone access to APRNs. By enhancing care access and engaging clients in their care, the IMCC serves as a reliable and professional healthcare network for patient-caregiver dyads who often receive suboptimal dementia care in mainstream healthcare.

### ONE-TWO PUNCH OF HIGH WAGES AND EMPOWERING PRACTICES FOR MAINTAINING CARE NETWORKS IN NURSING HOMES

Katherine Kennedy,[1] John R. Bowblis,[2] and Katherine M. Abbott[3], *1. Miami University, Department of Sociology and Gerontology, Oxford, Ohio, United States, 2. Miami University, Department of Economics, Farmer School of Business, Oxford, Ohio, United States, 3. Department of Sociology and Gerontology, Scripps Research Fellow, Miami University, 45056, Ohio, United States*

Stabilizing certified nursing assistant (CNA) employment is necessary for maintaining care networks and providing high quality of care for nursing home (NH) residents. This study's objective was to examine the relationship of high wages and empowerment practices on CNA retention. We used the 2015 Ohio Biennial Survey to construct a facility-level dataset of 547 NHs and estimated multivariable linear regressions. NHs that provided both high wages and high empowerment were associated with a 12.95 percentage-point improvement in the CNA retention rate (SE = 4.53, t-value = 2.86, p = 0.0045). High wages and a high empowerment score did not have significant effects individually (p > .05). Retention rates were similar between NHs that lacked high wages and scored low on the empowerment scale, and NHs that provided one at a high level but not the other. Implications for better retaining CNAs require multiple empowerment practices combined with high hourly wages.

### AGING ON THE AUTISM SPECTRUM: AN EXPLORATION OF SOCIAL NETWORKS AND IMPLICATIONS

Danielle Waldron,[1] Caitlin Coyle,[2] and John Kramer[3], *1. Department of Gerontology, University of Massachusetts Boston, Boston, Massachusetts, United States, 2. Center for Social and Demographic Research on Aging, Gerontology Institute, University of Massachusetts Boston, Boston, Massachusetts, United States, 3. Department of Occupational Therapy, University of Florida, Gainesville, Florida, United States*

Social isolation is associated with poor health and well-being in older adults. Little is known about isolation in persons aging on the Autism Spectrum (AS), a group with varied physical and mental health comorbidities. The purpose of this study was to explore social networks of adults aging on the AS. We conducted in-depth interviews (N=30) with adults on the AS (age 50+) and analyzed findings using a constant comparative method. Findings suggest that older adults on the AS struggle to build and maintain social networks over the life course, in large part, because of challenges with communication and trust. Implications of isolation include challenges with community supports and employment. We propose several social convoy models and intervention mechanisms to support this population--as their social networks narrow over time, and they face aging-related challenges without the buffer of strong social relations.

### MULTIDISCIPLINARY EMERGING PERSPECTIVES ON BUILDING AND MAINTAINING NETWORKS IN AGING

Eugenie Stephenson[1], *1. University of Maryland Baltimore County, Baltimore, Maryland, United States*

This presentation is a reflective piece on developing and staging of the inaugural Gerontology Student Workforce Day at the Georgia Capitol held in January 2016 through coordination with the state-level Council on Aging (GCOA: Georgia-Council-on-Aging). The aims of this initiative focused on bridging students' gerontology education and career aims with current legislative concerns for older adults at the state level through networking and advocacy efforts. We also sought out to highlight to state legislators the necessity to support gerontology education. Results of this networking engagement included educating state legislators on both the role of gerontology education to support the needs of older residents at the community-level and highlighting to both parties the impact of gerontology professionals on the state's workforce. As a result, we engaged gerontology students and early career aging professionals in high-impact networking opportunities focused on service and policy efforts with state legislators and local AAAs (area-agencies-on-aging).

### SESSION 2535 (SYMPOSIUM)

### INTEREST GROUP SESSION—AGING VETERANS: EFFECTS OF MILITARY SERVICE ACROSS THE LIFE COURSE: OPTIMIZING VETERAN AND CAREGIVER WELL-BEING THROUGH TECHNOLOGY

Chair: Christine E. Gould, *VA Palo Alto Health Care System, Palo Alto, United States*
Co-Chair: Julia Loup, *University of Alabama, Department of Psychology, Tuscaloosa, Alabama, United States*
Discussant: Laura O. Wray, *VA Center for Integrated Healthcare, Buffalo NY, United States*

Older Veterans account for more than half of Veterans receiving care through the VA. Many suffer from dementia, traumatic brain injuries, and mental health disorders, resulting in complex care needs, which may include need for caregiver support. Innovative approaches using technology have the potential to improve access to treatments that improve the well-being of older Veterans and their caregivers. This symposium will present data from three Veterans Health Administrative investigators who are examining the impacts of innovative technology-delivered interventions. First, Dr. Michelle Hilgeman will describe preliminary 6-month outcome findings from a telephone-delivered Care Consultation + Counseling (CC+C) intervention for Veterans with dementia and their caregivers. Second, Dr. Kaci Fairchild will detail the background, methodology, and preliminary results of The COACH Project (Combined Online Assistance for Caregiver Health). The COACH Project is a mobile-delivered intervention consisting of exercise and caregiver skills training designed to reduce caregiver burden in those caring for someone with cognitive impairment. Third, Dr. Christine Gould will describe the development and feasibility of the Geri-Mobile Health clinic, which helps older Veterans use VA mental health apps to meet their well-being and mental health goals. Dr. Gould will describe the clinic's personalized coaching method to support Veterans unfamiliar with apps and will share findings regarding the program's acceptance, feasibility, and preliminary outcomes. Dr. Laura Wray, the executive director of the VA Center for Integrated Healthcare, will serve as the discussant. She will comment on implementing these interventions in primary care settings and the potential barriers to their adoption.

### GERI-MOBILE HEALTH: DEVELOPMENT AND FEASIBILITY OF A PROGRAM TO HELP OLDER VETERANS USE VA MENTAL HEALTH MOBILE APPS

Christine E. Gould,[1] Flora Ma,[2] Ashley N. Scales,[2] Christine Juang,[2] Chalise Carlson,[2] Julia Loup,[3] and Erin Sakai[2], *1. VA Palo Alto Health Care System, Palo Alto,*

*United States, 2. VA Palo Alto, Palo Alto, California, United States, 3. University of Alabama, Tuscaloosa, Alabama, United States*

VA mental health self-management mobile applications (apps) teach evidence-based skills such as mindfulness and behavior activation. Older Veterans are likely to benefit from learning these skills, but many need assistance learning to use their mobile devices. To bridge this knowledge gap, we developed a clinical program (Geri-Mobile Health) and accompanying patient education materials to help older Veterans use apps to meet their mental health and well-being goals. The program provides personalized coaching support consisting of (1) teaching basic mobile device use; (2) selecting a goal-consistent app; and (3) encouraging behavior change using the app. The presentation will describe the preliminary outcomes and implementation process. Initial results suggest that the program is feasible, acceptable, and may increase mobile device proficiency for novice users. Additionally, we disseminated 1,418 sets of education materials to providers in 21 states. Challenges and successes at the local and national levels will be discussed.

### TECHNOLOGY-BASED INTERVENTIONS TO IMPROVE CAREGIVER WELL-BEING

Kaci Fairchild,[1] Shirit Kamil-Rosenberg,[2] Heather Taylor,[2] Peter Louras,[3] Blake Scanlon,[2] Jonathon Myers,[2] and Jerome Yesavage[3], *1. Sierra Pacific MIRECC at VA Palo Alto, Palo Alto, California, United States, 2. VA Palo Alto, Palo Alto, California, United States, 3. Palo Alto University, Palo Alto, California, United States, 3. VA Palo Alto / Stanford University School of Medicine, Palo Alto, California, United States*

Informal or unpaid care is the most common form of long-term care. Despite clear benefits for the care recipient, caregiving can have unintended physical and emotional consequences for caregivers. Traditional caregiver interventions are limited in scope, as they often focus on the emotional consequences of caregiving; however, the physiological effects of caregiving are equally deleterious to caregiver health. Exercise improves physical health, yet the demands of caregiving can limit participation in physical activity. Traditional gym-based interventions may not be feasible for many caregivers. Advances in technology present an opportunity to address these limitations, specifically in the areas of accessibility and acceptability. The Combined Online Assistance for Caregiver Health (COACH) program combines evidence-based skills training with physical exercise in a tablet-based intervention. Preliminary evidence for the physical and psychological benefits are promising; however, differential attrition rates are informative as to the acceptability of technology-based interventions among some caregivers.

### ADAPTING MINDFULNESS-BASED COUNSELING FOR THE TELEPHONE: A PILOT STUDY FOR CAREGIVERS AND VETERANS WITH DEMENTIA

Michelle M. Hilgeman,[1] Phoebe R. Block,[2] Teddy K. Bishop,[1] Kimberly Alexander,[1] and A. Lynn Snow[3], *1. Tuscaloosa VA Medical Center, Tuscaloosa, Alabama, United States, 2. The University of Alabama, Tuscaloosa, Alabama, United States, 3. Tuscaloosa VA Medical Center, The University of Alabama, Tuscaloosa, Alabama, United States*

Optimizing Dementia Care in Veterans with Dementia is a randomized, controlled, pilot study examining outcomes for Veterans and their caregivers at 6- and 12-months for two telephone-based interventions: a) Benjamin Rose Institute's (BRI) Care Consultation (CC), and b) CC + Counseling (CC+C). Counseling modules are integrated into the existing BRI CC framework using guided mindfulness-based skill-building exercises on various content domains (e.g., grief, identity, intimacy, stress management). Sixty-four caregivers and 47 Veterans (M = 74.3 years, MOCA Score M = 15.5) have been randomized in this ongoing pilot study. Caregivers are 91% female, 32% Black/African American, and 72% spouses. Preliminary implementation and 6-month outcome data is discussed (e.g., reaction to behavioral distress, mindfulness, depression, quality of life) using within-group paired samples t-tests for the 32 dyads randomized to CC+C. Lessons learned include strategies for adapting mindfulness-based approaches over the telephone to enhance access for Veterans and caregivers across geographic regions.

### SESSION 2540 (SYMPOSIUM)

### INTEREST GROUP SESSION—ENVIRONMENTAL GERONTOLOGY: PRECARIOUS AGING IN PLACE? CRITICAL PERSPECTIVES ON AGING IN CONTEXTS OF INSTABILITY

Chair: Jessica M. Finlay, *Social Environment and Health, Institute for Social Research, University of Michigan, Ann Arbor, Michigan, United States*
Co-Chair: Jarmin C. Yeh, *University of California, San Francisco, California, United States*

Population aging and longevity in an era of immense environmental instability raises concerns about the precarity of aging and insecurity in later life. From home- and neighborhood-level insecurities to uncertainties generated by climate change or broad economic and sociopolitical upheaval across the globe, the factors contributing to instabilities relevant to older populations are heterogeneous in scale and cause. This symposium focuses on understanding older people's needs and experiences in the context of unstable social, economic, political, and natural environments. The first paper investigates effects of socio-environmental disruption on the well-being, recovery, and resilience of older adults in Louisiana and Mississippi deeply affected by Hurricane Katrina. The second paper explores the confinement, exclusion, and loss of autonomy, as well as the creative negotiation and sociopolitical reclamation of space, among disabled older adults experiencing houselessness. The third paper discusses filmmaking with formerly homeless older adults as a method to engage marginalized individuals in community-based participatory research and better understand nuanced meanings of 'home'. The fourth paper explores how transportation and technology can serve as both facilitators and barriers to accessibility and social connectivity among ethnically diverse low-income older adults. Altogether, the papers critically discuss novel scholarship and applied research in environmental gerontology from the micro to macro scale. The symposium inspires methodological innovation and critical research directions, and informs place-based policymaking to address diverse contexts of aging in place.

### OLDER ADULT DISASTER RECOVERY FOLLOWING HURRICANE KATRINA

Alexis Merdjanoff,[1] David Abramson,[1]
Rachael Piltch-Loeb,[1] and Yoon Soo Park[2], *1. New York University, New York, New York, United States, 2. University of Illinois - Chicago, Chicago, Illinois, United States*

This study explores the effects of environmental disruption on older adult well-being, recovery and resilience following Hurricane Katrina. It is based upon the Gulf Coast Child and Family Health Study, a longitudinal cohort of 1,079 residents from Louisiana and Mississippi highly affected by the storm. Using five waves of data collected over the last 12 years, analyses examine the drivers of long-term recovery by age group, including factors such as household income, physical health, mental health, stable housing and social support. Path analyses compare the influence of these drivers on recovery among younger adults (18-54), the young-old (55-64), mid-old (65-74) and old-old (75+). Results demonstrate that each age group relies on specific factors to improve their recovery, and that only a small number of factors are critical for older adult recovery. Results can help identify points of intervention for disaster recovery planning that can facilitate long-term recovery for older adults.

### CHARACTERIZING THE EFFECTS OF HOSTILE ARCHITECTURE ON THE HEALTH GOALS OF HOUSELESS ELDERS

Ian M. Johnson[1], *1. University of Washington, Seattle, Washington, United States*

Over 12,000 residents of Seattle experienced houselessness in 2018—among them, 70% reported having health conditions, 17.5% were over 50, and over half do not access emergency housing services. Local governments increasingly use strategies aimed at deterring unhoused populations from using public space. This research aimed to characterize the effects of urban planning interventions on the health goals among older disabled adults experiencing houselessness. Agency-based focus groups were conducted with adults over 50 who self-identified as disabled and met the federal criteria for homelessness. Through participatory mapping methods, constituents identified places where opportunities and barriers toward achieving health goals were experienced. Findings indicate lived experiences of confinement, exclusion, and loss of autonomy as well as creative negotiation and reclamation of space. This research equips advocates and providers with spatial data to increase public awareness, enrich local advocacy efforts, and offer new methodologies for enhancing social work perspectives on place and aging.

### COMMUNITY-BASED PARTICIPATORY RESEARCH FILMMAKING WITH FORMERLY HOMELESS OLDER ADULTS

Victoria F. Burns[1], *1. University of Calgary, Calgary, Alberta, Canada*

This methodological paper discusses the process of co-creating a documentary film with seven formerly homeless older adults, highlighting some of the tensions carrying out community-based participatory research (CBPR). This paper is part of a larger study that explored 'finding home' through a series of individual and group audio and video-recorded interviews (including walk and drive alongs) with seven adults (aged 50+) with diverse homeless histories. In addition to the main findings, participants shared their experience of filmmaking and CBPR. Findings revealed four main tensions: 1) openness of sharing stories versus privacy and anonymity; 2) balancing participation/engagement and over-burdening; 3) negotiating interpersonal conflict and community building; and 4) ethical issues surrounding copyright and ownership of the film. Ultimately, we advocate for more CBPR film projects, as they not only provide a rich contextualized window into people's everyday lives but serve to advance the voices of marginalized populations beyond traditional academic circles.

### SOCIAL HEALTH, MOBILITY, AND TECHNOLOGY: ACCESSIBILITY WITHIN AGE-FRIENDLY COMMUNITIES

Haley B. Gallo,[1] Lia W. Marshall,[2] Lené Levy-Storms,[3]
Kathleen H. Wilber,[1] and Anastasia Loukaitou-Sideris[4],
*1. Leonard Davis School of Gerontology, University of Southern California, Los Angeles, California, United States, 2. Department of Social Welfare, University of California Los Angeles, Los Angeles, California, United States, 3. Department of Social Welfare, University of California Los Angeles, Department of Medicine/Geriatrics, University of California Los Angeles, Los Angeles, California, United States, 4. Department of Urban Planning, University of California Los Angeles, Los Angeles, California, United States*

To explore how access to transportation and technology/social media influence social connectivity among an ethnically diverse group of vulnerable low-income older adults, six focus groups were conducted (N=48) in English, Spanish, and Korean at a senior services agency. Qualitative thematic analyses revealed overarching themes that fit within the World Health Organization's Age-Friendly Domains of Livability. The sub-theme "barriers and facilitators to accessibility" ran through each of the overarching themes, demonstrating how specific factors of the built, social, and community health environments intersect to promote or hinder social connection. Although transportation and technology uses were linked to social engagement, challenges with the built environment and limited financial resources hindered older adults' abilities to remain engaged in their communities, both in-person and electronically. Age-Friendly initiatives must continue to consider the community-specific barriers and facilitators for older adults to remain physically and socially connected to the community.

### SESSION 2545 (SYMPOSIUM)

### REDUCING HEALTH DISPARITIES IN THE ERA OF VALUE-BASED CARE

Chair: Amit Kumar, *Northern Arizona University, Flagstaff, Arizona, United States*

Co-Chair: Elham Mahmoudi, *University of Michigan, Department of Family Medicine, Ann Arbor, Michigan, United States*
Discussant: Maricruz Rivera-Hernandez, *Brown University, Providence, Rhode Island, United States*

The US health care system is at a critical moment of transformation. The implementation of value-based models has made significant progress towards improving care quality and coordination, continuity of care and reducing cost. However, concerns have been raised regarding "cherry-picking" healthier people that may negatively impact patients with more complex needs and minority populations. Given that the US is becoming more diverse, there is a need for understanding the impact of social risk factors including ethnicity, immigration status, income and geography on health outcomes and issues of health care disparities. This panel brings together four studies that examine these phenomena in minority populations. These studies will provide novel insight regarding 1) healthcare utilization in Mexican-American Medicare beneficiaries and showing that social determinants of health are associated with a higher risk of hospitalization, emergency room admissions, and outpatient visits. 2) Mortality rates and predialysis care among Hispanics in the US, Hispanics in Puerto Rico, and Whites in the US demonstrating substantial disparities in access to recommended nephrology care for Hispanics in Puerto Rico; 3) Trends in age-adjusted mortality rates and supply of physicians in states with different nurse-practitioners regulation. 4) The impact of social risk factors on disenrollment from Fee-For-Service and enrollment in a Medicare Advantage plan in older Mexican-Americans. 5) Racial disparities in access to physician visits, prescription drugs, and healthcare spending among older adults with cognitive limitation. Studies in this panel will also discuss the effects of changes in care delivery and payment innovations in improving health equity.

### ROLE OF SOCIAL DETERMINANTS IN ENROLLMENT AND DISENROLLMENT IN MEDICARE INSURANCE PLANS IN OLDER MEXICAN AMERICANS

Amit Kumar,[1] Maricruz Rivera-Hernandez,[2] Lin-Na Chou,[3] Amol Karmarkar,[3] Yong-Fang Kuo,[3] and Kenneth J. Ottenbacher[3], *1. Northern Arizona University, Flagstaff, Arizona, United States, 2. Brown University, Providence, Rhode Island, United States, 3. University of Texas Medical Branch, Galveston, TX, Galveston, Texas, United States*

Objective: The objective of this study is to examine the association between social-medical risk factor with disenrollment from Medicare Fee-for-Service (FFS) and enrollment in a Medicare Advantage (MA) plan in Older Mexican Americans. Methods: The sample included older adults participating in the Hispanic Established Populations for the Epidemiologic Study of the Elderly linked with Medicare data. We used logistic regression to estimate odds ratios (OR) for the association of each sociodemographic and clinical factor with insurance plan switching. Results: FFS enrollees were more likely to speak Spanish, less educated, lower income, disability, and be dual eligible compared to MA enrollees. At 2-year follow up, older adults with social support had higher odds of switching from FFS to MA after controlling for all covariates (OR; 1.73, 95% CI: 1.11-2.69). Conclusion: Having social support from family and the community was strongly associated with disenrollment from FFS and transition to an MA plan.

### DIFFERENCES IN HOSPITALIZATIONS, ER ADMISSIONS, AND OUTPATIENT VISITS FOR MEXICAN-AMERICANS AGE 75 AND OLDER

Brian Downer,[1] Soham Al Snih,[2] Lin-Na Chou,[2] Yong-Fang Kuo,[2] Kyriakos Markides,[2] and Kenneth Ottenbacher[2], *1. University of Texas Medical Branch, Galveston, TX, Galveston, Texas, United States, 2. University of Texas Medical Branch, Galveston, Texas, United States*

Few studies have investigated the healthcare utilization of Mexican-American Medicare beneficiaries. We used data from 1,196 Hispanic-EPESE participants aged >75 years that has been linked with Medicare claims to describe the healthcare utilization of older Mexican-Americans and determine common reasons for hospitalizations. Participants were followed for two-years (eight-quarters). We estimated the probability of >1 hospitalization, emergency room (ER) admissions, and outpatient visits per quarter. The percentage of participants who had >1 hospitalizations, ER admissions, and outpatient visits for each quarter ranged from 10.6%-13.2%, 14.6%-19.5%, and 77.2%-80.5%, respectively. Twenty-three percent of hospitalizations were for circulatory conditions and 17% were for respiratory conditions. Older age (OR=1.26) and Spanish language (OR=1.51) were associated with hospitalizations. Women had higher odds than men to have an outpatient visit (OR=1.61). Greater education was associated with ER admissions (OR=0.72). Continued research is needed to identify social determinants and health characteristics associated with healthcare utilization among older Mexican-Americans.

### NEPHROLOGY CARE AND MORTALITY RATES AMONG PATIENTS WITH END-STAGE RENAL DISEASE IN PUERTO RICO AND THE UNITED STATES

Maricruz Rivera-Hernandez,[1] Shailender Swaminathan,[1] Rebecca Thorsness,[1] Yoojin Lee,[1] Rajnish Mehrotra,[2] Benjamin Sommers,[3] and Amal N. Trivedi[1], *1. Brown University, Providence, Rhode Island, United States, 2. University of Washington, Seattle, Washington, United States, 3. Harvard, Boston, Massachusetts, United States*

Hispanics with incidence of end-stage renal disease (ESRD) have shown lower mortality despites their high incidence rates; However, prior research has excluded Puerto Rico (PR). This study compared mortality rates and predialysis nephrology care among Hispanics in the US, Hispanics in PR, and Whites in the US with ESRD from 2006-2015. We identified 791,443 patients using the Renal Management Information System. The primary outcome was age-adjusted 1-year mortality beginning with the 91st day following dialysis initiation. Secondary outcomes were the presence of arteriovenous fistula or graft at dialysis initiation, and receipt of predialysis nephrology care. Despite higher rates of insurance coverage, we identified substantial disparities in access to recommended nephrology care between PR and the US. In addition, the adjusted absolute difference in mortality rates was higher for PR Hispanics. This finding indicates shortcomings in quality of care for Puerto Rico with serious chronic illness and complex care needs.

### NURSE PRACTITIONERS' PRESCRIPTIVE AUTHORITY AND THE RURAL URBAN DISPARITY IN MORTALITY RATES

Nasim B. Ferdows,[1] and Amit Kumar[2], *1. University of Southern California, Los Angeles, California, United States, 2. Northern Arizona University, Flagstaff, Arizona, United States*

Although nurse practitioners (NPs) might be the main providers of primary care to some communities, different states pursue different regulations for NP's practice authority. This study compared the trends in mortality rates and supply of physicians in states with different policies, using AHRF and CMF data. We categorized states based on their restrictive policies into: full, reduced, and restricted practice. We compared the trends in age-adjusted mortality rate and physician supply in rural and urban areas, and examined within-state changes in rural-urban difference in physician supply and mortality. Our results indicate that as the level of restrictive policy increased the rural-urban mortality gap increased while physician supply declined. Furthermore, regardless of increase or decrease in physicians supply disparity, rural-urban mortality disparity declined in full practice states, with a negative association between a decline in rural-urban physician supply disparity and decline in rural-urban mortality disparity in full or reduced practice states.

### RACIAL DISPARITIES IN ACCESS TO AND COST OF HEALTHCARE AMONG OLDER ADULTS WITH COGNITIVE LIMITATION: 2002 TO 2016

Elham Mahmoudi[1], *1. University of Michigan, Department of Family Medicine, Ann Arbor, Michigan, United States*

Using 2002-2016 Medical Expenditure Panel Survey, we examined racial/ethnic disparities in office-visits and prescription-drugs among individuals with cognitive limitation (CL). Medicare beneficiaries (65+) with CL (N=9,369) were included. We used generalized linear models. Prevalence of CL increased overtime among all racial/ethnic groups. Our findings indicate that 96% of Whites vs. 93% of Blacks had at least one office visit (diff=0.03; 95% CI:0.01-0.04). Whites had 2 (95% CI: 1.0-0.4) and 4 (95% CI: 2.5-6.0) more office visits compared with Hispanics and Asians; and used 4 (95% CI: 1-6.9), 5 (95% CI:1.0-9.3) and 6 (95% CI: 1.0-11.5) more prescriptions than their Blacks, Hispanics, and Asians, respectively. Whites had higher annual expenditures for office-visits compared with Asians ($889; 95% CI:409-1,368) and higher expenditures for prescriptions compared with Blacks ($484; 95% CI:$151-$816) and Asians ($546; 95% CI:$28-$1064), respectively. Disparities in care among older adults with CL may put vulnerable subpopulations at a higher risk.

### SESSION 2550 (PAPER)

### ROLE OF SOCIAL NETWORKS AND SUPPORT

### A CROSS-NATIONAL COMPARISON OF THE SOCIAL SUPPORT NETWORKS OF OLDER MEN AND WOMEN WHO LACK TRADITIONAL FAMILY TIES

Christine Mair,[1] and Kasey Knopp[1], *1. University of Maryland, Baltimore County, Baltimore, Maryland, United States*

Existing literature on "aging alone" focuses on potential lack of support to "kinless" older adults who do not have traditional family ties (e.g., child, spouse; Margolis & Verdery, 2018), as well as the ways in which childless or unpartnered older adults may construct non-kin networks of support (e.g., friendship; Djundeva et al., 2018; Mair, 2019). In addition, older men's and women's social networks vary, with women reporting more network growth than men and potentially lower family involvement (Schwartz & Litwin, 2018). Finally, patterns of support (e.g., family care, friend interactions) differ by country context. However, it is unknown if and how the social networks of older adults who lack traditional family ties may differ by gender, as well as what forms of cross-national variation exist in these patterns. Using data from the Survey of Health, Ageing and Retirement in Europe (SHARE, N=17 nations, N=53,247 adults aged 50+), we take advantage of a unique social support network module in this cross-national dataset to compare closeness, proximity, quality, and type of ties by gender among older childless and unpartnered men and women by country. Among those without traditional family ties, we find that older women may be advantaged in terms of social support compared to older men, but that this advantage varies by nation. We discuss the details and implications of these results regarding potential policy implications about the differential risks faced by older men and women who lack traditional family ties in various country contexts.

### AGE FRIENDLINESS OF COMMUNITIES CONTRIBUTES TO QUALITY OF LIFE

Charles Seguin,[1] Nadia Mullen,[1] Arne Stinchcombe,[2] Shawn Marshall,[3] Gary Naglie,[4] Mark Rapoport,[5] Bruce Weaver,[1] and Michel Bédard[1], *1. Lakehead University, Thunder Bay, Ontario, Canada, 2. Saint Paul University (Ottawa), Ottawa, Ontario, Canada, 3. University of Ottawa/ Ottawa Hospital, Ottawa, Ontario, Canada, 4. Baycrest Health Sciences/University of Toronto, Toronto, Ontario, Canada, 5. Sunnybrook Health Sciences/University of Toronto, Toronto, Ontario, Canada*

The World Health Organization (WHO) emphasized the importance of age-friendly communities in supporting quality of life for older adults. We aimed to determine the contribution of the age-friendliness of communities to quality of life in a sample of healthy older adults. We used data collected through a longitudinal study on drivers and ex-drivers. We used the World Health Organization Quality of Life instrument (WHOQOL-BREF; WHOQOL Group, 1998) to measure physical health, psychological health, social relationships, and environment. We used the Age-Friendly Survey (AFS; Menec & Nowicki, 2014) to measure 9 domains of participants' perceptions of community age-friendliness. We estimated 4 multivariable linear regression models. The dependent variables were the 4 domains of the WHOQOL-BREF. Each model had AFS as the focal independent variable and participants' age, gender, health status, and depression symptoms as control variables. Data from 171 participants were available; mean age was 83.2 years (SD=4.1), 61% were women. Most participants reported a good health status and few depression symptoms. The models explained between 18 and 27% of the variance in WHOQOL scores; community age-friendliness was a statistically significant variable in all models, accounting for 2-3% of the variance. The identification of factors that contribute to quality of life will serve as the foundation upon which policies and interventions to promote successful and healthy aging can be developed. Future work will require consideration of the specific aspects of communities that may affect quality of life the most and that have the most potential for modification.

### DISABLEMENT IN CONTEXT: DO SOCIAL NETWORKS MODERATE THE IMPACT OF INCREASING FUNCTIONAL IMPAIRMENT ON WELL-BEING?

William R. McConnell,[1] and Robbie Dembo[2], *1. Florida Atlantic University, Boca Raton, Florida, United States, 2. Brandeis University, Boston, Massachusetts, United States*

Older adults with disabilities face a higher risk of experiencing poor health and social isolation in later life. Prior research has shown that social factors such as supportive relationships can modify disablement trajectories and reduce the likelihood of negative outcomes. Although research has considered the functional benefits of relationships through examining mechanisms like social support provision, the effects of network structure on the disablement process are not well understood. This study examines multiple social network mechanisms to explain the link between disability, health, and social participation among older adults. We ask, do social networks characteristics mediate or moderate the effects of increasing functional impairment on health and social participation? We analyze longitudinal panel data from the National Social Life, Health, and Aging Project 2005 & 2010, including 2,261 adults aged 57-85. Respondents named 9,587 network members in 2005. We model indicators of health and social participation at 5-year follow-up using trajectories of functional impairment and social network characteristics. We find that larger, denser, and more supportive networks are associated with better health and more frequent social activity at follow-up. Furthermore, social network structure mediates the relationship between increasing functional impairment and health, but moderates the effect of impairment on social participation. For example, participants with more dense networks are more likely to maintain high social activity at follow-up, even at relatively high levels of impairment. This study demonstrates that functional impairments are not inherently disabling. Instead, personal and social resources can reduce the potential burdens of impairment in individuals' lives.

### FUNCTIONAL LIMITATION IN LATER LIFE: THE IMPACT OF SIPS, SOCIALIZATION, AND SADNESS

Rosanna Scott,[1] Chelsea Wiener,[1] and Daniel Paulson[1], *1. University of Central Florida, Orlando, Florida, United States*

Recent studies posit discrepant impacts of alcohol use on health outcomes. Potential reasons for contrasting results include: (1) selection bias involved in classifying individuals as "abstainers" or "drinkers," (2) unexamined demographic variables associated with alcohol use, and (3) unaddressed mechanisms of action. Given new studies identifying socialization as a mediator between alcohol use and health outcomes, this study examines social interaction and depressive symptoms, respectively, as serial mediators in the relationship between moderate alcohol use and functional limitation, while employing methods to reduce selection bias. HRS data from 2012 and 2014 were utilized (n=1,902); heavy drinkers, adults younger than 65, and respondents with inconsistent alcohol use from 2008 to 2014 were excluded. Hypotheses were evaluated using a longitudinal serial mediation model with bias-corrected bootstrapping. Results indicated that, in the context of demographic variables, medical burden, and previous functional limitation, the beneficial relationship between moderate alcohol use and future functional limitation is only present when considering social interaction and depressive symptoms as mediators, both individually and serially (variance accounted for=39.4%). There was no direct effect of moderate alcohol use on functional limitation outside the context of these mediators. Data indicate that previously suggested relationships between moderate drinking and reduced functional limitation are better explained through increased social interaction and subsequent reduced depressive symptoms. Results identify social interaction as an accessible treatment target to prevent/reduce depressive symptoms and functional limitation in later-life, and support increased assessment of IADLs in adults experiencing depressive symptoms to facilitate early treatment/prevention of functional limitation.

### LONELINESS FOLLOWING WIDOWHOOD: THE ROLE OF THE MILITARY AND SOCIAL SUPPORT

Brittany M. King,[1] Dawn Carr,[1] and Miles G. Taylor[1], *1. Florida State University, Tallahassee, Florida, United States*

Social support provides important benefits following widowhood. One context promoting social support throughout life may be the military, where benefits extend to both service members and their spouses. A substantial proportion of older men served in the military, so many widowed women today were married to veterans. We tested two hypotheses: 1) surviving military spouses will experience lower persistent loneliness following widowhood compared to their nonmilitary counterparts, and 2) this benefit is explained by increased emotional and structural social support. Our study uses the Health and Retirement Study (HRS) to examine changes in loneliness following widowhood among spouses of veterans and nonveterans. We used OLS regression and mediation tests to address our hypotheses. Overall, results supported our hypotheses. Widows of veterans reported lower levels of loneliness following widowhood compared to nonveteran widows (=-0.122; $p<0.05$). Emotional and structural social support mediated the relationship between veteran status of the deceased spouse and loneliness. Specifically, the beneficial effect of veteran status was reduced by almost 50% and became nonsignificant. Our findings suggest the military may facilitate lifelong cultivation of social support that flows not only to veterans but also to their families. These findings are suggest that the military may offer important opportunities to cultivate emotional and structural social supports that enhance the ability of veteran wives to more readily adjust to widowhood. Additionally, they emphasize the importance of having social support in later life when faced with adversity, as it seems to ameliorate some of the negative effects.

### SESSION 2555 (SYMPOSIUM)

### INTEREST GROUP SESSION—HEALTH BEHAVIOR CHANGE: USING DATA ABOUT PATIENT CHARACTERISTICS TO IMPROVE FALL PREVENTION

Chair: Gwen Bergen, *Centers for Disease Control and Prevention, National Center for Injury Prevention and Control, Division of Unintentional Injury Prevention, Atlanta, Georgia, United States*

Over one in four U.S. older adults (age 65+) reports falling each year with fall-related medical costs estimated at $50 billion. The American Geriatrics Society/British Geriatrics Society Clinical Practice Guideline for Prevention of Falls in Older Persons recommends that healthcare providers assess and manage their patients' fall risk. The Centers for Disease Control and Prevention's Stopping Elderly Accidents, Deaths, and Injuries (STEADI) initiative helps healthcare providers incorporate these guidelines by providing tools on how to screen, assess, and intervene to reduce risk. Evaluations of fall prevention have focused on the clinical process and outcomes. Understanding clinical activities is important in fall prevention but a better understanding of older adult characteristics that increase fall-risk, and attitudes that may affect their adoption of evidence-based interventions could improve the effectiveness of prevention strategies. The five presentations in this session include: 1. Demographic, health and functional characteristics of older adults with increased fall risk. 2. Caregivers of people with chronic conditions or disability as a group with increased fall risk. 3. The most effective and efficient ways of identifying older adults with increased fall risk. 4. Facilitators and barriers to older adults' adherence to evidence-based fall interventions. 5. Applying knowledge of older adult attitudes to improving an implementation of STEADI-based fall prevention. Multifactorial fall prevention strategies such as STEADI focus on the clinical aspects of fall prevention but their success depends on understanding and incorporating older adult characteristics and attitudes. The information presented in this session can inform fall prevention strategies and improve health.

### COMMON UNDERLYING FACTORS FOR KNOWN MODIFIABLE FALL RISKS

Gwen Bergen,[1] Ramakrishna Kakara,[2] Elizabeth Burns,[3] and Mark R. Stevens[3], *1. Centers for Disease Control and Prevention, National Center for Injury Prevention and Control, Division of Unintentional Injury Prevention, Atlanta, Georgia, United States, 2. Division of Unintentional Injury, National Center for Injury Prevention and Control, Oak Ridge Institute for Science and Educaton, Atlanta, Georgia, United States, 3. Centers for Disease Control and Prevention, Atlanta, Georgia, United States*

Certain demographics, health conditions, and functional limitations are associated with increased older adult falls. Health conditions and functional limitations are potentially modifiable and may have underlying factors in common. This study's objective is to understand whether health conditions and functional limitations related to increased fall risk have common underlying factors that could be useful in designing interventions. Factor analysis and multivariate logistic regression were used to analyze 2016 Behavioral Risk Factor Surveillance Survey data for adults aged 65+ years. About half of those who reported difficulty dressing (58%), difficulty running errands alone (53%), difficulty remembering (51%) and depression (48%) reported falling compared to 30% of the general older adult population. Two common factors of cognitive and physical limitations were identified and scales created for each. When controlling for demographic characteristics, both cognitive and physical limitations scales were significantly related to a higher odds of falling (Odds ratios=1.5, 1.4 respectively).

### FALLS AMONG CAREGIVERS AGE 45 AND OLDER IN 15 STATES, DC, AND PUERTO RICO, 2016

Erin D. Bouldin,[1] Vanna Labi,[1] Valerie Edwards,[2] and Lisa McGuire[2], *1. Appalachian State University, Boone, North Carolina, United States, 2. Centers for Disease Control and Prevention, Atlanta, Georgia, United States*

Family and friends who provide care to people with chronic conditions or disability (caregivers) are a critical component of community-based care in the US. Falls could negatively impact a caregiver's ability to provide care. In 2016, 15 states, DC, and Puerto Rico included the optional Caregiver Module on the Behavioral Risk Factor Surveillance System. We used these data to estimate fall and injurious fall prevalence among caregivers age ≥45 who had provided care for ≥2 years. Among 71,541 respondents, 11.8% provided care for ≥2 years. One-third of caregivers had fallen in the past year (16.6% once; 16.4% more than once), and 47.3% of caregivers who fell reported at least one resulted in an injury that limited activity or required treatment. Caregivers were significantly more likely to fall (p=0.02) and to experience injury (p=0.002) compared to non-caregivers. Falls are common among caregivers and potentially harmful to them and their care recipients.

### USING MODELING TO COMPARE THE ABILITY OF FALLS SCREENING TOOLS TO PREDICT FUTURE FALLS

Elizabeth R. Burns,[1] Vicki J. Pineau,[2] Rosalind Koff,[3] Sarah Hodge,[3] and Bess Welch[3], *1. Centers for Disease Control and Prevention, National Center for Injury Prevention and Control, Division of Unintentional Injury, Atlanta, Georgia, United States, 2. NORC, Bethesda, Maryland, United States, 3. NORC, Chicago, Illinois, United States*

The STEADI initiative recommends screening older adults for falls annually using either the 12-item "Stay Independent" or the "three-key questions" screening tools. Both tools ask about falling in the preceding year. However, the comparative predictability of each tool has not been assessed. In response, CDC and NORC, assessed both tools' ability to predict falls at six and twelve months. Adults 65+ (n=1900), were recruited from a nationally representative panel and were screened for fall risk at baseline using both tools and then followed for a year to determine if they fell. At baseline, 38% of older adults were categorized at-risk of falling based on the 12-item "Stay Independent" and 56% were considered at-risk based on the three-key questions. The history of falling question was excluded for the six month analyses. The "Stay Independent" identified 60% of fallers and the remaining two questions of the three-key questions identified 57% of fallers.

### UNDERSTANDING FACILITATORS AND BARRIERS TO PARTICIPATION IN EVIDENCE-BASED FALL-PREVENTION INTERVENTIONS

Janice Mark,[1] Gwen Bergen,[2] and Briana Moreland[2], *1. American Association of Colleges in Nursing, Washington DC, United States, 2. Centers for Disease Control and Prevention, Atlanta, Georgia, United States*

Prescribing evidence-based interventions based on older adults' modifiable risk factors is recommended to prevent falls. Older adults need to adhere to the prescribed intervention to successfully reduce risk. This study reports on a structured systematic review to understand patient attitudes to adherence to fall prevention interventions. A systematic search for publications from 2008-2018 identified 72 articles on patient attitudes toward exercise, physical therapy, medication management, podiatry, and vision care for fall prevention. Three reviewers coded facilitators and barriers based on a socio-ecological model of interpersonal, intrapersonal, community, and policy factors. Perceived susceptibility to falling and perceived effectiveness of the intervention were important factors across all fall prevention interventions. Physician prescribing and discussion facilitated exercise, medication changes, and physical therapy. For attitudes related to community and policy, the most reported barriers were transportation and cost. Information from this review can be used to improve the implementation of fall prevention interventions.

### OLDER ADULT FALLS PREVENTION: THREE ADOPTED CLINICAL STRATEGIES FROM EVALUATION OF THE CDC STEADI INITIATIVE

Jennifer Edwards,[1] Brittany Bickford,[2] Yvonne Johnston,[3] and Aaron Alford[1], *1. National Network of Public Health Institutes, Washington, District of Columbia, United States, 2. National Network of Public Health Institures, New Orleans, Louisiana, United States, 3. Binghamton University, Binghamton, New York, United States*

This evaluation examines patients' barriers and facilitators to adopting an evidence-based fall prevention strategy. Twenty-one patients were telephone interviewed. The purposive sample includes patients over age 65, screened as at risk for falls, and who received a referral for falls risk intervention. Seven themes emerged from the qualitative analysis of interview transcripts: 1. Behavioral Facilitators, 2. Personal Fall Experiences, 3. Informed Decision-making, 4. Providers, 5. Friends and Family, 6. Home Setting Facilitators, and 7. Risk Perception. Three opportunities were identified: 1. Develop an outpatient follow-up protocol, 2. Develop a falls screening public service announcement, and 3. Partner with the local Office for Aging to connect patients at risk with community programs such as Tai Chi. A systems approach involving the CDC, National Network of Public Health Institutes (NNPHI), Broome County Health Department, and an Upstate New York hospital system's outpatient practices was vital for the success of this evaluation.

### USING CAREERS IN AGING WEEK (CIAW) TO PROMOTE INTERGENERATIONAL COMMUNITY ENGAGEMENT

Carrie Andreoletti[1], *1. Central Connecticut State University, New Britain, Connecticut, United States*

This presentation will discuss how we used CIAW 2017 to promote intergenerational community engagement by collaborating with our office of Continuing Education and AARP Connecticut to host "#DisruptAging – A Conversation Across Professions, Perspectives, and Generations." Together we developed a half day program designed to build bridges among academic institutions, aging professionals, community collaborators, and consumers who are passionate about aging issues. We brought together a multi-generational audience of professors, students, professionals, and representatives of various community organizations to discuss ways to change the conversation around aging. This gave us an opportunity to highlight our gerontology programs and showcase our gerontology students, while students learned about aging-related careers, public policy initiatives, and organizations in Connecticut that support older adults and their families.

### SESSION 2560 (SYMPOSIUM)

### INTEREST GROUP SESSION—INTERGENERATIONAL LEARNING, RESEARCH, AND COMMUNITY ENGAGEMENT: USING INNOVATIVE INTERGENERATIONAL STRATEGIES TO BOOST CAREERS IN AGING

Chair: Joann M. Montepare, *Lasell College, Newton, Massachusetts, United States*
Discussant: Laura K. Donorfio, *University of Connecticut, Waterbury & Storrs, United States*

Populations are aging dramatically, and call for higher education to be more age-friendly and pave the way for career paths in aging. The Careers in Aging Week (CIAW) program sponsored annually by the Academy for Gerontology in Higher Education (AGHE) of GSA has been a core stimulus for building career interest – however, more could be done to strengthen and invigorate this effort. This symposium will show how intergenerational exchange can be used to mount interest in careers in aging and create new pipelines to gerontology programs in higher education. Examples of innovative approaches will demonstrate how career information can be communicated to students in more creative and compelling ways. The first paper will set the stage with an evidence-based overview of emerging areas for career development, and a presentation of career planning models to aid student understanding as to how to make aging career decisions. Two presentations will then focus on different aging-workforce initiatives aimed at building educational pipelines that connect high-school students with college students studying gerontology. In addition to highlighting the oft-overlooked population of high school students, attention will be given to the importance of including minority student populations in career development efforts. The final paper will describe the utility of broader intergenerational strategies that build bridges across students, educators, aging professionals, and community collaborators via campus career events. The discussant will bring these efforts together with an intergenerational programming lens that higher education can use to amplify awareness about the wide-range of career opportunities aging offers.

### PROMOTING EFFECTIVE CAREER DEVELOPMENT IN GERONTOLOGY THROUGH APPLIED INTERGENERATIONAL PROGRAMS

Margaret M. Manoogian[1], *1. Western Oregon University, Monmouth, Oregon, United States*

With the rise in global older adult populations, university programs need to produce an effective, gerontology-trained workforce (Silverstein & Fitzgerald, 2017). Career decision-making involves interactive learning (Super, 1990), as adults explore career options, engage in career learning, and understand curriculum integration within professional settings (Savickas, 2013). Gerontology faculty can utilize career planning models that integrate intergenerational engagement within the curriculum to aid student career decisions (Reardon, Lenz, Peterson, & Sampson, 2012). This paper provides an overview of a career planning model and highlights the ways intergenerational programs can be intentionally staged in research, service, and extracurricular domains to promote career planning and success in post-graduate employment. Data from our recent gerontology alumni survey including graduates since the program inception will be outlined to support the importance and success of developing strong applied intergenerational career programs in gerontology.

### TAKING THE HIGH (SCHOOL) ROAD: DEVELOPING INTERGENERATIONAL INTEREST FOR CAREERS IN AGING

Rona J. Karasik,[1] and Phyllis A. Greenberg[1], *1. Saint Cloud State University, Saint Cloud, Minnesota, United States*

Invisibility and ageism are ever-present challenges to developing a work-force commensurate with the expanding older population (Augustin & Freshman, 2016). Intergenerational and experiential endeavors (e.g., Simulations, Service-Learning, Careers in Aging Week) can counter stereotypes while highlighting gerontology career opportunities. Focusing workforce development efforts on the oft-overlooked population of high school students (Zhang, 2015) has numerous advantages, including: (1) identifying aging-related career paths prior to college; (2) reaching students eligible to become CNAs/PCAs; (3) uncovering potential benefits (e.g., scholarships, tuition reimbursements) agencies may offer to student-employees and (4) countering aging stereotypes. This presentation reports on a multi-faceted undertaking which includes collaborating with local service agencies on a regional aging-workforce initiative, bringing high-school students to a college campus for CIAW events, participating in local/regional career events focused on high school students, and developing/implementing experientially-rich learning opportunities such as a college-credit bearing introductory course on aging to be taught at the high school.

### USING INNOVATIVE INTERGENERATIONAL STRATEGIES TO BOOST CAREERS IN AGING

Roma S. Hanks[1], *1. University of South Alabama, Mobile, Alabama, United States*

Educational pipelines are effective in medical education with minority student populations. The University of South Alabama has a successful medical pipeline in the NIH/NIMHD-funded USA Center of Excellence – but no formal pipeline to support entry into careers in aging. In Gerontology and Geriatrics, the preponderance of pipelines focuses on students in advanced degree programs. The need remains largely unmet to inform young students about careers and research pathways in Gerontology before career and academic plans are established. The USA Gerontology Club initiated a student-led outreach to deliver information about careers in aging and academic programs in Gerontology and Geriatrics to high schools in communities with high health disparities. The program seeks to develop peer relationships with high school students to introduce them to careers in aging and related academic opportunities. The presentation includes barriers identified and development of a multi-phased, multi-disciplinary model leading to a formal pipeline for Gerontology.

### SESSION 2565 (SYMPOSIUM)

### INTEREST GROUP SESSION—REMINISCENCE, LIFE STORY AND NARRATIVE: RESEARCH AND PRACTICE: WISDOM OF THE ELDERS: ART, MEDICINE, NARRATIVES, AND POETRY

Chair: Ifeoma Nwankwo, *Vanderbilt University, Nashville, Tennessee, United States*
Discussant: Monika Ardelt, *University of Florida, Gainesville, Florida, United States*

The Wisdom of the Elders (WOE) is a series of autobiography production workshops conducted since 2012 through a collaboration among community, university, and municipal partners. Beginning in Murfreesboro, TN it has since been replicated in New Orleans, LA. The Wisdom of the Elders project has many elements, including life history interviews, creative writing workshops, and creative expressions that allowed elders to share their wisdom with younger generations. Our focus was to understand how personal characteristics and historical/environmental events interact to influence healthy aging. Later work extended to interviewing older hospitalized patients in order to gain insight into intergenerational themes. The interdisciplinary team has also used poetry and reflection to train healthcare professionals and caregivers to use active listening strategies to better understand how patients cope with life-changing illnesses, and how they incorporate the challenges of those illnesses into rich, fulfilling lives. WOE has been supported by the Mellon Foundation, the Meharry-Vanderbilt Community Engaged Research Core, and the Department of English, the College of Arts and Science Dean's Office, and the Chancellor's Higher Education Fellowship at Vanderbilt University. WOE has led to the formation of an interdisciplinary research and publication collaborative featuring distinguished clinicians, artists, and scholars from Creative Writing, Education, Geriatrics, Interprofessional Learning, Psychology, and Public Humanities.

### NARRATIVES ENHANCING TRAINEES' ATTITUDES TOWARD AGING

James S. Powers[1], *1. VANDERBILT UNIVERSITY SCHOOL OF MEDICINE, Nashville, Tennessee, United States*

We analyzed narratives from graduate students trained in aging themes who then interviewed older hospitalized adults about their life experiences. Three Discovery Fellows given a 90-minute training on open-ended interviewing, the medical care system, empathy, and aging themes derived from the Reframing Aging Program performed semi-structured interviews of 30-90 minutes. Subjects were hospitalized older veterans receiving care by an interdisciplinary geriatric rehabilitation team. Narratives were composed following the interviews and electronically uploaded to a secure site. Qualitative analysis was performed with each statement treated as a separate quote using a hierarchical coding system. A total of 774 codes were used to describe the themes present in 582 quotes. A conceptual framework from the narrative themes emerged with 5 groupings: 1) personal experience, 2) people important in life, 3) self-reflection, 4) medical condition, and 5) health maintenance. Concepts displayed included openness, compassion, and morality, as components of wisdom.

### WISDOM OF THE ELDERS, VOICES FROM OUR AMERICA

Ifeoma Nwankwo[1], *1. Vanderbilt University, Nashville, Tennessee, United States*

The fundamental goal of WOE was to determine whether and how autobiography production can enhance and provide valuable data about African American seniors' mental health, past and present. The second goal of WOE was to determine whether and how project partners could use the findings from WOE, particularly those about resilience and strategies for coping with challenging situations, as the basis for new interventions, studies, publications, curricula, and educational programs for learners across contexts, generations, levels, and disciplines. Outcomes are illustrated by rich examples of the genesis, history, products and pedagogy of the project.

### ILLNESS AND THE LANGUAGE OF POETRY

Destiny Birdsong[1], *1. Vanderbilt University, Nashville, Tennessee, United States*

Two interactive workshops were conducted: one for training healthcare professionals in nursing, PT, and pharmacy, and another for the continuing education for caregivers of HIV-positive patients. Active Listening and Visual Learning Strategies for Healthcare Professionals discussed the importance of using active listening skills to better hear, understand, and treat patients. Activities included viewing one WOE respondent's artwork and hearing a clip of the respondent's interview, followed by a facilitated discussion of how the respondent's story about her childhood shed light on her art. Additionally two published poems about bodies and illness were shared, with discussion of how each poem's circuitous narrative nature offered clues about the speaker's illness and outlook on life. Cultural Humility's activities focused on utilizing poetry to discuss how caregivers can use active listening strategies to better understand how patients cope with life-changing illnesses, and how they incorporate the challenges of those illnesses into rich, fulfilling lives.

### A QUALITATIVE ANALYSIS OF NARRATIVES CONTRIBUTED BY OLDER AFRICAN AMERICANS

David Schlundt,[1] and Kemberlee R. Bonnet[1], *1. vanderbilt University, Nashville, Tennessee, United States*

We analyzed 19 interviews of African Americans who came of age during desegregation. We approached the development of the coding system and analysis of coded transcripts using an iterative inductive/deductive approach guided theoretically by the social ecological framework and life course development theory. Major coding categories included time orientation, topics of discussion, emotions, habits and behaviors, family, community, race relations, and history. Analysis of the sorted and coded quotes led to the development of a conceptual framework for understanding how participants portrayed their life trajectories. Individuals' lives occurred in the context of societal institutions, the larger society, and culture. Life trajectories involve a series of changes in life circumstances and social relationships during which individuals engage with life goals such as making a living, staying healthy, coping with stressors, and maintaining satisfactory interpersonal relationships. Important themes associated with healthy aging included resilience, faith, forgiveness, and family.

### SESSION 3000 (SYMPOSIUM)

### AGEISM AT WORK: A THEORETICAL AND EMPIRICAL CONSIDERATION

Chair: Sarah A. Vickerstaff, *University of Kent, Canterbury, Kent, United Kingdom*
Co-Chair: Mariska van der Horst, *VU Amsterdam, Amsterdam, Netherlands*
Discussant: Debra A. Street, *State University of New York at Buffalo, Buffalo, New York, United States*

Ageism at work is becoming an increasingly popular research topic. It has been claimed that ageism is a serious threat to the extending working lives agenda that is prevalent in many Western countries, including the US and many countries in the EU. In this symposium, we consider this concept from a variety of perspectives in order to better understand what ageism is as well as how it affects older workers. To get a better grip on the concept, Sarah Vickerstaff and Mariska van der Horst look at the intersectionality of this concept, by assessing its relationships with gender and disability. Clary Krekula uses the concept of age coding to look at how both age normality and ageism are constructed. Jaap Oude Mulders assesses how age-related stereotypes translate into employment preferences of employers. Hannah Swift et al., testing social psychological theories, uses results from two experiments to analyse the role of ageism in recruitment practices. This symposium is inter-disciplinary combining sociologists, social psychologists and gerontologists and further combines quantitative (Jaap Oude Mulders), qualitative (Clary Krekula, Sarah Vickerstaff, and Mariska van der Horst) and experimental (Hannah Swift) methods. Together, the papers in this symposium show various dimensions of the concept 'ageism' and how it affects older workers in three European countries: Sweden, Netherlands, and the United Kingdom.

### DOING AGE AS MARGINALIZATION AND EXCLUSION: ON AGE CODING AND THE CONSTRUCTION OF AGE NORMALITIES AND AGEISM

Clary Krekula[1], *1. Karlstad University, Karlstad, Sweden*

Early definitions of ageism as prejudice, attitudes and discrimination tend to stress the cognitive aspects of the processes where groups of people are being marginalized and/or excluded because of their age. This differ from recurrent studies which emphazise social categorization such as age and gender as a dynamic social positioning practice and as a complex form of doing age as marginalization and exclusion. Based on qualitative interviews with eleven men between the ages of 56 and 74, who work with manual labour at a steel company in Sweden, this paper discusses the processes where age normality and ageism are constructed in parallel. Departing from the concept age coding; distinctive practices associating a context or a phenomenon with demarcated ages, it shows the disciplining dimension of ageism. The paper concludes with a reflection on the central position which ageism can have in neo-liberal governance by creating social insecurity among older workers.

### EMPLOYERS' AGE-RELATED NORMS, STEREOTYPES, AND AGEIST PREFERENCES IN EMPLOYMENT

Jaap Oude Mulders[1], *1. Netherlands Interdisciplinary Demographic Institute, The Hague, Netherlands*

Social norms about retirement timing and stereotypes about qualities of younger and older workers are pervasive, but it is unclear how they relate to employers' ageist preferences. Analyzing 2017 survey data from 960 Dutch employers, I study effects of employers' retirement age norms and age-related stereotypes on their preferences for younger or older workers in three employment decisions: (1) hiring a new employee; (2) offering training; and (3) offering a permanent contract. Higher retirement age norms are related to lower preferences for younger workers in all employment decisions. More positive views about older workers' soft qualities (such as reliability), but not about hard qualities (such as physical capacities), lead to managers being more favourable towards older workers for hiring and training, but not providing a permanent contract. The results show how ageist preferences of high-level organisational actors can influence outcomes in different employment decisions at the organisational level.

### GENDERED AGEISM: OLDER WORKERS' NARRATIVES ABOUT AGE

Sarah A. Vickerstaff,[1] and Mariska F. van der Horst[2],
*1. University of Kent, Canterbury, Kent, United Kingdom, 2. Vrije Universiteit Amsterdam, Amsterdam, Noord-Holland, Netherlands*

Existing research has highlighted that ageism in the workplace may take gendered forms with women 'never being the right age' (Duncan & Loretto, 2004). It is further known that individuals have internalised age stereotypes and self-stereotype when being older themselves, also referred to as stereotype embodiment. In this work place based study, through analysis of older workers talk, we examine the extent to which narratives of age differ by gender and work setting. The data base includes 185 participants in five different work settings and different kinds of jobs: blue collar, white collar, managerial, manufacturing and services sectors. Whilst many of the fears about 'being old' at work are common across women and men there are some distinct nuances related to the kind of work that people do and others that we argue are gender based.

### TO WHAT DEGREE IS AGEISM ACTUALLY DISABLEISM? A QUALITATIVE STUDY

Mariska van der Horst,[1] and Sarah Vickerstaff[2], *1. VU Amsterdam, Amsterdam, Netherlands, 2. University of Kent, Canterbury, England, United Kingdom*

Ageism has been identified as a possible threat to the extending working lives agenda that is prevalent in many Western countries. It recently became a popular topic of research, but is not yet well understood. In this article we explore to what degree ageism is actually hidden disableism. We suggest that not all ageism is likely to be disableism, but a large part is, with the difference that it is not (necessarily) about actual impairments, but expected impairments. Using data from a large case study based project in the UK (containing 185 participants), we further assess to what degree older workers link ageism to disableism in their own accounts of future work plans. We conclude that even though we would still expect ageism to affect employment of older workers, without disableism it is unlikely that ageism would be as detrimental to the employment of older workers as it is now.

### WHOM WOULD YOU HIRE? AGEISM IN ORGANIZATIONAL CONTEXTS

Hannah Swift,[1] Vanessa Dias,[1] and Dominic Abrams[1],
*1. University of Kent, Canterbury, England, United Kingdom*

People want to work at older ages, yet ageism and discrimination remain a barrier. Using theories of prejudice, social role theory, and conceptual models of age diversity in organisational contexts, we explore age-bias in hiring practices (Study 1) and how to reduce it (Study 2). Study 1 (N=150) investigated pro-youth bias in hiring practices and how this manifests depending on job/occupation. Study 2 (N=150) investigated whether pro-youth bias is reduced by manipulating organisational culture. In both studies, participants were given a fictional organisation, a job ad, and two applicants' profiles manipulated to represent men one each in their 30s and 50s. Study 1 supported the social role theory hypothesis: people match candidates to the age profile of the job. In the age-neutral job occupation participants chose equally between candidates. Study 2 supported the hypothesis that pro-youth bias can be mitigated when age-diverse nature of the organisational culture is made salient.

### SESSION 3005 (SYMPOSIUM)

### INTEREST GROUP SESSION—GERIATRIC EDUCATION: AGHE PROGRAM OF MERIT: PRACTICAL AND TECHNICAL APPLICATION INFORMATION AND ASSISTANCE

Chair: Marilyn R. Gugliucci, *University of New England College of Osteopathic Medicine, Biddeford, Maine, United States*

Co-Chair: Shannon D. Mathews, *Winston Salem State University, Winston Salem, North Carolina, United States*
Discussant: Pamela Elfenbein, *University of North Georgia, Oakwood, Georgia, United States*

At a time in education when it is important for programs at colleges and universities to be productive in teaching, student recruitment, and income generation, the AGHE Program of Merit review process can aid in advancing gerontology/geriatrics programs. The Program of Merit has expanded its review now Colleges of Osteopathic Medicine and any Health Professions program may apply for Program of Merit status – your institution need not be an AGHE member to apply. The POM designation provides gerontology programs and those health disciplines that include geriatrics/gerontology content in the curriculum with an AGHE "stamp of approval" which can be used to verify program quality to administrators, to lobby for additional resources, to maintain a quality program, to market the program, and to recruit prospective students into the program. This session will begin with the "Why," "What" and "How" of applying for Program of Merit and then provide time for small group consultation regarding the Program of Merit application. Preparation of the self-study document will be detailed by the experienced faculty members followed by the review process and timelines associated with application process. Free consultation available!

### AGHE PROGRAM OF MERIT: HEALTH PROFESSIONS PROGRAMS OPPORTUNITIES

Marilyn R. Gugliucci,[1] and Donna Weinreich[2], *1. University of New England College of Osteopathic Medicine, Biddeford, Maine, United States, 2. Western Michigan State University, Kalamazoo, Michigan, United States*

In 2015, the Program of Merit was expanded and adapted to implement a voluntary evaluation process for health professions programs that are choosing to integrate gerontology/geriatrics competencies in order to prepare students for working with older adults as well as their informal care partners. These programs are now eligible to apply for the Program of Merit designation. The Program of Merit for Health Professions Programs is based on the AGHE Standards and Guidelines for Gerontology/Geriatrics in Higher Education, Sixth Edition (2015), specifically Chapters 11 and 12. This session will provide the information and support systems in place for health professions programs to be recognized with the AGHE Program of Merit designation.

### AGHE PROGRAM OF MERIT: GERONTOLOGY DEGREE PROGRAMS, MINORS, AND CERTIFICATES

Shannon D. Mathews,[1] and Pamela Elfenbein[2], *1. Winston Salem State University, Winston Salem, North Carolina, United States, 2. University of North Georgia, Oakwood, Georgia, United States*

The Executive Committee of the Academy for Gerontology in Higher Education (AGHE), formerly known as the Association for Gerontology in Higher Education, approved a proposal to establish and implement a voluntary program of evaluation known as the Program of Merit (POM). The POM designation provides gerontology programs with an AGHE "stamp of approval," which can be used to verify program quality to administrators, to lobby for additional resources to maintain a quality program, to market the program, and to recruit prospective students into the program. In 2014, AGHE Gerontology Competencies for Undergraduate and Graduate Education© were established and have been integrated into this process. This session will outline the Program of Merit process and present the opportunities for gerontology program leaders to advance gerontology education.

### PROGRAM OF MERIT REVIEWS: THE REVIEWERS' PERSPECTIVE

Donna Weinreich[1], *1. Western Michigan University, Kalamazoo, Michigan, United States*

Each Program of Merit Application goes through a process of review. It is the same regardless if the application is for a gerontology program or a health professions program. In this session, the reviewer perspective will be shared and tips for how to complete an application to provide information in a format that aids the review will be shared.

### SESSION 3010 (PAPER)

### DEPRESSIVE DISORDERS IN OLDER ADULTS

### ASSOCIATIONS OF ADVERSE CHILDHOOD EXPERIENCES WITH DSM-5 DEPRESSIVE DISORDERS AND SUICIDE ATTEMPT IN OLDER ADULTS

Taeho Greg Rhee,[1] Lisa C. Barry,[1] George A. Kuchel,[1] David C. Steffens,[1] and Samuel Wilkinson[2], *1. University of Connecticut School of Medicine, Farmington, Connecticut, United States, 2. Yale University School of Medicine, New Haven, Connecticut, United States*

Adverse childhood experiences (ACEs) may have long-term effects on mental health. Using a life-course perspective, this study examines prevalence of ACEs and the associations of ACEs with depressive disorders and suicide attempt in US older adults. The study sample were those aged 65 and older who participated in the 2012-2013 National Epidemiological Survey on Alcohol and Related Conditions Wave III (n=5,806 unweighted). ACEs, the key independent variable, were assessed using validated measures and outcome variables included lifetime and past-year major depressive disorder (MDD) and dysthymia using DSM-5 criteria, and lifetime suicide attempt. We estimated national prevalence of ACEs in older adults and used multivariable-adjusted logistic regression analyses to assess the association between ACEs and the outcomes after adjusting for socio-demographics and clinical co-morbidities. Overall, 34.7% of older adults, representative of 14.3 million older adults nationwide, reported some form of ACEs. The most common type was parental psychopathology (20.8%), followed by neglect (14.8%), and physical/psychological abuse (8.4%) (non-mutually exclusive). Having experienced any ACEs was associated with higher odds of having a past-year MDD diagnosis (adjusted odds ratio [aOR]=1.77; 95% confidence intervals [CI]=1.36, 2.29). Similar results were found for other depressive disorders. ACEs were also associated with higher odds of having a lifetime suicide attempt (aOR=4.34; 95% CI=2.64, 7.14). In conclusion, ACEs may expose older adults to an increased risk for mood disorders and suicide attempts, even over long periods of time as seen in this sample. Reducing ACEs is an important public health goal that may yield long-term benefits.

### CHRONIC CONDITIONS, FUNCTIONAL LIMITATIONS, AND DEPRESSION IN OLDER ADULTS: ANALYSIS OF A LONGITUDINAL STUDY

Jyotsana Parajuli,[1] Diane Berish,[2] and Ying-Ling Jao[2], *1. The Pennsylvania State University, College of Nursing, State College, Pennsylvania, United States, 2. Pennsylvania State University, University Park, Pennsylvania, United States*

Background: Chronic conditions, functional limitations, and depression are highly prevalent in older adults. Evidence suggests the links between chronic conditions, functional limitations, and depressive symptoms separately. However, few studies have considered these three conditions together longitudinally. This study examined the longitudinal relationship between chronic conditions and depressive symptoms and evaluated the mediation effect of functional limitations on the relationship between chronic conditions and depressive symptoms in older adults. Methods: This study analyzed longitudinal data from the Health and Retirement Study collected in 2012 and 2014. Mediation analysis was used to examine the direct and indirect effects of chronic conditions and functional limitations measured at the year 2012 on depressive symptoms measured at the year 2014 controlling for demographics. Results: Results revealed that chronic conditions predicted depressive symptoms. Specifically, one additional chronic condition in 2012 corresponded to an increase of 0.35 in depressive symptoms in 2014 (p<.001). After adding functional limitations as a mediator, the direct effect was reduced to 0.26 and the indirect effect was .088 (p<.001). In other words, functional limitations explained approximately 25% of the direct effect of chronic diseases on depression. Discussion: Findings reveal the longitudinal impact of chronic conditions and functional limitations on depressive symptoms in older adults. Findings help identify the high-risk population of depressive symptoms and intervene early.

### GENDER DIFFERENCES IN DEPRESSIVE SYMPTOMS OF PATIENTS WITH END-STAGE LIVER DISEASE

Shirin O. Hiatt,[1] John Lee,[2] and Lissi Hansen[1], *1. Oregon Health & Science University School of Nursing, Portland, Oregon, United States, 2. Oregon Health & Science University, Portland, Oregon, United States*

Background/aims: Similar to many chronic diseases, depression is common in patients with end-stage liver disease (ESLD), although gender differences are less known. Understanding the burden of depression in this population may help identify at-risk patients who would benefit from early intervention. The purpose of this work, therefore, is to describe gender differences in depressive symptoms in patients with ESLD. Methods: Patient data were collected as part of a larger study (NINR: 1R01NR016017-01). Patients (≥ 21 years) diagnosed with ESLD from the outpatient hepatology clinics of two healthcare systems in Pacific Northwest completed Patient Health Questionnaire (PHQ-9). Survey data were analyzed using descriptive statistics. Results: Sample included 154 participants, 101 males (65.6%), average age 57 years (SD=10.92), and 53 females (34.4%), average age 55 years (SD=11.28). More than 75% of the sample (78% females and 77% males) reported at least mild depression (PHQ score ≥ 5); mean PHQ-9 scores were higher for males (M=9.26±5.86) than females (M=9.10±5.07), but were not statistically different (U=2396, p=0.99). There was no significant relationship between depression severity and gender [X2 (4, N=147)=1.90, p=0.594]. Conclusion: Our study showed a high prevalence of depression in patients. A higher percentage of females reported mild to moderate depression and had higher clinically significant levels of depression (PHQ-9 score ≥ 10) than males, indicating females may be at a greater risk for depression. Females may, therefore, gain greater benefit from interventions to improve depressive symptoms. Future studies should examine the benefit of interventions on depression severity in this patient population.

### STRENGTHENING LATE-LIFE DEPRESSION COLLABORATIVE CARE THROUGH COMMUNITY ENGAGEMENT: CARE PARTNERS INITIATIVE

Ladson Hinton,[1] Theresa J. Hoeft,[2] Stuart Henderson,[1] Melissa M. Gosdin,[1] Laura Rath,[3] and Jurgen Unutzer[2], *1. University of California Davis, Sacramento, California, United States, 2. University of Washington, Seattle, Washington, United States, 3. Archstone Foundation, Long Beach, California, United States*

Despite the availability of effective treatments for late life depression, many older adults with depression either do not access or fully engage in treatment. The goal of this study was to examine the feasibility and two-year outcomes from an Archstone Foundation funded Care Partners Initiative to strengthen depression care for adults 65 years of age and older. Seven sites throughout California implemented evidence-based collaborative care through partnerships between primary care organizations, community-based organizations (CBOs), and families of older adults with depression. Evaluation used a mixed-methods approach incorporating both qualitative and quantitative data. Of the seven sites, six formed partnerships between primary care clinics and CBOs and one site only focused on engaging family members in treatment. In the first two years, 274 patients were enrolled and rates of depression improvement were comparable to prior depression care effectiveness trials. Overall, 49% of patients at CBO sites interacted 3+ times with CBO staff/clinicians, while at the family site, 79% of patients had 3+ contacts including a family member. Using data from key informant interviews, focus groups, and site progress documents, seven core components were identified that facilitated successful implementation and delivery of partnered collaborative care, including three foundational components: strong stakeholder buy-in, effective patient engagement, and the promotion of depression treatment as a core value across organizations. Multiple complexities of partnering between primary care clinics and CBOs or families were identified. Challenges and lessons learned from this initiative will also be discussed.

### SESSION 3015 (SYMPOSIUM)

### DESIGNING STATE-BASED LTSS PROGRAMS IN CONTEXT OF UNIVERSAL FAMILY CARE AND EMANCIPATORY GERONTOLOGY

Chair: Benjamin Veghte, *National Academy of Social Insurance, Washington, District of Columbia, United States*
Co-Chair: Carroll Estes, *University of California, San Francisco, San Francisco, California, United States*
Discussant: Stacy Torres, *University of California, San Francisco, San Francisco, California, United States*

This symposium will present findings from a National Academy of Social Insurance study panel on Designing State-Based Social Insurance Programs for Long-Term Services and Supports, Paid Leave, and Affordable Child Care. The risk of needing to provide or receive care is universal. Policymakers in several states are now weighing the enactment of new social insurance programs to address the risk of needing long-term services and supports. The study panel has mapped out the key design choices such states would need to consider with regard to program structure, financing, integration with Medicaid, and implementation, and the implications of these choices for elders, people with disabilities, families, providers, and states. The symposium will also present the study panel's findings with regard to how long-term care benefits could be provided in the context of an integrated care program addressing three often interrelated caregiving risks: long-term services and supports, paid family and medical leave, and early child care and education: Universal Family Care. Finally, implications for emancipatory gerontology, including the impact of the current gaps in our care infrastructure on family caregivers and the care workforce, will be considered.

### UNIVERSAL FAMILY CARE: DECISION POINTS FOR STATES IN IMPROVING THE CARE INFRASTRUCTURE THROUGH SOCIAL INSURANCE

Benjamin Veghte,[1] Alexandra L. Bradley,[1] Marc Cohen,[2] Heidi Hartmann,[3] and Benjamin Veghte[4], *1. National Academy of Social Insurance, Washington, District of Columbia, United States, 2. University of Massachusetts, Boston, Boston, Massachusetts, United States, 3. Institute for Women's Policy Research, Washington, District of Columbia, United States, 4. Caring Across Generations, Washington, District of Columbia, United States*

This report presents policy options for a new social insurance program – akin to Social Security or Medicare – that would provide an integrated approach to protecting against the risk of needing to provide or receive care across the lifespan: Universal Family Care. The program would allow individuals and families to access caregiving supports at various points throughout the life course, from the arrival of a child to end-of-life care for a family member or oneself. The program covers three specific caregiving needs: long-term services and supports, paid leave, and early child care and education. This report identifies key design questions for states to consider in crafting a UFC program, outlines a range of vetted approaches, and describes the building blocks and tradeoffs involved. This analysis was developed during a year of deliberations by a Study Panel of 30 experts in care policies from a variety of perspectives.

### DESIGNING A STATE-BASED SOCIAL INSURANCE PROGRAM FOR LONG-TERM SERVICES AND SUPPORTS

Marc A. Cohen,[1] Benjamin Veghte,[2] Eileen J. Tell,[3] and Alexandra L. Bradley[2], *1. University of Massachusetts Boston Gerontology Institute, LeadingAge LTSS Center @ UMass Boston, Boston, Massachusetts, United States, 2. National Academy of Social Insurance, Washington, District of Columbia, United States, 3. ET Consulting, Belmont, Massachusetts, United States*

The fundamental LTSS financing problem today is the absence of an effective insurance mechanism. To achieve universal coverage, social insurance is required. This report identifies key design questions for states to consider in crafting an LTSS social insurance program, outlines a range of vetted approaches states could adopt, and describes the building blocks and tradeoffs associated with these options. This analysis was developed during a year of deliberations by a Study Panel of 15 experts in LTSS with a variety of perspectives. States must answer two critical first-order questions. First, who is the program seeking to help – only the elderly, or all people with disabilities? Only those who start paying into the program now, or current retirees as well? Second, how will the program be financed – through a payroll tax, an income tax, or some other dedicated revenue source? Additional considerations follow from these two overarching questions.

### CAREGIVING ACROSS GENERATIONS AND CULTURES

Valentine Villa[1], *1. UCLA, Los Angeles, California, United States*

Utilizing data from the California Health Interview Survey (CHIS) 2009, we examine caregiving activities among foreign born and US born Latinos, and US born Non-Hispanic whites ages 18 to 80 who are caring for an adult age 60 or older. Utilizing OLS regression, we first examine the relationship between race, ethnicity, nativity, and age and the amount of caregiving, types of caregiving, and use of long-term care (LTC) services among the population. Next we examine the health, mental health, and economic status of the three caregiver populations. The results find that middle aged and younger foreign born Latinos provide the greatest amount of care and also present with the greatest amount of economic challenges. US born middle- aged Latino caregivers also provide relatively high levels of care and also are more likely than other caregivers to experience health challenges including diabetes, obesity, depression and social isolation.

### SESSION 3020 (SYMPOSIUM)

### EFFECTS OF MUSCLE ALTERATIONS AND REHABILITATION ON MOBILITY IN CHRONIC CONDITIONS OF AGING

Chair: Monica C. Serra, *Emory University, Atlanta, Georgia, United States*

Age-related changes in muscle morphology and composition are associated with the development of mobility impairments, an increased risk for institutionalization, and mortality. While primary aging may account for some of these changes, the presence of aging related co-morbid conditions such as frailty, stroke, diabetes, and Parkinson's disease is associated with muscle fiber atrophy, increased intramuscular fat, changes in muscle fiber distribution, and vascular dysfunction that occurs in both large and small vessels. These changes ultimately result in impaired substrate delivery to the muscle, reduced capacity for muscle regeneration, and anabolic resistance leading to declines in balance, strength, and endurance. It is important to understand the biological underpinnings of the changes in muscle following each of these conditions to develop effective preventive measures and maximize restorative rehabilitation protocols. This session will discuss the changes in muscle from the fiber level (i.e., changes in innervation and muscle fiber type proportion) to the systemic level (i.e., changes in specific postural and locomotor muscles), and their relationship to mobility, balance, and function. We will describe techniques (i.e., imaging, EMG, and muscle biopsies) used to assess muscle changes and their applicability in rehabilitation research. Further, we will discuss clinical recommendations for exercise to preserve and enhance skeletal muscle in the aging adult.

### STROKE

Monica C. Serra[1], *1. Emory University, Atlanta, Georgia, United States*

Following stroke, the leading cause of disability in the United States, muscle alterations commonly occur. These alterations include gross atrophy and shift in fiber type, particularly in the paretic limb, which have lasting implications on gait ability. We will discuss the current evidence supporting a mechanism for these changes (i.e. physical inactivity, inflammation, oxidative stress, and decreased lower motor neuron activation) and how these factors differ between the paretic and non-paretic limbs. Further, we will discuss how various therapeutic exercise modalities can be used to modify or reverse skeletal muscle abnormalities and alter physical functioning post-stroke.

### PARKINSON'S DISEASE

Madeleine Hackney[1], *1. Emory University, Atlanta, Georgia, United States*

The risk of Parkinson's Disease (PD) increases with age as over 90% of individuals are diagnosed after the age of 50. Older individuals with PD face a compounding burden of reduced muscle power, decreased muscular endurance, and weakness related to disease specific processes such as an altered pattern of motor unit activation, rigidity and bradykinesia. Individuals with PD demonstrate a tendency towards type I fiber hypertrophy, and a greater heterogeneity for type II fibers. Despite the disease specific burden of PD, exercise results in improved mobility, balance, and movement initiation. Interventions like resistance training have resulted in strength gains, and other interventions, such as tango dancing may be particularly useful for balance improvements, as the movement involves multi-directional perturbations and whole-body coordination. Here, we will discuss the use of novel exercise interventions, such as resistance training and tango, to improve the muscle and mobility function of older adults with PD.

### FRAILTY

Odessa Addison[1], *1. Baltimore GRECC VAMHCS, Baltimore, Maryland, United States*

The ability to safely maintain mobility function with aging is critical as immobility and falls are among the top reasons for long-term care admissions. One potential cause for these functional deficits are muscle composition changes resulting in reductions in muscle mass, strength and power, ultimately contributing to the development of frailty. While the majority of work examining muscle composition and mobility changes with aging have focused on the quadriceps and ankle plantarflexor/dorsiflexor muscles, accumulating evidence suggests that deficits involving the proximal hip muscles may be particularly harmful to balance and mobility functions leading to falls, hip fractures, and frailty. We will discuss muscle changes that occur with aging and frailty, the implications on mobility, and the effects of potential exercise interventions on muscle structure and function as well as their ability to improve functional mobility.

### DIABETES MELLITUS

Steven J. Prior[1], *1. University of Maryland, Department of Kinesiology, College Park, Maryland, United States*

Nearly three-fourths of adults over 65 year of age are affected by impaired glucose tolerance or type 2 diabetes. Both aging and inactivity contribute to the numerous skeletal muscle changes that occur with insulin resistance and type 2 diabetes. These changes include reduced capillarization that can impaired glucose uptake and substrate delivery, resulting in metabolic abnormalities and metabolic inflexibility. These changes may ultimately contribute to reduced delivery of oxygen, nutrients, and hormones to the muscles leading to impairments in metabolism, muscle mass, and function. We will discuss current research on the role of vascular impairments and reduced skeletal muscle capillarization in the development of impaired muscle metabolism, fitness and function. Finally, we will discuss how exercise training may reverse these declines.

### SESSION 3025 (PAPER)

### ELDER ABUSE: IDENTIFICATION, INTERVENTION, AND STAFF PERSPECTIVES

### A UNIQUE MODEL TO ADDRESS ELDER ABUSE: THE OHIO ATTORNEY GENERAL'S ELDER ABUSE COMMISSION

Georgia Anetzberger,[1] and Amy Restorick Roberts[2], *1. Case Western Reserve University, Cleveland, Ohio, United States, 2. Miami University, Oxford, Ohio, United States*

This presentation will highlight Ohio's innovative model to address the prevalence, risk factors, and consequences of elder abuse and neglect. We will begin with an overview of the mission and duties of the recently codified Ohio Attorney General's Elder Abuse Commission. These include: (1) to raise awareness, improve education, and boost the level of research regarding elder abuse in Ohio, (2) to provide a forum for improving the elder justice system, and (3) to identify policy, funding, and programming recommendations to address elder abuse. Next, we will share findings from the Research Committee's first statewide survey on elder abuse research priorities. Completed by frontline practitioners, program administrators, advocates, researchers, and policy makers, the online survey received 459 responses from individuals across the state. Findings reflect gaps in the elder abuse literature, as respondents prioritized funding for research in how elder abuse can be prevented, and what programs/policies can best serve victims and their families after elder abuse has begun. We will conclude with recommendations regarding how the Elder Abuse Commission model may be adapted or modified to elevate awareness of elder abuse and elder abuse research in other parts of the country.

### IT'S ACTUALLY WORSE THAN IT LOOKS: IDENTIFYING ABUSE-RELATED INJURIES AMONG APS CLIENTS

Jeanine Yonashiro-Cho,[1] Zach Gassoumis,[2] Kathleen Wilber,[2] and Diana Homeier[3], *1. Keck School of Medicine of USC, Alhambra, California, United States, 2. Leonard Davis School of Gerontology, University of Southern California, Los Angeles, California, United States, 3. Keck School of Medicine of USC, University of Southern California, Los Angeles, California, United States*

While recent work has described elder abuse injuries seen in medical contexts, most abuse determinations are made by community-based health and social services practitioners in the field. Little is known about the types of injuries present among victims who do not seek medical care. The purpose of this study was to identify and describe injuries more likely to occur through abuse, rather than accidental injury, among older adults seen in non-medical settings. An observational, matched-comparison group design was used to compare findings among physically abused APS clients (n=61) with those from non-abused older adults (n=104) seeking usual-care in a Geriatrics clinic. Forensic nurse examiners conducted full-body examinations of subjects and collected data on injury diagnoses, locations, and characteristics. Descriptive statistics and bivariate tests of association were used to analyze differences in injury presentation between groups. Though 21.8% of APS clients had no observable injuries upon examination, as a group, they were more likely than non-abused elders to be injured ($p<0.05$) and had more injuries present ($p<0.01$). Abuse victims were also more likely to have at least one upper extremity abrasion ($p<0.05$) or a diagnosis of ecchymosis ($p<0.01$), swelling ($p<0.05$) or tenderness ($p<0.05$) in the head, neck, or maxillofacial region. Because physical abuse may not result in injury to victims, screening protocols are needed to improve abuse detection. The presence of injuries among older adults at-risk for abuse warrant further evaluation or queries from medical and social service providers, regardless of injury severity.

### SAFEGUARDING OLDER PEOPLE FROM FINANCIAL ABUSE: EXPERIENCES OF IRISH BANKS

Amanda A. Phelan,[1] Deirdre O'Donnell,[1] and Sandra McCarthy[1], *1. University College Dublin, Dublin, Ireland, Ireland*

Financial abuse is a significant issue for older populations and was identified as that most common form of maltreatment in a 2010 Irish prevalence study (Naughton et al. 2010). This study examined how abuse was experienced and responded to by staff in five Irish banks. A mixed method approach was used: online survey (n=898) amd semi structured interviews (n=25) Findings from the survey data demonstrate that more than half of the respondents (66.5%) had previously suspected a customer to be experiencing some form of financial abuse. There was a high index of suspicion to the five scenarios presented to the staff. Findings from the interviews demonstrate the complexity and wide variations of case experiences of bank managers and the National Safeguarding Committee. These include being financially abused in the context of undue influence, scams, fraud and some cases described the naivety or potential naivety of some customers who may have capacity challenges, engage with strangers through social engineering scams, share PINs or open bank accounts without fully understanding the consequences. All staff had some experience of a suspicion of financial abuse in older people and employed various strategies to respond to their suspicions. We focused our recommendations in two areas: Bank level responses-inter-sectorial collaboration, education and training, a vulnerable adult champion, having a choice of banking methods of engagement, direct client communication and enhancements within the Central bank of Ireland. Macro level responses were increasing public awareness, raising the profile of financial abuse as a crime and enhancing safeguarding legislation

### STAFF PERSPECTIVES ON COUNTERING STAFF-TO-RESIDENT MISTREATMENT IN LONG-TERM CARE FACILITIES

Melanie Couture,[1] Milaine Alarie,[1] and Sarita Israel[1], *1. Centre for research and expertise in social gerontology, CIUSSS West-Central Montreal, Montreal, Quebec, Canada*

In Canada, as well as in other countries, resident mistreatment is common in long-term care (LTC) facilities. In many situations, residents are mistreated by LTC staff. To address this problem, LTC facility managers and their employees must play an active role in the prevention as well as in the management of staff-to-resident mistreatment situations. However, it is still unclear what type of support they need to counter this type of mistreatment. Using an exploratory descriptive qualitative design, twenty-one managers and employees working in four different LTC facilities participated in semi-structured individual interviews. To allow participants to express themselves without risking self-incrimination or feeling pressured to report colleagues, vignettes depicting fictitious and common situations of staff-to-resident mistreatment were used as a conversation starter. Data analysis was performed using Miles, Huberman & Saldaña (2013) analytical method. Results show that participants think that staff-to-resident mistreatment is mainly caused by three staff characteristics: 1) not having the psychological profile to work in LTC facilities; 2) lack of training; and/or 3) being overworked. Consequently, participants believe that mistreatment prevention starts by improving employee selection practices to ensure candidates have adequate attitudes and training to work in LTC facilities. They also argue that staff should receive more training regarding mistreatment. Lastly, support interventions are suggested to prevent and address situations involving staff experiencing high levels of stress for personal or work-related reasons. This study shows that both individual and organisational measures are needed to fight against staff-to-resident mistreatment in LTC facilities.

### THE USE OF ELDERCARING COORDINATION FOR RESOLVING CASES INVOLVING OLDER ADULTS AND HIGH-CONFLICT FAMILY DYNAMICS

Pamela B. Teaster,[1] and Megan L. Dolbin-MacNab[1], *1. Virginia Tech, Blacksburg, Virginia, United States*

The Association for Conflict Resolution and The Florida Chapter of the Association of Family and Conciliation Courts developed a model of Eldercaring Coordination for use in guardianship/probate cases involving high-conflict family dynamics that interfere with the well-being and safety of an older adult, limit adherence to court orders, impede court processes, or detract from the efficacy of guardianship and other appointments by the court. Developed by 40 organizations and entities in the United States and Canada, Eldercaring Coordination focuses on improving family dynamics so that the older adult, family members, and other involved parties can better work together and with professionals to make thoughtful and informed decisions and to support each other during times of transition. The purpose of this research was to gather information about participant experiences with Eldercaring Coordination. A pre-post test design was employed in which data were collected from older adults or their surrogates, family members and other court-ordered participants, judges and court administrators, and the Eldercaring Coordinators themselves. Findings from post-tests of 23 judges and court administrators revealed that the most common advantages of Eldercaring Coordination were that the intervention prioritized the older adults' needs and improved family relationships. Post-test surveys from 17 Eldercaring Coordinators indicated some positive outcomes for older adults and their families, but also a need for enhanced authority, greater support from attorneys, and more cooperation from participants. Preliminary findings support the assertion that Eldercaring Coordination holds promise for intervening in high-conflict court cases involving older adults.

### SESSION 3030 (PAPER)

### EMOTIONS AND WELL-BEING IN LATER LIFE

### CHILDHOOD HAPPINESS, SELF-MASTERY, AND LATER-LIFE HEALTH

Haena Lee,[1] and Markus H. Schafer[2], *1. University of Michigan, Ann Arbor, Michigan, United States, 2. University of Toronto, Toronto, Ontario, Canada*

Considerable work has documented that positive childhood memories, especially childhood happiness, predict better health among young adults. However, it is not known whether growing up happy has enduring health consequences across the life course. Using two waves of the National Social Life, Health and Aging Project (2010-2011 and 2015-2016; N = 1,937), we investigate the relationship between childhood happiness and changes in physical, mental, and biological functioning in later life. Childhood happiness was retrospectively assessed using a question: "When I was growing up, my family life was always happy." Self-rated health, depressive symptoms, and frailty over a five-year period were examined to reflect changes in functional status. Childhood SES and living arrangement were examined to assess childhood sociodemographic background. Educational attainment, family support and strain, and self-mastery were considered as potential mediators. We find that, among other childhood factors, childhood happiness significantly predicts older adult health. Specifically, childhood happiness was associated with better self-rated health and lower depressive symptoms at follow-up, net of baseline health conditions. We did not find a relationship between frailty and childhood happiness. Unlike prior work, we found no significant effect of childhood SES on the measured outcomes. Associations between childhood happiness and self-rated health and depression were mediated by psychosocial resources including self-mastery and perceived social support from family members. This implies that growing up in nurturing, cherished family environment has the potential to cultivate social relationships and build resilience which could provide an important pathway to successful aging.

### DAILY EMOTIONS AND STRESS: AGE CHANGES AND DIFFERENCES

Jessica Blaxton,[1] Cindy Bergeman,[2] and Lijuan (Peggy) Wang[2], *1. Fontbonne University, St. Louis, Missouri, United States, 2. University of Notre Dame, Notre Dame, Indiana, United States*

Developmental processes differ between individuals (interindividual differences), fluctuate within them on a short-term basis (intraindividual variability), and change over time on a longer-term basis (intraindividual change; Nesselroade, 1991). We situate the relationship between stress and emotions in this process-oriented perspective by examining how the daily relationship between stress and negative affect (NA) as well as stress and positive affect (PA) change over time, while considering cross-sectional age and stress differences. Participants (N = 966) completed daily questionnaires assessing stress, NA, and PA. Three-level multi-level models depicted how cross-sectional age, within-person age changes, and global stress differences impact the daily stress-affect relationship. Findings illustrate that cross-sectional age and the aging process uniquely buffer the stress-NA relationship whereas global stress exacerbates it. Furthermore, older adults as well as adults with low global stress experience a weaker relationship between daily stress and PA as they age, but midlife adults and adults with high global stress experience a stronger relationship. These results depict differences in aging trajectories for both midlife and older adults and thus inform intervention and preventative care strategies aimed toward promoting emotional well-being, suggesting that targeting these strategies at the daily level can promote better stress regulation. Furthermore, we see that midlife adults and adults with greater global stress perceptions are most in need of these interventions, and encouraging these adults to maintain PA in the face of daily stress can be particularly beneficial.

### LINKING FAMILY TRANSITIONS AND LATER-LIFE DEPRESSION: DOES LIFE-COURSE SOCIOECONOMIC STANDING MATTER?

Claudia Recksiedler,[1] Boris Cheval,[2] Stefan Sieber,[2] Robert S. Stawski,[3] and Stephane Cullati[2], *1. German Youth Institute, Munich, Germany, 2. University of Geneva, Geneva, Switzerland, 3. Oregon State University, Corvallis, Oregon, United States*

Research documented the impact of marital transitions—particularly marital loss—on depression in old age, yet its severity depends multiple factors. Individuals' capability to cope with transitions depends on available resources and previous exposure to stressors, such as early-life adversity, which buffers or aggravates the impact of marital transitions on later-life depression. Although studies documented the pivotal link between early-life adversity and negative health trajectories, our study is the first attempt to examine whether early-life adversity influences the relationship between prospectively-tracked, later-life marital transitions and depression. We drew data from SHARE, which samples individuals aged 50+ across Europe (N = 13,258; 2004-2016). Using multilevel linear models, we found that women who became widowed had higher levels of depression compared to coupled and single women, but experienced lower increases in depression over time. After adjusting for early-life and adulthood SES, losing a partner remained significantly associated with depression. Life-course SES was associated with levels of depression, yet interactions between marital transitions and SES were not, with some exceptions: single women who reported difficulties in their ability to make ends meet experience higher increases of depression over time. Overall, results were similar for men. Interactions between family transitions and SES were again not significant, with a few exceptions for single men: those born in more childhood conditions, and those with high education, had lower levels of depression. We interpret and discuss our findings through the lens of life-course and stress-resiliency perspectives and in light of changing family dynamics for this age group.

### THE EXPERIENCE OF HAPPINESS IN LATE-LIFE REPARTNERING: SURPRISE AND DISAPPOINTMENT

Chaya Koren[1], *1. University of Haifa, Haifa, Israel, Israel*

Late life repartnering among those aged 65 and older is a phenomenon developing along with the increase in life expectancy. Although research indicates that older people are happier than adults at other life phases, the common lay person perception among the young as well as the old is that old age is associated with less happiness. Late life repartnering in Israel, culturally located between tradition and modernity, is not officially recognized as an option in old age. Exploring the experience of happiness within a social context that perceives late-life repartnering as the exception, using a naturalistic paradigm, has the potential for understanding lay persons perceptions of happiness. The aim of this qualitative research is to explore the experience of happiness in late-life repartnering relationships from a dyadic perspective of each and both partners. 20 couples (40 participants) functionally independent, aged 66-92 who entered their late-life repartnership at old age (men aged 65+; women aged 60+) after widowhood or divorce from a lifelong marriage raising a family, were interviewed separately. Interviews were recorded and transcribed verbatim. Data was analyzed using a dyadic interview analysis method. Findings indicate that happiness in late-life repartnering relationships include experiences of surprise and disappointment in three sub-themes: a. "A gift from heaven": Surprised of being happy; b. Disappointment not being happy; c. No surprise – No disappointment. Findings are discussed based on disappointment theory, and empirical literature on expectations and happiness. Implications are addressed.

### SESSION 3035 (SYMPOSIUM)

### EMPOWERING VULNERABLE GROUPS IN LATER LIFE: FOCUS ON THE ROLE OF ACTIVITIES, SOCIAL NETWORK, AND ROUND-THE-CLOCK CARE

Chair: An-Sofie Smetcoren, *Vrije Universiteit Brussel, Brussels, Belgium*
Discussant: G.A. Rixt Zijlstra, *Maastricht University, Care and Public Health Research Institute (CAPHRI), Department of Health Services Research, Maastricht, Netherlands*

Europe has been challenged with an intense rise of aging populations facing for example multiple chronic health problems, functional limitations and social and psychological challenges. With increasing age people may become vulnerable, nevertheless, they can still report high levels of well-being despite their deficits. Older adults' strengths and resources can balance negative experiences and increase positive well-being outcomes. These resources can be personal (e.g. have sufficient income) or stemming from the social environment of the older person (e.g. an involved social network). Hence, this symposium focusses on these strengths and resources and how they might (positively) affect the well-being of vulnerable groups ageing in place. The main objective of the symposium is to give insights into different aspects and strategies that can protect older adults against negative outcomes. Four different studies from Belgium will be presented: Sarah Dury starts with explaining the potential buffering predictor of leisure and civic activities, by uncovering the mechanisms underlying the relationship between multidimensional frailty and well-being. Lise Switsers examines if the absence of social and emotional loneliness can act as a buffer to maintain a good well-being for older adults at risk of frailty. An-Sofie Smetcoren examines how 'living in solidarity' in a co-housing project can contribute to ageing in place. Finally, Sylvia Hoens explores the experiences of the older care users and their informal caregivers with live-in migrant care workers and examines how this care can increase their well-being.

### SHAPING LIVING IN SOLIDARITY AMONG OLDER VULNERABLE PEOPLE IN BRUSSELS

An-Sofie Smetcoren,[1] and Liesbeth De Donder[2], *1. Vrije Universiteit Brussel, Brussels, Belgium, 2. Vrije Universiteit Brussel, Brussels, Brussels Hoofdstedelijk Gewest, Belgium*

This research explores how older people construct their view on 'living in solidarity'. The data was collected during a cohousing project (from construction till occupation). Co-creation sessions with residents and project coordinators were analyzed. 7 conditions and success factors were unraveled that deemed important to realize 'solidary housing': 1) The challenge to unite individual and collective needs; 2) Continuous task to engage (candidate-) residents, from early beginning; 3) A targeted selection of residents; 4) Maximizing the competences of older people, 5) Developing a group identity consciousness, 6) Involving experts providing support for group- and individual trajectories, 7) Creating a climate of confidence. Solidary housing appeared to be a nuanced and layered concept which should not be overidealized. Participants demonstrate what is realistic, and how it can be shaped in daily practice. By discussing and deciding on group issues, the project coordinators created a shared platform for implicit and explicit agreements.

### ARE LEISURE AND CIVIC ACTIVITIES REINFORCING WELL-BEING IN OLDER ADULTS AT RISK OF FRAILTY?

Sarah Dury,[1] Eva Dierckx,[2] liesbeth De Donder,[2] Deborah Lambotte,[2] Daan Duppen,[2] and lise switsers[2], *1. Vrije Universiteit Brussel, Brussels, Belgium, 2. Vrije Universiteit Brussel, Brussels, Brussels Hoofdstedelijk Gewest, Belgium*

A growing body of work suggest that leisure and civic activities may contribute to the understanding of healthy aging. Yet, only a limited number of studies have examined a less healthy population. Moreover, a broad array of leisure and civic activities tend to be lacking. This paper gives insight into the mechanisms underlying the associations between multidimensional frailty, and well-being with the moderating roles of leisure and civic activities. A two-wave interview survey from the D-SCOPE frailty program was derived using 441 participants aged 60 years and older residing in the Flanders region of Belgium. This study offers evidence that leisure and civic activities buffered the negative relationship between multidimensional frailty and well-being. Moreover, our study identified that for different frailty domains the buffering/moderating role of leisure and civic activities differs in relation to well-being.

### EXPERIENCES OF OLDER PEOPLE AND INFORMAL CAREGIVERS WITH LIVE-IN MIGRANT CARE WORKERS IN BELGIUM

Sylvia Hoens,[1] An-Sofie Smetcoren,[2] and Liesbeth De Donder[2], *1. Vrije Universiteit Brussel, Brussels, Belgium, 2. Vrije Universiteit Brussel, Brussels, Brussels Hoofdstedelijk Gewest, Belgium*

The increasing number of older people has a significant impact on the organization of care in European countries. Despite the availability of formal care services, adequate solutions are still missing and older people themselves search for alternative strategies to meet their care needs. For example, a recent tendency is to call upon help of migrant care workers. In Belgium, research concerning this often invisible care solution remains absent. Therefore, the study at hand explores the experiences of the older care users and their informal caregivers and examines how this care can increase their well-being. Eight in-depth interviews with older people who rely on live-in migrant care workers, five interviews with professionals and one focus group with experts have been conducted. This study found that live-in caregivers relieve the informal carers, guarantee the presence of permanent care 24/7 and enable older people to live longer at home.

### THE IMPORTANCE OF LOW SOCIAL LONELINESS FOR MAINTAINING GOOD WELL-BEING

Lise Switsers,[1] Liesbeth De Donder,[2] Eva Dierckx,[2] and Sarah Dury[2], *1. Vrije Universiteit Brussel, Brussels, Belgium, 2. Vrije Universteit Brussel, Brussel, Brussels Hoofdstedelijk Gewest, Belgium*

Older people are often confronted with dependence, death of spouse and other loss experience. Nevertheless, older adults generally experience a good well-being. This lack of age-related decline of subjective well-being has been named the 'paradox of ageing'. One possible explanation for this paradox can be found in the socio-emotional selectivity theory of Carstensen. Thus, we hypothesize that low emotional and/or low social loneliness can act as a buffer for the negative relationship between negative life events and well-being. We use data of the D-SCOPE project that includes 869 older community-dwelling adults at risk of frailty residing in Flanders. By means of regression moderating analyses the research gains insights into the relationships between older people and well-being where the absence of social loneliness is detected as a possible buffer against negative outcomes. The discussion develops the argument that the absence of loneliness is a crucial facet for maintaining a good well-being.

### SESSION 3040 (SYMPOSIUM)

### EXPLORING MECHANISMS OF AGING THROUGH LONGITUDINAL TRAJECTORIES: BIOLOGICAL, PHENOTYPIC, AND CLINICAL

Chair: Jennifer A. Schrack, *Johns Hopkins University, Baltimore, Maryland, United States*
Discussant: Luigi Ferrucci, *Translational Gerontology Branch, Intramural Research Program, National Institute on Aging, National Institutes of Health, Baltimore, Maryland, United States*

Over the past several decades, researchers have searched for early and accurate predictors of healthy aging, establishing metrics to potentially quantify the aging process based on functional performance, disease diagnosis, or other clinical tests. Using these metrics, efforts have been made to disentangle the concepts of "normal" versus "accelerated" aging to identify individuals transitioning into states of disease and disability, allowing for more targeted and effective treatment(s). Yet, many of the variables included in these indices have been chosen based on their availability using cross-sectional associations with chronological age. Such variables may reflect birth cohort differences and selective attrition and fail to accurately represent the mechanisms most representative of the aging process. Using data longitudinal data from the Baltimore Longitudinal Study of Aging and beyond, this symposium will present a conceptual framework of phenotypic aging and its relationship with biological aging, and trajectories of aging phenotypes using state-of-the-art measures collected over the past 10 years. Specifically, we will discuss new insights into trajectories of molecular (inflammatory and metabolic markers), physiological (energetics, body composition, brain atrophy), and clinical measures (gait speed and executive function), and discuss methodological considerations for combining these phenotypes into a measure that can be linked with biological aging measured using a systems approach to improve and refine future understanding of mechanisms of "normal" versus "accelerated" aging. Further development and application of the methodology presented in this symposium will highlight the importance of using longitudinal data to improve understanding of physical and cognitive trajectories with aging.

### SYSTEMS-LEVEL MODELING OF BIOLOGICAL AND MOLECULAR AGING CHANGES OVER TIME

Morgan E. Levine,[1] Perry Kuo,[2] Jennifer Schrack,[2] Eleanor M. Simonsick,[3] Susan Resnick,[3] Michelle Shardell,[3] and Luigi Ferrucci[3], *1. Yale University School of Medicine, New Haven, Connecticut, United States, 2. Johns Hopkins, Maryland, Maryland, United States, 3. National Institute on Aging, Baltimore, Maryland, United States*

Aging is associated with numerous changes at all levels of biological organization. Harnessing this information to develop measures that accurately and reliably quantify the biological aging process will require longitudinal modeling and incorporation of systems level approaches. We will describe applications of network modeling for longitudinal multi-system biomarker data. Using data from the Baltimore Longitudinal Study of Aging (BLSA) we are able to generate systems level models of biological and physiological function, and then demonstrate how these networks change with age. We will also link systems-level aging changes to hallmarks of aging, including epigenetic alterations, senescence, mitochondrial dysfunction, and proteostasis. Given the complexity of the biological aging process, modeling of systems dynamics over time will both lead to the development of better biomarkers of aging, and also inform our conceptualization of how alterations at the molecular level propagate up levels of organization to eventually influence morbidity and mortality risk.

### TRAJECTORIES OF PHENOTYPIC MARKERS OF AGING AS PRECURSORS TO FUNCTIONAL CHANGE

Jennifer A. Schrack,[1] Pei-Lun Kuo,[1] Eleanor M. Simonsick,[2] Susan M. Resnick,[2] Morgan Levine,[3] and Michelle Shardell[2], *1. Johns Hopkins University, Baltimore, Maryland, United States, 2. National Institute on Aging, Baltimore, Maryland, United States, 3. Yale University, New Haven, Connecticut, United States*

Delineating trajectories of aging phenotypes is essential to understanding mechanisms of clinical disease and disability. We investigated longitudinal changes in measures of body composition, energy expenditure, and brain volumes in >900 participants (age 67.0 (IQR: 57-77) years, 48.1% male) of the Baltimore Longitudinal Study of Aging using mixed effects regression models. Computed tomography-derived thigh muscle cross-sectional area declined 754.2 cm2 per decade at age 60 years (p<0.001) and 1294.3 cm2 at 75 years (p<0.001). Energy reserves, defined as a ratio of energy-cost-to-energy-capacity measured using indirect calorimetry, decreased 11.2% per decade at 60 years (p<0.001), and 16.8% at 75 years (p<0.001). MRI-derived measures of total brain volumes declined 41.6 cm3 per decade at 60 years (p<0.001) and 44.9 cm3 at 75 years (p<0.001). Linking these findings to biological and clinical measures of aging may contribute to more accurate assessment of phenotypic age.

### LONGITUDINAL CHANGE OF PHYSICAL AND COGNITIVE FUNCTIONS IN BLSA

Pei-Lun Kuo,[1] Morgan Levine,[2] Michelle Shardell,[3] Susan Resnick,[4] Eleanor M. Simonsick,[3] and Jennifer A. Schrack[1], *1. Johns Hopkins University Bloomberg School of Public Health, Baltimore, Maryland, United States, 2. Yale Center for Research on Aging (Y-Age), Department of Pathology, Yale School of Medicine, New Haven, Connecticut, United States, 3. Longitudinal Studies Section, Translational Gerontology Branch, National Institute On Aging , NIH, Baltimore, Maryland, United States, 4. Laboratory Of Behavioral Neuroscience, National Institute On Aging , NIH, Baltimore, Maryland, United States*

Optimally integrating metrics of aging requires evaluating the metrics' change with aging. We investigated longitudinal changes of physical and cognitive functions in the Baltimore Longitudinal Study of Aging. Usual gait speed declined -0.08 m/s (p<0.001) per decade at age 60 years, -0.10 m/s (p<0.001) per decade at 65 years, and -0.13 m/s (p<0.001) per decade at 70 years, after adjusting for sex and height. No sex difference of gait speed decline was observed after adjustment for height. Time to finish Trails B, an indicator of executive function, increased 11.3 seconds per decade at 60 years, 17.7 seconds (p<0.001) per decade at 65 years, and 24.1 seconds (p<0.001) per decade at 70 years, after adjusting for sex, education, and race. No sex difference of longitudinal decline in executive function was observed. Linking these findings to physiological measures may unveil an important mechanism of aging.

### ANALYTICAL CONSIDERATIONS OF DEVELOPING A PHENOTYPIC AGING MEASURE: THE CONCEPTUAL FRAMEWORK MUST COME FIRST!

Michelle Shardell,[1] Michelle Shardell,[2] Pei-Lun Kuo,[3] Jennifer Schrack,[3] Eleanor M. Simonsick,[2] Susan M. Resnick,[2] Morgan E. Levine,[4] and luigi Ferrucci[2], *1. Translational Gerontology Branch, Intramural Research Program, National Institute on Aging, National Institutes of Health, Baltimore, Maryland, United States, 2. National Institute on Aging, Baltimore, Maryland, United States, 3. Johns Hopkins University Bloomberg School of Public Health, Baltimore, Maryland, United States, 4. Yale School of Medicine, New Haven, Connecticut, United States*

We propose a latent structural model framework where phenotypic aging is a latent variable influenced by chronological age, genes and environment. Within this framework, phenotypic age influences aging-related outcomes and is reflected by latent domains of aging (body composition, energetics, homeostasis, and neural functioning) reflected by biomarkers. First, we validate the framework by selecting age-associated domain-specific biomarkers and assessing internal consistency and convergent construct validity (Cronbach's alpha). Using data from the Baltimore Longitudinal Study of Aging, within-domain Cronbach's alphas ranged from 0.80 to 0.92, supporting convergent construct validity. Second, we evaluate two broad methods for combining biomarkers into one phenotypic age measure customized to different objectives: 1) confirmatory factor analysis of chronological age-adjusted biomarkers to create a measure to identify pleiotropic genetic and environmental mechanisms, and 2) machine-learning methods to create a measure optimizing predictive and concurrent criterion validity. This framework will enable evaluation of candidate biological mechanisms of aging.

### SESSION 3045 (SYMPOSIUM)

### FINANCIAL LITERACY, RETIREMENT, AND BECOMING FINANCIALLY CAPABLE IN A DEVELOPING COUNTRY

Chair: Julian G. McKoy Davis, *Mona Ageing & Wellness Centre, Kingston, Jamaica*
Discussant: Denise Eldemire-Shearer, *Mona Ageing & Wellness Centre, Kingston, Jamaica*

Worldwide, 3.5 billion (67%) of older adults do not understand basic financial concepts. In the Caribbean, families and professionals alike are struggling to assist and financially support a rapidly ageing population. The concept of children as "pension guarantee" is widely practised throughout the Caribbean, but factors such as modernization, urbanization, migration, and a shift in societal norms pertaining to familial responsibilities, have left many older persons financially fending for themselves. Less than half of all Jamaicans have adequate retirement funds. People with limited financial resources can and do save, but often use strategies that are not advantageous. The stark reality of low pension coverage among older Jamaicans and austere working conditions among public and some private sector workers indicate growing economic, financial, and societal challenges. Jamaica's rapidly growing ageing population has resulted in both a workforce and a retirement financial crisis. This symposium provides an overview of the current policy and economic issues. We describe the design, implementation, and evaluation of an economic program to increase financial literacy and planning of adults nearing retirement age. To increase financial retirement literacy of Jamaicans with limited educational and financial resources, a peer-to-peer program was developed using a culturally adapted version of the FDIC Money Smart Program. A peer-to-peer approach has the potential to strengthen financial literacy and enhance retirement planning behaviours of underserved adults and avoid further burdening an already limited workforce. This project's findings can inform policy, shape program development, and guide implementation of similar programs in low resource countries.

### POLICY AND ECONOMIC INFLUENCES ON RETIREMENT, FINANCIAL LITERACY, AND ECONOMIC SECURITY IN JAMAICA

Julian G. McKoy Davis[1], *1. Mona Ageing & Wellness Centre, Kingston, Jamaica*

The changing pension landscape, as well as the accompanying privatization, marketization and individualization of the pension planning process, has resulted in inadequate and risky investment practices. Jamaica, like many other countries in the international community, has been engaged in pension reform. The main issues in the current dispensation of pension reform are in relation to pension adequacy to mitigate against longevity risk, low levels of pension coverage; with the aforementioned leading to the need to increase the number of persons contributing to the pension pool as well as possibly an increase in the value of pension contributions to account for inflation and investment risks. This paper describes the current legal and regulatory framework on pension reform in Jamaica within the context of population ageing and the National Financial Inclusion Strategy that targets persons who were previously underserved by the domestic financial system. Policy and programmatic recommendations are will be provided.

### RETIREMENT FINANCIAL LITERACY LEVELS AMONG JAMAICANS

Kayon Donaldson-Davis[1], *1. Mona Ageing & Wellness Centre, Kingston, Jamaica*

Although the general literacy rate (ability to read and write at a 6th-grade level or higher) of Jamaicans is 89%, the number of people who are adequately prepared financially is not comparable. Dissertation research findings revealed that 52% (n=203) of respondents had not received any financial education. Approximately 71% of respondents who reported high levels of financial distress and low financial well-being, had not received financial information about retirement planning. Results from the 2012 Social Status on the Elderly in Jamaica showed that 60.5% (n=1,716) of respondents had no pension and of those receiving money from abroad, most (75.0%, n=813) indicated that they received it only occasionally. Those who were self-employed, women and rural residents were most likely not to have a pension. This paper describes financial literacy levels among Jamaicans using two separate data sets – the 2012 Social Status on the Elderly and the 2018 Financial Preparation Study.

### APPLYING THE DIME APPROACH TO A FINANCIAL LITERACY PROGRAM

Lisa M. Brown[1], *1. Palo Alto University, Plo Alto, California, United States*

Financial literacy is defined as "the ability to use knowledge and skills to manage financial resources effectively for a lifetime of financial well-being." The Money Smart Peer-to-Peer Program (MSP) was developed by the FDIC for use with diverse populations. MSP was culturally adapted for use in Jamaica using the Johns Hopkins DIME process. Peer counselling programs consistently demonstrate that they are acceptable and effective with adults who have limited education and low-income by overcoming barriers that are typically encountered when people attempt to access unfamiliar services or guidance from a professional. Research conducted with the MSP program reveals that program participants were more likely to open and save using deposit accounts, use and adhere to a budget, and have increased confidence in their financial abilities 6 to 12 months after completing the course. Implementation of MSP increases the likelihood that people can retire with dignity and financial security.

### PILOTING THE MONEY SMART FINANCIAL LITERACY PROGRAM IN JAMAICA

Douladel Willie-Tyndale[1], *1. Mona Ageing & Wellness Centre, Kingston, Jamaica*

This paper describes the piloting of a culturally modified version of the FDIC Money Smart Peer-to-Peer Program. The pilot study examined the use of Mentorship Teams comprised of two persons one (60 years and over) and another (30 - 59 years) who collectively provided mentorship and financial education to four working-age adults. The pilot was designed with four (4) phases: Phase 1 included recruitment and training of Mentors, Phase 2 the assignment of Mentors and Mentees, Phase 3 application of the intervention, and Phase 4 the evaluation of outcomes. This paper will review the design and recruitment processes, modifications made to the existing Money Smart Peer-to-Peer Program for cultural sensitivity and financial system factors, implementation challenges (including barriers and facilitators) and lessons derived from initiation and program implementation.

### SESSION 3050 (SYMPOSIUM)

### FRAILTY AND THE EFFECTIVENESS AND SAFETY OF NEW GLUCOSE-LOWERING DRUGS IN OLDER ADULTS WITH TYPE 2 DIABETES

Chair: Dae H. Kim, *Hebrew SeniorLife, Boston, Massachusetts, United States*
Co-Chair: Elisabetta Patorno, *Brigham and Women's Hospital and Harvard Medical School, Boston, Massachusetts, United States*
Discussant: Medha Munshi, *Beth Israel Deaconess Medical Center, Boston, Massachusetts, United States*

Recently, several classes of non-insulin glucose-lowering drugs, such as dipeptidyl peptidase-4 inhibitors (DPP4i), glucagon-like peptide-1 receptor agonists (GLP1-RA), and sodium glucose co-transporter-2 inhibitors (SGLT2i), have been approved to treat type 2 diabetes. Clinical trials have shown that GLP1RA and SGLT2i confer cardiovascular benefit, while DPP4i do not have such benefit; these drugs do not seem to increase the risk of hypoglycemia. However, due to underrepresentation of older adults with frailty and lack of frailty assessment in clinical trials, little is known about how the effectiveness and safety of these drugs change across different levels of frailty. In this symposium, we present the real-world evidence from Medicare data April 2013-December 2016 on the utilization trends of newly approved diabetes drugs (Dr. Dave) and comparative effectiveness and safety of SGLT2i vs sulfonylurea (Dr. Pawar), SGLT2i vs DPP4i (Dr. Kim), and SGLT2i vs GLP1-RA (Dr. Patorno). The outcomes were 1) composite cardiovascular endpoint of mortality, myocardial infarction, stroke, or heart failure; and 2) severe hypoglycemia, defined as emergency department visits or hospitalizations due to hypoglycemia. We applied a validated claims-based frailty index (CFI) to estimate the treatment effectiveness and safety in non-frail (CFI<0.10), pre-frail (CFI 0.10-0.19), or frail individuals (CFI≥0.20). Following individual presentations, Dr. Munshi and presenters will lead an interactive discussion about clinical implications and methodological challenges in conducting geriatric pharmacoepidemiologic studies using Medicare data. This symposium will demonstrate the utility of CFI in claims-based pharmacoepidemiologic studies and provide health care providers with new evidence to tailor diabetes pharmacotherapy based on frailty.

### UTILIZATION TRENDS OF NEWLY APPROVED GLUCOSE-LOWERING DRUGS FOR TYPE 2 DIABETES

Chintan Dave,[1] Dae Kim,[2] and Elisabetta Patorno[1],
*1. Brigham and Women's Hospital, Boston, Massachusetts, United States, 2. Hebrew SeniorLife, Boston, Massachusetts, United States*

Using Medicare fee-for-service data from 2013-2015, we identified 3.2 million patients per year (mean [SD] age, 74.7 years [standard deviation, 7.2]) who were treated with glucose-lowering drugs for type 2 diabetes. Between 2013 and 2015, the proportion of patients treated with sulfonylureas declined from 27.4% to 25.1%; those using DPP4is (11.5% to 12.0%) and GLP1-RAs (1.8% to 2.4%) remained unchanged; those using SGLT2is increased from 0.2% to 1.9%. In the subgroup of patients initiating a glucose-lowering drug without prior use of the same class agent, the proportion of patients starting sulfonylureas (18.7% to 17.2% of initiators), DPP4is (16.0% to 15.0% of initiators), and GLP1-RAs (3.4% to 4.2% of initiators) changed little between 2013 and 2015, while those starting SGLT2is increased from 0.7% to 6.5% of initiators. In the Medicare population, we observed a persistently high use of sulfonylureas and a rapid uptake of SGLT2is among the newer classes.

### FRAILTY AND THE COMPARATIVE EFFECTIVENESS AND SAFETY OF SGLT2I AND SULFONYLUREA IN OLDER ADULTS WITH TYPE 2 DIABETES

Ajinkya Pawar,[1] elisabetta Patorno,[1] and Dae Kim[2],
*1. Brigham and Women's hospital, Boston, Massachusetts, United States, 2. Hebrew SeniorLife, Boston, Massachusetts, United States*

We conducted a 1:1 propensity score-matched retrospective cohort study of 70,826 patients with type 2 diabetes (mean age, 71.4 years [standard deviation, 5.0]) initiating a SGLT2i or a second-generation sulfonylurea in Medicare data. We estimated HRs (95% CIs) for a composite cardiovascular endpoint and severe hypoglycemia comparing the two treatments in the entire population and by the CFI-based frailty subgroups. Compared with sulfonylureas, SGLT2is were associated with lower rates of the composite cardiovascular endpoint (HR, 0.68 [95% CI, 0.62-0.75]) and severe hypoglycemia (0.43 [0.35-0.53]) over a mean follow-up of 9.5 months. The lower rate of composite cardiovascular endpoint for SGLT2i vs sulfonylureas was observed in pre-frail (0.68 [0.61-0.77]) and frail (0.64 [0.53-0.77]) subgroups, but not in non-frail subgroup (0.95 [0.59-1.54]). The rate of severe hypoglycemia was consistently lower for SGLT2i vs sulfonylureas across frailty subgroups (non-frail, 0.37 [0.12-1.16]; pre-frail, 0.45 [0.35-0.59]; frail, 0.40 [0.28-0.58]).

### FRAILTY AND THE COMPARATIVE EFFECTIVENESS AND SAFETY OF SGLT2I AND DPP4I IN OLDER ADULTS WITH TYPE 2 DIABETES

Dae H. Kim,[1] Ajinkya Pawar,[2] Seoyoung Kim,[2] and Elisabetta Patorno[2], *1. Hebrew SeniorLife, Boston, Massachusetts, United States, 2. Brigham and Women's hospital, Boston, Massachusetts, United States*

We conducted a 1:1 propensity score-matched retrospective cohort study of 83,432 patients with type 2 diabetes (mean age, 71.5 years [standard deviation, 5.0]) initiating a SGLT2i or a DPP4i in Medicare data. We estimated HRs (95% CIs) for a composite cardiovascular endpoint and severe hypoglycemia comparing the two treatments in the entire population and by the CFI-based frailty subgroups. Compared with DPP4i, SGLT2i were associated with a lower rate of the composite cardiovascular endpoint (HR, 0.70 [95% CI, 0.64-0.77]) and a similar rate of severe hypoglycemia (0.88 [0.71-1.07]) over a mean follow-up of 8.8 months. The rate of composite cardiovascular endpoint for SGLT2i vs DPP4i was consistently lower in pre-frail (0.71 [0.64-0.79]) and frail (0.67 [0.55-0.80]) subjects, but not in non-frail patients (0.98 [0.62-1.54]). The rate of severe hypoglycemia was not meaningfully different between SGLT2i and DPP4i (non-frail, 0.39 [0.12-1.16]; pre-frail, 0.83 [0.65-1.07]; frail, 1.13 [0.78-1.64]).

### FRAILTY AND THE COMPARATIVE EFFECTIVENESS AND SAFETY OF SGLT2I AND GLP1-RA IN OLDER ADULTS WITH TYPE 2 DIABETES

Elisabetta Patorno,[1] Ajinkya Pawar,[2] Seoyoung Kim,[2] and Dae Kim[3], *1. Brigham and Women's Hospital and Harvard Medical School, Boston, Massachusetts, United States, 2. Brigham and women's hospital, Boston, Massachusetts, United States, 3. hebrew SeniorLife, Boston, Massachusetts, United States*

We conducted a 1:1 propensity score-matched retrospective cohort study of 87,218 patients with type 2 diabetes (mean age, 71.5 years [standard deviation, 5.1]) initiating a SGLT2i or a GLP1-RA in Medicare data. We estimated HRs (95% CIs) for a composite cardiovascular endpoint and severe hypoglycemia comparing the two treatments in the entire population and by the CFI-based frailty subgroups. Compared with GLP1-RA, SGLT2i were associated with similar rates of the composite cardiovascular endpoint (HR, 0.94 [95% CI, 0.86-1.03]) and severe hypoglycemia (0.87 [0.71-1.07]) over a mean follow-up of 8.6 months. The rate of composite cardiovascular endpoint was not meaningfully different between SGLT2i and GLP1-RA across non-frail (1.33 [0.80-2.23]), pre-frail (0.96 [0.85-1.08]), and frail patients (0.87 [0.73-1.04]). Similarly, the rate of severe hypoglycemia was not meaningfully different between the two treatments among non-frail (0.97 [0.20-4.80]), pre-frail (0.83 [0.64-1.08]), and frail patients (0.95 [0.67-1.34]).

### SESSION 3060 (SYMPOSIUM)

### GETTING TO YES . . . I WILL RESPOND: CHALLENGES AND SUCCESSES SURVEYING AGING SERVICES PROVIDERS TO PRODUCE QUALITY DATA

Chair: Lauren Harris-Kojetin, *National Center for Health Statistics, Hyattsville, Maryland, United States*

Voluntary surveys of aging services providers are important data sources for research, quality improvement, and program evaluation efforts to inform evidence-based decision making. Ideally, provider surveys—a type of establishment survey—offer valuable information on providers and services users. However, decreasing survey response rates in recent years raise data quality concerns. This symposium highlights challenges leading to lower response rates (e.g., time constraints, skepticism, confidentiality concerns, getting to the correct respondent); specific data collection techniques tested, what did and did not work, and lessons learned. Although the surveys focus on long-term services and supports (LTSS) providers (e.g., assisted living) and services users (e.g., residents), the session is generalizable to other establishment surveys. Presenters bring extensive survey experience and diverse organizational perspectives—academic research center, national provider association, federal statistical agency, and research contractor. Over the years, the presenters have used their research network to share challenges and lessons learned with each other, which addresses the GSA conference theme, "Strength in Age: Harnessing the Power of Networks." The first presentation describes test results of a state survey protocol to obtain sampled resident information from assisted living providers. The second presentation examines approaches to increase provider participation in a quality improvement initiative. The third presentation discusses efforts to address response challenges in an on-going national survey of providers in two LTSS sectors. The session allows time for and facilitates interaction with audience members to share their insights and lessons learned.

### COLLECTING RESIDENT DATA FROM RESIDENTIAL CARE COMMUNITIES USING MAIL QUESTIONNAIRES: A NEW APPROACH

Ozcan Tunalilar,[1] Paula C. Carder,[1] Sarah Dys,[1] and Sheryl Elliott[1], *1. Portland State University, Portland, Oregon, United States*

Researchers who collect resident-level data from RCCs face several challenges. Conducting face-to-face, on-site interviews with administrators is costly and presents scheduling difficulties. Contacting administrators by telephone requires multiple attempts. Even when they are reached, they have limited time and might not share resident-level data. To overcome these difficulties, the current survey of Oregon RCCs combines a mail questionnaire with a self-administered sampling tool that allowed communities to select a random sample of their residents (two from each community). Pilot interviews with administrators indicated they were able to select a random sample of their residents easily, quickly, and accurately using this method. The feasibility and validity of this method were further tested by comparing results to community-level aggregate data collected using traditional mail questionnaires from RCCs (n=392). The similarities and differences between resident- and community-level data are discussed within the context of sampling design and mode of data collection from RCCs.

### LESSONS LEARNED FROM QUALITY IMPROVEMENT DATA COLLECTION FROM ASSISTED LIVING PROVIDERS

Lindsay Schwartz[1], *1. American Health Care Association/National Center for Assisted Living, Washington, District of Columbia, United States*

In 2012, the American Health Care Association/National Center for Assisted Living (AHCA/NCAL) launched the Quality Initiative, which was focused on four measurable goals: reduce hospital readmissions; reduce off-label use of antipsychotics; improve staff stability; and improve customer satisfaction. With this initiative, assisted living providers required measures to track their progress. In 2015, to enable benchmarking and comprehensive tracking, these measures were added to LTC Trend Tracker, AHCA/NCAL's online data collection portal. There have been challenges to getting providers to voluntarily submit data, including time, concerns about privacy, understanding the measures, and learning to use LTC Trend Tracker. We have worked consistently with providers to understand challenges of data submission and assist with lessening the burden such as enabling vendors to upload data. To increase participation, we have learned that measures should be easily understood; measures should be specific and important to providers and consumers; and education and training are important.

### EVALUATING ENHANCEMENTS TO ADDRESS SAMPLE DIVERSITY, RESPONDENT BURDEN, AND DIFFICULTY REACHING RESPONDENTS

Melissa Hobbs,[1] Melissa Hobbs,[2] Angela Greene,[2] Christine Caffrey,[3] Manisha Sengupta,[3] and Lauren Harris-Kojetin[3], *1. RTI International, RTP, North Carolina, United States, 2. RTI International, Research Triangle Park, North Carolina, United States, 3. NCHS, Hyattsville, Maryland, United States*

Every two years since 2012, the National Study of Long-Term Care Providers includes provider surveys with residential care communities (RCCs) and adult day services centers (ADSCs), via a multi-mode approach using Web, hard-copy questionnaires, and computer-assisted telephone interviewing. In each wave, we struggled to achieve target response rates. First, diversity among providers surveyed—e.g. RCC size, type of ADSC—presents unique challenges. For RCCs, small communities have lower response compared to larger ones. For ADSCs, how they define themselves (medically vs socially oriented) influenced their decision to participate. Second, respondents' perceived burden, particularly the time required to complete the survey, is a recurring concern especially for directors of multiple RCCs and ADSCs. Finally, reaching target respondents—directors, administrators or operators—is problematic. These challenges affect data quality. In this presentation, we share results of our efforts to enhance contacting and interviewing protocols intended to address low participation associated with these challenges.

### SESSION 3065 (SYMPOSIUM)

### PRESIDENTIAL SYMPOSIUM: HARNESSING SOCIAL NETWORKS TO OPTIMIZE ENVIRONMENTAL CONTEXTS FOR DIVERSE AGING EXPERIENCES

Chair: Philip A. Rozario, *Adelphi University, Garden City, United States*
Co-Chair: Emily Greenfield, *Rutgers University, New Brunswick, New Jersey, United States*
Discussant: Nancy Kusmaul, *University of Maryland, Baltimore County, Baltimore, Maryland, United States*

Social networks provide opportunities for engagement with others and structure the receipt and provision of emotional, instrumental, informational and appraisal support. Indeed scholars in this field have documented the importance of having strong social networks in influencing older adults' well-being and quality of life. The three papers in this symposium draw on the convoy model of social relations and ecological model to examine and better understand the micro, mezzo, macro contexts that shape and influence how older people engage with and benefit from their networks in three areas: low-income senior housing communities, urban areas specifically targeting older Latinos with dementia, and disaster preparedness in micropolitan counties in eastern Iowa. The first paper, a cross-sectional study focusing on social connections in senior housing communities, examines levels of social networks, engagement, support and loneliness and their relationship with well-being outcomes. The second paper, a community-based participatory research project, reports an intervention that seeks to train natural helpers in a predominantly Latino urban neighborhood to identify and refer older Latinos with dementia to bilingual assessment services. The third paper, synthesizing findings from interventions targeting network building at the individual and state levels as well as a community-based network analysis, presents ways to strengthen networks at the mezzo and macro levels as well as environmental contexts that enable better disaster preparedness for community-based older adults. These papers will consider practice, policy and research implications in strengthening social networks and engagement to optimize older adults' well-being in various settings.

### SOCIAL CONNECTIONS AND HEALTH AMONG OLDER ADULTS IN SENIOR HOUSING COMMUNITIES

Harry O. Taylor,[1] and Nancy Morrow-Howell[2], *1. Washington University in St. Louis, St. Louis, Missouri, United States, 2. Brown School, Washington University in St. Louis, St. Louis, Missouri, United States*

Our study describes social connections among residents in two low-income senior housing communities and then examines if these connections influence their well-being. Operationalization of social connections included social network size, informal social support, social engagement, and loneliness. The Convoy Model of Social Relations guides our study in identifying objective and subjective social connections and examining how they affect well-being. Most residents maintained active connections: 53% saw five or more family members in the previous month, 52% felt they could rely on their family members and over 60% participated in group and/or Church activities; however, 70% of residents were moderately or severely lonely. Greater loneliness and less informal social support were associated with worse self-rated physical health, worse mental health, and lower life satisfaction. This residential setting offers promise for developing interventions to decrease loneliness and strengthen social connections to improve residents' health and well-being.

### TAPPING NATURAL NETWORKS TO ADDRESS DEMENTIA IN A LATINO COMMUNITY

Caroline Gelman,[1] and Nancy Giunta[2], *1. Silberman School of Social Work, New York, United States, 2. Silberman School of Social Work, Hunter College, New York, New York, United States*

The need for health education regarding Alzheimer's disease and related dementias (ADRD), specifically designed for Latinos, has been well-documented. Many Latino older adults and their families delay seeking formal help for ADRD symptoms due to lack of information and access to culturally sensitive services. This paper presents preliminary findings of community-based participatory research to develop El Barrio SHARE, a culturally-tailored intervention tapping natural helpers (NHs) to address a need identified by community members in East Harlem, NY. It trains people who often interact with elders in the course of their work (e.g., hairdressers, bodega clerks), and are well-positioned to observe and detect ADRD-related problems and potentially link elders to relevant services. Findings from a process evaluation developing the 10-module NH training suggest that (a) participants seek information that debunks myths and stigma surrounding ADRD, and (b) the need for culturally-tailored, participant-centered interventions in marginalized communities is critical.

### STRENGTHENING PERSONAL AND COMMUNITY DISASTER SUPPORT NETWORKS OF OLDER ADULTS

Sato Ashida,[1] Lena Thompson,[2] and Erin L. Robinson[3], *1. College of Public Health, The University of Iowa, Iowa City, United States, 2. University of Iowa College of Public Health, Iowa City, Iowa, United States, 3. School of Social Work, Univesrsity of Missouri, Columbia, Missouri, United States*

The Ecological Model posits a multilevel approach to understanding of and strengthening social networks of community based older adults. We present findings from our intervention, network analysis, and efforts at influencing policy making at the state level to better prepare them for pre and post disaster and emergency situations. At the individual-level, we implemented the Disaster PrepWise program to help community-based older adults develop personal disaster plans. We found increases in personal emergency network size by an average of three non-familial individuals. At the community-level, we evaluated two disaster management networks in Eastern Iowa counties. We found strong collaborations in disaster planning and response among 44 governmental and community-based organizations, but weaker collaborations in supporting older residents, suggesting a need in this area. At the policy-level, we are developing a state-level network of organizations to address policy barriers to effectively support older Iowans.

### SESSION 3070 (PAPER)

### HEALTH CONCERNS OF LATIN OLDER ADULTS

### SOCIOCULTURAL VARIABILITY IN SELF-REPORTED COGNITIVE IMPAIRMENT AMONG OLDER LATINOS IN THE UNITED STATES

Marc A. Garcia,[1] David F. Warner,[1] and Catherine Garcia[2], *1. University of Nebraska, Lincoln, Nebraska, United States, 2. University of Southern California, Los Angeles, California, United States*

Cognitive impairment is a major public health concern in the United States. Research indicates cognitive impairment is higher for older U.S. Latinos than non-Latino whites, due in part to Latinos having longer life expectancy, lower educational attainment, and a higher prevalence of diabetes and cardiovascular disease. Prior studies on cognition have largely examined "Latinos" as a monolithic group. However, Latinos are heterogeneous in composition with unique socio-cultural characteristics based on nativity and country of origin. Accordingly, we used data from the 1997-2017 National Health Interview Survey (NHIS) to document age-specific trends in in self-reported cognitive impairment among US-born Mexican, foreign-born Mexican, island-born Puerto Rican, foreign-born Cuban, and non-Latino white adults aged 60 and older. Given the repeated cross-sectional nature of these data, we estimated hierarchical age period–cohort (HAPC) cross-classified random-effects model (CCREM) to isolate age trends in self-reported cognitive impairment across Latino subgroups and non-Latino whites. Results indicate significant heterogeneity among Latino subgroups, with island-born Puerto Ricans exhibiting the highest rates of cognitive impairment and foreign-born Cubans the lowest. Conversely, US-born and foreign-born Mexicans exhibited rates in between these two. All Latino subgroups statistically differed from non-Latino whites. Socio-demographic controls account for approximately 33%-45% of the disparity, but fully account for foreign-born Cubans and non-Latino whites differences. These findings indicate the importance of considering nativity and country of origin when assessing cognitive outcomes among older Latinos. Understanding minority and immigrant differences in cognitive impairment has implications for the development and implementation of culture-appropriate programs to promote healthy brain aging.

### STRENGTH IN SEEKING SUPPORT: OLDER LATINOS' ATTITUDES ABOUT CANCER

Iraida V. Carrion,[1] Tania Estapé,[2] Malinee Neelamegam,[3] Jane Roberts,[4] and Jorge Estapé[2], *1. University of South Florida School of Social Work, Tampa, Florida, United States, 2. FEFOC, Fundacion Contra El Cáncer, Barcelona, Spain, Spain, 3. University of South Florida College of Public Health, Tampa, Florida, United States, 4. University of South Florida Sarasota-Manatee, Sarasota, Florida, United States*

Given the growing Latino population 60 years and older, the current lack of relevant data, there is an urgent need to understand their attitudes about cancer to ensure effective prevention, intervention, and psycho-social care. A survey exploring attitudes about cancer was developed and administered in Spanish. Using convenience sampling (N = 168), univariate analysis was done to understand the study population's characteristics. Frequencies were assessed to understand participants' responses to questions on cancer-related attitudes. The effects of age, country of origin, length of stay in the U.S., and marital status were assessed using logistic regression. The participants had a mean age of 67.9 years, 65.5% were female, 56.5% were married or living with a partner, and 35.5% had tertiary education. Most respondents were from South America (46.7%), with a mean length of stay in the U.S. of 25.8 years. A high number (91.0%) indicated a preference to know if they had cancer, and 87.5% said that they would share their diagnosis with family and friends. Of the respondents, 80.4% felt that cancer patients should receive care from a psychologist and that cancer improves if a patient is lively and positive (82.6%). Compared to college-educated individuals, those with a high school education were less likely to choose to know about a cancer diagnosis (β=-1.92, p<0.01) or share it (β= 1.78, p<0.001). Attitudes about cancer vary depending on the educational level of older Latinos and may impact treatment decisions. These findings can enhance cancer information and education for older Latinos.

### THE EFFECTS OF CHRONIC PAIN AND LONELINESS ON FUNCTIONING AMONG LATINO AND WHITE OLDER ADULTS

David Camacho,[1] Maria P. Aranda,[2] Denise Burnette,[3] and Ellen Lukens[1], *1. Columbia University, New York, New York, United States, 2. University of Southern California, Los Angeles, California, United States, 3. Virginia Commonwealth University, Richmond, Virginia, United States*

The detrimental effects of loneliness and chronic pain on functioning in later life are well documented, yet there is little evidence of whether these patterns hold across racially diverse older adults. Guided by the Biopsychosocial Model of Assessment, Prevention, and Treatment of Chronic Pain, we used data from Waves 2 and 3 of the National Social Life, Health, and Aging Project (NSHAP) to examine the additive and interactive effects of loneliness and chronic pain on Elemental and Instrumental Activities of Daily Living (ADLs & IADLs) among a sample of 1046 Latino and White adults aged 50 and over. Using linear regression analyses, our final models (Adjusted R-squares: .316 & .304) included demographic characteristics, physical and mental health, medication, health behaviors and social factors. In this sample, approximately 33% experienced chronic pain, 50% reported at least transitory loneliness and 22% experienced both. Neither loneliness nor chronic pain was independently associated with functioning impairment. However, these two factors in combination were associated with lower scores on ADLs and I-ADLs. In addition, Latinos who reported chronic pain were more likely to report lower scores on ADLs only. Results highlight variations in the detrimental effects of loneliness and chronic pain for white and Latino elders. Findings suggest the need for interventions that address chronic pain and loneliness simultaneously. Future studies should examine how culturally-grounded experiences of loneliness and chronic pain may contribute to worsening of functioning among diverse groups of Latino elders.

### THE POWER OF CANCER KNOWLEDGE EXCHANGE AMONG OLDER LATINOS

Iraida V. Carrion,[1] Tania Estapé,[2] Malinee Neelamegam,[2] Jane Roberts,[3] and Jorge Estapé[2], *1. University of South Florida School of Social Work, Tampa, Florida, United States, 2. FEFOC, Fundacion Contra El Cáncer, Barcelona, Spain, Spain, 3. University of South Florida Sarasota-Manatee, Sarasota, Florida, United States*

Prior studies have indicated that older Latinos/as diagnosed with cancer experience social inequalities and other barriers due to their limited English language proficiency and access to health care (Fernández & Morales, 2007). In addition, for Latinos, a cancer diagnosis magnifies health disparities substantially (Gehlert & Colditz, 2011). Despite the impact of the cancer experience, Latinos manifest meaning-based beliefs and coping strategies in dealing with cancer diagnoses (Carrion, Nedjat-Haiem, Macip-Billbe, Black, 2017). However, little is known about older Latinos' (60 years and older) transmission of knowledge, beliefs, and attitudes to family members and friends. Understanding older Latinos' advice regarding cancer is essential, given their role in the transmission of knowledge. This study disseminates the latest qualitative findings on older Latinos/as and explores the perspectives shared with family members and friends by non-cancer participants. It explores the types of knowledge, beliefs, attitudes, and advice older Latinos provide to family members and friends about cancer. The data represent individuals without cancer (n=168) residing in the Greater Tampa Bay area. Latinos manifest meaning-based beliefs and coping strategies that assist in dealing with their cancer diagnoses and access to care. Recruitment occurred in community-based settings, with interviews conducted in Spanish and transcribed into English. Qualitative data were analyzed using a constant comparison method and coded in ATLAS.ti to identify emerging themes, including knowledge that a cancer diagnosis is beyond individual control and is in God's hands, careful choice of a doctor to ensure proper prevention and treatment, and a positive attitude despite the cancer diagnosis.

### THE ROLE OF CULTURE AND LANGUAGE IN DEPRESSION CARE EXPERIENCES AMONG OLDER LATINAS/OS AND HEALTH CARE PROVIDERS

Maria P. Aranda,[1] Janelle Christensen,[2] and Iris Aguilar[3], *1. University of Southern California, Los Angeles, California, United States, 2. Florida SouthWestern State College, Fort Meyers, Florida, United States, 3. USC Edward R. Roybal Institute on Aging, Los Angeles, California, United States*

Older Latinas/os face significant difficulties in accessing quality depression treatment due to cultural and language barriers. In our study, Programa Esperanza (a randomized behavioral trial), we conducted after-treatment, in-depth interviews with two groups: 21 low-income Latinas/os (55+) receiving psychosocial depression care (Problem Solving Treatment, and Psychoeducation) in a health care setting, and 22 staff (interventionists, supervisors) participating in study implementation. Speaking the same language was highlighted by both groups as an overarching factor in effective depression care, although matching providers and patients by country of origin was considered less a priority than a shared language. The mechanisms by which language serves as a facilitator of effective care were highlighted: fomenting rapport; facilitating the expression of feelings; shortening initial relationship-building; understanding nuanced words and linguistic expressions; etc. Similarly, we found that shared culture included themes around intervention uptake, decreased stigma, increased advocacy, enhanced identification of coping strategies, etc. Unlike patients, providers were more likely to speak in diverse narratives of "them," and "us." Given that depression is still a stigmatizing disorder in our society, asking for help and receiving quality care remain significant challenges for older persons in general, and older underrepresented minorities, in particular. Our work signals the importance of differentiating language from culture in intervention development for older, primarily Spanish-speaking Latinas/os with high medical comorbidity.

### SESSION 3075 (PAPER)

### MARKERS AND BIOMARKERS OF DEMENTIA

### BDNF GENOTYPE AND CHANGES IN WHITE MATTER HYPERINTENSITIES AND HIPPOCAMPAL MICROSTRUCTURE IN OLDER MEN AND WOMEN

C. Elizabeth Shaaban,[1] Andrea Metti,[1] Cindy Barha,[2] Kristine Yaffe,[3] and Caterina Rosano[4], *1. University of Pittsburgh Graduate School of Public Health, Pittsburgh, Pennsylvania, United States, 2. University of British Columbia, Vancouver, British Columbia, Canada, 3. UCSF School of Medicine, San Francisco, California, United States, 4. University of Pittsburgh, PIttsburgh, Pennsylvania, United States*

Brain-derived neurotrophic factor (BDNF) may protect against cerebral gray and white matter impairments in older age. The val66met genetic polymorphism of BDNF is recently emerging as an early marker of brain structural integrity. However, evidence is sparse, cross-sectional, and mostly in men. In a longitudinal cohort study of community-dwelling older adults (N=139, mean age=81.6, 58% female, 58% white, mean follow-up=3.4 years), we estimated the overall and sex-stratified effects of BDNF val66met polymorphism on changes in cognition and gray and white matter macro- and micro-structure. Annualized percent change was computed for volume of white matter (WM) hyperintensities and gray matter (GM), fractional anisotropy of normal appearing WM, and mean diffusivity (MD) of GM in whole brain and memory and executive control function networks. Significant associations were adjusted for variables differing by genotype (race, APOE4, diabetes, triglycerides, smoking). Compared to met carriers, val homozygotes had slower annual whole brain WMH accrual (median (IQR) 31.4% (61.7) vs. 60.7% (92.4)), stronger in women. Met carriers had slower annual accrual of hippocampal MD (median (IQR) 1.26% (0.92) vs. 1.85% (1.09) for right hippocampus, stronger for women, and 1.45% (1.06) vs. 1.97% (1.22) for left hippocampus, stronger for men) compared to val homozygotes. Associations were robust to covariates' adjustment. BDNF polymorphism was not associated with cognitive changes. BDNF polymorphism may help in early identification of those more likely to resist accrual of WMH and loss of hippocampal microstructural integrity, with effects varying by sex.

### DUAL DECLINE IN MEMORY AND GAIT UNIQUELY IDENTIFIES OLDER PERSONS AT HIGH RISK OF DEMENTIA

Qu Tian,[1] Susan Resnick,[1] Michelle Mielke,[2] Kristine Yaffe,[3] Caterina Rosano,[4] Eleanor M. Simonsick,[5] Stephanie Studenski,[6] and Luigi Ferrucci[7], *1. National Institute on Aging, Baltimore, Maryland, United States, 2. Mayo Clinic College of Medicine, Rochester, Minnesota, United States, 3. UCSF School of Medicine, San Francisco, California, United States, 4. University of Pittsburgh, PIttsburgh, Pennsylvania, United States, 5. Longitudinal Studies Section, Intramural Research Program, National Institute on Aging, Baltimore, Maryland, United States, 6. NIA, Longitudinal Studies Section, Baltimore, Maryland, United States, 7. Translational Gerontology Branch, Intramural Research Program, National Institute on Aging, National Institutes of Health, Baltimore, Maryland, United States*

Recent study has shown that incident dementia risk is higher among older persons who decline in both cognition and gait. This study assesses whether this relationship exists in multiple other aging populations. Data are from the Baltimore Longitudinal Study of Aging (n=662, 51% women, 22% blacks), Health, Aging and Body Composition Study (n=746, 51% women, 44% blacks), and Mayo Clinic Study of Aging (n=2771, 48% women, 0.3% blacks). Participants were at least 60, initially free of cognitive impairment, dementia, and dismobility (gait speed≤0.6m/s), with repeated measures of verbal memory and gait speed before dementia diagnosis (average follow-up 5.8-12.1 years). Within each cohort, participants were divided into four groups: memory decliners alone, gait decliners alone, dual decliners, or neither (healthy agers). Gait speed decline was defined as a loss of ≥0.05m/sec/year; memory decline was defined as cohort specific bottom slope tertile. Incident dementia risk was examined by Cox regression with healthy agers as reference, adjusted for sex, race, baseline age, gait speed and memory. Across studies, incident dementia ranged from 3% to 17%. Compared to healthy agers, memory decliners alone had 3.4 to 4.3 times higher risk for developing dementia ($p<0.01$). Gait decliners alone had 2.1-5.6 times higher risk for dementia ($p<0.05$). Dual decliners had 7.6-10.8 times the risk ($p<0.001$). Dual decline signifies a more rapid progression to dementia. These consistent findings suggest that dual decliners might be a potentially valuable target group for both preventive interventions and mechanistic studies. Whether dual decline identifies a particular subtype or multiple subtypes of dementia remains to be investigated.

### SURVIVAL OF PEOPLE WITH CLINICAL DIAGNOSIS OF DEMENTIA IN HONG KONG: A POPULATION-BASED STUDY

Hao Luo,[1] Yi Chai,[1] Jennifer Y.M. Tang,[2] and Gloria H.Y. Wong[1], *1. The University of Hong Kong, Hong Kong, Hong Kong, 2. Sau Po Centre on Ageing, The University of Hong Kong, Pok Fu Lam, Hong Kong*

Objectives: Studies on survival of people with clinical diagnosis of dementia can provide estimates of care outcomes of a health system and offer real-life insights on how to provide better support for the target population. This study aims to estimate survival from the point of recorded diagnosis of dementia, compared with people without dementia. Methods: This case-control study used data from Clinical Data Analysis and Reporting System (CDARS), a population-wide databased managed by Hong Kong Hospital Authority. All patients aged 60 years or over with a first-ever code for dementia from 2001 and 2010 (N=24,250) were matched with patients without dementia by sex and index date at a 1: 2 ratio. We adopted Cox proportional hazard model to estimate hazard ratios, with and without adjustment for age, sex, and comorbidities (diabetes, cardiovascular disease, hypertension, cerebrovascular disease, and high cholesterol). Results: A total of 5,847 patients have a diagnosis of Alzheimer's disease (AD), and 7,729 have vascular dementia (VaD). The median survival time, calculated based on the Kaplan-Meier estimator, for patients with dementia of any kind, AD, and VaD were 1163, 2448, and 1268 days, respectively. Compared with the control group, the raw and adjusted hazard ratios for dementia were 2.78 (95% CI, 2.71-2.84) and 1.14 (1.13-1.17), respectively. Conclusions: Median survival times were much lower than figures reported by other regions and in screened populations. The high risk of death may be an indicator of late diagnosis and hence call for promoting early diagnosis to ensure timely intervention.

### THE RELATIONSHIP OF APOE ε4 TO THE RELATIVE TIMES AND HAZARDS OF DEMENTIA

Danielle Powell,[1] Pei-Lun Kuo,[1] Jennifer A. Deal,[2] Rebecca Gottesman,[3] Priya Palta,[4] David Knopman,[5] and Alden L. Gross[6], *1. Johns Hopkins University Bloomberg School of Public Health, Baltimore, Maryland, United States, 2. Johns Hopkins University Bloomberg School of Public Health, Baltimore, Maryland, United States, 3. Johns Hopkins University, Baltimore, Maryland, United States, 4. Columbia University, New York, New York, United States, 5. Mayo Clinic, Rochester, Minnesota, United States, 6. Johns Hopkins Bloomberg School of Public Health, Baltimore, Maryland, United States*

Although APOE ε4 is an established risk factor for dementia, it is unclear whether it is associated with elevated risk or earlier symptom onset. Longitudinal study of 10,400 white-race individuals in the ARIC cohort followed from age 60 to incident dementia diagnosis, death, or censoring. All-cause dementia was defined using standardized algorithms incorporating longitudinal cognitive change, proxy report, and hospital or death certificate dementia codes. Death was ascertained via the National Death Index and death certificates. We used a parametric mixture of generalized gamma distributions to simultaneously estimate the distribution of event times and the proportion of individuals who experience each outcome (i.e., dementia and its competing risk, dementia-free death) by APOE ε4 status (≥ 1 allele vs. no alleles). Age-adjustment was through use of age as the time scale. APOE ε4 carrier status was associated with a doubling of the overall frequency of dementia incidence to 25% compared to 13% ($p < 0.001$) in non- ε4 carriers. The distributions of time to dementia was modified by APOE ε4 status ($p=0.007$): median time to dementia onset among APOE ε4 carriers was 81.9 years compared to 83.3 years in non-APOE ε4 carriers ($p = 0.005$). No differences in results were found by sex. APOE ε4 carrier status is associated with both elevated risk and earlier time to dementia onset. These findings clarify the causal role of APOE in dementia etiology, could help better identify at-risk subgroups, and may help facilitate better research recruitment.

### SESSION 3080 (SYMPOSIUM)

### MECHANISMS OF COGNITIVE AND NEUROLOGICAL AGING

Chair: Catherine Kaczorowski, *The Jackson Laboratory, Bar Harbor, Maine, United States*

This session will focus on recent advances in understanding how biological aging impacts neurological function and risk of developing age-related neurodegenerative diseases.

### MECHANISMS OF BRAIN REJUVENATION

Saul Villeda[1], *1. University of California San Francisco, San Francisco, California, United States*

A growing body of work has shown that systemic manipulations, such as heterochronic parabiosis and young blood administration, can partially reverse age-related cellular impairments and loss of cognitive faculties in the aged brain. These studies have revealed an age-dependent bi-directionality in the influence of the systemic environment indicating anti-aging factors in young blood elicit rejuvenation while pro-aging factors in old blood drive aging. It has been proposed that introducing anti-aging factors or mitigating the effect of pro-aging factors may provide effective strategies to rejuvenate aging phenotypes. Despite this potential, much is unknown as to the systemic and molecular mechanisms regulating anti-aging and pro-aging effects of blood-borne factors. I will discuss work from my research group that begins to provide mechanistic insight into the systemic and molecular drivers promoting rejuvenation in the aging brain.

### JUMP AROUND, JUMP AROUND: TRANSPOSABLE ELEMENT ACTIVATION IN NEURODEGENERATIVE TAUOPATHY

Bess E. Frost,[1] Wenyan Sun,[1] Hanie Samimi,[2] and Habil Zare[1], *1. University of texas health san antonio, San Antonio, Texas, United States, 2. Texas state university, San Marcos, Texas, United States*

Transposable elements, or "jumping genes," constitute ~45% of the human genome. We have identified transposable element activation as a key mediator of neurodegeneration in tauopathies, a group of disorders that are pathologically defined by deposits of tau protein in the brain. Cellular defenses that limit transposable element mobilization include 1) formation of silencing heterochromatin and 2) generation of piwi-interacting RNAs (piRNAs) that clear transposable element transcripts. Using genetic approaches in Drosophila models of tauopathy, we find evidence for a causal relationship between tau-induced heterochromatin decondensation and piRNA depletion, transposable element mobilization, and neurodegeneration. 3TC, an FDA-approved inhibitor of reverse transcriptase, suppresses transposable element mobilization and neuronal death in tau transgenic Drosophila. We detect a significant increase in transcripts of the human endogenous retrovirus class of transposable elements in postmortem human Alzheimer's disease brains. Our data identify transposable element activation as a conserved, pharmacologically targetable driver of neurodegeneration in tauopathy.

### MULTI-OMICS ANALYSIS IDENTIFIES GENE NETWORKS ASSOCIATED WITH COGNITIVE AGING AND ALZHEIMER'S DISEASE

Catherine Kaczorowski,[1] Sarah Heuer,[1] Chris Gaiteri,[2] and Catherine Kaczorowski[1], *1. The Jackson Laboratory, Bar Harbor, Maine, United States, 2. Rush University, Chicago, Illinois, United States*

Alzheimer's disease (AD) is the leading cause of age-related dementia, yet no treatment exists. AD is heterogeneous, and is resultant of the dysregulation of many genetic and biological processes. To decipher this complexity, we leveraged the first translational mouse population of AD to identify 15 gene networks related to individual differences in cognitive outcomes. Using QTL mapping, we also identified a novel putative driver of a module, Gstk1, highly conserved in humans that also significantly correlated with memory outcomes. Together, these transcriptional networks provide new mechanistic insight into the biological processes that regulate individual differences in cognitive function across a genetically diverse population. We could identify how demographics (age, sex, causal AD mutations) influence these modules and how they relate to cognitive outcomes. Finally, the high degree of conservation between our mouse modules to human modules reflects the translatability of our model to human AD, adding to its face validity.

### ASSESSING BRAIN VOLUMETRICS IN THE AGING BRAIN USING COMPUTED TOMOGRAPHY

ANDREI IRIMIA,[1] Alexander Maher,[1] and Kenneth Rostowsky[1], *1. University of Southern California, Los Angeles, California, United States*

Although broadly utilized, computed tomography (CT) has been superseded by magnetic resonance imaging (MRI) for the volumetric assessment of white matter (WM), grey matter (GM) and cerebrospinal fluid (CSF) in the aging human brain. Nevertheless, many scenarios remain where MRI is unavailable or discouraged; furthermore, in developing countries, CT can often be the only accessible imaging method for assessing brain structure in older patients with mild cognitive impairment or Alzheimer's disease. Thus, there is merit in developing effective approaches for the estimation of brain volumetrics using CT. Here, MRI and CT scans were acquired from 10 older adults [mean ($\mu$) ± standard deviation ($\sigma$) of age = 65 ± 7 yrs; 5 females]. To demarcate WM, GM and CSF from head CT, we developed a brain segmentation method based upon probabilistic, atlas-dependent classification. MRI-only segmentation was compared to CT-only segmentation; similarity was calculated through the Dice coefficient (DC). A normal distribution of DCs was found after contrasting the methods [$\mu \pm \sigma$ across participants: 85.5% ± 4.6% (WM), 86.7% ± 5.6% (GM) and 91.3% ± 2.8% (CSF)], suggesting a satisfactory capacity of CT to assess brain volumetrics. Sensitivity was adequate: WM, GM and CSF volumes were estimated within ~5%, ~4% and ~3% of their MRI-based values. There was no indication of volume over- or under-estimation with CT [$t(9) = 0.89$, $p > 0.80$]. These results facilitate the integration of CT-based brain volumetrics with MRI, thereby offering a wider range of methods for quantifying macroscale brain changes in neurodegenerative diseases.

### SESSION 3085 (SYMPOSIUM)

### PRECARITY AND LATER LIFE: UNDERSTANDING NEW INEQUALITIES AND RISKS IN LATER LIFE

Chair: Dale Dannefer, *Department of Sociology, Case Western Reserve University, Cleveland, Ohio, United States*
Co-Chair: Christopher Phillipson, *The University of Manchester, Manchester, United Kingdom*
Discussant: Dale Dannefer, *Department of Sociology, Case Western Reserve University, Cleveland, Ohio, United States*

This symposium addresses debates around the theme of precarity and its implications for understanding social and economic changes affecting the lives of older people. To date, the concept of precarity has been applied to several subpopulations by various academic disciplines but has yet to be systematically applied to later life. The symposium will give particular attention to the extent to which the lens provided by precarity can illuminate different types of inequalities experienced through the life course and reflected in public policies directed at older people. Chris Phillipson reviews theoretical perspectives relating to precarity, examining their potential contribution for the development of critical gerontology. His paper also considers the extent to which the concept of 'precarious ageing' offers a competing or complementary view to theories of 'active' and 'successful ageing'. Larry Polivka examines the growing precarity of life for older Americans emanating from austerity budgets and privatization of public services. The paper suggests that policies such as health care and long term care are in jeopardy, creating a glide path toward the extension of precarious employment into a precarious retirement for millions of older people. Wenxuan Huang examines how the focus on agency and other individual-level foci obscure understanding of social dynamics. Finally, Amanda Grenier draws on a scoping review of precarity to outline conceptual distinctions between frailty, vulnerability, and precarity. She presents reflections on what these concepts offer in terms of understandings of late life the study of disadvantage across the life course.

### PRECARITY AND AGING: THE SOCIAL CONSTRUCTION OF RISK IN LATER LIFE

Christopher Phillipson[1], *1. The University of Manchester, Manchester, United Kingdom*

This paper examines the growth of precarious lives in the context of policies which have marginalised the welfare state and priorities of social inclusion/security. Blackburn observes that ageing societies 'requires new welfare principles not their repudiation'. However, the reality has been the erosion of the social solidarity which gave the welfare state legitimacy. In Europe, one set of welfare and related institutions is being abandoned, replaced by precarious arrangements -- extended working lives, privatization of pensions, and greater demand for women especially) for unpaid work. This paper 1) draws upon perspectives from political economy and sociology to examine precarity, 2) considers 'precarious ageing' as a competing or complementary view to theories of 'active' and 'successful ageing' third, examines specific dimensions of precarity (work, resources, the environment, frailty), assessing prospects for an index of precarity and aging; finally, considers the public policy implications of the growth of old-age precarity .

### PRECARITY IN OLD AGE IN AMERICA

Larry Polivka[1], *1. Florida State University, Tallahassee, Florida, United States*

This presentation will analyze the growing precarity of life for older Americans emanating from austerity budgets and privatization of public services. As governments at every level continue to look for ways to reduce social and health spending and cut taxes they are also pursuing privatization through contracts with for-profit firms to provide regulated social and health services. The presentation will discuss how these trends toward greater precarity in old age relate to Streecks' concept of the Consolidation State in Western neo-liberal political economies. The Consolidation State serve the interest of capital, especially finance capital by implementing austerity budgets, preserving low taxes on wealth, prioritizing the financing of deficits/debt and privatizing public services and assets. Such policies put health care, long term care and other programs supporting older persons in jeopardy, creating a glide path toward the extension of precarious employment into a precarious retirement for millions of older people.

### AGENCY AS IDEOLOGY: PRECARITY AND DISEMBEDDEDNESS IN THE 21ST CENTURY LIFE COURSE

wenxuan huang[1], *1. Department of Sociology, Case Western Reserve University, Cleveland, Ohio, United States*

The "individualization" thesis has gradually merged into the discussion of increasing heterogeneity of the life course as well as growing inequality over historical time. As individuals are "disembedded" from both cultural traditions and more recently social institutions, individual agency has drawn revived interest in outlining "choice biography" that is seen as paramount to personal outcomes and even containing overcoming force against structure. This practice mutes the consideration of the ongoing forces of social structure that by their very nature continue to constitute individual selves and possibilities. The uncritical treatment of individual agency makes it problematic for the study of precarity, mystifying and obscuring the analysis of inequality-generating mechanisms, reducing them to the individual-level. We analyze current uses of the concept of agency in the life course research, and particularly in the areas of transition research, e.g., transition to adulthood/retirement, where individual agency is assumed to be most active.

### FRAILTY AND PRECARITY: UNTANGLING THE CONCEPTUAL PATHWAYS OF NEED AND DISADVANTAGE IN LATE LIFE

Amanda M. Grenier[1], *1. McMaster University, Health Aging and Society, Ontario, Canada, Canada*

The concepts of frailty and precarity circulate in social gerontology and studies of aging, with the former a dominant construct, and the latter emerging as a way of linking experiences, insecurities and risks. Although these concepts are used inter-changeably by some authors, their roots, key areas of focus and meanings differ. This paper considers the state of knowledge on frailty, and sets this against the uses of precarity. A After outlining a recent scoping review on precarity that revealed a high number of articles cross-referencing concepts of frailty and vulnerability. the paper distinguishes key aspects of frailty, vulnerability, and precarity. Situating qualitative experiences of each serves as a means to further explore similarities and differences. The paper concludes with reflections on what (if anything) each of these allied concepts may offer understandings of late life, and in particular, the study of disadvantage across the life course and into late life.

### SESSION 3090 (SYMPOSIUM)

### RESILIENCE AND HEALTH AMONG CHINESE OLDER ADULTS: FINDINGS FROM THE U.S. AND CHINA

Chair: Wei Zhang, *University of Hawaii at Manoa, Honolulu, Hawaii, United States*
Co-Chair: Bei Wu, *New York University, New York, New York, United States*
Discussant: David Matchar, *Duke-NUS Medical School, Singapore, Singapore, Singapore*

The new framework of resilient aging has gained its importance in recent years. This symposium provides new findings on resilience and health among the Chinese population. Using data collected among 430 Chinese older adults in Honolulu, the first presentation examines resilience as an explanatory mechanism linking neighborhood social environment and well-being. Results showed that neighborhood cohesion was positively related to psychological well-being and life satisfaction. Resilience contributed to a substantial portion of the associations. Using the same data, the second presentation examines the association between immigrant status and oral health related quality of life (OHQoL) and the moderating role of resilience. Findings showed that U.S.-born Chinese immigrant older adults had better OHQoL than their foreign-born Chinese American counterparts. Resilience was positively associated with OHQoL for the former but not for the latter. The third paper presents findings from the same dataset along with a survey of 800 older adults in Wuhan, China. The positive relationship between attitudes towards aging and self-rated health (SRH) was found to be moderated by resilience such that higher levels of resilience weakened this association substantially. Both the positive focal relationship and the moderating effect appeared to be stronger among participants in Honolulu. Using both datasets, the fourth paper investigates patterns of intergenerational transfer and their relationships with SRH as well as the meditating effect of resilience. Findings highlighted the beneficial health effects of receiving emotional support from adult children as well as the mediating role of resilience for older females in both study sites.

### NEIGHBORHOOD SOCIAL COHESION, RESILIENCE, AND WELL-BEING AMONG CHINESE OLDER ADULTS IN HONOLULU, HAWAII

Wei Zhang,[1] Sizhe Liu,[1] Keqing Zhang,[1] and Bei Wu[2], *1. University of Hawaii at Manoa, Honolulu, Hawaii, United States, 2. New York University, New York, New York, United States*

Few studies have examined the association of social environment and well-being among Chinese older adults, the fastest growing aging population across all racial/ethnic groups in the U.S. To address this gap, the current study aims to examine the associations of neighborhood social cohesion with psychological distress and life satisfaction as well as the mediating role of resilience and the moderating roles of gender and place of birth using data collected among 430 Chinese older adults in Honolulu. Results show that neighborhood cohesion was significantly associated with both distress and life satisfaction, with resilience being a significant mediator. The association between neighborhood cohesion and distress was moderated by birth place such that the protecting effects of neighborhood cohesion on distress were only salient for the U.S.-born. Our findings indicate the importance of a cohesive social environment in shaping well-being of U.S. Chinese older adults, the U.S.-born in particular, living in Hawai'i.

### IMMIGRATION, RESILIENCE, AND ORAL-HEALTH-RELATED QUALITY OF LIFE AMONG CHINESE AMERICAN OLDER ADULTS IN HAWAII

Bei Wu,[1] Yaolin Pei,[1] and Wei Zhang[2], *1. New York University, New York, New York, United States, 2. University of Hawaii at Manoa, Honolulu, Hawaii, United States*

Very few studies have compared oral health status between the US-born and foreign-born immigrant older adults. Using data collected among 430 Chinese older adults age 55+ residing in Hawai'i, we examined the association between immigrant status and oral health related quality of life (OHQoL) and the moderating role of resilience in linking the association. Controlling for some key covariates, our study results show that US-born Chinese immigrant older adults had better OHQoL than their foreign born counterparts. Factors such as higher level of education (graduate degree or higher), better self-reported health status and no significant tooth loss were related to better OHQoL. The association between immigrant status and OHQoL was moderated by resilience. Specially, resilience was positively and significantly associated with OHQoL among U.S.-born older adults but not among the foreign-born ones. Our findings indicate the importance of immigration and resilience in shaping oral health outcomes among older Chinese Americans.

### ATTITUDES TOWARD AGING, RESILIENCE, AND SELF-RATED HEALTH OF CHINESE OLDER ADULTS: FINDINGS FROM HONOLULU AND WUHAN

Keqing Zhang,[1] Wei Zhang,[1] Sizhe Liu,[1] and Bei Wu[2], *1. University of Hawaii at Manoa, Honolulu, Hawaii, United States, 2. New York University, New York, New York, United States*

A growing body of literature found that attitudes towards aging is related to individual's health among older adults. This paper aims to examine the relationship between negative attitudes towards aging and self-rated health among Chinese older adults living in Honolulu (N=400) and Wuhan (N=552) and explore the moderating roles of resilience and gender. Results showed that the positive relationship between attitudes towards aging and self-rated health was moderated by resilience such that higher levels of resilience weakened this association significantly and substantially, implying its stress-buffering role. Both the positive focal relationship and the moderating effect appeared to be stronger among older adults in Honolulu than their peers in Wuhan. For gender differences, the moderating effect of resilience was only significant for males in Honolulu and females in Wuhan, indicating that males in Honolulu and females in Wuhan may rely heavily on psychological resources such as resilience in coping with stressors.

### INTERGENERATIONAL TRANSFERS, RESILIENCE, AND SELF-RATED HEALTH: FINDINGS FROM HONOLULU AND WUHAN

Sizhe Liu,[1] Wei Zhang,[1] Keqing Zhang,[1] and Bei Wu[2], *1. University of Hawaii at Manoa, Honolulu, Hawaii, United States, 2. New York University, New York, New York, United States*

This study investigated the association between intergenerational transfer and self-rated health (SRH) using data collected from 323 older Chinese immigrants in Honolulu and 752 Chinese older adults in Wuhan. We also explored the mediating role of resilience and the moderating role of gender in linking the associations. Findings show, for both study sites, receiving greater emotional support was associated with increased levels of SRH and this association was partially explained by resilience for females but not for males. For country differences, receiving economic support was negatively associated with SRH whereas providing economic support was positively associated with SRH for males but not for females in Honolulu. In Wuhan, receiving housework support was negatively associated with SRH for females but not for males. These findings indicate that future studies need to consider country differences as well as the impact of acculturation when examining intergenerational transfers and health among Chinese older adults.

### SESSION 3095 (SYMPOSIUM)

### SOCIOCULTURAL DETERMINANTS OF RACIAL HEALTH DISPARITIES ACROSS THE LIFECOURSE

Chair: Nancy Chiles Shaffer, *National Institute on Aging, Baltimore, Maryland, United States*
Discussant: Carl V Hill, *NIH/National Institute on Aging, Bethesda, Maryland, United States*

Racial health disparities have been observed across the lifecourse. While some studies have attributed these disparities to underlying biomedical differences, a large body of research supports a sociocultural etiology. In this symposium we will demonstrate that observed racial differences in health outcomes are associated with sociocultural factors, and demonstrate effective strategies for assessing race and racial differences in qualitative and quantitative analyses. We will share an overview of health disparities and aging, highlighting the National Institute on Aging's Health Disparities Research Framework. After this introduction, four speakers will present scientific projects that exemplify the breadth and depth of research assessing health disparities and social determinants of health across the lifecourse. Dr. Brown will present qualitative work on how social determinants are positively addressed through exercise. Dr. Chiles Shaffer will present findings demonstrating the contribution of financial burden and lower education to racial differences in healthy aging. Dr. Noppert will present evidence highlighting the structural influences that underlie the association between education and metabolic health focusing on differences by race and gender. Dr. Thierry will present research on the association between neighborhood characteristics and telomere length with a focus on heterogeneity by race and urban/non-urban residence. Finally, Dr. Hill will provide an overview of the four presentations and facilitate questions and discussion.

### THE INTERSECT OF SOCIAL DETERMINANTS, MOTIVATION, AND BLACK WOMEN TRIATHLETES

Candace Brown[1], *1. Duke University, Durham, North Carolina, United States*

Social determinants of health are often adjacent to negative impacts on physical health due to the association of a sedentary lifestyle. Yet, knowledge is limited regarding how motivation to exercise, through sport, may relate to social determinants. This study aimed to understand the intersect of social determinants of Black women who exercise, by participating in triathlons, using the novel Motivations for Triathletes Interview Guide (MOTIG). The Organismic Integration Theory guided an assessment of social determinants among (N = 12) Black women from nine scales within the MOTIG. The subtheme 'physical awareness' supported Health Orientation while 'social support', 'camaraderie' and 'family', all supported the Affiliation scale. Results indicate that Black women who exercise through triathlon are motivated because certain social determinants are positively addressed. Future interventions directed toward sedentary women should consider how to positively reinforce individual and social determinants to increase motivation to exercise.

### THE HIGH COST OF SOCIOECONOMICS ON RACIAL DISPARITIES IN HEALTHY AGING

Nancy Chiles Shaffer[1], *1. National Institute on Aging, Baltimore, Maryland, United States*

Race-related differences in overall health across the agespan are well established; how differences manifest within a cohort selected for good functional status is unclear. Using data from Black and White older adults aged 70-79, in the Health, Aging and Body Composition (Health ABC) study, we created a healthy aging index (HAI) of mental health, fitness, lung capacity, bone mineral density, inflammation, and metabolic syndrome. We assessed if racial differences existed in HAI, the extent education, financial resources and stress attenuated any observed differences, and whether this varied by site (Memphis v. Pittsburgh). Blacks had lower HAI than whites, adjusted for age and site. Further adjustment for finances and education eliminated the effect of race in women and reduced the effect in men by 64%. A significant interaction between site and financial stress was observed. Future research should assess ways to reduce the harmful impact of low socioeconomic status on health.

### EDUCATIONAL ATTAINMENT AND YOUNG ADULT HEALTH IN THE UNITED STATES: DIFFERENCES BY RACE AND GENDER

Grace A. Noppert[1], *1. University of North Carolina at Chapel Hill, Chapel Hill, North Carolina, United States*

There is compelling evidence to suggest that educational disparities in health differ by both race and gender. This study examines the relationship between respondents' education and six health outcomes related to cardiometabolic and inflammatory outcomes using data from Wave IV of the National Longitudinal Study of Adolescent to Adult Health (ages 24-32 years; N = 13,458). We used logistic regression models to examine the relationship between education and the odds of each health outcome. Models were stratified by race and gender. We found that the association between education and each health outcome differed by race/ethnicity and gender. While among whites we observed an association between education and each health outcome, for blacks we observed no such associations. It may be that the benefits of education are particularly salient for those in more structurally advantaged positions, pointing to the continued need to address structural inequalities by both gender and race.

### EXAMINING THE ASSOCIATION BETWEEN NEIGHBORHOOD CHARACTERISTICS AND TELOMERE LENGTH BY RACE AND URBANICITY

Amy Thierry[1], *1. Xavier University of Louisiana, New Orleans, Louisiana, United States*

A dearth of research investigates socio-environmental mechanisms of health disparities. Therefore, this paper tests the association between neighborhood characteristics and telomere length (TL), a biomarker of cellular age, and examines if this relationship differs by race and urban residence. Data from US-born, non-Hispanic Black and White midlife and older adults (n=4,155) in the 2008 Health and Retirement Study was analyzed to test the relationship between TL and perceived neighborhood safety, cleanliness, and social cohesion. Linear regression models stratified by race included interaction terms between neighborhood characteristics and urban residence. Negative perceptions of neighborhood characteristics were associated with shorter TL in Blacks, with urban-dwelling Blacks having shorter TL than Blacks in less urban areas. Findings suggest that negatively perceived neighborhoods may be more detrimental to TL for urban compared to rural Blacks. Future research should elucidate the biobehavioral consequences of socially disadvantaged urban neighborhoods on healthy aging among Black adults.

### SESSION 3100 (SYMPOSIUM)

### ESPO/HEALTH SCIENCES: STRENGTH IN NUMBERS: LEVERAGING THE POWER OF NETWORKS FOR RECRUITING OLDER ADULTS AND CAREGIVERS

Chair: Karen O. Moss, *The Ohio State University, Columbus, United States*
Co-Chair: Anyah Prasad, *University of Massachusetts Boston, Boston, Massachusetts, United States*
Discussant: Fayron Epps, *Georgia State University, Atlanta, Georgia, United States*

Recruitment of older adults and/or their family caregivers for participation in research is challenging. This process is further complicated by population or procedure-specific factors that can impede recruitment efforts. The purpose of this symposium is to describe various recruitment challenges and solutions as well as strategies used to engage older adults and family caregivers in research. Dr. Glenna Brewster will present recruitment strategies used for a multisite telehealth intervention for caregivers of persons living with dementia. Dr. Todd Monroe will examine barriers and solutions for recruitment and retention of persons with Alzheimer's disease for neuroimaging studies. Dr. Karen Moss will share strategies used to recruit family caregivers of African American older adults with dementia for end-of-life research. Dr. Shiva Prasad will share strategies to recruit LGBT older adults to a study on exploring the idea of an LGBT online senior center, and Dr. Karen Rose will describe stigma associated with recruiting persons with Alzheimer's disease and other dementias, and their family caregivers, including best practices from the literature coupled with lessons learned from experiences. Shared strategies and solutions will assist researchers in identifying and addressing recruitment challenges and help to ensure recruitment of diverse groups of older adults and/or family caregivers for studies that use various research methodologies, including leveraging the power of professional and community-based networks. Increasing research participation will yield the knowledge necessary for improving health outcomes for older adults and their family caregivers living with serious chronic illnesses such as Alzheimer's disease and related dementias.

### RECRUITMENT OF FAMILY CAREGIVERS OF AFRICAN AMERICAN OLDER ADULTS WITH DEMENTIA

Karen O. Moss,[1] Kathy Wright,[2] Glenna Brewster,[3] Karen Rose,[4] Celia E. Wills,[2] and Todd Monroe[2], *1. The Ohio State University, Columbus, United States, 2. The Ohio State University, Columbus, Ohio, United States, 3. Emory University, Atlanta, Georgia, United States, 4. University of Tennessee, Knoxville, Knoxville, Tennessee, United States*

Multiple factors influence decision-making of family caregivers of African American older adults with dementia about participation in end-of-life care research. Existing literature on best practices for research recruitment includes little on recruitment strategies for this specific population. The purpose of this presentation is to analyze successful recruitment strategies used with family caregivers of African American older adults with dementia. Sixty-five caregivers of African American older adults with dementia were recruited over 11 months throughout a southeastern state from local communities and Program of All-Inclusive Care for the Elderly. Community partnership strategies such as community-based networking, purposively-targeted presentations, and leveraging of existing social networks provided a strong basis for successful recruitment. These strategies were credible to the participant population and effective in engaging both research participants and community partner stakeholders in working on shared goals of improving older adult and caregiver outcomes near the end of life.

### RECRUITING LGBT OLDER ADULTS FOR RESEARCH

Anyah Prasad,[1] Alice Fisher,[2] Michael Immel,[2] Kathrin Boerner,[1] Kamal Jethwani,[3] Timothy hale,[3] and Amanda Centi[3], *1. University of Massachusetts Boston, Boston, Massachusetts, United States, 2. Osher Lifelong Learning Institute, Boston, Massachusetts, United States, 3. Connected Health Innovation – Partners Health Care, Boston, Massachusetts, United States*

LGBT older adults constitute a rare population and so are methodologically difficult to recruit. Due to stigma, many of them may not disclose their sexual/gender identity, which makes it challenging for researchers to reach out to them. Due to history of discrimination, LGBT older adults may not trust researchers. The purpose of this presentation is to discuss strategies used to recruit LGBT older adults to a study on exploring the idea of an online senior center for LGBT older adults in Massachusetts. Building a rapport with community stakeholders, developing trust and having LGBT older adults themselves as part of the research team were important tools to help overcome these challenges. LGBT older adults are very diverse and focused efforts should be made to recruit them from various racial/ethnic backgrounds, rural areas; also, those who are not publicly open about their identity, and who are home bound due to restricted mobility.

### RECRUITING FAMILY CAREGIVERS OF PERSONS LIVING WITH DEMENTIA FOR PARTICIPATION IN AN ONLINE INTERVENTION

Glenna Brewster,[1] Fayron Epps,[2] Rachel Nash,[3] Patricia Griffiths,[4] Janice Phillips,[5] Joe Nocera,[2] Raj Shah,[5] and Kenneth Hepburn[6], *1. Emory University Nell Hodgson Woodruff School of Nursing, Atlanta, Georgia, United States, 2. Georgia State University, Atlanta, Georgia, United States, 3. Emory University, Atlanta, Georgia, United States, 4. Atlanta VA Medical Center, Decatur, Georgia, United States, 5. Rush University, Chicago, Illinois, United States, 6. Emory University Nell Hodgson Woodruff School of Nursing, Atlanta, Georgia, United States*

Responsibilities of caregiving for persons living with dementia make it challenging to participate in in-person research studies. Caregivers may be more willing to participate in studies that are online. This presentation will highlight recruitment strategies of a 4-site telehealth caregiver intervention for caregivers of persons living with dementia. Thus far, we have recruited 596 participants over the period of 2 years: 76, 189, 164 and 167 from each of the sites, respectively. Community partnership strategies such as presentations at churches and events organized by the Alzheimer's Association, and the Alzheimer's Disease Research centers, using a handshake protocol, and using social media sites such as Facebook and Twitter have all been effective at recruiting participants. Ongoing communication among the staff at different sites is also an important aspect of successful recruitment. These strategies have enabled recruitment to continue at a consistent rate and enabled the maintenance of relationships within the community.

### THE IMPACT OF DEMENTIA STIGMA IN RECRUITING PARTICIPANTS INTO RESEARCH STUDIES

Karen Rose,[1] and Ruth Palan Lopez[2], *1. University of Tennessee, Knoxville, Tennessee, United States, 2. University of Tennessee, Knoxville, Knoxville, Tennessee, United States*

Despite efforts to increase awareness and education about Alzheimer's disease and other dementias, persons with dementia and their family caregivers experience stigma. Dementia related stigma is associated with negative repercussions for those with the disease and their family caregivers. In our prior work, we identified shame as a mechanism by which stigma is enacted and results in isolating and delaying access to supportive services for family caregivers. As such, stigma may influence decisions to participate in research studies. Healthcare providers, friends and family, and society, in general, play roles that further perpetuate stigma in dementia. Best practices from the literature, coupled with our experiences and findings in recruiting persons with dementia and their family caregivers in to research studies will be examined.

### RECRUITING OLDER ADULTS WITH DEMENTIA FOR NEUROIMAGING RESEARCH: BARRIERS AND SOLUTIONS

Sebastian Atalla,[1] Kathy Wright,[1] Alison Anderson,[2] Karen O. Moss,[1] Ronald Cowan,[2] Ann McDaniel,[1] and Todd Monroe[1], *1. The Ohio State University, columbus, Ohio, United States, 2. Vanderbilt University, Nashville, Tennessee, United States*

Recruiting participants with dementia for magnetic resonance imaging (MRI) research is prone to inherent barriers including caregiver schedules, mistrust, transportation, expertise of MRI technologists, claustrophobia, and MRI safety requirements. This pilot study aimed to identify barriers and develop strategies for increasing recruitment and retention of persons with dementia in neuroimaging studies and to gauge improvements in recruitment outcomes associated with dedicated recruitment staff. Over a period of five years of active recruitment, a dedicated recruitment specialist and a full-time research assistant screened and enrolled an average of eight participants with dementia per month (8.16), of which, approximately two participants (1.97) successfully completed the MRI testing. The most common barrier was difficulty obtaining surgical records for MRI safety clearance. The most common solutions were thorough prescreening and maintaining a positive rapport with MRI technologists. Our study findings will assist others with engaging persons with dementia and their caregivers in MRI research.

### SESSION 3105 (PAPER)

### SUICIDE AND END OF LIFE

### CARING FOR OLDER INDIGENOUS PEOPLE WITH CO-MORBIDITIES AT END OF LIFE

Kathleen R. Mason,[1] Tess H. Moeke-Maxwell,[1] and Merryn Gott[1], *1. University of Auckland, Auckland, New Zealand*

The number of deaths among older Māori, the indigenous people of New Zealand, are expected to increase by 48% by 2030. Colonization has had a varied impact on Māori ways of being and end-of-life care has become more difficult. Many have become disenfranchised from their families, peoples, lands and culture. Pae Herenga, a for-Māori by-Māori with-Māori qualitative research project, investigated the traditional Māori end-of-life care customs that Māori families used while caring for someone who was dying. An online education resource was developed to support Māori families, their communities and the palliative care sector. Interviews were conducted with 60 Māori participants including older many people (aged over 70). The findings found that families rich in cultural knowledge were proficient in caring for a loved one at end-of-life irrespective of their social or economic position. Cultural care values such as unconditional love, companionship, reciprocity, supportive relationships and collective decision making safeguarded care preferences of the dying. Access to traditional knowledge and traditional healing practices, and an understanding of spirituality helped to strengthen and prepare the dying person, and their families, on the end-of-life journey. The study also found that those families connected to communities' rich in Māori cultural resources, such as knowledgeable older Māori people, were well supported by the community at end-of-life. This study highlights that Māori use of traditional care customs in all care settings can better support a 'good death' from a cultural perspective.

### END-OF-LIFE DECISION SUPPORT NEEDS OF DEMENTIA FAMILY CAREGIVERS

Jung Kwak,[1] and Lisa Geshell[2], *1. The University of Texas at Austin, Austin, Texas, United States, 2. School of Nursing, The University of Texas at Austin, Austin, Texas, United States*

One third of older adults die with dementia. At the end of life (EOL), persons with dementia require surrogate decision-makers, often their family caregivers, to make important EOL decisions. However, only a handful of evidence-based interventions exist to guide dementia caregivers in surrogate-decision making. In order to examine the acceptability and appropriateness of a decision coaching intervention developed for dementia caregivers, we conducted cognitive interviews (n=4), and one focus group (n=9) with dementia caregivers, and two focus groups with healthcare professionals (n=14) from a large healthcare system and a managed long-term care organization. Guiding questions for interviews and focus groups included: (1) types of decisions (what), and circumstances or triggers (when and how) that call for decision-making support by healthcare professionals, (2) barriers to families receiving decision-making support, and (3) decision support needs of family caregivers. All face-to-face interviews were audio-recorded, transcribed verbatim, and verified for accuracy. Content analysis was conducted to identify and organize themes and patterns emerging from the interview transcripts. Two main themes and subthemes emerged: (1) decision-making challenges and barriers: lack of advance care planning, caregivers' acquiescence with dementia progression and caregiving role, discontinuing life sustaining therapies, and lack of communication between providers; and (2) decision support for families: advance care planning at different stages of dementia, preparing caregivers for life after the patient's death, and providing adequate information about benefits and harms of treatment options specific to the practical concerns of patient and family caregivers. These findings provide implications for practice and future research.

### ETHNIC DIFFERENCES IN FACTORS INFLUENCING ATTITUDES TOWARD SUICIDE AND PHYSICIAN-ASSISTED SUICIDE AMONG ELDERS

Ruben Martinez[1], *1. Michigan State University, East Lansing, Michigan, United States*

This study examines differences in attitudes toward suicide and physician-assisted suicide in chronic pain scenarios among Latino and White elders. Face-to-face interviews were conducted with 204 elders at four community-based, outpatient care sites in the metropolitan area of San Antonio, Texas. In addition to ethnicity and the suicide-related questions, the interviews collected data on age, gender, marital status, education, income level, acculturation (Latinos only), depression, self-reported health status, daily living functioning, and religiosity/spirituality. In general, there were no ethnic group differences in attitudes, however, the factors associated with those attitudes varied between ethnic groups. Among Whites, attitudes toward suicide and toward physician-assisted suicide were significantly and negatively associated with religiosity. Among Latinos, depression was significantly and positively associated with attitudes toward suicide in chronic pain scenarios, while acculturation was significantly and negatively associated with attitudes toward physician-assisted suicide in chronic pain scenarios. This study's findings suggest that depression among Latino elders and religion among White elders are determinant factors of attitudes toward suicide in chronic pain scenarios. Future research is needed to confirm our findings with a more heterogeneous study sample, including Latinos from different countries of origin (e.g. Mexican Americans, Puerto Ricans, Cubans); and more heterogeneous ethnic groups in terms of socioeconomic status and educational level characteristics.

### LONELINESS AND SUICIDE IDEATION IN OLDER ADULTS: A LONGITUDINAL INVESTIGATION

Marnin J. Heisel[1], *1. The University of Western Ontario, London, Ontario, Canada*

Older adults have the highest rates of suicide globally, necessitating theory and research investigating suicide and its prevention in later-life. The experience of loneliness is significantly associated with depression, hopelessness, negative health outcomes, and mortality among older adults. Yet, relatively little research has focused on the role of loneliness in conferring suicide risk in later life. The purpose of the present study was thus to investigate the potential associations between loneliness and suicide ideation and behavior in a sample of community-residing older adults recruited into a larger two-year longitudinal study of psychological risk and resiliency to later-life suicide ideation. We specifically recruited 173 adults, 65 years or older, from community locations in a medium-sized Canadian city, for a study on "healthy aging." Participants completed measures of positive and negative psychological variables, including depression, loneliness, and suicide ideation at a baseline assessment, and again at 2-4 week, 6-12 month, and 1-2 year follow-up points. Findings indicated that loneliness (UCLA Loneliness Scale) was significantly positively associated with concurrent depression and suicide ideation, negatively associated with psychological well-being and perceived social support, and differentiated between participants who endorsed or denied having ever engaged in suicide behavior. Baseline loneliness also explained significant variability in the onset of suicide ideation over a 1-2 year period of follow-up, controlling for age, sex, and baseline depression and suicide ideation. These findings will be discussed in the context of the need for increased focus on psychosocial factors when assessing and intervening to reduce suicide risk in older adults.

### SESSION 3110 (SYMPOSIUM)

### THE ART OF CARING: EXPLORING HOW DEMENTIA CARE PARTNERS MANAGE CARE CHALLENGES AND FIND RESILIENCE

Chair: Amanda N. Leggett, *University of Michigan, Ann Arbor, Michigan, United States*
Discussant: Laura N. Gitlin, *Drexel University, Philadephia, Pennsylvania, United States*

Approximately 15 million Americans serve as family caregivers for a person with Alzheimer's disease or another form of age-related dementia and this care can take a physical and emotional toll. Understudied is the process of how families actually provide care in response to care challenges, and how to find respite and resilience amidst care challenges. This symposium considers how caregivers handle daily challenges related to dementia including activities of daily living, behavioral and psychological symptoms of dementia, general health and medical comorbidities. In addition to characterizing care partner's distinct styles of management (Leggett et al.) and knowledge and capacity to manage health care (Sadak et al.), the papers also provide perspective on positive aspects of care management such as the impact of respite on positive mood (Wylie et al.), the moderating role of relationship quality on responses to behavioral and psychological symptoms of dementia (Chunga et al.) and finally how caregivers' problem-related, self-growth, and help-related behaviors compose their resilience to care challenges (Zhou et al.). To conclude, our discussant Dr. Laura Gitlin will offer insight on cross-cutting implications across studies and offer perspective on how research, intervention science, and clinical practice may better account for caregiver management styles to promote growth and resilience in caregivers and their care partners with dementia.

### STYLE AND SUBSTANCE: A QUALITATIVE CHARACTERIZATION OF DEMENTIA CAREGIVING MANAGEMENT STYLES

Amanda N. Leggett,[1] Benjamin Bugajski,[1] Breanna Webster,[1] Brianna Broderick,[1] Daphne Watkins,[1] Laura Gitlin,[2] and Helen C. Kales[1], *1. University of Michigan, Ann Arbor, Michigan, United States, 2. Drexel University, Philadelphia, Michigan, United States*

Caring for a person living with dementia (PLWD) can take a physical and emotional toll, but understudied is the process of how family caregivers actually provide care (caregiver management styles). We interviewed 100 primary family caregivers regarding management of a recently experienced care challenge and values held which might impact care management decisions. Watkins' (2017) rigorous and accelerated data reduction (RADaR) technique was used to analyze qualitative data through open/focused coding, determining commonalities of style components/themes, and finally defining caregiving management styles. Style for a given caregiver emerged from enacted care strategies, caregiver's internal stances which informed their use of strategies, and broader engagement (or lack thereof) with the PLWD's lived experience/reality. Styles emerging from the analysis will be described including the direct, rigid "Just do it" style, and the flexible, empathic "Teamwork" style. Individualizing caregiver interventions and supports based on caregiver management style is an important future direction.

### PRECISION IN HEALTH CARE FOR DEMENTIA: DO CARE PARTNERS KNOW WHAT THEY DON'T KNOW?

Tatiana Sadak,[1] Emily Ishado,[1] and Soo Borson[1], *1. University of Washington, Seattle, Washington, United States*

Clinicians rely on care partners to provide health care at home for people with dementia, who typically have multiple chronic conditions in addition to progressive cognitive decline. We examined the accuracy of care partners' knowledge of care recipients' medical conditions and medications, using a benchmark of ≥ 80% match. Of 100 care partners of people with dementia who were recently hospitalized for a major medical illness, nearly all rated their knowledge as high, but about half did not correctly identify care recipients' medical conditions or know medications, and one fourth did not understand the purpose for which medications were given. A key predictor of poor objective knowledge was care partners' cognitive status. These findings highlight the importance of objective assessment of care partner knowledge and skills by clinicians who provide health care and advance care planning for people with dementia.

### TAKING A BREAK: DAILY RESPITE EFFECTS OF ADULT DAY SERVICE AS ACTUAL TIME AWAY FROM CAREGIVING

Molly J. Wylie,[1] Kyungmin Kim,[1] Yin Liu,[2] and Steven H. Zarit[3], *1. University of Massachusetts Boston, Boston, Massachusetts, United States, 2. Utah State University, Logan, Utah, United States, 3. Pennsylvania State University, University Park, Pennsylvania, United States*

Adult day service (ADS) can provide emotional and physical relief for caregivers of persons with dementia (PWD). Studies have examined differences between caregivers of service users and non-users; less known, however, is how actual hours away from caregiving responsibilities through using ADS impact caregivers' daily outcomes. Using daily diary data from 173 family caregivers whose relatives are using ADS (day N = 1,359), this study investigated within-person differences in respite hours across 8 consecutive days and how daily respite hours are associated with daily well-being (i.e., mood and health symptoms). On average, caregivers reported 7.12 respite hours on ADS days and 1.74 respite hours on non-ADS days. Multilevel models revealed that having more respite hours is associated with better positive mood, but not with negative mood and health symptoms – after controlling for ADS use. Further, when caregivers perceived more break time from caregiving responsibilities, they showed better positive mood.

### FAMILY CAREGIVERS' RESPONSES TO DAILY BEHAVIORAL SYMPTOMS OF DEMENTIA: THE MODERATING ROLE OF RELATIONSHIP QUALITY

Richard E. Chunga,[1] Yin Liu,[2] Kyungmin Kim,[1] and Steven H. Zarit[3], *1. University of Massachusetts Boston, Boston, Massachusetts, United States, 2. Utah State University, Logan, Utah, United States, 3. Pennsylvania State University, University Park, Pennsylvania, United States*

Providing care for persons with dementia (PWD) is frequently regarded as highly stressful, but how caregivers perceive care-related stressors depends on a variety of contexts. Research has demonstrated that relationship quality between the caregiver and receiver – as an important dyadic context – can influence the magnitude of this perceived distress. Using 8-day diary data from 173 family caregivers of PWD (day N = 1,359), this study examined the moderating effect of relationship quality on caregivers' stress responses to daily behavioral and psychological symptoms of dementia (BPSD), comparing within- and between-person effects. Multilevel models suggested differences in the association between BPSD occurrence and perceived distress of BPSD (i.e., negative within-person association, but positive between-person association). However, we found that both associations were moderated by relationship quality; that is, better dyadic relationship quality seemed to be protective against distress at both within- and between-person levels.

### BEHAVIORAL MARKERS OF RESILIENCE IN CARE PARTNERS OF PERSONS WITH DEMENTIA: A THEMATIC ANALYSIS FROM A SCOPING REVIEW

Yuanjin Zhou,[1] Avery O'Hara,[2] Emily Ishado,[2] Soo Borson,[3] and Tatiana Sadak[2], *1. University of Washington School of Social Work, Seattle, Washington, United States, 2. University of Washington School of Nursing, Seattle, Washington, United States, 3. University of Washington School of Medicine, Seattle, Washington, United States*

Caring for a person with dementia (PWD) requires commitment, flexibility, and resilience - the ability to endure and recover from stressors that arise during the caring process. However, it is unknown what behaviors can indicate resilience in care partners (CPs) of PWDs. We examined 46 peer-reviewed articles (1990 to 2018) that included measures or definitions of resilience in CPs of PWDs. Our goal is to identify resilience-related behaviors and create a behavior-based model/framework for CPs of PWDs. Three major themes emerged: (1) Problem-response behaviors (problem-identifying, problem-solving, problem-distancing, learning, and reflection); (2) self-growth behaviors (self-care, creative/spiritual activities, and developing/maintaining meaningful social relationships); (3) help-related behaviors (help-seeking and help-receiving). These findings informed the development of a behavior-based Care Partner Resilience (CPR) measure. Future steps in this research include evaluating to what extent behaviors in the CPR framework are associated with CPs' self-assessed resilience and can predict CPs' resilience following specific caregiving-related stressors.

### SESSION 3115 (SYMPOSIUM)

### THE POWER OF REVIEWS: HARNESSING RESEARCH ON COGNITIVE HEALTH

Chair: Kelly Quinn, *University of Illinois at Chicago, Chicago, Illinois, United States*
Discussant: Daniela B. Friedman, *University of South Carolina, Columbia, South Carolina, United States*

Networks harness powerful resources to tackle complex challenges. This symposium leverages the power of one such network to advance research in cognitive health. Using literature reviews, we seek to aid in the translation of knowledge on cognitive aging and health into practice. By documenting research successes and identifying gaps, we highlight areas in which important lines of research can be propelled forward. The papers together focus on three dynamics of research practice: communication among researchers, commonly-used research tools, and the need to develop valid and reliable measures. The first paper examines nomenclature that scientists use to describe cognitive health in their research reporting, and makes recommendations for alignment of important concepts. The second and third papers explore commonly-used tools in intervention research: one on the use of psychosocial assessments typically employed with individuals with ADRD, such as for depression and quality of life, and the other on tools used by educational and intervention programs to evaluate caregiver knowledge of dementia. Each paper suggests that consistent measurement practices would enhance interpretation of findings across studies. The final paper considers a promising research area, walkable neighborhoods and cognitive health, and argues for the importance of developing valid and reliable measures in emerging research. Taken together, these papers provide critical reflection on current practices in cognitive aging research and suggest directions in which to build future investigation. In addition, this collection of papers fulfills the clarion call of The Healthy Brain Roadmap for finding ways to improve the cognitive health of all adults.

### TERMS AND MEASURES OF COGNITIVE AGING AND COGNITIVE HEALTH: A SCOPING REVIEW

Christina E. Miyawaki,[1] Kelly Quinn,[2] Raina Croff,[3] Mia T. Vogel,[4] Basia Belza,[5] Anita M. Souza,[5] Minhui Liu,[6] and Valerie J. Edwards[7], *1. Graduate College of Social Work, University of Houston, Houston, Texas, United States, 2. University of Illinois at Chicago, Chicago, Illinois, United States, 3. Oregon Health & Science University, Portland, Oregon, United States, 4. Washington University in St. Louis, St. Louis, Missouri, United States, 5. University of Washington, Seattle, Washington, United States, 6. Johns Hopkins University, Baltimore, Maryland, United States, 7. Centers for Disease Control and Prevention, Atlanta, Georgia, United States*

The Healthy Brain Initiative: National Public Health Road Map to Maintaining Cognitive Health (2007) called on the research community to more widely disseminate its work on cognitive aging and cognitive health. However, communication beyond individual disciplines is complex. We identified terminology that social scientists use to describe cognitive aging and cognitive health among older adults, demonstrated how such terms are defined, and illustrated how these constructs are being measured. We searched terms such as Alzheimer* and dementia in studies between 2007 and 2018 (n=209). Geriatrics (n=95), neurology (n=81), psychiatry (n=65), and psychology (n=30) were most common disciplines; however, there was no consistency in how terms were used within and across disciplines. A detailed review of "cognitive impairment" and "mild cognitive impairment" demonstrated that formal definitions were provided infrequently and measurement of constructs ranged widely. The variability in terminology, definitions and measures reflects a need for greater specificity in research communication.

### OUTCOMES MEASUREMENT IN EARLY-STAGE MEMORY LOSS INTERVENTIONS: A SCOPING REVIEW

Anita Marie Souza,[1] Boeun Kim,[1] Christina Miyawaki,[2] Basia Belza,[1] Mee Kyung Lee,[1] Mia Vogel,[3] Frances Chu,[1] and Yan Su[1], *1. University of Washington/ School of Nursing, Seattle, Washington, United States, 2. University of Houston, Houston, Texas, United States, 3. Washington University, St Louis, Washington, United States*

Psychosocial and psychoeducational groups are widely recommended for individuals with early stage Alzheimer's disease and related dementias (ADRD). However, measurement challenges have hindered researchers' efforts to demonstrate the efficacy of these groups. The purpose of this scoping review was to identify common measurement tools used in interventions for individuals with early stage ADRD and to develop suggestions for future investigations. CINAHL, Embase, PsycINFO, and PubMed were searched; 102 studies were reviewed. Inclusion criteria were set to capture intervention studies that utilized quantifiable measures with participants over age 50. Eleven articles met inclusion criteria. The majority of studies (73%) employed randomized controlled trial designs. Sample sizes ranged from N=20-236. Most commonly measured outcomes included depression, self-efficacy, self-esteem, and quality of life, but there was little consensus on how to best measure these outcomes. Standardization of psychosocial assessment tools are needed for future intervention studies with early stage ADRD.

### A SCOPING REVIEW OF KNOWLEDGE EVALUATION INSTRUMENTS FOR FORMAL AND INFORMAL DEMENTIA CAREGIVER PROGRAMS

Nicholas V. Resciniti,[1] Weizhou Tang,[1] Masroora Tabassum,[1] Dana Al-Hasan,[1] Matthe C. Lohman,[1] Mindi Spencer,[1] and Daniela Friedman[1], *1. University of South Carolina, Columbia, South Carolina, United States*

With the increase in our older adult population there is a need for dementia training for informal and formal dementia caregivers. The objective of this scoping study was to assess dementia knowledge instruments utilized in educational programs and interventions intended for formal and informal dementia caregivers. Scoping review methodology was used to search PubMed, PsycInfo, CINAHL, and Web of Science with tailored database search terms. The search yielded 8,101 results, with 35 studies meeting inclusion criteria. Studies were conducted in eight countries, had varying study designs (RCTs=9, non-RCTs=6, one-group study design=20), and utilized previously published (19) and author-developed (16) instruments. Only two studies focused on minority populations. While author-developed instruments may be more relevant and time-saving, studies should strive to validate instruments or use previously published instruments to help standardize findings across studies and better understand the effects of education programs on caregiver knowledge.

### WALKABLE NEIGHBORHOODS AND BRAIN HEALTH IN LATER LIFE: A SCOPING REVIEW

Boeun Kim[1], *1. University of Washington, Seattle, Washington, United States*

Brain health is critical in independent living and well-being in later life. There have been growing interests in investigating the role of walkable neighborhoods in brain health. The purpose of this scoping review was to understand the influence of walkable neighborhoods on cognitive outcomes in older adults. The physical features of walkable neighborhoods and their measurements were examined. Database search identified 2957 peer-reviewed articles and eight studies (2012-2018) met inclusion criteria. Community facilities and land-use mix were consistently associated with cognitive outcomes across studies. The measures of walkable neighborhoods were under development and the measurement properties were not reported in most studies. Further research on the definition of walkable neighborhoods that accounts specifically for older adults with/without cognitive impairment is needed. Additionally, more rigorous, valid, reliable standardized instruments for measuring walkable neighborhoods, as well as a standard for evaluating the quality of instruments are warranted.

### SESSION 3120 (SYMPOSIUM)

### THE TIPPING POINT STUDY: DIGITAL DETECTION AND DECISION SUPPORT FOR OLDER ADULTS AND FAMILIES

Chair: Kimberly D. Shea, *University of Arizona, Tucson, Arizona, United States*
Co-Chair: Kimberly D. Shea, *University of Arizona, Tucson, Arizona, United States*

In 10 years, the United States will experience a “dependency” ratio of one working age adult (20-64 years old) to one non-working person (> 65 or 85 years old will comprise 19 million of the non-working people (US Census Bureau, 2008). Busy working adults will have to be vigilant to determine when to make life-changing decisions about health and safety issues for people that depend on them. Older adults have gradual and cumulative physical and/or psychological aging changes or can experience significant events. Knowing when to make a life-changing decision, such as when to intervene with independent living due to safety risks, is difficult even when situations have constant vigilance. Eventually, older adults experience a seemingly abrupt, sudden and absolute point where a life changing decision must be made. This is the Tipping Point. Health data, derived from unobtrusive wearable sensors, are algorithmically synthesized to provide critical information on impending concerns via an electronic portal will help the busy working adult to predict and prevent the Tipping Point. This application of precision health care results in targeted and personalized education thus avoiding a potentially catastrophic Tipping Point. This symposium provides insight into five aspects of the Tipping Point: 1) significance of identification, 2) theoretical foundation for environmental and cultural sensitivity, 3) feasibility outcomes from a Mexican American population, 4) methodology for synthesizing quantitative metrics from multivariate streams of data, 5) creation of a culturally sensitive electronic portal to display predictive information and education about consequences

### LAWTON’S THEORY OF PERSON-ENVIRONMENT FIT: THEORETICAL FOUNDATIONS FOR DETECTING TIPPING POINTS

Janice D. Crist,[1] Cheryl Lacasse,[2] Linda R. Phillips,[3] and Jian Liu[4], *1. University of Arizona, Tucson, Arizona, United States, 2. College of Nursing, The University of Arizona, Tucson, Arizona, United States, 3. Arizona Center on Aging, Tucson, Arizona, United States, 4. Department of Systems and Industrial Engineering , The University of Arizona, Tucson, Arizona, United States*

Caregiving families often experience “tipping points,” changes that forever alter their lives, such as a fall with a fractured femur. Tipping points for older adults can be conceptualized as an interaction between individuals and their environments. According to Lawton’s theory of person-environment fit (Lawton, 1983, 1985), physical and social environments and the person’s behavior are shaped by one another in a dynamic, ever-changing process. For older adults, the relationship between “environmental press,” or the mismatch between the person and his/her environment, and adaptation to that environment is mediated through one’s ability to cope. When stressors in health, cognition, or caregiver availability occur, environmental press may heighten, leading to a tipping point. In this paper the authors clarify how environmental press theory provides a foundation for studying early detection of impending tipping points and facilitating decisional support of families for choosing the right long-term support services at the right time.

### TIPPING POINTS PILOT STUDY: WEARABLE DEVICES AND ALGORITHMS

Rachel L. Peterson,[1] Kim Shea,[2] Kayla Luque,[2] Jessica Powell,[2] Jian Liu,[3] and Janice crist[2], *1. University of Arizona Center on Aging, Tucson, Arizona, United States, 2. University of Arizona College of Nursing, Tucson, Arizona, United States, 3. University of Arizona College of Engineering, Tucson, Arizona, United States*

Little is known about the use and acceptability of fitness watches (e.g. Fitbit) by diverse older adults, or how data from affordable (<$40) devices could be triangulated with self-report data to predict adverse health outcomes. We investigated interest and acceptability of fitness watch technology among Mexican American older adults via a week-long trial. Participants were asked to consistently wear the watches and complete daily diaries of activity, questionnaires, and a semi-structured interview. The watch data was triangulated with data from the daily diaries and questionnaires to validate its usefulness in developing algorithms that could detect important physiological transitions that lead to tipping points. Interview data was qualitatively analyzed and coded for barriers and facilitators of watch use and acceptability. Preliminary results suggest that participants are eager and willing to wear a fitness device. Participants reported interest in monitoring their health and using the device to track and improve physical activity.

### USING HEALTH INFORMATION FROM AFFORDABLE WEARABLE DEVICES FOR PREDICTING IMPENDING TIPPING POINTS

Jian Liu,[1] Beverly Heasley,[2] Janice D. Crist,[2] kimberly D. Shea,[2] Lorraine M. Martin Plank,[2] Linda R. Phillips,[1] and Rachel L. Peterson[3], *1. University of Arizona, Tucson, Arizona, United States, 2. University of Arizona College of Nursing, tucson, Arizona, United States, 3. University of Arizona College of Public Health, Tucson, Arizona, United States*

It is desirable to assess older adults' health status from their routine activities, identify tipping points based on quantitative metrics, and evaluate the potential risks. Such assessments based on infrequently measured, traditional physical performance instruments fall short of sensitivity as well as reliability. In this research, we investigate multiple types of data collected continuously from older adults wearing fitness watches, which are equipped with various wearable wireless sensors. We develop a methodology to fuse the information from multivariate data streams and define a new synthesized metric to detect significant physiological transitions that lead to tipping points. Both sensitivity- and robustness-analysis are conducted to evaluate the risks of miss-detection and false alarm. The detection results are cross-validated by the self-reported data from daily diaries and questionnaires.

### CREATING A PORTAL PLUS

Linda R. Phillips,[1] Beverly Heasley,[2] Janice Crist,[1] Cheryl Lacasse,[1] Lorraine Martin-Plank,[1] and jian liu[1], *1. University of Arizona, Tucson, Arizona, United States, 2. Enhancing Lives, LLC, Tucson, Arizona, United States*

Patient portals are popular health care tools. Few portals target caregiving families and few use predictive modeling to assist caregiving families make decisions about elder care options. Our goal is to incorporate the algorithms being developed for predicting tipping points in elder care to assist caregiving families visualize changes in older adults' activity patterns, understand possible implications for continued in-home safety, and access educational information and consultation. While many patients use portals, use is restricted among persons in racial and ethnic minorities, with lower educational levels and poorer health literacy, and with fewer economic resources. The following strategies help us address these issues. Our portal archives data from affordable, wearable technology. Portal access uses similarly technology. Our educational materials use techniques known to be appealing and effective with older individuals in unique groups, e.g., results displays; fotonovelas. Input from community members/users helps maintain sensitivity to cultural appropriateness and health literacy.

### THE SIGNIFICANCE OF IDENTIFYING A TIPPING POINT

Lorraine Martin-Plank,[1] Lori Martin-Plank,[2] Beverly Heasley,[3] Cheryl L. Lacasse,[4] Linda R. Phillips,[5] and Jian Liu[6], *1. University of Arizona, Tucson, Arizona, United States, 2. University of Arizona, College of Nursing, Pipersville, Pennsylvania, United States, 3. Arizona Center on Aging, Tucson, Arizona, United States, 4. University of Arizona, College of Nursing, Tucson, Arizona, United States, 5. University of Arizona, Center on Aging, Tucson, Arizona, United States, 6. University of Arizona, Department of Systems and Industrial Engineering, Tucson, Arizona, United States*

Defining a Tipping Point as seemingly abrupt, severe, and absolute vividly illustrates the end result of the consequence of not responding to impending events. Failure to detect an impending tipping point can result in unplanned consequences including falls, fractures, emergency care, a change in family caregiving situation, and a cascade of events leading to devastating permanent changes in chosen living arrangements or even death to the aging adult and the family caregiver. Proactive monitoring with a wearable device that can detect subtle changes in daily routines is a valuable tool, which caregiving families can use for decision support in accessing long term support services or planning for change in environment or level of care. Using this definition of tipping points and describing early changes can also be a valuable tool for clinicians when communicating potentially dangerous situations to the older adult and caregivers in the home or senior residential setting.

### SESSION 3125 (SYMPOSIUM)

### TURNING THE TIDE: PROMISING INITIATIVES IN GEROPSYCHOLOGY TRAINING AND RECRUITMENT

Chair: Katherine King, *William James College, Newton, Massachusetts, United States*

The field of geropsychology continues to attract insufficient numbers, and there is a growing sense of urgency among geropsychologists to turn the tide. As a result, a number of new research and programmatic initiatives have been started to better understand the problem and begin to generate solutions. This symposium will report on several such projects, including work from a number of early career geropsychologists. In this symposium, novel theoretical and pedagogical approaches to geropsychology training will be discussed. Graham explores how individuals develop an intention to work with older adults, and describes a new scale grounded in the theory of planned behavior. Dodson and Mlinac discuss bringing quality improvement into geropsychology training at all levels, while King, Rosowsky, and Jolson report on an experiential, intergenerational volunteer program used to demystify work with older adults and facilitate recruitment. Several presentations include the voices of students themselves. King, Rosowsky, and Jolson describe graduate student reactions to volunteering with older adults such as having previous negative beliefs about aging challenged. Bloom-Charette explores the experiences of geropsychology internship applicants, who tend to fare better than generalist applicants and feel well supported. Strong focuses on the problem of attracting individuals into academic geropsychology. In looking at participant responses to a recent webinar series on the topic, it is notable that a large number felt intimidated and overwhelmed by the prospect of entering academia. Taken together, projects in this symposium offer a variety of new directions for future geropsychology research, recruitment, and educational initiatives.

### CAREERS IN ACADEMIC GEROPSYCHOLOGY: STRENGTHENING THE PIPELINE

Jessica Strong,[1] Matthew Wynn,[2] Jennifer Moye,[1] and Brian Carpenter[2], *1. VA Boston Healthcare System, Boston, Massachusetts, United States, 2. Washington University St. Louis, St. Louis, Missouri, United States*

The shortage of clinical geropsychologists is further imperiled by the shortage of geropsychologists entering academia. The current study analyzed data collected during the implementation of a national webinar series (Advancing your Confidence as an Educator in Geriatrics and Gerontology). The development and implementation of the series has been previously reported. In the current study, we report on n=66 psychologists' and psychology trainees' responses to academia. Results included quantitative ratings of feelings towards imaging oneself in an academic role. Feelings rated included Excitement (41%), Intimidated (18%), Overwhelmed (30%), and Enthusiastic (30%), among others. These results were triangulated with open-ended qualitative responses on the frustrations and rewards of being an educator. Results are discussed in the context of the geropsychology pipeline shortage and implications for the future of clinical geropsychology. Suggestions for how to remedy this concern will be discussed.

### DEVELOPING COMPETENCE IN QUALITY IMPROVEMENT: A MODEL FOR GEROPSYCHOLOGY TRAINING

Lindsey Jacobs,[1] and Michelle Mlinac[2], *1. VA Boston Healthcare System, Brockton, Massachusetts, United States, 2. VA Boston Healthcare System, Boston, Massachusetts, United States*

Quality improvement (QI) efforts are imperative to ensuring patient-centered, safe, effective, timely, efficient, and equitable healthcare. QI training is well-established in the fields of geriatric medicine and nursing but is lacking in geropsychology. Clinical geropsychologists are in need of QI knowledge and skills, as the population they serve is often vulnerable to healthcare disparities due to complex medical, neurological, and/or psychiatric presentations, limited resources, and low social and/or instrumental support. This presentation will describe a developmental model of QI training, driven by initial pilot data from geropsychology trainees, analysis of geropsychology graduate program marketing materials, and expert opinion from geropsychologists who have QI implementation experience. Consistent with the Pikes Peak Competencies for Geropsychology Training, this model delineates aspirational QI knowledge and skills, as well as recommendations for incorporating QI training into geropsychology at progressing levels of competency from graduate school through independent clinical practice.

### GEROPSYCHOLOGY STUDENTS' EXPERIENCES OF THE INTERNSHIP MATCH AND IMPLICATIONS FOR TRAINING

Lisa Bloom-Charette[1], *1. ENRM Veterans Hospital, Bedford, Massachusetts, United States*

There has been little literature on the effectiveness of the clinical psychology internship match program within the specialty of geropsychology. This study reports student responses to a 2018 survey conducted by the Council of Professional Geropsychology Training Programs (CoPGTP). Students reported that they completed a mean of 18.4 (SD = 7.23) internship applications, and had a mean of 8.4 (SD = 2.3) interviews. Compared to generalists, geropsychology students applied to and interviewed at more programs, felt slightly less supported, but matched more frequently at their first choice site. All respondents matched to generalist rather than geropsychology programs, and all matched to their first ranked programs. The majority of students felt strongly supported, and noted that seminars, essay review, and the Pike's Peak training model were important resources. Reasons for not choosing geropsychology programs included geographical preferences and the belief that adequate geropsychology training can be obtained in a generalist track.

### PREDICTORS OF INTENTION TO WORK WITH OLDER ADULTS: THE CASE FOR STUDYING SOCIAL NORMS

Kirsten Graham[1], *1. Colorado State University, Fort Collins, Colorado, United States*

The expanding population of older adults, coupled with provider hesitance, is expected to result in a large service gap in the healthcare field. Research has largely focused on the impact of attitudes toward older adults and professional competency, with some recent explorations of social influences. There is currently no comprehensive measure that includes all of these areas. The present study outlines the development of the Working with Older Adults Scale (WOAS), which is grounded in the theory of planned behavior. Results indicated that the measure has an excellent factor structure and good internal reliability and construct validity. Intention to work with older adults was significantly predicted by attitudes, subjective norm, and perceived behavioral control, with subjective norm accounting for the greatest amount of variance. The WOAS offers new insights and ideas for future exploration of the service gap between older adults needs and professional availability across health service fields.

### DEVELOPING INTEREST IN GEROPSYCHOLOGY THROUGH INTERGENERATIONAL EXPERIENCE

Katherine King,[1] Erlene Rosowsky,[1] and Michelle Jolson[1], *1. William James College, Newton, Massachusetts, United States*

In order to develop interest in geropsychology, it is beneficial for students to have direct experiences with older adults. This presentation reports on the development of an innovative student volunteer group within a doctoral program in clinical psychology. In this group, students engage directly with older adults in the community. With the help of geropsychology faculty, students have developed and led intergenerational activities such as a panel discussion about dating, reminiscence using music, fraud recognition, and social media training. The group consists of 32 current students, along with 7 recent graduates. Community partners have provided overwhelmingly positive feedback. Students have reported benefits including personal satisfaction from intergenerational contact, learning and pleasure from hearing older adults' stories, and feeling appreciated. Reported challenges include communication issues and feeling unsure how to relate to people with dementia. Students also describe negative assumptions about aging that have changed as a result of their participation.

### SESSION 3130 (SYMPOSIUM)

### KENT AND KLEEMEIER AWARD LECTURES

Chair: Andrzej Bartke, *Southern Illinois University School of Medicine, Springfield, Illinois, United States*

The Donald P. Kent Award is given annually to a member of the The Gerontological Society of America who best exemplifies the highest standards of professional leadership in gerontology through teaching, service, and interpretation of gerontology to the larger society. The Kent lecture will feature an address by the 2018 recipient, Lewis Lipsitz, MD, Hebrew Senior Life. The Robert W. Kleemeier Award is given annually to a member of The Gerontological Society of America in recognition for outstanding research in the field of gerontology. The Kleemeier lecture will feature an address by the 2018 recipient, Keith Whitfield, PhD, Wayne State University.

### THE VIRTUE OF A LIFE OF COMPLEXITY: THE KEY TO HEALTHY AGING

Lewis Lipsitz[1], *1. Hebrew SeniorLife, Roslindale, Massachusetts, United States*

People often wish to simplify their lives as they age, hoping to lead a less complex, stress-free, and happy existence. However, the loss of complexity may actually endanger one's health. Aging is often associated with the pruning of work, family, and social networks in our external environment, and on a smaller scale, with the degradation of various anatomic structures and physiologic processes internally. This loss of complexity can impair our ability to perform activities of daily life or adapt to surgery or other stressors. We can quantify complexity using measures derived from the concept of fractals that describe patterns of behavior across different scales in space or time. Using these metrics we have shown that complexity loss is potentially reversible. This presentation will describe the measures, mechanisms, and consequences of complexity loss in different human systems, and interventions that can restore complexity and thereby improve functional health in older age.

### HOW NETWORKS POWERED MY CAREER STUDYING AGING IN AFRICAN AMERICANS

Keith E. Whitfield[1], *1. Wayne State University, Detroit, Michigan, United States*

This presentation will provide some insights about aging in African Americans I have drawn from the network of scholars in my 30 year journey studying aging in African Americans. This journey has been focused on understanding biobehavioral interplays among demographic, genetic, familial, and psychosocial factors to understand individual variability in health and cognitive aging in African Americans. The presentation will include insights my mentors imparted to me, the contributions of my peers and what I learned from and tried to teach to my mentees. I will also share how my contributions were fueled by funding of my research and interactions with the staff at NIA. I will use data from my Carolina African American Twin Study of Aging, the Baltimore Study of Black Aging and the Study of Longevity and Stress in African Americans to provide examples. I will conclude with some suggestions for the next generation of scholars.

### SESSION 3135 (SYMPOSIUM)

### POLICY SERIES: ADDRESSING LONELINESS AMONG OLDER ADULTS THROUGH RESEARCH, COMMUNITY PROGRAMS, AND A NEW FEDERAL POLICY AGENDA

Chair: Erica Solway, *Institute for Healthcare Policy and Innovation, University of Michigan, Ann Arbor, Michigan, United States*
Discussant: Brian W. Lindberg, *The Gerontological Society of America, Washington, District of Columbia, United States*

Millions of older adults experience feelings of loneliness. A growing body of research has found that chronic loneliness can impact memory, physical well-being, mental health, and life expectancy rivaling the impact on health outcomes of obesity and smoking. Loneliness has been found to impact memory, physical well-being, mental health, and life expectancy. In this session, GSA policy advisor Brian Lindberg will lead a data-driven discussion about who experiences loneliness and isolation and how we might create opportunities for connectedness through new areas of research, forward-thinking policies, and innovative community programs. Presenters include Erica Solway, associate director for the National Poll on Healthy Aging, who will highlight results from a poll conducted in October 2018 among a nationally representative sample of adults age 50 to 80 which found that more than one in three respondents felt a lack of companionships and more than one in four felt socially isolated. Then Catherine Spensley, Director of the Senior Division at Felton Institute, will describe lessons learned in developing and delivering culturally and linguistically appropriate programs and services that foster community and social connections among socially isolated, low income older adults in San Francisco. Finally, Andrew MacPherson, Principal at Healthsperien, LLC, and Director of the Coalition to End Social Isolation & Loneliness will describe stakeholder efforts to advocate for federal legislative and regulatory policy options to address the epidemic including increased funding for and access to supportive services, health care, technology, and public and private research initiatives.

### LONELINESS AND HEALTH AMONG OLDER ADULTS: RESULTS FROM THE UNIVERSITY OF MICHIGAN NATIONAL POLL ON HEALTHY AGING

Erica Solway,[1] John Piette,[2] Matthias Kirch,[2] Dianne Singer,[2] Jeffrey Kullgren,[2] and Preeti Malani[2], *1. Institute for Healthcare Policy and Innovation, University of Michigan, Ann Arbor, Michigan, United States, 2. University of Michigan, Ann Arbor, Michigan, United States*

In October 2018, the University of Michigan National Poll on Healthy Aging conducted an online survey using a nationally representative household sample of adults age 50 to 80. One in three respondents (34%) reported feeling a lack of companionship and 27% reported feeling isolated from others some of the time or often during the past year. Those with fair or poor self-reported physical health, mental health, or hearing were more likely to report feeling a lack of companionship or feel isolated as were those who reported less frequently engaging in healthy behaviors. More than one in four (28%)

reported social contact with people outside of their household once a week or less. Given the high prevalence of loneliness and its connection to poor health and health behaviors, research on this important issue and efforts to increase social engagement among older adults deserve increased attention.

### DEVELOPING A NEW FEDERAL POLICY AGENDA TO ADDRESS SOCIAL ISOLATION AND LONELINESS

Andrew MacPherson[1], *1. Coalition to End Social Isolation & Loneliness, Washington, District of Columbia, United States*

The Coalition to End Social Isolation and Loneliness convenes a diverse group of allied stakeholders, including consumer and patient groups, health plans, community-based organizations, private sector researchers and innovators, and others, to address the epidemic of social isolation and loneliness. The Coalition is developing and advocating for bipartisan federal policy solutions to provide individuals the support they need to be socially engaged. This session will describe the role of the Coalition in engaging stakeholders, promoting innovative research, and advocating for policy changes that combat the adverse consequences of social isolation and loneliness and advance approaches and practices that improve social connectedness for all Americans.

### ADDRESSING SOCIAL ISOLATION AND LONELINESS AMONG LOW-INCOME SENIORS IN SAN FRANCISCO

Catherine Spensley[1], *1. Felton Institute, San Francisco, California, United States*

Felton Institute has been working to eliminate isolation and loneliness in San Francisco since its inception as a community social service agency 130 years ago. Felton offers culturally and linguistically appropriate programs and services that foster community and social connections among socially isolated, low income older adults, including those living with serious mental illness and those residing in local long-term care facilities. Examples include workforce programs for older adults; the foster grandparent program; and the senior companion program which includes friendly visiting, senior counseling, and peer escort services. This session will highlight lessons learned from these successful programs as well as how they have led to the development of new initiatives focused on conducting outreach, providing trauma-informed services, offering wellness classes, and organizing meaningful activities including a choir, intergenerational gardening, arts and dance classes, and cultural exchange opportunities to isolated older adults living in poverty in San Francisco's Visitacion Valley.

### SESSION 3140 (PAPER)

### AGING IN PLACE

### AGING IN PLACE: REVISITING OLD ASSUMPTIONS ABOUT WHAT PEOPLE WANT AS THEY GROW OLD

Marc A. Cohen,[1] Robyn Stone,[2] and Ruth Katz[2], *1. University of Massachusetts Boston Gerontology Institute, LeadingAge LTSS Center @UMass Boston, Boston, Massachusetts, United States, 2. LeadingAge, Washington, District of Columbia, United States*

When people are asked how and where they would like to age, they overwhelmingly indicate they want to "age in place", that is, in their own homes. To support this desire, a range of home and community-based service options have been developed accompanied with major declines in the use of nursing homes and other institutional services. However, what if we've been asking the wrong question? Or asking the wrong people? Given that upwards of 70% of people turning age 65 will have a need for long-term services and supports (LTSS) and 52% will have significant need, the more relevant question is: how might you want to age in the presence of LTSS needs? In this research we explore the attitudes and expectations of a nationally representative sample of 1,200 "late Boomers" age 60 to 72 (instead of all adults) regarding how they want their life to look should they become physically or cognitively impaired and need LTSS. The analytic sample was derived from NORC's AmeriSpeak® Panel. We found that in the presence of significant LTSS need, many people -- 40% if physically disabled and 71% if cognitively impaired -- do not want to remain at home. More than anything else they value safety and do not want to burden their families. They are also concerned about feeling alone. Clearly, late boomers understand that there are circumstances where aging in place may not be right for them and implications for the way we invest resources in the service infrastructure.

### ASSESSMENT OF COMPARATIVE EFFECTIVENESS RESEARCH GAPS TO PROMOTE AGING IN PLACE: A STAKEHOLDER-DRIVEN APPROACH

Gyasi Moscou-Jackson,[1] Sindhura Gummi,[1] Steven B. Clauser,[1] and Neeraj K. Arora[1], *1. PCORI, District of Columbia, United States*

A priority for many older adults is to remain in their homes and communities. This paper describes the Patient-Centered Outcomes Research Institute's (PCORI) review of our investment in comparative effectiveness research (CER) that will advance the field of "aging in place." To inform our systematic evaluation, we first engaged our multi-stakeholder advisory panel of patients/caregivers, clinicians, health systems leaders, and payers to develop and refine a conceptual framework for research focused on aging in place among older adults. Key themes from stakeholders were: aging in place interventions should be patient-centered and align with the patients' needs and goals, studies should include individuals 55+ years who may be at risk for institutionalization, relevant interventions go beyond environmental modifications and include social support, healthcare (inclusive of palliative care), and personal care services. In addition, aging in place services should focus more on informal caregiver interventions and outcomes since informal caregivers play a major role in enabling older adults to age in place. We identified 14 PCORI-funded CER projects that will provide evidence on the most effective interventions to promote aging in place among older adults; the total investment is $113.4 million dollars. The portfolio is addressing decisional dilemmas faced by multiple stakeholders on a variety of topics including falls prevention, home-based palliative care, and community-based care models; however, several critical CER evidence gaps remain that need to be addressed in future funding investments and will be discussed during the presentation.

### DEVELOPMENT AND PSYCHOMETRIC TESTING OF THE PERSON-PLACE FIT MEASURE FOR OLDER ADULTS

Joyce Weil[1], *1. University of Northern Colorado, Colorado, United States*

Person-environment fit models are at the core of gerontological theory and practice. The Person-Place Fit Measure for Older Adults (PPFM-OA) was developed to capture key place domains arising from the expansion of aging in place and age-friendly concepts across the continuum of care, increasingly diverse older populations, and the changing meaning of place. The goal of this study was to create a validated measure to assess "fit". Data from in-depth interviews, focus groups, review of literature and existing comparable measures, and cognitive interviewing created an initial instrument of 90 items. Through the use of a Delphi panel of experts, 60 items were selected and tested in the final measure on Mechanical Turk (MTurk). Results from a sample of persons 60 and older are discussed. Findings from psychometric testing of the PPFM-OA measure are reported. These findings show the final, further-reduced-item version of the Person-Place Fit Measure for Older Adults has good internal consistency and validity. Both this measure and its development process can advance understanding and measurement of place models.

### OLDER ADULTS AND THE WORLD CAFé APPROACH: CROSS-GENERATIONAL INITIATIVES IN RESEARCH AND EDUCATION

Nuelle Novik,[1] Bonnie Jeffery,[1] and Tom McIntosh[1],
*1. University of Regina, Regina, Saskatchewan, Canada*

In Canada, numbers of older adults are considered to be increasing, and by 2036, it is expected that seniors will reach 25% of the total population. Since 2009, the Saskatchewan Population Health and Evaluation Research Unit (SPHERU) has developed an interdisciplinary approach to a community-based research program focused on rural older adults. The world café approach is recognized as collaborative and ideal for encouraging dialogue, sharing knowledge, and developing action plans. Set up like a café, four to six participants at each table engage in a series of three conversational rounds lasting approximately 20 minutes each. At the end of each round, participants move to different tables while the facilitator(s) remain at their original tables. We incorporated a world café approach in three distinct research projects, facilitating a total of five world café events. For each of these events, we also engaged with graduate and undergraduate students who were trained to serve as table facilitators. Participating students represented a variety of disciplines including social work, nursing, and gerontology. Older adults participating in the world café events reported positive experiences and appreciation for the opportunity to discuss new information. Student facilitators identified their participation as a "real life" learning and networking opportunity that enhanced their classroom experiences. Challenges identified included issues related to individual mobility, and issues related to noise and sound quality for those with hearing deficiencies. A community-based approach to research is effective when engaging with this population, and a word café event brings seniors directly into the discussion.

### THE RELEASE OF TECHNICAL QUALITY AND PATIENT EXPERIENCE STAR-RATINGS FOR HOME HEALTH AGENCIES AND AGENCY SELECTION

Margot L. Schwartz,[1] Momotazur Rahman,[1]
Kali S. Thomas,[2] R. Tamara Konetzka,[3] and Vincent Mor[1],
*1. Brown University School of Public Health, Providence, Rhode Island, United States, 2. Providence VA Medical Center, Providence, Rhode Island, United States, 3. University of chicago, Chicago, Illinois, United States*

To facilitate informed Home Health Agency (HHA) selection for the 3.5 million annual Medicare home health (HH) users, CMS introduced technical quality and patient experience summary star-ratings on the Home Health Compare website in July 2015 and January 2016. There is no information about the relationship between the introduction of these two unique sets of star-ratings and HHA selection. We utilized a conditional logit, discrete choice model, which accounts for all HHAs that each patient could have selected (their "choice-set") based on ZIP codes, to assess this relationship. We selected a random 5% sample (203,966 admissions) of new Medicare Fee-for-Service HH admissions that occurred in the year before, or the year after star-ratings were released. Star-ratings were obtained from the HH Compare website and categorized as low (=4 stars). We found the introduction of HHA star-ratings was associated with an increased likelihood of selecting an HHA with a high technical quality star-rating, and a decreased likelihood of selecting an HHA with a high patient experience star-rating. After controlling for each patient's choice-set, patients had 19% increased odds (OR 1.19, 95% CI:1.16,2.23) of selecting a high technical quality HHA and a 12% decreased odds (OR 0.88, 95% CI:0.84,0.92) of selecting a high patient experience HHA, compared to low quality HHAs. Findings suggest patients and referring providers may prioritize technical quality over patient experience. Policy-makers should provide resources to enable HH patients to utilize and interpret the two different HHA star-ratings.

### SESSION 3145 (SYMPOSIUM)

### AGING SUPPORT, HEALTH, AND WELL-BEING OF THE ELDERLY IN CHINA AND JAPAN

Chair: Emma Zang, *Duke University, Durham, North Carolina, United States*
Co-Chair: Yuan Zhang, *University of North Carolina at Chapel Hill, Chapel Hill, North Carolina, United States*

Countries in East Asia have the largest aging population in the world. The consequences of aging largely depend on whether it is accompanied by a healthy, active, and high-quality life. This symposium aims to gain a better understanding of aging support and determinants of health in the contexts of two major East Asian countries - China and Japan. We will present new research using data from the Fukui Longitudinal Caregiver Study (FLCS) in Japan, and two most important aging surveys in China – the China Health and Retirement Longitudinal Study (CHARLS) and the Chinese Longitudinal Healthy Longevity Survey (CLHLS), addressing critical topics including retirement, family care, social mobility, and mortality. Song and Smith investigate the impact of hukou change on mental health in later life. Zang examines the effect of a man's retirement on his wife's mental and physical health in China. Zhang et al. explore the determinants of mortality in China by conducting a comprehensive analysis of life-course conditions, community characteristics, biological and physical functioning, and disease burden. Zeng et al. compare demographic, socioeconomic, behavioral characteristics and health phenotypes of centenarians in China and Italy. Wakui et al. focus on the emergence of compound caregiving and the relationship of caregiving status to burden, depression, and social support in Japan. The cross-national comparisons will be informative regarding aging in various contexts. We will discuss the potential for further investigations using population-based aging data from different countries.

### A COMPARATIVE ANALYSIS ON HEALTHY LONGEVITY PHENOTYPES BETWEEN CHINESE AND ITALIAN CENTENARIANS

Huashuai Chen,[1] Yi Zeng,[2] Huashuai Chen,[2] and Yao Yao[3], *1. Duke University Medical Center, Durham, United States, 2. Medical School of Duke University, Durham, North Carolina, United States, 3. National School of Development, Peking University, Beijing, Beijing, China (People's Republic)*

This paper reviews and compares demographic, socioeconomic, behavioral (including diet) characteristics and heath phenotypes of centenarians in China and Italy. The results revealed that the interactions between familial longevity and any one of the three environmental factors (receipt of adequate medical care when ill as a child, number of living children, and household economic conditions) were significantly associated with the three health outcome indicators (IADL, self-rated life satisfaction, and anxiety-loneness) at old ages. We discovered that the effects of these environmental factors on the health outcome indicators were substantially stronger among elders who had no family history of longevity compared to centenarians' children who likely carry genes and/or inherited healthy behavior and better lifestyle from long-lived parents.

### A NEW FEATURE OF JAPANESE CAREGIVING? COMPOUND CAREGIVING OF OLDER ADULTS IN AN AGING SOCIETY WITH FEWER CHILDREN

Tomoko Wakui,[1] Tomoko Wakui,[2] Emily M. Agree,[3] and Ichiro Kai[4], *1. Tokyo Metropolitan Institute of Gerontology, Tokyo, Japan, 2. Tokyo Metropolitan Institute of Gerontology, Itabashiku, Tokyo, Japan, 3. Johns Hopkins University, Baltimiore, Maryland, United States, 4. The University of Tokyo, Bunkyoku, Tokyo, Japan*

Due to the combined effects of longevity of aging parents, fewer children, and caregiving traditions, family members face multiple caregiving responsibilities in Japan. This study examined the emergence of compound caregiving--providing care to multiple adults-- and the relationship of caregiving status to burden, depression, and social support. Data were from the Fukui Longitudinal Caregiver Study, a survey of family caregivers to older Japanese adults who received long-term care services. We analyzed data from 2,025 caregivers whose mean age was 63 years old. Results showed that 9.5% of caregivers provided care for more than two care recipients. Compared to single caregivers, compound caregivers were more significantly burdened. Compound caregivers who reported higher instrumental and informational support from live-in family and higher emotional support from friends showed significantly lower caregiving burden. We discuss how traditional caregiving norms and demographic changes lead to new needs for family support in Japan's aging society.

### EFFECT OF HUKOU MOBILITY ON DEPRESSION IN MID- AND LATER LIFE IN URBAN CHINA

Qian Song,[1] and James Smith[1], *1. RAND Corporation, Santa Monica, California, United States*

We investigate how the hukou system in China creates disparities in psychological well-being among rural-born urban residents in mid- and later-life, using China Health and Retirement Longitudinal Study (2013-2015) and its life history survey (2014). We differentiate merit- (education, employment), policy- (urbanization), and family-based rural-urban hukou converters, and our propensity score analyses show that merit-based (coef.=-0.14,p<0.01) and policy-based (coef.=-0.11,p<0.05) hukou conversion are psychologically protective of the hukou converters over those who still have rural hukou, but family-based conversion does not have an impact. Among all those who realized hukou mobility, those who did so in the period after 1998 has a relative disadvantage. The benefits of policy-based hukou conversion is most prominent for men, childhood converters, and those who realized hukou mobility between 1978 and 1998. Conversion at an older age puts women into disadvantage compared to an earlier age, though this is not the case for men.

### SOCIOECONOMIC, BIOLOGICAL, AND COMMUNITY ASSOCIATIONS WITH OLD-AGE MORTALITY IN CHINA

Yuan Zhang,[1] John A. Strauss,[2] Peifeng Hu,[3] yaohui Zhao,[4] and Eileen M. Crimmins[2], *1. University of North Carolina at Chapel Hill, Chapel Hill, North Carolina, United States, 2. University of Southern California, Los Angeles, California, United States, 3. UCLA, Los Angeles, California, United States, 4. Peking University, Beijing, Beijing, China (People's Republic)*

Determinants of mortality may differ depending on context. This study uses a nationally representative sample of persons aged 60 and over in China to determine if socioeconomic factors, early life conditions, community characteristics, biological and physical functioning, and disease burden predict four-year all-cause mortality. We employed a series of Weibull hazard models based on exact survival time to predict mortality. We find that current education and place of residence, physical functioning, uncontrolled hypertension, diabetes, cancer, a high level of systemic inflammation, and poor kidney functioning are strong predictors of mortality among older Chinese. We did not find linkages between early-life experiences or community infrastructure to mortality at older ages. Results from this study highlight the value of incorporating biological and performance measurements and the importance of social and historical context in studying old-age mortality.

### SPILLOVER EFFECTS OF A HUSBAND'S RETIREMENT ON A WOMAN'S HEALTH: EVIDENCE FROM URBAN CHINA

Emma Zang[1], *1. Duke University, Durham, North Carolina, United States*

This paper examines the effect that a man's retirement has on his wife's mental and physical health. I exploit the large increase in the probability of retirement at the legal retirement age for urban male wage earners in China as a natural experiment, using data from the China Health and Retirement Longitudinal Survey (CHARLS). I have implemented a fuzzy regression discontinuity design to compare the health outcomes of women whose husbands recently retired with those whose husbands are close to retire. My findings indicate that the retirement of her spouse improves physical and mental well-being of the woman, most likely by increasing the frequency of her social interactions and exercising. Although income and marital quality are less likely to be the main channels through which this positive spillover effect operates, decreased marital satisfaction or severe income constraints can be binding.

### SESSION 3150 (SYMPOSIUM)

### BEREAVEMENT IN A FAMILY CONTEXT

Chair: Jeffrey E. Stokes, *University of Massachusetts Boston, Boston, Massachusetts, United States*
Co-Chair: Kyungmin Kim, *University of Massachusetts Boston, Boston, Massachusetts, United States*
Discussant: Deborah Carr, *Boston University, Boston, Massachusetts, United States*

Bereavement is an impactful, often difficult experience for individuals throughout the life course. Moreover, bereavement experiences inherently involve wider family networks: The death of a spouse is often also the death of a parent, grandparent, or sibling, as well. The present symposium investigates a variety of different family loss experiences that individuals are exposed to in adulthood and older age, and situates such bereavement in a larger family context. Stahl explores how daily routines and sleep patterns can be altered by spousal bereavement, and assesses an intervention designed to improve widowed older adults' behaviors and, in turn, reduce their depressive symptomology. Kim and colleagues analyze the death of a parent in adulthood, examining the extent to which pre-loss relationship quality and relationship importance may predict post-loss symptoms of grief. Stokes and colleagues extend this intergenerational perspective, examining the death of a grandparent in adulthood, and whether adult grandchildren's relationships with their middle-generation parents – bereaved adult children themselves – impact their experiences of grief after loss. Focus is also paid to the influence of gender across all three generations. Lastly, Donnelly explores the cumulative consequences of experiencing multiple family deaths throughout the life course for adults' health trajectories. Together, these papers expand the scope of bereavement research to incorporate spousal, multigenerational, and cumulative loss experiences and their repercussions for midlife and older adults. As discussant, Carr will assess the contributions of these papers to theory and the literature, and highlight potential directions for future research concerning aging, families, and bereavement.

### FAMILY-MEMBER LOSS AS A CUMULATIVE BURDEN FOR HEALTH AMONG BLACK AND WHITE OLDER ADULTS

Rachel Donnelly[1], *1. Vanderbilt University, Nashville, Tennessee, United States*

The health consequences of multiple family member deaths across the life course has received less attention in the bereavement literature. Moreover, recent research shows that black Americans are more likely than white Americans to lose multiple family members. I analyze longitudinal data from the Health and Retirement Study (1992-2014) to assess how multiple family member losses across the life course are associated with declines in health among older adults. Findings suggest that multiple family losses prior to midlife are associated with a number of indicators of poor health (e.g., functional limitations, cardiometabolic health) and steeper declines in health as individuals age. Losses after midlife additionally undermine health declines for older adults. Thus, family member loss functions as a cumulative burden of stress across the life course that erodes health in mid- and later-life. Family loss disproportionately burdens black Americans and serves as a unique source of disadvantage for black families.

### DIGITAL MONITORING OF SLEEP, MEALS, AND EXERCISE AS A PREVENTIVE INTERVENTION FOR DEPRESSION IN BEREAVED SPOUSES

Sarah Stahl[1], *1. University of Pittsburgh, Pittsburgh, Pennsylvania, United States*

The death of a spouse brings profound change to bereaved survivors' lifestyle and daily routine. These changes disrupt circadian rhythms which, in turn, places individuals at high risk for depression. The purpose of this study is to examine the feasibility and acceptability of a 12-week behavioral intervention that targets the timing and regularity of sleep, meals, and physical activity via digital monitoring and motivational health coaching. Participants were 60+ years of age and assessed on intervention acceptability and adherence, depression symptoms (Hamilton Rating Scale for Depression) and the rest-activity rhythm, (a downstream indicator of the body's circadian rhythm (via actigraphic technology). The intervention was rated highly by participants (n=55); 88% were compliant in digital monitoring and 95% were retained. Depression symptoms declined from pre-to post-intervention; and the regularity of circadian rhythms increased. An intervention that targets the regularity of day- and nighttime activities may reduce depression in older spousally-bereaved adults.

### MIDDLE-AGED ADULTS' BEREAVEMENT RESPONSE TO PARENT DEATH: THE ROLE OF PRE-LOSS RELATIONSHIP QUALITY

Kyungmin Kim,[1] Jeffrey E. Stokes,[1]
Steven H. Zarit,[2] and Karen L. Fingerman[3], *1. University of Massachusetts Boston, Boston, Massachusetts, United States, 2. Pennsylvania State University, University Park, Pennsylvania, United States, 3. The University of Texas at Austin, Austin, Texas, United States*

The bereavement literature in adulthood has largely focused on spousal loss. Yet the death of a parent is an influential – and expected – loss experience in middle and later life. This study analyzed prospective data from two waves of the Family Exchanges Study (2008 and 2013) to explore adults' (N = 192; Mage = 56.76) experience of a recent parent death in the past 5 years, including grief responses and positive memories of the deceased parent. We examined how pre-loss relationships with the deceased parent (e.g., positive and negative relationship quality, relationship importance) are associated with different bereavement responses among the bereaved children. Findings showed that the levels of grief were higher for children who placed more importance on the parent prior to that parent's death. Positive relationship quality was associated with positive memories after a parent's death. However, negative relationship quality was not associated with any bereavement responses.

### DEATH OF A GRANDPARENT IN ADULTHOOD: GENDER, GRIEF, AND THE ROLE OF MIDDLE-GENERATION PARENTS

Jeffrey E. Stokes,[1] Kyungmin Kim,[1] and
Karen L. Fingerman[2], *1. University of Massachusetts Boston, Boston, Massachusetts, United States, 2. University of Texas at Austin, Austin, Texas, United States*

Although the death of a grandparent in adulthood is often an expected or "on-time" life event, this loss may still result in grief for adult grandchildren. Structural aspects of relationships, including gender of the grandparent, adult grandchild, and/or middle-generation parent, may affect the response such a loss elicits from adult grandchildren. Further, adult grandchildren's relationship quality and/or coresidence with middle-generation parents may also impact the effect of grandparent loss on adult grandchildren's grief. Using data from Wave 2 of the Family Exchanges Study (2013), we found that (a) grandsons report less grief than granddaughters, irrespective of grandparents' or middle-generation parents' gender; (b) relationship quality with and worry about middle-generation parents matter most for granddaughters and those who lost a maternal grandparent; and (c) worry about middle-generation parents matters most for bereaved grandchildren who coreside with middle-generation parents. Results highlight the intersection of gender and relationship quality in a multigenerational context.

### SESSION 3155 (SYMPOSIUM)

### BEYOND REPRODUCTION: EXPLORING DETERMINANTS OF FUNCTIONAL HEALTH AND WELL-BEING IN AGING WOMEN

Chair: Cassandra M. Germain, *North Carolina A&T State University, Greensboro, North Carolina, United States*

The goal of this symposium is to explore the various factors that influence functional health and general well-being in women during late life. We will explore 1) the role of depression on functional impairment in a group of diverse community dwelling women; 2) the relationship between chronic pain and depression, as well as strategies for intervention; 3) the desire for sexual intimacy among older women and it's relationship to wellbeing and 4) the importance of physical appearance in older women and its association with self confidence and wellbeing.

### ETHNICITY, DEPRESSION, AND FUNCTIONAL OUTCOMES IN OLDER WOMEN

Cassandra M. Germain[1], *1. North Carolina A&T State University, Greensboro, North Carolina, United States*

Women continue to have higher prevalence rates of functional impairment and depressive symptoms than men. In addition, women from certain ethnic groups experience disproportionately higher rates of ADL limitation, and are less likely to be screened for depression. The current study examines the association between race/ethnicity and depressive symptoms on functional limitations in Black, White and Hispanic women. We examined prevalence and adjusted odds of ADL limitations by race in n=9,846 women aged 50+ with low (CESD <4) and high (CESD =>4) depressive symptoms from the 2014 wave of the Health and Retirement Study. Overall, Black and Hispanic women had significantly higher rates of depression (p <.001) and ADL limitations (p<.001) than White women. Among those with high depressive symptoms, Black OR 1.98 [1.17, 3.34] and Hispanic OR 2.82 [1.42, 5.6] women have significantly higher rates of ADL limitations as compared to White women.

### AGING APPEARANCE AND ITS SIGNIFICANCE TO WELL-BEING

Naomi Woodspring[1], *1. University of the West of England, Bristol, United Kingdom*

Ageing is a profoundly embodied process, yet elder's concerns about appearance are perceived, by many, as trivial. Notions of appearance as a core human concern continues as a significant aspect throughout our lives. Self-presentation choices convey a sense of our identity. This paper is based on a qualitative study which aimed to explore current notions of beauty and age. A diverse group of postwar women (born between 1945 -1955) from the US and the UK were interviewed with a focus on their own self-presentation and the acts of seeing and being seen. This paper explores the some of the findings from this study. The majority of women, and the all women of colour, reported feeling more confident in their appearance and appreciative of other older women's appearance. This led to a more robust sense of well-being and suggests that age and appearance may be significantly linked to well-being.

### INTIMACY IN LATER LIFE: A POTENTIAL PATHWAY FOR IMPROVING WELL-BEING IN OLDER WOMEN

Renee J. Flores[1], *1. UT Houston McGovern Medical School, Houston, Texas, United States*

Despite benefits to overall health and well-being, healthcare professionals' knowledge and research is limited in regards to older women's sexuality and intimacy desires. There are barriers that impede fulfilling these desires and lack of understanding hinders ways to address this issue, which negatively affects the well-being of older women. A sexuality and intimacy survey of 29 women between the ages of 60-86 revealed that the majority were having sex at least once a month and expressed the desire to increase the frequency of sexual encounters. These data suggests that later-life sexuality and intimacy encounters are important for some women. Recognizing these desires could prompt responses that could greatly influence the quality of life in older women. A broader public health discussion needs to occur in order to promote awareness and optimize overall well-being.

### BELIEFS SURROUNDING THE PAIN AND DEPRESSION CYCLE AMONG OLDER AFRICAN AMERICAN WOMEN: FINDINGS FROM FOCUS GROUPS

Emerald Rivers,[1] Brittany Drazich,[1] Sarah Szanton,[1] Manka Nkimbeng,[1] and Janiece Taylor[1], *1. Johns Hopkins University, Baltimore, Maryland, United States*

Older African American women experience high rates of comorbid conditions and functional limitations that put them at risk of experiencing a cycle of pain and depressive symptoms. This cycle is often shaped by individual's behaviors, emotions, physical responses, and thoughts. Increased pain severity is associated with comorbid pain and depression making it essential for older African American women to communicate their experiences with these conditions. Hence, we explored older adult African American women's relevant beliefs, and identified strategies to address them in adapting the intervention, Get Busy Get Better. In three focus groups, we found that older African American women (mean age 60.7, n=11): (1) relied on companionship (emotions), (2) used physical activity strategies for pain and depression relief (behaviors), (3) had a general function reduction from pain (physical response), and (4) saw connections between depression and pain (thoughts). Thus, when adapting the intervention, strategies incorporate these four elements.

### SESSION 3160 (SYMPOSIUM)

### DISPARITIES OF DENTAL CARE AND CONSEQUENCE OF POOR ORAL HEALTH AMONG OLDER ADULTS IN THE U.S.

Chair: Yanyan Wu, *University of Hawai'i at Mānoa, Honolulu, Hawaii, United States*
Co-Chair: Wei Zhang, *University of Hawaii at Manoa, Honolulu, Hawaii, United States*
Discussant: Bei Wu, *New York University, New York, New York, United States*

Oral health is an essential part of staying healthy. Neglect of dental care may lead to tooth decay/ loss, poor nutrition, and affects individuals' quality of life. Over the past decades, dental care utilization has risen considerably, however, racial/ethnic and socioeconomic disparities still persist in the U.S. Additionally, poor oral health is a contributing factor to, and a consequence of chronic diseases such as cognitive impairment, diabetes and cardiovascular disease. Faced with the complex and intertwined health and social challenges, it's imperative to understand the disparities of dental care utilization and the relationships among oral health and chronic diseases so that effective policies and preventions can be implemented to improve quality of care. In this symposium, we present findings for older adults from diverse racial/ethnic populations in the U.S. We begin with results from two national-wide trend analyses: a 15-year review of dental care utilization and the evaluation of dental care performance over a 16-year period in nursing homes. The next study presents the barriers of dental care utilization in Hawaii. Finally, we present results of the negative effects of diabetes and poor oral health on cognitive function. Our studies address the disparities of dental care utilization among minority and under-represented ethnic groups as well as the connections between oral health and chronic conditions. Our results are helpful in educating policy makers and health practitioners about how to improve dental care and how dental care can be effectively integrated into chronic disease prevention and health promotion activities.

### DISPARITIES OF DENTAL CARE UTILIZATION AMONG WHITE, BLACK, HISPANIC, AND ASIAN FROM 2002 TO 2016

Yanyan Wu,[1] Wei Zhang,[1] and Bei Wu[2], *1. University of Hawaii at Mānoa, Honolulu, Hawaii, United States, 2. New York University, New York, New York, United States*

Utilizing eight waves (2002-2016) of data from the Behavioral Risk Factor Surveillance System (BRFSS), this study examines the disparities and trend of dental care utilization among dentate adults aged 50 years or older in the U.S. Findings reveal that there was a significant increase of dental care utilization among Asians and Blacks in older adults. The rates increased from 73% (95%CI: 69%-78%) to 81% (95%CI: 79%-84%) and 54% (95%CI: 52%-56%) to 60% (95%CI: 69%-62%) for Asians and Blacks respectively in the 75-79 age-group. Early in 2002, there was a linear decreasing pattern of dental care utilization with age. However, the 65-69 age-group became a turning point in 2018 with a lower rate of dental care utilization before 65-69 years of age and higher rate in the older age groups. Overall, Hispanics and Blacks had much lower rates of dental care utilization than that of Whites and Asians.

### THE QUALITY OF DENTAL CARE IN NURSING HOMES: VARIATION BY FACILITY AND MARKET CHARACTERISTICS

Zhiqiu Ye,[1] and Bei Wu[2], *1. University of Rochester Medical Center, Rochester, New York, United States, 2. New York University, New York, New York, United States*

Nursing home (NH) residents are disproportionately affected by poor oral health. But little we known about the root causes. We analyzed the 2000-2016 national inspection survey data for all certified-NHs (n=248,975 facility-years). Dental care performance was measured by two designated deficiency citations. Generalized estimating equation models were used to predict if the NH facility and market characteristics were associated with low performance. The rates of deficiency citation tripled from 1.2% in 2000 to 3.4% in 2016 ($p<0.001$) with substantial variation across states. NHs with more minority residents and poorer resources (higher share of Medicaid and lack of registered nurse), and NHs with high competing priorities (larger, for profit, chain-affiliated and urban locations) were more likely to receive deficiency citations. Residents in these facilities are at greater risks of poor oral health. This presentation will provide discussion on relevant policy and practice to improve dental care quality in nursing homes.

### THE MODERATING ROLE OF BIRTHPLACE ON DENTAL CLEANING SERVICE AMONG CHINESE OLDER ADULTS IN HAWAII

Sizhe Liu,[1] Wei Zhang,[1] Keqing Zhang,[1] and Bei Wu[2], *1. University of Hawaii at Mānoa, Honolulu, Hawaii, United States, 2. New York University, New York, New York, United States*

Regular dental cleaning is vital to maintaining good oral health. This study aims to identify socio-demographic characteristics that are associated with the use of dental cleaning services among Chinese older adults in Honolulu, Hawai'i. In addition, we examine if birth-place moderates these associations. The data for this study were collected from 398 Chinese older adults living in Honolulu. Results from multivariate logistic regressions showed that those who were married and with higher levels of education were more likely to have their teeth cleaned within the past year compared to those who were not married or with lower levels of education. These significant associations were only found salient for the foreign-born when the moderating role of birth place was accounted for in the model. These differences may be partially due to the impact of acculturation and the knowledge of oral health and dental services.

### THE IMPACT OF DIABETES AND POOR ORAL HEALTH ON COGNITION: IMPLICATIONS TO IMPROVE DENTAL CARE ACCESS

Bei Wu,[1] and Huabin Luo[2], *1. New York University, New York, New York, United States, 2. University of Alabama at Birmingham, Greenville, North Carolina, United States*

This study examined the joint effects of diabetes and poor oral health on cognitive function among older adults aged 60 years or older in the U.S. We analyzed data of 2,937 participants from the National Health and Nutrition Examination Survey (2011-2014). We investigated the interaction effects between diabetes and significant tooth loss, i.e. differences among the following four groups: 1=neither of the two conditions, 2=non-diabetic but with tooth loss, 3=diabetic but no tooth loss, and 4=both conditions. Significant interaction effects were found in our study. Having either diabetes or significant tooth loss was associated with lower cognitive function. When the two conditions were both present, the negative effects were much stronger than the total effects from either one of the two conditions. The additional loss of cognitive function resulting from multiple health conditions illustrates the importance of improving access to dental care for older adults in the U.S.

### SESSION 3165 (SYMPOSIUM)

### ENHANCING ACCESS AND QUALITY OF RURAL VETERANS' CARE THROUGH TELEMEDICINE IMPLEMENTATION

Chair: Aaron T. Seaman, *Iowa City VA Healthcare System, Iowa City, Iowa, United States*
Co-Chair: Samantha Solimeo, *Center for Access and Delivery Research and Evaluation, Primary Care Analytics Team, Iowa City VA Health Care System, Iowa City, Iowa, United States*
Discussant: Byron Bair, *VA Salt Lake City Healthcare System, Salt Lake City, Utah, United States*

Telemedicine, a promising approach for clinicians to provide care to patients who are unable to attend face-to-face encounters, has been embraced by the Veterans Health Administration to improve the delivery of specialty care to rural Veterans and their caregivers. Presenters in this symposium report on the potential and challenges of telemedicine in a variety of specialty care contexts. Stakeholder (Veteran, caregiver, and provider) perspectives are discussed. Hung et al.'s study examines the implementation of a teleconsultation intervention designed to connect geriatric teams with rural clinics, reporting on both patient outcomes and the challenges of implementation variability that teams encountered. Solimeo, et al.'s qualitative study of primary care providers' experience of an osteoporosis telemedicine clinic's outreach and care delivery demonstrates stakeholder acceptance as well as potential missed opportunities for patient and provider education. Findings from Nichols and Martindale-Adams' mixed methods evaluation of a telephone intervention to assist caregivers of older, rural-dwelling Veterans demonstrate the impact the intervention has had on participants' perceptions of and abilities to care for their family members and themselves. Hicken et al. present on the implementation of a videoconferencing intervention to provide in-home support to rural Veterans and their caregivers, reporting on both provider and patient/caregiver experiences of the intervention and its implementation. The four studies highlight the unique ways telemedicine can improve care and the necessity of including stakeholder perspectives across the implementation process.

### THE UNINTENDED CONSEQUENCES OF SEAMLESS IMPLEMENTATION OF A RURAL TELEMEDICINE CLINIC

Samantha Solimeo,[1] Melissa J. Steffen,[2]
Aaron Seaman,[3] and Karla L. Miller[4], *1. Center for Access and Delivery Research and Evaluation Primary Care Analytics Team, Iowa City VA Health Care System, Iowa City, Iowa, United States, 2. Veterans Rural Health Resource Center Central Region Center for Access and Delivery Research and Evaluation, Primary Care Analytics Team, Iowa City, Iowa, United States, 3. Carver College of Medicine, Iowa City, Iowa, United States, 4. Department of Internal Medicine, Rheumatology Section;, Salt Lake City, Utah, United States*

Osteoporosis is largely undetected and untreated in older men who are at-risk, with even lower rates of detection and treatment in rural communities. In this presentation we report the consequences for primary care providers (PCPs) of the implementation of an osteoporosis telemedicine clinic that targeted rural patients and is purposefully designed to minimize PCP workload. Analysis of qualitative interviews with PCPs who co-managed patients with the telemedicine clinic confirmed that implementation strategies used by the telemedicine clinic were successful at minimizing workload and had a positive impact on PCPs' work. However, the clinic's seamless approach may have contributed to unintended consequences of low PCP awareness of program care processes and selection criteria and a missed opportunity for PCPs to reinforce osteoporosis care goals in the primary care setting.

### A MULTI-SITE PROGRAM TO PROVIDE GERIATRICS TELECONSULTATION TO OLDER RURAL VETERANS (GRECC CONNECT)

William Hung,[1] Steven Barczi,[2] cathleen Colon-Emeric,[3] Michelle Rossi,[4] Stuti Dang,[5] Thomas Caprio,[6] and Sara Espinoza[7], *1. James J Peters VA Medical Center, Bronx, New York, United States, 2. Geriatric Research, education and Clinical Center, William S Middleton Memorial VA Medical Center, University of Wisconsin; Madison, Madison, Wisconsin, United States, 3. Duke University Geriatric Research; education and clinical center, durham vAMC, durham, North Carolina, United States, 4. Geriatric Research, education and clinical center, VA Pittsburgh healthcare system, pittsburgh, Pennsylvania, United States, 5. GRECC, Miami VAMC, Miami, Florida, United States, 6. Canandaigua VAMC, Canandaigua, New York, United States, 7. GRECC, San antonio VAMC, San antonio, Texas, United States*

Older Veterans living in rural areas often do not have access to geriatrics team care; rural frontline providers and teams may need support to address the needs of older adults with complex chronic conditions. GRECC Connect aims to link up geriatric teams at Geriatric Research, Education and Clinical Centers (GRECCs) and rural clinics to provide geriatric consultation remotely through clinical video telehealth (CVT) and other means. GRECC Connect is established in twelve GRECCs across the country with links to rural clinics in their catchment area; consultations led to identification and meeting of care needs of older adults with complex conditions, improving medication use and reducing older adults' need for travel to long distances for consultation. In this presentation, we review the experience of establishing connections with rural clinics, impact on older adult care and adaptations needed to address local needs and contexts.

### IMPROVING ACCESS AND OUTCOMES FOR RURAL CAREGIVERS USING THE TELEPHONE

Linda Nichols,[1] and Jennifer Martindale-Adams[2], *1. VA Medical Center, Memphis, Tennessee, United States, 2. Department of Preventive Medicine, Memphis, Tennessee, United States*

Many caregivers of older rural veterans have limited access to services. In 2017, VA's Office of Rural Health and Caregiver Support Program funded the Memphis Caregiver Center to deliver Resources for Enhancing All Caregivers Health (REACH VA) as a national behavioral intervention to improve access to care. To date, 438 caregivers of older Veterans have enrolled. REACH is a proven 4-session caregiver intervention, structured and easily replicated. Rural internet service is often problematic. Offering REACH by telephone provides many benefits, including access to service not available in the rural area, almost universal technology penetration, ease and familiarity of use, and removal of barriers of travel and care for the loved one. Through this enhanced service, caregivers of older veterans with dementia, PTSD, ALS, MS, and other conditions have shown statistically and clinically significant improvements in managing concerns and safety relating to their loved one and their own stress and coping.

### HOME VIDEO TELEHEALTH TO SUPPORT RURAL VETERAN CAREGIVERS

Bret Hicken,[1] Marren Grant,[1] Christopher Turner,[1] Christy Reynolds,[2] and Pamela Wright[2], *1. Veterans Rural Health Resource Center-SLC, Salt Lake City, Utah, United States, 2. VA Caregiver Support Line, Canandaigua, New York, United States*

VA offers multiple programs and services to support caregivers of US military Veterans. However, access for rural Veterans and caregivers is challenging due to distance from VA medical facilities. VA Video Connect (VVC) is a remote healthcare platform that enables Veterans to connect directly with VA clinicians through a secure, encrypted video connection. Rural caregivers and Veterans can participate in caregiver services through their own computer or another device from their home, reducing the need for travel to distant VA facilities. In 2018, VA's Caregiver Support Program and Office of Rural Health developed an implementation pilot to engage eighteen VA Caregiver Support Coordinators (CSCs) in using VVC to monitor and support 180 Veteran/caregiver dyads enrolled in VA's Program of Comprehensive Assistance for Family Caregivers. The presentation will provide an overview of each phase of implementation, report usability and outcome data from CSCs and caregivers, and discuss implications for broader implementation.

### SESSION 3170 (PAPER)

### EPIDEMIOLOGY OF AGING

### ACCELERATED CHILDHOOD SKELETAL AGING IS PROTECTIVE OF DEVELOPMENT OF SARCOPENIA IN LATER LIFE

Matthew J. Peterson,[1] and Andrew W. Froehle[2], *1. Campbell University, Buies Creek, North Carolina, United States, 2. Wright State University, Dayton, Ohio, United States*

Sarcopenia is an age-related loss of muscle mass and strength that has a multitude of adverse sequelae. Similar to other aging-related phenomenon, sarcopenia is likely the product of inputs that begin in utero and continue throughout the lifespan. We hypothesized that patterns of childhood skeletal growth predict sarcopenia status later in life. Data are from N=202 lifelong participants of the Fels Longitudinal Study (median lifetime visits=33). At the sarcopenia measure visit, participants were aged 65.8 + 10.3 years, 54% female, with body mass index of 27.5 + 4.9. Sarcopenia was defined using published sex-specific cutpoints from dual energy x-ray absorptiometry quantified appendicular lean mass/height2. Childhood skeletal age was calculated from serial hand-wrist radiographs (FELS method). Residual skeletal aging (RSA) was calculated as skeletal age minus predicted chronological age at peak height growth velocity during adolescence. RSA variance was similar in both sexes, with a range of -2 (delayed skeletal aging) to +2 years (accelerated skeletal aging). In older age, 6% of males and 22% of females exhibited sarcopenia. In multivariate logistic regression models controlling for age, self-reported physical activity, and grip strength (all measured at sarcopenia visit), accelerated RSA was protective of sarcopenia (Adjusted OR=0.58; 95% CI: 0.35-0.94). This is the first study to link childhood skeletal maturation to sarcopenia later in life. Biological pathways that explain this association likely include physiological, environmental, and genetic factors that facilitate communication between bone and muscle, and span the life course. Determining their influence is the next important step in this work.

### ARE THERE BIDIRECTIONAL ASSOCIATIONS BETWEEN COGNITION AND BALANCE ABILITY: EVIDENCE FROM A BRITISH BIRTH COHORT

Joanna M. Blodgett,[1] Rachel Cooper,[2] Daniel Davis,[1] Diana Kuh,[1] and Rebecca Hardy[1], *1. UCL, London, United Kingdom, 2. Independent author, London, United Kingdom*

Age-related changes in cognitive and balance abilities are well-established, as is their correlation with one another. There is, however, limited evidence regarding the directionality of associations and whether or not common biological processes may underlie their age-related declines. The main aim was to explore bidirectional associations between balance and cognitive abilities in mid-later life using data from the MRC National Survey of Health and Development, the 1946 British birth cohort (n=2735). Cognition was assessed at ages 43, 53, 60-64 and 69 with verbal memory and search speed tasks. One-legged standing balance time (eyes closed) was assessed at ages 53, 60-64 and 69. Two autoregressive cross-lagged models simultaneously assessed bidirectional associations of balance with verbal memory and search speed over time. Results suggest a unidirectional association between verbal memory and subsequent balance in both sexes, decreasing with age from 0.14 SD balance (95%CI: 0.10,0.17) per 1SD verbal memory to 0.06 (0.01,0.10) to 0.05 (0.01,0.09). Search speed at age 43 was associated with balance at age 53 [men: 0.11(0.06,0.16); women: 0.09 (0.03,0.13)]; additionally, in men, there was evidence of a bidirectional association between ages 60-64 and 69 [balance to search speed: 0.05 (0.00,0.10); search speed to balance: 0.09 (0.02,0.16)]. These findings support the notion that successful balance relies mainly upon cognitive processing to successfully integrate vestibular, visual and proprioceptive input with motor output. Including a cognitive component in balance and fall risk intervention programs could have an additive benefit in improving neural pathways involved in balance and thus reducing fall risk.

### COMPARING THE PROGNOSTIC VALUE OF GERIATRIC HEALTH INDICATORS: A POPULATION-BASED STUDY

Alberto Zucchelli,[1] Davide Vetrano,[2] Giulia Grande,[2] Amaia Calderon-Larranaga,[2] Laura Fratiglioni,[2] Alessandra Marengoni,[1] and Debora Rizzuto[2], *1. Department of Clinical and Experimental Sciences, University of Brescia, Brescia, Italy, 2. Aging Research Center, Department of Neurobiology, Care Sciences and Society, Karolinska Institutet and Stockholm University, Stockholm, Sweden*

Several indicators associated with poor outcomes in older persons have been developed, but a direct comparison of their accuracy is lacking. Knowing which indicator performs better in the prediction of specific outcomes could help health care providers to choose the most suitable one. We compared the accuracy in predicting different clinically-relevant outcomes of five indicators: frailty index (FI), frailty phenotype (FP), the Health Assessment Tool (HAT), walking speed (WS), and multimorbidity. Data from the Swedish National Study on Aging and Care in Kungsholmen, an ongoing population-based study including 3363 people 60+, were used. The ability of the five indicators to predict mortality (3- and 5-year), unplanned hospitalizations (1- and 3-year), and 2+ health provider contacts (6 months prior and after assessment) was compared using the area under the ROC curves (AUC). FI, WS, and HAT showed the best accuracy in the prediction of mortality (AUC for 3-year mortality: 0.84, 0.85, 0.87 respectively; AUC for 5-year mortality: 0.84, 0.85, 0.86 respectively; all $p < 0.05$). Unplanned hospitalizations were better predicted by the FI (AUC: 1-year 0.73; 3-year 0.72) and HAT (AUC: 1-year 0.73; 3-year 0.71).The most accurate predictor of multiple contacts with health providers was multimorbidity (AUC: 0.67; $p < 0.05$). All indicators, but multimorbidity, showed higher accuracy among older individuals (75+ years). Different indicators can be used to support physicians during their decision-making process. Some of these tools may also be used to forecast future use of health-care resources, including both hospital-based services and outpatient ones .

### GENOME-WIDE LINKAGE ANALYSIS IDENTIFIES A NOVEL LOCUS FOR LONGITUDINAL GAIT SPEED CHANGE WITH AGING

Adam J. Santanasto,[1] Mary K. Wojczynski,[2] Ryan K. Cvejkus,[3] Bharat Thyagarajan,[4] Kaare Christensen,[5] Nicole Schupf,[6] Ping An,[7] and Joseph M. Zmuda[3], *1. University of Pittsburgh, Pittsburgh, Pennsylvania, United States, 2. Department of Genetics Washington University in St. Louis, Saint Louis, Missouri, United States, 3. Department of Epidemiology, University of Pittsburgh; Pittsburgh, Pennsylvania, United States, 4. Department of Laboratory Medicine and Pathology, University of Minnesota; Minneapolis, Minnesota, United States, 5. Danish Aging Research Center, University of Southern Denmark, Odense C, Denmark, 6. Columbia University, Columbia University, United States, 7. St. Louis, Washington University, Missouri, United States*

Gait speed is an indicator of health and function with aging. The potential genetic contributions to gait speed and its decline with aging are not well characterized. We sought to better quantify the genetic contributions to and identify potential genes and genetic variants underlying change in gait speed among older adults. To accomplish these aims, we used data from 2379 individuals belonging to 509 families in the Long Life Family Study (mean age 64 ± 12, range 30–110 years; 45% men). Gait-speed was measured over 4 meters at baseline and after an average of 7±1.1 years. Quantitative trait linkage analyses were completed using pedigree-based maximum-likelihood methods with logarithm of the odds (LOD) scores > 3.3 indicating genome-wide significance. We also performed linkage analysis in the top 10% of families contributing to LOD scores to allow for heterogeneity among families (HLOD). Data were adjusted for age, sex, height and field center. At baseline, 26.9% of individuals had "low" gait-speed <1.0 m/s (mean: 1.1±0.2

m/s) and gait speed declined at a rate of -0.02±0.03 m/s per year (p<0.0001). Baseline and change in gait-speed were significantly heritable (h2 = 0.24-0.32, p<0.05). We did not find significant evidence for linkage for baseline gait speed; however, we identified a potentially novel locus for change in gait speed on chromosome 16p (LOD 4.2). A subset of 21 families contributed to this linkage peak (HLOD = 6.83). Sequence analysis of the chromosome 16 region may yield new insight on the biology of age-related mobility decline.

### TWELVE-YEAR CLINICAL TRAJECTORIES OF MULTIMORBIDITY IN OLDER ADULTS: A POPULATION-BASED STUDY

Davide Liborio Vetrano,[1] Albert Roso-Llorach,[2] Sergio Fernández,[2] Concepción Violán,[2] Graziano Onder,[3] Laura Fratiglioni,[4] Amaia Calderon-Larranaga,[4] and Alessandra Marengoni[5], *1. Aging Research Center - Karolinska Institutet, Stockholm, Sweden, Sweden, 2. Fundació Institut Universitari per a la recerca a l'Atenció Primària de Salut Jordi Gol i Gurina (IDIAPJGol), Barcelona, Spain, Spain, 3. Centro di Medicina dell'Invecchiamento, IRCCS Fondazione Policlinico "A. Gemelli" and Catholic University of Rome, Rome, Italy, 4. Aging Research Center, Department of Neurobiology, Care Sciences and Society, Karolinska Institutet and Stockholm University, Stockholm, Sweden, 5. Department of Clinical and Experimental Sciences, University of Brescia, Brescia, Italy*

The scarce knowledge of multimorbidity development hampers the effectiveness of clinical interventions. We aimed to identify multimorbidity clusters, trace their evolution in a cohort of older adults, and detect the clinical trajectories of single individuals as they move among clusters over 12 years. Population-based study including 2931 persons 60+ with ≥2 diseases participating in the SNAC-K study. A fuzzy c-means cluster algorithm was used to group participants by disease patterns at baseline and follow-ups. Migration from one cluster to another was tracked over time, and the association between the clusters and mortality was tested. At baseline 52% of participants were classified into five clinically meaningful disease clusters: psychiatric and respiratory (5%), heart (9%), respiratory and musculoskeletal (16%), cognitive and sensory impairment (10%), and eye diseases and cancer (11%). The remaining 48% of participants (unspecified group) were grouped in any cluster at baseline but greatly contributed to the other clusters at follow-ups. Multimorbidity clusters that included cardiovascular and neuropsychiatric diseases presented a significantly higher mortality risk (odds ratios ranging 1.58–6.00) than the group not part of any clusters. Clusters characterized by cardiovascular and neuropsychiatric diseases included 25% of the study population at baseline and 28% of participants at six years, and they accounted for 51% of deaths at six years and 57% of deaths at twelve years. Multimorbidity clusters and clinical trajectories of older adults with multimorbidity show great dynamism over time. The multimorbidity clusters and trajectories identified in this study may help identifying groups with similar needs and prognosis.

### SESSION 3175 (PAPER)

### EXERCISE INTERVENTIONS TO ADDRESS FUNCTIONAL DECLINE

### COGNITIVE PERFORMANCE, EXERCISE, AND AMYLOID BURDEN: A RANDOMIZED CONTROLLED TRIAL

Amber Watts,[1] Eric Vidoni,[2] Jill Morris,[2] Mark Perry,[2] David Johnson,[3] and Jeffrey Burns[2], *1. University of Kansas, Lawrence, Kansas, United States, 2. University of Kansas Medical Center, Kansas City, Kansas, United States, 3. UC Davis, Davis, California, United States*

Exercise is a promising strategy for prevention of Alzheimer's disease (AD). Amyloid neuroimaging can identify individuals at risk of developing AD prior to displaying symptoms. We screened adults (65+) with Florbetapir PET imaging and a comprehensive cognitive battery. We randomized 117 participants with normal cognition into a 52-week aerobic exercise program to examine the effects of aerobic exercise on cognitive performance. We compared an intensive exercise treatment group to a standard of care control group. Cognition was assessed at baseline, 26 weeks, and 52 weeks in the domains of verbal memory, visuospatial processing, attention, and executive function. Interim results on 87 participants show cardiorespiratory fitness improved in the exercise group vs. control group (t=3.66(81), p< .001). The degree of change in cardiorespiratory fitness did not differ between those with and without elevated amyloid (t=-0.37(81), p=.710). Greater improvements in cardiorespiratory fitness predicted better performance on cognitive tests including trailmaking test, Stroop test, and digit symbol substitution test, which did not differ by amyloid status. Elevated amyloid levels predicted lower cognitive scores in logical memory, space relations, and identical pictures test. Our findings suggest PET imaging is a valid marker of cognitive performance in non-impaired older adults, and that this pre-clinical amyloid status did not reduce the cognitive benefits of exercise for those who improved in cardiorespiratory fitness. Exercise interventions hold promise for cognitive maintenance among pre-symptomatic older adults with elevated amyloid levels. Finally, results highlight the importance of evaluating multiple cognitive domains which are associated differently with exercise and amyloid status.

### FOLLOW-UP OF A 6-MONTH LOW-TO-MODERATE INTENSITY VIRTUAL-HOME EXERCISE PROGRAM TO PREVENT FALLS

Machiko R. Tomita,[1] and Nadine M. Fisher[1], *1. University at Buffalo, Buffalo, New York, United States*

The aim of this study is to determine 3-month post effects of and adherence to a 6-month virtual-group exercise at home (V-GEAH) program, which offered low-to-moderate intensity exercise to community-dwelling older adults with past falls. The V-GEAH program converted solitary exercise to group exercise connecting participants via web-conference technology. A treatment group (n=25, 60 – 90 years old) exercised three times a week for 30-45 minutes each session. The program achieved 84.4% – 93.3% adherence, reducing fall risks. This study measured falls, balance confidence, lower extremity muscle strength and endurance, gait speed, stride length, and activities of daily living, and compared with baseline and posttest (at 6 months) data using Repeated measures ANOVA with contrasts. During the follow-up period, 40% of the treatment group exercised 2+ times/week (Adherer, or A), and 60% did 1 time/week or less (Non-adherer or NA). Half of NA joined a community exercise group and the rest did not do exercise due to pain in various body parts. None of A fell while 26.7% of NA and 20% of C did. At follow-up, the control group (C, n=25) showed no change or significant decline from posttest for all measures. A maintained gains made in the intervention period in all measures, but NA significantly lost strength in hamstrings, hip abduction, and quadriceps and hamstring endurance. These results indicate that low-to-moderate intensity exercise and technology use for providing visual instruction, regular monitoring and evaluation, and environments to increase participants' accountability are elements for successful home-based exercise programs.

### OUTCOMES OF FIT & STRONG! PLUS: A GROUP EXERCISE AND WEIGHT LOSS PROGRAM TO TREAT OSTEOARTHRITIS

Andrew DeMott,[1] and Susan Hughes[1], *1. University of Illinois at Chicago, Chicago, Illinois, United States*

Overweight older adults with osteoarthritis (OA) face increased risk for disability; however, no evidence-based programs target weight and OA simultaneously. Fit & Strong! (F&S!) is an 8-week evidence-based exercise program for persons with OA that improves lower extremity (LE) strength and mobility out to 18 months. F&S! Plus, a weight loss version of F&S! was tested against standard F&S! in a comparative effectiveness trial. Two and six-month trial outcomes were previously presented, this session will present maintenance outcomes at 12 and 18 months. This trial randomized 413 participants, 210 to F&S! and 203 to F&S! Plus. The mean sample age was 67.9, 86% female, and 92% African American. At 12 months, significant and marginally significant between-group differences favoring F&S! Plus were seen in several outcomes, including weight (p=.049), BMI (p=.04), waist circumference (p=.004), LE physical function (p=.09), 6-minute distance walk (mobility) (p=.08), 30-second chair stands (LE strength) (p=.46), and anxiety & depression (p=.08). At 18 months, only LE strength remained significantly improved for the F&S! Plus group (p=.045), however several within-group improvements remained statistically significant for both groups out to 18 months, including weight, BMI, waist circumference, LE pain & physical function, 30-second chair stands (LE strength), anxiety & depression, and self-efficacy for weight-management. F&S! Plus showed significant improvements over standard F&S! for several outcomes at the conclusion of the intervention (2 months) and many were maintained out to 12 months, with LE strength continuing to 18 months. Both groups showed significant within-group improvements out to 18 months.

### RELATIONSHIP OF CARDIOPULMONARY EXERCISE TESTING AND FIELD WALKING TESTS IN MILD-MODERATE ALZHEIMER'S DEMENTIA

Dereck L. Salisbury,[1] and Fang Yu[1], *1. University of Minnesota School of Nursing, Minneapolis, Minnesota, United States*

The purpose of this study was to investigate relationships among peak exercise parameters on 6-minute walk (6MWT) and shuttle walk tests (SWT), and laboratory-based cardiopulmonary exercise testing (CPET). These relationships have been established in cardiopulmonary patient populations, but not in community-dwelling older adults with mild-moderate Alzheimer's dementia (AD). This study is a cross-sectional analysis of the baseline data of 6MWT, SWT, and CPET from the FIT-AD Trial (n=88: 49 males [76.6 {7.0} years and MMSE 21.5{3.5}] and 39 females [77.3 {6.5} years and MMSE 22.1 {3.4}]). Peak values for each test included heart rate (HR), systolic blood pressure (SBP), and rating of perceived exertion (RPE). Peak oxygen assumption (VO2) was measured in the CPET. Peak walking distance (PWD) was measured for the 6MWT and SWT. CPET produced significantly higher peak HR (118.7 [17.5] vs. 106 [22.8] vs. 106 [18.8] bpm), RPE (16 [2.1] vs. 12 [2.3] vs. 11 [2.1]) and SBP (182 [23.7] vs. 156 [18.9] vs. 150 [16.9] mmHg) compared to the SWT and 6MWT respectively. PWD on SWT (240.4 [128.1] m) and 6MWT (364.3 [108.5] m) significantly correlated with peak VO2 (17.0 [4.3]ml/kg/min) on CPET (r=.44 and r=.43) respectively. Correlations of peak VO2 and PWD on SWT in persons with AD are considerably lower than what is seen for persons with cardiopulmonary diseases. This lower correlation seen in our sample may be due to shorter PWD on walking tests. Future research should focus how mobility affects correlation of peak values on these tests.

### RESPONDERS TO A PHYSICAL ACTIVITY COACHING PROGRAM IN COPD

Huong Q. Nguyen,[1] Amy Liu,[1] Janet Lee,[1] and Anny Xiang[1], *1. Kaiser Permanente Southern California, Pasadena, California, United States*

Rationale: Physical inactivity is associated with worse outcomes in COPD. There remains a critical gap regarding the real-world effectiveness of improving physical activity (PA) in large representative samples of older adults with COPD and its impact on hospitalizations. Methods: A pragmatic randomized trial was conducted to determine the effectiveness of a 12-month home-based physical activity coaching intervention (Walk On!, WO) compared to standard care (SC) in 2,707 patients at high risk for COPD exacerbations. The WO intervention included collaborative monitoring of steps, semi-automated step goal recommendations, individualized reinforcement, and peer/family support. This is a subgroup analysis of 321 patients who were randomized to WO and participated in the program, matched to SC patients based on their propensity scores (PS). Multivariate cox proportional hazards models were used to determine differences in all-cause hospitalizations in the 12-months following randomization. Results: WO patients with low PS (n=160) were matched to 888 low PS SC patients and 161 WO patients with high PS (n=161) were matched to 405 high PS SC patients. Characteristics of the cohort were: age: 72±10; 54% females; 74% Caucasian; FEV1% predicted: 61±23. WO-low PS patients had lower risk of all-cause hospitalizations compared to SC-low PS patients [HR:0.69, (95%CI, 0.50, 0.96), P=.03]. WO-high PS patients did not have significantly lower hospitalization risk compared to SC-high PS patients [HR:0.87, (95%CI, 0.64, 1.20), P=.40]. Conclusions:

Patients with COPD who had a lower propensity to participate in the physical activity coaching intervention had lower hospitalization risks compared to those with a higher participation propensity.

### SESSION 3180 (PAPER)

### FACTORS RELATED TO FAMILY CAREGIVING

### AN OLDER CARER, BUT STILL A CHILD: UNEXPECTED IDENTITIES OF OLDER ADULTS CARING FOR A NEAR-CENTENARIAN PARENT

Oscar Riberio,[1] Typhanie Macedo,[2] and Liliana Sousa[1], *1. University of Aveiro - Department of Education and Psychology | Center for Health Technology and Services Research (CINTESIS.UA), Aveiro, Portugal, 2. University of Aveiro - Department of Education and Psychology, Aveiro, Portugal*

According to the National Alliance for Caregiving (2015), the typical definition of an older adult caregiver points to a 79-year-old white female who cares for a close relative due to a long-term physical condition; in such cases the care-recipient is often a spouse, an adult child or a sibling, but not a parent. This cross-sectional qualitative study explores the experience of a group of fifteen children in their 70s who are main care-providers of their parents (mean age 98; range 95-105); it focuses on their overall caregiving experience with a particular emphasis on how they feel being still a child at such an advanced age. In-depth interviews were conducted and analyzed for recurrent themes using thematic analysis. Main findings revealed that although being in an overall positive experience, often socially exalted (proudness of having a parent alive/being in an unique situation), none had expected to be holding the identity of a child at their current age. These children's views of their ageing self were strongly embedded in their caregiving role, and some reported losing awareness of their own age and a mixture of feelings of being simultaneously a child, a parent and a grandparent. Role captivity (late life prison) and disregarding self-care shaped their current situation in terms of caregiving burden and altered life plans as older adults. This study adds to the limited available knowledge on very old caregiving dyads, and raises awareness on how personal and family identity may be shaped in older age by unforeseen family dynamics.

### HEALTH CARE PROVIDER ATTITUDES ABOUT INTEGRATING FAMILY CAREGIVERS INTO CLINICAL ENCOUNTERS

Joan M. Griffin,[1] Rachel Havyer,[2] Karen Schaepe,[2] Catherine Riffin,[3] and Lauren R. Bangerter[2], *1. Mayo Clinic, Rochester, United States, 2. Mayo Clinic, Rochester, Minnesota, United States, 3. Weill Cornell Medicine, New York, New York, United States*

The presence of family caregivers in clinical encounters is becoming more common with the aging of the US population and the continued shift of care responsibilities from health professionals in clinical settings to family caregivers at home. Patients accompanied to clinical encounters by caregivers are more likely to be older, sicker, and have lower health literacy. Research shows, however, that providers often do not initiate any caregiver participation and when they do, conversations center on relaying technical medical information rather than preferences and capacity to provide caregiving assistance. Little is known about provider perceptions of engaging caregivers in clinical encounters. Using data from 20 semi-structured interviews with physicians from primary and specialty care, we identified 3 inter-related themes about engaging caregivers in clinical encounters: 1) ambivalence about caregivers' role in clinical encounters; 2) trepidation about posing questions directly to caregivers; and, 3) beliefs that systemic barriers exist that inhibit integration of caregivers. Providers, especially in primary care encounters, chiefly view caregivers as sources of supplemental information or for absorbing or reinforcing clinical instructions for care at home. Providers also voiced concerns about the ethics of assessing caregiver capacity to provide assistance to the patient without having clinical authority to treat or adequate resources to provide to caregivers. Finally, providers identified structural barriers, including time constraints, for integrating caregivers into the clinical care team. Findings provide insight into provider attitudes on the caregivers' role, a perspective that is essential for understanding opportunities and challenges for implementing caregiver interventions in clinical settings.

### PROVIDING AND RECEIVING FAMILY CARE ACROSS THE LIFE COURSE: INSIGHTS FROM THE OLDEST OLD

Taylor Patskanick,[1] Julie Miller,[2] Chaiwoo Lee,[1] and Lisa D'Ambrosio[2], *1. Massachusetts Institute of Technology, Cambridge, Massachusetts, United States, 2. MIT AgeLab, Cambridge, Massachusetts, United States*

Unprecedented longevity comes with an increased need for providing and receiving care. A 2015 report estimated 39.8 million adults in the United States provided unpaid care to an adult in 2014 (NAC & AARP). Previous research has focused disproportionately on experiences of providing care to older adults, but little has explored experiences of providing care and receiving care among the oldest old. Adults aged 85 and older are likely to have provided care to an adult family member at some point in their lives, but now may be receiving care themselves. The presentation will report on findings from a mixed methods study investigating the experiences of providing and receiving care across the life course among a sample of the "oldest old." Data draw from focus groups and a survey with the MIT AgeLab Lifestyle Leaders, a bimonthly panel study of adults ages 85 and older. Findings suggest the Lifestyle Leaders had extensive experience providing care, particularly in older age. They most often cared for family members with long-term physical or cognitive conditions. Opinions on learning new technologies to help with caregiving and robot caregivers were mixed. The majority of the Lifestyle Leaders received regular help with at least one care task regardless of household composition or living situation. Many reported help had improved their health, but they felt like a burden to their caregivers. Even in later life, the Lifestyle Leaders had few ideas about who might take care of them if they needed care in the future.

### SUPPORTING FAMILY CAREGIVERS THROUGH PATIENT TRANSITIONS FROM HOSPITAL TO HOME: THE CARE ACT AND BEYOND

Joan M. Griffin,[1] Diane Holland,[2] Lauren R. Bangerter,[2] Cory Ingram,[2] Ellen Wild,[2] Erin Taylor,[2] Rachel Gentes,[2] and Catherine Vanderboom[2], *1. Mayo Clinic, Rochester, United States, 2. Mayo Clinic, Rochester, Minnesota, United States*

To date, nearly 40 U.S. states have passed the Caregiver Advise Record and Enable (CARE) Act to support family caregivers during transitions from hospital to home. These laws require hospitals to: 1) record designated family caregivers' name in a patient medical records during a hospital stay; 2) inform the caregiver when the patient will be discharged; 3) provide education and instructions on care tasks needed for post-hospital discharge. Transitions from hospital to home are often fraught with adverse event risk and poor continuity of care, especially for rural patients with progressive life-limiting conditions. The CARE ACT has potential to address healthcare system factors that are sometimes attributable to poor transitions in care. Our presentation focuses on preliminary findings from an intervention that provides teaching, guidance, and counseling to caregivers caring for an individual receiving palliative care in the hospital and transitioning home to a rural setting. Findings suggest that the CARE ACT may be vital, but not fully sufficient, for successful transitions. Additional targets for caregiver interventions to improve transitions for this subset of very ill patients include: 1) attend to caregivers' medical and non-medical needs which may impact their capacity to provide care; 2) advise clinical teams to communicate truthfully about prognosis and likely outcomes when creating post-hospital plans of care; 3) encourage caregivers to identify and engage services early in the transition; 4) urge caregivers to draw on multiple sources for social support. Findings can help optimize the CARE Act implementation, particularly in states with large rural populations.

### THE ASSOCIATION OF LONELINESS AND CHRONIC CLINICALLY SIGNIFICANT PAIN AMONG AFRICAN AMERICAN AND WHITE OLDER ADULTS

David Camacho,[1] Denise Burnette,[2] Maria P. Aranda,[3] and Ellen Lukens[1], *1. Columbia University, New York, New York, United States, 2. Virginia Commonwealth University, Richmond, Virginia, United States, 3. University of Southern California, Los Angeles, California, United States*

Loneliness and pain are significant public health problems in later life, yet limited research has examined how these factors interact among racially diverse older adults. Guided by the Biopsychosocial Model of Pain, we used data from Waves 2 and 3 of the National Social Life, Health, and Aging Project to investigate the relationship between loneliness and chronic pain among 1,102 African-American and White adults aged 50 and over. Using logistic regression analyses, our final models considered demographics, physical and mental health, functioning, medication, health behaviors and social factors. Approximately 32% of African Americans and 28% of Whites reported chronic loneliness. Compared to Whites African-Americans were 2.5 times more likely to experience chronic pain. Among White participants, loneliness was not associated with chronic pain; however, the interaction of being African-American and lonely was associated with decreased odds of chronic pain in main and gendered analyses. African American women were 4 times more likely than White women to report chronic pain. Our results address the objectives of the National Pain Strategy (2016) to elucidate the experiences of chronic pain among diverse elders in the US. Future work should seek a deeper understanding of loneliness and chronic pain among African Americans elders and how cultural dynamics may help explain our counter intuitive findings (e.g., "Superwoman Schema").

### THE INFLUENCE OF RECESSION HARDSHIPS ON THE PSYCHOSOCIAL WELL-BEING OF SANDWICHED AND FILIAL CAREGIVERS

Barbara Hodgdon,[1] and Jen D. Wong[1], *1. The Ohio State University, Columbus, Ohio, United States*

The 2007-2009 U.S. Great Recession impacted the lives of many families, and it has been documented that multigenerational households in the U.S. increased by 10% during this period. Given the vulnerability of providing care to multiple generations, there is a need to examine the influence of recession hardships on sandwiched caregiving and psychosocial well-being in the context of more normative caregiving (e.g., filial caregiving). Informed by the life course perspective, this study assessed the impacts of types of family caregiving (sandwiched and filial caregivers) on psychosocial well-being (e.g., affect, environmental mastery, and social actualization) and the moderating role of recession hardships (e.g., job loss, foreclosure). Sandwiched and filial caregivers (N=127; Mage=53; SD=11.02) from the Refresher Cohort of the Midlife in the United States (MIDUS) Survey provide information on demographics, recession hardships, family caregiving, and well-being. Results from regression analyses showed that sandwiched caregivers exhibited lower levels of positive affect and environmental mastery than filial caregivers. Moderation analyses showed that filial caregivers with lower recession hardships exhibited lower positive affect and social actualization when compared to sandwiched caregivers with lower recession hardships. Filial caregivers with lower recession hardships exhibited lower positive affect and social actualization than sandwiched and filial caregivers with greater recession hardships. These results illustrate the complexity of family caregiving in that providing care to multiple generations does not necessarily translate to lower levels of well-being. Study findings have the potential to inform programs that may promote sandwiched caregivers' well-being and support filial caregivers navigating financial disruptions.

### SESSION 3185 (SYMPOSIUM)

### HARNESSING THE NETWORK OF GERONTOLOGY IN HIGHER EDUCATION TO IMPROVE RECRUITMENT STRATEGIES

Chair: Jacqueline Eaton, *University of Utah, Salt Lake City, Utah, United States*
Discussant: Margaret B. Neal, *Portland State University, Portland, Oregon, United States*

While the workforce requires applicants trained to meet the needs of an aging population, many gerontology programs find recruiting students a challenge. Barriers to enrollment vary and are influenced by disciplinary silos, miscomprehension of terminology (such as "gerontology"), vague career paths, and limited resources. Program directors are challenged to identify innovative and new strategies that market training opportunities at a variety of levels and across colleges and departments. In an effort to harness the power of networks, it is our goal that this symposium fosters discussion in order to improve our communal approach to increase the number of individuals prepared for careers in aging. This symposium examines the recruitment experiences and challenges from the perspective of gerontology programs at four universities. Fruhauf (Colorado State University) explores the importance of university-community engagement in support of gerontology programs. Fauth and Liu (Utah State University) describe effective recruitment strategies for an interdisciplinary gerontology certificate housed within a department of Human Development and Family Studies. Eaton (University of Utah) presents the outcomes from a targeted initiative to increase early undergraduate enrollment in an interdisciplinary online gerontology minor. Finally, Yorgason (Brigham Young University) shares a systematic approach to leverage resources and fundraising that successfully increased program enrollment and faculty involvement throughout campus. Dr. Margaret B. Neal, an experienced educator and former director of the Institute on Aging at Portland State University, will facilitate discussion surrounding how we can mobilize our networks to strengthen, innovate, and expand approaches to recruitment in gerontology.

### THE ROLE OF ENGAGEMENT IN SUPPORTING AND LEVERAGING GERONTOLOGY AND GERIATRIC PROGRAMS

Christine Fruhauf[1], *1. Colorado State University, Fort Collins, Colorado, United States*

For nearly 20 years, gerontology and geriatric administrators and faculty have been challenged by managing "tough times" related to low enrollment and reduced or limited funding for their programs. At the same time, the aging population continues to increase and the need for highly trained individuals to work with and on behalf of older adults are needed in all sectors of the workforce. In this paper, I will build on previous empirical and theoretical work from AGHE and GSA Fellows as I integrate personal experience from my fifteen years at a land-grant university, whereby I serve as the coordinator of our undergraduate gerontology minor. In particular, in an effort to uplift the 2019 conference theme, I will organize my thoughts from the lens of university-community engagement, as I describe how to best harness networks to support and leverage gerontology and geriatrics programs.

### RECRUITMENT TO THE GERONTOLOGY CERTIFICATE: STRATEGIES FROM UTAH STATE UNIVERSITY

Elizabeth B. Fauth,[1] and Yin Liu[1], *1. Utah State University, Logan, Utah, United States*

There is a workforce shortage in age-related fields, in both medical and non-medical, and professional and nonprofessional employment levels. Jobs in aging-related fields are cited as growth careers, or in lists of "top ten growing jobs", yet academic training programs in gerontology are still facing low enrollment and other long-standing barriers. Utah State University offers an interdisciplinary gerontology certificate housed within the department of Human Development and Family Studies (HDFS). Recruitment to the certificate is most effective from two "feeder" classes: Adult Development and Aging (in HDFS), and via a guest lecture in Community Health (in Kinesiology and Health Sciences). Face-to-face meetings with academic advisors in other departments (e.g. Nutrition) has resulted in referrals to the certificate program. Office hours and drop-ins from interested students falls on the certificate coordinator, but is essential in recruitment. In sum, face-to-face recruitment is time intensive, but significantly more effective than online/print material.

### GROWING AN ONLINE GERONTOLOGY MINOR AT THE UNIVERSITY OF UTAH

Jacqueline Eaton[1], *1. University of Utah, Salt Lake City, Utah, United States*

The Gerontology Interdisciplinary Program at the University of Utah has a long history of offering certificates (48 years) and a Master of Science degree (25 years). Relatively new to our program is the minor in gerontology, first available in 2013. This paper will describe efforts to grow this program, barriers to enrollment, and plans for expansion. In 2018, the program developed a targeted strategy to increase student awareness of and enrollment in the minor. First, we focused on newly enrolled pre-nursing students through new student orientation presentations and registration support. The introductory course within the minor jumped in enrollment from 6 students in Fall of 2017 to 40 in Fall 2018. Students taking courses in the minor received follow-up support and instructions mid-semester to ease matriculation. Enrollment in the minor increased by 140%. Next, we plan to apply these strategies to a broader number of colleges and departments throughout campus.

### A SYSTEMATIC APPROACH TO SPREADING THE WORD ABOUT GERONTOLOGY TRAINING OPPORTUNITIES IN A UNIVERSITY SETTING

Jeremy B. Yorgason[1], *1. Brigham Young University, Provo, Utah, United States*

Training university students to work in professional gerontology settings is extremely important during an era when the number of older adults is increasing due to the Baby Boom cohort entering their later years. Efforts to reach students are critical given budget and enrollment challenges. Some university students find gerontology resources and training on their own, yet gerontology programs can do much to help students know of opportunities. In this paper, I will share methods that the gerontology program at my university has used to reach out to students and faculty across campus to encourage students to study gerontology. In the last 3 years, student enrollment in this gerontology minor has grown from 65 students housed in 3 colleges, to 275 students housed in 7 colleges. Faculty involvement has grown from a 7-faculty committee, to 61 faculty affiliates. The roles of university resources and fundraising will also be discussed.

### SESSION 3190 (SYMPOSIUM)

### INTEGRATION OF OBJECTIVE AND SUBJECTIVE EXPERIENCES OF HEALTH IN OLD AGE

Chair: Shannon T. Mejia, *University of Illinois, Urbana-Champaign, Champaign, Illinois, United States*
Co-Chair: Johanna Drewelies, *Humboldt University Berlin, Berlin, Germany, Germany*
Discussant: Cynthia Berg, *University of Utah, Salt Lake City, Utah, United States*

Life-span perspectives on adult development and aging highlight the processes of understanding and acting to support one's own health and well-being. Within this framework, subjective measures offer insight into the experience of objective measures of health. This symposium brings together four papers that highlight the overlap and discrepancy between subjective and objective measures of health and how these are shaped by individual development in different contexts and phases of adult development over various timescales. Mejía and colleagues consider the intraindividual dynamics of older adults' perceived and actual fall risk. Using 30 days of subjective and objective balance assessment, they illustrate individual differences in how awareness of fall-risk relates to physical activity on that day. Koffer and Kamarck use data from the Pittsburgh Healthy Heart Project to examine how reactivity of cardiovascular response and affective experience to daily experiences of stress changes with age. Drewelies and colleagues consider how objective aging feels. Using data from the Berlin Aging Study II, they examine how chronological and subjective age are linked to biological age in older adults. Gonzalez and colleagues use self-reported and behavioral data from an in-context biosocial research lab to comment on the theoretical and methodological implications of integrating objective and subjective measures of experience. The discussion by Berg integrates the four papers, highlights their theoretical and methodological contributions, and considers challenges and opportunities for subjective and objective inquiries of older adults' experiences of health.

### THE DAILY BALANCE PROJECT: AN INQUIRY INTO THE INTRAINDIVIDUAL DYNAMICS OF PERCEIVED AND ACTUAL FALL RISK

Shannon T. Mejia,[1] Katherine Hsieh,[2]
Jason Fanning,[3] and Jacob Sosnoff[4], *1. University of Illinois, Urbana-Champaign, Champaign, Illinois, United States, 2. University of Illinois, Urbana-Champaign, Urbana, Illinois, United States, 3. Wake Forest University, Winston-Salem, Illinois, United States, 4. University of Illinois, Urbana-Champaign, Urbana, Illinois, United States*

An accurate understanding of one's abilities and limitations allows adaptive response to the challenges that are faced in daily life. However, older adults may over or under estimate their actual abilities. The Daily Balance Project examined the intraindividual dynamics of older adults' perceived balance with objective measures of balance and physical activity. For 30 consecutive days, following a comprehensive fall risk assessment, 20 older adults rated their balance confidence (Activities Balance Confidence scale) at that moment and then performed five standardized balance assessments measured via smartphone accelerometer held to their chest. Physical activity was measured with an activity monitor. Baseline measurements of fall risk differentiated the extent of intraindividual variation and co-variation of balance and physical activity. For some participants, actual and perceived balance became more closely aligned as the study progressed. The implications of the findings for life-span perspectives on aging and fall prevention are discussed.

### LONGITUDINAL EVIDENCE FOR DISCREPANT CHANGES IN NEGATIVE AFFECT REACTIVITY AND BLOOD PRESSURE REACTIVITY WITH AGE

Rachel E. Koffer,[1] and Thomas W. Kamarck[1], *1. University of Pittsburgh, Pittsburgh, Pennsylvania, United States*

Both affective and blood pressure (BP) reactivity are associated with long term risk of chronic disease and mortality. Thus, understanding age-related changes in negative affect and BP responses to everyday demands is vital for promoting healthy aging. However, few studies have examined both psychological and BP reactivity simultaneously, which would provide more comprehensive understanding of regulatory processes at play. For the present study, 232 adults aged 50-70 years were assessed at baseline and 6 years later with ambulatory BP monitoring and momentary electronic diaries. Reactivity coefficients were output from multilevel models and used to test changes in negative affective and ambulatory BP reactivity to task demand, longitudinally. Results indicate that both systolic and diastolic BP reactivity increase with age, while negative affect reactivity does not change with age. Results are discussed in the context of life course theories of role strain and role changes and socioemotional theories of aging.

### FEELING YOUNGER, BEING YOUNGER: ASSOCIATIONS BETWEEN BIOLOGICAL AGE AND SUBJECTIVE AGE IN OLDER ADULTS

Johanna Drewelies,[1] Ilja Demuth,[2] Sandra Duezel,[3]
Gizem Hueluer,[4] Lars Bertram,[5]
Elisabeth Steinhagen-Thiessen,[2] and Denis Gerstorf[6], *1. Humboldt University Berlin, Berlin, Germany, Germany, 2. Charité – Universitätsmedizin, Berlin, Berlin, Germany, 3. Max Planck Institute for Human Development, Berlin, Berlin, Germany, 4. University of Zurich, Zurich, Zurich, Switzerland, 5. University of Lübeck, Lübeck, Schleswig-Holstein, Germany, 6. Humboldt University, Berlin, Berlin, Germany*

Subjective age has been shown to be a strong predictor of both subjective and objective health outcomes. However, little is known about the extent to which individuals' subjective age is related to one's biological age or not. In our study, we examine how subjective age relates to biological age—a comprehensive multi-indicator biomarker algorithm aggregating information of metabolic, cardiovascular, inflammatory, lung, and kidney functioning. We used data from 996 older adults from the Berlin Aging Study II (mean age = 68.40 years, range 60 to 85, 52% women) who provided information about chronological age, biological age, and subjective age. Multiple regression analyses revealed that subjective age was associated with biological age among older women with and without controls for age, education, and physician-observed comorbidity, but not older men. Our findings suggest that subjective age might provide unique insights into how biological age differs across adulthood and contributes to overall health.

### DISTINGUISHING SUBJECTIVE EXPERIENCE FROM OBJECTIVE FACTORS IN DECISION MAKING AND PERCEIVED EFFORT

RIchard Gonzalez,[1] Patricia Abbott,[1] James Ashton-Miller,[1] and Jacqui Smith[1], *1. University of Michigan, Ann Arbor, Michigan, United States*

We use self-reported and behavioral data from the HomeLab to comment on the theoretical and methodological implications of integrating objective and subjective measures of experience. To illustrate, we will focus on two domains that vary in the nature of objective and subjective measurements examined. One domain will be decision making where subjective measures include subjective probability and utility and the respective objective measures include probability and actual outcomes. The second domain will be activities of daily living where the subjective measure is perceived effort and the objective measures include various data from sensor such as EDA (arousal) and EMG (muscle contraction). The presentation will discuss the benefits of conducting such research in a realistic standardized context such as the HomeLab, which is a fully connected, fully functioning apartment set up as a standardized lab in order to study activities of daily living.

### SESSION 3195 (SYMPOSIUM)

### INTERVENING IN THE LONGEVITY NETWORK

Chair: Alexander Mendenhall, *University of Washington, Seattle, Washington, United States*
Co-Chair: George L. Sutphin, *University of Arizona, Tucson, Arizona, United States*

This session will focus on interventions to delay biological aging with the goal of in increasing lifespan and healthspan.

### TARGETING TRYPTOPHAN-KYNURENINE METABOLISM TO EXTEND LIFESPAN AND TREAT AGE-ASSOCIATED DISEASE

George L. Sutphin,[1] and George L. Sutphin[1], *1. University of Arizona, Tucson, Arizona, United States*

The kynurenine pathway, the major route for tryptophan catabolism, becomes dysregulated with age and in many age-associated pathologies in humans. Interventions targeting kynurenine metabolism are being pursued for neurodegeneration, cardiovascular disease, and chronic kidney disease. By manipulating kynurenine pathway enzymes and metabolites, we have extended lifespan up to 40% in Caenorhabditis elegans. Our most promising single target is the metabolite 3-hydroxyanthranilic acid dioxygenase (3HAA). Elevating physiological 3HAA by directly supplementing 3HAA or inhibiting the enzyme 3HAA dioxygenase (HAAO) extends worm lifespan by ~30% while reducing oxidative stress by directly degrading hydrogen peroxide. In rodents, anti-inflammatory activity of 3HAA improves outcomes in models of cardiovascular disease, asthma, and autoimmune encephalomyelitis. We are now beginning to validate our C. elegans work in mice and investigating a mechanistic model in which 3HAA acts to extend healthy lifespan by slowing age-associated accumulation of oxidative damage and repressing chronic inflammation.

### INCREASED LIFESPAN THROUGH ALTERED GCN4 / ATF-5 IN S. CEREVISIAE AND C. ELEGANS

Mark McCormick,[1] Christine Robbins,[1] Olivia Heath,[1] and Marissa Westenskow[1], *1. University of New Mexico Health Sciences Center, Albuquerque, New Mexico, United States*

In a whole-genome screen for deletions that increase lifespan in S. cerevisiae, we identified increased Gcn4 signaling as a mediator of increased lifespan. Gcn4 is a nutrient-responsive transcription factor whose entire pathway is functionally conserved from yeast through humans. Accumulation of uncharged tRNAs has been shown to upregulate Gcn4, and its mammalian ortholog, ATF4. Here we demonstrate that chemical inhibitors of tRNA synthetases significantly extend lifespan in both yeast and the nematode C. elegans, in a dose- and Gcn4-dependent manner.

### LEVERAGING DRUG-DRUG INTERACTIONS FOR PHARMACOLOGICAL EXTENSION OF HEALTHY LIFESPAN

Jan Gruber[1], *1. Yale-NUS, Singapore, Singapore*

Traditional approaches aimed at delaying or preventing age-dependent diseases view each disease as a distinct entity, resulting from separate pathophysiological chains of events. However, it is becoming increasing clear that even in adult animals there remains significant plasticity in terms of ageing trajectories and lifespan, suggesting that targeting ageing processes directly may be a promising alternative strategy. However, to date effects of even the most efficacious pharmacological interventions are smaller than those of ageing mutations, even when targeting the same ageing pathways. Interestingly, it has been shown that simultaneously targeting multiple ageing pathways can result in lifespan benefits that are synergistic (more than additive). We have recently shown that dramatic lifespan and healthspan extension can also be archived by leveraging interactions between drugs targeting distinct subsets of the gene-regulatory network controlling ageing of C. elegans. These interventions were highly efficacious, even when animals were treated only as adults.

### UNDERSTANDING AGING IN TERMS OF PHYSIOLOGICAL STATES

Alexander Mendenhall,[1] Nikolay Burnaevskiy,[1] Soo Yun,[1] and Bryan Sands[1], *1. University of Washington, Seattle, Washington, United States*

The "network" of homeostatic systems fails in distinct ways in individual isogenic animals during the aging process. We believe that understanding these distinct physiological states, the transitions between them, and how they relate to homeostatic system functions will allow us to better affect change in the aging process. Work in yeast showed that fixing an initial system failure, loss of vacuole acidification capacity, could increase cellular lifespan. Here we showed how the long-lived physiological state conferred by high expression of the hsp-16.2 promoter based lifespan/penetrance biomarker correlates with differences in the expression of other genes, and the structure and function of lysosomes. We found that vacuole acidification failure is not a major initial proximal cause of aging in C. elegans – at least not in their intestine cells.

### SESSION 3200 (PAPER)

### MARRIAGE, DIVORCE, AND SEXUAL HEALTH

### AFRICAN AMERICAN FAITH LEADERS IN FLINT, MICHIGAN, FACILITATING ACCESS TO SEXUAL HEALTH INFORMATION

Charles R. Senteio[1], *1. Rutgers University School of Communication and Information, New Brunswick, New Jersey, United States*

In 2006, to respond to the HIV pandemic, the Flint-based YOUR Center collaborated with the National Coalition of Pastors' Spouses to create an HIV Awareness and Prevention manual for African American faith communities. In 2006 this manual was transformed into Your Blessed Health (YBH), a community-level education and prevention intervention that provides HIV/STI health information to medically underserved communities throughout Michigan. Since then, YBH has trained over 200 faith leaders across 9 denominations in over 80 FBOs across Michigan to help disseminate information concerning the community-level impact of HIV. The YBH impact on congregants and community members has been detailed in peer-reviewed articles and presentations; however, the involvement of the faith leaders themselves has not been described. To address this gap in the literature, we conducted semi-structured interviews with African American faith leaders representing 20+ congregations in Flint who participated in YBH and welcomed the intervention into their congregations. Preliminary results show that faith leaders, whose median age is 65, embraced their role as conduits to health information in the interest of ministering to the "whole person". They remain committed to educating themselves, and their congregants, to providing health information for various health and wellness issues. Detailing this impact is critical because community-health education and promotion literature suggests that older adult faith leaders can facilitate access to sexual health information through faith-based organizations, and may possess untapped potential as sources of health information across various health conditions. Older adult faith leaders are vital sources of information for medically underserved communities.

### EXPLORING THE CAREGIVING CAREER: VARIATIONS IN SPOUSES' CONCERNS

Marie Y. Savundranayagam,[1] and Kaitlyn Terrana[1], *1. Western University, London, Ontario, Canada*

Caregiver identity theory posits that family caregivers' relationship identity changes across five phases of the caregiving career. As the career unfolds, the relationship identity changes from one comprised primarily of the spousal role (phase 1) to one comprised equally by spousal and caregiver roles (phase 3), and one comprised primarily by the caregiver role (phase 5). This study investigated whether spouses/partners' most important concerns about caregiving varied across the caregiving career. Participants included 135 caregivers of spouses/partners with a chronic condition. They were asked to identify their most important concern related to caregiving, along with demographic questions. Thematic analyses of their concerns yielded eight themes focused on the caregiver or dyad. Caregiver focused themes included burden, providing best possible care, worry about ability to care, physical health, financial concerns, and needing/managing help. Dyadic themes were communication and relational deprivation. Differences across the caregiving career were found in terms of ranked proportion of concerns. The most common concerns in phase 1 were equally distributed across communication, needing/managing help, and providing best possible care. Phase 2's most common concern was providing the best possible care. Phase 3's most common concern was worry about ability to care. The most common concerns in phase 4 were equally distributed across burden and worry about ability to care. Phase 5's most common concern was burden. Findings reveal there are differential concerns across the caregiving career that align with a greater focus on relational factors earlier in the career and caregiver burden later in the career.

### PERSONAL GROWTH AFTER GRAY DIVORCE: A LONGITUDINAL VIEW ON THE DISSOLUTION OF LONG-TERM MARRIAGES

Claudia Recksiedler,[1] Katharina Loter,[2] Oliver Arránz Becker,[2] and Pasqualina Perrig-Chiello[3], *1. German Youth Institute, Munich, Germany, 2. University of Halle-Wittenberg, Halle, Germany, 3. University of Bern, Bern, Switzerland*

Going through a divorce is one of the most stressful life events across the life course with far-reaching ripple effects for individuals' physical, mental, and often financial well-being. For some individuals, however, coping with marital dissolution and adapting to new social roles may lead to considerable gains in their reorientation in life and stimulate their personal development. This is captured in the concept of personal growth, which has been studied primarily in the context of becoming widowed, and through a cross-sectional lens. In our study, we track trajectories of personal growth after ending a long-term marriage of at least 15 years. We focus on divorce among adults in their second half of life because rates of grey divorce have been on the rise since the 1980s, yet longitudinal research on personal growth after marital dissolution remains sparse. We further examine whether trajectories of personal growth vary by gender, SES, reasons of divorce (e.g., sexual infidelity or drifted apart), and social support. Data stem from a Swiss panel on intimate relationships and marital dissolution among long-term married individuals conducted between 2012 and 2016 ($N = 530$). Random-effects group-specific growth curve models yielded similar—and slightly decreasing—trajectories of personal growth for divorced women and men over time. However, highly significant gender differences indicated that women showed higher levels of personal growth over the whole observation period. Because gender differences seemed to be fueled by social capital rather than economic assets, we discuss these results through the lens of a linked-lives perspective.

### RELIGIOUS DIFFERENCE, MARITAL SATISFACTION, AND PSYCHOLOGICAL WELL-BEING OF CHINESE MIDDLE-AGED AND OLDER ADULTS

Jia Li,[1] and Qi Wang[2], *1. The Hong Kong Polytechnic University, Hong Kong, Hong Kong, 2. University of Hong Kong, Hong Kong, Hong Kong*

Objectives: Religion plays an important role in people's individual and interpersonal life. Spousal religious difference is a potential risk factor of marital satisfaction, which will further influence people's psychological well-being. This study aims to explore the associations between spousal religious differences, marital satisfaction, and psychological well-being of Chinese middle-aged and older adults. We also investigated the gender differences in the captioned associations. Method: We adopted a sample of 1285 adults aged 45 and above from the China Health and Retirement Longitudinal Study (CHARLS). We conducted descriptive statistics, multiple regression models and a path analysis based on a general structural equation model (GSEM). Results: Spousal religious difference was only associated with wives' marital satisfaction. Marital satisfaction was associated with depression and life satisfaction for both genders, and wives' marital satisfaction had a stronger association with life satisfaction than husbands'. Wives' marital satisfaction mediated the relationship between spousal religious difference and their psychological well-being, including depression and life satisfaction. Discussion: This study calls for more further research on the individual and interpersonal outcomes of religiosity in middle-aged and older adults. Gender differences should be paid attention to in academic research, service provision and clinical settings.

### THE TRANSFORMATION OF LATE-LIFE SINGLEHOOD: EMERGENCE OF THE SOCIETY OF DIVORCEES

Torbjorn Bildtgard,[1] and Peter Öberg[2], *1. Stockholm University, Stockholm, Sweden, 2. University of Gävle, Gävle, Sweden*

More than four decades ago Lopata coined the concept "society of widows" to describe the gendered reality of late life singlehood, where widowed women were excluded from coupled social life and had to depend on other widows for social integration. We have coined the concept "society of divorcees" to describe the changing reality of late life singlehood. Swedish, American and EU census data and a national survey to Swedes 60-90 years old (n=1225; response rate 42%). Results show that more people enter later life as divorcees or become divorced at a high age. Among Swedes 60+ divorcees outnumber widowed people, and the incidence of late life divorce has more than doubled since the millennium in what has been called the grey divorce revolution. Many other Western countries follow the same demographic trend, posing important questions about late life singlehood. Based on two Swedish studies we will show that the structure of the late life single community is becoming less gender skewed as a consequence of the emerging society of divorcees, and that in this society relationship careers are increasingly complex, attitudes to repartnering increasingly liberal and partner sanctification seldom an issue. We conclude by discussing the consequences of the emerging society of divorcees for late life support structures.

### SESSION 3205 (SYMPOSIUM)

### MULTIPLE FACTORS INFLUENCING SUCCESSFUL AGING

Chair: Heather Fuller, *North Dakota State University, Fargo, North Dakota, United States*

Empirically-based theories on successful aging have emphasized the multidimensional nature of aging well, including physical health and functioning, cognitive and emotional well-being, and social connectedness and engagement (e.g., Rowe & Kahn, 1987, Depp & Jeste, 2006). Yet, the field is still continuing to discover, deeply characterize, and better understand the biopsychosocial mechanisms through which varying social, physical, or cognitive activities may influence unique domains of successful aging. The current symposium builds on this growing body of research by addressing factors supporting successful aging across multiple dimensions of well-being and among a diversity of samples ranging from urban to rural, West Coast to Midwest, and community populations to professional athletes. Webster and Antonucci examine the links between social engagement and successful aging among affordable senior housing residents. They found that more frequent participation in social activities was associated with increases in life satisfaction over time. Toyama and Fuller examine how social engagement and health affect aging well, finding that older adults' subjective health plays a more important role than objective health in maintaining social integration over time. Similarly, Turner describes the role of social networks and religiosity for health outcomes among aging NFL athletes. Finally, Casaletto and colleagues examined mechanisms underlying cognitive wellness as an aspect of successful aging. They found that engagement in both physical and cognitive activities independently support brain health and cognitive reserve in late-life. Taken together, these presentations provide a diverse and broad perspective on how varying factors influence the multiple dimensions of successful aging.

### LINKS BETWEEN SOCIAL ENGAGEMENT AND SUCCESSFUL AGING AMONG AFFORDABLE SENIOR HOUSING RESIDENTS

Noah J. Webster,[1] Noah Webster,[1] and Toni Antonucci[1], *1. University of Michigan, Ann Arbor, Michigan, United States*

We report on a study designed to integrate the convoy model of social relations with the successful aging model. The combined approach draws on the natural resources of interpersonal relations to promote successful aging thus minimizing risk of disease and disability, enhancing\maintaining mental and physical functioning, and facilitating continued engagement in life. We describe the MacHouse Affordable Housing Study, a field experiment conducted in an affordable senior housing community. The experiment involved a resident driven intensive intervention program designed to increase social activities and engagement. Based on an extensive needs assessment and interviews with the resident council as well as individual residents a program of activities was developed. This included group meals, an exercise program that included a walking group and an exercise class, communal gardens, computer classes and resident outings. Results indicate that more frequent participation in these activities was associated with increased life satisfaction over time.

### WHICH ASPECTS OF HEALTH PREDICT LATE-LIFE SOCIAL INTEGRATION OVER TIME?

Masahiro Toyama,[1] Masahiro Toyama,[1] and Heather R. Fuller[1], *1. North Dakota State University, Fargo, North Dakota, United States*

Associations between late-life social integration and health have been found to be reciprocal. The present study focuses on the direction of health predicting social integration as it is not yet fully understood how different aspects of health may affect social integration. Using two-wave data from a community-based sample (N = 413, mean age 80 at baseline), the present study investigates whether depressive symptoms, chronic health conditions, functional limitations, and self-rated health independently predicted multiple dimensions of social integration over two years. The results of multiple regression and path analyses indicated that self-rated health was the most consistent predictor for social integration over time as the other health measures predicted no or fewer dimensions of social integration. Subjective perception of health appeared to have greater implications for social integration over time than more objective health symptoms/conditions. These findings highlight the important role of subjective health for maintaining late-life social integration.

### RELIGIOUS ATTENDANCE, SOCIAL SUPPORT, AND SELF-RATED HEALTH IN FORMER NATIONAL FOOTBALL LEAGUE ATHLETES

Robert W. Turner II,[1] Robert Turner,[2] Amanda Sonnega,[3] Tim Cupery,[4] Evelyn Bush,[5] Teri Rosales,[6] and James S. Jackson[6], *1. The George Washington University, Washington, District of Columbia, United States, 2. Center on BioBehavioral Health Disparities Research, Duke University, Durham, North Carolina, United States, 3. Institute for Social Research, Ann Arbor, Michigan, United States, 4. Fresno State, Fresno, California, United States, 5. Fordham, Bronx, New York, United States, 6. University of Michigan, Ann Arbor, Michigan, United States*

Concern exists about the health and well-being of football players, yet little research exists on the psychosocial risk and protective factors of NFL athletes' well-being. This study assesses the role of religious attendance, social support, and self-rated health in former NFL athletes. Data comes from a stratified, random sample of 1,063 former NFL players. A set of nested linear regression models evaluated the relationship between self-rated health status and two indices of social support (family and friends) and attendance at religious services. Frequent attendance at religious services ($\beta=0.19$, $p<.01$), support from family ($\beta=0.06$, $p<.05$), and support from friends ($\beta=0.06$, $p<.01$) are positively and significantly related to better self-rated health. The ability to get out of the house did not affect these associations. However, the pain symptoms index fully accounted for any positive effect of family support and religious attendance in self-rated health.

### PHYSICAL VERSUS COGNITIVE ACTIVITY FOR SUCCESSFUL AGING: BRAIN MAINTENANCE OR COGNITIVE RESERVE?

Kaitlin Casaletto,[1] Kaitlin B. Casaletto,[2] Judy Pa,[3] Sarah Tom,[4] Miguel Arce-Renteria,[4] Amal Harrati,[5] Nicole Armstrong,[6] and Laura Zahodne[7], *1. University of California, San Francisco, San Francisco, California, United States, 2. UCSF, San Francisco, California, United States, 3. University of Southern California, Los Angeles, California, United States, 4. Columbia University, New York, New York, United States, 5. Stanford University, Stanford, California, United States, 6. NIH-NIA, Bethesda, Maryland, United States, 7. University of Michigan, Ann Arbor, Michigan, United States*

Mechanisms by which physical (PA) and cognitive (CA) activities promote healthy cognitive aging are unknown. We examined independent contributions of PA and CA to "brain maintenance" (MRI markers of brain integrity) versus "cognitive reserve" (better cognition than predicted by brain integrity) in two independent samples of non-demented older adults (UCSF n=344; UCD n=482). In UCSF, only PA was positively associated with white matter (WM) integrity, while CA attenuated the relationship between WM and cognition. This pattern suggests PA supports brain maintenance, while CA contributes to cognitive reserve. In UCD, CA was positively associated with total gray matter volume; PA was positively associated with age-related WM integrity, and attenuated the association between WM and cognition. This indicates that both PA and CA support brain maintenance, with PA more strongly related to cognitive reserve. There may be preferential, but overlapping pathways by which PA and CA maintain age-related brain and cognitive health.

### SESSION 3210 (SYMPOSIUM)

### MULTIMORBIDITY, MENTAL HEALTH, AND TERMINAL DECLINE IN LATER LIFE

Chair: Dorina Cadar, *University College London, London, United Kingdom*
Co-Chair: Lucy Stirland, *University of Edinburgh, Centre for Clinical Brain Sciences, Edinburgh, United Kingdom*
Discussant: Graciela Muniz Terrera, *University of Edinburgh, Edinburgh, United Kingdom*

The close interlink between physical and mental health outcomes has long been recognised in gerontological research. Mental-physical comorbidities – the presence of at least one physical health long term condition, and at least one mental health-related long term condition are common in older age individuals. Numerous studies have shown a positive association between the prevalence of multimorbidity and age so, as the population of older individuals in developed nations continues to grow, multimorbidity is likely to become increasingly higher in ageing populations. A major goal in current gerontological neuropsychology and neuroepidemiological research is to better understand how interindividual differences in cognitive and mental health in old age emerge. Cognitive reserve (a marker of brain resilience) may come into play when facing stressors that affect cognitive decline and mental health, such as suffering from chronic diseases. We present data from three different longitudinal studies of ageing i) the Lothian Birth Cohort of 1921, ii) PREVENT and iii) the English Longitudinal Study of Ageing from the United Kingdom. These studies are ideally placed to address key research questions related to mental ageing, psychological health, terminal decline and their determinants. We explored the following objectives: 1) to investigate the association between an increasing number of chronic physical conditions, medication and mental disorders 2) to assess the role of childhood intelligence and education on the terminal decline in later life 3) to investigate the associations between different markers of cognitive reserve and dementia

### ASSOCIATIONS BETWEEN CHRONIC PHYSICAL CONDITIONS, MEDICATIONS AND MENTAL HEALTH IN AN AGING COHORT

Lucy Stirland,[1] Lucy E. Stirland,[2] Tom C. Russ,[3] Craig W. Ritchie,[4] and Graciela Muniz Terrera[5], *1. University of Edinburgh, Centre for Clinical Brain Sciences, Edinburgh, United Kingdom, 2. Centre forClinical Brain Sciences, University of Edinburgh, Edinburgh, Scotland, United Kingdom, 3. Alzheimer Scotland Dementia Research Centre, Edinburgh, Scotland, United Kingdom, 4. Centre for Dementia Prevention, Edinburgh, Scotland, United Kingdom, 5. Centre for Clinical Brain Sciences ,University of Edinburgh, Edinburgh, Scotland, United Kingdom*

Older people increasingly live with multiple chronic conditions and medications. We explored their interactions with mental health in the PREVENT Dementia study participants. Using logistic and linear regression, we investigated the association between increasing self-reported chronic physical conditions and current medications with self-reported depression and anxiety disorder, and scores on the Center for Epidemiologic Studies Depression (CES-D) scale and Spielberger State-Trait Anxiety Inventory (STAI) state subtest. Among 210 participants, each additional condition was associated with increased odds of depression (adjusted OR, 95% CI: 1.41, 1.11-1.80; P=0.005) and anxiety (1.71, 1.35-2.21; P<0.001). Each additional medication was associated with depression (1.36, 1.07-1.73; P=0.010) but not anxiety. For each additional condition, CES-D scores increased by 0.62 (0.04-1.20, P=0.035) and for each medication, by 0.66 (0.12-1.21, P=0.017). There was no significant association between conditions or medications and STAI scores. These findings provide crucial information on the future brain health of these individuals.

### MARKERS OF COGNITIVE RESERVE AND DEMENTIA INCIDENCE IN THE ENGLISH LONGITUDINAL STUDY OF AGEING

Pamela Almeida-Meza,[1] Pamela Almeida-Meza,[2] Andrew Steptoe,[2] and Dorina Cadar[2], *1. University College London, London, United Kingdom, 2. Behavioural Science and Health, University College London, London, England, United Kingdom*

We investigated a multi-faceted index of cognitive reserve (CR), in relation to dementia incidence over 15 years follow-up in a representative sample of the English population. Data were 12,293 participants aged 50+ from the English Longitudinal Study of Ageing, dementia-free at baseline. CR was derived as a composite measure of education, occupation and leisure activities using a standardised questionnaire. From the overall sample, 603 participants developed dementia. Higher CR levels were associated with lower dementia risk (medium CR: HR 0.72, 95% CI 0.59-0.88, p=0.002 and high CR: HR 0.73, 95% CI 0.58-0.90, p=0.005) compared with lowest levels. These associations were independent of sex, marital status, wealth, smoking, depressive symptoms and poor physical health. Further individual analyses of CR sub-components showed that leisure activities (HR 0.72, 95% CI 0.56-0.91, p=0.007) were linked with reduced dementia risk, contributing to a higher CR and increased overall mental resilience.

### THE LONG ARMS OF CHILDHOOD INTELLIGENCE AND EDUCATION ON TERMINAL DECLINE: EVIDENCE FROM LOTHIAN BIRTH COHORT 1921

Dorina Cadar,[1] Dorina Cadar,[2] Annie Robitaille,[3] Alison Pattie,[4] Ian J. Deary,[5] and Graciela Muniz Terrera[6], *1. University College London, London, United Kingdom, 2. Behavioural science and Health, University College London, London, England, United Kingdom, 3. Department of Psychology, Université du Québec à Montréal, montreal, Quebec, Canada, 4. School of Philosophy, Psychology and Language Sciences, university of Edinburgh, edinburgh, Scotland, United Kingdom, 5. Centre for Cognitive Ageing and Cognitive Epidemiology, University of Edinburgh, Edinburgh, Scotland, United Kingdom, 6. Centre for Clinical Brain Sciences, University of Edinburgh, Edinburgh, Scotland, United Kingdom*

We investigated the heterogeneity of cognitive trajectories at the end of life by assigning individuals into groups according to their cognitive trajectories prior to death. Data were from the Lothian Birth Cohort of 1921. Growth mixture modelling was employed to identify groups of individuals with similar trajectories on the Mini-Mental State Examination in relation to time to death, accounting for childhood intelligence, education, hypertension, diabetes and cardiovascular disease. Two distinct groups of individuals (classes) were identified: a smaller class (18%) of individuals whose MMSE scores dropped linearly with about 0.5 points per year, and a larger group (82%) with stable scores across the study period. Childhood intelligence was associated with an increased probability of belonging to the stable class of cognitive functioning prior to death. These findings support a protective role of childhood intelligence, a marker of cognitive reserve, against the loss of cognitive function prior to death.

### SESSION 3215 (SYMPOSIUM)

### NEUROPROTECTIVE EFFECT OF APOE2: EVIDENCE AND IMPLICATION FOR COGNITIVE AGING

Chair: Paola Sebastiani, *Boston University, Department of Biostatistics, Boston, Massachusetts, United States*
Discussant: Nalini Raghavachari, *National Institutes of Health, Bethesda, Maryland, United States*

Apolipoprotein E is a glycoprotein mediator and regulator of lipid transport and uptake. The APOE4 allele has been associated with higher risk of Alzheimer's disease and of mortality, but the protective effect of the less prevalent APOE2 allele is less well established. This symposium will bring together experts in epidemiology, molecular biology, neurology and systems biology to clarify the role of APOE2 in longevity and cognitive health, to describe molecular targets of APOE alleles, and to suggest mechanisms of protection conferred by APOE2 using IPSc derived systems. Dr Seshadri will show the strong association between APOE2 and human longevity using data of 38,537 individuals of European ancestry. Mr Sweigart will examine longitudinal trajectories of cognitive function in participants of the Long Life Family Study and the New England Centenarian Study and show that carriers of the homozygote genotype of APOE2 have a significant slower rate of decline of their cognitive function compared to carriers of other genotypes. Dr Monti will present an association analysis of APOE genotype data with 4137 human proteins in serum of 222 New England Centenarian Study participants. The analysis discovered a signature of 16 proteins that associated with different APOE genotypes, and replicated in 3 independent studies. Dr. Ellerby will summarize her recent analyses that used transcription analysis of isogenic iPSCs with APOE2 and APOE4 homozygote genotypes differentiated into inhibitory GABAergic neurons to show that E2 inhibitory GABAergic neurons regulate genes involved in nuclear division, DNA integrity and DNA damage checkpoint.

### SURVIVAL ADVANTAGE OF APOE2

Sudha Seshadri[1], *1. UT Health San Antonio, San Antonio, United States*

Apolipoprotein E is a glycoprotein mediator and regulator of lipid transport and uptake. The APOE-ε4 allele has been associated with higher risk of Alzheimer's disease and of mortality, but the effect of the less prevalent APOE-ε2 on survival remains elusive. We aggregated data of 38,537 individuals of European ancestry (mean age 65.5 years; 55.6% women) from six large population-based cohorts to determine the association of APOE-ε2, with survival in the general population. During a mean follow-up of 11.7 years, 17,021 individuals died. Compared with homozygous APOE-ε3 carriers, APOE-ε2 carriers were at lower risk of death (hazard ratio,95% confidence interval: 0.94,0.90-0.99; P=1.1*10-2), whereas APOE-ε4 carriers were at increased risk (HR 1.17,1.12-1.21; P=2.8*10-16). Risk was lowest for homozygous APOE-ε2 (HR 0.89,0.74-1.08), and highest for homozygous APOE-ε4 (HR 1.52,1.37-1.70). Results did not differ by sex. The association was unaltered after adjustment for baseline LDL or cardiovascular disease. Larger, multiethnic collaborations are ongoing.

### REDUCED COGNITIVE DECLINE WITH THE APOE ε2/ε2 GENOTYPE IN THE LONG LIFE FAMILY STUDY AND NEW ENGLAND CENTENARIAN STUDY

Benjamin Sweigart,[1] Benjamin Sweigart,[2] Stacy L. Andersen,[3] Stephanie Cosentino,[4] Nicole Schupf,[5] Thomas T. Perls,[3] and Paola Sebastiani[2], *1. Boston University, Department of Biostatistics, Boston, Massachusetts, United States, 2. Department of Biostatistics, Boston University School of Public Health, Boston, Massachusetts, United States, 3. Department of Medicine, Boston University School of Medicine, Boston, Massachusetts, United States, 4. Department of Neurology, Columbia University, New York, New York, United States, 5. Department of Epidemiology, Columbia University Mailman School of Public Health, New York, New York, United States*

A growing body of evidence has suggested a protective effect on cognition of the ε2 allele of APOE. To determine if APOE ε2 is associated with protection against cognitive decline, we analyzed repeated measures of the Telephone Interview for Cognitive Status (TICS) from 2,933 Long Life Family Study subjects and 679 New England Centenarian Study subjects using a multivariable linear mixed effects model. The median age at first TICS administration was 73 (interquartile range [IQR] 64, 83). Subjects had a median of 3 TICS assessments (IQR 2, 4) and a median follow-up time of 5.0 years (IQR 2.9, 7.0). Carriers of the ε2/ε2 genotype had a significantly slower rate of decline in TICS score compared to the ε3/ε3 reference group (-0.05 points per annum for ε2/ε2 carriers compared with -0.15 points for ε3/ε3 carriers, p-value for difference 0.017). These results support a protective effect of the ε2 allele.

### GENOMIC ANALYSIS OF HUMAN ISOGENIC APOE IPSC-DERIVED INHIBITORY GABAERGIC NEURONS

Lisa Ellerby,[1] Sicheng Song,[2] Sean Mooney,[2] Stephen Scheeler,[3] and Swati Naphade[3], *1. The Buck Institute, Novato, California, United States, 2. University of Washington, Seattle, Washington, United States, 3. Buck Institute for Research on Aging, Novato, California, United States*

Isoforms of ApoE modify the risk for Alzheimer's disease (AD), cardiovascular disease and are also associated with exceptional longevity. Specifically, the ApoE E2 allele is associated with lower risk of AD-related neurodegeneration and with exceptional longevity, while the E4 allele is a major risk factor for AD and is associated with higher levels of Abeta deposition in the brain. The mechanisms modulating extended lifespan/healthspan mediated by E2 compared to E3 and E4 genotypes are not clear. One hypothesis is that the E2 allele is neuroprotective and compensates for neuronal dysfunction induced by misfolded protein expression in aging and disease. To understand the molecular basis of the protective effect of the E2 allele we performed transcriptomic analysis of isogenic iPSCs with E2E2 and E4E4 genotypes differentiated into inhibitory GABAergic neurons. Our analysis revealed that ApoE2 inhibitory GABAergic neurons regulate genes involved in nuclear division, DNA integrity and DNA damage checkpoint.

### A SERUM PROTEIN SIGNATURE OF APOE GENOTYPES IN CENTENARIANS

Stefano Monti,[1] Stefano Monti,[2] Paola Sebastiani,[3] Anastasia Gurinovich,[4] Toshiko Tanaka,[5] Lori L. Jennings,[6] David J. Glass,[6] and Thomas T. Perls[7], *1. Boston University, Boston, Massachusetts, United States, 2. Division Of Computational Biomedicine, Boston University School of Medicine, Boston, Massachusetts, United States, 3. Department of Biostatistics, Boston University School of Public Health, Boston, Massachusetts, United States, 4. Bioinformatics Program, Boston University, Boston, Massachusetts, United States, 5. Translational Gerontology Branch, National Institute on Aging, Baltimore, Maryland, United States, 6. Novartis Institutes for Biomedical Research, Cambridge, Massachusetts, United States, 7. Geriatrics Section, Department of Medicine, Boston University School of Medicine and Boston Medical Center, Boston, Massachusetts, United States*

The discovery of treatments to prevent or delay Alzheimer's disease is a priority. The gene APOE is associated with cognitive change and late onset Alzheimer's disease, and epidemiological studies have shown that the e_2 allele of APOE has a neuroprotective effect, and it is associated with increased longevity. We correlated APOE genotype data of 222 New England Centenarian Study participants, including 79 centenarians, 84 centenarian offspring and 55 carriers of APOE

e_2, with aptamer-based serum proteomics (SomaLogic technology) of 4783 human proteins corresponding to 4137 genes. We discovered a signature of 16 proteins that associated with different APOE genotypes, and replicated the signature in 3 independent studies. We show that the protein signature tracks with gene expression profiles in brains of late onset Alzheimer's disease vs. healthy controls. Finally, we show that seven of these proteins correlate with cognitive function changes. Therefore, targeting APOE e_2 molecularly may preserve cognitive function.

### SESSION 3220 (PAPER)

### PAIN AND PAIN MANAGEMENT

### AN EMOTION REGULATION THERAPY FOR LATER-LIFE PAIN: EVIDENCE OF EARLY TREATMENT EFFECTS

Emily Petti,[1] Dimitris Kiosses,[1] Lisa Ravdin,[1] Charles Henderson,[2] Lauren Meador,[1] Julianna Maisano,[1] and Cary Reid[1], *1. Weill Cornell Medicine, New York, New York, United States, 2. Cornell University, Ithaca, New York, United States*

Chronic pain (CP) is a common, morbid, and costly disorder in older adults. Guidelines encourage clinicians to employ non-pharmacologic therapies for its management, but current psychological interventions (e.g., CBT for pain) have modest treatment benefits and their effects are largely unknown in older cognitively impaired adults. We developed PATH-Pain, an emotion regulation therapy focused on reducing negative emotions and augmenting positive emotions. PATH-Pain is appropriate for use by older adults with CP, negative emotions, and a wide range of cognitive functioning. Treatment consists of 8 weekly individual sessions followed by 4 monthly booster sessions. One hundred older adults (ages 60+) with CP (≥ 3 months) and at least mild-to-moderate levels of negative emotions (per the Positive and Negative Affect Schedule) were randomized to receive PATH-Pain versus Usual Care (UC). Cognitive screening revealed that 44 participants were cognitively intact (Montreal Cognitive Assessment (MoCA) score ≥26), while 56 evidenced mild-to-moderate cognitive impairment (MoCA=16-25). Participants completed follow-ups at 5 (n=89) and 10 weeks (n=84), while 24-week assessments are ongoing. Examination of the treatment × time interaction in a repeated-measures mixed model indicate the presence of treatment effects. PATH-Pain (vs. UC) participants experienced significant reductions in pain intensity ($p<0.044$) and pain-related disability ($p<0.003$). Reductions in pain-related disability score were more pronounced among cognitively impaired individuals. The PATH-Pain group also demonstrated significant reductions in emotional suppression ($p<0.019$) and depression ($p<0.009$) scores. These results suggest that PATH-Pain is an effective treatment for the management of pain in cognitively intact and cognitively impaired older adults.

### CHRONIC PAIN CONTRIBUTES TO INJURIOUS FALLS IN COMMUNITY-DWELLING OLDER ADULTS

Yurun Cai,[1] Suzanne Leveille,[1] Ling Shi,[1] Tongjian You,[1] and Ping Chen[1], *1. University of Massachusetts Boston, Boston, Massachusetts, United States*

Fall injuries are a leading cause of death among older adults, and chronic pain has been identified as a fall risk factor. However, the potential impact of chronic pain on injurious falls is unknown. This prospective study examined the relation between chronic pain and injurious falls in a 4-year follow-up of community-dwelling older adults. The MOBILIZE Boston study recruited 765 older adults aged ≥70y living in the Boston area. Pain characteristics, including pain severity, pain interference, and pain location, were measured at baseline using the Brief Pain Inventory subscales and a joint pain questionnaire. Musculoskeletal pain distribution was categorized as "no pain", "single site pain", or "multisite pain". Injurious falls were ascertained in telephone interviews following reports of falls on the monthly fall calendar postcards. The overall rate of injurious falls was 35/100 person-years. Negative binomial models, adjusting for sociodemographics, BMI, chronic conditions, mobility difficulty, analgesic and psychiatric medications, and depression, showed that pain interference and pain distribution, but not pain severity, independently predicted injurious falls. Participants in the highest third of pain interference scores had a 53% greater risk of injurious falls compared to those in the lowest pain interference group (adj. IRR=1.53, 95% CI: 1.15, 2.05). Older adults with multisite pain had a 50% higher risk of injurious falls than those without pain (adj.IRR=1.50, 95% CI: 1.16, 1.93). Risk of injurious falls related to pain was stronger among women than men. Research is needed to determine effective strategies to prevent fall injuries among older adults with chronic pain.

### DEVELOPING A RAI-MDS 2.0 BEHAVIOR-BASED PAIN ASSESSMENT SCALE FOR LONG-TERM CARE RESIDENTS WITH ADVANCED DEMENTIA

Jennifer A. Knopp-Sihota[1], *1. Athabasca University, Athabasca, Alberta, Canada*

In Canadian and many international long-term care (LTC) facilities, pain assessment frequently relies on data from the Resident Assessment Instrument – Minimum Data Set 2.0 (RAI-MDS). The RAI-MDS produces a two-item scale, measuring both pain frequency and pain intensity. This scale correlates well with self-reported pain in cognitively intact LTC residents, but despite repeated testing, is less valid for use in residents with more advanced cognitive impairment who are unable to self-report their pain. In this study we aimed to develop and validate a behaviour-based pain assessment scale for long-term care residents using data available in the RAI-MDS. To construct our initial scale, we reviewed the literature and compiled a list of observable indicators of pain (e.g., grimacing) and linked these with 28 similar items available in the RAI-MDS. Using Delphi techniques, we further refined this to 20 items. We then evaluated the psychometric properties of our scale using two independent, representative samples, of urban LTC residents in Western Canada. Exploratory factor analyses were conducted in sample one (n=16,282) and confirmatory factor analyses (CFA) were then conducted in sample two (n=15,785) in order to test, and confirm, our model. A two-factor solution was identified grouping RAI-MDS items into subscales 1) change in status (e.g., new onset restlessness) and 2) behaviours (e.g., crying). Commonly recognized model fit indices were acceptable suggesting the adequacy of the two-factor solution. Results provide preliminary support for the use of behavioural-based pain assessment scale using RAI-MDS data. Further evaluation and validation of our scale is warranted.

### DO WARMED BLANKETS CHANGE PAIN, AGITATION, MOOD, OR ANALGESIC USE AMONG NURSING HOME RESIDENTS?

Christine Kovach[1], *1. Ovation Communities, Milwaukee, Wisconsin, United States*

Warmed blankets have not been empirically tested for use in long-term care. The purpose of this study was to describe the use of warmed blankets in a nursing home setting and to determine if use was associated with changes in pain, agitation, mood, or analgesic use. Short-term measures were compared from baseline to post warmed blanket use and longer term differences were compared between those receiving warmed blankets and a randomly selected comparison group. Excluded from eligibility were those using a transdermal drug, with an acute injury, acute inflammatory process, multiple sclerosis, open skin wound, or other condition that could be worsened by superficial heat. Measures included the Revised Faces Pain Scale, PAIN-AD scale, the Brief Agitation Rating Scale, and from the electronic medical record one month measures pain complaints, pain severity, and analgesic use. Long-term measures were taken from the electronic medical record. Of the 141 eligible residents, 24.1% (n = 34) received a warmed blanket over the one month study period. There were statistically significant decreases in both pain level and agitation between baseline, 20 minutes after application, and the subsequent shift assessments ($p < .001$). There were also long-term changes in the number of pain complaints ($p = .040$), severity of pain complaints ($p = .009$), and prn analgesic use ($p = .011$). There were no statistically significant differences between the treated group and comparison group on any long-term measures. Warmed blankets are a low-cost intervention with a high potential for bringing comfort to nursing home residents.

### PAIN AMONG OLDER MEXICANS: FINDINGS FROM THE MEXICAN HEALTH AND AGING STUDY

Sadaf A. Milani,[1] Rafael Samper-Ternent,[1] Martin Rodriguez,[1] and Rebeca Wong[1], *1. University of Texas Medical Branch, Galveston, Texas, United States*

In Mexico, palliative care and pain relief was recently added to the essential health services offered through Seguro Popular. Pain is more frequent in older adults, a growing segment of this population, and is a major contributor to decreased quality of life and increased morbidity. However, Mexico only has enough opioid analgesics to treat 36% of those in need. We used logistic regression models to examine correlates of pain using data from the 2012 wave of the Mexican Health and Aging Study, which includes Mexicans aged 50 and older (n=13,727). Overall, 38.2% of individuals reported that they often suffered from pain. Those who reported pain were more likely to be female (OR: 1.56; 95% CI: 1.41, 1.72), insured (OR: 1.17; 95% CI: 1.03, 1.33), live in a semi-rural locality (OR: 1.18; 95% CI: 1.04, 1.34), report their health as fair or poor (OR: 2.99; 95% CI: 2.73, 3.29), be a past smoker (OR: 1.17; 95% CI: 1.06, 1.29), have at least one ADL limitation (OR: 2.58; 95% CI: 2.27, 2.93), report depression (OR: 2.19; 95% CI: 2.02, 2.37), or report arthritis (OR: 2.74; 95% CI: 2.45, 3.07). Those who did not report pain were more likely to be widowed or have higher education. Diabetes, stroke, and cancer were not significantly associated with pain. Given that Mexico does not have the resources to treat over half of individuals living with pain, understanding the high burden of pain in this population is important to inform interventions and improve quality of life.

### SESSION 3225 (SYMPOSIUM)

### PALLIATIVE AND END-OF-LIFE CARE TO THE LGBT COMMUNITY

Chair: Gary L. Stein, *Wurzweiler School of Social Work-Yeshiva University, New York, New York, United States*
Co-Chair: Cathy Berkman, *Fordham University-Graduate School of Social Services, New York, New York, United States*

The lesbian, gay, bisexual, and transgender (LGBT) community experiences discrimination and stigma in accessing health care and social services – including palliative, hospice, and long-term care – across cultures and countries. Health care providers may fail to recognize, acknowledge or address disparities in care. Providers and institutions may be uncomfortable with gender and sexuality issues, and often fail to inquire about sexual orientation and gender identity. It is estimated that there are approximately 2.7 million LGBT adults in the U.S. age 50 and older and approximately 1.1 million age 65 and older. In the UK, an estimated 5-7% of the population identify as LGBT; there are between 600,000 and 840,000 LGBT adults aged 65+. With the projected increased number of older adults and improved treatments that extend the life of seriously ill individuals, even greater numbers of LGBT older adults, and their families, will require palliative and end-of-life in the coming years. Researchers in the US and UK have found that LGBT older adults living in the community and in long-term care facilities experience inadequate, disrespectful, and abusive care due to their sexual orientation and gender identity status. They fear being open about their identities, not receiving equal or safe treatment, and having their family of choice and designated surrogates disrespected or ignored by health care staff. This symposium will describe the experiences of LGBT individuals and their family members, and compare commonalities and differences faced by LGBT communities in the US and UK in accessing palliative and end-of-life care.

### EXPERIENCE OF LGBT PATIENTS AND FAMILY WITH HOSPICE AND PALLIATIVE CARE

Gary L. Stein,[1] and Cathy Berkman[2], *1. Wurzweiler School of Social Work-Yeshiva University, New York, New York, United States, 2. Graduate School of Social Service-Fordham University, New York, New York, United States*

This study examines the degree to which hospice and palliative care staff observe or perceive inadequate, disrespectful, or abusive care to LGBT patients and family members. A cross-sectional study using an online survey completed by 865 providers, including social workers, physicians, nurses, and chaplains. Among respondents, 55%

reported that LGB patients were more likely to experience discrimination at their institution than non-LGB patients; 24% observed discriminatory care; 65% reported that transgender patients were more likely than non-transgender patients to experience discrimination; 20% observed discrimination to transgender patients; 14% observed the spouse/partner of LGBT patients having their treatment decisions disregarded or minimized; and 13% observed the spouse/partner being treated disrespectfully. Findings reported also include: institutional non-discrimination policy, staff training, intake procedures, and comfort in assessing LGBT status. Implications for future research, policy, and practice will be presented.

### ACCESS TO HEALTH CARE FOR LGBT PEOPLE

Katherine Bristowe[1], *1. King's College London, London, England, United Kingdom*

LGBT people have higher risk of life-limiting illnesses and unmet needs when facing advanced illness.The ACCESSCare research programme aims to improve health and social care for LGBT people. ACCESSCare-A explored experiences of 40 UK LGBT people facing serious illness. Discrimination, heteronormative assumptions, and insensitivity influenced whether individuals disclosed relationships to professionals and place-of-care decisions. Professionals must go beyond anti-discrimination to proactive inclusion: 10 evidence-based recommendations were developed to improve care for LGBT people. Our sister study in Zimbabwe explored the healthcare experiences of key populations (LGBTI people, sex workers). Access to healthcare was dependent on conforming to 'sexual norms' and care was negatively affected by professionals' attitudes to key populations. There are two ongoing research projects in the ACCESSCare programme: ACCESSCare-B, a mixed-methods population-based comparative study of LGB and heterosexual bereavement outcomes; and ACCESSCare-C a qualitative study to develop communication guidance for professionals supporting LGBT people facing serious illness.

### END-OF-LIFE EXPERIENCES AND CARE NEEDS IN THE LIVES OF OLDER LGBT PEOPLE

Kathryn Almack[1], *1. School of Health and Social Work University of Hertfordshire, Hatfield, England, United Kingdom*

The socio-cultural and legal position of LGBT citizens varies across nations. However, even in the most liberal countries, an historical legacy of stigma impacts on older LGBT people's access to care. This paper draws upon the qualitative strand of a two-year UK project exploring the end of life experiences and care needs of older LGBT people. (N = 60 in-depth interviews with LGBT participants aged 60+). Findings highlight that the majority of respondents reported ways in which they manage their personal networks to minimize any vulnerability to discrimination. In planning or needing end-of-life-care, respondents identify new 'layers' of decisions about disclosing or hiding their sexual or gender orientation; informed by past experiences and fears about discrimination or exclusion from service providers. In conclusion, older LGBT people's histories and a legacy of stigma have ongoing profound influences on the means of support available to them at the end of life.

### SESSION 3230 (PAPER)

### PERCEPTIONS, STEREOTYPES, AND QUALITY OF LIFE

### CARE DEPENDENCY: EXPERIENCES AND EXPECTATIONS OF COMMUNITY-DWELLING OLDER ADULTS

Jan S. Jukema,[1] Sharon Oude Veldhuis,[1] Jacqueline Van Alphen,[2] Jopie Jorritsma,[1] and Frits De Lange[3], *1. Saxion University of Applied Sciences, Deventer, Netherlands, 2. Saxion University of Applied Sciences, Zwolle, Netherlands, 3. Protestant Theological University, Groningen, Netherlands*

"Not to be a burden" is a common phrase used by community-dwelling older adults in discussing their dependency on others in care for their daily life. This attitude may lead to conflicts with relatives, neighbors, or professionals when in their opinion, care is necessary and, ultimately, may result in unmet care needs. The goal of this study is to gain a better understanding of how older adults experience their increased dependency on others and to contribute to the development of an ethic of care. Thirty-two participants of a larger research sample (n=64) from a descriptive qualitative research were purposefully selected, resulting in an equal distribution of the following variables: gender, living situation, living with or without partner, and having children or not. From a multiphase qualitative analysis with five researchers, including two senior citizens four themes emerged: (1) relationships in the context of care; (2) experiences with giving, receiving and asking for care; (3) future perspectives towards receiving and asking for care; and (4) actual practices of caregiving and receiving. Our study clarifies how community-dwelling older adults deal with the changes in their dependency on others. The study results highlight particular dynamics which appear, at least, partly in contrast with current policy regarding care at home. Moreover, it contributes to an empirical refinement of the concepts of dependency and interdependency in an ethic of care. Further studies are needed to clarify the influential factors on asking for care in diverse groups of older adults and the response from their network.

### DOMAIN-SPECIFIC AGE STEREOTYPES AND ADULTS' PERCEIVED FUTURE TIME EXPERIENCES

Han-Jung Ko,[1] and Yen-Pi Cheng[2], *1. Central Michigan University, Mount Pleasant, Michigan, United States, 2. Independent Scholar, San Jose, California, United States*

Experiencing ageism has been shown to affect older adults' outlook for the future (Barber & Tan, 2018). However, ageism is a multi-faceted construct in addition to positive and negative age stereotypes. In this study, we examined to what extent domain-specific age stereotypes are related to different aspects of a person's perceived future time experiences. A total of 646 participants (aged 18 to 83) were recruited from a U.S. mid-Western public university for an online anonymous survey. Age stereotypes were assessed in eight life domains of family, friends, religion, leisure, lifestyle, finance, work, and health (Kornadt & Rothermund, 2011). Future time experiences were assessed in four aspects, including perceived openness and concreteness of future, acceptance of life's finitude, preoccupation with the past, and experiences of obsolescence (Brandtstädter & Wentura, 1994). Age stereotypes in the family domain were associated with one's perceived openness and concreteness of future and preoccupation with the past. The religion and financial domains were both associated with perceived openness and concreteness of future and acceptance of life's finitude. Moreover, the older the participants were, the more they accepted life's finitude. Surprisingly, none of the domains significantly predicted one's perceived future experiences of obsolescence. Unlike the common finding that people hold negative age stereotypes due to physical and mental declines, age stereotypes in the health domain did not predict any aspects of the future time experiences. Our findings highlight the importance of distinguishing ageism across domains in order to improve one's perceived future time experiences.

### LATER-LIFE LIVING ARRANGEMENTS OF CHILDLESS AMERICANS: A LIFETABLE APPROACH

James Raymo,[1] Xiao Xu,[2] Jersey Liang,[3] BoRin Kim,[4] and Mary Beth Ofstedal[3], *1. University of Wisconsin-Madison, Madison, Wisconsin, United States, 2. Yale University, New Haven, Connecticut, United States, 3. University of Michigan, Ann Arbor, Michigan, United States, 4. University of New Hampshire, Durham, New Hampshire, United States*

The large body of research on living arrangements at older ages pays little attention to the growing population of childless men and women. We begin to fill this gap by using data from the Health and Retirement Study (HRS) over a period of 14 years (2000-2014) to describe the number of years between ages 65-90 that childless Americans live in four different living arrangements: alone, with spouse, with others, and in a nursing home. Because the number of childless HRS respondents is not large (n = 835 in 2000), we first estimate sex-specific single decrement life tables by race/ethnicity and by educational attainment. We then use Sullivan's method to calculate living arrangement-specific life expectancy for each group, thus providing a comprehensive descriptive portrait of sociodemographic differences in living arrangements across older ages for childless Americans. Preliminary results show that differences in living arrangement-specific life expectancy by race/ethnicity and educational attainment primarily reflect group differences in mortality. The proportion of later life spent in different living arrangements is generally similar across racial/ethnic groups and education levels. This stands in contrast to large racial/ethnic and educational differences documented in earlier studies of older Americans with at least one living child. Results also show that the proportion (and years) of later life spent living alone is substantial, especially for women (over 50%). We discuss the potential implications of these findings with reference to both projected trends in the childless population and research on associations between living arrangements and health of childless older Americans.

### QUALITY OF LIFE AND PERCEIVED NEEDS AMONG OLDER ADULTS RECEIVING LONG-TERM SERVICES AND SUPPORTS

Eleanor Rivera,[1] Karen Hirschman,[1] and Mary Naylor[1], *1. University of Pennsylvania, Philadelphia, Pennsylvania, United States*

Long term services and supports (LTSS) are vital for older adults with physical and cognitive disabilities. LTSS can be provided in settings such as nursing homes, assisted living, or via community-based services. The aim of this study is to describe the perceived needs for older adults new to LTSS, examine whether those needs are met in the first three months of LTSS, and determine the relationship with quality of life (QoL). This secondary analysis included data from 470 older adults new to LTSS (average age: 81, 71% female, 51% white, 35% black, 20% Hispanic.) The main outcome of QoL was measured using a single item ("How would you rate your overall quality of life at the present time?"). Perceived needs included supportive equipment devices, transportation, physical therapy, and social activities. Analyses at baseline and three months included t-tests, ANOVAs and simple regression modeling. LTSS recipient reported needs at baseline were: 29% supportive equipment, 31% transportation, 20% physical therapy, and 25% social activities. Those who reported needs at baseline had a lower QoL than those who reported no needs (for all). At three months reported needs decreased by an average of 6% (range: 3%-10%). QoL ratings were associated with changes in physical therapy and social activities needs at three months. The implications of these results related to LTSS recipients' QoL in the first three months of services, with emphasis on physical therapy and social activities needs, is an opportunity to be more person-centered in delivery of care.

### SESSION 3235 (SYMPOSIUM)

### REFRAMING AGING: PRACTICES AND MEASURES TO HARNESS THE STRENGTH OF INTERGENERATIONAL NETWORK TIES

Chair: Shannon E. Jarrott, *The Ohio State University, Columbus, Ohio, United States*
Co-Chair: Karen Hooker, *Oregon State University, Corvallis, Oregon, United States*
Discussant: Nancy Morrow-Howell, *Washington University, St. Louis, Missouri, United States*

GSA and other agencies have undertaken to "Reframe Aging" to reduce pervasive global ageism, which has been associated with negative attitudes towards and disinterest in working with older adults and poor health in old age. Familial and non-familial intergenerational contact, that is between members of non-adjacent generations, provides valuable opportunities to reduce ageism through relationships. However, much of the research on intergenerational relationships focuses on attitudes, usually those young people hold about older adults and aging. We propose that attention should shift towards measuring the relationship and giving voice to both younger and older partners. Presenters will address a range of intergenerational relationships focusing on their measurement as a means to advance the Reframing Aging initiative, including examples, challenges, and recommendations. Jarrott will provide an overview of the Reframing Aging initiative and address measurement of non-familial intergenerational relationship. This will be complemented by Pillemer's focus on how universities can reframe aging through intergenerational network ties, including the Cooperative Extension System. Mendoza and Fruhauf will shift attention to familial connections, focusing on grandparent-grandchild caregiving relationships. Turner, Hooker, and Jarrott will present new efforts to develop a validated theory- and evidence-informed measure of intergenerational relationships that can be completed by young and old persons. Discussant Morrow-Howell, past GSA President, will address how a long history of diverse intergenerational solutions can support efforts to reframe aging through enhanced measurement tools and strategies.

### NON-FAMILIAL INTERGENERATIONAL NETWORK TIES: MOVING THE NEEDLE ON MEASUREMENT

Shannon E. Jarrott[1], *1. The Ohio State University, Columbus, Ohio, United States*

GSA and other professional organizations recognize the threat that ageism poses to the country's health and welfare, from its youngest to oldest citizens. Reframing Aging involves communication and outreach strategies to inform the conversation about aging and its implications. Non-familial intergenerational relationships can support the Reframing Aging initiative. By fostering positive, intentional, and mutually beneficial interactions, intergenerational exchange can achieve a variety of individual, relational, and community goals. Measures of their impact should reflect these goals; unfortunately, intergenerational network ties are typically represented by measures of young people's attitudes towards older adults. Practitioners, researchers, policy makers, and funders need additional indicators to document the best practices and potential impact of non-familial intergenerational programs. Reflecting recent systematic surveys of measurements and 20 years of intergenerational research, the current paper addresses challenges of measuring non-familial intergenerational relationships and presents select measures appropriate for common network ties.

### REFRAMING AGING: UNIVERSITIES AS AN ENGINE FOR EVIDENCE-BASED INTERGENERATIONAL APPROACHES

Karl Pillemer[1], *1. Cornell University, Ithaca, New York, United States*

Societal changes are decreasing opportunities for youth to engage with older adults. Geographical mobility, the digital divide, and the growth of age-segregated communities for older people increase age segregation. The lack of interaction can lead to negative attitudes and stereotypes among young and older people. A solution is increasing meaningful contact between youth and older adults. This presentation proposes that higher education can play a unique role in reframing aging through intergenerational programs. Colleges and universities can integrate innovative bodies of research and practice, foster more rigorous research designs to study the effectiveness of intergenerational programs; and discover new ways to provide youth with needed skills and knowledge on how to interact with older adults. The national Cooperative Extension System is presented as a model for how evidence-based practice in intergenerational programs can be translated to communities, with a focus on collaborative program design, evaluation, and broader uptake.

### REFRAMING INTERGENERATIONAL FAMILIAL RELATIONSHIPS THROUGH THE STUDY OF GRANDPARENT-GRANDCHILD CONNECTIONS

Nancy Mendoza,[1] A. Nancy Mendoza,[1] and Christine A. Fruhauf[2], *1. The Ohio State University, Columbus, Ohio, United States, 2. Colorado State University, Fort Collins, Colorado, United States*

Intergenerational relationships include non-familial and familial connections. Common familial bonds exist between grandparents and grandchildren. Although grandparent-grandchild connections have over 40 years of research, measurement and design gaps remain. With this paper, we will address new approaches to examining grandparent and grandchild relationships in an effort to understand how this connection impacts our attitudes on aging. Specifically, we will discuss the opportunities of approaching such relationships from a longitudinal perspective. The grandparent-grandchild relationship can span close to 30 years, and yet knowledge of relationship stability and change between individuals in these family roles is limited. We will highlight the conference theme by presenting how social network analysis (SNA) applied to empirical data of grandparents raising grandchildren can reframe aging's network ties. Further, future research using SNA with grandchildren will be addressed as a way to build on previous work, extending our knowledge of intergenerational relationships from the family perspective.

### ADVANCING THE MEASUREMENT OF INTERGENERATIONAL CONTACT: BEYOND ATTITUDES TOWARD AGING

Shelbie Turner,[1] Karen Hooker,[1] and Shannon E. Jarrott[2], *1. Oregon State University, Corvallis, Oregon, United States, 2. Ohio State University, Columbus, Ohio, United States*

In our presentation, we will offer insights into our process of creating and validating a comprehensive theory- and evidence- informed measure of intergenerational contact that expands beyond the measurement of age-related attitudes. While attitudinal shifts are an important construct related to intergenerational contact and its impact on ageism, efforts to "Reframe Aging" require a more nuanced understanding of the mechanisms by which intergenerational contact can have positive impacts on individuals, families, and communities. Intergenerational contact is dynamic; it varies both between- and within- people, dyads, and places, as well as over time. Our measure includes quantity and qualities of intergenerational contact, including the extent to which the contact is between family vs. non-family members. Unlike existing measures of intergenerational relationship, ours reflects young persons' and older adults' intergenerational relationships. A psychometrically valid instrument of intergenerational contact is an essential first-step for determining how aging can be reframed through intergenerational interactions.

### SESSION 3240 (SYMPOSIUM)

### RE-IMAGINING LTC: QUANTITATIVE AND QUALITATIVE RESULTS OF A NATIONAL MODIFIED DELPHI STUDY, IMPLICATIONS AND NEXT STEPS

Chair: Rosalie A. Kane, *DIvision Health Policy & Management, School of Public Health. University of Minnesota, Minneapolis, Minnesota, United States*

Discussant: Howard Degenholtz, *University of Pittsburgh, Pittsburgh, Pennsylvania, United States*

In 11/2016 Robert and Rosalie Kane began a 3-round Delphi study to re-imagine long-term care (LTC), , which took as a starting premise that LTSS in the United States fails to comport to the values and preferences of consumers. The Delphi study is "modified" from more typical Delphi designs because of 1) a sample sizes over 100, 2) an unusually broad topic--optimal LTC systems if not constrained by existing programs, financial arrangements and regulations; and 3) incorporation of new sample at each round. Round 1 asked respondents to rate and add to a list of values important to LTC< but largely was an open-ended request for respondents' ideas, Round 2 was fielded in 6/2018 with all data collection completed by 11/2018 (the delay partly due to Robert Kane's sudden death on March 6, 2017 and also the time needed to analyze, summarize and present the complex and detailed responses to the first round). Round 3, to be fielded in 4/2019., will provide participants with the ratings of values, principles and programmatic building blocks at Round Two, and the open-ended comment of respondents in explanation of their ratings. Each Round is analyzed cross-sectionally and can be considered a separate "virtual town square." Ellen McCreedy and Rosalie Kane, respectively, present quantitative and qualitative results from the first two rounds. Discussants will each comment briefly from their perspectives as 1) state LTC policy developer,2) LTC university-based researcher; 3) consumer advocate, followed by audience and presenter discussion of the implications of the findings.

### QUALITATIVE FINDINGS AND THEMES IN REIMAGINING LTC: RESULTS ROUND 1 AND 2 OF THE NATIONAL DELPHI STUDY

Rosalie A. Kane,[1] and Audrey Workman[2], *1. DIvision Health Policy & Management, School of Public Health. University of Minnesota, Minneapolis, Minnesota, United States, 2. U of MN School of Public Health, Minneapolis, Minnesota, United States*

From Round 1 we developed programmatic building blocks, which we classified as: housing suggestions; services suggestions; housing and/or technology heavy suggestions; policy or regulation suggestions;new philosophical approaches; and long-range social engineering. Besides the quantitatively ratings of the importance of each building block, respondents explained what they liked and disliked about each.. They frequently commented that environments rich in design features, amenities and activities would not be practical for low-income people. Respondents felt that some ideas would not be suitable for people with dementia because they would be insufficiently protected. Principles that seemed to be incompatible could be highly endorses; e.g., the principle that we prioritize people staying in their own homes and a principle that frail elderly persons living along should relocate to group residential settings to avoid social isolation. This paper concludes with a list of areas for further discussion by work groups.

### QUANTITATIVE RE-IMAGINING OF LTC : RESULTS FROM ROUNDS 1 AND 2 OF A NATIONAL DELPHI STUDY

Ellen McCreedy,[1] and Caleb Hoover[2], *1. Brown University, School of Public Health, Providence, Rhode Island, United States, 2. Hennepin County Medical Center, Mineapols, Minnesota, United States*

At Round , 110 participants answered an open text question about how they would redesign LTC if starting fresh without regulatory or financial constraints.. They also rated a list of values as to whether they were reflected in the respondents' suggestions. From analysis of Round 1 open text, principles for LTC were extracted and 20 programmatic building blockscreated. At Round 2, respondents rated the importance of the original value list (after Round 1 results were shared) and rated the principles and building blocks. This paper presents those findings and highlights inconsistencies in results: for example, both a universal LTC and a means-test benefit were endorsed. Participants preferred the term Long-Term Services and Support for the subject matter but no strong term was the favorite for service users. Alternate ways of displaying endorsement of individual items (mean value, different score cutoffs) resulted in the same most popular and least popular items.

### REFLECTIONS FROM THE PERSPECTIVE OF A STATE POLICY MAKER

LaRhae Knatterud[1], *1. Minnesota Department of Human Services, St. Paul, Minnesota, United States*

From her perspective as Director of Systems Transformation, Minnesota Department of Human Services, and her role as planner and policy analyst in state agencies, this presentation briefly comment on the implications of the findings. Using thepory of disruptive innvoation, she will suggest next steps for these results.

### REFLECTIONS FROM THE PERSPECTIVE OF AN ADVOCATE AND CASE MANAGER

Tracy Keibler[1], *1. APparentPlan, Eden Prairie, Minnesota, United States*

From her perspective as director of ApparentPlan, a nonprofit care agency to assist low income consumer of LTC, and as co-founder and director of the MN Long-Term Care Think Tank, an advocacy organization. Ms. Keibler will reflect on these findings and next steps.

### SESSION 3245 (PAPER)

### ROLES AND EXPERIENCES OF GRANDPARENTING

### GRANDPARENTHOOD AND RISK OF MORTALITY: FINDINGS FROM THE HEALTH AND RETIREMENT STUDY

Lea Ellwardt,[1] Karsten Hank,[2] and Carlos F. Mendes de Leon[3], *1. University of Cologne, Cologne, Germany, North-Rhine Westphalia, Germany, 2. University of Cologne – Institute of Sociology & Social Psychology, North-Rhine Westphalia, Germany, 3. University of Michigan School of Public Health, Ann Arbor, Michigan, United States*

Grandparenthood is a significant social role for older adults and may have important health implications. Parenthood itself has been associated with some protective health effects, although findings have been mixed. Whether grandparenthood is associated with important long-term health effects such as mortality is largely unknown. This study examines the grandparenthood-mortality nexus, and whether it is modified by gender and education. Longitudinal data from the Health and Retirement Study (HRS) were used, comprising twelve biennial follow-up waves from 1992 to 2014 with linked data on vital status derived from the National Death Index. Submodules assessed participants' family structure during follow-up. The sample included 24,325 participants aged > 51 years with at least one child. Cox proportional hazard models were used to test the association between grandparenthood and mortality risk with adjustment for socio-demographic variables, for social variables including characteristics of and contact with children, and for health variables, including measures of overall, functional and mental health. Stratified models assessed these associations separately by gender and education. Grandparenthood was associated with a substantially increased mortality risk in women (fully adjusted HR = 1.65; 95% CI 1.27-21.14), and increased with larger number of grandchildren. No significant association was found for men (fully adjusted HR=1.25; 95% CI 0.97-1.62). Mortality risks associated with grandparenthood were highest among grandparents with low levels of education. The findings are among the first to suggest a potential grandparenthood survival "penalty", especially for grandmothers. Higher levels of education appear to mitigate this negative survival effect among grandparents.

### GRANDPARENTING CHILDREN WITH DISABILITIES AND ITS IMPACT ON GRANDPARENT HEALTH

Ynesse Abdul-Malak,[1] and Madonna Harrington Meyer[2], *1. Colgate University, Hamilton, New York, United States, 2. Syracuse University, Syracuse, New York, United States*

Across the US, millions of grandparents are providing vital care for their grandchildren with disabilities when their adult children are in need of assistance and public programs do not provide needed supports. Research suggests the impact on grandparent physical health is mixed. This paper draws on in-depth interviews with 50 grandparents to explore how caring for grandchildren with disabilities shapes their physical wellbeing. We use life course perspective to assess the choices grandparents make from available resources and options at different stages of their lives and the effects on their health. We find in addition to providing routine care, helping with feeding, bathing, and dressing, some grandparents provide constant supervision and medically intense care, such as tending to feeding tubes, catheters, and oxygen lines. Many grandparents said care work has improved, or at least helped sustain, their overall level of fitness, while other grandparents find that care work has adverse impacts. Often there is too much chasing, bending, and lifting for their aging bodies. This paper suggests that stronger social programs for children with disabilities and the grandparents who step in to help them would decrease the negative effects of care work on grandparent health.

### GRANDPARENTS' ROLES IN THE CONTEXT OF ADVERSE CHILDHOOD EXPERIENCES: THE VOICES OF EMERGING ADULTS

Athena Chung Yin Chan,[1] Doris Leung,[2]
Bessie Chan,[2] and Grace Wing Ka Ho[2], *1. Department of Family Social Science, University of Minnesota, Saint Paul, Minnesota, United States, 2. School of Nursing, The Hong Kong Polytechnic University, Kowloon, Hong Kong*

The influence of grandparents, in the context of adverse childhood experiences (ACEs), is largely understudied. With strong kinship in Asian families, grandparents may provide a crucial resource to their grandchildren; not limited to those living together, but having close emotional proximity. This qualitative study used secondary analysis to explore the roles of grandparents, upon reflection of participants' childhood adversities. Semi-structured interviews were conducted with 19 emerging adults, between 18 and 24 years old in Hong Kong, China. Participants were eligible if they: (1) reported at least one ACE, namely, abuse (physical, emotional, sexual), neglect (physical, emotional), witnessing domestic violence, or a dysfunctional household (due to parental divorce/death, household member substance use, incarceration, mental illness); and (2) described their interactions with grandparents during the interviews. Participants were asked to think of a challenging time during their childhood, and strategies they used to overcome them. All interviews were audio-recorded and transcribed verbatim. Data regarding the interactions with grandparents were coded and analyzed by four researchers following interpretive description. Preliminary findings described four primary roles grandparents played in the context of ACEs, which were sometimes positive and/or negative. Grandparents were portrayed as being unique persons in participants' lives that influenced how they faced their childhood adversities. We will discuss how grandparents' stewardship may significantly shape cultural patterns of how families cope with ACEs. In particular, our findings, examined against literature, will discuss how grandparents may enhance resilience of young people with ACEs.

### NUTRITIONAL NEEDS AND PREFERENCES AMONG AFRICAN AMERICAN CUSTODIAL GRANDMOTHERS: A QUALITATIVE STUDY

Kellie E. Mayfield,[1] Deborah Whitley,[1] and Susan J. Kelley[1], *1. Georgia State University, Atlanta, Georgia, United States*

This presentation summarizes a qualitative analysis from focus groups with African American, urban dwelling grandmothers raising grandchildren in parent-absent households. Nutritional needs of custodial grandparents are an under explored area of research. Previous studies on custodial grandparents have acknowledged the physical, social, and familial burdens they endure as caregivers of their grandchildren. Limited financial support is a consistent concern. One manifestation of having scarce monetary resources is not being able to meet daily nutritional requirements. As a result, adverse health outcomes related to the onset of diet-related diseases (e.g., obesity, hypertension, diabetes) are too common, especially for custodial grandparents of color. The present study qualitatively explores grandmothers' (N=9) experiences and ideas about food choices/options, decisions about when and where to purchase food, and the involvement of grandchildren in food-related practices and traditions. Grandparent participants were recruited from a community-based intervention, a program that provides health and social support services to grandparents raising grandchildren in Atlanta. Each of the focus groups consisted of 4-6 custodial grandmothers , facilitated by a doctoral-level community nutritionist. The major themes summarized from the qualitative group interviews were framed within a feminist/race theoretical context. Dominant themes from the focus group encounters include traditional gender roles related to food purchase and preparation, prioritizing food options to meet family preferences, available/accessible urban-based food options, food knowledge deficits, and sustaining cultural identity and nutritional health. Findings suggest implications for food and health policy, community-level programming, and nutrition education interventions.

### PARENTAL ABSENCE AND GRANDPARENT CAREGIVING IN THE WAKE OF THE CONTEMPORARY AMERICAN OPIOID EPIDEMIC

Jessica Y. Ho[1], *1. University of Southern California, Los Angeles, California, United States*

The contemporary American drug overdose epidemic has wreaked havoc on individuals, families, and communities across the country. As increasing numbers of middle-aged adults become addicted to or die from drugs, grandparents may be called upon to provide care for their grandchildren. However, few studies examine the broader social impacts of the drug overdose epidemic. This paper fills this gap in the literature by examining whether the opioid epidemic has contributed to increased grandparental care provision and coresidence. I use data from the 2000 Census and the 2011-2015 American Community Surveys, and data on drug overdose mortality (a measure of the epidemic's severity) and socioeconomic indicators. I find consistent and robust associations between increases in drug overdose mortality and increases in: children's likelihood of experiencing parental absence from the household, grandparent-grandchild coresidence, and grandparental provision of custodial care. Fixed-effect estimates correspond to an additional 158,138 grandparents coresiding with grandchildren, and a 3% increase in the proportion of coresident grandparents who are primarily responsible for their grandchildren. These associations are stronger among younger (aged 30-64) versus older (aged 65+) grandparents. This paper leverages multiple measures and nationally-representative data to demonstrate that increases in precarious family arrangements, vulnerability, and the burden of caregiving among older adults are occurring in response to the opioid epidemic. These findings suggest that the epidemic is placing considerable and unexpected strains on older adults' financial and emotional resources at a time when they themselves may be requiring care, retiring from the labor force, and experiencing declines in health.

### SESSION 3250 (PAPER)

### SOCIOEMOTIONAL HEALTH AMONG ASIAN COMMUNITIES

### CORRELATES OF LONELINESS AMONG OLDER ASIAN IMMIGRANTS: A SYSTEMATIC REVIEW

Yihan Wang,[1] Bongki Woo,[2] and Nan Jiang[3], *1. Boston College, Boston, Massachusetts, United States, 2. University of Southern Carolina, Columbia, South Carolina, United States, 3. Columbia University, New York, United States*

Loneliness is a prevalent social concern among older adults, which calls for attention as a condition itself and its influence on mental and physical conditions. However, limited efforts have been made to understand loneliness, particularly among immigrant older adults. Guided by the ecological perspective, the present study contributes to the literature by providing a systematic review of the prevalence and individual-, household-, and community-level correlates of loneliness among Asian older immigrants, one of the fast-growing immigrant population. Following the PRISMA guideline, we systematically searched eight electronic databases to identify relevant empirical research articles. Of the 828 articles identified, ten articles met the inclusion criteria. Majority of these articles focused on older Chinese and Korean immigrants. On the individual level, migration grief, longer length of residence, and weaker ethnic attachment were linked to higher level of loneliness, indicating that immigration can be a challenging experience for later life well-being of Asian older adults. Other identified correlates include mental and functional impairment and worsening health changes. On the household level, while living alone was a commonly identified correlate of loneliness, those who live with family also reported loneliness when they have fewer interactions with their family members. On the community-level, smaller social network and lack of social support and interactions were correlates of loneliness. The findings of the present study are helpful for identifying older Asian immigrants who may be at risk of loneliness and implicates that the efforts to mitigate the loneliness need to be made at various ecological levels.

### FAMILY COHESION MODERATES THE RELATIONSHIP BETWEEN ACCULTURATION AND HEALTH AMONG OLDER CHINESE AMERICANS

Kaipeng Wang,[1] Anao Zhang,[2] Fei Sun,[3] and Rita X. Hu[4], *1. Texas State University, San Marcos, Texas, United States, 2. University of Michigan, Ann Arbor, Michigan, United States, 3. Michigan State University, East Lansing, Michigan, United States, 4. University of Michigan, Ann Arbor, Ann Arbor, Michigan, United States*

Migration and resettlement are major life events that affect immigrants' functioning and health status. Previous research has well-established the influence of acculturation and family cohesion on Chinese Americans' mental health and health behavior; however, the moderation effect of family cohesion on the relationship between acculturation and self-rated health – a robust measure of an individual's general health – has not been examined among this population. The purpose of this study is to examine the association between family cohesion, acculturation, and self-rated health among older Chinese Americans. Data came from a survey of 385 Chinese Americans aged 55 and older living in a large metropolitan area in Southwest America through face-to-face interviews. We used logistic regression to examine the association between acculturation, family cohesion, and self-reported health. In general, acculturation was significantly associated with higher odds of reporting excellent or good health after adjusting for demographic and psychosocial covariates; however, the association between acculturation and self-reported health differed by family cohesion. We found that acculturation was positively associated with self-reported health only among those with medium or high family cohesion, but not among those with low family cohesion. Findings highlighted the significance of involving family members and strengthening family support for providing acculturation services, such as language class and healthy literacy education, to older Americans. Family cohesion needs to be considered by health and mental health care providers for older Chinese Americans to further understand the resources and barriers that influence their health service use and health behaviors.

### GRIEF EXPERIENCE PATTERNS AMONG OLDER ADULTS IN RURAL CHINA: A LATENT PROFILE ANALYSIS

Haimin Pan[1], *1. Xiamen University, Xiamen, China, China*

Grief experiences among older adults in China are understudied, though a variety of negative bereavement outcomes have been delineated. The present work sought to explore grief patterns among Chinese older people in rural areas, as well as the factors influencing the bereavement results. Participants were 352 older residents who responded to a face-to-face interview and lived in rural areas in Zhejiang Province of China. Latent profile analysis (LPA) was used to identify subtypes of class membership in combing complicated grief (CG), depression, anxiety, and meaning in life. Afterwards, these subgroups were compared on demographic characteristics and meaning making variable. The LPA model best fitting the data was a three-class solution comprised of "adaptive" (n=235; 66.8% of the sample), "moderate maladaptive" (n=83; 23.6% of the sample), and "severe maladaptive" groups (n=34; 9.7% of the sample). Compared to the "severe maladaptive" group, participants in the "adaptive" group had better physical functioning, higher education and incomes levels, and less meaning making engagement, while participants in the "moderate maladaptive" group had longer bereavement duration, better physical functioning, and less meaning making activities. Relative to the "moderate maladaptive" group, participants who were adaptive to the loss possessed longer bereavement duration better physical functioning, higher education and incomes levels, and less meaning making engagement. Findings suggest three distinct patterns of bereavement outcomes among Chinese older adults. Multiple factors impacting the results were taken into consideration. Future replication is necessary to validate these subgroups, and professional services should be provided to bereaved older Chinese in need.

### MORE OR LESS? A DECADE'S TREND OF SOCIAL PARTICIPATION AMONG CHINESE OLDER ADULTS: BASED ON A LONGITUDINAL SURVEY

Chenxin Tan,[1] and Yun Zhou[1], *1. Department of Sociology, Peking University, Beijing, China*

Social participation is of great significance in healthy aging. While studies on social participation among Chinese elderly are growing, there is a lack of understanding the changes over time of the participation. Using datasets from the Chinese Longitudinal Healthy Longevity Survey (CLHLS), this paper presents a comprehensive analysis on a decade's trend of social participation among Chinese older adults. First, we use the method of Latent Class Analysis (LCA) to identify types of social participation; in this study, we concluded three types, no participation, the family-centered, and the society-oriented. Second, we examine the characteristics of the elderly by types of participation in terms of demographic, socioeconomic and health condition and analyze the changes in the characteristics over time. And third, we interpret the trend of social participation with broader social environment, or the fluctuant structural and institutional differences under the context of China's unique social system. Our general conclusion is that while the overall level of participation holds relatively steady, there is a dynamic micro progress and complex mechanisms in this long period. In addition, although both the family-centered participants and the society-oriented possess broader scopes of social participation, the related attributes are different across time. This paper contributes to our knowledge of life of the elderly under the circumstances of fast aging process in China.

### RECIPROCAL RELATIONSHIP BETWEEN COGNITIVE STATUS AND HIERARCHY FUNCTIONAL LOSS AMONG FRAIL OLDER ADULTS IN CHINA

Qian Sun,[1] Nan Jiang,[2] Nan Lu,[3] and Vivian W. Lou[4], *1. Hebei University of Business and Economics, Shijiazhuang, Hebei, China, 2. Columbia University, New York, United States, 3. Renmin University of China, Beijing, China, 4. The University of Hong Kong, Hong Kong, P.R.C., Hong Kong*

The present study aimed to determine the reciprocal relationship between cognitive status and the loss hierarchy of specific functional activities among frail older adults in China. Data were derived from a sample of 469 older adults who participated in both the 2010 and 2013 waves of the Longitudinal Study on Family Caregivers for Frail Older Adults Aged 75 or Above in Shanghai, China. A two-wave cross-lag analysis was used to examine the proposed model. In general, the results showed that cognitive status in 2010 was a significant predictor of dependence in activities of daily living (ADLs) in 2013. Specifically, cognitive status at baseline has significant effects on feeding [$\beta$ (SD) = -0.198 (0.043), $p < .001$], continence [$\beta$ (SD) = -0.172 (0.045), $p < .001$], bladder [$\beta$ (SD) = -0.159 (0.045), $p < .001$], toileting [$\beta$ (SD) = -0.119 (0.043), $p < .001$], hygiene [$\beta$ (SD) = -0.108 (0.044), $p < .05$], stairclimbing [$\beta$ (SD) = -0.101 (0.044), $p < .05$], and dressing in 2013 [$\beta$ (SD) = -0.100 (0.045), $p < .05$]). Furthermore, toileting and bathing in 2010 were significant risk factors of cognitive status in 2013 [toileting: $\beta$ (SD) = -0.146 (0.066), $p < .05$; bathing: $\beta$ (SD) = -0.113 (0.047), $p < .05$]. The findings not only expanded our understandings of the relationship between cognition and the hierarchy of functional loss, but also provided evidences for clinicians and service planners for anticipating the subsequent care and service needs of the elderly and their families.

### SESSION 3255 (SYMPOSIUM)

### SUSTAINING STAR-VA: EVALUATING SYSTEMIC OUTCOMES AND TEAM STRATEGIES FOR MANAGING BEHAVIOR SYMPTOMS OF DEMENTIA

Chair: Kim Curyto, *Center for Integrated Healthcare, VA Western New York Healthcare System, Batavia, New York, United States*

Discussant: Kimberly Van Haitsma, *Penn State, University Park, Pennsylvania, United States*

The Veterans Health Administration (VHA) has invested in the implementation and evaluation of STAR-VA, a Veteran-centered, interprofessional intervention for managing behavioral symptoms of dementia (BSD), with 86 Community Living Center (CLC nursing home) teams between 2013 and 2018. Results of a VHA Quality Enhancement Research Initiative (QUERI) partnered evaluation project are presented, including a multi-site interprofessional network created to collaborate on evaluation of the longitudinal impact of STAR-VA on CLC Veteran- and site-level outcomes, and to determine factors associated with sustained implementation and positive outcomes. The development and validation of a Minimum Data Set quality indicator of behavior symptoms of dementia (BSD) is presented. Characteristics of STAR-VA trained and untrained CLCs are presented, along with facilitators and barriers to sustained program implementation. Findings support the effectiveness of implementation of STAR-VA on decreased use of psychotropic medication. Qualitative outcomes demonstrate importance of having the appropriate staff, positive team relationships, supportive usual routines, and culture as critical for STAR-VA sustainability efforts. Results emphasize the importance of using a valid, routine measure of BSD to provide feedback to CLC teams, and to develop a sustainability intervention focused on addressing reported barriers to program sustainability through interprofessional networks. Both qualitative and quantitative outcomes will inform development and recommendations for an implementation and evaluation strategy for an outcome-driven, tailored intervention to support CLC teams in sustaining STAR-VA and to improve poor-performer and maintain high-performer outcomes. The potential benefit of our findings for other interprofessional behavioral nursing home interventions will be discussed.

### PARTNERED EVALUATION OF LONGITUDINAL IMPACT OF STAR-VA: IMPLICATIONS FOR PROGRAM SUSTAINABILITY

Michele J. Karel,[1] Lisa A. Minor,[2] jenefer M. Jedele,[3] Orna Intrator,[4] and Kim J. Curyto[5], *1. Office of Mental Health and Suicide Prevention, Veterans Health Administration, Washington, District of Columbia, United States, 2. Geriatrics and Extended Care, Department of Veterans Affairs, Washington, District of Columbia, United States, 3. Serious mental Illness Treatment Resource and Evaluation Center, Office of Mental Health and Suicide Prevention, Department of Veterans Affairs, Ann Arbor, Michigan, United States, 4. GECDAC at Canandaigua VA Medical Center and Department of Public Health Sciences, University of Rochester, Rochester, New York, United States, 5. Center for Integrated Healthcare, VA Western NY Healthcare System, Batavia, New York, United States*

Between 2013 and 2018, 86 Community Living Center (CLC) teams received STAR-VA training and consultation and implemented the intervention. Program evaluation has consistently demonstrated that STAR-VA Veteran training cases experience significantly decreased frequency and severity of target behaviors, symptoms of depression, anxiety, and agitation. VA Central Office leaders responsible for ongoing STAR-VA implementation and evaluation sought to understand the program's systemic impact on important outcomes, if outcomes are maintained, and factors influencing variation in sustained adherence to STAR-VA. This presentation will describe the rationale for and methods used to develop a valid quality improvement metric to assess behavioral symptoms of dementia, to compare Veteran- and site-level outcomes at trained and untrained CLCs, and to identify factors associated with sustained STAR-VA implementation. This partnered evaluation, combining quantitative and qualitative methodologies, is critical for informing operational strategies for promoting STAR-VA and supporting its sustained implementation in VA CLCs.

### AN MDS 3.0 BEHAVIORAL INDICATOR: RELIABILITY AND VALIDITY WHEN MEASURING BEHAVIOR SYMPTOMS OF DEMENTIA IN CLCS

Kim Curyto,[1] Jenefer M. Jedele,[2] Jennifer J. Mach,[3] David C. Mohr,[4] Laura O. Wray,[5] April Eaker,[6] and Orna Intrator[7], *7. GECDAC, Canandaigua VA Medical Center & Department of Public Health Sciences, University of Rochester, Rochester, New York, United States, 1. Center for Integrated Healthcare, VA Western New York Healthcare System, Batavia, New York, United States, 2. Serious Mental Illness Treatment Resource and Evaluation Center, Office of Mental Health and Suicide Prevention, Department of Veterans Affairs, Ann Arbor, Michigan, United States, 3. Serious mental Illness Resource and Evaluation Center, Office of Mental Health and Suicide Prevention, Department of Veteran Affairs, Ann Arbor, Michigan, United States, 4. VA HSR&D Center for Healthcare Organization and Implementation Research, Boston VA Healthcare System, Boston, Massachusetts, United States, 5. VA Center for Integrated Healthcare, VA Western NY Healthcare System (116N), Buffalo, New York, United States, 6. Center for Integrated Healthcare, VA Western NY Healthcare System, Buffalo, New York, United States*

Persons with dementia frequently demonstrate behavior symptoms of dementia (BSD), associated with poorer outcomes. A measure of BSD was created for routine use in VA Community Living Centers (CLCs). Reliability and validity of Minimum Data Set (MDS 3.0) behavior items was established using exploratory factor analysis and a multitrait, multimethod correlation matrix. 385 CLC residents with BSD were assessed using validated measures of BSD, depression, and anxiety, and team ratings of the frequency and severity of target behaviors identified for intervention. Factor analysis on MDS items closest to baseline resulted in two stable factors. MDS behavior factors related to validated clinical measures in predicted ways at baseline and post-intervention. MDS distress behavior factor sensitivity to change was evaluated by using change score correlations with validated clinical measures. The MDS distress behavior factor can be used routinely, evaluate the impact of intervention effectiveness, and provide quality improvement feedback.

### FACILITY CHARACTERISTICS, STAFF INJURY, AND DISRUPTIVE BEHAVIOR REPORTING RATES IN VA COMMUNITY LIVING CENTERS

David Mohr,[1] Kim J. Curyto,[2] Kevin W. McConeghy,[3] Jenefer M. Jedele,[4] and Kelly Vance[5], *1. Center for Healthcare Organization and Implementation Research, VA Boston Healthcare System, Boston, Massachusetts, United States, 2. VA Western NY Healthcare System, Batavia, New York, United States, 3. Centor of Innovation in Long-Term*

*Services and Support, Providence VA Medical Center, Providence, Rhode Island, United States, 4. Serious mental Illness Treatment Resource and Evaluation Center, Office of mental Health and Suicide Prevention, Department of Veterans Affairs, Ann Arbor, Michigan, United States, 5. Workplace Violence Prevention Program, Office of Mental Health and Suicide Prevention, Lexington, Kentucky, United States*

Facility characteristics' influence on staff injuries was evaluated from 2013-2018 for 105 VA CLCs. Nursing hours, nurse skill-level, resident case-mix (percent of residents with mental health or other conditions) and facility size were evaluated in a multivariable regression model. Overall the average injury rates per year were 2.7 (standard deviation 4.3) and 1.5 (2.7) in STAR-VA enrolled vs. never enrolled sites (p=0.04). Statistically significant predictors for higher staff injury rates included percent of residents with dementia, larger bed facilities, and more mental health employee coverage. Lower staff injury rates were associated with facilities with more short-stay residents. After adjustment for facility characteristics, STAR-VA sites were not an independent predictor for staff injury rates. Sites selected for enrollment in STAR-VA have higher overall injury rates which may be due to facility differences in size, staffing and proportion of residents with dementia. Implications for training and monitoring CLC sites will be discussed.

### EVALUATING THE IMPACT OF STAR-VA ON VETERANS' PSYCHOTROPIC MEDICATION USE IN CLCS

Kevin McConeghy,[1] Kim Curyto,[2] Jenefer M. Jedele,[3] Jennifer Mach,[4] Orna Intrator,[4] and Ilse R. Wiechers[5], *1. Center of Innovation and Long-Term Services and Support, Providence VA Medical Center, Providence, Rhode Island, United States, 2. VA western NY healthcare system, batavia, New York, United States, 3. Serious Mental Illness Treatment Resource and Evaluation Center, Ann Arbor, Michigan, United States, 4. Serious Mental Illness Resource and Evaluation Center, Office of Mental Health and Suicide Prevention, Department of Veteran Affairs, Ann Arbor, New York, United States, 4. , Geriatrics & Extended Care Data & Analyses Center (GEC DAC), Canandaigua VAMC, Canandaigua, New York, United States, 5. Office of mental health and suicide prevention, uS department of veteran affairs, menlo park state, California, United States*

The impact of STAR-VA on psychotropic drug use among residents with behavioral symptoms of dementia was evaluated through a difference-in-differences framework. STAR-VA residents enrolled 2013-2017 were evaluated longitudinally pre-post intervention. The primary outcome was the number of as needed administrations with an indication of 'anxiety' or 'agitation'. The analytical cohort included 214 training cases and 1,870 controls from untrained sites meeting eligibility criteria. STAR-VA cases were less white (48% vs. 54%), less black (11% vs. 14%), and had significantly longer median length of stay (830 vs. 261 days), respectively. STAR-VA cases had on average 3.5 as needed doses/month of psychotropic medication before the intervention and 1.7 after, controls averaged 1.8 doses/month. After adjustment for person-time-fixed effects, enrollment was associated with 55% (95% CI:30, 68) reduction or an average 0.8 as needed psychotropic doses/month. Findings demonstrate effectiveness in decreasing as-needed psychotropic drug use among CLC residents, supporting continued implementation of STAR-VA.

### VARIATIONS IN SUSTAINING IMPLEMENTATION OF STAR-VA: THE ROLE OF KNOWLEDGE RESERVOIRS AND OTHER FACTORS

Jennifer L. Sullivan,[1] Kim Curyto,[2] Omonyêlé l. adjognon,[3] Jacqueline Pendergast,[1] Laura O. Wray,[4] and Michele Karel[5], *1. Center for Healthcare Organization and Implementation Research (CHOIR), VA Boston Healthcare System, Boston, Massachusetts, United States, 2. vA western new york healthcare system, batavia, New York, United States, 3. center for healthcare organization and implementation research, boston VA healthcare system, boston, Massachusetts, United States, 4. VA Center for Integrated Healthcare, VA Western NY Healthcare System (116N), Buffalo, New York, United States, 5. Office of Mental Health and Suicide Prevention, Veterans Health Administration, Underhill, Vermont, United States*

Variation in STAR-VA sustainability across 20 trained VA Community Living Centers was explored using prospective qualitative methods utilizing the knowledge reservoirs framework including seven domains: People, Routines, Artifacts, Relationships, Information space, Culture, and Structure. We conducted directed content analysis of transcripts to identify facilitators and barriers of successful program sustainment. We found that people, usual routines, information sharing, and team relationships were the most often mentioned facilitators by CLC staff. Common reported barriers were people, team relationships, and work culture. Overlap was found in knowledge reservoirs acting as both facilitators and barriers at the same site, most often for people/teams, team relationships, and work culture. Results will be used to develop a sustainability intervention focused on addressing reported barriers. Most notably, a focus on having the appropriate team members, positive team relationships, usual routines, and a supportive work culture are critical for STAR-VA sustainability efforts.

### SESSION 3260 (SYMPOSIUM)

### THE ASPREE STUDY: DISABILITY-FREE SURVIVAL, UPDATED RESULTS, SUB-STUDIES, AND IMPLICATIONS FOR ASPIRIN USE

Chair: Anne M. Murray, *Berman Center for Outcomes and Clinical Research, Minneapolis, Minnesota, United States*
Co-Chair: John McNeil, *Department of Epidemiology & Preventive Medicine, Monash University, Melbourne VIC, Australia, Melbourne VIC, Australia, Australia*
Discussant: Basil Eldadah, *National Institute on Aging, NIH, Bethesda, Maryland, United States*

The NIA/NCI ASPREE (ASPirin in Reducing Events in the Elderly) Study was a landmark RCT of 19,114 healthy adults aged 70 (whites) and 65 (US minorities) in Australia and the US that demonstrated lack of effect of low dose aspirin (LDA:100 mg/d) on the novel primary end point of Disability- Free Survival (life free of disability and dementia) over a mean treatment of 4.7 years. Surprisingly, LDA was associated with a trend toward increased all cause mortality, driven by cancer deaths (results published NEJM September 2018). After the LDA intervention was halted in June 2017, ASPREE was extended as an observational cohort follow-on study, ASPREE-XT, to measure potential delayed LDA effects on ASPREE outcomes. The ASPREE study primary results will be summarized, and the rationale for and performance of the novel DFS geriatric outcome discussed. New results of the analysis of dementia as a secondary outcome will also be presented (both for overall dementia and Alzheimer's disease). We will also examine the unexpected increased all-cause mortality attributed to cancer deaths, despite no significant difference between groups for all incident cancer, and effects of LDA on incident metastatic disease. The important implications of the ASPREE results for prescribing LDA for primary prevention in health elderly will be discussed, and the ASPREE-XT study design and progress described. Lastly, the breadth of the ASPREE sub-studies including the Biobank, Brain Imaging studies and Genomics, and opportunities to access the rich ASPREE data and collaborate with ASPREE investigators will be reviewed.

### RATIONALE FOR ASPREE DISABILITY-FREE SURVIVAL PRIMARY OUTCOME AND OVERVIEW OF PRIMARY OUTCOME RESULTS

John McNeil[1], *1. Department of Epidemiology & Preventive Medicine, Monash University, Melbourne VIC, Australia, Melbourne VIC, Australia, Australia*

Disability-free survival (DFS), defined as survival free of disability and dementia was the primary outcome measure of the ASPREE clinical trial. As previously reported, there was no benefit of low dose aspirin on the primary end point of dementia, physical disability or death, but bleeding risks were increased. In total, 1,835 participants reached the primary endpoint, confirmed amongst approximately 3,000 who had triggered for one of the end-points. Dementia was the most labor intensive component of DFS. Several previous primary prevention aspirin studies had identified a reduction of vascular events counterbalanced by an increase in serious bleeding, leaving the question of net outcome to an intuitive decision. DFS was chosen because it balances the positive and negative effects of a preventive drug such as aspirin. It also encapsulates the primary purpose of a preventive drug in older people i.e., to prolong a healthy lifespan rather than prevent a defined disease.

### EFFECT OF INITIATING ASPIRIN ON CANCER EVENTS IN THE HEALTHY ELDERLY

Andrew T. Chan,[1] Peter Gibbs,[2] Jessica E. Lockery,[3] Galina Polekhina,[3] Suzanne G. Orchard,[3] Leslie Ford,[4] Asad Umar,[4] and john J. McNeil[3], *1. Clinical and Translational Epidemiology Unit, Massachusetts General Hospital, Boston, Massachusetts, United States, 2. The Walter & Eliza Hall Institute of Medical Research, Parkville VIC, Victoria, Australia, 3. Department of Epidemiology & Preventive Medicine, Monash University, Clayton VIC, Victoria, Australia, 4. Division of Cancer Prevention, National Cancer Institute, Bethesda, Maryland, United States*

In ASPREE, we previously reported a surprising increase in cancer-related deaths associated with initiating aspirin. We now report primary incident cancer events. Aspirin was not associated with risk of incident cancer (HR=1.04, 95% CI 0.95-1.14), including non-metastatic cancer (HR=0.99, 95% CI, 0.89-1.11) and colorectal cancer (HR=1.02; 95% CI, 0.81-1.30). However, risk of incident metastatic cancer was elevated with aspirin (HR=1.18; 95% CI,0.96-1.46), although this could be attributable to chance. In ASPREE, the increase in cancer deaths associated with initiation of aspirin was not accompanied by a significant increase in overall incident cancer after 4.7 years. However, there did appear to be an increase in the incidence of advanced cancer in the aspirin arm. These data support the possibility that aspirin may adversely affect short-term outcomes among elderly participants with undiagnosed cancers (e.g. prevalent tumors at enrollment or early incident tumors) and/or may have differential effects according to age.

### THE ASPREE STUDY: DEMENTIA DUE TO ALZHEIMER'S DISEASE OUTCOMES

Raj C. Shah[1], *1. Rush University, Chicago, Illinois, United States*

In the ASPREE clinical trial, aspirin 100mg daily in health older adults did not delay onset of dementia, a pre-specified secondary outcome over a period of 5 years. We examine whether low-dose aspirin versus placebo is related to incident dementia due to Alzheimer's disease. Older community-dwelling participants free of dementia, physical disability, and conditions requiring aspirin treatment were recruited (n=19,114). Participants were administered a cognitive test battery during follow-up and participants with suspected dementia underwent a more extensive dementia assessment. An expert international panel adjudicated dementia according to DSM-IV criteria, with sub-classification according to NIA-AA criteria. Over a median 4.7 years, 575 participants had a confirmed dementia diagnosis. In analysis of the 41% of cases classified as dementia probably due to Alzheimer's disease, no difference in the incidence between the treatment arms was found (HR=0.96, 95% CI =0.74, 1.24). Plans for continued assessment of cognition in ASPREE-XT will be presented.

### POTENTIAL IMPLICATIONS OF ASPREE ON ASPIRIN PRIMARY PREVENTION GUIDELINES

Anne M. Murray[1], *1. Berman Center for Outcomes and Clinical Research, Minneapolis, Minnesota, United States*

The 2016 USPSTF guidelines for aspirin to prevent CVD and colorectal cancer noted insufficient evidence to assess the balance of benefit and harm in those 70 and older. The long-awaited ASPREE trial, conducted in healthy elderly aged 70 and older (65 for US minorities), evaluated aspirin's effect on disability-free survival, a composite of death, dementia, or persistent physical disability. CVD and cancer were pre-specified secondary endpoints, positioning ASPREE's results to substantially inform the evidence gap noted in the USPSTF guidelines. Low-dose aspirin over 5 years did not lower CVD events or colorectal risk, but significantly increased bleeding. The ASPREE-XT observational follow-up study over the next 5-7 years will observe for potential legacy effects of aspirin on the primary and secondary outcomes of ASPREE, thus adding further evidence to define the risk-benefit profile of aspirin for primary prevention in healthy elderly.

### ASPREE SUB-STUDIES

Robyn L.. Woods,[1] John J. McNeil,[1] and Anne Murray[2],
*1. Monash University, Melbourne, Victoria, Australia,*
*2. Berman Center for Outcomes and Clinical Research, Minneapolis, Minnesota, United States*

To maximize opportunities provided by a large-scale clinical trial, we established sub-studies during the ASPREE trial to investigate specific areas of interest to the health of older persons. A biobank now stores multiple aliquots of blood, urine or saliva collected from >15,000 healthy ASPREE participants across the US and Australia, most at baseline (pre-randomization) and/or after 3 years post- randomization. Other sub-studies included ALSOP (ASPREE Longitudinal Study of Older Persons; sets of questionnaires administered every 2 years focused on medical or social issues) and neuroimaging projects with brain MRI and retinal vascular imaging to investigate anatomical changes linked with cognitive or cerebrovascular outcomes. Further sub-studies addressed the impact of aspirin on cerebral microhemorrhages, age-related macular degeneration, age-related hearing loss, severe sepsis, and falls and fractures. Biomarker analyses underway include plasma androgens in older women and DNA sequencing of the cohort to investigate contributions of genomics to aging health and disease.

### SESSION 3265 (SYMPOSIUM)

### THE GSA PRESIDENTIAL SYMPOSIUM — STRENGTH IN AGE: HARNESSING THE POWER OF NETWORKS

Chair: Harvey J. Cohen, *Duke University Medical Center, Durham, North Carolina, United States*

GSA is a network of disciplinary components each of which is a network composed of members. This large system possesses powerful emergent properties that are realized at many interdisciplinary interfaces. The GSA Presidential Symposium mirrors this by bringing together rapporteurs from our disciplinary presidential symposia who will summarize the major findings of these symposia. The Behavioral and Social Sciences presidential symposium explores the use of social networking by aging adults, the media and technologies utilized, and the impact of these trends on health and well-being. The Biological Sciences presidential symposium examines multiple layers of biological networks as predictors of systemic aging. The Social Research Policy and Practice presidential symposium dissects the reciprocal relationship between broad environmental contexts and social networks throughout life and ways in which this relationship can be used to optimize the aging experience. The Health Sciences presidential symposium reports on the improvement of perioperative care of older adults achieved through harnessing the partnership and collaboration of all disciplines involved in this care. The Academy of Gerontology in Higher Education presidential symposium discusses ongoing initiatives that build networks to shape age-friendly programs and policies at universities, international collaborations, and competency-based gerontology education. The Humanities and Arts presidential symposium investigates the synergy between museums and aging, both of which are represented as networks. A discussion between the presidential symposia rapporteurs with audience participation will ensue to identify common, overarching themes and to spark approaches to important, interdisciplinary problems in gerontology. • Kelly Niles-Yokum, Academy for Gerontology in Higher Education, Education Networks: Strengthening of Gerontology and Geriatrics through Connectivity • Kristine J. Ajrouch, Behavioral and Social Sciences Section, The Ties That Bind: The Influence of Social Media and Technology in the Lives of Older Adults • George L. Sutphin, Biological Sciences Section, Expanding the Geroscience Network • Luigi Ferrucci, Health Sciences Section, Optimizing Surgical Care for All Older Adults • Kate de Medeiros, Humanities and Arts Committee, Museums and Aging: Novel Network Opportunities to Support Optimal Aging • Emily Greenfield, Social Research Policy and Practice Section, Harnessing Social Networks to Optimize Environmental Contexts for Diverse Aging Experiences

### SESSION 3270 (POSTER)

### ASSISTED LIVING & CAREGIVING

### PROFESSIONAL QUALITY OF LIFE, LONELINESS, AND EMPATHY IN PROFESSIONAL CAREGIVERS

Janelle Beadle,[1] Julie Blaskewicz Boron,[1] and Julie Masters[1],
*1. University of Nebraska Omaha, Lincoln, Nebraska, United States*

Retaining professional caregivers is an ongoing challenge for home care agencies. Thus, understanding emotional factors associated with caregiver burnout is critical. While professional caregivers are at risk for burnout and loneliness, less is known about how these factors relate to empathy and professional quality of life. Professional caregivers (n=31) currently employed at two U.S. non-skilled home care agencies participated by completing several questionnaires. Surveys included: UCLA Loneliness Scale (loneliness), Interpersonal Reactivity Index (empathy), and the Professional Quality of Life Scale (compassion satisfaction, compassion fatigue, and burnout). Participants were adults (M=56.8, SD=11.5, Range=24-75 years; 90.3% female) who had obtained an Associates degree (education years: M=14.2, SD=1.7). Overall, participants reported average to high levels of loneliness (M=37.5, SD=9.8), and emotional (M=23.2, SD=3.7) and cognitive (M=20.3, SD=4.6) empathy. Caregivers generally reported positive feelings about their work (compassion satisfaction: M=42.8, SD=5.0) and low levels of burnout (M=17.1, SD=4.2). In contrast, they reported relatively high levels of compassion fatigue (M=17.7, SD=4.0). Burnout was positively associated with compassion fatigue (r=.7, p<.001), and loneliness (r=.5, p=.004), whereas a negative association was found between burnout and cognitive empathy (r=-.5, p=.008). Using a linear regression, the associations between compassion fatigue, loneliness, and cognitive empathy were examined in relation to burnout. This model was statistically significant (r2=.72; F change (3,21)=17.8, p<.001), but only compassion fatigue (p=.002) and cognitive empathy (p=.002) were significant predictors of burnout. This study highlights the importance of the contributing role of socioemotional factors to burnout experienced in a professional caregiving setting.

### FOOD INSECURITY AND COST-RELATED MEDICATION NON-ADHERENCE IN OLDER ADULTS: A SYSTEMATIC REVIEW

Lisa L. Boss,[1] and Shaunna Caouette[1], *1. UTHSC, Houston, Texas, United States*

Food insecurity (FI) is defined as having limited access to nutritional and safe foods due to lack of financial resources and is believed to negatively influence health outcomes. Older adults, in particular, face rising healthcare costs and may be forced to choose between purchasing prescribed medications and using their limited financial resources for basic needs, such as food. The purpose of this systematic review was to examine the relationship of food insecurity (FI) and cost-related medication non-adherence (CRN) in older adults living in the community setting. A comprehensive, electronic review of the literature was performed. Criteria for inclusion were original quantitative or qualitative research, written in English, human participants ≥60 years, and published from January 2000 through January 2019. The total number of studies included was six. Main findings from the studies largely indicate that FI and CRN are significantly and positively correlated in older adults living in American communities. Further, CRN increases with the severity of FI. Most participants in these six studies were female, non-Hispanic white, with an annual household income <20k, and with less than a high school education. Although preliminary evidence is promising, research with more rigorous design is warranted to better understand the relationship of FI and CRN in older adults, and to develop appropriate interventions and programs for this growing public health concern.

### INNOVATIONS IN HOME MODIFICATION RESEARCH: THE STATE OF THE ART

Emily Nabors,[1] Mindy Renfro,[2] Jon Pynoos,[1] Sarah L. Szanton,[3] Jon Sanford,[4] and Susan Stark[5], *1. University of Southern California, Los Angeles, California, United States, 2. Touro University Nevada, Henderson, Nevada, United States, 3. Johns Hopkins School of Nursing, Baltimore, Maryland, United States, 4. Georgia Tech, Atlanta, Georgia, United States, 5. Washington University School of Medicine in St. Louis, St. Louis, Missouri, United States*

The overwhelming preference of older adults is to stay in their homes for as long as possible (AARP). However, most housing lacks supportive features and presents barriers that jeopardize residents' ability to successfully age in place. Only 1% of houses have five key features to ensure accessibility: no-step entry, single-floor living, lever door handles, accessible electrical controls, and extra-wide doors and hallways (Harvard Joint Center for Housing Studies), making the vast majority unsuitable for persons who use wheelchairs and problematic for the growing number of people with activity limitations. Persons least likely to have such features in their homes need them the most: old-old, low income, frail, and residents in older housing stock. Although home modification can support people as their needs change and preclude the need to move, often to institutional settings, the majority of older adults lack these supports. Recent studies have demonstrated the role of home modification in health, safety, and cost effectiveness. This symposium will convene a panel of researchers to share evidence-base in home modification, recent cost-saving innovations including the CAPABLE Program, and policy change to improve service delivery.

### NOT ENJOYING THE MEAL WAS ASSOCIATED WITH DEPRESSIVE SYMPTOMS, DESPITE THE SIZE OF SOCIAL CONNECTIONS

Mai Takase,[1] Hiroshi Murayama,[2] Sayaka Hirukawa,[3] Tomoki Tanaka,[4] Sachiko Ono,[3] Sugimoto Minami,[3] and Mari Kimata[2], *1. The University of Tokyo, Institute of Gerontology, Bunkyo-ku, Tokyo, Japan, 2. Institute of Gerontology, The University of Tokyo, Tokyo, Japan, 3. The University of Tokyo, Bunkyo-ku, Tokyo, Japan, 4. Institute of Gerontology, The University of Tokyo, Japan, Tokyo, Japan*

Conventional studies report that the enjoyment of the meal is related to likelihood of contracting depressive mood. The Japanese assisted living facilities currently support seniors build social connections to maintain their health and well-being, but psychological feeling during mealtime is often left unquestioned. Because seniors engage in conversation with tablemates while dining, the feeling during mealtime should not be ignored. This study aimed to explore the relationship among social connection, enjoyment during mealtime, and depressive mood. A cross-sectional questionnaire study was performed for independent residents at assisted living facility in Kanagawa Prefecture, Japan. The size of social connection (the number of facility residents that one can easily talk to) and enjoyment during the meal were assessed by a single item, respectively. The 15-item Geriatric Depression Scale was used to measure depressive symptoms. The analysis included 190 questionnaires. A logistic regression analysis showed that enjoyment during mealtime was associated with less likelihood of depressive symptoms, but the size of social connection was not, after adjusting for sociodemographics and health conditions. Moreover, a significant interaction between social connection and enjoyment during mealtime was observed. This indicated that greater size of social connection was inversely related to depressive symptoms among those who enjoyed the meal; however, among those who did not enjoy the meal, the likelihood of depressive symptoms were stably higher despite the size of social connection. The findings suggested that along with helping seniors build social connection, care takers should focus on improving the dining environment of seniors.

### DEPENDENCY IN ACTIVITIES OF DAILY LIVING: THE ROLE OF MULTIDIMENSIONAL FRAILTY AND PROTECTIVE FACTORS

G.A. Rixt Zijlstra,[1] Anne van der Vorst,[1] Linda P.M. Op het Veld,[2] Nico De Witte,[3] Jos M.G.A. Schols,[1] and Gertrudis Kempen[1], *1. Maastricht University, Care and Public Health Research Institute (CAPHRI), Department of Health Services Research, Maastricht, Netherlands, 2. Zuyd University of Applied Sciences, Faculty of Health, Centre of Research Autonomy and Participation for Persons with a Chronic Illness, Heerlen, Netherlands, 3. Vrije Universiteit Brussel, Brussel, Belgium*

Most older adults prefer to "age in place" and maintain independent regarding activities of daily living (ADL). Dependency in ADL might be caused by frailty. This study explored the relationship between multidimensional frailty and ADL dependency, and if protective factors, derived from a systematic literature review, moderate this relationship. A longitudinal study with a 24-month follow-up was performed among 1,027 community-dwelling older adults. Multidimensional frailty was assessed with the Tilburg Frailty Indicator, and ADL dependency with the Groningen Activity Restriction Scale. Other measures included sociodemographic characteristics and seven protective factors against ADL dependency, such as physical activity and non-smoking. Logistic regression analyses showed that frail older people had a twofold risk of developing ADL dependency in comparison to non-frail older people after 24 months (OR = 2.12, 95% CI = 1.50-3.00). Analyses with interaction terms indicated that the selected protective factors against ADL dependency did not significantly moderate this relationship. Nonetheless, higher levels of physical activity and having sufficient financial resources decreased the risk of becoming ADL dependent in the overall sample (OR = 0.67, 95% CI = 0.46-0.98 and OR = 0.49, 95% CI = 0.35-0.71, respectively). In conclusion, multidimensional frail older people are at higher risk of developing ADL dependency and the studied factors against ADL dependency did not significantly moderate this relationship. To develop prevention strategies for ADL dependency and facilitate aging in place, future studies might explore the relationship between each specific frailty domain and ADL dependency, and the role of (other) moderating factors.

### LIVES WORSE THAN DEATH: ADULT CHILDREN ARE POOR AT ESTIMATING PARENTS' QUALITY-OF-LIFE VALUATIONS

Matthew Pichiello,[1] Meghan McDarby,[1]
Elissa K. Kozlov,[2] and Brian Carpenter[1], *1. Washington University in St. Louis, St. Louis, Missouri, United States, 2. Rutgers University, New Brunswick, New Jersey, United States*

Adult children often help older parents make medical decisions when their health is compromised. To do so in a way that respects parent values requires children to know how their parent views health states and consequent quality of life. The current study compared older parent and adult child valuations of quality of life in different health contexts. Families consisted of older parents (n = 37) and their adult children (n = 66). Parents rated perceived quality of life in 14 compromised health states on a scale from 1 (difficult but acceptable) to 5 (not worth living). Children estimated how their parent responded to each health state, yielding an index of their knowledge of parent perceptions. Overall, parents described all compromised health states as less acceptable than adult children thought they would, $t(99) = 2.19$, $p < .05$. Notably, parents believed situations that caused financial or emotional burden to their family were much less acceptable than their children estimated. Children were more knowledgeable about parent valuations for more extreme circumstances, such as living with a feeding tube. Within families, children demonstrated only slight knowledge about parent quality of life valuations (kappa = .081). Across the entire sample of families, there was a broad range of knowledge (kappas = -.181 – .351), but at best, knowledge was still only fair. Results from this study suggest that adult children may underestimate the impact of compromised health states on parent estimations of quality of life, which could affect collaborations on healthcare decisions.

### INFERRING ADL BEHAVIORS FROM HIGH DENSITY, AMBIENT, INTERNET OF THINGS SENSOR DATA USING MACHINE LEARNING

Susann M. Keohane,[1] and Scott N. Gerard[2], *1. IBM, Austin, Texas, United States, 2. IBM, Durham, North Carolina, United States*

To address the growing desire for older adults to age-in-place, there has been an influx of research and development in telehealth services that utilize smart home and ambient assisted living (AAL) technology. AAL technology is designed to care for, remotely monitor, and support healthy aging. The challenge with AAL technology is to accurately classify an older adults' activities and provide a baseline for detecting changes in physical and cognitive state. We employ (1) rules-based, (2) Latent Dirichlet Allocation (LDA) and (3) auto-encoders (a type of neural network) to infer activities of daily living and instrumented activities of daily living behavior from low-level Internet of Things sensor data. We apply our techniques to a large dataset over 5 older adults in independent living apartments, for over 5 months with 30 to 50 ambient sensors per apartment. Findings highlighted three main themes: (1) lessons learned, (2) an evaluation of the various approaches employed, and (3) a semi-automated methodology to label large volumes of raw sensor data.

### WOULD YOU RATHER BE AT HOME OR IN A HOME? AN EXPLORATION AND COMPARISON OF OLD-AGE SOCIAL CONNECTEDNESS

Melissa Berlin[1], *1. University of Michigan, Ann Arbor, Michigan, United States*

Aging-related changes challenge older adults' experience of social connectedness and increase their risk of perceived isolation. As seniors cope with these changes, they often face the question of whether to age in place or move to a senior living facility. While many studies explore these scenarios separately, there is little research comparing these two living arrangements. Therefore, this study examined the following questions: how do older adults experience social connectedness and perceived isolation? How does this experience vary between older adults living alone in private homes and those living in assisted living facilities? I conducted 16 qualitative interviews with middle-old and old-old women (ages 75+) who lived alone in a private home or in an assisted living facility in southeast Michigan. The themes that emerged revealed differences in three aspects of social connectedness; interactions, relationships and belonging. Community dwelling respondents' interactions were characterized by intentionality, with minimal investment in forging new or deeper relationships and an emphasis on belonging to the world in terms of awareness, contribution, and cognitive ability. Assisted living respondents' experiences where characterized by availability of interactions and casual relationships within the facility and an emphasis on belonging to the facility community, while positioning oneself between the status of resident and staff. Surprisingly, neither group reported a notable degree of perceived isolation. Both had adapted their social connectedness expectations to reflect their current situation. These findings have meaningful implications for older adults facing decisions about where to age, as well as for the communities that serve them.

### THAT'S MY CREATOR'S REALM: UNDERSTANDING AFRICAN AMERICAN ASSISTED LIVING RESIDENTS' END-OF-LIFE PREFERENCES

Candace L. Kemp,[1] Alexis A. Bender,[2] Tammie Quest,[3] Mary H. Coyle,[4] and Molly M. Perkins[4], *1. Georgia State University, Atlanta, Georgia, United States, 2. Emory University, Atlanta, Georgia, United States, 3. Emory, Atlanta, Georgia, United States, 4. Emory University School of Medicine, Atlanta, Georgia, United States*

Assisted living, one of the fastest growing formal long-term care options for older adults in the U.S., increasingly is a site for end-of-life care.. Most residents are non-Hispanic and white, yet African Americans reside in these settings and relatively little is known about their end-of-life preferences. In this paper, we present an analysis of data collected as part of a larger five-year mixed-methods NIA-funded study (R01AG047048) examining end of life in assisted living. We analyze longitudinal qualitative data collected over two years in a large (>90 beds) care community catering to African American older adults. Drawing on 850 hours of participant observation, in-depth interviews with 25 residents, and record review data, we seek to: (a) understand residents' end-of-life preferences; and b) identify how and why preferences vary. Guided by principles of grounded theory, our analysis shows that most preferred a death where "you go to sleep and never wake up." Yet, residents varied in their preferences for the timing and location of death, nature of end-of-life care, and use of advanced directives. Age, health, health literacy, perceived quality of life, and not wanting to be a burden all influenced preferences. For most, religious beliefs were a key factor shaping these preferences. Perceiving that end of life, including how, when, where one dies, and the nature of suffering and care, ultimately is their "creator's realm," led to the near universal conclusion: "I got no control over it." We discuss implications of these findings for improving end-of-life care for African American residents.

### SOCIAL CAPITAL AND HOME- AND COMMUNITY-BASED SERVICE UTILIZATION AMONG URBAN OLDER CHINESE ADULTS

Changmin Peng,[1] Jeffery A. Burr,[2] Kyungmin Kim,[2] and Nan Lu[3], *1. Umass Boston, Boston, Massachusetts, United States, 2. University of Massachusetts Boston, Boston, Massachusetts, United States, 3. Renmin University of China, Beijing, China*

Home- and Community-Based Services (HCBS) are increasingly important for older adults who want to maintain their independence and remain in their communities. Although HCBS systems have been developed widely in many western countries and in some countries in Asia, China is just beginning to grapple with its rapidly aging population by offering HCBS in a limited fashion. The purpose of this study was to investigate the relationship between structural (e.g., citizenship activities, volunteering) and cognitive (e.g., social trust, a sense of belonging) social capital and HCBS utilization among older Chinese adults. The study also examined the mediating effect of structural social capital for the the relationship between cognitive social capital and HCBS utilization. We frame the study within the Andersen behavioral model of health services utilization and argue that within this framework social capital is an enabling factor. We analyzed survey data from 456 community-dwelling older adults living in the Gusu district of the city of Suzhou, China in 2015. Structural equation modeling was used to test the hypothesized relationships. The results showed that both cognitive and structural social capital were significantly associated with HCBS utilization. Structural social capital also served as a mediator between cognitive social capital and HCBS utilization, even after controlling for sociodemographic characteristics and other relevant covariates. The findings supported the utility of employing Andersen's behavioral model and social capital theory for better understanding older Chinese adults' utilization of HCBS. Interventions for increasing social capital may be useful for improving HCBS utilization in Chinese urban communities.

### TESTING THE RELIABILITY AND VALIDITY OF THE RESIDENT SATISFACTION INDEX

Sarah D. Holmes,[1] Elizabeth Galik,[2] and Barbara Resnick[2], *1. University of Maryland Baltimore Doctoral Program in Gerontology, Baltimore, Maryland, United States, 2. University of Maryland School of Nursing, Baltimore, Maryland, United States*

Understanding residents' life satisfaction in assisted living (AL) is essential for creating supportive environments that are targeted toward the needs and desires of residents. Unfortunately, few measures have been developed and tested to evaluate residents' life satisfaction in AL. The purpose of this study was to test the reliability and validity of the Resident Satisfaction Index (RSI) which was designed to measure residents' life satisfaction in AL. Baseline data was used from a study testing the Dissemination and Implementation of Function Focused Care in AL. A total of 501 residents from 54 AL facilities were included in the sample. Based on Rasch analysis, there was evidence of internal consistency with an alpha coefficient of 0.95 and validity based on INFIT and OUTFIT statistics which ranged from 0.4 to 1.6. Item mapping showed the easiest item to endorse was item 10 which referred to perceptions of staff's kindness. The hardest item to endorse was item 17 which asked about staff's responsiveness to residents' needs. Differential item functioning (DIF) analysis was done to examine differences in item responses by age, gender, and cognitive status. The measure was equally appropriate for those with and without mild to moderate cognitive impairment and between males and females, 5 out of 22 items were answered differently based on gender. 117 (23%) participants scored so high in satisfaction that they could not be differentiated. Findings support the reliability and validity of the measure although we recommend adding more difficult items to better differentiate between residents.

### DEVELOPING AND TESTING AN ASSISTED LIVING ENVIRONMENT MODEL

Sarah D. Holmes,[1] Elizabeth Galik,[2] and Barbara Resnick[2], *1. University of Maryland Baltimore Doctoral Program in Gerontology, Baltimore, Maryland, United States, 2. University of Maryland School of Nursing, Baltimore, Maryland, United States*

The assisted living (AL) environment plays an important role in supporting residents' life satisfaction and helping them to age in place. Guided by ecological theory, the AL environment is multidimensional and has many interrelated components including staffing (e.g. direct care workers, nursing, activity staff), services provided (e.g. medical, mental health, pharmacy), amenities offered at the setting (e.g. beauty salon, computer room, exercise facilities), and built environment features (e.g. walkability). Moreover, evidence suggests that aspects of the AL environment can enhance or detract from the physical function, well-being, social engagement, and behavioral outcomes among residents. The purpose of this study was to develop and test an integrative AL environment measurement model that includes indicators of staffing, services, amenities, and the built environment. Baseline data was used from a study testing the Dissemination and Implementation of Function Focused Care in AL. A total of 54 AL facilities across three states were included in the sample. Settings ranged in size from 31 to 164 beds with an average size of 82.2 (SD=26.2) beds and the majority were for profit facilities (n=41, 74.5%). Structural equation modeling was used to test the proposed model. Results showed that the model fit the data (chi-squared/df=1.86, p<.05; CFI=.858, RMSEA=.126). Having an integrative AL environment measurement model will advance future research that explores the impact of the environment on resident outcomes. In addition, findings from this study can inform interventions and programs designed to modify AL environments to optimize residents' ability to age in place.

### REASONS FOR RELOCATING TO ASSISTED LIVING: THE PUSH, THE PULL, AND DECISIONAL CONTROL

Evan Plys[1], *1. University of Colorado School of Medicine, Denver, Colorado, United States*

Using the push-pull framework, this study describes reasons for relocation and self-reported decisional control in the move to assisted living (AL). A sample of 202 residents of 21 ALs responded to a semi-structured questionnaire regarding their relocation. Participants most commonly relocated from a private local residence (n = 80, 40%), hospital/rehab facility (n = 27, 13%), or private long-distance residence (n = 24, 12%). The most frequently reported pull reasons to relocate to an AL were: "security and safety" (n = 46), "closer to family or friends" (n = 43), and "appearance of the facility" (n = 40). The most frequently reported push reasons to relocate from a previous residence were: "health problems" (n = 94), "others planned the move" (n = 87), and "fear of an accident" (n = 53). On average, participants who moved from other ALs reported the most decisional control in the move (M = 3.94, SD = 1.47), while participants from hospitals/rehab facilities reported the lowest control (M = 2.48, SD = 1.42). On average, participants who relocated from other ALs reported the most pull factors (M = 2.67, SD = 1.15), while participants from independent living communities reported the most push factors (M = 2.53, SD = 1.46). Results suggest that current residents commonly cite safety as both a push and pull reason for relocating to their AL. In addition, reasons for relocation and decisional control varied based on previous residence, which may be useful for identifying AL residents at risk for relocation stress.

### SESSION 3275 (POSTER)

### CANCER & BIOBEHAVIORAL HEALTH

### HEALTH EXPERIENCES RESEARCH ON CANCER-RELATED FATIGUE AMONG OLDER ADULTS

Susan M. Hannum,[1] Katherine Smith,[1] Dori M. Beeler,[1] and Jill Owczarzak[2], *1. Johns Hopkins Bloomberg School of Public Health, Baltimore, Maryland, United States, 2. Johns Hopkins School of Public Health, Baltimore, Maryland, United States*

Among older cancer patients, cancer-related fatigue is a common chronic problem that may result in functional dependence and decreased quality of life. Currently, there exists no standard of care for managing fatigue among cancer patients, pressing the need for further research and the development of novel interventions. Our research followed the validated DIPEx (Database of Patient Experiences) methodology, which utilizes in-depth, narrative interviews to create a rich and broad archival resource of patient stories to highlight commonalities and differences in illness experiences. We interviewed 9 older women (aged 60+ years) who have been diagnosed with and treated for breast cancer (Stage I-IV (IV only if clinically-stable). Interviews were audio and/or video-recorded and transcribed for analysis of emergent themes using MAXQDA qualitative software. Four primary themes have emerged from the data: (1) fatigue is a distressing side effect of treatment for which patients do not feel adequately prepared; (2) information about fatigue and how to deal with it is not systematically provided within the oncology setting; (3) patients develop their own systems for managing fatigue and general energy levels (e.g., limiting activities, using blocks of time strategically, etc.); and (4) social support for fatigue varies. Education about cancer-related fatigue and its management represents an unmet need among older breast cancer survivors. The development and implementation of both clinician training initiatives and patient-facing educational and engagement interventions represent important next steps in supporting the care needs of cancer patients and survivors.

### CHANGES IN RELIGIOUSNESS AMONG OLDER ADULTS FOLLOWING CANCER DIAGNOSIS

Tonia K. Murphy,[1] Sara Wilton,[1] Amanda Blackmar,[1] Edward H. Thompson,[2] and Andrew Futterman[3], *1. Loyola University of Maryland, Baltimore, Maryland, United States, 2. College of the Holy Cross, Worcester, Massachusetts, United States, 3. Department of Psychology, Loyola University Maryland, Baltimore, Maryland, United States*

This study aims to identify the changes in individuals' religious beliefs after receiving a cancer diagnosis. Open-ended religious development interviews were completed with 278 older adults (aged 55 to 102) in the northeast United States. Of these 278, 16 of the interviewees had received a cancer diagnosis within the previous two years. These older adults who report a cancer diagnosis describe few changes in their overall religiousness. On the contrary, these individuals describe longstanding, generally consistent patterns of religiousness that began early in life, most often related to their family upbringing. While most of these cancer-diagnosed individuals have sustained their religious involvement over time, a few have rejected or substantially modified their religious involvement for reasons unrelated to their recent diagnosis (e.g., most often due to marrying someone of a different faith tradition). In addition, those who received a cancer diagnosis describe their religious involvement primarily in terms of its emotional and social reward. These individuals report little complexity of religious belief - little questioning, little doubt in religious doctrine. Instead they report a straightforward, deep, comforting religious belief. This is in contrast to frequent critical comments on religious belief and behavior reported by healthy older adults in our sample. Such findings are in keeping with previous research that describes a more extrinsically oriented religious involvement among older adults who are ill or vulnerable. We discuss these findings in light of both social selectivity theory (E.g., Carstensen, 1992) and stress and coping theory (Lazarus & lazarus, 2006).

### δ PREDICTS GENERAL PSYCHOPATHOLOGY

Donald R. Royall,[1] and Raymond F. Palmer[2], *1. Departments of Psychiatry, Medicine and Family & Community Medicine, The University of Texas Health Science Center at San Antonio, San Antonio, Texas, United States, 2. Department of Family & Community Medicine, The University of Texas Health Science Center at San Antonio, San Antonio, Texas, United States*

"δ" is a transdiagnostic omnibus dementia severity measure derived from general intelligence (i.e., Spearman's "g"). It can be estimated in any cognitive battery that contains a measure of Instrumental Activities of Daily Living (IADL). As dementia's essential cognitive impairment, δ may also explain the appearance of Behavioral and Psychological Symptoms of Dementia (BPSD). Alternatively, the latter may be associated with orthogonal domain-specific cognitive impairments, unrelated to δ and therefore to dementia. The δ homolog "dDx" was tested as a predictor of one year prospective BPSD among n = 723 participants in the Texas Alzheimer's Research and Care Consortium (TARCC). Twelve Neuropsychiatric Inventory (NPI-Q)-rated BPSD were themselves submitted to confirmatory factor analysis resulting in a well-fit bifactor model rating general psychopathology (p), positive (+) and negative (-) symptoms. dDx and orthogonal cognitive factors rating memory (MEM) and executive function (EF) were regressed onto prospective p, (+) and (-). dDx was strongly associated with p ($r = -0.59$, $p < 0.001$). MEM was associated only with (+) ($r = 0.14$, $p < 0.001$). EF was associated only with (-) ($r = -0.23$, $p < 0.001$). This is the first demonstration of p in the geriatric literature. p's strong association with δ suggests that general psychopathology arises from dementia itself. In contrast, (+) and (-) symptoms may arise from regional insults, e.g., to temporo-limbic and frontal circuits (respectively). No such regional pathology is likely to impact δ or p, as they are "indifferent" to their indicators and must derive from global Central Nervous System (CNS) features.

### LIVING ALONE, HAVING CHILDREN AND FRIENDS NEARBY, AND DAILY FRUIT AND VEGETABLE CONSUMPTION

Yeon Jin Choi,[1] Jennifer A. Ailshire,[1] and Eileen Crimmins[2], *1. University of Southern California, Los Angeles, California, United States, 2. Davis School of Gerontology, University of Southern California, Los Angeles, California, United States*

Fruit and vegetable consumption is associated with lower risk of chronic diseases and mortality. Despite the numerous health benefits, fruit and vegetable consumption of most older adults are below the daily recommendation. This paper aimed to investigate whether living alone and having children and friends nearby are associated with older adults' daily fruit and vegetable consumption using a nationally representative sample of older Americans. Daily fruit and vegetable consumption was measured using (1) daily serving and (2) daily recommendation (2 or more servings for fruits; 3 or more servings for vegetables). Poisson and logistic regression models were estimated using the HRS Health Care and Nutrition Study. The sample included 6,915 community-dwelling older adults. Older adults who were living alone had lower fruit and vegetable consumption and less likely to meet daily recommendation for vegetables, compared to those who were living with someone. Having friends nearby was positively associated with the outcomes, while having children nearby was associated with meeting daily recommendation for vegetables only among older adults living alone. Based on the findings, older adults who are living alone and do not have children and friends nearby may be at the risk of poor nutrition due to low levels of social support. Provision of help with grocery shopping (e.g., transportation, Supplemental Nutrition Assistance Program) and meal preparation (e.g., home-delivered meals) as well as more social opportunities that can improve social support network and encourage healthy eating (e.g., congregate meals) may increase daily fruit and vegetable consumption of older adults.

### SENIOR FRIENDLY PACKAGING AND PRODUCT TESTING: FEEDBACK FROM OLDER CONSUMERS

Lisa A. Hollis-Sawyer,[1] and Alison O'Neil[2], *1. Northeastern Illinois University, Chicago, Illinois, United States, 2. SeniorSelect, Atlanta, Georgia, United States*

By 2050, older adults ages 65 or older will account for 83.7 million people in the U.S. population (An Aging Nation: The Older Population in the United States, 2014). It is imperative that products and technologies are designed to accommodate age-related changes that older adults are likely to experience. Given this population surge of older adults, there is a growing interest in identifying consumer products that are usable for older adults or "senior friendly." Senior-friendly product testing (e.g., Senior Select®) focuses on the usability of various health and consumer products targeted to people with diminishment of any of the following: hearing, vision, taste, touch, smell, mobility & dexterity and / or mental acuity. A usability evaluation study was conducted in three senior living communities located in the Atlanta area. Twenty-nine participants ranged in age from 66 years old to 102 years old. Participants were shown a snack bar product and then asked to use the product themselves to perform a series of prepared tasks. After interacting with the product, participants were asked to share any comments that they had concerning the product. Issues of color contrast between the main packaging and the pull tab, easy of gripping and tearing the wrapper, the labeling of the nutrition information, and the package labeling (should refer to “adult” snack) were reported. Many respondents suggested that senior-friendly package design relates to their health and well-being. Implications toward a wide range of products for older adults of varying ability levels will be discussed.

### MATTERING AND MOBILITY: POSITIVE RELATIONS WITH OTHERS PROTECTS AGAINST FUNCTIONAL DECLINE IN AGING ADULTS

Hannah Baker,[1] Meghan Mitoraj,[1] and Elliot M. Friedman[1], *1. Purdue University, West Lafayette, Indiana, United States*

Social relationships are robust predictors of better health and greater longevity in aging adults. The current study examined a relatively understudied aspect of relationship quality - positive relations with others, a domain of eudaimonic well-being - and its independent associations with change in and incidence of mobility limitations in a sample of mid-life and older adults from the Midlife in the United States (MIDUS) study. Using data from all 3 waves of MIDUS, we examined the extent to which positive relations with others predicted smaller increases in limitations and reduced risk of onset of new limitations over a 18-20 follow-up period. Linear and logistic regression models, adjusted for demographic and health confounds, showed that higher positive relations scores predicted slower increase in limitations over time (p=.003) and reduced risk of incidence of new limitations (p=.01). These effects were also observed over and above the associations with more traditional structural and functional measures of social relationships, neither of which was significantly linked to changes in functional abilities. These results suggest that positive relations with others may act as a unique protective factor for functional decline, both in individuals with no initial functional limitations and in those with existing limitations. They also extend prior research on the potential health benefits of social relationships to include a social dimension of eudaimonic well-being. Finally, they suggest that vulnerability to functional limitations and the potential benefits of social connectedness extend to mid-life as well as older adults.

### CANCER, COGNITION, AND COMMUNICATION: A 2-DAY INTERPROFESSIONAL GERIATRIC PSYCHO-ONCOLOGY SYMPOSIUM

Natalie Gangai,[1] Ruth Manna,[1] Smita Banerjee,[2] Rosario Costas Muniz,[1] Christian Nelson,[2] and Beatriz Korc-Grodzicki[1], *1. Memorial Sloan Kettering Cancer Center, New York, United States, 2. Memorial Sloan Kettering Cancer Center, New York, New York, United States*

Background: Most cancer deaths are in patients older than 65 years. Healthcare professionals (HCPs) caring for older adults with cancer must be equipped with skills to manage cognitive related changes and the nuances of communication with patients and caregivers. Methods: A two-day interprofessional symposium was developed to increase knowledge regarding 1) chemotherapy-related cognitive changes; 2) distress, delirium, dementia and depression in older cancer patients; 3) communication with patients with cognitive deficits and their caregivers; 4) decision making capacity. Presenters include geriatric medicine, geriatric psychiatry, occupational therapy and legal, ethics and communication experts. Day one centered on didactics with a complex case interprofessional discussion. Day two comprised of a communication skills training consisting of three modules: Geriatrics 101, Communication and Cognitive Deficits and Shared Decision Making. Participants role-played with simulated older adult patients and caregivers. Knowledge, self-efficacy and satisfaction were assessed. Results: A total of 75 people attended day one and 33 people attended day two. Most attendees were white (74.4%) and female (85.4%). Nurses (36.6%), social workers (29.3%), physicians (14.6%), others (19.5%) were represented. Mean knowledge increased (t=-3.23, df (13), p<0.05) from pre (M=0.83) to post (M=0.96). Mean self-efficacy in communication skills increased significantly across the three modules from 3.33 to 4.51 on a 5-point Likert scale (t=-6.40, df=23, p<0.001). Discussion: This two-day symposium shows an increase of knowledge and self-efficacy among HCPs caring for older adults. Skills related to cognitive changes and communication are essential to providing patient-centered care and making shared decisions with older adults and their caregivers.

### PSYCHOCOGNITIVE REACTIVITY TO INSUFFICIENT SLEEP AND ITS ASSOCIATION WITH BODY MASS INDEX IN MIDDLE-AGED WORKERS

Taylor Drury,[1] Soomi Lee,[1] Orfeu M. Buxton,[2] and David M. Almeida[2], *1. University of South Florida, Tampa, Florida, United States, 2. Pennsylvania State University, University Park, Pennsylvania, United States*

Individuals tend to report more stressors on days after nights with fewer hours of sleep. There may be individual differences such that this negative sleep duration—stressor perception relationship is stronger for some than others, which may have implications for health outcomes. However, we know little about whether differences in stressor perception in response to insufficient sleep (“psychocognitive reactivity to insufficient sleep”) are associated with health outcomes such as body weight. This study examined whether psychocognitive reactivity to insufficient sleep were associated with body mass index (BMI) in midlife workers. We used a sample of 127 office workers (Mage=45.2±6.2) who participated in a daily diary study for 8 consecutive days as part of the Work, Family, and Health Study. Multilevel models tested whether daily number of stressors was predicted by previous nights’ sleep. We outputted within-person slopes of stressors regressed on sleep duration to predict BMI (kg/m2). Analyses adjusted for sociodemographic characteristics and mean stressors across days. On average, workers reported more stressors following nights with shorter sleep duration than usual (negative slope means higher reactivity). Compared to those with average reactivity (within ±½SD; reference), workers with higher reactivity (≤-½SD) had higher BMI (p<.05). The BMI of these workers fell in the obese range. This study is one of the first to report that middle-aged workers with higher psychocognitive reactivity to insufficient sleep may be at greater risk for obesity. Future interventions should focus on improving middle-aged workers’ sleep health to reduce next-day stressors and thereby improve their body weight.

### DOES PERCEIVED LONELINESS MATTER FOR DIVERSE OLDER MEN AND THEIR PROSTATE-SPECIFIC-ANTIGEN TESTING BEHAVIORS?

Tamara J. Cadet,[1] Shanna L. Burke,[2] Jamie Mitchel,[3] Kyaien Conner,[4] and Frances Nedjat-Haiem[5], *1. Simmons University, Boston, Massachusetts, United States, 2. Florida International University, Miami, Florida, United States, 3. University of Michigan School of Social Work, Ann Arbor, Michigan, United States, 4. University of South Florida, Tampa, Florida, United States, 5. New Mexico State University School of Social Work, Las Cruces, New Mexico, United States*

Evidence suggests loneliness is associated with poorer health practices and fewer health promoting behaviors, yet may be associated with greater use of the healthcare system. This dichotomy highlights the lack of understanding in the literature about the relationship between the experience of loneliness in early disease detection behaviors, such as prostate cancer screening and appropriate clinical interventions to support older men. Utilizing a series of logistic regression models, this investigation examined the relationship between loneliness and prostate cancer screening in 2008 and 2012 among White, Black, and non-Hispanic men, ages 50-74 years, (n= 4,875 for 2008 and 7.063 for 2012). The data source as the Health and Retirement Study. Findings indicate that White men were less likely to participate in PSA screening in 2008 if they felt left out or isolated. There was a reduced likelihood of screening among Black men who feel as though they have a lot in common with those around them in 2012. Utilizing approaches such as cognitive-behavioral therapy, motivational interviewing, and solution-focused practice, clinical social workers can have shared decision-making conversations to understand this phenomenon. Clinical social workers have unique training in the person-in-environment model that emphasizes the biological, psychological, social, and spiritual factors that influence behaviors such as cancer screening participation, that can help to understand men's experiences, feelings or needs related to cancer screening participation. Given the lack of focus on men's, this study provides formative data to test interventions to increase the well-being of older men.

### RESTING HEART RATE MODERATES THE RELATIONSHIP BETWEEN NEUROPSYCHIATRIC SYMPTOMS, MCI, AND ALZHEIMER'S DISEASE

Shanna L. Burke[1], *1. Florida International University, Miami, Florida, United States*

Little is known about how resting heart rate moderates the relationship between neuropsychiatric symptoms and cognitive status. This study examined the relative risk of NPS on increasingly severe cognitive statuses and examined the extent to which resting heart rate moderates this relationship. A secondary analysis of the National Alzheimer's Coordinating Center Uniform Data Set was undertaken, using observations from participants with normal cognition at baseline (13,470). The relative risk of diagnosis with a more severe cognitive status at a future visit was examined using log-binomial regression for each neuropsychiatric symptom. The moderating effect of resting heart rate among those who are later diagnosed with mild cognitive impairment (MCI) or Alzheimer's disease (AD) was assessed. Delusions, hallucinations, agitation, depression, anxiety, elation, apathy, disinhibition, irritability, motor disturbance, nighttime behaviors, and appetite disturbance were all significantly associated (p<.001) with an increased risk of AD, and a reduced risk of MCI. Resting heart rate increased the risk of AD but reduced the relative risk of MCI. Depression significantly interacted with resting heart rate to increase the relative risk of MCI (RR: 1.07 (95% CI: 1.00-1.01), p<.001), but not AD. Neuropsychiatric symptoms increase the relative risk of AD but not MCI, which may mean that the deleterious effect of NPS is delayed until later and more severe stages of the disease course. Resting heart rate increases the relative risk of MCI among those with depression. Practitioners considering early intervention in neuropsychiatric symptomology may consider the downstream benefits of treatment considering the long-term effects of NPS.

### METABOLITES ASSOCIATED WITH HIGH VERSUS LOW WALKING ABILITY AMONG COMMUNITY-DWELLING OLDER MEN AND WOMEN

Megan M. Marron,[1] Stacy G. Wendell,[1] George C. Tseng,[1] Robert M. Boudreau,[1] Adam J. Santanasto,[1] Clary Clish,[2] Joseph M. Zmuda,[3] and Anne B. Newman[1], *1. University of Pittsburgh, Pittsburgh, Pennsylvania, United States, 2. Broad Institute, Cambridge, Massachusetts, United States, 3. Department of Epidemiology, University of Pittsburgh; Pittsburgh, Pennsylvania, United States*

Low walking ability is highly prevalent with advanced age and associated with a higher risk of major adverse health outcomes. Metabolomics may help better characterize differences among older adults with vastly different walking abilities and provide insight into altered metabolic processes underlying age-related declines in physical functioning. Here, we sought to identify metabolites associated with high versus low walking ability using a nested case-control study of 120 community-dwelling adults ages 79-95 (40% men, 10% black) from the Cardiovascular Health Study (CHS) All Stars study. Participants with high versus low walking ability were matched one-to-one on age, gender, race, and fasting time. Using liquid chromatography-mass spectrometry, 569 metabolites were identified in overnight-fasting plasma. High versus low walking ability was defined as the best versus worst tertile of gait speed (≥0.9 versus <0.7 meters/second) and Walking Ability Index scores (7-9 versus 0-1). Ninety-six metabolites were associated with walking ability extremes (p<0.05, false discovery rate<30%), where 24% were triacylglycerols. Triacylglycerols containing mostly polyunsaturated fatty acids (e.g., omega-3) were higher, whereas those containing mostly saturated/monounsaturated fatty acids were lower among those with high versus low walking ability. Arginine and proline metabolism was a top pathway identified. Body mass index partly explained the association between a subset of metabolites and walking ability extremes. These findings may partly reflect pathways implicating modifiable risk factors including excess dietary lipids and lack of physical activity, which contribute to obesity and cause further alterations in metabolic pathways, potentially leading to age-related declines in walking ability in this cohort.

### AGE MODERATES THE ASSOCIATION BETWEEN ENGAGEMENT IN MINDFULNESS AND PSYCHOLOGICAL DISTRESS AMONG CANCER SURVIVORS

Anao Zhang,[1] Rita X. Hu,[2] Lydia Li,[1] and Kaipeng Wang[3], *1. University of Michigan, Ann Arbor, Michigan, United States, 2. University of Michigan, Ann Arbor, Ann Arbor, Michigan, United States, 3. Texas State University, San Marcos, Texas, United States*

Geriatric cancer survivors constitute up to 65% of the U.S. survivorship population, and their psychosocial wellbeing are increasingly recognized. Facing high risk of psychological distress, many cancer survivors engage in mindfulness to reduce psychological distress. However, the acceptance of mindfulness across age groups was rarely examined. Based on the Motivational Theory of Life Span Development, mindfulness practice actives survivors' secondary control strategies to better cope with the loss of primary control caused by cancer. In other words, older survivors may respond differently than their young counterparts when facing psychological distress. Therefore, it is important to examine to what extent engagement in mindfulness is different across age groups. Using the cross-sectional 2017 National Health Interview Survey (N=3,068), this study evaluated the association between engagement in mindfulness and psychological distress among cancer survivors and examined whether age moderates such relationship. Age significantly moderated the association between psychological distress and engagement in mindfulness, OR=0.97, p<0.05. The correlation coefficient between engagement in mindfulness and psychological distress were significantly greater among younger cancer survivors (<65 years old) than their older counter parts (65+), z=2.24, p<0.05. Consistent with previous literature and the Motivational Theory, this study found age moderates the relationship between psychological distress and mindfulness engagement, and this relationship was much stronger among younger cancer survivors comparing to their older counter parts. Therefore, geriatric cancer survivors' unique psychosocial challenges should be addressed in ways that are appropriate to their developmental characteristics.

### STRAIN AMONG CAREGIVERS OF OLDER BREAST CANCER PATIENTS

Janine A. Overcash,[1] Susan Fugett,[2] Alai Tan,[3] Jane Ginther,[3] and Nicole Williams[3], *1. The Ohio State University, College of Nursing, Columbus, Ohio, United States, 2. Stephanie Spielman Comprehensive Breast Center, The Ohio State University, Columbus, Ohio, United States, 3. The Ohio State University, Columbus, Ohio, United States*

Caregiver strain is the emotional and physical demands associated with providing care, particularly when the needs exceed the resources. The purpose of this presentation is to illustrate the predictors of strain among caregivers of older breast cancer patients. Relationships among caregiver strain (Modified Caregiver Strain Index), age, employment status, patient characteristics and scores on the comprehensive geriatric assessment (grip strength, Timed up & Go, Mini Nutritional Assessment, MiniCog, Geriatric Depression Scale, Pittsburgh Sleep Quality Index, and Brief Fatigue Inventory) are described. This cross sectional study included women who were diagnosed with breast cancer, over the age of 69 years, receiving any type of treatment and seeking an initial assessment in a geriatric oncology program.. Dyads (N=39) had a mean patient age of 77 and caregiver age of 64 years. Most patients were diagnosed with infiltrating ductal carcinoma 30 (76.9%), most had lumpectomy 23 (59%), and were stage 1 15 (38.5%). Most patients had one caregiver 22(56.4%) who worked full-time 12(30.8) and rated their health as very good 16 (25.4%). Increasing age of the caregiver was associated with less caregiver strain (r = -0.45, p=.02). Employment status of the caregiver was significantly related to caregiver strain -0.35 (p=.03). Caregivers employed full time 3.5 (p = .08), and part time 5.9 (p=0.3) experienced greater strain (mean±SD: 9.4±8.8) than those employed full-time (4.7±3.4). Patient depression, caregiver employment status and age of the caregiver are related to caregiver strain. Healthcare providers must assess for strain in cancer givers of cancer patients.

### GENETIC INFLUENCES ON LUNG FUNCTION CONTRIBUTE TO SUBSEQUENT AGE CHANGES IN MOTOR FUNCTION

Deborah G. Finkel,[1] and Marie Ernsth-Bravell[2], *1. Indiana University Southeast, New Albany, Indiana, United States, 2. Jönköping University, Jönköping, Sweden*

Researchers have striven to determine whether the age changes in physical and cognitive functioning are coincident, or does change in one domain precede functioning in the other. Dual change score models (DCSM) facilitate testing of hypotheses about temporal patterns of aging. Previous investigations in the Swedish Adoption/Twin Study of Aging (SATSA) indicate three directional effects: age changes in processing speed contribute to subsequent age changes in cognition, age changes in lung function contribute to subsequent age changes in processing speed, and age changes in motor function contribute to subsequent age changes in processing speed. In the current analysis we apply DCSM to twin data to examine the nature of the longitudinal relationship between motor functioning and lung function. Three motor functioning factors were created from 20 performance measures: balance, flexibility, and fine motor movement. Peak expiratory flow measured lung function. Participants were 829 adults aged 50-88 at the first of 9 waves of testing (mean = 4.4 waves) covering a 27-year follow-up period (mean = 13.1 years). Model comparisons indicated that genetic influences on decline in lung function contributed to subsequent decline in motor function. Combined with previous results, these results suggest a pathway that may start with age declines in lung function, which then contribute to declines in motor function, which in turn contribute to subsequent declines in processing speed and then cognitive decline. These data indicate that interventions focusing on improving or maintain lung function should have the added effect of maintaining motor and cognitive function.

### SESSION 3280 (POSTER)

### CARDIOVASCULAR DISEASE

### AUGMENTATION INDEX IS A PREDICTOR OF CEREBRAL BLOOD FLOW ACROSS GLOBAL GRAY MATTER IN THE ELDERLY

Adrian Noriega de la Colina,[1] Badji Atef,[2] Sven Joubert,[3] Louis Bherer,[4] Maxime Lamarre-Cliche,[5] Claudine Gauthier,[6] Julien Cohen-Adad,[7] and Hélène Girouard[8], *1. Research Centre of the IUGM, University of Montreal, Montréal, Québec, Canada, 2. NeuroPoly Lab, Institute of Biomedical Engineering, Polytechnique Montreal, Montréal, Québec, Canada, 3. Centre de recherche de l'Institut Universitaire de Gériatrie de Montréal, Montréal, Québec, Canada, 4. Montreal Heart Institute, Montreal, Quebec, Canada, 5. Institut de Recherches Cliniques de Montréal, Université de Montréal, Montreal, Quebec, Canada, 6. PERFORM Centre, Concordia University, Montreal, Quebec, Canada, 7. NeuroPoly Lab, Institute of Biomedical Engineering, Polytechnique Montreal, Montréal, Québec, Canada, 8. Department of Pharmacology and Physiology, Faculty of Medicine, Université de Montréal, Montréal, Québec, Canada*

Arterial stiffness and blood pressure (BP) are contributors to cognitive decline and dementia. Lower global cerebral blood flow (CBF) is one of the earliest manifestations of biological alterations linked to cognitive decline, nevertheless the best cardiovascular predictor of CBF in gray-matter (CBF-GM) remains to be identified. Our objective is to determine the best predictor of CBF-GM levels amongst cardiovascular parameters. Eigthy-four healthy participants between 60-80 years-old were evaluated. The measured parameters for arterial stiffness were the carotid-femoral pulse wave velocity (cf-PWV) and the augmentation index (Aix), measured by applanation tonometry (SphygmoCor). Mean systolic BP (SBP) was monitored over 24-hours and analyzed following Hypertension-Canada's guidelines(2018). The coefficient of variation for 24-hours SBP was calculated by dividing the standard deviation by the mean. Resting CBF-GM was quantified from pseudocontinuous arterial-spin-labeling using Neurolens 2.0(pcASL), and acquired on a 3T scanner (MAGNETOM Prisma-Fit). We created multiple linear regression models for each independent variable (cf-PWV, Aix, mean-SBP in 24 hours and the coefficient of variation of 24-hours SBP) using age, sex, schooling and body mass index as covariates. Multiple linear regression models demonstrated that two independent variables could predict CBF-GM levels: a)PWV (p=0.010) and b)Aix (p=0.006). In this cohort, we demonstrated that while PWV and Aix are both predictors of CBF-GM levels, it is Aix which has the highest predictive value and could be a useful tool to understand the interplay between lower CBF-GM and arterial stiffness. These results also indicate that Aix may be a good therapeutic target to preserve brain health and cognition.

### ASSOCIATION BETWEEN BLOOD PRESSURE AND COGNITIVE FUNCTION OF COMMUNITY-DWELLING OLDEST OLD IN OKINAWA, JAPAN

Yukihiro Namihira,[1] Takashi Tokashiki,[2] Akio Ishida,[3] Yusuke Ohya,[4] and Hiroko H. Dodge[5], *1. Department of Cardiovascular Medicine, Nephrology and Neurology, Graduate School of Medicine, University of the Ryukyus, Nakagami county, Okinawa, Japan, 2. Department of Neurology, National Hospital Organization Okinawa Hospital., Okinawa, Japan, Japan, 3. Department of Cardiovascular Medicine, Nephrology and Neurology, Graduate School of Medicine, University of the Ryukyus, Okinawa, Japan, Japan, 4. Department of Cardiovascular Medicine, Nephrology and Neurology, Graduate School of Medicine, University of the Ryukyus, Okinawa, Japan, 5. University of Michigan, Ann Arbor, Michigan, United States*

Background: Adults 80 years and older are the fastest-growing segment of the Japanese population and face a high risk of cognitive decline. There are some evidences connecting hypertension to cognitive decline. In mid-life hypertension is known to have influence the cognitive decline in older age. However, a few study have examined the association between hypertension or vascular stiffness and cognitive function among elderly over 80 years old. We analyzed the associations between vascular stiffness and cognitive function among relatively healthy community-dwelling non-demented oldest old. Method: Data came from the Keys to Optimal Cognitive Aging (KOCOA) study; an ongoing cohort of relatively healthy volunteers aged over 80 years old, living in Okinawa, Japan. In 2017, 105 non-demented (Clinical Dementia Rating < 1) subjects completed three kinds of examination for vascular function (75 % female, mean age (SD) 84.0 (3.0)). We categorized subjects into low and high cognitive function groups using Montreal Cognitive Assessment (MoCA) (25/26 as a cutpoint). Logistic regression models were used to examine the association between cognitive and vascular functions. Results: Narrower pulse pressure, an indicator of lower arterial stiffness, was associated with better cognitive function among subjects, after adjusting for gender, age, and education ($p \leqq 0.05$), although systolic and diastolic blood pressure were not. Conclusion: Our findings suggest that narrower pulse pressure is related with cognitive preservation. The present study supports the hypothesis that lower arterial stiffness is related with better cognitive function even among the oldest old.

### EFFECT OF INFLUENZA VACCINE ON TWEAK LEVELS IN THE ELDERLY: IMPLICATION IN CARDIOVASCULAR PROTECTION

Juliette Tavenier,[1] Min Ouyang,[2] Huifen Li,[3] and Sean X. Leng[3], *1. Clinical Research Centre, Copenhagen University Hospital, Hvidovre, Hvidovre, Denmark, 2. Department of Geriatrics, The Second Xiangya Hospital, Central South University, Changsha, Hunan, China, 3. Johns Hopkins University School of Medicine, Baltimore, Maryland, United States*

Substantial evidence suggests a protective effect of annual influenza immunization on cardiovascular diseases (CVD). The inflammatory mediator TNF-related weak inducer of apoptosis (TWEAK) is thought to be involved in the pathogenesis of CVD. We previously showed that administration of a standard-dose influenza vaccine reduced circulating TWEAK levels. This study aimed to test the hypothesis that a high-dose vaccine would have greater impact on TWEAK levels than the standard-dose. Two groups of participants matched on age and sex were included in the study. One group (n=25) received standard-dose vaccine during 2008-2009 season, the other group (n=25) received high-dose vaccine during 2014-2015 season. Soluble TWEAK (sTWEAK) levels were assessed using ELISA in serum samples collected immediately before vaccination and during the 4th week after vaccination. Vaccine-induced strain specific antibody titers were measured by hemagglutination inhibition assay. The participants had a mean age 86 years and 68% were women. Our preliminary results thus far demonstrated no statistically significant change in sTWEAK levels after vaccination in either group (Wilcoxon matched-pairs signed rank test: standard-dose group: median change [interquartile range]=11.2 [-92.2–197.1] pg/mL, p=0.72; high-dose group: -24.8 [-58.9–87.3] pg/mL, p=0.70). Pre-vaccination sTWEAK levels tended to be negatively associated with age (unadjusted linear regression of log2(x) transformed TWEAK levels on age: estimate [±SE]=-4.6% [±2.7%] change in TWEAK level per year, p=0.08). We continue to evaluate more pre- and post-vaccination samples. We have also begun exploring TWEAK expression by circulating immune cells (T and B lymphocytes and monocytes) and potential impact of influenza immunization in older adults.

### THE ASSOCIATION BETWEEN VITAMIN K STATUS AND CARDIOVASCULAR DISEASE IN OLDER ADULTS

M. Kyla Shea,[1] Daniel Weiner,[2] Gergory Matuszek,[1] Sarah L. Booth,[1] Stephen Kritchevsky,[3] Mary Cushman,[4] and Kathryn Barger[1], *1. Tufts University USDA Human Nutrition Research Center on Aging, Boston, Massachusetts, United States, 2. Tufts Medical Center, Boston, Massachusetts, United States, 3. Wake Forest School of Medicine, Winston-Salem, North Carolina, United States, 4. University of Vermont Larner College of Medicine, Burlington, Vermont, United States*

A role for vitamin K in cardiovascular disease (CVD) has been proposed because vitamin K-dependent proteins are present in vascular tissue. We evaluated the association between vitamin K status and incident CVD and mortality in older adults from the Health, Aging, and Body Composition Study (Health ABC), and conducted a replication analysis using the Multi-ethnic Study of Atherosclerosis (MESA). In both cohorts circulating phylloquinone (vitamin K1), measured from baseline fasting blood samples, was categorized as ≤0.5nM, >0.5-≤1.0nM, and >1.0nM. Multivariable Cox proportional hazards models assessed the association between circulating phylloquinone and risk of a composite of CVD and mortality. In Health ABC (n=1246, mean age 74 years, 57% female, 58% Caucasian), over a median 11.5 follow-up years, participants with ≤0.5 nM plasma phylloquinone (n=351) had a 27% higher risk for CVD and mortality compared to those with >1.0nM (n=457) [adjusted hazard ratio (95% confidence interval) (HR(95%CI)): 1.27(1.06-1.52)]. However, the risk for CVD and mortality did not differ between those with >0.5-≤1.0nM (n=438) and with >1.0nM plasma phylloquinone [HR(95%CI): 1.03(0.87-1.52)]. Serum phylloquinone was similarly associated with CVD and mortality in MESA, over a median 12.1 follow-up years (n=764, mean age 62 years, 54% female, 35% Caucasian) [HR(95%CI), compared to those with >1.0nM (n=368): <0.5nM (n=253): 1.54(1.03-2.32); 0.5-≤1.0nM (n=153): 1.23(0.76, 1.98)]. Lower circulating phylloquinone was associated with a higher CVD and mortality risk in two independent cohorts. Additional studies are needed to corroborate our findings and clarify if certain segments of the population can derive cardiovascular benefit from improving vitamin K status.

### CARDIOVASCULAR HEALTH AND SUCCESSFUL AGING: THE MULTI-ETHNIC STUDY OF ATHEROSCLEROSIS

Tzuo-Yun Lan,[1] Amy Krefman,[2] Michael Bancks,[3] Steven Shea,[4] Kiang Liu,[5] and Norrina Allen[5], *1. Institute of Hospital & Health Care Administration, National Yang-Ming University, Taipei, Taiwan, 2. Department of Preventive Medicine, Feinberg School of Medicine, Northwestern University, Chicago, Illinois, United States, 3. Wake Forest University School of Medicine, Winston Salem, North Carolina, United States, 4. Columbia University Mailman School of Public Health, New York, New York, United States, 5. Department of Preventive Medicine, Feinberg School of Medicine, Northwestern University, Chicago, Illinois, United States*

Individual risk factors have been shown to be associated with successful aging. However, the combined effect of behaviors and biomarkers on successful aging remains unclear. By using the Multi-Ethnic Study of Atherosclerosis (MESA) dataset, this study was to examine the association of AHA's Cardiovascular Health (CVH) with successful aging. A total of 1,597 who were followed from baseline (2000-2002) at age 49-64 through exam 6 (2016-2018) at age 65-80 were included. CVH, including smoking, body mass index (BMI), physical activity, diet, blood pressure, cholesterol, and blood glucose, was measured at baseline. The CVH score, ranging from 0-14, was divided into ideal (11–14), intermediate (9–10), and poor (0–8) groups. Normal or successful aging, defined as avoiding major disease (including cancer, cardiovascular, or severe lung or kidney diseases), no disability, high cognitive function, high physical functioning, and engagement with life, was assessed at exam 6. We compared the cumulative incidence of successful aging among three groups. Modified Poisson regression model was employed to estimate relative risk (RR) adjusting for age, gender, race, education, income, marital status, and alcohol consumption. Among study participants at baseline, 36% were in ideal, 39% in Intermediate, and 25% in poor CVH. By exam 6, only 18% met the criteria for successful aging. Compared with the poor group, the adjusted RRs (95 % CI) of successful aging for the intermediate and ideal groups were 1.78 (1.23-2.56) and 2.56 (1.79-3.67). Our data suggest that CVH in midlife is associated with successful aging in later life.

### READMISSION RISK BY INSURANCE TYPE FOR PATIENTS WITH HEART FAILURE IN SKILLED NURSING FACILITIES

Andrea E. Daddato,[1] Edward A. Miller,[2] Pamela Nadash,[2] Denise Tyler,[3] and Rebecca S. Boxer[4], *1. University of Colorado School of Medicine, Aurora, Colorado, United States, 2. University of Massachusetts Boston, Boston, Massachusetts, United States, 3. RTI International, Waltham, Massachusetts, United States, 4. Kaiser Permanente Institute for Health Research, Aurora, Colorado, United States*

Heart failure (HF) is a leading cause of potentially preventable hospital readmissions for Medicare beneficiaries from skilled nursing facilities (SNFs). This research seeks to determine if a HF patient's insurance type (Medicare Fee-for-Service (FFS) vs. Medicare Advantage (MA)) influences their risk for readmission within 30 days of hospital discharge to a SNF. MA beneficiaries receive benefits through managed care plans with restricted networks, but typically expanded benefits. This research is particularly timely in light of CMS' new penalties under the Protecting Access to Medicare Act (PAMA) directed at SNFs for 30-day rehospitalizations. Outcomes data on readmissions from a randomized controlled trial of HF Disease Management in SNFs conducted from 2014-2017 were used to evaluate the risk of readmission. Patients with HF receiving SNF care were enrolled and followed for 30 days from SNF admission. Patients were recruited from 29 primarily for-profit (93%) SNFs that contracted with an average of 4.07 (±5.48) MA plans. Of the 340 study participants followed, 62% had FFS Medicare coverage (n=212) and 38% had MA (n=128). In total, 23% (n=79) of patients experienced at least one readmission within 30 days of hospital discharge. FFS patients had a higher risk of rehospitalization within 30 days of hospital discharge than MA patients (25% vs. 20%), but the association between insurance type and rehospitalization was not statistically significant (p-value=0.177). Findings suggest that insurance type may be an important risk factor for rehospitalizations for patients with HF from SNF; however, a larger sample will need to confirm this relationship.

### CARDIOVASCULAR RISK MODERATES THE EFFECT OF RESISTANCE TRAINING ON PHYSICAL PERFORMANCE IN OLDER ADULT WOMEN

Lauren Marcotte,[1] Cindy Barha,[1] and Teresa Liu-Ambrose[1], *1. University of British Columbia, Vancouver, British Columbia, Canada*

We aimed to examine whether the Framingham Cardiovascular Risk Profile Score (FCRP) moderates the effect of progressive resistance training (RT) on mobility in older adult women. This is an exploratory analysis of a single-blind, 12-month randomized controlled trial in 155 omen, aged 65 to 75 years old, who were randomized to: 1x/week progressive RT; or 2x/week progressive RT program; or 2x/week balance and tone (BAT). At baseline and trial completion, mobility was measured using the Short Physical Performance Battery (SPPB). The SPPB is a composite measure of usual gait speed, standing balance, and sit to stand performance; scores < 9/12 are indicative of functional decline. Baseline 10-year cardiovascular risk was calculated using the FCRP. Participants were classified as either low risk (<16.5% FCRP score; LCVR) or high risk ≥16.5% FCRP score; HCVR). A complete case analysis (n=126) was conducted using a two-way analysis of covariance (ANCOVA) to evaluate the interaction effect of group by FCRP risk on SPPB scores at trial completion; baseline SPPB scores and age in years were entered as covariates. There was a significant interaction effect (F(1,126)=3.74, p=0.027). At trial completion, both 1x/RT and 2x/RT participants with HCVR demonstrated greater SPPB scores than those with LCVR (11.59 vs. 11.38 for 1x/week; 11.86 vs 11.46 for 2x/week). In contrast, BAT participants with HCVR demonstrated worse SPPB scores than those with LCVR (11.18 vs 11.66). Our data suggest that RT may be more efficacious for improving mobility in older women with higher cardiovascular risk than women with lower risk.

### SESSION 3285 (POSTER)

### CIVIC ENGAGEMENT AND LATE-LIFE EMPLOYMENT

### FACTORS INFLUENCING AMERICAN ELDERS' FORMAL CIVIC ENGAGEMENT: A SYSTEMATIC LITERATURE REVIEW

Haiping Chen,[1] and Fengzhi Ma[1], *1. Department of Sociology, Peking University, Beijing, China*

Summary: Today's American elders are increasingly being seen as an asset to society through civic engagement. A new challenge facing America now and in the years ahead is how to tap such asset. Formal organizations should play a leading role in institutionalizing American elders' civic engagement. Due to a structural lag between social changes and organizational practices, however, many organizations are not ready to engage the large number of American elders. To address this issue, formal organizations first need to know which factors affect American elders' civic engagement and then are able to come up with effective solutions. Although some studies have investigated the contributing factors of American elders' civic engagement, there are very few systematic syntheses of these factors from various different studies. To fill this gap, the authors conducted a mixed methods systematic literature review using meta-summary. Through electronic search of five databases and hand search of bibliographies, 22 articles were used in final analysis based on eligibility criteria, including 19 quantitative studies, two qualitative studies and one mixed methods study. Findings: The review identified six themes and 28 factors related to American elders' civic engagement. These themes encompassed socio-demographic factors (eight factors), health status (four factors), program characteristics (four factors), engagement opportunities (three factors), engagement outcomes (five factors) and social capital (four factors). Applications: Formal organizations are advised to develop relevant competencies to capture the influences of identified factors. Social workers are also required to develop multilevel competencies to better engage American elders with the organizational settings.

### A RISING TIDE LIFTS ALL BOATS: OLDER WORKER EMPLOYMENT RATES

Robert Harootyan[1], *1. Senior Service America Inc., Silver Spring, Maryland, United States*

U.S. population aging has been accompanied by aging of its workforce. One-fourth of workers are now 55+, a result of increasing labor force participation rates (LFPRs) of boomers. Older female LFPRs increased notably since 2000, now 34% (1.5 times greater than the 1947-2000 average). Older male LFPRs steadily declined for 50 years, but increased since 2000 (currently 45%). Despite higher LFPRs, many older workers are underemployed, unemployed or having trouble finding desired jobs. We conducted a pilot to test the hypothesis that areas with strong economic and overall-employment growth are more likely to have higher age 55+ LFPRs - i.e., strong overall demand should increase the likelihood that older persons are employed. Major metropolitan areas were selected because of their large labor pools and ACS age-specific labor force data. Of 318 large metros, we selected 14 whose 2017-2018 employment growth ranged from 0.7% to 3.3% (excluded were those with small negative change and those with concentrated special industries). Results: After calculating age-specific LFPRs from ACS five-year data and overall employment growth for each metro, we found a generally strong relationship between high growth and higher than average LFPRs for persons ages 55+. For example, two opposites: Dallas, with 3.2% employment growth, had age 55+ (five year intervals to 75+) LFPRs substantially higher than the national average (by 4-7 percentage points; significant at 0.05). But Pittsburgh, with 0.8% overall employment growth, had age 55+ LFPRs slightly lower than national average. Conclusion: A rising (economic/employment) tide lifts all boats, including older workers.

### CULTURAL MOTIVATION TO VOLUNTEER AMONG OLDER JEWISH ADULTS: AN EXPLORATORY STUDY

Eireann O'Dea,[1] and Andrew Wister[1], *1. Simon Fraser University, Vancouver, British Columbia, Canada*

The physical, mental, and social benefits for older adults who volunteer are well-documented. Absent from this area of research however, is an understanding of volunteer motivation and experiences among culturally diverse older adults. This study addresses this research gap by exploring the volunteer pathways, motivations, and experiences of Jewish older adults in Vancouver, BC, Canada. The Jewish community is notable for possessing high levels of social capital, indicated by close community ties and the large number of faith and culturally based organizations, including community centres, day schools, seniors' centres, and family service agencies, which provide many opportunities for older adults to volunteer. Despite this, they remain an understudied population. Semi-structured qualitative interviews were conducted with twenty-one older adult volunteers (age 55+), and two paid volunteer staff in the Jewish community. Theoretical concepts including social capital, generativity, and the life course perspective on aging were used to guide interview questions. Data analysis revealed three themes related to cultural motivation to volunteer: 1.) A desire to support the current and future generations of the Jewish community, 2.) To satisfy the "Jewish ethic" of giving back, and 3.) Experiences of discrimination (anti-Semitism) over the life course. Participants frequently volunteered for organizations that supported the infrastructure of the Jewish community. Findings indicate how cultural experiences and values may influence the decision to volunteer and the types of volunteer roles taken on by older adults. Further, they suggest the ways in which cultural and religious generativity may be expressed through volunteerism, a previously unexplored concept.

### CONTEMPORARY EARLY RETIREMENT FACTORS AND STRATEGIES TO ENCOURAGE AND ENABLE LONGER WORKING LIVES

Donna M. WILSON[1], *1. University of Alberta, Edmonton, Alberta, Canada*

Accelerating population aging is raising concern in many countries now about the availability of workers for essential work roles and responsibilities. A scoping research literature review was done to identify factors currently associated with early retirement and contemporary strategies to encourage and support longer working lives. Among 53 relevant articles, seven early retirement factors were revealed: Ill health, good health, workplace issues, the work itself, ageism, social norms, and having achieved personal financial or pension requirement criteria. Six solutions, none of which had been proven effective, were identified: Occupational health programs, workplace enhancements, work adjustments, addressing ageism, changing social norms, and pension changes. The evidence base on early retirement prevention is not strong, with qualitative research studies needed to gain a more in-depth understanding of early retirement influences and also mixed-methods studies needed to test early retirement prevention solutions for their effects, and particularly in the healthcare sector as healthcare needs typically rise with advanced ageing. Until more evidence is available, every healthcare and other organization should perform an early retirement risk assessment and identify current versus needed policies and programs to encourage and to enable more middle-aged and older people to work longer in life.

### RURAL RETIREMENT MIGRATION AND ACTIVE CITIZENSHIP: MOTIVATIONS, PRACTICES, AND PROCESSES

Rachel Winterton[1], *1. La Trobe University, Bendigo, Victoria, Australia*

Within rural settings, older adults play a significant role in ensuring community age-friendliness through their engagement as active citizens. However, the increasing heterogeneity of rural older adults is challenging expectations around how, and in what circumstances, this cohort will engage as active citizens. Given that rural retirement migration is a key contributor to this increasing heterogeneity, there is a need to understand the motivations for, and practices associated with active citizenship among older in-migrants. Drawing on qualitative data from two rural regions in Victoria, Australia, this paper draws on concepts from the rural citizenship literature to investigate how active citizenship practices of rural retirement migrants align with traditional rural codes of conduct. Semi-structured interviews were conducted with 39 rural retirement migrants (aged 56-76 years), which explored engagement in, and motivations for, active citizenship. Findings indicate that in line with traditional expectations around rural citizenship, most rural retirement migrants had assumed responsibilities associated with community governance, and engaged in protest aimed at defending the rights of rural people. However, conflict with traditional codes of conduct was observed in relation to both how active citizenship was enacted, and motivations for engagement. Additionally, rural retirement migrants highlighted barriers that had precluded their involvement as active citizens. These findings are discussed in relation to their implications for both the capacity of rural settings to meet the needs and expectations of older in-migrants, and the experience of ageing in place for resident older adults.

### POLICIES FOR PRESERVING THE WORK ACTIVITY OF PEOPLE IN RETIREMENT AGE IN HIGHER EDUCATION INSTITUTIONS IN BULGARIA

Zaharina A. Savova[1], *1. Medical College Yordanka Filaretova at Medical University - Sofia, Sofia, Bulgaria*

Demographically, aging of the world's population is a fact that requires the implementation and development of integrated, active policies for the inclusion of older people in economic and social life. In response to this challenge, an international UN plan for active aging was adopted in Madrid in 2002. By adopting this plan, all EU member states are committed to contribute to active aging by integrating the rights and needs of older people into national economic and social policies. The European Commission formulates the concept of active aging as adopting a healthy lifestyle, longer labor market participation, later retirement, and retention after retirement. In recent years managers of higher education institutions in Bulgaria have been pursuing a policy of preserving the labor and intellectual potential of retired research and teaching staff. There are different practices and activities aimed at preserving this valuable resource built for decades. This paper outlines the main activities and evaluation of the effectiveness of the Mentoring Program for Shared Experience and Knowledge. This pilot program was implemented over a five-year period from 2012 to 2017. It involved teachers in pre-retirement and retirement age working in higher education institutions. Monitoring and evaluation of the different forms of activities included in the program were made. The benefits for all participants have been derived. The results and the analysis made support the initial suggestion that the program is an opportunity for gradual withdrawal from work activity, preserving social well-being and successful adaptation to retirement from active labor activity.

### SESSION 3290 (POSTER)

### COGNITION

### AGE-RELATED DIFFERENCES IN INHIBITION INVESTIGATION OF SIMON AND FLANKER CONFLICTS IN ERPS

Rachel Scrivano,[1] and Paul Kieffaber[1], *1. William & Mary, Williamsburg, Virginia, United States*

It is unclear whether or not older adults experience more difficulty managing cognitive conflict by inhibiting distracting stimuli and/or ignoring irrelevant information than younger adults. A common procedure used to measure inhibitory function is through the use of congruent and incongruent stimuli. Specifically, past literature that used tasks like the Simon and flanker have found differing effects on reaction times and various event-related potential (ERP) amplitudes and latencies, suggesting that either inhibitory function is a unitary mechanism or multifaceted. Moreover, research exhibits uncertainty for whether or not age influences deficits to inhibitory function. Therefore, the present study sought to investigate these research questions by combining the Simon and flanker tasks into one unique task. The study's behavioral results indicate that older adults have greater difficulty with cognitive conflict in both Simon and flanker tasks due to significantly prolonged reaction times during incongruent trials. Furthermore, reaction time data posits that there are no significant age-related differences between the Simon and flanker task. This finding indicates that through the use of these tasks, inhibitory function is a unitary mechanism. In addition, preliminary electroencephalogram data shows that younger and older adults process the task's information similarly. These initial findings can further inhibition research through its use as benchmarks for the measurement of cognitive change and deficit identification in older adults.

### DO PSYCHOLOGICAL AND SOCIAL FACTORS MODERATE LINKS BETWEEN CARDIOVASCULAR HEALTH AND COGNITION IN LATER LIFE?

Jeremy B. Yorgason,[1] Melanie S. Hill,[1] Hailey Wellar,[1] Lance Erickson,[1] and Shawn Gale[1], *1. Brigham Young University, Provo, Utah, United States*

Cardiovascular health is related to cognition in later life (Samieri, 2018). Psychological factors, such as depressive symptoms, have been linked with cardiovascular health (Thomas, Kalaria, & O'Brien, 2004). Marital quality, an important indicator of social connection, has been linked with cardiovascular response (Seider et al., 2009), and both depression and marital satisfaction are linked with a quicker recovery from heart attacks (Keller, 1998). Depressive symptoms and marital quality may buffer links between cardiovascular health and cognitive functioning. The purpose of this study was to examine cardiovascular links with cognition, in connection with depressive symptoms and marital quality. Using data from 864 participants of the Life and Family Legacy study (Mean age = 61.78), we examined predictors from 2010 in relation to cognition measured in 2017/2018. Word recall and computation subscales of the Minnesota Cognitive Acuity Screen (MCAS) were used to assess cognitive functioning. Results from multiple regression models indicated that after controlling for age, gender, education, income, and marital status, having hypertension and higher depressive symptoms were predictive of word recall. Lower depressive symptoms were also predictive of higher computation scores. Depression did not moderate links between cardiovascular health and cognitive functioning. Among married participants (n=632), positive marital quality had no main effect nor moderating association with cardiovascular health predicting cognitive functioning. Further research is needed to better understand how biological, psychological, and social factors interact to affect cognition in later life. Longitudinal work should track these associations in context of cognitive changes with aging.

### SOMATIC COMPLAINTS, A KEY FACTOR OF DEPRESSIVE SYMPTOMS ASSOCIATED WITH SUBJECTIVE MEMORY

Wonjeong Haavisto,[1] Julie Blaskewicz Boron,[2] Sherry Willis,[3] and Warner Schaie[3], *1. University of Nebraska, Omaha, Nebraska, United States, 2. University of Nebraska at Omaha, Omaha, Nebraska, United States, 3. University of Washington, Seattle, Washington, United States*

Prior research found depressive symptoms and subjective memory to be associated with objective memory (OM) performance. One key factor of subjective memory, as measured by the Memory Functioning Questionnaire (MFQ), is General Frequency of Forgetting (GFF). However, few studies focused on identifying specific factors of depressive symptoms when examining associations between depressive symptoms, subjective memory and OM. Using structural equation modeling, cross-sectional associations of factors in the CES-D (depressive symptoms) to the MFQ (subjective memory) and OM were investigated in the Seattle Longitudinal Study (mean Age=72.39; SD=8.28; mean Education=15.12; SD=2.76; 58.4% female). Differential associations of the CES-D factors with the MFQ factors [n=389; RMSEA=.031; CFI=.973; TLI=.966] and the GFF subscales [n=389; RMSEA=.033; CFI=.971; TLI=.964] resulted. Only the CES-D somatic complaints factor was significantly associated with the GFF factor ($\beta$= -.45, p <.001), suggesting that people with more somatic complaints reported more memory concerns. The CES-D somatic complaints factor was negatively associated with the frequency of forgetting in daily life ($\beta$=-.36, p< .001) and forgetting while reading subscales ($\beta$= -.33, p < .001), indicating individuals that reported more somatic complaints experienced more frequent memory failures when performing daily activities and reading. Overall, a key CES-D factor, somatic complaints, emerged as influential to subjective memory, whereas there was no relation to OM. Further study of the longitudinal associations between the CES-D factors and subjective and objective memory is essential to determine the potential impact on memory deficits.

### LONGITUDINAL ASSOCIATIONS OF DEPRESSIVE SYMPTOM AND SUBJECTIVE MEMORY FACTORS WITH OBJECTIVE MEMORY PERFORMANCE

Wonjeong Haavisto,[1] Julie Blaskewicz Boron,[2] Sherry Willis,[3] and Warner Schaie[3], *1. University of Nebraska, Omaha, Nebraska, United States, 2. University of Nebraska at Omaha, Omaha, Nebraska, United States, 3. University of Washington, Seattle, Washington, United States*

Prior research demonstrated a history of depressive symptoms predicted oncoming memory deficits, and that self-evaluation of memory was associated with forthcoming memory difficulties. However, prior work lacks consistent consideration of multifaceted depressive symptoms in regards to longitudinal associations with objective memory (OM). Structural models were examined to determine how latent factors of depressive symptoms (via the CES-D) and SM factors predicted memory deficits at later time points when taking into account baseline OM performance [n=270; RMSEA=.034; CFI=.974; TLI=.965] in the Seattle Longitudinal Study (mean Age=70.33; SD=7.29; mean Education=15.30; SD=2.72; 61.9% female). The somatic complaints CES-D factor showed a significant longitudinal association with OM performance after seven years ($\beta$ = -.25, p < .05), while none of CES-D factors showed cross-sectional associations with the baseline OM. The general frequency of forgetting SM factor was positively associated with OM performance at baseline ($\beta$ = .26, p < .001), suggesting that those performing better at recalling words reported fewer memory problems. None of SM factors showed longitudinal associations with OM measured seven years later, indicating that self-evaluation of memory had no impact on future memory deficits. Overall findings suggested that a key CES-D factor, somatic complaints, was detected and that people endorsing more somatic issues experienced greater memory decline over a seven year period. Thus, extending prior work, the current study suggests that although both subjective memory and depressive symptom factors showed concurrent associations, only a specific factor of depressive symptoms, somatic complaints, was influential in regards to predicting later memory performance

### INVESTIGATING THE RELATIONSHIP BETWEEN SOCIAL SUPPORT AND COGNITION AS MEDIATED BY HEALTH AND POSITIVE AFFECT

Francesca Falzarano,[1] Karen L. Siedlecki,[1] and Timothy Salthouse[2], *1. Fordham University, Bronx, New York, United States, 2. University of Virginia, Charlottesville, Virginia, United States*

Decreased social networks are common in old age after major life events such as retirement, loss of loved ones, and declining health (Shankar et al., 2013). Diminished social ties are associated with increased feelings of loneliness and perceived isolation, which can have negative effects on cognition and physical health. The current study examines the relationship between social support (assessed via the Social Network Questionnaire) and overall cognitive performance (assessed as a latent construct comprising indicators that represent mean verbal episodic memory, processing speed, reasoning, and spatial visualization), and investigates positive affect and self-rated health as mediators of this relationship. The current study included 5,125 participants between the ages of 18-99 years from the Virginia Cognitive Aging Project (VCAP). Cross-sectional analyses were conducted using structural equation modeling. After controlling for age and education, results showed that a social support construct (comprising indicators representing each social network subscale) significantly and positively predicted cognitive performance (.59, p< .001). This relationship was reduced to .22 (p <.001) when positive affect was included as a mediator, and to .14 (p< .001) when self-rated health was included as a mediator. When the variables were included in a joint mediation model the relationship between social support and cognition was .20 (p < .001). Thus, health and positive affect are partial mediators of the relationship between social support and cognition and may help explain the relationship between social support and cognition. Furthermore, these findings provide additional evidence that social networks may play an important role in successful aging.

### EXAMINING THE RELATIONSHIP BETWEEN COGNITIVE FUNCTIONING AND SUBJECTIVE WELL-BEING ACROSS AGE

Francesca Falzarano,[1] Jillian Minahan,[1] Neshat Yazdani,[1] Karen L. Siedlecki,[1] and Timothy Salthouse[2], *1. Fordham University, Bronx, New York, United States, 2. University of Virginia, Charlottesville, Virginia, United States*

Higher levels of subjective well-being (SWB) are associated with myriad of positive outcomes, including better physical health. Several variables have been shown to predict SWB, including cognitive functioning. The relationship between aspects of SWB (positive affect, negative affect, and life satisfaction) and cognition were examined in participants (N = 5, 125) between the ages of 18- 99 years from the Virginia Cognitive Aging Project (VCAP). Participants completed a battery of cognitive tasks, including tests of verbal episodic memory, processing speed, reasoning, spatial visualization, and vocabulary. Cross-sectional analyses were conducted using structural equation modeling, using full information maximum likelihood estimation. In the models, the five latent cognitive constructs simultaneously predicted each of the SWB outcome variables separately. Age, education, gender, and self-rated health were included as covariates. Results show that reasoning was a significant unique predictor of negative affect (-.30), vocabulary was a significant unique predictor of positive affect (-.21), and spatial visualization was a significant unique predictor of life satisfaction (.21). Age moderation was examined by dividing the sample into three age groups (younger, middle-aged, and older). There was some evidence of age moderation. Namely, spatial visualization was a significant unique predictor of life satisfaction in the younger sample only. Reasoning and processing speed predicted negative affect in the younger group, whereas only reasoning predicted negative affect in the older group. In conclusion, in a large community-based sample spanning adulthood, there is evidence that cognition predicts aspects of SWB but there is variation across SWB outcome variables, and across age.

### DOES CHANGE IN COGSTATE MONTHLY COGNITIVE MONITORING OF OLDER ADULTS TRACK WITH 2-YEAR CHANGE IN GLOBAL COGNITION?

Taylor J. Atkinson,[1] and Ross Andel[1], *1. University of South Florida, Tampa, Florida, United States*

CogState is a repeatable, accessible online cognitive testing suite with evidence of low practice effects that could be used as a monitoring tool to detect cognitive decline early. We compared participants' change in CogState to change in the Telephone Interview for Cognitive Status (TICS). Participants (N = 41, age M = 75.5, 66% female) completed monthly CogState and two TICS assessments over two years. Reaction time on a psychomotor speed task, attention task, and working memory task, and accuracy on a memory task were assessed. A TICS difference score was calculated to measure change. Standardized scores were used. Covariates were age, sex, and education. Paired t-tests indicated that participants performed worse on the TICS the second time, p = .02, Cohen's d = 0.39, but better on their last working memory task, p = .007, Cohen's d = 0.45, and their last memory task, p = .001, Cohen's d = 0.56. Growth curve models indicated CogState memory and working memory scores improved over time, ps < .05, by 0.17 SD accuracy units and 0.16 SD speed units, respectively. There were no significant TICS difference score by time interactions, indicating that changes in CogState were not related to change in TICS. CogState monthly repeat assessment did not track with change in the TICS, indicating that participants may become more proficient in task performance with repeated testing even while global cognition worsens. Despite prior evidence of low practice effects, less frequent assessment may still be warranted to avoid losing sensitivity to change.

### EFFECTS OF MINDFUL FELDENKRAIS EXERCISES AND STRENGTH TRAINING ON COGNITIVE EXECUTIVE FUNCTION IN OLDER ADULTS

Gerhild Ullmann,[1] Yuhua Li,[2] and Meredith A. Ray[2], *1. University of Memphis, School of Public Health, Memphis, Tennessee, United States, 2. University of Memphis - School of Public Health, Memphis, Tennessee, United States*

Decline in cognitive function associated with aging is one of the greatest concerns of older adults and often leads to significant burden for individuals, families, and the health care system. This 3-arm randomized controlled trial (RCT) responds to the urgent need to identify strategies which can enhance and/or maintain cognitive vitality in older adults. The study is funded by the National Institute on Aging, and aims to examine the effects of both the mind-body exercise Feldenkrais and strength training on cognitive executive function in independent living older adults (N=90) age 65 to 85. Participants of the first wave (n=45) were randomized to a (1) Feldenkrais group, (2) strength training and (3) no-intervention control group. Intervention groups met twice a week for 12 weeks. Cognitive and physical performance measures of the NIH-Toolbox were used at baseline, post-intervention and at a 3-month follow-up. Results of changes in cognitive executive functions within and across groups will be presented. The findings will suggest if such interventions would be a viable low-cost option for older adults to maintain cognitive vitality and thereby impact the development of programs and guidelines for combatting decline in cognitive function.

### COGNITIVE FUNCTIONING AMONG BREAST CANCER SURVIVORS AND NON-CANCER PARTICIPANTS: EVIDENCE FOR SIMILARITIES

Giancarlo Pasquini,[1] Brent J. Small,[2] Jacqueline Mogle,[3] Martin Sliwinski,[4] and Stacey B. Scott[1], *1. Stony Brook University, Stony Brook, New York, United States, 2. University of South Florida, Tampa, Florida, United States, 3. Penn State University, University Park, Pennsylvania, United States, 4. Penn State University, Center for Healthy Aging, University Park, Pennsylvania, United States*

Breast cancer survivors may experience accelerated decline in cognitive functioning compared to same-aged peers with no cancer history (Small et al., 2015). Survivors may show important differences in mean-level performance or variability in cognitive functioning compared to those without a history of cancer (Yao et al., 2016). This study compared ambulatory cognitive functioning in a sample of breast cancer survivors and an age-matched community sample without a history of cancer

(n_cancer=47, n_non-cancer=105, age range: 40-64 years, M=52.13 years). Participants completed three cognitive tasks measuring working memory, executive functioning, and processing speed up to five times per day for 14 days. Results indicated no mean-level differences in cognitive performance on the three tasks between cancer survivors and those without cancer history (p's>.05). Unexpectedly, women without cancer history showed more variability than survivors on working memory but not on the other two tasks. Across both groups, those without a college education performed worse on executive functioning (B=-0.05, SE=0.03, p<.05) and working memory (B=0.94, SE=0.36, p<.05) compared to those that completed college. Additionally, older age was associated with slower processing speed (B=31.67, SE=7.44, p<.001). In sum, this study did not find mean-level group differences in cognitive functioning between cancer survivors and age-matched women without a history of cancer. Contrary to hypotheses, those without a history of cancer were more variable on working memory. Results suggested similarities in cognitive functioning in the two samples and that education and age are important predictors of cognitive functioning independent of cancer history.

### EXPLORING THE WITHIN-PERSON COUPLING RELATIONSHIP BETWEEN BLOOD PRESSURE AND COGNITION IN OLDER AFRICAN AMERICANS

DeAnnah R. Byrd,[1] Alyssa A. Gamaldo,[2]
Jason C. Allaire,[3] and Keith E. Whitfield[1], *1. Wayne State University, Detroit, Michigan, United States, 2. Penn State, University Park, Pennsylvania, United States, 3. NC State University, Raleigh, North Carolina, United States*

We examined the within-person relationship between systolic blood pressure (SBP) and cognition and whether this association is moderated by hypertension status and stress level. Analysis was conducted on 50 (39 women and 11 men) community-dwelling African Americans ranging in age from 50 to 80 years (M = 65.40, SD = 8.53). Participants' blood pressure and cognition (i.e., executive function, memory, perceptual speed, inductive reasoning, constructional praxis, and language) was assessed on 8 different occasions over a 2-week time period. Stress was measured at baseline using the Elderly Life Stress Inventory. Findings show on days systolic blood pressure increased above an individual's average level, their memory performance also tended to improve on that day. A significant 3-way interaction was further observed; such that on occasions when an individual's systolic blood pressure was above his or her personal average, their executive functioning performance (i.e., Letter Fluency test) was significantly better, particularly for participants who on average had a high SBP (or were hypertensive) and reported a history of high stress. These results suggest that higher levels of blood pressure are related to better cognitive performance among middle age to older African Americans. These counterintuitive findings may be attributed to the inclusion of a selective sample of resilient African Americans who have survived despite having adverse health conditions. This association may also arise from stress induced high effort coping, which is needed to perform well on cognitive tasks but also increases blood pressure.

### CHANGES IN COGNITIVE FUNCTION AMONG SENIOR AMERICANS: TRENDS AND DIFFERENCES BY RACE

Keqing Zhang,[1] Wei Zhang,[1] and Yanyan Wu[1], *1. University of Hawai'i at Mānoa, Honolulu, Hawaii, United States*

Improvements in health and increase in life expectancy have contributed to the increasing proportion of older population over the past century. It is estimated that by 2050, the number of older adults with cognitive impairments in the United States will increase by 2.5-4 fold, while age-specific rates remain constant. This paper uses data from 10 waves (1996-2014) of the Health and Retirement Study (N= 33213) to crystalize the trends in cognitive function changes and cognitive impairment rates in a nationally representative sample of older adults. OLS and logistic regressions are used to estimate the trends and determine the contribution of sociodemographic variables to decreasing trends in the prevalence of cognitive impairment over time. Results show that with the increase of age, the cognitive function of older adults decline in all races, after adjustment for age, gender, education, and other sociodemographic factors. Also, the annual decline rate of cognitive function is larger for African Americans and Hispanic Americans, while smaller for white and other races. A further investigation of the possibility of cognitive impairment reveals a different scenario: as individual ages, the Hispanic are the least likely to suffer from cognitive impairment, followed by the white, other and black. Improvements in educational level contribute to declines in cognitive impairment across all races, particular the Hispanic Americans. Race-specific findings suggest that future research need to take into account the racial diversity and possibly cultural influences when examining the cognitive functions of older adults.

### PURPOSE IN LIFE PROTECTS AGAINST COGNITIVE DECLINE AMONG OLDER ADULTS

Giyeon Kim,[1] Su Hyun Shin,[2] Monica Scicolone,[2] and
Patricia A. Parmelee[2], *1. Chung-Ang University, Seoul, Korea, Republic of, 2. The University of Alabama, Tuscaloosa, Alabama, United States*

Objective: This study examined whether having a sense of purpose in life protects against cognitive decline among older adults and whether purpose in life moderates the relationship between selected risk factors (age, sex, and race/ethnicity) and cognitive abilities. Methods: This was a longitudinal analysis of existing secondary data of adults (N = 11,557) aged 50 or older using the 2006 –2012 waves of the Health and Retirement Study. The study measured purpose in life, cognitive functioning score, and various covariates. Results: Growth curve modeling revealed that, after adjusting for covariates, purpose in life was positively associated with participants' total cognition scores. Purpose in life significantly moderated the relationship between age and race/ethnicity and cognitive decline. Further, purpose in life was a protective factor against cognitive decline for those who were older and black. There was no significant interaction between purpose in life and sex. Conclusion: Having a purposeful life protects against cognitive decline in older adults, and the associations varied by age and race/ethnicity, but not by sex. Potential ways to increase purpose in life are discussed in a clinical context.

### THE ROLE OF PRINT EXPOSURE IN SUPPORTING COGNITIVE ABILITY AMONG OLDER ADULTS

Xiaomei Liu,[1] Elizabeth A L Stine-Morrow,[1] and Giavanna S. McCall[1], *1. Beckman Institute and Educational Psychology, Urbana, Illinois, United States*

Reading is an activity that has long been thought to have beneficial effects on cognitive ability, however, little is known about the effects of long-term reading engagement on cognition among older adults. Pre-test data are reported from an ongoing study of the effects of a literacy-based intervention on cognition among older adults. Participants (66.7% female) ranging from 60 to 79 years of age with education ranging from 11 to 19 years were administered a battery of measures, including print exposure checklists, which are objective indices for assessing long-term reading-related activities, as well as cognitive assessments. A composite measure of print exposure was based on the Author Recognition Test, Magazine Recognition Test, Non-fiction Author Recognition Test, and Fiction Recognition Test (alpha=.92). Cognition was assessed in a comprehensive battery, including measures of verbal ability, working memory, episodic memory, and verbal fluency (alpha=.83). Print exposure was marginally related to self-reported time spent reading (r=.27, p.4), suggesting criterion-related validity. Age-related declines were not evident for any of our cognitive measures in this sample, t(46)'s.8, and there was a modest increase in reading engagement with age (r=.36, p=.01). Controlling for verbal ability, print exposure had a significant positive influence on global cognition (standardized beta=0.38, p=.01); however, these effects were specific to episodic memory and verbal fluency, t(46)>2, p<.05. These preliminary findings are suggestive of selective cognitive benefits from continued literacy engagement in reading in later life.

### ALCOHOL CONSUMPTION AND MEMORY: A LONGITUDINAL ANALYSIS OF RECIPROCAL IMPACTS ACROSS OLD AGE

Oliver K. Schilling[1], *1. Department of Psychological Ageing Research, Institute of Psychology, Heidelberg University, Heidelberg, Germany*

Research on the association of alcohol consumption with cognitive aging revealed mixed evidence: Whereas a u-shaped relationship has been found in many studies, suggesting that low to moderate alcohol consumption predicts more favorable cognitive outcomes than abstinence, other findings suggest that alcohol is a more linearly related risk factor for cognitive decline. These inconsistencies may partly be due to methodological variation in the statistical modeling of intraindividual changes in both, alcohol consumption and cognition across old age. The present study analyzed longitudinal change in and the mutual effects between alcohol consumption habits and verbal episodic memory (word list recall), using vector autoregressive (VAR) mixed models with nonlinear cross-lagged effects. Data from the English Longitudinal Study of Ageing was examined, including N=13388 aged 50+ (M=67.6, SD=9.25; 54.7% female), assessed at up to eight occasions with two-year follow-up intervals (2002/3–2016/17). The self-reported one-year frequency of alcohol drinking days (ADD) served as indicator of alcohol consumption. Basically, ADD predicted follow-up memory performance in a reverse u-shaped fashion, indicating best memory performance after moderate ADD, compared with both ends of the ADD continuum (i.e., drinking never vs. every day). Considering moderators, most notably age did not interact with cross-lagged effects, suggesting that those observed across an older age-range were not more (or less) vulnerable to effects of alcohol consumption on memory performance. Thus, this study adds further support for non-detrimental, if not beneficial, effects of moderate alcohol consumption on cognitive aging – regarding in particular age-related loss of episodic memory.

### AGE OF MUSIC ACQUISITION IMPACTS MEMORY FUNCTIONING IN OLDER ADULT MUSICIANS

Jessica V. Strong,[1] William Milberg,[1] Regina McGlinchey,[1] and Elizabeth Leritz[1], *1. VA Boston Healthcare System, Boston, Massachusetts, United States*

Music playing is an involved activity, activating many areas of the brain and relying on integration of multiple cognitive processes simultaneously. Older adult musicians have been found to experience some cognitive benefits compared to non-musicians, seemingly related to their musical training. However, we still do not understand what factors of musical training may be driving these differences. The current study sought to isolate age of acquisition from "dose" of playing (i.e., amount of time spent playing) to explore music learning as a skill acquired during a sensitive period. Participants (n=48) were middle aged and older adults who self-reported on musical experiences, demographics, and underwent extensive neuropsychological assessment of all major domains. The sample was divided into Early Age of Acquisition (≤9 years old), Late Age of Acquisition (>9), and Non-Musicians. Results showed that musicians who began formal training at the age of 9 or younger, had significantly higher scores on tests of verbal memory (California Verbal Learning Test – II: Immediate Recall – p = 0.04, partial $\eta 2$ = 0.14, Short-Delay Free Recall - p = 0.03, partial $\eta 2$ = 0.16, Long-Delay Free Recall - p = 0.03, partial $\eta 2$ = 0.15). Results are discussed in the context of a sensitive period for acquiring musical education, and implications of these results on cognitive aging.

### EFFECTS OF OBESITY ON EXECUTIVE FUNCTION AND MEMORY IN MIDDLE-AGED AND OLDER ADULTS

Andrew J. Fiscella,[1] and Ross Andel[1], *1. University of South Florida, Tampa, Florida, United States*

Obesity has been linked to multiple conditions including cardiovascular disease, cancer, and metabolic syndrome. While the relationship between obesity and these diseases is well-established, the relationship between obesity and changes in cognitive function is less clear. This study examined the effects of overweight and obese status on performance in tests of executive function and memory. It was hypothesized that overweight and obese individuals would perform worse than normal weight individuals. Data from 547 individuals who participated in the Midlife in the United States Refresher cohort were used. Body mass index (BMI) and waist circumference (WC) were employed as measures of obesity. Both variables were examined continuously and categorically (from NHBLI-defined cutoffs). Z-scores were computed for each cognitive test and averaged together to create separate composite scores for executive function and memory. When measuring BMI and WC continuously, higher BMI was significantly associated with lower executive function (B=-0.010, p=.029); no significant association between executive function and WC was found (p=.162). When measuring BMI and WC with clinical thresholds, participants with overweight or obese WC had significantly lower executive function (p=.030). No overall association between BMI-based thresholds and executive function was found (p=.682). Memory did not differ between overweight/obese participants and healthy weight controls with continuous or clinical threshold specifications. These findings suggest that: 1) obesity may relate to poorer executive function but not worse memory, and 2) the way in which obesity is measured reflects saliently in the results. Future researchers should consider conducting sensitivity analyses with different obesity criteria.

### RACIAL-ETHNIC DIFFERENCES IN THE EFFECTS OF POSITIVE AND NEGATIVE AFFECT, AND DEPRESSION ON COGNITIVE TRAJECTORIES

Soohyun Park,[1] Su Hyun Shin,[1] Rebecca S. Allen,[1] and Giyeon Kim[2], *1. The University of Alabama, Tuscaloosa, Alabama, United States, 2. Chung-Ang University, Seoul, Korea, Republic of*

Purpose of study: This study investigated (1) whether positive affect(PA), negative affect(NA), and depression are related to trajectories of cognitive functioning among older adults, (2) whether PA or NA could moderate the relationship between depression and cognitive trajectories, and (3) whether there are racial/ethnic differences in the relationships among PA, NA, depression and cognitive trajectories. Design and Methods: Growth-curve modeling was conducted using the sample of 10,289 individuals in the U.S. aged 50 or older from the 2006-2014 Health and Retirement Study. Racial/ethnic groups in this study were non-Hispanic Whites (NHW, n=8.009), African Americans (AA, n=1,428), Hispanics (n=611), and others (n=241). Results: After adjusting for covariates, PA showed positive effect, and depression had negative effect on cognitive functioning trajectories (p < .05, z = 8.76, 95% CI= 0.27, 0.43; p < .05, z = -6.38, 95% CI= -0.19, -0.10). Racial/ethnic minorities (i.e., AA, Hispanics, others) exhibited lower cognitive functioning over time compared to NHW. PA significantly moderated the effect of depression on cognitive trajectories (p < .05, z = - 8.04, 95% CI = -0.19, -0.11), and the protective effect of PA against cognitive decline was pronounced for AA (p < .05, z = 2.75, 95% CI = 0.10, 0.63). Conclusion: Findings suggest that PA may protect against cognitive decline in older adults, providing a buffer against the negative effect of depression or racial/ethnic minority status on cognitive trajectories. Potential intervention strategies are discussed to assist older adults in maintaining and improving PA to promote cognitive health.

### BEHAVIORAL INTERVENTIONS FOR COGNITION AND EVERYDAY FUNCTION: A SYSTEMATIC REVIEW

Briana N. Sprague,[1] Sara A. Freed,[1] Christina E. Webb,[2] Christine B. Phillips,[3] Jinshil Hyun,[4] and Lesley A. Ross[1], *1. The Pennsylvania State University, University Park, Pennsylvania, United States, 2. University of Texas at Dallas, Dallas, Texas, United States, 3. Arizona State University, Phoenix, Arizona, United States, 4. Albert Einstein College of Medicine, New York, New York, United States*

Behavioral interventions to improve cognitive function in older adults are widespread and can vary from theatre classes to cognitive training programs targeting one domain. However, the effectiveness in maintaining different cognitive domains varies greatly both across and within intervention types. To date, no systematic reviews have synthesized findings across more than a few types of interventions (e.g., cognitive vs. exercise). This systematic review examined nine types of behavioral interventions and the respective transfer to 18 cognitive domains and everyday function. The 2017 search yielded 75 unique eligible articles comprising of educational, theatre, mindfulness, cognitive, exercise, video game, and combination interventions. In general, there was limited evidence of consistent transfer from behavioral interventions to untrained cognitive domains. Few studies examined education, theatre, mindfulness, or video game interventions, leaving inconclusive results about their effect on cognitive function. Nine studies evaluated transfer to everyday function and found that both process- and strategy-based cognitive training conferred benefits up to 10 years posttest. These results suggest that while there is weak-to-moderate evidence of far transfer from behavioral interventions to untrained cognitive domains, it may be more important to examine far transfer to measures more indicative of older adult everyday life. Furthermore, it highlights the necessity to continue long-term follow-up. While there were notable limitations of the extant literature, including inconsistent use of terms such as active control or inadequate intervention description, there were strengths such as the recent implementation of factorial designs. Implications for future research and practice will be discussed.

### EFFECT OF PHYSICAL EXERCISE ON COGNITION IN ADULT DAY PARTICIPANTS

Bradford Stucki,[1] Ben Katz,[1] Jonathan Briganti,[1] Ila Schepisi,[1] and Shannon Jarrott[2], *1. Virginia Tech, Blacksburg, Virginia, United States, 2. The Ohio State University, Columbus, Ohio, United States*

Aerobic exercise has been demonstrated to be one of the most effective means of improving cognition in older adults. However, less is known about how exercise programs may improve cognition in older adults participating in Adult Day Service (ADS) programs. We analyzed a ten-year longitudinal data set from the Virginia Tech ADS center. We limited our analyses to individuals for whom we had two time points of the Mini-Mental Status Exam (MMSE) (n=142; average age = 78.48; 63 female, average days at center = 347; SD=432.71). Participants in the center completed approximately 30 minutes of physical exercise each day of attendance. The exercise regimen was largely composed of aerobic chair exercise, stretching, and lifting. Facilitator ratings of engagement with the exercise activity between the two test administrations were used to create an average engagement score for each participant. Multiple regression analyses were conducted using engagement as a predictor and change in MMSE as an outcome; no significant relationship was identified between exercise engagement and change in cognitive status. However, a moderation analysis conducted with diagnosis of Alzheimer's disease (AD) or dementia as a predictor, change in MMSE as an outcome, and exercise engagement as a moderator revealed a significant moderation effect (p = .001). Greater exercise engagement was associated with improvements on the MMSE, but only for individuals without a diagnosis of AD or dementia. Given that many ADS programs serve individuals both with and without AD or dementia, these findings may inform more personalized exercise interventions at ADS centers.

### SUBJECTIVE AGE AND COGNITIVE, PHYSICAL, AND PSYCHOSOCIAL HEALTH IN OLDER ADULTS

Briana N. Sprague,[1] Sara B. W. Troutman,[2]
Sara E. Bickhart,[1] Nathanael Jiya,[1] and Lesley A. Ross[1],
*1. The Pennsylvania State University, University Park, Pennsylvania, United States, 2. The Pennsylvania State University, The Pennsylvania State University, United States*

Subjective age predicts cognitive, physical, and psychosocial function in older adults independent of objective age. However, the stability of this association is unknown. The goals of this study were to determine whether (1) subjective age was stable across eight years and (2) if subjective age predicted cognitive, physical, and psychosocial function in older adults. This study used data from a nationally-representative longitudinal cohort study (Health and Retirement Study) of 3,084 older adults aged 65-96 measured biannually from 2008 – 2016 with at least two waves of data. The main predictor was the proportion of subjective age compared to one's objective age. The outcomes were dementia status (TICSm), physical function (grip strength, walk speed), and psychosocial function (Self-Perceptions of Aging). On average, the sample reported feeling 15% younger than their objective age. Among older adults who reported feeling older than the sample average, there were significant deficits in TICSm, walk speed, and self-perceptions of aging, but not in grip strength. Older adults who feel older than average have poorer cognitive, lower limb physical, and psychosocial function, and these deficits are exacerbated when those individuals feel even older compared to their own average. These results suggest that instability in subjective aging is particularly disruptive for those already at risk for poor aging, so interventions targeting the most vulnerable could promote healthy aging.

### STABILITY AND CHANGE IN DEPRESSIVE SYMPTOMS AND COGNITION FOR CENTENARIAN COHORTS

Gina Lee,[1] and Peter Martin[1], *1. Iowa State University, Ames, Iowa, United States*

The purpose of the study was to examine the stability and change of cognition and depression levels and how they predict each other over time. Participants of the Health and Retirement Study who survived to centenarian status (N = 331) were included in this study. The total cognition summary score and the CES-D summary score of depressive symptoms were used to conduct four cross-lagged regression analyses from wave 2 to wave 6. Age was used as a covariate. Results indicated that the stability coefficients for depressive symptoms and cognition from wave 2 to wave 6 were high. Depressive symptoms at wave 2 significantly predicted change in cognition at wave 3, whereas depressive symptoms at all other waves did not predict change in cognition in the next wave. Cognition did not predict changes in depressive symptoms for any wave. Age as a covariate predicted change in cognition in each following wave, particularly from wave 2 to wave 5. The coefficients without stability for depressive symptoms and cognition from wave 2 to wave 5 predicted each other significantly over time, except for the last wave. In conclusion, cognition and depressive symptoms predict each other over time, but they do not predict each other if stabilities are included in the analyses. Further research needs to examine the stability and change in depressive symptoms and cognition including more waves in order to examine whether cross-lagged effects fade or continue in very late life.

### THE ASSOCIATION OF OBJECTIVE FINANCIAL DECISION MAKING WITH FINANCIAL LITERACY, EDUCATION, AND MATH SKILLS

Preeti Sunderaraman,[1] Sarah Ho,[1] Yaakov Stern,[1] and Stephanie Cosentino[1], *1. Columbia University Medical Center, New York City, New York, United States*

Rationale: Financial decision-making (FDM) is a critical ability with implications across the adult life-span. In healthy adults, demographic and cognitive factors impact FDM. However, the impact of these factors on FDM has yet to be fully investigated. The aim of the current study was to understand the extent to which demographics (age, education, sex), financial literacy (crystallized ability), and mathematical ability (fluid ability) influence FDM. Participants and Methods: The sample, recruited from a larger ongoing study, consisted of 73 adults; mean age=61.31 (13.76) years, mean education=15.68 (2.61) years, 59.5% female, 58% Caucasian. FDM was measured using the Financial Competence Assessment Inventory, financial literacy using a standard set of 23 questions, and math skills using WAIS-III Arithmetic, WRAT-IV Math and Cognitive Reflection Test. Results: Only variables that were significantly associated with FDM in bivariate analysis were selected for the multiple regression analysis. After adjusting for multicollinearity, stepwise multiple regression analyses revealed that the overall model with 3 predictors (education, financial literacy, WAIS-III Arithmetic) was significant (F =23.64, p < .001) and explained 50.7% of the variance in FDM. Education and WAIS-III Arithmetic predicted FDM to a higher extent than financial literacy. Conclusions: The finding that education and fluid ability has a relatively higher impact on FDM as compared to crystallized ability is important. As one ages, fluid abilities decline more rapidly than crystallized abilities. This may be one explanation for why FDM ability worsens with age. To increase confidence in these findings, future research should test these models using age-stratified analyses.

### SUBJECTIVE MEMORY COMPLAINTS AND COGNITIVE PERFORMANCE: THE MODERATING ROLE OF DEPRESSIVE SYMPTOMS

Elizabeth A. Gallagher[1], *1. University of Massachusetts Boston, Boston, Massachusetts, United States*

Cognitive health is a rising public health concern in the U.S. Currently, approximately 5.7 million older adults suffer from Alzheimer's disease (AD), and by the year 2050 this number is expected to increase to 14 million. Subjective memory complaints (SMC) are shown to be an early indicator of cognitive decline, and accordingly included as a clinical criterion for diagnoses of MCI, an indicator of pre-dementia states, and a research criterion for AD diagnoses. Among older adults, depressive symptoms hinder the accuracy of memory self-ratings. However, there has yet to be consensus regarding the nature of how depressive symptoms may condition the relationship between SMC and cognitive performance. The aims of the present study are to both investigate whether SMC is related to episodic memory and to determine whether depressive symptoms act as a moderator for the relationship between SMC and episodic memory among older adults. This research used nationally representative sample of 8,123 older adults aged 65 and older who completed the Leave Behind Questionnaire in the 2012 and 2014 waves of the Health and Retirement Study. Linear regression was performed and results showed that there was a significant main effect of SMC on episodic memory performance, in that older adults with increased SMC have worse episodic memory. There was also a significant moderating effect of depressive symptoms, in that depressive symptoms cause older adults to underestimate their memory abilities. In order to use SMC as a tool for early detection efforts it is critical to understand these complex relationships.

### PERFORMANCE ON COGNITIVE FUNCTIONING RELATED TO SUCCESSFUL AGING

Neyda Ma. Mendoza Ruvalcaba,[1]
Elva Dolores Arias Merino,[2]
Maria Elena Flores Villavicencio,[3] and
Melina Rodriguez Díaz[3], *1. Universidad de Guadalajara CUTONALA, Guadalajara, Mexico, Mexico, 2. Universidad de Guadalajara CUCS, Guadalajara, Mexico, Mexico, 3. University of Guadalajara CUCS, Guadalajara, Mexico, Mexico*

Introduction The cognitive functioning, as a general measure, is a criterion commonly used to define and operationalize successful aging. (Project-Conacyt-256589) The aim of this study is to analyze cognitive function and its relationship with the successful aging in older adults. Methods Population based, random sample included n=401 community-dwelling older adults 60-years and older (mean age=72.51,SD=8.11 years,59.4% women). Cognitive functioning was assessed by a comprehensive battery including working memory(Digit Span Backward WAIS-IV), episodic memory, metamemory(self-report), processing speed(Symbol Digit WAIS-IV), attention(TMT-A), executive functioning(TMT-B), learning potential(RAVLT), language(FAS), visuospatial skills(Block Design WAIS-IV). Successful aging was operationalized in accordance with Rowe & Kahn definition (no important disease, no disability, physical functioning, cognitive functioning, and being actively engaged). Sociodemographic and health data were also asked. Data were analyzed in SPSSv24. Results In total 11.2% were successful agers and 11.4% had Mild Cognitive impairment. Global cognitive functioning was significantly related to the achievement of successful aging criteria. Specifically, the more successful agers showed a significant (p´s<.05) better performance on learning potential, working memory, metamemory, processing speed and attention. Executive functions were not related to successful aging criteria. None cognitive domain was related to the being actively engaged criteria. Better visuospatial skills were showed in older adults meeting the criteria of being free of disability and high physical functioning. Conclusion Knowledge generated by this study reveals the role of specific domains of cognitive functioning in successful aging, and sets a scenario to promote successful aging, through alternatives centered in the improvement of cognition in the older adults.

### COGNITION

### OLDER ADULTS WITH PREDIABETES EXPERIENCE ACCELERATED NEUROCOGNITIVE DECLINE

Joyla Furlano,[1] and Lindsay Nagamatsu[1], *1. Western University, London, Ontario, Canada*

Type II diabetes (T2D) is associated with neurocognitive decline beyond normative aging, and thus older adults with T2D are at high risk for developing dementia. However, the extent to which similar deficits occur in prediabetic older adults is not well understood. While few studies have shown that prediabetic older adults experience some cognitive decline, further research is needed to determine the specific cognitive domains affected and the degree to which this decline occurs. Moreover, structural and functional brain changes that may occur with these deficits is currently unknown in this population. Therefore, the aim of this study was to assess cognitive function and brain health in prediabetic older adults. We conducted a cross-sectional analysis of older adults (aged 60-80) with prediabetes (FPG 6.1-7.0 mmol/L) and healthy aged-matched controls, examining 1) cognitive performance, 2) functional brain activation as measured by fMRI, and 3) structural measures such as volume of the hippocampus. Based on our cross-sectional analysis, prediabetic older adults show impaired cognition (e.g., memory), as well as decreased hippocampal volume and activation. Therefore, we conclude that older adults with prediabetes experience brain decline, and could benefit from lifestyle interventions to prevent or delay the onset of such decline.

### AFFECTING COGNITION: HOW DO POSITIVE AND NEGATIVE AFFECT PREDICT THE FUTURE COGNITIVE PERFORMANCE OF MIDLIFE ADULTS

Jungmin Lee,[1] Sara B. W. Troutman,[2] Victoria Gertel,[1] Peter Molenaar,[1] and David M. Almeida[3], *1. The Pennsylvania State University, State College, Pennsylvania, United States, 2. The Pennsylvania State University, The Pennsylvania State University, United States, 3. The Pennsylvania State University, University Park, Pennsylvania, United States*

The purpose of the present study is to examine whether positive affect and negative affect can predict the changes in cognitive abilities, assessed by episodic memory and executive functioning, during the transition to late adulthood. Using the longitudinal data from the national survey of midlife development in the United States (MIDUS), this study implements a cross-lagged panel model to test the significance of two pathways; one from affect to cognition and the other in the opposite direction. The results show the three main findings: First, the relationship between affect and cognition is unidirectional in which only positive affect and negative affect during the first occasion significantly predict episodic memory in the second occasion. Second, regardless of the type of affect, higher levels of both positive and negative affect predicts worse episodic memory and this tendency is more prominent in older adults. The combined results emphasize the benefits of using affect as a predictor of changes in the specific types of cognition and suggest using the longitudinal model to account for complex relationships between these psychological constructs.

### EFFECT OF SELF-PACED WALKING ON REDUCING COGNITIVE DEMANDS OF WALKING WHILE TALKING

Hyeon Jung Kim,[1] Farahnaz Fallahtafti,[2] Jennifer M. Yentes,[1] Dawn Venema,[3] and Julie Blaskewicz Boron[2], *1. University of Nebraska Omaha, Omaha, Nebraska, United States, 2. University of Nebraska at Omaha, Omaha, Nebraska, United States, 3. University of Nebraska Medical Center, Omaha, Nebraska, United States*

Although walking is considered an automated process, internal and external factors can alter gait performance. Fear of falling leads to walking with a wider base of support and decreased walking speed, due to a combination of shorter stride lengths and/or stride time. Exacerbation of gait deficits have been reported under high cognitive load situations (HCLS, also known as dual-tasks). Talking on a phone is considered an increased HCLS over talking in-person due to visualization of the individual on the phone. The purpose of the study was to explore the added effects of walking while talking on a phone compared to talking in-person. Fifteen healthy older adult subjects (70.86±4.7yrs) performed three conditions while walking on a self-paced treadmill for ten minutes: (1)walking alone, (2)walking while talking in-person, (3)walking while talking on a phone. Mean stride length(SL), stride time(ST), and step width(SW) were compared using one-way, repeated-measures ANOVAs ($p=0.10$). Dual-task cost of walking while talking in-person and on a phone was calculated for each gait variable and compared with t-tests. Mean gait variables did not differ between conditions (SL $p=0.95$, ST $p=0.77$, SW $p=0.57$). Dual-task costs were not significantly different between talking conditions (SL $p=0.99$, ST $p=0.54$, SW $p=0.14$). Use of a self-paced treadmill allowed the subjects to perform in their "comfort zone", however, walking on a set-speed treadmill may force walking speed outside of the comfort zone, pushing one's "reserve", and revealing differences. Use of a self-paced treadmill better approximates daily life by providing the opportunity to make adaptations under HCLS.

### THE AGING BRAIN AND MOTOR LEARNING

Qu Tian,[1] Roger Mullins,[1] Abby Corkum,[1] David Reiter,[2] Daniel Pupo,[3] Eleanor M. Simonsick,[4] Dimitrios Kapogiannis,[1] and Stephanie Studenski[5], *1. National Institute on Aging, Baltimore, Maryland, United States, 2. Emory University, Atlanta, Georgia, United States, 3. Johns Hopkins Bloomberg School of Public Health, Baltimore, Maryland, United States, 4. Longitudinal Studies Section, Intramural Research Program, National Institute on Aging, Baltimore, Maryland, United States, 5. University of Pittsburgh, Pittsburgh, Pennsylvania, United States*

The effect of aging on motor learning is poorly understood. This study investigated response time and patterns of brain activation induced over the course of a bimanual motor learning task in three age groups. Twenty-two cognitively unimpaired participants (32%women) were grouped into Young (<35,n=6), Middle-Age (36-59,n=10), and Old (60+,n=6). A self-paced bimanual motor learning task was performed during fMRI. The task consisted of using 2 capital and 2 lower case letters in strings of 16 cues with 6 novel alternating with 6 repeated sequence blocks. To assess learning, a repeated measures ANOVA tested whether average time per slide differed over time between novel and sequence conditions. Voxel-wise changes in brain activation between novel and sequence conditions over time were examined using a within-subject repeated measures model. Faster initial time per slide was associated with younger age (p0.05). Old had increased brain activation in repeated sequence than novel conditions in right postcentral and superior parietal regions during the early half of the task compared to the second half (p0.05). We found behavioral evidence of motor learning in Middle-Age and Old, but not Young, perhaps because younger individuals performed quickly and learned sequence almost immediately. Among older individuals, sequence-specific learning in parietal regions challenges the view that it is mediated by only motor areas.

### QUADRICEPS STRENGTH IS ASSOCIATED WITH COGNITION IN OLDER ADULTS WITH CHRONIC STROKE

Kimberly Bennett,[1] Rachel A. Crockett,[2] Lisanne F. ten Brinke,[2] Jennifer C. Davis,[3] and

Teresa Liu-Ambrose[2], *1. University of British Columbia, Vancouver, Canada, 2. University of British Columbia, Vancouver, British Columbia, Canada, 3. University of British Columbia - Okanagan Campus, Kelowna, British Columbia, Canada*

Individuals who have suffered a stroke are at risk for developing cognitive impairment and dementia. Thus, it is important to identify modifiable risk factor for cognitive decline in this population. Among older adults without a history of stroke, greater muscle strength is associated with better cognitive function. Whether this relationship also exist in older adults with a history of stroke is not known. Thus, we aimed to examine whether cognition, as measured by both the Montreal Cognitive Assessment (MoCA) and the 13-item Alzheimer's Disease Assessment Scale-Cognitive (ADAS-Cog 13), is associated with lower extremity muscle strength in adults with chronic stroke (> 1 year post stroke). Ninety-one community-dwelling adults, aged 55 years and older, with chronic stroke were included in this analysis. Isometric strength of the quadriceps was measured bilaterally in kilograms. Two linear regression models were constructed to determine the independent association of quadriceps strength (mean kilograms of both legs) with: 1) MoCA; and 2) ADAS-Cog 13, after controlling for age, sex, and mood. Mean quadriceps strength was independently associated with both MoCA and ADAS-Cog scores, after accounting for age, sex, and mood. Specifically, quadriceps strength explained an additional 5.6% of the variable in MoCA scores; total variance explained by the model was 12.0%. For ADAS-Cog 13, quadriceps strength explained an additional 5.4% of the variance; total variance explained by the model was 16.5%. Our current cross-sectional results suggest that the maintenance of muscle strength may be important for cognitive health in older adults who have suffered a stroke.

### VISUAL IMPAIRMENT AND ENGAGEMENT IN COGNITIVELY STIMULATING ACTIVITIES

Bonnielin Swenor,[1] beatriz Munoz,[2] and Eleanor M. Simonsick[3], *1. The Wilmer Eye Institute, Johns Hopkins University School of Medicine, Baltimore, Maryland, United States, 2. The Wilmer Eye Institute, Johns Hopkins School of Medicine, Baltimore, Maryland, United States, 3. Longitudinal Studies Section, Intramural Research Program, National Institute on Aging, Baltimore, Maryland, United States*

We examined the relationship between visual impairment (VI) and engagement in cognitively stimulating activities using data from 924 participants in the Cognitive Vitality Sub-Study of the Health ABC Study. At Year 3 (baseline for these analyses), vision was assessed as: visual acuity (VA), contrast sensitivity (CS), and stereo acuity (SA). Participation in cognitively stimulating activities was determined based on responses to 12 questions (administered at Years 3, 5, 7, and 9) assessing frequency of participation ranging from none to daily. We calculated the total number of activities engaged in at least monthly. In cross-sectional analyses adjusted for age, race, and sex, impaired VA (≤20/40, 8%), CS (<1.55, 5%), and SA (<80 secs arc, 29%) was associated with participation in fewer cognitive activities (β=-0.54, 95% CI:-1.06, -0.03; β=-0.59, 95% CI:-0.12, 0.06; β=-0.40, 95% CI:-0.81, -0.18, respectively). Longitudinally, change per year in the number of activities differed by baseline participation levels. Those participating in ≥5 activities at baseline (population median) had a significant decline in the number of activities, irrespective of VI status. However, for those participating in <5 activities at baseline, the increase in these activities tended to be lesser in the VI than in non-VI groups, and for SA this increase was significantly lower for the impaired group (βimpaired=0.004; 95% CI:-0.05, 0.05; βnot-impaired=0.06; 95% CI: 0.03, 0.10; time x SA interaction p=0.0496). These data indicate that older adults with VI participate in fewer cognitive activities and the change in participation over time differs from than those without VI.

### ASSOCIATIONS BETWEEN COGNITIVE FUNCTION AND BIOACTIVE FOOD COMPOUNDS IN 100% WATERMELON JUICE

Amy Ellis,[1] Ann Bradford Hansen,[1] Navneet Baidwan,[2] Vinoth Aryan Nagabooshanam,[2] and Kristi Crowe-White[1], *1. The University of Alabama, Tuscaloosa, Alabama, United States, 2. University of Alabama at Birmingham, Birmingham, Alabama, United States*

Objectives: Decline in cognitive function and increases in inflammation and oxidative stress are part of normal aging. Watermelon contains numerous bioactive compounds including lycopene, arginine, and citrulline that exhibit both anti-inflammatory and antioxidant functionality. Thus, the objective of this study was to examine the effect of 100% watermelon juice supplementation on cognitive performance. Methods: A placebo-controlled, randomized, double-blind, crossover trial was conducted with postmenopausal women (n = 16, 60 + 4.1y). Participants initiated a low-lycopene diet during a one-week run-in period and adhered to this diet throughout the study. For four weeks, participants were randomized to consume either two 360 mL servings of pasteurized 100% watermelon juice or a placebo beverage. Following a two-week washout period, participants received the opposite beverage for an additional four weeks. Pre/post each intervention arm, fasting blood samples were collected, and cognitive tests were administered to assess various neurocognitive domains. Statistical analyses included mixed models and Spearman correlations. Results: Serum lycopene exhibited a significant treatment effect (p=0.002); however, lycopene was not correlated with any cognitive test. In contrast, no significant treatment effect was observed for serum arginine or citrulline, yet arginine was significantly inversely correlated with Digit Span Forward (p = 0.005, r = -0.547) and Letter Fluency (p = 0.024, r = -0.507). Conclusion: Despite research supporting the relationship between lycopene and enhanced cognition, lycopene was not related to improvements in cognitive performance in this study. Nevertheless, consumption of 100% watermelon juice may be beneficial for increasing circulating levels of this antioxidant.

### A SYSTEMATIC REVIEW OF RANDOMIZED CONTROLLED TRIALS OF COGNITIVE TRAINING INTERVENTIONS IN OLDER ADULTS

Ayse Malatyali,[1] Carol Reive,[1] and Christine Williams[1], *1. Christine E Lynn College of Nursing, Florida Atlantic University, Boca Raton, Florida, United States*

There has been a growing interest in cognitive training interventions for their potential effect of maintaining and promoting cognitive functioning in older adults. Rapid and significant changes in technology has had a significant impact on the design and assessment methods of cognitive training interventions. Investigating changes in brain networks and blood markers are relatively new approaches and sparsely examined in the literature. The purpose of this systematic literature review is to analyze the effect of cognitive training interventions on brain networks, blood markers and associated cognitive performance of healthy older adults. We conducted a comprehensive literature search on four databases, following PRISMA guidelines. Initially, 2426 citations were retrieved, and 251 full-text publications were evaluated in detail for eligibility. Fourteen randomized control trials were included in this review. Functional imaging analysis of brain networks showed significant activity changes primarily in the Default Mode Network. These changes were associated with improvement in memory, learning, attention, and affective performances. Also, there were activity changes in the Central Executive Network that were associated with improvement in reasoning, attentional control, innovative thinking, and processing speed. Training-induced changes have been observed in the brain-derived neurotrophic factor levels and the markers of antioxidative and anti-inflammatory regulatory mechanisms. Improvement in attention and memory performances were significantly related to these changes. Limitations of the studies included methodological inconsistencies, sampling issues, and the lack of long-term follow up assessment. Cognitive training appears to promote improvement and maintenance of cognitive functioning in healthy older adults.

### THE ASSOCIATION OF MILD TBI WITH EARLY ONSET DEMENTIA IN POST-9/11 VETERANS

Mary Jo Pugh,[1] Hari Krishna Raju Sagiraju,[1] Blessen Eapen,[2] Alicia A. Swan,[3] Margaret Wells,[4] Jason R. Soble,[5] and Janice C. Marceaux[4], *1. University of Utah, Salt Lake City, Utah, United States, 2. VA Greater Los Angeles Health Care System, Los Angeles, California, United States, 3. University of Texas San Antonio, San Antonio, Texas, United States, 4. South Texas Veteran Health Care System, San Antonio, Texas, United States, 5. University of Illinois College of Medicine, Chicago, Illinois, United States*

Evidence produced by studies using ICD-9 codes to identify dementia suggests that mild traumatic brain injury (mTBI) can accelerate age-related neurodegeneration and dementia risk. However, ICD-9 codes are unreliable in identifying early onset dementia (<65 years; EOD) in civilian (positive predictive value [PPV]=58%) and VA (PPV=28%). From 1,724 Veterans <65years of age with 2 or more dementia diagnoses based on ICD-9 codes recommended by VA Dementia Steering Committee, we validated dementia diagnoses in 153 randomly selected cases using medical chart abstractions and reviews by a neuropsychologist panel. We matched valid cases based on age, sex, race/ethnicity, year of entry to VA care, and branch of service to 2490 controls with no indicators of dementia or cognitive impairment. TBI severity was defined using multiple DoD and VA data sources. We also identified diagnoses for mental (e.g., depression, post-traumatic stress disorder, substance use disorders) and other medical conditions (e.g., stroke) associated with dementia. We used conditional logistic regression to examine the association of TBI severity with EOD controlling for comorbidity. After controlling for mental health and other comorbid conditions mild TBI (mTBI) was significantly associated with validated EOD [aOR( 95%CI): mTBI-4.5(2.4-8.9), moderate/severe TBI-21.3(8.4-54.3)]. Stroke, depression, PTSD, and headache were also associated with higher odds of EOD. These findings suggest that Veterans with mTBI are at risk for dementia, and clinicians should consider brief screening for cognitive dysfunction to ensure that they receive timely treatment to mitigate and address the impact of dementia on the individual, caregiver, family, and health care system

### PREDICTORS OF LONGITUDINAL COGNITIVE DECLINE AMONG COMMUNITY-DWELLING KOREAN OLDER ADULTS

Jinhee Shin,[1] and Eunhee Cho[2], *1. College of Nursing, Yonsei University, SOUTH KOREA, SEOUL, Korea, Republic of, 2. Professor, College of Nursing, Yonsei University, Seoul, Korea, Republic of*

Objective This study aims to explore predictors to longitudinal decline of cognitive function in old adults in Korean. Methods The data were derived from the information system of the Korean Longitudinal Study of Aging (KLoSA) which performed nationwide aging panel survey for adults aged over 45 years between 2006 and 2016. The sample consisted of 1,262 older adults who completed K-MMSE. Of the total 1,262 participants, 752 had normal cognition, 243 had mild cognitive impairment, and the rest 267 had dementia. Variables from diverse dimensions were derived from the KLoSA. The linear mixed models were used to predict and explain predictors affecting cognitive function decline over time. Results The ADL and IADL, depression, exercise, and social activity were time-varying variables significantly related to the cognitive function of the older adults. Over time, difference in change of the K-MMSE score between three groups was significant. Conclusions This study identified predictors influencing decrease of cognitive function over time in older adults in Korea. Tailored intervention needs to be developed and implemented in order to delay the cognitive function decline. Improving physical function through regular exercise, increasing social activity, and managing depression by early detection and treatment are recommended according to the cognitive function status. Keywords: old adults, cognitive function, K-MMSE, predictor

### IDENTIFYING COGNITIVE IMPAIRMENT WITH PRIMARY CARE IMPLEMENTATION OF THE BRIEF INTERVIEW FOR MENTAL STATUS

Karen L. Gilbert[1], *1. Alzheimer's Community Care, West Palm Beach, Florida, United States*

Overview: An estimated 5.7 million Americans are affected by Alzheimer's disease (AD; 2018 Alzheimer's disease facts and figures, 2018, p. 367). Cognitive impairment fails to be identified in the primary care setting as often as 76% of the time (Moyer, 2014, p. 793). Screening can identify patients with emerging impairment who might otherwise appear cognitively intact Grober, Wakefield, Ehrlich, Mabie & Lipton, 2017, p.191). Early identification of cognitive impairment promotes evaluation of treatable causes (Possin et al., 2018, p. 150), and access to early treatment for irreversible disease, facilitating future planning (Swallow, 2017, pp. 57, 63). Methods: This quantitative study's aim was to identify patients with occult cognitive impairment. After training staff in a Palm Beach County Florida primary care practice, the Brief Interview of Mental Status (BIMS) was administered to patients aged 45 years and older. Results: Seven of 120 screened patients, with no known AD diagnosis, scored as moderately impaired. One of these patients was 64 years of age, the remaining six ranged from age 71 to 93. Fourteen patients scored at the lowest range of "cognitively intact," eight were under age 65. Conclusion: Cognitive screening of primary care patients with no known diagnosis of AD identified approximately 7% scoring as moderately impaired; an additional 12% scored at the lowest range of "cognitively intact," suggesting a potentially emerging cognitive impairment warranting follow up evaluation for treatable causes, developing AD, or a related neurocognitive disorder.

### EFFECT OF STIGMA ON OUTCOMES OF A MEMORY TRAINING INTERVENTION

Graham J. McDougall,[1] Ian M. McDonough,[2] and Kyle Kraemer[2], *1. Florida State University, Tallahassee, Florida, United States, 2. University of Alabama, Tuscaloosa, Alabama, United States*

Cultural stereotypes equated with aging that emphasize decreasing competence and increasing forgetfulness can be threatening to older adults. Even brief exposure via entertainment media or the patronizing behavior of others may induce stigma in elders and thereby impair memory and executive functions. The sample was recruited for an RCT known as Senior WISE and conducted in Central Texas. The average age was 75 years and average of 14 years of education. Sex and minority status were consistent across groups. Data were analyzed using SPSS, v21. First, Pearson Rs were calculated between stigma (MIA Anxiety subscale) and memory outcomes at baseline. Stigma related significantly to Rivermead (RBMT), HVLT, and Memory Self-Efficacy (MSQ). Then, after controlling for the effects of trait anxiety, stigma explained a significant portion of the variance within scores on the RBMT ($\beta = -.139$, R2 MIA Change = .016, $p = .037$), HVLT ($\beta = -.145$ R2 Change = .017, $p = .032$), and MSQ-35 ($\beta = -.253$, R2 Change = .053, $p < .001$). Change in stigma was significantly associated with change in HVLT scores among those in the memory training group, $r(105) = -.228$, $p = .018$. Reductions in stigma were related to increases in HVLT score, $\beta = -.14$, $F(1, 201) = 3.021$, $p = .084$, R2 Change = .015; however, the overall regression model was not a good predictor of HVLT change, $F(4, 201) = 1.793$, $p = .132$, R2 = .034. Stigma is a high priority area of scientific inquiry and a self-fulfilling prophecy.

### OPTIC FLOW IMPROVES SPATIAL GAIT FUNCTIONING ESPECIALLY IN MEDIOLATERAL DIRECTION

Farahnaz Fallahtafti,[1] Hyeon Jung Kim,[1] Jennifer M. Yentes,[1] Dawn Venema,[2] and Julie Blaskewicz Boron[1], *1. University of Nebraska at Omaha, Omaha, Nebraska, United States, 2. University of Nebraska Medical Center, Omaha, Nebraska, United States*

Instances where multiple tasks are completed simultaneously are considered high cognitive load situations (HCLS, also called dual-task), potentially affecting gait performance in older adults. Walking while talking is a common HCLS that requires additional cognitive resources. Optic flow (OF) provides visual information about speed and direction of self-motion, and thus, may ameliorate gait deficits under HCLS. This study aimed to identify the effect of HCLS, as well as OF, on gait performance in older adults. The HCLS included walking while talking on the phone, compared to walking alone. Fifteen older adults (70.86±4.7yrs) underwent four experimental conditions: walking alone with(1) and without OF(2), as well as walking while talking with(3) and without OF(4). Step width, step length, and double support time were measured and examined with 2(HCLS) x 2(OF) repeated-measures ANOVAs. There was a main effect of OF; step width was narrower with OF compared to without OF ($p=0.048$). For step length, there was a significant interaction between HCLS and OF ($p=0.045$). Without OF, there were no differences in step length; however, with OF step length was significantly longer when walking alone compared to when walking while talking ($p=0.002$). Double support time was not affected by HCLS or OF. Considering younger adults have longer and narrower steps compared to older adults, OF may have enhanced step width regardless of HCLS and step length when walking only. Using OF in training programs designed for older adults, could be a potential factor to improve spatial gait function, more so in the mediolateral direction.

### EFFECTS OF COGNITIVE TRAINING ON WHITE MATTER MICROSTRUCTURE AND COGNITION IN OLDER ADULTS: A SYSTEMATIC REVIEW

Grace M. McPhee,[1] Luke A. Downey,[1] and Con Stough[1], *1. Swinburne University of Technology, Melbourne, Victoria, Australia*

Adults who remain cognitively active may be protected from age-associated changes in white matter (WM) and cognitive decline. To determine if cognitive activity is a precursor for WM plasticity, the available literature was systematically searched for Region of Interest (ROI) and whole-brain studies assessing the efficacy of cognitive training (CT) on WM microstructure using Diffusion Tensor Imaging (DTI) in healthy adults (> 40 years). Seven studies were identified and included in this review.

Results suggest there are beneficial effects to WM microstructure after CT in frontal and medial brain regions, with some studies showing improved performance in cognitive outcomes. Benefits of CT were shown to be protective against age-related WM microstructure decline by either maintaining or improving WM after training. These results have implications for determining the capacity for training-dependent WM plasticity in older adults and whether CT can be utilised to prevent age-associated cognitive decline. Additional studies with standardised training and imaging protocols are needed to confirm these outcomes.

### INVESTIGATING WALNUT CONSUMPTION AND COGNITIVE HEALTH IN A REPRESENTATIVE SAMPLE OF OLDER U.S. ADULTS

Nicholas J. Bishop,[1] and Krystle E. Zuniga[2], *1. Texas State University San Marcos, San Marcos, Texas, United States, 2. Texas State University, San Marcos, Texas, United States*

Population aging increases the need to identify modifiable risk factors of cognitive decline such as nutritional intake. Several nutrients found in walnuts appear to play a neuro-protective role, yet few studies examine whole walnut consumption or draw from representative longitudinal samples. We draw observations from the nationally-representative Health and Retirement Study and Health Care and Nutrition Study to investigate the association between walnut consumption and cognitive trajectories among older US adults. The analytic sample consisted of 6,639 adults age 50 and over in 2013, representing a population of 77,726,682 community-dwelling older adults. Walnut consumption was a categorical measure representing no consumption, moderate consumption (< one serving per week), or high consumption (≥ one serving per week). Indicators of cognitive function representing working memory (immediate and delayed word recall) and global cognitive function (Telephone Interview of Cognitive Status, TICS) were measured at 3 time points (2012, 2014, and 2016). Latent growth models were used to estimate each linear trajectory while adjusting for covariates and complex survey design. Walnut consumption was positively associated with word recall and global mental status at baseline, but was not associated with change over the four year observational window. For example, those with high walnut consumption had baseline TICS scores .89 units greater (SE = .17, p < .001) than those consuming no walnuts. These results indicate that walnut consumption appears to have a positive association with cognitive health, but walnut consumption does not appear to be associated with short-term change in the cognitive outcomes measured.

### PARALLEL DEVELOPMENT OF MEMORY, DISABILITY, AND COMORBIDITY IN U.S. ADULTS AGE 65 AND OLDER

Nicholas J. Bishop,[1] and Kaipeng Wang[2], *1. Texas State University San Marcos, San Marcos, Texas, United States, 2. Texas State University, San Marcos, Texas, United States*

Cognitive health, physical function, and chronic disease represent interdependent health outcomes that may exert influence on the course of each other's development. To investigate the association between baseline health in each domain and developmental change across domains, we estimated trajectories of working memory, mobility limitations, and comorbidity among US adults age 65 and older over 18 years. We drew observations from the nationally-representative Health and Retirement Study with an analytic sample consisting of 5,963 adults age 65 and over in 1998. Immediate word recall, an 11-item Nagi scale of mobility limitations, and a summary count of eight doctor-diagnosed chronic conditions were measured biennially from 1998 to 2016. Parallel-process quadratic growth models with individually-varying time scores were used to estimate non-linear trajectories of each health measure, allowing identification of associations between baseline health and developmental change in each health process at both earlier and later stages of older adulthood. All estimates adjusted for covariates, complex survey design, and missing data. Greater baseline immediate word recall was associated with less rapid increase in mobility limitations at earlier ages. More baseline mobility limitations were associated with faster increase in comorbidity at earlier ages. Greater baseline chronic conditions were associated with more rapid increase in mobility limitations at later ages. These results highlight the importance of conceptualizing health among older adults as an interdependent and developmental process and should help clinicians recognize that single-domain health status may influence the progression of other health outcomes at different stages of older adulthood.

### TUNING ENVIRONMENTAL LIGHTING TO IMPROVE SLEEP QUALITY AND COGNITIVE PERFORMANCE IN OLDER ADULTS

Nastaran Shishegar,[1] and Mohamed Boubekri[1],
*1. University of Illinois at Urbana-Champaign, Champaign, Illinois, United States*

Sleep is important for memory consolidation, hence the disruption of normal sleep patterns as a result of age-related changes in the circadian system could be one of the contributors to memory impairment among older adults. It is now well-established that light is the main environmental element that synchronizes circadian rhythms. An appropriate lighting condition can be considered as a non-pharmacological solution to improve the sleep quality of individuals and consequently their overall health and well-being. The present study investigates the effectiveness of two proposed whole-day lighting interventions (L1 and L2) applied by Tunable White Lighting Technology (TWLT) on sleep quality and cognitive performance in older adults. Both lighting interventions provide a high illuminance level (500 lux) in the morning and then the illumination is dimmed gradually throughout the day and reached 100 lux in the evening. However, while L1 offers a constant Correlated Color Temperature (CCT) of 2700K, during the L2 intervention, the CCT is changing in the range of 6500K – 2700K from morning towards evening. Fifteen healthy older adults (mean age = 73.2 years; 12F) participated in a 41-day counterbalanced crossover study. Participants were exposed to each lighting condition for 9 days. Actigraphy, standard questionnaires (PROMIS and PSQI), and tests (Trail Making Test (TMT) A & B and Digit Symbol Substitution Test (DSST)) were employed to measure sleep quality and cognitive performance before, during, and after lighting interventions. Significant improvements in sleep quality and cognitive performance were found for both lighting interventions with better outcomes for L2.

### MINDFULNESS AND COGNITIVE FUNCTION IN PATIENTS WITH COGNITIVE IMPAIRMENT

Magdalena I. Tolea,[1] Juyoung Park,[1] and James Galvin[1], *1. Florida Atlantic University, Boca Raton, Florida, United States*

Mental health benefits of mindfulness, the attribute of being aware and present in the moment, have long been acknowledged. Mindfulness has also been linked to improved cognitive performance and improvements in AD neuropathology (↓hippocampal atrophy, ↑brain connectivity) in MCI or early-stage AD patients. This study was designed to: investigate the relationship between mindfulness and cognitive function in a patient population with varying degrees of cognitive impairment; identify the specific mindfulness components that provide benefits; and explore differences by sex and disease severity. Patients (N=112; 43% female; 77.0±7.7yrs; 11% cognitively normal, 27% MCI, and 67% dementia) attending a university-based dementia clinic were administered the Applied Mindfulness Process Scale (AMPS) and underwent neuropsychological testing. Cognition was linearly regressed on AMPS with adjustment for age, gender, education, and disease stage, in the entire sample and stratified by sex and stage. In fully adjusted models, higher mindfulness was associated with lower AD8 scores (β=-0.05±0.02(p = 0.003)), better animal naming (AN)(β=0.11±0.04(p = 0.008)), and faster TMA times (β=-0.72±0.32(p=0.025)). All three mindfulness factors (F1=decentering; F2=positive emotional regulation; F3=negative emotional regulation) were significantly linked to AD8, while F3 was not predictive of AN, and F1 was not predictive of TMA. In addition, mindfulness significantly predicted subjective cognitive impairment (SCI) (βF2AD8=-0.18±0.07(p=0.011)) and TMA in men βTMA=-1.14±0.42(p=0.011); βF2TMA=-2.63±1.26(p=0.043); βF3TMA=-2.74±1.12(p=0.019) and dementia patients (βF1AD8=-0.19±0.08(p=0.021); βF2AD8=-0.14±0.07(p=0.044); βTMA=-0.09±0.51(p=0.039)); and AN in women ((βAN=0.12±0.06(p=0.047);βF2AN=0.34±0.16(p=0.036)) and MCI patients (βAN=0.13±0.06, p=0.033; βF3AN=0.36±0.16(p=0.035)). Our findings suggest that effectiveness of mindfulness-based interventions may be enhanced by a focus on emotional regulation and sex- and stage-specific cognitive targets.

### THE VALUE OF US: EXPRESSIONS OF TOGETHERNESS IN COUPLES WHERE ONE SPOUSE HAS DEMENTIA

Anna Swall,[1] Christine Williams,[2] and Lena Marmstål Hammar[3], *1. School of Education, Faculty of Health Sciences, School of Health and Social Studies, Falun, Sweden., Falun, Sweden, Sweden, 2. Christine E Lynn College of Nursing, Florida Atlantic University, Boca Raton, Florida, United States, 3. School of Education, Health and Society, Falun. Sweden, Karolinska Institute, Division of Nursing, Department of Neurobiology, Care Sciences, Stockholm, Sweden, Falun, Sweden*

Background: Living with dementia involves both illness and health and involves self-care and care by others. As most persons with dementia are living in their ordinary housing, dementia affects not only the person with the disease, but also the life of the family, commonly the partner. Research show that spouse carers feel like they are losing their partners due to an inability to share thoughts, feelings and experiences as a couple. Aim: The aim of the study was to describe spouse's experience of their togetherness when one spouse has dementia. Method: The sample consisted of eighteen recorded conversations between 15 persons with dementia and their spouses. The filmed conversations were transcribed verbatim and then analyzed using qualitative content analysis. Findings: One overarching theme arose from the data "Dementia preserved and challenged the value of "us". Being a couple trying to preserve a sense of togetherness and have the relationship they wished for could be seen as a challenge when one spouse was living with dementia. Conclusion: Based on our results, we suggest that practitioners should help couples to reinforce or strengthen their bonds as a couple to maintain well-being. Future studies should examine couplehood under differing conditions such as long versus short term relationships. Prior relationship quality may also be a factor influencing the sense of couplehood following a serious health challenge such as dementia.

### REAL-WORLD REPRESENTATIVENESS OF CANADIAN RESEARCH SUBJECTS WITH MILD COGNITIVE IMPAIRMENT

Vivian Huang,[1] David B. Hogan,[2] Zahinoor Ismail,[2] Colleen J. Maxwell,[3] Eric E. Smith,[2] and Brandy L. Callahan[2], *1. Ryerson University, Toronto, Ontario, Canada, 2. University of Calgary, Calgary, Alberta, Canada, 3. University of Waterloo, Waterloo, Ontario, Canada*

Studies of mild cognitive impairment (MCI) utilize stringent inclusion/exclusion criteria which may impact the generalizability of findings to the broader clinical population. We compared characteristics of MCI patients in a Canadian memory clinic in Calgary to MCI research participants in published Canadian studies to assess the representativeness of research samples. Clinic participants included 555 MCI patients from the Prospective Study for Persons with Memory Symptoms registry. Research participants included 4,981 individuals with MCI retained from a systematic literature review of 112 peer-reviewed empirical Canadian studies. Clinic patients and research participants were diagnosed with MCI using similar diagnostic criteria (i.e., from the NIA-AA, or Petersen criteria). Both samples were compared on baseline demographic variables, medical and psychiatric comorbidities, and global cognitive performance using chi-square tests and t-tests with weighted means. Diverse presumed causes were noted among clinic patients. Clinic patients were younger, more likely to be male, and more educated than research participants (ds: 0.22-0.98). Psychiatric disorders, traumatic brain injury, and sensory impairments were common in clinic patients (up to 83%), but participants with these conditions were excluded from approximately 80% of studies in the systematic review. Clinic patients performed significantly worse on two global cognitive assessments (ds: 0.53 – 1.27). Stringent eligibility criteria such as used in Canadian MCI research studies would exclude a considerable subset of MCI patients seen in our referral clinic. This may have contributed to the disparities between the clinical and research cohorts in the cognitive measures examined. The implications of these findings will be discussed.

### BILINGUAL SPEAKERS POSTPONE SYMPTOMS OF COGNITIVE DEFICIT IN PARKINSON'S DISEASE

Ladan Ghazi Saidi[1], *1. University of Nebraska at Kearney, Kearney, Nebraska, United States*

Maintaining cognitive abilities despite healthy aging, neurodegeneration or acute damage is known as cognitive reserve (Stern, 2002; Stern, et. al., 2018). There is evidence for a higher cognitive reserve in bilingual speakers (Kavé, et. al., 2008), mainly due to their improved executive functioning and attention. Thus, I hypothesized that patients with Parkinson's disease would manifest PD related cognitive symptoms later than monolinguals as a result of better compensation. The aim of this study is to explore how bilingualism affects cognitive abilities in PD patients with cognitive deficit. QPN publicly available database was used to analyze the data on PD patients with (PD-CD) and without (PD) cognitive deficit and their demographic information including the number of spoken languages. Monolingual PD and PD-CD patients were compared to their bi- and multilingual peers on their age of on-set of their cognitive decline as well as descriptive demographic information. The results showed that PD-CD patients who speak more than one language manifest symptoms of cognitive impairment at least three years later than their monolingual peers. These results bring evidence that life-long bilingualism contributes to a stronger cognitive reserve and better compensation in case of a neurodegenerative disorder such as PD.

### STORY CIRCLES AS A METHODOLOGY: A PILOT STUDY EXPLORING COGNITIVE PROBLEMS IN AGING

Logan A. Sweeder,[1] Nikki Hill,[1] Emily Whitaker,[1] Anushka Tiwari,[1] and William Doan[1], *1. The Pennsylvania State University, University Park, Pennsylvania, United States*

A Story Circle refers to a group of individuals in a comfortable social environment sharing personal experiences through stories to explore problems shared by a community and facilitate artistic representation of experiences of interest. In this pilot study, we examined the feasibility of Story Circles to facilitate qualitative inquiry of the experience of cognitive problems among older adults. A convenience sample of six cognitively intact, community-dwelling older adults (M=72.5; SD=5.09 years; 83% female) with self-reported cognitive complaints participated in a 90-minute Story Circle as well as a follow-up phone call. Each shared a personal story of experiencing a cognitive complaint and related these experiences to those shared by others in relation to a prompt provided by the group facilitator. Participants reported enjoying the Story Circle experience (M=8.5/10; 10 = extremely positive) and interest in participating in future Story Circles (M=9.3/10; 10 = extremely likely). Common themes included a sense of community established during the group that persisted after its conclusion as well as a normalization of the experience of occasional cognitive problems. Story Circles may be a useful data collection method to enhance understanding of complex phenomena within a social context.

### SESSION 3300 (POSTER)

### EDUCATION AND TRAINING

### THE IMPACT OF PARTICIPATION IN SOCIAL HEALTH NETWORKS

Anna Faul,[1] Mona Huff,[1] Samantha G. Cotton,[1] Pamela Yankeelov,[1] Joe D'Ambrosio,[1] and Megan Austin[1], *1. University of Louisville, Louisville, Kentucky, United States*

Stress and compassionate fatigue are common among graduate level students working in healthcare professions, however, few studies focus on preventative self-care and its' impact on these learners. As part the University of Louisville's Behavioral Health Workforce Enhancement Training Program (BHWET), graduate students are trained to work with older adults in rural communities. The focus of our BHWET program is to provide holistic, behavioral health care through our FlourishCare Network. As part of the student's weekly curriculum, an interdisciplinary group of learners from counseling psychology, social work and psychiatric nursing were invited to the attend the sessions were invited to participate in a 2-semester Microclinics and Health Matters course that was designed to promote self-care and harness the power of social networks to promote health. A total of 15 students completed the program. Biomarkers including BMI, Cholesterol, A1C, Blood Pressure were taken every week time the course was offered. Cortisol levels were taken every 4 months to measure stress levels. Across the initial 12 weeks of programming, there were positive outcomes for the participants in terms of either maintenance of healthy goals or biomarkers. Additionally, the program had an impact on the older adult clients that were being served by the students compared to students that did not participant. In a review of the plan of care items, which is central to our work with FlourishCare clients, plan of care items showed a stronger focus on connecting clients to social health interventions and a stronger connection to education about health-related content.

### FACTORS PROMOTING CONTINUED LIFELONG LEARNING: FOCUS ON THE PERSON-ENVIRONMENT FIT IN JAPAN

Chiharu Yasuzato,[1] and Keiko Katagiri[1], *1. Graduate School of Human Development and Environment, Kobe University, Kobe, Hyogo, Japan*

Lifelong learning of older people is categorized as social participation. Most studies on social participation have examined the motivation to start; however, those on continuing participation are few. This study aimed to identify the factors promoting continued lifelong learning among older adults, focusing on both personal and socio-relational factors. To do this, in-depth semi-structured interviews were conducted with 20 Japanese citizens, aged 60-75, with a learning experience of more than 3 years. Results showed that both personal and socio-relational factors matter. First, personal factors include older adults' past learning experience, access to learning in the present, and work status. Past learning experience relates to their perception as students and their memories of learning during school age. Regarding accessibility, classes within walking distance, for example, would help in continuation of learning, especially for people with health problems. People may choose to become involved in learning activities after retiring from the workforce and they have time to spend. Second, the importance of socio-relational factors was evident in how family supported older people by accepting how important learning was for them and offering them rides to the classes. Within the class, they can share information about the happenings and activities in their community, become mentors, and stimulate each other. The instructor enhances their enthusiasm to learn and provides a comfortable learning space. Therefore, the findings of this study suggest that while there is no single condition, a person-environment fit promotes older adults continued learning.

### PROMOTING AND ATTRACTING STUDENTS TO CAREERS IN AGING SERVICES

Melinda Heinz[1], *1. Upper Iowa University, Fayette, Iowa, United States*

Three student focus groups were conducted at residential, center, and online university locations (N = 15) to investigate interest and understanding of careers in gerontology and exposure to aging issues. Students majoring in health services administration, psychology, and human services were recruited and given an honorarium for participating. Sessions were recorded and transcribed with two researchers independently coding to identify themes. Center and online participants were more likely to be non-traditional students. Eighty-seven percent of participants were female, 13 percent were male. Ages ranged from 19 to 34 (M = 23.4). Eighty-seven percent were upperclassmen. This study is unique as most research has investigated aging issues with traditional aged students. Center students reported aging issues were discussed in courses outside of their majors, whereas residential students stated issues did not receive attention outside of gerontology classes. Online students stated discussions depended on the class. Common deterrents for not considering careers in gerontology were concerns about performing "physical cares" or coping with death anxiety. Few considered what a career in gerontology looked like outside of nursing homes. To increase awareness, some students felt "gerontology classes should be mandatory." Students felt taking field trips to modern nursing homes "changed their perspective," from medical model facilities. All participants reported little exposure to older adults or gerontology as a viable career path in high school. With the rapidly aging population, we suggest incorporating a "Careers in Aging" unit in high schools to increase awareness of gerontology opportunities.

### TRAINING SENIOR VOLUNTEERS TO IMPLEMENT A GROUP MUSIC INTERVENTION FOR PERSONS WITH DEMENTIA

Silvia Orsulic-Jeras,[1] Sarah Nicolay,[1] and Cristen Kane[2], *1. Benjamin Rose Institute on Aging, Cleveland, Ohio, United States, 2. RSVP of Lake County, Willoughby Hills, Ohio, United States*

The Retired Senior Volunteer Program (RSVP) is a national program with many regional offices that offers a variety of volunteer opportunities for seniors. Research demonstrates the importance of volunteering and social participation on the well-being, health and longevity of older individuals. Further, studies of RSVP volunteers have demonstrated marked improvement in their perceived physical, mental health and quality of life (McDonald et al., 2013). Little evidence exists, however, supporting the effectiveness of using senior volunteers to assist with or even lead activities programming for persons with dementia (PWDs). Seniors Making Connections through Music, a group program designed to engage persons in the moderate to advance stages of dementia, included the training of 21 RSVP volunteers to lead or assist with the program. Volunteers were mostly female (95%) and educated (80% college/advanced degree), with more than half reporting professional or personal experiences with dementia (62%). Data were gathered through structured interviews and post-session debrief meetings. Findings suggest volunteers felt they were able to use existing knowledge and experience, received good and helpful supervision, and reported increased knowledge of dementia after volunteering for the group. Other results summarize: Volunteer satisfaction with program, role, tools and materials used, the program's impact on the community, and willingness to volunteer for the program in the future. Programmatic features, training protocols, and the impact of using retired senior volunteers to strengthen program sustainability will be discussed. Volunteer testimonials and video demonstrations will also be shared.

### REDUCING OPIOID OVERDOSE RISK IN A COMMUNITY CARE COORDINATION CLINIC: RICHMOND HEALTH AND WELLNESS PROGRAM

Faika A. Zanjani,[1] Marshall Brooks,[1] Leland Waters,[2] Pamela Parsons,[1] and Patricia Slattum[1], *1. Virginia Commonwealth, Richmond, Virginia, United States, 2. Virginia Commonwealth University, Richmond, Virginia, United States*

Objective Opioid safety is increasingly important in the care of older adults due to higher risk for negative opioid-related outcomes related to higher prevalence of chronic pain, multimorbidity, polypharmacy, and age-associated changes in drug metabolism and elimination. Evidence-based practices for screening and safe opioid use for older adults are needed. Our project aims to develop, implement, and evaluate a care-coordination workflow and interprofessional clinical opioid misuse screening, support, and referral training to support older adult care. Methods Our research occurs in context of the Richmond Health and Wellness Program (RHWP), a community-based interprofessional care coordination initiative, with interprofessional faculty and students providing on-site integrated care to residents in low-income senior housing communities. Curriculum development, and interprofessional clinical faculty, peer support, and health professions student training have been conducted. Results will discuss the findings from the health professions student training. Findings The Opioid Overdose Risk Reduction Curriculum was delivered via Blackboard and in-person during student orientation. Pre (n=66)/Post (n=59) assessment indicated that after the training, there was an increase in knowledge, specifically change from 50% at baseline, 60% recognized Tramadol as an opioid, change from 80% to 97% understanding that MME represents Morphine Milligram Equivalent, and change from 62% to 93% understanding that 50 MME level greatly increases overdose risk. Only 20%, change from 60%, reported not being able to calculate MME. Conclusions Our findings indicate that opioid safety training within community care coordination is feasible. Future works needs to explore the impact on resident health as the workflow is implemented.

### THE ROLE OF IMPROVED SOCIAL SUPPORT FOR HEALTHY EATING IN A LIFESTYLE INTERVENTION: TEXERCISE SELECT

Aya Yoshikawa,[1] Matthew L. Smith,[1] Shinduk Lee,[1] Samuel D. Towne Jr.,[2] and Marcia G. Ory[3], *1. Center for Population Health and Aging, Texas A&M University, College Station, Texas, United States, 2. Health Management & Informatics, University of Central Florida, Orlando, Florida, United States, 3. Texas A&M Center for Population Health and Aging, College Station, Texas, United States*

Healthy diet is essential to the management of chronic conditions such as cardiovascular disease and diabetes. Research suggests an association between social support and dietary behavior, yet the relationship is not fully explored. The role of social support in nutrition consumption was examined among older participants in a group-based lifestyle enhancement program (Texercise Select) designed to improve dietary behaviors and physical activity and related supports. Factor analysis and structural equation modeling were performed using secondary data from a quasi-experimental study of participants who completed a baseline survey and three-month follow-up (Intervention group N = 211, comparison group N = 175). The majority of participants were age 70 years or older (Mean = 74.30, SD = 8.54), female (82.1%), and had at least two chronic conditions (63.5%). The two groups did not differ in baseline levels of nutrition intake or social support. Program participants improved in terms of intake of water and fruits/vegetables as well as social support. Structural equation models adjusting for the effect of baseline scores indicated that the intervention effect on fruits/vegetable intake ($\beta = 0.19$, $p < 0.001$) was partially mediated ($\beta = 0.03$, $p = 0.021$) by social support for planning and keeping dietary goals and reducing barriers to healthy eating (X2/df = 1.89; RMSEA = 0.04; CFI = 0.99; TLI = 0.99; SRMR=0.02). Findings suggest that programs designed to enhance social support may be effective in improving dietary behaviors among older adults. Future research should investigate various types of social support for promoting healthy diets.

### LATER-ADULTHOOD TRAUMA REENGAGEMENT IN VIETNAM VETERANS WITH PTSD: FINDINGS FROM A PROGRAM EVALUATION PROJECT

Kelly A. O Malley,[1] Patricia M. Bamonti,[2] Kirsten Graham,[2] Ronald Smith,[2] Jacqueline Gurevitch,[2] and Anica Pless Kaiser[3], *1. New England GRECC, Boston VA Healthcare System, Boston, Massachusetts, United States, 2. VA Boston Healthcare System, Boston, Massachusetts, United States, 3. National Center for PTSD, Behavioral Sciences Division VA Boston Healthcare System, Boston, Massachusetts, United States*

As Veterans age, they may experience an emergence or exacerbation of stress symptomatology. Later-Adulthood Trauma Reengagement (LATR) is an intervention that provides psychoeducation on the LATR process, teaches mindfulness and coping, facilitates trauma re-integration, and fosters meaning-making in late life. This program evaluation project evaluated the LATR group offered in the Geriatric Mental Health Clinic at VA Boston. Twenty-one Vietnam Veterans with PTSD symptomatology were referred to the group between 2017-2018. Fifteen completed the 10-week group, and 12 completed pre- and post-intervention assessments and offered feedback. At the conclusion of the group, Veterans reported a decrease in depressive and PTSD symptoms. Of the 4 Veterans who endorsed suicidal ideation at the start of the group, 3 reported a decrease post-group. Veterans endorsed slightly more positive appraisals of their military service. Veterans reported that the group offered support, connection, and a sense of belonging with other Veterans, helped increase coping, and increased insight into thoughts, feelings, and behaviors. A suggested improvement to the group was to offer more sessions. Eleven Veterans sought continued mental health services following the group; three newly sought trauma-focused interventions. Four Veterans reported that their trauma-related symptoms were resolved and declined referral. Veterans found the LATR group helpful as evidenced by reduction in symptoms and responses to open-ended questions. A small number sought additional trauma-focused treatment; others reported that their symptoms were resolved and did not require additional treatment. Findings support the continued offering of the LATR group within this clinical setting.

### CNA WORK IS BETTER LEARNED HANDS-ON: TRAINING CERTIFIED NURSING ASSISTANTS TO FEED RESIDENTS WITH DEMENTIA

Joy W. Douglas,[1] Seung Eun Jung,[1] Hyunjin Noh,[2] Amy Ellis,[1] and Christine Ferguson[1], *1. University of Alabama, Tuscaloosa, Alabama, United States, 2. The University of Alabama School of Social Work, Tuscaloosa, Alabama, United States*

In nursing homes across the United States, Certified Nursing Assistants (CNAs) provide essential mealtime assistance to residents with dementia who have difficulty feeding themselves. However, dementia-related training content in CNA programs can vary. In this qualitative study, we sought to understand the training provided to CNAs in Alabama, and to identify the ideal training modality for content related to feeding residents with dementia. Nine focus groups were conducted with 53 CNAs. Each participant had at least one year of working experience as a CNA caring for older adults. Focus groups were audio recorded and transcribed verbatim. Data were analyzed using the directed content analysis approach. Analyses revealed several key themes related to training CNAs to feed residents with dementia. Across focus groups, CNAs agreed that they needed additional training about feeding residents with dementia. They unanimously agreed that the best person to provide such training should be an experienced CNA, not a nurse or other healthcare provider. In terms of delivery, they preferred hands-on training and role playing. CNAs also emphasized that while some learning takes place in a didactic setting, the most valuable learning moments involve on-the-job experience in feeding residents, where they are mentored by seasoned CNAs. Findings from this study revealed the need for providing CNAs training on feeding residents with dementia. CNA training programs that includes hands-on activities mentored by seasoned CNAs could increase CNAs' ability to provide optimal meal assistance to nursing home residents with dementia.

### MOTIVATION TO LEARN, LITERACY, NUMERACY, AND LATER-LIFE ADULT EDUCATION PARTICIPATION IN THE U.S.

Takashi Yamashita,[1] Phyllis Cummins,[2] Roberto J. Millar,[1] Shalini Sahoo,[3] and Thomas J. Smith[4], *1. University of Maryland, Baltimore County, Baltimore, Maryland, United States, 2. Scripps Gerontology Center, Miami University, Oxford, Ohio, United States, 3. University of Maryland, Baltimore, Baltimore, Maryland, United States, 4. Northern Illinois University, DeKalb, Illinois, United States*

The objective of this study is to examine the associations between the motivation to learn, basic skills (i.e., literacy and numeracy), and organized formal and non-formal adult education and training (AET) participation among middle-aged and older adults in the U.S. Rapid technological advancement and globalization requires that adults engage in lifelong learning to actively participate in society. However, little is known about the roles of motivation to learn and basic skills in AET participation among aging adults in the U.S. We obtained restricted data from the 2012/2014 Program for International Assessment of Adult Competencies, and included adults aged 50 years and older (n = 2,580) in the analysis. Structural equation models were used to examine (1) any AET, (2) formal AET and (3) non-formal AET participation as a function of the latent construct of motivation to learn, literacy and numeracy scores (0 – 500), and covariates. Per the confirmatory factor analysis, the motivation to learn latent construct was a valid measure among the older adults. Results from the structural equation models showed that the motivation to learn ($b = 0.35$, $p < 0.05$), literacy ($b = 0.05$, $p < 0.05$) and numeracy ($b = 0.03$, $p < 0.05$) are all positive predictors of non-formal AET participation. However, only motivation to learn ($b = 0.47$, $p < 0.05$) is associated with formal AET participation. Findings from this study inform future interventions as well as policy changes to promote specific types of organized AET programs among the aging populations in the U.S.

### EDUCATIONAL CHALLENGES OF CHILDREN IN SKIPPED GENERATION HOUSEHOLDS IN NIGERIA

Bayode I. POPOOLA,[1]
FUNMI TOGONU-BICKERSTETH,[1] Joshua O. Aransiola,[2] AKINJIDE AKINTOMIDE,[1] and OPEYEMI EKUNDAYO[1], *1. OBAFEMI AWOLOWO UNIVERSITY, ILE IFE, OSUN, Nigeria, 2. Obafemi Awolowo University, Ile-Ife, Ile-Ife, Osun State, Nigeria*

The paper examined the challenges of accessing education by Nigerian children raised in an unusual family context, the skipped generation households. Specifically, it determined the proportion of Nigerian school-age children in skipped generation households enrolled in the formal school system and investigated the children's perception of the effect of living in skipped generation household on their education. The paper also ascertained regional differences in education challenges experienced by children in skipped generation households. The study adopted the descriptive survey design. The research procedure involved the collection of primary data through the administration of a survey questionnaire on 2144 indexed children from three purposively selected states representing the three major ethnic groups in Nigeria. The results showed that 88.2% of Nigerian children in skipped generation households were enrolled in the formal school system, and that significant regional variations existed in school attendance by children in skipped generation households. Also, a substantial majority (74.1%) of grandchildren reported that living in SGHs had no negative effect on their academic performance. The specific education challenges of school-going children in skipped generation households included having to do assignment alone, not getting enough time to study, and difficulty in paying school fee. The results further indicated that the education challenges experienced by children in skipped generation households were significantly different across the selected states which constitute the study area. The paper highlighted the need for government to improve the welfare of older adults in Nigeria, especially those who serve as custodial grandparents for their grandchildren.

### WHAT IT TAKES TO COMBAT DECLINE: LEARNING THROUGH AN INTERPROFESSIONAL STUDENT SENIOR PARTNERSHIP

Anne D. Katz,[1] and Judy Axonovitz[1], *1. USC Suzanne Dworak-Peck School of Social Work, Los Angeles, California, United States*

Healthcare is in need of a workforce with the knowledge and skills to meet the needs of our aging population. Seniors face social, mental, and physical issues as they move into the later stages of life. The Student Senior Partnership Program (SSPP) connects teams of students with healthy senior volunteers in the community. Through this partnership, students learn the strategies seniors implement to stay engaged and maintain their activities of daily living to combat decline and frailty. The SSPP focuses on increasing student's capability to assess older adults, and learn from their senior within an interprofessional team. Faculty provided training to the senior volunteers so they were prepared to function in a role as "teachers" to students from six professional disciplines (Medicine, Social Work, OT, PT, Dentistry, Pharmacy) through meaningful dialogue focused on physical well-being, relationships with family, friends, community, memory and cognition . Evaluation was performed using the Geriatrics Attitudes Scale (GAS). The pre and post survey (n=72) measured the reported capability to assess older adults and evaluate their attitudes toward the elderly. There were statistically significant improvements in six of nine assessment topics. The largest improvements were in the capability to assess resource gaps (mean=2.79 to 3.44 on a 5-point scale, $p<0.001$), mental status (3.03 to 3.57, $p<0.001$) and fall risk (2.99 to 3.50, $p<0.001$). Attitudes toward the elderly were measured using the Geriatrics Attitudes Scale (GAS). Overall, students improved during the program (mean=3.88 to 4.05 on a 5-point Likert scale, p<0.001).

### EDUCATION AND TRAINING

### ELECTRONIC NAVIGATION SYSTEMS' IMPACT ON OLDER DRIVER PERFORMANCE AND TRAINING TO OVERCOME UNFAMILIARITY

Anne E. Dickerson,[1] F. Dennis Thomas,[2] Lindsay Graham,[2] M.Chandler Coleman,[1] Richard Blomberg,[2] and Tim Wright[2], *1. East Carolina University, Greenville, North Carolina, United States, 2. Dunlap and Associates, Stamford, Connecticut, United States*

Technology may assist older adults in improving their driving performance and therefore driving safety. However, it is sometimes a distraction and some older adults avoid its use due to the complexity of learning the systems. This study examined how older drivers interacted with an electronic navigation system (e.g., GPS) and the extent to which it impacted driving performance on unfamiliar routes. It also examined three approaches to training older adults how to program the devices. In Phase 1, 80 older drivers navigated unfamiliar routes using a GPS or paper directions and completed destination entry tasks. In Phase 2, 60 older drivers completed one of three training conditions (video, video with hands-on, placebo) to examine the impacts of training on destination entry performance. Driving performance was improved with GPS over paper directions (p = .025), as evaluated by a driver rehabilitation specialized on counterbalanced standardized routes. Analyses also showed significant effects for familiarity for use of GPS (p=.035) and age group (60's versus 70's) (p<.001), but many drivers had difficulty entering destinations. In Phase 2, a main effect of training was found (p=.02) with using video and one-on-one training showing the best performance. This study demonstrates older drivers could benefit from the use of such devices when driving to unfamiliar destinations, but training is needed with hands-on training with a live instructor being the best.

### TWO STEPS FORWARD AND THREE STEPS BACK: STUDENTS' PERSPECTIVES ON LIVING AND WORKING WITHIN AN AGING SOCIETY

Catherine J. Tompkins,[1] Emily S. Ihara,[1] and Katelyn Bittinger[1], *1. George Mason University, Fairfax, Virginia, United States*

Each day, 10,000 Americans celebrate a 65th birthday, but there are still many young adults not choosing to enter a field that focuses on working with the older population. As part of a continued effort to understand low enrollments in a minor in aging studies and a graduate certificate program in gerontology, focus groups were held with students to explore why they are not choosing to learn more about one of the fastest growing sectors of the U.S. population. A total of 21 students participated in two focus groups. Students' majors varied but included social work, public health, nursing and communications. All of the students were between the ages of 18 and 22 except for one student who was 68 years old. Only 6 students had taken an aging class, but every student indicated that they were close to a family member, 60 years old or older. Two researchers coded and analyzed the focus group data for themes. Examples of the themes about older adults included being unwilling to change, having negative views toward millennials, and being hampered by technology. Themes relative to student perspectives included needing empathy and patience to work with older adults, assuming decline and lacking opportunities within their majors to take gerontology courses. Comparing views and perspectives of older adults decades ago by traditional college-aged students to current day perspectives resulting from this data will be discussed as well as successful strategies for increasing enrollments in gerontology programs.

### ADVANCING POLICY TO BUILD A BEHAVIORAL HEALTH WORKFORCE THAT ADDRESSES THE NEEDS OF OLDER ADULTS

Kathryn G. Kietzman,[1] Alina Palimaru,[2] and Janet C. Frank[1], *1. UCLA Center for Health Policy Research, Los Angeles, California, United States, 2. RAND Corporation, Santa Monica, California, United States*

California's Mental Health Services Act has infused funding for workforce education and training into the public mental health system. However, funding has not kept pace with an existing behavioral health workforce shortage crisis, the rapid growth of an aging population, and the historical lack of geriatric training in higher education for the helping professions. This study draws on findings from a recent evaluation of how older adults are served by California's public mental health delivery system, and a review of state planning documents and academic literature, to describe gaps and deficiencies in the workforce that serves older adults. While California has more than 80,000 licensed behavioral health professionals in a variety of disciplines, very few have specialized training in geriatrics. Across the U.S., there are fewer than 1,800 geriatric psychiatrists and only about 3% of medical students take any geriatrics electives during their training. Very few nurses (1%), psychologists (4%), or social workers (4%) have training in and/or specialize in geriatrics. Of additional concern in California is the lack of representation of ethnic and racial minorities, and rural/urban geographic disparities in the distribution of the behavioral health workforce. Recommendations for advancing policy change to improve the preparation and distribution of the geriatric behavioral workforce are presented to three distinct audiences: state policymakers and administrators; educational institutions, accrediting bodies, and licensing boards; and county mental health/behavioral health departments and their contracted providers.

### DEVELOPMENT OF AN OPIOID EDUCATION RESOURCE FOR OLDER CANCER PATIENTS

Ruth Manna,[1] Natalie Moryl,[2] Natalie Gangai,[1] Vivek Malhotra,[2] Jennifer Wang,[2] and Beatriz Korc-Grodzicki[1], *1. Memorial Sloan Kettering Cancer Center, New York, United States, 2. Memorial Sloan Kettering Cancer Center, New York, New York, United States*

Pain is a common problem in older cancer patients, estimated to affect 70% of those with advanced disease. As older adults live longer after diagnosis, the use and misuse of opioids will continue to rise. Gaps in available age-friendly opioid resources for patients were identified at a Comprehensive Cancer Center. An interprofessional team worked to develop a resource to educate older cancer patients and their caregivers regarding safe opioid use. Expert clinical opinions from Supportive Care, Anesthesia Pain, Nursing, and Geriatrics Services as well as patient observations informed drafts of the resource. A total of 22 older patients in three geriatric clinics were approached for a short interview (8 open questions) surrounding opioid understanding and concerns. The most stated concerns were fear of addiction, concern about the opioid epidemic, and potential unwanted side effects. There was an evident lack of awareness of what an opioid was or which one of the medications in their list was an opioid. The interviews underscored the need for the education resource to include names of opioids, address concerns about the opioid epidemic and signs of addiction. Language was added to describe safe use, storage and disposal of opioids. Special considerations in the older adult were emphasized. Links to additional information were provided. Finally, patient education experts reviewed the draft to adapt the language to be patient-friendly. Opioids are often effectively used in cancer pain management and older cancer patients warrant proper education. Patient perspectives are critical in the development of relevant patient education resources.

### A MIXED-METHODS EVALUATION OF A 20-YEAR SENIOR MENTOR PROGRAM FOR MEDICAL STUDENTS

Tina R. Kilaberia,[1] Edward Ratner,[2] and June Englund[3], *1. Minnesota State University, Mankato, Mankato, Minnesota, United States, 2. University of Minnesota - Twin Cities, Minneapolis, Minnesota, United States, 3. Augustana Care Organization, Minneapolis, Minnesota, United States*

The purpose of this mixed-methods evaluation is to demonstrate the success from the past 20 class years of continuous use of senior mentors to teach geriatrics to approximately 3400 Year 1 and Year 2 medical students in a home visit model. Using a pre-test post-test design (N=131), we evaluated student satisfaction, self-confidence in various geriatrics assessments, and performance on an Objective Structured Clinical Evaluation (OSCE). We additionally conducted a formal qualitative evaluation of students' open-ended reflections (N=97) across three years, from 2016-18, on what they learned. In addition, relying on the principles of community-based research, we collaborated with a volunteer senior on all aspects of this evaluation, and included a community of seniors at a large urban senior care high-rise to enhance our interpretive validity by asking seniors to reflect on a set of representative quotes for each qualitative theme. The senior mentor home visit model demonstrated high satisfaction, improved self-confidence in geriatric assessment, attainment of competencies, and improvement in attitude scores. With regard to the qualitative portion of the evaluation, six themes about learning were recognized: (1) how to perform an interview and exam in a home setting, (2) how life for seniors is different than students expected, (3) the value of physical infrastructure and amenities in senior housing, (4) the importance of senior's community, including family, neighbors, spiritual community, (5) challenges with aging, and (6) strengths among seniors in coping with such challenges while maintaining individual agency.

### ADVANCING PROGRAM REACH BY TAKING TRAINING ONLINE

Andrew DeMott,[1] Susan Hughes,[1] and Lesha Spencer-Brown[2], *1. University of Illinois at Chicago, Chicago, Illinois, United States, 2. National Recreation and Parks Association, Ashburn, Virginia, United States*

The National Recreation and Park Association (NRPA) is one of four national organizations to receive funding from CDC to disseminate arthritis-appropriate evidence-based interventions (AAEBIs). NRPA has partnered with Fit & Strong!, a CDC-approved AAEBI and National Council on Aging falls prevention program, to disseminate through local park and recreation agencies across the country. To meet the needs of this dissemination effort, Fit & Strong! adapted its in-person instructor training into an online curriculum which became available March 2018. After initial pilot-testing, Fit & Strong! created a five-module training curriculum using Moodle teaching software. The training curriculum uses slides, graphics, videos, and interactive questions to assess mastery. After completing the training, fidelity checks are performed on all instructors by having them video record a class session and share it with the Fit & Strong! team for review. After evaluating the videos, the team meets with the instructors by phone to debrief and advise on any needed improvements. Through this partnership, Fit & Strong! trained an initial cohort of 39 instructors across 18 states within a month's time. Training evaluation results have been positive. To date, over 900 participants have been reached. A second cohort of approximately 40 new instructors will be trained in Spring 2019. This presentation will review the processes of creating and executing the online training, potential cost and time-saving benefits to CBOs, instructor feedback, and the ease with which instructors can complete the training and offer the program anywhere across the country.

### STRENGTHENING A COMMUNITY HEALTHY AGING EDUCATION PROGRAM THROUGH CBO PARTNERSHIPS AND RCQI

Jeffrey P. Graupner,[1] Jason Molony,[1] Leah Kelemen,[1] Kedong Ding,[2] and Katherine Thompson[1], *1. University of Chicago, Chicago, Illinois, United States, 2. University of Chicago School of Social Service Administration, Chicago, Illinois, United States*

The SHARE Network (Supporting Healthy Aging Resources & Education) is a Geriatrics Workforce Enhancement Program serving older adults in a large urban, underserved community. For 4 years, one objective of the SHARE Network has been to increase health literacy while fostering positive change in older adults through "Healthy Aging" presentations. Led by geriatrics subject experts, these presentations cover a wide range of important health topics for older adults at a variety of community-based organization (CBO) partner locations. Rapid Cycle Quality Improvement (RCQI) efforts to enhance this programming include surveying audience members after a presentation, then sharing results with CBO partners at monthly meetings to review popular topics, participant demographics, quality of presentations, suggestions for improvement, etc. These recurring RCQI meetings have proven critical to maintaining a robust healthy aging curriculum and network engagement across a variety of organizations and interests. Analysis of participant survey data demonstrates the strength of QI efforts and includes a qualitative analysis of open-ended responses. To date, 153 presentations have been held with 2,704 attendees and 1,470 respondents, 86.3% of whom planned to make a health change after attending the presentation. Qualitative analysis of these behavior change plans reveals that 82.6% are proactive behaviors versus restrictive behaviors, and that diet, exercise, and lifestyle changes are most common. This program, and its RCQI approach, serves as a successful model for aligning the educational objectives and priorities of network partners, while ensuring relevancy and cultural competency in promoting positive healthy behaviors in the older adult community.

### IMPROVING THE PERSONAL CARE ASSISTANT WORKFORCE THROUGH TRAINING, DATA, AND TECHNOLOGY

Fuad Abujarad,[1] Sarah J. Swierenga,[2] Clare Luz,[2] Katherine Hanson,[2] Chris Curtin,[3] Zhehui Luo,[4] and Lauren A. Swanson-Aprill[5], *1. Yale University, New Haven, Connecticut, United States, 2. Michigan State University, East Lansing, Michigan, United States, 3. DIRECT CARE PROFESSIONAL TRAINING, Traverse City, Michigan, United States, 4. Department of Epidemiology and Biostatistics, Michigan State University, East Lansing, Michigan, United States, 5. Michigan Department of Health and Human Services, Aging and Adult Services Agency (AASA), Lansing, Michigan, United States*

Ensuring a quality personal care aide (PCA) workforce is critical to meeting the needs of an aging population. The Integrated Model for Personal Assistant Research and Training (IMPART) program is a PCA training and advocacy model designed to increase the number of qualified PCAs. A core component is an evidence-based, comprehensive PCA training program, Building Training…Building QualityTM (BTBQTM), supported by a robust data platform. This online system was designed to register, train and certify PCAs and Trainers, maintain a qualified PCA workforce database, and enhance the capacity to track IMPART process, performance, and impact measures. This innovative system was developed using a User-Centered Design approach, which includes four main user interfaces: Administrator, PCA, Trainer, and Public. It automates central administration functions, tracking, and reporting of training events, and standardizes and consolidates all training activities on a scalable and usable web-based technology platform. User acceptance testing of the tool and a usability evaluation with representative PCAs and trainers has been completed. Over 50 PCAs have completed BTBQTM modules and 22 new trainers have completed a new BTBQTM Trainer Certificate program during the tool development phase. The PCAs are already reporting substantially higher wages. Launching the new web-based data platform in April 2019 will make it possible for these programs to scale up for wide distribution. As more PCAs and trainers graduate, the number of qualified PCAs will increase exponentially. The data collected with this technology can inform responsible fiscal and policy decisions about resource allocation to support a stronger PCA workforce.

### EXPERIENCES AND BARRIERS TO SUCCESS FOR MID- AND LATER-LIFE COLLEGE STUDENTS: APPLYING A GERONTOLOGICAL LENS

Phyllis Cummins,[1] Annabelle Arbogast,[2] Kathryn McGrew,[3] and Peter Bahr[4], *1. Scripps Gerontology Center, Miami University, Oxford, Ohio, United States, 2. Miami University, Oxford, Ohio, United States, 3. Miami University, Scripps Gerontology Center, Oxford, Ohio, United States, 4. University of Michigan, Michigan, United States*

Adult students have emerged as a key population of interest within higher education as states and institutions strategize to meet postsecondary attainment goals. However, much of the previous research on non-traditional age college students has collapsed all students age 25 and older into a single category, glossing over important age and life stage differences. Using a gerontological lens, this paper examines experiences and barriers encountered by mid-and later-life (MLL) students (age 40 and older) attending community colleges. We report qualitative findings from a mixed-methods study of MLL students in Ohio community colleges, funded by the Institute of Education Sciences. Based on thematic analysis of interviews and focus groups with students, faculty, staff, and administrators at 23 colleges, we identify multiple dimensions of age and aging that each play a meaningful role in shaping MLL students' community college experiences and outcomes. Additionally, we provide an in-depth profile of MLL students—including their educational and work trajectories, reasons for enrolling, and experiences in community colleges—that can help colleges better recruit and serve this segment of the adult student population. MLL students face both unique and common barriers that colleges can address at the classroom, program, and institution levels. Implications for research, policy, and practice are discussed.

### SESSION 3310 (POSTER)

### END OF LIFE

### END-OF-LIFE SERVICES IN TRIBAL COMMUNITIES

Lori L. Jervis,[1] and Derrell W. Cox[1], *1. University of OKlahoma, Norman, Oklahoma, United States*

Terminally ill American Indians/Alaska Natives (AI/ANs) are less likely to receive hospice and palliative care than other racial/ethnic groups, with fewer than 1/3 receiving these services compared to over 45% of EuroAmericans (Johnson, 2013; NHPCO, 2017). While some AI/ANs believe that End of Life (EoL) services will hasten their deaths (Colclough & Brown, 2014), claims that Natives reject EoL services due to death taboos are likely overgeneralizations. Rather, extant studies point to barriers to access resulting from lack of financial resources and inadequate service infrastructure, especially in rural areas (Jervis, Jackson, & Manson, 2002; Kitzes & Berger, 2004; Kitzes & Domer, 2004; Weech-Maldonado et al., 2003). While these factors undoubtedly play a role in underutilization, our preliminary research suggests that other factors—such as a lack of tribally based EoL programs and the cultural mismatches that occur when non-Native programs attempt to deliver hospice services to Native clients—may discourage AIANs from seeking and/or retaining these services. In this presentation, we report on results from a nationwide telephone survey of the availability of EoL care across AIAN tribes. We also present findings from in-depth interviews with local service providers on the challenges and successes they experienced in providing EoL care to their AI clients in one tribal community. Together, these findings will add to our growing understanding of the factors that inhibit and facilitate EoL service utilization, and suggest possibilities for improving access.

### STRENGTHENING OLDER INDIGENOUS NEW ZEALANDERS AT END OF LIFE: WHAT ROLE DO HEALTH SERVICES PLAY?

Tess H. Moeke-Maxwell,[1] Kathleen R. Mason,[1] and Merryn Gott[1], *1. University of Auckland, Auckland, New Zealand*

Older indigenous people and their families draw on specific tribal care customs to support end-of-life care as these help to fortify and strengthen older people. New Zealand's health and palliative care services can either help or hinder families to utilise their care customs. The aim of the Pae Herenga study was to investigate the specific traditional care customs employed by older New Zealand Māori. This involved 60 face-to-face interviews with participants who had a life limiting illness (majority aged over 65), family carers, indigenous healers, spiritual practitioners, and health and palliative care professionals across four key geographical sites. Three digital story workshops involving 16 participants were also included. The study findings show that no matter what the older person's illness was, their cultural customs and protocols helped to fortify them and kept them spiritually safe at end-of-life. Hospitals and hospices helped families to action their customs by providing rooms large enough to host gatherings of thirty or more people; prayers, songs, speechmaking and communal sharing of food took place. However, incidences of racism, a lack of space, and a lack of support for indigenous plant medicines prevented the use of ancient traditional end-of-life care customs for older people. The findings suggest that health and palliative care services can help older indigenous people maintain their spiritual strength by providing them with culturally supportive care and environments equipped to host the dying and their families.

### FAMILY AND SOCIAL NETWORKS FOR AGE-RELATED PLANNING CONVERSATIONS: CHARACTERISTICS AND VARIABILITY

Emily K. Chen[1], *1. RAND Corporation, Arlington VA, Virginia, United States*

Informal advance care planning (IACP) – that is, conversations with surrogate decision-makers about wishes for health care or end-of-life preferences – have been identified as equally if not more important than legal documentation for achieving a high-quality end-of-life experience. Fairly high rates of adults report having had these conversations; this is especially true of people who are older, sicker, or who have had caregiving experience. However, relatively little is known about the content and characteristics of these conversations, such as who people are talking to, what triggers the conversation, and what is actually said. This paper reports findings from interview-based research that asked 38 middle-age and older adult respondents (ages 55 to 74) about conversations related to several areas of age-related planning, including planning for health care needs and wishes about end-of-life. The interactive interview protocol used the open-source EgoWeb software to elicit information about age-related planning conversations, family and social networks, and who within those networks served as conversation partners for the various topics. We will share results of the analysis of the networks and conversation topics. We found that some individuals more readily engage in discussions about future planning across topics than others, but that part of this is driven by the readiness of their family and friends to engage in these topics. This suggests that targeting individuals to increase rates of IACP has limitations when family and friends are resistant, and that dyadic interventions may be appropriate in some cases.

### CAREGIVER BURDEN, BENEFIT, AND PERCEPTIONS OF END-OF-LIFE CARE QUALITY

Elizabeth A. Luth,[1] and Teja Pristavec[2], *1. Weill Cornell Medicine, New York, New York, United States, 2. University of Virginia, Biocomplexity Institute and Initiative, Arlington, Virginia, United States*

End-of-life care quality (EOLCQ) gauges our success in providing quality care to dying individuals. EOLQC measures rely on reports from bereaved family members who provide care for dying loved ones, but analyses seldom account for how caregivers' experiences influence their EOLCQ perceptions. Caregivers frequently experience burden, which is linked to poor health outcomes and may negatively bias EOLCQ reports. Individuals may also perceive caregiving benefits that can offset deleterious burden effects, but potentially encourage overly positive EOLCQ perceptions. This paper links National Study of Caregivers (2011) and National Health and Aging Trends Study (2011-2016) data, using regression analysis and a sample of 380 EOL caregivers to examine how caregiving burden and benefits perceptions shape and moderate EOLCQ reports. Caregiving burden is unrelated to EOLCQ in adjusted models. Benefits are associated with marginally greater odds of being informed about the dying person's condition and reporting their personal care needs were met. Burden and benefits moderate these two measures. Despite benefits, low burden caregivers report they were informed about the dying person's condition with 90% probability. Regardless of burden, high benefits caregivers report the same with 90% probability. Low burden and benefits caregivers report met care needs with 90% probability. High burden and benefits caregivers have 90% probability of such reports. Given these reports are used in formal hospice care evaluations by CMS, additional research should explore why caregiving burden and benefit are associated with some EOLCQ measures and why individuals reporting high burden and benefits provide more positive EOLCQ appraisals.

### ADULT CHILDREN'S UNDERSTANDING OF PARENTS' CARE AND LIVING PREFERENCES AT END OF LIFE

Meghan McDarby,[1] Matthew Picchiello,[1] Elissa K. Kozlov,[2] Catherine Ju,[1] Dani Worthalter,[1] and Brian Carpenter[1], *1. Washington University in St. Louis, St. Louis, Missouri, United States, 2. Rutgers University, New Brunswick, New Jersey, United States*

Adult children who are uninformed about their parents' preferences for end-of-life care may not be prepared to advocate on their behalf when the circumstances arise. The purpose of the current analysis was to examine how well adult children understand their parents' end of life preferences. We analyzed responses from adult children (n = 70) of 40 older adults (65+) who participated in an intervention to improve family communication about end-of-life care. We compared children's and parents' responses on the same set of 6 questions about healthcare decision-making (e.g., "Which medical decision-maker has the final say?") and 4 questions about living preferences (e.g., "Would you want to move to a nursing home?"). We also examined demographic differences between children who had higher agreement (≥ 6/10 questions correct; n = 32) versus lower agreement (<6/10 questions correct; n = 38). On average, children provided the same response as their parents on approximately 5 out of 10 questions. Overall, adult children answered more questions correctly about living preferences compared to preferences about healthcare decisions ($t(69) = 6.59$, $p < 0.001$). In terms of demographic characteristics, there were no significant differences between children with higher and lower agreement with their parents' preferences on variables including gender, frequency of contact with parents, and living proximity to parents. Our results underscore the need for increased communication between adult children and their parents about topics likely to influence quality of care at end of life.

### END-OF-LIFE CARE PREFERENCES AND WELL-BEING IN LONG-TERM CARE RESIDENTS

Kelly Shryock,[1] Jacinta Dickens,[1] Anisha Thomas,[2] and Suzanne Meeks[1], *1. University of Louisville, Louisville, Kentucky, United States, 2. John Hopkins University, Baltimore, Maryland, United States*

Research on end-of-life care in nursing homes comes largely from the viewpoint of staff or family members. We examined patient perspectives on end-of-life care, preferences for care, and quality of life in long-term care settings. We hypothesized that fulfillment of the Self Determination Theory (SDT) needs of autonomy, competence, and relatedness would be related to better well-being and that the degree to which end-of-life care preferences are seen as possible in the setting would be related to SDT need fulfillment and well-being. Preliminary data, collected from older individuals at the end of life (over 55, presence of significant chronic disease, in long term care setting) (n= 72), demonstrated that autonomy, competence, and relatedness measures were moderately and significantly correlated with well-being as measured by life satisfaction, higher positive affect, lower negative affect, and overall quality of life measures The degree to which residents believed that their end-of-life care preferences could be honored in the setting was also significantly correlated with autonomy, competence, relatedness, positive affect, and psychological quality of life. These results are consistent with SDT and suggest that if long term care settings can promote autonomy, connection, and competence in making end of life decisions, possibly by discovering and fulfilling preferences for end of life care, individuals who end their lives on those settings have potential for greater satisfaction and happiness. These results suggest that SDT is a useful framework for ongoing research on how to improve the end of life experiences of older adults in long term care.

### PREVALENCE AND CORRELATES OF SYMPTOMS OF ANXIETY AND DEPRESSION AT THE VERY END OF LIFE

Elissa K. Kozlov,[1] Veerawat Phongtankuel,[2] and Cary Reid[2], *1. Rutgers University, New Brunswick, New Jersey, United States, 2. Weill Cornell Medicine, New York, New York, United States*

Rates of psychological symptoms for patients with serious illness is high, but there has been limited research investigating psychological symptoms at the very end of life. The aim of this study was to better understand the prevalence, intensity and correlates of psychological distress at the very end of life. This cross-sectional study utilized caregiver proxy interviews. Caregivers were contacted after their loved one recently died after being on home hospice and invited to participate in a brief interview with a trained research assistant. Patient, caregiver and hospice utilization data were also abstracted from electronic medical records. N = 351 caregivers were included in the study. According to caregivers, 46.4% of patients had moderate to severe anxiety, as assessed with a score of ≥4 on the Edmonton Symptom Assessment Scale (ESAS) and 43% had moderate to severe symptoms of depression in the last week of life. Symptoms of anxiety and depression were significantly associated with caregiver burden scores and inversely associated with the age of the patient. Psychological symptom management at the very end of life is essential to providing comprehensive hospice care. Our study revealed that nearly half of patients die with moderate to severe symptoms of anxiety and/or depression. Future research is needed to improve psychological symptom management at the very end of life in order to improve the quality of life for both patients and their families.

### FREQUENCY OF CONTACT AMONG FAMILY MEMBERS AND END-OF-LIFE CARE PLANNING

Meghan McDarby,[1] Elissa K. Kozlov,[2] and Brian Carpenter[1], *1. Washington University in St. Louis, St. Louis, Missouri, United States, 2. Rutgers University, New Brunswick, New Jersey, United States*

The purpose of the current analysis was to examine how contact between adult children and their older parents may relate to having end-of-life care conversations. We analyzed responses from adult children (n = 66) of 36 older adults (65+) who participated in an intervention to improve family communication. Children reported the frequency of their in-person and phone contact with parents. They also completed the Conversations about Care Arrangements Scale (alpha = 0.95), 8 items that measure the extent to which adult children have discussed plans about future care with their parents (1=have not talked at all, 5=talked extensively). We calculated a composite from all 8 items (potential range 5-40). Participants reported that they had, on average, not talked extensively with their parent about plans for future care ($M = 18.7$, $SD = 8.22$). Overall, 42.4% of children reported that they visited their parent one time or less per year. Children reported speaking on the phone with their parent an average of 4.74 times per week ($SD = 6.80$, range = 0-30) and initiating an average of 2.59 of those calls ($SD = 3.82$, range = 1-20). Frequency of weekly phone conversations between child and parent was significantly associated with having talked more extensively about future care plans ($r = 0.25$, $p < 0.05$), as was frequency of phone calls initiated by the adult child (r = 0.29, p < 0.05). Frequency of in-person visits to parents was not significantly associated with conversations. Infrequent contact may limit opportunities for care conversations.

### SESSION 3315 (POSTER)

### FAMILY & INTERGENERATIONAL RELATIONS II

### COULD CHASING GRANDKIDS KEEP US YOUNG? THE ASSOCIATION BETWEEN CUSTODIAL GRANDPARENT STATUS AND HEALTH

Danielle K. Nadorff,[1] Emily A. Williamson,[2] and Ian T. McKay[1], *1. Mississippi State University, Starkville, Mississippi, United States, 2. Mississippi State University, Mississippi State, Mississippi, United States*

There are approximately 7.2 million grandparents living with their grandchildren in the United States. Of these, roughly 2.5 million are skipped-generation households in which grandparents are solely responsible for meeting the needs of their grandchildren (U.S. Census, 2017). Previous research has established that custodial grandparents suffer from added strain and burden compared to their peers, which negatively impacts their health (Lo, M., Liu, Y., 2009). A decline in functional ability has a negative impact on not only the lives of these older adult grandparents but also their family members who are dependent upon them for care. The current study examines adults aged 65 and older using data from the American Community Survey 2016 to assess the extent to which raising one's grandchildren is associated with five areas commonly subject to decline in older adulthood: cognitive performance, self-care ability, ambulatory difficulty, hearing, and vision abilities. Hierarchical binary logistic regression analyses found that after controlling for the effects of age, sex, race, and income-to-poverty ratio, custodial grandparents (those who reported having primary responsibility for their grandchildren in their own home with no parents present) were significantly less likely than their peers to report experiencing any of these five disabilities. Details of each model and clinical implications will be discussed.

### HUSBAND'S EMOTIONAL SUPPORT PROVISION TO ADULT CHILDREN AND WIFE'S MARITAL SATISFACTION IN LATER LIFE

Heejeong Choi,[1] and Jaeeon Yoo[2], *1. Sungkyunkwan University, Sungkyunkwan University, Seoul, Korea, Republic of, 2. Korea Institute for Health & Social Affairs, Korea Institute for Health & Social Affairs, Sejong, Korea, Republic of*

The current study examined the association between intergenerational support exchange and marital satisfaction among older Korean couples. Prior work has not paid due attention to the fact that older parents and adult children often exchange various types of support in the context of marital relationships, and that provision or receipt of support could influence their marital relationships (see Lee, Zarit, Rovine, Biritt, & Fingerman, 2010; Polenick, Birditt, & Zarit, 2018, for exceptions). Using the 2008 Actual Living Condition of the Elderly and Welfare Need Survey (ALCEWNS), a nationally representative survey of community-dwelling adults 60 years and older, we evaluated the links between marital satisfaction and each spouse's reports of emotional and instrumental support provided to or received from adult children. For analyses, a series of actor-partner interdependence models were estimated. Findings revealed that wives' marital satisfaction was associated with their husband's exchange of emotional support with adult children. By contrast, husbands' marital satisfaction was unaffected by their wife's emotional support exchange with adult children. More specifically, wives were more satisfied with their marriage when their husband reported providing greater emotional support to adult children than receiving it from adult children. In addition, wives indicated higher marital satisfaction when the couple provided similar levels of emotional support to their children. Provision or receipt of instrumental support had no bearings on marital satisfaction of either spouse. Taken together, our findings highlight how older couples may evaluate their relationship quality in the Korean cultural context.

### THE PERSISTENCE OF TRADITIONAL VALUES: GRANDPARENTS REARING GRANDCHILDREN

Anne B. Edwards,[1] Tammy L. Henderson,[2] Crystal Niemeyer,[1] and Jessica L. Stanley[3], *1. Purdue University Northwest, Hammond, Indiana, United States, 2. Lamar University, Beaumont, Texas, United States, 3. University of Oklahoma, Tulsa, OK, United States*

The goal of this study is to examine how cultural values are preserved and transmitted by grandparents rearing grandchildren in one community in the southeast region of the Yukon-Koyukuk Census Area in Alaska. The eight participants (six females and two males) lived in a community in the Kusilvak Census Area, with ages ranging from 47 to 73 years old. Participants' took part in a semi-structured interview, which were then transcribed and coded into larger themes of 1) loss of traditional values, 2) continuing traditional values, 3) practicing traditional values, and 4) transmitting traditional values. The participants provided examples of how the cultural values that were strong at one point in their lives, were no longer exemplified in their community, and, in fact, behaviors that went against accepted values were seen. Participants spoke most often of how community members were cared for, how the community was valued over the individual, and the connections within families. The GRGs practiced those traditional values by caring, supporting, and loving the people in their families and communities, and by practicing humor and sharing with others. While this community has been influenced by modern ways of living currently found in the United States and Canada, it still remains relatively isolated from the technological and social influences that dominate what is considered "typical, modern" family life. The findings from this study illustrate the important roles that GRGs play in the persistence of cultural values, and the importance of incorporating these values in programs to assist this community.

### OBSERVING SUPPORT INTERACTIONS BETWEEN PARENTS AND CHILDREN IN ADVANCED AGE

Claudia Meystre,[1] Daniela Jopp,[2] Joëlle Darwiche,[1] Kathrin Boerner,[3] and Dario Spini[4], *1. University of Lausanne, Institute of Psychology, Lausanne, Vaud, Switzerland, 2. University of Lausanne, Lausanne,*

*Switzerland, Switzerland, 3. University of Massachusetts Boston, Boston, Massachusetts, United States, 4. University of Lausanne, Swiss National Centre of Competence in Research LIVES, Lausanne, Vaud, Switzerland*

Studies on support exchanges in older parent-child dyads have so far not used observational approaches. Rather, they have mostly relied on self-report/questionnaire approaches. However, support exchanges represent a dyadic phenomenon that goes beyond individual perspectives on the quality of support; thus, self-reports offer only a part of the picture and are subject to bias (e.g., memory bias). In contrast, observations are better suited to capture specific support behaviors and allow for studying the dyad "in action." Our study purpose was to examine mutual support during interactions between older adults and their children, and to investigate the links of support behaviors to relationship quality and health. Fifty dyads living in Switzerland, composed of individuals aged 70 and over and the child involved in their care, participated in a standardized interaction task: The parent described and discussed a personally challenging situation with the child for ten minutes; then roles were reversed. The videotaped interaction was analyzed using the Social Support Behavior Code Adapted for Elders (Meystre et al., submitted). Relationship quality and subjective health were assessed via standardized questionnaires. Various types of support were observed, ranging from positive to negative. Children provided more informational support than parents. Dyads with poor relationship quality showed more informational support. Better participant health was associated with fewer negative behaviors. In sum, observing the dyads in real time offered unique insights into interacting patterns of support among older parents and their children, and enabled us to capture the nature of their relationship above and beyond individual self-reports.

### BLOOD THICKER THAN WATER? STEPGAP IN INTERGENERATIONAL RELATIONSHIPS

Peter Öberg,[1] and Torbjorn Bildtgard[2], *1. University of Gävle, Gävle, Sweden, 2. Stockholm University, Stockholm, Sweden*

The increasing prevalence of ageing stepfamilies and stepchildren's potential to act as a source of support for older parents has prompted research about intergenerational cohesion in step-relationships. Previous research has hypothesized a qualitative gap (a step-gap) between step and biological relationships to the advantage of the latter. In this Swedish study we compare emotional closeness between older parents and adult children among parents (aged 66-79) who have had both biological and stepchildren, and children (aged X-Y) who have had both biological and stepparents. Qualitative interviews (n=24) of family histories including a hierarchical convoy model of family relationships were collected and analyzed. Results show that with few exceptions biological relationships are rated as emotionally closer than step-relationships, both by parents and adult children, supporting the step-gap hypothesis. While the older parents tend to deemphasize the importance of blood for their ratings, the adult children often emphasize the importance of blood. The difference is explained by a parental adherence to an ideology of equal treatment of children, while the adult children stress the importance of biology for their identity and belonging.

### ARE FAMILY RELATIONSHIPS A PROTECTIVE RESOURCE AMONG ADULTS WITH FUNCTIONAL IMPAIRMENT?

Richard E. Chunga,[1] Haowei Wang,[1] and Deborah Carr[2], *1. University of Massachusetts Boston, Boston, Massachusetts, United States, 2. Boston University, Boston, Massachusetts, United States*

Health-related declines that affect physical functioning are a common stressor among older adults. Functional impairment can take a toll on older adults' psychological well-being as it limits one's capacities to independently carry out meaningful daily activities. The extent to which impairment affects mental health may vary based on the levels of support and strain in one's personal relationships. Stress buffering perspectives suggest that support mitigates the detrimental psychological consequences of impairment, whereas stress amplification perspectives predict that strain will amplify these consequences. We use data from 2012 and 2016 waves of the Health and Retirement Study (N=3800) to explore: (a) the direct effects of functional limitation on depressive symptoms (CES-D); (b) the extent to which these associations are moderated by spouse, child, other relative, and friend support/strain; and (c) gender and marital status differences therein. Using lagged endogenous regression models, we find that impairment significantly increases depressive symptoms among men and women, and these effects are intensified by marital strain for both married men and women. However, buffering effects are found for women only, such that marital support mitigates against depressive symptoms in the face of current impairment. These results may reflect the gendered nature of marriage, where men with impairment uniformly benefit from marriage although women may experience protective effects of only in highly supportive unions. Results for other strain and support moderators also reveal gender differences, reflecting the distinctive ways that men and women interact with kin and friends over the life course.

### IS ME IMPORTANT TO US? ACTOR AND PARTNER EFFECTS OF SELF-ACCEPTANCE ON SPOUSAL SUPPORT

Kyuho Lee[1], *1. Daegu University, Gyeongsan, Korea, Republic of*

Marital relationship is influenced by substantial and perceived support exchange. Therefore, it is important to find predictors of support-giving and support-receiving. However, very few studies attempted to do so. As some previous studies reported, individuals' personality-related characteristics seem to play a significant role in the support exchange within couples. This study aimed to assess the effect of individuals' self-acceptance on support exchange within older couples. Data of 2,082 heterosexual older couples aged between 50 and 85 in 2006 from the Health and Retirement Study were assessed utilizing an actor-partner inter-dependence model and growth curve model. For the actor effects, both husbands' and wives' higher levels of self-acceptance predicted their own perception of the support from a spouse. For the partner effects as well, individuals' self-acceptance positively predicted their spouse's perception of the received support. Husbands' self-acceptance, however, was associated with a decrease in wives' perceived support. We discuss the possible mechanism with regard to older adults' self-acceptance and marital support. The findings of the current study contribute to the theories of spousal support and personality as well as in the practical settings of couple therapy and education.

### SPOUSAL ASSOCIATIONS IN MONTHLY REPORTS OF DISABILITY IN THE PRECIPITATING EVENTS PROJECT

Joan K. Monin,[1] Holly Laws,[2] Evelyne Gahbauer,[3] Terrence E. Murphy,[3] and Thomas M. Gill[3], *1. Yale School of Public Health, New Haven, Connecticut, United States, 2. University of Massachusetts Amherst, Amherst, Massachusetts, United States, 3. Yale School of Medicine, New Haven, Connecticut, United States*

While many prior studies have evaluated the antecedents and consequences of changes in disability, few have considered the social context. As nearly 60% of older adults currently live with a spouse or intimate partner, it is important to examine spousal influences on disability. This study examined spousal associations in self-reported disability using data from the Precipitating Events Project, an ongoing longitudinal study of 754 initially nondisabled community living adults age 70 and over who have had monthly assessments of functional status since 1999. We hypothesized that one spouse's level of disability would be associated with increases in the other spouse's subsequent disability. We used the Actor Partner Interdependence Model (APIM), a statistical modeling framework that accounts for the interdependence in two-person data and tests the associations of both self (actor) and partner influences on outcomes. We used multilevel, longitudinal APIMs to examine lagged associations in spouses' monthly reports of disability in 13 activities of daily living (e.g., walking a quarter mile, bathing) in the 37 married couples. As hypothesized, one partner's prior disability level was significantly associated with the other partner's (the actor's) subsequent disability level ($B = .674$, $SE = .012$, $p < .001$) after controlling for the actor's prior disability level. Also, when both couple members had higher levels of prior disability, they were particularly at risk of subsequent increases in disability ($B = .016$, $SE = .003$, $p < .001$). Incorporating partner disability level in modeling individuals' outcomes provides greater precision in predicting future disability levels.

### INCREASING THE POWER OF INTERGENERATIONAL NETWORKS: ADVANCING A NEW EVALUATION TOOL

Shannon Jarrott,[1] Lisa A. Juckett,[1] and Jill Juris Naar[2], *1. The Ohio State University, Columbus, Ohio, United States, 2. Appalachian State University, Boone, North Carolina, United States*

According to a 2018 national survey of intergenerational (IG) care providers, practitioners identified as their number one concern a need for evidence-informed evaluation tools to demonstrate their impact on older and younger participants. The Best Practices Checklist is a 14-item (yes/no) measure grounded in evidence of effective intergenerational strategies. Trained evaluators complete the checklist based on their observations of facilitators' behaviors during IG activities. Exploratory factor analysis (promax rotation) of the Checklist for 132 IG activities offers insight to factor structure and item construction. An adequate two-factor structure was achieved; seven Checklist items were retained with factor loadings greater than .39. Seven items were deleted due to non-variance, high missing data, or double loading across factors. Factors reflect dimensions of: (a) person-centered strategies (e.g. selecting activities based on participants' interests) and (b) creating a positive physical environment (e.g., grouping participants into intergenerational pairs or small groups). Findings indicate that a Best Practices Checklist with fewer items may offer a suitable tool for assessing the utilization of Best Practices during IG activities. Given the demand for IG evaluation tools, the 7-item BP Checklist can be a brief, easy-to-use measure that documents IG facilitators' implementation of evidence-informed practices. Its use could be especially helpful if connected to varied indicators of program effectiveness and participant outcomes.

### PARENT-CHILD RELATIONSHIPS AND SLEEP QUALITY AMONG CHINESE AGING PARENTS

Haowei Wang,[1] Kyungmin Kim,[1] Jeffrey Burr,[1] and Bei Wu[2], *1. University of Massachusetts Boston, Boston, Massachusetts, United States, 2. New York University, New York, New York, United States*

Relationships with adult children play an important role in older adults' well-being. However, little is known about the association between parent-child relations and aging parents' sleep quality, which is an emerging health issue that is closely related to individuals' physical and mental well-being in later life. With the largest aging population, China has experienced rapid changes of family structure and traditional norms regarding parent-child ties. This study focused on different dimensions of parent-child relationships (i.e., upward/downward financial and instrumental support, emotional closeness) in Chinese aging families. This study examined the association between parent-child relationships and older parents' sleep quality, comparing one-child and multiple-children Chinese families. Utilizing the 2014 wave of the Chinese Longitudinal Aging and Social Survey, we analyze data from 8,450 respondents (aged 60+) who had at least one living child. Descriptive analysis showed that parents with multiple children engaged in more intense financial exchanges, less frequent instrumental support, and lower levels of emotional closeness with their adult children compared to their counterparts with only one child. Logistic regression models revealed that older parents who received more instrumental support were more likely to report sleep difficulty in both one-child and multiple-children families. For parents with multiple children, the overall level of financial transfers from children was negatively associated with having sleep difficulties, while the variability of financial transfers across multiple children was positively associated with having sleep difficulty. Findings highlight the importance of considering family dynamics in studying sleep quality among Chinese older adults.

### GRANDPARENTS' PERSPECTIVES ON A MULTIGENERATIONAL DIGITAL HEALTH PHYSICAL ACTIVITY INTERVENTION

Marissa Kobayashi,[1] Rafael O. Leite,[1] Isabella Lugo,[1] Rachel Wetstone,[1] Dario Vanegas,[1] Sara M. St. George,[2] and Sara J. Czaja[3], *1. University of Miami, Miami, Florida, United States, 2. University of*

*Miami Miller School of Medicine, Miami, Florida, United States, 3. Weill Cornell Medicine, New York, New York, United States*

Studies examining associations among grandparents' involvement and grandchildren's lifestyle behaviors have been largely mixed or negative highlighting the need for interventions that help grandparents promote grandchildren's healthy behaviors. The current study explored older adults' interest in participating in a digital intervention with their grandchildren. As part of the intervention, grandparents and grandchildren would engage in weekly walks and use a mobile application to track their steps, photos and conversations. Twelve grandparents (63±6.5yrs; 75% female; 50% Hispanic) participated in qualitative interviews. Researchers asked open-ended questions to assess grandparents' relationships with their grandchildren, motivation to participate, and feedback on prototypes. A 10-item systems usability questionnaire was also administered. Three researchers independently analyzed interview transcripts using a rapid assessment approach and reached consensus on key themes. Grandparents described having positive relationships with their grandchildren and used texts to schedule time with them; family dynamics (conflicts, divorce) influenced the amount of time they spent together. Grandparents' motivation for participating in the intervention included the opportunity to enhance their relationship with their grandchild and improve their own health. Grandparents noted weekly walks would feasibly occur on the weekends given their grandchildren's competing activities. They were receptive to proposed weekly session topics (e.g., sports/hobbies, ancestry, humor) and suggested other topics to discuss during weekly walks, such as faith/religion, morality, safety, and nutrition. They strongly agreed or agreed that they would use the prototypes frequently and found them easy to use (83% and 92%, respectively). Results from this study will inform the next iteration of intervention prototypes.

### RACE DIFFERENCES IN THE CONSEQUENCES OF ADULT CHILDREN'S PROBLEMS ON MOTHERS' PSYCHOLOGICAL WELL-BEING

Catherine Stepniak,[1] J Jill Suitor,[1] Megan Gilligan,[2] Karl Pillemer,[3] and Marissa Rurka[4], *1. Purdue University, West Lafayette, Indiana, United States, 2. Iowa State, Ames, Iowa, United States, 3. Cornell University, Ithaca, New York, United States, 4. Purdue University, Lafayette, Indiana, United States*

Adult children's problems have been found to be strong predictors of older parents' psychological well-being, regardless of whether the sources of the problems are psychological or physical health, life circumstances outside of the children's control, or children's poor life decisions. Further, this pattern remains regardless of the number or proportion of offspring with problems, or whether children with problems were favored or disfavored by their parents. One important question that has not been addressed is whether the impact of children's problems differs in Black and White families. Race disparities in health and other life circumstances lead Black adult children to be at greater risk of experiencing problems than are their White counterparts. Thus, Black mothers are at greater risk of having adult children with problems; however, increased exposure does not necessarily lead to a stronger impact of children's problems on well-being. Alternatively, it can be argued that due to stronger kin networks and higher levels of religiosity, children's problems may have a weaker impact on Black than White mothers' well-being. In this paper, we use mixed-methods data collected from 101 Black mothers and 295 White mothers as part of the Within-Family Differences Study to explore differences in the impact of adult children's problems on mothers' depressive symptoms. Preliminary analyses of quantitative and qualitative data suggest that mothers' interpretations of the circumstances surrounding their children's problems, rather than support or type of problem, play a greater role in the impact of those problems on well-being in Black than White families.

### ATTITUDES TOWARD PARENTAL FINANCIAL SUPPORT OF YOUNG-ADULT CHILDREN

Radion Svynarenko[1], *1. University of Kentucky, Lexington, Kentucky, United States*

Studies have shown that Americans do not save enough for retirement because they prioritize providing support of their young-adult children over saving for retirement. Attitudes toward parental support has been largely overlooked in existing literature. Using a factorial vignette experimental design and a sample of 500 Americans of age 40 and older, this study investigated how manipulation of contextual factors changed endorsement of parental support. This study found that endorsement of parental support varied depending on the goal of support, whether it was to purchase a car, pay for school tuition, or to pay for down payment for a house. Thus, 67% of respondents endorsed parental financial assistance with purchasing a car, 44% endorsed down payment for a house, and only 38% endorsed paying for college tuition, reflecting overall social importance of these three elements in becoming an adult person. Gender of the child did not affect endorsement of parental financial support to adult children, indicating that there were no gender specific social expectations of who should receive more support from parents, daughters or sons. The major motives of parental support included (a) desire to be a "good parent" and to take responsibility for the child, (b) expectation that children would eventually pay back their parents, and (c) desire to make sacrifice for own children. Parental support may provide numerous benefits to both children and their parents; however, it is important to educate parents on ways to support their children without threatening their own financial needs in retirement.

### SESSION 3320 (POSTER)

### FAMILY CAREGIVING III

### A SYSTEMATIC REVIEW ON CAREGIVING FOR ABUSIVE FAMILY MEMBERS

Qiyi Zhang[1], *1. University of Pittsburgh, School of Social Work, Pittsburgh, Pennsylvania, United States*

While elder abuse perpetrated by caregivers has raised significant attention in the past 30 years, the well being of and support for caregivers of abusive or previously abusive family members remains little known. This population is large in scope, but greatly neglected in the scientific investigation of elder abuse or domestic violence. This systematic review aims to summarize conceptual discussions and empirical findings from existing peer-reviewed articles to shed light on how to understand, identify, and provide support for this group. I performed a search using MEDLINE, PsycINFO, Cochrane, CINAHL, EMBASE, Ageline, and Social Work Abstract, and examined reference lists of identified articles in the stage of full-text screening. Targeted articles are written in English, peer-reviewed, published between 1989 to 2019, and contribute significant knowledge to the study aims. The search concluded with four conceptual articles and six empirical articles. This review reveals a change in approaches in understanding the living situation of maltreated caregivers: previously an indicator of poor caregiving relationship for predicting caregiving outcomes, now it is increasingly acknowledged as a vulnerable group in need of special attention and support. Empirical studies showed that a significant percentage of caregivers have a history of being abused, and they suffered from worse mental health and responded more poorly to certain coping mechanisms than caregivers without those experiences. Stress and self-esteem were found to be possible mediators. Competing conceptual frameworks on this issue include: 1) abuse versus interpersonal violence; 2) life course perspective; 3) communal model versus exchange model of caregiving.

### SERVING UNDERSERVED VETERANS AND THEIR CAREGIVER: DEVELOPING TAILORED CULTURALLY RELEVANT INTERVENTIONS

I. Magaly Freytes,[1] Schmitzberger Magda,[1] Rivera-Rivera Naiomi,[2] Lopez Janet,[2] Mylott Daniela,[2] Motta-Valencia Keryl,[3] and Constance R. Uphold[4], *1. North Florida/South Georgia Veterans Health System, Gainesville, Florida, United States, 2. NF/SG VHS CINDRR, Gainesville, Florida, United States, 3. VA CHS, San Juan, PR, United States, 4. North Florida/South Georgia Veterans Health System, Gainesville, Florida, United States*

Family members of stroke survivors experience high rates of depression and burden. The majority of stroke survivors return to their homes and need assistance to perform activities of daily living. These demands coupled with the lack of preparedness for their new roles lead to a high risk for developing depression and other negative outcomes among caregivers. Studies indicate that Hispanic caregivers report higher levels of depression compared to others. However, no interventions have focused on this population. Our objective is to develop culturally-relevant interventions to help reduce disparities Hispanic Veterans post-stroke and their caregivers. We tailored our Spanish RESCUE intervention for the Puerto Rican population. The goal of the problem-solving telephone support & educational intervention is to reduce caregiver burden and depressive symptoms by teaching them a creative and optimistic approach to solving caregiving related problems. The intervention was developed to reflect specific characteristics of the target population. To enhance the cultural relevance of the intervention, we used recommendations from Key Stakeholders and guidelines from authoritative sources such as: 1) involving persons from the target population in all phases of the project; 2) emphasizing themes valued by the PR culture; 3) assuring that the language and wording of the materials is at appropriate reading level; 4) using certified translators and Spanish-speaking experts, and 5) having Hispanic research members, fluent in Spanish, and knowledgeable about the PR culture conduct the intervention and assessments. The intervention is currently been tested in a RCT.

### EXPLORING RACIAL-ETHNICITY DIFFERENCES IN SERVICES USED BY GRANDPARENTS RAISING GRANDCHILDREN

Tamar E. Shovali,[1] and Kerstin G. Emerson[2], *1. Eckerd College, St. Petersburg, Florida, United States, 2. University of Georgia, Athens, Georgia, United States*

Nearly three million grandparents in the US serve as primary caregivers for their grandchildren. Little research on formal service use and grandfamilies exists for Black and Hispanic populations. To begin to address this gap we conducted exploratory analyses using nationally representative estimates of characteristics and service accessibility of noninstitutionalized children living with grandparents from the 2013 National Survey of Children in Nonparental Care. Our goal was to understand differences in service use as a function of grandfamily race/ethnicity. We specifically explored grandparents' formal service count, financial services received, confidence in obtaining/using community services, and level of role preparation by race/ethnicity. We calculated descriptive statistics for these service variables for grandparents raising Hispanic, White, Black, and Other identified grandchildren (N = 892). On average, there was a minimal range for the number of formal services used (M range = 5.26 – 5.84, possible = 0 – 10 higher equals more services used), reported number of financial services (M range = 0.71 - 0.78, possible = 0 – 3 higher equals more financial services received), and confidence obtaining/using services (M range = 7.4—7.9, possible = 1 – 9 higher equal more confidence). Most prepared to take on the caregiving role were grandparents of White children (55%) followed by Black (21.6%), Other (12.3%), and Hispanic (11.1%) indicating that although grandparents in this sample report being confident and able to access formal services, grandparents of White children report being feeling more prepared to take on caregiving than grandparents of Black, Hispanic, and Other combined.

### GAMEPLAN4CARE: AN ONLINE TRANSLATION OF REACH II REFINED THROUGH EARLY STAKEHOLDER USABILITY TESTING

Jinmyoung Cho,[1] Alan B. Stevens,[1] Thomas Birchfield,[1] Sandhya Sanghi,[1] Ashley Vernon,[1] and Marcia G. Ory[2], *1. Baylor Scott & White Health, Temple, Texas, United States, 2. Texas A&M Center for Population Health and Aging, College Station, Texas, United States*

Despite a number of evidences on the implementation of multicomponent education and support programs to improve the lives of dementia caregivers, there is still a need to adapt these interventions to various implementation settings and preferences of a new generation of caregivers. In this study, we present the development and usability testing of a dementia caregiver support program, GamePlan4Care (GP4C), an online translation of the Resources for Enhancing Alzheimer's

Caregiver Health II (REACH II) intervention. The goal of the GP4C is to create an online family caregiver support system that would facilitate delivery of an evidence-based skills-training and support for dementia caregivers with the potential of both scalability and sustainability. GP4C includes the full breadth of REACH II education and skill-building materials, delivered within an automated, online platform with integrated support from a Dementia Care Specialist via telephone/video conferencing. Dementia caregivers, community agency staff, and other experts are involved in usability testing to ensure acceptability of this new approach to intervention delivery. The software development is completed and usability testing is currently underway. The feasibility and success of this new modality of intervention delivery will be made possible by an innovative intervention design supported by appropriate technical and content elements. We will also present the strategies employed to adapt the intervention to an online platform capable of supporting caregiver self-directed exposure to therapeutic content, the results of usability testing with approximately 32 caregivers, and feedback from other external stakeholders on the feasibility of this approach.

### A DECADE OF STROKE CAREGIVER RESEARCH: THE RESCUE CREST STUDY

Constance R. Uphold,[1] I. Magaly Freytes,[1] LeLaurin Jennifer,[2] Schmitzberger Magda,[1] Nathaniel Eliazar-Macke,[1] Tatiana Orozco,[1] S. Angelina Klanchar,[3] and Stuti Dang[4], *1. North Florida/South Georgia Veterans Health System, Gainesville, Florida, United States, 2. North Florida/South Georgia Veterans Health System, Gainesville, United States, 3. James A. Haley Veterans' Hospital, Tampa, Florida, United States, 4. Miami VA Healthcare System, Miami VA Healthcare System, Florida, United States*

Stroke caregivers need education and support to care for suddenly disabled family members. To address this need, we are conducting a nurse-led intervention, called RESCUE CREST, to teach caregivers problem-solving skills using an educational website and an online messaging center. In our first, preliminary study, we pre-tested an investigator-created, senior-friendly website called RESCUE (Resources and Education for Stroke Caregivers' Understanding and Empowerment) using focus groups and interviews with clinicians and caregivers. We refined the website based on additional surveys, a cognitive usability study, and Webtrends analysis. In the second study, we used a single-group pre- and post-test design to evaluate the content and outcomes of the problem-solving intervention. Third, we conducted a four-arm RCT (4-week intervention, 8-week intervention, attention control, standard care) to refine our methods. We will describe the lessons learned and results of our 10 years of stroke caregiver research that provide background for the RESCUE CREST study.

### SOCIAL ENGAGEMENT MEDIATES THE ASSOCIATION BETWEEN ROLE OVERLOAD AND NIGHTTIME AWAKENING OF DEMENTIA CAREGIVERS

Jiaming Liang,[1] Maria P. Aranda,[1] and Donald Lloyd[1], *1. University of Southern California, Los Angeles, California, United States*

Caregivers tend to report role overload and nighttime awakening given the burden of caregiving. Previous research has not explored the association between role overload and nighttime awakening among dementia caregivers. Social engagement has been found to be associated with physical and psychological health outcomes of caregivers. Thus, the present study aims to examine whether role overload is associated with nighttime awakening of dementia caregivers and whether social engagement will mediate the association. We conducted a cross-sectional study by using the 2015 National Health and Aging Trends Study (NHATS) and National Study of Caregiving (NSOC). Six-hundred-and-sixty-nine dementia caregivers were included in the analysis. Relevant factors were controlled as covariates including age, gender, race/ethnicity, etc. Nearly 45% of participants reported suffering from nighttime awakening at "some" or "more" nights within one month, among which half of them reported "almost" or "every" night. Role overload was found associated with caregivers' nighttime awakening ($\beta$=.135, 95%CI: .094 to.176, p<.001). The mediated model shows 7.4% of association between role overload and nighttime awakening could be explained by the mediation of social engagement ($\beta$= .010, 95%CI: 0.99x10-2 to 1.01x10-2, p<.05). The study suggests that dementia caregivers with high levels of role overload tend to experience nighttime awakening more than those who reported low role overload. A modest mediated effect indicates that role overload of dementia caregivers can predict their nighttime awakening partly through decreasing their social engagement. Other factors and longitudinal models shall be discussed to further explore the theoretical mechanisms of caregiver stress.

### HOW POLITICO-ECONOMIC SYSTEMS CAN SHAPE THE ATTITUDE TOWARD ELDERLY CARE: LESSONS FROM THE GERMAN REUNIFICATION

Freya Diederich,[1] Hans-Helmut König,[1] and Christian Brettschneider[1], *1. University Medical Center Hamburg-Eppendorf, Hamburg, Germany*

Migration flows have been rising over the past decades and are not expected to mitigate in the future. Migrants brings along values and preferences that were shaped by their origin countries, among those, their perceptions of how societies should care for the elderly. In this study, we examine how the attitude towards informal care is shaped by the politico-economic system an individual grew up in and if this attitude adjusts over time once an individual lives in a different system. After the fall of the Berlin Wall, Eastern Germans were exposed to the market-oriented western economy, a natural experiment that allows us to address these issues. By analyzing data from the German Family Panel (2009/10-2015/16), we assess differences in attitudes towards informal care among four birth cohorts that were born during the German separation (N=11,966) using random-effects models. We control for socio-demographic factors as well as the institutional and economic environment an individual lives in. The results reveal that, on average, older generations that grew up in East Germany exhibit up to a 6.7% (95% CI, 0.03-0.1) higher willingness to provide informal care to their parents than older generations that grew up in West Germany. There is no significant difference in younger birth cohorts. Attitudes do not significantly converge over the observed time horizon. The results provide evidence that individuals' attitude towards informal care is deeply shaped by the system they grew up in such that migration flows can influence the supply of informal care in the future.

### A HOME-VISIT PILOT INTERVENTION TO PROMOTE COMMUNICATION SKILLS AND WELL-BEING FOR DEMENTIA FAMILY CAREGIVERS

Jung-Ah Lee,[1] Priscilla Kehoe,[1] and Lisa Gibbs[2], *1. University of California Irvine, Irvine, California, United States, 2. University of California Irvine, Orange, California, United States*

Dementia takes a significant toll on caregivers resulting in their suffering from chronic stress and depression due to responsibility for care for persons with dementia (PWD). Behaviors of PWD could be aggravated by inappropriate responses by family caregivers such as correcting PWD's memories. The study purpose is to examine the feasibility of a home-visit-based intervention designed to promote communication skills with PWD and well-being in family caregivers. This pilot study used a single-arm experimental pre-post design to test the feasibility of 4 weekly home visits for 13 female family caregivers in Southern California (spouse, n=7; adult children, n=6; mean age=64.3, ranging 46-82). Trained home visitors used video scenarios for behavioral education for caregivers. All caregivers completed the entire home visit program. Significantly caregiver burden was decreased from baseline (M(SD)=51.38(4.58)) to follow-up at 5 weeks (M=43.31(5.67), Wilcoxon signed rank test: p=.04). Additionally, caregiver-reported PWD's negative behaviors were reduced from baseline to follow-up (Mbase=22.31(3.52), Mfolllowup=19.31(4.4), p=.13). There were other improvements (non-significant) in greater caregiver self-efficacy and less depressive symptoms from baseline to follow-up. Caregiver satisfaction with the intervention was high (M=4.6(0.65) of 5). Qualitatively, participants appreciated the home visits for educational sessions and welcomed the empathy provided. Caregivers expressed better communications and responsiveness to the PWDs. The results showed the home-visit-based caregiver intervention was feasible and had a potential effectiveness on reduction of caregiver burden and possibly on self-efficacy and well-being. A larger-scale study will be needed to demonstrate long term positive effects on caregiver interactive skills and their well-being.

### REASONS FOR GRANDFAMILY FORMATION: CHANGING PATTERNS OVER 15 YEARS

McKenzie Wallace,[1] Alexandra B. Jeanblanc,[1] and Carol M. Musil[1], *1. Case Western Reserve University, Cleveland, Ohio, United States*

As the number of grandparent-headed households continues to rise, it is vital to examine the changing impetus for the formation of grandfamilies. We compared the reasons for caregiving of custodial grandmothers from a current study (2017-2019: N=236) to a previous study (2002-2003: N=183) to determine whether reasons for caregiving have shifted over the 15 year period. Participants were asked to describe the reason for caregiving in an open ended question. The responses were coded into 17 categories. The percentage of participants who described each category as being a reason of caregiving were compared to determine any changes in reasons for caregiving. In both samples drug use and abandonment were the top reasons for caregiving. While abandonment shows no change (30% vs 28%), the current study shows a significant increase in drug use (40% vs 21%). The frequency at which child services was involved increased from 10% in the original sample to 28% in the current sample. Instances of violence in the home significantly increased from the original study (0.5%) to the current study (12%). Other reasons, child abuse, death of a parent, financial strain, parental health, and relationship strife, remained similar between the two samples. These data may represent an increase in family life events, but also greater willingness to disclose disruptive family life events. The substantial increase in drug use and violence as reasons for grandparents to become caregivers is concerning and suggests critical direction for clinical practice, research, and policy change to support grandparent caregivers.

### ALWAYS TRY TO DO MY BEST: A THEMATIC ANALYSIS OF CHINESE AMERICAN DEMENTIA CAREGIVERS

Jinyu Liu,[1] Bei Wu,[2] Ada Mui,[1] Yifan Lou,[3] Wenxing Wei,[1] and Jiyun Jiang[1], *1. Columbia University, New York City, New York, United States, 2. New York University, New York, New York, United States, 3. Columbia University, New York, New York, United States*

Objectives: Given the increasing prevalence of Alzheimer's disease and related dementia (ADRD) in the United States and the rapid growth of the older Chinese American population, many older Chinese Americans are expected to need intensive care because of cognitive impairment. Prior studies on Chinese ADRD caregivers lack comprehensive examinations from a life course perspective that emphasizes the importance of time, context, process and meaning on human development. Using the life course perspective, this study aims to identify challenges and strength of caregiving experience for this population. Methods: We conducted semi-structured face-to-face interviews with 28 Chinese family caregivers of persons with ADRD in New York City. Thematic analysis method was used to assess the interview data. Results: Seven life-course themes emerged from the data. In the domain of challenges, four themes were identified: (1) physical and emotional exhaustion, (2) limited understanding on cognitive health, (3) difficulty in accessing effective and culturally-sensitive health care services for care recipients, and (4) caregivers' inability to do self-care. Other three themes were found in the domain of strengths: (1) commitment to care due to cultural and religious values, (2) emotional closeness as resource to sustain caregiving, and (3) family support and cohesion. Conclusion: This study indicates that the life course perspective is an important lens to understand challenges and strengths of Chinese American caregivers. This study also suggests that health professionals could incorporate the life course perspective into assessment and intervention development when working with minority and immigrant ADRD family caregivers.

### PERCEIVED AVAILABILITY OF CARE AND SUPPORT AMONG OLDER ADULTS IN BANGLADESH

Farhana Ferdous Luna[1], *1. Syracuse University, Syracuse, New York, United States*

Care for older adults is most precarious in developing countries where poverty and weak state support systems have put the well-being of their older populations at risk. Bangladesh is one such nation characterized by excess poverty, poor health, high mortality rates, and illiteracy among its older adults. The lack of elder-friendly infrastructure presents another problem for aging well in Bangladesh. This study examined perceptions about the adequacy of care and support received by older Bangladeshis. A cross-sectional survey collected data from 100 older people who were purposively sampled. Results revealed that older people generally are not satisfied with support services from the government and feel that old-age care has historically declined. Inadequate care and support was cited both at family and state levels. Respondents expressed concern that earlier generations of older people were better taken care of than the present generation, and that the former received more respect than the latter. Factors related to perceived support deficits included poverty, widowhood, and migration of sons. In this patrilineal culture, widowed women in particular perceived themselves as disadvantaged in terms of care availability. We conclude by recommending that policies be designed to enhance care and support services for older people in Bangladesh, particularly the most vulnerable and marginalized among them.

### THE INTERSECTION OF RELIGION AND SES IN MANAGING CHRONIC CONDITIONS AMONG OLDER PERSONS IN NIGERIA

Kafayat O. Mahmoud[1], *1. University of Kansas, Lawrence, Kansas, United States*

Increased life expectancy in Nigeria has corresponded with higher rates of chronic diseases among older persons. Consequently, this is a new experience that older persons progressively have to deal with. In this study, I explored how religion and social support helped older persons cope with their chronic disease conditions, in light of the prevailing socio-cultural and economic circumstances in Nigeria. The research was conducted in two state-owned medical institutions, in a city in the North-Central part of Nigeria. In-depth, qualitative interviews were conducted among 19 purposively selected chronically ill persons aged between 50 years and over, during clinic days. The study revealed that religion is central to peoples' management of feelings of despair, and acceptance of chronic disease conditions, as well as their adherence to prescriptions. This is explained by the theme "God as the Bestower and Reliever." Also, some respondents perceived their coreligionists to be financially supportive. Although, some participants expressed that they depended on their families for their upkeep and emotional well-being, dire socio-economic conditions and lack of governmental support in chronic care meant that financial support was limited. This is explained by the theme "Times are Hard." Subsequently, most respondents bore a dual burden of coping with chronic conditions even as they were financially responsible for themselves and their families. This was particularly stressful because it meant that most respondents were constantly worried about being able to meet basic daily needs, as well as manage the financial costs of their treatments, which proved expensive to manage.

### INNOVATING CAREGIVER EDUCATION: INCLUDING COOPERATIVE EXTENSION IN RURAL CAREGIVING EDUCATION

Kristopher M. Struckmeyer,[1] Alex J. Bishop,[1] Gina Peek,[1] Paula Tripp,[1] and Sarah Gordon[2], *1. Oklahoma State University, Stillwater, Oklahoma, United States, 2. Arkansas Tech University, Russellville, Arkansas, United States*

Studies consistently report that caregivers under utilize resources, citing unawareness or inability to access programs as barriers to service utilization. Family and Consumer Science (FCS) educators within Extension have a unique blend of training that can help transform access to education for rural caregivers. Extension programming covers a wide range of topics, but few have implemented a curricular program or workshops to educate caregivers about caregiving issues. The current study involved a multi-state examination of innovativeness in caregiving program implementation among N = 216 FCS educators employed by the Cooperative Extension Service. Educators rated their perceptions of Extension's receptiveness to change and personal factors. Results indicated that more urban areas ($\beta = .19$, $p < .05$), fewer years in their current position ($\beta = -.23$, $p < .05$), and greater leadership self-efficacy ($\beta = .17$, $p < .05$) predicted educator innovativeness to implement new caregiver education programming. However, when personal factors were added to the model, only years in current position ($\beta = -.20$, $p < .05$) remained significant. Subjective age ($\beta = -.25$, $p < .01$) and social support ($\beta = .28$, $p < .01$) were also found to significantly predict educator innovativeness. Despite previous research, these results indicate that personal factors may have a greater influence on educators' innovativeness than organizational factors. These findings are critical when adopting and implementing a rural caregiver education programs through new organizational networks.

### PATIENTS' PAIN AND THEIR SPOUSAL CAREGIVERS' NEGATIVE AFFECT: THE MODERATING ROLE OF SELF-EFFICACY

Suyoung Nah,[1] Lynn Martire,[1] and Christina Marini[1], *1. The Pennsylvania State University, University Park, Pennsylvania, United States*

Spousal caregivers of chronic pain patients may experience high levels of negative affect, perhaps in part because they regularly witness patients' suffering. Yet, few studies have examined the relation between patients' chronic pain and spousal caregivers' negative affect. According to social cognitive theory, individuals' self-efficacy may modulate how much negative affect they experience in response to stressful situations. The purpose of this study was to test the hypothesis that spousal caregivers would report higher levels of negative affect on days when patients experienced higher levels of knee pain. We also tested the hypothesis that patients' and spouses' self-efficacy for managing pain would each buffer this positive association. A total of 144 knee osteoarthritis (OA) patients and their spouses completed baseline interviews and a 22-day diary assessment. Multilevel models indicated that patients' self-efficacy, but not spouses' self-efficacy, moderated the positive association between patients' pain and their spouses' negative affect, even after controlling for spouses' gender, age, and depressive symptoms. That is, spouses reported higher levels of negative affect on days when patients experienced more pain, but only among patients whose self-efficacy for managing pain was low. These findings suggest that patients' self-efficacy for managing pain may serve as a protective factor for their spousal caregivers' daily negative affect. Interventions targeting patients'

self-efficacy for managing pain may be beneficial for couples coping with knee OA.

### LONG-TERM OUTCOMES OF THE BENEFIT-FINDING GROUP INTERVENTION FOR ALZHEIMER'S FAMILY CAREGIVERS: A DOUBLE-BLIND RCT

Sheung-Tak Cheng[1], *1. The Education University of Hong Kong, Hong Kong, Hong Kong*

This study examines the long-term effects of benefit-finding on caregivers' depressive symptoms (primary outcome), and global burden, role overload, and psychological well-being (secondary outcomes). 96 Hong Kong Chinese caregivers of relatives with Alzheimer's disease were randomly assigned to receive the benefit-finding intervention (BFT) or one of two control conditions, namely, simplified psychoeducation (lectures only; SIM-PE) or standard psychoeducation (STD-PE). Caregivers received four biweekly one-to-one interventions of three hours each at their own homes. Participants and raters were blind to experimental assignment. We focused on outcomes measured at 4- and 10-month follow-ups. The trajectories of intervention effects were modeled by BFT x time and BFT x time2 interaction terms. Mixed-effects regression showed significant BFT x time2 interaction effects on depressive symptoms against both control conditions, suggesting diminishing BFT effects over time. Z tests showed that, compared with controls, BFT participants reported substantial reductions in depressive symptoms at 4-month follow-up (d = -0.85 and -0.75 vs. SIM-PE and STD-PE respectively). At 10-month follow-up, BFT was indistinguishable from STD-PE whereas a moderate effect was observed in the comparison with SIM-PE (d = -0.52). In addition, some inconsistent effects on role overload were observed but no effect was found for the other outcome variables. It is concluded that benefit-finding is an efficacious intervention for depressive symptoms in Alzheimer caregivers, with strong effects in the medium-term post-intervention and possible moderate effects in the long-term.

### THE MODERATING EFFECT OF RAISING ONE'S GRANDCHILDREN ON THE RELATION BETWEEN SLEEP AND DEPRESSION

Melanie Stearns,[1] and Danielle K. Nadorff[1], *1. Mississippi State University, Starkville, Mississippi, United States*

Recent evidence has shown that poor quality sleep is associated with depression, particularly among older individuals (Bao et al., 2017; Nadorff, Fiske, Sperry, & Petts, 2012). Moreover, given the high prevalence of depressive symptoms among older adults, it is important to identify possible risk factors of poor sleep quality. One possible risk factor is being a custodial grandparent (raising one's grandchildren), as increased caregiving responsivities are associated with increased depressive symptoms (Brand-Winterstein, Edelstein, & Bachner, 2018). Based upon these previous findings, the current study examines the effect of custodial status on the relation between sleep quality and depressive symptoms. The sample (N = 466) was a subset of individuals recruited in the second wave of the MIDUS biomarkers project completed in 2009 who answered the sleep, caregiving, and depressive symptoms variables of interest. Measures included the Center for Epidemiological Studies Depression Scale (CESD), the Pittsburgh Sleep Quality Index (PSQI), and a question regarding custodial grandparent status. The current study aimed to examine whether poor sleep quality might serve as a risk factor for experiencing depressive symptoms and how custodial grandparents might differ from other older adults. Moderation analyses were conducted using SPSS' Process macro on the sample. The interaction between global sleep quality and custodial grandparent status was significant in predicting depressive symptoms, t (1, 465) = 3.90, p = .04, such that custodial grandparents reported a stronger positive correlation between greater global sleep problems and depressive symptoms than non-custodial grandparents. Implications, future directions, and limitations are discussed.

### VALIDATION OF THE CAREGIVER REACTION SCALE IN A SAMPLE OF NON-HELP-SEEKING CAREGIVERS

Kelly A. O Malley,[1] Kelsey Bacharz,[2] and Sara H. Qualls[2], *1. New England GRECC, Boston VA Healthcare System, Boston, Massachusetts, United States, 2. University of Colorado Colorado Springs, Colorado Springs, Colorado, United States*

The Caregiver Reaction Scale (CRS) is a comprehensive measure of the family caregiving experience that assesses burden, family strains and positive aspects of caregiving (PAC). The CRS has been validated in sample of older adult help-seeking caregivers, but its validity and reliability in a non-help-seeking sample of caregivers was unknown. This study aimed to explore how well the CRS assesses the full caregiving experience in a younger non-help-seeking sample of family caregivers and to further evaluate the validity of the PAC subscales. A sample of non-help-seeking caregivers (N =452; Mage = 48.56, SD = 17.15) completed online questionnaires of burden, positive aspects of caregiving, and psychological well-being. All subscales of the CRS demonstrated very good internal consistency reliability ($\alpha \leq .88$). The PAC subscales of the CRS demonstrated medium to large positive correlations with a measure of positive aspects of caregiving ($r \geq .44$) and small to medium positive correlations with psychological well-being ($.25 \geq r \leq .42$). Burden subscales of the CRS had large positive correlations with another measure of burden ($r \geq .66$). Medium positive correlations were also found between family and job conflict subscales of the CRS and the burden measure ($r \geq .35$). CRS PAC subscales were negatively correlated with the burden measure ($r \leq -.13$). The CRS is a valid and reliable measure of the caregiving experience as evidenced by convergent and discriminant validity of CRS subscales and well validated measures of burden and positive aspects of caregiving.

### GRANDPARENT CAREGIVERS: THE RELATION BETWEEN SOCIAL NETWORKS AND RESILIENCE

Nancy Mendoza,[1] and Christine Fruhauf[2], *1. The Ohio State University, Columbus, Ohio, United States, 2. Colorado State University, Fort Collins, Colorado, United States*

Grandparents raising grandchildren experience multiple challenges as they take on the unexpected role of caring for their grandchildren, which usually occurs under stressful and stigmatizing conditions. Many of the challenges grandparents experience are well documented in the research. Less attention is given to understanding how a grandparent caregiver's social network changes when s/he becomes a caregiver and how her/his social network influences resilience. Thus, the purpose of this study was to use social network analysis (SNA) to examine the relation between social networks and resilience in grandparents raising their grandchildren. This was done by conducting face-to-face interviews with twenty grandparents raising grandchildren after they completed a survey measuring social support, social isolation, and resilience. The interview protocol included questions related to participants' social network, social support, and services. Prior to the interviews, using data from the surveys participants were identified as representing one of four resilience quadrants: resilient, maladaptive, competent, and vulnerable. Qualitative analysis of grandparent's social networks across groups indicated resilient grandparent caregivers' networks were structured in a way that provided more opportunities for the inflow of new information and resources. Whereas the proportion of professionals in maladaptive grandparent caregivers' networks tended to be less than for other networks. This could suggest that for grandparent caregivers, having professionals in one's network can be beneficial. Findings from the current study provide opportunities for future research such as identifying ways to help grandparent caregivers structure their social networks to promote resilience.

### IMPROVING SUPPORT FOR CAREGIVERS OF SERIOUSLY ILL OLDER VETERANS: STEPS TOWARD LAY NAVIGATION

Nathan A. Boucher[1], *1. Durham VA Medical Center, Health Services Research & Development, Durham, North Carolina, United States*

This session will discuss mid-stage findings from a five-year, federally-funded study to develop lay navigation supporting informal caregivers, often family/friends, of older Veterans with advanced stage illness. Caregivers of Veterans report numerous burdens in their caregiver role related to food, clothing, shelter, utilities, and transportation. Current programs focus on Veterans' needs rather than caregivers' needs. Few programs focus on practical needs that can be met with VA and community-based supports. Lay navigator programs may be used to support caregivers' social/practical needs. Lay navigation is used with patient populations, but models focused on caregivers do not readily exist. Dr. Boucher will discuss establishment of and input from the study's Stakeholder Advisory Board and data from sample of caregiver and Veteran interviews informing a lay navigation training curriculum and pilot intervention. Feedback from audience members will be encouraged in this session exploring quality improvements in caregiver support applicable to multiple health systems.

### SESSION 3325 (POSTER)

### FRAILTY AND SARCOPENIA

### PRESCRIPTION DRUG USE FOR PAIN AND FOR SLEEP AND INCIDENT FRAILTY IN OLDER ADULTS

Gulcan Cil,[1] Juyoung Park,[2] and Andrew W. Bergen[1], *1. Oregon Research Institute, Eugene, Oregon, United States, 2. Florida Atlantic University, Boca Raton, Florida, United States*

There is emerging evidence for association of polypharmacy with incident frailty. We performed a longitudinal study within the Health and Retirement Study (HRS) to address whether self-reported prescription drug use for pain and/or sleep (co-use or single use for pain or for sleep) influences incident frailty. We utilized data from the 2006–2014 waves of core and family member exit files in HRS to assign self-reported prescription drug use and sociodemographic and other drug use behavior variables as covariates and construct a Burden Model of frailty (≥ 0.2 ratio of positive/total indicators). We performed unadjusted and adjusted competing risk hazard model analysis with death as a competing risk. In a sample of 7,201 unique non-frail (at baseline) individuals (mean[SD] age 72[6.5] years, 54% female, 85% White, 12% African American, 7.3% Hispanic), prevalences of co-use and single-drug use for pain or for sleep were 2.2%, 14.9%, and 5.6%, respectively. Of 7,201 respondents, 2,723 (37.8%) became frail over the follow-up period and 713 (9.9%) died in non-frail state. The adjusted competing risk hazard model suggest that co-use and single use for pain or for sleep were associated with an increase in the risk of frailty by 92%, 58%, and 31%, respectively ($p < .001$), with statistically significant differences between all risk strata. Adjustment for baseline frailty score and selected chronic disease resulted in modest reductions in effect size with retention of significance. Validation of these initial findings should be undertaken with provider and pharmacy data to identify drug-, dosage-, and duration-specific risks.

### SCREENING FOR LOW SHORT PHYSICAL PERFORMANCE BATTERY SCORES: CAN GRIP STRENGTH AND SINGLE CHAIR STAND BE USEFUL?

Paulo H. Chaves[1], *1. Benjamin Leon Center for Geriatric Research and Education at Florida International University, Miami, Florida, United States*

The clinical value of low Short Physical Performance Battery (SPPB) scores for identification of older adults at-disability-risk who may benefit from structured intervention is well-established. Feasibility concerns – e.g., time, space constraints – are factors that often preclude SPPB implementation in clinical settings. We assessed whether grip strength (GS) and/or single chair stand (SCS), simple and highly feasible tests, could be useful for clinical identification of older adults with poor SPPB performance. Cross-sectional study using most recent data (Round 7) from the National Health and Aging Trends Study, which enrolled a large U.S. representative sample of Medicare beneficiaries 65 years and older (baseline round: 2011; yearly follow-ups). Nursing home residents were excluded. Sample size was 4,612. Outcome: poor SPPB performance (score <8). Low GS: <20 Kg (women) or <30 Kg (men), and able to do a SCS without use of arm (yes/no) were predictors. Logistic regression, areas under the curves (AUC), and accuracy statistics were computed. AUC for low GS was 0.66, and SCS inability was 0.68; when both tests were considered together, AUC increased significantly: 0.76. Among those SCS-unable (n=752), 95.6% had SPPB<8. A two-stage screening approach; i.e., detection of SCS inability first, followed by low GS in those SCS-able resulted in a net-sensitivity of 75.3%, and net-specificity of 83.5%. Sequential screening with SCS and GS testing might offer a case finding screening approach appealing to busy clinical settings from feasibility, accuracy, and/or efficiency perspectives for identification of older adults with low SPPB who may benefit from established interventions.

### HYPERGLYCEMIA AND INCIDENCE OF FRAILTY IN OLDER MEXICAN ADULTS LIVING IN RURAL AREAS

Betty Manrique Espinoza,[1] Ana Rivera Almaraz,[1] and Aaron Salinas Rodriguez[1], *1. National Institute of Public Health, Cuernavaca, Morelos, Mexico*

Hyperglycemia is the main characteristic of diabetes and is the result of an absolute or partial deficit in the production or action of insulin. Recent evidence suggests that hyperglycemia increases the risk of frailty. This issue is of great importance for the Mexican population given the high prevalence of diabetes, particularly in older adults. Our objective was to analyze the association between hyperglycemia and the incidence of frailty in a cohort of rural older adults in Mexico. Prospective cohort study with 600 rural older adults, with measurements made in 2009, 2013 and 2018. Fragility was defined using the proposal of Fried and colleagues. The determination of glycosylated hemoglobin was performed through the A1CNow® device, with capillary blood; hyperglycemia was defined considering the recommendation of the American Diabetes Association; where values greater than 6.5% (140 mg / dL) of glycosylated hemoglobin were considered hyperglycemia. We used an ordinal logistic regression model to analyze the relationship between hyperglycemia and incidence of frailty. In the baseline measurement (2009), 8.6% of older adults presented frailty. The incidence of frailty was 6.9%. After adjusting for health and sociodemographic characteristics, hyperglycemia was significantly associated with the incidence of frailty (RR = 2.24 P = 0.018). These findings allow us to determine that hyperglycemia is a prognostic factor of the incidence of frailty. Because frailty is preventable, future interventions for the prevention of frailty should consider the presence of hyperglycemia.

### DOES PHYSICAL FUNCTION RESPONSE TO INTENTIONAL WEIGHT LOSS IN OLDER ADULTS VARY BY RACE-ETHNICITY?

Daniel P. Beavers,[1] Rebecca Neiberg,[1] Kristen Beavers,[2] Dalane Kitzman,[1] Barbara M. Nicklas,[1] Stephen Messier,[2] Jack Rejeski,[2] and Stephen Kritchevsky[1], *1. Wake Forest School of Medicine, Winston-Salem, North Carolina, United States, 2. Wake Forest University, Winston-Salem, North Carolina, United States*

The purpose of this study is to explore whether the effect of weight loss on physical function in older adults varies by race/ethnicity. Individual level data from 1369 older, (67.7±5.4 years), obese (BMI: 33.9±4.4 kg/m2), adults (30% male, 21% African American) who participated in eight randomized controlled trials of weight loss were pooled. Studies were 5-6 months in duration and collected baseline demographic and pre/post gait speed (n=1296), short physical performance battery (SPPB; n=866), and grip strength (n=401) data. Treatment effects were generated by weight loss assignment [weight loss (WL; n=764) versus non-weight loss (NWL; n=605)], as well as categorical amount of weight change (high loss: >-7%, moderate loss: -7 to -3%, and weight gain/stability: <-3%). Analyses were adjusted for age, sex/gender, study, education, baseline BMI, and baseline value of the outcome measure of interest. Race/ethnicity stratified results were presented if the interaction term was p≤0.10. A race/ethnicity*weight loss assignment interaction was observed for gait speed (p=0.07), with African Americans experiencing greater weight loss-associated improvement (WL: 0.07±0.01 m/s versus NWL: 0.02±0.01 m/s; p=0.03) compared to Whites (WL: 0.08±0.01 m/s versus NWL: 0.07±0.01 m/s). A race/ethnicity*weight loss amount interaction was also observed for gait speed (p<0.01), with greater weight loss associated with greater improvement in both African Americans and Whites; although, gains were most apparent in African Americans experiencing high loss (0.12±0.02 m/s) compared to gain/stability (0.01±0.01 m/s). The beneficial effects of weight loss on gait speed appear greater in African Americans and are augmented with greater weight loss.

### OPERATIONALIZING THE FRAILTY INDEX BASED ON WEARABLE SENSOR TO ASSESS FUNCTIONAL PERFORMANCE IN OLDER ADULTS

Hung Nguyen,[1] Jacqueline Yang,[1] Mohsen Zahiri,[1] and Bijan Najafi[1], *1. Baylor College of Medicine, Houston, Texas, United States*

Frailty status is a well-known predictor of adverse health outcomes and functional performance. An assessment tool based on a wearable sensor was developed to quickly assess frailty using an upper extremity flexion and extension test. However, the current tool has relied on conventional frailty assessment to classify the frailty status of the participant. The aim of this study is to operationalize the frailty index based on wearable sensor to classify frailty status of older adults. 104 older adults were recruited for the study (age=78.6 ±9.7 years old). Participants were asked to perform a quick 20-second upper flexion and extension task while wearing a gyroscope on the wrist. A sensor-based frailty index (FI) was derived using parameters extracted from the sensor. Participants were also assessed using the Fried Phenotype Criteria (FC) and were classified into three groups: robust, pre-frail, and frail. Mean-shift clustering algorithm was used to operationalize the FI by identifying the cut-off point for each group. Grip strength and physical activity level were used as functional outcome measures. Regression analysis (r) was used to compare the correlation of the FC and FI with the identified metrics. Bivariate analysis show that grip strength was highly associated with the sensor-based frailty classification (r=-0.547) and FC (r =-0.503). The sensor-based classification was significantly associated with walking activity (r=-0.355). The results showed that the sensor-based frailty assessment tool could be used to quickly classify frailty status in older adults and eliminated the need for subjective and time-consuming evaluation.

### VALIDITY OF COMMUNITY-BASED FRAILTY CHECK-UP BY SENIOR VOLUNTEERS FOR PREDICTING ADVERSE HEALTH OUTCOMES

Tomoki Tanaka,[1] Kyo Takahashi,[2] Masahiro Akishita,[3] and Katsuya Iijima[4], *1. Department of Geriatric medicine, The University of Tokyo, Hongo,*

*Bunkyo-ku, Japan, 2. Institute of Gerontology, The University of Tokyo, Hongo, Bunkyo-ku, Japan, 3. Department of Geriatric Medicine, Graduate School of Medicine, the University of Tokyo, Tokyo, Japan, 4. Institute of Gerontology, The University of Tokyo, Tokyo, Japan*

Aim: For achieving healthy aging for all, multi-faceted frailty is serious problem in super-aged society such as Japan. We developed community-based frailty check-up program performed by trained senior volunteers. In this study, we aimed to validate the ability of the results of check-up to predict needing long-term support or care insurance or death in community-dwelling older population. Methods: A total of 1,536 older adults (mean age, 73.0±6.1 years; 74% women; non-eligible for long-term support or care) participated in the check-ups held from April, 2015 to March, 2018 in Kashiwa City, Japan. At check-ups cite, 21 items including nutrition, oral and physical functions, and social conditions were assessed; Outcome was needing long-term support or care insurance, or death from the day of check-ups until October, 2018. Results: During follow-up {median 678 days (inter-quartile range, 199-1263)}, 116 (7.6%) were newly needing for long-term support (n=50) or care (n=49), or death (n=18). The number of positive responses among 21 items was associated with decreased risks of outcome {age-sex adjusted hazard ratio (95% confidence interval), 0.87 (0.81-0.92)}. Compared those with > 18 positive responses (third tertile), individuals with < 14 positive responses (first tertile) were highly increased risks of outcome {age-sex adjusted hazard ratio (95% confidence interval), 2.44 (1.22-4.49)}. Conclusions: Community-based frailty check-ups program could predict the needing long-term support or care insurance or death in community-dwelling older population. The appropriate intervention for individuals with bad results of the check-up might contribute to serving as early prevention of multi-faceted frailty.

### EUROPEAN OLDER ADULTS FRAIL: FINDINGS FROM THE SURVEY OF HEALTH, AGEING AND RETIREMENT IN EUROPE (SHARE)

Giulia Manfredi,[1] Luís Midão,[2] Constança Paúl,[3] Clara Cena,[4] Mafalda Duarte,[5] and Elísio Costa[6],
*1. Department of Pharmaceutical Science and Technology, Via P. Giuria 9, I-10125 Turin, Italy, 2. ICBAS - Abel Salazar Institute of Biomedical Sciences, University of Porto, Porto, Portugal, Porto, Portugal, 3. Instituto de Ciências Biomédicas Abel Salazar, Universidade do Porto, Porto, Portugal, 4. Department of Pharmaceutical Science and Technology, Via P. Giuria 9, I-10125 Turin, Italy, Turin, Italy, 5. ISAVE – Superior Institute of Health, Amares, Braga, Portugal, ISAVE – Superior Institute of Health, Braga, Portugal, 6. UCIBIO/REQUIMTE, PORTO4AGEING - Competences Centre on Active and Healthy Ageing of the University of Porto, Faculty of Pharmacy of the University of Porto, Porto, Portugal, UCIBIO/REQUIMTE, Portugal*

Frailty is a geriatric multidimensional syndrome whose signs and symptoms are predictors of increased vulnerability to minor stress events and risk of adverse outcomes such as falls, fractures, hospitalisation, disability and death. In this work, we aimed to update the data of frailty status in European community dwelling population, based on the latest data released (wave 6) of SHARE database, and to study the impact of each criterion on frailty assessment. Frailty status was assessed applying a version of the Fried Phenotype operationalised for SHARE. We included all participants who answered all the questions used in a frailty assessment and who disclosed their gender and, further, whose age was 50 or more. Our final sample was 60816 individuals. Of these, the mean age was 67.45 ± 9.71 years; 38497 (56.4%) were female. The overall prevalence of pre-frailty was 42.9% (ranging from 34.0% in Austria to 52.8% in Estonia) and frailty was 7.7% (ranging from 3.0% in Switzerland to 15.6% in Portugal). Pre-frailty and frailty prevalence increased along age and were more frequent among women. Regarding the five criteria considered on frailty assessment, exhaustion seems to be the criterion that contributes most to frailty status, followed by low activity, weakness, loss of appetite and slowness. With this work, we demonstrated that more than 50% of the 50+ European population are pre-frail/frail, which must be considered when designing interventions to reduce/postpone/mitigate the progression of this condition, reducing the burden associated with it.

### ADAMO INDOOR MOBILITY, PHYSICAL FRAILTY, AND AUTONOMY IN OLDER ADULTS: A MEDIATION MODEL

Alberto Rainoldi,[1] Lorenzo M. Donini,[2] Paolo R. Brustio,[3] Anna Mulasso,[3] Eleonora Poggiogalle,[2] Gianluca Zia,[4] Luca C. Feletti,[5] and Susanna Del Signore[6],
*1. University of Turin, Italy - Neuro Muscular Function Research group, School of Exercise and Sport Sciences, Department of Medical Sciences, Torino, Italy, 2. Sapienza University, Rome, Italy, 3. University of Turin - NeuroMuscularFunction research group School of Exercise and Sport Sciences Department of Medical Sciences, Torino, Italy, 4. Bluecompanion LTD, London, England, United States, 5. Caretek srl, Torino, Italy, 6. Bluecompanion LTD, United Kingdom, France*

Physical frailty represents a clinical condition among older adults leading to adverse health outcomes, such as autonomy loss. To evaluate physical frailty in older adults, adopting information and communication technologies (ICT) may be useful. ADAMO (Caretek S.r.l.) is a care-watch accelerometer that allows to measure mobility in a non-intrusive way (Magistro et al., 2018) providing wider information on individual general health (Mulasso et al., in press). The aim of this study was to evaluate the relationship between indoor mobility, physical frailty and autonomy in a sample of Italian older adults. Methods: Thirty-two volunteers (age 65–84 years; women 56.2%) participated in the study. All wore ADAMO care-watch continuously over a 7-day period. The number of steps indoor was the main endpoint. Fragmented daily mobility was estimated. Physical frailty and autonomy were measured using the Tilburg Frailty Indicator (physical components) and the Groningen Activity Restriction Scale, respectively. Results: Significant inverse correlations were observed between number of steps and autonomy, and number of steps and physical frailty. Conversely, a significant direct correlation was observed between physical frailty and autonomy. Additionally, mediation analysis demonstrated full mediation effect of physical frailty between the number of steps and autonomy. Our results imply that high indoor mobility per se can reduce physical frailty and consequently helps to maintain autonomy. Conclusions: Indoor mobility captured by ADAMO accelerometer may be an important indicator of physical frailty and autonomy. ADAMO may be used as a non-intrusive telemonitoring solution to capture relevant information on individual general health in aged people.

### A RANDOMIZED CONTROLLED TRIAL OF METFORMIN FOR FRAILTY PREVENTION: STUDY DESIGN AND BASELINE CHARACTERISTICS

Sara E. Espinoza,[1] Nicolas Musi,[1] Chen-pin Wang,[2] and Joel Michalek[3], *1. Barshop Institute for Longevity and Aging Studies, University of Texas Health Science Center, San Antonio, Texas, United States, 2. University of Texas Health Science Center, Texas, United States, 3. University of Texas Health Science Center, San Antonio, Texas, United States*

Inflammation and insulin resistance are major predictors of frailty. Here we describe the study design of an ongoing double-blind, randomized controlled trial of metformin for frailty prevention. Subjects are adults aged 65+ years with pre-diabetes assessed by 2-hour oral glucose tolerance test (OGTT). Those who are frail (Fried criteria) are excluded. Participants are randomized to metformin (maximum dose of 2,000 mg/day) vs. placebo and followed for 2 years. The primary outcome is frailty (category and score); secondary outcomes are physical performance and function (short physical performance battery, 6-minute walk, lower extremity strength), systemic and skeletal muscle tissue inflammation, muscle insulin signaling, insulin sensitivity (insulin clamp), glucose tolerance (OGTT), and body composition (dual-energy x-ray absorptiometry). Safety assessments occur every 3 months; frailty, systemic inflammation, and OGTT are assessed at baseline and every 6 months, and insulin clamp with muscle biopsies are assessed at baseline and every 12 months. To date, 51 subjects have been randomized; 120 completers are planned. Mean age is 73.4 ±5.7 years, 43% are female, and 39% Hispanic. Mean BMI is 30.5 ±5.5 kg/m2, waist circumference is 105 ±13.1 cm, fasting glucose is 102.3 ±8.8 mg/dL, Hemoglobin A1c is 5.8 ±0.3, and glucose at 2 hours during OGTT is 168.5 ±20.4 mg/dL. Metformin is being examined in this study as a potential therapeutic agent to prevent frailty in older adults with pre-diabetes. Findings from this trial may have future implications for the screening and potential treatment of pre-diabetes in older patients with metformin for the prevention of frailty.

### HAS FRAILTY SCORE AND FRAILTY LETHALITY CHANGED OVER TIME? HARMONIZATION OF NHANES COHORTS FROM 1999 TO 2016

Joanna M. Blodgett,[1] Kenneth Rockwood,[2] and Olga Theou[2], *1. UCL, London, United Kingdom, 2. Dalhousie University, Halifax, Nova Scotia, Canada*

Positive advances in life expectancy, healthcare access and medical technology have been accompanied by an increased prevalence of chronic diseases and substantial population ageing. How this impacts changes in both frailty level and subsequent mortality in recent decades are not well understood. We aimed to investigate how these factors changed over an 18-year period. Nine waves of the National Health and Nutrition Examination Survey (1999-2016) were harmonized to create a 46-item frailty index (FI) using self-reported and laboratory-based health deficits. Individuals aged 20+ were included in analyses (n=44086). Mortality was ascertained in December 2015. Weighted multilevel models estimated the effect of cohort on FI score in 10-year age-stratified groups. Cox proportional hazard models estimated if two or four-year mortality risk of frailty changed across the 1999-2012 cohorts. Mean FI score was 0.11±0.10. In the five older age groups (>40 years), later cohorts had higher frailty levels than did earlier cohorts. For example, in people aged 80+, each subsequent cohort had an estimated 0.007 (95%CI: 0.005, 0.009) higher FI score. However, in those aged 20-29, later cohorts had lower frailty [$\beta$=-0.0009 (-0.0013, -0.0005)]. Hazard ratios and cohort-frailty interactions indicated that there was no change in two or four-year lethality of FI score over time (i.e. two-year mortality: HR of 1.069 (1.055, 1.084) in 1999-2000 vs 1.061 (1.044, 1.077) in 2011-2012). Higher frailty levels in the most recent years in middle and older aged adults combined with unchanged frailty lethality suggests that the degree of frailty may continue to increase.

### DOES GENDER INFLUENCE RISK FOR ORTHOSTATIC HYPOTENSION IN OLDER ADULTS WITH SARCOPENIA?

Melissa J. Benton,[1] Amy L. Silva-Smith,[1] and Jefferson M. Spicher[1], *1. University of Colorado Colorado Springs, Colorado Springs, Colorado, United States*

Older adults with sarcopenia may be at risk for unstable postural blood pressure due to diminished lean mass that plays a role in maintaining fluid volume. Males have greater lean mass, so risk may be mediated by gender. We compared postural blood pressure changes in older men (77.1 ± 2.0 years; n = 15) and women (79.6 ± 2.0 years; n = 13) with sarcopenia before and after an overnight fast. Sarcopenia was defined using the Lean Mass Index (males ≤ 19.0 kg/m2; females ≤ 15.0 kg/m2). Body composition was measured using multi-frequency bioelectrical impedance, and blood pressure was measured lying, sitting, and standing. On Day 1 (normally hydrated) there were significant drops in systolic blood pressure, with an overall decrease of -9.1 ± 2.2 mmHg (p < 0.001) between lying and standing. On Day 2 (overnight fast), postural changes were more profound, with an overall decrease of -14.1 ± 2.8 mmHg (p < 0.001). However, when compared by gender, postural changes between lying and standing remained significant but did not differ between men and women (Day 1: men -8.9 ± 2.5 vs. women -9.3 ± 2.5 mmHg; Day 2: men -14.6 ± 4.6 vs. women -13.6 ± 3.1 mmHg). On both days diastolic blood pressure remained stable. In this group of older adults, significant decreases in postural systolic blood pressure were observed in the early morning fasted condition, increasing the risk for orthostatic hypotension (drop in systolic blood pressure -20.0 mmHg). Interestingly, gender did not influence risk.

### RACIAL AND ETHNIC DIFFERENCES BETWEEN GRIP STRENGTH AND FUNCTIONAL LIMITATIONS: RESULTS FROM NHATS 2010-2014

Elizabeth Vasquez,[1] Ana R. Quiñones,[2] and Lenore Gensburg[3], *1. University at Albany, SUNY, Albany,*

*New York, United States, 2. Family Medicine, Oregon Health & Science University, Portland, Oregon, United States, 3. University at Albany, New York, United States*

Current research regarding grip strength highlights the robustness of grip strength as a predictor of morbidity and mortality. The aim of this study was to evaluate the association of grip strength over four years with functional limitations among racially/ethnically diverse older adults. We analyzed National Health and Aging Trends Study (NHATS) data 2010-2014. Our sample included 4,413 adults > 65 years old. Functional limitation was defined as a sum of difficulty performing eight ADL/IADLs (range 0-8) at each wave. Grip strength was measured using a digital hand dynamometer and readings were recorded in kilograms (kg) (maximum of 32 kg for men and > 20 kg for women). We estimated stratified linear regression models by race/ethnicity and age, and adjusted for BMI, education, and gender. The majority of the sample was between 65-79 years of age (64%), 55.1% were female and the average BMI was 27.5. We found that differences in ADL/IADL limitations increased and grip strength decreased over the four year period of observation. We also found racial/ethnic differences between waves 1 and 4 with greater ADL/IADL limitations for Hispanics with lower grip strength scores compared to non-Hispanic whites. There were racial/ethnic differences in the association between grip strength and ADL/IADL over time in Non-Hispanic blacks and Hispanics when compared to Non-Hispanic whites. This is an important issue to address since loss of muscle strength in older adults may lead to several negative outcomes such as limited activities of daily living which may affect older adults differentially based on race/ethnicity.

### ASSOCIATION BETWEEN FRAILTY AND DEVELOPMENT OF COGNITIVE IMPAIRMENT: EVIDENCE FROM THE HEALTH AND RETIREMENT STUDY

Nicholas V. Resciniti,[1] Jaleel McNiel,[1] and Matthew Lohman[1], *1. University of South Carolina, Columbia, South Carolina, United States*

Research has shown there is currently an increasing prevalence of cognitive impairment and dementia in older adults. To date, there remains a paucity of research to explain this increase and research on early markers and risk factors are warranted. This study aims to assess the association of cognitively normal older adults who are frail and the development of cognitive impairment four years later. Data from the Health and Retirement Study – a nationally representative sample of older US adults – was used from 2004-2008 for individuals 65 and older (n=8,377). Frailty was categorized by using Fried's phenotype model: individuals were grouped into frail, pre-frail, and robust. Cognitive impairment – a composite score that assessed memory recall and global mental status – was classified as scoring eight or less on a 35-point scale. After restricting to cognitively healthy individuals, logistic regression with weights was used to assess the association between frailty status and the development of cognitive impairment four years later. The model was adjusted for baseline age, gender, race, education years, smoking status, and chronic health issues (high blood pressure, diabetes, cancer, lung disease, heart disease, stroke, psychiatric problems, and arthritis). Frail individuals, compared to those who were robust, had increased odds of cognitive impairment (OR=1.74; 95% CI: 1.48-2.16), after fully adjusting. Evidence from this study suggest that frail individuals are more likely to become cognitively impaired over time. This provides a potential pathway of intervention to help delay or prevent the development of cognitive impairment in older US adults.

### IMPACT OF HIGH BODY MASS INDEX ON FRAILTY AND MORTALITY IN MIDDLE-AGED AND OLDER ADULTS

Kulapong Jayanama,[1] Olga Theou,[2] Judith Godin,[3] Leah Cahill,[4] and Kenneth Rockwood[2], *1. Chakri Naruebodindra Medical Institute, Faculty of Medicine Ramathibodi Hospital, Mahidol University, Samut Prakan, Thailand, 2. Dalhousie University, Halifax, Nova Scotia, Canada, 3. Division of Geriatric Medicine, Dalhousie University & Nova Scotia Health Authority, Halifax, Nova Scotia, Canada, 4. Department of Medicine, Dalhousie University, Halifax, Nova Scotia, Canada*

Obesity is associated with higher risk of metabolic diseases. How body mass index (BMI) relates to mortality across frailty levels is controversial. We investigated the association of high BMI with frailty, and their effects on mortality. We included 36,583 participants aged ≥50 years from the 1999-2006 National Health and Nutrition Examination Survey (NHANES) cohorts (7,372) and 29,211 participants aged ≥50 years from wave 1 (2004) of Survey of Health Ageing and Retirement in Europe (SHARE). BMI was categorized as: normal: 18.5-24.9 kg/m2, overweight: 25-29.9, obese I: 30-34.9 and obese II+III: >35. A frailty index (FI) was constructed excluding nutrition-related items using 36 items for NHANES and 68 items for SHARE. Mortality data were obtained until 2015. All analyses were adjusted for educational, marital, working and smoking status. In participant aged 50-65 years, higher BMI was associated with greater frailty. Being obese level II+III increased mortality risk in male participants aged 50-65 years with FI≤0.1 [NHANES (hazard ratio (HR) 2.10, 95%CI 1.17-3.79); SHARE (2.35,1.14-4.87)]. In males aged >65 years with FI>0.3, being overweight and obese (any level) decreased mortality risk. In females aged 50-65 years, higher BMI was not associated with mortality across all frailty levels. BMI and frailty were cross-sectionally associated. The subsequent mortality impact differed by age, sex, and frailty. Obesity was not associated with mortality in middle-aged females, regardless of the degree of frailty. In males, obesity was harmful in those who were fit in middle age and protective in moderately/severely frail older ones.

### AN INNOVATIVE PLATFORM BASED ON WEARABLE SENSOR TO QUANTIFY FRAILTY PHENOTYPES

He Zhou,[1] and Bijan Najafi[1], *1. Baylor College of Medicine, Houston, Texas, United States*

This study evaluated an innovative wearable sensor based platform (instrumented trail-making task, iTMT) to quickly quantify frailty phenotypes, without the need of walking test. 61 older adults (age=72.8 ± 9.9years, BMI=27.4±4.9kg/m2) were recruited and assessed by Fried Frailty Criteria to determine frailty phenotypes. All subjects participated the iTMT test by standing in front of a computer, wearing a wearable sensor on the front lower shin. The sensor tracked the subject's ankle movement and projected it on a computer-screen as a moving cursor at real-time. The subject rotated the ankle joint to navigate the cursor to reach 5 indexed circles (including numbers 1-to-3 and letters A&B placed in random orders) in the alternative order of numbers and letters. The iTMT required coordination of brain and body movement, testing subject's cognitive-motor function. The sensor quantified ankle-rotation biomechanics during the test. All subjects completed the iTMT with average time less than 3 minutes. The ankle-rotation velocity generated during the test distinguished between the presence and absence of Slowness phenotype (Cohen's effect size d=1.40, p<0.001). The decline of ankle-rotation velocity determined the presence of Exhaustion phenotype (d=0.98, p=0.003). The ankle-rotation power generated during the test determined the presence of Weakness phenotype (d=1.38, p<0.001). The ankle-velocity variability determined the presence of Inactivity phenotype (d=0.90, p<0.001). This study demonstrated the feasibility and validity of the iTMT to quantify frailty phenotypes. This new platform is time-efficient and doesn't require walking test. It's more practical for routine assessment in small and busy clinics among patients with mobility limitation.

### UNDERSTANDING AGING AND FRAILTY WITH A PREDICTIVE NETWORK MODEL

Spencer Farrell,[1] Arnold Mitnitski,[2]
Kenneth Rockwood,[1] and Andrew Rutenberg[3], *1. Dalhousie University, Halifax, Nova Scotia, Canada, 2. Dalhousie University, Department of Medicine, Halifax, Nova Scotia, Canada, 3. Dalhousie University, Department of Physics, Halifax, Nova Scotia, Canada*

Health deficits are age-related binary health issues (typically self-reported disabilities) that accumulate with age. Acquiring a deficit makes an individual more frail and susceptible to other associated deficits. We model this process as a network of health deficits that interact with each other. Mortality depends on an individual's current deficits and their age. The model is trained with self-reported data from the Canadian Study of Health and Aging (CSHA) or the National Health and Nutrition Examination Survey (NHANES). The model generates longitudinally data for synthetic-individuals with frailty trajectories and mortality resembling the observed data. We verify this by comparing the prevalence of individual deficits, correlations between deficits, and predicted death ages with test data. Our trained model performs well on all of these measures. Our model informs our understanding of aging by providing an interaction network representing the associations between pairs of deficits. Our model can generate the frailty trajectories of individuals starting from a set of deficits at a given age. This can extrapolate the trajectories of observed individuals to older ages and enables "inducing" or "treating" deficits to understand the effects of individual deficits or sets of deficits on health.

### RELATIONSHIP OF PREOPERATIVE COMPREHENSIVE GERIATRIC ASSESSMENT TO HEALTH STATUS IN OLDER ADULTS

Ji Yeon Lee,[1] Kwang Joon Kim,[1] Chang Oh Kim,[1] and Kyung Hee Lee[1], *1. Yonsei University, Seoul, Korea, Republic of*

Although comprehensive geriatric assessment has been widely used in surgical older adults, its relationship to health status has not been fully identified. This study aimed to examine the relationships of preoperative comprehensive geriatric assessment to frailty and length of stay. This was a descriptive study based on multi-professional health assessments found in electronic medical records. Study participants were 150 older adults in a neurosurgical department. The comprehensive geriatric assessment was comprised of nutrition, functional status, physical activity, depression, cognition, and basic items such as the Timed Up and Go test, grip strength, and self-rated health. Frailty level and length of stay were dependent variables which represented health status. The result showed that instrumental activities of daily living, physical activity, nutrition, self-rated health, and cognition were significantly associated with frailty. Specifically, comparing robustness with pre-frail and frail level, worseness in the instrumental activities of daily living, self-rated health, physical activity, and nutrition were associated with frailty. With progression of frailty level from pre-frail to frail, the worse score in the cognitive function and self-rated health were associated with frailty. In addition, more depressive symptoms, postoperative complications, and prolonged in the Timed Up and Go test were associated with lengthened hospital stay. Older adults with worsened status in physical, emotional, or cognitive function tended to be frail and stay longer in the hospital. Clinicians need to pay attention to the subcomponents of the comprehensive geriatric assessment and are encouraged to implement it to improve health status of surgical older adults.

### COMPARATIVE PERFORMANCE OF FOUR CLAIMS-BASED FRAILTY MEASURES IN THE UNITED STATES MEDICARE DATA

Dae H. Kim,[1] Elisabetta Patorno,[2] Ajinkya Pawar,[3]
Hemin Lee,[3] Sebastian Schneeweiss,[3] and Robert Glynn[3], *1. Hebrew SeniorLife, Boston, Massachusetts, United States, 2. Brigham and Women's Hospital and Harvard Medical School, Boston, Massachusetts, United States, 3. Brigham and Women's Hospital, Boston, Massachusetts, United States*

Background: There has been increasing effort to measure frailty in the United States Medicare data. The performance of claims-based frailty measures has not been compared. Methods: This retrospective cohort study included 2,326 community-dwelling Medicare beneficiaries who participated in the 2008 assessment of the Health and Retirement Study. The claims-based frailty measures developed by Davidoff, Faurot, Segal, and Kim were compared against clinical measures of frailty (gait speed, grip strength) using correlation coefficients and health outcomes (e.g., mortality, hospitalization, activities-of-daily-living disabilities) over 2 years using C-statistics. Results: The Davidoff, Faurot, Segal, and Kim indices were negatively correlated with gait speed (-0.19, -0.33, -0.37, and -0.37, respectively), but age and sex adjustment variably attenuated the correlation to -0.17, -0.22, -0.18, and -0.33, respectively. The corresponding correlation coefficients with grip strength were -0.17, -0.27, -0.35, and -0.24, which attenuated to -0.09, -0.14, -0.05, and -0.23 after age and sex adjustment, respectively. The models that included age, sex, and each of Davidoff, Faurot, Segal, and Kim indices showed

C-statistics of 0.67, 0.71, 0.71, 0.75 for mortality (versus C-statistic for age and sex: 0.66); 0.59, 0.64, 0.63, 0.70 for hospitalization (versus C-statistic for age and sex: 0.58); and 0.64, 0.63, 0.63, 0.70 for activities-of-daily-living disabilities (versus C-statistic for age and sex: 0.61), respectively. Conclusions: The choice of a claims-based frailty measure results in a meaningful variation in the identification of frail older adults at high risk for adverse health outcomes. Claims-based frailty measures that included demographic variables offer limited risk adjustment beyond age and sex.

### EFFECT OF CHAIR YOGA ON FRAILTY IN OLDER ADULTS WITH LOWER EXTREMITY OSTEOARTHRITIS: RANDOMIZED CLINICAL TRIAL

Juyoung Park,[1] Zuyun Liu,[2] Edgar R. Vieira,[3] and Patricia Liehr[1], *1. Florida Atlantic University, Boca Raton, Florida, United States, 2. Department of Pathology, Yale School of Medicine, New Haven, Connecticut, United States, 3. Department of Physical Therapy, Florida International University, Miami, Florida, United States*

This study examined whether chair yoga (CY) could reduce severity of frailty in community-dwelling older adults with lower extremity osteoarthritis (OA). Participants were randomly assigned to CY or health education program (HEP) at each of two sites and attended twice-weekly 45-minute sessions for 8 weeks. Data were collected at baseline and 4 and 8 weeks. For primary analysis, followed by Rockwood's suggesiton, 97 deficits/variables measuring OA symptoms, physical function, balance, fatigue, depression, social activities, and life satisfaction were used to construct a frailty index, ranging from 0 to 1. Fewer deficits/variables were used to construct three alternative versions of the index. Linear mixed-effects models with random intercept were used to analyze longitudinal repeated outcome measures. A total of 112 participants (n = 63 CY, n = 49 HEP; 75.3[7.5] years; 76% female, 46% Hispanic) completed the study. After adjusting for site, cohort effect, and baseline of frailty, there was no significantly greater decline in frailty in the CY group compared to the HEP group (between-group difference, -0.019; 95% CI, -0.063 to 0.025) or the trend of changes in the index (p for interaction = .489). Additional adjustment for baseline characteristics (age, gender, ethnicity, marital status, living alone, health status, pain medication) did not change results substantially. Secondary analysis of three alternative versions of the index indicated similar nonsignificant changes. Thus, an 8-week CY intervention did not reduce severity of frailty in older adults with lower extremity OA. A longer duration of CY with a larger sample size is needed.

### THE ASSOCIATION BETWEEN LOWER EXTREMITY FUNCTION, FRAILTY, AND LOW-MILEAGE DRIVER STATUS AMONG OLDER ADULTS

Christopher L. Crowe,[1] Howard Andrews,[1] Lisa J. Molnar,[2] David W. Eby,[3] Carolyn DiGuiseppi,[4] David Strogatz,[5] Guohua Li,[6] and Thelma J. Mielenz[6], *1. Columbia University Mailman School of Public Health, New York, New York, United States, 2. University of Michigan Transportation Research Institute, Ann Arbor, Michigan, United States, 3. University of Michigan Transportation Research Institute and Center for Advancing Transportation Leadership and Safety, Ann Arbor, Michigan, United States, 4. Colorado School of Public Health, Aurora, Colorado, United States, 5. Bassett Healthcare Network, Cooperstown, New York, United States, 6. Columbia University Mailman School of Public Health, New York, New York, United States*

The crash rate per mile driven among older adults is higher than that of most age groups and comparable to that of the youngest, most inexperienced drivers. The low-mileage bias posits that the elevated rate among older adults results from an increased rate among those who accrue the fewest annual miles. This study evaluated whether low physical capacity among older drivers, measured by the National Health and Aging Trends Study (NHATS) Expanded Short Physical Performance Battery (SPPB) and Fried's frailty phenotype, increases the risk of being low-mileage drivers. Data were collected for 2,990 older drivers via questionnaires and assessments in addition to 61,528 person-months of driving data. Multivariable log-binomial regression was used to estimate risk ratios. Those with fair and good function had 0.53 (95% CI: 0.40-0.69) and 0.60 (0.47-0.78) times the risk of driving fewer than 3,000 miles/year and 0.45 (0.26-0.77) and 0.48 (0.32-0.72) times the risk of driving fewer than 1,865 miles/year, respectively, compared to those with poor function. For an increase from not frail to pre-frail and from pre-frail to frail, the risk of driving fewer than 3,000 or 1,865 miles/year increased 1.36 (1.11-1.65) or 2.38 (1.63-3.46) times, respectively. Having low physical capacity is associated with an increased risk of low annual mileage. Given the known association between low-mileage driver status and increased crash rates and the modifiable nature of the risk factors examined in this study, interventions aimed at improving physical capabilities may lead to an improvement in safety among older drivers.

### THE ELECTRONIC FRAILTY INDEX BASED ON THE COMPREHENSIVE GERIATRIC ASSESSMENT: DEVELOPMENT AND TESTING

Betty Chinda,[1] Katayoun Sepehri,[2] Macy Zou,[2] Mckenzie Braley,[2] Antonina Garm,[3] Grace Park,[4] Kenneth Rockwood,[5] and Xiaowei Song[2], *1. Department of Biomedical Physiology and Kinesiology, Simon Fraser University, Burnaby, British Columbia, Canada, 2. Health Research and Innovation, Surrey Memorial Hospital, Fraser Health Authority, Surrey, British Columbia, Canada, 3. Community Actions and Resources Empowering Seniors, Fraser Health Authority, British Columbia, Canada, Canada, 4. Community Actions and Resources Empowering Seniors, Fraser Health Authority, Surrey, British Columbia, Canada, 5. Division of Geriatric Medicine, Dalhousie University, Halifax, Nova Scotia, Canada*

Frailty is characterized by loss of biological reserves across multiple systems and associated with increased risks of adverse outcomes. A Frailty Index (FI) constructed using items from the Comprehensive Geriatric Assessment (CGA) has been validated in geriatric medicine settings to estimate the level of frailty. Traditionally, the CGA used a paper form and the CGA-based FI calculation was a manual process. Here, we reported building of an electronic version of the assessment on personal computers (PC), i.e., standalone eFI-CGA, to benefit frailty assessment at points of care. The eFI-CGA

was implemented as a software tool on the WinForms platform. It automated the FI calculation by counting deficits accumulation across multiple domains assessing medical conditions, cognition, balance, and dependency of activities of daily living. Debugging, testing, and optimization were performed to enhance the software performance with respect to automation accuracy (processing algorithm), friendly user interface (user manual and feedback), and data quality control (missing data and value constraints). Systematically-designed simulation dataset and anonymous real-world cases were both applied. The optimized assessment tool resulted in fast and convenient conductance of the CGA, and a 100% accuracy rate of the eFI-CGA automation for up to four decimals. The stand-alone eFI-CGA implementation has provided a PC-based software tool for use by geriatricians and primary and acute care providers, benefiting early detection and management of frailty at points of care for older adults.

### A GENETIC ALGORITHM-BASED APPROACH TO OPTIMIZE THE CONSTRUCTION OF A FRAILTY INDEX

Alberto Zucchelli,[1] Alessandra Marengoni,[1] Debora Rizzuto,[2] Amaia Calderon-Larranaga,[2] Graziano Onder,[3] Laura Fratiglioni,[2] and Davide Vetrano[2], *1. Department of Clinical and Experimental Sciences, University of Brescia, Brescia, Italy, 2. Aging Research Center, Department of Neurobiology, Care Sciences and Society, Karolinska Institutet and Stockholm University, Stockholm, Sweden, 3. Centro di Medicina dell'Invecchiamento, IRCCS Fondazione Policlinico "A. Gemelli" and Catholic University of Rome, Rome, Italy*

The frailty index (FI) is a reliable prognostic indicator based on an individual clinical and functional deficits, which is strongly associated with poor outcomes. We hypothesize that an optimization algorithm may help to select the best candidate deficits to generate a highly-predictive FI. We aimed to optimize the predictive accuracy (area under the curve; AUC) of a FI employing a "genetic algorithm", an iterative meta-heuristic that selects and recombines the most accurate FIs among randomly-generated ones. We used data from 3363 individuals aged 60+ enrolled in the Swedish National Study on Aging and Care in Kungsholmen (SNAC-K). To avoid overfitting, the algorithm was run on a randomly-chosen subsample (70%) of 10 imputed datasets. About 825,000 FIs were built, evaluated, and recombined. The best genetic algorithm-based FI (ga-FI) was compared in terms of 3- and 6-year mortality prediction with a clinically-generated FI (c-FI) in the remaining 30% of the data. Ga-FI showed better AUCs in comparison to the c-FI, overall and in all age and sex subsamples. Several sensitivity analyses were carried out. The major AUC improvement was seen among participants aged <75 [3-year mortality AUC: 0.83 vs 0.63; p<0.001]; 6-year mortality AUC: 0.76 vs 0.63; p<0.001], while smaller differences were seen among participants aged ≥75 [3-year mortality AUC: 0.86 vs 0.84; p=0.216; 6-year mortality AUC: 0.84 vs 0.81, p=0.017]. The genetic algorithm is a feasible method to optimize the construction of a highly performant FI that might be used to assess health comprehensively both in clinical and research settings.

### MULTIDIMENSIONAL FRAILTY SCORE IS SUPERIOR TO PREDICT COMPLICATIONS AFTER SURGERY THAN CONVENTIONAL RISK FACTORS

Jung-Yeon Choi,[1] Kwang-il Kim,[1] Hee-won Jung,[1] Cheol-Ho Kim,[1] Sung-Bum Kang,[1] Ho-Seong Han,[1] and HyungHo Kim[1], *1. Seoul National Bundang Hospital, Bundang-gu, Seongnam-si, Korea, Republic of*

Frail older adults are at increased risk for postoperative morbidity compared with their robust counterparts. We compared predictive utility of multidimensional frailty score (MFS) with physical performance parameters or conventional risk stratification indicators to identify postoperative complication in older surgical patients. From January 2016 to June 2017, 648 older surgical patients (age≥ 65) were included for analysis. The MFS was calculated through comprehensive geriatric assessment (CGA). Grip strength and gait speed were measured preoperatively. The primary outcome was postoperative complication (eg, pneumonia, urinary tract infection, delirium, acute pulmonary thromboembolism, and unplanned ICU admission). Secondary outcome was 6-months all-cause mortality. Sixty-six (10.2%) patients experienced postoperative complications and 6-months mortality was 3.9% (n=25). Grip strength, gait speed, MFS and ASA classification could predict postoperative complication but only MFS (Hazard Ratio = 1.564, 95% CI, 1.283-1.905, p < 0.001) could predict 6-months mortality after full adjustment. MFS (C index = 0.747) had superior prognostic utility than age (0.638, p value = 0.008), grip strength (0.566, p value < 0.001) and ASA classification (0.649, p value = 0.004). MFS only had additive predictive value on both age (C-index of 0.638 (age) vs 0.754 (age +MFS), p = 0.001) and ASA classification (C index of 0.649 (ASA) to 0.762 (ASA + MFS), p < 0.001) for postoperative complication, but gait speed or grip strength had no statistical additive prognostic value on both age and ASA classification.

### DOES PHYSICAL FUNCTION RESPONSE TO INTENTIONAL WEIGHT LOSS IN OLDER ADULTS VARY BY SEX-GENDER?

Kristen Beavers,[1] Rebecca Neiberg,[2] Daniel P. Beavers,[2] Eliza Dewey,[1] Dalane Kitzman,[2] Stephen Messier,[1] Jack Rejeski,[1] and Stephen Kritchevsky[2], *1. Wake Forest University, Winston-Salem, North Carolina, United States, 2. Wake Forest School of Medicine, Winston-Salem, North Carolina, United States*

The purpose of this study is to explore whether the effect of intentional weight loss on physical function in older adults varies by sex/gender. Individual level data from 1369 older, (67.7±5.4 years), obese (BMI: 33.9±4.4 kg/m2), adults (30% male, 21% African American) who participated in eight randomized controlled trials of weight loss were pooled. All studies were 5-6 months in duration and collected baseline demographic and pre/post gait speed (n=1296), short physical performance battery (SPPB; n=866), and grip strength (n=401) data. Treatment effects were generated by weight loss assignment [weight loss (WL; n=764) versus non-weight loss (NWL; n=605)], as well as categorical amount of weight change (high loss: >-7%, moderate loss: -7 to -3%, and weight gain/stability: <-3%). Analyses were adjusted for age, race/ethnicity, study, education, baseline BMI, and baseline value of the outcome measure of interest. Sex/gender stratified results were presented if the interaction term was p≤0.10. A sex/gender*weight loss assignment interaction was observed for SPPB (p=0.07), with women experiencing greater weight loss-associated improvement in SPPB score (WL: 0.42±0.08 versus NWL: 0.10±0.09; p=0.02) compared to men (WL: 0.30±0.11 versus NWL: 0.30±0.13). A sex/gender*weight loss amount interaction was observed for grip strength (p=0.05), with no difference observed across categories in women; however, greatest grip strength improvement was seen in men experiencing moderate weight loss compared to high loss and weight gain/stability categories. Weight loss-associated improvement in SPPB score is greater in women than men; grip strength gains in men are greatest among those achieving moderate weight loss.

### THE ASSOCIATION OF SUBCLINICAL HYPERTHYROIDISM WITH FRAILTY

Nalini S. Bhalla,[1] Karyne Vinales,[1] Janet Fawcett,[1] Ming Li,[1] Richa Bhattarai,[2] and Sherman M. Harman[1], *1. Phoenix VA Health Care System, Phoenix, Arizona, United States, 2. Banner University Medical Center-Phoenix, Phoenix, Arizona, United States*

The relevance of subclinical hyperthyroidism in the elderly has not been clearly defined. We studied whether the reported association of low TSH with frailty is an indicator of subclinical hyperthyroidism as assessed by free T3 levels. In a retrospective chart review of patients seen between January 2017 and December 2018 at the Phoenix VA Medical Center, we identified 100 patients aged ≥60 years with at least 2 low TSH measurements (<0.5 µIU/ml) and a free T3 measurement within 6 months of the measured TSH and 50 sex- and age-matched controls (TSH 0.5-5.0 µIU/ml). Patients with exogenous or clinical hyperthyroidism were excluded. We used a deficit accumulation approach evaluating 31 factors, to create a frailty index between 0 and 1 for each patient. The higher the FI, the more likely (p<0.001) it was that patients had expired in the interim. Patients with low (0.31±0.11 µIU/mL) vs. normal (1.84±0.84 µIU/mL) TSH had higher mean FI compared to controls (0.25±0.12 vs. 0.15±0.07, p<0.001). TSH significantly predicted frailty score (p<0.0001) independent of age. However, lower TSH was not associated with higher free T3 or free T4 levels. There was a nonsignificant inverse association of free T3 levels with FI (P = 0.09), which disappeared when adjusted for age. Similar to prior studies, low TSH predicted frailty. However, neither free T3 nor free T4 predicted low TSH or frailty index, suggesting that the association of low TSH with frailty is not due to subclinical hyperthyroidism, but perhaps to effects of comorbidities on TSH secretion.

### SESSION 3330 (POSTER)

### HEALTH AND MENTAL HEALTH CARE: USE AND ACCESS

### A FRAMEWORK FOR CARE TRANSITIONS FOR OLDER ADULTS WITH COMPLEX HEALTH CONDITIONS

Paul Stolee,[1] Jacobi B. Elliott,[1] Kerry Byrne,[1] Joanie Sims-Gould,[2] Catherine Tong,[1] Bert Chesworth,[3] Mary Egan,[4] and Dorothy Forbes[5], *1. University of Waterloo, Waterloo, Ontario, Canada, 2. University of British Columbia, Vancouver, British Columbia, Canada, 3. Western Health Sciences, London, Ontario, Canada, 4. University of Ottawa, Ottawa, Ontario, Canada, 5. University of Alberta, Edmonton, Alberta, Canada*

For older adults with complex health conditions, transitions between care settings are common and a major risk to quality of care and patient safety. Care transition interventions have shown positive impacts on continuity of care and health service use, however, most require additional human resources (e.g., transition coach), focus on one transition or "handoff", and provide support for individual patients without addressing underlying challenges of health system integration. We sought to develop a framework for system-level enhancements to care transitions for older adults. We report a secondary framework analysis of an ethnographic investigation (the "InfoRehab" project) of care transitions for older persons who had experienced a hip fracture. The ethnographic study involved interviews, observations, and document reviews for 23 patients, 19 family caregivers, and 92 health care providers. Data were collected at each transition point (1-4/patient) along the care continuum, at three Canadian sites (large urban, mid-size urban, rural). Our framework analysis followed the approach described by Gale et al. (2013), using as cases 12 peer-reviewed papers which had reported InfoRehab results. Two researchers coded findings from each paper, then developed an analytical framework of eight themes by consensus; these include: patient involvement and choice, family caregiver involvement, patient complexity, health care provider coordination, information sharing, documentation, system constraints, and relationships. NVivo 11 was used to index findings into these themes and to generate a matrix. We are working with system stakeholders, including patients and caregivers, to apply this framework in the development of improved systems for care transitions.

### DECISION-MAKING AND DISPARITIES IN QUALITY OF CHOSEN PLANS AMONG RACIAL-ETHNIC GROUPS IN MEDICARE ADVANTAGE

Maricruz Rivera-Hernandez,[1] and Kristy L. Blackwood[2], *1. Brown University, Providence, Rhode Island, United States, 2. The Warren Alpert Medical School of Brown University, Providence, United States*

Limited research regarding decision-making in Medicare Advantage (MA), which now disproportionally serves racial and ethnic minorities (~45% Hispanics and 30% African-Americans), has been conducted. Without understanding the extent to which vulnerable groups select low quality plans with high out-of-pocket costs (OOPC) and what factors influence this selection, these beneficiaries could continue to be adversely affected. The objective of this study is to understand plan choice decision-making process and differences in quality and OOPC of chosen plans between racial and ethnic minorities enrolled in MA. We used 2015 national data from Medicare and conducted in-depth interviews with 25 MA enrollees. African-Americans were enrolled in plans with higher drug deductibles and lower OOPC. In addition, Hispanics and African-Americans enrollment in high quality plans were lower by 10% and 5%, respectively, regardless whether mean OOPC were 100 or 1000 (P<.05). Our interviews highlighted issues regarding financial literacy, choice overload and complexity. These seniors did not use Plan Finder from Medicare and obtained information from insurance companies. Most seniors were confused about insurance terminology and expressed poor computer literacy. Among them, there was a prevailing sentiment that more expensive plans are better. Our findings could inform the Medicare program, and vulnerable populations who would benefit from plans that maximize quality of care with lower out-of-pocket spending. Finally, this information could contribute to state organizations' future efforts such as ensuring quality of health services for older adults including. Overall, this research provides new evidence about an increasingly important part of our publicly funded health system.

### RATES OF HOSPITALIZATION AND EMERGENCY DEPARTMENT VISITS AND THE QUALITY OF NURSING HOMES IN THE U.S.

Ian Breunig,[1] Qing Zheng,[1] Alan White,[1] Christianna Williams,[1] and Allison Muma[1], *1. Abt Associates, Inc., Durham, North Carolina, United States*

CMS strives to reduce costs and improve care for nursing home (NH) residents by reducing acute care transfers. We used a national database of Medicare claims and the Minimum Data Set to build NH stays from July 2017 through June 2018 and identify dates of hospital admissions and emergency department visits without hospitalization (ED) among all residents. We calculated rates of 30-day re-hospitalization and ED among short-stay (rehabilitation) residents, and the number of hospitalizations or ED per long-stay resident day (LSRD), then examined associations with NH Five-Star ratings (data.medicare.gov) and other provider characteristics available from Medicare administrative data. We identified 1.79 million short-stays and 898,290 long-stays at 15,576 NHs. Nationally, the 30-day re-hospitalization rate is 22.6%, the short-stay ED rate is 12.0%, there was one hospitalization every 561 LSRD (1.8 per 1000 LSRD), and there was one ED every 617 LSRD (1.6 per 1000 LSRD). Median facility rates were 22.3% (IQR=17.8%, 27.1%) for 30-day re-hospitalizations, 12.0% (IQR=8.7%, 16.1%) for short-stay EDs, 1.6 hospitalizations per 1000 LSRD (IQR=1.1, 2.3), and 1.4 ED per 1000 LSRD (IQR =0.9, 2.2). Higher rates were strongly associated with lower Five-Star ratings, particularly staffing ratings, and larger, for-profit, non-hospital facilities; even after risk-adjustment. NH variation and associations with provider characteristics suggest it is possible to further reduce acute care transfers. CMS incorporated these measures into the Five-Star rating system, providing greater transparency for residents and possibly incentivizing NHs to improve through competition. Future research should monitor success or identify the need for other avenues to improve.

### IMPLEMENTING A NEW MODEL IN PRIMARY CARE FOR OLDER CANADIANS LIVING WITH FRAILTY

Jacobi B. Elliott,[1] Joanie Sims Gould,[2] Susie Gregg,[1] Catherine Tong,[1] and Paul Stolee[1], *1. University of Waterloo, Waterloo, Ontario, Canada, 2. University of British Columbia, Vancouver, British Columbia, Canada*

Primary care may be the best place within the health system to coordinate care for older persons, but at present, it is poorly equipped to do so. Effective models for complex patients require appropriate targeting, patient/caregiver engagement, and care coordination. A large national project aims to co-design and implement a model in primary care that includes risk-stratification, patient engagement and care coordination techniques for older adults. This presentation focuses on the process of implementation in primary care. Grounded in the Consolidated Framework for Implementation Research, researchers worked with nine primary care sites in three Canadian provinces. Project implementation was completed in two phases. Pre-implementation: Interviews with providers (n=25) and older adults (n=8) were conducted to understand current practices and plan for implementation. Implementation: Researchers worked with sites to train staff and support implementation. Monitoring of the implementation process included Interviews with providers (n=20) and field notes. Data were analyzed using directed coding, following the framework. A number of learnings emerged: buy-in was required from the entire team, teams provided meaningful information to guide implementation, contributing to a sense of ownership, and it was important that intervention components were tailored to the needs at each site. Ongoing and frequent discussions with the team was necessary. Scheduling meetings and training sessions for providers was challenging due to the length of time away from direct patient care. A new primary care model for older adults living with frailty was implemented. Lessons from this project will be used to guide future implementation and spread.

### TRANSGENDERED OLDER ADULTS IN LONG-TERM CARE: PREPARING FOR THE RISING TIDE

Jean Henry,[1] and Susan K. Patton[1], *1. University of Arkansas, Fayetteville, Arkansas, United States*

Gender non-conforming older adults are more likely to be without traditional support systems in place; many may need to turn to nursing homes for long-term care (LTC). Little information is available about the experiences of LGBT older adults in these settings. Research questions: What is known about the experiences of transgender (TG) older adults in LTC? What evidence-based recommendations exist to guide LTC administrators in providing quality care to TG residents? What are key future research areas? Method: Systematic review of extant literature, using databases: PubMed, Medline, Psych Info, CINAHL, Academic Search Complete, and ProQuestCentral. Key Results:RQ1) Published research in transgender healthcare consists primarily of case reports, and retrospective and cross-sectional studies; minimal literature specifically on TG individuals; combining LGBT carries risk of minimizing or equating the TG experience to that of gays and lesbians. RQ2) Recommendations: take proactive approach; focus on awareness of relevant laws and regulations; establish non-discriminatory and inclusive environments, policies, and procedures; regular staff training, monitoring, and evaluation. RQ3) Need exists for quantitative and qualitative research into all aspects of the TG experience in LTC. Key areas include: lived experience of the TG in LTC; beliefs, attitudes, values and practices of LTC staff; administrative challenges and responses. Conclusions: Transgender and gender non-conforming individuals frequently experience discrimination in healthcare. Lack of cultural and clinical competence, and discrimination and bias by providers, create barriers to quality healthcare. Healthcare professionals and systems must be proactive in preparing for the inevitable growth of this population in the LTC setting.

### THEORY-DRIVEN ROLE MODEL STORIES' EFFECTIVENESS IN IMPROVING PALLIATIVE CARE KNOWLEDGE AMONG A DIVERSE POPULATION

Deborah Hoe,[1] Anna Rahman,[1] Kayla Johari,[1] and Susan Enguidanos[1], *1. University of Southern California, Los Angeles, California, United States*

Palliative care, specialized medical care for seriously ill people, has been demonstrated to reduce pain and symptoms while increasing satisfaction with health care. Yet, national surveys show that less than 10% of people are aware of palliative care, and among these, many believe it to be synonymous with hospice. The purpose of this study is to determine the effectiveness of 3-minute long theoretically-driven role model video stories at improving knowledge of palliative care. We recruited 161 adults age 50 and older from senior centers, assisted living, and other community-based sites. Pretest-posttest study design was employed and each participant completed 20 question surveys about palliative care knowledge, intent to seek palliative care, and perceptions about the videos. We also recorded participants' opinions of the videos. Regression analysis was conducted to test the effectiveness of the role model video stories and qualitative analysis to elicit the major themes in participants' opinions. Overall, palliative care knowledge score (max=13) improved from an average of 4.64 to 9.99 (t=11.99, p<0.001). Two-sample t-test revealed no significant difference in change of score by race. Regression analysis revealed that belief in the role models as real people and participants with 12 years of education significantly predicted higher change in knowledge scores. Conversely, being non-white and widowed were significant predictors of lower changes in score. Qualitative analysis of participants' perspectives revealed two major positive themes: authenticity and informativeness of videos. This study suggests that theoretically-driven role model video stories may be an effective strategy to improve palliative care knowledge.

### PROVIDER-IDENTIFIED BARRIERS TO PALLIATIVE CARE FOR MEDICAID PATIENTS

Susan Enguidanos,[1] Anna Rahman,[1] Deborah Hoe,[1] and Kate Meyers[2], *1. University of Southern California, Los Angeles, California, United States, 2. California Health Care Foundation, Oakland, California, United States*

In January 2018, California enacted Senate Bill 1004, which requires Medicaid (or Medi-Cal in California) managed care providers to offer home-based palliative care (HBPC) to their seriously ill patients. Since then, enrollment in HBPC has been lower than projected, which means many across the state continue to suffer without the pain and symptom management and psychosocial support available from a palliative care team. This study elicited clinician-perceived barriers to access to HBPC by Medi-Cal patients. We conducted a qualitative study comprising 25 individual interviews with a range of healthcare leaders and practitioners. Interviews were audio-recorded and transcribed verbatim. Using a grounded theory approach, we analyzed transcripts to determine primary themes. Our findings identified a myriad of access barriers to HBPC for the Medi-Cal population, including lack of physician knowledge about HBPC programs and benefits, a physician office structure that hampers the provision of HBPC education (i.e., one physician per office), cultural and language barriers among both physicians and patients, physicians' lack of time, and competing demands on physicians. Providers also identified patient-related barriers, including cultural mismatch between HBPC providers and patients, trust issues related to the health-care system, and the complex challenges facing some patients such as lack of adequate and safe housing, behavioral health problems, and limited access to services that meet basic needs. These findings underscore the need for multiple approaches to increase physician education and awareness of HBPC and HBPC Medi-Cal benefits, develop culturally appropriate HBPC services, and develop programs that improve patients' palliative care health literacy.

### EXAMINING LONGITUDINAL HEALTHCARE UTILIZATION, OUTCOMES, AND SATISFACTION IN PRE-FRAIL OLDER ADULTS

Ivana A. Vaughn,[1] Nicole M. Marlow,[2] Kalyani Sonawane,[3] Roger B. Fillingim,[4] and Rebecca J. Beyth[5], *1. The New York Academy of Medicine, New York, New York, United States, 2. University of Florida College of Public Health and Health Professions, Gainesville, Florida, United States, 3. The University of Texas Health Science Center at Houston School of Public Health, Houston, Texas, United States, 4. Pain Research and Intervention Center of Excellence, University of Florida, Gainesville, United States, 5. Geriatric Research Education and Clinical Center, North Florida South Georgia Veterans Health System,, Gainesville, Florida, United States*

Frailty is a medical syndrome occurring in nearly 60% community dwelling older adults and could have insidious origins in middle-adulthood that manifest predominantly as decline and multi-morbidity. Identification of pre-frail states in adults could potentially reduce its impact in late-life. The study used data from the Health and Retirement Study (HRS) to: 1) compare long-term healthcare utilization between pre-frail and non-frail patients, 2) compare risk of pain progression, functional decline, and mortality between pre-frail and non-frail patients, and (3) compare satisfaction with healthcare, self-perceptions of aging and satisfaction with life between pre-frail and non-frail patients. The primary predictor, pre-frailty phenotype, was based on the Paulson-Lichtenberg Frailty Index (PLFI), a validated HRS-version of the Fried criteria. Additional covariates included sociodemographics, comorbidities, smoking status, sleep quality, health status, and body mass index. Statistical analyses consisted of descriptive statistics, univariate analysis, negative binomial regression with log-link function, logistic regression, generalized linear modeling and Cox regression. Pre-frailty status increased the incidence of hospitalizations (IRR: 1.23; 95%CI: 1.18-1.28) and doctor's visits (IRR: 1.18; 95%CI: 0.15-1.22); increased the risk of pain progression (HR: 1.61, 95%CI: 1.53-1.69), functional decline (HR: 1.77, 95%CI: 1.67-1.87), and all-cause mortality (HR: 1.09, 95%CI:1.03-1.16); lowered odds of healthcare satisfaction (OR: 0.79, 95%CI: 0.62-0.99) and lowered satisfaction with aging ((β: -0.23 95%CI: -0.36- (-0.10)) and satisfaction with life (β: -0.27 95%CI: -0.44- (-0.11)). Frailty syndrome is highly prevalent and having a better understanding of its influence on health outcomes at intermediate pre-frail states could provide insight into reducing manifestations in later life.

### MULTIDIMENSIONALLY HETEROGENEOUS HEALTH LATENT CLASSES AND HEALTHCARE UTILIZATION FOR OLDER CHINESE

Linglong Ye,[1] Jiecheng Luo,[1] Ben-Chang Shia,[2] and Ya Fang[1], *1. School of Public Health, Xiamen University, Xiamen, China, 2. School of Management, Taipei Medical University, Taipei, Taiwan*

Objectives: Based on a multidimensional perspective, this study aimed to assess the heterogeneous health latent classes of older Chinese, and further examined the effects of health latent classes and associated factors on healthcare utilization. Methods: Data came from the Chinese Longitudinal Healthy Longevity Survey in 2014. Latent class analysis was adopted to identify heterogeneous health latent classes by health indicators of physical, psychological, and social dimensions. Two-part models were used to evaluate the impact of health latent classes and socio-demographic factors on outpatient and inpatient utilization. Results: Among 2,981 participants aged 65 and over without missing health indictors, four health latent classes were identified and labeled as "Lacking Socialization" (10.4%), "High Comorbidity" (16.7%), "Frail Group" (7.7%), and "Relatively Healthy" (65.1%). Among 1,974 participants with complete information, compared with the Relatively Healthy group, those in the Lacking Socialization group costed more inpatient expenditure (p-value =0.02). Those in the High Comorbidity and Frail groups tended to use healthcare services and costed more outpatient expenditure (all p-value <0.01). After controlling for health latent classes, the effects of age, gender, marital status, education, residence area, occupation, and health insurance on healthcare utilization were significant. Conclusions: Four heterogeneous health latent classes were identified by multidimensional health, and had significant effects on healthcare utilization. After controlling for health latent classes, different effects of socio-demographic factors on healthcare utilization were found. It enhances our understanding of heterogeneous health and complex healthcare demands in older Chinese, and is valuable for improving healthcare resource allocation targeted for healthy aging.

### WHAT MATTERS FOR COMPLETION OF ADVANCE DIRECTIVES AMONG AMERICAN OLDER ADULTS?

Yifan Lou,[1] and Jinyu Liu[2], *1. Columbia University, New York, New York, United States, 2. Columbia University, New York City, New York, United States*

Background: Most previous studies consider advance directives as one single outcome, which conceals possible variations of individuals' decisions on two different advance directives documents–living will (LW) and durable power of attorney for healthcare (PA). To advance the knowledge on advance planning among older adults in the US, this study examined how health status and education are associated with completions of LW and PA and whether such associations vary by age and race. Methods: Data are from the 2016 wave of Health and Retirement Study. Health status was indicated by chronic condition and ADL and IADL functional limitations. Logistic regression model was used to examine how the completions of LW and PA are associated with health and education variables respectively. Interaction terms were created to test the moderating effects of race and age. Results: The analysis results show that older adults with higher IADL functional limitation and more education were more likely to complete completing PA or LW. Being white and higher age will increase the probability for an older adult to complete PA, whereas the older adults with heart problem were more likely to complete LW. The association between IADL/ADL and PA was stronger in white than other racial groups, and the association between IADL and LW was stronger in young-old than the oldest-old. Conclusion: The findings highlight the importance of examining the completions of two advance directive documents respectively and indicate the necessity of developing distinct and concrete strategies to promote the completions of PA and LW.

### ELDER VOICES NETWORK: PARTNERING WITH OLDER PEOPLE AND THEIR CAREGIVERS TO AVOID MEDICAL HARM

Molly Ranahan,[1] Mary Brennan-Taylor,[1] Michael Richbart,[1] Collin Clark,[1] Ryan Gadzo,[2] Ranjit Singh,[1] and Robert Wahler[1], *1. University at Buffalo, Buffalo, New York, United States, 2. Erie County Department of Senior Services, Buffalo, New York, United States*

Team Alice, named after an older adult in our community who died as a result of medication harm, is an interdisciplinary team of prescribers, pharmacists, educators, advocates, and researchers with a mission to protect older people from medication-related harm across the continuum of care. In 2019, Team Alice partnered with the Erie County Department of Senior Services, older people, and caregivers to form the Elder Voices Network (EVN) as a vehicle for patient-driven deprescribing in the Western New York region. The objective of this presentation is to detail the planning and implementation of critical components of EVN's formation, including outreach and engagement, funding development, community partnerships, roles and communication, and decision-making. Case study results demonstrate the capacity of community-based participatory research (CBPR) to empower older people and caregivers with knowledge, skills, and tools to promote self-advocacy across the system. Presenters will also discuss recommendations useful for future patient engagement initiatives.

### CHANGES IN THE MEDICARE HOME HEALTH CARE MARKET: THE IMPACT OF ACA REIMBURSEMENT POLICY

Jamila M. Torain,[1] Joan Davitt,[2] Charlotte L. Bright,[3] Nancy Miller,[4] Sarah Chard,[5] and Denise Orwig[1], *1. University of Maryland, Baltimore School of Medicine, Baltimore, Maryland, United States, 2. University of Maryland, Baltimore, Maryland, United States, 3. University of Maryland, Baltimore, Baltimore, Maryland, United States, 4. The University of Maryland Baltimore County, Baltimore, Maryland, United States, 5. UMBC, Baltimore, Maryland, United States*

This study evaluated the effect of recent ACA changes to Medicare Home Health Care (HHC) reimbursements on the mix of agencies and staff in the HHC market. We used Provider of Services (POS) data and logistic regression, to determine which agency characteristics were associated with the likelihood of exiting the HHC market and likelihood of decreasing staff before (n=13,878) and after (n=13,702) implementation of the ACA-mandated reimbursement cuts. Free standing agencies had 1.35 times the odds of exiting from the HHC market post ACA cuts. There were no differences in the odds of exiting the HHC market between for-profit and non-profit agencies. Agencies in the New York, Atlanta, and Chicago regions had a greater likelihood of exiting the HHC market post ACA cuts. Small agencies had two times the odds of exiting (aOR= 2.09) and agencies with one or more branch had less than half the odds of exiting (aOR= 0.46) from the HHC market. The average number of all staff was similar before and after the ACA cuts; however, office staff and home health aides experienced the greatest decrease in number. Agencies that were for-profit, free-standing, small, and/or with one or more branch were more likely to decrease staff post the ACA cuts. Agencies in the New York, Atlanta, Chicago, Dallas and Kansas regions were more likely to decrease staff. Overall, the reimbursement cut effects varied by geographic region and had greater impact on more vulnerable agencies and staff that were non-skilled.

### HOME AND COMMUNITY HEALTHCARE FOR FRAIL OLDER ADULTS: A PATIENT, CAREGIVER, AND PRACTITIONER PERSPECTIVE

Andrew McDonald,[1] Rowena Rizzotti,[2] Joanna Rivera,[3] Grace Park,[4] Antonina Garm,[5] Ryan D'Arcy,[3] and Xiaowei Song[3], *1. Health Research and Innovation, Surrey Memorial Hospital, Fraser Health, Surrey, British Columbia, Canada, 2. Health and Technology District, Surrey, British Columbia, Canada, 3. Health Research and Innovation, Surrey Memorial Hospital, Fraser Health Authority, Surrey, British Columbia, Canada, 4. Community Actions and Resources Empowering Seniors, Fraser Health Authority, Surrey, British Columbia, Canada, 5. Community Actions and Resources Empowering Seniors, Fraser Health Authority, British Columbia, Canada, Canada*

Frailty and the decline in ability to maintain independent living may be forestalled through discussions with healthcare providers and seniors about managing health at home. In addition, the use of technology in supplementing doctors' visits to assess frailty progression may be easily adopted by some but not others. We conducted this qualitative study to elucidate the context in which seniors access care at home and current barriers to independent living, from the perspectives of both seniors and practitioners. Pre-approved discussion questions were administered to two audio-recorded focus group sessions of 14 participants. The first group were community-dwelling older adults and informal caregivers, while the second consisted of healthcare practitioners. Group members were sampled to include a range of health backgrounds and levels of technological expertise. Thematic analysis with NVivo Software was used to parse out key discussion topics from the audio transcripts. The caregiver/patient group emphasized the stigma of frailty and age-related isolation, desiring transparency and advocacy from care teams. Practitioners/researchers discussed the importance of a holistic biopsychosocial approach to frailty management and the need for standardized frailty measurement. Patients/caregivers used health-tracking devices at home and were more optimistic about telehealth/videoconferencing than practitioners. Awareness of contextual factors surrounding "aging in place" and what aspects of care are valued by patients and practitioners is key to advancing home health and paving the way for new evidence-based services in the home.

### MEDICARE IS THE LAST HOLDOUT: EXAMINING THE IMPACT OF MEDICARE POLICY ON OLDER ADULTS' ACCESS TO MENTAL HEALTH CARE

Matthew C. Fullen,[1] Jonathan Wiley,[1] Amy Morgan,[1] Gerard Lawson,[1] and Jyotsana Sharma[1], *1. Virginia Tech, Blacksburg, Virginia, United States*

Medicare is the primary insurance provider for approximately 59 million Americans, and the number of beneficiaries is expected to surpass 80 million by 2030. Currently, Medicare regulations allow psychiatrists, psychologists, clinical social workers, and psychiatric nurses to provide mental health services. These providers were last updated in 1989 with passage of the Omnibus Budget Reconciliation Act of 1989. Since that time, the mental health marketplace has changed dramatically, and Medicare beneficiaries are unable to access care from approximately 200,000 graduate-level mental health professionals with similar training to eligible Medicare providers. There is evidence that this Medicare mental health coverage gap (MMHCG) impacts both providers and beneficiaries. For example, some beneficiaries may begin treatment only to have services interrupted, or stopped altogether, once the provider is no longer able to be reimbursed by Medicare. We surveyed 6,550 members of the American Counseling Association, including 3,815 who identified themselves as practicing counselors. These individuals work in diverse contexts (e.g., community mental health agencies, private practice, and integrated care settings). Survey results indicated that a significant number of practicing counselors turn away or refer Medicare beneficiaries who seek mental health care due to the MMHCG. In-depth interviews were also completed with eight licensed mental health professionals who detailed the challenges they and their clients experienced. Participants perceived a discrepancy between Medicare's intended aims to promote health and provider restrictions that were confusing and frustrating to navigate. Participants concluded that the MMHCG has a negative impact on older adult clients.

### THE USE OF SELF-MANAGEMENT TECHNOLOGY AND HEALTH CARE UTILIZATION IN LATER LIFE

Darren Liu,[1] Takashi Yamashita,[2] Betty Burston,[3] and Jennifer Keene[3], *1. Des Moines University, Des Moines, Iowa, United States, 2. University of Maryland, Baltimore County, Baltimore, Maryland, United States, 3. University of Nevada Las Vegas, Las Vegas, Nevada, United States*

Health-related websites and applications are useful tools for self-management in health care settings. However, the limited accessibility due to the lack of technology-related skills can be a "digital divide" issue that particularly affects older adults. To better understand the role of such tools in later life, this study examined the use of internet-based technology in relation to health care service utilization. A nationally representative samples of adults age 55 years and older (n = 1,157) were obtained from the health behaviors module of the 2014 Health and Retirement Study (HRS) in the U.S. We analyzed the use of health management websites and apps and its association with health care service utilization measured by the number of doctor visits during the last two years before the interviews. Negative binomial regression was used to model the number of doctor visits as a function of the health management technology use and other covariates. Results revealed that the level of technology use ($b = 0.41$, $p < 0.05$) was significantly associated with a 50% greater number of doctor visits. Such technology use was significantly more common among adults with good health than those with poorer health outcomes (17.32% & 9.60%, respectively, $p < 0.05$). The findings suggest that healthy adults take advantage of available internet-based self-management tools and proactively utilize health care services more often than individuals who can be categorized as "unhealthy". There is a continuing need for research on introducing strategies that can ultimately reduce health disparities by decreasing the digital divide.

### SESSION 3335 (POSTER)

### HEALTH RISK BEHAVIORS

### ADVERSE CHILDHOOD EXPERIENCES AND ALCOHOL-IMPAIRED DRIVING IN LATER LIFE

Shalini Sahoo,[1] and Paul Sacco[1], *1. University of Maryland-Baltimore, Baltimore, Maryland, United States*

Adverse Childhood Experiences (ACEs) are associated with a range of negative behavioral health outcomes in adulthood. Those with ACEs may use alcohol as a component of long-term coping, increasing risk of alcohol-impaired driving. Associations between ACEs and alcohol-impaired driving are relatively understudied. Using the 2012/2013 data from the National Epidemiologic Survey on Alcohol and Related Conditions (NESARC), logistic regression models examined the relationship between five types of ACEs (e.g. child abuse and neglect) and lifetime alcohol-impaired driving among a representative sample of American adults aged between 18 to 90 years (N = 36,309). ACEs were positively associated with lifetime alcohol-impaired driving for adults under age 50 (witnessed intimate partner violence (OR = 1.624, $p < 0.001$), physical abuse (OR = 1.723, $p < 0.001$), sexual abuse (OR = 1.651, $p < 0.001$), physical neglect (OR = 1.571, $p < 0.001$), and emotional neglect, ($P > 0.05$). We found similar positive associations between ACEs and impaired driving among adults aged 50 and over (witnessed intimate partner violence (OR = 1.398, $p < 0.05$), physical abuse (OR = 1.751, $p < 0.001$), sexual abuse (OR = 1.690, $p < 0.001$), physical neglect (OR = 1.455, $p < 0.001$), and emotional neglect, $p > 0.05$). Among adults under age 50, ACEs were associated with past-year alcohol-impaired driving, but this relationship was not seen in adults aged 50 and over. Findings suggest that the effect of ACEs on alcohol-impaired driving is in younger adulthood when alcohol use and driving occur most.

### SUICIDE TRENDS AND DISPARITIES AMONG OLDER ADULTS IN THE UNITED STATES, 2008-2017

Sanae El Ibrahimi,[1] Yunyu Xiao,[2] and Matthew L. Smith[3], *1. University of Nevada, Las Vegas, Las Vegas, Nevada, United States, 2. New York University, Silver School of Social Work, New York, New York, United States, 3. Center for Population Health and Aging, Texas A&M University, College Station, Texas, United States*

Background: Suicide ranks within the top fifteen causes of death among adults 55 and older in the United States and is a growing concern in the face of social isolation and other end-of-life issues. This study examined differences and trends in suicide rates and methods among older adults in the U.S. Methods: Suicide mortality rates from 2008-2017 were derived from the Multiple Cause of Death files in the CDC's WONDER database. Suicide deaths were identified from the underlying causes of death using ICD-10 codes. Age-adjusted death rates (per 100,000) were calculated. Older adults were grouped into four age categories: 55-64, 65-74, 75-84, and 85+ years. Percent change in suicide rates between 2008-2017 were examined, which were then stratified by gender and top suicide methods. Results: Suicide rates increased by 16% among adults 55 years of age and older from 2008 to 2017 (15.4 vs 17.8 per 100,000 respectively). In 2017, the suicide rate among older adults was 27% higher than the general population (14.0 per 100,000). Suicide rates were significantly higher among men relative to women for those ages 85+ (14:1 ratio of males-to-females). However, females in the 65-74 age group experienced the highest increase of suicide rate (41%) compared to other females or males across age groups. The most common method of suicide was firearms, followed by poisoning and suffocation. Suffocation had the highest increase over time (37%). Conclusion: Rising suicide rates among older adults suggest the need for tailored intervention strategies that address upstream suicide-related risk factors.

### PATTERN OF HEALTH-RELATED BEHAVIORS AND RESOURCES AS PREDICTOR OF MEDICAL CARE AND MORTALITY RISK AMONG OLDER ADULTS

Dongmei Zuo,[1] and Merril D. Silverstein[2], *1. Aging Studies Institute (ASI), Syracuse University, Syracuse, New York, United States, 2. Syracuse University, Syracuse, New York, United States*

This study investigates the patterns and consequences of a wide range of health-related behaviors and resources that include health-compromising behaviors, health-promoting behaviors, preventive health behaviors, and health risks coping resources. We aim to identify the empirically-derived subgroups of individuals with unique profiles of health behaviors and resources to determine how subgroup membership predicts health outcomes and medical care utilization four years later. Data derived from 5,067 respondents in the 2010 and 2014 waves of the Health and Retirement Study. Latent class analysis was used to define classes based on 13 indicators in the 2010 wave, which also provided sociodemographic and health-related covariates. Outcomes were measured over 4 years. Six latent subgroups were identified: "Best Behavior/Resources", "Low Social Support ", "Low Physical Activity", "High Substance Abuse", "Low Preventive Tests", and "Low Governmental Health Insurance". Compared with the "Best"

group, older adults identified as "Low Physical Activity" and "High Substance Abuse" were found to have higher mortality risks and a lower likelihood of seeing doctors and less nursing home nights; older adults with the lowest level of receiving flu shots, cholesterol and cancer screen test ("Low Preventive Tests") reported a less likelihood of seeing doctors; respondents in "Low Governmental Health Insurance" subgroup were associated with a lower likelihood of hospital stay and more nursing home nights. Results suggest that distinct groups of older individuals characterized by their health behaviors and resources provide a basis for identifying the high-risk segment of the older population for intervention.

### PATTERNS AND DETERMINANTS OF POLYSUBSTANCE USE AMONG RETIRED POPULATION IN A U.S. NATIONALLY REPRESENTATIVE SAMPLE

Tzung-Shiang Ou,[1] Yi-Han Hu,[2] Hsien-Chang Lin,[2] and Su-Wei Wong[1], *1. INDIANA UNIVERSITY, Bloomington, Indiana, United States, 2. Indiana University Bloomington, Bloomington, Indiana, United States*

Polysubstance use in the U.S. has been a public health concern. The prevalence of substance use among middle-aged and older adults is predicted to increase by 50% by 2020. Previous studies revealed retirement could serve as a risk factor for substance use as this population was known to be more susceptible to mental health issues. However, patterns and determinants of polysubstance use among retired population remained understudied. The purpose of this study was to investigate the patterns and determinants of polysubstance use among retired adults aged 50 and older. This study extracted 3,019 retired participants from the 2017 National Survey on Drug Use and Health study. Polysubstance use was defined as the use of two or more substances, including alcohol, tobacco, marijuana, and painkiller misuse, in the past month. Weighted multinomial logistic regression model was conducted to examine the associations between utilization of mental health treatment and poly-use of substances. The findings suggested 52.0% of retired adults used at least one substance in the past month, where 17.0% used substances concurrently; 15% co-used alcohol, tobacco, and marijuana; 8.6% co-used tobacco and marijuana. Females were less likely to poly-use substances (RRR=0.37, $p<.001$) than males. Those who had received mental health treatment in the past year were more likely to co-use more than two substances in relative to substance non-users in the past month (RRR=1.71, $p<.05$). Retirement plan incorporating behavioral intervention and early detection of mental health issues are warranted to reduce polysubstance use among the retired population in the U.S.

### SOCIODEMOGRAPHIC DISADVANTAGE, LIVING WITH A SMOKER, AND OBESITY IN MIDDLE-AGED AND OLDER WOMEN

Carole K. Holahan,[1] Charles J. Holahan,[1] Sangdon Lim,[1] Yen T. Chen,[1] and Daniel A. Powers[1], *1. University of Texas at Austin, Austin, Texas, United States*

Although sociodemographic disadvantage is a recognized risk factor for obesity, the potential role of living with a smoker in this relationship has been unexamined. This study investigated: (a) the association between sociodemographic disadvantage and living with a smoker, and (b) the role of living with a smoker in partially explaining the link between sociodemographic disadvantage and obesity. The study used limited access data from the Women's Health Initiative Observational Study obtained from NHLBI. Participants were 91,888 women ranging in age from 50 to 79; 6,527 participants reported living with a smoker. Analyses were cross-sectional. Logistic regression analyses examined paths in the proposed model; bias-corrected bootstrapped confidence intervals tested indirect effects. All analyses controlled for age, marital status, and participants' current smoking status. Results demonstrated a significant association ($p < .001$) between sociodemographic disadvantage and living with a smoker across three measures of disadvantage (for low education, low income, and Black ethnicity, ORs were 1.95, 2.10, and 2.63, respectively), as well as between living with and smoker and obesity (OR = 1.71). Moreover, the unstandardized indirect effect (CIs are in brackets) from sociodemographic disadvantage to obesity through living with a smoker was statistically significant for all three measures of disadvantage (for low education, low income, and Black ethnicity, indirect effects = .05 [.04, .06], .06 [.05, .06], and .07 [.06, .08], respectively). These findings underscore the need for innovative household-level interventions for disadvantaged families living with a smoker integrating smoking- and obesity-prevention efforts. This project was supported by the NIH/NCI (R03CA215947).

### SESSION 3340 (POSTER)

### LIFE COURSE & DEVELOPMENTAL CHANGE

### AGING ACROSS THE LIFE COURSE: RESEARCH COLLECTIONS AVAILABLE FROM THE NATIONAL ARCHIVE OF COMPUTERIZED DATA ON AGING

James McNally,[1] and Kathryn Lavender[1], *1. University of Michigan, Ann Arbor, Michigan, United States*

The creation and maintenance of sustainable data archives can be challenging but it offers clear advantage. Properly curated data can be used by multiple researchers, testing a variety of hypotheses and increasing the return on investment to the expensive process of data collection. Having an internally managed archival system also provides greater control and autonomy in the equitable distribution of data resources, insuring all researchers have full use of the data for original research, teaching and new directions once the data leaves the control of the local investigator's control. This poster reviews the advantages of having a local strategy geared toward the preservation and sharing of gerontological research data. Using the National Archive of Computerized Data on Aging (NACDA) as a working example, the poster offers an overview of collections at NACDA. Using our metadata tools and variable search database, NACDA can identify studies in its collections that examine aspects of aging and health among adults during their lifecourse. Many of the studies are longitudinal or repeat measure cross-sectional studies. We are also able to identify studies which focus on aging that are not maintained by NACDA but which are available to interested researchers. Using a strategy of archival preservation combined with a strong focus on productive research and outreach, NACDA has amassed data and metadata covering a wide array of studies worldwide that address the aging lifecourse. Because our collections are multinational, we share these data at no cost to interested users worldwide

### OLDER ADULTS' HEALTH AND SOCIAL ENGAGEMENT: THE MEDIATING ROLE OF SUBJECTIVE LIFE EXPECTANCY

Dahee Kim,[1] and Kyuho Lee[2], *1. Iowa State University, Ames, Iowa, United States, 2. Daegu University, Gyeongsan, Republic of Korea*

Older adults' mental and physical health is likely to limit social engagement, but their perception of how much time they have left, according to the socio-emotional selectivity theory, might influence it as well. The aim of the research is to investigate the mediating effect of subjective life expectancy (SLE) on the pathways from older adults' mental health and functional limitation to volunteering and contacts with close relationships. The current research used data of 5,285 older adults aged 50 to 75 from the Health and Retirement Study collected in 2014. Structural equation modeling was performed to investigate the direct effect of older adults' depressive symptoms and functional limitation on volunteering and contact with close relationships. Predictors' indirect effects via SLE was also assessed. The results indicated that older adults' higher depressive symptoms and functional limitations significantly decreased volunteering time and frequency of contacts with close relationships. Older adults' SLE attenuated the effects of depressive symptoms and functional limitations on their volunteering time and frequency of contact with close relationships. The findings describe the mechanism of how older adults engage in volunteering and contact with close relationships through their perception of remaining time. Further, this research highlights SLE as a motivator for encouragement of older adults' social engagement.

### PHYSICAL AND SOCIAL ACTIVITIES AND COGNITIVE CHANGES AMONG OLD-OLD ADULTS: RESULTS FROM 3-YEAR FOLLOW-UP PERIOD

Yoshiko L. Ishioka,[1] Midori Takayama,[1] and Ikuko Sugawara[2], *1. Keio University, Yokohama, Kanagawa, Japan, 2. University of Tokyo, Bunkyo, Tokyo, Japan*

The association between activity engagement and late-life cognitive function is considered to depend on the characteristics of the activity, the cognitive processes it involves, and the life stage of participants. A better understanding of this association is required to comprehend cognitive function in old age. The present study examined the association between baseline activity engagement and cognitive changes across a 3-year period among old-old adults. We extracted data for 873 Japanese community-dwelling participants from data of the Keio-Kawasaki Aging Study. We assessed cognitive performance thrice (at baseline, 1.5-year follow-up, and 3-year follow-up) using a short version of the Mini-Mental Status Examination. For the subsequent analyses, we used three measures of cognitive function: total score, orientation, and concentration, which showed diverse individual differences. We measured the frequency of physical activity and social group participation at baseline. Using conditional latent growth curve models, we examined which baseline activity was associated with the three measures of cognitive function over 3 years. Greater physical activity was significantly related to higher rate of orientation, after adjusting for age and education ($\beta = -.261$, $p < .001$). Social activity was significantly related to rates of higher total cognitive score ($\beta = -.276$, $p < .001$) and higher orientation ($\beta = -.207$, $p < .001$). These findings suggest that the association between activity engagement and late-life cognitive function among old-old adults varies by activity type and cognitive domain.

### LEISURE, LONGITUDINALLY: PARTNER DISAGREEMENTS ABOUT LEISURE DECREASE OVER THE LIFE COURSE

Jill Juris Naar,[1] and Shelbie Turner[2], *1. Appalachian State University, Boone, North Carolina, United States 2. Oregon State University, Corvallis, Oregon, United States*

Leisure is a major context within which older couples interact, and researchers have recently called for more longitudinal data analysis exploring how leisure-related couple interactions change over the life course. Several waves of the Midlife in the United States (MIDUS) study include a single-item question asking respondents how much they disagree with their spouse or partner about leisure activities. Given the longitudinal nature of MIDUS, the variable offers great utility to explore shifts in leisure-related couple interactions over the life course. Utilizing longitudinal data from Wave 1 (1995-1997), 2 (2004-2006), and 3 (2013-2015) of the MIDUS study, we explored how leisure-related partner disagreement changed with increased age (age range = 20-93). We first ran an unconditional multilevel model, which revealed that 68% of the variation in leisure-related spousal disagreement was attributed to within-person differences over time, justifying our analysis of longitudinal within-person change. An age-based growth curve model then revealed that leisure-related partner disagreements decreased linearly over the life course (Estimate = -0.01, SE = 0.001, $p<.0001$). Men reported more leisure-related partner disagreements than women at age 20 ($p = 0.002$). But men's reported disagreements decreased over the life course at a faster rate than did women's reported disagreements ($p = 0.03$), so that from ages 70-93, men reported less disagreements than women. To our knowledge, this is the first longitudinal study to explore leisure-related couple disagreements over an extended period of time (20 years). The significance of our results sheds light on the value of longitudinal research on leisure.

### FACE-TO-FACE AND FACEBOOK ACROSS THE LIFESPAN: SOCIAL INTERACTIONS BUFFER STRESS ON MOOD, BUT ONLY FOR ADOLESCENTS

Yin Liu,[1] Elizabeth B. Fauth,[1] Myles Maxey,[1] and Troy Beckert[1], *1. Utah State University, Logan, Utah, United States*

Social support serves as a protective factor, buffering stress in both adolescents and adults, however Socioemotional Selectivity Theory suggests developmental differences in stress reactivity and social support. It is unclear how modern forms of social contact, such as social media buffer stress, and the extent to which this differs across the lifespan. We utilized ecological momentary data to examine the moderating effects of age and two distinct types of social contacts the person had experienced in prior hours (frequency of face-to-face, or social media contacts) on the association between daily stress and momentary mood. Participants were recruited initially through Amazon.com's Mechanical Turk (adolescents referred by a parent). A total of 119 adolescent (n = 44; Agemean= 15.73) and middle-aged/older adult participants (n = 75; Agemean= 59.67) provided momentary data three times a day, on three consecutive days, every two weeks, for up to 12 weeks. Multi-level models showed significant 3-way interactions between stress appraisal of avoiding an argument, age group, and frequency of social contact via face-to-face (β = 1.698, se = 0.542, p = .002) and social media (β = 3.341, se = 0.984, p = .001). Older adults experienced better mood than adolescents. When avoiding an argument was appraised as more stressful, both age groups displayed worse mood. Whereas high levels of recent social contact (both face-to-face and social media) seemed to exacerbate the impact of this stressor on poorer mood for older persons, high levels of recent social contact, particularly social media, had stress-buffering benefits for adolescents.

### RETROSPECTIVE LIFE HISTORY SURVEY REVEALED AUTOBIOGRAPHICAL MEMORY BUMP

Marina Larkina,[1] Lindsey Meister,[2] and Jacqui Smith[2],
*1. Institute for Social Research, University of Michigan, Ann Arbor, Michigan, United States, 2. University of Michigan, Ann Arbor, Michigan, United States*

The reminiscence bump is a well-documented autobiographical memory phenomenon characterized by middle-aged and older adults reporting a disproportionate number of memories from adolescence and early adulthood (Rubin, Wetzler, & Nebes, 1986). It is typically assessed through either cue word or important memory techniques. The Life History Mail Survey (LHMS) in the Health and Retirement Study affords unique data to investigate this phenomenon among a representative US sample of older adults. At the beginning of the LHMS, participants (N=3088, M age=70, range 50-107) completed a calendar noting the important things that happened to them in seven life decades, starting with ages 0-9 and ending by ages 70-79 (or their actual age). For each life period, we coded the number of events respondents reported. We observed significantly more memories reported for the age decade 20-29, compared with other life periods (80% vs 47-66%). Our results are consistent with previous findings in the autobiographical memory literature. Follow-up analyses evaluated existing theoretical accounts of the bump, such as cultural life script theory which suggests that life events occur in a specific order and are characterized by a prototypical life course. For example, we determined whether respondents' sociodemographic characteristics, such as age cohort, gender, marital and educational histories (information available in LHMS) influenced the size and temporal location of the reminiscence bump. We also analyzed the content of reported important life events to investigate whether types of events included in each decade of life are consistent with the cultural life script account of the phenomenon.

### EARLY-LIFE ADVERSITY AND ADULT MENTAL AND BEHAVIORAL HEALTH OUTCOMES: AN INTERSECTIONAL APPROACH

Chioun Lee,[1] Lexi Harari,[1] and Jennifer Coons[2],
*1. University of California-Riverside, Riverside, California, United States, 2. California State University, Fullerton, California, United States*

There is much research confirming the link between experiencing early life adversities (ELAs) and adverse health outcomes in adulthood. Further, experiencing co-occurring ELAs rather than one ELA appears to be the rule rather than the exception, and leads to poorer health outcomes. However, information on how ELAs cluster and differentially harm the health of different intersectional groups is lacking. The stress process model suggests that intersectional configurations of race and gender are differentially exposed to ELAs and as a result, some groups suffer worse outcomes than others. We examine the risks of experiencing different clusters of ELAs—low childhood socioeconomic status (SES), family instability, and child abuse—among four intersectional groups (white men, white women, Black men, Black women). We also investigate whether experiencing these ELAs is responsible for the association between having a particular racial and gender configuration and adverse mental and behavioral health outcomes. Data come from a subsample of the Midlife in the U.S. Study (n = 2,076). Black men and women have the highest risk of experiencing all three ELA configurations. Men, regardless of race, have a higher risk of experiencing low SES while women are more vulnerable to low SES/family instability and all three ELAs. Black men and women appear to suffer the worst mental health outcomes, while Black and white men experience more drug/alcohol abuse than their female counterparts. ELAs, especially the co-occurrence of all three ELAs, partially mediate most of these associations, but more so for women than men.

### INFLUENCING FACTORS OF SUBJECTIVE AGE: FINDINGS FROM THE KUSATSU LONGITUDINAL STUDY ON AGING AND HEALTH

Tomoko Ikeuchi,[1] Satoshi Seino,[1] Yu Taniguchi,[1]
Miki Narita,[1] Takumi Abe,[1] Hidenori Amano,[1]
Akihiko Kitamura,[1] and Shoji Shinkai[1], *1. Tokyo Metropolitan Institute of Gerontology, Tokyo, Japan*

Background: Subjective age (SA) has been found to be a biopsychosocial marker of aging, yet little is known about factors that influence SA development. This study examined factors influencing SA using longitudinal data of community-dwelling older Japanese. Methods: Data drawn from the Kusatsu Longitudinal Study were collected during annual health check-ups in 2017 and 2018 from participants (aged 65-95) who completed all the measurement items used for this analysis (N=981). SA was indexed by asking participants to specify in years how old they felt. Proportional discrepancy scores ((subjective age - chronological age)/chronological age ×100) were calculated to indicate younger or older SAs and used as a dependent variable. As influencing factors of SA, chronological age, sex, years of schooling, history of smoking, cognitive function (using MMSE scores, range 14-30 at baseline), depressive symptoms, physical function (gait speed), and social function (employment status) were examined. Analyses were performed with random-effects GLS regression models. Results: Significant partial regression coefficients were found for cognitive function (0.48%, CI: 0.18, 0.79), years of schooling (-0.42%, CI: -0.69, -0.15), depressive symptoms (0.32%, CI: 0.11, 0.53), and chronological age (-0.18%, CI: -0.30, -0.68). Implications: This study found that older age and longer years of schooling were associated with younger SA, while better cognition and depressive symptoms were linked to older SA. Better cognition being associated with older SA was inconsistent with existing studies. This may be due in part to the association of better cognition and the level of satisfaction influenced by awareness of age-related physical/social changes.

### ADVANCING OUR UNDERSTANDING OF ELDERHOOD: A NARRATIVE REVIEW

Jenny Inker,[1] Tracey Gendron,[1] and Marshall Brooks[2], *1. Virginia Commonwealth University, Richmond, Virginia, United States, 2. Virginia Commonwealth, Richmond, Virginia, United States*

There are relatively few explorations of later life in the peer reviewed gerontological literature that holistically embrace the duality of potential and decline. This is in striking contrast to a growing body of non-scholarly literature, frequently authored by elders, displaying deep interest in the phenomenon of elderhood, i.e. the holistic, lived experience of later life. We conducted a narrative review with the aim of describing the state of the science with regard to the bio-psycho-social-spiritual experience of elderhood. Following a search of multiple databases for English language, peer reviewed articles published from 2000-2017, we identified 24 articles in the disciplines of gerontology, anthropology, psychology, the humanities, and spirituality studies, reflecting elderhood in Eastern and Western cultures. While the articles offered no shared operational definition of elderhood, nor applied any unifying conceptual or theoretical structures, several common themes emerged. These included the description of elderhood as both inward facing (inner development) and outward facing (social contributions of elders). Numerous articles also recognized that ageism socially mediates the experience of elderhood, resulting in a failure of social systems and structures to recognize or provide opportunities for lifelong growth in later years, including a lack of mentors and role models for individuals transitioning into elderhood. This review demonstrates that there is a compelling need for the discipline of gerontology to strengthen our understanding of the phenomenon of elderhood by leading on the development and implementation of theoretically driven empirical research into the subject of the holistic, lived experience of later life.

### TRAJECTORIES OF POSITIVE AND NEGATIVE AFFECT: A COORDINATED ANALYSIS OF 11 LONGITUDINAL SAMPLES

Emily D. Bastarache,[1] Eileen K. Graham,[2] Ryne Estabrook,[2] Anthony Ong,[3] Andrea Piccinin,[4] Scott Hofer,[4] Avron Spiro III,[5] and Daniel K. Mroczek[2], *1. Northwestern University, Evanston, Illinois, United States, 2. Northwestern University, Chicago, Illinois, United States, 3. Cornell University, Ithaca, New York, United States, 4. University of Victoria, Victoria, B.C., United States, 5. Boston University, Boston, Massachusetts, United States*

This study assessed age-graded change in positive and negative affect over decades of the lifespan. We conducted a coordinated integrative data analysis (IDA) using data from 11 longitudinal samples, comprising a total of 74076 respondents, spanning the ages of 11 to 106. Positive and negative affect were measured using the CES-D in 8 studies, the PANAS in 3 studies, and the MIDI scale in the MIDUS with three to eleven measurement occasions across studies. To assess and compare the extent and nature of change in affect over time across studies, analyses were coordinated, deploying identical multi-level growth models on each dataset. The curvilinear models suggested PA was best characterized by an inverted U-shaped trajectory, peaking in the mid-to-late 50s, while change in NA was best described by a U-shaped curve, bottoming out in the late 60s. We also found measure-related differences in the proportion of variance in affect attributable to within- or between person differences; The majority of the variability in CES-D-assessed affect was attributable to within-person differences over time, while the variability in PANAS-assessed affect was predominantly attributable to between-person differences. Overall, the results did not support steady improvement of emotional experience over the entire life-course as previous studies have suggested, but show promise for midlife when PA peaks and NA bottoms out. This study demonstrates the value of coordinated conceptual replications, resolving some of the mixed findings in the literature regarding age-graded change in affect and enhancing the current understanding of the longitudinal affect phenomenon.

### SOCIAL CHARACTERISTICS OF THE FOURTH AGE

John Pothen,[1] Emily C. Dore,[1] and Ellen Idler[1], *1. Emory University, Atlanta, Georgia, United States*

How can we differentiate distinct phases of aging in later life? Theorizations of the third and fourth age posit that later life often involves a time of continued growth and increased opportunity (the “third age”) as well as a time marked by growing cognitive, physical, and social losses (the “fourth age”). In contrast to population-based definitions that place this transition around the age of 80, a person-based definition using frailty as a marker offers more sensitivity by focusing on ability and agency instead of age alone. In this study, we apply both definitions in order to examine the social characteristics of the fourth age. Using a nationally representative sample of adults over the age of 65 from from the National Health and Aging Trends Study (NHATS) seventh round (n=6,312) we find that the population-based definition overestimates the number of adults in the fourth age (2,834 vs 569; p<0.001). Additionally, social network patterns observed when comparing adults above and below the age of 80 - increased rates of including a daughter or son and a decreased rate of including a friend - are not seen when comparing adults who do and do not meet criteria for frailty. Our findings suggest that common understandings of the social characteristics of the oldest old - understandings with important implications for policy and the promotion of human dignity - may be biased by focusing on age alone as a marker of change instead of ability and agency.

### IDENTIFYING THE TRAJECTORIES OF MULTIMORBIDITY AND THEIR CORRELATES AMONG KOREAN OLDER ADULTS

Sun Ah Lee,[1] Hey Jung Jun,[1] Susanna Joo,[1] and Hye Won Chai[2], *1. Yonsei University, Seoul, Korea, Republic of, 2. The Pennsylvania State University, University Park, Pennsylvania, United States*

Multimorbidity, the co-existence of two or more chronic diseases, has become prevalent among the older population. This study focused on identifying different patterns of multimorbidity trajectories across older adulthood and examining their predictors. We used six waves of the Korean Longitudinal Study of Aging (KLoSA), a nationally representative longitudinal data collected every two years from 2006 to 2016. The sample was older adults aged 65 years and older in 2006 (N=1,668). Multimorbidity was measured as the self-reported number of medically-diagnosed chronic diseases, and Growth Mixture Modeling was used to examine multimorbidity trajectories. Identified patterns of multimorbidity trajectories were then used as outcome variables in multinomial logistic regression models. Independent variables were socio-demographic, psychological, health-related behavioral and interpersonal factors at baseline. At Wave1, 76% of the sample had no or one chronic disease and 24% had two or more. At Wave6, 49% had none or one and 51% had two or more. Results identified four patterns of multimorbidity trajectory: “maintaining-low” (59.1%; reference), “maintaining-high” (7.3%), “moderately increasing”(26.4%), and “rapidly increasing” (7.2%). In terms of the correlates of these patterns, female older adults and respondents with higher depressive symptoms were more likely to be in the “maintaining-high” group. In addition, respondents who had less frequent meetings with friends, neighbors or relatives were more likely to be in the “rapidly increasing” group. The findings suggest that there are distinct patterns of multimorbidity trajectories across older adulthood, and interventions focusing on depressive symptoms or social engagement may be useful in preventing the increase in multimorbidity.

### WAR COHORT DIFFERENCES IN MILITARY SERVICE APPRAISALS AND HOMECOMING EXPERIENCES

Dylan Lee,[1] Soyoung Choun,[1] Maria Kurth,[1] Hyunyup Lee,[2] and Carolyn M. Aldwin[2], *1. OSU Oregon State University, Corvallis, Oregon, United States, 2. Oregon State University, Corvallis, Oregon, United States*

Nearly all the research on appraisals of military service and homecoming experiences have been done on World War II veterans. However, Spiro et al. (2016) hypothesized that there were war cohort differences in military experiences that could affect life-long adaptation. For example, Boscarino et al. (2018) found that Vietnam veterans reported less welcoming homecoming experiences than OEF/OIF/OND veterans. We examined war cohort differences among OEF/OIF/OND, Persian Gulf, and Vietnam combat veterans in military service appraisals and homecoming experiences. We used pilot data from Veterans Aging: Longitudinal studies in Oregon (VALOR) from an online survey. The sample included male and female combat veterans (Mage = 58.1, SD = 12.0, range = 35-83, 30.5% female): 39 from the OEF/OIF/OND, 68 from the Persian Gulf War, and 60 from the Vietnam War cohorts. Comparable to earlier studies (e.g., Aldwin et al., 1994), combat veterans were surprisingly much more likely to endorse desirable appraisals than the undesirable ones, with each of the 14 desirable appraisals endorsed by over 90% of the veterans. Fewer endorsed the undesirable experience items; the most common was separation from loved ones and loss of friends. Most also reported positive homecoming experiences. Contrary to expectations, ANOVAs revealed that there were no significant differences in appraisals of desirable and undesirable military service experiences, nor in homecoming experiences among the war cohorts. In this small sample, military experiences were perceived similarly among combat veterans despite differences in wartime experiences. Most felt that positive experiences resulted from their desirable military service.

### COHORT DIFFERENCES IN CHANGES IN LIFE SATISFACTION AMONG OLDER JAPANESE

Takeshi Nakagawa,[1] and Erika Kobayashi[2], *1. National Center for Geriatrics and Gerontology, Aichi, Japan, 2. Tokyo Metropolitan Institute of Gerontology, Tokyo, Japan*

Life span research has been interested in how sociocultural contexts shape individual development and aging processes. Empirical studies have reported that later cohorts show higher levels of well-being. However, more recent studies indicate that cohort differences are not sustained in very late life. The present study examined whether cohort differences in well-being, as measured by life satisfaction, are observed in the young-old and old-old, and further explored potential determinants of cohort differences. Using longitudinal data from a nationally representative study of older Japanese, conducted from 1987—2002, we compared earlier- and later-born cohorts in the young-old (N = 874; age 60—65; year of birth: 1922—1927 and 1931—1936) and old-old (N = 1,022; age 70—80; year of birth: 1907—1917 and 1919—1929), respectively. To control for covariates, we used case-matched groups based on age and gender. Results revealed that later cohorts exhibited higher levels of life satisfaction in both age groups. In the young-old, life satisfaction declined across cohorts. In the old-old, life satisfaction remained stable among earlier cohorts but declined among later cohorts. Socioeconomic, social, and health factors at the individual level and methodological factors (i.e., number of observations) did not fully explain the cohort differences in both age groups. Our results suggest that historical increases in levels of well-being are observed in late life, but that these improvements do not hold in very late life. Future studies should consider potential societal factors behind observed cohort differences in well-being.

### SESSION 3345 (POSTER)

### LONG TERM CARE: ORGANIZATIONAL AND STAFFING ISSUES

### KNOWLEDGE MANAGEMENT AND THE ADOPTION OF CULTURE CHANGE INITIATIVES BY HIGH-MEDICAID-CENSUS NURSING HOMES

Larry Hearld,[1] Akbar Ghiasi,[1] Jeffery Szychowski,[1] and Robert Weech-Maldonado[1], *1. University of Alabama at Birmingham, Birmingham, Alabama, United States*

Culture change represents an organizational transformational process to become person-centered, through staff and resident empowerment. Culture change initiatives have been associated with fewer health-related deficiency citations and better psychosocial outcomes. Knowledge management, defined as the process of creating or locating knowledge, and managing the dissemination of knowledge within and between organizations, has been shown to be associated with the adoption of innovations such as culture change initiatives. This study examines the relationship between knowledge management activities of high Medicaid census (70% or higher) nursing homes (NHs) and the adoption of culture change initiatives. This study used facility survey data from approximately 324 nursing home administrators (30% response rate) from 2017- 2018, merged with data from LTCFocus, Area Health Resource File, and Medicare Cost Reports. Binary logistic regression models revealed that the probability of adopting a culture change initiative was 0.12 higher for facilities reporting a one-unit higher level of knowledge management activities. Additional interaction analysis revealed that knowledge management activities were associated with a greater likelihood of adopting a culture change initiative for NHs where the director had been in his/her position fewer years. Similarly, higher levels of overall knowledge management activities were significantly associated with greater adoption of culture change initiatives at intermediate levels of nurse retention. Results suggest that knowledge management activities may help high Medicaid NHs acquire and mobilize informational resources in ways that can support the adoption of patient-centered initiatives. These activities may be particularly effective in nursing homes with leadership and nursing staff instability.

### THE NATURE OF PEER WORKPLACE INCIVILITY AND BULLYING AMONG RESIDENTIAL CARE AIDES IN LONG-TERM CARE

Heather A. Cooke,[1] Kaitlin Murray,[2]
Jennifer Baumbusch,[1] and Lisa Kelly[3], *1. University of British Columbia, Vancouver, British Columbia, Canada, 2. Simon Fraser University, Vancouver, British Columbia, Canada, 3. Sienna Senior Living, Vancouver, British Columbia, Canada*

Residential care aides (RCAs; unregulated workers also known as certified nursing assistants or personal care assistants) provide much of the hands-on care in long-term residential care (LTRC). While many RCAs report being exposed to violent or aggressive acts from residents, we know little about their exposure to incivility and bullying from their colleagues. This is a significant knowledge gap as increased workplace incivility and bullying is associated with specific gender-dominated fields, hierarchical and stressful work environments, and low job autonomy, all of which characterize LTRC. Drawing on data from a critical ethnography examining workplace incivility and bullying among RCAs in a rural, not-for-profit care home, this presentation explores the types of incivility and bullying encountered by RCAs, and the contextual factors impacting their experiences with such behaviors. To date, more than 50 hours of participant observation, and 20 in-depth interviews with RCAs, licensed practical nurses, support staff, and management have been conducted. Findings illustrate the pervasiveness of incivility; while bullying acts (repeated, hostile behaviors intended to undermine, humiliate or injure) were rare, incivility (low-intensity acts with an ambiguous intent to harm) was an almost daily occurrence. Commonly-occurring behaviors included ignoring and refusing co-worker's requests for help, social exclusion, acting impatient with, blaming and criticizing co-workers, and insisting on getting one's own way. Chronic staffing shortages, staffing arrangements, and workload issues exacerbated RCAs' experiences. Findings provide an important first step in understanding the nature of workplace incivility and bullying in LTRC.

### THE RELATIONSHIP BETWEEN ORGANIZATIONAL CULTURE AND FINANCIAL PERFORMANCE AMONG HIGH-MEDICAID NURSING HOMES

Robert Weech-Maldonado,[1] Akbar Ghiasi,[1]
Ganisher K. Davlyatov,[1] Justin C. Lord,[2] and
Jane Banaszak-Holl[3], *1. University of Alabama at Birmingham, Birmingham, Alabama, United States, 2. Louisiana State University at Shreveport, Shreveport, United States, 3. Monash University, Melbourne, Victoria, Australia*

This study examines the relationship between organizational culture and financial performance of high Medicaid census (70% or higher) nursing homes (NHs). Based on the Competing Values Framework, there are four types of organizational culture: clan culture (friendly working environment); adhocracy culture (dynamic/creative working environment); market culture (results-based organization); and hierarchy culture (formalized/structured work environment). This study used facility survey data from approximately 324 nursing home administrators (30% response rate) from 2017- 2018, merged with secondary data from LTCFocus, Area Health Resource File, and Medicare Cost Reports. The dependent variable consisted of the operating margin, while the independent variable comprised type of organizational culture. Control variables were organizational (ownership, chain affiliation, size, occupancy rate, and payer mix), and county-level factors (Medicare Advantage penetration, income, education, unemployment rate, poverty, and competition). Multivariable regression was used to model the relationship between organizational culture type and financial performance. Regression results show that compared to a market culture, a hierarchy culture was associated with an 11.8 % lower operating margin, a clan culture with a 10.6% lower operating margin, and a non-dominant culture with 11.4% lower operating margin. Organizational culture is associated with financial performance among high Medicaid facilities, with market cultures outperforming other organizational cultures. Given increasing competition in the nursing home market and declining resources for high Medicaid nursing homes, facilities with a more external orientation and focus on results may be able to perform better financially. Future research should examine the effect of organizational culture on quality of care.

### SUPPORTING NURSING HOME STAFF THROUGH PERSON-CENTERED CARE PRACTICES

Diana White,[1] Sarah Dys,[2] Jaclyn Winfree,[1]
Serena Hasworth,[1] and Ozcan Tunalilar[1], *1. Institute on Aging, Portland, Oregon, United States, 2. Portland State University, Portland, Oregon, United States*

Policies and practices have increasingly focused on person-centered care (PCC) to improve quality of life for long-term care residents and staff. Adequate staffing has been a consistent barrier to implementing and sustaining PCC practices. The purpose of this paper is to explore the association between job satisfaction and PCC practices. This research was conducted in a stratified random sample of 33 Oregon nursing homes which were representative in terms of quality, profit/nonprofit ownership, and urban/rural location. Data were collected from 415 staff who completed the staff assessment of person-directed care, direct care worker job satisfaction scale, turnover intention, and organizational belongingness. Consistent with other research, job satisfaction is significantly and negatively correlated with turnover intention (r=-.66) and positively associated with belongingness (r=.66). It is also significantly correlated with scales related to five PCC practices: personhood, autonomy, knowing the person, individualized care, and relationships. Regression analyses examined how these five aspects of PCC practices were associated with 1) job satisfaction and 2) number of deficiencies. Perceptions of practices to support autonomy, personhood, and relationships were associated with higher ratings of job satisfaction among staff. In general, those reporting these practices were in place at least half of the time or with at least half of the residents, showed significantly greater positive associations with job satisfaction (p<.05). Only lower staff reports of autonomy practices were associated with higher deficiencies (p<.05). Findings from this research suggest that supporting PCC practices benefit staff through increased job satisfaction and potentially reduced turnover.

### EVACUATING OR SHELTERING IN PLACE DURING A DISASTER: HOW ASSISTED LIVING ADMINISTRATORS MAKE THE DECISION

Lindsay J. Peterson,[1] Kathryn Hyer,[2] David Dosa,[3] Joseph June,[4] Debra J. Dobbs,[2] and Kali S. Thomas[5], *1. University of South Florida School of Aging Studies, Tampa, Florida, United States, 2. University of South Florida, Tampa, Florida, United States, 3. Brown University School of Public Health, Providence, Rhode Island, United States, 4. School of Aging Studies, University of South Florida, Tampa, Florida, United States, 5. Providence VA Medical Center, Providence, Rhode Island, United States*

The decision to evacuate or shelter in place during a natural disaster such as a hurricane is complicated and poses risks to long-term care residents. While research has documented the difficulty of the evacuation decision for nursing home administrators, little is known about how assisted living residence (ALR) administrators make this decision. This is a concern given the physical and cognitive impairment level of many ALR residents, the increasing number of ALRs in the U.S., and the frequency of natural disasters. The purpose of this paper was to explore the factors that influenced whether assisted living administrators evacuated their ALRs for Hurricane Irma, a large hurricane that made landfall on Florida's Southwest coast in September, 2017. This qualitative study used semi-structured interviews and focus groups with ALR owners or administrative staff (N=60) with questions including how they prepared for Hurricane Irma, their experiences during the hurricane, including whether they evacuated or sheltered in place, and lessons learned. The sample includes small (< 25 beds) and large ALRs in the multiple Florida counties affected by the hurricane. A content analysis approach was used. Atlas.ti version 7 was used for initial and axial coding. Prevalent themes included "emergency management planning", "logistics", "pressure", "storm characteristics," and "staffing". The results of this study have implications for long-term care policy and training, potentially leading to changes in how ALR leaders prepare for and respond to disasters to improve the safety of residents.

### ORGANIZATION OF WORK AND DECISION-MAKING OF PROFESSIONALS WORKING IN NURSING HOMES IN CATALONIA (SPAIN)

Montserrat Gea-Sánchez,[1] Alvaro Alconada-Romero,[1] Roland Pastells-Peiró,[1] Filip Bellon,[1] Lourdes Teres-Vidal,[2] Joan Blanco-Blanco,[1] and Katherine McGilton[3], *1. Faculty of Nursing and Physiotherapy, University of Lleida, Lleida, Spain, 2. Col legi Oficial d'infermeres i infermers de Lleida, Lleida, Spain, 3. KITE, Toronto Rehab, University Health Network, Toronto, Ontario, Canada*

The long term care environment demands specific requirements of the staff, namely that they provide a holistic approach to care. Providing holistic care leads to complex decision-making processes which go beyond just finding a solution to a specific health problem. It requires that staff are able to respond to the diverse needs expressed by the residents and which, in many cases, are only identified through the relationship that professionals have with them. However, this relationship that staff establishes with the resident often leads to burden for these professionals. The researchers sought to identify characteristics of the nursing homes that lead to positive outcomes for staff. The study involves collecting questionnaires (n=132) and conducting semi structured interviews (n=35) in 9 Catalan nursing homes with number of staff, utilizing a quantitative questionnaire and semi-structured interviews. Practices in organisations that led to positive outcomes for staff included coordinated care that includes processes of support for staff, effective communication and decision making practices, clear responsibilities for staff, and utilization of care plans. Effective long term care practices can favour both patient care and professional practice in residences for elderly.

### A CASE STUDY: THE INVISIBLE LABOR OF CULTURALLY SENSITIVE CARE

Mai See Thao,[1] Odichinma C. Akosionu,[2] Heather Davila,[3] and Tetyana P. Shippee[2], *1. Medical College of Wisconsin, Milwaukee, United States, 2. University of Minnesota, Minneapolis, Minnesota, United States, 3. VA Boston Healthcare System, Boston, Massachusetts, United States*

Nursing homes are increasingly becoming more racially/ethnically diverse yet racial disparities in resident's quality of life and quality of care continue to persist. One reason for these disparities is lack of culturally-sensitive care and racial/ethnic similarity between residents and staff. This study examines a case of a high proportion minority nursing home with racially/ethnically diverse staff to understand how shared culture among direct care staff and residents may influence care delivery. We used three months of participant observation, supplemented by in-depth qualitative interviews with 8 Hmong residents and 5 Hmong staff to explore the labor of culturally sensitive care in a large, urban NH. We discovered four themes: 1) Culturally sensitive care was often equated to fulfilling language needs for residents who didn't speak English. 2) Hmong staff members had to take the initiative to inform non-Hmong staff members how to care for Hmong residents. 3) Hmong staff members also had to communicate the culture of NH care and its limitations to Hmong residents and their families. 4) Hmong staff members have to advocate for the culturally relevant needs of Hmong residents. The findings of this case study illuminate that having staff members from diverse cultural backgrounds and meeting language needs of residents does not reflect the everyday practices of culturally sensitive care. This type of emotional labor can also result in higher levels of burn-out for staff of color. Additional research into what constitutes culturally sensitive care to NH residents and staff is needed.

### THE IMPORTANCE OF CREATING SHARED EXPECTATIONS FOLLOWING NURSING HOME DISCHARGE

Whitney L. Mills,[1] Nathan Kerr,[2] and Kelsey Simons[3],
*1. Center for Innovation in Long-Term Services and Supports, Providence VA Medical Center, Providence, Rhode Island, United States, 2. Uniformed Services University of the Health Sciences, North Bethesda, Maryland, United States, 3. VISN 2 Center of Excellence for Suicide Prevention, Canandaigua, New York, United States*

Nursing home stays often represent a time of change for residents and their social networks (e.g., family, friends, and other social supports). Nursing home residents may be experiencing exacerbations of medical conditions, changes in physical functioning, and/or cognitive impairment. These changes often preclude the resident from returning to their pre-admission level of functioning, resulting in the need for adjustments for both residents and their social network. However, nursing home staff and residents/caregivers frequently have different expectations for post-discharge functioning. This may be the result of inadequate or unclear communication on the part of nursing home staff and/or residents and caregivers being unable or unwilling to internalize the information. The lack of shared expectations can leave residents and caregivers unprepared, creating unanticipated caregiving burdens for the social network and unwanted outcomes for the resident. We conducted interviews with 14 resident/caregiver dyads who experienced care transitions from VA nursing homes to examine expected and actual functioning and activities post-discharge. Using a qualitative content analysis approach, we identified several themes including marital strain related to mismatched expectations, impact of physical changes on Veterans' social functioning, and differences between planned and actual post-discharge activities. We will discuss the importance of clear communication about expectations throughout the nursing home stay. We will also provide suggestions for improving discharge care planning to ensure creation of shared expectations and timely communication to allow residents and their social networks to adequately prepare for nursing home discharge.

### A QUALITATIVE STUDY OF NURSING HOME STAFF CONCEPTUALIZATION OF EVERYDAY DECISION-MAKING CAPACITY

Whitney L. Mills,[1] Mark E. Kunik,[2] Lea Kiefer,[3]
Hannah Curren-Vo,[1] Amy Mochel,[1] and Aanand Naik[4],
*1. Center for Innovation in Long-Term Services and Supports, Providence VA Medical Center, Providence, Rhode Island, United States, 2. Baylor College of Medicine, Houston, Texas, United States, 3. Michael E. DeBakey VA Medical Center, Houston, Texas, United States, 4. Center for Innovations in Quality, Effectiveness and Safety, Houston, Texas, United States*

Because nursing homes are both a residence and a treatment setting, care providers are faced with the challenge of balancing resident autonomy and safety on a daily basis. While there are standardized approaches for determining a capacity to make larger decisions such as providing consent for medical procedures, there are virtually no methods for assessing capacity to make everyday decisions (e.g., food choices, smoking, navigating outside the nursing home). While it is easier for staff to prevent residents from making decisions they deem risky, to truly offer person-centered care, it is important to support a resident's right to make decisions if they have the capacity to do so. Currently, little is known about how nursing home staff conceptualize and determine everyday decision-making capacity and how that information is used in care planning. To understand the current processes and language nursing home staff use when considering a resident's decision-making capacity, we conducted interviews with 37 staff at two Veterans Affairs (VA) Community Living Centers (VA-operated nursing homes; CLCs). Using qualitative content analysis, we coded the transcribed interviews and identified several overarching themes: autonomy vs. safety, communication (e.g., pathways, with caregivers, with residents), determining capacity (e.g., information gathering, assessment, assumptions, indicators, interdisciplinary team member roles, referrals), interventions (e.g., legal and staff-led), and terminology used. We will describe how the findings from this study can be used to tailor development and adaptation of tools to help nursing home staff assess resident everyday decision-making capacity and to incorporate the results into person-centered care approaches.

### ORGANIZATIONAL CHARACTERISTICS OF HIGHLY PERSON-CENTERED UNITS IN SWEDISH NURSING HOME

Anders B. Sköldunger,[1] and Annica Backman[2],
*1. Department of Nuring, Umeå, Sweden, 2. Departement of Nursing Umea University, Umea, Sweden*

The movement from an institutional model of care towards a person-centred care as the gold standard of practice is now guiding the provision of care services in nursing homes around the world. The organizational context of care has been described as a determining factor for the extent to which staff can offer person-centred care. However, few studies have empirically investigated which factors that defines nursing home units as being person-centred. Providing information about organizational characteristics would therefor provide insight into an organizational context with capacity to enhance a person-centred care. Thus, the aim was to explore factors of nursing homes with high vs. low person-centred care with focus on organizational variables. The study was based on a cross-sectional national survey, and data on 4831 residents, 3605 staff, and facility variables were collected in 2014. Descriptive statistics and regression modelling were used to analyze the data. The preliminary results showed that characteristics of highly person-centred units were; dementia specific units and units with fewer number of beds. No significant differences were seen between private and public nursing homes in terms of degree of person-centred care. Person-centred units was characterized by managers supporting staff to provide individualized care based on the resident's needs, as well as staff receiving supervision of a reg. nurse in the direct care. These findings can be seen as facilitators ' for person-centred care, suggesting several contextual and organizational elements of significance for enhancing person-centred practice.

### THE INFLUENCE OF STAFF RETENTION ON NURSING HOME QUALITY

Nicholas Castle,[1] Kathryn Hyer,[2] and John A. Harris[3], *1. WVU, Morgantown, United States, 2. University of South Florida, Tampa, Florida, United States, 3. University of Pittsburgh, Pittsburgh, Pennsylvania, United States*

The association of retention of Nurse Aides (NAs) with nursing home quality of care is examined. Retention is defined as staff continuously employed in the same facility for a defined period of time. Deficiency citations were used as quality indicators. Data used came from a survey of nursing home administrators, the Certification and Survey Provider Enhanced Reporting (CASPER) data, and the Area Resource File. All of the data was from 2015, and included 3,550 facilities. Analyses included negative binomial regression and multivariate logistic regression models (using GEE). The analytic modeling included staffing variables (turnover, agency use, staffing levels), facility factors (size, ownership, occupancy rate), and market characteristics (competition, Medicaid rates). The average number of deficiency citations was significantly lower ($p<.01$) in facilities with the higher levels of NAs consistently employed for one year or more. The same was found for facilities with the higher levels of NAs consistently employed for two years or more. While the average number of deficiency citations, the quality of care grouping of deficiency citations, and J, K, L deficiency citations were all significantly lower ($p<.01$) in facilities with the higher levels of NAs consistently employed for three years or more. Staff retention has been promoted as potentially influential based on little empirical evidence. The findings provide some justification for the importance of NA retention.

### LACK OF POLICIES, TRAINING, AND SPECIAL CARE UNITS FOR OBESITY CARE IN PENNSYLVANIA NURSING HOMES

John A. Harris,[1] and Nicholas Castle[2], *1. University of Pittsburgh, Pittsburgh, Pennsylvania, United States, 2. WVU, Morgantown, United States*

It is unclear how nursing homes in the U.S. prepare for the specific needs of residents with obesity at a population level in terms of equipment availability, policies, staff training, and special care units. Using a mail survey of Directors of Nursing (DON) to 420 Pennsylvania Nursing Homes in 2017 and 2018, we examined the reported presence of obesity-specific equipment availability, organizational policies, staff training, and special care units. We compared the presence of these adaptation approaches by whether the DON strongly agreed that obesity was a problem for resident and staff safety using $\chi 2$ tests. One hundred fifty-one surveys were returned and included in the analysis (response rate of 36%). 80.7% of respondents were, on average, very concerned when asked about 11 resident medical, functional, relational, and staff-related safety outcomes (e.g., pressure ulcers, hospital readmissions, social isolation, and staff injury). DONs reported reduced equipment availability in nursing homes for obesity-specific beds (66%), walkers (34%), bedside commodes (30%), and gowns (28%). The presence of obesity-specific organizational policies (44%), staff training (26%), and special care units (7%) was limited. DON strong agreement with obesity-related resident and staff safety issues was significantly associated with obesity-specific bed availability ($p=0.04$) but was not significantly associated with obesity-specific organizational policies ($p=0.17$), staff training ($p=0.51$), and special care units ($p=0.09$). Despite a high concern for resident and staff safety related to obesity care expressed by DONs, there is little appropriate nursing home organizational response as measured by policies, staff training or special care units.

### STAFF PERCEPTIONS OF INVOLUNTARY NURSING HOME CLOSURE AND RELOCATION PROCESSES

Raven H. Weaver,[1] Karen A. Roberto,[2] Nancy Brossoie,[2] and Pamela B. Teaster[2], *1. Washington State University, Pullman, Washington, United States, 2. Virginia Tech, Blacksburg, Virginia, United States*

Involuntary nursing home closures happens infrequently, but when they do occur, they impact residents, their family, and facility staff. During the transition, residents' care needs are of primary concern, yet few studies have examined the centrality of the actions of staff to residents' relocation adjustment. This paper examined staff perceptions of the involuntary relocation process for 132 residents after a facility lost its Medicaid certification because of low quality performance. Interviews were conducted with 34 staff (e.g., administrators, nurses, social workers) from 21 receiving facilities. Using content analysis, we identified challenges that hindered relocation and affected resident/family experiences. Receiving facility staff perceived undue distress and hardship on residents and family members because of inadequate notification about the situation. Limited, untimely, and poor communication led to residents being uninformed or unprepared for moving. The efficiency and effectiveness of the resident discharge process was also viewed as unacceptable. Minimal documentation in residents' charts hampered the coordination of resident moves. Receiving facility staff offered recommendations for decertified facilities and receiving facilities to improve the relocation experience including the need for open communication, thoughtful and early engagement in the process, and transparent and timely interactions. Findings suggest that staff are well-positioned for active involvement in the relocation process and should facilitate deliberate and strategic planning, decision-making, and communication with residents and their relatives. Resident-centered policies are needed to improve the involuntary relocation process and give voice to remaining/receiving staff, both of whom are integral to residents' support system.

### DEMENTIA CARE FOR RESIDENTS IN ASSISTED LIVING: PERSPECTIVES OF STAFF RESPONDING TO BEHAVIORAL EXPRESSIONS

Debra J. Dobbs,[1] Sheryl Zimmerman,[2] Anna Beeber,[2] Paula Carder,[3] Stephanie Miller,[2] Jennifer Hodgkinson,[4] and Julia Thorp[2], *1. University of South Florida, Tampa, Florida, United States, 2. Cecil G. Sheps Center for Health Services Research, Chapel Hill, North Carolina, United States, 3. Portland State University Institute on Aging, Portland, Oregon, United States, 4. Cecil G. Sheps Center for Health Services Research, Chapel Hill, United States*

Background: An estimated 42% of persons who live in assisted living (AL) have dementia. The majority of these individuals express behaviors (such as agitation, aggression, and wandering) that indicate a mismatch between their ability to cope and demands in the social and physical environment. In some cases, AL staff are able to successfully address those behaviors and in other cases they are not. This study explores behavioral expressions of persons with dementia residing in AL, strategies used to address those behaviors, and residents' behavioral results, as reported by 251 AL healthcare supervisors across seven states. We also examine what differentiates situations deemed successful from situations that were not successful. Methods: Qualitative interviews conducted with healthcare supervisors revealed cases of successful and unsuccessful strategies for addressing severe/disruptive behavioral expressions of persons with dementia residing in AL. During initial analysis, a data-driven conceptual model was developed to identify common structural domains within and across responses, which ranged from recognizing antecedents to final discharge from the AL community. Additionally, content analysis was applied to identify themes. Results: A minority (<5%) of reports indicated that staff recognized antecedents to behaviors, or noted including residents' families in addressing behaviors. The majority of both successful and unsuccessful cases referenced the use of medications to address behaviors, and a notable proportion (10%) referenced professional psychiatric assessment. Discussion: Findings suggest the benefit of helping staff identify antecedents of behavioral expressions, and the important role of psychiatric assessment for AL residents who experience agitation, aggression, and similar behaviors.

### DIRECT CARE WORKERS EMPLOYED BY PRIVATE HOUSEHOLDS: A DISTINCT LABOR FORCE

Christopher M. Kelly,[1] Jerome Deichert,[1] and Lyn Holley[2], *1. University of Nebraska at Omaha, Omaha, Nebraska, United States, 2. University of Nebraska, Omaha, United States*

Purpose: This study tracks the growing number of direct care workers (DCWs) employed by private households and describes the differences between this often ignored labor force and DCWs employed by agencies. Design and Methods: Data were from the 1% Public Use Microdata Sample (PUMS) of the 2000 and 2017 American Community Survey (ACS). Logistic regression was used to compare demographic and employment characteristics of DCWs employed by private households and DCWs employed by agencies, which include outpatient care centers, home health care services, and individual and family services. Results: Between 2000 and 2017, the number of DCWs employed by private households in the U.S. increased 32% and the majority of this growth was since 2007. Compared to DCWs employed by agencies, DCWs employed by private households were more likely to be over age 65, white, unmarried, have higher educational attainment, be more likely to be in poverty, receive health insurance from Medicare or direct-pay. DCWs employed by private households were less likely to be under age 25, nonwhite, Hispanic, speak a language other than English, work year-round and full-time, receive health insurance from an employer or through Medicaid, and have a disability. Implications: DCWs employed by private households represent a small, but growing proportion of the long-term care (LTC) workforce in the U.S. Further, these workers are distinct within the LTC workforce. This has important implications both for DCWs and for families, particularly those with limited LTC options due to location, financial resources, family support, or other factors.

### STAFFING AND CARE QUALITY OF NURSING HOMES IN KOREA

Hee Seung Lee[1], *1. National Health Insurance Service, Health Insurance Policy Research Institute, Wonju, Korea, Republic of*

Staffing has been regarded as the most important factor for the quality of care service in nursing homes. Korea introduced Long-Term Care Insurance (LTCI) in 2008. The payment system of LTCI has incentivized LTC facilities based on the staffing level of LTC facilities. This study aims to investigate whether staffing is associated with quality of care. The effect of staffing on care quality was assessed using ordered logit analysis. Staffing data in 2015 were retrieved from claim data in the National Health Insurance Service. The publicly reported care service quality grade in 2015 was used as a proxy for care quality. Staffing of registered nurses (RN) and social workers were strongly associated with the care quality. As the number of RNs per residents additionally increased, the LTC facilities were more likely to receive better grades (OR=16851.54, p<0.000). The effect of social workers' staffing was significant for the care service quality, even though the effect size of smaller than that of RNs (OR=345.87, p<0.000). However, staffing in other professions such as nurse assistants (NA) and personal care workers (PCW) was insignificantly associated with care quality. The effect of staffing on service quality might not be profession-neutral. RN staffing affects most in care quality in Korea. Still, the possibility remains that PCWs or NAs could serve for indirect care services such as cooking or cleaning because of short staffing in indirect care services. This finding could be considered when designing financial incentives for nursing homes in Korea as well as other countries.

### ORGANIZATIONAL AND POLICY FACTORS INFLUENCING FEEDING IN RESIDENTS WITH DEMENTIA: NURSING ASSISTANTS' EXPERIENCES

Joy W. Douglas,[1] Seung Eun Jung,[1] Hyunjin Noh,[2] Amy Ellis,[1] and Christine Ferguson[1], *1. University of Alabama, Tuscaloosa, Alabama, United States, 2. The University of Alabama School of Social Work, Tuscaloosa, Alabama, United States*

In the United States, Certified Nursing Assistants (CNAs) are critical in providing direct care to nursing home residents with dementia, which includes the challenging task of feeding residents. Guided by the Social Ecological Model (SEM), this qualitative study aimed to gain an in-depth understanding of organizational and policy constructs that CNAs encounter when feeding residents with dementia. Using purposive sampling, nine semi-structured focus groups were conducted with 53 CNAs. Each participant had at least one year of experience working as a CNA with older adults. Focus groups were audio recorded and transcribed verbatim. Data were analyzed using the directed content analysis. Factors that emerged were organized into organizational and policy categories within the SEM levels. CNAs identified organizational barriers such as exclusion from the interdisciplinary team, inability to meet resident needs and wants due to budgetary constraints, and inadequate staffing to function efficiently. Organizational facilitators included teamwork, interdisciplinary assistance, and varying dining styles and meal times to accommodate resident needs. Policy-related barriers included funding concerns, staffing ratios, and frustration with unrealistic regulations and state inspections. These results suggest that organizational and policy factors have a large influence on the ease of feeding nursing home residents with dementia. Involving CNAs in interdisciplinary collaboration, resident-centered accommodations, and subtler state inspection behaviors could improve the mealtime experience for both residents and CNAs. Careful attention to these factors may enhance facilitators and minimize barriers to improve the feeding experience of CNAs and residents with dementia.

### OLDER ITALIANS' VIEWS OF AND EXPERIENCES WITH IMMIGRANT CAREWORKERS

Katia Vecchione,[1] and Anne Barrett[2], *1. University of Trento, Trento, Italy, 2. Florida State University, Tallahassee, Florida, United States*

Population aging has led, in many countries, to new care arrangements to meet the growing need. In Italy, with the second oldest population in the world, family members, especially women, provide the majority of care; however, paid immigrants are increasingly filling in where families cannot. Known as "badanti," most of these careworkers are middle-aged women from Eastern Europe. Although some research examines this phenomenon, it focuses exclusively on careworkers – not those receiving their care. Addressing this gap, my paper examines older Italians' attitudes toward and experiences with immigrant careworkers, using interviews with 20 nursing home residents and 20 senior center participants. Analyses reveal polarized views of "badanti, with more positive views found among those with personal experience receiving their care. I find that negative attitudes are shaped by three broader cultural discourses about aging, as well as immigration. Attitudes are influenced by views of independence and autonomy -- core values perceived to be threatened by badanti. Relatedly, attitudes are influenced by the centrality of space and home, which again are viewed as challenged by badanti's presence. Negative views of badanti also are shaped by dominant discourses regarding immigrants, who are viewed as threats to security, particularly regarding one's belongings. Such beliefs and values influence older adults' willingness to accept help from careworkers and its effectiveness -- knowledge of which can help create better care scenarios.

### THE SHARED MEANING OF COMPASSION FATIGUE AMONG REGISTERED NURSES WORKING IN SKILLED NURSING FACILITIES

Marlene Steinheiser[1], *1. Infusion Nurses Society, Norwood, Massachusetts, United States*

The purpose of this hermeneutic interpretive phenomenology study was to describe the shared meaning of compassion fatigue (CF) among registered nurses (RNs) who work in skilled nursing facilities (SNFs). The specific aims were to describe: 1) contributors (triggers, situation, or patient characteristics) that cause symptoms of compassion fatigue, 2) associated physical and emotional symptoms, and 3) the short-term outcomes of unresolved compassion fatigue impacting nurses and patient care. CF can negatively impact patient outcomes, is associated with decreased quality of patient care, and can be a reason why nurses leave the profession. Eight participants were interviewed three times each, while concurrent data analysis helped to formulate mutual understanding of the phenomenon and informed subsequent interviews. Self-reflection, journaling, record keeping, and use of direct quotes enhanced trustworthiness. Four shared meanings were abstracted:1) I feel conflicted and that causes my CF; 2) physical and emotional manifestations of CF; 3) CF is infused in every aspect of my life; 4) we are trying to cope with CF. The participants shared their central desire to make a difference in the lives of their patients, which was of paramount importance. When participants felt they were unable to make the desired difference, they began to develop symptoms of CF. Symptoms were compounded when they experienced frequent patient deaths. A resiliency program specifically addressing the needs of SNF nurses, incorporating individuals and their organizations, could positively impact the nurses' professional quality of life. Future research is needed to better understand CF and interventions specific to SNF nurses.

### CARING RELATIONSHIP DEVELOPMENT IN THE NURSING HOME IN SHANGHAI: A DYADIC QUALITATIVE STUDY

Lin Chen,[1] Minzhi Ye,[2] and Qiang Zhu[3], *1. Fudan University, Shanghai, China, 2. Benjamin Rose Institute on Aging, Cleveland, Ohio, United States, 3. Fudan University, Shanghai, China, China*

Caring relationships between older residents and nurse aides are fundamental in terms of service delivery in nursing homes. However, little is known for the nuanced dynamics of this relationship in China. The purpose of this study is to explore how caring relationships develop between older residents and nurse aides in the nursing home setting in urban China. Informed by the dyadic perspective, this study illustrates the development process and relational nuances by simultaneously eliciting residents' and nurse aides' perceptions. This qualitative study purposively sampled 20 matched resident-nurse aide dyads (N= 40) in a government-sponsored nursing home in Shanghai. Participants participated in semi-structured, in-depth interviews from January to June 2017. Thematic analysis was performed. The findings reveal that the caring relationship began with nursing home assignment and primarily focused on instrumental assistance. Gradually, emotional involvement grew within dyads and reciprocity emerged. Based on different dyadic perceptions, this study conceptualized four types of caring relationships: (a) parent-child alike, (b) mutually respectful, (c) solo performance, and (d) reasonably detached. The findings suggest that residents and nurse aides could have different views on caring relationships, which further influenced the relationship development. The four types of caring relationships shared some similar traits while differentiating from some of the common types of interactions found in the existing nursing evidence across the world. Chinese filial tradition also influenced the relationship dynamics.

### PERCEPTIONS OF EMPOWERMENT AMONG HOME CARE AIDES

Sandra S. Butler,[1] and Nancy Kusmaul[2], *1. University of Maine, Orono, Maine, United States, 2. University of Maryland, Baltimore County, Baltimore, Maryland, United States*

Workforce issues in eldercare are of growing concern with the dramatic aging of our population and the growing need for personal care assistance. Due to difficult job conditions, recruitment and retention of direct care workers have presented challenges throughout the country both in facility-based and community-based care. The empowerment of workers has been theorized as one vehicle for improving job satisfaction among healthcare workers. Models of structural and psychological empowerment have been explored as factors to reduce job strain among healthcare workers in institutional settings, but have not been well examined in the home care setting. This paper will report on an exploratory study of perceptions of empowerment among home care aides (HCAs) in two states. In-person and telephone semi-structured interviews were conducted with 12 HCAs ranging in age from 20 to 64; eight were white and U.S. born and four were black and African born. All 12 study participants reported positive aspects of empowerment in their home care work (e.g., receiving information and good support, feeling competent, having an impact on their clients' lives, and finding the work meaningful), and most felt autonomous in their work and believed they had the resources they needed to do the job. Nonetheless, negative aspects of empowerment were also frequently described (e.g., poor support, constrained information and resources, limited autonomy due to regulations, and few opportunities for advancement). This paper will explore and interpret these seemingly contradictory findings, with a discussion of implications for recruitment and retention of this valuable workforce

### SESSION 3350 (POSTER)

### MEASUREMENT AND RESEARCH METHODS

### PATH ANALYSIS OF EDUCATION AND DISEASE BURDEN IN DEMENTIA VULNERABILITY

Sarah B. Hubner,[1] Hyeon Jung Kim,[1] and Julie Blaskewicz Boron[1], *1. University of Nebraska at Omaha, Omaha, Nebraska, United States*

When considering the various extrinsic variables that may affect disease vulnerability, it is valuable to study temporal ordering of factors to identify areas for disease intervention efforts. This study sought to inform improvement of networks for the purposes of education and health by attempting to better define the causal ordering of ethnicity, age, gender, education, disease burden, and dementia diagnosis with the Aging, Demographics, and Memory Study, a sub-study of the Health and Retirement Study. Participants and/or proxies self-reported total number of chronic conditions, subsequently regarded as disease burden. Assessments occurred over four waves; participants were not reassessed after dementia diagnosis. The current study categorized participants as demented (n=414), identified in any wave, or normal (n=117), identified in the final wave. Cognitively-impaired-not-demented and deceased participants were not considered due to lack of diagnosis. Cross-sectional weighting was used. A path model was developed; ethnicity, age, and gender were antecedent to education, and education was casually ordered before disease burden, which was antecedent to dementia diagnosis. A series of logistic and linear regression analyses were conducted. Results revealed that being non-white ($\Sigma\beta=0.643$, all p's<.001), of older age ($\Sigma\beta=0.250$, all p's<.001), female ($\Sigma\beta=0.180$, all p's<.001), and having increased disease burden ($\Sigma\beta=0.118$, p<.001) all demonstrated a positive total effect on dementia diagnosis. Conversely, more years of education ($\Sigma\beta=-0.245$, p<.001) had a negative total effect on diagnosis. The education to disease burden pathway was non-significant. Ultimately, these results may indicate a need for dementia interventions that target those with low education or high disease burden.

### CHALLENGES AND IMPERFECT SOLUTIONS FOR COGNITIVE SCREENING WITH OLDER IMMIGRANTS

Lisa Willoughby,[1] Chuleeporn Pusopa,[1] Sattha Prakobchai,[1] Paweena Meekanon,[1] Spondita Goswami,[1] Hisako Matsuo,[1] and Theodore K. Malmstrom[1], *1. Saint Louis University, St. Louis, Missouri, United States*

Cultural diversity among older adults is increasing and with this comes challenges in health care needs, including the detection of cognitive impairment. Cognitive impairments manifests in many ways, with early symptoms often difficult to detect. Detecting cognitive dysfunction is typically facilitated with brief, portable screening tools. Scores on screening tools may be influenced by culture, education, and verbal abilities; in particular, these are acute issues for screening older immigrants from linguistically and culturally diverse backgrounds. The consequences of improper screening are high and, as such, finding practical, cost-effective solutions is of critical importance. In this project, we qualitatively examined the usability of different cognitive screening tools with the ultimate goal of improving the detection and classification of cognitive dysfunction among older adult immigrants. We extended our previous work by piloting adaptation guidelines for the Saint Louis University Mental Status (SLUMS) exam for use with linguistically and culturally diverse persons. We recruited 23 older immigrants (6 non-English speaking) and 14 U.S. born participants to explore the usability and test-retest reliability of the SLUMS exam administered with and without the adaptation guidelines. Our attempts to improve the flexibility of the SLUMS exam did not achieve the level of success as anticipated. Although this pilot work had low power, when coupled with our past work on developing adaptation guidelines, sheds critical light on the layered complexity that arises at the intersections of education, culture, race, gender, socioeconomic status, and intercultural interactions and the resulting potential directions for future work will be discussed.

### MEASUREMENT EQUIVALENCE OF THE POSITIVE AND NEGATIVE AFFECT SCHEDULE BETWEEN YOUNG-MIDDLE-AGED AND OLDER ADULTS

Jeongwoo Lee,[1] and En-Jung Shon[2], *1. Case Western Reserve University, Cleveland, Ohio, United States, 2. Miami University, Oxford, Ohio, Oxford, Ohio, United States*

A Short form of the Positive and Negative Affect Schedule (PANAS-SF) has been widely used to measure of affect in diverse cultural groups. Limited studies have been evaluated the measurement equivalence test of PANAS-SF in diverse age groups. This study examined whether parameters in the measurement model (two-factor model: positive and negative affect) is equivalent across the two age generations (young-middle aged: <65 years [n=1,122]; older adults : ≥65 years [n=1,817]). The sample was obtained from the 2012 Health and Retirement Study and Multiple Group Analysis was performed. The five items of determined, enthusiastic, inspired, alert, and excited reflected positive affect; and the five items of afraid, upset, scared, nervous, and distressed reflected negative affect. The configural model reported acceptable fit (X2= 904.98 [df = 64, p < .001], X 2/ df =14.14, CFI =.93, GFI=.94, RMSEA=.06 [90% CI=.06 - .07]). When all factor loadings were constrained, it indicated measurement non-invariance status between young-middle aged and older adults (ΔX 2 = 56.03, Δdf = 8, p< .001, CFI=.93, ΔCFI=.004). Given findings of non-invariance on the full constrained model, the invariance test of each factor loading was performed additionally. Majority of negative items (Afraid, upset, scared, and nervous) and several positive items (determined and excited) were nonequivalent between the two groups. Variances in the measure between two age groups raise a number of issues for future research on affect assessment, suggesting cautious using of PANAS-SF in older adults.

### AGING, TASK DIFFICULTY, AND EFFORT: A META-ANALYSIS

Ryan Best,[1] Laure Freydefont,[1] and Alexandra M. Freund[1], *1. University of Zurich, Zurich, Switzerland*

Tasks of increasing difficulty require increasing levels of cognitive engagement from participants. The costs associated with cognitive engagement rise with age in response to normative cognitive decline. Additionally, previous studies have shown an interaction between age and task difficulty, with age differences in effort expenditure increasing along with task demands. Motivational accounts of effort allocation predict the opposite relationship, where increased task difficulty in the face of declining cognitive abilities result in disengagement among older adults, comparatively lowering their effort expenditure relative to younger adults that remain committed to the task. The current study quantitatively reviews the available literature on age and effort expenditure across tasks of increasing difficulty. An initial meta-analysis found no age differences in effort across task difficulty, but inspection of the significantly heterogeneous effect sizes indicated that measurement domain might account for some of the variance found between the effect sizes. A second, post-hoc meta-analysis was conducted, recoding effect sizes giving preference to subjective measures. Subsequent moderator variable analyses found that the combined effect of age and domain of effort measurement explained a sufficient portion of the variance across effect sizes. When using physiological measures, effort was not found to differ across task difficulty for either age group. Alternatively, when measured subjectively, effort was reported to greatly increase (>1 standard deviation) with difficulty, with a larger increase in younger adults. Results are discussed in terms of effort mobilization across adulthood and the importance of measurement domain in the interpretation of results.

### REACTIVITY TO THE MEASUREMENT OF PSYCHOLOGICAL WELL-BEING IN OLDER ADULTS

Steve Amireault,[1] Elliot M. Friedman,[1] and Mary K. Huffman[1], *1. Purdue University, West Lafayette, Indiana, United States*

Although it is known that measurement reactivity can yield medium-sized effects (Cohen's d = .50) on anxiety, its effects on psychological well-being (PWB) measures (e.g., purpose in life, personal growth) are unknown. The aim of this study was to evaluate reactivity to the measurement of PWB in older adults. Ninety-four adults aged 60 ≥ years (mean = 75) were recruited from a fitness center, retirement home and community center in Indiana. All participants received a questionnaire via postal mail at baseline (T1) and 2-3 weeks later (T2). Using block randomization (block size = 16), older adults were randomly allocated (1:1 ratio) to one of two conditions: PWB measures assessed at T1 and T2; and PWB measures were at T2 only. Purpose in life and personal growth were assessed using Ryff's PWB scales. Multiple imputation analysis was conducted to account for attrition at T2 (18%), and all participants were analyzed according to the condition they were originally assigned. ANCOVA, controlling for recruitment sites, revealed that purpose in life (p = .03), but not personal growth (p = .08), T2 scores were on average lower when PWB was measured twice compared to those when PWB was measured once. The detected difference in purpose in life T2 scores was of medium size (d [95%CI] = - .50 [-.95, -.04]). This finding suggests that initial or later scores might be biased – a problematic finding because if ignored, measurement reactivity has the potential to affect conclusions drawn from gerontological research that focuses on PWB.

### DEVELOPMENT AND VALIDATION OF THE TESTAMENTARY CAPACITY MEASURE AMONG OLDER ADULTS AND PATIENTS WITH DEMENTIA

Christopher M. Nguyen,[1] Cady Block,[2] John Linck,[3] and Natalie Denburg[4], *1. The Ohio State University Wexner Medical Center, Columbus, Ohio, United States, 2. The Ohio State Wexner Medical Center, Columbus, Ohio, United States, 3. University of Oklahoma Health Sciences*

Center, Oklahoma City, Oklahoma, United States, 4. University of Iowa Hospitals and Clinics, Iowa City, Iowa, United States

As the growth of the population aged 65 and older is projected to be one of the most substantial demographic trends in history, geriatricians and other professionals working with older adults will be regularly consulted for opinion regarding an individual's testamentary capacity (Brenkel et al., 2018). For individuals with severe cognitive and psychiatric impairment, the reduced capacity to make decision is evident. However, testamentary capacity among individuals with mild or moderate cognitive impairment has been mixed: some patients with mild cognitive impairment may be incapable of will making, while other patients with moderate cognitive impairment may have testamentary capacity (Spar & Garb, 1992). While several measures of testamentary capacity have been developed, these instruments are either not available for clinicians or lacks the sophistication of a comprehensive assessment (Marson, Huthwaite, & Herbert, 2004; Papageorgiou et al., 2018). With increasingly complex modern family structures (e.g., multiple marriages and stepchildren from these relationships), together with a projected largest transfer of wealth in human history about to occur in the next 30 years (Havens and Schervish, 2003), more standardized assessment procedures for testamentary capacity will be valuable for clinicians working with geriatric populations. In this study, healthy community-dwelling older adults and patients with Major Neurocognitive Disorder due to Alzheimer's disease were recruited to participate in a validation study of a proposed testamentary capacity measure. Preliminary findings and implications are discussed.

### METHODOLOGICAL STRATEGIES AND CHALLENGES FOR EVALUATING PARTICIPANT VIEWS ON DEMENTIA RESEARCH RECRUITMENT

Laura Block,[1] Shannon Mullen,[1] Gay Thomas,[1] Betty Kaiser,[1] and Andrea Gilmore-Bykovskyi[1], *1. University of Wisconsin-Madison School of Nursing, Madison, Wisconsin, United States*

There is an urgent need to expand enrollment in clinical Alzheimer's disease (AD) research. Current recruitment methods for AD research predominantly identify patients from primary/specialty clinic settings, potentially creating barriers for individuals unconnected to care. In response to these challenges, the 2018 National Strategy for Research Recruitment and Participation has called for the development of an applied science of recruitment to inform best strategies. However, progress in this area is hindered by methodological challenges to accurately measure AD patient/caregiver participant views on research participation. The objective of this presentation is to report methodological strategies developed to during a prospective qualitative study to investigate AD patient/caregiver views on acute care research recruitment and participation. Participants included patients with dementia (N=2) who had recently been hospitalized and/or their informal caregivers (N=15). We engaged in iterative development and revision of data collection approaches (i.e. semi-structured questions, audiovisual tools, interview guidance) through collaboration with a Community Advisory Board (CAB). Detailed memos were generated to document interview-related challenges, successes and revisions. Therapeutic misconception in delineating research from clinical care was common during interviews regardless of prior research participation. Interview strategies that focus on lived experiences, remove ambiguity from hypothetical recruitment scenarios, and incorporate supportive visual tools that clarify processes around recruitment improved data collection. Challenges included the lack of a common, shared language around recruitment, which was addressed through CAB guidance and input. In conclusion, thoughtful collaboration with community/lay advisers can successfully inform and data collection methods used in applied recruitment research.

### THE INFLUENCE OF THE UNIVERSAL TWO-CHILD POLICY ON CHINA'S FUTURE AGING AND POPULATION

CAN JIA,[1] and Handong li[2], *1. Systems Science, Beijing Normal University, Beijing, China, China, 2. Beijing Normal University, Beijing, China*

China's aging situation is becoming more and more prominent, and both the people and the government are facing unprecedented pressure of providing for the aged. For this reason, the Chinese government began implementing a new family planning policy for couples to have two children since 2016 (referred to as "universal two-child policy"). In order to explore the impact of the newly released policy, our research is based on the sixth census of China. And first, we use the cohort-component method and a Leslie matrix to construct the population prediction model. Considering some certain unique factors in China, such as the significant urban-rural dual structure and the household registration system and so on, we divide the total fertility rate into urban and rural areas which fully reflects the characteristics of China's family planning policy. Then we predict and analyze the number and structure of China population between 2011 and 2050 based on the three scenarios of high, medium and low. And the results show that the Chinese population will present an inverted pyramid structure, and the population structure will continue to deteriorate. Besides, we adapt three indicators to analyze the aging trend in China, namely, the old-age coefficient, the population aging index, and the social dependency ratio. And the three indicators of China will continue to grow under the universal two-child policy with different changing rate, which means, the newly released policy will not change China's aging population growth trend and the severity of China's aging.

### YOU'LL SEE: YOUNGER RESEARCHERS INTERVIEWING OLDER PARTICIPANTS

Sarah Jen,[1] Yuanjin Zhou,[2] and Mijin Jeong[1], *1. University of Kansas School of Social Welfare, Lawrence, Kansas, United States, 2. University of Washington School of Social Work, Seattle, Washington, United States*

In qualitative research, similarities and differences between the participant and researcher influence the research process and dynamics. Specifically, the age difference between older participants and relatively younger qualitative researchers is a common, but under-examined dynamic requiring nuanced, reflexive analysis. Using a life course conceptual framing, this study explored age-related participant-researcher dynamics in interviews from two qualitative studies of older women's sexual experiences in later life. Participants included 25 women whose ages ranged from 55 to 93 and both studies were completed by the same researcher, a relatively younger woman (age 23 and 28 at times of data collection). A thematic analysis revealed three primary themes: 1) taking care - participants took care of the researcher by offering advice, asking about the researcher's life, and expressing hopes for a positive future, 2) expertise – varied expertise was demonstrated by the researcher (e.g. substantive and scholarly) and participants (e.g. life experience), and 3) researcher growth - the researcher's interviewing tactics shifted between the two studies (e.g. use of validation rather than consolation in response to aging-related concerns), indicating a shift in perceptions of aging and later life. Findings indicate that older women participants and younger women researchers are bound together through the life course, by shared gendered experiences, the fact that one will eventually become the other, and the mutual sharing of expertise and caring. Gerontology researchers must actively reflect on the impact of their own identities and aging perceptions on the interviewing process in order to enhance rigor in qualitative research.

### THE TRUTH ABOUT AGE DIFFERENCES IN PERFORMANCE ON VARYING LENGTHS OF LIKERT-TYPE TEST ITEMS

Julie Lutz,[1] Aaron Metzger,[1] Nicholas A. Turiano,[1] Rachael Spalding,[1] Emma Katz,[1] and Barry Edelstein[1], *1. West Virginia University, Morgantown, West Virginia, United States*

Guidelines for self-report assessment with older adults emphasize the use of shorter Likert-type or agree/disagree response formats to reduce cognitive load (e.g., Yesavage et al., 1983). However, these suggestions are not founded on empirical studies directly comparing younger and older adults' responses on different scales. Thus, the current study tested differential responding on varying Likert-type response scale lengths between younger, middle-aged, and older adults. Participants completed three versions of the International Personality Item Pool (IPIP) Neuroticism scale with 3, 5, and 7 Likert-type response scale lengths in counterbalanced orders with other questionnaires between versions. Six multi-group confirmatory factor analyses (CFAs) assessed measurement invariance across scale lengths and age groups. Invariance of convergent validity networks was also assessed with multi-group CFAs of the associations between the IPIP and measures of depression, anxiety, anger, worry, and affect. The final sample consisted of 835 adults (327 18-44; 279 45-64; and 229 65 or older) via Amazon Mechanical Turk. Measurement invariance was supported in analyses by age within each scale length and by scale length within each age group, indicating that response patterns across all scale lengths and age groups did not significantly differ. Analyses of convergent validity also supported invariance, suggesting that responses across all scale lengths and age groups reflect the same underlying construct. This study indicates that, among community-dwelling adults, shortened response scale lengths do not yield significantly different or more valid responses for older adults compared to younger adults.

### A MULTIDIMENSIONAL MODEL FOR POLYPHARMACY MEASUREMENT IN OLDER ADULTS: EVIDENCE FROM THE HEALTH RETIREMENT STUDY

Rebecca Bendayan,[1] Ewan Carr,[1] Alex D. Federman,[2] and Richard J Dobson[1], *1. Dept. Biostatistics and Health Informatics, Institute of Psychiatry, Psychology & Neuroscience (IoPPN), King's College London, London, United Kingdom, United Kingdom, 2. Division of General Internal Medicine, Department of Medicine, Icahn School of Medicine at Mount Sinai, New York, United States*

Polypharmacy is associated with increased health care costs and adverse health outcomes. Traditional research on polypharmacy uses dichotomous measures which overlook its multidimensional nature. We propose a new approach to grouping older adults based on the number and type of medications taken as well as other indicators of polypharmacy. Data was extracted from 1328 respondents of the 2007 Prescription Drug Survey (a sub-study of the Health Retirement Study) who were between 50 and 70 years old and taking ≥1 medication each month. Latent class analysis was carried out with the optimal number of classes assessed based on relative model fit (AIC, adjusted BIC) and interpretability. Latent classes were formed based on the number of medications, drug types, duration of medication intake, side effects, and presence of chronic health conditions. A four-class model was selected based on model fit and interpretability of the solutions. Although there was some overlap when we compared our model with standard cut-offs for polypharmacy (i.e., 'high polypharmacy' classes were more likely to take 5+ and 9+ medications), chi-square tests showed significant differences between our latent classes and cut-offs based on 5+ [$X2 = 894$; $p<0.001$] and 9+ medications [$X2 = 398$; $p<0.001$]. Among individuals taking <5 medications, our model differentiated two distinct types of 'low polypharmacy' based on the types of drugs reported. Our proposal to incorporate a multidimensional assessment of polypharmacy considers the wider context of medication use and chronic health in older age, moving beyond crude medication counts.

### COGNITIVE INTRAINDIVIDUAL VARIABILITY TO MEASURE INTERVENTION EFFECTIVENESS: BALTIMORE EXPERIENCE CORPS TRIAL

Allison A. Bielak,[1] Christopher R. Brydges,[1] Michelle C. Carlson,[2] Ryan M. Andrews,[3] and George W. Rebok[2], *1. Colorado State University, Fort Collins, Colorado, United States, 2. Johns Hopkins Bloomberg School of Public Health, Baltimore, Maryland, United States, 3. Leibniz Institute for Prevention Research and Epidemiology – BIPS, Bremen, Germany*

Studies investigating the effectiveness of intervention programs on cognitive ability in older adults are equivocal; however, these studies generally focus on traditional measures of cognition, and therefore may miss some improvements by not utilizing alternate measures. We evaluate the potential for intraindividual variability in cognitive speed

(IIV), a demonstrated sensitive indicator of cognitive functioning, to be used as an index of cognitive plasticity from an intervention. The current study evaluated whether older adults in a school volunteering program showed a reduction in IIV, compared to a low-activity control group over two years of exposure. Non-demented aging older adults (n = 336) participated in the Baltimore Experience Corps Trial, an evaluation of a volunteering program conducted at elementary schools designed to increase older adults' physical, cognitive, and social engagement. Participants completed a cognitive battery that included a computerized Stroop task at baseline and after 12 and 24 months. Participants who complied at the 80th percentile or above showed a significant reduction in IIV at 24 months, with an additional trend of improved IIV with increased compliance to the treatment protocol, both at 12 months, and at 24 months. Men specifically also showed significant dose-dependent improvements after 12 months. The Experience Corps program resulted in an improvement in cognitive performance as measured by IIV. Analyzing previously collected data with non-traditional measures of cognition, such as IIV, may be a potentially fruitful and cost-effective method for understanding how interventions impact cognition in aging populations.

### THE EFFECT OF COGNITIVE TESTING ON STATE ANXIETY AND CORTISOL REACTIVITY IN YOUNGER AND OLDER ADULTS

MacKenzie L. Hughes,[1] Ann Pearman,[1] and Shevaun D. Neupert[2], *1. Georgia Institute of Technology, Atlanta, Georgia, United States, 2. North Carolina State University, Raleigh, North Carolina, United States*

Although it is well established that stress is negatively associated with cognitive functioning, less is known about age differences in the effects of stressors and anxiety on state anxiety and physiological reactivity (i.e., changes in cortisol). The current study examined state anxiety and cortisol reactivity during a series of cognitive tasks in a sample of younger (n=26) and older (n=29) adults. Participants completed the State-Trait Anxiety Inventory prior to cognitive testing and provided six salivary cortisol samples throughout one testing session: two cortisol samples prior to cognitive testing, three samples during testing, and one sample after testing. Six cognitive tasks were administered that measured attention span, declarative memory, and processing speed. Results indicated a significant interaction effect of age by time with younger adults' cortisol linearly decreasing during the testing session and older adults' cortisol showing a quadratic trend. A second interaction was found between age and state anxiety whereby older adults who reported more anxiety had higher cortisol levels during the cognitive testing session than both the older adults who reported low levels of anxiety and the younger adults. Only age (not cortisol or anxiety) was significantly related to cognitive performance. Results from this study suggest that standard cognitive testing could be anxiety producing for older adults, particularly for those who are already anxious. Future investigations should examine age-related differences in the processes linking anxiety and cortisol to specific types of performance, such as memory and attention.

### SESSION 3355 (POSTER)

### MEDICATION ISSUES

### EMERGING MODELS OF CARE: THE IMPACT OF PHARMACIST-LED MEDICATION MANAGEMENT IN A TRANSITIONAL CARE PROGRAM

Ebony Andrews,[1] Travonia Brown-Hughes,[1] Ronald Lyon,[1] Shanea D. Parker,[1] and Brad Lazernick[2], *1. Hampton University, Hampton, Virginia, United States, 2. Senior Services of Southeastern Virginia, Norfolk, Virginia, United States*

Transitional care programs have emerged as successful models of care in which to reduce cost and improve health outcomes. However, few transitional care models have directly incorporated the expertise of the pharmacist as an integral member of the care coordination team. Therein lies an inherent limitation of many community-based transitional care programs, the underutilization of pharmacist during all stages of the care transition process. In 2013, the Hampton Roads Care Transitions Project (HRCTP), a partnership between Senior Services of Southeastern Virginia Area Agency on Aging in Norfolk, VA and Hampton University School of Pharmacy, was established. The goal of the HRCTP is to provide medication management services to reduce preventable hospital readmissions for adults 60 years of age and older with targeted diagnoses. Pharmacists work in collaboration with social workers who act as HRCTP care transition coaches. Between May 2017- October 2018, 678 patients were enrolled in the HRCTP. The hospital readmission rate among patients with targeted diagnoses was reduced by 55.3% with an absolute percentage point reduction of 9.9% and estimated savings amount per avoided readmission of $14,400. Patients who participated in the HRCTP showed a 14% increase in the Patient Activation Assessment indicating an improvement in self-managing efficacy. 93% of patients/caregivers indicated they felt more confident in their ability to manage their health, and 91% expressed satisfaction with the program. The program has proven effective in assisting seniors to remain in their home, reducing hospitalizations, promoting health, increasing patient satisfaction, and reducing healthcare cost.

### A SIMPLE INTERVENTION TO REDUCE ANTICHOLINERGIC DRUG USE WHILE ATTENDING A GERIATRIC DAY HOSPITAL

Costa Apostolides[1], *1. Dalhousie University, Halifax, Nova Scotia, Canada*

Attendance at a Geriatric Day Hospital has previously been shown to reduce both the overall number of medications and the number of anticholinergic medications of patients. In the present study, patients enrolled in a Geriatric Day Hospital program from January to February 2019 were divided into a control and intervention group. Anticholinergic medications in the intervention group were flagged by highlighting them in the patient chart and alerting the attending clinician, whereas no alerts were provided in the control group. Anticholinergic load was calculated using the Anticholinergic Cognitive Burden (ACB) and Drug Burden Index (DBI) scores. In comparing admit versus discharge medications in the intervention group, both the mean number of overall medications

(10 vs. 9.7) and anticholinergic medications (3.5 vs. 3.1) was reduced; this was not the case in the control group, where the mean number of overall medications remained the same (11.92) and the mean number of anticholinergic medications increased (3.83 vs. 3.92). More significantly, in comparing admit versus discharge scores, both the mean ACB and DBI scores were reduced in the intervention group, but in the control group both the ACB and DBI scores either remained the same or increased at the time of discharge. This clearly shows that a simple intervention (highlighting anticholinergic medications in the patient chart) can have a clinically beneficial outcome of reducing these harmful medications in patients. With approximately 50% of the older population taking at least one anticholinergic drug, the importance of reducing anticholinergic burden cannot be overemphasized.

### THE OPIOID EPIDEMIC IN LONG-TERM CARE: A STAFF PERSPECTIVE

Rachael Hemmert,[1] Gabriella E. Dull,[1] and Linda S. Edelman[1], *1. University of Utah, Salt Lake City, Utah, United States*

Opioid-based analgesic therapy is a common treatment for moderate to severe pain among long term care (LTC) residents. It has been estimated that 60% of LTC residents have an opioid prescription. Of these, 14% use opioids as part of a long term pain management strategy. LTC residents are particularly vulnerable to opioid misuse, exhibiting higher rates of adverse drug events. However, addressing pain, polypharmacological needs and resident well-being in the LTC setting is challenging. More research and education regarding opioid use in LTC is needed. The Utah Geriatric Education Consortium conducted interprofessional focus groups with LTC partners to 1) determine educational needs of staff regarding opioid use, and 2) gather qualitative data about the pain management experiences of staff when working with residents and families. Staff identified the following training needs: pain manifestation and assessment; certified nurse assistant education on opioid use; non-pharmacological options for pain management. Review of staff's perception of the intersection of opioids, family and staff in a LTC setting revealed that 1) family is concerned about opioid use; 2) conversely, staff may not see opioid use as a problem; and 3) non-pharmacological options for pain management are often costly and unavailable to those in LTC. Identifying educational needs of LTC staff will help guide the development of educational materials and provide baseline data for future assessments of the impact of opioid education on long-term care patient outcomes.

### TRENDS IN OPIOID USE IN LONG-TERM CARE NURSING HOME RESIDENTS WITH DEMENTIA

Hemalkumar B. Mehta,[1] Yong-Fang Kuo,[1] Jordan Westra,[1] Mukaila Raji,[1] and James S. Goodwin[1], *1. The University of Texas Medical Branch, Galveston, Texas, United States*

We examined opioid use in long-term care nursing home residents with dementia. This retrospective cohort study used Minimum Data Set linked Medicare data, 2011-2016, and included long-term care episodes for residents 65+ years who survived 100+ days each year (592,211 episodes for 256,207 residents). Cognitive status at first annual assessment was classified as none/mild, moderate and severe impairment. Overall opioid use, prolonged opioid use (prescription supply 90+ days) and long-acting opioid use were identified from Medicare part D. Descriptive statistics were used to describe opioid use by cognitive impairment. Cochrane Armitage trends test was used to determine trends in opioid use. 114,622 (19%) patients had severe and 129,257 (22%) had moderate dementia. Overall opioid (none/mild=15.4%, moderate=13.9%, severe=9%), prolonged opioid (none/mild=5.2%, moderate=4.5%, severe=3.2%) and long-acting opioid use (none/mild=1.1%, moderate=0.9%, severe=0.3% ) were lower in patients with advanced dementia. Opioid use was significantly higher in females and Whites and varied by states. Substantial increase was found in overall opioid and prolonged opioid use from 2011 to 2016, with greater increase in none/mild and moderate dementia patients. For example, prolonged opioid use increased by 69% in none/mild and 71% in moderate dementia patients compared to 52% in severe dementia patients ($p<0.0001$). Long-acting opioid use decreased, with a greater decline in none/mild (69%) and moderate (71%) dementia patients compared to severe dementia patients (58%) ($p<0.0001$). Contrary to decreasing opioid use in community setting, overall and prolonged opioid use increased in nursing home residents. Future studies should identify the reasons behind increased use.

### CHALLENGES FACED BY OLDER PERSONS IN USING PRESCRIPTION MEDICATION LABELS: WHAT NEEDS TO CHANGE?

Rahul Malhotra,[1] Sumithra Suppiah,[1] Yi Wen Tan,[1] and PROMISE Study Group PROMISE Study Group[1], *1. Duke-NUS Medical School, Singapore, Singapore*

In Singapore, while many older people cannot read English, prescription medication labels (PMLs) are predominantly dispensed in English. This qualitative study documented the challenges faced and solutions employed by users (i.e. older Singaporeans) and dispensers (i.e. pharmacy staff) of PMLs. In total, 30 in-depth interviews were conducted; 20 were equally divided between older Singaporeans (≥60 years) who could read English and those with limited/no English reading ability, and 10 were conducted with pharmacy staff across 6 polyclinics. The audio-taped interviews were transcribed verbatim and analysed thematically. The interviews with older Singaporeans and pharmacy staff revealed similar challenges in using PMLs. The first challenge related to reading and understanding PMLs by older people, mainly due to their limited English proficiency (LEP) or illiteracy. Consequently, older Singaporeans often relied on family members, domestic workers or pharmacy staff to help them interpret PMLs. Specifically, to address LEP, pharmacy staff reported translating PML instructions verbally and also handwriting them on PMLs. For illiterate patients, pharmacy staff reported drawing illustrations on PMLs to communicate key medication information. The second challenge related to PML readability, due to small font size. To address this, pharmacy staff routinely re-wrote medication information on PMLs in larger handwriting. Such improvised solutions by pharmacy staff to address the challenges faced by older Singaporeans in using PMLs indicate a pressing need for system-level improvements to PMLs. Improvements such as standardised and legible bilingual medication instructions and/or pictograms would appreciably facilitate medication counselling and allow for better understanding of PMLs by older Singaporeans.

### AN EXPLORATION OF HOME CARE NURSES' EXPERIENCES IN DEPRESCRIBING OF MEDICATIONS FOR OLDER ADULTS IN THE COMMUNITY

Winnie Sun[1], *1. University of Ontario Institute of Technology, Oshawa, Ontario, Canada*

The aim of this study is to explore the barriers and enablers of deprescribing from the perspectives of home care nurses, as well as to conduct a scalability assessment of an educational plan to address the learning needs of home care nurses about deprescribing. This study employed an exploratory qualitative descriptive research design, using scalability assessment from two focus groups with a total of eleven home care nurses in Ontario, Canada. Thematic analysis was used to derive themes about home care nurse's perspectives about barriers and enablers of deprescribing, as well as learning needs in relation to deprescribing approaches. Home care nurse's identified challenges for managing polypharmacy in older adults in home care settings, including a lack of open communication and inconsistent medication reconciliation practices. Additionally, inadequate partnership and ineffective collaboration between inter-professional healthcare providers were identified as major barriers to safe deprescribing. Further, home care nurses highlighted the importance of raising awareness about deprescribing in the community, and they emphasized the need for a consistent and standardized approach in educating healthcare providers, informal caregivers, and older adults about the best practices of safe deprescribing. Nurses in home care play a vital role in medication management and, therefore, educational programs must be developed to support their awareness and understanding of deprescribing. Study findings highlighted the need for the future improvement of existing programs about safer medication management through the development of a supportive and collaborative relationship among the home care team, frail older adults and their informal caregivers.

### PATTERNS OF PSYCHOACTIVE MEDICATION USE IN COMMUNITY-DWELLING OLDER ADULTS IN THE UNITED STATES IN 2015

Rashmita Bajracharya,[1] and Danya Qato[2], *1. University of Maryland, Baltimore, Maryland, United States, 2. University of Maryland Baltimore, School of Medicine, Maryland, United States*

Per the 2015 Beer's Criteria, most psychoactive medications are identified as potentially inappropriate for use in older adults as this population is especially vulnerable to the potential adverse effects associated with psychoactive medications, including sedation, anticholinergic effects, and falls. Past studies found increasing use of psychoactive medications in community-dwelling older adults; however, patterns of use by other sociodemographic, socioeconomic, and clinical subgroups have not been explored. This is a cross-sectional analysis of 2015 Medical Expenditure Panel Survey in a sample of 6122 older adults (60-85 years). We utilized Andersen's Behavioral Model of Health Services Utilization to guide logistic regression model development and estimated odds ratios (OR) with 95% confidence intervals (CI) to quantify the association between psychoactive use and predisposing(sex and race); enabling(marital status, education, poverty, insurance); and need-based[multimorbidity and activities of daily living (ADL) limitations] factors. Over 30% of older adults in the U.S. reported taking a psychoactive medication in 2015. Prevalence of use was significantly higher in women (35.9%), the unmarried(34.1%), low-income(35.7%), white(34.0%), multimorbid (32.0%), and ADL limitation groups (45.9%) compared to men, married, high-income, other races, not multimorbid, and no ADL limitations groups, respectively. Female sex [OR=1.62(1.38-1.91)], low-income [OR=1.30(1.04-1.6)], multimorbidity [OR=3.2(2.6-3.9)], and ADL limitations [OR=2.2(1.7-2.8)] were identified as independent predictors of psychoactive use. There is differential use of psychoactive medications by sociodemographic, socioeconomic, and clinical factors. Given the increased complexity of pharmacotherapy regimens, especially in those with multimorbidity and ADL limitations, improved efforts aimed at prudent use of psychoactive medications should be intensified.

### WHAT IS THE EVIDENCE BASE FOR NONPHARMACEUTICAL INTERVENTIONS TO DECREASE USE OF PSYCHOTROPIC MEDICATIONS?

German Alarcon Garavito,[1] and Kathleen Abrahamson[2], *1. Universidad Nacional de Columbia, Bogata, Colombia, 2. Purdue University, West Lafayette, Indiana, United States*

Psychotropic medications (anti-depressants, anti-anxiolytics, anti-psychotics, others), have been targeted as a class of medications that can be reduced among residential long-term care (LTC) residents with proper environmental intervention. Reduction of psychotropic medications has the potential to increase resident health/well-being and decrease costs. However, residential LTC facilities face numerous challenges in reducing psychotropic medications, including but not limited to behavior and communication difficulties in advanced dementia, lack of staff training/awareness of needed environmental modifications, and use of medications to manage the cascade of side-effects that occur with polypharmacy and multi-morbidity in the LTC population. A developed literature exists surrounding non-pharmaceutical interventions to reduce psychotropic use in residential LTC. The goal of this current study was to synthesize that body of literature through a systematic review, and categorize findings in a way that is meaningful to direct-care nursing staff. The search of 13 databases identified 14 studies published since 2007 that focused specifically upon residential care and nurse-led non-pharmaceutical interventions. Identified study designs were experimental (5), observational (3), and systematic reviews/meta-analyses from within 8 countries. Findings were categorized based upon type of intervention (educational, environmental, sensorial, care-approach, physiological, and social), as well the target population. Successful interventions were most commonly targeted toward staff and residents, less commonly toward family. The most common approach was education and change in care-approach, with physiological interventions being the least common. We will discuss the implications for nursing staff surrounding the available evidence, and pro/cons of implementing interventions within different organizational settings based upon the study findings.

### IMPACT OF A COLLABORATION WITH CLINICAL PHARMACISTS ON OSTEOPOROSIS TREATMENT RATES

Silvina Levis,[1] Violet Lagari,[1] Maribel Garcia,[1] Sue Kwok,[1] and Martha Salazar[1], *1. Miami Veterans Affairs Healthcare System, Miami, Florida, United States*

Despite the availability of effective drugs to treat osteoporosis, many patients remain untreated and at high risk for developing a fracture. To improve treatment rates, we included clinical pharmacists in the management of patients with osteoporosis. Over 6 months, 30 days after bone mineral density (BMD) results became available to the ordering physician, all patients with an abnormal BMD were evaluated for management according to guidelines if they had not been approached for care. One of three clinical pharmacists discussed patients with the team that included an endocrinologist and a geriatrician. The team made recommendations and pharmacists followed-up by calling patients and prescribing medications. After excluding those who were already on treatment or did not have an indication, 87 patients qualified: 57 (66%) had T-score ≤-2.5, 19 (22%) had osteopenia and high FRAX score, and 11 (12%) osteopenia by BMD and a fragility fracture. After 30 days, the ordering physicians had treated 32/87 (37%) of patients with an indication: 26/57 (46%) patients with T-score ≤-2.5, 1/19 (2%) with high FRAX, and 5/11 (50%) with fractures. After the pharmacists' intervention, an additional 33/87 (38%) patients were on treatment: 16 with T-score ≤-2.5; 14 with high FRAX, and 3 with fractures; 6 patients were unreachable, 9 declined, and 6 were referred to endocrinology for work-up. By the end of the 6-month period, 75% of patients with an indication received osteoporosis treatment. These results suggest that an osteoporosis intervention employing clinical pharmacists as part of a multidisciplinary team effectively improves osteoporosis treatment rates.

### A TRIAL TO IMPROVE MEDICATION SAFETY IN OLDER ADULTS: RECRUITMENT CHALLENGES HAVE GENERALIZABILITY IMPLICATIONS

Kathryn Anzuoni,[1] Terry Field,[2] Kathleen Mazor,[1] Yanhua Zhou,[1] Timothy Konola,[1] Alok Kapoor,[2] Lawrence Garber,[3] and Jerry Gurwitz[1], *1. Meyers Primary Care Institute, a joint endeavor of University of Massachusetts Medical School, Reliant Medical Group, and Fallon Health, Worcester, Massachusetts, United States, 2. University of Massachusetts Medical School, Worcester, Massachusetts, United States, 3. Reliant Medical Group, Worcester, Massachusetts, United States*

For older adults, the transition from hospital to home is a high-risk period for adverse drug events, functional decline, and hospital readmission. Randomized trials of interventions to improve this transition must recruit potential subjects immediately after hospital discharge, when people are recovering and tired. Within a randomized trial assessing the impact of a pharmacist home visit to provide medication assistance immediately post-discharge, we determined whether individuals who enrolled were comparable to those who were invited but did not enroll, and described reasons for not enrolling. Individuals ≥50 years of age discharged from the hospital and prescribed a high-risk medication were eligible. We attempted to recruit individuals by phone within 3 days of discharge, and recorded reasons for not enrolling. Of 3,606 eligible individuals reached, 3,147 (87%) declined, 361 (10%) were enrolled, and 98 (3%) were initially recruited but did not complete a consent form. Individuals ≥80 years of age (odds ratio 0.45, CI 0.25, 0.78) and those with an assigned visiting nurse (odds ratio 0.64, CI 0.48, 0.85) were least likely to enroll. Among those who provided a reason for declining (2,473) the most common reason given was the belief they did not need medication assistance (22%). An additional 332 (13%) declined because they were receiving visiting nurse services. Recruiting older adults recently discharged from the hospital is difficult and may under-enroll the oldest individuals, limiting the ability to generalize findings across older patient populations. Researchers planning RCTs among newly discharged older adults may need creative approaches to overcome resistance.

### SESSION 3360 (POSTER)

### MINORITY & DIVERSE POPULATIONS II

### DIABETES, SELF-EFFICACY TOWARD DIABETES PREVENTIVE BEHAVIORS, AND DEPRESSIVE SYMPTOMS AMONG KOREAN AMERICANS

Soyeon Cho[1], *1. City University of New York-New York City College of Technology, Brooklyn, New York, United States*

Type 2 diabetes is a largely preventive chronic disease, which requires persevering self-management by maintaining healthy life style. Prevalence of Type 2 diabetes among Asian Americans are rapidly increasing, yet little is known about Asian Americans' self-efficacy towards diabetes preventive behaviors. Thus, the present study examined self-efficacy on diabetes preventive behaviors (DPB) as a potential mediator in the association between diabetes and depressive symptoms among older Korean Americans. Data were driven from a cross-sectional study of 235 community-dwelling Korean American older adults (aged 60 and older) in 2013. The direct significant relation between diabetes and depressive symptoms became insignificant after self-efficacy on DPB was introduced, which demonstrates a full mediation effect of self-efficacy on DPB. Results suggest that even in the presence of diabetes, mental well-being such as depression of older adults can be maintained by having competence in self-management of their own health.

### AGE, EXPERIENCE OF RACIAL MICROAGGRESSIONS, AND DISTRESS

Shayla Thompson,[1] Broderick Sawyer,[1] and Suzanne Meeks[1], *1. University of Louisville, Louisville, Kentucky, United States*

Racial microaggressions are a common form of racial discrimination consisting of subtle or interpersonal slights. Racial microaggressions are linked to various kinds of psychological distress in younger adults, but have not been studied across the lifespan. We examined the relationship of racial microaggressions with psychological distress and anger rumination among younger and older adults identified as racial or ethnic minorities. We hypothesized that age would moderate the relationship between racial microaggressions and psychological distress and anger rumination, that is, the relationship would be weaker for older than for younger adults. Participants were recruited from Amazon Mechanical Turk and were compensated $1 for their participation. Preliminary tests of the hypotheses (N=220), using multiple regression analyses to test for moderation, failed to support the hypothesis that age would mitigate the impact of microaggressions on symptom severity. Both age and microaggressions were related to psychological distress and anger rumination, but contrary to prediction, older adults showed more exacerbation of distress in the face of microaggressions than younger adults. The results also differed by gender and ethnic groups, suggesting the importance of examining intersectional experiences of race, gender, and age in response to discrimination. These cross-sectional findings lend support to the importance of considering both subtle and overt discriminatory experiences in understanding the mental health challenges for minority groups in the U.S., but more work is needed to examine the intersection of ethnicity with other demographic variables, and to understand how the lifelong experiences of discrimination may shape older adults' vulnerability, well-being, and resilience.

### A 20-YEAR COHORT STUDY OF LIVING ARRANGEMENTS AND COGNITIVE DECLINE IN THE MEXICAN AMERICAN POPULATION

Phillip A. Cantu[1], *1. University of Texas at Austin, Austin, Texas, United States*

Backgrounds/Objectives: The prevalence of dementia in the elderly Latino population is expected to significantly increase from around 200,000 cases in 2000 to as many as 1.3 million cases in 2050. This demographic trend has important consequences for options in care and living arrangements. Very little is known about how of cognitive impairment trajectories co-vary with support available to Mexican Americans. We examine the relationship between living arrangements and the social support of individuals with dementia. Methods: Using data from nine waves from the 23 years of the Hispanic EPESE (n=3,952), we examine trajectories of cognitive functioning and family and social support. We first describe the change in Mini Mental Status Examination (MMSE) scores for survivors from Wave 1 (1993/1994) to Wave 9 (2015/2016), n=255. Growth Mixture Modeling (GMM) is then used to assess how changes in MMSE scores are distributed among living arrangements for individuals living independently compared to household extension (living with others) using the full sample. Results: Analyses reveal different trajectories in MMSE score. 12% (n=27) of the sample had no decrease, while the remaining (88%) were split between moderate decline (60% n=136, 1-10 point decline in MMSE) and severe decline (28% n=62 >10 point decline In MMSE). Changes in living arrangement over the same period show that 89% of individuals who move from independent living into extended household experienced cognitive decline. Conclusions: This study provides new information on how cognitive trajectories are associated with living arrangements. We discuss implications for improving community-based interventions for Latino family caregivers.

### RACE DIFFERENCES IN ALLOSTATIC LOAD AMONG BLACK AND WHITE MEN: DOES AGE MATTER?

Roland J. Thorpe,[1] Hossein Zare,[2] Paul Archibald,[3] Marino A. Bruce,[4] Keith Norris,[5] and Keith E. Whitfield[6], *1. Johns Hopkins Bloomberg School of Public Health, Baltimore, Maryland, United States, 2. Sr. Programmer, JHU, Baltimore, Maryland, United States, 3. Morgan State University, Baltimore, Maryland, United States, 4. Vanderbilt University, Nashville, Tennessee, United States, 5. UCLA, Los Angeles, California, United States, 6. Wayne State University, Detroit, Michigan, United States*

Although Black-White disparities in health and mortality among men persist, there is a paucity of work focusing on race differences in physiological dysregulation of biological processes resulting from the cumulative impact of stressors among men. The purpose of this study was to assess potential race differences in Allostatic Load (AL) among adult men and if such differences vary by age. Data were drawn from the 1999-2010 NHANES, and the study population included 2700 non-Hispanic Black (NHB) and 19930 Non-Hispanic White (NHW) born in US. AL was derived by summing across cardiovascular, metabolic, and inflammatory biomarkers considered to be high risk, resulting in a count variable ranging from 0 to 9. Race was based on self-report. Age was categorized: 18-24, 25-44, 45-64, and 65 years and older. Negative binomial regression was used to examine the relationship between race and AL score. Models included education, marital status, family income, health insurance and self-reported health. Adjusting for potential confounders, NHB men had a higher AL score ((incidence rate ratio (IRR) = 1.06, 95% confidence interval (CI) 1.01, 1.11) than NHW men. NHB men 25-44 years old had a higher AL score than (IRR = 1.14, 95% CI;1.04, 1.24) than their NHW peers. No race differences with respect to AL score were observed among the other age groups. Race differences in AL vary by age categories. Efforts to improve longevity should focus on developing age-tailored health promoting strategies to reduce stress among Black men during early adulthood.

### MIGRATION, MOBILITY, AND PURSUIT OF GOOD OLD AGES: NARRATIVES OF OLDER PUERTO RICAN ADULTS WHO MIGRATE

Brooke V. Jespersen[1], *1. Case Western Reserve University, Cleveland, Ohio, United States*

Migration research has articulated "regimes of mobility," or multi-scalar movements (within and across households, communities, and borders) that are interconnected and embedded in unequal power relations. Research on late-life migration has been limited by: (1) simplistic conceptualizations of mobility as adaptive or traumatic; and (2) a focus on transnationalism. The migration of older adults between Puerto Rico and US mainland presents a new frontier for examining mobility in aging. Puerto Rico's population is rapidly aging and out-migrating. Moreover, as US citizens, Puerto Ricans experience no legal restrictions on migration typical of transnationalism. Yet little is known about their migration patterns and associated narrated meanings. I conducted semi-structured interviews and participant observation among older Puerto Ricans who migrated to the US

mainland in late-life. Preliminary findings suggest that older Puerto Ricans negotiate competing definitions of "good" old ages based on residential context. They report migrating to the mainland to pursue "good" old ages defined in material terms, namely access to social and medical services. Post-migration, however, older Puerto Ricans report experiences of confinement and loneliness, due to language barriers and familial separation. In narrating hopes for the future, they describe an alternative "good" old age in Puerto Rico, emphasizing belonging and familial connection. As older Puerto Ricans negotiate multiple definitions of "good" old ages through circular mobility, the social and economic inequalities which first necessitated migration reproduce disadvantage in the new location. This study highlights the need to conceptualize multi-scalar mobilities that intersect with inequality to shape aging among migrant populations.

### MEASUREMENT EQUIVALENCE OF THE KESSLER 6 PSYCHOLOGICAL DISTRESS SCALE FOR OLDER ASIAN IMMIGRANT SUBGROUPS

En-Jung Shon[1], *1. Miami University, Oxford, Ohio, Oxford, Ohio, United States*

The Kessler 6 (K6) Psychological Distress Scale is a well-known screening instrument to screen for psychological distress of general population. While some studies (e.g., Mitchell & Beals, 2011) concluded that the K6 was appropriate for capturing psychological distress of diverse racial/ethnic groups, other studies (e.g., Andersen et al., 2011) reported that it was less successful in screening for psychological distress of diverse racial/ethnic groups. Few studies conducted measurement equivalence test across older Asian immigrant subgroups. Using Multiple Group Analysis, this study examined whether parameters of the single factor model (items: nervous, hopeless, restless or fidgety, so depressed, everything was an effort, and worthless) is equivalent across the two Asian immigrants (≥65 years; Chinese [n=175] and Korean [n=300] immigrants). Data were generated from the California Health Interview Survey. The configural model showed good fit (X2=41.70 [df=16, p<.001], X 2/df=2.61, CFI=.98, GFI=.97, RMSEA=.06 [90% CI=.04-.08], and SRMR=.04). When all factor loadings were constrained, it indicated measurement non-invariance status between Chinese and Koran (ΔX 2=17.86, Δdf=5, p=.003, CFI=.972, ΔCFI=.009). Given findings of non-invariance on the full constrained model, the invariance test of each factor loading was performed additionally. It was focused on evaluating which items were similar or different across the two groups. The three items, 'hopeless,' 'restless,' and 'depress,' were significantly nonequivalent between the two groups. Clinicians/researchers should aware of the potential risk for misclassification when they try to screen for psychological distress in older Chinese or Korean immigrants. Professionals should pay attention to cross-cultural comparability when interpreting results from the K6.

### DISABILITY IN PRISON ACTIVITIES OF DAILY LIVING AND DEPRESSION IN OLDER PRISONERS: A PROSPECTIVE STUDY

Lisa C. Barry,[1] Dorothy Wakefield,[2]
David C. Steffens,[1] and Yeates Conwell[3], *1. University of Connecticut School of Medicine, Farmington, Connecticut, United States, 2. UCONN Center on Aging, Farmington, Connecticut, United States, 3. University of Rochester School of Medicine, Rochester, New York, United States*

The U.S. prison population is aging; more persons are being incarcerated in the second half of life and are aging "in place." In the first prospective study to evaluate older prisoners' mental health (Aging INSIDE), we determined if disability in activities of daily living specific to prison, prison activities of daily living (PADLs), predicts depression in this vulnerable population. To date, 134 older prisoners (age ≥50) sentenced at 9 Connecticut correctional facilities completed in-person interviews (baseline and one-year follow-up). A score of ≥10 on the 9-item Physician Health Questionnaire (PHQ-9) indicated depression. Participants were considered to have PADL disability if they reported any of the following as "very difficult" or "cannot do": climbing on/off the top bunk (34%), cleaning their cell (5%), hearing orders (6%), walking while wearing handcuffs (33%) or shackles (34%), standing in line for medications (4%), and walking to chow (5%). Participants were mean age 57.0±6.6 years (range 50-79 years), racially diverse (43% White, 38% Black, 19% Hispanic/Other), 69 (50%) had PADL disability, and 35 (25%) were depressed at follow-up. Using logistic regression and controlling for gender, number of chronic conditions, lifetime suicide attempt, and baseline depression, baseline PADL disability was associated with depression one year later (OR = 3.41; 95%CI = 1.16, 9.97). As depression is a strong risk factor for subsequent suicide, and given the high rate of suicide among older prisoners in the U.S., these preliminary results indicate that PADL assessment may offer a simple means of identifying older prisoners at risk of depression.

### SOCIOCULTURAL DETERMINANTS IN PAIN PERCEPTION AND MANAGEMENT AMONG OLDER CHINESE AMERICANS

Simona Kwon,[1] Jazmine Wong,[1] Janet Pan,[1]
Andrew Rosenberg,[1] Germaine Cuff,[1] and Myint Aye[2],
*1. NYU School of Medicine, New York, New York, United States, 2. NYU Langone Hospital Brooklyn, Brooklyn, New York, United States*

Background: Chinese Americans make up the largest Asian American subgroup in the US. Data from a large health system indicate that older Chinese Americans experience lower satisfaction in pain management after surgery compared to all other racial/ethnic groups. Objective: To understand pain experience among older Chinese American patients to improve pain satisfaction strategies Methods: A mixed methods study was conducted, including: 1. A scoping review of the peer-reviewed published literature; 2) face-to-face survey; and 3) qualitative interviews. 14 Chinese American postsurgical patients >65 years of age were recruited for the survey and interview with a trained bilingual Community Health Worker. Questions from the Survey on Disparities in Quality of Healthcare and Kleinman's Explanatory Model of Illness guided the data collection tools. Results: The 31 studies identified in the review were largely observational; none assessed pain control or management interventions for older Chinese Americans. Most participants reported experiencing a language barrier that hindered healthcare staff communication during hospital stay. Even with an interpreter, limited English proficient patients reported lower understanding of health information compared to those who did not need interpretation. Ideas of "pushing through" pain, perceiving physicians as "busy people," and mismatch in pain assessment tools contributed to pain attendance delay. Facilitators to care included family support, culturally and linguistically-tailored tools, and availability of cultural remedies. Conclusions: This mixed-methods study identified key themes including socio-cultural barriers and facilitators to effective pain care and management. Findings will inform tools and resources to better capture and address pain management in Chinese Americans.

### MIDDLE-AGED AND OLDER LATINOS' SATISFACTION OF BAILAMOS LATIN DANCE PROGRAM

Guilherme M. Balbim,[1] Susan Aguinaga,[2] Isabela G. Marques,[3] Jacqueline Guzman,[4] David X. Marquez,[1] and Priscilla Vasquez[5], *1. University of Illinois at Chicago, Chicago, Illinois, United States, 2. University of Illinois at Urbana-Champaign, Urbana, Illinois, United States, 3. University of Victoria, Victoria, British Columbia, Canada, 4. University of Illinois at Urbana-Champaign, Urbana-Champaign, Illinois, United States, 5. University of California San Diego, California, San Diego, California, United States*

Older Latinos engage in low levels of leisure-time physical activity (LTPA). Dance is a culturally appropriate activity which can be used to increase LTPA levels. We examined middle-aged and older Latinos' satisfaction with the revised BAILAMOS Latin dance program. Healthy and low actives middle-aged and older Latinos (Mage = 64.89±7.08) were randomized to a 4-month dance program (n=167) or health education (n=166). The dance program consisted of four Latin dance styles (Merengue, Bachata, Cha Cha Cha, and Salsa). Classes were held twice a week for one hour. A total of 113 participants completed the program. Participants completed a program evaluation about the 4-months program regarding time, duration, settings, instructor, and overall satisfaction. Items were evaluated on a 1 (strongly disagree/very bad) to 4 (strongly agree/excellent) Likert agreement scale. A total of 73 participants evaluated the 4-month dance program. Participants evaluated the program adequacy agreeing or strongly agreeing as far: time, duration and setting (96-98%); instructor's enthusiasm, quality of instructions, and eager to help (96-100%); dance program's progression and enjoyment (93-96%); difficulty level (59%). Participants reported they intended to keep dancing by themselves (93%) and would recommend the program to friends and family (98%). Many participants (88%) reported feeling physically excellent or good as a result of the program, 95% found the program excellent or good, and 100% thought the program was worth their time. Overall, the BAILAMOS program evaluation demonstrated high participants' acceptability and satisfaction. Those results can promote sustained LTPA and provide initial evidence to translation into community settings.

### THE EFFECTS OF IMMIGRANT STATUS ON WELL-BEING AMONG OLDER ADULTS BY RACE-ETHNICITY: A MULTI-GROUP ANALYSIS

Heehyul Moon,[1] Hyesook Kim,[2] Sunshine Rote,[1] and William E. Haley[3], *1. University of Louisville, Louisville, Kentucky, United States, 2. Daegu University, Daegu, South Korea, Korea, Republic of, 3. University of South Florida, Tampa, Florida, United States*

Although prior researchers have decried the lack of research on racial/ethnic minority older adults, they have been less vocal about the gaps in research concerning the ways in which immigrant status and race/ethnicity affect their well-being. Thus, we examined the role of immigrant status on the stress coping process by race/ethnicity using the Transactional Model of Stress and Coping. The multi-group analysis function in structural equation modeling was used to determine whether the stress coping process was equivalent across three racial/ethnic groups (Non-Hispanic White (NHW), Non-Hispanic Black(NHB), and Hispanic) by immigrant status using the Round 1 of the National Health and Aging Trends Study (NHATS, (U.S.-born= 4,799, foreign-born=612)). We found that immigrant status and race/ethnicity may have complex effects on the stress coping process. For example, the total effects of being an immigrant were significantly associated with more stressors, less resources, and worse physical health. Except NHW, the total effects of being immigrant were associated with higher levels of depression and anxiety. With respect to the direct and indirect effect of immigrant status in the three groups, the Hispanic group has a larger effect of immigrant status on stressors, resources, depression/anxiety and physical health than their NHW and NHB counterparts. The results indicated that immigrant racial/ethnic minority older adults were more likely to have higher levels of depression and anxiety than the U.S.-born except for NHW. Immigrant status will require special attention in both assessment and management of depression/anxiety among racial/ethnicity minority older adults.

### EXPERIENCES OF DISCRIMINATION ARE ASSOCIATED WITH DECREASED FUNCTIONAL ABILITY IN AFRICAN IMMIGRANT OLDER ADULTS

Manka Nkimbeng,[1] Karen Bandeen-Roche,[2] Hae Ra Han,[1] and Sarah L. Szanton[1], *1. Johns Hopkins School of Nursing, Baltimore, Maryland, United States* Roland Thorpe[2], *2. Johns Hopkins Bloomberg School of Public Health, Baltimore, Maryland, United States*

Discrimination impacts functional health outcomes of African Americans and other racial/ethnic minorities in the United States; yet this is understudied in African immigrants whose population has risen by 137% since 2000. We examined the relationship between discrimination and physical function with a convenience sample of first-generation African immigrants age 50+ recruited through community-based organizations (N=124). Discrimination was measured with the Everyday Discrimination scale with higher scores indicating more experiences of discrimination (range=0-23). High versus low levels of discrimination were categorized at the mean. Physical function was measured using the PROMIS Physical Function measure with high scores indicating greater functional ability (range=11-50). Raw function scores were converted to standardized T-scores with a population mean of 50 and standard deviation (SD) of 10. Linear regression was used for analyses. Mean age of the sample was 61.4(SD=7.9) years. About two-thirds (63%) were female, more than half (52.4%) immigrated in search of better opportunities and half of the sample had high levels of discrimination. The mean function score was 44.2(SD=8.3) indicating that this sample had functional ability 6 points less than the population average. After adjusting for demographic and migration factors, the mean physical function score was 2.5 points lower (b=-2.53, 95% CI= -5.04, -0.01) for participants with more experiences compared to those with fewer experiences of discrimination. In conclusion, discrimination was associated with poor physical function in African immigrant older adults after adjusting for covariates. Longitudinal studies of discrimination and physical functioning should be pursued in more diverse, larger samples of African immigrants.

### TóMALO SUAVE (TAKE IT EASY): HOW LATINO OLDER ADULTS PERCEIVE OF POSITIVE AGING

Lissette M. Piedra,[1] Melissa Howe,[2] John Ridings,[3] Yadira Montoya,[2] and Kendon Conrad[4], *1. University of Illinois at Urbana Champaign, Urbana, Illinois, United States, 2. NORC at the University of Chicago, Chicago, Illinois, United States, 3. The Salvation Army Evanston Corps Community Center, Evanston, Illinois, United States, 4. the School of Public Health at the University of Illinois at Chicago, Chicago, Illinois, United States*

With the help of the Positive Aging of Latinos Study (PALS) steering committee (N = 20), we used concept-mapping methods to learn what Latino older adults (N = 101) consider important for aging well, positively, and successfully. We used data from nine focus groups (six Spanish, three English) to generate an unabridged list of 171 statements that described what positive and successful aging meant to participants. The PALS steering committee reviewed the statements, assisted with the translation and back translation of items in Spanish, eliminated vague and duplicate statements, and approved a final list of 85 statements. Next, Latino older adults thematically sorted (n = 35) and rated (n = 93) the 85 statements (using a 1-5 scale; higher values indicate greater importance). These data were used to produce a concept map for how participants conceptualized positive and successful aging. The final map consisted of 11 clusters nested within 4 overarching regions. Region 1 [Self-Sufficiency] contains clusters of items which address "Stability" and "Independence." Region 2 [Healthy Behaviors] includes clusters with items related to "Staying Healthy" and "Avoiding Trouble." Region 3 [Perspectives on Life] encompasses four clusters of items that address mindsets: "Tómalo Suave (Take it Easy)," "Outlook on Life/Self-Care," "Emotional Well-being," and "Maturing." Region 4 [Convivir (To coexist)] features indictors of interrelatedness such as "Social & Community Engagement," "Coping & Adjustment," and "Family Relationships." These findings provide insights into how Latino older adults conceive of positive aging, which could be useful when designing culturally sensitive programming for Latino seniors.

### UNIMAGINED FUTURES: THE PARADOX OF FAMILISM AND ELDERCARE AMONG AGING LATINOS IN THE CHICAGOLAND AREA

Melissa Howe,[1] Alexis Howard,[1] Wendy Hsieh,[2] and Lissette M. Piedra[2], *1. NORC at the University of Chicago, Chicago, Illinois, United States, 2. University of Illinois at Urbana-Champaign, Urbana, Illinois, United States*

Scholars of gerontology highlight the ways aging varies cross-culturally. Whereas North Americans tend to describe "successful aging" as the maintenance of social and physical independence, Latin Americans tend to view aging as a natural process of social transition. In this study, we conducted a content analysis of nine focus groups (N =101) and 20 interviews with Latino older adults in the Chicagoland area to examine how they characterize successful aging and view the health declines that accompany aging. We found that Latino older adults often used rhetoric associated with "successful aging," which tended to emphasize the maintenance of independence and physical functioning. Even immigrant respondents employed this language, suggesting that descriptions of "good old age," may be more culturally transferable than previously thought. At the same time, the cultural values of respeto and familismo also emerged. Regardless of the participant's nativity status, centrality of family and the importance of respect represented constant sources of support. Still, adherence to these values came with considerable drawbacks for those intensely focused on self-sacrifice for the sake of their families. Taken together, "successful old age" was defined by the participants as one in which a person maintains physical independence in the context of an interdependent, kin-focused, social life. This paradoxical combination of valuing independence and familial interdependence produced a number of benefits and challenges for Latino adults as they transitioned into to older adulthood.

### HEALTHY LIFE EXPECTANCY OF OLDER HISPANICS: THE INFLUENCE OF NEIGHBORHOOD CHARACTERISTICS

Kerstin G. Emerson,[1] Anqi Pan,[1] Hanwen Huang,[1] and Kyriakos Markides[2], *1. University of Georgia, Athens, Georgia, United States, 2. University of Texas Medical Branch, Galveston, Texas, United States*

Research consistently shows a survival advantage among Hispanics, despite a worse health profile. The goal of this study was to calculate disability free life expectancy for older Hispanics in the United States, and to explore any difference by neighborhoods. We used data from the Wave 5 (2004-5) of the Hispanic Established Population for the Epidemiological Study of the Elderly (Hispanic EPESE), linked to vital status data through 2016. We used Sullivan's method to create disability free life expectancy (DFLE) estimates, and to calculate the ratio of life expectancy without disability to life expectancy with disability. These estimates were compared across neighborhood characteristics using Census FIPS data. All neighborhood characteristics were cut into tertiles and significance testing compared high versus low. The average age of the sample was 82 (range 75-109), a majority female (62%), non-married (57.5%), and born in the US (56%). Results showed that neighborhood Hispanic density, poverty, and percent linguistically isolated were not statistically significant for disability free life expectancy estimates. However, disability free life expectancy was higher in neighborhoods with higher density of immigrants, compared to neighborhoods with lower density of immigrants. This was statistically significant for all age groups 75 until age 88. These results suggest that for very old Mexican Americans living in the southwest, neighborhood effects are not significant predictors of disability free life expectancy. The exception is for the neighborhood immigrant density. This suggests that the healthy immigrant effect may maintain even in older ages. Policy and practice implications will be discussed.

### SESSION 3365 (POSTER)

### PAIN: SYMPTOMS AND MANAGEMENT

### PAIN AND DEPRESSION AFFECT SELF-REPORTED STRESS RATINGS IN ADULTS WITH AND WITHOUT FIBROMYALGIA

Ha M. Nguyen,[1] Kristen Phillips,[1]
Barbara J. Cherry,[1] and Laura Zettel-Watson[1], *1. California State University, Fullerton, Fullerton, California, United States*

Fibromyalgia (FM) is a chronic pain condition characterized by tenderness, fatigue, stiffness, joint pain, and physical and emotional distress. Depression is common, as well, and stress may be perceived as more severe. The current study examines perceived stress in adults ages 50 and older with and without FM. It was hypothesized that individuals with FM and/or depression would subjectively rate stressors as more severe compared to those without. Ninety-four participants (53% with FM, 78% female) aged 50 to 93 (M = 67.72, SD = 9.26) were administered an updated version of the Social Readjustment Rating Scale (SRRS) to assess amount of stress experienced in the past year. The difference between the SRRS pre-determined values and participants' subjective ratings was calculated. Lower difference scores indicated that self-reported severity exceeded standardized values. Hierarchical linear regression analyses revealed that older adults and men were less likely to report exaggerated stressor severity ($p < .05$). Controlling for age and gender, individuals with FM were significantly more likely to report stressor severity far above standardized severity scores ($p < .05$). Results also revealed that both depression and chronic pain impact stress ratings, but impact is significantly greater for depression ($p < .001$). When controlling for depression, FM impact is no longer significant, suggesting that depression is a stronger predictor of subjective stress. The findings emphasize the importance of stress management and lifestyle changes to reduce the likelihood of depression and stress perception in individuals experiencing chronic pain.

### PAIN THAT LIMITS PHYSICAL ACTIVITY AFFECTS COGNITION IN ADULTS

Amanda F. Elliott,[1] and Ann Horgas[1], *1. University of Florida, Gainesville, Florida, United States*

Reduced cognitive skills have been observed in adults with chronic pain. The purpose of this study was to explore the relationship between pain and cognition in adults utilizing a large national sample. This was a cross-sectional study employing the 2015 wave of the Behavioral Risk Factor Surveillance System (BRFSS). A total of 134,058 adults had complete data on the variables of interest for this study. Half of the sample (50%; n = 66,479) reported having joint symptoms or arthritis that was physically limiting and 16% (n = 21,976) reported having difficulty concentrating, remembering, or making decisions. Twelve percent (n = 16,537) of this sample reported having both physically limiting pain and difficulty concentrating and remembering. Chi-square analyses reveal a statistically significant association between pain and cognition in this sample [X2(1, N= 134,058) = 6925.5, p<.01], with a small to medium effect size (phi=0.227). This study provides support that pain is associated with difficulty concentrating and remembering in adults. Pain is a common persistent symptom among older adults and its effect on cognitive functioning should be noted. Effective pain treatment strategies are warranted to help reduce the cognitive burden of chronic pain. In turn, in older adults who are experiencing concentration or memory problems, health care providers should assess the individual's pain as a possible contributor. Additional studies assessing both pain and cognition are warranted, especially looking at the relationship of these conditions over time.

### NUTRITIONAL RISK IS ASSOCIATED WITH LOW BACK PAIN AMONG OLDER ADULTS: RESULTS FROM THE UAB STUDY OF AGING

David R. Buys,[1] Marion W. Evans,[1] Richard E. Kennedy,[2]
Julie Locher,[2] Katie Buys,[2] and Cynthia J. Brown[2],
*1. Mississippi State University, Starvkille, Mississippi, United States, 2. University of Alabama at Birmingham, Birmingham, Alabama, United States*

Poor nutritional status is associated with adverse health outcomes across the life course, affecting older adults' ability to maintain overall well-being, limiting physical strength, and affecting mobility. International research has demonstrated associations between nutritional risk and general musculoskeletal pain; however, no research has explored relationships between nutritional risk and low back pain. Using the University of Alabama-Birmingham Study of Aging, we examined this relationship among 1000 community-dwelling older Alabamians (65+years). We used the DETERMINE Checklist, a well-validated nutritional risk assessment and assessed presence and severity of low back pain over the past 4 weeks. We completed univariate and bivariate analysis and multivariate logistic regression, adjusting for factors significant in the bivariate analyses: sex, body mass index, depression, and co-morbidities. More than half of the participants were at nutritional risk (55.2%). In multivariate analyses, one point increases in nutritional risk were associated with a 14% increase in the likelihood of low back pain 95% CI (1.087,1.213); in categorical analyses, moderate nutritional risk and high nutritional risk were associated with an increase in likelihood of low back pain [46% (95% CI 1.07,2.02) and 164% (95% CI 1.80,3.94), respectively]. While this cross-sectional analysis should be interpreted cautiously, it further emphasizes the importance of nutritional health for older adults. Clinicians treating patients with low back pain or nutritional risk may consider assessing for the other condition. When nutritional risk is detected, clinicians should refer to services such as counseling with a registered dietitian or to a social worker for assistance identifying community-based nutritional supports.

### FEAR AND DYNAMIC INFLAMMATORY RESPONSE TO MOVEMENT AMONG OLDER ADULTS WITH LOW BACK PAIN: A FEASIBILITY ANALYSIS

Corey Simon,[1] Francis Keefe,[1] Gregory Hicks,[2]
Carl Pieper,[3] and Cathleen Colon-Emeric[3], *1. Duke*

*University, Durham, North Carolina, United States, 2. University of Delaware, Newark, Delaware, United States, 3. Duke University Medical Center, Durham, North Carolina, United States*

Low back pain (LBP) is a prevalent cause of disability, with higher prevalence among older adults. However, the level of LBP disability cannot be explained by either pain intensity or LBP pathology (bone degeneration). We hypothesize that older adults with LBP are susceptible to disability based on the combination of two factors: fear of movement and dynamic inflammatory response to movement (DIR). In this feasibility study, we are measuring these two factors among 30 older adults aged 60-85 with LBP. Participants attend a laboratory session to complete four performance tasks: 30-second chair rises, seated trunk rotation, standing forward reach, and Six-Minute Walk. Fear of movement is tested using the Tampa Scale for Kinesiophobia and Situational Catastrophizing Questionnaire. DIR is tested by drawing blood via a peripheral venous catheter before and after performance tasks, and assessing changes in six inflammatory markers: IL-6,8,10; TNF-alpha, c-reactive protein, and substance P. LBP disability is assessed using the Late-life Functional Disability Instrument (LLFDI) and 7-day ecological momentary assessment (EMA) via smartphone application. Participants have tolerated all laboratory testing procedures and have adequately reported LBP disability in the home and community using EMA. We will test for exploratory associations between fear of movement, DIR, and LBP disability measures using ordinary least squares regression analyses. The overarching goal of this research line is to determine if the combination of fear of movement and DIR are an actionable clinical risk phenotype of downstream LBP disability among older adults.

### A CRITICAL REVIEW OF PAIN MANAGEMENT TRAINING PROGRAMS FOR FAMILY CAREGIVERS

Nai-Ching Chi,[1] Emelia Barani,[2] and Ying-Kai Fu[1], *1. University of Iowa, Iowa city, Iowa, United States, 2. University of Washington, Seattle, Washington, United States*

Many American family caregivers (FCs) manage hospice and palliative patients' pain at home. However, FCs encounter many barriers to pain management due to inadequate training. Although some studies have designed pain management training programs for caregivers, the content and durations vary greatly. Thus, there is a lack of consistent recommendation on how to educate caregivers on pain management. The purpose of this study is to critically evaluate existing training programs to inform clinical practice and future program design. A literature review was conducted to search available articles published before June 2018 in databases including PubMed, CINAHL, PsycINFO, and Scopus. Search strategies used index and keyword methods. The inclusion criteria were peer-reviewed, research studies published in English that evaluated a pain management education or training for family caregivers. Twenty-seven studied were included. All studies improved either patients' outcomes (e.g. pain intensity, hospital visits) or caregivers' outcomes (e.g. knowledge, quality of life). Most studies (85%) had research teams provide caregivers with pain management education via multiple face-to-face training sessions and written booklet, while some studies (15%) had practicing nurses used videophones, web-based platforms, and telehealth to enhance the collaboration of care and pain management with caregivers. Providing adequate pain management training can improve patients' and caregivers' outcomes. However, in-person training programs are not practical for busy and overwhelmed caregivers. Researcher-delivered training sessions are not clinically adoptable. Future studies should develop pain management interventions that allow nurses to coach caregivers during routine visits and enhance their communication and collaboration with caregivers.

### IDENTIFY THE BARRIERS THAT FAMILY CAREGIVERS FACE IN MANAGING PATIENTS' PAIN: A LITERATURE REVIEW

Nai-Ching Chi,[1] Ying-Kai Fu,[1] Emelia Barani,[2] and Elizabeth Summerson[1], *1. University of Iowa, Iowa city, Iowa, United States, 2. University of Washington, Seattle, Washington, United States*

Most Americans enrolled in palliative and hospice care receive care at home and thus rely on their family caregivers (FCs) to manage their pain. However, FCs encounter many barriers to pain management. Previous studies focused on barriers faced by FCs of cancer patients. However, as palliative and hospice care evolves, the populations and diagnoses become diverse, and more barriers have been identified. Hence, the purpose of this study is to comprehensively investigate FCs' barriers to pain management. A literature review was conducted to search available articles published before June 2018 in databases including PubMed, CINAHL, PsycINFO, and Scopus. Search strategies used index and keyword methods. The inclusion criteria were peer-reviewed, research studies published in English that explored barriers that FCs faced in managing pain. Eighty-six studied were identified: 76% of the studies focused on cancer pain and 6% focused on dementia pain. The identified barriers included: (1) caregivers' limited knowledge in drug/non-drug pain management and verbal/non-verbal pain assessment; (2) caregivers' issues (e.g. function, fear of analgesic, misbeliefs in pain management); (c) caregivers' organizational skills (treatment recording and tracking); (d) patients' issues (e.g. inability to verbalize pain); (e) communication issues with care teams. This is one of the few literature reviews that comprehensively investigate the barriers that family caregivers experience beyond cancer pain management. The results can be used to develop a screening questionnaire for palliative and hospice providers to assess and resolve FCs' barriers to pain management, thereby improving the quality of pain management and patients' and FCs' outcomes.

### BURDEN OF POTENTIALLY INAPPROPRIATE MEDICATIONS AMONG OLDER VETERANS WITH PAIN AND DEMENTIA

Heather Allore,[1] Danijela Gnjidic,[2] and Ling Han[3], *1. Yale University, New Haven, Connecticut, United States, 2. The University of Sydney, Sydney, NSW, Australia, 3. Yale School of Medicine, New Haven, Connecticut, United States*

Among 115,869 Veterans ≥65 years old with a history of moderate to severe musculoskeletal pain (numerical pain intensity rating ≥4) admitted to Veterans Health Administration hospitals in fiscal year 2013, we tested whether medication use is potentially inappropriate (defined using the 2015 Beers Criteria; categorized as central nervous system (CNS) or other drugs) according to Alzheimer's Disease and Related Dementias (ADRD) status based on ICD-9 codes. We used Poisson regression to estimate the association of the number of CNS Beers Criteria drugs (anticholinergics, antidepressants, antipsychotics, benzodiazepines, sedative hypnotics, pain medications, and opioids) and other Beers Criteria drugs according to ADRD status. The mean age of the cohort at the index hospital admission was 74.5 (SD 8.2), with 19.0% having ADRD and a Charlson comorbidity index of 3.9±3.0. After adjusting for age, sex, race/ethnicity, marital status, body mass index, 16 diagnostic criteria for pain and chronic conditions, mental health conditions and use of other system Beers Criteria drugs, we found that the mean number of CNS Beers Criteria drugs were 25% higher among Veterans with ADRD, with an adjusted risk ratio (aRR) of 1.25 (95% CI 1.23, 1.27; P <.0001). On the other hand, the mean number of other Beers Criteria drugs appeared to be 2% lower among ADRD group compared with inpatients without ADRD (aRR: 0.98 (95% CI: 0.97, 0.99), p<0.004). Clinicians need to be aware of potential side effects of using CNS Beers drugs in people with ADRD and whether there are alternatives to their use.

### PAIN, AFFECT, AND OBJECTIVE ACTIVITY LEVELS IN OLDER ADULTS WITH OSTEOARTHRITIS

Emily A. Behrens,[1] Jason A. DeCaro,[1] Dylan M. Smith,[2] and Patricia A. Parmelee[1], *1. The University of Alabama, Tuscaloosa, Alabama, United States, 2. Stony Brook University, Stony Brook, New York, United States*

This research examined the relation between physical activity, pain, and mood among older adults with osteoarthritis (OA). Physical activity is associated with long-term maintenance of function in persons with chronic pain (Dunlop et al., 2014), but less is known about the association between objective measures of activity and transient mood states. Therefore, we captured the activity and mood levels of 218 older adults with knee OA over a seven-day period. Wrist and waist accelerometers captured small and large motor movements. Self-reported momentary pain and affect were collected through phone calls four times daily. We examined average and peak activity levels over the 4-hour windows between self-reports. Cross-sectionally, there was no association between momentary pain and activity. Average large motor movement was positively associated with positive affect and negatively associated with negative affect. Analyses revealed one association between affect and average previous activity; small motor movements predicted greater positive affect. Peak levels of both movements predicted greater positive affect, but only peak wrist activity predicted negative affect. Peak small motor movement at the previous call was associated with both positive and negative affect. These results provide insight into the unique contributions of small and large motor activity to mood and pain states. It appears that average large motor movements and prior small motor activity may have the greatest impact on momentary affect. Further study of distinct activity types and mood will be important for understanding and improving the quality of life among individuals diagnosed with OA (Supported by R01-AG041655).

### CAN HEART DISEASE SELF-MANAGEMENT EDUCATION ALSO REDUCE PAIN INTERFERENCE IN AFRICAN AMERICAN OLDER ADULTS?

Mary R. Janevic,[1] Kristi Allgood,[1] Jessica Ramsay,[1] Cainnear Hogan,[1] Rebecca Courser,[1] and Cathleen M. Connell[1], *1. University of Michigan School of Public Health, Ann Arbor, Michigan, United States*

Many older adults with heart disease, especially those from vulnerable populations, live with chronic pain. Self-management strategies for reducing cardiovascular risk (e.g., physical activity, stress reduction) are also recommended for management of chronic pain. It is not known, however, whether self-management education focused on heart health has a beneficial "side effect" on pain-related outcomes. We explored this possibility using data from a randomized controlled trial (n=405) of Take Heart, a group intervention adapted for low-income, African American adults 50 and over with heart disease or significant risk factors. We first assessed the sample prevalence of high-impact chronic pain; i.e., substantial pain-related disability in one or more life domains (National Pain Strategy, 2016). Next, we assessed whether participation in Take Heart resulted in decreased pain interference (PROMIS-29). One-third of participants (n=131) met criteria for high impact chronic pain. Mean pain interference T-score in the entire sample at baseline was 58.1 (SD=9.9), indicating a score one standard deviation greater than population average. Compared to controls, intervention participants had a greater, but non-significant, improvement in pain interference (0.7 vs. -0.8; p=.11). Overall, findings demonstrate that African American adults with cardiovascular conditions have a high burden of comorbid pain. This pain was not greatly improved by an intervention that taught key chronic disease self-management skills but did not address pain specifically. Future research can test whether incorporating pain-management content into heart disease education has a stronger impact on improving pain outcomes -- which may, in turn, promote adherence to behaviors to reduce cardiovascular risk.

### IS THERE A RELATIONSHIP BETWEEN PAIN AND FEAR OF FALLING IN OLDER ADULTS LIVING IN LONG-TERM CARE FACILITIES?

Edgar R. Vieira,[1] Diego Tavares,[2] Particia Nobrega,[2] and Alvaro Maciel[2], *1. Department of Physical Therapy, Florida International University, Miami, Florida, United States, 2. Graduate Program in Physical Therapy, Federal University of Rio Grande do Norte, Natal, Rio Grande do Norte, Brazil*

Fear of falling is common in older adults and it is associated with multiple factors such as gait and balance issues, difficulties in activities of daily living, visual impairment, and frailty. Unfortunately, fear of falls increases the risk of falls as opposed to protect from falls. Pain can impair mobility, affects activities of daily living, and may also be associated with fear of falling but no studies have evaluated this potential association. The objectives of this study were to evaluate if there was an association between pain and fear of falling in older adults living in long-term care facilities. One hundred and eight older adults living in long-term care facilities participated in the study. The mean age was 79±7 years, and 65% of the participants were women. The participants completed the Geriatric Pain Measure (GPM) questionnaire for multidimensional pain assessment (scores range from 0 to 42), and the Falls Efficacy Scale International (FES-I) for fear of falling assessment (scores range from 16 to 64). The data was analyzed using multiple linear regression. Forty-five percent of the participants had chronic pain (≥3 months) and 18% had acute pain (<3 months). Pain scores were 29±31. Pain was associated with an increase of 3 to 7 points (out of 64 max) in the FES-I. The prevalence of pain in long-term care residents was high, and pain was associated with increased fear of falling.

### DEVELOPING A PAIN IDENTIFICATION AND COMMUNICATION TOOLKIT FOR FAMILY CAREGIVERS OF PERSONS WITH DEMENTIA

Catherine Riffin,[1] Karl Pillemer,[2] Keela Herr,[3] Emily Petti,[1] and Cary Reid[1], *1. Weill Cornell Medicine, New York, New York, United States, 2. Cornell University, Ithaca, New York, United States, 3. University of Iowa, Iowa City, United States*

Best practice guidelines have emphasized the importance of routine pain assessment of older persons with dementia (PWD), yet pain remains severely underdetected and undermanaged in this population. Training family caregivers in observational pain assessment and subsequent communication of the assessment results to a healthcare provider has the potential to help improve pain management among PWD. The goal of this presentation is to describe the approach to developing, refining, and pilot testing the Pain Identification and Communication Toolkit (PICT), a novel intervention to help family caregivers recognize pain symptoms in PWD and communicate those symptoms to healthcare providers. Guided by self-efficacy theory and empirical research on dementia and pain communication, this presentation will detail the approach to developing the PICT intervention manual and delineate its major components, including modules that prepare caregivers to: a) recognize and differentiate pain from dementia symptoms, b) administer an observational pain assessment tool, c) communicate effectively about pain symptoms, and d) plan steps to take when pain is detected. The presentation will report results from the process by which preliminary versions of the PICT manual were refined, including iterative field-testing with a sample of racially and ethnically diverse caregivers of community-dwelling PWD and healthcare providers. Results suggest that the development of PICT represents a useful step in addressing the underdetection and undermanagement of pain in PWD, and can pave the way for future intervention research on caregivers' initiation of pain-related communication with healthcare providers.

### CHRONIC PAIN AND ASSOCIATED RISK OF COGNITIVE IMPAIRMENT AMONG MIDDLE-AGED AND OLDER ADULTS

Yi-Han Hu,[1] and Hsien-Chang Lin[1], *1. Indiana University Bloomington, Bloomington, Indiana, United States*

Chronic non-cancer pain (CNCP) is an emerging health issue among the older population. Not only did the CNCP prevalence increase gradually in past decades, but also it may cause difficulties in cognitive processing and social and emotional functioning. However, evidence for the associations between CNCP and incident mild cognitive impairment (MCI) and Alzheimer's disease and related dementias (ADRDs) is inconsistent and insufficient. Using the administrative claims data from health insurance companies from January 2007 to December 2017, this prospective cohort study investigated the impact of CNCP on the risks of developing MCI and ADRDs among adults aged 50 and older. To reduce potential selection bias, the propensity-score matched cohort design was applied for selecting comparable CNCP and non-CNCP patients at the beginning of the follow-up. Time-dependent Cox proportional-hazards regression models were conducted to estimate the hazard ratios (HRs) of incident MCI/ADRDs, adjusting for baseline sociodemographics and time-dependent medical conditions. Of 236,782 patients with/without CNCP, 342 individuals (0.14%) developed MCI and 1,183 patients (5.0%) had been diagnosed with one type of ADRDs during the follow-up. After adjusting confounders, CNCP patients had a 42% increased MCI risk (HR=1.42; 95% CI=1.14-1.76) and a 20% increased ADRDs risk (HR=1.20; 95% CI=1.07-1.34) relative to non-CNCP patients. Our findings indicate that CNCP is associated with incidences of MCI and ADRDs. Early diagnosis of CNCP and CNCP management may prevent cognitive impairment among middle-aged and older adults. Future studies are warranted to explore the potential effects of pain treatments on restoring cognitive function of CNCP patients.

### SESSION 3370 (POSTER)

### SUCCESSFUL AGING

### I GOT THIS! SUCCESSFUL AGING THROUGH THE MAINTENANCE OF AUTONOMY

Alexandria G. Nuccio,[1] and Ashely M. Stripling[2], *1. Nova Southeastern University, Davie, Florida, United States, 2. Nova Southeastern University, Fort Lauderdale, United States*

As America ages, an increased interest has been placed on understanding the development and maintenance of autonomy in later life. This is of particular importance given the impact of autonomy on vitality, well-being, and mood within older adults (Warner et al., 2011). However, the research examining which aspects of autonomy directly impact successful aging remains underexplored. As such, the current study utilizes the Functional Autonomy Measurement System (SMAF) to better understand which facets of autonomy promote life satisfaction in older adults. Data included assessments of the Functional Autonomy Measurement System and data was derived from the Survey of Midlife in the US Database (MIDUS 3). Participants were primarily White/Caucasian (88.7%) and female (54.0%); with a mean age of 63.64 years (SD=11.35). A series of hierarchical multiple linear regressions revealed that higher levels of Mental Functions predicted increased life satisfaction scores in models adjusted for age, sex, race, marital status, and education ($F=54.152, p<0.001$) and that higher levels of Communication (F=37.553,p<0.001), Activities of Daily Living (F=33.495,p<0.001), Mobility (F=37.311,p<0.001), and Instrumental Activities of Daily Living (F=8.630,p<0.001) also predicted increases in life satisfaction scores but to a lesser extent. The present findings suggest that higher levels of autonomy, both physically and mentally, result in higher satisfaction with life; with perceptions of cognitive independence producing the highest levels. Implications of the current findings include increased understanding of the multifaceted nature of autonomy, and provision of recommendations which could increase autonomous behavior in later life, thus increase life satisfaction and promote successful aging.

### RESILIENCE AND POST-TRAUMATIC GROWTH: EXPLORING THE PERCEPTIONS OF GAINS AFTER ADVERSITY IN OLDER ADULTS

Lauren M. Bouchard,[1] and Lydia K. Manning[2], *1. Concordia University, River Forest, Illinois, United States, 2. Concordia University Chicago, River Forest, Illinois, United States*

Resilience has been consistently shown across the literature as a protective factor in terms of aging successfully. Resilience is defined as a process of adjustment and adaptation, where painful life experiences can result in accumulative positive outcomes such as greater life meaning, hopefulness, and spiritual transcendence (Ramsey, 2012). These outcomes are also mentioned in the separate but related construct of "post-traumatic growth," defined as positive outcomes (i.e. self-perception, improved interpersonal relationships, and a changed philosophy on life) which emerge after traumatic experiences (PTG; Tedschi & Calhoun, 1996). This study explored older adults perceptions on adaptation in regards to adverse life situations. Our findings indicate some participants were more likely to espouse resiliency and post-traumatic growth related explanations while others participants articulated difficulty in seeing the benefit related to the challenges they had faced. Similarly, participants faced a range of challenges from everyday stress to major life traumas, which also shaped perceptions of their own growth. Participants also indicated a range of orientations toward growth after adversity including denial, reluctance, acceptance, and optimism. Our results also suggest key differences in these constructs while they also remain similar and complementary in terms of our participants lives and stories. Our study also provides limitations and future directions in operationalizing PTG and resilience in the gerontological literature.

### ROLE MODEL OF AGING AND ATTITUDES TOWARD AGING IN ASIAN AMERICANS

Seojung Jung,[1] and Daniela Jopp, *1. SUNY at Old Westbury, Old Westbury, New York, United States, 3. University of Lausanne, Lausanne, Switzerland, Switzerland*

While having a role model for successful aging may have a beneficial influence and promote successful aging throughout the course of one's life, very little empirical research has examined role models for successful aging, particularly among Asian Americans. The current study examined Asian Americans' role models for successful aging and their link with attitudes toward own aging with a sample of 135 individuals aged 18 to 74 years (Mage = 36.76, SD = 14.73; female, 71.9%). Participants were asked whether they had a specific role model for successful aging and, if they had, the reasons why the role models were chosen. Participants' open-ended answers were then analyzed using qualitative content analysis using a coding scheme drawn from a prior study on role models for successful aging (Jopp et al., 2016). The categories most often mentioned were public figures, followed by parents, other relatives, non-specific persons, grandparents, non-family acquaintances, and self. Participants with at least one family role model were more likely to have positive attitudes toward aging, and this effect was strong even after controlling for ethnic identity, filial obligation, self-rated health, and other demographic variables. The present study suggests that Asian Americans identify role models for successful aging both within their immediate families and among public figures, while family role models are more likely to promote the development of positive attitudes toward own aging. The implications for understanding Asian Americans' role models on motivating, promoting, and facilitating positive aging processes will be discussed.

### DON'T JUST GO THROUGH LIFE, GROW THROUGH LIFE: RESILIENCE AND ITS EFFECT ON SUCCESSFUL AGING

Alexandria G. Nuccio,[1] and Ashely M. Stripling[2], *1. Nova Southeastern University, Davie, Florida, United States, 2. Nova Southeastern University, Fort Lauderdale, United States*

Resilience is commonly recognized as a protective factor that increases an individual's ability to adapt positively to adversity or change. Given the rates of change, which occur in mid and late life, understanding resilience within this population is of particular importance. Yet to date, the direct mechanisms through which resiliency functions remains underexplored in this population. As such, the current investigation seeks to examine the mechanism through which lifelong resiliency may affect the lives of older adults in order to promote successful aging. Data including assessments of lifelong resiliency (measured by an individual's opinion that life has been a continuous process of learning/changing/growing), environmental mastery (Ryff, 1989), instrumental activities of daily living (IADLs), mood (e.g. depression and self-rated emotional health), and subjective physical health were derived from the Survey of Midlife in the US Database (MIDUS 3). Participants were primarily White/Caucasian (88.7%) and female (54.9%); with a mean age of 63.64 years (SD=11.35). A series of hierarchical multiple linear regressions revealed that resiliency predicted better physical (F=43.448,p<0.001) and emotional health (emotional health (F=46.515,p<0.001), depression (F=17.665,p<0.011)), as well as increased environmental control (IADLs (F=109.992,p<0.001), and Environmental Mastery (F=64.686,p<0.001)) in models adjusted for known correlates (i.e. age, sex, race, marital status, education). The present findings suggest that high levels of resilience increase not only perceptions of the world (subjective environmental mastery, physical and mental health) but also direct outcomes (IADLs). Implications of the current finding include elucidation of the concept of resiliency, as well as, recommendations for increasing resiliency in late life.

### WHICH CAME FIRST, WELL-BEING OR PHYSICAL HEALTH? A LONGITUDINAL INVESTIGATION IN MID- TO LATE LIFE

Tarah Raldiris,[1] and Joseph Dzierzewski[1], *1. Virginia Commonwealth University, Richmond, Virginia, United States*

As the United States population continues to age, focus has turned toward understanding and promoting positive aging processes. However, positive aging is not only about maintaining physical health, but also about maintaining and improving psychological health. Though previous research has found well-being to be predictive of physical health outcomes, research has yet to examine the temporal associations between these variables. The aim of the current study was to begin to disentangle these temporal associations by investigating how well-being and physical health relate over the course of nearly two decades in a nationwide sample of adults (N = 7,419, Mage = 46.38 at Time 1). The current study employed a cross-lagged panel SEM design across three time points to investigate if well-being, operationalized as purpose in life and personal growth, predicted future self-rated physical health. Data from the Midlife in the United States (MIDUS) study was analyzed from 1995, 2004, and 2014. Cross-lagged analyses revealed well-being was significantly predictive of future physical health, and physical health was also significantly predictive of future well-being. However, the magnitude of the path loadings indicated well-being was a stronger predictor of future physical health than physical health was of future well-being. Thus, these results suggest that personal growth and purpose in life may be particularly important for the promotion of physical health as individuals age. Future research should investigate if these predictive associations apply to all age-groups, as well as for individuals who self-identify as caregivers.

### MEANING IN LIFE AMONG THE OLD-OLD

Polina Ermoshkina,[1] Eva Kahana,[1] and Boaz Kahana[2], *1. Case Western Reserve University, Cleveland, Ohio, United States, 2. Cleveland State University, Cleveland, Ohio, United States*

Older adults, who view their lives as meaningful demonstrate better physical and mental health (Krause, 2007). However, the voices and the experiences of the old-old about what gives meaning to their lives have rarely been explored. This descriptive study examined key components of having meaning in life among independently living old-old migrants to the sunbelt. The sample consisted of 27 women and 18 men (N=45), with the mean age of 88.5 (SD=3.75) and the median annual income of $22,400, who participated in the Florida Retirement Study (Kahana et al., 2002). In response to the question "What gives the greatest meaning to your life at present?" family was reported as the primary source of meaning for the vast majority (40) of the participants. This is consistent with Tornstam's (1997) theory of gerotranscendence reflected in a decreased sense of self-centeredness and greater connection to other generations. Men were more likely to list spouse as the primary source of meaning in life, followed by family, while women referred more generally to family. Health was equally important for men and women, followed by close friendships reported by 22 participants. This finding is consistent with Carstensen's (2003) socioemotional selectivity theory suggesting that with age, the meaning of relationships changes and superficial relationships fade away. For those, who reported being very religious (7) Christian faith and attending church comprised key determinants. A transcendent, rather than materialistic view of life was illuminated by the fact that only one participant reported money as the greatest meaning in life.

### IS COMBINED TRAINING MORE EFFECTIVE THAN SINGLE-DOMAIN TRAINING: A RANDOMIZED CONTROLLED TRIAL WITH OLDER ADULTS

Soledad Ballesteros,[1] Jennifer Rieker,[1] josé M. Reales,[1] julia Mayas,[1] María Pilar Jiménez,[1] Antonio Prieto,[2] and Pilar Toril[1], *1. Universidad Nacional de Educación a Distancia, Madrid, Spain, Spain, 2. Centro Cardenal Cisneros (Alcalá de Henares), Madrid (Spain), Spain*

Previous research suggests that both cognitive training and physical exercise help to maintain brain health and cognitive functions that decline with age. The main objectives of this four-arms RCT are (1) to investigate the synergetic effects of a group-based multidomain training program that combines cognitive video-game training with physical exercise, in comparison to those produced by cognitive training combined with physical control activity, physical training combined with cognitive control activity, or a combination of both control activities; (2) to investigate in a memory-based task switching task whether event Related Potential (ERP) latencies of the P2 component are shorter, and N2 and P3b components are enhanced after training; and (3) to find out whether possible enhancements persist after a 3-month period without training. One hundred and twenty participants will be randomly assigned to one of the four combinations of cognitive training and physical exercise. The cognitive component will be either video-game training (cognitive intervention, CI) or video games not specifically designed to train cognition (cognitive control, CC). The physical exercise component will either emphasize endurance, strength, and music-movement coordination (exercise intervention, EI) or stretching, toning and relaxation (exercise control, EC). This RCT will investigate the short and long-term effects of combined multi-domain training compared to cognitive training and physical training alone, on executive control and memory functions of healthy older adults, in comparison with the performance of an active control group. This trial is an ongoing project started in 2018. Trial registration: Clinicaltrials.gov ID: NCT03823183; https://register.clinicaltrials.gov/

### RESILIENT AGING ROUNDTABLE: AN EVALUATION OF A BRIEF COMMUNITY PSYCHOEDUCATION DISCUSSION GROUP

Andrea June,[1] and Meghan Marty[2], *1. Central Connecticut State University, New Britain, Connecticut, United States, 2. Rose City Geropsychology, LLC, Portland, Oregon, United States*

Increased aging resilience levels are associated with many positive outcomes for older adults including improved quality of life, increased coping and adaptation, and decreased depressive symptoms (Earvolino-Ramirez, 2007; Fullen & Gorby, 2016; Hicks & Conner, 2014; (Sharpley, Bitsika, Wootten, & Christie, 2014). However, very few resilience promotion programs are described in the literature. The purpose of the present study was to evaluate a brief, community-based psychoeducation group designed to enhance aging resilience. Participants were recruited through a private mental health practice focused on serving older adults in the Pacific Northwest. Nine participants (M age = 71; 78% female, 100% non-Hispanic white; 100% with some college) completed the pre- and post-assessment measures: An adapted 9 item version of the Communicative Ecology Model of Successful Aging (CEMSA; Fowler, Gasiorek, & Giles, 2015) and the Groningen Ageing Resilience Inventory (GARI; van Abbema et al., 2015). The discussion group consisted of six 90-minute meetings every-other-week, facilitated by a licensed clinical psychologist. Each meeting focused on a different topic related to psycho-social aspects of aging and included understanding ageism, embracing change, creating meaning, normal and "successful" aging, and strengthening social ties. Although not statistically significant, initial results showed lower post-assessment mean scores on the CEMSA indicating lower levels of aging uncertainty, negative attributions, and pessimism as well as higher post-assessment mean scores on the GARI indicating higher perceived resilience. Moreover, 77.7 % of the group agreed or strongly agreed that they had learned a lot from the group. Future directions will be discussed.

### IDENTIFYING PREDICTORS OF SURVIVAL TO 100 IN OKLAHOMA USING CENTENARIAN BIOGRAPHIES

Nadia Firdauysa,[1] Jyoti Bhatta,[1] and Alex J. Bishop[1],
*1. Oklahoma State University, Stillwater, Oklahoma, United States*

The purpose of this study was to identify key predictors of centenarian survival in Oklahoma. Data originated from N = 607 centenarian biographies maintained within Oklahoma Centenarians, Inc. historical records database. Biographies were analyzed and coded for demographic content. IBM/SPSS 23.0 was then used to compute linear regression analyses to examine the association of predictor variables sex, race, education, cohort, and longevity secret relative to days of survival. Only race (std. B = .10, $p < .05$) and cohort (std. B = -.11, $p < .01$) emerged as significant predictors of overall survivorship. Non-white centenarians live longer than their White-Caucasian counterparts; whereas earlier-born cohorts have shorter survival. Closer examination of these findings revealed that non-White centenarians have historically lived an average of 300.38 days longer than their White counterparts; whereas later born cohorts have historically an average of 48.10 days shorter than earlier-born cohorts. Despite the fact that sex and subjective longevity secrets failed to yield any significance, further inspection revealed two interesting highlights. First, centenarian males have historically lived an average of 147.89 days less than female centenarians. Second, centenarians who cite God as the secrete to their longevity have historically lived 100.23 fewer days than centenarians who attribute their longevity to something else. Results have implications to further understanding the interplay of race and human longevity, as well as variables attributed to improved survivorship across successive cohorts. Further discussion relative to health practices and policies to improve longevity in states like Oklahoma will be further highlighted.

### SUCCESSFUL AGING AND HEALTHCARE SERVICE UTILIZATION IN EAST ASIA: NATIONAL COMPARISONS OF CHINA, KOREA, AND JAPAN

Jinmyoung Cho,[1] Takeshi Nakagawa,[2] and Dannii Yeung[3],
*1. Baylor Scott & White Health, Temple, Texas, United States, 2. National Center for Geriatrics and Gerontology, Aichi, Japan, 3. City University of Hong Kong, Hong Kong, Hong Kong*

As the older population increases and lives longer, the demand for healthcare has been increased dramatically. To date, it is unknown whether older people's healthcare utilization varies between countries and how it relates to successful aging. Using Rowe and Kahn's model, we examine cross-national differences in the relationship between successful aging and healthcare service utilization in East Asia. Harmonized datasets at baseline from China Health and Retirement Longitudinal Study (CHARLS), Korean Longitudinal Study (KLoSA), and Japanese Study on Aging and Retirement (JSTAR) were used. Including 7,651 participants (aged 65-75 years), successful agers were identified using Rowe and Kahn's criteria (i.e., no disease, no disability, high cognitive function, and active engagement). Healthcare service utilization includes hospital visit, number of hospital stay, number of nights per hospital stay, regular medical center visit, number of medical center visits, and possession of private insurance in previous year. Generalized linear models showed that successful agers' healthcare service utilization is significantly different from non-successful agers (e.g., OR=2.19, $p<.001$ for regular medical center visits), and Korean and Chinese healthcare service utilization is different from Japanese (e.g., OR=0.44 and OR=10.18 for Chinese and Korean number of medical center regular visits, respectively, $p<.001$). Furthermore, the number of nights in hospital among Chinese and Korean successful agers tend to be greater than that of Japanese successful agers (OR=2.93 and OR=1.99 for Chinese and Korean successful agers, respectively, $p<.001$). This study indicates cross-national variations in pattern of healthcare service utilization between successful and non-successful agers in East Asia.

### THE EFFECT OF DISPOSITIONAL OPTIMISM ON SUCCESSFUL AGING OF OLDER ADULTS AND THEIR PARTNERS

Gina Lee,[1] and Kyuho Lee[2], *1. Iowa State University, Ames, Iowa, United States, 2. Daegu University, Gyeongsan, Korea, Republic of*

The purpose of the study was to examine how dispositional optimism of an older adult influences both the individual's and the spouse's successful aging. Data from the Health and Retirement Study were included in this analysis. The age of participants ranged from 18 to 104 (M = 67.91, SD = 11.26). The successful aging components included low levels of depressive symptoms and low levels of difficulties in activities of daily living, subjective health, and cognition. A structural equation model was computed including optimism as a latent variable and four components of successful aging for older adults and their spouses, all from the same wave in 2014. The results of the study revealed that higher dispositional optimism of older adults significantly predicted lower depressive symptoms, lower difficulties with activities of daily living, better cognitive function, and higher subjective health, for older adults and their spouses. The results, in conclusion, support the notion that dispositional optimism not only plays a significant role in well-being for oneself but also benefits the partner's well-being as well. Further research need to include other components of successful aging, such as social engagement, loneliness, and life satisfaction. In addition, including covariates, such as gender, educational attainment, race/ethnicity, and household income, will also further examine the effect of optimism above and beyond the demographic factors.

### GRANDPARENTS CARING FOR GRANDCHILDREN: HOW AGE AND AGING IMPACT PROVISION OF CHILDCARE

MaryBeth A. Apriceno,[1] Stacey B. Scott,[1] and Sheri Levy[1], *1. Stony Brook University, Stony Brook, New York, United States*

The economic need for dual-income households has contributed to more grandparents providing childcare for their grandchildren. Research on these grandparents has examined their life satisfaction, health, and spare time. Little work to date has examined how cross-sectional differences in grandparents' age may contribute to when they begin providing childcare or how their increasing age while caregiving influences when they reduce or stop providing childcare. Using Health and Retirement Study data, we identified 5.38% of participants (N=516) who reported providing at least one hour of childcare for their grandchildren per wave (range=1-9,996) between 2004 and 2014. The resulting sample ranged from 44-88 years of age (M=59.78, SD=7.75) when they first reported providing childcare; 48.8% were retired during the study period. Using multilevel modeling, we tested age and retirement as predictors of individual differences in initial amount of childcare (intercept) and change in childcare (slope). Cross-sectionally, no significant differences in childcare hours were observed based on the age at which grandparents began caregiving or retirement status. Longitudinally, however, as grandparents got older during the study, hours of childcare decreased by 48 hours per year (B=-48.368, t(1584)=-6.55, p<.0001) relative to when they starting providing childcare. These results suggest grandparent childcare providers represent an age diverse group managing multiple roles and responsibilities who continue caregiving well into older adulthood. Future research on grandparents who provide childcare for their grandchildren should observe these individuals over time to better understand how aging impacts provision of care and to examine how aging may moderate previous cross-sectional findings.

### SOCIAL VALUE OF THE ELDER PUMI MINORITY IN CHINA

Meng ran Gao[1], *1. Peking University, Beijing, China*

Social status of the elderly nowadays declines rapidly in China. As anthropologist Margaret Mead considered, post-figurative culture leading in contemporary society and the source of knowledge are from youth. The value of the elderly has been overlooked. However, in Pumi, one of the smallest ethnic minority groups in northwestern Yunnan Province of China, it is common that senior residents have high social status. This study examines the social values the Pumi elderly have by systematic analysis and participation observation methods. Based on data collected in a Pumi village during a 6-month fieldtrip, we conclude that Pumi elderly enjoy a high status in the community. They occupy core positions in all important ceremonies, such as religious activities and other daily activities including hospice. Factors behind the special old-age care phenomenon are Pumi's history and its culture. The special culture has united the group members together and enhanced individual development with community social capital. It is clear that respecting elderly does not only contribute the transformation of ethnical knowledge but also enhance community cohesiveness. Evaluating the role of the elderly should not only from economic perspective, but also from the holistic perspective of social culture, so as to reconsider the importance of the elderly to our society.

### SENIOR MODELS: SUCCESSFUL AGING AND SERIOUS LEISURE

Sanghee Lee,[1] Jaeyoon Bae,[1] Sua Im,[1] and Jinmoo Heo[1], *1. Yonsei University, Seoul, Korea, Republic of*

Serious leisure involves productive engagement and commitment in leisure activities. Literature shows that participating in serious leisure is associated with physical and mental health benefits of older adults. The behavior of senior modeling reflects serious leisure engagement that might offer a new insight useful in understanding successful aging. We explored the experience of senior models as a form of serious leisure. Using selective optimization with compensation as well as serious leisure framework, we attempted to identify how senior modeling activity contributes to successful aging. This study used in-depth interviews using purposeful sampling, and data were collected over two months in 2019. The participants were 31 senior models (average age = 67). The analysis resulted in three themes which contained characteristics of serious leisure as well as selective optimization with compensation: identifying new possibilities, serious engagement, and rewards from meaningful experiences. This study demonstrated various experiential characteristics associated with modeling as a form of serious leisure. Through selection, optimization, and compensation process, the participants seemed to achieve successful aging. We found that senior model experiences promoted active lifestyle, health benefits, and interpersonal relationships. To our knowledge, this is the first exploration of the experience of senior modeling activity. Consistent with existing literature, our study provides evidence of the significant role of serious leisure in later life. We suggest that senior modeling program holds promise as an effective way for older adults because it can be used as a self-care approach and community programs not only in Korea, but at various locations.

### THE UPSIDE OF GETTING OLD: TESTING A MODEL OF OLDER AGE AND BETTER EMOTIONAL WELL-BEING

Jenna Wilson,[1] JoNell Strough,[1] and Natalie Shook[1], *1. West Virginia University, Morgantown, West Virginia, United States*

Older adults often report better emotional well-being than younger adults. Socioemotional Selectivity Theory posits that with age, reduced future time perspective motivates prioritizing emotional well-being in the present moment (Carstensen, 2006). Mindfulness, a receptive attention to experiences as they occur (Brown & Ryan, 2003), and savoring, the ability to regulate positive feelings in the moment (Bryant, 2003), are present-oriented processes associated with greater well-being. Recent evidence indicates that greater mindfulness in part accounts for age differences in positive affect (Shook et al., 2017). The current study investigated whether older age is associated with a greater present-oriented time perspective, which in turn is related to greater savoring and mindfulness, thus statistically accounting for older adults' better well-being. Participants (N = 888, 20-88 years, Mage = 46.37, SD = 15.20) recruited via MTurk completed an online survey. Data were analyzed using structural equation modeling. The model provided an adequate fit to the data (CMIN/DF = 2.94, CFI = 0.985, RMSEA = 0.048). Older age was associated with greater present-oriented time perspective, and present-oriented time perspective was associated with greater savoring and mindfulness which, in turn, were associated with better emotional well-being. Alternative models were tested, but did not significantly improve model fit. Findings suggest that there may be benefits for younger adults' well-being if they learned to be more present focused, savor the moment, and be more mindful like older adults. Thus, present-oriented time perspective may be an important factor for healthy aging.

### DISCRIMINATION, STRESS, AND MORTALITY AMONG BLACKS AND WHITES

Soyoung Choun,[1] Sungrok Kang,[2] Hyunyup Lee,[1] and Carolyn M. Aldwin[1], *1. Oregon State University, Corvallis, Oregon, United States, 2. Korea Military Academy, Seoul, Korea, Republic of*

Mortality rates have declined significantly in the past decades. However, Case and Deaton (2015) showed that middle-aged white Americans with lower education levels have increasing mortality rates. Although some have suggested that stress is an important factor in both this and in racial/ethnic disparities in mortality, relatively few studies have examined vulnerability to stress and mortality, and typically have examined only one type of stress. We examined racial/ethnic and gender differences in different types of stressors, from everyday discrimination, to lifetime trauma, as well as differential mortality risk due to stress vulnerability. Using data from the Health and Retirement Study (HRS), the sample consisted of 6,810 (Mage=68.9 years, SD=10.1) who completed the Psychosocial Questionnaire (PQ) in 2006; mortality was assessed to 2014. Blacks were higher on most stressors except for lifetime trauma. Women reported higher level of financial strain but lower levels of everyday discrimination and lifetime trauma than men. Controlling for demographics and self-rated health, Cox proportional hazard models revealed that everyday discrimination, financial strain, SLEs, lifetime trauma were significantly associated with the risk of mortality. There were no significant racial/ethnic differences in mortality risk. However, interaction effects showed that whites had higher mortality risk with lifetime trauma than Blacks, while those with lower education had higher mortality risk for SLEs. This supports the idea that lower education whites may be more susceptible to some types of stressors, providing a possible mechanism for Case and Deaton's finding (2015) of increasing mortality risk in this group.

### PREDICTORS OF MORTALITY RISK AMONG MEDICARE ADVANTAGE ENROLLEES

Soyoung Choun,[1] Lan Doan,[1] Diana J. Govier,[1] Karen Hooker,[1] Carolyn Mendez-Luck,[1] Shelbie Turner,[1] and Veronica L. Irvin[1], *1. Oregon State University, Corvallis, Oregon, United States*

Overall all-cause mortality rates have declined significantly in past decades among individuals aged 65 and above in every racial and ethnic group. We explored demographic, overall health, and disability development as predictors of mortality in Medicare beneficiaries enrolled in Medicare Advantage plans. We used data from the 2014-2018 Medicare Health Outcomes Survey, a nationally representative panel survey with a two-year follow-up, administered by the Centers for Medicare and Medicaid Services. Our sample consisted of 1,273,494 community-dwelling adults aged 65 and older (Mage = 74.5 years, age range: 65-109 years) enrolled in Medicare Advantage plans. Mortality was assessed over a 2-year follow-up period. We used Cox proportional hazards regression analysis to predict risk of all-cause mortality by demographics, self-rated health, chronic health conditions, smoking status, and activities of daily living (ADLs). Among all participants, the mortality rate was 7.0% (n = 88,058) at 2-year follow-up. Advanced age and being male were significantly associated with greater risk of mortality, while higher levels of education and income were inversely associated with mortality. Controlling for other factors, white adults had higher mortality risk than black or African American, Hispanic, and Asian older adults. Individuals who were unmarried, had lower self-rated health, had more chronic health conditions, smoked, and had more ADL limitations had higher mortality risk. Our findings suggest that sustained health and better functional capacity are important elements in decreasing the risk of mortality in older adults.

### DOES PERSONALITY PREDICT PATTERNS OF AGING? LONGITUDINAL FINDINGS FROM THE VA NORMATIVE AGING STUDY

Ritwik Nath,[1] Carolyn M. Aldwin,[1] Soyoung Choun,[1] Maria Kurth,[1] and Avron Spiro III[2], *1. OSU Oregon State University, Corvallis, Oregon, United States, 2. Boston University, Boston, Massachusetts, United States*

Previous research using the Veterans Affairs Normative Aging Study identified four patterns of aging using group-based multi-trajectory modelling to identify joint changes in life satisfaction (LS) and functional health (FH) (Nath et al., 2018a,b). The purpose of the present study was to examine whether personality traits predicted these four patterns: impaired (stable low LS and FH), normal (decreasing LS and FH), optimal (high LS, decreasing FH), and successful aging (high LS and FH). The sample consisted of 992 NAS men who provided 3,853 observations (M=2.81, SD=1.54, range 1–8) between 1987 and 2010 (Mage=62.31, SDage=7.50, range 44-86 in 1987). Multinomial logit regression analysis with robust estimation controlled for marital, employment, and self-rated health status, using optimal aging as the reference group. Compared to the optimal aging group, neuroticism predicted membership to the normal aging (RR=1.61, CI=1.16–2.22) and the successful aging groups (RR=.55, CI=.38–.79). Optimism predicted membership to all groups, with lower risk ratios to the impaired aging group (RR=.11, CI=.04–.33) and normal aging group (RR=.57, CI=.42–.79), and higher risk ratio to the optimal aging group (RR=1.96, CI=1.35–2.85). Extraversion only predicted membership in the impaired aging group (RR=.27, CI=.12–.63) indicating high levels of social isolation. Thus, personality may play an important role in determining the patterns of aging.

### SESSION 3375 (POSTER)

### WHAT'S NEW IN GERONTOLOGICAL EDUCATION

### EXPECTATIONS AND VALUE OF EDUCATION OF STUDENTS OF THE MASTER IN GERONTOLOGY OF THE UNIVERSITY OF GUADALAJARA

Martha Elena Vazquez Arias,[1] and
Elva Dolores Arias Merino[1], *1. Centro Universitario de Ciencias de la Salud. Universidad de Guadalajara, Guadalajara, Jalisco, Mexico*

For Institutions of Higher Education, it's important to know better the expectations and the value of education of their students to plan and offer successful postgraduate educational services The purpose of this project is to identify and analyze the expectations and the value of education of students of the Master in Gerontology at the University of Guadalajara, The data collection was carried out through a questionnaire divided into two parts. The first one contemplated socio-demographic characteristics of the participants such as: name, age, marital status and coexistence with older adults. In the second part, the Value of Education Scale (Battle & Wigfield, 2003) was applied, which measures different aspects of the expectations and the value that graduate students give to education. 25 students participated. 64% (16) were women and 36% (9) were men, with an age range of 24 to 53 years. Single people (80%) and 56% coexist with older adults every day. Highest scores: Intrinsic Values "I like to learn from people who are experts in their field" 4.96. Value of Achievement "graduate studies have a great value for me" 4.64. Value of Utility "I want to complete my postgraduate studies to acquire knowledge and experience" 4.80. Cost Perceived "I think in the end it will be worth the postgraduate despite all the work and effort." 4.96. We consider a guarantor of success for our students, their high expectations and the value they give to their education that make them stand out and gain knowledge every day.

### TACKLING THE OPIOID EPIDEMIC: A STATE-WIDE INITIATIVE TO DECREASE OPIOID PRESCRIBING

Jasleen K. Chahal,[1] and Kimberly Northrip[1], *1. UK HealthCare CECentral, Lexington, Kentucky, United States*

More than 500,000 preventable deaths annually have been attributed to the growing opioid epidemic and increased opioid prescribing rates by providers (CDC, 2016). A report by the American Medical Association (AMA) found that only 50% of physicians have taken continuing professional development (CPD) activities on managing pain with opioid alternatives (2016). In 2012, Kentucky passed a comprehensive law regulating the prescribing of controlled substances and requiring ongoing CPD in opioid prescribing and abuse. UK HealthCare CECentral partnered with the Kentucky Office of Drug Control Policy (KODCP) to provide CPD training. This study utilized retrospective learner data collected from CECentral between 2012 – 2017. Since the initiative began in 2012, CECentral has provided this training to 8,893 individuals: 5,877 physicians, 1,527 APNs, and 831 other health professionals. This initiative has resulted in more than 6,000 total participants committing to various forms of clinical practice change to help combat the opioid crisis. Between 2012 and 2016, these commitments resulted in an increase in Buprenorphine/Naloxone prescriptions and prescribers requesting controlled substance reports on their patients. An overall decrease in opioid prescriptions was also shown to be a result of this Kentucky opioid initiative. Results of the study indicate that partnerships between state agencies and CPD providers can lead to practice improvements that address public health concerns. Future research should focus on the efficiency and effectiveness of CPD training and such collaborations as public health initiatives.

### THE SAGE SYMPOSIUM: A MODEL FOR HANDS-ON INTERACTIVE CONTINUING EDUCATION

Becky Powers[1], *1. South Texas Veterans Health Care System Geriatric Research Education and Clinical Center, San Antonio, Texas, United States*

Current graduate and postgraduate medical education has minimal geriatric and palliative care curricular requirements, leaving Continuing Education (CE) programs poised to fill a critical educational niche. The San Antonio Geriatric and Palliative Education (SAGE) Symposium was a 3 day long interprofessional CE conference for providers caring for older adults. SAGE addressed geriatric knowledge, skill, and attitude deficits in practicing providers by incorporating: 1) a community and provider based needs assessment, 2) active skills sessions culminating in a health fair, and 3) multimedia based reflective exercises. Needs Assessment (Knowledge): A video needs assessments of older adults were performed using a convenience sampling methodology in 13 non-healthcare public locations in each quadrant of the city. 23 respondents were interviewed before reaching thematic saturation with 3 main themes: geriatric syndromes, patient-provider relationships, and support. Content areas for the course were derived from the needs assessment. Skills Sessions (Skills): In addition to standard plenary sessions, multiple active breakout session taught attendees common geriatric skills. On the last day of the conference, attendees applied these skills under supervision at a senior community health fair. Reflective Exercises (Attitudes): Videos, poems, and artwork with themes on aging were displayed during conference breaks. Attendees received CE credit for electronically submitting short reflections to each multimedia piece. Reflections were compiled and reported back to the group at the end of the conference. By changing the traditional CE conference format to an interactive experience, the SAGE Symposium was able to address knowledge, skills, and attitudes towards aging in its attendees.

### INTERGENERATIONAL EMPLOYEE NETWORK AGEISM: COLLABORATIVE INTERVENTION TRAINING CREATES ATTITUDE MODIFICATIONS

Heath D. Harllee,[1] Amanda Noah,[1] and Becky P. Knight[1], *1. University of North Texas, Denton, Texas, United States*

Lack of positive attitudes towards aging has shown to cause challenges within intergenerational networks in employment situations. These can include job satisfaction, intrinsic motivation to work, and ageism subjectivity as underlying determinants and consequences. The collaborative intervention pilot training program goals were two-fold: 1) To expose and understand ageism as a discriminatory action. 2) To create a more positive social dynamic network in a diverse workplace in regard to general expectations of ageism. Two team-based learning intervention programs were created in order to increase collaborative awareness of ageism and were presented to a medium size intergenerational department staff (N=64) as part of a professional development series on equity, diversity and inclusion. Through three multidimensional self-help training activities, learning was done individually, within similar age employee groups, and within intergenerational employee groups. Participants were able to discuss and express general understandings and expectations of aging and learning tools such as intergenerational reactivity and emotion regulation strategies were presented. Within survey responses at the completion of the trainings, key findings showed that respondents had a better understanding of ageism (76%) and felt better equipped to work within an employment team of diverse ages (71%). Additionally, the subject matter of this pilot training program resulted in re-conceptualized positive aging (61%). Future implications and goals for the program include interventions to further increase positive intergenerational understanding and workplace generational inclusiveness.

### CROSS-SECTOR COLLABORATION TO BUILD DEMENTIA-FRIENDLY BUSINESSES: ENGAGING STUDENTS IN PUBLIC ISSUES IN AGING

Lesa L. Huber,[1] Amanda Mosier,[2] and Victoria Abramenaka-Lachheb[3], *1. Indiana University School of Public Health, Bloomington, Indiana, United States, 2. IUI Health, IU Health, United States, 3. Indiana University, Bloomington, Indiana, United States*

Despite a nationwide campaign to build dementia friendly communities, increasing awareness and engagement with programs like Dementia Friendly Businesses remains a challenge for local champions. To create welcoming environments for people living with dementia (PLWD), communities may benefit from cross-sector collaboration. Fundamental to collaborative efforts is the conviction that individuals, families, businesses, organizations, and institutions each play a role in reducing the stigma associated with dementia and facilitating welcoming environments. This exploratory study considers the outcomes of a cross-sector collaboration between a university, a hospital, individual community members, and students to build awareness of the need for new norms that reduce stigma for people living with dementia. We measured change at each ecological level. Through this collaboration, 61 local businesses and organizations are now DFB certified, 350 individuals have participated in training, and 150 students have joined 12 community trainers to advocate for dementia friendly spaces. Students' survey responses show that 75% agree or strongly agree that the project engaged them in a meaningful real world experience and advocating for the need for dementia friendly spaces. PLWD provide qualitative data on feeling welcomed in the community. The university and hospital co-created an online open access dementia friendly training taken by 89 people in 6 months. 150 community members came together for a day-long workshop to build capacity and innovate solutions. Implications of the project suggest that changing cultural norms about dementia requires creative solutions that meaningfully engage individuals, organizations, institutions, and communities to collaborate across multiple sectors

### THE USE OF CREATIVITY WITH CLIENTS WITH DEMENTIA TO ENHANCE STUDENT LEARNING AND POSITIVE PERCEPTIONS

Megan Foti,[1] and Michelle Ghaul[2], *1. Stockton University, Galloway, New Jersey, United States, 2. Fox Rehabilitation, Galloway, New Jersey, United States*

Funding and productivity demands combined with curative approaches of medicine can make working with older adults less appealing to health professionals (Samra et al, 2013 George et al, 2013). It is essential to recognize and address such perceptions to promote the provision of quality, humanistic healthcare. Educators can impact perceptions by facilitating innovative opportunities for interaction with older adults, especially those in dementia care. Creative therapies, such as art, music, and storytelling provide opportunities for reminiscence and self-expression and have been proven to yield potential psychosocial benefits for people with dementia including enhanced well-being, lessened cognitive decline, decreased anxiety and depression, and improvements in memory, social interaction, orientation, and cognitive functioning. (Phillips et al, 2010; Subramanium, Trentham, n.d.; Woods, & Whitaker, 2013). Similarly, these interventions yield benefits for facilitators such as increased comfort and higher levels of humanistic care for clients with dementia (George et al, 2013, & 2014). The proposed presentation will provide evidence related to the benefits of creative therapies for people with dementia and highlight methods for integrating this into healthcare curricula in addition to supporting evidence and positive outcomes.

### EXPLORING DEATH AND DYING WITH HEALTHCARE STUDENTS

Megan Foti,[1] and Stacy Cassel[1], *1. Stockton University, Galloway, New Jersey, United States*

Although death is a universal experience, many avoid discussing or learning more about the topic (Mak, 2011). However, healthcare professionals are expected to be knowledgeable, resourceful, and professional within their scopes of practice, but oftentimes avoid end of life topics (Ramvi & Gripsrud, 2017). Students also report concerns when working with clients at the end of life expressing fear of how they will handle an encounter with a dying client (Ek et al., 2014). Therefore, professionals need didactic and personal preparation to be more comfortable with discussions related to end of life treatment and planning (Kumar et al, 2013). Evidence supports that students can benefit from self-reflective and narrative exercise with older adults that challenge their perspectives on end of life. Students who are able to openly discuss death, dying, illness and loss express a desire to learn more about working with the older adult population and increased sensitivity to the beliefs and attitudes of older adults (Butler & Baghi, 2008; Nelson et al., 2018). In order to provide students with an opportunity to explore end of life topics, professors designed an educational module which included activities such as writing a living will, discussing end of life topics with older adults, and critically reflecting on their experiences. This proposed poster will highlight current background literature relevant to end of life topics, methods for integrating end of life topics into academic curricula, and student perceptions related to end of life topics as shared in their reflections.

### KNOWLEDGE OF END-OF-LIFE CARE, LIFE-SUSTAINING TREATMENT, AND ADVANCE DIRECTIVES IN NURSING STUDENTS

Jooyoung Cheon[1], *1. Sungshin Women's University, Seoul, Korea, Republic of*

Knowledge of end-of-life (EOL) care and being able to make the appropriate decisions for patients who are dying is important for nursing students, who will soon play a critical role in EOL decision-making among patients and their caregivers. Accordingly, the purpose of this study was to examine the level of knowledge of EOL care, life-sustaining treatment, and advance directives among nursing students in South Korea. This cross-sectional descriptive study was conducted from December 2017 to February 2018. Data were collected from 220 undergraduate nursing students and analyzed using descriptive statistics, t-tests, one-way ANOVA, and a post hoc test with the SPSS 19.0 program. The score for knowledge of EOL care was 7.8 out of 11 points, for knowledge of life-sustaining treatment was 4.6 out of 6 points, and for knowledge of advance directives was 7.0 out of 9 points. There were significant differences in knowledge of EOL care scores by year of study, experiences in clinical practicum education, and experiences of caring for dying patients. Knowledge of life-sustaining treatment significantly differed by year of study, experiences in clinical practicum education, experiences of caring for and observing dying patients during clinical practicum education, and perceived self-rated health. There were significant differences in knowledge of advance directive scores by year of study, satisfaction with nursing major, experiences in clinical practicum education, and experiences of caring for and observing dying patients during clinical practicum education. Further studies should develop educational intervention programs that improve knowledge of EOL care, life-sustaining treatment, and advance directives.

### THE IMPACT OF DEMENTIA TRAINING ON HOSPITAL STAFF'S KNOWLEDGE AND ATTITUDES

Julia Schneider,[1] Birgit Teichmann,[1] and Andreas Kruse[2], *1. Network Aging Research, University of Heidelberg, Heidelberg, Baden-Wurttemberg, Germany, 2. Institute of Gerontology, University of Heidelberg, Heidelberg, Baden-Wurttemberg, Germany*

Older people with dementia are more frequently hospitalized and more strongly associated with negative outcomes. We examined the acceptance and the effect of a dementia training on attitudes and knowledge of the hospital staff. In the current study, we utilized a mixed-methods research design to examine a clinic group of six hospitals in Germany. Besides semi-structured interviews, we collected quantitative data with a questionnaire given before and three-months after the training. The questionnaire contained German translated versions of the Knowledge in Dementia (KIDE) Scale and the Dementia Attitudes Scale (DAS-D) to assess changes in attitudes. The participant population (N=60) consisted of nurses (n=35, 58%), medical assistants (n=13, 22%) and other medical professions. Satisfaction with the training was predominantly positive, 92% would recommend the training to their colleagues. At baseline, a small but significant correlation between the standardized questionnaires KIDE and DAS-D was evident (r(60)=.357, p=.005). The participants (n=32) showed a more positive attitude in the post-test (M=5.39, SD=0.64) than in the baseline-test (M=5.19, SD=0.66). This difference was significant (t(31)=-2.434, p=.021). However, we did not find any significant effects on the KIDE. The reason for this may be the use of a standardized questionnaire, which does not reflect the increase in knowledge, or there has been no increase in knowledge of dementia. The results are based on a small sample size. However, they have demonstrated that dementia training can positively influence attitudes toward people with dementia. A significant increase in knowledge was expected but could not be demonstrated.

### IMPROVING COLLEGE STUDENTS' ATTITUDES TOWARD AGING POPULATIONS: USING CBT-BASED GERONTOLOGICAL PEDAGOGY

Qiwei Li,[1] Heath Harllee,[1] and Becky P. Knight[1], *1. University of North Texas, Denton, Texas, United States*

The U.S. is facing a shortage of aging-related professionals. Lack of positive attitudes among undergraduate students towards the older population may restrict them from participating as aging professionals. However, research majorly focused on the attitudes among students already in the medical training fields instead of improvement of the attitudes among undergraduate students who potentially will devote themselves to be aging professionals. We seek to enhance attitudes toward the aging population through gerontological coursework at the undergraduate level. The introductory course is based on Cognitive Behavioral theory (CBT) which emphasizes to unlearn false concepts. Therefore, the class exposes students to positive aging images in contemporary films and literature demonstrating concepts such as successful aging, retirement, gender issues, and aging as minorities, etc. Students review their perceptions of the aging population at the beginning and end of the semester, presenting five words that best describe the aging images. The variances of occurrence of negative to positive descriptions are analyzed as an evaluation of the class. The class also stimulates civic responsibilities of the students toward the aging population with concepts such as generational equities by conducting team-based discussions. The results report increased positive words and improved attitudes on in-class team projects and post-class evaluations. The implication of the course outcomes is that discussing and exposing the positive images, and justifying typical aging-related behaviors significantly improves students' attitudes toward the aging population, and may encourage undergraduate students to select an aging profession. Qualitative evaluation of the course will be conducted in future semesters.

### RESIDENTIAL HOMES FOR THE DYING: AN UNTAPPED RESOURCE FOR TEACHING PATIENT- AND FAMILY-CENTERED END-OF-LIFE CARE

Chantelle Sharpe,[1] and Carol Weisse[2], *1. Connecticut College, New London, Connecticut, United States, 2. Union College, Schenectady, New York, United States*

Clinical training opportunities in end-of-life care are lacking, especially in home settings where death is expected and supported as a natural process. The Community Action, Research and Education (CARE) program provides students who are interested in healthcare a better understanding the challenges of providing end-of-life care. Over 8 weeks, undergraduate students serve as surrogate family members providing care to hospice patients in residential homes for the dying. Additionally, students engage with a formal curriculum by completing online learning modules each emphasizing different skills for providing end-of-life care. This study analyzed data from three cohorts of undergraduate students (n = 21) who participated in the CARE Program. Analyses from assessment surveys revealed that students reported improved knowledge and skills, including enhanced bedside education and training and increased ability to care for someone at the end-of-life after completion of the program. Also, 95% (n = 20) of students over the three cohorts reported that the formal coursework enhanced skills and training related to bedside care. Previous research has examined end of life training in a professional school setting, but the focus was on care in an institutional or facility setting (Billings et al., 2010; Supiano, 2013). The CARE program is a model for experiential learning in a home setting that provides a special lens to the dying experience in a holistic, patient and family centered way.

### THE EFFECT OF PERCEIVED APPRECIATION ON CAREGIVER WELL-BEING: A TEST OF EQUITY THEORY

Kylie Meyer,[1] Frank Puga,[1] and Carolyn E. Pickering[1], *1. University of Texas Health Science Center San Antonio, San Antonio, Texas, United States*

Equity theory suggests that equal (reciprocal) exchange of support in social relationships leads to better outcomes for members. However, in caregiving, exchange of support may become unbalanced due to heightened instrumental support from caregivers (CG). Imbalance may be exacerbated in dyads where the care recipient (CR) has dementia, since cognitive changes can attenuate expression of social support. One way social support is demonstrated in through show of appreciation. We used data from National Study on Caregiving (NSOC) to test whether CGs who did not feel appreciated by CRs were more likely to experience depression in the future. To accomplish this, we applied lagged dependent variable (LDV) models to the 2011 and 2015 NSOC waves (N=150). CGs who felt appreciated by the CR in the 2011 wave had 0.22 times the odds of being depressed in 2015 as those who did not feel appreciated by the CR (CI 0.07 to 0.68). This effect appears to exist primarily among CGs to people with dementia. In stratified models, we found CGs to people with dementia in 2015 had 0.04 times the odds of being depressed in 2015 if they previously felt appreciated by the CR (CI 0.003 to 0.40). A statistically significant effect was not observed among those assisting someone without depression (OR=0.65, CI 0.22 to 1.91). Results suggest that CGs to people with dementia who feel appreciated have less risk of experiencing depression. Findings have applications for future interventions for caregiver wellbeing, such as enhancing perceived appreciation (e.g., cognitive restructuring, communication training).

### STUDYING AGING ABROAD: TWO INTENSIVE COURSES AT DREXEL UNIVERSITY

Kristine A. Mulhorn,[1] Jesse F. Ballenger,[1]
Katherine F. Clark,[1] and F Yoshinaga[1], *1. Drexel University, Philadelphia, Pennsylvania, United States*

The model for short-term study abroad courses---called Intensive Courses Abroad (ICA) is one where various topics can be covered under a single theme over a 10-to-14 day travel timeframe. Students participate in pre-departure assignments, a daily schedule in the host country with meaningful visits followed by group discussions, and a final presentation upon return to the U.S. In both examples presented, the topic is aging. In one, there is a visit to Japan, focusing on aging, technology and culture. Students consider design and lifestyle implications of an aging society. The course includes visits to a geriatric rehabilitation facility and to a residential facility that employs various robots and other technology. Students explore a remote community and its design challenges for an aging society. Participation in a community survey to address a question they plan to pursue for their final project, such as the role of technology in society, how we understand the aging process, and how culture defines aging. In the second course, global aging frames discussions on the way Chile is adopting mechanisms to address chronic conditions associated with aging, including dementia. In the course, students learn about the social and political context. Students observe various ways Chile has adopted innovative approaches to address dementia care and various community-level interventions. Students will reflect in journals regularly and give a presentation about the ways the country is facing the challenges of an aging society where more than 30% of the population is projected to be over 60 by 2050.

### SESSION 3380 (SYMPOSIUM)

### BEING RESPECTED AND ADMIRED IN OLD AGE: DYNAMICS OF SOCIAL STATUS AND AGING

Chair: David Weiss, *Leipzig University, Leipzig, Germany*
Co-Chair: Jennifer A. Bellingtier, *Friedrich Schiller University Jena, Jena, Germany*
Discussant: Manfred Diehl, *Colorado State University, Fort Collins, Colorado, United States*

Social status - the standing of a person or group in the social hierarchy - is part and parcel of social life and a significant determinant of cognition and behavior. Status hierarchies are basis of virtually all human and primate societies and assign different roles and privileges to its members. However, the dynamic nature of social status and the underlying mechanisms in old age are not well understood. Therefore, this symposium addresses questions of how social status is influenced by aging-related changes in roles, life events, self-concept, and images of aging and how social status shapes in response personality, subjective age, prosocial behavior, performance, and well-being in old age. Bellingtier and colleagues examined objective and subjective social status and their connections to subjective age, attitudes towards aging, and awareness of age-related changes. Zhang shows paradoxical association between aging stereotypes and prosocial behaviors toward older adults. Barber and Hamel investigated how stereotypes of reduced physical competence in old age affect the gait performance on easy and difficult tasks. Weiss and colleagues take a cross-cultural perspective on the different sources of social status in China, Germany, and the US showing that generations in contrast to age groups represent a source of high social status in later life. Finally, Kornadt investigates the dynamic interplay of changes in social roles and personality in old age. Together, these presentations enlarge our understanding of the dynamics of social status in old age.

### KEEPING UP WITH THE JONESES: SOCIOECONOMIC STATUS AND SUBJECTIVE PERCEPTIONS OF AGING

Jennifer A. Bellingtier,[1] Alaina N. English,[2] and Shevaun D. Neupert[2], *1. Friedrich Schiller University Jena, Jena, Germany, 2. North Carolina State University, Raleigh, North Carolina, United States*

Money may not buy happiness, but can it buy a more positive outlook on aging? Past research examining the associations between indicators of socioeconomic status (SES) and subjective perceptions of aging have been mixed with some finding greater socio-economic resources predict more positive aging perceptions, whereas others find no connection. We examine objective (i.e., income and education) and subjective social status (i.e., MacAurthur ladder) and their connections to subjective age, attitudes towards aging, and awareness of age-related changes using hierarchical multiple regression analyzes. Participants (n =296, age range 60-90) completed survey measures online. Results indicate minimal connection between income, education, and aging perceptions. However, perceiving oneself to be higher in social standing compared to one's community was consistently related to more favorable perceptions of aging. Higher community standing may indicate favorable development, fewer stressors, or more resources compared to others, which could contribute to more positive perceptions of aging.

### MULTIPLE SOURCES OF SOCIAL STATUS IN OLD AGE: THE ROLE OF AGE GROUPS AND GENERATIONS

David Weiss,[1] and Xin Zhang[2], *1. Leipzig University, Leipzig, Germany, 2. Peking University, Beijing, Beijing, China (People's Republic)*

This cross-cultural study compared attitudes towards age and generational groups across the life span in China, Germany, and the US including N = 1302 participants between 18 and 86 years of age. We asked younger, middle-aged, and older respondents to rate either age (e.g., adolescents, young adults, middle-aged adults, and older, adults) or generational groups (e.g., Millennials, Generation X, Baby Boomer, and Silent Generation) on various characteristics. Results demonstrate that across all three cultures older age groups were perceived consistently less positive and more negative, whereas older generations were perceived as significantly more positive and less negative. Our results suggest that generations in contrast to age groups represent a source of high social status in later life providing a sense of respect, value, and admiration. Thus, social status can be derived from multiple sources and older adults can draw upon alternative social status domains (their generation) when confronted with loss.

### PARADOXICAL ASSOCIATION BETWEEN AGING STEREOTYPES AND PROSOCIAL BEHAVIORS TOWARD OLDER ADULTS

Xin Zhang,[1] Cai Xing,[2] and Ming Liu[3], *1. Peking University, Beijing, China, 2. Renmin University of China, Beijing, Beijing, China (People's Republic), 3. Peking University, Beijing, Beijing, China (People's Republic)*

According to stereotype content model, older adults were perceived as low in competence (but high in warmth). Studies have demonstrated that such negative stereotypes could affect older adults significantly. However, it remained unclear how younger adults could be influenced, especially during intergenerational interactions, i.e., would positive or negative aging stereotypes promote more prosocial behaviors toward older adults. 104 younger adults were randomly assigned to three aging stereotype conditions (i.e., negative, neutral vs. positive), and they were then introduced to play two prosocial tasks (i.e., social value orientation and ultimatum game), in which they were imagined to play with either a younger or an older adults. It was found that younger adults exhibited more prosocial tendencies toward older partners than that to younger partners in both tasks. Moreover, activation of a negative aging stereotype could make younger adults behave more prosocially to the older partners in the social value orientation task.

### SOCIAL ROLES AND PERSONALITY IN LATER LIFE

Anna E. Kornadt,[1] and Anna E. Kornadt[2], *1. Bielefeld University, Bielefeld, Germany, 2. Bielefeld University, Bielefeld, Nordrhein-Westfalen, Germany*

Despite considerable stability of the Big Five personality traits, there is evidence for personality plasticity and change across the lifespan. In younger years, the investment in social roles, such as entering worklife or starting a family has been shown to drive personality change. With regard to personality in later life, the investigation of social roles has so far been neglected. A questionnaire was developed to assess a large number of social roles that can be assumed in the second half of life. N = 306 participants aged 50 to 86 years reported on their social roles and rated their personality traits. Results show that assuming and investing in certain social roles (e.g. friend, retiree, volunteer) mediated the effects of age on the Big Five, especially for the oldest participants and in the domains openness and extraversion. The findings support the importance of social roles for personality also in later life.

### THE EFFECTS OF AGE-BASED EVALUATIONS ON OLDER ADULTS' GAIT VARIABILITY

Sarah Barber,[1] Kate Hamel,[2] Carl Ketcham,[2] and Kristy Lui[2], *1. Georgia State University, Atlanta, Georgia, United States, 2. San Francisco State University, San Francisco, California, United States*

Older adults are stereotyped as being slow, weak, and frail. In this study we examined how these stereotypes about age-related physical decline affect older adults' walking performance. Healthy, community-dwelling older adults were asked to walk at their own comfortable pace along a 24' temporospatial-measuring walkway 10 times. For some participants this was done with a normal-base of support (i.e., usual gait). However, for other participants this was done with a narrow-base of support (i.e., walking within a path of 15 cm outlined by tape). Walking tasks were done either in the presence or absence of a negative age-based evaluation. Results showed that the negative age-based evaluations were associated with greater stride-to-stride variability, particularly for participants who felt less confident in their abilities. Given that gait variability is a predictor of falling, this raises the possibility that negative age-based evaluations can produce concerns that are an intrinsic risk factor for falls.

### SESSION 3385 (SYMPOSIUM)

### CELL NON-AUTONOMOUS MECHANISMS OF AGING

Chair: Scott Leiser, *University of Michigan, Ann Arbor, Michigan, United States*

It is now recognized that biological aging can be affected both positively and negatively by intercellular communication. This symposium will focus on recent discoveries related to cell non-autonomous mechanisms of aging.

### CELL NON-AUTONOMOUS SEROTONIN SIGNALING MEDIATES STRESS RESISTANCE AND LONGEVITY

Scott Leiser,[1] Hillary Miller,[1] and Shijiao Huang[1], *1. University of Michigan, Ann Arbor, Michigan, United States*

The ability of organisms to perceive and respond to their environment is crucial to their long-term survival. Recent studies in model organisms identify signaling pathways that perceive environmental stress and cell non-autonomously modify systemic physiology. These pathways often originate in the neurons, where key cells monitor the external environment for changes including food availability, air-quality, and the presence of dangerous toxins. Our previous work identified a key role for serotonin signaling in the induction of flavin-containing monooxygenase-2 (fmo-2) downstream of hypoxic signaling. fmo-2 expression is necessary and sufficient to promote stress resistance and longevity downstream of multiple genetic pathways, making it a useful tool for identifying key components of these pathways. Our current data defines environments, pathways, and signaling molecules that induce fmo-2 and subsequently increase lifespan. Our resulting data define key roles for serotonin signaling and fmo-2 that rely upon the perception of oxygen and food.

### NEURONAL FGF-21 SIGNALING: A SENSOR OF DIETARY PROTEIN

Cristal Hill,[1] and Christopher Morrison[1], *1. Pennington Biomedical Research Center, Baton Rouge, Louisiana, United States*

Our data demonstrates that dietary protein restriction increases energy expenditure and improves glucose homeostasis, and that this effect is largely mediated by the metabolic hormone fibroblast growth factor 21(FGF21). Considering that the central nervous system (CNS) is acknowledged as a major regulator of both energy and glucose homeostasis, we have extended our studies to identify the tissue site mediating these FGF21-dependent effects via dietary protein restriction. In this study, mice with dysfunctional FGF21-signaling in either the CNS or adipose tissue were fed a control or low protein (LP)-diet to assess changes in body weight and metabolic endpoints. Our data show that LP diet increased energy expenditure and reduced body weight in control littermates, but these effects were lost in mice bearing CNS-specific deletion of Klb. These data highlight a liver to brain FGF21-signal as the first known neuroendocrine mechanism to explain the coordinated metabolic changes induced by dietary protein restriction.

### INCREASE IN HSP25 EXTENDS LIFESPAN AND IMPROVES RESPONSE TO TAU TOXICITY THROUGH A CELL, NON-AUTONOMOUS MECHANISM

Karl Rodriguez[1], *1. UT Health San Antonio, San Antonio, Texas, United States*

The accrual of aggregation-prone cytotoxic proteins underlies neural pathologies seen in aging, Alzheimer's disease and other dementias. Recent evidence indicates that heat shock protein 25kDa (HSP25) interacts with tau. To demonstrate a causal role for HSP25 in these pathologies, we overexpressed HSP25 protein in worms. This manipulation led to an increase in life span. Moreover, the longevity-effect was associated with increased expression of genes downstream of the SKN-1/Nrf2 stress-response transcription factor. HSP25 over-expression also reduces aggregate pathology and extends lifespan in a C. elegans neuronal-specific, aggregate-prone tau model . We propose that over-expression of HSP25 could provide protection from protein aggregation induced neurodegeneration. However, it is not yet clear whether this HSP25 effect could be efficaciously provided exogenously by other cell types. Thus, we will test whether increased peripheral HSP25 will reduce protein aggregation and stimulate a global Skn-1 stress-response pathway, reduce toxicity in neurons, and improve health outcomes.

### RESTORATION OF HYPOXIA SIGNALING IMPROVES AGING-ASSOCIATED LOSS OF SKELETAL MUSCLE REGENERATIVE POTENTIAL

Kodi Udeh,[1] Bin Li,[2] Yori Endo,[1] Adriana Panayi,[1] Dharaniya Sakthivel,[1] Ron Neppl,[1] and Amy Wagers[3], *1. Brigham and Women's Hospital, Harvard Medical School, Boston, Massachusetts, United States, 2. Brigham and Women's Hospital, Division of Plastic Surgery, Boston, Massachusetts, United States, 3. Harvard Stem Cell Institute, Cambridge, Massachusetts, United States*
Idranil Sinha[1]

Skeletal muscle retains the ability to regenerate throughout life, but this decreases significantly with aging.

The present study investigates whether aging-associated loss of muscle hypoxia signaling limits regenerative potential. Utilizing young (3 months) and old (22-24 months) mice, skeletal muscle from old mice exhibited a 40% decline in the cross-sectional area (CSA) of newly regenerating fibers following cryoinjury at day 10 (p < 0.01) post-injury as compared to young. Focused PCR array demonstrated a greater than 3-fold decline in expression of the majority of hypoxia signaling genes. In particular, aryl hydrocarbon receptor nuclear translocator (ARNT), which is required for downstream hypoxia signaling and the transcription of hypoxia response genes, is 5-fold lower for both gene expression (p < 0.01) and protein levels (p < 0.01) in old versus young mice. To determine the effects of ARNT on muscle regeneration, we utilized a genetically modified mouse which results in an 80% decrease in ARNT gene expression following activation, specifically in skeletal muscle. Compared to littermate controls, mice with a muscle specific knockdown of ARNT (mKO ARNT) exhibit a 30% decline in regenerating fiber sizes at day 10 (p < 0.01) following cryoinjury, without any loss of regenerative potential in FACS isolated satellite cells ex vivo. Administration of a pharmacologic hypoxia activator, ML228, induced a 30% increase in regenerating fiber CSA in both old mice and mKO ARNT mice (p < 0.01) as compared to treatment with vehicle control. These data suggest hypoxia signaling declines with aging in skeletal muscle and activation of hypoxia signaling may promote regeneration.

### SESSION 3390 (PAPER)

### DEMENTIA IN THE LONG-TERM CARE SETTING

### MEDICATION DISCREPANCIES AND COMMUNICATION ERRORS DURING NURSING HOME INTAKE: A MIXED-METHODS ANALYSIS

Mark E. Patterson,[1] Sandra Bollinger,[2] and Janice Foust[3], *1. University of Missouri-Kansas City School of Pharmacy, Kansas City, Missouri, United States, 2. Health Priorities, Inc., Cape Girardeau, Missouri, United States, 3. University of Massachusetts-Boston, Boston, Massachusetts, United States*

Older adults experience high medication discrepancy rates during transitions from inpatient to nursing home settings. Dosage changes and multiple prescribers increases the risk of inaccurate handoffs and creates challenges for medication reconciliation at nursing home intake. Our objectives were to 1) Characterize medication discrepancies occurring at nursing home intake and 2) Identify resident and medication related factors associated with medication discrepancies. Demographics, comorbidities, medications, discrepancy types and location were prospectively collected over 9-months. Chi-square tests were used to determine factors associated with discrepancies. A focus group of nurse practitioners, pharmacists, and administrators from four long-term care facilities was convened to discuss medication reconciliation challenges at resident intake. Thematic analysis was used to determine key themes. 22%, 12%, and 3% of residents experienced one, 2 to 5, or six or more discrepancies, respectively. The most prevalent discrepancies were omission (34%), frequency (20%), and therapeutic duplication (13%) occurring in analgesics, respiratory and genitourinary medications. 44% of discrepancies occurred between nursing homes and hospitals and 39% involved the community pharmacy. The most significant risk factors for discrepancies included age over 70, Charlson comorbidity indices over 7, readmission to nursing homes, or the prescribing of at least 17 medications. Staff faced challenges of delayed and/or inaccurate data, incompatible documentation forms and inefficient workflows for resolving discrepancies. Residents at greatest risk for medication discrepancies require additional attention during admission medication reconciliation to prevent errors. Nursing home intake and medication reconciliation workflow needs to be improved with data sharing technology to increase accuracy and efficiency.

### OUTCOMES OF DISCONTINUING CHOLINESTERASE INHIBITORS IN NURSING HOME RESIDENTS WITH SEVERE DEMENTIA

Joshua D. Niznik,[1] Xinhua Zhao,[1] Meiqi He,[1] Sherrie Aspinall,[1] Joseph Hanlon,[1] David Nace,[1] Joshua Thorpe,[2] and Carolyn Thorpe[2], *1. University of Pittsburgh, Pittsburgh, Pennsylvania, United States, 2. University of North Carolina Eshelman School of Pharmacy, Chapel Hill, North Carolina, United States*

Some clinical guidelines advocate for the withdrawal of cholinesterase inhibitors (ChEIs) in patients with severe dementia. However, there have been no studies of the outcomes of deprescribing ChEIs specifically in patients with severe dementia, and concerns about subsequent worsening of behavioral symptoms may serve as a barrier to ChEI discontinuation. Our objective was to evaluate the impact of deprescribing ChEIs on aggressive behaviors and depression severity in older nursing home (NH) residents with severe dementia. We conducted a retrospective cohort study using Medicare claims, Part D prescriptions, Minimum Data Set (MDS) v3.0, Area Health Resource File, and Nursing Home Compare, for non-skilled NH residents aged 65+ with severe dementia receiving AChEIs with ≥2 MDS assessments in 2016 (n=30,788). The Aggressive Behavior Scale (ABS) and the Patient Health Questionnaire (PHQ-9) evaluated aggression and depression, respectively. Marginal structural models with inverse probability of treatment weights evaluated the association of deprescribing with outcomes, accounting for time-dependent confounding. The sample was primarily white (78.7%), female (76.6%), >80 years old (77.6%), and 22.8% were deprescribed ChEIs. In adjusted models, deprescribing was not associated with aggression (0.002 point increase in ABS, p=0.90) or depression (0.04 point increase in PHQ-9, p=0.50). Deprescribing ChEIs in NH residents with severe dementia did not lead to an increase in aggressive behaviors or depression severity. Our findings provide insight into the potential risks and benefits associated with deprescribing ChEIs and help inform decision-making in patients with severe dementia.

### THE IMPACT OF MUSIC AND MEMORYSM ON RESIDENT MOOD, BEHAVIORS, AND USE OF MEDICATIONS IN NURSING HOMES

Debra Bakerjian,[1] Kristen Bettega,[1] Ana Marin-Cachu,[1] Leslie Azzis,[1] and Sandra Taylor[2], *1. Betty Irene Moore*

*School of Nursing at UC Davis, Sacramento, California, United States, 2. University of California, Davis, Sacramento, California, United States*

The numbers of Americans with dementia are projected to increase by 44.8% by 2025. Many of these patients are cared for in nursing homes (NH), 70% of NH residents with dementia have been reported to have significant behavioral or psychiatric symptoms (BPSD) that are often challenging to manage. Historically, the first lines of treatment for BPSD has been antipsychotic medications; however, serious adverse effects have been associated with these drugs. Our main aim was to study the effects of Music and MemorySM (M&M), a personalized music program, on improving behaviors and reducing antipsychotics and other medications in residents with dementia in participating NHs. This 3-year, quasi-experimental, mixed methods study used a cluster, randomized design in three phases. We used the Qualtrics Research Suite to create and disseminate a baseline survey and a 4-part quarterly survey thereafter. The quarterly survey collected select resident MDS data including diagnoses, medication use, pain, falls, mood and behaviors; how M&M was being implemented; resident use of M&M; organization level information. We also downloaded NH 5-Star quality rating quarterly. A total of 265 NH and 4,109 residents participated in the study. We found the odds of antipsychotic use declined by 11%, antianxiety medications by 17%, and antidepressants by 9% per quarter. The odds of residents exhibiting aggressive behaviors declined by 20% per quarter, depressive symptoms by 16% and residents reporting pain by 17%. Our findings indicate that M&M provides substantial benefit to NH residents, particularly in reduction of psychoactive medications and improving mood and behaviors.

### TRANSITIONAL CARE OUTCOMES IN VETERANS RECEIVING POST-ACUTE CARE IN A SKILLED NURSING FACILITY

Robert Burke,[1] Anne Canamucio,[1] Thomas Glorioso,[2] Anna Baron,[3] and Kira Ryskina[4], *1. Center for Health Equity Research and Promotion, Corporal Crescenz VA Medical Center, Philadelphia, Pennsylvania, United States, 2. Center of Innovation for Veteran-Centered and Value-Driven Care Rocky Mountain Regional VA Medical Center, Aurora, Colorado, United States, 3. Colorado School of Public Health, University of Colorado Anschutz Medical Campus, Aurora, Colorado, United States, 4. Division of General Internal Medicine, Department of Medicine, University of Pennsylvania Perelman School of Medicine, Philadelphia, Pennsylvania, United States*

More than 200,000 Veterans transition between hospital and skilled nursing facility (SNF) annually. Capturing outcomes of these transitions has been challenging because older adult Veterans receive care at VA and non-VA hospitals, and four different kinds of SNFs: VA-owned and -operated Community Living Centers (CLCs), VA-contracted community nursing homes (CNHs), State Veterans Homes (SVHs), and non-VA community SNFs. We used a novel data source which concatenates VA, Medicare, and Medicaid data into longitudinal episodes of care for Veterans, to calculate the rate of adverse outcomes associated with the transition from hospital to SNF in all enrolled Veterans age 65 and older undergoing this transition 2012-2014. The composite primary outcome included Emergency Department (ED) visits, rehospitalizations, and mortality (not in the context of hospice) within 7 days of hospital discharge to SNF. We used multivariable logistic regression to adjust for Veteran and hospital characteristics and hospital random effects. In the 388,339 Veterans discharged from 1502 hospitals in our sample, we found more than 4 in 5 Veteran transitions (81.7%) occurred entirely outside the VA system. The overall 7-day outcome rate was 10.7%. After adjustment, VA hospitals had lower adverse outcome rates than non-VA hospitals (OR 0.80, 95% CI 0.74-0.86). VA hospital-CLC transitions had the lowest adverse outcome rates; in comparison, non-VA hospital-CNH (OR 2.51, 95% CI 2.09-3.02) and non-VA hospital-CLC (OR 2.25, 95% CI 1.81-2.79) had the highest rates. These findings raise important questions about the VA's role as a major provider and payer of post-acute care in SNF.

### SESSION 3395 (PAPER)

### ECONOMICS AND DISPARITIES IN LATER LIFE

### INCREMENTAL COST AND READMISSIONS ASSOCIATED WITH COLECTOMY PATIENTS UNDERGOING HOME HEALTH SERVICES

Neil Kamdar,[1] and Elham Mahmoudi[2], *1. University of Michigan, Ann Arbor, Michigan, United States, 2. University of Michigan, Department of Family Medicine, Ann Arbor, Michigan, United States*

Home health services has been considered a recommended form of post-discharge management of older patients undergoing surgery. In this study, we examined total 30-day episode payments and 30-day readmission rates for those who were and were not utilizers of home health services following colectomies. We utilized administrative claims data (2012-2017) from the Michigan Value Collaborative across 77 hospitals in Michigan. Post-acute care includes skilled nursing facility, inpatient/outpatient rehabilitation, emergency department visits, home health services, and other outpatient visits. All payments were risk adjusted using generalized linear models using Hierarchical Condition Categories, age, sex, insurance type, and prior 6-month payments. Controlling for selection bias for those utilizing home health services, we performed propensity score matching at a caliper of 0.001 without replacement. We identified 15,531 colectomies with 4,397 (28.3%) utilizing home health services within 30 days. There were 4,197 home health users and non-users with home health users having significantly higher adjusted 30-day episode payments ($33,254 vs. $30,463, 95% CI Diff: ($2,097, $3,486)). While there were only slight differences in adjusted index surgical base payments between home health users ($21,531, 95% CI: ($21,279, $21,783) and non-users ($20,703, 95% CI: ($20,409, $20,998)), there were substantially larger post-discharge payments for home health users ($3,617 vs, $1,839, 95% CI Diff: ($1,597, $1,959)). Colectomy patients utilizing home health services had a higher readmission rate (21.5% vs. 15.6%, p <0.0001). Colectomy patients utilizing home health services are more resource intensive in the post-discharge period. Recommendation for home health services increases costs and readmission.

### RACIAL DISPARITIES IN RURAL-URBAN MORTALITY GAP IN THE UNITED STATES: A 24-YEAR LONGITUDINAL STUDY

Nasim Ferdows,[1] Soroosh Baghban Ferdows,[2] and Amit Kumar[3], *1. University of Southern California, Los Angeles, California, United States, 2. Istanbul Technic U, Istanbul, Turkey, Turkey, 3. Northern Arizona University, Flagstaff, Arizona, United States*

Although overall life expectancy in the US has improved rapidly over the course of the 20th century and the racial gap in all-cause mortality has declined in recent decades, geographical disparities in mortality have increased in the last three decades. This research aims to study racial and geographical disparities by comparing the race and sex-specific mortality trends of the US rural and urban populations. We created a longitudinal county level analytic file of the US population 65 years and older, over the period of 1968 to 2015 obtained from CDC-WONDER and Area Health Resources Files. First, we used an OLS regression of age-adjusted mortality rate onto year indicators interaction with race and gender to depict the race and sex-specific trend in age-adjusted mortality rates. We also estimated the change in in mortality rate over time, for each race and gender, relative to values in 1968. Finally, we estimated race and sex specific trend in rural-urban mortality gap using state fixed effects regression. Our results indicate that racial gap in mortality rates has only declined in urban areas. Mortality rates of the whites in rural areas declined more rapidly than their Black counterparts, resulting in a gap that has been widening in the last three decades. The racial gap has increased considerably for males residing in rural counties not adjacent to an urban county. Thus, racial disparity in mortality has increased in rural areas, with a considerable widening between white and black male population living in the more remote rural areas.

### UNDERSTANDING NON-MEDICAL COSTS FOR HEALTH CARE: EVIDENCE FROM INPATIENT CARE FOR OLDER PEOPLE IN CHINA

Wei Yang[1], *1. King's College London, London, United Kingdom*

Non-medical costs can constitute a substantial part of total health care costs, especially for older people. Costs associated with carers, travel, food and accommodation for family members accompanying and caring for older people during their medical visits can be hefty. This study seeks to examine the effects of non-medical costs on catastrophic health payments and health payment-induced poverty among older people in rural and urban China. Using data from the China Health and Retirement Longitudinal Survey 2015, this study finds that inpatient costs account for a significant proportion of household expenditure, and non-medical costs can account for approximately 18% of total costs. That share is highest for those who belong to the lowest wealth groups. Non-medical costs increase the chances of older people incurring catastrophic health payments and suffering from health payment-induced poverty. Such effects are more concentrated among the poor than the rich. The results also show that the rural population are more likely to incur catastrophic health payments and suffer from health payment induced poverty compared to the urban population. This paper urges policy makers to consider reimbursing the non-medical costs of patient care, improving health care systems in general and for the rural populations specifically.

### WEALTH PROFILES AND VARIATIONS BY GENDER AND RACE: A GROWTH-MIXTURE MULTIGROUP COMPARISON

Yu-Chih Chen,[1] and Nancy Morrow-Howell[1], *1. Washington University in St. Louis, St. Louis, Missouri, United States*

Wealth is fundamentally affected by various life course characteristics. However, little is known about the role of life course factors in shaping wealth trajectories in later life. This study explored how the longitudinal profiles of wealth varied by gender and race (white and non-white populations). Data came from the 2004-2014 Health and Retirement Study with 16,189 older adults aged 51 and older. With corrections for clustered effect within household, this study used growth mixture modeling (GMM) to identify the longitudinal patterns of wealth, and how these profiles varied by these two important life course attributes. The model began with a separate GMM model for race and gender to investigate the optimal latent class model. These results were combined using multi-group approach to incrementally examine the gender and race invariance using configural (same form), structural (same trajectory mean), dispersion (same trajectory variance), and distributional (same latent class size) test. Results identified four distinct wealth profiles—Stable high, Low and increasing, Stable low, and High but decline—for each race and gender category. The multigroup GMM analyses revealed that the wealth profiles varied by gender and race, but the degrees of variation differed a great deal, with results supporting a dispersion model for gender but a configural model for race. Results indicate that race has a stronger effect in shaping wealth development compared to gender. The findings suggest that understanding wealth disparities in later life could be facilitated by examining how wealth varies by gender and race.

### WHAT ARE THE PATHWAYS TO RETIREMENT: DO HEALTH AND COHORT MATTER?

Fang-Yi Huang,[1] and Min Li[2], *1. Trinity Washington University, College Park, Maryland, United States, 2. University of Florida, Gainesville, Florida, United States*

Studies about retirement often neglect ethnic identity. Yet, racial and ethnic inequality plays an important role in retirement decisions. For instance, minorities are less likely to continue working in old age beyond the normal retirement age because of some individual factors such as poor or fair health, cognitive impairment, functional limitation (Green, 2005; Ghilarducci & Moore, 2015; Mudrazija, 2010), physical limitations (Radl, 2014; Solem et al., 2016; Pienta & Hayward, 2002), retirement benefit and eligibility of pension (Radl, 2014; Honig, 1996). This research examined the influence of ethnicity on whether working for people aged above 60 in Taiwan. In addition to testing the pathways to retirement, we scrutinized how occupational sector mediates the likelihood of ethnic effect on retirement. After comparing two cohorts from the dataset "Taiwan Longitudinal Study in Aging", we found that working in public sector, white-collar workers, being a Mainlander, being non-married, higher education, self-reported bad health and functional limitation are all associated with the higher likelihood of retirement. Finding suggested that the incentive structure of the public pension for public sector workers, who are mostly

Mainlanders, could contribute to the higher likelihood of retirement. However, the magnitude of mediator is much larger for the older cohort than the younger cohort. The study not only could benefit cumulative advantage approach by analyzing East Asia data but also bring policy implication, that retirement policy makers should reduce public pension incentive for public sector workers to delay the retirement age.

### SESSION 3400 (SYMPOSIUM)

### ENVIRONMENTALLY SUSTAINABLE DIET AND RECYCLING THERAPY FOR OLDER ADULTS' WELL-BEING AND HEALTHCARE EXPENDITURES

Chair: Hsinyi Hsiao, *Tzu Chi University, Hualien, Taiwan, Taiwan*
Discussant: Aaron Hagedorn, *University of Southern California, Los Angeles, California, United States*

Due to the rapid climate change in recent years, global communities have seen an increased frequency of large-scale natural and man-made disasters. Acknowledging extravagant lifestyles and wasteful consumption of natural resources as prominent factors leading to intensified stress placed on the biosphere and ecosystems, many older adults with chronic medical conditions or disabilities in Taiwan have promoted environmental sustainability through two ways: 1) participating a recycling program in their local communities by collecting, sorting, and reclaiming reusable resources that are regenerated into eco-friendly blankets for disaster survivors and 2) practicing plant-based diet to promote healthy lifestyle and mitigate climate change. These older participants of environmentally sustainable initiatives believe the interconnectedness among humanity, the environment, and sustainable lifestyles. To understand association between the recycling therapy and older adults' well-being a quasi-experimental design with 72 people age 60 and older (recycling group vs. non-recycling group) and photovoice were used to examine longitudinal effects of the recycling therapy on physical and mental health. To examine the effects of plant-based diet on dementia and health care cost ten-year data from Taiwan's National Health Insurance Database were used by two studies to compare risk of dementia and medical expenditure between vegetarian and non-vegetarian groups among older adults in Taiwan. Four presentations in the symposium will discuss positive outcomes of environmental volunteering on older adults' physical and mental wellness and longitudinal effects of a cohort with environmental-friendly diet on dementia risk and lower actual health care expenditures.

### COMPASSION-BASED ENVIRONMENTAL VOLUNTEERISM AMONG OLDER ADULTS: A CASE STUDY OF TZU CHI RECYLING PROGRAM

Hsinyi Hsiao,[1] Chiu-Tien Hsu,[2] Lei Chen,[3] Jinli Wu,[4] Pao-Sheng Chang,[4] Chin-Lon Lin,[5] Miing-Nan Lin,[6] and Tin-Kwang Lin[6], *1. Tzu Chi University, Hualien, Taiwan, Taiwan, 2. National Chung Chen University, Chiayi, Chiayi, Taiwan (Republic of China), 3. University of California, Los Angeles, Los Angeles, California, United States, 4. University of Southern California, Los Angeles, California, United States, 5. Hualien Tzu Chi Hospital, Hualien 970, Hualien, Taiwan (Republic of China), 6. Dalin Tzu Chi Hospital, Dalin, Chiayi, Taiwan (Republic of China)*

To promote environmental sustainability and mitigate climate change that causes numerous families to suffer from natural disasters, a group of older residents' volunteer to recycle usable materials by setting up recycling stations in their communities and transform recycled bottles into eco-friendly blankets for disaster survivors globally. This study examined long-term effects of the peer-led Tzu Chi Recycling Program (TCRP) on older adults' compassion, psychological and physiological well-being. Using a quasi-experimental design, 1-year longitudinal data were collected from older adults at recycling stations (intervention group n = 36) and community centers (control group n = 36) in rural areas in Southern Taiwan. Findings from two-way repeated analysis of variance show that TCRP significantly improved older adults' self-compassion, compassion for others, depression, hostility, happiness, and hypertension. Older adults built resilience through environmental volunteering in the TCRP as an environmental sustainability model for health promotion and social good

### THE IMPACT OF A PLANT-BASED DIETARY PATTERN ON DEMENTIA RISK: A PROSPECTIVE COHORT STUDY.

Ming-Nan Lin,[1] Tina H. Chiu,[2] Chiao-Erh Chang,[3] and Ming-Nan Lin[1], *1. Dalin Tzu Chi Hospital, Dalin, Chiayi, Taiwan (Republic of China), 2. Fu Jen Catholic University, New Taipei, New Taipei, Taiwan (Republic of China), 3. National Taiwan University, Taipei, Taipei, Taiwan (Republic of China)*

High antioxidants and phytochemicals from plant foods may protect against cognitive decline, while saturated fats from animal based foods considered as risk factors have been associated with dementia. Plant-based diets with limited animal products have been shown to improve glycemic control and reduce diabetes risk. The study aims to examine the association between vegetarian diet and dementia risk in a prospective cohort study. Quantitative data collected from 12,062 participants of Tzu Chi Vegetarian Study from 2005 to 2014 in Taiwan. Data included self-administered questionnaire on demographics, medical history, life-style, and a 57-item food frequency questionnaire (FFQ), National Health Insurance Database and the National Death Registry. Findings show that compared with non-vegetarians, vegetarians had 38% lower risk of dementia (HR = 0.62, 95% CI = 0.39, 0.98) after adjusting for age, sex, education, marital status, physical activities, smoking, alcohol drinking, hypertension, diabetes, hyperlipidemia, and ischemic heart diseases.

### ASSOCIATION BETWEEN ENVIRONMENTALLY FRIENDLY DIET AND LOWER HEALTH CARE EXPENDITURES

Chin-Lon Lin,[1] Tina H. Chiu,[2] Jen-Hung Huang,[3] and Ming-Nan Lin[1], *1. Dalin Tzu Chi Hospital, Min-Sheng, Chiayi, Taiwan (Republic of China), 2. Fu Jen Catholic University, Xinzhuang, New Taipei, Taiwan (Republic of China), 3. Hualien Tzu Chi Hospital, Hualien, Hualien, Taiwan (Republic of China)*

Vegetarian diets have decreased incidence rates of chronic non-communicable diseases that have been associated with burden on medical systems worldwide. Previous studies propose a relationship between meat consumption and high medical expenditure; however, there is a dearth in the literature with strong evidence supporting the hypothesis between two factors. Using the data from Taiwan's National Health Insurance Database this study is the first one comparing medical expenditure between vegetarian and non-vegetarian groups among older adults in Taiwan. In the National Health Insurance Database there were 2127 vegetarians matched with 4254 non-vegetarians based on age and gender from 2005 to 2014. Vegetarian diet is associated with approximately 10 – 15% lower total and outpatient medical expenditure. Vegetarians had lower medical expenditure incurred by diabetes and metabolic diseases, mental diseases, and genitourinary diseases. Implication for future research will be discussed at the presentation.

### SESSION 3405 (SYMPOSIUM)

### EPIGENETICS OF STRESS AND ADAPTATION ACROSS HEALTHSPAN AND LIFESPAN

Chair: Luigi Ferrucci, *Translational Gerontology Branch, Intramural Research Program, National Institute on Aging, National Institutes of Health, Baltimore, Maryland, United States*
Co-Chair: Shabnam Salimi, *University of Maryland Baltimore School of Medicine, Baltimore, Maryland, United States*
Discussant: Luigi Ferrucci

Any stimulus that endangers body integrity (stressor) results in an adaptive response to resolve stressful state and determine adaptive or and maladaptive responses. Both chronic extrinsic and intrinsic stressors can produce long-lasting, epigenetic changes in various organs that can eventually result in accelerated changes in bio-physio-pathology. There is initial evidence that stress response involves mechanisms of the epigenetic basis of adaptation and stress response to biological aging and chronic diseases. With aging, homeostasis stability declines causing augmented vulnerability to the external and internal stress. Individuals in whom vulnerability trespass a certain threshold experience accelerated aging and deterioration of health and/ or "secondary aging" phenomena such as premature mortality. Because of substantial heterogeneity of the rate of decline in homeostatic stability, there is inter-individual variability in the age of appearance of chronic diseases and the increased risk of disability and mortality. Thus, tools for the quantification of a stress response would be clinically valuable. Therefore, this symposium suggests approaches to study the epigenetic basis of molecular adaptations across various age, organs' health-span, and life-span.

### DNA METHYLATION AT THE CROSSROADS OF PHYSIOLOGICAL RESPONSE TO STRESS AND PATHOLOGICAL BIOLOGY

Shabnam Salimi, *University of Maryland Baltimore School of Medicine, Baltimore, Maryland, United States*

It is likely that the dimension and the scope of stress perception are different in different body systems conditioning their specific rates of aging. Moreover, in addition to between-individual variabilities in body response to stress, there is inter-organs variability in adaptations resulting in pleiotropy of phenotypes evolved with aging. Epigenetic measures may be considered possible readouts of the extent and proxy of the adaptive response to intrinsic and/or extrinsic stress in body organs. It is thus important to disentangle the epigenetic basis of stress, adaptation, and pathology. In this symposium we discuss a combination of the specific DNA methylation patterns relate to the diseases as an intrinsic stressor and adaptation responses imposed to bodily systems, and also accelerated body organ morbidities. We explain the static and dynamic DNA methylation differentiation over time as the "epigenetic code" of accelerated pathology across various age using longitudinal data.

### EPIGENETICS MARKERS OF AGING AND EXPOSURES

Joanne Murabito[1], *1. Boston University, Boston, Massachusetts, United States*

Widespread changes to the epigenome occur with aging. DNA methylation is one of the most commonly studied epigenetic mechanisms, reflects lifetime exposures that impact aging, and is associated with age-related disease risk. Many longitudinal cohort studies have existing cross-sectional or longitudinal DNA methylation data along with genotype and expression data permitting investigation of relationships between DNA methylation markers, exposures, and disease. The data can be leveraged to conduct large epigenome-wide association studies (EWAS) of aging and age-related disease to identify DNA methylation biomarkers and lead to insights into novel biologic pathways for development of interventions to delay aging. DNA methylation age measures robustly predict chronologic age and associate with both healthspan and lifespan. During the workshop, examples from cohort studies and the CHARGE consortium will be presented.

### ENVIRONMENTAL EPIGENETICS AND AGING

Andrea Baccarelli[1], *1. Columbia, Mailman School of Public Health, New York, New York, United States*

The human epigenome is a flexible, environmental sensitive component of human biology that changes over time. Multiple studies have identified prospective changes in epigenetic marks that indicate that the epigenome ages as we grow older. These changes have been leveraged to create multiple indicators of age that may also predict mortality and age-related disease. There is ongoing research to determine the extent to which age-related epigenomics changes are inherent to cell biology and/or driven by lifestyle and environmental factors. In this presentation, I will review the current evidence derived from human aging studies and potential contributions to human health and disease. I will discuss the source of data, methodological challenges for large human studies, limitations and possible future directions.

### EPIGENETIC CLOCKS OF COMPUTED TOMOGRAPHY MEASURES OF FATTY ORGANS

Ake T. Lu,[1] and Steve Horvath[2], *1. Department of Human Genetics, University of California, Los Angeles, Los Angeles, California, United States, 2. UCLA, Los Angeles, California, United States*

DNA methylation (DNAm) based biomarkers collectively known as "epigenetic clock" yielding accurate measure of age in any tissue across the entire life course have associated with a large host of age-related conditions. To study their implications in metabolic syndromes, we analyzed computed tomography (CT) derived measures of adiposity with 1) three widely-used clocks: Horvath, Hannum, and Levine (PhenoAge), and a newly developed clock DNAmGrimAge, and 2) seven DNAm based plasma proteins, the components comprising the DNAmGrimAge. Age-adjusted DNAmGrimAge and several age-adjusted DNAm plasma proteins including plasminogen activation inhibitor 1 (PAI-1) stand our followed by adrenomedullin and leptin. Fatty liver (r ≤ 0.41, P ≤ 2.9E-37) and excess visceral adipose tissue fat (r ≤0.42, P ≤ 1.5E-41) are the most significant CT-based measures of DNAmPAI-1 and DNAmGrimAge. Furthermore, age-adjusted DNAmGrimAge and age-adjusted DNAmPAI-1 continued showing the most significant relationship with lifestyle factors including healthy diet and educational attainment in expected directions.

### SESSION 3410 (PAPER)

### GENDER-CENTERED TOPICS IN AGING

### GENDER DIFFERENCES IN WIDOWHOOD IN THE SHORT RUN AND LONG RUN: FINANCIAL AND EMOTIONAL WELL-BEING

Jialu L. Streeter[1], *1. Stanford University, Stanford, California, United States*

This paper addresses the question of whether differences in financial and emotional wellbeing after the death of one's spouse vary systematically with gender, using the Health and Retirement Study data between 1992 and 2014. Results show that all else being equal, widows have 22% less income than widowers, and a nine percentage points increase in their likelihood of falling into poverty. The income gap attributable to gender is considerably large for those newly widowed. The gap shrinks by half in the next ten years in widowhood but widens again substantially after the ten-year mark. The study suggests that men are protected from income losses to a higher degree than women when they lose their spouse, and the survivor benefits of the Social Security and private pension plans do not necessarily cushion widows from this risk. We also find that, in widowhood, men experience more negative affects (e.g., depression and loneliness), while women face more challenges with somatic symptoms (e.g., restless sleep). The emotional gender gaps vary with widowhood durations. In the year of their spousal losses, men suffer significantly more than women from negative affects, in particular, loneliness. Though women score better than men in affects in the short run, they are prone to having more somatic symptoms in the long run, especially restless sleep. The results indicate that men and women need different types of emotional support and attention at different times of widowhood.

### GENERAL AND GENDER-SPECIFIC DYNAMICS DETERMINING THE HEALTH STATUS OF WORKING INFORMAL CAREGIVERS

Abby Schwartz,[1] Leanne J. Clark-Shirley,[2] and G. Rainville[2], *1. East Carolina University, School of Social Work, Greenville, North Carolina, United States, 2. AARP, Washington, District of Columbia, United States*

As the gender divide among family caregivers closes, it is important to understand whether and how men and women differently experience caregiving. For example, literature suggests that employed family caregivers experience burden and health outcomes differently than unemployed caregivers, but less is known about how these factors affect men and women differently. Using data from Caregiving in the U.S., 2015 (source: AARP and NAC), this study reliably modelled the effect of multiple threats to good health within the caregiving role (e.g. physical, financial, and emotional strain). In the analysis, several moderated relationships were observed using data from 816 working caregivers. In the full sample, the relationship between objective caregiving burden (hours of care and counts of ADLs/IADLs) and self-reported health status was altered by financial strain. In the high burden condition, relatively poor health was progressively related to increasing levels of financial strain controlling for traditional covariates. In separate analyses for males and females, this moderated relationship was discovered to be limited to female caregivers. Physical and emotional strain did not moderate the relationship between burden and health. Several covariates related to employment conditions (e.g., caregiver-friendly workplace policies) behaved differently across models and are presented and discussed in relation to financial strain as a determinant of caregiver health. These findings shed light on gender-based differences in caregiver outcomes, and suggest that interventions aimed at assessing and improving caregiver health should account for the financial strain experienced particularly by women.

### THE CENTRALITY OF GENDER EXPRESSION FOR TRANSGENDER ELDERS AND CONCEPTUALIZATIONS OF SUCCESSFUL AGING

Steffany Sloan,[1] and Jacquelyn Benson[1], *1. University of Missouri-Columbia, Columbia, Missouri, United States*

Successful aging is a construct regularly addressed in the gerontological literature, most typically referred to as living in the best physical and cognitive health possible, experiencing the least amount of disease, and engaging actively within one's social environment. There are, however, conceptually distinct aspects of aging for older adults who identify as transgender, particularly given lifelong experiences of marginalization and stigma. In order to identify factors transgender older adults consider most relevant to successful aging, a Theory-Generating Qualitative Meta-synthesis was conducted. The study utilized systematic methods and thematic analysis with qualitative data from empirical studies focusing on transgender aging experiences, particularly drawing upon data that amplified the transgender older adult point of view. Findings of this study indicate that although trans older adults do not minimize unique challenges inherent to aging as a gender minority, successful aging rests on the relief and resilience offered by authentic gender expression. As such, data from this study informed the development of a model of successful aging that centralizes the importance of gender expression. Understanding the central role of gender expression in later life can be especially impactful for social and health service providers, as it emphasizes gender affirming experiences as highly salient to one's own assessment of quality aging. Furthermore, conceptualizing aging for transgender elders in a way that prioritizes the importance of identity can aid in preventing an unnecessarily deficits-based perspective for service providers, and supports the necessity of amplifying a culturally specific understanding of successful aging.

### YOU HAVE TO BE STRONG TO GET OLD: MIDDLE-AGE CAREGIVING, WISDOM DEVELOPMENT, AND LIFE PLANNING AMONG OLDER ADULT WOMEN

Kathryn Hartikka[1], *1. University of Florida, Gainesville, Florida, United States*

This study explores women's development of wisdom in older adulthood resulting from caregiving of aging parents and spouses during their middle age years. As a way to grasp the complexity of middle age care work influencing wisdom development in older adulthood, this study used in-depth case analysis with in-depth interviews of three older adult women from the Silent Generation, ranging in birth years from 1933 to 1944. Women self-identified wisdom through themes such as compassion, knowledge attainment, and resilience resulting from their care work. This study also captured the significance of their caregiving in middle age as a turning point in their lives, marking their physical health and capabilities as notably stronger compared to their present selves in older adulthood. These women also illustrated increased awareness of their own mortality through caregiving. Jointly, these women self-identified their roles as caregivers as uniquely female roles. While all noted being confronted with care work as unexpected in their life path, these women believed in having a natural inclination to nurture and care, coupled with social pressures, leading to their ultimate caregiving. This study unearthed deeper meanings these women had for their lives resulting from caregiving, manifesting as knowledge gained about life, health, and aging. Thus, inspiring their own decision-making as they approach even older adulthood experiences such as dependency and death. This study ultimately illustrates the importance of caregiving on women's lives: marking their middle age, increasing their wisdom development, and influencing their life planning as they age.

### SESSION 3415 (PAPER)

### HEALTH AND SOCIAL SERVICE INTERVENTIONS

### INFLUENCE OF HEALTH AND SOCIAL SUPPORTS ON OUTCOMES POST-REHABILITATION FOR HIP FRACTURE SURGERY: SYSTEMATIC REVIEW

Katherine McGilton,[1] Shirin Vellani,[1] Melanie Bayly,[2] Elizabeth Tanjong-Ghogomu,[3] Andrea Iaboni,[4] Julie Lapenskie,[5] Vivian Welch,[6] and Abeer Omar[7], *1. KITE, Toronto Rehab, University Health Network, Toronto, Ontario, Canada, 2. Canadian Centre for Health and Safety in Agriculture (CCHSA), University of Saskatchewan, Saskatoon, Saskatchewan, Canada, 3. Bruyère Research Institute, University of Ottawa, Ottawa, Ontario, Canada, 4. University Health Network, Toronto, Ontario, Canada, 5. Bruyere Research Institute | Institut de recherche Bruyere Ottawa Hospital Research Institute | Institut de recherche de l'Hôpital d'Ottawa, Ottawa, Ontario, Canada, 6. Centre for Global Health, Bruyère Research Institute, Ottawa, Ontario, Canada, 7. KITE, Toronto Rehab, University Health network, Toronto, Ontario, Canada*

Background: Older adults who sustain hip fractures encounter physical and functional decline after discharge from inpatient rehabilitation. Currently, a synthesis of literature is lacking on health and social supports that may impact outcomes in the community-dwelling older adults, post-discharge from rehabilitation. Methodology: We conducted a systematic review to a) evaluate how health and social supports influence outcomes for older adults and their caregivers following inpatient rehabilitation post-hip fracture surgery, and b) identify the factors that affect their impact on outcomes. We searched Medline, CINAHL, Embase, Emcare, Psychinfo, and Ageline for publications between 2000 and 2018. We followed Cochrane Handbook methods to screen titles and abstracts, appraise quality, collect data and synthesize results. Results: A total of 3364 articles were retrieved, and 34 studies were included for final synthesis, including 24 randomized control trials and 10 observational studies. Most studies excluded persons with moderate or severe cognitive impairment. Interventions can be broadly categorized as either comprehensive care delivered by interdisciplinary teams focusing on exercise, nutrition and fracture prevention; or exercise sessions delivered by health professionals, trained instructors or volunteers. Interventions involving interdisciplinary teams demonstrated moderate improvement of mobility and functional ability in the first 3 months. However, the longitudinal effects of interventions were not realized for all. Conclusion: This review provides evidence of the effectiveness of health and social supports provided to older adults post-hip fracture. We are uncertain of the applicability to people with cognitive decline due to exclusion from most studies. Implications for practice and research will be discussed.

### MLTSS AND AGING IN PLACE: HOW PERSONAL CARE SERVICES HELP AVOID NURSING FACILITY ADMISSIONS OF OLDER ADULTS

Lycia Tramujas Vasconcellos Neumann,[1] and Patricia I. Documet[1], *1. University of Pittsburgh, Pittsburgh, Pennsylvania, United States*

Older adults value community living and prefer to "age in place", which increased the need for long-term care to be provided at home. Recently, a capitated option for Medicaid beneficiaries, managed long-term services and supports (MLTSS), has become popular. This evaluation research study investigated 1) the effectiveness of attendant care services to avoid long-term institutionalization of older adults when provided as part of an MLTSS program, and 2) the effect of the type of attendant care services on long-term institutionalization. Using enrollment and claims data of 491 community-dwelling older adults enrolled in an MLTSS program for at least six months, multivariate regression models analyzed the association between long-stay nursing facility (LSNF) admissions and the use of attendant care services. Findings confirmed the hypothesis that those receiving attendant care services are less likely to have LSNF admissions and that as the dosage increases, the odds of LSNF decreased. The type of attendant care services also influenced the results. Participants who used only self-directed AC services were 93% less likely to have an LSNF admission than those receiving no AC services, and 23.5% less likely to have this outcome than those who only received agency attendant care services. In addition, the use of other home and community-based services are also significantly associated with LSNF admissions. These findings have important research and practical implications at the program and policy level, as MLTSS programs spread across the country and aim to "rebalance" the LTSS system.

### PATTERNS OF UNMET NEEDS AND STRENGTHS AMONG STROKE PATIENTS AND THEIR INFLUENCE ON OUTCOMES

Amanda Woodward,[1] Anne K. Hughes,[1] Mat Reeves,[1] Michele Fritz,[1] Paul Freddolino,[1] Constantinos Coursaris,[1] and Sarah J. Swierenga[1], *1. Michigan State University, East Lansing, Michigan, United States*

The transition home after a stroke is a particularly challenging time for patients and caregivers. There is extensive literature on unmet needs among stroke patients, but there are fewer studies that examine the strengths and resources of stroke patients and how these may influence their subsequent recovery. This study uses data from the Michigan Stroke Transitions Trial (MISTT), a pragmatic 3-arm clinical trial that tested the efficacy of case management against usual care. Two intervention groups (n=160) received up to 90 days of services from a social work case manager. A complete biopsychosocial assessment was conducted approximately 5 days after discharge. Latent class analysis was used to identify different classes based on six indicators of unmet needs and twelve indicators of strengths. Four homogenous classes were identified in the final model. Class 1 (n=56, 35%) included patients with few needs and strong social support, but few other strengths. Class 2 (n=46, 29%) had few needs and a high level of strengths across all indicators. Class 3 (n=39, 24%) has moderate needs related to mental health, non-stroke physical health, and finances, but few strengths. Class 4 (n=19, 12%) has moderate needs related to social support, non-stroke related physical health, and finances, but with moderate strengths related to indicators like coping, cognition, and insight. Class membership was related to short-term quality of life. Understanding the combination of needs and strengths has potential implications for services provided during care transitions as well as the policies and funding mechanisms that support those services.

### THE IMPACT OF FOOD INSECURITY ON THE HEALTH AND WELL-BEING OF OLDER ADULTS: THE GROUPS MOST AT RISK

Lauren E. Popham,[1] and Erin McGovern[1], *1. National Council on Aging, Arlington, Virginia, United States*

Older adults who experience food insecurity (4.6 million) often have worse health outcomes. Food insecure older adults consume less nutrients, which puts them at greater risk of developing chronic diseases. They are at increased risk of falls due the impact of poor nutrition on muscle mass, bone density, and balance. Low-income older adults are often forced to choose between buying groceries and paying other bills. The Supplemental Nutrition Assistance Program (SNAP) plays an important role in reducing food insecurity. SNAP enables older adults to buy the nutritious food they need, while freeing up resources to pay for everyday things to meet their health needs such as prescription drugs. Research shows that medication adherence increases when low-income older adults enroll in SNAP. Despite the beneficial impact of enrolling in SNAP, it's estimated that 55% of eligible adults age 60 and older are not participating in this critical program. To understand which older adults are missing out on SNAP, the National Council on Aging engaged researchers at Leading Age LTSS Center at UMass Boston to analyze data from the 2014 Health and Retirement Study. The results show that some of the most vulnerable older adult populations are less likely to participate in SNAP even though they are eligible (i.e., Hispanic, age 75 and older, those who are not utilizing healthcare, etc.). The findings suggest that more targeted outreach to these groups is needed to ensure that the most vulnerable populations of older adults access this critical benefit.

### THE IMPACT OF THE OPIOID EPIDEMIC ON OLDER ADULTS: FINDINGS FROM A NATIONAL SURVEY OF COMMUNITY-BASED ORGANIZATIONS

Kathleen A. Cameron,[1] Lauren E. Popham,[1] and Angelica Herrera-Venson[1], *1. National Council on Aging, Arlington, Virginia, United States*

The National Council on Aging (NCOA) conducted a national survey of community-based organizations (CBOs) in early 2019 to better understand how older adults, people with disabilities, and their caregivers are affected by the opioid epidemic and identify new resources and tools needed by CBOs to better serve their community needs. Specifically, the survey asked about the extent to which CBOs' service delivery and level of effort has changed as a result of the opioid epidemic; unique issues reported by this population, directly or indirectly resulting from opioid misuse by them or loved ones; how organizations screen and refer older adults and individual with disabilities for support associated with opioid misuse; how organizations may be connecting with local or state initiatives addressing the opioid epidemic, or forming strategic partnerships to respond to emerging client needs; and pinpoint gaps in resources that may help organizations to more effectively respond to these issues. Over 200 organizations, representing urban, suburban and rural communities, responded to the survey and included senior centers, area agencies on aging, Senior Health Insurance Assistance Programs, as well as health care organizations. Seventy percent of organizations report spending more effort to address the needs of older adults/caregivers who are adversely affected by opioid misuse/abuse since 2 years ago. Common health and financial concerns, current strategies related to screening, partnership development, and educational programming as reported by CBOs will be presented. This session will include a discussion of opportunities to assist CBOs address the opioid-related needs of their older adult clients.

### SESSION 3420 (PAPER)

### ISSUES IN ACUTE CARE

### ACUTE CARE UTILIZATION IN OLDER ADULTS LIVING UNDIAGNOSED OR UNAWARE OF DEMENTIA

Halima Amjad,[1] David L. Roth,[1] Jin Huang,[1] Jennifer L. Wolff,[2] and Quincy M. Samus[3], *1. Johns Hopkins University School of Medicine, Baltimore, Maryland, United States, 2. Johns Hopkins University, Maryland, United States, 3. The Johns Hopkins University, Baltimore, Maryland, United States*

Most individuals with dementia are undiagnosed or they/their families are unaware of the diagnosis. Implications of dementia diagnosis and awareness are poorly understood. Our objective was to determine whether undiagnosed dementia or unawareness increases risk of hospitalization or emergency department (ED) visits, outcomes with recognized risk in diagnosed dementia. We linked National Health and Aging Trends Study (NHATS) data to fee-for-service Medicare claims for 4,311 community-living participants in the nationally representative cohort. We assessed probable versus no dementia using validated NHATS dementia criteria, undiagnosed versus diagnosed using Medicare claims, and aware versus unaware using NHATS self or proxy report of diagnosis. Cox proportional hazards models evaluated hospitalization and ED visit risk by time-varying dementia diagnosis and awareness status, adjusting for sociodemographic characteristics, functional impairment, medical comorbidities, and prior hospitalization. Compared to no dementia, persons with dementia who were unaware but diagnosed had greater risk of hospitalization (HR 1.66, 95% CI 1.26-2.19) and ED visits (HR 1.63, 95% CI 1.28-2.08). Persons unaware but diagnosed also had greater risk compared to persons aware and diagnosed (hospitalization HR 1.34, 95% CI 0.98-1.82; ED HR 1.38, 95% CI 1.05-1.83). Persons with undiagnosed dementia demonstrated hospitalization risk similar to persons with no dementia (HR 1.02, 95% CI 0.79-1.31) and similar or potentially lower than persons aware and diagnosed (HR 0.82, 95% CI 0.61-1.10); ED visit findings were similar. Results suggest that being unaware of dementia diagnosis may affect healthcare utilization. Strategies to improve communication and understanding of dementia could potentially reduce hospitalizations and ED visits.

### FAMILY CAREGIVER FACTORS AND HOSPITALIZATION IN DISABLED OLDER ADULTS WITH AND WITHOUT DEMENTIA

Halima Amjad,[1] John Mulcahy,[2] Judith D. Kasper,[3] Julia Burgdorf,[3] David L. Roth,[1] Kenneth Covinsky,[4] and Jennifer L. Wolff[5], *1. Johns Hopkins University School of Medicine, Baltimore, Maryland, United States, 2. Johns Hopkins University Bloomberg School of Public Health, Baltimore, Maryland, United States, 3. Johns Hopkins Bloomberg School of Public Health, Baltimore, Maryland, United States, 4. University of California San Francisco, San Francisco, California, United States, 5. Johns Hopkins University, Maryland, United States*

Older adults with disabilities commonly rely on family caregivers' help, yet effects of caregiver factors on patient outcomes are poorly understood. Within this population, dementia is common. Our objective was to evaluate the association between caregiver factors and risk of hospitalization in disabled older adults with and without dementia. We examined 2,589 community-living older adults with mobility/self-care disability and their primary family caregiver in four waves of the National Long-Term Care Survey and National Health and Aging Trends Study. We used Cox proportional hazards models to examine risk of one-year, Medicare claims-derived, all-cause hospitalization as a function of caregiver factors, adjusting for older adult characteristics (sociodemographics, comorbidities, healthcare utilization) and survey year, considering dementia a characteristic of interest. Among disabled older adults, 38% were hospitalized over one year, and 31% had probable dementia. Hospitalization rates were similar for older adults with and without dementia (39.5% and 37.3% respectively); dementia was not associated with hospitalization risk (HR 1.09, 95% CI 0.95-1.26). Older adults demonstrated greater risk of hospitalization if their caregiver was male (HR 1.31, 95% CI 1.10-1.56), new to caregiving (HR 1.61, 95% CI 1.27-2.04 for < 1 year versus ≥ 4 years), or helped with healthcare tasks (HR 1.21, 95% CI 1.04-1.41). The association between most caregiving factors and hospitalization risk did not differ by dementia status. Results suggest that strategies to reduce hospitalization in older adults with disabilities could target select caregivers using similar strategies in populations with and without dementia.

### HOSPITAL CARE USE IN OLDER ADULTS: THE ROLE OF PSYCHOLOGICAL AND SOCIAL FACTORS

VIVIANE STRAATMANN,[1] Serhiy Dekhtyar,[2] Bettina Meinow,[1] Laura Fratiglioni,[2] and Amaia Calderon-Larranaga[2], *1. Aging Research Center - Karolinska Institute, Stockholm, Sweden, Sweden, 2. Aging Research Center, Department of Neurobiology, Care Sciences and Society, Karolinska Institutet and Stockholm University, Stockholm, Sweden*

Although older people's health status is the main determinant of healthcare use, there has been little research on how psychosocial factors relate to healthcare utilization. We explored the extent to which psychological and social aspects predict the use of hospital care in an older Swedish population. 2867 people ≥60 years from the Swedish National study on Aging and Care in Kungsholmen (SNAC-K) were followed from baseline (2001-2004) for four years. We created standardized indexes of psychological well-being, and social well-being. Binomial negative mixed models were used to estimate the association of psychological and social indexes with hospital care use (i.e. unplanned hospital admissions [UHA], 30-day readmissions [30DR] and length of stay [LOS]). Individuals with a psychological well-being score above the median had less UHA (IRR 0.43, 95%CI 0.20-0.93) and lower LOS (IRR 0.18, 95% 0.06-0.58), even after full adjustment. High levels of social well-being were also protective for UHA and LOS in the minimally adjusted model, but not after adjusting by life style and personally traits. Relative to individuals with poor well-being on both indexes, those with rich psychological and poor social well-being had reduced hospital care use (IRR 0.44 95%CI 0.24-0.84; IRR 0.23, 95%CI 0.08-0.67, respectively), and even further in those with rich psychological and social well-being (IRR 0.33 95%CI 0.14-0.75; IRR 0.10, 95% 0.02-0.45, respectively). No statistically significant association was found with 30DR. Provided the importance of psychosocial aspects in predicting UHA and LOS, targeting the former could be a strategy for reducing healthcare use and, eventually, costs.

### IMPLEMENTING A GERIATRIC FRACTURE PROGRAM IN A PLURALISTIC ENVIRONMENT REDUCES LENGTH OF STAY AND TIME TO SURGERY

Sonja L. Rosen,[1] Kathy Breda,[1] Carol Lin,[1] Jeanne Black,[1] Jae Lee,[1] Aaron Chiang,[1] Mark Vrahas,[1] and Brad Rosen[1], *1. Cedars-Sinai Medical Center, Los Angeles, California, United States*

Geriatric-orthopaedic co-management models have been demonstrated to improve patient outcomes, but are typically implemented in closed, non-pluralistic medial systems. The Cedars-Sinai Geriatric Fracture Program (GFP) was developed through collaboration amongst a multi-disciplinary group. Cedars-Sinai is an academic medical center with a pluralistic medical staff that includes faculty, several hospitalist groups, and private practitioners. The GFP was introduced in July 2018 as a quality improvement pilot to provide standardized treatment for geriatric fracture patients. We hypothesized GFP enrollment would reduce time to surgery (TTS) and length of stay (LOS). Geriatric fracture patients were prospectively enrolled from July -December 2018. The Wilcoxon Rank- Sum test was used to compare TTS and LOS between the two patient groups. A $p < 0.05$ was considered significant. 190 operative fractures in patients over 65 years-old were prospectively followed.56 (30%) were enrolled in the GFP, 54 (28%) were admitted to other hospitalist groups (OH), and 80 (42%) were managed by their primary care physician (PCP). There were no demographic differences between groups. Patients enrolled in the GFP had a significantly shorter LOS compared to the OH and PCP groups (4 days v 5 days v 5 days, $p = 0.039$) as well as a significantly shorter TTS (19.7hrs v 22.4 hrs vs 23.3 hrs, $p = 0.037$). Our data shows that a multi-disciplinary geriatric fracture program can be successfully implemented in a complex pluralistic environment resulting in improved patient metrics. Adherence to evidence-based protocols and close multidisciplinary teamwork are critical to program success.

### IN-HOSPITAL NURSE CARE CONTINUITY: DOES IT MATTER?

Orly Tonkikh,[1] Anna Zisberg,[2] and Efrat Shadmi[1], *1. University of Haifa, Haifa, Israel, 2. The Cheryl Spencer Department of Nursing Faculty of Social Welfare and Health Science, University of Haifa, Israel, Haifa, Isreal, Israel*

In-hospital cognitive decline affects up to 40% of hospitalized older adults and is associated with post-hospitalization worsening of medical and functional status. Studies pointed to the substantial role of the interpersonal relationship between older adults with cognitive impairment and the nurses who care for them. We investigated the association between nursing interpersonal continuity and cognitive outcomes in a cohort of 646 older adults aged 70 or older admitted to internal units for non-disabling conditions. Cognitive decline was defined as at least one point decline in the Short Portable Mental Status Questionnaire from at admission to discharge assessments. Nursing interpersonal continuity was measured using continuity of care index (CoC). CoC assesses the extent of different nurses assigned to take care of each patient during the hospital stay (2 shifts per day) and ranges from 0 (none of the nurses is the same) to 0.4 (highest feasible score according to full time standard shift plan and length of stay (LOS)). Multivariate logistic regression showed that achieving 25% of the highest feasible in-hospital nursing CoC was associated with lower odds of cognitive decline (OR=0.67, 95% CI=0.47-0.97), controlling for age, sex, premorbid activities of daily living status, at admission cognitive status, comorbidities, severity of illness and LOS. This study shows that in-hospital nursing continuity is negatively associated with older adults' cognitive decline, even in low-continuity levels. Future studies should investigate in-hospital continuity patterns and interventions maintaining continuity in larger and more heterogenic samples.

### SESSION 3425 (SYMPOSIUM)

### IT'S THE LITTLE THINGS THAT COUNT: IMPLICATIONS OF DAILY EXPERIENCES FOR WELL-BEING AND BIOLOGICAL INDICATORS

Chair: Kira S. Birditt, *Institute for Social Research, University of Michigan, Ann Arbor, Michigan, United States*

Middle-age and older adults vary widely in their physical health. This symposium describes studies that identify diverse daily experiences that account variation in health using multiple indicators of well-being (self-reported, biological). Fingerman et al. assessed daily TV viewing among older adults and found that more frequent television watching was associated with poorer physical health, worse health behaviors, and less energy expenditure (via actical watch). Leger et al., examined links between daily affect and sleep. Greater variability in daily positive affect is associated with fewer hours of sleep and greater morning tiredness even after adjusting for mean levels of affect. Luong et al. examined links between daily stress and affect. Interpersonal stressors were associated with greater affect reactivity than non-interpersonal stressors and links were reduced among older adults. Birditt et al. assessed links between daily social interactions and cardiovascular reactivity. More frequent social interactions and negative social interactions were associated with increased heart rate and links varied by gender and race. Polenick et al. examined links between daily social interactions and salivary DHEA-S (a marker of the stress response). Positive interactions predicted greater DHEA-S over the course of the day and links between negative interactions and DHEA-S varied by age group such that younger individuals appeared to be more reactive. These studies offer important clues regarding how daily experiences get under the skin to influence health and well-being.

### TELEVISION VIEWING AND HEALTH IN LATE LIFE

Karen Fingerman,[1] Yee To L. Ng,[1] and Meng L. Huo[1], *1. The University of Texas at Austin, Austin, Texas, United States*

Television viewing is a risk factor for poor physical and cognitive health. Yet, we know little about associations between current health and frequency of television viewing throughout the day. This study examined associations between multiple assessments of physical and cognitive well-being and television viewing. Participants (N = 313) from the Daily Experiences and Well-being Study completed an initial interview assessing their health and well-being. They also wore an Electronically Activated Recorders (EAR) to capture sound in the environment and an Actical to measure physical activity for 5 days. Coders rated the audiofiles for television viewing. Multilevel models revealed that participants who spent a greater proportion of time tuned into television had: higher BMI, poorer health, expended less energy, were more sedentary, and reported drinking more alcohol and eating more junk food. We discuss findings with regard to potential reciprocal influences between television viewing and poor health.

### AFFECT VARIABILITY AND SLEEP: EMOTIONAL UPS AND DOWNS ARE RELATED TO A POORER NIGHT'S REST

Kate A. Leger,[1] Susan Charles,[2] and Karen Fingerman[3], *1. University of Kentucky, Lexington, Kentucky, United States, 2. UC Irvine, Irvine, California, United States, 3. University of Texas Austin, Austin, Texas, United States*

Emotional experience is strongly related to physical health. Yet, fluctuations in daily emotional experience, known as affect variability, have been less examined. It is unknown how affect variability throughout the day is related to sleep, a critical health behavior. The current study examines this relationship in an ecological momentary assessment of 277 older adults. Regression models indicate that greater variability in daily positive affect is associated with fewer hours of sleep ($b = -0.648$, $p = .04$) and greater morning tiredness ($b = 0.67$, $p = .006$) even after adjusting for mean levels of affect. Although greater negative affect variability is associated with worse sleep quality ($b = -0.77$, $p = .02$) and greater morning tiredness ($b = 0.91$, $p = .004$), these relationships disappear once mean negative affect is added into the model. Findings support models describing the downside in the fragility of positive affect.

### AGE DIFFERENCES IN CONCORDANCE BETWEEN LAB AND DAILY LIFE STRESS RESPONSES

Gloria Luong,[1] and Carla M. Arredondo[1], *1. Colorado State University, Fort Collins, Colorado, United States*

The literature is mixed with respect to how stress reactivity changes with age. Previous studies have overlooked contexts, ignoring whether stressors occurred in the laboratory or in daily life. The Health and Daily Experiences (HEADE) study includes 126 younger and older adults who completed both laboratory stressors and ecological momentary assessments (EMA) of affect and stressor experiences in daily life. We found that the laboratory stressor elicited the greatest levels of negative affect reactivity (i.e., larger increases in negative affect) and positive affect reactivity (i.e., larger reductions in positive affect) compared to the two types of daily life stressors. Interpersonal stressors were associated with greater negative and positive affect reactivity compared to non-interpersonal stressors in daily life. Younger adults exhibited greater stress reactivity than older adults. Together, these findings support age-related reductions in stress reactivity. Implications for understanding stressor-health links are discussed.

### MATTERS OF THE HEART: DAILY SOCIAL INTERACTIONS AND CARDIOVASCULAR REACTIVITY IN MIDDLE AND OLD AGE

Kira S. Birditt,[1] Angela Turkelson,[1] Meaghan Mones,[1] Kayvan Najarian,[2] and Richard Gonzalez[1], *1. Institute for Social Research, University of Michigan, Ann Arbor, Michigan, United States, 2. Computational Medicine, University of michigan, ann Arbor, Michigan, United States*

Social ties are essential for survival but the mechanisms accounting for this link are unclear. This study examined links between daily interpersonal experiences and cardiovascular reactivity. A total of 34 participants (aged 40 to 80) completed ecological momentary assessment surveys every three hours for 4 days and wore a device that assessed heart rate (HR) and heart rate variability (HRV). Multilevel models revealed that a greater number of social interactions and negative social interactions predicted increased HR. Links between social interactions and cardiovascular reactivity varied by gender and race. A greater number of interactions and negative interactions predicted increased HRV among men and not women. A greater number of social interactions predicted increased HR among Black individuals and White women but not White men. Thus, social interactions appear to get under the skin via the cardiovascular system but in unique ways that vary by gender and race.

### DAILY SOCIAL INTERACTIONS AND BIOLOGICAL STRESS REACTIVITY: ARE THERE AGE DIFFERENCES?

Courtney A. Polenick,[1] Steven H. Zarit,[2] and Kira S. Birditt[1], *1. University of Michigan, Ann Arbor, Michigan, United States, 2. Pennsylvania State University, University Park, Pennsylvania, United States*

Older people experience fewer negative social interactions and report less anger and stress when faced with interpersonal tensions. Little is known, however, about age differences in biological responses to social interactions. We evaluated how salivary DHEA-S, a key indicator of stress reactivity, is associated with daily positive and negative social interactions among midlife and older adults. Participants were drawn from the Daily Health, Stress, and Relationship Study, which includes 93 adults age 40 to 95 who completed 14 days of daily diary interviews and provided saliva samples on four of those days. Multilevel models showed that people had higher DHEA-S on days in which they reported more positive interactions. Older respondents were less reactive to negative interactions relative to younger respondents. These findings indicate that positive social interactions may benefit biological stress reactivity regardless of age, whereas older adults are more resilient to the adverse effects of negative social interactions.

### SESSION 3430 (PAPER)

### LGBTQ AND AGING

### CULTURALLY COMPETENT CARE FOR OLDER SEXUAL MINORITY ADULTS: A SYSTEMATIC REVIEW FOR HEALTHCARE DELIVERY

Debra Saliba,[1] Sarah MacCarthy,[1] Biayna Darabidian,[1] and Marc N. Elliott[1], *1. RAND Corporation, Santa Monica, California, United States*

Recent attention to culturally competent care has largely overlooked the needs of older LGB adults. To address this, we conducted a systematic literature review and make recommendations for how the healthcare workforce can reduce sexual-orientation-based disparities. We searched PubMed, PsycINFO & CINAHL for manuscripts 1/1/10-6/19/18 (n=799), deduplicating, dually-screening abstracts (n=80), reviewing full-text articles (n=44), and classifying relevant articles (n=27) into five domains of cultural competency and associated recommendations: 1) Physical environment: display pictures with older same-sex couples and LGB-identified symbols; 2) Education/staffing: expand to include older-specific LGB issues, especially for key conditions (e.g., cancer, dementia,) and hire LGB-identified administrative/clinical staff; 3) Inclusive language and communication: review terminology on forms, electronic health records, and used with patients to ensure a broad range of terms (e.g., partner/spouse) and note older LGB may have more limited understanding/comfort with terminologies (e.g., self-identify as 'something else' instead of 'gay/lesbian' or 'bisexual'); 4) Patient histories: discuss how factors particular to their sexual orientation (e.g., level of outness) may affect their support networks; 5) Subgroup differences: consider specific health concerns by sexual minority subgroups (e.g., healthy weight for lesbian women, HIV for gay men, and negative health outcomes for bisexual adults related to their simultaneous isolation from sexual minority and heterosexual communities) and note additional challenges based on characteristics such as race/ethnicity and urbanicity. Cutting across these domains are the ways in which local and national policies affect healthcare access and surrogacy (e.g., legality of same-sex partners to obtain health insurance, participation in medical decision making/visitation).

### IMPROVING HEALTH CARE OF LGBT OLDER ADULTS: INTERDISCIPLINARY PROJECT ENHANCES HEALTH OUTCOMES

Noell L. Rowan,[1] Stephanie D. Smith,[1] Tamatha Arms,[1] and Kris L. Hohn[1], *1. University of North Carolina Wilmington, Wilmington, North Carolina, United States*

To date, there is a dearth of interdisciplinary simulation education and research that involves LGBT older adults within schools of social work and nursing. The purpose of this mixed method study was to examine the use of an intervention among social work and nursing students to determine if lecture and simulations impacted their health-related knowledge and cultural sensitivity/awareness of health provisions with LGBT older adults. Interprofessional faculty created lecture and interdisciplinary simulations with actual members of the older LGBT communities using simulation clinic/lab and health care scenarios. An adapted survey with permission from Grubb et al (2013) was used to include quantitative and qualitative measures of cultural awareness with LGBT populations. Pre-Post test data were analyzed using Generalized Linear Models in SAS software. Results indicated that the intervention positively changed perceptions and increased knowledge among (N=90; 32 social work; 58 nursing) allied health students. Statistically significant change experiences in their work with LGBT individuals were noted to positively alter their beliefs about sexuality, gender identity, and sexual development (Agree to Strongly Agree, $X2(1)=26.51$, $p<0.001$). Qualitative findings include four primary themes about how gender identity and sexual orientation influences health: (a) bias of health care providers, (b) access to quality care, (c) specific health care needs, and (d) health risks of LGBT older adults. As older adults continue to be the largest population needing health care, it is imperative that professionals are trained to give culturally sensitive health care and demonstrate this competency in their practice and interpersonal interactions with clients.

### LGBT OLDER ADULTS IN RURAL SOUTHERN APPALACHIA: PERCEPTIONS OF CURRENT AND FUTURE FORMAL SERVICE NEEDS

Kelly A. Williams,[1] and Emily Dakin[1], *1. Appalachian State University, Boone, North Carolina, United States*

Older sexual and gender minority adults in rural communities face challenges in accessing formal health, mental health, and long-term care services (Butler, 2017; Koch & Knutson, 2016; Stein et al., 2010). Formal service providers in rural Southern Appalachia are more likely to have conservative values that are closely linked to their religious beliefs (Keefe, 2005). Some may be opposed to gender non-conformity and same-sex relationships or marriage, making it wise for LGBT older adults in rural contexts to carefully select formal service providers and settings (Willging et al., 2006). Barriers to accessing formal services for LGBT older adults residing in rural contexts include few LGBT-inclusive service providers and facilities, transportation, cost, and health insurance (Butler, 2017). When faced with the prospect of long-term care, older LGBT adults are more likely to conceal their sexual or gender identity due to fears of being mistreated (Brotman et al., 2003). This session will present results of a qualitative study examining experiences, concerns, and recommendations regarding formal services among 11 LGBT older adults residing in rural southern Appalachia. Several of the participants described experiencing discrimination from local service providers. A number of participants were fearful about the perceived lack of LGBT-inclusive services in the area and expressed that they would consider leaving the area if their own or their partner's health declined. Many participants expressed the need for local provider education about the needs of LGBT older adults. The presenters will discuss the implications for research and for health, social, and long-term care services.

### SOCIAL ISOLATION AMONG LGBT OLDER ADULTS: LESSONS LEARNED FROM A PILOT FRIENDLY-CALLER PROGRAM

Angie Perone,[1] Berit Ingersoll-Dayton,[1] and Keisha Watkins-Dukhie[2], *1. University of Michigan, Ann*

*Arbor, Ann Arbor, Michigan, United States, 2. SAGE Metro Detroit, Detroit, Michigan, United States*

Lesbian, gay, bisexual, and transgender (LGBT) older adults face heightened risks of social isolation, given decades of discrimination. Research on telephone buddy programs with non-LGBT participants have proved predominantly unsuccessful at addressing social isolation and have found the greatest success with same-age matches. However, evidence suggests that LGBT adults may actually benefit from telephone buddy programs and in ways uniquely different from other groups. This article shares lessons learned from 30 participants across a 12-month pilot program that matched LGBT older adults to both LGBT and non-LGBT volunteer callers of various ages. One-third of participants identified as African American or Black. This project employed community-based participatory action research to identify, execute, and evaluate the program. Data includes information from questionnaires and telephone interviews prior to and during the program. In contrast to other research, data here revealed strong support for intergenerational matches. LGBT older adults of color especially benefited from program referrals and matches with/from LGBT adults of color, regardless of age. While the project aimed to capture two groups (LGBT older adults experiencing isolation and volunteer callers), the project revealed a third group: LGBT adults at risk of social isolation. This third group usually emerged among the "volunteer" callers but also identified concerns and risk factors for social isolation. The program also revealed unexpected benefits to both LGBT and non-LGBT volunteers, including less loneliness and a stronger sense of community. This article concludes with recommendations for developing similar programs to reduce social isolation in the LGBT community.

### THE MANIFESTATION OF MULTI-LEVEL STIGMA IN THE LIVED EXPERIENCES OF TRANSGENDER OLDER ADULTS

Vanessa Fabbre,[1] and Eleni Gaveras[1], *1. Washington University in St. Louis, St. Louis, Missouri, United States*

Transgender and gender nonconforming (TGNC) older adults experience disparities in mental health outcomes when compared to non-TGNC sexual minority older adults. Stigmatizing experiences are thought to influence these outcomes, but little is known about this process. Recent conceptualizations of stigma draw attention to multiple levels – individual, interpersonal, and structural – experienced by TGNC people of all ages. To explore how multi-level stigma manifests in the lives of TGNC older adults, we conducted a two-phase qualitative content analysis of in-depth biographical interviews with 88 TGNC adults aged 50 and older, from across the United States. Data were obtained from the photography and interview project To Survive on This Shore. Our interpretive analyses suggest that TGNC older adults' development and well-being are impacted by multiple levels of stigma, which are dynamic and unpredictable, resulting in constant awareness of a changing social environment. Individual level stigma is experienced as ongoing vigilance about aspects of oneself that break gender norms, which is also marked by self-imposed social isolation and fears about accessing older adult services. At the interpersonal level, TGNC older adults navigate unpredictable interpersonal relationships, which manifest as fluctuating levels of love, acceptance, strain, and exclusion. Structural stigma manifests in the awareness of stigmatizing policies and systems but also in the conscious action of TGNC older adults to resist these structures. TGNC older adults promote supportive structural responses to stigma to both improve conditions for younger generations while also reducing experiences of individual and interpersonal stigma for themselves.

### SESSION 3435 (PAPER)

### LONG-TERM CARE II

### EFFECTS OF A NEW VALUE-BASED REIMBURSEMENT SYSTEM ON EXPENDITURES AND CARE QUALITY IN MINNESOTA NURSING HOMES

Zachary Hass,[1] Valerie Cooke,[2] and Greg Arling[1],
*1. Purdue University, West Lafayette, Indiana, United States, 2. Minnesota Department of Human Services, St. Paul, Minnesota, United States*

Although several states have implemented Value Based Reimbursement (VBR) systems in long-term care; little is known of their impact. In 2016, Minnesota passed new VBR legislation to tie payment to quality with increased funding earmarked for nursing and other care-related services. We evaluated the effect of the policy change on care-related expenditures and measures of care quality. Data sources were cost reports and quality measures for the years 2013-2017 from 348 Minnesota nursing homes. Analysis consisted of descriptive tables, time plots, and linear growth curve modeling. We found increased expenditures for nursing and care-related services, particularly nursing assistants, during the first year; while quality metrics did not appear to be impacted by the policy change. Some differentiation was seen across facilities in their response to the policy change based on occupancy rate, rural-urban continuum code, attachment to a hospital, and resident acuity. The lack of an improvement in care quality might be attributable to VBR design as the quality incentive affected very few facilities. Additionally, there is the challenge of improving quality metrics even with additional resources. The legislature is currently considering changes to VBR policies in response to the report from this study.

### HUMAN RESOURCE MANAGEMENT PRACTICES, ORGANIZATIONAL CULTURE, AND NURSE STAFFING TURNOVER

Robert Weech-Maldonado,[1] Akbar Ghiasi,[1]
Ganisher K. Davlyatov,[1] Justin C. Lord,[2] and
Kent Rondeau[3], *1. University of Alabama at Birmingham, Birmingham, Alabama, United States, 2. Louisiana State University at Shreveport, Shreveport, United States, 3. University of Alberta, Edmonton, Alberta, Canada*

This study examines whether nursing homes' (NHs) human resource management (HRM) practices and organizational culture are associated with nursing staff turnover. HRM practices are classified into traditional (employment selection tests, formal performance appraisal systems, and realistic job previews); employee-centered (flexible work hours and job sharing); and high involvement (incentive based/merit pay and self-managing teams). Organizational culture consists of four types: clan culture (friendly working environment); adhocracy culture (dynamic/creative working environment); market culture (results-based organization); and hierarchy culture (formalized/structured work environment). This study used facility survey data from approximately 324 NH administrators (30% response rate) from 2017- 2018, merged with secondary data from LTCFocus, Area Health Resource File, and Medicare Cost Reports. The dependent variables consisted of RN, LPN, and CNA turnover rates (% voluntarily quit), while the independent variables comprised HRM practices and type of organizational culture. Control variables consisted of organizational (ownership, chain affiliation, size, occupancy rate, and payer mix) and county-level factors (Medicare Advantage penetration, income, education, unemployment rate, poverty, and competition). Generalized linear model results show that every unit increase in high-involvement HRM practices is associated with a reduction of 6%, 4%, and 2% in RN, LPN, and CNA turnover rates, respectively. Also compared to hierarchical cultures, nursing homes with a clan culture are associated with a reduction of 62%, 49%, and 33% in RN, LPN, and CNA turnover rates, respectively. HRM practices and organizational cultures that promote employee participation, engagement, and empowerment have the potential to reduce nurse staffing turnover rates among underresourced nursing homes.

### NOT JUST HOW MANY BUT WHO IS ON SHIFT: THE IMPACT OF WORKPLACE INCIVILITY AND BULLYING AMONG RCAS ON RESIDENT CARE

Heather A. Cooke,[1] Kaitlin Murray,[2] and
Jennifer Baumbusch[1], *1. University of British Columbia, Vancouver, British Columbia, Canada, 2. Simon Fraser University, Vancouver, British Columbia, Canada*

Much of the literature examining the link between care quality and staffing in long-term residential care focuses on staffing ratios and staffing mix; that is, how many staff are on shift. Far less attention has been devoted to exploring the impact of staff members' workplace relationships, or who is on shift, on care quality. Of increasing concern is the potential for peer incivility and bullying to disrupt the respectful, collaborative and effective working relationships considered key to residential care aides' (RCAs) care provision. This paper draws on data collected from a critical ethnography examining workplace incivility and bullying in a rural, not-for-profit care home. To date, more than 50 hours of participant observation, and 20 in-depth interviews with RCAs, licensed practical nurses, support staff, management and residents have been conducted. Thematic analyses identified three key themes: impact on resident safety; cutting corners; and impact on resident agitation and anxiety. Impact on resident safety highlights how incivility and bullying can result in non-adherence to two-person lift policies and procedures. Cutting corners outlines how RCAs' relationships with their co-workers dictates to what extent they provide the requisite care to a resident for whom another RCA is responsible. Impact on resident agitation and anxiety focuses on residents' reactions to the tensions that emerge between RCAs as a result of incivility and bullying. Findings highlight how peer incivility and bullying may indirectly influence certain quality indicators (e.g., pressure sores, psychotropic medication use) thereby offering additional insight into the staffing-care quality link.

### USING A UNIVERSAL SATISFACTION SCORE IN LONG-TERM CARE SETTINGS

Nicholas Castle,[1] Lindsay Schwartz,[2] and David Gifford[2],
*1. WVU, Morgantown, United States, 2. American Health Care Association/National Center for Assisted Living, Washington, District of Columbia, United States*

The CoreQ (not an acronym) consists of a limited number of satisfaction items (3-4 items, depending on setting) that are used to create an overall satisfaction score for long-term care facilities. This measure has been used in assisted living (AL) and skilled nursing facilities (SNFs) and has been endorsed by the National Quality Forum (NQF). Briefly, the development and psychometric testing of the CoreQ will be described, including the rationale for producing an overall satisfaction score and correlation with important quality indicators like Five-Star. Using data collected over the past 3 years, comprising more than 100,000 respondents, the use of the CoreQ measure will be described. For example, the CoreQ scores are used in MA to allow providers to benchmark their performance. The use of the scores in this way will be discussed including how providers have used the scores for quality improvement. Some states have elected to use CoreQ in pay for performance and other state initiatives. A case study of how New Jersey uses CoreQ with SNFs will be presented, including distribution of scores and addressing data collection challenges. CoreQ can be utilized as a short customer satisfaction measure to allow providers to benchmark their performance, residents and families in decision-making, and states and others to use for accountability.

### VARIABILITY IN ACCESS TO VA'S AID AND ATTENDANCE PENSION BENEFIT: A MIXED-METHODS STUDY

Kali S. Thomas,[1] Emily Corneau,[1] Stefanie Gidmark,[1]
Taylor Rickard,[1] and Susan Allen[2], *1. Providence VA Medical Center, Providence, Rhode Island, United States, 2. Brown School of Public Health, Providence, Rhode Island, United States*

The Veterans Benefit Administration's (VBA) Aid and Attendance enhanced pension benefit (A&A) is available to older, low-income Veterans who require assistance meeting their daily needs. However, reports indicate that A&A is underutilized with only 1/3 of eligible Veterans receiving this benefit. The objective of this mixed methods study is to characterize the variability in A&A enrollment across VA Medical Centers (VAMCs) and determine factors attributable to the variation. Using VA administrative data, we calculated the rate of enrollment in A&A among Veterans receiving pension. We then purposefully sampled 16 Chiefs of Social Work at VAMCs with the highest (n=7) and lowest (n=9) rates of enrollment. Interviews were transcribed, coded, and analyzed using conventional qualitative research methods. The rate of enrollment in A&A varies from <1% to 23% across VAMCs. VAMCs that had higher rates of enrollment were larger and more likely to be located in the South and Mid-Atlantic regions. Respondents at sites with low rates of enrollment indicate that education around the eligibility criteria is needed for VAMC staff. They also report that outreach to Veterans about this benefit is limited. Respondents at VAMCs with high rates of enrollment indicate that the relationships with VBA and Veterans Service Organizations facilitates access. Universally, respondents viewed the A&A benefit positively and note that it helps meet Veterans' long-term care needs. As the Veteran population continues to age, it is important that VA ensure equal access to A&A for eligible Veterans. Implications of these findings and next steps will be discussed.

### SESSION 3440 (SYMPOSIUM)

### INTEREST GROUP SESSION—GEROSCIENCE: METHODS FROM BENCH TO POPULATION SCIENCE TO INFORM CONSTRUCTION OF GEROSCIENCE CLINICAL TRIALS

Chair: Jason L. Sanders, *Brigham and Women's Hospital, Boston, Massachusetts, United States*
Discussant: Anne B. Newman, *University of Pittsburgh, Pittsburgh, Pennsylvania, United States*

We are on the cusp of a revolution in aging science. It has matured to the point where geroscience trials will test interventions in humans which alter aging mechanisms to lengthen healthspan and possibly lifespan. This goal is unprecedented in clinical trial design, and it requires retooling the clinical trial toolbox. Traditionally, trials are constructed around a single disease; interventions target a narrow part of a defined biological pathway involving only one molecule, tissue, or organ; events are well known intermediate endpoints and clinically-defined hard outcomes; and follow up may be short and historically informed based on prior trials. Geroscience trials by design target aging mechanisms which, when altered, are likely to have pleiotropic effects that modify several biologic pathways; efficacy and safety signals may require integration across multiple levels of biologic organization; intermediate endpoints are not agreed upon; and follow up timelines are undefined. In this symposium, we provide guidance on the design of geroscience trials using examples that span from bench to population science. Dr. LeBrasseur will discuss screening senolytic compounds across models of age-associated decline and advancing their candidacy as interventions. Dr. Justice will detail a framework for biomarker selection in geroscience trials, focusing on a trial of metformin as an example. Dr. Sanders will illustrate how observational data can inform phenotype use in clinical trials. Dr. Levine will explain translating omics data for use in geroscience trials, focusing on epigenomics. We expect additional discussion to hasten development of well-designed geroscience trials.

### USING OBSERVATIONAL DATA TO INFORM CANDIDATE PHENOTYPES FOR GEROSCIENCE TRIALS

Jason L. Sanders,[1] Alice Arnold,[2] Robert Boudreau,[3] Stephen Kritchevsky,[4] and Anne Newman[3], *1. Brigham and Women's Hospital, Boston, Massachusetts, United States, 2. University of washington, Seattle, Washington, United States, 3. University of pittsburgh, Pittsburgh, Pennsylvania, United States, 4. wake forest school of medicine, Winston-Salem, North Carolina, United States*

Geroscience trials will manipulate aging mechanisms which may have pleiotropic effects and alter multiple biologic processes and clinical outcomes. Determining an intervention's efficacy and safety will require measuring several aspects of aging and intermediate endpoints with less regard to specific diseases. Picking the right measurements will significantly impact a trial's cost-effectiveness and chance of success. Observational studies are ideal resources to test candidate phenotypes before investing in trials. We present a decade's worth of results from the Cardiovascular Health Study as examples of using observational data to inform measurement in geroscience trials. Specifically, we illustrate the underlying theory, construction, operational characteristics, and inter-relationships of candidate phenotypes spanning circulating biomarkers, tissue and organ structure, and functional status, all of which can be used in geroscience trials depending on the intervention's target and predicted outcome.

### SENOLYTIC DRUGS: DISCOVERY, TRANSLATION, AND APPLICATION

Nathan LeBrasseur[1], *1. Robert and Arlene Kogod Center on Aging, Mayo Clinic, Rochester, Minnesota, United States*

Diverse forms of molecular and cellular stress trigger senescence, a state of growth arrest in proliferation-competent cells that is often accompanied by a robust secretory phenotype. Senescent cell burden increases with age in multiple tissues and, plausibly, contributes to the pathogenesis of age-related diseases and geriatric syndromes. Discovery science efforts have identified druggable targets in senescent cells, including key nodes in anti-apoptosis pathways, that distinguish them from non-senescent counterparts and enable pharmacological approaches for their selective elimination. The therapeutic potential of senolytic interventions to improve health- and lifespan has been supported by translational research studies, including murine models of aging, atherosclerosis, osteoporosis, neurodegeneration, pulmonary fibrosis, and frailty. These studies have informed the design of first-in-human clinical trials of senolytic drugs, which have recently begun. The objective of this lecture is to highlight both the progress and challenges of advancing interventions targeting senescent cells from bench to bedside.

### BIOMARKER STRATEGIES FOR GEROSCIENCE-GUIDED CLINICAL TRIALS

Jamie N. Justice,[1] George A. Kuchel,[2] Nir Barzilai,[3] and Stephen Kritchevsky[1], *1. Wake Forest School of Medicine, Winston-Salem, North Carolina, United States, 2. university of connecticut, Farmington, Connecticut, United States, 3. Albert Einstein College of Medicine, Bronx, New York, United States*

Significant progress in the biology of aging and animal models supports the geroscience hypothesis: by targeting biological aging the onset of age-related diseases can be delayed. Geroscience investigators will test this hypothesis in a multicenter clinical trial, to determine if interventions on biological aging processes can prevent accumulation of multiple age-related diseases and aging phenotypes in older adults. Prodigious activity is underway to develop markers of biological aging, but currently there is no aging biomarker consensus to support geroscience-guided clinical trial outcomes.

We convened an expert committee to establish a framework for selection of blood-based biomarkers, emphasizing: feasibility/reliability; aging relevance; ability to predict clinical trial outcomes; and responsiveness to intervention. We applied this framework and identified a short-list of blood-based biomarkers with potential use in multicenter trials on aging. We review progress on efforts to test these candidate biomarkers of aging and development of biomarkers strategy for geroscience-guided clinical trials.

### DEVELOPMENT OF EPIGENETIC MEASURES FOR GEROSCIENCE CLINICAL TRIALS

Morgan E. Levine[1], *1. Yale University School of Medicine, New Haven, Connecticut, United States*

One of the major goals of the NIA is to oversee development of biomarkers of aging. In recent years, DNA methylation has emerged as a promising avenue from which to quantify biological age. We and others have shown that these measures track age across various tissues and cells, and further deviations between chronological and "epigenetic age" have been shown to confer risk for various aging outcomes. However, the usefulness of these measures will depend on both their modifiability and ability to capture known targetable hallmarks of aging. Using DNA methylation data from cell line experiments, we have recently generated epigenetic predictors of cellular senescence for both human and mouse that when assessed in vivo from bulk samples, show age-related increases and are associated with aging outcomes. In moving forward, measures such as these may serve as promising surrogate endpoints for assessing efficacy of senolytic drugs and/or other anti-aging therapeutics.

### SESSION 3445 (SYMPOSIUM)

### MOBILIZING COMMUNITY PARTNERSHIPS TO ENHANCE HEALTH AND REDUCE INEQUITIES IN MULTICULTURAL COMMUNITIES

Chair: Daniel S. Gardner, *Silberman School of Social Work, Hunter College, CUNY, New York, New York, United States*
Discussant: Nancy Giunta, *Silberman School of Social Work, Hunter College, CUNY, New York, New York, United States*

Community-based gerontological research plays an indispensable role in identifying and addressing the strengths, intersectionalities, and socio-structural inequities that shape the lives of older adults in multicultural communities around the world. This symposium highlights the innovative, global scholarship of Silberman Aging: A Hartford Center of Excellence in Diverse Aging, as the Center begins its sixth year. Through community-based research and academic-community collaborations, Center researchers examine challenges affecting the health and wellbeing of diverse and often marginalized aging communities in North America, West Africa, and East Asia. The first paper describes and evaluates a CBPR project that trains community-based natural helping networks to identify and refer older adults with dementia in East Harlem, NY. The second study explores the perceptions and strategies of community-based primary care physicians in Ulaanbaatar, Mongolia in dealing with elder abuse and neglect. The third takes a population health approach to the relationship between social capital and health among older adults in Ghana. Fourth, preliminary results from an evaluation of a nationwide training initiative that promotes cultural-competencies among aging services providers working with LGBT elders. Finally, we present findings from a CBPR study examining barriers to palliative care among racially and ethnically-diverse community-dwelling older adults with serious illness. Although substantively and methodologically varied, these studies all demonstrate the importance of social networks in health in later life, and underscore the value of community-based research that supports collaboration, empowers communities, and ultimately transforms practice and policy to better meet the diverse needs of older adults around the globe.

### EXPLORING PALLIATIVE CARE DISPARITIES IN RACIALLY AND ETHNICALLY DIVERSE COMMUNITY-DWELLING OLDER ADULTS

Daniel S. Gardner,[1] and Meredith Doherty[2], *1. Silberman School of Social Work, Hunter College, CUNY, New York, New York, United States, 2. the Graduate Center, CUNY, New York, New York, United States*

Despite the growth and recognized benefits of palliative care for people with serious illness and their families, there are significant racial and ethnic disparities in access to and utilization of services, particularly among older adults living in impoverished, medically-underserved communities. This paper presents preliminary findings from a mixed-method, CBPR study exploring the experiences, supportive care needs, and service use of diverse older adults living with serious illness in an urban, medically-underserved community in the U.S. Systematic analyses of focused, semi-structured interviews with 45 older adults identified cultural, environmental, financial, and structural barriers to palliative care, and identified the critical importance of familial, social, spiritual, and formal networks of support in coping with serious illness and associated symptoms. The investigators describe implications for practice and policy that addresses palliative care disparities, and strategies for engaging with communities to extend culturally-sensitive palliative care to diverse, community-dwelling older adults and their social networks.

### MOBILIZING NETWORKS TO ADDRESS DEMENTIA IN A LATINO COMMUNITY

Caroline Gelman,[1] and Nancy Giunta[2], *1. Silberman School of Social Work, New York, United States, 2. Silberman School of social Work, Hunter College, CUNY, New York, New York, United States*

Many Latino older adults delay seeking help for symptoms of Alzheimer's Disease or Related Dementia (ADRD) due to substantial barriers to services. Community-based Natural helpers (NHs) can increase health-related knowledge and can serve as full partners in health education and promotion. This paper presents the process and product of the first phase of a community-based participatory research study to develop a culturally-tailored intervention increasing knowledge about ADRD and services in East Harlem, NY. We describe the results of the initial survey and development of El Barrio SHARE, an intervention that recruits and trains community residents to provide information and referrals about dementia, tapping into natural community networks of people (hairdressers, bodega clerks, mail carriers) who interact with and have longstanding relationships with older adults in the course of their work. NHs are well-positioned to observe and detect problems, and can link elders to relevant, culturally-sensitive resources, accessible support, and treatment.

### DETECTION OF ELDER ABUSE BY FAMILY PHYSICIANS IN MONGOLIA: AN EXPLORATORY STUDY

Ariunsanaa Bagaajav[1], *1. The Graduate Center, CUNY, New York, New York, United States*

Elder abuse (EA) is a significant global social problem that jeopardizes the health and wellbeing of older adults around the world. Health providers can play a pivotal role in detection of EA and accessing resources and interventions. In Mongolia, where elder abuse is not widely recognized, family physicians (FPs) are particularly critical, providing frequent contact and home visits free of charge to community-dwelling older adults. However, little is known about FP knowledge and engagement with potential victims of EA. We interviewed 12 FPs from Ulaanbaatar participated via Skype about their knowledge of and responses to EA, using Grounded Theory data collection and analysis. All respondents reported encountering at least one case of EA in practice, and described the creative strategies they used to engage older patients, detect abuse, and prevent further harm. We present significant implications for guidelines for identification and prevention of EA in primary health care.

### SOCIAL CAPITAL AND HEALTH AT OLDER AGES IN GHANA

Kojo Paul Ayernor[1], *1. The Graduate Center, CUNY, New York, New York, United States*

Significant increases in life expectancy and declining fertility confirms that population ageing is fast becoming a reality in several West African nations, and the demographic transition is expected to continue well into this century. This study examines the association between social capital, self-rated health, and depression among older adults aged 50 years and over in Ghana. It draws on a small scale, yet nationally representative longitudinal data from the Global Ageing Study (SAGE-WHO, 2003-2007). Social capital is conceptualized through four dimensions: personal control, generalized trust, safety in the community and free expression. Although there were not significant findings on social capital and depression, results demonstrated significant associations between social capital and self-rated health. The relationship between social capital and self-rated health suggests the need to extend and expand upon research regarding the relationship between social capital, health, and well-being in later life in aging African communities.

### KNOWLEDGE OF LGBT CULTURAL SENSITIVITY AMONG AGING SERVICE PROVIDER NETWORKS

Austin Oswald,[1] Daniel S. Gardner,[2] Tim R. Johnston,[3] Sabretta G. Alford,[2] and Nancy Giunta[2], *1. The Graduate Center, CUNY, New York, New York, United States, 2. Silberman School of social Work, Hunter College, CUNY, New York, New York, United States, 3. SAGE, Los Angeles, California, United States*

The population of lesbian, gay, bisexual, and transgender (LGBT) adults aged 50 or above is rapidly aging, posing distinct challenges to aging service providers who work to meet their complex health and social needs. In 2011, Services and Advocacy for GLBT Elders (SAGE), home of the National Resource Center on LGBT Aging (NRC), developed and implemented a suite of trainings designed to sensitize aging service providers to the needs and experiences of LGBT elders. This descriptive study examines baseline (pre-test) survey data from individuals trained between 2013 and 2018 (N=10,500). Training participants represent all states throughout the U.S. and vary widely in race/ethnicity, age, and job title. Preliminary results indicate that knowledge measured prior to trainings shows substantial variation across geographic regions, suggesting the need for more geographically targeted approaches to training delivery. Implications for practice, research, and policy with LGBT adults in later life will be discussed.

### SESSION 3450 (SYMPOSIUM)

### POLICY SERIES: NEW FAMILY CAREGIVING RESEARCH TO SUPPORT POLICY AND PRACTICE CHANGE

Chair: Jean C. Accius, *AARP, Washington, District of Columbia, United States*
Discussant: Heather Young, *Betty Irene Moore School of Nursing - UC Davis, Sacramento, California, United States*

Families are the most important source of support to older adults. Today, there is growing recognition of the escalating complexity of family caregiving. Family caregivers are increasingly carrying out health-related tasks with little training or preparation, as well as continuing to provide the majority of long-term services and supports (LTSS) at home. Providing care in the context of rapidly changing health care and LTSS systems can have a significant impact on the family members who provide care and take a significant toll, emotionally, physically, and financially. Studies commonly show that family caregivers report learning complex tasks by trial and error and worry about making a mistake. This symposium highlights new caregiving research from the AARP Public Policy Institute. The first paper will present new research on the increasing complexity of the challenges facing family caregivers, such as managing multiple medications, wound care, and interaction with the health care system. The second paper will present new data on the economic value of the unpaid contributions of family caregivers in the United States. The third paper will examine the growing importance of family caregiving on the public policy agenda, and describe recent policy developments that recognize and explicitly support caregiving families. Recommendations for policy and practice change to address caregiving needs will be examined in all papers. Our discussant will identify key implications from this research for policymakers and practitioners, and potential drivers for developing a better system of family support at the federal and state levels.

### HOME ALONE REVISITED: FAMILY CAREGIVERS PROVIDING COMPLEX CARE

Susan Reinhard[1], *1. AARP, Washington, District of Columbia, United States*

In 2012, the Home Alone report brought to light the 46 percent of family caregivers that perform complex medical/

nursing tasks. This presentation highlights new research conducted as part of a second Home Alone study to look closer at the family caregivers who perform these complex tasks with a specific focus on key medical/nursing tasks, including incontinence care, special diets, as well as certain sub-populations (e.g., multicultural communities, men, Millennials). An analysis of the increasing complexity of the challenges facing family caregivers who perform medical/nursing tasks in addition to assisting with instrumental activities of daily living and activities of daily living, will be shared. Presenters will also discuss its findings and the implications for individuals, their family caregivers and the healthcare providers who work with them.

### ARE POLICY AND PRACTICE CHANGE TO SUPPORT FAMILY CAREGIVERS ON THE RISE?

Lynn F. Feinberg[1], *1. AARP Public Policy Institute, Washington, District of Columbia, United States*

The challenges and pressures of family caregiving for older relatives or friends are a reality of daily life. Better recognition of and support for family caregivers has emerged as a national health, economic and social priority that can no longer be ignored. Unlike previous generations, most (60%) family caregivers work at a paying job today in addition to caring for older relatives with a serious health condition or disability. Building a better system of care for older adults means changes in workplaces as well as health care and LTSS settings. This paper presents an analysis of the key challenges that family caregivers face, and highlights policy developments and practice change at the federal and state levels since 2015. The presentation addresses the importance of person- and family-centered care in everyday practice, and discusses the road ahead to change the culture of care.

### THE ECONOMIC VALUE OF FAMILY CAREGIVING: NEW NATIONAL ESTIMATES AND STATE VARIATION

Ari Houser,[1] and Ari Houser[1], *1. AARP Public Policy Institute, Washington, District of Columbia, United States*

The economic value of family caregiving, by any measure, dwarfs actual expenditures on formal long-term services and supports (LTSS). This presentation discusses new estimates of the number of caregivers, intensity of caregiving, and the total economic value of family caregiving in 2017 in the United States, and in every state, the District of Columbia, Puerto Rico, and the Virgin Islands, based on a meta-analysis of recent nationally representative surveys of family caregivers. Previous analyses of this type have found that the total economic value of family caregiving has increased steadily from $350 billion in 2005 to $470 billion in 2013. State variation in the incidence, intensity, and economic value of caregiving will be discussed, and key predictors of this variation will be identified.

### SESSION 3455 (SYMPOSIUM)

### NIH PATHWAYS TO PREVENTION WORKSHOP: RESEARCH GAPS FOR LONG-TERM DRUG THERAPIES FOR FRACTURE PREVENTION

Chair: Lyndon Joseph, *National Institute on Aging, Bethesda, Maryland, United States*
Co-Chair: Faye Chen, *NIAMS/NIH, Bethesda, Maryland, United States*

The NIH Pathways to Prevention Workshop was held on October 30-31, 2018 to present scientific evidence, as well as physician and patient perspectives to better understand the benefits and harms of drug therapies for osteoporosis fracture prevention. Osteoporotic fractures lead to substantial morbidity, mortality, and economic costs. The underlying medical condition, is a skeletal disorder characterized by compromised bone strength predisposing to increased fracture risk. Several medications approved by the U.S. Food and Drug Administration to prevent osteoporotic fractures have been effective when taken by people who are at high risk of fracture. These include bisphosphonates , denosumab, teriparatide, estrogens, and selective estrogen-receptor modulators. However, rare but serious adverse events, such as atypical femoral fractures and osteonecrosis of the jaw associated with bisphosphonates have raised questions regarding the safety of their use. There is limited evidence on the benefits and harms of long-term osteoporosis drug therapy, including the timing and duration of drug discontinuation or drug holidays. It is not clearly known which patients will benefit or may be harmed from continued drug intervention, who should and should not be put on drug holiday, how to identify such patients and properly treat them, and how to predict their outcomes. Key scientific areas covered by the workshop included: the benefits and risks of osteoporotic drugs with short-term and long-term use and factors that influence outcomes; the impact of drug discontinuation and drug holidays on outcomes; and patient and clinician factors that impact the use of and adherence to osteoporotic drugs.

### CURRENT STATE OF SCIENCE ON DRUG THERAPIES FOR OSTEOPOROTIC FRACTURE PREVENTION

Sundeep Khosla[1], *1. Mayo Clinic, Rochester, Minnesota, United States*

During the workshop, experts discussed the state of the science. The U.S. Food and Drug Administration (FDA) has approved several medications to prevent osteoporotic fractures. These include bisphosphonates (BPs), denosumab, teriparatide, estrogens, and selective estrogen-receptor modulators. BPs are a first line of pharmacologic treatment for most women and men who have osteoporosis. Despite the efficacy of short-term use of BPs and other osteoporosis medications, treatment rates are low and appear to be decreasing. This talk would describe the problem/epidemiology of fracture prevention in the United States; the importance of drug therapy and their effectiveness; the limited evidence regarding long-term use of drug therapies; the questions surrounding the application of drug holidays; and the complex physician and patient factors that impact the use of and adherence to osteoporotic drugs.

### REPORT ON SYSTEMATIC EVIDENCE REVIEW

Howard Fink[1], *1. Minneapolis VA Health Care System, Minneapolis, Minnesota, United States*

During the workshop, results from a systematic evidence review prepared by an Agency for Healthcare Research and Quality Evidence-based Practice Center to inform the workshop were presented, focusing on long term osteoporosis drug treatment. This talk will describe the goals of the systematic review, which addressed four questions: (1) What are the effects of long-term (>3 years) osteoporosis drug treatment versus control on risks of incident fractures and harms; (2) Do effects of long-term osteoporosis drug treatment vary as a function of patient, bone, or osteoporosis drug characteristics; (3) Among individuals receiving osteoporosis drug treatment to prevent fracture, what are the benefits and harms of continuing versus discontinuing treatment (e.g., osteoporosis drug holiday); and (4) Do outcomes of drug discontinuation or drug holidays vary as a function of patient, bone, or osteoporosis drug characteristics?

### WORKSHOP PANEL REPORT ON EVIDENCE GAPS AND RESEARCH OPPORTUNITIES

Heather Allore[1], *1. Yale University, New Haven, Connecticut, United States*

Although varied in treatment regimens and outcomes assessment, trials show safe and effective osteoporosis drug therapies up to 5 years in reducing the incidence of vertebral fractures. Trials provide evidence mainly for white postmenopausal women, while other populations (e.g., men, spectrum of race and ethnicities, residents in facilities, and people with advanced and multiple comorbid conditions) were absent or underrepresented. Evidence for osteoporosis drug therapies is lacking for people who meet neither bone mineral density nor fracture criteria for osteoporosis but are at elevated risk due to other health, genetic, or medication use factors. Fewer studies have shown that some osteoporosis drug therapies reduce the incidence of non-vertebral, including hip fractures. Notably, the workshop participants emphasized the importance of fracture on patient morbidity and survival; however, the trial results presented no data on non-fracture patient outcomes or sequelae of fractures, such as functional status, mobility, hospitalizations, and nursing home placement.

### THE NIH PATHWAYS TO PREVENTION PROGRAM

Keisha L. Shropshire[1], *1. NIH Office of Disease Prevention, Bethesda, Maryland, United States*

The National Institutes of Health Office of Disease Prevention (ODP) Pathways to Prevention (P2P) Program identifies research gaps, methodological and scientific weaknesses in selected scientific areas; suggests research needs; and moves the field forward through an unbiased, evidence-based assessment of a complex public health issue. P2P workshops are designed for topics that have incomplete or underdeveloped research and that have a need for a synthesis and critical assessment of the published literature. This talk will explain the purpose of this P2P workshop on use of drug therapies for osteoporotic fracture prevention within the larger context of NIH efforts to promote prevention research.

### SESSION 3460 (SYMPOSIUM)

### PREPARATION FOR FUTURE IN OLD AGE: WHY AND WHY NOT?

Chair: Helene H. Fung, *Chinese University of Hong Kong, Hong Kong, Hong Kong*

With population aging, many people can expect to spend 30 or more years in old age. The five papers included in this symposium aim at shedding light on whether and how to make plans for old age, using data from the "Aging as Future" Project. First, Park and Hess used data spanning across adulthood from Germany, Hong Kong and the USA to examine how changes experienced in domains of functioning and the importance attached to these domains influenced preparations for old age. Next, de Paula Couto and Rothermund, examining Germans aged 40-90 years, pointed out that prescriptive age stereotypes might be the main drive for why people make preparations for age-related changes. The remaining three papers use qualitative data to qualify the above quantitative findings. Adamson and Ekerdt interviewed older Midwest US residents. They observed that SES greatly impacted how older adults perceived and made plans for their future. The final two papers examined how rural vs. urban contexts might affect preparations for future. Liou interviewed older adults in rural Tainan and found that their ideal old age was one about no future preparation, at least not about making plans for themselves (called "tranquil life"). Ho and colleagues, in contrast, found that for older Chinese residing in urban Hong Kong, not preparing for the future (called "time freeze") was negatively related to physical and psychological well-being. The symposium will end with an overall discussion on future research directions on whether and how to make plans for old age.

### THE IMPACT OF PERCEIVED IMPORTANCE AND RECENT CHANGES ON PREPARATION FOR OLD AGE

Jeongsoo Park,[1] and Thomas M. Hess[1], *1. North Carolina State University, Raleigh, North Carolina, United States*

This study focused on identifying factors that motivate individuals to make provisions for their old age. We hypothesized that the motivation to make preparations would be associated with the importance attached to a specific domain of functioning along with the extent to which the individual experienced recent change within that domain. We examined how perceived changes in five different domains of functioning (social relationships, leisure, finances, work, health) during the last four years and the importance attached to each domain affected preparation for old age. Participants aged 35 to 85 (Germany: N=811; Hong Kong: N=482; US: N=515) were part of the Aging as Future Study. Across domains, perceptions of positive change and high importance were related to more engagement in preparations, as was being older. The strength of these effects as well as moderating influences varied across domains and cultures, reflecting potential differences in values and social/cultural supports.

### ECONOMIC INEQUALITY AND FUTURE THOUGHT AMONG OLDER ADULTS

Erin M. Adamson,[1] David J. Ekerdt,[1] and Erin M. Adamson[1], *1. University of Kansas, Lawrence, Kansas, United States*

Cumulative advantage theory sees inequality extending into later life. Does inequality also extend to the imagination of one's future and desires for life yet to come? We draw from semi-structured interviews with 55 individuals residing in Midwest cities to explore differences in talk about finances and expectations for longevity, fulfillment, and "active" pursuits. Addressing personal futures, participants' desires for longevity varied. Lower SES individuals discussed not having expected to live to their current age (or much beyond it) and not wanting to live a long time. Higher SES individuals, by contrast, expressed confidence that they could afford care no matter how long they lived. Higher SES participants often described future leisure goals whereas lower SES participants tended not to name leisure goals, or they named activities they desired but could not afford. For low SES individuals, active pursuits also were limited by diseases disproportionately affecting poor Americans, such as diabetes.

### DO NOT BECOME A BURDEN: ACTIVATION AND DISENGAGEMENT PRESCRIPTIVE AGE STEREOTYPES

Clara de Paula Couto,[1] and Klaus Rothermund[1], *1. Friedrich-Schiller University Jena, Jena, Thuringen, Germany*

Prescriptive age stereotypes encompass activation (active-aging) and disengagement expectations (succession-consumption-identity). We investigated whether activation and disengagement represent opposite stereotypes or whether they exemplify the overarching norm that older adults should not become a burden to other people and society. Based on data of the Ageing-as-Future project (N=743 German participants, 40-90 years old) our findings support the idea that activation and disengagement represent a single superordinate prescriptive age stereotype: (a) items assessing prescriptive age stereotypes form a single factor comprising activation and disengagement, (b) activation and disengagement show an increase in the strength of personal endorsement over the lifespan, demonstrating an internalization of these stereotypes as people become older, and (c) relations to reference variables show that internalized prescriptive stereotypes are more strongly associated with preparation for age-related changes, reflecting an internalization of the norm that one should take individual responsibility for their age rather than enjoying life in old age.

### BESIDES ACTIVE AGING? A TRANQUIL LIFE AFTER RETIREMENT

Shyhnan Liou[1], *1. National Cheng Kung University, Tainan, Taiwan, Taiwan*

This research explores how people think of the time plan after their retirement in rural area in Taiwan. fifty-nine participants in rural communities were interviewed. The findings show patterns of a tranquil life after retirement in three aspects which represent the philosophy mixing in Taiwan (Taoism, Buddhism, and Confucianism). 1). Taking things as they are and following the mandate of heaven(Taoism). 2). the retirement life will not be affected by social changes, and feeling that the pace of life in the society has not changed (Buddhism). 3) When talking about "Future aspirations" and "Future ideal life", they show concern to their children and posterity. (Confucianism). The findings of tranquil life contribute an alternative way of active ageing with considering the culture. The implications of tranquil life are discussed in the design of elderly education program, technology development to enhance social interaction, and culturally motivated ageing and wisdom.

### A FREEZE IN TIME: PERCEPTION AND EXPERIENCE OF AMERICAN AND HONG KONG CHINESE OLDER ADULTS

Tinky Oi Ting Ho,[1] Helene Hoi-Lam Fung,[2] Vivian Hiu Ling Tsang,[2] Angel Yee-lam Li,[2] David J. Ekerdt,[3] and Hansol Kim[3], *1. The Chinese University of Hong Kong, Hong Kong, Hong Kong, 2. The University of Hong Kong, Hong Kong, Not Applicable, Hong Kong, 3. The University of Kansas, Lawrence, Kansas, United States*

According to self-continuity model, older adults are less likely to distinguish between the present and future, relative to younger adults. This mixed method design study aims at examining whether older adults perceive future as an infinite extension of present (i.e. "time freeze") and investigating whether it is associated with life satisfaction, perceived control and perceived changes in future. 30 older adults from the US (aged 60-85, M = 78.4) and Hong Kong (aged 60-85, M =71.4) completed a structured interview and a survey. Findings revealed that 43% of Americans and 83% of Hong Kongers were experiencing 'time freeze'. Individuals with a lower level of time freeze held more vivid and positive images of the future, and were achieving life goals actively, whereas individuals with a higher level of time freeze had comparatively more vague and neutral future views, and focused more on maintaining the current lifestyle.

### SESSION 3465 (SYMPOSIUM)

### PRETTY GOOD PRACTICES: GERIATRICS WORKFORCE ENHANCEMENT PROGRAMS AND LIFELONG DISABILITIES

Chair: Catherine Taylor, *Rhode Island Geriatric Education Center, University of Rhode Island, Kingston, Rhode Island, United States*
Discussant: Kelly Munly, *Penn State Altoona, Altoona, Pennsylvania, United States*

Growing old with lifelong disabilities is a recent reality that is catching healthcare providers unprepared. While there's little extant federal or state public policy on aging with lifelong disabilities, and aging, disability, and healthcare systems lack a history of intersystem collaboration, Geriatrics Workforce Enhancement Programs (GWEPs) can lead the way in developing curricula, training, policy, and standards to respond. The GWEPs can intervene to create meaningful intersystem knowledge and skills and better prepare providers. Two GWEPs are filling the best practices void, operationalizing de facto public policy and "pretty good" practices to improve care for individuals with lifelong disabilities. In metro Richmond, VA, the GWEP at the Virginia Geriatric Education Center (VGEC) has built on the successful Area Planning and Services Committee on Lifelong Disabilities (APSC) intersystem partnership to provide this expertise. In Rhode Island, the RI Geriatric Education Center (RIGEC) has aligned its GWEP Alzheimer's disease supplemental funding with other federally funded programs to build dementia capability into the systems that support adults with intellectual or developmental disabilities (I/DD). RIGEC incorporated expertise previously gained through the University of Rhode Island's CMS-funded LivingRIte Innovation, which established pilot centers to support individuals with I/DD living with dementia and other chronic conditions, through novel person-centered approaches. This symposium examines how the two GWEPs expanded upon a foundation of previous efforts to serve older adults with lifelong disabilities, the methods by which they built and fostered effective networks, the resulting system improvements, and suggested strategies to move from "pretty good" to best practices.

### COMMON PURPOSE: NETWORKING INITIATIVES TO FOSTER DEMENTIA-CAPABLE CARE FOR LIFELONG DISABILITIES

Catherine Taylor,[1] Christine Gadbois,[2] Sandra L. Fournier,[3] Phillip G. Clark,[4] and Faith Helm[4], *1. Rhode Island Geriatric Education Center, University of Rhode Island, Kingston, Rhode Island, United States, 2. CareLink, Providence, Rhode Island, United States, 3. Neighborhood Health Plan of RI, Smithfield, Rhode Island, United States, 4. Rhode Island Geriatric Education Center, Kingston, Rhode Island, United States*

As individuals with I/DD live longer, Alzheimer's disease is on the rise, particularly in individuals with Down syndrome. Practitioners have recognized that the I/DD system does not possess the expertise to provide appropriate care for this population as it ages. A series of federally funded initiatives in Rhode Island – supported by the Centers for Medicare and Medicaid Services (CMS), the Administration for Community Living (ACL), and the Health Resources and Services Administration (HRSA) -- have begun to equip health care and direct care professionals to meet the needs of individuals with I/DD and AD. Using a "connector" model, the GWEP at RIGEC has woven together these efforts, aligned program goals from disparate funders, built connections between the Aging Network and the disability system, and worked with the National Task Group on Intellectual Disabilities and Dementia Practices (NTG) to create sustainable resources for the health care and direct care workforces.

### COMMUNITY PARTNERSHIPS: A MODEL FOR LIFELONG DISABILITIES AND DEMENTIA CARE

Faith Helm,[1] Catherine Taylor,[2] and Phillip G. Clark[2],
*1. Rhode Island Geriatric Education Center at the University of Rhode Island, Kingston, Rhode Island, United States, 2. Rhode Island Geriatric Education Center, Kingston, Rhode Island, United States*

In 2015 the Rhode Island Geriatric Education Center (RIGEC) was one of 44 organizations funded by HRSA to implement the Geriatrics Workforce Enhancement Program (GWEP). A primary objective was to develop and deliver Alzheimer's Disease and Related Dementias (ADRD) education to patients, families, caregivers, and health professionals, with a focus on special populations. Concurrently, Seven Hills Rhode Island, a nonprofit agency that serves people with disabilities, received a grant from the Administration for Community Living (ACL) to provide education and resources to health professionals and caregivers of people with I/DD. As goals for both projects aligned closely, they worked together, fostering a strong partnership, amplifying the opportunity to offer high-quality educational programs and reach target audiences. Lessons learned from this networked approach are critical to informing sustained improvements to the I/DD and health care systems in subsequent GWEP projects.

### COMMUNITY NETWORKS ADDRESSING AGING WITH LIFELONG DISABILITIES

Edward F. Ansello[1], *1. Virginia Commonwealth University, Richmond, Virginia, United States*

Adults aging with lifelong developmental disabilities (I/DD: Down syndrome and other intellectual disabilities, cerebral palsy, etc.) continue to pose a challenge to the Aging Network, developmental services, and healthcare systems. Some funded projects, model programs, and episodic initiatives have helped advance intersystem collaboration but there is still scant federal or state public policy specific to aging with lifelong disabilities. The Area Planning and Services Committee (APSC), a product of three consecutive AoA grants on building and testing intersystem cooperation/collaboration between these systems, is a partnership of community based organizations and academe operating in metro Richmond since 2003. Its successful hands on, interdisciplinary management process has implemented needs assessments, statewide training, staff development, dementia awareness, etc., and been a model for the Virginia Geriatric Education Center (VGEC) Plenary overseeing all GWEP programs. The APSC history informs VGEC GWEP's incorporating I/DD content into faculty development programs, microlearning, ECHO, and other practitioner training.

### SESSION 3470 (SYMPOSIUM)

### RAISING OUR VOICES: THE BENEFITS OF AN INTERGENERATIONAL CHOIR FOR PEOPLE WITH DEMENTIA AND THEIR CARE PARTNERS

Chair: Debra J. Sheets, *University of Victoria, Victoria, British Columbia, Canada*
Discussant: Theresa A. Allison, *University of California, San Francisco, San Francisco, California, United States*

This interdisciplinary symposium focuses on the Voices in Motion (ViM) choir, a novel social intervention to address issues of stigma and social isolation among older adults with dementia and their caregivers. ViM is an intergenerational choir for community-dwelling older adults with dementia (PwD) and their caregivers. Local high school students participated in the choir and added to the lively social interactions. Two professionally directed ViM choirs were fully implemented in 2018-2019 with a public performance in the Fall and Spring seasons. This symposium brings together multiple methodologies to investigate the effects of choir participation on cognition, social connections, stigma, and quality of life for the dyads. Results in the individual papers demonstrate the positive impact of choir participation on dyads (n=26) for measures that includecognition (MacDonald), well-being and quality of life (Sheets), and social connections (Smith). Taken as a whole, the papers indicate that this social intervention offers an effective non-pharmacological alternative approach for older adults with dementia. Choir participation has important and significant impacts on psycho-social well-being and quality of life. The body of evidence presented points to the importance of intergenerational programs that are dementia-friendly and that support meaningful participation by older adults with dementia in the broader community. Discussion focuses on implications for social policy with attention on the replication and sustainability of the program.

### NOTEWORTHY: THE IMPACT OF A CHOIR FOR OLDER ADULTS WITH DEMENTIA ON THE WELL-BEING AND QUALITY OF LIFE OF CAREGIVERS

Debra J. Sheets,[1] Stuart W. MacDonald,[1] Andre Smith,[1] and Mary Kennedy[1], *1. University of Victoria, Victoria, British Columbia, Canada*

Informal caregivers provide 80% of the care needed to support community-dwelling older adults with dementia. Over time caregivers often face adverse effects on their health, quality of life and well-being; particularly those caring for someone with dementia. This study examines the impact of participation in the Voices in Motion (ViM) choir on caregiver burden, mood and quality of life. A measurement burst approach was used to investigate intraindividual variability on key psychosocial and health indicators. Results indicate that choir participation significantly improves caregiver well-being (e.g. mood, burden) and quality of life. Findings suggest that choirs offer significant caregiver support and respite. The discussion focuses the public policy and on the potential economic implications which suggests a shift is needed in the services available to older adults with dementia and their caregivers.

### THE MAGIC OF MUSIC FOR IMPROVING PSYCHOLOGICAL HEALTH FOR THOSE WITH DEMENTIA AND THEIR CAREGIVERS

Stuart MacDonald,[1] Debra J. Sheets,[1] Andre P. Smith,[1] Mary Kennedy,[1] and Sandra Hundza[1], *1. University of Victoria, Victoria, British Columbia, Canada*

Arts-based interventions for person's with dementia and their caregivers represent an inexpensive, non-invasive, and non-pharmacological intervention with the potential to improve psychological function as well as reduce healthcare costs. The paper presents an overview of Voices in Motion (ViM), and the impact of this social-cognitive intervention on changes in psychological function for those with dementia and their caregivers (current n=26 dyads). Choir rehearsals were held on a weekly basis, and included a social discussion component. A range of outcomes (neuropsychological and physiological function, neural activation) were assessed using an intensive repeated measures design that facilitates both between- and within-person analyses, including nuanced evaluation of whether psychological function improves post intervention relative to an individual's personal average, yielding a conservative within-person test of the benefits of intervention. Discussion focuses on the promise of such interventions for mitigating dementia symptoms and facilitating the psychological health of caregivers.

### THE EMERGENCE AND BENEFITS OF SOCIAL RELATIONSHIPS IN TWO COMMUNITY-BASED DEMENTIA CHOIRS

Andre Smith,[1] Debra J. Sheets,[2] Mary Kennedy,[2] Tara Erb,[2] Ruth Kampen,[2] and Min Zhou,[2] Chandra Berkan Hozempa,[2] and Stuart W. MacDonald[2], *1. Department of Sociology, University of Victoria, Victoria, British Columbia, Canada, 2. University of Victoria, Victoria, British Columbia, Canada*

Community choir participation for persons with dementia (PwD) confers benefits to health and well-being, including the benefit of socializing which can reduce feelings of loneliness and social isolation. Using the concept of social capital, this study examines the degree to which two intergenerational Voices in Motion choirs facilitate the development of social relationships between PwD, caregivers, and high school students. Data collection involved interviews with 17 dyads of PwD and caregivers, completion of a social relationship questionnaire, and focus groups with a total of 29 high school students. The results show a gradual increase in the level of interactions between all participants, with students in particular interacting more frequently with PwD. Over time, trust and reciprocity emerged within the choirs as more people shared information about themselves. Students' understanding of dementia changed over time as they learned to appreciate PwD as unique human beings with rich life stories and experiences.

### SYSTEMATIC CHANGES IN GAIT PURSUANT TO CHOIR PARTICIPATION FOR CAREGIVERS AND PERSONS WITH DEMENTIA

Sandra Hundza,[1] Stuart W. MacDonald,[1] Debra J. Sheets,[1] and Andre P. Smith[1], *1. University of Victoria, Victoria, British Columbia, Canada*

Choir interventions confer psychological benefits for persons with dementia (PwD) and their caregivers. However, less is known about whether physiological function also exhibits improvements pursuant to such social-cognitive interventions. The present study, based upon a subsample of the Voices in Motion (ViM) project, explored whether participation in an intergenerational choir results in systematic improvements in gait velocity (indexed using a GAITRite computerized walkway) for both informal caregivers (n=14; 71.4% female) and PwD (n=14; 64.3% female). Longitudinal burst data from the first of three cohorts spanning 4 assessments over 3.5 months was analysed using multilevel modeling. Whereas caregivers exhibited significant improvements ($p<.05$) in gait velocity, PwD showed no improvement. Ongoing analyses are exploring additional cohorts, and whether improvements in gait dynamically covary with reductions in comorbidities (e.g., neuropsychological function, caregiver burden, depressive affect). These results underscore the potential of choir for facilitating both psychosocial and physiological function for caregivers and PwD.

### SESSION 3475 (SYMPOSIUM)

### RESILIENCE, COPING, AND HEALTH IN MIDDLE TO LATE LIFE

Chair: Roland J. Thorpe, *Johns Hopkins Bloomberg School of Public Health, Baltimore, Maryland, United States*
Discussant: Keith E. Whitfield, *Wayne State University, Detroit, Michigan, United States*

Stress and resilience are two factors that are receiving attention as key determinants that can provide insights that underlie the deleterious effects on the overall health and well-being of individuals by influencing behavioral and biological processes. This symposium contains a collection of papers seeking to address the influence of resilience and coping on health outcomes in middle to late life adults. Tobin and Thorpe identified profiles of psychosocial resilience and examined their association with allostatic load (AL) among 283 Black men in the Nashville Stress and Health Study. Using Latent class analysis (LCA), individuals in the high resilience class had the greatest odds of high AL; high resilience worsened physical health for older but not younger Black men. Tan and colleagues explored satisfaction across life domains and correlates of satisfaction across domains in 93 Black adults. The authors report that higher satisfaction was associated with less education, less financial strain, lower depressive symptoms, and better self-rated physical health. Nguyen examined the association between everyday discrimination and generalized anxiety disorder (GAD) and whether church-based relationships buffer the negative effects of everyday discrimination on GAD among older African Americans. Using data from 670 African American respondents age 55 and older from the NSAL, the author reports, that significant interactions indicated that frequent contact with church members and high levels of subjective closeness to church members buffered against the negative effects of discrimination on GAD. These presentations collectively will bolster our knowledge of how stress and resilience impacts health disparities.

### DO PSYCHOSOCIAL RESOURCES CONFER PHYSIOLOGICAL RISK? EXAMINING ALLOSTATIC LOAD BLACK MEN ACROSS THE LIFE COURSE

Courtney S. Thomas Tobin,[1] and Roland J. Thorpe Jr[2], *1. UCLA Fielding School of Public Health, Los Angeles, California, United States, 2. Department of Health, Behavior, and Society, Baltimore, Maryland, United States*

Research suggests positive psychosocial resources promote resilience, although this has been underexplored among Black men. The present study identified profiles of psychosocial resilience and examined their association with allostatic load (AL) among young, middle-aged, and older Black men. Data come from 283 Black men in the Nashville Stress and Health Study. Latent class analysis (LCA) identified resource profiles comprised of eight psychosocial resources across four categories (coping strategies, sense of control, racial identity, social support). Logistic regression was used to estimate the odds of high AL (5+ high-risk indicators) across classes. LCA indicated three latent classes: low (33%), moderate (26%), and high (41%) psychosocial resources. Unexpectedly, individuals in the high resilience class had the greatest relative odds of high AL; high resilience worsened health for older but not younger Black men. Findings suggest elevated levels of resources resilience may undermine physical health in this population.

### CHURCH RELATIONSHIPS, DISCRIMINATION, AND GENERALIZED ANXIETY DISORDER AMONG OLDER AFRICAN AMERICANS

Ann W. Nguyen[1], *1. Jack, Joseph and Morton Mandel School of Applied Social Sciences, Case Western Reserve University, Cleveland, Ohio, United States*

The African American church has played a major role in African American communities, and church relationships represent an important stress-coping resource for older African Americans. This study examined 1) the association between everyday discrimination and generalized anxiety disorder (GAD) and 2) whether church-based relationships buffer the negative effects of everyday discrimination on GAD among older African Americans. Logistic regression analyses were conducted using data from 670 African American respondents age 55 and older from the National Survey of American Life: Coping with Stress in the 21st Century. More frequent experiences of everyday discrimination was associated with higher odds of meeting criteria for GAD. Significant interactions indicated that frequent contact with church members and high levels of subjective closeness to church members buffered against the negative effects of discrimination on GAD. Interventions that focus on the use of church members for support capitalize on a major strength among older African Americans.

### CORRELATES OF LIFE SATISFACTION AMONG MIDDLE-AGED AND OLDER BLACK ADULTS

Shyuan Ching Tan,[1] Alyssa Gamaldo,[2] Angie L. Sardina,[3] and Ross Andel[4], *1. The Pennsylvania State University, University Park, Pennsylvania, United States, 2. The Pennsylvania State University, State College, Pennsylvania, United States, 3. University of North Carolina Wilmington, Wilmington, North Carolina, United States, 4. University of South Florida, Tampa, Florida, United States*

This study explored satisfaction across life domains (e.g., family, daily life, health, finances, city of residence) and correlates of satisfaction across domains. Black adults (n=93, age range=55-80) completed the domains of life satisfaction scale and measures of sociodemographic factors, personality, and mental/physical health. Participants' satisfaction was highest for home condition, but lowest for health. Univariate analyses of variance demonstrated better life satisfaction in the oldest-old (80+) than the youngest-old (55-64; $p<.05$), particularly in the domains of daily life/leisure, current financial situation, and total household income. Linear regression models suggested that higher satisfaction was associated with less education, less financial strain, lower depressive symptoms, and better self-rated physical health, although the pattern of results varied by domain. Satisfaction may increase with advancing old age, at least in some life domains. It can also vary across life domains and unique factors likely relate to satisfaction in each life domain.

### SESSION 3480 (PAPER)

### SOCIAL ENGAGEMENT AND SUPPORT

### CHANGES IN SOCIAL SUPPORT AND THE INFLUENCE OF DRIVING CESSATION

Brittany M. King[1], *1. Florida State University, Tallahassee, Florida, United States*

Driving cessation has been shown to be a potentially isolating transition in life, with important implications for mental health, social isolation, and social support. Older adults who live alone are vulnerable to social isolation in the context of driving cessation. Although some research has examined the association between driving cessation and certain kinds of social engagement activities, no research has specifically examined changes in social support, particularly among older adults most vulnerable to social isolation – those who live alone . The present study addresses this gap, using data drawn from the 2006-2014 waves of the Health and Retirement Study (HRS) to examine how social support changes in the context of driving cessation among older adults who live alone (N=412). This study specifically focuses on instrumental and emotional social support, and how different sources of the support (children, friends, and other family) are influenced by loss of driving. I use a series of ordinary least squares regression (OLS) to examine four-year changes in various forms of social support among those who live alone, comparing those who lose the ability to drive relative to their continuously driving counterparts. Preliminary results indicate that driving cessation is associated with decline in perceived instrumental support of friends (-0.984, p<01) for older adults who live alone. However, these effects did not extend to children or other family members. These results suggest that loss of driving may perpetuate vulnerabilities facing individuals who live alone by leading to lower levels of perceived support from non-family members.

### INVESTIGATING SOCIAL NETWORKS OF OLDER SINGAPOREAN LEARNERS: THE MIXED-METHODS SOCIAL NETWORK APPROACH

Pei-Chun Ko[1], *1. National University of Singapore, Singapore, Singapore*

Lifelong learning has been regarded as an important factor of promoting active engagement in later life for researchers and policy makers. Most of the studies tend to illustrate old learners as a homogeneous and self-resilient group of people to engage in lifelong learning. Few studies address older learners' social capital in affecting their decision of engagement and in sustaining their motivation. The study documented the existing social networks of older Singaporeans in lifelong learning programs and illustrated how social networks contributed their participation in learning. The mixed methods consist of in-depth interviews and two network instruments (Name Generator and Position Generator) based on 30 older Singaporeans (between 50 and 79 years old) who attended lifelong learning courses between 2016 and 2018. Interviews are transcribed and analyzed. The network instruments of are quantified and visualized. The findings show that older learners' networks included a mixture of social ties from family and friends. Learners' closeness with network members and their living arrangement with them influenced learners' involvement in learning and future planning. Single respondents who had more non-kin members in the networks reported to be more active due to their weak ties. Overlapping networks among couple learners increase the spousal support for learning. Learners who had wider ranges of social resources are associated with their interest in learning activities. The study suggests that advocating lifelong learning needs to take older adults' networks into considerations as networks represent the social forces that influence their decisions and motivations.

### IS SOCIAL ENGAGEMENT RELATED TO FEWER HOSPITALIZATIONS IN COMMUNITY-DWELLING OLDER ADULTS?

Bryan D. James,[1] Raj C. Shah,[2] Melissa Lamar,[3] Lisa L. Barnes,[3] Robert S. Wilson,[3] David A. Bennett,[3] and Julie A. Schneider[3], *1. Rush Alzheimer's Disease Center, Chicago, Illinois, United States, 2. Rush University, Chicago, Illinois, United States, 3. Rush Alzheimer's Disease Center, Chicago, Illinois, United States*

We tested the hypothesis that more socially engaged older adults experience fewer hospitalizations. Data came from 1,153 older adults (72.4% female, mean age 80.8, 14.6 years education at baseline), enrolled in the Rush Memory and Aging Project, with survey data linked to Medicare claims records (mean 5.0 [SD=3.1] years of Medicare coverage after study baseline). Linear regression models were fit with annual rate of hospitalization as outcome with terms for age, sex, and education. Engaging in more social activities (est=-0.16, SE=0.05, p=0.002) and larger life space (est=-0.08, SE=0.03, p=0.005) were associated with a lower rate of hospitalization, while a higher level of loneliness (est=0.18, SE=0.06, p=0.002) was associated with greater rate of hospitalization; size of social networks (est=-0.01, SE=0.01, p=0.069) was not associated with hospitalization. When examined separately by admission type, the same significant associations were found for nonelective (emergency and urgent) hospitalizations, but not for elective hospitalizations. After further adjusting for marital status, baseline levels of depressive symptoms, chronic medical conditions, physical activity, and ADL disabilities, only social activities were significantly related to hospitalization rate (total and nonelective). Adjusting for disability attenuated these associations the most, indicating that functional status may confound the relationship between social engagement and hospitalization. More research is necessary to determine if social engagement in older age can proactively help to keep older adults out of the hospital, or alternatively whether level of social engagement is a marker for functional status or other health factor that is directly related to risk of hospitalization.

### SOCIAL ENGAGEMENT AND DEPRESSIVE SYMPTOMS IN OLDER AFRICAN AMERICANS: A 6-YEAR CROSS-LAGGED PANEL ANALYSIS

James Muruthi,[1] and J. Tina Savla[2], *1. University of Oregon, Eugene, Oregon, United States, 2. Virginia Tech, Blacksburg, Virginia, United States*

Although previous studies have extensively investigated the cross-sectional relationship between social engagement and depressive symptoms in late life, longitudinal studies have produced mixed results. Furthermore, studies on the associations between these two concepts among aging African Americans are few. Using a sample of 1688 older African Americans adults from waves 1 and 7 of the National Health and Aging Trends Study (60% women; Average age = 77 years), the present study investigates the longitudinal associations between social engagement (an index from scores on visiting friends and family, attending religious services, attending religious services, participating in group activities, and going out for enjoyment) and depressive symptoms across seven years. Structural equation modeling was used to test cross-lagged relationships between the variables.

Findings suggest that social engagement at baseline significantly predicted subsequent depressive symptoms and social engagement. Depressive symptoms at baseline, however, were not significantly associated with subsequent social engagement. These findings suggest that low social engagement in older African Americans is directly associated with increased depressive symptoms over time, but not vice versa. The implications of these findings are discussed in relation to the barriers of social engagement for older African Americans and its effects on their mental health.

### WHEN CLOSE TIES LIVE FAR AWAY: PATTERNS AND PREDICTORS OF GEOGRAPHIC NETWORK RANGE AMONG OLDER EUROPEANS

Haosen Sun,[1] and Markus H. Schafer[1], *1. University of Toronto, Toronto, Ontario, Canada*

Using the Survey of Health, Ageing and Retirement in Europe (SHARE, Wave 6 in 2015), this paper examines the structure of older adults' core discussion networks in terms of their geographical outreach. We also examine how far respondents live from their friends, and how such a connection is conditioned by the presence of a proximate child in the network. Findings suggest that older adults in Northern Europe are more likely to have a confidant at mid- and long-range (5-25km and >25km, respectively) than seniors in Central Europe, while their counterparts from Eastern and Southern Europe are less likely to identify a discussant out of their 5km radius. This pattern persists when focusing only on non-kin members of one's network. However, having a nearby child confidant does not affect the probability of being connected to friends at variant distances in North Europe, while it does predict a lower likelihood of having close-by (0-5km) and long-distance (>25km) friends in Eastern and Southern regions. Other significant predictors of one's geographical network reach, such as education, financial standing, cognitive ability, computer skills, and car ownership are also discussed and compared across European regions.

### SESSION 3485 (SYMPOSIUM)

### STAKEHOLDER PERSPECTIVES ON DESIGN, IMPLEMENTATION, AND SUSTAINMENT OF SERVICES FOR OLDER VETERANS

Chair: Samantha Solimeo, *Center for Access and Delivery Research and Evaluation Primary Care Analytics Team Iowa City VA Health Care System, Iowa City, Iowa, United States*
Co-Chair: Bret Hicken, *Veterans Rural Health Resource Center-SLC, Salt Lake City, Utah, United States*

A majority of United States Veterans are older adults, compelling healthcare systems such as Veterans Health Administration to attend to their unique needs when designing and implementing programs for workforce development and service delivery. In this symposium authors will present findings from four studies examining how older Veterans' needs and preferences affect implementation and sustainment in a variety of settings. Presenters demonstrate how: 1) understanding Veterans' perspectives and preferences for measuring functional status may inform the improvement of care coordination in the primary care setting; 2) the role of population characteristics in implementation of geriatric patient centered medical home teams (i.e., GeriPACTs); 3) the interaction of patient, provider, and delivery system information needs in limiting sustainment of diverse initiatives to improve osteoporosis screening and management for Veterans; and 4) the factors affecting transferability and sustainment of rural and geriatrics-focused quality improvement initiatives beyond local settings. Beyond their focus on how older adults' needs are reflected or shape implementation, the studies illustrate the application of qualitative data to clinical practice and workforce development.

### OLDER VETERANS' PERSPECTIVES ON MEASURING FUNCTIONAL STATUS IN VA PRIMARY CARE CLINICS

Francesca Nicosia,[1] Malena J. Spar,[2] Alicia Neumann,[3] Molly C. Silvestrini,[2] Maureen Barrientos,[3] and Rebecca T. Brown[4], *1. San Francisco VA Health Care System, San Francisco, California, United States, 2. San Francisco VA Medical Center Division of Geriatrics; San Francisco, California, United States, 3. Division of Geriatrics, San Francisco, California, United States, 4. Corporal Crescenz VA Medical Center Division of Geriatric Medicine, Philadelphia, Pennsylvania, United States*

Little is known about older adults' perspectives on measuring functional status (i.e., ability to perform basic and instrumental activities of daily living). This study used a qualitative design to understand older Veterans' perspectives on measuring function in primary care settings. Thematic analysis of interviews conducted with 28 Veterans ≥65 years and 5 caregivers from one VA Medical Center identified several themes including: 1) importance and relevance of discussing function; 2) preferences for assessment method (e.g., provider- or self-assessment;) and 3) wording of questions (i.e., needing help vs. having difficultly). These findings suggest that effective approaches to measuring function must consider patient preferences on content and format and ensure that measurement is used to inform care. We applied these findings to develop an interprofessional intervention to improve functional status measurement for older Veterans in primary care.

### WE ARE NOT CURING A LOT OF THINGS, BUT WE ARE CARING: GERIPACT PATIENT NEEDS SHAPE TEAM NEEDS

Samantha Solimeo,[1] Melissa J. Steffen,[2] Ellen E. Gardner,[3] Omonyele Adjognon,[4] and Marlena Shin,[4] Jennifer Moye,[5] and Jennifer L. Sullivan[6], *1. Center for Access and Delivery Research and Evaluation Primary Care Analytics Team Iowa City VA Health Care System, Iowa City, Iowa, United States, 2. Center for Access & Delivery Research & Evaluation (CADRE) Primary Care Analytics Team, Iowa City, Iowa, United States, 3. Carver College of Medicine, Iowa City, Iowa, United States, 4. Center for Healthcare Organization & Implementation Research, Boston, Massachusetts, United States, 5. New England Geriatric Research, Education, and Clinical Center Department of Psychiatry; Harvard Medical School, Boston, Massachusetts, United States, 6. Center for Healthcare Organization and Implementation Research, Boston*

*University School of Public Health, Boston, Massachusetts, United States*

This study used a qualitative observational design to identify the team-, clinic-, and system-level resources necessary for effective geriatric medical home (i.e., GeriPACT) implementation and to differentiate the needs of GeriPACT compared to traditional PACT. Analysis of 80 interviews conducted with team members from 8 geographically dispersed GeriPACTs identified needs that may be unrecognized by primary care leadership, including: clinical space to accommodate caregivers and patients with impaired visual, mobility, cognitive, or hearing acuity; greater utilization of caregiver support programs and social workers to facilitate aging-in-place; age-sensitive clinical reminders; team member continuity and direct phone lines to reduce patient anxiety; and longer standard appointment lengths to reflect clinical complexity. In contrast to traditional primary care teams, GeriPACTs are not simply "PACTs for older adults": GeriPACT members articulate population-specific resources that require support from facility leadership to accommodate the complex, age- clinical and social resources needed to support aging-in-place.

### CLINICIAN-PERCEIVED CHALLENGES OF OSTEOPOROSIS CARE IN AN INTEGRATED HEALTHCARE DELIVERY SYSTEM

Aaron T. Seaman,[1] Melissa J. Steffen,[2] Karla Miller,[3] and Samantha Solimeo[4], *1. Iowa City VA Healthcare System, Iowa City, Iowa, United States, 2. Veterans Rural Health Resource Center Central Region Center for Access and Delivery Research and Evaluation Primary Care Analytics Team, Iowa City, Iowa, United States, 3. Veterans Rural Health Resource Center Western Region Department of Internal Medicine, Rheumatology Section Division of Rheumatology; University of Utah School of Medicine, Salt Lake City, Utah, United States, 4. Veterans Rural Health Resource Center Central Region, Center for Access and Delivery Research and Evaluation Primary Care Analytics Team, Iowa City, Iowa, United States*

The burden of osteoporosis, both on the health care system and individuals, is high. Despite this, a high percentage of patients with or at risk of osteoporosis are not identified, screened and treated appropriately. Delivering osteoporosis care to at-risk patients is complicated by a fractured health care delivery system. In this presentation, we present data from interviews with VA clinicians in order to identify challenges of osteoporosis care within the VA health care system. While clinicians reported initiating a range of bone health care delivery interventions, they identified challenges that inhibited long-term sustainability: 1) low prioritization of bone health among national and facility leadership; 2) fragmentation of clinical responsibility and care delivery; and 3) barriers endemic to the osteoporosis care delivery system. Our results indicate that, even within an integrated health care delivery system, significant coordination challenges exist.

### SPREAD AND SUSTAINMENT OF QUALITY IMPROVEMENTS IN VA RURAL PRIMARY CARE CLINICS

Josea Kramer,[1] Claire O'Hanlon,[2] Joe Douglas,[3] Michael N. Mitchell,[3] Michael McClean,[4] Shawn Clarke,[5] and Carol Callaway-Lane[6], *1. VA Greater Los Angeles Healthcare System GRECC, Los Angeles, California, United States, 2. Center of Innovation, Los Angeles, California, United States, 3. Geriatric Research, Education and Clinical Center, Sepulveda, California, United States, 4. Center of Innovation, Geriatric Research, Education and Clinical Center, Sepulveda, California, United States, 5. Geriatric Research, Education and Clinical Center, Sepulveda, California, United States, 6. Geriatric Research, Education and Clinical Center, Nashville, Tennessee, United States*

The VA has invested in developing the skills of its primary care workforce through the longitudinal Geriatric Scholars Program. Now in its 11th year, the program has increased career satisfaction and job retention, standardized provider behaviors, improved clinical decision-making and reduced dispensing of potentially inappropriate medications. The program consists of: intensive coursework in geriatrics; workshop in quality improvement (QI); and initiation of a micro QI projects in the Scholar's clinic. Electives enable learners to tailor the program to self-identified gaps in knowledge, skills and competencies. This presentation focuses on the sustainment and spread of these QI projects based on a recent survey of Scholars. Differences between rural and urban QI projects are compared. Commonality among rural QI projects is explored based on topic, team composition, and the types of efficiencies gained in clinical and/or organizational processes to improve care for older Veterans living in rural areas.

### SESSION 3490 (SYMPOSIUM)

### THE APPLICATION OF MASS OBSERVATION DATA IN SOCIAL RESEARCH

Chair: Paul Kingston, *University of Chester, Chester, United Kingdom*

Responding to the opportunities and challenges of an ageing world the University of Chester established the Centre for Ageing, Mental Health and Veterans' Studies in 2013 to provide research, consultancy and education, with the aim of promoting innovation in health and social care services for older people. This symposium brings together researchers from a wide range of disciplines and career stages, to explore the utility of Mass observation data in social research in the field of gerontology. The Mass Observation Project, established in 1937, documents the lives of ordinary people living in the UK, and explores a wide range of social issues. The symposium comprises four separate papers. The Methodological Relevance of Mass Observation Data: This preliminary overview will outline the mass observation archive, highlighting challenges and issues encountered utilising the data produced in social research. Personal Narratives of Ageing: This paper presents personal narratives reflecting on the ageing process, and growing older in the UK. The Health Impact of Scams: This presentation will offer new and alternate insights into 'scams' and the health effects of fraud on older people, using data from the mass observation directive commissioned by the centre. Perceptions of Dementia: This paper presents a perspective on the public knowledge and understanding about dementia not previously considered, where respondents have written openly about their own experiences, and reflected on their perception of the wider public's knowledge and understanding about dementia.

### THE METHODOLOGICAL RELEVANCE OF MASS OBSERVATION DATA

Charlotte L. Eost-Telling,[1] Paul Kingston,[1] Louise Taylor,[1] and Jan Bailey[1], *1. University of Chester, Chester, England, United Kingdom*

The Mass Observation Project, established in 1937, documents the lives of ordinary people living in the UK, and explores a wide range of social issues. The Project distributes a set of written questions ("Directives") to a panel of 500 members of the British public ("Observers") three times each year; "Observers" respond in writing. From the initial commissioning of a "Directive" to data becoming available for analysis takes between four to six months. This approach offers researchers an opportunity to capture in-depth qualitative data from individuals with a range of demographic backgrounds who live across the UK. As there are no word limits on "Observers'" responses and they remain anonymous, a "Directive" often yields rich, high-quality data. Additionally, compared with alternative methods of collecting large volumes of qualitative data from a heterogeneous population, commissioning a "Directive" is cost-effective in terms of time and resource.

### PERCEPTIONS OF DEMENTIA

Paul Kingston,[1] Louise Taylor,[1] Charlotte Eost-Telling,[1] and Jan Bailey[1], *1. University of Chester, Chester, England, United Kingdom*

This paper considers narratives of 143 respondents ("Observers") to a Mass Observation Project Directive exploring individuals' perceptions of dementia. Perceptions of dementia held by "Observers" with experience of dementia and those without differed sharply. "Observers" with experience of dementia offered insight into living with and caring for a person with dementia, and the impact this had on their lives and personal relationships. Whereas, "Observers" with no direct experience of dementia focused more on common disease symptoms such as memory loss and reflected idealised views of care. "Observers" often feared being diagnosed with dementia themselves. This suggests education to facilitate care planning and ameliorate fears held by the public is required.

### PERSONAL NARRATIVES OF AGING

Louise Taylor,[1] Jan Bailey,[1] Paul Kingston,[1] and Charlotte Eost-Telling[1], *1. University of Chester, Chester, England, United Kingdom*

This presentation reflects on self-written narratives from respondents to a mass observation directive, focusing on the experiences of growing older. Narrative methods are theoretically and methodologically diverse, and are helpful in social research to understand events or happenings in human lives. This data presents accounts from a heterogeneous sample in the form of self-penned responses. These experience-centred narratives bring stories of personal understanding into being by means of the first person description of past, present, future or imaginary experiences. This presentation will focus on the findings with reference to physical and mental impacts, both real and anticipated. We will also explore themes arising from the data including gender differences, age-cohort effects and stigma. The data can be used to inform Health and Social Care education and practice, particularly in co-producing appropriate person-centred services with older people.

### THE HEALTH IMPACT OF SCAMS

Jan Bailey,[1] Paul Kingston,[1] Louise Taylor,[1] and Louise Eost-Telling[1], *1. University of Chester, Chester, England, United Kingdom*

This presentation will offer new and alternate insights into 'scams' and the health effects of fraud on older people. It reports data captured from a Mass Observation Project "Directive" focusing on scams and their impact on individuals. Eighty "Observers'" aged 50 and over responded to the "Directive". Responses indicate that falling victim to a scam may have negative impacts on individuals' mental wellbeing, self-esteem and relationships with others. Data analysis also identified that fear of victimisation can also affect individuals, resulting in worry, anxiety and maladaptive coping strategies. Offering a sociology of health perspective, we will focus is on these health impacts of scams and the legitimisation of the issue as a socio-political problem. We will also highlight additional important areas for consideration, such as the absence of a common understanding of the concept and nomenclature of 'scam', and the 'vagaries of scams' by presenting a typology of scams.

### SESSION 3495 (SYMPOSIUM)

### UTILIZING EFFECTIVE MENTORING STRATEGIES FOR RESEARCH SUCCESS: NYU SUMMER SCHOLARS PROGRAM

Chair: Meredeth Rowe, *University of South Florida, Tampa, Florida, United States*
Co-Chair: Tracey Yap, *Duke University, Durham, North Carolina, United States*
Discussant: Bei Wu, *New York University, New York, New York, United States*

Since 1998 the Hartford Institute for Geriatric Nursing has offered a summer program designed to accelerate the development of research projects and trajectories for young scientists. This interprofessional program brings faculty and scholars together from across the globe representing all healthcare disciplines. In the week long program, scholars benefit from expert presentations and daily mentoring sessions with experts in gerontological research. In this symposium, scholars will discuss strategies they have used to successfully move from ideas for research, to development of proposals, conduct of research projects and planning career trajectories. We will present differing mentoring strategies and available mentoring opportunities for gerontological-focused scientists. Each scholar will present their work with topics including optimal medication management to sustain home placement: use of adaptive dance to improve mood and function in persons with Alzheimer's disease; facilitating positive attitudes toward older adults in nursing students; the use of mindfulness to reduce symptoms of urinary incontinence; and, a novel intervention to reduce pressure ulcers in the nursing home.

### HOW CLINICAL SETTING AND EXPERIENCES INFLUENCE NURSING STUDENTS' ATTITUDES TOWARD OLDER ADULTS

Susan Hovey[1], *1. Illinois State University, Normal, Illinois, United States*

This study is significant because older healthcare consumers continue to rise with estimations that nearly 72.1 million persons in the United States will be over the age of 65 by 2030. A fundamental question remains, will the future nursing workforce possess the attitude and knowledge to competently provide age-friendly care to older adults. The aim of this study explores how clinical setting, previous experiences with older adults, and previous work experiences in long-term care settings influence the attitudes of first year prelicensure nursing students toward this population. Six baccalaureate nursing schools from a Midwest state in the United States participated in this descriptive, cross-sectional, correlational study. One hundred and nine participants who completed their first clinical experience participated in the study. An understanding of this experience may provide nurse educators with insight into how to design clinical learning activities so nursing students' acquire interest in care of older adults.

### IMPACT OF ADAPTED DANCE ON MOOD AND PHYSICAL FUNCTION AMONG ALZHEIMER'S DISEASE ASSISTED LIVING RESIDENTS

Crystal Bennett[1], *1. University of West Florida, Pensacola, Florida, United States*

Neuropsychiatric secondary symptoms and altered physical function are prevalent among persons with Alzheimer's Disease and related dementia disorders (ADRD) which increase healthcare costs and caregiver burden. Adapted dance is a promising intervention that may improve these symptoms and physical function. The purpose of this study is to test whether 12 weeks of adapted dance (60 min 2x/week) improves agitation, physical function, and reduces caregiver burden. An experimental crossover design will be used. ADRD residents with a Montreal Cognitive Assessment score between 6-26, Timed up and go of <20 seconds, Cohen-Mansfield Agitation Inventory Score (CMAI) of >15, and do not use oxygen or assistive device will be eligible to participate. Outcomes will be assessed at baseline and every 4 weeks during each study arm for 24 weeks. Measures include CMAI and the Neuropsychiatric Inventory-Clinician Scale for agitation; Short Physical Performance Battery for physical function; and Zarit Burden Interview for caregiver burden.

### SAFER MEDICATION MANAGEMENT FOR HOME-DWELLING OLDER ADULTS

Henk Verloo,[1] Armin von Gunten,[2] Boris Wernli,[3]
Marie SANTIAGO-DELEFOSSE,[4]
Maria Manuela MARTINS,[5] and Filipa Pereira[6],
*1. University of Applied sciences and Arts Western Switzerland, Switzerland, Switzerland, 2. Service of Old Age Psychiatry, Lausanne University Hospital, Prilly, Vaud, Switzerland, 3. FORS, Swiss Centre of Expertise in the Social Sciences, University of Lausanne, Expert in Database Processing, Lausanne, Vaud, Switzerland, 4. University of Lausanne, Lausanne, Vaud, Switzerland, 5. Institute of Biomedical Sciences Abel Salazar, University of Porto, Porto, Porto, Portugal, 6. University of Applied Sciences and Arts Western Switzerland, Sion, Valais, Switzerland*

Taking several medications at the same time can lead to adverse effects and dangerous situations for home-dwelling older adults with chronic conditions. Accurate medication management can be a difficult challenge, especially for people living at home. However, little research has been carried out into the experience of older adults and their informal caregivers with medication management. The aim of the study is, first, to identify factors that can cause undesirable side effects and make taking multiple medications potentially dangerous for home-dwelling older adults. Second, the study will investigate the experiences of this group of patients with medication management. Third, the role of both professional and informal caregivers will be examined. Recommendations will be made on how to improve the safety of medication management for home-dwelling older adults with chronic conditions and should help to prevent the adverse effects and dangerous situations that can lead to hospitalization, institutionalization or premature death

### LONG-TERM EFFECTS OF MINDFULNESS ON URGE URINARY INCONTINENCE IN OLDER ADULT WOMEN: ANALYZING RCT RESULTS AT 6 MONTHS

Katarina Friberg Felsted[1], *1. University of Utah, Salt Lake City, Utah, United States*

Urge urinary incontinence is a condition estimated to cost $82 billion by 2020. Innovative treatments are needed, particularly in the older adult population. A prior combined feasibility study and randomized controlled trial examined six feasibility determinants and five preliminary efficacy outcomes of treating urge urinary incontinence in older adult women (N=25; average age=74 years) utilizing an 8-week mindfulness-based stress reduction (MBSR) intervention compared with the health enhancement program (HEP), which is an active comparison modality specifically validated to be used alongside MBSR in scientific research. Feasibility and preliminary efficacy results were reported at GSA in 2018. This 2019 presentation relays the preliminary efficacy results at 6-month follow up. Outcomes include symptom severity, symptom bother, perceived stress, perceived self-efficacy, and rate and trajectory of change. Future research is needed in the form of a multi-site trial to provide a larger sample with greater diversity.

### SESSION 3500 (SYMPOSIUM)

### UTILIZING TECHNOLOGY NETWORKS TO SUPPORT SOCIAL NETWORKS FOR PEOPLE AGING WITH DISABILITY

Chair: Elena T. Remillard, *Georgia Institute of Technology, Atlanta, Georgia, United States*
Co-Chair: Wendy Rogers, *University of Illinois, Urbana-Champaign, Champaign, Illinois, United States*
Discussant: Sarah Ruiz, *National Institute on Disability, Independent Living, and Rehabilitation Research, Washington, D.C., United States*

A growing number of new smart, internet-enabled technologies from smart phone applications, to teleconferencing, to the Internet of Things (IoT), provide great promise and potential to support successful aging-in-place for people with long-term disabilities. This symposium highlights ongoing research at the TechSAge Rehabilitation Engineering Research Center to identify technology needs and develop/adapt new technologies to promote independence, health, and participation of this population. To understand user needs, Harris et al. will present findings from a large-scale interview study with older adults with long-term vision and mobility disabilities (N=120) that explored specific task-based challenges with community activities (e.g., going to entertainment events, volunteering) as well as solutions and strategies to overcome them. Koon et al. will present findings on perceived facilitators and barriers to using digital assistants (e.g., Amazon Alexa) to facilitate a variety of everyday tasks at home, from shopping to communicating with others, among adults aging with mobility disabilities. Levy et al. will discuss findings from research driving the creation of augmented reality tools that can enable individuals to experience how IoT devices, such as smart thermostats and lightbulbs, could be used within the context of one's own abilities and home. Mitzner et al., will describe the development of a Tele Tai Chi intervention for older adults with long-term mobility disabilities that employs teleconferencing software to translate an in-person, evidence-based class to an online, social experience. TechSAge Program Officer, Sarah Ruiz (National Institute on Disability, Independent Living, and Rehabilitation Research), will serve as the discussant.

### A TELEWELLNESS APPROACH TO REDUCE BARRIERS TO GROUP EXERCISE FOR ADULTS AGING WITH MOBILITY DISABILITY

Tracy L. Mitzner,[1] and Elena T. Remillard[1], *1. Georgia Institute of Technology, Atlanta, Georgia, United States*

Group exercise classes have the potential to provide physical, cognitive, and emotional health benefits, through the physical activity performed as well as the social interaction among class participants. Substantial barriers exist for adults aging with lower-body mobility disabilities to engage in group exercise classes, including lack of transportation to classes, inaccessible buildings where classes are held, and lack of appropriate modifications offered for this population of older adults. Just as telehealth interventions have reduced barriers to healthcare, telewellness interventions can reduce barriers to engaging in wellness activities, such as group exercise classes. We will discuss a research study employing teleconferencing software to translate an evidence-based group tai chi class for adults aging with lower-body mobility disabilities. We will present the adaptation requirements identified to test the efficacy of a telewellness intervention for improving increasing social interaction and positive health behaviors (i.e., physical exercise frequency) for adults aging with disabilities.

### SUPPORTING AGING IN PLACE WITH THE INTERNET OF THINGS: MEETING CHALLENGES OF USE THROUGH AUGMENTED REALITY TOOLS

Laura Levy,[1] and Maribeth Gandy[2], *1. Georgia Institute of Technolopgy, Atlanta, Georgia, United States, 2. Georg, Atlanta, Georgia, United States*

Internet of Things (IoT) devices (including smart thermostats, lightbulbs, and door locks) have the potential to greatly enhance independence and promote aging-in-place among older adults with mobility disabilities. However, these devices require extensive information technology expertise to select, configure, use, and adapt to meet one's needs and this creates considerable barriers to their adoption, acceptance, and utilization. Meanwhile, increasingly available consumer augmented reality (AR) technologies can enable individuals to experience how IoT function and are used within the context of one's own abilities and home. This may provide potential users an effective means of overcoming barriers to adoption, acceptance, and utilization of IoT to support aging in place. We present preliminary results from a participatory design study of older adults with mobility disabilities on the use of AR on a smart phone device to support IoT understanding.

### IDENTIFYING COMMUNITY PARTICIPATION CHALLENGES FOR ADULTS AGING WITH MOBILITY AND VISION DISABILITIES

Maurita T. Harris,[1] Lyndsie M. Koon,[1] Elena T. Remillard,[2] and Wendy A. Rogers[1], *1. University of Illinois at Urbana Champaign, Champaign, Illinois, United States, 2. Georgia Institute of Technology, Atlanta, Georgia, United States*

There are growing numbers of older adults with mobility and vision disabilities acquired in early to mid-life who are a part of a population described as "aging with disability". For these individuals, the addition of normative age-related declines (e.g., vision loss, arthritis) on top of a long-term disability can create extensive barriers to community participation. We present findings on activity challenges with community participation among older adults with long-term vision and mobility disabilities (N=120) from the Aging Concerns, Challenges, and Everyday Solution Strategies (ACCESS) interview study. Results provide detailed insights on the specific task-based challenges experienced when engaging in one's community (e.g., going to entertainment events, doing activities with a group or organization, and participating in religious services and activities) as well as the solutions and strategies employed to overcome those challenges. Findings provide guidance for the design of supportive technologies that promote participation and independence for this understudied population.

### VOICE-ACTIVATED DIGITAL ASSISTANTS: PERCEPTIONS FROM NOVICE USERS WITH LONG-TERM MOBILITY DISABILITY

Lyndsie M. Koon,[1] Kenneth Blocker,[2] and Wendy Rogers[3], *1. University of Illinois, Urbana-Champaign, Illinois, United States, 2. University of Illinois at Urbana Champaign, Champaign, Illinois, United States, 3. University of Illinois, Champaign, Illinois, United States*

Voice-activated digital assistants (e.g., Amazon Echo, Google Home) are an emerging technology that have great potential to provide support for adults aging with a long-term mobility disability. Digital assistant technologies allow the user to perform a variety of everyday tasks and activities through voice interactions. Such tasks may include environmental control (e.g., turning on/off lights, voice-activated temperature control); supporting self-health management

(e.g., providing medication reminders, encouraging physical activity engagement); and fostering opportunities for social engagement (e.g., messaging/calling others, playing games remotely). This presentation will focus on the perceived facilitators and barriers to digital assistant use in the home among adults aging with mobility disabilities. The findings provide design guidelines and insight for intervention implementation for the use of these technologies for the target population.

### SESSION 3505 (SYMPOSIUM)

### DECONSTRUCTING THE MODEL-MINORITY MYTH OF U.S. ASIANS: DETERMINANTS AND CONSEQUENCES OF ELDER MISTREATMENT

Chair: XinQi Dong, *Rutgers Institute for Health, Health Care Policy and Aging Research, New Brunswick, United States*
Co-Chair: Melissa Simon, *Northwestern University, Chicago, Illinois, United States*

Elder mistreatment (EM) is increasingly recognized as a global health concern. Among U.S. minority and immigrant populations, the social contexts and psychological consequences associated with EM remain poorly understood. Further population-based epidemiological studies using standard EM measures are required to advance the field. To address this gap and to challenge prior assumptions regarding Asian populations, this purpose of this symposium is to improve our understanding of EM epidemiology in an older minority population. Data were drawn from the Population-based Study of Chinese Elderly in Chicago (PINE), a longitudinal, representative, population-based study of 3,157 community-dwelling Chinese older adults in the greater Chicago area. Session 1 will examine the transmission between child mistreatment, intimate partner violence, and EM. Session 2 will take a typology approach to capture the multifaceted family relationships, and will further examine which family typologies were associated with greater likelihood of EM, while which typologies were protective against EM. Session 3 will explore the positive and negative aspects of social support from spouse, family, and friends in relationship to EM subtypes, including psychological, physical, financial and sexual mistreatment, and caregiver neglect. Session 4 will examine the relationship between broad, moderate, and strict definitions of EM and likelihood of experiencing anxiety. Last, Session 5 will explore the differential relationships between EM subtypes and depressive symptoms. In summation, this symposium challenges popular conceptions of the "model minority myth" and aims to increase the practical and clinical relevance of EM epidemiology in community, research, healthcare, and policy settings.

### CYCLE OF VICTIMIZATION: CHILD MALTREATMENT, INTIMATE PARTNER VIOLENCE, AND ELDER ABUSE IN A U.S. CHINESE POPULATION

Bei Wang,[1] and XinQi Dong[2], *1. Institute for Health, Health Care Policy, & Aging Research, Rutgers University, New Brunswick, New Jersey, United States, 2. Rutgers University, New Brunswick, New Jersey, United States*

Victims of violence might have higher risks of revictimization, but this has been insufficiently examined among older populations. The present study used cross-sectional data among 3,157 U.S. Chinese older adults in Chicago, Illinois. Multiple logistic regression analyses were used to examine the relationships among subtypes (psychological, physical/sexual, financial exploitation, caregiver neglect) of child maltreatment (CM), intimate partner violence (IPV), and EM. Violence experiences were positively associated. CM psychological was positively associated with IPV psychological (OR 7.60, 95% CI 4.29-13.45) and EM psychological (OR 3.79, 95% CI 2.20-6.51). CM physical/sexual was positively associated with IPV physical/sexual (OR 1.86, 95% CI 1.02-3.38) and IPV physical/sexual was positively associated with EM physical/sexual (OR 8.54, 95% CI 3.53,20.64). EM financial exploitation was positively associated with all types of CM and IPV, whereas EM caregiver neglect has no significant association with any CM or IPV. Clinical and policy implications of the findings will be discussed.

### TYPOLOGY OF FAMILY RELATIONSHIP AND ELDER MISTREATMENT IN A U.S. CHINESE POPULATION

Mengting Li,[1] Man Guo,[2] Meredith Stensland,[3] Merril Silverstein,[4] and XinQi Dong[5], *1. Rutgers, The State University of New Jersey, New Brunswick, New Jersey, United States, 2. University of Iowa, Iowa City, Iowa, United States, 3. University of Texas at Austin, Austin, Texas, United States, 4. Syracuse University, Syracuse, New York, United States, 5. Rutgers, The State University of New Jersey, New Brunswick, New Jersey, United States*

Early research on family relationship and Elder Mistreatment (EM) often focused on one or two indicators of relations. A typology approach that capture the complexity and variation of relations is a useful tool to understand the association between multifaceted family relationship and EM. EM was measured by a modified Vulnerability to Abuse Screening Scale. Latent Class Analysis was used to construct family typologies, evaluating structural, associational, functional, affectual, and normative aspects of family relationship. Logistic regression was used. Unobligated ambivalent (OR, 1.90; 95%CI, 1.54-2.34) and detached (OR, 1.78; 95%CI, 1.32-2.42) typologies were associated with greater risk of EM, while tight-knit (OR, 0.34; 95%CI, 0.27-0.44) typology was associated with lower risk of EM. Unobligated ambivalent typology, featured by high intergenerational closeness and conflict, was prevalent among US Chinese immigrants, and associated with greater likelihood of EM. Culturally customized social services were suggested to reduce intergenerational ambivalence and prevent EM for immigrants.

### SOURCES SOCIAL SUPPORT AND SUBTYPES OF ELDER MISTREATMENT AMONG CHINESE OLDER ADULTS: FINDINGS FROM THE PINE STUDY

Shenglin Zheng,[1] Stephanie Bergren,[1] and XinQi Dong[1], *1. Rutgers University, New Brunswick, New Jersey, United States*

To examine the relationships between positive social support (PSS) and negative social support (NSS) from different sources and subtypes of EM, we used the data from a representative sample of 3,157 Chinese older adults aged 60 years or older in Chicago. Subtypes of EM include psychological mistreatment, physical mistreatment, financial exploitation, and caregiver neglect. Higher PSS from spouse and family members were less associated with lower likelihood to experience any of four self-reported EA subtypes. Higher PSS from friends was associated with lower likelihood of caregiver neglect. Increased levels of NSS from spouse and family members were associated with higher likelihood of psychological mistreatment, financial exploitation, and caregiver neglect. A significant association was also found between NSS from friends and psychological mistreatment. This study demonstrates the positive and negative aspects of social contexts in relationship to EM. Future longitudinal studies are needed to examine causal relationships.

### ELDER MISTREATMENT SUBTYPES AND ANXIETY: DO DEFINITIONS MATTER?

Jieyang Zheng,[1] Stephanie Bergren,[1] and XinQi Dong[1], *1. Rutgers University, New brunswick, New Jersey, United States*

Elder mistreatment (EM) and the magnitude of its relationship to anxiety may vary depending on definitional criteria. We leveraged data from the PINE Study, a study of 3,157 Chinese older adults in Chicago. EM was measured by 56 items on psychological, physical and sexual mistreatment, caregiver neglect and financial exploitation subtypes. Least restrictive, moderately restrictive, and most restrictive definitions of EM were constructed. Symptoms of anxiety were measured by the Hospital Anxiety and Depression Scale. Least restrictive (OR, 1.94; 95%CI, 1.57-2.40), moderately restrictive (OR, 1.56; 95%CI, 1.22-1.99), and most restrictive (OR, 1.39; 95%CI, 1.07-1.79) definitions of EM were all significantly associated with the likelihood of experiencing any anxiety symptoms. The magnitude of associations between EM and anxiety symptoms vary based on strictness of the EM definition. Future research should explore the potential causal relationships between EM and anxiety through longitudinal data.

### ELDER MISTREATMENT AND DEPRESSIVE SYMPTOMS AMONG OLDER CHINESE AMERICANS

Ying-Yu Chao,[1] Yu-Ping Chang,[2] and XinQi Dong[3], *1. Rutgers School of Nursing, Newark, United States, 2. The State University of New York, University at Buffalo, Buffalo, New York, United States, 3. Rutgers University, New Brunswick, New Jersey, United States*

This study aimed to examine the association between different types of elder mistreatment and depressive symptoms among U.S. Chinese older adults. Data were from the Population Study of Chinese Elderly in Chicago (PINE). Participants were 3,157 Chinese older adults who were 60 years and over (mean age = 72.8). Logistic regression analyses were performed. The results showed that participants with overall mistreatment (OR, 2.11; 95% CI, 1.83-2.43), psychological mistreatment (OR, 2.12; 95% CI, 1.78-2.51), physical mistreatment (OR, 1.82; 95% CI, 1.10-2.99), and financial exploitation (OR, 1.33; 95% CI, 1.11 – 1.60) were more likely to report more depressive symptoms. There was no significant association between sexual mistreatment and depressive symptoms (p = 0.07). Longitudinal studies are needed to obtain a more comprehensive understanding of the pathways between elder mistreatment and depressive symptoms.

### SESSION 3510 (PAPER)

### EDUCATIONAL STRATEGIES TO ADDRESS NEEDS OF AN AGING POPULATION

### CAPTURING IMPACT ON STUDENTS PARTICIPATING IN AGING IN PLACE: A PROGRAM TO ENHANCE GERIATRIC EDUCATION

Stephen T. Smilowitz,[1] Elizabeth O'Toole,[2] Diana L. Morris,[3] Todd Fennimore,[4] Cynthia Booth-Lord,[1] Elizabeth Smilovich-Fine,[1] David M. Rosenberg,[1] and Patricia A. Thomas[1], *1. Case Western Reserve University School of Medicine, Cleveland, Ohio, United States, 2. MetroHealth/Case Western Reserve University School of Medicine, Cleveland, United States, 3. Case Western Reserve University Frances Payne Bolton School of Nursing, Cleveland, United States, 4. Case Western Reserve University, Cleveland, Ohio, United States*

Curricula to enhance healthcare students' geriatric training has been lacking. Therefore, we developed AIP, an interprofessional (IP) community-based curriculum, in which IP student teams visit community-dwelling older adults. Using established instruments did not capture personal and professional changes experienced by students. Thus, an additional method using qualitative analyses of students' six post-visit reflections over 15 weeks was employed to evaluate students 'experiential learning. A grounded theory approach was used to describe students' growth in geriatric proficiencies related to participation in the January-April 2017 AIP program . By program completion, 21 students had submitted 111 reflective essays. An interdisciplinary panel reviewed a sample of reflections and developed an initial coding system, which was then systematically applied to the whole via QSR-NVivo. Seventy-three distinct codes across 111 student essays generated 2515 occurrences. Prevalent themes, revealed by frequency analysis, and themes with remarkable trendlines yielded fifteen central themes. Students became attuned to their client's life-world (n=185) as demonstrated by four central themes: 1) isolation, loneliness, and depression (n=44); 2) risks of fall (n=19); 3) loss of function/control (n=98); and, 4) importance of socializing in care (n=24). This attunement informed interactional intentionality (n=284), which shaped interactions with their client (n=207). From these authentic encounters, students described learning about 1) myself; 2) current and future practice; 3) team dynamics; and 4) my client as an older person. Systematic analysis of student reflections revealed student growth attributable to AIP. This evaluation approach should be further assessed in geriatric curricula.

### INTERPROFESSIONAL SKILLS AND CULTURAL AWARENESS OF STUDENTS CARING FOR OLDER ADULTS WITH MULTIPLE CHRONIC CONDITIONS

Kathryn M. Daniel,[1] and Mary Lou Bond[1], *1. University of Texas at Arlington, Arlington, Texas, United States*

Based on rapidly increasing numbers of older adults and growing populations of culturally diverse citizens, we developed and provided an interprofessional education program for graduate nursing and social work students that focused on the delivery of care to older adults and veterans with multiple chronic conditions. The students participated in multiple activities together over a year long period. Activities included an introductory IPE day with all students together and simulated patient cases that had required roles for the students' specialties. Knowledge and skills of interprofessional collaborative practice were measured with the Interprofessional Self Assessment Competencies (IPEC) and Assessment of Interprofessional Team Collaboration Scale (AITCS). We also measured attitudes toward cultural differences using the Cultural Awareness Scale (CAS). The entire project was repeated a second year with a second cohort of students. Overall, our students were better able to understand and work collaboratively with other health care professionals after participating in this program. Regarding cultural awareness, both NP and SW students were more aware of and comfortable with cultural differences after participating in the program. After one year of experience, we wanted to know more about our students' skills and attitudes. Focus groups were added to the design to dive deeper into the students' perceptions about which activities were most impactful and recommendations for future IPE activities. This paper will describe these data and implications for future planning of more effective interprofessional and cultural programming for students caring for older adults and veterans.

### PURSUING AGE-FRIENDLY UNIVERSITY PRINCIPLES AT A MAJOR UNIVERSITY: LESSONS IN GRASSROOTS ORGANIZING

Clare C. Luz[1], *1. Michigan State University, East Lansing, Michigan, United States*

Rapid population aging presents opportunities for higher education to address major aging-related public issues facing society that have a direct impact on students, faculty, and both local and global communities. Students in virtually all disciplines will be working within the context of an aging society post-graduation and need to be prepared as they make career choices and enter the workforce. Further, faculty and staff are not only aging themselves but may be caregivers, which has an impact on health, income and productivity. Michigan State University (MSU) is now addressing these needs through a new program guided by Age-Friendly University (AFU) principles called AgeAlive that grew out of five years of grassroots organizing. Large-scale, research-intensive institutions present special challenges to pursuing AFU status but the lessons learned by AgeAlive may help any organization that wishes to become more age friendly. This session will review AgeAlive's path to a recognized program with a clear vision, a strategic plan, two crosscutting goals including AFU designation, and five focus areas with initiatives in each area. Tools to help achieve these goals include an inventory of aging-related activity on campus and a virtual hub for networking and information exchange. Key steps in the program development process will be described as will recommendations related to choosing a model, cultivating champions, making decisions based on data, and building infrastructure. It will allow others to understand what challenges they may face and potential approaches to minimizing and overcoming these challenges in their own AFU journey.

### SESSION 3515 (SYMPOSIUM)

### POLICY SERIES: ELDER ABUSE AND THE OPIOID EPIDEMIC IN RURAL AMERICA

Chair: Brian W. Lindberg, *The Gerontological Society of America, Washington, District of Columbia, United States*
Discussant: Robert Blancato, *Matz, Blancato & Associates, Washington, District of Columbia, United States*

Misuse of opioids is a national crisis affecting the social and economic welfare of communities throughout the U.S. and is particularly rampant in rural America. Older adults are far too frequently excluded from consideration of those who are affected by the opioid epidemic. While rural older adults may not suffer the highest per capita rate of opioid overdose deaths, they are deeply affected by the problem. In their youth and middle adulthood, many older adults used their bodies for labor. At older ages, they experience multiple chronic conditions and high rates of chronic pain for which opioids and related prescription and non-prescription drugs are often the treatment of choice. Also and far too frequently, older people become easy targets for abuse by persons needing resources to feed their addiction. This symposium focuses on elder abuse associated with opioid and related substance misuse. Zanjani's presentation provides the context of rurality and drugs and alcohol as a precursor to elder abuse. The second paper by Teaster and colleagues examines trends in APS cases of elder abuse in which the perpetrator is a substance user and identifies perpetrator and victim characteristics predictive of different types of substantiated abuse. Using APS case notes, Roberto and colleagues characterize cases of elder abuse in rural Kentucky in which the perpetrator used opioids and related substances. Robert Blancato and Brian Lindberg will discuss presenters' collective findings by weaving together concepts of rurality, addiction, and elder abuse and recommending strategies for prevention, intervention, and policy.

### RURALITY, ALCOHOL, AND OPIOID USE AS PRECURSOR TO ELDER ABUSE

Faika Zanjani[1], *1. Virginia Commonwealth University, Richmond, Virginia, United States*

Pain, alcohol, and opioid risk management is an increasingly important issue in the care of the older adult population. Older adults are at higher risk for pain and the negative outcomes associated with opioid and alcohol use due to higher prevalence of multimorbidity, polypharmacy, and the age-associated changes in metabolism and elimination. Evidence-based approaches for pain and substance use management exist and are effective for older adults, but underutilized and have limited availability in rural areas. Therein, undermanagement of pain and substance use becomes a risk factor for elder abuse. Improved dissemination and implementation in practice, as well as training for health professionals and the community is needed to better realize and prevent negative health outcomes from pain, alcohol, and opioid use. Community education and training tools for health professionals will be discussed, with suggestions for community initiatives to address pain, alcohol, and opioid risk management to prevent elder abuse.

### ELDER ABUSE AMID THE OPIOID EPIDEMIC: APS CASES IN RURAL AMERICA

Pamela B. Teaster,[1] Karen A. Roberto,[2] and Jyoti Savla[2], *1. Virginia Tech, Blacksburg, Virginia, United States, 2. Center for Gerontology, Virginia Tech, BLACKSBURG, Virginia, United States*

Older adults are hidden victims of the opioid crises, suffering abuse at the hands of those who seek resources to support their addiction. Using APS data from 2015-2017 provided by the Kentucky Department for Community-Based Services, we used a logistic regression model with robust standard errors to examine whether substantiated cases of elder abuse were associated with opioid misuse by perpetrators. Overall, 9% of the 462 substantiated cases over the three-year period involved perpetrators were substance users. The percentage of these cases rose from 5% in 2015 to 13% in 2016 before dropping to 7% of elder abuse cases in 2017. Opioid use was most prevalent among perpetrators of financial abuse of older adults with cognitive and/or physical care needs. The current study offers a first look at empirical linkages between opioid misuse and elder abuse and revealed consistencies across cases that call for further investigation.

### ELDER ABUSE AND THE OPIOID CRISIS: PERPETRATORS WHO ARE SUBSTANCE USERS

Karen A. Roberto,[1] and Pamela B. Teaster[2], *1. Virginia Tech, Blacksburg, Virginia, United States, 2. Center for Gerontology, Virginia Tech, BLACKSBURG, Virginia, United States*

Substance abuse, particularly the diversion/abuse of prescription drugs along with illicit opioid deviates by alleged perpetrators has been identified as is a risk factor for elder abuse. The purpose of this study was to characterize cases of elder abuse substantiated by APS in which the perpetrator used opioids and related substances. Guided by the Contextual Theory of Elder Abuse, we conducted a within-case/across-cases thematic analysis of Kentucky APS caseworkers' notes on 40 substantiated cases of elder abuse. Financial exploitation was the most commonly identified type of abused associated with perpetrators who abuse opioids. Findings revealed that most cases of elder abuse occurred when the perpetrators' substance abuse intersected with employment status, complex family relationships, and a history of altercations with the law. Findings provide new insights into a more elaborate conception of the ways in which the opioid epidemic is contributing to the perpetration of elder abuse today.

### SESSION 3520 (PAPER)

### EMPLOYMENT IN LATER LIFE

### EXCLUSION SPOTTER: APPLYING ADVANCES IN AI TO IDENTIFY AGEISM IN ONLINE JOB POSTING

Nicola Palmarini,[1] Lee Martie,[1]
Mattie F. Wasiak,[2] and Gaoyuan Zhang[1], *1. IBM Research, Cambridge, Massachusetts, United States, 2. Massachusetts Institute of Technology, Cambridge, Massachusetts, United States*

The landscape in which employers and candidates interact is changing as more job adverts are pushed online. Employment platforms (e.g., Indeed and LinkedIn) are now among the primary mechanisms for job posting, job search, and initial negotiations. Through such job platforms, a single job advert can now reach millions of people around the world. This exposure of a job advert has obvious benefits for the employer, but this exposure also has the power to alienate and exclude large portions of society. In particular, the word choice of a single job advert can, perhaps unintentionally, exclude thousands of people by their personal traits (e.g., gender or race). Age is a particular trait that garners more attention as ageism is often cited in the literature as going overlooked, not understood, and generally escaping social awareness. To begin tackling this problem, with the purpose of supporting older adults and enabling their contribution to society, we applied advances in AI to create a tool, called Exclusion Spotter, that gives feedback to recruiters and employers on which words in their advert are possibly excluding people by age. We applied Exclusion Spotter to 3660 job adverts, clustered by 372 job titles. We found a significant difference (p=.02) in the number of age-related words for engineering related positions versus all other job titles. Among 47 engineering related titles we matched 47.37 age related words per title and 2.8 per advert. Among the other 325 titles we matched 24.37 age related words per title and 2.1 per advert.

### HOW CAN IMPROVING EMPLOYMENT PROSPECTS OF UNEMPLOYED OLDER WORKERS BE EXPLAINED?

Per H. Jensen[1], *1. Center for Comparative Welfare Studies, Aalborg University, Nordjylland, Denmark*

Until recently employment prospects of older workers have been rather poor. In recent years, however, the duration of unemployment among older workers has diminished, meaning that older workers are more frequently hired by employers. Changing employment prospects of older workers are no doubt framed by a decrease in overall unemployment. The aim of this paper, however, is to shed light on the emergence of new inclusive mechanisms by answering three inter-related research questions: (1) where are the job openings for unemployed seniors? (2) How have unemployed seniors been recruited? (3) Why do companies hire older workers. Using Denmark as a test case findings show that job openings are rather frequent in branches with tight labor markets and that characteristics of companies and management are important; for instance, the older the average age of management the higher the inclination to hire unemployed older workers. Findings furthermore show that mouth-to-mouth recommendation and the internet are the most used recruitment channels, while the public employment service is less used. Finally, findings show that qualification, stability and experience are the most reported reasons as to why employers hire unemployed older workers are. The paper is based on a survey with 2,525 valid respondents, response rate: 25.

### ORGANIZATIONAL PRACTICES FOR THE AGING WORKFORCE: DEVELOPMENT AND VALIDATION OF THE LATER-LIFE WORK INDEX

Max R. Wilckens,[1] Anne M. Wöhrmann,[1]
Jürgen Deller,[1] and Mo Wang[2], *1. Leuphana University Lüneburg, Lüneburg, Germany, 2. University of Florida, Gainesville, Florida, United States*

Most developed countries face an ageing workforce. Extended working lives require organizations to employ older individuals successfully. It is, however, widely untapped, which organizational practices drive successful employment of an older and age-diverse workforce, because adequate measure are missing. We hence develop the Later Life Work Index (LLWI) as a multi-faceted measure for researchers and practitioners, combining the detailed level of assessment relevant in practice with thorough conceptual coverage. We build on an empirically derived taxonomy of organizational practices developed by Wöhrmann et al. (2018). Proposed taxonomy is based on qualitative expert interviews and consists of nine dimensions covering age-inclusive organizational climate and leadership, as well as practices regarding work design, health management, individual development, knowledge management, transition to retirement, continued employment options, and health and retirement coverage. Within a first study, we developed an inventory to operationalize the intentionally broad LLWI construct. Items were iteratively developed and pretested with 30-42 German human resource managers. The final inventory consists of 100 Likert scaled, sufficiently reliable items. Within a second study among 600 managers and older workers in Germany, we confirmed the factor structure proposed by the LLWI, and ensured construct validity regarding similar scales (convergent validity) and individual level health and motivation outcomes (criterion validity). Discriminant validity is shown among the index dimensions, and regarding positive and negative affect. This paper enhances the understanding and quantitative assessment of organizational practices for later life work. We further plan to reduce the number of items to increase practicability of the measure.

### SELF-EFFICACY AND TEAM LEADER EQUITY MATTER: A STUDY OF ACTIVE AGING AT WORK

Mi Sun Choi,[1] Holly Dabelko-Schoeny,[1] Mo Yee Lee,[1] and Alicia Bunger[1], *1. The Ohio State University, Columbus, Ohio, United States*

Many older Americans have decided to remain in the labor market beyond the traditional retirement age, suggesting the need for companies to consider human resource initiatives to retain and support the aging workforce. Applying active aging concepts, which emphasize older adults' active roles through participation in social and economic activities for healthy later life, to the workplace could be helpful for developing programs that enhance the health, well-being, and work outcomes of older workers. Despite the expected benefits of active aging at work for older workers' overall well-being, little research has been conducted on what personal and team factors impact on outcomes of active aging at work; what mechanism exists in the links between factors and outcomes in the contemporary workplace. The current study tested the validity of an active aging framework using the Age and Generations Study data. We analyzed responses of 508 American workers aged 50 and older using structural equation modeling. Results showed that perceived self-efficacy was a strong predictor of engagement, mental health, and performance, whereas perceived leader equity predicted only engagement. Also, work engagement was powerful mechanism for promoting older adults' mental health; engagement mediated the relationship between perceived self-efficacy and the relationship between leader equity and mental health. The findings highlight how important it is for employers to invest in human capital, suggesting human resource programs should focus on strategies that target older adults' engagement through tailored self-efficacy programs and diversity leadership training programs. Such attempts would contribute to the well-being of older workers.

### SESSION 3525 (SYMPOSIUM)

### GENDER-SPECIFIC APPROACHES TO HEALTHY AGING WITH HIV

Chair: Tonya N. Taylor, *SUNY Downstate Medical Center, Brooklyn, New York, United States*
Discussant: Ruth Finkelstein, *The Brookdale Center on Aging, Hunter College, New York City, United States*

Older adults with HIV (OAH) evidence a significant burden of disease, characterized by high frequencies of non-HIV related comorbidities that result in multimorbidity and polypharmacy decades earlier than non-HIV infected older adults. Commonly observed comorbidities among OAH 50 years and older include cardiovascular disease, hypertension, diabetes, osteoporosis, and cancers. Geriatric conditions such as frailty, functional impairment, chronic inflammation, and cognitive dysfunction are also prevalent. Poverty, unemployment, housing, and food insecurity, as well as stigma-driven discrimination, persistent social isolation and high rates of depression, can make health-related challenges associated with aging overwhelming, resulting in a diminished capacity for self-care. Older men and women with HIV; however, are aging differently due to gender-specific differences in comorbidity profiles, polypharmacy burden, social networks, and intersectional stigmas (HIV/AIDS, ageism, racism, sexism, classism, homophobia, and transphobia). This symposium explores the impact of gender on healthy aging among OAH. Dr. Brennan-Ing and colleagues describe specific strategies for achieving healthy aging among female OAH in NYC and Oakland, CA. Dr. Rubtsova and colleagues explore multiple meanings of "successful aging" among older women in Atlanta and Brooklyn, and the role of resilience that empowers them as long-term HIV survivors. Dr. Taylor qualitatively examines the impact of biopsychosocial factors on notions of "Healthy HIV Aging" among older gay, bisexual and heterosexual men with HIV. Dr. Ruth Finkelstein will discuss the public health implications for developing equitable and gender-specific and intersectional HIV prevention and wellness interventions and suggest future areas of inquiry to improve HIV outcomes across the care continuum.

### INTERSECTIONAL DIFFERENCES IN HEALTHY AGING WITH HIV

Tonya N. Taylor[1], *1. SUNY Downstate Medical Center, Brooklyn, New York, United States*

Intersecting stigmatized social identities (i.e., old, Black, gay, HIV+) and structural forms of privilege and oppression (ageism, racism, homophobia, and HIV stigma) can contribute to poor psychological wellbeing and clinical outcomes among older men with HIV (OMH). Using data from 6 focus groups and 15 interviews with 45 gay, bisexual and heterosexual OMH in Brooklyn, NY and inductive thematic analysis methods, we explored the impact of heteronormative gender roles and ideologies associated with masculinity on healthy aging with HIV. We found that changing physical and sexual function, appearance and growing financial expectations created threats to masculinity, fueling fears of perceived weakness, internalized feelings of shame, depression, anticipated loss of social status (partner loss), and loss of independence and autonomy. These findings suggest that normative gender beliefs, a key social determinant of men's health, aging and intersectional stigma combined undermine psychosocial support and wellbeing and self-care needed to achieve healthy aging.

### OLDER WOMEN WITH HIV: STRATEGIES FOR HEALTHY AGING

Mark Brennan-Ing,[1] Liz Seidel,[2] Rebecca Erenrich,[3] Stephen E. Karpiak,[2] Ryann N. Freeman,[4] Megan Johnson Shen,[5] Chelsie O. Burchett,[5] and Eugenia L. Siegler[5], *1. Brookdale Center for Healthy Aging, Hunter College, The City University of New York, New York, New York, United States, 2. ACRIA Centers at GMHC, new York, New York, United States, 3. ACRIA Centers at GMHC, San Francisco, California, United States, 4. Hennepin County Human Services & Public Health Department, Minneapolis, Minnesota, United States, 5. Weill Cornell Medical Center, new York, New York, United States*

Women over 50 represent one-quarter of U.S. older adults with HIV, but we know little about how these women face the challenges of aging. As part of a larger study, we conducted two focus groups with racially diverse older women with HIV in New York City (n = 9) and Oakland, CA (n=11). Discussions concerned strategies for healthy aging. Transcripts were analyzed using inductive thematic methods. Women in both groups shared that they had close connections to family members and the importance of family support for motivating them to take care of their health. Some took proactive steps to stay healthy through nutrition and exercise, although the cost of gym memberships was a barrier. Many were lonely and needed socialization opportunities. Some suggested exercise classes could help to both maintain health and bolster social connections. Implications of these findings for developing programs for older women with HIV will be discussed.

### HIV MADE ME STRONGER: CONCEPTIONS OF SUCCESSFUL AGING AMONG OLDER WOMEN LIVING WITH HIV

Anna A. Rubtsova,[1] Tonya Taylor,[2] Gina Wingood,[3] Igho Ofotokun,[4] Deborah Gustafson,[2] and Marcia Holstad[4], *1. Emory University Rollins School of Public Health, Atlanta, Georgia, United States, 2. SUNY Downstate Medical Center, Brooklyn, New York, United States, 3. Mailman School of Public Health, Manhattan, New York, United States, 4. Emory University, Atlanta, Georgia, United States*

Little is known about successful aging (SA) among older (50+) women living with HIV (OWLH). Therefore, the purpose of this qualitative study was to examine subjective understandings of SA among OWLH. Participants were OWLH enrolled in the Women's Interagency HIV Study (WIHS) and those not enrolled in WIHS: 8 in Atlanta, GA (3 WIHS and 5 non-WIHS) and 9 in Brooklyn, NY (6 WIHS and 3 non-WIHS). Semi-structured interviews ranged from 30 to 120 minutes. Several themes emerged in participants' definitions of SA, e.g. self-care, taking HIV medications, and being resilient ("HIV made me stronger"). Both WIHS and non-WIHS participants emphasized life course perspective in their definitions of SA -- women viewed their aging successful as a more stable phase of life in contrast to hardships they experienced while being younger (e.g., drug use, incarceration). Data collection efforts are ongoing and will allow further characterization of SA among this population.

### SESSION 3530 (SYMPOSIUM)

### GRECCS AS A NETWORK TO ENHANCE THE VA PRIMARY CARE WORKFORCE

Chair: Josea Kramer, *VA Greater Los Angeles Healthcare System GRECC, Los Angeles, California, United States*
Discussant: Marianne Shaughnessy, *Veterans Health Administration Geriatrics and Extended Care Services, Office Geriatrics Programs, Washington, District of Columbia, United States*

The VA Geriatric Scholars Program is a workforce development program to integrate geriatrics into primary care in all VA clinics. The program has increased career satisfaction among clinicians, improved quality of care for aging Veterans, changed provider prescribing behaviors and improved patient outcomes. The core curriculum is an intensive course in geriatrics, an intensive workshop in quality improvement (QI) and a micro QI project to demonstrate application of new knowledge to local needs of patients and clinic processes; this is a longitudinal program and alumni continue to develop skills over time through a series of elective activities that tailor learning to self-identified gaps in career development and training. The program is a collaboration among the Geriatric Research Education and Clinical Centers (GRECC), centers of excellence that have been leveraged to create a network of expertise and creativity to develop the core and elective educational activities. This symposium focuses on recent outcomes of three educational components that improve quality of care for older Veterans. The Palo Alto GRECC developed an intensive course in gerontological psychology to enhance the skills and competencies of psychologists leading to greater job effectiveness. The Bronx GRECC developed an interdisciplinary training program for all clinical and non-clinical staff in rural clinics, leading to improved recognition of functional and safety concerns among older veterans. The Tennessee Valley GRECC developed the core QI component and many of these micro-QI projects have been sustained, spread or recognized by VA as Promising Practices.

### OVERVIEW: THE VA GERIATRIC SCHOLARS PROGRAM FOR PRIMARY CARE WORKFORCE DEVELOPMENT

Josea Kramer,[1] Joe Douglas,[2] Shawn Clarke,[3] and Luis Melendez[4], *1. VA Greater Los Angeles Healthcare System GRECC, Los Angeles, California, United States, 2. GLA GRECC, Golden, Colorado, United States, 3. VA*

*Greater Los Angeles Healthcare System GRECC, Sepulveda, California, United States, 4. VA Greater Los Angeles Healthcare System, Sepulveda, California, United States*

The VA has invested in developing the skills of its primary care workforce through the longitudinal Geriatric Scholars Program. The program consists of core components --- intensive course in geriatrics, intensive workshop in quality improvement (QI) and initiation of a micro QI projects in the Scholar's clinic; electives allow learners to tailor the program to self-identified gaps in knowledge, skills and competencies. The program has demonstrated direct impacts of continuing education through a workforce development process that enhances skills and competencies at a pace and selection that meets clinicians' self-identified gaps in training. Now in its 11th year, the program has been shown to increase career satisfaction and job retention, standardize provider behaviors, improve clinical decision-making and reduce dispensing of potentially inappropriate medications. This symposium further explores the impact of the program on individual clinicians and on clinical teams.

### AN EVALUATION OF THE VA RURAL INTERDISCIPLINARY TEAM TRAINING PROGRAM: OUTCOMES AND IMPLICATIONS

Judith L. Howe,[1] Josea Kramer,[2] Nancy Weintraub,[2] Eve Gottesman,[3] Regina Richter,[4] and Andrew Bean[3], *1. James J Peters VA Medical Center, GRECC, Bronx, New York, United States, 2. Greater Los Angeles Health Care System GRECC, North Hills, California, United States, 3. James J. Peters GRECC, Bronx, New York, United States, 4. David Geffen School of Medicine, UCLA, North Hills, California, United States*

The rural interdisciplinary team training (RITT) program has provided in-person training for almost 2000 VA providers and staff at 125 rural clinics since 2011. The multimodal workshop, accredited for 6.5 hours for a number of disciplines, focuses on the recognition of common issues facing older Veterans, red flags prompting further assessment, how to administer screening instruments and team-based approaches for improving patient outcomes. Participants develop an improvement action plan improvement project based on common challenges faced by clinic patients. A program evaluation found an increase in geriatrics knowledge and a modest improvement in teamwork after the education program. It also found that participants self-identified an enhanced ability on average to use red flags after the 6.5 hour training in areas such as polypharmacy, falls and caregiver stress. The action plans were often not implemented at follow-up. The evaluation results will be discussed, as well as challenges, limitations, and implications.

### THE EFFECT OF GEROPSYCHOLOGY TRAINING ON JOB EFFECTIVENESS AMONG VA PSYCHOLOGISTS

Ashley Scales,[1] Rachel Rodriguez,[2] Christine Gould,[1] Jeffrey Gregg,[2] Joung Huh,[3] and Josea Kramer[4], *1. VA Palo Alto Health Care System, Palo Alto, California, United States, 2. Durham VA Health Care System, Durham, North Carolina, United States, 3. San Francisco VA Health Care System, San Francisco, California, United States, 4. VA Greater Los Angeles Health Care System, Sepulveda, California, United States*

Providing training to assist employees with excelling in their job role may increase their job effectiveness, which then translates to improvements in an organizations' performance measures. In 2018, the Geriatric Scholars Psychology Program measured whether a multi-day course influenced the Psychologists' perceived job effectiveness using modified questions from Godat and Brigham's Reaction Measure. Ninety-two percent agreed the training assisted them with identifying and overcoming obstacles; helped them set goals to increase problem solving at their designated facilities; boosted their confidence in fulfilling their job duties; and the training should be made available to other employees who provide care to older Veterans on a regular basis. Findings from the 3-month follow-up assessment demonstrated that the positive effect on job effectiveness was sustained. Discussion will explore aspects of the course that may be key in improving perceived effectiveness and consider novel approaches to enhance this outcome.

### SPREAD OF QUALITY IMPROVEMENT PROJECTS IMPLEMENTED IN A GERIATRICS WORKFORCE DEVELOPMENT PROGRAM

Claire E. OHanlon,[1] Claire E. O'Hanlon,[2] Debra Saliba,[3] Joseph Douglas,[4] Michael N. Mitchell,[5] Carol Callaway-Lane,[6] and Josea Kramer[7], *1. VA Greater Los Angeles Healthcare System, Los Angeles, California, United States, 2. Veterans Affairs Center for the Study of Healthcare Innovation, Implementation & Policy, Los Angeles, California, United States, 3. Veterans Affairs Center for the Study of Healthcare Innovation, Implementation & Policy University of California; Los Angeles Borun Center for Gerontological Research, Los Angeles, California, United States, 4. Geriatric Research Education Clinical Center, VA Greater Los Angeles Healthcare System (contractor), North Hills, California, United States, 5. Veterans Affairs Center for the Study of Healthcare Innovation, Implementation & Policy, North Hills, California, United States, 6. Veterans Affairs Tennessee Valley Healthcare System, Geriatric Research Education Clinical Center, Nashville, Tennessee, United States, 7. Veterans Affairs Greater Los Angeles Healthcare System Geriatric Research Education Clinical Center Division of Geriatric Medicine, David Geffen School of Medicine at UCLA, North Hills, California, United States*

This project characterizes spread of quality improvement (QI) projects initiated in the U.S. Department of Veterans Affairs (VA) Geriatric Scholars Program (GSP) workforce development program. This mixed methods study analyzes a recent cross-sectional survey of GSP participants and program-level data on participant characteristics and QI project topics. We surveyed 578 scholars who had completed all program requirements to that point, and still worked for VA; 207 (35%) responded. The majority of respondents who had been in the program for at least six months (70%) reported sustainment of their QI project beyond initial implementation and nearly a third (30.4%) reported any spread beyond their own care team. QI project topics spanned many domains and percent of projects reporting spread varied across domains from 0% to 67%. A workforce development capstone activity in which participants demonstrate substantive and methodological competency can foster bottom-up QI activities in a large, diverse health care system.

### SESSION 3535 (PAPER)

### HEALTH CARE SERVICES AND INTERVENTIONS

### DAYS AND NIGHTS OF DYADS: EXAMINING BEHAVIORAL INTERDEPENDENCE IN CO-RESIDING COUPLES USING ACTIGRAPHY

Lyndsey M. Miller,[1] Zachary Beattie,[1] Nora Mattek,[1] Nicole Sharma,[1] Katherine Wild,[1] and Jeffrey Kaye[1], *1. Oregon Health & Science University, Portland, Oregon, United States*

Physical activity and sleep are behaviors that contribute to overall health, and are notoriously challenging to improve. For individuals co-residing as a couple, there is growing recognition of the importance of the interdependence between individuals' behaviors (e.g. the physical activity and sleep habits of one's partner has an influence on one's own sleep and physical activity). Yet, studies examining this phenomenon have primarily been conducted among young adults and have either used self-report or episodic measures. The purpose of this study was to examine physical activity and sleep as interdependent behaviors within older adult couples. Using intensive longitudinal data (continuously collected over 35 days) from actigraph watches worn by 54 dyads (mean age in years 72.4 ± 7.1), we examined daily step counts and nightly sleep duration (total of 3780 observations) as a dyadic daily process. Results from multilevel process models for distinguishable dyads (Mplus version 8.2: Muthén & Muthén, 1998-2018) indicated that step count and sleep duration were interdependent processes at the within-dyad level (ps < 0.001). At the between-dyad level, on days with a typical number of steps, on average dyad members' sleep duration was 7.55 hours [95% CI 4.27 - 10.79 hours], and across dyads, partners' sleep duration and step counts were also interdependent processes (ps < 0.05). In order to improve the physical activity and sleep of older adults who co-reside with a partner, these results suggest that targeting the dyad, rather than the individual, may be necessary.

### FEASIBILITY OF SCREENING AND MANAGING CAREGIVER BURDEN AND DEPRESSIVE SYMPTOMS DURING PATIENTS' CLINIC VISITS

Ranak Trivedi,[1] Rashmi Risbud,[2] Manali Patel,[3] Steven Asch,[2] and Karl Lorenz[2], *1. Center for Innovation to Implementation, VA Palo Alto Health Care System, Menlo Park, California, United States, 2. VA Palo Alto Health Care System, Menlo Park, California, United States, 3. Stanford University, Stanford, California, United States*

Half of cancer caregivers experience depression, caregiver burden, or stress, yet less than a third have discussed their needs with anyone. Identifying this vulnerable population is challenging since caregivers only interact with the healthcare system in service of the patients. Our objectives were: 1) To test the feasibility of screening cancer caregivers for burden and depressive symptoms during patients' clinic visits; and 2) To test the feasibility of a brief counseling session for caregivers who screened positive for either. Caregivers of patients with head and neck cancers were recruited from cancer clinic waiting rooms at Palo Alto VA and Stanford. Caregivers completed the PHQ-9 (depressive symptoms), and Zarit Burden Inventory-Short Form (caregiver burden). Participants screening positive for burden (>16) and/or depressive symptoms (>9) were provided psychoeducational resources and the choice to attend 1 brief counseling session with a clinical psychologist. Of the 50 participants who completed the surveys, 36 (72%) were women and 30 (60%) were significant others. Mean scores for depressive symptoms and caregiver burden were 6.29±5.01 and 11.02±8.62, respectively. Twenty participants screened positive for depressive symptoms (n=9) or caregiver burden (n=11); 3 screened positive for both. Of those who screened positive, only 4 indicated an interest in counseling. Main reason for refusal was lack of time, or that they were already receiving mental health care. Screening caregivers at patient visits is feasible and convenient. However, connecting those in need to mental health resources may be more challenging.

### MENTAL AND PHYSICAL HEALTH EFFECTS OF EVERYDAY DISCRIMINATION TRAJECTORIES

Collin Mueller,[1] and Carlos Tavares[2], *1. Duke University, Durham, North Carolina, United States, 2. Lafayette College, Easton, Pennsylvania, United States*

How do perceived discrimination trajectories impact health of Black, Latinx, and White adults ages 50 and older? Few researchers have sought to discern between the health effects of perceived discrimination measured cross-sectionally versus longitudinally. We aim to address this gap by leveraging newly available 3-wave panel data. We analyze 3 waves of data from the Health and Retirement Study (HRS) (n=2830 individuals ranging in age from 50 to 84, observed from 2008-2016). We first estimate group-based trajectories of everyday discrimination using finite mixture models. Second, we use multinomial logistic regression to estimate the likelihood of group membership in one of the identified trajectories of everyday discrimination based on psychosocial and demographic covariates. Third, we estimate the effects of group membership in one of these trajectories on four health outcomes: functional mobility, self-rated health, a count of chronic health conditions, and depressive symptoms. We identify three latent group-based trajectories of perceived discrimination. Black Americans are more likely than Latinx or White Americans to be members of latent trajectories characterized by higher levels of perceived discrimination over time. Membership in higher-discrimination groups over time is associated with worse physical and mental health profiles. Including measures of trajectory membership fully mediates the relationship between cross-sectional perceived discrimination and chronic conditions. Levels of discrimination decline across cohorts, suggesting that younger individuals perceive higher levels of everyday discrimination than their older counterparts.

### ORGANIZATIONAL READINESS TO CHANGE AND NURSING HOME SAFETY: RESULTS FROM A NATIONAL SURVEY

Christine W. Hartmann,[1] Emma Quach,[2] Shibei Zhao,[1] Valerie Clark,[1] Sarah McDannold,[1] Pengsheng Ni,[3] and Lewis Kazis[3], *1. Bedford VA Medical Center, Bedford,*

*Massachusetts, United States, 2. VA Center for Healthcare Organization and Implementation Research, Bedford, Massachusetts, United States, 3. Boston University School of Public Health, Boston, Massachusetts, United States*

In nursing homes, safety climate (employee attitudes and beliefs about safety) is a key contributing factor to safety and a potential leverage point for improvement. Yet relatively little is known about how contextual factors such as organizational readiness to change affect safety climate. We sampled employees from 56 Department of Veterans Affairs (VA) Community Living Centers (CLCs—nursing homes) and conducted an anonymous, cross-sectional web-based survey using the previously validated CLC Employee Survey of Attitudes about Resident Safety (CESARS) and the Organizational Readiness to Change Assessment instrument. From hierarchical mixed random effects regression models, we calculated intraclass correlation coefficients (ICC) as the proportion of CLC-level variance over the sum of CLC-level plus residual variance. Each of the CESARS' 7 safety climate domains was a dependent variable in separate models; employee- and CLC-level factors were independent variables. The survey had a 26% response rate; 1,397 respondents. Mean ORCA scores (1-5 scale, higher better) was 3.3. We began with models containing only employee-level variables. ICC values ranged from 2.34% to 9.85%, suggesting substantial variation in CESARS outcomes. As we dropped insignificant variables and added CLC-level variables to the models, the ICC decreased over 2% in six models, suggesting organizational-level variables accounted for substantial variability. The only independent variable with a significant effect in all 7 models was organizational-level: organizational readiness to change. Unlike many other organizational-level variables, organizational readiness to change is potentially amenable to low-cost interventions such as communication and teamwork interventions, providing viable opportunities to efficiently improve nursing home care.

### STORIES OF TRAUMA AND RECONCILIATION OF WORLD WAR II VETERANS

Hanna K. Ulatowska,[1] Tricia Santos,[1] Diane Walsh,[1] Jilliane Lagus,[1] Mitchell Pruett,[1] and Sara Aguilar[1], *1. The University of Texas at Dallas, Dallas, United States*

The present qualitative study examined the reconciliation of trauma experienced by 55 World War II veterans (22 aeronautical crew members, 27 non-pilot combatants, and 6 veterans with dementia) demonstrated via testimonial language within a semi-structured interview. The research team considered themes of language coherence as they relate to veteran experiences of trauma and reconciliation. Trauma literature documents the importance of personal narratives in both identifying and reconciling traumatic experiences. This study examined morals and values of participants, traumatic experiences either lived or witnessed, and reconciliation of trauma as demonstrated by the coherence of participants' linguistic and paralinguistic communication. Linguistic analysis included the use of evaluative and emotional language; linguistic devices such as crowding, topic maintenance, and humor; and lessons learned from trauma and the reconciliation process. Prosody was analyzed as a paralinguistic indicator of trauma and reconciliation using audio recordings of semi-structured interviews. The primary findings revealed that highly coherent language is present among participants with distinct content when comparing episodes from youth and reflections of experience in old age. The unique differences demonstrated overall strength of veterans' narrative identity throughout their lives. Strength of identity and coherence of language indicated adequate reconciliation of traumatic events. Reconciliation of trauma was also evident in veterans' participation in the study and generative behavior described in testimonial language.

### SESSION 3540 (SYMPOSIUM)

### IMMUNITY AND AGING—THE HUMAN FACE

Chair: Bérénice A. Benayoun, *University of Southern California, Leonard Davis School of Gerontology, Los Angeles, California, United States*

### AGE INDUCES AN EMERGENCE FROM MELANOMA DORMANCY

Mitchell Fane[1], *1. The Wistar Institute, Philadelphia, Pennsylvania, United States*

Melanoma cell dormancy is regulated by intrinsic cues that maintain a slow-cycling state in cells and by extrinsic interactions with stromal and immune components of the microenvironment. A key factor is that a significant lapse of time occurs between initial diagnosis, and metastatic recurrence of dormant cells. During this time, the organism ages and age is a poor prognostic indicator for cancer. We have found that melanoma cells form lung metastases more efficiently in aged mice and remain dormant in young mice. Analysis of the immune-microenvironment of the lung reveals that healthy young and aged mice have little difference in infiltration of immune subpopulations; however aged tumor bearing mice have significantly increased immunosuppressive MDSCs and Tregs and decreased CD4+ and CD8+ t-cells in the lung compared with young mice. Our data indicates that aging induces an immunosuppressive lung microenvironment which allows immune-evasion and an emergence from melanoma dormancy.

### SEX-DIMORPHISM IN THE GENOMIC REGULATION OF AGING

Bérénice A. Benayoun,[1] Ryan A. Lu,[2] and Nirmal K. Sampathkumar[2], *1. University of Southern California, Leonard Davis School of Gerontology, Los Angeles, California, United States, 2. University of Southern California, Los Angeles, California, United States*

The current cohort of human supercentenarians reveals a surprising predictor for achieving such an exceptional longevity: being female. Indeed, out of 34 living supercentenarians, 33 are women. We obtained samples from 4 and 20 months old female and male mice. Our data indicates that cytokine levels are differentially regulated with age in males vs. females, with pro-inflammatory cytokines specifically upregulated in the serum of old males, but not females. Because of the central role of macrophages in inflammation and their infiltration in tissues with age, we have generated RNA-seq from purified macrophages of aging animals. Female macrophages displayed ~7-20-fold more transcriptional remodeling with aging than males. Pathways specifically downregulated in females with aging included lysosome, inflammation and phagolysosome. Consistently, our data shows that aged female, but not male macrophages, display decreased phagocytic efficiency. Our results support the notion that there are differences in aging trajectories in female vs. male mice.

### DYNAMICS OF HUMAN MUCOSAL-ASSOCIATED INVARIANT T CELL REPERTOIRES ACROSS THE HUMAN LIFE SPAN

Liyen Loh,[1] Martha Lappas,[2] Jane Crowe,[3] Paul G. Thomas,[4] Sidonia Eckle,[2] Dale I. Godfrey,[2] Katherine Kedzierska,[2] and Jeremy C. Crawford[4],
*1. Department of Microbiology and Immunology, The University of Melbourne at the Peter Doherty Institute or Infection and Immunity, Melbourne, Australia,*
*2. Department of Microbiology and Immunology, University of Melbourne, at the Peter Doherty Institute for Infection and Immunity, Melbourne, Victoria, Australia,*
*3. Deepdene Surgery, Deepdene, Victoria, Australia,*
*4. Department of Immunology, St Jude Children's Research Hospital, Memphis, Tennessee, United States*

Mucosal-associated invariant T (MAIT) cells are innate-like lymphocytes and are important for immune responses against bacterial and viral infections. While MAIT cells are known to undergo marked numerical changes with age in humans, our understanding of how these cells alter during these different phases across the human lifespan is largely unknown. Here we investigated MAIT cells from umbilical cord, children, young-adults and elderly. Functional analyses across 18-90 y/o adults showed that their MR1-dependent polyfunctionality was robust throughout old age. Strikingly, elderly MAIT cells displayed upregulated basal inflammatory cytokines, which were reduced to the level of young-adult MAIT cells in the absence of the aged environment. T cell receptor αβ analyses of MAIT cells across the human lifespan showed narrowing with age and large clonal TCRαβ expansions in elderly. These data suggest that MAIT cells in the elderly display remarkable plasticity, highlighting MAIT cells as key players in aged immune responses.

### A LONGEVITY PROMOTING FACTOR THAT SUPPRESSES IMMUNITY AND HEALTHSPAN

Arjumand Ghazi,[1] Francis Amrit,[1] Nikki Naim,[1] Ramesh Ratnappan,[1] and Julia Loose,[1] Guoqiang Wang,[2] Monica Driscoll,[2] and Judith Yanowitz[1], *1. University of Pittsburgh School of Medicine, Pittsburgh, Pennsylvania, United States, 2. Rutgers University, Piscataway, New Jersey, United States*

A positive correlation exists between stress resistance and longevity, but emerging evidence suggests that lifespan and stress endurance are physiologically distinct. A major challenge in aging biology has been identifying factors that play distinct roles in these closely coupled processes because genes that promote longevity often enhance stress resistance. Here, we demonstrate that TCER-1, the Caenorhabditis elegans homolog of the human transcription elongation and splicing factor, TCERG1, has discrete and opposite effects on lifespan and stress resistance. We previously identified tcer-1 as a gene that promotes longevity in germline-less C. elegans and reproductive fitness in wild-type animals. Surprisingly, tcer-1 mutants exhibited exceptional resistance against multiple biotic and abiotic stressors, including infection by the human opportunistic pathogen Pseudomonas aeruginosa. Conversely, TCER-1 overexpression increased susceptibility to infection. TCER-1 acted cell non-autonomously to both enhance longevity and repress immunity. Interestingly, TCER-1 inhibited immunity only during the fertile stages of life and not in post-reproductive adults. Elevating its levels ameliorated the fertility loss that follows infection, suggesting that TCER-1 may repress immunity to augment fecundity. Mechanistically, TCER-1 acts through the inhibition of the conserved kinase, PMK-1, as well as through repression of PMK-1-independent, novel antibacterial factors critical for innate immunity. Overall, our data establish key roles for TCER-1 in coordinating immunity, longevity and fertility, and reveal the molecular mechanisms that distinguish length of life from functional aspects of aging.

### SESSION 3545 (SYMPOSIUM)

### IMPLEMENTING THE 4MS IN PRIMARY CARE: BUILDING AN AGE-FRIENDLY HEALTH SYSTEM

Chair: Ellen Flaherty, *Dartmouth-Hitchcock Medical Center, Lebanon, New Hampshire, United States*
Discussant: Terry Fulmer, *The John A. Hartford Foundation, New York, New York, United States*

The Age Friendly Health Systems initiative is a culture change movement funded by the John A. Hartford Foundation in collaboration with the Institute for Health Care Improvement. Transforming clinical training environments into integrated geriatrics and primary care systems to become Age-Friendly Health Systems must incorporate the principles of value-based care and alternative-payment models. This symposium will discuss how the implementation of the Geriatric Interprofessional Team Transformation in Primary Care (GITT-PC) model and the Reducing Avoidable Facility Transfer Model (RAFT) in primary care will improve patient outcomes focused on the 4M's of the Age Friendly Health System. The success of the GITT-PC model focuses on 4 Medicare reimbursable services including the Annual Wellness Visit, Transitional Care Management, Chronic Care Management and Advance Care Planning. The RAFT model focuses on What Matters Most to residents of long term care facilities and reduces ED visits and hospital transfers through elicitation of goals of care and 24 hour virtual support from an interprofessional geriatric team.

### REDUCING AVOIDABLE FACILITY TRANSFERS: THE RAFT MODEL

Daniel Stadler[1], *1. Dartmouth-Hitchcock Medical Center, Lebanon, New Hampshire, United States*

Reducing Avoidable Facility Transfers (RAFT) is a Dartmouth-developed program that identifies and honors "what matters most" to patients residing in skilled nursing facilities in a value-based, sustainable way. RAFT aims to reduce avoidable facility transfers of older adults from long-term care and post-acute care facilities to emergency departments (ED). Key components of RAFT presently include (1) systematically eliciting goals of care for all skilled nursing facility residents, (2) translating these goals into orders using the Physician Orders for Life-Sustaining Treatment form, (3) documenting patient wishes about hospitalization, and (4) ensuring that these wishes inform decision-making during acute crises. Data from a pilot program, begun in 2016 with three rural skilled nursing facilities in collaboration with the Dartmouth-Hitchcock Medical Center geriatric practice, showed a 35% reduction in monthly ED transfers, a 30.5% reduction in monthly hospitalizations, and a 50.7% reduction in monthly ED and hospitalization-related charges.

### THE 4MS IN PRIMARY CARE: THE GERIATRIC INTERPROFESSIONAL TEAM TRANSFORMATION IN PRIMARY CARE (GITT-PC) MODEL

Daniel Moran[1], *1. Dartmouth-Hitchcock Medical Center, Lebanon, New Hampshire, United States*

GITT-PC is a Dartmouth GWEP developed program that builds on lessons learned from the original GITT program funded by the John A. Hartford Foundation in the 1990s. GITT-PC improves delivery of healthcare to older adults in primary care by training healthcare professionals in team functioning, rapid cycle QI, and evidence based geriatric practice. The program capitalizes on the role of nursing and other healthcare disciplines. To maximize sustainability, it focuses on Medicare-reimbursable visits, including: the Annual Wellness Visit (AWV), Chronic Care Management (CCM), Advance Care Planning (ACP), and Transitional Care Management (TCM). The model's standardized approach to implementation begins with practice assessments and two trainings. Practices participate in a data-driven, virtual learning collaborative with monthly data collection and learning sessions. Since 2015, GITT-PC has been implemented in 14 sites in northern New England, 10 sites in upstate New York, and nationally through five other GWEP awardees across the country.

### DEVELOPING A COMMUNITY-BASED COMPREHENSIVE FALLS PREVENTION PROGRAM IN COLLABORATION WITH PRIMARY CARE

Dawna Pidgeon[1], *1. Dartmouth-Hitchcock Medical Center, Lebanon, New Hampshire, United States*

Reducing falls in older adults requires a comprehensive screening program, a systems approach to refer those at risk and an evidence based community falls prevention programs. The Dartmouth Centers for Healthy & Aging has been the recipient of 2 Association of Community Living (ACL) Falls Prevention grants. This has enabled the development of a robust program for falls screening both in primary care and through community based balance screening events called "Balance Days". At risk individuals receive coaching, based on the principles of motivational interviewing, focusing on enrolling in either "Matter of Balance" or "Tai Chi Quan: Moving for Better Balance". Through the ACL grant we have built significant capacity across New England for these programs. This talk will focus on the "secret sauce" of implementing a robust community based falls prevention program in partnership with primary care.

### SESSION 3550 (SYMPOSIUM)

### LESSONS LEARNED FROM NURSING HOME CIVIL MONETARY PENALTY PROJECTS

Chair: Diana L. Sturdevant, *University of Oklahoma, Fran and Earl Ziegler College of Nursing, Oklahoma City, Oklahoma, United States*
Discussant: Kathleen C. Buckwalter, *University of Oklahoma, Fran and Earl Ziegler College of Nursing, Oklahoma City, Oklahoma, United States*

Nursing homes must comply with numerous federal/state regulations to receive Medicare and Medicaid funding. Failure to comply with these regulations can result in deficiency citations, and depending on the severity of the deficiency, a resulting Civil Monetary Penalty (CMP). Through the Centers for Medicare and Medicaid Services (CMS) Civil Monetary Penalty Reinvestment Program, CMP funds are reinvested to support activities that benefit nursing home residents and that protect or improve their quality of life or quality of care. This symposium presents some of the unique challenges, successes, failures, and surprise findings from CMP-funded nursing home quality improvement projects in two, predominantly rural Midwestern states: Oklahoma and Kansas. Dr. Williams presents findings of a pilot-study testing an adaptation of a successful family caregiver telehealth support intervention in the nursing home setting and implications for future research. Dr. Sturdevant shares successes, challenges, and unanticipated results from the "It's Not OK to Fall" project, a comprehensive, 3 year fall prevention project implemented in Oklahoma nursing homes. Lastly, Ms. Round's paper describes the implementation and findings of a Long-term Care Leadership Academy aimed at improving leadership and team building skills of three levels of nursing home staff, including Administrators/Directors' of Nursing, RN/LPN charge nurses and certified nursing assistants. Discussant, Dr. Kathleen Buckwalter Ph.D., FAAN, RN, will discuss how principles of nursing home culture change provides a common framework for these projects and conclude by offering suggestions on how promotion of these principles might improve the quality of care provided by nursing homes.

### IT'S NOT OK TO FALL: CHALLENGES AND SUCCESSES OF A NURSING HOME FALL PREVENTION PROJECT

Diana L. Sturdevant,[1] Barbara Carlson,[1] and Teri Round[2], *1. University of Oklahoma, Fran and Earl Ziegler College of Nursing, Oklahoma City, Oklahoma, United States, 2. University of Oklahoma, Fran and Earl Ziegler College of Nursing, Yukon, Oklahoma, United States*

This study pilot-tested a 12-week, comprehensive, resident-centered fall prevention program aimed to lower falls in nursing home residents in Oklahoma. Staff from 52 nursing homes received a training on evidence-based fall prevention strategies and fall-risk assessment. Content was present using motivational scenarios that encouraged situational problems solving. Rate of falls, including falls with major injury) were collected for 3 months before (roll-in), during (treatment), and following (sustainability). Nursing homes completing the project (n=29) showed significant decline in falls, including falls with major injury. Staff acceptance and project implementation varied across nursing homes and was related to organizational policies that did not adapt care based on residents needs on any given day. Adaptability, not just knowledge, is important for ensuring the safety of residents. Future work will focus on the role of leadership in promoting more open systems for delivering care.

### NOT READY FOR PRIME TIME: A FAILED STUDY TESTING A TECHNOLOGICAL INTERVENTION IN NURSING HOME CARE

Kristine N. Williams,[1] and Clarissa Shaw[2], *1. University of Kansas Medical Center, Kansas City, Kansas, United States, 2. University of Iowa, College of Nursing, Iowa City, Iowa, United States*

This pilot-study tested an adaptation of a successful family caregiver telehealth support intervention in nursing home settings. The goal was to support staff in managing challenging behaviors of residents with dementia. Nursing homes were provided with an iPad mini equipped with a special application to video record staff-resident challenging care situations. Videos were uploaded to a secure site for review by dementia care experts who provided feedback to improve care. Despite efforts to engage staff, only four videos were submitted. Factors including privacy, workload, and fear of documenting abuse contributed to implementation failure. The same intervention was successfully implemented in the home setting; differences in engagement and utilization will be discussed. Understanding the unique environments of nursing home dementia care is needed to successfully implement technology-based interventions to improve care. Evaluation of factors predicting the failure of this intervention may inform future research.

### CHANGING NURSING HOME CULTURE: LONG-TERM CARE LEADERSHIP ACADEMY

Teri Round,[1] and Diana Sturdevant[2], *1. University of Oklahoma, Fran and Earl Ziegler College of Nursing, Oklahoma City, Oklahoma, United States, 2. University of Oklahoma, Fran and Earl Ziegler College of Nursing, McAlester, Oklahoma, United States*

Nursing homes that fostered open communication and teamwork were more likely to change their practices and adopt the “It’s Not OK to Fall” program. This pilot study evaluated the feasibility and acceptance of a team-building approach for developing leadership skills to three groups of coworkers: Administrators/Directors of Nursing, charge nurses, and certified nursing assistants (CNA) employed by nursing homes in Oklahoma. Each coworker group received one day of job specific leadership training, with another one half-day session where all levels engaged in team-building exercises. Participant satisfaction with course content ranged from agree-to-strongly agree. All stated that they could apply the leadership strategies at their facility. Administrators/Directors of Nursing found tools for tracking turnover/retention and strategies for improving staff communication helpful. Charge nurses and certified nursing assistants seldom viewed themselves as leaders, found coworker group communication very fragmented, and felt least knowledgeable about nursing home care best practices.

### SESSION 3555 (PAPER)

### LONG-TERM CARE III

### A CHANGING INDUSTRY IN A CHANGING WORLD: LONG-TERM SERVICES WHERE ARE YOU GOING?

Robert A. Applebaum,[1] Matt Nelson,[2] Jane Straker,[1] A. Katherine Harrington,[2] and John R. Bowblis[3], *1. Miami University, Scripps Gerontology Center, Oxford, Ohio, United States, 2. Scripps Gerontology Center at Miami University, Oxford, Ohio, United States, 3. Department of Economics, Miami University, Oxford, Ohio, United States*

From its name to the type and setting of care provided, the world of long-term services and supports has changed dramatically in the last two decades. Using 26 years of longitudinal data from the state of Ohio this presentation describes how the long-term services system is different from the one that existed in the early 1990’s. Data come from 13 biennial surveys of Ohio nursing homes and residential care facilities (95% response rate) and comprehensive resident and home care participant data on user characteristics and utilization rates. Findings show large changes in where services are provided and who receives services. For example, Ohio, as has most other states, has changed the ratio of its older population using Medicaid long-term care, going from 91% nursing home users in 1993, to more than half using home and community-based services in 2017. At the same time the sheer number of admissions to Ohio nursing homes increased from 70,000 to more than 220,000 and Medic are admissions increasing from 30,000 to 145,000, painting a picture of today’s nursing home as a short-term care provider. These massive changes indicate an industry in transition. What will this mean for the future of the home care and nursing home industries? What will tomorrow’s system of long-term services and supports look like? Building on more than two decades of findings the presentation will tackle the question of where long-term services is going in the future.

### BARRIERS AND FACILITATORS TO IMPLEMENTING COMMUNITY FIRST CHOICE

Lisa Beauregard,[1] and Edward A. Miller[1], *1. University of Massachusetts Boston, Boston, Massachusetts, United States*

Community First Choice is a program within the Affordable Care Act that encourages states to expand Medicaid home and community-based services (HCBS). Specifically, this Medicaid state plan benefit provides states with an additional 6% federal match to promote greater rebalancing of long-term services and supports. Through Community First Choice, states can offer services that assist with activities of daily living, instrumental activities of daily living, and health-related tasks. The program is optional for states, and, to date, eight states have pursued Community First Choice. The purpose of this study is to understand the barriers and facilitators to implementing Community First Choice in two states. Data was collected through semi-structured interviews with individuals involved in HCBS policy nationally and in Maryland and Texas, including government bureaucrats, consumer advocates, and provider representatives. The results suggest that communication with the Centers for Medicare and Medicaid Services, the enhanced federal match, and leveraging existing HCBS infrastructure facilitated implementation. Maryland and Texas encountered challenges implementing Community First Choice because of constraints posed by existing HCBS programs, ambitious timelines, limited staff resources, and insufficient engagement with external stakeholders. The findings suggest that implementing Community First Choice is a large undertaking, and states should ensure they have enough time and sufficient staffing for the implementation process. States should also understand how implementing Community First Choice will impact existing HCBS offerings and how leveraging HCBS infrastructure can facilitate implementation. The lessons from implementing Community First Choice can be informative to other states pursuing or contemplating this program.

### PUBLIC REPORTING OF NH ANTIPSYCHOTIC USE: CHANGES IN THE REPORTING OF EXCLUSIONARY DIAGNOSES?

Judith A. Lucas,[1] John R. Bowblis,[2] Mary Lynn Davis-Ajami,[3] Kosali Simon,[3] and Miyeon Song[4], *1. Seton Hall University, South Orange, New Jersey, United States, 2. Miami University, Oxford, Ohio, United States, 3. Indiana University, Bloomington, Indiana, United States, 4. Texas A&M University, College Station, Texas, United States*

Antipsychotics (APMs) are commonly used off-label to control behavioral symptoms of dementia in nursing home (NH) residents despite FDA warnings. As part of the Centers for Medicare and Medicaid Services' (CMS) partnership to reduce the use of APMs in long-stay NH residents with dementia, CMS began publicly reporting an APM quality measure on the NH Compare website in July of 2012. This measure proxies for potentially "inappropriate" use by excluding residents with diagnoses in which APMs may be appropriate. While APM use as declined, some question if the quality measure creates an incentive to change documentation of exclusionary diagnoses to show improvements in the publicly reported APM quality measure. These exclusionary diagnoses include: Huntington's disease, Tourette syndrome, and schizophrenia. Using Minimum Data Set (MDS) assessments from long-stay NH residents in freestanding NHs (2011-2015), we utilized a difference-in-differences framework to compared the proportion of assessments with exclusionary diagnoses in NHs that were required to report the APM quality measure (treatment) to NHs that were exempt from reporting the measure (control). We also stratified the results by grouping residents under and over 65 year of age. We find no change in exclusionary diagnoses immediately following the implementation of public reporting. However, starting in 2014, the use of exclusionary diagnoses increased in the treatment group, especially among residents aged 65+. While APM use declined for all groups over the study period, our results indicate that the reduction in APM use may not be as large as expected, especially after 2014.

### RELATIONSHIP BETWEEN FAMILY INVOLVEMENT IN CARE PLANNING AND ESTABLISHING AN ADVANCE DIRECTIVE

Ellen McCreedy,[1] lacey Loomer,[1] Jennifer Palmer,[2] Angelo Volandes,[3] Susan Mitchell,[4] and Vincent Mor[1], *1. Brown University, School of Public Health, Providence, Rhode Island, United States, 2. Hebrew SeniorLife, Institute for Aging Research, Roslindale, Massachusetts, United States, 3. Harvard Medical School, Boston, Massachusetts, United States, 4. Hebrew SeniorLife, Institute for Aging Research, Boston, Massachusetts, United States*

The purpose of this research is to understand the effect of family participation in care planning assessments on the use of advance directives for newly admitted nursing home (NH) residents. This is a retrospective cohort study using data from 115 nursing homes involved in a pragmatic randomized clinical trial testing a video intervention for advance care planning (versus usual care). Data sources included the electronic health record and the Minimum Data Set (MDS). Competing risks regression analyses estimated the cumulative incidence of establishing an advance directive, after accounting for death, discharge from NH, and time censoring (12-month observation window). 18,978 residents admitted to eligible NHs without an advance directive (full code) between April 2, 2016 and August 31, 2017 were followed for one year. 11,905 (63%) died or were discharged without an advance directive; 4,155 (22%) remained in the NH without an advance directive; and 2,918 (15%) established an advance directive. Median time to establishing an advance directive was 75 days (SD: 72, 79). After adjusting for competing risks and resident factors known to be associated with advance directive use, each care planning assessment involving a family member was associated with a 6% increase in the cumulative incidence advance directive use. Involving family members of NH residents in routine care planning assessments may reduce the amount of futile care received by NH residents. As we test interventions to improve advance care planning for NH residents, focus should be placed upstream on the first 90 days after admission.

### TECHNOLOGY IN LONG-TERM CARE: DOES IT FACILITATE THE WORKFORCE OR IMPROVE SATISFACTION AND RETENTION?

Susan A. Chapman,[1] Joanne Spetz,[1] and Jacqueline Miller[1], *1. University of California, San Francisco, San Francisco, California, United States*

Emerging technological advances hold potential for individuals and caregivers to help an aging population in the home or long term care settings and has the potential to alter workforce needs and potentially mitigate the rising workforce demand. The purpose of the proposed research was to research available technologies that may facilitate, replace, or enhance recruitment, training, and retention of the LTC workforce. This qualitative study included environmental scan and literature search identifying 62 companies meeting criteria for potential impact on the workforce. Categories included wearables, robots, sensors/alerts, health/social data collection and tracking, family/caregiver communication services, online care management, online worker training, and facility or home health staffing systems. Thematic analysis findings of telephone interviews with 12 national and international companies included: 1) most development focused on software rather than hardware, 2) company founders generally had a tech background and start up experience along with personal family caregiving experiences, 3) data collected in the home often did not connect directly to the health care team, 4) payment was generally out of pocket to individuals or facilities under subscription services or contracts 5) worker retention improved when system allowed better client to worker matching, more control over shift scheduling, and more efficient staffing. There was little rigorous research on the impacts for care and services or which will have the greatest potential impact on the workforce providing direct care. Reimbursement from federal and private payers is minimal to date yet demand for government payment may grow.

### SESSION 3560 (PAPER)

### METHODS, THEORY, AND INNOVATION

### A BAYESIAN ANALYSIS OF EVIDENCE IN SUPPORT OF THE NULL HYPOTHESIS IN GERONTOLOGICAL PSYCHOLOGY (OR LACK THEREOF)

Christopher Brydges,[1] and Allison A. Bielak[1], *1. Colorado State University, Fort Collins, Colorado, United States*

Objective: Non-significant p values derived from null hypothesis significance testing do not distinguish between true null effects or cases where the data are insensitive in distinguishing the hypotheses. This study aimed to investigate the prevalence of Bayesian analyses in gerontological psychology, a statistical technique that can distinguish between conclusive and inconclusive non-significant results, by using Bayes factors (BFs) to reanalyze non-significant results from published gerontological research. Method: Non-significant results mentioned in abstracts of articles published in 2017 volumes of ten top gerontological psychology journals were extracted (N = 409) and categorized based on whether Bayesian analyses were conducted. BFs were calculated from non-significant t-tests within this sample to determine how frequently the null hypothesis was strongly supported. Results: Non-significant results were directly tested with Bayes factors in 1.22% of studies. Bayesian reanalyses of 195 non-significant t-tests found that only 7.69% of the findings provided strong evidence in support of the null hypothesis. Conclusions: Bayesian analyses are rarely used in gerontological research, and a large proportion of null findings were deemed inconclusive when reanalyzed with BFs. Researchers are encouraged to use BFs to test the validity of non-significant results, and ensure that sufficient sample sizes are used so that the meaningfulness of null findings can be evaluated.

### GENERATIVITY ACROSS ADULTHOOD: THE EFFECTS OF AGE AND AFFECT

Niccole A. Nelson,[1] and Cindy Bergeman[1], *1. University of Notre Dame, Notre Dame, Indiana, United States*

Generativity, or investment in the next generation, is heavily implicated in successful aging. Due to its benefits to both the individual and society, researchers have attempted to identify factors that contribute to generativity. Most studies on generativity, however, have been conducted in cross section, limiting established relations to those occurring between individuals, not within them. The identification of true within-person effects is vital, as between-person relations do not accurately describe within-person processes. One factor that may contribute to generativity within individuals is positive affect (PA), as experiencing PA seems to broaden individuals' resources, and ultimately build human flourishing. This process may also be moderated by age or change over time. To test these ideas, the current study applied multilevel modeling to ten years of data from N = 1,117 mid-to-later life individuals. A main effects model indicated between-person effects such that younger individuals, as well as individuals with higher global PA, tended to report higher generativity, on average, than older individuals and individuals with lower global PA, respectively. Next, considering within-person effects, individuals tended to report higher generativity during years they experienced more PA. Finally, a two-way interaction effect indicated an age-related difference in a within-person process: although midlife individuals reported higher generativity during years they experienced more PA, later-life individuals did not show this effect. Theoretical implications, including age-graded goal strivings and the broaden-and-build theory, will be discussed.

### IMPACT OF THE BE EPIC: A PERSON-CENTERED COMMUNICATION INTERVENTION FOR HOME CARE WORKERS

Marie Y. Savundranayagam,[1] Kristine N. Williams,[2] Shalane Basque,[1] JB Orange,[1] Marita Kloseck,[1] Vicki Schmall,[3] and Karen Johnson[4], *1. Western University, London, Ontario, Canada, 2. University of Kansas Medical Center, Kansas City, Kansas, United States, 3. Aging Concerns, West Linn, Oregon, United States, 4. McCormick Dementia Services, London, Ontario, Canada*

The current study assessed the impact of Be EPIC, an innovative, evidence-informed and theoretically-grounded 6-week person-centered communication intervention for personal support workers (PSWs) caring for persons with dementia. Be EPIC focuses on [E]nvironment contexts for using [P]erson-centered communication, while considering client relationships ([I] matter too), and [C]lients' abilities, life history and preferences during routine care. A pre- post-Be EPIC comparative design included an intervention (n=13) and a 6-week waitlist control group (n=10) who completed the same communication-related questionnaire. A Two-Way Mixed ANOVA showed a significant group by time interaction for perceived communication skill ($F(1, 21) = 4.67$, $p = .042$, $\eta p2= .18$). Simple main effects analysis showed that participants who completed Be EPIC reported feeling more confident in communicating with persons with dementia (Mpre = 13.46; SD = .76; Mpost = 16.31, SD = .85). There was no significant change in the control group. Similarly, there was a significant group by time interaction for perceived helpfulness of communication strategies ($F(1, 21) = 6.23$, $p = .021$, $\eta p2 = .23$). Simple main effects analysis showed that participants who completed Be EPIC reported significant increases in the helpfulness of effective communication strategies (Mpre = 36.92; SD = 3.42; Mpost = 43.15, SD = 3.21), with no significant change among controls. Findings indicate that Be EPIC enhanced PSWs' confidence in communicating with persons with dementia and enhanced their perception of the helpfulness of effective communication strategies.

### IMPLEMENTATION OF PATIENT PRIORITIES CARE WITHIN A VA GERIATRICS CLINIC

Jennifer Freytag,[1] Lilian Dindo,[1] Angela Catic,[2] Aanand Naik,[1] and Mary Tinetti[3], *1. Center for Innovations in Quality, Effectiveness and Safety, Houston, Texas, United States, 2. Michael E DeBakey VA Medical Center, Houston, Texas, United States, 3. Yale University, New Haven, Connecticut, United States*

Patient Priorities Care (PPC) is an approach to decision-making for older adults with multiple chronic conditions (MCC). PPC trains facilitators to have structured conversations with patients to identify their priorities (the outcomes that matter most given what care they are willing/able to do or receive). Clinicians then align care to achieve patient priorities rather than focusing on multiple single-disease guidelines. We piloted PPC in a VA geriatrics clinic and compared it to usual care (UC) for multimorbid adults. This retrospective cohort study (n=36 PPC, 36 UC) describes changes made by clinicians after Veterans with MCC had facilitated conversations in a VA geriatric clinic. UC Veterans were matched by prognosis, same primary clinician, and timeframe. Coders used a standardized rubric to assess documented care within medical records. Changes to care examined include medications added/removed, referrals made/avoided, self-care recommendations, and recommendations for social engagement. Although PPC and UC patients were seen by the same clinicians, patients receiving PPC had fewer added medications (mean difference -.47, $t(70)=-1.99$, $p=.05$); received more recommendations for self-care aligned with priorities (mean difference .25, $t(69)=2.14$, $p=.003$); received more recommended consultations with desired care, including podiatry, transportation, and dermatology (mean difference .55, $t(70)=2.06$, $p=.01$), and more recommendations for care and services to facilitate social interactions ($p<.0001$). PPC produced documented changes in care that better align with patient priorities within the routine care workflow of a busy geriatrics clinic. Our results provide evidence that structured priorities conversations change the way clinicians provide care for older adults with MCC.

### UNCOVERING DEEPER MEANING: APPLYING MULTIPLE METHODS OF DATA ANALYSIS IN NARRATIVE INQUIRY WITH OLDER ADULTS

Rebecca Meraz,[1] Kathryn Osteen,[2] and Jocelyn S. McGee[3], *1. Baylor University Louise Herrington School of Nursing, Dallas, Texas, United States, 2. Baylor University, Dallas, Texas, United States, 3. Baylor University, Waco, Texas, United States*

Personal narrative is at the heart of how humans share information, represent identity, and convey ideas. Using narrative inquiry, researchers may gain some insight into an individual's personal understanding of the events in their life. Although many narrative investigations report themes from study data, there is no single, well defined approach to data analysis in narrative research. We describe how applying multiple methods of systematic evaluation to narrative data leads to a deeper and more holistic understanding of told stories about medication-taking decisions among older heart failure (HF) patients. In this paper, we share a more holistic interpretation of the decision making process by applying Riessman's thematic, structural, and performance analysis provided a method for analyzing the data beyond the spoken words. Data was transcribed verbatim then arranged according to the essential discourse between the participant and researcher, the intact story, and the story elements needed to facilitate each method of evaluation. The data analysis process outlined in this article contributes to the academic discourse and knowledge supporting the use of multiple methods of systematic evaluation to uncover deeper meaning in narrative data. In clinical practice, care should be guided by a deeper and holistic understanding of the decisions patients make about whether to take or not take a HF medication.

### SESSION 3565 (SYMPOSIUM)

### OUT AND ABOUT? PARTICIPATION IN OUT-OF-HOME ACTIVITIES AND PLACES AMONG PEOPLE WITH DEMENTIA IN MULTIPLE COUNTRIES

Chair: Habib Chaudhury, *Simon Fraser University, Vancouver, British Columbia, Canada*
Co-Chair: Louise Nygard, *Karolinska Institute, Stockholm, Sweden*
Discussant: Malcolm Cutchin, *Wayne State University, Detroit, Michigan, United States*

Maintaining participation in activities and places in the community can be challenging for people with dementia in the face of cognitive decline. These challenges could be related to driving cessation, accessing transit, ability to navigate in the community, socially uncomfortable situations and functionally difficult tasks. Consequently, people with dementia are at risk of progressively becoming isolated from the community and being unable to meaningfully age-in-place in the home and community. Familiar and responsive neighborhood built environmental features, public awareness of dementia and appropriate support, dementia-friendly transit systems, are among the potential socio-physical environmental factors to foster maintenance of participation and activities for persons living with dementia. Empirical literature exploring levels of participation outside the home, wayfinding and orientation issues, and the nature and prevalence of challenging circumstances for older adults with dementia in the community is scarce. This session will present four papers in this area based on empirical projects conducted in Sweden, Switzerland, U.K. and Canada. Three of these projects used the recently developed Participation in ACTivities and Places OUTside the Home (ACT-OUT) questionnaire to generate evidence on the levels of participation and perceptions of risks outside the home for people with and without dementia. A presentation will also summarize the theoretical bases for the ACT-OUT research discussed in the other presentations. One study will focus on the influence of Everyday Technology on participation patterns. Implications of the research findings to advance theoretical understanding, generate evidence-base and impact policy and practice, along with future research directions will be considered.

### ACCESS TO EVERYDAY ACTIVITIES IN PUBLIC SPACE - VIEWS OF PEOPLE WITH DEMENTIA

Anna Brorsson,[1] Louise Nygard,[1] and annika Ohman[2], *1. Division of Occupational Therapy, NVS, Karolinska*

*Institutet, Huddinge, Stockholms Lan, Sweden, 2. Division of Occupational Therapy, Department of Social Welfare Studies. Linkoping University, Norrkoping, Ostergotlands Lan, Sweden*

People with dementia value staying active and continuing with their everyday lives in public space, however there is a lack of knowledge about how they experience accessibility, problematic situations and how to manage these situations. The aim is to illuminate experiences of accessibility in public space in people with dementia with focus on places, activities and problematic situations. A Grounded theory approach was used in the thesis with multiple data collection methods (interviews, focus group interviews, observations and visual methods). Findings show that having access to everyday activities at different places in the neighbourhood was very important for the participants when they perceived themselves as being a part of the society and being active and independent persons. Engaging in familiar activities in familiar places was important. However, their activity radii in the community became smaller. The findings inspired the development of the questionnaire Participation in Activities and Places Outside Home.

### PARTICIPATION OUTSIDE HOME FOR PEOPLE LIVING WITH DEMENTIA: DO THEY REALLY EXPERIENCE A SHRINKING WORLD?

Isabel Margot-Cattin,[1] Nicolas Kühne,[2] Catherine Ludwig,[3] Louise Nygard,[4] and Anders Kottorp[5], *1. University of Applied Sciences and Arts of Western Switzerland (HES-SO), Haute école de travail social et de la santé (EESP), Lausanne, Vaud, Switzerland, 2. University of Applied Sciences and Arts of Western Switzerland, Lausanne, Vaud, Switzerland, 3. University of Applied Sciences and Arts of Western Switzerland, Geneva, Geneve, Switzerland, 4. Karolinska Institutet, Huddinge, Stockholms Lan, Sweden, 5. Malmö University, Malmö, Skane Lan, Sweden*

Accessing places outside home where activities are performed provides both benefits (e.g. participation in activities), and challenges (e.g. finding one's way) for people with cognitive deficits. Participation in these places appears to depend on various factors such as the living situation of the person, availability of commodities and supporting social networks, or preserved ability to drive. However, clear patterns of participation remain scarcely documented. This study addresses the need for understanding of participation outside home among people with dementia through the places they visit. The aim is to describe how the outside world may be shrinking for them. People with and without dementia (n=70), aged 65+, were interviewed using the Participation in ACTivities and Places OUTside the Home (ACT-OUT) questionnaire across Switzerland. Results show that people with dementia participate significantly less in commercial, social and cultural places, but visit medical care places at a higher level, than those without dementia.

### EVERYDAY TECHNOLOGY: A USEFUL SERVANT BUT DANGEROUS MASTER FOR PARTICIPATION IN SOCIETY?

Sophie N. Gaber,[1] Sophie N. Gaber,[2] Louise Nygard,[2] Anna Brorsson,[2] Anders Kottorp,[3] Sarah Wallcook,[2] Georgina Charlesworth,[4] and Camilla Malinowsky[2], *1. Karolinska Institutet, Stockholm, Stockholm Lans, Sweden, 2. Division of Occupational Therapy, Department of Neurobiology, Care Sciences and Society, Karolinska Institutet, Stockholm, Stockholms Lan, Sweden, 3. Faculty of Health and Society, Malmo University.Division of Occupational Therapy, Department of Neurobiology, Care Sciences and Society, Karolinska Institutet, Malmo, Skane Lan, Sweden, 4. Research Department for Clinical, Education, and Health Psychology., London, England, United Kingdom*

With an increasingly technological society comes an assumed ability to use Everyday Technologies (ET) in order to participate in activities and places in public space, e.g. operating ticket machines to access public transport. This study addresses a mismatch between a growing dependency on ET and evidence that people with dementia experience increased challenges using ET. The aim is to explore how ET-use and perceived risk relate to participation in public space, among people with and without dementia. People with dementia and without dementia, aged 55+, were interviewed using questionnaires including the Participation in ACTivities and Places OUTside the Home questionnaire, across Sweden (n=69) and the UK (n=128). The Swedish and UK findings show small but significant associations between total participation in places within public space, and i) ET-use, and ii) perceived risk in public space. Furthermore, people with dementia participated in fewer places within public space than those without dementia.

### PARTICIPATION IN THE COMMUNITY AMONG PEOPLE WITH AND WITHOUT DEMENTIA: DESTINATIONS AND PERCEPTIONS OF CHALLENGES

Habib Chaudhury,[1] Tanveer Mahal,[2] and Kishore Seetharaman[1], *1. Simon Fraser University, Vancouver, British Columbia, Canada, 2. University of Sydney, Sydney, New South Wales, Australia*

Older adults with dementia face challenges in their outdoor mobility and there are concerns of their not being able to continue going outside for everyday activities and social participation. The focus of this study was to identify patterns of visits to community destinations and activities, and perceptions of risks. Interviews were conducted with 59 adults (aged 54-84) with (n=29) and without (n=30) dementia using the Participation in ACTivities and Places OUTside the Home (ACT-OUT) questionnaire in Vancouver, Canada. Findings indicate that participants with dementia had abandoned visiting a few places over time (e.g., bank, cemetery, buildings of worship), whereas there were no change in participation in taking transit to destinations such as supermarkets, entertainment and cultural places. However, in some cases, companions or partners of persons with dementia indicated that they were prone to getting anxious when left alone in public places and were at high risk of getting lost.

### SESSION 3570 (SYMPOSIUM)

### PAVING THE WAY FORWARD IN HOUSING WITH SERVICES: THE RIGHT CARE, RIGHT PLACE, RIGHT TIME PROGRAM

Chair: Edward A. Miller, *University of Massachusetts Boston, Boston, Massachusetts, United States*

Co-Chair: Pamela Nadash, *University of Massachusetts Boston, Boston, Massachusetts, United States*
Discussant: Alisha Sanders, *LeadingAge, Washington, D.C., United States*

Older people living in congregate environments are obvious beneficiaries of supportive services. The potential for prevention is clear, particularly among low-income elders living in subsidized housing; it is this group that is at high risk for significant healthcare and other costs, and it is this group that suffers considerably from a fragmented healthcare system. Policymakers have long seen the advantages of reaching this population, but most existing housing with services programs have focused more on social than health-related supports. The Right Care, Right Place, Right Time initiative (R3) was launched in July 2017 to demonstrate the value of supportive services to seniors living independently in affordable housing in the Greater Boston area, while reducing health care costs. The R3 program consists of two on-site wellness teams, including a wellness nurse and wellness coordinator. Each team is responsible for about 200 participants across two housing sites. The R3 evaluation included both quantitative and qualitative components. The quantitative component entails pre/post comparison as well as a control group analysis, focusing on various health and health utilization outcomes. The qualitative component includes key informant interviews examining program development and implementation and focus groups capturing the resident experience. The purpose of this symposium is for evaluation team members to report on the experiences of program participants, administrators/staff, housing managers/staff, and community partners with the R3 program, and to assess program impact. Edward Miller and Pamela Nadash will serve as chair and co-chair, respectively; Alisha Sanders as the discussant.

### LESSONS FROM THE RESIDENT EXPERIENCE WITH THE R3 PROGRAM

Edward A. Miller,[1] Pamela Nadash,[1] Elizabeth Simpson,[1] Natalie Shellito,[1] and Marc Cohen[1], *1. University of Massachusetts Boston, Boston, Massachusetts, United States*

Understanding the resident experience is a critical step to creating a sustainable and replicable model of affordable resident-centered housing with supportive services programs. This study thus draws lessons from focus groups with participants in the R3 program for designing and implementing such initiatives in affordable senior housing. Findings indicate that the R3 program brings value to residents: they benefit from reliable information on health-related issues, as well as emotional support and assistance with accessing appropriate care. By focusing on prevention and ensuring timely access to services, findings suggest how the intervention could promote seniors living independently longer and lower health system costs. Results also suggest ways to improve the effectiveness of housing with services programs, including providing clarity regarding the purpose of the program, its components and staffing, building trust between program staff and residents, addressing concerns about privacy and confidentiality, and implementing a multipronged marketing and promotion strategy.

### R3 PROGRAM MANAGEMENT AND WELLNESS TEAM VIEWS ON BARRIERS AND FACILITATORS TO IMPLEMENTING THE R3 PROGRAM

Natalie Shellito,[1] Pamela Nadash,[1] Edward Miller,[1] Elizabeth Simpson,[1] and Marc Cohen[1], *1. University of Massachusetts Boston, Boston, Massachusetts, United States*

Implementing programs such as R3, which adds health-focused supportive services into senior housing sites, can be complicated. This study aimed to understand program management and wellness team views on barriers and facilitators to implementation. Semi-structured interviews were conducted with managers, social workers, and wellness nurses who implemented the R3 program. Facilitating factors included monthly phone calls between wellness team members and program participants, which strengthened relationships and provided valuable information; top-level management support, which was critical in building community partnerships; and daily ambulance reports from local emergency responders, which provided actionable information about participants. Barriers included the need for more wellness team time at individual intervention sites, challenges connecting R3 staff with participants' hospitals and insurance companies, and refining the technological approach used to facilitate work flow and information exchange. Although obstacles were encountered during implementation, the findings provide support for the beneficial effects of enhanced services within senior housing.

### THE ROLE OF HOUSING MANAGEMENT AND STAFF IN ADMINISTERING THE R3 PROGRAM

Elizabeth Simpson,[1] Edward Miller,[1] Pamela Nadash,[1] Taylor Jansen,[1] Yan Lin,[1] and Marc Cohen[1], *1. University of Massachusetts Boston, Boston, Massachusetts, United States*

Existing staff and management are central players in integrating enhanced services into affordable senior housing. This study describes the experience of housing providers in the implementation and operations of the R3 program. Semi-structured interviews were conducted with executives and direct service staff across the four intervention sites. Results indicate that staff served an important role in facilitating resident recruitment by operating as trusted sources of information about the R3 program. Top-level support for R3, acculturating R3 staff to the housing site, developing communication and data systems, and integrating new and existing staff were seen as crucial to the success of the program. Benefits noted by housing staff included freedom to redirect one's energies/focus, production of actionable data/insights, reductions in resident turnover, and the addition of a nurse to the onsite services team. Housing management/staff experience with R3 can serve as a guide to moving to an enhanced services model.

### COLLABORATION WITH FIRST RESPONDERS IN R3: THE CRITICAL ROLE OF COMMUNITY PARTNERS IN HOUSING WITH SERVICES

Pamela Nadash,[1] Edward Miller,[1] Elizabeth Simpson,[1] and Natalie Shellito[1], *1. University of Massachusetts Boston, Boston, Massachusetts, United States*

Because seniors represent a rising proportion of Emergency Medical Services (EMS) provider activity, there is a growing focus on determining how EMS providers can better serve the aging population. Multifamily senior housing properties benefit from reductions in resident turnover; EMS providers aim to improve community health and minimize unnecessary and low-priority callouts. Developing partnerships between the two service sectors can help both meet their individual goals while reducing health system costs. Drawing on experience with the R3 initiative, this study describes how a partnership between EMS providers and supported housing sites has led to reductions in ambulance transfers to hospitals and reinforced falls reduction programs within senior housing. The R3 program represented an effort to work closely with EMS providers and, by doing so, provides an example of how collaboration between the two sectors can work to the advantage of both parties by identifying residents who would benefit from intervention.

### THE IMPACT OF THE R3 PROGRAM ON SERVICE UTILIZATION, COSTS, AND QUALITY OF LIFE

Marc A. Cohen,[1] Jennifer Hefele,[2] Edward Miller,[2] Pamela Nadash,[2] Natalie Shellito,[2] and Elizabeth Simpson[2], *1. University of Massachusetts Boston Gerontology Institute, LeadingAge LTSS Center @UMass Boston, Boston, Massachusetts, United States, 2. University of Massachusetts Boston, Boston, Massachusetts, United States*

Relatively few housing with services evaluations employ rigorous multiple group quasi-experimental designs rather than simple before/after research designs. This study employs a pre-post experimental design with a comparison group to analyze whether the R3 program led to reductions in certain key utilization measures, including emergency department visits, inpatient hospitalizations, ambulance usage, and skilled nursing facility admissions, over 18 months. Also examined is whether resident quality of life indicators changed over the period. Data derive from assessments with 410 residents in four intervention sites and 227 residents in five comparison group sites. Also obtained was data on emergency department transfers from first responder services and Medicare fee-for-service data from the local quality improvement organization. Results suggest that the program has positive implications for service utilization, costs, and quality of life. Early detection and intervention inherent in the R3 program may improve resident quality of life while lowering service utilization and costs.

### SESSION 3575 (SYMPOSIUM)

### PERSONALITY AND COGNITIVE AGING

Chair: Eileen K. Graham, *Northwestern University, Chicago, Illinois, United States*
Co-Chair: Stacey B. Scott, *Stony Brook University, Stony Brook, New York, United States*
Discussant: Avron Spiro III, *Boston University, Boston, Massachusetts, United States*

Understanding when, how, and for who, cognitive decline occurs is essential to understanding how to optimize quality of life among aging adults. It is well known that there is large variation in cognitive change: the pace and direction of change differs greatly across individuals. Personality traits are one key factor that account for some of these individual differences. Individuals with high levels of certain characteristics are more or less likely to engage in lifestyle behaviors that may put them at greater or less risk of decline over time. The goal of our symposium is to present novel research in this area and discuss the implications for understanding personality and cognitive decline. First, Scott and colleagues will demonstrate a novel approach to personality measurement, and the extent to which there is longitudinal measurement invariance in these measures. This is an important first step in the study of change processes. Second, Terracciano and Sutin will test associations between personality traits and verbal fluency in aging adults, and whether these associations replicate across multiple large panel studies. Third, Graham and colleagues will investigate trajectories of cognitive decline, specifically whether personality is associated with decline both before and after a diagnosis of dementia. Fourth, James and colleagues will discuss the extent to which personality is associated with discordances between dementia diagnosis and neuropathology. All talks will focus on open science, reproducibility, replicability, and generalizability, consistent with GSA's efforts toward these goals. Discussant Avron Spiro will contextualize these new findings and propose next steps.

### EVERYDAY MARKERS OF PERSONALITY IN ADULTHOOD

Stacey B. Scott,[1] Andreas Neubauer,[2] Joshua Smyth,[3] and Martin Sliwinski[3], *1. Stony Brook University, Stony Brook, New York, United States, 2. DIPF, Frankfurt, Hessen, Germany, 3. Penn State, University Park, Pennsylvania, United States*

Personality should manifest everyday as behaviors, affective states, and thoughts. Trait personality measures require retrospection and appraisal, processes affected by cognitive changes. Ecological momentary assessments (EMA), however, may identify sensitive everyday personality markers. We analyzed data from 178 individuals aged 20-79 who completed 3 EMA measurement bursts. Each burst, participants rated positive affect (PA), negative affect (NA), negative thoughts (URT), total social interactions, and interaction pleasantness up to 5x daily for 7 days. We tested for measurement invariance across bursts in a confirmatory factor analysis using 4 indicators of Neuroticism (N; a trait measure of N from BFI and 3 EMA-based indicators: mean NA, standard deviation NA, mean URT) and 4 indicators of Extraversion (E; trait E from BFI and mean PA, mean interactions, mean pleasantness). Strict measurement invariance held, indicating that the association among these indicators remained stable across the 18 month observation period.

### PERSONALITY TRAITS AND VERBAL FLUENCY IN 10 COHORTS

Antonio Terracciano,[1] and Angelina R. Sutin[1], *1. Florida State University, Tallahassee, Florida, United States*

Personality traits are associated with cognitive outcomes across the lifespan, including cognitive function in young adulthood and risk of cognitive impairment and dementia in old age. This study examined the association between the Five Factor Model personality traits and verbal fluency in 10 cohorts (11 samples) that totaled more than 90,000 participants (age range 16-101). Meta-analysis indicated that participants who scored lower in Neuroticism, and higher in Extraversion, Openness, and Conscientiousness retrieved more words, independent of age, gender, and education. These associations were consistent across semantic and letter fluency tasks. Moderation analysis indicated that the associations between personality and semantic fluency were stronger in older samples (except for Openness) and among individuals with lower education. This pattern suggests that these associations are stronger in groups vulnerable to cognitive impairment and dementia. Personality traits have pervasive associations with fluency tasks that are replicable across samples and age groups.

### ASSOCIATIONS BETWEEN PERSONALITY TRAITS AND COGNITIVE RESILIENCE IN OLDER ADULTS

Eileen K. Graham,[1] Bryan James,[2] and Daniel K. Mroczek[1], *1. Northwestern University, Chicago, Illinois, United States, 2. Rush University, Chicago, Illinois, United States*

There are considerable individual differences in the rates of cognitive decline across later adulthood. Personality traits are one set of factors that may account for some of these differences. The current project explores whether personality traits are associated with trajectories of cognitive decline, and whether the associations are different before and after a diagnosis of dementia. The data will be analyzed using linear mixed effects regression. Across these goals is a focus on replicability and generalizability. Each of these questions will be addressed in four independent longitudinal studies of aging (EAS, MAP, ROS, SATSA), then meta-analyzed, thus providing an estimate of the replicability of our results. This study is part of a registered report of existing data that is currently under stage 1 review.

### ASSOCIATIONS BETWEEN PERSONALITY TRAITS AND COGNITIVE RESILIENCE IN OLDER ADULTS

Bryan D. James,[1] Daniel K. Mroczek,[2] and Eileen K. Graham[2], *1. Rush Alzheimer's Disease Center, Chicago, Illinois, United States, 2. Northwestern University, Chicago, Illinois, United States*

There is often discordance between brain pathology and dementia diagnosis. Some individuals maintain cognitive function throughout their lives but show high burden of neuropathology after death (e.g. amyloid plagues, neurofibrillary tangles, vascular disease, Lewy bodies, and/or TDP-43). Conversely, some demonstrate significant decline and receive a dementia diagnosis, while showing minimal neuropathology at autopsy. The current study seeks to understand these resilience/vulnerability profiles, with a focus on individual differences. That is, are individuals with certain personality characteristics (e.g. high openness, low neuroticism) more/less likely to have cognitive resilience or vulnerability? Using psychosocial and autopsy data from the Rush Memory and Aging Project and the Religious Orders Study, this study uses a resilience index based on residuals derived from regressing global cognition on global pathology, then entering personality traits as predictors of cognitive resilience. The analysis plan will be submitted to the Journal of Gerontology's special issue on pre-registration of existing data.

### SESSION 3580 (SYMPOSIUM)

### PSYCHOLOGICAL AND SOCIAL FACTORS ASSOCIATED WITH SLEEP HEALTH ACROSS ADULTHOOD

Chair: Soomi Lee, *University of South Florida, Tampa, Florida, United States*
Discussant: Orfeu M. Buxton, *Pennsylvania State University, University Park, Pennsylvania, United States*

Sleep is associated with all-cause mortality, cardiovascular disease, cognitive impairment, as well as daily social interactions and productivity. Studies often have focused on sleep duration only, lacking the ability to comprehensively understand the importance of age-related changes in varied facets of sleep health. Moreover, psychological and social factors that may be associated with sleep health in adulthood are still poorly understood. This symposium showcases contemporary endeavors towards understanding how diverse indicators of sleep health relate to psychological and social factors across adulthood. Paper 1 uses perceived job discrimination as a social stressor to test associations between perceived job discrimination and sleep health (difficulty falling/staying asleep, excessive daytime sleepiness, sleep duration) among working women. Paper 2 examines the relationship between personality traits and self-reported and actigraphy-measured sleep health (sleep duration, sleep quality, sleep latency, insomnia symptoms, wake-after-sleep-onset). Paper 3 uses daily diary data to examine the link between pain and sleep health (sleep disturbances, napping) in older adults' everyday lives and test moderating effect of social support. Paper 4 examines sleep health (sleep latency, feeling unrested) as a mechanism linking physical activity and cognitive function. These papers use different project datasets that include diverse populations of middle-aged and older adults, such as the Sister Study, Midlife in the United States Study, and Daily Experiences and Well-being Study. At the end of these presentations, Dr. Buxton will discuss their theoretical and methodological contributions, and consider challenges and opportunities for future research.

### PERCEIVED JOB DISCRIMINATION AND SLEEP HEALTH AMONG WORKING WOMEN: FINDINGS FROM THE SISTER STUDY

Soomi Lee,[1] Anne-Marie Chang,[2] Dale P. Sandler,[3] Orfeu M. Buxton,[2] and Chandra L. Jackson[4], *1. University of South Florida, Tampa, Florida, United States, 2. Pennsylvania State University, State College, Pennsylvania, United States, 3. NIEHS, Durham, North Carolina, United States, 4. National Institutes of Health, Durham, North Carolina, United States*

Job discrimination is a social stressor that may lead to sleep health disparities in workers; however, limited research has examined the relationship, especially with specified sources of job discrimination. Using longitudinal data from the Sister Study, we tested the associations of perceived job discrimination (due to race, sex, age, and health conditions) with sleep health among working women (n=26,085). Among those without sleep difficulty at Time 1, race- and age-specific job discrimination was associated with increased odds of new onset sleep difficulty at Time 2. Moreover, among those without excessive sleepiness at Time 1, sex-, age-, and health-specific job discrimination predicted new onset of excessive sleepiness at Time 2. There was no association with sleep duration. We also found a dose-response relationship such that those who experienced job discrimination due to ≥3 reasons had greater odds of developing a sleep problem. Results suggest sleep health disparities emanating from the workplace.

### PERSONALITY AND SELF-REPORTED AND ACTIGRAPHY-MEASURED SLEEP HEALTH IN ADULTHOOD

Nasreen A. Sadeq,[1] Nasreen A. Sadeq,[1] Soomi Lee,[1] Alyssa Gamaldo,[2] and David M. Almeida[2], *1. University of South Florida, Tampa, Florida, United States, 2. The Pennsylvania State University, University Park, Pennsylvania, United States*

Personality may be associated with sleep health, however, the majority of existing studies rely on self-reported measures of sleep (often focusing on sleep duration). The purpose of this study is to examine the associations between Big Five personality traits and self-reported and actigraphy measured sleep. This study included 3928 participants and a subsample of 441 participants from the Midlife in the United States study. Linear regressions were used to analyze the relationships between personality traits and sleep. Neuroticism was associated with more frequent actigraphy-measured waking after sleep onset, and several self-reported measures of sleep quality, including shorter sleep duration, longer sleep latency, and a greater number of insomnia symptoms. Agreeableness was associated with shorter actigraphy-measured sleep duration and more self-reported insomnia symptoms. Our findings support an association between Neuroticism and poor sleep, and suggest that Agreeableness may be associated with worse sleep health.

### DOES RECEIVING SUPPORT HURT? PAIN AND SLEEP IN OLDER ADULTS' EVERYDAY LIVES

Meng Huo,[1] Jamie l. Fuentecilla,[1] kate Leger,[2] and Karen l. Fingerman[1], *1. The University of Texas at Austin, Austin, Texas, United States, 2. university of kentucky, Lexington, Kentucky, United States*

Pain is prevalent in late life and may cast negative impacts on older adults' sleep. We examined this link in older adults' everyday lives and asked whether receiving support on days when older adults had pain improved or worsened their sleep. We drew on the Daily Experiences and Well-being Study; over 300 adults aged 65+ reported on their pain, sleep and social support received throughout each day across 5 days. Multilevel models revealed that older adults in greater pain were more likely to nap throughout the day and to incur sleep disturbances at night. Older adults who slept better at night reported less pain the next day. The link between pain and sleep disturbances was stronger on days when older adults received support compared to days when they did not. This study adds to the literature regarding pain and sleep and explores what roles social factors play in this link.

### SLEEP QUALITY AS A MECHANISM LINKING PHYSICAL ACTIVITY AND COGNITIVE FUNCTIONING

Alycia N. Bisson,[1] and Margie E. Lachman[1], *1. Brandeis University, Waltham, Massachusetts, United States*

Modifiable health behaviors, such as physical activity and sleep quality are important for cognition throughout life. A growing body of research also suggests that engaging in enough physical activity is important to sleeping well. One recent study found that sleep efficiency mediates the relationship between physical activity and cognition. It is still unknown whether other metrics of sleep quality are mediators. The present study tested mediation in the second wave of the Midlife in the United States (MIDUS) study. Using the PROCESS macro for SPSS, we found that those who were more physically active fell asleep faster, and had better executive functioning. In addition, those who were more physically active reported waking up fewer times during the night, and had better executive functioning and self-rated memory. Discussion will focus on the moderating role of gender and distinctions between findings with different measures of sleep, physical activity, and cognition.

### SESSION 3585 (SYMPOSIUM)

### RACIAL DISPARITIES IN COMMUNITY- AND INSTITUTION-BASED LONG-TERM SERVICES AND SUPPORTS

Chair: Chanee D. Fabius, *Johns Hopkins University, Baltimore, Maryland, United States*

Long-term services and supports (LTSS) are services provided to individuals with functional limitations and chronic conditions who need assistance to perform daily activities such as bathing, dressing, preparing meals, and administering medications, and can be provided in community settings via services such as home health, as well as institutions such as nursing homes. Racial disparities are persistent across systems of LTSS, with older adults of color receiving lower quality care and experiencing worse health outcomes than their white counterparts. Given the increasing diversity of the aging population, and the need to ensure equity in quality and health outcomes in LTSS, there is a greater need for more understanding of how experiences of care vary across multiple settings for diverse groups of older adults and the people who help them. This symposium will feature 5 presentations that provide novel insight regarding racial disparities in community- and institution-based LTSS. We focus on racial differences in functional needs and disparities among those receiving home health services and living in nursing homes. Individual presentations will describe 1) race and gender differences in physical functioning needs of older adults; 2) disparities in home health quality across racially diverse and low income geographic areas; 3) racial disparities in nursing home residents overtime; 4) racial and ethnic disparities in rates of 30-day rehospitalization from skilled nursing facilities among Medicare Fee-For-Service and Medicare Advantage patients; and 5) the impact of the unequal burden of care provided to minority nursing home residents by staff of color.

### RACE AND GENDER DIFFERENCES IN PHYSICAL FUNCTIONING AMONG COMMUNITY-DWELLING OLDER ADULTS

Chanee D. Fabius,[1] Lauren J. Parker,[1] and Roland J. Thorpe Jr.[1], *1. Johns Hopkins University, Baltimore, Maryland, United States*

Prior work has demonstrated that there are race and gender disparities in the prevalence of need for assistance with tasks such as self-care, mobility, and household activities. Research has historically shown that older black Americans and women experience greater prevalence of physical functioning declines. It is unclear whether these differences persist among those receiving assistance. Using data from the 2015 National Health and Aging Trends Study (NHATS), a nationally representative study of Medicare beneficiaries aged 65 and older, and after adjusting for covariates, black men received less assistance with self-care and mobility activities, and white and black women received more help with mobility and household activities, compared to white men. Findings are critical to advancing our understanding of the needs of vulnerable older adults receiving assistance. More research is needed to understand the implications of these differences on long-term services and supports provided by both informal and formal caregivers.

### THE ROLE OF GEOGRAPHIC-LEVEL RACE AND SOCIOECONOMIC FACTORS ON ACCESS TO HIGH QUALITY MEDICARE HOME HEALTH

Shekinah A. Fashaw,[1] and Kali Thomas[1], *1. Brown University, Providence, Rhode Island, United States*

Prior research suggests that neighborhoods with predominantly minority and low-income individuals have decreased access to high quality hospitals, primary care physicians, and nursing homes. The purpose of this study is to determine if low quality Home Health Agencies (HHAs) are concentrated within more disadvantaged neighborhoods, while high quality HHAs cluster in more affluent neighborhoods. We characterize neighborhoods by racial and ethnic composition, and the proportion of residents below the federal poverty line; and HHAs according to their star ratings. We conduct a national, observational descriptive study using data from the 2017 HH Compare and American Community Survey. Predominantly black neighborhoods are served by the highest proportion of unrated and low-rated HHAs, while predominantly white neighborhoods have the highest proportion of high quality HHAs. This study signals and potential explanation for why minorities and duals receive care from lower quality HHAs. Such knowledge may help inform reimbursement and incentive practices.

### RACIAL DISPARITIES IN NURSING HOME RESIDENTS: QUALITY OF LIFE: DOES THE GAP PERSIST OVER TIME?

Tetyana P. Shippee,[1] Weiwen Ng,[2] John Bowblis,[3] Yinfei Duan,[2] Odichinma Akosionu,[1] and Mark Woodhouse[1], *1. University of Minnesota, Minneapolis, Minnesota, United States, 2. Dr, Minneapolis, Minnesota, United States, 3. University of Miami Ohio, Oxford, Ohio, United States*

The proportion of racial/ethnic minorities in nursing homes (NHs) has increased steadily in recent years. This study longitudinally examines minority NH residents' quality of life (QoL); a key measure of overall well-being. We used unique data from Minnesota annual QoL interviews (2011-2015), merged with resident and facility characteristics to model QoL. Mixed models with various resident and facility level controls, facility random effects, and both fixed and random effects for year were fit to estimate the effect of being a minority and living in a high-proportion minority facility on QoL. While white residents' unadjusted QoL scores remained stable over time, scores for minority residents declined. In full models, white residents in low-minority facilities consistently had the highest QoL scores while minority residents in high-minority facilities had the lowest scores. More policy attention is needed to address these persistent and possibly widening racial disparities, with targeted attention needed for high-minority facilities.

### RACIAL DISPARITIES IN 30-DAY READMISSION RATES AMONG MEDICARE PATIENTS DISCHARGED TO SKILLED NURSING FACILITIES

Maricruz Rivera-Hernandez,[1]
Maricruz Rivera-Hernandez,[1] Momotazur Rahman,[1] Vincent Mor,[1] and Amal N. Trivedi[1], *1. Brown University, Providence, Rhode Island, United States*

The 30-Day All-Cause Readmission Measure is part of the Skilled Nursing Facility Value-Based Purchasing (SNFVBP) beginning 2019. The objective of the study was to characterize racial and ethnic disparities in 30-day rehospitalization rates from SNF among fee-for-service (FFS) and Medicare Advantage (MA) patients using the Minimum Data Set. The American Health Care Association risk-adjusted model was used. The primary independent variables were race/ethnicity and enrollment in FFS and MA. The sample included 1,813,963 patients from 15,412 SNFs across the US in 2015. Readmission rates were lower for whites. However, MA patients had readmission rates that were ~1 to 2 percentage points lower. In addition, we also found that African-Americans had higher readmission rates than whites, even when they received care within the same SNF. The inclusion of MA patients could change SNF penalties. Successful efforts to reduce rehospitalizations in SNF settings often require improving care coordination and care planning.

### (UN)EQUAL BURDEN OF CARE: STAFF PERSPECTIVES ON NURSING HOME CARE AND QUALITY OF LIFE FOR RESIDENTS OF COLOR

Odichinma C. Akosionu,[1] Tetyana P. Shippee,[1] Heather Davila,[2] Mai See Thao,[3] Moses Waiswa,[1] and Tricia Skarphol[1], *1. University of Minnesota, Minneapolis, Minnesota, United States, 2. VA Boston Healthcare System, Boston, Massachusetts, United States, 3. Medical College of Wisconsin, Wauwatosa, Wisconsin, United States*

Racial disparities in quality of care (QoC) and quality of life (QoL) for nursing home (NH) residents persist even as the proportion of minorities is significantly increasing. Staff of color are a growing part of the long-term care workforce and staffing is a key component for delivering quality care. This study looks at staff (n=60) perspectives on resident QOL through semi-structured interviews, using thematic analysis in six Minnesota high proportion minority NHs. Key findings show that staff of color are concerned about the QoC and QoL residents of color experience, and take extra steps to provide care that goes beyond addressing their clinical needs. This agency of providing extra care is a factor in burnout among staff of color. More research on how this unequal burden of care impacts QoC/QoL is important to address the disproportionate role that staff of color play in reducing disparities in resident QoC and QoL.

### SESSION 3590 (PAPER)

### REFINING CONCEPTS IN FRAILTY

### A NETWORK ANALYSIS OF FRAILTY USING DATA FROM THE MEXICAN HEALTH AND AGING STUDY

Ricardo Ramírez-Aldana,[1] Carmen García-Peña,[1] Luis Miguel Gutiérrez-Robledo,[1] Lorena Parra-Rodriguez,[1] Juan Carlos Gómez-Verján,[1] and Mario Ulises Pérez-Zepeda[2], *1. Instituto Nacional de Geriatria, Mexico City, Mexico, 2. Instituto Nacional de Geriatria, Ciudad de Mexico, Mexico*

Frailty remains a challenge in the aging research area with a number of gaps in knowledge still to be filled. New approaches to its study have been proposed, including the one discussed in this article. We tested frailty as through the use of graphical probabilistic models (bayesian networks) with empirical data. Data from the Mexican Health and Aging Study (main data 2012, mortality 2015) was used. Frailty was operationalized with a 35-deficit frailty index (FI). Analyzed nodes were the deficits, plus death and the total score of the FI. The edges, or ties, linking those nodes (set E) were obtained through structural learning, and an undirected discrete graph G (V, E) associated with a discrete graphical probabilistic model (Markov network) was derived. Structural learning was possible through hill-climbing (hc) and PC algorithms. Analyses were performed for the whole population and tertiles of the total FI score. The number of connections within nodes increased according to the tertile level of the total FI score. Groups of interconnected deficits increased as the FI score raised. Almost all deficits related to mobility were interconnected and death was not the most connected node. Frailty behaves as a nonlinear system under the looks of a complex network. Further research should aim to identify the nature of the interactions observed. This could contribute to the development of a conceptual framework that would allow specific interventions to mitigate the consequences of frailty in older adults to be developed.

### IMPACT OF AN INTENSIVE MULTIDOMAIN LIFESTYLE INTERVENTION ON DEFICIT ACCUMULATION FRAILTY INDICES

Felicia Simpson,[1] Nicholas M. Pajewski,[2] Alain M. Bertoni,[2] Frank Ingram,[1] Barbara M. Nicklas,[2] Stephen Kritchevsky,[2] Daniel Ojeranti,[1] and Mark M. Espeland[2], *1. Winston-Salem State University, Winston-Salem, North Carolina, United States, 2. Wake Forest School of Medicine, Winston-Salem, North Carolina, United States*

Background: Type 2 diabetes and obesity increase accumulation of health deficits over time and may accelerate biological aging. It is unknown whether multidomain lifestyle interventions can mitigate against this. Methods: Within a large, randomized controlled clinical trial of intensive lifestyle intervention (ILI) including caloric restriction, increased physical activity, dietary counseling, and risk factor monitoring compared with diabetes support and education (DSE) we examined the trajectory of frailty across 8 years. We used two complementary frailty index (FI) definitions, one modeled on work in the Systolic Blood Pressure Intervention Trial; the other including additional deficits related to aging with obesity and type 2 diabetes mellitus. Differences between intervention groups and the consistency of these across clinical subgroups were assessed with re-randomization tests. Results: Data from 4859 adults (45-76 years at baseline, 59% female) were analyzed. Random assignment to ILI was associated with lower FI scores throughout 8 years of follow-up ($p<0.001$), over which time mean differences between intervention groups averaged 5.8% and 5.4% for the two indices. At year 8, the percentages of participants categorized as frail ($FI>0.21$) were lower among ILI (39.8% and 54.5%) compared with DSE (42.7% and 60.9%) for the two indices (both $p<0.001$). Intervention benefits were relatively greater for individuals who were older, not obese, and without history of cardiovascular disease at baseline. Conclusions: Eight years of multidomain lifestyle intervention slows the accumulation of health deficits over time in overweight or obese adults with type 2 diabetes.

### PREDICTING ADVERSE OUTCOMES IN ED PATIENTS USING THE CLINICAL FRAILTY SCALE AND A FRAILTY INDEX

Kenneth Rockwood,[1] Mohammad Pulok,[2] Alex van der Valk,[2] and Olga Theou[1], *1. Dalhousie University, Halifax, Nova Scotia, Canada, 2. Nova Scotia Health Authority, Halifax, Nova Scotia, Canada*

Our aim was to use the Comprehensive Geriatric Assessment (CGA) database to investigate whether the Clinical Frailty Scale (CFS) measuring the baseline state, and a Frailty Index (FI) based on a CGA (current state, with acute illness) can predict adverse outcomes in acutely ill Emergency Department (ED) patients. It contains CFS and FI scores on 1028 ED patients referred to internal medicine at the Halifax Infirmary between 2009-2019 (Mage 80.69 ± SD 8.28, range 57-103; 54.9% female). The mean scores were 0.44±0.14 (FI) and 5.58±1.66 (CFS). Most patients (72%) arrived via ambulance. The average length of stay was 27.0±20.5 hours. Overall, 22% were discharged home, and 63.5% had died by December 2017 with a mean survival time of 1.98±2.01 years. Controlling for age, sex, and Canadian Triage Acuity Score, the odds ratio (95% Confidence Interval) of being discharged home and the hazard ratio (95% Confidence Interval) for mortality was 0.94 (0.92-0.95) and 1.02 (1.02-1.03), respectively per 0.01-point increase in FI. For the CFS, using score ≤4 as the reference, the odds ratio and the hazard ratio were 0.70 (0.42-1.16) and 2.02 (1.51-2.69), respectively for the CFS 5 group, 0.47 (0.27-0.81) and 2.72 (2.05-3.61), respectively for the CFS 6 group, and 0.38 (0.21-0.70) and 4.67 (3.51-6.20), respectively for the CFS 7-9 group. Even controlling for acuity, both the CFS and the FI independently predict adverse outcomes in ED patients. These add prognostic information to the routinely collected ED assessments, and establish targets for care plan based on recovery to baseline.

### SOCIAL COHESION AFFECTS THE ASSOCIATION BETWEEN FRAILTY AND DISABILITY IN COMMUNITY-DWELLING OLDER ADULTS

Boqin Xie[1], *1. Fudan University School of Nursing, Shanghai, China, China*

The relationships between physical frailty and perceived neighborhood social cohesion (PNSC) and functional disability among community-dwelling older adults are poorly understood. This study aims to (1) examine the associations of frailty and PNSC with disability; and (2) evaluate low PNSC as a risk factor in the association between frailty and disability. A sample of 1645 older adults using multistage sampling method in Shanghai were randomly selected in this cross-sectional study. Frailty operationalized as Cardiovascular Health Study criteria (OR=2.4, 95%CI 1.16-4.96 for pre-frailty; OR=7.28, 95%CI 3.37-15.73 for frailty) and PNSC measured as Neighborhood Cohesion Scale (OR=1.81, 95%CI 1.23-2.67) were independently associated with basic and instrumental activities of daily living disability. A significant interaction of frailty and PNSC on disability (F (2, 66)=4.31, P=.014) was found, using a two-way analysis of covariance (ANCOVA). Compared to robust individuals with high PNSC, pre-frailty with high PNSC was not significantly associated with disability while pre-frailty with low PNSC was associated with approximate 4-fold increased prevalence of disability (OR=3.87, 95%CI 1.46-10.24, p=.006). Frailty with high PNSC was associated with higher likelihood of disability (OR=6.47, 95%CI 2.35-17.87) and frail individuals with low PNSC stood out with 10-fold increased prevalence of disability (OR=9.94, 95%CI 3.50-28.26). All analyses were controlled for demographical and clinical covariates. Our results suggest high level of social cohesion serves as a buffer against the impact of physical frailty on functional disability. These findings notably imply to the development of interventions for older frail adults from the neighborhood perspective.

### SESSION 3595 (PAPER)

### RESEARCH METHODS AND MEASUREMENT OF COGNITIVE IMPAIRMENT

### FEASIBILITY AND ACCEPTABILITY OF DETECTING COGNITIVE IMPAIRMENT AND DEMENTIA IN PRIMARY CARE PRACTICES

Rachel O'Conor,[1] Julia Yoshino Benavente,[1] Laura Curtis,[1] Marina Arvanitis,[1] Richard Gershon,[1] and Michael Wolf[1], *1. Northwestern University, Chicago, Illinois, United States*

As the age of the US population increases, so does cognitive impairment (CI); therefore early detection of CI is critical for ensuring its appropriate management. As part of a NINDS Consortium to detect CI and dementia in primary care (DetectCID), we are implementing and evaluating a brief 2-step CI detection paradigm (MyCog), that can be delivered in clinics with diverse populations via the electronic health record (step 1) and iPad (step 2). We conducted focus groups with 25 clinicians and administrative leaders from academic and community primary care practices to 1) understand how CI is being assessed, and 2) evaluate the feasibility of implementing the MyCog paradigm into existing primary care workflows. Several key themes emerged from the discussions. No proactive detection strategy for CI was regularly used outside of the Medicare Annual Wellness Visits (AWV); variable assessments including the Minicog, MoCA, or MMSE were used to fulfill the AWV requirement. Regarding the feasibility of our MyCog Paradigm, our 2-step process was positively received, with the brief case-finding step 1 satisfying AWV requirements and replacing the longer assessments currently being used. Clinicians preferred that step 2 be self-administered due to limited clinician time for wellness visits, and highlighted logistical challenges such as room availability and storage and maintenance of the iPad. Overall, clinicians felt that the identification of CI was valuable and supported standardization, but indicated regular case finding was unlikely without clear guidance on clinical decision-making.

### GEOGRAPHIC DISPARITIES IN COUNTY-LEVEL PREVALENCE OF ALZHEIMER'S DISEASE ACROSS THE UNITED STATES

Mackenzie E. Fowler,[1] Christina DiBlasio,[1] Michael Crowe,[1] and Richard E. Kennedy[1], *1. University of Alabama at Birmingham, Birmingham, Alabama, United States*

Small-area geographic disparities in health care delivery have been observed across multiple disorders, but remain poorly studied for Alzheimer's disease (AD) and other dementias. While national and state estimates of the prevalence and incidence of AD are available, estimates across finer geographic regions offer an opportunity to tailor programs to the needs of the local population. We estimated prevalence of AD at the county level across the continental United States. We used prevalence rates of AD by age category and race among Medicare fee-for-service beneficiaries published by the Centers for Disease Control (CDC). These prevalence rates were projected onto bridged-race county-level population data for 2017 from the National Center for Health Statistics, with empirical Bayes spatial smoothing to reduce variability in rates due to small population sizes. Estimated prevalence of AD varied more than threefold across counties, from a low of 51.8 per 1,000 persons in Loving County, Texas to a high of 169.6 per 1,000 persons in Kalawao County, Hawaii. Higher prevalence of AD was seen in the Southeastern and Midwestern United States. However, we observed specific counties with low prevalence of AD within regions with high prevalence of AD, and vice versa. These small-area geographic variations may provide vital information about social and environmental influences on dementia care, yet little data have been available to date. Understanding geographic disparities in prevalence will be critical for addressing practice variation in the prevention and diagnosis of dementia.

### MISCLASSIFICATION IN DEMENTIA DATA IN THE CARDIOVASCULAR HEALTH STUDY: A PROBABILISTIC BIAS ANALYSIS

Michelle Odden,[1] Alice Arnold,[2] Anne B. Newman,[3] Adina Zeki Al Hazzouri,[4] Lindsay Miller,[5] Oscar Lopez,[6] Kenneth Mukamal,[7] and Andreea Rawlings[8], *1. Stanford University, Stanford, California, United States, 2. University of Washington, Seattle, Washington, United States, 3.*

*University of Pittsburgh, Pittsburgh, Pennsylvania, United States, 4. Columbia University, New York, New York, United States, 5. Oregon State University, Corvallis, Oregon, United States, 6. University of Pittsburgh, Pittsburgh, Pennsylvania, United States, 7. Beth Israel Deaconess Medical Center, Boston, Massachusetts, United States, 8. Kaiser Permanente Center for Health Research, Portland, Oregon, United States*

Data routinely collected in observational studies from phone calls, medical records, and death certificates can be used to categorize dementia, though there may be misclassification. We applied probabilistic bias analyses to assess the magnitude, direction, and uncertainty of the error due to misclassification of dementia data in the Cardiovascular Health Study (CHS). We categorized dementia among all participants (73,284 person-years) using medications, ICD-9 codes, use of proxy, and death certificates, and compared to the gold standard adjudicated dementia in the CHS Memory Studies (28,250 person-years). Using the gold standard, positive (PPV) and negative predictive values (NPV) of dementia categorization were estimated within strata defined by age and sex. In probabilistic bias analyses, we reclassified participants from the full study using estimated PPVs and NPVs in 5,000 replicates. We estimated the hazard ratio (HR) of dementia associated with age, race, sex, hypertension, diabetes, and APOE4 genotype in this bias analysis and compared these results to those using original data. ICD-9 codes had low specificity and were excluded in further analyses. The NPV was differential by sex (66% for females and 79% for males) and race (51% for blacks, 60% for whites). In bias analysis, the HR for black race was attenuated from 2.81 (95%CI:1.36-5.80) to 1.23 (95%CI:1.14-1.33). The estimate for hypertension was statistically significant only in bias analysis. Estimates and inferences for the other covariates were modestly different. Differential misclassification may lead to important biases of risk factors, but can be recognized and addressed using probabilistic bias analyses.

### PROMIS COGNITIVE FUNCTION AND CONCERN SCALES: LINGUISTIC VALIDATION IN AMERICAN SIGN LANGUAGE FOR DEAF OLDER ADULTS

Poorna Kushalnagar[1], *1. Gallaudet University, Washington, District of Columbia, United States*

Objective: To culturally and linguistically validate PROMIS Cognitive Functions and Concerns Scales in American Sign Language (PROMIS-ASL) for use with deaf older adults over 50. Methods: We used the standard procedures developed at the U.S. National Center for Health Statistics Cognitive Survey Laboratory to culturally adapt and translate items from the cognitive scales of the Patient Reported Outcomes Measurement Information System (PROMIS). We describe cultural adaptation and linguistic translation procedures led by a team of primarily deaf investigators. Using multidimensional exploratory or confirmatory factor analyses, we identify or confirm the items most likely to comprise each subset. Once the PROMIS-ASL version is finalized, we will compute test-retest reliability using ICC (intraclass correlation coefficient) from two-way random effects ANOVA models. Results: We produced an accessible patient reported outcomes cognitive measure in American Sign Language and a culturally appropriate set of items that are relevant to the experiences of deaf older adult over 50 users of accessible technology and services. Conclusions: The final PROMIS-ASL product with cognitive domain will be distributed for public use.

### USING SERIAL TRICHOTOMIZATION WITH NEUROPSYCH MEASURES TO INFORM DECISIONS ON FITNESS TO DRIVE AMONG OLDER ADULTS

Stephanie Yamin,[1] Valerie Ranger,[1] Arne Stinchcombe,[1] Frank Knoefel,[2] and Michel Bédard[3], *1. Saint Paul University (Ottawa), Ottawa, Ontario, Canada, 2. Bruyere Continuing Care, Ottawa, Ontario, Canada, 3. Lakehead University, Thunder Bay, Ontario, Canada*

Older adults report that driving provides a sense of independence and wellbeing. For some older adults, driving cessation becomes necessary due to their health status having an impact on their ability to drive safely. Decisions related to driving cessation are difficult and often left to the clinical judgement of primary care physicians. There is an interest in developing a method that could help assist physicians in making that determination. To date, there is no neuropsychological test that produces an acceptable level of sensitivity and specificity allowing for the determination of an individual's fitness to drive. Serial trichotomization involves classifying drivers as either pass, fail or indeterminate based on cutpoints that leads to 100% sensitivity and specificity. The purpose of this study was to examine the serial trichotomization method using four common neuropsychological tests (i.e., 3MS, Trails A & B, clock drawing). Sensitivity and specificity for each test were established using a medical expert's clinical judgement. Charts of 105 patients at a tertiary memory disorders clinic were reviewed and data related to neuropsychological test scores and clinical judgement around fitness to drive were abstracted. After applying the trichotomization, 38.1% of the sample were classified as unfit to drive, 36.1% were classified as indeterminate, and 25.8% were classified as fit to drive. This study adds to the growing body of literature supporting the use of serial trichotomization to streamline decision-making about fitness to drive.

### SESSION 3600 (PAPER)

### SOCIAL DETERMINANTS OF HEALTH

### COMMUNITY-LEVEL SOCIAL DETERMINANTS OF MORTALITY IN OLDER ADULTS: AN ASSESSMENT OF RHODE ISLAND CITIES AND TOWNS

Steven A. Cohen,[1] Julia Broccoli,[1] Heather O'Neill,[2] and Mary L. Greaney[1], *1. University of Rhode Island, Kingston, Rhode Island, United States, 2. PixelsforHumans, East Greenwich, Rhode Island, United States*

Addressing the causes of place-based health disparities among older adults have focused on understanding social determinants of health on a large geographic level, such as region, state, or county. However, there is a growing realization for the need to understand how place-based characteristics at smaller geographic areas relate to population health and contribute to successful aging. The purpose of this study was to assess the magnitude of the associations between place-based social determinants and life expectancy (LE)

among older adults and related measures. Methods: LE at age 50 (LE50) and age 65 (LE65), and the age-specific mortality rate (ASMR) for ages 65+ (ASMR65) were calculated from mortality data (2009-2011) from the Rhode Island (RI) Department of Health (RIDoH) using abridged life table methods for each RI city/town. City/town-specific LE and ASMR were linked to the US Census, RIDoH, and other databases that include social determinants: demographics, household composition, wealth, education, environment, food insecurity, crime, transportation, and rural-urban status. Bivariate and partial correlations were examined between city/town-level LE50, LE65, and ASMR65. Results: LE50, (range: 29.3-34.0 years) was most strongly associated with the percent of the population with at least a bachelor's degree (r=0.652, p<0.001), per capita income (r=-0.632, p < 0.001), and percent multigenerational households (r=-0.489, p=0.003). The associations between both LE65 and ASMR65 and examined social determinants were more attenuated, however. Discussion: These results highlight substantial place-based disparities in mortality and potentially addressable social determinants that could improve population health for older adults and reduce place-based disparities among neighboring communities.

### COPING STRATEGIES AS MODERATORS OF NEGATIVE HEALTH IMPACTS FROM RELOCATION AT VERY OLD AGE

Bjorn Slaug,[1] and Oliver K. Schilling[2], *1. Department of Health Sciences, Faculty of Medicine, Lund University, Lund, Sweden, 2. Department of Psychological Ageing Research, Institute of Psychology, Heidelberg University, Heidelberg, Germany*

Relocation at very old age is a major life event that may have profound psychological impact. The loss of a home environment that has shaped important aspects of the life course can have negative health impacts, such as lower life satisfaction, depressive symptoms, loss of perceived autonomy and functional independence. However, from an ecological theory perspective, implying that patterns of health and well-being are impacted by a dynamic interplay of personal and environmental factors unfolding throughout the life course, the individual's adaptive repertoire and resources influence how the individual manages and copes with such major life events as relocation to a new housing environment. To study if coping strategies moderated negative health impacts from relocation at very old age, we utilized longitudinal data of older community-living people from Sweden and Germany who had relocated at some point over a nine-year period (N=79, aged 80+ at baseline). A mixed model approach, adjusting for age at time of relocation, was used to analyze moderating effects of different coping strategies, defined according to Staudinger, Freund & Smith (1995). We found pro-active coping strategies such as reminding oneself of previous ability to solve problems, to significantly moderate negative effects on perceived functional independence and resilient strategies such as letting things just have their course, to significantly moderate negative effects on life satisfaction. We found no significant moderation of depressive symptoms. These results suggest that individual disposition to use different coping strategies can moderate the impact on health that relocation at very old age has.

### INTERVENTIONS FOR LONELINESS AMONG OLDER ADULTS IN FACILITIES: A SYSTEMATIC REVIEW

Nicolas Quan,[1] and Matthew Lohman[1], *1. University of South Carolina, Columbia, South Carolina, United States*

Loneliness affects an estimated 1 in 3 older adults ages 65 and over in America. According to a 2017 report by AARP, a lack of meaningful social contact among older adults is associated with $6.7 billion dollars of federal healthcare spending. Research suggests that interventions such as social facilitation and skills development may decrease loneliness; however, the effectiveness of such interventions for older adults living in long-term care facilities is unclear. Articles matching search criteria were collected from PubMed, PsycInfo and Web of Science from 2009 to 2019. Inclusion criteria were: 1) intervention studies, 2) individuals age >= 65, 3) participants living in a long-term care facility such as a nursing home, assisted-living, or hospice facility. Randomized controlled trials, quasi-experimental and single-group studies were included. Title and abstract screening, as well as full text extraction followed PRISMA guidelines. A total of 16 articles that met inclusion criteria were identified. The interventions included video chatting with family members, human-volunteer interaction, human-robot interaction, humor therapy, a reminiscent radio program, laughter therapy and gardening education. Fourteen studies demonstrated a statistically significant decrease in loneliness from baseline to post-intervention. Laughter therapy showed the greatest reduction in loneliness. Diversity of intervention types and loneliness measures meant we could not estimate a pooled measure of effectiveness. Results suggest that there are several effective interventions to reduce loneliness among older adults in facilities; however, lack of standardized measures and high-quality studies limits comparisons between intervention types and generalizability to different populations.

### MULTIPLE CHRONIC CONDITIONS AND ACCELERATED AGING IN PEOPLE EXPERIENCING HOMELESSNESS

Julie Zuniga,[1] Whitney Thurman,[2] and Dong Eun Jang[1], *2. University of Texas at Austin College of Pharmacy, Austin, Texas, United States, 1. The University of Texas at Austin School of Nursing, Austin, Texas, United States*

The median age of the U.S. homeless population is increasing, and PEH often experience accelerated aging compared to the general population. Moreover, PEH have disproportionately high rates of chronic illness, psychological distress, and poor health status. The purpose of this study was to investigate associations between morbidity, psychological distress, and healthcare utilization in PEH. The specific aims of this study were to: 1) determine the prevalence of multiple chronic conditions in PEH, 2) analyze whether prevalence varies by level of psychological distress, and 3) analyze associations between age, health conditions, and psychological distress. We used data from the 2010 National Health Interview Survey. Analysis included descriptive statistics, t-tests, chi-square tests, ANOVA, and correlations. Homelessness was operationalized using the variable from the NHIS asking if the individual had spent >24 hours homeless in the past year. Measures included demographics, 11 chronic conditions, and psychological distress. PEH (n = 1809) were majority male (68.9%), White (66%), and unmarried (61.1%). Mean age of

PEH was significantly higher than housed individuals (43.5 vs. 38.4 years, p < .000). Chi-square tests indicated that the prevalence of hepatitis, heart disease, emphysema, arthritis, epilepsy, and asthma were significantly higher (p < .01) in the PEH group than in the general population. Psychological distress differed significantly by morbidity. The relatively young mean age and the high burden of morbidity suggest that aging may be progressing more rapidly in PEH than previously recognized. Future research is needed to investigate the trajectory of aging in this population.

### SESSION 3605 (SYMPOSIUM)

### SOCIAL ENGAGEMENT IN OLD AGE: IMPACTS OF AGING UNDER INTERPERSONAL, MORAL, AND POLITICAL CONTEXTS

Chair: Minjie Lu, *Sun Yat-Sen University, Guangzhou, Guangdong, China*
Discussant: Xin Zhang, *Peking University, Beijing, China*

To promote successful aging, social engagement has been encouraged among older adults. Yet, thus far, research on older adults' social engagement have been preoccupied with the close and intimate relationships between older adults and their families, friends, or caretakers. Little attention is being paid to how older adults may engage in peripheral social networks, civic activities, or public affairs. This symposium features four presentations that investigate older adults' social engagement in these contexts. First, with the focus on interpersonal interaction with strangers, Yi Lu and colleagues examine the age differences in trustworthiness perception of unfamiliar faces. The second and third presentations focus on moral and political contexts. Minjie Lu will present the divergent impacts of age on the cognitive evaluations and emotional responses towards moral issues. Wong and Fung analyze older adults' engagements in political discussions and actions and the factors that may promote these engagements. Last but not least, Nicole Fung and colleagues will present findings that generativity among older adults can promote sense of meanings and death acceptance, demonstrating the beneficial effects of social engagement in old age. Finally, Zhang will provide closing discussions about the implications and future directions of these presentations.

### VIEWING TIME AND FACIAL TRUSTWORTHINESS PERCEPTIONS: GIVE IT A SECOND THINK MAY NOT WORK FOR OLDER ADULTS

Yi Lu,[1] Xu Ye,[1] Yin Xiaowei,[1] and Zhang Xin[1], *1. School of Psychological and Cognitive Sciences, Peking University, Beijing, Beijing, China (People's Republic)*

Older adults tend to rate higher on trustworthiness of unfamiliar faces than younger adults. Contrary to the notion that it would be beneficial for them to have enough time to process, cognitive control theory suggests that older adults' facial trustworthiness evaluation increases over time, making them more vulnerable to fraud. The present study aimed at exploring age differences in trustworthiness perception of unfamiliar faces, and how viewing time could impact. A 2 (age: old vs. young) ×2 (viewing time: 500 ms vs. 3000 ms) ×2 (facial trustworthiness: control vs. low trustworthiness) factorial design was conducted. As expected a significant three-way interaction revealed that viewing time only influenced older adults' facial trustworthiness evaluation, and only when given shorter viewing time did older adults exhibit accurate facial trustworthiness ratings as younger adults. These findings suggest that a second think in facial perception may not work for older adults.

### MORAL GAIN OR DECAY? EXAMINING AGE-RELATED CHANGES IN MORAL JUDGMENT

Minjie Lu,[1] and Helene H. Fung[2], *1. Sun Yat-Sen University, Guangzhou, Guangdong, China, 2. The Chinese University of Hong kong, shatin, Not Applicable, Hong Kong*

The present study investigates age-related changes in moral judgment. In particular, we examined both cognitive and affective dimensions of morality in contributing to moral punishment. One hundred and twenty participants (aged from 22 to 75) recruited from Mturk were presented with 10 moral transgression stories (e.g. lying, harming), and reported their wrongness judgment, moral conviction, emotional experience, and moral punishment. Results revealed divergent patterns on the relationships between age and the evaluations on cognition and emotion. In terms of cognitive evaluation, compared to younger adults, older adults perceived immoral acts as more wrong and considered their stands as more connected to their moral conviction. However, older adults reported less intense negative emotions (anger, disgust, contempt), suggesting they were less aroused by immoral acts. In terms of moral punishment, age was negatively correlated with punishment, and this correlation was mediated by the age-related decrease in negative emotions.

### AGE DIFFERENCES IN POLITICAL ENGAGEMENT: ROLE OF SELF-RELEVANCE

Tze Kiu Wong,[1] and Helene H. Fung[2], *1. The Chinese University of Hong Kong, Hong Kong, Hong Kong, 2. Department of Psychology, The Chinese University of Hong Kong, Hong Kong, Not Applicable, Hong Kong*

Previous studies usually found that older people are less politically engaged than younger adults, especially when considering political behavior other than voting. The current study extends the Selective Engagement hypothesis (Hess, 2014) to political engagement. 81 younger adults and 79 older adults rated 8 issues on self-relevance and their willingness to engage in political discussion, arguments and collective action on each issue. The predicted moderating effect of self-relevance was not found, but older people indeed are more willing to discuss ($B = 0.07$, $p = 0.027$) and argue with others on more self-relevant issues ($B = 0.06$, $p = 0.031$). Perceived cost of collective action was found to be a moderator, such that self-relevance was less important than other factors for high-cost actions ($B = -0.016$, $p = 0.013$). The current research sheds light on potential ways to increase older adults' engagement in social issues.

### BE GENERATIVE TO ACCEPT DEATH: ROLE OF MEANING IN LIFE MODERATED BY AGE AND PERCEIVED IMPACT

Nicole Long Ki Fung,[1] Steven Tsun-wai Chu,[2] and Helene Hoi-lam Fung[2], *1. the Chinese University of Hong Kong, Hong Kong, Hong Kong, 2. The Chinese University of Hong Kong, Hong Kong, Not Applicable, Hong Kong*

Meaning-management theory (MMT) suggests living a meaningful life leads to higher death acceptance. This paper investigates how generativity, i.e. the intention to bring benefits to the next generation, can affect death acceptance through achieving meaning in life (MIL). 343 participants in Hong Kong (aged 18-90) filled in a questionnaire as part of "Age(ing) as Future" project. Generativity positively correlates with death acceptance. MIL fully mediates the effect of generativity on death acceptance (r(343)=.132, p=.014). The effect of generativity on MIL might differ by age and perceived influences of generative acts. As speculated, the mediation is moderated by Age and Transformational Future Time Perspective (TFTP; generative impact that expands future time perspective). In older adults with lower TFTP, generativity no longer predicts MIL and the mediation was nonsignificant. The results provide empirical support to MMT and emphasise the importance of perceived impact and the needs in different developmental stage.

### SESSION 3610 (SYMPOSIUM)

### THE BLUES AND OLDER MINORITY MUSICIANS: MORE THAN JUST MUSIC XXVI

Chair: John N. Migliaccio, *Maturity Mark Services Co., White Plains, New York, United States*

This 26th annual symposium again showcases a worldwide musical genre in a regional setting with local performers. Texas Blues has emanated from a pantheon of talented artists beginning in the earliest days of Roots and Blues music to the present day, and Austin has become the epicenter of this talent and music. With legendary classic blues musicians from the early 2oth century to emerging younger musicians who re-energize and re-invent this uniquely American musical genre, to legendary music labels and venues like Antone's which continue to engage blues music artists of all ages, Austin continues to be the home of Texas Blues. Lifetime Achievement Award -winning 89-year-young, Miss Lavelle White epitomizes the resilience and energy interchange of both the performer and the music, and their mutual contribution to longevity and continued engagement. Still performing after 70 years, she has influenced generations of younger prominent blues performers and continues to appear weekly at local blues mecca Antone's along with her Grammy Award winning band and is preparing her fourth album release. This session will celebrate her music, her artistry, and her continued success as an older blues performer. A visit to a local blues venue later in the evening will allow for a true appreciation of the blues music scene in Austin.

### OLDER BLUES MUSICIANS

Michael Marcus[1], *1. Consultants for Community Resources, Raleigh, N.Carolina, United States*

Older Blues musicians have provided inspiration to generations of younger musicians as well as their own contemporaries.

### OLDER BLUES MUSICIANS

Debra J. Sheets[1], *1. University of Victoria, Victoria, British Columbia, Canada*

Older female musicians face challenges and advantages from longevity

### SESSION 3615 (SYMPOSIUM)

### VIEWS ON AGING: NEW PERSPECTIVES FOR THEORY AND RESEARCH

Chair: Verena Klusmann, *University of Konstanz, Konstanz, Germany*
Co-Chair: Anna E. Kornadt, *Bielefeld University, Bielefeld, Germany*

Over the past 20 years, research on views on aging has substantiated their importance for successful development and sustained quality of life over the full length of the life span. However, a deep understanding of the origins of views on aging and the underlying processes of their lifespan development and manifestation is lacking. Since 2017, the scientific network "Images of Aging" funded by the German Research Foundation (http://www.health.uni-konstanz.de/images-of-aging) assembles national and international renowned experts in the field. The network engages in empirical clarifications on both the distinctness and validity of the construct (contribution of Klusmann et al.) as well as in critically reviewing terminology and measures of views on aging (contribution of Notthoff et al.). The network aims to help clarifying the dynamic interplay of determinants and outcomes in the context of health (contribution of Wolff et al.) as well as disentangling intra- and intergenerational stereotypic perceptions (contribution of Kornadt et al.). Both of these are understudied issues with highly practical implications for two of the largest demographic challenges: shaping the coexistence of generations as well as providing adequate health care supply. Integrating both pertinent theoretical approaches and empirical findings the network regards views on aging under a lifespan perspective. Recently, it suggested three core principles of views on aging regarding lifelong bio-psycho-social development, their multidimensional nature, and their impact across life. These considerations provide a background for an integrative discussion of the symposium's contributions.

### SUBJECTIVE AGING: SOMETHING UNIQUE OR JUST ANOTHER EXPRESSION OF GENERAL SELF-BELIEFS?

Verena Klusmann,[1] Svenja M. Spuling,[2] Catherine E. Bowen,[3] Anna E. Kornadt,[4] and Eva-Marie Kessler[5], *1. University of Konstanz, Konstanz, Germany, 2. German Center of Gerontology, Berlin, Berlin, Germany, 3. Independent Researcher Scientific Network Images of Aging, Vienna, Wien, Austria, 4. Bielefeld University, 33615, Nordrhein-Westfalen, Germany, 5. Medical School Berlin, 12247, Berlin, Germany*

Using data from the German Ageing Survey (adults aged 40–85), this study tested the convergent and discriminant validity of subjective aging measures by comparing three different measures of subjective aging with one another and relating them to established measures of general self-beliefs (optimism, self-efficacy, subjective health) and subjective well-being (depression, affect). Correlations between subjective aging measures ranged from −.61 (amongst general self-perceptions of aging measures) to −.09, with subjective age being least related to the other measures. The highest overlap was observed between optimism and global self-perceptions of aging (.69) and it was for these global self-perceptions that the highest amount of variance could be explained by correlates in a regression analysis (R-square=.55). In contrast, only 10% of variance could be explained for subjective age. Our results underline the merit of taking the multidimensional nature of subjective aging into account since global measures appear less distinct from general personality traits.

### THE ASSESSMENT OF VIEWS ON AGING: A REVIEW OF SELF-REPORT MEASURES AND INNOVATIVE EXTENSIONS

Nanna Notthoff,[1] Ann-Kristin Beyer,[2] Anne Blawert,[3] Martina Gabrian,[4] and Verena Klusmann[5], *1. Leipzig University, Leipzig, Germany, 2. Charité Universitätsmedizin Berlin, Berlin, Berlin, Germany, 3. Friedrich-Alexander University of Erlangen-Nürnberg, Nuremberg, Bayern, Germany, 4. Independent Researcher Scientific Network Images of Aging, Frankfurt, Hessen, Germany, 5. University of Konstanz, Konstanz, Baden-Wurttemberg, Germany*

Individual views on aging refer to either older people in general or to a person's own age and aging. Classical approaches seem to hardly map the multidimensional, multidirectional, and highly individual nature of experiences as well as the malleability of perceptions of aging. For this paper, we reviewed existing measures of views of aging. These were categorized based on eight dimensions which were defined by an expert panel and based on current characterizations of views on aging in the literature. Results on 96 instruments call for a strengthening of the affective and the behavioral components in contrast to the apparently cognitive focus of the measures and argue for a stronger emphasis on the developmental nature of views on aging (time references, changes). This is particularly important when aiming to study the lifelong dynamics of views on aging. The suitability of innovative extensions will be discussed.

### DO SERIOUS HEALTH EVENTS CHANGE HOW WE VIEW OUR OWN AGING? ON THE ROLE OF CARDIOVASCULAR EVENTS

Julia K. Wolff,[1] Svenja M. Spuling,[2] Ann-Kristin Beyer,[3] Maja Wiest,[4] and Susanne Wurm[1], *1. Friedrich-Alexander-Universität Erlangen-Nürnberg, Nuremberg, Bayern, Germany, 2. German Centre of Gerontology, Berlin, Berlin, Germany, 3. Charité - Universitätsmedizin Berlin, Berlin, Berlin, Germany, 4. Freie Universität Berlin, Berlin, Berlin, Germany*

Several studies have demonstrated beneficial effects of views on aging (VoA) on health, while the reverse relationship is seldom in focus. Serious health events (e.g., myocardial infarction) are life-threatening and remind individuals of the finitude of life possibly changing their VoA. The present study investigates the effect of cardiovascular events (CVE) on longitudinal changes in VoA using pooled data of three waves of the German Ageing Survey (2008, 2011, 2014, age-range: 40-95 years). To account for alternative explanations, individuals without CVE were matched to the individuals with CVE (n = 202) using a propensity-score-matching approach. Individuals who experienced a CVE showed more adverse changes in three VoA indicators (aging associated with physical losses, ongoing development, felt age) than individuals without CVE. Results show that CVE can change how we view our own aging which in turn affects future health changes. Following a CVE people may benefit from promoting positive VoA.

### YOUNG PEOPLE FEEL WISE, OLD PEOPLE FEEL ENERGETIC: COMPARING AGE STEREOTYPES AND SELF-EVALUATIONS ACROSS ADULTHOOD

Anna E. Kornadt,[1] Catherine E. Bowen,[2] Svenja M. Spuling,[3] and Maja Wiest[4], *1. Bielefeld University, Bielefeld, Germany, 2. Independent Researcher, Vienna, Wien, Austria, 3. German Centre of Gerontology (DZA), Berlin, Berlin, Germany, 4. Free University Berlin, Berlin, Berlin, Germany*

Using questionnaire data from the MIDUS study (N=6.325) we examined the extent to which people in their late 20s, 40s, and 60s think that positive stereotypic "old" and "young" characteristics describe themselves, their age peers, and other age groups. A constellation of "old" characteristics (e.g., wise, caring, calm) was seen as more descriptive of older adults, while a constellation of "young" characteristics (e.g., healthy, energetic) was seen as more descriptive of younger adults. Self-evaluations were highly positive and largely consistent across age groups. Compared to their age peers, younger adults saw themselves as having as many positive "young" characteristics but more positive "old" characteristics whereas older adults saw themselves as having more positive "young" characteristics but fewer positive "old" characteristics. The results support the stability of the aging self despite the existence of age stereotypes and the role of negative age stereotypes as a frame of reference for making self-evaluations.

### SESSION 4000 (SYMPOSIUM)

### AGING IN LATIN AMERICA: A FOCUS ON MIDDLE-INCOME COUNTRIES

Chair: Catherine Garcia, *University of Southern California, Los Angeles, California, United States*
Discussant: Maria P. Aranda, *University of Southern California, Los Angeles, California, United States*

Population aging is occurring rapidly across Latin America, a region that includes some of the world's most racially, ethnically, and culturally diverse populations. Aging in this region is occurring in a context of high levels of poverty and income inequality, which has implications for disease risk, cognitive health, and overall well-being. This symposium focuses on Mexico and Colombia, two of Latin America's largest middle-income countries, which have recently undergone rapid epidemiological and demographic transitions. The papers in this symposium examine a variety of health dimensions among older Latinos that include physiological functioning, cognition, and psychological and physical well-being. García uses the Mexican Health and Aging Study (MHAS) and the Health and Retirement Study (HRS) to examine biomarkers known to predict health risk among Mexican-origin populations: Mexico-born living in Mexico, Mexico-born living in the U.S., and U.S.-born Mexican-Americans. Saenz examines the importance of education on late-life cognitive ability among Mexicans using data from the MHAS Cognitive Aging Ancillary Study. Using data from the Colombian Survey of Health, Well-Being, and Aging (SABE-Colombia), Ailshire examines variation in biological risk across key subgroups of the population. Osuna uses data from the Colombian National Quality of Life Survey (ENCV) to determine if social and economic inequalities are reflected in unequal health and well-being among older adults. Results highlight which Latin American populations have increased risk for poorer health, which merit further research and policy attention. The findings highlight the importance of understanding health and well-being in the rapidly growing older adult populations of Latin America.

### BIOLOGICAL RISK PROFILES IN THE OLDER MEXICAN POPULATION

Catherine Garcia,[1] Joseph Saenz,[1] Jennifer A. Ailshire,[1] Rebecca Wong,[2] and Eileen M. Crimmins[1], *1. University of Southern California, Los Angeles, California, United States, 2. University of Texas-Medical Branch, Galveston, Texas, United States*

Research examining biological risk is critical given that both the Mexican and U.S. populations are aging. Biomarkers can help us understand underlying disease patterns among Mexican-origin individuals in Mexico and the U.S. to help inform disease-prevention efforts for these populations. Using data from the 2012 Mexican Health and Aging Study and the 2010/2012 Health and Retirement Study, we examine seven biomarkers known to predict health risk: systolic and diastolic blood pressure, pulse rate, total cholesterol, HDL cholesterol, glycosylated hemoglobin, and C-reactive protein. Logistic regression models, controlling for age and sex, are used to predict high-risk for each biomarker among Mexico-born Mexicans, Mexico-born Mexican-Americans, and U.S.-born Mexican-Americans. Results show that Mexico-born Mexicans exhibit higher biological risk for systolic blood pressure, pulse rate, low HDL cholesterol, glycosylated hemoglobin, and inflammation than Mexico-born and U.S.-born Mexican-Americans. Additionally accounting for socioeconomic status and health behaviors did not explain differences in high-risk among Mexican-born Mexicans.

### ENVEJECIMIENTO Y ESTRATOS: SOCIOECONOMIC INEQUALITY IN AGING IN COLOMBIA

Margarita Osuna,[1] and Jennifer A. Ailshire[1], *1. University of Southern California, Los Angeles, California, United States*

Colombia has the highest level of income inequality among Latin American countries, which likely translates into disparities in the aging experience. This study uses data on adults ages 60 and older from the 2017 National Survey of Quality of Life to examine socioeconomic stratification in physical health and psychological well-being. Colombians are assigned to estratos that reflect their residential location as well as social and economic position. Compared to those in the lowest estrato, older adults in the middle and high estratos are less likely to report having sensory impairment or difficulty with daily activities. They are also 1-2 times more likely to report feeling happy and calm. Those in the highest estratos are less likely to report feeling worried. Results suggest there is tremendous variation in the aging experience across socioeconomic strata and that older adults in the lowest strata are particularly disadvantaged with respect to health and well-being.

### THE DIRECT AND INDIRECT EFFECTS OF EDUCATION ON LATE-LIFE COGNITIVE ABILITY IN MEXICO

Joseph Saenz,[1] Eileen M. Crimmins,[1] and Elizabeth Zelinski[1], *1. University of Southern California, Los Angeles, California, United States*

Education and cognitive ability are closely associated. Less is known regarding mechanisms of this association. We evaluate direct effects of education on cognition and indirect effects through health and socioeconomic status (SES) in Mexico. We analyze adults age 50+ from the 2016 Mexican Cognitive Aging Ancillary Study (n=2,042). We constructed latent variables of visual and verbal cognitive abilities. Using structural equation modeling, we estimated direct effects of education on cognition and indirect effects through SES (income and wealth), and health (chronic conditions and health behaviors). Small, yet statistically significant, indirect effects of education on cognition through income, wealth, and stroke (for visual ability) and through stroke (for verbal ability) were observed. However, the majority of the association between education and cognitive ability (90% and 96% for visual/verbal cognitive ability, respectively) was not explained SES or health. Interventions to reduce disparities in late-life cognitive ability should address educational disparities in early-life.

### VARIATION IN BIOLOGICAL RISK AMONG OLDER COLOMBIANS BY AGE, GENDER, AND EDUCATIONAL ATTAINMENT

Jennifer A. Ailshire,[1] and Margarita Osuna[1], *1. University of Southern California, Los Angeles, California, United States*

Recent rapid aging in Colombia raises questions about the health status of older adults, but there has been very little research in this population. This study examines variation in biological risk by age, gender, and educational attainment using data from the 2015 SABE-Colombia, a nationally representative survey of Colombians ages 60 and older. Levels of cholesterol (total, HDL, and LDL), triglycerides, glucose, and hemoglobin were measured from whole blood and clinical cut-points were used to determine high-risk on each indicator. The five metabolic indicators were summed to create a total risk score; 58% of older adults had a score of 1 or more. Those ages 80 and older and women had lower risk, as did those with at least primary education. These patterns were also observed for high-risk on hemoglobin (13% prevalence), an indicator of anemia. Most older Colombians have some biological risk, but this varies by key subgroups.

### SESSION 4005 (PAPER)

### CHRONIC DISEASE AND MULTIMORBIDITY MANAGEMENT

### ENGAGEMENT AND EFFECTIVENESS OF USING MOBILE APP FOR DIABETES SELF-MANAGEMENT AMONG OLDER ADULTS

Mei-Lan Chen,[1] Shinyi Wu,[2] Pey-Jiuan Lee,[3] and Haomiao Jin[3], *1. Byrdine F. Lewis College of Nursing and Health Professions, Georgia State University, Atlanta, Georgia, United States, 2. University of Southern California, Los Angeles, California, United States, 3.*

*Suzanne Dworak-Peck School of Social Work, University of Southern California, Los Angeles, California, United States*

Mobile health applications (app) have shown to be beneficial for chronic disease management. However, few studies assessed older adults' engagement in tracking self-management activities with app functions and effectiveness on improving their diabetes outcomes. This study investigated tracking patterns of each app function (blood sugar, blood pressure, diet, exercise, medication adherence etc.) in a graphic-based aging-friendly diabetes self-management app (IMTOP app) and associated the patterns with changes in HbA1c, self-care behavior, diabetes empowerment, and health promotion. The sample included 334 community-dwelling older adults with type 2 diabetes in Taiwan (mean age 64.57 ± 6.64 years) participated in the IMTOP training course that designed to motivate and train older adults with diabetes to use mobile tablets and apps. We performed trajectory analyses using SAS TRAJ procedure to identify distinct classes of individuals following similar longitudinal patterns on absence or presence of weekly app use for each individual app function. The relationships between the app engagement class memberships and 4- and 8-month diabetes health outcomes were assessed using an econometric regression analysis approach. The results showed the degree of app engagement on any single function was significantly and positively correlated with diabetes self-care scale scores (all $p < .05$). Only the engagement on the blood sugar function had statistically significant association with HbA1c improvements ($p < .05$). The app use was not associated with diabetes empowerment or health promotion. The study findings suggest any app function engagement significantly improved older adults' overall self-management but blood sugar tracking is critical to improve HbA1c.

### INCREASED PHYSIOLOGICAL VARIABILITY PREDICTS DECLINING HEALTH AND CRITICAL TRANSITIONS IN HEMODIALYSIS PATIENTS

Alan A. Cohen,[1] Yuichi Nakazato,[2] Tomoko Sugiyama,[2] Diana L. Leung,[3] Véronique Legault,[1] and Anne-Marie Côté[1], *1. University of Sherbrooke, Sherbrooke, Quebec, Canada, 2. Hakuyukai Medical Corporation, Saitama-shi, Japan, 3. Yale University, New Haven, Connecticut, United States*

Increased variability in levels of several individual biomarkers has been shown to predict adverse outcomes, particularly in hemodialysis patients, for whom time series data is often available. Here, we evaluate the feasibility of using multivariate approaches to quantify global physiological variability as a potential predictor of adverse outcomes. We used data on 588 deaths and 1196 hospitalisations across ~38,000 visits of 591 hemodialysis patients at a Quebec hospital, as well as data on frailty and mortality in 580 patients assessed 20+ times within a one-year period at a hospital in Saitama, Japan. We use two approaches: principal components analysis (PCA) of the coefficients of variation (CVs) of the individual biomarkers over the previous year, and Mahalanobis distance (MD) of the biomarker profile relative to the same profile at the previous time point. We show that both methods provide substantial prediction of both impending mortality and impending hospitalisation, with hazard ratios across the 95% quantile range of the indices varying between 1.5 and 3.5 ($p<0.0001$). Each unit change on the first PCA axis (PC1) increased frailty odds by 2.34 (95% CI: 1.21-4.52). PCA performed substantially better than MD. CVs of various biomarkers were consistently positively correlated, and PC1 was a good predictor of frailty, mortality, and hospitalisation. Overall, these results confirm that complex physiological integration can break down, resulting in loss of homeostatic control and increasing variability, as predicted by complex systems theory. The resulting indices provide a predictive signal of impending critical health transitions, with both theoretical and clinical implications.

### MULTIMORBIDITY PATTERNS ARE DIFFERENTLY ASSOCIATED WITH DEPRESSION IN MIDDLE-AGED AND OLDER CHINESE

Shan-Shan Yao,[1] Gui-Ying Cao,[1] Zi-Shuo Chen,[2] Zi-Ting Huang,[1] and Beibei Xu[3], *1. Department of Epidemiology and bio-statistics School of Public Health, Peking University, Beijing, China, 2. Peking University, Beijing, China, 3. Peking University Medical Informatics Center, Beijing, China*

The associations of multiple physical conditions with depression are still unclear. This study examined the relationship between physical multimorbidity patterns and depression among middle-aged and older Chinese. Patterns of physical multimorbidity were identified using Exploratory Factor Analysis (EFA) among 21,933 participants ≥ 45 years from 2011 to 2015. Multiple logistic regressions were performed to assess the associations between multimorbidity, multimorbidity patterns (factor scores) and depression for each age group (45-60 years vs. ≥60 years). The overall prevalence of multimorbidity was 40% and it was higher among participants with depression (54%) than those without depression (33%). Middle-aged (OR: 1.45; 95%CI 1.16–1.80) and older (OR: 1.85; 95%CI 1.62–2.11) adults with multimorbidity were more likely to have depression compared with those without multimorbidity. Five multimorbidity patterns were identified: cardio-metabolic, respiratory, splanchnic, cardio-cerebrovascular, and tumor-and-degenerative. Middle-aged participants with higher respiratory pattern score had a higher odds to have depression (OR: 1.59; 95%CI 1.15–2.21). Among older adults, higher cardio-metabolic pattern score was significantly associated with lower odds of depression (OR: 0.78; 95% CI 0.63–0.97), while higher respiratory (OR: 1.32; 95%CI 1.04–1.68), splanchnic (OR: 1.22; 95%CI 1.01–1.47) and tumor-and-degenerative pattern scores (OR: 1.86; 95%CI 1.42–2.43) were all found to be significantly associated with higher risk of depression. The associations between physical multimorbidity patterns and depression differ by age. Future studies are needed to investigate the temporal nature of how physical multimorbidity patterns may induce depression and the underlying mechanisms.

### TRANSLATING RESEARCH TO PRACTICE: USING CHANGE MODEL TO IMPROVE SUSTAINABILITY OF HEALTH ALERTS FOR CHRONIC ILLNESS

Kari R. Lane,[1] Marilyn J. Rantz,[1] and Marjorie Skubic[1], *1. University of Missouri, Columbia, Missouri, United States*

Chronic illness is the primary reason for hospitalization and rehospitalization in the US today. Nearly 1/3 of older adults have 3 or more chronic illnesses. Chronic illnesses require significant self-management or management by nursing staff. This paper highlights the use of a change model to assist in sustaining nursing interventions in assisted living environments. We utilized embedded sensors measuring heart rate, respiratory rate, time in bed, restlessness in bed, and gait parameters to manage chronic illness. The embedded sensors use an algorithm to signify when a measure has changed for a resident, based on the past 2 weeks of data. Early health messages are emailed or texted to nursing staff. Nursing staff can use these messages as tools to further assess the resident's condition. It was important to revisit the education, hold the staff accountable, phone in suggestions/reinforcement of what the alerts meant, and provide positive messages. This interdisciplinary study has been deployed in 6 assisted living settings (n=386) (facility-wide) in the midwest. We used a wait-list control group (n=482) of facilities awaiting sensor installation. Outcome variables included length of stay, hospitalizations, falls, and medication changes. Results included a decrease in all outcome variables length of stay 1.98 years longer (F=3.67; p=0.003); hospitalizations (F=2.15; p=0.048); falls (F=1.899; p=0.012); and medication changes (F=3.9; p=0.0008) when compared to the control group. We feel these results may benefit other clinicians in the future when implementing new protocols and practices.

### USING CHRONIC DISEASE SELF-MANAGEMENT TO ENHANCE PATIENT-PROVIDER PARTNERSHIPS

Brea Case,[1] Angela M. Zell,[2] and Joan Ilardo[2], *1. Michigan State University College of Human Medicine, Grand Rapids, Michigan, United States, 2. Michigan State University College of Human Medicine, East Lansing, Michigan, United States*

The Partners in Aging Strategies and Training (PAST) project employed a bilateral approach to educate both healthcare professionals and consumers. Our theory is that improved health outcomes are attained by teaching healthcare providers and consumers how to engage better with each other, especially when consumers use the skills learned in community-based programs, such as self-management and healthy lifestyle choices. PAST activities provided an integrated educational program for healthcare providers and older adult patients, their families and caregivers to learn skills that enhance their ability to form productive patient-provider partnerships. We used three types of training: 1) multi-disciplinary health professions and primary care provider continuing-education face-to-face workshops and webinars; 2) older adult patient and caregiver workshops, resource materials; and 3) reverse marketing comprised of sending information to physicians whose patients attended a workshop that included the topics covered in the workshops and the patients' three- to six-month action goals. We found that physicians who attended the grand rounds presentations were very receptive to the 'nuts and bolts' approach to things like doing a quick mobility assessment, effectively communicating with patients, health literacy, and referring patients to community-based non-medical services and supports. We conducted seven types of evidence-based workshops. Over 90% of participants gave permission to send a letter to their physician to tell them they attended the workshop. We used pre-post confidence scales based on each workshop's learning objectives to measure changes in workshop participants' self-management confidence. There was positive change in confidence for all seven workshops.

### SESSION 4010 (SYMPOSIUM)

### DIVERSITY IN HOSPICE AND END-OF-LIFE EXPERIENCES: THE INFLUENCE OF CHRONIC DISEASE AND SOCIOCULTURAL FACTORS

Chair: David Russell, *Visiting Nurse Service of New York, New York, New York, United States*
Co-Chair: Elizabeth A. Luth, *Weill Cornell Medicine, New York, New York, United States*
Discussant: Ruth Masterson Creber, *Weill Cornell Medical College, New York, New York, United States*

Hospice provides supportive and palliative services to persons nearing the end-of-life. Use of the Medicare hospice benefit has grown to cover nearly half of all Medicare decedents. Even more notably, hospice agencies now serve patients with a diverse range of terminal conditions, including those not traditionally served by hospices, such as dementia and heart failure. In addition to expanded use of hospice care by patients with multiple types of chronic disease, demographic transitions in the United States over the last several decades have also led to increased use of hospice services among patients with diverse socio-cultural and linguistic backgrounds. Limited research has identified the unique experiences of patients enrolled in hospice who have diagnoses of dementia and heart failure, or explored how socio-cultural factors act to influence the course and outcomes of hospice care. This symposium features interdisciplinary collaborations between academic researchers and clinical practitioners at a large non-profit hospice agency in a multicultural urban environment. These collaborations, which draw on multiple theoretical perspectives and research methodologies, shed new light on patient experiences in hospice and identify opportunities for improving care and comfort at end-of-life. Presentations will include an exploration of the unique symptoms and experiences of hospice patients with heart failure, an evaluation of a clinical program for heart failure hospice patients, an exploration of collaborative goal setting between patients-providers, and an examination of cultural health capital as it relates to race/ethnic and socioeconomic disparities in hospitalization among hospice patients, and factors for disenrollment among hospice patients with dementia.

### SOCIAL AND CULTURAL FACTORS FOR HOSPICE CARE OUTCOMES: PERSPECTIVES OF PATIENTS, CAREGIVERS, AND PROVIDERS

David Russell,[1] Dawon Baik,[2] Lizeyka Jordan,[1] Frances Dooley,[1] and Ruth M. Masterson Creber[3], *1. Visiting Nurse Service of New York, New York, New York, United States, 2. Columbia University, New York, New York, United States, 3. Weill cornell Medical College, New York, New York, United States*

Use of hospice services in the U.S. has grown to cover an expanding number of patients with varying conditions and demographic characteristics. Notably, hospice agencies increasingly serve patients with diverse socio-cultural and linguistic backgrounds. Limited research has explored how these factors act to influence the course and outcomes of hospice care, or their role in shaping race/ethnic and socioeconomic disparities in burdensome outcomes like acute hospitalization. This presentation uses the theoretical lens of Cultural Health Capital to explore how socio-cultural factors affect patient-provider interactions within the home hospice setting. Qualitative interviews were conducted with both providers (N=32) and patients/caregivers (N=7) at a large not-for-profit hospice agency in New York City. Themes identified from these interviews included prior knowledge and familiarity with hospice, family dynamics and social support, and linguistic and cultural barriers to care. Findings indicate the need for greater attention to socio-cultural influences on interactional dynamics within home hospice.

### RISK FACTORS FOR DISENROLLMENT AMONG HOME HOSPICE PATIENTS WITH DEMENTIA

Elizabeth A. Luth,[1] and David Russell[2], *1. Weill Cornell Medicine, New York, New York, United States, 2. Visiting Nurse Service of New York, New york, New York, United States*

Hospice delivers care to a substantial and growing number of individuals with primary and comorbid dementia diagnoses. Dementia diagnosis and racial/ethnic minority status are risk factors for hospice disenrollment. However, little research examines racial/ethnic disparities and other risk factors for hospice disenrollment among hospice patients with dementia. This paper uses multinomial logistic regression to explore sociodemographic and functional status risk factors for hospice disenrollment among 3,949 home hospice recipients with primary or comorbid dementia. Results indicate that patients with a primary dementia diagnosis, racial/ethnic minority groups, and those higher functional status have elevated risk of disenrollment due to hospitalization, disqualification, and electively leaving hospice care. Additional research is needed to understand why primary dementia diagnosis and underrepresented racial/ethnic status are associated with multiple kinds of hospice disenrollment so that hospice practice can be tailored to respond to the needs of these individuals.

### CARE MANAGEMENT CHALLENGES AND PROGNOSTIC TOOLS FOR HEART FAILURE PATIENTS IN HOSPICE

Ruth Masterson Creber,[1] Lizeyka Jordan,[2] Dawon Baik,[3] and David Russell[4], *1. Weill Cornell Medical College, New York, New York, United States, 2. Visiting Nurse Service of New York, New York, New York, United States, 3. Columbia University School of Nursing, New York, New York, United States, 4. Appalachian State University, Boone, North Carolina, United States*

Heart failure (HF) patients enroll in hospice at lower rates despite their worse prognosis. This multi-method study explores the characteristics and challenges associated with caring for HF patients. Data from qualitative interviews with hospice providers (n=32) and quantitative records (N=1,114) were used to identify care management issues and prognostic tools. Hospice providers described HF patients unique and often unpredictable symptomatology, their limited understanding and discordant hospice expectations, and difficulties managing symptoms at home. Providers also highlighted HF patients use of assistive medical devices and complex medication regimens. Palliative Performance Scale (PPSv2) scores at hospice enrollment were found to be strongly associated with hospice survival (AUC: 7 days=0.80; 14 days=0.77) and live discharge risk (PPSv2 50-70% AOR=5.68 [CI=3.66-8.79]). Findings underscore the need for specially-tailored trainings and protocols for providers to prevent unplanned discharges and support HF patients at end-of-life.

### THE DEVELOPMENT AND IMPLEMENTATION OF A CARDIAC HOME HOSPICE PROGRAM FOR ADVANCED STAGE HEART FAILURE PATIENTS

Lizeyka Jordan,[1] Ruth Masterson Creber,[2] David Russell,[3] and Dawon Baik[4], *1. Visiting Nurse Service of New York, New York, New York, United States, 2. Weill Cornell Medicine, New York, New York, United States, 3. Appalachian State University, Boone, North Carolina, United States, 4. Columbia University, New York, New York, United States*

Heart failure (HF) patients encounter distressing symptoms at the end-of-life including dyspnea, edema, and fatigue. Left untreated, these symptoms increase risk for hospice disenrollment. This presentation used the RE-AIM framework to examine a cardiac home hospice program for HF patients. Qualitative interviews with hospice providers (N=32) and quantitative medical record data were used to examine the program. Reach—1,183 HF participants were served between 2013-2016. Effectiveness—Enrollment of HF patients in the hospice program increased from 7.9% to 9.5% after the cardiac protocol was implemented. Adoption—implementation was spearheaded by a clinical champion aware of the challenges of HF symptom management. Implementation— additional support services (i.e., paramedicine, infusion services, cardiac comfort medication kits) were incorporated in the cardiac protocol to better manage complex clinical cases in the home. Maintenance—Reinforcing factors include ongoing training for nursing staff and a 3.5-hour introduction module providing information about HF case management and symptoms/treatments.

### PERSPECTIVES ON SETTING GOALS OF CARE AMONG HOSPICE PATIENTS WITH HEART FAILURE, THEIR FAMILIES, AND HEALTHCARE TEAM

Dawon Baik,[1] David Russell,[2] Lizeyka Jordan,[3] Frances Dooley,[3] and Ruth Masterson Creber[4], *1. Columbia University, New York, New York, United States, 2. Appalachian State University, Visiting Nurse Service of new York, Boone, North Carolina, United States, 3. Visiting Nurse Service of New York, New York, New York, United States, 4. Weill Cornell Medicine, New York, New York, United States*

Older adults with heart failure (HF) face many end-of-life care issues. Shared decision making (SDM) in hospice is an important process that allows HF patients and their family caregivers to discuss their preferences on goals of care (GOC) with their healthcare team. Yet, little research has explored how the values and preferences of HF patients and their family are integrated into their care plans through SDM process. This presentation examines facilitators and barriers to setting GOC among hospice HF patients. Qualitative interviews were conducted with HF patients/family caregivers

(n=7) and providers (n=32) at a large not-for-profit hospice agency. Several facilitators emerged: building trust, active listening, helping patients and family caregivers understand hospice and prognosis. Barriers included acceptance, family conflict, language discordance between patients and providers and lack of communication about care transition. Findings confirmed the need for individually-tailored goal-setting approaches to navigating the end-of-life trajectory among HF patients.

### SESSION 4015 (SYMPOSIUM)

### FACETS OF CLOSE, ROMANTIC, AND INTIMATE RELATIONSHIPS IN LATER LIFE

Chair: Karolina Kolodziejczak, *Humboldt University Berlin, Berlin, Germany*
Co-Chair: Denis Gerstorf, *Humboldt University Berlin, Berlin, Germany*
Discussant: Karen Rook, *University of California, Irvine, Irvine, California, United States*

Research on the role of close social relationships for physical health and well-being in later life has received increased attention over the past decades. Yet, we are still only beginning to understand potentially underlying mechanisms such as joint goals and affectionate touch. Likewise, we also know little about the relevance of particular social facets such as the role of friends and the nature of sexuality. In this symposium, we have compiled four empirical projects that showcase current and future endeavors to address some of these long-standing questions. Ungar et al. use dyadic data from older couples to examine how shared goals with the partners and positive illusions about these joint goals relate to goal progress and relationship satisfaction. Zhaoyang and Martire analyze long-term longitudinal dyadic data from older couples to examine if and how the frequency of affectionate touch between partners predicts physical health, well-being, and relationship satisfaction five years later. Fiori et al. make use of three-wave longitudinal data from a large and representative US sample to examine the unique roles that close social ties and weaker social ties have independently of one another for age-related changes in two central aspects of affective experience. Kolodziejczak et al. use time-lag data from two cohorts of adults in late midlife to capture historical changes in the perceived importance of sexuality and the evaluation of one's sex life. Karen Rook will integrate the insights gained from these four papers, discuss their potential and limitations, and consider directions for future research.

### JOINT GOALS IN OLDER COUPLES: ASSOCIATIONS WITH GOAL PROGRESS AND RELATIONSHIP SATISFACTION

Nadine L. Ungar,[1] Victoria I. Michalowski,[1] Stella Bähring,[1] Denis Gerstorf,[2] Maureen C. Ashe,[3]
Kenneth M. Madden,[3] and Christiane A. Hoppmann[3],
*1. University of British Columbia, Vancouver, British Columbia, Canada, 2. Humboldt University Berlin, Berlin, Berlin, Germany, 3. University of British Columbia and Center for Hip Health and Mobility, Vancouver, British Columbia, Canada*

Goals often involve close others such as spouses, but we know little about how this helps or hinders goal progress and what couple consequences arise. To examine these questions, we investigate associations between joint goals, goal progress, and relationship satisfaction by applying multilevel modeling to data from 119 couples (50% female; Mage=71 years). Participants listed their most important goals and reported if they wanted to achieve these together with their partner (self-rated joint goals). 85% self-reported at least one joint goal. Two raters classified goals as "joint" if both partners mentioned the same goal. Positive illusions–i.e., older adults thinking a goal was joint although it was not reported by the spouse–were frequent. Number of joint goals was related to increased goal progress but only for those with low positive illusions, whereas positive illusions were linked to higher relationship satisfaction. We discuss theoretical and practical implications of our findings.

### AFFECTIONATE TOUCH AND RELATIONAL, MENTAL, AND PHYSICAL WELL-BEING IN OLDER COUPLES: A NATIONAL LONGITUDINAL STUDY

Ruixue Zhaoyang,[1] and Lynn M. Martire[2], *1. Center for Healthy Aging, The Pennsylvania State University, University Park, Pennsylvania, United States, 2. Human Development and Family Studies, The Pennsylvania State University, University Park, Pennsylvania, United States*

Recent theories suggest that non-sexual physical contact with close others plays a key role in promoting health and well-being in adulthood. However, the impact of non-sexual physical contact in later life, especially the affectionate touch between romantic partners, has been largely unexplored. Using two waves of dyadic data (N=953 couples, Mage=71 years) from National Social Life, Health, and Aging Project (NSHAP), we examined whether shared affectionate touch between spouses prospectively predicted both partners' relational, mental and physical well-being five years later, independent of sex activity. Dyadic analyses results indicated that frequency of shared affectionate touch with the partner predicted increases in spouses' own relationship satisfaction, life satisfaction and mental health, but not in physical health, over five years. No interpersonal (i.e., partner) effect of shared affectionate touch was found. Findings underscore the unique role of non-sexual physical contact between spouses in promoting relational and mental well-being for older couples.

### THE STRENGTH OF WEAKER TIES: HAVE WE BEEN IGNORING A RESOURCE FOR AGING ADULTS?

Katherine L. Fiori,[1] Oliver Huxhold,[2]
Noah J. Webster,[3] and Toni C. Antonucci[3], *1. Adelphi University, Garden City, New York, United States, 2. German Centre of Gerontology, Berlin, Berlin, Germany, 3. Institute for Social Research Room, University of Michigan, Ann Arbor, Michigan, United States*

The purpose of this study was to examine links between changes in social ties (close ties and weaker ties) and changes in positive and depressed affect across three waves in a large, representative sample of U.S. adults aged 40 and over (N = 802). Using trivariate dual-change score models, we found that a greater number of weaker ties was associated with higher numbers of close ties over time, and that the number of weaker ties was more strongly predictive of positive age-related changes in both aspects of well-being (i.e., less depressed affect and more positive affect) than the number of close ties. Our findings imply that focusing investment on the outer circles may have the unintended benefit of compensating for losses in the inner circle, and that contrary to popular theoretical orientations, weaker ties may offer older adults an avenue for both promoting positive affect and decreasing negative affect.

### COHORT DIFFERENCES IN THE IMPORTANCE OF SEXUALITY AND EVALUATION OF ONE'S SEX LIFE IN LATE MIDLIFE

Karolina Kolodziejczak,[1] Johanna Drewelies,[1] Dorly J. Deeg,[2] Martijn Huisman,[3] and Denis Gerstorf[1], *1. Humboldt University Berlin, Berlin, Berlin, Germany, 2. Amsterdam UMC, Vrije Universiteit Amsterdam, Amsterdam, Noord-Holland, Netherlands, 3. Vrije Universiteit Amsterdam, Amsterdam, Noord-Holland, Netherlands*

Age-related declines in multiple aspects of sex life are well documented, but we know little about historical change in key sexuality facets. We examine cohort differences in the perceived importance of sexuality and the evaluation of one's sex life among middle-aged adults. We compare data from 55 to 64-year-olds in the Longitudinal Aging Study Amsterdam (LASA) obtained in 1992–1993 (n = 718) vs. 2012–2013 (n = 860). Results revealed that later-born adults perceive sexuality as more important than their earlier-born peers. Effect sizes were small at the sample level (d < .15), but substantial for particular subpopulations (women without partner: d = .56). In zero-order models, later-born adults evaluated their sex life as slightly less pleasant, but differences did not hold when covarying relevant individual and cohort difference factors. We conclude that historical changes in late-midlife sexuality are multifaceted and discuss theoretical and practical implications of our findings.

### SESSION 4020 (PAPER)

### FUNCTIONAL STATUS AND MOBILITY

### LIFE-SPACE MOBILITY AS A PREDICTOR OF MEDICARE UTILIZATION AMONG COMMUNITY-DWELLING OLDER MEXICAN AMERICANS

Jason P. Johnson,[1] Lin-Na Chou,[1] Yong-Fang Kuo,[1] Kenneth Ottenbacher,[1] and Soham Al Snih[2], *1. University of Texas Medical Branch, Galveston, Texas, United States, 2. University of Texas Medical Branch at Galveston, Galveston, Texas, United States*

Hispanics are a large and growing group of older adults, with higher rates of morbidity and disability than other racial/ethnic groups. Mexican Americans make up more than half of this population and are well represented in the Hispanic Established Populations for the Epidemiologic Study of the Elderly (HEPESE) survey, a longitudinal study of community-dwelling older Mexican Americans. The University of Alabama Birmingham Life-Space Assessment (LSA) is a measure assessing patterns of functional mobility in and around the home, neighborhood, and community. This study addresses the gap in research of life-space mobility and healthcare utilization with linked insurance claims data. Four hundred eight participants with 1-year continuous Medicare enrollment from wave 7 (2010) of the HEPESE were linked with Medicare claims. Logistic regression analysis was used to estimate the odds ratio of hospitalization and ER admissions. Negative binomial regression was used to estimate the rate ratio of physician visits. LSA score ranges 0 to 120, with higher scores indicating greater life-space mobility; LSA was analyzed as a 10-point decrease or dichotomously as restricted ≤59 or not restricted ≥60. A restricted LSA score among older Mexican American Medicare beneficiaries was associated with OR of 2.73 for hospitalizations (95% CI= 1.18-6.31). In addition, a 5-point decline in LSA score was associated with OR of 1.12 for hospitalizations (95% CI= 1.04-1.22). LSA score was not significantly associated with ER admission or physician visit. Interventions aimed to increase mobility in the home and the community may reduce the risk of hospitalizations in this population.

### POST-TRANSPLANT FUNCTIONAL STATUS TRAJECTORY AMONG ADULT KIDNEY TRANSPLANT RECIPIENTS

Nadia M Chu,[1] Zhan Shi,[1] Christine Haugen,[1] Dorry Segev,[1] and Mara McAdams-DeMarco[2], *1. Department of Surgery, Johns Hopkins School of Medicine, Baltimore, Maryland, United States, 2. Department of Epidemiology, Johns Hopkins Bloomberg School of Public Health, Baltimore, Maryland, United States*

Frailty and disabilities are highly prevalent among kidney transplant (KT) recipients, but are not routinely measured in KT recipients. The Karnofsky Performance Scale (KPS) is a clinically perceived measure used to evaluate patient's ability to manage daily activities, but little is known about its post-KT trajectories and its relationship to frailty and disability in KT recipients. We leveraged a cohort of 159,992 adult KT recipients from SRTR (1/2005-6/2018) and a cohort of 1,106 adult KT recipients from a prospective cohort study on aging and KT with recorded KPS (range 10%-100% integers). In each separate cohort, we used mixed effects models to assess differences in trajectories of KPS post-KT. In 159,992 KT recipients in SRTR, the mean unadjusted KPS score was 88.34% (95%CI: 88.28%, 88.40%) and declined at a rate of -0.59%/year (95%CI: -0.61%, -0.57%) post-KT, such that by 2-years post-KT the average was 87.00% (95%CI: 86.94%, 87.05%). Age at KT was associated with steeper decline in KPS (p0.05). KPS is a measure of functional status distinct from frailty, ADL, IADL, and SPPB at KT admission that declines with older age post-KT. Older KT recipients should be monitored closely for declines in physical function, and potentially undergo prehabilitation to improve functional status post-KT.

### PREFRONTAL CORTICAL ACTIVITY DIFFERENCES WHILE DUAL-TASK WALKING IN OLDER ADULTS WITH IMPAIRED MOBILITY

Manuel E. Hernandez[1], *1. University of Illinois at Urbana-Champaign, Urbana, Illinois, United States*

Mobility impairments are prevalent in older adults. Whereas walking had traditionally been viewed as an autonomous process, evidence over the last decade has shown that cognitive processes such as attention and executive function have a significant impact on gait function in older adults. However, the exact neural mechanisms underlying difficulties in the control of mobility in older adults remains an open question. We examine the changes in the executive control of mobility in older adults with mobility impairments using functional near-infrared spectroscopy, as operationalized by performance in the community balance and mobility scale (CB&M). We hypothesized that prefrontal cortical (PFC) activity increases would be higher in older adults with mobility impairments, compared with older adults without mobility impairment, as dual-task walking difficulty increased. Older adults with (n=10, mean±SD age: 77±8 years, 8 females, CB&M= 58±12) and without mobility impairment (n=14, mean±SD age: 63±9 years, 11 females, CB&M= 87±6) were recruited from the local community. Dual-task walking was performed at a comfortable pace, while the difficulty of the concurrent cognitive task was increased using the modified Stroop test. PFC activity was measured using measures of oxygenated hemoglobin across the PFC. Older adults with mobility impairments demonstrated disproportionate increases in PFC activity, in comparison to those without mobility impairments, as the difficulty of the concurrent cognitive task increased (P<.001), even after controlling for age. In conclusion, these data suggest that older adults with mobility impairments may require greater attentional resources than those without mobility impairments when concurrently performing thinking and walking tasks.

### TARGETED TRANSCRANIAL DIRECT CURRENT STIMULATION IMPROVES DUAL-TASK WALKING PERFORMANCE IN OLDER ADULTS

Brad Manor,[1] Junhong Zhou,[1] On-Yee Lo,[1] Racheli Katz,[2] Marina Brozgol,[2] Pablo Cornejo Thumm,[2] Giulio Ruffini,[3] and Jeffrey M. Hausdorff[4], *1. Hinda and Arthur Marcus Institute for Aging Research, Harvard Medical School, Roslindale, Massachusetts, United States, 2. Tel Aviv Sourasky Medical Center, Tel Aviv, Israel, 3. Neuroelectrics Corporation, Cambridge, Massachusetts, United States, 4. Center for the study of Movement, Cognition and Mobility, Neurological Institute, Tel Aviv Sourasky Medical Center, Israel, Tel Aviv, Israel*

In older adults, the ability to walk while engaged in an unrelated cognitive task (i.e., dual tasking) depends upon activation of both motor and cognitive brain networks. Noninvasive transcranial direct current stimulation (tDCS) can facilitate the excitability of specific brain regions and their connected neural networks. In this multi-site, randomized controlled within-subject cross-over study, we tested the effects of single, 20-minute sessions of tDCS targeting 1) the primary motor cortex (M1), 2) the left dorsolateral prefrontal cortex (dlPFC, a primary region subserving cognitive function), 3) both M1 and left dlPFC, or 4) neither region (sham). Forty-eight older adults free of overt illness or disease (mean±SD age=75±6 years, 35 women) completed four study visits at least 72 hours apart, during which dual task gait was assessed before and after tDCS administration. Stimulation was delivered using the Starstim™ system (Neuroelectrics Corp) and the same array of six gel electrodes to ensure double-blinding. Participants were successfully blinded to tDCS condition and reported no unexpected tDCS side effects. Repeated-measures ANOVAs adjusted for age and sex revealed that the dual task cost to gait speed was smaller (i.e., better and closer to zero) following tDCS that targeted both M1 and the left dlPFC, as well as the left dlPFC alone, compared to all other time points (condition-time interaction: F=3.0, p=0.04). The dual task costs following these two types of stimulation were similar. These results suggest that noninvasive facilitation of cognitive-motor brain network excitability leads to acute improvement in dual task performance in older adults.

### TRANSITIONING HOME AFTER STROKE: IMPROVING PHYSICAL HEALTH AND PATIENT ACTIVATION

Anne K. Hughes,[1] Amanda Woodward,[1] Paul Freddolino,[1] Michele Fritz,[1] Constantinos Coursaris,[1] Sarah J. Swierenga,[1] and Mat Reeves[1], *1. Michigan State University, East Lansing, Michigan, United States*

While the majority of stroke patients will return home after being hospitalized, this transition is physically and emotionally challenging. We developed a social work based case management program to address these challenges. The Michigan Stroke Transitions Trial (MISTT), a pragmatic 3-arm clinical trial tested the effects of the case management program on its own and combined with technology against usual care in patients recovering from stroke. Patients from three Michigan hospitals were randomized to one of three groups upon discharge to home. The two treatment groups received services from a social work case manager via home visit and telephone. One treatment group also was given training and access to a curated stroke website developed for MISTT. The intervention lasted up to 90 days and data was collected via telephone at 7 and 90 days. Quality of life and patient activation were the primary outcomes, measured by the PROMIS Global 10, and the Patient Activation Measure (PAM), respectively. We compared treatment efficacy by comparing the change in outcomes between the three groups (N=265) using a difference-in-differences (D-in-D) analysis. The mean change in PROMIS scores for the social work + technology group was significantly higher than both the social work only group (difference= +2.4; 95%CI=0.46, 4.34; p=0.02) and usual care (difference= +3.4; 95%CI=1.41, 5.33; p<0.001). The mean change in PAM scores for the social work + technology group was significantly higher than the social work only group (+6.7; 95%CI=1.26, 12.08; p=0.02) and marginally higher than usual care (+5.0; 95%CI=-0.47, 10.52; p=0.07).

### SESSION 4025 (SYMPOSIUM)

### GOING FOR THE WIN-WIN-WIN: HARNESSING THE POWER OF SENIOR VOLUNTEERISM TO ADDRESS DEMENTIA CARE AND PROMOTE HEALTH

Chair: Quincy M. Samus, *The Johns Hopkins University, Baltimore, Maryland, United States*
Co-Chair: Joseph E. Gaugler, *University of Minnesota - School of Public Health, Division of Health Policy and Management, Minneapolis, Minnesota, United States*

Discussant: George W. Rebok, *Johns Hopkins Bloomberg School of Public Health, Baltimore, Maryland, United States*

The public health implications of Alzheimer's disease or related dementias (ADRDs) are significant and have placed considerable pressure on the U.S. healthcare system. Training and mobilizing a critical mass of volunteers to address unmet dementia care needs may be a potent, scalable, and cost efficient approach to address gaps in dementia care and to support family caregivers. Further, by engaging older volunteers to do this work and remain in productive and impactful post-retirement roles, additive population health benefits may be possible. This session will focus on ways we might harness the power of senior volunteers to meet the public health challenges associated with ADRD. Presentations will draw from three innovative community-based projects that utilize senior volunteers to support and enhance health in aging and dementia care. Dr. Carlson will provide an update on the evaluation and scaling Experience Corps, an intergenerational program that engages senior volunteers to work in elementary schools. Dr. Gaugler will discuss the Porchlight Project, a new multicomponent training approach for senior volunteers in Minnesota to enhance dementia care capabilities and support to underserved older persons. Dr. Samus will introduce the MEMORI Corps program, a novel activity-based companion care program for home-residing persons with ADRD delivered by trained senior volunteers. Given the current and impending shortages in the geriatric work force and family caregivers, respectively, innovative and readily available long term service and support options are needed to offset potential care gaps. The current session proposes the novel incorporation of volunteers as one solution to do so.

### MAKING ENGAGEMENT MEANINGFUL THROUGH ORGANIZED ROUTINE INTERACTION (MEMORI) CORPS TRIAL: AN INTRODUCTION

Quincy M. Samus,[1] Laura N. Gitlin,[2] Michelle Carlson,[1] George Rebok,[1] Deirdre Johnston,[1] Jeannie-Marie Leoutsakos,[1] and Betty Black[1], *1. The Johns Hopkins University, Baltimore, Maryland, United States, 2. Drexel University, Philadelphia, Pennsylvania, United States*

This presentation will review the design, methods, and early lessons learned from the Making Engagement Meaningful through Organized Routine Interaction (MEMORI) Corps trial recently funded by NIA. This trial is evaluating the feasibility and efficacy of the MEMORI Corps program, a novel 12-week activity-based companion care model designed to mobilize and equip senior volunteers to deliver individualized, evidence-based activity programming to persons with dementia PWD living at home and offer family CGs needed respite. The intervention synthesizes and adapts prior evidence-based work from the Tailored Activities Program® (an activity-based intervention persons with dementia), Experience Corps® (an intergenerational civic engagement program that engages senior volunteers to work in elementary schools), and MIND at Home® (a home-based dementia care coordination program) to simultaneously address unmet respite care needs of family CGs, provide PWD structured meaningful activities and social engagement, and provide meaningful engagement and peer support opportunities for senior volunteers.

### THE PORCHLIGHT PROJECT: PARTNERING WITH VOLUNTEERS TO ENHANCE COMMUNITY-BASED DEMENTIA CARE AND OUTREACH

Joseph E. Gaugler,[1] Christina Rosebush,[2] Gabriela Bustamante,[2] Jeri Schoonover,[3] Roxanne Jenkins,[4] Nicole Bauer,[3] Lisa Beardsley,[3] and Laura Rowe[3], *1. University of Minnesota - School of Public Health, Division of Health Policy and Management, Minneapolis, Minnesota, United States, 2. Division of Environmental Health Sciences, School of Public Health, University of Minnesota, Minneapolis, Minnesota, United States, 3. Lutheran Social Service of Minnesota, St. Paul, Minnesota, United States, 4. Lutheran Social Service of Minnesota, Saint Paul, Minnesota, United States*

Families often remain unaware of long-term services and supports (LTSS) that could help to mitigate the negative effects of Alzheimer's disease and related dementias (ADRDs). Approaches that: a) identify community-residing older persons with potential memory impairment; b) assist their families in navigating the healthcare system; and c) facilitate the identification of appropriate community-based LTSS could result in more effective management of ADRD. The Porchlight Project is a multicomponent training approach for lay volunteers in Minnesota (i.e., Senior Companions) that enhances their capability to deliver dementia care and support to underserved older persons in need. Mixed methods analysis of qualitative and quantitative data among 20 Senior Companions and up to 25 persons with ADRD and their family caregivers suggest the potential success of the Porchlight Project, as well as areas to refine and enhance prior to large-scale evaluation throughout Minnesota.

### THE IMPACT OF MEANINGFUL VOLUNTEER ENGAGEMENT IN AGING ADULTS: THE BALTIMORE EXPERIENCE CORPS TRIAL

Michelle C. Carlson,[1] and George Rebok[2], *1. Johns Hopkins Bloomberg School of Public Health, Baltimore, Maryland, United States, 2. The Johns Hopkins University, Baltimore, Maryland, United States*

Experience Corps was designed to embed cognitive, social, and physical activity into volunteer service by training older adults to serve in neighborhood elementary schools as mentors of children in Kindergarten-3 for 15 hours a week over two academic years. We incorporated cognitive activities through the intentional design of a variety of roles in reading, math, library support, and positive communication. Socially, volunteers engage with other volunteers, teachers, and children, and functional walking 3-4 days/week to and from as well as within the schools. The Baltimore Experience Corps Trial (BECT) is the largest randomized controlled trial (N = 702) examining the impact of volunteer engagement on cognitive functions in cognitively intact older adults, over sampling African Americans (91%) who have twice the risk of Alzheimer's disease as whites. Findings will be summarized and demonstrate the dose-dependent cognitive, psychosocial, lifestyle activity, and brain benefits of volunteering for up to two years.

### SESSION 4030 (PAPER)

### HEALTH AND HEALTH PROMOTION

### HOME CARE AIDE-LED RESISTANCE EXERCISE FOR MEDICAID HOME AND COMMUNITY-BASED SERVICES CLIENTS

Margaret Danilovich,[1] Laura Diaz,[1] Jody Ciolino,[2] William Healey,[1] Gail Huber,[3] and Daniel Corcos[1], *1. Northwestern, Chicago, Illinois, United States, 2. Northwestern University, Chicago IL, United States, 3. Northwestern University, Chicago, Illinois, United States*

Home Care Aides (HCAs) assistance is the most common care provision to help clients with activities of daily living, but exercise is not currently provided, despite well-established health benefits. We developed a resistance exercise intervention using a stakeholder panel of physical therapists, HCAs, and clients. Stakeholders suggested a mobile application that played exercise videos. We then enrolled 128 HCA-client dyads (93% african american & 79% female) and randomized home care aides to lead the intervention with their clients in addition to usual care for 6 months or continue usual care. Our aims were to 1) evaluate the feasibility of the intervention and 2) determine the effects of the intervention on client physical performance, frailty classification, self-reported health, and strength. 96% of HCAs reported the training session successfully prepared them to lead exercise and we had no adverse events. There was high attrition rates among dyads (28.13%) at 6 months which was worse among HCAs (55%). We found no statistically significant differences between study arms. However, the range of completed sessions was 1-34 out of 52 possible sessions. Program evaluation indicated high satisfaction among clients and HCAs (8.9 & 8.6/10). HCA attrition limited client participation, technology did not facilitate exercise completion, and client motivation was a barrier to adherence. Results show it is feasible to train HCAs to lead exercise, however, given high turnover in the HCA profession, alternative implementation models should be studied. While the app use was an innovative way to engage older adults in exercise, technology challenged HCAs.

### INTERNAL MIGRATION AND HEALTH CHANGES: A LONGITUDINAL STUDY OF CHINESE ADULTS IN MID AND LATER LIFE

LI Gao,[1] Zheng Wu,[2] and Shu z. Li[3], *1. School of Public Policy and Administration, Xi'an, Shaanxi, China, 2. Department of Gerontology, Vancouver, Canada, 3. Institute for Population and Development Studies, Xi'an, Shannxi, China*

Objectives: This study examines the effects of internal migration on health status and health changes among middle-aged and older migrants in China. Methods: Using longitudinal data from the 2011-2015 China Health and Retirement Longitudinal Study (CHARLS), this study compares non-migrants with those of recent migrants and earlier migrants in regard to their self-rated health and mental health. OLS and a series of fixed effects models were conducted to examine the effects of migration on health status and health changes. Results: Compared with non-migrants, earlier migrants report better self-rated health but no difference in depression. Our findings demonstrate that recent migrants show better self-rated health changes than non-migrants. In addition, for recent migrants, there are significant changes in self-rated health among rural-to-urban migrants and rural-to-rural migrants, while urban-to-rural migrants and urban-to-urban migrants are not significantly different from non-migrations. Discussion: There are associations between internal migration and self-rated health in China. The effects of migration on health appear to differ by the type of migration. Those who migrated from rural area are mostly likely to be affected by migration. However, migrants from urban area are less affected.

### ONE HUNDRED MILLION HEALTHIER LIVES: MEASURING WHAT MATTERS

Kathleen A. Cameron,[1] and Angelica Herrera-Venson[2], *1. National Council on Aging, Arlington, Virginia, United States, 2. National Council on Aging, Arlington, Virginia, United States*

The rise of chronic disease, an aging population, inequity in health care access and outcomes, and new technology demand a fundamentally different approach to fostering health—one that brings sectors together to address the social and behavioral determinants of health. To address these concerns, NCOA is leading the Aging Hub of the 100 Million Healthier Lives (100MLives), an initiative convened by the Institute for Healthcare Improvement. 100MLives is a collaboration of change agents across sectors working to transform how the world thinks and acts to improve health, well-being, and equity across the lifespan. NCOA is collaborating with national and local organizations, as well as federal agencies to support a nationwide ecosystem for measuring, fostering, and bringing to scale innovations that address social and behavioral determinants of health. The Aging Hub is committed to consistently measuring what matters (adult well-being assessment-AWA), learn what programs and services within the aging network improve a person's life, and work with partners to scale those programs. The initiative encourages use of the Adult Well-Being Assessment, a validated 7-question survey that asks individuals to rate their overall well-being; financial, physical, mental, and spiritual health; and social support. Shared use of these outcome measures can make it easier for the aging network to demonstrate the impact of their programs and services. Join NCOA to learn about progress to date, including a report on AWA and demographic data collected by the Baltimore County Department with over 10,000 older adults and how data is being used to improve service delivery.

### PERSPECTIVES OF OLDER ADULT STAKEHOLDERS ON THE IMPACT OF COMMUNITY-BASED EXERCISE

Lekaa Elhajjmoussa,[1] Allison M. Mays,[2] Katrina B. Rosales,[2] and Sonja L. Rosen[3], *1. Cedars Sinai Medical Group, Beverly Hills, California, United States, 2. Cedars-Sinai, Los Angeles, California, United States, 3. Cedars-Sinai Medical Center, Los Angeles, California, United States*

This focus-group study aims to identify the perceptions of older adults (>50 years) who participated in community-based exercise classes as part of the Leveraging

Exercise to Age in Place (LEAP) Study. LEAP enrollees participate in community-based classes that include Tai Chi, EnhanceFitness, Arthritis Exercise, and the Healthier Living Workshops. Nine LEAP participants attended a focus group at Cedars-Sinai Medical Group. The focus group included quantitative and qualitative questions in both a verbal discussion format and a written questionnaire that examined the effects of liaisons, social connections, and incurred changes as a result of participating in LEAP classes. Participants endorsed liaisons within the healthcare system, including physician referrals and communication with a community health coach, as aiding in their decision to participate in health classes. Participants cited positive changes in their physical states, such as increases mobility and decreases in pain, and positive psychological changes, such as increases in energy and socialization, post LEAP completion. Among the ideas and critiques noted by the focus group attendees was the desire to have LEAP classes become part of communities permanently, and to have longer exercise class session duration. These findings suggest that direct merging of liaisons within the healthcare system and community-based exercise programs for older individuals, such as those offered by LEAP, is an effective way to positively influence older patient outcomes both physiologically and psychologically. These results call for future research that focuses on how healthcare systems and community programs can work together to maximize positive patient outcomes for older individuals.

### SESSION 4035 (SYMPOSIUM)

### HEALTH IMPLICATIONS OF SOCIAL ROLES AND ROLE TRANSITIONS IN MIDLIFE AND LATER LIFE

Chair: Kelly E. Cichy, *Kent State University, Kent, Ohio, United States*
Co-Chair: Athena Koumoutzis, *Kent State University, Kent, Ohio, United States*

Demographic and social trends shape the timing, nature, and implications of social roles and transitions. With increased life expectancy and a changing world, expectations for work and retirement and the need for informal and formal caregiving continue to evolve. Families are also more heterogeneous and the population is becoming increasingly more racially/ethnically diverse. These changes underscore the need for research that focuses on the varied social roles individuals occupy in midlife and later adulthood and the implications of these roles for health and well-being. The current symposium features research that explores multiple roles, including romantic partner, grandparent, and employee/retiree, caregiver/care recipient while attending to individual differences in how these roles and transitions are associated with physical and mental health outcomes. Garcia, Donnelly, and Umberson utilize dyadic diary data from midlife men and women in gay, lesbian, and heterosexual marriages to consider how exposure and reactivity to daily stress varies across union types. Rickenbach and colleagues examine longitudinal changes in health and well-being associated with being a caregiving and non-caregiving grandparent. Cichy and Koumoutzis examine racial differences in the associations between providing care to a spouse/parent and daily health and well-being among African Americans and European Americans. Savla, Roberto, and Sands classify community-living older adults based on their care needs while considering the type of care they receive, predictors of this care, and its implications for care recipients' health. Finally, Stawski and colleagues examine how mental, physical, and cognitive health change as a function of the transition to and through retirement.

### RACIAL DIFFERENCES IN THE DAILY EXPERIENCES OF AFRICAN AMERICANS AND EUROPEAN AMERICANS PROVIDING CARE

Kelly E. Cichy,[1] and Athena Koumoutzis[1], *1. Kent State University, Kent, Ohio, United States*

African Americans often report lower caregiver burden, however, few studies consider the broader daily context of African American caregivers' lives. This study examines racial differences in the associations between providing care for a spouse or parent and daily health and well-being among African Americans and European Americans, including how other daily stressors moderate these associations. During eight days of interviews, respondents aged 34 to 84 years (N = 1,931) from the National Study of Daily Experiences (NSDE II) reported on their daily stressors, negative affect (NA), physical symptoms, and whether or not they provided support to a spouse or parent with a disability. Controlling for demographics, on caregiving days, NA was higher than on non-caregiving days ($p < .05$) for all respondents. On caregiving days with no work stressors, African Americans only reported more physical symptoms than on caregiving days with work stressors ($p < .05$). Implications will be discussed.

### EXPOSURE AND EMOTIONAL REACTIVITY TO DAILY STRESS IN SAME-SEX AND DIFFERENT-SEX MARRIAGES

Michael Garcia,[1] Rachel Donnelly,[2] and Debra Umberson[2], *1. Population Research Center, Austin, Texas, United States, 2. Population Research Center, The University of Texas at Austin, Austin, Texas, United States*

Recent work exploring links between stress processes and well-being within marriage suggest that women may be at an increased risk for exposure and emotional reactivity to daily stress. However, studies have focused primarily on heterosexual couples, raising questions concerning whether and how these gendered patterns might unfold differently for men and women in same-sex marriages. In the present study, we analyze 10 days of dyadic diary data from 756 midlife men and women in 378 gay, lesbian, and heterosexual marriages to consider how exposure and emotional reactivity to daily stress may differ across union types. We find that women are exposed to more daily stressors than men and that this exposure is especially detrimental to the well-being of women in different-sex marriages. These findings highlight the need to include same-sex couples when exploring gendered linkages between daily stress processes and well-being within marriage.

### LONG-TERM COSTS OF GRANDPARENT CAREGIVING: RESULTS FROM THREE WAVES OF THE MIDLIFE IN THE UNITED STATES STUDY (MIDUS)

Elizabeth Rickenbach,[1] Elizabeth H. Rickenbach,[1] Chih-Chien Huang,[1] Jessica Y. Allen,[2] and Kelly E. Cichy[3],

1. Saint Anselm College, Manchester, New Hampshire, United States, 2. Birmingham-Southern College, Birmingham, Alabama, United States, 3. Kent State University, Kent, Ohio, United States

Cross-sectional studies reveal the health burden of grandparent caregiving. Still, longitudinal, research is needed to understand how grandparent caregiving compromises grandparents' long-term health. Using three waves of data from the Midlife in the United States Study (MIDUS), we examined sociodemographic factors, health and well-being outcomes between caregiving (CG) and non-caregiving (NCG) grandparents. By wave 3, 12.8% (n = 234) were CG. CG were younger, more likely female, and had lower income and education. MANCOVA adjusted for age, gender, education, and number of children revealed CG reported poorer physical and emotional well-being (e.g. higher depression, anxiety, lower life satisfaction, greater morbidity); CG were consistently less healthy than NCG across all three waves. Lower income and less healthy older adults are more likely to become grandparents, and they remain less healthy over time. Policies and resources to assist grandparents, particularly low-income and vulnerable older adults who are caring for grandchildren, are needed.

### A NATIONAL PROFILE OF FRAIL OLDER ADULTS WITH INSUFFICIENT CARE AND MISMATCHED SUPPORT

Jyoti Savla,[1] Karen A. Roberto,[1] and Laura Sands[1], *1. Virginia Tech, Blacksburg, Virginia, United States*

Older adults differ widely both in the care they require and who they rely upon for care. We use data from the National Health and Aging Trends Study (2011; N=3,265; MAge [SD] = 77 [7.74] years, 62% women) to classify community-living older adults based on their care needs and the various informal and formal providers of care. We also examine the type of care they receive, predictors of this care, and its implications on their health. Older adults with a co-residing caregiver were more likely to report that their needs were not being met (OR = 1.67; 95% CI=1.15–2.42), compared to those who received informal care and paid support. Moreover, older adults who needed help with self-care activities, but received help with household activities were more likely to report unmet needs (OR = 1.55; 95% CI=1.13–2.12). Results are discussed in light of sociodemographic factors differences and mismatched support.

### AGING-RELATED CHANGES IN MENTAL, PHYSICAL, AND COGNITIVE HEALTH: THE IMPACT OF THE RETIREMENT TRANSITION

Robert S. Stawski,[1] and Kelly D. Chandler[1], *1. Oregon State University, Corvallis, Oregon, United States*

Retirement is an important transition in later life, associated with changes in social roles. It is unclear, however, whether the retirement transition modifies aging-related changes in mental, physical, and cognitive health. Using data from the Health and Retirement Study, we examined changes in depressive symptoms, self-rated health, and memory prior to, at, and after the retirement transition among 6,830 participants (Ages=50-97, 58% female) assessed biennially up to 10 times from 1992-2010. Preliminary results indicate a sudden and significant increase in depressive symptoms and decreases in self-rated health and memory at the transition to retirement (ps<.05). These effects increased among individuals retiring at older ages (ps<.01). Further, aging-related increases in depressive symptomatology became faster after retirement (p<.01). Aging-related decreases in self-rated health and memory were unchanged by the transition. Discussion will focus on the contribution of transitions to understanding trajectories of mental, physical, and cognitive health in later life.

### SESSION 4040 (SYMPOSIUM)

### HEALTH TRAJECTORIES OVER TIME IN THE ATHLOS PROJECT: FINDINGS FROM MULTIPLE COHORTS

Chair: Matthew Prina, *King's College London, London, United Kingdom*

The ATHLOS (Ageing Trajectories of Health: Longitudinal Opportunities and Synergies) project is a consortium of 15 partners across Europe who are working together to understand patterns of healthy ageing trajectories, and to seek the factors that determine those patterns, in a harmonised dataset of 17 international cohort studies of ageing. During this symposium we will be presenting some of the work that has recently been carried out within this project. The symposium will consist of four talks: the first talk will introduce the project, and describe the preliminary work that took place within the first few years of the project, and the challenges faced by the consortium. The second talk will focus on the harmonisation process and on the development of the health metric, an indicator used to measure healthy ageing in this project. The third talk will focus on inequalities in healthy ageing, specifically investigating the impact of education and wealth across cohorts. Finally, in the last talk we will describe the role of lifestyle behaviours (specifically physical activity, smoking and alcohol consumption) and their impact on healthy ageing trajectories.

### AN INTRODUCTION TO THE ATHLOS PROJECT: AGEING TRAJECTORIES OF HEALTH: LONGITUDINAL OPPORTUNITIES AND SYNERGIES

Matthew Prina,[1] Demosthenes Panagiotakos,[2] Martin Prince,[3] Martin Bobak,[4] Warren Sanderson,[5] Serguei Scherbov,[5] Jose L. Ayuso-Mateos,[6] and Jose Maria Haro[7], *1. King's College London, London, United Kingdom, 2. Harokopio University, Athens, Attiki, Greece, 3. Global Health Institute, King's College London, London, England, United Kingdom, 4. Department of Epidemiology and Public Health, University College London, London, England, United Kingdom, 5. International Institute for Applied Systems Analysis, World Population Program, Wittgenstein Centre for Demography and Global Human Capital, Laxenburg, Niederosterreich, Austria, 6. Centro de Investigación Biomédica en Red de Salud Mental, Madrid, Madrid, Spain, 7. Parc Sanitari Sant Joan de Déu, Barcelona, Catalonia, Spain*

ATHLOS is a 5-year project, funded by the European Union's Horizon 2020 Research and Innovation Program. Its aim is to achieve a better understanding of healthy ageing, utilising longitudinal data from existing cohort studies. The measure of healthy ageing used within ATHLOS is based on the definition used by the World Health Organization as the ongoing process of developing and maintaining functional ability to enable well-being in older age. The first step of the project was to harmonise 17 community based cohort studies of ageing, covering 38 countries over the world and over 411,000 individuals. In this talk we will discuss the work of the different work packages of the project, including a description of the existing evidence on risk factors of healthy ageing.

### CREATION OF A COMMON METRIC OF HEALTH STATUS IN THE HARMONIZED DATASET OF THE ATHLOS PROJECT

Albert Sanchez-Niubo,[1] Francisco Felix Caballero,[2] Christina Daskalopoulou,[3] Javier de la Fuente,[4] Alejandro de la Torre,[4] Iago Giné Vazquez,[5] Yu-Tzu Wu,[3] and Matthew Prina[3], *1. Parc Sanitari Sant Joan de Déu, Universitat de Barcelona, Barcelona, Spain, 2. Centro de Investigación Biomédica en Red de Salud Mental, CIBERSAM, Madrid, Madrid, Spain, 3. Social Epidemiology Research Group. Health Service and Population Research Department, Institute of Psychiatry, Psychology & Neuroscience, King's College London, London, England, United Kingdom, 4. Department Preventive Medicine and Public Health, Universidad Autónoma de Madrid, Madrid, Madrid, Spain, 5. Parc Sanitari Sant Joan de Déu, Baecelona, Catalonia, Spain*

Although life longevity has increased across the world, evidence suggests some heterogeneity of the ageing process across individuals. To investigate different ageing patterns, the ATHLOS project harmonised data from 411,000 individuals across 17 existing cohort studies. The harmonised dataset provides comparable information on functioning measures, cognition, mental health, sociodemographic and lifestyle behaviours. To measure the process of healthy ageing across time and cohorts, we employed a Bayesian Multilevel Item Response Theory(IRT) and created a common metric of health status by using items of functioning. The IRT measurement model includes parameters describing the difficulty and discriminatory power of each item. We adopted the Bayesian Multilevel framework as it allows item parameters to vary among studies and the simultaneous estimation of all parameters under a Markov Chain Monte Carlo(MCMC) method. Finally, we assessed the predictive validity of the metric against mortality by performing a Receiver Operating Characteristic(ROC) curve analysis.

### INEQUALITIES IN HEALTHY AGING: THE DIFFERENTIAL IMPACT OF EDUCATION AND WEALTH ACROSS COHORT STUDIES

Yu-Tzu Wu,[1] Christina Daskalopoulou,[1] Graciela Muniz Terrera,[2] Martin Prince,[3] and Matthew Prina[3], *1. Social Epidemiology Research Group, Health Service and Population Research Department, Institute of Psychiatry, Psychology & Neuroscience, King's College London, London, England, United Kingdom, 2. Centre For Clinical Brain Sciences, University Of Edinburgh, Edinburgh, Scotland, United Kingdom, 3. Global Health Institute, King's College London, London, England, United Kingdom*

Several studies have investigated longitudinal changes in health status and functional ability but few have examined whether inequalities in healthy ageing varied across different countries. The aim of this study is to investigate trajectories of health metric scores (generated in previous symposium abstract) over the ageing process and examine the impact of education and wealth on the trajectories across eight cohorts in the ATHLOS consortium (N=135,828) using multilevel regression modelling. After adjusting for age, gender and study, higher levels of education (9.52; 95% CI: 9.30, 9.74) and wealth (8.06; 95% CI: 7.84, 8.28) were associated with higher baseline scores but had minimal impacts on decline rates. These effect sizes varied across different cohort studies and the inequality gradient was found to be strongest in the Health Retirement Study from US. Future research may investigate potential mechanisms which might explain the differential impact of education and wealth in different societies.

### THE IMPACT OF LIFESTYLE BEHAVIORS ON HEALTHY AGING TRAJECTORIES: THE ATHLOS PROJECT

Christina Daskalopoulou,[1] Yu-Tzu Wu,[1] Artemis Koukounari,[2] Graciela Muniz Terrera,[3] Stefanos Tyrovolas,[4] Demosthenes Panagiotakos,[5] Martin Prince,[6] and Matthew Prina[1], *1. Social Epidemiology Research Group, Health Service and Population Research Department, Institute of Psychiatry, Psychology & Neuroscience, King's College London, London, England, United Kingdom, 2. Faculty of Epidemiology and Population Health, London School of Hygiene & Tropical Medicine, London, England, United Kingdom, 3. Centre for Dementia Prevention, Centre for Clinical Brain Sciences, University of Edinburgh, Edinburgh, England, United Kingdom, 4. Parc Sanitari Sant Joan de Déu, Universitat de Barcelona, Barcelona, Catalonia, Spain, 5. Department of Nutrition and Dietetics, School of Health Science and Education, Harokopio University, Athens, Attiki, Greece, 6. Global Health Institute, King's College London, London, England, United Kingdom*

The number of people above 60 years old will double by 2050. There is a considerate variability in the health status of older people. The identification of the different trajectories that people follow as they grow older constitutes one of the aims of the ATHLOS project. In the current study, we created a metric of health in the four available waves (2001, 2003, 2012, 2015) of the Mexican Health and Aging Study (MHAS) by employing Bayesian multilevel Item Response Theory. Growth mixture modelling indicated that older Mexicans (n=14,143) age by following four distinct pathways (i.e. high-stable, moderate-stable, low-stable, decliners). Adherence to healthy lifestyle behaviours (i.e. physical activity, non-smoking, limited alcohol consumption) was associated with better health trajectories. Preliminary analyses in the ATHLOS harmonised dataset also suggest that older people age by following four distinct pathways. The impact of lifestyle behaviours within the harmonised dataset will be investigated and also presented.

### SESSION 4045 (PAPER)

### HYPERTENSION AND CARDIOVASCULAR DISEASE

### COMPARISON OF A FRAILTY INDEX WITH CARDIOVASCULAR RISK SCORES IN PREDICTING CARDIOVASCULAR DISEASE MORTALITY

Dustin S. Kehler,[1] Olga Theou,[1] and Kenneth Rockwood[1], *1. Dalhousie University, Halifax, Nova Scotia, Canada*

We compared the predictive and discriminative ability of frailty with traditional cardiovascular risk scores to estimate 10-year cardiovascular disease (CVD) mortality risk. Individuals aged 20-79 years old from the National Health and Nutrition Examination Survey who were free from CVD were included (n= 32,066). A 33-item frailty index (FI) which excluded CVD and diabetes-related variables was calculated. We calculated the Framingham Disease Risk (FDR) Hard Coronary Heart Disease and General CVD risk scores, the American Heart Association/American College of Cardiology (AHA/ACC) atherosclerotic cardiovascular disease risk equation, and the European Systematic Coronary Risk Estimation tool. A total of 322 individuals died (1.0%) from CVD. There was a low correlation between the FI and CVD risk scores (spearman's r= 0.19-0.33; p<0.0001) and a weak to strong correlation between CVD risk scores (spearman's r=0.19-0.88; p<0.0001). The competing-risks hazard ratio for CVD mortality for every 1% increase in the FI was 1.040 (95% CI: 1.032-1.048; p<0.0001) in an age and sex-adjusted model. The FI was independently predictive of CVD mortality when the other CVD risk scores were added to the model. The area under the receiving operating characteristic (ROC) curve was 0.800 (95% CI: 0.789-0.808; p<0.0001) for the FI. ROC values for the CVD risk scores ranged from 0.710 (95% CI: 0.700-0.721; p<0.0001) for the AHA/ACC risk score to 0.779 (95% CI: 0.770-0.789; p<0.0001) for the FDR General CVD risk score. An FI calculated with non-CVD and diabetes variables can predict 10-year CVD mortality risk independently of traditional CVD risk scores.

### FRAILTY STATUS, COGNITIVE IMPAIRMENT, AND THE EFFECTS OF INTENSIVE BLOOD PRESSURE CONTROL

Kathryn E. Callahan,[1] Maryjo Cleveland,[1] Mark Supiano,[2] Jeff Williamson,[1] and Nicholas M. Pajewski[1], *1. Wake Forest School of Medicine, Winston-Salem, North Carolina, United States, 2. University of Utah School of Medicine Center on Aging, Salt Lake City, Utah, United States*

Background: Frailty associates with cognitive decline and incident dementia in older adults. The Systolic Blood Pressure Intervention Trial (SPRINT) has highlighted blood pressure (BP) control as a potentially modifiable risk factor for cognitive impairment. Using data from SPRINT, we explore whether frailty status, based on a frailty index (FI), prospectively associates with mild cognitive impairment (MCI) and dementia, and whether the effect of intensive BP control on these outcomes varies by frailty status. Methods: SPRINT randomized participants to either to an systolic BP goal of <120 mmHg (intensive treatment) or a goal of <140 mmHg (standard treatment). We used Cox regression to model the association of the FI with MCI and dementia, and to conduct subgroup analyses by frailty status for the effect of intensive treatment. Results: We include 9307 participants, with the majority categorized as pre-frail (0.100.21, 38.0%). Adjusting for age, sex, race/ethnicity, education, and treatment group, a 0.1 increase in the FI was associated with increased risk for MCI (Hazard Ratio (HR) = 1.42, 95% CI: 1.29, 1.58) and dementia (HR = 1.80, 95% CI: 1.56, 2.08). There was weak evidence of an interaction between frailty status and intensive treatment for the composite outcome of MCI and dementia (p=0.03), with a beneficial effect of intensive treatment in pre-frail participants (HR=0.71 95% CI: 0.58, 0.89), and a largely null effect in frail participants (HR=0.98, 95% CI: 0.82, 1.18). Conclusions: Frailty status may modify the effect of intensive BP control on MCI and dementia.

### HYPERTENSION TREATMENT IN U.S. LONG-TERM NURSING HOME RESIDENTS WITH AND WITHOUT DEMENTIA

Kenneth Boockvar,[1] Wei Song,[2] Sei Lee,[3] and Orna Intrator[2], *1. Icahn School of Medicine at Mount Sinai, New York, New York, United States, 2. University of Rochester, Rochester, New York, United States, 3. University of California, San Francisco, San Francisco, California, United States*

Background: Treatment of hypertension in individuals with dementia has benefit-harm tradeoffs. The objectives of this study were to describe patterns of antihypertensive treatment in U.S. nursing home (NH) residents and determine the association between intensity of antihypertensive treatment and outcomes important for persons with dementia. Methods: Medicare claims and Minimum Data Set (MDS) assessments were used to capture information on dementia, hypertension, comorbid conditions, cognitive function, activities of daily living (ADLs), and hospitalizations in Medicare-enrolled long-term care residents in 2013. Intensity of hypertension treatment was defined as number of antihypertensive drugs received on the date of an index MDS according to Medicare part D claims. Outcomes ascertained were hospitalizations, ADL decline, and mortality within 180 days of the index MDS. Results: A total of 262,285 residents had both hypertension and dementia. Residents were 85.7 years old on average and 77% female. At baseline, 30%, 40%, and 30% were receiving 0, 1, and 2+ antihypertensive medications, respectively. In multivariable logistic regression models, increased intensity of antihypertensive treatment was associated with increased hospitalization among residents with dementia (OR 1.03 per added medication; 95%CI 1.01-1.04), but decreased hospitalization among residents without dementia (OR .98 per added medication; 95%CI .97-1.00; p-value for interaction < .0001). In residents with and without dementia, increased intensity of antihypertensive treatment was associated with increased hospitalization for cardiovascular diseases, but small decreases in ADL decline and mortality. Conclusion: Study findings suggest that long-term NH residents with hypertension and dementia may not experience benefit from more intensive blood pressure control.

### PHENOTYPES OF CARDIOVASCULAR DAMAGE AND THE RELATIONSHIP WITH COGNITIVE DECLINE: LATENT CLASS ANALYSIS

Lindsay Miller,[1] Chenkai Wu,[2] Calvin Hirsch,[3] Oscar Lopez,[4] Mary Cushman,[5] and Michelle Odden[6], *1. Oregon State University, Corvallis, Oregon, United States, 2. Duke Kunshan University, Kunshan, Jiangsu, China, 3. University of California, Davis, Davis, California, United States, 4. University of Pittsburgh, Pittsburgh, Pennsylvania, United States, 5. University of Vermont, Colchester, Vermont, United States, 6. Stanford University, Stanford, California, United States*

The association between subclinical cardiovascular disease (SCVD) and cognitive function is well established, however, current tools for addressing subgroups of SCVD have focused on the overall burden of disease. Identifying risky combinations of characteristics may lead to a better understanding of pathophysiologic changes that underlie cognitive decline. Participants included 5,072 older adults from the Cardiovascular Health Study without cardiovascular disease at baseline and followed for 6 years. Using latent class analysis, we identified cardiovascular damage phenotypes based on vascular (internal intima-media thickness, ankle-arm index, white matter grade, and brain infarctions), cardiac (major echocardiogram abnormalities, ST2, and N-terminal pro-brain natriuretic peptide) and inflammatory (interleukin-6, galectin-3, and cystatin C) markers. Using the maximum probability assignment rule, participants were assigned to phenotypes based on the highest posterior probability of membership. We used adjusted linear mixed effects models to evaluate the association between phenotype and cognitive decline, measured annually using the Modified Mini-Mental State Exam. The analysis yielded 5 prevalent phenotypes: healthy (65%), cardiac (10%), inflammatory (10%), multisystem morbidity (11%), and vascular (4%). The vascular phenotype had the greatest rate of decline at 0.88 points per year (95% CI= -1.33, -0.44), followed by the multisystem morbidity phenotype ($\beta$=-0.72, 95% CI= -1.07, -0.38), the inflammatory phenotype ($\beta$=-0.67, 95% CI= -0.95, -0.38), and the cardiac phenotype ($\beta$=-0.45, 95% CI= -0.70, -0.21) compared to the healthy phenotype. Among patterns of cardiovascular damage, vascular damage appears to have the greatest influence on cognitive decline. Identification of these phenotypes may give rise to more informed clinical interventions.

### SESSION 4050 (PAPER)

### LONG-TERM CARE IV

### A REVIEW OF THE LONG-TERM CARE INSURANCE PROGRAMS IN 15 PILOT CITIES IN CHINA

Yan Lin,[1] Edward A. Miller,[2] Marc A. Cohen,[3] Pamela Nadash,[2] and Peng Du[4], *1. Department of Gerontology, University of Massachusetts, Boston, Boston, Massachusetts, United States, 2. University of Massachusetts Boston, Boston, Massachusetts, United States, 3. University of Massachusetts Boston Gerontology Institute, LeadingAge LTSS Center @UMass Boston, Boston, Massachusetts, United States, 4. Institute of Gerontology, Renmin University of China, Beijing, China*

China is now making all the efforts to solve the problem of who pays the bill for the rapidly increasing long-term care services. Since 2016, 15 cities in China have begun their pilot programs in long-term care insurance. Each city designed its own program. Some cities finance their long-term care services from medical insurance funding solely. Others supplement it with individual and/or employer contributions. This study documents the nature and extent of implementation of long-term care insurance across the 15 pilot cities to draw lessons for subsequent implementation nationally. This study used qualitative methods, including document review and key informant interviews with long-term care insurance administrators, medical insurance administrators, service providers in different settings and families and individuals who use long-term care services. Results reveal considerable variation in the specific attributes of the long-term care insurance programs implemented across the 15 participating cities, with respect to such characteristics as the target population, policy coverage, and payment methods, etc. Results also shed light on the achievements and challenges in implementing the long-term care insurance program. This study's examination of long-term care insurance adoption in 15 Chinese cities serves as an important base with which to inform future national long-term care insurance design and implementation. It suggests that successful adoption of long-term care insurance across China will require administering jurisdictions to anticipate and address policy bottlenecks and implementation barriers that might otherwise inhibit program impact and effectiveness in addressing the growing long-term care needs of China's aging population.

### AN EMPIRICAL TYPOLOGY OF CULTURE CHANGE IMPLEMENTATION IN NURSING HOMES

Yinfei Duan,[1] and Christine Mueller[2], *1. School of Nursing, University of Minnesota, Minneapolis, Minnesota, United States, 2. University of Minnesota, Minneapolis, Minnesota, United States*

The implementation of culture change (CC) practices has been increasing in U.S. nursing homes (NHs) with varying success. No study has examined the typology of CC implementation across NHs using empirical data. This study aimed to identify an empirical typology of CC implementation and to examine NH organizational characteristics associated with types of CC implementation. An online CC survey using a validated instrument was sent to administrators of 363 NHs in Minnesota and 78 administrators completed the survey. No significant difference in NH characteristics was found between participants and non-participants. Using a hierarchical cluster analysis based on scores of five CC domains, this study identified three distinctive clusters. Overall, NHs most frequently engaged in resident-centered care and environment transformation and least frequently implemented family/community engagement practices. While NHs in cluster 1 (20.8%) performed best in all CC domains, they were distinguished from others by more frequency of family/community engagement, staff empowerment and staff leadership practices. NHs in cluster 3 (41.7%) were distinguished by overall lower engagement of CC practices in all domains. While NHs in cluster 2 (37.5%) and cluster 1 were comparable in environment transformation and resident-centered care, NHs in cluster 2 and cluster 3 were similar in their low family/community engagement. NHs in cluster 3 had significantly lower registered nurse retention, and lower mental health and social service staffing compared to NHs in cluster 2. This typology provides insight into the variations in implementing CC practices across NHs and pragmatic implications for promoting deep and extensive CC.

### CONSISTENT ASSIGNMENT: AN UPDATE ON THE QUALITY IMPACT

Nicholas Castle,[1] and John A. Harris[2], *1. WVU, Morgantown, United States, 2. University of Pittsburgh, Pittsburgh, Pennsylvania, United States*

The association of consistent assignment of Nurse Aides (NAs) with nursing home quality indicators is examined. Consistent assignment is defined as the same caregivers consistently caring for the same residents almost (80% of their shifts) every time they are on duty. Data used came from a survey of nursing home administrators, Nursing Home Compare, the Certification and Survey Provider Enhanced Reporting (CASPER) data, and the Area Resource File. All of the data was from 2015, and included 3,550 facilities. Several multivariate logistic regression models (using GEE) were used, including staffing variables (turnover, agency use, staffing levels), facility factors (size, ownership, occupancy rate), and market characteristics (competition, Medicaid rates). An average of 77% of nursing homes reported using at least some level of consistent assignment; although some were at low levels. In the multivariate analyses, accepted levels of consistent assignment were used. Turnover and family satisfaction quality were significantly ($p<.01$) better in facilities with the highest levels of consistent NA assignment. 7 of the 9 Quality Measures and 3 of the 5 Five-Star measures examined were significantly ($p<.01$) better in facilities with the highest levels of consistent NA assignment. Consistent assignment has developed as a preferred practice in nursing homes based on little empirical evidence. The findings presented here provide substantial justification for the use of this staffing practice for NAs.

### STATE PERFORMANCE ON HOME- AND COMMUNITY-BASED SERVICES EXPENDITURES UNDER THE BALANCING INCENTIVES PROGRAM

Regina A. Shih,[1] Esther M. Friedman,[2] Bonnie Ghosh-Dastidar,[3] and Jose Escarce[4], *1. RAND, Arlington, Virginia, United States, 2. RAND Corporation, Santa Monica, California, United States, 3. RAND, Santa Monica, California, United States, 4. UCLA, Los Angeles, California, United States*

Medicaid has been at the forefront of policy efforts to "rebalance" long-term services and supports (LTSS) from institutional toward home- and community-based services (HCBS). The Balancing Incentives Program (BIP), authorized under the Affordable Care Act, sought to increase spending on HCBS relative to total LTSS among states that were spending less than 50% of total Medicaid LTSS dollars on HCBS each year. BIP's benchmark for states to spend more than 50% of LTSS on HCBS was meant to improve states' infrastructure to ensure an equitable, consumer-friendly process to access HCBS. For the first time, we quantified how states performed on HCBS expenditures. We analyzed state-level HCBS expenditure data for states eligible for BIP (n=35) from 2008 to 2015, which includes several years both before and after BIP implementation. We examined the effect of BIP on HCBS expenditures as a percent of total LTSS spending using fixed-effects modeling which controls for stable characteristics of states and years. We find that BIP states increased HCBS spending relative to non-BIP states, and this effect continued to grow over our study period. In the first year after BIP implementation, BIP states increased spending by 1.8% relative to non-BIP states. The difference in spending between BIP and non-BIP states is even larger in subsequent years (4.8% in year 2, 5.3% in year 3, and 5.8% in year 4). Results indicate that BIP prompted increased state-level spending that continued to rise several years after initial implementation of BIP.

### THE STUDY OF KNOWLEDGE AND ATTITUDE ABOUT LONG-TERM CARE AMONG HISPANICS

Ganesh Baniya,[1] and Ami Moore[1], *1. University of North Texas, Denton, Texas, United States*

Attitudes towards long-term care can help policymakers tailor policies considering different racial and ethnic experiences of the elderly population. The theory of Proactive Coping can help elderly people to better prepare for aging and minimize stressors related to aging by identifying potential sources of stress before they occur and help gather resources and skills for successful aging. The data from the "Long-term care in America: views on who should bear the responsibilities and costs of care" (2017) study showed that Hispanics generally perceived their health to be in a better status and Hispanic women perceived that they had better health compared to males. Similarly, Hispanic males generally thought that they would not need assistance at old age whereas women anticipated that they would require assistance at old age. Similarly, there was a gender difference on who should bear the caregiving responsibility. More Hispanic women thought it would be their responsibility to provide care than males. Women were more prepared than males to provide the care needed to family members of friends. In regards to financial preparation, males reported being more financially capable than females to bear expenses during the old age. Similarly, women were more likely to solely depend on using governmental assistance such as Medicare and Medicaid during old age for needed care. Most of the respondents thought that the US was not well prepared to meet the needs of the aging population and suggested that the government needs to do more before it would be too late.

### SESSION 4055 (PAPER)

### METHODOGICAL ISSUES IN THE STUDY OF AGING

### A SEARCH FOR MEANINGFUL PATIENT AND PUBLIC INVOLVEMENT IN GERONTOLOGICAL QUALITATIVE DATA ANALYSIS

Barbara Hanratty,[1] Rachel Stocker,[1] and Katie Brittain[2], *1. Newcastle University, Newcastle upon Tyne, United Kingdom, 2. Northumbria University, Newcastle, United Kingdom*

Patients and the public are involved in health and social care research more than ever before. Much effort has been put into developing patient and public involvement (PPI), and promoting co-production of research with patients and the public. Yet there is little guidance for researchers on how to involve PPI partners in the research process, or how involvement can be judged as meaningful. This presentation has its origins in the attempts of one research team to question and navigate a way of involving PPI in long term care research. In this presentation, we describe our model of collaborative qualitative data analysis with PPI partners, in a study exploring primary care services for older adults living in long-term care facilities in England. Anonymised interview transcript excerpts were presented in written, audio, and role-play format to our PPI partners. PPI partners derived meaning from interview data, identifying, confirming and critiquing emerging themes. Their input at this critical stage of the study deepened our initial analysis and prompted the research team to new and different interpretations of the data. This talk addresses ways of engaging PPI partners in innovative ways during data analysis, and offers other researchers some questions, challenges and potential principles for effective practice. We conclude that in areas such as long term care, with multiple stakeholders and a dynamic environment, effective PPI may be flexible, messy and difficult to define.

### COMPOSITE MEASURES FOR MULTIDIMENSIONAL SOCIAL EXCLUSION: AN APPLICATION TO THE IRISH LONGITUDINAL STUDY ON AGEING

Sinead Keogh,[1] Stephen O' Neill,[2] and Kieran Walsh[2], *1. National University of Ireland Galway, Galway, Ireland, Ireland, 2. NUI Galway, Galway, Ireland, Ireland*

The measurement of the complex, multidimensional and dynamic concept of old-age social exclusion has been constrained due to theoretical and methodological challenges as well as a reliance on secondary data sources not designed to collect social exclusion indicators. Limitations in measuring social exclusion in later life hinder the expansion of our empirical and conceptual understanding of social exclusion. In this paper, we seek to address these limitations by developing a composite measure of old-age social exclusion using three methods: 1) normalisation through re-scaling with linear aggregation, 2) a sum-of-scores approach with an applied threshold and, 3) classification and regression trees (CART), a machine learning approach. Using the conceptual framework of old-age exclusion presented by Walsh et al., (2017), these three approaches are applied empirically with data from Wave 1 of The Irish Longitudinal Study on Ageing (TILDA). The measures are assessed in terms of their ability to explain a validated measure of psychological well-being. Results suggest that despite the challenges associated with secondary data and measurement techniques that implicitly measure social exclusion, the newly proposed composite measure computed using CART performed better than the other two measures which are more prevalent in the literature.

### HUMAN RESOURCES PROFESSIONALS' PERCEPTIONS OF AGE-FRIENDLY EMPLOYMENT

Mi Sun Choi,[1] Holly Dabelko-Schoeny,[1] Katie White,[1] and Marisa Sheldon[1], *1. The Ohio State University, Columbus, Ohio, United States*

Many researchers have attempted to find alternative ways to address the challenges of the aging workforce. Applying the concept of age-friendly to work settings may be a promising strategy to promote older adults' well-being in the workplace. However, age-friendly employment is an abstract concept, and empirical research identifying its features is lacking. Also, few studies adequately represented the diverse characteristics of different industries and stakeholder's perceptions of age-friendly work environments. This study used focus group data and the Delphi method, along with existing literature to create an instrument to measure age-friendliness in diverse work environments. In partnership with a senior employment organization serving central Ohio, six human resources (HR) professionals participated in a focus group discussion and 10 HR professionals participated in a two-round Delphi study. In the focus group discussion, HR professionals identified flexibility, mobility, ergonomics, health benefits, and respect as age-friendly aspects in the workplace. We developed 37 items within five subcategories: accommodation, development, maintenance, utilization, and inclusion. In the first round, HR professionals assessed the importance of each item, presenting training opportunities for employees of all ages as the most important practice; four weeks later they examined the revised 33 items in regard to the feasibility of their real-world implementation, showing 100% agreements among experts in career advice, opportunities for promotion, involving decision making, educating age discrimination, and formal acknowledgment. The second phase of this study will include scale evaluation to confirm its reliability and validity with a broader number of HR professionals and older adults.

### SURVIVING THE COMPLEXITY: USING GROUNDED THEORY TO UNDERSTAND KINSHIP CAREGIVING

Catherine J. Tompkins[1], *1. George Mason University, Fairfax, Virginia, United States*

The role of a caregiver often goes beyond the task of caring for someone who is dependent in managing activities of daily living. Children are dependent on others to care for them due solely to their age and maturity; others are dependent due to chronic ailments or short-term disabilities. Regardless of why someone is dependent, the caregiving relationship is complex. This paper focuses on a grounded theory, developed and applied to understand the complexities of kinship caregiving. The literature continues to support the identified needs of kinship caregivers (Tompkins, 2015; Lee, Clarkson-Hendrix, & Lee, 2016). To understand the unique needs of kinship families, the following grand tour question was asked: What is it like for you to live within a kinship caregiving household? The theory was developed over several years based on observational data and 15 interviews with grandparent caregivers and at least one of the grandchildren they were raising. The theory, Surviving the Complexity, is a survival process of taking on the caregiving role and doing one's best in spite of multiple obstacles. Surviving the complexity consists of three stages: rescuing, taking-on and role reversal. The theory identifies and explains emotional, relationship and situational complexity within kinship families. Hope and denial are factors of emotional complexity: "It's not that she (my daughter) does not love him (the child), she is just unable to right now. She will get better." Theory development and further application of the theory will be discussed.

### HEALTHY RESIDENCE IN RICHARDSVILLE: A PHOTOVOICE PROJECT ON AGING IN PLACE

Terri Lewinson,[1] and Gaynell M. Simpson[2], *1. Georgia State University, School of Social Work, Atlanta, Georgia, United States, 2. University of Alabama, Tuscaloosa, Alabama, United States*

Based on AARP's domains of community livability, this presentation presents findings from a photovoice study of residents in an urban community in Atlanta, Georgia. Richardsville (pseudonym) is one of many communities in Atlanta's inner city that has transformed from a blighted, long-derelict area to a hotbed of high rent apartment units, mixed with quarter-million-dollar-plus homes. With property values skyrocketing and rents soaring, it has becoming increasingly difficult for longtime, older residents of this historically African-American community to remain in the vicinity. Richardsville Senior Residences, an affordable rental unit, was built to provide housing for a mix of incomes and ages and retain longtime residents in Atlanta's neighborhoods. Therefore, from this qualitative study, we share about residents' motivations for moving into Richardsville Senior Residences and their perceived livability of the environment that influence healthy aging in place.

### PATHWAYS TO RESILIENCE: TRANSFORMING TRAUMA THROUGH THE POWER OF NETWORKS

Connie Corley,[1] Maureen Feldman,[2] and Scott Kaiser[3], *1. Fielding Graduate University, Santa Barbara, California, United States, 2. Los Angeles Pierce Community College, Woodland Hills, California, United States, 3. University of Southern California, Los Angeles, California, United States*

Resilience has been examined in various age groups, initially focused on vulnerable children and more recently in enhancement of resilience in various age groups and in response to trauma. Based on studies of resilience in Holocaust survivors and intergenerational engagement to promote resilience in former gang members and isolated older adults, Corley's 3E model of Experience, Expression and Engagement is discussed in terms of multiple studies and the implications for forming and strengthening networks in communities at risk. This includes initiating creative coalition-building endeavors to address loneliness in residential settings for older adults evolving from a project funded to the Motion Picture and Television Fund in Los Angeles from the AARP Foundation.

### DEVELOPING TRAUMA-INFORMED APPROACHES IN PUBLIC HOUSING COMMUNITIES FOR OLDER PERSONS

Sharon Bowland,[1] and Becky Knight[1], *1. University of North Texas, Denton, Texas, United States*

In a study using a grounded theory approach with trauma survivors living in a mixed-age public housing community in Louisville, KY, twenty-five older women (50+) identified their main concern as housing safety. The study underlined the importance of community-level effects on health and well-being. Additionally, almost all participants identified that they had multiple experiences of interpersonal trauma across the life course. There may be increased negative health effects for older women living in public housing based on a history of interpersonal trauma. First, we will examine how women living in the community coped with their safety concerns. Second, we will explore policy implications of older trauma survivors living in communities that may exacerbate their trauma histories due to an ongoing lack of safety. Finally, we will consider how implementing trauma-informed approaches in public housing can potentially lead to improved health and well-being for those living with complex trauma.

### SESSION 4060 (SYMPOSIUM)

### MOVING BEYOND INDIVIDUAL COPING TO DEVELOPING SYSTEMS-LEVEL INTERVENTIONS FOR COMMUNITY-DWELLING ADULTS

Chair: Terri Lewinson, *Georgia State University, School of Social Work, Atlanta, Georgia, United States*
Discussant: Sharon Bowland, *University of North Texas, Denton, Texas, United States*

According to AARP, the number of Americans aged 65 and older will grow to 90.5 million, which is double the count in 2010. Along with the growth of the aging population, we can also anticipate more diverse older adult communities due to increased immigration and evolving, complex family constellations. Two major concerns for aging adults is managing increasing geriatric conditions that affect mobility/function and chronic morbidities, while also trying to afford quality housing, the environmental context within which older adults spend most of their time. Urban planners and human service providers must remain abreast of the housing-health needs of this aging population and prepare aging-friendly communities that foster resident resilience and address "environmental, economic, and social factors that influence the health and wellbeing of older people" (AARP, 2017). In this symposium, panelists one present findings from a qualitative study to explore collective trauma and resilience narratives among residents in Flint, Michigan. Panelists two share findings from a community-based participatory study that explores the intricate network of relationships among residents as a core indicator of livability in senior housing. Panelists three share strategies for strengthening networks to reducing resident isolation and loneliness. Lastly, the fourth set of panelists discuss policy implications and trauma-informed approaches needed for residents with a history of interpersonal trauma.

### COMMUNITY MEETINGS AS SITES OF RESILIENCE AND TRAUMA: INVESTIGATING ADVOCACY IN FLINT, MICHIGAN

Tam Perry,[1] and Jessica C. Robbins[1], *1. Wayne State University, Detroit, Michigan, United States*

During the ongoing water crisis in Flint, Michigan, residents continue to express their voices in community meetings. In this study of older adults, the research team conducted participant observation of such meetings in addition to interview older adults. This presentation highlights themes of trauma and resilience as found in the collective; we illustrate the knowledge gained by researchers about the challenging environmental contexts in which study participants are navigating and how narratives that are both personal and collective co-emerge. We examine how these narratives illustrate concerns of health and wellbeing from a life course perspective and index relationships of residents to their spaces. We conclude by offering lessons learned on investigating community meetings as a way of ensuring research is "community based."

### SESSION 4065 (SYMPOSIUM)

### NEW FINDINGS ON WORK AND FAMILY USING IPUMS DATA

Chair: Phyllis Moen, *University of MInnesota, Minneapolis, Minnesota, United States*
Discussant: James Raymo, *University of Wisconsin-Madison, Madison, Wisconsin, United States*

The IPUMS Data in Aging Research symposium will showcase aging research that is possible using freely available population-level data accessible via IPUMS. This session will feature papers that use IPUMS data in novel ways to examine aging-related topics including living arrangements, widowhood and divorce, paths toward retirement, intergenerational effects of interventions on later life outcomes, and time use of caregivers. In addition to the common threads of work and family, these papers all represent innovative contributions to aging research based on census and nationally-representative survey data. The potential value of these types of data for studying aging may not be immediately apparent to all researchers. By combining papers on an array of topics from a variety of data sources, this symposium highlights exemplar papers that demonstrate the types of novel research possible using public use census and survey data.

### SHORT-TERM U.S. PATHS TOWARD RETIREMENT: SIMILARITIES AND DIFFERENCES ACROSS AGE-GENDER-CLASS INTERSECTIONS

Sarah Flood,[1] Phyllis Moen,[1] sarah flood,[1] and Janet Wang[1], *1. University of Minnesota, Minneapolis, Minnesota, United States*

Existing knowledge about retirement transitions comes from studies of cohorts living through demographic, technological, social and economic environments, and private sector and public policy regimes that are very different from those of today. The Boomer cohort now transitioning to retirement is more educated, healthier, and more engaged in paid work than their parents or grandparents at the same ages. How is the large, diverse Boomer cohort (in their 50s, 60s, and 70s) navigating leaving the labor force compared to the Silent Generation preceding them? How similar or different across cohorts are patterned yet heterogeneous short-term workforce pathways, in terms of the timing, sequencing, and voluntariness of working hours, work participation, and exits, including subjective retirement? We use linked Current Population Survey (CPS) panel data (over 16 months) to capture change, complexity, heterogeneity, and inequities in even short-term work/retirement dynamics across intersections of age, gender, and class.

### THE INTERGENERATIONAL EFFECTS OF THE NEW DEAL WORK RELIEF PROGRAMS ON LATE-LIFE OUTCOMES: AN 80-PLUS-YEAR FOLLOW-UP STUDY

Sepideh Modrek[1], *1. San Francisco State University, San Francisco, California, United States*

Evidence from multiple disciplines suggests that early-life conditions and environments affect outcomes across the life course. However, less is known about the effects of policy interventions targeted to adults and communities that may have intergenerational consequences on exposed children. In this study, we undertake novel data linkages to examine the effects of the New Deal work–relief programs on long-term health, disability and mortality outcomes of children born between 1920-1940. We first link the American Manufacturing Cohort (AMC) workforce backward to their childhood census records to capture parental and community exposure to New Deal work-relief programs. We then test the hypothesis that employment in New Deal work-relief programs is associated with lower levels of chronic disease, lower rates of disability and delayed mortality for both the children in benefitting households and children in non-benefit households living in areas that received greater amounts of New Deal funding.

### INFLUENCES OF CAREWORK INTENSITY ON DAILY TIME USE PATTERNS OF CAREGIVERS

Liana C. Sayer[1], *1. University of Maryland Department of Sociology, College Park, Maryland, United States*

With the aging of the Baby Boom generation, increasing numbers of older adults require assistance in their daily lives and most help comes from family members. Delays in childbearing mean many adult elder care providers are simultaneously raising children. Although past research has documented disparities in psychological distress and financial costs, less is known about the social costs resulting from elder caregiving and how this varies by parental status. We examine the social costs of elder caregiving by comparing elder and child care configurations to investigate three questions. First, do the daily time use patterns of elder caregivers differ by parental status? Second, do the daily time use patterns of elder caregivers differ by caregiving intensity? Third, does caregiving intensity moderate associations of elder caregiving and parental status on daily time use? We address these questions using nationally representative time diary data from the 2011-2017 American Time Use Survey (ATUS).

### 'TIL DEATH DO US PART? DECLINING WIDOWHOOD AND RISING GRAY DIVORCE, 1980-2017

Susan L. Brown,[1] and I-Fen Lin[1], *1. Bowling Green State University, Bowling Green, Ohio, United States*

Roughly one-third of dissolutions among married persons aged 50 and older occur through divorce rather than widowhood, reflecting the rising gray divorce rate and lengthening life expectancies. We use data from the 1980 Vital Statistics and the 2017 American Community Survey (ACS) to estimate the divorce and widowhood rates among married individuals (aged 50+) in 1980 and 2017 to track how much the widowhood rate has declined and the divorce rate has risen. In 1980, women's widowhood rates exceeded their divorce rates at all ages. For men, the rate of divorce outpaced the rate of widowhood through ages 50-54. By 2017, divorce rates were higher for women through ages 55-59 and for men through ages 60-64, coinciding with the growth in gray divorce. We also examine subgroup variation in the 2017 patterns and the sociodemographic correlates of having experienced divorce versus widowhood during the past year using the ACS data.

### HOUSEHOLDS AND LIVING ARRANGEMENTS OF OLDER PERSONS AROUND THE WORLD

Yumiko Kamiya,[1] Yumiko Kamiya,[1] and Sara Hertog[1], *1. United Nations, New York, New York, United States*

The household living arrangements of older persons – whether living alone, with a spouse or partner, with their children or in multi-generational households – can be an important factor associated with their health, economic status and overall well-being. Understanding the patterns and trends in older persons' living arrangements is thus relevant for global efforts to achieve the sustainable development goals, in particular those targeting poverty, hunger and health. The United Nations Database on the Households and Living Arrangements of Older Persons 2018 presents evidence drawn from 672 unique data sources, including census and survey microdata samples archived at IPUMS-International and household rosters from Demographic and Health Surveys, among other sources. The resulting dataset describes older persons' households across 147 countries or areas, representing approximately 97 per cent of persons aged 60 or over globally.

### SESSION 4070 (SYMPOSIUM)

### NOVEL DATA AND APPROACHES TO THE STUDY OF HEALTH AND AGING IN NSHAP

Chair: Linda J. Waite, *University of Chicago, Chicago, Illinois, United States*

The National Social Life, Health, and Aging Project (NSHAP) is a longitudinal, population-based study that seeks to improve an understanding of the well-being of older, community-dwelling Americans. It accomplishes this by affording researchers a wide range of high quality measures that enable examining interactions among physical health and illness, medication use, cognitive function, emotional health, sensory function, health behaviors, social connectedness, sexuality, and relationship quality. The panelists in this symposium use NSHAP data to shed light on previously un- or underexplored aspects of health during aging. Kaufman et al. use interviewer ratings of respondents' skin shade along with respondents' individual experiences of discrimination, neighborhood racial composition, and other factors to characterize heterogeneity in the racial experience and how heterogeneity relates to health inequities. Riley integrates information on respondents' residential region at birth and in older age to show that older adults who left the South are less healthy than those who stay in the South, and that social embeddedness helps to explain the health benefits for those who stay. Huang et al. take advantage of rich structural and functional social connectedness data to show that self-reported hearing impairment is associated with depth but not breadth of social connections. Huisingh-Scheetz et al. capitalizes on performance measures of gait speed and chair stands obtained at each wave to examine whether repeated measures improve the ability to predict loss of independence in activities of daily living. Discussant will discuss the importance, strengths, and weaknesses of these papers, and consider implications for future research.

### THE MULTIPLE DIMENSIONS OF THE RACE EXPERIENCE AND ASSOCIATIONS WITH HEALTH IN OLDER ADULTS

Jerry Kaufman[1], *1. University of Chicago, Chicago, Illinois, United States*

Race is experienced along a number of dimensions. In the United States, education, family background (e.g., parents' education), skin shade, experiences of racial discrimination, neighborhood racial composition, state/region of birth, and interracial marriage help to define the "race experience." Many of these factors have been individually associated with adverse outcomes for African Americans relative to Whites, but little research has examined how these factors cohere within individuals. Using a national survey of African American and White older adults, we employed latent class analysis and, in preliminary analyses, identified three clusters of individuals who were characterized by unique race experiences. We then assessed and determined that these clusters were also unique in their differential associations with health outcomes. This data-driven approach will provide insight into the profiles of individuals whose race experience contributes to health inequities among older Americans.

### THE DIFFERENTIAL INFLUENCE OF REGIONAL CONTEXT ON LATER-LIFE HEALTH AND MORTALITY

Alicia Riley[1], *1. University of Chicago, Chicago, Illinois, United States*

This study examines regional disparities in later life health from a life course perspective. To sort out when and how region influences health over the life course, I focus on the sharp contrast between the South and the rest of the U.S. in health and mortality. I draw on data from the National Life Health and Aging Project (NSHAP), a nationally representative sample of community-dwelling older adults in the U.S., to estimate the differential risk of multiple health outcomes and mortality by regional trajectory. I find that older adults who leave the South are worse off in multiple outcomes than those who stay. I also find evidence of a protective health effect of community cohesion and dense social networks for the Southerners who stay in the South. My results suggest that regional trajectory influences health in later life through its associations with socioeconomic status, access to healthcare, and social rootedness.

### DISTRIBUTION, PREDICTORS, AND CLINICAL RELEVANCE OF 5-YEAR CHANGE IN FRAILTY MEASURES

Megan Huisingh-Scheetz,[1] Kristen Wroblewski,[1] Mark Ferguson,[1] Elbert Huang,[1] Linda Waite,[1] and L. P. Schumm[1], *1. University of Chicago, Chicago, Illinois, United States*

Implementing frailty assessment into routine clinical practice is a priority. Gait speed and performance on 5 repeated chair stands are two measures of frailty. We face a number of clinical implementation challenges: (1) We lack normative data for U.S. older adults and (2) The clinical relevance of change in frailty measures is unclear. The National Social Life, Health and Aging Project dataset allows an examination of the distribution of 3-meter gait and 5-repeated chair stands times as well as 5-year change in these measures in a nationally-representative, community-dwelling older adult sample. Dr. Huisingh-Scheetz will describe demographic predictors of change in these measures as well as determine whether baseline plus 5-year change in these measures predicts loss of independence in activities of daily living (ADLs).

### SESSION 4075 (SYMPOSIUM)

### PARTNERING IN GERIATRICS WORKFORCE ENHANCEMENT PROGRAMS: MODELS TO ENHANCE COLLABORATION AND ENGAGEMENT

Chair: Leland Waters, *Virginia Commonwealth University, Richmond, Virginia, United States*
Discussant: Nina Tumosa, *Health Resources and Services Administration, Rockville, Maryland, United States*

To achieve their healthcare system transformational goals to improve care for older adults, Geriatrics Workforce Enhancement Programs (GWEPs) facilitate the building of strong relationships among academia, community-based organizations, and primary care networks. Each GWEP develops strategies to formalize collaborations and build sustainable networks to meet program goals while addressing partner needs. Unique models from four GWEPs addressing stakeholder engagement are described, and factors facilitating collaboration are explored. One GWEP achieves mutual goals by collaborating with statewide coalitions that have a history of successful partnerships. Another GWEP achieves programmatic goals through an "all-in" interprofessional model called the Plenary. A third GWEP has capitalized on a shared complex outcome that requires multi-level stakeholder engagement to support aging in place. The final GWEP has coopted the resource exchange model as a conceptual foundation in order to enhance collaboration. Themes emerging from these four models include: (1) the enhancement of interpersonal relationships through communication, trust, and engagement; (2) the importance of commitment to the overall partnership itself; (3) the critical component of resource sharing and synergy across projects; and (4) strategies for sustainability in the face of changes and challenges across healthcare systems. Given the complex nature of person-centered interventions in geriatrics, it truly takes a village to develop and provide services for a heterogeneous, targeted population. This symposium emphasizes key elements of the structures and processes of these transformational GWEP villages.

### FACTORS FACILITATING COLLABORATION AND ENGAGEMENT: BUILDING AND SUSTAINING STATEWIDE COALITIONS

Cristine B. Henage,[1] Ellen C. Schneider,[2] Ellen Roberts,[2] Vicki Tilley,[3] and Jan Busby-Whitehead[3], *1. University of North Carolina, Chapel Hill, North Carolina, United States, 2. The University of North Carolina at Chapel Hill, Chapel Hill, North Carolina, United States, 3. The University of North Carolina at Chapel Hill School of Medicine, Chapel Hill, North Carolina, United States*

Sustaining collaboration across multiple community-based organizations (CBOs) creates synergies and economies of scale to support age-friendly communities beyond the provision of direct services any single CBO can achieve. The Carolina Geriatrics Workforce Enhancement Program (CGWEP) created and sustained multiple statewide coalitions focused on geriatrics syndromes. More than 290 CBOs, including Area Health Education Centers, social services programs and nongovernmental organizations, meet quarterly to form linkages, promote education and build infrastructure to support rural and underserved older adults. Shared governance with pooled resources has been achieved because of a long history of partnership, mutually beneficial relationships, flexibility, and frequent communication. The strength of the partnership is evidenced by continued growth in number of CBOs, number of sponsored events, and number of referrals to CBOs. Two coalitions, focused on falls prevention and mental health respectively, have been adopted by partners and sustained beyond grant funding.

### STAKEHOLDER ENGAGEMENT IN THE PLENARY AS A MODEL FOR PROFESSIONAL-COMMUNITY PARTNERSHIPS

Edward F. Ansello,[1] Sarah A. Marrs,[1] and Leland H. Waters[1], *1. Virginia Commonwealth University, Richmond, Virginia, United States*

The Virginia Geriatric Education Center (VGEC), a consortium of four Virginia universities, directs all initiatives in its Geriatrics Workforce Enhancement Program (GWEP) through an "all-in" interprofessional model called the Plenary. Both the structure and the process of the Plenary can serve as a model for building and maintaining successful, interdisciplinary, and intersystem partnerships that work toward shared goals. In addition to faculty and staff from the four institutions who represent nine health professions, representatives from CBOs also serve on the Plenary and attend in-person meetings twice monthly to engage in a continuous, democratic, and hands-on PDSA (Plan-Do-Study-Act) cycle to improve GWEP programs. This allows our community partners to be engaged in all components of identifying and addressing unmet needs in current and emerging interprofessional gerontology and geriatrics training, increasing CBO's stake in the overall success of the GWEP beyond their specific involvement. Team science principles guide program improvement and growth.

### CUSTOMIZING STAKEHOLDER ENGAGEMENT TO SUPPORT AGING IN PLACE

Elyse Perweiler,[1] Jennifer DeGennaro,[2] Sherry Pomerantz,[2] Lisa Bodenheimer,[2] Marilyn Mock,[3] and Margaret Avallone[4], *1. Rowan School of Osteopathic Medicine, Stratford, New Jersey, United States, 2. Rowan University School of Osteopathic Medicine, Stratford, New Jersey, United States, 3. Fair Share Housing Inc., Northgate II, Camden, New Jersey, United States, 4. Rutgers University School of Nursing-Camden, Camden, New Jersey, United States*

Geriatrics infusion and transformation of community-based settings to support "aging in place" is complex. It requires a customized approach that engages multiple stakeholders who are invested in systems redesign and process change. Rowan University School of Osteopathic Medicine's NJ Geriatrics Workforce Enhancement Program, in partnership with an affordable housing facility operated by Fair Share Housing/Northgate II, and Rutgers University School of Nursing (RSoN), implemented a Resident Health Risk Assessment (RHRA) tool as part of an interprofessional community-based training experience for health professions students. The goal was to identify health risks that impact a resident's ability to age in place and implement a person-centered intervention plan. Multi-level stakeholder engagement and ongoing rapid cycle quality improvement catalyzed changes in structure and process for all partners. Results included refinement of an interprofessional clinical rotation, introduction of competency attainment into the orientation process, and resource reallocation to support data collection.

### THE RESOURCE EXCHANGE NETWORK: A MODEL FOR TRANSFORMATIVE CHANGE IN GERIATRIC CARE

Phillip G. Clark,[1] Phillip Clark,[1] Christine Ferrone,[1] Faith Helm,[1] and Alexandra Morelli[1], *1. University of Rhode Island, Kingston, Rhode Island, United States*

Transformational efforts to redesign the care system for older adults call for the development of novel partnership models incorporating academic institutions, primary care networks, and community-based organizations. The Rhode Island Geriatric Workforce Enhancement Program (RI-GWEP) has utilized the "resource exchange model" to develop innovative, interdisciplinary, and integrated projects combining educational resources, clinical expertise, programmatic experience, and impact evaluation. With particular emphasis on the challenges of developing interprofessional education spanning the traditional gaps between disciplines and departments, this presentation emphasizes the critical conditions, components, and capacities of such a collaborative network. These include: (1) mission dominance, (2) barter exchange, (3) partnership investment, and (4) interpersonal relationships. RI-GWEP's experience provides insights into the conceptual foundation of geriatrics networks and specific, concrete examples of projects incorporating these principles. Implications for the development of networks in other settings include consideration of particular: (1) projects, (2) people, (3) places, (4) personalities, and (5) possibilities.

### SESSION 4080 (PAPER)

### TECHNOLOGY, THERAPY, AND TREATMENT OF VARIOUS HEALTH CONDITIONS

### EVALUATION OF ART ON THE BRAIN, A HEALTH APP FOR OLDER ADULTS, IN LONG-TERM CARE

Elizabeth P. Howard,[1] TAMMY RETALIC,[2] and JESSICA ROGAN[3], *1. CONNELL SCHOOL OF NURSING, BOSTON COLLEGE, CHESTNUT HILL, Massachusetts, United States, 2. HEBREW SENIORLIFE, BOSTON, Massachusetts, United States, 3. LIFE ENHANCEMENT, HEBREW SENIORLIFE, BOSTON, Massachusetts, United States*

ArtOn the Brain is a mobile application that incorporates art history education, play and socialization for older adults. This pilot project examined its health and social impact among long term care residents. A convenience sample of 48 residents, 60+ years, with borderline intact to moderate cognitive impairment were recruited. Residents with mild or better cognitive status used the app individually on a tablet while the remaining participated in therapist-led group sessions. One-hour intervention occurred twice weekly. Primary outcomes, health and well-being, were measured with 5Q-5D-5L and Warwick-Edinburgh Mental Well-being tools at baseline and after 6 weeks. Secondary outcomes were mood, cognition, function and overall satisfaction with activity options and personal relationships. Pre-post paired t-test analyses of data from EQ-5D-5L and Warwick tools did not show a significant difference. There were no notable declines with cognitive performance, functional ability and depression post-intervention. Four activity options and three personal relationship items had statistically significant improvements post-intervention. Activity options addressed engagement in enjoyable activities on weekends and evenings, and opportunity to, spend time with other like-minded residents and explore new skills, interests. Personal relationships items included engagement in activities helpful to others and having people to do things with. ArtOn the Brain is a novel application suitable for long term care residents. While 6 week intervention did not show improvement in overall health and well-being, there were significant improvements with residents' reported engagement in activity options and improvement of personal relationships. Longitudinal evaluations are needed to examine changes in health status and well-being.

### INSTAPALS: FACILITATING INTERGENERATIONAL CONTACT THROUGH TECHNOLOGY

Ashley Lytle,[1] and Nancy Nowacek[1], *1. Stevens Institute of Technology, Hoboken, New Jersey, United States*

Using the traditional framework of Pen Pals, Instapals was a project that facilitated 1-to-1 intergenerational relationships through daily exchanges on Instagram for 30 days. Although communication channels have exploded in the past 10 years in large part to social platforms and digital technologies, the diversity of daily social interactions has decreased. More and more, society has become siloed by age, interest, and belief. Building off intergroup contact theory, Instapals was designed to encourage positive intergenerational contact between younger (undergraduate students) and older adults (individuals 65+) and challenge ageist beliefs. Intergenerational social exchanges occurred both on Instagram and during three in-person meetings. Among undergraduate students, attitudes and stereotypes toward older adults and aging were assessed at three timepoints (before meeting their older adult partner, during a mid-point evaluation, and at the end of the project). Quantitative analyses demonstrate a decrease in aging anxiety, a decrease in psychological concerns about the aging process, and a greater endorsement of positive perceptions toward one's older adult partner. Qualitative analyses of written responses from students (collected before meeting their older adult partner and at the end of the project) revealed that the Instapals project helped students challenge ageist attitudes and stereotypes and was perceived to be a positive experience overall. Implications suggest that positive intergenerational contact can be facilitated and enhanced through the use of technology. Future research should explore whether attitudes, stereotypes, and self-perceptions of aging changed among older adults as well as other technological mechanisms for facilitating intergenerational contact.

### MECHANISM OF ACTION: AGE DIFFERENCES IN RESPONSE TO ACCEPTANCE AND COMMITMENT THERAPY FOR CHRONIC PAIN

Julie L. Wetherell,[1] Matthew Herbert,[1] and Niloofar Afari[1], *1. VA San Diego Healthcare System, San Diego, California, United States*

A recent randomized comparison of Acceptance and Commitment Therapy (ACT) vs. Cognitive-Behavioral Therapy for chronic pain found a clear age interaction effect, such that older adults benefitted more from ACT. In a subsequent study comparing ACT delivered in person to ACT delivered via telehealth to a sample of veterans (N=128, mean age 51.9, SD 13.3, range 25-89), we found no significant age by modality interactions, suggesting that older veterans responded as well as younger people did to telehealth delivery. Consistent with our previous findings, we found a trend for older adults to experience greater reduction in pain interference (p = .051) and significantly greater reduction in pain severity (p = .001) than younger adults following ACT. In younger veterans, change in pain acceptance from baseline to posttreatment was related to change in pain interference from baseline to 6-month follow-up (r = -.38), but change in pain interference from baseline to posttreatment was not related to change in pain acceptance from baseline to follow-up (r = .14), suggesting that, consistent with the ACT model, increased pain acceptance at posttreatment was related to reduced pain interference at follow-up. By contrast, in older veterans, both correlations were significant and of comparable magnitude (rs = -.43 and -.46, respectively), providing no support for the idea that change in pain acceptance drove change in pain interference. Overall, our findings suggest that ACT may work better in older adults with chronic pain than in younger adults, but via a different mechanism.

### SHINGLES VACCINE UPTAKE AMONG OLDER ADULTS: IDENTIFYING EARLY, LATE, AND NON-ADOPTERS

Hyewon Kang,[1] Eileen Crimmins,[2] and Jennifer A. Ailshire[1], *1. University of Southern California, Los Angeles, California, United States, 2. Davis School of Gerontology, University of Southern California, Los Angeles, California, United States*

Although a shingles vaccine (Zostavax) has been available since 2006, vaccination uptake has been slow. As a newly approved shingles vaccine (Shingrix) became available in 2018, understanding factors affecting acceptance and timing of the original vaccine would be useful in establishing effective strategies for greater immunization. Using the Health and Retirement study, we examined individual-level and area-level characteristics of early and late vaccine adopters, and those who were not vaccinated between 2006 and 2016. Early adopters were those who got vaccinated during the four year window after the approval of the vaccine; late vaccine adopters were those who got vaccinated from 2010 to 2016. Early adopters (13.5%) and late adopters (18.5%) comprised 32% of the sample, leaving two-thirds unvaccinated. Regression results suggest that those who received the vaccine were more likely to be socioeconomically advantaged, covered by insurance, socially active, healthy, to use other preventive vaccines, and to live in a region with more vaccinated people. Early adopters were more likely to be highly educated, affluent, and more conscientious compared to late adopters. Utilization of influenza vaccine and living in the region with the highest level of vaccination were found to be significant factors predicting early vaccine uptake. Our findings highlight the importance of outreach efforts and health care access in increasing vaccination rates.

### WHAT DETERMINES THE USE OF MEANING FOCUSED COPING IN DEMENTIA CAREGIVING?

Doris Yu,[1] and Sheung Tak cheng[1], *1. The Education University of Hong Kong, Hong Kong, Hong Kong*

Meaning-focused coping has been identified as an important factor contributing to more positive adjustment and health outcomes for family caregivers of persons with dementia. Yet, there is less evidence about what determines the use of this coping strategy. Based on the Meaning Making Model, this quantitative exploratory study identified the relationship between meaning focused coping and intrinsic motivation towards caregiving, quality of relationship between the care dyads and level of religiosity. Two hundreds and five family caregivers of PWD were recruited from a geriatric clinic from March 2018 – Feb 2019. A battery of questionnaires including the Meaning-Focused Coping Scale, Relationship Quality Scale, the Duke University Religion Index and Intrinsic Motivations to Care was administered in face-to-face interview. By using hierarchical regression analysis to control the effects of caregivers' demographic profile, caregiving history, clinical severity of dementia, and level of neuro-psychiatric symptoms, the results indicated that a higher intrinsic motivation to caregiving ($\beta$ = 1.457, p = 0.044), better dyadic relationship quality ($\beta$ = 0.768, p = 0.004) and a higher level of religiosity ($\beta$ = 0.969, p = 0.001) are independently related to a higher likelihood of using meaning-focused coping. The findings shift the paradigm of caregiver support from a deficit model to helping them to appraise the meaning of caregiving in dyadic relational, spiritual and motivational perspectives. The application of strength-based and person-centered approach to caregiver supportive program will be discussed.

### SESSION 4085 (SYMPOSIUM)

### USING MOBILE TECHNOLOGY TO ELUCIDATE PSYCHOSOCIAL AND COGNITIVE FUNCTION IN OLDER ADULTS

Chair: Nelson A. Roque, *Pennsylvania State University, Center for Healthy Aging, University Park, Pennsylvania, United States*
Co-Chair: Jinshil Hyun, *Albert Einstein College of Medicine, Bronx, New York, United States*

Discussant: Stacey B. Scott, *Stony Brook University, Stony Brook, New York, United States*

Ambulatory methods (AM) improve the reliability and ecological validity of cognitive assessments, and help to elucidate psychological influences through concurrent reports of pain, stress, and other psychosocial outcomes. Ecological momentary assessment (EMA) involves sampling of daily experiences in natural settings, including completing cognitive assessments, and answering questions related to, for example, social interactions and sleep. The purpose of this symposium is to present innovative methods and results, exploring questions at the intersection of intensive longitudinal data collection, cognition, and psychosocial influences, using data from two EMA studies, the Einstein Aging Study (EAS) and the Effects of Stress on Cognitive Aging, Physiology, and Emotion (ESCAPE) Study. The EAS (ages >= 70) and ESCAPE (ages 25 - 65) protocols, ask participants to complete an annual 14-day EMA measurement burst. A unique value of these methods is the ability to explore effects from moment-to-moment (or day-to-day; within-person effects) as we will present. We will also contrast these with conventional analyses of between-person differences, typical of clinic and in-person studies. Dickens (using ESCAPE data) examines end-of-day perceived stress and anticipation of next-day stress in predicting sleep quality. Hyun and colleagues (using EAS data) discuss the effects of affectionate physical touch on mitigating pain and emotional distress. Using a model-based cluster analysis approach (with EAS data), Roque unpacks differences in psychosocial factors, as a function of cognitive status risk groups. Stacey Scott will discuss these papers in the context of using ambulatory methods to improve the characterization of risk status in older adults.

### MODEL-BASED CLUSTER ANALYSES OF COGNITION FOR UNPACKING SUBGROUP DIFFERENCES IN PSYCHOSOCIAL OUTCOMES

Nelson A. Roque,[1] and Martin J. Sliwinski[1], *1. Pennsylvania State University, Center for Healthy Aging, University Park, Pennsylvania, United States*

We forward a methodological approach, using model-based cluster analyses, and ambulatory assessments of cognition (2 indicators from each task), to derive subgroups of interest for tailored clinical follow-up in a longitudinal framework. Community dwelling adults were asked to complete 14 consecutive days of ecological momentary assessments (EMAs) using smartphones, including measures of cognitive performance, and self-reported physical and mental health outcomes (e.g., stress, memory complaints, depression, pain). A stable four-cluster solution emerged, labelled as: (1) a high-risk cognitive group (13%; most memory complaints, slowest performing, more memory errors); (2) subjective risk group (42%; highest levels of somatic and cognitive complaints); (3) normative aging (28%; intermediate cognitive performance -- speed/accuracy); (4) super-cognitive agers (17%; fastest speed, best memory). In conclusion, these findings highlight the potential of a cluster-based approach for risk classification, uncovering different profiles of poor performance that may represent different etiologies.

### AFFECTIONATE PHYSICAL TOUCH MITIGATES PAIN AND EMOTIONAL DISTRESS IN OLDER ADULTS

Jinshil Hyun,[1] Richard B. Lipton,[1] Ruixue E. Zhaoyang,[2] Jennifer E. Graham-Engeland,[2] Jelena M. Pavlovic,[3] and Martin J. Sliwinski[2], *1. Albert Einstein College of Medicine, Bronx, New York, United States, 2. The Pennsylvania State University, University Park, Pennsylvania, United States, 3. Albert Einstein College of Medicine, Bronx, New York, United States*

Although research suggests that social interactions can decrease pain and emotional distress, it is unclear what produces these salubrious effects. We examined whether older adults experienced lower pain and emotional distress after two types of social interactions (affectionate physical contact and non-physical pleasant interactions) using data from the Einstein Aging Study (N=193, age=70-92). Participants completed a 14-day ecological momentary assessment protocol via which they reported the quality of recent social interaction, types of physical touch, levels of current stress, negative affect, and pain intensity five times a day. Multilevel models indicated that, following affectionate physical contact, individuals reported low levels of current pain intensity, negative affect, and stress (ps<.05). Following a pleasant non-physical interaction, individuals reported low negative affect (p<.05); pleasant interactions did not predict current pain or stress. Results highlight the potential unique utility of affectionate physical contact versus mere pleasant social interactions in older adults' daily lives.

### DAILY EFFECTS OF PERCEIVED AND ANTICIPATED STRESS ON SUBJECTIVE SLEEP QUALITY

Chelsea N. Dickens,[1] and Martin J. Sliwinski[2], *1. Pennsylvania State University, University Park, Pennsylvania, United States, 2. Pennsylvania State University, Center for Healthy Aging, University Park, Pennsylvania, United States*

Prior studies of stress and sleep have postulated that anticipation of future stress may have a greater impact on sleep quality than stress experienced during the day. We examined this possibility using data collected over 14 consecutive days. Participants (Npersons=257, age=25-65) rated each evening how stressful the day was and how stressful they expected the next day would be. Each morning, participants rated their subjective sleep quality. After adjusting for the effect of the previous night's sleep, on days when individuals reported feeling more stressed, they also reported not sleeping as well that night. However, expectations of stress the next day did not have an effect on sleep quality, suggesting that stress experienced during the day impacts sleep quality more so than anticipation of future stress. Despite previous findings that older age is associated with sleep complaints, age did not act as a moderator in our analysis.

### SESSION 4090 (SYMPOSIUM)

### CLINICAL TRIALS IN GEROSCIENCE

Chair: Matt Kaeberlein, *University of Washington, Seattle, Washington, United States*
Discussant: Matt Kaeberlein, *University of Washington, Seattle, Washington, United States*

This session will focus on clinical trials in geroscience.

### CELLULAR SENESCENCE AS A THERAPEUTIC TARGET FOR GEROSCIENCE-GUIDED CLINICAL TRIALS

Jamie N. Justice,[1] Anoop M. Nambiar,[2] Tamar Tchkonia,[3] Nathan K. LeBrasseur,[3] Nicolas Musi,[4] Stephen B. Kritchevsky,[5] and James L. Kirkland[3], *1. Wake Forest School of Medicine, Winston-Salem, North Carolina, United States, 2. University of Texas Health Sciences Center at San Antonio and South Texas Veterans Health Care System, San Antonio, Texas, United States, 3. Robert and Arlene Kogod Center on Aging, Mayo Clinic, Rochester, Minnesota, United States, 4. Barshop Institute for Longevity and Aging Studies, Center for Healthy Aging, University of Texas Health Sciences Center at San Antonio and South Texas Veterans Health Care System, San Antonio, Texas, United States, 5. Wake Forest School of Medicine, Winston-Salem, North Carolina, United States*

The geroscience hypothesis holds that targeting fundamental mechanisms of aging has the potential to prevent or reduce severity of multiple age-related diseases. Cellular senescence is a key mechanism that may be driving disease in human aging, including Idiopathic pulmonary fibrosis (IPF), a progressive, ultimately fatal, senescence-associated disease. Importantly, cellular senescence may be targeted therapeutically. Senolytic agents are drugs that selectively induce senescent cell apoptosis by transiently disabling anti-apoptotic pathways. Selective ablation of senescent cells using the senolytic drug combination dasatinib plus quercetin (DQ) alleviates IPF-related dysfunction in bleomycin-administered IPF mouse model. We conducted the first-in-human trial of senolytics in IPF patients, and our data indicate that short-term, intermittent administration of DQ may alleviate physical dysfunction that accompanies IPF in human aging, including clinically-meaningfully improvements in mobility ($p<0.05$). This geroscience-guided clinical feasibility study supports evaluation of senolytics in larger randomized, controlled trials of cellular senescence-associated age-related diseases.

### TORC1 INHIBITION AS AN IMMUNOTHERAPY TO DECREASE INFECTIONS IN THE ELDERLY

Joan Mannick[1], *1. resTORbio, Boston, Massachusetts, United States*

Inhibition of TORC1 has extended lifespan in multiple preclinical species. Thus drugs inhibiting TORC1 may have therapeutic benefit for aging-related conditions in humans. An aging-related condition that improves with TORC1 inhibition in old mice is immunosenescence (the aging-related decline in immune function). Immunosenescence leads to increased rates of infections including respiratory tract infections (RTIs) in the elderly. In two Phase 2 clinical trials in over 900 elderly subjects, the TORC1 inhibitor RTB101 was observed to improve immune function and decrease the incidence of RTIs. Decreasing the incidence of RTIs is important because RTIs are the fourth leading cause of hospitalization in people age 65 and above. Based on these findings, two Phase 3 studies are underway to determine whether RTB101 given for 16 weeks during winter cold and flu season decreases relative to placebo the percentage of elderly subjects with illnesses associated with RTIs.

### AGING INTERVENTIONS GET HUMAN: CAN WE EXTEND HEALTHSPAN?

Brian Kennedy[1], *1. National University of Singapore, Not applicable, Singapore*

Understanding biologic aging will afford opportunities for novel interventions to enhance human healthspan. If ageing can be slowed, the effect would be simultaneous protection from many of the chronic diseases. One strategy is to use animal model organisms to find common pathways that modulate ageing and then to seek methods for their human manipulation. The TOR pathway is one point of convergence and a clinically approved drug targeting the TOR kinase, rapamycin, extends murine lifespan and healthspan. Many more small molecules are being added to the list of anti-ageing compounds. Here, I use examples of interventions to conceptualize how agents extending healthspan might improve human health. We are entering a stage in aging research where it is imperative to test ageing interventions in humans and several strategies are contemplated. The potential to directly impact human healthspan is emerging from ageing research and this approach, if successful, will have global impact.

### AGING TREATMENT BY COUNTERACTING INTRINSIC DNA DAMAGE AND IMMUNOSENESCENCE

Andrei Gudkov

Progressive systemic poisoning by gradually accumulated damaged cells has been proposed as a major contributor to mammalian aging. Our preclinical studies support the hypothesis that this process results from a failure of innate immunity-mediated eradication of cells with DNA damaged by intrinsic mechanisms caused by the epigenetic desilencing of endogenous retroelements. This model suggests two translational approaches to improve the counteract accumulation of damaged cells: (i) by pharmacological suppression of LINE1 reverse transcriptase activities – the main driver of expansion of "retrobiome" and the trigger of damaged cell-associated inflammation and (ii) by counteracting immunosenescence by innate immunity stimulation. Preclinical proofs of concept have been obtained for both using nucleoside reverse transcriptase inhibitors and immunostimulators acting via TLR5 activation. Preparations for clinical testing of these agents in the context of age-related pathologies is in underway.

### SESSION 4095 (SYMPOSIUM)

### ACTIVITY SELECTION AND ENGAGEMENT IN OLD AGE: MOTIVATIONAL AND GOAL-BASED INFLUENCES

Chair: Thomas M. Hess, *North Carolina State University, Raleigh, North Carolina, United States*
Discussant: Christopher Hertzog, *Georgia Institute of Technology, Atlanta, Georgia, United States*

Research from a variety of perspectives has emphasized the central role played by activity in supporting a variety of positive outcomes in later life. For example, participation in activities that place demands on personal resources has been shown to be beneficial in promoting brain, cognitive, and physical health. From another perspective, older adults may also engage in certain activities to promote specific outcomes (e.g., emotional) in service of psychological well-being. Such findings highlight the adaptive significance of activity selection and engagement processes. Using a variety of approaches, the presentations in this symposium focus specifically on goal-based and motivational factors that may facilitate or impede such processes. Moored and colleagues examine adaptive characteristics—including motivational ones—of individuals whose activity patterns are protective against dementia. Using longitudinal data from the Health and Retirement Study, Lothary and colleagues explore the degree to which intrinsic motivation to engage cognitive resources mediates the effect of personal resources (e.g., physical and emotional health) on participation in challenging everyday activities. Growney and colleagues present research demonstrating that subjective perceptions of difficulty affect decisions to engage in challenging activities, but that such perceptions may reflect biases associated with negative aging attitudes as opposed to actual effort expenditure. Finally, Lind and Isaacowitz examine selection associated with affective aspects of the activities, finding that both middle-aged and older adults exhibited similar biases toward positive activities in congruence with emotion-regulation goals, though age differences were observed in non-affective aspects of the activities. The discussion by Hertzog will highlight common themes.

### AGING STEREOTYPES MODERATE THE RELATIONSHIP BETWEEN COSTS AND DISCOUNTING

Claire Growney,[1] Erica L. O'Brien,[1] Jesse DeLaRosa,[1] and Thomas M. Hess[1], *1. North Carolina State University, Raleigh, North Carolina, United States*

Aging is associated with normative declines in cognitive resources that increase the costs associated with mobilizing resources in cognitively demanding activities. Selective Engagement Theory (Hess, 2014) hypothesizes that changes in costs influence the motivation to engage in such activities in everyday life. We used an economic discounting task to examine the relationship between both objective estimates (systolic blood pressure responses) and subjective estimates (NASA Task Load Index) of cognitive costs in 78 older adults' (ages 64-85) decisions to engage in more or less demanding activities. Perceptions of costs were meaningfully tied to actual costs (SBP), but further influenced by personal or primed attitudes about aging. Interestingly, decisions to engage in demanding activities—as reflected in discounting decisions—were less influenced by effort expenditure in the activity than by perceptions of difficulty. These results underscore the role that negative stereotypes play in undermining motivation to engage in potentially beneficial activities.

### EXAMINING WHETHER LIFESTYLE ACTIVITY PATTERNS PREDICT DEMENTIA INCIDENCE AMONG COMMUNITY-DWELLING OLDER ADULTS

Kyle Moored,[1] Kyle Moored,[2] Jeanine Parisi,[2] and Michelle Carlson[2], *1. Johns Hopkins, Baltimore, United States, 2. Johns Hopkins University, Baltimore, Maryland, United States*

Engagement in lifestyle activities can be neuroprotective, but it remains unclear what aspects of engagement are most beneficial. Examining activity patterns may better characterize both quantitative (e.g., number) and qualitative (e.g., characteristic/motivational) differences in engagement. We used a novel, latent class analysis (LCA) to characterize subgroups with distinct activity patterns and examined whether they have differential risk of incident dementia. We compared these findings to models including standard activity frequency and variety metrics. Using the Ginkgo Evaluation of Memory Study (N=3,069), we fit Cox regressions of each activity metric on time to dementia, adjusting for intervention group and demographics. For the LCA, we derived group/class indicators for Cox regression. Variety predicted incident dementia and will be compared to LCA activity metrics in predicting risk. Activity metrics that are most protective against dementia inform intervention design. Unlike standard activity metrics, LCA may further identify subgroups with common motivations to sustain activity.

### MOTIVATION AS A MECHANISM: THE LONGITUDINAL RELATIONSHIP BETWEEN PERSONAL RESOURCES AND ACTIVITY ENGAGEMENT

Allura F. Lothary,[1] Tara L. Queen,[2] and Thomas M. Hess[1], *1. North Carolina State University, Raleigh, North Carolina, United States, 2. University of North Carolina at Chapel Hill, Carrboro, North Carolina, United States*

Past research has demonstrated an association between health and cognitive resources, intrinsic motivation, and activity participation in older adulthood, both cross-sectionally and from a daily perspective (e.g., Queen & Hess, 2018; Hess et al., 2018). This highlights the potential importance of motivation as a mediator of the impact of changing personal resources on engagement in cognitively beneficial activities. This study expanded on prior research by examining these relationships longitudinally in a large representative sample of adults over 50. Specifically, we used data from the Health and Retirement Survey (N = 5600) to create two 4-year longitudinal assessments, with multi-level structural equation modeling used to test the mediating role of motivation on everyday activity engagement. Consistent with expectations from Selective Engagement Theory (Hess, 2014), motivation served as a partial mediator of the impact of changing resources on engagement, with the effect being selective based on the cognitive demands of the activity.

### THE ROLE OF AGE AND EMOTION REGULATION IN CHOICE OF EMOTIONAL ACTIVITY IN EVERYDAY LIFE

Majse Lind,[1] and Derek Isaacowitz[1], *1. Northeastern University, Boston, Massachusetts, United States*

Situation selection is a form of activity selection focused on the affective tone of potential choices. We investigated this type of emotional activity selection in everyday life during a longitudinal multi-burst study of middle-aged and older adults. In each burst, participants were asked to complete six phone-based assessments per day across five consecutive days, including a situation selection question about what type of emotional situation they would want to engage in at the time. They also reported on various emotion regulation strategies. Both age groups favored positive activities, but older adults preferred positive-deactivated activities whereas middle-aged adults favored positive-activated activities. Both middle-aged and older adults reported more attention towards positive relative to negative thoughts. Greater attention to positive thoughts was associated with the tendency to select more positive activities across age groups. Both age and emotion regulation strategy use may therefore contribute to choice of emotional activities in everyday life.

### SESSION 4100 (SYMPOSIUM)

### CHARACTERIZING THE PATHWAYS UNDERLYING THE ASSOCIATION BETWEEN PSYCHOLOGICAL WELL-BEING AND HEALTH

Chair: Eric S. Kim, *Harvard T.H. Chan School of Public Health, Boston, United States*
Discussant: Anthony Ong, *Cornell University, Ithaca, New York, United States*

As populations age, identifying factors that foster the maintenance of health is crucial for improving the health and well-being of older adults. Yet, most psychological, biomedical, and public health efforts have focused on reducing harmful risk factors. While the risk management approach has contributed greatly to prevention and treatment programs, our aging society continues to grapple with the steadily rising tide of chronic conditions. Expanding the focus to include upstream, health-promoting psychosocial assets may help inform a more comprehensive response effort. Mounting research suggests that different dimensions of psychological well-being are uniquely associated with reduced risk of chronic conditions. However, the mechanisms underlying these associations remain understudied. This symposium presents 4 studies evaluating potential mechanisms. The first talk presents research evaluating how a spouse's level of optimism may be uniquely associated with an individual's cognitive health over time (above and beyond that own individual's level of optimism). A second talk, draws upon a multi-burst daily diary study and focuses on affective stress response as a potentially modifiable target that could explain the health benefits of optimism. A third talk evaluates how baseline levels purpose in life might be associated with repeated measures of five key health behaviors over time. A fourth talk discusses results from a longitudinal-burst daily diary study determining the reciprocal relationships among optimism, pain interference, and goal-directed activity among older women who experience pain. Overall, these studies add to the growing research on psychological well-being and physical health by providing evidence around potential biobehavioral pathways.

### THE ASSOCIATION BETWEEN SENSE OF PURPOSE IN LIFE AND REPEATED MEASURES OF HEALTH BEHAVIORS OVER TIME

Eric S. Kim,[1] Koichiro Shiba,[2] and Laura Kubzansky[2], *1. Harvard T.H. Chan School of Public Health, Boston, United States, 2. Harvard T.H. Chan School of Public Health, Boston, Massachusetts, United States*

Although a stronger sense of purpose in life has been associated with reduced risk of chronic conditions and mortality, potential pathways underlying these associations remain understudied. In the present study, we tested if a higher baseline sense of purpose in life was associated with maintenance of recommended levels of five health behaviors. Prospective data included 13,771 adults from the Health and Retirement Study, who were assessed up to six times across an average of 9 years. In mixed models that adjusted for sociodemographic factors, those in the highest quartile of purpose, compared to those in the lowest quartile, had a higher likelihood of not smoking (RR=1.04; 95% CI: 1.03–1.06), remaining physically active (RR=1.42; 95% CI: 1.33–1.51), drinking moderate amounts of alcohol (RR=1.21; 95% CI: 1.13–1.29), not suffering from sleep problems (RR=1.29; 95% CI: 1.24–1.34), and maintaining healthy BMI (RR=1.09; 95% CI: 1.06–1.11) over follow-up.

### IS THE PATH EASIER ON THE BRIGHTER SIDE? OPTIMISM AND DAILY STRESS PROCESSES ACROSS 16 YEARS

Lewina O. Lee,[1] Avron Spiro,[2] Daniel K. Mroczek,[3] and Laura D. Kubzansky[4], *1. Boston University School of Medicine, Boston, Massachusetts, United States, 2. VA Boston Healthcare System, Boston, Massachusetts, United States, 3. Northwestern University Feinberg School Of Medicine and Weinberg College of Arts & Sciences, Chicago, Illinois, United States, 4. Harvard T.H. Chan School of Public Health, Boston, Massachusetts, United States*

Accumulating evidence supports optimism as a health asset, yet little is known about underlying pathways. This study evaluates the long-term influence of optimism on exposure and affective reactivity to daily stressors. The sample comprised 233 community-dwelling men who completed a validated measure of optimism in 1986 (age: M=59, SD=6), and participated in up to three 8-day daily diary studies between 2002-10. Daily stressor occurrence, end-of-day positive affect (PA) and negative affect (NA) were assessed nightly in the diary studies. Results from multilevel structural equation modeling showed that optimism was unrelated to affective stress reactivity. However, higher optimism preceded lower overall NA and higher overall PA. Lower daily stressor exposure mediated the association from optimism to lower NA (indirect effect: B=-0.26, 95% Bayesian CI: -0.48, -0.09), but it did not account for the optimism-PA association. Our findings add to knowledge on pathways by which optimism promotes affective well-being in old age.

### THE ASSOCIATION BETWEEN ACTOR-PARTNER OPTIMISM AND COGNITIVE ABILITY AMONG OLDER COUPLES

William J. Chopik,[1] Jeewon Oh,[1] and Eric S. Kim[2], *1. Michigan State University, East Lansing, Michigan, United States, 2. Harvard T. H. Chan School of Public Health, Boston, Massachusetts, United States*

Optimism has been found to be associated with physical health and interpersonal well-being. Spouses also play an important role on people's health especially in late life. Yet, little is known about how a spouse's optimism might be associated with an individual's cognitive health. This study examined how actor and partner optimism in couples are associated with cognitive ability. Results showed positive associations between actor optimism and cognitive ability (.03 ≤ rs ≤ .17), and partner optimism and cognitive ability (.03 ≤ rs ≤.04), which mostly persisted over time. Further, partner optimism moderated actor optimism. Although highly optimistic people had higher cognitive ability regardless of partner's optimism (r = .02, p = .22), people particularly benefitted from being married to an optimist (r = .05, p < .001). These results suggest that we need to consider the context of spousal relationships when understanding optimism and cognitive health in older-adulthood.

### OPTIMISM AND PAIN INTERFERENCE IN AGING WOMEN

Stephanie T. Judge,[1] Jodi L. Clasey,[2]
Leslie J. Crofford,[3] and Suzanne C. Segerstrom[1],
*1. Department of Psychology, University of Kentucky, Lexington, Kentucky, United States, 2. Department of Kinesiology and Health Promotion, University of Kentucky, Lexington, Kentucky, United States, 3. Departments of Medicine and Pathology, Microbiology, and Immunology, Vanderbilt University School of Medicine, Nashville, Tennessee, United States*

Pain limits individuals' ability to engage in activities that promote well-being. This longitudinal-burst daily diary study tested reciprocal relationships among pain, optimism, pain interference, and activity in older women. Multilevel models tested between- and within-person relationships among these variables. Pain best predicted interference (person: γ001 = .227, SE = .022, p < .0001; wave: γ010 = .267, SE = .014, p < .0001; day: γ100 = .246, SE = .010, p < .0001); optimism best predicted activity (γ002 = .684, SE = .101, p < .0001). In linear regression models, baseline optimism (sr2 = 0.560, p < .0001), less interference (sr2 = 0.064, p < .0001), and more activity (sr2 = 0.015, p = .013) predicted higher end-of-study optimism. Ultimately, more optimistic women were significantly more active than less optimistic women, and less interference and more activity promoted increased optimism, creating a virtuous cycle that enhances well-being among older women.

### SESSION 4105 (SYMPOSIUM)

### COGNITIVE AGING IN OLDER MINORITY POPULATIONS: LONGITUDINAL EVIDENCE FROM U.S. CHINESE OLDER ADULTS

Chair: XinQi Dong, *Rutgers Institute for Health, Health Care Policy and Aging Research, New Brunswick, United States*
Co-Chair: Melissa Simon, *Northwestern University, Chicago, Illinois, United States*

The increasing diversity in the aging population warrants systematic investigations regarding ethnic differences related to cognitive aging and ethnicity-unique risk factors. However, due to the great paucity of population-based longitudinal data on cognitive aging in racial/ethnic minority populations, our knowledge in this area remain limited. The purpose of this symposium is, therefore, to examine various psychological, socio-cultural, and physical factors associated with cognitive aging among U.S. Chinese older adults, representing one of the biggest and fastest growing older minority populations nationally. Using longitudinal data from a population-based prospective cohort study, namely The Population Study of ChINese Elderly in Chicago (PINE) with a sample size of 3,157, this symposium presents findings from five research projects. Session 1 investigates the relationship between psychological well-being and change of cognitive function over four years. Session 2 and 3 examine the relationships between two socio-cultural factors and cognitive function. Specifically, session 2 investigates the associations between immigration-related factors and the incidence of cognitive impairment. Session 3 explores the relationship between cognitive function and Tai-Chi practice. Session 4 and 5 examine the relationships between two physical health indicators and cognitive aging. Specifically, session 4 examines the relationship between physical function and change of cognitive function over two years. Session 5 explores the association between body mass index and cognitive function decline over two years. Taken together, this symposium aims to further our knowledge of cognitive aging among ethnically/culturally diverse populations. The research findings will identify unique factors related to cognitive aging in older minority populations.

### PSYCHOLOGICAL WELL-BEING AND CHANGE OF COGNITIVE FUNCTION OVER 4 YEARS

Dexia Kong,[1] Dexia Kong,[2] and XinQi Dong[2], *1. Rutgers University, New Brunswick, New Jersey, United States, 2. Rutgers University, The State University of New Jersey, New Brunswick, New Jersey, United States*

This study aims to examine the relationship between psychological-wellbeing (including depressive symptoms, loneliness, anxiety, hopelessness, and stress) and cognitive function during a 4-year period among U.S. Chinese older adults. Data were from 2,300 PINE participants who completed in-home interviews at baseline, 2-year and 4-year follow-up. Cognitive function was assessed by five individual cognition tests at baseline and follow-up interviews. Mixed-effects regression models were conducted. A trend of decline in global cognition and multiple cognitive domains was observed. The study findings showed that depressive symptoms (Estimate= -0.03, p<0.001), anxiety (Estimate = -0.02, p<0.001), hopelessness (Estimate = -0.01, p<0.01), and stress (Estimate = -0.01, p<0.001) were associated with poorer global cognitive function at baseline. Baseline psychological well-being was not associated with changes in cognitive function during a 4-year period. Significant predictors of rate of change in cognitive function will be discussed. Potential explanations and implications of these findings will be presented.

### GENDER DIFFERENCES IN THE PREVALENCE AND INCIDENCE OF COGNITIVE IMPAIRMENT: DOES IMMIGRATION MATTER?

Fengyan Tang,[1] Fengyan Tang,[1] Ke Li,[1] Iris Chi,[2] and XinQi Dong[3], *1. University of Pittsburgh, Pittsburgh, Pennsylvania, United States, 2. University of Southern California, Los Angeles, California, United States, 3. Rutgers University, The State University of New Jersey, New Brunswick, New Jersey, United States*

Using two waves of the PINE data, this study examined gender difference in the associations between immigration-related factors and cognitive impairment (CI). CI was assessed by the Chinese Mini-Mental State Examination (C-MMSE). CI prevalence was determined by C-MMSE < 18 at baseline; incidence was the percentage of the respondents whose C-MMSE > 18 at baseline but dropped below 18 at Wave 2. We found 7.62% CI prevalence and 5.12% incidence rate. Women were more likely than men to have CI, consisting of 77.06% and 75.20% among persons with CI at two time points. Older Chinese women were generally disadvantaged in cognition and overall health compared with older men. Yet immigration experience does not link to CI for both men and women after controlling the well-established effects of age and education. Future research needs to investigate what biological and contextual factors earlier in life are predictive of late-life CI risk.

### THE ASSOCIATION BETWEEN COGNITIVE FUNCTION AND TAI-CHI: FINDINGS FROM THE PINE STUDY

Ying-Yu Chao,[1] Ying-Yu Chao,[2] Peijia Zha,[2] and Xinqi Dong[3], *1. Rutgers School of Nursing, Newark, United States, 2. Rutgers School of Nursing, Newark, New Jersey, United States, 3. Rutgers Institute for Health, Health Care Policy and Aging Research, New Brunswick, New Jersey, United States*

The study examined the association between cognitive function and Tai Chi practice among older Chinese Americans. Data from the Population Study of Chinese Elderly in Chicago (N=3,157) was analyzed by multivariate logistic regression. Cognitive function was assessed by global cognition, episodic memory, executive function, working memory, and Chinese Mini-Mental State Examination (C-MMSE). The results showed that cognitive function was significantly associated with Tai Chi practice. Participants with higher scores of global cognition (OR = 1.44, 95% CI: 1.20–1.73, p = 0.00), episodic memory (OR =1.27, 95% CI: 1.10–1.47, p = 0.00), executive function (OR = 1.017, 95% CI:1.00–1.03, p = 0.01), working memory (OR = 1.07, 95% CI: 1.02–1.12, p = 0.01), and C-MMSE (OR = 1.06, 95% CI: 1.03–1.09, p = 0.00) were more likely to practice Tai Chi. This study demonstrated that Tai Chi may benefit cognitive function in Chinese older adults.

### PHYSICAL FUNCTION AND LONGITUDINAL CHANGES IN COGNITIVE FUNCTION

Jinjiao Wang,[1] and Jinjiao Wang[2], *ork, United States* Dexia Kong,[3] and XinQi Dong[3], *1. University of Rochester, Rochester, New York, United States, 2. University of Rochester School of Nursing, Rochester, New Y, 3. Rutgers University, The State University of New Jersey, New Brunswick, New Jersey, United States*

Among 2,038 older Chinese adults in the U.S., we examined the relationship between physical function (Short Performance Physical Battery [SPPB], [instrumental] activities of daily living [ADL/IADL] limitations) at baseline (2011-2013) and changes in cognitive function in the two-year follow-up (2013-2015). Cognitive function was measured by the East Boston Memory Test (EBMT), the Digit Span Backwards assessment (DSB), the Symbol Digit Modalities Test (SDMT), and the Mini-Mental State Examination (MMSE). During the two-year follow-up, 41.8%-50.88% of the participants decreased in cognitive function and 32.88%-44.8% increased. In linear regression that adjusted for baseline cognitive function, education, age, and other covariates, baseline SPPB and ADL/IADL limitations were significantly associated with changes in cognitive function in the two-year follow-up (SPPB: $\beta$EBMT=0.0149, $p<0.05$; $\beta$DSB=0.0253, $p>0.05$; $\beta$SDMT=0.2742, $p<0.01$; $\beta$MMSE=0.1070, $p<0.001$; ADL/IADL limitations: $\beta$EBMT= -0.0401, $p<0.0001$; $\beta$DSB= -0.0410, $p<0.05$; $\beta$SDMT= -0.3027, $p<0.01$; $\beta$MMSE= -0.2566, $p<0.0001$). This suggests that better physical function predicts positive changes in cognitive function.

### BODY MASS INDEX AND COGNITIVE FUNCTION DECLINE

Yingxiao Hua,[1] Yingxiao Hua,[2] Dexia Kong,[2] and XinQi Dong, *1. Rutgers Institute for Health, Health Care Policy and Aging, New Brunswick, New Jersey, United States, 2. Rutgers University, The State University of New Jersey, New Brunswick, New Jersey, United States*

Body composition has been proposed as an important modifiable risk factor of cognitive decline in multiple epidemiological studies. However, the relationship between body mass index (BMI) and cognitive function remains controversial and conflicting in diverse populations. This study aims to investigate the association between BMI and cognitive decline in U.S. Chinese older adults. Classifications of BMI are based on Asian criteria recommended by WHO (underweight: BMI<18.5, normal weight: 18.5≤BMI<23, overweight: 23≤bmi<27.5, obesity: bmi≥27.5). Logistic regression models were conducted. Compared with normal-weight participants, underweight participants were more likely to experience decline in episodic memory (OR=1.68, p=0.002) and work memory (OR=1.38, p=0.05). Being overweight and obesity were not associated with cognitive function decline. The findings indicate that underweight could potentially be a risk factor of cognitive function decline among U.S. Chinese older adults. Perspective studies may further investigate the association between weight loss and cognitive decline for the development of prevention strategies.

### SESSION 4110 (PAPER)

### COGNITIVE FUNCTIONING AMONG DIVERSE POPULATIONS

### CHRONIC DISEASES IN MIDDLE-AGED ADULTS WITH SUBJECTIVE COGNITIVE DECLINE: UNITED STATES, 2015-2017

Christopher A. Taylor,[1] Erin D. Bouldin,[2] and Lisa C. McGuire[3], *1. Centers for Disease Control and Prevention, Atlanta, Georgia, United States, 2. Appalachian State University, Boone, North Carolina, United States, 3. CDC, Atlanta, Georgia, United States*

While adults aged 65 years and older are most at risk for chronic conditions, studies show that middle-aged adults aged 45–64 years also have growing numbers of comorbid chronic diseases. Regardless of age, managing chronic conditions requires decision-making abilities to manage treatments effectively. Symptoms of memory loss and confusion may impair a person's ability to manage their health. This study examined chronic conditions in persons with subjective cognitive decline (SCD), defined as the self-reported experience of increased memory problems or confusion. Behavioral Risk Factor Surveillance System data from 2015–2017 were used to define SCD and disease status for eight chronic conditions (heart disease, stroke, cancer, arthritis, asthma, depression, diabetes, and chronic obstructive pulmonary disease) for adults 45–64 years from 49 states, District of Columbia, and Puerto Rico that collected data on cognitive decline. Among adults aged 45–64 years, 10.8% reported SCD. Among those with SCD, 77.4% had at least one chronic disease compared to 47.1% of those without SCD. Those with SCD had a higher prevalence for all eight conditions compared to those similarly-aged without SCD. Adults with at least one chronic condition were more likely to discuss their symptoms of SCD with a health care professional (54.2%) compared to those with no chronic conditions (30.3%). Poor management of chronic conditions can result in increased health care costs and might worsen existing symptoms of confusion and memory problems. Self-care interventions for chronic disease management should consider the importance of an individual's cognitive status, including SCD.

### DISCERNING STATUS: SUBJECTIVE SOCIAL POSITION AND COGNITIVE HEALTH AMONG OLDER ADULTS IN RURAL SOUTH AFRICA

Lindsay C. Kobayashi,[1] Guy Harling,[2] Meagan T. Farrell,[3] Ryan G. Wagner,[4] and Lisa F. Berkman[3], *1. Georgetown University, Washington, District of Columbia, United States, 2. University College London, London, United Kingdom, 3. Harvard Center for Population and Development Studies, Cambridge, Massachusetts, United States, 4. University of the Witwatersrand, Johannesburg, South Africa*

Rapid population aging in high absolute poverty settings, such as much of South Africa, demands new research on the social context factors that affect cognitive aging in these settings. We investigated the relationships between subjective social position within one's village and cognitive function and impairment, with the rationale that psychosocial stress induced by low relative social position may affect cognitive aging outcomes independently of absolute socioeconomic conditions. Data were from the population-representative HAALSI study of 5,059 adults aged 40+ in rural Agincourt, South Africa. Subjective social position was assessed using the MacArthur Network social ladder, which asks respondents to indicate how high up a ladder they stand, relative to others, in their village. Cognitive function was a composite z-score of time orientation and word recall tests; scores ≤1.5 standard deviations (SD) below the mean indicated cognitive impairment. Twenty percent of those on the bottom rung had cognitive impairment, declining to 2% on the top rung. In regression models adjusted for age, sex, country of birth, education, literacy, marital status, employment, and asset-based household wealth, each ladder rung increase was associated with an 0.05 SD increase in cognitive z-score (95% CI: 0.04-0.06), and a 17% decrease in odds of cognitive impairment (OR=0.83; 95% CI: 0.79-0.88); these associations were greater in magnitude than those for years of education with these cognitive outcomes. Future work should examine if these relationships persist longitudinally, and investigate the mechanisms of these relationships, including village-level contextual factors that may contribute to subjective social position in this setting.

### EARLY IDENTIFICATION OF COGNITIVE IMPAIRMENT AMONG VULNERABLE OLDER ADULTS LIVING AT HOME

Maria T. Brown,[1] and Sharon A. Brangman[2], *1. Syracuse University, Syracuse, New York, United States, 2. SUNY Upstate Medical University, Syracuse, New York, United States*

African Americans are twice as likely as Caucasians to develop Alzheimer's disease and related dementias (AD/D), are at greater risk of frailty if they have AD/D, and are less likely to seek a diagnosis before experiencing a crisis. This collaborative community-based project aimed to evaluate the processes and outcomes of adding a Mini-Cog© screening to existing Area Agency on Aging (AAA) assessments performed by a neighborhood advisor in a lower-income African American neighborhood in a mid-sized northeastern city. AAA clients receive initial in-home screenings to assess their need for income-related services to help them continue living safely in the community, but are not screened for potential cognitive issues that may exacerbate or be masked by the very conditions that AAAs aim to address. This pilot was designed to introduce a mechanism for the early identification of previously undetected cognitive impairment, provide referrals for comprehensive cognition screening at a Center of Excellence for Alzheimer's Disease (CEAD), and increase access to needed health and community support services. We hypothesized that adding the Mini-Cog© to existing AAA assessments would increase the early identification of vulnerable older African Americans with or at risk of AD/D. Process outcomes included challenges encountered when engaging community caregivers on the project team, learning to interpret Mini-Cog© results, the labor-intensive nature of scheduling Mini-Cog© assessments with existing clients, addressing client stigma and fear of AD/D, and client resistance to comprehensive testing. This presentation reviews process and outcome evaluation data from the pilot, and discusses implications for expanding the intervention.

### PROMOTING KNOWLEDGE OF COGNITIVE DYSFUNCTION IN MINORITY COMMUNITY-DWELLING OLDER ADULTS

Koshy Alexander,[1] Ruth Manna,[1] Natalie Gangai,[1] Rosario Costas Muniz,[1] and Beatriz Korc-Grodzicki[1], *1. Memorial Sloan Kettering Cancer Center, New York, United States*

Minorities and older adults represent an intersection of populations that are particularly vulnerable to suboptimal care. The US population is aging and growing more diverse. Dementia afflicts minority populations more than Caucasians. Many people in the community with mild cognitive impairment (MCI) and Alzheimer's disease do not recognize cognitive, functional or behavioral impairment as abnormal. Several barriers to treatment exist within both the older and minority populations. The Health Resources and Services

Administration (HRSA), is the primary Federal agency for improving health and achieving health equity. Queens County in New York City is a multicultural, ethnically diverse urban area with 48% of its population being foreign born. Funded by a grant from HRSA, in collaboration with the South Asian Council for Social Services (SACSS), the Geriatric Resource Interprofessional Program (GRIP) at Memorial Sloan Kettering Cancer Center (MSKCC) developed and conducted educational lectures on "Memory Loss & Dementia" at 7 different community centers in Queens. Between November 2015 and January 2019 eight sessions were conducted. A total of 182 people attended. Their mean age was 68, 59% were women. 71% were born in South Asia. Consecutive interpretation of the lecture was performed; the written materials were translated beforehand. Pre and post-test questionnaires were administered to measure understanding of the topic, and qualitative comments collected. The developmental process, results of the study and challenges we faced in developing and applying the educational initiative will be presented.

### RACE, DEATH OF A SIBLING, AND DEMENTIA RISK AT LATE ADULTHOOD

Minle Xu[1], *1. The University of Texas at Austin, Austin, United States*

The death of a family member is a stressful life event that compromises health and well-being. Considerable research has documented the detrimental health effects of parental, child, and spousal death. However, much less is known with regard to the health consequences of sibling death especially in late adulthood. Relationship with sibling is one of longest and intimate social relationships and the death of a sibling can be a devastating life event especially for older adults as they are more vulnerable to adverse effect of stress. As sibling death is more prevalent in late adulthood, it is important to examine whether sibling death increases risks of dementia which has become a public health concern due to its deleterious effects on individuals, families, and societies. Therefore, this study investigates the association between sibling death and dementia incidence in later life by using longitudinal data from the Health and Retirement Study. Results from discrete-time hazard models show that respondents who experienced the death of a sibling between 1992 and 2000 are more likely to develop dementia during follow-up. This positive association between the death of a sibling and dementia incidence remains unchanged after accounting for respondents' health status before sibling death and shared family social status during childhood. Further analyses indicate that psychological distress, health behaviors, and health status cannot explain the relationship between sibling death and dementia incidence. In addition, the association of sibling death with dementia incidence is similar for non-Hispanic whites and non-Hispanic blacks.

### SESSION 4115 (SYMPOSIUM)

### EFFECTS OF SOCIAL SUPPORT ON PERSONS' NEUROLOGICAL WELL-BEING:IMPLICATIONS FROM CROSS-CULTURAL STUDIES

Chair: Nan Jiang, *Columbia University, New York, United States*
Co-Chair: Jing Wang, *Duke University School of Nursing, Durham, North Carolina, United States*

The impact of social determinants on health is well documented. Among them, social support has emerged as one of the most important old ideas being revisited from a new perspective. Social support refers to perceived supportive resources from an individual's social engagement or social network. Recently, the concept of social support has become the subject of intense discussion, as it represents a mechanism by which interventions lead to health improvement. Neuropsychological disorders, on the other hand, represent a large burden on worldwide health. Research has shown that older adults with neurological conditions are embedded in social structures that may affect their outcomes, but not enough attention has been paid to the potential effect of social support on many neurological conditions. Using data collected from studies across cultures that have sought to understand neurological well-being worldwide, this symposium will present evidence of the relationship between social support and neurological conditions among older adults and explore how these mechanisms of social support extend the understanding of health disparities in old-age neurological wellbeing. A discussant will draw out common themes from these papers and connect them with the broader literature on the effect of social support on neurological well-being. We propose that social support constitutes an integral part of medical care for older adults with neurological conditions. This symposium will generate insights to help clinical practitioners more effectively design their social support interventions.

### THE IMPACT OF INTERGENERATIONAL SUPPORT ON DEPRESSIVE SYMPTOMS AMONG OLDER MEXICAN AMERICANS: DOES GENDER MATTER?

Yaolin Pei,[1] Zhen Cong,[2] and Bei Wu[3], *1. New York University, New York, New York, United States, 2. The University of Texas at Arlington, Arlington, Texas, United States, 3. NYU Rory Meyers College of Nursing, New York, New York, United States*

The study examined gender differences in the impact of living alone and intergenerational support on depressive symptoms among Mexican American older adults. The sample included 335 parent-adult child dyads which were nested within 92 Mexican American respondents in a city in West Texas. Each respondent reported their specific relationships with each child. The results from clustered regression showed that men provided and received less intergenerational support than women, but their depressive symptoms were more susceptible to living alone and different types of intergenerational support. Factors such as living alone, and receiving instrumental support were related to higher levels of depressive symptoms among Mexican American older men than among in their female counterparts, whereas emotional closeness with children was associated with lower level of depressive symptoms in men than in women. The findings can be used to develop and target a gender-specific approach for depression interventions among older Mexican Americans.

### RISK AND RESILIENCY IN THE RELATIONSHIP BETWEEN WIDOWHOOD AND DEPRESSIVE SYMPTOMS AMONG OLDER MEXICAN AMERICANS

Zhen Cong,[1] Yaolin Pei,[2] and Bei Wu[2], *1. School of Social Work, University of Texas at Arlington, Arlington, Texas,*

*United States, 2. NYU Rory Meyers College of Nursing, New York, New York, United States*

This study investigated the association between widowhood and depressive symptoms and the extent to which the association is contingent upon immigration status, functional limitations, financial strains, and intergenerational support, among older Mexican Americans. A sample of 344 parent-child dyads reported by 83 older adults in a city of West Taxes completed the measures for socioeconomic status and depressive symptoms. Clustered regression analysis showed that widowhood elevated the risk of depressive symptoms. Living with functional limitations, having more children and residing in the same city with children exacerbated the adverse effects of widowhood on depressive symptoms. Residing in the same city with children increased the detrimental effects of widowhood on the depressive symptoms among men, whereas this pattern did not appear among women. The findings highlight the heterogeneity of depressive symptoms among the widowed Mexican American older adults.

### LONGITUDINAL INTERRELATIONSHIP BETWEEN SENSORY LOSS, SOCIAL SUPPORT, LONELINESS, AND COGNITION

Shaoqing Ge,[1] Wei Pan,[2] Bei Wu,[3] Brenda Plassman,[4] and Eleanor S. McConnell[2], *1. Duke University School of Nursing, North Carolina, United States, 2. Duke University School of Nursing, Durham, North Carolina, United States, 3. NYU Rory Meyers College of Nursing, New York, New York, United States, 4. Duke Department of Neurology, Durham, North Carolina, United States*

This study aims to understand the roles that psychosocial factors play on the longitudinal associations between sensory (including hearing and vision) loss and cognitive decline. Specifically, we hypothesized that (1) loneliness mediates the associations between sensory loss and cognitive decline; and (2) social support moderates the associations between sensory loss and cognitive decline. We used longitudinal parallel process (LPP) modeling with data from the Health and Retirement Study (HRS) and the Aging, Demographics, and Memory study (ADAMS). Age variable centered at its mean age of 82. In the most parsimonious model, loneliness fully mediated the associations between vision loss and the average cognitive status at age 82 ($p < .05$). Social support moderated the associations between vision loss and the average cognitive status at age 82 ($\beta = .14$, $p < .05$). No moderation or mediation effect was found for the psychosocial factors on the associations between hearing loss and cognition.

### COMMUNITY-LEVEL SOCIAL CAPITAL AND COGNITIVE FUNCTION AMONG OLDER ADULTS IN FIVE LOW- AND MIDDLE-INCOME COUNTRIES

Nan Jiang,[1] Nan Lu,[2] and Bei Wu[3], *1. Columbia University, New York, United States, 2. Renmin University of China, Beijing, Beijing, China (People's Republic), 3. NYU Rory Meyers College of Nursing, New York, New York, United States*

This study aims to investigate which community social capital components are associated with a higher likelihood of cognitive functions across economically and culturally distinctive low- and middle-income countries (LMICs). We used cross-sectional survey data from the World Health Organization's Study on global AGEing and adult health (SAGE) 2007-2010. Associations between community-level social capital indicators and global cognitive scores were examined using ordinary least squares regressions and random-effects meta-analyses. The pooled analysis and meta-analyses of within-country effects indicated that trust in neighbors and coworkers were positively associated with cognitive functions for all these countries, whereas the significant effect of perceived neighborhood safety was only found in South Africa and China. Community participation approached a null effect only in South Africa. This finding explains how community social capital may contribute to better cognitive function through community environments, heath systems, and availability of public resources.

### SESSION 4120 (SYMPOSIUM)

### EXPLORING THE MULTIDIMENSIONAL BIOPSYCHOSOCIAL PATHWAYS LINKING SOCIAL STATUS AND HEALTH AMONG OLDER ADULTS

Chair: Heather R. Farmer, *Duke University, Durham, North Carolina, United States*
Co-Chair: Amy Thierry, *Xavier University of Louisiana, New Orleans, Louisiana, United States*
Discussant: Linda A. Wray, *Pennsylvania State University, University Park PA, United States*

An abundant literature has documented the social patterning of health, where those with lower social status experience poorer outcomes relative to those with higher status. This symposium examines how social status (e.g., age, race/ethnicity, gender, and SES) impacts various aspects of midlife and older adults' lives and their psychological and physical health. The research presented in this symposium lend support to utilizing a biopsychosocial framework for understanding mechanisms of health and aging. First, Heather Farmer et al. will explore race and gender differences in elevated C-reactive protein (CRP), a marker of inflammation linked to poor acute and chronic outcomes, using data from the Health and Retirement Study (HRS). Linda Wray and Amy Thierry will use HRS data to test whether race/ethnicity and sex interact to produce unequal outcomes in functional status. Jen Wong et al. will utilize data from the Midlife in the United States (MIDUS) survey to investigate the moderating influences of age, gender, marital status, and social support on caregiving and psychological well-being. Collin Mueller and Heather Farmer will use HRS data to examine how perceptions of unfair treatment are associated with healthcare satisfaction and self-rated health across Black, Latinx, and White subpopulations. Taken together, this work highlights the need for a comprehensive approach to better address physical and mental health disparities over the life course. After attending this session, participants will have a stronger understanding of how social status shapes important outcomes in older adults' lives and some of the mechanisms responsible for these variations.

### RACIAL DIFFERENCES IN ELEVATED C-REACTIVE PROTEIN AMONG U.S. OLDER ADULTS

Heather R. Farmer,[1] Heather R. Farmer,[1] Linda A. Wray,[2] Hanzhang Xu,[1] Ying Xian,[1] Neha Pagidipati,[1] Eric D. Peterson,[1] and Matthew E. Dupre[1], *1. Duke University, Durham, North Carolina, United States, 2. Pennsylvania State University, University Park, Pennsylvania, United States*

C-reactive protein (CRP) is a marker of inflammation linked to numerous acute and chronic conditions. Studies have not considered racial differences in elevated CRP among older adults at the national level. We investigate racial differences in elevated CRP and the socioeconomic, psychosocial, behavioral, and physiological factors that contribute to these differences overall and by gender using a nationally-representative prospective cohort of 14,700 non-Hispanic black and white participants in the Health and Retirement Study followed from 2006 to 2014. Random effects logistic regression models showed that blacks were more likely to have elevated levels of CRP than whites. In men, the racial differences in elevated CRP were attributed to a combination of socioeconomic, psychosocial, and behavioral factors. In women, the racial differences in elevated CRP were primarily attributable to physiological factors. The findings from this work have potentially important implications for clinical practice and interventions targeting vulnerable segments of the population.

### RACE-ETHNICITY, GENDER, AND DISABILITY: A TEST OF STRESS DISABLEMENT PROCESS MODEL AND INTERSECTIONALITY THEORY

Linda A. Wray,[1] Linda A. Wray,[2] and Amy D. Thierry[3], *1. Pennsylvania State University, University Park PA, United States, 2. Pennsylvania State University, University Park, Pennsylvania, United States, 3. Xavier University, New Orleans, Louisiana, United States*

Decades of studies demonstrate that: physical disability differs widely by race/ethnicity, sex, and achieved social status in midlife and older adults; health conditions explain part or all of the differences by race/ethnicity and/or sex; but disparities by race/ethnicity and/or sex often persist beyond controls for health conditions. Using longitudinal HRS data on 16,280 adults and sequential logistic regression models, Wray and Thierry test if those links are additive or interactive and if achieved social status attenuates remaining disparities. Initial models support past studies indicating that impairments in strength, mobility, and ADLs are greater for females and race/ethnic minority adults. Fully adjusted models, including terms for the intersection of race/ethnicity, sex, and health conditions and controlling for other health and social status factors, reveal differential impacts on reported impairments; and that achieved social status only partly attenuates the disparities. More research is warranted to understand underlying mechanisms that produce health disparities.

### LIFE COURSE EXAMINATION OF GLOBAL AND DAILY PSYCHOSOCIAL IMPACTS OF FAMILY CAREGIVERS IN MIDLIFE AND LATE ADULTHOOD

Jen D. Wong,[1] Jen D. Wong,[1] Yetty Shobo,[2] and Barbara T. Hodgdon[1], *1. The Ohio State University, Columbus, Ohio, United States, 2. Virginia Board of Health Professions, Richmond, Virginia, United States*

Family members often serve as informal caregivers for the first line of care. The complexity of family caregiving suggests the need to examine the personal and environmental resources that contribute to caregivers' psychosocial well-being. Informed by the life course perspective, this study investigates the impacts of providing care to a family member on global and daily psychosocial well-being, and the moderating influences of age, gender, marital status, and social support. The sample consists of 1449 (M=55.99, SD=9.31) participants from Midlife in the United States (MIDUS-II: Main and Diary) survey. Regression and multilevel models results indicated greater global negative affect and daily stressors in caregivers as compared to non-caregivers. In line with the positive correlates of caregiving, caregivers reported greater daily positive events. Age, gender, and marital status significantly moderated the associations between caregiving and well-being. Findings showed that services aimed at family caregivers should take into account of personal resources.

### RACIAL-ETHNIC DISPARITIES IN HEALTHCARE SATISFACTION

Collin Mueller,[1] and Heather Farmer[1], *1. Duke University, Durham, North Carolina, United States*

This paper explores how perceptions of unfair treatment shape healthcare satisfaction across race/ethnicity. We investigate the overall impact of life course exposure to healthcare discrimination on current healthcare satisfaction across race/ethnicity among a sample of midlife and older Black, Latinx, and White Americans age 50+ in the Health and Retirement Study. We then test whether everyday healthcare discrimination mediates the impact of major medical discrimination on healthcare satisfaction, controlling for sociodemographic factors, mental and physical health characteristics, functional status, life course stress exposure, and lifetime and everyday discrimination in contexts beyond healthcare settings. Black Americans had poorer healthcare satisfaction than White Americans. Everyday discrimination in healthcare settings mediated a modest amount of the relationship between lifetime healthcare discrimination and healthcare satisfaction, and this association varied in strength across White, Black, and Latinx Americans. Results underscore the need for future work identifying and addressing mechanisms shaping healthcare satisfaction.

### SESSION 4125 (SYMPOSIUM)

### GENDER AND HEALTH IMPLICATIONS OF EXTENDING WORKING LIFE: CROSS-NATIONAL PERSPECTIVES

Chair: Aine Ni Leime, *National University of Ireland, Galway Ireland, Galway, Ireland, Ireland*
Co-Chair: Debra A. Street, *State University of New York at Buffalo, Buffalo, New York, United States*

This symposium addresses the issue of extended working life policy by considering the influence of gender and health on the experiences of older workers. In response to population ageing, policies designed to extend working life have been introduced in many countries. These policies include raising state pension age and linking the amount of state pensions more closely to years spent in paid employment. Such policies tend to be undifferentiated by gender or health status – in most countries, state pension age has been raised to the same age for men and women. Yet, research evidence indicates that women in all countries are disadvantaged in relation to employment at older ages and pensions. There are also health inequalities for older workers, depending on their occupation and whether they are in precarious or secure employment. Extended working life is of pressing societal concern. This symposium brings together the work of a group of leading international scholars who have been researching and reflecting on its implications in a forthcoming book on the topic across 34 countries. The symposium begins with an overview and analysis of the empirical landscape of older employment and pension policy by Martina Rasticova and Jim Ogg; Paper 2 offers a discussion of the theoretical perspectives and policy debates across 34 countries by Clary Krekula; there will be an analysis of extended working life policy in Ireland by Aine Ni Leime and a final presentation synthesising policy recommendations and mapping future research directions in extended working life by Debra Street.

### GENDER, HEALTH, AND EXTENDED WORKING LIFE IN IRELAND

Aine Ni Leime[1], *1. National University of Ireland, Galway Ireland, Galway, Ireland, Ireland*

This presentation is based on a forthcoming book chapter which analyses policies, statistical evidence and qualitative data to investigate the gender and health implications of Extended Working Life policy in Ireland. The qualitative data is from a study conducted in 2018 that investigated attitudes to extended working life and experiences of late life work among sixty older workers, 30 men and 30 women in three different occupations, health care workers/cleaners, teachers and academic faculty.The data were analysed using a lifecourse approach. Workers in physically-demanding occupations, those in precarious employment and women were found to be more likely to be disadvantaged in relation to options for extending their working lives. It is recommended that policies be modified to address the disadvantages faced by these groups of workers.

### PROBLEMS AND PROSPECTS FOR CURRENT POLICIES TO EXTEND WORKING LIVES

Debra A. Street,[1] and Debra A. Street[1], *1. State University of New York at Buffalo, Buffalo, New York, United States*

Current policy debates fluctuate between "extending working life" and "delaying retirement"—assuming policies that reflect different conceptual approaches are identical. This presentation uses a different analytic strategy, conceptualizing later life work policies as representing distinctive approaches to consideration of work conditions/income security for older workers. Using data from over 30 countries, I discuss main trends in extended working life policies (mainly in the EU) and the gender and health implications for current and future workers. We find that policies committed to "extending working life"—supporting adequate/meaningful employment for later life work—are enacted rarely, but with potentially positive effects for the health and wellbeing of older workers of either gender. However, "delaying retirement" policies, which dominate the political landscapes of most of the country-specific policies we consider, reproduce or exacerbate gender inequalities and health risks for vulnerable older workers.

### PUTTING THE CONTEXT BACK INTO THE DEBATE ON EXTENDED WORKING LIFE

Clary Krekula[1], *1. Karlstad University, Karlstad, Sweden*

Policy on extended working life has tended to focus on individuals. The debate has to a great extent described older people as the problem and their current retirement trends as problematic as well as selfish, uninformed, out-dated and a threat to welfare provision and benefits. This depicts the political initiatives as a phenomenon disconnected from social, political and economic trends. This presentation reintroduces the context, by locating the role of policies to extend working lives as forming part of a neoliberal policy agenda. Starting from the understanding that policies are proactive measures which focus on some aspects and play down others, this paper analyses international policies from the EU and OECD and also government policies from 34 European countries. The results draw attention to the narrow and contradictory ways in which the issue is often framed, and how this relegates related new inequalities to the background.

### EXTENDED WORKING LIFE POLICIES: INTERNATIONAL GENDER AND HEALTH PERSPECTIVES, EMPIRICAL AND POLICY LANDSCAPE

Martina Rasticova,[1] and Jim Ogg[2], *1. Mendel University, Brno. Czech Republic, Brno, Czech Republic, 2. French National Pension Fund (Caisse nationale d'assurance vieilliesse), Paris, Ile-de-France, France*

As populations age, extending the working life appears to be widely accepted and promoted by governments (OECD 2006; 2017). Without exception, all countries with modern economies have responded in one way or another to the financial challenges arising from increased life expectancy and ageing populations. Policies to extend working life are ubiquitous, each based on the premise that unsustainable pension systems must be reformed, and public spending reduced. Although there are diverse perspectives on extended working life, gender and health consistently prevail as key dimensions. To date, policies extending working life have not taken sufficiently into account these two dimensions. A clear example in the case of gender concerns the shift towards equality in retirement ages between men and women. In this presentation, we set the stage by presenting the empirical and policy landscapes across 34 countries that characterise the trend of extended working life from gender and health perspectives.

### SESSION 4130 (PAPER)

### HEALTH CARE AND HOSPITALIZATION

### AN INFLAMMATORY SIGNATURE OF POSTOPERATIVE DELIRIUM

Sarinnapha Vasunilashorn,[1] Long H. Ngo,[1] Simon Dillon,[1] Hasan Otu,[2] Bridget Tripp,[2] Sharon Inouye,[3]

Towia A. Libermann,[4] and Edward R. Marcantonio[1], *1. Beth Israel Deaconess Medical Center, Boston, Massachusetts, United States, 2. University of Nebraska-Lincoln, Lincoln, Nebraska, United States, 3. Harvard Medical School Aging Brain Center, Boston, Massachusetts, United States, 4. Beth Israel Deaconess Medical Center, Boston, Massachusetts, United States*

Delirium is a common, morbid, and costly geriatric syndrome, yet its pathophysiology remains poorly understood. In a nested matched case-control study within the Successful Aging after Elective Surgery (SAGES) study, a cohort of adults age ≥70 without dementia undergoing major non-cardiac surgery, we previously identified inflammatory proteins to be associated with delirium. Using the entire SAGES cohort, the current study examines the independent associations of these inflammatory proteins with postoperative delirium. Plasma was collected preoperatively (PREOP) and on postoperative day 2 (POD2). Neuroinflammatory marker chitinase-3-like protein [CHI3l1 or YKL-40]; PREOP and POD2) and systemic inflammatory markers interleukin [IL]-6 (POD2 only) and C-reactive protein (CRP; PREOP and POD2) were measured using enzyme-linked immunosorbent assays. Generalized linear models were used to determine the independent (multivariable) associations between the inflammatory markers, measured in sample-based quartiles (Q). All models adjusted for age, sex, baseline cognition, surgery type, Charlson comorbidity index, and medical complications. Among the 555 patients (mean age 77 years, standard deviation, SD 5.2), 58% were female and 86% underwent orthopedic surgeries. Postoperative delirium occurred in 24%. High YKL-40 PREOP and IL-6 at POD2 (Q4 vs. Q1) were significantly associated with an increased risk of delirium: relative risk (RR) [95% confidence interval (CI)] 2.2[1.1-4.4] and 2.7[1.3-5.7], respectively. CRP (PREOP and POD2) was not significantly associated with delirium ($p=0.37$ and $p=0.73$, respectively). This work underscores the importance of inflammation (YKL-40 and IL-6) in the pathophysiology of postoperative delirium.

### FACTORS ASSOCIATED WITH MORTALITY AMONG LONG-TERM CARE RESIDENTS TRANSITIONING TO AND FROM EMERGENCY DEPARTMENTS

Kaitlyn C. Tate,[1] Colin Reid,[2] Patrick McLane,[3] Garnet E. Cummings,[4] Brian H. Rowe,[4] Greta G. Cummings,[4] and CLEAR Outcomes: OPTIC Research Team[4], *1. University of Alberta, Edmonton, Alberta, Canada, 2. University of British Columbia, Kelowna, British Columbia, Canada, 3. Alberta Health Services, Edmonton, Alberta, Canada, 4. University of Alberta, Edmonton, Alberta, Canada*

Studies examining risk of death during acute care transitions have highlighted potential predictors of death during transition. However, they have not closely examined the relationships and directional effects of organizational context, care processes, resident demographics and health conditions on death during transition. By employing structural equation modeling, we aimed to 1) identify predictive factors for residents who died during transitions from long term care (LTC) to emergency departments (EDs) and back; 2) examine relationships between identified organizational, process and resident factors with resident death during these transitions; and 3) identify areas for further investigation and improvement in practice. We tracked every resident transfer from 38 participating LTC facilities to two included EDs in two Western Canadian provinces from July 2011 to July 2012. Overall, 524 residents were involved in 637 transfers of whom 63 residents (12%) died during the transition. Sustained dyspnea (in both LTC and the ED), sustained change in level of consciousness (LOC) and severity measured by triage score were direct and significant predictors of resident death during transition. The model fit the data, ($x2 = 83.77$, $df = 64$, $p = 0.049$) and explained 15% variance in resident death. Dyspnea and change in LOC in both LTC and ED needs to be recognized regardless of primary reason for transfer. More research is needed to determine the specific influences of LTC ownership models, family involvement in decision-making, LTC staff decision-making on resident death during transition, and interventions to prevent pre-death transfers.

### LONG-TERM CARE FACILITY VARIATION IN THE INCIDENCE OF PNEUMONIA AND INFLUENZA HOSPITALIZATIONS

Elliott Bosco,[1] Andrew Zullo,[1] Kevin McConeghy,[2] Patience Moyo,[1] Robertus van Aalst,[3] Ayman Chit,[3] Vincent Mor,[1] and Stefan Gravenstein[1], *1. Brown University School of Public Health, Providence, Rhode Island, United States, 2. Center of Innovation and Long-Term Services and Support, Providence VA Medical Center, Providence, Rhode Island, United States, 3. Sanofi Pasteur, Swiftwater, Pennsylvania, United States*

Pneumonia and influenza (P&I) increase morbidity and mortality among older adults, especially those residing in long-term care facilities (LTCFs). Facility-level characteristics may affect P&I risk beyond resident-level determinants. However, the relationship between facility characteristics and P&I is poorly understood. We therefore identified potentially modifiable facility-level characteristics that might influence the incidence of P&I across LTCFs. We conducted a retrospective cohort study using 100% of 2013-2015 Medicare claims linked to Minimum Data Set 3.0 and LTCF-level data. Short-stay (<100 days) and long-stay (≥100 days) LTCF residents aged ≥65 were followed for the first occurrence of hospitalization, LTCF discharge, Medicare disenrollment, or death. We calculated LTCF risk-standardized incidence rates (RSIRs) per 100 person-years for P&I hospitalizations by adjusting for over 30 resident-level demographic and clinical covariates using hierarchical logistic regression. The final study cohorts included 1,767,241 short-stay (13,683 LTCFs) and 922,863 long-stay residents (14,495 LTCFs). LTCFs with lower RSIRs had more Physician Extenders (Nurse Practitioners or Physician's Assistants) among short-stay (44.9% vs. 41.6%, $p<0.001$) and long-stay residents (47.4% vs. 37.9%, $p<0.001$), higher Registered Nurse hours/resident/day among short-stay and long-stay residents (Mean (SD): 0.5 (0.7) vs. 0.4 (0.4), $p<0.001$), and fewer residents prescribed antipsychotics among short-stay (21.4% (11.6) vs. 23.6% (13.2), $p<0.001$) and long-stay residents (22.2% (14.3) vs. 25.5% (15.0), $p<0.001$). LTCF characteristics may play an important role in preventing P&I hospitalizations. Hiring more Registered Nurses and Physician Extenders, increasing staffing hours, and reducing antipsychotic use may be modifiable means of reducing P&I in LTCFs. Funding provided by Sanofi Pasteur.

### READMISSION FOR HEALTHCARE-ACQUIRED INFECTIONS: DOES PATIENT DISPOSITION MATTER?

Geoffrey J. Hoffman,[1] Lillian Min,[1] Haiyin J. Liu,[1] and Lona Mody[1], *1. University of Michigan School of Nursing, Ann Arbor, Michigan, United States*

Both common and preventable, healthcare-acquired infections (HAI) are nevertheless associated with high risk for hospital readmission. However, whether these infection-related readmissions are more common among older adults discharged from the hospital to a nursing facility as opposed to home is unknown. We used 2013-14 HCUP data and multivariable logistic regression models to retrospectively examine the relationship of patient disposition (home, nursing facility, home health care) with an unplanned readmission for the same HAI observed at the index admission, among older Medicare beneficiaries, controlling for patient sociodemographics, comorbidity score, and length of stay during index hospitalization. Of 8.4 million index admissions, 323,332 (3.9%) involved an index HAI, of which 15,870 (4.9%) resulted in a linked HAI readmission. HAI readmissions were more common for Clostridium difficile infections (4.0%) and urinary tract infections (UTI, 2.3%) than for ventilator-acquired pneumonia (1.4%) or surgical site infections (1.1%) (p<0.001). Being discharged home or to home health care, compared to a post-acute care setting, was associated with increased odds (OR: 1.63 and 1.62, p<0.001) of HAI readmission, particularly for patients with higher comorbidity scores. For home discharges, HAI readmission risk was doubled for patients with the most compared to fewest comorbidities while nursing facility discharges were equally protective across comorbidity levels. We conclude that Clostridium difficile and UTIs result in higher risk for readmission than other HAIs. Patients discharged to nursing facilities are protected from readmission. Further research into identifying modifiable mechanisms for HAI readmission, in order to improve post-hospital care of infection at home, is needed.

### RESILIENCY PHENOTYPES FOLLOWING HIP FRACTURE IN OLDER ADULTS

Cathleen Colon-Emeric,[1] Heather E. Whitson,[1] Carl Pieper,[1] Richard Sloane,[1] Denise Orwig,[2] Kim Huffman,[1] Daniel C. Parker,[1] and Jay S. Magaziner[3], *1. Duke University Medical Center, Durham, North Carolina, United States, 2. University of Maryland, Baltimore, Maryland, United States, 3. University of Maryland Baltimore School of Medicine, Baltimore, Maryland, United States*

Defining common patterns of recovery after an acute health stressor (resiliency phenotypes) has clinical and research implications. We examined groups of patients with similar recovery patterns across 10 outcomes following hip fracture to determine the most important predictors of resiliency group membership. This study is a secondary analysis of three prospective cohort studies. Participants, community-dwelling adults aged >65 with recent surgical repair of a hip fracture (n=541), were recruited from eight hospitals near Baltimore and followed for up to one year. Self-reported function and activity measures were collected using validated scales at baseline, 2, 6, and 12 months. Physical performance tests were administered at all follow-up visits. Stressor characteristics, co-morbidities, psychosocial and environmental factors were collected at baseline, and latent class profile analysis was used to identify resiliency phenotypes and logistic regression models to identify associated factors. Three resiliency phenotypes had similar patterns across the 10 outcome measures and were defined as “high resilience” (n=163, 30.1%), “medium resilience” (n=242, 44.7%), and “low resilience” (n=136, 25.2%). Recovery trajectories for outcome measures were plotted for each resiliency group. Self-reported pre-fracture function was by far the strongest predictor of resilience group membership (AUC 0.84). Demographic factors, co-morbidities, stressor characteristics, environmental factors, and psychosocial characteristics were less predictive, but several factors remained significant in a fully adjusted model (AUC 0.88). These three resiliency phenotypes have immediate utility for clinical decision-making. They can be measured in future studies with a more parsimonious set of variables, and may prove useful for understanding mediators of physical resilience.

### SESSION 4135 (PAPER)

### ISSUES IN HOME CARE AND CAREGIVING

### ARE MEDICAL FOSTER HOMES SERVING VETERANS WHO HAVE SIMILAR FUNCTIONAL IMPAIRMENT AS TYPICAL NURSING HOME RESIDENTS?

Cari Levy,[1] Kate Magid,[2] Chelsea Manheim,[3] Kali S. Thomas,[4] Leah M. Haverhals,[5] and Debra Saliba[6], *1. VA Rocky Mountain Regional VA Medical Center, Aurora, Colorado, United States, 2. Seattle/Denver Center of Innovation, VA Eastern Colorado Health Care System, Aurora, Colorado, United States, 3. Rocky Mountain Regional Medical Center, Aurora, Colorado, United States, 4. Providence VA Medical Center, Providence, Rhode Island, United States, 5. Seattle-Denver Center of Innovation, Denver, Colorado, United States, 6. RAND Corporation, Santa Monica, California, United States*

The Veterans Health Administration’s (VHA’s) Medical Foster Home (MFH) program was developed as a community-based alternative to institutional care. This study compares the clinical and functional characteristics of Veterans in the VHA MFH program to residents in nursing homes to understand whether MFHs substitute for nursing home care or serve a population with different care needs. All data were derived from Minimum Data Set (MDS) 3.0 assessments. Nurses collected MDS assessments from Veterans (n=92) in 4 MFHs between April 2014-December 2015. Data for nursing home residents were from a national nursing home dataset of residents with an annual MDS assessment in 2014 (n=818,287). We found that MFH Veterans were more likely to be male, have higher functional status, and perform more activities of daily living (ADLs) independently relative to nursing home residents (p<0.01 for all comparisons). Yet, a similar proportion of MFH Veterans and nursing home residents required total assistance in 9 of the 11 measured ADLs. Cognitive impairment, neurological comorbidity, and psychiatric comorbidity were similar in both cohorts; however, MFH Veterans were more likely to have traumatic brain injury (p<0.01), higher Patient Health Questionnaire (PHQ)-9 depression scores (p=0.04) and less likely to have anxiety (p=0.05). Our results suggest there are two distinct MFH populations, one with lower-care needs and another with Veterans completely dependent in performing ADLs. Given these findings, MFHs may be an ideal setting for both low-care nursing home residents with less functional impairment as well as residents with higher care needs who desire community-based long-term care.

### FUNCTIONAL STATUS ASSOCIATED WITH RISK OF READMISSION DURING HOME HEALTH CARE FOR PATIENTS WITH DEMENTIA

Sara Knox,[1] Brian Downer,[2] Allen Haas,[3]
Addie Middleton,[4] and Kenneth Ottenbacher[3], *1. MGH Institute of Health Professions, Boston, Massachusetts, United States, 2. University of Texas Medical Branch, Galveston, TX, Galveston, Texas, United States, 3. University of Texas Medical Branch, Galveston, Texas, United States, 4. Medical College of South Carolina, Charleston, South Carolina, United States*

Approximately 14.0% of Medicare beneficiaries are readmitted to a hospital within 30-days of home health admission. Individuals with dementia account for 30% of all home health care admissions and are at high-risk for rehospitalizations. Our primary objective was to determine the association between functional status and social support at admission to home health and 30-day potentially preventable readmissions (PPR) during home health care. We conducted a retrospective cohort study of 124,119 Medicare beneficiaries receiving home health (7/2013 – 6/2015) and diagnosed with dementia (ICD-9 codes). Approximately 65% of participants were over the age of 81, 61% were female, and 80% were Caucasian. The primary outcome was 30-day PPR during home health. OASIS items were used to create mobility, self-care, social support, and cognition categories. The overall rate of 30-day PPR was 7.6% (95% CI: 7.4-7.7) but varied by patient and health care utilization characteristics. After adjusting for sociodemographic and clinical characteristics, the odds ratios (OR) for the most dependent score quartile versus the most independent was 1.68(1.56,1.80 95% CI) for mobility, 1.78 (95% CI: 1.66-1.91) for self-care, and 1.10(95%CI: 1.03-1.17) for social support. The OR for impaired versus intact cognition was 1.12 (95% CI: 1.05-1.20). Impaired functional and cognitive status as well as limited social support at admission to home health care are associated with increased risk of PPR for individuals with dementia. Future research is needed to determine if strategies targeted at mobility and self-care can decrease PPR during home health for individuals with severe dementia.

### HOME HEALTH CARE AS A PREDICTOR OF SURVIVAL AMONG PEOPLE LIVING WITH ALZHEIMER'S DISEASE AND RELATED DEMENTIAS

Olga F. Jarrín,[1] Abner Nyandege,[2] and Irina B. Grafova[1],
*1. Rutgers, The State University of New Jersey, New Brunswick, New Jersey, United States, 2. Rutgers University, New Brunswick, New Jersey, United States*

Over the past 10 years, dementia care has been shifting to the community; however, there are significant regional and sociodemographic differences in the use of formal home health care services. Does the use of home health care improve survival and other outcomes among people diagnosed with dementia? The aim of the study was to determine the individual, societal, and health systems predictors of survival after a diagnosis of dementia. Using linked Medicare administrative, claims, and assessment data (N = 4,349,565); we found that home health care significantly reduced risk of death for males, but not females. This effect was strongest among older adults between the ages of 65 and 80. While men who were living alone or in a congregate/assisted living environment benefited the most (O.R. = 0.87) the effect was also strong for men living with a spouse or other caregiver (O.R. = 0.90). These findings suggest home health care may provide a survival advantage for men through the provision of rehabilitative and supportive services, as well as patient and family caregiver education. Further research is needed to understand if sex based survival differences are associated with the intensity of home health care services provided or social determinants of health.

### SNF TO HOME: OUTCOMES OF THE SNF RE-ENGINEERED (RED) DISCHARGE PROCESS

Lori L. Popejoy,[1] Yan Wang,[1] David Mehr,[1]
Amy A. Vogelsmeier,[1] Bonnie Wakefield,[1]
Colleen M. Galambos,[2] and Greg Petroski[1], *1. University of Missouri, Columbia, Missouri, United States, 2. University of Wisconsin Milwaukee, Milwaukee, Wisconsin, United States*

Re-engineered (RED) Discharge Process has been effective in hospitals but little tested in SNFs. This study tested the effect of an 18-month adapted RED implementation in four Midwestern SNFs on 30, 60, 180 day hospital, SNF, and emergency department (ED) readmissions. The sample included 1026 SNF residents discharged to the community during 2013 (n = 526) and 2015 (n = 500). We used Medicare claims Data and Minimum Data Set (3.0) File to identify SNF admissions and readmissions to SNF, acute care, or ED. Data were linked across all four facilities by year and compared using appropriate statistics for descriptive, comparison, and regression tests. Residents discharged in 2013 had statistically significantly higher Charlson Comorbidity Scores 4.65 vs 3.97 in 2015 (p < 0.01), while those discharged in 2015 had significantly worse cognitive status, mood, and ADL self-performance. Overall, rates and number of 30, 60-day, 180 rehospitalization, SNF readmission, ED visits with rehospitalization were lower for all 2015 compared to 2013, but not statistically significantly different, except for the number of 180-day rehospitalizations in 2015. Likewise, the only statistically significantly different result was in the regression model was 180 day rehospitalizations. However, in the two facilities that had the best uptake of the intervention, numbers of readmissions were lower at 60 and 180 days (p=.0106 and .0013, respectively for regression results). SNF RED may be a useful tool for SNFs to use to reduce rehospitalizations after discharge; however, benefit depends on the degree of adoption of the intervention.

### THE EFFECTS OF THE NAMASTE CARE FAMILY PROGRAM ON QUALITY OF LIFE OF PEOPLE WITH ADVANCED DEMENTIA

Jenny T. van der Steen,[1] Karlijn J. Joling,[2] Anneke L. Francke,[2] Wilco P. Achterberg,[3] and Hanneke J.A. Smaling[2], *1. Leiden University Medical Center, Leiden, Netherlands, 2. Amsterdam University Medical Centers, Amsterdam, Netherlands, 3. Leiden University Medical Center, Leiden, Netherlands*

People with advanced dementia often die in nursing homes. Family caregivers frequently feel that their loved one's quality of life and dying is suboptimal. The daily Namaste Care Family program -derived from the US Namaste Care program- involves family caregivers and integrates personalized care with meaningful activities for people with advanced dementia. A cluster-randomized controlled trial (December 2016 - December 2018) examined effects of the Namaste Care Family program on resident and family caregiver outcomes. Ten Dutch nursing homes implemented Namaste Care Family for 117 residents, while nine nursing homes provided usual care for 114 residents in the study. Nursing staff assessed quality of life over the last week with the Quality of Life in Late-Stage Dementia (QUALID, the primary resident outcome measure). Research assistants observed discomfort during the sessions with the Discomfort Scale-Dementia of Alzheimer Type (DS-DAT). Assessments were at baseline and after 1, 3, 6, and 12 months. We found a significant difference in QUALID score at 12 months favoring quality of life in the intervention group. Further, the intervention group showed less signs of discomfort at 3, 6, and 12 months compared with the control group. The Namaste Care Family program can improve quality of life of people with advanced dementia in the long run. These study findings support sustained implementation of the daily program in nursing homes. Further analyses of effects on the other outcomes will include blinded DS-DAT assessments, more secondary outcome measures and family caregiver outcomes.

### SESSION 4140 (SYMPOSIUM)

### LATE-LIFE SUICIDE: IDENTIFYING PREVENTION TARGETS AND MOBILIZING COMMUNITIES TO MANAGE RISK

Chair: Alexandria R. Ebert, *West Virginia University, Morgantown, United States*
Co-Chair: Emily S. Bower, *VISN Center of Excellence for Suicide Prevention, Canandaigua, New York, United States*
Discussant: Yeates Conwell, *University of Rochester School of Medicine, Rochester, New York, United States*

Adults aged 70 and older have the highest suicide rates in most countries across the globe (World Health Organization, 2014). Prevalence may increase as the Baby Boomers transition into older adulthood (Phillips, 2014). Disease and disability increase risk for late-life suicide, thus identifying opportunities to intervene within the network of services utilized by older adults with illness and disability could reduce suicide risk. This symposium will explore the impact of disease and disability on late-life suicide risk, and present novel ideas for intervention and prevention. Emphasizing the conference theme, we will discuss prevention points spanning networks of care, including mental health and social services, and long-term care. Dr. Bower will frame the scope of the problem by presenting findings of age-stratified associations between physical illness and suicide attempt among older veterans using secondary data from a retrospective case-control study of veterans. Ms. Lutz will present findings on the relationship between disability and suicidal thoughts from a clinical trial of problem-solving therapy. Dr. Lane will discuss management and identification of suicide risk factors during the transition from independent living to long-term care through the lens of a case-series. Dr. Fullen will present preliminary findings from a novel, randomized controlled trial of a community-based suicide prevention training program for nutrition service volunteers. Dr. Yeates Conwell, Co-Director of the Center for the Study and Prevention of Suicide and Director of the Geriatric Psychiatry Program at the University of Rochester Medical Center, will serve as the discussant.

### PHYSICAL CONDITIONS ASSOCIATED WITH SUICIDE ATTEMPTS: DO RISKS DIFFER AMONG OUR OLDER VETERANS?

Emily S. Bower,[1] Kelsey Simons,[1] Patrick G. Walsh,[1] Lisham Ashrafioun,[1] Kimberly A. Van Orden,[2] and Todd M. Bishop[1], *1. VISN Center of Excellence for Suicide Prevention, Canandaigua, New York, United States, 2. University of Rochester School of Medicine, Rochester, New York, United States*

Physical illness confers risk for late-life suicide, yet few studies report whether risks differ with older age and among Veterans. We examined age-stratified associations between physical illness and suicide attempt among Veterans 65+ years (n=8452, 97% male) from a larger retrospective case-control study that utilized secondary data from the Veterans Affairs Corporate Data Warehouse and Suicide Prevention Applications Network. Controls were matched by age, sex, and mental health treatment utilization. Risk estimates for 15 conditions and a combined comorbidity score were stratified by young-old (65-74), middle-old (75-84), and oldest-old (85+), adjusting for age and sex within strata. Neurodegenerative disorder (ORs=4.5, 6.0, 6.5) or dementia (ORs=5.0, 5.7, 4.4) diagnosis within 180 days conferred the highest risks across young-, middle-, and oldest-old. Cerebrovascular disorder was associated with higher risk among the oldest-old versus young-old (ORs=6.1 vs 2.2). Findings differ from reported risks for suicide death. Illness may be experienced differently across later-life.

### UTILIZING NUTRITION SERVICES PROVIDERS TO PREVENT LATE-LIFE SUICIDE: IMPLEMENTING A RANDOMIZED, CONTROLLED TRIAL

Matthew C. Fullen,[1] Mary Chase Mize,[2] and Laura R. Shannonhouse[2], *1. Virginia Tech, Blacksburg, Virginia, United States, 2. Georgia State University, Atlanta, Georgia, United States*

A challenge in preventing late-life suicide is identifying and responding to persons-at-risk prior to a suicide attempt. Distressed older adults are less likely to turn to a mental health professional, meaning that community-based prevention strategies are vitally important to comprehensive prevention frameworks. Due to their "natural helper" role, nutrition services (NS) volunteers may be well-positioned to identify suicide warning signs and respond accordingly. Unfortunately, there is a lack of systematic, empirically-tested evaluations of the effectiveness of community-based strategies to prevent older adult suicide, including the use of NS volunteers. To remedy this, the authors partnered with several home- and community-based service organizations to measure the impact of training nutrition services volunteers in suicide prevention skills. The authors will present preliminary findings from this federally-funded randomized, controlled trial of suicide prevention training (i.e., ASIST; safeTALK) on late-life suicidality and its correlates.

### SUICIDE RISK ASSESSMENT AND MANAGEMENT IN THE CONTEXT OF TRANSITION TO LONG-TERM CARE

Douglas W. Lane[1], *1. VA Puget Sound Healthcare System, Geriatrics and Extended Care Svc, Tacoma, Washington, United States*

The transition from independent living to living in a supported care facility can create significant emotional stress for the older person undergoing the move. From the perspective of suicide risk management, it is vital to appreciate the potential increase in risk associated with this period of adjustment. In this case-based talk, we will discuss key issues in assessing and managing suicide risk in this context. Factors in risk management to be discussed include the person's social/family context, baseline psychological status, coping assets, and intraindividual values and diversity characteristics. Emphasis will be placed on the need for active assessment, mobilization of resiliency factors, and monitoring during the phase of adjustment. The need to ensure coordination of care across settings will be discussed as well. Finally, we will identify the ways in which the transition can be undermined by ageism on the part of professionals and/or the person's loved ones.

### CHANGES IN FUNCTIONAL DISABILITY AND SUICIDAL IDEATION ASSOCIATED WITH PSYCHOTHERAPY FOR DEPRESSION

Julie Lutz,[1] Sherry Beaudreau,[2] Ruth Morin,[3] David Bickford,[4] J. Craig Nelson,[4] and Scott Mackin[4], *1. West Virginia University, Morgantown, West Virginia, United States, 2. Sierra Pacific Mental Illness Research Education and Clinical Center, VA Palo Alto Health Care System, Palo Alto, California, United States, 3. San Francisco VA Medical Center, San Francisco, California, United States, 4. University of California, San Francisco Department of Psychiatry, San Francisco, California, United States*

Given the association between functional disability and suicidal ideation (SI) in late life (Lutz & Fiske, 2017), this study examined associations between functional disability and SI among older adults receiving problem solving therapy (PST) for depression. PST is a promising intervention for SI (e.g., Gustavson et al., 2016) and has been shown to be effective in reducing functional disability among older adults with depression (e.g., Choi et al., 2014). Regression analyses with adults age 65-91 (n=65) found that level of SI (Geriatric Suicide Ideation Scale) at baseline was not significantly associated with change in SI from pre- to post-treatment. However, lower baseline disability (WHODAS-II; $\Delta R2$=.08, p=.022) and greater reduction in disability ($\Delta R2$=.12, p=.004) were significantly associated with greater reduction in SI after controlling for age and baseline SI. These results suggest that treatments that decrease disability may be particularly advantageous for reducing SI in older depressed adults.

### SESSION 4145 (PAPER)

### LIFE COURSE AND DEVELOPMENTAL CHANGE IN MIDDLE AND LATE LIFE

### CHANGES IN OLDER ADULTS' INFORMAL CARE NETWORKS

I-Fen Lin,[1] and Hsueh-Sheng Wu[1], *1. Bowling Green State University, Bowling Green, Ohio, United States*

Many older adults rely on informal care networks to overcome challenges in life and maintain well-being. The composition and function of the informal care network may change as existing caregivers leave and new caregivers join the network over time. The majority of prior studies on caregiving to older adults are based on cross-sectional data and thus cannot examine changes in older adults' informal care networks. Although some have followed older adults' informal caregivers over time, they usually focus on primary caregivers, rather than the entire informal care network longitudinally. The newly available panel data on a nationally representative sample of caregivers from the National Study of Caregiving (NSOC) provide an excellent opportunity for researchers to understand how older adults' informal care networks change over time and what factors relate to discontinuation of care. Using the NSOC 2015 and 2017, we found that 70% of older adults (N = 1,395) experienced changed in informal care networks within two years. Only a small portion of spouses (6%) discontinued giving care to older adults, whereas 21% adult children, 56% other kin, and 77% nonkin stopped caregiving by 2017. We further examined how older adults' needs for support, caregivers' resources and constraints, and caregiving experiences were associated with discontinuation of care. This study is expected to advance gerontological research by broadening our understanding of informal caregiving in late life and providing practical implications on how to sustain informal care.

### CUMULATIVE DIS/ADVANTAGE AND HEALTH TRAJECTORY IN LATE LIFE: A COMPARISON BETWEEN FOUR COUNTRIES

Peiyi Lu,[1] and Mack Shelley[1], *1. Iowa State University, Ames, Iowa, United States*

Cumulative Dis/Advantage (CDA) theory concerns how societal structure influences individual developmental and health trajectory across the life span, but few studies have applied CDA in the international setting with gender comparisons. This study provides a cross-national perspective to test CDA in explaining health inequality between developing and developed countries. Cross-sectional harmonized data from the international Health Retirement Study-series (United States, China, Mexico, and England) in 2013-2014 were used (n=97,978). Four health indicators were included: self-reported health, depressive symptoms, functional ability, and memory. Sociodemographic information and health behaviors were used as covariates. Regression models were fitted to examine the moderation roles of country and gender in health trajectory in later life. Results indicate self-reported health and mental health remained steady while functional ability and memory declined across late life span. Older Chinese and Mexican respondents had poorer health status than their British and American counterparts consistently except for memory in the Mexican data. Cumulative health gaps between developing and developed countries existed only for functional ability. Females in the four countries had poorer health than their male counterparts except their memory status. We conclude that CDA explains only the temporal decline of functional ability and its increasing gaps in later life between countries. Health inequality between countries could be attributed to the limited availability of healthcare and social resources in developing countries. However, other health status, including general health and mental health, depend more on individuals' intrinsic capacity and human agency.

### HARDWIRED BIOLOGY AND LIGHT-BULB MOMENTS: DIVERGENT DISCOURSES AND LIFE TRAJECTORIES OF OLDER BISEXUAL WOMEN

Sarah Jen[1], *1. University of Kansas School of Social Welfare, Lawrence, Kansas, United States*

Older bisexual women report a less positive sense of their sexual identity, less belonging in LGBTQ communities, and worse mental health outcomes compared to lesbian counterparts. These patterns are consistent with those identified among younger bisexual cohorts and appear to be connected to how bisexual identities are perceived and experienced; however, sexual identities take on unique meaning by gender and age and across historical contexts. To explore how older bisexual women construct and make meaning out of bisexual identities, this study applied a Foucauldian discursive and critical feminist conceptual framing to examine semi-structured interviews with bisexual women ages 60 and older (N=13). Findings reveal two divergent groups of women, the Early Emergers and Mature Migrators, who differ in their constructions of bisexuality and the timing of their first experienced attractions to other women. While the Early Emergers construct bisexuality as a stable, "hardwired" biological concept, the Mature Migrators challenge this narrative by emphasizing the fluidity of sexuality through discourses of migration spurred by "light bulb" moments in which they first recognized their attractions to women. This study illustrates the contributions of discourse analysis in revealing nuanced constructions of life course histories as well as the need for acknowledgment of life context in research and practice with older bisexual individuals. Scholars and practitioners must intentionally critique and contribute to discourses of bisexuality in later life.

### PERCEIVED DISCRIMINATION, ANGER CONTROL, AND INFLAMMATION IN MIDLIFE: THE ROLE OF EARLY-LIFE ADVERSITY

Chioun Lee,[1] Jennifer Coons,[2] and Lexi Harari[3], *1. University of California-Riverside, Riverside, California, United States, 2. California State University, Fullerton, California, United States, 3. University of California-Riverside, Riverside, California, United States*

Early life adversity has severe consequences for adult biological health particularly in minority group individuals. Two ways in which it may be possible to reduce these negative consequences on adult health are individual differences in perceived discrimination due to early life adversities and learning internal skills (i.e. anger control) to help cope with early life adversities and perceived discrimination. The current study utilized 2,118 participants (55% female) from the MIDUS Projects. Early life adversities included three constructs: low socioeconomic status, family instability, and abuse (sexual, physical, and emotional). The best-fitting model from the latent class analyses revealed four distinct groups: 1) no early life adversities group, 2) low socioeconomic status only group, 3) low socioeconomic status and family instability group, and 4) all three early life adversities group. Minority groups were more likely to reside in the all three early life adversities group. Perceived discrimination was measured via two pathways: lifetime discrimination and daily discrimination. Anger control (one measure of an internal skill one learns to cope with early life adversities and perceived discrimination) was assessed with an anger-control scale. Inflammation markers were used as an indicator of biological health. Experiencing more early life adversities was related to greater inflammation and this relationship was partially explained via the pathway of greater early life adversities, greater perceived lifetime/daily discrimination, worse anger control, and greater inflammation. The findings support the need for a more holistic measure of early life adversities as it has a clear impact on adult inflammation.

### THE RELATIONSHIP OF THE TRAJECTORY OF COMORBIDITY ON FUNCTIONAL STATUS IN A COMMUNITY SAMPLE OF ELDERS

Carl Pieper,[1] Barrett Bowling,[2] and Gerda Fillenbaum[3], *1. Duke University Medical Center, Durham, North Carolina, United States, 2. Duke University Medical Center, Department of Medicine, Durham, North Carolina, United States, 3. Duke University Medical Center, Center on Aging, Durham, NC, United Arab Emirates*

The Duke Established Populations for the Epidemiologic Study of the Elderly (EPESE) study is a 1:7 random sample elderly over 65YO at baseline living in the Piedmont NC, followed yearly for 10 years beginning in1986. There were 4162 participants at baseline of which slightly over 1100 were still alive and responding at year 10. The participants self-reported on 5 comorbidities (cancer, high BP, diabetes, stroke and heart attack) at each year, as well as 4 functional scales - Katz, Rosow-Breslau, Nagi, and IADL scales. Using Mixed Models, the slope (trajectory) and intercept (level) of comorbidity over 10 years was estimated for each person, and correlated with the function scales in the final year. Correlations of the final comorbidity trajectory with the 4 functions levels ranged between 0.12 and 0.18, the trajectory with function ranged between 0.15 and 0.27. All p-values<0.0001. The first Canonical Correlate of function with comorbidity was 0.28 (p<0.0001). Slope and intercept were related jointly to each outcome and multivariately (p<0.001 for the joint effects, controlling for race, gender, age, and years of education. The multimorbidity relationship with function differed by race (omnibus p-value for the raceXmultimorbidity interaction p=0.03 (by Wilk's Lambda), controlling for the demographics). This method is an example of combining longitudinal and cross-sectional outcomes and shows that the change in number of comorbidities is associated with functional status level.

### SESSION 4150 (PAPER)

### LONELINESS AND ISOLATION IN LATER LIFE

### DEVELOPING EVIDENCE-BASED INSTRUMENTS TO TALK ABOUT LONELINESS

Eric Schoenmakers[1], *1. Fontys University of Applied Sciences, Eindhoven, Netherlands*

People, professionals and non-professionals, lonely and non-lonely, experience difficulties in talking about loneliness. Yet, talking about loneliness is important. Without conversation about loneliness, it is impossible to identify lonely persons and to assist them in a tailor-made way. The aim of this research project is to create evidence-based instruments to make talking about loneliness easier. From 2016 to 2019 (ongoing) six researchers held about 60-70 interviews with lonely persons, discussing feelings of loneliness and coping behavior. After the first 23 interviews with lonely older adults, a qualitative analysis on how to discuss loneliness was performed, resulting in a format for talking about loneliness and a topic list to help in conversations. These instruments were tested in the remaining interviews with lonely older adults and students, after which the format and topic list were updated. The format for talking about loneliness discusses guidelines for the inter human relationship, how to bring up the topic of loneliness, and what to discuss, e.g. feelings, timing, coping, consequences and taboo. 'What to discuss' is also addressed in the topic list. It is important to not be eager to help. Often, lonely people have felt so for a long time and considered many coping options. This illustrates complexity. Trying to 'solve' loneliness with oversimplified suggestions makes people feel that their situation is not taken seriously. These instruments emphasize the importance of true listening. The instruments can be used to train professionals and volunteers who want to discuss loneliness with lonely people.

### DRIVING CESSATION AND SOCIAL ISOLATION IN OLDER ADULTS

Weidi Qin,[1] Xiaoling Xiang,[2] and Harry O. Taylor[3], *1. Case Western Reserve University, Cleveland, Ohio, United States, 2. University of Michigan, Ann Arbor, Michigan, United States, 3. Washington University in St. Louis, St. Louis, Missouri, United States*

This study aimed to investigate the impact of driving cessation on social isolation in older adults. Data came from the National Health and Aging Trends Study round 1 through round 6 surveys. Study sample consisted 6,916 Medicare beneficiaries aged 65+ who were eligible drivers at baseline. Social isolation measure was based on social domains concerning marriage, family and friends, church participation, and club participation. The impact of driving cessation on social isolation was assessed using mixed-effects ordered logistic regression and piece-wise regression. In multivariable mixed-effects ordered logistic regression, past-year non-drivers had a two-fold increase in the odds of being in a higher social isolation category (OR=2.1, p<.001). The odds of social isolation were higher among persons aged 85-89 (OR=1.40, p<.001) or 90+ (OR=1.99, p<.001) as compared with those aged 65-69, among men as compared with women (OR=2.54, p<.001), among persons living alone (OR=3.95, p<.001), with elevated depressive symptoms (OR=1.54, p<.001), and with possible (OR=1.39, p<.001) or probable dementia (OR=1.51, p<.001). The odds of social isolation decreased significantly as education and family income levels increased. Functional limitation was associated with higher odds of social isolation. Piecewise regression analysis showed social isolation score increased by 0.08 points (p=.024), indicating short-term impacts of incident driving cessation. Driving cessation is associated with higher risk of social isolation in older adults. Interventions to provide alternative transportation resources and reduce social isolation among older adults may improve public health impact by targeting older adults who recently stopped driving.

### PRODUCTIVE ACTIVITIES AND LONELINESS AMONG JAPANESE MIDLIFE AND OLDER ADULTS

Yang Wang,[1] Yihan Wang,[2] Florian Kohlbacher,[3] and Ernest Gonzales[4], *1. University of Illinois at Urbana-Champaign, Urbana, Illinois, United States, 2. Boston College, Boston, Massachusetts, United States, 3. The Economist Intelligence Unit, Economist Corporate Network, North Asia, The Economist Group, Tokyo, Japan, 4. New York University, Silver School of Social Work, New York, New York, United States*

Background: Loneliness among older population is a public health concern shared worldwide. Using the motivational theory for life-span development, this study examined the associations between loneliness (social and emotional) and productive activities among midlife and older adults in Japan. Methods: The Japanese National Data on Lifestyle and Mental Health, a nationally representative sample of midlife and older adults (2011, N=1,575), were used to examine how employment, volunteering, helping family and friends, and informal caregiving was associated with social and emotional loneliness, controlling for multiple risk and protective factors. Results: Family caregiving was related to more social loneliness. Working, helping family, and volunteering were related to less emotional loneliness, while family caregiving was related to more emotional loneliness. Japanese male caregivers reported more social isolation compared to female caregivers. Face-to-face interactions reduced emotional loneliness among caregivers. Discussion: Findings underscored the nuanced difference of social loneliness and emotional loneliness. Social policies that advance productive aging should recognize "unintended consequences" and aim to protect older adults from social and emotional loneliness. Counselling services and social support programs specifically for Japanese male caregivers are warranted.

### SOCIAL ISOLATION MEASUREMENT AND OLDER ADULT HEALTH: A REVIEW

Janet S. Pohl[1], *1. Betty Irene Moore School of Nursing at University of California Davis, Sacramento, California, United States*

The objectives were to examine social isolation research literature, investigate reports of associations with health, and explore the numerous approaches to operationalize social isolation in gerontological research. While associated with negative health outcomes and mortality, the interpretation of social isolation research is hampered by a lack of conceptual clarity and the use of numerous ad hoc measures of the concept. A systematic search was conducted for published empiric studies regarding social isolation health outcomes in older adult samples. The electronic databases: Medline, CINAHL, and PsycINFO were utilized. Reports including social isolation as an independent variable and health outcomes at the individual level were extracted. Of 2,614 studies initially identified, 14 met study criteria. Study outcomes recognized smoking cessation, sleep disruption, inadequate diet, risk for malnutrition, health-related quality of life, subjective well-being, cognitive function, psychological distress, depression, functional decline, stroke, myocardial infarction, and mortality as related to social isolation. Measurement strategies revealed numerous definitions of social isolation reporting to evaluate objective and subjective social isolation, loneliness, engagement, social disconnectedness, and perceived isolation. Measures utilized: eight ad hoc, three versions of the Lubben Social Network Scale, two versions of the Social Network Index, and one question from the Rand Social Battery. Continuing to develop knowledge regarding the predictive power of social isolation on health is important for the care of older adults. Distinguishing social isolation from related but distinctly different social concepts will facilitate the forward movement of the science. Reliably measuring social isolation will enable the comparison of results across studies.

### THE INFLUENCE OF LONELINESS AND RURAL RESIDENCE ON DEPRESSION IN LATER LIFE

Na Sun,[1] Cassandra Hua,[1] Xiao Qiu,[2] and J. Scott Brown[1], *1. Miami University, Oxford, Ohio, United States, 2. Miami University, Oxford, United States*

Loneliness is associated with depression among older adults. Limited research has examined the role of rurality in relationship to loneliness and depression; the extant research has mixed findings. The socioemotional selectivity theory states that as people age the quality of relationships become more important than the quantity (English & Carstensen, 2016). Individuals in rural areas may have a low quantity of relationships but deeper social ties within the community; thus, they may be less likely to become depressed over time. The association between loneliness and depression may be amplified for people in non-rural areas because they are surrounded by other people but lack close relationships that are most important during the aging process. This study examines the effect of living in rural areas on loneliness on predicting baseline depression and loneliness, as well as changes in these outcomes over time. Data are from the 2006-2014 waves of Health Retirement Study. Regression models examine the relationship between depression loneliness and rural residence controlling for health conditions and demographic characteristics. Latent curve models examine the disparity in trajectories of loneliness and depressive symptoms by urban and rural residence. Older adults who feel lonely ($p<.001$) and in urban areas ($p<.0.05$) are more likely to be depressed. Furthermore, the effect of loneliness on depression is weakened by rural residence ($p<.05$). It is salient to understand the protective effect of rural residency on depression among older adults in the U.S. We discuss implications for policy.

### SESSION 4155 (PAPER)

### POLICIES TO INCREASE ACCESS AND ADDRESS DEPENDENCY

### HETEROGENEITY IN MULTIDIMENSIONAL DEPENDENCY IN OLDER ADULTS IN MEXICO: A LATENT CLASS ANALYSIS APPROACH

Kely Rely,[1] Delfino Vargas-Chanes,[1] Carmen García-Peña,[2] Luis Miguel Gutiérrez-Robledo,[2] Rebeca Wong,[3] and Ricardo Ramírez-Aldana[2], *1. National Autonomous University of Mexico, Mexico City, Mexico, 2. Instituto Nacional de Geriatria, Mexico City, Mexico, 3. University of Texas Medical Branch, Galveston, Texas, United States*

Background: The number of older adults expected to increase over the coming decades, the public health impact in this population may be substantial, and a greater understanding of the structure underlying risk factor presentation as a potential source of heterogeneity is critical. Objective: Identify and characterize profiles of dependency status in a population of dependent elderly individuals. Methods: The present study is based on the first wave of the Mexican Health Aging Study (MHAS). We included subjects aged 50 or older (n = 13,463 respondents interviewed in 2001). We performed Latent Class Analysis on four domains in older adults' indicators (physical, psychological, economic and social) to identify distinct classes of dependency profiles. We used LCA to group individuals into homogenous categories of dependency based on observed domains of multidimensional dependency. Multivariable logistic regression was conducted to examine the sociodemographic characteristics associated with each profile. Results: A 4-class solution based on cognitive performance at baseline was the best-fitting model. We characterized the four distinct classes of dependency profiles: active older adult, low, moderate and severe dependency that encompassed multiple dimensions of dependence. Using the "active older adult" class as the reference group, severe dependency, low dependency, and moderate dependency class were more likely to contain females, low education level and poor quality of life 3) the moderate dependency class was less likely to contain cigarette smoking and alcohol user. Conclusions: This study suggests that dependency do not follow a uniform adjustment pattern during the aging process, which reconciles inconsistent previous findings.

### INNOVATIVE POLICIES AND TECHNOLOGIES TO INCREASE ACCESS TO HEARING AIDS FOR ADULTS

Michael Yong,[1] Amber Willink,[1] Catherine McMahon,[2] Bradley McPherson,[3] Carrie L. Nieman,[4] Nicholas Reed,[1] and Frank Lin[1], *1. Johns Hopkins University, Baltimore, Maryland, United States, 2. Macquarie University, Sydney, Australia, 3. The University of Hong Kong, Hong Kong, China, 4. Johns Hopkins University School of Medicine, Department of Otolaryngology-HNS, Baltimore, Maryland, United States*

As the proportion of older adults in the world's total population continues to grow, the deleterious downstream health economic outcomes of age-related hearing loss are steadily becoming more prevalent. While recent research has shown that age-related hearing loss is the single greatest modifiable risk factor for dementia, the rate of hearing aid use remains low in many countries across the globe. Reasons for poor uptake are multifactorial and likely involve a combination of factors, ranging from increasing costs of hearing aid technology to lack of widespread insurance coverage. This paper aims to first identify the current state of hearing aid access across the world using eight representative countries as examples. We then provide recommendations on how to facilitate greater access to hearing aids for consumers by addressing areas in regulation, technology, reimbursement, and workforce.

### STRENGTH IN AGE-FRIENDLY HEALTH SYSTEMS: AN INNOVATIVE INTEGRATED INTERPROFESSIONAL MODEL

Teri Kennedy[1], *1. The University of Kansas Medical Center, Kansas City, Kansas, United States*

This paper presents an innovative conceptual approach to health care policy for older adults: the Age-Friendly Health Systems Integrated Interprofessional Model. In 2017, the John A. Hartford Foundation and Institute for Healthcare Improvement, in partnership with the American Hospital Association and Catholic Health Association of the United States, advanced the concept of an Age-Friendly Health System. This initiative is designed to respond to the needs of a burgeoning U.S. older adult population, expected to double from 2012 to 2050, largely due to the aging of Baby Boomers and increased life expectancy. These Baby Boomers will demand a well-coordinated, communicative health system responsive to their values and preferences. In an Age-Friendly Health System, all older adults receive the best possible care, without care-related harms, and with satisfaction of care received. Essential elements include what matters, mentation, mobility, and medications, with a focus on patient-directed, family-engaged care. While a solid framework for improving healthcare for older adults, this model is further strengthened by incorporating the essential elements of person-, family-, and community-centered approaches to care; interprofessional team-based competencies, and Quadruple Aim outcomes. This enhanced model, referred to as the Age-Friendly Health System Integrated Interprofessional Model, combines elements essential to quality healthcare within the framework of an Age-Friendly Health System. This paper will present the original Age-Friendly Health System framework, the proposed Age-Friendly Health System Integrated Interprofessional Model, then compare and contrast each model's essential principles. Implications for adoption of this enhanced model for policy, education, and practice will be explored.

### THE IMPACT OF MANDATED COGNITIVE ASSESSMENTS ON ALZHEIMER'S DISEASE AND RELATED DEMENTIA DIAGNOSIS RATES

Johanna Thunell,[1] and Julie Zissimopoulos[1], *1. University of Southern California, Los Angeles, California, United States*

Although no cure for Alzheimer's disease exists, early diagnosis allows clinicians to detect reversible causes of memory loss, inform pharmacologic treatment options that may delay cognitive decline, and inform patients about clinical trial opportunities. It allows patients to communicate medical, legal, financial, living, and end-of-life desires. Barriers to diagnosis include low public awareness of early symptoms, stigma and misconceptions about the disease that delay seeking medical assistance. Provider-related barriers include low recognition of cognitive impairment and/or insufficient training in dementia diagnosis, and reimbursement issues. The new annual wellness visit (AWV) benefit available to Medicare Part B beneficiaries may reduce some of these barriers. The Patient Protection and Affordable Care Act of 2010 mandated an AWV that, along with routine preventive services, included for the first time, a cognitive screen at each visit. In this study, we analyze the effect of the introduction of the AWV benefit on Alzheimer's disease and related dementias (ADRD) diagnoses rates, average age at diagnosis and geographic dispersion of diagnoses. We use the 100% sample of Medicare claims and regression discontinuity to estimate the impact of the legislation enactment. Preliminary analyses show immediate and increasing take-up in AWV visits after the policy went into effect. The total number of preventative exams claims went from about 2,500 to 27,000 immediately after the policy and continued to grow in subsequent months. We also observe an increase in ADRD incidence in the months immediately following ACA, from about 14,000 claims in December 2010 to about 16,000 in January 2011.

### SESSION 4160 (PAPER)

### SUCCESSFUL AGING

### A SELF-RELIANT UMBRELLA: DEFINING SUCCESSFUL AGING AMONG THE OLD-OLD (80+) IN SHANGHAI

Lin Chen,[1] Minzhi Ye,[2] and Eva Kahana[3], *1. Fudan University, Shanghai, China, 2. Benjamin Rose Institute on Aging, Cleveland, Ohio, United States, 3. Case Western Reserve University, Cleveland, Ohio, United States*

The Chinese old-old (80+) population has steadily increased in recent years; however, limited studies have examined how they age. The purpose of this study is to explore how the old-old in urban China define successful aging. Guided by grounded theory, community-dwelling old-old individuals participated in semi-structured, in-depth interviews (N= 97). Participants identified self-reliance as the goal of successful aging, which was supported by four proactive behaviors, including physical activity, financial security, community connectedness, and willing acceptance of reality. These four proactive behaviors were conceptualized to constitute the ribs of an umbrella model offering a canopy to protect the pole of self-reliant successful aging. This study offers new insight in understanding dynamic and nuanced ways that the old-old in urban China age successfully and their valiant efforts to maintain an ideal status.

### COMMUNITY CAPACITY AND SOCIAL PARTICIPATION AMONG COMMUNITY-DWELLING MIDDLE-AGED AND OLDER CHINESE

Min Li,[1] and Fang-Yi Huang[2], *1. University of Florida, Gainesville, Florida, United States, 2. Trinity Washington University, College Park, Maryland, United States*

Objectives. This study investigates how social participation of the aging population is associated with the community capacity, measured by the number of amenities and organizations within the community. Method. Using nationally representative survey data from the China Health and Retirement Longitudinal Study (2011), this study examines the availability of community amenities and organizations in rural and urban areas, and investigates the associations between community capacity and social participation among the middle aged and older Chinese using multilevel analysis. Results. The results of this study indicate that both community amenities and community organizations are positively associated with the social participation of the middle-aged and older Chinese. Additionally, the association between community organizations and the frequency of formal social participation is stronger among urban communities than rural ones, even after controlling for the individual-level socioeconomic status and health conditions. Conclusion. This study highlights the importance of building the community capacity by developing community-based grassroots organizations to promote the social engagement and participation of the aging population.

### EFFECTS OF PRODUCTIVE ENGAGEMENT ON SUBJECTIVE HEALTH AMONG OLDER ADULTS WITH SENSORY IMPAIRMENTS

Othelia E. Lee,[1] and Junghyun Park[2], *1. University of North Carolina Charlotte, Charlotte, United States, 2. New York University, NY, New York, United States*

Background: Productive engagement becomes significant protective factors in healthy aging. Yet, subgroups of older adults with age-related vision and hearing impairments lack access to various activities , suggesting that unequal ability to participate in productive aging is a major public health and health-disparities concern. Methods: Older adults experiencing age-related vision and hearing impairments were drawn from the 2015-2017 National Survey on Drug Use and Health (n=2,164). Perceived health status (good vs. poor) was outcome measures used in multivariate logistic regression. Two aspects of productive engagement was considered: 1) employment status (unemployed vs employed) and 2) regular religious service attendance as tools to build social capital in their faith-based communities. Gender, race, marital status, educational attainment, poverty, urbanization, obesity, chronic disease, hospitalization, binge drinking, cigarette smoking, and difficulty with mobility were considered as covariates. Results: Working older adults with sensory loss were more likely to perceived good health status, compared to their unemployed counterparts (OR=2.46, $p<.05$). Religious service attendance also became protective factors for health (OR=1.60, $p<.01$). Of the covariates, higher educational attainment, White race, having one chronic disease, hospitalization, smoking, drinking, and mobility challenges appeared to affect the health status. Conclusions/Implications: Study findings implied the needs to identify late-life engagement through work and participation in faith-based community as a major public health issue. Given the barriers and disincentives to the productive engagement of older adults in this culture, healthcare providers should provide programs promoting employment and religious attendance.

### PROMOTION OF CULTURAL GENERATIVITY TO SUPPORT SUCCESSFUL AGING IN ALASKA NATIVE COMMUNITIES

Steffi M. Kim,[1] and Jordan P. Lewis[1], *1. University of Alaska Anchorage, Anchorage, Alaska, United States*

The psychological construct of generativity encompasses a person's motivation to leave a legacy for future generations by investing in acts that will outlive the self (Ericson, 1950). Lewis and Allen (2017) outlined the importance of generativity within AN cultures (caring for the future of our youth) and expanded Erikson's western-based definition by adding the indigenous cultural generativity component to describe and incorporate Alaska Native specific cultural elements. These culturally based elements involve giving back to family and community including guidance and teaching of future generations (Lewis & Allen, 2017). Generativity within western societies accentuates independent achievements and successes more than the notion to care for future generations. This study is part of a larger community-based, exploratory, study between researchers and communities to explore successful aging that included 42 Alaska Native Elders in the Norton-Sound subregion, 21 Alaska Native Elders from the Aleutian Pribilof Islands, and 26 Elders from the Bristol Bay region. Qualitative interviews explored the participant's life, influences on aging well, and their aging process. Thematic analysis was used to investigate the impact of generativity on successful aging was used to establish codes and main themes based on the three different cultural regions of Alaska. The findings suggest that generativity promotes successful aging. Elders who live in communities that promote community engagement, support family and school activities, and maintain and/or revitalize culture and traditions reported increased emotional well-being. Results can guide communities to incorporate or support cultural activities that promote generative activities and meaningful engagement which fosters successful aging.

### SUCCESSFUL AGING IN ALASKA NATIVE ELDERS ACROSS DIVERSE REGIONS

Steffi M. Kim,[1] Eric Wortman,[2] and Keri Boyd[1], *1. University of Alaska Anchorage, Anchorage, Alaska, United States, 2. UW School of Medicine, Anchorage, Alaska, United States*

Existing conceptualizations of successful aging are mainly based on Western cultures, ignoring the inclusion or exploration of culturally-relevant knowledge within the experience of successful aging. Lewis (2011) drew on the experiences of Elders and identified four elements of Eldership (successfully aging elders) in the Bristol Bay region of Alaska: "a) emotional well-being, b) community engagement, c) spirituality, and d) physical health" (p. 544). Given the unique and distinct environmental locations of this study, this presentation builds upon Lewis previous research and will highlight similarities and differences of Alaska Native successful aging between three rural geographic areas of Alaska. 42 Alaska Native Elders were interviewed from the Norton-Sound subregion, 21 Alaska Native Elders from the Aleutian Pribilof Islands, and 26 Elders from the Bristol Bay region. A community-based, exploratory, qualitative research methodology was used to allow for the collaboration of researchers and communities as equal partners. Qualitative interviews explored the participant's life, influences on aging well, and their aging process. Thematic analysis was used to establish codes and main themes based on the three different cultural regions of Alaska. Results argue for the expansion and emphasize on social components, historical perspectives, and the importance of place (cultural and geographic differences), as well as generativity and gerotranscendence. Findings will be used to develop community-specific health promotion and prevention programs to help Elders find meaningful activities that promote health and teach individuals to cope with aging-related changes.

### SESSION 4165 (SYMPOSIUM)

### THE CLINICAL TRANSLATION OF SENSOR TECHNOLOGY TO IMPROVE PROVIDER AND PATIENT CARE: METHODS AND ACCEPTABILITY

Chair: Megan Huisingh-Scheetz, *University of Chicago, Chicago, Illinois, United States*
Co-Chair: Qianli Xue, *Department of Medicine, School of Medicine, Johns Hopkins University, Baltimore, Maryland, United States*
Discussant: Jennifer A. Schrack, *Johns Hopkins University, Baltimore, Maryland, United States*

Sensor-based technologies are rapidly emerging and are capable of collecting objective, dynamic, and high resolution health data not captured in the clinical setting. However, the precise clinical applications of such devices are not yet well delineated; extensive challenges to their implementation remain. The objectives of this symposium are to highlight a) opportunities for sensor technology use in clinical practice, b) implementation challenges reported by key stakeholders, and c) an NIH/VA-sponsored initiative to create an open technology research platform to improve aging technology research. Dr. Young will discuss the novel application of wearable sensors for maintaining proper posture/position during patient transfer including the generation of sensor metrics defining proper lifting technique and body mechanics. Dr. Huisingh-Scheetz will report analytic strategies for identifying frailty using wrist-worn accelerometry data collected in the free-living environment in the NIA-supported National Social Life, Health and Aging Project dataset. She will report her work relating hourly activity and between/within subject hourly activity variance to frailty. Ms. Blinka will report qualitative feedback collected from patients, caregivers, and healthcare providers about their perspectives on the utility and challenges of using sensor technology in a clinical context. Dr. Kaye will discuss ongoing developments addressing challenges to implementing technology use in clinical care, with particular attention to the Collaborative Aging Research using Technology (CART) initiative supported by the NIH and VA. Collectively, these presentations will advance sensor technology to improve healthcare delivery.

### A FEASIBILITY STUDY: THE DEVELOPMENT OF WEARABLE TECHNOLOGY FOR INJURY PREVENTION AMONG DIRECT CARE WORKERS

Yuchi Young,[1] Yuchi Young,[1] Mitch Leventhal,[1] Jonathan Muckell,[2] Peter E. Raymond,[3] Fred Erlich,[4] and Christopher Paynter[5], *1. SUNY at Albany, Albany, New York, United States, 2. SUNY at Albany, Engineering & Computer engineering, Albany, New York, United States, 3. The New Bureau, New York, New York, United States, 4. Living Resources, Inc., Albany, New York, United States, 5. SUNY at Albany, Albany, New York, United States*

Objectives: 1) create metrics for lifting techniques and transferring mechanisms, 2) calibrate sensors for data collection 3) identify potential injurious posture among home health aides (HHAs) while transferring patients. Participants: 7 HHAs and a physical therapist. Interview and sensor data were collected. Outcome variables included improper lifting techniques and improper body mechanisms. Obesity of HHAs was associated with worse scores of body mechanics ($p < 0.0001$), while fear of injury with better body mechanics ($p < 0.0001$). GEE results identified that twisting the spine during transfers (OR = 6.3; 95% CI: 1.09–36.7) and not using a wide support base when lifting from supine to sitting (OR= 6.0, 95% CI: 2.03–17.7) were associated with improper lifting technique and body mechanics. Results show it is viable to use sensor technology to collect HHAs' data to design intervention for injury prevention. A larger-scale study is needed to validate the results.

### NEW ACCELEROMETRY PATTERNS IN FRAILTY: HOURLY ACTIVITY AND VARIANCE.

Megan Huisingh-Scheetz,[1] Kristen Wroblewski,[1] Linda Waite,[1] Elbert Huang,[1] Donald Hedeker,[1] and L. P. Schumm[1], *1. University of Chicago, Chicago, Illinois, United States*

Wearable sensors may improve our ability to identify frailty in the community. Frailty has been historically defined, in part, by reduced average activity; however, new analytic methods of aggregate, free-living accelerometry data suggest that frailty may be more fully characterized above and beyond reduced average activity. Using mixed-effect regression models of awake hourly activity from the National Social Life, Health and Aging Project dataset, we have shown that frail adult activity is most reduced in the morning relative to pre- and non-frail adults rather than the afternoon or evening. High residual between- and within-subject activity variance in this model prompted further study of activity variance. A follow-up analysis using a mixed-effect location-scale model of hourly activity data revealed that increasing frailty in older adults is associated with greater between-subject as well as within-subject hourly activity variability, particularly in the morning and afternoon. Study implications and future directions will be discussed.

### DEVELOPING A SENSOR-BASED MOBILE APPLICATION FOR IN-HOME FRAILTY ASSESSMENT: A QUALITATIVE STUDY

Marcela D. Blinka,[1] Brian Buta,[1] Kevin Bader,[2] Casey L. Hanley,[2] Nancy Schoenborn,[3] Matthew McNabney,[3] and Qian-Li Xue[1], *1. Center on Aging and Health, Division of Geriatric Medicine and Gerontology, Johns Hopkins University School of Medicine, Baltimore, Maryland, United States, 2. The Johns Hopkins Applied Physics Laboratory, Laurel, Maryland, United States, 3. Division of Geriatric Medicine and Gerontology, Johns Hopkins University School of Medicine, Baltimore, Maryland, United States*

Frailty is an important concept in the care of older adults, and there is great interest in incorporating user-friendly frailty assessments into research and clinical settings. In-home, sensor-based technologies may provide a more dynamic, sensitive, and accurate assessment of frailty measures. To investigate user perspectives for use of sensor-based technologies and mobile applications, we held five focus groups with community-dwelling older adults (n= 10), their informal caregivers (n=9), and medical professionals (n=8). We used qualitative inductive analysis to organize thematic content. Caregivers and care-recipients viewed the early identification of frailty as beneficial, but highlighted the need for secure data infrastructure and clear demonstration of how frailty assessment would improve care. They also expressed concerns that technology-based communication could reduce in-person interactions. Medical providers noted the utility of objective data for difficult conversations with caregivers of frail patients, but worried about resources for analyses and interpretation of sensor-based health information.

### COLLABORATIVE AGING RESEARCH USING TECHNOLOGY: NEW PATHWAYS FORWARD

Jeffrey Kaye,[1] Zachary Beattie,[1] Nicole Sharma,[1] Thomas Riley,[1] Lisa Silbert,[1] Lisa Barnes,[2] Sarah Czaja,[3] and Hiroko Dodge[1], *1. Oregon Health & Science University, Portland, Oregon, United States, 2. Rush University, Chicago, Illinois, United States, 3. Weill Cornell Medical College, New York, New York, United States*

A profusion of technologies and protocols have been developed to more effectively assess and deliver care to older adults with cognitive impairment, challenged health and declining function. These technologies take advantage of important developments in sensing and pervasive computing, wearable technologies, mobile and wireless communications, and "big data" analytics. Despite great promise challenges remain to realizing their full potential and achieving wider uptake and dissemination in research and practice. This presentation will review and provide an overview of major technologies, their integration, and their use-cases, as well as key challenges present in the current landscape. The presentation will highlight ongoing developments addressing these challenges with particular attention to the Collaborative Aging Research using Technology (CART) initiative supported by the NIH and VA, an initiative directed toward providing an open technology research platform to be used by diverse investigators across the U.S. to facilitate and improve aging research using technology.

### SESSION 4170 (PAPER)

### UPDATES ON CURRICULAR INNOVATIONS IN GERONTOLOGY EDUCATION

### AFU PRINCIPLES IN ACTION: ENGAGING STUDENTS THROUGH HANDS-ON AGE-RELATED ACTIVITIES

Cassandra Barragan,[1] and Stephanie Wladkowski[1], *1. Eastern Michigan University, Ypsilanti, Michigan, United States*

The Age-Friendly University (AFU) Initiative is a global network of universities working to embrace and promote the growing population of older adults (OAs) on campuses. Integrating inter-generational learning is a proven benefit to share knowledge (Gerpott, Lehmann-Willenbrock, & Velopel, 2017) and to mutually benefit both older and more traditional learners (Pstross, Corrigan, Knopf, et al., 2017). To thoughtfully develop AFU initiatives on their campus, one midwestern university created an educational activity for students to better understand the needs of OAs. This presentation will cover results of this activity and offer suggestions for aging-focused learning activities. In winter 2019, 23 undergraduate students from 5 disciplines participated in a guided sensory activity with 5 Masters in Social Work (MSW) students that simulated impaired vision, hearing, and dexterity. Afterwards, using the AARP walking audit, they walked campus to understand challenges those with limitations might face. Students then completed a guided reflection and thought of ways to advocate for anyone with physical challenges, both off and on campus. This activity resulted in several successful learning outcomes and provided concrete experiences, establishing grounds to think about advocacy in a practical way. First, the undergraduate students presented their experiences at a campus-wide activism and advocacy event. They aimed to 1) increase awareness of the challenges those with visual and physical challenges and 2) promote the AFU initiatives. MSW students further analyzed their experience from a policy perspective and presented to the AFU steering committee with recommendations to influence policy in alignment with the AFU principles.

### KNOWLEDGE OF AGEISM AND ATTITUDES ABOUT AGING AS A CORE COMPETENCY FOR HEALTH PROFESSIONALS

Sarah Marrs,[1] Tracey Gendron,[1] Leland Waters,[1] Jenny Inker,[1] and Maddie McIntyre[1], *1. Virginia Commonwealth University, Richmond, Virginia, United States*

Senior mentoring programs have been established that provide medical students exposure to a community-dwelling older adult mentor with whom they meet multiple times throughout the program. The goal of these programs is to expose students to healthy older adults, increase knowledge of geriatrics, and prepare them to care for an aging population. However, even while participating in a senior mentoring program, health professions students still demonstrate some discriminatory language towards older adults (e.g., Gendron, Inker, & Welleford, 2018). In fact, research suggests ageist practices occur, intentionally or not, among health professions in disciplines such as medicine, nursing, and social work and even within assisted and long-term care facilities (e.g., Bowling, 1999; Dobbs et al., 2008; Kane & Kane, 2005). We evaluated a senior mentoring program to gauge the impact of a new pedagogical approach and to gain a deeper understanding of the learning gained in relation to ageism and elderhood. This qualitative content analysis explored first-year medical students' opinions of their own aging and attitudes towards caring for older adults. Students (n = 216) participating in a brief curriculum model of a senior mentoring program responded to the following open-ended prompts before and after the program: 1) How do you feel about your own aging?; 2) How do you feel about working with older adult patients after you complete your medical training? Responses suggest that students' views of their own aging and views towards towards working with older patients are positively impacted by their experiences in the senior mentoring program.

### LEARNING LIFE COURSE CONCEPTS THROUGH FILM

Lisa C. Hall[1], *1. Missouri State University, Springfield, Missouri, United States*

This paper explains how students learned foundational concepts through viewing a commercial film. The students (mostly social, behavioral, and biomedical sciences majors) were enrolled in a survey course for the gerontology major in a Program of Merit-certified Bachelor of Science program. Elements of the life course (transitions, counter-transitions, trajectory, age norms and timetables, sequence, duration, and social clock), and types of aging (biological, psychological, social, chronological, functional and subjective) are evident in the 2008 fictional film, The Curious Case of Benjamin Button. An adaptation of F. Scott Fitzgerald's short story by the same name, the film chronicles a man's life who was born physiologically old and dies as an infant with dementia. A multi-phased, active learning approach was used in which students (1) read about the abstract concepts from a textbook, (2) viewed the film while simultaneously identifying and applying concepts through notetaking, reflective writing prompts, and class discussion, (3) defined concepts and critically analyzed the contents in a written assessment, and (4) recalled definitions of concepts, eight weeks later, in a multiple choice final exam. Over three-quarters of students scored 80% or higher on the written assessment. Students under the active learning approach demonstrated higher recall on the final exam compared to students who were under the previous approach of standard lecture. Though these findings indicate that active learning through film is effective, they were realized retrospectively while attempting to improve a course's learning outcomes. A planned, cross-sectional study could better account for variability and yield more conclusive results.

### USING AN INTERGENERATIONAL RELATIONSHIP TEACHING STRATEGY AND REFLECTION IN ON-GROUND AND ONLINE COURSES

Christine B. Thurlow[1], *1. Research College of Nursing, Kansas City, Missouri, United States*

Contact or experiences with older adults by college-level students is varied in nature and duration but is often reported as minimal and negative. By pairing groups from different generations, these relationships may influence established attitudes, beliefs, and behaviors of the groups. Research has shown that intergenerational relationships between older adults and college students through interviews within gerontology courses may influence college students' views on aging and older adults towards the more positive. Specific steps in the development of an intergenerational relationship teaching strategy begin with locating an elder to interview and guidance for how relationship development can change from beginning to end. Role play may help prepare for first interview. Interview questions correspond to course content. Reflections are represented via discussion board if online, and group process forums if on-ground. Intergenerational relationship strategies can be flexible in the amount of contact time with the elder and the student. Reflection strategies can be utilized in several ways: discussion board, journal entries, presentations to classmates, and a culminating paper/PowerPoint. The opportunity for students to tell stories of their own past experiences, relationships with their elders, care and concerns for their elders, and how personal views about older adults and aging may be changing supports the effectiveness of this teaching strategy. Further research into the amount of time needed for effective intergenerational relationship development between student and elder may assist in course planning. Need clearer understanding of the benefits for the elder mentors and if their views about young people have been altered.

### SESSION 4175 (SYMPOSIUM)

### THE GUT MICROBIOME AND AGING

Chair: Christy S. Carter, *University of Alabama at Birmingham, Birmingham, Alabama, United States*
Co-Chair: Michal Masternak, *University of Central Florida, Orlando, Florida, United States*
Discussant: Thomas W. Buford, *University of Alabama at Birmingham, Birmingham, Alabama, United States*

The human intestinal tract (i.e., "gut") is inhabited by over 100 trillion microorganisms; including over 1000 species of known bacteria. These organisms have co-evolved with humans over millennia to live together for mutual benefit. Though long overlooked in considerations of human health and disease treatment, gut microorganisms are highly involved in numerous metabolic reactions which influence normal host physiology. A variety of biologic, medical, and lifestyle factors appear to contribute to gut dysbiosis in late-life, and interventions specifically designed to target these factors may be useful in restoring microbial balance. Evidence from both clinical and preclinical studies suggests that gut dysbiosis is related to age-related inflammation as well as age-related conditions including frailty, Alzheimer's disease, and perhaps even longevity. Crosstalk between the gut and multiple organ systems (brain, heart, muscle etc.) may lead to the development of age-related diseases and loss of physiological function, although the signals are not well understood. In this symposium we address the broad topic of the Gut Microbiome and Aging by presenting evidence from multiple model systems (mice, rats and monkeys) and provide a forum to discuss critical areas of research for moving forward.

### THERAPEUTIC DELIVERY OF ANG(1-7) VIA GENETICALLY MODIFIED PROBIOTIC: A DOSING STUDY

Christy S. Carter[1], *1. University of Alabama at Birmingham, Birmingham, Alabama, United States*

Aging is associated with loss of diversity in gut microbiota leading to dysbiosis; a condition linked with cognitive/physical frailty. Age-related health benefits have been ascribed to the renin-angiotensin system (RAS), mediated via the angiotensin-converting-enzyme-2 (ACE2)/angiotensin (1-7) or Ang(1-7) axis. A genetically modified probiotic secreting Ang(1-7) or GMP-A, targeting the gut, has been described for diabetes and hypertension; however, we are the first to provide proof of concept for its use in aging. We report that GMP-A increases circulating levels of Ang(1-7) when orally-administered over 4 weeks in aged F344/BN rats. Ongoing experiments examine GMP-A impact on distal tissues (muscle/brain). GMP-A provides translational advantages including ease of administration, production cost, and burden for regulatory approval.

### THE INFLUENCE OF LIFE-EXTENDING MUTATION AND DIETARY INTERVENTION ON GUT MICROBIOTA

Michal Masternak,[1] Denise S. Wiesenborn,[2]
Augusto Schneider,[1] Till Strowig,[3]
Karl –Herbert Schafer,[4] and Eric Galvez[3], *1. University of Central Florida, Orlando, Florida, United States, 2. Burnett School of Biomedical Sciences, Orlando, Florida, United States, 3. Helmholtz Centre for Infection Research, Braunschweig, Sachsen-Anhalt, Germany, 4. University of Applied Sciences, Zweibrucken, Rheinland-Pfalz, Germany*

The gastrointestinal microbiota represents a large and complex ecological system of different microorganisms. Recently, there is an increasing interest in the impact of microbiota on development of different age-related diseases. We tested the changes of gut microbiota during development in long-living Ames dwarf (df/df) mice and we compared the effects of this life-extending mutation with the impact of calorie restriction (CR). Importantly, the analysis of microbiome showed significant differences in the ratio of Bacteroidetes and Firmicutes when comparing df/df and normal (N) mice ($p<0.001$). The LefSe analysis showed distinct microbiome distribution between CR and ad libitum (AL) feeding regimen in N animals ($p<0.004$), yet there was lack of similar changes in response to CR in df/df mice. In summary, our study showed significant genotype impact on gut microbiota and we showed that life-extending CR regimen provide divergent effects on gut microbiota in N when comparing with df/df mice.

### NONHUMAN PRIMATE GUT MICROBIOME: IMPACT OF AGE AND CALORIE RESTRICTION

Ricki Colman,[1] and Federico Rey[1], *1. University of Wisconsin, Madison, Wisconsin, United States*

The human microbiome is composed of bacteria, archaea, viruses and eukaryotic microbes that reside in and on our bodies, the largest community of which is in the gut. Although the functions of the gut microbiota are not fully understood, they are known to play an essential role in immune, endocrine, and metabolic functions. To begin to understand the relationship between the gut microbiome, aging, and adult-onset, moderate calorie restriction in rhesus macaques (Macaca mulatta), we collected fecal samples at one timepoint from a total of 52 macaques for 16S rRNA gene analyses. Samples were taken from 20 males and 20 females across the natural macaque age range and from 6 males and 6 females enrolled in the long-term study of aging and calorie restriction at the Wisconsin National Primate Research Center. Preliminary data show that, like humans, NHPs exhibit a large interindividual variation in microbiota composition despite well-controlled environmental conditions.

### SESSION LB935 (LATE BREAKING POSTER)

### LATE BREAKING POSTER SESSION I

### USING NEURAL NETWORK TO UNCOVER CELL TYPES THROUGH SNRNA SEQUENCING

Fahad Paryani,[1] and Vilas Menon[1], *1. Columbia University Vagelos College of Physicians and Surgeons, New York, New York, United States*

The advent of single-nucleus RNA-sequencing (snRNAseq) has allowed for the exploration of genetic signatures of the numerous cells in the brain. In particular, snRNAseq data can provide new insights into how many neurodegenerative diseases, such as Alzheimer's Disease, alter cells in the brain. One major challenge with analyzing snRNAseq data is the lack of a systematic way to classify the various cell types across different datasets. To address this challenge, we developed a general classifier ("DeepSeq") that uses state-of-the-art deep learning approaches. We trained our model on multiple snRNAseq datasets derived from post-mortem brain tissue in individuals with and without clinical diagnosis of Alzheimer's Disease from the ROSMAP cohorts. The two snRNAseq datasets contained 70,064 nuclei and 170,275 nuclei. The two studies employed different clustering techniques, and identified 44 and 18 putative cell types. To map these disparate cluster identities across datasets, we extracted the most relevant genes and trained two separate networks, one on each dataset. We then validated each classifier separately on the holdout cells. The resulting classifier accuracy were 87% and 94%. To map clusters across datasets, we then applied each classifier to the other dataset. Both classifiers yielded mappings that reflected the overall biology, correctly categorizing the nuclei into broad and fine cell type classes. Although validation on additional datasets would expand the generality of this approach, our results show that DeepSeq is an easily implementable classification tool that can assign identity to nuclei in new snRNAseq datasets without the need for preprocessing or cross-batch alignment.

### PATHOLOGICAL TAU ABORTS CELL CYCLE ACTIVATION BY MIMICKING EPITHELIAL-MESENCHYMAL-TRANSITION IN NEURONS

Roseann V. Phan,[1] Adrian Beckmann,[2] and Bess E. Frost[3], *1. Howard University, Washington, District of Columbia, United States, 2. University of Texas San Antonio, San Antonio, Texas, United States, 3. University of Texas Health San Antonio, San Antonio, Texas, United States*

Alzheimer's disease (AD) is an irreversible neurodegenerative disorder which is characterized by neurofibrillary tau tangles and amyloid-ß plaques. Our laboratory uses tau transgenic Drosophila to rapidly test hypotheses along with human brain samples in order to ensure that our work is relevant to clinical endeavors. Using this approach, we identified a neurodegenerative pathway whereby pathological tau over-stabilizes filamentous actin (f-actin), leading to disrupting aberrant nuclear pleomorphisms and decondensation of heterochromatic DNA. Due to the neuronal phenotypes observed in tau-transgenic Drosophila and reentry of post-mitotic neurons into the cell cycle, we hypothesized that tau perturbs the cellular program that maintains terminal neuronal differentiation. Based on RNA-sequencing, we identified prospero as the most differentially expressed and downregulated transcript in tau transgenic flies. Prospero regulates genes that promote and maintain terminal neuronal differentiation. At the protein level, prospero is significantly reduced in tau transgenic flies at 10 days old. We find that over stabilization of f-actin or over-expression of moesin, a cytoskeletal protein known to participate in tumor progression and metastasis, depletes prospero. Using genetically manipulated prospero target genes we find a connection to neurodegeneration. These targets include a PKC, tep4, and knot-suggesting that broad cellular processes are affected by this reduction. Overall, our findings suggest that pathological tau causes cell cycle re-entry by disrupting transcription factors governing terminal neuronal differentiation as well as over-stabilization of f-actin being the driving loss of terminal neuronal differentiation and casually associated with neuronal death.

### MICRORNA REGULATORS OF THE SENESCENCE TRANSCRIPTOME

O'Wayne Rodney,[1] Myriam Gorospe,[2] and Kotb Abdelmohsen[2], *1. Howard University, Washington, District of Columbia, United States, 2. National Institute on Aging, Baltimore, Maryland, United States*

Cellular senescence is a state of indefinite growth arrest triggered in response to sublethal stresses such as telomere shortening, DNA damage, oxidative injury, oncogene activation, and hypoxia. Compared with proliferating cells, senescent cells are enlarged, display heterochromatic DNA foci, and express distinct subsets of proteins, including the enzyme β-galactosidase (β-gal). Previously, we identified transcriptome signature of senescent cells. We asked if these transcripts might be regulated by microRNAs (miRNAs). To address this question, we identified six miRNAs (miR-129-5p, -19a-3p, -128-3p, -124-3p, -340-5p, and -27b-3p) as potential regulators of subsets of transcripts differentially expressed during senescence. RT-qPCR analysis indicated that miR-129-5p, -19a-3p, -128-3p, -124-3p, and -340-5p were downregulated in senescent cells. We modulated these miRNAs in proliferating WI-38 fibroblasts and found that miRNA antagomirs did not show significant changes in β-gal activity. Interestingly, however, overexpression of miR-124-3p or miR-340-5p increased β-gal activity. We conclude that despite the decrease of miR-124-3p and miR-340-5p in senescent cells, their overexpression enhanced senescence as indicated by β-gal activity. Future analyses will focus on the mechanisms through which these miRNAs induce senescence and their physiologic and pathologic impacts in vivo.

### TRANSGENIC MOUSE MODELS OF ALZHEIMER'S DISEASE

Charnae A. Henry-Smith,[1] and Xianlin Han[2], *1. Howard University, Washington, D.C., United States, 2. Sam and Ann Barshop Institute for Longevity and Aging Studies, San Antonio, Texas, United States*

Alzheimer's disease is a progressive brain disease that slowly destroys memory and thinking skills. Alzheimer's is characterized by an increase in Aβ plaques , and tau tangles. Neurons in the brain have axons covered in myelin sheath that connect microglia and astrocytes. The myelin sheath is composed of about 70% lipid composition; Sulfatide contributing to 30% overall. Sulfatide changes the morphology of primary microglia to their activated form. To study the role of microglia activation and sulfatide levels, three different mouse models were created: APP KI mice, CST Whole Body Ko mice, and cCST (conditional) KO. In order to create the genotype of the APP KI mice, a breeding mouse line was created. The APP KI gene had to be introduced in Plp1-Cre and cCST KO crossed mice to receive a working mouse model. During the duration of breeding for the APP KI mice, a preliminary experiment was performed on the CST KO mice. These mice were given the PLX3397 diet with the aim to remove the microglia and to see the effect of Aβ plaques. The PLX3397 will reduce the microglia targeting the CSF1R. After consuming the diet, the mice were harvested to collect tissues from the brain and spinal cord. Lipidomics and immunohistology were performed. In conclusion, we will continue the breeding of the CST flox/flox / Plp1-Cre / APP KI mice, and the drug dosage and treatment to be used in our APP KI mice will be based on preliminary data from our CST mice.

### THE ROLE OF CAPRIN-1 PROTEIN DYSREGULATION IN SYNAPSE DECLINE LEADING TO PROGRESSION OF TAUOPATHIES

Caitlyn Fastenau,[1] Helen Cifuentes,[2] Grant Kauwe,[2] and Tara Tracy[2], *1. University of California, Davis, Davis, California, United States, 2. Buck Institute for Research on Aging, Novato, California, United States*

Many neurodegenerative diseases are characterized by accumulation of proteins such as tau, a microtubule stabilization protein. Toxic tau forms tangles and affects neuronal synapse function, an early step of neurodegeneration. To focus on synapse function, we highlighted Caprin-1 protein. Through gene ontology, Caprin-1 is related to RNA granule proteins, important for transport and local translation in dendrites. Caprin-1 is of interest for neurodegeneration because it is a memory related protein, transports mRNA in RNA granules, and knock out mice demonstrate memory deficits. As we age, the performance of local translation in the dendrites is compromised and leads to synapse dysfunction. We hypothesize Caprin-1 binds to Tau and becomes disrupted. This leads to the dissolution of RNA granules, inhibition of mRNA transport in dendrites, suppression of translation, and failure of synapse. Early western blot data showed reduced Caprin-1 in PS19 Tau+, supporting our model that Caprin-1 is disrupted in disease models. Through immunohistochemistry, we investigated the localization of Caprin-1 in the mouse hippocampus. We observed Caprin-1 localization to dendrites of CA1 neurons in the hippocampus. Furthermore, Caprin-1 exhibited colocalization with Rps6, an RNA granule marker. This suggests Caprin-1 associates with RNA granules in mouse hippocampus. Finally, we investigated the localization of Caprin-1 in human iPSC-derived neurons. Similar to the mouse hippocampus, we observed localization of Caprin-1 to dendrites of human neurons. In future directions, we will examine whether pathogenic tau alters the association of Caprin-1 with RNA granules and the mechanisms by which pathogenic tau negatively effects synapse function.

### EXERCISE ACUTELY IMPROVES COGNITION IN HEALTHY OLDER ADULTS: THE ROLE OF AROUSAL

Pearl N. Cummins,[1] James Kent,[2] Timothy Weng,[3] Vincent Magnottta,[2] Gary Pierce,[2] and Michelle Voss[4], *1. San Diego State University, San Diego, California, United States, 2. University of Iowa, Iowa City, Iowa, United States, 3. University of Texas, Austin, Texas, United States, 4. University of Iowa, Iowa City, United States*

Previous researchers have reported that aerobic exercise improves cognition in older adults; however, few researchers have examined the role of arousal on improvements in cognition after exercise. The purpose of this study was to understand how changes in arousal acutely affect changes in cognitive performance after a single session of light compared to moderate intensity aerobic exercise. Cognitively normal older adults (N = 34) were enrolled in a randomized controlled trial where they were asked to complete the N-back task with faces, a cognitive task used to test working memory, in an fMRI scanner. On separate days, the task was completed before and 15 to 20 minutes after light and moderate intensity exercise. An intervention was also completed, but our question focuses on the acute effects of exercise rather than training. Arousal was measured before and after exercise through a questionnaire and a direct measure of physiological activation of the sympathetic nervous system with galvanic skin response (GSR). On average, resting GSRs decreased from pre- to post-exercise scan; however, the change was not statistically significant. The decrease in arousal after light exercise indicated that older adults had decreased sympathetic activity after both light and moderate intensity exercise. By contrast, N-back task performance improved most after moderate compared to light intensity exercise. Together, evidence that sympathetic activity tended to decrease generally for both intensities, whereas cognitive improvements were more specific, suggests that changes in arousal at rest were not a critical factor connecting exercise and improved working memory in this study.

### INVESTIGATING THE ROLE OF AHR IN MEDIATING SEX DIFFERENCES OF AGING MACROPHAGES

Evelyn Navar,[1] Bérénice A. Benayoun,[2] Nirmal Sampathkumar,[2] and Jisoo Chae[2], *1. University of California at Davis, Davis, California, United States, 2. University of Southern California, Leonard Davis School of Gerontology, Los Angeles, California, United States*

"Inflamm-aging" describes a state of chronic low-grade inflammation which occurs with age in the absence of infection. This process is related to many chronic age-related diseases. Aryl hydrocarbon receptor (Ahr), is a transcription factor that is thought to decrease inflammation, and decrease of Ahr with aging only in females was previously observed in a macrophage RNA-seq with aging. Based on this, I hypothesized that 1) Ahr expression will decrease with age in female cells; and 2) phagocytic activity and Ahr expression in macrophages will increase when exposed to estrogens (E2). To test these hypotheses, Ahr signaling was quantified by RT-qPCR in aging male and female mice BMDMs, and in macrophages that were treated with E2. I also performed a phagocytosis assay on macrophages treated with E2. I found a significant downregulation of Ahr in old female BMDMs. Ahrr (Ahr Repressor) was significantly downregulated in both old female and males with aging. Arnt (Ahr Nuclear Translocator) did not significantly change with aging. The qPCR performed on the E2 treated cells showed no significant trend for Ahr regulation. Finally, the phagocytosis assay revealed an overall increase in phagocytosis activity in cells treated with estrogen. Our hypotheses were supported by data showing a decrease in Ahr expression with age and increase in phagocytosis activity in estrogen treated cells. The RT-qPCR results for the E2 treated cells did not support our hypothesis, but could stem from a relatively short exposure time for estrogen.

### PRIVACY AND SECURITY FOR TELEHEALTH DEVICES

London Thompson,[1] and Csilla Farkas[2], *1. Claflin University, Columbia, South Carolina, United States, 2. University of South Carolina, Columbia, South Carolina, United States*

In this research, we study the privacy and security capabilities provided by telehealth devices. Our aim is to evaluate how vulnerable these popular devices are in the presence of malicious cyber attackers. As older adults increasingly rely on telehealth devices, it is crucial that cybersecurity aspects of these devices are clearly communicated to them. Moreover, older adults frequently lack the technical expertise to evaluate the security and privacy capabilities of the devices. The lack of control over telehealth devices is a major concern for older adults. Older adults view certain limitations within these devices as decreasing their privacy and security. These limitations include the lack of control over accepting calls, taking screenshots, and assigning access privileges. For large scale adaptation of telehealth devices by older adults, it is crucial that these devices not only satisfy their intended purpose but also exhibit user friendly features and strong security and privacy capabilities. Modeling cyber threats against telehealth devices is not studied sufficiently . Malicious actors may compromise telehealth devices and create further threats to security and privacy of the users. In this research, we studied the cyber threats against telehealth devices. We built a threat model that ranks cyber threats based on their impact. We investigated how the operating system of popular devices supports access control. We found that none of the current technologies support location-based access control. We claim that this represents a major limitation and that supporting location-based access control is necessary to ensure users' privacy in their own home.

### WALKING AWAY FROM LONELINESS: THE MEDIATING ROLE OF SOCIAL ISOLATION

Natalie Shellito,[1] and Nidya Velasco Roldan[1], *1. University of Massachusetts Boston, Boston, Massachusetts, United States*

This study examines the relationship between physical activity (PA) and loneliness among older adults. Participation in walking enables individuals to come into contact with other people, thus social isolation may mediate the relationship between walking and loneliness. The study uses participants from the Leave Behind Questionnaire of the 2016 data wave of the Health and Retirement Study with a sample size of 6,157. The dependent variable, loneliness, is measured using the 11-item UCLA Loneliness Scale and the independent variable, walking, is measured as participants who walk 20 minutes or more per day. The mediator, social isolation, is measured using a standardized 9-item score, including closeness and frequency of contact with children, friends, and other family members, and participation in group activities. We analyzed the effect of walking on loneliness and the role of social isolation as a mediator of that relationship using structural equation modeling. Our results suggest that walking is significantly associated with lower levels of both social isolation (B=-.10) and loneliness (B=-.05). As well, there is a positive association between social isolation and loneliness, as social isolation increases, so does loneliness (B=0.31). Moreover, results from the mediation analysis using bootstrapping suggest that social isolation partially mediates the relationship between walking and loneliness (B=-.03). Our findings confirm the benefits of PA on wellbeing. This research provides evidence that suggests establishing walking programs may decrease the risk of loneliness. Future interventions concentrated on lowering social isolation through PA among older adults should consider the opportunity to reduce loneliness.

### THE IMPACT OF FUNCTIONAL DISABILITY ON HOSPITALIZATION SPENDING IN SINGAPORE

Cynthia Chen[1], *1. Saw Swee Hock School of Public Health, Singapore, Singapore, Singapore*

Singapore is one of the fastest-aging populations due to increased life expectancy and lowered fertility. Lifestyle changes increase the burden of chronic diseases and disability. These have important implications for social protection systems. The goal of this paper is to model future functional disability and healthcare expenditures based on current trends. To project the health, disability and hospitalization spending of future elders, we adapted the Future Elderly Model (FEM) to Singapore. The FEM is a dynamic Markov microsimulation model developed in the US. Our main source of population data was the Singapore Chinese Health Study (SCHS) consisting of 63,000 respondents followed up over three waves from 1993 to 2010. The FEM model enables us to investigate the effects of disability compounded over the lifecycle and hospitalization spending, while adjusting for competing risk of multi-comorbidities. Results indicate that by 2050, 1 in 6 older adults will have at least one ADL disability and 1 in 3 older adults will have at least one IADL disability, an increase from 1 in 12 elders and 1 in 5 elders respectively in 2014. The highest prevalence of functional disability will be in those aged 85 years and above. Lifetime hospitalization spending of elders aged 55 and above is US$24,400 (30.2%) higher among people with functional disability compared to those without disability. Policies that successfully tackle diabetes and promote healthy living may reduce or delay the onset of disability, leading to potential saving. In addition, further technological improvements may reduce the financial burden of disability.

### SELF-PERCEPTION OF AGING AND ASSOCIATED CHARACTERISTICS AMONG OLDER ADULTS

Timothy L. Barnes,[1] Shirley Musich,[1] Shaohung Wang,[1] Sandra Kraemer,[2] and Charlotte Yeh[3], *1. Optum, Eden Prairie, Minnesota, United States, 2. UnitedHealthcare, Minneapolis, Minnesota, United States, 3. AARP Services, Inc., Washington, District of Columbia, United States*

Positive self-perception of aging has been linked to better physical and psychosocial health outcomes among older adults. Negative self-perception of aging has been associated with poorer health consequences including depression, limited mobility, and mortality. Despite significant findings, the comprehensiveness and quality of self-perception of aging research still warrants further investigation, especially when identifying factors for intervention. Using a large random stratified sample of AARP Medicare Supplement insured members, age≥65 years, with continuous coverage for ≥12 months, self-perception of aging and various sociodemographic, medical, and psychosocial characteristics were examined using Chi-square and multivariate logistic regression models. Self-perception of aging was measured using the five-item Attitudes Towards Own Aging subscale. Characteristics of interest included age, gender, health status, resilience, purpose, optimism, social network, physical activity, depression, falls, vision, hearing, oral health, and sleep quality. Propensity weighting was used to adjust for potential survey non-response bias. Of weighted survey respondents (N=14,046), 59% exhibited a positive self-perception, while 41% exhibited a negative self-perception. Respondents with a positive self-perception were more likely to be healthier, younger (<75 years), more active (≥3days), less depressed, have more diverse social networks, higher resilience, and purpose. Negative self-perception was associated with poorer health, older age, depression, and poorer vision, hearing, oral health, and sleep quality. The strongest characteristics associated with positive self-perception were purpose, resilience, physical activity, and social networks. Depression and sleep quality were the strongest characteristics associated with negative self-perception. Interventions targeting these characteristics could be beneficial in promoting positive self-perception of aging and health over time.

### THE CURRENT SITUATION AND INFLUENTIAL FACTORS OF FRAILTY IN ELDERLY PATIENTS WITH DIABETES IN CHINA

Fumin Dai,[1] Wenwen Jia,[1] and Mengdie Jiang[2], *1. Henan Provincial People's Hospital, Zhengzhou, Henan province, China, 2. Henan University, Zhengzhou, Henan, China*

Patient with diabetes may increase the incidence of frailty. Frailty cause chronic inflammation and insulin resistance. The purpose of this study is to investigate the current situation of frailty and its influencing factors in elderly patients with diabetes. Totally 300 elderly patients with diabetes were selected from a tertiary hospital in Zhengzhou of China via convenience sampling method , and were investigated by self-designed general information questionnaire , TFI (Tilburg Frailty Indicator) and SDSCA(Scale of Diabetes Self-care Activities). Totally 296 valid questionnaires were collected. A total of 137 elderly patients with diabetes suffered from frailty and the prevalence was 46.3%. The mean score of total frailty was (5.26±2.87) and the scores for each dimension were as follows: physical frailty (2.79±2.08), psychological frailty (1.40±0.94) and social frailty (1.07±0.75). Multiple linear regression showed that comorbidity, self-management behavior, glycosylated hemoglobin, educational degree, polypharmacy and smoking were the major influential factors (P<0.05). The prevalence of frailty in elderly patients with diabetes was at a high level, containing different degrees of physical, psychological and social frailty. Medical staff should attach great importance to the assessment of frailty among elderly patients with diabetes, take targeted and holistic interventions timely to prevent or delay the development of frailty.

### A QUALITATIVE STUDY ON THE COGNITION AND EXPERIENCES MOBILITY DEVICES USE IN KNEE OSTEOARTHRITIS PATIENTS OF CHINA

Fumin Dai,[1] Mengdie Jiang,[2] and Wenwen Jia[1], *1. Henan Provincial People's Hospital, Zhengzhou, Henan province, China, 2. Henan University, Zhengzhou, Henan, China*

The benefits of mobility devices for knee Osteoarthritis Patients include reducing burden of knee joint, enhancing confidence and increasing autonomy, yet many who might benefit from using mobility devices do not use them. The purpose of this study is to explore the cognition and using experiences about mobility devices use in patients with knee osteoarthritis of China. Naturalistic inquiry research was adopted, 15 patients with knee osteoarthritis were recruited and Semi-structured interviews were conducted. Data were analyzed based on conventional content analysis methodology. Two themes of using experiences about mobility devices were extracted, including positive feelings, negative feelings. Cognition about mobility devices use included light consciousness, incorrect attitude. Mobility devices have a positive effect on knee osteoarthritis patients, but some patients lack sufficient understanding and face many problems in the use of mobility devices. Policy Support, greater physician involvement, positive peer modules, and safe, visually appealing devices would promote greater acceptance and satisfaction of mobility devices with knee osteoarthritis patients.

### DESCRIBING A CONCEPT OF NURSE INTUITION AND IMPLICATIONS FOR DEMENTIA CARE IN NURSING HOMES

Rebekah Perkins,[1] and
Katherine Supiano'katherine.supiano@hsc.utah.edu' kather[1], *1. University of Utah, Salt Lake City, Utah, United States*

The purpose of this concept analysis was to explore nurse intuition historically and in current literature, appraise the value of nurse intuition to research and practice, and discuss implications for practice in caring for residents with behavioral and psychological symptoms of Dementia (BPSD) in nursing homes. To date, no research has examined the presence or utility of nurse intuition in the nursing home setting. A conceptual analysis using pragmatic utility was chosen and based on the work of Walker and Avant (2005). In the literature, nurse intuition is characterized by attributes of knowledge not preceded by inference, knowledge that is holistic in nature, independent of linear thinking, and drawn from synthesis instead of analysis. Nurse intuition is based on preconditions of experience, empathy, limited information, and limited time to make vital decisions about patient care. Studies on nurse intuition have been criticized for their lack of rigor and empirical evidence of the effect of nurse intuition on positive patient outcomes. The attributes of intuitive nursing practice have important implications in nursing applied to residents with BPSD. The nursing home nurse with extensive educational and experiential knowledge is well-situated to understand the complex, changing needs of residents exhibiting various forms of BPSD in an effort to communicate their needs. Future studies on nurse intuition should focus on early education in dementia care, nurse residency-mentor programs to enhance intuitive thinking in the management of BPSD, and more empirical studies on the use of intuition in the context of dementia care.

### MITOCHONDRIAL-DERIVED PEPTIDE, SHLP2, A NOVEL PROTECTIVE FACTOR IN PARKINSON'S DISEASE

Su Jeong Kim,[1] Anjali Devgan,[1] Hemal H. Mehta,[1] and Pinchas Cohen[1], *1. Leonard Davis School of Gerontology, University of Southern California, Los Angeles, California, United States*

Mitochondrial DNA (mtDNA) variants are associated with a wide range of diseases of aging, from diabetes to Alzheimer's, as well as with longevity itself. However, to date, little work has thoroughly examined the functional roles of mtDNA variants in such age-related diseases or the therapeutic potential of mitochondrial-derived peptides (MDPs) in these conditions. Our lab hypothesizes that mtDNA SNPs could affect MDPs, and we recently showed that a mtDNA SNP is associated with reduced circulating levels of an MDP called humanin and with cognitive decline. How other mtDNA SNPs affect MDPs and disease risk has yet to be analyzed. Remarkably, a recent paper showed a mtDNA SNP (m.2158 T>C) reduces the risk of Parkinson's disease (PD). Of note, this SNP changes lysine (K) 4 to arginine (R) of a MDP called SHLP2, which is encoded by the 16S rRNA region of the mtDNA. SHLP2 acts as a neuroprotective factor and as a metabolic regulator. We hypothesized that K4R SHLP2 – produced by individuals who carry mtDNA m.2158 T>C – is a protective factor for Parkinson's disease. Cycloheximide-treated pulse-chase experiments additionally showed that K4R SHLP2 is more stable than WT SHLP2. WT SHLP2 has a polyubiquitination whereas K4R SHLP2 diminish the polyubiquitination. K4R SHLP2 more potently inhibits PD toxin (MPP+) induced apoptosis in neuronal cells. K4R SHLP2 reverse the mitochondrial membrane potential loss and mitochondria respiration defect in TFAM heterozygous knockout MEFs. Altogether, SHLP2 has the therapeutic potential as a precision medicine in PD.

### "WITH AGE COMES WISDOM:" A QUALITATIVE REVIEW OF ELDER PERSPECTIVES ON HEALTHY AGING IN THE CIRCUMPOLAR NORTH

Britteny M. Howell,[1] and Jennifer R. Peterson[1],
*1. University of Alaska Anchorage, Anchorage, Alaska, United States*

Cross-cultural research has shown marked variation in health outcomes across the world's older adult populations. Indeed, older adults in the Circumpolar North experience a variety of health disparities. Because aging is a biological process rooted in sociocultural context, there exists great variation in the ways older adults define and experience healthy, or "successful," aging in their communities. The aim of this analysis was to synthesize qualitative research among older residents (aged 50+ years) in the Circumpolar North to identify a definition of healthy aging common in the region. A thorough review was conducted across a variety of academic search databases for peer-reviewed, qualitative studies conducted among community-dwelling older adults. The search strategy initially identified 194 articles; 23 articles met the inclusion criteria. Included studies were coded and analyzed using Grounded Theory to examine underlying themes of healthy aging in the Circumpolar North. The findings reveal the importance older adults place on respect, their relationship to the land, and psychosocial resilience into multidimensional models of healthy aging. We present a complex concept map demonstrating how healthy aging perspectives fit together into a multidimensional model of health in the Circumpolar North. This research also highlights the need for increased translational research with populations in the Circumpolar North that are under-represented in the literature.

### AGING AND FEELING CURIOUS: A TIME-SAMPLING STUDY

Li Chu,[1] and Helene H. Fung[2], *1. The Chinese University of Hong Kong, Shatin, New Territories, Hong Kong, 2. Chinese University of Hong Kong, Hong Kong, Hong Kong*

Curiosity is commonly defined as "the desire for new information and experience." While curiosity has been associated with numerous positive outcomes (e.g., improved well-being, better cognitive performance and longer life expectancy, some studies suggested that curiosity declined with age. However, very few studies actually attempt to examine why curiosity may be lower among older adults. Moreover, scholars disagreed on "why" people feel curious. According to the dual process theory (Spielberger & Starr, 1994), curiosity is induced by optimal level of uncertainty and anxiety with the desire to reduce these aversive feelings. However, the personal growth facilitation model (Kashdan, Rose, & Fincham, 2002) posits that people are curious intrinsically for one's own growth, which is associated with positive affects. Therefore, the present study aims to examine age differences in the affective profile of feeling curious by comparing the momentary affective experience of curiosity between younger and older adults. In this study, we conducted a 2-week time-sampling study with 78 younger adults (age 19-29) and 79 older adults (age 60-85) from Hong Kong. Multilevel modeling analyses demonstrated a positive relationship between curiosity and positive emotions for both younger ($\beta$=.29, p<.01) and older adults ($\beta$=.70, p<.01). Interestingly, anxiousness was positively associated with younger adults' curiosity ($\beta$=.09, p=.01) but not for older adults ($\beta$=.06, p=.29). Our study supported both theories, but suggested that one may be more dominant among older adults. These findings have important implications for future interventions to reduce anxiousness to encourage older adults to keep an open-minded attitude towards novelties.

### RACE AND ETHNICITY DISPARITIES IN SUBJECTIVE COGNITIVE DECLINE

Nia Reed,[1] Christopher A. Taylor,[1] Benjamin Olivari,[2] Karen Wooten,[3] and Lisa C. McGuire[4], *1. Centers for Disease Control and Prevention, Atlanta, Georgia, United States, 2. CDC, Atlanta, Georgia, United States, 3. DB Consulting, Silver Springs, Maryland, United States, 4. Centers for Disease and Prevention, Alzheimer's Disease and Healthy Aging Program, Atlanta, Georgia, United States*

Alzheimer's disease (AD) is the most common form of dementia. Subjective cognitive decline (SCD) is the self-reported experience of worsening or more frequent confusion or memory loss and it is one of the earliest noticeable symptoms of AD. Data from respondents aged 45 years and older to the Centers for Disease Control and Prevention's Behavioral Risk Factor Surveillance System Cognitive Decline module were examined to identify race and ethnicity disparities in SCD. This module was administered by 49 participating states, District of Columbia, and Puerto Rico from 2015-2018. Data were analyzed using SAS statistical software and methods that accounted for survey design and weighted data. Prevalence of SCD by race/ethnicity with 95% confidence intervals (CI) was calculated. Among adults aged 45 years and older, one in nine (10.8%; CI=10.5-11.2) non-Hispanic white adults experienced SCD. In comparison, among adults aged 45 years and older, one in nine (11.2%; CI=9.8-12.7) Hispanic, one in eight (13.2%; CI=12.0-14.3) African American/black, and one in five (19.6%; CI=16.0-23.2) American Indian/Alaska Native (AI/AN) adults experienced SCD. These numbers are expected to increase significantly over time, especially for some minority groups. More specifically, Hispanics and African Americans are expected to constitute a large proportion of older adults in the coming decades. There are implications in how communities are reached with respect to awareness of cognitive decline (this includes AI/AN adults, as well). Race and ethnicity disparities in SCD may be influenced by differences in chronic diseases and other risk factors that are also disparate between communities.

### ETHNIC DIFFERENCES IN THE RELATIONSHIP BETWEEN SOCIAL CAPITAL AND PSYCHOLOGICAL DISTRESS IN OLDER CALIFORNIANS

Nenette A. Caceres,[1] Qais Alemi,[1] Larry Ortiz,[1] and Carl Stempel[2], *1. Loma Linda University, Loma Linda, California, United States, 2. California State University, East Bay, Hayward, California, United States*

Seniors aged 65 and older are at great risk of psychological distress given their functional decline, which is known to limit participation and engagement in community life. The purpose of this study is to examine whether higher indices of social capital have a positive impact on the mental health of older, ethnic Californians. We conducted a secondary analysis of data for 7,485 Californians 65 and older from the 2016 California Health Interview Survey (CHIS). A principal components analysis generated two social capital measures; one measuring safety and social cohesion, the other civic engagement. Hierarchical linear regression analyses were conducted to assess the independent effects of social capital subscales on the severity of psychological distress as measured by the Kessler-6 (K6).Respondents were on average moderately distressed, with small yet significantly higher K6 scores observed among African Americans, Asians, and Native Americans. The addition of our social capital variables in subsequent steps resulted in little yet significant change in explaining psychological distress ($\Delta R2$ = .02,

$p < .001$) with only neighborhood safety and social cohesion being inversely associated with K6 ($\beta = -.15$, $p < .001$). The interaction between ethnicity and neighborhood safety and social cohesion resulted in non-significant associations with K6 scores for all ethnic minority subgroups; however, for African Americans the relationship with psychological distress actually increased significantly ($\beta = .24$, $p < .001$). Our findings suggest that specific types of social capital may be helpful in remediating psychological distress for certain ethnic minority groups.

### INCREASED C3 IN THE AGING BRAIN PROMOTES INFLAMMATORY TRANSITION IN ENDOTHELIAL CELLS

Nicholas E. Propson,[1] Alexandra Litvinchuk,[1] Ethan R. Roy,[1] Bianca Contreras,[1] Wei Cao,[1] and Hui Zheng[1], *1. Baylor College of Medicine, Houston, Texas, United States*

Innate immunity has been implicated in normal aging, and age-related disease. The connection between age-related neuroinflammation and change in brain vasculature prior to disease onset remains poorly understood. The complement pathway is an established mediator of neuroinflammation, and increased complement C3 is seen in the aging brain. Thus, we asked whether C3 can promote changes in brain vasculature. We found age dependent increase of brain C3 levels in C57BL/6J mice. Furthermore, we found an increase in expression of adhesion molecule VCAM-1 in endothelial cells (ECs) of the cortex and hippocampus, which was rescued in aged C3a receptor null (C3ar1-/-) mice and aged C3a receptor (C3aR) antagonist treated mice. We confirmed these results by qPCR analysis for Vcam1 in sorted ECs. Human brain microvascular endothelial cells (HBMECs) treated with C3a showed increased expression of VCAM-1, but not other adhesion molecules. Sorted ECs from C3ar1-/- mice challenged with LPS confirmed these findings. Furthermore, C3aR signaling in ECs showed increased blood-brain barrier (BBB) permeability using trans-endothelial electrical resistance (TEER), and BBB impermeable dye injections. HBMECs treated with C3a revealed mis-localization of VE-Cadherin, followed by reduction in protein level when analyzed by immunofluorescence, which promotes increased barrier permeability. As a functional consequence of VCAM-1 expression and increased BBB permeability we found aged mouse brains have increased peripheral lymphocyte (CD45+/CD11b-) infiltration, which was reduced in a C3aR dependent manner. In conclusion, our work suggests there is a strong relationship between C3 expression and vascular C3aR contributing to a functional transition in endothelial cells during aging.

### RETURNING THE FAVOR: EXPECTATIONS OF CAREGIVING RECIPROCITY AND DEPRESSIVE SYMPTOMS AMONG GRANDPARENTS

Cindy N. Bui[1], *1. University of Massachusetts Boston, Boston, Massachusetts, United States*

This study draws upon social capital and intergenerational reciprocity concepts to better understand how grandparents' depressive symptoms are related to their provision of grandchild care, within the context of their expectations regarding adult children reciprocating caregiving needs in the future. Analyses used the 2014 Health and Retirement Study dataset. The sample consisted of 9,612 grandparents, 2,595 of whom were providing grandchild care. Linear regression models were used to analyze how depressive symptoms were influenced by grandchild care provision and expectations of future care from adult children. Future care is measured as expectations from (1) any adult child, and (2) from the same adult child for whom the older parent provides grandchild care. Provision of grandchild care was not significantly related to grandparents' number of depressive symptoms. Among grandparents who provided grandchild care, both expecting any adult child and expecting the same adult child were associated with reporting fewer depressive symptoms. Expecting any adult child to provide future care showed a stronger effect than expecting the same adult child to provide future care. The results suggest that expectations of general reciprocity within the family system, rather than specific dyadic reciprocity, may be more important for a caregiving grandparent's emotional well-being. Providing grandchild care while expecting future care from adult children can indicate a sense of social capital within an intergenerational family system. Expecting support reciprocity from adult children may be a protective factor that allows caregiving grandparents to feel more secure about their future care needs, and consequently, less depressed.

### HIPPOCAMPAL GLUTAMATE MODULATION DURING MEMORY ENCODING: ASSOCIATION WITH AGE AND SUBFIELD VOLUMES

Chaitali Anand,[1] Roya Homayouni,[1] Qijing Yu,[1] Sruthi Ramesh,[1] Dalal Khatib,[1] Cheryl Dahle,[1] Jeffrey Stanley,[1] and Naftali Raz[1], *1. Wayne State University, Detroit, Michigan, United States*

Hippocampal glutamatergic activity plays a pivotal role in memory consolidation, including the ability to form novel associations that declines with age. To test whether glutamatergic dysfunction may underpin age-related memory declines, we examined in vivo age differences in hippocampal glutamate modulation during encoding of associations, and its relationship with hippocampal subfield volumes. Proton functional magnetic resonance spectroscopy was performed on 32 young (25.1±2.8 years; 18 females) and 16 older (65.9±2.7 years; 7 females) adults to measure changes in hippocampal (randomly assigned right or left) glutamate during an object-location paired association learning task (with 12 cycles of encoding-retrieval epochs). Volumes of the dentate gyrus&CA3, CA1, subiculum, and entorhinal cortex were manually measured from T2-weighted MRI images. Memory performance differed between the age-groups [$F(1, 46)=8.56$, $p<.01$], with the older attaining a lower asymptote [$t(46)=2.93$, $p<.05$] compared to the younger. Age differences in glutamate were observed only during encoding (age-group x epoch: $F(3,137)=5.28$, $p<.01$), and varied over the epochs. Young adults showed increased glutamate during the first four encoding epochs of each cycle, with levels remaining high thereafter. Old adults evidenced a decrease in glutamate during the first four epochs, and a slow, sustained ramping-up afterwards. Including both age-groups, the maximum change in glutamate, calculated using the maximum and minimum levels during encoding, was positively associated with CA1 [$F(2,39)=4.28$, $p<.05$] and the dentate gyrus&CA3 volume [$F(2,39)=4.4$, $p<.05$], after correcting for multiple comparisons. Glutamate modulation specific to encoding may underlie age-related memory declines and be related to selected hippocampal subfield volumes.

### PURPOSE AND NUMBER OF CHRONIC HEALTH CONDITIONS AMONG OLDER ADULTS

Janis Sayer,[1] Jennifer L. Smith,[1] Cate O'Brien,[1] Joseph G. Bihary,[1] Dugan O'Connor,[1] and Ajla Basic[1], *1. Mather LifeWays Institute on Aging, Evanston, Illinois, United States*

An overwhelming three-quarters of persons age 65 and over have multiple chronic health conditions (Gerteis et al., 2014). With a growing population of older adults, understanding the factors that predict health and reduce the risk of chronic disease is critical. Recent evidence finds that a high sense of purpose- "the belief that one's life is purposeful and meaningful" (Ryff & Keyes, 1995, p. 720)- is associated with positive health outcomes among older adults. This study investigated the association between purpose and number of chronic conditions among older adults, and whether the relationship depended on age. The study included 6148 older adults (mean age=83.8) who participated in a larger study on wellness. Participants completed a survey that included a measure of sense of purpose and questions about chronic health conditions. Data were analyzed controlling for demographics, optimism, pessimism, social contact, BMI, physical activity, and smoking. Lower levels of purpose were significantly associated with higher numbers of chronic conditions. There was a significant interaction between purpose and age, such that relatively younger older adults with high levels of purpose had fewer chronic conditions. There was no relationship between purpose and number of chronic conditions for the oldest adult participants. The results add new findings to the body of research that demonstrates that sense of purpose is associated with chronic disease. As sense of purpose is modifiable, interventions that increase purpose among older adults, with an emphasis on the youngest-old, should be developed and implemented.

### TRANSITION TO A MORE EVEN DISTRIBUTION OF PROTEIN INTAKE IS ASSOCIATED WITH ENHANCED FAT LOSS IN OBESE OLDER ADULTS

Samaneh Farsijani,[1] Jane A. Cauley,[1] Adam J. Santanasto,[1] Nancy W. Glynn,[2] Robert M. Boudreau,[1] and Anne B. Newman[1], *1. University of Pittsburgh, Pittsburgh, Pennsylvania, United States, 2. University of Pittsburgh, Department of Epidemiology, Pittsburgh, Pennsylvania, United States*

Background: Optimization of intentional weight loss in obese older adults, through preferential fat mass reduction, is challenging, as the concomitant lean mass loss may exacerbate sarcopenia. Here, we assessed whether changes in within-day protein intake distribution are related to improvements in body composition in overweight/obese older adults during a hypocaloric and exercise intervention. Methods: Thirty-six community-dwelling, overweight-to-obese (BMI 28.0-39.9 kg/m2), sedentary older adults (aged 70.6±6.1 years) were randomized into either physical activity plus successful aging health education (PA+SA; n=15) or physical activity plus weight loss (PA+WL; n=21) programs. Body composition (by CT and DXA) and dietary intake (by three-day food records) were determined at baseline, 6-month, and 12-month follow-up visits. Within-day protein distribution was calculated as the coefficient of variation of protein ingested at breakfast [5:00–10:59], lunch [11:00–16:59] and dinner [17:00–1:00]. Secondary analysis was performed to determine associations between changes in protein intake distribution and body composition. Results: In both groups, baseline protein intake was skewed towards dinner. The pattern of protein intake changed towards a more even within-day distribution in PA+WL, but it remained unchanged in PA+SA. Transition towards a more even pattern of protein intake was independently associated with a greater decline in BMI ($P<0.05$) and abdominal subcutaneous fat ($P<0.05$) in PA+WL. However, changes in protein CV were not associated with weight loss in PA+SA. Conclusion: Our results show that mealtime distribution of protein intake throughout the day was associated with improved weight and fat loss under hypocaloric diet combined with physical activity.

### CHILDREN CAREGIVERS' EXPERIENCES OF OLDER ADULTS WITH DEMENTIA IN CARE TRANSITION: A QUALITATIVE SYSTEMATIC REVIEW

Rungnapha Khiewchaum[1], *1. Sinclair School of Nursing, University of Missouri, Columbia, Columbia, Missouri, United States*

Background/Purpose: Young and adult children have experienced caring for people who have been diagnosed with dementia. Caregiving needs affect family members who become the primary caregivers in care transition from hospital to home. This study aims to synthesize primary qualitative research on the experiences of children caregivers of older adults with dementia. Method: This is a systematic review describing young and adult child caregivers' experiences in caring for patient with dementia in home-based care. Data sources were published literature written in English from CINAHL, Scopus, PubMed, and PsychoINFO (published from January 1976 to October 2018). The thematic synthesis approach was also applied to generate theory generating meta-synthesis research (TGMS). and to describe the process of caring for demented patients by caregivers. Result: Eight primary studies reporting 388 potential studies were included. Four themes emerged: 1) well-being which included encouraging and destructive well-being; 2) role transition which included positive or negative role transformations; 3) caregiver needs which included medical and nursing information or knowledges and health care services/community services; and 4) the challenge of dementia which included symptoms of dementia which were impairing. Conclusion The findings of this meta-synthesis study support evidence of well-being among adult children in caring for people with dementia in transition phases. We present thematic synthesis that could be useful to professionals working with caregivers and patients with dementia. We suggest that research importance should shift towards the development and evaluation of care transitions intervention, especially professionals preparing support after diagnosis.

### ANGER, LOVE, SADNESS: DO EMOTION WORDS HAVE THE SAME SEMANTIC MEANING ACROSS AGE-GROUPS?

Madeline J. Nichols,[1] Jennifer A. Bellingtier,[2] and Frances Buttelmann[3], *1. Furman University, Greenville,*

*South Carolina, United States, 2. Friedrich Schiller University Jena, Jena, Germany, 3. Friedrich-Schiller-Universität, Jena, Thüringen, Germany*

Every day we use emotion words to describe our experiences, but past research finds that the meanings of these words can vary. Furthermore, historical shifts in language use and experiential knowledge of the emotions may contribute to age-differences in what these emotion words convey. We examined age-related differences in the valence, arousal, and expression connoted by the words anger, love, and sadness. We predicted age-related differences in the semantic meanings of the words would emerge such that older adults would more clearly differentiate the positivity/negativity of the words, whereas younger adults would report higher endorsement for the conveyed arousal and expression. Participants included American and German older adults (N=61; mean age=68.98) and younger adults (N=77; mean age=20.77). Using the GRID instrument (Swiss Center for Affective Sciences, 2013), they rated each emotion word for its valence, arousal, and expression when used by a speaker of the participant's native language. Across emotions and dimensions, older adults were generally more moderate in their understanding of emotion words. For example, German older adults rated anger and sadness as suggesting the speaker felt less bad and more good than the younger adults. American older adults rated love as connoting the speaker felt more bad and less good than younger adults. Arousal ratings were higher for German younger, as opposed to older, adults. Cultural differences were most pronounced for sadness such that German participants gave more moderate answers than American participants. Overall, our research suggests that there are age-related differences in the understanding of emotion words.

### LIPOPOLYSACCHARIDE BINDING PROTEIN IS ASSOCIATED WITH LOWER CVD RISK IN OLDER ADULTS

Lisa Roberts,[1] and Thomas W. Buford[1], *1. University of Alabama at Birmingham, Birmingham, Alabama, United States*

Intestinal (i.e. "gut") permeability may be related to cardiovascular disease (CVD) risk, but biomarkers for gut permeability are limited and associations with CVD risk are unknown – particularly among older adults. This cross-sectional study aimed to determine if serum biomarkers related to gut permeability [intestinal fatty acid-binding protein (iFABP)] and bacterial toxin clearing [cluster of differentiation 14 (CD14), lipopolysaccharide binding protein (LBP)] are associated with CVD risk among older adults. Older adults ($n = 38$, $72.0 \pm 7.1$ years old) were stratified by CVD risk category (Adult Treatment Panel: moderate, high-moderate, high). One-way ANOVAs determined differences in each biomarker by risk category, and associations with risk score were evaluated via linear regression and/or Pearson correlations. LBP ($p < 0.001$), but not iFABP and CD14 (p's > 0.05), was significantly different between CVD risk categories. Post-hoc tests indicated LBP was higher in the moderate risk compared to both higher risk categories ($p < 0.005$). LBP was a significant predictor of 10-year CVD risk ($\beta = -0.629$; $p = 0.001$) and evaluation of individual components in the risk score demonstrated a moderate, positive correlation of LBP with total cholesterol ($r = 0.326$, $p = 0.046$). Higher circulating concentrations of LBP were associated with lower CVD risk among older adults. Further, total cholesterol was positively associated with circulating levels of LBP. Importantly, cholesterol assists LBP in clearing bacterial toxins from circulation by transporting toxins for removal. These data suggest LBP may be a key component in reducing CVD risk in older adults.

### MUSIC IN THE LIFE COURSE: A FOCUS ON DEMENTIA

Amanda Couve,[1] and Joseph A. Kotarba[2], *1. Encompass Health - Hospice, San Antonio, Texas, United States, 2. Texas State University, San Marcos, Texas, United States*

The music experiences of Baby Boomers (born 1946 to 1965) continue to evolve as they age, with the Beatles and the Blues remaining key features. Baby boomers grew up in the post-World War II period when the variety of media technologies and outlets grew tremendously. Baby boomers continue this trend to experience and enjoy music through media such as theme cruises, community center activities, formal concerts, TV music awards programs, iPhones, and house concerts. The value of music in caring for the increasing number of Baby Boomers living with dementia is also increasing. For example, music can help patients recall pleasant and calming moments in their past, while helping enhance body movement and balance through dance. The audience's' takeaways will be ideas for creative marketing of music to the elderly; for integrating music into senior living situations, and for celebrating technology's contributions to the culture of aging.

### THE ROLE OF FOXO3 TRANSCRIPTION FACTOR IN ALZHEIMER'S DISEASE PATHOLOGY

Shuqi Du,[1] Laure Maneix,[1] Qinghao Zhang,[1] Ying-Wooi Wan,[1] and Hui Zheng[1], *1. Baylor College of Medicine, Houston, Texas, United States*

Alzheimer's disease (AD) is a progressive and degenerative brain disease and age is one of its strongest risk factors. Aging is a complex process but it is reasonable that a delayed aging process may lower the risk of AD or postpone its pathogenesis. Studies in C. elegans revealed that the activity of DAF-16 is required for the life span extension in many long-lived strains. The mammalian homologs for DAF-16 are the Forkhead box O (FOXO) transcription factors. FOXO3, one of the members in the FOXO family, has been identified in several studies as a susceptibility gene for human longevity. I found that the expression level of Foxo3 in the mouse brain decreases with age or in an AD mouse model. To further study the role of Foxo3 in AD, I generated Foxo3 conditional knockout mice which depletes Foxo3 in neural cells. These mice have reactive astrogliosis in the cortex and also upregulation of some astrocytes specific markers like Gfap and Aqp4. In vitro culture of primary astrocytes from the knockout mice shows impaired respiratory capacity in the Seahorse mito stress test, which corresponds to the expression level change of metabolic genes like Acot1. When we bred the knockout mice with 5xFAD, a mouse model for amyloid pathology. I found increased plaque load and core plaque size in the cortex of the mice with Foxo3 deficiency. This study shows the critical function of FOXO3 in maintaining brain homeostasis and provide new insights into AD pathogenesis and drug discovery.

### FACTORS INFLUENCING HEALTH BEHAVIORS OF KOREAN OLDER ADULTS WITH CORONARY ARTERY DISEASE

So Young Shin,[1] and Ji Mi Mun[2], *1. Inje University, Busan, Korea, Republic of, 2. Busan Veterans Hospital, Busan, Korea, Republic of*

Purpose: The purpose of this study was to examine the effects of disease-related knowledge, depression, and family support on health behaviors of older patients with coronary artery disease. Methods: The subjects were 139 older patients with coronary artery disease who had visited the internal medicine outpatient clinic at one general hospital located in metropolitan city B, Korea. A set of self-reported questionnaire was administered to assess general characteristics, disease-related knowledge, depression, family support, and health behaviors of the subjects. Collected data was analyzed using descriptive statistics, t-tests, one-way ANOVA, Pearson correlation coefficients, and multiple regression. Results: The mean (±SD) age of the subjects was 70.86 (±4.70) years. Health behaviors of the subjects had significant negative correlations with disease-related knowledge ($r=-.17$, $p=.050$) and depression ($r=-.32$, $p<.001$) while having a significant positive correlation with family support ($r=.67$, $p<.001$). In the final multiple regression analysis, factors influencing health behaviors of subjects were medication intake status ($\beta=-.17$, $p=.009$), depression ($\beta=-.15$, $p=.017$) and family support ($\beta=.61$, $p<.001$). The explanatory power of the subjects' medication intake status, disease-related knowledge, depression and family support on health behaviors was 48.9% ($F=33.97$, $p<.001$). Conclusion: Medication intake status, depression, and family support had significant influences on health behaviors of older patients with coronary artery disease. Improvements in medication intake, depression, and family support for older patients with coronary artery disease may be beneficial for their health behaviors, and ultimately, have a positive effect on their recovery from the disease and well-being.

### DEPENDENT INDEPENDENCE: REFRAMING AGING AND CAREGIVING AFTER CHINA'S ONE-CHILD POLICY

Chen Chi[1], *1. University of Chicago, Chicago, Illinois, United States*

Global aging has led to substantive demands for caregiving. In China, the country's birth control policy and recent economic downturn have exacerbated the situation. Given the decreasing care they have received, older Chinese online users 65-74 years of age from single-child families, however, have demonstrated more positive attitudes than negative ones—expressing satisfaction and speaking highly of their adult children's filial care. Why older Chinese come to appreciate their adult children's filial performance? Building upon the concept of "regeneration" (Cole and Durham, 2007), I propose the term "dependent independence" to highlight the mutually constitutive parent-adult children relations in China's single-child families. Employing a relational approach to aging and care in China, I analyze online posts from China's most populous information-sharing platform, Zhihu. As the major cause of the care crisis in China, the One-Child Policy, I argue, creates the solution at the same time by modernizing Chinese families such that new care values and preference, specifically affective bonds and independence, have become dominant. I first demonstrate that this sample of older Chinese adults have shifted care preferences from physical support to affective bonds. I then analyze their reformulation of care values from servility to older adults to adult children's independence and individual success. Revealing the changing values and preference of caregiving and family relations in China, this paper reminds us of what kind of future we aspire to, and what kind of values we cling to no matter as an adult child or an older adult.

### HOW A RURAL COMMUNITY ADAPTS TO AN AGING POPULATION USING AN ALLIANCE

Briana Sisofo,[1] and Anne R. Asman[2], *1. University of Utah, Salt Lake City, Utah, United States, 2. University of Utah, Department of Psychiatry, Salt Lake City, Utah, United States*

Older adult populations in rural America are growing more rapidly than ever before, but most do not have the facilities and resources to support the growth. Good health and quality of life are the driving forces in these communities. However, they need assistance in restructuring their support for older adult services and education, housing, social engagement opportunities, and care for aging-in-place. Summit County Utah is a federally recognized rural community and may act as a functioning model for recognizing and addressing these aging issues for other rural areas within the United States. The Summit County Aging Alliance (SCAA) is a unique union of multiple federal, state and local agencies and, concerned and influential citizens who are helping to create the programs and services needed such as the submission of grants, the reassessment of the senior center, the creation of open communication between county and city politicians, connection of locals with key stakeholders, and growth to geriatric medicine in the area. SCAA is one of the five priorities of the Summit County Health Department Mental Wellness Alliance. SCAA focuses on the issues that are crucial for an older adult to sustain a healthy lifestyle in a rural community.

### TELLING THE STORY OF ADULT PROTECTIVE SERVICES: CALIFORNIA'S IDENTIFICATION, SERVICES, AND OUTCOMES MATRIX

Pi-Ju (. Liu,[1] Zachary Hass,[1] Kendon Conrad,[2] Karen Conrad,[3] Jarmin C. Yeh,[4] Madelyn Iris,[5] Sara Stratton,[6] and Andrew Butler[7], *1. Purdue University, West Lafayette, Indiana, United States, 2. the School of Public Health at the University of Illinois at Chicago, Chicago, Illinois, United States, 3. University of Illinois at Chicago, Chicago, Illinois, United States, 4. University of California, San Francisco, California, United States, 5. Northwestern University, Evanston, Illinois, United States, 6. San Francisco Adult Protective Services, San Francisco, California, United States, 7. University of California, Berkeley, Berkeley, California, United States*

Adult Protective Services (APS) is responsible for investigating reports of abuse, exploitation, and neglect among vulnerable adults. California's APS program investigates approximately 15% of all abuse, neglect, and exploitation reports in the country. Once abuse or neglect is substantiated, caseworkers design and implement a service plan for clients to reduce future risk; however, APS intervention effectiveness have not been extensively investigated. In partnership with San Francisco and Napa APS, risk and harm of abuse were measured by type using standardized items from the Identification, Services, and Outcomes Matrix, which is derived from the validated Elder Abuse Decision Support Short Form during case investigation (before APS interventions) and at case closure (after APS interventions). Data from 1,472 older adults' (on average 78 years old; 57% females) served by APS during the six-month pilot demonstration showed the reduction of risk/harm in self-neglect (p<.001), neglect (p<.001), emotional abuse (p<.001), physical abuse (p=.002), and financial abuse (p<.001) after APS interventions. Effective interventions differ by type of abuse such that caregiver support (b=-.98, p<.10), emergency assistance (b=-1.14, p<.05), and client engagement (b=-1.85, p<.05) decreased self-neglect risk/harm; client engagement (b=-2.24, p<.05) decreased neglect by others risk/harm; case management services (b=-1.17, p<.05) decreased physical abuse risk/harm; and financial planning services (b=-3.99, p<.05) decreased financial abuse risk/harm. No services were identified as effective for emotional abuse. Since effective services differed by type of abuse, it is important to consider the etiology of abuse before implementing the services needed by older adults to effectively decrease harm/risk.

### DETERMINING THE PREVALENCE OF SARCOPENIA IN AN INPATIENT GERIATRIC POPULATION USING EWGSOP2 OR FNIH DEFINITIONS

Matthew M. Scholl,[1] Elena Volpi,[2] and Rachel R. Deer[2], *1. University of Texas Medical Branch, Galveston, Texas, United States, 2. UTMB, Galveston, Texas, United States*

Sarcopenia has been recognized as a progressive and generalized skeletal muscle disorder leading to loss of strength, muscle mass, and function. It is associated with an increased likelihood of adverse outcomes like falls, fractures, physical disability, and mortality. International consensus groups continue providing new definitions and clinical cut-off points despite over a decade of work in this area. Thus, the purpose of this research was to determine the prevalence of sarcopenia using two of the most current operational definitions (Foundation of NIH Sarcopenia Project (FNIH) and the European Working Group on Sarcopenia in Older Persons 2 (EWGSOP2)). Our cohort of acutely hospitalized older adults was formed from combining data from two randomized controlled trials and one cross-sectional observational study. Testing during hospitalization included measures of: demographics, body composition (DEXA), physical function tests, psychological wellbeing and independence questionnaires, and chart review. These were used to analyze the cohort according to three main groupings of low physical performance, low muscle strength, and low lean mass. We compared multiple tests and cutoffs for each of the three groupings under the FNIH and EWGSOP2 definitions, which varied 3% for low lean mass up to 48% for tests of low physical performance. After examining the efficacy of each cutoff, we evaluated the differences between FNIH and EWGSOP2. In our cohort, the prevalence of sarcopenia was 15.79% by EWGSOP2 and 13.59% by FNIH. The groupings within FNIH and EWGSOP2 were found to be near identical across almost all measures despite the definitions' discrepancies in cutoff points.

### A TELEHEALTH DELIRIUM COACHING INTERVENTION FOR FAMILY CAREGIVERS OF OLDER ADULTS WITH ALZHEIMER'S DISEASE

Deborah A. D'Avolio,[1] Andra Opalinski,[1] and David Newman[1], *1. Florida Atlantic University, Boca Raton, Florida, United States*

The purpose of this study is to develop and evaluate the feasibility of a telehealth coaching intervention for delirium prevention among family caregivers (FCs) of community-dwelling older adults with dementia. This study used an explanatory mixed methods design in which survey data was augmented with semi-structured interviews. A purposive sample of 20 older adult dyads participated. The intervention consisted of 6-weeks of telephone coaching sessions. FCs conducted daily delirium assessments. We employed correlations and GLM to investigate the relationships between variables and the outcomes. Results: The model showed a statistically significant positive correlation between the Human Connection Scale and the SF-36 pretest domain of general health (r= .47, p= .04). There were statistically significant positive correlations between the Human Connection Scale and the SF-36 posttest domains of physical functioning (r= .54, p= .014) and general health (r= .76, p<.001). There were a small positive changes in mean scores on each domain between the pre- and post-test scores on the SF-36. The most impressive findings came from FCs identification of delirium using the FAM-CAM. These participants had no history of delirium but 6 of 20 (30%) reported at least one episode of delirium. The qualitative data revealed that FGs found weekly coaching sessions beneficial and supportive. The results suggests that the intervention has a meaningful impact on how we assess delirium in the community and warrants further study.

### ANGIOTENSIN (1-7) EXPRESSING LACTOBACILLUS DOSE-DEPENDENTLY BENEFITS THE GUT-BRAIN AXIS IN AGED RATS

Yi Sun,[1] Qiuhong Li,[2] Amrisha Verma,[2] Christy Carter,[1] and Thomas W. Buford[1], *1. University of Alabama at Birmingham, Birmingham, Alabama, United States, 2. University of Florida, Gainesville, Florida, United States*

Aging is associated with gut dysbiosis – a condition linked with altered central nervous system function (i.e the "gut-brain axis"). Age-related health benefits have been ascribed to the renin-angiotensin system (RAS), mediated partially via the angiotensin (1-7) or Ang(1-7) axis. This pre-clinical study explored dosing of a genetically modified probiotic expressing Ang(1-7) – which we previously showed to induce dose-dependent increases in circulating Ang(1-7) – in modulating the gut-brain axis. Twenty-nine male F344BN rats were randomized at 24 months of age to receive oral gavage of Ang(1-7) Lactobacillus paracasei (LP) zero (control), one, three, or seven times/week over 28 days. At day 29, samples of feces, serum and pre-frontal cortex (PFC) were collected. Microbiome taxonomic analysis of fecal samples was performed via 16S-based PCR. Serum samples were analyzed for tryptophan and downstream metabolites via LC-MS. PFC was evaluated for mRNA expression of select inflammatory cytokines. PCoA revealed that groups differed in the overall fecal microbiota community structure as determined by Unweighted UniFrac. Indices of alpha-diversity, including richness and phylogenetic diversity, displayed significant group differences – with the most dramatic effects observed in the 3-times/week group. Compared to control, serum serotonin and 2-Picolinic Acid were significantly increased in the 3-times/week group. The 3-times/week regimen also significantly reduced COX2, IL1β, and TNFα mRNA expression, and 7-times/week reduced COX2 and IL1β expression in PFC. Therefore, we conclude that short-term treatment with Ang(1-7) LP dose-dependently benefits the gut-brain axis in aged rats, with 3-times/week appearing to be the optimal dosing regimen.

### GAIT UNSTEADINESS AS AN INDICATOR OF COGNITIVE STATUS IN INDIVIDUALS WITH PERIPHERAL NEUROPATHY

Gu Eon Kang,[1] Jacqueline Yang,[1] He Zhou,[1] Changhong Wang,[1] Bareera Akhtar,[1] Brian xu,[1] Guillermo Beckmann,[1] and Bijan Najafi[1], *1. Baylor College of Medicine, Houston, Texas, United States*

Neuropathic individuals are at risk of falls, however potential impact of cognitive impairment in neuropathic individuals is not well-understood. Since cognitive impairment is considered an independent risk factor for falls, knowing its potential, additional impact may help better understand underlying mechanism of risk of falls in neuropathic individuals. We aimed to investigate stride-to-stride variability in neuropathic individuals with cognitive impairment (NP-Cog-Impaired) and without cognitive impairment (NP-Cog-Intact) during normal and dual-task walking. Neuropathic symptoms and cognitive status was measured using maximum vibration perception threshold (VPTmax) in the feet and the Montreal Cognitive Assessment (MoCA), respectively. We analyzed data from 19 NP-Cog-Impaired (8 men; 68.5±9.1 years; 29.0±6.2 kg/m2; VPTmax=27.2±12.1 volts; MoCA=19.6±2.4) and 25 NP-Cog-Intact (15 men; 66.5±9.1 years; 31.3±5.9 kg/m2; VPTmax=26.3±12.7 volts; MoCA=25.6±1.6). We collected movement data using five inertial sensors (LEGSysTM, BioSensics LLC, Watertown, MA) attached on the shanks, thighs and lower back. We used previously validated algorithm to calculate coefficient of variations (CV) of stride velocity and stride length. CV of stride velocity and stride length were significantly greater for the NP-Cog-Impaired group (11.07±5.22% and 7.31±3.20%, respectively) than for the NP-Cog-Intact group (7.31±3.20% and 4.81±2.80%, respectively) for dual-task walking but not for normal walking. Between normal and dual-task walking, CV of stride velocity and stride length increased 43.2% (significantly) and 46.4% (marginally), respectively, from normal walking to dual-task walking for the NP-Cog-Impaired group but not for the NP-Cog-Intact group. Results suggest that cognitive impairment may be an additional risk factor of falls in neuropathic individuals.

### PREDICTIONS OF RISK TOLERANCE ACROSS DISEASE SCENARIOS AMONG OLDER ADULTS

Catherine Ju,[1] and Brian Carpenter[1], *1. Washington University in St. Louis, St. Louis, Missouri, United States*

Advance care planning requires older adults to contemplate what kind of medical care they would prefer if faced with serious illness. Those decisions involve weighing risks of medical treatments with quality of life. The purpose of this project was to explore older adults' tolerance for risk in medical treatment scenarios. Thirty-three adults over age 60 (mean age = 74.9, SD = 8.9) were presented with four medical scenarios reflecting increasingly severe diseases (urinary incontinence, kidney failure, dysphagia, Alzheimer disease). For each, they were asked whether they would accept a treatment with increasing risk of death (5%, 50%, 95%). Participants also recorded demographic characteristics and completed a physical and mental health measure. Older adults were more willing to accept hypothetical treatments with lower risk of death (Cochran's Q = 27.8 - 39.0, p's < .001), and they were more willing to accept treatments when faced with more severe diseases (Cochran's Q = 8.7 - 29.8, p's < .05). There were no significant associations between risk tolerance and demographic and health characteristics. These results suggest that older adults are diverse in their willingness to accept medical treatments risks, at least when presented with hypothetical medical scenarios. Medical professionals and family members who collaborate with older adults should be aware of this diversity of preferences and check assumptions based in demographic and health characteristics. Given these nuances, interventions to promote communication about medical preferences are essential to patient-centered advance care planning and medical decision making.

### COMMUNITY-BASED SERVICES AND OLDER ADULTS: A COMBINATION IN MENTAL HEALTH PROMOTION

GLAUCIA SALGADO,[1] and Sharon Koehn[1], *1. SIMON FRASER UNIVERSITY, Vancouver, British Columbia, Canada*

Mental health promotion among older adults has been considered an important goal by the World Health Organization (WHO, 2017). Mental health has been understood as not necessarily the absence of mental illness, but in fact, points on a continuum that although are not mutually exclusive, and it may intersect at times (Cowen, 1991; Keyes & Westerhof, 2012). With such complex health component, medical practices—although extremely critical in many cases—are often one factor of in the equation. Other practices, such as positive relationships among individuals and measures to tackle isolation are relevant and successful when planning practice for mental health promotion (Wister & McPherson 2014; Newall & Menec, 2019). A study done at a community-based seniors service in Vancouver – Canada shows that these spaces are considered a critical resource for visible minority older adults. Two focus groups were conducted at a community-based senior service with visible minority older adults between ages 55 to 80 years old. Results show that visible minority older adults strongly rely on this sector to maintain the connection with the society, and to the services provided in the wider community. Community-based seniors service provide opportunities for social inclusion and interactions, learning new things, and it has an inverse association with feeling isolated and lonely at home—a constant issue stated in the research. These findings indicate the critical role of this sector in ameliorating and promoting the mental health of visible minority older adults.

### ADVANCE CARE PLANNING COMMUNICATION: AN INTERACTIVE WORKSHOP WITH ROLE-PLAY FOR STUDENTS AND PRIMARY CLINICIANS

Ben A. Blomberg,[1] Catherine Quintana,[1] Jingwen Hua,[1] Leslie Hargis-Fuller,[1] Jeff Laux,[1] and Margaret Drickamer[1], *1. University of North Carolina, Chapel Hill, North Carolina, United States*

There is a need for increased clinician training on advance care planning (ACP). Common barriers to ACP include perceived lack of confidence, skills, and knowledge necessary to engage in these discussions. Furthermore, many clinicians feel inadequately trained in prognostication. There is evidence that multimodality curricula are effective in teaching ACP, and may be simultaneously targeted to trainees and practicing clinicians with success. We developed a 3-hour workshop incorporating lecture, patient-oriented decision aids, prognostication tools, small group discussion, and case-based role-play to communicate a values-based approach to ACP. Cases included discussion of care goals a patient with severe COPD and one with mild cognitive impairment. The workshop was delivered to 4th year medical students, then adapted in two primary care clinics. In the clinics, we added an interprofessional case applying ACP to management of dental pain in advanced dementia. We evaluated the workshops using pre-post surveys. 34 medical students and 14 primary care providers participated. Self-reported knowledge and comfort with ACP significantly improved; attitudes toward ACP were strongly positive both before and after. The workshop was well received. On a seven-point Likert scale, (1=Unacceptable, 7=Outstanding), the median overall rating was 6 ("Excellent"). In conclusion, we developed an ACP workshop applicable to both students and primary clinicians. We saw improvements in self-reported knowledge and comfort with ACP, though long-term effects were not studied. Participants found the role-play especially valuable. Most modifications for primary care clinics focused on duration rather than content. Future directions include expanding the interprofessional workshop content.

### HEALTHCARE ENCOUNTERS AS PREDICTORS OF DEATH ANXIETY IN OLDER ADULTS

Todd D. Becker[1], *1. University of Maryland, Baltimore, Baltimore, Maryland, United States*

Literature suggests 10–15% of Americans experience death anxiety (DA). While DA is a response to heightened death awareness, research has not fully explored the link between DA and healthcare utilization. 2012 Health and Retirement Study data were used. Three binary logistic regressions analyzed hospitalization, ER admission, nursing facility placement, and outpatient visits' abilities to predict DA (0 = "none," 1 = "at least some") over the past week. Model 1 included demographics and all four types of healthcare encounters. One item from the Beck Anxiety Inventory assessed DA. Most subjects in the sample (N = 7,185) were married (n = 4,302), and White (n = 5,479). Fifteen percent reported ER admissions, 24.6% reported hospitalizations, 1.1% reported nursing facility placements, and 91.5% reported outpatient visits. Regression results from each model showed nursing facility placement and outpatient visits to be statistically significant predictors of DA across all models (OR ranges = 2.23–2.38 and 1.23–1.25 respectively, $p < .05$ in all models). Hospitalization predicted DA in Models 1 (OR = 1.30, $p = .004$) and 2 (OR = 1.46, $p < .001$). Model 3 showed ER admission (OR = 1.52, $p < .001$) predicted DA. Results from each model indicated individuals who experienced nursing facility placements exhibited double the odds of experiencing DA, compared to those who did not. Outpatient visits were the weakest predictor of DA in each model. DA's association with healthcare utilization provides implications for providers. Future research should explore the relation between DA and health outcomes.

### NEUROTICISM PREDICTS INFORMANT-REPORTED COGNITIVE DECLINE THROUGH HEALTH BEHAVIORS

Rachel D. Best,[1] Patrick J. Cruitt,[1] and Patrick L. Hill[1], *1. Washington University in St. Louis, St. Louis, Missouri, United States*

Our current study attempts to better understand the relationship between personality and cognitive decline. In this study, we analyzed whether health behaviors act as mediating variables for the relationship between personality and cognitive decline. Additionally, we were interested to see how personality influences different health behaviors, and which health behaviors in particular are predictive of cognitive decline. In addition to analyzing the composite score of health behaviors in relation to personality and cognitive decline, we analyzed each of its four components (wellness maintenance, accident control, traffic risk, and substance abuse; Vickers, Conway & Hervig, 1990). To measure cognitive decline, we used the Ascertain Dementia Eight Item Scale, an informant-report screening measure. Personality has consistently been linked to cognitive decline (Curtis, Windsor & Soubelet, 2015; Chapman, Duberstein, Tindle, Sink, Robbins, Tancredi & Franks, 2013; Low, Harrison & Lackersteen, 2013), but has not yet been analyzed with an informant report measure. Informant-report may be more reliable than self-report when measuring cognitive decline because participants who exhibit cognitive impairment may not be equipped or willing to report about their own cognitive ability. We found that neuroticism significantly predicted informant-reported cognitive decline and that this relationship was mediated by health behaviors, specifically, wellness maintenance. Wellness maintenance was the only category of health behavior that predicted informant-reported cognitive decline. Surprisingly, conscientiousness was unrelated to informant-reported cognitive decline as were extraversion, agreeableness, and openness to experience.

### OLDER AND MORE MINDFUL? ASSOCIATIONS OF MINDFULNESS CHARACTERISTICS AND WELL-BEING VARY WITH AGE

Leeann Mahlo,[1] and Tim D. Windsor[1], *1. Flinders University, Adelaide, South Australia, Australia*

Research examining how mindfulness confers benefits for well-being is in its infancy. Furthermore, few studies have considered the positive effects of mindfulness on psychological functioning from a lifespan perspective. The present study aimed to examine a recently proposed model of mindfulness and whether the importance of the proposed mechanisms for well-being varied as a function of age. A community-based sample of 623 participants aged between 18 and 86 years (M = 48.78, SD = 16.74) was recruited via an internet-based research platform. Participants completed questionnaire measures of mindful characteristics (i.e., present-moment attention, nonjudgment, interoception, acceptance, nonattachment, and decentering), flexible goal adjustment, and well-being. Parallel mediation analyses using bootstrapping showed that both present-moment attention and nonjudgment provided significant pathways to (a) well-being through acceptance, nonattachment, and decentering; and (b) flexible goal adjustment through nonattachment and decentering. Furthermore, most aspects of mindfulness were positively associated with age. Conditional process analyses revealed that the direct relationships between (1) present-moment attention and well-being, (2) nonjudgment and well-being, and (3) decentering and flexible goal adjustment were significant for adults from around age 40 and became stronger with increasing age. The findings provide preliminary support for a recently proposed model of mindfulness and suggest that present-moment attention, nonjudgment, and decentering may become especially important for well-being across the second half of life. In particular, these aspects of mindfulness may represent psychological qualities that require a relatively modest investment of physiological and cognitive resources and can be targeted in interventions designed to enhance well-being in later adulthood.

### PREDICTION OF THE NUMBER OF AND CARE COSTS FOR DISABLED OLDER ADULTS FROM 2020 TO 2050 IN URBAN AND RURAL AREAS IN CHINA

liangwen zhang,[1] and Ya Fang[2], *1. Center for Aging Health Research, School of Public Health, Xiamen University, Xiamen, China, China, 2. School of Public Health, Xiamen University, Xiamen, China*

Disability for the elderly has become a crucial policy concerns in rapidly aging Asia counties, especially in China. This study aimed to predict the trend of the number of and care costs for disabled older adults from 2020 to 2050 in urban and rural areas in China. Population Administration Decision Information System was used to predict the population of China by urban and rural areas and age group from 2020 to 2050. Monte Carlo simulation and Policy Simulation Model were used to estimate the number and care costs of disabled elderly between urban and rural areas, based on the Chinese latest census data, statistical yearbook, and national survey database. The total disabled population rises rapidly from 43.75 million in 2020 to 91.4 million in 2050, of which 69.7% were urban adults. Compared with the values in 2020, the growth rates of the adults with mild, moderate and severe disabilities were 108%, 104% and 120% in 2050, respectively. The value were 167% and 39% in urban and rural areas, respectively. By 2050, the total care costs increase from 538.0 billion yuan in 2020 to 8530.8 billion yuan, of which 80.2% occurs in urban areas. The predicted results indicate that the numbers and care costs for disabled older adults increase sharply from 2020 to 2050, especially in urban areas of China. It provided a series of evidence for the establishment of the long-term care insurance system in China.

### A COHORT LONGITUDINAL STUDY OF SOCIAL CAPITAL AND DEPRESSIVE SYMPTOMS IN THE WISCONSIN LONGITUDINAL STUDY

Kyle A. Carr[1], *1. Boston College, Chestnut Hill, Massachusetts, United States*

This study examined the association between the two dimensions of social capital, structural and cognitive, and depression, as well as investigating their within- and between-effects. Using the Wisconsin Longitudinal Study, I applied a multi-level 2-wave longitudinal analysis, over a 7-year period, to examine these two dimensions of social capital influence on individual's depressive symptoms at both the between- and within person levels. Results suggest both dimensions of social capital are negatively related with levels of depressive symptoms for individuals. The within-person changes for both self-efficacy and sense of belonging were larger than the estimates of between-effects, while trust and structural social capital effects were equal. These findings add to the growing body of literature examining depressive symptoms in late life, while also providing evidence for policymakers to hone in on key areas that can address depressive symptoms with social capital interventions.

### NUTRITIONAL INTAKE AND STATUS AND PHYSICAL FUNCTION OF OLDER ETHNIC MINORITIES: A LONGITUDINAL STUDY

Evans A. Asamane,[1] Carolyn A. Greig,[1] and Janice L. Thompson[1], *1. School of Sport, Exercise and Rehabilitation Sciences, University of Birmingham, Birmingham, West Midlands, United Kingdom*

There are limited longitudinal data regarding nutritional intake and status, and physical function in community-dwelling ethnically diverse older adults. This study explored these variables and their relationship at baseline (n=100) and after 8-months (n=81) among community-dwelling ethnically diverse older adults (≥60 years) in Birmingham, United Kingdom. Multiple pass 24-hour dietary recalls and the Mini Nutritional Assessment-Short Form assessed nutritional intake and status, respectively. The Short Physical Performance Battery(SPPB) and handgrip strength measured physical function. Linear and multinomial regression analyses were used to predict the relationship between nutritional intake, status and physical function. Mean(SD) age was 70(8.1) years (60% male), with 62% of the sample being obese. Significant decreases in intakes of vitamin B6(0.88-0.77mg/day, p=0.014); vitamin B1(0.73-0.63mg/day, p=0.029); iron(6.16-5.85mg/d, p=0.045); folate(113.23-106.66µg/d, p=0.043); and magnesium(154.54-144.59mg/d, p=0.031) occurred over time. At both timepoints, across sexes, daily intakes of all micronutrients except vitamin B12, phosphorus and manganese were below the Recommended Nutrient Intakes. There were significant declines in SPPB scores(Z=-4.01, p<0.001) and nutritional status(Z=-2.37,p=0.018) over time. At baseline, younger age, better nutritional status, and higher vitamin D and fibre intakes were associated with higher SPPB scores. At follow-up, higher baseline SPPB scores (OR=0.54 95% CI:0.35, 0.81) were associated with reduced decline in nutritional status. The observed declines in nutritional status and physical function, and the inadequate nutrient intakes in the absence of weight loss within eight months pose serious challenges to healthy ageing. There is an urgent need to re-evaluate and tailor appropriate dietary advice for this population to support them to age healthily.

### THE PREVALENCE OF ORTHOSTATIC HYPOTENSION AMONG HOSPITALIZED OLDER ADULTS AT RISK FOR FALLING

Denise L. Lyons,[1] and Kathleen Schell[2], *1. Christiana Care Health system, Newark, Delaware, United States, 2. University of Delaware, Newark, Delaware, United States*

Orthostatic hypotension (OH) may cause falls in hospitalized older adults. OH is a sustained drop of at least 20 mm Hg for systolic blood pressure or at least 10 mm Hg for diastolic blood pressure when changing position from supine to sitting, sitting to standing, or supine to standing. A recent systematic review revealed an inconsistent relationship between OH and falls. Orthostatic vital signs (OVS) measurement is often included in fall prevention initiatives. Some experts suggest that the time required to collect OVS and the possibility of measurement inaccuracy by nurses make this bedside screening unnecessary. The study aims were to determine: 1) the prevalence of OH, 2) if those older adults with documented OH experienced falls, and 3) the influence of medications known to be associated with OH and falls. Medication categories included antihypertensives, dopamine agonists, antipsychotics and antidepressants. A retrospective chart review was conducted on a convenience sample of 8,474 older adults on two Acute Care of the Elderly units at a large health system in the mid-Atlantic between 2015 and 2018. Results were determined using contingency tables and Chi-square analysis. More complex relationships were pursued using log linear models. The overall OH prevalence was 46.9% at some point during their hospital stay. Over the three years, 68 patients of whom 62% were hypotensive (p=.034). There was no statistical association of OH with medications or co-morbidities. The results demonstrate that although prevalent in almost half of this sample, orthostatic hypotension did not lead to falls.

### EMBEDDING DEMENTIA-FRIENDLY TRAINING IN A HEALTH CARE SYSTEM

Ellen C. Schneider,[1] Maureen Dale,[2] Krista Wells,[1] John Gotelli,[3] Ellen Roberts,[4] Cristine B. Henage,[1] and Jan Busby-Whitehead[4], *1. University of North Carolina at Chapel Hill, Chapel Hill, North Carolina, United States, 2. University of North Carolina at Chapel Hill, Division of Geriatric Medicine, Chapel Hill, North Carolina, United States, 3. UNC Hospitals - Hillsborough Campus, Hillsborough, North Carolina, United States, 4. University of North Carolina at Chapel Hill, Division of Geriatric Medicine, Chapel Hill, North Carolina, United States*

Alzheimer's disease, the most common form of dementia, is now the 6th leading cause of death in the United States, affecting one in ten people over the age of 65. With our country's rapidly aging population, and age being the primary known risk factor for dementia, the number of people with dementia is expected to increase from 5.8 million in 2019 to 14 million in 2050. People with dementia are hospitalized more often and have prolonged stays, poorer outcomes, higher costs, and increased readmission rates. Hospital employees have expressed the desire to have specialized training to learn how to more effectively communicate with and provide better care to patients with dementia to minimize adverse outcomes and increase patient satisfaction. To better address these identified patient and hospital employee needs, the University of North Carolina's Center for Aging and Health (UNC CAH) is disseminating hospital-specific dementia-friendly training to four hospitals within the UNC Health Care System. The training is being delivered via online modules and follow-up didactic sessions to over 4,000 clinical and non-clinical staff who interact with patients. To monitor outcomes, pre and post training data is being collected on dementia patients' length of stay, readmission rates, and falls. The pilot project was conducted in 2019, and results of the pilot will be presented in the poster. The dementia-friendly hospital training initiative will prepare hospitals to provide better care for people with dementia, which should lead to improved health outcomes and more positive experiences for patients, caregivers, and hospital employees.

### THE IMPACT OF NEIGHBORHOOD CONNECTEDNESS ON LIFE SATISFACTION AND SPIRITUAL EXPERIENCES ACROSS THREE AGE GROUPS.

Stephen J. Fogle,[1] and Christopher M. Kelly[1], *1. University of Nebraska - Omaha, Omaha, Nebraska, United States*

This study operationalized the third dimension of Rowe & Khan's Successful Aging model, social engagement, as neighborhood connectedness. We examined 2820 older adults in the MIDUS III dataset to assess the impact of neighborhood connectedness on life satisfaction and daily spiritual experiences. A composite scale for neighborhood connectedness (Cronbach = .745) was created. Linear regression analysis was undertaken for life satisfaction on daily spiritual experience, neighborhood connectedness, neighborhood environment and age controlling for gender, co-habitation, income, and disability. Regression analysis was also conducted for daily spiritual experience on the same variables. Analysis for each outcome variable was run three times to explore changes across three age groups of older adults (55-69, 70-85, and 86-100). Results of regression analysis found frequency of daily spiritual experience was a substantial and significant predictor of life satisfaction for all age groups ($\beta$= .211, $\beta$= .191, $\beta$= .208) Additionally, regression analysis revealed a higher level of neighborhood connectedness was the most powerful predictor of daily spiritual experience across all age groups ($\beta$= .329, $\beta$= .312, $\beta$= .327) This study demonstrates the applicability of operationalizing the Successful Aging model's social engagement dimension as neighborhood connectedness. This study also contributes evidence of the impact of daily spiritual experience on life satisfaction. Finally, the study supplies promising new evidence linking neighborhood connectedness with spiritual well-being.

### MEASURING THE EFFECTS OF A CYCLING PROGRAM ON QUALITY OF LIFE OF OLDER ADULTS LIVING IN LONG TERM CARE

Victoria Cotnam,[1] and Aleksandra Zecevic[1], *1. Western University, London, Ontario, Canada*

Cycling Without Age (CWA) is a program offered in long-term care (LTC) homes around the world that allows older adults who are unable to ride a bicycle the pleasure of a bike ride again. Two residents sit in the front bench seat of a trishaw, and a volunteer bike pilot pedals the bike. A variety of anecdotal benefits have been reported and no study has rigorously measured the effects of this program. The purpose of this research is to measure the effects of the CWA program on happiness and quality of life of LTC home residents, through observation of an existing program in a Canadian LTC home. A total of 24 residents were purposefully recruited in a biking group (n=23) who were biked twice a week for 12 weeks, and a strolls group (n=16) who went for outdoors walks or wheelchair rides for the same period of time. Data on pain, cognition, social engagement, and aggressive behaviour was harvested from the Resident-Assessment Instrument – Minimum Data Set (RAI-MDS). Happiness was measured pre and post all bike rides and strolls using a visual analogue scale, and the LTC QoL assessment was used to assess QOL. Findings show that biking group scored higher on happiness after bike rides compared to before, as well as compared to strolls. Bike group QOL scores are higher at the end of the 12 weeks than were strolls group. In summary, CWA shows potential to increase QOL and happiness of residents living in LTC.

### KEY LEARNING FOR TWELVE UNECOM MEDICAL STUDENTS IMMERSED FOR 48 HOURS IN AN ACUTE CARE HOSPICE HOME DURING 2017-18

Emily Tamimie,[1] and Marilyn R. Gugliucci[1], *1. University of New England College of Osteopathic Medicine, Biddeford, Maine, United States*

Introduction: Most US medical schools are not able to provide practical experiences in end-of-life or palliative care. The University of New England College of Osteopathic Medicine Learning by Living 48 Hour Hospice Home Immersion (HHI) Project provides intense learning for second through fourth year medical students. Students are immersed into an acute care 18-bed in-patient hospice home for 48 hours to provide patient care, family support, and post-mortem. Students work with an Interprofessional staff team and independently. Methods: The HHI utilizes qualitative ethnographic/autobiographic research designs. Two key research questions include: (1) What is it like for ME to live in the Hospice Home for 48 hours? and (2) What will I take from this experience to my future as a practitioner?" Each student writes a journal during the three research stages; pre-fieldwork; fieldwork; post-fieldwork. Twelve 2nd year medical student journals randomly selected from 2017-18 immersions (N=24) were analyzed 5/2019-7/2019. Detailed qualitative manual and content analysis utilizing established interrater-reliability procedures were conducted on 300+ pages of data (UNECOM Morgane Student Research Fellowship). Results: Of the many themes identified, three key themes were notable for all 12 students for question one: Religion/Spirituality; Acceptance; and Reactions to Death. Key takeaways for question two included being: (a) able to have conversations about death; (b) at peace with death; (c) present with death, and knowing death is a part of life. Conclusion: Each student experienced his/her immersion differently, but all expressed this was a life-altering project providing critical education on hospice and end-of life care.

### A TYPICAL WEEK WITH MILD COGNITIVE IMPAIRMENT: A PHOTO-ELICITATION STUDY

Jenny Wool,[1] and Brenna N. Renn[2], *1. University of Washington, Seattle, Washington, United States, 2. University of Washington School of Medicine, Seattle, Washington, United States*

Mild cognitive impairment (MCI) is an important precursor to dementia syndromes and carries with it both public and personal health significance, yet affected individuals may experience stigma, fear, and reluctance to participate in research or access services. Identifying the experience of people with MCI may help develop research agendas, interventions, and other supports to better match patients' needs. To this end, we conducted photo-elicitation interviews with 11 community-dwelling adults aged 57-79 years with diagnosed MCI. Interviews took place remotely using teleconferencing software to reduce access barriers. Each semi-structured interview used 5-10 participant-generated photographs to elicit the experience of living with MCI, barriers to daily activities, and facilitators and supports. Interviews were transcribed, coded, and analyzed using Dedoose software. Qualitative analysis revealed themes of important activities, including physical activity, social engagement, and cognitive stimulation. Barriers presented by MCI included difficulty with former routines (e.g., cooking, finances), reduction of activities, and perceived stigma or fear of disclosure. Facilitators of daily activities included increased use of new strategies and environmental supports (e.g., calendars, smartphones), in addition to social and familial support. Multiple participants noted that the diagnosis of MCI led to opportunities for inner reflection and seeking a sense of inner calmness. Incorporating participant-generated images aided in data collection and facilitated discussion of sensitive topics with a cognitively impaired sample. Clinicians and researchers should support engagement in meaningful activities, assess barriers to important daily activities, and consider support to match the experience and needs of those with MCI.

### DO BLUEBERRIES ACTUALLY IMPROVE COGNITIVE PERFORMANCE? AN ANALYSIS OF PUBLICATION BIAS IN PUBLISHED RESEARCH

Christopher Brydges,[1] and Laura Gaeta[2], *1. Colorado State University, Fort Collins, Colorado, United States, 2. California State University, Sacramento, Sacramento, California, United States*

A recent published systematic review (Hein et al., 2019) found that consumption of blueberries could improve memory, executive function, and psychomotor function in healthy children and adults, as well as adults with mild cognitive impairment. However, attention to questionable research practices (QRPs; such as selective reporting of results and/or performing analyses on data until statistical significance is achieved) has grown in recent years. The purpose of this study was to examine the results of the studies included in the review for potential publication bias and/or QRPs. p-curve and the test of insufficient variance (TIVA) were conducted on the 22 reported p values to test for evidential value of the published research, and publication bias and QRPs, respectively. The p-curve analyses revealed that the studies did not contain any evidential value for the effect of blueberries on cognitive ability, and the TIVAs suggested that there was evidence of publication bias and/or QRPs in the studies. Although these findings do not indicate that there is no relationship between blueberries and cognitive ability, more high-quality research that is pre-registered and appropriately powered is needed to determine whether a relationship exists at all, and if so, the strength of the evidence to support this association.

### A RANDOMISED FEASIBILITY STUDY TO REDUCE SITTING IN OLDER ADULTS UNDERGOING ORTHOPAEDIC SURGERY

Justin A. Aunger,[1] Colin T. Greaves,[1] Evans A. Asamane,[1] Edward T Davis,[2] Anna C. Whittaker,[3] and Carolyn A. Greig[4], *1. University of Birmingham, Birmingham, West Midlands, United Kingdom, 2. The Royal Orthopaedic Hospital NHS Foundation Trust, Birmingham, West Midlands, United Kingdom, 3. University of Stirling, Stirling, Scotland, United Kingdom, 4. School of Sport, Exercise and Rehabilitation Sciences, University of Birmingham, Birmingham, West Midlands, United Kingdom*

Osteoarthritis is a prevalent condition in older adults that causes many patients to require a hip or knee replacement in order to improve quality of life and reduce pain. Reducing sedentariness prior to surgery may aid in improving physical function and post-operative outcomes. Thus, we performed a pragmatic randomised controlled feasibility study with 2:1 allocation into intervention or usual care in Birmingham, UK. The intervention involved multiple techniques to reduce sedentary behaviour, including motivational interviewing and action planning. The primary outcome was feasibility, assessed using mixed methods. We included exploratory measures to inform a future definitive trial, such as ActivPal3 objective measurement of physical activity and sedentariness, Short Physical Performance Battery (SPPB), Basic Psychological Needs questionnaire, and serum cardiometabolic biomarkers. Assessments were at baseline, one-week pre-surgery, and six-weeks post-surgery. We recruited 35 participants aged 64-87 years eight weeks before hip or knee arthroplasty. The study was found to be feasible with some modifications. Within-group comparisons showed that the intervention group significantly improved their physical function (SPPB) score by 0.71 points (95% CI 0.07 to 1.36, $p=0.032$), a clinically significant increase. Additionally, those in the intervention group retained to post-surgery trended towards a mean sedentary time reduction of 66 min.d-1 ($p=0.497$). In this older mobility-limited surgical population it is feasible to use behavioural techniques to displace sedentary time to activity. Future trials should further assess the effect of reducing sedentariness on health in larger samples of older adults with osteoarthritis undergoing orthopaedic surgery.

### GIVE A PIECE OF YOUR MIND: A CONTENT ANALYSIS OF A FACEBOOK SUPPORT GROUP FOR DEMENTIA CAREGIVERS

Jenny A. Lagervall,[1] Madeline R. Lag,[1] Sophie Brickman,[1] Rebecca E. Ingram,[1] and Leilani Feliciano[1], *1. University of Colorado Colorado Springs, Colorado Springs, Colorado, United States*

The online environment offers individuals a means of obtaining information, support, and social connection. Older adults are growing users of the internet1. Online support groups (e.g., Facebook groups) have been found to provide health-related information and encourage mental well-being2. They may be particularly advantageous for caregivers of individuals diagnosed with dementia, as it is difficult to leave loved ones with dementia alone. However, the mechanisms by which online support groups engage caregivers, and the content of support, have yet to be explored. In the current study, content from 100 posts from a private Facebook caregiver support group were evaluated for gender of post author, relationship to the person receiving care, distress, emotional tone, grief reaction, caregiver burden, and coping strategy. Results indicated that caregiver distress was associated with the presence of grief reactions, negative emotional tone, and higher caregiver burden. Utilizing venting as a coping strategy was associated with higher caregiver burden, similar to what is observed in a traditional in-person support group. Online communication for caregivers may provide an indication of caregivers' psychological well-being, as specific coping strategies and grief reactions indicated higher levels of caregiver burden and distress. Research on interventions for dementia caregivers may benefit from a focus on online social support as a means of accessing caregivers and treatment delivery.

### HOW ARE SOCIAL ISOLATION AND LONELINESS RELATED TO COGNITIVE FUNCTIONING AMONG CHINESE OLDER ADULTS?

Rumei Yang,[1] HAOCEN WANG,[2] Eunjin L. Tracy,[3] Linda S. Edelman,[4] Katherine Sward,[5] George Demiris,[6] and Gary Donaldson[7], *1. School of Nursing Nanjing Medical University, Nanjing, Jiangsu, China, 2. University of Wisconsin-Madison, Madison, Wisconsin, United States, 3. University of Utah, SLC, Utah, United States, 4. University of Utah, Salt Lake City, Utah, United States, 5. University of Utah College of Nursing, SLC, Utah, United States, 6. University of Pennsylvania School of Nursing, Philadelphia, Pennsylvania, United States, 7. University of Utah School of Medicine, SLC, Utah, United States*

Older adults have increased risk of social isolation, loneliness, and cognitive functioning decline, but the relationships among these factors are not conclusive. We used the 2011 and 2012 waves of the harmonized China Health and Retirement Longitudinal Study to: 1) measure the association between social isolation and cognitive functioning among Chinese older adults within their cultural context, and 2) investigate the potential mediation mechanism of loneliness on this association. Specifically, we applied a multiple indicator multiple cause approach to determine whether the construct of social isolation is well defined by four indicators (social activity engagement, weekly adult children contact, caregiving for grandchildren, and living alone). We used structural equation modeling to examine the direct and indirect effects among variables of interest. The results demonstrated that three indicators of social isolation were significantly associated with cognitive functioning ($\beta$ =-0.26 to -0.28, all $ps<0.05$). The indirect effect of social isolation on cognitive functioning through loneliness was significant ($\beta$ = -0.15, $p<0.05$), indicating loneliness was an important mediator. After controlling for the indirect effect of loneliness, the direct effect of social isolation on cognitive functioning remained significant ($\beta$ =-0.83, $p<0.05$), suggesting a partial mediation effect. Our study confirms that social isolation contributes to cognitive functioning decline among Chinese older adults and that loneliness plays a mediating role. The findings suggest maintaining social relations and coping with feelings of loneliness are beneficial to older adults' cognitive functioning.

### THE IMPACT OF ALIVIADO DEMENTIA CARE HOSPICE EDITION TRAINING PROGRAM ON HOSPICE STAFF'S DEMENTIA SYMPTOM KNOWLEDGE

Catherine E. Schneider,[1] Alycia A. Bristol,[2] Ariel Ford,[1] Shih-Yin Lin,[2] and Abraham A. Brody, *1. New York University, New York, New York, United States, 2. NYU Rory Meyers College of Nursing, New York, New York, United States*

A lack of high quality dementia training for healthcare workers is a key barrier to effective care for persons with dementia (PWD), a vulnerable and increasing population across the care continuum. Hospice agencies in particular are underprepared to care for this population, although annually about 17% of hospice patients have a primary diagnosis of dementia and an additional 28% as a comorbidity. Aliviado Dementia Care-Hospice Edition is an interdisciplinary, evidence-based quality improvement program developed to assist hospice interdisciplinary teams in caring for PWD and their caregivers. Interdisciplinary hospice team members in two agencies were enrolled in online training modules, which addressed multiple areas including pain, behavioral and psychological symptoms of dementia (BPSD), and working with caregivers. They were also provided a toolkit to integrate training in daily practice. Changes in knowledge, confidence and attitudes were tested before and after training and paired t-tests were utilized to evaluate the program's effect. Thirty-five individuals completed the program and pre/post tests. Paired t-tests showed clinically and statistically significant increases in knowledge, attitudes and confidence in five of 10 domains including depression knowledge and confidence and BPSD knowledge, confidence and interventions. The greatest increase was in using BPSD interventions (18.5% increase, p-value: 0.0002), depression confidence (15.9% increase, p-value: 0.006) and BPSD confidence (12.6% increase, p-value: 0.02). Aliviado is an evidence-based, systems-level intervention shown to improve clinical knowledge, attitudes and confidence in treating pain and BPSD in PWD. This training could be used to produce systems-level practice change for hospice interdisciplinary team members serving PWD.

### GENDER AND BELIEFS ABOUT SUCCESSFUL AGING IN EASTERN NEPAL

Xiao Qiu,[1] Na Sun,[1] Sara J. McLaughlin,[1] Janardan Subedi,[1] Suman S. Thapa,[2] Mohan K. Shrestha,[3] Matthew Johnson,[4] and Sarah Williams-Blangero[4], *1. Miami University, Oxford, Ohio, United States, 2. Tilganga Institute of Ophthalmology, Kathmandu, Kathmandu, Nepal, 3. Tilganga Institute of Ophthalmology, Kathmandu, Nepal, 4. The University of Texas Rio Grande Valley, Brownsville, Texas, United States*

Gender shapes opportunities and experiences over the life course, which may influence beliefs about what it means to age successfully. In Nepal, a developing nation in South Asia, women and girls have historically had fewer social and economic opportunities than their male counterparts. To understand how gender may shape beliefs about successful aging, adult members of the Jiri population in eastern Nepal were asked to rate the importance of health-related (e.g., longevity), psychological (e.g., satisfaction with life), and social (e.g., support of family and friends) elements of successful aging (n = 1479; 52.9% female; 49.0% age 18-39, 33.1% age 40 to 59, 17.8% age 60 and over). Each of the 13 elements was rated as very important by over two thirds of the sample. Few gender differences in beliefs were observed; however, results of logistic regression analysis indicate that the odds of Jiri women endorsing longevity (OR = 0.75, p = 0.02) and life satisfaction (OR = 0.65, p = 0.02) as very important to successful aging were significantly lower than for men, controlling for age, education, and presence of illness. While more similarities than differences in beliefs about successful aging were observed by gender, the extent to which socially-defined roles and expectations may be responsible for observed differences should be explored in future research.

### EFFECTS OF FUNCTIONAL BRAIN NETWORKS AND WHITE MATTER DISEASE ON MOBILITY OF OLDER ADULTS IN AN EXERCISE INTERVENTION

Blake R. Neyland,[1] Robert Kraft,[1] Mary Lyles,[1] Stephen Kritchevsky,[1] Paul J. Laurienti,[1] Barbara M. Nicklas,[1] and Christina Hugenschmidt[1], *1. Wake Forest School of Medicine, Winston-Salem, North Carolina, United States*

Declining mobility is associated with increased accumulation of white matter hyperintensities (WMH). However, a high WMH burden is not always accompanied by impaired mobility. Our previous work demonstrates that some variance in mobility may be explained by brain network connectivity. Here, we extended this work by measuring WMHs and brain networks in older adults participating in a lifestyle intervention. The Short Physical Performance Battery (SPPB) and resting state functional magnetic resonance imaging (fMRI) were collected before and after a 5-month caloric restriction plus aerobic exercise intervention in 57 obese, sedentary adults aged 65-78. Participants were categorized based on median splits of baseline SPPB scores and WMH burden: Expected Healthy (EH: low WMH, SPPB≥11, n=16), Expected Impaired (EI: high WMH, SPPB≤10, n=17), Unexpected Healthy (UH: high WMH, SPPB≥11, n=12), and Unexpected Impaired (UI: low WMH, SPPB≤10, n=12). Graph theory-based methods were used to characterize brain networks and compare the four groups. At baseline, the somatomotor cortex community structure (SMC-CS) was less consistent in EI (p=0.05) and UI (p=0.23) compared to EH. The EI (mean=1.25, p=0.003) and UI (mean=1.57, p=0.001) significantly improved their SPPB scores following the intervention. Although both groups had equivalent SPPB scores, SMC-CS was less consistent in the UH than EH (p=0.16). However, UH displayed a significant (p=0.004) increase in second-order connections to the precuneus compared to EH. These data suggest that studying brain networks could improve the understanding of the development of mobility disability and the CNS contributions to mobility independent of white matter disease.

### IMPROV FOR CARE: TEACHING CAREGIVERS IMPROVISATION IMPROVES MOOD AND SENSE OF BURDEN

Ruth Almen,[1] and Jessica Z. Kirkland Caldwell[2], *1. Cleveland Clinic Nevada Lou Ruvo Center for Brain Health, Las Vegas, Nevada, United States, 2. Cleveland Clinic Lou Ruvo Center for Brain Health, Las Vegas, Nevada, United States*

There is a growing need for novel intervention for caregivers of family members with dementia. Improv for Care is a six-week program designed to teach improvisation ("improv") skills to caregivers to practice flexible communication, build social support, and process the demands of caregiving through humor and play. This study aimed to examine changes in caregiver depression (Beck Depression Inventory-II), perception of burden (Zarit Burden Interview), qualitative experiences related to caregiving, and their cared-for person's neuropsychiatric symptoms (Neuropsychiatric Inventory Questionnaire). Fifteen caregivers completed questionnaires before and after the Improv for Care program. Wilcoxon signed rank tests for related samples revealed significant declines in both caregivers' depressive symptoms, $Z = -2.64$, $p = .008$, and sense of burden, $Z = -2.16$, $p = .031$, after completing the program. Caregivers reported that their loved ones' neuropsychiatric symptoms increased during the course of the intervention, $Z = -2.10$, $p = .036$, though associated distress did not also increase, $Z = -1.12$, $p = .265$. Of the 15 caregivers, 12 completed follow up questionnaires three months after course completion, which showed that their post-intervention reduction in depressive symptoms remained stable, $Z = -1.02$, $p = .306$. The Improv for Care program shows promise as an intervention for caregivers to improve stress, mood, and coping skills.

### YOU DON'T KNOW WHAT YOU DON'T KNOW: A SURVEY OF CAREER MENTORING NEEDS IN CLINICAL GEROPSYCHOLOGY

Rachael Spalding,[1] Brenna N. Renn,[2] Rebecca S. Allen,[3] Jennifer Birdsall,[4] and Barry Edelstein[1], *1. West Virginia University, Morgantown, West Virginia, United States, 2. University of Washington School of Medicine, Seattle, Washington, United States, 3. The University of Alabama, Tuscaloosa, Alabama, United States, 4. N/A, N/A, United States*

Mentorship carries the potential to improve the placement, persistence, and success of those working in geriatric mental health. We sought to explore the career preferences and perceived barriers to obtaining desired jobs in the field of clinical geropsychology using an online survey of trainees and etablished professional geropsychologists. This cross-sectional observational cohort study recruited 96 respondents .Both trainees ($n = 42$) and professional geropsychologists ($n = 54$) completed an online survey. Trainees endorsed a variety of characteristics to describe their ideal jobs in geropsychology; particularly interdisciplinary teamwork and jobs in medical settings, palliative/hospice care facilities, long-term care, and geriatric outpatient care. The most commonly endorsed perceived barrier to trainees' ideal jobs was the location of the position. Responses from professional geropsychologists elucidated factors that influenced their first job and resources that were helpful, or would have been helpful, during their job search process. The majority of respondents described the role of mentoring in career development, specifically with skill development, decision-making assistance, and personal support. Mentorship is instrumental throughout one's training and career and offers instrumental and emotional support in job-searching and defining one's career. Results should be considered with respect to training and retention of clinical geropsychologists given the workforce shortage, particularly in academia.

### THE EFFECT OF COGNITIVE TRAINING AND PHYSICAL ACTIVITY PROGRAM IN COGNITIVE FUNCTION OF ELDERLY FACILITY RESIDENTS

Chien-Ning Tseng[1], *1. Department of Nursing, Oriental Institute of Technology, Nil, Taiwan*

This study evaluated the effectiveness of a combined cognitive training (CT) and physical activity (PA) intervention in improving cognitive function for institutionalized older residents with cognitive impairment. An experimental design with pre/post-test evaluations in a double-blind assessments at three points (baseline, T0; post-treatment, T1; 8-weeks-follow-up, T2), conducted an 8-week-CTPA intervention. Participants ($N=134$) were recruited from 12 institutions. Centers were randomly assigned into wait-list control, treatment I or treatment II groups. Treatment I group (low frequency) underwent combined 30-minutes sessions of individual-non-computer-based multi-domain CT (twice a week) with 30-minutes-group-chair-based PA (3 times a week). Treatment II group underwent the same protocol as Treatment I group, but with high frequency, 5 days per week for both CT and PA. The primary outcome, Cognitive Assessment Screening Instrument(CASI) total scores showed significant improvement in the treatment I and treatment II groups at T1-T0 and T2-T0, compared to the wait-list control group($10.55\pm9.60$, $12.75\pm11.64$, $-8.01\pm6.61$, $p=0.000$; $8.32\pm7.81$, $11.75\pm10.19$, $-7.11\pm5.78$, $p=0.000$), however there were no significant differences between two treatment groups. In CASI's nine sub-domains, all the mean difference between groups were also significant at T1-T0 and T2-T0 (all $p<0.05$). The two treatment groups only significantly differ on CASI-ORIENT domain at T2-T0 ($p=0.02$). The findings revealed that a combined CT-and-PA intervention have positive immediate (T1-T0) and delayed (T2-T0) effects in cognitive function for older institutional residents with cognition-impairment. The two treatment groups did not show dose-response relationship. Even more, the low frequency intervention was more effective on several domains than high frequency intervention did.

### FROM PERSONAL TO GLOBAL: CULTIVATING YOUR GERONTOLOGICAL VOICE

Leah M. Janssen[1], *1. Miami University & Scripps Gerontology Center, Oxford, Ohio, United States*

From intro-level undergraduates to advanced graduate students in gerontology, understanding and developing a "gerontological voice" is often an elusive concept and challenging process to articulate. How we view, understand, and give voice to aging-related issues is influenced by a number of different factors: Micro-level factors (e.g., personality, personal experiences with older adults, and personal interests and values); meso-level factors (e.g., coursework in aging and academic major/discipline); and, macro-level factors (e.g., societal and cultural values, media, activism, and advocacy). As an adaptable teaching exercise for all levels of students, this poster presents a novel framework to support students' exploration and cultivation of their unique gerontological voice. Through this three-level scaffolded discovery, students across disciplines strengthen their awareness and understanding of their distinct voice and build confidence to activate around aging-related issues pertinent to their specific interests and passions. From personal to global, this guided introspective exercise provides an opportunity for students to focus and embed their voice into the larger tapestry of gerontology, an inclusive community rich with perspective and diversity. Encouraging the development of students is an essential part of building social capital which is instrumental to the creation of strong networks to serve older adults through a multitude of disciplines.

### FINANCIAL HARDSHIP IN OLDER CHINESE CANCER SURVIVORS

Mingzhu Su,[1] Nengliang Yao,[1] and Xiaojie Sun[1],
*1. Shandong University, Jinan, Shandong, China*

Purpose: To estimate the proportion of Chinese cancer survivors experienced financial hardship and then examine whether older age was associated with financial hardship. Methods: We surveyed 965 cancer survivors 30 to 64 years of age and 643 cancer survivors age >=65 years in China. Cancer survivors were asked whether (1) they have borrowed money because of cancer, its treatment, and lasting effects of treatment and (2) they have forgone some cancer-related medical care because of cost. Multi-variable logistic regression models were used to examine factors associated with financial hardship. Results: About 44% of cancer survivors older than 65 borrowed money because of cancer, and 18% had borrowed more than 20,000 CNY (about 2,900USD, the disposable personal income in China in 2015 was about 22,000 CNY). In contrast, 54% of younger patients (P<0.01) had cancer-related debts, and 32% had to borrow more than 20,000 CNY. About 11% of cancer survivors have forgone cancer care in both age groups. The logistic regression analyses show that being 65 or older was 43% less likely to report cancer-related debts than younger patients (OR=0.57, 95% CI: 0.44-0.73). Among older cancer survivors, those who were older than 75, female, and had Urban Employee Medical Insurance and higher family income were less likely to report financial hardship. Conclusion: Older cancer survivors in China experience significant financial hardship, but not as striking as younger patients. Additional research is needed to analyze whether the finding is associated with the Chinese family structure and traditional filial piety culture.

### EVALUATING ITEM NONRESPONSE IN A LIFE HISTORY CALENDAR: AN ANALYSIS OF MEMORY EFFECTS

Mengyao Hu,[1] Roberto Melipillán,[2] Xinyu Zhang,[1] and Jacqui Smith[1], *1. University of Michigan, Ann Arbor, Michigan, United States, 2. UNIVERSIDAD DEL DESARROLLO, Concepción, Chile*

Memory decline contributes to response inaccuracy and can produce item missing data, especially in retrospective surveys with older adults. Event history calendars, or the life grid approaches, are commonly used to obtain retrospective life history data. As indicated in previous literature, this approach can assist respondents' memory retrieval. Despite its wide use, the important issue of item nonresponse due to memory effects in life grid questions has received little attention. Autobiographical memory (AM) research has shown that there are two interconnected long-term memory systems: episodic memories of event details from specific remote times in an individual's life; and semantic memories of the important facts and themes that define an individual's life history. Episodic and semantic AM may introduce different levels of difficulty in retrieving memory and thus contribute to different levels of missing data. This study examines the effects of both item-level predictors (e.g., types of memories) and respondent-level predictors (e.g., cognitive status, age, and health status) on the likelihood of item missing data in life grid questions. We analyzed missing data in the 2017 Health and Retirement Study (HRS) Life History Mail Survey (n = 3,844), using multilevel logistic regression. The results revealed higher rates of item missing for episodic memories, and that overall respondents' cognitive status was significantly associated with their likelihood of providing item missing data. Recent residential information was better recalled than childhood information. These results have implications for life course analysis of exposures linked to residential histories.

### SEX IN NURSING HOMES? A PRELIMINARY ANALYSIS OF INTERVIEWS WITH COMMUNITY-DWELLING ADULTS

Rachael Spalding,[1] Emma Katz,[1] and Barry Edelstein[1],
*1. West Virginia University, Morgantown, West Virginia, United States*

Most older adults living in long-term care settings (LTCs) indicate that expressing their sexuality is important to them (Doll, 2013). However, negative views of late-life sexuality persist in the United States (Robinson & Molzahn, 2007), particularly among nursing staff in LTCs. Staff often express discomfort regarding residents' sexual lives (Bouman, Arcelus, & Benbow, 2007), despite the fact that LTCs are residents' homes where private behaviors such as sexual activity might be expected to occur. Little is known about the general public's attitudes towards sexual behaviors in LTCs. Attitudes of LTC residents' family members is particularly important, as they are most likely to visit residents and to care about their quality of life, in turn informing facility policies and management. In this study, we took preliminary steps toward gathering this information by focusing on attitudes of community-dwelling adults. Using an iterative approach, we conducted semi-structured interviews with community-dwelling adults (n = 9; age range = 18 – 65 years) regarding their beliefs about romantic relationships and sexual behaviors among LTC residents. Major themes were identified through thematic content analysis. Participants indicated favorable attitudes towards residents' sexual expression as a means of combatting loneliness and fostering emotional intimacy. Other themes included residents' rights to privacy, potential risks of sexual behavior, and the need to consider how individuals may differ in their ability to consent to sexual activity. This data is intended to inform the development of a measurement tool for use with LTC residents' family members.

### PERCEIVED CONTROL AND CORTISOL STRESS REACTIVITY: VARIATIONS BY AGE, RACE, AND FACETS OF CONTROL

Jin-Hui Wen,[1] and Nancy L. Sin[2], *1. University of British Columbia, British Columbia, Canada, 2. University of British Columbia, Vancouver, British Columbia, Canada*

Greater perceived control is associated with better aging-related health outcomes, and these associations have previously been shown to differ based on sociodemographics. Physiological stress responses—including cortisol reactivity to stressors—may underlie the link between perceived control and health. The goal of this study was to evaluate the associations of perceived control and its facets (personal mastery and perceived constraints) with cortisol reactivity to acute laboratory stressors, in addition to the moderating roles of age and race. Participants (N = 737) ages 25-75 completed a perceived control questionnaire and two lab-based stress tasks. Salivary cortisol was collected pre- and post-stressor exposure. The results showed no main effects of perceived control, personal mastery, nor perceived constraints on salivary cortisol reactivity to stressors. However, age and race moderated the association between perceived constraints and post-stressor cortisol level, adjusting for baseline cortisol, sociodemographics, and health covariates. Among white participants, younger adults who reported higher constraints had elevated cortisol responses compared to those who reported lower constraints, whereas constraints were unrelated to cortisol reactivity among midlife and older adults. Among black participants, perceived control and its subscales were unrelated to cortisol, regardless of age. These findings suggest that older age buffers against the association between constraints and stress reactivity, but this buffering effect is only evident for white participants. Future research on the role of perceived control in stress and health should consider the importance of racial differences, facets of control, and age variations.

### THE MEDIATING EFFECTS OF LIFESTYLES AND HEALTH CAPABILITIES ON THE RELATIONSHIP BETWEEN AGING AND LIFE SATISFACTION

Dongwook Cho[1], *1. Alcorn State University, Lorman, Mississippi, United States*

The importance of life satisfaction has been getting significant attention as the older population has increased rapidly. There are many studies to examine the relationship between older adults' past and current lifestyles and life satisfaction levels that resulted to be either positively or negatively associated with. It is also well documented in the literature that aging is negatively associated with life satisfaction level due to the decrease in health capabilities. However, little study has been researched the direct and indirect relationships among advancing age, lifestyles, health capabilities, and life satisfaction levels. The purpose of the study was to examine the mediating effects of lifestyles and health capabilities on the relationship between aging and older adults' life satisfaction levels. A total of 290 older adults completed the self-administered questionnaire of lifestyles and life satisfaction levels and their health capability assessments were evaluated at three clinical research centers in the U.S. Multiple regression test was utilized to analyze the data. The results showed the direct effects of advancing age [B =1.196, t=2.608, p=.010] and engagement in exercise or physical activity [B=-2.684, t=-3.071, p=.002] on life satisfaction levels among older adults. However, there were no mediating effects of lifestyles and health assessments on the relationship between age difference and life satisfaction levels among older adults. The results suggest the advancing age itself would be the strongest factor in older adults' life satisfaction. Additionally, the findings also suggest that exercise and physical activity can enhance older adults' life satisfaction levels as a supplemental factor.

### EXPERIENCES AND FACILITATORS OF FALLS PREVENTION AMONG ETHNICALLY DIVERSE OLDER ADULTS: A QUALITATIVE STUDY

Ladda Thiamwong,[1] and Norma E. Conner[2], *1. College of Nursing, University of Central Florida, Orlando, Florida, United States, 2. University of Central Florida, Orlando, Florida, United States*

Background: Falls increase as people age and decrease the quality of life. Even though fall interventions have received great attention, fall incidence rates have still arisen. In order for older adults to reap the benefits of evidence-based fall interventions, a challenge of implementation in the real world and right context must be met. Understanding experiences, facilitators, and barriers of fall prevention among four major ethnic groups in the Unites States could be extremely valuable. Objective: The aim of this study was to describe experiences and highlight facilitators and barriers on fall and fear of falling interventions among ethnically diverse community-dwelling older adults. Methods: Four ethnically specified (African American, Asian, Hispanic and Non-Hispanic White) focus groups were conducted. A total of 28 older adults and four family caregivers were interviewed. Interviews covered experiences on falls and fear of falling, attitudes, factors, consequences, risk assessment, and interventions. Data were organized and analyzed with the NViVo software. Results: Falls related experiences and behaviors were multifaceted and varied. Three themes related to falls experiences and behaviors were identified, 1) falls prevention versus fear of falling amplification; 2) role identity, culture and family considerations; and 3) take care of you, take care of me. Facilitators of fall prevention were integration of individual learning within a group meeting, providing appropriate assistive devices and promoting environmental safety. Barriers were inconsistent fall risk assessments, low fall risk awareness and acknowledgment, and balance and visual impairment.

### DEVELOPMENT OF A TAILORED EDUCATIONAL PROGRAM TO ASSESS FALL RISK AND PREVENT FALLS FOR DIVERSE OLDER ADULTS.

Ladda Thiamwong,[1] and Norma E. Conner[2], *1. College of Nursing, University of Central Florida, Orlando, Florida, United States, 2. University of Central Florida, Orlando, Florida, United States*

Background: There is limited data on personal use fall prevention programs, and the relationship of race and ethnicity on fall risk awareness, personal beliefs, behavior change, and response to intervention. Objective: The aim of this study was to develop an educational program to prevent falls for ethnically diverse older adults. This program will be a culturally attuned program that values diversity and seeks to eliminate words and behaviors that might be discriminatory based on racial/ethnic or cultural identity. Methods: Three steps were used to develop the program: 1) constructing content domains; 2) generating the program draft; and 3) judging the program domain and content. The content domains were constructed based on data from a conventional content analysis of four focus groups from older participants (n=28) and their family caregivers (n=4), and individual in-depth interviews from health care providers (n=8). We generated the program outline with three response choices. Eight older participants and two health care providers rated it. Results: The program consisted of risk assessment, outreaching and raising awareness and knowledge. Risk assessment: all participants suggested that risk assessment should consists of objective and subjective measures. Outreaching: participants agreed that group-teaching and individual learning by peer coaching based on their culture, new blasts, brochures, and family-based approaches were the best outreaching methods that they preferred. They identified that raising awareness and knowledge should include the following topics: performing physical activity with fall risk awareness, medication management, visual care, behavioral adaptation with appropriate accessories /equipment, and environmental safety.

### EASILY ACCESSIBLE EVALUATIVE INFORMATION COULD FACILITATE OLDER ADULTS' (BUT NOT ALL) TRUSTWORTHINESS JUDGEMENT

Linlin Chen,[1] and Xin Zhang[2], *1. School of Psychological and Cognitive Sciences, Peking University, Beijing, China, 2. Peking University, Beijing, China*

Facial appearance served great function in social interactions, especially for older adults in making trustworthiness judgements. Previous literatures have consistently shown that when making trustworthiness judgements older adults tended to rely more on facial cues rather than behaviors, due to declines in cognition. However, one question remains unsolved, whether older adults could make accurate trustworthiness judgements if evaluative information (with minimal memory load) is easily accessible. Sixty younger adults (YAs) and sixty older adults (OAs) were recruited, and asked to make investment decisions for different brokers in ninety-six trials. In each trial, brokers' facial appearance (trustworthy and untrustworthy looking) and different behavioral evaluative information (good: Ninety percent positive evaluations, neutral: Fifty percent positive evaluations, bad: Ninety percent negative evaluations) were displayed simultaneously on screen to facilitate investment decisions. Brokers' facial appearances and behaviors were set to be independent to each other. The results indicated that YAs' and the majority of OAs' proportions of correct investment increase, gradually reaching a stable high correction rate, although OAs needed more trials than did YAs. The findings extended prior work by suggesting that both OAs and YAs had similar abilities to distinguish different brokers according to easily accessible evaluative information. However, and surprisingly, a small subgroup of OAs (with low economic status) still had a lower correction rate even after ninety-six trials, suggesting that they could not distinguish brokers based on their evaluations at all, who might be at risk for fraud.

### CAREGIVERS OF OLDER ADULTS: AN ANALYSIS OF THE PCORI FUNDED RESEARCH PORTFOLIO

Marissa Rurka,[1] Carly P. Khan,[2] Rachel Witsaman,[2] Steven Susana-Castillo,[3] Sindhura Gummi,[4] Gyasi Moscou-Jackson,[4] Steven B. Clauser,[4] and Neeraj K. Arora[5], *1. Purdue University, Lafayette, Indiana, United States, 2. Patient-Centered Outcomes Research Institute, Washington, District of Columbia, United States, 3. Yale School of Public Health, New Haven, Connecticut, United States, 4. PCORI, District of Columbia, United States, 5. Patient-Centered Outcomes Research Institute, District of Columbia, United States*

A family-centered approach to care is vital, and caregivers play an important role in patient-centered care for older adults. This analysis of the Patient-Centered Outcomes Research Institute's (PCORI) portfolio of clinical comparative effectiveness research (CER) trials explores the extent to which caregiving for older adults is a focus within our funded studies and examines how these studies incorporate interventions and outcomes related to caregivers. Of 116 studies in the portfolio with a caregiving component, only 35 studies focus on caregivers of older adults. Approximately half of these studies (16) were not focused on a specific disease, but rather included older adults with a variety of diseases. Caregivers were the target of a delivered intervention in 18 studies. Among these studies, all but one included caregivers as part of a multicomponent intervention. The most common intervention components were caregiver training (14 studies) and inclusion of caregivers in the delivery of health services, notably coordination of care (17), home visits (9), integrated care (9), multidisciplinary care teams (9), and clinical decision tools (8). Caregiver-focused outcomes were assessed in 26 studies. The most frequently assessed domains include measures of health and well-being (most commonly psychosocial status; n=20), evaluation of care (most commonly satisfaction; n=8), and health behavior (most commonly attitudes; n=6). In general, given stakeholder interest in family-centered research on older adults, future CER research should include caregivers and/or compare interventions focused solely on the unique needs of caregivers of older adults. Inclusion of caregiver-related outcomes should also be promoted.

### LANGUAGE DIFFERENCE AS A PREDICTOR OF OUTPATIENT FOLLOW-UP AMONG MIDLIFE SPANISH-SPEAKING PATIENTS

Daniel Jimenez,[1] Steven Figiel,[2] Jerad Moxley,[3] Pablo Radusky,[4] and Dante Durand[1], *1. University of Miami Miller School of Medicine, Miami, Florida, United States, 2. Lakeview Behavioral Health, Norcross, Georgia, United States, 3. Weill Cornell Medicine, New York, New York, United States, 4. Universidad de Buenos Aires Psychology Research Institute, Buenos Aires, Argentina*

The purpose of this study is to determine if psychiatrists' level of Spanish proficiency impacts patient adherence with follow-up mental health care. We hypothesize that increased Spanish proficiency will increase the likelihood of follow up mental health care. Data were collected via retrospective chart review of Spanish-speaking patients at an adult outpatient center. Our final sample included 201 patients with an average age of 46.5 years. Spanish speaking ability of the clinician was categorized using the standard set by the US Census bureau. The dependent variable of presence or absence of follow-up was dichotomized to whether or not the patient came back for a visit within 6 months of initial evaluation. Odds ratios (ORs) were calculated for each independent variable. Results indicate that Spanish proficiency significantly predicted a follow-up visit. Additionally, patients who were married and those who received psychotherapy were more likely to have a repeat visit. The significance of these results lies in that most research up to date has focused on the English proficiency of the patient as a factor for decreased use of mental health services. Although, effective strategies have been designed to reduce the language barrier in mental health care, Spanish-speaking patients still express a strong preference for bilingual providers. These results may allow identifying which patients are at higher risk of not adhering with follow-up visits. This provides a possibility to anticipate and prevent dropouts from treatment and to implement strategies to support patients that experience a greater burden of social and psychiatric disadvantages.

### A MIXED METHODS SYSTEMATIC REVIEW OF COMMUNITY-BASED GROUPS FOR SOCIAL ISOLATION AND LONELINESS IN OLDER PEOPLE

Brenda A. Hayanga,[1] Dylan Kneale,[2] and Ann Phoenix[3], *1. University College London, England, United Kingdom, 2. The Evidence for Policy and Practice Information and Co-ordinating Centre, Institute of Education, University College London, England, United Kingdom, 3. Thomas Coram Research Unit, Institute of Education, University College London, England, United Kingdom*

In the UK, many older people from minoritised ethnic groups are vulnerable to social isolation and loneliness. Yet, we know little about which interventions are effective for them. With existing systematic reviews of social isolation and loneliness lacking a theory-based framework of their life-course experiences, we set out to address this gap. This review aims to explore the effectiveness and suitability of community-based group interventions (CBGIs) for social isolation and loneliness in older people. The decision to focus on CBGIs was based on findings from an exploratory study of the friendship networks of older people and narrative interviews with older minoritised people living in the UK. The findings suggested that community groups of shared interests/backgrounds were protective of social isolation and loneliness. To address the objectives, we searched for randomised controlled trials and process evaluations of CBGIs published in English, which included older people living in countries with membership to the Organisation for Economic Co-operation and Development. We identified 4791 studies, 36 of which were eligible for inclusion. In this poster, we present the preliminary findings of this mixed-methods systematic review, which seeks to not only assess whether CBGIs are effective but also to understand the underlying processes that make interventions (in)effective. As this review is guided by findings from two exploratory studies with older people from minoritised ethnic groups, it takes into account their life-course experiences. It is the results of reviews such as this that can produce generalisable findings which are directly applicable to policy.

### GENDER DIFFERENCES IN THE ASSOCIATION BETWEEN MODIFIABLE RISK FACTORS AND FINANCIAL HARDSHIP

Gillian Marshall,[1] William Bryson,[2] Ola Rostant,[3] and Sarah Canham[4], *1. University of Washington, Tacoma, Washington, United States, 2. Oregon Health and Sciences University, Portland, Oregon, United States, 3. University of Michigan - Institute for Social Research, Ann Arbor, Michigan, United States, 4. University of Utah, Salt Lake City, Utah, United States*

Objective: To identify associations between modifiable risk factors (cigarette smoking, alcohol consumption, and obesity) and financial hardship (difficulty paying bills, food insecurity and medication need) among middle-aged and older Americans in a nationally representative sample. Methods: This was a cross-sectional study of 8,212 persons age 50 years and older who completed the core 2010 Health and Retirement Study survey and the psychosocial questionnaire. We ran separate multinomial logistic regressions to assess the association of three modifiable risk factors and three different financial hardship indicators. Results: Adjusting for all covariates, compared to men of normal weight, men who were obese had a 1.4 greater odds of difficulty paying their bills (95% CI: 1.08-1.76); former smokers had a 1.8 greater odds of being food insecure (95% CI: 1.05-2.95); current smokers were twice as likely to be food insecure (95% CI: 1.21-3.73); Compared to women who never smoked, current smokers had a 1.5 greater odds of having difficulty paying their bills (95% CI: 1.11-2.02); current smokers had a 1.8 greater odds of being food insecure (95% CI: 1.13-2.91); and women who were obese had a 1.5 greater odds of reducing medication due to cost (95% CI: 1.11, 2.02). Conclusion: Our findings contribute to the literature on health behaviors and financial hardship by highlighting the cyclical nature between different indicators of socioeconomic status, modifiable risk factors, and poor health outcomes among middle-aged and older adults. Furthermore, findings highlight how modifiable risk factors may culminate in financial hardship in later life.

### FALL PREVENTION PROGRAMS FOR RURAL COMMUNITY DWELLING OLDER ADULTS RESULTS IN IMPROVED BALANCE

Robin E. McAtee,[1] Ronni Chernoff,[2] Kathryn Packard,[3] Whitney Thomasson,[4] Laura Spradley,[4] and Cynthia Mercado[4], *1. University of Arkansas for Medical Sciences, Little Rock, Arkansas, United States, 2. Univ Arkansas for Medical Sciences, Little Rock, Arkansas, United States, 3. UAMS, Hot Springs, Arkansas, United States, 4. University of Arkansas for Medical Sciences, Little Rock, Arkansas, United States*

More than one in four older adults (OA) in the US fall each year and 20 to 30% suffer moderate to severe injuries such as head trauma or hip fractures. Falls are both a medical and financial burden that can be significantly reduced by lifestyle interventions. One of the main strategies to help reduce these statistics is evidence-based fall prevention classes which help to build muscle, improve balance, and increase participants' confidence in fall control. A goal of the Arkansas Geriatric Workforce Enhancement Program (HRSA grant U1QHP28723) is to deliver community-based programs that improve health outcomes and quality of life. Evidence-based preventive health training is a major way to meet this goal. Two of these evidence-based trainings are Tai Chi (TC) and A Matter of Balance (AMOB), multi-week classes that help to improve balance and reduce fall risk. This study took place at scheduled community-based classes, where demographic and balance data were obtained. The aim was to determine the difference in the balance of community-dwelling OA as measured by Balance Tracking System® before and after AMOB and TC courses. Both groups showed positive changes in their mean balance percentage with the AMOB class having a higher Mean ± SD (24.3 ± 21.0) vs. the TC participants (4.0 ± 29.7). The One-Way Analysis of Variance showed statistically significant difference in the AMOB class over those in the TC class, $F(1, 32) = 5.280$, $p < 0.05$. The Cohen's $d = 0.789$ indicates a large effect between the two groups.

### RACIAL DIFFERENCES IN NON-COGNITIVE SYMPTOMS IN ALZHEIMER'S DISEASE AND CAREGIVER DEPRESSION

Weizhou Tang[1], *1. University of South Carolina, Columbia, South Carolina, United States*

Behavioral and psychological symptoms (BPS) represent a heterogeneous group of non-cognitive symptoms occurring in persons with Alzheimer's disease (PwAD), and they are often associated with negative outcomes for AD caregivers. Evidence indicates differences in caregivers' mental health across race/ethnic groups. However, there is a lack of research that compares racial differences in BPS in PwAD. This study aims to compare racial differences in BPS in PwAD and caregiver depression. The study analyzed data collected from the South Carolina AD Registry in 2010. The survey used in the interview included measures of caregiver depression, caregiver burden, PwAD's non-cognitive symptoms, caregiving competence, caregiver distress, and demographics. The final analysis focused on 635 African-American (n=313) and white (n=322) caregivers. Mann-Whitney U-tests, Chi-square tests, and multiple linear regression were conducted. Among all PwAD, higher percentage of whites than African Americans exhibited apathy/indifference (67.52% vs 52.44%, p=.0001), depression/dysphoria (61.54% vs 44.59%, p<.0001), and anxiety (45.08% vs 29.64%, p<.0001). In terms of both frequency and severity of BPS, whites had significantly higher BPS score (Mean=35.49, SD=24.75) than African Americans (Mean=28.13, SD=23.97; p<.0001). Mean comparisons indicated significant group differences in caregiver depressive symptoms between white caregivers (mean=11.89, SD=6.90) and African-American caregivers (mean=9.41, SD=5.77). However, there were no racial differences in the relationship between BPS in PwAD and caregiver depression. The findings of this study highlight the importance of developing more effective and targeted treatment options and therapies for neuropsychiatric symptoms and delivering cultural relevant education programs/interventions to ethnic groups.

### PEER TO PEER SUPPORT AND HEALTH CARE UTILIZATION AMONG COMMUNITY-DWELLING OLDER ADULTS

Elizabeth A. Jacobs,[1] Rebecca Schwei,[2] Scott Hetzel,[3] Jane Mahoney,[4] and KyungMann Kim[5], *1. The University of Texas at Austin Dell Medical School, Austin, Texas, United States, 2. University of Wisconsin-Madison School of Medicine and Public Health, Madison, Wisconsin, United States, 3. University of Wisconsin-Madison Biostatistics & Medical Informatics, Madison, Wisconsin, United States, 4. University of Wisconsin School of Medicine and Public Health, Madison, Wisconsin, United States, 5. University of Wisconsin-Madison School of Medicine and Public Health, Madison, Wisconsin, United States*

The majority of older adults want to live and age in their communities. Some community-based organizations (CBOs) have initiated peer-to-peer support services to promote aging in place but the effectiveness of these programs is not clear. Our objective was to compare the effectiveness of a community-designed and implemented peer-to-peer support program vs. access to standard community services, in promoting health and wellness in vulnerable older adult populations. We partnered with three CBOs, one each in California, Florida, and New York, to enroll adults 65 > years of age who received peer support and matched control participants (on age, gender, and race/ethnicity) in an observational study. We followed participants over 12 months, collecting data on self-reported urgent care and emergency department visits and hospitalizations. In order to account for the lack of randomization, we used a propensity score method to compare outcomes between the two groups. We enrolled 222 older adults in the peer-to-peer group and 234 in the control group. After adjustment, we found no differences between the groups in the incidence of hospitalization, urgent and emergency department visits, and composite outcome of any health care utilization. The incidence of urgent care visits was statistically significantly greater in the standard community service group than in the peer-to-peer group. Given that the majority of older adults and their families want them to age in place, the question of how to do this is highly relevant. Peer-to-peer services may provide some benefit to older adults in regard to their health care utilization.

### HOW TO STEADI YOUR PATIENTS WITH THE COORDINATED CARE PLAN TO PREVENT OLDER ADULT FALLS AND EVALUATION GUIDE

Janice A. Mark[1], *1. Centers for Disease Control and Prevention, Atlanta, Georgia, United States*

Falls are common, costly, and the leading cause of fatal and nonfatal injuries for older Americans. Reports show that fall death rates are increasing. Healthcare providers play an important role in fall prevention but few talk to their patients about falls. This lack of communication demonstrates the need for more physician-initiated fall prevention. The Centers for Disease Control and Prevention (CDC) created the Stopping Elderly Accidents, Deaths, and Injuries (STEADI) initiative to help providers talk to their patients about falls. Specifically, CDC's new STEADI-based fall prevention program, the Coordinated Care Plan to Prevent Older Adult Falls (CCP) and Evaluation Guide for Older Adult Clinical Fall Prevention Programs can assist healthcare providers in integrating and evaluating new fall prevention programs that screen older adults for fall risk, assess patients' modifiable fall risk factors, and implement evidenced-based fall prevention interventions (e.g., medication management, physical therapy). The CCP offers guidance for incorporating a STEADI-based fall prevention program including how to engage leadership, integrate with existing clinic workflow and electronic health records, and strategies on how to obtain reimbursement for fall prevention. The Evaluation Plan offers details on how to engage stakeholders, collect data, interpret findings and how to share results for maximum impact. Both documents were based on lessons learned from successful implementation of STEADI-based programs in primary care. A STEADI-based program in New York found fewer fall-related hospitalizations among at-risk patients who received a fall prevention care plan compared to at-risk patients who did not receive a care plan.

### RECONSIDERING PROTECTIVE FACTORS OF SUICIDALITY IN OLDER ADULTHOOD: RESULTS FROM THE CDC NVDRS NATIONAL DATASET

Kyle L. Bower,[1] and Kerstin G. Emerson[1], *1. University of Georgia, Athens, Georgia, United States*

Existing literature comprehensively addresses the issue of suicide; however, there is still much to be understood concerning this phenomenon in later life. For instance, suicide rates are particularly high for older agricultural workers, yet there remains considerable ambiguity concerning the factors contributing to this public health crisis. We conducted exploratory analyses with the Centers for Disease Control (CDC) National Violent Death Reporting System (NVDRS) 2003-2017 dataset. We identified a sample of individuals who committed suicide while working in the agricultural sector (N= 2,106). We coded "farmer" as anyone who worked in the agricultural sector at their time of death, or prior. The majority (>90%) of the sample was Caucasian male, and approximately 38% was age 65 and older. We also found that 40% of farmers were married (or in a domestic partnership) at the time of death. Specifically, of those 65 and older, 26% were married at the time of death (second largest majority as 70% were widowed). This finding is particularly interesting as marriage or long-term partnerships have been shown in the literature to be a protective health factor in later life, especially for older men. Our findings regarding marital status suggest that having a significant other may not be the most critical relationship for suicide prevention. Future mental health interventions should explore alternative social connections as a means to identify more effective methods of preventing suicide in this vulnerable population.

### STEADI-RX: ADAPTING CDC'S STEADI INITIATIVE FOR USE IN COMMUNITY PHARMACIES

Elizabeth R. Burns,[1] Stefanie Ferreri,[2] Jessica Robinson,[3] and Chelsea Refroe[4], *1. Centers for Disease Control and Prevention, National Center for Injury Prevention and Control, Division of Unintentional Injury, Atlanta, Georgia, United States, 2. University of North Carolina Eshelman School of Pharmacy, Chapel Hill, North Carolina, United States, 3. UNC Eshelman School of Pharmacy, Chapel Hill, North Carolina, United States, 4. University of Tennessee Health Science Center College of Pharmacy, Memphis, Tennessee, United States*

Falls among older adults (age 65 and older) cause roughly 3 million emergency department visits and $50 billion in medical costs annually. The Centers for Disease Control and Prevention (CDC) developed the Stopping Elderly Accidents, Deaths, and Injuries (STEADI) initiative to increase clinical fall prevention. STEADI encourages screening for fall risk annually, assessing modifiable risk factors, and intervening to reduce risk using effective strategies like medication management. Medication management is critical given new data on the increased use of medications linked to falls. In 15 years, opioid use among older adults increased from 15% to 35% and anticonvulsant use tripled from 4% to 14%. To address the issue, CDC partnered with the University of North Carolina who 1) adapted STEADI tools for use in community pharmacies and 2) piloted and assessed their use in 31 North Carolina pharmacies. The adapted tools and processes were branded STEADI-Rx. Community pharmacists following the STEADI-Rx initiative, screen older patients for fall risk in the pharmacy, perform a medication review to identify medications that could increase fall risk, and intervene to reduce risk by sharing information with the patients' healthcare providers. Providers can then optimize medications to reduce fall risk and improve health. STEADI-Rx tools include 1) a community pharmacy algorithm, outlining how pharmacists can conduct fall risk screening, assessment, and care coordination; 2) a community pharmacy fall risk checklist; and 3) communication materials to help pharmacists share information with patients' providers. STEADI-Rx was launched in July 2019. More information is available at www.cdc.gov/STEADI.

### GENE-OBESOGENIC ENVIRONMENT INTERACTIONS ON BODY MASS INDICES ACROSS RACE AND SEX AMONG OLDER ADULTS

Mika D. Thompson,[1] Catherine M. Pirkle,[1] Fadi Youkhana,[1] and Yanyan Wu[1], *1. University of Hawai'i at Mānoa, Honolulu, Hawaii, United States*

Gene-obesogenic environment interactions influence body mass index (BMI) across the life-course; however, limited research examines how these interactions may differ by race and sex. Utilizing mixed-effects models, we examined interaction effects of polygenic risk score (PGS) generated from 97 single nucleotide polymorphisms, and environmental factors, including age and physical activity, on measured longitudinal BMI from the Health and Retirement Study (HRS). HRS is a population representative survey study of older adults aged 50-years or older in the U.S. This study used a sub-sample of genotyped Black (N=1,796) and White (N=4,925) males and females. The association between PGS and mean BMI weakened as individuals aged among White males (Pinteraction=0.038) and White females (Pinteraction=0.054). The mean BMI difference between the highest and lowest PGS quintiles was 4.25 kg/m2 among 50-year old White males but 3.11 kg/m2 among the 70-year old's, i.e. a decrease of 1.14 kg/m2 (95%CI: -0.06,2.65) over 20 years. Similarly, the decrease among 50- and 70-year old White females was 1.33 kg/m2 (95%CI: 0.07,3.45). Additionally, the association between physical activity and BMI was stronger among White females with higher PGS (Pinteraction=0.038). Vigorous physical activity

(compared to never) was associated with a 1.71 kg/m2 (95%CI: 1.08,2.35) decrease in mean BMI among those in the highest PGS quintile, compared to a 0.80 kg/m2 (95%CI: 0.32,1.27) decrease among those in the lowest. Overall, we found unique interaction effects across race and sex within an older population; findings reinforce the importance of physical activity among those with an elevated genetic risk.

### FALLS IN MID-LIFE: A SCOPING LITERATURE REVIEW

Aleksandra Zecevic,[1] Daniella Bozzo,[1] Alison Stirling,[2] and Helene Gagne[3], *1. Western University, London, Ontario, Canada, 2. Ontario Neurotrauma Foundation, Fall Prevention Community of Practice, Toronto, Ontario, Canada, 3. Ontario Neurotrauma Foundation, Toronto, Ontario, Canada*

The implications of falls of middle-aged adults (40-64 years) on falling in late life (65+) have not received much attention. The patterns of falling over the lifespan require more research. The purpose of this scoping literature review was to answer: What is known about falls occurring in mid-life? and How falls in mid-life relate to falling over the adult lifespan? A six-stage scoping literature review framework by Levac et al. (2010) was followed. A total of 5,136 titles were identified in CINAHL, EMBASE and MEDLINE using MeSH terms for accidental fall, middle-aged and longitudinal studies. Inclusion/exclusion criteria narrowed the search to 30 full-text research articles and nine gray literature sources that were charted. Literature on falls in mid-life produced five discrete themes: two distinct populations of fallers, prevalence rates, fall-related injuries, causes of falls, and risk factors for falling. The two groups of fallers were the general population (falls prevalence 8.7-35.8%) and the special population of mid-life adults living with chronic health conditions (falls prevalence 26% for diabetes and 32.3% intellectual disabilities). Middle-aged adults had a higher proportion of injurious falls (11.5-30%) compared to older adults and extrinsic risk factors were the most frequent causes of falling (83.3%). For special populations, the risk of falls was frequently attributed to intrinsic factors. In conclusion, falls in mid-life require further exploration to establish patterns over the adult lifespan, determine influence of chronic diseases, establish clear fall incidences and risk factors, and determine if current falls prevention interventions are appropriate for mid-life adults.

### THE ETHNIC-SPECIFIC EFFECTS OF BEHAVIORS ON INCIDENT GOUT WITHIN A LARGE MULTIETHNIC COHORT OF OLDER ADULTS

Mika D. Thompson,[1] Catherine M. Pirkle,[1] Yanyan Wu,[1] Fadi Youkhana,[1] Robert V. Cooney,[1] and Lynne R. Wilkens[2], *1. University of Hawai'i at Mānoa, Honolulu, Hawai'i, United States, 2. University of Hawai'i Cancer Center, Honolulu, Hawai'i, United States*

Gout, a common rheumatic disease in older adults, disproportionately affects Pacific Islander and Black populations, relative to Whites; however, the ethnic-specific incidence and determinants remain severely understudied within these groups. We examined gout incidence and the effects of behavioral factors, including diet, physical activity, and smoking, on incident gout within a large multiethnic population of older adults from the Multiethnic Cohort Study linked to approximately 20 years of Medicare gout claims. Using samples of Black (N=15,660), Native Hawaiian (N=7,600), Japanese (N=32,923), Latino (N=21,793), and White (N=29,129) participants, we conducted multiple Cox regressions, producing hazard ratios (HR) and 95% confidence intervals (CI). Native Hawaiians had the highest incidence of gout (9.97 per 1,000 person-years), followed successively by Black, Japanese, White, and Latino participants. 3+ alcoholic drinks/day was associated with an increased risk, especially among Blacks (HR:1.55, 95%CI:1.23,1.94). Current smoking was associated with increased risk among Blacks (HR:1.28, 95%CI:1.10,1.49) and Japanese (HR:1.17, 95%CI:1.03,1.32). Better dietary quality tertiles were associated with a decreased gout risk for most ethnic groups, with the largest effect observed among Whites (HR[Q1vsQ3]:0.70, 95%CI:0.63,0.78), while vitamin C was weakly associated with a decreased risk of gout only among Native Hawaiians (HR:0.85, 95%CI:0.75,0.98) and Japanese (HR:0.91, 95%CI:0.85,0.98). Overall, notable ethnic differences were observed in both gout incidence and effects of modifiable behaviors. As the first study to examine the incidence and longitudinal determinants of gout utilizing several large samples of underrepresented ethnic groups, our findings offer crucial insights that may improve precision in preventing and treating gout.

### A SURVEY OF FALLS AND ASSOCIATED FACTORS AMONG OLDER COMMUNITY-DWELLERS

Meng-Ling WU,[1] Shang-Yu Wu,[2] and Chien-Ning Tseng[3], *1. Oriental Institute of Technology, New Taipei, Taiwan, 2. Guoxian Chinese Medicine Clinic, New Taipei City, Taiwan (R.O.C.), Taiwan, 3. Department of Nursing, Oriental Institute of Technology, Nil, Taiwan*

After surveying elderly people in northern Taiwan with a structured questionnaire to collect basic information and experience of falls in the past year. In total, 250 valid questionnaires were gathered. The SPSS software package used in statistical analysis of data and a p-value <0.05 was considered to reflect statistical significance by Chi-square ($\chi 2$) test. In this study, 176 female accounted for 70.4%; 213 people living with family accounted for 85.2%; 68 people aged 66-70 years old accounted for 27.2%; 85 people with public education at the elementary level accounted for 34%. 156 people with falling experience accounted for 62.3%; 60 people who fell home accounted for 38.5%; 74 people who fell during outdoor activities accounted for 47.4%; 63 people fell in walking, cycling and sports, accounting for 85.1%. In addition, there is a significant correlation between residents' medication and fall experience; Such as antihypertensive drugs, hypoglycemic agents, and analgesics, are more statistically significant about fall; and taking sedative, muscle relaxation, Chinese medicine, there is no statistically significant. This shows that it is very important to prevent the fall of the elderly and the safety of the elderly during outdoor activities. In addition to the need to increase the muscles and strength of the elderly, it is also necessary to strengthen the outdoor space and drug education for the elderly.

### MINDFULNESS TRAINING FOR CAREGIVERS OF ALZHEIMER PATIENTS: PRELIMINARY RESULTS OF A RANDOMIZED CONTROLLED TRIAL

MIRIAM HURTADO POMARES,[1] DANIEL MENDIALDUA CANALES,[2] PAULA PERAL GÓMEZ,[3] CRISTINA ESPINOSA SEMPERE,[3] IRIS JUÁREZ LEAL,[3] DESIREÉ VALERA GRAN,[3] EVA Mª NAVARRETE MUÑOZ,[1,3] and ALICIA SÁNCHEZ PÉREZ[3], *1. UNIVERSIDAD MIGUEL HERNÁNDEZ DE ELCHE, Elche (Alicante), Spain, 2. Miembro de la Orden del Interser, San Juan de Alicante (Alicante), Spain, 3. UNIVERSIDAD MIGUEL HERNÁNDEZ DE ELCHE, San Juan de Alicante (Alicante), Spain*

Mindfulness Based Health Care (MBHC) provides meditation techniques that may help to mitigate negative impact of dementia caregiving. We conducted a pilot randomized controlled trial with two parallel groups including 42 caregivers of Alzheimer patients (21 pairs). MBHC group learned the practice of mindfulness meditation once a week through 8 classes; control group did not receive any therapy. Anxiety and depression symptoms were the primary outcomes assessed in two occasions, i.e. at baseline and the end of the program, using the Hospital Anxiety and Depression scale respectively. Neuropsychiatric disorders of Alzheimer patients were also measured with Neuropsychiatric Inventory-Questionnaire. The details of the randomized controlled trial were described in clinicaltrial.gov NCT03858283. In this abstract, we presented the results of a pilot study conducted with 42 caregivers. Robust linear regression using MM-type estimator was performed to evaluate the changes in neuropsychiatric disorders in patients and anxiety and depression symptoms in caregivers adjusting for Score of Scale Global Deterioration. We observed that, compared to the control group, the MBHC group showed a tendency to a reduction in the total score Neuropsychiatric Inventor ($\beta$=-5.20; CI 95%: -10.47; 0.07) and the depression symptoms in the caregivers ($\beta$=-2.30; CI 95%: -5.43; 0.83). However, no changes were observed in the anxiety symptoms of the caregivers. In conclusion, the results of this pilot study suggested a positive effective on the reduction of neuropsychiatric symptoms in patents as well as on the depression in the caregivers. Nevertheless, these findings should be confirmed in a further complete study.

### DIABETES AS A RISK FACTOR FOR MILD COGNITIVE IMPAIRMENT IN OLDER RESIDENTS OF RURAL WEST TEXAS

Gabriela Arandia,[1] Annette Boles,[1] Veronica Lopez,[1] and Volker Neugebauer[1], *1. Texas Tech University Health Sciences Center - Garrison Institute on Aging, Lubbock, Texas, United States*

While a growing body of evidence suggests a link between diabetes and Alzheimer's disease, few studies have examined the impact of diabetes on mild cognitive impairment, the precursor to Alzheimer's disease, especially among older, rural, and ethnically diverse populations. Using data from Project FRONTIER (Facing Rural Obstacles to Healthcare Now Through Intervention, Education, & Research), a longitudinal cohort aging study in rural West Texas, the aim of this study was to compare the risk for mild cognitive impairment among participants who, according to blood sugar levels, were pre-diabetic/diabetic versus normal. This study uses baseline and 3-year follow-up data from a subsample (recruited from Cochran County) of the larger, four-county sample of Project FRONTIER. The study sample (n=206) ranged from 40 to 87 years old (mean age: 58.3 + 11.7 years old), was predominantly female (73.3%), White (88.4%), with slightly over half self-reporting as Hispanic (51.0%). Logistic regression results revealed that those who had prediabetes/diabetes had 1.81 times the risk for developing mild cognitive impairment compared to those who had normal blood sugar levels. These findings indicate the need for earlier intervention for improved diabetes prevention, self-management, and control (diet, physical activity, treatments) to help offset the development of mild cognitive impairment, which could progress to Alzheimer's disease later in life. More research is needed to confirm the link between pre-diabetes/diabetes and mild cognitive impairment in other populations and settings.

### TECHNOLOGY USE IN RURAL DEMENTIA CARE: PERSPECTIVES FROM FAMILY CAREGIVERS, PWD, AND SERVICE PROFESSIONALS

Lucas R. Prieto,[1] Fei Sun,[1] Wenwu Zhang,[1] and Anastasia Kononova[1], *1. Michigan State University, East Lansing, Michigan, United States*

Purpose: The use of technology in dementia care has shown promising benefits for both people living with dementia and their family caregivers, however, little is known regarding how technology is used among families affected by dementia who reside in rural communities. The purpose of this study was to explore technology use and barriers among people living with dementia, family caregivers, and service professionals who live in rural areas of Michigan. Methods: This study was based upon focus group data from six groups of family caregivers (n=32); one group of people living with early stages of dementia (n=4), and one group of service professionals (n=4) recruited from rural counties in Michigan. Results: Technology use included assisting caregiving tasks (e.g., monitoring a wandering care recipient), facilitating treatment (e.g., access treatment through telemedicine), and providing social connection and support. Themes related to strategies included addressing educational needs of young older and old-older caregivers, providing step-by-step toolkits, and collaborating with multi-sectors (e.g., public libraries, grocery stores, and churches). Discussion: Findings suggest a great need to facilitate technology literacy and competency for rural family caregivers to access caregiving resources. To effectively reach out to this population, technology methods such as local TV news network, radio, and newspaper are still beneficial. Health and social service professionals should consider collaborations with public service institutions (e.g. libraries) and faith-based organizations to include educational workshops about technology in their curriculum and training programs for dementia family caregivers.

### PSYCHOSOCIAL PREDICTORS OF THE GENDER DIFFERENCE IN DEPRESSION OF OLDER ADULTS IN THE ORANJ BOWL PANEL

Christine V. Ferri,[1] Kaite Yang,[2] and Joan Girgus[3], *1. Stockton University, Atlantic City, New Jersey, United*

*States, 2. Stockton University, Galloway, New Jersey, United States, 3. Princeton University, Princeton, New Jersey, United States*

Recent reviews show that the gender difference in depressive symptoms that emerges in adolescence persists in older adults (Girgus, Yang & Ferri, 2017; Salk, Hyde & Abramson, 2017). However, researchers have yet to explain why this gender difference occurs in older adults and if it is for the same reasons as in younger ages. The present study used data collected from 3008 participants over the age of 60 (M=66.45, SD=4.36) from the baseline wave of the ORANJ BOWL, a longitudinal study based in New Jersey (Pruchno, Wilson-Genderson, Rose, & Cartwright, 2010). Depressive symptoms were measured with the CES-D. Six psychosocial predictors of the gender difference in depression were analyzed: functional ability, social support, perceived financial comfort, income level, marital status, and living alone. Women reported significantly more depressive symptoms compared to men ($p<.001$). Women had significantly lower functional ability, physical health, financial comfort, income level, but more social support than men (all p's<.02). Women were more likely to live alone and to be separated, divorced, or widowed (all p's<.001). Significant interactions for gender x functional ability, gender x social support, and gender x living alone were found in predicting depression using hierarchical linear regression. Living alone and poor function were more detrimental for men than women. Low social support was more detrimental for women than men. Understanding the gender difference in depressive symptoms will allow for targeted screening and preventative interventions for women and men at highest risk for depression.

### ORGANIZATIONAL AND OLDER ADULT VOLUNTEER PERSPECTIVES ON ROLE CONFLICT MANAGEMENT STRATEGIES

Jennifer A. Crittenden,[1] and Sandra S. Butler[2], *1. UMaine Center on Aging, University of Maine, Bangor, Maine, United States, 2. University of Maine, Orono, Maine, United States*

Older adults are increasingly occupying multiple life roles, including working, caregiving, and volunteering, creating the opportunity for role conflict. Such conflict occurs when stress and strain created by the demands of multiple life roles outstrips an individual's resources to successfully manage such demands. A two-phase research study was recently completed with 1,697 RSVP volunteers drawn from 55 RSVP program sites across the country (Phase I) with a follow-up survey of RSVP programs conducted with 17 sites (Phase II). Grounded in role theory, the Phase I volunteer survey explored role conflict in addition to self-reported strategies used to mitigate the experience of role conflict. The Phase II program survey gathered responses from volunteer managers and staff about the strategies used by their older adult volunteers to avoid and address role conflict. Based on findings from both surveys, caregivers engaged the following strategies in order to minimize role conflict: obtaining respite care, and volunteering alongside their care recipient. Worker-specific strategies focused largely on time management and included volunteering during off-work hours and completing time-limited volunteer assignments. While a high level of convergence was noted between volunteer manager and volunteer perspectives, two themes emerged from the volunteer survey that were not identified in program survey responses: seeking volunteer opportunities that leverage similar skills and experiences across roles and seeking volunteer opportunities that provide a different experience from that of other roles. Implications for future research and volunteer management strategies will be discussed.

### PREVALENCE AND PREDICTORS OF STATIN USE IN LONG-STAY NURSING HOME RESIDENTS WITH LIFE-LIMITING ILLNESSES

Deborah S. Mack,[1] and Kate L. Lapane[2], *1. University of Massachusetts Medical School, Graduate School of Biomedical Sciences, Department of Population and Quantitative Health Sciences, Worcester, Massachusetts, United States, 2. University of Massachusetts Medical School, Department of Population and Quantitative Health Sciences, Worcester, Massachusetts, United States*

Statins are one of the most commonly prescribed medications in the United States. While statin use has been studied extensively in the general population, national data on statin use in US nursing homes do not exist. This study estimated the point prevalence of statin use on September 1, 2016 and identified predictors of statin use in nursing home residents with life limiting illness. We conducted a cross-sectional analysis using national MDS 3.0 data linked to Medicare claims. We identified 424,312 long-stay residents with life limiting illnesses defined as a palliative care consultation (ICD-10 Z51.5), prognosis <6 months on MDS, the Veterans Health Administration palliative care index (PCI), or a diagnosis of a serious illness (e.g., cancer, stroke, heart failure, etc.). Poisson models accounted for clustering of residents within facilities. Overall, 34% were on statins which varied by age (65-75 years: 44.1%; >75 years: 31.5%). The strongest positive predictor of statin use was hyperlipidemia, while coronary artery disease and stroke were only marginally predictive across age. The strongest negative predictors were a palliative care consultation or a prognosis <6 months, while PCI was not strongly associated with use. A substantial proportion of long stay nursing home residents with life limiting illnesses continue statin therapy despite evidence of net harm. Efforts to deprescribe statins in the nursing home setting may be warranted. These findings can be used to help identify and target missed opportunities to reduce the therapeutic burden and improve end-of-life care for the US nursing home population.

### CAREGIVER PREFERRED STRUCTURE FOR A TELEHEALTH-BASED INTERVENTION ONE-TO-ONE VERSUS GROUP

Sabrina J. Shofner,[1] Glenise McKenzie,[2] David LaFazia,[3] Loriann McNeill,[4] Natasha Spoden,[5] Nora Mattek,[5] Linda Teri,[6] and Allison Lindauer[7], *1. OHSU, Portland, Oregon, United States, 2. Oregon Health & Science University, Portland, Oregon, United States, 3. University of Washington, Seattle, Washington, United States, 4. Multnomah County, Portland, Oregon, United States, 5. Oregon Health & Science University, Portland, Oregon, United States, 6. University of Washington, Seattle, Washington, United States, 7. Oregon Alzheimer's Disease Research Center, Portland, Oregon, United States*

Caring for a person with Alzheimer's disease and related dementias (ADRD) can be stressful. Support programs are available for caregivers, but distance and cost present barriers to participation. Our mixed-methods study explored consumer acceptability and preliminary efficacy of a telehealth-based caregiver intervention, Tele-STAR. Caregivers in Tele-STAR met one-to-one with a consultant over eight weeks, via video conferencing, to address behavioral symptoms of dementia. We measured the effect of the intervention on caregiver reactivity to behavioral symptoms. Focus groups were used to assess acceptability. The literature suggests that stressed caregivers drop out of support programs. Thus, in the focus groups we asked about preference for intervention mode (one-to-one versus group). We hypothesized that more stressed caregivers would prefer the one-to-one mode. Data were analyzed using paired t-tests for the quantitative data, and a phenomenological lens for the qualitative data. Of the thirteen enrolled caregivers, twelve completed the study. Significant improvement was found in caregiver reactivity (p=0.001). Twelve caregivers participated in focus groups, in which they reported that the intervention helpful and they valued the therapeutic relationships with the consultants. Few had difficulty with the technological interface. Most liked the one-to-one mode of the intervention, but were open to a hypothetical group-based option. However, more stressed caregivers preferred a one-to-one intervention over a group intervention (p=0.06). While one-to-one interventions tend to be preferred by caregivers, they are expensive. Our findings suggest that one-to-one telehealth-based interventions should be reserved for more stressed caregivers who need intensive support with managing behavioral symptoms of dementia.

### EDUCATIONAL OUTREACH IN ALZHEIMER'S DISEASE AMONG OLDER AFRICAN AMERICANS

Ashley R. Shaw,[1] Briana Bright,[1] Jaime Perales Puchalt,[1] Eric Vidoni,[2] Gabriela Amparan,[3] and Broderick Crawford[4], *1. University of Kansas Alzheimer's Disease Center, Fairway, Kansas, United States, 2. University of Kansas Medical Center, Kansas City, Kansas, United States, 3. University of Kansas Alzheimer's Disease Center, Fairway, Kansas, United States, 4. NBC Community Development Corporation, Kansas City, Kansas, United States*

Alzheimer's disease (AD) is a growing public health problem that continues to disproportionally impact African Americans. African Americans are twice as likely to be afflicted with AD compared to non-Latino Whites. However, continued lack of inclusion of African Americans in clinical research trials may reduce the generalizability of future treatments. We investigated how culturally tailored prevention education impacted knowledge, attitudes, and beliefs of AD among older African Americans. We also assessed how culturally tailored prevention education impacted participation in clinical research trials among older African Americans. Researchers delivered "Aging with Grace," a culturally tailored dementia program to community and faith-based organizations. Demographic information, knowledge of AD, and beliefs of clinical research trials were collected using pre- and post-surveys. In addition, information from community members interested in enrolling in a clinical research study was acquired. A total of 66 community members attended "Aging with Grace" from March to August 2019. 32% of participants perceived an increase in AD knowledge. Most participants (89.1%) believed that more African Americans should participate in research and 29 (44%) expressed interest in enrolling in clinical trials (observational – 73.2%, prevention – 68.2%, treatment – 24.4%). Most participants (93.1%) rated the presentation highly informative and 78% reported that the presentation was very applicable to their daily life. Overall knowledge of AD and interest in participating clinical trials improved with culturally tailored education. Future research should explore ways of enhancing knowledge and participation to enhance inclusion in prevention and treatment trials.

### THE INTERNET IS A BOY'S CLUB?: ANALYZING GENDER DIFFERENCES IN COMPUTER & INTERNET ADOPTION BY OLDER ADULTS

Derek A. Wilson[1], *1. University of Kansas, Lawrence, Kansas, United States*

The realization of technology's exponential advancement has been noted in recent years. With ever advancing technologies becoming more integrated in our everyday lives, we must adapt and learn to utilize these new technologies in order to maintain a presence in society. One group that has been stereotyped as struggling to adopt and learn the processes involved with advancing technologies is that of the older adult population. However, previous literature tends to suggest that there are also gendered differences in the adoption of different forms of modern and advancing technologies among older adults. While some articles state that older adult women have been reported to use social media than their male counterparts, conflicting previous literature states that older adult women use newer technologies less, but are more creative in their uses. With these conflicting reports, there comes a need for proper analyses on the gendered use of modern technology among older adults. Using data from the 2015 Current Population Survey (CPS) Computer and Internet Use Supplement, different demographic factors are analyzed for influences on use of technologies. The older adult age cohorts analyzed include the Baby Boomer and the Silent Generation age cohorts. This analysis includes of controlling for additional factors such as regional residence, educational attainment, and other social location variables. Contrary to much of the existing literature, there is little difference in the adoption of computer and internet technologies by older adults. Additionally, the analyses are performed on the Generation X and Millennial age cohorts for comparison.

### SOCIAL ISOLATION AND COGNITIVE FUNCTIONING AMONG OLDER JAPANESE ADULTS

Shohei Okamoto,[1] Erika Kobayashi,[2] and Jersey Liang[3], *1. Keio University, Minato-ku, Tokyo, Japan, 2. Tokyo Metropolitan Institute of Gerontology, Tokyo, Japan, 3. University of Michigan, Ann Arbor, Michigan, United States*

This research aimed to assess the relationship between social isolation and cognitive functioning among older Japanese adults, thereby expanding the relevant literature in two main ways. First, we estimated a social isolation score to incorporate objective measurements of social isolation into a subjective measurement. Second, a panel data analysis was utilised to consider the change in the social isolation score and time-invariant unobserved heterogeneity. Data were derived from the National Survey of the Japanese Elderly, a survey of a sample of older Japanese adults aged 60 to 99 in waves 3 through 7, which contain unified information of social isolation. The sample included 4,889 observations (1,836 individuals) for men and 6,621 observations (2,433 individuals) for women. The predicted isolation score was obtained by a random-effects ordered logistic regression (i.e., regressing a subjective feeling of isolation on variables regarding social interaction, social support, and social engagement). The association of cognitive functioning with the isolation score was estimated by a fixed-effects ordinary least squares regression, controlling for age, socioeconomic variables, health conditions, and time fixed-effects. We found that increased isolation was associated with a deterioration in cognitive functioning, both for men (coefficient: 0.66, robust standard error [SE]: 0.30) and women (coefficient: 0.90, SE: 0.26). Findings of this research highlight the importance of actions aimed at inhibiting social isolation for the prevention of cognitive decline. This approach is potentially beneficial for developing measurements of both subjective and objective social isolation and estimating the longitudinal relationship between social isolation and cognitive functioning.

### INEQUALITY IN COMMUNITY HEALTH SERVICES USE IN ISRAEL: A COMPARISON BETWEEN ELDER JEWS AND ARABS

Ariela Lowenstein,[1] and Sigal Pearl Naim[2], *1. University of Haifa, Haifa, Israel, 2. Yezreel Valley Academic College, Yezreel Valley, Israel*

Population aging is an important social and public health issue globally. However, increase in longevity causes physical frailty and disability for many elders, which might lead to independence loss and impact quality of life. This increases health services usage and leads to higher costs of medical treatments. Data show that higher socio-economic status and accessibility to health services might reduce inequality in service use and impact mortality rates and quality of life. Also, that improved socio-economic status and population accessibility to Health services may stem from inner health system factors, as well as those related to the patients. Among minorities lower usage of formal professional services, including health services, are often related to cultural differences and many times to lower technological level, which are not considered by service providing organizations. Thus, lack of attention to service using minorities' needs may cause a gap between potential consumers to services use. Israel is a multi-cultural society with mixed population of Jews and Arabs. Currently, Arabs comprise 20.9% and Jews 74.3%. However, the rate of disabled Jews is 16% compared to 31% among older Arabs.

### SERUM 25(OH)D SIGNIFICANTLY IMPACTS ALZHEIMER'S DISEASE IN OLDER ADULTS LIVING IN LONG-TERM CARE COMMUNITIES.

Robert A. Churchill,[1] Ronna Robbins,[2] Nalini Ranjit,[3] Sara Sweitzer,[1] and Margaret Briley[1], *1. The University of Texas at Austin, Austin, Texas, United States, 2. The University of Texas at Austin, Austin, Texas, United States, 3. The University of Texas Health Science Center at Houston School of Public Health in Austin, Austin, Texas, United States*

6.08 million Americans suffer from Alzheimer's Disease (AD), with some estimating diagnoses will reach 15.0 million by 2060. Age is the strongest risk factor for AD, and the prevalence of AD among older adults necessitates investigation into preventable risk factors. 25-hydroxyvitamin D [25(OH)D] deficiency is more prevalent in older adults than other age demographics. Research shows a strong correlation between deficient 25(OH)D serum levels (≤20ng/ml) and AD diagnosis. The association with insufficient (≤30 ng/ml) levels remains unclear. Older adults (age > 65 yo) of five LTC communities in Texas participated in the cross-sectional study. One-year medical history and demographics were abstracted from medical records using double-blinded data abstraction and entry. Blood draws measured 25(OH)D serum levels. Adjusted logistic regression models examined if insufficient 25(OH)D serum levels (≤30 ng/ml) are associated with AD diagnosis. Confounders were total daily vitamin D supplementation, BMI, race, gender, age, years in community, and diagnosis of liver and renal disease. Participants (n=174, mean age: 83 yo) consisted of 63% female and 89% Caucasian. Fifty five percent had insufficient serum 25(OH)D levels (mean level: 32.6 ng/ml; mean supplementation rate: 1,138 IU per/d), and 20% had diagnosis of AD. 25% had both insufficient serum levels and AD, while 12.6% had adequate serum levels and AD. Those with insufficient 25(OH)D serum levels had elevated odds (OR=2.8; CL: 1.14, 7.02; p=0.024) of having AD after adjusting for confounders. Insufficient serum 25(OH)D levels (≤30 ng/ml) are associated with increased diagnoses of AD, indicating the importance of adequate levels among LTC residents.

### DIFFERENTIAL EFFECTS OF STATINS ON COGNITION IN WOMEN AT RISK FOR ALZHEIMER'S DISEASE

Tonita E. Wroolie,[1] Siena Roat-Shumway,[2] Katie Watson,[2] and Natalie Rasgon[2], *1. Stanford University, School of Medicine, Department of Psychiatry & Behavioral Sciences, Stanford, California, United States, 2. Stanford University, School of Medicine, Stanford, California, United States*

It is well established that the apolipoprotein epsilon 4 allele (APOE4) and being female are risk factors for late onset Alzheimer's disease (AD) and declines in verbal learning and memory are early cognitive symptoms of conversion to AD. Because of conflicting findings regarding the effects of statins on cognition, this study examined statin use with respect to verbal learning and memory by APOE4 status in a sample of cognitively unimpaired women at risk for AD. Neuropsychological, statin use, and APOE4 data were utilized as a secondary analysis from an ongoing longitudinal study at the Banner Alzheimer's Institute in Arizona. Subjects were cognitively unimpaired women aged 47-75 with a family history of probable AD in at least one first-degree relative. Neuropsychological outcome variables included total learning, immediate memory, and delayed memory scores from the Rey Auditory Verbal Learning Test (RAVLT). Statin use was defined by use of a cholesterol lowering drug at study enrollment. APOE4 status was defined by presence of at least one APOE4 allele. Linear regression analyses were conducted to determine existence of interactions between statin use and APOE4 status on cognition. Statistically significant interactions were found between statin use and APOE4 status in RAVLT total learning and immediate memory. Statin use in women APOE4 non-carriers was associated with better verbal learning and immediate memory performances whereas statin use in women APOE4 carriers was associated with worse performances on these same tasks. Conclusions. Findings suggest that sex and APOE4 status may be important factors in consideration of statin use.

### COMPREHENSIVE CARE FOR JOINT REPLACEMENT MODEL: POST-ACUTE CARE AND PREFERRED PROVIDER NETWORKS

Cynthia A. Gruman,[1] Amy Cowell,[2] Kathryn Palmisano,[2] Shannon Rogers,[2] and Laura Dummit[1], *1. The Lewin Group, Falls Church, Virginia, United States, 2. The Lewin Group, Eden Prairie, Minnesota, United States*

The Comprehensive Care for Joint Replacement (CJR) model, implemented by the Centers for Medicare & Medicaid Services in 2016, is a randomized, controlled trial that tests the effect of holding a hospital accountable for payments and quality of all services provided to lower extremity joint replacement (LEJR) patients during an episode of care. The newly released results include 147,923 LEJR episodes that were initiated by 733 hospitals in 67 randomly selected metropolitan statistical areas. The objective of this presentation is to explore changes to the care pathway using results from a mixed-methods analytic approach including triangulation of findings from analysis of Medicare claims, hospital survey and hospital and associated provider interview data. Hospitals reported implementing notable changes over the past two years including hiring navigators, changes to therapy protocols, and direct discharge home. Hospital interviewees described efforts to strengthen relationships with PAC providers including the investment of resources into the development of preferred PAC provider networks. As a result of these changes, the average number of SNF days decreased by 2.3 days more for CJR episodes than for control group episodes from the baseline to the intervention period ($p<0.01$). Changes in two of nine complexity measures indicated a statistically significant relative decrease in CJR patients' functional status at SNF admission. The relative increases in CJR patients' average early-loss activities of daily living (ADLs) scores ($p<0.05$) and motion scores ($p<0.10$) suggest an increase in patients with greater needs were discharged to a SNF relative to the control group.

### WHAT DOES SUCCESS MEAN IN THE CONTEXT OF ELDER ABUSE INTERVENTION FROM THE PERSPECTIVE OF VICTIMS?

David Burnes,[1] Jessica Hsieh,[1] Clara Scher,[2] Paula Zanotti,[3] Chelsie O. Burchett,[3] Jo Anne Sirey,[3] and Mark Lachs[4], *1. University of Toronto, Toronto, Ontario, Canada, 2. Weill Cornell Medicine, White Plains, New York, United States, 3. Weill Cornell Medicine, New York, New York, United States, 4. Weill Cornell Medical College / New York Presbyterian Hospital, New York, New York, United States*

Adult protective services (APS) and other community-based agencies respond to hundreds of thousands of elder abuse cases each year in the United States; however, little is known about what constitutes success in the context of elder abuse response intervention. This study explored the meaning of elder abuse intervention success from the perspective of victims themselves toward the development of a victim-centric taxonomy of outcomes. Guided by a phenomenological qualitative methodology, this study conducted in-person, semi-structured interviews with a sample of elder abuse victims (n = 30) recruited from APS in the states of Maine, New York, and California, as well as a community-based elder abuse social service program in New York City. To enhance trustworthiness, two researchers independently analyzed transcript data to identify key transcript statements into themes. Outcomes of success were identified across broad domains related to the victim, perpetrator, victim-perpetrator relationship, family system, and home environment. Specifically, common themes represented outcomes related to victim safety, autonomy, social support, and state of mind; perpetrator independence and accountability; and victim-perpetrator separation. For decades, the field of elder abuse has struggled to understand how to define success in the context of community-based intervention from a client-centered perspective. The taxonomy developed in this study provides a comprehensive and conceptually organized range of successful outcomes to serve as infrastructure for the development of meaningful intervention outcome measures. This study represents one of the largest efforts to understand and integrate the perspectives and needs of victims into elder abuse intervention practice/research to date.

### VIRTUAL ONLINE COMMUNITIES FOR AGING LIFE EXPERIENCE (VOCALE) APPROACH: PILOT STUDIES

Oleg Zaslavsky,[1] Annie T. Chen,[1] Andrew Teng,[1] Shih-Yin Lin,[2] Soojeong Han,[1] Frances Chu,[1] and George Demiris[3], *1. University of Washington, Seattle, Washington, United States, 2. NYU Rory Meyers College of Nursing, New York, New York, United States, 3. University of Pennsylvania School of Nursing, Philadelphia, Pennsylvania, United States*

Emerging evidence suggests behavioral strategies focusing on symptom management can reduce frailty symptoms and improve quality of life. Unfortunately, these interventions are rarely scalable for implementation in geriatric care. Contemporary online technologies have tremendous potential for addressing this need. We developed and pilot tested an approach entitled Virtual Online Community for Aging Life Experience (VOCALE). The approach had two stages. In the first stage, we piloted the use of a Facebook platform to engage older adults with frailty symptoms in ten-week moderated discussions on health-related topics. In the second study, we used data from stage one to develop a prototypical persona of a person with frailty symptoms. The persona was then incorporated into an eight-week Facebook intervention informed by problem solving therapy to facilitate self-management in another group of older adults with frailty symptoms. The results from both rounds showed that it was feasible to recruit, engage, and retain persons ages 69-92 into virtual online community interventions. Attrition ranged from 25% to 33% in rounds one and two. In both rounds, we observed positive trends of change in health measures such as general health self-efficacy, disease self-efficacy, and health literacy. Throughout the studies, older adults shared multiple posts concerning their experience with age-related symptoms and described their self-management practices.

These projects, which leveraged a common social media platform, demonstrated preliminary efficacy of an online intervention for frailty management. If confirmed, this approach might provide a viable model for other medically complex geriatric conditions where self-management is essential.

### ASSOCIATION BETWEEN WALKING ENERGETICS AND FRAGMENTED PHYSICAL ACTIVITY IN MID-TO-LATE LIFE

Fangyu Liu,[1] Amal A. Wanigatunga,[1] Pei-Lun Kuo,[2] Vadim Zipunnikov,[1] Eleanor M. Simonsick,[3] and Jennifer A. Schrack[4], *1. Johns Hopkins Bloomberg School of Public Health, Baltimore, Maryland, United States, 2. Johns Hopkins University Bloomberg School of Public Health, Baltimore, Maryland, United States, 3. Longitudinal Studies Section, Intramural Research Program, National Institute on Aging, Baltimore, Maryland, United States, 4. Johns Hopkins University, Baltimore, Maryland, United States*

Physical activity becomes increasingly fragmented with age, and may be an early marker of functional decline. Energy regulation has been linked with functional decline, yet whether the energy needed for walking, a common type of physical activity, is related to fragmentation of physical activity remains unknown. The study population included 493 participants aged 50-93 years from the Baltimore Longitudinal Study of Aging. Energetic measures included the energetic cost of usual-paced overground walking (ml/kg/m), the average energy expended (ml/kg/min) during a rapid-paced 400-m walk, and a cost-to-capacity ratio between the energy expended during 5-min treadmill walk (0.67 m/s, 0% grade) and the energy expended during the 400-m walk. Activity fragmentation was extracted from accelerometer data collected over ≥3 valid days and quantified via an active-to-sedentary transition probability (ASTP). Associations between the energetic measures and ASTP were assessed using multivariate linear regression models. Interactions between energetics and total daily physical activity, quantified as total log-transformed activity counts (TLAC), were also assessed. After adjusting for TLAC, demographics, body composition and comorbidity, higher cost-to-capacity ratio was associated with 3.51% greater fragmented physical activity ($p=0.005$). Energetics by TLAC interactions revealed that lower rapid-paced walking energy expenditure and higher cost-to-capacity ratio were only significantly associated with greater fragmentation in the most sedentary participants ($p<0.01$ for both). Our results suggest that deterioration of walking efficiency may manifest as a more fragmented physical activity profile, especially among sedentary adults. Future longitudinal studies to understand whether declining walking efficiency predicts the onset and progression of activity fragmentation are warranted.

### THE IMPORTANCE OF HSP-25 IN CAENORHABDITIS ELEGANS LONGEVITY

Niaya James,[1] Jessica L. Scheirer,[2] and Karl Rodriguez[3], *1. Howard University, washington, District of Columbia, United States, 2. UT Health Science Center, San Antionio, San Antionio, Texas, United States, 3. Sam and Ann Barshop Institute for Longevity and Aging Research, San Antonio, Texas, United States*

Karl A. Rodriguez's laboratory at the University of Texas Health Science Center, San Antonio, Texas, is interested in the role of small heat shock proteins in the proteostasis network and aging using the model organism, Caenorhabditis elegans. Molecular chaperones facilitate protein folding and improve the degradation activity of the proteasome and autolysosome hence decreasing disease-associated aggregates. Previous work in rodents have shown an increase in expression levels of the small heat shock protein 25 (HSP-25) correlates with maximum lifespan potential. To further explore the role of HSP-25 in C. elegans, two HSP-25 knock-out strains were exposed to a one-hour heat stress, heat shock, and two non-heat stress conditions.

### SESSION LB1545 (LATE BREAKING POSTER)

### LATE BREAKING POSTER SESSION II

### NEUROPSYCHOLOGICAL ASSESSMENT OF POSTERIOR CORTICAL ATROPHY: A CASE STUDY

Amy Albright,[1] John Burkhardt,[1] Catherine Ikard,[1] and Anne Halli-Tierney[2], *1. University of Alabama, Tuscaloosa, Alabama, United States, 2. The University of Alabama College of Community Health Sciences, Tuscaloosa, Alabama, United States*

The following case study examines the presentation of Mr. Fraser*, an older adult African American male diagnosed with Posterior Cortical Atrophy (PCA) following neuropsychological evaluation. PCA is a rare variant of Alzheimer's Disease (AD) that results in visuospatial and perceptual deficits. Unlike other forms of neurocognitive degeneration, PCA tends to present at a relatively young age and may progress rapidly. There is currently a lack of studies examining PCA from a neuropsychological perspective, which may contribute to low awareness of this condition, as well as delayed diagnosis. It has been estimated that approximately 5% of patients with AD exhibit the PCA variant, implying that this a rare but serious condition. The following case study focuses on Mr. Fraser, a 65-year-old who was referred for neuropsychological assessment to assess his cognitive functioning. Mr. Fraser was administered a comprehensive assessment battery, and his overall results were suggestive of severe deficits in delayed memory and visuospatial skills. In the case of Mr. Fraser, these observed deficits, along with identification of visual complaints noted by his geriatrician, ultimately led to a diagnosis of PCA. While this was supported by neurological testing, the DSM-5 does not currently recognize PCA as a diagnosis. As a result, Mr. Fraser was given a diagnosis of possible AD with potential PCA, which may contribute to underestimates of the prevalence of this disorder. Future research and practice should focus on common neuropsychological presentations of this condition. *Identifying information changed in accordance with HIPAA guidelines

### NINE YEAR CHANGES IN PREVALENCE OF COGNITIVE IMPAIRMENT IN THE CZECH REPUBLIC

Dominika Seblova,[1] Marie Kuklova,[3] Miloslav Kopecek,[3] and Pavla Cermakova, *1. Columbia*

*University, Taub Institute for Research in Alzheimer's Disease and the Aging Brain - The Gertrude H. Sergievsky Center, New York, New York, United States*
Carol Brayne[2], 2. *Department of Public Health and Primary Care, Cambridge Institute of Public Health, University of Cambridge, Cambridge, United Kingdom*
Vendula Machu[3], 3. *National Institute of Mental Health, Klecany, Czech Republic*

Studies from North America and Western Europe suggest stable or declining trends in impaired cognition. Nevertheless, data on changes in cognitive health from Central and Eastern Europe are largely lacking. Therefore, we aimed to examine changes in the age-specific prevalence of cognitive impairment in the Czech Republic, a country in Central Europe. To this aim we used two samples from the population-based Czech Survey on Health, Ageing and Retirement in Europe (SHARE). Age-specific prevalence of cognitive impairment (defined based on scores in verbal fluency, immediate recall, delayed recall and temporal orientation) was compared between participants in wave 2 (2006/2007; n=1,107) and wave 6 (2015; n=3,104). Logistic regression was used to estimate the association between wave and cognitive impairment, step-wise adjusting for sociodemographic and clinical characteristics. Multiple sensitivity analyses, focusing on alternative operationalisations of relative cognitive impairment, impact of missing cognitive data and survival bias, were carried out. The most conservative estimate suggested that the age-specific prevalence of cognitive impairment declined by one fifth, from 11% in 2006/2007 to 9% in 2015. Decline was observed in all sensitivity analyses. Multivariate decomposition for nonlinear models was used to examine which predictors explain the change in prevalence. Reduction in physical inactivity, control of high blood cholesterol and increases in length of education were the main predictors contributing to decline in cognitive impairment. In conclusion, our findings are in line with those found in North America and Western Europe even though countries in Central and Eastern Europe, including Czech Republic, have poorer risk profiles.

### "HE EXPLAINED IT TO ME AND I ALSO DID IT MYSELF": HOW OLDER ADULTS GET HELP WITH DIGITAL TECHNOLOGY

Amanda E. Hunsaker,[1] Minh Hao Nguyen,[1] Jaelle Fuchs,[1] Teodora Djukaric,[1] Larissa Hugentobler,[1] and Eszter Hargittai[1], 1. *University of Zurich, Zurich, Switzerland*

Older adults comprise a highly heterogeneous group that engages with digital media in varying ways, therefore a large variation in technology support needs is likely. This study examines the nature of support for using digital media among older adults. We conducted in-depth qualitative interviews with older adults (age 59+) in Hungary, the Netherlands, and Switzerland (N=58) in 2019 exploring: (1) whether and how older adults receive support in using digital media; and (2) older adults' perceptions of whether the support they receive meets their needs. We began with open coding, then conducted consensus meetings to identify themes and coding schemes, and wrote memos to share findings and ensure reliability across coders. We find that older adults voice a highly varying range of need for technical support as well as varying instances of both receiving and not receiving technical help. Participants report receiving help from different informal (e.g. spouses) and formal (e.g. computer classes) sources. However, support may not be immediate, posing challenges for older adults who depend on the availability of their support sources. Importantly, we also find that there are older adults who are quite self-sufficient in the ways they use digital technology. For older adults needing support, greater access to community-based support may help those without satisfactory options in their own social circle. Given our findings that older adults can have great ease with solving technology-related problems, peer-driven support networks where older adults can offer support to others may be an effective approach to providing digital technology guidance.

### DEVELOPMENT AND EVALUATION OF THE NURSES IMPROVING CARE FOR HEALTHSYSTEM ELDERS (NICHE) LONG-TERM CARE PROGRAM

Jennifer Pettis,[1] Alycia A. Bristol,[2] Abraham A. Brody,[3] Mattia J. Gilmartin,[1] Catherine O. D'Amico,[4] Sherry Greenberg,[5] and Eileen M Sullivan-Marx[6], 1. *New York University Rory Meyers College of Nursing, Nurses Improving Care for Healthsystem Elders (NICHE), New York, New York, United States,* 2. *NYU Rory Meyers College of Nursing, New York, New York, United States,* 3. *NYU Rory Meyers College of Nursing, New York, United States,* 4. *NICHE -- NYU Meyers College of Nursing, New York, New York, United States,* 5. *Seton Hall University College of Nursing, South Orange, New Jersey, United States,* 6. *Rory Meyers College of Nursing, New York University, New York, New York, United States*

Nurses Improving Care for Healthsystem Elders (NICHE) is a geriatric care model that positions nurses as leaders to address the unique care needs of older adults. From 2016 to 2019, we developed and piloted the NICHE-LTC program. NICHE-LTC program includes the three interrelated components of 1). the Geriatric Resource Nurse (GRN) and Geriatric Certified Nursing Assistant (GCNA) clinical leadership roles; 2) research-based clinical protocols and assessment tools; and 3). staff development, quality improvement (QI), and care coordination models to build clinician and organizational capacity in geriatrics. We report the results of a summative program evaluation of the NICHE-LTC program. We collected data on organizational and participant demographics, facility enrollment and retention, program completion rates, clinical quality improvement project plans, and participant satisfaction data from 369 individuals working in 79 facilities participating in NICHE-LTC program from January 2016 to February 2019. The majority of participants (80%) reported a positive learning outcome after completing NICHE-LTC program. Additionally, 80% reported they agreed or strongly agreed that their knowledge and ability to implement quality improvement initiatives in their facilities improved. The NICHE-LTC supported facilities ability to move from process-oriented QI priorities to patient-based outcome QI priorities such as falls, pain management, and managing behaviors of dementia. Over the three-year pilot period, NICHE-LTC program maintained an 89.4% annual retention rate. The high annual retention rate suggests that members value the program as an approach to address the clinical quality and nursing workforce development needs in LTC settings.

### AN INTEGRATED TRANSITIONAL CARE MODEL FOR OLDER ADULTS WITH MULTIMORBIDITY AND DEPRESSIVE SYMPTOMS

Maureen Markle-Reid,[1] Carrie McAiney,[2] Rebecca Ganann,[1] Kathryn Fisher,[1] Amy Bartholomew,[1] McElhaney Janet,[3] Gauthier Alain,[4] and Amiram Gafni[1], *1. McMaster University, Hamilton, Ontario, Canada, 2. University of Waterloo, Waterloo, Ontario, Canada, 3. Health Sciences North, Research Institute, Medical Sciences Division, Northern Ontario School of Medicine, Sudbury, Ontario, Canada, 4. Laurentian University, Sudbury, Ontario, Canada*

This pragmatic randomized controlled trial examined the implementation, effectiveness and costs of a nurse-led transitional care intervention to improve hospital-to-home transitions for 127 older adults (≥ 65 years) with depressive symptoms and multimorbidity in three Ontario communities. Participants were randomly allocated to receive the intervention plus usual care (n=63) or usual care alone (n=64). The intervention included an average of 5 in-home visits and 6 phone calls from a Registered Nurse (RN) over a 6-month period. The RN provided system navigation, patient education, medication review, and management of depressive symptoms and chronic conditions. Implementation outcomes included engagement rate, intervention dose, and feasibility of intervention implementation. Effectiveness outcomes included quality of life, depressive symptoms, anxiety, social support, and health and social service use and costs. Participants were an average of 76 years and had an average of 8 chronic conditions. Findings suggest that the intervention was feasible and acceptable to participants and providers. Intention-to-treat analyses using ANCOVA models showed no statistically significant group differences for the outcomes. However, the upper 95% confidence interval for the mean group difference showed greater clinically significant improvements in physical functioning in the intervention group. Quantile regression showed that the intervention may result in greater improvements in physical functioning for individuals with low to average physical functioning values compared to the control group. The intervention may also result in higher levels of perceived social support for individuals with a range of social support values. No statistically significant group differences were observed for service use or costs.

### PET VISITATION AS NARRATIVE CARE FOR OLDER ADULTS IN THERAPEUTIC SETTINGS

Jill Yamasaki,[1] and Kelley Murfin[2], *1. University of Houston, Houston, Texas, United States, 2. MD Anderson Cancer Center, Houston, Texas, United States*

A growing body of research highlights the physiological and psychosocial benefits of pet visitation programs in therapeutic settings. These programs utilize the profound connection between humans and animals to promote holistic healing, foster greater quality of life, and influence meaningful communication between patients and providers. For older adults in hospitals or long-term care, these benefits are often correlated with moments of pleasure, comfort, relaxation, and entertainment. The current study builds on this prior knowledge by examining pet visitation programs as a novel form of narrative care that can also help preserve biographical continuity and promote the sharing of lived stories. We worked with two volunteer pet visitation programs in Houston and one in Los Angeles. Our research included a variety of ethnographic methods, including participant observation; informal interviews with providers, patients (or residents, depending on the context), and their families; semi-structured interviews with volunteers; and discourse review of organizational materials. We employed a method of constant comparison to identify and thematically analyze recurrent patterns of behavior and overarching meanings across the data. Three primary themes emerged from the data: (a) compassion, (b) connection, and (c) response. Collectively, the presence of pets prompted stories and behaviors that foster healing relationships characterized by empathy and mutual understanding between patients (or residents), family members, and providers. Pet visitation programs facilitate storied conversations, increased autonomy, and alternative ways of knowing that promote greater understandings of the patient's (or resident's) psychosocial context and biographical history, leading to more personalized care and improved well-being.

### META-ANALYSIS OF INTERVENTIONS TO REDUCE SEDENTARY BEHAVIOR AMONG OLDER ADULTS

Jennifer A. Otmanowski,[1] Sheri A. Rowland,[2] Pamela S. Cooper,[3] and Jo-Ana D. Chase[4], *1. University of Missouri-Columbia, Concord, Michigan, United States, 2. University of Nebraska Medical Center, Lincoln, Nebraska, United States, 3. Cooper Editorial and Scientific Consulting, Columbia, Missouri, United States, 4. University of Missouri, Columbia, Missouri, United States*

ABSTRACT BODY Sedentary behavior (SB) is associated with substantial health risks such as increased risk of cardiovascular mortality, diabetes, and cognitive and physical functioning decline. Older adults are particularly at risk as they are the most sedentary population. The purpose of this meta-analysis was to determine the overall effects of interventions designed to reduce SB among older adults. A comprehensive literature search of online databases, bibliographies, and author searches located published and unpublished studies. Included studies tested interventions to reduce SB time, were written in English, and focused on community-dwelling adults age 60 years or older. Data were extracted on sample, study design, and intervention characteristics using an investigator-developed tool. Study effect sizes were synthesized using a random effects model. Heterogeneity of effects across studies was examined; however, moderator analyses were not conducted due to the small number of included studies. Of the 2,408 reviewed citations, 22 reports were included representing 17 distinct studies, eight of which were included in the two-group post-test meta-analysis (n= 1,024 participants). Interventions overall modestly reduced SB time among older adults (d=-.25, 95% CI [-.50, .00], p=.05); however, significant heterogeneity of effect size was observed across studies (Q=22.34, p<.01). Our findings demonstrate a need for more research targeting SB reduction in this high-risk population. Future research should include measures of breaks in sedentary time and types of SB

(e.g., watching TV, reading) which are also critical indicators of health risk. Moreover, further exploration of relationships between health outcomes and SB intervention effects is needed.

### MAILED SURVEYS WITH NONAGENARIANS - CHALLENGES AND LESSONS FROM THE VITALITY 90+ STUDY

Marja Jylha,[1] Jani Raitanen,[1] Kristina Tiainen,[1] Pauliina Halonen,[1] and Linda Enroth[1], *1. Tampere University, Gerontology Research Center, Tampere, Finland*

Reliable population-based data on health, functioning and quality of life among very old people are scarce because only during the last decades this age group has grown to be an important segment of population, and because data collection among the oldest old is challenging. Due to poor health, problems in hearing and vision, cognitive decline, and institutionalization, very old individuals may not be able to participate in research studies, or, the information they give may not be reliable. In the Vitality 90+ Study, the whole population aged 90+ in the Tampere area, Finland, has been investigated six times since 2001. Mailed surveys have been conducted in years 1995, 1996, 1998, 2001, 2003, 2007, 2010, 2014, and 2018. In each data collection, the response rate has been ca 80%. The questionnaires and the wording of the questions have been identical in each survey round, which provides data for investigating time trends in health, functioning, and quality of life. Linkages with national population and care registers are used for studying mortality and care use. In this poster, we analyze the impact of 1) exhaustive base data, 2) the questionnaire, 3) including institutionalized individuals and proxy answers, on the findings and on the quality and reliability of the data. We conclude that mailed surveys can be a feasible method of data collection among very old people, but only in favorable local circumstances and with great efforts from the research group.

### PILOT OUTCOMES OF A COMMUNITY-BASED FALLS PREVENTION PROGRAM TARGETING OLDER ARAB AMERICANS

Lan Yao,[1] Suha Kridli,[1] and Amne Talab[2], *1. Oakland University, Rochester, Michigan, United States, 2. Arab Community Center for Economic and Social Services, Dearborn, Michigan, United States*

Arab American (AA) is the 3rd largest ethnic population in the state of Michigan. Previous studies found that Michigan Arabs were less healthy than the general population in Michigan. Older AAs have higher mortality risk than non-Arab and non-Hispanic Whites, particularly due to chronic diseases. Community-based programs are an effective approach to prevent disease and injury, improve health, and enhance quality of life. While evidence for functional gains resulting from Tai Chi exercise is accumulating, there is little research and support for its feasibility and effectiveness that target older AAs, who are not culturally related to Tai Chi. Participants in this report were 8 older female AAs (mean age 62.4±3.2, range 58-66) who sought services at a not-for-profit Arab Community Center, which aims to enable and empower residents and communities to lead informed, productive and culturally sensitive lives. A certified Tai Chi instructor led the classes using Yang-style Tai Chi moves. The participants completed a 12-week twice-weekly 1-hour Tai Chi classes and post-program focus group discussion, held in a classroom of the Community Center. Post-intervention improvement in timed up & go test (p>.05), comfortable & fast gait speed (p>.05), unipedal stance time (p>.05) were observed. Themes identified from focus group supported Tai Chi's benefits in balance and salient mental health benefits. The pilot data provides preliminary evidence for therapeutic gains resulting from Tai Chi practices. Agencies serving older AAs play important roles by creating and promoting evidence-based health promotion practices to address the growing needs among older adults.

### THE ASSOCIATION OF PAIN WITH PAST AND FUTURE SELF-CONTINUITY

Gillian Fennell,[1] Abby Pui Wang Yip,[2] Cary Reid,[3] and Corinna Loeckenhoff[2], *1. University of Southern California, Los Angeles, California, United States, 2. Cornell University, Ithaca, New York, United States, 3. Weill Cornell Medicine, New York, New York, United States*

Qualitative research on chronic pain patients' subjective experiences has documented feelings of discontinuity between present and past selves due to changes in physical functioning and social roles. This investigation is the first to test the relationship between pain and self-continuity quantitatively and does so across two samples: Study 1 involved an adult community sample (n = 230, aged 18-87) and Study 2 involved a sample of older chronic pain patients (n = 145, aged 45-94). We explored potential differences for proximal versus distant selves and past versus future selves. In both studies, pain magnitude was negatively associated with average self-continuity (ps <.05), although the effect was selectively driven by future self-continuity in Study 1 (p < .01) and past self-continuity in Study 2 (p < .01). Additionally, in Study 2, recency of pain onset was negatively associated with past self-continuity (p < .001), but not with future self-continuity (p = .47). These findings suggest that chronic pain may be detrimental to self-continuity, with some variability linked to magnitude and chronicity of the pain. Health care providers may want to monitor their patients for feelings of disconnectedness with past and future selves. Future research is needed to identify therapeutic strategies that promote a continuous sense of self in spite of pain-related challenges.

### PREDICTORS OF SUICIDE IDEATION AMONG OLDER GHANAIAN WOMEN: RESULTS FROM THE STUDY ON GLOBAL AGEING AND ADULT HEALTH

James Muruthi,[1] Alicia DeLouize,[2] Richard Biritwum,[3] Nirmala naidoo,[4] Paul Kowal,[5] and Josh Snodgrass[2], *1. University of Oregon, Eugene, Oregon, United States, 2. University of Oregon, Oregon, United States, 3. University of Ghana, Acrra, Ghana, 4. WHO, Geneva, SD, Switzerland, 5. The University of Newcastle, School of Medicine and Public Health, Faculty of Health and Medicine, Australia*

The World Health Organization (WHO) estimates that 79% suicides occur in low- and middle-income countries (LMICs) in 2016 (WHO, 2018) putting a spotlight on the topic of suicide in these countries. While the rates are highest among individuals ages 15 to 29 years, suicide affects individuals, families, and communities throughout the lifespan. The topic of suicide among older adults, especially those living in LMICs, has unfortunately received limited attention. Using a sample of 228 women with depression from the first wave of the WHO's Study on global AGEing and adult health (SAGE, Ghana), the present study sought to identify predictors of suicide ideation among aging African women. Binominal logistic regression results revealed that wealth (negatively) and health (negatively) were associated with suicide ideation in the sample; indicating that the women who were poor and unhealthy were more likely to have suicidal thoughts. Implications of the findings for aging Ghanaian women will be addressed.

### ATTITUDES, EXPECTATIONS, KNOWLEDGE AND INTENTIONS REGARDING ORAL HEALTH: PERCEPTIONS OF OLDER ADULTS

Virginia M. Hardgraves,[1] Jean Henry,[2] and Susan K. Patton[2], *1. University of Arkansas Fayetteville, Fayetteville, Arkansas, United States, 2. University of Arkansas, Fayetteville, Arkansas, United States*

Many adults in today's aging cohort are maintaining their teeth into their advanced years. The advantages of fluoridated water, dental insurance, greater awareness of preventive oral healthcare, and more restorative dental services, have made this possible. The demand for oral health care services will be greater and more complex than that of previous generations. Evidence of a link between oral health and overall health underscores the need to better integrate dental care into the healthcare system. The aim of this study was to better understand these issues from the perspective of older adults (N = 26) 65 years of age and older and living independently. Semi structured interviews guided by the behavioral constructs of the Reasoned Action Approach Theory were conducted. Results from the qualitative analysis revealed five themes: 1) Difficulties accessing dental care, 2) Stoic independence, 3) Taking care of your mouth as part of overall health, 4) Relationships affecting oral health related quality of life, and 5) Supporting roles. The findings demonstrate a need to increase oral health literacy in the older adult population with attention to reducing modifiable risk factors. Understanding these behaviors and the current level of oral and overall health knowledge from the perspective of older adults, is vital to helping these individuals' transition into increasing levels of dependency with a high level of oral health related quality of life. Public health program planning can use this information to help older adults prepare for the transitions that come with healthy aging.

### BACTERIAL PNEUMONIA IS A POSSIBLE RISK FACTOR FOR ORAL CANDIDIASIS IN OLDER ADULTS: A RETROSPECTIVE COHORT STUDY.

Masato Nakajima,[1] Yojiro Umezaki,[1] Masahiro Yamaguchi,[1] Michiko Makino,[1] Nao suzuki,[2] Masahiro Yoneda,[1] Takao Hirofuji,[1] and Hiromitsu Morita[1], *1. Department of General Dentistry, Fukuoka Dental College, Fukuoka, Japan, 2. Department of Preventive and Public Health Dentistry, Fukuoka Dental College, Fukuoka, Japan*

Both aspiration pneumonia and oral candidiasis are caused by opportunistic infection of intraoral commensals and have many similar risk factors linked to oral health in older adults. Candida albicans forms biofilms with respiratory bacteria such as Klebsiella pneumoniae and Pseudomonas aeruginosa. The aim of our study was to determine the relationship between bacterial pneumonia, including aspiration pneumonia, and oral candidiasis in older patients who were hospitalized with several systemic diseases in a community-based acute care hospital without a dental unit. We retrospectively analyzed 228 older patients (male: 105, female: 123) using multiple logistic regression. The mean age of the patients was 81.3 (SD: 11.1) years. Forty-four patients were oral candidiasis positive, and 78 patients suffered from bacterial pneumonia, including aspiration pneumonia. Results showed that bacterial pneumonia had the strongest statistical relationship with oral candidiasis (p=0.000, OR: 5.173, 95 % CI: 2.368–11.298). This was followed by poor oral hygiene (p=0.001, OR: 6.095, 95 % CI: 2.003–18.545) and severe dry mouth (p=0.043, OR: 2.507, 95 % CI: 1.031–6.098). Other conventional risk factors for oral candidiasis, such as diabetes mellitus, denture wearer, dysphagia, malnutrition, requiring care, use of inhalation steroids, were not statistically significant in this study. Fifteen species of bacteria, including Klebsiella pneumoniae and Pseudomonas aeruginosa, were detected by pharyngeal sputum culture in 24 of 31 patients who were diagnosed with both oral candidiasis and bacterial pneumonia. In conclusion, bacterial pneumonia is a possible new risk factor for oral candidiasis in older adults.

### PSYCHOSOCIAL CHARACTERISTICS AND DEPRESSIVE SYMPTOMATOLOGY AMONG MEXICAN OLDER ADULTS

L. Paige B. Downer,[1] and Rebeca Wong[1], *1. The University of Texas Medical Branch, Galveston, Texas, United States*

The incidence of mental health problems, specifically depression, are increasing among older adults. Previous research identified psychosocial characteristics related to mental resiliency, such as increased conscientiousness and internal locus of control, as contributing to improved health behaviors and better physical and mental health outcomes. This study identifies the association between these psychosocial traits and depressive symptomatology among Mexican adults aged 50 and older using Wave 4 of the Mexican Health and Aging Study (2015). We hypothesize that psychosocially 'strong' older Mexican adults, those with higher levels of conscientiousness and an internal locus of control, will report fewer depressive symptoms; we theorize that gender moderates this relationship. Older Mexican adults' mental health status was measured through depressive symptomatology using a 9-item version of the Centers for Epidemiologic Studies-Depression (CES-D) scale. Conscientiousness was measured using a 6-item sub-dimension of the "Big 5" personality scale. Locus of control was measured using an 8-item scale adapted from Rotter (1966). Multivariable linear regression was performed and adjusted for socio-demographic characteristics and comorbid chronic health conditions. Depressive symptoms were lower with higher age and formal education; however, older adults in rural environments who are divorced/

separated or widowed exhibit higher depressive symptoms. The association between conscientiousness and depressive symptoms differed by gender. Locus of control and conscientiousness influenced depressive symptomatology in men, however, only conscientiousness impacted women's depressive symptoms. Continued research is needed to assess psychosocial changes over the life-course as they contribute to resilience from adverse life-events that influence mental health in old age.

### THE EFFECTS OF A DECADE OF CHANGE IN THE YOUNG-OLD POPULATION IN A MARGINAL COMMUNITY IN JAPAN

Yuichi Watanabe[1], *1. Musashino University, Nishitokyo, Tokyo, Japan*

Population aging is occurring throughout the world, with an estimated 82 countries expected to have more than 20% of their population age 65 or older by 2050. Communities in which these older adults live face significant challenges in maintaining residential services. Japan is on the leading edge of this phenomenon. In Japan, marginal communities are defined as those where over 50% of the population is 65 years of age or older and face difficulties maintaining the traditional mutual support system in the community. The old-old population might have vulnerabilities because of a lack of resources for daily living. Documenting the changes in these communities over time may help to identify solutions to how to be an inclusive community. we examine the effects of a decade of change using surveys completed in a marginal community in Japan in 2009 and 2019. Data were collected from a cohort of 65 to 74 year olds in 2009 (n=45) and 75 to 84 year olds in 2019 (n=26) in one marginal community. Analysis by Fisher's exact test shows decreasing the exchange of information and decreasing discussion of the future of the community with family and relatives over time. In 2009, respondents surveyed brought up issues such as the shortage of care services and lack of bank facilities, while in 2019 these issues were not mentioned. Results suggest that decisions made regarding the viability of those communities should include input from the elderly to optimize the effects of aging in place.

### GAIT SPEED AND CHILDHOOD-TO-MIDLIFE BRAIN HEALTH: RETHINKING GAIT

Line Rasmussen,[1] Avshalom Caspi,[1] Terrie Moffitt,[1] Harvey J. Cohen,[2] Miriam C. Morey,[3] and Leah Richmond-Rakerd[1], *1. Dept. of Psychology and Neuroscience, Duke University, Durham, North Carolina, United States, 2. Duke University Medical Center, Durham, North Carolina, United States, 3. Durham Veterans Affairs Health Care System, Durham, North Carolina, United States*

Background: Gait speed is a well-known predictor of functional decline and mortality in older adults, but little is known about the origins of gait speed earlier in life. We tested the hypothesis that slow gait reflects accelerated biological aging already at midlife, as well as poor neurocognitive functioning in childhood and childhood-to-midlife cognitive decline. Methods: Prospective study of the population-representative Dunedin Study birth cohort (n=1,037), followed to age 45 (until April 2019). We measured age-45 gait speed in 904 (90.7%) participants and tested associations with key life course factors. Results: The mean (SD) gait speeds (m/s) were: usual: 1.30 (0.17); dual task: 1.16 (0.23); and maximum: 1.99 (0.29). Among midlife adults, those with more physical limitations (β -0.27; P<.001), poorer physical functions (β 0.24–0.36; all P<.001), accelerated biological aging across multiple organ systems (β -0.33), older facial appearance (β -0.25), smaller brain volume (β 0.15), more cortical thinning (β 0.09), smaller cortical surface area (β 0.13), and more white matter hyperintensities (β -0.09) had slower gait speed, all P<.05. Participants with lower IQ in childhood (β 0.34) and midlife (β 0.38) and who exhibited childhood-to-midlife cognitive decline (β 0.10) had slower gait speed at midlife, all P<.01. Adults with poorer neurocognitive function as early as age 3 had slower gait in midlife (β 0.26; P<.001). Conclusion: Adults' gait speed is more than an indicator of geriatric functional status, it is also an index of midlife aging and lifelong brain health.

### MEMORY FUNCTIONING AND CAREGIVING: CONTINUITY, CONNECTEDNESS AND METAPHORS IN ALASKA NATIVE PERCEPTIONS

Rosellen M. Rosich,[1] and Maria Crouch[2], *1. University of Alaska Anchorage, Center for Human Development, Anchorage, Alaska, United States, 2. University of Alaska Anchorage, UAA-UAF PhD in Clinical-Community Psychology with a Rural and Indigenous Emphasis, Wasilla, Alaska, United States*

Biomedical models often define dementia in a negative and diachronic manner, which shape Western, cultural understandings and approaches. However, utilizing a critical gerontological approach has allowed the current study to explore Alaska Natives (i.e., adults who hold "Elder" status and are 50 years or older) perception of memory decline, and the stresses imposed upon caregiving when a Western biomedical model of dementia is utilized. Multitudinous research demonstrates definitions and intersections of health, illness, ethnicity, and family are not universal. Subsequently, it is critical to examine these sociocultural concepts from diverse cultural belief systems and imperative to examine historical processes impacting these constructs to identify specific risk and protective factors regarding holistic health. Recent qualitative data analysis from an exploratory study of Alaska Native Elder's perception of memory functioning and dementia has yielding themes that are consistent with previous research on indigenous culture. However, themes of continuity, connectedness, spirituality, intergenerational transmission, traditional belief systems, and barriers to cultural continuity such as oppression and historical trauma, are being filtered through Alaska Natives unique cultural lens. This cultural lens allows Alaska Natives to utilize positive metaphors for memory functioning and dementia embedded within their belief systems and these are distinct from Western biomedical definitions. The poster proposed will highlight themes recently uncovered from thematic analysis, code book development, and code matrices as well as present the positive, culturally adaptive and congruent representations that Alaska Native Elder's utilize in understanding memory changes and forms of dementia that both explain and transcend biomedical models.

### DOES PAIN PREDICTS FRAILTY AMONG OLDER ADULT AMERICANS: FINDINGS FROM THE NATIONAL HEALTH AND AGING TRENDS STUDY.

Jaspreet k. Sodhi,[1] Lin-Na Chou,[1] and Soham Al Snih[2], *1. The University of Texas Medical Branch, Galveston, Texas, United States, 2. University of Texas Medical Branch at Galveston, Galveston, Texas, United States*

Musculoskeletal pain is highly prevalent among older adults and is one of the common causes of disability. The objective of this study was to examine the effect of pain on becoming frail among American older adults over 6 years of follow-up. We studied 5,229 participants aged ≥ 65 years from the National Health and Aging Trends Study (2011-2017) who were non-frail at baseline. Key variables included pain, socio-demographic characteristics (age, gender, race/ethnicity, marital status, and education), depression, comorbidities, and body mass index. The outcome variable was frailty assessed using the frailty phenotype, defined as meeting three or more of the following criterions: shrinking, weakness, exhaustion, slowness, and low physical activity. General estimation equation model was fitted to test the effect of pain on frailty over time. Prevalence of pain in American older adults was 48.9% at baseline. The prevalence of frailty ranged from 6.7 % at baseline to 7.4 % at wave 6 among those with pain. The odds ratio (OR) of becoming frail overtime was 1.07 (95% CI 1.02 – 1.12) over time. The OR of becoming frail over time as a function of pain was 1.76 (95% CI 1.51-2.05), after controlling for all covariates. Other predictor factors of becoming frail were being ≥ 75 years, having one or more comorbid conditions, and with high depressive symptoms. Participants with higher level of education were less likely to become frail. These findings suggest that early treatment of pain may reduce frailty and improve the quality of life in this population

### EXPLORING HOW "TELE-SAVVY" INFLUENCES SELF-CARE BEHAVIORS FOR DEMENTIA FAMILY CAREGIVERS

Fayron Epps,[1] Elizabeth Maloch,[1] Patricia Griffiths,[2] and Ken Hepburn[1], *1. Emory University, Atlanta, Georgia, United States, 2. Emory University School of Medicine, Atlanta, Georgia, United States*

Tele-Savvy is a 3-arm randomized controlled trial (RCT) of a psychoeducation intervention that equips dementia family caregivers with the knowledge and skills they need to provide care to their person, while also caring for themselves. This RCT is currently underway, with cohorts rotating through over a period of 12 months. The purpose of this presentation is to explore the effectiveness of Tele-Savvy (active) versus Healthy Living Intervention (attention control) or usual care (waitlist) on self-care behaviors among dementia family caregivers. We conducted semi-structured interviews with 16 caregivers after their initial participation in either the active, attention control, or usual care groups. Interviews elicited caregivers' perceptions regarding the program's influence on their self-care behaviors and engagement in self-care activities. The overall emerging theme for the family caregivers who participated in the Tele-Savvy and Healthy Living programs was "increased awareness of self-care activities". Family caregivers in the Healthy Living program spoke mainly about engaging more in physical activities and improving their nutrition by eating healthier and keeping track of foods. Across all study groups, social engagement and having a support system were common self-care activities. Various limitations to engaging in self-care activities while in enrolled in the study included time and caregiving responsibilities. Suggestions were made for more respite services to become available to allow for family caregivers to engage in self-care activities. These findings provide researchers and practitioners with pertinent information to develop and refine programs for family caregivers to improve their self-care practices.

### THE IMPACT OF "TELE-SAVVY" ON THE CAREGIVING EXPERIENCE OF DEMENTIA FAMILY CAREGIVERS: QUALITATIVE FINDINGS

Fayron Epps,[1] Elizabeth Maloch,[1] Patricia Griffiths,[2] and Ken Hepburn[1], *1. Emory University, Atlanta, Georgia, United States, 2. Emory University School of Medicine, Atlanta, Georgia, United States*

Tele-Savvy is a 3-arm randomized controlled trial (RCT) of a psychoeducation intervention that equips family caregivers of people living with dementia with the knowledge and skills they need to provide care to their person, while also caring for themselves. This RCT is currently underway, with cohorts rotating through over a period of 12 months. The purpose of this presentation is to describe the effectiveness of Tele-Savvy (active) versus Healthy Living Intervention (attention control) or usual care (wait-list) on the caregiving experience among dementia family caregivers. We conducted semi-structured interviews with 16 caregivers at the 6 month time point and after their initial participation in either the active, attention control, or usual care groups. Interviews elicited caregivers' perceptions regarding the program's influence on their caregiving experience. Caregivers who participated in Tele-Savvy reported positive experiences such as recognizing that their experience has become more pleasurable and happier. They also shared that the program expanded their knowledge and allowed them to become patient while implementing caregiving strategies learned. Majority of caregivers who participated in the Healthy Living program indicated that nothing changed related to their caregiving experience. Half of the caregivers in the usual care group, expressed frustration and unhappiness, while the others expressed no change in their caregiving experience. Results suggest that psychoeducation caregiver interventions are meaningful and can enact positive changes in their outlook on caregiving. Thus, results of this study may guide future policies and further development of caregiver programs and interventions.

### EFFECTS OF AN INTERGENERATIONAL EXERCISE BUDDY PROGRAM ON PARTICIPANTS

Eun hae Kim,[1] Kyong Hee Chee,[1] Clay DeStefano,[2] and Anna Broome[3], *1. Texas State University, San Marcos, Texas, United States, 2. Greater San Marcos Area Seniors Association/Price Center, San Marcos, Texas, United States, 3. SMTX Yoga, San Marcos, Texas, United States*

Structured social support may enhance the benefits of an exercise class for participants. This study examined the effects of an intergenerational exercise buddy program on participant well-being. A convenience sampling was used to recruit participants from Central Texas (N = 51): 34 were community-dwelling adults aged 65 and above, and 17 were university students aged 18-25 years. The study used a pretest-posttest, quasi-experimental design and focus group interviews. Randomly selected 18 older-adult participants were paired with young-adult participants as an exercise buddy for each other to attend 8 weekly Tai Chi or Chair Yoga classes at a community center. Meanwhile, 16 older-adult participants formed a control group attending different exercise classes without young-adult buddies. The survey results show that, compared to the control group, the intervention group (n = 35) had significantly greater satisfaction with life ($p < .09$ ) and a more positive attitude towards aging ($p < .01$) after attending exercise classes with their buddies. In focus group interviews, the participants most frequently mentioned that commitment to their buddies as a key factor for class attendance. The participants typically perceived that their buddies were pleasant and did not judge or treat them based on their age. They stressed the positive effects of building relationships with all involved in the program, including the exercise instructors. Meanwhile, control-group participants wished that they, too, had buddies. Although the study should be replicated with a larger sample, its findings suggest that an intergenerational exercise program offers added benefits for participants.

### SLEEP QUALITY AND DEPRESSIVE SYMPTOMS IN LATER-LIFE: CROSS-SECTIONAL EXAMINATION OF COGNITIVE MECHANISMS

David Brush,[1] Daniel Paulson,[1] Manuel Herrera Legon,[1] Nicholas James,[1] Jennifer Scheurich,[1] and Brittany Stevenson[1], *1. University of Central Florida, Orlando, Florida, United States*

Sleep quality relates to depressive symptom endorsement. The mechanisms relating these variables are not clearly elucidated, though inhibitory control and rumination are believed to play key roles. The current study aims to elucidate the relationship between sleep quality and depressive symptoms by examining the moderated mediating effect of inhibitory control and rumination. The sample included 41 community-dwelling older adults (age 70 and older). Measures included the Pittsburg Sleep Quality Inventory, a Stroop task (inhibitory control), the Ruminative Responses Scale, and the Geriatric Depression Scale. A series of bootstrapped models were employed to test hypotheses using a stepped approach. Poorer sleep quality was associated with higher rumination and depressive symptoms; however, these associations were no longer significant among older adults with higher inhibitory control. The association between sleep quality and depression was fully attenuated by rumination, and inhibitory control significantly moderated the association between sleep quality and rumination in the final model. Among community-dwelling older adults, the association between sleep quality and depression is mediated by rumination, and this effect is mitigated by inhibitory control. As such, these findings suggest that inhibitory control may be a relevant target for intervention in older adults with poor sleep quality, rumination, and depressive symptoms.

### TRANSCRIPTOME ALTERATIONS ASSOCIATED WITH AGE-RELATED DECLINE IN PHYSICAL FUNCTION.

Ted G. Graber,[1] Rosario Marota,[2] Jill Thompson,[2] Steve Widen,[2] and Blake Rasmussen[3], *1. East Carolina University, Department of Physical Therapy, Greenville, North Carolina, United States, 2. University of Texas Medical Branch, Department of Nutrition and Metabolism, Galveston, Texas, United States, 3. University of Texas Medical Branch, Department of Nutrition and Metabolism, Galveston, Texas, United States*

One inevitable consequence of the effect of age on our bodies is the graduated deterioration of physical function and exercise capacity, driven, in part by the adverse effect of age on muscle tissue. Our primary purpose was to determine the relationship between patterns of gene expression in skeletal muscle and loss of physical function. We hypothesized that some genes that change expression with age would correlate with functional decline, or conversely with preservation of function. Male C57Bl/6 mice [adults (6-7 months old, n=9), older (24-25 months old, n=9), and elderly (28+ months of age, n=9) were tested for physical ability using a comprehensive functional assessment battery [CFAB, a composite scoring system: comprised of the rotarod (overall motor function), grip strength (fore-limb strength), inverted cling (4-limb strength/endurance), voluntary wheel running (activity rate/volitional exercise), and treadmill tests (endurance)]. We extracted RNA from the tibialis anterior muscles, ran RNAseq to examine the transcriptome using an Illumina NextSeq 550, comparing adults (n=7) to older (n=7) and elderly mice (n=9). Age resulted in gene expression differences of 1.5 log2 fold change or greater ($p<0.01$) in 46 genes in the older mice and in 252 genes in the elderly (both compared to adults). Current ongoing work is examining the physiological relevance of these genes to age-related loss of physical function. We are in the process of using linear regression to determine which of the genes with age-related changes in expression are associated ($R>0.5$ and $p<0.05$) with functional status as measured by CFAB.

### TEACHING MOTIVATIONAL INTERVIEWING OF OLDER ADULTS IN AN INTERPROFESSIONAL EDUCATION SETTING: A PILOT STUDY

Nina L. Tamashunas,[1] and Elizabeth Smilovich-Fine[1], *1. Case Western Reserve University School of Medicine, Cleveland, Ohio, United States*

Motivational interviewing (MI), a person-centered method of strengthening personal motivation for change, improves health behaviors (i.e. blood pressure control) in older adults. However, literature lacks exploration of teaching MI in interprofessional and geriatric settings. We sought to pilot and assess the utility of an MI curriculum for an interprofessional group of second-year graduate students (medical, physician assistant, nursing, and speech pathology) working with older adults. The 5-week curriculum included a 2-hour interactive workshop, a geriatric standardized patient (SP), and small group meetings with a geriatric community member. We employed a pretest/posttest design to assess perceived importance and confidence using a 5-point Likert scale and knowledge acquisition using a modified version of the

Motivational Interviewing Knowledge and Attitudes Test. Feedback from students on curricular design was obtained via a post-curriculum survey. Of the 17 students enrolled, 100% (17/17) completed the pretest and posttest and 94% (16/17) completed the post-curriculum survey. Previous MI training ranged from 0 to 12 hours. Students felt more confident in their MI skills (p<0.05) after the curriculum. Aggregate knowledge scores did not achieve statistical significance. Feedback indicated that students enjoyed practicing with SPs and processing feedback and would have preferred more student diversity and a more advanced curriculum. While limited by sample size, this pilot demonstrated that a brief geriatric-specific curriculum was well received and improved student confidence in MI skills. Larger studies should explore tailoring teaching methods of MI knowledge and skills in a diverse learner population working with older adults to promote health behavior change.

### SOCIODEMOGRAPHIC AND HEALTH DISPARITIES IN USE OF PREVENTIVE HEALTHCARE SERVICES

Terese Sara Hoej Joergensen,[1] Heather Allore,[2] Corey Nagel,[3] and Ana R. Quiñones[4], *1. Oregon Health and Science University, Portland, United States, 2. Yale University, New Haven, Connecticut, United States, 3. University of Arkansas for Medical Sciences, Little Rock, Arkansas, United States, 4. Family Medicine, Oregon Health & Science University, Portland, Oregon, United States*

Introduction: Older adults experience multiple health problems necessitating medical care. However, studies have shown that healthcare is not equally accessible for older adults in the US. In 2011, Medicare introduced Annual Wellness Visits to improve access to preventive healthcare including influenza vaccination. Objectives: Ascertain whether sociodemographic factors, multimorbidity, and other health indicators pose a barrier for older adults to access Annual Wellness Visits and influenza vaccination. Methods: We analyzed data from the 2012 and 2014 waves of the Health and Retirement Study linked with Medicare records. 4,858 older adults aged 65+ years were included in Conditional Random Forests to identify the most important predictors of Annual Wellness Visits and influenza vaccination during this two-year period. The predictors included: age, sex, race/ethnicity, partnered, geographical region, wealth, educational level, Medicaid coverage, body mass index, activities and instrumental activities of daily living, proxy interview, cognitive impairment, dementia diagnosis, and multimorbidity. Results: In total, 1,142 (23.6%) older adults had an Annual Wellness Visit and 3,316 (68.4%) older adults received an influenza vaccination. 11.9% were non-Hispanic black, 6.3% were Hispanic, with a median of 6 chronic conditions and 16.9% had dementia. The most important predictors of Annual Wellness Visits were region, wealth, dementia diagnosis, and race/ethnicity. The most important predictors of influenza vaccination were multimorbidity, race/ethnicity, educational level, and wealth. Conclusion: The importance of geographical region for Annual Wellness Visits suggests that the service has not been adopted equally throughout the US, whereas multimorbidity is the most important factor for receiving influenza vaccination.

### CREATIVE STORIES FROM A MEMORY CARE COMMUNITY: VALUES, NORMS, IDENTITIES, AND EXPERIENCES

Kyong Hee Chee,[1] Olga Gerhart,[1] Seoyoun Kim,[1] and Sara Caldwell[2], *1. Texas State University, San Marcos, Texas, United States, 2. Texas State University, San Marcos, United States*

As an arts-based, creative storytelling program for persons living with Alzheimer's Disease and related dementias (ADRD), TimeSlips involves a facilitator showing a picture to participants, who then exercise their imagination to create a story. The program has shown to benefit participants' well-being, possibly because of the opportunity to express themselves. Although they may reflect participants' values and identities, the content of such stories had not been the focus of investigation. The aim of this study is, therefore, to identify major themes of such stories through a qualitative content analysis. We implemented a creative storytelling program at Silverado Onion Creek Memory Care Community (currently, The Auberge) in Austin, and offered 6 weekly sessions with 4 small groups of residents. A total of 26 residents participated in the study, creating 24 collective stories in total. Three researchers first open-coded these stories and then met to reach consensus concerning the themes that emerged. Ten themes were identified: family values, generativity, religious reference, reference to love, reference to home, cultural norms, uncertainty and worries, positivity, negativity, and dissonance and disagreements. The first 6 themes represent the values, beliefs, and norms of the participants, with the remaining 4 reflecting their personal identities, personalities, and experiences. The findings suggest that they continue to value families and religion, care about others, and make judgements about people, things, and circumstances that they face. Researchers, practitioners, and care partners can benefit from "listening to" creative storytellers more closely to learn about their opinions, expectations, and preferences.

### USABILITY OF AN ONLINE DISCUSSION PLATFORM: DEVELOPMENT OF A CODING SCHEME

Soojeong Han,[1] Andrew Teng,[2] Shih-Yin Lin,[2] Frances Chu,[1] George Demiris,[3] Oleg Zaslavsky,[1] and Annie T. Chen[1], *1. University of Washington, Seattle, Washington, United States, 2. NYU Rory Meyers College of Nursing, New York, New York, United States, 3. University of Pennsylvania School of Nursing, Philadelphia, Pennsylvania, United States*

Online discussion platforms have the potential to support or even improve older adults' well-being. Nonetheless, potential health benefits are often shaped by the usability of such platforms. Improving usability is imperative to maximize these potential benefits. As a part of a qualitative analysis of a study of an online community for older adults with frailty symptoms, we developed and refined a coding scheme targeting usability. The scheme was derived by reviewing contemporary literature on user experience, usability, and health information technology in older adults. Our review revealed challenges to apply commonly used terms to summarize our qualitative data. For example, the concept of perceived usability has different meanings and definitions in existing frameworks as they pertain to user engagement and technology adoption (specifically, the Technology

Acceptance Model) than usability in an online discussion context for older adults. Because none of the meanings of usability fully encompassed the breadth of this concept for older users, we developed a coding scheme that is practical and captures a broad range of older adults' perceptions of usability. Through qualitative analysis of the online discussion content using the newly developed coding scheme, new themes emerged such as confusing layout (e.g., difficulty in locating discussion boxes), insufficient instruction or training (trouble posting discussions), unwanted results (e.g., pressed a wrong key), and memory issues and cognitive burden. This presentation describes the process of developing a coding scheme, illustrates nuances of meanings in concepts related to usability, and presents preliminary results of our qualitative analysis.

### SOCIABILITY AMONG PERSONS LIVING WITH DEMENTIA IN A CREATIVE GROUP STORYTELLING CONTEXT

Kyong Hee Chee,[1] Seoyoun Kim,[1] Olga Gerhart,[1] and Sara Caldwell[2], *1. Texas State University, San Marcos, Texas, United States, 2. Texas State University, San Marcos, United States*

Considering healthcare costs related to Alzheimer's Disease and related dementias, shifting attention to the relatively malleable abilities of persons living with dementia holds promise for improving their well-being while reducing care burden. Defined as the ability to successfully interact with others, social intelligence is found to benefit well-being. Nevertheless, no known prior study has examined social intelligence among persons living with dementia. The purpose of this study is, therefore, to fill this gap by identifying the themes of social intelligence in this group. We used video-recorded data from an arts-based, creative group storytelling program (TimeSlips) that we implemented at Silverado Onion Creek Memory Care Community (currently, The Auberge) in Austin. The program is designed for persons living with dementia and involves a facilitator encouraging participants to use their imagination to collectively create a story from a staged picture. We offered 6 weekly sessions with 4 small groups of their residents (N = 26) in fall 2018 and spring 2019, and videotaped the sessions. Three researchers open-coded how participants interacted during storytelling sessions, and then met to reach consensus concerning verbal and non-verbal indicators of social intelligence. Major themes that emerged from our analysis are social awareness, initiating social interactions, and social diplomacy. Our findings suggest that those with lower cognitive function scores do not necessarily lack sociability. These findings add to social intelligence and dementia literatures, with potential implications for future research that can investigate the relationship between sociability and well-being among persons living with dementia.

### DEMENTED OLDER ADULT'S SALIVARY CORTISOL LEVELS AND PHYSICAL STRESS INDEX AS STRESS INDICATOR

Eunji Kwon,[1] and Eunhee Cho[2], *1. Yonsei university, Seoul, Korea, Republic of, 2. Professor, College of Nursing, Yonsei University, Seoul, Korea, Republic of*

Demented older adults experience many internal and external stress inducers that are thought to be a source of behavioral and psychological symptoms of dementia(BPSD). The purpose of this study was to compare the stress index among older adults through salivary cortisol levels and physical stress index. This study was cross-sectional design, including 139 participants who recruited until May of this year(104 demented older adults who visited hospital outpatient neurology and 35 non-demented older adults as control group). The physical stress index was measured by heart rate variability and salivary cortisol levels(4 samples/day, 1 days). Salivary cortisol levels were measured at four times after wake up, after breakfast, before dinner and after dinner. The data were analyzed using independent t-test and generalized estimating equations. In salivary cortisol levels measured after wake up, the demented older adults reported about 1.5 times higher than non-demented older adults(p=.042). And the salivary cortisol levels measured after breakfast were about 2.3 times higher in the demented older adults than in control groups(p=.002). Accordingly, the results can be concluded that demented older adults have higher stress levels than control groups in the morning. Also the physical stress index through heart rate variability(HRV) in the demented older adults(6.30±0.65) had higher than control groups(6.00±0.55, t=2.45, p=.016). There are significant differences in salivary cortisol levels and physical stress index between demented older adults and control groups. As stress inducers affects BPSD for the demented older adults, nursing intervention should be tailored to proper way based on their stress inducers.

### THE ASSOCIATION OF MORTALITY WITH DEMENTIA IN VETERANS ENROLLED IN A MEMORY DISORDERS CLINIC: THE EFFECT OF FRAILTY

Aakashi Shah,[1] Raquel Apracio-Ugarriza,[1] Amar Morani,[1] Mercedes Rodriguez-Suarez,[2] and Jorge G. Ruiz[3], *1. Department of Public Health, University of Miami Miller School of Medicine, Miami, Florida, United States, 2. Miami VAHS Mental Health Service, Miami, Florida, United States, 3. Miami VAHS Geriatric Research, Education and Clinical Center, Miami, Florida, United States*

Dementia is a syndrome of deterioration in cognition and ability to perform everyday activities. Frailty, a state of vulnerability to stressors leading to increased morbidity, mortality and utilization is a determinant of dementia. The aim was to determine if dementia leads to increased mortality in Veterans and whether frailty affects this association. We conducted a retrospective cohort study of 308 Veterans enrolled in VA memory disorders clinic during 2016-2019. Dementia was diagnosed based on complete clinical assessments, brain imaging and neuropsychological testing. A 44–item frailty index (FI) was constructed using demographics, comorbidities, medications, laboratory tests, and activities of daily living. Patients were divided into non-Frail (FI<0.21) and Frail (FI≥0.21). After adjusting for age, race, ethnicity, income, education, substance abuse, BMI, comorbidities, hospitalizations, medication use, the association of dementia with mortality was assessed using Cox proportional hazards regression. Patients were 55.2% White, 74% non-Hispanic, and the mean age was 74.4 ± 8.3 years. 113 patients were diagnosed with dementia out of which 27 died. Over a median follow-up period of 526 days (Interquartile Range: 431.5 days), there were 27 deaths. There was a significant and positive association of dementia with mortality significant even after all adjustments, HR=2.65 (95% CI: 1.02-6.92), p: 0.045. After subgroup analysis, there was no significant association between mortality and dementia according to frailty status. Our study results suggest that dementia is associated with a higher risk for mortality in Veterans at a Memory Disorders clinic. Frailty did not modify the effect.

### IMPROVED HEALTHSPAN AND LIFESPAN WITH LATE ONSET PHARMACOLOGICAL OR DIETARY INTERVENTIONS IN MICE

Sarah Mitchell,[1] Michael MacArthur,[1] Alice Kane,[2] Margaret Torrence,[1] Brendan Manning,[1] and James mItchell[1], *1. Harvard T.H. Chan School of Public Health, Boston, Massachusetts, United States, 2. Paul F. Glenn Center for Biology of Aging Research at Harvard Medical School, Boston, Massachusetts, United States*

While late-onset dietary (e.g. calorie restriction) or pharmacological (e.g. rapamycin) interventions can extend longevity in rodents, whether or not they can be used to reverse or forestall onset of aging-related symptoms (i.e. frailty) remains untested. Here, we employed methionine restriction (MR), a dietary intervention associated with weight loss and longevity extension, or a fumagillin derivative, ZGN-1062, as a pharmacological agent associated with weight loss across multiple species. 21mo Male and female C57BL/6 mice were randomized to one of three groups: control, 0.1% MetR, or ZGN-1062 (1.5mg/kg). There was no difference in frailty index (FI) amongst groups at baseline (21 mo age). MetR and ZGN-1062 treatments resulted in modest weight loss independent of sex. After 6 months, FI increased consistent with reduced healthspan in control males (0.23+/-0.01 to 0.34+/-0.01 A.U, p<0.0001) and females at this age (0.19+/-0.03 to 0.24+/-0.01, p<0.0001). Male MetR and ZGN-1062 mice had significantly lower FI scores after 6 mo of treatment (MetR: 0.28+/-0.04, p<0.0001; ZGN-1062: 0.29+/-0.05, p=0.0002). While female mice were not significantly different from controls, they were, overall, less frail than males at 26 months, suggesting sexual dimorphism in the timing frailty onset in mice. Although the end points of this study have not been reached (survival studies are ongoing), the data obtained to date suggest that late-life treatments can improve healthspan markers, at least in male mice. Such treatments include a pharmacological intervention associated with weight loss, which may be a more practical therapeutic strategy towards mitigation of age-related healthspan decline than dietary restriction-based interventions.

### EFFECTS OF A 6-WEEK TASK SPECIFIC POWER TRAINING WITH AND WITHOUT COGNITIVE TRAINING AMONG OLDER ADULTS WITH SLOW GAIT

Elisa F. Ogawa,[1] Rebekah Harris,[2] Joseph DeGutis,[3] Rachel Ward,[2] Jennifer Brach,[4] Ildiko Halasz,[5] and Jonathan Bean[1], *1. New England GRECC, VA Boston Healthcare System, Boston, Massachusetts, United States, 2. NE GRECC, VA Boston Healthcare System, Boston, Massachusetts, United States, 3. Department of Psychiatry, Boston, Massachusetts, United States, 4. School of Health and Rehabilitation Sciences, University of Pittsburgh, Pittsburgh, Pennsylvania, United States, 5. Department of Medicine, VA Boston Healthcare System, Boston, Massachusetts, United States*

Task-specific power training (InVEST) targets leg power and mobility skills that are beneficial for treating slow gait speed for older adults. This study investigated the efficacy of a short-term InVEST training on leg power, mobility performance, and gait characteristics and further examine whether the addition of cognitive training would augment the impact on the outcomes. Mobility limited community-dwelling older Veterans age ≥65 years were recruited. Participants were randomly assigned to either InVEST training (n=10) or InVEST+cognitive training (n=11). Training occurred 3 times per week for 6 weeks. Sessions were either 70 minutes (InVest+cognitive training) or 40 minutes (InVEST) in duration. Leg power, mobility performance (Short Physical Performance Battery), and gait characteristics (gait speed, stance time, step width, swing time, step length and their variabilities under single-task, simple and complex dual-task walking conditions) were evaluated. Twenty-one men with mean age 76±7 years completed the study and 86% were of white race. Among all participants, clinically relevant and statistically significant improvements in leg power, mobility performance, and gait characteristics (gait speed, step length, stance time under all three gait conditions) were observed. There were no statistically significant or clinically relevant group differences among any of the outcomes based on cognitive training status. Short-term InVEST training led to clinically meaningful improvements in leg power, mobility performance, and gait characteristics. These findings add to the body of evidence supporting the benefits of InVEST training on mobility and do not support the contention that mixed modes of training (cognitive and physical) may augment mobility outcomes.

### ASSOCIATION BETWEEN NEIGHBORHOOD CHARACTERISTICS AND DEMENTIA: AN ECOLOGICAL ANALYSIS

Dana M. AlHasan,[1] Matthew Lohman,[1] Maggi Miller,[1] Jana A. Hirsch,[2] and Bo Cai[1], *1. University of South Carolina, Columbia, South Carolina, United States, 2. Drexel University, Philadelphia, Pennsylvania, United States*

Research has examined the relationship between neighborhood environments and cognitive decline, yet few have investigated the role of neighborhood characteristics specifically on dementia. This ecologic study examined the geographic distribution of dementia incidence and investigated ecologic associations between census-tract neighborhood characteristics and diagnosed dementia case incidence from 2010-2014 in the South Carolina (SC) Alzheimer's Disease Registry. Analyses took place on the census-tract level (n=1089) with population ≥1. Neighborhood measures came from the Decennial Census, American Community Survey, Rural Commuting Area Code, and County Health Rankings. To estimate the ecologic association between neighborhood characteristics and dementia incidence, we conducted a zero-inflated, negative binomial mixed-effects model. The overall age-sex standardized dementia incidence rate was 2,885.4 per 100,000 people per census tract from 2010-2014 in SC

(95% CI: 2857-2913.7). In an adjusted model, neighborhood characteristics had a significant association with dementia incidence. Rural and small urban census tracts had 62% (IRR=0.38; 95% CI= 0.27-0.54) and 58% (IRR=0.42; 95% CI= 0.29-0.60) lower dementia incidence, respectively, among ≥50 years old compared to urban census tracts while adjusting for neighborhood median-household income, racial composition, commute time, and age structure. The results from this study show a negative relationship between rural neighborhoods and dementia, contrary to previous findings. However, lower access to care in rural neighborhoods can result in lower detection rates and thus could present a reporting bias. Future research should investigate additional census-tract neighborhood characteristics (e.g. green space, pollution rates or psychosocial stress) that contribute to lower dementia rates in rural areas.

### HIGH THROUGHPUT YEAST REPLICATIVE LIFESPAN SCREEN UNCOVERS HISTONE DEACETYLASE HDA AS NOVEL REGULATOR OF AGING

Ruofan Yu,[1] Xiaohua Cao,[1] and Weiwei Dang[1], *1. Baylor College of Medicine, Houston, Texas, United States*

In this work, we set out to develop a high throughput screening method, SEBYL (SEquencing Based Yeast replicative Lifespan screen), in order to identify new aging regulators in budding yeast. By utilizing SEBYL on yeast knockout collection, we were able to identify 285 long-lived gene deletions, of which a significant portion was proven to have extended lifespan by previous classical experiments. To demonstrate the ability of our method to discover new genes and pathways involved in aging process, we focused on characterizing one newly identified long-lived candidate emerged from the screening, histone deacetylase complex HDA, and found it regulates aging through mediating stress response pathways, especially DNA damage stress response. Presence of HDA complex inhibits expression of trehalose metabolism genes, which act as stress protectant. When HDA complex is mutated, trehalose genes are de-repressed, enhancing stress response and eventually promotes longevity. In summary, we conclude SEBYL to be time and energy saving, robust, and suitable for discovery of aging regulating genes using various preexisting yeast mutant collection resource.

### THE EFFICACY OF CASE-BASED INSTRUCTION ON STUDENT ATTITUDES TOWARDS PAIN AND OPIOID RISK ASSESSMENT IN OLDER ADULTS

Aarushi Aggarwal,[1] Sandra Sanchez-Reilly,[2] Michael J. Mader,[3] and Jeanette Ross[4], *1. UT Health San Antonio, Long School of Medicine, San Antonio, Texas, United States, 2. South Texas Veterans Health Care System, San Antonio, Texas, United States, 3. South Texas Veterans Health Care System, Dept of Research, San Antonio, Texas, United States, 4. University of Texas Health Science Center San Antonio, San Antonio, Texas, United States*

A survey of US medical schools found that only 10-12 hours of the 4 year curriculum are dedicated to instruction in pain management in older adults. Since chronic pain afflicts 100 million Americans, and older adults have a higher risk of prescription medication misuse, there is urgency regarding proper education.This study evaluates the success of case-based instruction on the topics of pain management and opioid risk assessment, with a goal of increasing instructional hours in a format other than didactic. 200 fourth-year medical students were split into groups of 20, with 2 instructors in each room. A survey was administered pre/post workshop asking participants to rate 10 statements using a Likert scale. The 2 hour workshop involved case studies to work through while using a created pain instructional card.The case-based instruction format demonstrated high efficacy in shaping the beliefs and personal evaluations of medical students. Prior to the workshop, only 34% of students were positive about their pain assessment abilities. 9.5% were positive about their opioid conversion skills, and only 4% were positive about opioid risk assessment skills. After the workshop, these positive evaluations increased to 97%, 95%, and 92% respectively. The McNemar test proved these findings to be statistically significant ($p<.0001$). Case-based instruction with small-group discussion is a reliable tool in teaching medical trainees about pain management and opioid risk assessment in adults aged 65 and older. This workshop needs to be run with geriatric/palliative care residents to evaluate clinical incorporation of session concepts through resident charting.

### CURIOSITY TOWARDS ROBOT: THE ROLE OF AGE AND PERSONAL RELEVANCE

Li Chu,[1] and Helene H. Fung[2], *1. The Chinese University of Hong Kong, Shatin, New Territories, Hong Kong, 2. Chinese University of Hong Kong, Hong Kong, Hong Kong*

Being curious has various physical, social and psychological benefits. However, theories like the socioemotional selectivity theory suggest that information seeking goals tend to be overshadowed by emotionally meaningful goals with age. Personality and social psychology research also found consistent decline of curiosity in later adulthood. In contrast, selective engagement theory propose that people simply become more selective on where they allocate their cognitive resources as they age. Particularly, older adults tend to invest in things that have personal relevance. Yet, few studies have explored the interaction between age and personal relevance in the context of information seeking tendencies. We conducted a pre-test-post-test experiment with 50 younger (age 19-34) and 50 older adults (age 60-78). Participants were invited to learn about a robot (Vector by Anki) and freely interact with the robot for about 30 minutes. Questionnaires were filled before and after the interaction. Our results confirmed previous findings that older adults showed lower level of trait curiosity than younger adults ($F(1, 98)=21.94$, $p<.001$, $\Delta R2=$). However, older adults actually showed higher level of state curiosity towards robot than their younger counterparts ($F(1, 96)=21.94$, $p<.001$). Moreover, there was a marginally significant interaction effect of personal relevance ($p = .06$). Tukey's post-hoc test revealed that older adults who perceived increased relevance ($M=5.39$) after the interaction were significantly more curious than younger adults who also perceived increased relevance ($M=4.51$, $p=.02$), but there was no significant age difference when they perceived decreased relevance. Present study offers insights on promoting curiosity among older adults.

### COST-EFFECTIVENESS ANALYSIS OF THE COLLABORATIVE STEPPED CARE INTERVENTION FOR LATE-LIFE DEPRESSION

Shiyu Lu,[1] Gloria H.Y. Wong,[1] Terry Lum,[2] and Tianyin Liu[1], *1. The University of Hong Kong, Hong Kong,*

*Hong Kong, 2. Department of Social Work and Social Administration, PokFuLam, Hong Kong*

Late-life depression is a burden on society because it is costly and have a significant adverse effect on the quality of life. The aim of this study is to evaluate the cost-effectiveness of the collaborative stepped care intervention for depression among community-dwelling older adults compared to care as usual from a societal perspective. The intervention was piloted from 2016-2019 in Hong Kong. The study used a two-armed quasi-experimental design. Eventually, 412 older people were included (314 collaborative stepped care, 98 care as usual). Baseline measures and 12-month follow-up measures were assessed using questionnaires. We applied the 5-level EQ-5D version (EQ-5D-5L) and the Client Service Receipt Inventory (CSRI) respectively measuring quality-adjusted life-year (QALY) and health care utilization. The average annual direct medical cost in the intervention group was USD 6,589 (95% C.I., 4,979 to 8,199) compared to US$ 6,167 (95% C.I., 3,702 to 8,631) in the care as usual group. The average QALYs gained was 0.036 higher in the collaborative stepped care group, leading to an incremental cost-effectiveness ratio (ICER) of US$ 11,722 per QALY, lower than the cost-effectiveness threshold suggested by The National Institute for Health and Clinical Excellence. The study showed that collaborative stepped care was a cost-effective intervention for late-life depression over service as usual.

### THE RELATIONSHIP OF FRAILTY, FEAR OF FALLING, AND DEPRESSION WITH HRQOL IN HIGH-RISK OLDER ADULTS

Michelle A. McKay,[1] Linda Copel,[1] and Catherine Todd-Magel[1], *1. Fitzpatrick College of Nursing, Villanova University, Villanova, Pennsylvania, United States*

One in four older adults fall every year. Falls result in negative outcomes including decreased health-related quality of life (HRQoL). Frailty, fear of falling, depression, and HRQoL are not routinely screened in high-risk community-dwelling older adults. Continued study of modifiable fall risk factors is warranted due to varied reported prevalence rates, inconsistent definitions and the persistent high rate of falls resulting in poor HRQoL. The purpose of the study was to determine the relationship between frailty, fear of falling, and depression with physical and mental functioning and well-being measures of HRQoL in community-dwelling older adults 55 years of age and older. A cross-sectional correlational design and chart review were conducted. The sample consisted of 84 primarily African American (81%) nursing home eligible members of the Program for All-Inclusive Care for the Elderly (PACE) program. Data were analyzed with correlational statistics, multiple linear, and hierarchical regression models. Physical functioning and well-being measures were significantly decreased when compared to the general population. Increased frailty, fear of falling, and depression were associated with decreased physical and mental well-being. In the regression model, frailty and fear of falling were significant predictors of decreased physical functioning and well-being, and depression was a significant predictor of decreased mental functioning and well-being. This study provides clarification of the relationship between frailty, fear of falling, and depression with HRQoL in high-risk older adults. Screening for common modifiable risk factors can assist in the development of targeted interventions and treatments to improve HRQoL in high-risk older adults.

### PREDICTORS OF HEALTH-RELATED QUALITY OF LIFE AND RECOVERY AMONG OLDER ADULTS WITH SERIOUS MENTAL ILLNESS

Anjana Muralidharan,[1] Clayton H. Brown,[2] and Richard W. Goldberg[3], *1. Veterans Affairs Capitol Healthcare Network, Mental Illness Research Education and Clinical Center, Baltimore, Maryland, United States, 2. University of Maryland School of Medicine, Baltimore, Maryland, United States, 3. Veterans Affairs Capitol Healthcare Network, Baltimore, Maryland, United States*

Older adults with serious mental illness (i.e., schizophrenia spectrum disorders and affective psychoses) exhibit marked impairments across medical, cognitive, and psychiatric domains. The present study examined predictors of health-related quality-of-life and mental health recovery in this population. Participants (N=211) were ages 50 and older with a chart diagnosis of serious mental illness and a co-occurring medical condition, engaged in outpatient mental health services at a study site. Participants completed a battery of assessments including subtests from the Repeatable Battery for the Assessment of Neuropsychological Status (RBANS), the 24-Item Behavior and Symptom Identification Scale (BASIS-24), the 12-Item Short-Form Health Survey (SF-12), and the Maryland Assessment of Recovery Scale (MARS). Multiple linear regression analyses, with age, race, gender, and BMI as covariates, examined number of current medical conditions, RBANS, and BASIS as predictors of quality-of-life and recovery. Significant predictors of physical health-related quality-of-life ($R$-squared=.298, $F(9,182)=8.57$, $p<.0001$) were number of medical conditions ($\beta=-1.70$, $p<.0001$), BASIS-Depression/Functioning ($\beta=-4.84$, $p<.0001$), and BASIS-Psychosis ($\beta=2.39$, $p<.0008$). Significant predictors of mental health-related quality-of-life ($R$-squared=.575, $F(9,182)=27.37$, $p<.0001$) were RBANS ($\beta=0.03$, $p=.05$), BASIS-Depression/Functioning ($\beta=-6.49$, $p<.0001$), BASIS-Relationships ($\beta=-3.17$, $p<.0001$), and BASIS-Psychosis ($\beta=-1.30$, $p=.03$). Significant predictors of MARS ($R$-squared=.434, $F(9,183)=15.56$, $p<.0001$) were BASIS-Depression/Functioning ($\beta=-4.68$, $p=.002$) and BASIS-Relationships ($\beta=-9.44$, $p<.0001$). To promote holistic recovery among older adults with serious mental illness, integrated interventions are required. For example, to improve physical health-related quality-of-life, one should target depression and psychotic symptoms as well as medical illness burden. To improve mental health-related quality-of-life, depression symptoms and interpersonal functioning may be key targets, as well as neurocognitive function.

### BROODING MODERATES THE RELATIONSHIP BETWEEN CEREBROVASCULAR BURDEN AND VASCULAR DEPRESSION

Manuel Herrera Legon,[1] and Daniel Paulson[1], *1. University of Central Florida, Orlando, Florida, United States*

Objective: The vascular depression hypothesis posits that cerebrovascular burden confers risk for late-life depression. Though neuroanatomical correlates of vascular depression (prefrontal white matter hyperintensities) are well established, little is known about cognitive correlates; the identification of which may suggest therapeutic targets. Aims of this study are to examine the hypothesis that the relationship between cerebrovascular burden and depressive symptoms is moderated by brooding, a type of rumination. Method: A sample of 52 community-dwelling, stroke-free, individuals over the age of 70, without history of severe mental illness or dementia completed the Ruminative Responses Scale, and provided self-report (cardiac disease, hypertension, diabetes, high cholesterol) CVB data. The Geriatric Depression Scale was used to assess depressive symptomatology. Results: Results of a bootstrapped model were that self-reported measures of CVB predicted depressive symptomatology. This relationship was significantly moderated by brooding. Among older adults, those who self-reported high CVB and medium to elevated levels of rumination experienced disproportionately more depressive symptomatology. Conclusions: These findings suggest that brooding rumination may be one correlate of the vascular depression syndrome. Future research should examine neuroanatomical correlates of rumination among older adults, and further explore brooding as a therapeutic target for those with late-life depression.

### SMART HOME TECHNOLOGY FOR OLDER ADULTS WITH MOBILITY DISABILITIES: POTENTIAL AND CHALLENGES

Kenneth A. Blocker,[1] Lyndsie M. Koon,[2] Travis Kadylak,[1] Widya A. Ramadhani,[1] Roshanak Khaleghi,[1] Christopher Kovac,[1] Ramavarapu S. Sreenivas,[1] and Wendy A. Rogers[1], *1. University of Illinois at Urbana-Champaign, Champaign, Illinois, United States, 2. University of Illinois, Urbana-Champaign, Illinois, United States*

Recently, there has been a significant expansion in the number of smart and connected technologies for assisting individuals with a variety of tasks within the home. Examples include digital home assistants (e.g., Amazon Echo), smart lights, smart plugs, robotic vacuums, as well as a multitude of other devices. Such technologies hold the potential to support independence for older adults with long-term mobility disabilities, as they may experience challenges engaging in daily activities. The aim of the current study was to utilize a comprehensive approach with an interdisciplinary team to improve understanding of how to integrate smart technology into older adults' homes. We focused on identifying functionality that would be useful to them, understanding their perceptions, and developing instructional support. We conducted interviews among older adults with, and without, long-term mobility disabilities to better understand their attitudes towards digital assistants, identify needs for instructional support, and test the usability of our instructional protocol. The overall goal of this research is to improve understanding of older adults' perceptions of these technologies and identify usability challenges within the home. The instructional protocol offers support by reducing the identified barriers to initial adoption and continued use to promote aging-in-place and improving overall quality of life for older adults with long-term mobility disabilities.

### BARRIERS TO ORAL HEALTH IN THE OLDER POPULATION

Kavita Sharma,[1] Lynn Tepper,[2] and Carol Kunzel[2], *1. Columbia University, College of Dental Medicine, New York, United States, 2. Columbia University, College of Dental Medicine, NY, United States*

A strong challenge is posed for patients and their caretakers by the growing need for promoting oral healthcare for this population, as research substantiates the connection between oral health and systemic health. This study identified the major barriers to providing optimal oral care to the older population. Fifty patients aged 60 and over visiting the Columbia University College of Dental Medicine Clinic were administered a questionnaire which reflected possible barriers to oral health care. Statistical analysis of data revealed that the top three barriers in order of relevance were the (1) cost of treatment, (2) anxiety, and (3) transportation. The youngest old (60-69) indicated that the lack of time and conflict with work schedules were additional barriers, while the older sample (70+) experienced obstacles due to disability and illness. When gender differences were analyzed, transportation was the most significant as a barrier among males, and fear/anxiety was most significant for females. Ethnically, non-Hispanics indicated that (1) shortage of time, (2) anxiety, and (3) lack of social/physical support were significant barriers. Findings indicated that even those with Medicaid insurance coverage believed that the cost of dental treatment and caregiving responsibilitieswere major barriers to seeking care. Conversely, even those without dental insurance indicated that disability and illness were barriers to seeking care. This pilot study highlighted various barriers to oral health care and highlighted the need for intervention to address barriers, such as social services, expanded Medicaid coverage, and transportation assistance, to ultimately improve access to optimal oral health care.

### LONG TERM OUTCOMES OF THE IN-HOSPITAL MOBILITY INTERVENTION (WALK FOR) IN A SAMPLE OF OLDER ADULTS

Anna Zisberg,[1] Maayan Agmon,[2] Efrat Shadmi,[3] Efrat Gil,[4] Yehudit Hait,[5] Nurit Gur-Yash,[6] and Ksenya Shulyaev[1], *1. The Cheryl Spencer Department of Nursing Faculty of Social Welfare and Health Science, University of Haifa, Israel, Haifa, Isreal, Israel, 2. Department of Nursing, Haifa University, Haifa, Israel, 3. University of Haifa, Haifa, Israel, 4. Clalit Health Services, Haifa, Israel, 5. HaEmek Medical Center, Clalit Health Services, Afula, Israel, 6. Oranim, College of Educational, Israel., Oranim, Israel*

Evaluation of in-hospital mobility programs is usually short-term. To examine the sustainability of Walk-FOR (Walk for Outcome and Recovery), an in-hospital mobility program in internal-medicine older (70+) patients, we conducted a quasi-experimental pre-post four-group comparative study. Walk-FOR incorporated policies encouraging patients to walk more than 900 steps/day and addressed conditions limiting patients' in-hospital mobility. Self-reported mobility was assessed in intervention (N=159), control (N=154) and two-year follow-up groups: previous-intervention (N=75) and non-intervention (N=95) units. Two-years post-implementation, in previous-intervention units 82.7% of patients reported walking at least twice a day outside their room, similarly to the within-implementation intervention phase (81.2%, p=ns) and significantly more than in the control group (57.2%, $p<.0001$). No differences in walking were found between intervention and non-intervention units (84.2%, p=ns) two-years post-implementation. Multivariate analysis compering 4 study groups applying logistic regression with covariance of age, sex, walking and function ability at admission, comorbidities and length of stay demonstrated similar results. Patients from intervention units two years after it implementation had a higher odds of walking at least twice a day outside their room (OR=3.82, 95% CI 1.636-8.899, p=0.002) then patients from the same units before intervention. Logistic regression didn't show significant differences between probability of walking at least twice a day outside their room in the group evaluated immediately after intervention implementation and two-years letter. Also there were no significant difference between not-intervention and intervention units two-years post-intervention. Walk-FOR is a sustainable practice and tends to spread to additional hospital-units probably due to hospital leadership and organizational commitment.

### DISPARITIES IN DEPRESSION AMONG CHINESE OLDER ADULTS WITH NEURODEGENERATIVE DISEASES

Zhichao Hao,[1] qingyi Li,[1] and Nicole Ruggiano[1], *1. University of Alabama, Tuscaloosa, Alabama, United States*

Depression is a major health issue among older adults, and it exerts negative impacts on them physically and mentally. In turn, various factors facilitate or impede the occurrence of depression, socially, economically and culturally. At the same time, neurodegenerative diseases have become a leading cause of death and disability worldwide. In China, the incidence rate of Parkinson's disease among older adults aged 65 and older is 1.7%, which means 100,000 new cases occur each year, more than 2.5 million in total. Meanwhile, 3.21% of incidence rate, more than 8 million older adults aged 65 and older with Alzheimer's Disease and Related Dementia (AD/RD) makes China become the largest and fastest-growing area of AD/RD in the world. Around 2050, Chinese older adults with AD/RD will exceed 20 million. However, little is known about the extent that to which older adults with Parkinson's or AD/RD in China will suffer from depression. This study was conducted on the latest wave (2011-2014) of the Chinese Longitudinal Healthy Longevity Survey (CLHLS, 1998-2014). The sample included 334 Chinese older adults aged 65 and older with neurodegenerative diseases (Parkinson's or AD/RD). A univariate and binomial hierarchical logistic regression were performed. Result showed that 13.5% (n = 45) participants reported depression. Several covariates were significantly correlated with the occurrence of depression, including: co-residence of interviewee, activity level, level of chronic diseases, self-reported health status and Instrumental Activity of Daily Life. Implications for research, policy, and practice are discussed.

### AGE-RELATED ELEVATED CD4+ T HELPER 17 CELL RESPONSE PROMOTES PROSTATE CANCER CELL GROWTH, MIGRATION, AND INVASION

Qiuyang Zhang,[1] Sen Liu,[1] Bing Zhang,[1] Elizabeth Norton,[2] S. Michal Jazwinski,[1] Oliver Sartor,[2] Chad Steele,[2] and Asim B. Abdel-Mageed[1], *1. Tulane University School of Medicine, New Orleans, Louisiana, United States, 2. Tulane University School of Medicine, New Orleans, United States*

Age is the most important risk factor for prostate cancer (PCa). But, how age contributes to PCa remains unknown. Interleukin-17 (IL-17) -producing CD4+ T helper 17 (Th17) cells play a critical role in inflammatory diseases. It is often elevated in aging humans and mice, however, whether aging affects Th17 cell function and subsequent PCa risk increase is unclear. In this study, we investigated the role of CD4+ T cells in PCa cell growth during the aging process. Splenic T cells were isolated and purified into CD4+CD25- T cells from young and old mice, then cultured in the presence of plate-bound anti-CD3/anti-CD28. Four days later, the cells were re-stimulated with PMA and ionomycin in the presence of brefeldin A for 4 hours and then were collected and used for flow cytometry and/or qPCR. The supernatant (conditioned media) from young and old cultures was collected and used in subsequent experiments. Flow and qPCR results showed that 17-producing T cells and associated cytokines were significantly increased in old mice compared to young mice. When PCa cell lines (LNCaP, DU-145, and PC3) were treated by the conditioned media for 48 and 72 hours. The cell proliferation, migration, and invasion, as well as the activation of NF-B signaling in PCa cells, were significantly increased after exposure to the conditioned media from aged mice, compared to that from young mice. These results indicated that age-related CD4+ Th17 cell responses are elevated in mice in the aging process and play an important role in PCa growth.

### DISPARITIES IN DEPRESSION AMONG CHINESE OLDER ADULTS

Zhichao Hao,[1] qingyi Li,[1] and Nicole Ruggiano[1], *1. University of Alabama, Tuscaloosa, Alabama, United States*

Depression has become one of the major health issues among older adults. In turn, various factors can facilitate or impede the occurrence of depression, socially, economically and culturally. Around 2050, China will have 487 million older adults or nearly 35 percent of the total population. Due to the different background of society, economy and culture, what explanations and knowledge can China provide based on present experiences and practices to help better understand depression among older adults from more comprehensive way? This study was conducted on the latest wave (2011-2014) of the Chinese Longitudinal Healthy Longevity Survey (CLHLS, 1998-2014). The sample included 7,107 Chinese older adults age from 65 to 117 years in China. A binomial hierarchical logistic regression was performed to examine the likelihood of having depression among older adults predicted by geographic characteristics, quality of life, chronic diseases, personal community services, social community services, and demographic variables including gender, age, and current marital status. Analysis indicated that approximately 10% of Chinese older adults in the sample reported depression. Compared to female and young-old adults (age 65-74), males (OR=.636, p<.001) and oldest-old adults (age 95+) (OR=.822, p<.001) were less likely to have depression. Older adults who lived in rural areas (OR=.681, p<.001) showed less likelihood of having depression. Older adults who had better life quality (OR=.553, p<.001) revealed less likely to have depression. Having social services in the community (OR=.908, p<.05) significantly lowered the likelihood of having depression among Chinese older adults. Implications for research, policy, and practice are discussed.

### SEPARATED KIN: RURAL-URBAN MIGRATION AND MENTAL HEALTH TRAJECTORIES OF OLDER ADULTS LEFT BEHIND IN RURAL CHINA

Zhiyong Lin[1], *1. University of Maryland, College Park, Maryland, United States*

As the processes of urbanization and globalization have intensified across the world, a burgeoning literature has documented the impact of emigration on the health of family members left behind in emigrant communities. Although the association between children's migration and parental well-being is well documented, few have examined the health implications of children's migration in the milieu of multiple children and further differentiated between children's short-term and long-term migration. Therefore, I argue that it is not the geographic locality of a single child but the composition of all children's location that matters. I further suggest that the impact of children's migration on parental wellbeing is conditioned on the duration of children's migration. Using a six waves longitudinal data (2001-2015) collected in rural China, this paper compares mental health (measured as depressive symptoms) trajectories of old adults (aged 60 and older) across different compositions of local and migrant children over a 14-year span. Results from growth curve models show that parents having more migrant children relative to local children experience a more rapid increase in depressive symptoms. In addition, older adults who have their most children migrate away for three or more waves of data have experienced the steepest rate of increase in depressive symptoms. These findings provide new evidence to support the life course processes of mental health disparities among older adults from the perspective of intergenerational proximity.

### ASSOCIATION BETWEEN HOME HEALTH SERVICE USE AND FACILITY ADMISSION IN OLDER ADULTS WITH AND WITHOUT DEMENTIA

Jinjiao Wang,[1] Thomas V. Caprio,[1] Adam Simning,[2] jingjing Shang,[3] Yeates Conwell,[4] Fang Yu,[5] and Yue Li[6], *1. University of Rochester, Rochester, New York, United States, 2. . University of Rochester Medical Center, Department of Psychiatry, NY, Rochester, New York, United States, 3. Columbia University School of Nursing, New York, New York, United States, 4. University of Rochester School of Medicine, Rochester, New York, United States, 5. University of Minnesota School of Nursing, Minneapolis, Minnesota, United States, 6. University of Rochester, Department of Public Health Sciences, NY, Rochester, New York, United States*

This study is a secondary analysis of the Outcome and Assessment Information Set (OASIS) and administrative billing records of 6,153 adults ≥ 65 years old who received home health (HH) from a not-for-profit HH agency in upstate New York in CY 2017. We evaluated the association between the use of home health services (HH) with the hazard of unplanned facility admissions among Medicare patients with and without Alzheimer's disease and related dementia (ADRD). Outcome was time from HH start of care to an unplanned facility admission of any type, including the hospital, nursing home, or rehabilitation facility. Independent variables included weekly intensity (visits/week, hours/week) of each discipline, including skilled nursing (SN), physical therapy (PT), occupational therapy (OT), social work (SW), and home health aide (HA), separately. ADRD was identified by diagnosis (ICD-10 codes) and cognitive impairment (M1700, M1710, M1740 [OASIS]). In multivariable Cox Proportional hazard models that adjusted for time-varying effects of HH intensity, receiving the highest intensity of SN (3.3 visits of 2.78 hours per week) and PT (2.5 visits of 2 hours per week) was related to up to a 54% and 86% decrease, respectively, in the hazard of unplanned facility admission among patients with ADRD (n=1,525), and decreases of 56% and 90% respectively among patients without ADRD (n=4,628). This is the first study in the United States showing that receiving a sufficient amount and appropriate mix of HH services was associated with substantially reduced risk of unplanned facility admission among patients with ADRD by up to 86%.

### RELATIONSHIP OF DEMENTIA AND MEDICAID ELIGIBILITY WITH FACILITY ADMISSIONS IN MEDICARE HOME HEALTH PATIENTS

Jinjiao Wang,[1] Thomas V. Caprio,[1] Helena Temkin-Greener,[2] Xueya Cai,[2] jingjing Shang,[3] and Yue Li[2], *1. University of Rochester, Rochester, New York, United States, 2. University of Rochester, Department of Public Health Sciences, NY, Rochester, New York, United States, 3. Columbia University School of Nursing, New York, New York, United States*

This study was a secondary analysis of the Outcome and Assessment Information Set (OASIS) and administrative billing records of 6,153 adults ≥ 65 years old who received home health (HH) from a not-for-profit HH agency in upstate New York between 01/01/2017 and 12/31/2017. We examined the relationships of dementia and Medicare-Medicaid dual eligibility with unplanned institutional admission (i.e. to hospital, nursing home, or inpatient rehabilitation facility) among these HH recipients. Dementia was identified by ICD-10 codes and OASIS items (M1700, M1710, M1740). We also used OASIS record to identify dual eligible status (M0150) and unplanned facility admission (M2410 [occurrence], M0906 [date], M2430 [reason]). Time-to-facility admission was defined as the number of days from HH start date to the facility admission date. The rate of having an unplanned facility admission was 14.2% among Medicare-only patients without dementia, 15.8% among dual eligible patients without dementia, 16.7% among Medicare-only patients with dementia, and 39.3% among dual eligible patients with dementia ($p<0.001$). In the multivariable Cox proportional hazard model of time-to-facility admission adjusting for patient covariates, dually eligible patients with dementia were more than twice as likely as Medicare-only patients without dementia to have an unplanned facility admission (Hazard Ratio=2.35, $p=0.006$). This is the first study that identified synergistic effects of having both dementia and Medicare-Medicaid dual eligibility on increasing the risk of healthcare facility admission in the Medicare HH population in the United States. Policies should ensure that appropriate and sufficient HH services be provided for dually eligible patients with dementia.

### DEMENTIA IMMERSION SIMULATION EXPERIENCE (DISE): ELICITING EMPATHY, IMPROVING COMMUNICATION SKILLS IN DEMENTIA

Gabriela Zaragoza,[1] Jaime Cruz-Martinez,[2] Paula Reilly,[3] Jeanette Ross,[4] Michael J. Mader,[5] and

Lyda C. Arevalo-Flechas[6], *1. The University of Texas Health Science Center San Antonio, San Antonio, Texas, United States, 2. The Long School of Medicine at UT Health San Antonio, San Antonio, Texas, United States, 3. Health Careers High School, San Antonio, Texas, United States, 4. University of Texas Health Science Center San Antonio, San Antonio, Texas, United States, 5. South Texas Veterans Health Care System, Dept of Research, San Antonio, Texas, United States, 6. South Texas Veterans Heath Care System - Geriatrics and Extended Care Service, San Antonio, United States*

Dementia awareness training alone does not improve care or outcomes for patients living with dementia. Effective dementia education programs for family caregivers and healthcare providers can lead to improved care practices and patient outcomes. The Dementia Immersion Simulation Experience (DISE) is a face-to-face 2-hour educational program that includes simulation, videos, a virtual reality station, group debriefing, and a didactic session delivered by faculty with dementia caregiving expertise. The purpose of this project was to evaluate the effectiveness of DISE in a group of 48 interdisciplinary healthcare providers, trainees and administrative staff. A program evaluation and pre and post knowledge questionnaires were administered. Prior to the activity, the mean score of all participants was 8.85. After the activity, the mean score was 10.1 ($p<0.0001$). 35.4% of all participants were well informed on dementia before DISE and 70.8% were well informed after the activity ($p <0.0005$). Qualitative analysis of the comments section of the program evaluation showed that 95% of the participants mentioned empathy for those living with dementia. Participants rated DISE on a scale of 1 (Strongly Disagree) to 5 (Strongly Agree) across ten categories, covering objectives, relevance, effectiveness, and value of the learning experience. Over 95% of respondents agreed or better (score = 4 or 5) with each evaluation statement and at least 85% strongly agreed with each statement. The evaluation scores are further evidence of an effective program. DISE is an effective tool to teach and support family caregivers, healthcare workers, and healthcare professionals and trainees.

### CHILDHOOD SOCIOECONOMIC STATUS AND SENSE OF CONTROL OVER COGNITIVE AGING: DO GENES MODERATE?

Addam S. Reynolds,[1] and Emily Greenfield[2], *1. Rutgers University School of Social Work, New Jersey, United States, 2. Rutgers University, New Brunswick, New Jersey, United States*

Individuals who lack a sense of control over cognitive aging (SOC-CA) believe little can be done to optimize their cognitive functioning. While prior research indicates that higher SOC-CA is a protective factor against age-related cognitive decline, few studies have examined predictors of change in SOC-CA. To address this gap, we used data from the Midlife in the United States (MIDUS) study. Guided by prior research on linkages between socioeconomic status (SES) and control beliefs, we examined childhood SES as an early life course influence on changes in SOC-CA. The analytic sample consisted of 663 White participants, ages 34 to 81, who were interviewed in 2004 and approximately nine years later. SOC-CA was measured by using three items from the Personality in Aging Context scale, and childhood SES encompassed retrospective reports of parental education and occupational status. A hierarchical linear model was estimated, which modeled SOC-CA at baseline, as well as change over the study period, controlling for gender, age, ancestry, and adult SES. While childhood SES was not associated with SOC-CA at baseline nor over time, a statistically significant gene-environment interaction was found over the 9-year study period. Specifically, participants who scored high on a polygenetic measure for cognitive ability and reported high childhood SES demonstrated a faster rate of decline in SOC-CA. These findings indicate that inter-individual differences stemming from early life influence people's SOC-CA as they age. Overall, results suggest the importance of subgroup differences within efforts to engage individuals in preventive measures to optimize healthy brain aging.

### FAMILY CONFLICT: A WELL KEPT SECRET IN LATINO DEMENTIA CAREGIVING

Gabriela Zaragoza,[1] Lyda C. Arevalo-Flechas,[2] Bertha E. Flores,[3] and Janna Lesser[4], *1. The University of Texas Health Science Center San Antonio, San Antonio, Texas, United States, 2. South Texas Veterans Heath Care System - Geriatrics and Extended Care Service, San Antonio, United States, 3. UT Health San Antonio, San Antonio, Texas, United States, 4. UT Health San Antonio School Of Nursing, San Antonio, Texas, United States*

The number of Latino older adults living with Alzheimer's disease and related dementias (ADRD) could increase more than six-fold, from fewer than 200,000 in 2004 to more than 1.3 million by 2050 . One-third of Hispanic households report having at least one family caregiver (36%). 63% of Latino family caregivers are in high burden situations compared to 51% of non-Latino caregivers. Although in recent years research with Latino caregivers has increased, studies examining the family as a unit in a culture in which familismo is a central value, remain limited. The purpose of this study was to describe the experience of Latino families caring for loved ones living with ADRD along the illness trajectory. Group interviews were conducted with families caring for individuals in different stages of dementia. Interviews were conducted in English or Spanish, transcribed verbatim, and analyzed in the source language by bilingual investigators. In contrast to the numerous positive aspects of caregiving reported by individual Latino caregivers in previous studies, preliminary analysis of family group interviews revealed predominantly negative perceptions of the family caregiving experience. Families reported conflict between family members, feelings of resentment, lack of family member involvement, and uneven distribution of caregiving duties. The complex nature of fulfilling family duties as dictated by familismo appears to have idealistic and realistic consequences for families. Further exploration of Latino family dynamics in the context of dementia caregiving is needed.

### PERCEPTIONS, ACCEPTABILITY, EXPECTATIONS, AND CONCERNS OF SMART HOME TECHNOLOGIES AMONG OLDER ADULTS

Amy Guan,[1] Hannah C. Dannewitz,[1] Lauren Stratton,[1] Jennifer Margrett,[1] Walter R. Boot,[2]

Neil H. Charness,[2] and Balaji Narasimhan[1], *1. Iowa State University, Ames, Iowa, United States, 2. Florida State University, Tallahassee, Florida, United States*

Optimal aging in place has become a common preference among older adults to maintain identity and independence, thus smart home technologies are increasingly utilized to achieve these goals. However, disconnect may exist between potential technological benefit and perceptions of acceptability and usability (Lee & Coughlin, 2015). We assessed perceptions of adults aged 50+ (range 50-90 years) to analyze their priorities and ultimate acceptability of smart home technology. Data were collected through surveys, focus groups, and case study interviews. Three major themes emerged regarding smart home utilization: benefits, concerns, and expectations. Participants endorsed smart home technologies (e.g., sensors, telehealth devices) and identified benefits, such as the promotion of optimal aging (e.g., maintaining independence, staying active, safety). However, responses also reflected concerns about privacy, ease of use, and amount of control. Expectations regarding smart homes included more mobility, efficiency, and safety within the home. One participant described technology as having "options [that] are exhausting, but also exciting." Survey responses (n=30) were analyzed to understand participants' familiarity with smart home technologies, including: nanotechnology (10.7%), smart showers (42.9%), home sensors (70.4%), telehealth (74.1%), smart appliances (71.4%), personal sensors (81.5%), and voice-activated devices (96.4%). Additionally, respondents indicated their willingness to implement these technologies to maintain and/or improve their daily functioning: nanotechnology (53.8%), smart showers (28.6%), home sensors (66.6%), telehealth (81.5%), smart appliances (40.0%), personal sensors (55.5%), voice-activated devices (64.3%). Discussion focuses on the priorities and needs older adults express regarding technology utilization and the implications for person-centered design and implementation of future smart home technologies.

### PARENTING ALL OVER AGAIN : HISPANIC "ABUELITAS" RAISING GRANDCHILDREN IN THE CONTEXT OF SUBSTANCE ABUSE DISORDERS

Bertha E. Flores,[1] Lyda C. Arevalo-Flechas,[2] and Martha Martinez[3], *1. UT Health San Antonio, San Antonio, Texas, United States, 2. South Texas Veterans Heath Care System - Geriatrics and Extended Care Service, San Antonio, United States, 3. University of Texas Health San Antonio School of Nursing, San Antonio, Texas, United States*

Around 7 million grandparents in the U.S live with grandchildren under 18 and 39% have primary caregiving responsibilities. In Texas, more than 313,499 children under age 18 live with a grandparent who is responsible for them. Hispanic grandparents are disproportionately more likely to care for grandchildren compared to non-Hispanic whites. In San Antonio, Texas, 36.6 % of grandparents are the primary caregivers for their grandchildren. Parental substance abuse disorders have been identified as one of the causes prompting child removal from parental custody. To understand the experience of custodial Hispanic grandmothers raising their grandchildren, three focus groups were conducted in English and Spanish by three bilingual investigators. Twenty-three grandmothers, mean age 60, caring for an average of 3.2 children, 2 months to 17 years of age participated in the focus groups. The narratives were transcribed and analyzed in the source language. The following overarching themes were identified: Family is family, parenting all over again, this is a struggle but a blessing, what did I do wrong? , fear of losing the children to foster care, I do not trust anybody with my children, financial and legal challenges, role captivity, aging as a limitation, and hope for the future. The findings from the study contribute to the body of knowledge necessary to foster urgent policy changes aimed at supporting these grandparents who feel unjustly treated by human services agencies and the legal system. Programs to support custodial Hispanic grandmothers need to be linguistically congruent and culturally competent.

### CARDIAC TROPONIN T MEDIATED AUTOIMMUNE RESPONSE AND ITS ROLE IN SKELETAL MUSCLE AGING

Tan Zhang,[1] Xin Feng,[2] Bo Feng,[1] Juan Dong,[1] Karen Haas,[1] Barbara M. Nicklas,[1] Osvaldo Delbono,[1] and Stephen Kritchevsky[1], *1. Wake Forest School of Medicine, Winston-Salem, North Carolina, United States, 2. Wake Forest School of Medicine, Winston-Salem, United States, 3. Wake Forest University, Winston-Salem, North Carolina, United States*

Cardiac troponin T (cTnT), a key component of contractile machinery essential for muscle contraction, is also expressed in skeletal muscle under certain conditions (e.g. neuromuscular diseases and aging). We have reported that skeletal muscle cTnT regulates neuromuscular junction denervation preferentially in fast skeletal muscle of old mice. Here, we further report that cTnT is also enriched within some myofibers, and/or along microvascular walls in old mice fast skeletal muscle. Strikingly, immunoglobulin G (IgG), together with markers of complement system activation, cell death (necroptosis or apoptosis), and macrophage infiltration, were all found to be co-localized with cTnT and IgG in those areas. In addition, elevated cTnT and IgG are associated with lower dystrophin expression on muscle fiber membrane, lower muscle capillary density, and reduced muscle performance (wire hanging test). Using purified recombinant TnT proteins, we confirmed that only cTnT, but not slow or fast skeletal muscle TnT1 or TnT3, was detected by immunoblot using sera from old (but not young) mice with pre-determined elevated cTnT and IgG in their skeletal muscle, indicating the existence of anti-cTnT autoantibodies in sera (previously found in human blood) and skeletal muscle of old mice. Immunoblotting further revealed that the age related changes in skeletaI muscle cTnT and IgG are more prominent in fast skeletal muscle than in slow. Importantly, elevated cTnT and IgG were also detected in skeletal muscles from 4 older adults (65-70 yrs, IMFIT). Our finding suggests a novel autoimmune mechanism mediated by cTnT that underlies age related skeletal muscle abnormalities and dysfunction.

### AGE-RELATED DECLINES IN SOCIAL COGNITIVE PROCESSES OF OLDER ADULTS

W.Quin Yow,[1] Jiawen Lee,[1] and Xiaoqian Li[1], *1. Singapore University of Technology & Design, Singapore, Singapore*

Despite current literature suggesting that various social cognitive processes seem to be impaired in late adulthood, e.g., processing of social gaze cues, the trajectory decline of social cognition in late adulthood is not well understood (e.g., Grainger et al., 2018; Paal & Bereczkei, 2007). As part of a multi-institutional research project, we began to systematically investigate whether there is age-related decline in older adults' ability to infer others' mental states, integrate multiple referential cues, and identify emotional states of others using prosodic cues. Sixteen older adults aged 71-85, of which 9 were cognitively healthy and 7 with mild-to-moderate dementia, and 7 younger adults aged 19-37 underwent three tasks. In a theory-of-mind story task, participants answered true/false questions about the beliefs of the protagonists in the stories. A cue integration task assessed participants' ability to integrate the experimenter's gaze and semantic cues to identify a referent object. In an emotion-prosody task, participants judged whether the speaker sounded happy or sad in low-pass filtered audio. Non-parametric tests revealed that younger adults outperformed both groups of older adults (both ps=.001) in inferring the protagonists' beliefs in the stories. Younger adults were also better and more accurate than both groups of older adults in integrating cues to identify the referent object and in using prosodic cues to identify emotional states respectively (ps<.001). Both groups of older adults did not differ significantly from each other in the tasks. These findings provide emerging and important insights into the decline of social cognitive processes in late adulthood.

### FACILITY AND RESIDENT LEVEL DIFFERENCES: ANTIBIOTIC USE IN NURSING HOMES FOR RESIDENTS WITH ADVANCE DEMENTIA

Meghan Hendricksen,[1] Daniel Habtemariam,[2] and Susan Mitchell[2], *1. Hebrew SeniorLife, Marcus Institute for Aging Research, Boston, Massachusetts, United States, 2. Hebrew SeniorLife, Institute for Aging Research, Boston, Massachusetts, United States*

Previous studies have shown that there is a high frequency of antibiotic use in NH for advance dementia patients. However, research has shown limited clinical benefit from antimicrobial use for this population, and antimicrobial exposure increases colonization with drug-resistant bacteria in nursing homes. The aim of this study was to identify NH and resident level characteristics associated with antibiotic use for patients with advance dementia. Using data from an ongoing cluster RCT in 28 Boston NHs; Trial to Reduce Antimicrobial use in Nursing home residents with Alzheimer's disease and other Dementias (TRAIN-AD), testing a program intervention to improve management of infections in advanced dementia. These data are taken from baseline measurements 2 months prior to intervention, and individual nursing home residents with advance dementia are units of analysis (n = 425). We ran multivariable logistic regression model with antibiotic use as the outcome, adjusting for clustering at NH level, with NH (#beds, profit status, staffing, #cognitively impaired, etc.) and individual patient characteristics (age, gender, race, etc.) as independent variables. Analyses found residents were more likely to receive antibiotics if they resided in nursing homes that employed less intense infectious disease practices prior to baseline (AOR = 2.34; 95% CI 1.08, 5.05), and full-time nurse practitioners or physician assistants (AOR= 3.68; 95%CI 1.49, 9.04). Female patients also had higher odds of receiving antibiotics (AOR=2.16; 95%CI1.10, 4.67). These findings provide potential insight into the importance of education regarding stringent infectious disease practices for practitioners, particularly for patients with advanced dementia.

### AUTOMATED MEASUREMENT OF MUSCLE DENSITY ON COMPUTED TOMOGRAPHY (CT) PREDICTS ALL-CAUSE MORTALITY IN OLDER ADULTS

Leon Lenchik,[1] Ryan Barnard,[1] Robert D. Boutin,[2] Stephen B. Kritchevsky,[1] Ashley A. Weaver,[1] and Fang-Chi Hsu[1], *1. Wake Forest School of Medicine, Winston-Salem, North Carolina, United States, 2. UC Davis School of Medicine, Sacramento, California, United States*

The purpose was to examine the association of paraspinous muscle density (CT surrogate of myosteatosis) with all-cause mortality in 6803 men and 4558 women, age 60-69 years (mean age 63.6) in the National Lung Screening Trial. Our fully-automated machine learning algorithm: 1) selected the appropriate CT series, 2) chose a single CT image at the level of T12 vertebra, 3) segmented the left paraspinous muscle, and 4) recorded the muscle density in Hounsfield Units (HU). Association between baseline muscle density and all-cause mortality was determined using Cox proportional hazards models, adjusted for age, race, body mass index, pack years of smoking, and presence of diabetes, lung disease, cardiovascular disease, and cancer at enrollment. After a mean 6.44 ± 1.06 years of follow-up, 635 (9.33%) men and 265 (5.81%) women died. In men, lower muscle density on baseline CT examinations was associated with increased all-cause mortality (HR per SD = 0.90; CI = 0.83, 0.99; p=0.03). Each standard deviation (7.8 HU) decrease in muscle density was associated with a 10% increase in mortality. In women, the association did not reach significance.

### ORAL CANCER SCREENING IN NON-INSTITUTIONALIZED OLDER ADULTS LIVING IN RURAL COMMUNITIES OF SOUTHEAST GEORGIA

SWAHA PATTANAIK,[1] Bettye Apenteng,[1] Adrienne L. Cohen,[2] Georgia Dounis,[3] and Raymona Lawrence[1], *1. Jiann Ping Hsu College of Public Health, Georgia Southern University, Statesboro, Georgia, United States, 2. Georgia Southern University, Statesboro, Georgia, United States, 3. University of Nevada, Las Vegas, Las Vegas, Nevada, United States*

The older population in the United States is growing at an unprecedented rate. Oral diseases such as oral cancer can affect physical, psychological, and social well-being in older adults. Oral cancer screening can prevent development of the disease in high-risk individuals. The purpose of this research was to assess determinants of preventive oral health behavior including oral cancer screening in noninstitutionalized older adults living in rural/medically underserved communities of southeast Georgia. A mixed methods sequential explanatory design was used. Surveys were administered to 206 individuals aged 50 and older. Phone interviews were conducted with 22 individuals from the survey sample and 11 key informants. The majority of the participants (83.01%) said they had never been examined for oral cancer by a doctor or a dentist. Those who correctly recognized the most common sign of oral cancer were about three times more likely (OR=3.75; 95% CI: 1.04 – 13.50) to have had an exam for oral cancer (p=0.04). The survey participants who lived alone were more likely (OR = 4.39; 95% CI = 0.95 – 20.26) to have been examined for oral cancer (p = 0.05). During the interview, older adult participants rarely mentioned oral cancer with regards to an unhealthy mouth. The interview participants revealed that living alone gave them more time to pay attention to their health. For the older adults, prevention of oral diseases was grounded in the autonomy in their own behaviors, while the key informants saw more macro community and systems- level factors as the solution.

### INVESTIGATING THE INVERSE RELATIONSHIP BETWEEN SMOKING AND PARKINSON'S: EVIDENCE FROM A MULTIETHNIC COHORT

Fadi Youkhana,[1] Yanyan Wu,[1] Catherine M. Pirkle,[1] Eric Hurwitz,[2] Andrew Grandinetti,[1] Alan Katz,[1] and Lynne R. Wilkens[1], *1. University of Hawai'i at Mānoa, Honolulu, Hawai'i, United States, 2. University of Hawaii at Manoa, Hawaii, United States*

Parkinson's disease (PD) is the second most common neurodegenerative disease in the United States with more than 50,000 new cases annually. Studies have reported an inverse relationship between smoking status and the risk for PD. Current smoking status, the number of pack-years smoked, and the lifetime duration of smoking have all been shown to have a lower risk for PD compared to non-smokers. However, studies exploring smoking behaviors in a multiethnic cohort with an ample sample size of PD cases to analyze smoking differences between men and women are rare. Using the Multiethnic Cohort (MEC), our study included 680 self-reported cases of PD from total sample of 98,191 Blacks, Latinos, Japanese, Native Hawaiians, and Whites from Hawaii and Los Angeles surveyed in 2003-2007. Stratified by sex, we conducted a cross-sectional logistic regression analysis to examine the odds of developing PD by various smoking indicators. Overall, current smokers had the lowest risk for PD (OR=0.46, 95%CI 0.27-0.76) compared to non-smokers. The odds of developing PD gradually decreased as the number of years of smoking increased with participants that smoked for 50 years or more having the lowest odds of developing PD (OR= 0.41, 95%CI 0.22-0.78) compared to non-smokers. Using a multiethnic cohort, our analyses further supported the inverse association between PD and smoking status, as well as the number of years of smoking. Future studies are necessary to investigate the possible genetic modulation on the relationship between tobacco and PD.

### DISCUSSING SPIRITUAL HEALTH IN PRIMARY CARE IN ENGLAND

Ishbel Orla Whitehead[1], *1. Newcastle University, Newcastle, United Kingdom*

The organisation that regulates doctors and family physicians' professional body in the UK both require doctors to consider patients' spiritual health, especially towards the end of life. Discussion of spiritual health can encapsulate positive aspects of patients' lives, and may be valuable for older people as physical and mental health decline. Tools are available for doctors to structure discussion of spiritual health in consultations but anecdotal reports suggest that this seldom happens. This study aimed to understand the barriers to GPs discussing spiritual health and their knowledge and views of current tools, particularly the HOPE tool by Anandarajah and Hight. Narrative literature review using systematic methods and mixed methods investigation into current practice, An online survey was conducted with 177 family physicians in England, investigating how doctors define spiritual health, their comfort with the topic, and their knowledge and acceptability of the HOPE tool, using patient vignettes. Definitions of spiritual health were heterogeneous, within three themes: self-actualisation and meaning; transcendence and relationships beyond self; and expressions of spirituality. Doctors felt more comfortable discussing spiritual health after a patient-led cue. Introduction of the HOPE tool increased doctors' comfort with the topic. Discordance between doctor and patient beliefs and cultural backgrounds influenced views and practice. Concerns about regulator disapproval was a major barrier to discussions. Spiritual health does not appear to be a routine part of family practice in the UK. Tailored education, containing a structured tool such as HOPE, with regulatory approval, may help overcome barriers to discussion of spiritual health.

### BARRIERS AND SOLUTIONS TO IMPROVE AGE-RELATED SERVICE ACCESS IN UTAH: A QUALITATIVE STUDY

Alex T. Schiwal,[1] and Elizabeth B. Fauth[2], *1. Center for Persons with Disabilities - Utah State University, Logan, Utah, United States, 2. Utah State University, Logan, Utah, United States*

Utah is projected to be in the top 10 states for growth in the aging population, but it is among the most rural. Local and regional contexts guide policy and practice, and these perspectives will inform solutions as more older adults require services in rural and other under-served areas in the coming decades. Guided by Bronfenbrenner's Process-Person-Context-Time model, this study used a qualitative participatory research orientation involving stakeholders in Utah's aging service system in order to identify local barriers and solutions to accessing rural aging services. The stakeholders included service providers, caregivers, older adults, state-administrators, and other community members. There were 3 male and 7 female participants ranging in age from 40 to 80. Thematic analysis revealed that communities faced barriers common to rural areas (local service insufficiencies, distance and time concerns, systemic issues such as healthcare and ageism, finances - both personal and programmatic were deemed a recurrent barrier, in addition to transportation issues. However, participants reported assets in rural areas, such as a strong sense of belonging in the community and creative problem solving. Solutions for improving access to age-related services included strategies for making information more available, publicized, and centralized and increasing access to telehealth or internet-delivered services and health information. These barriers and solutions were nested across the levels of context in Bronfenbrenner's model, with both person, time, and in interactions (processes) having influence, but localized analysis of the barriers is necessary to ensure that the solutions are appropriate in a specific context.

### USING SPATIAL ANALYSIS TO OPTIMIZE DISASTER PREPAREDNESS FOR FRAIL OLDER ADULTS

Megan Bond,[1] and Jessica L. Tice[1], *1. Florida Department of Elder Affairs, Tallahassee, Florida, United States*

The Florida Department of Elder Affairs (DOEA) provides programs and services for over 65,300 older people and adults with disabilities. These individuals are uniquely vulnerable and may be disrupted, displaced, and disoriented during the natural disasters common to Florida. DOEA clients are homebound or dependent upon community-based services to provide supervision or direct assistance to perform basic self-care. Often, clients are unable to complete personal care independently, which makes sheltering in place a unique challenge, yet they can only evacuate with special transportation and support arrangements in the shelter. It is critical to DOEA to accurately predict clients likely to be seriously affected by storms, plan for relocation before an event, and arrange for the provision of extended care after. DOEA responded by utilizing ArcGIS mapping software to join client residence locations to evacuation zone polygons and developed a methodology to prioritize clients with personal and functional barriers to evacuation. Proven during Hurricane Michael (2018), local emergency managers were able to use this tool to complete wellness checks on survivors before outside aid arrived. This initiative is evolving with the challenges posed by each storm season. Hurricane Dorian (2019) required the addition of latitude and longitude of client locations for when traditional street navigation became unavailable. Importing and overlaying primary data on secondary emergency management resources is a strategy that could be replicated by other organizations that have similar needs to reconcile individual locations in context of local threats, making this methodology transferrable to other disaster and flood-prone communities.

### HEPATIC AGO2-MEDIATED RNA SILENCING REGULATES SYSTEMIC GLUCOSE METABOLISM.

Kazutoshi Murakami[1], *1. Kurashiki Central Hospital, Kurashiki, Okayama, Japan*

Obesity causes various metabolic complications, including insulin resistance and T2DM. However, currently, we have a limited understanding of the pathophysiology in the development of these processes. It's generally considered that obesity develops when energy intake chronically exceeds total energy expenditure. The liver is a major organ for energy consumption, then mRNA translation accounts for the majority of energy expenditure in liver. While RNA silencing regulates mRNA translation, it's unclear if RNA silencing regulates glucose metabolism. To investigate the role of RNA silencing in glucose metabolism, we focused on Argonaute 2 (Ago2), which is the main component of RNA-induced silencing complex that carries out RNA silencing. By generating liver-specific Ago2-deficient (L-Ago2 KO) mice, we revealed Ago2 regulates the maturation process of metabolic disease related miRNAs (MD-miRNAs), that silence genes critical for glucose metabolism. In addition, Ago2-deletion enhances ATP consumption associated with mRNA translation. Consequently, inactivation of hepatic Ago2 protect from diet-induced glucose intolerance in mice. Then, to further investigate if these molecular mechanisms are still activated in "older mice", we employed 34-week of age mice and analyzed. Around this age, despite having the similar body weight, L-Ago2 KO mice fed HFD exhibited lower blood glucose and serum insulin levels. Consistently, expression levels of MD-miRNAs were decreased in the liver of L-Ago2 KO mice fed HFD. These results suggest that hepatic Ago2 function is continuously activated in "older mice" fed HFD, leading to enhanced biogenesis of MD-miRNAs and reduction of their target mRNAs, and these alterations are associated with systemic glucose metabolism.

### VALIDATING THE CARE PREFERENCE ASSESSMENT OF SATISFACTION TOOL TO MEASURE QUALITY OF CARE IN NURSING HOMES

Caroline Madrigal,[1] Kimberly VanHaitsma,[2] Jacqueline Mogle,[2] Donna Fick,[2] Dennis Scanlon,[2] Katherine M. Abbott,[3] and Liza Behrens[4], *1. U.S. Department of Veterans Affairs, Providence, Rhode Island, United States, 2. The Pennsylvania State University, University Park, Pennsylvania, United States, 3. Miami University, Oxford, Ohio, United States, 4. University of Pennsylvania, Philadelphia, Pennsylvania, United States*

The Institute for Healthcare Improvement's Triple Aim calls for measures of the 'patient care experience' to understand and improve the quality of care delivery. But, quality measures in the nursing home (NH) historically lack the resident perspective. Measuring whether residents are satisfied with the fulfillment of their care preferences using the Care Preference Assessment of Satisfaction Tool (ComPASS) has been encouraged nationally by the Centers for Medicare and Medicaid (CMS); however, the ComPASS has not been validated as a measure of the resident care experience. The purpose of this study was to compare ComPASS to the Ohio NH Resident Satisfaction Survey (a widely accepted quality measure for reimbursement). We examined 196 resident responses from 28 NHs in Pennsylvania using multilevel modeling to account for dependencies in the data (residents in the same NH may respond similarly compared to residents from different NHs). Residents were 81.2 years old (SD= 11.1), female (70.4%), and white (80.1%). Residents with higher scores on the ComPASS reported significantly higher levels of satisfaction with care (B=2.94, SE B=0.59, $p<0.000$). Results from this study support the potential use of ComPASS to measure, track, and improve the quality of NH care. Using ComPASS aligns with CMS's Section F of the Minimum Dataset, an assessment of residents' preferences which promotes the delivery of more person-centered care. Ultimately, ComPASS can help benchmark the quality of the resident care experience across facilities which aids staff, facilities, policy-makers, and NH-shoppers in improving decision-making and care delivery.

### ASSOCIATION BETWEEN GAIT SPEED MEASURED USING A WEARABLE DEVICE AND SARCOPENIA

Min-gu Kang,[1] Kwang-il Kim,[2] Joon Koo Kang,[1] Seong-Ji Kang,[3] Hye-Kang Roh,[4] and Hwa-Young Jung[4], *1. Department of Internal Medicine, Chonnam National University Bitgoeul Hospital, Gwangju, Korea, Republic*

*of, 2. Seoul National Bundang Hospital, Bundang-gu, Seongnam-si, Korea, Republic of, 3. Graduate School of Health Science and Management, Yonsei University, Seoul, Korea, Republic of, 4. WELT Corp., Ltd, Seoul, Korea, Republic of*

As slow gait speed is a major feature of frailty and a diagnostic criterion of sarcopenia, gait speed measurement is widely used. Nowadays, with development of wearable devices, it is possible to measure daily-life gait speed without additional effort just by wearing the device. It is meaningful to measure daily-life gait speed and to analyze the association between the speed and sarcopenia. Participants were men over 50 years of age who visited the university hospital. Daily-life gait speed was checked using a smart belt (WELT) for 4 weeks. Afterwards, a survey about past medical history, usual gait speed measurement, handgrip strength measurement, and dual energy X-ray absorptiometry were performed. A total of 217,548 daily-life gait speed measurement data were analyzed for 106 participants. The mean daily-life gait speed was 1.23 ± 0.26 m/s. The mean age was 71.1 ± 7.6, and daily-life gait speed was significantly slower as people get older. ($P<0.001$) Additionally, weekday gait speed (1.23 ± 0.26 m/s) was significantly faster than weekend gait speed (1.22 ± 0.26 m/s). ($P<0.001$) Participants with sarcopenia (1.15 ± 0.25 m/s) had significantly slower mean daily-life gait speed than normal subjects (1.23 ± 0.26 m/s). ($P<0.001$) In analyzing factors related to gait speed, age and skeletal muscle mass of lower limbs were significantly associated with mean daily-life gait speed. Additional information about the gait speed can be obtained by measuring daily-life gait speed, and the daily-life gait speed has a significant association with the skeletal muscle mass of lower limbs.

### NATURALISTIC DRIVING BEHAVIOR AS A NEUROBEHAVIORAL MARKER OF PRECLINICAL ALZHEIMER'S DISEASE

Ganesh Babulal[1], *1. Washington University School of Medicine (St. Louis), St. Louis, Missouri, United States*

Decline in driving skills begins in preclinical AD, when an older adult remains cognitively normal, but the underlying disease process has begun. Preclinical AD is detectable among cognitively normal individuals using molecular biomarkers: positron emission tomography (PET) imaging and cerebrospinal fluid (CSF). The aim of this prospective, longitudinal study is to determine whether naturalistic driving behavior using in-vehicle dataloggers can distinguish older adults with (n=36) and without preclinical AD (n=134). Driving data was calculated as mean/month for several variables (number of trips/day, trip length, trip time, speeding, and hard-braking) for participants followed between one to 46 months. Using stepwise logistic regression, the area under the receiver operating curve (AUC) and 95% confidence interval for these five variables was 0.73 (0.63-0.79) in distinguishing those with and without preclinical AD via amyloid imaging. When age, gender, race, and education were added, the model improved: 0.80 (0.72-0.88). Finally, when apolipoprotein ε4 allele (APOε4), obtained via blood or saliva, was added to the model, accuracy improved: 0.84 (0.77-0.89). Similar results were found using CSF biomarker tau/A$\beta$42: AUCs (95% CI) were 0.68 (0.58-0.79) for driving variables alone, 0.77 (0.69-0.86) for driving variables and demographics, and 0.87 (0.80-0.94) driving variables, demographics, and apolipoprotein ε4 allele. These promising findings suggest that naturalistic driving behavior can predict those with and without preclinical AD. The AUC is further improved with demographics and APOε4, an easily obtainable genetic biomarker. This model may be used in clinical/research settings as a screen or adjunct for diagnostics and prognostics purposes.

### YES, CHANGING HOW YOU TEACH DOES MAKE A DIFFERENCE

Edmund H. Duthie,[1] Kathryn Denson,[1] Deborah Simpson,[2] Steven Denson,[1] and Amanda Szymkowski[1], *1. Medical College of Wisconsin, Milwaukee, Wisconsin, United States, 2. Aurora UW Medical Group, Milwaukee, Wisconsin, United States*

Perceptions of an educational experience's value impact learning. "Hands-on" activities promote deeper learning and retention. Educators may jettison more poorly rated sessions, not having time for perceived content revisions based on evaluation data. We sought to determine if simply changing the sequence of a session's activities, using the same content, improved learner evaluations. Using a session focused on application of resources for dementia patient caregivers, we provided two versions of the same content to 2 groups of clinicians. In session version #1 (V1), participants were asked about caregiver stresses and barriers and then viewed two video triggers of a dementia patient and a stressed family caregiver. Participants then identified the caregiver's struggles and recommended resources. At the session's end they were provided with a Geriatric Fast Fact (GFF) (www.geriatricfastfacts.com) that hyperlinked to a variety of evidence-based resources by topic. In session version #2 (V2), only the content was flipped. The GFF was presented prior to the video, with clinicians were then tasked to identify best resources using the GFF. The V2 cohort rated the session higher than V1 cohort on a 4-point scale (1= Excellent, 4= Poor). Overall quality of learning plan (V1 =1.4 ; V2 =1.3); Would you recommend the session to peers (V1 = 1.5; and V2 =1.2) and Overall course evaluation (V1 = 1.5; V2. = 1.4) all improved. Using learner evaluations to revise the sequence of the same content was an effective educational strategy. Don't throw the baby out with the bathwater!

### INFLUENCE OF DEPRESSION, SOCIAL SUPPORT AND MEANING IN LIFE ON SUICIDAL IDEATION OF OLD ADULTS HEMODIALYSIS PATIENTS

HYEBEEN SIM,[1] and Jinhee Shin[2], *1. Kyung Hee university, Kangbuk samsung hospital, Seoul, Korea, Republic of, 2. College of Nursing, Yonsei University, SOUTH KOREA, SEOUL, Korea, Republic of*

Purpose: This study is to identify how depression, social support and meaning in life influence to suicidal ideation of home based Korean old adult renal dialysis patients and the relating factors according to their general characteristics. Methods: This descriptive correlative study was conducted through a organized and structured self-administrated questionnaire and 120 sampled home based old adult renal dialysis patients. Collected data was analyzed by t-tests, ANOVA, Pearson's correlation coefficient and multiple regression analysis using SPSS/WIN 24.0. Results: Findings revealed that; 1) The degrees of suicidal ideation were significantly different among groups according to the marital status, drinking and smoking history. 2) Pearson's correlation coefficient revealed a significant association among the suicidal ideation, depression, social support and meaning in life. 3) Multiple regression analysis showed depression, social support and meaning in life were related to factors. Conclusion: Based on the findings of this study, health professionals should provide home based Korean old adult renal dialysis patients with proper management of suicidal ideation as well as its relating factors, depression, social support and meaning in life. Especially, it needs to implement suicidal ideation management and self-help group program to home based old adult renal dialysis patients.

### BIOLOGICAL AGE INFLUENCES HEART FAILURE PATHOGENESIS

Kathleen Woulfe,[1] Jacob Zumo,[2] Ross Cook,[3] Benjamin McNair,[3] Jacob Schlatter,[3] Emily Schmitt,[3] Vikram Chhatre,[3] and Danielle Bruns[3], *1. University of Colorado-Denver, Aurora, Colorado, United States, 2. University of Washington School of Medicine, WWAMI Regional Medical Education, Seattle, Washington, United States, 3. University of Wyoming, Laramie, Wyoming, United States*

Heart failure (HF) impacts patients of all ages and is an enormous public health problem. Historically, HF has been treated with a single, multi-purpose approach, despite the observation that biological differences such as age influence HF pathogenesis and therapeutic outcomes. We hypothesized that HF pathogenesis differs across the life-course, a hypothesis which we tested with a mouse model of cardiac dysfunction at three distinct stages of life. C57BL/6 mice at pediatric (5 weeks), adult (3-5 months), and old (18 months) ages were treated with a mini-osmotic pump that eluted isoproterenol (ISO; 30mg/kg/hour) for six days. As expected, cardiovascular morbidity and mortality were significantly worse in the old group. Both pediatric and adult underwent hypertrophic remodeling, as evident by higher LV weight relative to tibia length (TL). However, ISO exposure did not increase LV/TL in old mice. We performed RNA-sequencing to understand pathways and genes differentially regulated by age. 119, 1515, and 33 genes were significantly differentially expressed in pediatric, adult, and old mice exposed to ISO, respectively. Of these, only 2 transcripts were upregulated in response to ISO across all three ages. Expression of pro-fibrotic mediators differed across the life-course, with adults inducing a pro-fibrotic transcriptional program ($\alpha$-smooth muscle actin, fibronectin, collagen, periostin) that was attenuated in old and absent in pediatric animals. Our data clearly demonstrates that pediatric, adult, and aged hearts activate distinct molecular remodeling in response to ISO, highlighting the significance of age as a biological variable in HF pathogenesis.

### SOCIAL NETWORK INFLUENCES ON SENSE OF CONTROL AND ATTRIBUTED DIGNITY IN OLDER AGE: RESEARCH RESULTS

Raeann G. LeBlanc,[1] and Cynthia Jacelon[1], *1. University of Massachusetts Amherst, Amherst, Massachusetts, United States*

The purpose of this study was to explore the relationship between the functions of individual social networks, defined here as social support, and the outcomes of sense of control and attributed dignity among a sample of older people living with multiple chronic conditions. This study integrated an explanatory sequential (Quan/Qual) mixed methods design. Descriptive statistics were used to describe social networks. Bivariate correlations and regression statistics were used to examine the relationships of social network support (MOS-Social Support Scale) with the dependent variables of sense of control (Wallhagen Revised PCQ Questionnaire) and attributed dignity (Jacelon Attributed Dignity Scale). Open-ended interviews and thematic analysis were used to expand understanding of the quantitative findings. A cross-sectional sample of eighty-nine community dwelling older people living with multiple chronic health conditions participated. Social support, as a function of one's social network, predicted the outcome of sense of control ($\beta = .33$, $p \leq .01$) and attributed dignity ($\beta = .44$, $p \leq .001$). Correlation statistics and regression models substantiated positive relationships of social supports' influence on perceived sense of control and attributed dignity. Thematic analysis, based on open-ended interviews (n=12), expanded on the nuances of social influences on sense of control and attributed dignity in managing chronic health conditions through the themes "learning to ask for help", "only a phone call away" and "smaller circles". This research proposes new ways of understanding the relationships between perceptions of social support, sense of control and attributed dignity in later life in managing health.

### AGING ALTERS STEROID HORMONE METABOLISM AND EXACERBATES LOWER URINARY TRACT DYSFUNCTION IN MICE

Teresa T. Liu,[1] and William A. Ricke[1], *1. University of Wisconsin - Madison, Madison, Wisconsin, United States*

Benign prostatic hyperplasia (BPH) is a disease of aging that impacts 50% of men in their 50s and 90% of men in their 80s. While rodent models have been invaluable in the study of lower urinary tract dysfunction (LUTD) associated with human disease, many studies recapitulate aspects of aging, steroid hormone fluctuations, and/or inflammation without using aged mice that would better correspond to the age range of patients. In this study, we examine the impact of age in the hormone-induced mouse model of LUTD, so we can better understand the contribution of age to disease initiation and progression. We've discovered that aged mice exhibit a level of LUTD that is further exacerbated by hormone implantation when compared to both treated and untreated younger mice. Examination of circulating levels of androgens and estrogens indicate an alteration in steroid hormone metabolism with age, suggesting an altered nuclear receptor activation within disease. Epigenetic modifications have been associated with normal aging, including an increase in DNA methylation to alter gene expression. Examination of the proximal promoter of a steroid enzyme gene, CYP7B1, responsible for the degradation of 3$\beta$-diol (an ER$\beta$ ligand) within the prostate shows an age-mediated increase in methylation. With this, there is a corresponding decrease in CYP7B1 gene expression in the aged mice. Taken together, this suggests the altered steroid hormone environment seen in LUTD is further exacerbated due to normal aging-related epigenetic modifications that need to be considered for better treatment efficacy.

### COMBATTING LONELINESS: SERVICE USE AMONG RURAL FAMILY CAREGIVERS OF PERSONS WITH DEMENTIA

Emily K. Hoyt,[1] Karen A. Roberto,[2] and Jyoti Savla[2], *1. Virginia Polytechnic Institute and State University, Blacksburg, Virginia, United States, 2. Virginia Tech, Blacksburg, Virginia, United States*

According to a 2018 AARP study, 42% of unpaid caregivers experience loneliness. While findings across multiple studies suggest that caregivers experience loneliness either because they lack intimacy in close relationships (i.e., emotional loneliness) or they feel disconnected from their social network (i.e., social loneliness), little is known about how aspects of dementia caregiving influence loneliness, particularly among rural caregivers. The purpose of this study was to examine the association between in-home service use and caregivers experience with both types of loneliness. Eighty-eight co-residing dementia caregivers in rural Appalachia (Mean Age = 68 years; 91% White; 58% Spouses) completed telephone interviews that included questions about their use of formal services and perceptions of emotional and social loneliness. More than half (58%) of the caregivers accessed 1 to 4 formal services. Regression models revealed that caregivers who experienced greater social loneliness were more likely to access personal care services (p=0.013) and respite services (p=0.004) compared to caregivers who experienced less social loneliness. Further, caregivers who experienced greater emotional loneliness were also more likely to access personal care (p=0.028) and respite (p=0.039) services compared to caregivers who experienced lower emotional loneliness. These associations remained robust even after controlling for relationship to the PwD (spouse vs. non-spouse). Findings suggest that beyond assisting with the care of the PwD, the use of formal services may help family caregivers manage loneliness and relieve social isolation. Discussion will focus on the importance of service accessibility and use for the health and psychological well-being of rural family caregivers.

### ART TO LIFE: THE IMPACT OF AN EXPERIENTIAL ARTS PROGRAM ON ENGAGEMENT IN PERSONS LIVING WITH DEMENTIAS

Candice Reel[1], *1. The University of Alabama, Tuscaloosa, Alabama, United States*

The Art to Life (ATL) program aims to improve the quality of life for persons living with dementia (PWD) through art therapy, intergenerational contact with college students, and life story preservation within an adult day service. This poster will present the results of an ongoing program evaluation to determine the effects of the intervention on PWDs' engagement in (1) communication with others and (2) art/creative activity. A two-member analysis team independently coded ethnographic field notes utilizing operational definitions of PWDs' observed behavior during momentary time sampling, and recording events of communication and art engagement using the modified ENGAGE measure (Hartmann et. al, 2017). Results across sessions (N=97) reveal communication engagement (M=28.30, SD=13.36) significantly exceeded art engagement (M=9.86, SD=5.56), t (96)=20.85, p=0.001). These results suggest that engagement in reminiscence via intergenerational contact is a fundamental feature in comparison to art and creative activity within the ATL program. Exploratory qualitative content analysis of ethnographic field notes by a three-member coding team identified two key themes within the communication engagements. These emergent themes included validation of personhood and reminiscence of family ties. More studies are needed to determine if the scope and reach of intergenerational interventions may be increased through the nationwide translation of the ATL program.

### AGING SEXUAL STEREOTYPES AND SEXUAL EXPRESSION IN MID- AND LATER LIFE: EXAMINING THE STEREOTYPE MATCHING EFFECT

Maggie Syme,[1] and Tracy Cohn[2], *1. Kansas State University, Manhattan, Kansas, United States, 2. Radford University, Radford, Virginia, United States*

Ageist sexual stereotypes are culturally embedded and may prohibit midlife and older adults from achieving sexual wellness when internalized over the life course (i.e., stereotype embodiment), which was examined in the current study. A cross sectional, convenience sample of 972 adults aged 50 and older was recruited online via a crowdsourcing platform. Participants completed an online survey assessing aging sexual stigma and their participation in a spectrum of sexual and intimate behaviors. Two hierarchical linear regressions examined study hypotheses predicting a) sexual and b) intimate behaviors among middle age and older adults, while accounting for several known covariates (e.g., education, relationship status, health). Results suggest that older age ($\beta = -.24$, $p < .001$), being a woman ($\beta = -.29$, $p < .001$), and higher levels of aging sexual stigma ($\beta = -.30$, $p < 0.001$) were associated with less sexual activity ($F(19, 945) = 32.51$, $p < .001$, R2 = 0.40). For intimate behaviors, older age ($\beta = -0.14$, $p < .05$) and higher levels of aging sexual stigma ($\beta = -0.24$, $p < .001$) were significantly associated with lower levels of intimate activity ($F(19, 945) = 39.80$, $p < .001$, R2 = 0.45). Contrary to expectations, neither gender nor age cohort moderated the effect of aging sexual stigma. Ageist sexual stereotypes appear to affect individual sexual health and wellness via internalized beliefs. Future studies should focus on the potential malleability of aging sexual stigma beliefs, and at what point(s) in the life course they are modifiable.

### SOCIAL MEDIA RESOURCES FOR BILINGUAL CAREGIVERS OF STROKE SURVIVORS

Sandra Sanchez-Reilly,[1] Laura M. Reilly-Sanchez,[2] Valeria Restrepo,[3] Marcos I. Restrepo,[1] Jeanette Ross,[4] and Michael J. Mader[1], *1. South Texas Veterans Health Care System, San Antonio, Texas, United States, 2. Health Careers High School, San Antonio, Texas, United States, 3. Antonian High School, San Antonio, United States, 4. University of Texas Health Science Center San Antonio, San Antonio, Texas, United States*

Background: Stroke survivors experience long-term disability also affecting informal caregivers (ICG). With current technology, social media might be the only way for ICG to gain training/access support. What resources are available for

ICG of older adults who survived stroke (OASS)? Objective: To identify/analyze types of bilingual social media resources available to ICG of OASS Methods: Facebook data was bilingually collected (Spanish), including most popular groups and pages based on search engines containing terms such as stroke, CVA, caregiver. Similar numbers of groups (35 English vs. 52 Spanish) and pages (32 English vs. 34 Spanish) were analyzed. Data included pages and groups' information, numbers-of-likes, type-of-organization and resources provided. Results: English-Facebook resources were more popular for pages and groups (3820/2010 vs. 190/7; p<0.001), Spanish resources were present, but with little activity among ICG. Majority of Spanish posts came from experts and English posts related to offering services or raising community awareness. Among both languages, pages provided resources related to social support (81%), improving caregiver skills (35%), advocacy (100%-English vs.56%-Spanish, p<0.001) and research news (84%-English vs.41%-Spanish, p<0.001). For English-ICG, more opportunities for live chats, messaging and inspirational messages were found (22-44% vs.3-9%, p<0.001). Conclusions: ICG of OASS could access Facebook resources to support multiple areas of caregiving including retrieving social support, gaining skills, learning new stroke-science findings and encountering live chats while getting inspired. Some resources are more available to English-ICG. Stroke-supporting organizations must consider using social media as crucial platforms to access bilingual resources and improve quality-of-life for ICG and OASS.

### THE ASSOCIATION OF FRAILTY WITH MCI AND DEMENTIA IN A MEMORY DISORDERS CLINIC FOR OLDER VETERANS

Aakashi Shah,[1] Amar Morani,[1]
Mercedes Rodriguez-Suarez,[2]
Raquel Apracio-Ugarriza,[1] and Jorge G. Ruiz[3], *1. Department of Public Health, University of Miami Miller School of Medicine, Miami, Florida, United States, 2. Miami VAHS Mental Health Service, Miami, Florida, United States, 3. Miami VAHS Geriatric Research, Education and Clinical Center, Miami, Florida, United States*

Frailty, a state of increased vulnerability to stressors due to multiple physiological dysfunction is associated with mild cognitive impairment (MCI) as well as dementia and may moderate its progression. Frailty and cognitive decline are highly prevalent in the Veteran population. The aim of our study was to determine whether frailty is associated with MCI and dementia in older Veterans at a Memory Disorders Clinic. We performed a cross-sectional study of 308 Veterans enrolled in VA Memory Disorders Clinic during 2016-2019. MCI and dementia were diagnosed based on complete clinical assessment including cognitive testing, brain imaging and neuropsychological testing. A 44–item frailty index (FI) was constructed using potential variables (demographics, comorbidities, number of medications, laboratory tests, and activities of daily living). Binomial logistic regression was run using MCI and dementia as outcome variable and frailty status (frail and non-frail) as independent variable. Age, race, marital status, ethnicity, median household income, education, comorbidities, BMI, history of substance abuse, smoking, alcohol, hospitalizations, anticholinergic use, and utilization were considered as covariates. The mean age was 74.43± 8.31 years. 43.2% population was frail (FI>0.21) and 56.8% was non-frail (FI≤0.21). The number of Veterans with MCI and dementia was 114 and 113 respectively. Frailty was significantly and positively associated with dementia (OR: 2.29 95%CI:1.25-4.21, p=0.007) but there was no association with MCI (OR: 1.06 95%CI: 0.605-1.886, p=0.820). Our study results suggest that frailty might not be associated with MCI but has significant association with dementia in a group of Veterans at a Memory Disorder Clinic.

### DYNAMIC, BIDIRECTIONAL LONGITUDINAL RELATIONSHIP BETWEEN LEISURE ACTIVITY ENGAGEMENT AND COGNITIVE PERFORMANCE

Nicole Armstrong,[1] Sarah Tom,[2] Miguel Arce Renteria,[3] Kaitlin Casaletto,[4] Jennifer Weuve,[5] Eleanor M. Simonsick,[6] Jennifer Manly,[7] and Laura B. Zahodne[7], *1. National Institute on Aging, Baltimore, Maryland, United States, 2. Department of Neurology, Columbia University Medical Center, New York, New York, United States, 3. Taub Institute for Research on Alzheimer's Disease and the Aging Brain, New York, New York, United States, 4. University of California, San Francisco, San Francisco, California, United States, 5. Department of Epidemiology, Boston University School of Public Health, Boston, Massachusetts, United States, 6. Longitudinal Studies Section, Translational Gerontology Branch, National Institute On Aging, NIH, Baltimore, Maryland, United States, 7. University of Michigan, Ann Arbor, Michigan, United States*

Engagement in leisure activities, i.e., intellectual, social, and physical activities, may reduce the risk of incident dementia, yet little is known about the longitudinal, dynamic relationship between overall leisure activity engagement and cognition in older adulthood. Using data from a survey measure of 13 leisure activities, e.g., doing unpaid volunteer work and playing cards, games, or bingo, and a neuropsychological battery collected concurrently over 14 years from 2,259 multi-ethnic participants (mean age of 76.0 years) in the Washington Heights-Inwood Columbia Aging Project, we used a parallel process latent growth curve model of trajectories of both leisure activity engagement and cognitive z-scores (global cognitive performance, language, memory, and visuospatial ability). Estimates were adjusted for baseline age, years of education, sex, race/ethnicity, recruitment year, occupation (unskilled, skilled, and housewife), and baseline income. More baseline activity engagement (range, 0-13, higher indicating more engagement) was associated with higher baseline cognitive performance, i.e., global cognitive performance (estimate=0.129, standard error, SE=0.017, p<0.001), language (estimate=0.146, SE=0.020, p<0.001), memory (estimate=0.141, SE=0.025, p<0.001), and visuospatial ability (estimate=0.111, SE=0.020, p<0.001). Decline in leisure activity engagement were associated with decline in global cognitive performance (estimate=0.002, SE=0.000, p<0.001), language (estimate=0.002, SE=0.000, p<0.001), memory (estimate=0.002, SE=0.001, p<0.001), and visuospatial ability (estimate=0.001, SE=0.000, p=0.001). While both level and change in overall leisure activity engagement and cognitive performance were correlated, level of one did not predict change in the other. Similar relationships were found when examining leisure activity categories. This suggests a dynamic, bidirectional relationship between leisure activity engagement and cognitive performance among older adults.

### FACTORS INFLUENCING EPISODIC MEMORY IN SUBJECTIVE COGNITIVE DECLINE: AN IMPLICATION FOR DEMENTIA PREVENTION

Sangwoo Ahn[1], *1. University of Tennessee College of Nursing, Knoxville, Tennessee, United States*

Episodic memory is typically affected early in the process of Alzheimer's disease. Little is known about factors affecting episodic memory in subjective cognitive decline (SCD). The purpose of this study was to identify vascular and neuropsychiatric risk factors associated with episodic memory changes in older adults with SCD. Using the National Alzheimer's Coordinating Center-Uniform Data Set, the relationship between baseline modifiable risk factors and episodic memory changes was analyzed using linear mixed-effects regression models. The study included a total of 1,401 subjects with SCD (mean ages: 74.0±8.2 years, 67.5% females, 84.2% White, mean follow-up period: 4.1±2.4 years). In univariate adjusted model, statistically significant coefficients on main effect or interaction with time were selected and entered into multivariate model, which was adjusted mutually for chosen independent variables and for all covariates. Reference in the final model was subjects without 1) hypercholesterolemia, 2) cigarette smoking history, and 3) depression. Those with hypercholesterolemia and former smokers had 0.024 and 0.035 points higher episodic memory scores than reference at baseline with similar rate of score changes between each group and reference over time, respectively. Current smokers scored 0.081 points lower than reference at baseline with similar rate of change over time between groups. Despite no difference at baseline, the score of depressed subjects decreased by 0.014 points a year compared to reference. It is important to manage current smoking and depression for older adults with SCD. Further research needs to identify which levels of cholesterol and smoking have a protective effect on episodic memory.

### COGNITIVE AND SUBJECTIVE EFFECTS OF OXYCODONE IN OLDER ADULTS WITH HEALTHY AND UNHEALTHY ALCOHOL PATTERNS

Monique Cherrier,[1] Gregory Terman,[1] Danny Shen,[1] Laura Shireman,[1] Andrew Saxon,[2] Tracy Simpson,[2] Preetma Kooner,[1] and Katie Wicklander[1], *1. University of Washington, Seattle, Washington, United States, 2. VA Puget Sound Health Care System, Seattle, Washington, United States*

Chronic unhealthy levels of alcohol use, may predispose adults to use illicit substances and/or modify their response to prescribed medications, such as pain medications. We examined the cognitive and side effect response of older adults who met criteria for healthy and unhealthy alcohol drinking patterns after exposure to 10mg of oxycodone. Using a human laboratory model, eligible participants were characterized on cognitive, side effect measures and cold-pressor pain test (CPT) at baseline and repeated 90 minutes, 3 and 5 hours post dosing (10mg oxycodone). Blood samples were taken at regular intervals to measure drug levels. One-hundred twenty-five adults completed the study day, eighty participants with heavy alcohol consumption and 45 with healthy. Middle age (MA) group had a mean age of 51 (11.2) years, older adults (OA) 72 (4.2) years. Between group (unhealthy vs healthy drinkers, middle age vs older adult) comparisons for cognitive performance indicate a significant decline at 90 min. However, MA and OA heavy alcohol consumers evidenced less decline on sustained attention (D2) and working memory, but more decline on a measure of balance (berg). Anti-nocioceptive effects were greatest in healthy (MA,OA) in comparison to heavy, however there were no differences on pupil miosis. Subjective rating of side effects were rated more severe in the OA unhealthy group compared to MA and healthy. These findings indicate unhealthy alcohol consumption attenuates the impact of opioid medication. Results indicate that alcohol consumption patterns should be considered when using opioids in older and middle age adults.

### ASSESSING THE ADEQUACY OF SOCIAL SECURITY RETIREMENT BENEFITS ACROSS RACE-ETHNICITY, GENDER, AND AGE OF RETIREMENT

Jin H. Kim[1], *1. Northeastern Illinois University, Chicago, Illinois, United States*

This research assessed the adequacy of Social Security retirement benefits across race-ethnicity, gender, and age of retirement, and in turn, whether the differing levels of benefit adequacy have any relation to mortality risk. Prior studies generally find that a replacement rate of between 70 to 80 percent of prior earnings would likely allow a worker to maintain his or her standard of living in retirement since various work-related expenses are reduced or eliminated at the point of transition. As such, the current study used panel data from the 1996 - 2016 waves of the Rand version of the Health and Retirement Study to 1) determine earnings replacement rates for non-Hispanic White, non-Hispanic Black, and Hispanic males and females in the first period of retirement, and 2) to examine whether earnings replacement rates are associated with mortality risk in a Cox regression model. The findings revealed that for those retiring at age 65 or later, Hispanic females and White males had the lowest earnings replacement rates at 39.3% and 40.7%, respectively. For those retiring before age 65, Hispanic males and White males had the lowest earnings replacement rates at 30.3% and 26.7%. Although replacement rates should indeed be lower for high earners due to Social Security's progressive benefit formula, the low replacement rates determined for Hispanic males and females were unexpected. Moreover, mortality risk was found to be significantly associated with earnings replacement rates in the final model, but the combination of race-ethnicity and gender still showed a stronger relation.

### DISCOVERING STAFFING ISSUES AND EXPERIMENTING WITH STAFFING LEVELS IN DUTCH NURSING HOMES

Marleen Prins,[1] Janne van Erp,[1] Ceciel Heijkants,[1] Erica de vries,[1] Ramona Backhaus,[2] Sil Aarts,[1] Hilde Verbeek,[3] and Astrid van der schot[4], *1. Maastricht University, Faculty of Health Medicine and Life Sciences, CAPHRI, Department of Health Services Research, Maastricht, Netherlands, 2. Maastricht University, Maastricht, Netherlands, 3. Department of Health Services Research, Care and Public Health Research Institute*

*(CAPHRI), Faculty of Health, Medicine and Life Sciences, Maastricht, Netherlands, 4. Netherlands Institute of Mental Health and Addiction (Trimbos-institute), Department on Aging, Utrecht, Netherlands*

Background: In 2017, the Dutch National Health Care Institute developed a quality framework describing what high quality nursing home care entails, aimed to improve nursing home care. The current study focuses on evaluating whether Dutch nursing homes comply to the framework, specifically regarding norms about staffing and formation of care teams (skill mix and educational level). Care teams that were experimenting with new staffing levels were monitored to evaluate what changes in staffing occurred and which obstructing or promoting factors they experienced. Methods: Quantitative data about staffing and team characteristics were gathered. Further, qualitative data about motives for wanting to change, the change approach, obstructing and promoting factors and evaluation of changes was collected. Telephone interviews were held at baseline, after 3 months and after 6 months. Thirty-two teams participated in the study. Results: Challenges for making changes in staffing consisted of attracting new care staff, dealing with sickness leave, communication within and between teams, communication with informal carers and combining care for and having attention for well-being of residents in the daily work routine. Additionally, teams wanted to better adjust the skill mix of staff to the needs of residents. Conclusion: For the formation of care teams, there seems to be no 'one-size fits all' approach. A quantitative norm that applies to all nursing homes in the Netherlands as described in the quality framework (e.g. a minimum of two care professionals for eight residents during intensive moments of care) is therefore not always the route to high quality care.

### EXAMINING THE NEEDS OF PEOPLE WITH DEMENTIA AND FAMILY CARERS DIRECTLY AFTER THE DIAGNOSIS

Claudia van der Velden,[1] Iris van Asch,[1] and Elsemieke van belzen[1], *1. Netherlands Institute of Mental Health and Addiction (Trimbos-institute), Department on Aging, Utrecht, Netherlands*

Most often the diagnosis dementia gives people clarification, but at the same time it raises questions and causes a range of emotions. According to professionals, support for people with dementia and family carers (dyads) nowadays is mainly focused on later-stage-dementia instead on early-stage. Besides, this support is mostly about practical/medical topics and less about emotional/psychological or social topics. Studies show that it is a complex process dyads to cope with the diagnosis in their daily life directly after diagnosis. The aim of the present study was to examine the specific needs of dyads in order to support them in the early-stages after diagnosis. Focus groups, interviews and a literature-review were performed to determine these needs. Together with professionals, the findings were translated into a conversation guide, which helps professionals to have a structured dialogue on practical/medical, emotional/psychological and social topics to support dyads in an early-stage after diagnosis. An evaluation is conducted by observations and interviews with dyads and a work-session with professionals. People with dementia, their caregiver(s) and professionals experienced these conversations as very positive. Dyads indicated the type of counsellor (accessible, warm and open), timing of the conversation and topics about 'not making the diagnosis too heavy' and 'sharing the diagnosis with others' as important. The professionals were also very positive about the conversations. Important themes for them were focusing on the more personal, holistic and preventive approach in the conversations.

### E-LEARNING FOR IMPROVING PERSON-CENTERED ATTITUDE IN PROFESSIONALS WORKING WITH PEOPLE WITH DEMENTIA: PILOT STUDY

Claudia van der Velden[1], *1. Netherlands Institute of Mental Health and Addiction (Trimbos-institute), Department on Aging, Utrecht, Netherlands*

Healthcare professionals working with people with dementia (PwD) have increasingly been moving towards a person-centered care (PCC) attitude. Several studies have showed positive results of PCC on quality of life of PwD. Also, it shows positive effects on self-esteem and work-satisfaction of professionals. An effective way to educate professionals in PCC is by using online-learning-tools. We developed an e-learning - in co-creation with end-users - focusing on well-being and challenging behavior of PwD. The interactive e-learning supports healthcare professionals in developing a PCC-attitude, using practical videos and exercises. In the current pilot-study, the e-learning is evaluated. To date, 32 professionals working in Dutch care homes from different care organizations participated in the study and completed the e-learning. In addition, they filled in online questionnaires before and after completing the e-learning. The questionnaires include the Approach to Dementia Questionnaire, Dementia Knowledge, Person Centered Care, Sense of Competence in Dementia Care Questionnaire. Post-measurement also included questions about satisfaction with the e-learning and user-friendliness. The final participants are currently completing their post-measurement questionnaires and final results are expected in October 2019. Preliminary data-analysis shows promising results. Although no significant effects were found, two-thirds of the participants indicated they had more knowledge about dementia, could better deal with challenging behavior and understood better how their own behavior and actions influence the behavior of PwD. The user-friendliness was also positively evaluated. Preliminary results suggest that the e-learning helps professionals in their approach to PwD and indicate that participants have positive experiences with the e-learning.

### THE FRAILTY INDEX CONSTRUCTED USING FEATURE SELECTION METHODS IS THE BEST MEASURE OF BIOLOGICAL AGE

Sangkyu Kim,[1] Jessica Fuselier,[1] David Welsh,[2] Katie E. Cherry,[3] Leann Myers,[1] and S Michal Jazwinski[1], *1. Tulane University Health Sciences Center, New Orleans, Louisiana, United States, 2. Louisiana State University Health Sciences Center, New Orleans, Louisiana, United States, 3. Louisiana State University, Baton Rouge, Louisiana, United States*

No single biomarker can reliably represent the complexity of aging. One way to overcome this shortcoming is to aggregate multiple biomarkers into a composite index. The frailty index (FI), which is simply the proportion of accumulated deficits among a set of various health markers, reflects functional abilities and risks of adverse outcomes. Furthermore, the FI accounts for the variation in mortality among individuals of the same chronological age (CA). Thus, the FI is a reliable measure of biological age (BA). Unlike the FI, other popular BA-estimating algorithms use CA directly as a biomarker or indirectly to derive model parameters. However, genetic, pharmaceutical, and intervention studies have shown that aging is delayable or reversible, indicating that CA is not the direct cause of aging. The popular Klemera-Doubal (K-D) method proposes two equations for BA estimation: BE uses CA to derive equation parameters, and BEC directly incorporates CA as an additional biomarker. BA estimates by the K-D method, especially by BEC, have been shown to outperform CA. Using Louisiana Healthy Aging Study (LHAS) data, we constructed an FI from a battery of health items selected using machine learning methods for their ability to predict mortality. We compared the FI with CA and the two K-D BA estimates and found that the FI was the better predictor of mortality, especially among nonagenarians. The results were replicable with the FI calculated from different sets of selected health items using NHANES and HRS datasets. These results demonstrate the FI as the best-performing measure of BA.

### CHARACTERISING POLYPHARMACY IN THE VERY OLD: FINDINGS FROM THE NEWCASTLE 85+ STUDY

Laurie Davies,[1] A Kingston,[1] Adam Todd,[2] and Barbara Hanratty[1], *1. Newcastle University, Newcastle upon Tyne, United Kingdom, 2. School of Pharmacy, Newcastle University, England, United Kingdom*

Polypharmacy is potentially harmful and under-researched amongst the fastest growing section of society, the very old (85+). This study aimed to characterise polypharmacy using data from the Newcastle 85+ Study – a prospective cohort of people living in north east England who turned 85 in 2006 (n=845). The prevalence of polypharmacy was examined using cut-points of 0, 1, 2-4, 5-9 and ≥10 medicines – so-called 'no polypharmacy', 'monotherapy', 'minor polypharmacy', 'polypharmacy' and 'hyperpolypharmacy.' Crosstabulations and Upset plots identified the most frequently prescribed medicines and medication combinations within these categories, at age 85. Mixed effects models assessed whether gender and socioeconomic position were associated with prescribing changes over time (age 85.5-90.5 years). Polypharmacy (49.6%) was more common than minor polypharmacy (24.6%) and hyperpolypharmacy (16.0%). Within these categories, cardiovascular, non-opioid analgesic and gastrointestinal medications were most frequently prescribed. Medication combinations were many and varied. Aspirin and statins were most commonly co-prescribed amongst people with minor polypharmacy and polypharmacy. Non-opioid analgesics, statins, aspirin and loop diuretics were most common in hyperpolypharmacy. Medications varied by gender and socioeconomic status. Nitrates and oral anticoagulants were more frequently prescribed for men. Bisphosphonates, analgesics and antidepressants were more common in women. Cardiovascular medications, including loop diuretics, were more frequently prescribed for socioeconomically disadvantaged people (25th centile Index of Multiple Deprivation (IMD)), with tricyclic antidepressants and selective beta-2 agonists more common amongst the least disadvantaged (>75th centile IMD). By highlighting prescribing patterns, this study informs our understanding of how polypharmacy may contribute to adverse outcomes in later life.

### USABILITY ASSESSMENT OF A SENSOR-CONTROLLED DIGITAL GAME FOR OLDER ADULTS WITH HEART FAILURE

Kavita Radhakrishnan,[1] Christine Julien,[2] Matthew O'Hair,[2] Catherine Fournier,[2] Grace Lee,[2]
Thomas Baranowski,[3] and Miyong T. Kim[2], *1. School of Nursing, the University of Texas at Aust, Austin, Texas, United States, 2. The University of Texas Austin, Austin, Texas, United States, 3. Houston, TX, Houston, Texas, United States*

The inability of older persons with heart failure (HF) to self-manage has contributed to poor health outcomes. Our team from nursing, digital game design, and mobile computing developed an innovative sensor-controlled digital game (SCDG) called 'Heart Mountain' to offer a portable, and enjoyable tool to facilitate engagement in HF self-management. We installed the SCDG application, which featured older adult game avatars on the participants' smartphones. The SCDG utilized data from an activity tracker and weight scale to trigger game rewards, knowledge content and messages based on participants' real-time behaviors. In this study we assessed the usability of a SCDG prototype with 10 HF older adults in Central Texas. Observations on the usability of the SCDG app by older adults were noted on a usability heuristics checklist. Acceptance and satisfaction were collected by an open-ended survey guided by Intrinsic Motivation Inventory after a week of playing the game. Participants (60% males, 60% white, ages 63-84) were able to play the game and use the devices after a training session that lasted for 15 minutes. We will present results on participants' ease of use of the SCDG app, satisfaction with the knowledge content, quizzes and rewards features of the SCDG, and perceptions on acceptance and satisfaction with the SCDG for heart failure self-management. Our project will generate insights on designing digital gaming solutions that are acceptable to older adults and can be applied to improve self-management of chronic diseases like heart failure.

### POLYGENETIC SCORE IS ASSOCIATED WITH T2D; EVIDENCE FROM THE HEALTH AND RETIREMENT STUDY

Fadi Youkhana,[1] Yanyan Wu,[1] Mika Thompson,[1] and Catherine M. Pirkle[1], *1. University of Hawai'i at Mānoa, Honolulu, Hawai'i, United States*

Type 2 diabetes (T2D) is a complex chronic disorder influenced by genetic and environmental factors. Studies that use a combined polygenetic score (PGS), calculated based on the number of risk alleles an individual may have, are rarely applied to a representative national sample. We used data from the Health and Retirement Study (HRS), a nationally representative study of older U.S. adults 50-years or older to examine the impact of PGS and behavioral risk factors (education, poverty ratio, BMI, smoking status, alcohol consumption and physical activity) with incident T2D. We used ethnic-straitifed Poisson generalized estimating equation (GEE) models with robust standard errors to estimate prevalence ratios (PRs) and risk ratios (RRs). Our sample included genotyped Black (N=2,823) and White (N=11,178) men and women.The highest PRs for T2D were among those in the 5th PGS quintile in both Whites

(PR=2.24, 95%CI 1.89, 2.65, P-value <0.0001) and Blacks (PR=1.73, 95%CI 1.28,2.33, P-value 0.0003). The highest risk for T2D was among obese Whites (RR=3.35, 95%CI 2.93,3.82, P-value <0.0001) and Blacks (RR=1.60, 95%CI 1.28, 2.00, P-value <0.0001). Our findings found associations between PGS and T2D as well as some lifestyle factors among both Black and White individuals in a nationally representative sample with similar patterns in age, physical activity and poverty ratio. Our study supports the importance of including modifiable and non-modifiable life-style factors in the analysis of risk alleles for T2D to continue addressing the disparities between T2D risk between race/ethnicity groups

### CROSS-GENERATIONAL UNDERSTANDING OF AGEISM AND ITS IMPACT ON PERSONAL-PUBLIC HEALTH

Diana Mayo,[1] Thomas M. Meuser,[2] and Regula H. Robnett[2], *1. University of New England, Biddeford, Maine, United States, 2. University of New England, Portland, Maine, United States*

In 2016, the World Health Organization (WHO) declared a call to combat ageism, labeling it "pervasive" and having "profound consequences on older adults' health and well being." This study explored generational differences in understanding the WHO's definition of ageism, between baby boomers (ages 65-72) and silent generation members (ages 78-85), as well as the perceived impact on personal and public health outcomes. A focus group protocol built around the WHO framing of ageism was administered to boomer (n=18) and silent generation members (n=11). Discussion was transcribed, reviewed in depth by each research team member, and themes were extracted by consensus. Members of both cohorts initially denied effects of ageism, stating that they reject discriminatory behavior; later sharing explicit examples of ageism's negative impact on their lives. Boomers conflated the words "ageism" and "aging", perhaps implying a lack of awareness of the terms and the issues as presented by WHO. A central finding was that older adults in both groups experienced economic and health care disparities due to their age. In both groups, perceived perpetrators of discriminatory behavior were found in various environments including places of employment, healthcare sites, restaurants, public transportation, retirement communities, and at home among family and care services. Our results are critical to understanding what environments to target for public health intervention efforts, which will include establishing future education and training for people of all ages to help society learn about ageism, and to advocate for inclusive and equitable treatment of older adults in the community.

### SCHIZOPHRENIA EPIGENETIC AGING PATTERNS REFLECT ALTERED MORTALITY AND CANCER RISKS

Albert T. Higgins-Chen,[1] Christiaan Vinkers,[2] Marco P. Boks,[3] and Morgan E. Levine[4], *1. Department of Psychiatry, Yale School of Medicine, New Haven, Connecticut, United States, 2. Amsterdam UMC, Amsterdam, Netherlands, 3. University Medical Center Utrecht Brain Center, Utrecht, Utrecht, Netherlands, 4. Yale University School of Medicine, New Haven, Connecticut, United States*

Schizophrenia (SZ) is associated with large increases in all-cause mortality, high smoking rates, and elevated levels of age-associated proteins—suggesting individuals with SZ may experience accelerated rates of biological aging. Yet surprisingly, multiple previous studies found no association between SZ and biological age using Horvath's epigenetic clock, a well-recognized and validated biomarker of aging based on DNA methylation (DNAm) levels. However, numerous epigenetic clocks have been developed to date, many of which are better indicators of differential lifespan and healthspan than the original Horvath clock. Thus, we hypothesize that these epigenetic clocks may be better proxies for the presumed accelerated aging rate in SZ. Here we investigate 14 epigenetic clocks using three publicly available DNAm datasets from whole blood, comparing SZ to non-psychiatric controls (NPC). In all data sets, we find SZ age acceleration in three clocks previously shown to be most predictive of age-related morbidity and mortality risk. In contrast, two clocks developed to capture mitotic rate are decelerated in SZ, consistent with low cancer rates despite smoking observed in epidemiological studies of SZ. We use these clocks to investigate the determinants of altered aging in SZ, such as smoking, alcohol, BMI, age-associated proteins, blood cell composition, and psychotropic medications. Principal component analysis suggests mortality clock acceleration, mitotic clock deceleration, and medication effects are independent phenomena in SZ. Our study demonstrates the importance of studying the various epigenetic clocks in tandem and highlights their potential utility for understanding how mental illness influences long-term outcomes including cancer and early mortality.

### DO PERSONALITY TRAITS INFLUENCE PERCEPTIONS OF COGNITIVE CHANGE IN COMMUNITY DWELLING OLDER ADULTS?

Thomas M. Meuser,[1] and Regula H. Robnett[1], *1. University of New England, Portland, Maine, United States*

Recent research has linked personality traits and risk for cognitive impairment in advancing age. Associations with neuroticism are particularly robust. Both longstanding and recent elevations may predict dementia. Other traits – conscientiousness and openness to experience – also show unique associations. These findings derive mainly from large sample population studies and smaller clinical investigations. Relevance to the general population is unclear. We investigated the "big five" personality traits and cognition in 232 community-dwelling adults (73% female, 97% Caucasian, mean age 72 years). Scores on a self-report screen for dementia – the AD8 – framed the sample: 77% scored 0 points, no dementia; 23% scored 2+, possible dementia. Age and personality were independent variables in a binary logistic regression with AD8 status as dependent. All predictors but one, extraversion, were significant ($p < .05$), suggesting that personality traits may influence perceptions of cognitive change. Higher agreeableness and neuroticism predicted possible dementia status on the AD8, whereas higher openness and conscientiousness predicted normal cognition. Interestingly, most in the AD8 positive group (70%) denied having "more problems with memory than most" on the Geriatric Depression Scale. These perceptions would seem incompatible, especially for true positive cases. Our findings suggest that the role of personality in dementia screening (and, perhaps, diagnosis) may be more nuanced than indicated in other studies. Longstanding traits and present perceptions are both elements of the evaluative process, as much as test scores and reported history. Our findings speak to the value of a person-centered, context-aware approach in cognitive screening.

### IMPORTANCE OF DIAGNOSTIC IMAGING FOR INTERPRETATION OF SELF-REPORTED SURVEYS OF SHOULDER FUNCTION

Derik L. Davis,[1] Ranyah Almardawi,[2] Brock Beamer,[2] Rao Gullapalli,[1] Alice Ryan,[3] and Michael Terrin[1], *1. University of Maryland School of Maryland, Baltimore, Maryland, United States, 2. Baltimore VA Medical Center GRECC, Baltimore, Maryland, United States, 3. University of Maryland Baltimore School of Medicine, Baltimore, Maryland, United States*

Rotator cuff (RC) tear is highly prevalent in older adults. The American Shoulder and Elbow Surgeon (ASES) survey, which quantifies subjective self-reported shoulder function, was originally validated in adults <60 years, and more recently is suggested to be valid in adults ≥60 years. We tested the hypothesis that ASES score (1) discriminates between adults 60—85 years with and without RC tear and (2) correlates with self-reported health quality and objective shoulder measures. Cross-sectional study: forty-two community-dwelling-older-adult volunteers (mean age, 69.4 ± 5.7 years; range, 61-84 years; male, 45%) with no history of RC surgery completed shoulder magnetic resonance imaging (MRI), shoulder forward flexion (FF) and abduction (ABD) range-of-motion (ROM) testing; and ASES, shoulder-pain and SF-36 (pain/physical function) surveys. Four groups (group-1:-pain,-tear,n=14; group-2:+pain,-tear,n=4; group-3:-pain,+tear,n=12; group-4:+pain,+tear,n=12) were compared using one-way ANOVA with ad hoc pairwise comparisons and Spearman Rank Order Correlation (rho). Age, Charlson co-morbidity index, and SF-36 pain/physical function were not appreciably different among all groups. ASES score (p<0.001), FF-ROM (p=0.032) and ABD-ROM (p=0.018) comparing all groups. ASES score: group-1 versus group-4 (p<0.001); but no difference between group-1 versus group-3 (p=0.999), and group-2 had lowest ASES score. ASES score correlated with SF-36 physical function (rho=0.47,p=0.002), SF-36 pain (rho=0.34,p=0.028), FF-ROM (rho=0.63,p<0.001), and ABD-ROM (rho=0.66,p<0.001). Our results suggest that additional research on ASES score in older adults is needed. Although valid, interpretation of ASES score in older adults should be approached cautiously in studies without shoulder diagnostic imaging tests, since painless RC tears and painful shoulders without RC tear are not rare.

### HEALTH CARE PRIORITIES OF OLDER KOREAN IMMIGRANTS: THROUGH THE LENS OF LIFE COURSE THEORY

Hyeyoung K. Park,[1] Sharron L. Docherty,[2] Cristina C. Hendrix,[2] Ruth A. Anderson,[3] and Kimberly S. Johnson[1], *1. University of Massachusetts Amherst College of Nursing, Amherst, Massachusetts, United States, 2. Duke University School of Nursing, Durham, North Carolina, United States, 3. University of North Carolina at Chapel Hill School of Nursing, Chapel Hill, North Carolina, United States*

More than half of Korean Americans living in the US are immigrants, these immigrants hold unique cultural perspectives, including collectivism and filial piety that originates from Korean culture. Every older adult has life experiences and background that build and shape their own wishes and values for their health care goals. Thus, a qualitative descriptive study was conducted using the Life Course Theory as a guiding framework to examine older Korean immigrants' health care goals and the influence of their life courses. Twenty six interviews from 13 participants were analyzed using content thematic analysis. Study rigor was ensured by audit trail, peer debriefing, and prolonged engagement. Data were organized under five overarching themes: health care priorities, time, location, linked lives, and turning point. Older Korean immigrants valued painlessness and being independent as health care goals (Health care priorities). They experienced a dynamic historical period in Korea before immigrating to the US (Time). Once they reached the US, they were disconnected from their social support and traditional values (Location). Children and Korean churches constitute older Korean immigrants' primary support system once in the US (Linked lives). Their tumultuous life experiences contributed to their current perspectives on health care goals and priorities (Turning point). In studies of older immigrant populations, it is important to acknowledge individual differences while simultaneously understanding the general life history and cultural background behind individuals' values and perspectives. Life course approach provides both a contextual understanding of older adults' backgrounds and the trajectories of their individual life courses.

### ASSOCIATIONS BETWEEN SMOKING CONSUMPTION, TOBACCO CESSATION ATTITUDES, AND AGE AMONG LATINO HEALTHCARE PROVIDERS

Belén Hervera,[1] Lisette Irarrázabal,[2] Lilian Ferrer,[2] and Rosina Cianelli[1], *1. University of Miami, Miami, Florida, United States, 2. Pontificia Universidad Católica de Chile, Santiago, Chile*

Hospitalization is a good opportunity to offer smoking cessation programs to smokers. Healthcare providers´ (HCP) tobacco consumption and cessation attitudes are known to affect the provision of cessation interventions. Lesser known are Latino HCP's tobacco intervention attitudes. This study aimed to examine the associations between tobacco cessation attitudes (TCA), levels of consumption, and demographics among Latino HCP's. A quantitative, correlational, cross-sectional design was used. 66 HCP's working in a public hospital in Santiago, Chile self-reported demographics (age, gender, profession), tobacco consumption, and TCA. TCA's include questions regarding Acceptability of Brief Counseling (ABC), belief whether smoking is harmful for patients, and duty to aid patients quit smoking. Majority of HCP's (34 years old, 83% female, 58.5% technical nurses, 38.5% nurses, 3.1% Kinesiologists) did not consume tobacco (67%). Pearson's correlation revealed that greater HCP age was significantly associated with less belief that smoking is harmful for their patients (r = -.36, p. = .004). ABC (M = 22, SD = 5.5) was positively associated with the belief that smoking is harmful for patients (r = .306, p = .016) and duty to help patients quit smoking (r = .574, p = .000). Findings provide evidence that HCP's TCA's are important factors to consider during implementation of a brief counseling for tobacco cessation. Further research should focus on increasing HCP's acceptability of providing cessation care to their patients. Specifically, tailoring education and interventions by age might serve useful to address the differences in TCA's which may subsequently influence their tobacco cessation practices.

### EFFECT OF VISION AND HEARING IMPAIRMENT ON LIFE SPACE MOBILITY AMONG OLDER MEXICAN-AMERICANS

Viridiana Saenz Monsivais,[1] Brian Downer,[2] Mukaila Raji,[1] and Yong-Fang Kuo[1], *1. University of Texas Medical Branch, Galveston, Texas, United States, 2. University of Texas Medical Branch, Galveston, TX, Galveston, Texas, United States*

Background: Poor vision and hearing can restrict an older adult's life space mobility (LSM). Our objective was to examine if poor vision and hearing differentially impact the decline in LSM over a 2-year period among older Mexican-Americans. Methods: Data came from waves 7 (2010/11) and 8 (2012/13) of the Hispanic EPESE. Our final sample included 452 participants aged >79 years. Participants who said they could not recognize a person across the street, room, or arms-length were classified as having poor vision. Participants who said they could not understand a normal voice in a quiet room were classified as having poor hearing. Participants who decreased >10 points on the Life-Space Assessment were classified as having a decline in LSM. Logistic regression was used to estimate the odds for a decline in LSM according to poor hearing and vision, controlling for baseline demographic and health characteristics. Results: Poor vision was associated with 2.70 (95% CI=1.37-5.62) greater odds for a decline in LSM. This association varied according to depressive symptoms. Poor vision was significantly associated with a decline in LSM for participants without high depressive symptoms (OR=5.04, 95% CI=2.00-15.5), but not for participants with depression (OR=0.63, 95% CI=0.12-3.13). The association between poor hearing and decline in LSM was not significant (OR=0.68, 95% CI=0.36-1.27). Conclusions: Poor vision is a risk factor for decline in LSM, especially for older Mexican-Americans who do not have high depressive symptoms. Poor hearing does not appear to be a risk factor for a decline in LSM for older Mexican-Americans.

### PERCEIVED PHYSICAL FATIGABILITY PREDICTS ALL-CAUSE MORTALITY: THE LONG LIFE FAMILY STUDY

Nancy W. Glynn,[1] Theresa Gmelin,[1] Sharon W. Renner,[2] Robert M. Boudreau,[3] Mary Feitosa,[4] Stacy L. Andersen,[5] Kaare Christensen,[6] and Anne B. Newman[3], *1. University of Pittsburgh, Department of Epidemiology, Pittsburgh, Pennsylvania, United States, 2. University of Pittsburgh Graduate School of Public Health, Pittsburgh, Pennsylvania, United States, 3. University of Pittsburgh, Pittsburgh, Pennsylvania, United States, 4. Washington University School of Medicine, St Louis, Missouri, United States, 5. Boston University School of Medicine, Boston, Massachusetts, United States, 6. Danish Aging Research Center, University of Southern Denmark, Odense C, Denmark*

Fatigability, the likelihood of fatigue with lower versus higher levels of exertion, is associated with declines in physical function and disability and related to fitness. Thus, fatigability may be a good predictor of mortality. We examined this relationship in the Long Life Family Study (LLFS), an international family cohort enriched for longevity and their spousal controls. We measured perceived physical fatigability at Visit 2 (2014-2017) using the Pittsburgh Fatigability Scale (PFS, 0-50 with higher score=greater fatigability). We identified deaths by family members notifying field centers, reporting during annual phone follow-up, or finding an obituary when unable to reach. Otherwise, we censored participants at most recent contact date when confirmed alive. Covariates included age, sex, and self-reported physical activity using the Framingham Physical Activity Index. We adjusted all analyses for field center and family structure. Participants alive ≥60 years (range 60-108, mean 73.6±10.5) and completed the PFS (N=2,326) at Visit 2 were predominantly white (99.5%) and female (55.1%). Post-Visit 2, 195 (8.4%) died during mean 2.5±1.0 years of follow-up. Age-adjusted PFS score was 7.7 points greater (p<.0001) for those who died (19.8) compared to alive (12.1). Using Cox Proportional-Hazard modeling, each 5-point greater PFS score was associated with 31% (HR: 1.31, 95% CI 1.18,1.43) higher all-cause mortality rate adjusted for covariates listed above. Further adjustment for comorbidities did not attenuate association. PFS's perceived physical fatigability score may be a useful self-report clinical tool to predict higher risk of mortality among older adults when objective measures of fitness and function are unavailable.

### HOW DOES PARENT-CHILD RELATIONSHIP AFFECT CARE? FOCUSING ON MOTHER-DAUGHTER CAREGIVING

Ayako Baba[1], *1. the University of Tokyo, Tokyo, Japan*

OBJECTIVE: Long-term caregiver (child)/care-recipient (parent) relationships have both positive and negative effects on care. However, the mechanism of that impact is unclear. This study aimed to explore how parent–child relationships affect care and which aspects cause those effects. METHOD: Five hundred thirty-four adult children who were caring for or had cared for their parents at home completed the scales of parent–child psychological independence, the acceptance of care, care attitude, and care burden. Data were analyzed using a pass analysis with multiple group structural equation modeling to identify the relationship between parent–child psychological independence, acceptance of care, care attitude, and care burden, and the care dyad difference of the models. RESULT: 1) "Reliable relationship with parent" in parent–child psychological independence affected "resignation" and "understanding actively" in acceptance of care. 2) "Psychological individuation from the parent" in parent–child independence affected all subscales of care attitudes. 3) "Resistance" and "understanding actively" in acceptance of care and "auto-pilot" in care attitude affected care burden. 4) In mother–daughter caregiving, "resistance" and "resignation" had stronger effects on "auto-pilot" whereas "utilization of resource" and "flexible response" in care attitude and "resistance" had weaker effects on care burden. CONCLUSION: The relationship between long-term parent–child relationship and care were revealed. In some points, daughters who were caring for or had cared for their mothers had a different model from other care dyads. These results suggest that child caregivers should be supported mentally in accordance to their difficult points and dyads.

### USING ECHO TO SUPPORT DEMENTIA DIAGNOSIS IN THE PRIMARY CARE NETWORK

Allison Lindauer,[1] Katherine Wild,[2] Andrew Natonson,[3] Miriam Wolf,[4] Nora Mattek,[3] and Deborah Messecar[5], *1. Oregon Alzheimer's Disease Research Center, Portland, Oregon, United States, 2. OHSU, Portland, Oregon, United States, 3. Oregon Health & Science University, Portland, Oregon, United States, 4. Oregon ECHO Network,*

*Portland, Oregon, United States, 5. OHSU School of Nursing, Portland, Oregon, United States*

Approximately 60% of those with dementia do not carry a diagnosis, undermining patient participation in clinical trials and family access to support. Under-diagnosis is driven by lack of knowledge about dementia, stigma, clinician inexperience and therapeutic nihilism. To address clinician-based contributors to under-diagnosis, we developed and implemented "Dementia 360," a telementoring program modeled on the ECHO (Extension for Community Health Outreach) framework. Remote participants (n=67) learned about the diagnostic process, pharmacological management, family support and dementia-related resources. The video-conference-based one-hour sessions occurred weekly over 2 months. Instruction was provided by a multi-disciplinary faculty team with extensive clinical experience. Didactic presentations were followed by case studies offered by participants. Physicians, nurses, psychologists and social workers from 40 organizations participated, of which 62% were from medically underserved rural clinics. Participants were administered pre- and post-program questionnaires about their level of confidence in assessing and treating individuals with memory loss and dementia-related behavioral symptoms. Of the 54 clinicians who completed pre-intervention confidence assessments, 30 completed post-assessment. The clinicians had significantly increased confidence in diagnosing and treating dementia and managing behavioral symptoms of dementia (p ranging from .0002 to .003). Qualitative feedback from focus groups was generally positive, for example, "Knowing the diagnosis criteria and steps to take to rule out other diagnoses will help me more accurately diagnose and rule out dementia for my patients." Our findings suggest that delivering case-based education via ECHO has potential to increase clinician workforce confidence in diagnosing and managing dementia.

### URINARY MARKERS OF OXIDATIVE STRESS CORRESPOND TO INFECTION AND AGING IN WILD CHIMPANZEES

Nicole A. Thompson,[1] Emily Otali,[2] Zarin Machanda,[3] Martin Muller,[4] Richard Wrangham,[5] and Melissa Emery-Thompson[1], *1. University of New Mexico, Department of Anthropology, Albuquerque, New Mexico, United States, 2. Kibale Chimpanzee Project, Fort Portal, Uganda, 3. Tufts University, Department of Anthropology, Medford, Massachusetts, United States, 4. University of New Mexico, Department of Anthropology, Albuquerque, United States, 5. Harvard University, Department of Human Evolutionary Biology, Cambridge, United States*

Oxidative stress (OS) plays a central role in aging and results from a variety of stressors, making it a powerful measure of health and a way to examine phylogenetic variation in life history. However, few urinary OS markers have been examined under field conditions, particularly in primates, and their utility to non-invasively monitor acute vs. chronic conditions is poorly understood. In this study, we examined variation in 5 urinary markers of oxidative damage and protection under 5 validation paradigms in 37 wild, adult chimpanzees living in the Kibale National Park, Uganda. We used 925 urine samples to conduct both cross-sectional and within-individual analyses of responses to acute infection and variation with age. Markers of damage (8-OHdG, F-isoprostanes, MDA-TBARS, and neopterin) and total antioxidant capacity were generally positively correlated with one another. Within individuals, all markers responded to at least one if not both types of acute infection. Markers of damage also varied with age, particularly in individuals near death. Unlike in human and rodent tissues, DNA damage in urine decreased with age, both across and within individuals near death, suggesting a potential decline in DNA repair and/or metabolic rate during senescence. Our results suggest that OS can be measured using field-collected urine and may be useful for both short- and long-term indicators of health. Our results further confirm that using multiple markers and longitudinal sampling within individuals is the most productive approach for studies that seek to determine the role of OS in health and lifespan in long-lived organisms.

### MOVE UP STUDY RESULTS: WEIGHT LOSS POSITIVELY AFFECTS HEALTH-RELATED QUALITY OF LIFE BUT NOT DEPRESSIVE SYMPTOMS

Elizabeth M. Venditti,[1] Robert M. Boudreau,[2] Anne B. Newman,[2] Michelle E. Danielson,[2] Lori A. Kieffer,[2] Judith R. Rager,[3] and Steven Albert[2], *1. Department of Psychiatry, University of Pittsburgh School of Medicine, Pittsburgh, Pennsylvania, United States, 2. The University of Pittsburgh, Pittsburgh, Pennsylvania, United States, 3. University of Pittsburgh Graduate School of Public Health, Pittsburgh, Pennsylvania, United States*

Obesity is prevalent among older adults as are increases in depressive symptoms and declines in health-related quality of life (HRQOL). Healthy weight loss and mitigating mild depressive symptoms (MDS) and HRQOL could have critical public health significance. The Mobility and Vitality Lifestyle Program (MOVE UP) led by Community Health Workers delivered 32 healthy aging/weight management group sessions over 13 months. Data from 240 participants were evaluated to assess program impact on CES-D (20-item) depressive symptom and SF-36 HRQOL scores. Participants were 88% female, 28% black/other race, 42% ≥ college-educated. Mean (SD) age was 67.6 (4.1) and BMI was 34.7 (4.7). At baseline, average CES-D score was 7.9 (7.2) and 27.9 % (N = 67) had MDS, scoring 17.1 (6.2). Results show significant mean (SD) weight change of -12.7 (13.3) lb from baseline ($p<0.0001$). Overall, CES-D mean (SD) score change was -0.4 (6.7) ($p=0.33$); participants with MDS had an average CES-D decrease of -4.4 (7.8) points ($p<0.0001$). Further, HRQOL improved significantly in all realms, particularly the physical component score ($p<0.0001$). SF-36 (SD) total score improved +1.1 (7.6), mental + 2.1 (11.7), and physical + 5.0 (16.7). Regression analyses (age/sex adjusted) demonstrate that for each 5 lb of weight loss there was an average (SEM) 3.35 (1.49) point increase in SF-36 total score ($p=0.03$). The mitigation of depressive symptoms in the MDS subgroup was not significantly associated with weight loss but may reflect other positive effects of the intervention experience. Conversely, positive HRQOL changes appear to be driven strongly by weight loss.

### POLICY MODIFICATION, CLINICIAN TRAINING, AND ENROLLMENT OF COMMUNITY-DWELLING OLDER ADULTS TO ADOPT A MODEL OF CARE

Sandra L. Spoelstra[1], *1. Grand Valley State University, Grand Rapids, Michigan, United States*

Partnering with a Medicaid Home and Community Based waiver, we tests implementation strategies on adoption and sustainability of a model of care to support aging-in-place, intervening on individual capabilities and the home environment. Knowledge-to-Action model underpins the 2-arm (usual care + internal facilitation versus additive external facilitation) 3-year randomized (sites) trial statewide using an implementation strategy bundle (relationship/coalition/team building; education; interdisciplinary care/coordination; facilitation; and audit/feedback). Consolidated Framework for Implementation Research guides examining characteristics (site/RN-OT-SW/beneficiary), clinician attitude/self-efficacy, leadership/readiness to implement, and policy on adoption and sustainability; and intervention impact on beneficiary outcomes. Linear models will be used to analyze fidelity, Poisson and bootstrapping gage adoption, sustainability, fidelity, and level of facilitation. Champions (internal facilitators/supervisors; N=57) were engaged and 33.3% (n=19) completed online training (1.5 hours; 7.0 [SD 2.3] understood role; 6.8 [SD 1.8] would use training: 1-10 [lo-hi]); 100% (N=19) attended monthly coalition-building meetings. RN-OT-SWs were recruited (mail); 608 of 685 (88.8%) consented and completed online education (5.5 hours; Certified). Policymakers included quality incentives in contracts (10/1/2019) with sites for >95% certified RN-OT-SWs. Beneficiaries (N=12,000) were recruited (mail); 5% (n=608) did not participate (16.1% [n=98] poor cognition; 11.5% [n=70] nursing home/assisted living; 7.1% [n=43] no needs; 5.9% [n=36] too sick; 5.2% [n=32] many caregivers; 5.1% [n=31] inability to improve]; no English/hospitalized/other). Stages of Implementation Completion and fidelity data (September/October) will be reported. Adoption of models of care to support aging-in-place in Medicaid settings are a challenge, and this research harnesses a network (university/policymaker/supervisors/waiver sites-clinicians/beneficiaries) to improve care for vulnerable older adults.

### THE RELATIONSHIP BETWEEN SUBJECTIVE AGE, CHRONOLOGICAL AGE, AND COGNITIVE FUNCTIONING IN OLDER ADULTS.

Emily P. Morris,[1] and Laura B. Zahodne[1], *1. University of Michigan, Ann Arbor, Michigan, United States*

Prior longitudinal research suggests that younger subjective age (i.e., feeling younger than your chronological age) predicts better subsequent cognitive functioning and lower dementia incidence independent of chronological age. However, no research has investigated interactions between subjective age and chronological age. This study examined whether older adults with more youthful subjective age performed better on cognitive evaluations than those with older subjective age and whether associations differed as a function of chronological age. Data from 1,047 older adults aged 65 and older from the Health and Retirement Study's 2016 Harmonized Cognitive Aging Project were analyzed. Separate linear regressions estimated associations between subjective age and factor scores corresponding to five cognitive domains: executive function, episodic memory, language, visuospatial functioning, and processing speed. Covariates included sociodemographic characteristics and chronic disease burden. Interaction terms tested whether chronological age modified associations between subjective age and cognition. In the whole sample, younger subjective age was associated with better language. Significant interactions for all five domains revealed that associations were stronger and statistically significant for participants at the oldest chronological ages. The predictive value of subjective age may be highest among the oldest adults. These cross-sectional findings suggest that the oldest adults are most vulnerable to the detrimental effects of feeling older than one's chronological age, cognitive difficulties are more relevant for subjective age perceptions for the oldest adults, and/or subjective age better reflects consequential physiological deterioration in that group. Future longitudinal research is needed to elucidate these possibilities.

### FACTORS RELATED TO QUALITY OF LIFE OF OLDER PEOPLE IN TEHRAN IN 2019

Ali Darvishpoor Kakhki,[1] Zhila Abedsaeedi,[1] and Patricia Davidson[2], *1. Shahid Beheshti University of Medical Sciences, Tehran, Iran (islamic Republic Of), 2. Johns Hopkins University, Baltimore, Maryland, United States*

Aging is accompanied by a variety of challenges and tensions that have consequences for quality of life in older people. Optimizing quality of life is a key goal for active aging. This study was conducted to describe factors and quality of life of older people in the city of Tehran. This descriptive, correlational study was conducted on a sample of 290 older people above age 60 recruited in 10 public parks in five regions of Tehran in 2019. A socio-demographic questionnaire and instrument on Quality Of Life of Older People (QOL-OP) were used for data collection. Content validity and Cronbach's alpha were used for evaluation of the validity and reliability of questionnaires. Data were analyzed with SPSS software. Fifty-one percent of older people were male and 49% male with a mean age of 69.52(± 7.11) years. The mean scores for quality of life domains ranged from 57.61(±7.11) for family integrity, to 64.73(±7.50) for spiritual well-being on a 100-point scale. The scores on health-related quality of life domains were influenced significantly by characteristics of age, gender, marital status, housing status, income source, treatment insurance, supplementary treatment insurance, education level, living persons, number of offspring, and annual physician referral rates. The findings of this study showed that quality of life of older people was lower than expected and related to a number of factors. Monitoring modifiable factors such as treatment insurance and non-modifiable factors will help us to preserve or improve quality of life of older people.

### DETECTING PRE-FRAILTY STATUS: COMPARISON OF CLINICAL JUDGMENTS AND THE PAULSON LICHTENBERG FRAILTY INDEX.

Yi-Ling Hu,[1] and Heather A. Fritz[1], *1. Wayne State University, Detroit, Michigan, United States*

Nearly 50% of U.S. elders are prefrail and at risk for frailty. Identifying prefrail elders and escalating care could attenuate frailty progression. Screening tools are seldom used in practice. Thus, clinical judgment may be a realistic way to ensure widespread frailty screening. No studies, however, have assessed the validity of clinicians' judgment in identifying prefrail elders. This study explored the level of agreement between clinical judgments of frailty status and status categorizations made using the validated Paulson Lichtenberg Frailty Index (PLFI). Older Blacks (n = 202) recruited from a primary care clinic were first categorized as healthy, pre-frail, or frail using the PLFI. Next, geriatric physicians and nurses categorized participants into one of the same categories based on clinical judgment. Clinicians could use medical records to make determinations. We used Cohen's Kappa to determine the level of agreement of both approaches. We used descriptive statistics to explore if any of the 5 PLFI indicators explained discordant categorizations. Of the 202 participants (mean age: 76.7 8.6), 52 (26%) were prefrail and 57 (28%) were frail based on the PLFI. Physicians' judgments aligned with the PLFI in 43% of prefrail and 65.7% of frail cases. Nurse judgments aligned with the PLFI in 43.9% of prefrail and 17% of frail cases. There was slight to fair agreement between clinical judgments and PLFI (physicians Cohen's $\kappa = .23$; Nurses Cohen's $\kappa = .59$). No specific PLFI indicators independently explained discordant categorizations. Findings suggest that clinical judgments did not align well with PLFI categorizations.

### SARCOPENIA ACCORDING TO BRAZILIAN AND EWGSOP 2 CUTOFFS: DATA FROM SARCOS

Julia M. Menezes,[1] Angela T. Paes,[1] and Alberto Frisoli[2], *1. Faculdade Israelita de Ciências da Saúde Albert Einstein, São Paulo, São Paulo, Brazil, 2. Universidade Federal de São Paulo, São Paulo, São Paulo, Brazil*

Cutoff values for lean mass and muscle strength are still controversial in the diagnosis of sarcopenia. The use of European, American and Asian consensus outside these regions may lead to important diagnostic errors. We hypothesized that there are significant differences between the cutoff points from Brazil and Europe in older people. This is a cross-sectional analyses of 502 older adults from SARCOS study, conducted at São Paulo - Brazil. All subjects underwent DXA analyses of total body. Lean mass was obtained from appendicular lean mass by height2 and muscle strength by dynamometer of dominant hand. The Brazilian cutoff points were based on 25th percentile by gender. The European ones were from EWGSOP 2. Agreement was assessed by the Kappa coefficient. The mean age was 78.39 ± 7.08 years old and 277 (55.18%) individuals were women. Among the ethnic groups, 339 (67.53%) were caucasian, 145 (28.88%) afrodescendants and 18 (3.59%) asians. The Brazilian cutoffs for muscle strength were 26 kg for men and 16 kg for women (equivalent to EWGSOP2); while those for lean mass were significantly lower, 6.56 kg/m2 vs. 5.56 kg/m2, respectively. The prevalence by EWGSOP 2 was higher than that obtained by the Brazilian cutoff points (20.32% vs 14.14%, p <0.001), even though these criteria presented Kappa = 0.792; p <0.001. Considering these disparities, 6 out of 100 subjects are considered sarcopenic by European criteria and not by the Brazilian cutoffs. There are significant differences in sarcopenia cutoffs between Brazil and Europe, and this cause important diagnostic variations.

### CUSTODIAL GRANDPARENTS: AN ANALYSIS OF PARENTING AND ITS IMPACT ON HEALTH

Karen C. Clark,[1] Kari R. Lane,[2] and Linda Bullock[3], *1. Georgia State University, Atlanta, Georgia, United States, 2. University of Missouri, Columbia, Missouri, United States, 3. University of Missouri Columbia, Columbia, Missouri, United States*

In the United States, there are 2.7 million grandparents raising grandchildren without a biological parent present (U.S. Census, 2014). Caring for grandchildren can present challenges and stressors to custodial grandparents as they find themselves adjusting to this unanticipated role. Despite the growing knowledge base related to custodial grandparents, there has been limited research into the relationship between parenting self-efficacy and psychological well-being. This study was guided by the Parenting Self-Efficacy Theory derived from Bandura's Social Cognitive Theory. The purpose of this study was to examine the relationship between parenting self-efficacy and psychological well-being (anxiety and depression) among custodial grandmothers. Additionally, self-reported general health was examined to determine if it moderated the relationship between parenting self-efficacy and psychological well-being. Sixty-eight custodial grandmothers recruited across the United States participated in the study. Their mean age was 58 years old; 57% were Caucasian, 35% African American, and 3% Hispanic. With a mean income of $26,000.00, most were retired (32%) or working full-time (29%). Participants responded to psychometrically sound instruments measuring anxiety, depression, general health and parenting self-efficacy. Findings indicated parenting self-efficacy scores were not significantly associated with anxiety scores (r = .029; p = .816) or depression scores (r= -.207; p = .090) among participants. Furthermore, general health did not moderate a relationship between parenting self-efficacy, anxiety (R2= .030; p= .5753) or depression (R2= .051; p= .3376). Further research is needed to determine whether the role of parenting self-efficacy in custodial grandparents. Implications for policy will also be discussed

### EXPLORING PROMISING PRACTICE MODELS FOR HOUSING OLDER PERSONS EXPERIENCINGHOMELESSNESS

Christine Walsh,[1] Sarah L. Canham,[2] Joe Humphries,[3] Victoria F. Burns,[1] Natasha Dharshi,[1] Tamara Sussman,[4] and Jackson Hangar[4], *1. University of Calgary, Calgary, Alberta, Canada, 2. Simon Fraser University, Vancouver, British Columbia, Canada, 3. Simon Fraser University, Burnaby, British Columbia, Canada, 4. McGill University, Montreal, Quebec, Canada*

The numbers of older persons experiencing homelessness (OPEH) is on the rise globally Yet housing and shelter options that support the varied and complex needs of this population are scarce. In order to understand effective solutions for housing OPEH, it is critical to explore promising practices that support aging in the right place for OPEH. In an effort to inform this critical gap, 100 OPEH and service providers were purposefully selected and invited to attend one of three World Café workshops held in three major urban cities in Canada: Vancouver, Calgary, and Montréal. Participants engaged in facilitated discussions aimed at supporting knowledge exchange and generating dialogue about gaps, opportunities and promising local housing options. Thematic analyses of audiotaped deliberations revealed three themes: 1) The limited nature of current housing options and programs in each locality; 2) The importance of supporting integrative housing models that increase access to formal health and social support staff, transportation, and income supports; and 3) The significance of supporting sustainability, by conducting regular program evaluations, increasing public awareness of homelessness issues, and involving multi-sector stakeholders. Findings highlight how meeting the unique health and psychosocial needs of OPEH requires a nuanced understanding of the development, design, and sustainability of effective housing options. World Café dialogues revealed that identifying and sustaining existing promising practice models provides an avenue to supporting aging in the right place for OPEH.

### INTERACTIONS BETWEEN GENES FROM AGING-RELATED PATHWAYS: IMPACT ON HUMAN LONGEVITY

Svetlana Ukraintseva,[1] Matt Duan,[2] Deqing Wu,[2] Konstantin Arbeev,[2] Alexander Kulminski,[3] Joseph M. Zmuda,[4] Kaare Christensen,[5] and Anatoliy Yashin[1], *1. Duke University, Durham, North Carolina, United States, 2. Duke University, Durham, United States, 3. Duke University, Durham, North Carolina, United States, 4. Department of Epidemiology, University of Pittsburgh; Pittsburgh, Pennsylvania, United States, 5. Danish Aging Research Center, University of Southern Denmark, Odense C, Denmark*

Role of genetic interactions (GxG) in human longevity remains poorly understood. We hypothesized that GxG between genes from biologically connected pathways involved in aging may impact longevity. To test this hypothesis, we selected 53 candidate genes from the aging-related pathways (IGF-1/AKT/FOXO3A, TP53/P21/P16, and mTOR/S6K mediated) that are known to jointly influence outcomes of cell responses to stress and damage, such as apoptosis, senescence, growth/proliferation, and autophagy. We evaluated the effects of interactions between SNPs in these genes on longevity in LLFS and CARe data. RESULTS: The IGF1R, PPARGC1A and BCL2 genes were consistently involved in top GxG effects (p<10-6) on survival in the oldest old (85+ and 95+). One SNP, rs2970870 in PPARGC1A gene, was broadly involved in significant interaction effects on survival 96+ (p<10-7) when paired with SNPs in IGF1R and NFKB1 genes. This SNP individually was associated with survival with nominal significance only; therefore, it would have not been selected in a GWAS. We conclude that interactions between genes from aging-related pathways that regulate cell responses and resilience to damage may have major impact on human longevity and contribute to its genetic heterogeneity. The research was supported by the NIA/NIH grants R01AG062623, U19AG063893, P01AG043352.

### SOCIAL FRAILTY IN RECENTLY RELOCATED SEMI-INDEPENDENT OLDER ADULTS

Suzanne Dupuis-Blanchard,[1] Catherine Bigonnesse,[1] Danica Maillet,[1] Odette Gould,[2] Melissa Andrews,[3] and France Légaré[4], *1. Univeristé de Moncton, Moncton, New Brunswick, Canada, 2. Mount Allison University, Sackville, New Brunswick, Canada, 3. Dalhousie University, Halifax, Nova Scotia, Canada, 4. Université de Laval, Québec, Québec, Canada*

Although most older adults live outside of care institutions, not all seniors choose to live in traditional family homes. Among those who relocate, some relocate too early while others are pre-frail or frail when they relocate. Social frailty – the interaction between social vulnerability and frailty – could contribute to these untimely relocations. The goal of this study was to inform the concept of social frailty by examining a population of semi-independent older adults who recently relocated to a continuum of care community. The objectives of this study were to: 1) understand the influence of the social determinants of health on the relocation process; 2) explore whether relocation increases or reduces social frailty; and 3) measure the level of post-relocation frailty in study participants. This mixed method study combined semi-structured interviews on the relocation process, the frailty identification tool PRISMA-7, and socio-demographic surveys. Twenty-nine recently relocated seniors were recruited with the assistance of a Citizens' Advisory Committee along with advertisements, presentations, information booths, and word of mouth. Qualitative descriptive thematic analysis and descriptive statistical analyses were used to examine the relationship between frailty, socio-demographic variables and relocation. Findings indicated that several social determinants contributed to frailty and that relocation into a continuum of care community could mitigate some aspects of social frailty. A conceptual framework on the influence of social frailty on relocation is discussed. More research is needed to inform the concept of social frailty and to better understand the impact of social factors on frailty.

### THE EFFECT OF ACTIVITIES OF DAILY LIVING ON FAMILY CAREGIVER SOCIAL ISOLATION

Tony Gallanis[1], *1. Framily Health, Boston, Massachusetts, United States*

Social isolation has been shown to associate with negative health outcomes including depression and stress. For family caregivers of older adults, the demands on the caregiver often are associated with increasing feelings of loneliness and decreased social contact. The degree to which the caregiver's social isolation is related to the complexity of the caregiving situation remains unknown. Through a cross-sectional analysis of 526 family caregivers from the Family Caregiver Alliance client record database, an association has been established between care recipient functional decline and caregiver social isolation. Social isolation was measured through the Lubben Social Network Scale and functional decline was measured through ADL/IADL reporting. Covariates controlled for in the analysis included caregiver ethnicity, duration of caregiving, adult child status, caregiver education, care recipient income, and hours per week caregiving. Family caregivers of care recipients with higher functional decline experienced elevated odds of social isolation as compared to family caregivers of care recipients with little to no functional decline. The results from this study highlight the need for medical personnel and non-profit actors to anticipate social isolation as a risk factor for family caregivers of older adults given the care recipient is experiencing functional decline.

### WHAT CONSUMERS SAY ABOUT HOSPICES IN ONLINE REVIEWS

Valeria Cardenas,[1] Anna N. Rahman,[2] Mekiayla Singleton,[3] YuJun Zhu,[2] and Susan Enguidanos[1], *1. University of Southern California Leonard Davis School of Gerontology, Los Angeles, California, United States, 2. University of Southern California, Los Angeles, California, United States, 3. Leonard Davis School of Gerontology, University of Southern California, Los Angeles, California, United States*

Until recently, consumers have had limited resources to assess quality of hospices agencies, contributing to growing numbers of consumers turning to online review sites, such as Yelp. Yet little is known about the content of hospice Yelp reviews and how these relate to recently available Center for Medicare and Medicaid Services'

Hospice Compare site data. No study has examined Yelp hospice reviews and compared the themes identified in Yelp reviews to the topics addressed by CMS's HC measures. To better understand how consumers perceive hospice care, we drew a purposeful sample of 67 hospices in California. Researchers used grounded theory to identify themes and categories within the hospice reviews. Each of two teams of two researchers independently coded the reviews and then met to compare coding and reconcile discrepancies until 100% consensus was reached. We coded a total of 692 consumer Yelp reviews, identifying 15 themes and grouping them under five overarching thematic categories: patient/caregiver-provider relationship; clinical care; agency competency; staff professionalism; and medical equipment and supplies. We found that overall Yelp comments were positive. The most frequently mentioned Yelp themes in hospice reviews were compassionate, caring staff; patient/family gratitude; and staff responsiveness. There was considerable overlap between the themes captured in HC caregivers survey items and Yelp. However, Yelp reviews cover a greater number and more diverse themes than those measures reported on the CMS Hospice Compare site. We recommend that consumers consider both HC and online review sites such as Yelp when evaluating a hospice.

### PHYSICAL ACTIVITY IN EARLY AND MIDDLE ADULTHOOD PREDICTS LATER-LIFE MEMORY TRAJECTORIES VIA HEALTH PATHWAYS

Zarina Kraal,[1] and Laura B. Zahodne[1], *1. University of Michigan, Ann Arbor, Michigan, United States*

Physical inactivity measured during late-life is a modifiable risk factor for dementia, but many studies use concurrent assessments with limited longitudinal follow-up. Less is known regarding life course exposure to physical inactivity. Physical activity patterns at different ages may make independent contributions to dementia risk, which would point to multiple critical periods for intervention. Using Health and Retirement Study Life History Mail Survey data (N=4,396), latent growth curves tested whether retrospectively-reported activity in early (18-29 years) and middle (40-49 years) adulthood predicted later-life memory trajectories over 18 years (mean age at study entry = 60.56 ± 5.44; mean follow-up = 13.27 ± 4.03 years). Total metabolic equivalents were computed from reports of moderate and vigorous physical activity. Biennial memory performance was modeled from study entry (between 1996 and 2014) to 2014. Self-reported physical and mental health at study entry were modeled as independent mediators. Models were adjusted for age at study entry, sex, education, race, ethnicity, childhood socio-economic status, year of study entry, and year of mail survey enrollment. More physical activity at ages 18-29 and 40-49 were independently associated with better initial memory, but not subsequent memory change. The association between physical activity at ages 40-49 and initial memory was partially mediated by better mental and physical health. These observational results support the possibility that physical activity interventions during multiple stages of the adult life course might be effective at lowering dementia risk. In particular, mid-life physical activity may have broad effects on later mental, physical, and cognitive health.

### CONSCIENTIOUSNESS, ACTIVITY ENGAGEMENT AND MOMENTARY AFFECT IN OLDEST-OLD ADULTHOOD

Tim D. Windsor,[1] Rachel G. Curtis,[2]
Christiane A. Hoppmann,[3] and Mary A. Luszcz[1], *1. Flinders University, Adelaide, South Australia, Australia, 2. University of South Australia, Adelaide, South Australia, Australia, 3. University of British Columbia, Vancouver, British Columbia, Canada*

Participation in meaningful activities may be particularly important for late life well-being. We examined associations of moment to moment variability in meaningful activity engagement with positive and negative affect in the daily lives of oldest-old adults. Moderating effects of conscientiousness on meaning-affect associations were also examined considering recent theorising that late life declines in conscientiousness could reflect adaptive self-regulatory processes. Participants were 73 adults aged 84 and above from the Australian Longitudinal Study of Aging Daily Life Time-Sampling (ADuLTS) study, who provided self-report data on activity engagement (including ratings of meaning and degree of challenge associated with activities) and affect on five occasions per day over seven days. Within-person variability in meaningful activity engagement was associated with positive and negative affect; however, these associations were conditional upon the extent to which activities were rated as challenging. Specifically, positive affect tended to be lower on occasions when activities were rated as less meaningful, but also more challenging. Similarly, negative affect was rated as lower on occasions when activities were regarded as more meaningful, and at the same time less challenging. Participants who were higher in conscientiousness reported higher overall positive affect, and associations of higher conscientiousness with lower momentary negative affect were evident on occasions when activities were rated as more challenging. Engagement in meaningful activity is associated with higher positive, and lower negative affect in late life, with these associations dependent on the extent to which activities are challenging. Findings are discussed in the context of self-regulatory perspectives on adaptation.

### ANALYSIS OF THE EFFECTS OF SOCIAL CAPITAL ON SMOKING BEHAVIOR AMONG OLDER ADULTS IN CHINA

Xiaojie Sun,[1] and Linlin Yuan[2], *1. Shandong University, Jinan, Shandong, China, 2. Shandong Maternal and Child Health Hospital, Jinan, China*

By 2030, China will become the most aging country in the world. At the same time, China is the world's largest producer and consumer of tobacco, also the largest victim of tobacco. Tobacco exposure is one of the most important risk factors for many chronic non-communicable diseases among older adults. Based on the data from China Health Retirement Longitudinal Study(2011&2015), we aim to analyze the effects of base-period social capital on current smoking behaviors among Chinese older adults of 60 and above (N=7686) with univariate analysis and ordered logit model. Resluts show that, older adults with high social trust (OR=0.783) preferred to choose not to smoke ; those who had emotional support (OR=0.933) and financial support (OR=0.967) would be more possible to choose not to smoke; and older adults were more likely to choose heavy smoking if they had two or more types of social participation (OR=1.223). This study demonstrates that social trust and social support are the protective factors of smoking behavior among Chinese older adults, while social participation was a risk factor. The Chinese government has launched "the 2030 Plan for healthy China" to promote people's healthy behaviors, and this study will provide good evidence for actions aiming at reducing smoking behaviors among Chinese older adults.

### SESSION LB2570 (LATE BREAKING POSTER)

### LATE BREAKING POSTER SESSION III

### 30-DAY READMISSION IN PATIENTS WITH CARDIOVASCULAR DISEASE: DO PATIENTS KNOW THEIR RISK BEFORE DISCHARGE?

HANZHANG XU,[1] Heather R. Farmer,[2] Bradi Granger,[3] and Matthew Dupre[3], *1. Duke University School of Medicine, Durham, North Carolina, United States, 2. Duke University, Durham, North Carolina, United States, 3. Duke University School of Nursing, Durham, North Carolina, United States*

Cardiovascular disease (CVD) is the leading cause of disability and death in the United States, and older adults with CVD are at a high risk of readmission after discharge. This study examined whether patients' perceived risk of readmission at discharge was associated with actual 30-day readmissions in patients with CVD. A standardized survey and electronic health records (EHR) were used to collect sociodemographic, psychosocial, behavioral, and clinical data on patients admitted to the Duke Heart Center (n=730). Prior to discharge, patients were asked their perceived likelihood of returning to the hospital for an unplanned/emergency visit within 30-days. Logistic regression models were used to examine all-cause 30-day readmission among patients who perceived low versus high readmission risk. Nearly 1-in-3 patients (31.4%) perceived high-risk of readmission at the time of discharge. Life stressors, poor self-rated health, and ADL limitations were associated with perceptions of high-risk. Patients who perceived high-risk had significantly higher subsequent readmissions compared with low-risk (23.3% vs. 15.6% p=0.016). Among patients who perceived low-risk of readmission, those who were widowed, had inadequate health literacy, and reported difficulty accessing care exhibited a higher likelihood of being readmitted. In those perceiving a high-risk, nonwhites and those with poor self-rated health, difficulty accessing care, and prior hospitalizations in the past year were significantly more likely to be readmitted. These findings have important implications for identifying CVD patients at high risk of readmission within 30 days after discharge, particularly older adults who may lack adequate resources (e.g., social support, literacy, access to care).

### PREMIGRATION, DISPLACEMENT, AND POSTMIGRATION EXPERIENCES OF LONELINESS AMONG HMONG OLDER ADULTS

Cindy Vang,[1] and Michael Sieng[2], *1. University of Minnesota-Twin Cities School of Social Work, Saint Paul, Minnesota, United States, 2. Arizona State University, Tempe, Arizona, United States*

Available evidence highlights the detrimental impact of loneliness on the mental and physiological health of older adults. While immigrant older adults report higher prevalence for experiencing loneliness compared to native born older adults, minimal research has simultaneously explored the premigration, displacement, and postmigration experiences of loneliness among older adults with a refugee history. This study aimed to explore loneliness in these three phases among Hmong older adults, an ethnic minority group resettled in the United States as refugees over 40 years ago. Drawing on data from a constructivist grounded theory study guided by an intersectionality framework, the first author interviewed 17 community-dwelling Hmong older adults age 65 and older residing in Fresno and Sacramento, California. Two coders coded and analyzed the transcribed interviews. Findings revealed negative, disruptive, and discriminatory experiences underscored by systems of oppression grounded on the social, political, psychological, and cultural context of each phase. Influencing factors that contributed to loneliness were identified as: trust, loss, aging-related issues, isolation, sense of community, access to cultural community, instability, violence, and cultural adjustments. In specific phases, particular influencing factors were more evident and persistent in producing loneliness. Some influencing factors remained a problem for participants across all phases. With the unprecedented growth of refugees all over the world, this study highlights the need for more research, practice, and policy focused on the context of the refugee experience to gain greater insight into their loneliness experiences and expand the notion of loneliness as an individual experience.

### FACTORS AFFECTING BEHAVIORAL AND PSYCHOLOGICAL SYMPTOMS IN OLDER ADULTS WITH DEMENTIA

HWANG SINWOO,[1] and Eunhee Cho[2], *1. Yonsei university, Seoul, Korea, Republic of, 2. Professor, College of Nursing, Yonsei University, Seoul, Korea, Republic of*

The purpose of this study was to identify factors predicting behavioral and psychological symptoms of dementia(BPSD) in older adults with Dementia. This is a cross-sectional study, recruiting 157 participants from neurology general hospital as study subjects. Data collection was performed from June 2018 to May 2019. BPSD were classified using a modified version of the Cohen-Mansfield agitation inventory(CMAI), which are physically non-aggressive behaviors(PNAB), physically aggressive behaviors(PGAB), verbally non-aggressive behaviors(VNAB), verbally aggressive behaviors(VAGB). The Cornell scale for depression in dementia(CSDD), korea activity of daily living scale(K-ADL), korean mini-mental state examination(K-MMSE), activity and sleep time through using actigraphy for 2weeks, salivary melatonin and cortisol level at 4 times a day done after waking up, after breakfast, before and after dinner, and medication were measured as influencing factors. The generalized linear mixed model analyses indicated that VNAB and VAGB were associated with severe depression($p<0.01$, respectively), low melatonin level at the after waking up ($p<0.05$, respectively), and high melatonin level before dinner(p<0.05, respectively). On the PNAB and PGAB, severe depression(p<0.05, respectively) and high activity(p<0.05, respectively) showed great influences. These findings suggest that developing of intervention of BPSD must be started with detecting depression, ADL. Considering the factors of each type of symptom, tailoring an individual approach is recommended. In addition, this study identified that the activity through actigraphy and salivary melatonin measurement are useful tools to examine BPSD. It can be helpful in the objective evaluation of BPSD.

### RETIREMENT TIMING AND POST-RETIREMENT HEALTH: QUASI-EXPERIMENTAL EVIDENCE FROM DENMARK

Jeevitha Y. Qvist[1], *1. Department of Political Science, Aalborg University, Aalborg, Denmark*

A growing concern among policy makers in the European welfare states is that the proportion of the population in the working age has decreased over the last decades. In response to these demographic trends, many European countries have introduced reforms that roll back welfare policies that enables early retirement in order to sustain the current standards of living. However, scholars have voiced the concern that reforms which prevent early retirement could cause a rise in health inequality in old age because some people are not able to extend their working life. There are two contradictory views on post-retirement health. Retirement can either be seen as a kind of identity crisis, leading to less motivation to maintain health or retirement can be seen as a health preserving transition, enabling individuals to relieve stress and be more aware of their health. So far, empirical evidence on the effect of retirement timing on post-retirement health is inconclusive about the causal nature of this relationship. To estimate the causal effect of retirement timing on post-retirement health, this paper uses month of birth variation in incentives to postpone early retirement in the cohort born in 1939 that was created by a reform of the Danish retirement legislation, which the government introduced in 1999. The results suggest that people who retire at the age of 60 have more adverse health outcomes in old age than people who retire later, but this difference does not appear to be caused by differences in retirement timing.

### HEALTHY AGEING FOR ALL? SOCIOECONOMIC CHANGES OVER TIME IN DISABILITY-FREE AND INDEPENDENT LIFE EXPECTANCIES

Holly Q. Bennett,[1] Fiona E. Matthews,[1] Louise Robinson,[1] Lynne Corner,[1] A Kingston,[1] and Carol Jagger[1], *1. Newcastle University, Newcastle upon Tyne, United Kingdom*

Background: Previous research using cross-sectional data shows widening inequalities in disability-free life expectancy (DFLE) by socioeconomic status (SES) in the UK. We aimed to understand the underlying transitions of DFLE and years dependency free (DepFLE) using longitudinal data from the Cognitive Function and Ageing Studies (CFAS I and CFAS II). Methods: Two large population based studies of those aged 65+ in three centres (Newcastle, Nottingham, Cambridgeshire) interviewed at baseline in 1991 (CFAS I) and 2011 (CFAS II) with follow up two years later. Disability was measured using difficulty in activities of daily living (ADL), and dependency by time between help required for ADLs. SES was based on area deprivation categorised into study specific tertiles. Transitions between disability or dependency states and death were modelled using Interpolated Markov Chain software. Results: Between 1991 and 2011, DFLE and disabled life expectancy (DLE) at age 65 increased for men in every SES group, with men being less likely to become disabled or die, and more likely to recover, in 2011 than 1991 across SES groups. For the most disadvantaged women, DFLE was similar, and DLE increased, whilst for the remaining women DFLE increased and DLE was similar. For women probability of recovery increased and probability of death from disability decreased but probability of becoming disabled decreased only for the most advantaged. DepFLE patterns across time were similar but more pronounced. Conclusion: Preventive measures should focus on reducing the disability and dependency onset in the most disadvantaged to ensure inequalities do not widen further.

### CEREBRAL ATROPHY CORRESPONDING TO TOOTH LOSS IN ELDERLY INDIVIDUALS

Yojiro Umezaki,[1] Rui Egashira,[2] Michiko Makino,[1] and Toru Naito[2], *1. Department of General Dentistry, Fukuoka Dental College, Fukuoka, Japan, 2. Fukuoka dental college, Fukuoka, Japan*

Recent advances in the aging of Japanese society, have meant that the chance of encountering dementia patients in dental clinics is dramatically increasing. Many studies have shown that the brain volume decreases along with the progression of dementia. Although previous studies have reported a relationship between tooth loss or periodontitis and the onset of dementia, the pathological mechanisms underlying this association have not been elucidated. In this study, we focused on the relationship between the oral condition and brain atrophy to discuss how to adequately deal with dementia patients. This study included fifteen participants who underwent brain MRI (magnetic resonance imaging). We obtained information on the oral condition, lifestyle, cognitive function and brain atrophy. The cognitive function was assessed using the Mini-Mental State Examination (MMSE). MR images of each patient were analyzed with the Voxel-based Specific Regional Analysis System for Alzheimer's Disease to quantitate the degree of brain atrophy. The study population included 4 male and 11 female patients. The mean age was 75.9 years. The mean number of present teeth was 15.0. The median MMSE score was 26. The degree of atrophy of the whole brain was significantly correlated with the number of present teeth (r=-0.72, p<0.05) and the presence of a daily exercise habit (r=-0.66, p<0.05). This study showed that the number of present teeth could be an indicator reflecting the progress of dementia. Preserving the teeth as well as the acquisition of a regular exercise habit might be important for preventing dementia.

### HEALTHCARE ACCESS AND SOCIAL ENGAGEMENT OF OLDER MINORITY ADULTS: EXPERIENCES NAVIGATING TRANSPORTATION BARRIERS

December Maxwell,[1] Rebecca L. Mauldin,[1] and Dennis Kao[2], *1. The University of Texas at Arlington, Arlington, Texas, United States, 2. Carleton University, School of Social Work, Ottawa, Ontario, Canada*

Transportation is vital in the daily lives of older adults and provides access to health care services and health enhancing activities, such as social engagement. Disparities in mobility exist for older African American and Hispanic adults compared to non-Hispanic Whites, including higher likelihood of driving cessation at an earlier age and having a higher risk for reduced life space. This poster presents findings from a qualitative analysis of data from the Using Geo-Ethnography to Explore the Spatial Accessibility of Health Services for Aging Minorities Study (GeoSAS), a mixed methods study of older minority adults in Houston, TX. Using interpretive phenomenological analysis, the transcripts of semistructured interviews with 23 older adults (13 African American and 10 Hispanic; 17 female; mean age = 71.3 yrs, SD = 6.3 years) were analyzed to address the research question: What are the mobility experiences and perceptions of minority older adults regarding healthcare access and social engagement? Based on an ecological systems theoretical framework, we found reciprocal influences of (1) healthcare systems and transportation utilization and (2) participants' health and well-being, mobility, and social engagement. Support from family members and financial capacity were critical for participants' mobility. Implications of this research include educating health care providers about patients' transportation experiences and barriers, optimizing social support to increase mobility, and addressing systematic disparities in transportation access to enhance health and well-being for older minority adults.

### VALIDATING SILICONE WRISTBANDS TO MEASURE PESTICIDE EXPOSURES AMONG OLDER ADULTS -- PROOF-OF-CONCEPT STUDY

Marianne Chanti-Ketterl,[1] Brenda L. Plassman,[1] Christine Parks,[2] Nicholas Herkert,[3] Julie Fleenor,[4] Sharon Zhang,[3] and Heather Stapleton[3], *1. Duke University, Durham, North Carolina, United States, 2. Epidemiology Branch, National Institute of Environmental Health Sciences, Research Triangle Park, North Carolina, United States, 3. Nicholas School of the Environment, Duke University, Durham, North Carolina, United States, 4. Department of Psychiatry and Behavioral Sciences, School of Medicine, Duke University, Durham, North Carolina, United States*

Silicone wristbands have been used to measure exposure to pesticides and other chemicals among children and younger farm workers, but not in older adults. Thus, we aimed to examine exposure to pesticides using silicone wristbands in a small cohort of older adults living on agricultural land, with variable contact with fields and pesticides. We also investigated correlations between pesticide levels on wristbands and urinary pesticide metabolites. Organophosphate (OPH) pesticides and several organochlorines were measured in wristbands worn by 15 males age 70+ (10 farmers using pesticides and 5 non-farmers with no recent pesticide use). Wristbands were worn continuously for 5-days. End-of-day urine samples were collected on days 1-3-5. Spearman correlations and Wilcoxon Scores were calculated. Five pesticides were quantified in the wristbands and detection frequencies ranged from 40-90%. In urine,12 OPH metabolites were quantified, but only 5 were detected in >50% of the samples. None of 5 urinary herbicides were detected. Imputation was performed by dividing minimum-detect by square-root-2. Malathion was only detected in farmers compared to non-farmers. Correlations between OPH urinary metabolites and wristband were examined but only two were significant and were negative in direction. Notably, organochlorine DDE on the wristbands was significantly correlated with 3 OPH metabolites. These unexpected relationships, based on small numbers, suggest a need to replicate this work in a larger study sample to explore potential for confounding or mixtures in future studies of pesticides and health in older farmers.

### MACHINE LEARNING ANALYSIS OF MOUSE FRAILTY FOR PREDICTION OF BIOLOGICAL AGE AND LIFE EXPECTANCY

Alice Kane,[1] Michael B. Schultz,[1] Sarah Mitchell,[2] Michael MacArthur,[2] James Mitchell,[2] Susan E. Howlett,[3] Michael S. Bonkowski,[4] and David Sinclair[1], *1. Paul F. Glenn Center for Biology of Aging Research at Harvard Medical School, Boston, Massachusetts, United States, 2. Harvard T.H. Chan School of Public Health, Boston, Massachusetts, United States, 3. Dalhousie University, Halifax, Canada, 4. Department of Dermatology, The Feinberg School of Medicine, Northwestern University, Evanston, Illinois, United States*

In mammals, the lack of accurate biomarkers for biological age is a current limitation to identifying novel aging interventions. Molecular biomarkers including DNA methylation hold promise but are invasive and currently expensive. The Frailty Index (FI) quantifies the accumulation of health-related deficits and is fast, cheap, and non-invasive. Studies have demonstrated that FI correlates with age and mortality risk in mice and humans. However, the FI has not been modelled to directly predict biological age or life expectancy. We tracked aging male C57BL/6 mice until their natural deaths, scoring them longitudinally with the FI. We find that FI score correlates with and is predictive of age and that some but not all parameters of the FI are individually well-correlated with age. To better predict chronological age, we performed an elastic net regression on the FI termed FRIGHT (Frailty Inferred Geriatric Health Timeline) Age. FRIGHT Age is a strong predictor of age ($r2=0.73$, median error=47.5 days), but is not superior to chronological age at predicting life expectancy. To better predict mortality, we built a random forest model termed the AFRAID (Analysis of Frailty and Death) score, which predicted survival at multiple ages ($r2=0.375$, median error = 46.4 days). The FRIGHT and AFRAID models were responsive to chronic treatment with enalapril (30mg/kg/day), an angiotensin converting enzyme inhibitor that extends healthspan, and methionine restriction, a dietary intervention that extends healthspan and lifespan. Our findings underscore the value of assessing non-invasive biomarkers for aging research and may help speed the identification of aging interventions.

### WHITE MATTER INTEGRITY UNDERLYING SUBSYNDROMAL DEPRESSION SYMPTOMS IN DEMENTIA CAREGIVERS

Stephen Smagula,[1] Tales Santini,[2] Sarah Stahl,[2] Tamer Ibrahim,[2] Charles Reynolds,[2] Richard Schulz,[2] Layla Banihashemi,[2] and Liang Zhan[2], *1. Department of Psychiatry, School of Medicine, University of Pittsburgh,*

*Pennsylvania, United States, 2. University of Pittsburgh, Pittsburgh, Pennsylvania, United States*

Past research shows that major depression is associated with lower white matter integrity in fronto-limbic and other areas. But it is not known whether the integrity of these white matter connections is associated with subsyndromal depression symptoms, a marker of risk for major depression, in family dementia caregivers (dCGs) who reported stress. If specific aspects of white matter integrity are related to depression symptoms in this high-risk group, this could provide a biomarker of vulnerability or target for treatment. Participants included 41 dCGs (average age=69, standard deviation=6.4), who underwent a 7 Tesla 64-direction (12-minute) diffusion-weighted imaging sequence. Analyses compared dCGs with (n=20) and without (n=21) subsyndromal depression symptoms (nine-item Patient Health Questionnaire scores ≥5). Using fractional anisotropy (FA), we assessed differences in the integrity of 11 white matter aspects implicated in prior studies of major depression. We found that caregivers with subsyndromal depression had lower FA in tracts connecting to the posterior cingulate cortex (Cohen's D=-0.9, p-value=0.006, FDR=0.03) and in white matter connecting the dorsolateral prefrontal cortex with the rostral cingulate (Cohen's D=-1.2, p-value=0.0005, FDR=0.006). Thus, differences in the integrity of white matter (and related functions) reaching the posterior cingulate (autobiographical memory/planning) and connecting dorsolateral prefrontal and rostral cingulate regions (emotion re-appraisal) may contribute to depression vulnerability in dCGs. These observations require contextualizing further (e.g., assessing roles of depression history and other risk factors) for their meaning to be fully elucidated. Potentially, relationships between known risk factors (e.g., subjective stress) and depression emerge from or drive changes in white matter.

### CARDIAC MYBP-C IN C57BL/6 MICE: THE EFFECTS OF AGE

Amanda Pinheiro,[1] Ted G. Graber,[2] and LaDora V. Thompson[1], *1. Boston University, Boston, Massachusetts, United States, 2. East Carolina University, Department of Physical Therapy, Greenville, North Carolina, United States*

Aging is a known contributor to cardiovascular dysfunction. It is well-established that with age there are functional changes in the heart; yet, the proteins responsible for maintaining sarcomere integrity are not well understood during the aging process. A key protein, cardiac myosin binding protein C (cMyBP-C), contributes to the structural integrity and the regulation of actomyosin interactions. To date, little is known about the effects of aging on cMyBP-C. Therefore, the first step in evaluating this sarcomere protein was to determine the expression of cMyBP-C in cardiac tissue across the lifespan. Using ten C57BL/6 male mice per age group (adult (6-7 months), old (22-25 months), and very old (≥29 months)), body and heart mass were determined. Next a portion of the cardiac tissue was homogenized, and protein concentration was determined (BCA assay). The protein samples were probed for cMyBP-C with MYBPC3 (Abcam, #ab133499) by Western Blot. One-way ANOVA was performed to evaluate differences between groups. Results indicated there was an increase in heart mass with age, but relative to body weight there was no significant difference between the three age groups. Western blot analysis revealed no significant age-related difference in the expression of cMyBP-C. Although there was no change in expression levels, it is not possible to rule out cMyBP-C as a contributor to age-related cardiac dysfunction because phosphorylation is known to play a critical role in the function of cMyBP-C. Thus, further investigation of the phosphorylation status of cMyBP-C is needed and is ongoing.

### EXAMINING COMMUNITY-BASED SERVICES DISCONNECTS IN LATE OLD AGE: PATHS FOR REACH THROUGH THE COMMUNICATION ECOLOGY

Carrie Leach,[1] and Julie Novak[2], *1. Institute of Gerontology, Wayne State University, Detroit, Michigan, United States, 2. Wayne State University, Detroit, Michigan, United States*

As potentially eligible recipients continue to increase in number, understanding service system utilization and barriers can help ensure very old adults can access support from their communities when needed. A communication disjuncture between seniors and community-based service providers was revealed through a multi-year, county-wide older adult needs assessment utilizing data from 1,870 individuals. In response, officials convened a post assessment team that formed the three-person Community Advisory Group (CAG, all ≥69 years) who participated in this community-based participatory research (CBPR) study. This applied, qualitative study, guided by an ecological health communication research framework, conducted multilevel examinations of interactions among older adults and their social environment. Twenty in-depth, face-to-face, semi-structured interviews (mean = 82.5 years) were conducted based on a critical threshold of understanding achieved via researcher immersion in the community preceding this study, data collected, and CAG insight revealed through collaborative analysis. Communication Infrastructure Theory helped to reveal how participants' diminishing social network interrelated with the communication environment acted to impede connections to services. In addition to utilization impediments, enabling elements of the communication infrastructure were identified so those resources might be leveraged to bridge the senior-provider divide. Findings from this study suggest new outreach approaches for connecting to older adults through their communication ecology. The findings add to the growing convergence of evidence that calls for improved communication with older adults to minimize poor interactions that hinder accessing resources that may enhance their social, emotional, and physical well-being.

### UNDERSTANDING DEMENTIA PREVALENCE AND HEALTH CARE USE PATTERNS IN RURAL NORTH CAROLINA

Amanda N. Grant,[1] Tsai-Ling Liu,[2] Nigel L. Rozario,[2] Deanna A. Mangieri,[2] Jennifer M. Woodward,[2] and Yhenneko J. Taylor[2], *1. University of North Carolina at Charlotte, Charlotte, North Carolina, United States, 2. Atrium Health, Charlotte, North Carolina, United States*

Rural and remote communities have limited access to high quality dementia care, prompting a need for innovative solutions to meet the health care needs of affected older adults. As part of a study aimed at implementing a telehealth intervention for primary care patients with dementia in two rural North Carolina counties, we examined baseline dementia prevalence and compared health care use between patients with and without dementia. Electronic health records from January 2018 to December 2018 were examined for 2,288 patients aged 65 or older. A zero-inflated Poisson regression model was used to compare healthcare use between patients with and without dementia adjusting for patients' demographic and clinical characteristics. Dementia prevalence was 8.7% based on diagnosis codes. Most patients with dementia were women (70%), not married (55%), Medicare-insured (78%), and had more comorbidities (mean: 2±2) than non-dementia patients. Dementia patients had a significantly higher number of primary care visits, emergency department visits, inpatient visits, and preventable hospitalizations than patients without dementia (risk ratio = 1.1, 1.8, 2.18, and 1.3, respectively; all P< 0.05). Dementia burden was higher among women and use of acute care services by patients with dementia in this rural setting was higher than patients without the disease, similar to urban settings. These findings suggest opportunities to improve care coordination and access to resources to help reduce the need for acute care services among patients with dementia and can help tailor interventions to address the health care needs of this group.

### POST DISCHARGE WALKING ACTIVITY AND 30-DAY READMISSION IN OLDER ADULTS

Noah Kemp,[1] Rachel R. Deer,[1] Elena Volpi,[1] and Steve Fisher[1], *1. UTMB, Galveston, Texas, United States*

The Centers for Medicare and Medicaid Services has determined high rates of unplanned 30-day readmission to be an indicator of substandard care. More research is needed to identify strong, objective markers of readmission risk. The purpose of this analysis was to investigate the utility of average steps per day as a biomarker in determining the 30-day readmission risk of recently discharged older adults. 133 men and women, aged 65 and older, who were capable of walking on their own, recently hospitalized with an acute illness, and discharged to home were given a StepWatch Activity Monitor and monitored for up to 30 days following discharge. Average steps per day and clinical characteristics were assessed and compared with 30-day readmission. 20 of 133 participants were readmitted within 30 days. Those who were readmitted took significantly fewer steps per day overall: 4412 vs. 5948, p=0.027, and had significantly longer stays in the hospital: 4.50 vs. 2.90 days, p=0.002. Survival analysis of our sample, grouped by tertile of mean daily steps, while not statistically significant, p=0.093, demonstrated a trend toward greater probability of readmission over the 30 days post discharge for those who were in the lowest tertile. Walking activity appears to be a moderate predictor of readmission risk. A more extensive study must be conducted to better understand the relationship of walking activity after discharge and readmission.

### THE ASSOCIATION OF A FRAILTY INDEX AND INCIDENT DELIRIUM IN HOSPITALIZED VETERANS

Andrea Y. Sillner,[1] Robert McConeghy,[2] Caroline Madrigal,[3] Deborah J. Culley,[4] Rakesh C. Arora,[5] and James Rudolph[6], *1. The Pennsylvania State University, University Park, Pennsylvania, United States, 2. Veteran's Health Administration, Providence, Rhode Island, United States, 3. U.S. Department of Veterans Affairs, Providence, Rhode Island, United States, 4. Brigham and Women's Hospital, Boston, Massachusetts, United States, 5. University of Manitoba, Winnipeg, Manitoba, Canada, 6. Providence RI VA, Providence, Rhode Island, United States*

Frailty is an accumulation of deficits that helps identify patients who are vulnerable to stressors. Acute illness and hospitalization are stressors that may result in delirium. Delirium is significant in older adults, resulting in increased hospital stays, institutionalization, morbidity, and mortality. This study aimed to determine if a frailty index (FI), calculated on hospital admission, was associated with the development of incident delirium. An FI was built on an accumulation of deficits model which included assessments of cognition, physical function, and medical comorbidities for a cohort of 218 patients admitted to a Veteran Affairs medical facility. The FI was calculated as a proportion of possible deficits (range 0-1; higher scores indicate increased frailty). Delirium was assessed daily by expert clinician interview. Participants were, on average, 71 years (SD=9.53), white (92.7%), and male (91.7%). Participants were grouped using FI ranges as non-frail (FI<0.25; 26%), pre-frail (FI=0.25-0.35; 39%), and frail (FI>0.35; 35%). Incident delirium was more likely to occur in those who were frail (29.3%, p=0.001), compared to those who were pre-frail (20.9%) or non-frail (3.6%). The association of FI and incident delirium remained after adjustment for age, education, and other demographics (pre-frail: adjusted OR=5.64, 95%CI; 1.23, 25.99; frail: adjusted OR=6.80, 95%CI; 1.38, 33.45). Continued data analysis will include an AUC model to demonstrate robustness of the FI. The results from this study support the use of frailty assessments at hospital admission to identify patients at high risk of delirium and in need of additional clinical support and interdisciplinary resources.

### CHALLENGES FOR BLIND AND VISION IMPAIRED USERS OF A VISUAL QUESTION ANSWERING TOOL: IMPLICATIONS FOR AGING ADULTS

Nathan Davis,[1] Bo Xie,[1] and Danna Gurari[1], *1. The University of Texas at Austin, Austin, Texas, United States*

Recent technological developments provide individuals with vision impairment the transformative ability to upload pictures they take and promptly receive descriptions from remote workers. This study aimed to: identify challenges for visually impaired individuals to use such technology to obtain health-related information and provide recommendations for crowd-workers and the future development of assistive artificial intelligence (AI) design. In spring and summer of 2019, we analyzed 265 images of medication packages submitted by users of a visual question answering (VQA) application called VizWiz -- a smartphone application that provides near realtime assistance to visually impaired users by employing crowd-workers. We developed a 4-category coding scheme to analyze image quality, with two independent coders achieving excellent intercoder reliability (85%-95%). Of the 265 images, we found less than half were legible (46%), contained clear indicators for information sought (40%), or had minimum background noise (40%); while only a small percentage contained complete information (6%). Through thematic analysis of the data, we also highlight seven challenges with queries submitted by vision impaired users . Based on our findings, we make recommendations for the future design of VQA technologies, such as VizWiz, for visually impaired users. We also suggest that there is both great need and potential for user-centered design research to significantly enhance such assistive technologies. While this study did not focus exclusively on data submitted by aging adults, many VizWiz users are, in fact, aging adults, and such assistive technologies have strong implications for the design of assistive technology for this age group.

### COMPARISON OF A FRAILTY RISK SCORE AND COMORBIDITY FOR EARLY REHOSPITALIZATION USING ELECTRONIC HEALTH RECORD DATA

Deborah A. Lekan,[1] Thomas P. McCoy,[2] Marjorie Jenkins,[3] Somya Mohanty,[4] and Prashanti Manda[4], *1. UNC-Greensboro School of Nursing, Greensborol, North Carolina, United States, 2. University of North Carolina at Greensboro, Greensboro, North Carolina, United States, 3. Cone Health, Greensboro, North Carolina, United States, 4. University of North Carolina-Greensboro, Greensboro, North Carolina, United States*

Frailty is a clinical syndrome of impaired homeostasis and decreased physiologic reserve and resilience resulting in diminished ability to recover from stressors. In the hospital setting, barriers to adoption of popular frailty assessments make them impractical for widespread use. Improving quality and costs associated with hospitalization has motivated using data from the electronic health record (EHR) to identify patients at risk for adverse outcomes such as early readmission. Patient-level factors such as frailty and comorbidity may signal high readmission risk. In this retrospective study and secondary analysis of EHR data, we investigated Frailty Risk Scores (FRS) in models that included sociodemographic, comorbidity, and laboratory data for early 3-, 7-, and 30-day unplanned readmission. Study data were collected from a health system in the Southeastern U.S. on adults >50 years with an inpatient stay of >24 hours, 2013-2017. Exclusions included planned readmission and in-hospital mortality. The FRS was constructed using ICD-10-CM codes mapped for symptoms, syndromes, and laboratory values. Cox and logistic regression were conducted to examine associations with readmission. Area under the receiver operating characteristic curve (AUC) quantified accuracy. The sample was 53% female and 73% non-Hispanic White (N=55,778). About one-third took at least 7 prescribed medications (34%) and average length of stay was 4.3 days (max=103.6). FRS was a significant predictor of readmission for almost all models, independently of three comorbidity indices (range AUC=.850-.854 for 3-day, .809-.813 for 7-day, and .757 to .768 for 30-day). Frailty and comorbidity are independently associated with early rehospitalization.

### THE EFFECT OF SOCIAL SUPPORT ON QUALITY OF LIFE OF PERSONS WITH COGNITIVE IMPAIRMENT

Wilma E. Afunugo,[1] and Rafael Samper-Ternent[2], *1. University of Texas Medical Branch at Galveston, Galveston, Texas, United States, 2. University of Texas Medical Branch, Galveston, Texas, United States*

According to the Alzheimer's Association, 5.6 million Americans age 65 and older are living with Alzheimer's Disease. Since pharmacological treatments have yet to be developed, we want to determine whether the amount and quality of social support influence the quality of life (QoL) of persons with dementia so they can lead active and purposeful lives. We analyzed data from 22,030 individuals aged 50+ from the 2010 Health and Retirement Study cohort. The dependent variable, QoL, was measured as self-rated health. The main independent variable, cognitive status, was obtained through direct and proxy interview measurements of cognition. For social support, a composite score including the number of social contacts/close relationships and perceived social support/strain was created. Lastly, several covariates were included. Longitudinally, we examined how QoL changed between 2010 and 2012 using 3 stepwise regression models. Model 1 found those with normal cognition have lower odds of poor QoL vs. those with cognitive impairment (OR = 0.38, p <.0001), number of relationships and perceived social support decreases the odds of poor QoL (p = 0.003, p <.0001), while social strain increases the odds of poor QoL (p <.0001). Model 3 revealed similar findings but also, persons with comorbidities have increased odds of poor QoL (p <.0001), while persons with better function have decreased odds of poor QoL (p <.0001). In conclusion, these results can be used to design interventions to improve social support and reduce social strain, which can also improve QoL for dementia caregivers.

### PENSION AND SUBJECTIVE WELL-BEING AMONG OLDER ADULTS IN URBAN CHINA: THE ROLE OF SOCIAL PARTICIPATION

haojun jiang,[1] and Iris Chi[2], *1. nanjing agricultural university, nanjing, China, 2. University of Southern California, Los Angeles, California, United States*

This study highlighted the relationship and interaction mechanism among pension, social participation and subjective well-being of older adults in urban China, which provided cross-cultural evidence for theories, and had significant implications for social security policy. It examined the relationship between pension and subjective well-being (i.e., life satisfaction, depression) among older adults in urban China. It also assessed the mediating and moderating effects of social participation (i.e., three types of activity participations including labor activity participation, political activity participation, voluntary activity participation; and the variation of activity participation) in the linkage between pension and subjective well-being. The data came from the 2014 China Longitudinal Aging Social Survey (CLASS), a national, large-scale survey of a representative community aging sample (60 years and older) in urban China (N=6907). The study used hierarchical regression analysis and structural equation modeling methods. The results showed that pension could improve the subjective well-being of the older adults, specifically, pension enhances life satisfaction and reduces the depression of older adults. Both of three types of activity participation and the variation of activity participation were a significant moderator in the relationship between pension and subjective well-being. Besides, both labor activity participation and variation of activity participation were also a partial mediator in the relationship between pension and subjective well-being.

### DEVELOPMENT AND VALIDATION OF THE ANTIPSYCHOTIC MEDICATION RISK SCORE FOR NURSING HOME RESIDENTS

Lorraine J. Phillips,[1] Greg Petroski,[2] and Nancy Birtley[2], *1. University of Delaware, Newark, Delaware, United States, 2. University of Missouri, Columbia, Missouri, United States*

Prevalence rates of antipsychotic medication (APM) use in U.S. long-stay nursing home (NH) residents, excluding those with approved diagnoses, range from 7.2% to 20.7%; Missouri's rate is 18.6%. This study developed an APM risk score for NH residents using variables from the Minimum Data Set 3.0 (MDS 3.0) assessment. Data from the most recent Missouri MDS 3.0 assessment, excluding admission and discharge, for each long-stay NH resident from November 2017–December 2018 were used to create development (n= 30,893) and validation (n= 7,651) data sets. Potential predictors of APM use were entered in a logistic regression model with variable selection via the least absolute shrinkage and selection operator (LASSO). In a final step, only variables with odds ratios > 1.2 were retained. A weighted score was created by assigning points relative to the maximum coefficient [10*βi /max (β)] and rounded to integer values. APM rates were 17.29% and 17.70% in the development and validation data, respectively. The final model included 14 demographic and clinical indicators; assigned points (1-10) summed for total score (0-50). Areas under receiver operator characteristic curves were 0.801 and 0.798 for the development and validation models, respectively. Youden's index cut-point = 8, with sensitivity of .70 and specificity of .75. Our findings demonstrate it is possible to predict with good accuracy a NH resident's risk of APM use. Identifying residents at increased risk of receiving an APM, perhaps inappropriately, could position NH staff to proactively design and deliver nonpharmacological interventions individualized to each resident's needs and preferences.

### DEATH ANXIETY AND FINANCIAL DECISION-MAKING IN AGING: A STUDY FROM THE HUMAN CONNECTOME PROJECT AGING (HCP-A)

Timothy K. Ly,[1] Mirella Diaz-Santos,[1] Liam Campbell,[1] Marcela Caldera,[1] Taylor Kuhn,[1] and Susan Bookheimer[1], *1. University of California, Los Angeles, Los Angeles, California, United States*

While research addressing late-life death anxiety (the fear of death or the dying process) has focused on end-of-life care decision-making, few have studied the effect of late-life death anxiety on financial decision-making. This is particularly relevant to financial decision-making as older adults are more vulnerable to fraud and deception. The aim of this study was to determine how age and death anxiety affect financial decision-making in a sample of older adults of 60-93 years of age (N = 102), who participated in the HCP-A project at UCLA. To study this relationship, we used a delayed reward discounting task to model financial decision-making, where higher rates of discounting indicate a greater preference for immediate, smaller monetary rewards and lower rates of discounting indicate more future-oriented planning. To account for age-related cognitive decline, cognitive functioning was assessed using the NIH Toolbox. We hypothesized that the presence of death anxiety will increase discounting of future rewards in older adults. Results from a univariate ANOVA showed an interaction between age, death anxiety, and delayed reward discounting. Specifically, older adults with self-reported death anxiety showed greater preference for immediate, smaller monetary rewards. By controlling for cognition, these findings suggest that death anxiety moderates decision-making in late-life adults and may add to our understanding of why older adults are more susceptible to financial abuse. These results suggest a need to consider death anxiety as a moderating variable when developing and implementing policies and services that are geared towards older adults.

### USE OF SERVICES BY PEOPLE LIVING ALONE WITH COGNITIVE IMPAIRMENT: A SYSTEMATIC REVIEW

Amy Rosenwohl-Mack,[1] Anna Chodos,[1] Sarah Dulaney,[2] Min-Lin Fang,[3] Jennifer Merrilees,[2] Leslie Dubbin,[4] and Elena Portacolone[1], *1. University of California, San Francisco, San Francisco, California, United States, 2. Memory and Aging Center, University of California, San Francisco, San Francisco, California, United States, 3. Education and Research Services, UCSF Library, University of California, San Francisco, San Francisco, California, United States, 4. Department of Social & Behavioral Sciences, School of Nursing, University of California, San Francisco, San Francisco, California, United States*

At least one third of older adults with dementia live alone in the United States. Living alone may represent an opportunity to maintain independence and autonomy, while remaining in a familiar home environment. However, living alone with cognitive impairment is also associated with health risks and unmet needs. No systematic reviews on this population have been published. We systematically reviewed research on use of healthcare and long-term services and supports (LTSS) by people living alone with cognitive impairment. Following PRISMA guidelines, we searched six electronic databases for studies reporting quantitative findings on use of services by people living alone with cognitive impairment; 33 studies met inclusion criteria. Nine countries were represented, all high-income economies. Race/ethnicity data was reported in just five studies, and only one included a majority of racial/ethnic minorities. Overall, people living alone with cognitive impairment appear to use health services at similar or lower rates compared to those living with others; however, LTSS use is higher among people living alone. Representation of non-white participants was poor, but the evidence available suggests that among racial/ethnic minorities with cognitive impairment, there is no difference in LTSS use between those living alone and living with others. Findings highlight inconsistencies in access to and use of essential services by older adults living alone with cognitive impairment. As the populations of the US and other high-income countries become both older and more diverse, with increasing numbers living alone, researchers and service providers must consider the specific needs and preferences of this population.

### THE EFFECTS OF SOCIAL NETWORK ON RESILIENCE OF COMMUNITY-DWELLING OLDER ADULTS LIVING ALONE

Sangmi Park,[1] Tae Hui Kim,[2] Soyeon Choi,[2] Kyuwon Lee,[2] Jisoo Jung,[2] and Myounghee Hong[2], *1. Wonju Severance*

*Christian Hospital, Wonju, Korea, Republic of, 2. Wonju Severance Christian Hospital, Wonju, Gangwon, Korea, Republic of*

Resilience is one of the components for successful aging and is related to wellbeing in late life. Studies have shown that older people living alone have low resilience. However, most of these studies were mainly conducted on unhealthy participants. The aim of this study is to examine the factors that contribute to resilience of healthy older adults living alone. Older people living alone who are not subject to public health care service provided to the economically or physically challenged or depressed people were recruited. Data collected from 295 participants were used to conduct hierarchical multiple regression analyses, controlling demographic characteristics, level of cognitive and physical functions, and emotional status. A self-reported questionnaire, UCLA Loneliness Scale, Lubben Social Network Scale(LSNS), and Multidimensional Individual and Interpersonal Resilience Measure(MIIRM) were used to measure study variables. A hierarchical model accounted for 48.8% of the variance in resilience. In model 1(demographics), the religion($\beta$=.178, p<.001) and the perceived economic status($\beta$=-.176, p<.001) variables were significantly related to resilience. The subjective health($\beta$=-.109, p=.038) in model 2(level of function) and the loneliness($\beta$=-.379, p<.001) in model 3(emotional status) had a significant effect on resilience. In model 4, the size($\beta$=-.115, p=.029) and the frequency($\beta$=.160, p=.003) of social networks significantly predicted resilience. The results showed that protecting older adults' social networks could lead to promote their health and wellbeing. What can be inferred from this finding is that even though the members are small, the social network they often have contact with is important for the resilience of older adults living alone.

### CANCER: ARE OFFSPRING OF LONG-LIVED SIBLINGS BOTH ROBUST AND RESILIENT?

Angéline Galvin,[1] Jacob Krabbe Pedersen,[2] Svetlana Ukraintseva,[3] Thomas T. Perls,[4] Mary K. Wojczynski,[5] and Kaare Christensen[6], *1. Epidemiology, Biostatistics, and Biodemography, Department of Public Health, University of Southern Denmark, Odense, Denmark, 2. The Danish Aging Research Center, Department of Public Health, University of Southern Denmark, Odense, Denmark, 3. Center for Population Health and Aging, Duke University, Durham, North Carolina, United States, 4. Boston University School of Medicine, Boston, Massachusetts, United States, 5. Department of Genetics, Washington University in St. Louis, Saint Louis, Missouri, United States, 6. Danish Aging Research Center, University of Southern Denmark, Odense C, Denmark*

Background: The mechanisms underlying clustering of longevity in families are unclear. We have previously shown a low cancer incidence in offspring of long-lived siblings, i.e. cancer robustness. Here we test whether such offspring are also more resilient in terms of survival after cancer diagnosis. Methods: Identification of offspring from long-lived families was undertaken in three nationwide, consecutive Danish studies (DOS, GeHA, LLFS). Cancer cases were identified through linkage with the Danish Cancer Registry. Each offspring cancer case was matched with two control cancer cases from the 5% random sample of the Danish population. Matching criteria were birth year, sex, year of diagnosis and cancer site. The main outcome was overall survival. Factors studied were sociodemographic, health-related and cancer-related. Survival analyses were performed using stratified Cox proportional hazards models based on the matching data. Results: Among the 5,377 offspring of the 634 families, 465 offspring of long-lived siblings with first primary cancer were included, along with 930 controls. Offspring of long-lived siblings had a significantly better survival than controls (HR=0.64 95%CI=[0.52-0.78]). The association attenuated only slightly after adjustment of marital status, education, Charlson Comorbidity Index, and number of prescribed drugs (HR=0.66 95%CI=[0.54-0.81]). Conclusion: Our results suggest that in addition to being more robust to cancer risk, offspring of long-lived siblings are also more resilient to cancer after its diagnosis and show better overall survival compared to individuals with cancer from general Danish population. Funding: The LLFS study is funded by the US National Institute on Aging / National Institutes of Health.

### BIOLOGICAL AGING IS ASSOCIATED WITH INCREASED MONOCYTE INFLAMMATORY ACTIVITY IN OLDER ADULTS

Juliette Tavenier,[1] Line JH Rasmussen,[1] Morten B Houlind,[1] Aino L Andersen,[1] Anne Langkilde,[1] Jan Nehlin,[1] Janne Petersen,[1] and Ove Andersen[1], *1. Clinical Research Centre, Copenhagen University Hospital, Hvidovre, Hvidovre, Denmark*

Chronic inflammation is thought to play a central role in biological aging. However, the causes of chronic inflammation are not fully elucidated. We hypothesized that a dysregulation in monocyte inflammatory activity may contribute to chronic inflammation and biological aging. There are no validated methods for Biological Age (BA) estimation. Therefore, we also hypothesized that older adults with a recent ED (Emergency Department) admission had a higher BA compared to age-matched older adults without a recent ED admission. Two groups of older adults were enrolled: a "high BA"-group who were discharged from the ED four weeks preceding data collection (n=52), and a "low BA"-group consisting of age and sex matched participants without ED admission within the two years preceding data collection (n=52). We assessed NF-κB phosphorylation (Ser529) and NLRP3 inflammasome levels in monocytes using flow cytometry staining of whole blood. Preliminary analyses showed that participants had a median age of 74.8 (IQR: 70.7–82.0) years, 48% were women. Participants in the high-BA group had reduced lower body strength (30 seconds chair stand test p=0.02 and 4 meters gait speed p=0.001) and cognitive function (Digit Symbol Substitution Test p=0.001 and Trail Making Test p=0.002) compared to the low-BA group. Monocytes of participants in the low BA group had lower constitutive p-NF-κB (p< 0.0001) and NLRP3 (p=0.0001) median fluorescence intensity compared to the high BA group. Increased monocyte inflammatory activity assessed by p-NF-κB and NLRP3 was associated with a higher BA. We will investigate associations between monocyte inflammatory activity and markers of chronic inflammation.

### THE IMPACT OF AN EDUCATION INTERVENTION: IMPROVING COMMUNICATION BETWEEN OLDER ADULTS AND HEALTH CARE PROVIDERS

Janna Heyman,[1] Linda White-Ryan,[2] and Peggy Kelly[3], *1. Fordham University, New York, United States, 2. Fordham University, New York, New York, United States, 3. Fordham University, West Harrison, New York, United States*

As people age, ensuing physical and psychological problems can increase, which makes it paramount to be comfortable discussing medical needs with health care professionals, particularly in light of the danger associated with misunderstanding medication use and combining alcohol with prescriptions and/or over the counter medications (National Institute on Aging, 2018). National studies found that about 40 percent of adults ages 65 and older drink alcohol and often do not understand the dangers of combining alcohol with medications (National Institute for Alcohol and Alcohol Abuse, 2008). An educational intervention was developed with a team of expert physicians, nurses, pharmacists and social workers who work in gerontology to focus on improving communication and addressing alcohol and medication use for older adults. A randomized controlled trial was conducted to assess whether the educational intervention improved older adults' comfort in communicating with their health care providers, as well as their knowledge of the concomitant use of alcohol and prescription and over-the-counter (OTC) medications. Results of a MANCOVA showed that those in the intervention group showed larger increases in scores on communication with their health providers and knowledge about the implications of combining alcohol with prescription drugs than those in the control group (Wilks' Lamda=.808, $F(3,76)=6.039$, $p=.001<.05$). In addition, linear regression models showed that the intervention was significantly associated with participants' knowledge of the implications of combining alcohol with prescription drugs. The coefficient across models was approximately 1.00, which represented a substantial increase given the average score of 6.5.

### EFFECTIVENESS OF MULTICOMPONENT EXERCISE INTERVENTIONS IN DEMENTIA PATIENTS: A SYSTEMATIC REVIEW

Oscar Ribeiro,[1] Flavia Borges-Machado,[2] Nádia Lima,[3] Paulo Farinatti,[2] and Joana Carvalho[2], *1. University of Aveiro - Department of Education and Psychology | Center for Health Technology and Services Research (CINTESIS. UA), Aveiro, Portugal, 2. Research Centre in Physical Activity, Health and Leisure, Faculty of Sport Science, University of Porto, Porto, Portugal, 3. Laboratory of Physical Activity and Health Promotion, University of Rio de Janeiro, Rio de Janeiro, Brazil*

Multicomponent training (MT) combines aerobic, strength, postural, and balance exercises, and has been presented as a promising intervention strategy for dementia due to its potential influence in treating symptoms and/or delaying the disease progression in addition to its intrinsic health benefits. This study aims to systematize evidence on how effective MT is in what concerns physical fitness, cognition, and functionality in dementia patients. Four databases (PubMed, WoS, Scopus & SportDiscus) were systematically searched to locate potential trials through March 2019. A total of 2,312 records were identified and a final set of 17 manuscripts reviewed; of these, only 6 satisfied all inclusion/exclusion criteria: peer-reviewed studies performed with humans aged 60+ years; interventions exclusively MT conducted with clinically diagnosed dementia patients; controlled trials (randomized or not). Cochrane Collaboration's tool was used to estimate risk of bias. Samples sizes ranged from 27 to 170 participants; MT programs lasted between 4 weeks up to 12 months, took place from a daily basis to twice a week, and sessions ranged from 30 to 60min. Routine medical care was the most frequent intervention offered to control groups. In overall, studies revealed that MT benefits agility/balance, gait speed and strength. Evidence remains unclear regarding MT effectiveness on increasing cognitive function and ADL performance, although maintenance and more pronounced decline on control groups were reported. MT may be an important non-pharmacological strategy to enhance physical conditioning on dementia patients, but further evidence is needed for acknowledging its benefits in specific cognitive abilities and ADL performance.

### EXECUTING AN ENVIRONMENTAL SCAN OF UNIVERSITY AGE-FRIENDLINESS: FINDINGS FROM A MIXED METHODS STUDY

Andrea G. Zakrajsek,[1] Sarah Masinda,[1] Amanda Simon,[1] and Cassandra Barragan[1], *1. Eastern Michigan University, Ypsilanti, Michigan, United States*

Internationally, universities are recognizing the importance of understanding and enhancing age as a component diversity and inclusion efforts through the Age-Friendly University (AFU) initiative. This session will describe an environmental scan of "age-friendliness" that one AFU university designed. The overall aims of the project include: (1) exploring how stakeholders understand age-friendliness, (2) identifying current efforts and opportunities that exist within the university, and (3) gathering data that describes the perception of barriers that older learners encounter at the university. This presentation will be used to discuss a mixed-methods study that included interviews and a survey of performance and importance ratings of the international AFU principles. Twenty-eight participants were purposefully recruited from divisions across a campus of a regional university to participate in in-depth interview data collection with the research team. Qualitative thematic findings that emerged through a constant comparative method of analysis of interview transcripts include: Existence of Age-Inclusivity Barriers and Opportunities for Change, Need for Intentionality in Age-Friendly Efforts, and Importance of Connections. Furthermore, AFU principle performance and importance ratings were descriptively analyzed in order to prioritize university efforts to enhance inclusion initiatives related to age.

### PHYSICAL ACTIVITY ATTENUATES AGE DIFFERENCES IN CHANGE IN PERCEIVED PHYSICAL FATIGABILITY

Yujia (Susanna) Qiao,[1] Theresa Gmelin,[2] Robert M. Boudreau,[3] Stacy L. Andersen,[4] Stephanie Cosentino,[5] Kaare Christensen,[6] Mary K. Wojczynski,[7] and Nancy W. Glynn[8], *1. University of Pittsburgh, Pisstburgh, United States, 2. University*

*of Pittsburgh. Department of Epidemiology, Pittsburgh, Pennsylvania, United States, 3. University of Pittsburgh, Pittsburgh, Pennsylvania, United States, 4. Boston University School of Medicine, Boston, Massachusetts, United States, 5. Columbia University, New York, New York, United States, 6. Danish Aging Research Center, University of Southern Denmark, Odense C, Denmark, 7. Department of Genetics, Washington University in St. Louis, Saint Louis, Missouri, United States, 8. University of Pittsburgh, Department of Epidemiology, Pittsburgh, Pennsylvania, United States*

Lower physical activity is cross-sectionally associated with greater fatigability; whether such a relationship holds for longitudinal changes in fatigability is under-studied. We examined this question in offspring (≥60 years, range 60-93y, 99.7% white; 53.2% female) enrolled in the Long Life Family Study, a two-generation cohort enriched for exceptional longevity and their spousal controls. At Visit 2 (2014-2017), we measured self-reported physical activity (PA) with the Framingham Physical Activity Index (dichotomized by median value: less active <37 MET-hrs/wk and more active ≥37 MET-hrs/wk). Perceived physical fatigability was assessed using the Pittsburgh Fatigability Scale (PFS, 0-50) at Visit 2 and repeated during a follow-up contact 2.7±0.92 years later. We constructed a repeated-measures linear mixed-effect model to examine the effect of PA on longitudinal change in PFS by median age (younger <70y; older ≥70y) adjusted for family structure, field center, follow-up time, sex, and self-rated health. We found a strong dose-response relationship of PFS scores across the four age/PA groups (ptrend<0.001). Specifically, older/less active (N=310) participants had the highest annual PFS increases of 0.37 points/yr (p<0.001) while those older/more active (N=340) had annual increases of 0.17 points/yr (p=0.03). Younger/less active (N=371) participants had annual PFS increases of 0.09 points/yr (p=0.008); those younger/more active (N=341) had annual decreases (improvement) of 0.18 points/yr (p<0.001). Although annual PFS changes were modest, our findings indicate physical activity attenuated age differences in these trajectories. Physical activity is emerging as a potential target for intervention aimed at reducing fatigability - an important risk factor in the disability pathway.

### IMPROVEMENT IN FINE MOTOR CONTROL IN DEMENTIA ELDERLY FROM A COMPUTERIZED TOUCHSCREEN TRAINING PROGRAM

W.Quin Yow,[1] Hui-Ching Chen,[1] and Tharshini Lokanathan[1], *1. Singapore University of Technology & Design, Singapore, Singapore*

Dementia, a prevalent ageing disease, affects both the higher brain function and motor function, particularly finger movements (Chan, Haber, Drew, & Park, 2014). Task-based finger-tapping speed on a touchscreen device has been used as an assessment criterion to identify patients with deteriorating cognitive abilities (Gualtieri & Johnson, 2005; Cipriani, Bianchetti, & Trabucchi, 2006). As part of a larger project, we designed a computerized cognition intervention program and examined whether the intervention program would improve the finger-tapping speed of the dementia vis-à-vis the cognitively-healthy elderly. Ten mild-to-moderate dementia elderly (aged 83± 5.6) and 8 cognitively healthy elderly (aged 78±6.1) participated in a computerized intervention program where they played cognitive games on touch-screen tablet for about 30-45 minutes per session over two weeks. Participants' touch interaction data over six sessions were collected and analyzed. Using a linear mixed-effect model for analysis, we found that in the 1st session, the touch performance of the dementia elderly was significantly worse than that of the cognitively-healthy elderly (b=-0.172, Z=-2.311, p<.05). By the 6th session, the dementia elderly had significantly improved their touch performance (b=-0.171, Z= -8.042, p<.001) such that their touch performance was now comparable to the cognitively-healthy elderly (b=-0.064, Z=-0.874, p=.393). Overall, our preliminary results suggested that after participating in 6 sessions of our computerized cognitive intervention program, the dementia elderly showed significant improvement in their fine motor movement as measured by their finger-tapping speed. The improved finger-tapping speed serves as a first step toward slowing down the cognitive decline of the dementia elderly.

### PREPARATION FOR THE END OF LIFE AND PERCEIVED QUALITY OF CARE PRIOR TO HOSPICE REFERRAL: A SURVEY OF CAREGIVERS

Laura Prater,[1] Phillip L. Wachowiak,[2] Masami Otsubo,[3] and Seuli Bose-Brill[4], *1. The Ohio State University Comprehensive Cancer Center – Arthur G. James Cancer Hospital and Richard J. Solove Research Institute, Columbus, Ohio, United States, 2. Ohio State University College of Medicine, Columbus, Ohio, United States, 3. Division of General Internal Medicine, The Ohio State University Wexner Medical Center, Columbus, Ohio, United States, 4. The Ohio State University College of Medicine, Department of General Internal Medicine, Columbus, Ohio, United States*

Lack of preparation for the end of life (EOL) can lead to poor outcomes for caregivers. The purpose of this study was to learn if perceived quality of EOL care and communication provided by the primary clinician prior to hospice referral is associated with caregivers' perceived preparedness to deal with medical emergencies/complications at the EOL. We conducted a survey of caregivers of patients who died and were referred to hospice in the prior year. Perceived quality, was measured by the Quality of End of Life Care (QEOLC) scale, and "preparedness" was a binary measure asking if caregivers felt unprepared to deal with medical emergencies and/or complications related to the care of the patient at the EOL. We performed a bivariate analysis using a two-sample t-test to determine if QEOLC scores were greater among those indicating they were prepared compared to those indicating they were not prepared. We received a response rate of over 22.8% (49/215). Mean EOL scale scores were 82.0 (SD=3.8) for those reporting that they did not feel unprepared for medical emergencies/compilations that arose near the EOL, and 68.2 (SD=7.3) for those who reported that they did feel unprepared. Results show that the mean difference approached significance (t=1.85; p=0.07). Although results only approached significance, EOL care from clinicians involved prior to hospice enrollment may be perceived as higher quality among those who felt prepared to handle emergencies/complications. This finding suggests that caregivers highly value early EOL education and communication aiding in preparation for emergencies/complications arising after hospice referral.

### CIRCADIAN PATTERNS OF FREE-WHEEL RUNNING IN YOUNG AND OLD MICE

Carly Hibbs,[1] Musharraf Yusifova,[1] Benjamin McNair,[1] Danielle Bruns,[1] and Emily Schmitt[1], *1. University of Wyoming, Laramie, Wyoming, United States*

The mammalian circadian clock operates on a 24-hour cycle and regulates physiological, endocrine, and metabolic responses to changes in the environment. Aging disrupts this circadian process, increasing risk for development of age-associated diseases. Free-wheel running is not only an indicator of circadian rhythm, but also a strong predictor of survival from age-related diseases (i.e. cardiovascular disease). Thus, understanding the impact of age on free-wheel running can lead to a better understanding of disease progression. We analyzed free wheel running in both male and female C57BL/6J mice at young (3-6 months) and old (18-21 months) ages exposed to standard 12h light/dark cycle. Running wheel data was recorded hourly for 10 days. As expected, young female mice ran more than male mice, and old mice ran less than young mice. Regulation of wheel running demonstrated that older mice of both sexes had a delayed start time in activity patterns. Young mice began running immediately at lights off (signaling the start of their active period) and ran consistently throughout the dark phase with peak activity in the first 2 hours. In contrast, older mice had a delayed response to light with peak activity not occurring until hours 4-6 of the dark cycle and nightly activity ending 2 hours before lights on. Ongoing work will assess the central (brain) and peripheral (muscle, cardiac) regulation of free-wheel running in aging. Together, we demonstrate the importance of studying molecular mechanisms underlying circadian misalignment in older individuals to identify ways to combat age-associated disease with circadian misalignment.

### THE IMPORTANCE OF MAINTAINING PHYSICAL ACTIVITY FOR TRANSITIONS BETWEEN COGNITIVE STATES: A COORDINATED ANALYSIS

Tomiko Yoneda,[1] Jonathan Rush,[1] Nathan A. Lewis,[1] Jamie E. Knight,[1] Jinshil Hyun,[2] Andrea Piccinin,[3] and Graciela Muniz Terrera[4], *1. University of Victoria, Victoria, British Columbia, Canada, 2. Albert Einstein College of Medicine, New York, New York, United States, 3. University of Victoria, Victoria, B.C., United States, 4. University of Edinburgh, Edinburgh, United Kingdom*

Although existing research shows that physical activity (PA) protects against cognitive decline, it is unclear if maintenance of PA throughout older adulthood influences the timing of onset or transitions through cognitive states. Further understanding of modifiable lifestyle factors that protect against cognitive changes characteristic of both normal aging and pathological aging, such as Alzheimer's disease and other dementias, is imperative. Data were drawn from fourteen longitudinal studies of aging from Europe and America (total N=53,069). Controlling for demographics and chronic conditions, multi-state models were independently fit between datasets to investigate the impact of PA (computed based on Metabolic Equivalent of Task Method) on the likelihood of transitioning through three cognitive states, while also accounting for death as a competing risk factor. Random effects meta-analysis of transition probabilities indicated that more PA was associated with a reduced risk of transitioning from normal cognition to mildly impaired cognition (HR=0.90, CI's=0.84, 0.97, p=0.007) and death (HR=0.24, CI's=0.06, 0.92, p=0.04), as well as an increased likelihood of transitioning from severe impairment back to mild impairment (HR=1.09, CI's=1.01, 1.17, p=0.03). Engagement in national minimum recommendations for PA (~150 minutes/week) increased total life expectancy for 70 year old males and females by 4.08 and 5.47 years, respectively. These results suggest that engaging in at least 150 minutes of physical activity per week in older adulthood contributes to delays in onset of mild cognitive impairment, substantially increases life expectancy, and may also diminish the symptoms that contribute to poor cognitive performance at the severely impaired stage.

### HEAVY ALCOHOL CONSUMPTION IN MIDLIFE IS ASSOCIATED WITH ACCELERATED BRAIN AGING SIX YEARS LATER

Riki E. Slayday,[1] Carol E. Franz,[2] Sean N. Hatton,[2] Linda K. McEvoy,[2] Michael J. Lyons,[3] and William S. Kremen[2], *1. San Diego State University, San Diego, California, United States, 2. University of California, San Diego, San Diego, California, United States, 3. Boston University, Boston, Massachusetts, United States*

Excessive alcohol consumption is associated with cognitive decline, exacerbated brain atrophy, and dementia in older adults, but associations with midlife brain health are less well understood. We hypothesized that heavy drinkers would have older-looking brains in late midlife. We examined alcohol consumption at mean age 56 (range 51-59) in 364 men from the Vietnam Era Twin Study of Aging (VETSA) and their predicted brain age at mean age 62 (range 56-67). We created five midlife alcohol consumption groups based on drinks consumed over the past two weeks: never, former, light (1-14), moderate (15-28), and heavy (>28). Participants underwent structural magnetic resonance imaging at mean age 62. Predicted brain age was measured using the Brain-Age Regression Analysis and Computation Utility software (BARACUS). Models adjusted for age, scanner, race/ethnicity, SES, smoking, health, depressive symptoms, alcohol dependence, general cognitive ability at age 20, and non-independence of twins within pairs. Heavy drinkers had a significantly older predicted than chronological brain age (M= 5.93, SE= 0.88) compared to each of the other four groups (p's < 0.05). There were no significant differences among the never (M= 2.88, SE= 0.98), former (M= 2.76, SE= 0.74), light (M= 3.00, SE= 0.94), or moderate (M= 5.93, SE= 0.88) consumption groups. Heavy alcohol consumption at age 56 was associated with an approximately 3-year greater predicted brain age difference at age 62. There was no evidence of protective effects of light/moderate drinking over non-drinking. The neurotoxic effects of excessive alcohol may exacerbate brain aging in late midlife.

### LIVING-APART-TOGETHER RELATIONSHIPS: EXPLORING OLDER ADULT BLACK WOMEN'S PERSPECTIVES ON CAREGIVING EXCHANGES

Nytasia Hicks[1], *1. Scripps Gerontology Center, Miami University, Oxford, Ohio, United States*

Recent research suggests that the preference for living-apart-together (LAT) relationships, where individuals are committed to one another but reside in separate households, has increased among older adults. However, older adult LAT couples prefer not to exchange care to maintain autonomy. In this study, we examine future expectations of spousal caregiving exchanges among older adult black women in LAT relationships. Eleven black women ages 60-74 (married and unmarried) completed two semi-structured phone interviews about future spousal caregiving expectations as to receiving and providing emotional, financial, and physical support. Regarding providing spousal care, findings from this qualitative descriptive study suggest that participants were consistent in their intent to provide emotional support, yet divided in visualizing themselves providing both financial and physical support for a spouse. As to receiving care from a spouse, participants envisioned their spouses providing emotional support, but responses were inconsistent on the expectation of spouses providing financial and physical support. Participants anticipate receiving emotional and physical support from their adult children and/or close family members instead of spouses. Autonomy, level of commitment, and the exchange of health information were also identified as core concepts. This study has implications for applications to different intimate relationships regarding living arrangements, to professionals supporting caregiving families, and to further studies of family gerontology.

### HEALTH BEHAVIORS ASSOCIATED WITH SUBJECTIVE COGNITIVE DECLINE IN OLDER ADULTS

Kristi M. Wisniewski,[1] and Elizabeth M. Zelinski[2],
*1. University of Southern California, Los Angeles, California, United States, 2. Davis School of Gerontology, University of Southern California, Los Angeles, California, United States*

Participation in risky health behaviors can increase the potential for cognitive decline. Smoking, alcohol consumption, and minimal physical activity are modifiable risk factors associated with worse performance on cognitive assessments; however, the relationship between subjective cognitive decline (SCD) and risky practices has not been assessed. As a potential early indicator of cognitive impairment, SCD may serve as a screening measure for dementia. The Behavioral Risk Factor Surveillance System is an annual, self-reported telephone survey of Americans that includes fifteen core and twenty-five optional sections. The present study included Behavioral Risk Factor Surveillance System participants age 45 or older who completed the core and cognitive decline modules in 2015 (n=147,243). Roughly 11% of participants endorsed worsening memory in the previous year. Logistic regression examined the impact of smoking, drinking, and inactivity on self-reported cognitive decline. Current or former smokers had greater odds of endorsing cognitive decline compared to those who never smoked (OR=1.4; 95% CI: 1.27-1.52). Individuals who consumed at least one alcoholic beverage in the previous month had lower SCD odds compared to non-drinkers (OR=0.8; 95% CI: 0.72-0.87). Respondents who engaged in little to no physical activity had greater odds of endorsing cognitive decline compared to active respondents (OR=1.4; 95% CI: 1.31-1.57). Individuals who endorsed cognitive decline engaged in unhealthy habits such as smoking or inactive lifestyles; however, low to moderate alcohol consumption may be beneficial for cognitive functioning.

### "THEY DON'T TELL YOU ANYTHING": ETHICS OF PRIVACY AND SOCIAL RELATIONSHIPS IN ASSISTED LIVING

Emma Cooke,[1] Molly M. Perkins,[1] Patrick Doyle,[2] Kathy Kinlaw,[3] Kevin Wack,[3] and Ann E. Vandenberg[1],
*1. Division of General Medicine and Geriatrics, Emory University School of Medicine, Atlanta, Georgia, United States, 2. Center for Innovative Care in Aging, Johns Hopkins School of Nursing, Baltimore, Maryland, United States, 3. Emory University Center for Ethics, Atlanta, Georgia, United States*

The Health Insurance Portability and Accountability Act was developed to ensure patient privacy. Yet in assisted living (AL), social connectedness—which is associated with positive sense of self and well-being—may conflict with privacy regulations restricting information sharing. These regulations, while intended to protect, rely on a traditional conception of autonomy that foregrounds self-determination, freedom of choice, and freedom from outside interference, rather than a relational definition that acknowledges dependency, interdependence, and care relationships. We sought to identify health information sharing practices in AL that help or hinder residents' ability to maintain a positive sense of self. We conducted a thematic analysis with secondary data (61 interviews with residents and their informal and formal caregivers, 916 hours of ethnographic observation) from one large (125 beds) AL community in Atlanta enrolled in a 5-year NIA-funded end-of-life study (5R01AG047408). We examined these data to determine how health information is shared in AL, and the valence of different sharing practices. Findings showed that exchanging information about shared life stage and health circumstances built community within AL. Conversely, receiving partial or inadequate health information frustrated residents. Medical information could be inferred from environmental cues, but many residents felt these cues harmfully "medicalized" social space. Negotiating privacy boundaries required staff and resident compromise. These results indicate divergence between AL policies intended to preserve privacy, and resident values emphasizing social relationships and bonding. A relational perspective may be valuable in developing alternatives for residents wishing to share health information with peers.

### YY1-MEDIATED PROMOTER-SUPER ENHANCER INTERACTIONS REGULATE MSC MULTIPOTENCY DURING REPLICATIVE AGING

Luyang Sun,[1] Brenna McCauley,[1] and Weiwei Dang[1],
*1. Baylor College of Medicine, Houston, Texas, United States*

Mesenchymal stem cells (MSC) are versatile in stem cell therapy and regenerative medicine, due to its capacity of in-vitro expansion and multilineage differentiation. However, how the MSC's functions and the underlying epigenomic features are affected by aging and cellular senescence remains poorly understood. Here we used culture expanded human MSC isolated from umbilical cord tissue as an ex vivo model to characterize the chromatin 3D conformational changes associated with aging and senescence and correlate these changes to the changes in MSC stem cell functions during aging. By comparing late passage (P15) vs early passage (P5) MSC, no fundamental changes were found on the large-scale chromatin spatial structures including compartment switching and TAD boundaries. However, when focused on super enhancers, which act as major cis-regulatory hubs associated with lineage-specific functions, a large number of significant promoter-super enhancer interaction changes were discovered. Further examination revealed that YY1, a key regulator of promoter-enhancer looping, was highly enriched in these promoters and their corresponding super-enhancers. The expression of the genes involved in such changes is positively correlated with the variation of promoter-super enhancer interaction, indicating that the spatial communications between promoter and super-enhancer positively regulate gene expression. GO-term and TFBS enrichment analysis showed that these genes involved in cell cycle, adipocyte and osteoblast differentiation. Collectively, these results suggest that YY1-mediated promoter-super enhancer looping is an important regulatory mechanism for MSC multipotency during replicative aging.

### PREVENTING FALLS AMONG OLDER FALLERS: LIVE-LIFE

Sarah L. Szanton,[1] Lindy Clemson,[2] Minhui Liu,[3] Laura N. Gitlin,[4] David L. Roth,[3] Melissa D. Hladek,[3] Sarah LaFave,[1] and Marianne Granbom[5], *1. Johns Hopkins University, Baltimore, Maryland, United States, 2. University of Sydney, Sydney, Australia, Australia, 3. Johns Hopkins University School of Nursing, Baltimore, Maryland, United States, 4. Drexel University, Philadephia, Pennsylvania, United States, 5. Lund University, Lund, Sweden, Sweden*

OBJECTIVES: To evaluate whether a fall prevention intervention, adapted from the LiFE program, reduces fall risk in older adults who have previously fallen. DESIGN: Randomized controlled pilot trial SETTING: Participants' homes INTERVENTION: LIVE-LIFE is an occupational therapy delivered fall prevention intervention that integrates strength and balance training into daily habits in 8 visits over 12 weeks. The intervention also provides 1) up to $500 in home safety changes prioritized by the participants 2) vision contrast screening and referral, and 3) personalized fall risk medication recommendations to Primary Care Providers (PCP) from a Pharmacist. This multi-component intervention was compared to a control condition consisting of CDC fall prevention materials and an individualized fall risk summary. MEASUREMENT: Primary outcome: Fall risk measured by Timed Up and Go (TUG) and Tandem stand. Secondary outcomes: Falls efficacy, feasibility and acceptability of the intervention. RESULTS: The sample of 37 people was 65% female, 65% white and an average 77 years old. Two were lost to follow up (95% retention). Compared to the control group, the mean of each outcome improved in the intervention. The LIVE-LiFE intervention had a large effect size (1.1) for amount of time study participants could hold a tandem stand, a moderate effect (0.5) in falls efficacy, and a small effect (0.1) in the TUG. CONCLUSION: LIVE-LIFE was acceptable to participants, feasible to provide, and averaged large to small effect sizes. Simultaneously addressing preventable fall risk factors is feasible and should be investigated due to the growing population at risk for falls.

### FRAILTY IN HEALTHY OLDEST OLD: CHARACTERIZING THE FRAILTY INDEX OF SUPER-SENIORS

Betty Chinda,[1] Xiaowei Song*,[2] Shirromi Sarveswaran,[1] Kenneth Rockwood,[3] and Angela Brooks-Wilson*[4], *1. Department of Biomedical Physiology and Kinesiology, Simon Fraser University, Burnaby, British Columbia, Canada, 2. Health Research and Innovation, Surrey Memorial Hospital, Fraser Health Authority, Surrey, British Columbia, Canada, 3. Dalhousie University, Halifax, Nova Scotia, Canada, 4. Genome Sciences Centre, BC Cancer, Vancouver, British Columbia, Canada*

People at advanced ages often have multiple comorbidities and high frailty. We characterized frailty in "Super-Seniors", individuals 85 or older who have never been diagnosed with cancer, cardiovascular or lung disease, diabetes or dementia. Super-Seniors were enrolled in the Vancouver Healthy Aging Study that consisted of Phase1 (2004-2007; n=486; age=88.6±3.1 years; female=67.5%) and Phase2 (2014-2019; n=167; age=89.2±3.8 years; female=65.3%). A frailty index (FI) that assesses the accumulation of health deficits was calculated as the proportion of deficits present over those considered (here, 30). The FI distribution patterns, mean, median, 99% limit values, relationship to age, and sex differences were analyzed. The FI of Super-Seniors is right-skewed, with a mean of 0.19±0.09 (median=0.17; limit=0.54) in Phase1 and 0.22±0.08 (median=0.21; limit=0.47) in Phase2. Most Super-Seniors (79% and 61% in Phases 1 and 2) had ≤8 of the 30 deficits; FI≤0.24. The FI increased with age (r's=0.29 and 0.24); women showed a higher mean FI than men. Data demonstrated the known and consistent characteristics of the FI. The Super-Seniors, who are healthier than the general population of oldest old, have a significantly lower FI that is more typical of individuals aged about 65. The low FI of these healthy oldest old is consistent with their health and high physical and cognitive function, and underscores their suitability for study as a healthy aged group. Further research will investigate how the FI of Super-Seniors is related to lifestyle and genetic factors and health outcomes.

### SHORT-TERM INTRAINDIVIDUAL DYNAMICS OF HEART RATE AND COGNITIVE FUNCTIONING IN OLDER ADULTS

Jonathan Rush,[1] Tomiko Yoneda,[1] Rebecca Venditelli,[1] Jamie E. Knight,[1] Nathan A. Lewis,[1] Philippe Rast,[2] and Scott Hofer[3], *1. University of Victoria, Victoria, British Columbia, Canada, 2. University of California, Davis, Davis, California, United States, 3. University of Victoria, Victoria, B.C., United States*

Short-term intraindividual variability in cognition can provide insights into the underlying processes that cannot be captured by only examining mean levels (MacDonald et al., 2003). This variability is often dependent on a number of individual or contextual characteristics. Thus, modeling the heterogeneity of the within-person variance and including meaningful covariates to account for why individuals are more variable on some occasions than others is an alternate way to understand process. The present study utilized a 14-day intensive measurement design to examine the effects of fluctuations in daily heart rate on variability in cognitive performance. Fifty-five older adults (Mage = 70.1 years)

completed daily cognitive tasks and measures of well-being, while also wearing an accelerometer to capture physical activity, sleep, and continuous heart rate. Data were examined with a mixed effects location scale model (Hedeker et al., 2008; Rast et al., 2012), which incorporates both an individual's measured level (location) and their variability around that level (scale). Both the location and the scale are permitted to be random such that on any given occasion an individual may deviate from their typical level and they may also deviate from their typical amount of variability. In this way, the location scale model allows for the heterogeneity of the variance in cognition to be modeled and accounted for with other covariates. Results revealed that there were significant amounts of variability in daily heart rate and cognition across 14 days. The impact of heart rate variability on cognitive performance will be discussed.

### PREDICTING FALLS IN PARKINSON'S DISEASE

Ingrid A. Pretzer-Aboff,[1] and Ronald. K. Elswick[1],
*1. Virginia Commonwealth University School of Nursing, Richmond, Virginia, United States*

People with Parkinson's disease (PD) have a proclivity to falling. Early identification and treatment of PD patients with a high risk of falling is important to decrease morbidity, mortality, and improve quality of life. We compared functional performance tests and balance tests in people with PD to determine which tests predict fallers. Sixty participants were recruited, mean age 71.4 y.o. (range 55 – 89), 43 were male, and 34 were identified as fallers (defined as having fallen at least twice in the past year). A logistic regression model was built to determine which functional performance and balance tests would best predict fallers. Predictors in the model included Hoehn and Yahr stage of disease, Unified Parkinson Disease Rating Scale (UPDRS), Barthel Index, Timed Up and Go (TUG), Tinetti Balance Assessment, Berg Balance Scale, and results from a force plate that recorded sway in both static and dynamic conditions (open eyes and closed eyes). Correlations among predictor variables were assessed for multicollinearity and were less than 0.8. Using both a forward and backward stepwise approach, the best prediction model included Tinetti Balance Total score only. ROC analysis yielded an area under the curve of 74% with a cutoff of 13 which had a diagnostic accuracy of 68.8% with an 83% specificity and 56% sensitivity. Given that the cost of treatment for an injurious fall far exceeds preventative measures a clinician may opt to use a cut off of 14 when using the Tinetti Balance Assessment given an 70% specificity and 68% sensitivity.

### IDENTIFYING DIGITAL BIOMARKERS OF DEMENTIA-RELATED AGITATION: PRELIMINARY RESULTS FROM THE MODERATE STUDY

Wan-Tai M. Au-Yeung,[1] Lyndsey M. Miller,[1]
Zachary Beattie,[1] Christina L. Reynolds,[1] and Jeffrey Kaye[1],
*1. Oregon Health & Science University, Portland, Oregon, United States*

Agitation is a challenging dementia-related behavioral symptom and disconcertingly leads to an undesirable increase in pharmaceutical intervention. Agitation is primarily assessed subjectively by time-impoverished caregivers or clinicians, who may not be able to provide comprehensive or sensitive enough reporting to detect early signs of agitation or identify its precipitants. The purpose of the MODERATE (Monitoring Dementia Related Agitation Using Technology Evaluation) study is to characterize dementia-related agitation and its potential catalysts using wearables and sensors installed in participants' private living quarters within memory care (skilled nursing) facilities. Preliminary data from seven episodes of agitation–reported by nursing staff and treated by pharmaceutical intervention–were captured and characterized with an integrated network consisting of research-grade actigraphy, passive infrared presence sensors, door contact sensors, ambient environmental sensor boards, and bed sensor mats. Behaviors (e.g. pacing, space transitions) during 8-hour nursing shifts that included episodes of agitation were compared with 8-hour nursing shifts without any episodes of agitation. The environmental conditions (temperature, lighting, humidity, and decibel level) during agitated periods and non-agitated periods were also compared. There was a statistically significant difference between agitated periods and non-agitated periods for the number of space transitions in the living room ($p<0.01$) and for the measured amount of ambient light and humidity ($p <0.05$). Passive monitoring of behavioral activity is a promising method of unobtrusive, early, and objective detection of dementia-related agitation, expanding the window of opportunity for identifying factors associated with behavioral change and delivering appropriate evidence-based interventions.

### ASSESSING PREFERENCES FOR COMMUNICATING WITH TECHNOLOGY: COMMUNITY-BASED PROVIDER PERSPECTIVES

Andrea Y. Sillner,[1] Kimberly VanHaitsma,[1]
Rachel Wion,[1] and Marie Boltz[1], *1. The Pennsylvania State University, University Park, Pennsylvania, United States*

Miscommunication during older adults' care transitions from hospital to community-based settings (e.g. home health) can lead to adverse events. Effective use of technology assisted communication (TAC) may help to remedy miscommunication surrounding care transitions. Care providers in community-based settings are well-positioned to provide insight on the feasibility and current use of TAC. The purpose of this research was to determine contextual factors (i.e., intrapersonal, interpersonal, environmental) that influence the use of TAC in the home health setting from the perspective of community-based direct care providers and administrators. Focus groups were conducted with direct care providers and c administrators from two different settings – rural and urban/suburban. Content analysis was used to determine themes. Participants indicated that there are many barriers for older adults' use of TAC such as low interest, fear of technology, knowledge gaps, and lack of access to technology. However, others embraced the use of TAC and technology in the community-based care. Additionally, certain forms of TAC, such as text and email, may be better for communicating with informal caregivers. Some direct care providers indicated they were not allowed or encouraged to use certain TAC with patients due to potential security concerns. The community-based care administrators highlighted the importance of TAC but did indicate that use can be limited due to liability and HIPAA concerns. These findings provide important insight for both determining how to best implement TAC for older adults in community-based care settings and aiding in the development of a tool for measuring preferences.

### FORMAL AND DAILY CARE DECISION-MAKING INVOLVEMENT IN AFRICAN-AMERICAN DEMENTIA DYADS

Kalisha Bonds,[1] MinKyoung Song,[2] Karen Lyons,[3] and Martha Driessnack[2], *1. Emory University, Atlanta, Georgia, United States, 2. Oregon Health & Science University, Portland, Oregon, United States, 3. Boston College William F. Connell School of Nursing, Chestnut Hill, Massachusetts, United States*

Decision-making involvement (e.g., verbal and/or nonverbal communication) of persons with dementia (PWD) has been associated with quality of life of PWDs and their caregivers, underscores personhood, and reduces ethical dilemmas for caregivers regarding the PWD's care. Yet, no study has explored the decision-making involvement in formal and daily care of both members of African-American dementia dyads (i.e., African-American PWDs and their African-American caregivers), limiting our understanding of how these dyads navigate decision-making during the dementia trajectory. This study took a closer look through in-depth, semi-structured interviews with African-American dementia dyads as they reflected on their decision-making surrounding formal and daily care. A pilot study of five dyadic interviews, each averaging 45 minutes, was completed. We used a combination of quantitative content analysis, decision-making matrices and I-poems created from I-statements of the dyad regarding their decision-making involvement. Decision-making matrices (i.e., diagrams of the degree of sharing, the balance of power within the dyad, and the final decision maker in formal and daily care) were constructed across interviews. The pairing of traditional analyses with the novel use of I-poems traces participants' sense of self, ensuring their voice is retained. There was agreement within all five dyads regarding the final decision maker(s) in formal and daily care. Between dyads, daily decision-making involvement was led by African American PWDs; whereas, formal care decision-making involvement of African American PWDs varied. Findings highlight the importance of a deeper understanding of formal and daily care decision-making involvement within and between African-American dementia dyads and potential clinical implications.

### WHAT IS AGEISM?: A REVIEW AND ANALYTICAL CRITIQUE OF CURRENT MEASURES OF AGEISM

Jerin Lee,[1] Jenna Wilson,[1] and Natalie Shook[1], *1. West Virginia University, Morgantown, West Virginia, United States*

The past two decades have been marked by a rapidly aging population in the U.S. (U.S. Census Bureau, 2018), making prejudicial attitudes toward older adults (i.e., ageism) and the impact of such attitudes more relevant. Understanding ageism is necessary to change institutionalized beliefs and reduce prejudice toward older adults. However, it requires the availability of valid and reliable measures of ageism. The purpose of the present research was to: (1) provide an analytical review of three existing self-report measures of ageism (i.e., Fabroni Scale of Ageism [FSA]; Relating to Older People Evaluation [ROPE]; Ambivalent Ageism Scale [AAS]); and (2) examine the reliability and convergent validity of these ageism measures. A total of 473 undergraduate students completed the FSA, ROPE, and AAS online. The results indicated that the FSA, subscales of the ROPE (i.e., positive and negative ageism), and subscales of the AAS (i.e., benevolent and hostile ageism) were generally positively associated with one another, with two exceptions. First, positive ageism was negatively correlated with the FSA. Second, positive ageism was not significantly correlated with hostile ageism. Importantly, there was notable variability in the magnitude of the correlations between the measures, as correlations were mostly weak to moderate in magnitude (rs ranged from -.13 to .65). These associations are below the recommended threshold of $r = \pm.70$ for convergent validity (Carlson & Herdman, 2012), suggesting conceptual problems with current ageism measures as they do not appear to reflect a common construct, which has practical implications for future theoretical and empirical work.

### AN ANALYSIS OF C-REACTIVE PROTEIN AND COGNITIVE FUNCTION IN OLDER CHINESE ADULTS

Xi Pan,[1] and Ye Luo[2], *1. Texas State University, San Marcos, Texas, United States, 2. Clemson University, Clemson, South Carolina, United States*

Recent evidence suggests that inflammatory markers may reflect cerebral disease mechanisms related to dementia. However, whether and how inflammation is associated with cognitive function in older Chinese is unknown. The current study is to analyze the association between high-sensitivity C-reactive protein (CRP), an indicator of chronic inflammation, and domain-specific cognitive function (i.e., mental status, memory, and overall cognition) among older adults aged 65 years and over in China. Data of the study (n=2,438) were selected from the 2015 China Health and Retirement Longitudinal Study (CHARLS) and were analyzed using linear regression models. Results of the study show that for every 1.00 mg/L increase in CRP, the scores of mental status and overall cognition both decreases by 0.03 unit and higher levels of CRP are observed in men than women. The findings might be useful in interpreting the biological mechanisms of dementia and add value in dementia prevention and health promotion for the general older population.

### LOSS, SLEEP, AND HEART RATE VARIABILITY: GENDER DIFFERENCES

Hye Won Chai,[1] Dylan J. Jester,[2] Soomi Lee,[3] and Susanna Joo[4], *1. The Pennsylvania State University, University Park, Pennsylvania, United States, 2. School of Aging Studies, University of South Florida, Tampa, Florida, United States, 3. University of South Florida, Tampa, Florida, United States, 4. Yonsei University, Seoul, Korea, Republic of*

Death of a significant other is consistently found to have a detrimental effect on cardiovascular functioning, and such relationship may be stronger when loss is accompanied by low-quality sleep. Using data from the Biomarker project of Midlife in the United States study (n=1,310), we examined whether quality-of-sleep has an additive effect on the relationship between loss and heart rate variability (HRV). Loss was measured as losing someone close within a year of data collection, and was categorized based on the respondents' relationship with the deceased. Relationship was categorized as: immediate family, relative, and friend. Quality-of-sleep was measured using the Pittsburgh Sleep Quality scale. Results showed that the associations among loss, sleep, and HRV differed by gender. For women, losing an immediate family was associated with worse HRV and this did not differ by quality-of-sleep. For men, death of an immediate family was associated with worse HRV only among those with poorer quality sleep. These results suggest that low-quality sleep may indicate psychophysical vulnerability for men who experienced loss, which may relate to their lower capacity for physiological adaptation.

### PATHWAYS TO A GOOD LIFE? MULTIPLE SOCIAL ROLES IN ADULTHOOD AND MENTAL WELL-BEING IN LATER LIFE IN EUROPE

Christian Deindl,[1] and Miriam Engels[2], *1. Heinrich-Heine-University Düsseldorf, Centre for Health and Society, Düsseldorf, Nordrhein-Westfalen, Germany, 2. Heinrich-Heine-University Düsseldorf, Centre for Health and Society, Düsseldorf, Germany*

The connection between employment, family life and health is well documented. Job demands and family obligations are divergent responsibilities and can be a constant source of conflict. The resulting role strain can have a long lasting impact on mental health. Using data from SHARE and ELSA, we take a life course perspective and look at patterns of employment history from the age of 25 to 40 combined with partnership and fertility history of 17,189 men and 23,266 women in 22 European countries. Sequence analysis combined with cluster analysis shows a clear picture of five dominant states in our sample: Stable work and family, stable work without family, working single parent, working childless couples, and being non employed. This pattern is similar for men and women. We use path models to distinguish the impact of childhood conditions on such life course patterns and the direct and indirect impact of employment and family life on mental health. Women who did not combine work and family roles, (work without family, family without work) reported higher levels of depression in comparison with women who combined work and family. Non-working women and single mothers also experienced indirect effects on depression through their economic situation. Unemployed men or men without family reported higher levels of depression. Unemployment and being a single father also have an indirect impact on depression via economic conditions and health. Moreover, such results also differ between countries, with lower employment rates reducing role strain for women, but not so for men.

### RELATIONSHIP BETWEEN CARE CHALLENGE TYPE AND PSYCHOSOCIAL OUTCOMES IN FAMILY CAREGIVERS OF PERSONS WITH DEMENTIA

Clarissa Shaw,[1] Kristine N. Williams,[2] Maria Hein,[3] Carissa Coleman,[2] and Yelena Perkhounkova[3], *1. University of Iowa College of Nursing, Iowa City, Iowa, United States, 2. University of Kansas Medical Center, Kansas City, Kansas, United States, 3. The University of Iowa, Iowa City, Iowa, United States*

Enhancing dementia care is a public health priority and supporting family caregivers of persons living with dementia (PLWD) is a critical need. This poster reports the relationships between the types of care challenges reported by family caregivers and their scores on psychosocial measures. Family caregivers (N=83) participating in the FamTechCare clinical trial identified three top priority care challenges and completed a series of measures (i.e., burden, depression, sleep quality, and reaction to dementia behaviors) at baseline. Priority care challenges were classified using the 10-category Technology-supported Dementia Care Typology. Three of the categories (i.e., behavioral and psychological symptoms of dementia [BPSD], activities of daily living [ADL], and disease expectations [DE]) were reported by an adequate number of caregivers in order to test relationships with psychosocial measures using the Kruskal-Wallis Test. Caregivers reporting 2 or 3 BPSD challenges had higher burden (p=.007), more depression (p=.022) and worse sleep quality (p=.020) compared to those reporting 0 or 1 care challenges related to BPSD. In comparison, caregivers with 2 or 3 challenges related to DE (e.g., PLWD memory loss) had less burden (p=.008), less depression (p=.030), and better sleep quality (p=.042), compared to those reporting 0 or 1 challenge related to DE. Caregivers identifying 2 or 3 care challenges related to ADLs also reported higher levels of depression (p=.036). Dementia caregivers face vast caregiving responsibilities. Caregivers facing BPSD challenges report greater burden and depression. These results reinforce the need for tailored interventions to assist family caregivers in the managing varied care challenges.

### IDENTIFYING SOCIAL MEDIA RESOURCES AVAILABLE TO CAREGIVERS OF ALS PATIENTS

Sandra Sanchez-Reilly,[1] Sireesha Vutukuri,[2] Paula Reilly,[3] Isabela S. Ross,[4] Andrea Mejia,[5] and Jeanette Ross[6], *1. South Texas Veterans Health Care System, San Antonio, Texas, United States, 2. The University of Texas Health Science Center at San Antonio, San Antonio, Texas, United States, 3. Health Careers High School, San Antonio, Texas, United States, 4. International School of the Americas, San Antonio, Texas, United States, 5. Colegio Mayor de Nuestra Senora del Rosario School of Medicine, Bogota, DE, Colombia, 6. University of Texas Health Science Center San Antonio, San Antonio, Texas, United States*

Background: Amyotrophic Lateral Sclerosis (ALS) is a progressive neurodegenerative disorder that causes muscle weakness, disability, and death, with survival of 3-5 years and affecting all populations including Hispanics. ALS patients are mostly cared for by family caregivers (FCG) who experience burden, psychological distress and impaired quality-of-life. Thus, FCG may not leave their homes to access resources. Social media might be a way to accessing support, but little is known about quality/quantity of AL- FCG resources. Research Objectives: To identify and categorize types of bilingual resources available to ALS-FCG in Facebook. Methods: We used Facebook both groups (public and closed groups) and pages and collected numbers-of-likes, organization type and resource provided. Bilingual search terms (Spanish) included ALS/ALS-caregiver. Results: 24 pages/31 groups in English vs. 37 pages/29 groups in Spanish. English resources had more likes/members (3510/790 vs.410/95; p<0.001) and were more active (60+ posts in recent month). Community resources and provision of social support were similarly shared through groups in both languages; advocacy was more prominent among Spanish groups (69%vs.39%, p<0.001). English pages significantly provided more social support (96% vs.65%, p<0.001), more caregiver skills (67%vs.49%, p<0.001), inspirational roles (88% vs.59%, p<0.001). Spanish pages and groups were better in providing news/research to FCG (59%vs.16%, p<0.001). Implications for research, policy or practice: Facebook provides numerous useful bilingual resources to ALS-FCG. FCG may receive social support, inspiration, training, news/research and advocacy. Further interventions need to actively improve the role of social-media in supporting bilingual ALS FCG and use it as a platform of dissemination.

### PALLIATIVE CARE... WHAT'S THAT? : MEDICAID PATIENT-IDENTIFIED BARRIERS TO PALLIATIVE CARE

Deborah Hoe,[1] Yu-Hsuan Wang,[2] Kate Meyers,[3] and Susan Enguidanos[1], *1. University of Southern California, Los Angeles, California, United States, 2. Leonard Davis School of Gerontology, University of Southern California, Los Angeles, California, United States, 3. California Health Care Foundation, Oakland, California, United States*

Multiple studies demonstrate most consumers do not know about palliative care. And, since January 2018, California's Medi-Cal Managed Care patients have been eligible for palliative care services under Senate Bill 1004 (SB 1004). Yet, the uptake of palliative care services has been underwhelming. The purpose of this study is to explore patient-centered barriers to palliative care. We recruited 27 adult Medicaid patients suffering from advanced cancer, chronic obstructive pulmonary disease, congestive heart failure, or liver disease, from community-based sites in Los Angeles, and conducted semi-structured qualitative interviews. Each participant was asked questions to elicit their knowledge about, and perspectives on, palliative care as well as their preferred communication approaches for receiving a referral to palliative care. The interviews were audio-recorded and transcribed verbatim. We used a grounded theory approach to guide our analysis of primary themes. Our findings indicated that the barriers to palliative care referrals among this population included lack of knowledge about palliative care and available services; the reliance on, and trust in, primary care physicians for information; language and cultural barriers; and patient believing they are neither old enough nor sick enough to need palliative care. This population also preferred direct contact, with in-person consultations more favorable than telephone calls. These findings emphasize the critical role primary care physicians play in advocating for safety net patients and the necessity for culturally sensitive education about palliative care. Promoting knowledge and understanding of palliative care among both primary care physicians and consumers is vital to ensuring access to care.

### INFLAMMATORY DIET PATTERN AND COGNITIVE FUNCTION IN 5 EUROPEAN COUNTRIES OVER 3-YEARS FOLLOW-UP

Patricia O. Chocano-Bedoya,[1] Bruno Vellas,[2] Reto W. Kressig,[3] Endel J. Orav,[4] Walter Willett,[5] and Heike A. Bischoff-Ferrari[1], *1. Centre on Aging and Mobility, University of Zurich and City Hospital Waid, Zurich, Switzerland, 2. Institut des Sciences du Cerveau, de la Cognition et du Comportement de Toulouse (ISCT), Toulouse, France, 3. Felix Platter Hospital · University Center for Medicine of Aging Basel, Switzerland, Basel, Switzerland, 4. Department of Biostatistics, Harvard T.H. Chan School of Public Health, Boston, Massachusetts, United States, 5. Department of Nutrition, Harvard T.H. Chan School of Public Health, Boston, Massachusetts, United States*

Diet patterns associated with low chronic inflammation may modulate cognitive decline. We investigated an empirical dietary pattern (EDP) associated with inflammation in five European countries and its association with cognitive changes over 3-years. This prospective study included 2157 community dwelling-seniors 70 years and older, followed for 3 years as part of DO-HEALTH, a randomized clinical trial. At baseline, participants completed a food frequency questionnaire and C-Reactive Protein (CRP) and interleukin-6 (IL-6) was measured. We used the Montreal Cognitive Assessment (MoCA) every year of the study. Based on reduced rank regression, we estimated a dietary pattern associated with CRP and IL-6. To evaluate the association between the EDP and cognitive changes over time, we used repeated measure linear regression models adjusting for age, total calories, BMI, study center, time, alcohol intake, education, physical activity, presence of depression symptoms, hypertension, diabetes or heart disease. The EDP was characterized by higher intakes of red and organ meat, refined grains, legumes, poultry and white fish, and lower intakes of coffee, tea, ginger, nuts and cheese. In multivariate adjusted models, participants with lowest adherence to the EDP (range -7.3 to -0.3) increased their MoCA scores 0.7 points over three years whereas those with highest adherence (range 0.4-10.1) increased their MoCA scores only by 0.2 points (p=0.01). In conclusion, a low inflammatory diet was associated with better cognitive function over time among adults ≥70 years from five European countries. This finding supports the role of diet in the promotion of cognitive health among older adults.

### CO-DESIGNING CANCER SCREENING INTERVENTIONS WITH PEOPLE OVER 55 YEARS OLD AT RISK FOR LUNG CANCER

Leah Tuzzio,[1] Lorella Palazzo,[1] Sarah Brush,[1] Kelly Ehrlich,[1] Melissa Anderson,[1] Hongyuan Gao,[1] and Karen Wernli[1], *1. Kaiser Permanente Washington Health Research Institute, Seattle, Washington, United States*

In 2014, the US Preventive Task Force recommended annual lung cancer screening with low dose CT (LDCT) for adults aged 55 to 80 years old with significant smoking history. Although screening reduces lung cancer mortality, the leading cause of cancer mortality in the US, adherence to screening follow-up remains low. In a human-centered design qualitative study, health services researchers and eight adults over 55 years old from Kaiser Permanente Washington who had recently had an LDCT participated in two co-design sessions. We elicited barriers, facilitators and design principles to develop multilevel interventions that aim to improve adherence to ongoing LDCT. In the initial discussion, participants identified four key areas for improvements to adherence: a) reminders for scheduling and appointments, b) knowledge about tests and follow-up, c) convenience in location and scheduling, and d) financial and non-financial incentives. In a second session, participants referenced patient personas and sketched storyboards, a comic strip-like format showing steps in a journey, to describe different ways to help patients return for LDCTs. Through qualitative analysis, we identified ten elements to consider incorporating in multilevel interventions: versatility (e.g., multiple reminder options), social support (e.g., families, peers), individualization (e.g., tailoring to patient needs), feelings (e.g., fear, relief), knowledge (e.g., harms/benefits, expectations), responsibility (e.g., who is accountable for reminders), continuity (e.g., clear pathway to adherence), consistency (e.g., same messages), cadence (e.g., rhythm of messages), and acknowledgment (e.g., recognition of screening completion). Next steps are to incorporate feedback from clinical stakeholders and develop multilevel interventions for further testing.

### BEING WOMEN AND BEING DEMENTIA CAREGIVERS: SELF-CONCEPTS IN THE MAKING OF VIETNAMESE FEMALE CAREGIVERS

Trang T. Nguyen[1], *1. Center for Studies of Displaced Populations, Tulane University, New Orleans, Louisiana, United States*

In Vietnam, the majority of dementia caregivers are women. They play multiple social roles, and confront role conflicts and caregiving burdens with insufficient social supports. Dementia caregiving alters their self-concepts, or who they think they are. This paper aims to explore self-concepts of Vietnamese female caregivers of older relatives with Alzheimer's Disease (AD). In total, 21 face-to-face, semi-structured interviews, including six follow-up interviews, with 13 Vietnamese female caregivers of older patients with AD were conducted. These 13 caregivers were from 44 to 71 years old, mostly spouses of the patients with AD (n = 8), and retired (n = 9). Thematic coding procedure and the program MaxQDA12 were used for data analysis. Results show that the self-concepts of female caregivers in dementia care were complex, contextualized, and manifested in different aspects. First, self-concepts of these female caregivers were the outcome of the interactions between the guided-self and the performed-self. Their guided-self was the self that their social norms and cultural traditions told them about who they should be, while their performed-self was the self they demonstrated to the outside world. The mismatch between these two types of self caused distress among caregivers. Second, caregivers' self-concept was the combination of the three key types of the self: the moral-self (a filial daughter or a responsible wife); the feminine-self (a patient and graceful women); and the worthy-self (a devoted and helpful caregiver). Understanding Vietnamese women's self-concepts associated with their sociocultural context will better inform the development of support programs for them.

### LOSS NEGATIVELY IMPACTS A HEALTHY LIFE IN US ADULTS: FINDINGS FROM THE HEALTH AND RETIREMENT STUDY

Justin B. Ingels,[1] and Toni Miles[2], *1. College of Public Health, University of Georgia, Athens, Georgia, United States, 2. University of Georgia College of Public Health, Athens, Georgia, United States*

Previous research reports that the loss of a loved one increases the risk of mortality and physical and mental health problems. Using data from the 2004 to 2014 waves of the Health and Retirement Study, we estimate the years of healthy life (YHL) from 2004 to death for each respondent. YHL is based on the combination of years lived between 2004 and 2014, a projection of years beyond 2014, and self-rated health. Regression models stratified by age and gender were developed with the loss of a parent or spouse as the primary exposure and YHL as the dependent variable. Annual estimates of the total YHL lost associated with bereavement were based on these regression analyses and US Census data. Models reveal a strong dose-relationship between YHL lost and the number of losses. In total, the annual YHL lost associated with loss in US adults between 50 and 84 years of age is estimated at 2.0 and 1.6 million for men and women, respectively. Nearly three-fourths of the annual YHL lost are associated with adults younger than 65. Interaction analyses suggest that increasing physical activity has the greatest impact on reducing YHL lost in those with the greatest number of losses, one to two YHL per person. Understanding the full impact of loss on the lives of adults is an important step toward framing loss as a public health issue, especially for middle-aged adults. Results suggest that physical activity should be an important aspect of bereavement interventions.

### ASSESSMENT OF A MICROPLATE SYSTEM FOR MEASURING INDIVIDUAL REAL-TIME RESPIRATION IN SMALL MODEL ORGANISMS OF AGING

Ashley N. Turner,[1] Jessica M. Hoffman,[1] Mickie L. Powell,[1] Melissa J. Sammy,[2] Douglas R. Moellering,[2] Tim R. Nagy,[2] Steven N. Austad,[1] and Daniel L. Smith[2], *1. Department of Biology, University of Alabama at Birmingham, Birmingham, Alabama, United States, 2. Department of Nutrition Sciences, University of Alabama at Birmingham, Birmingham, Alabama, United States*

The ability to measure oxygen consumption rates of a living organism in real-time provides an indirect method of monitoring dynamic changes in metabolism reflecting organismal level mitochondrial function. In this study, we assessed the Loligo Systems microplate system for measuring individual respiration in small organisms. This included adult nematodes (Caenorhabditis elegans, N2), zebrafish embryos (Danio rerio, AB), and adult fruit flies (Drosophila melanogaster, w1118). Organisms were placed inside 80 µL glass chambers on a 24-well microplate atop a 24-channel optical fluorescence oxygen reading device. Adult nematodes and zebrafish embryos were in liquid culture, M9 buffer and egg water respectively, and the adult flies were in room air. The microplate and reader were placed inside an incubator for temperature control. A silicone gasket with a thin liner was used to seal the chambers. Reference standard oxygen consumption (respiration) of single and multiple adult nematodes (n=1–4 animals/well), zebrafish embryos (n=1–4 animals/well), and adult flies (n=1–2 animals/well) in the microplate system were achieved. Significant differences across numbers of animals/well and by sex were observed. Validation experiments of the oxygen consumption rates measured in C. elegans in parallel with Seahorse extracellular flux (XF) experiments are underway. The Loligo Systems microplate system offers a non-invasive, non-destructive method to measure real-time respiration in smaller organisms. These data provide preliminary evidence for utility of the system for a variety of biomedical applications that relate to organismal and mitochondrial function/dysfunction, including research in the basic biology of aging in these highly-utilized, pre-clinical, genetic model organisms.

### PERSONAL SELF-MONITORING DEVICES TO IMPROVE SLEEP AMONG OLDER PEOPLE: A FEASIBILITY STUDY

Raeann G. LeBlanc,[1] Maral Torossian,[1] Paige Czarnecki,[1] Rebecca Spencer,[2] Cynthia Jacelon,[1] and Jenna Marquard[1], *1. University of Massachusetts Amherst, Amherst, Massachusetts, United States, 2. University of Massachusetts, Amherst, Massachusetts, United States*

Chronic sleep disturbances reduce physical and mental health and affect over 8 million people age 65 years and older in the United States. There is evidence that use of a wearable Personal Self-Monitoring Device (PSMD) may improve sleep self-management in young adult populations. Feasibility of PSMD use for older individuals has not been explored and was the goal of this study. Persons age 65 years and over with self-reported sleep disturbances were recruited in a local community and were asked to wear a commercial PSMD for a 4-week period. To assess whether such an intervention may be feasible, outcomes included consent rate, study completion rate, data download interpretation, identification of a sleep self-management goal, improved knowledge about sleep, and improved sleep. Twenty-six persons (12 males and 14 females) were recruited over 3 months, out of a total of 33 expressing interest. Mean age=72, SD=4.99. Ninety-two percent of participants completed the study and reported improved awareness of sleep patterns and identified a sleep goal. Total sleep time was M=7 hours 14 minutes, SD=40 minutes; total restful sleep time was M=4 hours 33 minutes, SD=1 hour 22 minutes. In conclusion, sleep self-management with the use of a PSMD is feasible and of interest among persons in the young-old age category (65-74 years). There is potential for the use of PSMD among older people with the goal of improved sleep self-management. Future studies for sleep health self-management and interventions using personal sleep monitoring are recommended.

### EVERYDAY MEMORY IN OLDER AND YOUNGER ADULTS: OUTSOURCING, SOCIALLY-DISTRIBUTED REMEMBERING & CONCEPTUALIZATION

Emily Lustig,[1] Christopher Hertzog,[1] Ann Pearman,[1] Kirsten Reynolds,[1] and Aiman Waris[1], *1. Georgia Institute of Technology, Atlanta, Georgia, United States*

Qualitative interview data about everyday remembering within the context of older adults ages 62-83, (N= 27, M=69.5, SD=5.72) and younger adults ages 18-24 (N=29, M=21.2, SD= 1.77) lives were collected and analyzed using constructivist grounded theory methods. This study sought to compare the processes used by these individuals in their pursuit of everyday memory-demanding goals and their conceptualization of these methods. Older adults typically reported importance beliefs that guided memory-supportive behaviors for things like social engagements or medical appointments, whereas younger adults reported important information as being primarily school-related. There were major differences in the execution and conceptualization for remembering critical information. Younger adults engaged in a form of socially-distributed cognition, wherein they relied on and outsourced remembering to technology and other people via apps. Interestingly, younger adults relied on others to remind them about coursework, extra-curricular activities, and social obligations via social communication platforms (e.g. GroupMe), text messages, and shared calendar alerts. Very few of the younger adults sampled were responsible for reminding others, but relied on the social altruism of their peers who were responsible for disseminating mass reminders. Conversely, technological outsourcing was not as prevalent in the older adults interviewed and only a few shared that they received similar reminders via text or email. Of the few cases that did outsource, a small subset did, however, engage in these processes within small groups or pairs, wherein a friend or significant other reminded them about social gatherings or names but in a much smaller proportion, comparatively.

### IMPACT OF SOCIAL SUPPORT AND STRAIN ON DEPRESSIVE SYMPTOMS: MARITAL STATUS AND GENDER DIFFERENCES

Deborah Carr,[1] and Yeonjung Jane Lee[2], *1. Boston University, Boston, Massachusetts, United States, 2. University of Hawaii, Honolulu, Hawaii, United States*

Social relationships are a well-established correlate of late-life well-being. Extensive research finds social support is associated with fewer depressive symptoms, yet few studies distinguish fine-grained types of support from spouse, children, other family and friends, nor whether these linkages differ by gender and marital status. Studies exploring coarse associations between support and well-being may conceal gender and marital status differences. We use data from two waves of the Health and Retirement Study (HRS; 2006 and 2010) to study fine-grained linkages between diverse types of relationship strain and support and depressive symptoms (CESD) among adults aged 51+. The results show that the association between support/strain and depressive symptoms varies based on the source of support. For instance, among married/partnered older adults, spousal support is negatively associated with depressive symptoms whereas friend strain is positively associated with depressive symptoms. Among widowed respondents, friend support is negatively associated with depressive symptoms. These marital status patterns differed by gender however, such that the impact of friend strain on depressive symptoms was especially large for divorced men. Our results suggest that no single form of social support (or strain) is uniformly protective (or distressing), so services and interventions to enhance late-life mental health should more fully consider older adults' social location, including gender and marital status. For current cohorts of older adults, who have lower rates of marriage and childbearing than their predecessors, it is critically important to understand both the levels and impacts of alternative sources of support from other kin and friends.

### LONG-TERM EFFECT OF A MULTICOMPONENT INTERVENTION ON PHYSICAL PERFORMANCE AND FRAILTY IN OLDER ADULTS

Gahee Oh,[1] Il-Young Jang,[2] Heayon Lee,[2] Hee-Won Jung,[3] Eunju Lee,[2] and Dae Hyun Kim[4], *1. Harvard T.H. Chan*

*School of Public Health, Boston, Massachusetts, United States, 2. Division of Geriatrics, Department of Internal Medicine, Asan Medical Center, University of Ulsan College of Medicine, Seoul, Korea, Republic of, 3. Seoul National University Hospital, Seoul, Korea, Republic of, 4. Division of Gerontology, Beth Israel Deaconess Medical Center, Harvard Medical School, Boston, Massachusetts, United States*

Multicomponent interventions improve physical function and frailty in older adults, but their long-term benefit remains uncertain. We report the 30-month outcomes of a 24-week multicomponent intervention versus usual care in community-dwelling older adults. This prospective non-randomized study was conducted in 383 older Koreans (mean age, 76.8 years; female 72.3%) living alone or receiving medical aid in rural communities. Of these, 187 received a 24-week multicomponent intervention that consisted of group exercise, nutritional supplements, depression management, deprescribing, and home hazard reduction. The remaining 196 individuals received usual care. After 1:1 propensity score matching, we compared the short physical performance battery (SPPB) score (0-12 points), frailty phenotype scale (0-5 points), and deficit-accumulation frailty index (0-1) at 6, 18, and 30 months. Restricted mean survival time was estimated for death and institutionalization-free survival time at 30 months. The intervention group had higher SPPB scores than the comparison group at 6 months (difference 3.2; 95% CI, 2.5-3.8), 18 months (1.2; 95% CI, 0.5-1.9), and 30 months (1.1; 95% CI, 0.5-1.8). They had lower frailty phenotype scale (-0.6; 95% CI -0.9 to -0.3) and frailty index (-0.04; -0.07 to -0.02) only at 6 months, but similar scores at 18 and 30 months. Death and institutionalization-free survival time over 30 months was 28.5 months (95% CI, 27.7-29.3) in the intervention group versus 24.2 months (95% CI, 22.3-26.1) in the comparison group (difference, 5.2 months; 95% CI, 3.0-7.3). The 24-week multicomponent intervention showed sustained improvement in physical function for 30 months, but only temporary reduction in frailty.

### ELDER FAMILY FINANCIAL EXPLOITATION: EXPERIENCES WITH SOCIAL SERVICES

Tina R. Kilaberia,[1] Emma Fuhrman,[2] Marlene Stum,[3] and Iris Freeman[4], *1. University of Minnesota - Twin Cities, Minneapolis, Minnesota, United States, 2. Minnesota State University, Mankato, Mankato, Minnesota, United States, 3. University of Minnesota, St. Paul, Minnesota, United States, 4. Mitchell Hamline School of Law, St. Paul, Minnesota, United States*

Elder family financial exploitation (EFFE) has attracted the attention of scholars and professionals across disciplines. This qualitative study examines the experiences of help seeking by non-perpetrator family members with a focus on the role of social services. 15 in-depth interviews were examined in which social services were mentioned as being involved. Findings provide insight into the role and involvement of social services, whether wishes expressed by victims to participants made a difference in help-seeking, and gaps experienced. Participants described social services professionals as those who (1) received reports of exploitation; (2) provided education and served as a liaison with families; (3) conducted assessments, including cognitive assessment of elders; and (4) acted as connectors to other systems. In some cases, when elders were assertive about their wishes, they had results such as reporting exploitation or transferring power of attorney to non-perpetrator family members. In other cases, elders were prevented from taking such action because of undue influence by perpetrators, disregard of their wishes, due to being uninformed, or opposing helpful family members. Participants explained experienced gaps in two ways: by attributing responsibility to social services in terms of failure to believe victims, do meaningful cognitive assessments, and navigate family dynamics. On the other hand, participants were not able to clearly ascribe responsibility, and questioned whose fault it was, suggesting opportunities for improved systems functioning. Recommendations for improving the role of social services in addressing the help-seeking needs of concerned family members coping with EFFE follow.

### DIGITAL INEQUALITY IN OLDER ADULTS' ONLINE SOCIAL ENGAGEMENT AND SOCIAL CAPITAL

Minh Hao Nguyen,[1] Amanda E. Hunsaker,[1] and Eszter Hargittai[1], *1. University of Zurich, Zurich, Switzerland*

The increasing popularity of social media and other online communities offers new possibilities for older adults to stay socially connected. This study examines the relationship of older adults' online social engagement and bonding as well as bridging social capital based on a survey of over 1,000 adults aged 60 and over. Social bonding refers to support obtained from existing strong social ties while social bridging is creating connections across varied social networks. We estimated three multi-stage regression models to examine these relationships when controlling for sociodemographic factors, as well as Internet experiences and skills. We then extended the regression models with Internet skills as a moderator. Findings show that older adults who engage more often in specific online social activities (i.e., asking questions on social media, looking at photos of family members/others) enjoy greater bridging social capital (both in offline and online contexts) than those who do so less often. Furthermore, Internet skills moderate the relationship between online social engagement and social capital. Specifically, older adults with greater Internet skills benefit relatively more from engaging in specific online social activities more often with respect to online social bridging. These results imply that digital inequalities may put older adults who are less skilled in using the Internet at a disadvantage when it comes to building social capital from online social engagement. Thus, while social media has potential positive implications for well-being among older adults, the current manifestation of this does not suggest equitable distribution of those benefits across different older users.

### THE EFFECTS OF SIMULATION-BASED EMPATHY ENHANCEMENT PROGRAM FOR CARER OF THE ELDERLY ON COUNSELLING

Sangmi Park,[1] Tae Hui Kim,[2] Tae Rim Um,[2] Kyuwon Lee,[2] Jisoo Jung,[2] Myounghee Hong,[2] Soyeon Choi,[2] and Areum Han[3], *1. Wonju Severance Christian Hospital, Wonju, Korea, Republic of, 2. Wonju Severance Christian Hospital, Wonju, Gangwon, Korea, Republic of, 3. Yonsei University, Wonju, Gangwon, Korea, Republic of*

Empathy of the caregiver can influence both the caregiver's performance and the receiver's enhanced life. The aim of this study is to examine whether Simulation-based Empathy Enhancement program for the Carer of the Elderly (SEE-C) is effective in increasing care receivers' session satisfaction and positive emotional change. We developed SEE-C by modifying the Dementia Live(TM) program and adding with a brief mindfulness. The effect on counselling was assessed using the Session Evaluation Questionnaire (SEQ), which is self-report tool asking the client about their experience with the session just ended. A total of 100 older adults living alone were interviewed by caregivers who experienced SEE-C (n=12) and by non-experienced (n=12). Participants in this study were randomly assigned to each of the two caregiver groups, and were interviewed about demographics, health and emotional status, and lifestyle using the same protocols. Analysis of covariance was conducted, controlling variables of age of subjects and caregivers' months of career, which were found to differ significantly between the two groups. Among the four subcategories of SEQ, the experimental group reported significantly higher scores than the control group in three subcategories of session-depth (F(1, 96)=9.647, P=.002), session-smoothness (F(1, 96)=13.699, p<.001), emotion-positive (F(1, 96)=18.056, p<.001), with the exception of emotion-alertness (F(1, 96)=0.366, p=.546). These results suggest that SEE-C could have a positive impact on interviewing the elderly in terms of improving the capacity of the interviewer and raising the satisfaction of the interviewee.

### USE OF A HOME SYMPTOM MANAGEMENT ZONE TOOL IN GERIATRIC PATIENTS UNDERGOING CANCER TREATMENT

Mary F. Baker,[1] and Debra Shockey[2], *1. Virginia Commonwealth University School of Nursing, Richmond, Virginia, United States, 2. Virginia Commonwealth University, Richmond, Virginia, United States*

Adult oncology practices are caring for a growing number of older adults with cancer. Evidence suggests that patients with cancer have higher emergency department (ED) utilization, particularly in patients 65 years of age and older and in those whose symptoms are poorly controlled. The ED is not the most appropriate nor most cost-effective option for addressing urgent care needs of patients undergoing cancer treatment. This quality improvement project is being conducted in a community medical oncology practice during usual cancer treatment education. Older adult patients starting or changing cancer treatment are presented with a zone tool (green-yellow-red) to inform decision-making at home regarding level of severity of current symptoms and recommended actions (e.g., you are stable, you need to call the office, you need to go immediately to the ED). The tool has been distributed to 28 patients with a goal of at least 30 by 9/20/19. Outcome measures include the number of ED visits in the intervention group compared to a pre-intervention sample of older adults from the same medical oncology office. Other outcome measures are acceptability of zone tool among clinical staff and patients. Preliminary data suggest suggest acceptability among staff and patients; and ease of use among clinical staff. Tracking of visits to the ED in the sample population and surveys of patients and staff are ongoing through October 2019.

### DELIRIUM SEVERITY AND COGNITIVE OUTCOMES

Sarah A. Welch,[1] E. Wesley Ely,[1] and Jin H. Han[2], *1. Vanderbilt University Medical Center, Nashville, Tennessee, United States, 2. Department of Emergency Medicine, Department of Medicine, Vanderbilt University Medical Center, Nashville, Tennessee, United States*

Delirium is heterogeneous and can vary by severity. The impact of its severity is unclear. This prospective cohort study enrolled emergency department (ED) patients who were > 65 years old and admitted to the hospital. Delirium severity was determined by the Confusion Assessment Method for the Intensive Care Unit Severity (CAM-ICU-S) Scale measured at enrollment. This scale ranges from 0 (no symptoms) to 7 (most severe). Premorbid and 6-month cognition were determined using the Informant Questionnaire on Cognitive Decline in the Elderly (IQCODE) which ranges from 1 to 5 (severe cognitive impairment). Multiple linear regression was performed to determine if delirium severity was associated with 6-month function and cognition adjusted for pre-illness IQCODE, baseline functional status, comorbidity burden, severity of illness, and central nervous system diagnosis. Two-factor interactions were incorporated to determine if pre-illness cognition modified the relationship between delirium severity as measured by the CAM-ICU-S and 6-month cognition. A total of 228 older patients were enrolled in the ED and of these, 105 were delirious. Median (interquartile range) CAM-ICU-S scores was 2 (0, 5). In patients with intact pre-illness cognition, a point increase in the CAM-ICU-S significantly increased the 6-month IQCODE by 0.06 (95%CI: 0.01 to 0.12) points. In patients with impaired pre-illness cognition, there was no significant association between the CAM-ICU-S and 6-month IQCODE. Thus delirium severity is associated with poorer 6-month cognition, but this association is more prominent in those with intact pre-illness cognition.

### USING SOMATOSENSORY GAMES TO IMPROVE HEALTH AND SOCIAL ENGAGEMENT OF TAIWANESE OLDER ADULTS IN A COMMUNITY

Duan-Rung Chen,[1] Ya-Mei Chen,[1] and Winston Tseng Tseng[2], *1. National Taiwan University, Taipei, Taiwan, 2. School of Public Health, California, United States*

Objective. Playing games has become a new way to enhance the physical activity, quality of life, social engagement of older adults. This study aims to conduct a 6-month somatosensory game program, inviting older adults to play Microsoft Xbox Kinect games and study whether games can bring benefits to them. Methods. A total of 70 community-dwelling older adults (35 as experimental group, 35 as controls) were recruited. The experiment group played somatosensory games twice per week in a local health center. These games contained three types of categories: 1) Tournament games (for upper limb and lower limb); 2) Single games (for aerobic exercise and muscle training);3) Puzzle games (for collaboration and group dynamics): Results. After 6 months, in the experiment group, the body mass index decreased from 23.45 to 23.29 (p<0.03). In muscular endurance category, 30 second chair rise jumped from 18 to 23.07 (p<0.0001). And 2 min leg lifting increased from 119.48 to 137.75 (p<0.001). In the flexibility category, back scratch test from right hand on top and left hand on top both improved (p<0.026; p<0.46, respectively). Trend analysis indicates that the improvements in 30 second chair rise (p<0.0001) and 2 min leg lifting (p<0.001) is linear. The intensity of social interactions increased noticeably in the experiment group. The SF8 health survey also revealed that experimental group perceived lower level of bodily pain. No significant improvement in the control group in all categories. Conclusion. Playing somatosensory games regularly may help older adults physically and socially.

### EXTERNAL HOME DISREPAIR ASSOCIATED WITH OBJECTIVE SLEEP DISTURBANCES IN LOW-INCOME OLDER ADULTS WITH DISABILITIES

Safiyyah M. Okoye,[1] Nancy Perrin,[2] Sarah Szanton,[3] and Adam P. Spira[4], *1. Johns Hopkins University Bloomberg School of Public Health, Baltimore, Maryland, United States, 2. Johns Hopkins University, Baltimore, Maryland, United States, 3. Johns Hopkins School of Nursing, Baltimore, Maryland, United States, 4. Johns Hopkins Bloomberg School of Public Health, Baltimore, Maryland, United States*

Sleep disturbances are linked to poor health, loss of independence and mortality in older adults. Rates of poor sleep are higher among socioeconomically disadvantaged older adults. Understanding how environmental factors may affect sleep in this population could lead to interventions to improve sleep-related health outcomes. We determined cross-sectional associations of home and neighborhood conditions with sleep parameters, measured by wrist actigraphy, in 136 low-income, predominantly African-American older adults with disabilities. Primary predictors were third-party-rated objective indicators of disrepair or disorder based on: 1) inside-home conditions (e.g., evidence of pests, tripping hazards, clutter); 2) outside-home conditions (e.g., broken windows, crumbling foundation); and 3) neighborhood conditions (e.g., litter, graffiti, vacant buildings). Outcomes were actigraphic total sleep time (TST; total number of minutes in bed spent asleep), wake time after sleep onset (WASO; total number of minutes spent awake after initially falling asleep), and sleep efficiency (SE; % of time in bed spent asleep). Presence of one or more outside-home conditions indicating disrepair or disorder was associated with 36.3-minutes shorter TST, 18.1-minutes more WASO, and 4.7% lower SE (all p <0.05). Conditions inside the home and of the neighborhood were not associated with sleep. These preliminary findings suggest that among low-income older adults with disabilities, external-home disrepair is associated with objectively measured WASO, TST, and SE. External-home disrepair may affect sleep through physical, psychosocial and behavioral pathways. Further research should examine longitudinal associations between external-home conditions and objectively measured sleep in socioeconomically disadvantaged older adults.

### EVALUATING AN INTERVENTION TO PREVENT SELF-NEGLECT IN VULNERABLE ADULT HEALTHCARE PATIENTS

Miriam Rose,[1] Farida Ejaz,[1] Courtney Reynolds,[1] and Minzhi Ye[1], *1. Benjamin Rose Institute on Aging, Cleveland, Ohio, United States*

Alleged self-neglect is the most commonly reported type of abuse to Adult Protective Service (APS) agencies nationwide. Researchers, healthcare practitioners and APS staff in Texas collaborated on a project funded by the U.S. Administration for Community Living to develop and evaluate an intervention to prevent self-neglect in older and/or disabled adults. Nineteen primary care clinics in a large healthcare system were randomized to intervention (intensive case management over a four-month period) and control (usual healthcare) groups. Patients with risk factors for self-neglect in these clinics were randomly selected, and 480 patients consented to participate in the study. Baseline EMR data indicated the most common risk factors for self-neglect included depression (54% of these participants), dependence in activities of daily living (28%), and dementia (27%). Social workers conducted a home visit with 287 intervention clinic patients, identified their needs, developed a care plan, and followed up regularly with patients. Based on the Adult Self-Neglect Assessment, 61% were identified as having concerns related to self-neglect. Their most frequently identified areas of need for help were food assistance/nutrition (54%), functional limitations (40%), social isolation (36%), home modifications (34%), and mental health issues (32%). Along with public assistance, and referrals to home- and community-based services, findings are promising for preventing patients from becoming self-neglecting in the future. Only 7 intervention clinic participants were reported by project staff to APS for suspected abuse, neglect or exploitation during the 16-month study. This type of multi-disciplinary collaboration can inform development of evidence-based innovations in practice and policy.

### INTEGRATING HEALTH IT INTO CLINICAL WORKFLOW FOR DEMENTIA CARE: PROVIDER AND CAREGIVER PERSPECTIVES

Nicole Ruggiano,[1] Ellen Brown,[2] and Peter Clarke[2], *1. University of Alabama, Tuscaloosa, Alabama, United States, 2. Florida International University, Miami, Florida, United States*

Each year, more than 5.5 million people in the U.S. are affected by Alzheimer's disease and related dementias (AD/RD), most of whom reside within the community, receive ongoing care from family caregivers, and medically managed by primary care providers. However, AD/RD caregiving and care is often compromised and costly, with medical costs projected to increase from $203 billion in 2013 to $1.2 trillion in 2050. A significant barrier to AD/RD care relates to collecting the health information needed for clinical decision making, due to patients' communication problems associated with AD/RD, insufficient time to collect relevant patient information from caregivers during the medical visit, and lack of communication with in-home health and support providers. To address these issues, the research team has developed CareHeroes, a multi-functional web and mobile app designed to increase the quality of communication about patient symptoms between the caregivers and providers of people with dementia. This study integrates qualitative findings from two studies evaluating CareHeroes that involved: (a) interviews and a focus group with AD/RD caregivers and (b) focus groups with clinical staff at two memory clinics. Findings indicate that caregivers want technologies that can inform clinical decision making on a regular basis while providers emphasize the use of information technologies that prepare patients and caregivers for upcoming medical appointments. Implications for practice, research, and policy are discussed.

### MULTIMORBIDITY RESILIENCE IN COMMUNITY-RESIDING OLDER ADULTS: MEASUREMENT AND HEALTH OUTCOMES

Yingzhi Xu,[1] Eleanor McConnell,[1]
Tingzhong (Michelle) Xue,[1] and Kirsten Corazzini[2],
*1. Duke University, Durham, North Carolina, United States,*
*2. University of Maryland School of Nursing, Baltimore, Maryland, United States*

Multimorbidity is widespread, costly, and associated with a range of deleterious outcomes; it affects an estimated 67-80% of older adults. This study tests the validity of a multimorbidity resilience index developed in a Canadian sample of older adults by Wister et al., (2018), with a U.S.-based sample, using National Social Life, Health, and Aging Project (NSHAP) data, and draws upon the index to investigate the effects of resilience on outcomes over time. We mapped Wister et al.'s (2018) index to NSHAP measures, and assessed cross-sectional associations with health outcomes, using logistic regression. To assess the effects of resilience on health outcomes over time, we estimated mixed models of the relationships between resilience on outcomes over a 5-year interval. Total resilience was consistently associated with improved outcomes, including pain level (OR=.51, CI .41-.64); reduced utilization (OR=.45, CI .33-.60); improved mental health (OR=9.13, CI 6.20-13.44); self-rated physical health (OR=6.97, CI 4.76 10.19); and sleep quality (OR=3.66, CI 2.76-4.86). Longitudinal model results indicate change in multimorbidity resilience and number of chronic diseases predict ($\alpha$=.001) pain level and self-rated physical health. Effects were moderated by socio-demographic factors. Our findings validate Wister et al.'s (2018) resilience index in a U.S. sample, supporting the importance of this measure to capture core components of older adults' capacity to sustain well-being in the context of living with multiple, chronic conditions. Results from the longitudinal models provide beginning insights into the effects of resilience on symptom experience and perceived health over time, highlighting potential levers for change.

### PERIPHERAL ARTERIAL DISEASE AND SEDENTARY TIME IN OLDER ADULTS

Dottington Fullwood,[1] Mary McDermott,[2] and
Todd M. Manini[1], *1. University of Florida, Gainesville, Florida, United States, 2. Northwestern University, Chicago, Illinois, United States*

Peripheral artery disease (PAD) is associated with increased rates of physical disability in older adults, yet few interventions exist to reduce this risk. Intermittent claudication, exertional calf symptoms that resolve within 10 minutes of rest, is the classic symptom for PAD, but many people with PAD are absent of these symptoms. Ankle brachial index (ABI) is a non-invasive measure that identifies the presence and severity of lower extremity arterial obstruction due to atherosclerosis. We studied whether abnormal ABI is associated with increased time spent in sedentary behavior in a large sample of community-dwelling older men and women (70-89 years) enrolled in the Lifestyle Interventions and Independence for Elders (LIFE) study. Older adults underwent an ABI test and then wore a tri-axial accelerometer on the hip for up to seven days. Total accumulated sedentary time and sedentary time spent in bout lengths of 10minutes or more, 30 minutes or more, and 60 minutes or more were calculated. ABI values, divided into PAD (<.90, n=156) and non-PAD (0.90 - 1.40, n=960), were evaluated in covariate-adjusted regression models adjusting for age, body mass index, comorbidity presence, gender and smoking. Older adults with PAD had significantly higher total accumulated time spent in sedentary behavior than those without PAD (13.1 minutes per day, p<0.02). No associations were found with longer bout lengths of sedentary time. These results suggest that older adults with PAD accumulate more time in shorter bouts of sedentary behavior. Future interventions may consider targeting short sedentary bout-lengths for reducing PAD symptoms.

### HEAT KILLED LB. PARACASEI OR CELL WALL LIPOTEICHOIC ACID AMELIORATES AGE-RELATED LEAKY GUT AND INFLAMMATION

Shaohua Wang,[1] Shokouh Ahmadi,[2] Ravinder Nagpal,[2]
Shalini Jain,[3] Sidharth Mishra,[2] Stephen Kritchevsky,[2]
Dalane Kitzman,[2] and Hariom Yadav[3], *1. Wake Forest School of Medicine, Winston-Salem, United States, 2. Wake Forest School of Medicine, Winston-Salem, North Carolina, United States, 3. Wake Forest Medical Center, Winston-Salem, North Carolina, United States*

Increased inflammation associated with leaky gut is a major risk factor for morbidity and mortality in older adults; however, successful preventive and therapeutic strategies are not available to ameliorate these conditions. In this study, we demonstrate that a human-origin Lactobacillus paracasei D3-5 strain (D3-5), even when dead, extended life span of C. elegans. In addition, feeding D3-5 to older mice (>79 weeks) prevented high fat diet (HFD)-induced metabolic dysfunction and decreased leaky gut and inflammation, which were associated with improved physical and cognitive function. D3-5 feeding significantly increased mucin production and proportionately, the abundance of mucin-degrading bacteria Akkermansia muciniphila was also increased. Mechanistically, we show that the cell wall of D3-5 contains lipoteichoic acid (LTA), which enhanced mucin (Muc2) expression via TLR-2/p38-MAPK pathway, which in turn reduced age-related leaky gut and inflammation. This study indicates that the D3-5 and its LTA can prevent/treat age-related leaky gut and inflammation.

### MAPPING COLLABORATION RELATIONSHIPS IN AGE-FRIENDLY COMMUNITIES INITIATIVES

Althea Pestine-Stevens[1], *1. School of Social Work, Rutgers University, New Brunswick, United States*

Age-friendly initiatives (AFIs) convene stakeholders throughout a community to improve social and built environments for long lives. Despite rapid growth in AFIs worldwide, research on how AFIs operate, sustain, and impact their communities has been slow to develop. This poster presents a new social network analysis (SNA) survey instrument, which can be used to advance research on AFIs by identifying key relationships and activities that drive collaborative community change processes. The survey asks a representative from each organizational member of an AFI coalition to select "partner" organizations with whom they have worked on AFI goals. Respondents then select from a list of activities in which they engage with each partnering organization. The questions regarding collaboration activities were developed based on theories of inter-sectoral and community-wide collaboration, SNA studies of collaboration in health prevention networks, and qualitative interviews with leaders of an established AFI coalition in Upstate New York. This tool was administered with respondents from 18 organizations comprising the New York coalition. Administration of the pilot indicated that the questions were acceptable and feasible for participants to complete. Analysis of the data through SNA software (UCINET) yielded visual maps to understand dimensions of the AFI's inter-organizational network. Local government offices and nonprofits emerged as central network nodes for connecting stakeholders. Findings also indicated denser networks around lower-intensity collaboration activities, such as sharing information, relative to higher-intensity activities, such as sharing financial resources. Implications of the tool for future research on the development of AFIs across diverse community contexts are discussed.

### QUANTIFYING THE BURDEN OF HOSPITALIZED DAYS IN MEDICARE BENEFICIARIES WITH MULTIMORBIDITY

Melissa Y. Wei,[1] Nicholas Tilton,[1] and Kenneth J. Mukamal[2], *1. Division of General Medicine, University of Michigan, Ann Arbor, Michigan, United States, 2. Division of General Medicine, Beth Israel Deaconess Medical Center and Harvard Medical School, Brookline, Massachusetts, United States*

Multimorbidity predicts several health outcomes including physical and cognitive functioning and mortality. Multimorbidity also predicts healthcare burden, but this has not been studied using a patient-centered measure that weights conditions by their impact on physical functioning. Health and Retirement Study participants were continuously enrolled in Medicare Parts A/B 1-year before and after the 2012-2013 HRS interview. Medicare claims were used to compute ICD-coded multimorbidity-weighted index (MWI-ICD) by summing physical functioning-weighted conditions. Given excess observations of zero hospital days (78.1%), we used zero-inflated Poisson regression to examine the association between multimorbidity and hospitalized days. First, logit models predicted membership into the zero-coded "no hospitalizations" group. Second, Poisson models predicted hospital days for participants not in the zero-coded group. We converted adjusted regression coefficients to odds ratios to report odds of zero hospitalized days. To compare model fit between MWI-ICD and simple disease count we used AICs. The final sample N=5201 participants had mean age 77.6+/-11.6 years, MWI-ICD 16.5+/-11.6, and 1.9+/-6.0 (range 0-90) hospitalized days. Each 1-point increase in MWI-ICD was associated with 4.3% decreased odds of zero hospitalized days (OR=0.96, 95%CI: 0.95-0.96) and 2% increased number of expected hospitalized days (IRR=1.02, 95%CI: 1.01-1.03) over one year in adjusted models. MWI-ICD had a lower AIC than simple disease count. Multimorbidity measured with MWI-ICD was associated with a decreased odds of zero hospitalized days and an increased number of expected hospitalized days. Multimorbidity contributes greatly to patient burden through increased hospitalization and is better captured through an index weighting conditions to physical functioning.

### VALIDATION OF A BRIEF SCREEN TO IDENTIFY PERSONS WITH DEMENTIA AT RISK FOR BEHAVIORAL DISTURBANCE

Tracy L. Evans,[1] Amber Amspoker,[1] Mark E. Kunik,[1] and Srijana Shrestha[2], *1. Baylor College of Medicine, Houston, Texas, United States, 2. Wheaton College, MA, Norton, United States*

Per current guidelines, clinical assessment of persons with dementia (PWD) should include potential causes of behavioral and psychiatric problems including pain, depression, and caregiver-patient relationship quality. Many validated assessment tools are available; however, administering a battery of instruments is not practical in most clinical settings. Objectives of this secondary analysis are to 1) evaluate the construct validity of brief screens (1-3 questions each) for pain, depression, and relationship strain by examining their associations with validated measures (Geriatric Depression Scale, Modified Philadelphia Pain Scale, Zarit Burden Interview, Mutuality Scale) and medication use and 2) evaluate the predictive validity of each individual screen and the screens as a set (number positive) by examining their associations with frequency of disruptive behaviors on the Revised Memory and Behavior Problem Checklist. PWDs (n=228) were included in the original trial if the PWD or the caregiver endorsed one or more of the three screens. There was evidence of good convergent and discriminate validity for each individual screen (p's < 0.01). Although only the relationship screen was individually associated with frequency of disruptive behaviors ($p < 0.00010$), the total number of screens endorsed was positively associated with this frequency ($F(2,225) = 5.50$, $p = 0.005$). In this sample, the brief screening questions showed good construct and predictive validity. Further studies are needed to determine if they can be used to identify patients with depression, pain, and/or caregiver-patient relationship problems in the clinical setting.

### FEASIBILITY OF ONLINE SYNCHRONOUS CAREGIVER DEMENTIA COACHING FOR REJECTION-OF-CARE BEHAVIORS

Rita A. Jablonski,[1] Winstead Vicki,[1] Giovanna Pilonieta,[1] and David Geldmacher[1], *1. University of Alabama at Birmingham, Birmingham, Alabama, United States*

Problem: Two-thirds of family caregivers of persons living with dementia have encountered rejection-of-care behavior, usually during assistance with activities of daily living. Purpose: To describe the feasibility of an online videoconferencing platform to help caregivers prevent and reduce ROC behavior. Design: Quasi-experimental. Sample: Twenty-six family caregivers: 54% female, 77% white, 62% spouses (31% wives, 31% husbands), mean age 65 years, and college-educated (92%). Their care recipients were 61% female, 77% white, mean age of 76 years, and college-educated

(88%). Procedure: Family caregivers who endorsed problematic ROC behaviors in their care recipients participated in six online, individual, synchronous, sequential, and weekly 1-hour coaching sessions. We measured general burden (Zarit Burden Inventory) and the frequency, severity, and associated distress of responsive behaviors (Neuropsychiatric Inventory Questionnaire). Data collection intervals were before coaching (baseline), immediately after the final session (Time 1), and six weeks (Time 2) and 12 weeks (Time 3) after the final session, respectively. Results: Caregivers reported less overall distress scores at Time 2 compared to baseline: 13.58 (SD 6.44) versus 17.42 (SD 6.90), t=2.56, p=0.017). Distress scores returned to baseline by Time 3. Caregivers reported less severe ROC behavior at Time 2 which was not statistically significant. Burden remained unchanged throughout the 24 weeks. Conclusion: Online caregiver coaching that targets ROC behavior is feasible. Qualitative review of the encounters suggests that a longer period of intervention and an outcome measure more sensitive to ROC effects on activities of daily living may be needed in future studies.

### ATTACHMENT SECURITY AND DEPRESSIVE SYMPTOMS IN OLDER-ADULT MARRIAGES

Sumaiyah U. Syed,[1] and Joan K. Monin[2], *1. Yale University, New Haven, Connecticut, United States, 2. Yale School of Public Health, New Haven, Connecticut, United States*

Attachment theory emphasizes attachment security, providing and receiving communication of safety and emotional support, as one of the most fundamental needs in close relationships across the lifespan. Having an insecure attachment style, anxious or avoidant attachment, has been related to depressive symptoms in mostly young adult marriages. This study examined the interpersonal associations between attachment anxiety, attachment avoidance, and depressive symptoms in 98 older adult couples, using self-report measures. The Actor Partner Interdependence model was used to analyze the data. Results show that one partner's anxious attachment was significantly positively associated with their own greater depressive symptoms (β=2.10, p=0.000). This effect was stronger for husbands than for wives (β=1.13, p=0.002). Results remained when controlling for age, physical functioning, marital length, and socio-demographics. There were no significant cross-partner associations. Findings suggest that attachment anxiety may be particularly impactful for depressive symptoms in husbands.

### ABO GENE AND AGING-RELATED OUTCOMES IN OVER 379,000 EUROPEAN-DESCENT PARTICIPANTS OF UK BIOBANK

Chia-Ling Kuo,[1] Ziwei Pan,[2] Luke C. Pilling,[3] George A. Kuchel,[4] and David Melzer[5], *1. University of Connecticut, Farmington, Connecticut, United States, 2. University of Connecticut Health, Farmington, Connecticut, United States, 3. University of Exeter, Exeter, United Kingdom, 4. University of Connecticut School of Medicine, Farmington, Connecticut, United States, 5. University of Exeter, Exeter, Devon, United Kingdom*

Genetic variants associated with multiple traits are potential targets to delay aging. Drugs supported by genetic evidence are twice as likely to succeed in human trials. Single nucleotide polymorphisms (SNPs) in the ABO gene were reported by genome-wide associations studies, associated with breast cancer, coronary artery disease, stroke, and type II diabetes. To evaluate the potential of ABO gene as a target for aging intervention, we conducted a phenome-wide association study (PheWAS) to associate the genotype-derived blood types (based on two SNPs in the ABO gene) with a wide range of aging-related outcomes. The genotype-derived blood type distribution (41% A, 9% B, 3% AB, and 47% O) is similar to that reported by the UK National Health Service (39% A, 10% B, 3% AB, and 48% O). The blood type was not associated with parental lifespan or extreme parental longevity. Non-O types had modestly lower risk of hypertension than O type but higher risk of type II diabetes and pancreatic cancer (e.g., OR= 1.37, 95% CI: 1.17 to 1.59 comparing A to O). Additionally, "A" type had modestly higher risk of breast cancer than other types. "A" allele (in A or AB type) was associated with lower heel bone mineral density, alkaline phosphatase (e.g., 0.41 standard deviation lower in A than that in O, 95% CI: -0.42 to -0.40), and hemoglobin concentration, but higher HbA1c, direct LDL, and cholesterol. Blood types with A allele(s) are less favored than other blood types, which however are adversely associated with some aging traits.

### SEX HORMONES AND ARTHRITIS IN A LONG-LIVED ANIMAL MODEL, THE ELEPHANT

Daniella E. Chusyd,[1] Janine Brown,[2] Tim R. Nagy,[3] Timothy Griffin,[4] Virginia Kraus,[5] Janet Huebner,[5] David Allison,[6] and Steven N. Austad[7], *1. Indiana University, Bloomington, Indiana, United States, 2. Smithsonian Conservation Biology Institute, Front Royal, Virginia, United States, 3. Department of Nutrition Sciences, University of Alabama at Birmingham, Birmingham, Alabama, United States, 4. Oklahoma Medical Research Foundation, Oklahoma City, Oklahoma, United States, 5. Duke University, Durham, North Carolina, United States, 6. Indiana University, Bloomington, Indiana, United States, 7. Department of Biology, University of Alabama at Birmingham, Birmingham, Alabama, United States*

Arthritis affects women more so than men, particularly after menopause. Sex hormones may play a role in arthritis development, yet this is still unclear. Elephants have a similar lifespan to humans and develop arthritis and joint issues (musculoskeletal impairments (MI)), but do not undergo menopause. Interestingly, many female zoo elephants do not exhibit normal ovarian cycles, closely resembling that in post-menopausal women. Objectives: 1) examine ovarian cyclicity status with MI (Asians: N=84; Africans: N=83); 2) determine whether gonadotropin and sex hormones are reduced in cycling elephants with MI (Asians: N=16; Africans: N=26); and 3) evaluate whether serum CTXI, a marker of type 1 collage degradation, is associated with MI in elephants (Asians: N=40; Africans: N=32). We used longitudinal serum and health data on ovarian cycling and non-cycling zoo African and Asian elephants with and without MI. Serum progestagen, leutinizing hormone, follicle stimulating hormone, and CTXI concentrations were measured. Cyclicity status was based on progestagen patterns. Zoo veterinarians classified the elephant with MI based on clinical exams and observations. In both Asian and African elephants, MI was significantly associated with cyclicity status (P=0.015, 0.032, respectively), but not independent of age (P=0.251, 0.091, respectively). There were no significant differences in any hormone concentration, during either the luteal or follicular phase, between elephants with or without MI (P>0.135). There were no significant differences in CTXI concentrations between age- and cycling-matched elephants with and without MI (P>0.957). Preliminary results do not provide compelling evidence that gonadotropin and sex hormones influence MI development in elephants.

### VERIFICATION OF PAIN MEDICATION ADHERENCE IN OLDER ADULTS USING INTERACTIVE VOICE REMINDERS

Marcia Shade,[1] Kyle Rector,[2] and Kevin Kupzyk[1], *1. University of Nebraska Medical Center College of Nursing, Omaha, Nebraska, United States, 2. University of Iowa, Iowa City, Iowa, United States*

Adherence to analgesics needs to be monitored to ensure optimal pain management and avert adverse events among older adults. mHealth reminders may encourage adherence behavior, but it is unclear if medication use ensues following the reminder. The purpose of this study was to trial the use of medication event monitoring to verify the initiation of scheduled pain medication after an mHealth reminder. Methods: N=15 adults 55 and older created Google Assistant reminders to take their scheduled pain medication and write in a pain diary. A sub sample of n= 5 participants used a Medication Event Monitoring System Cap with their scheduled pain medications over 4 weeks. Data were collected on demographics, pain severity, and medication adherence. Descriptive statistics were performed. Results: Five women with ages ranging from 56-80 years, reported pain in multiple body locations. Pain severity on average was rated at 4 and at its worst 7; with pain relief ranging from 50-90%. Adherence percentages ranged from 82% to 100%. The overall latency was M = 55 min, SD = 100 min. The average latencies varied among the 5 participants; the shortest average time was 17 minutes and the longest average time was 4.5 hours. Only 15% of pain medications were taken within 5 minutes and 64% within 30 minutes of the interactive voice assistant reminder. Conclusions: It is important to ensure a behavioral intervention promotes the desired outcome. Medication event monitoring systems may help to identify non adherent behaviors when using mHealth interventions to promote pain medication adherence.

### ADJUDICATION PILOT STUDY OF CAUSE OF DEATH IN THE LONG LIFE FAMILY STUDY

Habibatou Diallo,[1] Joanne Murabito,[2] Anne B. Newman,[3] Thomas T. Perls,[1] Diane Ives,[3] and Larry Honig[4], *1. Boston University Medical Campus, Boston, Massachusetts, United States, 2. Boston University, Boston, Massachusetts, United States, 3. University of Pittsburgh, Pittsburgh, Pennsylvania, United States, 4. Columbia University, New York, New York, United States*

Background: Death certificate inaccuracy increases at older ages. The Long Life Family Study (LLFS) utilizes a physician adjudication committee to review the death certificate, medical records and a family narrative about cause of death. We report here the adjudication process and the prevalent underlying causes of death for a subsample of those who have died so far. Methods: We first describe the adjudication process. There were ~1,250 deaths in LLFS. We report underlying causes of death for a subset of proband generation subjects enrolled and evaluated by two LLFS study centers. Results: As of May 2019, we have adjudicated 190 deaths (98 male, 92 female) . Mean age 95 years (range 81-105 years). Top 5 causes of death for men: cancer (13%), coronary heart disease (CHD, 13%), dementia (13%), "other" (11%) and "unknown" (9%) and for women: dementia (21%), valvular heart disease (14%), coronary heart disease (12%), unknown (12%) and other (9%). Rate of death due to dementia was greater in women compared to men (CHI2 =7.33, p=0.006). Conclusions: In this pilot study, a significantly greater proportion of women died due to dementia compared to men. At least some portion of this difference may be due to the observation that women are known to survive chronic aging-related diseases more than men and thus have a greater opportunity to die from dementia at advanced ages. An additional cause to consider includes clinicians' gender bias in ascribing diagnoses in the medical records that were relied upon as part of the adjudication process.

### EVALUATION OF SMART BELT HIP PROTECTION IN A SKILLED NURSING FACILITY

Rebecca J. Tarbert,[1] and Wamis Singhatat[1], *1. Active Protective Technologies, Inc, Fort Washington, Pennsylvania, United States*

Hip fractures impact > 300,000 US older adults yearly resulting in 70,000 deaths and are expected to gain in numbers with the rising population. Residents of skilled nursing facilities are among the highest at risk of sustaining hip fractures due to increased risk of falls and frailty. These most vulnerable stand to experience the most serious results of hip fracture with > 50% resulting in total dependency and/or death. Care providers are focused on providing an environment of safety with implementation of traditionally utilized fall prevention measures. Unfortunately, the maintenance of safety in this high-risk population often comes at the price of limiting independent mobility. The utilization of passive hip protector padding for those recognized as being at high risk of hip fracture can decrease the risk of hip fracture by 82%; however, challenges to adherence of hip protectors limit the effectiveness of this widely utilized measure. Emerging technology in the form of a smart belt was evaluated in a skilled nursing setting to offer insight into efficacy and user adherence. The smart belt is capable of sensing when the wearer is experiencing a motion that would likely result in a fall onto the hip, deploy an anatomically conforming airbag and alert caregivers that a fall has occurred. The embedding of the hip protection technology into care planning led to daily patient utilization totaling over 3000 hours. Specific findings of the user derived motion and experience will be articulated through case studies and illustration of the technology captured motion data.

### HARNESSING VOICE-ASSISTED TECHNOLOGY AND IN-HOME SENSORS TO MANAGE OLDER ADULT HEALTH: A USER PREFERENCE STUDY

Erin L. Robinson,[1] Geunhye Park,[2] Shradha Shalini,[3] Trevor Levins,[3] Kari R. Lane,[3] Marjorie Skubic,[3] and

Brianna Markway[4], *1. University of Missouri, School of Social Work, Columbia, Missouri, United States, 2. University of Missouri-Columbia, Columbia, Missouri, United States, 3. University of Missouri, Columbia, Missouri, United States, 4. University of Missouri, College of Engineering, Columbia, Missouri, United States*

In recent years voice-assisted technologies, such as the Amazon Echo Show and Google Home, have been harnessed to help older adults manage their health. However, little is known about the use of such technologies in combination with in-home sensor systems to help older adults age in place. Therefore, this research explored user preferences of older adults and a designated family member/friend in using voice-assisted technologies to retrieve in-home sensor-generated health information, such as fall risk and other early indicators of health changes. Seventeen dyad interviews were conducted with known pairs of older adults (Mean age=75; 56% female) and a family member/friend (Mean age=64; 89% female). Participants were given a description of the technology and its capabilities, and then were instructed to interact with each device using a prepared scenario. Participants asked each device health-related questions to elicit pre-programmed information for the respective scenarios and provided user experience feedback for each device. At the end of the interview, participants completed a speech recognition test for each device and a technology acceptance survey. Overall acceptance of the technology was high, and participants believed that using voice-assisted technologies to retrieve sensor-generated health information would be beneficial in managing their health or providing care to a family member/friend. However, advantages and disadvantages exist with each device and the Google Home generally performed better on the speech recognition test for each dyad pair. These findings provide valuable insight about older adults' preferences (as well as family members'/friends) in using voice-assisted technologies to manage their health.

### BINGE EATING AMONG OLDER WOMEN: PREVALENCE RATES AND HEALTH CORRELATES ACROSS THREE UNIQUE SAMPLES

Lisa S. Kilpela,[1] Carolyn B. Becker,[2] Francesca Gomez,[2] Keesha Middlemass,[3] Sara E. Espinoza,[4] and Nicolas Musi[4], *1. UT Health San Antonio, San Antonio, Texas, United States, 2. Trinity University, San Antonio, Texas, United States, 3. Howard University, Washington, District of Columbia, United States, 4. Barshop Institute for Longevity & Aging Studies, University of Texas Health Science Center, San Antonio, Texas, United States*

Emerging research indicates that older women struggle with disordered eating more frequently than once thought. Among older women, binge eating (BE; consuming unusually large amounts of food in one siting while feeling a loss of control) appears to be the most common form of disordered eating. Notably, BE is associated with significant medical morbidity, including metabolic dysfunction and chronic pain. Aging-related experiences, such as sleep disruptions following menopause, mood changes and psychosocial stressors, may increase risk for BE. This study comprises a 3-sample comparison of BE prevalence and health correlates among older women. We gathered self-reported frequencies of BE in three separate samples of older women, using three different methods and validated measures. Sample 1: N = 185 women aged 60-83; collected online via snowball sampling and Amazon MTurk; 86% White. Sample 2: N = 100 women aged 55-79; collected online via snowball sampling; 72% White; 50% Masters/Doctoral Degree; 72% married. Sample 3: N = 64 women aged 66+ living with food insecurity, collected in person at local food pantries; 65% Hispanic, 16% African American; 39% disabled status; 48% < high school/GED; 47% household income < $10,000/year. Per DSM-5 frequency criterion of BE at least weekly, we found prevalence rates ranging from 19%-26.5% across the samples; correlates included elevated negative mood, worry, and BMI, and less nutritious food consumption. Across three very different samples in terms of race/ethnicity, education, and food security status, we found consistent rates of self-reported BE at least weekly (roughly 1/5). Implications will be discussed.

### LATINO HOSPICE PATIENTS' INVOLVEMENT IN CARE DECISIONS AND PLANNING: FACTORS THAT SHAPE PARTICIPATION PREFERENCES

Susanny J. Beltran[1], *1. University of Central Florida, School of Social Work, Orlando, Florida, United States*

Patient participation in care decisions is a primary tenet of patient-centered care and has been emphasized in policies and programs shaping clinical practice. Patient self-determination and involvement is associated with improved outcomes, including patient satisfaction and compliance. However, studies exploring Latino patients' care involvement preferences find a cultural preference for limited autonomy and shared or family-based decision-making. This study describes terminally-ill older Latinos and their proxies' preferences regarding involvement in decision-making and care planning following a referral to hospice care. Semi-structured interviews were conducted with 13 hospice-enrolled Latinos 65 or older, or their proxies. The sample is predominantly female (54% of all patients; 80% of proxies) and Mexican (77%). The average age of all patients was 82 years, and of proxies 54. Patients interviewed had an average of 7 years of education and proxies an average of 11 years. Patients were receiving care in a variety of settings, including inpatient hospice units, nursing homes, and community. Interviews were transcribed verbatim in the original language and coded thematically using an inductive approach. A second coder was used to code a Spanish and an English transcript (15% of the data), and a high level of interrater reliability was obtained (k = 0.85). Findings elucidate the paths through which culture, religion, and trust in provider shape preferences for limited autonomy and planning, confirming previous findings. However, this was not replaced by a preference for family-based decision-making. Findings highlight the importance of Latinos having access to culturally-competent primary care providers and fostering lasting relationships.

### NEUROPATHOLOGY AND GAIT SPEED DECLINE IN OLDER ADULTS: THE ATHEROSCLEROSIS RISK IN COMMUNITIES (ARIC) STUDY

Kevin J. Sullivan,[1] Michael Griswold,[1] Timothy Hughes,[2] Christina E. Hugenschmidt,[2] Samuel Lockhart,[2] Thomas Mosley,[1] Rebecca Gottesman,[3] and Gwen Windham[1], *1. University of Mississippi Medical*

*Center MIND Center, Jackson, Mississippi, United States, 2. Wake Forest School of Medicine, Winston-Salem, North Carolina, United States, 3. Johns Hopkins University, Baltimore, Maryland, United States*

Neuropathological markers including amyloid-beta (Aβ) have been implicated in mobility decline in older adults, but no studies have examined the relationship between these markers and longitudinal change in gait speed in a racially diverse community-based sample. In the multi-site prospective ARIC study, a subsample of participants (n=1,978, mean age=76.3, 28.5% black) underwent brain MRI at Visit 5 (2011-13). Of these, 343 participants (mean age=75.9, 42.6% black) completed PET scans using the tracer florbetapir to estimate global brain Aβ. We investigated the relationship between four neuropathological markers [white matter hyperintensities (WMH; log2cm3), infarcts (present/absent), brain atrophy (log2cm3), and global Aβ (log2SUVR)] with cross-sectional usual pace gait speed (cm/s) over 4 meters, and change in gait speed through Visits 6 (2016-17) and 7 (2018-19). Linear regression models were adjusted for age, site, sex, education, BMI, intracranial volume, and all race interactions. Cross-sectionally, slower gait was associated with higher WMH volume (β=-2.16, 95%CI: -2.92, -1.39), infarcts (β=-5.81, 95%CI: -7.86, -3.76), and brain atrophy (β=-16.39, 95%CI: -21.07, -11.71). Longitudinally, only higher WMH volume was statistically associated with gait speed decline (β=-0.14, 95%CI: -0.28, -0.01). Global Aβ was not statistically associated with gait speed cross-sectionally (β=-.269, 95%CI: -8.11, 7.57) or longitudinally (β=-1.16, 95%CI: -2.94, 0.62). There were no significant interactions with race. Detrimental relations of cerebral small vessel disease to mobility and mobility decline were observed across race in this diverse sample. The magnitude of the Aβ association with gait speed decline was high, although not statistically significant in the smaller PET subsample.

### EXAMINING THE ROLE OF ADVERSITY AND POSITIVE LIFE EVENTS IN THE RELATION BETWEEN GRATITUDE AND WELL-BEING

Omar E. Staben,[1] Frank Infurna,[1] Kevin Grimm,[2] and Suniya Luthar[1], *1. Arizona State University, Tempe, Arizona, United States, 2. Arizona State University, Tempe, United States*

Character strengths are emerging as a key outcome of interest in midlife and old age. One key avenue that has been largely unexplored is what the key antecedents are and the moderating role of adversity and positive life events experiences. The limited current research on the topic has examined the direct relations among character strengths and well-being, whereas less is known regarding the role of negative and positive experiences, which may provide a better understanding of what contributes to character strengths. This study explores whether major life adversities (i.e. personal, family, work related) and positive life events (i.e. job promotion, engagement, vacations) experiences are associated with character strengths— namely gratitude, and well-being. We use data from a sample of participants in midlife (n=362, ages 50-65) who completed monthly online surveys for a period of two years. Multilevel models showed that greater adversity was associated with poorer well-being, whereas positive life events were predictive of higher overall well-being. Individuals' experience of fewer positive life events was associated with stronger increases in well-being when individuals expressed more gratitude. Conversely, adversity was associated with increasing well-being when individuals expressed more gratitude. Collectively, our findings provide evidence for the role of adversity and positive life experiences to the extent that character strengths have the potential to shape the course of development in adulthood. Our discussion focuses on the potential links that underlie our findings and how they can inform interventions aimed at mitigating the consequences of adversity.

### LIVING WITH MULTIPLE MYELOMA: SELF-MANAGEMENT STRATEGIES

Matthew R. LeBlanc,[1] Thomas W. LeBlanc,[1] Bryant L. Ashley,[2] Kathryn I. Pollak,[1] Donald E. Bailey,[1] and Sophia K. Smith[1], *1. Duke University School of Nursing, Durham, North Carolina, United States, 2. University of North Carolina Chapel Hill School of Nursing, Chapel Hill, North Carolina, United States*

Multiple myeloma (myeloma), is an incurable cancer of the plasma cells that affects many older adults. Over 30,000 new diagnoses and over 12,000 deaths are attributed to myeloma annually in the United States, where the median age of diagnosis is 69 years old. Dramatic improvements in survival over the past fifteen years have transformed myeloma into a chronic disease for many. The disease and its toxic, ongoing treatment lead to significant challenges for patients. In this study we explore the self-management strategies patients use to address the challenges of living with myeloma through semi-structured one-on-one interviews with myeloma patients and clinicians. Fifteen myeloma patients and ten myeloma clinicians were interviewed between September 2017 and September 2018. Self-management strategies emerged in five major categories; managing uncertainty, finding emotional strength, seeking support, medication management, and activity management. The care of MM patients has made great strides as new and more effective treatments have extended survival for many patients. Effective self-management strategies are critical in addressing the challenges of this increasingly chronic disease. Our study explores the ways myeloma patients address the many challenges they face due to their disease and its' treatment. Findings from this study could inform the development of interventions to optimize and support patients living with myeloma self-management.

### WHAT MAKES A NURSING HOME FEEL LIKE HOME? RESIDENT PERSPECTIVES

Diana Cater,[1] Serena Hasworth,[1] Diana White,[2] Ozcan Tunalilar,[2] and Jaclyn Winfree[2], *1. Portland State University Institute on Aging, Portland, Oregon, United States, 2. Portland State University, Portland, Oregon, United States*

Providing an environment that feels like home is increasingly a programmatic goal in nursing homes (NH), yet few NH studies have explored "home" as a concept through a large number of residents' voices. In the current study, 294 residents living in 32 randomly selected NH in Oregon were asked if it felt like home. We followed up with "what makes it feel like home?" or "what would make it feel more like home?" Open-ended responses were classified via open coding. Six major themes emerged: relationships, meaningful possessions, quality of care, personal autonomy, physical environment, and the impossibility of it feeling like home. Overall, 26% percent of residents found that their current setting felt like home. Among residents who perceived their current setting as feeling like home, the most prominent theme was relationships with people at the nursing home (45% of responses). Residents also emphasized the importance of having family or other meaningful relationships from outside the nursing home (22% of all responses) for an environment that was home. Among those who responded that the nursing home did not feel like home, 32% reported that nothing could replace their home. Results indicate that NH can become home environments for their residents and provides additional evidence that relationship building, personal autonomy, and physical environment are likely critical pillars for creating and sustaining feelings of home. Future research will examine variations in responses described here, including resident or facility characteristics, and length of stay.

### INCIDENCE OF COGNITIVE IMPAIRMENT AMONG U.S. CHINESE OLDER ADULTS: DOES SOCIAL ENGAGEMENT MATTER?

Dexia Kong,[1] and XinQi Dong[1], *1. Rutgers University, New Brunswick, New Jersey, United States*

The increasing diversity in U.S. aging population warrants improved understanding of risk factors of cognitive aging in minority populations. This study presents the prevalence of incident cognitive impairment (CI) among U.S. Chinese older adults; and the relationship between social engagement and incident CI. Data were obtained from the Population-based Study of Chinese Elderly in Chicago, a prospective cohort study of Chinese older adults. Baseline (collected between 2011 and 2013) and one subsequent wave of data (collected between 2013 and 2015) were used in analyses (N=2,713). Social engagement was measured by the frequency of participation in social and cognitive activities (range=0-65). Cognitive function was assessed by a battery of 5 validated instruments. Incidence of CI was defined as having a follow-up cognition score lower than 1.5 standard deviations below the mean baseline cognition score. Logistic regression analyses were conducted. Nearly 6% of the sample reported incident CI. Chinese older adults who are more socially-engaged had a lower likelihood of developing CI (odds ratio [OR] 0.94, 0.92-0.96). The relationship was consistent across cognitive domains, including episodic memory (OR 0.95, 0.92-0.97), working memory (OR 0.92, 0.88-0.95), and perceptual speed (OR 0.95, 0.92-0.98). Furthermore, older age (OR 1.12, 1.09-1.15), and lower education (OR 0.91, 0.87-0.96) were associated with incident CI. No significant association was observed between gender, income, marital status, household size, acculturation, medical morbidities, depressive symptoms, and incident CI. The findings highlight the importance of social engagement in cognitive aging. Discrepancies with prior literature and implications of these findings will be discussed.

### UNDERSTANDING ACCEPTANCE OF HEALTHCARE TECHNOLOGY BY OLDER ADULTS: IMPLICATIONS FOR ADOPTION

Maurita T. Harris,[1] and Wendy A. Rogers[1], *1. University of Illinois at Urbana Champaign, Champaign, Illinois, United States*

Healthcare Technology (HCT) can support older adults as it is not uncommon for them to be managing one or more chronic diseases at a time. Thus, understanding older adults' willingness to use HCTs can guide introduction of new technologies to help with their health management. The purpose of the research is to understand what influential factors emerged when older adults considered using new HCTs and how well current models of technology acceptance represented these factors. Twenty-three older adults (age 65-84) with hypertension completed a semi-structured interview to gain insight into these factors. During the interview, participants were first presented with a scenario to imagine and one of three HCTs (blood pressure monitor, electronic pillbox, and multifunctional healthcare robot) to consider. The qualitative coding identified: (a) facilitators: perceived advantages, easy to use, familiar, useful, and advice acceptance from a healthcare provider; (b) barriers: good for others, not good for me, disadvantages, and unfamiliar; and (c) transition factors that can lead to acceptance: with advice acceptance from a healthcare provider. These findings provide recommendations which can inform dissemination of new HCTs. Recommendations include: highlight the facilitators when introducing new HCT, understand the barriers and transition factors to give support where needed, and include the care network (i.e., people knowledgeable about the HTC and health conditions) to recommend the technology.

### EFFECTS OF SOCIAL SUPPORT NETWORK ON AMERICAN OLDER ADULTS' MEMORY FUNCTIONS

Ethan Siu Leung Cheung,[1] and Kedong Ding[2], *1. Columbia University, New York City, New York, United States, 2. Community Counseling & Mediation NYC, New York, New York, United States*

Background: Previous studies have found older adults' cognitive functions are strongly associated with their social networks, including memory. Yet, few studies have explored the influences of specific social network members, such as siblings and children. Further, little studies examined the impact of the size of older adults' social networks. Hence, this study aimed to investigate how older adults' relationships with their spouses, siblings, and children, as well as the size of their social networks, affect American older adults' memory functions. Methods: Using the 2018 data from NHATS, 5547 samples were included. We adopted a multiple logistic regression model to test the impact of social support network sizes, and how associations of social support networks varied between spouses, siblings, and children. All models were calibrated for age, gender, education, income, and race/ethnicity. Results: Analysis showed that higher socioeconomic status (more education and without Medicaid), being female, and younger age were associated with increased odds of having good self-rated memory functions. Older adults with larger social support networks (>=3 individuals) were more likely to have better self-rated memory function (adjusted odds ratio, 1.182, $p<0.05$), while holding other variables. Having a spouse also increased odds of higher self-rating memory function, in contrast to having children. Conclusion: This study highlighted the importance of having a larger social network size for older adult's memory function and indicated the necessity of developing intervention programs to expand older adults' social network size, especially for those with lower socioeconomic status.

### WHAT'S MOTIVATION GOT TO DO WITH IT? USING LATENT PROFILE ANALYSIS FOR BIOMARKERS & PHYSICAL ACTIVITY IN OLDER ADULTS

Elizabeth Teas,[1] Jay Kimiecik,[2] Rose Marie Ward,[2] and Kyle Timmerman[2], *1. Purdue University, West Lafayette, Indiana, United States, 2. Miami University, Oxford, Ohio, United States*

Heart disease is prevalent among older adults. The aim of this study was to a) identify different health behavioral motivation profiles among older adults; and b) investigate if these profiles differed in physical activity and cardiometabolic risk factors. Data on 79 participants (mean age = 68.76 years) was collected. Participants' degree of intrinsic/extrinsic motivation for diet and exercise was assessed using intuitive eating and self-determination scales. Cardiometabolic risk factors included inflammation and blood lipids. Latent profile analysis was used to identify the optimal number of groups and one-way ANOVAs assessed group differences on the variables of interest. Three profiles were found to best represent the data. The most self-determined, or most intrinsically motivated, group comprised the highest number of participants. In line with Self-Determination Theory, this group demonstrated the highest levels of objective and self-reported physical activity as well as the lowest inflammation and most optimal cholesterol measures. The group with the lowest intuitive eating and high identified exercise regulation scores exhibited the worst outcomes among the three groups. The results suggest that among older adults, different types and levels of motivation for diet and exercise can coexist and interact, and these differences produce varying health outcomes. If supported by future work, these findings can inform practitioners in developing more specific and tailored interventions relevant to older adults based on their motivational profile.

### IMPROVING THE MEASUREMENT OF EXERCISE SELF-EFFICACY TO INFORM INNOVATIVE REHABILITATION TREATMENTS

Patricia Bamonti,[1] Rebekah Harris,[2] Jennifer Moye,[3] Kelly Doherty,[3] and Jonathan Bean[4], *1. VA Boston Healthcare, Boston, Massachusetts, United States, 2. NE GRECC, VA Boston Healthcare System, Boston, Massachusetts, United States, 3. VA Boston Healthcare, Boston, Massachusetts, United States, 4. New England GRECC, VA Boston Healthcare System, Boston, Massachusetts, United States*

Exercise self-efficacy (ESE) is a consistent determinant of exercise behavior but barriers to ESE remain unexplored in medically-complex older adults. This study explored 1) concordance between Physical Therapist's (PT's) and patients' rating of their confidence to exercise; and 2) Whether patient demographic and clinical variables (e.g., depression) were related to confidence ratings. Data were collected as part of a clinical demonstration project, "Live Long Walk Strong," a PT intervention to prevent mobility decline. Patients (N=35) had a mean age of 78.26 +/- 11.12, were 86% male, 80% White, and manifested an average of 5.63+/-1.96) chronic conditions. They had an average of 3.46+/-5.00, falls in the previous year and a mean M=4.97 (SD=2.96), Geriatric Depression Scale score of 4.97+/-2.96. Patients and PT's rated exercise confidence (1= not at all to 5 = extremely). Participants' and PT's confidence ratings of the patient were highest for exercise in the clinic, followed by at home, and continuing after PT, and were moderately correlated (r = .41-.52, p < .001). PT confidence was associated with younger age (r =.48, p < .001) and lower depression (r = -.35, p = < .05), whereas these variables were not significantly associated with patient confidence. In qualitative analysis patients cite barriers to exercise: (1) physical health such as pain, fatigue, balance/previous falls, weakness; (2) memory and cognition; (3) time; (4) past experiences with PT; (5) poor social support; (6) low self-efficacy. In sum, older adults and PT's generally agree in their confidence ratings, but barriers to ESE differ.

### BUILDING AN AGE-FRIENDLY UNIVERSITY: AN INTERGENERATIONAL CAREGIVER EDUCATION PROGRAM

Becky Cordes,[1] Clare C. Luz,[1] Fei Sun,[1] and Jaewon Lee[2], *1. Michigan State University, East Lansing, Michigan, United States, 2. Michigan State University, East Lansing, United States*

A rapidly aging U.S. population means an increased need for supportive services that allows aging in place. Eighty percent of such support is provided by unpaid family caregivers, many of whom are juggling multiple obligations. There is clear evidence that mental health risks of caregiving include social isolation, depression and identity loss, and potentially "caregiver burnout". This study focuses on the lesser known impact of caregiving on college students who care for older family members and if their experiences differ from older caregivers. Presumably, students face additional stressors related to their age and school demands. Further, we posited that learning more about the aging process and interfacing with older retirees who are family caregivers through an intergenerational educational program would benefit both populations. Hosted by a campus organization, AgeAlive, which is dedicated to building an Age-Friendly University, the team's specific aims included: 1. Conducting focus groups to identify concerns among college student and retiree family caregivers related to aging, caregiving, dementia, and intergenerational relationships. 2. Developing and piloting an intergenerational, educational program with the objective of addressing these concerns, and 3. Evaluating the program's impact on measurable outcomes such as perceptions of aging, caregiver stress, and knowledge of resources. Focus group findings indicate that there are both shared concerns and divergent perspectives that provide opportunities for cross-generational learning. All program participants reported improved knowledge of aging, communication skills, and resources that will have a direct, positive impact. Lessons learned can guide development of supportive services for student and retiree caregivers.

### THE EFFECTS OF HEARING LOSS AND MOBILITY DECLINE ON COGNITIVE FUNCTION OF OLDER ADULTS

Ryota Sakurai,[1] Hisashi Kawai,[2] Shuich Yanai,[2] Hunkyung Kim,[2] Hirohiko Hirano,[2] Kazushige Ihara,[3] Shuichi Obuchi,[2] and Yoshinori Fujiwara[2], *1. Tokyo Metropolitan Institute of Gerontology, Itabashi, Tokyo, Japan, 2. Tokyo Metropolitan Institute of Gerontology,*

*Tokyo, Japan, 3. Hirosaki University Graduate School of Medicine, Aomori, Japan*

Background: Both hearing loss and mobility decline are well-known risk factors of cognitive impairment among older adults. However, the effects of the accumulation of these functional impairments are still unclear. Thus, the present study examined whether the interactive effects of hearing loss and poor gait performance contribute to cognitive impairments. Methods: Hearing loss and gait performance were assessed in 716 community-dwelling older adults at baseline. Pure-tone audiometry was conducted to determine hearing loss at 1 (i.e., low tone) and 4 kHz (i.e., high tone). Poor gait performance was defined as the lowest quartile (fourth quartile) of age- and sex-appropriate mean gait velocity. Participants were then classified into four groups according to the presence of hearing loss and poor gait performance. Cognitive function was assessed using MMSE and MoCA at baseline and four years later. Results: Older adults who had either hearing loss (low or high tone) or poor gait performance showed lower MMSE and MoCA scores at baseline. Multiple regression models showed that hearing loss and poor gait performance at baseline were significantly associated with decreased cognitive function at follow-up. Among older adults with low tone hearing loss, absence of slow gait was not associated with decreased cognitive function. Conclusions: Our results indicate the possibility that hearing loss and slow gait synergistically increase the risk of cognitive impairment. The results also suggest that the effect of slow gait on cognition exceeds the effects of hearing loss, indicating the importance of maintaining mobility in late life.

### ASSOCIATION BETWEEN THE NIH TOOLBOX COGNITION BATTERY AND GAIT IN OLDER ADULTS

Kate Hamel,[1] Sarah Barber,[2] Carl Ketcham,[1] and Kristy Lui[1], *1. San Francisco State University, San Francisco, California, United States, 2. Georgia State University, Atlanta, Georgia, United States*

The relationship between older adults' gait and cognition has been well-studied, however there is little consensus regarding a best set of measures to assess cognition. One option that has not been previously examined is the NIH Toolbox Cognition Battery (NIHT-CB), which was developed to be used across the lifespan and across research disciplines. This study examined the relationships between the seven subtests of the NIHT-CB, Trail-Making Tests A and B, and temporospatial measures of gait. One hundred sixty-seven healthy, community-dwelling older adults (115 females, 73.4 ± 4.5 years) completed these cognitive measures and also walked at their self-selected pace back-and-forth five times along a temporospatial-measuring walkway. The mean and coefficient of variation were calculated for each gait variable (stride length, width, time and velocity; stance/swing time and % of stride). After controlling for potential confounders (height, weight, age, sex, education, self-efficacy, health, exercise and falls history), executive function measures were typically the most significant cognitive predictors. More specifically, the Dimensional Change Card Sort task was the best predictor of temporal measures and stride velocity (all ps < 0.001) and the Trail-Making Test Part B was the best predictor for variability measures (five of eight p-values < 0.001). Interestingly, stride length and also stance and swing % of stride were strongly related to a measure of language - Picture Vocabulary (all ps < 0.006). The NIHT-CB appears to be a useful tool for studies of gait in older adults, particularly when used in conjunction with Trail-Making Test B.

### EVERYDAY ACTIVITIES AND DEPRESSIVE SYMPTOMS: DOES GENDER MATTER?

Celeste Beaulieu,[1] and Jeffrey E. Stokes[1], *1. University of Massachusetts Boston, Boston, Massachusetts, United States*

Previous research has suggested that informal socializing can be beneficial for mental health, whereas prior findings concerning solitary activities and mental health have been equivocal. Activity theory posits that involvement in activities – particularly social activities – can improve adults' self-concept and self-esteem, leading to improved well-being. Solitary activities may perform the same function, though without any social reinforcement. However, social engagement and mental health may both vary by gender. Thus, we examined associations of informal socializing and solitary activities with depressive symptoms among 13,387 respondents of the 2012/2014 waves of the Health and Retirement Study, and further assessed potential gender differences. Results revealed that both informal socializing and solitary activities were significantly associated with lower depressive symptoms when analyzed separately. However, when both types of activities were modeled simultaneously, only informal socializing remained significant. Further, stratified analyses revealed that informal socializing was a significant predictor of depressive symptoms among women but not men, although these coefficients were not significantly different from each other. Overall, findings suggest that both informal socializing and solitary activities may be beneficial for mental health, yet results were clearly stronger for informal socializing. Socializing may benefit mental health not only by bolstering one's self-concept, but also by linking adults with social ties and support networks that are instrumental for well-being in mid- and later life. Moreover, gender differences in effects were minimal and largely non-significant, indicating that activity involvement can bolster mental health for men and women alike.

### AN ASSESSMENT OF GERIATRIC TTRAINING AND SUPPORT NEEDS FOR CAREGIVERS AND HEALTH CARE PROFESSIONALS IN RURAL TEXAS

Susanna Luk-Jones,[1] Jennifer J. Severance,[1] Doni Green,[2] Donald Smith,[3] and Roslin Jose[1], *1. University of North Texas Health Science Center, Fort Worth, Texas, United States, 2. North Central Texas - Area Agency on Aging, Arlington, Texas, United States, 3. United Way's Area Agency on Aging of Tarrant County, Fort Worth, Texas, United States*

Of the 254 counties in Texas, 69% are rural, and three out of every four counties are designated as whole or partial Primary Care Health Professional Shortage Areas. Rural counties in Texas have a higher proportion of older adults compared to metropolitan counties, and rural older adults with Alzheimer's Disease and their caregivers face unique challenges of limited access to healthcare and lower earnings, resulting in more health-related problems. As part of a HRSA Geriatrics Workforce Enhancement Program, an academic medical center, two Area Agencies on Aging in North Texas, and an Alzheimer's Association Chapter partnered to expand access to evidence-based programs into surrounding rural counties for older adults and caregivers of persons with Alzheimer's Disease. An interdisciplinary workgroup developed focus group questionnaires for older adults, caregivers, and health care providers in rural areas to identify perceived needs, barriers to accessing services, and strategic partnerships. The North Central Texas Council of Governments conducted 11 focus groups in late 2018 and early 2019. Of these, seven consisted of family members caring for persons with memory loss. Four consisted of professionals who treat persons with memory loss. Transcription and thematic analysis identified key themes of training needs (both providers' and laypersons'), resource needs, providers' best practices, barriers to quality care, and other support needs. Practice implications of the findings include cross-sector partners and integrating telehealth platforms for program delivery. Collaboration between academic and community partners can expand access to evidence-based programs for rural and other underserved communities and address areas of need.

### EXPLORING VIDEO CHAT FOR SOCIAL ENGAGEMENT IN OLDER ADULTS WITH AND WITHOUT COGNITIVE IMPAIRMENT

Lydia T. Nguyen,[1] Qiong Nie,[2] Elizabeth Lydon,[1] Dillon Myers,[3] Alan Gibson,[4] Chantal Kerssens,[5] Raksha A. Mudar,[1] and Wendy A. Rogers[1], *1. University of Illinois at Urbana-Champaign, Champaign, Illinois, United States, 2. University of Illinois Urbana-Champaign, Illinois, United States, 3. OneClick.chat, Philadelphia, Pennsylvania, United States, 4. OneClick.chat, Philadelphia, Pennsylvania, United States, 5. Georgia Institute of Technology, Atlanta, Georgia, United States*

Social engagement technologies have the potential to benefit health and quality of life in older adults with and without mild cognitive impairment (MCI). However, technologies are rarely designed to accommodate the interests, capabilities, and limitations of these populations. In the current study, we focused on examining the potential of video chat to socially engage older adults with and without MCI by providing opportunities to link people with shared interests. Eight cognitively normal older adults (Mage: 73.3 years) and five with MCI (Mage: 70.0 years) completed a four-week experiential field trial using a novel online video chat platform called OneClick. System Usability Scale scores at both pre- and post-assessment revealed that OneClick was easy to use for older adults with and without MCI, however individuals with MCI experienced more technical issues and required additional assistance to use the system. Pre- to post- comparisons of questionnaire data revealed positive changes for the Quality of Life, Friendship/Social Isolation, and Loneliness scales in both groups. Of the 13 participants, five cognitively normal and four individuals with MCI reported that they would be interested in continuing to use the video chat system at home to connect with family and friends or to discuss topics of mutual interests. Overall, all participants enjoyed using the video chat system as a means for social engagement and showed trends for social health and quality of life benefits. This field trial illustrates the potential for video chat to provide social engagement opportunities for older adults with and without cognitive impairment.

### GERIATRIC WORKFORCE ENHANCEMENT PROGRAM: EDUCATIONAL OUTCOMES OF DEMENTIA CAREGIVER CONFERENCES IN RURAL UTAH

Kara B. Dassel,[1] Rand Rupper,[1] Katherine Supiano,[1] Troy Andersen,[1] Jorie Butler,[1] Jacqueline Telonidis,[1] and Linda S. Edelman[1], *1. University of Utah, Salt Lake City, Utah, United States*

The University of Utah was awarded federal funds to establish a Geriatrics Workforce Enhancement Program (GWEP) focused on integrating geriatric training into 20 long-term care centers across the state of Utah. One specific objective of the GWEP was to: Provide community-based dementia education to 500 community participants from four geographically diverse urban and rural locations. In year four of the project, an interdisciplinary academic and community-based team planned and implemented dementia caregiver conferences in two rural areas in Utah that included American Indian (AI) tribal communities. Educational topics included an overview of dementia, engaging in goals of care discussions, managing caregiver stress, and finding local resources. Demographic surveys and pre-post modified 15-item version of the Alzheimer's Disease Knowledge Scale (ADKS; Carpenter et al., 2009) were administered. A total of 148 participants attended the conferences. Participants were primarily female (76.6%), white (84.2%), and self-identified as family/informal caregivers (35.0%). Of the participants, 17.8% self-identified as AI. Overall, there were no significant differences on the pre- and post ADKS scores (mean scores of 12.45 and 12.5 out of 15 possible points, respectively). However, AI ADKS mean scores were lower (10.2 and 10.5, respectively; range 9-13) than white participants (12.7 for both, respectively; range 12-13) reflecting a greater need for ADRD education in AI tribal communities. The conferences were well-received with 82.5% of attendees said that the conference met their educational needs and 83.9% reported that the information would improve the type of care they provide to someone with dementia.

### LAY YOUR BURDENS DOWN: DEFERRED RELIGIOUS COPING AS A MARITAL STRESS BUFFER FOR OLDER AFRICAN AMERICAN COUPLES

Antonius D. Skipper[1], *1. Winston-Salem State University, Winston-Salem, North Carolina, United States*

Although growing bodies of research explore the dynamics of minority families, few consider the African American family from a strengths-focused perspective. Stressors that threaten familial stability, such as financial strain, health disparities, and sporadic employment, disproportionately affect African American families and contribute to high rates of dissolution. In response, African American families often rely on religion as a source of coping and resilience. While existing literature adequately captures the frequency of religious-based responses to stress, opportunities to examine the nuances and underlying processes of religious coping for African American families exist. This study addresses the need to move beyond the broad measures of religiosity and religious coping, in exchange for a more in-depth exploration of how various forms of religious coping, specifically deferred coping, impact well-being. Deferred religious coping is characterized as a complete reliance on a higher power during a time of stress. Thirty-five older African American couples (N=70 individuals), representing 11 states in the U.S., were interviewed regarding the dynamics of deferred religious coping in the marital dyad. Following the digital recording and transcription of the narrative data, the interviews were analyzed with an open coding procedure consistent with grounded theory and Numeric Content Analysis (Marks, 2015). Analyses reveal that nearly 75% of the couples interviewed utilized deferred religious coping in response to stressors that could threaten marital stability. Further, salient themes include: (1) The Three-Party, Divine Triangle of Marriage, (2) Deferring Health Problems Reduces Worry, and (3) A Healthy Work-Family-Prayer Balance. Implications for practice are also discussed.

### ALCOHOL USE SCREENING AND INTEGRATED BEHAVIORAL HEALTH IN GERIATRIC PRIMARY CARE IN THE DEEP SOUTH

Bailey Lanai,[1] Deanna Dragan,[1] Rebecca S. Allen,[1] Anne Halli-Tierney,[2] Dana Carroll,[1] and Amy Albright[1], *1. The University of Alabama, Tuscaloosa, Alabama, United States, 2. The University of Alabama College of Community Health Sciences, Tuscaloosa, Alabama, United States*

This longitudinal behavioral health surveillance and integrated care project aims to assess physical and mental health and substance use in a geriatric primary care setting. Approximately 230 patients (mean age = 76; 74% female; 16% African American) attending an interdisciplinary geriatrics clinic in Alabama have taken part in baseline behavioral health screenings since 2014. Behavioral health measures include cognitive status, self-reported mood, subjective and objective health literacy, and alcohol use. All measures are administered by clinical psychology graduate students. Patients had an average of 5.83 medical diagnoses. Only 26.2% of patients had scores indicating cognitive functioning within normal limits; 32.6% had scores indicative of mild neurocognitive disorder, and 41.2% had scores indicative of dementia. Over 80% of patients had adequate self-reported health literacy; however, measurements of objective health literacy indicated a significant number of individuals have difficulty following medical directions independently. Over 30% of patients reported clinically significant levels of depression or anxiety, and 16.5% of patients reported at least one indicator of hazardous alcohol use. Specifically, 50.7% of patients consume alcohol on at least a yearly basis with 38.2% endorsing at least one problematic drinking behavior and 11.6% scoring in the clinically significant range for alcohol misuse. Moreover, 22.7% report use of opioid pain medication. The results of this study demonstrate that routine hazardous alcohol use screening as one component of integrated behavioral health care within geriatric primary care increases detection of hazardous alcohol use among older adults.

### FAMILY DEMENTIA CAREGIVER RECRUITMENT STRATEGIES IN LONG-TERM CARE FACILITIES

Olimpia Paun,[1] Ben R. Inventor,[1] Louis Fogg,[1] Hugh Vondracek,[1] and Ilse Salinas[1], *1. Rush University College of Nursing, Chicago, Illinois, United States*

Recruitment of dementia caregivers whose family members reside in long-term care facilities (LTCFs) poses unique challenges as it traditionally relies on the assistance of facility administrators. The purpose of this presentation is to examine and evaluate new recruitment strategies to determine their effectiveness in an ongoing Stage I randomized clinical trial testing the effects of a Chronic Grief Management Intervention, Video-streamed (CGMI-V) on caregivers' mental health (grief, depression, anxiety) and facility-related outcomes (conflict with staff, satisfaction with care). A total of 144 caregivers will be randomly assigned to the CGMI-V or to the minimal treatment conditions. The initial recruitment plan was to build on already-established relationships with more than 35 LTCFs that helped recruit in a previous study. The usual approach was to offer written materials and onsite presentations about the study to facility staff and to dementia family caregivers of facility residents. Within the first six months, recruitment efforts yielded less than a dozen participants, thus we had to refine our approach. Revised recruitment strategies included the adoption of resources from the National Institute on Aging's ADORE (Alzheimer's and Dementia Outreach, Recruitment, and Engagement) and ROAR (Recruiting Older Adults into Research) platforms. This new approach included online study advertising on NIH and Alzheimer's Association research study repositories and advertising on parent institution's on-hold messaging system. Adoption of these new strategies is yielding an increase in participant screening and enrollment. Results are pending.

### PREDICTORS OF MEDICAL, SOCIAL SERVICE, AND MENTAL HEALTH USE AMONG PEOPLE WITH DEMENTIA AND THEIR CAREGIVERS

Srijana Shrestha,[1] Amber Amspoker,[1] Tracy L. Evans,[2] Melinda Stanely,[2] Jessica Freshour,[3] Sheila Richey,[2] and Mark E. Kunik[2], *1. Wheaton College, MA, Norton, Massachusetts, United States, 2. Baylor College of Medicine, Houston, Texas, United States, 3. Michael E. DeBakey VA Medical Center, Houston, Texas, United States*

People with Alzheimer's disease use more medical services (Eaker et al., 2002) and are admitted to inpatient facilities at higher rates (Zhu et al., 2015) than normal controls. In addition, social services provide support for caregivers and are associated with positive outcomes for care-recipients and their caregivers (Neville, Beattle, Fielding, & MacAndrew, 2014). Despite high level of need, utilization of mental health services and social agencies for caregiver support remains low (Goodarzi, Mele, Roberts & Holroyd-Leduc, 2017; Weber, Pirraglia & Kunik, 2011). Following the Andersen and Newman model (1973), we examined whether predisposing factors (i.e., age of the caregiver and type of PWD-caregiver relationship), needs (i.e., memory impairment, disruptive behaviors, depression, anxiety, pain, functional impairment, caregiver burden, total number of prescribed medications), and enabling factors (i.e., PWD and caregiver income, quality of the PWD-caregiver relationship) differentially predicted the presence of medical, social, and mental health service use. A total of 228 dyads (PWD and the caregiver) were included. We examined each PWD and caregiver characteristic individually (univariate models) and then as a unique predictor of each of the three service use outcomes (multivariate models). A greater number of medications uniquely predicted higher medical service use, greater pain severity and PWD income were uniquely associated with higher social service use, and a greater number of medications and increased memory impairment predicted more mental health service use. These results show that distinct factors predict use of different types of service use among PWD and their caregivers.

### ELECTRONIC MEDICAL RECORD ALGORITHM TO IDENTIFY UNDIAGNOSED (PRE)DIABETES: A CASE STUDY FROM HAWAI'I

David Stupplebeen,[1] Tetine L. Sentell,[2] Lance Ching,[3] Blythe Nett,[4] Hermina Taylor,[5] Tiffany Lemmen,[5] and Catherine M. Pirkle[2], *1. University of Hawaii at Manoa, Honolulu, Hawaii, United States, 2. Office of Public Health Studies, University of Hawaii at Manoa, Honolulu, Hawaii, United States, 3. Hawaii Department of Health, SEEO, Honolulu, Hawaii, United States, 4. Hawaii Department of Health, Honolulu, Hawaii, United States, 5. The Queen's Health System, Honolulu, Hawaii, United States*

An estimated one-quarter of United States' older adults (≥65 years) have diabetes (DM) while half have prediabetes (PreDM). Timely diagnosis can prevent disease progression, but significant proportions of PreDM/DM are undiagnosed. Among Hawai'i adults, one-third of diabetes and two-thirds of prediabetes cases are undiagnosed; rates for older adults are unknown. Algorithms integrated into Electronic Medical Records (EMR) may improve care by identifying probable undiagnosed cases in patient panels using clinical/laboratory measures. We assessed one algorithm developed by the Hawai'i Department of Health that identified individuals overdue for screening or with Pre/DM using the records of 20,362 adult patients (51.33% were >65) from a major state health system. 6,371 (31.3%) patients were excluded from analysis; they had no HbA1c screening in the past year or were overdue for screening (70%) based on standard guidelines. Of the remaining 13,991 patients, 7317 were older adults; 6130 (84%) had a PreDM (50.6%) or DM (33.2%) HbA1c value; the rest were controlled or false-positive. Of those older adults with probable PreDM/DM, 38.6% were undiagnosed. Adults >65 were significantly more likely to be flagged with undiagnosed PreDM compared to their younger counterparts (58 versus 54%, p<.001). Notably, 61% of older men flagged with PreDM were undiagnosed. Of the 5,737 patients identified with DM, 22% of those 65 were undiagnosed. Given the recognized high burden of diabetes among older adults, results indicate substantial missed opportunities for the prevention and early treatment of this condition as identified by an EMR algorithm.

### CHANGE IN PHYSICAL FUNCTION, SWALLOWING, AND NUTRITIONAL RISK IN PEOPLE WITH PARKINSON'S DISEASE OVER 4 YEARS

Dara L. LoBuono,[1] Sarah Sarah Dobiszewski,[1] Alison Tovar,[1] Skye N. Leedahl,[1] Matthew J. Delmonico,[1] Furong Xu,[1] Mahler Leslie,[1] and Ingrid E. Lofgren[1], *1. University of Rhode Island, Kingston, Rhode Island, United States*

Parkinson's disease, a neurodegenerative disease, impacts physical function, swallowing, and nutritional risk, but research has not examined how these health markers change longitudinally in one study. This study was an observational, longitudinal study with assessments conducted every 6 months over 4 years. The assessments included: the short physical performance battery (SPPB) (scores 75)), and weight in pounds. A one-way repeated measures ANOVA compared outcomes variables at baseline, years 2 and 4. Partial eta square are reported for effect size. Eight participants, baseline mean age 67.1±4.0 years and time since diagnosis=8.1±7.5 years, were assessed. At baseline, 3 participants exhibited possible mobility issues (SPPB=9.9±1.8), 2 participants exhibited a possible swallow issue (swallow speed=15.7±9.8mL/s), and 7 were at possible or at nutrition risk (64.0±13.8). At year 4, 5 participants exhibited possible mobility issues (SPPB=8.1±3.2, ($\eta p2$=0.5), 4 participants exhibited a possible swallow issue (swallow speed=13.4±10.4 ml/s, $\eta p2$=0.3), and there was a decline in perceived swallow function (77.3±12.4 vs. 74.8±16.2, $\eta p2$=0.5). Six participants were at possible or at nutrition risk. Participants experienced weight declines from baseline to year 4 (178.5±31.4 vs.168.8±24.9, $\eta p2$=0.5). The demonstrated decline in physical and swallow functioning, presence of nutrition risk, and experienced weight loss suggests that interdisciplinary intervention strategies may need to be designed and tested in this population.

### CHILD ABUSE AND ADULT MENTAL HEALTH: DOES GENDER MATTER?

Patricia M. Morton,[1] Blakelee Kemp,[2] and Frass Ahmed[3], *1. Wayne State University, Detroit, Michigan, United States, 2. Syracuse University, Syracuse, New York, United States, 3. University of Michigan, Ann Arbor, Michigan, United States*

Numerous studies have demonstrated that child abuse is associated with poor adult mental health, but few have investigated the extent to which the frequency of different types of abuse increase mental health conditions, especially at the nexus of gender. The present study examines whether parental abuse frequency and abuse perpetrator have distinct effects for men and women on three mental health outcomes—depressive symptoms, generalized anxiety, and global self-reported mental health. Data came from three waves of the National Survey of Midlife Development in the United States (MIDUS), comprising a baseline sample of 3,032 adults aged 25-74. Estimating a series of mixed effects models revealed that maternal abuse and frequent abuse during childhood were associated with poorer adult mental health during our 20-year observation period, net of childhood and adult risk factors. Specifically, maternal emotional abuse raised the risk of depression, anxiety, and lower self-rated mental health, and was more strongly associated with depression and anxiety for women than men. Compared to adults who did not experience parental abuse during childhood, adults who experienced frequent emotional and physical abuse by either parent were more likely to experience depression and anxiety and report lower ratings of mental health in adulthood. Frequent child abuse was more strongly associated with anxiety for women than men. These results demonstrate that gender differences in adult mental health have early-life antecedents. Future research investigating the long-term mental health consequences of child abuse should consider the type and magnitude of abuse as well as the perpetrator.

### SERVICE PROVIDERS' PERSPECTIVES OF UNDERREPORTING ABUSE OF OLDER ADULTS IN ALBERTA, CANADA

Carla R. Liepert,[1] Christine Walsh,[1] Kerstin Roger,[2] Donna Goodridge,[3] and Michelle Ranville[4], *1. University*

*of Calgary, Calgary, Alberta, Canada, 2. University of Manitoba, Winnipeg, Manitoba, Canada, 3. University of Saskatchewan, Saskatoon, Saskatchewan, Canada, 4. A & O: Support Services for Older Adults, Winnipeg, Manitoba, Canada*

Minimal research has investigated disclosure/non-disclosure of abuse of older adults. To address this gap, this exploratory, qualitative study gathered the perspectives on reporting of elder abuse from 10 service providers working with elder abuse survivors across Alberta. Face-to-face and online interviews were conducted, audio-recorded, transcribed, and thematically analyzed. Four major barriers to abuse disclosure for older adults were identified. First, complex parent/adult-child relationships reduce the likelihood of disclosure among older adults who are experiencing abuse perpetrated by a family member; the corollary is that disclosure for non-familial perpetrators results in higher rates of disclosure. Second, older adults residing in rural and remote locations face greater barriers to disclosure compared to those residing in urban/suburban settings. Third, older adults are unlikely to report even if they recognize that they are in a situation of abuse, as a consequence of their internalized ageist beliefs that policing and social services agencies have more pressing needs to address than their well-being. Finally, local policing bodies and legal authorities may inadequately serve older adults facing abuse, particularly in cases of non-physical abuse, due to prioritized client and community needs. This study highlights the need for additional supports and service provision for vulnerable older adults in Alberta, specifically for those residing in rural and remote regions and those dependent on family members. It also points to the critical need for a greater understanding and awareness of violence against older adults among the general public and those tasked with ensuring the safety and well-being of older adults.

### CHILD ABUSE AND ADULT MENTAL HEALTH: DOES GENDER MATTER?

Patricia M. Morton,[1] Blakelee Kemp,[2] and Frass Ahmed[3], *1. Wayne State University, Detroit, Michigan, United States, 2. Syracuse University, Syracuse, New York, United States, 3. University of Michigan, Ann Arbor, Michigan, United States*

Numerous studies have demonstrated that child abuse is associated with poor adult mental health, but few have investigated the extent to which the frequency of different types of abuse increase mental health conditions, especially at the nexus of gender. The present study examines whether parental abuse frequency and abuse perpetrator have distinct effects for men and women on three mental health outcomes—depressive symptoms, generalized anxiety, and global self-reported mental health. Data came from three waves of the National Survey of Midlife Development in the United States (MIDUS), comprising a baseline sample of 3,032 adults aged 25-74. Estimating a series of mixed effects models revealed that maternal abuse and frequent abuse during childhood were associated with poorer adult mental health during our 20-year observation period, net of childhood and adult risk factors. Specifically, maternal emotional abuse raised the risk of depression, anxiety, and lower self-rated mental health, and was more strongly associated with depression and anxiety for women than men. Compared to adults who did not experience parental abuse during childhood, adults who experienced frequent emotional and physical abuse by either parent were more likely to experience depression and anxiety and report lower ratings of mental health in adulthood. Frequent child abuse was more strongly associated with anxiety for women than men. These results demonstrate that gender differences in adult mental health have early-life antecedents. Future research investigating the long-term mental health consequences of child abuse should consider the type and magnitude of abuse as well as the perpetrator.

### SERVICE PROVIDERS' PERSPECTIVES OF UNDERREPORTING ABUSE OF OLDER ADULTS IN ALBERTA, CANADA

Carla R. Liepert,[1] Christine Walsh,[1] Kerstin Roger,[2] Donna Goodridge,[3] and Michelle Ranville[4], *1. University of Calgary, Calgary, Alberta, Canada, 2. University of Manitoba, Winnipeg, Manitoba, Canada, 3. University of Saskatchewan, Saskatoon, Saskatchewan, Canada, 4. A & O: Support Services for Older Adults, Winnipeg, Manitoba, Canada*

Minimal research has investigated disclosure/non-disclosure of abuse of older adults. To address this gap, this exploratory, qualitative study gathered the perspectives on reporting of elder abuse from 10 service providers working with elder abuse survivors across Alberta. Face-to-face and online interviews were conducted, audio-recorded, transcribed, and thematically analyzed. Four major barriers to abuse disclosure for older adults were identified. First, complex parent/adult-child relationships reduce the likelihood of disclosure among older adults who are experiencing abuse perpetrated by a family member; the corollary is that disclosure for non-familial perpetrators results in higher rates of disclosure. Second, older adults residing in rural and remote locations face greater barriers to disclosure compared to those residing in urban/suburban settings. Third, older adults are unlikely to report even if they recognize that they are in a situation of abuse, as a consequence of their internalized ageist beliefs that policing and social services agencies have more pressing needs to address than their well-being. Finally, local policing bodies and legal authorities may inadequately serve older adults facing abuse, particularly in cases of non-physical abuse, due to prioritized client and community needs. This study highlights the need for additional supports and service provision for vulnerable older adults in Alberta, specifically for those residing in rural and remote regions and those dependent on family members. It also points to the critical need for a greater understanding and awareness of violence against older adults among the general public and those tasked with ensuring the safety and well-being of older adults.

### UPDATING AND TESTING THE PASRR SCREEN IN KANSAS: REAL WORLD IMPLICATIONS

Carrie L. Wendel-Hummell,[1] Hyun Kang,[1] Darcy Sullivan,[1] Alan O'Neal,[1] Lora Swartzendruber,[1] Marion Boyd,[1] Tracey LaPierre,[1] and John Poggio[1], *1. University of Kansas, Lawrence, Kansas, United States*

Kansas is updating the PASRR (Preadmission Screen and Resident Review) Level 1 screen per new guidance from the

PASRR Technical Assistance Committee (PTAC), via a partnership between the State and university researchers. PTAC has directed states to screen for undiagnosed serious and persistent mental illness (SPMI) and also recommends screening for substance related disorders. Stakeholders were engaged through advisory workgroups and a content validity expert panel. These activities led to the creation of a revised PASRR Level-1 screen, but stakeholders also raised several concerns. PASRR law does not require Level-1 assessors to have professional training in mental health diagnoses or treatment, yet new guidelines asks them to screen for undiagnosed SPMI. Further, there are apparent discrepancies between these new guidelines and PASRR Level-2 criteria. Finally, current information management systems are not equipped to handle the higher security protocols associated substance use disorders. The draft instrument was tested with a sample of 103 nursing facility applicants by trained PASRR assessors and inter-rater reliability (IRR) was tested via a standardized vignette with 14 trained PASRR assessors. Only 3% of actual NF applicants were identified as possibly having an undiagnosed SPMI and only 43% of assessors correctly identified symptoms of a suspected SPMI in the standardized vignette, indicating poor validity and reliability in assessing for undiagnosed SPMI during the Level-1 screen. New PASRR guidelines may better ensure that nursing facility residents receive appropriate care for SPMI, however, there are many challenges to ensuring an accurate screen and supporting successful implementation.

### DIETARY INTAKE AND SELF-REPORTED NUTRITION CONCERNS OF PEOPLE WITH PARKINSON'S AND THEIR INFORMAL CAREGIVERS

Dara L. LoBuono,[1] Kyla Shea,[1] Sarah Sarah Dobiszewski,[1] Alison Tovar,[1] Skye N. Leedahl,[1] Furong Xu,[1] Mahler Leslie,[1] and Ingrid E. Lofgren[1], *1. University of Rhode Island, Kingston, Rhode Island, United States*

Dietary recommendations for managing Parkinson's disease (PD) can be confusing for people with PD (PwPD) and their informal caregivers (ICGs) who often have the responsibility to buy and prepare foods. PwPD (63-78 years of age) and their ICGs (39-75 years of age) completed two 24-hour dietary recalls and semi-structured, dyadic interviews which were conducted to gather information about dyads' (n=9) nutrition concerns. Calorie, macro- and micronutrient intake were averaged over two days and compared to the National Academy of Sciences' dietary reference intakes. Independent t-tests and the Mann-Whitney U tests compared PwPD and ICG. Interviews were audio-recorded, transcribed verbatim, and analyzed for preliminary themes. Mean calorie intake was 1766.6±658.5 kcal/d; the majority of calories were from carbohydrates. Calorie and nutrient intake between PwPD and ICG did not differ. All participants were below recommendations for fiber (17.2±7.9g/d) and potassium (2213.3±890.0mg/d) and exceeded recommendations for sodium (2741.2±1396.9mg/d) and added-sugars (59.5 ±38.3g/d). More than half of participants (55.6%) agreed an eating plan to manage PD symptoms is important and that a nutrition consultation would be helpful. Emerging themes from qualitative interviews include: nutrition concerns related to PD symptoms, managing other conditions and PD, perceptions regarding diet quality, impact of diet on PD symptoms, and complementary medicine. In conclusion, there were no differences in dietary intake between PwPD and ICG, and participants presented with lack of adherence to recommended dietary reference intakes. Sub-optimal dietary intake and self-reported nutrition concerns strongly suggest including both nutrition professionals and ICGs when providing care for PwPD.

### HAND GRIP STRENGTH ASSESSMENT IN A HOME-DELIVERED MEAL PROGRAM: FEASIBILITY AND PRELIMINARY FINDINGS

Heather L. Hutchins-Wiese,[1] and Sarah E. Walsh[1], *1. Eastern Michigan University, Ypsilanti, Michigan, United States*

Declining hand grip strength is associated with adverse health outcomes and is a relatively quick and easy-to-administer functional assessment; however, grip strength is not routinely assessed in home-delivered meal (HDM) programs. The purpose of this sub-study was to test if grip strength assessment is feasible and useful in a HDM population. Among HDM clients (n=258) who completed health assessments between 2016 and 2018, a voluntary convenience sample of 34 HDM clients (23 women, 11 men) participated in the grip strength assessment sub-study. Sub-study participants were younger (72.2+/-7.35 vs. 77.0+/-10.50 years), with no other significant differences compared to the full sample of HDM participants. The average maximum grip strength was 21.99+/-6.97 kg for the dominant hand; 16 clients were categorized as having normal, 6 intermediate, and 12 weak grip strength. Normative categories for grip strength vary by gender because men typically have higher grip strength measures than women. In this population, more men were categorized as having weak grip strength compared to women (X (2, 34) =11.44, p=.03). In men, hand grip strength tended to be lower in those who reported a fall in the previous 6 months compared to those who did not report a fall (20.8+/-7.01 vs 29.3+/-6.32, p=.079). The gender differences and 8 kg difference in grip strength relative to reported falls are clinically meaningful in this vulnerable population. Future research is needed with a larger sample of HDM clients to confirm these preliminary findings.

### THE RELATIONSHIP BETWEEN THE EXPERIENCE OF SIGNIFICANT HISTORICAL EVENTS AND COGNITIVE FUNCTION AMONG OLDER ADULTS

Stephanie M. Bergren,[1] and Gabriella Dong[1], *1. Rutgers, The State University of New Jersey, New Brunswick, New Jersey, United States*

Research has found relationships between experiencing stressful events and lower cognitive function in late life. However, there is little research about the cumulative experiences of significant historical events and cognitive function. Historical events may be unique compared to other life events due to their potential distal relationship to the individual. This study aims to examine the relationship between experiencing significant historical events and cognitive function among Chinese older adults. Data were drawn from the PINE Study, a cohort study of 3,126 US Chinese older adults collected from 2017-2019. Participants were asked if they experienced the Japanese invasion, famine, Great Leap Forward, Vietnam War, Cultural Revolution, and the

Tian'anmen Square Protests. A composite score of 0-6 was calculated to count the number experienced. Cognitive function was measured through global cognition, episodic memory, working memory, processing speed, and Chinese MMSE. Linear and quantile regression were performed. Among the participants, 1908 (61.04%) were female with mean age of 75.33 (SD=8.22) years. The average number of historical events experienced was 2.36 (SD=1.44). After adjusting for covariates, every one additional historical event experience was associated with better global cognition (b=0.26, SE=0.009, p<.01), episodic memory (b = 0.045, SE=0.012, p<.001), and processing speed (b=0.383, SE=0.135, p<.01). Number of historical events was not significantly associated with working memory or C-MMSE. The positive relationship between historical events and some cognitive domains suggests a potential resilience effect after experiencing historical events. Future research should examine whether participants found events stressful and whether there are differential relationships to cognitive function.

### EPIGENETIC PROFILES OF ALZHEIMER'S DISEASE

Kyra Thrush,[1] and Morgan E. Levine[2], *1. Yale University, New Haven, Connecticut, United States, 2. Yale University School of Medicine, New Haven, Connecticut, United States*

Although age is highly correlated with incidence of Alzheimer's Dementia (AD), the field continues to lack a clear understanding of how either normal and/or pathological aging processes drive neurodegeneration. As such, there remains a clear lack of valid and reliable clinical biomarkers to predict that disease's future development and severity. Epigenetic age based on DNA methylation (DNAm) in brain have been shown to relate to AD neuropathology and cognitive decline. However, they were not initially designed as AD biomarkers. We hypothesized that supervised and unsupervised machine learning techniques (e.g. network analysis, clustering, and regressed-based techniques) could be used to build composite scoring variables from DNAm data that are predictive of AD progression. This work analyzes the methylation of 3 brain regions (cerebellum (CBM), prefrontal cortex (PFC), striatum (ST))—totaling 1,047 brain methylation samples. The samples contain neuropathologically confirmed AD cases and controls, and is enriched for APOE4+ carriers. Detailed subject-level information concerning cognitive measures, lifestyle choices, medications, and neuropathology at death were also considered. Based on epigenome-wide association study (EWAS), we identified a CpG in AIMP2 that is a robust predictor of AD-related phenotypes. Using network analysis, we have also identified co-methylation modules that relate to multifactorial AD phenotypes. Following validation, we intend to follow-up on the biological processes and molecular pathways associated with these epigenetic signatures. In moving forward, predictors of AD diagnosis and prognostication have major implications for early detection and treatment of this major age-related disease.

### CONTEMPORARY CORE JOB FUNCTIONS OF NURSING HOME SOCIAL SERVICES DEPARTMENT IN THE UNITED STATES

Amy L. Lemke,[1] and Mercedes Bern-Klug[2], *1. University of Iowa School of Social Work, Iowa City, Iowa, United States, 2. University of Iowa, Iowa City, Iowa, United States*

Nursing home social services departments (NH SS-Ds) are involved in a myriad of duties i.e., from care manager, patient advocate to counselor. But it is unclear how much variation there is across departments. Begging the question, what are the most common core functions of NH SS-Ds in the United States? A nationally representative cross-sectional sample of 922 NH SS-Ds completed a survey on-line or via mail. They were asked to indicate the extent their department was involved in 46 job functions. The listing of functions was inspired by the literature and pilot tested. The most common core functions would include those whereby at least 2/3s of the respondents would report "always/usually" being involved. 32 of the 46 functions were rated as "always/usually" and therefore qualified as the most common core functions. The five most common were discussing with staff the discharges of long-term as well as short-stay residents; arranging services for residents returning to the community; creating care plans; and mediating issues between residents. There were only two items whereby less than 10% of departments were "always/usually" involved – working with volunteers and helping feed residents. This survey tool successfully represented core SS-D functions as remarkable similarity across SS-Ds in the US existed. Responsibilities associated with care planning and care transitions were heavily featured, thus reflecting recent federal guidelines changes. The study's results are pertinent to researchers, educators as well as nursing home administrators for role clarification and identification of training domains.

### TRAUMA THROUGH THE LIFE CYCLE IN AN IMMIGRANT POPULATION

Gabriella Dong,[1] and Stephanie M. Bergren[1], *1. Rutgers, The State University of New Jersey, New Brunswick, New Jersey, United States*

The majority of studies on traumatic events have focused on either children or younger adults, while traumatic events in older adults have not been sufficiently investigated. Older immigrants encountered a wide range of traumatic events across the life span, before and after immigration, in the origin and host countries. This study aims to provide a descriptive epidemiology of lifetime traumatic events in older Chinese Americans. The data were drawn from the Population Study of Chinese Elderly in Chicago (PINE) in 2017-2019, with a sample size of 3,126. Traumatic events were evaluated by natural disasters, personal and historical events. After examining the lifetime prevalence of natural disasters, we found typhoon (64.46%) has the highest prevalence, followed by earthquake (39.81%) and tornado (7.25%). In terms of personal events, death of a loved one (69.78%) was the most prevalent, followed by robbery (12.57%), physical assault (5.36%), fire (5.29%), divorce (5.16%), cancer (5.10%), falsely accused (2.15%), homeless (1.57%), sexual assault (0.99%), and imprisonment (0.74%). In addition, 18.91% of women experienced abortion and 11.25% of women experienced miscarriage,. With respect to historical events, most participants experienced the Cultural Revolution (73.27%), the Great Leap Forward (62.71%), and famine (60.01%). A small proportion experienced the Japanese invasion of China (27.14%), Tiananmen Square protests (7.86%), and the Vietnam war (4.78%). In our sample, women were more likely than men to encounter traumatic life events. Further studies could examine the influence of cumulative exposure to natural disasters, personal events and historical events on health outcomes of older immigrants.

### FACTORS ASSOCIATED WITH HEALTH-RELATED QUALITY OF LIFE IN KOREAN ELDERLY BY REGION

Jungmi Yun,[1] Yeongsuk Lee,[2] and Hyun-Ju Lee[2], *1. Graduate School, College of Nursing, Pusan National University, Busan, Korea, Republic of, 2. College of Nursing, Catholic University of Pusan, Busan, Korea, Republic of*

There have been inequalities in healthcare indices in the elderly by regions in Korea, and this gap is increasing. To improve their health-related quality of life (HRQoL), it is essential to identify the cause of the regional health gap and to work out solutions. This study aimed to analyze the factors associated with HRQoL in the Korean population aged over 65 years by region. Integrated raw data from the 2017 Korea Community Health Survey (n = 57,317), a cross-sectional study, were utilized. Based on the reduced Social-Ecological Model, the dependent variable was HRQoL and individual, interpersonal, and community factors were independent variables. Data were analyzed by complex-samples descriptive methods, and complex-samples general linear model (CSGLM). In both regions, individual factors (gender, age, household income, education level, drinking, BMI, sleep duration, the number of chronic illness, economic status, subjective health status, and physical activity), interpersonal factors (neighbor and friend contact frequency, religious activity, fellowship activity), and community factors (satisfaction with safety level, public transportation, and medical service) were commonly associated with HRQoL of elderly people ($p < .05$). In urban areas, household type and charity activity were associated with HRQoL, in contrast, neighboring help was associated with HRQoL in rural areas ($p < .05$). Based on the results of this study, multi-dimensional efforts considering personal, social, and environmental factors are necessary to improve HRQoL of the elderly. Furthermore, it implies that efforts should be made to ensure health equity through social support, or improvement of community factors considering regional characteristics.

### CUMULATIVE ADVERSITY AND COGNITIVE FUNCTION AMONG OLDER ADULTS

Gabriella Dong,[1] and Mengting Li[1], *1. Rutgers, The State University of New Jersey, New Brunswick, New Jersey, United States*

The majority of studies on traumatic life events focus on posttraumatic stress disorder and depression, while less is known whether the cumulative exposure to traumatic events over the life course will deteriorate cognitive function. This study aims to investigate the association between lifetime traumatic events and cognitive function in an immigrant population. The data were drawn from the Population Study of Chinese Elderly in Chicago (PINE). Face-to-face interviews were conducted with a sample of 3,126 U.S. Chinese older adults in 2017-2019. Twelve types of traumatic events were assessed: physical assault, residential fires, sexual assault, miscarriage, abortion, imprisonment, being falsely accused, divorce, death of a loved one, being robbed, experiencing cancer, and being homeless. Cognitive function was measured through global cognition, episodic memory, working memory, processing speed, and Mini-Mental State Examination (MMSE). Linear regression was performed. In our sample, the maximum traumatic events experienced by one participant are eight types. Older adults who experienced one additional personal event were associated with higher global cognition (b=0.101, SE=0.012), episodic memory (b=0.130, SE=0.016), working memory (b=0.151, SE=0.034), processing speed (b=1.709, SE=0.178), and MMSE (b=0.124, SE=0.057), while controlling for age, gender, income, and education. In contrast with earlier studies, we identified the positive relationships between traumatic events and cognition. Older adults who had prior experience with stressful life events could demonstrate an advantage over those without such an experience. Further studies could investigate how individuals would respond to stressful life events, and how their resilience mechanism would promote cognitive function.

### HOUSING COSTS BURDEN AND SELF-RATED HEALTH: PRELIMINARY FINDINGS FROM A U.S. SAMPLE

Ji Hyang Cheon,[1] John Cagle,[1] and Amanda Lehning[1], *1. University of Maryland, Baltimore, Maryland, United States*

Self-rated health is a multidimensional construct that includes not only physical health but also emotional and social well-being. Previous research has demonstrated that multiple factors contribute to individual self-rated health, including income. Because income is a somewhat limited indicator of older adults' financial circumstances, alternative measures such as housing cost burden may enhance our understanding of contributors to self-rated health. Further, because homeowners and renters may have a different attachment to their home and neighborhood, homeownership may moderate the association between housing cost burden and self-rated health. This study examined these relationships using data from 3,212 older adults in round 7 (2017) of the National Health & Aging Trends Study. Findings from multiple linear regression models indicate that the housing cost burden is associated with lower self-rated health, and this association is stronger for renters compared to homeowners. The findings indicate the potential for reduced housing cost burden to have a positive effect on health. The poster will conclude with practice and policy implications, including the potential benefits of expanding rental assistance programs to older adults who may not meet current income requirements but are experiencing high housing cost burden, as well as research implications, including the need for longitudinal approaches.

### REFLECTIONS FROM HOMELAB: DEVELOPING TASKS TO MEASURE UPPER-BODY FUNCTION IN A SIMULATED HOME

Alicia G. Carmichael,[1] Maren Wisniewski,[1] Karen Nielsen,[2] Natalie M. Leonard,[1] Marianthie Wank,[1] Vineet Raichur,[1] and RIchard Gonzalez[1], *1. University of Michigan, Ann Arbor, Michigan, United States, 2. Georgia State University, Atlanta, Georgia, United States*

Homelike research spaces provide a unique context for studying older adulthood by blurring the boundaries between the participant's own home and traditional laboratories. Such intermediary spaces hold promise for developing measures of functional performance with increased ecological validity. While "home labs" are becoming more accessible at institutions around the world, the research design process in these spaces contains hidden challenges that can be a barrier to entry for the uninitiated. We identify and reflect on these challenges through the lens of a recent protocol built to assess upper-body performance among older adults during activities of daily living. The U-M HomeLab served as a proving ground for four example tasks: opening a water bottle, sorting pills, tying an apron, and hanging laundry. The evolution of each task is traced through ideation, testing, and refinement, culminating in a pilot among nine community-dwelling volunteers aged 61 to 72 with upper-body pain. Based on this experience, we recommend that designers of naturalistic tasks in homelike environments give special consideration to (1) feasibility, (2) scorability, and (3) safety while carefully balancing standardization against verisimilitude. In turn, each of these elements must be grounded not only in the context of the facility itself but also in the population using that facility. Among older adults with upper body pain, considerations included remaining cognizant of fall risk, anticipating and capturing compensatory behaviors, tailoring task difficulty for a wide range of physical ability, and accounting for the impact of historically gendered divisions of labor on task performance.

### DIETARY INTAKE AND NUTRITIONAL RISK AMONG OLDER AMERICANS

Yeon Jin Choi,[1] Jennifer A. Ailshire,[1] and Eileen Crimmins[2], *1. University of Southern California, Los Angeles, California, United States, 2. Davis School of Gerontology, University of Southern California, Los Angeles, California, United States*

A suboptimal diet and nutritional deficiency are among the leading causes of chronic diseases (e.g., cardiovascular diseases, metabolic syndrome, cancer, and osteoporosis), morbidity, and mortality. The objective of this study is to assess dietary intake and nutritional risk among older Americans. The dietary intake of 15 food and nutrients that are closely associated with the risk of poor health was assessed based on the dietary guidelines and nutritional goals for older Americans using a nationally representative sample of older adults (N=7,737) in the Health and Retirement Study Health Care and Nutrition Survey. The average consumption of most food and nutrients was out of the optimal range. For example, older men and women consumed 1.32-1.35 cups of dairy products and 1.23-1.29 ounces of whole grains, which is less than half of the suggested amount. The average consumption of sodium, on the other hand, was over 12 times greater than suggested dietary recommendation for older men and about 10 times greater for older women. The nutritional risk index (range: 0–15) was created by summing the number of dietary risk factors (not meeting the dietary guidelines and nutritional goals), the index scores for older men and older women were 11.05 (SD=2.31) and 10.09 (SD=2.60) respectively, suggesting the high level of nutritional risk. A healthy diet should be encouraged to prevent chronic diseases and improve the health of older adults. Nutritional education may be an effective way to promote a healthy diet.

### ALTERATIONS IN TARGETED METABOLOMICS PRIOR TO UNINTENTIONAL WEIGHT LOSS IN OLDER ADULTS

Mary Elizabeth Baugh,[1] Olga R. Ilkayeva,[2] James R. Bain,[2] Michael J. Muehlbauer,[2] Megan M. Marron,[3] Anne B. Newman,[3] Samaneh Farsijani,[3] and Stephen Kritchevsky[1], *1. Wake Forest School of Medicine, Winston-Salem, North Carolina, United States, 2. Sarah W. Stedman Nutrition and Metabolism Center, Duke Molecular Physiology Institute, Durham, North Carolina, United States, 3. University of Pittsburgh, Pittsburgh, Pennsylvania, United States*

In older adults, unintentional weight loss (UWL) is associated with poor outcomes, but its pathophysiology remains poorly understood. We sought to identify potential biomarkers of UWL using targeted metabolomics, including 8 conventional metabolites, 45 acylcarnitines, and 15 amino acids. We identified individuals from the Cardiovascular Health Study All Stars with UWL (n=40) or weight stability (WS; n=40) from Years 9 to 11. Participants had WS through Year 8. UWL was defined as experiencing >6% weight loss from Years 9 to 11 and self-reporting that loss as unintentional. Mean plasma metabolite concentrations measured in Year 9 were compared between individuals with UWL or WS between Years 9 and 11. The strongest signals in metabolomic differences between individuals going on to experience UWL versus WS were observed among the branched-chain amino acids, valine (236.54 ± 54.43 vs. 215.79 ± 32.69 μM, 95%CI: -40.81, -0.70) and isoleucine/leucine (159.09 ± 36.53 vs. 142.75 ± 23.78 μM, 95%CI:-30.10, -2.59); lactate (1.23 ± 0.44 vs. 1.00 ± 0.57 μM, 95%CI: -0.45, -0.001); histidine (35.69 ± 5.33 vs. 38.62 ± 4.86 μM, 95%CI: 0.65, 5.20); the medium-chain acylcarnitine octenoyl carnitine (C8:1) (0.23 ± 0.10 vs. 0.29 ± 0.14 μM, 95%CI: 0.01, 0.12); and long-chain acylcarnitine myristoyl carnitine (C14) (0.04 ± 0.01 vs. 0.03 ± 0.01 μM, 95%CI: -0.01, -0.002). These findings suggest altered branched-chain amino acid and fatty acid metabolism and increased oxidative stress and inflammation may be evident before individuals undergo UWL. Further investigation of these pathways may reveal novel preventive or treatment strategies for UWL.

### SELF-ACCEPTANCE BUFFERS NEGATIVE SOLITUDE-PHYSICAL ACTIVITY LINKS IN OLD AGE

Jody Mielcarski,[1] Peter Graf,[1] Maureen C. Ashe,[1] and Christiane A. Hoppmann[1], *1. University of British Columbia, Vancouver, British Columbia, Canada*

Loneliness is positively associated with a number of negative psychological and health outcomes. Solitude, a related yet distinct phenomenon, can have positive or negative ramifications depending on the context. As older adults spend significant time in solitude, there is particular need to investigate the effects of solitude on the health of this specific segment of the population. This study investigated everyday life associations between solitude and obstacles to physical activity as well as resources for overcoming these obstacles in order to determine whether and for whom solitude is negatively or positively associated with physical activity. Multilevel modelling was used to analyze data from 138 community-dwelling adults aged 65 years and older. Participants completed three daily questionnaires over a period of ten days concerning social context, activities and obstacles, as well as managing obstacles. Preliminary analyses using a subset of participants with complete data (N = 93) indicate that participants reported more physical activity obstacles when they were in solitude. This only applied to participants low in self-acceptance. Furthermore, self-acceptance was also positively associated with the extent to which individuals who had experienced an obstacle (N = 71) managed to overcome it. Further analyses will examine accelerometry-based movement information as well as the role of additional resources (e.g. living with others) and vulnerability factors (loneliness, anxiety).

### MITOCHONDRIAL DNA VARIANT C2639T IS AN APOE4 RESILIENCE FACTOR

Brendan Miller,[1] Su Jeong Kim,[2] Junxiang Wan,[2] Hemal H. Mehta,[2] Kelvin Yen,[2] and Pinchas Cohen[2], *1. University of Southern California, Los Angeles, California, United States, 2. Leonard Davis School of Gerontology, University of Southern California, Los Angeles, California, United States*

The APOE4 allele is the greatest genetic risk factor for sporadic Alzheimer's disease, yet select APOE4 carriers remain cognitively intact and become centenarians due to unclear reasons. In order to identify resilience genes for APOE4 carriers, we (1) sequenced whole mitochondrial DNA in a centenarian cohort, (2) searched for differentially expressed genes in the temporal cortex of APOE4 carriers, and (3) experimentally simulated the effects of a novel mitochondrial DNA variant that confers APOE4 resilience. The mitochondrial DNA variant, C2639T, is highly enriched in centenarians and APOE4 carriers, which changes the third amino acid of the mitochondrial-derived peptide humanin from proline to serine (humanin P3S). In addition, APOE4 carriers differentially expressed 127 genes in the humanin genetic network that map back to mitochondrial function. Therefore, we experimentally characterized the relationship between humanin, centenarian-enriched humanin P3S, and APOE. We found that humanin is a novel APOE binding partner, humanin P3S binds APOE nearly 15 times greater than wild type humanin, and humanin P3S modifies the APOE4 metabolic profile.

### USE IT TOO MUCH AND LOSE EVERYTHING? THE EFFECTS OF HOURS OF WORK ON HEALTH

Colin McKenzie,[1] Kei Sakata,[2] and Shinya Kajitani[3], *1. Keio University, Tokyo, Japan, 2. Australian Institute of Family Studies, Southbank, Victoria, Australia, 3. Kyoto Sangyo University, Kyoto-shi, Kyoto-fu, Japan*

We examine the causal impact of working hours on various health outcomes of Australian men aged 40 and over. To capture the potential non-linear dependence of health status on working hours, the models for health outcomes include working hours and its square. We deal with the potential endogeneity of working hours by using the instrumental variable estimation technique using instruments based on the age for pension eligibility. A non-linear causal effect of working hours on health is confirmed. For males working relatively moderate hours (up to 20–24 hours for a week), an increase in working hours has a positive impact on health, but thereafter an increase in working hours has a negative impact on health. The results also indicate that there is a non-linear dependence of working hours on the pension eligibility age, and also a non-linear dependence of health outcomes on the pension elgibility when this last relationship is treated as a "reduced form" relationship.

### EXPOSURE TO FINE PARTICULATE MATTER, GLOBAL COGNITIVE PERFORMANCE, AND EMOTIONAL DISTRESS IN OLDER WOMEN

Andrew J. Petkus,[1] Xinhui Wang,[1] Diana Younan,[1] Mark M. Espeland,[2] JoAnn E. Manson,[3] Eric Whitsel,[4] Susan Resnick,[5] and Jiu-Chiuan Chen[1], *1. University of Southern California, Los Angeles, California, United States, 2. Wake Forest School of Medicine, Winston-Salem, North Carolina, United States, 3. Brigham & Women's Hospital/ Harvard University, Boston, Massachusetts, United States, 4. University of North Carolina, Chapel Hill, North Carolina, United States, 5. National Institute on Aging, Baltimore, Maryland, United States*

Among older adults, exposure to ambient PM2.5 (particulate matter with aerodynamic diameter <2.5 μm) has been associated with more rapid decline in cognitive performance and greater emotional distress. However, the interrelationship between PM2.5 exposure, emotional distress, and global cognitive decline is unexamined. We examined whether long-term PM2.5 exposure affects global cognitive ability and emotional distress in 5,982 older women (baseline age 70.6 ± 3.8 years) from the Women's Health Initiative Memory Study. PM2.5 exposure for the three-years prior to baseline was estimated at each participant's residence via a national kriging model. Using structural equation models (SEM), a z-score standardized latent factor consisting of items from the 6-item CESD and the SF-36 Emotional Well-Being scale was constructed to estimate emotional distress at baseline and one-year follow-up. Individual-specific trajectories of global cognitive performance (Modified-Mini Mental State Examination; 3MS) were also estimated. All effects reported were adjusted for multiple demographic, lifestyle, and clinical variables. Higher PM2.5 exposure was associated with lower baseline 3MS performance (β= -.159 per IQR=3.38 μg/m3; 95% CI = -.276 to -.042), which was associated with increased emotional distress over the subsequent year (β= -.011; 95% CI= -.017 to -.004). A statistically significant indirect association of PM2.5 on changes in emotional distress via worse baseline 3MS performance (β=.002; 95% CI = .001 to .004) was present. In contrast, no statistically significant association between PM2.5 on baseline emotional distress occurring prior to declines in 3MS performance was present. PM2.5 neurotoxicity may contribute to global cognitive decline, which precedes increased emotional distress.

### THE INFLUENCE OF HUMOR AND SPIRITUALITY ON THE RESILIENCY OF COMMUNITY-DWELLING OLDER ADULTS

Bonnie L. Kenaley,[1] Zvi D. Gellis,[2] Eun hae Kim,[3] and Kimberly Mclive-Reed[2], *1. Boise State University, Boise, Idaho, United States, 2. University of Pennsylvania, Philadelphia, United States, 3. Texas State University, Texas, United States*

Older adults are confronted with many distinct challenges, which require the use of various coping mechanisms to maintain psychological balance, including humor and spirituality (Bonanno et al., 2012; Koenig, 2012). This study examined the influence of humor and spirituality on resiliency of 156 (age 60 years and older) community-dwelling members of an Osher Lifelong Learning Institute located in the western region of the United States who completed a pen and paper and electronic newsletter surveys. The majority of the sample used humor to cheer themselves when feeling depressed, were amused by the absurdities of life, used humor to feel better and to cope with problems, and believed their humorous outlook prevented them from being upset or depressed. Almost three-quarters of the sample looked to a spiritual force for strength, support, and guidance, 58% worked together with a spiritual force and less than 39% thought about how their lives were part of a larger spiritual force. In the final hierarchical regression model (F (5, 143, = 8.895, p .000), only spirituality (beta = -.238, p < .001) and humor (beta = .444, p < .000) were statistically significant; whereas age, gender and living with another were not statistically significant. The findings suggests that humor along with spirituality are two internal resources that promote resiliency in older adults. Humor infused in informal interactions and planned activities as well as spiritual support may contribute to the promotion and enhancement of resiliency in community-dwelling older adults.

### FACTORS ASSOCIATED WITH HOSPITAL-TO-HOME DISCHARGE: USING A DECISION TREE ANALYSIS

Ji Yeon Lee,[1] Kwang Joon Kim,[1] and Chang Oh Kim[1], *1. Yonsei University, Seoul, Korea, Republic of*

Because the number of older adults discharged home is increasing, a comprehensive geriatric assessment (CGA) becomes a helpful tool to make a decision about discharge destination. This study aimed to examine the predictors related with discharge destination using the component of CGA. We used decision tree analysis with the Classification and Regression Trees algorithm. Study participants were older adults over 65 years old who were admitted to geriatric department at a university-affiliated hospital in Korea. The older adults were assessed by multi-professional team at the initial stage of their hospitalization and received blood tests and the CGA. A total of 184 patients was included. The mean age was 84.6 years old and 61.6% were female. The analysis revealed arm circumference and level of frailty were significant predictors of home discharge. Specifically, 90.1% of patients had discharged home if their upper arm circumference was more than 18.3 centimeters, and 36.4% was discharged home if it was less than 18.3 centimeters. Among older adults with the arm circumference more than 18.3 centimeters, 57% of robust older adults and 91.7% of prefrail or frail older adults were discharged home. These robust older adults had relatively better daily living ability, and discharged to other facilities rather than home for rehabilitation. As many frail older adults are discharging to their own home, clinicians need to pay more attention to them. Also it is encouraged to perform the CGA, which includes nutritional assessment in it, for inpatient older adults.

### DO THE PRESENCE OF ADULT CHILDREN AND THEIR MARITAL STATUS MATTER FOR ACCESS TO THE PUBLIC LONG-TERM CARE SYSTEM?

Tomoko Wakui,[1] Suguru Okubo,[2] Nanako Tamiya,[3] Taeko Watanabe,[4] Tatsuro Ishizaki,[1] and Ichiro Kai[5], *1. Tokyo Metropolitan Institute of Gerontology, Tokyo, Japan, 2. BMS Yokohama, Yokohama, Kanagawa, Japan, 3. Department of Health Services Research, Faculty of Medicine, University of Tsukuba, Tsukuba, Ibaraki, Japan, 4. Department of Health Services Research , Faculty of Medicine, University of Tsukuba, Tsukuba, Ibaraki, Japan, 5. The University of Tokyo, Tokyo, Japan*

Knowing how the presence of family affects access to the public long-term care system is important for evaluating the adequacy of the system. This study examined the relationship between the presence of adult children and their marital status, and access to a public system by examining the gap between self-reported care needs and the official certification as needing care under the Japanese public long-term care insurance system. Data from Japan's 2016 Comprehensive Survey of Living Conditions were used. A total of 23,466 older adult claimants, aged 65 years and older were analyzed. Outcomes were whether or not claimants were officially certified as needing care under the system, and the relationship of the presence of both live-in and live-out children and their marital status were examined controlling for claimants' age, gender, education, financial status, and physical and cognitive conditions. Females comprised 64.8% of the sample, and the average age was 83 years (SD=7.8). The percentage of claimants living with a single or married child were 25.2% and 26.9%, respectively, and 60.1% were parents of children who lived independently. The percentage who were officially certified as needing care was 68.5%. Logistic regression analysis revealed that claimants with a live-in child were less likely to be officially certified as needing care, and claimants with live-in a single child were less likely to be officially certified compared with those with a married child. Live-in single children may provide long-term care by themselves, and the excess burden on them needs to be further investigated.

### AMERICAN INDIAN FEMALE CAREGIVERS: STRESS AND RESILIENCE

Felina Cordova-Marks,[1] Robin Harris,[1] Nicolette Teufel-Shone,[2] Beatrice Norton,[3] Ann Mastergeorge,[4] James Cunningham,[1] and Lynn Gerald[1], *1. University of Arizona, Tucson, United States, 2. Northern Arizona University, Flagstaff, United States, 3. Hopi Office of Aging and Adult Services, Kykotsmovi, United States, 4. Texas Tech University, Lubbock, United States*

Background/Introduction: Increased stress has been found to be a part of the caregiving experience. However, how stress is handled is important. Resilience has been shown to decrease stress in non-caregivers. There is a lack of information about American Indian (AI) caregiver stress. In this study, we seek to investigate if resilience acts as a stress buffer in this population of AI female caregivers.Methods: The Hopi Adult Caregiver Survey was conducted in 2017 with 44 female Hopi caregivers. Resilience and stress scale questions were asked as well as variables potentially affecting these. Resilience as measured by the Connor Davidson Resilience Scale-10 (CD-RISC) and stress as measured by the Perceived Stress Scale-10 scores were calculated as well as categorical levels of higher and lower stress/resilience. Variables possibly associated with each were assessed using linear regression analyses. Results: Forty-four female caregivers were surveyed. The overall mean stress score for caregivers was 17.9 Â± 6.2 on the PSS. For difference between higher and lower stress, expectation of females to be caregivers, number of times using a traditional healer/traditional medicine person, number of caregiver difficulties, self-perceived health rating, self-perceived changes to eating habits since becoming a caregiver were significant. Average sum resilience score was 28.7 Â± 6.2 on the CD-RISC. In linear regression, it was found that with an increase in the resilience score, stress score decreased.Discussion: In these caregivers, resilience acts as a stress buffer. Increasing resilience and countering factors that decrease resilience may reduce stress experienced by caregivers.

### SESSION LB3620 (LATE BREAKING POSTER)

### LATE BREAKING POSTER SESSION IV

### FILIAL PIETY AND RELATIONSHIP QUALITY AMONG OLDEST-OLD PARENTS AND OLDER-ADULT CHILDREN IN KOREA: A DYADIC APPROACH

Kyuho Lee,[1] Joohong Min,[2] and Gyounghae Han[3], *1. Daegu University, Gyeongsan, Korea, Republic of, 2. Jeju National University, Jeju, Korea, Republic of, 3. Seoul National University, Seoul, Korea, Republic of*

Objectives: Research has consistently reported the importance of adult children's filial piety in maintaining relationship quality with their older-adult parents, especially in Asian countries due in large part to the influence of Confucianism. However, less attention has been paid to its effects in their later relationship—oldest-old parents and older-adult children, and very few studies employed a dyadic approach. Methods: In the current study, we examined the effects of actor- and partner effects of the attitudes toward filial piety on intergenerational relationship quality. Data from 105 dyads of very old parents, 81 to 97 years old (M = 87.9, SD = 2.8) and their older-adult children, 65 to 72 years old (M = 65.9, SD = 1.2) were utilized for the analyses. Results: Results showed that children's attitudes toward filial piety were positively related to the relationship quality reported by both old children (i.e., actor-effects, $\beta = .64$, $p < .001$) and oldest-old parents (i.e., partner-effects, $\beta = .63$, $p = .001$). The effect of actor's and partner's attitudes toward filial piety on relationship quality was not significant. Discussion: Findings suggest that children's attitudes toward filial piety, as compared to parents, may play a more important role in the quality of relationships between oldest-old parents and their older-adult children in South Korea.

### DAILY INTERACTIONS WITH THE VERY OLD PARENTS AND OLD CHILDREN: CONCORDANCE IN REPORTS AND RELATIONSHIP TYPE

Joohyun Kim,[1] and Joohong Min[2], *1. Seoul National University, Seoul, Korea, Republic of, 2. Jeju National University, Jeju, Korea, Republic of*

Objectives: Studies have been increased on daily lives of older adults; however, little is known about daily interactions between parents and children, especially both are in their later lives. Utilizing parent-child dyadic data, this study aims to investigate the joint daily interactions of the very old parents and their old children with focus on the relationship type and differences in parent's and child's report. Methods: A sample of 105 parents (aged ≥ 80) and 105 children (aged ≥ 65) were interviewed to assess frequency on daily interactions (e.g., contact by phone or email, talking, eating and going out together). Structural Equation Modeling was used to examine differences in amounts of daily interactions by relationship type (e.g., mother-daughter, mother-son and mother-in-law – daughter-in-law). Results: Both parents and children reported high level of daily interactions; however, frequency of watching TV and going out together were relatively lower, compared to other forms of daily interactions. Parents and children showed high concordance in reports; 84% of dyads had the same reports in interactions (absolute mean discrepancy score < 0.1). Mother-daughter relationship reported significantly higher frequency on daily interactions, comparted to mother-son relationship. No significant differences were found between the mother-daughter dyads and the mother-in-law and daughter-in-law dyads. Discussion: Findings suggest that very old parents and old children tend to have high levels of daily interactions, with high concordance in reports. It also highlights its importance of considering relationship type in the dynamic of daily lives and experiences between very old parents and old children.

### HEALTH PROMOTION FOR PEOPLE WITH EARLY-STAGE DEMENTIA: A QUASI-EXPERIMENTAL STUDY

Ingelin Testad,[1] Martine Kajander,[1] Anne Torsvik Henriksen,[1] Målfrid Meling,[1] Vigdis Vagle,[1] and Martha Therese Gjestsen[1], *1. Centre for Age-related Medicine - SESAM, Stavanger University Hospital, Stavanger, Norway*

With the limited advancements in medical treatment, there is a growing emphasis on supporting people with early-stage dementia adjusting to their diagnosis and improve their quality of life. This study aimed to evaluate the effects and experiences of people with early-stage dementia attending a 12-week Health Promotion, consisting of 2-hour sessions at weekly intervals focusing on understanding the progression of dementia, promoting physical activity, nutrition, coping, relationships, home- and travel safety, medication and communication with health care providers. Quantitative and qualitative assessments were conducted at baseline and follow-up 1-2 months post intervention, including cognition, self-rated health and depressive symptoms and individual short interviews. A total of 90 persons with dementia participated in this study. The results demonstrated a stable cognitive function, measured by Mini Mental State Examination, of people with dementia during the 4 month follow-up, and a significant improvement in self-rated health measured by EQ VAS (95% CI 0.2 to 7.6, p=0.037). Depressive symptoms measured by Cornell Scale for Depression in Dementia, demonstrated a decline by one point, which is an improvement in depressive symptoms, although not significant. 32 of the 90 qualitative interviews with participants and their carers were included and analyzed with systematic text condensation. The results demonstrated that the intervention was well received by the participants, learning about dementia, practical strategies and focusing on remaining resources was particularly highlighted as well as improved family communication. In conclusion, the intervention had beneficial effects on the cognitive function, self-rated health and depressive symptoms of people with dementia.

### THE APOE-ε4 ALLELE AND AGE SYNERGISTICALLY DRIVE DISEASE PROGRESSION IN ALZHEIMER'S DISEASE

Luca Kleineidam,[1] Andrea R. Zammit,[2] Alyssa DeVito,[3] Richard B. Lipton,[4] Oliver Peters,[5] Alfredo Ramirez,[6] Michael Wagner,[7] and Graciela Muniz Terrera[8], *1. Department of Neurodegenererative Diseases and Geriatric Psychiatry, University Hospital Bonn, Bonn, Germany, 2. Department of Neurology, Albert Einstein College of Medicine, Bronx, New York, United States, 3. Department of Psychology, Louisiana State University, Baton Rouge, Louisiana, United States, 4. Department of Neurology, Albert Einstein College of Medicine, Bronx, New York, United States, 5. Department of Psychiatry, Charité Berlin, Berlin, Germany, 6. Department of Psychiatry, University of Cologne, Medical Faculty, Cologne, Germany, 7. Department of Neurodegeneration and Geriatric Psychiatry, University Hospital Bonn, Bonn, Germany, 8. University of Edinburgh, Edinburgh, United Kingdom*

The Apolipoprotein E (APOE)-ε4 allele is the strongest genetic risk factor for Alzheimer's disease (AD) and other neurodegenerative dementias. Cross-sectional case-control studies suggest that the effect of APOE-ε4 decreases in old age. However, since APOE- ε4 is associated with mortality, these studies might be prone to bias due to selective survival. Therefore, we used multi-state-modeling in longitudinal cohort studies to examine the effect of APOE-ε4 on the transition through cognitive states (i.e. cognitively normal, mild cognitive impairment (MCI) and dementia) while taking death as a competing risk into account. Results from the German AgeCoDe study (n=3000, aged 75-101 years) showed that APOE-ε4 increases the risk for cognitive deterioration in all disease stages. Contrary to results from cross-sectional studies, the effect of APOE-ε4 on the transition from MCI to dementia increased with increasing age (HR=1.044, 95%-CI=1.001-1090). The direction of this effect was confirmed in a smaller sample from the Einstein Aging Study (n=744, HR=1.032, 95%-CI=0.949-1.122). To examine the pathophysiological basis of these results, generalized additive models were used to study AD biomarkers in the liquor of 1045 patients with MCI or AD-dementia. Here, increased amyloid (Abeta1-42) pathology was associated with increased tau pathology (pTau181), consistent with the amyloid-cascade-hypothesis. Interestingly, higher age and presence of the APOE-ε4 synergistically lowered the amount of amyloid required to exacerbate tau pathology (interaction p=0.012). Taken together, our results suggest that the effect of APOE-ε4 on disease progression increases with advancing age. An altered neuroinflammatory response to neurodegeneration should be further explored as potential underlying mechanism.

### ELDER ABUSE AND NEGLECT IN URBAN AND RURAL AREAS IN CHINA: PREVENTION STRATEGIES

Chen Chen,[1] Liu Yue,[2] and Hong Mi[3], *1. Institute for Population and Development Studies, Zhejiang University, Hangzhou, Zhejiang, China, 2. Institute for Population and Development Studies, Hangzhou, Zhejiang Province, China, 3. School of Public Affairs, Zhejiang University, Hangzhou, Zhejiang Province, China*

With acceleration of the ageing population globally, more and more governments are concerned about the potential increase in elder abuse and neglect (EA/N). Recently the National Office for Ageing and the provincial offices for ageing conducted a survey of 224,352 Chinese over the age of 60 years using household interviews to assess economy, health, service, social participation, culture, rights protection, livable environment, etc. Author's analysis of this data shows that 54% of the elderly people interviewed experienced physical and mental abuse or intimidation, and 6.95% of them felt that their legal rights were violated. Data also supports that the occurrence of EA/N was significantly correlated to self-care ability, economic status, and urban and rural regions of the elders. The researcher will discuss the practice and policy implications for the prevention of EA/N.

### SMOKING BEHAVIOUR: PATTERNS AND COSTS OF HEALTH SERVICE USAGE USING HAGIS AND LINKED ADMINISTRATIVE HEALTH DATA

Elaine Douglas,[1] and David Bell[2], *1. University of Stirling, Stirling, Scotland, United Kingdom, 2. University of Stirling, Stirling, United Kingdom*

The associations between smoking and health are well documented. Using the Healthy Ageing In Scotland (HAGIS) survey linked to the administrative Scottish National Health Service (NHS) records this study analyses health service resource usage by older people according to self-reported smoking status. Individual level smoking status (current, ex-smoker, or never smoked), socio-demographic characteristics (age, gender, level of deprivation) and subjective health are sourced from people aged 50+ across Scotland using HAGIS. These responses are then linked to NHS Scottish Morbidity Records to analyse variation in health service usage as measured by the total number of days spent in hospital (daycases and inpatient stays), number of stays, and mean length of stay. Costs are then assigned by medical speciality. We use a two-part model to analyse the i) the probability of having been hospitalised at all, and ii) the quantum of resource usage and its associated cost for those who have been in hospital. Our study provides a conceptual and empirical framework for the associative relationship between smoking status and actual (rather than self-reported) health service usage and expenditure. This study demonstrates the insights to be gained from the linkage of individual survey responses to administrative health service data on resource usage and costs, and discusses the implications for health policy.

### MEANINGFUL ENGAGEMENT AND QUALITY OF LIFE AMONG ASSISTED LIVING RESIDENTS WITH DEMENTIA: EMERGENT FINDINGS

Joy Ciofi,[1] Candace L. Kemp,[1] Alexis A. Bender,[2] Elisabeth O. Burgess,[3] Jennifer C. Morgan,[1] Fayron Epps,[2] Patrick Doyle,[4] and Molly M. Perkins[5], *1. Georgia State University, Atlanta, Georgia, United States, 2. Emory University, Atlanta, Georgia, United States, 3. Gerontology Institute, Georgia State University, Atlanta, Georgia, United States, 4. Center for Innovative Care in Aging, Johns Hopkins School of Nursing, Baltimore, Maryland, United States, 5. Division of General Medicine and Geriatrics, Emory University School of Medicine, Atlanta, Georgia, United States*

This poster provides an overview of the aims, methods, and emergent findings from an ongoing five-year NIA-funded project (R01AG062310) examining meaningful engagement and quality of life among assisted living (AL) residents with dementia. The overall goal of this project is to determine how opportunities for meaningful engagement can best be recognized, created, and maintained for individuals with different dementia types and varying levels of functional ability. Guided by grounded theory, this qualitative study will involve 12 diverse AL communities in and around Atlanta, Georgia, USA. Presently, our interdisciplinary team is collecting data in four communities using ethnographic observations, semi-structured interviews, and resident record review. We are studying daily life in each community, following 30 resident participants, and actively recruiting and interviewing their formal and informal care partners. Based on ongoing analysis, we offer key emergent findings. First, meaningful engagement is highly individualized and dynamic. Differing personal interests, along with wide variations in cognitive and physical abilities, can present challenges for AL community staff and other care partners when trying to recognize what constitutes meaningful engagement for residents. Second, multiple complex factors interplay to shape the experience of meaningful engagement among persons living with dementia, such as personal characteristics, care partner background and training, AL community design and philosophy, and state/corporate regulations. Finally, flexibility and 'meeting the resident where they are at' appear to be critical to identifying and fostering meaningful engagement for persons living with dementia. We discuss the implications of these preliminary findings for translation, dissemination, and future research.

### FINDING THE ELDERS WHO STAYED- CONDUCTING OUTREACH IN THE AFTERMATH OF HURRICANE MICHAEL

Jessica L. Tice,[1] and Megan Bond[1], *1. Florida Department of Elder Affairs, Tallahassee, Florida, United States*

The Florida Department of Elder Affairs (DOEA) provides programs and services for over 65,300 older people and adults with disabilities. These individuals are uniquely vulnerable and may be displaced, and/or disoriented during natural disasters. DOEA clients are dependent upon community-based services to provide supervision or assistance to perform basic self-care, which often makes sheltering in place alone a danger to their health and well-being. During Hurricane Michael (2018) many older adults who previously were independent sought help for many issues including property damage, utility interruption, food and medicine scarcity, and physical or mental health problems associated with the storm and its aftermath. In normal conditions, DOEA identifies older populations via Census tracts and then conducts outreach events to inform the public how to access social services. However, after the widespread displacement post-storm, traditional outreach approaches were insufficient. A method was needed to remove areas that were rendered uninhabitable and find who remained in place. DOEA identified viable neighborhoods by overlaying property damage locations on base layers of Census tracts with concentrations of older adults and polling places with high percentage of age 60+ voter participation in the subsequent November election. Then in partnership with Feeding Florida, we provided information and registration assistance via local food distribution sites in those areas. This methodology of overlaying Division of Emergency Management property damage records and voter participation records against publicly available Census tract files is a strategy that could be replicated by other disaster and flood-prone communities or organizations that have similar needs.

### INCREASING VULNERABILITY AMONG OLDER AMERICANS ACT HOME-DELIVERED NUTRITION SERVICE CLIENTS

Kristen N. Robinson,[1] and Heather L. Menne[2], *1. Social & Scientific Systems, Inc., Silver Spring, Maryland, United States, 2. RTI International, Washington, District of Columbia, United States*

Older Americans Act (OAA) programs are designed to help frail and vulnerable older adults remain in their homes through the provision of long-term services and supports. Administrative data from the Administration for Community Living (ACL) show that older adults receiving OAA services are three times more likely to live below the poverty level (33.0%) as compared with all older adults (9.2%). In addition, they are almost twice as likely to be living alone (45%) as compared with the general population (28%). Using data from the recently released 2018 National Survey of Older Americans Act Participants, we examine the largest program administered by ACL, the OAA Nutrition Program, to see if the economic vulnerability of home-delivered nutrition service clients has changed over the past 10 years. Results from this study show that recipients of home-delivered nutrition services are more in need of low-cost or free meals in 2018 than they were in 2008 due to a 24% increase in Medicaid eligibility, 41% increase in those who report not having enough money or food stamps to buy food, and 101% increase in those who report receiving food stamps. This increase in economic need may be due to a demographic shift in the marital status and living arrangements of older adults, specifically the 75-84 age group. The increase in the percentage of older adults who are divorced, live alone, and have low income has made the home-delivered nutrition services program even more important today than it was a decade ago.

### THE INFLUENCE OF CUSTODIAL STATUS ON MARITAL AFFECTUAL SOLIDARITY AND MENTAL HEALTH AMONG GRANDPARENTS.

Eunbea Kim,[1] Danielle K. Nadorff,[2] Rachel Scott,[3] and Ian T. McKay[2], *1. Mississippi State University, Mississippi State, United States, 2. Mississippi State University, Starkville, Mississippi, United States, 3. Mississippi State, Mississippi State, United States*

Increased life expectancy and the diversity of family structure have resulted in a substantial rise in the number of families with grandparents as the main caregivers (e.g. custodial grandparents). The structures of these families affect the well-being of all family members. After middle age, psychological well-being is associated with marital relationship quality, and raising one's grandchildren is a known source of strain to relationships. The current study examined adults aged 40 and older (M age = 57.6 yr, 53% female) using a nationwide sample from MIDUS to assess the extent to which custodial grandparenting status influences marital affectual solidarity, depressive symptoms, life satisfaction, and perceived stress. Measures included the Center for Epidemiological Studies Depression Index, Spousal Affectual Solidarity, Satisfaction with Life Scale, and Perceived Stress Scale. Marital affectual solidarity was significantly related to custodial status and psychological well-being, and there were significant differences in marital relationship quality and psychological well-being between custodial grandparents and non-custodial grandparents. However, custodial status failed to moderate the relation between marital affectual solidarity and mental health. Although other factors surrounding custodial grandparents likely affect their marital relationship and mental health, these results suggest that grandparents raising grandchildren are under particular strain in their marriages and are in need of targeted interventions to ameliorate stress and depressive symptoms. These findings will inform the need for more research and supportive educational programs on family relationships and the psychological health of custodial grandparents.

### SEQUENTIAL IMPLEMENTATION AS A LEARNING HEALTH ORGANIZATION: THE EQUIPPED GERIATRIC MEDICATION SAFETY MODEL

Ann E. Vandenberg,[1] Michelle Kegler,[2] Susan Hastings,[3] Ula Hwang,[4] and Camille Vaughan[1], *1. Division of General Medicine and Geriatrics, Emory University School of Medicine, Atlanta, Georgia, United States, 2. Rollins School of Public Health, Emory University, Atlanta, Georgia, United States, 3. Department of Medicine, Duke University School of Medicine, Durham, North Carolina, United States, 4. Departments of Emergency Medicine and Geriatrics, Icahn School of Medicine at Mount Sinai, New York, New York, United States*

A learning health organization (LHO) is one that systematically integrates internal data and experience with external evidence to improve internal healthcare practice. Yet collaborative research networks implementing evidence-based interventions across sites with the goal of widespread dissemination are also effectively LHOs. The EQUIPPED (Enhancing Quality of Prescribing Practices for Older Adults Discharged from the Emergency Department) network formed to address an important public health issue: potentially inappropriate medications (PIMs) prescribed to older adults at discharge from hospital Emergency Departments (ED). EDs nationwide serve increasing numbers of older adults but lack clinical decision support to avoid prescribing PIMs associated with adverse events including hospitalization and death. The EQUIPPED geriatric safety program was adapted from the VA and implemented sequentially at three different academic institutions sharing the same electronic health record (Epic)(AHRQ R18HS24499). Implementation challenges, solutions, and innovations informed successive iterations. Using the Replicating Effective Programs framework, we conducted a process evaluation using data from implementation team focus groups (n=3), meeting minutes (n=98 hours), and organizational profiles (n =3) to understand how organizations working together within a research network build an intervention package for program scale-up. We present structural characteristics of the three organizations, implementation steps as they developed across three sites, and the resulting process protocol and a prototype toolkit. Lessons learned include having multiple internal champions at the intervention site, observing workflow pre-intervention, and streamlining data collection with a relational database and visualization software. Insights from the EQUIPPED experience can serve as a model for other systems and collaborative networks.

### LIFESPAN AGE-RELATED DIFFERENCES IN THE REGIONAL WHITE MATTER MICROSTRUCTURE OF THE HUMAN CORPUS CALLOSUM

John Lynn,[1] Chaitali Anand,[1] Muzamil Arshad,[2] Dalal Khatib,[1] Jeffrey Stanley,[1] and Naftali Raz[1], *1. Wayne State University, Detroit, Michigan, United States, 2. Saint Joseph Hospital, Chicago, Illinois, United States*

The corpus callosum (CC) connects homologous cortical structures across hemispheres and is the largest white matter tract in the human brain. Post-mortem studies suggest that CC myelination begins in infancy, reaches a plateau in the middle age, and declines in the later years. The latter is accompanied by myelin disruption and reduction in fiber density and diameter, i.e. changes in intra-/extracellular water space. We used multi-echo T2 imaging to estimate, via multi-exponential T2 relaxation of water, the myelin water fraction (MWF), a direct proxy for myelin content, and geometric mean T2 (geomT2IEW) that reflects water in the intra-/extracellular space, to investigate age differences in five CC regions covering its anterior to posterior span in 395 healthy individuals (7-85 years; 161M+235F). The general linear model analysis of MWF showed main effects of age and age-squared conditioned on interactions by CC region. Univariate polynomial regressions on three age groups (7-29, 30-59, and 60-85 years) revealed the overall quadratic association between age and MWF as mainly driven by the positive linear relationship in the youngest group and minimal differences in the remainder of the lifespan, save for two weak negative linear associations in the anterior/middle CC body. With geomT2, a main linear effect of age, and significant interactions between age and age-squared by region were observed. The positive linear association was especially prominent in the regions with greater fiber density. The results are consistent with CC myelination into adulthood and decreased axonal density and diameter but not prominent myelin degeneration in elderly individuals.

### A MUSIC AND MINDFULNESS INTERVENTION FOR PERSONS WITH DEMENTIA AND THEIR CAREGIVERS

Punam G. Risal,[1] Debra J. Dobbs,[2] Soomi Lee,[2] Jennifer Bugos,[2] Sheikh I. Ahamed,[3] William Monaco,[2] William Patterson,[4] and Hongdao Meng[2], *1. School of Aging Studies, University of South Florida, Tampa, Florida, United States, 2. University of South Florida, Tampa, Florida, United States, 3. Marquette University, Milwaukee, United States, 4. Digital Media Innovations and eLearning Enterprises, Tampa, Florida, United States*

The number of older adults with Alzheimer's Dementia is projected to increase by 28.6% in Florida by 2025. Cost-effective non-pharmacological interventions targeting both persons with dementia (PWD) and their family caregivers (FCs) are urgently needed. This small pilot study tested the feasibility of a music and mindfulness intervention in an assisted living community (ALC) in Tampa, FL. Methods: We used a mixed-methods single group pre-post study design. To date, a total of 5 PWD and 3 FCs were enrolled, and all completed the 4-week intervention (12 sessions for PWD and 4 sessions for FCs). Outcome measures include behavioral symptoms and sleep quality for PWD, and mindfulness, sleep quality and perceived stress for FCs. Outcome data were collected via survey questionnaires and focus group interviews (FCs and staff). Results: Preliminary analysis showed that the intervention is well-received by PWD, FCs, and ALC staff. PWD participants experienced a reduction in behavioral symptoms post-intervention (Cohen's d=0.33). FC participants reported increased mindfulness and reduced stress. In focus group interviews, both FCs and staff corroborated the positive findings in residents' mood and behaviors. Barriers to large scale implementation include intervention time commitment (ALC staff) and scheduling difficulties due to work (FCs). Both FCs and ALC staff recommended the continuation of the intervention and making it available to all memory care residents. Based on the initial results, additional testing of the intervention is currently in progress (10 PWD) and updated results will be reported. Implications for conducting community-based non-pharmacological interventions will be discussed.

### AGING DIMINISHES THE DIRECT ASSOCIATION BETWEEN BRAIN ACTIVATION AND POSTURAL CONTROL DURING THE N-BACK TASK

Azizah J. Jor'dan,[1] Ikechukwu Iloputaife,[2] and Brad Manor[3], *1. VA Boston Healthcare System, Boston, Massachusetts, United States, 2. Hebrew SeniorLife, Marcus Institute for Aging Research, Boston, Massachusetts, United States, 3. Hinda and Arthur Marcus Institute for Aging Research, Harvard Medical School, Boston, Massachusetts, United States*

Aging diminishes the control of standing posture, which relies upon the capacity to activate brain regions involved in cognitive-motor function. Our prior work shows that impaired cerebral blood flow (CBF) regulation within the middle cerebral artery (MCA) in response to the N-Back executive function task is linked to worse walking performance in older adults. However, the effects of aging on the relationship between CBF regulation and postural control during the N-Back task is unknown. Sixteen young (27 years) and 15 older participants (76 years) stood upright and completed the N-Back (i.e., control [Identify X, IdX] and an experimental condition [2-Back]) presented on a screen while CBF and postural sway were simultaneously recorded. CBF was recorded using transcranial Doppler. Sway was recorded using a lumbar motion sensor. Elliptical area, root mean square (RMS), distance, velocity, and acceleration were computed. There were no group differences in sway outcomes ($p>0.37$). Young participants had higher CBF during the IdX and 2-Back compared to older participants ($p<0.001$). Within the young, and not within older participants, those with lower CBF during performance of the 2-Back exhibited greater elliptical area ($\beta=-0.67$, $p=0.03$) and RMS ($\beta=-0.68$, $p=0.03$), faster acceleration ($\beta=-0.78$, $p=0.02$), and longer distance ($\beta=-0.64$, $p=0.04$). There were no associations between CBF and sway outcomes in either group during the IdX. These results suggest that dual-task sway performance is directly linked to CBF in younger adults, and furthermore, this link is diminished in older adults. These results underpin the need for cognitive-motor interventions in older adults.

### ATTITUDES AND PREFERENCES FOR ROBOT-LED PIANO COGNITIVE TRAINING: FEASIBILITY IN MILD COGNITIVE IMPAIRMENT

George Mois,[1] Bailley Collette,[1] Lisa M. Renzi-Hammond,[2] Laura Boccanfuso,[3] Aditi Ramachandran,[4] Paul Gibson,[5] Kerstin G. Emerson,[1] and Jenay M. Beer[1], *1. University of Georgia, Athens, Georgia, United States, 2. Institute of Gerontology The University of Georgia, Athens, Georgia, United States, 3. Van Robotics, Columbia, United States, 4. Van Robotics, Columbia, South Carolina, United States, 5. Applied Universal Dynamics, Corp., Loretto, Minnesota, United States*

Cognitive training has been shown to improve neural plasticity, increase cognitive reserve and reduce the risk of dementia in older adults. Specifically, learning to play the piano has been shown to be an engaging, multimodal form of cognitive training. However, accessing this form of cognitive training can pose a challenge for older adults. Socially assistive robots present a unique opportunity to increase access to user-tailored piano learning cognitive training. The present study utilized a robot-led four-week piano lesson feasibility intervention for older adults with mild cognitive impairment ($N = 11$; $M= 74.64 \pm 6.02$ years of age; 72.72% female; 90.1% White/Caucasian). Cognitive Status was assessed during screening via the Telephone Interview for Cognitive Status, and after screening via the Mini-Mental State Exam and the CNS Vital Signs computerized test suite to measure cognitive domain-specific functioning. Perceptions and acceptance of the robot were measured using the Robotic Social Attributes Scale (RoSAS) and Technology Acceptance Scale. Cognitive function improved after four weeks of training in the verbal memory, executive function, reaction time and cognitive flexibility domains, and in the computed neurocognitive index score ($p<0.05$). Survey data and qualitative interviews show that participants perceived the robot instructor as socially engaging, competent, useful, and easy to use. These results provide insight into the potential of SARs to facilitate cognitive training in the form of piano lessons, as well as recommendations for creating a suitable robot instructor for this application.

### KYNURENINE, AN ENDOGENOUS AHR AGONIST, UPREGULATES CXCL12- AND HDAC3-TARGETING MIRNAS INHIBITING OSTEOGENESIS

Ahmed M. Elmansi,[1] Nada Eisa,[1] Dmitry Kondrikov,[1] Sadanand Fulzele,[2] Meghan mcGee-Lawrence,[3] Mark Hamrick,[3] Carlos Isales,[3] and William D. Hill[1], *1. Medical University of South Carolina, Charleston, South Carolina, United States, 2. Department of Orthopaedic Surgery, Medical College of Georgia, Augusta University, Augusta, Georgia, United States, 3. Augusta University, Augusta, Georgia, United States*

Osteoporosis is among the most debilitating disorders for aging men and women. Mechanisms underlying loss of bone mass and impaired fracture healing in aged population are not completely understood. Our lab and others reported increased levels of the oxidized tryptophan metabolite, kynurenine (Kyn), in bone marrow mesenchymal stem cells (BMSCs) and interstitial fluid. Here, we report that Kyn significantly inhibits osteogenic differentiation of BMSCs likely via activating aryl hydrocarbon receptor (AhR). KYN significantly downregulated mRNA levels of pro-osteogenic CXCL12 axis, including CXCL12, and its two main receptors CXCR4 and ACKR3. Secreted protein levels of CXCL12 were significantly reduced with KYN, and were rescued upon the use of AhR antagonist 3',4'-dimethoxyflavone (DMF). Moreover, KYN upregulated levels of pro-aging and CXCL12-targeting miRNAs miR-29b-1-5p and miR-141-3p. Additionally, KYN significantly inhibited mRNA and protein levels of the epigenetic enzyme Hdac3 which is responsible for activating osteogenesis and inhibiting adipogenesis in BMSCs. Using mutagenesis and luciferase assays, we show evidence that miR-141-3p and miR-29b-1-5p directly target CXCL12 3'-UTR. We also show that miR-29b-1-5p directly targets Hdac3 explaining the significantly increased acetylation levels of H4 with KYN treatment. Finally, we show that mRNA levels of both KYN receptor AhR, and KYN-producing enzyme IDO-1 are regulated by CXCL12. We believe this data explains a novel mechanism of the bone aging phenotype and provides multiple potential clinical intervention targets for controlling osteoporosis.

### LATENT DYADIC CHANGE MODEL OF SPOUSAL SIMILARITY IN PHYSIOLOGICAL MARKERS OF AGING

Shannon T. Mejia,[1] and RIchard Gonzalez[2], *1. University of Illinois, Urbana-Champaign, Champaign, Illinois, United States, 2. University of Michigan, Ann Arbor, Michigan, United States*

Older adult populations are known for their diversity in health. Within couples, however, population studies have documented exceptional levels of spousal similarity in health and health behaviors. The presence of spousal similarity in health among older adults suggests a process of convergence, yet few studies have examined this phenomenon longitudinally. We present a latent dyadic change model to estimate the extent to which couples' similarity in grip strength, Cystatin C, and lung function--indicators of frailty/physiologic reserve. The model is a longitudinal extension of the latent dyadic model, where husbands' and wives' markers of health are parsed into variance that is attributed to the couple and individual levels. Change in the biomarkers of aging is then estimated at the couple and individual levels, resulting in estimates of husbands' change, wives' change, and shared change. We illustrate our model using physiological data from the Health and Retirement Study, a nationally representative panel study of individuals aged 51+ in the United States (3,500+ eligible couples). At the individual level, grip strength and lung function decreased, whereas Cystatin C increased for both husbands and wives. The shared change parameter estimated 16% to 25% of the change in markers of aging existed at the couple level. This suggests, consistent with convergence, that similarities in markers of aging at T2 were due to shared processes of change. Shared processes held after adjusting for indicators of partner selection. The latent dyadic change model offers a methodology to examine change in couples' shared processes over time.

### SPOUSAL DRIVING CESSATION AND VOLUNTEERING: MODERATING ROLE OF CAREGIVING AND DEPRESSION

Angela L. Curl,[1] Christine M. Proulx,[2] and Teresa M. Cooney[3], *1. Miami University, Oxford, Ohio, United States, 2. University of Missouri, Columbia, Missouri, United States, 3. University of Colorado Denver, Denver, Colorado, United States*

Driving cessation is a normative transition in later adulthood, yet previous research shows that having a spouse who ceases to drive, even when you are still able to, negatively impacts one's own engagement (i.e., formal and informal volunteering). Little is known about the conditions under which volunteer engagement might vary after a spouse stops driving. We used longitudinal data from 10 waves (1998–2016) of the Health and Retirement Study (HRS) to examine whether depressive symptoms and caregiving demands moderate the association between a spouse's driving cessation and one's own formal and informal volunteering. Respondents were included if, at baseline, both spouses participated in HRS, both were age 65+, and both were still driving. Respondents were dropped at the time of their own driving cessation, to focus specifically on the impact of spousal driving cessation. Multilevel model results for 1,370 husbands and 1,368 wives show that moderation occurred only for wives who were still driving. After controlling for sociodemographic factors, physical health, and cognitive ability, husbands' driving cessation negatively impacted formal volunteering but only for wives who were primary ADL and IADL caregivers for their spouses. Further, husbands' driving cessation negatively impacted informal volunteering for wives who reported relatively high levels of depressive symptoms. Results suggest the importance of contextual factors like caregiving engagement/needs and psychological wellbeing, especially for wives, when examining the role of spousal driving cessation in partners' volunteer engagement, and highlight the need for additional research on the relationship between spousal driving cessation and volunteering for husbands.

### GENETIC PREDISPOSITION TO ACCELERATED BIOLOGICAL AGES PREDICTED BY BIOCHEMICAL MARKERS

Chia-Ling Kuo,[1] Luke C. Pilling,[2] Zuyun Liu,[3] and Morgan E. Levine[4], *1. University of Connecticut, Farmington, Connecticut, United States, 2. University of Exeter, Exeter, United Kingdom, 3. Department of Pathology, Yale School of Medicine, New Haven, Connecticut, United States, 4. Yale University School of Medicine, New Haven, Connecticut, United States*

Biological ages predicted by biochemical markers (biomarkers) outperform other measures in predicting a variety of aging outcomes. Several have been developed in recent studies, and there is evidence that each may independently predict mortality. While the included biomarkers are disease-associated, it is unclear what aspects of aging are captured. We aimed to understand and quantify genetic predisposition to accelerated biological ages, determined based on two measures, PhenoAge (9 biomarkers plus chronological age, Levine et al. 2018) and BioAge (7 biomarkers plus chronological age, Levine 2013). We performed genome-wide scans using the

UK Biobank data (n=107,460 for PhenoAge, n=98,446 for BioAge). The SNP-based (single nucleotide polymorphism) heritability estimates were 14.45% and 12.39% for PhenoAge and BioAge, respectively. Both shared the strongest signal in the APOE region, with opposite associations with e2 and e4 alleles. e2 was associated with younger BioAge but older PhenoAge. e4 was associated with older BioAge but younger PhenoAge. BioAge was highly genetically correlated with its element of systolic blood pressure (rg=0.84) and the genetic correlation between PhenoAge and red blood cell distribution width was 0.65. Previous genome-wide association study findings of the top hits suggest that BioAge mostly captures cardiac aging but PhenoAge has more to do with inflammatory aging. The results are consistent with SNP clusters by associations with a broad range of aging traits, including an independent cluster with SNPs near the APOE. Genetic risk scores will be created to quantify the genetic predisposition and will be tested for associations with numerous aging traits.

### EVERYDAY ETHICS AND THE UNSEEN MORAL LANDSCAPE OF ASSISTED LIVING

Candace L. Kemp,[1] Jason Lesandrini,[2] Clifvette Webb,[1] Elisabeth O. Burgess,[3] and Jennifer C. Morgan[1], *1. Georgia State University, Atlanta, Georgia, United States, 2. WellStar Health System, Atlanta, Georgia, United States, 3. Gerontology Institute, Georgia State University, Atlanta, Georgia, United States*

Assisted living (AL) communities are increasingly popular long-term care settings where people live, work and visit, and where social relationships and care, including end-of-life care, are negotiated. In the context of daily life, residents, their family members and friends, AL care workers and other staff, external care providers, and volunteers, continually encounter one another and make choices about a range of matters. AL is fraught with uncertainty and conflict about values, especially given residents' cognitive and physical frailty. These value-laden issues have important implications for both resident and care partners' quality of life. Yet, almost no research has examined ethics in this dynamic and complex care environment. We seek to address this important knowledge gap by examining AL through an ethical lens. Drawing on our research and practice experiences, we present a conceptual model that situates everyday ethics within multiple multi-levels of moral decision-making factors and systems involving individuals, the care setting and surrounding community, social norms, and the broader regulatory context. We provide an overview of AL's moral landscape and present examples of three everyday ethical issues. We first examine informed consent as it pertains to sexual encounters involving residents with dementia. Next, we consider boundary and role issues inherent in care process and relationships. Finally, we discuss resident privacy as an area for value uncertainty in everyday life within AL. We conclude by emphasizing the need for in-depth and systematic identification of ethical issues in AL and development of management strategies for applied practice.

### AFFORDABLE SENIOR HOUSING AND HEALTH AMONG LOW-INCOME OLDER IMMIGRANTS: THE ROLE OF SERVICE USES

Jihye Baek,[1] Oejin Shin,[2] Sojung Park,[1] BoRin Kim,[3] and Byeongju Ryu[1], *1. Brown School, Washington University in St. Louis, Saint Louis, Missouri, United States, 2. University of Illinois at Urbana-Champaign, Urbana-Champaign, Illinois, United States, 3. University of New Hampshire, Durham, New Hampshire, United States*

Older immigrants in affordable senior housing face a unique set of challenges due to their demographic, social, economic, and cultural diversity. Existing knowledge about health among this unique but increasing aging subgroup population is extremely limited. Focusing on older immigrants subgroups (Asian and Russian older adults) in affordable senior housing in St.Louis, MO, this study aimed to examine to what extent different ethnic minority elders' health varies by their uses of services available in the housing. Data came from the survey interviews at a subsidized independent senior housing in St. Louis (n=136). Hierarchical multiple regressions were used to examine ethnic differences in self-rated health and the role of services for the health of low-income ethnic minor elders in senior housing. Compared to the non-immigrants (White/African older adults), Asian (b=0.67, p<.05) and Russian residents (b=0.89, p<.05) were likely to have lower self-rated health. Interestingly, for both ethnic groups, they report a better self-rated health when they use supportive daily service (i.e. e.g. meal delivery, transportation, housekeeping and others) (b= -0.84, p<.05 for Asian, b=- 0.90, p<.05.for Russian) and social service (e.g. recreational, wellness, and exercise programs) (b= -0.73, p<.05 for Asian, b=- 0.83, p<.05.for Russian). Our findings point to an important role of services for the health of low-income ethnic minor elders in senior housing. As the first attempt to examine services that explicitly focus on ethnic minority elders, our study provides meaningful implications for future research on the health and service needs for older immigrant populations in senior housing.

### MEN WITH PARKINSON'S MAY HAVE GREATER DISEASE BURDEN IN ASPECTS OF COGNITIVE AND PSYCHOSOCIAL FUNCTION THAN WOMEN

Madeleine E. Hackney,[1] Eeshani Singh,[2] Ella Leeth,[2] Allison Bay,[3] and Liang Ni[3], *1. Department of Medicine, Emory University School of Medicine, Atlanta, Georgia, United States, 2. Emory University College of Arts and Sciences, Atlanta, Georgia, United States, 3. Emory University School of Medicine, Atlanta, Georgia, United States*

Early in PD, women may experience a more benign disease course than man. Limited research has examined differences between men and women with moderate, treated PD with a mean duration >5 y. Retrospective analyses were performed on data collected from studies, conducted 2011–2019, that assessed motor, cognitive and psychosocial function in 199 people with PD (women=72). We compared performance using univariate analyses, adjusting for age, housing type and education. Men and women patients were not different in PD stage (Stage Mdn= 2, IQR=.5), age (mean±SD; 69.1± 8.9 y), education (16.4 ± 2.3 y), number of medications (5.9±4.1), comorbidities (3.4±1.8), physical function, nor time with PD (6.6±4.6 y). Women were more likely to live in assisted living or senior residences (p=.005). Men gave more correct answers in subtraction, (8.8 ± 4.0 vs. 6.4±3.7; p<.001) but sexes did not differ in percent correct. On the MDS-UPDRS, Men exhibited more burden in subjectively rated non-motor (13.4 ±

7.6 vs. 10.7±7.3; p=.013), and motor experiences of daily living (16.9±8.9 vs. 10.6±7.1; p<.001) and motor symptoms (34.1 ± 12.1 vs. 31.8±12.2; p=.014). Men performed worse at inhibition (6.4±4.6 vs. 7.8 ± 5.0; p=.014) but made fewer errors on inhibition/switching (7.0±3.9 vs.7.8±4.4; p=.05). Men had higher depression scores: 12.5±8.9 vs. 9.4±7.8; p=.016. No differences in performance on spatial cognition were noted. Men with moderate PD were more depressed, had worse motor and cognitive function, non-motor and motor experiences of daily living and motor symptoms than women. Sex-tailored therapies may reduce differences in performance between sexes.

### APOLIPOPROTEIN E, LEUKOCYTE TELOMERE LENGTH AND MEMORY IN EXCEPTIONALLY LONG-LIVED FAMILIES

Adiba Ashrafi,[1] Megan Barker,[1] Larry Honig,[1] Joseph Lee,[1] Stacy L. Andersen,[2] Joseph M. Zmuda,[3] Mary K. Wojczynski,[4] and Stephanie Cosentino[1], *1. Columbia University, New York, New York, United States, 2. Boston University School of Medicine, Boston, Massachusetts, United States, 3. Department of Epidemiology, University of Pittsburgh; Pittsburgh, Pennsylvania, United States, 4. Department of Genetics, Washington University in St. Louis, Saint Louis, Missouri, United States*

Exceptional aging has heritable components. One genetic risk factor for cognitive aging may be Apolipoprotein E (APOE), but it is unclear to what extent APOE relates to cognitive aging versus risk of Alzheimer's disease. Cognitive aging may also be influenced by leukocyte telomere length (LTL), posited to be a marker of "biological age". We examine the relationship between APOE, LTL, and memory in aging. For APOE, effects of ε4 (ε3ε4/ε4ε4) and ε2 (ε2ε3/ε2ε2) versus the more common ε3ε3 referent genotype on episodic (EM) and working memory (WM) were examined, comparing longevous families to the general population. Participants belonged to a multi-generational, international cohort (Long Life Family Study) including relatives from long-lived families and spouse-controls. 3,654 participants with valid memory, APOE, and telomere data at baseline were included. Regression analyses were stratified by age group and relative status, adjusting for sex, education, and country. Among controls, ε2 was associated with better WM (p<0.05) in those aged 70-79. In relatives, ε2 was linked to better EM (p<0.05) in those 60-69. Within ε2 carriers, longer LTL related to higher EM/WM for those <60, but lower EM/WM among those 60-69 (p<0.05). In relatives, ε4 was linked to worse EM, but better WM in those <50. Within ε4 carriers ≥80, longer LTL related to poor EM/WM. Thus, APOE related differently to distinct memory functions, and such associations varied by familial longevity and age. LTL demonstrated both positive and negative associations with memory functions depending on APOE status and age group.

### IS THERE AN ASSOCIATION BETWEEN OBSTRUCTIVE SLEEP APNEA AND FRAILTY IN OLDER VETERANS WITH DIABETES?

Jahan E. Mahjabin,[1] Dhanya Baskaran,[2] Christopher Lewis,[3] Valeria C. Baldivieso,[3] William Wohlgemuth,[2] and Jorge G. Ruiz[4], *1. University of Miami Miller School of Medicine, Miami, Florida, United States, 2. Miami VA, Miami, Florida, United States, 3. Jackson Memorial Hospital, Miami, Florida, United States, 4. Miami VAHS Geriatric Research, Education and Clinical Center, Miami, Florida, United States*

Obstructive Sleep Apnea is a highly prevalent disease, where incidence increases with age. Individuals with chronic diseases such as diabetes and obesity are at risk of OSA increasing the risk of frailty. A retrospective chart review was conducted to study the association between OSA and frailty in older diabetic Veterans. Baseline polysomnography data for 91 patients ≥ 65 years was obtained from the electronic health records at the Miami VA Medical Center. Patients were screened for frailty from January 2016 to August 2017, and followed until October 2018. Patients were then dichotomized into frail (Frailty Index (FI) ≥.21) and non-frail (robust FI =<.10 and pre-frail FI ≥.10, <.21) groups. The mean participant age is 70.9 years, with (SD) of 4.8. The mean age for the frail group is 71.1 years, with a SD of 5.2. Mean age for the non-frail group is 70.5 years, with a SD of 4.2. Linear regression demonstrated a significant positive linear relationship between BMI (t=2.096 p-value= .039) and the frailty index. In binomial logistic regression, adjusting for covariates, BMI was associated with increased apnea severity (OR=1.139, 95% CI= 1.044-1.241), p=.003. However, no significant association was found between FI and apnea severity. The severity of OSA based on the Apnea-Hypopnea Index had no significant association with frailty status. However, the study demonstrated a significant association between obesity and frailty, where higher BMI coincided with higher frailty. Increasing BMI coincided with increased severity of OSA, suggesting that BMI acts as a possible confounder between frailty and OSA.

### QUALITY IMPROVEMENT IN LTC: EFFECTIVENESS OF MONTESSORI-BASED ACTIVITY PROGRAMMING IN VA COMMUNITY LIVING CENTER

Thomas Chacko,[1] Kim Curyto,[2] Michelle M. Hilgeman,[3] and Virginia Keleher[4], *1. University at Buffalo, New York, United States, 2. Center for Integrated Healthcare, VA Western New York Healthcare System, Batavia, New York, United States, 3. Tuscaloosa VA Medical Center, Tuscaloosa, Alabama, United States, 4. Tuscaloosa VA Medical Center, Alabama, United States*

Montessori-based Activity Programming (MAP) was adapted for Veterans Affairs (VA) Community Living Centers (CLCs) and aims to increase independence and meaningful engagement in residents with cognitive impairment. The Montessori model prioritizes offering choice, knowing and harnessing a resident's abilities, and enabling them to carry out purposeful roles and activities. Any perceived deficit in cognitive functioning is "circumvented" by preparing the environment to support maximum independence. The implementation of MAP-VA in VA Western NY CLC involved 3 lodges, 52 staff, 16 champions, and 65 CLC residents. Standardized implementation measures demonstrated improvements over six months in five domains assessing development of a resident-directed community. Hypothesized outcomes included improved national percentile quality improvement (QI) rankings related to psychological symptoms and medications (e.g., depressive symptoms and use of antipsychotic/antianxiety medications)

and physical functioning (e.g., less falls and ability to move independently). Scores six months prior to the implementation of MAP-VA (April, 2018 to September, 2018) were compared with scores during six months of implementation post training (November, 2018 to March, 2019). Compared to pre-intervention QI measures related to psychological symptoms, a clinically meaningful trajectory of symptom decrease was observed with rankings during implementation (e.g., depressive symptoms, amount of antipsychotic medications). Likewise, compared to pre-intervention QI rankings regarding physical functioning, post-training rankings showed a trajectory of improvement (e.g., help with ADLs, ability to move independently). Implementation of the MAP-VA intervention demonstrates preliminary evidence for improvement in QI measures related to psychological symptoms and physical functioning. Implications for QI efforts in VA CLCs will be presented.

### ENGAGING AFRICAN AMERICAN FAMILIES IN END OF LIFE DISCUSSIONS: CHALLENGES AND FACILITATORS

Pamela Z. Cacchione,[1] Le'Roi L. Gill,[2] and Justine Sefcik[3], *1. University of Pennsylvania School of Nursing, Senior Fellow , Leonard Davis Institute of Health Care Economics, Philadelphia, Pennsylvania, United States, 2. Mercy LIFE West Philadelphia, Philadelphia, Pennsylvania, United States, 3. University of Pennsylvania, Philadelphia, Pennsylvania, United States*

Our previous study, African American Preferences Around End of Life, identified that AA Elders wanted to talk to their family about their preferences, but their family tended to avoid discussing end of life topics. We found that African American families often have a difficult time broaching the subject of end of life for a variety of emotional, cultural and religious reasons. Therefore, the purpose of this qualitative descriptive study was: To better understand the challenges and facilitators that influenced end of life conversations within the African American family. Methods: In this qualitative descriptive study, we interviewed 15 AA family caregivers of older adults. Participants were family members of older adults enrolled in an urban Program of All-inclusive Care for the Elderly. Individual interviews lasted on average 50 minutes. Data analysis was completed using conventional content analysis. Results: The majority of participants were between 55 - 65 years of age and adult children of the AA older adult. Two themes emerged for challenges: I'm not comfortable and We just don't talk about it. For facilitators again, two themes emerged: Another person took the initiative (e.g. health care provider led the conversation) and participants' previous experience with death led them to initiate EOL conversations. In addition, three participants reported that after participating in the interview they planned to talk to their loved one to find out their end of life preferences. The results of this study provide insight into how health care providers can facilitate these important end of life preferences conversations.

### SURVEILLANCE PROGRAM IDENTIFIES HEALTH OUTCOMES FOR FORMER WORKERS

Ashley Golden,[1] Jeffery Miller,[1] Zachariah Hubbell,[2] Sara Howard,[1] and Donna Cragle[1], *1. Oak Ridge Associated Universities, Oak Ridge, Tennessee, United States, 2. Oak Ridge Associated Universities, Arvada, Colorado, United States*

According to the US Bureau of Labor Statistics, the average age of retirement is 62. While many retirees may have employer-provided or other access to healthcare, others have limited access to affordable care until full Medicare eligibility at 65. Regardless of access, retirees with toxic occupational exposures may not have providers with specialized knowledge of tests or diagnoses for exposure-related health conditions, especially those with long-latency. The National Supplemental Screening Program for U.S. Department of Energy Former Workers is described here as a nationwide program providing recurring (every 3 years) integrated health screenings designed to identify both occupational and non-occupational conditions in the context of exposure so that early identification can enable appropriate and timely diagnoses and treatments to improve health outcomes. Since September 2005, there has been 18,518 initial exams for former workers, of whom 5,461 returned for rescreening exams through April 2019. The average age of those returning was significantly younger at initial exam (63.4 years) compared to those who did not return (65.1 years). The most common occupational condition was noise-induced hearing loss not attributable to natural, age-related loss (67%). Rare and long-latency occupational health conditions, such as asbestosis or silicosis, were identified at rates expected (1-4%). The most common non-occupational condition was elevated body mass index (BMI>25, 77.3%), followed by hypertension (20.7%), of which 50% had no prior knowledge or clinical diagnosis. In conclusion, occupational health surveillance programs can provide value for identifying non-occupational health conditions and as a supplementary source of health information and care.

### MIDLIFE HEALTH FACTORS AND LATER-LIFE COGNITIVE DISEASE: RESULTS FROM THE 1998-2014 HEALTH AND RETIREMENT STUDY

Benson Wu,[1] Mohammad Usama Toseef,[2] Wassim Tarraf,[2] and Hector M. González[3], *1. Department of Neurosciences at UC San Diego School of Medicine, La Jolla, California, United States, 2. Wayne State University, Detroit, Michigan, United States, 3. University of California, San Diego, San Diego, California, United States*

Understanding lifecourse determinants of older-age health outcomes is indispensable for resources planning and optimizing public health in light of continued gains in longevity in the US and worldwide. Data increasingly points to midlife health and modifiable risk factors as critical targets for improving older-age health outcomes and mitigating potential cognitive impairment and disease. We used 16-years of biennial data (1998-2014) from the Health and Retirement Study (unweighted-n=6,724), to examine how a comprehensive battery of midlife (age 50-64 years) health measures (disability, physical function, comorbid conditions, and self-reported health) affect cognitive status (using Langa-Weir criteria: Normal, Cognitively Impaired Not Dementia (CIND), and Dementia) and death 16-years later. Additionally, we test for racial/ethnic and gender modifications in the effects of these conditions on the outcomes of interest. We used survey multinomial logistic regression models adjusting for predisposing sociodemographic factors, health-enabling economic characteristics and health behaviors. Relative risk ratios (RRR) across all unadjusted models varied from 1.36-4.84 and 1.36-3.31 for those with dementia and who died in 2014 respectively, suggesting worse health outcomes in midlife are associated with higher dementia/mortality risk in later-life. After covariates-adjustment, comorbidities (RRR=1.15[1.04,1.27]) and Self-reported Health (RRR=1.36[1.22,1.52]) were associated with CIND, and attenuation was particularly pronounced for IADLS (RRR=3.15[2.25,4.43]) and Fine Motor Skills (RRR=1.94[1.46,2.57]) for individuals with dementia in 2014. Neither sex nor race/ethnicity modified these associations. Modifying the midlife health profile of US adults can yield important public health savings and reductions in structural and social health burdens through extenuating the prevalence of dementias and reducing excess mortality.

### PROBLEMS AND PROSPECTS OF TECHNOLOGICAL AND BIOPHYSIOLOGICAL STUDIES OF TECHNOSTRESS IN MID-LIFE ADULTS

Elizabeth Mack,[1] Shelia Cotten,[1] Chu-Hsiang Chang,[1] and Wenda Bauschpies[1], *1. Michigan State University, East Lansing, Michigan, United States*

Long-term exposure to stress places people at risk for chronic diseases including but not limited to obesity, Type-2 diabetes, and heart disease. Various aspects of technology use are associated with stress. Known as technostress, this unique stress is characterized by individuals' inability to cope with demands generated by computer-related technologies. To date, studies on technostress have focused on young adults and older adults, with an emphasis on self-reported indicators of both technology use and stress. This study differs from prior work in two ways. One, it examines technology use and stress in mid-life adults (50-64), an understudied population in research on technostress. This segment of the population is important because their technostress may negatively affect their successful transition into older adulthood. Second, we use three types of data to elucidate the linkages between technology use and stress: (1) self-reported survey measures of technology use and stress; (2) objective measures of technology use from tracking applications, and (3) biophysiological measures of stress. The study focuses on smartphone use, which was the most commonly used technology by mid-life adults on both weekdays and weekends based on our initial results (N=40). The goal of this pilot study is to highlight the problems and prospects of conducting technostress research through the utilization of multiple data collection modes: self-report, tracking applications (apps), and biophysical indicators.

### DEMENTIA WITHOUT BORDERS: BUILDING COMMUNITY CONNECTIONS TO REDUCE STIGMA AND FOSTER INCLUSION

Alison Phinney,[1] Marigrace Becker,[2] Lee Burnside,[2] and Gloria Puurveen[1], *1. University of British Columbia, Vancouver, British Columbia, Canada, 2. University of Washington, Seattle, Washington, United States*

BACKGROUND: The concept of social citizenship is gaining traction in the field of dementia studies, but as a practical tool to guide development of supports and services, it remains poorly understood. A one year project to promote collaboration between University of Washington in Seattle and University of British Columbia in Vancouver addressed this very question. Activities were undertaken so these communities could know each other better, with researchers, service providers and people with dementia connecting to share knowledge and expertise. PURPOSE: The project culminated with a public festival to put into practice and share some of what was learned over the year. METHODS: People with dementia and care partners helped plan "Dementia Without Borders", held at an international park straddling the border between Seattle and Vancouver. 150 people came from the US and Canada, including many people with dementia, family members and friends. The day began with a community walk and gift exchange, followed by a meal and creative activities including poetry readings, music, an art exhibit, and quilt making. RESULTS: Evaluation was overwhelmingly positive with people expressing a sense of hope and belonging. For some, it was their first time to speak openly about having dementia, and meeting others in this space was a joy-filled experience. CONCLUSIONS: This project has leveraged the symbolic power of an international border to raise awareness of the importance of social connection for people with dementia. We further explore how the notion of "dementia without borders" extends theoretical and practical understanding of social citizenship.

### EFFECTS OF RESISTANCE EXERCISE TRAINING ON ENDOTHELIAL FUNCTION AND MUSCLE PERFUSION IN OLDER ADULTS WITH DIABETES

Amanda Randolph,[1] Tatiana Moro,[2] Adetutu Odejimi,[1] Blake Rasmussen,[1] and Elena Volpi[3], *1. UNIVERSITY OF TEXAS MEDICAL BRANCH, Galveston, Texas, United States, 2. Sealy Center on Agyng, Galveston, Texas, United States, 3. UTMB, Galveston, Texas, United States*

BACKGROUND: Sarcopenia contributes to frailty, disability, and dependence in older adults, and is accelerated by Type 2 Diabetes Mellitus (T2DM). In addition to its direct role in increasing muscle mass, progressive resistance exercise training (PRET) may also reduce sarcopenia by improving endothelial function and muscle perfusion. METHODS: Fifteen older adults with uncomplicated and well-controlled T2DM participated in a PRET program 3 times weekly for 3 months. Prior to and immediately following the intervention, flow-mediated dilation testing was performed to assess large vessel endothelial function via ultrasound and muscle perfusion via near-infrared spectroscopy (NIRS). RESULTS: Preliminary ultrasound data from 9 subjects show a significant increase (5.21% to 8.73%, p=0.0448) in percent flow mediated dilation (%FMD), suggesting a modest improvement in endothelial function after 3 months' PRET. Preliminary NIRS data from 7 subjects showed no significant changes in oxygen saturation or reperfusion rates as a result of the intervention. CONCLUSION: Our preliminary data indicate that, in older adults with T2DM, 3 months' PRET is associated with modestly improved endothelial function in large vessels (as demonstrated by a significant increase in %FMD), but does not appear to be associated with improvements in muscle perfusion

### EMERGING ADULT CAREGIVERS: PERCEPTIONS OF OLDER ADULT CARE RECIPIENTS, QUALITY OF CONTACT, AND AGEISM

Anastasia E. Canell,[1] and Grace Caskie[1], *1. Lehigh University, Bethlehem, Pennsylvania, United States*

Approximately 12-18% of family caregivers to older adults in the U.S. are 18-25 years old (i.e., emerging adulthood), yet minimal research has focused on this subgroup of caregivers (Levine, 2005; Smyth, Blaxland, & Cass, 2011). Individuals' perceptions of an older adult's social role relate to their attitudes toward older adults as a group (Hummert, 1999; Kite & Wagner, 2002). However, whether perceptions that emerging adult caregivers hold of older adults are specific to the social role of "care-recipient" has not been studied. A sample of 210 informal caregivers (ages 18-25) were surveyed to collect qualitative responses regarding perceptions of an older adult care-recipient (age 65+) and to assess quality of contact with the care-recipient and ageist attitudes. Participants were asked to provide five adjectives describing their older adult care-recipient. Approximately 43% provided a set of adjectives in which 80%-100% were coded as positive adjectives (e.g., "active", "wise"); similarly, half of the sample's adjective sets contained 0%-25% negative adjectives (e.g., "helpless", "obnoxious"). The quality of contact with the care-recipient was significantly correlated ($p<.001$) with the percentage of positive ($r=.47$) and negative ($r=-.49$) adjectives. Scores on the Fraboni Scale of Ageism were also significantly correlated ($p<.01$) with the percentage of positive ($r=-.19$) and negative ($r=.20$) adjectives. Overall, these emerging adult caregivers had generally positive perceptions of their older adult care-recipients, and these perceptions reflected the positive quality of contact with the care-recipient. Less ageist attitudes' relationship with more positive and less negative perceptions may have implications for experiences within a caregiving dyad.

### AMINO ACIDS PREDICT COGNITION BEYOND CLINICAL METABOLIC MARKERS: A MACHINE LEARNING APPROACH

Jessie Alwerdt,[1] Yuan Tian,[2] Andrew D. Patterson,[3] and Martin Sliwinski[1], *1. Penn State University, Center for Healthy Aging, University Park, Pennsylvania, United States, 2. Penn State University, Huck Institutes of the Life Sciences, University Park, Pennsylvania, United States, 3. Penn State University, The Department of Veterinary and Biomedical Sciences, University Park, Pennsylvania, United States*

Prior work has suggested that metabolic disorders increase the risk for cognitive decline. Further, studies have identified amino acids (AAs) as potential biomarkers for dementia and diabetes. This study examines AAs and metabolic clinical markers (MCM) as predictors of cognition (Processing Speed (SOP), Working Memory (WM), Fluid (Gf) and Crystallized Intelligence (Gc)). The sample included 241 middle-aged adults from Bronx, NY. Predictors included age, gender, education, ethnicity, smoking, having diabetes, glucose, insulin, triglycerides, diastolic, and systolic blood pressure (BP), and cholesterol. AAs and associated derivatives were obtained from serum using NMR-based metabolomics. Analyses were conducted for each cognitive domain using repeated cross-validation random forests and lasso regressions. Overall, all models had acceptable cross-validation mean squared error except for WM. Several MCMs were specific to each cognitive domain, such as lower triglycerides and glucose associated with better SOP and higher systolic BP associated with better Gc while none were identified for Gf. The Gf model had the least number of AAs with lower serine associated with better FI. Two AAs, higher histidine and alanine, were associated with better SOP. Further, higher alanine, valine, isoleucine, serine, methionine, betaine, and moderate tyrosine were associated with better Gc. These results indicate that AAs were specific to each cognitive domain and ranked similar or higher in importance as several MCMs These results suggest that further investigation of AAs alongside associated MCMs is needed to assess the metabolic contribution to cognitive performance. Such research will help identify specific metabolic targets relating to cognition.

### METABOLOMICS PROFILING OF MUSCLE FAT DEPOSITION IN AGING: RESULTS FROM THE HEALTH ABC STUDY

Samaneh Farsijani,[1] Megan M. Marron,[1] Iva Miljkovic,[1] Mary E. Baugh,[2] Stephen Kritchevsky,[3] and Anne B. Newman[1], *1. University of Pittsburgh, Pittsburgh, Pennsylvania, United States, 2. Wake Forest School of Medicine, Winston-Salem, United States, 3. Wake Forest School of Medicine, Winston-Salem, North Carolina, United States*

Background: Age-related inter-muscular fat (IMF) deposition is associated with poor physical function in those with preserved/high muscle mass. However, the heterogeneity of IMF in aging is poorly understood. We used a semi-targeted metabolomics approach to: 1) determine the metabolites associated with IMF infiltration in aging; and 2) establish a model to predict IMF using the metabolome. Methods: We performed a cross-sectional analysis of 314 African-American men (age: 69-79 years) from the Health ABC study at baseline. Mid-thigh IMF area (cm2, by CT) and 350 plasma metabolites (by liquid-chromatography/mass spectrometry) were measured. Correlation analysis was performed to determine metabolites associated with IMF. An IMF prediction model was calculated, using regression analysis with 10-fold cross-validations on random halves of the population with metabolites, age and weight as predictors. Results: Of 161 metabolites correlated with IMF ($P<0.05$), 34 remained significant after adjusting for age, weight, physical activity, medications, smoking and multiple comparisons (false discovery rate ≤0.25). IMF-associated metabolites were primarily lipids (76%) and amino acids (15%). Most metabolites were positively correlated with IMF (94%), with the exception of mevalonic acid (from fatty acids sub-class) and glutamine (from organic-acids) which were negatively associated with IMF. IMF-associated metabolites predicted 49% of the variability in the actual IMF in the test set of the random half of the population (50%, n= 144). Conclusion: Identification of the unique metabolomics features associated with IMF may improve our understanding of key biological alterations of muscle during aging.

### EVALUATING GERIATRIC WORKFORCE DEVELOPMENT NEEDS AMONG HOSPITALS USING NICHE BENCHMARKING DATA

Roy A. Thompson,[1] Loretta Matters,[2] Kirsten Corazzini,[3] and Eleanor McConnell[2], *1. Duke*

*University School of Nursing, Durham, North Carolina, United States, 2. Duke University, Durham, North Carolina, United States, 3. University of Maryland School of Nursing, Baltimore, Maryland, United States*

The Nurses Improving Care for Health System Elders (NICHE) program aims to improve geriatric care competencies for improved care quality. A quantitative descriptive design utilizing secondary data analysis was done to evaluate geriatric workforce enhancement efforts in one acute healthcare system. Data were collected using the Geriatric Institutional Assessment Profile (GIAP) from 2008 and 2013. The GIAP measures perceived professional issues (disagreements among staff and families, limited access to geriatric services, vulnerability to legal action, intensity and burden of behavioral problems) on a Likert scale from best=0 to poor=10. Staff perception of the Geriatric Care Environment was scored by the GIAP as: age sensitive care delivery (0-40), institutional values (0-28), resource availability (0-32) and capacity for collaboration (0-12). Higher scores on the Geriatric Care Environment reflected improvements. Independent sample t-tests examined changes in baseline scores. Post-NICHE implementation, compared to peer hospitals by teaching status and bed size in 3 hospitals there were significantly ($p<0.05$) improved scores for: access to geriatric services (2.79-3.21), burden of behavioral problems (2.40-3.15), aging sensitivity care delivery (26.05-29.53), institutional values (18.85-19.59) and resource availability (19.51-19.97). Peer hospitals had significantly ($p<0.05$) better scores for: disagreements among staff about treatment of older adults (1.63-1.94) and capacity for collaboration (7.72-7.99). Findings indicate improvement in perceived professional issues and need for improvement in the geriatric care environment and care redesign to progress to becoming an Age-Friendly health system. This was an initial step in a health system to improve care quality through health workforce development.

### PERSONS LIVING WITH COGNITIVE IMPAIRMENT SHARE THEIR VIEWS ON TECHNOLOGY

Susan Wehry,[1] and Regula H. Robnett[2], *1. University of New England College of Osteopathic Medicine, Biddeford, Maine, United States, 2. University of New England, Portland, Maine, United States*

The purpose of this study was to examine the experience of adults living with cognitive impairments and that of their care partners with digital technology including current use of, ease with and openness to using smart assistive technologies (SATs). SATs for older adults with (and without) cognitive impairments have become increasingly commonplace. Research on various digital devices has focused primarily on supporting users' independence and care partner concerns for safety and security. Our qualitative, interview-based research project provided digital devices chosen by participants to address a specific personal goal. Interviews were conducted in the home and set-up assistance was provided during the initial interview. At the conclusion of the trial period, a second interview was conducted in the home. We describe the participants' commendations for, expectations of, and frustrations with current technology as well as recommendations for potential, helpful digital technology. Current technology offers great promise but a disconnect between the design of digital technologies and the needs and wishes of the end-user still exists. This study will help inform additional user-driven application SATs, including those aimed at enhancing enjoyment and a higher quality of life.

### TOGETHER THROUGH DEMENTIA: MEASURING RELATIONSHIP SATISFACTION FOR VETERANS WITH DEMENTIA AND THEIR SPOUSES

Julia Loup,[1] Phoebe Block,[1] Nathalie Rieder,[2] Kimberly Alexander,[3] Teddy K. Bishop,[3] A. Lynn Snow,[3] and Michelle M. Hilgeman[3], *1. University of Alabama, Department of Psychology, Tuscaloosa, Alabama, United States, 2. Indiana University of Pennsylvania, Department of Psychology, Indiana, Pennsylvania, United States, 3. Tuscaloosa VA Medical Center, Tuscaloosa, Alabama, United States*

Current and prior relationship quality can have significant impacts on the mental and physical health of individuals with dementia (IWD) and their spousal caregivers (CGs). Yet, marital satisfaction is not often assessed. This poster examines use of the Dyadic Adjustment Scale (DAS), a widely-researched marriage counseling tool, for contextualizing interpersonal factors beyond established measures of quality-of-life (QoL), burden, and depression. Community-dwelling spousal CGs ($n = 49$) and Veterans with dementia ($n = 37$) completed self-report measures including dyadic relationship satisfaction/distress, mutuality, QoL, depression, burden, and the IWD's cognitive status. Descriptive statistics, bivariate correlations, and regressions were performed. Scores on the DAS significantly correlated with CG QoL (.567, $p < .001$), CG depression (-.525, $p < .001$), CG burden (-.428, $p = .002$), IWD's cognitive status (.355, $p = .034$), and IWD social engagement (-.424, $p = .011$). IWD-reported DAS scores were positively correlated with IWD QoL (.381, $p = .024$), CG QoL (.340, $p = .043$), and IWD subjective health (.360, $p =.031$). Regression analysis showed CG DAS ($b = .188$, $\beta = .464$, $p = .002$) and IWD social engagement ($b = -2.806$, $\beta = -.37$, $p = .012$) are significantly predictive of CG QoL; $F(2, 32) = 15.865$, $p < .001$; $R2 = .495$. Findings suggest that the DAS provides important relationship quality insights and may improve QoL needs assessments for caregiving support and respite services, including CG willingness to continue in the CG role. Examining the DAS longitudinally could also inform intervention delivery as dementia severity progresses.

### CULTURE-RELATED TASK DIFFICULTIES AND NEGATIVE CONSEQUENCES FOR CAREGIVERS FROM DIVERSE COMMUNITIES

Rachel Schaffer,[1] David Bass,[1] Sara Powers,[1] Jenna McDavid,[2] and Ocean Le[2], *1. Benjamin Rose Institute on Aging, Cleveland, Ohio, United States, 2. Diverse Elders Coalition, New York, New York, United States*

The Diverse Elders Coalition, in partnership with its six member organizations and the Benjamin Rose Institute on Aging, completed a national survey of 840 family and friend caregivers from diverse racial, ethnic, and sexual orientation communities to understand their unique caregiving issues and challenges. Data from a subsample of 404 caregivers identifying as Hispanic/Latino, Asian, Southeast Asian or from multiple ethnicities were examined to determine the relationship between difficulties performing culture-related care tasks (i.e., assisting with immigration issues and language barriers) and a variety of caregiver outcomes. Regression analysis controlling for background and context characteristics showed caregivers experiencing more culture-related task difficulties had significantly higher levels of several negative caregiving consequences, including health strain (B=.19, p=.001), relationship strain (B=.17, p=.005), work strain, (B=.24, p=.000) isolation (B=.17, p=.002), and symptoms of depression (B=.29, p=.000). Moreover, higher levels of strain and depression among caregivers who reported high levels of culture-related task difficulties ranged from 26%-54%. More difficulties with culture-related tasks also were significantly related to dissatisfaction with support resources, including lower ratings of the quality of care receivers' healthcare (B=-.20, p=.001), and lower satisfaction with support they and their care receivers received from family and friends (B=-.17, p=.006 and B=-.16, p=.011, respectively). Results suggest caregivers from diverse communities struggling with culture-related tasks experience more negative consequences of caregiving and less helpful social support. Service providers working with caregivers from diverse communities should assess for culture-related task difficulties, recognizing these problems may be a window into a variety of adverse caregiving effects.

### HEARING LOSS AND SOCIAL ISOLATION: THE ROLE OF NEIGHBORHOOD SOCIAL COHESION AND DISORDER

Sol Baik,[1] and Amanda Lehning[2], *1. University of Maryland, Baltimore, Baltimore, Maryland, United States, 2. University of Maryland, Baltimore, Maryland, United States*

Social isolation is a critical public health issue that socially isolated individuals are at increased risk for mortality and deteriorated health. Those who acquire hearing loss in later life experience difficulties with communication, potentially leading to social isolation. However, less is known about the social consequences of age-related hearing loss, and few studies have assessed the influence of environmental factors on hearing loss and social isolation. The aims of this study are to examine: (1) the association between hearing loss and social isolation of older adults over time, and (2) the moderating effects of perceived neighborhood social cohesion and disorder on this relationship. We analyzed 2,080 community-dwelling Medicare beneficiaries aged 65 or above from Round 1 to 3 of National Health and Aging Trends Study. We conducted random coefficient models, entering hearing loss as a random coefficient. Older adults with hearing loss were less socially isolated than those without hearing loss. However, the effect of hearing loss on social isolation varied depending on perceived neighborhood social cohesion. Older adults with hearing loss who reported high neighborhood cohesion had significantly lower social isolation compared to those without hearing loss, while those with hearing loss who perceived low social cohesion had significantly higher social isolation. Our findings suggest neighborhood social cohesion can serve as a potential protective factor for older adults with hearing loss. This poster will propose neighborhood-level interventions that could supplement other services for those with hearing loss, such as assistive devices that are rarely covered by health insurance.

### RELATIONSHIPS BETWEEN PERSONAL NEEDS AND THE ENVIRONMENT: A STUDY OF ABUSE AND SELF-NEGLECT

Minzhi Ye,[1] Farida Ejaz,[1] and Miriam Rose[1], *1. Benjamin Rose Institute on Aging, Cleveland, Ohio, United States*

Estimates of self-neglect among older and/or disabled adults are much higher than the estimated 10% of older adults in the U.S. who experience physical or emotional abuse, neglect, and exploitation by others. It is not clear how the social environment affects these vulnerable adults who become self-neglecting. This study uses network analysis and a GIS approach to explore patterns of needs and environmental risks for adult healthcare patients who had risk factors for self-neglect. Sources of data included face-to-face interviews, self-neglect risk factors (e.g., depression, dependency in activities of daily living, etc.) from electronic medical records, and neighborhood information from census block data. More than two-thirds of the 480 study participants reported an average income of less than $1,360 monthly, and 89% self-identified as Hispanic or Latino. Using ArcGIS Pro, respondents' geocoded addresses were matched to mapped neighborhood census information. The maps showed that most respondents live in Hispanic-dominated communities, in neighborhoods with crime rates above average and median household income of less than $49,066/year. These neighborhoods were probably resource-poor and had spatial inequalities. Using network analysis, the study found that the at-risk patients' most frequently reported needs (e.g., food assistance/nutrition, functional limitations, social isolation) appear clustered together, demonstrating that people had multiple needs. The study findings suggest that practitioners and policymakers must not only provide a range of services to help disadvantaged groups, but also focus specifically on offering services in neighborhoods where low-income minority groups reside and there is a lack of community resources.

### END-OF-LIFE CARE DURING THE NATIONAL OPIOID CRISIS: A NATIONAL SURVEY OF HOSPICE PROVIDERS

John Cagle,[1] Mary Lynn McPherson,[2] Jodi Frey,[2] Paul Sacco,[3] Orrin Ware,[2] and Jack Guralnik[4], *1. University of Maryland, Baltimore, Maryland, United States, 2. University of Maryland, School of Social Work, Baltimore, Maryland, United States, 3. University of Maryland-Baltimore, Baltimore, Maryland, United States, 4. University of Maryland Baltimore School of Medicine, Baltimore, Maryland, United States*

No national data exist on drug shortages, missing medications, opioid diversion, and opioid diversion prevention in hospice. We conducted a national survey of hospices (administered June-September, 2018). We randomly selected 600 hospices to survey representatives about: (1) care for patients/families with substance use disorder (SUD) (2) drug shortages; (3) instances of drug diversion; and, (4) drug disposal practices. Surveys were conducted by phone and online. Sample weights were used to adjust for non-response. A total of 371 hospices completed surveys (response rate=62%), 63% of which were administered by phone. Half (50%) of agencies were mid-sized (26-100 patients) and non-profit. Two thirds (66%) of hospices reporting that medications either "never" or "rarely" go missing. On average, there were 0.80 reported cases of confirmed diversion per agency within the past 90 days. Although a majority of hospices (78%) screen patients for SUD, only 43% screen informal caregivers. Just under half (42%) of hospices reported drug shortages over the past year. A minority (8%) of hospices stopped prescribing certain medications altogether due to concerns about diversion. 52% of hospices reported that employees are not allowed to dispose of medications after a home death. Agency representatives estimated that, after a home death, unused opioids were left in the home 32% of the time. On average, hospices have nearly one case of opioid diversion per quarter. Hospices are experiencing medication shortages and restrictions on medication disposal. Changes are needed in policy and practice to address these challenges.

### COMPARISON OF CAREGIVERS FROM DIVERSE COMMUNITIES WHO IMMIGRATED TO OR WERE BORN IN THE US

Lauren Stratton,[1] David Bass,[2] Rachel Schaffer,[2] Sara Powers,[2] Ocean Le,[3] and Jenna McDavid[3], *1. Iowa State University, Ames, Iowa, United States, 2. Benjamin Rose Institute on Aging, Cleveland, Ohio, United States, 3. Diverse Elders Coalition, New York, New York, United States*

The Diverse Elders Coalition, in partnership with its six member organizations and the Benjamin Rose Institute on Aging, completed a national survey of 840 family and friend caregivers from diverse racial, ethnic, and sexual orientation communities to understand their unique caregiving issues and challenges. Data from a subsample of 369 caregivers identifying as Hispanic/Latino, Asian, Southeast Asian or multiple ethnicities were analyzed to understand similarities and differences between caregivers born in the US and who immigrated to the US. The Stress Process Conceptual Model guided selection of characteristics used for comparative analysis. Results of logistic regression revealed that caregivers born in the US were younger (B=-.08, p<.001), had higher educational degrees (B=.42, p<.001), and higher incomes (B=.34, p=.002). They assisted care receivers with more health-related tasks (B=.27, p=.013), but fewer culture-related tasks (B=-.51, p=.002); reported higher levels of strain in their relationship with care receivers (B=.66, p=.038); and were less satisfied with the quality of care receivers' healthcare (B=-.62, p=.042). In terms of reasons for being a caregiver, there were no significant differences in cultural commitment to caring for older family members, however those born in the US were more likely to report providing care because it was more convenient for them than for other family and friends (B=.99, p=.002). Understanding the needs of diverse caregivers has implications for healthcare and service providers, such as providing training on diverse needs. Additionally, the differences between US born and immigrant caregivers highlights implications on the dynamic between caregivers and their care receiver.

### HIGH RUMINATION AND LOW AMINO ACIDS ARE ASSOCIATED WITH AN INCREASED RISK OF METABOLIC SYNDROME

Jessie Alwerdt,[1] Yuan Tian,[2] Andrew D. Patterson,[3] and Martin Sliwinski[1], *1. Penn State University, Center for Healthy Aging, University Park, Pennsylvania, United States, 2. Penn State University, Huck Institutes of the Life Sciences, University Park, Pennsylvania, United States, 3. Penn State University, The Department of Veterinary and Biomedical Sciences, University Park, Pennsylvania, United States*

Metabolic syndrome (MetS) is an increasing epidemic worldwide. Identifying modifiable behaviors that intersect with the association between MetS and associated metabolites could result in alternative methods to prevent those at risk for MetS. Here we investigate the moderation of ruminating thought processes between metabolites and MetS. Rumination has been linked to exacerbating the physiological response of stress and increasing the risk for poor health outcomes, such as hypertension. Data consisted of 180 middle-aged adults from Bronx, NY. MetS was calculated based on the NIH guidelines using waist, triglycerides, high-density lipoproteins, blood pressure, and glucose. Using NMR-based metabolomics, 26 serum metabolites were obtained. The Rumination-Reflection Questionnaire measured rumination of thoughts. Interactions between rumination and each metabolite were conducted with logistic regressions (e.g., Valinexrumination). Overall, significant moderation occurred with the greatest effect involving different levels of phenylalanine, betaine, creatine, and isoleucine with higher rumination in relation to the increased probability for MetS. The greatest risk for MetS was in those who were high ruminators and had low values of these AAs. Therefore, within those who are high ruminators, an increase in these AAs may be beneficial in improving the risk for MetS. Further, in those who are low ruminators, minimal moderation occurred. AAs disturbance has been linked with MetS in past studies, as well as in mental health. These results suggest that ways of handling thoughts that are intertwined with everyday stress may exacerbate these associations and could benefit with modification.

### PERSISTENCE OF ACCELERATED VASCULAR AGING FOLLOWING THERAPY IN OLDER CANCER SURVIVORS

Barbara W. Carlson,[1] Melissa Craft,[1] Rachel Funk-Lawler,[2] Karla Keepper,[1] Wajeeha Razaq,[3] and Anna Csiszar[4], *1. Fran and Earl Ziegler College of Nursing, The University of Oklahoma Health Sciences Center, Oklahoma City, Oklahoma, United States, 2. Department of Psychiatry, University of Oklahoma Health Sciences Center, Oklahoma City, Oklahoma, United States, 3. Stephenson Cancer Center, The University of Oklahoma Health Sciences Center, Oklahoma City, Oklahoma, United States, 4. Reynolds Oklahoma Center on Aging, The University of Oklahoma Health Sciences Center, Oklahoma City, Oklahoma, United States*

Chemotherapy destroys cells indiscriminantly and releases proinflammatory factors into the bloodstream that can adhere to endothelial cells (ECs); resulting in phenotypical changes consistent with "accelerated vascular aging". This pilot study examined associations between markers of EC integrity, vascular aging, and cognition in 15 female breast cancer survivors 12–18 months after chemotherapy (median age: 57 years) and 2 non-cancer controls (58/59 years). EC integrity was evaluated using frequency-dependent electrical impedance (Z4000Hz) and levels of apoptosis (caspase-3/7), inflammation (NFkB activation), and oxidative stress (NRF2 activation) in cultured human endothelial cells (EC) exposed to the subject's serum. Vascular aging was characterized by low serum insulin growth factor ([IGF1a] <85mg/dL) and laterality in cerebral oxygenation (Lat-FLOX), an in-vivo marker of altered cerebral blood flow (near-infrared spectroscopy). Z4000Hz were higher in cells treated with serum from survivors (520-1100 ohms) than controls (400-450 ohms). Higher Z4000Hz was associated with higher amounts of EC oxidative stress (rNRF2= -.55), inflammation (rNFkB= .43), apoptosis (rcapsase=.76), and Lat-FLOX (r=.39). Higher Z4000Hz (r= -.48) and Lat-FLOX (r= -.66) were associated with lower cognitive function, as measured by the Montreal Cognitive Assessment (MOCA). At lower Z4000Hz, combined Lat-FLOX and low IGF1a characterized persons with cognitive impairment (MOCA < 26). These findings suggest that proinflammatory factors related to cancer and/or its treatment persist for months following active treatment. Cognitive symptoms occur when EC damage results in alterations in cerebral blood flow. With depletion of growth factors, like IGF-1, these symptoms may occur at lower levels of EC damage.

### IMPACT OF AGING POPULATIONS ON MUNICIPAL EMERGENCY MEDICAL SERVICES

Nidya Velasco Roldan,[1] Caitlin E. Coyle,[2] Michael Ward,[1] and Jan Mutchler[3], *1. University of Massachusetts Boston, Massachusetts, United States, 2. Gerontology Institute, University of Massachusetts Boston, Boston, Massachusetts, United States, 3. University of Massachusetts Boston, Boston, Massachusetts, United States*

The services that residents require from their local governments vary depending on the demographics of their populations. While municipalities have long sought to consider how changes in the young population may impact their school system needs, few systematic considerations have been developed relating to how aging populations may impact municipal service provision. This study aims to address this issue by focusing on demands on emergency services at the municipal level. Using data from the Massachusetts Ambulance Trip Record Information System (MATRIS) we explore the association between emergency medical services (EMS) demand and population age-structure. The data shows an overrepresentation of older people among EMS users. People age 65 and older represent 16% of Massachusetts' population but account for 31% of the transported emergent calls —e.g., 911 calls— and 60% of the scheduled transports. Results from the OLS regression analysis suggest that communities with larger shares of older residents have significantly higher numbers of EMS calls. The type of community and other age-related community features such as the percentage of older residents living alone and the percentage of older population dually eligible for Medicare and Medicaid are also significantly associated with the number of EMS calls. Contrary to our expectations, other resources available in the community such nursing homes or assisted living facilities were not significantly associated with number of EMS calls. Our research indicates that if growth in the older population occurs as projected, the demand placed on the EMS system by older populations will grow considerably in coming decades.

### AGE-ASSOCIATED INCREASE IN KYNURENINE INHIBITS AUTOPHAGY AND PROMOTES SENESCENCE IN BONE MARROW STEM CELLS

Dmitry Kondrikov,[1] Ahmed Elmansi,[1] Xing-ming Shi,[2] Sadanand Fulzele,[3] Meghan mcGee-Lawrence,[2] Mark Hamrick,[2] Carlos Isales,[2] and William D. Hill[1], *1. Medical University of South Carolina, Charleston, South Carolina, United States, 2. Augusta University, Augusta, Georgia, United States, 3. Department of Orthopaedic Surgery, Medical College of Georgia, Augusta University, Augusta, Georgia, United States*

Aging is characterized by progressive decline of tissue functionality and age-related accumulation of cellular and molecular damage leading to multiple pathological conditions including osteoporosis and increased fracture rates. Bone marrow mesenchymal stem cells (BMSCs) play an essential role in bone development and regeneration with their ability to undergo differentiation into osteogenic, chondrogenic, myogenic, and adipogenic cell lines cell lines. Proliferation rate of MSC is declined with ages leading to misbalance between bone resorption and osteogenesis. A recently identified age-related change in bone and bone marrow is an accumulation of tryptophan metabolite, kynurenine (KYN), catalyzed by indoleamine-2,3-dioxygenase (IDO) or free-radical oxidation. We previously reported that KYN suppresses autophagy in BMSC. We now investigated the effect of KYN on BMSC cellular function. In vitro treatment of murine BMSC isolated from 18 month old mice with kynurenine disrupted autophagy suppressing autophagic flux. KYN treatment also induces senescence in BMSC marked by increase in SA-beta-galactosidase activity as well as, increased expression of senescence marker p21. Inhibition of Aryl Hydrocarbon Receptor (AhR) by AhR inhibitors significantly reduced β-galactosidase activity increase and blocked p21 expression elevation suggesting that KYN induces senescence in BMSC through the AhR pathway. Interestingly, KYN treatment failed to up-regulate beta-gal activity in BMSC isolated from 6 month-old mice suggesting that KYN induction of senescence maybe potentiated with aging. Together those data support the idea that KYN shifts the homeostatic balance of BMSC during prolonged stress or in aging through downregulating survival autophagic pathway in favor of driving BMSCs to senescence.

### DOES THE NEWS MEDIA FOSTER A HOSTILE ENVIRONMENT FOR OLDER ADULTS: A CONTENT ANALYSIS OF NEWS ARTICLES

Erfei Zhao[1], *1. University of Southern California, Los Angeles, California, United States*

The media has become increasingly more agenda-driven and politically biased in recent years. This research explores whether the major news outlets fosters an environment of negative sentiment towards the older adult population over a 4-year-period from 2016-2019. The paper uses a content analysis approach. 199 news articles that include keywords such as "older adults", "seniors" or "elderly" are randomly selected among those published in the first week of March in 2016, 2017, 2018 and 2019 from the online archives. Main attitudes towards the older adults are extracted and categorized. In the 4-year span, regression analysis shows that negative attitudes are apolitical and are found in almost two-thirds (64%) of the 199 news articles in both liberal and conservative news outlets, suggesting that the current media creates a ubiquitously hostile environment for older adults. However, more liberal news outlets tend to produce more articles with positive messages about the older adults ($p<0.05$), and more conservative news outlets tend to create more content that reinforces negative stereotypes of older adults as being vulnerable and less productive ($p<0.01$). The high percentage of negative attitudes and the ubiquitous negative attitudes in news organizations, in spite of their political leaning, suggests that the media environment has some influence on the news content. Political biases of the organization are associated to numbers of articles with positive messages and negative stereotyping. Promoting professional development and self-regulating mechanisms in journalism could aid in reducing ageism perpetuated in the media.

### BEST PRACTICE CAREGIVING: DIFFERENCES AND GAPS AMONG DEMENTIA SUPPORT PROGRAMS FOR FAMILY & FRIEND CAREGIVERS

Julie H. Rentsch,[1] David Bass,[1] Kathy Kelly,[2] Katie Maslow,[3] Alyssa Ciancibello,[1] Leah Eskenazi,[2] and Rachel Schaffer[1], *1. Benjamin Rose Institute on Aging, Cleveland, Ohio, United States, 2. Family Caregiver Alliance, San Francisco, California, United States, 3. Gerontological Society of America, Washington, District of Columbia, United States*

Family members and friends are the main providers of care for persons living with dementia. However, dementia caregivers are at greater risk than other caregivers of experiencing negative caregiving consequences. Despite the development of evidence-based programs to support dementia caregivers, few health or social service organizations offer any of these programs due, in part, to a lack of knowledge about their availability. Best Practice Caregiving is a newly launched website where professionals can get detailed information about these programs. Data collected to develop Best Practice Caregiving are analyzed for a sample of 42 evidence-based dementia caregiving programs to describe similarities and differences among programs including gaps in assistance available from these programs. Results show 64% of programs are delivered to caregivers only while the remaining are delivered to the caregiver and/or persons with dementia. Nearly half (43%) of the 42 programs are delivered in-person, 38% by phone, with 17% delivered all or in part online. Most programs are delivered by professionals (86%) followed by trained lay leaders (40%) and self-guided (12%). Most programs (95%) provide assistance with coping with illness/caregiving and the relationship of the dyad. Fewer than half of the programs assist caregivers with issues regarding finances (45%), end-of-life care (43%), and medical care (40%). Data from 233 delivery organizations show the most common challenge was getting caregivers to accept and complete the program (86%). Delivery sites reported more success with funding the program (mean=8.2 on a scale of 1-10) than with marketing and recruiting participants (mean=6.7).

### DATABASE FOR INDICES OF AGING IN NONHUMAN PRIMATES

Joseph W. Kemnitz[1], *1. University of Wisconsin-Madison, Madison, Wisconsin, United States*

The Primate Aging Database (PAD) is a multi-centered, relational database of biological variables in aging, captive monkeys and apes containing approximately one million data points for body weight, blood chemistry and hematology, for male and female subjects over time (https://primatedatabase.org). More than forty species are currently represented, primarily chimpanzees, macaques and common marmosets. Metadata include housing environment, social context and diet. Life history information for each species is also provided. Data in PAD is gathered from various research facilities, sanctuaries and zoos. PAD has recently been extensively revamped to enhance ease of use. Tools for data visualization and analysis in multiple formats are included. PAD has been useful for exploring biomarkers of aging in primates and for examining physiological dysregulation in aging across primate species. It also provides age-specific normative values that are valuable in clinical veterinary medicine. New data are being added to PAD, including additional subjects and variables, and additional contributors are solicited. (Supported by contract HHSN2711201800025C from the National Institute on Aging to CleMetric Data Analytics and Management, LLC.)

### ALTERED TRYPTOPHAN DEGRADATION LINKS CHRONIC INFLAMMATION TO FUNCTIONAL DECLINE & FRAILTY IN MICE AND HUMANS

Reyhan Westbrook,[1] Tae Chung,[2] Jacqueline Lovett,[3] Chris Ward,[4] Mohammed Khadeer,[3] Ruin Moaddel,[3] Jeremy Walston,[5] and Peter Abadir[1], *1. Division of Geriatric Medicine and Gerontology, Johns Hopkins University School of Medicine, Baltimore, Maryland, United States, 2. Department of Physical Medicine and Rehabilitation, Johns Hopkins University School of Medicine, Baltimore, Maryland, United States, 3. National Institute on Aging, National Institutes of Health, Baltimore, Maryland, United States, 4. Department of Orthopedics, University of Maryland School of Medicine, Baltimore, Maryland, United States, 5. Johns Hopkins University School of Medicine, Baltimore, Maryland, United States*

Chronic inflammation is associated with frailty and functional decline in older adults but the molecular mechanisms of this linkage are not well understood. We sought to examine metabolic and physiologic states associated with aging and frailty by analyzing the composition of metabolites in the blood of a population of community dwelling young, and older adults. Serum inflammatory cytokines and demographic and physiological covariates were collected in a set of community-dwelling adults age 20-97 (n=166). We then used LC/MS technology to profile 121 metabolites from five substance classes. Associations of the cytokines and metabolites with grip strength, walking speed, falls and outcomes were assessed in young, robust, pre-frail and frail participants. Age and frailty status positively correlated with IL6, TNFα, TNFαR1, IL1β ($p<0.0001$). Analysis of metabolites revealed significant alterations in tryptophan degradation pathway with aging and frailty. Among the top metabolites to correlate with age and frailty status were kynurenine ($p<0.0001$) and the kynurenine/tryptophan ratio ($p<0.0001$). The kynurenine/tryptophan ratio also tightly correlated with serum inflammatory cytokines TNFαR1 ($p<0.0001$) and IL-6 ($p<0.0001$). Higher kynurenine/tryptophan levels were associated with weaker grip strength and slower walking speed, even after adjusting for age, gender, BMI and blood pressure. Further dissection of the pathway revealed the accumulation of 3-hydroxykynurenine, a cytotoxic and neurotoxic intermediate from the kynurenine pathway, with frailty. The increased levels of cytotoxic and neurotoxic molecules in this pathway may in part explain the link between inflammation and cognitive and physical decline in frailty.

### ASSOCIATION BETWEEN GERIATRIC HOME VISIT AND EMERGENCY ROOM OR HOSPITAL UTILIZATION: A RETROSPECTIVE STUDY

Xin Zhang,[1] and Paul Takahashi[1], *1. Mayo Clinic, Rochester, Minnesota, United States*

Homebound older adults tend to have more medical comorbidities, higher risk of mortality, and higher healthcare utilization compared to non-homebound, community dwelling older adults in the U.S. Preventative primary care home visit have been shown to reduce hospitalizations and total healthcare costs in this population. Through a retrospective study, we aim to explore characteristics associated with ED and hospital utilization in patients who have received a home visit. A total 608 subjects, 70% female and median age of 86 years, were involved. A 184 (30%) of homebound subjects were hospitalized or visited the ED in 90 days. Comparing those with hospitalization or ED and those without, there was no significant difference in sex, age, race, marital status, or advance care planning. A 74% of those with 90 day ED/hospitalization had prior ED visit within 1 year of home visit compared to 59% of those without (p=0.0004). In addition, 58% of those with 90 day ED/hospitalization had prior hospitalization within 1 year of home visit compared to 44% of those without (p=0.0015). They also had significantly higher number of total medical comorbidities (median 4.5 vs 4, p=0.02). Our study suggests that prior healthcare utilization and medical comorbidity burden may be better predictors of 90 day hospitalization or ED use in geriatric patients who have received a home visit. Advance care planning and age did not significantly differ between the two groups in our study. Further studies should be performed to validate our findings in a prospective manner.

### COPING BEHAVIORS THAT INCREASE STRESS AMONG OLDER ADULTS

G. Rainville,[1] Alicia R. Williams,[1] and Lona Choi-Allum[1], *1. AARP, Washington, District of Columbia, United States*

This study examined the efficacy of a series of 28 behaviors (e.g., comfort eating, attending worship services, getting a massage, etc.) in moderating the perception of stress among older adults. First, 28 individual behaviors were assessed to determine whether they buffered or exacerbated the impact of an objective stress measure (i.e., the count of stressful life events) on perceptions of stress (measured using Cohen's Perceived Stress Scale [PSS]). A full sample analysis used data from 1,000 randomly selected U.S. adults age 40 and older, but subsequent analyses explored coping behaviors for two age groups—those age 40 to 59 and those 60 and older. In the full sample analysis, multiple moderating conditions were noted including stress-buffering for worship service attendance, recreational shopping, and getting a massage. Also among the full 40+ sample, stress-exacerbation was noted for social media use and coping by "overreacting to things." Factor analysis (employing a polychoric correlation matrix) reduced the 28 individual behaviors into 9 clusters comprised of related behaviors and representing a general coping approach. Looking within the age groups, significant stress-buffering was limited to those age 60 and older for two coping approaches—a "Self-Care and Travel" approach and an Inspirational approach (e.g., praying, attending church, etc.). For both age subgroups there was no coping approach, not even the hedonistic "blowing off steam" approach, that was found to exacerbate the impact of stressful life events on the perception of stress.

### RELATION BETWEEN ERECTILE DYSFUNCTION AND AMNESTIC MILD COGNITIVE IMPAIRMENT ACROSS TWO TIME POINTS

Richard Vandiver,[1] Michael J. Lyons,[1] and Kristy Cuthbert[1], *1. Boston University, Boston, Massachusetts, United States*

Previous research by the Vietnam Era Twin Study of Aging (VETSA) demonstrated an association between erectile dysfunction (ED) and cognitive functioning. That finding supports a hypothesis that cardiovascular dysfunction may underlie both ED and problems in cognitive functioning. The purpose of the current research was to extend these findings by investigating a putative association between ED and amnestic and non-amnestic mild cognitive impairment (MCI). MCI is of particular interest because of its relationship with Alzheimer's disease and other dementing illnesses. VETSA is a longitudinal study of twins who served in the US military during the Vietnam conflict (N= 960) consisting of data collected at age 20 (enlistment), age 55 (VETSA 1), and 61 (VETSA 2). The results of the current analyses show that ED is related to both amnestic MCI (p=.032) and non-amnestic MCI (p=.009) at VETSA 1. At VETSA 2, however, the relationship between ED and non-amnestic MCI was no longer significant (p=.751) while the relationship between ED and amnestic MCI was stronger (p=.001). These results are consistent with ED and MCI sharing, to some extent, a common etiology. Vascular dysfunction, which is associated with both ED and MCI, is a plausible mechanism responsible for the observed relationship. These results also highlight the potential role that may be played by ED as an early indicator of cognitive impairment and, perhaps, pre-symptomatic AD.

### KEY DIMENSIONS OF OASIS, AN OLDER-ADULT DRIVEN MODEL OF AGING-IN-PLACE IN CANADA

Vincent DePaul,[1] Catherine Donnelly,[1]
Simone Parniak,[1] and Oasis Study Collaborative[2],
*1. Queen's University, Kingston, Ontario, Canada, 2. Oasis Study Collaboration, Ontario, Canada*

Oasis Senior Supportive Living (Oasis) is an active aging-in-place model created by older adults in a naturally occurring retirement community, such as an apartment building. The program is member-driven so that participating older residents determine the programming and services that best address their needs. The first Oasis program was established in an apartment building in Kingston, Canada and has been running for more than ten years. Preliminary evaluations of the Oasis program demonstrate that its members report feeling more socially connected, are more physically active, and have increased nutrition as a result of participation.

In-depth interviews were conducted with Oasis members and key program stakeholders to identify the core dimensions of the Oasis program that has led to its success in supporting active aging in place. Interviews were audio-recorded and transcribed verbatim. Thematic analysis was used to identify, analyze and report themes. Four themes emerged: (1) nutrition, social and physical activities as critical programming pillars; (2) the importance of active member participation and decision-making; (3) the need for onsite support to facilitate programming; and (4) Oasis as a family. These findings highlight the need for programming that is designed for and by older adults. Supporting older adults to come together and form community is key to healthy and active aging. Identification of these elements is critical to modelling Oasis in other community contexts. Oasis is currently being expanded to seven new communities across Ontario using a participatory action research approach.

### BLOOD PRESSURE TRENDS PRECEDING DEMENTIA: A STUDY OF HEART FAILURE PATIENTS

Connor Wilson,[1] Amanda Pangle,[1] Jeanne Wei,[1] and Gohar Azhar[1], *1. Donald W. Reynolds Institute on Aging, UAMS, Little Rock, Arkansas, United States*

Blood pressure and perfusion of the brain are central components of neurological health that are often influenced by heart failure. This retrospective case-control study analyzed blood pressure changes preceding the diagnosis of dementia in patients over the age of 60. Blood pressures were obtained from the date of dementia diagnosis, and then one year and five years before diagnosis. Study "controls" were age-matched patients without dementia, using the mean age of dementia diagnosis as the first data point. Over the five-year period preceding diagnosis, 67.2% with dementia showed decreasing systolic pressure compared to 46.6% of patients without dementia. Similarly, 62.7% with dementia showed a decreasing systolic pressure over the one-year period, compared to 50.0% of those without dementia. Additionally, there was a significant difference ($p < 0.001$) in the dementia rates between African American and Caucasian subgroups (55.0% African Americans vs. 31.0% Caucasians). Patients with dementia were more likely to have decreasing blood pressure trends than age-matched patients without dementia and appeared to have significantly lower blood pressures one year before the diagnosis. It is crucial that providers are cognizant of these trends and risk factors for dementia as they manage blood pressures in geriatric patients.

### SMOOTHING THE MOVE FROM POST-ACUTE TO HOME CARE FOR OLDER CARDIAC PATIENTS: A SOCIAL WORK TRANSITIONS INITIATIVE

Orah R. Burack,[1] Jessica Auerbach-Burgoon,[1] Sandra Mundy,[1] and Verena R. Cimarolli[2], *1. The New Jewish Home, New York, New York, United States, 2. LeadingAge LTSS Center @UMass Boston, Washington, District of Columbia, United States*

Transitioning across medical settings (e.g. from hospital to post-acute (PA) or PA to homecare (HC)) is a difficult time with numerous challenges, as critical information passes across sites, new systems are quickly established, and caretakers change. Older cardiac heart failure (CHF) patients, often with comorbidities and having fewer social supports, are especially vulnerable to rehospitalizations at that time. This study examines the impact of a Social Work Transitions (SWT) intervention, designed to ease older cardiac patients' transition from a PA to HC setting, on rehospitalization rates. The SWT model for CHF patients was developed in a large healthcare system with a continuum of services for older adults including PA and HC. Once a patient enters PA from the hospital a transitions social worker (SW) remains the patient's primary support and contact through PA discharge and the transition to HC. In HC, that same SW ensures needed services occur, conducts home visits, and provides additional follow-up via phone calls. Study 1: compared HC rehospitalization rates of CHF patients receiving SWT (N=28) with those not receiving SWT (N=26). This natural control group arose during the initial program months, as SW turnover occurred and some CHF patients were not accompanied by a transitions SW. SWT patients had half the rehospitalizations (25%) as controls (54%). Study 2 tracked 30 day rehospitalizations rates for the first 17 study months (N=257). Program rehospitalization rates (16.7%) were below the CMS benchmark (21%). These findings support using the SWT program to prevent unnecessary rehospitalizations in CHF patient.

### BEYOND BINGO: THE IMPACT OF ACTIVITY ENGAGEMENT IN AN ALL-AFRICAN AMERICAN ASSISTED LIVING COMMUNITY

Inneke L. Vargas,[1] Alexis A. Bender,[2] Candace L. Kemp,[3] and Molly M. Perkins[4], *1. Wichita State University, Wichita, Kansas, United States, 2. Emory University, Atlanta, Georgia, United States, 3. Georgia State University, Atlanta, Georgia, United States, 4. Division of General Medicine and Geriatrics, Emory University School of Medicine, Atlanta, Georgia, United States*

More than one million older adults reside in assisted living (AL) communities in America. This figure is projected to double by the year 2030. It is typical for residents in these communities to have cognitive and physical impairments requiring differing levels of care. Due in part to these impairments, it is vital to the health and well-being of residents to participate in meaningful recreational activities. This secondary analysis of ethnographic observations totaling 818 hours and semi-structured interviews with 25 residents enrolled in an NIA-funded study (5R01AG047408) explores in depth barriers and facilitators to activity engagement in a large (90+ bed) moderate-income, all-African-American AL community located in a large urban city in the southern US. Residents range in age from 59 to 103 (mean = 85) and are predominantly female. We linked our findings from thematic analysis to six domains of quality palliative care identified by the 2018 National Consensus Project (NCP) Guidelines for Quality Palliative Care: cultural, physical, psychological, social, structures and processes, and spiritual aspects of care. Key barriers include limitations related to staffing (a low staff-to-resident ratio and high staff turnover) and activities that do not adequately address needs of residents with varying interests and abilities. A robust daily devotion and other activities that incorporate culturally relevant music are activities highly valued by most residents. Implications of these findings contribute to a larger effort to create positive change in the structures and processes of care in AL and can inform best practices for palliative care within these communities.

### THE HEALTHY AGING INDEX AND ITS ASSOCIATION WITH MORTALITY IN OLDER MEXICAN AND EUROPEAN AMERICANS

Vinutha Ganapathy,[1] Mini E Jacob,[1] Meghan I Short,[1] Mitzi M Gonzales,[1] Claudia Satizabal Barrera,[1] Sudha Seshadri,[2] Chen-pin Wang,[3] and Helen P Hazuda[4], *1. Glenn Biggs Institute for Alzheimer's and Neurodegenerative Diseases, U T Health San Antonio, San Antonio, Texas, United States, 2. UT Health San Antonio, San Antonio, United States, 3. University of Texas Health Science Center, Texas, United States, 4. U T Health San Antonio, San Antonio, Texas, United States*

Mexican Americans (MA) have higher morbidity compared to European Americans (EA); however, the mortality rate remains lower (Hispanic Paradox). The Healthy Aging Index (HAI) captures clinical and subclinical morbidity in older adults and is useful in examining ethnic differences in mortality for a given disease burden. We assessed the association between baseline HAI and all-cause mortality over 12 years of follow-up among older MAs (n=394) and EAs (n=355) in the San Antonio Longitudinal Study of Aging (SALSA) and examined differences between ethnic groups. HAI incorporates non-invasive measures (systolic blood pressure, forced vital capacity, creatinine, fasting plasma glucose (FPG), Mini-Mental State Exam). Missing baseline data for HAI components and covariates were imputed using multiple imputations. Proteinuria was used instead of creatinine due to non-availability. Scores of 0, 1, 2 were given from lowest to highest tertile HAI categories; diagnosis of diabetes, hypertension, and renal failure were included in the highest tertiles. Cox proportional hazards models estimated the association between HAI and mortality, adjusting for confounders. After adjusting for age, gender, education, income, BMI, smoking and ethnicity, HAI was independently associated with mortality (HR 1.25 (1.16-1.35), p-value <0.0001). We found no interaction effect between HAI and ethnicity on mortality ((p-value for interaction = 0.78). In the SALSA sample, HAI is a predictor of mortality after adjusting for confounders in both MAs and EAs. The absence of a significant HAI*ethnicity interaction effect further demonstrated that HAI works equally well as a predictor of mortality in both MAs and EAs.

### DEVELOPING RURAL INSIGHTS FOR BUILDING SUSTAINABLE AGE-FRIENDLY COMMUNITIES INITIATIVES

Elizabeth McCrillis,[1] Mark Skinner,[2] and Amber Colibaba[2], *1. Trent University, Ontario, Canada, 2. Trent University, Peterborough, Ontario, Canada*

Researchers have questioned the focus on describing features of preliminary age-friendly implementation and the absence of program evaluations or analyses of long-term implementation. This lack of knowledge inadvertently simplifies unique pathways to age-friendly sustainability, preventing researchers from conducting in-depth, retrospective examinations of age-friendly and post-age-friendly perspectives. Seeking to address this critique, this research examines the challenges to rural age-friendly program sustainability, and the factors that may help committees overcome these barriers. Data were collected through a succession of qualitative studies, including a provincial age-friendly program evaluation and a series of studies examining sustainability in rural initiatives. Eighty in-depth interviews with age-friendly leaders and older participants from 27 rural Canadian programs were conducted, seeking knowledge about programs' development and implementation. Key findings include the conceptualization of an implementation gap between early development and long-term viability, the important role played by individual communities, the challenges of capacity and jurisdictional fragmentation, and the inability of rural age-friendly programs to tackle bigger picture issues such as housing and transportation given their necessarily limited scope and reach. Implications relevant for research and practice suggest that drawing on individual, community, and jurisdictional factors will maximize the success and sustainability of rural age-friendly programs, thereby extending the reach and scale of programs to more directly affect older people. From this, we conclude that the sustainability and success of rural age-friendly programs would benefit from consistent, renewable government funding that considers factors relevant to overcoming the implementation gap and challenges created by jurisdictional fragmentation and de-emphasizing community individuality.

### RESEARCH FOR ACTION: IDENTIFYING SERVICE NEEDS OF MONGOLIAN OLDER ADULTS

Uyanga Batzogs,[1] Kathryn Braun,[1] Margaret Perskinson,[1] and Tyran M. Terada[1], *1. University of Hawaii, Honolulu, Hawaii, United States*

Although adults aged 60 years and older currently represent 6% of the total population of Mongolia, they are projected to increase to 19% by 2050. More than 21% of Mongolians live below the poverty line. Social security payments represent the main source of income for many retirees. With little government funding, eldercare services are limited, creating a large gap between service needs and availability. Before an effective system of eldercare can be developed, in-depth understanding of older adults' needs and resources is required. A mixed methods design was used. Four-hundred twenty-seven Mongolians aged 55 years and older were surveyed. Two focus groups with 10 older adults and eight in-depth interviews with senior center stakeholders were conducted between June 2019 and August 2019. Descriptive statistics were run to determine frequencies of participants' service needs. Linear regression examined the relationship between age groups, service needs, and ability to pay for services. Focus group and interview transcripts were analyzed for underlying themes. Findings indicated high service needs among older adults. Retirement homes, assisted living, home care, and places to socialize were identified as the most needed services. Lack of services, employment opportunities, and income sources concerned them the most. Ability to pay for services was negatively correlated with age groups. Qualitative analysis yielded two themes: importance of services and lack of resources. Further research on starting and sustaining networks of supportive services for older adults living in Mongolia is needed.

### THE SEARCH STUDY: A QUALITATIVE INQUIRY INTO DIVERSE STAKEHOLDER PERSPECTIVES ON HEALTHY AGING ACROSS CHICAGOLAND

Mushira Khan,[1] Karen Graham,[1] Tarisha Washington,[2] Abigail Kim,[2] Raj C. Shah,[3] and Crystal M. Glover[2], *1. Rush Alzheimer's Disease Center, Illinois, United States, 2. Rush Alzheimer's Disease Center, Chicago, Illinois, United States, 3. Rush University, Chicago, Illinois, United States*

Older adults face increased risk of chronic diseases of aging such as Alzheimer's dementia and other adverse age-related outcomes. However, the conceptualization of healthy aging and how age-related issues are addressed in community-based structures, particularly among racial and ethnic minorities, remain poorly understood, especially from the Stakeholder perspective. Stakeholders, such as faith leaders and members of community-based organizations, engage in regular negotiations to advance health equity in their communities by partnering and collaborating with older adults and their families as well as other local and federal organizations. The Stakeholder Engagement in Aging Research and Community Health (SEARCH) Study employs multiple research methods to illuminate Stakeholders' perspectives on barriers and facilitators to healthy aging in diverse communities. This presentation highlights findings from in-depth, qualitative interviews with Stakeholders (N=37) serving African American, Latinx, and South Asian older adults. Emergent themes suggest that systemic racism, stigmatization, limited health literacy, and cultural beliefs serve as barriers to healthy aging across groups. Within groups, Stakeholders report precarious immigration status and fragile and fragmented life situations as barriers among Latinx older adults, while acculturative stress presents a challenge to healthy aging in South Asian older adults. Food insecurity and neighborhood factors such as exposure to violence and socio-economic disadvantages act as barriers among African American older adults. Conversely, religious faith and spirituality, familial support, and culturally-congruent care serve as facilitators across groups. The findings from this study underscore the continued need for intersectional, inclusive, and culturally-informed approaches to supporting healthy aging within diverse communities.

### HEART FAILURE, ARRHYTHMIAS AND DEMENTIA IN THE "OLDEST OLD" OF SOUTH CENTRAL UNITED STATES

Brandon Duke,[1] Gohar Azhar,[1] Amanda Pangle,[1] Priya Mendiratta,[1] and Jeanne Wei[1], *1. Donald W. Reynolds Institute on Aging, UAMS, Little Rock, Arkansas, United States*

Heart failure and dementia are common age-related conditions. Heart failure and associated comorbidities of hypertension and arrhythmias may impact cognition. Retrospective data of 311 patients averaging 98 (±3.2) years who received treatment at the University of Arkansas for Medical Sciences were analyzed for diagnoses, prescribed medications and health conditions relevant to heart failure, hypertension, arrhythmias and dementia. 74% of the subjects were white, non-Hispanic, 24% were African American, and 2% were of unknown ethnicity. 83% were women and 17% were men. Only 251 (81%) of the reviewed charts had blood pressures recorded, of whom 43% (n=114) had systolic pressures >140mmHg. Furthermore, 50% (n=156) of patients had heart failure, and 29% (n=89) had dementia. Of those with dementia, 35% had an arrhythmia. For those without a diagnosis of dementia or any treatment for dementia, 25% had an arrhythmia. Heart failure and arrhythmias have not been well studied as an etiological factor for dementias. In our cohort, the presence of heart failure diagnoses was not different in those with dementia versus those without dementia. However, more patients with dementia had arrhythmias versus those without dementia, suggesting that arrhythmias may contribute to cognitive decline, even in the oldest old. Approximately 70% of the arrhythmias were atrial fibrillation. We did not have data on the management of these arrhythmias and whether anticoagulants were being used appropriately, especially for atrial fibrillation. Nevertheless this highlights the importance of close management of arrhythmias for maintaining cognitive health in older adults.

### LOW GLUCOSE ENHANCES THE CYTOPROTECTIVE EFFECT OF METFORMIN AGAINST DOXORUBICIN-INDUCED CYTOTOXICITY

Fathima Ameer,[1] Xiaomin Zhang,[1] Gohar Azhar,[1] Yingni Che,[1] and Jeanne Wei[1], *1. Donald W. Reynolds Institute on Aging, UAMS, Little Rock, Arkansas, United States*

Metformin, an oral anti-diabetic drug, is currently being investigated for its anti-aging properties and has also been used as adjunct therapy in cancer. Cancer is a disease of aging. Type 2 diabetes is also prevalent in older adults. We wanted to test the hypothesis that metformin could protect normal cells during chemotherapy treatment under different glucose conditions. We used C2C12 myoblast cells to study cellular bioenergetics, variations in gene expressions, and biochemical alterations induced by metformin and pegylated liposomal doxorubicin (L-Doxo) under low glucose (2.7 mM or 50 mg/dL) and normal physiologic glucose (5.5 mM, or 100 mg/dL) conditions. Using confocal microscopy, we noted that treatment of C2C12 cells with 30 μg/mL L-Doxo under low glucose and normal physiologic glucose conditions induced cellular defects. Furthermore, L-Doxo treatment dysregulated the expression of mitochondrial fission and fusion genes, which may influence transformation of the network's connectivity. L-Doxo treatment significantly reduced mitochondrial oxygen consumption rate (OCR) and extracellular acidification rate (ECAR). However, pre-treatment with 100 nM metformin provided protection against L-Doxo-induced damage and increased cell viability and ATP levels in cells even under low glucose conditions. Our data provide further evidence by which low dose metformin exerts protective effects against L-Doxo, a chemotherapeutic drug, under low glucose conditions. Metformin appears to act via AMPKα, Raptor, and SRF, and has significant cellular protective effects that may be useful in cancer and/or aging.

### CHOREOGRAPHING INTERACTION WITHIN THE U-M HOMELAB: CONSIDERATIONS FOR AN OLDER ADULT POPULATION

Natalie M. Leonard,[1] Marianthie Wank,[1] Karen Nielsen,[2] Alicia G. Carmichael,[1] Maren Wisniewski,[1] Vineet Raichur,[1] and RIchard Gonzalez[1], *1. University of*

*Michigan, Ann Arbor, Michigan, United States, 2. Georgia State University, Atlanta, Georgia, United States*

Drawing from the lens of architecture, designing a research study for a controlled, simulated environment requires the consideration of three primary types of interaction involving people and infrastructure. These three types include the interface between and among the respondent(s) and the researcher(s), the interface between and among the people playing these roles and the infrastructure surrounding them (inclusive of self-report measures, sensing technology, furniture, etc.), and the interface between and among the various types of study infrastructure. The flow of a study across these interfaces becomes a form of choreography, with implications for protocol adherence, reproducibility, and data quality. Recently, a pilot study assessing an older adult population's upper-body performance was our first iteration of research utilizing a simulated environment: the U-M HomeLab, an ADA-accommodating, one-bedroom apartment built within the basement of a large research facility. Nine participants, aged 61 to 72, with self-reported upper-body weakness completed a series of tasks resembling activities of daily living, such as lifting laundry baskets and vacuuming. By back-tracking through our development of this pilot study, we illustrate how considerations of interface play a significant role across every stage of study design, incorporating aspects of wayfinding, dialogue, safety, acclimation, and visibility that are relevant to an older adult population. From these reflections, considerations of interface inform a "check-list" for simulation choreography, providing guiding questions for assessing these types of interactions while iterating through study design.

### PATIENT PREFERENCES FOR LEVEL OF HEALTH LITERACY IN APPS FOR CHRONIC DISEASE SELF-MANAGEMENT

Mary Louise Goodyear,[1] Juan J. Toral-Garcia,[1] Amarilis Acevedo,[2] Drenna Waldrup-Valverde,[3] and Raymond Ownby[2], *1. Department of Psychiatry and Behavioral Medicine, Nova Southeastern University, Fort Lauderdale, Florida, United States, 2. Nova Southeastern University, Fort Lauderdale, Florida, United States, 3. Nell Hodgson Woodruff School of Nursing, Atlanta, Georgia, United States*

In spite of expert recommendations that written material should be provided at a level of health literacy that matches that of the person receiving it, there have been few studies of matching. In this study we evaluated the utility of a new strategy to assess patients' preference for information at different difficulties and assessed the relation of their preference to measured health literacy and health locus of control (LOC). We measured health literacy in participants then asked them to choose between pairs of texts with the same content but at the 3rd, 6th, or 8th-grade levels. Statistical analyses assessed the relation of participants' health literacy to their preference as well as personal characteristics. Participants ($n = 155$) were 84 women and 71 men aged from 40 to 82 years (mean=57; 136 African Americans and 19 whites). Participants had clear preferences: those with lower levels of health literacy preferred texts at the 3rd grade level and those with higher levels preferred more difficult texts. Preference was not related to age, gender or race but was to education and health literacy ($p < 0.05$). Persons who chose more difficult texts reported higher levels of internal health locus of control ($t [144] = 2.68$, $p = 0.01$). A predictive analytic model using education and preference resulted in 80% correct classification of participants. Using this model may be a simple way to match information presentation to patients' level of health literacy. Further research on this strategy is needed.

### BENEFICENCE, COMPANIONSHIP AND FINANCES AS MOTIVATORS FOR PARTICIPATION IN INTERGENERATIONAL HOME SHARING

Cary Sweeney,[1] and Rachel K. Bell[1], *1. University of California, Berkeley Retirement Center, Berkeley, California, United States*

In January 2019, the UC Berkeley Retirement Center began piloting Berkeley Home Match (BHM), a program that matches graduate students who need affordable housing with retirees who live near campus and have an extra room. BHM seeks to address affordable housing challenges for retirees and students while creating meaningful relationships. While some data exists for home sharing programs, less is known about the motivators and outcomes of retirees and students participating in these programs. In addition to decreasing the financial burden of housing, Social Exchange Theory suggests the built-in reciprocity of intergenerational home sharing may facilitate meaningful relationships. The purpose of this program evaluation was to understand the motivating factors for participation in a University-based home sharing program. Applicants (N=35) rated factors that influenced their decision to participate using a 7-point scale (1="Not Influential", 7="Very Influential"). On average, homeowners (N=6) rated "helping a student" 5.2, "income" 5.2, and "companionship" 3.6, while home seekers (N=29) rated "helping an older adult" 5.03, "rent price" 6.1, and "companionship" 4.76. Surprisingly, ratings for "helping a student" and "helping a senior" were the same or close to ratings for "income" and "rent prices," suggesting beneficence could be a key motivator and interdependence a mechanism for relationship development. Additionally, students' rating for "companionship" was higher than expected, indicating an openness to the relationship. These findings as well as other factors impacting home matching will be discussed, including participant demographics, reduced housing cost, housing characteristics, and long-term program feasibility.

### THE UNIQUE IMPORTANCE OF RELATIONSHIP QUALITY FOR COMMUNITY-DWELLING OLDER ADULTS

Ashley M. Tate,[1] and Lynn Martire[1], *1. The Pennsylvania State University, University Park, Pennsylvania, United States*

Social networks can directly influence the health and well-being of older adults. Some work has suggested that network growth is associated with increased well-being. However, little is known about how the quality of relationships with confidants may be associated with better psychological well-being over and above the number of confidants. We aimed to test the hypothesis that feeling closer to confidants would be associated with lower anxiety and fewer depressive symptoms above and beyond the influence of the number of confidants as well as the number of children and grandchildren. To test this hypothesis, we collected data during face-to-face interviews with 131 community-dwelling adults who were between the ages of 58 and 94. Participants' gender, age, marital status, self-rated health, and cognitive function were included as covariates in the models. In line with predictions, regression analyses showed that average closeness with confidants predicted significantly lower reports of anxiety ($p < .05$) and depressive symptoms ($p < .001$). Additionally, the number of confidants was not significantly associated with anxiety or depressive symptoms. Interestingly, having a greater number of children and grandchildren was associated with increased anxiety symptoms. These results extend previous work by suggesting that the quality of the relationship with confidants is more important for psychological well-being than the number of confidants. Future work should test these associations longitudinally so that directionality can be inferred.

### ECOLOGICAL MOMENTARY ASSESSMENT OF ASSOCIATIONS AMONG HIGH AND LOW AROUSAL AFFECT AND COGNITIVE HEALTH

Eric S. Cerino,[1] and Martin Sliwinski[2], *1. Pennsylvania State University, University Park, Pennsylvania, United States, 2. Penn State University, Center for Healthy Aging, University Park, Pennsylvania, United States*

Negative affect (NA) and positive affect (PA) vary from moment-to-moment and these variations are associated with cognitive health. Past work has primarily focused on valence (negative/positive), however, largely ignoring the potential import of arousal (high/low). We address this gap by assessing the impact of high and low arousal NA and PA on daily cognition. A sample of 238 older adults (Mage=77.30 years, SD=5.14, Range=70–90) completed mobile surveys up to four times daily for 14 days. Participants reported current levels of high and low arousal NA and PA and performed processing speed and working memory tasks. For processing speed, there were significant within-person affect by age interactions. Moments when low arousal NA was higher than usual were associated with slower processing speed (Est.=0.87, SE=0.44, $p<.05$), and this effect was amplified in older participants (Est.=1.69, SE=0.60, $p<.01$). Moments when high arousal PA was higher than usual were associated with faster processing speed (Est.=-0.81, SE=0.40, $p<.05$), and this effect was amplified in younger participants (Est.=-1.81, SE=0.56, $p<.01$). For working memory, a significant within-person high arousal PA by age interaction emerged (Est.=0.001, SE=0.00, $p=.046$) such that moments when high arousal PA was higher than usual were marginally associated with worse working memory performance only among older participants (Est.=0.004, SE=0.002, $p=.06$). Results suggest momentary increases in low arousal NA and high arousal PA may confer greatest risk to daily cognitive health among older adults with more limited capacity and/or cognitive resources, whereas affective influences may be more facilitative among comparatively younger adults.

### AGE, FUTURE TIME PERSPECTIVE, AND EVERYDAY PHYSICAL ACTIVITY AND NUTRITION IN COUPLES POST STROKE

Elizabeth Zambrano Garza,[1] Theresa Pauly,[2] Wolfgang Linden,[1] Maureen C. Ashe,[1] Rachel Murphy,[1] Kenneth Madden,[1] and Christiane A. Hoppmann[1], *1. University of British Columbia, Vancouver, British Columbia, Canada, 2. University of British Columbia, Vancouver, Canada*

Physical activity and fruit/vegetable consumption are recommended to help prevent and manage cardiovascular disease. Yet, most people struggle to meet physical activity and nutrition guidelines. This study examined the role of age and future time perspective for these two health behaviors using repeated daily life assessments as well as accelerometry-based step counts from 70 persons living with the effects of stroke and their partners (50% female, M age=69 years). Consistent with previous research, older age and living with stroke were associated with taking fewer steps in everyday life but also with consuming more fruit and vegetables. Furthermore, participants who viewed their future as being filled with many opportunities took more daily steps and ate more fruit and vegetables than participants low in future opportunities. Further analyses will examine dyadic associations in these two health behaviors as well as partner factors that may facilitate or hamper the engagement of the behaviors. It is important to analyze these relationships to gain further insight into the effects partners have on each other.

### CRYPTIC TRANSCRIPTION IS ASSOCIATED WITH AGE IN MAMMALIAN STEM CELLS

Brenna McCauley,[1] Luyang Sun,[1] and Weiwei Dang[1], *1. Baylor College of Medicine, Houston, Texas, United States*

Aging is a multifaceted process that challenges organisms with stresses resulting from the dysregulation of cellular processes. Unsurprisingly, given how tightly regulated it is under normal conditions, transcription is one of the key pathways disrupted during aging. Indeed, dysregulation of transcription contributes to the activation of transposable elements, the loss of cellular identity, and decreased stem cell potency with age. Our previous work identified intragenic cryptic transcription (CT) as a novel type of age-associated transcriptional dysregulation that limits the lifespan of yeast and worms. Continuing this work, we show for the first time that CT increases with age in mammalian stem cells. Increased CT is associated with disrupted chromatin structure, particularly with the reduction of H3K36me3, a histone modification known to inhibit CT throughout eukaryotes. We propose that an age-associated reduction in H3K36me3 in actively transcribed gene bodies drives disruption of chromatin structure in these regions, resulting in an open chromatin state. This open chromatin state is permissive for the entry of RNA Pol II, which can then initiate transcription from within the gene body. These aberrant cryptic transcripts may contribute to the pathological load of mammalian aging.

### VALUE AND INFORMATION NEEDS FOR DEMENTIA FAMILY CAREGIVERS: CONSIDERATIONS DURING END-OF-LIFE DECISION MAKING

Karen E. Schlag,[1] Lorie Kmetz,[1] David Burrows,[1] and Jung Kwak[1], *1. The University of Texas at Austin, Austin, Texas, United States*

Family caregivers often make key end-of-life care decisions for their relatives. For those caring for persons with dementia (PWDs), a third of older decedents, making end-of-life decisions as a surrogate is especially challenging. Notably, few evidence-based interventions exist to support caregivers in this capacity. Guided by the Ottawa Decision Support Framework which recognizes three determinants of informed decisions - information, value clarity, and support, the current study identifies key value considerations and information needs among family caregivers as they weigh decisions regarding hospice enrollment and artificial nutrition and hydration (AHN) for PWDs. One focus group (n=7) and four individual interviews (n=4) were conducted with dementia family caregivers. All face-to-face and telephone interviews were audio-recorded, transcribed verbatim, and verified for accuracy. Thematic analysis (Braun & Clarke, 2006) was conducted to identify and organize themes. Two main themes and subthemes emerged: 1. Caregivers expressed hospice-related values including having enough knowledge about hospice treatments for both Alzheimer's and new symptoms, having caregiver support services, considering family needs, and weighing the extent the PWD can engage with others meaningfully and remain at home. 2. Caregivers shared AHN-related values including clearly understanding AHN treatments, services and risks for the PWD considering the patient's functional status. Participants' information needs reflected their priority of practical needs being met. These findings offer implications for how to design decision support tools and interventions that provide practical and specific information on the benefits and risks of hospice and AHN for PWDs and caregivers.

### SOCIAL DETERMINANTS OF HEALTH DRIVE EMERGENCY ROOM AND HOSPITAL USE BY DUAL ELIGIBLE AND DISABLED PENNSYLVANIANS

Sih-Ting Cai,[1] Howard Degenholtz,[1] and Hayley Germack[1], *1. University of Pittsburgh, Pittsburgh, Pennsylvania, United States*

The study examined correlates and consequences of social determinants of health risk factors (SDoH) among dual eligible aged and disabled individuals; Pennsylvania is transitioning this population into a managed care plan with responsibility for care coordination and incentives to prevent hospitalization and nursing home placement. Medicaid and Medicare claims were used to identify people with SDoH based on ICD-10 codes in 2016 in four domains: economic insecurity, life stressors, physical dependence, and potential health hazards. Of 281,918 people, 38.6% had one or more SDoH. Among people with severe mental illnesses (SMI; schizophrenia, psychosis, major depressive disorder, or bipolar disorder), the prevalence of SDoH was 57.9%. Of people with one or more SDoH, 42% visited the ED, compared to only 32% of people with no SDoH. Economic insecurity (OR 1.68; CI 1.59-1.78), life stressors (OR 1.39; CI 1.29-1.48), physical dependence, (OR 2.01; CI 1.97-2.06), and potential health hazards (OR 1.52; CI 1.47-1.56) were independently associated with risk of hospitalization, controlling for age, gender, race, SMI, chronic conditions and disability. The introduction of diagnosis codes for SDoH under ICD-10 has facilitated identifying individuals with deficits that might increase health care use above and beyond their underlying health status. Although the prevalence of these risk factors as captured in diagnosis data is likely an underestimate, the strong association with subsequent ED use and hospitalization lends credence to these indicators. Medicare and Medicaid claims data can be used to identify people with SDoH and target interventions to prevent downstream health services use.

### UNIQUE TRANSCRIPTOME OF AGE-ASSOCIATED VISCERAL FAT-RESIDENT GAMMA DELTA T CELLS

Marlene E. Starr[1], *1. University of Kentucky, Lexington, Kentucky, United States*

Gamma delta T cells (Tγδ) are a unique group of immune cells that perform both adaptive and innate functions. We recently identified a population of Tγδ cells which show an age-dependent expansion in the visceral fat of both mice and humans. However, little is known regarding the role of these cells in the fat. The purpose of this study was to begin delineating their role by: (1) comparing the gene expression profile of visceral fat-resident Tγδ to conventional T cells (Tconv) and to circulating Tγδ, and (2) identifying age-dependent changes in gene expression within the visceral fat-resident Tγδ population. Tγδ and Tconv were magnetically purified from blood and visceral fat of young and aged mice. Using NanoString technology, we found that overall transcriptomes of Tγδ in fat and blood were strikingly different. Transcriptomes of Tγδ and Tconv within the fat were more similar, but distinct with the former having high representation of pathways related to inflammation, cytokine and chemokine signaling, and macrophage function and the latter having high representation of pathways related to T- and B-cell function and TNF superfamily. Within the Tγδ population we identified significant ($>1.8$-fold change, $p<0.01$) age-associated differences in expression for 8 upregulated genes (C6, Cxcl13, Prg2, Il5ra, Ctla4, Marco, Ccl8, Il10) and 10 downregulated genes (Col4a1, Xcl1, Col1a1, Cfd, Col3a1, Lbp, Thbs1, Klrd1, Il6st, Ccl17). These genes will guide further research aimed at understanding the role of these cells and how their age-associated changes contribute to chronic inflammation, an underlying component of multiple age-related diseases.

### FLAVIN-CONTAINING MONOOXYGENASES (FMOS): LONGEVITY-PROMOTING ENZYMES OR ATHEROSCLEROSIS RISK FACTOR?

Ryan J. Rossner,[1] and Matt Kaeberlein[1], *1. University of Washington, Seattle, Washington, United States*

Since their discovery in 1970, flavin-containing monooxygenases (FMOs) have been studied as Phase 1 xenobiotic metabolizing enzymes that act on sulfur- and nitrogen-containing small molecules. In 2015, we demonstrated that C. elegans (worm) fmo-2 was not only necessary for hypoxic response and dietary restriction-mediated lifespan extension, but was also sufficient to extend lifespan when overexpressed. Consistenet with a conserved role for FMOs as longevity-promoting enzymes, mouse hepatic Fmo3 transcript is highly upregulated by numerous major lifespan extending interventions. A contrasting series of reports, however, have described mammalian Fmo3-mediated production of trimethylamine N-oxide (TMAO) as a risk factor for atherosclerosis and other major diseases. My thesis research aims to define the regulation and function of worm fmo-2 using genetic and biochemical approaches. My data thus far support the hypothesis that fmo-2 acts on sulfur amino acid pathway intermediates to promote longevity and healthspan.

### THE RELATIONSHIP BETWEEN COGNITION, FUNCTIONAL ABILITIES, AND THE LATENT DEMENTIA PHENOTYPE AMONG CENTENARIANS

Jonathan Sober,[1] John L. Woodard,[1] L. Stephen Miller,[2] Adam Davey,[3] Peter Martin,[4] and Leonard Poon[2], *1. Wayne State University, Detroit, Michigan, United States, 2. University of Georgia, Athens, Georgia, United States, 3. University of Delaware, Newark, Delaware, United States, 4. Iowa State University, Ames, Iowa, United States*

Adequate assessment of cognitive abilities and functional capacity is essential for a diagnosis of dementia. However, cognition is only moderately related to functional status, and this relationship is poorly understood among centenarians, a group of older adults with high risk for dementia. A bifactor structural equation model can be used to delineate the variance attributed to dementia-specific related cognitive changes (i.e., the latent variable delta) and the variance due to general intelligence (i.e., g'). This study aimed to determine the validity of delta as a marker of cognitive decline among centenarians. It was hypothesized that delta was correlated with cognitive status, functional abilities and, dementia severity. Overall, 244 community dwelling centenarians (Mage = 100.58, 84.8% female) were recruited through the Georgia Centenarian Study, a population-based study of octogenarians and centenarians from northern Georgia. Older adults were administered measures of cognition and a self-report measure of functional abilities. Latent variable scores (i.e., g' and delta) were modeled and correlated with standard global cognitive screening measures (i.e., MMSE) and measures of dementia severity. Results indicate that delta was significantly correlated with functional ability and cognitive abilities. Consistent with our hypotheses, delta was also significantly related to dementia severity. Overall, estimates of the latent dementia phenotype, delta, were significantly related to cognitive and functional abilities among centenarians, providing validation of delta as a useful index of dementia severity.

### ADULT FOSTER HOME OWNERS' PERSPECTIVES ON REWARDS AND CHALLENGES OF OPERATING AN ADULT FOSTER HOME

Sheryl Elliott,[1] Sarah Dys,[2] Jaclyn Winfree,[3] and Paula Carder[4], *1. The Institute on Aging at Portland State University, Portland, Oregon, United States, 2. Institute on Aging, Portland, Oregon, United States, 3. Portland State University, Portland, Oregon, United States, 4. Portland State University Institute on Aging, Portland, Oregon, United States*

Adult foster homes (AFHs) are small, residential settings providing older adults and persons with disabilities an alternative to nursing facilities and larger residential care settings. Some groups, including individuals with Alzheimer's disease and related dementias, are well served by smaller settings. Although AFHs are common throughout the US, research on this setting is scant and dated. This study summarizes four years of qualitative data from Oregon AFH owners' (N=726) responses to open-ended questions about the challenges and rewards of owning and operating an AFH. Content analysis of 924 comments indicate that providing resident care (42%), finding the work meaningful and "a life calling" (21%), developing a family-like connection with residents (15%), and working at home (8%) were the most commonly reported rewards. The most frequently described challenges included caring for residents with multiple chronic health conditions—including those with difficult behaviors (17%), difficulty hiring and retaining qualified caregivers (15%), low Medicaid reimbursement rates (14%), and adhering to administrative rules (14%). Results highlight AFH providers' personal satisfaction with caring for and establishing connections with residents, and challenges associated with residents' increasingly complex care needs, Medicaid reimbursement rates, and attitudes about state regulations. Although AFHs are licensed by states, they are subject to federal regulations, including the 2014 Centers for Medicare & Medicaid Services home and community-based services ruling. The new regulations, Oregon administrative rules, Medicaid reimbursement rates, and caregiver supply are presented to contextualize AFH owner comments and regulatory considerations.

### EXPLORING OLDER ADULTS' EXPERIENCES IN CARE TRANSITIONS

Flora Vieira Zamora,[1] Michelle Silver,[1] Whitney Berta,[1] Michelle Nelson,[1] and Angela Hamilton[1], *1. University of Toronto, Toronto, Ontario, Canada*

Older adults are Canada's largest growing demographic. Later adulthood is frequently accompanied by increased comorbidities, resulting in more people living with chronic conditions for longer portions of their lives and requiring care across multiple settings. These individuals are also the most susceptible to challenges within health care systems, especially during vulnerable times such as care transitions, which can be challenging due to issues of care integration and coordination. A scoping review was conducted to explore the experiences of older adults transitioning through various levels of care. The main themes found included personal realizations, social connectedness, importance of navigating the system and recommendations for the future. During care transitions, older adults must carefully consider their personal circumstances and limitations and often accept a new baseline, thus, adapting their lives and activities to match possible limitations. Older adults indicate the need for strong social networks, accessible and available services, as well as effective communication, information, education and engagement during care transitions. Issues with care transitions can be exacerbated in smaller communities, where resources and services may be limited. As such, this scoping review is the foundation for an ongoing systematic review which aims to summarize what is known about care transitions for older adults living in small and rural communities. By better understanding the different interacting factors that might influence care transitions for older adults living in small communities, important and sustainable changes can be identified and implemented to ensure that care transitions for older adults are safe, positive and empowering.

### EXPECTATIONS, ROLES, AND EXPERIENCES OF GRANDPARENT-CAREGIVERS OF CHILDREN WITH RARE DISEASE

Jasmine Manalel,[1] Betina Hollstein,[2] and Laura M. Koehly[3], *1. National Institutes of Health, Bethesda, Maryland, United States, 2. University of Bremen, Bremen, Germany,*

*Germany, 3. National Human Genome Research Institute, National Institutes of Health, Bethesda, Maryland, United States*

Grandparenting can be a rewarding and health-promoting experience for older adults. However, grandparent-caregivers often experience greater stress and poorer health relative to non-caregiving grandparents. Further, little is known about grandparents caring for a child with a rare, chronic illness. This study aimed to extend knowledge of the expectations, roles, and experiences of grandparents providing care to a child affected with an inherited metabolic condition. The sample included 23 grandparent-mother dyads from the Inherited Diseases, Caregiving, and Social Networks Study. The grandparent sub-sample ranged from 49 to 79 years of age (Mage = 64), the majority were female (83%) and married (74%), and almost half (48%) were retired. Social network assessments were analyzed to determine concordance between mother- and grandparent-reports of grandparents' role in the child's caregiving network. Fifteen mother-grandparent dyads (65%) agreed on grandparents' role in the child's network, with the majority of those (93%) considering the grandparent to be very close and important (versus less close or excluded from the caregiving network). Grandparents whose self-reports of their role in their child's caregiving networks were consistent with mother-reports appeared more likely to report that they spend enough time caregiving than those whose reports were inconsistent. Content analysis of grandparents' interviews provided supporting information about the joys and regrets of their grandparenting experience and perspectives on caregiving expectations. This research leverages multi-informant social network and qualitative data to illuminate grandparents' role in the caregiving networks of chronically ill children and adaptation to non-normative grandparenting experiences.

### ADULT DAY HEALTH SERVICES ATTENDANCE IS LINKED TO A REDUCTION IN LONELINESS

Wingyun Mak,[1] Orah R. Burack,[1] and Joann P. Reinhardt[1], *1. The New Jewish Home, New York, New York, United States*

Loneliness is the subjective experience of inadequate social contact, and it is linked to numerous detrimental psychological and medical outcomes like depression, cognitive decline, sleep fragmentation, metabolic syndrome, diminished immune functioning, and morbidity. Older adults with cognitive impairment and/or other comorbidities are at greater risk for loneliness due to diminishing social roles, functional status, and death of peers. They are often encouraged to attend adult day services to engage in an environment of socialization and supported activities, and in the case of adult day health services, additional medical services such as physical therapy, skilled nursing care, medical management, and nutritional counseling. In this study we examined whether attending adult day health services (ADHS) at least once a week would be associated with decreased levels of loneliness across time. Our data came from a sample of older adults attending ADHS in New York City from 2018-2019 who scored five or greater on the Nursing Facility Level of Care Index, which is a score derived from assessments of cognition, communication and vision, mood and behavior, functional status, continence, and nutritional status from the Uniform Assessment System in New York (UAS-NY). We found that attendance was linked with fewer reports of loneliness across time, $\sum 2(1, N=563) = 21.33$, $p<.001$. These results highlight the importance of attending adult day health services for people with a complex health status and the potential role ADHS may play in reducing loneliness in a vulnerable population.

### DISPARITIES IN HEALTH STATUS, HEALTH BEHAVIORS AND PREVENTIVE HEALTH SERVICES OF OLDER ADULTS WITH DIABETES

Sang Ah Chun[1], *1. University at Albany, SUNY, Albany, New York, United States*

The prevalence of diabetes among older adults has increased substantially and health complications resulting from diabetes have significant adverse effects on health status of older adults. While diabetes cannot be cured, it can be managed successfully with healthy lifestyle choices. The purpose of this study is to examine the disparities in health status, health behaviors, and preventive health services for older adults with diabetes. This study used data from the 2018 Behavioral Risk Factor Surveillance System. The sample included older adults 50 and over. Health behaviors included exercise, smoking, and heavy alcohol drinking. Preventive health services included dental visit, flu shot, and colorectal cancer screening. Chi-Square analysis and weighted multivariate logistic regression was performed. Not surprisingly, older adults with diabetes were significantly more likely to be in poor health than those without diabetes. Compared to non-diabetic group, older adults with diabetes were more likely to have had no exercise in the previous month. Interestingly, more older adults with diabetes reported having visited dentist, had flu shot and colonoscopy than those without diabetes. In both groups, older adults who presented health behaviors and received preventive health services were more likely to report good health compared to those who did not. The results suggest that further efforts are needed to address the health disparities for older adults with diabetes. Given the risk of comorbidities and its complications for older adults with diabetes, further research should be directed toward designing better health promotion programs and policies for older adults with diabetes.

### COMPENSATION EFFECT OF MORTALITY: A CHALLENGE TO LIFE EXTENSION

Natalia S. Gavrilova,[1] and Leonid Gavrilov[1], *1. NORC at the University of Chicago, Chicago, Illinois, United States*

In order to develop genuine anti-aging interventions it is important to find the best estimate of the aging rate in humans, which is often measured as a slope parameter of the Gompertz law. The compensation effect of mortality (CEM), refers to mortality convergence, when higher values for the slope parameter are compensated by lower values of the intercept parameter (initial mortality) in different populations of a given species. The age of this convergence point is called the "species-specific life span". Due to CEM, factors associated with life span extension are usually accompanied by paradoxical increase in actuarial aging rate. We evaluated the stability of CEM by analyzing the United Nations abridged life tables for 241 countries and regions and estimating parameters of the Gompertz-Makeham model using method of non-linear regression in the age interval 30-80 years. We found that the species-specific lifespan is equal to 94.5 ± 0.5 years, which is the same as reported in the past for years before the 1960s: 95 ± 3 years (Gavrilov, Gavrilova, 1991). Thus, the convergence point of CEM is stable despite significant mortality decline over past 50 years and is not affected by factors decreasing mortality at younger ages. Populations deviating from CEM with apparently slow aging (with both slow actuarial aging rate and low intercept parameter) have been identified. The existence of CEM in mice (ITP data) allowed us to find interventions that are able to both extend lifespan and slow the actuarial aging rate giving promise for radical life extension.

### SOCIAL ISOLATION, LONELINESS, AND CAREGIVER BURDEN AMONG PAID AND UNPAID CAREGIVERS OF HOMEBOUND OLDER ADULTS

Amy Y. Sun,[1] Emily Finkelstein,[1] and Karin Ouchida[2], *1. Weill Cornell Medicine, New York, New York, United States, 2. Weill Cornell Medicine, New York, United States*

Caregivers of homebound older adults may have high levels of burden and more vulnerability to social isolation and loneliness, given that their care recipients are more physically frail and isolated. Existing literature has not fully investigated differences between paid and unpaid caregiver burden or their experiences of social isolation. We interviewed paid (n=21) and unpaid family caregivers (n=22) of homebound older adults in a hospital-affiliated geriatric house call program. We used validated survey instruments to measure social isolation, loneliness, and caregiver burden, and semi-structured interviews to solicit qualitative data. In our sample, 42% of caregivers helped with 5+ ADLs and 58% with 5+ iADLs. Using the Caregiver Burden Inventory, burden types between caregivers were compared with chi-squared tests. Compared to paid caregivers, unpaid family caregivers experienced more "developmental" burden such as "missing out on life" ($p<0.01$). Paid caregivers exhibited more "time" burden, such as "not having a minute's break from caregiving responsibilities" ($p<0.01$). 44% of caregivers were considered socially isolated according to the Berkman-Syme Social Index. However, using the UCLA 3-item Loneliness Scale, few caregivers felt lonely (14%). Thematic analysis revealed that family caregivers desired support groups but time pressures limited their participation (23%). Interestingly, smart phones were regularly cited as a tool for alleviating loneliness for paid caregivers when alone on the job (19%), a novel finding. Findings suggest that caregivers of the chronically ill and physically isolated may be at particular risk of social isolation. Network based social support interventions may mitigate some of these vulnerabilities.

### COMPLEMENT CONTRIBUTES TO ALZHEIMER'S DISEASE-INDUCED SYNAPSE DECLINE IN THE MURINE RETINA

Fenge Li,[1] Danye Jiang,[1] and Melanie Samuel[1], *1. Baylor College of Medicine, Houston, Texas, United States*

Alzheimer's disease (AD) is among the most debilitating form of cognitive impairment in aged patients. Synapse deficits are thought to be a central trigger of neural miswiring and brain dysfunction in AD. However, the pathways that control synapse connectivity remain largely unknown. The retina is an easily accessible system with two distinct synapse layers and three cellular layers comprised of distinct neural types. In this study, we leveraged this system to assess synapse and cell integrity in the APPNLGF amyloid-beta AD mouse model. We showed that the expression of the complement component C3 is significantly increased in APPNLGF retina synapses, and that there is a significant decline of several synapse-associated markers by RT-PCR. These mice also display disorganized horizontal cell processes and visual function deficits. These results suggest that complement may drive AD-related changes in the synaptic and functional properties of the retina, which could serve as assessable preclinical biomarkers for AD. In ongoing studies, we are testing whether and how complement regulates synapse refinement and shapes retina synapse specificity in AD.

### MICROGLIA MAY INSTRUCT SYNAPTIC FATE VIA SIRPα IN MOUSE RETINA

Danye Jiang,[1] and Melanie Samuel[1], *1. Baylor College of Medicine, Houston, Texas, United States*

As we age, our nervous system undergoes many deleterious alterations: cognitive and sensory functions decrease while the risk of disease increases. Synapses are responsible for neural information processing, and the decline of these structures via microglia-mediated remodeling is thought to underline many age-related neural changes. However, the molecular pathways responsible for microglia-mediated synapses removal in development and old age remain unknown. To begin to elucidate these pathways, we leveraged the precisely organized murine retina where neurons form synapses in distinct lamina. Using this system, we screened 102 lacZ reporter lines available through the Knockout Mouse Project (KOMP) and uncovered a unique synapse regulatory candidate, SIRPα. We show that SIRPα is present in microglia prior to synapse formation but becomes selectively enriched in neural synapse terminals as these connections mature. Further, the levels of SIRPα decrease in the context of age-related neural decline. In ongoing studies, we are testing the hypothesis that neuronal SIRPα regulates its receptor CD47 to modulate refinement by microglial SIRPα. Together, these studies will resolve the molecular cues through which microglia prune synapses in development and dissect how these programs may go awry in the context of aging and disease.

### PEOPLE LIVING WITH PARKINSON DISEASE WITH AND WITHOUT FREEZING: DIFFERENCES USING THE NEURO-QOL MEASUREMENT SYSTEM

Kerri Rawson,[1] Ryan Duncan,[1] Tamara DeAngelis,[2] Timothy Nordahl,[2] Jim Cavanaugh,[3] Gammon Earhart,[1] and Theresa Ellis[2], *1. Washington University School of Medicine, St. Louis, Missouri, United States, 2. Boston University, Boston, Massachusetts, United States, 3. University of New England, Portland, Maine, United States*

Parkinson disease (PD) is the second most common neurodegenerative disease and approximately half of those diagnosed with PD will experience freezing of gait (FOG). FOG is a severe motor disturbance that prevents stepping despite the intention to do so and may be associated with anxiety, decreased cognitive functioning, depression, and poorer quality of life. In this study, we administered the short-form

Quality of Life in Neurological Disorders (Neuro-QOL) measurement system to 43 people with PD (28 non-freezers and 15 freezers) and determined freezing status using the New Freezing of Gait-Questionnaire. Freezers had greater motor severity as measured with the MDS-UPDRS-III (p =.003) and poorer balance using the Mini-BESTest (p = .017). Data from the Neuro-QOL battery indicated freezers had greater difficulty with Lower Extremity Function (p = .019) and Upper Extremity Function (p = .027). Freezers also had poorer scores on the Positive Affect and Well-Being (p = .005), Satisfaction with Social Roles and Activities (p = .005), and Stigma (p = .026) scales, but less difficulty on the Communication scale (p = .005) than non-freezers. There were no differences between freezers and non-freezers on the Ability to Participate in Social Roles and Activities, Anxiety, Cognitive Function, Depression, Emotional and Behavioral Dyscontrol, Fatigue, or Sleep Disturbance scales. These findings suggest freezers are less likely to have a positive outlook and satisfaction with their daily lives in combination with poorer functioning. Interventions that target freezing with the potential to improve functioning may result in better quality of life among freezers.

### HOUSING CONTEXT AND FUNCTIONAL HEALTH AMONG LOWER INCOME OLDER ADULTS

Noah J. Webster[1], *1. University of Michigan, Ann Arbor, Michigan, United States*

The characteristics of where older adults live have strong links with disability. Although lower income older adults experience disability at higher rates, less is known about the link between housing characteristics and functional health in this group. A within group comparison among this population is needed to understand how aspects of this vulnerable subgroup's housing context are associated with health outcomes. The present study examines the association between housing and functional health among a U.S. nationally representative sample of independent living (i.e., not living in nursing homes or assisted living facilities) lower income adults age 65+. Using data from round one of the National Health and Aging Trends Study, a sub-sample of N=2,865 lower income (<$15,000 in the past year) older adults was selected for analysis. Regression analyses indicate that lower income older adults living in multiunit buildings reported better functional health compared to those in other housing contexts (e.g., free-standing homes). This link also significantly varied by age and gender. Living in multiunit housing was associated with better functional health among those age 90+, not associated among those age 80-90, and was negatively associated among those age 65-79. In terms of gender, the link between multiunit housing and better functional health was only significant among women. Findings highlight variation in health across lower income older adults' housing contexts. Potential explanatory mechanisms (e.g., social isolation) will be discussed. Such information can inform senior housing policy regarding best approaches to providing housing for older adults that optimizes and promotes independent living.

### ELDER SEXUAL ABUSE: A REVIEW OF LEGALLY MANDATED APPROACHES TOWARD PREVENTION AND FUTURE IMPLICATIONS

Michelle Hand[1], *1. The Ohio State University, Columbus, Ohio, United States*

Rape stereotypes involve the assault of young women due to sexual desire, resulting in the exclusion of older adults from sexual violence research, policies, and interventions, suggesting a need to further knowledge in this area (Bows & Westmarland, 2015). Debates also persist on the prevalence and nature of elder sexual abuse (ESA), where it occurs, and its most common perpetrators (Bows, 2018). Thus a systematic scoping review was conducted to explore the nature and prevalence of ESA as well as mandated prevention and intervention strategies to guide practice, policy, research and education on prevention. Eligible sources were research-based and focused on the nature of ESA along with legal prevention and/or intervention mandates. In total, 38 peer-reviewed articles and reports were screened in for review, obtained from AgeLine, EBSCO, Clinical Key Flex, PubMed, Google, and Google Scholar databases. Findings suggest while consensus has not been reached (Bows, 2018), ESA estimates range from 0.2% to 7% of U.S. elder abuse cases, yet actual rates are likely much higher due to underreporting (Cannell et al., 2014). Additionally, results suggest ESA most often occurs in nursing homes, predominantly perpetrated by staff or residents (Ramsey-Klawsnik et al., 2008). Still, ESA remains underreported despite several mandated approaches to prevention and intervention (Payne, 2010). Thus findings demonstrate a need for reliable estimates of prevalence, location as well as common victim-perpetrator relations and awareness of required steps toward prevention and intervention (Payne, 2010). Beyond this, transdisciplinary efforts are needed to yield effective training, education, and culturally appropriate resources.

### USEFUL FIELD OF VIEW AND DRIVING SIMULATION ACROSS TWO AGE GROUPS

Rebecca A. Dunterman,[1] Robert C. Intrieri,[2] and Marisa Guernsey[2], *1. Auburn University, Auburn, Alabama, United States, 2. Western Illinois University, Macomb, Illinois, United States*

The Insurance Information Institute (2017) reports that drivers aged 65 and older have the second highest rate of fatal car crashes. Research with the useful field of view (UFOV) assessment has predicted crashes in older drivers (Ball, 2006). "UFOV is defined as the area from which an individual can extract information quickly without head or eye movement" (Posit Science, 2019). Research demonstrates that older drivers are limited by poorer vision, divided attention and the inability to ignore distractions, and slower reaction time to critical stimuli (Owsley et al. 1998). As a result UFOV is an effective variable in assessing driver safety. We hypothesized that older compared to younger drivers would be less likely to inhibit attention to task irrelevant visual stimuli while engaged in a simulated driving task. Participants were community dwelling older adults and students recruited from a research pool and through word of mouth. Participants completed a series of demographic and health questions, Snellen visual acuity test a series of cognitive measures (e. g., Trails 1 and 2, digit symbol, digit span) and the UFOV assessment. Participants completed a driving simulation task while information on driving performance: number of collisions, speed limit deviations, turn signal use, time spent tailgating another vehicle, and braking reaction times. ANOVA demonstrated that as hypothesized, younger participants had significantly lower UFOV risk scores (p = .000). Older adults' (M = 2.15, SD = .945) and younger adults' (M = 1, SD = 0).

### RATINGS OF PERCEIVED EXERTION: PREDICTING MOBILITY DISABILITY AND RESPONSE TO PHYSICAL ACTIVITY IN OLDER ADULTS

Erta Cenko,[1] Thomas M. Gill,[2] Nancy W. Glynn,[3] Marco Pahor,[1] Peihua Qiu,[1] Vincenzo Valiani,[4] Lu You,[1] and Todd M. Manini[1], *1. University of Florida, Gainesville, Florida, United States, 2. Yale School of Medicine, New Haven, Connecticut, United States, 3. University of Pittsburgh, Department of Epidemiology, Pittsburgh, Pennsylvania, United States, 4. University of Bari, Bari, Italy*

Ratings of perceived exertion (RPE) during exercise are linked to several physiological indices and are often elevated in older adults. This study evaluated the association between RPE of walking and incident major mobility disability (MMD) as well as response to a physical activity (PA) program. Older adults (n=1633) at-risk for mobility impairment were randomized to a structured PA or health education (HE) program. During a 400m walk, participants rated exertion as "none", "light", "somewhat hard" or "hard". An MMD event was defined as the inability to complete the 400m walk. Transitions between RPE states and an MMD event—when RPE was not collected— were assessed over the follow-up (every 6 months for an average of 2.6 years). Participants rating their exertion as "hard" at baseline 400m walk had nearly 3-fold higher risk of MMD compared with those rating as "light" (HR: 2.61, 95%CI: 2.19-3.11). During follow-up, the PA group was 25% more likely to transition from "light" to "hard" RPE (1.25, 1.05-1.49), but was 27% (0.73, 0.55 – 0.97) less likely to transition from a "hard" RPE to MMD than the HE group. Additionally, the PA group was more likely to transition from an MMD event to a "hard" RPE (2.09, 1.38-3.17) than the HE group (i.e. recovery). Older adults rating "hard" effort during a standardized walk test were at increased risk of MMD. A structured PA program increased transition from light to hard effort, which may reflect greater capacity to perform the test and increased recovery from an MMD event.

### STARTING THE CONVERSATION: RESULTS FROM AN INTERPROFESSIONAL WORKSHOP ABOUT ADVANCE CARE PLANNING

Stacy L. Barnes,[1] Susan Breakwell,[1] and Jordan Cannon[2], *1. Marquette University, Milwaukee, Wisconsin, United States, 2. Medical College of Wisconsin, Milwaukee, Wisconsin, United States*

Advance care planning is more than documenting end of life medical decisions; it should be the beginning of an important and ongoing conversation about personal values, goals, and preferences. Health profession students must be prepared to have these conversations and overcome any existing barriers to effective patient-provider communication. To this end, a multidisciplinary planning team from Marquette University and the Medical College of Wisconsin developed a 2-hour workshop directed at students in the health professions. It was designed to be highly interactive, including guided self-reflection, drawing, writing, videos, small group work, case presentations, and large group discussion. The workshop was scheduled to coincide with National Healthcare Decisions Day (April 16th). A total of 149 students participated, representing the disciplines of medicine, nursing, physician assistants, counseling psychology, speech pathology, and biomedical sciences. Feedback from both students and faculty was overwhelmingly positive, indicating interest and need for this type of program. A post-event questionnaire, which included a retrospective pre/post-test, assessed learners' gains in knowledge and self-efficacy. Significant (< 0.001) gains were found on all measured items. Data from an electronic follow-up survey suggested the majority of participants took additional actions steps related to advance care planning in the month following the workshop. In conclusion, this is a low-cost, replicable workshop that aligns with current recommendations for advanced care planning (IOM, 2015), is well received by students and faculty in the health professions, and may serve as a springboard for increasing the number of advance care planning conversations.

### "THEY SHOULD PUT ALZHEIMER'S GROUPS IN THE CHURCHES": A SYNTHESIS OF AFRICAN AMERICAN CAREGIVERS' LIVED EXPERIENCES

Erin R. Murphy,[1] Destony Brooks,[1] Julie Bryant,[1] Noelle L. Fields,[2] and Ling Xu[2], *1. University of Texas at Arlington, School of Social Work, Arlington, Texas, United States, 2. University of Texas at Arlington, Arlington, Texas, United States*

Alzheimer's disease and related dementias (ADRD) are challenging chronic health conditions that disproportionately impact African Americans. Caring for a family member with ADRD can be a taxing experience that impacts the mental, social, and physical realms of the caregiver's life. Chronic fatigue and high levels of anxiety, depression, and agitation have all been associated with caregiving. The extant literature on caregivers is limited by being conducted primarily in settings with White participants, excluding the cultural attitudes and values that may impact caregiver experience. As part of a larger, mixed-methods team studying the impact of an innovative psychoeducational intervention, the researchers conducted a qualitative interpretive meta-synthesis (QIMS) to better understand the experiences and perceptions of African Americans who care for family members with ADRD. A QIMS was chosen as the methodology for this study because of its ability to create a more holistic understanding of the phenomenon, while maintaining the integrity of the original studies. An exhaustive literature search yielded 1,285 potentially relevant studies. Studies were compared across a priori inclusion criteria. Findings of this study indicate that overall knowledge of ADRD is relatively low among caregivers and participants are unsure of how to access educational materials. Synthesis of these studies also indicate a need for incorporating spiritual well-being into caregiving services. Results of this study may help social workers and other health care professionals to better understand cultural perceptions of the disease and how to better provide psychoeducational interventions related to the specific needs of African American caregivers.

### EXPANDING BENEVOLENT AGEISM: MEASURING EXPERIENCES OF OLDER ADULTS

Jennifer F. Sublett,[1] and Toni L. Bisconti[1], *1. The University of Akron, Akron, Ohio, United States*

Benevolent ageism has recently been recognized as a form of patronizing treatment that older adults experience because of the kind and incompetent age stereotype proposed by the Stereotype Content Model. However, there is limited research that examines older adults' experiences with patronizing treatment. The aim of this study was to conceptualize benevolent ageism based on older adults' experiences with items from an existing measure of ageism, the Ambivalent Ageism Scale, and additional items created by us that expand the measurement of benevolent ageist behaviors. In an internet-based sample of older adults who were 65 years old and older (N =135), the benevolent subscale of the Ambivalent Ageism Scale with our additional 10 items demonstrated excellent reliability ($\alpha$ = .90). An exploratory factor analysis cleanly yielded a 4-factor solution that mirrored previous findings, (1) hostile ageism, (2) unwanted help, (3) cognitive assistance/protection, while introducing a new factor of (4) condescending endearment. The findings from this study have widened the scope with which ageism is viewed by examining older adults' experiences with ageism and conceptualizing characteristics of benevolence that older adults may face due to the widespread belief that they are kind and incompetent. The validation of a scale measuring individuals' experiences with ageism will provide insight as to whether older adults experience ageist behaviors that people report endorsing and if older adults receive unnecessary offers of help. A recipient's perspective of ageism will aid in the understanding of the insidious and benevolent characteristics of ageism within society.

### DNA METHYLATION CLOCKS IN MOUSE

Margarita Meer,[1] and Vadim Gladyshev[1], *1. Brigham and Women's Hospital and Harvard Medical School, Boston, Massachusetts, United States*

One of important limiting factors in aging research is the time required to measure the effect of an intervention on lifespan. But this situation is now changing due to a recent discovery of DNA methylation-based markers (DNAm clocks). We developed a whole lifespan multi-tissue DNAm clock for mice with R2 =0.89. We also carried out comparative analyses of the available mouse DNAm clocks (single- or multi-tissue, based on different number of sites, based on one genomic locus or multi-loci). In general, tissue specific clocks are more accurate than muti-tissue clocks. We applied these tools to a variety of experimental systems, ranging from interventions to rejuvenation approaches, and analyzed various mouse tissues and public datasets. We further applied DNAm clocks to newly sequenced sets of blood and liver samples. Multi-loci blood clock outperforms other clocks when applied to blood samples, and the liver and multi-tissue clocks show similar precision on liver.

### EXPERIENCES WITH SHARED DECISION-MAKING AND ADVANCED CARE PLANNING AMONG OLDER SPANISH-SPEAKING LATINOS

Yelba M. Castellon-Lopez,[1] and Gery Ryan[2], *1. University of California, Los Angeles, Los Angeles, California, United States, 2. RAND, Santa Monica, California, United States*
O.Kenrik Duru,[1] and Gerardo Moreno[1]

Advance care planning (ACP) helps individuals clarify their values and preferences for future care, and to communicate their wishes to loved ones, surrogate decision-makers and healthcare providers in advance of when they become unable to make healthcare decisions. Older Spanish-speaking Latino adults have among the lowest rates of advance directives a form of ACP. Shared decision-making (SDM) interventions have significantly improved outcomes for disadvantaged patients. SDM has proven to be particularly beneficial in ethnic minority populations with low literacy and low socioeconomic status groups. The aim of this study was to pilot an online SDM module delivered in Spanish to better understand experiences with ACP among older Spanish-speaking Latino adults. We recruited a sample of older Latino adults ages 56-81 who were low-income and primarily Spanish-speaking (N=20). Sixty-five percent of the sample was female. Participants were asked to complete an SDM module delivered by a physician to discuss ACP. The online module was developed by Healthwise © a national leader in SDM. Qualitative interviews were conducted to examine experiences with SDM and ACP. SDM improved awareness about ACP options and facilitated conversations with family members among participants. Results from the study demonstrated a four-step process for engaging participants in ACP including: Awareness, Initial conversations, Conversations with medical provider, and Formalizing. Interviews informed opportunities for culturally tailored interventions along different points on the continuum. This study highlights important opportunities to better understand the process-based steps and targeted interventions needed to address disparities in ACP among Spanish-speaking low-income Latinos.

### BENEVOLENT AGEISM: THE CORRELATES OF OVERACCOMMODATION TOWARDS OLDER ADULTS

Jennifer F. Sublett,[1] and Toni L. Bisconti[1], *1. The University of Akron, Akron, Ohio, United States*

Using the Stereotype Content Model as a framework for understanding ageism, our two objectives are (1) examining the predictive utility of benevolent ageism on well-being outcomes and (2) identifying conditional relationships between sex, perceived age, benevolent ageism, and well-being outcomes. In a snowball sample of 150 older adults who were 65 years old and older, we examined sex, perceived age, ageism, environmental mastery, and depression. Our benevolent ageism scale is an expanded version of the Ambivalent Ageism Scale that included additional items of accommodation created by us. Environmental mastery and depression were assessed by standard, internally valid, measures. Using regression analyses, we found that benevolent ageism predicted depression above and beyond hostile ageism. Additionally, benevolent ageism uniquely predicted environmental mastery for men, whereas hostile ageism uniquely predicted environmental mastery and depression for women. Finally, perceived age was a better predictor of well-being than chronological age. It is essential to consider how benevolent ageism relates to well-being due to the tenets of the Stereotype Content Model. Additionally, delineating the ways that sex and perceived age contribute to double jeopardy vs. crisis competence in the face of benevolence will lead to a more intricate understanding of the paths in which overaccommodative behaviors relate to well-being in older adulthood.

### HEALTH PERSONALITY, HEALTH ACTIVATION, AND GENERAL HEALTH

Nicholas Cone,[1] Angelica Jasper,[1] and Peter Martin[1],
*1. Iowa State University, Ames, Iowa, United States*

The purpose of this investigation was to examine associations between health personality and health activation as predictors of general health. Participants from the study included 3907 individuals 65 years of age and older from AARP® Medicare Supplement Plans insured by UnitedHealthcare Insurance Company. The participants completed a survey including the Health Personality Assessment, the Consumer Health Activation Index, and a single-item assessment of self-rated health. Structural equation modeling determined how health personality predicted health activation, and health activation in turn predicted general health. The hypothesized model fit without direct paths from health personality to general health was not optimal. In a second step, we added direct paths from health openness, health neuroticism, and health conscientiousness to general health. The final model fit was then excellent, x2 (df=2) = 18.26, $p < .01$, RMSEA = .05, CFI = .99. Health neuroticism and health openness were negatively related to health activation, which indicated participants with high health neuroticism and health openness scores were less health activated. Conversely, higher health agreeableness and conscientiousness were associated with more activation. Pathways from health personality via health activation to general health were tested for mediation. Health activation significantly mediated relationships between health neuroticism, health openness, health agreeableness and health conscientiousness to general health. These findings support health activation accounting for some of the associations between health personality and general health. Health neuroticism, health openness, health agreeableness, and health conscientiousness were more closely connected to health activation than health extraversion.

### AFFECTIVE NEUROPSYCHIATRIC SYMPTOMS MAY BE EARLY SIGNS OF ALZHEIMER'S DISEASE IN NON-DEMENTED OLDER ADULTS

Jung Y. Jang,[1] and Daniel A. Nation[2], *1. University of Southern California, Los Angeles, California, United States, 2. University of California, Irvine, Irvine, California, United States*

The current study sought to investigate the association between affective neuropsychiatric symptoms (aNPS: depression, anxiety, apathy, irritability), Alzheimer's disease (AD) cerebrospinal fluid (CSF) biomarkers profiles, and the risk of progression to dementia in non-demented older adults. Participants consisted of 763 individuals with normal cognition (CN) (mean age = 73.73 ± 6.68) and 617 with mild cognitive impairment (MCI) (mean age = 73.19 ± 7.40) at baseline, who were enrolled in the Alzheimer's Disease Neuroimaging Initiative (ADNI). Latent class analyses (LCA) identified three subgroups of older adults within CN and MCI, respectively, showing distinct patterns of the neuropsychiatric inventory (NPI) domains. Results indicated that the subgroup with higher probabilities of aNPS had elevated risk of progression to dementia (HR = 3.18, 95% CI [1.70, 5.94] in CN, HR = 1.79, 95% CI [1.01, 3.16] in MCI), adjusting for age, sex, and Apolipoprotein E e4 (APOE4) carrier status. Subgroups did not differ in their profiles of AD CSF biomarkers. Findings suggest that aNPS might be symptoms of secondary disease processes in the brain, lowering the threshold for AD pathophysiology to manifest clinically in CN and MCI. The current study highlights the importance of assessment and interventions for emotional and behavioral symptoms in non-demented older adults.

### HOW THE IMPACT OF CHRONIC PAIN ON COGNITION VARIES BY POLYGENIC RISK SCORE (PRS)

Jieun Song[1], *1. University of Wisconsin-Madison, Institute on Aging & Waisman Center, Madison, Wisconsin, United States*

While prior research has found associations between chronic pain and cognition and genetic risk of cognitive decline, little research examined moderating effects of genetic risk on the association between chronic pain and cognition. This study investigate whether genetic risk of accelerated cognitive decline, assessed by polygenic risk score (PRS) of Alzheimer disease (AD), moderates the association between severe chronic pain and cognitive decline. The data are drawn from Midlife in the US (MIDUS), a survey of a nationally representative sample of US adults. The analytic sample consists of two groups: 201 individuals who reported severe chronic pain (116 women, 85 men) and 404 individuals without severe chronic pain (215 women, 189 men) who completed MIDUS 2 (2004-06) and MIDUS 3 (2013-14) surveys and participated in biomarker data collection. The findings showed that men who suffered from severe chronic pain were more vulnerable to genetic risk of cognitive decline than men who did not experience severe chronic pain. Specifically, men who suffered from severe chronic pain and had higher level of PRS of AD experienced a greater decline of episodic memory than men who experienced chronic pain with lower level of PRS of AD. This association was not found in women sufferers. For both men and women who did not have chronic pain, cognitive change was not a function of the level of genetic risk of cognitive decline. Findings suggest that genetic risk of cognitive decline would be manifested contingent on life circumstances as well as gender of individuals.

### RECRUITING COMMUNITY-DWELLING LIVE-ALONE PERSONS WITH DEMENTIA: AN EXPLORATION OF FIVE GATEKEEPER DOMAINS

Laura Girling,[1] and Kate de Medeiros[2], *1. University of Maryland, Baltimore County, Baltimore, Maryland, United States, 2. Miami University, Oxford, Ohio, United States*

Although recruiting persons with dementia into research is challenging enough, finding those who live-alone in the community is even more difficult. Consequently, live-alone persons with dementia are often overlooked and/or deliberately excluded from inquiry despite calls for more inclusive approaches to dementia research. Based on enrollment strategies from an interview-based protocol recruiting 120 live-alone persons with dementia, our National Institute on Aging- funded study identified five domains of gatekeepers imperative to gaining access to community-dwelling, live-alone persons with dementia: 1) housing (e.g., service coordinators), 2) data proprietors (e.g., regulatory specialists), 3) institutional (e.g., review boards), 4) kin (including fictive kin), 5) clinical (e.g., medical providers, clinician practices). In addition, gatekeeper domains are multilayered and serve distinct roles in both facilitating and hindering access to and enrollment of this under-researched vulnerable population. Analysis of our recruitment efforts contribute significant insights into how the dementia research community may engage the various domains of community gatekeepers, providing direction for current and future social science research.

### VIDEODINING IN OLDER ADULTS AGING IN PLACE: A FEASIBILITY AND ACCEPTABILITY STUDY

Laura K. Barre,[1] Sarah Coupal,[1] and Tara Young[1],
*1. Cornell University, Ithaca, New York, United States*

Loneliness and a loss of commensality contribute to the decline in nutritional status observed in older adults. The use of video chatting while dining, i.e. "VideoDining", provides an opportunity for older adults to eat with another person virtually while dining at home. We tested the acceptability and feasibility of VideoDining in older adults receiving Meals on Wheels (MOW) and explored whether it changed meal intake. Participants were recruited from a rural county in NY and ate their MOW meal while VideoDining with a companion diner at a different location. To assess acceptability, we conducted semi-structured qualitative interviews with each participant and companion diners completed a written survey. The amount of the VideoDining meal consumed was compared to usual intake from three days of food records. 140 MOW clients were contacted,13 agreed to participate and 10 completed the VideoDining experience. Barriers to participation included being uncomfortable with the technology, lack of internet service and illness. Participants were 80% female, 100% white, and all lived alone. Average meal length was 39 minutes and 40% ate more than usual, 30% ate the same, and 30% ate less. Reasons for eating less included being nervous and eating when not their usual mealtime. All participants reported they would VideoDine again and companion diners rated the overall experience a 9.2 out of 10. Older adults are able to VideoDine with a new acquaintance and have a positive experience. Further study is needed to determine if VideoDining can increase dietary intake and decrease loneliness in older adults.

### MISSING VALUE IMPUTATION VIA GRAPH COMPLETION IN QUESTIONNAIRE SCORES FROM PERSONS WITH DEMENTIA

Chen Kan,[1] Won Hwa Kim,[1] Ling Xu,[1] and Noelle L. Fields[1], *1. University of Texas at Arlington, Arlington, Texas, United States*

Background: Questionnaires are widely used to evaluate cognitive functions, depression, and loneliness of persons with dementia (PWDs). Successful assessment and treatment of dementia hinge on effective analysis of PWDs' answers. However, many studies, especially pilot ones, are with small sample sizes. Further, most of them contain missing data as PWDs skip some study sessions due to their clinical conditions. Conventional imputation strategies are not well-suited as bias will be introduced because of insufficient samples. Method: A novel machine learning framework was developed based on harmonic analysis on graphs to robustly handle missing values. Participants were first embedded as nodes in the graph with edges derived by their similarities based on demographic information, activities of daily living, etc. Then, questionnaire scores with missing values were regarded as a function on the nodes, and they were estimated based on spectral analysis of the graph with a smoothness constraint. The proposed approach was evaluated using data from our pilot study of dementia subjects (N=15) with 15% data missing. Result: A few complete variables (binary or ordinal) were available for all participants. For each variable, we randomly removed 5 scores to mimic missing values. With our approach, we could recover all missing values with 90% accuracy on average. We were also able to impute the actual missing values in the dataset within reasonable ranges. Conclusion: Our proposed approach imputes missing values with high accuracy despite the small sample size. The proposed approach will significantly boost statistical power of various small-scale studies with missing data.

### PHYSICAL THERAPY STUDENTS' ATTITUDES TOWARDS WORKING WITH PEOPLE WITH DEMENTIA AFTER VARIOUS LEARNING EXPERIENCES

Jennifer L. Vincenzo,[1] and Holly Bennett[1], *1. University of Arkansas Medical Sciences, Fayetteville, Arkansas, United States*

Working with people with dementia (PWD) can be challenging even for the most seasoned health professionals. Hence, teaching health professional students how to effectively work with this patient population is of importance. Two cohorts (n=43; aged 23-36 years) of Graduate Physical Therapy students participated in multi-modal learning experiences geared towards working with PWD within a geriatrics course. Modules included: 1) online lectures and readings followed by a team based learning activity, 2) 3 hours of 'positive approaches to care' along with a simulated experience of performing Activities of Daily living and Instrumental Activities of Daily Living of PWD, and 3) one-on-one interactions during both lunch and dinnertime with at least three PWD residing in a state veteran's home. The Dementia Attitudes Scale (DAS) was used to measure attitudes of students at baseline and following each activity. Repeated measures analysis of variance revealed a significant increase in positive attitudes of students working with PWD across each activity (98.2 +/- 10.5 baseline) with the most positive attitudes noted after interactions with PWD in a state veteran's home (111.2 +/- 15.0), [F (2.0, 83.8) = 19.4, p < .01, partial eta^2 = .32]. However, this difference was not significant when controlling for students who had previous experience interacting with PWD . In conclusion, Doctor of Physical Therapy students' attitudes towards PWD improve with different learning experiences, with the greatest improvements after one on one interactions with PWD if the student did not have prior experience interacting with PWD.

### SOCIAL RELATIONS AND FRAILTY TRAJECTORY IN LATER ADULTHOOD: EVIDENCE FROM HEALTH AND RETIREMENT STUDY

Ji Hyun Lee[1], *1. Florida State University, Tallahasssee, Florida, United States*

Frailty is a state of heightened vulnerability due to the cumulative declines across multiple physiological systems. Growing attention is given to identifying social environmental factors associated with the risk of frailty. It is not yet known how different aspects of social relationships (structure and quality) are linked to frailty, and whether the strength and directions of links may differ by relationship types. Current study aims to 1) to identify sub-populations that follows distinctive trajectory of frailty and 2) to examine the multidimensional social relationship predictors of frailty trajectory. Data came from six waves of the Health and Retirement Study (2006-2016). Sample was older adults aged over 65 (n = 8,892; Mage = 74, SD = 6.96). Frailty index was created using 32 items each wave. Network size, frequency of contact, support, and strain with each relationship type (spouse, children, family, friends) was measured at baseline. Growth mixture model identified three distinctive subpopulations of older adults who share similar frailty trajectory, namely the average, high, and steep increase frailty group. Multinomial logistic regression results showed that frequent contact with friends were associated with lesser frailty. Perceived strain with the spouse, children, and family members all had additive influence on the membership to the higher or steep increase frailty group, compared to the average frailty group. Total network size or perceived support from ties were not significant factors for frailty progression. Interventions can target friendships and stress with kin members as modifiable factors to reduce the risk of frailty progression.

### SYSTEMS BIOLOGY OF HUMAN AGING - NETWORK MODEL 2019

John D. Furber[1], *1. Legendary Pharmaceuticals, Gainesville, Florida, United States*

This network schema is presented to aid in conceptualizing the many processes of aging, the causal chains of events, and the interactions among them, including feedback and vicious cycles. Contemplation of this network suggests promising intervention points for therapy development. This diagram is maintained on the Web as a reference for researchers and students. Content is updated as new information comes to light. www.LegendaryPharma.com/chartbg.html At first glance the network looks like a complicated web. However, as a conceptual summary, in one view, we can see how the many biogerontological processes relate to each other. Importantly, examination of these relationships allows us to pick out reasonably plausible causal chains of events. Within these chains, we can see age-related changes or accumulations that appear to be promising targets for future therapy development. Especially harmful is damage to the body's regeneration and repair systems, because they normally repair damage to other structures and systems. The many observable signs of human senescence have been hypothesized by various researchers to result from several primary causes. Inspection of the biochemical and physiological pathways associated with age-related changes and with the hypothesized causes reveals several parallel cascades of events that involve several important interactions and feedback loops. This network model includes both intracellular and extracellular processes. It ranges in scale from the molecular to the whole-body level. Effects due to externalities, lifestyle, environment, and proposed interventions are highlighted around the margins of the network.

### BUILDING H.O.U.S.E. (HEALTHY OUTCOMES USING A SUPPORTIVE ENVIRONMENT) FOR LGBTQ OLDER ADULTS

Amy Rosenwohl-Mack,[1] Matt Beld,[2] Meredith Greene,[3] Karyn Skultety,[4] Leslie Dubbin,[5] Madeline B. Deutsch,[6] and Jason D. Flatt[1], *1. University of California, San Francisco, San Francisco, California, United States, 2. Institute for Health & Aging, School of Nursing, University of California, San Francisco, San Francisco, California, United States, 3. School of Medicine, University of California, San Francisco, San Francisco, California, United States, 4. Openhouse, San Francisco, California, United States, 5. Department of Social & Behavioral Sciences, School of Nursing, University of California, San Francisco, San Francisco, California, United States, 6. Center of Excellence for Transgender Health, University of California, San Francisco, San Francisco, California, United States*

Lesbian, gay, bisexual, transgender, and queer (LGBTQ) older adults face unique challenges in finding affordable, inclusive, and supportive housing. These challenges may be due to discrimination, income disparities, and higher rates of health problems compared to cisgender heterosexual seniors. To our knowledge, this is the first longitudinal study of the health and wellbeing of older adults who move into LGBTQ-welcoming, affordable senior housing. Participants completed a brief baseline survey at the time of their housing lottery application. Questions focused on physical, psychological, and social health and current health service use. We calculated descriptive statistics on health status at baseline. 184 participants completed the baseline survey, mean age was 68 years (SD 5.2), and nearly 75% reported an annual income under $30,000. Almost half reported a diagnosis of hypertension, 40% depression, 27% anxiety, and 25% HIV/AIDS. Around 70% reported their health as good to excellent, 21% fair, and 9% poor or very poor. However, 58% reported their physical activities were at least somewhat limited by their physical health, 43% reported difficulties with balance or walking, and 32% reported memory problems. Nearly 3% had been admitted into the hospital and 10% had visited the emergency room in the past 30 days. In terms of social wellbeing, 63% felt isolated from others at least some of the time. In summary, LGBTQ older adults seeking affordable senior housing report relatively good health, although they also experience functional and social difficulties. New forms of housing that are explicitly LGBTQ-welcoming may help address these health challenges.

### CORRELATES OF MENTAL HEALTH PROBLEMS AMONG OLDER AFRICAN AMERICANS WITH CHRONIC ILLNESS

Eunkyung Yoon,[1] and Susie Spence[1], *1. Jackson State University, Jackson, Mississippi, United States*

Prior epidemiologic studies have established that high rates of comorbidity of diabetes and depression is common. The aim of this study is to examine prevalence and correlates of mental health conditions among older African Americans with chronic health conditions The sample (n=1,399) from the first round of the NHATS includes older African Americans living in their community: 60% women, 35% married, 39% less than HS, 57% from the South. A two-way MANCOVA was conducted to determine the effect of diabetes and high blood pressures on four DV (no interest, depressed, anxious, worry) while controlling for age. Data indicated that 37% are with diabetes and 80% with high blood pressure and showed that 10% no interest, 4.4% depressed, 3.6% anxious, and 6.4% constantly worry for nearly every day. The main effects of high blood pressure [Wilk's $\Lambda$ = .983, F= 2.322, p =.010, $\eta$ 2 = .015)] and diabetes [Wilk's $\Lambda$ = .975, F= 3.526, p =.010, $\eta^2$ = .024] indicate significant effect on combined mental health DV. The covariate significantly influenced the combined MH DV [Wilks' =.983, F = 3.697, p = .003]. Univariate ANOVA results showed that only anxiety and worry were significantly affected by diabetes and high blood pressure except "depressed" and "no interest" These results partially support that older African Americans with chronic illness are at high risk for general mental health. However, anxiety and worry were more prevalent than just 'be depressed'. Greater attention should be paid to specific mental expressions of minority older patients.

### PROGRESSIVE CARE OF AN AGING WOMAN: INTERACTIVE VIDEO SIMULATION CASES SPANNING 15 YEARS

Laurie M. Kennedy-Malone,[1] Karen Amirehsani,[2] Rachel Zimmer,[3] and Joshua Borders[4], *1. School of Nursing, UNCG, Greensboro, North Carolina, United States, 2. School of Nursing, UNCG, Greensboro, United States, 3. Sticht Center for Healthy Aging and Alzheimer's Prevention, Winston-Salem, United States, 4. School of Nursing, UNCG, North Carolina, United States*

As a means of enhancing experiential educational opportunities for adult-gerontology nurse practitioner students who are prepared to manage the complex care of older adults, interactive simulation videos were developed using the eLearning authoring tool H5P to create learning experiences for students that can be used either in face to face classroom experiences or embedded in learning management systems. H5P is a web-based authoring tool that helps faculty build interactive course content. H5P activities provide instant feedback to students, allowing them to self-assess their understanding of the dynamic video simulation case. With funding through the Health Resources and Service Administration Advanced Nursing Education Workforce grant, four video simulation cases were developed that address emerging chronic care conditions in an older women who aged 15 years presenting initially with signs of hypothyroidism, progressed to early frailty, through moderate dementia and eventually along with her daughter face end of life health care issues. Partnering with the university instructional design experts, nurse practitioner faculty created questions that were inserted throughout the video as a means of keeping students engaged in problem-solving and decision making. A faculty handbook that described the case scenario with the interactive questions with suggested discussion questions was developed for each video simulation. The adult-gerontology primary care nurse practitioner competencies addressed in each case are identified in the handbook. Recommendations for the interactive question format will be presented and QR codes with access to direct viewing of the videos will be presented on the poster.

### QUALITY IMPROVEMENT IN DEMENTIA CARE: EFFECTIVENESS OF IMPLEMENTING STAR-VA IN A VA COMMUNITY LIVING CENTER

Thomas Chacko,[1] Kim Curyto,[2] Marc Maller,[3] and Connie Eddy[3], *1. University at Buffalo, New York, United States, 2. Center for Integrated Healthcare, VA Western New York Healthcare System, Batavia, New York, United States, 3. VA Western NY Healthcare System, New York, United States*

Staff Training in Assisted Living Residences adapted for Veterans Affairs (STAR-VA) is an evidence-based, interdisciplinary behavioral intervention to manage challenging Behavioral Symptoms of Dementia (BSD) at VA Community Living Centers (CLCs). Components of STAR-VA included creating realistic expectations for individuals with dementia, effective communication, using a behavioral problem-solving approach, and increasing resident-centered pleasant events. STAR-VA was implemented at VA Western New York CLC to help staff manage Veteran BSD. The purpose of the study was to examine the effectiveness of STAR-VA implementation in 17 CLC Veterans with Dementia who demonstrated BSD distressing to themselves, other Veterans and/or staff and documented in 2017-2018. STAR-VA was facilitated by the behavioral coordinator and nurse champion in partnership with the CLC behavior team during weekly rounds and as needed modeling and debriefing. The team included the medical provider, recreation therapist, social worker, and all levels of nursing. On average, the intervention involved four assessments over 75 days. Outcome measures at baseline and post-intervention included assessments of target behavior frequency and severity, direct care tracking of behaviors, and the Minimum Data Set distress behavior indicator. Compared to baseline scores, clinically meaningful reduction was documented using team assessment and direct care ratings of BSD frequency and severity, and overall inappropriate utilization of antipsychotic medication. CLC clinical staff tailored implementation of STAR-VA to be feasible and effective. Ongoing evaluation of STAR-VA implementation using routine measures promotes measurement based care, providing feedback to the team to improve care quality.

### AGE DIFFERENCES IN THE CHARACTERISTICS AND HABITS OF 2019 BIKE WEEK ATTENDEES IN DAYTONA BEACH, FLORIDA

Felicia V. Wheaton,[1] and Desmond Brown[1], *1. Bethune-Cookman University, Daytona Beach, Florida, United States*

Older adults are an important demographic group in the tourism sector, particularly in Florida. This study assessed age-group differences in the characteristics and habits of 2019 Daytona Bike Week attendees, as evidence indicates the average age of bike week visitors is rising. Aging Studies and Hospitality undergraduate students at Bethune-Cookman University interviewed 335 participants using Google Forms on their mobile phones. Results from chi-square tests indicate that there were significant differences between younger (20-39 years), middle-aged (40-59 years) and older adults (60+ years) in terms of gender and income, expenditures on accommodations, type of accommodations, nights spent, and first-time attendance. A greater percentage of older bike week attendees were male and higher income, and were less likely to be first-time attendees. Older adults also tended to spend more on accommodation and stayed longer, and were more likely to stay in Air BnBs, rental homes or a second home/condo compared with younger and middle-aged adults, but were less likely to stay in a hotel/motel or with friends/family. No significant differences were found in expenditures on food, alcohol, gas, entertainment or shopping. Findings suggest that older bike week attendees are important contributors to bike week tourism, particularly with respect to accommodations.

### U-EXCEL'S "@ HOME" PROGRAM: STAFF SUPPORTED BALANCE EXERCISE INTERVENTION AND ADHERENCE FOR LIFE CARE RESIDENTS

Marilyn R. Gugliucci,[1] Erica Robertson,[2] Ashley Cronkright,[2] and Sujaay Jagannathan[1], *1. University of New England College of Osteopathic Medicine, Biddeford, Maine, United States, 2. University of New England College of Osteopathic Medicine, Maine, United States*

Introduction: The University of New England College of Osteopathic Medicine U-ExCEL Program was established in 2006 and specializes in older adult fitness and balance programming. Falls account for $54 billion costs in direct and Indirect costs. Methods: This randomized control single blinded pilot project included an 8 week intervention to measure the impact of supported consistent individual balance programming in individuals' apartments for a select group of older adults residing in a life care living environment. Twenty residents (75-92 y/o) were recruited, however 12 participants (6 intervention/6 control group) participated in the study. The remaining 8 participants were pulled from the wait list as attrition occurred. Demographic data collection and 6 validated assessments were conducted at baseline and at study completion. The intervention group conducted the Balancing Act (Falls Prevention) Program 3 times/week with social support. The control group only received social support. Data analysis included descriptive statistics, SAS 5.1 was used for non-parametric Mann-Whitney U Test (Wilcoxon Rank Sum Test); a repeated measures ANOVA was also conducted. Results: The effects of the intervention (Balancing Act Program) on Oxygen Saturation ($p=0.009$), Wong Baker Score ($p=0.008$), and the Rapid Assessment of Physical Activity (RAPA) 2 ($p=0.008$) test were statistically significant. The effect of the intervention on all other variables was not statistically significant including validated balance measures. Conclusion: Quantitative measures failed so show significant improvement in balance from the start to the end of the intervention; however improvements were experienced and expressed by the intervention group. Social Support is necessary for adherence.

### FEASIBILITY OF A TABLET DESIGNED FOR OLDER ADULTS TO FACILITATE TELEMEDICINE VISITS

Carla Perissinotto,[1] Cai Zhang,[1] Tim Oseau,[2] Danielle Balik,[2] Caroline Sou,[1] Claire Burnight,[3] and Kerry Burnight[2], *1. University of California, San Francisco, San Francisco, California, United States, 2. Grandpad, Minnetonka, Minnesota, United States, 3. USC, Los Angeles, California, United States*

Introduction: By incorporating age-friendly technology into the care of older adults with high utilization, providers can address complex care needs more efficiently and urgently. Age-friendly technology has the potential to alleviate loneliness that occur with homebound older adults and affect healthcare outcomes. Specific Aims: 1) To evaluate the feasibility of integrating age-friendly telemedicine in a homebased primary care setting using. 2) To evaluate the effects of engaging older adults with age-friendly technology to address loneliness. Methods: UCSF Geriatric Medicine provided GrandPads to 21 patients ages 60 to 94 over a one year period. All research participants were community residing, predominantly low income, and homebound. There was no charge to the patients for the GrandPad. Providers who used the GrandPad with their patients were queried on their experiences. Patient questionnaires included a telemedicine readiness survey, satisfaction and UCLA 3-item loneliness scale. GrandPad tablets are commercially available and underwent an evaluation with the IT Security Risk Assessments team. Results: 100% of providers who used grandpad felt was easy to use and efficient. 83% felt the platform was easier than other platforms. 83% of patients felt the grandpad helped them connect to their providers and 82.5% felt the grandpad was easy to use and 78% felt more connected as a result of using grandpad. Conclusion: Patients and providers are satisfied with an older adults friendly tablet and it can easily be implemented in health care settings. Grandpads may also help older adults feel more connected.

### MAKING OLD BONES: QUALITY IMPROVEMENT IN OSTEOPOROSIS MANAGEMENT IN A GERIATRIC PRIMARY CARE CLINIC

Meredith Gilliam,[1] and Sabrina Vereen[1], *1. University of North Carolina, Chapel Hill, North Carolina, United States*

Osteoporotic fractures and their sequelae are a leading cause of morbidity and mortality in older adults. In the United States, nearly 50% of white women and 20% of black women and white men will suffer a fragility fracture in his or her lifetime. Osteoporosis medications reduce the risk of major fragility fracture by 31-62%, but numerous care gaps exist, including screening rates as low as 1-47% and treatment rates as low as 16-30% even after a fracture has already occurred. From January to August 2019, we conducted a multi-faceted quality improvement project at a university hospital-based geriatric primary care clinic, with a goal of improving our rates of osteoporosis screening and treatment. We designed and tested electronic health record-based registries of eligible patients, and developed patient outreach workflows and physician "inreach" workflows. We piloted a bone health clinic. While we did not meaningfully affect the rate of osteoporosis screening, our efforts resulted in an increase in treatment of osteoporosis from 49% to 53%. Documentation of osteoporosis decision making among eligible patients improved from 66% to 80%. In our clinic, ongoing barriers to evidence-based osteoporosis management include competition for time with other medical issues, patient mistrust of medications, and the complexity of decision making around osteoporosis in older adults with polypharmacy and limited life expectancy. Future work must balance the broad application of treatment guidelines via population health tools with the need to individualize treatment decisions for each patient's overall health and goals of care.

### THE EFFECT OF LANGUAGE ON PREPARATION FOR OLD AGE: EVIDENCE FROM CROSS-CULTURAL SURVEY AND TWITTER DATA

Amber Xuqian Chen,[1] and Helene H. Fung[1], *1. The Chinese University of Hong Kong, Hong Kong, Hong Kong*

We aimed to further investigate the linguistic-savings hypothesis (Chen, 2013) in the field of aging, which maintains that when languages grammatically divide the future and the present (e.g. English and Czech), speakers tend to behave less future-oriented than those speaking languages that do not mark future tense (e.g. German and Chinese). In the 2018 wave of Aging as Future Project, 2,042 participants from the United States, Germany, Czech Republic, Hong Kong and Taiwan (18-93 year, Mean age= 55.47, 55.61% female) completed online questionnaires. The results supported the hypothesis that people speaking future-less languages tended to perceive the timing of preparation for old age closer to the present in terms of finance, living arrangement, nursing care, and loneliness, they also took action earlier and performed more relevant activities. Furthermore, the association between language and preparation timing was more salient in older adults than younger counterparts. And path analysis revealed that time discounting was a significant predictor (P=0.049) for the future-oriented behavior. Hence, speakers of futureless languages will view the future as temporally closer to the present, causing them to discount the future less and prepare for old age actively. Using LIWC 2017, we then analyzed community-level of future orientation with 80 million Tweets across countries and replicated our principal result through that usage of future-oriented languages partly predicted prevalence of health behaviors. The findings indicate that language not only shape people's own future-oriented outcomes, through decreasing time discounting, but also influence population health as a whole.

### THE ROLE OF RELIGIOSITY IN DEPRESSIVE SYMPTOMS AMONG CHINESE OLDER ADULTS

Ru Jia,[1] Dexia Kong,[1] and XinQi Dong[1], *1. Rutgers University, New Brunswick, New Jersey, United States*

This study aims to examine the relationship between religiosity and depressive symptoms in a large cohort of community-dwelling U.S. Chinese older adults living in the Greater Chicago area, which has received relatively little research attention. Cross-sectional self-report data was obtained from the Population Study of Chinese Elderly in Chicago between 2011 and 2013 (N=3,157). Depressive symptoms were measured by the nine-item Patient Health Questionnaire (PHQ-9). A score of 5 and above indicated the presence of clinically significant depressive symptoms. Logistic regression analyses were conducted to examine the association between religiosity and depressive symptoms. Out of 3,157 participants, 20.3% participants had a score of or above 5 on PHQ-9. 35.4% reported religiosity as being "important" (24.7%) and "very important" (10.7%); 16% reported attending organized religious services at least once a month (3.1% reported once a month; 12.3% reported once a week; 0.6% reported almost every day); 23% reported having religious services at home at least once a month (10.3% reported once a month; 3.2% reported once a week; 9.5% reported almost daily). Results showed that recognizing religiosity as important is significantly negatively associated with depressive symptoms (odds ratio [OR]=0.94, 95% confidence interval [CI]=0.89-0.99). However, no significant associations between depressive symptoms and religious activity attendance or religious service at home were observed. Findings suggest that senses of belonging and life meaning may help reduce depressive symptoms, rather than the religious activities per se. Future interventions could reduce depressive symptoms of U.S. Chinese older adults through religiosity.

### DEPRESSION AND COGNITIVE DECLINE OF OLDER ADULTS IN THE TRANSITION TO WIDOWHOOD

Feng Zhao,[1] Peter Martin,[1] and Gina Lee[1], *1. Iowa State University, Ames, Iowa, United States*

This study examined the trajectories of depression and cognitive function in the transition to widowhood and investigated the temporal and causal relationship between these two closely related constructs. Respondents were 1,822 widowed adults aged 51 to 91 from a restructured data set (Wave 3 to Wave 12) of the Health and Retirement Study. The results of cross-lagged panel analysis indicated a bidirectional relationship between depressive symptoms and cognition decline, but the effects of cognitive impairment at earlier time points on later depression were larger than the effects of previous depressive symptoms on later cognition. The latent growth curve analysis showed that the cognitive function declined over time, whereas the initial level of depressive symptoms first increased following widowhood and gradually decreased over time. Significant negative associations were found between the initial levels of depression and cognitive function ($p < .001$) and between the rates of change of these two variables ($p = .025$). Older adults tended to have lower initial level of cognitive function and they showed faster cognitive decline over time. Female respondents were more likely to report more depressive symptoms and higher cognitive function. White respondents were more likely to report fewer depressive symptoms and higher levels of cognitive function. Higher levels of education were protective for one's cognitive function but not for depressive symptoms. The study highlighted the reciprocal relationship between depression and cognitive function following widowhood and pointed out that accelerated cognitive decline may precede elevated levels of depression.

### THE ASSOCIATIONS BETWEEN PERSONAL EVENTS AND COGNITIVE FUNCTION: DOES GENDER MAKE A DIFFERENCE?

YINGXIAO HUA,[1] and Gabriella Dong[1], *1. Rutgers, The State University of New Jersey, New Brunswick, New Jersey, United States*

Recent studies suggested stressful personal events were associated with lower cognitive function and that men and women have different responses to stressful events. There is additionally ample evidence supported gender difference in cognitive function in later age. However, research regarding whether gender would moderate the relationship between personal events and cognitive function are limited. Our data were retrieved from 3,126 US Chinese older adults in Chicago between 2017 to 2019. Personal events were measured by a composite score (range 0-28) of all lifetime events including fire, physical assault, rob, sexual assault, divorce, death, cancer, homeless, imprisonment, false accuse, miscarriage and abortion. Global cognition was constructed by mean of z scores from five cognition tests, covering episodic memory, working memory, executive function and C-MMSE test. Stepwise linear regressions were performed to test the association. After adjusting for social demographic confounders, personal events score was positively associated with global cognition (b=0.018, SE=0.007, p=0.013), and significant interaction term of gender and global cognition was found (b=0.019, SE=0.009, p=0.041). The study found the protective effect of undergoing personal events on cognitive function in an aging population, and it was stronger among women. The findings highlighted the important role of gender in the relationship. Identifying mechanisms underlying this may provide gender-specific information for prevention of cognition decline among older adults. Future studies could explore gender difference in short-term and chronic stressful personal events, respectively, to improve understandings of how traumatic personal events impact cognitive function.

### WHAT FAMILY CAREGIVERS THINK AND FEEL WHEN PROXY ASSESSING FROM DIFFERENT PERSPECTIVES

PATRICIA EGAN[1], *1. University of Wisconsin-Madison, Madison, Wisconsin, United States*

Dementia family caregivers are routinely enlisted as proxy assessors of care recipient quality of life (QOL). Proxy assessment is not ideal because proxy assessments differ systematically from self-assessments and the assessment process can elicit negative affect from family caregivers. Prompting adoption of the care recipient's perspective can enhance assessment congruence and may improve the emotional experience for assessors. This study explored family caregivers' cognitive and affective experiences during QOL proxy assessments made from both their own and care recipients' perspectives. Thirty-six dementia family caregivers were recruited from senior service agencies. Subjects completed the Quality of Life-Alzheimer Disease (QOL-AD), Caregiver Version using standard instructions to assess QOL across thirteen domains of their care recipient's life without specifying the perspective to be used. Subjects were next asked to repeat the QOL-AD with instructions to adopt the perspective of their care recipient, as they imagined it to be. Subjects were then interviewed about what they thought and felt during each proxy assessment experience. Content analysis indicated that spontaneous perspective shifts and response shifts frequently occurred. Most subjects (91.7%) reported changed thinking for one or more QOL-AD domains when they were prompted to switch perspectives. Over half (61.12%) reported changed affect when switching perspectives and 90.9% of those experiencing changed affect reported affective improvement. Little or no affective change was reported by 38.89%. Findings suggest awareness of perspective can enhance clinical interpretation of proxy assessed QOL and can inform clinical response to dementia family caregivers who experience negative emotions while proxy reporting QOL.

### DOES EXPOSURE TO INFORMATION ABOUT DEMENTIA CHANGE STIGMA? AN EXPERIMENTAL STUDY

Fan Zhang,[1] and Sheung-Tak Cheng[1], *1. The Education University of Hong Kong, Hong Kong, Hong Kong*

Objectives: Educational programs on dementia may backfire, as recipients could feel more negatively about people with dementia after exposure to the alarming symptoms (e.g., behavioral and psychological symptoms of dementia, or BPSD). This study aimed to investigate whether such exposures had any effect on stigma. Methods: 200 adults aged 18-83 years were randomly assigned to three groups. The first group read vignettes describing fictitious older adults with memory impairment. The second group read the same vignettes that were expanded to include descriptions of BPSD (i.e., memory impairment cum BPSD). After reading the vignettes, both groups answered questions about stigma, while the third group directly responded to this questionnaire without reading any vignette (i.e., not exposed to experimental manipulation). ANOVA was performed to analyze the effect of experimental manipulation, as well as that of age, education, whether having relatives with dementia, and belief about treatability of dementia. Results: At posttest, the level of stigma was moderate and was comparable across the three groups, suggesting that exposures to information about cognitive and behavioral symptoms did not change people's stigmatizing attitude. The absence of group effect in stigma did not vary by age, education, whether having a relative with dementia, or belief about prognosis. Only the main effects of age and education were significant, where younger and least educated participants reported higher stigma. Conclusion: There was no evidence that stigma would be affected by exposure to information about symptoms of dementia, including the more disturbed ones (i.e., BPSD).

### UNDERSTANDING BARRIERS TO PALLIATIVE CARE USE AMONG ASIAN AMERICANS: A SYSTEMATIC REVIEW

Min Kyoung Rhee,[1] Yuri Jang,[1] Mona Liu,[1]
William E. Haley,[2] and Susan Enguidanos[1], *1. University of Southern California, Los Angeles, California, United States, 2. University of South Florida, Tampa, Florida, United States*

Despite the well-documented clinical and public health impacts of palliative care on older patients' end of life experiences, racial and ethnic disparities in palliative care use still remain. However, empirical evidence on the sources of disparities is lacking, particularly for Asian Americans. Given the rapid growth and increasing cultural diversity of the older Asian population in the United States, a systematic review was conducted to provide a comprehensive overview of the best available evidence on the barriers to palliative care use among Asian Americans. Search was conducted using six electronic databases (PubMed, CINAHL, PsycINFO, EMBASE, Cochrane Library, and Google Scholar), and all procedures of the study have been guided by Preferred Reporting Items for Systematic Reviews and Meta-Analysis Protocols (PRISMA-P). A total of 23 studies published in the U.S. since 1998 were included. Individual level barriers to palliative care service use among Asian Americans identified by the study included lack of palliative care knowledge, lack of insurance, cultural beliefs, language barrier, attitude toward death, family centered decision-making. Provider level barriers included lack of training and experience about palliative care, patient-provider discordance in language and culture, and absence of policy or guidance for providers. Although racial disparities in palliative care for Asian Americans were evident, Asian Americans were underrepresented in palliative research. Future research effort should be directed toward the development of culturally sensitive palliative care services and policies that reduce racial and ethnic specific barriers.

### AGE DIFFERENCES IN PSYCHOSOCIAL AND HEALTH OUTCOMES AMONG ADULTS EXPERIENCING HOMELESSNESS

Carolyn L. Turturro,[1] Rosalie Otters,[1] and Zeferin Turturro[2], *1. University of Arkansas at Little Rock, School of Social Work, Little Rock, Arkansas, United States, 2. Auburn University, Auburn, Alabama, United States*

Homelessness is a critical social problem for this nation. It takes a tremendous toll on individuals and families who live without stable shelter, adequate food, and personal security, as well as strains the societal resources. The mean age of death for people experiencing homelessness has been reported to be age 50, far less than the 76 years that is the average for Arkansas. This study investigates the sociodemographic, behavioral and health-related data of 331 adults experiencing homelessness in Central Arkansas. Data was collected by structured interviews during recent homeless counts. Participants ranged in age from 18 to 70, with quartiles established for ages 18-35, 36-45, 46-55 and 56 –70. Sociodemographic comparisons of the groups included education, income, months homeless, work and veteran status, behavioral variables included alcohol use and drug abuse, whereas health-related comparisons included reported diabetes, heart disease, diabetes, high blood pressure, heart disease, problems with hearing, vision, walking as well as mental health including anxiety and depression; in addition, medication procurement and available assistance from family and friends were compared. Preliminary results suggest significant differences, as well as some similarities, among the four age quartiles. Analyses are on-going.

### RESILIENCE OF CHINESE OLDER COUPLES LIVING WITH CHRONIC ILLNESSES

Esther O. Chow[1], *1. City University of Hong Kong, Hong Kong, China, Hong Kong*

Applying theories of lifelong psycho-social growth and life course principles of human development, older couples generally possess rich and valuable resources from diverse aspects of life. However, empirical evidence, on whether these supports are fully transferred to benefit themselves as they age, remains uncertain due to inevitable physiological decline and increasing caregiving strains. The present study explicated the underlying processes of the older Chinese couples in forming a dyadic resilience model as they age with chronic illnesses. Using in depth interviews, 15 pairs of older Chinese couples were interviewed twice through home visits. The conversations were transcribed and analyzed using thematic analysis. Eight themes related to resilience and challenges of spousal caregiving were identified: 1) insights and acceptance of the chronicity of illnesses, 2) a sense of purpose and commitment as being the spouse in providing care for each other, 3) mastering skills and resources to handle the duties, 4) mixed feelings when the spouse was functioning changes, 5) realizing the importance of self-care and life-management, 6) cultivating positive meanings amidst difficult circumstances, 7) accepting things that cannot change, and cherishing on those aspects which are still functioning well, 8) working closely with other formal and informal support for common good. Findings have both practice and policy implications to health and social care practitioners in understanding different patterns of resilience as experienced by older Chinese couples in mitigating the developmental challenges from individual, relational and societal levels, and research implications in examining actor-partner interdependence practice in addressing caregiving challenges.

### PROVIDING QUALITY ASSURANCE IN ONLINE GERONTOLOGY COURSES THROUGH A PEER REVIEW NETWORK

Louise M. Murray,[1] Enoch Park[2], *1. University of Maryland Baltimore County, Erickson School of Aging Studies, Baltimore County, Maryland, United States, 2. University of North Carolina at Charlotte, Charlotte, North Carolina, United States*

In health care and senior housing and care, the challenge of meeting diverse regional workforce needs is increasingly important in higher education. Educators within gerontology are rising to this challenge using multiple platforms, including online education (Carter, Solberg, & Solberg, 2017; Nadash, Miller, Porell, Birchander, Glickman, & Burr, 2014). This poster presents the life cycle of a traditional face to face introductory level graduate gerontology course which was transitioned to a fully online course. This transition was achieved using a structured course development process based on nationally recognized online course quality standards by Quality Matters (QM). Utilizing a team approach, the process incorporated faculty (subject matter experts and course developers), instructional designers, and professional staff in distance education, faculty teaching support, and online learning specialists. QM standards serve to assure quality online instruction, with the goal of providing students with a positive and successful online learning experience. Analysis of this case highlights the role of Certified (QM) peer reviewers who have expertise in Gerontology and can serve as the connector of online gerontology courses offered within programs, between institutions, and globally. Recommendations are presented to improve the pedagogical quality of online courses to attract and retain students in gerontology and aging studies courses. The case for building an initial network of online course peer reviewers to strengthen online teaching and learning within the gerontology profession will be made.

### THE ASSOCIATION BETWEEN PHYSICAL FRAILTY AND DEPRESSIVE SYMPTOMS: DOES SOCIAL SUPPORT MATTER?

Shuting Liang,[1] Dexia Kong,[2] and XinQi Dong[2], *1. Institute for Health, Health Care Policy & Aging Research, New Brunswick, New Jersey, United States, 2. Rutgers University, New Brunswick, New Jersey, United States*

This research will present the association between physical frailty and depressive symptoms among U.S. Chinese older adults, and the extent to which social support moderates the relationship. Cross-sectional data were obtained from the Population Study of Chinese Elderly in Chicago collected between 2011 and 2013 (N=3,157). Physical frailty was assessed by the Short Performance Physical Battery (range=0-15). A cut-off point of 6 was used to define physical frailty as suggested by prior research. Depressive symptoms were assessed by the nine-item Patient Health Questionnaire. Social support was measured by a scale assessing positive support and negative strain from spouse, family members, and friends. Logistic regression analyses with interaction terms were conducted. In our sample, 1,682 (54.3%) had depressive symptoms, and 16.1% had physical frailty. Having physical frailty was positively associated with depressive symptoms (Odds Ratio [OR] 1.15, 1.11-1.18). Additionally, female gender (OR 1.39, 1.20-1.61), education (OR 1.03, 1.01-1.04), and chronic conditions (OR 1.18, 1.12-1.25) were positively associated with depressive symptoms. Social support (OR 0.85, 0.83-0.87) and children (OR=0.92, 0.87-.97) were negatively associated with depressive symptoms. Furthermore, family members (OR 0.96, 0.94-0.98) and friends (OR 0.96, 0.94-0.98) has moderating effect on the relationship between physical frailty and depressive symptoms. However, the interaction between social support from spouse and physical frailty was not significant. The findings highlight the interconnections among physical frailty, social support, and depressive symptoms. Intervention strategies focusing on social support may have the potential to reduce depressive symptoms among frail U.S. Chinese older adults.

### DO INTRA-PROXY GAPS EMERGE WHEN FAMILY CAREGIVERS ASSESS QOL FROM DIFFERENT PERSPECTIVES?

PATRICIA EGAN[1], *1. University of Wisconsin-Madison, Madison, Wisconsin, United States*

Dementia family caregivers are routinely enlisted as proxy assessors of care recipients' quality of life (QOL). This study explored whether prompts to change perspective during QOL assessment could elicit an intra-proxy gap. The intra-proxy gap was hypothesized to be any difference between those assessments made from the caregiver's own perspective and those made from the adopted perspective of the care recipient, as the care giver imagined it to be (Pickard and Knight, 2005). Thirty-six dementia family caregivers were recruited from senior service agencies. Subjects completed the Quality of Life-Alzheimer Disease (QOL-AD), Caregiver Version under two conditions: First, from an unprompted perspective and second, from the adopted perspective of the care recipient, as the family caregiver imagined it to be. T-testing indicated intra-proxy gaps emerged for eleven of the QOL-AD's thirteen domains. For these domains, QOL scores were higher when assessed from the care recipient's perspective, as the family caregiver imagined it to be. The sample was then repeatedly bisected using caregivers' personal, relational, and health factors. T-testing indicated that family caregivers' personal factors were associated with intra-proxy gaps across more QOL-AD domains than their relational or health factors were. Three personal factors, being of older age, having more empathetic concern, and having more empathetic distress, were associated with intra-proxy gaps more frequently than other personal factors were. Findings suggest that clinicians should be alert for perspective employed by proxy assessors and for the possibility of intra-proxy gaps. Recognition of these gaps could help improve interpretation of QOL scores.

### SENIOR COMMUNITY CENTER AS A SOCIAL ENGAGEMENT PLATFORM FOR OLDER ADULTS IN NEPAL: AN ADAPTATION OF WESTERN CONCEPT

Sabita Shrestha,[1] and Tina Colson[2], *1. Southeastern Louisiana University, Hammond, Louisiana, United States, 2. Southern Illinois University, Carbondale, Illinois, United States*

Older adults around the world are living longer. Similarly, in Asian countries longevity of older adults have significantly altered the demographics shift as well as the cultural landscape and needs of the society. These changes have compounded with challenges and needs as a community grapples with how to best take care of aging population. Nepal, a developing country, is also faced with a similar demographic shift among the geriatric population. This shift has brought challenges and needs to communities such as health care, daily living needs, social support systems, economic needs, etc. The geriatric population will require social support systems as they age. Historically, older adults have relied on traditional family support systems for their care and needs maintaining cultural values and norms which may burden immediate or extended family members. Recently, traditional family structures along with social support systems are breaking away from their family trees due to community advancement and modernization, and many are leaving for better economic opportunities. This trend has left many older adults alone in social isolation. Despite challenges in the community, Nepal government doesn't offer infrastructure for social engagement for older adults. One solution to prevent isolation and loneliness is to establish "senior community centers" (western based concept) for social engaging older adults. Based on ecological framework, this presentation proposes a need of "Senior Community Centers" for older adults where they can become involved in social engagements and receive social supports outside traditional family support systems; thus, optimizing their health and well-being.

### DUAL IMPACTS ON MEANING IN LIFE IN OLDER PERSONS VOLUNTEERING FOR ELDERLY MENTAL HEALTH PROGRAM

Dara K.Y. Leung,[1] Joyce Sing,[1] Lesley Sze,[1] Jessica Tang,[1] Shiyu Lu,[1] Tianyin Liu,[1] Gloria H.Y. Wong,[1] and Terry Lum[2], *1. The University of Hong Kong, Hong Kong, Hong Kong, 2. Department of Social Work and Social Administration, PokFuLam, Hong Kong*

Volunteering provides sense of meaning in life. The impact of volunteering on different dimensions of meaning in life and the mechanisms explaining the effects have been rarely researched. This study examined the effects and the mechanism of a formal volunteering program for mental health in older persons, including training, service provision, and supervision, on two dimensions of meaning in life — presence of meaning and searching for meaning — among senior volunteers. A mixed method study was conducted. 103 volunteers (average age=63.3±6.6) completed assessments at three time points: before and after the training, and one-year after service provision. They self-assessed Meaning in Life Questionnaire (MLQ) and reported time use in different tasks. 26 of them participated in focus groups discussing their experience in the program. Volunteers' search for meaning differed between time points ($F(1.87,173.81)=3.20$, $p<.01$)

while presence of meaning persisted. Search for meaning reduced from before the training to after service provision (p<.05) as revealed by post-hoc tests. Proportion of home visit during service provision explained 2.7% of the variance of presence of meaning before and after service provision ($R2=0.05$, $F(6,74)=1.376$, $p<.05$). Findings from focus groups revealed that application of trained skills and building trusting relationship with their clients via home visits are sources of meaning. Formal volunteering may have dual impacts on meaning in life in older age: reducing search for meaning and maintaining presence of meaning. For senior volunteers, being able to apply what they learn and building social connects are the key factors for attaining meaning.

### FALL PREVENTION IN RURAL COMMUNITIES OF NORTH DAKOTA

Melissa L. OConnor,[1] Jane Strommen,[1] Philip Estepp,[1] Megan Hay,[1] Heather Fuller,[1] Rachel Grace,[1] and Sean Brotherson[1], *1. North Dakota State University, Fargo, North Dakota, United States*

Fall-related injuries are common sources of morbidity and mortality for adults aged 65 and older. Thus, interventions for preventing falls can have substantial public health benefits. One promising fall prevention program is the Stepping On program, which involves seven community-based workshops. In these workshops, older adults learn about risk factors for falls, as well as safety-conscious behaviors. Stepping On has been offered in several states, and outcomes have been positive. However, research in rural areas has been lacking. To address this issue, the current study examined 508 older adults who participated in the Stepping On program across 53 rural communities in North Dakota through May of 2019. Most participants were female (82%), with an average age of 79 years (range 65-98). Participants completed baseline and post-test assessments of their knowledge regarding health, mobility, and safety issues. At baseline, 46% of participants reported falling at least once during the previous year. Repeated-measures ANOVAs showed that participants had improved significantly in the following areas at post-test: understanding how vision influences safety; knowledge of balance and strength exercises; recognizing hazards in the home; choosing safe footwear; confidence in mobility; understanding how medications affect fall risk; and the importance of bone health ($p<0.001$ for all). Just 14% of participants reported falling during the year following the workshops, and 62% felt that the program reduced their risk of falls "to a big extent." These findings suggest that the Stepping On program is feasible to administer in rural areas and benefits older adults in such communities.

### OASIS STUDY: ACTIVITY PATTERNS OF OLDER ADULTS LIVING IN FIVE AGING IN PLACE COMMUNITIES

Vincent DePaul,[1] Catherine Donnelly,[1] Simone Parniak,[1] and Oasis Study Collaborative[2], *1. Queen's University, Kingston, Ontario, Canada, 2. Oasis Study Collaboration, Ontario, Canada*

Background: The Oasis program is a model of aging-in-place that targets social connectedness, physical activity and nutritional wellness through member-driven programming. Oasis, first established in an apartment building in Kingston Ontario, has recently been expanded to 6 new communities across Ontario in a participatory action research study. The purpose of this poster is to describe the physical activity patterns of five unique Oasis communities (Original and 4 new) and explore the impact of personal, environmental, and Oasis program characteristics on these patterns. Methods: Participants were recruited from Oasis communities in 3 market-priced apartments, 1 subsidized apartment, and 1 mobile-home park. Participants wore the ActivPAL3 activity monitor for 7 days. Mobility was measured using the Timed Up and Go. Programming was described by type (e.g. social, exercise, nutrition), frequency, and timing of programming. Results: Participants included 70 older adults aged 79.8(min 62, max 97), community mean age ranged from 66.2 – 83.5 years. TUG score 11.6(SD 4.9) (community range 10.5 to 13.7 s). Average daily step count was 5800(SD 2835) steps, with communities ranging from 4685 to 6472 steps/day. An average of 604(community range 236 – 1056) of steps were taken at a healthy pace(100 steps/min). Only 27% of participants took the recommended 7000 steps/day (with community rates ranging from 9.5% to 37.5%). Conclusions: Older adults within these aging in place communities demonstrated low to moderate levels of physical activity, with activity patterns differing across communities. Impact of community make up and characteristics on activity patterns will be presented.

### PERCEIVED SOCIAL ISOLATION AND HEALTH-RELATED QUALITY OF LIFE IN HEAD AND NECK CANCER

Janet H. VanCleave,[1] and Jane M. Fall-Dickson[2], *1. NYU Meyers College of Nursing, New York, New York, United States, 2. Georgetown University School of Nursing & Health, Washington, D.C., United States*

Patients treated for head and neck cancer may experience impaired eating and talking that may affect their ability to undergo social activities. We conducted a secondary data analysis to explore: 1) prevalence of perceived social isolation, and 2) association between perceived social isolation and health-related quality of life (HRQoL) in patients with head and neck cancer. Data were collected during a clinical usefulness study of the Electronic Patient Visit Assessment (ePVA), a valid, reliable web-based patient-reported symptom measure for head and neck cancer. The study population consisted of 56 patients recruited during or after treatment for head and neck cancer. Perceived social isolation data were collected using the ePVA. HRQoL data were collected using the European Organization for Research and Treatment of Cancer (EORTC) QLQ-C30, a valid measure frequently used in the head and neck cancer population. Data analysis consisted of descriptive statistics and Student's T-Test. The study population consisted primarily of persons > 60 years (mean age = 61.5 + 12), male (68%), White (77%), and receiving surgery, chemotherapy, radiation therapy or combination of these treatments (70%). Among participants, 36% reported that their current health situation negatively affected their social activities. Reasons for perceived social isolation included fatigue, feeling ill. Statistical analysis found that perceived social isolation was significantly associated with deceased HRQoL ($t=5.3$, $p<.001$). We conclude that participants in this sample treated for head and neck cancer were at risk for perceived social isolation, which has previously been reported to negatively influence cancer treatment outcomes.

**CAREGIVERS: PURPOSEFUL AGING LOS ANGELES (PALA) SURVEY OF CITY AND COUNTY EMPLOYEES**

Iris Aguilar,[1] Donald A. Lloyd,[1] Laura Trejo,[2] and Maria P. Aranda[3], *1. USC Edward R. Roybal Institute on Aging, Los Angeles, California, United States, 2. Los Angeles Department of Aging, Los Angeles, California, United States, 3. University of Southern California, Los Angeles, California, United States*

The City and County of Los Angeles Age-Friendly Cities initiative, Purposeful Aging Los Angeles (PALA), 2017 needs assessment included an online survey of the workforce expected to implement age friendly policies and programs. The study aim was to describe the rate and characteristics of family caregivers within in the City and County workforce (employees). The overall response rate was 6.5% (City 4.5%, County 7.5%) for a final sample size of 9,071. The family caregiver category was derived from the following survey questions: 1) Are you or someone else in your home caring for a family member or friends who are experiencing problems/thinking that is affecting their ability to work or live a normal life? and 2) Are you a family caregiver? The analysis revealed that 34% of all respondents were family caregivers and of those, 53% cared for adults and 12% cared for children under the age of 18 (minors). Of the 53% caregiving for adults, 40% cared for someone with memory/thinking problems including but not limited to dementia (cognitive impairment). 15% of caregivers of minors were also taking care of an adult with cognitive impairment. The current caregiving research literature suggests that family caregivers experience higher rates of depression, anxiety, and social isolation due to the caregiving demands. Caregiving demands have the potential to create work-family conflicts that are important for employers to consider when designing wellness programs, and other supportive programs. It is critical to identify ways to address caregiving implications on social services, healthcare, and the environment.

Innovation in Aging
cite as: *Innovation in Aging*, 2019, Vol. 3, No. S1, 1022
doi:10.1093/geroni/igz052

THE GERONTOLOGICAL SOCIETY OF AMERICA®

OXFORD

Addendum

### Addendum to the Abstract Book of the GSA 2019 Annual Scientific Meeting

The following abstract is part of Session Number 2055, Symposium titled, "Gaps and Opportunities To Improve Access To Healthcare for Older Rural Veterans":

### SESSION 2055 (SYMPOSIUM)

### GAPS AND OPPORTUNITIES TO IMPROVE ACCESS TO HEALTHCARE FOR OLDER RURAL VETERANS

### ENGAGING RURAL VETERANS' PERCEPTIONS OF SURVIVORSHIP CARE FOR HEAD AND NECK CANCER

Aaron T. Seaman[1], Julia Friberg[2], Jenna L. Adamowicz[3], Nicholas Kendell[3], Nitin A. Pagedar[3], M. Bryant Howren[4] and Alan J. Christensen[3]

[1]Iowa City VA Healthcare System, Iowa City, Iowa, [2]Center for Comprehensive Access and Delivery Research and Evaluation, Iowa City, Iowa, [3]University of Iowa, Iowa City, Iowa, [4]Rural Health Resource Center, Iowa City, Iowa

The purpose of this study was to investigate 1) rural patients' perceptions of their own rurality and its effects on experience of head and neck cancer survivorship, and 2) potential barriers and facilitators to survivorship care within an integrated health care delivery system of the Veterans Health Administration (VHA). Data from qualitative interviews with Veterans who have a history of head and neck cancer are presented to understand the complex ways that rurality impacts cancer survivorship. Head and neck cancer survivors must contend with specific challenges resulting from their risk factors and treatment, including access to complex medical follow up, long-term physical and psychological effects of treatment, and tobacco- and alcohol-related comorbidities. While integration within the VHA facilitates coordination of specialty and primary care and the transfer of medical information, the use of community care in rural areas presents coordination challenges, especially for survivors with comorbidities.

©The Author 2019. Published by Oxford University Press on behalf of The Gerontological Society of America.
This is an Open Access article distributed under the terms of the Creative Commons Attribution License (http://creativecommons.org/licenses/by/4.0/), which permits unrestricted reuse, distribution, and reproduction in any medium, provided the original work is properly cited.

### Index Participant

A

Aaltonen, M.: 2430
Aarts, S.: LB1545-414
Abadir, P.: LB3620-364
Abbate, L. M: 630
Abbott, K. M: 690, LB1545-397
Abdel-Mageed, A.: LB1545-377
Abdelmohsen, K.: LB935-347
Abdul-Malak, Y.: 3245
Abe, T.: 3340-204
Abedsaeedi, Z.: LB1545-435
Abell, J. G: 730, 1045
Abrahamson, K.: 1400-265, 3355-254
Abramenaka-Lachheb, V.: 3375-306
Abrams, L. R: 915-286
Abramson, T. A: 900-249, 2430-302
Abujarad, F.: 3305-109
Abushakrah, J.: 1285
Accius, J. C: 3450
Acevedo, A.: LB3620-379
Achterberg, W.: 4135
Acierno, R.: 1055, 2225, 2365-107
Acker, L.: 2405-231
Acton, G.: 1395-247, 2395-208
Adamek, M. E: 880-200, 905-251, 910-273
Adams, C. M: 815-15
Adams, I.: 845-117
Adams, J.: 2325-26
Adamson, E. M: 3460
Adamson, J.: 2395-213
Adams-Price, C. E: 915-291, 2200
Addison, O.: 630, 3020
Adelnia, F.: 820-24
Adeniji, D. O: 2405-240, 2430-299
Adusei-Asante, K.: 2430-295
Afari, N.: 4080
Affuso, O.: 2410-260
Afolabi, M.: 640, 1355-149
Afunugo, W. E: LB2570-332
Ågård, P.: 1210
Agarwal, M.: 2395-217
Aggarwal, A.: LB1545-366
Agmon, M.: 2315-7
Agmon, M.: LB1545-375
Agniel, D.: 2250
Agree, E. M: 880-202
Aguila, E.: 750
Aguilar, I.: 3070, LB3620-444
Aguilar, S.: 3535
Aguinaga, S.: 3360-266
Aguirre, A.: 850-134
Aguirre, A.: 895-230
Ahamed, S. I: LB3620-327
Ahmadi, S.: LB2570-387
Ahmed, B.: 1325-74
Ahmed, F.: LB2570-423
Ahn, H.: 1402-313
Ahn, S.: LB1545-411
Ahuja, R.: 2325-23
Aiken Morgan, A. T: 1485
Ailshire, J. A: 1315-53, 1345-106, 1401-289, 3275-17, 4000, 4080, LB2570-436
Aizenstein, H.: 825-45
Aizenstein, H.: 2360-94
Ajrouch, K.: 1210, 2455
Akhtar, B.: LB935-381
Akintomide, A.: 3300-100
Akinyemi, A.: 1355-149
Akishita, M.: 820-27, 3325-157
Akosionu, O. C: 2390-200, 3345-217, 3585
Akushevich, I.: 825-60
Akushevich, I.: 2240, 2370-132
Al- heizan, M. O: 2385-187, 2400-226
Al Snih, S.: 660, 850-135, 2385-171, 4020, LB1545-349
Aladeokin, T.: 1315-56
Alahmari, S.: 1310-39
Alain, G.: LB1545-336
Alarcon Garavito, G.: 3355-254
Alarie, M.: 3025
Albert, S.: LB1545-432
Albrecht, J.: 2370-130
Albright, A.: LB1545-332, LB2570-418
Albright, T.: 500
Alconada-Romero, A.: 3345-216
Aldwin, C. M: 3340-209, 3370-301
Alemi, Q.: LB935-363
Alessi, C.: 2395-215, 2420-273
Alexander, E.: 2390-196
Alexander, K.: LB3620-351
Alexander, K.: 4110
Alexander, N.: 530
Alfred, D. L: 2270
AlHasan, D. M: LB1545-364
Ali, I.: 2360-83
Ali, S.: 910-273
Ali, T.: 925-337
Aliyu, K.: 1355-149
Allaire, J. C: 3290-53
Allen, J. R: 920-310
Allen, J.: 1310-36
Allen, N.: 1315-48
Allen, N.: 3280-34
Allen, P. D: 815-12, 855-146
Allen, R. S: 535, 870-183, 1400-264, 2130, 2405-231, 3290-60, LB935-405, LB2570-418
Allen, S.: 3435
Allgood, K.: 3365-280
Allison, D.: LB2570-394
Allison, T. A: 3470
Allore, H.: 1395-248, 3365-278, 3455, LB1545-356
Allred, C. D: 820-32
Allred, K. F: 820-32
Allsopp, R.: 830-72
Almack, K.: 3225
Almardawi, R.: LB1545-424
Almeida, B.: 695
Almeida, D. M: 825-53
Almeida, D. M: 2375-148
Almeida, D. M: 3275-21
Almeida, D. M: 3295-69
Almeida-Meza, P.: 3210
Almen, R.: LB935-404
Aloiau, A.: 840-105
Alpaugh, W.: 840-112
Alston, R.: 2360-100
Alvarez, C.: 2325-28
Alves, S.: 2365-113
Alwerdt, J.: LB3620-347
Amano, H.: 3340-204
Amano, T.: 845-118, 1300-8
Ambale-Venkatesh, B.: 820-34
Ameer, F.: LB3620-377
Ames, A.: 1345-110, 2355-73
Amireault, S.: 2410-255, 3350-238
Amirehsani, K.: LB3620-421
Amjad, H.: 1170, 3420
Amorim, C. F: 2360-87
Amparan, G.: LB935-437
Amspoker, A.: LB2570-390
An, P.: 2370-125, 3170
An, Q.: 500
An, R.: 2360-100
An, S.: 2375-138
An, Y.: 2370-129
Anand, C.: LB935-366, LB3620-326
Anand, P.: 1515
Andel, R.: 590, 1365-177, 1380-196, 3290-59
Andersen, A.: LB2570-339
Andersen, O.: LB2570-339
Andersen, S. L: 2060, 2345-55, LB1545-428, LB2570-343, LB3620-337
Andersen, T.: LB2570-416
Anderson, J. G: 895-238

Anderson, J. A: 2340-47
Anderson, K. K: 2385-190
Anderson, K. A: 885-207, 1385-214
Anderson, L.: 860-161
Anderson, M.: LB2570-368
Anderson, R.: 765
Anderson, R. A: LB1545-425
Andersson, M. A: 885-209
Andreoletti, C.: 2435-307, 2560
Andrew, M.: 565
Andrew, N.: 2390-195
Andrews, E.: 3355-247
Andrews, H.: 3325-171
Andrews, M.: LB1545-441
Andrews, R. M: 3350-245
Anetzberger, G.: 1055, 1265, 2365-107, 3025
Ang, N.: 1365-166
Ang, S.: 2310
Angel, J. L: 750, 1025
Angel, N.: 920-307
Angel, R. J: 750
Angell, B.: 910-274
Anglem, J.: 640
Annoni, G.: 820-31
Ansello, E. F: 3465, 4075
Anthony, D.: 880-197
Antolini, L.: 820-31
Antoniades, J.: 2325-26
Antonucci, T. C: 800, 925-337, 1130, 1195, 1350-121, 2455
Anunsen, R. A: 1285
Anzuoni, K.: 3355-256
Apchemengich, I.: 2410-258
Apenteng, B.: LB1545-391
Apostolides, C.: 3355-248
Applebaum, R. A: 1220, 3555
Apracio-Ugarriza, R.: LB1545-361
Apriceno, M. A: 3370-295
Aranda, M. P: 1355-138, 3070, 3180, 3320-138, 4000, LB3620-444
Arandia, G.: LB935-431
Aransiola, J. O: 890-220, 1355-149, 3300-100
Araujo, L.: 880-201
Arbeev, K.: 825-60, LB1545-440
Arbisi, P.: 1390-236
Arbogast, A.: 3305-110
Archibald, P.: 3360-260
Archuleta, W. P: 2425-290
Ardelt, M.: 2200, 2565
Arendacs, R.: 2315-3
Arevalo-Flechas, L. C: LB1545-382
Argueta, I.: 1402-295
Argueta, J.: 1402-295
Arias Merino, E.: 3290-67
Arias Merino, E.: 3375-302
Arieli, R.: 925-322
Arif, A.: 1345-117
Arling, G.: 3435
Arms, T.: 3430
Armstrong, J.: 565
Armstrong, N.: LB1545-410
Arnold, A.: 3595
Arora, K.: 1100
Arora, N. K: 3140, LB935-416
Arora, R. C: LB2570-329
Arránz Becker, O.: 3200
Arshad, M.: LB3620-326
Arvan, T.: 820-28
Arvanitis, M.: 3595
Aryan Nagabooshanam, V.: 3295-74
Asamane, E. A: LB935-390
Asamane, E. A: LB935-398
Asante, S.: 1400-250
Asch, S.: 3535
Ascha, M.: 895-227
Asgari, M.: 1195
Ashcraft, A. S: 2395-207
Ashe, M. C: LB2570-438, LB3620-383
Ashida, S.: 3065
Ashihara, T.: 1305-23
Ashley, B. L: LB2570-403
Ashrafi, A.: LB3620-337
Asman, A. R: LB935-376
Aspinall, S.: 3390
Astell, A.: 2325-30
Astuti, R. D: 915-287
Atakere, D.: 2080
Atalla, S.: 3100
Atef, B.: 3280-29
Atkins, J.: 1180
Atkinson, E.: 830-68
Atkinson, T. J: 3290-50
Atlas, L. S: 915-280
Attoh-Mensah, E. K.: 825-49
Auerbach, D. I: 755
Auerbach-Burgoon, J.: LB3620-370
Aufill, J.: 2045
Augsburger, M.: 1330-88
August, K. J: 1315-58
Aunger, J. A: LB935-398
Austad, S.: 2230, LB2570-371
Austen, A.: 905-255
Austin, M.: 1345-107
Austin, M.: 3300-90
Austin, N.: 2360-101
Au-Yeung, W. M: LB2570-357
Avanzino, L.: 1402-304
Avent, E. S: 1355-150, 2375-150
Averett, P.: 1380-191
Aversa, Z.: 835-84
Avila, K.: 2360-101
Avis, N. E: 1455
Awosanya, A.: 2310
Awuviry-Newton, K.: 2410-251
Axonovitz, J.: 3300-101
Aye, M.: 3360-265
Ayernor, K.: 3445
Ayyaswami, V.: 1402-309
Azhar, G.: LB3620-369
Azzis, L.: 3390

### B

B. Ferdows, N.: 2545
Baba, A.: LB1545-429
Babulal, G.: LB1545-399
Baccarelli, A.: 3405
Bacharz, K.: 2375-143, 3320-149
Bachelar-Murphy, M.: 2355-74
Bachner, Y.: 1160
Backhaus, R.: 2020, LB1545-414
Backhouse, D.: 1510
Backman, A.: 635, 3345-220
Backus, D.: 900-245
Bacsu, J. R: 2355-62
Badana, A.: 2270, 2355-69, 2375-141
Bae, J.: 3370-297
Baek, J.: LB3620-335
Baeuerle, E.: 820-39, 825-56
Bagaajav, A.: 3445
Baghban Ferdows, S.: 3395
Bahr, P.: 3305-110
Baidwan, N.: 3295-74
Baier, R.: 705
Baik, D.: 4010
Baik, S.: LB3620-353
Bailey, D. E: LB2570-403
Bailey, J.: 3490
Bailey, P. E: 2365-109
Bain, J. R: LB2570-437
Bair, B.: 3165
Bajracharya, R.: 3355-253
Bakas, T.: 1350-120
Baker, H.: 3275-19
Baker, L. D: 850-132
Baker, M. F: LB2570-379
Baker, S. J: 1385-206
Baker, T. A: 1130, 1390-231, 2080, 2425-283
Bakerjian, D.: 3390
Balaswamy, S.: 1070, 1500
Balbim, G. M: 2155, 3360-266
Baldivieso, V. C: LB3620-338
Baldwin, P.: 890-222
Baldwin, P.: 1075
Bales, C. W: 2070, 2380-167
Balik, D.: LB3620-425
Ball, K.: 1360-156
Ballenger, J. F: 3375-314
Ballesteros, S.: 3370-290
Balmuth, A.: 660, 1375-184
Bamonti, P. M: 1390-221, 2255, 3300-97
Bamonti, P.: LB2570-409
Banaszak-Holl, J.: 3345-213
Bancks, M.: 3280-34
Bandeen-Roche, K.: 820-34, 830-75, 3360-268
Banerjee, K.: 1390-231

Banerjee, S.: 3275-20
Bang, E.: 1400-250
Bangerter, L. R: 880-193
Bangerter, L. R: 2325-25, 3180
Banh, A.: 825-44
Banham, V.: 2430-295
Banihashemi, L.: LB2570-324
Baniya, G.: 4050
Bansal, A.: 2360-83
Barani, E.: 3365-277
Baranowski, T.: LB1545-419
Barber, S.: 3380, LB2570-412
Barbieri, V.: 815-9
Barca, M.: 1330-86
Bardach, S. H: 2270
Bardo, A. R: 1325-73, 1385-215, 1400-259, 2010
Bardutz, H. A: 855-153
Barger, K.: 3280-33
Barha, C.: 715, 920-313, 3075, 3280-36
Barker, M.: LB3620-337
Barker, R. O: 1290, 2395-213
Barker, W.: 2355-66
Barmon, C.: 2435-307
Barnard, R.: LB1545-390
Barnes, L. L: 3480
Barnes, S. L: LB3620-404
Barnes, T.: 1015, LB935-355
Barnett, A. E: 2435-310
Barnett, M. D: 815-15, 2340-44, 2425-288
Baron, A.: 3390
Barooah, A.: 1405-323
Barr, E.: 705
Barr, R. A: 2135, 2290
Barragan, C.: 1420, 4170, LB2570-342
Barre, L. K: LB3620-414
Barrett, A.: 1380-193, 2340-48, 3345-229
Barrett, E.: 920-299
Barrientos, M. E: 900-246
Barry, L. C: 915-295, 3010, 3360-263
Barry, R. A: 870-186, 1365-168
Barsness, S.: 505
Bartholomew, A.: LB1545-336
Bartke, A.: 835-83, 3130
Bashian, H. M: 815-10, 2340-41
Bashore, A.: 835-87
Basic, A.: 2340-33, LB935-367
Baskaran, D.: LB3620-338
Basnet, P.: 2395-208
Basque, S.: 3560
Bass, D.: LB3620-352
Bastarache, E. D: 3340-206
Basu, T.: 2010
Batchelor, F.: 1385-206
Bates, E.: 1402-300
Batey, S.: 1400-267
Batsis, J. A: 2070
Battalio, S.: 855-148
Batty, G.: 730
Batzogs, U.: LB3620-374
Baudouin, C.: 1320-64
Bauers, C.: 2385-173
Baugh, M. E: LB3620-348
Baugh, M.: LB2570-437
Bauman, A.: 855-147
Baumbusch, J.: 3345-212, 3435
Baumel, Z. J: 915-284
Bauschpies, W.: LB3620-343
Bay, A.: LB3620-336
Bayliss, E.: 1401-291
Bayly, M.: 3415
Bazzano, L.: 1330-87
Beach, S. R: 620, 1055
Beadle, J.: 3270-1
Beam, C.: 925-317
Beamer, B.: LB1545-424
Bean, J.: 2255, LB1545-363, LB2570-409
Beasley, J.: 1205
Beattie, E.: 505, 850-130
Beattie, Z.: 3535, LB2570-357
Beatty Moody, D. L: 545
Beaulieu, C.: 915-288, 1380-201, LB2570-413
Beauregard, L.: 910-261, 3555
Beavers, D. P: 3325-175
Beavers, K.: 3325-175
Bechshoeft, R.: 820-30
Beck, M. S: 2380-162
Beck, S.: 1115
Becker, C. B: LB2570-399
Becker, H.: 1395-247
Becker, M.: LB3620-344
Becker, T. D: LB935-385
Beckert, T.: 3340-201
Beckett, M.: 520, 2250
Beckham, J.: 1330-83
Beckmann, A.: LB935-346
Beckmann, G.: LB935-381
Bédard, M.: 1410-331, 2550, 3595
Bednarcyzk, M.: 635
Beeber, A.: 3345-224
Beeler, D. M: 3275-14
Beer, J. M: 1401-287, LB3620-329
Beer, J.: 1350-132
Behar, R.: 2355-66
Behrens, E. A: 3365-279
Behrens, L.: LB1545-397
Bekelman, D.: 1401-291
Belanger, E.: 880-194
Beld, M.: LB3620-419
Beleno, R.: 2325-31
Belfer, G.: 885-204
Belfort, T.: 850-141
Bell, D.: 2430-303, LB3620-320
Bell, G.: 870-183
Bell, J.: 2295
Bell, R. K: LB3620-380
Bell, T.: 1175
Bell, T. R: 1360-156
Bell, V.: 1401-273
Bellander, T.: 850-137
Bellingtier, J. A: 3380, LB935-370
Bellon, F.: 3345-216
Belsky, D.: 820-41
Belsky, D. W: 1430
Belsky, D.: 2240, 2370-117
Beltran, S. J: LB2570-400
Belza, B.: 1405-320
Benavidez, G.: 2410-248
Benayoun, B. A: 3540, LB935-351
Bendayan, R.: 1395-244, 3350-244
Bender, A. A: 885-211
Bender, A. A: 2305, 3270-9, LB3620-321
Bender, F.: 910-269, 1320-62
Bene, M. R: 830-77
Bengtson, V.: 1475
Bennett, C.: 920-307, 3495
Bennett, D. A: 3480
Bennett, H. Q: LB2570-319
Bennett, H.: LB3620-416
Bennett, J.: 930-343
Bennett, K.: 3295-72
Bennett, R.: 915-298
Benson, J.: 3410
Benson, W. F: 1505
Benton, D.: 1355-150, 1405-319, 2375-150
Benton, M. J: 840-105, 3325-162
Berg, C.: 1315-48, 3190
Bergeman, C.: 3030, 3560
Bergen, A. W: 3325-152
Bergen, G.: 2555
Bergeron, C. D: 1300-5, 1380-190
Bergh, S.: 1330-86
Bergquist, S. H: 2385-176
Bergren, S. M: LB2570-428
Berg-Weger, M.: 1020, 1385-214, 1530
Berish, D.: 850-133, 3010
Berkman, C.: 3225
Berkman, L. F: 4110
Berkowsky, R. W: 1401-275
Berlin, M.: 3270-8
Bernacki, R. E: 870-181
Bernard, M. A: 2135, 2290
Bern-Klug, M.: 1480, LB2570-430
Berrett-Abebe, J.: 755
Berridge, C.: 1525
Berta, W.: LB3620-391
Bertolotti, M.: 515
Bertoni, A. M: 3590
Bertrand, K.: 2345-55
Bertrand, R. M: 2395-212
Best, C.: 2355-63
Best, J. R: 565, 715, 1510, 2360-97
Best, R. D: LB935-386
Best, R.: 3350-237
Bettega, K.: 3390
Betz, J.: 2495
Bewernitz, M. R: 890-217
Beyth, R. J: 3330-184

Bhalla, N. S: 3325-176
Bhargava, S.: 1175
Bhatta, J.: 1385-212, 2415-266, 3370-292
Bhatta, T.: 895-239, 1400-258
Bhattarai, R.: 3325-176
Bhaumik, A.: 500
Bherer, L.: 3280-29
Bhutkar, A.: 835-92
Bibbo, J.: 1090
Bickhart, S.: 3290-63
Biderman, A.: 1160
Bielak, A. A: 3350-245, 3560
Bienvenu, B.: 610
Biery, N.: 2380-156
Bigonnesse, C.: LB1545-441
Bihary, J. G: 2340-33, LB935-367
Bildtgard, T.: 3200, 3315-124
Binder, E.: 2210
Binette, J. V: 1465
Birchfield, T.: 3320-136
Birditt, K. S: 3425
Birdsall, J.: LB935-405
Birdsong, D.: 2565
Birg, J.: 815-9
Biritwum, R.: LB1545-342
Birkeland, R.: 1385-210, 2375-140
Birtley, N.: LB2570-334
Bischoff-Ferrari, H. A: LB2570-367
Bisconti, T. L: 1380-200, LB3620-406
Bishop, A. J: 645, 1385-212, 1401-285, 1475, 2415-266, 3320-145, 3370-292
Bishop, C. E: 2480
Bishop, N. J: 3295-83
Bishop, T.: LB3620-351
Biss, R.: 2360-98
Bisset, E.: 820-36
Bisson, A. N: 2410-257, 3580
Biswal, S.: 2315-4
Bittinger, K.: 3305-103
Bitto, A.: 2090
Bjarnadottir, R.: 1402-294, 2370-120
Black, J.: 3420
Black, K.: 595
Black, M.: 1305-24
Black, S.: 2405-231
Blackmar, A.: 3275-15
Blackwell, T.: 820-22
Blackwood, J.: 1360-161
Blackwood, K. L: 3330-178
Blair, E.: 2385-183
Blair, J.: 2430-298
Blancato, R.: 760, 1230, 3515
Blanchard, J.: 890-213
Blanco, E.: 2375-153
Blanco-Blanco, J.: 3345-216
Blaskewicz Boron, J.: 3270-1, 3290-47, 3295-80, 3350-234
Blaxton, J.: 3030
Bleijenberg, N.: 675
Bleijlevens, M.: 2380-164
Blieszner, R.: 1025, 1315-51
Blinka, M. D: 4165
Bliwise, D.: 1510
Block, C.: 845-117, 3350-239
Block, L.: 3350-240
Block, P.: LB3620-351
Blocker, K. A: LB1545-373
Blodgett, J. M: 3170, 3325-161
Bloemen, E.: 2370-118
Blomberg, B. A: LB935-384
Blomberg, R.: 3305-102
Blondet, R.: 2495
Bloom-Charette, L.: 3125
Blozis, S.: 920-314
Blue, B.: 830-81
Bluethmann, S. M: 2050
Boateng, A.: 915-289
Bobitt, J.: 1100
Boccanfuso, L.: LB3620-329
Bock, J.: 1390-223
Boddu, P.: 1402-310
Bodenheimer, L.: 885-204
Boerner, K.: 2235, 2340-37, 3315-123
Bogosian, C.: 695
Bohn, L.: 1360-162
Bohnen, N.: 820-38
Boker, S.: 2005
Boks, M. P: LB1545-422
Boles, A.: LB935-431
Bolkan, C.: 1305-26, 2035, 2355-80, 2385-189
Bollinger, S.: 3390
Bolllag, W.: 830-73
Bolognesi, M.: 820-31
Bolstad, C. J: 670, 915-291
Boltz, M.: 1255, 2215, 2315-3, LB2570-358
Bombelli, S.: 820-31
Bond, M.: 3510
Bond, M.: LB1545-395
Bond, M.: LB3620-322
Bonds, K.: LB2570-359
Bonilla, H. J: 820-25
Bonkowski, M. S: LB2570-323
Bonner, A.: 795
Bonner, T.: 1490
Bonner, T.: 2405-234
Bonner Cozad, A.: 1465
Boockvar, K.: 4045
Booker, S.: 775, 2080
Bookheimer, S.: LB2570-335
Bookwala, J.: 1140
Boolani, A.: 1380-190
Boone, A.: 2315-8
Boot, W. R: 620, 1060, 1270, 1525, 2110, LB1545-385
Boot, W.: 2060, 2265, 2475
Booth, K.: 2315-4
Booth, S. L: 715, 3280-33
Booth-Lord, C.: 875-187, 3510
Boparai, M.: 635
Borders, J.: LB3620-421
Borges-Machado, F.: LB2570-341
Borromeo, G. L: 2380-159
Borza, T.: 1330-86
Boscardin, J.: 2315-1
Bosco, E.: 4130
Bose-Brill, S.: LB2570-345
Boss, L. L: 3270-2
Boston, N. C: 915-298
Bostrom, A.: 1405-320
Bostrom, M.: 1365-179
Bosworth, H.: 1330-83
Botor, N.: 730
Botoseneanu, A.: 1395-248, 2370-124
Bott, N.: 1402-300
Boubekri, M.: 3295-84
Bouchard, L. M: 1315-44, 3370-285
Boucher, N.: 520
Boucher, N. A: 3320-151
Boudreau, R. M: 565, 1425, 2370-125, 3275-24, LB935-368, LB1545-428, LB2570-343
Bouldin, E. D: 920-304, 1030, 2555, 4110
Boutin, R. D: LB1545-390
Bouweraerts, A. G: 2360-91
Bowblis, J. R: 2400-220, 2480, 3555
Bowblis, J. R: 3555
Bowen, P.: 2410-260
Bower, E. S: 910-275, 4140
Bower, K. L: LB935-424
Bowers, B.: 1155, 2110, 2525
Bowland, S.: 1265, 4060
Bowles, K. H: 1170
Bowling, B.: 4145
Bownes, E.: 640
Boxer, R. S: 1401-291, 2385-180, 3280-35
Boyd, K.: 4160
Boyd, M.: LB2570-425
Boyken, L.: 880-189
Bozzo, D.: LB935-427
Brach, J.: LB1545-363
Bradley, E. S: 820-29
Brady, S.: 660, 1375-184
Bragg, R. T: 835-100
Braithwaite, S.: 825-43
Braley, M.: 820-28, 3325-172
Branco, K. J: 1300-9
Brandhorst, S.: 1310-43
Brandt, C.: 2370-119
Brangman, S. A: 4110
Branson, S.: 1090
Bratzke, L.: 2315-2
Braun, K.: LB3620-374
Brauner, D.: 910-258
Bravo, V.: 1365-171
Brayne, C.: LB1545-333
Breakwell, S.: LB3620-404
Breckman, R.: 2225
Breda, K.: 3420
Brem, R.: 765

Brennan-Ing, M.: 2485, 3525
Brennan-Taylor, M.: 3330-187
Brettschneider, C.: 3320-139
Breunig, I.: 3330-179
Brewster, G.: 1355-142
Brewster, G. S: 1510
Brewster, G.: 2375-149, 3100
Brewster, K. K: 2495
Breytspraak, L.: 2340-36
Brick, T.: 2005
Brickman, A.: 2495
Brickman, S.: LB935-399
Bridges, K.: 680, 1465
Briganti, J.: 3290-62
Bright, B.: LB935-437
Bright, C. L: 3330-188
Brijnath, B.: 2325-26
Briley, M.: 2360-86, LB935-441
Brill, S.: 710
Bristol, A. A: 870-184, LB935-401, LB1545-335
Bristowe, K.: 3225
Brittain, K.: 1290, 4055
Broccoli, J.: 1400-251, 3600
Brodaty, H.: 655
Brody, A. A: 870-184, 1495, 2395-215, LB935-401, LB1545-335
Broese van Groenou, M.: 2430-292
Bromet, E.: 825-52
Bronskill, S. E: 555
Brooks, D.: LB3620-405
Brooks, M.: 3300-94, 3340-205
Brooks-Wilson, A.: LB2570-354
Broome, A.: LB1545-352
Brorsson, A.: 3565
Brossoie, N.: 3345-223
Brothers, A. F: 2040
Brotherson, S.: LB3620-441
Brown, C.: 1485, 3095
Brown, C. H: 2360-92
Brown, C. H: LB1545-371
Brown, C. J: 1400-255
Brown, C. J: 1425, 3365-274
Brown, D.: LB3620-423
Brown, E.: LB2570-384
Brown, J.: 805, 4150
Brown, J.: LB2570-394
Brown, J.: 865-168
Brown, J. A: 1270
Brown, L.: 1050
Brown, L.: 2390-202
Brown, L. M: 2065, 2450, 3045
Brown, M. T: 4110
Brown, M. J: 2485
Brown, P. P: 1485
Brown, R.: 1305-33
Brown, R. P: 1390-223
Brown, S.: 1365-166
Brown, S. L: 4065
Brown-Hughes, T.: 3355-247
Browning, C.: 2325-26
Broyles, I. H: 2385-190
Brozgol, M.: 4020
Bruce, M. A: 1145, 3360-260
Brummett, C.: 1425
Brunet, A.: 1245
Bruns, D.: LB1545-402
Bruns, D.: LB2570-346
Brunson, K.: 1380-192
Brush, D.: LB1545-353
Brush, S.: LB2570-368
Brustio, P. R: 3325-159
Bryan, C.: 1355-147
Bryant, J.: LB3620-405
Brydges, C. R: 3350-245
Brydges, C.: 3560, LB935-397
Bryson, W.: LB935-419
Buchman, A.: 1402-303
Buck, H. G: 1330-93
Buckwalter, K. C: 3550
Bueno, T.: 1390-222
Buford, T. W: 4175, LB935-371
Bugos, J.: LB3620-327
Bui, C. N: 915-288, 1380-201, 2385-179, LB935-365
Bull, S.: 1401-291
Bullock, L.: LB1545-438
Bulow, J.: 820-30
Bültmann, U.: 660
Bunger, A.: 3520
Burack, O. R: LB3620-370
Burack, O. R: LB3620-393
Burant, C. J: 890-216, 915-293, 1320-63
Burchett, C. O: LB935-444
Burgdorf, J.: 1110, 1170, 2045, 2270, 3420
Burgess, E. O: 920-312, LB3620-321
Burgunder, A.: 2155
Burhenn, P.: 855-146
Burholt, V.: 2105
Burke, R.: 3390
Burke, S. L: 1390-237, 2355-66, 3275-23
Burkhardt, J.: LB1545-332
Burnaevskiy, N.: 830-78
Burnes, D.: 2225, 2365-108, LB935-444
Burnett, J.: 2365-114
Burnette, D.: 885-212, 2450, 3070, 3180
Burnight, C.: LB3620-425
Burnight, K.: LB3620-425
Burning, M.: 830-68
Burns, E. R: 2555, LB935-425
Burns, J.: 3175
Burns, V. F: 2540, LB1545-439
Burnside, L.: LB3620-344
Burr, J. A: 3270-10
Burr, J.: 915-288, 1065, 1095, 1360-163, 3315-129
Burrows, D.: LB3620-385
Burston, B.: 3330-191
Burzynska, A. Z: 590
Busby-Whitehead, J.: LB935-392
Butler, A.: LB935-377
Butler, J.: LB2570-416
Butler, S. S: 910-257, 3345-233, LB935-434
Buttelmann, F.: LB935-370
Buurman, B.: 1365-176
Buxton, O. M: 3275-21, 3580
Buys, D. R: 1425, 3365-274
Buys, K.: 3365-274
Bybee, S.: 500
Byers, A.: 1390-235
Bygrave, D.: 655
Byles, J.: 2410-251
Byrd, D. R: 2455, 3290-53
Byrne, K.: 3330-177

C

Cabin, W. D: 910-256, 1375-189, 1400-269
Cabrera, L. Y: 2455
Cabrero Castro, E.: 2360-104
Cacchione, P. Z: LB3620-340
Cacciottolo, M.: 825-50
Caceres, N. A: LB935-363
Cadar, D.: 1045, 3210
Cadet, T. J: 1390-237, 2385-174, 3275-22
Cadogan, M.: 2395-215
Caffeé, L.: 2420-276
Caffrey, C.: 910-268
Caffrey, C.: 2390-203, 2395-214
Cagle, J.: LB2570-434, LB3620-355
Cahill, L.: 3325-165
Cai, B.: LB1545-364
Cai, S.: LB3620-386
Cai, X.: LB1545-381
Cai, Y.: 3220
Cajita, M.: 2355-79
Calamia, M. R: 1345-116
Caldera, M.: LB2570-335
Caldera, S.: 910-259
Calderon-Larranaga, A.: 3170, 3325-173, 3420
Caldwell, S.: LB1545-357
Callahan, B. L: 3295-87
Callahan, K. E: 4045
Calvillo, M.: 825-61
Calvo, R.: 2280
Camacho, D.: 3070, 3180
Camell, C.: 835-96
Cameron, K. A: 3415, 4030
Camp, L.: 1401-271
Campbel, T.: 1400-265
Campbell, L.: LB2570-335
Campbell, M.: 1315-48
Campbell, m.: 825-43
Campbell, R. J: 860-157, 2365-112
Campione, J. R: 2045
Campitelli, M. A: 555
Canamucio, A.: 3390
Canell, A. E: 815-10, LB3620-346
Canham, S.: LB935-419
Canham, S. L: LB1545-439

Cannell, M. B: 930-341
Canning, D.: 1400-261
Cannizzaro, K. A: 2385-173
Cannon, J.: LB3620-404
Cannon, M. L: 810-2, 2450
Cantave Burton, C.: 1465
Cantu, P. A: 3360-259
Cao, G.: 1330-77, 2370-137, 4005
Cao, J.: 1305-21
Cao, W.: 825-47, LB935-364
Cao, X.: LB1545-365
Caouette, S.: 3270-2
Capistrant, B. D: 2380-155
Caprio, T. V: 1530, LB1545-380
Carberry, K.: 850-134
Cardenas, V.: LB1545-443
Carder, P.: 1305-27, 2305, 3345-224, LB3620-390, 2470
Carlson, B. W: LB3620-358
Carlson, M. C: 830-75, 2095, 2170, 3350-245, 4025
Carlson, M.: 890-213
Carmel, S.: 1160
Carmichael, A. G: LB2570-435, LB3620-378
Carpenter, B.: 535, 3270-6, 3310-119, LB935-382
Carpenter, J. G: 605
Carr, D. C: 1160, 1400-252, 2550
Carr, D.: 1065, 1190, 1300-3, 1440, 2205, 3150, 3315-125, LB2570-374
Carr, E.: 3350-244
Carr, K. A: LB935-389
Carramusa, C.: 1360-153
Carrasquillo, O.: 1402-301
Carreno, J.: 1402-312
Carrero-Tagle, M.: 2430-300
Carrico, C.: 870-186, 1365-168, 1530
Carrion, I. V: 3070
Carroll, D.: LB2570-418
Carstensen, L.: 865-165, 925-323
Carter, C. S: 4175
Carter, C.: LB935-380
Carter, M. W: 2370-121, 2390-198
Carvalho, J.: LB2570-341
Casaletto, K.: 3205, LB1545-410
Casanova, G.: 2425-281
Casanova, R.: 1402-296
Case, B.: 4005
Casey, A. S: 655
Casey, D.: 920-299
Cashdan, E.: 2325-24
Casillas, A.: 1401-291
Caskie, G.: 815-10, 1380-199, 2340-41, LB3620-346
Caspi, A.: LB1545-347
Cassel, S.: 3375-308
Castellon-Lopez, Y. M: LB3620-408
Castle, N.: 3345-222, 3435, 4050
Castle, S.: 530, 920-303
Cater, D.: LB2570-404
Catic, A.: 850-145, 3560
Catlin, C.: 1390-221
Cattoretti, G.: 820-31
Caudle, B.: 850-132
Cauley, J. A: 820-22, LB935-368
CAVALCANTI, F.: 2360-85
Cavaleiro, A.: 2325-28
Cavanaugh, J.: LB3620-399
Caves, K.: 530, 1402-308
Cawthon, P. M: 820-22
Cena, C.: 3325-158
Cenko, E.: LB3620-403
Cereatti, A.: 1402-304
Cerino, E. S: 1195
Cerino, E. S: LB3620-382
Cermakova, P.: LB1545-333
Chacko, S.: 1310-37
Chacko, T.: LB3620-339
Chae, J.: LB935-351
Chahal, J. K: 505, 3375-303
Chai, H.: 1360-159, 3340-208
Chai, H.: LB2570-362
Chai, Y.: 3075
Chamberlain, S.: 2430-293
Champion, J. D: 2395-208
Chan, A. T: 3260
Chan, A.: 715, 865-167, 2360-96
Chan, A.: 3245
Chan, B.: 3245
Chan, J. L: 715
Chan, L.: 1515
Chan, P.: 1510, 2360-97
Chan, R.: 915-294
Chan, S.: 895-233, 1330-85
Chan, T.: 1402-295
Chang, C.: LB3620-343
Chang, E.: 815-17, 2225
Chang, E.: 1170
Chang, M.: 2370-131
Chang, S.: 1375-186
Chang, S.: 1365-173
Chang, Y.: 2375-146
Chang, Y.: 920-312
CHANG, Y.: 895-234
Chang, Y.: 1330-90
Chanti-Ketterl, M.: LB2570-322
Chao, N. J: 2345-56
Chao, Y.: 920-312
Chao, Y.: 1330-90, 3505, 4105
Chaparro, M.: 895-228
Chapman, A.: 1055
Chapman, S. A: 3555
Chappell, A.: 1385-203
Chard, S.: 3330-188
Charlton, J.: 1410-331
Charness, N. H: 620, 1130
Charness, N.: 1270
Charness, N. H: LB1545-385
Chase, J. D: 1170, LB1545-338
Chase, J.: 920-300
Chasteen, A. L: 815-5
Chau, A.: 2425-286
Chaudhari, N.: 825-61
Chaudhury, H.: 1401-292
Chaudhury, H.: 3565
Chaves, P. H: 3325-153
Chavoix, C.: 825-49
Che, Y.: LB3620-377
Chee, K.: LB1545-352
Cheesman, K. L: 2415-265
Chen, A.: LB3620-427
Chen, A. T: LB935-445, LB1545-358
Chen, C.: 1297, LB3620-319
Chen, C.: 1405-319
Chen, C.: LB935-354
Chen, D.: 1300-7, LB2570-381
Chen, E. K: 2235, 3310-113
Chen, F.: 3455
Chen, G.: 2375-152
Chen, H.: 3285-37
chen, h.: 805
Chen, H.: 3145
Chen, H.: LB2570-344
Chen, J.: 615
Chen, J.: LB2570-441
Chen, L.: 2400-224
Chen, L.: 1340-102, 3345-231, 4160
Chen, L.: LB935-415
Chen, M.: 920-312, 4005
Chen, M.: 825-42
Chen, N.: 2360-94
Chen, P.: 900-246
Chen, P.: 1320-63
Chen, P.: 3220
Chen, R.: 830-72
Chen, S.: 1405-322
Chen, X.: 1400-262
Chen, X.: 885-210
Chen, Y.: 910-267, 1300-7, 2385-182, LB2570-381
Chen, Y. T: 3335-196
Chen, Y.: 2030, 3395
Chen, Z.: 2030
Chen, Z.: 1330-77, 2370-137
Chen, Z.: 4005
Cheng, G.: 865-167
Cheng, J.: 855-151
Cheng, J.: 895-230
Cheng, K. G: 2375-145
Cheng, M.: 880-199
Cheng, S.: 4080
Cheng, S.: 845-120, 3320-147, LB3620-432
Cheng, Y.: 3230
Cheon, J.: LB2570-434
Cheon, J.: 3375-309
Chernoff, R.: LB935-420
Cherrier, M.: LB1545-412
Cherry, B. J: 3365-272
Cherry, D.: 1355-138

Cherry, K. E: 815-12, 1345-116, 2065, LB1545-417
Chess, E.: 845-116
Chesworth, B.: 3330-177
Cheung, E.: LB2570-407
Cheung, W.: 2360-90
Cheval, B.: 3030
Chhatre, V.: LB1545-402
Chi, C.: LB935-375
Chi, I.: 2475, LB2570-333
Chi, N.: 3365-277
Chia, B.: 885-210
Chia, C.: 835-99
Chiang, A.: 3420
Chiang, T.: 910-267, 1300-7
Chien, S.: 1401-288
Chiles Shaffer, N.: 3095
Chilton, R. L: 920-302
Chinchankar, M. N: 830-71
Chinda, B.: 820-28
Chinda, B.: 3325-172, LB2570-354
Ching, J.: 715
Ching, L.: LB2570-421
Chiodo, L.: 930-342
Chippendale, T.: 855-148
Chiriboga, D.: 1325-70, 2140, 2405-235
Chirinos, C. L: 2355-66
Chisholm, L.: 520
Chit, A.: 4130
Chiu, C.: 805
Chiu, R.: 1345-115
Chiu, T.: 1345-115
Cho, D.: LB935-412
Cho, E.: 840-111, 3295-77, LB1545-360, LB2570-317
Cho, J.: 645, 865-171, 1325-69
Cho, J.: 2300, 3320-136, 3370-293
Cho, J.: 925-332
Cho, S.: 915-282, 1390-227
Cho, S.: 2405-244, 3360-257
Cho, Y.: 1395-241
Choate, S.: 2385-192
Chocano-Bedoya, P. O: LB2570-367
Chocron, S.: 2090
Chodos, A.: 900-246, LB2570-336
Chodosh, J.: 1297, 2395-215
Choi, E.: 850-129, 1401-284
Choi, H.: 3315-121
Choi, J.: 855-151
Choi, J.: 3325-174
Choi, M.: 3520, 4055
Choi, M.: 1525, 2410-252, 2450
Choi, N. G: 1100, 2385-170
Choi, S.: 2355-79
Choi, S.: 865-162
Choi, S.: LB2570-337
Choi, W.: 1395-242
Choi, Y.: 910-279, 3275-17, LB2570-436
Choi, Y.: 2310
Choi-Allum, L.: LB3620-366
Chong, M.: 2360-95
Chopik, W. J: 550, 4100
Chopra, A.: 885-204
Chou, C.: 2395-216
Chou, L.: 850-135, 2385-171, 4020, LB1545-349
Choun, S.: 3340-209, 3370-301
Chow, E. O: LB3620-435
Chow, S. W: 895-231
Choy, J.: 2355-77
Christ, S. L: 1395-246
Christensen, J.: 3070
Christensen, K.: 2370-125, 3170, LB1545-428, LB2570-338
Christian, T. J: 520
Chu, C. H: 2360-98
Chu, F.: LB935-445, LB1545-358
Chu, L.: LB935-361, LB1545-367
Chu, N.: 830-75, 2380-168, 4020
Chui, C.: 1345-115
Chukwurah, Q. C: 895-228
Chukwurah, Q. C: 1330-87
Chun, A.: 895-231
Chun, H.: 2325-22
Chun, S.: LB3620-394
Chung, J.: 1525
Chung, K.: 2385-182
Chung, S.: 2405-235
Chung, T.: LB3620-364
Chunga, R. E: 3110
Chunga, R. E: 3315-125
Church, S.: 1335-97
Churchill, L. C: 895-230
Churchill, R. A: LB935-441
Chusyd, D. E: LB2570-394
Ciancibello, A.: LB3620-362
Cianelli, R.: LB1545-426
Ciarleglio, A.: 2495
Cicala, D.: 1365-166
Cichy, K. E: 4035
Cifuentes, H.: LB935-349
Cil, G.: 3325-152
Cimarolli, V. R: 2015, 2520, LB3620-370
Ciofi, J.: LB3620-321
Ciolino, J.: 4030
Cirillo, N.: 915-298
Civitella, F.: 1402-312
Clare, L.: 650
Clark, B.: 1410-330
Clark, C.: 3330-187
Clark, D.: 820-19
Clark, J.: 1320-62
Clark, K. C: LB1545-438
Clark, K. F: 3375-314
Clark, M. A: 740
Clark, P.: 2220
Clark, P. G: 4075
Clark, S.: 2370-118
Clark, V.: 3535
Clarke, A.: 895-230
Clarke, C.: 2360-100
Clarke, G. D: 835-94
Clarke, M.: 825-53
Clarke, P.: LB2570-384
Clarke, P. J: 600
Clark-Shirley, L. J: 680, 3410
Clauser, S. B: 3140, LB935-416
Clay, O. J: 2410-260
Clayton, Z. S: 835-89
Clegg, A.: 1360-165
Clemency, B. M: 2385-181
Clement, F.: 880-199
Clemson, L.: LB2570-353
Cleveland, M.: 4045
Clevenger, C.: 840-107, 850-125
Clish, C.: 3275-24
Clouston, S.: 825-52
Cobb, R. J: 1050, 1450
Cochran, K.: 2185
Cochrane, B. B: 1455
Coffey, T.: 1380-190
Coggan, A. R: 820-18
Cohen, A. L: LB1545-391
Cohen, A. A: 525, 4005
Cohen, C. C: 2370-135
Cohen, D.: 1360-160
Cohen, H.: 1300-13
Cohen, H. J: 2345-56, 3265, LB1545-347
Cohen, M. A: 865-163, 3015, 3140, 3570, 4050
Cohen, P.: 2510, LB935-359, LB2570-439
Cohen, R.: 710
Cohen, S. A: 1170, 1400-251, 3600
Cohen-Adad, J.: 3280-29
Cohen-Mansfield, J.: 710
Cohn, T.: LB1545-407
Coldiron, A. M: 815-15
Coldiron, A. M: 2340-44
Cole, L.: 850-138
Cole, T. R: 1225
Coleman, C.: LB2570-364
Coleman, M.: 3305-102
Colibaba, A.: 560, LB3620-373
Collaborative, O.: LB3620-368
Collette, B.: LB3620-329
Collins, S.: 910-265
Colman, R.: 4175
Colmenares, C.: 835-91
Colon-Emeric, C.: 3365-275, 4130
Colson, T.: LB3620-439
Cominato, M.: 1515
Commandeur, D. T: 2360-105
Condon, S. E: 1385-217
Cone, N.: 915-296, LB3620-410
Cong, Z.: 2450, 4115
Connell, C. M: 705, 3365-280
Connelly, C.: 915-297
Conner, K.: 3275-22
Conner, N. E: LB935-413
Conrad, K.: LB935-377
Conrad, K.: 3360-269, LB935-377
Conte, F.: 860-158
Contreras, B.: LB935-364

Convery, K.: 870-184
Conwell, Y.: 3360-263, 4140, LB1545-380
Cook, R.: LB1545-402
Cook, W.: 565
Cooke, E.: LB2570-351
Cooke, H. A: 3345-212, 3435
Cooke, V.: 3435
Coolidge, F. L: 915-283
Coon, D.: 2300
Cooney, R. V: LB935-428
Cooney, T. M: LB3620-332
Coons, J.: 3340-203, 4145
Cooper, G.: 1395-243
Cooper, P. S: LB1545-338
Cooper, R.: 3170
Copel, L.: LB1545-369
Coppard, L.: 1345-111
Corazzini, K.: 780, 2020
Corazzini, K.: LB2570-385, LB3620-349
Corbett, D.: 1402-296, 2410-264
Corcos, D.: 4030
Cordes, B.: LB2570-410
Cordova-Marks, F.: LB2570-445
Corey, K. L: 840-109
Corkum, A.: 3295-71
Corley, C.: 4060
Corley, C.: 855-153
Corley, R.: 1150
Corneau, E.: 3435
Cornejo Thumm, P.: 4020
Cornelison, L.: 2515
Cornell, C.: 1365-179
Cornell, P. Y: 1375-187, 2470
Corner, L.: LB2570-319
Cortes, T. A: 1530
Cosentino, S.: 3290-65
Cosentino, S.: LB2570-343, LB3620-337
Costa, E.: 1402-307, 3325-158
Costacou, T.: 565
Costas Muniz, R.: 635, 1365-174, 3275-20, 4110
Costlow, K.: 915-281
Côté, A.: 4005
Cothran, F.: 2295
Cotnam, V.: LB935-394
Cotten, S.: 1060, LB3620-343
Cotton, S. G: 1345-107, 1365-167, 2355-76, 3300-90
Coughlin, J.: 660, 1375-184
Coulourides Kogan, A. M: 520
Coupal, S.: LB3620-414
Coursaris, C.: 3415, 4020
Courser, R.: 3365-280
Cousins, K. A: 850-136
Couture, M.: 2045, 3025
Couve, A.: LB935-372
Covinsky, K.: 1170, 2010, 2315-1, 3420
Cowell, A.: LB935-443
Cowley, A.: 1315-47
Cox, B.: 1385-217, 2415-265
Cox, C.: 800
Cox, D. W: 3310-111
Cox, J.: 640
Coyle, C. E: 910-265, 1445, 2385-179, LB3620-359
Coyle, E.: 920-310
Coyle, M. H: 3270-9
Coyne, S.: 1401-277
Craft, M.: LB3620-358
Cragle, D.: LB3620-341
Crandall, J.: 1402-315
Crawford, B.: LB935-437
Cremer, S.: 2380-164
Crespi, E.: 2355-80
Crimmins, E.: 710, 1315-53, 1400-262, 1401-289, 2260, 2345-57, 3275-17, 4080, LB2570-436
Crist, J. D: 3120
Crittenden, J.: 910-257, 1305-32, LB935-434
Crockett, R. A: 715, 920-313
Crockett, R.: 2215
Crockett, R. A: 2360-97, 3295-72
Croff, R. L: 2405-241
Croker, J.: 1100
Cronkright, A.: LB3620-424
Cross, S. H: 870-182
Crouch, M.: LB1545-348
Crowe, C. L: 3325-171
Crowe, M.: 1360-156, 2410-260, 3595
Crowe-White, K.: 3295-74
Crowley, M.: 1350-129
Crowther, M. R: 640, 2405-231, 2500
Cruitt, P. J: LB935-386
Crumley-Branyon, J.: 815-5
Crutzen, R.: 565
Cruz-Martinez, J.: LB1545-382
Crystal, S.: 910-274
Crystal, S.: 2205
Csiszar, A.: LB3620-358
Cuff, G.: 3360-265
Cui, C.: 835-99
Cullati, S.: 3030
Culley, D. J: LB2570-329
Culminskaya, I.: 820-33
Cummings, G. E: 4130
Cummings, G. G: 2315-15, 2395-219, 4130
Cummings, S. R: 820-22, 830-80
Cummins, P. N: LB935-350
Cummins, P.: 510, 1285, 1325-71, 1400-259, 1401-290, 3300-99, 3305-110
Cunningham, J.: LB2570-445
Curl, A. L: 860-160, LB3620-332
Curran, S. P: 830-64
Curren-Vo, H.: 3345-219
Curtin, C.: 2415-268
Curtin, C.: 3305-109
Curtis, L.: 3595
Curtis, R. G: LB1545-445
Curyto, K.: 3255
Curyto, K.: 3255, LB3620-339
Cushman, M.: 3280-33
Cushman, M.: 4045
Cutchin, M.: 745, 2105, 3565
Cuthbert, K.: LB3620-367
Cvejkus, R. K: 2370-136, 3170
Czaja, S. J: 550, 620, 1055, 1130, 1295, 3315-130
Czarnecki, P.: LB2570-372
Czirr, E.: 825-43

D

Da Silva, J.: 1390-222
Da Silva, R.: 2360-87
Dabelko-Schoeny, H.: 885-207, 1305-28, 3520, 4055
Daddato, A. E: 2385-180, 3280-35
D'Agostino, C.: 825-51
Dahle, C.: LB935-366
Dai, F.: LB935-356
Dakin, E.: 3430
Dale, M.: LB935-392
Dallo, F.: 2355-64, 2455
Dalton, H. M: 830-64
Dalton, J. E: 835-86
Damberg, C.: 2250
D'Ambrosio, J.: 1345-107, 1365-167, 2355-76, 3300-90
D'Ambrosio, L.: 660, 1375-184, 3180
D'Amico, C. O: LB1545-335
D'Amico, D.: 820-20
Damman, M.: 865-170
Dang, S.: 840-108
Dang, S.: 1320-61
Dang, S.: 1330-94, 1402-301
Dang, S.: 2350-59
Dang, S.: 3320-137
Dang, W.: LB1545-365, LB2570-352, LB3620-384
Daniel, K. M: 3510
Daniela, M.: 3320-134
Danielson, M. E: LB1545-432
Danilovich, M.: 880-189
Danilovich, M.: 2385-185
Danilovich, M.: 4030
Dannefer, D.: 2205, 3085
Dannewitz, H.: 2390-202
Dannewitz, H. C: LB1545-385
D'Antonio, P. M: 1230, 2285
Darabidian, B.: 3430
D'Arcy, R.: 3330-189
Darin-Mattsson, A.: 515
Darling, N.: 1350-128

Darvishpoor Kakhki, A.: LB1545-435
Darwiche, J.: 3315-123
Darzins, P.: 1410-331
Das, S.: 1401-271
Daskalopoulou, C.: 4040
Dassel, K. B: 500, 735, LB2570-416
Daum, C. H: 2325-30
Dave, C.: 3050
Dave, P.: 1310-39
Davey, A.: LB3620-389
Davidson, E. J: 670
Davidson, P.: LB1545-435
Davies, L.: LB1545-418
Davila, H.: 910-258, 2390-200, 3345-217
Davila, V. S: 2385-185
Davis, D.: 3170
Davis, D. L: LB1545-424
Davis, E.: LB935-398
Davis, E. M: 2355-67
Davis, J. C: 565, 1510, 2360-90, 3295-72
Davis, J.: 1402-310, 2340-36
Davis, M.: 835-87
Davis, N.: LB2570-330
Davis, T.: 2435-306
Davis-Ajami, M.: 3555
Davitt, J.: 3330-188
Davlyatov, G. K: 910-262, 2395-218, 3345-213, 3435
D'Avolio, D. A: LB935-379
Davy, P.: 830-72
Dawson, N. T: 920-308
D'Cunha, N. M: 705
de Bruin, S.: 635
De Donder, L.: 1305-22
De Gomensoro, E.: 1135
de Jong, L. D: 730
De Lange, F.: 3230
de Man-van Ginkel, J. M: 2380-164
de Medeiros, K.: 2200, LB3620-413
de Paula Couto, C.: 3460
De Vilmorin, C.: 2310
de Vivo, I.: 830-72
de Vries, E.: LB1545-414
de Wit, N.: 675
De Witte, N.: 3270-5
Deal, J. A: 2380-166, 2425-291, 2495, 3075
DeAngelis, T.: LB3620-399
Deardorff, W.: 665
DeCaro, J. A: 3365-279
Deeg, D.: 2430-292
Deer, R. R: LB935-378, LB2570-328
Degenholtz, H.: 690, 1220, 2305, 2480, 3240, LB3620-386
DeGennaro, J.: 885-204
deGrauw, X.: 2385-186
DeGutis, J.: LB1545-363
Deichert, J.: 3345-225
Deimling, G.: 915-293
Deindl, C.: LB2570-363
Dekhtyar, S.: 515, 3420
Del Din, S.: 1402-304
del Rosario, K. E: 2340-45
Del Signore, S.: 1402-298, 3325-159
DeLaTorre, A.: 1305-16
Delbaere, K.: 565
Delbono, O.: 820-25, LB1545-387
Delgado, F.: 890-223, 1095
Deller, J.: 3520
Delmonico, M. J: LB2570-422
DeLouize, A.: LB1545-342
DeLuca, J. A: 820-32
DeMaio, F.: 1300-6
DeMaria, J.: 925-329
Dembo, R.: 2550
Dembosky, J.: 2250
Demiris, G.: 1080, 2310, LB935-400, LB1545-358
DeMott, A.: 3175, 3305-107
Denburg, N.: 3350-239
Denny, P.: 855-150
Denson, K.: LB1545-400
Denson, S.: LB1545-400
Dentino, A.: 1365-171
DePaul, V.: LB3620-368
Depenbrock, A.: 2360-92
DerAnanian, C.: 890-223, 1095
Derby, C.: 845-124, 2445
DeRosa, N.: 1310-38
Desai, L.: 2380-157
Descatoire, P.: 825-49
Despins, L. A: 820-37
DeStefano, C.: LB1545-352
Detwiler, B.: 1380-199
Deutsch, A.: 2385-190
Deutsch, M. B: LB3620-419
Devgan, A.: LB935-359
DeVito, A.: LB3620-318
Dewey, E.: 3325-175
Dhakal, U.: 1305-18
Dharshi, N.: LB1545-439
Dhokai, N.: 610
Di Gessa, G.: 1350-130
Di Stefano, M.: 1410-331
Diallo, H.: LB2570-396
Dian, L.: 565
Dias, C.: 1350-131
Diaz, L.: 880-189, 4030
Diaz Moore, K.: 1305-16
Diaz-Santos, M.: LB2570-335
Diaz-Valdes Iriarte, A. E: 865-174
DiBartolo, M. C: 900-242
DiBartolo, M.: 2360-102
DiBlasio, C.: 2315-14, 3595
Dick, A. W: 2395-217
Dickens, C. N: 4085
Dickens, J.: 3310-117
Dickerson, A. E: 1410-330, 3305-102
Dicocco, C.: 810-3
Dieckmann, N.: 840-104, 1360-160
Diederich, F.: 3320-139
Diehl, M.: 3380
Dieker, J.: 2375-143
DiGasbarro, D.: 845-119
DiGuiseppi, C.: 3325-171
Dijkstra, N. E: 675
Dillon, S.: 825-48, 4130
DiMaria-Ghalili, R.: 2070
Diminich, E. D: 825-52
Dindo, L.: 850-145, 3560
Ding, K.: 3305-108, LB2570-407
Ding, K.: 830-73
DiNitto, D. M: 2385-170
Disco, M.: 1305-24
Dixit, V.: 835-96
Dixon, M.: 2310
Dixon, R.: 1360-162
Djukaric, T.: LB1545-334
Do, H.: 1401-285
Doan, L.: 2405-237, 3370-300
Doan, W.: 3295-89
Dobbins, S.: 1330-91
Dobbs, D. J: 1260, 1405-325, 2065, 2435-311, 3345-224, LB3620-327
Dobiszewski, S.: LB2570-422
Dobson, R.: 1395-244, 3350-244
Docherty, S. L: LB1545-425
Documet, P. I: 3415
Dodge, H. H: 1195, 3280-30
Doh, D.: 2430-295
Doherty, K.: LB2570-409
Dolansky, M. A: 895-240
Dolbin-MacNab, M. L: 1350-129, 3025
Doll, G.: 2515
Doll, J.: 860-158
Domingue, B.: 1430
Donaldson, G.: LB935-400
Donaldson-Davis, K.: 3045
Donelan, K.: 755
Dong, C.: 1402-301
Dong, G.: LB2570-428, LB3620-430
Dong, J.: LB1545-387
Dong, T.: 1340-103
Dong, X.: 615, 1330-90, 2010, 3505, 4105, LB2570-405, LB3620-428
Donini, L. M: 1402-298, 3325-159
Donnellan, K. M: 1505
Donnelly, C.: LB3620-368
Donnelly, R.: 2280, 3150
Donnenfeld, E.: 1320-64
Donohue, J. M: 565
Donorfio, L. K: 900-248, 2560
Donovan, E.: 2420-272
Dore, E. C: 3340-207
Dorigatti, J.: 820-35, 830-77
Dorman, H.: 535, 1400-264
Doron, I.: 1055
Dorr, D. A: 1395-248
Dorsey, S.: 2385-186
Dosa, D.: 1405-325, 3345-215

Doshi, S.: 2410-261
Dotan, E.: 855-146
Douglas, E.: 2430-303, LB3620-320
Douglas, J. W: 2390-201, 2395-211, 3300-98, 3345-228
Douglas, R. A: 2340-48
Douglas, S.: 710
Dounis, G.: LB1545-391
Dourado, M.: 850-141
Dow, B.: 915-290
Dowling, M.: 850-144
Downer, B.: 850-135, 1360-158, 2545, 4135, LB1545-427
Downer, L. B: LB1545-345
Downey, L. A: 3295-81
Doyle, P.: LB2570-351, LB3620-321
Dozier, M. E: 670
Dragan, D.: 2130, LB2570-418
Drahota, A. K: 2380-161
Drake, C.: 2385-180
Drewelies, J.: 3190
Drickamer, M.: LB935-384
Driessnack, M.: LB2570-359
Driscoll, L.: 915-288
Driscoll, R.: 835-99
Drobot, A. C: 2390-202
Drummond, N.: 850-126
Drury, T.: 3275-21
D'Silva, J.: 880-194
D'Souza, N.: 1402-312
Du, P.: 4050
Du, P.: 545
Du, S.: LB935-373
Du, X.: 2385-192
Du, Y.: 2165
Du Bois, A.: 920-303
Duan, M.: 825-60, LB1545-440
Duan, Y.: 910-276, 1405-324, 4050
Duarte, M.: 1402-307, 3325-158
Dubbin, L.: LB2570-336, LB3620-419
Dubin, S.: 1345-110
Duchowny, K. A: 820-22
Dufour, S.: 1395-245
Duggan, A.: 2385-178
Duggleby, W.: 2430-293
Duke, B.: LB3620-376
Dulaney, S.: LB2570-336
Dull, G. E: 3355-249
Dummit, L.: LB935-443
Duncan, R.: LB3620-399
Dunkle, R. E: 890-222, 1075, 1345-111
Dunn, D.: 1350-120
Dunterman, R. A: LB3620-402
Dunterman, R. A: 815-14
Dupre, M.: LB2570-315
Dupuis-Blanchard, S.: LB1545-441
Durand, D.: LB935-417
Duru, O.: LB3620-408
Dury, S.: 575, 3035
Duthie, E. H: LB1545-400
Dutta, P.: 820-29
Dys, S.: 1305-27, 3345-214, LB3620-390
Dziedzic, P.: 2420-277
Dzierzewski, J.: 1390-239, 2420-272, 2425-282, 3370-288

E

Eagen, T. J: 855-148
Eaker, M.: 1410-330
Eapen, B.: 3295-76
Earhart, G.: LB3620-399
Earl, J.: 865-166
Eaton, J.: 1370-180, 3185
Ebert, A. R: 2355-78, 4140
Eby, D. W: 1410-326, 3325-171
Eckstrom, E.: 1360-160
Eddy, C.: LB3620-422
Edelman, L. S: 3355-249, LB935-400, LB2570-416
Edelstein, B.: 885-203, 3350-243, LB935-405
Edwards, A. B: 3315-122
Edwards, D. F: 2385-187, 2400-226
Edwards, E.: 610
Edwards, J.: 2555
Edwards, V. J: 1030
Edzards, S.: 815-15, 2340-44
Egan, M.: 3330-177
Egan, P.: LB3620-431
Egashira, R.: LB2570-320
Egbert, A. R: 1510, 2215
Ehrlich, J. R: 2495
Ehrlich, K.: LB2570-368
Ehsan, A.: 925-321
Eisa, N.: LB3620-330
Eisel, S. L: 1390-230
Eismann, M.: 660
Ejaz, F.: 1385-207, LB2570-383, LB3620-354
Ekerdt, D. J: 1375-188, 1490
Ekundayo, O.: 890-220, 3300-100
El Ibrahimi, S.: 3335-193
Eldadah, B.: 3260
Eldemire-Shearer, D.: 2065, 3045
Elfenbein, P.: 3005
Elhajjmoussa, L.: 4030
Eliazar-Macke, N.: 3320-137
Elkins, J.: 1360-155, 2045
Eller, M.: 1401-279
Ellerby, L.: 3215
Elliott, A. F: 3365-273
Elliott, E. M: 1345-116
Elliott, J. B: 3330-180
Elliott, K.: 900-248
Elliott, M. N: 520, 2250, 3430
Elliott, M.: 925-337
Elliott, S.: LB3620-390
Ellis, A.: 2390-201, 2395-211, 3295-74, 3300-98, 3345-228
Ellis, K.: 2325-26
Ellis, T.: LB3620-399
Ellison, C.: 2420-274
Ellor, J. W: 1475
Ellwardt, L.: 3245
Elman, A.: 2225, 2370-118
Elmansi, A.: 835-100
Elmansi, A. M: LB3620-330
Elmansi, A.: LB3620-360
Elswick, R. K: LB2570-356
Elton, J.: 1350-124
Ely, E.: LB2570-380
Emerman, J.: 1540
Emerson, K. G: 1300-12, 3320-135, 3360-271, LB935-424, LB3620-329
Emery-Thompson, M.: LB1545-431
Emery-Tiburcio, E.: 635
Emlet, C.: 1415, 2285
Enayati, M.: 820-37
Endo, Y.: 820-40
Engel, I. J: 2425-283
Engels, M.: LB2570-363
Engineer, C.: 2370-121, 2390-198
Englund, J.: 3305-106
Enguidanos, S.: 910-270, 1315-59, 1340-101, 3330-183, LB1545-443, LB2570-366, LB3620-433
Enroth, L.: LB1545-339
Ensrud, K. E: 820-22
Enzler, C. J: 2365-114
Eost-Telling, C. L: 3490
Eppler Hattab, R.: 1055
Epps, F.: 1510, 3100
Epps, F.: LB1545-350, LB3620-321
Erb, T.: 2270
Erbes, C.: 1390-236
Erickson, K. I: 715
Erickson, L.: 3290-45
Erik, B.: 1402-311
Eriksdotter, M.: 2215
Ermoshkina, P.: 3370-289
Ernst, J. S: 1000
Ernst, M. E: 870-180
Ernsth Bravell, M.: 2380-160
Ernsth-Bravell, M.: 3275-28
Erraguntla, M.: 1095
Errett, N.: 1405-320
Erving, C. L: 1390-233
Erwin, M. J: 815-12
Escarce, J.: 4050
Eskenazi, L.: LB3620-362
Espana, M.: 2405-239
Espeland, M. M: 850-132, 3590, LB2570-441
Espinosa Sempere, C.: LB935-430
Espinoza, S. E: 3325-160, LB2570-399
Esposito, M. H: 600
Estabrook, R.: 3340-206
Estabrooks, C.: 2430-293
Estapé, J.: 3070

Estapé, T.: 3070
Estepp, P.: LB3620-441
Estes, C.: 3015
Estrazulas, J.: 1402-312
Eto, M.: 820-27
Ettema, R.: 2380-158
Eugene, D.: 2385-192
Evans, D. S: 830-80
Evans, D. S: 1120
Evans, M. W: 3365-274
Evans, T. L: LB2570-390
Evans, W. J: 820-22
Everaars, B.: 675
Ewen, H. H: 680
Eymundsdottir, H.: 2370-133
Ezeah, P. C: 880-190

F

Fabbre, V.: 3430
Faber, M. A: 1285
Fabius, C. D: 1110, 1375-187, 2270, 2405-234, 3585
Faghani Hamadani, T.: 2400-222
Fairchild, K.: 2535
Faizah, F.: 2400-230
Falck, R. S: 1510
Fales, K.: 2313
Fallahtafti, F.: 3295-80
Fall-Dickson, J. M: LB3620-443
Falzarano, F.: 655, 2520, 3290-49
Fane, M.: 3540
Fanelli, S.: 1315-55
Fang, B.: 1355-143, 2375-152
Fang, J.: 2385-175
Fang, M.: LB2570-336
Fang, Y.: 910-271, 3330-185, LB935-388
Fang, Y.: 2425-286
Fani, S.: 895-231
Farah, K.: 1420
Farina, A.: 1385-214
Farinatti, P.: LB2570-341
Farkas, C.: LB935-352
Farmer, H. R: 4120, LB2570-315
Farrell, M. T: 4110
Farrell, S.: 3325-167
Farrell-Savage, K. R: 2380-161
Farsijani, S.: LB935-368, LB2570-437, LB3620-348
Fashaw, S. A: 3585
Fast, J.: 2430-293
Fastenau, C.: LB935-349
Faul, A.: 1345-107, 1365-167, 2075, 2355-76, 3300-90
Faul, J.: 710, 1395-240, 2260, 2345-57
Fauth, E. B: 650, 3185, 3340-201, LB1545-394
Fawcett, J.: 3325-176
Fazeli, P.: 1360-156
Fazio, S.: 1160
Feaster, D. J: 1395-246
Fedarko, N.: 515
Federman, A. D: 1395-244, 3350-244
Fedus, D.: 2435-307
Feehan, L.: 1510
Feger, D. L: 2410-259
Feinberg, L. F: 1110, 3450
Feitosa, M.: 1310-42, LB1545-428
Feletti, L. C: 1402-298, 3325-159
Feliciano, L.: LB935-399
Felix, C.: 2265
Felix, L.: 2380-161
Felpeto, A.: 1315-45
Fendrich, M.: 2410-258
Feng, B.: LB1545-387
Feng, X.: LB1545-387
Fennell, G.: LB1545-341
Fennimore, T.: 3510
Fenstermacher, E. A: 815-9
Ferdous, F.: 2355-60
Ferdows, N.: 3395
Ferguson, C.: 2390-201
Ferguson, C.: 2395-211
Ferguson, C.: 3300-98, 3345-228
Fernandes, K.: 2360-87
Fernandez, J.: 1305-15
Fernández, S.: 3170
Fernández Ardèvol, M.: 580
Ferraro, K. F: 720, 730, 1420
Ferras, N.: 1402-301
Ferreira, E.: 1390-222
Ferrer, L.: LB1545-426
Ferrer, R.: 2340-45
Ferreri, S.: LB935-425
Ferri, C. V: LB935-433
Ferris, T.: 2185
Ferrucci, L.: 820-24, 835-99, 1180, 2290, 3040, 3075, 3405
Fetherstonhaugh, D. M: 505, 2395-210
Fick, D.: LB1545-397
Field, T.: 3355-256
Fielding, R.: 2190
Fields, N. L: 910-263, 1305-28, 2365-111, LB3620-405
Fierro, L. A: 815-15, 2425-288
Figiel, S.: LB935-417
Figueroa, A. C: 1380-202
Filho, B.: 2360-85
Fillenbaum, G.: 4145
Fillingim, R. B: 3330-184
Fillo, K. T: 2395-212
Finch, C. E: 825-50, 830-64
Finchum, T.: 1385-212, 2415-266
Fine-Smilovich, E.: 875-187
Fingerman, K.: 925-316, 1350-134, 2160, 3425
Fink, H.: 3455
Finkel, D. G: 2380-160, 3275-28
Finkelstein, E.: LB3620-396
Finkelstein, R.: 800, 3525
Finlay, J. M: 600, 1401-278, 2170, 2540
Finlayson, E.: 2115, 2315-1
Fiocco, A.: 820-20
Fiorentino, L.: 2420-273
Fiori, K. L: 4015
Firdauysa, N.: 1385-212, 2415-266, 3370-292
Fiscella, A. J: 3290-59
Fisher, A.: 830-71
Fisher, K.: LB1545-336
Fisher, N. M: 3175
Fisher, S.: LB2570-328
Fitzgerald, T.: 880-197
Flaherty, E.: 2075, 3545
Flanagan, J.: 1355-148
Flatt, J. D: 895-238, LB3620-419
Fleenor, J.: LB2570-322
Floegel, T.: 2410-249
Flood, K.: 2315-4
Flood, S.: 4065
Flores, B. E: LB1545-384
Flores, R. J: 1225, 3155
Flores Villavicencio, M.: 3290-67
Fogg, L.: LB2570-419
Fogle, S. J: LB935-393
Foley, K.: 915-289
Folz, R.: 545
Fong, T. G: 825-48
Fontes, F.: 2360-85
Forbes, D.: 3330-177
Ford, A.: LB935-401
Ford, K.: 1390-228
Fornalski, N.: 915-298
Forrester, S. N: 1400-260
Fortinsky, R. H: 2045, 2210, 2490
Fortner, J.: 900-245
Fossum, S.: 1410-329
Foster, M. J: 1360-151
Foti, M.: 3375-308
Fournier, C.: LB1545-419
Foust, J.: 3390
Fowler, M. E: 2315-14, 3595
Fox, A.: 1355-139
França, M.: 1390-222
Francke, A.: 4135
Franco, Y.: 850-129
Francois, E.: 2405-241
Frank, A. K: 1300-11
Frank, J. C: 3305-104
Franke, K.: 1365-168
Frankenstein, L.: 885-208
Franz, C. E: LB2570-348
Franzosa, E. C: 1155
Fraser, K.: 1395-245
Fratiglioni, L.: 515, 3170, 3325-173, 3420
Frazier, C.: 1390-233
Frazier, J.: 610
Freddolino, P.: 3415, 4020
Fredriksen Goldsen, K.: 1085, 1415, 2285
Freed, S. A: 1410-328
Freed, S.: 3290-61
Freedman, V. A: 2495
Freeman, I.: LB2570-376
Freeman, J.: 1350-124
Freeman, W. M: 820-25
Freiberger, C.: 890-217
Freijomil, F.: 2360-102
French, M.: 1505
Frenk, S.: 2355-70
Freshour, J.: LB2570-420

Freund, A. M: 3350-237
Frey, J.: LB3620-355
Freydefont, L.: 3350-237
Freytag, J.: 850-145, 3560
Freytes, I.M.: 840-108, 3320-137
Friberg Felsted, K.: 3495
Friedland, L.: 1135
Friedman, D. B: 3115
Friedman, E. M: 3275-19, 3350-238
Friedman, E. M: 4050
Friedmann, E.: 705
Frisoli, A.: LB1545-437
Fritz, H. A: 1360-164, LB1545-436
Fritz, M.: 3415, 4020
Froehle, A. W: 3170
Frolinger, T.: 825-57
Frook, G.: 1055
Frost, B. E: 3080, LB935-346
Fruhauf, C.: 1355-139, 1401-291, 1460, 3185, 3320-150
Frutos, B.: 2380-156
Fu, R.: 1400-265
Fu, Y.: 3365-277
Fuchs, J.: LB1545-334
Fugett, S.: 3275-26
Fuhrman, E.: LB2570-376
Fujita, K.: 830-63
Fujiwara, Y.: 855-154, 895-226, LB2570-411
Fullen, M. C: 3330-190, 4140
Fuller, H.: 1140, 3205, LB3620-441
Fuller, P.: 2405-241
Fuller-Thomson, E.: 2440
Fullwood, D.: 2410-264, LB2570-386
Fulmer, T.: 795, 3545
Fulzele, S.: LB3620-330
Fung, C.: 2420-273
Fung, H. H: 2425-286, 3460, LB935-361, LB1545-367, LB3620-427
Fung, N.: 3605
Funk, L. M: 2225
Funk-Lawler, R.: LB3620-358
Furber, J. D: LB3620-418
Furlano, J.: 3295-68
Fuselier, J.: LB1545-417
Futterman, A.: 2340-47, 2415-268, 3275-15

### G

Gaber, S. N: 3565
Gadzo, R.: 3330-187
Gaeta, L.: LB935-397
Gaffy, E.: 915-290
Gafni, A.: LB1545-336
Gagen, T. M: 1305-20
Gagne, H.: LB935-427
Gagnon, S.: 1410-329
Gahbauer, E.: 3315-127
Gaillot, S.: 2250
Gaines, B. E: 1260
Gaini, M.: 1360-154
Galambos, C. M: 1480, 4135
Galdamez, G.: 2320-19
Gale, S.: 3290-45
Galea, S.: 1345-116
Galenbeck, E.: 1402-314
Galik, E.: 920-301, 1255, 2390-194, 2525, 3270-11
Gallager, I.: 825-43
Gallagher, E. A: 915-288, 1380-201, 3290-66
Gallanis, T.: LB1545-442
Gallardo, E.: 820-18
Gallegos, C.: 1300-8
Gallo, H. B: 1405-319, 2540
Galvan, V.: 825-44, 835-98, 1310-38, 1520
Galvin, A.: LB2570-338
Galvin, J.: 3295-85
Gamaldo, A.: 2420-277
Gamaldo, A. A: 3290-53
Gamaldo, C.: 2420-277
Gambert, S.: 1402-309
Gammonley, D. L: 910-269
Ganai, S.: 1360-161
Ganann, R.: LB1545-336
Ganapathy, V.: 835-91, LB3620-372
Gangai, N.: 635, 1365-174, 3275-20, 3305-105, 4110
Ganz, N.: 1402-303
Gao, H.: LB2570-368
Gao, L.: 4030
Gao, M.: 3370-296
Gao, Q.: 1515
Garbarino, J. T: 900-244
Garbarino, V. R: 825-56
Garber, L.: 3355-256
Garcia, A.: 2380-163
Garcia, C.: 3070, 4000
Garcia, D.: 835-83
Garcia, M. A: 3070
Garcia, M.: 3355-255
Garcia, M.: 2280, 4035
Garcia, Y.: 1350-120
García, I.: 1305-16
García-Peña, C.: 3590, 4155
Garcia-ptacek, s.: 2215
Gardenhire, D. S: 920-312
Gardiner, P.: 570
Gardner, D. S: 3445
Garland Baird, L.: 1395-245
Garm, A.: 3325-172, 3330-189
Garner, K. K: 920-311, 2365-106
Garonzik Wang, J.: 2380-168
Garrett, S.: 545
Garrison, B.: 1340-99, 1475
Gartz, R.: 1410-330
Gassoumis, Z.: 910-270, 2320-19, 2370-118, 2375-150, 2385-193, 2465, 3025
Gately, M.: 1295
Gaudet Hefele, J. L: 740
Gaugler, J. E: 885-207, 1005, 1385-214, 1401-278, 1495, 2325-21, 2355-71, 2375-140, 2490, 4025
Gauthier, C.: 3280-29
Gauthier-Beaupré, A.: 2360-98
Gaveras, E.: 3430
Gavrilov, L.: LB3620-395
Gavrilova, N. S: LB3620-395
Gazit, E.: 1402-303
Gazzola, J.: 2360-85
Ge, M.: 2095
Ge, S.: 4115
Gea-Sánchez, M.: 3345-216
Gebelin, K. D: 1150
Gebremariam, M.: 905-251
Gee, N. R: 705
Geirsdottir, O.: 2370-131
Geirsdottir, O.: 2370-133
Gelb, B.: 885-212
Geldmacher, D.: LB2570-391
Gell, N. M: 920-304, 2360-89
Gellis, Z. D: LB2570-442
Gelman, C.: 3065, 3445
Gendron, T.: 2285, 3340-205, 4170
Gensburg, L.: 3325-163
Gentes, R.: 3180
Georgousopoulou, E. N: 705
Gepshtein, S.: 500
Gerald, L.: LB2570-445
Gerard, F.: 2385-178
Gerard, S. N: 3270-7
Gerhard, L.: 1235
Gerhart, O.: LB1545-357
Germack, H.: LB3620-386
Germain, C. M: 2380-167, 3155
Gershon, R.: 3595
Gerstorf, D.: 2275, 4015
Gertel, V.: 3295-69
Geshell, L.: 3105
Getz, M. A: 2435-305
Geva, A.: 1402-293
Ghag, C.: 715
Ghaul, M.: 3375-307
Ghazi, A.: 3540
Ghazi Saidi, L.: 3295-88
Ghiasi, A.: 910-262, 2395-218, 3345-213, 3435
Ghosh-Dastidar, B.: 4050
Gibb, Z.: 2385-191
Gibbs, L.: 3320-140
Gibson, A.: LB2570-415
Gibson, A.: 1305-14, 1340-99, 2060, 2450
Gibson, P.: LB3620-329
Gideon, C.: 1320-63
Gidmark, S.: 3435
Gifford, D.: 3435
Gil, A.: 2360-87
Gil, E.: LB1545-375
Gilbert, K. L: 3295-78
Gilbert, S. B: 1270
Giles, G. M: 2385-187, 2400-226

Gill, L. L: LB3620-340
Gill, T. M: 1400-262, 3315-127, LB3620-403
Gillespie, P.: 920-299
Gillespie, R.: 2425-289
Gillespie, S.: 910-275
Gilliam, M.: LB3620-426
Gilligan, M.: 2035, 2160, 2365-110, 3315-131
Gills, J.: 1402-300
Gilmartin, M. J: LB1545-335
Gilmore-Bykovskyi, A.: 3350-240
Gilpin, A.: 1390-232
Ginis, P.: 1402-304
Ginther, J.: 3275-26
Ginting, M. L: 2360-95
Giordani, B.: 500
Giordano, L.: 520
Gioscia-Ryan, R. A: 835-89
Girgus, J.: LB935-433
Girling, L.: LB3620-413
Girouard, H.: 3280-29
Gitlin, L. N: 770, 885-207, 1005, 1495, 2355-79, 3110, LB2570-353
Giuliano, R.: 825-53
Giunta, N.: 3445
Gjestsen, M.: LB3620-317
Gladyshev, V.: LB3620-407
Glaser, K.: 1350-130
Glass, A. P: 2150, 2430-298
Glazner, G.: 1075
Gleason, K.: 1401-291
Glenn, J. M: 1402-300
Glicksman, A.: 1070, 1210, 1500, 2150
Glorioso, T.: 3390
Glover, C. M: 2155, LB3620-375
Glynn, N. W: 1240, LB935-368, LB1545-428, LB2570-343, LB3620-403
Glynn, R.: 3325-169
Gmelin, T.: 1240, LB1545-428, LB2570-343
Gnanasekaran, G.: 2315-13
Gnjidic, D.: 3365-278
Godfrey, M.: 1360-165
Godin, J.: 565, 3325-165
Godina, S. L: 2170
Godoy, M.: 2310
Goel, R.: 2360-99
Goeman, D.: 2325-26
Gogol, M.: 930-344, 1490
Goktas, S. O: 815-17
Goldberg, R. W: LB1545-371
Golden, A.: LB3620-341
Golden, R.: 2385-174
Goldgof, D.: 1310-39
Goldman, T.: 695
Goldsmith, S.: 1365-179
Goler, T.: 895-239
Goltz, H. H: 1380-190
Golub, J. S: 2495
Gomez, F.: LB2570-399
Gomez-Orozco, C.: 1330-94
Gomez-Orozco, C.: 1402-299
Gómez-Verján, J.: 3590
Gonyea, J. G: 1010
Gonzales, E.: 4150
Gonzales, M.: LB3620-372
Gonzalez, A. L: 1320-64
Gonzalez, R.: 3190, LB2570-435, LB3620-331
González, H. M: 860-159, LB3620-342
Gonzalez-Marquez, A.: 890-224
Goodridge, D.: LB2570-424
Goodwin, J. S: 3355-250
Goodyear, M.: LB3620-379
Gooneratne, N.: 920-305
Gorbunova, V.: 525
Gordon, S.: 3320-145
Gore, S.: 1320-65
Gorenko, J. A: 2340-40
Gorges, R.: 2480
Gorospe, M.: 835-99
Gorospe, M.: LB935-347
Gorzelle, g.: 500
Gorzelle, G.: 2355-63
Gosdin, M. M: 3010
Goswami, S.: 3350-235
Gotelli, J.: LB935-392
Goto, J.: 925-319
Gott, M.: 2270, 3105, 3310-112
Gottesman, R.: 2495, 3075, LB2570-401
Gotwals, A.: 1230
Gould, C. E: 1390-238, 1401-286, 2535
Gould, O.: LB1545-441
Goulet, J. L: 2370-119
Govier, D. J: 3370-300
Gozalo, P. L: 520, 740, 2325-21
Graber, T. G: LB1545-354, LB2570-325
Graboski-Bauer, A.: 890-224
Grabowski, D. C: 1375-185
Grace, R.: LB3620-441
Gracner, T.: 2395-217
Graf, A. S: 1380-195
Graf, P.: LB2570-438
Grafova, I. B: 4135
Graham, C.: 1210, 2150
Graham, E. K: 585, 3340-206, 3575
Graham, G.: 915-293
Graham, K.: LB3620-375
Graham, K.: 3125
Graham, K.: 3300-97
Graham, L.: 3305-102
Gramlich, C.: 850-140
Granbom, M.: LB2570-353
Grande, G.: 850-137, 3170
Grandinetti, A.: LB1545-392
Granger, B.: LB2570-315
Grant, A. N: LB2570-327
Grasselli, C.: 820-31
Gratton, C.: 840-114
Graupner, J. P: 3305-108
Gravenstein, S.: 4130
Graves, J. L: 1240
Gray, D. A: 820-18
Gray, J. A: 1365-172
Gray, M.: 1402-300
Gray, S.: 855-147
Gray-Miceli, D. L: 2360-103
Greaney, M. L: 1170, 1400-251, 3600
Greaves, C. T: LB935-398
Green, A.: 545
Green, C.: 2365-114
Green, D.: LB2570-414
Greenberg, J. S: 2375-148
Greenberg, P. A: 815-4, 900-248
Greenberg, S.: LB1545-335
Greene, M.: LB3620-419
Greene, N. A: 1300-13
Greene, R. R: 1300-13
Greenfield, E.: 595, 720, 2150, 2440, 3065, LB1545-383
Greenfield, J. C: 845-116
Greenwood-Hickman, M.: 570
Greer, J.: 910-263
Gregg, S.: 3330-180
Gregory, M. A: 1310-40
Greig, C. A: LB935-390
Greig-Custo, M. T: 2355-66
Grenier, A. M: 905-254, 3085
Grice, J.: 2415-266
Griffin, J. M: 880-193, 3180
Griffin, S.: 2425-282
Griffin, T.: LB2570-394
Griffiths, P. C: 840-107, 850-125, 1360-155
Griffiths, P.: LB1545-350
Grimaldi, A.: 2430-297
Grimm, K.: 855-150
Grimm, K.: LB2570-402
Grinshteyn, E.: 2365-113
Griswold, M.: LB2570-401
Gross, A.: 830-75
Gross, A. L: 2265
Gross, A.: 2370-129
Gross, A. L: 2410-259, 3075
Gross, E. Z: 2365-112
Gross, M.: 1402-293
Growney, C.: 1385-216, 4095
Gruber, J.: 3195
Gruber, M.: 2325-25
Gruber-Baldini, A.: 820-21, 1402-311, 2210, 2370-122
Grudzien, A.: 1390-237
Gruman, C. A: LB935-443
Grunwald, O.: 575, 865-170
Gruss, V.: 2270
Gu, D.: 1515
Guaita, A.: 2425-281
Guan, A.: LB1545-385

Gubrium, J. F: 1185
Gudkov, A.: 4090
Gudnason, V.: 2370-133
Guernsey, M.: LB3620-402
Guerra, L.: 2075
Guest, M. A: 2500
Gugliucci, M. R: 1370-181, 3005, LB935-395, LB3620-424
Guidoboni, G.: 820-37
Gullapalli, R.: LB1545-424
Gummi, S.: 3140, LB935-416
Gunn, L.: 860-156
Gunzler, D. D: 835-86
Guo, G.: 1297
Guo, M.: 615, 2010
Guo, M.: 1400-261
Gupta, R.: 2250
Guralnik, J.: 570
Guralnik, J.: 820-21, LB3620-355
Gurari, D.: LB2570-330
Gurevitch, J.: 1390-221, 3300-97
Gurinovich, A.: 835-92, 1120
Gurvich, T.: 2120
Gurwitz, J.: 3355-256
Gur-Yash, N.: 2315-7, LB1545-375
Gusmano, M.: 1065
Gutierrez, C.: 1365-171
Gutiérrez-Robledo, L.: 3590, 4155
Guzman, J.: 3360-266

### H

Ha, J.: 2375-148
Haaga, J.: 2290
Haas, A.: 850-139, 4135
Haas, K.: LB1545-387
Haavisto, W.: 3290-47
Habtemariam, D.: LB1545-389
Hackett, P.: 2355-62
Hackney, A.: 705, 1090
Hackney, M.: 920-307
Hackney, M.: 3020
Hackney, M. E: LB3620-336
Hadad, A.: 1402-303
Haderlein, T.: 1170
Hadley, E.: 1120, 2290
Hagedorn, A.: 3400
Hagemann, L.: 1355-145
Hagg, S.: 625
Haghani, A.: 830-64
Hahn, J. S: 2100
Hahn, S. J: 735
Hahnel, T.: 1385-218
Hait, Y.: LB1545-375
Hajat, A.: 855-152
Halasz, I.: LB1545-363
Hale, A.: 1402-314
Hale, L.: 2420-274
Hale Gallardo, J.: 2405-238
Haley, W. E: 1170, 1250, 1355-136, 2355-69, 2270, 2375-141, 2435-311, 3360-267, LB3620-433
Hall, C. B: 845-124
Hall, J.: 2370-118
Hall, K. S: 530, 630, 1330-83
Hall, L.: 2365-112
Hall, L. O: 1310-39
Hall, L. C: 4170
Halli-Tierney, A. D: 870-183
Halli-Tierney, A.: 900-243
Halli-Tierney, A.: 1315-56, LB1545-332, LB2570-418
Halonen, P.: LB1545-339
Halpern, D.: 1305-24
halpern, J.: 2010
Halpin, S. N: 2385-176
Halvorsen, C. J: 1540
Ham, C. R: 2385-186
Hambarsoomian, K.: 2250
Hamdani, M.: 1320-64
Hamel, K.: LB2570-412
Hamers, J.: 2020
Hamilton, A.: LB3620-391
Hammond, N. G: 1380-198
Hampstead, B. M: 500
Hamrick, M.: 830-73, 835-100, LB3620-330
Han, A.: 1365-178, LB2570-378
Han, C.: 1400-258
Han, D.: 2400-223
Han, G.: LB3620-315
Han, H.: 3360-268
Han, H.: 3325-174
Han, J. H: LB2570-380
Han, J.: 1245
Han, L.: 2370-119, 3365-278
Han, S.: 1250
Han, S.: LB935-445
Han, S.: LB1545-358
Han, X.: LB935-348
Han, Y.: 500
Han, Y.: 1355-144
Hancock, C.: 1035, 2025
Hand, M.: LB3620-401
Handing, E.: 805
Handy, D.: 1345-107
Handy, T. C: 715
Hangar, J.: LB1545-439
Hank, K.: 3245
Hanks, R. S: 2560
Hanlon, J.: 3390
Hanna, G. P: 610
Hannon, P.: 2410-256
Hannum, S. M: 3275-14
Hanratty, B.: 1290, 1325-67, 2395-213, 4055, LB1545-418
Hansen, A.: 3295-74
Hansen, L.: 3010
Hanson, K.: 3305-109
Hanson, L.: 520
Hanson, L.: 2300
Hansson, I.: 575
Hantman, S.: 1300-13, 1375-183
Hao, N.: 1115
Hao, Z.: LB1545-376
Haran, J. P: 820-29
Harari, L.: 3340-203, 4145
Hardgraves, V. M: LB1545-343
Hardin, H. K: 1330-75
Harding, M.: 2365-106
Hardland, J.: 1335-97
Hardy, R.: 3170
Harfmann, E.: 1355-142
Harfmann, E.: 2375-149
Hargis-Fuller, L.: LB935-384
Hargittai, E.: LB1545-334, LB2570-377
Harling, G.: 4110
Harllee, H.: 3375-311
Harllee, H. D: 3375-305
Harlow-Rosentraub, K.: 1345-111
Harman, S. M: 3325-176
Harmon, A.: 1305-21
Harootyan, L. K: 2085
Harootyan, R.: 1085, 2085, 3285-38
Harralson, T.: 915-281
Harrati, A.: 1430
Harrington, A.: 510, 3555
Harrington, A.: 505
Harrington, C. C: 2505
Harrington, E.: 1401-285
Harrington Meyer, M.: 3245
Harris, A. P: 1405-321
Harris, J. A: 3345-222, 4050
Harris, M. T: 3500, LB2570-406
Harris, M. L: 850-127
Harris, R.: LB1545-363, LB2570-409
Harris, R.: LB2570-445
Harris, S. M: 2340-47
Harris, T. B: 1425
Harris-Kojetin, L.: 3060
Harrison, K.: 1305-33, 2010
Harrison, T. C: 920-314
Hartikka, K.: 3410
Hartmann, C. W: 740, 3535
Hartz, S.: 1335-97
Harvath, T.: 2295
Hasan, N.: 2400-230
Hasebe, M.: 895-226
Hasegawa, M.: 830-63
Hasnain, M.: 2270
Hass, Z.: 3435, LB935-377
Hassett, A.: 1425
Hassouneh, D.: 840-104
Hastings, S.: LB3620-325
Hastings, W. J: 2370-117
Hasworth, S.: 910-277
Hasworth, S.: 3345-214
Hasworth, S.: LB2570-404
Hata, C.: 2390-199
Hathaway, D.: 2410-254
Hatton, S. N: LB2570-348
Hatzifilalithis, S.: 905-254
Hauer, K.: 920-306
Haugen, C.: 2380-168, 4020
Hausdorff, J. M: 1402-304, 4020
Haverhals, L. M: 1345-105, 1402-314, 4135

Haviland, A.: 2250
Havyer, R.: 3180
Hawkins, B.: 505
Hawkins, N.: 2385-175
Hawkley, L.: 2275
Hay, M.: LB3620-441
Hayanga, B. A: LB935-418
Hayden, K.: 805
Hayslip, B.: 925-333
Hayslip, B.: 1460
Hayslip, B.: 2375-147
Hayward, M. D: 1200
Hazuda, H.: LB3620-372
He, M.: 3390
He, W.: 1325-74
Healey, W.: 4030
Hearld, L.: 3345-211
Heckman Coats, A. L: 2435-312
Hedeker, D.: 2400-221
Hees, E.: 550
Heffner, K. L: 670
Heflin, M. T: 1305-24
Heijkants, C.: LB1545-414
Hein, M.: LB2570-364
Heinz, M.: 645, 2390-205, 3300-92
Heisel, M. J: 3105
Heisler, C. J: 1265
Heitkamp, R.: 1335-95
Heldmann, P.: 920-306
Helgeson, V.: 1315-48
Helm, F.: 3465
Helphrey, J. H: 815-15, 2425-288
Helvik, A.: 1330-86
Hemmady, S. A: 2410-256
Hemmert, R.: 3355-249
Henage, C. B: 4075, LB935-392
Henderson, C.: 3220
Henderson, S.: 3010
Henderson, T. L: 3315-122
Hendricksen, M.: LB1545-389
Hendrix, C. C: LB1545-425
Hendry, L.: 1315-49
Henkens, K.: 660, 865-170
Henry, J.: 3330-181, LB1545-343
Henry-Smith, C. A: LB935-348
Heo, J.: 2410-246, 3370-297
Hepburn, K.: 710, 840-107, 850-125, 1510, LB1545-350
Heppner, A.: 690
Herbert, M.: 4080
Herd, A.: 2395-210
Herd, P.: 720
Herkert, N.: LB2570-322
Herman, H.: 2510
Hermesch, A.: 690
Hermida, R.: 910-274
Hernandez, A.: 2310
Hernandez, D.: 2395-215
Hernandez, D. F: 1330-93
Hernandez, M. E: 4020
Hernandez, S. C: 1330-84
Hernandez, S. F: 1310-38
Hernandez-Lujan, D.: 1345-105
Hernandez-Tejada, M.: 1055, 2365-107
Herr, K.: 3365-282
Herr, N.: 815-9
Herrera Legon, M.: LB1545-353
Herrera-Venson, A.: 3415, 4030
Herron, R.: 2105, 2225
Hershey, D.: 865-166
Hertzog, C.: 4095, LB2570-373
Hervera, B.: LB1545-426
Hess, T. M: 1385-216, 2275, 2340-34, 4095
Hetzel, S.: LB935-422
Heyman, J.: LB2570-340
Heyn, P. C: 770, 1445
Hiatt, S. O: 3010
Hibbs, C.: LB2570-346
Hicken, B.: 2055, 3165, 3485
Hicks, G.: 3365-275
Hicks, H. J: 500, 2410-250
Hicks, N.: LB2570-349
Hicks Patrick, J.: 890-221
Hicks Patrick, J.: 925-334, 1065, 1475
Higgins, M.: 850-125
Higgins, M.: 850-128
Higgins-Chen, A. T: LB1545-422
Hilgeman, M. M: 2535, LB3620-339
Hill, C.: 545
Hill, C.: 1050, 1450, 2240, 3095
Hill, C.: 3385
Hill, J. E: 865-168
Hill, J.: 865-168
Hill, M. S: 865-168, 1401-277, 3290-45
Hill, N.: 1175
Hill, N.: 3295-89
Hill, P. L: LB935-386
Hill, R.: 1355-148
Hill, T. D: 2420-274
Hill, W. D: 830-73
Hill, W. D: 835-100
Hill, W. D: LB3620-330
Hillel, I.: 1402-304
Hilsabeck, R.: 850-134
Hincapie, D.: 1315-45, 1330-82
Hines, D.: 850-144
Hinton, L.: 3010
Hirano, H.: 855-154
Hirano, H.: 895-232
Hirano, H.: LB2570-411
Hirayama, R.: 880-202, 2375-151
Hirofuji, T.: LB1545-344
Hirsch, C.: 4045
Hirsch, J. A: LB1545-364
Hirschman, K.: 840-109, 915-289, 1080, 2215, 3230
Hirukawa, S.: 3270-4
Hladek, M. D: 725, LB2570-353
Ho, G.: 3245
Ho, J. Y: 3245
Ho, S.: 3290-65
Ho, S.: 2360-95
Ho, T.: 3460
Hobbs, M.: 3060
Höbler, F.: 850-142
Hochberg, M.: 820-21
Hodes, R.: 2135, 2290
Hodgdon, B.: 3180
Hodgkinson, J.: 3345-224
Hodgson, N.: 605, 915-289, 1160, 2215, 2420-276, 2490
Hoe, D.: 3330-183, LB2570-366
Hoeft, T. J: 3010
Hoek, L. J: 785
Hoens, S.: 3035
Hofer, S.: 3340-206, LB2570-355
Hoffman, G. J: 4130
Hoffman, J. M: 2230, LB2570-371
Hoffman, R.: 820-18
Hogan, C.: 3365-280
Hogan, D. B: 3295-87
Hohn, K. L: 3430
Hojfeldt, G.: 820-30
Holahan, C. K: 1395-247, 3335-196
Holahan, C. J: 3335-196
Holland, D.: 3180
Holland, E.: 1320-64
Holley, L.: 2500, 3345-225
Hollinda, K.: 2325-30
Hollis-Sawyer, L. A: 1380-197, 3275-18
Hollstein, B.: LB3620-392
Holm, L.: 820-30
Holmes, A.: 2360-101
Holmes, H. M: 1225
Holmes, S.: 1255
Holmes, S. D: 3270-11
Holt, H.: 1505
Höltge, J.: 1330-88
Homan, K. J: 720
Homayouni, R.: LB935-366
Homeier, D.: 3025
Hommelhoff, S.: 1385-218
Honda, T.: 1370-180
Hong, J.: 2375-148
Hong, M.: 1340-100, 2140, 2405-240
Hong, M.: LB2570-337
Hong, Y.: 1360-151
Honig, L.: LB2570-396, LB3620-337
Hoogland, A. I: 1390-230
Hooker, K.: 1195, 1350-125, 2405-237, 3235, 3370-300
Hooker, S.: 890-223, 1095
Hooyman, N.: 2313
Hopfgartner, I.: 2390-206
Hopp, F. P: 1000
Hoppmann, C. A: LB1545-445, LB2570-438, LB3620-383
Horgas, A.: 3365-273
Horhota, M.: 815-5
Horowitz, A.: 2520
Hossain, M.: 2400-230
Hou, S.: 1040, 1500, 2150
Houlind, M.: LB2570-339
Hounsell, C.: 695

Houser, A.: 3450
Houston, A.: 1355-142
Houston, A. M: 2375-149
Hovey, S.: 3495
Howard, A.: 3360-270
Howard, E. P: 4080
Howard, S.: LB3620-341
Howard, S.: 1402-310
Howard, V.: 1170
Howe, J. L: 2220, 3530
Howe, M.: 3360-270
Howell, B. M: 1325-66, 2225, LB935-360
Howlett, S. E: 820-36, LB2570-323
Hoy, C.: 855-147
Hoyle, J.: 1345-117
Hoyle, J.: 2375-139
Hoyt, E. K: LB1545-405
Hsiao, H.: 3400
Hsieh, J.: 905-255
Hsieh, J.: LB935-444
Hsieh, W.: 3360-270
Hsiung, G.: 715, 920-313
Hsu, C.: 565, 715, 2215, 2360-97
Hsu, E.: 2345-53
Hsu, F.: LB1545-390
Hsu, J.: 1310-37
Hsu, J.: 2360-82
HSU, S.: 1075
Hsu, W.: 2155
Hu, M.: LB935-409
Hu, R. X: 1350-121, 2495, 3250, 3275-25
Hu, S.: 890-219, 1330-89
Hu, Y.: 3335-195, 3365-283
Hu, Y.: LB1545-436
Hua, C.: 4150
Hua, J.: LB935-384
Hua, P.: 840-115
Hua, Y.: 4105
Hua, Y.: LB3620-430
Huang, A.: 2425-291
Huang, C.: 905-250
Huang, F.: 1390-225
Huang, F.: 1400-257
Huang, F.: 3395, 4160
Huang, H.: 1300-12, 3360-271
Huang, J.: 1170, 2280, 3420
Huang, N.: 890-219
Huang, V.: 820-20, 3295-87
Huang, W.: 3085
Huang, Y. A: 2380-163
Huang, Y.: 2370-128
Huang, Z.: 1330-77, 2370-137, 4005
Hubbard, E.: 1330-91
Hubbell, Z.: LB3620-341
Huber, G.: 4030
Huber, H.: 835-94
Huber, L. L: 900-241, 1370-182, 1401-271, 3375-306
Huber, N.: 825-43
Hubner, S. B: 3350-234
Hudson, J.: 1015
Huebner, J.: LB2570-394
Hueluer, G.: 590
Huff, M.: 1345-107, 3300-90
Huffman, K.: 4130
Huffman, M. K: 2410-255, 3350-238
Hugenschmidt, C.: LB935-403
Hugenschmidt, C. E: LB2570-401
Hugentobler, L.: LB1545-334
Hughes, A. K: 3415, 4020
Hughes, M. L: 3350-246
Hughes, M. L: 700
Hughes, S.: 1401-273
Hughes, S.: 3175, 3305-107
Hughes, T. F: 1360-153
Hughes, T.: LB2570-401
Huh, T.: 1390-238
Huisingh-Scheetz, M.: 4070, 4165
Hummer, R.: 2280
Humphries, J.: LB1545-439
Hundza, S.: 2360-105, 3470
Hung, L.: 1060, 1401-292
Hung, M.: 660
Hung, W.: 3165
Hunsaker, A. E: LB1545-334, LB2570-377
Huo, M.: 725, 925-316, 1250, 1350-134, 3580
Hurtado Pomares, M.: LB935-430
Hurwitz, E.: LB1545-392
Husa, R.: 2405-238
Hussong, S. A: 825-44, 835-98
Hutchins, D.: 850-140
Hutchins-Wiese, H. L: LB2570-427
Huynh, A.: 1170
Hwang, S.: 2355-69
Hwang, U.: LB3620-325
Hwang, Y.: 915-289
Hyer, K.: 1365-177, 1405-325, 1530, 2220, 2400-220, 3345-221
Hyun, J.: 3290-61, 4085, LB2570-347

### I

Iaboni, A.: 2355-61, 3415
Iacob, E.: 500
Iapalucci, M.: 2415-269
Ibrahim, S.: 915-293
Ibrahim, T.: LB2570-324
Idler, E.: 3340-207
Igarashi, A.: 895-225
Igarashi, H.: 1350-123
Ighodaro, E.: 2270
Ihara, E. S: 3305-103
Ihara, K.: LB2570-411
Iijima, K.: 895-232, 3325-157
Ikard, C.: LB1545-332
Ikeuchi, T.: 3340-204
Ilardo, J.: 1305-29, 4005
Ilkayeva, O. R: LB2570-437
Iloputaife, I.: LB3620-328
Im, K.: 2435-309
Im, S.: 3370-297
In, L.: 2120
Inagaki, A.: 895-225
Infurna, F. J: 1215, 1350-129
Infurna, F.: LB2570-402
Ingber, M. J: 2385-190
Ingels, J. B: LB2570-370
Ingersoll-Dayton, B.: 3430
Ingram, C.: 3180
Ingram, E.: 1330-80
Ingram, F.: 3590
Ingram, R. E: LB935-399
Inker, J.: 505, 815-11, 3340-205, 4170
Inoue, M.: 1070, 1260
Inouye, S.: 2245, 4130
Intrator, O.: 4045
Intrieri, R. C: 815-13, LB3620-402
Inventor, B. R: LB2570-419
Iqbal, A.: 890-218
Iqbal, U.: 825-57
Irani, E.: 725
Irarrázabal, L.: LB1545-426
Irimia, A.: 825-61, 3080
Iris, M.: LB935-377
Irizarry-Arroyo, N. E: 1390-230
Irvin, V. L: 2405-237, 3370-300
Isaacowitz, D. M: 1385-209, 2110
Isales, C.: 830-73, 835-100, LB3620-330
Isbel, S.: 705
Ishida, A.: 3280-30
Ishikawa, Y.: 1400-256
Ishioka, Y.: 1345-109
Ishioka, Y. L: 3340-199
Ishizaki, T.: LB2570-444
Ismail, N.: 2360-96
Ismail, Z.: 3295-87
Israel, S.: 3025
Israel, S. M: 915-292
Issachar, G.: 1402-293
Iveniuk, J.: 1345-108
Ives, D.: LB2570-396
Iyer, S.: 1390-238

### J

Jablonski, R. A: LB2570-391
Jabson, J.: 895-238
Jacelon, C.: 930-342, 1395-249, LB1545-403, LB2570-372
Jachim, S.: 830-67
Jackson, E.: 1400-267
Jackson, L.: 1400-267
Jackson Stoeckle, R.: 795
Jacob, M.: LB3620-372
Jacobs, E. A: 930-343, LB935-422
Jacobs, J. M: 2315-9
Jacobs, L.: 535, 1355-145, 3125, 1390-221
Jacquelin, B.: 2430-302
Jagannathan, S.: LB3620-424

Jagger, C.: 685, 1200, LB2570-319
Jahoor, F.: 1310-37, 2190
Jain, I.: 2090
Jain, S.: LB2570-387
James, B.: 1390-230
James, B. D: 3480, 3575
James, D.: 2315-4
James, N.: LB-935-447
James, N.: 1355-140, LB1545-353
Janet, L.: 3320-134
Janet, M.: LB1545-336
Janevic, M. R: 705, 775, 3365-280
Jang, A.: 1402-305
Jang, D.: 3600
Jang, H.: 640
Jang, I.: LB2570-375
Jang, J.: 2360-83
Jang, J.: 600
Jang, J. Y: LB3620-411
Jang, K.: 1350-126
Jang, Y.: 1325-70, 2140, 2405-235, LB3620-433
Janssen, L. M: LB935-407
Jao, Y.: 850-133, 3010
Jarosek, S.: 2155
Jarrett, T.: 2385-176
Jarrín, O. F: 850-143, 910-274, 4135
Jarrott, S. E: 1205
Jarrott, S.: 2405-239
Jarrott, S. E: 3235
Jarrott, S.: 3290-62, 3315-128
Jarvis, L.: 530, 1402-308
Jason, K.: 1055, 1160
Jasper, A.: LB3620-410
Jayanama, K.: 3325-165
Jazwinski, S.: LB1545-417
Jazwinski, S.: LB1545-377
Jeanblanc, A.: 890-216
Jeanblanc, A. B: 2385-177, 3320-141
Jean-Louis, G.: 2325-29
Jeffery, B.: 1515, 2355-62, 2430-296, 3140
Jehu, D. A: 2360-90
Jen, S.: 3350-242, 4145
Jenkins, M.: LB2570-331
Jenkins Morales, M.: 910-260
Jenney, K. N: 2360-86
Jennifer, L.: 3320-137
Jennings, B. M: 840-107, 850-125
Jennings, J.: 925-324
Jennings, S. C: 630
Jensen, A. C: 1401-277
Jensen, C. J: 505, 2300
Jensen, J.: 2365-106
Jensen, P. H: 3520
Jeon, S.: 1305-33
Jeon, Y.: 505
Jeong, M.: 3350-242
Jervis, L. L: 3310-111
Jespersen, B. V: 3360-261
Jester, D. J: 1355-136, 1365-177, 2400-220, LB2570-362
Jette, N.: 555
Jia, C.: 3350-241
Jia, R.: LB3620-428
Jia, W.: LB935-356
Jiang, D.: LB3620-397
Jiang, H.: LB2570-333
Jiang, J.: 3320-142
Jiang, M.: LB935-356
Jiang, N.: 1340-103, 3250, 4115
Jicha, G. A: 2270
Jim, H. S: 1390-230
Jimenez, D.: LB935-417
Jimenez, D.: 2520
Jimenez, E.: 2425-284
Jimenez, E. E: 1170
Jiménez, M.: 3370-290
Jin, H.: 4005
Jing, B.: 2315-1
Jiwani, H.: 2315-15
Jiya, N.: 3290-63
Joergensen, T.: LB1545-356
Johansson, B.: 2215
Johansson, L.: 2380-160
Johari, K.: 3330-182
Johnson, B. P: 2315-6
Johnson, D.: 3175
Johnson, I. M: 2540
Johnson, I.: 1315-46
Johnson, J. P: 4020
Johnson, J. E: 765
Johnson, J.: 2215
Johnson, J.: 1160
Johnson, K.: 3560
Johnson, K. S: 520
Johnson, K. J: 2430-299
Johnson, K. S: LB1545-425
Johnson, K.: 2395-207
Johnson, M.: LB935-402
Johnson, M. R: 2345-55
Johnson, M. L: 895-237
Johnson, N. L: 1380-199
Johnson, R. E: 880-189
Johnson, S.: 2355-62
Johnson, T.: 815-4
Johnson, T.: 1315-52
Johnson, V.: 1380-195
Johnson-Lawrence, V.: 1450
Jolejole, K.: 730
Joling, K.: 4135
Jones, C.: 1405-322
Jones, C. A: 920-315
Jones, G.: 1180
Jonnakuti, S. T: 1310-34
Jonsdottir, M.: 2370-133
Jonsson, P. V: 2370-131
Joo, H.: 2385-175
Joo, S.: 1360-159, 3340-208, LB2570-362
Jopp, D.: 1385-213, 2340-37, 3315-123, 3370-286
Jordan, L.: 1170, 4010
Jordan, M.: 840-108
Jor'dan, A. J: LB3620-328
Jorritsma, J.: 3230
Jose, R.: LB2570-414
Joseph, L.: 545, 3455
Josephson, K.: 2420-273
Joubert, S.: 3280-29
Ju, C.: 3310-116, LB935-382
Juang, C.: 1390-238, 1401-286
Juárez Leal, I.: LB935-430
Juckett, L.: 1205
Juckett, L. A: 3315-128
Judge, S. T: 4100
Jukema, J. S: 3230
Julien, C.: LB1545-419
Jun, H.: 1360-159, 3340-208
Jun, J.: 1300-4
June, A.: 2435-307, 3370-291
June, J.: 1405-325, 3345-215
Jung, H.: 3325-174, LB2570-375
Jung, H.: LB1545-398
Jung, J.: LB2570-337
Jung, M.: 2340-51
Jung, S.: 3370-286
Jung, S.: 645, 2390-201, 2395-211, 3300-98, 3345-228
Jurkeviciute, M.: 1402-298
Jurkowski, E. T: 1335-95
Justice, J. N: 835-89, 1450, 2530, 3440, 4090
Jutkowitz, E.: 2325-21, 2490
Jylha, M.: LB1545-339
Jyothi, K.: 1402-310

K

Kaasalainen, S.: 910-264, 1395-245
Kaczorowski, C.: 3080
Kadylak, T.: LB1545-373
Kaeberlein, M.: 825-59, 830-79, 1245, 2230, 4090, LB3620-388
Kagotho, N.: 2405-239
Kahana, B.: 890-218, 1400-258, 3370-289
Kahana, E.: 890-218, 1400-258
Kahana, E.: 1401-276
Kahana, E.: 3370-289, 4160
Kai, I.: 880-202, LB2570-444
Kail, B. L: 2280
Kaiser, B.: 3350-240
Kajander, M.: LB3620-317
Kajitani, S.: LB2570-440
Kallmyer, B.: 1160
Kalmijn, M.: 660
Kamat, A.: 835-93
Kamdar, N.: 1300-10, 2010, 2325-32, 3395
Kamesawa, A.: 925-319
Kamiya, Y.: 4065
Kammerer, C. M: 1310-42
Kampen, R.: 2270
Kamper, H.: 2385-173
Kan, C.: LB3620-415
Kane, A.: 820-36, LB1545-362, LB2570-323
Kane, C.: 3300-93
Kane, K.: 895-230
Kane, R. A: 3240
Kang, B.: 1105
Kang, C.: 820-33

Kang, G.: 1402-297, 2360-99, LB935-381
Kang, H.: 1315-53, 4080
Kang, H.: 1300-2, 1375-188
Kang, H.: LB2570-425
Kang, H.: 2360-82
Kang, J.: 2430-294
Kang, J.: LB1545-398
Kang, M.: LB1545-398
Kang, S.: LB1545-398
Kang, S.: 2395-216
Kang, S.: 3325-174
Kang, S.: 3370-299
Kang, Y.: 1315-57
Kanne, G. E: 1305-24
Kanyat, R.: 1380-201
Kao, D.: LB2570-321
Kaplan, A.: 2410-258
Kaplan, D. B: 1075
Kapogiannis, D.: 3295-71
Kapoor, A.: 3355-256
Karandikar, S.: 2405-239
Karasik, R. J: 735, 900-248, 2560
Karel, M. J: 2015, 3255
Karikari, G.: 900-241, 1370-182
Karikari-Martin, P.: 2385-184
Karim, H.: 825-45
Karim, H.: 2360-94
Karl, J.: 2420-270
Karmarkar, A.: 2385-171
Karnick, C.: 1365-174
Karpecki, P.: 1320-64
Kartin, D.: 855-152
Karus, D. G: 2430-300
Karvonen-Gutierrez, C.: 855-148, 2445
Kasahara, S.: 2390-199
Kaskie, B. P: 1100, 2470
Kasper, J. D: 1170, 3420
Katagiri, K.: 2425-278
Katagiri, K.: 3300-91
Katlic, M.: 2115
Katsumata, A.: 2320-16
Katz, A.: LB1545-392
Katz, A. D: 3300-101
Katz, B.: 3290-62
Katz, B.: 2500
Katz, E.: 3350-243, LB935-410
Katz, G. R: 2395-212
Katz, M.: 1170
Katz, M.: 845-124, 1175
Katz, R.: 4020
Katz, R.: 3140
Katz, S.: 580
Kaufman, B. G: 870-182
Kaufman, D.: 2325-30
Kaufman, J.: 4070
Kaufmann, C. N: 2125
Kaup, M.: 2515
Kauwe, G.: LB935-349
Kavanagh, K.: 835-87
Kawai, H.: 855-154, LB2570-411
Kaye, J.: 1401-282, 2060, 2405-241, 3535, 4165, LB2570-357
Kaye, L.: 910-257
Kayed, R.: 825-46
Kazis, L.: 740, 3535
Kazlauckas, N.: 1385-210
Kcomt, L.: 1000
Keefe, B.: 1390-224
Keefe, F.: 3365-275
Keenan, B. E: 2380-161
Keene, J.: 3330-191
Keepper, K.: LB3620-358
Kegler, M.: LB3620-325
Kehat, O.: 1402-293
Kehler, D. S: 4045
Kehoe, P.: 3320-140
Keibler, T.: 3240
Keiser, S.: 2215
Keleher, V.: LB3620-339
Kelemen, L.: 3305-108
Kelfve, S.: 865-178
Keller, A. O: 840-103
Keller, J.: 820-37
Keller, K.: 820-36
Kellett, J.: 705
Kellett, K. G: 1305-25
Kelley, J.: 1145
Kelley, S. J: 3245
Kelly, C. S: 1315-58
Kelly, C.: 1335-95
Kelly, C. M: 915-284, 3345-225, LB935-393
Kelly, K.: LB3620-362
Kelly, L.: 3345-212
Kelly, O. J: 1315-55
Kelly, P.: LB2570-340
Kelly, R.: 820-28
Kemeny, M. E: 505
Kemeny, M. E: 850-140
Kemnitz, J. W: LB3620-363
Kemp, B.: 720, LB2570-423
Kemp, C. L: 3270-9, LB3620-321
Kemp, N.: LB2570-328
Kempen, G.: 565, 3270-5
Kenaley, B. L: LB2570-442
Kennedy, A.: 890-222, 1075
Kennedy, B.: 4090
Kennedy, E.: 830-73
Kennedy, J.: 1402-295
Kennedy, K.: 2530
Kennedy, R. E: 1425, 2315-4, 3365-274, 3595
Kennedy, T.: 4155
Kennedy-Malone, L.: 2315-8
Kennedy-Malone, L. M: LB3620-421
Kenney, K. C: 2370-123
Kent, J.: LB935-350
Kent, M.: 2325-26
Kent de Grey, R.: 1315-48
Kenya, S.: 1402-301
Keogh, S.: 2105, 4055
Keohane, S. M: 3270-7
Kerns, R. D: 2370-119
Kerr, N.: 3345-218
Kerssens, C.: LB2570-415
Kertai, M. D: 2240
Kerwood, R.: 810-2
Keryl, M.: 3320-134
Kessler, V. L: 1305-18
Ketcham, C.: LB2570-412
Khadeer, M.: LB3620-364
Khaleghi, R.: LB1545-373
Khan, A. M: 600
Khan, C. P: LB935-416
Khan, M.: LB3620-375
Khan, N.: 2400-230
Khatib, D.: LB935-366, LB3620-326
Khatri, N.: 2400-222
Kheifets, V.: 825-43
Khiewchaum, R.: LB935-369
Khoo, Y.: 2010
Khosla, S.: 3455
Kiefe, C. I: 1400-260
Kiefer, L.: 3345-219
Kieffaber, P.: 3290-44
Kieffer, L. A: LB1545-432
Kiel, J. J: 1400-261
Kietzman, K. G: 3305-104
Kiflezghi, M.: 830-79
Kikutani, T.: 895-232
Kilaberia, T. R: 3305-106, LB2570-376
Kilgore, D.: 2120
Kilpela, L. S: LB2570-399
Kim, A.: LB3620-375
Kim, A.: 2410-246
Kim, B.: 3115
Kim, B.: 2215
Kim, B.: 1305-30, 1350-122, 3230
Kim, B.: LB3620-335
Kim, C.: 3325-168, LB2570-443
Kim, C.: 3325-174
Kim, D.: 1402-305
Kim, D. H: 3050, 3325-169
Kim, D.: LB2570-375
Kim, D.: 1350-119, 3340-198
Kim, E. S: 1425, 4100
Kim, E.: LB1545-352
Kim, E.: LB2570-442
Kim, E.: LB3620-324
Kim, E.: 1402-305
Kim, E.: 2355-80
Kim, G.: 1310-35, 1325-73, 1340-100, 1390-232, 3290-60
Kim, H.: 830-74
Kim, H.: 1375-188
Kim, H.: LB2570-411
Kim, H.: 710
Kim, H.: 3295-80, 3350-234
Kim, H.: 3360-267
Kim, H.: 3325-174
Kim, H.: 1415
Kim, J.: 1305-16
Kim, J.: 925-319
Kim, J. H: LB1545-413
Kim, J.: 1365-172
Kim, J.: 2420-276
Kim, J.: 1305-19
Kim, J.: LB3620-316
Kim, J.: 830-74
Kim, K.: 3325-168, LB2570-443
Kim, K.: 3325-174, LB1545-398
Kim, K.: 1340-100
Kim, K.: 2410-246
Kim, K.: LB935-422

Kim, K.: 2340-37, 3150, 3270-10, 3315-129
Kim, M.: 2380-156
Kim, M.: 925-327, 1390-234
Kim, M.: 2140, LB1545-419
Kim, M.: 2315-6
Kim, S.: LB1545-417
Kim, S.: LB1545-357
Kim, S. M: 1405-318, 4160
Kim, S.: LB935-359, LB2570-439
KIM, S.: 840-111
Kim, T.: LB2570-337
Kim, T.: 1365-178
Kim, W.: LB3620-415
Kim, Y. K: 2340-37
Kimata, M.: 3270-4
Kimberly, L. L: 885-212
Kimiecik, J.: LB2570-408
Kindratt, T.: 2355-64
King, B.: 2315-2
King, B.: 2410-262
King, B. M: 2550, 3480
King, G.: 1402-310
King, J. W: 2110, 2455
King, K.: 3125
King McLaughlin, J. B: 845-116
Kingston, A.: 1290, 1325-67, LB1545-418, LB2570-319
Kingston, A.: 685
Kingston, P.: 3490
Kinlaw, K.: LB2570-351
Kiosses, D.: 3220
KIP, K. E: 1330-93
Kirch, M.: 705, 2155
Kirchin, D.: 2225
Kirkland, S.: 565
Kirkland Caldwell, J. Z: LB935-404
Kishimoto, K.: 900-248
Kitamura, A.: 3340-204
Kitamura, S.: 895-225
Kitner-Triolo, M. H: 705
Kitzman, D.: 3325-175, LB2570-387
Kivnick, H. Q: 1185, 2145, 2200
Kiyoshi-Teo, H.: 1360-160
Klanchar, S.: 3320-137
Klaus, J.: 1330-81
Klein, E. M: 885-211
Kleineidam, L.: LB3620-318
Kler, S. E: 1305-33
Klima, D. W: 2360-101
Klimstra, M.: 2360-105
Klinger, C.: 905-255
Kloseck, M.: 2355-75, 3560
Klunk, W.: 825-45
Klusmann, V.: 3615
Kmetz, L.: LB3620-385
Knatterud, L.: 3240
Kneale, D.: LB935-418
Knepple Carney, A.: 1345-112
Knight, B. P: 855-149, 3375-311
Knight, B. G: 1315-49
Knight, E. L: 825-53
Knight, J. E: LB2570-347
Knoefel, F.: 3595
Knopman, D.: 3075
Knopp, K.: 2550
Knopp-Sihota, J. A: 3220
Knox, S.: 850-139, 4135
Ko, H.: 3230
Ko, P.: 3480
Kobayashi, E.: 3340-210, LB935-439
Kobayashi, L. C: 4110
Kobayashi, M.: 3315-130
Kochan, N. A: 655
Koehly, L. M: 925-331, LB3620-392
Koehn, S.: LB935-383
Koepp, R. M: 2015
Koeuth, S.: 885-207
Koffer, R. E: 3190
Kogan, L.: 1090
Kohlbacher, F.: 4150
Kolanowski, A. M: 2525
Kolodziejczak, K.: 4015
Kondash, M.: 810-2
Kondolf, K.: 2130
Kondrikov, D.: 835-100, LB3620-330
Konety, B.: 2380-155
Konetzka, R.: 910-258
Konetzka, R.: 2480, 3140
Kong, D.: 4105, LB2570-405, LB3620-428
Kong, J.: 1250, 1440
König, H.: 3320-139
Konnert, C.: 2340-40
Konola, T.: 3355-256
Kononova, A.: LB935-432
Konopka, A. R: 835-88
Konrad, T.: 780
Koon, L. M: 3500, LB1545-373
Kooner, P.: LB1545-412
Kopecek, M.: LB1545-333
Kopera-Frye, K.: 890-224, 1285
Koppel, S.: 1410-331
Korc-Grodzicki, B.: 635, 1365-174, 3275-20, 3305-105, 4110
Koren, C.: 3030
Kornadt, A. E: 2040, 3380, 3615
Koroukian, S.: 1395-243
Kostiuk, R.: 1390-224
Kotarba, J. A: LB935-372
Koumoutzis, A.: 4035
Kovac, C.: LB1545-373
Kovach, C.: 3220
Kovaleva, M. A: 840-107, 850-125, 2530
Kovalik, J.: 715
Kowal, P.: 2410-251, LB1545-342
Kozlov, E. K: 3270-6, 3310-119
Kraal, Z.: LB1545-444
Krabbe Pedersen, J.: LB2570-338
Kraemer, K.: 3295-79
Kraemer, S.: 1315-60, 1400-268, 2420-270, LB935-355
Kraft, R.: LB935-403
Kramer, J.: 3485, 3530
Kraus, V. B: 820-41
Kraus, V.: LB2570-394
Kravchenko, J.: 2240, 2370-132
Krefman, A.: 3280-34
Krekula, C.: 3000, 4125
Kremen, W.: 1425, LB2570-348
Kreps, G. L: 1401-276
Kresevic, D. M: 895-227, 915-293, 1320-63
Kressig, R. W: LB2570-367
Kridli, S.: LB1545-340
Krieger, N. I: 835-86
Kritchevsky, S.: 805, 2085, 3280-33, 3325-175, 3590, LB935-403, LB1545-387, LB1545-390, LB2570-387, LB3620-348
Krok-Schoen, J. L: 1315-55
Krook, M.: 1345-113
Krousel-Wood, M.: 1315-52
Krueger, S.: 1385-219
Kruger, T. M: 680, 735, 2440
Kruisbrink, M.: 565
Krus, G.: 915-283
Kruse, A.: 3375-310
Kuai, Z.: 825-42
Kube, E.: 2055
Kuchel, G. A: 3010, LB2570-393
Kuh, D.: 3170
Kuhn, T.: LB2570-335
Kuipers, A. L: 1310-42
Kuipers, A. L: 2370-136
Kuklova, M.: LB1545-333
Kulibert, D.: 2355-78
Kulkarni, A. S: 2530
Kullgren, J.: 705, 2155
Kulminski, A.: 820-33, 2370-125, LB1545-440
Kumar, A.: 830-68
Kumar, A.: 2545, 3395
Kumar, P.: 1310-37
Kunik, M. E: 2385-170, 3345-219, LB2570-390
Kunkel, S. R: 1305-18, 2385-183
Kunzel, C.: LB1545-374
Kuo, A.: 835-94
Kuo, C.: 1180, LB2570-393, LB3620-333
Kuo, N.: 2385-182
Kuo, P.: 2370-129, 3040, 3075, LB935-446
Kuo, T.: 2400-223
Kuo, Y.: 2155
Kuo, Y.: 850-135
Kuo, Y.: 2345-53, 2385-171, 3355-250, 4020, LB1545-427
Kupzyk, K.: LB2570-395
Kurth, M.: 815-13, 2055, 3340-209, 3370-301
Kurucz, S.: 2270
Kushalnagar, P.: 3595
Kusmaul, N.: 1480, 3065, 3345-233

Kuy, K.: 515
Kuzmik, A.: 2315-3
Kwak, J.: 3105, LB3620-385
Kwan, R.: 840-106
Kwoh, K.: 915-293
Kwok, S.: 3355-255
Kwok, W.: 2400-227
Kwon, E.: 840-111, LB1545-360
Kwon, E.: 2030
Kwon, S. C: 850-131
Kwon, S.: 3360-265

L

Labreche, T.: 850-142
Labunskyy, V.: 1115
Lach, H. W: 1020
Lachance, C.: 2380-161
Lachman, M. E: 700, 890-214, 915-285, 925-320, 2410-257
Lachs, D.: 2370-118
Lachs, M.: 2225, LB935-444
LaCroix, A. Z: 570, 1455
Laditka, J. N: 860-156, 1345-117, 2375-139
Laditka, S. B: 860-156, 1345-117, 2375-139
LaFave, S.: LB2570-353
LaFazia, D.: LB935-436
Laffer, A.: 500
Laffer, A.: 2410-250
Laflamme, E.: 1300-6
Lafontaine, C.: 580
Lafranchise, E. A: 1390-230
Lag, M. R: LB935-399
Lagacé, M.: 1300-5
Lagari, V.: 3355-255
Lagervall, J. A: LB935-399
Lagus, J.: 3535
Lai, A.: 1390-235
Lai, D.: 1515, 2030
Lai, Y.: 1401-288
Laico, A.: 920-310
Laird, R.: 1320-62
Laksari, K.: 820-26
Lam, B.: 1395-246
Lam, J.: 1385-215
Lam, J.: 2010
Lamar, M.: 3480
Lamarre-Cliche, M.: 3280-29
Lambert, D.: 1305-25
Lamming, D. W: 1520
Lampkin, C.: 2420-271
Lampraki, C.: 1385-213, 2340-49
Lan, T.: 3280-34
Lanai, B.: LB2570-418
Landes, S. D: 1445
Landry, G. J: 1510
Lane, D. W: 4140
Lane, K. R: 4005
Lane, K. R: LB1545-438, LB2570-398
Langa, K.: 2010
Langa, K.: 2325-29
Lange Maia, B. S: 1300-6, 2445
Langkilde, A.: LB2570-339
Langley, J.: 610
Lapane, K. L: LB935-435
Lapenskie, J.: 3415
Lapham, S.: 655
LaPierre, T.: LB2570-425
Lares, G.: 2495
Larkina, M.: 3340-202
Lasri, N.: 1402-293
Lassale, C.: 730
Latham, N.: 2210
Latham-Mintus, K.: 1150, 2410-261
Latimer, A.: 1340-99
Lau, D.: 910-268
Laude, T.: 730
Lauderdale, S. A: 1401-281
Lauer, S.: 855-150
Laurienti, P. J: LB935-403
Lautenschlager, N.: 915-290
Laux, J.: LB935-384
Lavacot, J.: 1330-84
Lavayen, S.: 895-231
Lavender, K.: 3340-197
Lawley, M.: 1095
Lawrence, C.: 2405-241
Lawrence, R.: LB1545-391
Laws, H.: 3315-127
Lawson, G.: 3330-190
Lazernick, B.: 3355-247
Le, O.: LB3620-352
Leach, C.: LB2570-326
Leach, C.: 2050
Leal, I.: 930-339
LeBlanc, M. R: LB2570-403
LeBlanc, R. G: 930-342, 1090
LeBlanc, R. G: LB1545-403, LB2570-372
LeBlanc, T. W: LB2570-403
LeBrasseur, N.: 830-67, 835-84, 2510, 3440
Lederman, S.: 2510
Lee, A.: 895-227
Lee, C.: 630
Lee, C.: 3180
Lee, C.: 1440, 3340-203, 4145
Lee, C.: 2410-246
Lee, D. J: 1395-246
Lee, D.: 3340-209
Lee, E.: LB2570-375
Lee, G.: 915-296, 3290-64, 3370-294, LB3620-429
Lee, G.: LB1545-419
Lee, H.: 3030
Lee, H.: LB2570-375
Lee, H. Y: 2140
Lee, H.: 3345-226
Lee, H.: 3325-169
Lee, H.: 2140
Lee, H.: 2430-294
Lee, H.: LB2570-432
Lee, H.: 925-336
Lee, H.: 3340-209, 3370-299
Lee, J.: 3420
Lee, J.: LB2570-410
Lee, J.: 3175
Lee, J.: 2235, 2280, 2365-110
Lee, J.: 3350-236
Lee, J.: LB2570-360
Lee, J.: 880-199
Lee, J.: LB3620-417
Lee, J.: 2215, 3325-168, LB2570-443
Lee, J.: LB1545-388
Lee, J.: 3010
Lee, J.: LB3620-337
Lee, J.: 3320-140
Lee, J.: 3295-69
Lee, K.: 910-278
Lee, K.: 1300-4
Lee, K.: 1365-173
Lee, K.: 3315-126, 3340-198, 3370-294
Lee, K.: LB3620-315
Lee, K.: 2215
Lee, K.: 3325-168
Lee, K.: LB2570-337
Lee, L.: 1305-32
Lee, L. O: 1130, 4100
Lee, L.: 1315-56
Lee, L.: 2410-260
Lee, M.: 3520
Lee, O. E: 4160
Lee, P.: 4005
Lee, S.: 3370-297
Lee, S.: 825-61
Lee, S.: 4045
Lee, S.: 635
Lee, S.: 3300-96
Lee, S.: 880-197
Lee, S. R: 1400-266
Lee, S.: 3275-21, 3580, LB2570-362, LB3620-327
Lee, S.: 3340-208
Lee, Y.: LB2570-432
Lee, Y.: LB2570-374
Lee, Y.: 1300-4, 2405-245
Lee, Y.: 1305-23
Lee, Y.: 1400-267
Lee, Y.: 2340-42
Lee, Y.: 1350-126, 2375-138
Lee, Y.: 1395-242
Lee, Y.: 1395-241
Leech, A. L: 1225
Leedahl, S. N: 905-253, LB2570-422
Lees-Haggerty, K.: 795
Leeth, E.: LB3620-336
Lefler, L. L: 920-311
Légaré, F.: LB1545-441
Legault, V.: 4005
Leger, K. A: 3425
Leggett, A. N: 3110
Lehning, A.: 745, LB2570-434, LB3620-353
Leinenweber, M.: 1350-123
Leiser, S.: 2085, 3385
Leist, A. K: 1400-263
Leite, R. O: 3315-130
Leiter, R. E: 870-181
Lekan, D. A: 2315-8, LB2570-331
Lekhak, N.: 895-239
Lemke, A. L: LB2570-430
Lemmen, T.: LB2570-421

Lenchik, L.: LB1545-390
Lendon, J. P: 910-268
Leng, I.: 805
Leng, S. X: 3280-32
Lenox, M. B: 1315-45, 1330-82
Lensing, S. Y: 920-311
Leon, T.: 2365-109
Leonard, N. M: LB2570-435, LB3620-378
Lepore, M. J: 740
Leritz, E.: 3290-58
Lesandrini, J.: LB3620-334
Leslie, M.: LB2570-422
Lesser, J.: LB1545-384
Lette, M.: 635
Leung, A.: 840-106, 1345-115, 2060
Leung, D.: 2400-227, 2425-287, LB3620-440
Leung, D. L: 2230, 4005
Leung, D.: 3245
Leutwyler, H. C: 1330-91
Leveille, S.: 565, 3220
Levine, M. E: 625, 1400-262, 2230, 2260, 3040, 3440, LB1545-422, LB2570-429, LB3620-333
Levins, T.: LB2570-398
Levis, S.: 3355-255
Levy, B.: 2225
Levy, C.: 1402-314, 4135
Levy, L.: 3500
Levy, S.: 3370-295
Lewinson, T.: 4060
Lewis, C.: LB3620-338
Lewis, J. P: 1405-318, 1425, 2405-243, 4160
Lewis, N. A: LB2570-347
Li, B.: 820-40
Li, C.: 1515
Li, C.: 835-94
Li, C.: 885-210
Li, C.: 1330-89
Li, C.: 1040
Li, C.: 2385-171
Li, D.: 1330-89
Li, F.: LB3620-397
Li, G.: 3325-171
Li, H.: 3350-241
Li, H.: 805
Li, H.: 3280-32
Li, J.: 1515
Li, J.: 2415-267
Li, J.: 3200
Li, J.: 885-210
Li, J.: 920-305
LI, J.: 920-305
Li, J.: 2420-276
Li, L.: 1510
Li, L.: 2030
Li, L.: 855-151, 1350-121, 2400-229, 3275-25
Li, M.: 615, 2010, 3505, LB2570-433
Li, M.: 830-65
Li, M.: 1390-225
Li, M.: 3395, 4160
Li, M.: 3325-176
Li, P.: 845-120
Li, Q.: LB1545-376
Li, Q.: LB935-380
Li, Q.: 855-149, 3375-311
Li, S. z: 4030
Li, W.: 660
Li, W.: 895-230, 1070, 1500
Li, X.: 2313
Li, X.: LB1545-388
Li, Y.: 860-155
Li, Y.: 2375-146
Li, Y.: 1390-235
Li, Y.: LB1545-380
Li, Y.: 810-1, 1297
Li, Y.: 3290-51Lim, M. A
Liang, H.: 835-91
Liang, J.: 1305-30, 1350-122, 3230, LB935-439
Liang, J.: 3320-138
Liang, S.: LB3620-437
Liao, H.: 925-323, 1385-218
Libermann, T. A: 825-48, 4130
Liberty, A.: 2315-7Lichtenberg, P.
Liddle, J.: 850-130
Liddle, J.: 2395-213
Liebzeit, D.: 2315-2
Liehr, P.: 3325-170
Liepert, C. R: LB2570-424
Ligus, K.: 2430-297
Lim, E.: 740, 915-288, 2310
Lim, S.: 850-131
Lim, S.: 3335-196
Lima, J.: 820-34
Lima, J. C: 740
Lima, K.: 860-158
Lima, N.: LB2570-341
Lin, C.: 3420
Lin, C.: 3400
Lin, F.: 2380-166, 2425-291, 2495, 4155
Lin, H.: 3335-195, 3365-283
Lin, I.: 4145
Lin, J.: 925-331
Lin, J.: 905-250
Lin, L.: 845-122
Lin, M.: 3400
Lin, S.: 910-267
Lin, S.: 870-184, LB935-401, LB1545-358
Lin, X.: 925-320, 1060
Lin, Y.: 4050
Lin, Y.: 895-234
Lin, Z.: LB1545-379
Linck, J.: 3350-239
Lind, M.: 4095
Lindauer, A.: 840-104, LB935-436, LB1545-430
Lindberg, B. W: 695, 1230, 1530, 2085, 2285, 2460, 3135, 3515
Lindeman, C.: 850-126
Lindeman, D.: 2295
Linden, W.: LB3620-383
Lindsey, J.: 1195
Liou, C.: 2000, 2390-204
Liou, S.: 3460
Lippe, M. E: 900-243
Lipsitz, L.: 1402-302, 3130
Lipson, A. R: 710
Lipton, R. B: LB3620-318
Litvinchuk, A.: LB935-364
Litzelman, K.: 1355-135
Liu, A.: 3175
Liu, C.: 2270
Liu, C.: 1310-37
Liu, D.: 1040, 1325-73, 1400-259, 3330-191
Liu, F.: LB935-446
Liu, H. J: 4130
Liu, H.: 2340-43
Liu, H.: 1355-137
Liu, J.: 1401-288
Liu, J.: 3120
Liu, J.: 2400-229, 3320-142, 3330-186
Liu, K.: 3280-34
Liu, L.: 2325-30
Liu, M.: 2475
Liu, M.: 920-305, 1105, 1510, LB2570-353
Liu, M.: LB3620-433
Liu, P.: LB935-377
Liu, S.: LB1545-377
Liu, S.: 3090, 3160
Liu, T. T: LB1545-404
Liu, T.: 915-294, 2400-227, 2425-287, LB1545-368, LB3620-440
Liu, T.: 2395-216
Liu, T.: LB2570-327
Liu, W.: 2355-74
Liu, X.: 2400-225
Liu, X.: 3290-56
Liu, X.: 805
Liu, Y.: 3340-201
Liu, Y.: 2313
Liu, Y.: 820-35, 830-70, 835-97
Liu, Y.: 890-214, 915-285
Liu, Z.: 1400-262, 2230, 3325-170, LB3620-333
Liu-Ambrose, T.: 565, 715, 920-313, 1510, 2215, 2360-90, 3280-36, 3295-72
Livingston, D.: 930-341
Ljungman, P.: 850-137
Lloyd, D.: 3320-138
Lloyd, D. A: LB3620-444
Lo, H.: 1330-85
Lo, O.: 1402-302, 4020
LoBuono, D. L: LB2570-422
Locher, J.: 1425, 2070, 3365-274
Lockhart, S.: LB2570-401
Lodi-Smith, J.: 585
Loeckenhoff, C.: LB1545-341
Loeser, R. F: 715
Loew, D.: 2365-107
Loewenstein, D.: 1055
Lofgren, I. E: LB2570-422
Loggia, G.: 825-49
Loh, K.: 855-146
Loh, L.: 3540
Lohman, M.: 3325-164, 3600, LB1545-364
Lohr, s.: 825-43
Lohrmann, D. K: 900-241, 1370-182
Loi, S.: 915-290
Loika, Y.: 820-33
Lokanathan, T.: LB2570-344
Loomer, l.: 3555
Looper, M.: 920-315

Lopez, O.: 3595, 4045
Lopez, T.: 1305-25
Lopez, V.: LB935-431
López-Ortega, M.: 750
Lor, M.: 775
Lord, J. C: 910-262, 2185, 2395-218, 3345-213, 3435
Lorenz, K.: 3535
Losinski, G.: 500, 2410-250
Loter, K.: 3200
Lothary, A. F: 2340-34, 4095
Lou, V. W: 1345-115, 1355-137, 2000, 3250
Lou, Y.: 1340-104, 3320-142, 3330-186
Loup, J.: 1401-286, 2535, LB3620-351
Louras, P.: 2390-196
Lourenço, M.: 1390-222
Lovett, J.: LB3620-364
Lowder, Y. P: 2345-56
Lowenstein, A.: LB935-440
Loza, R.: 2380-157
Lu, A. T: 625, 3405
Lu, J.: 1297
Lu, M.: 3605
Lu, N.: 1340-103, 1435, 3250, 3270-10
Lu, P.: 4145
Lu, S.: 2400-227, 2425-287, LB1545-368, LB3620-440
Lu, Y.: 3605
Lu, Z.: 845-117Lubieniecki, K.
Lucas, J. A: 3555
Lucas, L.: 855-146
Lucero, R.: 1402-294, 2370-120
Luchsinger, J.: 2495
Luci, K.: 1355-145
Ludlow, N.: 880-199
Ludwig, R.: 585
Luetke-Stahlman, H.: 2325-25
Luft, B.: 825-52
Lugo, I.: 3315-130
Lui, K.: LB2570-412
Lukens, E.: 885-212, 3070, 3180
Luk-Jones, S.: LB2570-414
Lukkahatai, N.: 920-305
Lum, H. D: 1335-97, 2385-173
Lum, T.: 845-121, 1345-115, 2400-227, 2425-287, LB1545-368, LB3620-440
Luna, F.: 3320-143
Lundy, J.: 1020
Luo, H.: 3075
Luo, J.: 3330-185
Luo, Y.: 1401-270
Luo, Y.: LB2570-361
Luo, Z.: 3305-109
Luong, G.: 3425
Luppi, F.: 1515
Luster, J.: 540
Lustig, E.: LB2570-373
Luszcz, M. A: LB1545-445
Luth, E. A: 1160, 3310-115, 4010
Luthar, S.: LB2570-402
Luther, S.: 2370-119
Lutz, J.: 3350-243, 4140
Luxenberg, J.: 2010
Luz, C.: 3305-109
Luz, C. C: 3510, LB2570-410
Ly, M.: 825-45
Ly, T. K: LB2570-335
Lydon, E.: LB2570-415
Lyles, M.: LB935-403
Lynch, S.: 805
Lynn, J.: LB3620-326
Lyon, R.: 3355-247
Lyons, D. L: LB935-391
Lyons, K.: 1350-120, LB2570-359
Lyons, M. J: LB2570-348, LB3620-367
Lytle, A.: 4080
Lyytinen, K.: 1401-276

**M**

M Khan, K.: 565
Ma, C.: 1400-262
Ma, C.: 2380-165
Ma, F.: 3285-37
Ma, F.: 1401-286, 2390-196
Ma, M.: 2115
Ma, R.: 2395-219
Macaluso, S.: 875-188
MacArthur, M.: LB1545-362, LB2570-323
MacCarthy, S.: 3430
MacDonald, S.: 2360-105, 3470
Macedo, T.: 3180
Machanda, Z.: LB1545-431
Machu, V.: LB1545-333
Maciel, A.: 3365-281
Mack, D. S: LB935-435
Mack, E.: LB3620-343
MacKenzie, B.: 895-231
Mackey, D.: 2380-161
Mack-Minniefield, R.: 1305-24
Maclagan, L. C: 555
MacNamara, M.: 1090
MacPherson, A.: 1230, 3135
Macro, J.: 830-65
Madden, K.: 565, 920-300
Madden, K.: LB3620-383
Mader, M. J: LB1545-366
Madero, E.: 1402-300
Madigan, J.: 2355-80
Madjaroff, G.: 1401-280
Madole, J.: 1430
Madrigal, C.: LB1545-397, LB2570-329
Maercker, A.: 1330-88
Magaziner, J. S: 820-21, 2210, 4130
Magda, S.: 3320-137
Magid, K.: 4135
Magnottta, V.: LB935-350
Magruder, K.: 2365-111
Mahjabin, J. E: LB3620-338
Mahlo, L.: LB935-387
Mahmoud, K. O: 3320-144
Mahmoudi, E.: 1300-10, 2010, 2325-32, 2545, 3395
Mahoney, E. K: 1235
Mahoney, J.: LB935-422
Mahoney, J. R: 2360-84
Mahoney, K. J: 1235
Maiden, R. J: 2100, 2500
Mailick, M. R: 2375-148
Maillet, D.: LB1545-441
Mair, C.: 745, 2550
Maisano, J.: 3220
Mak, W.: LB3620-393
Makino, M.: LB1545-344, LB2570-320
Malani, P.: 705, 2155
Malatyali, A.: 3295-75
Maleku, A.: 2405-239
Malhotra, R.: 865-167, 2310, 3355-251
Malhotra, V.: 3305-105
Maller, M.: LB3620-422
Mallya, J. U: 2360-96
Malmstrom, T. K: 3350-235
Maloch, E.: LB1545-350
Maloof, M.: 2215
Malta, S.: 915-290
Mama, S. K: 2050
Manalel, J.: LB3620-392
Manda, P.: LB2570-331
Mandi, D.: 2495
Maneix, L.: LB935-373
Manfredi, G.: 3325-158
Mangieri, D. A: LB2570-327
Manheim, C.: 4135
Manini, T. M: 1402-296, 2410-264, LB2570-386, LB3620-403
Manly, J.: LB1545-410
Manna, R.: 635, 1365-174, 3275-20, 3305-105, 4110
Mannick, J.: 4090
Manning, B.: LB1545-362
Manning, C.: 2355-67
Manning, L. K: 1315-44, 3370-285
Manoogian, M. M: 1285, 1350-123, 2560
Manor, B.: 530, 1402-302, 4020, LB3620-328
Manrique Espinoza, B.: 3325-154
Mansfield, J.: 1390-230
Manson, J. E: LB2570-441
Mantantzis, K.: 2275
Manthorpe, J.: 850-138
Manuel-Apolinar, L.: 1310-40
Mao, W.: 615, 1435
Marcantonio, E. R: 825-48, 4130
Marceaux, J. C: 3295-76
Marcelli, C.: 825-49
Marchese, C.: 1410-329
Marcinek, D.: 835-101
Marcione, N.: 920-303

Marcoe, J.: 1401-282
Marcotte, L.: 3280-36
Marcucio, R.: 820-19
Marcum, C. S: 925-331
Marcum, Z. A: 565
Marcus, E.: 2315-9
Marcus, K.: 900-245
Marcus, M.: 3610
Mardini, M. M: 1402-296, 2410-264
Marello, M. M: 890-221, 925-334
Marengoni, A.: 3170, 3325-173
Margot-Cattin, I.: 3565
Margrett, J.: 1270, 2390-202, LB1545-385
Marin-Cachu, A.: 3390
Marini, C. M: 2420-275
Marini, C.: 3320-146
Marino, V. R: 1355-136, 2435-311
Mark, J.: 2555
Mark, J. A: LB935-423
Markey, C. H: 1315-58
Markides, K.: 750, 850-135, 1360-158, 3360-271
Markle-Reid, M.: 1395-245, LB1545-336
Marks, T.: 2385-187, 2400-226
Markt, S.: 1395-243
Markwardt, S.: 1395-248
Markway, B.: LB2570-398
Marlow, N. M: 3330-184
Marmstål Hammar, L.: 3295-86
Marolf, A. J: 835-88
Marota, R.: LB1545-354
Marquard, J.: LB2570-372
Marques, E.: 1402-307
Marques, I. G: 3360-266
Marquez, D. X: 2155, 3360-266
Marras, C.: 555
Marron, M. M: 1425, 3275-24, LB2570-437, LB3620-348
Marrs, S.: 4170
Marsack-Topolewski, C. N: 2505
Marsh, J.: 1315-52
Marshall, G.: LB935-419
Marshall, S.: 1410-331, 2550
Marsiske, M.: 2265
Marszalek, J.: 2340-36
Martie, L.: 3520
Martin, J.: 2395-215, 2420-273
Martin, J. G: 1365-171
Martin, K. J: 1401-281
Martin, P.: 915-296, 925-322, 1350-119, 2440, 3290-64, LB3620-389
Martin, R.: 2315-14
Martinez, L.: 905-255
Martinez, M.: LB1545-386
Martinez, R.: 3105
Martínez, J.: 890-213
Martino, S.: 2250
Martin-Plank, L.: 3120
Martire, L.: 2420-275, 3320-146, LB3620-381
Marty, M.: 3370-291
Marumoto, M.: 2000
Marx, K. A: 885-207, 1005
Maryanna, K.: 845-117
Masinda, S.: LB2570-342
Masliah, E.: 2290
Maslow, K.: 1295, LB3620-362
Mason, K. R: 2270, 3105, 3310-112
Mason, T.: 1330-93
Massimo, L. M: 605
Massimo, L.: 850-136
Mast, B. T: 845-119
Mastergeorge, A.: LB2570-445
Masternak, M.: 835-83, 4175
Masters, J.: 3270-1
Masterson Creber, R.: 4010
Matchar, D.: 715, 3090
Mateus, C.: 2310
Mathews, M.: 2250
Mathews, S. D: 3005
Matook, D.: 1402-295
Matsuda, Y.: 1305-23
Matsunobu, K.: 1390-229
Matsuo, H.: 2010, 2405-238, 3350-235
Matsuura, S.: 1390-220
Mattek, N.: 1401-282, 3535, LB935-436, LB1545-430
Matters, L.: LB3620-349
Matthews, F.: 1200
Matthews, F. E: 1325-67, 2370-134, LB2570-319
Mattos, M.: 2355-67
Matuszek, G.: 3280-33
Mauldin, R. L: 910-278, 1305-15, LB2570-321
Maust, D.: 2155
Mavandadi, S.: 1330-81
Mavroeidi, A.: 730
Mawhorter, S.: 1345-106
Maxey, M.: 3340-201
Maxfield, M.: 2355-68
Maxwell, C. J: 555, 3295-87
Maxwell, D.: LB2570-321
Mayas, j.: 3370-290
Maydan, G.: 710
Mayfield, K. E: 3245
Mayo, D.: LB1545-421
Mays, A. M: 4030
Mazor, K.: 3355-256
Mazula, D.: 835-84
Mazumder, H.: 2400-230
Mazzola, E.: 870-181
Mazzola, P.: 820-31
Mazzone, J. A: 2390-196
Mc Gee, S.: 1330-88
McAdams-DeMarco, M.: 2380-168, 4020
McAiney, C.: 1395-245, LB1545-336
McAlindon, T. E: 715
McAtee, R. E: LB935-420
McCall, G. S: 3290-56
McCarron, H. R: 1385-210, 2355-71
McCarthy, M. J: 1350-120
McCarthy, S.: 3025
McCaskill, G.: 2410-260
McCaughey, D.: 880-199
McCauley, B.: LB2570-352
McCauley, B.: LB3620-384
McCollister, K.: 1395-246
McConeghy, K.: 3255, 4130
McConeghy, R.: LB2570-329
McConnell, E. S: 520, 780
McConnell, E.: LB2570-385, LB3620-349
McConnell, W. R: 2550
McCormick, M.: 3195
McCoy, T.: 2315-8, LB2570-331
McCrae, C.: 670
McCreedy, E.: 705, 3240, 3555
McCrillis, E.: LB3620-373
McCulloch, T.: 920-315
McDannold, S.: 740, 3535
McDarby, M.: 3270-6, 3310-119
McDavid, J.: LB3620-352
Mcdermid, R.: 820-28
McDermott, M.: LB2570-386
McDevitt, K.: 905-252
McDonald, A.: 3330-189
McDonald, L.: 905-255
McDonough, I. M: 3295-79
McDougall, G. J: 3295-79
McDowd, J.: 1402-310, 2340-36
McDuffie, D. L: 2405-231
McEvoy, L.: 1455, LB2570-348
McFadden, S.: 2355-78
McFall, G.: 1360-162
McGarry, B. E: 1375-185
McGee, J. S: 2425-289, 3560
mcGee-Lawrence, M.: 830-73, 835-100, LB3620-330
McGhan, G.: 840-112, 880-199
McGilton, K.: 850-142, 3345-216, 3415
McGinley, J. M: 880-195, 2385-181
McGlinchey, R.: 3290-58
McGory Russell, M.: 2115
McGough, E.: 855-152
McGovern, E.: 3415
McGrew, K.: 3305-110
McGuinness, C.: 2340-40
McGuire, L. C: 1030, 1400-264, 1505, 4110
McGuire, L. C: LB935-362
McIlveen, H. A: 2355-75
McIlwain, A. S: 900-245
McIntosh, T.: 1515, 2355-62, 2430-296, 3140
McIntyre, M.: 4170
McKay, H.: 855-147
McKay, I. T: 3315-120, LB3620-324
McKay, M. A: LB1545-369

McKee, M.: 2010
McKenzie, C.: LB2570-440
McKenzie, G.: LB935-436
McKibbin, C.: 870-186, 1365-168, 2075
McKinney, R. E: 900-243
McKneely, K.: 1345-116
McKoy Davis, J. G: 3045
McKune, A. J: 705
McLane, P.: 4130
McLaughlin, S. J: LB935-402
McLin, C. L: 2340-50
McLinden, D.: 2225
Mclive-Reed, K.: LB2570-442
McMahan, E. A: 2310
McMahon, C.: 4155
McMahon, E. E: 2315-6
McMahon, M.: 2310
McManus, D. D: 1400-260
McMullen, T.: 2385-190
McNair, B.: LB1545-402, LB2570-346
McNally, J.: 3340-197
McNamara, S.: 1190
McNeil, J.: 870-180, 3260
McNeill, L.: LB935-436
McNiel, J.: 3325-164
McPhee, G. M: 3295-81
McPherson, B.: 4155
McPherson, M.: LB3620-355
McPhillips, M. V: 2420-276
McSweeney-Feld, M. H: 2065
Mdaki, K. S: 830-70
Meador, L.: 3220
Meekanon, P.: 3350-235
Meekes, W.: 730
Meeks, S.: 845-119, 1190, 2390-195, 3310-117, 3360-258
Meer, M.: LB3620-407
Meghani, S. H: 840-109
Mehegan, L.: 2340-39
Mehlsen, M.: 1385-205
Mehr, D.: 4135
Mehri, N.: 510
Mehta, H.: LB935-359, LB2570-439
Mehta, H. B: 3355-250
Mehta, N.: 915-286
Meinertz, N. R: 725, 2365-110
Meinhardt, A. J: 1240
Meinow, B.: 3420
Meister, L.: 3340-202
Mejia, A.: LB2570-365
Mejia, S. T: 550, 1350-125, 3190, LB3620-331
Melekin, A.: 2395-214
Melekis, K. A: 2320-20
Meling, M.: LB3620-317
Melipillán, R.: LB935-409
Melzer, D.: 1180, LB2570-393
Mendenhall, A.: 830-78, 3195
Mendes de Leon, C.: 925-337
Mendes de Leon, C. F: 3245
Mendez, J.: 1365-166
Mendez-Luck, C.: 2405-237, 3370-300
Mendialdua Canales, D.: LB935-430
Mendiratta, P.: LB3620-376
Mendoza, N.: 3235, 3320-150
Mendoza Ruvalcaba, N.: 3290-67
Menezes, J. M: LB1545-437
Meng, H.: LB3620-327
Mengel-From, J.: 1310-42
Menne, H. L: LB3620-323
Menon, V.: LB935-345
Meraz, R.: 3560
Mercado, C.: LB935-420
Merdjanoff, A.: 2540
Merrilees, J.: LB2570-336
Mertz, K.: 820-30
Meshoulam, I.: 1055
Messecar, D.: LB1545-430
Messi, M.: 820-25
Messier, S.: 3325-175
Metti, A.: 3075
Metzelthin, S.: 2380-164
Metzger, A.: 3350-243
Meucci, M. R: 1170
Meuser, T. M: LB1545-421
Meyer, K.: 1355-150, 2375-144, 2465, 3375-313
Meyers, K.: 3330-183, LB2570-366
Meyers, M.: 885-204
Meystre, C.: 2340-49, 3315-123
Mezuk, B.: 2410-252
Mi, H.: LB3620-319
Miao, H.: 1402-313
Michalek, J.: 3325-160
Michalowski, E.: 2435-307
Miclau, T.: 820-19
Micoli, T.: 2155
Midão, L.: 1402-307, 3325-158
Middlemass, K.: LB2570-399
Middleton, A.: 850-139, 4135
Mielcarski, J.: LB2570-438
Mielenz, T. J: 3325-171
Mielke, M.: 3075
Miescier, L.: 2385-184
Migliaccio, J. N: 3610
Mikkelsen, M. B: 1385-205
Milanez, M.: 2495
Milani, S. A: 3220
Milberg, W.: 3290-58
Miles, T.: 1000, LB2570-370
Miljkovic, I.: 2370-125
Miljkovic, I.: 2370-136, LB3620-348
Millar, R. J: 1325-71
Millar, R. J: 1345-118
Millar, R. J: 1400-259, 1401-290, 3300-99
Miller, B.: LB2570-439
Miller, E. A: 910-261, 1065, 1095, 2385-180, 3280-35, 3555, 3570, 4050
Miller, J.: 3555
Miller, J.: LB3620-341
Miller, J.: 835-84
Miller, J.: 660, 1375-184, 3180
Miller, K.: 1150
Miller, L.: LB3620-389
Miller, L.: 3595, 4045
Miller, L.: 2380-169
Miller, L. M: 3535, LB2570-357
Miller, M.: LB1545-364
Miller, M. G: 2380-167
Miller, N.: 3330-188
Miller, S.: 3345-224
Miller, S. C: 740
Miller, T.: 2435-312
Miller, V. J: 1480
Millett, G.: 820-20
Milligan, C.: 2310, 2430-301
Mills, W. L: 910-275, 3345-219
Milosevic, M.: 1402-306
Min, J.: LB3620-315
Min, L.: 4130
Min, S. N: 1400-252
Minahan, J.: 2080
Minahan, J.: 655, 3290-49
Minami, S.: 825-43
Minami, S.: 3270-4
Mingo, C. A: 770, 1160
Minkman, M.: 1365-176
Minster, R.: 1310-42
Minton, D. M: 835-88
Mion, L.: 845-117
Mireles-Cabodevila, E.: 2315-13
Mirelman, A.: 1402-304
Mirza, R. M: 905-255
Mishra, S.: LB2570-387
Mitchel, J.: 3275-22
Mitchell, G.: 850-130
Mitchell, J.: LB1545-362, LB2570-323
Mitchell, L. L: 1390-236, 1401-278, 2325-21
Mitchell, M.: 2395-215, 2420-273
Mitchell, S.: LB1545-362, LB2570-323
Mitchell, S.: 3555, LB1545-389
Mitchell, U.: 2280
Mitnitski, A.: 820-23, 825-55, 3325-167
Mitoraj, M.: 3275-19
Mittelman, M.: 2155
Mittlieder, C.: 895-236
Mitzner, T. L: 3500
Miyawaki, C. E: 1025, 2000, 3115
Mize, D.: 895-229
Mizuno, A.: 825-45
Moaddel, R.: LB3620-364
Mobley, T.: 835-100
Mochel, A.: 3345-219
Modrek, S.: 4065
Mody, L.: 4130

Moeke-Maxwell, T. H: 2270, 3105, 3310-112
Moellering, D. R: LB2570-371
Moen, P.: 4065
Moffat, S.: 1270
Moffatt, E.: 1325-67
Moffitt, T.: LB1545-347
Mogle, J.: 1175, 3290-52
Mogle, J.: LB1545-397
Mohaimin, S.: 850-131
Mohanty, S.: LB2570-331
Mohler, J.: 820-26
Mohr, D.: 3255
Mois, G.: 1401-287
Mois, G.: LB3620-329
Mojadam, A.: 2315-6
Mokhtari, B. K: 815-16, 2425-288
Molenaar, P.: 3295-69
Molhman, J.: 1401-281
Molnar, L. J: 1410-331, 3325-171
Molony, J.: 3305-108
Molony, S. L: 2315-5
Molton, I.: 855-148
Monaco, W.: LB3620-327
Mondadori, R.: 835-83
Monin, J. K: 815-17, 3315-127, LB2570-392
Moninger, S.: 1402-308
Monroe, T.: 845-117
Monserud, M.: 1150
Montano, A. L: 1365-169
Monteiro, A.: 930-339
Montemuro-Rode, L.: 1340-99
Montepare, J. M: 1420, 2220, 2560
Montero-Odasso, M.: 2095
Monti, S.: 835-92, 3215
Montoro-Rodriguez, J.: 1460
Montoya, Y.: 2155, 3360-269
Moo, L.: 1295, 2055
Moody, M.: 1315-47
Moon, H.: 2355-69, 3360-267
Mooney, A.: 865-166
Moore, A.: 1330-76
Moore, A.: 4050
Moore, C. S: 2395-212
Moore, J. M: 2340-44, 2425-288
Moore, K. J: 915-290
Moore, S. E: 1330-75, 1395-243
Moore, S. C: 1425
Moore, T.: 2395-212
Moore, Z. D: 1380-194
Moored, K.: 4095
Moorman, S. M: 720, 2235
Moorthi, R.: 820-18, 2410-261
Mor, V.: 705, 3140, 3555, 4130
Morales, X.: 2495
Moran, D.: 3545
Morani, A.: LB1545-361
Morano, C.: 880-192
Morehouse, S.: 1380-192
Morello, R.: 825-49
Moreno, G.: 2380-157, LB3620-408
Morey, M. C: 1330-83, 2345-56, LB1545-347
Morgan, A.: 3330-190
Morgan, B.: 870-179
Morgan, J. C: 2305, LB3620-321
Morgan, L.: 2395-219
Morgan, T. E: 825-50, 830-64
Morhardt, D.: 710, 2155
Mori, C.: 2340-40
Morita, H.: LB1545-344
Moro, T.: LB3620-345
Morris, D. L: 875-187
Morris, D. L: 3510
Morris, E. P: LB1545-434
Morris, J.: 3175
Morrow-Howell, N.: 845-118, 1420, 2030, 3235, 3395
Morthland, M.: 2310
Morton, P. M: LB2570-423
Moryl, N.: 3305-105
Moscou-Jackson, G.: 3140, LB935-416
Mosier, A.: 3375-306
Mosley, T.: 2495, LB2570-401
Moss, K. O: 3100
Moss, M. S: 1185
Mota, M.: 895-235
Mota, T. M: 2395-212
Motel-Klingebiel, A.: 865-178
Mouchawar, A.: 840-105
Moulder, R.: 2005
Mouton, P. R: 1310-39
Moxley, J.: LB935-417
Moye, J.: LB2570-409
Moyle, W.: 505, 1405-322
Moyo, P.: 4130
Mroczek, D. K: 2110, 3340-206
Mroz, T.: 855-148
Mrug, S.: 1360-154
Mtsunaga, H.: 895-226
Mudar, R. A: LB2570-415
Mudrazija, S.: 880-191
Mudzonga, C.: 860-160
Muehlbauer, M. J: LB2570-437
Mueller, C.: 1470, 4050
Mueller, C.: 2410-263, 3535, 4120
Muggy, T.: 2370-135
Mui, A.: 1405-316, 3320-142
Mukamal, K.: 3595
Mukamal, K. J: LB2570-389
Mulasso, A.: 3325-159
Mulcahy, J.: 1170, 3420
Mulhorn, K. A: 3375-314
Mullen, N.: 2550
Mullen, S.: 3350-240
Muller, M.: LB1545-431
Mullins, R.: 3295-71
Muma, A.: 3330-179
Mun, J.: LB935-374
Mundy, S.: LB3620-370
Muniz, P.: 1390-222
Muniz Terrera, G.: 3210, LB2570-347, LB3620-318
Munkhtogoo, D.: 2385-182
Munly, K.: 1445, 3465
Munoz, b.: 3295-73
Munoz, E.: 925-338
Munoz, L.: 895-231
Munshi, M.: 3050
Muppidi, N.: 825-45
Murabito, J.: 1525, 3405, LB2570-396
Murakami, K.: LB1545-396
Muralidhar, K.: 1320-61, 1330-94, 1402-299, 2350-59
Muralidharan, A.: LB1545-371
Muramatsu, N.: 1095
Murata, W.: 830-63
Murawski, P.: 855-150
Murayama, H.: 885-205
Murayama, H.: 3270-4
Murfin, K.: LB1545-337
Murphy, E. R: LB3620-405
Murphy, I.: 1305-28
Murphy, J.: 2345-58
Murphy, R.: LB3620-383
Murphy, T. E: 3315-127
Murphy, T. K: 3275-15
Murray, A. M: 870-180, 3260
Murray, K.: 3345-212, 3435
Murray, L. A: 2390-205
Murray, L. M: LB3620-436
Muruthi, J.: 3480, LB1545-342
Musi, N.: 820-39, 830-76, 835-91, 1310-41, 3325-160, LB2570-399
Musich, S.: 1315-60, 1400-268, 2420-270, LB935-355
Musil, C. M: 890-216, 2385-177, 3320-141, 1350-129, 1460
Mutchie, H. L: 2370-122
Mutchler, J.: 860-155, LB3620-359
Muzaale, A.: 2380-168
Muzzy, W.: 1055, 2225, 2365-107
Myers, D. R: 2425-289
Myers, D.: LB2570-415
Myers, L.: LB1545-417

### N

Naar, J.: 2435-314, 3315-128, 3340-200
Nabholz, L.: 2365-106
Nabors, E.: 3270-3
Nace, D.: 3390
Nadash, P.: 910-266, 1405-323, 2385-180, 3280-35, 3570, 4050

Nadorff, D. K: 2200, 3315-120, 3320-148, LB3620-324
Nadorff, M. R: 915-291
Nagamatsu, L.: 3295-68
Nagel, C.: 1395-248, LB1545-356
Nägga, K.: 2215
Naglie, G.: 1410-329, 2550
Nagpal, R.: LB2570-387
Nagy, T. R: LB2570-371
Nah, S.: 3320-146
Naidoo, N.: LB1545-342
Naik, A.: 850-145, 3345-219, 3560
Naim, S.: LB935-440
Naiomi, R.: 3320-134
Naito, T.: LB2570-320
Najafi, B.: 1402-297, 2360-99, 3325-166, LB935-381
Nakagawa, T.: 865-171, 1325-69, 3340-210, 3370-293
Nakajima, M.: LB1545-344
Nakajo, K.: 895-232
Nakamura, J.: 1540
Nakamura, M.: 820-19
Nakayama, R.: 925-319
Nakazato, Y.: 4005
Nambisan, P.: 1401-276
Namihira, Y.: 3280-30
Namkung, E.: 640, 1300-3
Narasimhan, B.: 1270, LB1545-385
Narita, M.: 3340-204
Naruse, T.: 895-225
Naseh, M.: 1390-237
Nash, P.: 1355-150, 1420, 2375-150
Nath, R.: 3370-301
Nathanielsz, P.: 835-94
Nation, D. A: LB3620-411
Natonson, A.: LB1545-430
Naumovski, N.: 705
Navar, E.: LB935-351
Navarette Muñoz, E.: LB935-430
Navarro, E.: 1402-296
Naylor, M.: 2215, 3230
Neal, M. B: 3185
Nearing, K. A: 1335-97, 2385-173
Nedjat-Haiem, F. R: 1000
Nedjat-Haiem, F.: 3275-22
Needham, B.: 925-337
Neelamegam, M.: 3070, 3075
Neese-Todd, S.: 910-274
Neff, L.: 1350-132
Nehlin, J.: LB2570-339
Nehrkorn-Bailey, A. M: 890-221, 925-334
Neiberg, R.: 3325-175
Neiberg, R.: 3325-155
Neiva, C. M: 895-235
Nelson, C. L: 1380-196
Nelson, C.: 3275-20
Nelson, E. L: 2340-47
Nelson, H.: 2390-198
Nelson, H.: 710, 2345-57
Nelson, M.: 870-180
Nelson, M.: 3555
Nelson, M.: LB3620-391
Nelson, N. A: 3560
Nemoto, Y.: 895-226
Neppl, R.: 820-40
Nerella, S.: 2410-264
Nett, B.: LB2570-421
Nettlefold, L.: 855-147
Neubauer, N.: 2325-31
Neugebauer, V.: LB935-431
Neumann, A.: 900-246
Neupert, S. D: 700, 2040
Neupert, S. D: 3350-246
Newell, J.: 920-299
Newman, A. B: 565, 1425, 3275-24, 3440, 3595, LB1545-432, LB2570-396, LB3620-348, LB935-368
Newman, D.: LB935-379
Newman, M.: 635
Newsom, J. T: 1395-248
Newton, N. J: 1140
Neyland, B. R: LB935-403
Ng, B.: 2360-88
Ng, H.: 2400-223
Ng, I. H: 2360-95
Ng, J.: 2250
Ng, R.: 2360-96
Ng, T.: 715
Ng, W.: 910-276, 1405-324, 2390-200
Ng, Y.: 925-316, 1350-134
Ngo, L. H: 825-48, 4130
Ngo, S.: 2315-1
Nguyen, A. W: 2405-232, 3475
Nguyen, C. M: 3350-239
Nguyen, H. M: 3365-272
Nguyen, H.: 1402-297, 2360-99, 3325-156
Nguyen, H. Q: 3175
Nguyen, L. T: LB2570-415
Nguyen, M.: LB1545-334, LB2570-377
Nguyen, Q.: 1345-116
Nguyen, T. T: LB2570-369
Ni, L.: LB3620-336
Ni, P.: 3535
Ni Leime, A.: 560, 865-169, 4125
Nichols, J.: 2430-298
Nichols, K.: 1320-64
Nichols, L.: 3165
Nichols, M. J: LB935-370
Nicholson, H.: 2425-284
Nicklas, B. M: 3325-155, 3590, LB935-403, LB1545-387
Nickols-Richardson, S.: 2360-100
Nicolay, S.: 3300-93
Nicosia, F.: 3485
Niculescu, I.: 2355-61
Nie, J.: 1310-41
Nie, Q.: 1445, LB2570-415
Nielsen, K.: LB2570-435, LB3620-378
Nieman, C. L: 4155
Niemeyer, C.: 3315-122
Nightingale, G.: 855-146
Nijpels, G.: 635
Niles-Yokum, K.: 735, 1265
Nishide, K.: 1305-23
Nishimoto, M.: 895-232
Nishino, A.: 1305-23
Nishita, C. M: 2325-22
Niznik, J. D: 3390
Nkimbeng, M.: 3360-268
Noah, A.: 3375-305
Noblitt, J. A: 1380-193
Nobrega, P.: 3365-281
Noel, O. R: 1390-226
Noguchi-Watanabe, M.: 895-225
Noh, H.: 1260, 1315-56, 2390-201, 2395-211, 3300-98, 3345-228
Noh, J.: 820-32
Nonaka, K.: 895-226
Noppert, G. A: 3095
Nordahl, T.: LB3620-399
Noriega de la Colina, A.: 3280-29
Norman, S.: 2380-168
Norris, K.: 3360-260
Northrip, K.: 505, 3375-303
Northrup-Snyder, K.: 1360-160
Norton, B.: LB2570-445
Norton, E.: LB1545-377
Notthoff, N.: 3615
Novak, J.: LB2570-326
Novik, N.: 1515, 2355-62, 2430-296, 3140
Nowacek, N.: 4080
Nsobundu, C. K: 1360-151
Nuccio, A.: 1380-194, 1405-321
Nuccio, A. G: 3370-287
Nunes, T.: 2325-28
Nwakasi, C. C: 805
Nwankwo, I.: 2565
Nyandege, A.: 850-143, 4135
Nygard, L.: 3565

### O

O Malley, K. A: 880-193, 2325-25, 3300-97, 3320-149
O' Neill, S.: 4055
Oakes, C. E: 2435-307
Öberg, P.: 3200, 3315-124
O'Brien, C.: 2340-33, LB935-367
O'Brien, T.: 2410-254
Obuchi, s.: 855-154, LB2570-411
Ochsner, K.: 1385-219
OConnor, M. L: LB3620-441
O'Connor, D.: 2340-33, LB935-367
O'Conor, R.: 3595
Odd, K.: 865-177
Odden, M.: 3595, 4045
O'Dea, E.: 3285-39
Odejimi, A.: LB3620-345

O’Donnell, D.: 2385-178, 3025
Ofstedal, M.: 1305-30, 1350-122, 3230
Ogawa, E. F: 2255
Ogawa, E. F: LB1545-363
Ogino, R.: 925-319
Ogita, A.: 830-63
Ogletree, A. M: 1315-51
Oh, G.: 2340-35
Oh, G.: LB2570-375
OH, H.: 1395-242
Oh, P. A: 1010, 1305-32
O’Hair, M.: LB1545-419
OHanlon, C. E: 3530
O’Hanlon, A. M: 2340-50
Ohya, Y.: 3280-30
Ojeranti, D.: 3590
Okamoto, S.: LB935-439
Okata, J.: 925-330
Okoye, S. M: LB2570-382
Okubo, S.: LB2570-444
Oladimeji, A.: 890-218
Olde Rikkert, M.: 1402-304
Oleskiewicz, D.: 925-325
Olinger, S.: 2435-310
Oliva, C.: 2325-31
Olivari, B.: LB935-362
Oliveira, M.: 2360-87
Oliveira, R. F: 895-235
Ologhobo, T.: 1365-179
Olsen, B.: 2385-188
Olson, A.: 825-46
Oluwaleimu, O.: 890-220
Olzmann, A.: 1355-142
Olzmann, A.: 2375-149
Omar, A.: 3415
Onder, G.: 3170, 3325-173
O’Neal, A.: LB2570-425
O’Neil, A.: 3275-18
O’Neill, D.: 1490
O’Neill, H.: 3600
Ong, A.: 3340-206
Ong, A.: 4100
Ono, S.: 3270-4
Op het Veld, L.: 3270-5
Opalinski, A.: LB935-379
OPTIC Research Team, C.: 4130
O’Rand, A.: 2205
Orange, J.: 2355-75, 3560
Orav, E. J: LB2570-367
Ornelas, E. X: 925-335
Ornstein, K.: 840-115, 1260, 2280
Orozco, T.: 840-108, 3320-137
Orr, M. E: 820-39, 825-56
Orsak, B.: 835-91
Orsulic-Jeras, S.: 1160, 3300-93
Ortega, F.: 1402-312
Ortega, J.: 2360-91
Ortenberg, E. Kwee: 1125
Ortiz, L.: LB935-363
Orwig, D.: 820-21, 2370-122, 3330-188
Orwig, D.: 4130
Orwoll, E. S: 820-22
Orwoll, E.: 1120
Ory, M. G: 635, 3300-96, 3320-136
Orzeck, P.: 2045
Osawa, Y.: 820-24
Oseau, T.: LB3620-425
Oslin, D.: 1330-81
Ossenfort, K. L: 1385-209
Ostbye, T.: 865-167
Osteen, K.: 3560
O’Sullivan, R.: 2500
Osuna, M.: 4000
Oswald, A.: 3445
Otali, E.: LB1545-431
Otieno, D.: 2130
Otmanowski, J. A: LB1545-338
O’Toole, E.: 875-187, 3510
Otsubo, M.: LB2570-345
Otsuki, T.: 1305-23
Ottenbacher, K.: 850-135
Ottenbacher, K.: 850-139, 2385-171, 4020, 4135
Otters, R.: LB3620-434
Ottmar, H.: 1355-142, 2375-149
Otu, H.: 825-48, 4130
Ou, A.: 1515
Ou, T.: 3335-195
Ouchida, K.: LB3620-396
Oude Mulders, J.: 865-172, 3000
Oude Veldhuis, S.: 3230
Oura, M.: 820-27
Ouyang, M.: 3280-32
Overcash, J. A: 3275-26
Overholser, J. C: 1330-84
Owczarzak, J.: 3275-14
Owen, D. C: 2395-207
Ownby, R.: LB3620-379
Owusu, C.: 1395-243
Oyetunji-Alemede, C. O: 890-220
Ozer, E.: 1402-312

### P

Pace, G. T: 890-222, 1075, 1345-111
Pachana, N. A: 850-130
Packard, K.: LB935-420
Paes, A. T: LB1545-437
Pagnini, F.: 2340-38
Pahlen, S.: 1425
Pahor, M.: LB3620-403
Pais, I.: 1515
Pajewski, N. M: 3590, 4045
Palazzo, L.: LB2570-368
Palekar, N.: 825-52
Palimaru, A.: 3305-104
pallaki, m.: 1320-63
Palmarini, N.: 1515, 3520
Palmer, J.: 3555
Palmer, L. A: 2385-190
Palmer, R. F: 1360-158, 3275-16
Palmer, T.: 2310
Palmisano, K.: LB935-443
Palta, P.: 3075
Pan, A.: 1300-12, 3360-271
Pan, H.: 3250
Pan, J.: 3360-265
Pan, X.: LB2570-361
Pan, Y.: 895-233
Pan, Z.: LB2570-393
Panayi, A. C: 820-40
Panesar, M.: 855-150
Pangle, A.: LB3620-369
Papenkov, M.: 2155
Papp, K. K: 875-187
Parajuli, J.: 3010
Parayil, M.: 930-341
Parekh, R.: 1500, 2405-239
Park, C.: 2385-175
Park, D.: 880-192
Park, E.: LB3620-436
Park, E.: 2340-51
Park, G.: LB2570-398
Park, G.: 3325-172, 3330-189
Park, H.: 640
Park, H. K: LB1545-425
Park, H.: 890-215
Park, J.: 3460
Park, J.: 4160
Park, J.: 3295-85, 3325-170
Park, N.: 1325-70, 2405-235
Park, S.: LB2570-337
Park, S.: 515, 845-118, 2030, 2430-294, LB3620-335
Park, S.: 1310-35, 3290-60
Park, S.: 2405-245
Park, Y.: 910-258
Parker, B. M: 925-328
Parker, D. C: 820-41, 4130
Parker, D.: 505
Parker, L.: 1450
Parker, S. D: 3355-247
Parks, C.: LB2570-322
Parks, J.: 835-87
Parmelee, P. A: 915-281, 1095, 1385-217, 1400-264, 2375-142, 2415-265, 3290-55, 3365-279
Parniak, S.: LB3620-368
Parra-Rodriguez, L.: 3590
Parsons, P.: 2425-280, 3300-94
Paryani, F.: LB935-345
Pasinetti, G. M: 825-57
Pasquini, G.: 3290-52
Pasta, P. M: 1380-200
Pastells-Peiró, R.: 3345-216
Pastva, A. M: 2345-56
Patel, K.: 1305-33
Patel, K.: 570
Patel, M.: 3535
Patel, P.: 830-65
Patel, P.: 1360-164
Patorno, E.: 3050, 3325-169
Patrick, J.: 1345-112
Patskanick, T.: 3180
Pattanaik, S.: LB1545-391
Patterson, A. D: LB3620-347
Patterson, M. E: 3390
Patterson, W.: LB3620-327

Patton, S. K: 3330-181, LB1545-343
Paudel, A.: 2390-194
Paul, S.: 710
Paúl, C.: 880-201
Paúl, C.: 1402-307
Paúl, C.: 2325-28
Paúl, C.: 3325-158
Paules, S.: 2325-28
Paulson, D.: 1320-62, 1355-140, 2550, LB1545-353
Paulson, H.: 500
Pauly, T.: 2275, LB3620-383
Paun, O.: LB2570-419
Pavão, R.: 1390-222
Pavon, J. M: 1402-308
Pawar, A.: 3050, 3325-169
Payer, C.: 2390-206
Payne, M.: 2380-167
Peacock, E.: 1315-52
Pearman, A.: 3350-246, LB2570-373
Pearsall, V.: 835-84
Pearson-Littlethunder, J.: 1385-208
Pebole, M.: 1330-83
Pedersen, N.: 1215
Peek, G.: 3320-145
Pei, Y.: 1105, 2340-43, 4115
Peitz, L.: 2365-110
Pel-Littel, R. E: 1365-176
Penate, C.: 2385-188
Pence, B.: 835-85
Pendergrast, C.: 1405-320
Peng, C.: 3270-10
Peral Gómez, P.: LB935-430
Perales Puchalt, J.: LB935-437
Perego, R.: 820-31
Peremen, Z.: 1402-293
Perez, A. G: 605
Perez, E.: 1390-239, 2420-272
Perez, K.: 2375-140
Perez, V. I: 1520
Pérez-Zepeda, M.: 565, 3590
Perion, J.: 1355-146, 2355-73
Perissinotto, C.: LB3620-425
Perkhounkova, Y.: LB2570-364
Perkins, M. M: 885-211, 2485, 3270-9, LB2570-351, LB3620-321
Perkins, R.: 2325-24, LB935-358
Perkinson, M. A: 2220
Perkovic, S.: 2400-222
Perls, T. T: 835-92, 1120, 2345-55, LB2570-338
Perone, A.: 3430
Perrig-Chiello, P.: 3200
Perrin, N.: LB2570-382
Perry, M.: 3175
Perry, T.: 4060
Perskinson, M.: LB3620-374
Perweiler, E.: 885-204, 4075
Perzynski, A. T: 835-86
Pessoa-Filho, D.: 895-235
Pestine-Stevens, A.: LB2570-388
Peters, O.: LB3620-318
Petersen, J.: LB2570-339
Peterson, C. M: 1385-210, 2375-140
Peterson, J. R: 1325-66, LB935-360
Peterson, L. J: 1405-325, 3345-215
Peterson, M.: 1395-240
Peterson, M. J: 3170
Peterson, N. L: 2280
Peterson, R. L: 3120
Peterson, T. L: 1350-133
Petkus, A. J: LB2570-441
Petropoulos, A.: 2045
Petroski, G.: 4135, LB2570-334
Petrovsky, D. V: 670, 1160, 1435, 2215, 2420-276, 2530
Petti, E.: 3220, 3365-282
Pettis, J.: 2395-212, LB1545-335
Petty, R.: 1315-52
Pfund, G. N: 585
Pham, A.: 850-126
Pham, T.: 885-210
Pham, T. N: 850-136
Phan, R. V: LB935-346
Phelan, A. A: 2385-178, 3025
Philips, K. L: 1330-84
Phillips, C.: 1150, 3290-61
Phillips, J. R: 2065, 2425-284, 2450
Phillips, K.: 3365-272
Phillips, L. R: 3120
Phillips, L. J: LB2570-334
Phillipson, C.: 510, 3085
Phinney, A.: LB3620-344
Phipps, K.: 2385-185
Phoenix, A.: LB935-418
Phongtankuel, V.: 3310-118
Piao, Y.: 835-99
Picchiello, M.: 3310-116
Piccinin, A.: 3340-206, LB2570-347
Pichiello, M.: 3270-6
Pichora-Fuller, M.: 850-142
Pickering, A. M: 825-62
Pickering, C. E: 2375-144, 2465, 3375-313
Pidgeon, D.: 3545
Piedra, L. M: 3360-270
Pieper, C.: 3365-275, 4130, 4145
Pierce, G.: LB935-350
Pierce, L.: 1355-147
Pierce, L.: 1355-146
Pierpaoli Parker, C.: 2420-277
Pietrobon, R.: 2240
Pietzak, L.: 2385-185
Pike, J.: 1160
Pillai, V.: 2250
Pillemer, K.: 1250, 2085, 3235, 3315-131, 3365-282
Pilling, L. C: 1180, LB2570-393, LB3620-333
Pilonieta, G.: LB2570-391
Pinheiro, A.: LB2570-325
Pinto, J.: 2425-291
Pinto Pereira, S.: 685
Pirkle, C. M: LB935-426, LB1545-392
Pirkle, C. M: LB2570-421
Pisegna, J.: 1315-55
Pitt, J.: 830-81
Pizzi, L. T: 1005
Pizzini, J.: 835-93
Plassman, B. L: LB2570-322
Platt, J.: 880-197
Platt, J.: 2170
Platts-Mills, T.: 795
Pleasants, D.: 850-132
Pless Kaiser, A.: 3300-97
Plocher, T.: 1385-214
Ploeg, J.: 1395-245
Plotkin, E.: 855-146
Plotzke, M.: 520
Plude, D.: 1125
Plys, E.: 3270-13
Podolskiy, D.: 625
Poey, J.: 2515
Poggio, J.: LB2570-425
Poggiogalle, E.: 1402-298, 3325-159
Pohl, J. S: 2295, 4150
pohnert, A.: 895-240
Polekhina, G.: 870-180
Polenick, C. A: 725, 3425
Polivka, L.: 1220, 3085
Polk, B. K: 1385-207
Pollak, K. I: LB2570-403
Polson, C.: 2425-289
Polter, E.: 2380-155
Pomilio, C.: 825-54
Poon, L.: 925-322, LB3620-389
Pope, C. N: 1360-156
Pope, N.: 1305-14
Popejoy, L. L: 4135
Popescu, M.: 820-37
Popham, L. E: 3415
Popoola, B. I: 3300-100
Popovich, A.: 2425-280
Portacolone, E.: 2010, 2215, LB2570-336
Porter, H.: 505
Porter Starr, K. N: 2070, 2380-167
Porterfield, S. L: 2030
Portz, J. D: 1080, 1401-291
Post, K.: 1355-148
Potapragaa, N. R: 2385-176
Pothen, J.: 3340-207
Potter, K.: 1090
Poulin, L.: 2105
Powell, D.: 3075
Powell, M. L: LB2570-371
Power Cyr, J.: 840-112
Powers, B.: 1335-96, 3375-304

Powers, D. A: 3335-196
Powers, J. S: 2565
Powers, S.: LB3620-352
Prabhu, A.: 1402-309
Pradeep Kumar, D.: 820-26
Pradiee, J.: 835-83
Prakobchai, S.: 3350-235
Prasad, A.: 3100
Prater, L.: LB2570-345
Prescott, C. A: 655
Presnell, A.: 2310
Pretter, S.: 2430-300
Pretzer-Aboff, I. A: LB2570-356
Price, N.: 1120
Prieto, A.: 3370-290
Prieto, L. R: LB935-432
Prina, M.: 4040
Prins, M.: 840-110, LB1545-414
Prior, S. J: 3020
Pristavec, T.: 1160, 3310-115
Prizment, A.: 710, 2345-57
Proff, K.: 2325-23
PROMISE Study Group: 3355-251
Promislow, D.: 1245
Propson, N. E: LB935-364
Proulx, C. M: LB3620-332
Pruchno, R.: 1315-51, 2520
Pruett, M.: 3535
Pruitt, A.: 2405-241
Prusak, A.: 2415-269
Prvu-Bettger, J.: 630
Pu, C. T: 870-181
Pud, D.: 2315-7
Puga, F.: 2375-144, 3375-313
Pugh, M.: 3295-76
Pulok, M.: 3590
Pupo, D.: 3295-71
Puro, N.: 2185
Pusopa, C.: 3350-235
Putnam, M.: 1235
Puurveen, G.: LB3620-344
Pynoos, J.: 3270-3

### Q

Qato, D.: 3355-253
Qian, X.: 2125
Qiao, Y.: LB2570-343
Qin, W.: 4150
Qin, X.: 2155
Qiu, P.: LB3620-403
Qiu, X.: 4150, LB935-402
Quach, E.: 740, 3535
Quach, L.: 1360-163, 2255
Qualls, S. H: 1100, 2375-143, 3320-149
Quan, N. S: 885-211
Quan, N.: 3600
Quest, T.: 3270-9
Quinn, C. C: 530, 1402-311
Quinn, K.: 1401-283, 3115
Quiñones, A. R: 1395-248, 1450, 3325-163, LB1545-356
Quintana, C.: LB935-384
Qvist, J. Y: LB2570-318

### R

Rabinovitch, P.: 835-101
Radhakrishnan, K.: 1395-247, LB1545-419
Radusky, P.: LB935-417
Raftery, J. P: 2380-161
Rager, J. R: LB1545-432
Raghavachari, N.: 3215
Rahman, A. N: 1340-98
Rahman, A.: 3330-183
Rahman, A.: 3330-182
Rahman, A. N: LB1545-443
Rahman, M.: 3140
Rahman-Filipiak, A. M: 500
Raichur, V.: LB2570-435, LB3620-378
Rainoldi, A.: 1402-298, 3325-159
Rainville, G.: 1401-279, 2340-39, 2420-271, 3410, LB3620-366
Raitanen, J.: LB1545-339
Raj, M.: 880-197
Raji, M.: 850-135, 3355-250, LB1545-427
Rajpara, A.: 915-289
Raldiris, T.: 3370-288
Ram, N.: 2005
Ramachandran, A.: LB3620-329
Ramadhani, W. A: LB1545-373
Ramel, A.: 920-309, 2370-126
Ramesh, S.: LB935-366
Ramirez, A.: LB3620-318
Ramírez-Aldana, R.: 3590, 4155
Ramos, A. M: 1315-45
Ramsay, J.: 3365-280
Ranahan, M.: 3330-187
Randall, G. K: 1475
Randolph, A.: LB3620-345
Ranger, V.: 3595
Ranjit, N.: 2360-86, LB935-441
Ranka, S.: 2410-264
Rantz, M. J: 4005
Ranville, M.: LB2570-424
Rapoport, M.: 1410-329, 2550
Rapp, S. R: 850-132
Rasgon, N.: LB935-442
Rashidi, P.: 2410-264
Rasmussen, B.: LB1545-354
Rasmussen, B.: LB3620-345
Rasmussen, L.: LB1545-347
Rasmussen, L.: LB2570-339
Rast, P.: LB2570-355
Rasticova, M.: 4125
Ratcliffe, S. J: 2360-103
Rath, L.: 1355-150
Rath, L.: 2375-150, 3010
Ratner, E.: 3305-106
Ravdin, L.: 3220
Raveis, V. H: 2430-300
Ravi, K. E: 1305-28
Ravyts, S.: 1390-239
Ravyts, S.: 2420-272
Ravyts, S.: 2425-282
Rawlings, A.: 2495, 3595
Rawson, K.: LB3620-399
Ray, M. A: 3290-51
Raymo, J.: 1305-30, 1350-122, 3230, 4065
Rayner, J.: 505
Raz, N.: LB935-366, LB3620-326
Razaq, W.: LB3620-358
Reales, j.: 3370-290
Rebok, G. W: 590, 1125, 2265, 2410-259, 2425-291, 3350-245, 4025
Reckrey, J. M: 840-115, 1155
Recksiedler, C.: 3030, 3200
Rector, J.: 2005
Rector, K.: LB2570-395
Reed, C.: 815-16, 2425-290
Reed, M.: 850-142
Reed, N.: 925-318
Reed, N.: LB935-362
Reed, N.: 665, 2380-166, 2495, 4155
Reed, P. S: 2385-191
Reel, C.: LB1545-406
Reese-Melancon, C.: 1401-285
Reeves, M.: 3415, 4020
Refroe, C.: LB935-425
Regier, N. G: 1005
Reid, C.: 1315-57, 3220, 3310-118, 3365-282, LB1545-341
Reid, C.: 2315-15, 2395-219, 4130
Reilly, P.: LB1545-382
Reilly, P.: LB2570-365
Reilly-Sanchez, L. M: LB1545-408
Reinhard, S.: 3450
Reinhardt, J. P: 2015, LB3620-393
Reinhardt, R.: 1315-45, 1380-194
Reitelseder, S.: 820-30
Reiter, D.: 3295-71
Reive, C.: 3295-75
Rejeski, J.: 3325-175
Religa, D.: 2215
Rely, K.: 4155
Remillard, E. T: 3500
Ren, Y.: 2345-56
Renfro, M.: 3270-3
Renn, B. N: LB935-396
Renner, S. W: 1240, LB1545-428
Reno, J.: 1305-16
Renteria, M.: LB1545-410
Rentsch, J. H: LB3620-362
Renzi-Hammond, L. M: LB3620-329
Resciniti, N. V: 3115, 3325-164

Resnick, B.: 705, 920-301, 1255, 2315-3, 2390-194, 2525, 3270-11
Resnick, S. M: 2370-129
Resnick, S.: 3075, LB2570-441
Restorick Roberts, A.: 1480, 3025
Restrepo, M. I: LB1545-408
Restrepo, V.: LB1545-408
Retalic, T.: 4080
Rey, S.: 1150
Reyes-Ortiz, C.: 2365-115
Reynolds, A. S: LB1545-383
Reynolds, C. A: 925-338, 1150, 1425
Reynolds, C.: LB2570-324
Reynolds, C. L: LB2570-357
Reynolds, C.: LB2570-383
Reynolds, K.: LB2570-373
Reynolds, L.: 680
Rhee, M.: LB3620-433
Rhee, M.: 1325-70
Rhee, M.: 2405-235
Rhee, T.: 3010
Rhodes, J.: 1170, 2280
Rhoten, N.: 2360-101
Ribeiro, O.: LB2570-341, 645, 880-201, 3180
Ricamato, A.: 900-243
Richards, C.: 1385-204
Richards, l.: 660
Richardson, S.: 865-168
Richbart, M.: 3330-187
Richey, S.: LB2570-420
Richman, K.: 2380-154
Richmond-Cullen, c.: 705
Richmond-Rakerd, L.: LB1545-347
Rickard, T.: 3435
Ricke, W. A: LB1545-404
Rickenbach, E.: 4035
Ridings, J.: 3360-269
Rieder, N.: LB3620-351
Rieker, J.: 3370-290
Riffin, C.: 1110, 3180, 3365-282
Riley, A.: 4070
Rincon, J.: 835-83
Ring, L.: 1070, 1210
Rippon, I.: 650
Risal, P. G: LB3620-327
Risbud, R.: 3535
Ritchie, C.: 1305-33
Ritenour, D.: 520
Rivera, E.: 3230
Rivera, J.: 3330-189
Rivera Almaraz, A.: 3325-154
Rivera-Burciaga, A.: 1365-171
Rivera-Hernandez, M.: 2545, 3330-178, 3585
Rivers, E.: 3155
Rizzotti, R.: 3330-189
Rizzuto, D.: 850-137, 3170, 3325-173
Roach, K.: 775
Roat-Shumway, S.: LB935-442
Robayo, C.: 2355-66
Robbins, J. C: 2405-236
Robbins, L.: 1365-179
Robbins, R.: 2325-29
Robbins, R.: 2360-86, LB935-441
Roberge, B.: 755
Robert, M. N: 885-211
Robert, S.: 910-260
Roberto, K. A: 1025
Roberto, K. A: 3345-223
Roberto, K. A: 3515
Roberto, K. A: LB1545-405
Roberts, A.: 1325-74
Roberts, E.: LB935-392
Roberts, J.: 3070
Roberts, L. C: 515
Roberts, L.: 1365-179
Roberts, L.: LB935-371
Roberts, M.: 1402-293
Roberts, T.: 850-131
Roberts, T.: 1365-179
Robertson, E.: LB3620-424
Robinson, E. L: 2485, LB2570-398
Robinson, J.: LB935-425
Robinson, K. N: LB3620-323
Robinson, L.: LB2570-319
Robinson, S. A: 2255
Robinson, T.: 2115
Robison, J.: 1065, 1305-25, 2430-297
Robnett, R. H: LB1545-421, LB3620-350
Rockwood, K.: 565, 820-23
Rockwood, K.: 3325-172
Rockwood, K.: 3325-167, 3590, 4045, LB2570-354
Rodney, O.: LB935-347
Rodriguez, A.: 2345-53
Rodriguez, D.: 815-16, 2425-288
Rodriguez, K.: 830-69, 3385
Rodriguez, K.: LB935-447
Rodriguez, M.: 3220
Rodriguez, M.: 2355-66
Rodriguez Díaz, M.: 3290-67
Rodriguez-Suarez, M.: LB1545-361
Roes, M.: 785
Rogan, J.: 4080
Roger, K.: LB2570-424
Rogers, N.: 685, 1200
Rogers, R.: 830-65
Rogers, S.: LB935-443
Rogers, W.: 550, 620, 1130, 3500, LB1545-373, LB2570-406
Roggio, A.: 1402-311
Rogina, B.: 830-65
Rogowski, J.: 2360-103
Roh, H.: LB1545-398
Roh, S.: 1300-4, 2405-245
Rollins, J. A: 830-66
Roman, C.: 925-317
Romano, J.: 1402-301
Rome, V.: 2395-214
Romero, K.: 2345-56
Rondeau, K.: 3435
Rook, K.: 925-325, 1400-255, 4015
Root, E. Z: 1380-199
Roque, N. A: 4085
Rosa, B.: 895-235
Rosales, K. B: 4030
Rosano, C.: 805, 820-38, 2360-94, 3075
Rosati, R. J: 850-143
Rose, J.: 1395-243
Rose, K.: 3100
Rose, M.: 1385-207, LB2570-383, LB3620-354
rose, t.: 895-229
Rose III, D. W: 2425-290
Rosemond, C.: 520
Rosen, B.: 3420
Rosen, S. L: 3420, 4030
Rosen, T.: 795, 2225, 2370-118
Rosenberg, A.: 3360-265
Rosenberg, D. M: 875-187, 3510
Rosenberg, D. E: 570
Rosenberg, D.: 920-304, 2410-256
Rosenthal, M.: 910-274
Rosenthal, R.: 2115
Rosenwohl-Mack, A.: LB2570-336, LB3620-419
Rosich, R. M: LB1545-348
Roso-Llorach, A.: 3170
Ross, C.: 525
Ross, I. S: LB2570-365
Ross, J.: 925-328, 1335-96, LB1545-366, LB2570-365
Ross, L.: 1410-328
Ross, L.: 1150, 1410-327, 3290-63
Ross, S.: 900-247
Rosselli, M.: 2355-66
Rosser, B.: 2380-155
Rossi, D.: 2385-185
Rossman, M.: 835-89
Rossner, R. J: LB3620-388
Rosso, A.: 820-38, 2170, 2360-94
Rostant, O.: LB935-419
Rostgaard, T.: 1445
Rostowsky, K.: 825-61
Rote, S.: 750, 3360-267
Roth, D. L: 1170, 1250, 2270, 2280, 3420, LB2570-353
Rothberg, Z.: 2405-241
Rothermund, K.: 2040
Round, T.: 3550
Rourk, R.: 2315-8
Rouse, H. J: 2355-72
Roush, R.: 1490
Rousseau, J.: 850-134
Rowan, N. L: 3430
Rowe, B. H: 4130
Rowe, M.: 3495
Rowland, S. A: LB1545-338
Rowles, G. D: 1185

Rowley, S.: 810-2
Roy, E.: 825-47
Roy, E. R: LB935-364
Royall, D. R: 1360-158, 3275-16
Rozario, N. L: LB2570-327
Rozario, P. A: 3065
Rubtsova, A. A: 3525
Rudolph, J.: 705
Rudolph, J.: LB2570-329
Ruffini, G.: 4020
Ruggeri, M.: 1515
Ruggiano, N.: 880-193, 2325-25, LB1545-376, LB2570-384
Ruiz, D.: 1330-94, 1402-299
Ruiz, J. G: LB1545-361, LB3620-338
Ruiz, M.: 895-230
Ruiz, S.: 3500
Rupper, R.: LB2570-416
Rurka, M.: 3315-131, LB935-416
Rush, J.: LB2570-347
Russell, C.: 2410-254
Russell, D.: 1015
Russell, D.: 4010
Russell, J.: 825-59, 2230
Russell, M.: 915-290
Russell, S.: 2395-213
Rustin, P.: 2045
Rutenberg, A.: 825-55
Rutenberg, A.: 3325-167
Rutherford, B.: 2495
Rutledge, M.: 1315-47
Ryan, A.: 820-21, LB1545-424
Ryan, G.: LB3620-408
Rybarczyk, B.: 2425-282
Ryff, C.: 1425
Ryskina, K.: 3390
Ryu, B.: LB3620-335
Ryu, H.: 1365-173
Ryu, J.: 2410-246

### S

Sá, C.: 1402-307
Saad-Lessler, J.: 2380-154
Sabatini, S.: 2040
Sacco, P.: 3335-192, LB3620-355
Saccon, T.: 835-83
Sachdev, P.: 655
Saczynski, J. S: 1400-260
Sadak, T.: 3110
Sadamitsu, K.: 2385-188
Sadarangani, T.: 1205
Sadeq, N. A: 3580
Saenz, J.: 4000
Saenz Monsivais, V.: LB1545-427
Safi, N.: 830-64
Sagiraju, H.: 3295-76
Sagna, A.: 1330-79
Sagong, H.: 1402-305
Sahoo, S.: 1325-71, 1400-259, 1401-290, 3300-99, 3335-192
Sakai, A.: 830-63
Sakai, E.: 925-319
Sakai, E.: 1401-286
Sakaniwa, R.: 1045
Sakata, K.: LB2570-440
Sakka, M.: 895-225
Sakurai, R.: 895-226, LB2570-411
Sala, L.: 820-37
Salari, S. L: 2365-116
Salas, R.: 2420-277
Salazar, M.: 3355-255
Saldana, S.: 1402-296
Salem, G.: 920-303
Salerno, E.: 2050
Salgado, G.: LB935-383
Saliba, D.: 520, 3430, 4135
Salimi, S.: 820-21, 3405
Salinas, I.: LB2570-419
Salinas Rodriguez, A.: 3325-154
Salisbury, D. L: 3175
Salmon, A. B: 820-35, 830-70, 835-88
Salmon, C.: 885-210
Salomons, T.: 905-255
Salthouse, T.: 655, 3290-49
Šaltytė -Benth, J.: 1330-86
Saltzman, B. S: 2370-123
Samadani, A.: 1402-306
Samanez-Larkin, G. R: 1485
Sammy, M. J: LB2570-371
Sampathkumar, N.: LB935-351
Samper-Ternent, R.: 3220, LB2570-332
Sampson, L.: 1345-116
Samsi, K.: 850-138
Samuel, L.: 515
Samuel, M.: LB3620-397
Samuel, M.: LB3620-398
Samus, Q. M: 2490, 3420, 4025
Sanchez Arenas, R.: 1310-40
Sanchez-Niubo, A.: 4040
Sánchez Pérez, A.: LB935-430
Sanchez-Reilly, S.: LB1545-366, LB2570-365
Sanders, A.: 3570
Sanders, J. L: 1425, 3440
Sandlin, A. M: 815-16, 2340-44
Sands, B.: 830-78
Sands, L. P: 1095, 1190, 1315-51
Sanford, J.: 3270-3
Sanghi, S.: 3320-136
Santana, C.: 895-235
Santanasto, A. J: 2370-136, 3170, 3275-24, LB935-368
Santangelo, K. S: 835-88
Santiago, E. D: 825-52
Santiago, H. J: 2415-268
Santini, T.: LB2570-324
Santos, E. J: 1265
Santos, P. M: 2325-28
Santos, T.: 3535
Santos, Z.: 895-235
Sappington VandeWeerd, E.: 1402-293
Sapra, M.: 1355-145
Sarhadi, K.: 1402-301
Sartor, O.: LB1545-377
Sarveswaran, S.: LB2570-354
Sathavarodom, N.: 835-91
Satiroglu, R.: 920-310
Satizabal Barrera, C.: LB3620-372
Sato, T.: 895-232
Saunders, K. C: 2395-212
Saunders, M.: 1435
Saunders, P.: 610
Sautter, J. M: 2355-70
Savage, A.: 880-192
Savla, J.: 1025, 1095, 1315-51, 1355-145, 3480, 4035, LB1545-405
Savova, Z. A: 3285-43
Savundranayagam, M. Y: 2355-75, 3200, 3560
Sawchuk, K.: 580
Sawyer, B.: 3360-258
Sawyer, J.: 815-15, 2340-44, 2425-288
Saxon, A.: LB1545-412
Sayer, J.: LB935-367
Sayer, L. C: 4065
Scala-Foley, M.: 2385-183
Scales, A.: 1401-286, 3530
Scales, K.: 2305
Scanlon, D.: LB1545-397
Scerpella, D.: 1005
Schaeffer, J.: 1015
Schaepe, K.: 3180
Schafer, M.: 830-67, 835-84
Schafer, M. H: 885-209, 925-329, 1345-108, 3030, 3480
Schaffer, R.: LB3620-352
Schaie, W.: 3290-47
Scharre, D.: 845-117
Scheidt, R.: 2145
Scheirer, J. L: 830-69, LB935-447
Schell, B. R: 820-29
Schell, K.: LB935-391
Schepens Niemiec, S. L: 890-213
Schepisi, I.: 3290-62
Scher, C.: LB935-444
Scherer, A. M: 540
Scheurich, J.: LB1545-353
Schiaffino, M. K: 2345-58
Schiffman, R.: 2410-258
Schiller, M.: 1365-166
Schilling, O. K: 3290-57, 3600
Schiltz, N.: 1395-243
Schinka, J.: 2355-72
Schino, R.: 1515
Schiwal, A. T: LB1545-394
Schlag, K. E: LB3620-385
Schlatter, J.: LB1545-402
Schlundt, D.: 2565
Schmall, V.: 3560
Schmidt, B. R: 920-314
Schmitt, E.: LB1545-402, LB2570-346
Schneeweiss, S.: 3325-169
Schneider, A.: 835-83
Schneider, C. E: 870-184, LB935-401

Schneider, E. C: 810-3, LB935-392
Schneider, J.: 3375-310
Schneider, J. A: 3480
Schoemann, A.: 2410-249
Schoenmakers, E.: 1285, 4150
Scholl, M. M: LB935-378
Schols, J.: 3270-5
Scholte op Reimer, W.: 1365-176
Schoonhoven, L.: 2380-158
Schork, N. J: 1120
Schrack, J. A: 820-24, 2360-92, 2370-129, 2485, 3040, 4165, LB935-446
Schroeder, E.: 1320-63
Schuler, E.: 1350-131
Schulman, D. J: 1402-306
Schultz, M. B: LB2570-323
Schulz, R.: 1055, LB2570-324
Schumacher, J. G: 2315-11
Schumann-Bard, P.: 825-49
Schupf, N.: 2370-125, 3170
Schuurmans, M. J: 2380-158
Schwartz, A.: 2505, 3410
Schwartz, L.: 2305, 2470, 3060, 3435
Schwartz, M. L: 740, 3140
Schwei, R.: LB935-422
Schwertner, E.: 2215
Scicolone, M.: 2375-142, 3290-55
Sciegaj, M.: 1235
Scogin, F.: 2310
Scott, C.: 1355-141
Scott, R.: 1385-203
Scott, R.: LB3620-324
Scott, R.: 2550
Scott, S. B: 825-52, 3290-52, 3370-295, 3575, 4085
Scott, T. L: 850-130
Scrivano, R.: 3290-44
Seals, D.: 835-89
Seaman, A. T: 2055, 3165, 3485
Seaman, K. L: 585
Sears, J.: 2355-69
Sebastiani, P.: 835-92, 2345-55, 3215
Seblova, D.: LB1545-333
Seckin, G.: 1401-273
Secnik, J.: 2215
Seetharaman, K.: 1010
Sefcik, J. S: 605
Sefcik, J.: 915-289
Sefcik, J. S: 2215
Sefcik, J.: LB3620-340
Segal, D. L: 840-105, 915-283, 1390-226
Segal-Gidan, F.: 1100
Segel-Karpas, D.: 700
Segev, D.: 2380-168, 4020
Seguin, C.: 2550
Sehgal, S.: 2120
Seibel, K.: 2405-236
Seino, S.: 3340-204
Sekhar, R. V: 1310-37, 2190, 2485
Sell, C.: 2440
Sendroiu, I.: 515
Sengupta, M.: 2390-203
Senteio, C. R: 3200
Sentell, T. L: LB2570-421
Sepehri, K.: 3325-172
Seplaki, C. L: 1330-92
Serra, M. C: 3020
Serrano, I.: 905-253
Seshadri, S.: 3215, LB3620-372
Sesso, J.: 820-34
Severance, J. J: 900-247, 1530, LB2570-414
Shaaban, C.: 3075
Shacham, E.: 1380-190
Shade, M.: LB2570-395
Shadmi, E.: 3420, LB1545-375
Shah, A.: LB1545-361
Shah, A.: 2375-140
Shah, A. A: 2310
Shah, A.: 2430-300
Shah, K.: 2495
Shah, R. C: 710, 1300-6, 3260, 3480, LB3620-375
Shah, S.: 1360-161
Shahid, F.: 2155
Shake, M.: 1402-315
Shakya, S.: 690
Shalev, I.: 2370-117
Shalini, S.: LB2570-398
Shang, j.: LB1545-380
Shank, S.: 825-53
Shapkin, K.: 840-112
Shardell, M.: 820-24, 3040
Sharifian, N.: 745
Sharma, J.: 3330-190
Sharma, K.: LB1545-374
Sharma, N.: 1401-282, 3535
Sharpe, C.: 3375-312
Sharrett, A.: 830-75, 2495
Shaughnessy, M.: 3530
Shavit, Y. Z: 865-165, 925-323
Shaw, A. R: LB935-437
Shaw, C.: LB2570-364
Shea, K. D: 3120
Shea, K.: LB2570-426
Shea, M.: 715, 3280-33
Shea, S.: 3280-34
Sheehan, O. C: 1170, 2280
Sheets, D. J: 2060, 3470, 3610
Sheldon, M.: 1305-28, 4055
Shelley, M.: 2390-202, 4145
Shellito, N.: 3570, LB935-353
Shen, D.: LB1545-412
Shen, H.: 880-196
Shen, S.: 2360-100
Sheng, W.: 1401-285
Shepherd, G.: 1320-62
Shepherd-Banigan, M.: 880-194
Shepley, M.: 1010
Sherlala, R. A: 1310-42
Sherman, A. M: 1140
Sherman, L. D: 2405-234
Sherrill, C.: 835-87
Shi, J.: 1355-141
Shi, L.: 3220
Shi, X.: 830-73, LB3620-360
Shi, Z.: 4020
Shia, B.: 3330-185
Shih, R. A: 4050
Shim, H.: 1060, 1401-289
Shim, J. K: 1520
Shimabukuro, M.: 830-72
Shin, J.: 3295-77, LB1545-401
Shin, O.: 2430-294
Shin, O.: LB3620-335
Shin, S.: LB935-374
Shin, S.: 1310-35, 3290-60
Shinkai, S.: 3340-204
Shippee, T. P: 910-258, 1405-324, 2155, 2390-200, 3345-217, 3585
Shirai, K.: 1045
Shireman, L.: LB1545-412
Shirmohammadi, F.: 830-64
Shiroma, K.: 2165
Shishegar, N.: 3295-84
Shockey, D.: LB2570-379
Shoemaker, V.: 850-142
Shofner, S. J: LB935-436
Shojaei, A.: 1320-64
Shon, E.: 3350-236, 3360-262
Shook, N.: 3370-298, LB2570-360
Short, M.: LB3620-372
Shovali, T. E: 3320-135
Shrestha, M. K: LB935-402
Shrestha, S.: LB3620-439
Shrestha, S.: LB2570-390
Shrira, A.: 700
Shropshire, K. L: 3455
Shryock, K.: 3310-117
Shuey, K.: 2205
Shulyaev, K.: 2315-7, LB1545-375
Shumaker, E. M: 535
Shumaker, S. A: 850-132
Shuman, S. K: 1435
Shusterman, G.: 2385-193
Siden, R.: 895-230
Sidrak, H.: 925-338
Sieber, S.: 3030
Siebert, J.: 2040
Siedlecki, K. L: 655, 3290-49
Siegel, E.: 2295
Siegel, M.: 900-249
Sieng, M.: LB2570-316
Sierra, F.: 2290
Sikoki, B.: 915-287
Sillito, C.: 2365-116
Sillner, A. Y: LB2570-329
Sills, J.: 2390-196
Silva, D.: 505
Silva-Smith, A. L: 3325-162
Silver, J.: 1315-52
Silver, M.: 1150, 2030, LB3620-391
Silverman, M. A: 2495

Silverstein, M.: 2160, 3335-194
Sim, H.: LB1545-401
Sima, C.: 2410-258
Simmons, E.: 2315-4
Simning, A.: 1330-92
Simning, A.: LB1545-380
Simões Neto, J.: 850-141
Simon, A.: LB2570-342
Simon, C.: 3365-275
Simon, K.: 3555
Simon, M.: 3505, 4105
Simons, K.: 910-275, 1480, 3345-218
Simonsick, E. M: 515, 705, 820-24, 1240, 1455, 2370-129, 3075, 3295-73, LB935-446, LB1545-410
Simpson, D.: LB1545-400
Simpson, E.: 915-288, 1065, 3570
Simpson, F.: 3590
Simpson, T.: LB1545-412
Sims Gould, J.: 855-147, 3330-180
Sims-Gould, J.: 3330-177
Sin, N. L: LB935-411
Sinclair, D.: LB2570-323
Sing, J.: 2400-227, 2425-287, LB3620-440
Singer, D.: 705, 2155
Singh, E.: LB3620-336
Singh, K. K: 2090
Singh, P.: 1402-306
Singh, R.: 3330-187
Singh, T.: 1360-157
Singhatat, W.: LB2570-397
Singleton, M.: 910-270, LB1545-443
Singsank, K.: 855-148
Sinha, I.: 820-40
Sinwoo, H.: 840-111, LB2570-317
Sion, K.: 785
Sioutas, C.: 825-51, 830-64
Sirey, J. 2430-302, LB935-444
Sisofo, B.: LB935-376
Sison, S.: 2380-168
Sivanesathurai, R.: 850-131
Sivers-Teixeira, T.: 2385-188
Sjöberg, L.: 515
Skanderson, M.: 2370-119
Skelton, D. A: 730
Skinner, M.: 560, 2105, LB3620-373
Skipper, A. D: LB2570-417
Sköldunger, A.: 635
Sköldunger, A. B: 3345-220
Skrajner, M.: 500, 2355-63
Skubic, M.: 820-37, 4005, LB2570-398
Skufca, L.: 1401-279
Skultety, K.: LB3620-419
Slattum, P.: 3300-94
Slaug, B.: 3600
Slayday, R. E: LB2570-348
Slindee, L.: 1315-60, 1400-268
Sliwinski, M.: 3290-52, LB3620-347
Sloan, C. J: 2375-148
Sloan, J.: 870-179
Sloan, S.: 3410
Sloane, R.: 2380-167, 4130
Slosser, A.: 870-186
Smaga, S.: 1335-95
Smagula, S.: LB2570-324
Smaling, H.: 4135
Small, B. J: 2355-72, 3290-52
Smalls, B.: 1315-47
Smetcoren, A.: 1305-22, 3035
Smilovich-Fine, E.: 3510, LB1545-355
Smilowitz, J.: 2395-215
Smilowitz, S. T: 3510
Smit, L. C: 675
Smith, A.: 2270, 3470
Smith, A. B: 1350-127
Smith, C.: 825-58
Smith, D. L: LB2570-371
Smith, D.: LB2570-414
Smith, D. M: 1095, 1385-217, 2415-265, 3365-279
Smith, E. E: 3295-87
Smith, G.: 865-162
Smith, G. C: 1350-129
Smith, J.: 720, 925-332, 3340-202, LB935-409
Smith, J. L: 2340-33, LB935-367
Smith, K.: 3275-14
Smith, K.: 1535
Smith, L. N: 815-15, 2340-44, 2425-288
Smith, L.: 2060
Smith, M. L: 635, 770, 1380-190, 2360-103, 3300-96, 3335-193
Smith, R.: 3300-97
Smith, S. K: LB2570-403
Smith, S. A: 1402-300
Smith, S. D: 3430
Smith, T. J: 3300-99
Smith, T.: 895-227
Smith, V.: 880-194
Smith-Ray, R. L: 1360-157
Snodgrass, J.: LB1545-342
Snow, A.: LB3620-351
Snyder-Mackler, N.: 765, 1430
Soares, J.: 895-235
Sober, J.: LB3620-389
Soble, J. R: 3295-76
Sodhi, J. k: LB1545-349
Soh, Y.: 1400-261
Sohn, Y.: 850-131
Solimeo, S.: 3165, 3485
Solinge, V.: 575
Sollid-Gagner, C.: 2395-219
Solow, A.: 915-298, 1330-82
Solway, E.: 540, 705, 2155, 3135
Somerville, C.: 910-265, 2385-179
Son, B.: 820-27
Sonawane, K.: 3330-184
Song, H.: 2395-207
Song, J.: LB3620-412
Song, M.: 710, 840-107, 850-125
Song, M.: LB2570-359
Song, M.: 3555
Song, Q.: 3145
Song, S.: 2165
Song, W.: 4045
Song, X.: 820-28, 3325-172, 3330-189
Song, X.: LB2570-354
Song, Y.: 1340-102
Song, Y.: 2420-273
Song, Z.: 2345-55
Sonnega, A.: 2325-29
Sonnier-Netto, L.: 2435-314
Sorbero, M.: 2395-217
Sörensen, S.: 2130, 2235
Sorkpor, S.: 1402-313
Sorrentino, V. A: 2410-257
Sotelo, V.: 870-184
Soto, M.: 1515
Sou, C.: LB3620-425
Sousa, L.: 3180
Souza, A.: 3115
Spahr, M.: 515
Spalding, R.: 885-203, 3350-243, LB935-405
Spears, E.: 2405-234
Speirs, C.: 2340-40
Spence, M.: 1325-74
Spence, S.: LB3620-420
Spencer, D.: 2225
Spencer, R.: LB2570-372
Spencer-Brown, L.: 3305-107
Spensley, C.: 3135
Sperber, N. R: 1110
Spetz, J.: 755, 3555
Spicher, J. M: 3325-162
Spiers, G. F: 1325-67
Spilsbury, K.: 1290, 2020
Spini, D.: 1385-213, 3315-123
Spira, A. P: LB2570-382
Spiro III, A.: 3340-206, 3370-301, 3575
Spoden, N.: LB935-436
Spoelstra, S. L: LB1545-433
Spradley, L.: LB935-420
Sprague, B. N: 1150, 3290-63
Sreenivas, R. S: LB1545-373
Srikumaran, U.: 2360-83
St. George, S. M: 3315-130
St. John, P.: 870-185
St. Louis, R. M: 1410-331
Staben, O. E: LB2570-402
Stadler, D.: 3545
Stagg, B. C: 2495
Stahl, S.: 3150, LB2570-324
Stallard, E.: 820-33, 825-60
Stanback, B. M: 2435-308
Stanely, M.: LB2570-420
Stange, K.: 1401-276

Stanley, A.: 900-243
Stanley, J.: LB935-366, LB3620-326
Stanley, J. T: 815-8, 1385-204
Stanley, J. L: 3315-122
Stanmore, E. K: 730
Stapleton, H.: LB2570-322
Stark, S.: 3270-3
Starr, L. T: 840-109
Starr, M. E: LB3620-387
Statler, A.: 1395-243
Statz, T. L: 1385-210, 2375-140
Stavrinos, D.: 1410-327
Stawski, R. S: 1350-125, 3030, 4035
Stearns, M.: 3320-148
Steedley, M.: 1055
Steele, C.: LB1545-377
Steele, J. C: 1385-203
Steeves-Reece, A.: 1305-27
Steffens, D. C: 3010, 3360-263
Stein, G. L: 3225
Steiner, V.: 1345-110, 1355-147, 2355-73
Steinheiser, M.: 3345-230
Steinman, B. A: 895-236
Steinman, L.: 810-3
Stempel, C.: LB935-363
Stengel, J.: 2415-268
Stensland, M.: 2010
Stephenson, E.: 2530
Stepniak, C.: 3315-131
Steptoe, A.: 730, 2370-128
Sterling, M.: 1155
Stern, Y.: 3290-65
Sterns, H. L: 2100
Stessman, J.: 2315-9
Stevens, A. B: 2300, 3320-136
Stevens, G.: 2385-188
Stevens, J. D: 1445
Stevenson, B.: LB1545-353
Steward, A.: 905-252
Stewart, J.: 2360-102
Still, C. H: 770
Stinchcombe, A.: 1380-198, 1410-329, 2550, 3595
Stine-Morrow, E.: 3290-56
Stirland, L.: 3210
Stirling, A.: LB935-427
Stocker, R.: 2395-213, 4055
Stockmeier, C. A: 1330-84
Stohrer, H.: 800
Stokes, J. E: 725, 1315-54, 1380-201, 3150, LB2570-413
Stolee, P.: 3330-180
Stone, K. L: 2125
Stone, L. E: 915-283, 1390-226
Stone, P. W: 2395-217
Stone, R.: 1155, 2015, 2515, 3140
Stoop, A.: 635
Stough, C.: 3295-81
Stow, D.: 1200
Stoyles, S.: 1360-160
Straatmann, V.: 3420
Straker, J.: 2385-183, 3555
Strasser, I.: 2390-206
Strath, S.: 2410-258
Stratton, L.: 2390-202, LB1545-385, LB3620-356
Stratton, S.: LB935-377
Strecher, V.: 1425
Street, D. A: 3000, 4125
Streeter, J. L: 3410
Streiff, J.: 1401-271
Stripling, A. M: 915-298, 1380-192, 1405-321, 3370-287
Strogatz, D.: 3325-171
Strommen, J.: LB3620-441
Strong, J.: 535, 3125
Strong, J. V: 3290-58
Strotmeyer, E.: 565, 2445
Strough, J.: 3370-298
Stroustrup, N.: 1115
Struble, L. M: 850-127
Struckmeyer, K. M: 3320-145
Stubbings, G. M: 825-55
Stucki, B.: 3290-62
Studenski, S.: 705, 820-38
Studenski, S.: 2360-94
Studenski, S.: 3075
Studenski, S.: 3295-71
Stum, M.: 2035, 2235, LB2570-376
Stuppard, R.: 835-101
Stupplebeen, D.: LB2570-421
Sturdevant, D. L: 3550
Su, M.: LB935-408
Suarez, J.: 850-134
Subedi, J.: LB935-402
Sublett, J. F: LB3620-406
Suchting, R.: 2365-114
Suchy, Y.: 1315-48
Sugawara, I.: 1345-109, 3340-199
Sugiyama, T.: 4005
Suitor, J.: 2160, 3315-131
Sullivan, D.: LB2570-425
Sullivan, J. L: 3255
Sullivan, K. J: LB2570-401
Sullivan, M.: 1535
Sullivan-Marx, E.: LB1545-335
Sultana, A.: 2400-230
Sultana, A.: 2360-98
Summerson, E.: 3365-277
Sun, A. Y: LB3620-396
Sun, F.: 500, 2313, 3250, LB935-432, LB2570-410
Sun, H.: 3480
Sun, J.: 1297
Sun, L.: LB2570-352
Sun, L.: LB3620-384
Sun, N.: 4150, LB935-402
Sun, Q.: 3250
Sun, W.: 3355-252
Sun, X.: LB935-408, LB1545-446
Sun, Y.: LB935-380
Sun, Y.: 820-32Sung, A. D
Sunderaraman, P.: 3290-65
Super, N.: 2325-23
Supiano, K.: 2325-24
Supiano, K.: 500, LB2570-416
Supiano, K.: LB935-358
Supiano, M.: 4045
Suppiah, S.: 3355-251
Surachman, A.: 1440
Susana-Castillo, S.: LB935-416
Sussman, J. C: 2385-173
Sussman, T.: 910-264, LB1545-439
Sutherland, L.: 890-222
Sutherland, L.: 1075
Sutphin, G. L: 3195
Suwalski, M. W: 1360-157
Suzuki, H.: 855-154
Suzuki, N.: LB1545-344
Suzuki, R.: 1360-161
Svynarenko, R.: 3315-132
Swall, A.: 3295-86
Swan, A. A: 3295-76
Swann, L.: 2370-134
Swanson-Aprill, L. A: 3305-109
Sward, K.: LB935-400
Swartzendruber, L.: LB2570-425
Sweeder, L. A: 3295-89
Sweeney, C.: LB3620-380
Sweigart, B.: 2345-55, 3215
Sweitzer, S.: 2360-86, LB935-441
Swenor, B.: 665, 3295-73
Swierenga, S. J: 3305-109, 3415, 4020
Swift, A.: 870-185
Swift, H.: 3000
Switsers, L.: 3035
Syed, S. U: LB2570-392
Syme, M.: 1485, 2515, LB1545-407
Szanton, S. L: 515
Szanton, S.: 920-305
Szanton, S. L: 1510, 3270-3
Szanton, S. L: 3360-268, LB2570-353
Szanton, S.: LB2570-382
Sze, L.: 2400-227, 2425-287, LB3620-440
Szkody, E.: 915-292
Szychowski, J.: 3345-211
Szymkowski, A.: LB1545-400

### T

Taani, M. H: 2410-258
Taeckens, A.: 845-116
Taffet, G. E: 2190
Taguchi, A.: 885-205
Taguchi, J.: 520
Taitel, M.: 1360-157
Takahashi, K.: 3325-157
Takahashi, P.: LB3620-365
Takaoka, M.: 895-225
Takase, M.: 925-330, 3270-4
Takata, Y.: 2405-237
Takayama, M.: 1345-109
Takayama, M.: 3340-199
Talab, A.: LB1545-340
Talebzadeh, A.: 2360-98
Talley, K. M: 2380-155
Talwar, S.: 2420-276
Tamashunas, N. L: LB1545-355

Tamayo, L.: 2250
Tamimie, E.: LB935-395
Tamiya, N.: LB2570-444
Tan, A.: 3275-26
Tan, C.: 3250
Tan, P.: 2360-96
Tan, S.: 3475
Tan, Y.: 3355-251
Tanaka, T.: 895-232, 3270-4
Tanaka, T.: 3325-157
Tanaka, T.: 830-63
Tang, F.: 2345-54
Tang, F.: 640, 4105
Tang, J.: 915-294, 1345-115, 2425-287, 3075
Tang, J.: LB3620-440
Tang, V. L: 2315-1
Tang, W.: LB935-421
Taniguchi, Y.: 3340-204
Tanjong-Ghogomu, E.: 3415
Tanksley, B.: 795
Tanzi, R.: 825-51
Tarabochia, D. S: 2430-304
Tarbert, R. J: LB2570-397
Tarbi, E.: 870-179
Tardif, S.: 525
Tarleton, E.: 2360-89
Tarraf, W.: 860-159, 1360-164, LB3620-342
Tarter, R.: 840-104
Tasnim, S.: 2400-230
Tate, A. M: LB3620-381
Tate, K. C: 2315-15, 2395-219, 4130
Tate, R. B: 870-185
Tavares, C.: 3535
Tavares, D.: 3365-281
Tavares, J. L: 865-163, 1095
Tavener, M.: 2410-251
Tavenier, J.: 3280-32, LB2570-339
Tay, P. K: 2360-96
Tayade, A. S: 630
Taylor, C.: 2410-249
Taylor, C.: 3465
Taylor, C. A: 1030
Taylor, C. A: 1315-55
Taylor, C. A: 4110, LB935-362
Taylor, E.: 3180
Taylor, H. O: 1145, 3065, 4150
Taylor, H.: LB2570-421
Taylor, J. L: 775
Taylor, L.: 2365-106
Taylor, L.: 3490
Taylor, M. G: 1160, 1400-252, 2550
Taylor, P.: 510, 1540
Taylor, S.: 3390
Taylor, T. N: 2485, 3525
Taylor, Y. J: LB2570-327
Teas, E.: LB2570-408
Teaster, P. B: 1265, 2035, 2430-293, 3025, 3345-223, 3515
Teichmann, B.: 3375-310
Teixeira, D.: 2360-87
Teixeira, L.: 880-201
Tejada, L.: 1380-197
Telonidis, J.: LB2570-416
Temkin-Greener, H.: LB1545-381
Templeman, M.: 2435-311
ten Brinke, L. F: 715, 3295-72
Ten Cate, D.: 675, 2380-158
Teng, A.: LB935-445, LB1545-358
Teno, J.: 520
Tepper, L.: LB1545-374
Terada, T. M: LB3620-374
Teres-Vidal, L.: 3345-216
Terhune, K. N: 680
Teri, L.: 1415, LB935-436
Terman, G.: LB1545-412
Terracciano, A.: 3575
Terrana, K.: 3200
Terrin, M.: LB1545-424
Terry, E.: 775
Testad, I.: LB3620-317
Teuber, I.: 2340-47
Teufel-Shone, N.: LB2570-445
Thao, M.: 3345-217
Thapa, S. S: LB935-402
Theng, Y.: 885-210
Theou, O.: 565, 3325-165, 3590, 4045
Thiamwong, L.: 2360-88, LB935-413
Thierry, A.: 1485, 3095, 4120
Thill, L.: 920-314
Thoma, M. V: 1330-88
Thomas, A.: 3310-117
Thomas, F.: 3305-102
Thomas, G.: 3350-240
Thomas, K. S: 705, 1305-33, 1375-187, 2470, 3140, 3345-215, 3435, 4135
Thomas, K.: 2265
Thomas, M.: 1535
Thomas, P. A: 725, 875-187, 3510
Thomas Tobin, C. S: 1050, 3475
Thomasson, W.: LB935-420
Thomeer, M.: 2160
Thompson, E. H: 870-185, 2415-268, 3275-15
Thompson, H. J: 2310
Thompson, J. L: LB935-390
Thompson, J.: LB1545-354
Thompson, K.: 3305-108
Thompson, L. V: LB2570-325
Thompson, L.: LB935-352
Thompson, M. D: LB935-426
Thompson, M.: LB1545-420
Thompson, N. A: LB1545-431
Thompson, R. A: LB3620-349
Thompson, S.: 3360-258
Thorp, J.: 3345-224
Thorpe, C.: 3390
Thorpe, J.: 3390
Thorpe, R. J: 1050, 1145, 1450, 3360-260, 3360-268, 3475
Thorpe, S.: 1401-281
Thorsdottir, I.: 2370-131
Throckmorton, C.: 1402-308
Thrush, K.: LB2570-429
Thunell, J.: 4155
Thurlow, C. B: 4170
Thurman, W.: 3600
Thyagarajan, B.: 710, 2260, 2345-57, 2370-125, 3170
Thyne, K.: 820-35, 830-77, 835-97
Tiainen, K.: LB1545-339
Tian, J.: 1410-328
Tian, Q.: 3075, 3295-71
Tian, Y.: LB3620-347
Tice, J. L: LB1545-395, LB3620-322
Tilton, N.: LB2570-389
Timmerman, K.: LB2570-408
Tinetti, M.: 3560
Tipirneni, R.: 540
Titler, M.: 850-127
Tiwari, A.: 3295-89
Tkatch, R.: 1015
Todd, A.: LB1545-418
Todd, C.: 730
Todd, O. M: 1360-165
Todd-Magel, C.: LB1545-369
Tofthagen, C.: 1330-93
Togonu-Bickersteth, F.: 890-220
Togonu-Bickersteth, F.: 1355-149
Togonu-Bickersteth, F.: 3300-100
Tokashiki, T.: 3280-30
Tolea, M. I: 3295-85
Tom, S.: LB1545-410
Tomita, M. R: 925-326, 3175
Tompkins, C. J: 3305-103, 4055
Tong, C.: 3330-180
Tong, H.: 2030
Tonkikh, O.: 3420
Toosizadeh, N.: 820-26
Torain, J. M: 3330-188
Toral-Garcia, J. J: LB3620-379
Toril, P.: 3370-290
Torossian, M.: 1395-249, LB2570-372
Torrence, M.: LB1545-362
Torres, G. R: 730
Torres, S.: 1070, 1210
Torres, S.: 3015
Torres-Gil, F.: 2400-224
Torsvik Henriksen, A.: LB3620-317
Toseef, M.: LB3620-342
Tovar, A.: LB2570-422
Towne Jr., S. D: 3300-96
Towns, J.: 2405-241
Townsend, A.: 1390-227
Toyama, M.: 3205
Toye, C.: 505

Toyokawa, N.: 1350-128, 2000
Toyokawa, T.: 1350-128
Tracy, E.: LB935-400
Tracy, T.: LB935-349
Trageser, K.: 825-58
Tramm, G.: 1385-205
Tramujas Vasconcellos Neumann, L.: 3415
Tran, V.: 1330-82
Trapuzzano, A.: 920-308
Travison, T.: 1402-302
Trejo, L.: LB3620-444
Trinh-Shevrin, C.: 850-131
Triolo, F.: 515
Tripp, B.: 825-48, 4130
Tripp, P.: 3320-145
Trivedi, A.: 2325-21
Trivedi, R.: 1170, 3535
Troutman, S.: 3290-63, 3295-69
Troy, L. M: 715
Troya, Y.: 900-246
Trubenstein, B. P: 1150
Trudeau, S. A: 1295
Tsai, H.: 2385-182
Tsai, Y.: 805
Tsay, T.: 2360-92
Tse, M.: 1345-115
Tseng, C.: 2380-157
Tseng, C.: LB935-406
Tseng, G. C: 3275-24
Tseng, T.: 1040
Tseng, W.: LB2570-381
Tsotsoros, C.: 865-166
Tsukada, N.: 2320-16
Tu, Y.: 1300-7
Tu, Y.: 1401-288
Tuchaai, E.: 820-32
Tulner, L.: 1365-175
Tumosa, N.: 1020, 2075, 4075
Tunalilar, O.: 910-277, 3060, 3345-214, LB2570-404
Tung, K.: 2375-152
Turiano, N. A: 1440, 3350-243
Turner, A. N: LB2570-371
Turner, J. R: 815-8
Turner, K. W: 1315-50
Turner, S.: 1350-125, 3235, 3340-200, 3370-300
Turner II, R. W: 2325-29, 3205
Turney, I. C: 2455
Turturro, C. L: LB3620-434
Tuzzio, L.: LB2570-368
Tworek, G.: 1330-82
Tyler, D.: 2385-180, 3280-35
Tzeng, H.: 2385-172

U

Uchida, H.: 1390-220
Udeh, K.: 820-40, 3385
Ukraintseva, S.: 825-60, 1310-42, 2370-132, LB1545-440, LB2570-338
Ulack, C.: 850-134
Ulatowska, H. K: 3535
Ullah, M.: 1310-34
Ullmann, G.: 3290-51
Um, T.: LB2570-378
Umberson, D.: 2160, 2280
Umezaki, Y.: LB1545-344, LB2570-320
Ungar, N. L: 4015
Ungar, R.: 1015
Unson, C. G: 865-164
Unutzer, J.: 3010
Upchurch, G.: 1305-24
Upenieks, L.: 515, 725
Upenieks, L.: 925-329
Upenieks, L.: 1345-108
Uphold, C. R: 840-108, 3320-137
Urban, K.: 2385-193
Urbanek, J.: 820-24, 2360-92
Ureña, S.: 1400-252
Utz, R.: 500

V

Vadlamani, A.: 2370-130
Vagle, V.: LB3620-317
Vailati Riboni, F.: 2340-38
Valdez, H.: 890-223, 1095
Vale, M. T: 1380-200
Valencia, A. P: 835-101
Valencia, G.: 835-101
Valencia, W.: 1320-61, 1330-94, 1402-299, 2350-59
Valenzano, D.: 525
Valera Gran, D.: LB935-430
Valiani, V.: LB3620-403
Valigosky, M.: 1345-110
van Aalst, R.: 4130
Van Alphen, J.: 3230
van Asch, I.: LB1545-415
van belzen, E.: LB1545-415
van de Pol, M.: 1365-176
van der Horst, M.: 3000
Van Der Most, R.: 1135
van der schot, A.: LB1545-414
van der Steen, J. T: 4135
van der Valk, A.: 3590
van der Velden, C.: 840-110, LB1545-415
van der Vorst, A.: 3270-5
Van Dussen, D. J: 1360-153
van Erp, J.: LB1545-414
Van Haitsma, K.: 785, 845-119, 2525, 3255
van Hout, H.: 635
Van Houtven, C. H: 880-194, 1110
Van Orden, K.: 910-275
Van Skike, C. E: 825-44, 1310-38
Van Vleet, S. C: 2340-44
van Weert, J.: 1365-176
Vanajan, A.: 660
VanCleave, J. H: LB3620-443
Vandenberg, A. E: LB2570-351, LB3620-325
Vandenbosch, J.: 2145
Vanderboom, C.: 3180
Vandiver, R.: LB3620-367
Vanegas, D.: 3315-130
Vang, C.: LB2570-316
VanHaitsma, K.: LB1545-397, LB2570-358
Vardarli, E.: 920-310
Varey, S.: 2310
Vargas, I.: LB3620-371
Vargas-Chanes, D.: 4155
Vasquez, E.: 2345-54, 2370-124, 3325-163
Vasquez, P.: 3360-266
Vasunilashorn, S.: 825-48, 4130
Vattigunta, S.: 2360-83
Vaughan, C.: LB3620-325
Vaughan, M. W: 2385-190
Vaughn, I. A: 3330-184
Vayndorf, E.: 830-81
Vazquez Arias, M.: 3375-302
Vecchione, K.: 3345-229
Veerbeek, M.: 840-110
Vega-Lopez, S.: 890-223, 1095
Vega-Soto, S.: 890-223, 1095
Veghte, B.: 3015
Velasco Roldan, N.: 2385-179, LB935-353
Velasco Roldan, N.: LB3620-359
Velez Ortiz, D.: 2400-228
Vellani, S.: 3415
Vellas, B.: LB2570-367
Velsey, K.: 2360-90
Venditelli, R.: LB2570-355
Venditti, E. M: LB1545-432
Venema, D.: 3295-80
Verbeek, H.: 785, LB1545-414
Vereen, S.: LB3620-426
Verloo, H.: 3495
Verma, A.: LB935-380
Vernon, A.: 3320-136
Versey, S.: 595
Vest, L.: 2215
Vetrano, D.: 515, 3170, 3325-173
Vetrano, D.: 3170
Viana, J.: 1402-307
Vickers, J. K: 2315-4
Vickerstaff, S. A: 510, 3000
Vicki, W.: LB2570-391
Victor, C.: 650, 1535
Videon, T. J: 850-143
Vidoni, E.: 3175, LB935-437
Vieira, E. R: 1402-312, 2360-85, 3325-170, 3365-281
Vieira Zamora, F.: LB3620-391
Vigen, C.: 890-213
Viger, M.: 2355-62
Villa, V.: 3015
Villeda, S.: 3080
Vinales, K.: 3325-176
Vincenti, V. B: 1055, 2035
Vincenzo, J. L: LB3620-416
Vinkers, C.: LB1545-422
Violán, C.: 3170

Virnig, B.: 2390-200
Visaria, A.: 865-167
Vitaliano, P.: 1345-113
Vitiello, M. V: 1510
Vivek, S.: 710, 2345-57
Viviano, N. A: 920-301
Vivoda, J.: 1305-21
Vo, T.: 925-338
Voaklander, D.: 850-126
Voelkner, A. R: 2340-41
Vogel, C. E: 915-295
Vogelsmeier, A. A: 4135
Voisine, M.: 2345-55
Volandes, A.: 3555
Volpi, E.: LB935-378, LB2570-328, LB3620-345
von Humboldt, S.: 930-339
Vondracek, H.: LB2570-419
Voss, M.: 660
Voss, M.: LB935-350
Vrahas, M.: 3420
Vutukuri, S.: LB2570-365

### W

Wachowiak, P. L: LB2570-345
Wack, K.: LB2570-351
Wadsworth, S.: 1150
Wagen, B.: 930-343
Wagg, A.: 850-126
Wagner, M.: LB3620-318
Wagner, R. G: 4110
Wagner, S.: 825-51
Wagstaff, R. A: 1315-49
Wahler, R.: 3330-187
Waite, L. J: 2425-291, 4070
Wakefield, B.: 4135
Wakefield, D.: 2045, 3360-263
Wakui, T.: 880-202, 2375-151, 3145, LB2570-444
Waldron, D.: 2185, 2530
Waldrop, D. P: 855-150, 880-195, 2385-181
Waldrup-Valverde, D.: LB3620-379
Wales, K.: 2410-251
Walker, C.: 2270
Walker, D.: 2355-63
Walker, L.: 1325-74
Walker, M.: 925-328
Wall, R.: 1401-282
Wallace, D.: 2315-8
Wallace, F.: 635
Wallace, L. M: 2095
Wallace, M.: 890-216, 2385-177, 3320-141
Wallace, S.: 850-134
Walsh, C.: LB1545-439, LB2570-424
Walsh, C.: 1350-132
Walsh, D.: 3535
Walsh, D.: 910-257
Walsh, E.: 2415-269
Walsh, K.: 560, 2105, 4055
Walsh, S. E: 895-237, LB2570-427
Walston, J.: 820-34, LB3620-364
Walton, R.: 1400-267
Wan, J.: LB2570-439
Wan, Y.: LB935-373
Wang, B.: 825-47
Wang, B.: 615, 3505
Wang, C.: 2360-99, LB935-381
Wang, C.: 3325-160, LB3620-372
Wang, F.: 1355-144, 2405-232
Wang, G.: 2385-175
Wang, H.: 835-93
Wang, H.: 2410-262, LB935-400
Wang, H.: 915-297, 1315-54, 3315-129
Wang, J.: 3305-105
Wang, J.: 4115
Wang, J.: 4105, LB1545-380
Wang, J.: 740
Wang, K.: 1320-61, 1330-94, 1402-299, 2350-59
Wang, K.: 500, 3250
Wang, K.: 3275-25
Wang, K.: 3295-83
Wang, L.: 3030
Wang, L.: 910-271
Wang, M.: 3520
Wang, Q.: 2415-267, 3200
Wang, S.: LB2570-387
Wang, S.: 1315-60, 1400-268, 2420-270, LB935-355
Wang, S. Y: 1390-232
Wang, X.: 910-269
Wang, X.: LB2570-441
Wang, Y.: 4135
Wang, Y.: 4150
Wang, Y.: 810-1
Wang, Y.: 3250, 4150
Wang, Y.: 2340-43
Wang, Y.: 910-267
Wang, Y.: 910-272
Wang, Y.: 890-219
Wang, Y.: 1340-101, LB2570-366
Wang, Z.: 1355-137
Wang, Z.: 2435-312
Wang, Z.: 820-25
Wanigatunga, A. A: 1402-296, 2125, 2360-92, LB935-446
Wank, M.: LB2570-435, LB3620-378
Wanka, A.: 575
Warburton, J.: 1385-206
Ward, C.: LB3620-364
Ward, D. V: 820-29
Ward, M.: LB3620-359
Ward, R.: LB1545-363
Ward, R.: LB2570-408
Ward, S.: 710
Ward, S.: 2425-280
Warden, K.: 1360-161
Ware, O.: LB3620-355
Waris, A.: LB2570-373
Warner, D. F: 3070
Warraich, H.: 870-182
Warren-Clem, K.: 2385-174
Washburn, A. M: 2505
Washington, K. T: 1080
Washington, T.: LB3620-375
Washington, T. R: 770, 1160, 1401-287, 1485
Wasiak, M. F: 3520
Waszynski, C.: 2315-5
Watanabe, T.: LB2570-444
Watanabe, Y.: LB1545-346
Watanabe, Y.: 895-232
Waters, L.: 1530, 3300-94, 4075, 4170
Watkins, J.: 1055
Watkins-Dukhie, K.: 3430
Watson, K.: LB935-442
Watts, A.: 500, 2410-250, 3175
Waycott, J.: 1385-206
Weaver, A. A: LB1545-390
Weaver, B.: 1410-329, 2550
Weaver, R. H: 1305-26, 2385-189, 2435-314, 3345-223
Webb, C.: 3290-61
Webb, C.: LB3620-334
Weber, K.: 2375-143
Webster, B. A: 1350-129, 1460
Webster, N. J: 745, 3205, LB3620-400
Weech-Maldonado, R.: 910-262, 2185, 2250, 2395-218, 3345-213, 3435
Wehlandl, E.: 2360-101
Wehr, H.: 810-2
Wehry, S.: LB3620-350
Wei, J.: LB3620-369
Wei, L.: 2370-128
Wei, M. Y: LB2570-389
Wei, S.: 780
Wei, W.: 3320-142
Weil, J.: 2505, 3140
Weimert, J.: 2360-101
Weiner, B.: 520
Weiner, D.: 3280-33
Weinreich, D.: 2100, 3005
Weintraub, S.: 830-76
Weir, M.: 2380-157
Weiss, D.: 3380
Weisse, C.: 3375-312
Welch, S. A: LB2570-380
Welch, V.: 3415
Wellar, H.: 3290-45
Wells, K.: LB935-392
Wells, M.: 3295-76
Wells, Y.: 2395-210
Welsh, D.: LB1545-417
Wen, J.: LB935-411
Wendel-Hummell, C. L: LB2570-425
Wendell, S. G: 1425, 3275-24
Weng, T.: LB935-350
Werner, C.: 920-306
Wernher, I. A: 2325-27
Wernli, K.: LB2570-368
West, J. S: 805, 2410-263
West, W. G: 2380-155
Westbrook, R.: LB3620-364
Westra, J.: 3355-250
Wetherell, J. L: 4080
Wetle, T.: 880-194

Wetstone, R.: 3315-130
Weuve, J.: LB1545-410
Wharton, T. C: 1320-62
Wharton, T.: 2313
Wharton, W.: 895-238
Wheaton, F. V: 1400-254, LB3620-423
Wheeler, V.: 2370-136
Whitaker, E.: 3295-89
White, A.: 740, 3330-179
White, D.: 910-277, 3345-214, LB2570-404
White, H.: 1305-24
White, K.: 1305-28, 4055
White, R.: 1400-257
White, T.: 830-67, 835-84
Whitehead, B.: 2410-247
Whitehead, I.: LB1545-393
Whitehouse, C. R: 1170
White-Ryan, L.: LB2570-340
Whitfield, K. E: 3130, 3290-53, 3360-260, 3475
Whiting, C.: 1030, 1230
Whitlatch, C.: 1160, 2245
Whitley, D.: 3245
Whitlow, R.: 2405-231
Whitmire, M.: 760
Whitsel, E.: LB2570-441
Whitson, H. E: 2240, 4130
Whitson, J.: 835-101
Whittaker, A. C: LB935-398
Whittier Eliason, S.: 2385-193
Wicklander, K.: LB1545-412
Widen, S.: LB1545-354
Wiener, C.: 2550
Wierenga, K.: 1330-75
Wiese, L. K: 2500
Wilber, K.: 2375-150, 3025
Wilckens, M. R: 3520
Wild, E.: 3180
Wild, K.: 1401-282, 3535, LB1545-430
Wiley, J.: 3330-190
Wilkens, L. R: LB935-428, LB1545-392
Wilkerson, T.: 2425-280
Wilkinson, L. R: 885-209
Wilkinson, S.: 3010
Wilks, S. E: 2385-192
Willcox, B. J: 830-72
Willcox, C.: 830-72
Willemse, B.: 840-110
Willett, W.: LB2570-367
Williams, A.: 910-278
Williams, A. R: LB3620-366
Williams, B.: 2425-282
Williams, C.: 740, 3330-179
Williams, C.: 3295-86
Williams, K. A: 3430
Williams, K. N: 850-133, 1495, 2355-74, 3550, 3560, LB2570-364
Williams, N.: 3275-26
Williams, R.: 1490
Williams, S.: 915-290
Williams, W.: 930-343
Williams-Blangero, S.: LB935-402
Williams-Farrelly, M.: 730
Williamson, E. A: 3315-120
Williamson, J.: 4045
Willie-Tyndale, D.: 3045
Willink, A.: 665, 4155
Willis, S.: 2265, 2410-259, 3290-47
Willoughby, L.: 2405-238
Willoughby, L.: 3350-235
Wilmoth, J. M: 1145, 2055, 2375-145
Wilson, C.: LB3620-369
Wilson, D. A: LB935-438
Wilson, D. M: 3285-40
Wilson, J.: 885-203, 3370-298, LB2570-360
Wilson, K.: 1380-198, 2250
Wilson, L. M: 2380-156
Wilson, R. S: 3480
Wilson, S. J: 2275, 2420-275
Wilson, T. L: 2385-183
Wilson-Frederick, S.: 2250
Wilton, S.: 3275-15
Winblad, B.: 2215
Winchester, B. S: 1505
Windham, G.: LB2570-401
Windsor, T. D: LB935-387, LB1545-445
Winfree, J.: 910-277, 3345-214, LB2570-404, LB3620-390
Wingood, M.: 2360-89
Winningham, R.: 2310
Winterton, R.: 560, 3285-41
Wion, R.: LB2570-358
Wisniewski, K. M: LB2570-350
Wisniewski, M.: LB2570-435, LB3620-378
Wister, A.: 3285-39
Witkowski, S.: 715
Witsaman, R.: LB935-416
Wittich, W.: 850-142
Wladkowski, S.: 4170
Wohlgemuth, W.: LB3620-338
Wöhrmann, A. M: 3520
Wojczynski, M. K: 1310-42, 3170, LB2570-338, LB3620-337
Wolf, M.: 3595
Wolf, M.: LB1545-430
Wolf-Ostermann, K.: 785, 2020
Wolfe, A.: 920-310
Wolfe, J.: 2360-101
Wolfe, R.: 870-180
Wolff, J. L: 1110, 1170, 1495, 2045, 2325-25, 3420
Wolff, J. K: 3615
Wolfram, A.: 2415-269
Wong, A.: 1390-229
Wong, C.: 2360-96
Wong, G.: 2400-227
Wong, G. H. Y: 845-121, 915-294, 2425-287, 3075, LB1545-368, LB3620-440
Wong, J.: 850-131, 3360-265
Wong, J. D: 3180, 4120
Wong, R.: 3220, 4155, LB1545-345
Wong, R.: 2355-81
Wong, S.: 3335-195
Wong, T.: 3605
Woo, B.: 3250
Woo, J.: 2425-286
Wood, K. A: 1350-134
Woodard, J. L: LB3620-389
Woodham, S.: 1170
Woodhouse, M.: 2155
Woods, N. F: 1455
Woods, R. L: 870-180, 3260
Woods, T.: 1365-168
Woodspring, N.: 1385-211, 3155
Woodward, A.: 3415, 4020
Woodward, J. M: LB2570-327
Woog, S.: 780
Wool, J.: LB935-396
Wooten, K.: LB935-362
Worthalter, D.: 3310-116
Wortman, E.: 2405-243, 4160
Woulfe, K.: LB1545-402
Wrangham, R.: LB1545-431
Wray, L. O: 2015, 2535
Wray, L. A: 4120
Wright, E.: 1330-81
Wright, K. D: 845-117
Wright, R.: 515
Wright, R. S: 655
Wright, T.: 3305-102
Wright-Freeman, K.: 780
Wroolie, T. E: LB935-442
Wu, B.: 1105, 1297, 1340-103, 1435, 2340-43, 3090, 3160, 3315-129, 3320-142, 3495, 1300-1
Wu, B.: LB3620-342
Wu, C.: 805, 4045
Wu, C.: 820-32
Wu, D.: 825-60, LB1545-440
Wu, H.: 4145
Wu, J.: 2475
Wu, M.: LB935-429
Wu, S.: LB935-429
Wu, S.: 910-267
Wu, S.: 2475, 4005
Wu, S.: 910-267
Wu, Y.: 3160, 3290-54, LB935-426, LB1545-392
Wu, Y.: 4040
Wu, Z.: 4030
Wyatt-Brown, A.: 1185
Wylie, M. J: 3110
Wyman, M. F: 2015

### X

Xiang, A.: 3175
Xiang, X.: 2495, 4150
Xiao, Y.: 3335-193
Xie, B.: 2165, LB2570-330
Xie, B.: 3590
Xu, B.: 1330-77, 2370-137, 4005
Xu, B.: LB935-381
Xu, F.: 1170, LB2570-422
Xu, H.: 1105
Xu, H.: LB2570-315
Xu, H.: 2400-229
Xu, L.: 910-263, 2340-46, 2365-111, LB3620-405
Xu, M.: 4110
Xu, S.: 915-297
Xu, X.: 1305-30, 1350-122, 3230
Xu, X.: 840-106
Xu, Y.: LB2570-385
Xu, Y.: 895-233
Xue, L.: 565
Xue, Q.: 830-75, 2095, 4165
Xue, T.: LB2570-385

### Y

Yadav, H.: LB2570-387
Yaffe, K.: 3075
Yamaguchi, M.: LB1545-344
Yamaguchi, T.: 885-205
Yamaguchi, Y.: 830-63
Yamamoto-Mitani, N.: 895-225
Yamasaki, J.: LB1545-337
Yamashita, T.: 510, 1325-73, 1385-215, 1400-259, 1401-290, 2010, 3300-99, 3330-191
Yamauchi, K.: 830-63
Yamin, S.: 840-114, 3595
Yan, E.: 1355-143, 2320-17, 2375-152
Yan, M.: 1305-17
Yan, S.: 1315-58
Yanai, S.: LB2570-411
Yancura, L.: 1460
Yang, B.: 2370-121, 2390-198
Yang, E.: 1365-173
Yang, F.: 825-42
Yang, F.: 1515
Yang, H.: 830-74
Yang, H.: 2410-246
Yang, J.: 3325-156
Yang, J.: LB935-381
Yang, J.: 865-176, 1380-191
Yang, K.: LB935-433
Yang, M. S: 2400-221
Yang, M.: 2385-182
Yang, R.: LB935-400
Yang, W.: 1375-186, 3395
Yang, X.: 2005
Yang, X.: 705
Yang, Y.: 925-319
Yankeelov, P.: 1345-107, 1365-167, 2355-76, 3300-90
Yao, L.: LB1545-340
Yao, N.: LB935-408
Yao, S.: 1330-77, 2370-137, 4005
Yap, T.: 3495
Yarbro, J.: 835-85
Yarbrough, M.: 2270
Yashin, A.: 825-60, 2370-132, LB1545-440
Yashkin, A.: 825-60
Yashkin, A.: 2370-132
Yasunaga, M.: 855-154
Yasuzato, C.: 3300-91
Yates, T.: 1355-145
Yazdani, N.: 3290-49
Ye, L.: 3330-185
Ye, M.: 1500, 3345-231, 4160, LB2570-383, LB3620-354
Ye, Z.: 1300-1, 3160
Yeager, K.: 1510
Yee, B.: 2140
Yefimova, M.: 2465
Yeh, C.: 665, 1015, 1315-60, 1400-268, 1425, 2380-166, 2420-270, LB935-355
Yeh, C.: 835-93
Yeh, J. C: 595, 2540, LB935-377
Yeh, S.: 1401-288
Yen, K.: LB2570-439
Yentes, J. M: 3295-80
Yeom, H.: 2340-51
Yeung, D.: 865-171, 1325-69, 3370-293
Yeung, K.: 895-233
Yi, E. G: 880-200
Yin, C.: 1315-50
Yin, L.: 1095
Yip, A.: LB1545-341
Ylitalo, K.: 2410-248, 2445
Yonashiro-Cho, J.: 2370-118
Yonashiro-Cho, J.: 3025
Yoneda, M.: LB1545-344
Yoneda, T.: LB2570-347
Yong, M.: 4155
Yoo, J.: 3315-121
Yoon, E.: LB3620-420
Yoon, H.: 615, 1325-70
Yoon, J.: 1325-72
Yoon, J.: 1402-305
Yorgason, J. B: 865-168, 1401-277, 3185, 3290-45
York, A. M: 2305
Yoshikawa, A.: 3300-96
Yoshinaga, F.: 3375-314
Yoshino Benavente, J.: 3595
You, L.: LB3620-403
You, T.: 3220
You, Y.: 1340-102
Youkhana, F.: LB935-426, LB1545-392
Younan, D.: LB2570-441
Young, H.: 2295, 2440, 3450
Young, M. A: 1305-18
Young, T.: LB3620-414
Young, Y.: 2155, 2385-182, 4165
Yous, M.: 1395-245
Yousefi Nooraie, R.: 2130
Yow, W.: LB1545-388, LB2570-344
Yu, C.: 1330-85
Yu, D.: 845-120, 4080
Yu, F.: 3175, LB1545-380
Yu, G.: 825-45
Yu, H.: 1355-146
Yu, H.: 910-267, 1300-7
Yu, K.: 1060, 2475
Yu, Q.: LB935-366
Yu, R.: LB1545-365
Yu, S.: 1345-116
Yu, T.: 2385-182
Yu, W.: 1402-302
Yu, Y.: 825-42
Yuan, L.: LB1545-446
Yue, L.: LB3620-319
Yuen, B.: 2360-95
Yun, J.: 890-215
Yun, J.: LB2570-432
Yun, S.: 830-78
Yun, S. W: 2355-68
Yuri, A.: 925-319
Yusifova, M.: LB2570-346

### Z

Zaccaria, D.: 2425-281
Zahiri, M.: 1402-297, 2360-99, 3325-156
Zahodne, L. B: LB1545-410
Zaia Rodrigues, A.: 820-25
Zaidel, C.: 2420-270
Zakrajsek, A. G: LB2570-342
Zakrajsek, J. S: 1410-326
Zambrano Garza, E.: LB3620-383
Zammit, A. R: LB3620-318
Zang, E.: 3145
Zaninotto, P.: 730, 2370-128
Zanjani, F. A: 2425-280, 3300-94
Zanjani, F.: 3515
Zanotti, P.: LB935-444
Zaragoza, G.: LB1545-382
Zare, H.: 3360-260
Zaslavsky, O.: LB935-445, LB1545-358
Zauszniewski, J.: 890-216
Zavala, C. M: 655
Zazove, P.: 2010
Zebrak, K.: 2045
Zecevic, A.: LB935-394
Zechner, M.: 2435-306
Zeisel, J.: 500
Zeisel, J.: 2355-63
Zeki Al Hazzouri, A.: 3595
Zelalem, A.: 905-251
Zelinski, E. M: 925-317, 1401-284, LB2570-350
Zell, A. M: 1305-29, 4005
Zelner, J.: 925-337
Zeng, Y.: 910-271
Zettel-Watson, L.: 3365-272
Zgibor, J. C: 565

Zhai, F.: 1515
Zhan, L.: LB2570-324
Zhang, A.: 3250, 3275-25
Zhang, B.: LB1545-377
Zhang, C.: LB3620-425
Zhang, C.: 825-42
Zhang, F.: 845-120
Zhang, F.: LB3620-432
Zhang, G.: 3520
Zhang, J.: 845-123
Zhang, K.: 3090, 3290-54
Zhang, L.: LB935-388
Zhang, L.: 2115
Zhang, N.: 820-39, 830-76
Zhang, Q.: LB935-373
Zhang, Q.: LB1545-377
Zhang, Q.: 3320-133
Zhang, Q.: 1355-137
Zhang, S.: LB2570-322
Zhang, S.: 700
Zhang, T.: LB1545-387
Zhang, T.: 820-28
Zhang, W.: 3090, 3160, 3290-54
Zhang, W.: 1400-253, 2370-124
Zhang, W.: 1395-247
Zhang, W.: LB935-432
Zhang, X.: LB3620-377
Zhang, X.: 2375-146, 3380, 3605, LB935-415, LB3620-365
Zhang, X.: LB935-409
Zhang, X.: 830-67
Zhang, Y.: 1405-317, 1515
Zhang, Y.: 845-121
Zhang, Y.: 2165
Zhang, Y.: 3145
Zhang, Y.: 1425, 2315-14
Zhang, Z.: 2400-229
Zhao, E.: LB3620-361
Zhao, F.: LB3620-429
Zhao, L.: 2340-46
Zhao, L.: 2385-184
Zhao, S.: 740, 3535
Zhao, X.: 3390
Zhao, X.: 1355-137
Zhaoyang, R.: 4015
Zheng, D.: 1395-246
Zheng, H.: 825-47, LB935-364
Zheng, J.: 3505
Zheng, M.: 2400-225
Zheng, Q.: 740
Zheng, Q.: 1350-122
Zheng, Q.: 3330-179
Zheng, S.: 3505
Zheng, Y.: 1360-162
Zheng, Z.: 1395-242
Zhong, C.: 1402-313
Zhou, H.: 2360-99, 3325-166, LB935-381
Zhou, J.: 2410-256
Zhou, J.: 1402-302, 4020
Zhou, Y.: 3355-256
Zhou, Y.: 500
Zhou, Y.: 3110, 3350-242
Zhou, Y.: 3250
Zhu, H.: 1402-302
Zhu, Q.: 3345-231
Zhu, Y.: 1315-59, LB1545-443
Zia, G.: 1402-298, 3325-159
Zidar, D. A: 835-86
Ziegler, U.: 1402-315
Zigler, M.: 835-89
Zijlstra, G.: 565, 3035, 3270-5
Zimmer, R.: LB3620-421
Zimmerman, S.: 520, 3345-224
Zipunnikov, V.: LB935-446
Zisberg, A.: 2315-7, 3420, LB1545-375
Zissimopoulos, J.: 4155
Zmora, R.: 1385-210, 1401-278, 2355-71
Zmuda, J. M: 1425, 2370-125, 3170, 3275-24, LB1545-440, LB3620-337
Zou, M.: 820-28, 3325-172
Zubatsky, M.: 1020
Zucchelli, A.: 3170, 3325-173
Zulka, L.: 590
Zullo, A.: 4130
Zumo, J.: LB1545-402
Zuniga, J.: 3600
Zuniga, K. E: 3295-82
Zúñiga, F.: 2020
Zuo, D.: 3335-194
Zurlo, K.: 2030
Zwakhalen, S. M: 2380-164
Zweig, R.: 915-280
